# Urban-rural distinction of potential determinants for prediabetes in Indonesian population aged ≥15 years: a cross-sectional analysis of Indonesian Basic Health Research 2018 among normoglycemic and prediabetic individuals

**DOI:** 10.1186/s12889-020-09592-7

**Published:** 2020-10-06

**Authors:** Frans Dany, Rita Marleta Dewi, Dwi Hapsari Tjandrarini, Julianty Pradono, Delima Delima, Kambang Sariadji, Sarwo Handayani, Nunik Kusumawardani

**Affiliations:** 1 Centre for Research and Development of Biomedical and Basic Health Technology, National Institute of Health Research and Development (NIHRD), Ministry of Health, Jakarta Pusat, Jakarta Indonesia; 2 Centre for Research and Development of Public Health Efforts, National Institute of Health Research and Development (NIHRD), Ministry of Health, Jakarta Pusat, Jakarta Indonesia; 3 Centre for Research and Development of Health Resources and Services, National Institute of Health Research and Development (NIHRD), Ministry of Health, Jakarta Pusat, Jakarta Indonesia

**Keywords:** Prediabetes, Determinants, Urban, Rural, Indonesia

## Abstract

**Background:**

Based on Basic Health Research (RISKESDAS) conducted by Ministry of Health, Indonesia, prediabetes prevalence tends to increase from 2007 until 2018. The numbers are relatively higher in rural than those in urban area despite of small discrepancies between the two (~ 2–4%). The purpose of this study was to identify urban-rural differences in potential determinants for prediabetes in Indonesia.

**Methods:**

This analysis used secondary data collected from nationwide Health Survey in 2018. Respondents were aged ≥15 years who met inclusion criteria of analysis with no history of diabetes mellitus. Prediabetes criteria followed American Diabetes Association 2019. Multiple logistic regression was also employed to assess the transition probability of potential determinants for prediabetes in urban and rural Indonesia.

**Results:**

Up to 44.8% of rural respondents were prediabetics versus their urban counterparts at 34.9%, yet non-response bias was observed in the two. Young adults aged 30 years were already at risk of prediabetes. Urban-rural distinction for marital status and triglyceride level was observed while other determinants tended to overlap across residence. Several modifiable factors might contribute differently in both population with careful interpretation.

**Conclusions:**

The minimum age limit for early prediabetes screening may start from 30 years old in Indonesia. Urban-rural distinction for marital status and triglyceride level was observed, yet non-response bias between the two groups could not be excluded. A proper model for early prediabetes screening need to be developed from a cohort study with adequate sample size.

## Background

Increasing trend of diabetes mellitus (DM) as one of important non-communicable diseases (NCDs) globally is attributable to many factors, one of which being lifestyle change. One of important determinants contributing to blood glucose intolerance is prediabetes. It is estimated that one third of prediabetes (which include impaired fasting and glucose tolerance) cases will develop into diabetes [1, 2]. According to Basic Health Research (Riset Kesehatan Dasar, Riskesdas) conducted by Ministry of Health, the prevalence of impaired glucose tolerance, particularly in urban area underwent dynamic changes from 10.2% in 2007 to 29.9% in 2013 and declined to 28.8% in 2018. Meanwhile, proportion of impaired fasting glucose shifted from 36.6% in 2013 to 26.3% in 2018. In 2007, fasting blood glucose was not measured in Riskesdas and the same analysis criteria was employed in 2013 and 2018 [3–5].

Interestingly, prediabetes numbers in Indonesia rural areas were relatively higher than those in urban. In 2013, proportion of impaired fasting glucose (IFG) in rural area was recorded at 38.2% vs 34.9% in urban. While proportion of impaired glucose tolerance (IGT) were not too much different between both (29.8% in rural vs 29.9% in urban). In 2018, IFG & IGT prevalence in rural was still higher than that in urban (27.7% vs 25.1% for IFG and 33.1% vs 28.8% for IGT) if the same analysis criteria of Riskesdas 2013 was implemented [4, 5]. When operational definition of American Diabetes Association (ADA) 2019 was used for 2018 data [6–8], those figures become smaller, yet the numbers in rural areas were still higher (14.0% vs 12.3% for IFG and 21.9% vs 17.9% for IGT) [5]. This implied a serious public health problem since metabolic diseases began to predominate in rural areas. Urbanisation influence in rural areas were reported in several studies and accounted for increasing prevalence of diabetes [9, 10]. Some determinants played as significant contributors for prediabetes. Some others were not statistically important but they may act as key confounders and hence, could not be excluded from the logistic regression model. These determinants or factors associated with prediabetes were but not limited to life style habits such as diet pattern, smoking, sedentary lifestyles, obesity, hypertension and dyslipidemia [11–14]. Urban-rural differences for prediabetes and diabetes were also demonstrated and shown to provide consideration in developing strategy towards controlling diabetes mellitus, specific for each residence category [9, 15, 16]. Hence, the aim of this study was to identify urban-rural distinction of potential determinants for prediabetes based on information gathered in Riskesdas 2018. The results from this investigation are expected to develop an early prediabetes screening algorithm specific for urban and rural population, which will be validated by discrimination analysis for each logistic regression model.

## Methods

This is a cross-sectional analysis using secondary data collected from Basic Health Research (Riset Kesehatan Dasar, RISKESDAS) which was conducted by Ministry of Health, Republic of Indonesia in March 2018. RISKESDAS is a nationally representative household health survey that has been conducted every 3 years since 2007 (in 2007, 2010 and 2013) by the National Institute of Health Research and Development (NIHRD), Ministry of Health, Indonesia [3–5]. Participants were selected using a multistage systematic random sampling method. The first stage identified groups of census blocks and designated them as primary sampling units (PSUs). The second stage used a probability proportional to enrolment size design to identify a census block from each PSU. From the master frame of 720,000 census blocks (CB) obtained in 2010 Indonesian population survey by The Indonesian Central Bureau of Statistics or Badan Pusat Statistik (BPS), 180,000 were selected as the sampling frame using Probability Proportional to Size (PPS) method (see Additional file 3). Another PPS considering urban-rural distribution proceeded with linear systematic sampling: implicit stratification at the level of 30,000 CB, followed by implicit stratification at the household level. The third stage comprised systematic random sampling of 10 census buildings from each census block. One household from each census building was randomly chosen at the fourth stage. All household members (defined as those staying in the premises for the past 6 months or more and having the same financial source for foods) of each selected household were asked to participate in the survey. The socioeconomic level was determined by BPS in which household assets as well as average income and expenditure were taken into account before categorizing wealth index into 5 categories (lowest, lower-middle, middle, upper-middle and highest).

Up to 2500 census blocks across 26 provinces with 1446 urban and 1054 rural sites were sub-sampled in representing national level for biomedical data collection. The RISKESDAS and SUSENAS data were then merged and weighted accordingly.

In this analysis, predictors were sociodemographic variables (age, gender, formal education level for each respondents aged > 5 years and for those aged ≤5 years, their educational level were represented by their father or head of household, occupational status for each respondents aged ≥10 years. As for those aged < 10 years, their occupational status were represented by their father or head of household. Others include marital status, socioeconomic level and knowledge of access to nearby health facilities), life-style related factors (diet pattern, smoking, physical activity level, alcohol consumption), measurements (waist circumference, hypertension diagnosis from systolic and diastolic blood pressure), level of serum lipid profile (total cholesterol, high-density lipoprotein/HDL, direct low-density lipoprotein LDL direct, triglycerides). As for the binary outcome variable was prediabetes status with those having normal plasma glucose as the reference group.

### Ethical consideration

Ethics and permissions for conducting this study followed the Ethical Approval for RISKESDAS 2018 from Komisi Etik Penelitian Kesehatan, Badan Penelitian dan Pengembangan Kesehatan (Ethical Committee of Health Research, NIHRD, Ministry of Health, Republic of Indonesia) No. LB.02.01/2/KE.267/2017.

### Primary data collection from basic Health Research survey in 2018

Selected respondents from all age groups (from newborn until elders) representing national level across 26 Indonesian provinces, originally in 1446 urban sites and 1054 rural sites. This subsampling in this study was part of the larger sampling frame determined by BPS (see Additional file 1: Figure S1). Selected enumerators from public health background (mostly fresh undergraduates) had had to participate in standardized training/workshops before they participated in the primary data collection. The trained enumerators then performed interview using validated questionnaires (see Additional file 4) in Bahasa Indonesia and measurement (blood pressure for those aged ≥18 years old, body weight for all age group, body height/length for all age group, waist circumference for respondents aged ≥15 years old except for pregnant women, mid-upper arm circumference for reproductive age women 15–49 years old or pregnant women) in household. The respondents were contacted more than once should data confirmation or completion could not be fulfilled in one visit. All interviews and measurements in respective household and biomedical testing were observed, assessed and validated by public health experts in selected areas. A makeshift laboratory in nearby local health facilities was set up to facilitate sample collection and point-of-care test (haemoglobin level using®Hemocue 201 Hb + for all age group, fasting blood glucose and 2-h oral glucose tolerance test using®Accuchek Performa for those aged ≥15 years old), helped and supervised by medical professionals. Inclusion criteria for sample collection were good general health status, agreed and had given their written informed consent declared by themselves if respondents were 15 years above or represented by their parents if they were below 15 years old. Subjects were excluded if they had history of blood coagulation abnormality or idiopathic thrombocytopenic purpura (ITP), heart diseases, consuming thrombolytic agents or steroids, and/or decline to participate in the study. A general examination by physicians was conducted and informed consent was reassured prior to on-site laboratory test and sample collection. Serum specimen was collected and sent to National Laboratory in Jakarta in order to check its lipid profile (total cholesterol, direct LDL, HDL and triglycerides) using enzymatic assay with®Roche. Health indicators stated in questionnaire encompassing sociodemographics, life-style habits, measurement and biomedical parameters were chosen after being reviewed internally and externally by public health experts. This process involved the exclusion of body mass index (BMI) variable from analysis by external reviewers due to potential multicollinearity with waist circumference and redundancy in reflecting nutritional status. The exclusion of variables by external reviewers also applied to self-reported questionnaire (SRQ) assessing mental health since this indicator was focused on depression assessment and not specifically designed for addressing stress.

Prediabetes definition criteria refer to American Diabetes Association (ADA) 2019 which include one or more fo the followings [7, 8]:

Impaired fasting glucose (IFG): fasting blood glucose 100–125 mg/dl with normal oral glucose tolerance test (OGTT) results, which is < 140 mg/dl or impaired glucose tolerance (IGT): oral glucose tolerance test (OGTT) in range 140–199 mg/dl, yet with normal fasting blood glucose < 100 mg/dl or both IFG and IGT.

In addition to prediabetes definition, analysis for other variables considers the following as reference: Physical activity level follows guidelines proposed by WHO [17]. Lipid profile cut-offs refer to the National Cholesterol Education Program Adult Treatment Panel (NCEP-ATP) III [18] and American Association of Clinical Endocrinologist (AACE) 2017. The respective cut-offs of lipid were as follows: total cholesterol was normal if < 200 mg/dl and abnormal when ≥200 mg/dl, normal LDL at < 130 mg/dl. As for HDL, normal level for men if its titer ≥40 mg/dl and normal HDL level for women if ≥50 mg/dl. While normal triglyceride level below 150 mg/dl is considered normal [19]. Blood pressure (as proxy for macrovasular dysfunction) values for systole and diastole adopt those described in Joint National Committee (JNC) VIII (2014). Hypertension is established if systolic blood pressure ≥ 140 mmHg and/or diastolic blood pressure ≥ 90 mmHg [20]. WHO criteria for Asia-Pacific (2000) was implemented pertaining to waist circumference [21]. As for smoking behaviour, Brinkmann index and amount of cigarettes smoked per day were included [22–24]. Last but not least, the criteria for harmful alcohol consumption by National Institute on Alcohol Abuse and Alcoholism (NIAAA) referring to Dietary Guidelines for Americans 2015–2020 were selected [25].

### Subject criteria for secondary data analysis

Respondents were aged ≥15 years with no history of diabetes mellitus who underwent biomedical procedures of random plasma glucose, fasting plasma glucose and oral glucose tolerance test. Missing data were already handled in primary data, mainly by confirming related information to enumerators responsible for data collection.

### Statistical analysis

Secondary data were weighted and adjusted by age and sex. Quantitative variables were checked for its normal distribution. Their mean differences were assessed using independent t test with complex samples or Mann-Whitney U test in case of non-normal distribution. Cut-off point classification (using SPSS) employed visual binning method for continuous data and clustered graphs for categorical variables. Composite measurement of dietary pattern was evaluated using factor analysis with principal component method, eigenvalue cut-off point > 1, loading factor > 0,5 with varimax rotation. Main predictors were dietary pattern, history of smoking, physical activity, anthropometric and blood pressure measurement and results of biomedical POCT with sociodemographic factors as potential confounders. Meanwhile, the dependent variable was prediabetes status (normoglycemia referred as normal glucose tolerance or NGT versus prediabetes). Early selection of potential factors associated with prediabetes was performed using univariate logistic regression. Complex sample technique was employed in descriptive cross-tabulations as well as multivariate logistic regression analysis by taking into account any possible multicollinearity, confounders, interaction effect and goodness-of-fit. The logistic model was also evaluated regarding their discrimination predictability by means of receiver operating curve (ROC) analysis. Population attributable fraction was calculated for selected modifiable determinants by using ‘punafcc’ package of Stata. In this analysis, final results are presented as *odds ratio* (OR) with 95% confidence interval. Significance is determined at *p value* < 0.05. The above statistical procedures were performed using SPSS version 16 dan Stata version 9.

## Results

There was substantial sample reduction in urban and rural groups (see Additional file 1: Figure S1), which may account for result differences between the two. From total of 19,684 subjects after our final selection process, the weighted proportion of prediabetes was higher in Indonesian rural with 44.8% versus 34.9% in urban (see Additional file 1: Figure S2). Proportion of prediabetics according to sociodemographic characteristics was consistently higher in rural population as compared to urban ones. As shown in Additional file 2: Table S1, the proportion of prediabetes in rural population was higher than that in urban counterpart (45.2% vs 37.0%). Prediabetic subjects were observed more in women, aged ≥30 years, low-education level, working either as farmer, fisherman, unemployed or other jobs, married, low-socioeconomic level and interestingly, easier access to health services. Those who only completed their formal education up to elementary school (6 years in Indonesia) were deemed at the low-education level. Meanwhile, respondents coming from the lowest and lower-middle level of household income and wealth index were included in the low-socioeconomic group.

Overview of continuous variable distribution between both residences are shown in Additional file 2: Table S2. Respondents with prediabetes tended to be older as compared to normal blood glucose group. Waist circumference, total cholesterol, LDL level and diastolic blood pressure were shown to have strong statistical significance among all possible pairwise comparisons and their corresponding values in prediabetes category were higher than those in NGT. In general, urban-rural differences were observed for age, waist circumference, lipid profiles and blood pressure.

The logistic regression analysis results presented potential determinants or predictors associated with prediabetes. The odds ratio were adjusted by sex besides residence category and reported accordingly in Additional file 2: Table S3-S4. From these two tables, several determinants were found to be significant both in urban and rural people, and other variables were not, such as fruit and vegetable intake, smoking and alcohol consumption. When observed separately and gender was also taken into consideration, some factors showed different results between the two residences, for example lipid profiles and risky diet pattern.

In this study, most respondents were women and women have higher risk of central obesity but central obesity were shown have no significant association after model-fitting in rural women population. This information triggered us to speculate its relationship with difference of physical activity level between urban and rural women and performed additional Mann-Whitney U test of physical activity between the two. The results of this test was shown in Additional file 1: Figure S3 where rural women tended to engage in more physical activities than their urban counterparts.

The discrimination analysis for four logistic models (urban men, urban women, rural men, rural women) were varied, particularly when it comes to sensitivity, specificity and percentage of correct classification (see footnote for Table S3-S4 in Additional file 2). The overall numbers for sensitivity were higher for rural group while higher specificity was observed in urban ones.

Last but not least, adjusted proxy of population attributable fraction for cross-sectional study in Additional file 2: Table S5 showed that several variables potentially contributing to prediabetes reduction were applied both in urban and rural, while remaining factors, notably grilled food diet and several lipid parameters, exhibit some contrast.

## Discussion

The overall proportion of prediabetics in this study was above 39% (see Additional file 1: Figure S2) and was higher than that described in other Asian countries [26–28]. This can be explained by inclusion criteria implemented in our study. Those with diabetes mellitus were not included and hence cannot be considered as the overall prediabetes prevalence for Indonesia population in 2018 since this analysis focused on identifying potential determinants only for prediabetes between residence category. From characteristic distribution point of view, all sociodemographic variables presented in Table S1 were significantly different between urban and rural group with the overall prediabetes proportion of 45.2% in rural versus 37.0% in urban.

Investigations over urban-rural differences of risk factors for non-communicable diseases particularly diabetes dan prediabetes are not something new and have been carried out in several studies [9, 16, 29]. Nevertheless, the data are still limited with regard to national representativeness for some developing countries with heterogenuous characteristics in Asia, including Indonesia. This analysis made use of informations gathered via Basic Health Survey (Riskesdas), a nationwide cross-sectional study conducted by Indonesian Ministry of Health in 2018.

There were some distinct characteristics between urban and rural shown in this study (see Additional file 2: Table S1-S2) and other investigations regarding risk factor for diabetes in general which already shifted over the last few years and began to affect both areas due to rural-urban migration [9, 15, 30]. In Additional file 2: Table S1, there is no significant relationship for access to health services, which is different from what we hypothesized earlier that it might lead to urban-rural discrepancies of prediabetes risk. This information signalled other main factors, notably lifestyle and environmental conditions, played a vital role to more extent [31]. Marital status was portrayed as protective factor in urban population, suggesting the importance of physical and psychological support in maintaining healthy lifestyle from spouse [32, 33]. In this study, significant variables affecting all groups based in residence and sex are the followings: age ≥ 30 years above, hypertension (140/90 mmHg) and low level of education. Another key predictor of prediabetes, central obesity, was found in almost all categories except for rural women. A possible conjecture is that rural women tended to do more physical activities than their urban counterparts and this finding was significantly different using Mann-Whitney U test (see Additional file 1: Figure S3) which is in line with findings asserted in other investigations [9, 34]. Indeed, there were strong associations between physical activity level and obesity reported, yet relatively no urban-rural distinctions were observed unless other variables, such as diet pattern, are included.

As for fruit and vegetable consumption habits, there is no significant association with regard to prediabetes which may be attributable to interview bias [35]. Also, detailed diet recall was not implemented. A relationship between prediabetes and consumption of inherently high-glycemic index fruit or vegetables may be identified from such recall since types of fruit and vegetables based on their sugar content has been shown to affect blood glucose metabolism, lipid profile and blood pressure [36, 37]. On the other hand, sugary-salty-fatty food was expectedly proved to be risk factors except for urban women. The possible conjecture for this is that higher demand in urban working environment rendered them to pay attention to their self-body image and hence, promoting several approaches to achieve ideally standard posture [38]. Interestingly, risky diet pattern like grilled and processed food with preservatives was portrayed significant for urban men. It is possible that those respondents ate this type of food together with sugary-salty-fatty type quite common. Combining different kinds of meal on a daily basis was already proved to yield various relationships with several health parameters [39, 40]. Surprisingly, sports and carbonated beverages did not significantly account for prediabetes. This finding may be contradictory to other investigations, which may be attributable to downsize of cross-sectional analysis [41].

Lipid profile as one reflection of food metabolism also similarly applied in this evaluation. Total cholesterol potentially put urban men at risk of prediabetes which may be contributed by grilled and processed food. High level of LDL affected urban women and this might be due to multiple factors, such low physical activity, poor diet habit and stress. However, low HDL level was observed in all women, regardless of their residency. Again, bad meal composition low physical activity in urban and possibly stress seemed to result in this association, as shown in other studies [42–44].

On the other hand, high level of serum triglyceride was demonstrated to be potential determinants in rural population only, suggesting segregation of diet pattern between two residences. Since triglyceride level is one of parameters employed in establishing metabolic syndrome besides central obesity, high systolic and diastolic blood pressure, abnormal fasting blood glucose and low HDL, the likelihood of metabolic syndrome (MS) in some respondents still remains. The mechanism of MS arising from insulin resistance and enhanced adiposity is closely related to that of impaired blood glucose tolerance [45]. Nevertheless, analysing MS would be beyond the scope of this research.

When it comes to smoking behaviour and alcohol consumption, we found no significant relationship with risk of prediabetes. It should be noted that large discrepancies of samples between reference and case groups may give rise to statistical insignificance [46]. Moreover, alcoholic consumption is not part of daily habit for Indonesians and concentrated in specific populations and regions, particularly in east region. Therefore, clustered analysis using subjects belonging to that group would be more appropriate.

From perspectives of population attributable fraction (PAF), central obesity, hypertension, sugary-salty-fatty diet and high triglyceride level affected both populations (see Additional file 2: Table S5). Suprisingly, LDL level ≥ 130 mg/dl only accounted for prediabetes in rural group while remaining lipid profiles (total cholesterol, HDL) significantly contributed in urban citizens. Variation of risky diet pattern seemed to be the main culprit for this discrepancies [47, 48]. This is not surprising since other variations of urban-rural risk factor differences have also been reported in other developing countries [13, 15, 16, 30, 47, 48]. Yet, the probability of other key factors such as rural-urban migration and physical stress associated with sleep disturbance cannot be excluded and might contribute considerably to higher proportion of prediabetes in rural people [9, 15, 49, 50].

One must also carefully interprete the results of this analysis in the context of several limitations. We realized that the “non-response” bias was hard to minimize in this analysis given considerable reduction in sample size notably in urban group (see Additional file 1: Figure S1). Therefore, urban-rural distinction as well as the overall prediabetes prevalence in the two groups still need further confirmation in a proper longitudinal study. The predictive potential of logistic regression model as reflected from discrimination evaluation was not satisfactorily accurate. In addition, there is still lack of causal relationship explained by a cross-sectional study compared to cohort design [51]. Moreover, due to instrument constraints, the detailed family history for diabetes was also not explored and hence, should be provided in future investigations. As for PAF calculation, odds ratio (OR) instead of relative risk (RR) was used due to restrictive nature of cross-sectional study. OR as proxy for RR in a cross-sectional study using ‘punafcc’ package was already implemented in several studies, which remains potentially debatable [52–54]. Nevertheless, using OR as approximation for PAF was the most optimal effort we could do and thus, these findings must be vigilantly assessed. Although fasting plasma glucose level measurement and oral glucose tolerance test are sufficient to establish criteria for prediabetes, it would be better if HbA1c level measurement can be carried out instead of blood glucose in future studies since its value represents more accurate status of blood glucose control [55]. Besides, comprehensive diet recall should be utilized to minimize bias in evaluating association between meal intake and risk of food-related metabolic diseases. Despite these drawbacks, this study showed that Indonesian population were already at risk of prediabetes even if they were still young adult. Early screening for prediabetes in community health centers need to be developed since a separate approach for prediabetes prevention and management is not included in the main Indonesia health coverage system at least when this article is written. It is important that specific algorithm for prediabetes detection based on several significant risk factors and intervention be implemented to minimize progression of prediabetes into diabetes, like the use of machine-learning based model developed by Choi SB et al. [56] This effort can be further explored in, but not limited to, development of potential specific biomarkers [57].

Despite the limitations, this was one of the very first investigations using integrated data both from nationwide survey conducted by Ministry of Health and Indonesian census data by BPS. The results of Riskesdas 2018 were different from those conducted in 2013 since 2018 data followed the sampling frame developed by BPS for SUSENAS. This was our effort in supporting the ‘One Data’ policy proposed by the current Indonesian Government. In addition, up to four logistic regression models were created and evaluated by discrimination analysis (ROC, sensititivity, specificity, predictive value) in order to illustrate better potential predictors for prediabetes. The least recommendation we can give is that the minimum age limit for early prediabetes screening in Indonesian population may start from 30 years old based on our regression analysis. Considering the bias between urban and rural group due to progressive reduction of respondents, further cohort study with adequate sample size may be carried out to develop algorithm of early prediabetes screening using significant contributors from these models.

## Conclusions

The minimum age limit for early prediabetes screening may start from 30 years old in Indonesia. Urban-rural distinction for marital status and triglyceride level was observed, yet non-response bias between the two groups could not be excluded. Other variables tend to affect both populations. Promoting heathy lifestyle for those with notable risk factors (obesity, hypertension, hypertriglyceridemia) and low education level in a creative way is important. This can be achieved using digital technology in forms of machine learning-based applications to make it more attractive for risky population. Given the sample bias in this analysis, a proper model for early prediabetes screening need to be developed from a longitudinal study like cohort with adequate sample size and detailed instrument for diet recall. Other important factors such as family history, sleep, commuting or urban-rural shifting, utilization of local community-based health center should also be included in the future analysis. Other possibility of developing specific genetic biomarkers for prediabetes can also be considered since most of known risk factors nowadays are still common across region.

## Supplementary information

**Additional file 1: **Additional figure file. List of Figure Titles, Legends and Figures. a. Depicting title and figure as printscreen embedded in Word for **Figure S1.** describing flow chart of sampling stages for urban-rural cross-sectional analysis, taken from Riskesdas 2018 data. **Figure S1.** has no legends. b. Depicting title, legends and figure as printscreen embedded in Word for **Figure S2.** describing weighted proportion of respondents with non-prediabetes and prediabetes, splitted for urban and rural population. c. Depicting title, legends and figure as printscreen embedded in Word for **Figure S3.** describing urban-rural difference for physical activity level in women, analyzed using Mann-Whitney U test.

**Additional file 2: **Additional table file. Tables and Table Footnotes a. Depicting **Table S1.** and its footnotes for sociodemographic characteristic of studied respondents. b. Depicting **Table S2.** and its footnotes for mean difference of age, waist circumference, lipid profile and blood pressure between normal and prediabetics in urban and rural respondents, analyzed using complex sample technique for comparing means. c. Depicting **Table S3.** for results of logistic regression for urban population, adjusted by gender as well as its footnotes describing results of discrimination analysis (Receiver Operating Curve analysis) for each gender logistic model. d. Depicting **Table S4.** for results of logistic regression for rural population, adjusted by gender as well as its footnotes describing results of discrimination analysis (Receiver Operating Curve analysis) for each gender logistic model. e. Depicting **Table S5.** and its footnotes for population attributable fraction (PAF) for selected determinants between urban and rural population using ‘punafcc’ package of Stata.

**Additional file 3.** Supp1 File. STATISTIK KESEJAHTERAAN RAKYAT (WELFARE STATISTICS) 2018. This is a pdf version for the National Socio-Economics Survey (SUSENAS, Survei Sosial Ekonomi Nasional) March 2018, a nationwide survey conducted periodically by Badan Pusat Statistik (BPS-Statistics Indonesia) every March and September. SUSENAS provides data related to social welfare in household units across Indonesia. In this report, a brief explanation of sampling frame, which was adopted in RISKESDAS 2018, is presented in page 5. The questionnaire VSEN18.K used in this survey can also be seen in page 277–297. The data collected by this VSEN18.K were used to measure wealth index for each individual assessed respectively in their household. The full report of SUSENAS 2018 as well as VSEN18.K questionnaire can also be accessed through this link: https://www.bps.go.id/publication/download.html?nrbvfeve=ODFlZGUyZDU2Njk4YzA3ZDUxMGY2OTgz&xzmn=aHR0cHM6Ly93d3cuYnBzLmdvLmlkL3B1YmxpY2F0aW9uLzIwMTgvMTEvMjYvODFlZGUyZDU2Njk4YzA3ZDUxMGY2OTgzL3N0YXRpc3Rpay1rZXNlamFodGVyYWFuLXJha3lhdC0yMDE4Lmh0bWw%3D&twoadfnoarfeauf=MjAyMC0wOS0xOCAxMjozMDoxMg%3D%3D.

**Additional file 4. **Supp2 File. LAPORAN NASIONAL RISKESDAS 2018. This is a pdf version for the Official Report of Indonesian Basic Health Research 2018, a nationwide survey conducted by Ministry of Health, Indonesia every 5 years. In addition to showing the main results of the study, this report also provides attachment of survey tools used for data collection, including the questionnaires. The questionnaires used in the survey can be seen in these pages: a. page 595–602 as Attachment 2 (**Lampiran 2**) for Kuesioner Rumah Tangga (Questionnaire for Household Unit) and. b. page 603–626 as Attachment 3 (**Lampiran 3**) for Kuesioner Individu (Questionnaire for Individuals/Respondents). The Official Report of RISKESDAS 2018 as well as its attachment including questionnaires used in the National Survey can also be accessed through this link: http://labdata.litbang.kemkes.go.id/images/download/laporan/RKD/2018/Laporan_Nasional_RKD2018_FINAL.pdf.

## Data Availability

The datasets generated during and/or analysed during the current study are not publicly available due to the restricted policy of author’s institution, which is implemented in order to prevent misuse of data manipulation and data sharing violation beyond authorized legitimation, but the data may be available from the Data Management Laboratory of NIHRD, Ministry of Health. Republic of Indonesia on reasonable request with prior officially written permission.

## Abbreviations

AACE: American Association of Clinical Endocrinologists
ADA: American diabetes association
CB: Census Blocks
DM: Diabetes mellitus
HDL: High-density lipoprotein
IFG: Impaired fasting glucose
IGT: Impaired glucose tolerance
JNC: Joint National Committee
LDL: Low-denisty lipoprotein
MS: Metabolic syndrome
NCD(s): Non-communicable disease(s)
NCEP ATP-III: National Cholesterol Education Program Adult Treatment Panel
NGT: Normal glucose tolerance
OGTT: Oral glucose tolerance test
