# Supplementary material for: Urban-rural distinction of potential determinants for prediabetes in Indonesian population aged ≥15 years: a cross-sectional analysis of Indonesian Basic Health Research 2018 among normoglycemic and prediabetic individuals
Source: BMC Public Health. 2020 Oct 6;20:1509. doi: 10.1186/s12889-020-09592-7 (PMC7539503; doi:10.1186/s12889-020-09592-7)
Supplement: Supplementary file 1 — Additional file 1: Additional figure file. List of Figure Titles, Legends and Figures. a. Depicting title and figure as printscreen embedded in Word for Figure S1. describing flow chart of sampling stages for urban-rural cross-sectional analysis, taken from Riskesdas 2018 data. Figure S1. has no legends. b. Depicting title, legends and figure as printscreen embedded in Word for Figure S2. describing weighted proportion of respondents with non-prediabetes and prediabetes, splitted for urban and rural population. c. Depicting title, legends and figure as printscreen embedded in Word for Figure S3. describing urban-rural difference for physical activity level in women, analyzed using Mann-Whitney U test. [file 12889_2020_9592_MOESM1_ESM.docx]

**List of Figure Titles, Legends and Figures**

**Figure Titles**

**Figure 1**. Flow chart of sampling stages for urban-rural cross-sectional analysis, taken from Riskesdas 2018 data. Census block was used as primary sampling unit. *Source of information from raw primary data and the Official National Report of Riskesdas 2018.^5^

**Figure 2**. Weighted proportion of prediabetes in urban and rural subjects (excluding those with diabetes).

**Figure 3**. Urban-rural difference for physical activity level in women. Significance obtained using Mann-Whitney U test.

**Legends**

**No legends for Figure 1**

**Legends for Figure 2**

**Left Pie (N=9702)**

Rose pie = weighted proportion of urban population with prediabetes at 34.9% (95% CI : 33.6-36.2)

Blue pie = weighted proportion of urban population with normal glucose tolerance (NGT) at 65.1% (95% CI : 63.8-66.4)

**Right Pie (N=10162)**

Light yellow pie = weighted proportion of rural population with prediabetes at 44.8% (95% CI : 43.4-46.2)

Light purple pie = weighted proportion of rural population with NGT at 55.2% (95% CI : 53.8-56.6)

**Legends for Figure 3**

Green bar = proportion of those with good physical activity

Orange bar = proportion of those with low physical activity

*p value* for physical activity difference between urban and rural women was <0.0001

**Figure 1**


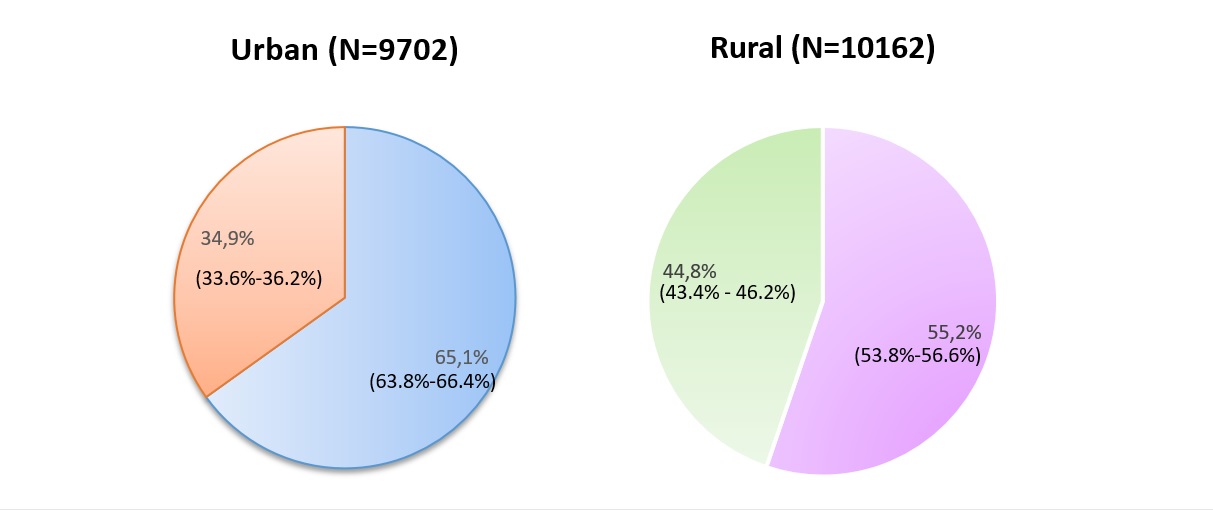


**Figure 2**


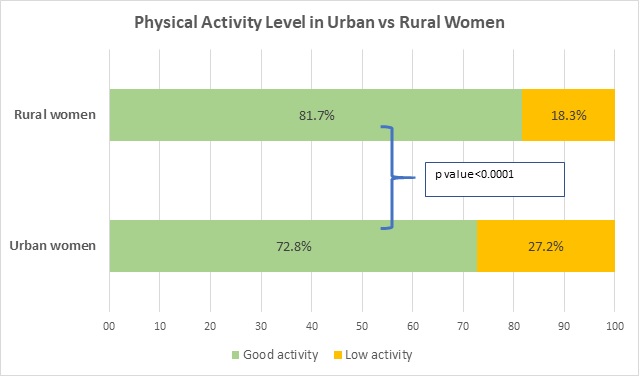


**Figure 3**
