# Supplementary material for: Urban-rural distinction of potential determinants for prediabetes in Indonesian population aged ≥15 years: a cross-sectional analysis of Indonesian Basic Health Research 2018 among normoglycemic and prediabetic individuals
Source: BMC Public Health. 2020 Oct 6;20:1509. doi: 10.1186/s12889-020-09592-7 (PMC7539503; doi:10.1186/s12889-020-09592-7)
Supplement: Supplementary file 2 — Additional file 2: Additional table file. Tables and Table Footnotes a. Depicting Table S1. and its footnotes for sociodemographic characteristic of studied respondents. b. Depicting Table S2. and its footnotes for mean difference of age, waist circumference, lipid profile and blood pressure between normal and prediabetics in urban and rural respondents, analyzed using complex sample technique for comparing means. c. Depicting Table S3. for results of logistic regression for urban population, adjusted by gender as well as its footnotes describing results of discrimination analysis (Receiver Operating Curve analysis) for each gender logistic model. d. Depicting Table S4. for results of logistic regression for rural population, adjusted by gender as well as its footnotes describing results of discrimination analysis (Receiver Operating Curve analysis) for each gender logistic model. e. Depicting Table S5. and its footnotes for population attributable fraction (PAF) for selected determinants between urban and rural population using ‘punafcc’ package of Stata. [file 12889_2020_9592_MOESM2_ESM.docx]

**Tables and Table Footnotes**

**Table 1** **Sociodemographic characteristic of studied respondents based on selection analysis criteria in Indonesia 2018**

|  | **Urban (n=9702)** | **Rural (n=10162)** |  |
| --- | --- | --- | --- |
| **Variables** | **Prediabetes (%)^a^** | **Prediabetes (%)^a^** | ***p-value*** |
| **Gender** |  |  |  |
| Men | 32.1 (30.7-33.6) | 39.9 (38.5-41.1) | <0.001 |
| Women | 40.4 (39.2-41.7) | 49.5 (48.2-50.8) | <0.001 |
| **Age, years** |  |  |  |
| 15-29 | 21.8 (20.1-23.5) | 31.2 (29.2-33.3) | <0.001 |
| ≥30 | 41.8 (40.7-42.9) | 48.6 (47.5-49.7) | <0.001 |
| **Formal education** |  |  |  |
| High | 32.9 (31.7-34.0) | 37.2 (35.6-38.8) | <0.001 |
| Low | 44.3 (42.7-45.9) | 49.4 (48.2-50.6) | <0.001 |
| **Occupation** |  |  |  |
| Employee, students, entrepreneur, labour, driver | 32.0 (30.8-33.3) | 38.8 (36.9-40.6) | <0.001 |
| Farmers, fishermen, others, unemployed | 42.5 (41.1-43.9) | 47.6 (46.4-48.7) | <0.001 |
| **Marital status** |  |  |  |
| Single/divorced | 32.6 (30.8-34.3) | 42.6 (40.5-44.8) | <0.001 |
| Married | 38.7 (37.6-39.9) | 45.9 (44.8-46.9) | <0.001 |
| **Socioeconomic level** |  |  |  |
| Low income | 38.4 (36.7-40.1) | 46.9 (45.6-48.2) | <0.001 |
| Mid-high income | 36.3 (35.2-37.5) | 42.9 (41.5-44.4) | <0.001 |
| **Knowledge about access to health services** |  |  |  |
| Easy | 36.4 (35.1-37.7) | 44.9 (43.0-46.9) | <0.001 |
| Difficult | 37.7 (36.3-39.1) | 45.3 (44.2-46.4) | <0.001 |
| **TOTAL** | **37.0 (36.1-38.0)** | **45.2 (44.2-46.2)** | <0.001 |

^a^Percentages are expressed in non-weighted proportion along with their 95% confidence interval in brackets. Statistical significance determined by independent t test.

**Table 2** **Mean difference of age, waist circumference, lipid profile and blood pressure between normal and prediabetics in urban and rural respondents**

|  | **Urban (n=9702)** | |  | **Rural (n=10162)** | |  |
| --- | --- | --- | --- | --- | --- | --- |
| **Variables** | **Normal** | **Prediabetes** | ***p-value*** | **Normal** | **Prediabetes** | ***p-value*** |
| Age (years) | 39.07  (38.65-39.50) | 46.05  (45.48-46.62) | <0.001 | 41.73  (41.24-42.21) | 47.08  (46.57-47.59) | <0.001 |
| Waist circumference (cm) | 79.76  (79.39-80.13) | 83.29  (82.81-83.77) | <0.001 | 76.42  (76.08-76.76) | 78.46  (78.00-78.93) | <0.001 |
| Total cholesterol (mg/dl) | 176.33  (175.21-177.46) | 187.51  (186.02-189.00) | <0.001 | 173.24  (172.02-174.46) | 181.92  (180.55-183.30) | <0.001 |
| HDL (mg/dl) | 48.12  (47.79-48.44) | 48.19  (47.76-48.62) | 0.837 | 47.85  (47.51-48.19) | 47.89  (47.50-48.29) | 0.815 |
| LDL (mg/dl) | 118.64  (117.67-119.60) | 127.11  (125.83-128.40) | <0.001 | 116.10  (115.12-117.09) | 122.48  (121.29-123.67) | <0.001 |
| Triglycerides (mg/dl) | 116.87  (114.66-119.08) | 130.83  (126.87-135.95) | <0.001 | 115.11  (112.82-117.41) | 124.56  (121.56-127.56) | <0.001 |
| Systolic blood pressure (mmHg) | 126.26  (125.62-126.93) | 134.85  (133.92-135.77) | <0.001 | 128.14  (127.49-128.78) | 135.28  (134.45-136.11) | <0.001 |
| Diastolic blood pressure (mmHg) | 82.91  (82.53-83.29) | 85.83  (85.32-86.34) | <0.001 | 81.98  (81.60-82.35) | 84.77  (84.29-85.24) | <0.001 |

Note: Values are expressed in means along with their 95% confidence interval in brackets. Independent t-test with complex sample technique was employed between normal and prediabetes group to get the *p* value.

**Table 3** **Potential determinants of prediabetes from multiple logistic regression in urban Indonesia, 2018**

|  | **Urban Men^a^** | | | | **Urban Women^b^** | | | |
| --- | --- | --- | --- | --- | --- | --- | --- | --- |
| **Variabel** | **OR *adjusted*** | **95% CI** | | ***p-value*** | **OR *adjusted*** | **95% CI** | | ***p-value*** |
| Age 15-29 yrs | 1 |  |  |  | 1 |  |  |  |
| Age ≥ 30 yrs | 2.154 | 1.636-2.835 | | <0.001 | 1.890 | 1.565-2.284 | | <0.001 |
| No central obesity | 1 |  |  |  | 1 |  |  |  |
| Central obesity | 1.635 | 1.341-1.993 | | <0.001 | 1.233 | 1.081-1.408 | | 0.002 |
| Normotension | 1 |  |  |  | 1 |  |  |  |
| Hypertension | 1.499 | 1.258-1.785 | | <0.001 | 1.326 | 1.152-1.525 | | <0.001 |
| Sugary-salty-fatty diet (factor score) | 1.150 | 1.063-1.244 | | 0.001 | NA | NA | | NA |
| Grilled & processed food (factor score) | 1.125 | 1.035-1.224 | | 0.006 | NA | NA | | NA |
| Normal HDL | 1 |  |  |  | 1 |  |  |  |
| Low HDL | 1.189 | 1.001-1.412 | | 0.049 | 1.170 | 1.020-1.343 | | 0.025 |
| Total cholesterol < 200 mg/dl | 1 |  |  |  | NA | NA | | NA |
| Total cholesterol ≥200 mg/dl | 1.292 | 1.070-1.562 | | 0.008 | NA | NA | | NA |
| LDL < 130 mg/dl | NA | NA | | NA | 1 |  |  |  |
| LDL ≥130 mg/dl | NA | NA | | NA | 1.208 | 1.016-1.436 | | 0.032 |
| Employee, students, entrepreneur, labour, driver, maids | 1 |  |  |  | 1 |  |  |  |
| Farmer, fisherman, others, unemployed | 1.321 | 1.111-1.569 | | 0.002 | 1.352 | 1.182-1.546 | | <0.001 |
| High formal education | 1 |  |  |  | 1 |  |  |  |
| Low formal education | 1.241 | 1.048-1.468 | | 0.012 | 1.299 | 1.130-1.493 | | <0.001 |
| Single/divorced | 1 |  |  |  | 1 |  |  |  |
| Married | 0.773 | 0.609-0.982 | | 0.035 | 0.854 | 0.731-0.999 | | 0.048 |
| Low income | NA | NA | | NA | 1 |  |  |  |
| Mid-high income | NA | NA | | NA | 0.839 | 0.722-0.974 | | 0.022 |

Note: NA is not applicable after fitting adjustment of logistic regression model.

**^a^**Discrimination as determined using ROC analysis: AUC of 0.6450, sensitivity of 10.1%, specificity of 95.6%, PPV 53.8%, NPV 69.3% and % of correct classification of 68.3%

**^b^**Discrimination as determined using ROC analysis: AUC of 0.6552, sensitivity of 34.8%, specificity of 81.2%, PPV 55.7%, NPV 64.7% and % of correct classification of 62.5%

**Table 4** **Potential determinants of prediabetes from multiple logistic regression in rural Indonesia, 2018**

|  | **Rural Men^c^** | | | | **Rural Women^d^** | | | |
| --- | --- | --- | --- | --- | --- | --- | --- | --- |
| **Variabel** | **OR *adjusted*** | **95% CI** | | ***p-value*** | **OR *adjusted*** | **95% CI** | | ***p-value*** |
| Age 15-29 yrs | 1 |  |  |  | 1 |  |  |  |
| Age ≥ 30 yrs | 1.617 | 1.247-2.096 | | <0.001 | 1.843 | 1.563-2.174 | | <0.001 |
| No central obesity | 1 |  |  |  | NA | NA | | NA |
| Central obesity | 1.607 | 1.252-2.062 | | <0.001 | NA | NA | | NA |
| Normotension | 1 |  |  |  | 1 |  |  |  |
| Hypertension | 1.388 | 1.195-1.611 | | <0.001 | 1.366 | 1.203-1.552 | | <0.001 |
| Sugary-salty-fatty diet (factor score) | 1.108 | 1.028-1.194 | | 0.008 | 1.080 | 1.009-1.157 | | 0.026 |
| Normal HDL | NA | NA | | NA | 1 |  |  |  |
| Low HDL | NA | NA | | NA | 1.184 | 1.039-1.350 | | 0.011 |
| LDL < 130 mg/dl |  |  |  |  | 1 |  |  |  |
| LDL ≥130 mg/dl |  |  |  |  | 1.202 | 1.058-1.364 | | 0.005 |
| Triglyceride < 150 mg/dl | 1 |  |  |  | 1 |  |  |  |
| Triglyceride ≥150 mg/dl | 1.201 | 1.014-1.423 | | 0.034 | 1.253 | 1.059-1.482 | | 0.009 |
| Employee, students, entrepreneur, labour, driver, maids | 1 |  |  |  | NA | NA | | NA |
| Farmer, fisherman, others, unemployed | 1.262 | 1.086-1.466 | | 0.002 | NA | NA | | NA |
| High formal education | 1 |  |  |  | 1 |  |  |  |
| Low formal education | 1.307 | 1.122-1.522 | | 0.001 | 1.234 | 1.072-1.421 | | 0.003 |
| Low income | NA | NA | | NA | 1 |  |  |  |
| Mid-high income | NA | NA | | NA | 0.814 | 0.716-0.925 | | 0.002 |

Note: NA is not applicable after fitting adjustment of logistic regression model.

**^c^**Discrimination as determined using ROC analysis: AUC of 0.6181, sensitivity of 21.8%, specificity of 88.8%, PPV 56.5%, NPV 63.1% and % of correct classification of 62.1%

**^d^**Discrimination as determined using ROC analysis: AUC of 0.6216, sensitivity of 62.3%, specificity of 55.8%, PPV 58.0%, NPV 60.1% and % of correct classification of 59.0%

**Table 5 Population attributable fraction for selected determinants between urban and rural population in Indonesia, 2018**

| **Determinants** | **PAF Urban (%)** | ***p-value* PAF Urban** | **PAF Rural (%)** | ***p-value* PAF Rural** |
| --- | --- | --- | --- | --- |
| Central obesity | 12.55 (8.74-16.21) | <0.001 | 6.05 (3.13-8.88) | <0.001 |
| Hypertension | 13.16 (9.61-16.58) | <0.001 | 11.82 (8.66-14.87) | <0.001 |
| Sugary-salty-fatty diet | 18.02 (8.49-26.56) | <0.001 | 18.18 (8.25-27.04) | <0.001 |
| Sports drink & carbonated beverages | 31.99 (-2.37-54.81) | 0.065 | 18.65 (-11.20-40.49) | 0.196 |
| Grilled and processed food | 31.84 (11.09-47.75) | 0.005 | 10.41 (-12.21-28.48) | 0.338 |
| Total cholesterol ≥ 200 mg/dl | 5.76 (1.15-10.16) | 0.015 | 0.48 (-3.55-4.36) | 0.811 |
| LDL ≥ 130 mg/dl | 2.55 (-3.47-8.22) | 0.398 | 5.54 (1.21-9.69) | 0.013 |
| Low HDL (<40 mg/dl for men; <50 mg/dl for women) | 5.93 (2.02-9.68) | 0.003 | 3.02 (-1.06-6.93) | 0.145 |
| Triglyceride ≥ 150 mg/dl | 3.38 (0.24-6.43) | 0.035 | 4.12 (1.58-6.60) | 0.002 |

Note: numbers were percentages along with their 95% confidence interval in brackets.

Analysis with Stata package ‘punafcc’ after running logistic regression which include age, sex, occupation, education level, marital status and wealth index as controlling variables.
