# Supplementary material for: Urban-rural distinction of potential determinants for prediabetes in Indonesian population aged ≥15 years: a cross-sectional analysis of Indonesian Basic Health Research 2018 among normoglycemic and prediabetic individuals
Source: BMC Public Health. 2020 Oct 6;20:1509. doi: 10.1186/s12889-020-09592-7 (PMC7539503; doi:10.1186/s12889-020-09592-7)
Supplement: Supplementary file 3 — Additional file 3. Supp1 File. STATISTIK KESEJAHTERAAN RAKYAT (WELFARE STATISTICS) 2018. This is a pdf version for the National Socio-Economics Survey (SUSENAS, Survei Sosial Ekonomi Nasional) March 2018, a nationwide survey conducted periodically by Badan Pusat Statistik (BPS-Statistics Indonesia) every March and September. SUSENAS provides data related to social welfare in household units across Indonesia. In this report, a brief explanation of sampling frame, which was adopted in RISKESDAS 2018, is presented in page 5. The questionnaire VSEN18.K used in this survey can also be seen in page 277–297. The data collected by this VSEN18.K were used to measure wealth index for each individual assessed respectively in their household. The full report of SUSENAS 2018 as well as VSEN18.K questionnaire can also be accessed through this link: https://www.bps.go.id/publication/download.html?nrbvfeve=ODFlZGUyZDU2Njk4YzA3ZDUxMGY2OTgz&xzmn=aHR0cHM6Ly93d3cuYnBzLmdvLmlkL3B1YmxpY2F0aW9uLzIwMTgvMTEvMjYvODFlZGUyZDU2Njk4YzA3ZDUxMGY2OTgzL3N0YXRpc3Rpay1rZXNlamFodGVyYWFuLXJha3lhdC0yMDE4Lmh0bWw%3D&twoadfnoarfeauf=MjAyMC0wOS0xOCAxMjozMDoxMg%3D%3D. [file 12889_2020_9592_MOESM3_ESM.pdf]

Katalog/*Catalog*: 4101002

STATISTIK KESEJAHTERAAN RAKYAT
WELFARE STATISTICS
2018

https://www.bps.go.id

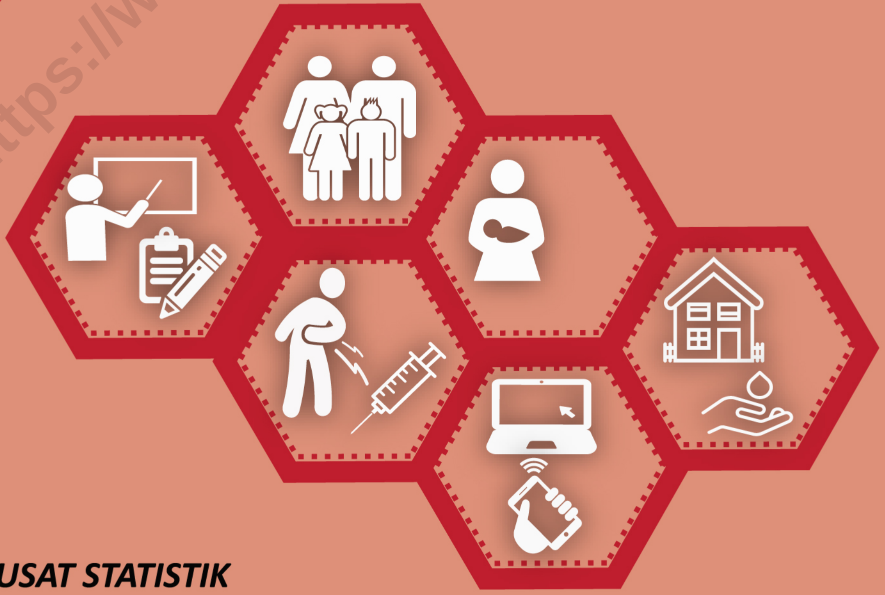

BADAN PUSAT STATISTIK
BPS-STATISTICS INDONESIA

STATISTIK KESEJAHTERAAN RAKYAT
WELFARE STATISTICS
2018

https://www.bps.go.id

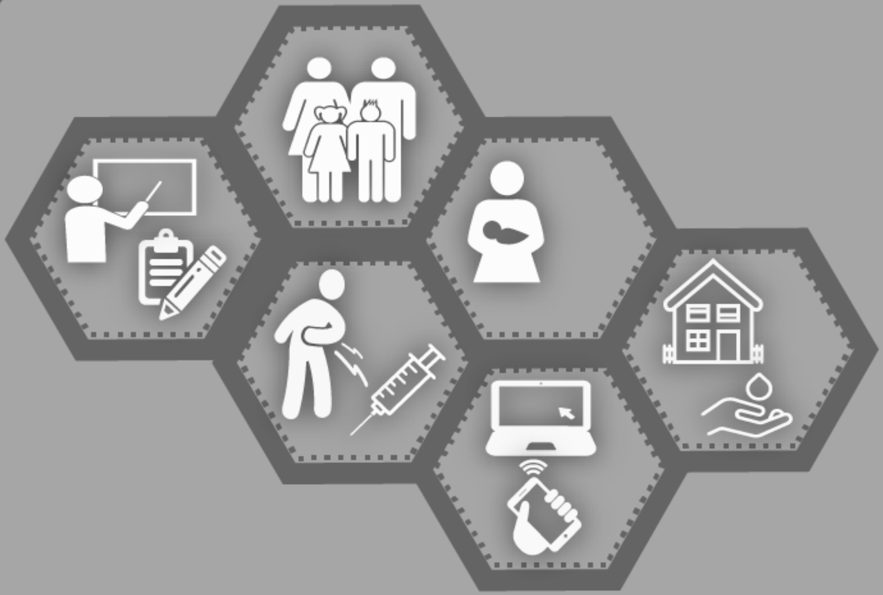

STATISTIK KESEJAHTERAAN RAKYAT
WELFARE STATISTICS
2018

ISSN: 2502-7492
Nomor Publikasi/*Publication Number*: 04210.1814
Katalog/*Catalog*: 4101002
Ukuran Buku/*Book Size*: **25,7x18,2 cm**
Jumlah Halaman/*Total Page*: **xxxiv+297 halaman/***pages*

Naskah/*Manuscript*:
Subdirektorat Statistik Rumah Tangga
Subdirectorate of Household Statistics

Penyunting/*Editor*:
Subdirektorat Statistik Rumah Tangga
Subdirectorate of Household Statistics

Gambar Kulit/*Cover Design*:
Subdirektorat Statistik Rumah Tangga
Subdirectorate of Household Statistics

Diterbitkan oleh/*Published by*:
©Badan Pusat Statistik/*BPS-Statistics Indonesia*

Dicetak oleh/*Printed by*:
CV. Dharmaputra

Dilarang mengumumkan, mendistribusikan, mengomunikasikan, dan/atau menggandakan sebagian atau seluruh isi buku ini untuk tujuan komersial tanpa izin tertulis dari Badan Pusat Statistik
It is prohibited to announce, distribute, communicate, and/or copy part or all of this book for commercial purposes without permission from BPS-Statistics Indonesia

https://www.bps.go.id

KATA PENGANTAR

Tujuan Pembangunan Berkelanjutan (*Sustainable Development Goals,* SDGs) merupakan agenda global dengan membawa semangat bahwa dampak positif pembangunan harus dapat dinikmati oleh semua pihak tanpa ada satupun yang tertinggal, *no one left behind*. Semangat pembangunan berkelanjutan dalam kerangka kerja SDGs sejalan dengan tujuan pembangunan nasional yaitu memberikan kesejahteraan untuk seluruh rakyat Indonesia. Dalam upaya mencapai target SDGs maupun target tujuan pembangunan nasional yang tertuang dalam RPJMN maupun Nawacita, diperlukan dukungan data yang kredibel dan *up to date*. Publikasi statistik kesejahteraan rakyat merupakan perwujudan tanggung jawab BPS sebagai lembaga yang bertugas menyediakan data-data statistik dasar sebagai masukan bagi perencanaan maupun evaluasi pembangunan.

Statistik kesejahteraan rakyat yang disajikan dalam publikasi ini bersumber dari hasil Survei Sosial Ekonomi Nasional (Susenas) yang merupakan survei berbasis rumah tangga. BPS menyelenggarakan Susenas secara periodik setiap tahun pada bulan Maret dan September. Publikasi ini menyajikan data-data hasil Susenas yang dilaksanakan pada bulan Maret 2018 terhadap 300.000 rumah tangga sampel.

Susenas mengumpulkan data mengenai kependudukan, pendidikan, kesehatan, fertilitas dan keluarga berencana, perumahan, teknologi informasi dan komunikasi, tindak kejahatan, dan perlindungan sosial. Data-data tersebut disajikan pada tingkat nasional dan provinsi sehingga memungkinkan keterbandingan antar wilayah. Publikasi ini diharapkan dapat memenuhi kebutuhan pengguna data akan data-data sosial ekonomi.

Kami mengucapkan terima kasih dan penghargaan kepada semua pihak yang telah berpartisipasi dalam pengumpulan data hingga penyusunan publikasi ini. Kami senantiasa mengharapkan kritik dan saran yang membangun untuk perbaikan publikasi ini di masa mendatang.

Jakarta, November 2018
KEPALA BADAN PUSAT STATISTIK
REPUBLIK INDONESIA

Dr. Suhariyanto

https://www.bps.go.id

PREFACE

Sustainable Development Goals (SDGs) is a global agenda coming with a spirit that positive impact of development process should benefit all population with no one left behind . The spirit of sustainable development within the SDGs framework is in line with the national development objective of providing prosperity for all Indonesians. In an effort to achieve the target of SDGs as well as the target of national development goal as stated in RPJMN and Nawacita, credible and up to date data support is required. Publication of welfare statistics is a manifestation of BPS-Statistics Indonesia responsibility as an institutionwho's main duty is providing basic statistical as input for development planning and evaluation.

Welfare statistics that is used to represent social welfare are derived from households based survey that is National Socio-Economics Survey (Susenas). BPS-Statistics Indonesia held Susenas periodically on March and September. The data provided in this publication is the result of the March 2018 Susenas from 300,000 samples of households.

Susenas provide data about demography, education, health, fertility and family planning, housing, information and communication technology, crime, and social protection. The data presented at national and provincial level in order to allow comparability across regions. This publication is hoped to meet the need data consumers on socio-economic data.

We would like to express our thanks and appreciation to everyone who participated in bringing this publication. We would like to receive criticism and constructive suggestions to improve this publication in the future.

Jakarta, November 2018
BPS-STATISTICS INDONESIA

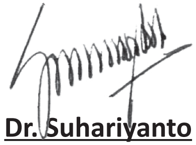

Dr. Suhariyanto
Chief Statistician

https://www.bps.go.id

DAFTAR ISI/*TABLE OF CONTENTS*

KATA PENGANTAR/*PREFACE* .... iii-iv

DAFTAR ISI/*TABLE OF CONTENTS* v

DAFTAR TABEL/*LIST OF TABLES* vii

SINGKATAN/*ABBREVIATION* xxv

STATISTIK KUNCI/*KEY STATISTICS* xxix

BAB I. **PENJELASAN UMUM/***EXPLANATORY NOTES* 3

BAB II. **KEPENDUDUKAN/***DEMOGRAPHY* 11

BAB III. **PENDIDIKAN/***EDUCATION* 47

BAB IV. **KESEHATAN/***HEALTH* 95

BAB V. **FERTILITAS DAN KELUARGA BERENCANA/***FERTILITY AND FAMILY PLANNING* 135

BAB VI. **PERUMAHAN/***HOUSING* 163

BAB VII. **TEKNOLOGI INFORMASI DAN KOMUNIKASI/***INFORMATION TECHNOLOGY AND COMMUNICATION* 201

BAB VIII. **LAIN-LAIN/***OTHERS* 219

ESTIMASI SAMPLING ERROR/*SAMPLING ERROR ESTIMATES* 243

KUESIONER VSEN18.K/*QUESTIONNAIRE VSEN18.K* 277

https://www.bps.go.id

https://www.bps.go.id

DAFTAR TABEL/*LIST OF TABLES*

II. KEPENDUDUKAN/*DEMOGRAPHY*

2.1.	**Persentase Penduduk menurut Kelompok Umur (5 Tahunan), Daerah Tempat Tinggal, dan Jenis Kelamin, 2018**<br>*Percentage of Population by Age Group (5 year), Urban Rural Classification, and Sex, 2018*	13
2.2.	**Persentase Penduduk menurut Provinsi, Daerah Tempat Tinggal, dan Jenis Kelamin, 2018**<br>*Percentage of Population by Province, Urban Rural Classification, and Sex, 2018*	14
2.3.	**Persentase Penduduk Berumur 7-24 Tahun menurut Provinsi, Daerah Tempat Tinggal, dan Jenis Kelamin, 2018**<br>*Percentage of Population Aged 7-24 Years by Province, Urban Rural Classification, and Sex, 2018*	15
2.4.1.	**Persentase Penduduk di Daerah Perkotaan menurut Provinsi, Jenis Kelamin, dan Kelompok Umur, 2018**<br>*Percentage of Population in Urban Area by Province, Sex, and Age Group, 2018*	16
2.4.2.	**Persentase Penduduk di Daerah Perdesaan menurut Provinsi, Jenis Kelamin, dan Kelompok Umur, 2018**<br>*Percentage of Population in Rural Area by Province, Sex, and Age Group, 2018*	17
2.4.3.	**Persentase Penduduk di Daerah Perkotaan dan Perdesaan menurut Provinsi, Jenis Kelamin, dan Kelompok Umur, 2018**<br>*Percentage of Population in Urban and Rural Area by Province, Sex, and Age Group, 2018*	18
2.5.	**Rasio Jenis Kelamin dan Angka Beban Ketergantungan menurut Provinsi dan Daerah Tempat Tinggal, 2018**<br>*Sex Ratio and Dependency Ratio by Province and Urban Rural Classification, 2018*	19
2.6.1.	**Persentase Penduduk Berumur 10 Tahun ke Atas di Daerah Perkotaan menurut Provinsi, Jenis Kelamin, dan Status Perkawinan, 2018**<br>*Percentage of Population Age 10 Years and Over in Urban Area by Province, Sex, and Marital Status, 2018*	20
2.6.2.	**Persentase Penduduk Berumur 10 Tahun ke Atas di Daerah Perdesaan menurut Provinsi, Jenis Kelamin, dan Status Perkawinan, 2018**<br>*Percentage of Population Aged 10 Years and Over in Rural Area by Province, Sex, and Marital Status, 2018*	22
2.6.3.	**Persentase Penduduk Berumur 10 Tahun ke Atas di Daerah Perkotaan dan Perdesaan menurut Provinsi, Jenis Kelamin, dan Status Perkawinan, 2018**<br>*Percentage of Population Age 10 Years and Over in Urban and Rural Area by Province, Sex, and Marital Status, 2018*	24
2.7.1.	**Persentase Penduduk Berumur 15-49 Tahun di Daerah Perkotaan menurut Provinsi, Jenis Kelamin, dan Status Perkawinan, 2018**<br>*Percentage of Population Aged 15-49 Years in Urban Area by Province, Sex, and Marital Status, 2018*	26
2.7.2.	**Persentase Penduduk Berumur 15-49 Tahun di Daerah Perdesaan menurut Provinsi, Jenis Kelamin, dan Status Perkawinan, 2018**<br>*Percentage of Population Aged 15-49 Years in Rural Area by Province, Sex, and Marital Status, 2018*	28
2.7.3.	**Persentase Penduduk Berumur 15-49 Tahun di Daerah Perkotaan dan Perdesaan menurut Provinsi, Jenis Kelamin, dan Status Perkawinan, 2018**<br>*Percentage of Population Aged 15-49 Years in Urban and Rural Area by Province, Sex, and Marital Status, 2018*	30

https://www.bps.go.id

2.8.1.	**Persentase Penduduk Berumur 15-19 Tahun di Daerah Perkotaan menurut Provinsi, Jenis Kelamin, dan Status Perkawinan, 2018**<br>*Percentage of Population Aged 15-19 Years in Urban Area by Province, Sex, and Marital Status, 2018*	32
2.8.2.	**Persentase Penduduk Berumur 15-19 Tahun di Daerah Perdesaan menurut Provinsi, Jenis Kelamin, dan Status Perkawinan, 2018**<br>*Percentage of Population Aged 15-19 Years in Rural Area by Province, Sex, and Marital Status, 2018*	33
2.8.3.	**Persentase Penduduk Berumur 15-19 Tahun di Daerah Perkotaan dan Perdesaan menurut Provinsi, Jenis Kelamin, dan Status Perkawinan, 2018**<br>*Percentage of Population Aged 15-19 Years in Urban and Rural Area by Province, Sex, and Marital Status, 2018*	34
2.9.1.	**Persentase Penduduk Berumur 0-17 Tahun di Daerah Perkotaan menurut Provinsi dan Kepemilikan Akta Kelahiran dari Kantor Catatan Sipil, 2018**<br>*Percentage of Population Aged 0-17 Years in Urban by Province and Birth Certificate Ownership from The Civil Registration Office, 2018*	35
2.9.2.	**Persentase Penduduk Berumur 0-17 Tahun di Daerah Perdesaan menurut Provinsi dan Kepemilikan Akta Kelahiran dari Kantor Catatan Sipil, 2018**<br>*Percentage of Population Aged 0-17 Years in Rural by Province and Birth Certificate Ownership from The Civil Registration Office, 2018*	36
2.9.3.	**Persentase Penduduk Berumur 0-17 Tahun di Daerah Perkotaan dan Perdesaan menurut Provinsi dan Kepemilikan Akta Kelahiran dari Kantor Catatan Sipil, 2018**<br>*Percentage of Population Aged 0-17 Years in Urban and Rural by Province and Birth Certificate Ownership from The Civil Registration Office, 2018*	37
2.10.1.	**Persentase Penduduk Berumur 0-4 Tahun di Daerah Perkotaan menurut Provinsi dan Kepemilikan Akta Kelahiran dari Kantor Catatan Sipil, 2018**<br>*Percentage of Population Aged 0-4 Years in Urban by Province and Birth Certificate Ownership from The Civil Registration Office, 2018*	38
2.10.2.	**Persentase Penduduk Berumur 0-4 Tahun di Daerah Perdesaan menurut Provinsi dan Kepemilikan Akta Kelahiran dari Kantor Catatan Sipil, 2018**<br>*Percentage of Population Aged 0-4 Years in Rural by Province and Birth Certificate Ownership from The Civil Registration Office, 2018*	39
2.10.3.	**Persentase Penduduk Berumur 0-4 Tahun di Daerah Perkotaan dan Perdesaan menurut Provinsi dan Kepemilikan Akta Kelahiran dari Kantor Catatan Sipil, 2018**<br>*Percentage of Population Aged 0-4 Years in Urban and Rural by Province and Birth Certificate Ownership from The Civil Registration Office, 2018*	40
2.11.	**Persentase Penduduk Berumur 10 Tahun ke Atas Berstatus Kawin yang Pasangannya Biasa Tinggal di Rumah Tangga menurut Provinsi, Daerah Tempat Tinggal, dan Jenis Kelamin, 2018**<br>*Percentage of Population Aged 10 Years and Over who Married and Their Spouses Usually Live in The Same Households by Province, Urban Rural Classification, and Sex, 2018*	41
2.12.	**Persentase Penduduk Berumur 5 Tahun ke Atas yang Mempunyai Nomor Induk Kependudukan (NIK) menurut Provinsi, Daerah Tempat Tinggal, dan Jenis Kelamin, 2018**<br>*Percentage of Population Aged 5 Years and Over Who Have National Identity Number by Province, Urban Rural Classification, and Sex, 2018*	42
2.13.	**Persentase Penduduk Berumur 17 Tahun ke Atas yang Mempunyai Nomor Induk Kependudukan (NIK) menurut Provinsi, Daerah Tempat Tinggal, dan Jenis Kelamin, 2018**<br>*Percentage of Population Aged 17 Years and Over Who Have National Identity Number by Province, Urban Rural Classification, and Sex, 2018*	43
2.14.	**Persentase Penduduk Berumur 0-4 Tahun yang Mempunyai Nomor Induk Kependudukan (NIK) menurut Provinsi, Daerah Tempat Tinggal, dan Jenis Kelamin, 2018**<br>*Percentage of Population Aged 0-4 Years Who Have National Identity Number by Province, Urban Rural Classification, and Sex, 2018*	44

https://www.bps.go.id

III. PENDIDIKAN/*EDUCATION*

3.1.1. **Persentase Penduduk Berumur 15 Tahun ke Atas di Daerah Perkotaan menurut Provinsi, Jenis Kelamin, dan Kemampuan Membaca dan Menulis, 2018** 51
Percentage of Population Aged 15 Years and Over in Urban Area by Province, Sex, and Literacy, 2018

3.1.2. **Persentase Penduduk Berumur 15 Tahun ke Atas di Daerah Perdesaan menurut Provinsi, Jenis Kelamin, dan Kemampuan Membaca dan Menulis, 2018** 52
Percentage of Population Aged 15 Years and Over in Rural Area by Province, Sex, and Literacy, 2018

3.1.3. **Persentase Penduduk Berumur 15 Tahun ke Atas di Daerah Perkotaan dan Perdesaan menurut Provinsi, Jenis Kelamin, dan Kemampuan Membaca dan Menulis, 2018** 53
Percentage of Population Aged 15 Years and Over in Urban and Rural Area by Province, Sex, and Literacy, 2018

3.2.1. **Persentase Penduduk Berumur 15-24 Tahun di Daerah Perkotaan menurut Provinsi, Jenis Kelamin, dan Kemampuan Membaca dan Menulis, 2018** 54
Percentage of Population Aged 15-24 Years in Urban Area by Province, Sex, and Literacy, 2018

3.2.2. **Persentase Penduduk Berumur 15-24 Tahun di Daerah Perdesaan menurut Provinsi, Jenis Kelamin, dan Kemampuan Membaca dan Menulis, 2018** 55
Percentage of Population Aged 15-24 Years in Rural Area by Province, Sex, and Literacy, 2018

3.2.3. **Persentase Penduduk Berumur 15-24 Tahun di Daerah Perkotaan dan Perdesaan menurut Provinsi, Jenis Kelamin, dan Kemampuan Membaca dan Menulis, 2018** 56
Percentage of Population Aged 15-24 Years in Urban and Rural Area by Province, Sex, and Literacy, 2018

3.3.1. **Persentase Penduduk Laki-Laki dan Perempuan Berumur 5 Tahun ke Atas di Daerah Perkotaan menurut Provinsi dan Status Pendidikan, 2018** 57
Percentage of Male and Female Population Aged 5 Years and Over in Urban Area by Province and Education Status, 2018

3.3.2. **Persentase Penduduk Laki-Laki dan Perempuan Berumur 5 Tahun ke Atas di Daerah Perdesaan menurut Provinsi dan Status Pendidikan, 2018** 58
Percentage of Male and Female Population Aged 5 Years and Over in Rural Area by Province and Education Status, 2018

3.3.3. **Persentase Penduduk Laki-Laki dan Perempuan Berumur 5 Tahun ke Atas di Daerah Perkotaan dan Perdesaan menurut Provinsi dan Status Pendidikan, 2018** 59
Percentage of Male and Female Population Aged 5 Years and Over in Urban and Rural Area by Province and Education Status, 2018

3.3.4. **Persentase Penduduk Laki-Laki Berumur 5 Tahun ke Atas di Daerah Perkotaan menurut Provinsi dan Status Pendidikan, 2018** 60
Percentage of Male Population Aged 5 Years and Over in Urban Area by Province and Education Status, 2018

3.3.5. **Persentase Penduduk Laki-Laki Berumur 5 Tahun ke Atas di Daerah Perdesaan menurut Provinsi dan Status Pendidikan, 2018** 61
Percentage of Male Population Aged 5 Years and Over in Rural Area by Province and Education Status, 2018

3.3.6. **Persentase Penduduk Laki-Laki Berumur 5 Tahun ke Atas di Daerah Perkotaan dan Perdesaan menurut Provinsi dan Status Pendidikan, 2018** 62
Percentage of Male Population Aged 5 Years and Over in Urban and Rural Area by Province and Education Status, 2018

3.3.7. **Persentase Penduduk Perempuan Berumur 5 Tahun ke Atas di Daerah Perkotaan menurut Provinsi dan Status Pendidikan, 2018** 63
Percentage of Female Population Aged 5 Years and Over in Urban Area by Province and Education Status, 2018

3.3.8. **Persentase Penduduk Perempuan Berumur 5 Tahun ke Atas di Daerah Perdesaan menurut Provinsi dan Status Pendidikan, 2018** 64
Percentage of Female Population Aged 5 Years and Over in Rural Area by Province and Education Status, 2018

https://www.bps.go.id

3.3.9.	**Persentase Penduduk Perempuan Berumur 5 Tahun ke Atas di Daerah Perkotaan dan Perdesaan menurut Provinsi dan Status Pendidikan, 2018**<br>*Percentage of Female Population Aged 5 Years and Over in Urban and Rural Area by Province and Education Status, 2018*	65
3.4.1.	**Persentase Penduduk Laki-Laki dan Perempuan Berumur 7-24 Tahun di Daerah Perkotaan menurut Provinsi dan Status Pendidikan, 2018**<br>*Percentage of Male and Female Population Aged 7-24 Years in Urban Area by Province and Education Status, 2018*	66
3.4.2.	**Persentase Penduduk Laki-Laki dan Perempuan Berumur 7-24 Tahun di Daerah Perdesaan menurut Provinsi dan Status Pendidikan, 2018**<br>*Percentage of Male and Female Population Aged 7-24 Years in Rural Area by Province and Education Status, 2018*	67
3.4.3.	**Persentase Penduduk Laki-Laki dan Perempuan Berumur 7-24 Tahun di Daerah Perkotaan dan Perdesaan menurut Provinsi dan Status Pendidikan, 2018**<br>*Percentage of Male and Female Population Aged 7-24 Years in Urban and Rural Area by Province and Education Status, 2018*	68
3.4.4.	**Persentase Penduduk Laki-Laki Berumur 7-24 Tahun di Daerah Perkotaan menurut Provinsi dan Status Pendidikan, 2018**<br>*Percentage of Male Population Aged 7-24 Years in Urban Area by Province and Education Status, 2018*	69
3.4.5.	**Persentase Penduduk Laki-Laki Berumur 7-24 Tahun di Daerah Perdesaan menurut Provinsi dan Status Pendidikan, 2018**<br>*Percentage of Male Population Aged 7-24 Years in Rural Area by Province and Education Status, 2018*	70
3.4.6.	**Persentase Penduduk Laki-Laki Berumur 7-24 Tahun di Daerah Perkotaan dan Perdesaan menurut Provinsi dan Status Pendidikan, 2018**<br>*Percentage of Male Population Aged 7-24 Years in Urban and Rural Area by Province and Education Status, 2018*	71
3.4.7.	**Persentase Penduduk Perempuan Berumur 7-24 Tahun di Daerah Perkotaan menurut Provinsi dan Status Pendidikan, 2018**<br>*Percentage of Female Population Aged 7-24 Years in Urban Area by Province and Education Status, 2018*	72
3.4.8.	**Persentase Penduduk Perempuan Berumur 7-24 Tahun di Daerah Perdesaan menurut Provinsi dan Status Pendidikan, 2018**<br>*Percentage of Female Population Aged 7-24 Years in Rural Area by Province and Education Status, 2018*	73
3.4.9.	**Persentase Penduduk Perempuan Berumur 7-24 Tahun di Daerah Perkotaan dan Perdesaan menurut Provinsi dan Status Pendidikan, 2018**<br>*Percentage of Female Population Aged 7-24 Years in Urban and Rural Area by Province and Education Status, 2018*	74
3.5.	**Angka Partisipasi Sekolah (APS) Penduduk Berumur 5-18 Tahun menurut Daerah Tempat Tinggal, Jenis Kelamin, dan Kelompok Umur, 2018**<br>*School Enrollment Ratio (SER) of Population Aged 5-18 Years by Urban Rural Classification, Sex, and Age Group, 2018*	75
3.6.	**Angka Partisipasi Murni (APM) Penduduk menurut Daerah Tempat Tinggal, Jenis Kelamin, dan Jenjang Pendidikan, 2018**<br>*Net Enrollment Ratio (NER) of Population by Urban Rural Classification, Sex, and Educational Level, 2018*	76
3.7.1.	**Persentase Penduduk Laki-Laki dan Perempuan Berumur 15 Tahun ke Atas di Daerah Perkotaan menurut Provinsi dan Ijazah/STTB Tertinggi yang Dimiliki, 2018**<br>*Percentage of Male and Female Population Aged 15 Years and Over in Urban Area by Province and The Highest School Certificate Owned, 2018*	77
3.7.2.	**Persentase Penduduk Laki-Laki dan Perempuan Berumur 15 Tahun ke Atas di Daerah Perdesaan menurut Provinsi dan Ijazah/STTB Tertinggi yang Dimiliki, 2018**<br>*Percentage of Male and Female Population Aged 15 Years and Over in Rural Area by Province and The Highest School Certificate Owned, 2018*	78
3.7.3.	**Persentase Penduduk Laki-Laki dan Perempuan Berumur 15 Tahun ke Atas di Daerah Perkotaan dan Perdesaan menurut Provinsi dan Ijazah/STTB Tertinggi yang Dimiliki, 2018**<br>*Percentage of Male and Female Population Aged 15 Years and Over in Urban and Rural Area by Province and The Highest School Certificate Owned, 2018*	79

https://www.bps.go.id

3.7.4. **Persentase Penduduk Laki-Laki Berumur 15 Tahun ke Atas di Daerah Perkotaan menurut Provinsi dan Ijazah/STTB Tertinggi yang Dimiliki, 2018** 80
Percentage of Male Population Aged 15 Years and Over in Urban Area by Province and The Highest School Certificate Owned, 2018

3.7.5. **Persentase Penduduk Laki-Laki Berumur 15 Tahun ke Atas di Daerah Perdesaan menurut Provinsi dan Ijazah/STTB Tertinggi yang Dimiliki, 2018** 81
Percentage of Male Population Aged 15 Years and Over in Rural Area by Province and The Highest School Certificate Owned, 2018

3.7.6. **Persentase Penduduk Laki-Laki Berumur 15 Tahun ke Atas di Daerah Perkotaan dan Perdesaan menurut Provinsi dan Ijazah/STTB Tertinggi yang Dimiliki, 2018** 82
Percentage of Male Population Aged 15 Years and Over in Urban and Rural Area by Province and The Highest School Certificate Owned, 2018

3.7.7. **Persentase Penduduk Perempuan Berumur 15 Tahun ke Atas di Daerah Perkotaan menurut Provinsi dan Ijazah/STTB Tertinggi yang Dimiliki, 2018** 83
Percentage of Female Population Aged 15 Years and Over in Urban Area by Province and The Highest School Certificate Owned, 2018

3.7.8. **Persentase Penduduk Perempuan Berumur 15 Tahun ke Atas di Daerah Perdesaan menurut Provinsi dan Ijazah/STTB Tertinggi yang Dimiliki, 2018** 84
Percentage of Female Population Aged 15 Years and Over in Rural Area by Province and The Highest School Certificate Owned, 2018

3.7.9. **Persentase Penduduk Perempuan Berumur 15 Tahun ke Atas di Daerah Perkotaan dan Perdesaan menurut Provinsi dan Ijazah/STTB Tertinggi yang Dimiliki, 2018** 85
Percentage of Female Population Aged 15 Years and Over in Urban and Rural Area by Province and The Highest School Certificate Owned, 2018

3.8.1. **Persentase Penduduk 0-6 Tahun di Daerah Perkotaan menurut Provinsi dan Keikutsertaan Pendidikan Pra Sekolah, 2018** 86
Percentage of Population Aged 0-6 Years in Urban Area by Province and The Participation of Pre-School Education, 2018

3.8.2. **Persentase Penduduk 0-6 Tahun di Daerah Perdesaan menurut Provinsi dan Keikutsertaan Pendidikan Pra Sekolah, 2018** 87
Percentage of Population Aged 0-6 Years in Rural Area by Province and The Participation of Pre-School Education, 2018

3.8.3. **Persentase Penduduk 0-6 Tahun di Daerah Perkotaan dan Perdesaan menurut Provinsi dan Keikutsertaan Pendidikan Pra Sekolah, 2018** 88
Percentage of Population Aged 0-6 Years in Urban and Rural Area by Province and The Participation of Pre-School Education, 2018

3.9.1. **Persentase Penduduk Berumur 0-6 Tahun yang Pernah/Masih Mengikuti Pendidikan Prasekolah di Daerah Perkotaan menurut Provinsi dan Jenis Pendidikan Pra Sekolah, 2018** 89
Percentage of Population Aged 0-6 Years who Has Ever/Still Attended Pre-School Education in Urban Area by Province and Type of Pre-School Education, 2018

3.9.2. **Persentase Penduduk Berumur 0-6 Tahun yang Pernah/Masih Mengikuti Pendidikan Prasekolah di Daerah Perdesaan menurut Provinsi dan Jenis Pendidikan Pra Sekolah, 2018** 90
Percentage of Population Aged 0-6 Years who Has Ever/Still Attended Pre-School Education in Rural Area by Province and Type of Pre-School Education, 2018

3.9.3. **Persentase Penduduk Berumur 0-6 Tahun yang Pernah/Masih Mengikuti Pendidikan Prasekolah di Daerah Perkotaan dan Perdesaan menurut Provinsi dan Jenis Pendidikan Pra Sekolah, 2018** 91
Percentage of Population Aged 0-6 Years who Has Ever/Still Attended Pre-School Education in Urban and Rural Area by Province and Type of Pre-School Education, 2018

https://www.bps.go.id

IV. KESEHATAN/*HEALTH*

4.1.	**Persentase Penduduk yang Mempunyai Keluhan Kesehatan selama Sebulan Terakhir menurut Provinsi, Daerah Tempat Tinggal, dan Jenis Kelamin, 2018**<br>*Percentage of Population Who Had Health Complaint during the Last Month by Province, Urban Rural Classification, and Sex, 2018*	97
4.2.	**Angka Kesakitan menurut Provinsi, Daerah Tempat Tinggal, dan Jenis Kelamin, 2018**<br>*Morbidity Rate by Province, Urban Rural Classification, and Sex, 2018*	98
4.3.	**Persentase Penduduk yang Mempunyai Keluhan Kesehatan dan Berobat Jalan dalam Sebulan Terakhir menurut Provinsi, Daerah Tempat Tinggal, dan Jenis Kelamin, 2018**<br>*Percentage of Population Who Had Health Complaint Which Treated Outpatient during the Last Month by Province, Urban Rural Classification, and Sex, 2018*	99
4.4.1	**Persentase Penduduk yang Mempunyai Keluhan Kesehatan tetapi Tidak Berobat Jalan dalam Sebulan Terakhir di Daerah Perkotaan menurut Provinsi dan Alasan Utama Tidak Berobat Jalan, 2018**<br>*Percentage of Population Who Had Health Complaint but Not Inpatient during the Last Month In Urban Area by Province and Main Reason Not Inpatient, 2018*	100
4.4.2.	**Persentase Penduduk yang Mempunyai Keluhan Kesehatan tetapi Tidak Berobat Jalan dalam Sebulan Terakhir di Daerah Perdesaan menurut Provinsi dan Alasan Utama Tidak Berobat Jalan, 2018**<br>*Percentage of Population Who Had Health Complaint but Not Inpatient during the Last Month In Rural Area by Province and Main Reason Not Inpatient, 2018*	101
4.4.3.	**Persentase Penduduk yang Mempunyai Keluhan Kesehatan tetapi Tidak Berobat Jalan dalam Sebulan Terakhir dI Daerah Perkotaan dan Perdesaan menurut Provinsi dan Alasan Utama Tidak Berobat Jalan, 2018**<br>*Percentage of PopulationWho Had Health Complaint but Not Inpatient during the Last Month In Urban and Rural Area by Province and Main Reason Not Inpatient, 2018*	102
4.5.1.	**Persentase Penduduk yang Berobat Jalan dalam Sebulan Terakhir di Daerah Perkotaan menurut Provinsi dan Tempat Berobat Jalan, 2018**<br>*Percentage of Population Who Were Treated as Outpatient during the Last Month in Urban Area by Province and Place of Outpatient, 2018*	103
4.5.2.	**Persentase Penduduk yang Berobat Jalan dalam Sebulan Terakhir di Daerah Perdesaan menurut Provinsi dan Tempat Berobat Jalan, 2018**<br>*Percentage of Population Who Were Treated as Outpatient during the Last Month in Rural Area by Province and Place of Outpatient, 2018*	104
4.5.3.	**Persentase Penduduk yang Berobat Jalan dalam Sebulan Terakhir di Daerah Perkotaan dan Perdesaan menurut Provinsi dan Tempat Berobat Jalan, 2018**<br>*Percentage of Population Who Were Treated as Outpatient during the Last Month in Urban and Rural Area by Province and Place of Outpatient, 2018*	105
4.6.	**Persentase Penduduk yang Menggunakan Jaminan Kesehatan untuk Berobat Jalan dalam Sebulan Terakhir menurut Provinsi, Daerah Tempat Tinggal, dan Jenis Kelamin, 2018**<br>*Percentage of Population Who Used Health Insurance for Outpatient during the Last Month by Province, Urban Rural Classification, and Sex, 2018*	106
4.7.1.	**Persentase Penduduk yang Memiliki Jaminan Kesehatan di Daerah Perkotaan menurut Provinsi dan Jenis Jaminan Kesehatan, 2018**<br>*Percentage of Population Who Have Health Insurance in Urban Area by Province and Type of Health Insurance, 2018*	107
4.7.2.	**Persentase Penduduk yang Memiliki Jaminan Kesehatan di Daerah Perdesaan menurut Provinsi dan Jenis Jaminan Kesehatan, 2018**<br>*Percentage of Population Who Have Health Insurance in Rural Area by Province and Type of Health Insurance, 2018*	108

https://www.bps.go.id

4.7.3.	**Persentase Penduduk yang Memiliki Jaminan Kesehatan di Daerah Perkotaan dan Perdesaan menurut Provinsi dan Jenis Jaminan Kesehatan, 2018**<br>*Percentage of Population Who Have Health Insurance in Urban and Rural Area by Province and Type of Health Insurance, 2018*	109
4.8.	**Persentase Penduduk yang Pernah Rawat Inap dalam Setahun Terakhir menurut Provinsi, Daerah Tempat Tinggal, dan Jenis Kelamin, 2018**<br>*Percentage of Population Who Had Have Hospitalized during the Last Year by Province, Urban Rural Classification, and Sex, 2018*	110
4.9.1.	**Persentase Penduduk yang Rawat Inap dalam Setahun Terakhir di Daerah Perkotaan menurut Provinsi dan Tempat Rawat Inap, 2018**<br>*Percentage of Population Who Were Inpatient during the Last Year in Urban Area by Province and Place of Inpatient, 2018*	111
4.9.2.	**Persentase Penduduk yang Rawat Inap dalam Setahun Terakhir di Daerah Perdesaan menurut Provinsi dan Tempat Rawat Inap, 2018**<br>*Percentage of Population Who Were Inpatient during the Last Year in Rural Area by Province and Place of Inpatient, 2018*	112
4.9.3.	**Persentase Penduduk yang Rawat Inap dalam Setahun Terakhir di Daerah Perkotaan dan Perdesaan menurut Provinsi dan Tempat Rawat Inap, 2018**<br>*Percentage of Population Who Were Inpatient during the Last Year in Urban and Rural Area by Province and Place of Inpatient, 2018*	113
4.10.	**Persentase Penduduk yang Menggunakan Jaminan Kesehatan untuk Rawat Inap dalam Setahun Terakhir menurut Provinsi, Daerah Tempat Tinggal, dan Jenis Kelamin, 2018**<br>*Percentage of Population Who Used Health Insurance for Inpatient during The Last Year by Province, Urban Rural Classification, and Sex, 2018*	114
4.11.1.	**Persentase Penduduk yang Pernah Rawat Inap di Daerah Perkotaan menurut Provinsi, Jumlah Hari Rawat Inap, dan Rata-Rata Lama Rawat Inap (Hari), 2018**<br>*Percentage of Population Who has Hospitalized during the Last Month in Urban Area by Province, Number of Inpatient Days, and Total Inpatient Average (Days), 2018*	115
4.11.2.	**Persentase Penduduk yang Pernah Rawat Inap di Daerah Perdesaan menurut Provinsi, Jumlah Hari Rawat Inap, dan Rata-Rata Lama Rawat Inap (Hari), 2018**<br>*Percentage of Population Who has Hospitalized during the Last Month in Rural Area by Province, Number of Inpatient Days, and Total Inpatient Average (Days), 2018*	116
4.11.3.	**Persentase Penduduk yang Pernah Rawat Inap di Daerah Perkotaan dan Perdesaan menurut Provinsi, Jumlah Hari Rawat Inap, dan Rata-Rata Lama Rawat Inap (Hari), 2018**<br>*Percentage of Population Who has Hospitalized during the Last Month in Urban and Rural Area by Province, Number of Inpatient Days, and Total Inpatient Average (Days), 2018*	117
4.12.	**Persentase Penduduk 5 Tahun ke Atas yang Merokok Tembakau selama Sebulan Terakhir menurut Provinsi, Daerah Tempat Tinggal, dan Kebiasaan Merokok, 2017***<br>*Percentage of Population 5 Years or Over Who Smoked Tobacco during the Last Month by Province, Urban Rural Classification, and Smoking Habit, 2017**	118
4.13.1.	**Persentase Penduduk 5 Tahun Keatas yang Merokok selama Sebulan Terakhir di Daerah Perkotaan menurut Provinsi dan Jumlah Batang Rokok yang Dihisap per Minggu, 2017***<br>*Percentage of Population 5 Years and Over Who Smoke during the Last Month in Urban Area by Province and the Number of Cigarettes Smoked per Week, 2017**	120

https://www.bps.go.id

4.13.2.	**Persentase Penduduk 5 Tahun Keatas yang Merokok selama Sebulan Terakhir di Daerah Perdesaan menurut Provinsi dan Jumlah Batang Rokok yang Dihisap per Minggu, 2017*** *Percentage of Population 5 Years and Over who Smoke during the Last Month in Rural Area by Province and the Number of Cigarettes Smoked per Week, 2017**	121
4.13.3	**Persentase Penduduk 5 Tahun Keatas yang Merokok selama Sebulan Terakhir di Daerah Perkotaan dan Perdesaan menurut Provinsi dan Jumlah Batang Rokok yang Dihisap per Minggu, 2017*** *Percentage of Population 5 Years and Over who Smoke during the Last Month in Urban and Rural Area by Province and the Number of Cigarettes Smoked per Week, 2017**	122
4.14.1.	**Persentase Penduduk Umur 0-59 Bulan (Balita) yang Mempunyai Kartu Imunisasi di Daerah Perkotaan menurut Provinsi dan Jenis Kelamin, 2017** *Percentage of Population Aged 0-59 Months (Under Five Years) Having Immunization Card in Urban Area by Province and Sex, 2017**	123
4.14.2.	**Persentase Penduduk Umur 0-59 Bulan (Balita) yang Mempunyai Kartu Imunisasi di Daerah Perdesaan menurut Provinsi dan Jenis Kelamin, 2017** *Percentage of Population Aged 0-59 Months (Under Five Years) Having Immunization Card in Rural Area by Province and Sex, 2017**	124
4.14.3.	**Persentase Penduduk Umur 0-59 Bulan (Balita) yang Mempunyai Kartu Imunisasi di Daerah Perkotaan dan Perdesaan menurut Provinsi dan Jenis Kelamin, 2017*** *Percentage of Population Aged 0-59 Months (Under Five Years) Having Immunization Card in Urban and Rural Area by Province and Sex, 2017**	125
4.15.	**Persentase Penduduk Umur 0-59 Bulan (Balita) yang Pernah Mendapat Imunisasi menurut Provinsi, Daerah Tempat Tinggal dan Jenis Imunisasi, 2017*** *Percentage of Population Aged 0-59 Months (Under Five Years) Who Ever Been Immunized by Province, Urban Rural Classification, and Type of Immunization, 2017**	126
4.16.	**Persentase Penduduk Umur 0-59 Bulan (Balita) yang Mendapat Imunisasi Lengkap menurut Provinsi, Daerah Tempat Tinggal, dan Jenis Kelamin, 2017*** *Percentage of Population Aged 0-59 Months (Under Five Years) Who Have Been Complete Immunized by Province, Urban Rural Classification, and Sex, 2017**	128
4.17.	**Persentase Penduduk Umur 0-23 Bulan (Baduta) yang Pernah Diberi ASI menurut Provinsi, Daerah Tempat Tinggal, dan Jenis Kelamin, 2017*** *Percentage of Population Aged 0-23 Months (Under Two Years) Ever Been Breastfeeding by Province, Urban Rural Classification, and Sex, 2017**	129
4.18.	**Persentase Penduduk Umur 0-23 Bulan (Baduta) yang Masih Diberi ASI menurut Provinsi, Daerah Tempat Tinggal, dan Jenis Kelamin, 2017*** *Percentage of Population Aged 0-23 Months (Under Two Years) Still Having Breastfeeding by Province, Urban Rural Classification, and Sex, 2017**	130
4.19.	**Persentase Penduduk Umur 0-23 Bulan (Baduta) yang Pernah Diberi ASI menurut Provinsi, Daerah Tempat Tinggal, dan Rata-rata Lama Pemberian ASI (Bulan), 2017*** *Percentage of Population Aged 0-23 Months (Under Two Years) Who Ever Been Breast Feeding by Province, Urban Rural Classification, and Average Duration of Breast Feeding (Months), 2017**	131
4.20.	**Persentase Penduduk Umur 0-23 Bulan (Baduta) menurut Provinsi, Daearah Tempat Tinggal, dan Pemberian Makanan/Cairan Tambahan dalam 24 Jam Terakhir, 2017*** *Percentage of Population Aged 0-23 Months (Under Two Years) by Province, Urban Rural Classification, and Weaning Food/Liquid Receiving Status in the Last 24 Hours, 2017**	132

https://www.bps.go.id

V. FERTILITAS DAN KELUARGA BERENCANA/*FERTILITY AND FAMILY PLANNING*

5.1.	**Persentase Perempuan yang Pernah Kawin Berumur 10 Tahun ke Atas menurut Provinsi, Daerah Tempat Tinggal, dan Umur Perkawinan Pertama, 2018**<br>*Percentage of Ever Married Female Aged 10 Years and Over by Province, Urban Rural Classification, and Age at First Marriage, 2018*	139
5.2.	**Persentase Perempuan Berumur 15-49 Tahun yang Pernah Hamil menurut Provinsi, Daerah Tempat Tinggal, dan Umur Saat Hamil Pertama Kali, 2017***<br>*Percentage of Ever Pregnant Female Aged 15-49 Years by Province, Urban Rural Classification, and Age at First Pregnancy, 2017**	140
5.3.1.	**Persentase Perempuan Berumur 15-49 Tahun Pernah Kawin yang Pernah Melahirkan dalam 2 Tahun Terakhir di Daerah Perkotaan menurut Provinsi dan Tempat Melahirkan Anak Lahir Hidup yang Terakhir, 2018**<br>*Percentage of Ever Married Female Aged 15-49 Years Who Have Given Birth in the Last Two Years in Urban Area by Province and Place of Last Live Birth, 2018*	141
5.3.2.	**Persentase Perempuan Berumur 15-49 Tahun Pernah Kawin yang Pernah Melahirkan dalam 2 Tahun Terakhir di Daerah Perdesaan menurut Provinsi dan Tempat Melahirkan Anak Lahir Hidup yang Terakhir, 2018**<br>*Percentage of Ever Married Female Aged 15-49 Years Who Have Given Birth in the Last Two Years in Rural Area by Province and Place of Last Live Birth, 2018*	142
5.3.3.	**Persentase Perempuan Berumur 15-49 Tahun Pernah Kawin yang Pernah Melahirkan dalam 2 Tahun Terakhir di Daerah Perkotaan dan Perdesaan menurut Provinsi dan Tempat Melahirkan Anak Lahir Hidup yang Terakhir, 2018**<br>*Percentage of Ever Married Female Aged 15-49 Years Who Have Given Birth in the Last Two Years in Urban and Rural Area by Province and Place of Last Live Birth, 2018*	143
5.4.1.	**Persentase Perempuan Berumur 15-49 Tahun Pernah Kawin yang Pernah Melahirkan dalam 2 Tahun Terakhir di Daerah Perkotaan menurut Provinsi dan Penolong Proses Kelahiran Terakhir, 2018**<br>*Percentage of Ever Married Female Aged 15-49 Years Who Have Given Birth in the Last Two Years in Urban Area by Province and Last Birth Attendant, 2018*	144
5.4.2.	**Persentase Perempuan Berumur 15-49 Tahun Pernah Kawin yang Pernah Melahirkan dalam 2 Tahun Terakhir di Daerah Perdesaan menurut Provinsi dan Penolong Proses Kelahiran Terakhir, 2018**<br>*Percentage of Ever Married Female Aged 15-49 Years Who Have Given Birth in the Last Two Years in Rural Area by Province and Last Birth Attendant, 2018*	145
5.4.3.	**Persentase Perempuan Berumur 15-49 Tahun Pernah Kawin yang Pernah Melahirkan dalam 2 Tahun Terakhir di Daerah Perkotaan dan Perdesaan menurut Provinsi dan Penolong Proses Kelahiran Terakhir, 2018**<br>*Percentage of Ever Married Female Aged 15-49 Years Who Have Given Birth in the Last Two Years in Urban and Rural Area by Province and Last Birth Attendant, 2018*	146
5.5.	**Persentase Perempuan Berumur 15-49 Tahun Pernah Kawin yang Pernah Melahirkan dalam 2 Tahun Terakhir menurut Provinsi, Daerah Tempat Tinggal, dan Berat Badan dari Anak Lahir Hidup yang Terakhir Ketika Dilahirkan, 2017***<br>*Percentage of Ever Married Female Aged 15-49 Who Have Given Birth in the Last Two Years by Province, Urban Rural Classification, and the Weight of Last Live Birth Child , 2017**	147

https://www.bps.go.id

5.6.	**Persentase Perempuan Berumur 15-49 Tahun Pernah Kawin yang Pernah Melahirkan dalam 2 Tahun Terakhir menurut Provinsi, Daerah Tempat Tinggal, dan Status Inisiasi Menyusui Dini (IMD) , 2017***<br>*Percentage of Ever Married Female Aged 15-49 Years Who Have Given Birth in the last two years by Province, Urban Rural Classification, and Initiation of Breast Feeding Status, 2017**	148
5.7.	**Persentase Perempuan Berumur 15-49 Tahun yang Pernah Kawin menurut Provinsi, Daerah Tempat Tinggal, dan Status Penggunaan Alat KB atau Cara Tradisional untuk Menunda atau Mencegah Kehamilan, 2018**<br>*Percentage of Ever Married Female Aged 15-49 by Province, Urban Rural Classification, and The Used of Contraception or a Traditional Method to Prevent or Delay Pregnancy Status, 2018*	149
5.8.1.	**Persentase Perempuan Berumur 15-49 Tahun Pernah Kawin yang Tidak Menggunakan Alat KB atau Cara Tradisional untuk Menunda atau Mencegah Kehamilan di Daerah Perkotaan menurut Provinsi dan Alasan Utama Tidak Menggunakan Alat KB atau Cara Tradisional, 2017***<br>*Percentage of Ever Married Female Aged 15-49 Who Currently Not Using Contraception or Traditional Method to Prevent or Delay Pregnancy in Urban Area by Province and The Main Reason Not Using Contraception or Traditional Method, 2017**	150
5.8.2.	**Persentase Perempuan Berumur 15-49 Tahun Pernah Kawin yang Tidak Menggunakan Alat KB atau Cara Tradisional untuk Menunda atau Mencegah Kehamilan di Daerah Perdesaan menurut Provinsi dan Alasan Utama Tidak Menggunakan Alat KB atau Cara Tradisional, 2017***<br>*Percentage of Ever Married Female Aged 15-49 Who Currently Not Using Contraception or Traditional Method to Prevent or Delay Pregnancy in Rural Area by Province and The Main Reason Not Using Contraception or Traditional Method, 2017**	151
5.8.3.	**Persentase Perempuan Berumur 15-49 Tahun Pernah Kawin yang Tidak Menggunakan Alat KB atau Cara Tradisional untuk Menunda atau Mencegah Kehamilan di Daerah Perkotaan dan Perdesaan menurut Provinsi dan Alasan Utama Tidak Menggunakan Alat KB, 2017***<br>*Percentage of Ever Married Female Aged 15-49 Who Currently Not Using Contraception or Traditional Method to Prevent or Delay Pregnancy in Urban and Rural Area by Province and The Main Reason Not Using Contraception or Traditional Method, 2017**	152
5.9.1.	**Persentase Perempuan Berumur 15-49 Tahun yang Pernah Kawin di Daerah Perkotaan menurut Provinsi dan Alat KB atau Cara Tradisional yang Sedang Digunakan, 2018**<br>*Percentage of Ever Married Female Aged 15-49 Years in Urban Area by Province and Type Contraception or Traditional Method Currently Used, 2018*	153
5.9.2.	**Persentase Perempuan Berumur 15-49 Tahun yang Pernah Kawin di Daerah Perdesaan menurut Provinsi dan Alat KB atau Cara Tradisional yang Sedang Digunakan, 2018**<br>*Percentage of Married Female Aged 15-49 Years in Rural Area by Province and Type Contraception or Traditional Method Currently Used, 2018*	154
5.9.3.	**Persentase Perempuan Berumur 15-49 Tahun yang Pernah Kawin di Daerah Perkotaan dan Perdesaan menurut Provinsi dan Alat KB atau Cara Tradisional yang Sedang Digunakan, 2018**<br>*Percentage of Married Female Aged 15-49 Years in Urban and Rural Area by Province and Type Contraception or Traditional Method Currently Used, 2018*	155
5.10.1.	**Persentase Perempuan Berumur 15-49 Tahun yang Pernah Kawin yang Menggunakan Alat KB Modern di Daerah Perkotaan menurut Provinsi dan Tempat Memperoleh Alat KB Modern yang Terakhir Kali, 2017***<br>*Percentage of Married Female Aged 15-49 Years Using Modern Contraception in Urban Area by Province and Place Obtains The Last Modern Contraception, 2017**	156
5.10.2.	**Persentase Perempuan Berumur 15-49 Tahun yang Pernah Kawin yang Menggunakan Alat KB Modern di Daerah Perdesaan menurut Provinsi dan Tempat Memperoleh Alat KB Modern yang Terakhir Kali, 2017***<br>*Percentage of Married Female Aged 15-49 Years Using Modern Contraception in Rural Area by Province and Place Obtains The Last Modern Contraception, 2017**	157

https://www.bps.go.id

5.10.3. **Persentase Perempuan Berumur 15-49 Tahun yang Pernah Kawin yang Menggunakan Alat KB Modern di Daerah Perkotaan dan Perdesaan menurut Provinsi dan Tempat Memperoleh Alat KB Modern yang Terakhir Kali, 2017*** 158
*Percentage of Married Female Aged 15-49 Years Using Modern Contraception in Urban and Rural Area by Province and Place Obtains The Last Modern Contraception, 2017**

5.11. **Persentase Perempuan Berumur 15-49 Tahun yang Pernah Kawin dan Sedang Menggunakan Alat/Cara KB dan Pernah Berhenti/Berganti Alat/Cara KB menurut Provinsi dan Daerah Tempat Tinggal, 2017*** 159
*Percentage of Ever Married Female Aged 15-49 Which Currently Used Contraception and Ever Stopped/Changed Contraception by Province and Urban Rural Classification, 2017**

VI. PERUMAHAN/*HOUSING*

6.1. **Rata-Rata Jumlah Keluarga dalam Bangunan Sensus/Rumah menurut Provinsi dan Daerah Tempat Tinggal, 2018** 165
Average of Total Family per Dwelling Unit by Province and Urban Rural Classification, 2018

6.2. **Persentase Rumah Tangga menurut Provinsi, Daerah Tempat Tinggal, dan Status Kepemilikan Bangunan Tempat Tinggal yang Ditempati, 2018** 166
Percentage of Households by Province, Urban Rural Classification, and Ownership Status of The Dwelling, 2018

6.3. **Persentase Rumah Tangga menurut Provinsi, Daerah Tempat Tinggal, dan Luas Lantai Rumah (m^2), 2018** 167
Percentage of Households by Province, Urban Rural Classification, and Floor Area (m^2), 2018

6.4. **Persentase Rumah Tangga menurut Provinsi, Daerah Tempat Tinggal, dan Luas Lantai per Kapita (m^2), 2018** 168
Percentage of Households by Province, Urban Rural Classification, and Floor Area per Capita (m^2), 2018

6.5.1. **Persentase Rumah Tangga di Daerah Perkotaan menurut Provinsi dan Bahan Bangunan Utama Atap Rumah Terluas, 2018** 169
Percentage of Households in Urban Area by Province and The Main Material of The Widest Part of the Roof, 2018

6.5.2. **Persentase Rumah Tangga di Daerah Perdesaan menurut Provinsi dan Bahan Bangunan Utama Atap Rumah Terluas, 2018** 170
Percentage of Households in Rural Area by Province and The Main Material of The Widest Part of Roof, 2018

6.5.3. **Persentase Rumah Tangga di Daerah Perkotaan dan Perdesaan menurut Provinsi dan Bahan Bangunan Utama Atap Rumah Terluas, 2018** 171
Percentage of Households in Urban and Rural Area by Province and The Main Material of The Widest Part of the Roof, 2018

6.6.1. **Persentase Rumah Tangga di Daerah Perkotaan menurut Provinsi dan Bahan Bangunan Utama Dinding Rumah Terluas, 2018** 172
Percentage of Households in Urban Area by Province and The Main Material of The Widest Part of Walls, 2018

6.6.2. **Persentase Rumah Tangga di Daerah Perdesaan menurut Provinsi dan Bahan Bangunan Utama Dinding Rumah Terluas, 2018** 173
Percentage of Households in Rural Area by Province and The Main Material of The Widest Part of Walls, 2018

6.6.3. **Persentase Rumah Tangga di Daerah Perkotaan dan Perdesaan menurut Provinsi dan Bahan Bangunan Utama Dinding Rumah Terluas, 2018** 174
Percentage of Households in Urban and Rural Area by Province and The Main Material of The Widest Part of Walls, 2018

6.7.1. **Persentase Rumah Tangga di Daerah Perkotaan menurut Provinsi dan Bahan Bangunan Utama Lantai Rumah Terluas, 2018** 175
Percentage of Households in Urban Area by Province and The Main Material of The Widest Part of The Dwelling Floor, 2018

6.7.2. **Persentase Rumah Tangga di Daerah Perdesaan menurut Provinsi dan Bahan Bangunan Utama Lantai Rumah Terluas, 2018** 176
Percentage of Households in Rural Area by Province and The Main Material of the Widest Floor, 2018

6.7.3. **Persentase Rumah Tangga di Daerah Perkotaan dan Perdesaan menurut Provinsi dan Bahan Bangunan Utama Lantai Rumah Terluas, 2018** 177
Percentage of Households in Urban and Rural Area by Province and The Main Material of The Widest Floor, 2018

https://www.bps.go.id

6.8.	**Persentase Rumah Tangga menurut Provinsi, Daerah Tempat Tinggal, dan Kepemilikan serta Penggunaan Fasilitas Tempat Buang Air Besar, 2018**<br>*Percentage of Households by Province, Urban Rural Classification, and The Owned and The Use of Toilet Facility, 2018*	178
6.9.1.	**Persentase Rumah Tangga* di Daerah Perkotaan menurut Provinsi dan Jenis Kloset yang Digunakan Rumah Tangga, 2018**<br>*Percentage of Households in Urban Area by Province and Type of Toilet Used by Households, 2018*	179
6.9.2.	**Persentase Rumah Tangga* di Daerah Perdesaan menurut Provinsi dan Jenis Kloset yang Digunakan Rumah Tangga, 2018**<br>*Percentage of Households in Rural Area by Province and Type of Toilet Used by Households, 2018*	180
6.9.3.	**Persentase Rumah Tangga* di Daerah Perkotaan dan Perdesaan menurut Provinsi dan Jenis Kloset yang Digunakan Rumah Tangga, 2018**<br>*Percentage of Households in Urban and Rural Area by Province and Type of Toilet Used by Households, 2018*	181
6.10.1.	**Persentase Rumah Tangga* di Daerah Perkotaan menurut Provinsi dan Tempat Pembuangan Akhir Tinja, 2018**<br>*Percentage of Households in Urban Area by Province and Final Disposal of Feces, 2018*	182
6.10.2.	**Persentase Rumah Tangga* di Daerah Perdesaan menurut Provinsi dan Tempat Pembuangan Akhir Tinja, 2018**<br>*Percentage of Households in Rural Area by Province and Final Disposal of Feces, 2018*	183
6.10.3.	**Persentase Rumah Tangga* di Daerah Perkotaan dan Perdesaan menurut Provinsi dan Tempat Pembuangan Akhir Tinja, 2018**<br>*Percentage of Households in Urban and Rural Area by Province and Final Disposal of Feces, 2018*	184
6.11.1.	**Persentase Rumah Tangga* di Daerah Perkotaan menurut Provinsi dan Sumber Air Utama yang Digunakan Rumah Tangga untuk Minum, 2018**<br>*Percentage of Households in Urban Area by Province and Main Source of Drinking Water Consumed by Household, 2018*	185
6.11.2.	**Persentase Rumah Tangga* di Daerah Perdesaan menurut Provinsi dan Sumber Air Utama yang Digunakan Rumah Tangga untuk Minum, 2018**<br>*Percentage of Households in Rural Area by Province and Main Source of Drinking Water Consumed by Household, 2018*	186
6.11.3.	**Persentase Rumah Tangga* di Daerah Perkotaan dan Perdesaan menurut Provinsi dan Sumber Air Utama yang Digunakan Rumah Tangga untuk Minum, 2018**<br>*Percentage of Households in Urban and Rural Area by Province and Main Source of Drinking Water Consumed by Household, 2018*	187
6.12.	**Persentase Rumah Tangga dengan Sumber Air Minum dari Sumur Bor/Pompa, Sumur Terlindung, Sumur Tak Terlindung, Mata Air Terlindung, dan Mata Air Tak Terlindung menurut Provinsi, Daerah Tempat Tinggal, dan Jarak ke Tempat Penampungan Limbah/Kotoran/Tinja Terdekat, 2018**<br>*Percentage of Households Using Artesian Well/Pump, Protected Well, Unprotected Well, Protected Spring, and Unprotected Spring as Source of Drinking Water by Province, Urban Rural Classification, and the Distance to the Nearest Final Disposal Site of Feces, 2018*	188
6.13.	**Persentase Rumah Tangga menurut Provinsi, Daerah Tempat Tinggal, dan Cara Memperoleh Air Minum, 2018**<br>*Percentage of Households by Province, Urban Rural Classification, and How to Get The Drinking Water, 2018*	189
6.14.1.	**Persentase Rumah Tangga di Daerah Perkotaan menurut Provinsi dan Sumber Air Utama yang Digunakan Rumah Tangga untuk Memasak/Mandi/Cuci/dll, 2018**<br>*Percentage of Households in Urban Area by Province, and The Main Source of Water Used by Household for Cooking/Taking Bath/Washing/etc, 2018*	190
6.14.2.	**Persentase Rumah Tangga di Daerah Perdesaan menurut Provinsi dan Sumber Air Utama yang Digunakan Rumah Tangga untuk Memasak/Mandi/Cuci/dll, 2018**<br>*Percentage of Households in Rural Area by Province and The Main Source of Water Used by Household for Cooking/Taking Bath/Washing/etc, 2018*	191

https://www.bps.go.id

6.14.3.	**Persentase Rumah Tangga di Daerah Perkotaan dan Perdesaan menurut Provinsi dan Sumber Air Utama yang Digunakan Rumah Tangga untuk Memasak/Mandi/Cuci/dll, 2018**<br>*Percentage of Households in Urban and Rural Area by Province and The Main Source of Water Used by Household for Cooking/Taking bath/Washing/etc, 2018*	192
6.15.	**Persentase Rumah Tangga dengan Sumber Air untuk Memasak/Mandi/Cuci/dll dari Sumur Bor/Pompa, Sumur Terlindung, Sumur Tak Terlindung, Mata Air Terlindung, dan Mata Air Tak Terlindung menurut Provinsi, Daerah Tempat Tinggal, dan Jarak ke Tempat Penampungan Limbah/Kotoran/Tinja Terdekat, 2018**<br>*Percentage of Households Using Artesian Well/Pump, Protected Well, Unprotected Well, Protected Spring, and Unprotected Spring as Source of Water for Cooking/Taking Bath/Washing/etc by Province, Urban Rural Classification, and the Distance to the Nearest Final Disposal Site of Feces, 2018*	193
6.16.	**Persentase Rumah Tangga menurut Provinsi, Daerah Tempat Tinggal, Sumber Air Minum Bersih, Sumber Air Minum Layak, dan Akses Air Layak, 2018**<br>*Percentage of Households by Province, Urban Rural Classification, Source of Clean Drinking Water, and Source of Decent Drinking Water, and Access of Decent Water, 2018*	194
6.17.	**Persentase Rumah Tangga menurut Provinsi, Daerah Tempat Tinggal, dan Sumber Penerangan Utama, 2018**<br>*Percentage of Households by Province, Urban Rural Classification, and The Main Source of Lighting, 2018*	195
6.18.1.	**Persentase Rumah Tangga di Daerah Perkotaan menurut Provinsi dan Bahan Bakar Utama yang Digunakan untuk Memasak, 2018**<br>*Percentage of Households in Urban Area by Province and Type of Fuel Mainly Used for Cooking, 2018*	196
6.18.2.	**Persentase Rumah Tangga di Daerah Perdesaan menurut Provinsi dan Bahan Bakar Utama yang Digunakan untuk Memasak, 2018**<br>*Percentage of Households in Rural Area by Province and Type of Fuel Mainly Used for Cooking, 2018*	197
6.18.3.	**Persentase Rumah Tangga di Daerah Perkotaan dan Perdesaan menurut Provinsi dan Bahan Bakar Utama yang Digunakan untuk Memasak, 2018**<br>*Percentage of Households in Urban and Rural Area by Province and Type of Fuel Mainly Used for Cooking, 2018*	198

VII. TEKNOLOGI INFORMASI DAN KOMUNIKASI/*INFORMATION TECHNOLOGY AND COMMUNICATION*

7.1.	**Persentase Penduduk Berumur 5 Tahun ke Atas yang Menggunakan Telepon Seluler (HP)/Nirkabel dalam 3 Bulan Terakhir menurut Provinsi, Daerah Tempat Tinggal, dan Jenis Kelamin, 2018**<br>*Percentage of Population Aged 5 Years and Over who Use Cellular Phone (HP) during The Last 3 Months by Province, Urban Rural Classification, and Sex, 2018*	203
7.2.	**Persentase Penduduk Berumur 5 Tahun ke Atas yang Memiliki Telepon Seluler (HP)/Nirkabel dalam 3 Bulan Terakhir menurut Provinsi, Daerah Tempat Tinggal, dan Jenis Kelamin, 2018**<br>*Percentage of Population Aged 5 Years and Over in Urban Area who Had Cellular Phone (HP) during The Last 3 Months by Province, Urban Rural Classification, and Sex, 2018*	204
7.3.	**Persentase Penduduk Berumur 5 Tahun ke Atas yang Menggunakan Komputer (PC/Desktop, Laptop/Notebook, Tablet) dalam 3 Bulan Terakhir menurut Provinsi, Daerah Tempat Tinggal, dan Jenis Kelamin, 2018**<br>*Percentage of Population Aged 5 Years and Over who Used Computer (PC/Desktop, Laptop/Notebook, Tablet) during The Last 3 Months by Province, Urban Rural Classification, and Sex, 2018*	205

https://www.bps.go.id

7.4.	**Persentase Penduduk Berumur 5 Tahun ke Atas yang Mengakses Internet (Termasuk Facebook, Twitter, BBM, Whatsapp) dalam 3 Bulan Terakhir menurut Provinsi, Daerah Tempat Tinggal, dan Jenis Kelamin, 2018**<br>*Percentage of Population Aged 5 Years and Over who Accessed Internet (Including Facebook, Twitter, BBM, Whatsapp) during The Last 3 Months by Province, Urban Rural Classification, and Sex, 2018*	206
7.5.1.	**Persentase Penduduk Berumur 5 Tahun ke Atas di Daerah Perkotaan yang Mengakses Internet dalam 3 Bulan Terakhir menurut Provinsi, Jenis Kelamin, dan Alat yang Digunakan untuk Mengakses Internet, 2017***<br>*Percentage of Population Aged 5 Years and Over in Urban Area Who Accessed Internet during The Last 3 Months by Province, Sex, and Media Used to Access The Internet, 2017**	207
7.5.2.	**Persentase Penduduk Berumur 5 Tahun ke Atas di Daerah Perdesaan yang Mengakses Internet dalam 3 Bulan Terakhir menurut Provinsi, Jenis Kelamin, dan Alat yang Digunakan untuk Mengakses Internet, 2017***<br>*Percentage of Population Aged 5 Years and Over in Rural Area Who Accessed Internet during The Last 3 Months by Province, Sex, and Media Used to Access The Internet, 2017**	208
7.5.3.	**Persentase Penduduk Berumur 5 Tahun ke Atas di Daerah Perkotaan dan Perdesaan yang Mengakses Internet dalam 3 Bulan Terakhir menurut Provinsi, Jenis Kelamin, dan Alat yang Digunakan untuk Mengakses Internet, 2017***<br>*Percentage of Population Aged 5 Years and Over in Urban and Rural Area Who Accessed Internet during The Last 3 Months by Province, Sex, and Media Used to Access The Internet, 2017**	209
7.6.1.	**Persentase Penduduk Berumur 5 Tahun ke Atas di Daerah Perkotaan yang Mengakses Internet dalam 3 Bulan Terakhir menurut Provinsi dan Tempat Mengakses Internet, 2017***<br>*Percentage of Population Aged 5 Years and Over in Urban Area Who Accessed Internet during The Last 3 Months by Province and Places Accessing The Internet, 2017**	210
7.6.2.	**Persentase Penduduk Berumur 5 Tahun ke Atas di Daerah Perdesaan yang Mengakses Internet dalam 3 Bulan Terakhir menurut Provinsi dan Tempat Mengakses Internet, 2017***<br>*Percentage of Population Aged 5 Years and Over in Rural Area Who Accessed Internet during The Last 3 Months by Province and Places Accessing The Internet, 2017**	211
7.6.3.	**Persentase Penduduk Berumur 5 Tahun ke Atas di Daerah Perkotaan dan Perdesaan yang Mengakses Internet dalam 3 Bulan Terakhir menurut Provinsi dan Tempat Mengakses Internet, 2017***<br>*Percentage of Population Aged 5 Years and Over in Urban and Rural Area Who Accessed Internet during The Last 3 Months by Province and Places Accessing The Internet, 2017**	212
7.7.1.	**Persentase Penduduk Berumur 5 Tahun ke Atas di Daerah Perkotaan yang Mengakses Internet dalam 3 Bulan Terakhir menurut Provinsi dan Tujuan Mengakses Internet, 2017***<br>*Percentage of Population Aged 5 Years and Over in Urban Area Who Accessed Internet during The Last 3 Months by Province and The Purpose for Accessing The Internet, 2017**	213
7.7.2.	**Persentase Penduduk Berumur 5 Tahun Ke Atas di Daerah Perdesaan yang Mengakses Internet dalam 3 Bulan Terakhir menurut Provinsi dan Tujuan Mengakses Internet, 2017***<br>*Percentage of Population Aged 5 Years and Over in Rural Area Who Accessed Internet during The Last 3 Months by Province and The Purpose for Accessing The Internet, 2017**	214

https://www.bps.go.id

7.7.3. **Persentase Penduduk Berumur 5 Tahun ke Atas di Daerah Perkotaan dan Perdesaan yang Mengakses Internet dalam 3 Bulan Terakhir menurut Provinsi dan Tujuan Mengakses Internet, 2017*** 215
*Percentage of Population Aged 5 Years and Over in Urban and Rural Area Who Accessed Internet during The Last 3 Months by Province and The Purpose for Accessing The Internet, 2017**

7.8. **Persentase Rumah Tangga yang Memiliki Telepon Rumah/PSTN dan Komputer/Laptop menurut Provinsi dan Daerah Tempat Tinggal, 2018** 216
Percentage of Household with Fixed Line Telephone (PSTN) and Computer/Laptop by Province and Urban Rural Classification, 2018

VIII. LAIN-LAIN/*OTHERS*

8.1. **Persentase Penduduk yang Menjadi Korban Kejahatan Pencurian, Penganiayaan, Pencurian dengan Kekerasan, Pelecehan Seksual, atau Lainnya sejak 1 Januari – 31 Desember 2017 menurut Provinsi, Daerah Tempat Tinggal, dan Jenis Kelamin** 221
Percentage of Population Being Victims of Crime, Theft, Violence, Sexual Assault, or Others during January 1st – December 31st by Province, Urban Rural Classification, and Sex

8.2.1. **Persentase Rumah Tangga yang Membeli/Menerima Beras Miskin (Raskin)/Beras Sejahtera (Rastra) dalam 4 Bulan Terakhir di Daerah Perkotaan menurut Provinsi dan Jumlah Raskin yang Dibeli/Diterima (Kg), 2018** 222
Percentage of Household That Bought/Received Rice for The Poor (Raskin/Rastra) during The Last 4 Months in Urban Area by Province and Amount of Rice for The Poor Bought/Received (Kg), 2018

8.2.2. **Persentase Rumah Tangga yang Membeli/Menerima Beras Miskin (Raskin)/Beras Sejahtera (Rastra) dalam 4 Bulan Terakhir di Daerah Perdesaan menurut Provinsi dan Jumlah Raskin yang Dibeli/Diterima (Kg), 2018** 223
Percentage of Household That Bought/Received Rice for The Poor (Raskin/Rastra) during The Last 4 Months in Rural Area by Province and Amount of Rice for The Poor Bought/Received (Kg), 2018

8.2.3. **Persentase Rumah Tangga yang Membeli/Menerima Beras Miskin (Raskin)/Beras Sejahtera (Rastra) dalam 4 Bulan Terakhir di Daerah Perkotaan dan Perdesaan menurut Provinsi dan Jumlah Raskin yang Dibeli/Diterima (Kg), 2018** 224
Percentage of Household That Bought/Received Rice for The Poor (Raskin/Rastra) during The Last 4 Months in Urban and Rural Area by Province and Amount of Rice for The Poor Bought/Received (Kg), 2018

8.3.1. **Persentase Rumah Tangga yang Membeli/Menerima Beras Miskin (Raskin)/Beras Sejahtera (Rastra) dalam 4 Bulan Terakhir di Daerah Perkotaan menurut Provinsi, Harga Raskin/Rastra Per Kg, dan Harga Rata-Rata Per Kg (Rp), 2018** 225
Percentage of Household that Bought/Received Rice for The Poor (Raskin/Rastra) during the Last 4 Months in Urban Area by Province, Price of Rice for The Poor Per Kg, and Price of Average Per Kg (Rupiahs), 2018

8.3.2. **Persentase Rumah Tangga yang Membeli/Menerima Beras Miskin (Raskin)/Beras Sejahtera (Rastra) dalam 4 Bulan Terakhir di Daerah Perdesaan menurut Provinsi, Harga Raskin/Rastra Per Kg, dan Harga Rata-Rata Per Kg (Rp), 2018** 226
Percentage of Household that Bought/Received Rice for The Poor (Raskin/Rastra) during the Last 4 Months in Urban Area by Province, Price of Rice for The Poor Per Kg, and Price of Average Per Kg (Rupiahs), 2018

8.3.3. **Persentase Rumah Tangga yang Membeli/Menerima Beras Miskin (Raskin)/Beras Sejahtera (Rastra) dalam 4 Bulan Terakhir di Daerah Perkotaan dan Perdesaan menurut Provinsi, Harga Raskin/Rastra Per Kg, dan Harga Rata-Rata Per Kg (Rp), 2018** 227
Percentage of Household that Bought/Received Rice for The Poor (Raskin/Rastra) during the Last 4 Months in Urban and Rural Area by Province, Price of Rice for The Poor Per Kg, and Price of Average Per Kg (Rupiahs), 2018

https://www.bps.go.id

8.4.	**Persentase Rumah Tangga yang Membeli/Menerima Beras Miskin (Raskin)/Beras Sejahtera (Rastra) dalam 4 Bulan Terakhir menurut Provinsi, Daerah Tempat Tinggal, dan Harga Raskin Per Kg (Rp), 2018**<br>*Percentage of Household that Bought/Received Rice for The Poor (Raskin/Rastra) during The Last 4 Months by Province, Urban and Rural Classification, and Price of Rice for The Poor Per Kg (Rupiahs), 2018*	228
8.5.1.	**Persentase Rumah Tangga yang Menerima Kredit dalam Setahun Terakhir di Daerah Perkotaan menurut Provinsi dan Jenis Kredit, 2018**<br>*Percentage of Household that Received Credit during The Last Year in Urban Area by Province and Type of Credit, 2018*	229
8.5.2.	**Persentase Rumah Tangga yang Menerima Kredit dalam Setahun Terakhir di Daerah Perdesaan menurut Provinsi dan Jenis Kredit, 2018**<br>*Percentage of Household that Received Credit during The Last Year in Rural Area by Province and Type of Credit, 2018*	230
8.5.3.	**Persentase Rumah Tangga yang Menerima Kredit dalam Setahun Terakhir di Daerah Perkotaan dan Perdesaan menurut Provinsi dan Jenis Kredit, 2018**<br>*Percentage of Household that Received Credit during The Last Year in Urban and Rural Area by Province and Type of Credit, 2018*	231
8.6.	**Persentase Rumah Tangga yang Menerima Program Indonesia Pintar (PIP) dalam Bulan Agustus 2016 - Maret 2017 menurut Provinsi dan Daerah Tempat Tinggal, 2018**<br>*Percentage of Household That Received Scholarship for The Smart Indonesia Program (PIP) from August 2016 through March 2017 by Province and Urban Rural Classification, 2018*	232
8.7.	**Persentase Rumah Tangga yang Menerima Program Indonesia Pintar (PIP) dalam Bulan Agustus 2017 - Maret 2018 menurut Provinsi, Daerah Tempat Tinggal, dan Jenis PIP, 2018**<br>*Percentage of Household Member who Received Smart Indonesia Program (PIP) from August 2017 through March 2018 by Province, Urban Rural Classification, and Type of PIP, 2018*	233
8.8.1.	**Persentase Rumah Tangga yang Memiliki atau Menerima Jaminan Sosial dalam Setahun Terakhir di Daerah Perkotaan menurut Provinsi dan Jenis Jaminan Sosial, 2018**<br>*Percentage of Household Who Had or Received The Following Social Insurance during The Last One Year in Urban Area by Province and Type of Social Insurance, 2018*	234
8.8.2.	**Persentase Rumah Tangga yang Memiliki atau Menerima Jaminan Sosial dalam Setahun Terakhir di Daerah Perdesaan menurut Provinsi dan Jenis Jaminan Sosial, 2018**<br>*Percentage of Household Who Had or Received The Following Social Insurance during The Last One Year in Rural Area by Province and Type of Social Insurance, 2018*	235
8.8.3.	**Persentase Rumah Tangga yang Memiliki atau Menerima Jaminan Sosial dalam Setahun Terakhir di Daerah Perkotaan dan Perdesaan menurut Provinsi dan Jenis Jaminan Sosial, 2018**<br>*Percentage of Household Who Had or Received Social The Following Social Insurance during The Last One Year in Urban and Rural Area by Province and Type of Social Insurance, 2018*	236
8.9.	**Persentase Rumah Tangga yang Menerima Kartu Perlindungan Sosial (KPS)/Kartu Keluarga Sejahtera (KKS) menurut Provinsi, dan Daerah Tempat Tinggal, 2018**<br>*Percentage of Household that Received Social Security Card (KPS) / Card for Family Welfare (KKS) by Province and Urban Rural Classification, 2018*	237
8.10.1.	**Persentase Rumah Tangga di Daerah Perkotaan dengan Kepemilikan Aset menurut Provinsi dan Jenis Aset yang Dimiliki, 2018**<br>*Percentage of Households in Urban Area with Assets by Province and Type of Assets Owned, 2018*	238

https://www.bps.go.id

8.10.2. **Persentase Rumah Tangga di Daerah Perdesaan dengan Kepemilikan Aset menurut Provinsi dan Jenis Aset yang Dimiliki, 2018** 239
Percentage of Households in Rural Area with Assets by Province and Type of Assets Owned, 2018

8.10.3. **Persentase Rumah Tangga di Daerah Perkotaan dan Perdesaan dengan Kepemilikan Aset menurut Provinsi dan Jenis Aset yang Dimiliki, 2018** 240
Percentage of Households in Urban and Rural Area with Assets by Province and Type of Assets Owned, 2018

IX. ESTIMASI *SAMPLING ERROR*/*SAMPLING ERROR ESTIMATES*

9.1. ***Sampling Error*** **Persentase Persentase Penduduk yang Mempunyai Keluhan Kesehatan selama Sebulan Terakhir menurut Provinsi dan Daerah Tempat Tinggal, 2018** 245
Sampling Errors Percentage of Population Who Had Health Complaint during the Last Month by Province and Urban Rural Classification, 2018

9.2. ***Sampling Error*** **Persentase penduduk berumur 0-17 Tahun yang Tidak Memiliki Akte Kelahiran dari Kantor Catatan Sipil menurut Provinsi dan Daerah Tempat Tinggal, 2018** 246
Sampling Errors Percentage Population Aged 0-17 Year Old who Does Not Have a Birth Certificate from a Civil Registry Office by Province and Urban Rural Classsification, 2018

9.3. ***Sampling Error*** **Persentase Penduduk yang Pernah Rawat Inap dalam Setahun Terakhir menurut Provinsi dan Daerah Tempat Tinggal, 2018** 247
Sampling Errors Percentage of Population Who Had Have Hospitalized during the Last Year by Province and Urban Rural Classsification, 2018

9.4. ***Sampling Error*** **Persentase Penduduk yang Memiliki Jaminan Kesehatan Jamkesda menurut Provinsi dan Daerah Tempat Tinggal, 2018** 248
Sampling Errors Percentage of Population Who Have Health Insurance Jamkesda by Province and Urban Rural Classsification, 2018

9.5. ***Sampling Error*** **Persentase Penduduk Berumur 5 Tahun ke Atas yang Tidak/Belum Pernah Sekolah menurut Provinsi dan Daerah Tempat Tinggal, 2018** 249
Sampling Errors Percentage Population Aged 5 Years and Over who Not Schooling/Never Attended School by Province and Urban Rural Classsification, 2018

9.6. ***Sampling Error*** **Persentase Penduduk Berumur 5 Tahun ke Atas yang Masih Bersekolah di SD/MI/Paket A menurut Provinsi dan Daerah Tempat Tinggal, 2018** 250
Sampling Errors Percentage Population Aged 5 Years and Over who Attending Primary School by Province and Urban Rural Classsification, 2018

9.7. ***Sampling Error*** **Persentase Penduduk Berumur 5 Tahun ke Atas yang Masih Bersekolah di SMP/MTs/Paket B menurut Provinsi dan Daerah Tempat Tinggal, 2018** 251
Sampling Errors Percentage Population Aged 5 Years and Over who Attending Junior School by Province and Urban Rural Classsification, 2018

9.8. ***Sampling Error*** **Persentase Penduduk Berumur 5 Tahun ke Atas yang Masih Bersekolah di SMA/SMK/MA/Paket C menurut Provinsi dan Daerah Tempat Tinggal, 2018** 252
Sampling Errors Percentage Population Aged 5 Years and Over who Attending Senior High School by Province and Urban Rural Classsification, 2018

9.9. ***Sampling Error*** **Persentase Penduduk Berumur 5 Tahun ke Atas yang Masih Bersekolah di Diploma I s.d Universitas menurut Provinsi dan Daerah Tempat Tinggal, 2018** 253
Sampling Errors Percentage Population Aged 5 Years and Over who Attending Diploma I to University by Province and Urban Rural Classsification, 2018

https://www.bps.go.id

9.10.	***Sampling Error*** **Persentase Perempuan Berumur 15-49 Tahun yang Pernah Melahirkan dan Tempat Melahirkan Anak Lahir Hidup yang Terakhir Di Rumah Sakit/RS Bersalin menurut Provinsi dan Daerah Tempat Tinggal, 2018**<br>*Sampling Errors Percentage of Ever Give Birth Female Aged 15-49 Years and Place of Last Live Birth At Hospital/Maternity Hospital by Province and Urban Rural Classsification, 2018*	254
9.11.	***Sampling Error*** **Persentase Perempuan Berumur 15-49 Tahun yang Pernah Melahirkan dan Tempat Melahirkan Anak Lahir Hidup yang Terakhir Di Rumah Bersalin/Klinik menurut Provinsi dan Daerah Tempat Tinggal, 2018**<br>*Sampling Errors Percentage of Ever Give Birth Female Aged 15-49 Years and Place of Last Live Birth At Maternity Clinic/Clinic by Province and Urban Rural Classsification, 2018*	255
9.12.	***Sampling Error*** **Persentase Perempuan Berumur 15-49 Tahun yang Pernah Melahirkan dan Penolong Proses Kelahiran Terakhir Dokter Kandungan menurut Provinsi dan Daerah Tempat Tinggal, 2018**<br>*Sampling Errors Percentage of Ever Give Birth Female Aged 15-49 Years and Last Birth Attendant by Obstetrician by Province and Urban Rural Classsification, 2018*	256
9.13.	***Sampling Error*** **Persentase Perempuan Berumur 15-49 Tahun Pernah Kawin yang Pernah Melahirkan dan Penolong Proses Kelahiran Terakhir Bidan menurut Provinsi dan Daerah Tempat Tinggal, 2018**<br>*Sampling Errors Percentage of Ever Married Female Aged 15-49 Years Who Have Given Birth and Last Birth Attendantby Midwife by Province and Urban Rural Classsification, 2018*	257
9.14.	***Sampling Error*** **Persentase Perempuan Berumur 15-49 Tahun yang Pernah Kawin dan Pernah Menggunakan Alat KB atau Cara Tradisional untuk Menunda atau Mencegah Kehamilan menurut Provinsi dan Daerah Tempat Tinggal, 2018**<br>*Sampling Errors Percentage of Ever Married Female Aged 15-49 and The Ever Used of Contraception or a Traditional Method to Prevent or Delay Pregnancy by Province and Urban Rural Classification, 2018*	258
9.15.	***Sampling Error*** **Persentase Perempuan Berumur 15-49 Tahun yang Pernah Kawin dan Sedang Menggunakan Alat KB atau Cara Tradisional untuk Menunda atau Mencegah Kehamilan menurut Provinsi dan Daerah Tempat Tinggal, 2018**<br>*Sampling Errors Percentage of Ever Married Female Aged 15-49 and The Currently Use of Contraception or a Traditional Method to Prevent or Delay Pregnancy by Province and Urban Rural Classification, 2018*	259
9.16.	***Sampling Error*** **Persentase Perempuan Berumur 15-49 Tahun yang Pernah Kawin dan Tidak Menggunakan Alat KB atau Cara Tradisional untuk Menunda atau Mencegah Kehamilan menurut Provinsi dan Daerah Tempat Tinggal, 2018**<br>*Sampling Errors Percentage of Ever Married Female Aged 15-49 and The Never Used of Contraception or a Traditional Method to Prevent or Delay Pregnancy by Province and Urban Rural Classification, 2018*	260
9.17.	***Sampling Error*** **Persentase Penduduk Berumur 5 Tahun ke Atas yang Menggunakan Telepon Seluler (HP)/Nirkabel dalam 3 Bulan Terakhir menurut Provinsi dan Daerah Tempat Tinggal, 2018**<br>*Sampling Errors Percentage of Population Aged 5 Years and Over Who Use Cellular Phone (HP) during The Last 3 Months by Province and Urban Rural Classification, 2018*	261
9.18.	***Sampling Error*** **Persentase Penduduk Berumur 5 Tahun ke Atas yang Memiliki Telepon Seluler (HP)/Nirkabel dalam 3 Bulan Terakhir menurut Provinsi dan Daerah Tempat Tinggal, 2018**<br>*Sampling Errors Percentage of Population Aged 5 Years and Over Who Had Cellular Phone (HP) during The Last 3 Months by Province and Urban Rural Classification, 2018*	262

https://www.bps.go.id

9.19. ***Sampling Error*** **Persentase Penduduk Berumur 5 Tahun ke Atas yang Mengakses Internet (Termasuk Facebook, Twitter, BBM, Whatsapp) dalam 3 Bulan Terakhir menurut Provinsi dan Daerah Tempat Tinggal, 2018** 263
Sampling Errors Percentage of Population Aged 5 Years and Over Who Accessed The Internet (Including Facebook, Twitter, BBM, Whatsapp) during The Last 3 Months by Province and Urban Rural Classification, 2018

9.20. ***Sampling Error*** **Persentase Rumah Tangga yang Sumber Air Minumnya Sumur Tak Terlindung menurut Provinsi dan Daerah Tempat Tinggal, 2018** 264
Sampling Errors Percentage of Households that Source of Drinking Water were Unprotected Well by Province and Urban Rural Classification, 2018

9.21. ***Sampling Error*** **Persentase Rumah Tangga yang Sumber Air Minumnya Mata Air Tak Terlindung menurut Provinsi dan Daerah Tempat Tinggal, 2018** 265
Sampling Errors Percentage of Households that Source of Drinking Water were Unprotected Spring by Province and Urban Rural Classification, 2018

9.22. ***Sampling Error*** **Persentase Rumah Tangga yang Tempat Pembuangan Akhir Tinjanya di Tangki Septik menurut Provinsi dan Daerah Tempat Tinggal, 2018** 266
Sampling Errors Percentage of Households that Final Disposal of Feces were on Septic Tank by Province and Urban Rural Classification, 2018

9.23. ***Sampling Error*** **Persentase Rumah Tangga yang Tempat Pembuangan Akhir Tinjanya di IPAL menurut Provinsi dan Daerah Tempat Tinggal, 2018** 267
Sampling Errors Percentage of Households that Final Disposal of Feces were on IPAL by Province and Urban Rural Classification, 2018

9.24. ***Sampling Error*** **Persentase Rumah Tangga yang Tempat Pembuangan Akhir Tinjanya di Pantai/tanah lapang/kebun/lainnya menurut Provinsi dan Daerah Tempat Tinggal, 2018** 268
Sampling Errors Percentage of Households that Final Disposal of Feces were on Beach/field/garden/other by Province and Urban Rural Classification, 2018

9.25. ***Sampling Error*** **Persentase Rumah Tangga yang Membeli/Menerima Beras Miskin (Raskin) dalam 4 Bulan Terakhir menurut Provinsi dan Daerah Tempat Tinggal, 2018** 269
Sampling Errors Percentage of Household That Bought/Received Rice for The Poor (Raskin) during the Last 4 Months by Province and Urban Rural Classification, 2018

9.26. ***Sampling Error*** **Persentase Rumah Tangga yang Menerima Program Indonesia Pintar (PIP) menurut Provinsi dan Daerah Tempat Tinggal, 2018** 270
Sampling Errors Percentage of Household That Received Smart Indonesia Program (PIP) by Province and Urban Rural Classification, 2018

9.27. ***Sampling Error*** **Persentase Rumah Tangga menurut Provinsi, Akses Air Layak, dan Daerah Tempat Tinggal, 2018** 271
Sampling Errors Percentage of Household by Province, Access of Decent Water, and Urban Rural Classification, 2018

9.28. ***Sampling Error*** **Persentase Rumah Tangga yang Menerima Kartu Perlindungan Sosial (KPS)/Kartu Keluarga Sejahtera (KKS) dan Dapat Menunjukkan Kartu menurut Provinsi dan Daerah Tempat Tinggal, 2018** 272
Sampling Errors Percentage of Household That Received Social Security Card (KPS)/Card for Family Welfare (KKS) and Can Show The Cards by Province and Urban Rural Classification, 2018

9.29. ***Sampling Error*** **Persentase Rumah Tangga yang Menerima Kartu Perlindungan Sosial (KPS)/Kartu Keluarga Sejahtera (KKS) dan Tidak Dapat Menunjukkan Kartu menurut Provinsi dan Daerah Tempat Tinggal, 2018** 273
Sampling Errors Percentage of Household That Received Social Security Card (KPS)/Card for Family Welfare (KKS) and Can not Show The Cards by Province and Urban Rural Classification, 2018

https://www.bps.go.id

https://www.bps.go.id

SINGKATAN/*ABBREVIATION*

BAB II. KEPENDUDUKAN — ***CHAPTER II. DEMOGRAPHY***

KK	:	Kartu Keluarga	*Family Card*
KTP	:	Kartu Tanda Penduduk	*Identity Card*
NIK	:	Nomor Induk Kependudukan	*National Identity Number*

BAB III. PENDIDIKAN — ***CHAPTER III. EDUCATION***

APS	:	Angka Partisipasi Sekolah	*School Enrollment Ratio*
APM	:	Angka Partisipasi Murni	*Net Enrollment Ratio*
BKB	:	Bina Keluarga Balita	*Mother's Programme*
MA	:	Madrasah Aliyah	*Islamic Senior High School*
MAK	:	Madrasah Aliyah Kejuruan	*Islamic Vocational Senior High School*
MI	:	Madrasah Ibtidaiyah	*Islamic Primary School*
MTs	:	Madrasah Tsanawiyah	*Islamic Junior High School*
PAUD	:	Pendidikan Anak Usia Dini	*Early Childhood Education*
PT	:	Perguruan Tinggi	*University*
SD	:	Sekolah Dasar	*Primary School*
SMP	:	Sekolah Menengah Pertama	*Junior High School*
SMA	:	Sekolah Menengah Atas	*Senior High School*
SMK	:	Sekolah Menengah Kejuruan	*Vocational Senior High School*
TA	:	Tahun Ajaran	*School Year*

https://www.bps.go.id

BAB IV. KESEHATAN — ***CHAPTER IV. HEALTH***

ASABRI	:	Asuransi Angkatan Bersenjata Republik Indonesia	*Insurance for Indonesian Army Member*
ASI	:	Air Susu Ibu	*Breast Feeding*
Askes	:	Asuransi Kesehatan	*Health Insurance*
Baduta	:	Bawah Dua Tahun	*Under Two Years of age*
Balita	:	Bawah Lima Tahun	*Under Five Years of age*
BCG	:	Bacillus Calmette Guerin	*Bacillus Calmette Guerin*
BPJS	:	Badan Penyelenggara Jaminan Kesehatan	*Organizing Board for Health Guarantee*
DPT	:	Difteri, Pertusis, Tetanus	*Difteri, Pertusis, Tetanus*
Jamkesmas	:	Jaminan Kesehatan Masyarakat	*Health Guarantee for People*
Jamsostek	:	Jaminan Sosial Tenaga Kerja	*Social Insurance for Labour*
PBI	:	Penerima Bantuan Iuran	*Aid Acceptant*
Puskesmas	:	Pusat Kesehatan Masyarakat	*Public Health Center*
Pustu	:	Pusat Kesehatan Masyarakat Pembantu	*Subsidiary Health Center*

https://www.bps.go.id

BAB V. FERTILITAS DAN KELUARGA BERENCANA — ***CHAPTER V. FERTILITY AND FAMILY PLANNING***

AKDR	:	Alat Kontrasepsi Dalam Rahim	*Intra Uterine Device*
IMD	:	Inisiasi Menyusui Dini	*Early Initiation of Breastfeeding*
IUD	:	Intra Uterine Device	*Intra Uterine Device*
KB	:	Keluarga Berencana	*Family Planning*
MOP	:	Metoda Operasi Pria	*Vasektomy*
MOW	:	Metoda Operasi Wanita	*Tubektomy*
MUYAN	:	Mobil Unit Pelayanan	*Vehicle Service Unit*
PPKBD	:	Pembantu Pembina Keluarga Berencana Desa	*Nurture Support Family Planning Village*
TKBK	:	Tim Keluarga Berencana Keliling	*Family Planning Team*
TMK	:	Tim Medis Keliling	*Medical Mobile Team*

BAB VI. PERUMAHAN — ***CHAPTER VI. HOUSING***

MCK	:	Mandi, Cuci, Kakus	*Public bathing, Washing, Toilet Facilities*
SPAL	:	Saluran Pembuangan Air Limbah	*Sewerage*
LPG	:	Liquid Petroleum Gas	*Liquid Petroleum Gas*
PLN	:	Perusahaan Listrik Negara	*The National Electric Company*
KG	:	Kilogram	*Kilogram*

BAB VII. TEKNOLOGI INFORMASI DAN KOMUNIKASI — ***CHAPTER VII. INFORMATION TECHNOLOGY AND COMMUNICATION***

BBM	:	Blackberry Messenger	*Blackberry Messenger*
HP	:	Handphone	*Handphone*
PC	:	Personal Computer	*Personal Computer*
Ponsel	:	Telepon Seluler	*Cellular Phone (Handphone)*
PSTN	:	Public Switched Telephone Network	*Public Switched Telephone Network*

BAB VIII. LAIN-LAIN — ***CHAPTER VIII. OTHERS***

BSM	:	Bantuan Siswa Miskin	*Aid for Poor Students*
KIP	:	Kartu Indonesia Pintar	*Smart Indonesian Card*
KKS	:	Kartu Keluarga Sejahtera	*Family Welfare Card*
KPS	:	Kartu Perlindungan Sosial	*Social Security Card*
Raskin	:	Beras Miskin	*Rice for the Poor*
PNPM	:	Program Nasional Pemberdayaan Masyarakat	*National Program for Community Empowerment*
KUB	:	Kelompok Usaha Bersama	*Business Group*
KUBE	:	Kelompok Usaha Bersama	*Business Group*
KUR	:	Kredit Usaha Rakyat	*People Business Credit Program*

https://www.bps.go.id

https://www.bps.go.id

STATISTIK KUNCI, 2014-2018
KEY STATISTICS, 2014-2018

**Rincian**/*Description*	2014	2015	2016	2017	2018
(1)	(2)	(3)	(4)	(5)	(6)
**KEPENDUDUKAN/***DEMOGRAPHY*					
**Rasio Jenis Kelamin/***Sex Ratio*	101,01	101,00	101,00	100,97	100,96
**Angka Beban Ketergantungan/***Dependency Ratio*	50,88	49,81	49,32	48,42	48,23
**Persentase Anak Berumur 0-17 Tahun yang Memiliki Akte Kelahiran** *Percentage of Children Aged 0-17 Years Who Had Birth Certificate from The Civil Registration Office*	77,72	79,92	81,68	83,33	83,55
**Persentase Anak Berumur 0-4 Tahun yang Memiliki Akte Kelahiran** *Percentage of Children Aged 0-4 Years Who Had Birth Certificate from The Civil Registration Office*	71,23	72,65	72,53	73,18	71,92
**PENDIDIKAN/***EDUCATION*					
**Angka Partisipasi Sekolah (APS) Formal dan Nonformal Penduduk Berumur 7-12 Tahun** *Formal and Non Formal School Enrollment Ratio (SER) of Population Aged 7-12 Years*	98,92	99,09	99,09	99,14	99,22
**Angka Partisipasi Sekolah (APS) Formal dan Nonformal Penduduk Berumur 13-15 Tahun** *Formal and Non Formal School Enrollment Ratio (SER) of Population Aged 13-15 Years*	94,44	94,72	94,88	95,08	95,36
**Angka Partisipasi Sekolah (APS) Formal dan Nonformal Penduduk Berumur 16-18Tahun** *Formal and Non Formal School Enrollment Ratio (SER) of Population Aged 16-18 Years*	70,31	70,61	70,83	71,42	71,99
**Angka Partisipasi Murni (APM) Formal dan Nonformal SD** *Formal and Non Formal Net Enrollment Ratio (NER) of Primary School*	96,45	96,70	96,82	97,19	97,58
**Angka Partisipasi Murni (APM) Formal dan Nonformal SMP** *Formal and Non Formal Net Enrollment Ratio (NER) of Junior High School*	77,53	77,82	77,95	78,40	78,84
**Angka Partisipasi Murni (APM) Formal dan Nonformal SMA** *Formal and Non Formal Net Enrollment Ratio (NER) of Senior High School*	59,35	59,71	59,95	60,37	60,67
**KESEHATAN/***HEALTH*					
**Persentase Penduduk yang Mempunyai Keluhan Kesehatan selama Sebulan Terakhir** *Percentage of Population Who Had Health Complaint During The Last one Month*	29,22	30,35	28,53	28,62	30,96
**Persentase Penduduk yang memiliki Jaminan Kesehatan** *Percentage of Population that Had Health Insurance*	-	50,55	55,04	59,41	68,63

https://www.bps.go.id

STATISTIK KUNCI, 2014-2018
KEY STATISTICS, 2014-2018

**Rincian**/*Description*	**2014**	**2015**	**2016**	**2017**	**2018**
(1)	(2)	(3)	(4)	(5)	(6)
**FERTILITAS DAN KELUARGA BERENCANA/***FERTILITY AND FAMILY PLANNING*					
**Persentase Wanita Pernah Kawin Usia 10 Tahun dengan Umur Perkawinan Pertama < 16 Tahun** *Percentage of Ever Married Women Aged 10 Years and Over with Age at First Marriage is < 16 Years*	11,21	8,24	15,87	14,18	15,66
**Wanita 15-49 Tahun dan Berstatus Kawin yang Sedang Menggunakan/Memakai Alat/Cara KB** *Percentage of Women Age 15-49 Years who Currently were Using Contraceptive*	61,74	72,62	67,61	55,28	55,50
**PERUMAHAN/***HOUSING*					
**Persentase Rumah Tangga Dengan Luas Lantai Per Kapita ≤ 7,2 m²** *Percentage of Households with per Capita Floor Area ≤ 7,2 m²*	10,71	10,05	9,33	8,47	8,94
**Persentase Rumah Tangga dengan Atap Terluas Genteng** *Percentage of Households with the Widest Roof Tile*	59,24	58,96	58,77	58,22	57,08
**Persentase Rumah Tangga dengan Dinding Terluas Tembok** *Percentage of Households with the Widest Stone Wall*	70,87	71,73	73,18	74,62	75,57
**Persentase Rumah Tangga dengan Luas lantai Terluas Tanah** *Percentage of Households with the Widest Land Floor*	7,20	6,77	6,29	5,52	5,10
**Persentase Rumah Tangga dengan Fasilitas Tempat Buang Air Besar Sendiri** *Percentage of Households with Own Defecation Facility*	71,52	74,34	75,93	77,84	79,35
**Persentase Rumah Tangga dengan Jenis kloset leher angsa** *Percentage of Households with Swan Trine Closet Facility*	86,08	87,55	90,48	92,57	93,50
**Persentase Rumah Tangga dengan Tempat pembuangan akhir tinja dengan tangki septik** *Percentage of Households with Final Disposal of Feces through Septic Tank*	64,57	54,97	68,13	77,43	78,69
**Persentase Rumah Tangga dengan Sumber air minum bersih** *Percentage of Households with Source of Clean Drinking Water*	66,77	70,08	70,63	71,27	72,99
**Persentase Rumah Tangga dengan Sumber air minum layak** *Percentage of Households with Source of Decent Drinking Water*	39,31	43,05	41,73	38,60	39,16
**Persentase Rumah Tangga dengan Sumber penerangan Listrik** *Percentage of Households with Electricity as the Source of Lighting*	97,01	94,44	94,93	98,14	98,51

https://www.bps.go.id

STATISTIK KUNCI, 2014-2018
KEY STATISTICS, 2014-2018

**Rincian**/*Description*	**2014**	**2015**	**2016**	**2017**	**2018**
(1)	(2)	(3)	(4)	(5)	(6)
**TEKNOLOGI INFORMASI DAN KOMUNIKASI /***INFORMATION TECHNOLOGY AND COMMUNICATION*					
**Persentase Rumah Tangga yang Memiliki Telepon Rumah/PSTN**<br>*Percentage of Household who Had Fixed Line telephone (PSTN)*	5,54	4,01	3,49	3,23	2,61
**Persentase Rumah Tangga yang Memiliki Komputer/Laptop**<br>*Percentage of Household who Had Computer/Laptop*	17,30	18,71	19,14	19,11	20,05
**LAIN-LAIN/***OTHERS*					
**Persentase Rumah Tangga yang menjadi Korban kejahatan**<br>*Percentage of Households who became the Victims of Crime*	1,06	1,00	0,93	1,22	1,18
**Persentase Rumah Tangga dengan PIP**<br>*Percentage of Households who Received PIP*	-	18,88	11,79	9,72	11,11
**Persentase Rumah Tangga dengan Raskin**<br>*Percentage of Households Who had access to Raskin*	51,12	42,61	44,32	35,93	34,74
**Harga rata-rata Raskin per kg (rupiah)**<br>*The Average price of one Kg Raskin (Rupiahs)*	2 181	2 285	2 048	2 079	1 497
**Persentase Rumah Tangga yang menerima kredit usaha**<br>*Percentage of Households Who Received Business Credit*	8,17	12,66	14,10	24,14	28,73
**Persentase Rumah Tangga yang menerima KPS/KKS**<br>*Percentage of Households Who Received KPS/KKS*	-	21,04	17,10	14,48	13,87

https://www.bps.go.id

BAB/*CHAPTER* 1

PENDAHULUAN

INTRODUCTION

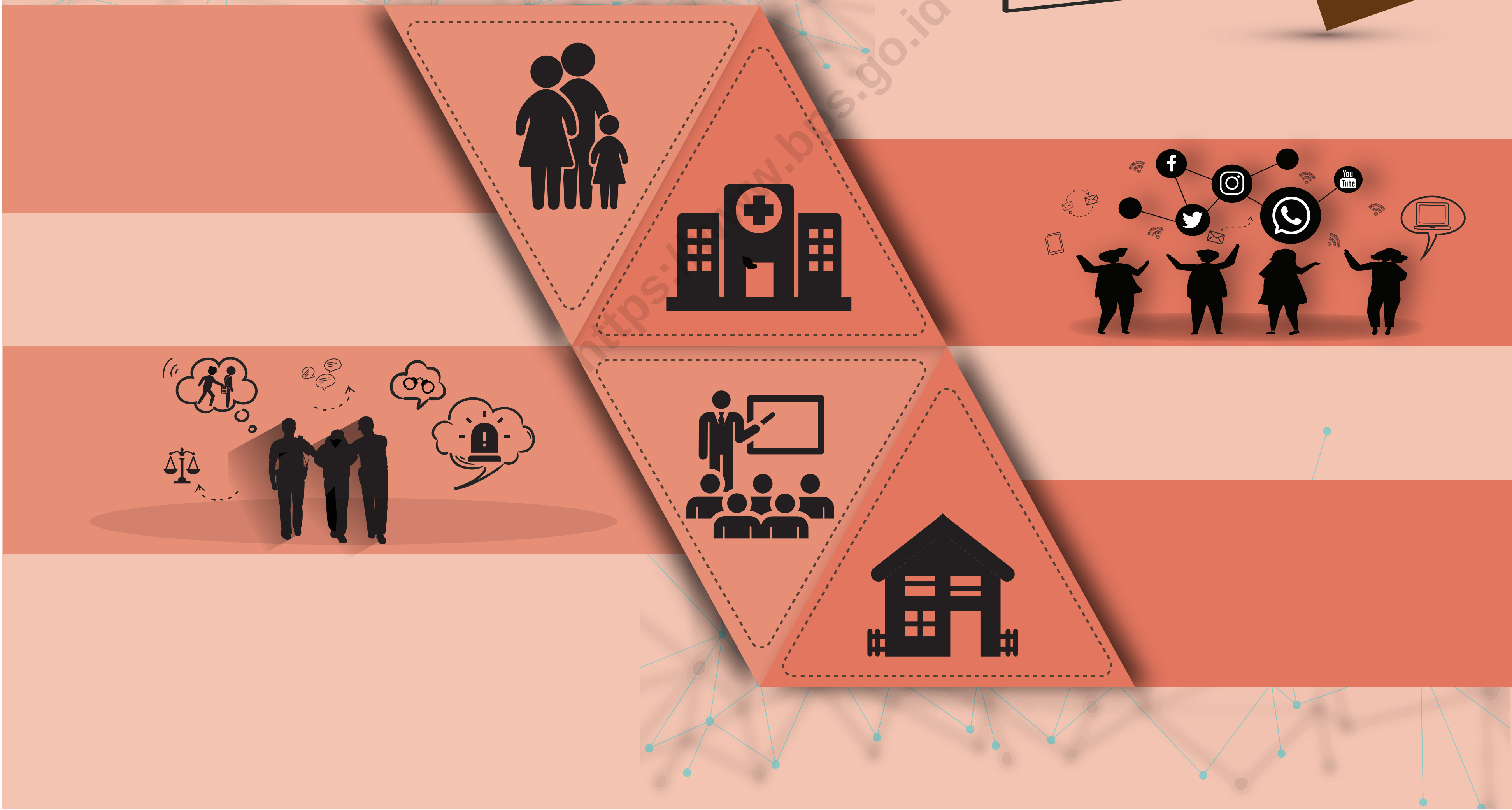

https://www.bps.go.id

https://www.bps.go.id

I. PENJELASAN UMUM/*EXPLANATORY NOTES*

1.1. PENDAHULUAN

1.1.1. Umum

Monitoring terhadap hasil-hasil pembangunan mutlak diperlukan untuk melihat sejauh mana pembangunan yang telah dilaksanakan bermanfaat bagi peningkatan kesejahteraan rakyat, sehingga program-program pembangunan berikutnya dapat lebih optimal. Survei Sosial Ekonomi Nasional (Susenas) yang diselenggarakan oleh BPS merupakan salah satu sumber informasi untuk mendapatkan gambaran mengenai kondisi sosial ekonomi masyarakat. Mulai tahun 2015, pengumpulan data Susenas Kor dilaksanakan pada Bulan Maret. Data Kor yang disajikan dalam publikasi ini estimasinya mencakup hingga level kabupaten/kota.

Informasi mengenai kondisi sosial ekonomi masyarakat yang telah dikumpulkan melalui Susenas, digunakan sebagai dasar untuk memperoleh berbagai indikator pencapaian kesejahteraan rakyat. Indikator tersebut meliputi: angka partisipasi sekolah dan angka melek huruf untuk bidang pendidikan; angka morbiditas, pemanfaatan fasilitas kesehatan, jaminan kesehatan, pemberian ASI pada baduta, dan imunisasi untuk bidang kesehatan, dan penolong persalinan; umur perkawinan pertama, partisipasi KB, dan rata-rata jumlah anak yang dilahirkan untuk bidang fertilitas dan KB; kondisi tempat tinggal, sumber air untuk minum, memasak, mandi dan mencuci untuk bidang perumahan; kepemilikan HP, akses internet dalam pemanfaatan teknologi informasi, serta bantuan/program pemerintah untuk kesejahteraan masyarakat.

1.1. INTRODUCTION

1.1.1. General

Monitoring of development results is absolutely necessary to see the extent to which development has been carried out is useful for the improvement of public welfare, so that the next programs can be more optimized. The National Socio-Economic Survey (Susenas) held by the BPS is one source of information to get descriptions of the socio-economic conditions of society. Starting in 2015, Susenas Core data collection held in Maret. The core data presented in this publication, the estimation covers up to district/municipality level.

Information about the socioeconomic conditions that have been collected through Susenas is used as the basis for the achievement of welfare indicators. The indicators include: school enrollment and literacy rates for education; morbidity, utilization of health facilities, health insurance, Batuta breastfeeding, and immunization for health, and birth attendants; the age of first marriage, participation of family planning program, and the average number of children born to the field of fertility and family planning; living conditions, a source of water for drinking, cooking, bathing and washing to the areas of housing; ownership of HP, internet access in the use of information technology. and aid/government programs for the welfare of society.

https://www.bps.go.id

Masih banyak indikator-indikator lain yang dapat dihasilkan dari Susenas, namun karena indikator yang disajikan dalam publikasi ini telah dianggap cukup mewakili berbagai bidang dan kondisi sosial ekonomi masyarakat, maka tidak semua indikator tersebut dipublikasikan. Indikator-indikator khusus maupun variabel yang terdapat pada kuesioner kor yang tidak disajikan dalam publikasi ini dapat diakses atau diperoleh melalui kemasan media komputer maupun mengolahnya dari data mentah (raw data).

There are many other indicators that can be generated from Susenas, but because of the indicators presented in this publication has been adequately represent the various fields of economic and social conditions of society, not all indicators are published. Specific indicators and variables contained in the core questionnaire which are not presented in this publication can be accessed or obtained through computer media package or process it from the raw data.

1.1.2. Sistematika Penyajian

1.1.2. Order of Presentation

Data yang disajikan dalam publikasi ini seluruhnya berasal dari data Kor Susenas Maret 2018 (Daftar VSEN2018.K), contoh kuesioner dicantumkan pada lampiran.

The data presented in this publication are entirely derived from the March Susenas Core 2018 data (VSEN2018.K). The questionnaire specimens are listed on appendix.

Publikasi ini terdiri dari penjelasan dan tabel. Penjelasan terdiri dari gambaran umum, metode survei yang meliputi ruang lingkup, kerangka sampel, rancangan penarikan sampel, metode pengumpulan data, dan pengolahan data. Tabel pada publikasi ini terdiri dari tabel data kependudukan, pendidikan, kesehatan, fertilitas dan keluarga berencana, perumahan, teknologi informasi dan komunikasi, dan tabel lain-lain.

This publication consists of annotations and tables. Explanation consists of a general overview, survey methods that include the scope, sampling frame, sampling design, data collection methods, and data processing. The table on this publication consists of data tables on population, education, health, fertility and family planning, housing, information technology and communication, and others table.

https://www.bps.go.id

1.2. METODE SURVEI

1.2.1 Ruang Lingkup

Susenas 2018 dilaksanakan di seluruh provinsi di Indonesia (34 provinsi) dengan ukuran sampel 300.000 rumah tangga yang tersebar di 514 kabupaten/kota di Indonesia. Sampel tidak termasuk rumah tangga yang tinggal dalam blok sensus khusus dan rumah tangga khusus seperti asrama, penjara, dan sejenisnya tetapi rumah tangga yang berada di blok sensus biasa. Seluruh rumah tangga sampel tersebut dicacah dengan kuesioner VSEN2018.K.

Data yang dihasilkan cukup representatif untuk disajikan sampai dengan tingkat kabupaten/kota namun tidak dapat dibedakan menurut daerah tempat tinggal (perkotaan/perdesaan).

1.2.2. Kerangka Sampel

Kerangka sampel induk atau sampling frame induk kegiatan Susenas, Sakernas, dan SUPAS 2015 adalah sekitar 180.000 blok sensus (25 persen populasi) yang ditarik secara PPS size rumah tangga SP2010 dari master frame blok sensus. Selanjutnya untuk kegiatan Susenas didefinisikan sebagai berikut:

1. Kerangka sampel tahap pertama adalah daftar blok sensus biasa SP2010.
2. Kerangka sampel tahap kedua adalah daftar 25 persen blok sensus SP2010 yang sudah ada kode stratanya. 25 persen blok sensus ini disebut sampling frame induk.
3. Kerangka sampel tahap ketiga adalah daftar rumah tangga hasil pemutakhiran di setiap blok sensus terpilih.

1.2. SURVEY METHODOLOGY

1.2.1. Coverage

Susenas 2018 was carried out in all Indonesia’s provinces (34 provinces) with a sample size 300,000 households in 514 district/municipalities, excluding households belonging to specific census block and specific households such as orphanage, residence hall, dormitory, hostel, prison, military barracks, and the like even if they are located in ordinary block census. All sample households were enumerated using VSEN2018.K .

The Susenas data were large enough to produce district/municipality level estimates so long as no urban rural classification distinction was made.

1.2.2. Sampling Frame

The sample frame is the parent or the parent frame sampling Susenas, Sakernas, and SUPAS 2015 is approximately 180,000 census blocks (25 percent of the population) were withdrawn by PPS SP2010 household size of the master frame of census block. Further to Susenas activities are defined as follows:

1. *The first phase sample frame is ordinary census block SP2010.*
2. *The second stage sample frame is a list of 25 percent of census block SP2010 existing strata code. 25 percent of census block is called the sampling frame parent.*
3. *The third phase sample frame is the result of updating the list of households in each selected census block.*

https://www.bps.go.id

1.2.3. Desain Sampel

A. Estimasi Kabupaten/Kota

Sampel dipilih dengan metode *two stages one phase stratified sampling*:

Tahap 1: Memilih 25 persen blok sensus populasi secara *Probability Proportional to Size* (PPS), dengan *size* jumlah rumah tangga hasil SP2010 di setiap strata.

Tahap 2: Memilih sejumlah n blok sensus sesuai alokasi secara *systematic* di setiap strata urban/rural per kabupaten/kota per strata kesejahteraan.

Tahap 3: Memilih 10 rumah tangga hasil pemutakhiran secara *systematic sampling* dengan *implicit stratification* menurut pendidikan tertinggi yang ditamatkan Kepala Rumah Tangga (KRT).

B. Estimasi Provinsi

Sampel untuk Susenas estimasi provinsi merupakan subsampel dari Susenas estimasi kabupaten/kota dan dipilih menggunakan metode *two stages stratified sampling* seperti berikut:

Tahap 1: Memilih 7.500 blok sensus secara systematic sampling dari 30.000 blok sensus estimasi kabupaten/kota sesuai alokasi dan mempertimbangkan distribusi sampel per strata di tingkat kabupaten/kota.

Tahap 2: Memilih 10 rumah tangga hasil pemutakhiran secara *systematic* sampling dengan *implicit stratification* pendidikan tertinggi yang ditamatkan kepala rumah tangga.

1.2.3. Sample Design

A. Estimation of District/Municipality

The sample was selected by two stages one method of stratified sampling phase:

Step 1: Choose 25 percent of the population census block Probability Proportional to Size (PPS), the number of household size in each stratum SP2010.

Step 2: Selecting a number n corresponding census block by systematic allocation in each stratum of urban/rural per district/municipality per strata welfare.

Step 3: Selecting 10 households by systematic sampling results updates with implicit stratification according to the highest education attained KRT.

A. Estimation of Provinces

Susenas sample to estimate the province is a sub sample of Susenas estimate of districts/cities and selected using stratified sampling method of two stages as follows:

Step 1: Choose 7,500 census block by systematic sampling of the estimated 30,000 census blocks districts/cities in accordance allocation and consider the distribution of samples per strata at the district/ municipality.

Stage 2: Selecting 10 households by systematic sampling results updates with implicit stratification highest education attained by head of household.

https://www.bps.go.id

1.2.4. Metode Pengumpulan Data

Tahun 2018, pengumpulan data Susenas Kor dilaksanakan pada Bulan Maret 2018. Jumlah sampel total sampel Susenas Kor sebanyak 300.000 rumah tangga.

Pengumpulan data dari rumah tangga terpilih dilakukan melalui wawancara tatap muka antara pencacah dengan responden. Untuk pertanyaan-pertanyaan dalam kuesioner yang ditujukan kepada individu diusahakan agar individu yang bersangkutan yang menjadi responden. Keterangan tentang rumah tangga dikumpulkan melalui wawancara dengan kepala rumah tangga, suami/istri kepala rumah tangga atau anggota rumah tangga lain yang mengetahui karakteristik yang ditanyakan.

1.2.5. Pengolahan Data

Proses pengolahan data meliputi tahap perekaman data, pemeriksaan konsistensi antar-isian dalam kuesioner sampai dengan tahap tabulasi, sepenuhnya dilakukan dengan menggunakan komputer. Sebelum tahap ini dimulai, terlebih dahulu dilakukan cek awal atas kelengkapan isian daftar pertanyaan, penyuntingan terhadap isian yang tidak wajar, termasuk hubungan keterkaitan (konsistensi) antara satu jawaban dengan jawaban yang lainnya. Proses perekaman data dilakukan di BPS kabupaten/kota.

Dari target 300.000 rumah tangga sampel, hanya 295.155 rumah tangga sampel yang dinyatakan bersih dan dapat diolah faktor pengali/ penimbang menggunakan penduduk tengah tahun 2018 untuk estimasi kabupaten/kota.

1.2.4. Data Collection Method

In 2018 the collection of data Susenas core held in March 2018. The sample size of Susenas core amount 300,000 households.

The collection of data from selected households was carried out by interview approach where the enumerator meets face to face with the respondent. For such questions in the questionnaire as correspond to individuals an effort was made to incite the individual to become respondent. Data on household characteristics were collected by interviewing household head, his or her spouse, or other household members who knew about the characteristics being asked.

1.2.5. Data Processing

Data processing--starting from data entry, consistency check between items in the questionnaire, to tabulation--was completely done using computer. However, before computer processing was done, a preliminary check was made to make sure whether questionnaire items were filled in properly, and to eliminate unseemly figures including consistency between data items were already checked. Data entry was done in BPS districts/ municipalities.

From 300,000 households target sample, only 295,155 households sample remains clean and can be processed. Weighting used 2018 mid-year population estimates for districts/municipalities

https://www.bps.go.id

BAB/CHAPTER 2

KEPENDUDUKAN
DEMOGRAPHY

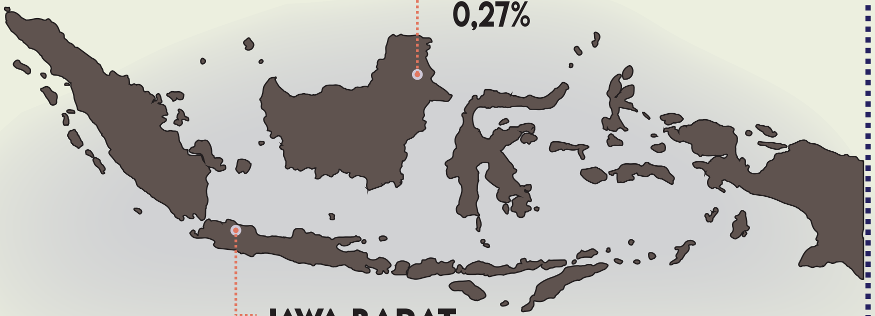

KALIMANTAN UTARA
persentase penduduk **terendah**
lowest *percentage of population*
0,27%

JAWA BARAT
persentase penduduk **tertinggi**
highest *percentage of population*
18,36%

RASIO JENIS KELAMIN
Sex Ratio

101

Terdapat **101** penduduk **laki-laki** diantara **100** penduduk **perempuan**

There are 101 male residents among 100 female residents

49,76% | 50,24%

RASIO KETERGANTUNGAN
Dependency Ratio

48,23

0-14 tahun *years*: 26,77%
15-64 tahun/*years*: 67,46%
65 tahun ke atas *years and over*: 5,77%

Setiap 100 penduduk usia produktif (15-64 tahun) menanggung 48 penduduk usia tidak produktif (0-14 tahun dan 65 tahun ke atas)

Every 100 productive residents (15-64 years) bear 48 unproductive residents (0-14 years and 65 years and over)

STATUS PERKAWINAN
Penduduk berumur 10 tahun ke atas
Marital Status *of population 10 years and over*

Belum kawin/*single* 32,20%
Kawin/*married* 59,49%
Cerai hidup/*divorced* 2,01%
Cerai mati/*widowed* 6,31%

Wilayah dengan persentase tertinggi penduduk berumur 10 tahun ke atas berstatus **kawin** ialah **Provinsi Bengkulu**

The region with the highest percentage of population aged 10 years and over with married status is Bengkulu Province

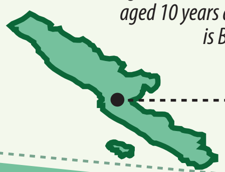

62,62%

https://www.bps.go.id

https://www.bps.go.id

https://www.bps.go.id

PENJELASAN TEKNIS

1. **Penduduk Indonesia** adalah semua orang yang berdomisili di wilayah teritorial Indonesia selama 6 bulan atau lebih dan atau mereka yang berdomisili kurang dari 6 bulan tetapi bertujuan menetap.

2. **Rasio Jenis Kelamin** adalah perbandingan antara banyaknya penduduk laki-laki dengan banyaknya penduduk perempuan pada suatu daerah dan waktu tertentu. Biasanya dinyatakan dengan banyaknya penduduk laki-laki untuk 100 penduduk perempuan.

3. **Angka Beban Ketergantungan** adalah perbandingan antara jumlah penduduk berumur 0-14 tahun ditambah dengan jumlah penduduk 65 tahun ke atas (keduanya disebut dengan bukan angkatan kerja) dibandingkan dengan jumlah penduduk usia 15-64 tahun (angkatan kerja).

4. **Belum kawin** adalah status dari mereka yang pada saat pencacahan belum terikat dalam perkawinan.

5. **Kawin** adalah status dari mereka yang terikat perkawinan pada saat pencacahan, baik tinggal bersama maupun terpisah. Dalam hal ini yang dicakup tidak saja mereka yang kawin sah secara hukum (adat, agama, negara, dan sebagainya) tetapi juga mereka yang hidup bersama dan oleh masyarakat sekelilingnya dianggap sebagai suami isteri.

TECHNICAL NOTES

1. ***The population of Indonesia*** *are all residents of the entire of Indonesia who have stayed for six months or longer, and those who intended to stay more than six months even though their length of stay is less than six months.*

2. ***Sex ratio*** *is the ratio of males population to females population in a given area and time, usually expressed as the number of males for every 100 females.*

3. ***Dependency Ratio*** *is the ratio of population aged less than 15 years and 65 years and over (considered the unproductive age) to the population aged between 15 and 64 years (productive age), multiplied by 100.*

4. ***Single*** *is marrital status when survey/cencus held is not in relationship of married.*

5. ***Married*** *is a status for those who on the enumeration date were bound by marriage, regardless of whether they are living together or not. This includes those who by law (e.g. tradition, religion, state, etc.) are formally married but also those who live together and are regarded by their community as husbands and wives.*

6. **Cerai hidup** adalah status dari mereka yang hidup berpisah sebagai suami isteri karena bercerai dan belum kawin lagi. Dalam hal ini termasuk mereka yang mengaku cerai walaupun belum resmi secara hukum. Sebaliknya, tidak termasuk mereka yang hanya hidup terpisah tetapi masih berstatus kawin, misalnya suami/isteri ditinggalkan oleh isteri/suami ke tempat lain karena sekolah, bekerja, mencari pekerjaan, atau untuk keperluan lain. Wanita yang mengaku belum pernah kawin tetapi pernah hamil, dianggap cerai hidup.

7. **Cerai mati** adalah status dari mereka yang ditinggal mati oleh suami/istrinya dan belum kawin lagi.

8. **Pernah Kawin** adalah status dari mereka yang pada saat pencacahan status perkawinannya kawin, cerai hidup, atau cerai mati.

9. **Akta kelahiran** adalah surat tanda bukti kelahiran yang dikeluarkan oleh kantor catatan sipil.

10. **Nomor Induk Kependudukan (NIK)** adalah nomor identitas penduduk yang bersifat unik atau khas, tunggal dan melekat pada seseorang yang terdaftar sebagai penduduk Indonesia.

*6. **Divorced** is a category for those who divorced their husbands or wives and have not yet remarried. Including those who have divorced without a formal divorce procedure and women who have not been married but have been pregnant. Excluded are husband and wives who are separated temporarily due to work, training, seeking work, etc.*

*7. **Widowed** is a status for those whose husbands or wives were deceased and had not yet remarried.*

*8. **Ever Married** is a status for those marital status at the time of enumeration, was either married, divorced, or widowed.*

*9. **The birth certificate** is proof of birth issued by the civil registration office.*

*10. **Population Identification Number (NIK)** is a social security number that is unique or distinctive, single and attached to a person who is registered as a resident of Indonesia.*

https://www.bps.go.id

Tabel **2.1.** **Persentase Penduduk menurut Kelompok Umur (5 Tahunan), Daerah Tempat Tinggal, dan Jenis Kelamin, 2018**
Table *Percentage of Population by Age Groups (5 years), Urban Rural Classification, and Sex, 2018*

**Kelompok Umur** *Age Groups*	**Perkotaan/***Urban*			**Perdesaan/***Rural*			**Perkotaan+Perdesaan/***Urban+Rural*		
	**Laki-laki** *Male*	**Perempuan** *Female*	**Laki-laki+ Perempuan** *Male+Female*	**Laki-laki** *Male*	**Perempuan** *Female*	**Laki-laki+ Perempuan** *Male+Female*	**Laki-laki** *Male*	**Perempuan** *Female*	**Laki-laki+ Perempuan** *Male+Female*
(1)	(2)	(3)	(4)	(5)	(6)	(7)	(8)	(9)	(10)
0-4	8,97	8,64	8,80	9,27	8,99	9,13	9,11	8,80	8,95
5-9	8,95	8,65	8,80	9,60	9,25	9,43	9,25	8,92	9,08
10-14	8,47	8,18	8,33	9,43	9,03	9,23	8,91	8,56	8,74
15-19	8,41	8,27	8,34	8,54	8,01	8,27	8,47	8,15	8,31
20-24	8,63	8,54	8,59	7,72	7,42	7,57	8,22	8,03	8,13
25-29	8,33	8,25	8,29	7,60	7,64	7,62	8,00	7,98	7,99
30-34	7,99	8,04	8,01	7,29	7,46	7,37	7,67	7,78	7,72
35-39	7,90	8,02	7,96	7,43	7,61	7,52	7,69	7,84	7,76
40-44	7,23	7,27	7,25	6,97	7,06	7,01	7,11	7,18	7,14
45-49	6,66	6,62	6,64	6,53	6,55	6,54	6,60	6,59	6,60
50-54	5,57	5,68	5,62	5,60	5,73	5,66	5,58	5,70	5,64
55-59	4,60	4,63	4,62	4,67	4,81	4,74	4,63	4,71	4,67
60-64	3,36	3,37	3,37	3,66	3,68	3,67	3,50	3,51	3,50
65-69	2,21	2,28	2,25	2,48	2,58	2,53	2,33	2,42	2,37
70-74	1,34	1,60	1,47	1,55	1,84	1,70	1,44	1,71	1,57
75+	1,37	1,96	1,66	1,69	2,34	2,01	1,52	2,13	1,82
**Jumlah/Total**	100,00	100,00	100,00	100,00	100,00	100,00	100,00	100,00	100,00

Sumber/*Source*: Susenas Maret 2018/*The March 2018 Susenas*

https://www.bps.go.id

Tabel *Table* **2.2.** **Persentase Penduduk menurut Provinsi, Daerah Tempat Tinggal, dan Jenis Kelamin, 2018**
Percentage of Population by Province, Urban Rural Classification, and Sex, 2018

**Provinsi** *Province*	**Perkotaan/***Urban*			**Perdesaan/***Rural*			**Perkotaan+Perdesaan/***Urban+Rural*		
	**Laki-laki/***Male*	**Perempuan/***Female*	**Jumlah** *Total*	**Laki-laki/***Male*	**Perempuan/***Female*	**Jumlah** *Total*	**Laki-laki/***Male*	**Perempuan/***Female*	**Jumlah** *Total*
**(1)**	**(2)**	**(3)**	**(4)**	**(5)**	**(6)**	**(7)**	**(8)**	**(9)**	**(10)**
Aceh	50,18	49,82	100,00	49,82	50,18	100,00	49,93	50,07	100,00
Sumatera Utara	49,79	50,21	100,00	50,03	49,97	100,00	49,90	50,10	100,00
Sumatera Barat	49,72	50,28	100,00	49,81	50,19	100,00	49,77	50,23	100,00
Riau	51,11	48,89	100,00	51,43	48,57	100,00	51,30	48,70	100,00
Jambi	50,61	49,39	100,00	51,22	48,78	100,00	51,02	48,98	100,00
Sumatera Selatan	50,27	49,73	100,00	51,15	48,85	100,00	50,82	49,18	100,00
Bengkulu	50,11	49,89	100,00	51,40	48,60	100,00	50,97	49,03	100,00
Lampung	50,37	49,63	100,00	51,57	48,43	100,00	51,22	48,78	100,00
Kepulauan Bangka Belitung	51,57	48,43	100,00	52,44	47,56	100,00	51,97	48,03	100,00
Kepulauan Riau	50,83	49,17	100,00	51,56	48,44	100,00	50,94	49,06	100,00
DKI Jakarta	50,15	49,85	100,00	-	-	-	50,15	49,85	100,00
Jawa Barat	50,68	49,32	100,00	50,60	49,40	100,00	50,66	49,34	100,00
Jawa Tengah	49,51	50,49	100,00	49,68	50,32	100,00	49,59	50,41	100,00
DI Yogyakarta	49,80	50,20	100,00	48,44	51,56	100,00	49,43	50,57	100,00
Jawa Timur	49,54	50,46	100,00	49,17	50,83	100,00	49,37	50,63	100,00
Banten	50,87	49,13	100,00	51,21	48,79	100,00	50,97	49,03	100,00
Bali	50,53	49,47	100,00	49,82	50,18	100,00	50,29	49,71	100,00
Nusa Tenggara Barat	48,42	51,58	100,00	48,65	51,35	100,00	48,54	51,46	100,00
Nusa Tenggara Timur	50,10	49,90	100,00	49,35	50,65	100,00	49,52	50,48	100,00
Kalimantan Barat	50,15	49,85	100,00	51,28	48,72	100,00	50,90	49,10	100,00
Kalimantan Tengah	51,45	48,55	100,00	52,69	47,31	100,00	52,21	47,79	100,00
Kalimantan Selatan	50,76	49,24	100,00	50,65	49,35	100,00	50,70	49,30	100,00
Kalimantan Timur	52,03	47,97	100,00	53,19	46,81	100,00	52,42	47,58	100,00
Kalimantan Utara	52,55	47,45	100,00	53,63	46,37	100,00	53,00	47,00	100,00
Sulawesi Utara	50,48	49,52	100,00	51,54	48,46	100,00	51,01	48,99	100,00
Sulawesi Tengah	50,18	49,82	100,00	51,41	48,59	100,00	51,07	48,93	100,00
Sulawesi Selatan	49,09	50,91	100,00	48,70	51,30	100,00	48,86	51,14	100,00
Sulawesi Tenggara	50,27	49,73	100,00	50,27	49,73	100,00	50,27	49,73	100,00
Gorontalo	49,26	50,74	100,00	50,63	49,37	100,00	50,10	49,90	100,00
Sulawesi Barat	49,19	50,81	100,00	50,44	49,56	100,00	50,14	49,86	100,00
Maluku	50,14	49,86	100,00	50,63	49,37	100,00	50,42	49,58	100,00
Maluku Utara	50,66	49,34	100,00	51,15	48,85	100,00	51,01	48,99	100,00
Papua Barat	52,42	47,58	100,00	52,81	47,19	100,00	52,65	47,35	100,00
Papua	53,65	46,35	100,00	52,19	47,81	100,00	52,60	47,40	100,00
**Indonesia**	**50,21**	**49,79**	**100,00**	**50,27**	**49,73**	**100,00**	**50,24**	**49,76**	**100,00**

Sumber/*Source*: Susenas Maret 2018/*The March 2018 Susenas*

https://www.bps.go.id

Tabel **2.3.** **Persentase Penduduk Berumur 7-24 Tahun menurut Provinsi, Daerah Tempat Tinggal, dan Jenis Kelamin, 2018**
Table *Percentage of Population Aged 7-24 Years by Province, Urban Rural Classification, and Sex, 2018*

**Provinsi** *Province*	**Perkotaan/***Urban*			**Perdesaan/***Rural*			**Perkotaan+Perdesaan/***Urban+Rural*		
	**Laki-laki/***Male*	**Perempuan/***Female*	**Jumlah** *Total*	**Laki-laki/***Male*	**Perempuan/***Female*	**Jumlah** *Total*	**Laki-laki/***Male*	**Perempuan/***Female*	**Jumlah** *Total*
**(1)**	**(2)**	**(3)**	**(4)**	**(5)**	**(6)**	**(7)**	**(8)**	**(9)**	**(10)**
Aceh	50,93	49,07	100,00	50,96	49,04	100,00	50,95	49,05	100,00
Sumatera Utara	50,66	49,34	100,00	51,47	48,53	100,00	51,05	48,95	100,00
Sumatera Barat	49,74	50,26	100,00	51,23	48,77	100,00	50,57	49,43	100,00
Riau	51,02	48,98	100,00	51,77	48,23	100,00	51,47	48,53	100,00
Jambi	49,72	50,28	100,00	51,50	48,50	100,00	50,93	49,07	100,00
Sumatera Selatan	50,91	49,09	100,00	51,41	48,59	100,00	51,22	48,78	100,00
Bengkulu	50,55	49,45	100,00	52,88	47,12	100,00	52,10	47,90	100,00
Lampung	50,83	49,17	100,00	51,92	48,08	100,00	51,58	48,42	100,00
Kepulauan Bangka Belitung	50,71	49,29	100,00	52,27	47,73	100,00	51,45	48,55	100,00
Kepulauan Riau	50,40	49,60	100,00	52,95	47,05	100,00	50,76	49,24	100,00
DKI Jakarta	50,06	49,94	100,00	-	-	-	50,06	49,94	100,00
Jawa Barat	50,82	49,18	100,00	51,41	48,59	100,00	50,97	49,03	100,00
Jawa Tengah	50,78	49,22	100,00	51,42	48,58	100,00	51,09	48,91	100,00
DI Yogyakarta	51,47	48,53	100,00	51,72	48,28	100,00	51,53	48,47	100,00
Jawa Timur	50,70	49,30	100,00	51,07	48,93	100,00	50,88	49,12	100,00
Banten	50,82	49,18	100,00	52,10	47,90	100,00	51,21	48,79	100,00
Bali	51,26	48,74	100,00	51,95	48,05	100,00	51,48	48,52	100,00
Nusa Tenggara Barat	50,91	49,09	100,00	51,11	48,89	100,00	51,02	48,98	100,00
Nusa Tenggara Timur	50,53	49,47	100,00	50,81	49,19	100,00	50,74	49,26	100,00
Kalimantan Barat	50,26	49,74	100,00	51,56	48,44	100,00	51,12	48,88	100,00
Kalimantan Tengah	50,67	49,33	100,00	51,97	48,03	100,00	51,46	48,54	100,00
Kalimantan Selatan	51,28	48,72	100,00	51,49	48,51	100,00	51,39	48,61	100,00
Kalimantan Timur	51,44	48,56	100,00	52,24	47,76	100,00	51,71	48,29	100,00
Kalimantan Utara	52,14	47,86	100,00	54,15	45,85	100,00	52,98	47,02	100,00
Sulawesi Utara	51,09	48,91	100,00	52,51	47,49	100,00	51,78	48,22	100,00
Sulawesi Tengah	49,92	50,08	100,00	51,95	48,05	100,00	51,36	48,64	100,00
Sulawesi Selatan	50,90	49,10	100,00	50,91	49,09	100,00	50,90	49,10	100,00
Sulawesi Tenggara	51,57	48,43	100,00	50,66	49,34	100,00	51,02	48,98	100,00
Gorontalo	50,31	49,69	100,00	51,16	48,84	100,00	50,83	49,17	100,00
Sulawesi Barat	51,10	48,90	100,00	51,09	48,91	100,00	51,10	48,90	100,00
Maluku	50,59	49,41	100,00	51,43	48,57	100,00	51,07	48,93	100,00
Maluku Utara	50,80	49,20	100,00	51,43	48,57	100,00	51,25	48,75	100,00
Papua Barat	52,44	47,56	100,00	52,03	47,97	100,00	52,20	47,80	100,00
Papua	53,49	46,51	100,00	52,74	47,26	100,00	52,94	47,06	100,00
**Indonesia**	**50,77**	**49,23**	**100,00**	**51,46**	**48,54**	**100,00**	**51,08**	**48,92**	**100,00**

Sumber/*Source*: Susenas Maret 2018/*The March 2018 Susenas*

https://www.bps.go.id

Tabel *Table* **2.4.1.** **Persentase Penduduk di Daerah Perkotaan menurut Provinsi, Jenis Kelamin, dan Kelompok Umur, 2018**
Percentage of Population in Urban Area by Province, Sex, and Age Groups, 2018

Provinsi *Province*	Laki-laki/*Male*				Perempuan/*Female*				Laki-laki+Perempuan/*Male+Female*			
	0-14	15-64	65+	Jumlah *Total*	0-14	15-64	65+	Jumlah *Total*	0-14	15-64	65+	Jumlah *Total*
(1)	(2)	(3)	(4)	(5)	(6)	(7)	(8)	(9)	(10)	(11)	(12)	(13)
Aceh	30,35	66,34	3,30	100,00	29,31	66,34	4,35	100,00	29,83	66,34	3,82	100,00
Sumatera Utara	30,05	66,16	3,79	100,00	28,73	66,54	4,73	100,00	29,39	66,35	4,26	100,00
Sumatera Barat	29,95	65,32	4,73	100,00	28,22	65,97	5,81	100,00	29,08	65,65	5,27	100,00
Riau	29,83	67,34	2,83	100,00	30,00	66,81	3,19	100,00	29,91	67,08	3,01	100,00
Jambi	25,98	69,85	4,18	100,00	26,31	69,53	4,16	100,00	26,14	69,69	4,17	100,00
Sumatera Selatan	27,85	68,03	4,11	100,00	26,97	68,36	4,66	100,00	27,41	68,20	4,39	100,00
Bengkulu	27,26	69,62	3,12	100,00	27,86	68,51	3,63	100,00	27,56	69,07	3,38	100,00
Lampung	27,36	68,19	4,45	100,00	26,87	68,30	4,82	100,00	27,12	68,25	4,63	100,00
Kepulauan Bangka Belitung	26,23	69,38	4,38	100,00	27,05	67,69	5,25	100,00	26,63	68,57	4,80	100,00
Kepulauan Riau	29,83	67,87	2,30	100,00	30,46	67,20	2,35	100,00	30,14	67,54	2,32	100,00
DKI Jakarta	25,39	70,56	4,05	100,00	24,38	71,09	4,54	100,00	24,89	70,82	4,29	100,00
Jawa Barat	26,64	68,64	4,72	100,00	26,08	68,46	5,47	100,00	26,36	68,55	5,09	100,00
Jawa Tengah	24,67	68,09	7,24	100,00	22,93	68,32	8,75	100,00	23,79	68,20	8,01	100,00
DI Yogyakarta	22,45	70,18	7,36	100,00	21,04	69,79	9,18	100,00	21,74	69,99	8,27	100,00
Jawa Timur	23,25	70,12	6,63	100,00	21,76	70,24	8,00	100,00	22,50	70,18	7,32	100,00
Banten	27,13	69,96	2,91	100,00	26,98	69,75	3,28	100,00	27,06	69,85	3,09	100,00
Bali	23,90	70,46	5,64	100,00	23,26	69,94	6,81	100,00	23,58	70,20	6,22	100,00
Nusa Tenggara Barat	30,34	64,99	4,67	100,00	27,24	67,49	5,26	100,00	28,74	66,28	4,98	100,00
Nusa Tenggara Timur	31,94	64,17	3,88	100,00	30,18	65,46	4,36	100,00	31,06	64,82	4,12	100,00
Kalimantan Barat	28,17	67,44	4,39	100,00	27,43	67,65	4,92	100,00	27,80	67,54	4,65	100,00
Kalimantan Tengah	26,88	70,02	3,10	100,00	27,67	69,34	2,99	100,00	27,27	69,69	3,04	100,00
Kalimantan Selatan	27,41	69,18	3,41	100,00	27,09	68,61	4,31	100,00	27,25	68,90	3,85	100,00
Kalimantan Timur	26,63	70,20	3,17	100,00	27,42	69,25	3,33	100,00	27,01	69,74	3,25	100,00
Kalimantan Utara	28,01	68,19	3,80	100,00	29,92	66,96	3,12	100,00	28,92	67,61	3,48	100,00
Sulawesi Utara	24,42	70,08	5,50	100,00	24,44	68,67	6,89	100,00	24,43	69,38	6,19	100,00
Sulawesi Tengah	26,31	69,62	4,07	100,00	26,89	68,63	4,49	100,00	26,60	69,13	4,28	100,00
Sulawesi Selatan	28,90	66,67	4,43	100,00	26,70	67,31	5,99	100,00	27,78	67,00	5,22	100,00
Sulawesi Tenggara	32,25	64,05	3,70	100,00	30,68	65,34	3,97	100,00	31,47	64,70	3,84	100,00
Gorontalo	26,92	68,72	4,36	100,00	25,78	69,32	4,90	100,00	26,34	69,02	4,63	100,00
Sulawesi Barat	32,00	64,20	3,81	100,00	30,22	64,83	4,95	100,00	31,10	64,52	4,38	100,00
Maluku	31,03	65,23	3,73	100,00	30,12	65,92	3,96	100,00	30,58	65,57	3,85	100,00
Maluku Utara	30,02	67,02	2,96	100,00	28,86	67,37	3,77	100,00	29,45	67,19	3,36	100,00
Papua Barat	28,78	69,08	2,14	100,00	30,46	67,17	2,37	100,00	29,58	68,17	2,25	100,00
Papua	26,73	70,73	2,54	100,00	29,72	67,80	2,49	100,00	28,11	69,37	2,52	100,00
**Indonesia**	**26,40**	**68,68**	**4,92**	**100,00**	**25,46**	**68,70**	**5,84**	**100,00**	**25,93**	**68,69**	**5,38**	**100,00**

Sumber/*Source*: Susenas Maret 2018/*The March 2018 Susenas*

https://www.bps.go.id

Tabel *Table* **2.4.2.** **Persentase Penduduk di Daerah Perdesaan menurut Provinsi, Jenis Kelamin, dan Kelompok Umur, 2018**
Percentage of Population in Rural Area by Province, Sex, and Age Groups, 2018

**Provinsi** *Province*	**Laki-laki/***Male*				**Perempuan/***Female*				**Laki-laki+Perempuan/***Male+Female*			
	**0-14**	**15-64**	**65+**	**Jumlah** *Total*	**0-14**	**15-64**	**65+**	**Jumlah** *Total*	**0-14**	**15-64**	**65+**	**Jumlah** *Total*
(1)	(2)	(3)	(4)	(5)	(6)	(7)	(8)	(9)	(10)	(11)	(12)	(13)
Aceh	32,19	64,01	3,79	100,00	30,75	64,47	4,78	100,00	31,47	64,24	4,29	100,00
Sumatera Utara	34,34	61,67	3,99	100,00	32,99	61,75	5,27	100,00	33,67	61,71	4,63	100,00
Sumatera Barat	31,53	62,97	5,49	100,00	30,30	62,81	6,89	100,00	30,92	62,89	6,19	100,00
Riau	31,06	65,98	2,96	100,00	31,76	65,05	3,20	100,00	31,40	65,53	3,08	100,00
Jambi	27,83	68,09	4,08	100,00	28,01	67,67	4,31	100,00	27,92	67,89	4,19	100,00
Sumatera Selatan	29,09	66,55	4,37	100,00	29,11	65,88	5,01	100,00	29,10	66,22	4,68	100,00
Bengkulu	28,50	66,86	4,64	100,00	28,60	66,33	5,07	100,00	28,55	66,60	4,85	100,00
Lampung	28,12	66,57	5,31	100,00	28,28	65,98	5,74	100,00	28,20	66,29	5,52	100,00
Kepulauan Bangka Belitung	26,49	69,36	4,14	100,00	28,18	67,24	4,58	100,00	27,30	68,35	4,35	100,00
Kepulauan Riau	29,24	66,26	4,50	100,00	30,91	64,57	4,52	100,00	30,05	65,44	4,51	100,00
DKI Jakarta	-	-	-	-	-	-	-	-	-	-	-	-
Jawa Barat	26,79	66,78	6,43	100,00	26,02	66,65	7,33	100,00	26,41	66,72	6,87	100,00
Jawa Tengah	24,81	67,01	8,18	100,00	23,19	67,21	9,61	100,00	23,99	67,11	8,90	100,00
DI Yogyakarta	22,71	66,22	11,08	100,00	20,72	65,95	13,33	100,00	21,68	66,08	12,24	100,00
Jawa Timur	23,27	68,95	7,77	100,00	21,57	68,96	9,47	100,00	22,41	68,96	8,63	100,00
Banten	30,41	65,63	3,97	100,00	30,09	65,32	4,59	100,00	30,25	65,48	4,27	100,00
Bali	24,20	67,65	8,14	100,00	22,78	67,53	9,69	100,00	23,49	67,59	8,92	100,00
Nusa Tenggara Barat	31,56	63,40	5,04	100,00	28,59	65,97	5,44	100,00	30,04	64,72	5,24	100,00
Nusa Tenggara Timur	36,96	58,07	4,97	100,00	35,00	59,35	5,64	100,00	35,97	58,72	5,31	100,00
Kalimantan Barat	29,64	66,08	4,28	100,00	29,53	65,94	4,53	100,00	29,59	66,01	4,40	100,00
Kalimantan Tengah	27,32	69,45	3,23	100,00	28,61	67,85	3,54	100,00	27,93	68,69	3,37	100,00
Kalimantan Selatan	28,81	67,23	3,96	100,00	28,58	66,39	5,03	100,00	28,70	66,82	4,48	100,00
Kalimantan Timur	27,53	68,58	3,89	100,00	29,43	66,90	3,67	100,00	28,42	67,79	3,79	100,00
Kalimantan Utara	29,81	66,23	3,96	100,00	31,32	65,21	3,47	100,00	30,51	65,76	3,74	100,00
Sulawesi Utara	26,43	67,15	6,42	100,00	26,50	66,19	7,31	100,00	26,46	66,68	6,85	100,00
Sulawesi Tengah	29,64	65,58	4,78	100,00	29,56	65,66	4,77	100,00	29,60	65,62	4,77	100,00
Sulawesi Selatan	30,15	63,99	5,86	100,00	27,41	65,11	7,48	100,00	28,74	64,56	6,69	100,00
Sulawesi Tenggara	34,92	60,88	4,20	100,00	33,09	62,09	4,82	100,00	34,01	61,48	4,51	100,00
Gorontalo	28,90	66,65	4,45	100,00	28,23	66,57	5,20	100,00	28,57	66,61	4,82	100,00
Sulawesi Barat	31,92	64,32	3,76	100,00	31,21	64,52	4,28	100,00	31,57	64,42	4,02	100,00
Maluku	35,19	60,65	4,17	100,00	34,65	60,54	4,80	100,00	34,92	60,60	4,48	100,00
Maluku Utara	34,76	61,60	3,64	100,00	34,07	62,23	3,70	100,00	34,42	61,91	3,67	100,00
Papua Barat	31,15	66,22	2,63	100,00	33,13	64,63	2,24	100,00	32,08	65,47	2,45	100,00
Papua	32,08	66,53	1,39	100,00	32,33	66,54	1,13	100,00	32,20	66,53	1,27	100,00
**Indonesia**	**28,30**	**65,99**	**5,72**	**100,00**	**27,27**	**65,97**	**6,76**	**100,00**	**27,79**	**65,98**	**6,23**	**100,00**

Sumber/*Source*: Susenas Maret 2018/*The March 2018 Susenas*

https://www.bps.go.id

Tabel *Table* **2.4.3.** **Persentase Penduduk di Daerah Perkotaan dan Perdesaan menurut Provinsi, Jenis Kelamin, dan Kelompok Umur, 2018**
Percentage of Population in Urban and Rural Area by Province, Sex, and Age Groups, 2018

Provinsi *Province*	Laki-laki/*Male*				Perempuan/*Female*				Laki-laki+Perempuan/*Male+Female*			
	0-14	15-64	65+	Jumlah *Total*	0-14	15-64	65+	Jumlah *Total*	0-14	15-64	65+	Jumlah *Total*
(1)	(2)	(3)	(4)	(5)	(6)	(7)	(8)	(9)	(10)	(11)	(12)	(13)
Aceh	31,61	64,75	3,64	100,00	30,30	65,05	4,65	100,00	30,96	64,90	4,14	100,00
Sumatera Utara	32,08	64,04	3,89	100,00	30,73	64,29	4,98	100,00	31,40	64,16	4,43	100,00
Sumatera Barat	30,84	64,00	5,16	100,00	29,38	64,20	6,41	100,00	30,11	64,11	5,79	100,00
Riau	30,56	66,53	2,91	100,00	31,04	65,76	3,19	100,00	30,80	66,15	3,05	100,00
Jambi	27,24	68,65	4,11	100,00	27,46	68,27	4,26	100,00	27,35	68,47	4,19	100,00
Sumatera Selatan	28,63	67,09	4,27	100,00	28,31	66,81	4,88	100,00	28,47	66,96	4,57	100,00
Bengkulu	28,10	67,75	4,15	100,00	28,36	67,05	4,59	100,00	28,23	67,41	4,37	100,00
Lampung	27,90	67,04	5,06	100,00	27,85	66,68	5,46	100,00	27,88	66,87	5,26	100,00
Kepulauan Bangka Belitung	26,35	69,37	4,27	100,00	27,57	67,49	4,94	100,00	26,94	68,47	4,59	100,00
Kepulauan Riau	29,75	67,64	2,61	100,00	30,52	66,83	2,65	100,00	30,13	67,24	2,63	100,00
DKI Jakarta	25,39	70,56	4,05	100,00	24,38	71,09	4,54	100,00	24,89	70,82	4,29	100,00
Jawa Barat	26,68	68,16	5,17	100,00	26,06	67,99	5,95	100,00	26,37	68,07	5,55	100,00
Jawa Tengah	24,74	67,56	7,70	100,00	23,06	67,78	9,17	100,00	23,89	67,67	8,44	100,00
DI Yogyakarta	22,52	69,13	8,35	100,00	20,95	68,73	10,32	100,00	21,73	68,93	9,34	100,00
Jawa Timur	23,26	69,57	7,17	100,00	21,67	69,63	8,70	100,00	22,45	69,60	7,95	100,00
Banten	28,08	68,70	3,21	100,00	27,87	68,47	3,65	100,00	27,98	68,59	3,43	100,00
Bali	24,00	69,52	6,48	100,00	23,10	69,12	7,79	100,00	23,55	69,32	7,13	100,00
Nusa Tenggara Barat	31,00	64,14	4,87	100,00	27,96	66,68	5,36	100,00	29,44	65,45	5,12	100,00
Nusa Tenggara Timur	35,80	59,49	4,72	100,00	33,91	60,74	5,35	100,00	34,84	60,12	5,04	100,00
Kalimantan Barat	29,15	66,53	4,32	100,00	28,81	66,53	4,66	100,00	28,98	66,53	4,49	100,00
Kalimantan Tengah	27,16	69,67	3,18	100,00	28,24	68,44	3,32	100,00	27,68	69,08	3,25	100,00
Kalimantan Selatan	28,16	68,14	3,70	100,00	27,88	67,42	4,69	100,00	28,02	67,79	4,19	100,00
Kalimantan Timur	26,93	69,66	3,41	100,00	28,07	68,48	3,44	100,00	27,48	69,10	3,43	100,00
Kalimantan Utara	28,77	67,36	3,87	100,00	30,50	66,24	3,26	100,00	29,58	66,83	3,58	100,00
Sulawesi Utara	25,43	68,61	5,96	100,00	25,45	67,45	7,10	100,00	25,44	68,04	6,52	100,00
Sulawesi Tengah	28,72	66,69	4,58	100,00	28,80	66,51	4,69	100,00	28,76	66,60	4,64	100,00
Sulawesi Selatan	29,63	65,11	5,26	100,00	27,12	66,02	6,86	100,00	28,34	65,58	6,08	100,00
Sulawesi Tenggara	33,90	62,09	4,01	100,00	32,17	63,33	4,50	100,00	33,04	62,71	4,25	100,00
Gorontalo	28,15	67,44	4,41	100,00	27,27	67,65	5,08	100,00	27,71	67,54	4,75	100,00
Sulawesi Barat	31,94	64,29	3,77	100,00	30,97	64,59	4,44	100,00	31,45	64,44	4,11	100,00
Maluku	33,46	62,55	3,99	100,00	32,75	62,80	4,45	100,00	33,11	62,67	4,22	100,00
Maluku Utara	33,42	63,14	3,45	100,00	32,57	63,71	3,72	100,00	33,00	63,42	3,58	100,00
Papua Barat	30,19	67,38	2,43	100,00	32,04	65,67	2,29	100,00	31,06	66,57	2,37	100,00
Papua	30,57	67,72	1,72	100,00	31,63	66,88	1,50	100,00	31,07	67,32	1,61	100,00
**Indonesia**	**27,26**	**67,46**	**5,28**	**100,00**	**26,28**	**67,46**	**6,25**	**100,00**	**26,77**	**67,46**	**5,77**	**100,00**

Sumber/*Source*: Susenas Maret 2018/*The March 2018 Susenas*

https://www.bps.go.id

Tabel *Table* **2.5.** **Rasio Jenis Kelamin dan Angka Beban Ketergantungan menurut Provinsi dan Daerah Tempat Tinggal, 2018**
Sex Ratio and Dependency Ratio by Province and Urban Rural Classification, 2018

Provinsi *Province*	Rasio Jenis Kelamin/*Sex Ratio*			Angka Beban Ketergantungan/*Dependency Ratio*		
	Perkotaan *Urban*	Perdesaan *Rural*	Perkotaan+Perdesaan *Urban+Rural*	Perkotaan *Urban*	Perdesaan *Rural*	Perkotaan+Perdesaan *Urban+Rural*
(1)	(2)	(3)	(4)	(5)	(6)	(7)
Aceh	100,71	99,27	99,72	50,73	55,67	54,08
Sumatera Utara	99,16	100,11	99,60	50,71	62,05	55,85
Sumatera Barat	98,87	99,23	99,07	52,33	59,01	55,99
Riau	104,55	105,87	105,33	49,07	52,61	51,16
Jambi	102,48	104,98	104,17	43,50	47,30	46,06
Sumatera Selatan	101,07	104,72	103,35	46,63	51,01	49,35
Bengkulu	100,43	105,74	103,98	44,79	50,15	48,35
Lampung	101,51	106,48	104,98	46,53	50,86	49,55
Kepulauan Bangka Belitung	106,49	110,26	108,22	45,85	46,30	46,05
Kepulauan Riau	103,40	106,44	103,82	48,06	52,80	48,71
DKI Jakarta	100,60	-!	100,60	41,20	-	41,20
Jawa Barat	102,76	102,41	102,67	45,88	49,89	46,90
Jawa Tengah	98,05	98,72	98,38	46,62	49,01	47,78
DI Yogyakarta	99,21	93,94	97,76	42,89	51,34	45,07
Jawa Timur	98,19	96,73	97,49	42,49	45,02	43,68
Banten	103,55	104,95	103,95	43,16	52,72	45,79
Bali	102,14	99,30	101,17	42,45	47,94	44,26
Nusa Tenggara Barat	93,87	94,74	94,34	50,87	54,51	52,80
Nusa Tenggara Timur	100,39	97,43	98,10	54,28	70,30	66,34
Kalimantan Barat	100,61	105,24	103,66	48,05	51,49	50,31
Kalimantan Tengah	105,98	111,37	109,26	43,49	45,57	44,77
Kalimantan Selatan	103,08	102,64	102,85	45,15	49,66	47,52
Kalimantan Timur	108,46	113,64	110,15	43,38	47,51	44,72
Kalimantan Utara	110,73	115,66	112,76	47,91	52,07	49,63
Sulawesi Utara	101,96	106,36	104,13	44,13	49,96	46,97
Sulawesi Tengah	100,73	105,81	104,37	44,66	52,39	50,15
Sulawesi Selatan	96,44	94,92	95,55	49,26	54,89	52,49
Sulawesi Tenggara	101,10	101,07	101,08	54,57	62,66	59,46
Gorontalo	97,08	102,56	100,40	44,88	50,13	48,05
Sulawesi Barat	96,83	101,76	100,57	54,99	55,24	55,18
Maluku	100,54	102,55	101,71	52,50	65,03	59,56
Maluku Utara	102,68	104,71	104,13	48,84	61,53	57,68
Papua Barat	110,15	111,90	111,19	46,69	52,74	50,22
Papua	115,77	109,17	110,96	44,15	50,30	48,55
**Indonesia**	**100,84**	**101,10**	**100,96**	**45,59**	**51,56**	**48,23**

Sumber/*Source*: Susenas Maret 2018/*The March 2018 Susenas*

https://www.bps.go.id

Tabel *Table* **2.6.1.** **Persentase Penduduk Berumur 10 Tahun ke Atas di Daerah Perkotaan menurut Provinsi, Jenis Kelamin, dan Status Perkawinan, 2018**
Percentage of Population Age 10 Years and Over in Urban Area by Province, Sex, and Marital Status, 2018

**Provinsi** *Province*	**Laki-laki/***Male*					**Perempuan/***Female*				
	**Belum Kawin** *Single*	**Kawin** *Married*	**Cerai Hidup** *Divorced*	**Cerai Mati** *Widowed*	**Jumlah** *Total*	**Belum Kawin** *Single*	**Kawin** *Married*	**Cerai Hidup** *Divorced*	**Cerai Mati** *Widowed*	**Jumlah** *Total*
**(1)**	**(2)**	**(3)**	**(4)**	**(5)**	**(6)**	**(7)**	**(8)**	**(9)**	**(10)**	**(11)**
Aceh	45,55	52,07	0,84	1,54	100,00	34,99	52,44	2,41	10,16	100,00
Sumatera Utara	43,85	52,86	1,14	2,15	100,00	35,51	52,82	2,15	9,52	100,00
Sumatera Barat	41,67	55,83	1,07	1,43	100,00	33,73	54,70	2,75	8,81	100,00
Riau	41,03	55,76	1,45	1,77	100,00	32,70	57,84	2,28	7,18	100,00
Jambi	39,53	56,73	1,99	1,75	100,00	31,88	57,14	3,00	7,98	100,00
Sumatera Selatan	41,79	54,78	1,21	2,22	100,00	33,36	55,63	2,22	8,79	100,00
Bengkulu	39,35	57,87	1,64	1,15	100,00	31,09	59,12	2,21	7,58	100,00
Lampung	39,25	57,53	1,25	1,96	100,00	30,88	58,19	2,17	8,75	100,00
Kepulauan Bangka Belitung	36,93	58,66	2,03	2,38	100,00	28,05	62,62	2,17	7,17	100,00
Kepulauan Riau	38,91	58,81	1,29	0,99	100,00	32,91	60,10	2,15	4,84	100,00
DKI Jakarta	38,40	57,61	1,31	2,69	100,00	30,85	56,85	3,23	9,07	100,00
Jawa Barat	37,86	58,17	1,78	2,18	100,00	28,39	59,47	2,98	9,16	100,00
Jawa Tengah	36,77	58,90	1,28	3,05	100,00	26,57	58,68	2,50	12,26	100,00
DI Yogyakarta	38,00	57,41	1,30	3,29	100,00	28,74	57,79	2,57	10,90	100,00
Jawa Timur	34,87	60,36	1,77	3,00	100,00	24,87	59,59	2,88	12,67	100,00
Banten	39,24	57,70	1,24	1,82	100,00	30,21	59,48	2,77	7,54	100,00
Bali	36,23	59,88	1,29	2,59	100,00	29,54	60,82	1,51	8,13	100,00
Nusa Tenggara Barat	39,36	56,01	2,53	2,11	100,00	29,94	55,67	5,03	9,36	100,00
Nusa Tenggara Timur	49,95	46,72	0,76	2,57	100,00	41,93	47,56	2,67	7,83	100,00
Kalimantan Barat	40,61	55,90	1,51	1,98	100,00	32,35	56,88	2,03	8,74	100,00
Kalimantan Tengah	38,69	57,96	1,14	2,22	100,00	29,44	61,39	2,49	6,68	100,00
Kalimantan Selatan	38,44	56,99	2,39	2,18	100,00	29,12	58,01	2,77	10,09	100,00
Kalimantan Timur	39,59	56,66	1,77	1,99	100,00	30,20	60,65	2,49	6,66	100,00
Kalimantan Utara	41,61	53,98	2,43	1,98	100,00	31,52	59,28	2,81	6,39	100,00
Sulawesi Utara	38,31	56,72	1,63	3,34	100,00	29,36	59,15	2,86	8,64	100,00
Sulawesi Tengah	41,79	54,36	1,47	2,38	100,00	35,17	54,11	2,83	7,90	100,00
Sulawesi Selatan	43,72	52,31	1,49	2,48	100,00	36,65	50,77	3,09	9,49	100,00
Sulawesi Tenggara	44,48	52,68	1,55	1,30	100,00	37,08	53,45	2,53	6,94	100,00
Gorontalo	40,88	54,56	1,32	3,24	100,00	32,67	55,13	2,84	9,36	100,00
Sulawesi Barat	42,66	54,38	1,77	1,18	100,00	36,11	52,40	2,71	8,79	100,00
Maluku	46,73	49,60	0,73	2,94	100,00	40,24	48,77	3,03	7,96	100,00
Maluku Utara	44,49	51,39	1,97	2,15	100,00	37,11	51,84	2,71	8,33	100,00
Papua Barat	45,72	51,31	1,27	1,70	100,00	36,02	55,29	2,32	6,37	100,00
Papua	45,13	51,89	0,92	2,06	100,00	34,88	57,42	2,12	5,58	100,00
**Indonesia**	**38,60**	**57,47**	**1,52**	**2,41**	**100,00**	**29,51**	**57,97**	**2,75**	**9,77**	**100,00**

Sumber/*Source*: Susenas Maret 2018/*The March 2018 Susenas*

https://www.bps.go.id

Lanjutan Tabel/*Table Continued* 2.6.1.

Provinsi<br>*Province*	Laki-laki+Perempuan/*Male+Female*				
	Belum Kawin<br>*Single*	Kawin<br>*Married*	Cerai Hidup<br>*Divorced*	Cerai Mati<br>*Widowed*	Jumlah<br>*Total*
(1)	(12)	(13)	(14)	(15)	(16)
Aceh	40,27	52,26	1,62	5,85	100,00
Sumatera Utara	39,65	52,84	1,65	5,87	100,00
Sumatera Barat	37,62	55,25	1,93	5,19	100,00
Riau	36,94	56,78	1,86	4,42	100,00
Jambi	35,74	56,93	2,49	4,84	100,00
Sumatera Selatan	37,58	55,20	1,71	5,50	100,00
Bengkulu	35,24	58,49	1,92	4,34	100,00
Lampung	35,09	57,86	1,71	5,34	100,00
Kepulauan Bangka Belitung	32,65	60,57	2,10	4,68	100,00
Kepulauan Riau	35,96	59,45	1,71	2,89	100,00
DKI Jakarta	34,62	57,23	2,27	5,88	100,00
Jawa Barat	33,18	58,82	2,37	5,63	100,00
Jawa Tengah	31,59	58,79	1,90	7,73	100,00
DI Yogyakarta	33,33	57,60	1,94	7,13	100,00
Jawa Timur	29,79	59,97	2,33	7,91	100,00
Banten	34,80	58,57	1,99	4,63	100,00
Bali	32,92	60,35	1,40	5,33	100,00
Nusa Tenggara Barat	34,44	55,83	3,83	5,89	100,00
Nusa Tenggara Timur	45,93	47,14	1,72	5,21	100,00
Kalimantan Barat	36,49	56,39	1,77	5,35	100,00
Kalimantan Tengah	34,22	59,62	1,79	4,38	100,00
Kalimantan Selatan	33,84	57,50	2,58	6,08	100,00
Kalimantan Timur	35,10	58,57	2,11	4,22	100,00
Kalimantan Utara	36,87	56,47	2,61	4,05	100,00
Sulawesi Utara	33,87	57,92	2,24	5,97	100,00
Sulawesi Tengah	38,47	54,23	2,15	5,15	100,00
Sulawesi Selatan	40,09	51,52	2,31	6,08	100,00
Sulawesi Tenggara	40,78	53,06	2,04	4,12	100,00
Gorontalo	36,71	54,85	2,09	6,35	100,00
Sulawesi Barat	39,30	53,37	2,25	5,08	100,00
Maluku	43,49	49,19	1,88	5,45	100,00
Maluku Utara	40,83	51,61	2,34	5,22	100,00
Papua Barat	41,14	53,19	1,76	3,91	100,00
Papua	40,43	54,42	1,47	3,68	100,00
**Indonesia**	**34,05**	**57,72**	**2,14**	**6,09**	**100,00**

Sumber/*Source*: Susenas Maret 2018/*The March 2018 Susenas*

https://www.bps.go.id

Tabel **2.6.2.** **Persentase Penduduk Berumur 10 Tahun ke Atas di Daerah Perdesaan menurut Provinsi, Jenis Kelamin, dan Status Perkawinan, 2018**
Table *Percentage of Population Age 10 Years and Over in Rural Area by Province, Sex, and Marital Status, 2018*

**Provinsi** *Province*	**Laki-laki/***Male*					**Perempuan/***Female*				
	**Belum Kawin** *Single*	**Kawin** *Married*	**Cerai Hidup** *Divorced*	**Cerai Mati** *Widowed*	**Jumlah** *Total*	**Belum Kawin** *Single*	**Kawin** *Married*	**Cerai Hidup** *Divorced*	**Cerai Mati** *Widowed*	**Jumlah** *Total*
**(1)**	**(2)**	**(3)**	**(4)**	**(5)**	**(6)**	**(7)**	**(8)**	**(9)**	**(10)**	**(11)**
Aceh	43,42	53,90	0,85	1,84	100,00	31,58	54,27	2,35	11,80	100,00
Sumatera Utara	41,71	54,75	0,97	2,57	100,00	32,44	55,22	1,83	10,51	100,00
Sumatera Barat	39,16	57,56	1,31	1,98	100,00	27,96	57,32	3,62	11,10	100,00
Riau	38,35	59,06	1,01	1,58	100,00	28,51	63,29	1,63	6,58	100,00
Jambi	35,81	60,85	1,23	2,11	100,00	25,49	64,16	1,84	8,51	100,00
Sumatera Selatan	36,21	60,69	1,08	2,02	100,00	25,41	64,17	2,01	8,40	100,00
Bengkulu	33,62	62,80	1,25	2,33	100,00	23,55	66,58	1,94	7,93	100,00
Lampung	34,26	62,21	1,26	2,27	100,00	23,20	66,11	1,86	8,83	100,00
Kepulauan Bangka Belitung	37,59	58,58	1,62	2,22	100,00	25,96	64,89	2,30	6,85	100,00
Kepulauan Riau	39,56	57,16	1,11	2,17	100,00	26,12	63,32	2,11	8,45	100,00
DKI Jakarta	-	-	-	-	-	-	-	-	-	-
Jawa Barat	32,85	63,55	1,62	1,99	100,00	21,70	65,12	2,89	10,30	100,00
Jawa Tengah	32,30	63,83	1,02	2,85	100,00	20,84	64,71	1,92	12,54	100,00
DI Yogyakarta	27,10	68,67	1,10	3,12	100,00	18,43	65,03	2,62	13,92	100,00
Jawa Timur	30,56	64,79	1,55	3,10	100,00	19,24	64,06	2,68	14,02	100,00
Banten	38,41	57,68	1,84	2,07	100,00	25,47	61,67	3,06	9,81	100,00
Bali	31,23	64,82	1,10	2,85	100,00	24,51	64,58	1,33	9,58	100,00
Nusa Tenggara Barat	35,99	60,11	1,52	2,38	100,00	23,86	62,71	4,31	9,13	100,00
Nusa Tenggara Timur	44,35	52,40	0,46	2,79	100,00	34,53	53,83	2,31	9,33	100,00
Kalimantan Barat	36,77	59,67	0,85	2,72	100,00	25,53	64,18	1,72	8,57	100,00
Kalimantan Tengah	35,55	60,96	1,57	1,91	100,00	23,83	67,72	1,89	6,56	100,00
Kalimantan Selatan	35,25	59,92	2,20	2,63	100,00	23,37	62,19	3,58	10,86	100,00
Kalimantan Timur	38,90	56,15	2,19	2,75	100,00	26,18	64,78	1,88	7,16	100,00
Kalimantan Utara	41,36	55,26	1,68	1,70	100,00	28,75	63,98	1,71	5,56	100,00
Sulawesi Utara	33,73	61,19	1,68	3,40	100,00	22,99	65,93	1,73	9,35	100,00
Sulawesi Tengah	36,30	58,93	1,95	2,81	100,00	25,71	63,19	2,41	8,68	100,00
Sulawesi Selatan	37,90	57,69	1,70	2,72	100,00	29,22	56,35	2,98	11,45	100,00
Sulawesi Tenggara	37,81	58,62	1,61	1,96	100,00	28,25	60,49	2,48	8,79	100,00
Gorontalo	35,58	60,44	1,69	2,29	100,00	27,15	62,95	2,23	7,67	100,00
Sulawesi Barat	39,97	56,06	1,71	2,26	100,00	31,60	58,01	2,69	7,70	100,00
Maluku	41,97	53,98	0,98	3,07	100,00	33,69	56,28	2,20	7,83	100,00
Maluku Utara	39,85	56,34	1,22	2,59	100,00	31,32	59,68	2,11	6,88	100,00
Papua Barat	41,57	54,45	0,93	3,05	100,00	32,25	59,44	2,25	6,07	100,00
Papua	38,29	57,40	0,82	3,49	100,00	29,25	63,49	1,18	6,08	100,00
**Indonesia**	**35,35**	**60,80**	**1,32**	**2,53**	**100,00**	**24,51**	**62,49**	**2,37**	**10,63**	**100,00**

Sumber/*Source*: Susenas Maret 2018/*The March 2018 Susenas*

https://www.bps.go.id

Lanjutan Tabel/*Table Continued* **2.6.2.**

**Provinsi** *Province*	**Laki-laki+Perempuan/***Male+Female*				
	**Belum Kawin** *Single*	**Kawin** *Married*	**Cerai Hidup** *Divorced*	**Cerai Mati** *Widowed*	**Jumlah** *Total*
**(1)**	**(12)**	**(13)**	**(14)**	**(15)**	**(16)**
Aceh	37,44	54,09	1,60	6,86	100,00
Sumatera Utara	37,05	54,99	1,40	6,56	100,00
Sumatera Barat	33,50	57,44	2,48	6,59	100,00
Riau	33,58	61,11	1,31	4,00	100,00
Jambi	30,77	62,46	1,53	5,24	100,00
Sumatera Selatan	30,95	62,39	1,54	5,13	100,00
Bengkulu	28,72	64,64	1,59	5,05	100,00
Lampung	28,91	64,10	1,55	5,44	100,00
Kepulauan Bangka Belitung	32,09	61,56	1,94	4,41	100,00
Kepulauan Riau	33,09	60,13	1,59	5,19	100,00
DKI Jakarta	-	-	-	-	-
Jawa Barat	27,32	64,32	2,25	6,11	100,00
Jawa Tengah	26,50	64,28	1,47	7,75	100,00
DI Yogyakarta	22,59	66,78	1,89	8,74	100,00
Jawa Timur	24,77	64,41	2,13	8,69	100,00
Banten	32,09	59,63	2,44	5,84	100,00
Bali	27,85	64,70	1,22	6,24	100,00
Nusa Tenggara Barat	29,68	61,46	2,97	5,88	100,00
Nusa Tenggara Timur	39,35	53,13	1,40	6,12	100,00
Kalimantan Barat	31,29	61,87	1,27	5,57	100,00
Kalimantan Tengah	30,04	64,14	1,72	4,10	100,00
Kalimantan Selatan	29,39	61,04	2,88	6,69	100,00
Kalimantan Timur	33,01	60,15	2,05	4,80	100,00
Kalimantan Utara	35,57	59,27	1,69	3,47	100,00
Sulawesi Utara	28,53	63,48	1,71	6,28	100,00
Sulawesi Tengah	31,17	61,00	2,17	5,66	100,00
Sulawesi Selatan	33,40	56,99	2,36	7,25	100,00
Sulawesi Tenggara	33,03	59,55	2,04	5,37	100,00
Gorontalo	31,40	61,69	1,96	4,95	100,00
Sulawesi Barat	35,80	57,03	2,20	4,97	100,00
Maluku	37,87	55,12	1,58	5,43	100,00
Maluku Utara	35,68	57,97	1,66	4,69	100,00
Papua Barat	37,20	56,79	1,55	4,46	100,00
Papua	33,99	60,30	0,99	4,72	100,00
**Indonesia**	**29,94**	**61,64**	**1,85**	**6,57**	**100,00**

Sumber/*Source*: Susenas Maret 2018/*The March 2018 Susenas*

https://www.bps.go.id

Tabel *Table* **2.6.3.** **Persentase Penduduk Berumur 10 Tahun ke Atas di Daerah Perkotaan dan Perdesaan menurut Provinsi, Jenis Kelamin, dan Status Perkawinan, 2018**
Percentage of Population Age 10 Years and Over in Urban and Rural Area by Province, Sex, and Marital Status, 2018

Provinsi *Province*	Laki-laki/*Male*					Perempuan/*Female*				
	Belum Kawin *Single*	Kawin *Married*	Cerai Hidup *Divorced*	Cerai Mati *Widowed*	Jumlah *Total*	Belum Kawin *Single*	Kawin *Married*	Cerai Hidup *Divorced*	Cerai Mati *Widowed*	Jumlah *Total*
(1)	(2)	(3)	(4)	(5)	(6)	(7)	(8)	(9)	(10)	(11)
Aceh	44,10	53,32	0,84	1,74	100,00	32,65	53,70	2,37	11,28	100,00
Sumatera Utara	42,86	53,73	1,06	2,35	100,00	34,10	53,93	2,00	9,97	100,00
Sumatera Barat	40,27	56,79	1,20	1,74	100,00	30,54	56,15	3,23	10,08	100,00
Riau	39,43	57,73	1,19	1,66	100,00	30,23	61,06	1,89	6,82	100,00
Jambi	37,00	59,53	1,48	2,00	100,00	27,57	61,87	2,22	8,34	100,00
Sumatera Selatan	38,27	58,51	1,13	2,09	100,00	28,45	60,92	2,09	8,55	100,00
Bengkulu	35,47	61,20	1,38	1,95	100,00	26,06	64,10	2,03	7,81	100,00
Lampung	35,71	60,85	1,26	2,18	100,00	25,53	63,71	1,95	8,81	100,00
Kepulauan Bangka Belitung	37,24	58,62	1,84	2,30	100,00	27,09	63,65	2,23	7,02	100,00
Kepulauan Riau	39,00	58,57	1,26	1,17	100,00	31,95	60,55	2,14	5,35	100,00
DKI Jakarta	38,40	57,61	1,31	2,69	100,00	30,85	56,85	3,23	9,07	100,00
Jawa Barat	36,56	59,57	1,74	2,13	100,00	26,65	60,94	2,95	9,46	100,00
Jawa Tengah	34,59	61,31	1,15	2,95	100,00	23,78	61,61	2,22	12,39	100,00
DI Yogyakarta	35,11	60,40	1,24	3,25	100,00	25,88	59,80	2,58	11,74	100,00
Jawa Timur	32,82	62,47	1,67	3,05	100,00	22,17	61,73	2,78	13,32	100,00
Banten	39,00	57,69	1,41	1,89	100,00	28,86	60,10	2,85	8,18	100,00
Bali	34,56	61,54	1,23	2,67	100,00	27,82	62,11	1,45	8,63	100,00
Nusa Tenggara Barat	37,56	58,20	1,99	2,25	100,00	26,71	59,41	4,65	9,24	100,00
Nusa Tenggara Timur	45,69	51,04	0,53	2,74	100,00	36,26	52,37	2,40	8,98	100,00
Kalimantan Barat	38,06	58,40	1,07	2,47	100,00	27,89	61,65	1,83	8,63	100,00
Kalimantan Tengah	36,75	59,82	1,40	2,03	100,00	26,04	65,22	2,12	6,61	100,00
Kalimantan Selatan	36,74	58,55	2,29	2,42	100,00	26,06	60,24	3,20	10,50	100,00
Kalimantan Timur	39,36	56,49	1,91	2,25	100,00	28,91	61,99	2,29	6,82	100,00
Kalimantan Utara	41,50	54,52	2,11	1,86	100,00	30,38	61,21	2,36	6,05	100,00
Sulawesi Utara	36,02	58,95	1,66	3,37	100,00	26,25	62,46	2,31	8,98	100,00
Sulawesi Tengah	37,82	57,67	1,82	2,69	100,00	28,46	60,56	2,53	8,46	100,00
Sulawesi Selatan	40,35	55,43	1,61	2,62	100,00	32,31	54,03	3,02	10,64	100,00
Sulawesi Tenggara	40,40	56,32	1,59	1,70	100,00	31,67	57,76	2,50	8,07	100,00
Gorontalo	37,62	58,18	1,55	2,65	100,00	29,34	59,85	2,47	8,34	100,00
Sulawesi Barat	40,59	55,67	1,72	2,01	100,00	32,68	56,66	2,69	7,96	100,00
Maluku	44,00	52,11	0,87	3,02	100,00	36,52	53,04	2,55	7,89	100,00
Maluku Utara	41,20	54,90	1,44	2,46	100,00	33,04	57,36	2,29	7,31	100,00
Papua Barat	43,27	53,17	1,07	2,50	100,00	33,81	57,73	2,28	6,19	100,00
Papua	40,27	55,80	0,85	3,08	100,00	30,80	61,82	1,44	5,94	100,00
**Indonesia**	**37,13**	**58,97**	**1,43**	**2,47**	**100,00**	**27,26**	**60,00**	**2,58**	**10,15**	**100,00**

Sumber/*Source*: Susenas Maret 2018/*The March 2018 Susenas*

https://www.bps.go.id

Lanjutan Tabel/*Table Continued* **2.6.3.**

**Provinsi** *Province*	**Laki-laki+Perempuan/***Male+Female*				
	**Belum Kawin** *Single*	**Kawin** *Married*	**Cerai Hidup** *Divorced*	**Cerai Mati** *Widowed*	**Jumlah** *Total*
**(1)**	**(12)**	**(13)**	**(14)**	**(15)**	**(16)**
Aceh	38,34	53,51	1,61	6,54	100,00
Sumatera Utara	38,45	53,83	1,53	6,19	100,00
Sumatera Barat	35,33	56,47	2,23	5,97	100,00
Riau	34,95	59,35	1,53	4,17	100,00
Jambi	32,37	60,68	1,84	5,11	100,00
Sumatera Selatan	33,44	59,69	1,60	5,27	100,00
Bengkulu	30,86	62,62	1,70	4,82	100,00
Lampung	30,74	62,25	1,60	5,41	100,00
Kepulauan Bangka Belitung	32,39	61,03	2,03	4,56	100,00
Kepulauan Riau	35,55	59,54	1,69	3,22	100,00
DKI Jakarta	34,62	57,23	2,27	5,88	100,00
Jawa Barat	31,66	60,25	2,34	5,76	100,00
Jawa Tengah	29,10	61,46	1,69	7,74	100,00
DI Yogyakarta	30,41	60,09	1,93	7,57	100,00
Jawa Timur	27,40	62,09	2,23	8,28	100,00
Banten	34,03	58,87	2,12	4,98	100,00
Bali	31,20	61,82	1,34	5,64	100,00
Nusa Tenggara Barat	31,91	58,83	3,37	5,89	100,00
Nusa Tenggara Timur	40,90	51,71	1,48	5,91	100,00
Kalimantan Barat	33,06	60,00	1,44	5,49	100,00
Kalimantan Tengah	31,66	62,39	1,75	4,21	100,00
Kalimantan Selatan	31,47	59,38	2,74	6,41	100,00
Kalimantan Timur	34,41	59,09	2,09	4,41	100,00
Kalimantan Utara	36,33	57,63	2,23	3,81	100,00
Sulawesi Utara	31,23	60,67	1,98	6,12	100,00
Sulawesi Tengah	33,24	59,08	2,17	5,51	100,00
Sulawesi Selatan	36,19	54,70	2,34	6,76	100,00
Sulawesi Tenggara	36,03	57,04	2,04	4,89	100,00
Gorontalo	33,47	59,02	2,01	5,50	100,00
Sulawesi Barat	36,63	56,17	2,21	5,00	100,00
Maluku	40,28	52,57	1,71	5,44	100,00
Maluku Utara	37,19	56,11	1,86	4,84	100,00
Papua Barat	38,82	55,31	1,64	4,23	100,00
Papua	35,81	58,64	1,13	4,43	100,00
**Indonesia**	**32,20**	**59,49**	**2,01**	**6,31**	**100,00**

Sumber/*Source*: Susenas Maret 2018/*The March 2018 Susenas*

https://www.bps.go.id

Tabel 2.7.1. **Persentase Penduduk Berumur 15-49 Tahun di Daerah Perkotaan menurut Provinsi, Jenis Kelamin, dan Status Perkawinan, 2018**
Table *Percentage of Population Aged 15-49 Years in Urban Area by Province, Sex, and Marital Status, 2018*

**Provinsi** *Province*	**Laki-laki/***Male*					**Perempuan/***Female*				
	**Belum Kawin** *Single*	**Kawin** *Married*	**Cerai Hidup** *Divorced*	**Cerai Mati** *Widowed*	**Jumlah** *Total*	**Belum Kawin** *Single*	**Kawin** *Married*	**Cerai Hidup** *Divorced*	**Cerai Mati** *Widowed*	**Jumlah** *Total*
(1)	(2)	(3)	(4)	(5)	(6)	(7)	(8)	(9)	(10)	(11)
Aceh	47,67	51,19	0,91	0,24	100,00	33,62	61,62	2,33	2,43	100,00
Sumatera Utara	46,32	52,25	1,05	0,39	100,00	35,46	59,91	2,00	2,62	100,00
Sumatera Barat	45,16	53,59	0,99	0,27	100,00	33,91	62,58	2,29	1,21	100,00
Riau	41,67	56,54	1,49	0,30	100,00	29,07	66,81	2,42	1,70	100,00
Jambi	41,85	55,86	2,03	0,26	100,00	30,00	65,01	3,28	1,71	100,00
Sumatera Selatan	44,38	53,76	1,48	0,38	100,00	32,73	63,34	2,30	1,62	100,00
Bengkulu	40,96	57,41	1,46	0,17	100,00	29,61	66,97	2,22	1,20	100,00
Lampung	42,14	55,95	1,44	0,47	100,00	30,09	65,37	2,63	1,90	100,00
Kepulauan Bangka Belitung	37,28	59,59	2,74	0,38	100,00	24,32	72,28	2,20	1,20	100,00
Kepulauan Riau	36,20	62,18	1,20	0,41	100,00	27,42	69,20	2,33	1,05	100,00
DKI Jakarta	41,18	56,90	1,32	0,60	100,00	31,07	63,77	3,21	1,96	100,00
Jawa Barat	39,95	57,67	2,00	0,37	100,00	26,75	68,24	3,11	1,91	100,00
Jawa Tengah	42,47	55,78	1,34	0,41	100,00	27,90	67,68	2,41	2,02	100,00
DI Yogyakarta	44,41	54,13	1,11	0,35	100,00	32,06	64,03	2,63	1,28	100,00
Jawa Timur	39,57	58,18	1,87	0,37	100,00	25,66	69,35	3,07	1,92	100,00
Banten	40,29	57,90	1,49	0,32	100,00	27,87	67,84	2,65	1,64	100,00
Bali	38,91	59,23	1,38	0,48	100,00	29,12	67,94	1,47	1,47	100,00
Nusa Tenggara Barat	40,04	56,41	2,89	0,65	100,00	27,83	65,66	4,85	1,67	100,00
Nusa Tenggara Timur	52,30	46,48	0,76	0,45	100,00	41,95	52,83	3,26	1,96	100,00
Kalimantan Barat	42,53	55,53	1,65	0,29	100,00	31,16	64,23	2,29	2,32	100,00
Kalimantan Tengah	38,61	59,75	1,19	0,46	100,00	25,40	71,02	2,27	1,31	100,00
Kalimantan Selatan	39,87	57,01	2,52	0,60	100,00	27,06	68,08	2,91	1,94	100,00
Kalimantan Timur	40,82	56,92	1,69	0,58	100,00	27,05	68,78	2,74	1,43	100,00
Kalimantan Utara	43,02	54,15	2,46	0,38	100,00	28,00	67,55	2,92	1,52	100,00
Sulawesi Utara	42,97	54,52	1,59	0,92	100,00	29,30	66,23	3,07	1,40	100,00
Sulawesi Tengah	45,31	52,66	1,51	0,51	100,00	34,68	60,69	2,89	1,74	100,00
Sulawesi Selatan	46,26	51,60	1,65	0,49	100,00	36,43	58,47	3,24	1,86	100,00
Sulawesi Tenggara	44,77	53,47	1,54	0,22	100,00	34,85	60,77	2,78	1,60	100,00
Gorontalo	43,85	53,78	1,46	0,90	100,00	32,96	62,47	2,82	1,74	100,00
Sulawesi Barat	42,65	55,38	1,84	0,13	100,00	34,65	61,16	1,64	2,55	100,00
Maluku	47,35	51,06	0,60	0,99	100,00	39,26	55,28	3,39	2,06	100,00
Maluku Utara	44,88	52,60	2,21	0,31	100,00	35,11	59,23	3,50	2,16	100,00
Papua Barat	46,54	51,60	1,26	0,59	100,00	32,60	62,91	2,37	2,13	100,00
Papua	48,44	49,71	1,00	0,85	100,00	32,11	63,73	1,96	2,19	100,00
**Indonesia**	**41,52**	**56,43**	**1,64**	**0,42**	**100,00**	**28,93**	**66,38**	**2,82**	**1,87**	**100,00**

Sumber/*Source*: Susenas Maret 2018/*The March 2018 Susenas*

https://www.bps.go.id

Lanjutan Tabel/*Table Continued* **2.7.1.**

Provinsi<br>*Province*	Laki-laki+Perempuan/*Male+Female*				
	**Belum Kawin**<br>*Single*	**Kawin**<br>*Married*	**Cerai Hidup**<br>*Divorced*	**Cerai Mati**<br>*Widowed*	**Jumlah**<br>*Total*
**(1)**	**(12)**	**(13)**	**(14)**	**(15)**	**(16)**
Aceh	40,66	56,39	1,62	1,33	100,00
Sumatera Utara	40,87	56,09	1,53	1,51	100,00
Sumatera Barat	39,52	58,09	1,64	0,74	100,00
Riau	35,50	61,57	1,94	0,99	100,00
Jambi	36,03	60,36	2,64	0,97	100,00
Sumatera Selatan	38,60	58,52	1,89	1,00	100,00
Bengkulu	35,34	62,14	1,84	0,68	100,00
Lampung	36,16	60,63	2,03	1,18	100,00
Kepulauan Bangka Belitung	31,06	65,68	2,48	0,78	100,00
Kepulauan Riau	31,87	65,64	1,76	0,73	100,00
DKI Jakarta	36,13	60,33	2,27	1,28	100,00
Jawa Barat	33,44	62,88	2,55	1,13	100,00
Jawa Tengah	35,13	61,77	1,88	1,22	100,00
DI Yogyakarta	38,29	59,04	1,86	0,81	100,00
Jawa Timur	32,57	63,80	2,47	1,15	100,00
Banten	34,17	62,80	2,06	0,97	100,00
Bali	34,10	63,51	1,43	0,97	100,00
Nusa Tenggara Barat	33,63	61,27	3,92	1,18	100,00
Nusa Tenggara Timur	47,11	49,67	2,02	1,21	100,00
Kalimantan Barat	36,83	59,89	1,97	1,31	100,00
Kalimantan Tengah	32,19	65,22	1,71	0,87	100,00
Kalimantan Selatan	33,58	62,45	2,71	1,26	100,00
Kalimantan Timur	34,21	62,61	2,19	0,99	100,00
Kalimantan Utara	35,90	60,50	2,68	0,92	100,00
Sulawesi Utara	36,30	60,23	2,31	1,16	100,00
Sulawesi Tengah	40,07	56,62	2,19	1,12	100,00
Sulawesi Selatan	41,25	55,10	2,46	1,19	100,00
Sulawesi Tenggara	39,79	57,13	2,17	0,91	100,00
Gorontalo	38,37	58,16	2,15	1,33	100,00
Sulawesi Barat	38,58	58,32	1,74	1,36	100,00
Maluku	43,35	53,15	1,98	1,52	100,00
Maluku Utara	40,05	55,88	2,85	1,22	100,00
Papua Barat	39,96	56,94	1,78	1,32	100,00
Papua	41,04	56,06	1,44	1,46	100,00
**Indonesia**	**35,26**	**61,38**	**2,22**	**1,14**	**100,00**

Sumber/*Source*: Susenas Maret 2018/*The March 2018 Susenas*

https://www.bps.go.id

Tabel **2.7.2.** **Persentase Penduduk Berumur 15-49 Tahun di Daerah Perdesaan menurut Provinsi, Jenis Kelamin, dan Status Perkawinan, 2018**
Table *Percentage of Population Aged 15-49 Years in Rural Area by Province, Sex, and Marital Status, 2018*

**Provinsi** *Province*	**Laki-laki/***Male*					**Perempuan/***Female*				
	**Belum Kawin** *Single*	**Kawin** *Married*	**Cerai Hidup** *Divorced*	**Cerai Mati** *Widowed*	**Jumlah** *Total*	**Belum Kawin** *Single*	**Kawin** *Married*	**Cerai Hidup** *Divorced*	**Cerai Mati** *Widowed*	**Jumlah** *Total*
**(1)**	**(2)**	**(3)**	**(4)**	**(5)**	**(6)**	**(7)**	**(8)**	**(9)**	**(10)**	**(11)**
Aceh	44,26	54,56	0,80	0,38	100,00	28,33	65,90	2,47	3,30	100,00
Sumatera Utara	41,74	56,61	1,05	0,61	100,00	29,26	66,19	2,16	2,39	100,00
Sumatera Barat	41,08	57,47	1,06	0,39	100,00	25,24	69,50	3,55	1,71	100,00
Riau	36,90	61,70	1,09	0,31	100,00	22,20	74,86	1,74	1,21	100,00
Jambi	35,68	62,28	1,47	0,57	100,00	20,12	76,04	2,02	1,83	100,00
Sumatera Selatan	35,53	62,95	1,28	0,24	100,00	20,21	75,88	2,10	1,80	100,00
Bengkulu	32,98	64,99	1,52	0,50	100,00	17,75	78,89	1,99	1,37	100,00
Lampung	34,85	63,24	1,42	0,49	100,00	18,12	78,47	1,88	1,53	100,00
Kepulauan Bangka Belitung	38,22	59,40	1,81	0,57	100,00	21,24	74,71	2,94	1,12	100,00
Kepulauan Riau	39,07	60,01	0,69	0,23	100,00	18,32	78,28	1,69	1,71	100,00
DKI Jakarta	-	-	-	-	-	-	-	-	-	-
Jawa Barat	34,53	63,29	1,87	0,31	100,00	17,78	77,50	3,05	1,67	100,00
Jawa Tengah	36,58	61,70	1,33	0,39	100,00	19,20	76,74	2,14	1,92	100,00
DI Yogyakarta	32,16	66,24	1,39	0,20	100,00	18,32	77,62	2,24	1,81	100,00
Jawa Timur	34,37	63,24	1,76	0,63	100,00	17,31	77,71	2,68	2,30	100,00
Banten	38,35	58,98	2,15	0,51	100,00	19,63	74,54	3,44	2,40	100,00
Bali	33,38	64,60	1,55	0,47	100,00	23,21	74,44	1,28	1,08	100,00
Nusa Tenggara Barat	34,74	63,37	1,60	0,29	100,00	18,34	75,50	4,00	2,16	100,00
Nusa Tenggara Timur	43,51	55,41	0,44	0,64	100,00	29,24	65,56	2,80	2,40	100,00
Kalimantan Barat	36,34	62,07	0,80	0,80	100,00	20,13	76,03	1,98	1,85	100,00
Kalimantan Tengah	34,42	63,30	1,71	0,57	100,00	17,42	79,66	1,75	1,17	100,00
Kalimantan Selatan	34,59	62,31	2,52	0,57	100,00	17,87	75,95	3,66	2,52	100,00
Kalimantan Timur	39,88	57,04	2,23	0,85	100,00	20,59	75,94	1,96	1,52	100,00
Kalimantan Utara	42,15	55,67	1,50	0,68	100,00	23,28	74,25	1,83	0,64	100,00
Sulawesi Utara	35,30	62,28	1,91	0,51	100,00	19,17	77,22	1,93	1,68	100,00
Sulawesi Tengah	34,94	62,06	2,24	0,76	100,00	19,86	75,97	2,63	1,54	100,00
Sulawesi Selatan	38,85	58,92	1,73	0,50	100,00	26,55	67,94	3,15	2,36	100,00
Sulawesi Tenggara	34,33	63,45	1,58	0,64	100,00	21,27	74,53	2,58	1,62	100,00
Gorontalo	34,76	62,60	1,85	0,78	100,00	22,55	73,63	2,36	1,46	100,00
Sulawesi Barat	38,67	58,97	1,84	0,52	100,00	25,87	69,67	2,64	1,81	100,00
Maluku	40,85	57,70	0,85	0,61	100,00	28,74	66,64	2,57	2,04	100,00
Maluku Utara	36,34	61,90	1,19	0,57	100,00	24,23	71,68	2,37	1,73	100,00
Papua Barat	39,71	58,34	0,82	1,14	100,00	25,16	70,17	2,43	2,24	100,00
Papua	34,04	62,86	0,95	2,15	100,00	21,51	73,39	1,25	3,85	100,00
**Indonesia**	**36,63**	**61,36**	**1,48**	**0,53**	**100,00**	**20,83**	**74,66**	**2,50**	**2,02**	**100,00**

Sumber/*Source*: Susenas Maret 2018/*The March 2018 Susenas*

https://www.bps.go.id

Lanjutan Tabel/*Table Continued* **2.7.2.**

**Provinsi** *Province*	**Laki-laki+Perempuan/***Male+Female*				
	**Belum Kawin** *Single*	**Kawin** *Married*	**Cerai Hidup** *Divorced*	**Cerai Mati** *Widowed*	**Jumlah** *Total*
**(1)**	**(12)**	**(13)**	**(14)**	**(15)**	**(16)**
Aceh	36,25	60,27	1,64	1,85	100,00
Sumatera Utara	35,55	61,36	1,60	1,49	100,00
Sumatera Barat	33,21	63,44	2,30	1,04	100,00
Riau	29,78	68,08	1,40	0,74	100,00
Jambi	28,09	68,99	1,74	1,18	100,00
Sumatera Selatan	28,10	69,22	1,68	1,00	100,00
Bengkulu	25,58	71,75	1,75	0,93	100,00
Lampung	26,78	70,59	1,64	0,99	100,00
Kepulauan Bangka Belitung	30,28	66,55	2,34	0,83	100,00
Kepulauan Riau	29,21	68,69	1,17	0,93	100,00
DKI Jakarta	-	-	-	-	-
Jawa Barat	26,26	70,30	2,46	0,98	100,00
Jawa Tengah	27,87	69,24	1,73	1,16	100,00
DI Yogyakarta	25,12	72,04	1,83	1,02	100,00
Jawa Timur	25,74	70,57	2,22	1,47	100,00
Banten	29,23	66,56	2,78	1,43	100,00
Bali	28,32	69,50	1,41	0,77	100,00
Nusa Tenggara Barat	26,16	69,72	2,86	1,27	100,00
Nusa Tenggara Timur	36,22	60,59	1,65	1,54	100,00
Kalimantan Barat	28,44	68,87	1,38	1,31	100,00
Kalimantan Tengah	26,44	70,98	1,73	0,85	100,00
Kalimantan Selatan	26,40	68,99	3,08	1,53	100,00
Kalimantan Timur	30,93	65,81	2,10	1,16	100,00
Kalimantan Utara	33,44	64,25	1,66	0,66	100,00
Sulawesi Utara	27,58	69,43	1,92	1,07	100,00
Sulawesi Tengah	27,62	68,81	2,43	1,14	100,00
Sulawesi Selatan	32,55	63,54	2,46	1,45	100,00
Sulawesi Tenggara	27,76	69,03	2,08	1,13	100,00
Gorontalo	28,75	68,04	2,10	1,11	100,00
Sulawesi Barat	32,32	64,28	2,24	1,16	100,00
Maluku	34,89	62,10	1,70	1,31	100,00
Maluku Utara	30,38	66,71	1,77	1,14	100,00
Papua Barat	32,92	63,86	1,57	1,65	100,00
Papua	27,97	67,96	1,09	2,97	100,00
**Indonesia**	**28,80**	**67,95**	**1,99**	**1,27**	**100,00**

Sumber/*Source*: Susenas Maret 2018/*The March 2018 Susenas*

https://www.bps.go.id

Tabel 2.7.3. **Persentase Penduduk Berumur 15-49 Tahun di Daerah Perkotaan dan Perdesaan menurut Provinsi, Jenis Kelamin, dan Status Perkawinan, 2018**
Table *Percentage of Population Aged 15-49 Years in Urban and Rural Area by Province, Sex, and Marital Status, 2018*

Provinsi *Province*	Laki-laki/*Male*					Perempuan/*Female*				
	Belum Kawin *Single*	Kawin *Married*	Cerai Hidup *Divorced*	Cerai Mati *Widowed*	Jumlah *Total*	Belum Kawin *Single*	Kawin *Married*	Cerai Hidup *Divorced*	Cerai Mati *Widowed*	Jumlah *Total*
(1)	(2)	(3)	(4)	(5)	(6)	(7)	(8)	(9)	(10)	(11)
Aceh	45,37	53,47	0,83	0,33	100,00	30,03	64,53	2,43	3,02	100,00
Sumatera Utara	44,24	54,23	1,05	0,49	100,00	32,69	62,72	2,07	2,52	100,00
Sumatera Barat	42,92	55,72	1,03	0,33	100,00	29,20	66,34	2,98	1,48	100,00
Riau	38,84	59,60	1,25	0,30	100,00	25,03	71,54	2,02	1,41	100,00
Jambi	37,69	60,19	1,65	0,47	100,00	23,37	72,41	2,43	1,79	100,00
Sumatera Selatan	38,82	59,53	1,36	0,29	100,00	25,01	71,08	2,18	1,73	100,00
Bengkulu	35,60	62,50	1,50	0,39	100,00	21,74	74,87	2,07	1,31	100,00
Lampung	37,02	61,07	1,43	0,48	100,00	21,82	74,42	2,11	1,64	100,00
Kepulauan Bangka Belitung	37,72	59,50	2,30	0,47	100,00	22,90	73,39	2,54	1,16	100,00
Kepulauan Riau	36,58	61,89	1,13	0,39	100,00	26,28	70,34	2,25	1,13	100,00
DKI Jakarta	41,18	56,90	1,32	0,60	100,00	31,07	63,77	3,21	1,96	100,00
Jawa Barat	38,62	59,05	1,97	0,36	100,00	24,54	70,51	3,10	1,85	100,00
Jawa Tengah	39,64	58,62	1,34	0,40	100,00	23,74	72,01	2,28	1,97	100,00
DI Yogyakarta	41,54	56,97	1,17	0,31	100,00	28,71	67,35	2,54	1,41	100,00
Jawa Timur	37,17	60,52	1,82	0,49	100,00	21,77	73,25	2,89	2,10	100,00
Banten	39,76	58,20	1,67	0,37	100,00	25,67	69,63	2,86	1,84	100,00
Bali	37,19	60,90	1,43	0,48	100,00	27,25	70,00	1,41	1,35	100,00
Nusa Tenggara Barat	37,25	60,09	2,21	0,46	100,00	22,84	70,83	4,40	1,93	100,00
Nusa Tenggara Timur	45,77	53,12	0,52	0,59	100,00	32,42	62,38	2,92	2,29	100,00
Kalimantan Barat	38,41	59,87	1,09	0,63	100,00	23,97	71,92	2,09	2,02	100,00
Kalimantan Tengah	36,02	61,95	1,51	0,53	100,00	20,60	76,22	1,96	1,22	100,00
Kalimantan Selatan	37,09	59,80	2,52	0,59	100,00	22,24	72,20	3,30	2,25	100,00
Kalimantan Timur	40,51	56,96	1,87	0,67	100,00	24,99	71,06	2,49	1,46	100,00
Kalimantan Utara	42,66	54,78	2,06	0,50	100,00	26,10	70,26	2,48	1,16	100,00
Sulawesi Utara	39,23	58,30	1,75	0,72	100,00	24,46	71,49	2,52	1,53	100,00
Sulawesi Tengah	37,94	59,34	2,03	0,69	100,00	24,25	71,44	2,71	1,60	100,00
Sulawesi Selatan	42,06	55,75	1,69	0,50	100,00	30,81	63,86	3,19	2,14	100,00
Sulawesi Tenggara	38,52	59,45	1,57	0,47	100,00	26,70	69,03	2,66	1,61	100,00
Gorontalo	38,27	59,20	1,70	0,83	100,00	26,67	69,21	2,54	1,57	100,00
Sulawesi Barat	39,60	58,14	1,84	0,43	100,00	27,99	67,62	2,40	1,99	100,00
Maluku	43,69	54,80	0,74	0,77	100,00	33,35	61,66	2,93	2,05	100,00
Maluku Utara	38,97	59,03	1,51	0,49	100,00	27,61	67,82	2,72	1,86	100,00
Papua Barat	42,54	55,54	1,00	0,91	100,00	28,29	67,12	2,40	2,19	100,00
Papua	38,19	59,07	0,96	1,78	100,00	24,31	70,85	1,44	3,41	100,00
**Indonesia**	**39,37**	**58,59**	**1,57**	**0,47**	**100,00**	**25,38**	**70,00**	**2,68**	**1,94**	**100,00**

Sumber/*Source*: Susenas Maret 2018/*The March 2018 Susenas*

https://www.bps.go.id

Lanjutan Tabel/*Table Continued* **2.7.3.**

Provinsi *Province*	Laki-laki+Perempuan/*Male+Female*				
	Belum Kawin *Single*	Kawin *Married*	Cerai Hidup *Divorced*	Cerai Mati *Widowed*	Jumlah *Total*
(1)	(12)	(13)	(14)	(15)	(16)
Aceh	37,67	59,01	1,63	1,68	100,00
Sumatera Utara	38,48	58,46	1,56	1,50	100,00
Sumatera Barat	36,08	61,01	2,00	0,91	100,00
Riau	32,12	65,41	1,62	0,84	100,00
Jambi	30,69	66,16	2,03	1,11	100,00
Sumatera Selatan	32,06	65,18	1,76	1,00	100,00
Bengkulu	28,83	68,55	1,78	0,84	100,00
Lampung	29,62	67,57	1,76	1,05	100,00
Kepulauan Bangka Belitung	30,70	66,09	2,41	0,80	100,00
Kepulauan Riau	31,53	66,04	1,68	0,75	100,00
DKI Jakarta	36,13	60,33	2,27	1,28	100,00
Jawa Barat	31,68	64,70	2,53	1,09	100,00
Jawa Tengah	31,65	65,35	1,81	1,19	100,00
DI Yogyakarta	35,13	62,15	1,85	0,86	100,00
Jawa Timur	29,40	66,94	2,36	1,30	100,00
Banten	32,83	63,82	2,25	1,10	100,00
Bali	32,28	65,39	1,42	0,91	100,00
Nusa Tenggara Barat	29,70	65,71	3,36	1,23	100,00
Nusa Tenggara Timur	38,98	57,83	1,74	1,45	100,00
Kalimantan Barat	31,31	65,80	1,58	1,31	100,00
Kalimantan Tengah	28,68	68,74	1,72	0,86	100,00
Kalimantan Selatan	29,81	65,88	2,90	1,40	100,00
Kalimantan Timur	33,14	63,65	2,16	1,05	100,00
Kalimantan Utara	34,89	62,04	2,26	0,81	100,00
Sulawesi Utara	32,09	64,68	2,12	1,11	100,00
Sulawesi Tengah	31,26	65,24	2,36	1,13	100,00
Sulawesi Selatan	36,31	59,90	2,46	1,34	100,00
Sulawesi Tenggara	32,58	64,26	2,12	1,04	100,00
Gorontalo	32,51	64,17	2,12	1,20	100,00
Sulawesi Barat	33,81	62,87	2,12	1,21	100,00
Maluku	38,59	58,18	1,82	1,40	100,00
Maluku Utara	33,37	63,36	2,10	1,17	100,00
Papua Barat	35,86	60,97	1,66	1,51	100,00
Papua	31,59	64,67	1,19	2,55	100,00
**Indonesia**	**32,42**	**64,26**	**2,12**	**1,20**	**100,00**

Sumber/*Source*: Susenas Maret 2018/*The March 2018 Susenas*

https://www.bps.go.id

Tabel *Table* **2.8.1.** **Persentase Penduduk Berumur 15-19 Tahun di Daerah Perkotaan menurut Provinsi, Jenis Kelamin, dan Status Perkawinan, 2018**
Percentage of Population Aged 15-19 Years in Urban Area by Province, Sex, and Marital Status, 2018

**Provinsi** *Province*	**Laki-laki/***Male*			**Perempuan/***Female*			**Laki-laki+Perempuan/***Male+Female*		
	**Belum Kawin** *Single*	**Kawin** *Married*	**Jumlah** *Total*	**Belum Kawin** *Single*	**Kawin** *Married*	**Jumlah** *Total*	**Belum Kawin** *Single*	**Kawin** *Married*	**Jumlah** *Total*
**(1)**	**(2)**	**(3)**	**(4)**	**(5)**	**(6)**	**(7)**	**(8)**	**(9)**	**(10)**
Aceh	100,00	0,00	100,00	99,12	0,88	100,00	99,57	0,43	100,00
Sumatera Utara	99,85	0,15	100,00	97,82	2,18	100,00	98,85	1,15	100,00
Sumatera Barat	100,00	0,00	100,00	97,72	2,28	100,00	98,84	1,16	100,00
Riau	99,17	0,83	100,00	97,27	2,73	100,00	98,25	1,75	100,00
Jambi	99,45	0,55	100,00	96,27	3,73	100,00	97,91	2,09	100,00
Sumatera Selatan	99,76	0,24	100,00	95,67	4,33	100,00	97,74	2,26	100,00
Bengkulu	99,03	0,97	100,00	95,63	4,37	100,00	97,43	2,57	100,00
Lampung	98,83	1,17	100,00	94,46	5,54	100,00	96,59	3,41	100,00
Kepulauan Bangka Belitung	99,25	0,75	100,00	91,77	8,23	100,00	95,60	4,40	100,00
Kepulauan Riau	98,74	1,26	100,00	97,63	2,37	100,00	98,22	1,78	100,00
DKI Jakarta	99,04	0,96	100,00	96,10	3,90	100,00	97,57	2,43	100,00
Jawa Barat	99,41	0,59	100,00	93,35	6,65	100,00	96,43	3,57	100,00
Jawa Tengah	99,53	0,47	100,00	95,80	4,20	100,00	97,70	2,30	100,00
DI Yogyakarta	99,61	0,39	100,00	99,38	0,62	100,00	99,49	0,51	100,00
Jawa Timur	99,17	0,83	100,00	93,81	6,19	100,00	96,51	3,49	100,00
Banten	100,00	0,00	100,00	96,54	3,46	100,00	98,28	1,72	100,00
Bali	99,05	0,95	100,00	95,89	4,11	100,00	97,50	2,50	100,00
Nusa Tenggara Barat	96,33	3,67	100,00	90,72	9,28	100,00	93,54	6,46	100,00
Nusa Tenggara Timur	99,80	0,20	100,00	93,75	6,25	100,00	96,76	3,24	100,00
Kalimantan Barat	99,49	0,51	100,00	95,72	4,28	100,00	97,59	2,41	100,00
Kalimantan Tengah	98,65	1,35	100,00	89,60	10,40	100,00	94,09	5,91	100,00
Kalimantan Selatan	99,00	1,00	100,00	93,29	6,71	100,00	96,22	3,78	100,00
Kalimantan Timur	98,67	1,33	100,00	94,09	5,91	100,00	96,43	3,57	100,00
Kalimantan Utara	98,14	1,86	100,00	90,41	9,59	100,00	94,39	5,61	100,00
Sulawesi Utara	98,24	1,76	100,00	92,01	7,99	100,00	95,24	4,76	100,00
Sulawesi Tengah	99,22	0,78	100,00	94,75	5,25	100,00	97,09	2,91	100,00
Sulawesi Selatan	98,53	1,47	100,00	94,53	5,47	100,00	96,55	3,45	100,00
Sulawesi Tenggara	99,57	0,43	100,00	93,19	6,81	100,00	96,41	3,59	100,00
Gorontalo	98,64	1,36	100,00	96,24	3,76	100,00	97,45	2,55	100,00
Sulawesi Barat	99,29	0,71	100,00	94,29	5,71	100,00	96,85	3,15	100,00
Maluku	98,98	1,02	100,00	95,91	4,09	100,00	97,48	2,52	100,00
Maluku Utara	99,93	0,07	100,00	93,22	6,78	100,00	96,53	3,47	100,00
Papua Barat	98,73	1,27	100,00	98,57	1,43	100,00	98,65	1,35	100,00
Papua	99,10	0,90	100,00	92,51	7,49	100,00	96,04	3,96	100,00
**Indonesia**	**99,31**	**0,69**	**100,00**	**94,87**	**5,13**	**100,00**	**97,12**	**2,88**	**100,00**

Sumber/*Source*: Susenas Maret 2018/*The March 2018 Susenas*

https://www.bps.go.id

Tabel **2.8.2.** **Persentase Penduduk Berumur 15-19 Tahun di Daerah Perdesaan menurut Provinsi, Jenis Kelamin, dan Status Perkawinan, 2018**
Table *Percentage of Population Aged 15-19 Years in Rural Area by Province, Sex, and Marital Status, 2018*

**Provinsi** *Province*	**Laki-laki/***Male*			**Perempuan/***Female*			**Laki-laki+Perempuan/***Male+Female*		
	**Belum Kawin** *Single*	**Kawin** *Married*	**Jumlah** *Total*	**Belum Kawin** *Single*	**Kawin** *Married*	**Jumlah** *Total*	**Belum Kawin** *Single*	**Kawin** *Married*	**Jumlah** *Total*
**(1)**	**(2)**	**(3)**	**(4)**	**(5)**	**(6)**	**(7)**	**(8)**	**(9)**	**(10)**
Aceh	99,60	0,40	100,00	94,03	5,97	100,00	96,88	3,12	100,00
Sumatera Utara	98,74	1,26	100,00	94,66	5,34	100,00	96,78	3,22	100,00
Sumatera Barat	99,56	0,44	100,00	94,19	5,81	100,00	96,99	3,01	100,00
Riau	99,25	0,75	100,00	93,99	6,01	100,00	96,76	3,24	100,00
Jambi	98,33	1,67	100,00	86,09	13,91	100,00	92,27	7,73	100,00
Sumatera Selatan	97,83	2,17	100,00	85,98	14,02	100,00	92,15	7,85	100,00
Bengkulu	97,30	2,70	100,00	85,46	14,54	100,00	91,70	8,30	100,00
Lampung	99,30	0,70	100,00	86,43	13,57	100,00	93,23	6,77	100,00
Kepulauan Bangka Belitung	97,95	2,05	100,00	86,54	13,46	100,00	92,63	7,37	100,00
Kepulauan Riau	99,28	0,72	100,00	91,09	8,91	100,00	95,80	4,20	100,00
DKI Jakarta	-	-	-	-	-	-	-	-	-
Jawa Barat	98,87	1,13	100,00	83,74	16,26	100,00	91,58	8,42	100,00
Jawa Tengah	99,11	0,89	100,00	86,57	13,43	100,00	93,07	6,93	100,00
DI Yogyakarta	100,00	0,00	100,00	96,01	3,99	100,00	98,13	1,87	100,00
Jawa Timur	97,73	2,27	100,00	81,07	18,93	100,00	89,64	10,36	100,00
Banten	98,51	1,49	100,00	84,62	15,38	100,00	92,05	7,95	100,00
Bali	99,01	0,99	100,00	88,41	11,59	100,00	93,74	6,26	100,00
Nusa Tenggara Barat	96,17	3,83	100,00	74,70	25,30	100,00	85,73	14,27	100,00
Nusa Tenggara Timur	99,09	0,91	100,00	93,20	6,80	100,00	96,22	3,78	100,00
Kalimantan Barat	97,19	2,81	100,00	83,03	16,97	100,00	90,34	9,66	100,00
Kalimantan Tengah	98,07	1,93	100,00	79,93	20,07	100,00	89,29	10,71	100,00
Kalimantan Selatan	99,01	0,99	100,00	85,83	14,17	100,00	92,71	7,29	100,00
Kalimantan Timur	99,21	0,79	100,00	87,98	12,02	100,00	93,95	6,05	100,00
Kalimantan Utara	99,84	0,16	100,00	86,97	13,03	100,00	93,49	6,51	100,00
Sulawesi Utara	97,64	2,36	100,00	87,87	12,13	100,00	93,03	6,97	100,00
Sulawesi Tengah	96,47	3,53	100,00	81,16	18,84	100,00	89,13	10,87	100,00
Sulawesi Selatan	98,06	1,94	100,00	88,72	11,28	100,00	93,53	6,47	100,00
Sulawesi Tenggara	97,42	2,58	100,00	82,95	17,05	100,00	90,14	9,86	100,00
Gorontalo	97,96	2,04	100,00	83,75	16,25	100,00	91,16	8,84	100,00
Sulawesi Barat	97,91	2,09	100,00	84,45	15,55	100,00	91,45	8,55	100,00
Maluku	98,56	1,44	100,00	92,37	7,63	100,00	95,66	4,34	100,00
Maluku Utara	96,88	3,12	100,00	85,92	14,08	100,00	91,46	8,54	100,00
Papua Barat	97,40	2,60	100,00	88,71	11,29	100,00	93,33	6,67	100,00
Papua	98,60	1,40	100,00	87,25	12,75	100,00	93,29	6,71	100,00
**Indonesia**	**98,46**	**1,54**	**100,00**	**86,42**	**13,58**	**100,00**	**92,67**	**7,33**	**100,00**

Sumber/*Source*: Susenas Maret 2018/*The March 2018 Susenas*

https://www.bps.go.id

Tabel 2.8.3. **Persentase Penduduk Berumur 15-19 Tahun di Daerah Perkotaan dan Perdesaan menurut Provinsi, Jenis Kelamin, dan Status Perkawinan, 2018**

Table 2.8.3. *Percentage of Population Aged 15-19 Years in Urban and Rural Area by Province, Sex, and Marital Status, 2018*

**Provinsi** *Province*	**Laki-laki/***Male*			**Perempuan/***Female*			**Laki-laki+Perempuan/***Male+Female*		
	**Belum Kawin** *Single*	**Kawin** *Married*	**Jumlah** *Total*	**Belum Kawin** *Single*	**Kawin** *Married*	**Jumlah** *Total*	**Belum Kawin** *Single*	**Kawin** *Married*	**Jumlah** *Total*
**(1)**	**(2)**	**(3)**	**(4)**	**(5)**	**(6)**	**(7)**	**(8)**	**(9)**	**(10)**
Aceh	99,72	0,28	100,00	95,67	4,33	100,00	97,74	2,26	100,00
Sumatera Utara	99,32	0,68	100,00	96,36	3,64	100,00	97,88	2,12	100,00
Sumatera Barat	99,75	0,25	100,00	95,82	4,18	100,00	97,82	2,18	100,00
Riau	99,22	0,78	100,00	95,34	4,66	100,00	97,36	2,64	100,00
Jambi	98,71	1,29	100,00	89,46	10,54	100,00	94,16	5,84	100,00
Sumatera Selatan	98,56	1,44	100,00	89,78	10,22	100,00	94,30	5,70	100,00
Bengkulu	97,91	2,09	100,00	89,03	10,97	100,00	93,72	6,28	100,00
Lampung	99,16	0,84	100,00	89,13	10,87	100,00	94,29	5,71	100,00
Kepulauan Bangka Belitung	98,60	1,40	100,00	89,29	10,71	100,00	94,16	5,84	100,00
Kepulauan Riau	98,83	1,17	100,00	96,69	3,31	100,00	97,84	2,16	100,00
DKI Jakarta	99,04	0,96	100,00	96,10	3,90	100,00	97,57	2,43	100,00
Jawa Barat	99,28	0,72	100,00	90,97	9,03	100,00	95,21	4,79	100,00
Jawa Tengah	99,33	0,67	100,00	91,41	8,59	100,00	95,47	4,53	100,00
DI Yogyakarta	99,71	0,29	100,00	98,62	1,38	100,00	99,17	0,83	100,00
Jawa Timur	98,48	1,52	100,00	87,86	12,14	100,00	93,26	6,74	100,00
Banten	99,54	0,46	100,00	93,15	6,85	100,00	96,42	3,58	100,00
Bali	99,04	0,96	100,00	93,36	6,64	100,00	96,23	3,77	100,00
Nusa Tenggara Barat	96,25	3,75	100,00	82,41	17,59	100,00	89,45	10,55	100,00
Nusa Tenggara Timur	99,27	0,73	100,00	93,35	6,65	100,00	96,36	3,64	100,00
Kalimantan Barat	97,98	2,02	100,00	87,57	12,43	100,00	92,87	7,13	100,00
Kalimantan Tengah	98,29	1,71	100,00	83,74	16,26	100,00	91,13	8,87	100,00
Kalimantan Selatan	99,00	1,00	100,00	89,51	10,49	100,00	94,42	5,58	100,00
Kalimantan Timur	98,86	1,14	100,00	92,10	7,90	100,00	95,60	4,40	100,00
Kalimantan Utara	98,82	1,18	100,00	89,00	11,00	100,00	94,02	5,98	100,00
Sulawesi Utara	97,96	2,04	100,00	90,10	9,90	100,00	94,21	5,79	100,00
Sulawesi Tengah	97,38	2,62	100,00	85,64	14,36	100,00	91,76	8,24	100,00
Sulawesi Selatan	98,26	1,74	100,00	91,20	8,80	100,00	94,81	5,19	100,00
Sulawesi Tenggara	98,32	1,68	100,00	87,17	12,83	100,00	92,75	7,25	100,00
Gorontalo	98,23	1,77	100,00	88,80	11,20	100,00	93,65	6,35	100,00
Sulawesi Barat	98,24	1,76	100,00	86,86	13,14	100,00	92,76	7,24	100,00
Maluku	98,73	1,27	100,00	93,89	6,11	100,00	96,43	3,57	100,00
Maluku Utara	97,80	2,20	100,00	88,21	11,79	100,00	93,02	6,98	100,00
Papua Barat	97,95	2,05	100,00	92,94	7,06	100,00	95,59	4,41	100,00
Papua	98,73	1,27	100,00	88,62	11,38	100,00	94,01	5,99	100,00
**Indonesia**	**98,92**	**1,08**	**100,00**	**91,11**	**8,89**	**100,00**	**95,11**	**4,89**	**100,00**

Sumber/*Source*: Susenas Maret 2018/*The March 2018 Susenas*

https://www.bps.go.id

Tabel **2.9.1.** **Persentase Penduduk Berumur 0-17 Tahun di Daerah Perkotaan menurut Provinsi dan Kepemilikan Akta Kelahiran dari Kantor Catatan Sipil, 2018**
Table *Percentage of Population Aged 0-17 Years in Urban by Province and Birth Certificate Ownership from The Civil Registration Office, 2018*

**Provinsi** *Province*	**Memiliki Akta Kelahiran** *Have a Birth Certificate*	**Tidak Memiliki Akta Kelahiran** *Do Not Have a Birth Certificate*	**Tidak Tahu** *Do Not Known*	**Jumlah** *Total*
**(1)**	**(2)**	**(3)**	**(4)**	**(5)**
Aceh	92,94	6,96	0,11	100,00
Sumatera Utara	78,12	21,17	0,71	100,00
Sumatera Barat	89,76	9,79	0,44	100,00
Riau	82,15	17,42	0,43	100,00
Jambi	95,51	4,42	0,08	100,00
Sumatera Selatan	92,39	7,55	0,06	100,00
Bengkulu	93,36	6,32	0,32	100,00
Lampung	88,89	10,89	0,22	100,00
Kepulauan Bangka Belitung	97,26	2,61	0,13	100,00
Kepulauan Riau	93,36	6,56	0,09	100,00
DKI Jakarta	96,23	3,61	0,16	100,00
Jawa Barat	83,23	16,58	0,19	100,00
Jawa Tengah	95,15	4,64	0,21	100,00
DI Yogyakarta	97,21	2,75	0,04	100,00
Jawa Timur	90,57	9,10	0,33	100,00
Banten	85,69	14,26	0,04	100,00
Bali	93,19	6,79	0,02	100,00
Nusa Tenggara Barat	86,32	12,99	0,69	100,00
Nusa Tenggara Timur	77,07	22,59	0,33	100,00
Kalimantan Barat	92,54	6,86	0,60	100,00
Kalimantan Tengah	90,60	8,88	0,52	100,00
Kalimantan Selatan	91,99	7,83	0,18	100,00
Kalimantan Timur	93,47	6,36	0,17	100,00
Kalimantan Utara	93,09	6,44	0,47	100,00
Sulawesi Utara	88,76	10,85	0,40	100,00
Sulawesi Tengah	80,18	19,09	0,73	100,00
Sulawesi Selatan	90,57	9,16	0,27	100,00
Sulawesi Tenggara	86,67	12,63	0,70	100,00
Gorontalo	94,09	5,77	0,14	100,00
Sulawesi Barat	87,50	11,53	0,97	100,00
Maluku	82,40	17,18	0,43	100,00
Maluku Utara	91,71	7,85	0,44	100,00
Papua Barat	82,11	16,59	1,30	100,00
Papua	78,52	21,05	0,43	100,00
**Indonesia**	**88,31**	**11,42**	**0,27**	**100,00**

Sumber/*Source*: Susenas Maret 2018/*The March 2018 Susenas*

https://www.bps.go.id

Tabel *Table* **2.9.2.** **Persentase Penduduk Berumur 0-17 Tahun di Daerah Perdesaan menurut Provinsi dan Kepemilikan Akta Kelahiran dari Kantor Catatan Sipil, 2018**
Percentage of Population Aged 0-17 Years in Rural by Province and Birth Certificate Ownership from The Civil Registration Office, 2018

**Provinsi** *Province*	**Memiliki Akta Kelahiran** *Have a Birth Certificate*	**Tidak Memiliki Akta Kelahiran** *Do Not Have a Birth Certificate*	**Tidak Tahu** *Do Not Known*	**Jumlah** *Total*
(1)	(2)	(3)	(4)	(5)
Aceh	85,64	14,21	0,15	100,00
Sumatera Utara	66,49	33,15	0,35	100,00
Sumatera Barat	82,06	17,52	0,42	100,00
Riau	73,29	26,27	0,44	100,00
Jambi	88,88	10,88	0,25	100,00
Sumatera Selatan	86,78	12,93	0,29	100,00
Bengkulu	89,36	10,41	0,23	100,00
Lampung	87,83	11,86	0,31	100,00
Kepulauan Bangka Belitung	92,68	7,21	0,11	100,00
Kepulauan Riau	90,58	9,00	0,42	100,00
DKI Jakarta	-	-	-	-
Jawa Barat	75,91	23,70	0,39	100,00
Jawa Tengah	92,47	7,31	0,21	100,00
DI Yogyakarta	97,91	2,00	0,10	100,00
Jawa Timur	81,32	18,43	0,25	100,00
Banten	56,40	42,77	0,82	100,00
Bali	85,33	14,50	0,17	100,00
Nusa Tenggara Barat	74,04	25,16	0,81	100,00
Nusa Tenggara Timur	53,12	46,60	0,27	100,00
Kalimantan Barat	76,80	22,47	0,73	100,00
Kalimantan Tengah	77,27	22,28	0,45	100,00
Kalimantan Selatan	86,55	13,38	0,08	100,00
Kalimantan Timur	89,83	9,48	0,69	100,00
Kalimantan Utara	84,79	14,32	0,89	100,00
Sulawesi Utara	82,28	17,42	0,30	100,00
Sulawesi Tengah	71,28	28,25	0,47	100,00
Sulawesi Selatan	84,60	15,09	0,31	100,00
Sulawesi Tenggara	78,42	21,30	0,27	100,00
Gorontalo	87,71	12,19	0,10	100,00
Sulawesi Barat	83,75	15,84	0,41	100,00
Maluku	68,03	31,50	0,47	100,00
Maluku Utara	70,41	29,02	0,57	100,00
Papua Barat	63,40	36,20	0,40	100,00
Papua	22,02	76,53	1,45	100,00
**Indonesia**	**78,15**	**21,48**	**0,37**	**100,00**

Sumber/*Source*: Susenas Maret 2018/*The March 2018 Susenas*

https://www.bps.go.id

Tabel *Table* **2.9.3.** **Persentase Penduduk Berumur 0-17 Tahun di Daerah Perkotaan dan Perdesaan menurut Provinsi dan Kepemilikan Akta Kelahiran dari Kantor Catatan Sipil, 2018**
Percentage of Population Aged 0-17 Years in Urban and Rural by Province and Birth Certificate Ownership from The Civil Registration Office, 2018

Provinsi *Province*	Memiliki Akta Kelahiran *Have a Birth Certificate*	Tidak Memiliki Akta Kelahiran *Do Not Have a Birth Certificate*	Tidak Tahu *Do Not Known*	Jumlah *Total*
(1)	(2)	(3)	(4)	(5)
Aceh	87,85	12,01	0,14	100,00
Sumatera Utara	72,29	27,18	0,53	100,00
Sumatera Barat	85,35	14,22	0,43	100,00
Riau	76,78	22,78	0,44	100,00
Jambi	90,95	8,86	0,19	100,00
Sumatera Selatan	88,82	10,98	0,20	100,00
Bengkulu	90,66	9,08	0,26	100,00
Lampung	88,14	11,58	0,29	100,00
Kepulauan Bangka Belitung	95,12	4,77	0,12	100,00
Kepulauan Riau	92,95	6,91	0,14	100,00
DKI Jakarta	96,23	3,61	0,16	100,00
Jawa Barat	81,33	18,43	0,24	100,00
Jawa Tengah	93,84	5,95	0,21	100,00
DI Yogyakarta	97,40	2,54	0,06	100,00
Jawa Timur	86,18	13,53	0,29	100,00
Banten	76,61	23,11	0,28	100,00
Bali	90,49	9,44	0,07	100,00
Nusa Tenggara Barat	79,65	19,60	0,75	100,00
Nusa Tenggara Timur	58,17	41,54	0,29	100,00
Kalimantan Barat	81,95	17,36	0,69	100,00
Kalimantan Tengah	82,33	17,19	0,47	100,00
Kalimantan Selatan	89,03	10,85	0,12	100,00
Kalimantan Timur	92,23	7,43	0,35	100,00
Kalimantan Utara	89,51	9,83	0,65	100,00
Sulawesi Utara	85,45	14,20	0,35	100,00
Sulawesi Tengah	73,69	25,77	0,54	100,00
Sulawesi Selatan	87,04	12,67	0,29	100,00
Sulawesi Tenggara	81,47	18,10	0,43	100,00
Gorontalo	90,08	9,80	0,12	100,00
Sulawesi Barat	84,63	14,83	0,54	100,00
Maluku	73,59	25,96	0,45	100,00
Maluku Utara	75,98	23,48	0,54	100,00
Papua Barat	70,75	28,49	0,75	100,00
Papua	36,32	62,49	1,19	100,00
**Indonesia**	**83,55**	**16,13**	**0,32**	**100,00**

Sumber/*Source*: Susenas Maret 2018/*The March 2018 Susenas*

https://www.bps.go.id

Tabel *Table* **2.10.1.** **Persentase Penduduk Berumur 0-4 Tahun di Daerah Perkotaan menurut Provinsi dan Kepemilikan Akta Kelahiran dari Kantor Catatan Sipil, 2018**
Percentage of Population Aged 0-4 Years in Urban by Province and Birth Certificate Ownership from The Civil Registration Office, 2018

**Provinsi** *Province*	**Memiliki Akta Kelahiran** *Have a Birth Certificate*	**Tidak Memiliki Akta Kelahiran** *Do Not Have a Birth Certificate*	**Tidak Tahu** *Do Not Known*	**Jumlah** *Total*
(1)	(2)	(3)	(4)	(5)
Aceh	83,36	16,42	0,22	100,00
Sumatera Utara	62,61	37,03	0,36	100,00
Sumatera Barat	79,27	20,65	0,07	100,00
Riau	65,40	34,18	0,43	100,00
Jambi	89,50	10,37	0,13	100,00
Sumatera Selatan	84,14	15,84	0,02	100,00
Bengkulu	81,50	18,49	0,00	100,00
Lampung	78,77	21,13	0,11	100,00
Kepulauan Bangka Belitung	92,23	7,64	0,12	100,00
Kepulauan Riau	83,80	16,08	0,13	100,00
DKI Jakarta	90,53	9,23	0,24	100,00
Jawa Barat	74,32	25,52	0,16	100,00
Jawa Tengah	89,48	10,41	0,11	100,00
DI Yogyakarta	93,47	6,53	0,00	100,00
Jawa Timur	80,98	18,73	0,28	100,00
Banten	73,85	26,07	0,08	100,00
Bali	81,20	18,80	0,00	100,00
Nusa Tenggara Barat	69,57	29,74	0,69	100,00
Nusa Tenggara Timur	51,84	47,68	0,48	100,00
Kalimantan Barat	82,80	16,70	0,50	100,00
Kalimantan Tengah	77,85	21,57	0,58	100,00
Kalimantan Selatan	82,65	17,30	0,05	100,00
Kalimantan Timur	84,62	15,23	0,15	100,00
Kalimantan Utara	84,34	15,10	0,55	100,00
Sulawesi Utara	72,77	26,90	0,33	100,00
Sulawesi Tengah	62,21	37,79	0,00	100,00
Sulawesi Selatan	78,58	21,00	0,42	100,00
Sulawesi Tenggara	69,58	29,88	0,54	100,00
Gorontalo	84,59	15,09	0,32	100,00
Sulawesi Barat	72,42	27,58	0,00	100,00
Maluku	54,84	44,93	0,24	100,00
Maluku Utara	76,29	22,60	1,10	100,00
Papua Barat	62,65	36,33	1,02	100,00
Papua	58,87	40,83	0,30	100,00
**Indonesia**	**78,23**	**21,55**	**0,21**	**100,00**

Sumber/*Source*: Susenas Maret 2018/*The March 2018 Susenas*

https://www.bps.go.id

Tabel *Table* **2.10.2.** **Persentase Penduduk Berumur 0-4 Tahun di Daerah Perdesaan menurut Provinsi dan Kepemilikan Akta Kelahiran dari Kantor Catatan Sipil, 2018**
Percentage of Population Aged 0-4 Years in Rural by Province and Birth Certificate Ownership from The Civil Registration Office, 2018

**Provinsi** *Province*	**Memiliki Akta Kelahiran** *Have a Birth Certificate*	**Tidak Memiliki Akta Kelahiran** *Do Not Have a Birth Certificate*	**Tidak Tahu** *Do Not Known*	**Jumlah** *Total*
**(1)**	**(2)**	**(3)**	**(4)**	**(5)**
Aceh	72,93	27,01	0,07	100,00
Sumatera Utara	46,44	53,42	0,15	100,00
Sumatera Barat	68,37	31,49	0,14	100,00
Riau	57,30	42,29	0,42	100,00
Jambi	76,82	23,04	0,14	100,00
Sumatera Selatan	73,89	25,92	0,20	100,00
Bengkulu	78,20	21,59	0,21	100,00
Lampung	75,61	24,21	0,18	100,00
Kepulauan Bangka Belitung	84,00	16,00	0,00	100,00
Kepulauan Riau	76,83	22,93	0,24	100,00
DKI Jakarta	-	-	-	-
Jawa Barat	65,77	33,79	0,44	100,00
Jawa Tengah	85,63	14,19	0,18	100,00
DI Yogyakarta	94,92	5,08	0,00	100,00
Jawa Timur	71,56	28,17	0,27	100,00
Banten	48,49	50,44	1,07	100,00
Bali	69,24	30,76	0,00	100,00
Nusa Tenggara Barat	51,67	47,94	0,38	100,00
Nusa Tenggara Timur	27,67	72,11	0,21	100,00
Kalimantan Barat	62,02	37,71	0,28	100,00
Kalimantan Tengah	64,22	34,89	0,89	100,00
Kalimantan Selatan	72,78	27,01	0,21	100,00
Kalimantan Timur	79,33	19,06	1,61	100,00
Kalimantan Utara	68,97	30,76	0,27	100,00
Sulawesi Utara	60,40	39,24	0,37	100,00
Sulawesi Tengah	50,37	49,48	0,16	100,00
Sulawesi Selatan	66,34	33,40	0,27	100,00
Sulawesi Tenggara	57,27	42,59	0,14	100,00
Gorontalo	70,85	29,05	0,10	100,00
Sulawesi Barat	67,00	32,96	0,04	100,00
Maluku	36,49	63,18	0,33	100,00
Maluku Utara	48,28	51,37	0,35	100,00
Papua Barat	45,57	54,22	0,20	100,00
Papua	18,34	79,58	2,08	100,00
**Indonesia**	**64,58**	**35,10**	**0,33**	**100,00**

Sumber/*Source*: Susenas Maret 2018/*The March 2018 Susenas*

https://www.bps.go.id

Tabel *Table* **2.10.3.** **Persentase Penduduk Berumur 0-4 Tahun di Daerah Perkotaan dan Perdesaan menurut Provinsi dan Kepemilikan Akta Kelahiran dari Kantor Catatan Sipil, 2018**
Percentage of Population Aged 0-4 Years in Urban and Rural by Province and Birth Certificate Ownership from The Civil Registration Office, 2018

**Provinsi** *Province*	**Memiliki Akta Kelahiran** *Have a Birth Certificate*	**Tidak Memiliki Akta Kelahiran** *Do Not Have a Birth Certificate*	**Tidak Tahu** *Do Not Known*	**Jumlah** *Total*
(1)	(2)	(3)	(4)	(5)
Aceh	76,12	23,76	0,12	100,00
Sumatera Utara	54,51	45,24	0,25	100,00
Sumatera Barat	73,07	26,82	0,11	100,00
Riau	60,51	39,07	0,42	100,00
Jambi	80,86	19,00	0,14	100,00
Sumatera Selatan	77,59	22,28	0,13	100,00
Bengkulu	79,26	20,59	0,14	100,00
Lampung	76,52	23,32	0,16	100,00
Kepulauan Bangka Belitung	88,34	11,59	0,07	100,00
Kepulauan Riau	82,93	16,93	0,14	100,00
DKI Jakarta	90,53	9,23	0,24	100,00
Jawa Barat	72,17	27,60	0,23	100,00
Jawa Tengah	87,61	12,25	0,14	100,00
DI Yogyakarta	93,84	6,16	0,00	100,00
Jawa Timur	76,57	23,15	0,28	100,00
Banten	66,35	33,27	0,38	100,00
Bali	77,33	22,67	0,00	100,00
Nusa Tenggara Barat	59,87	39,61	0,52	100,00
Nusa Tenggara Timur	32,89	66,84	0,27	100,00
Kalimantan Barat	68,78	30,87	0,35	100,00
Kalimantan Tengah	69,38	29,84	0,77	100,00
Kalimantan Selatan	77,34	22,53	0,13	100,00
Kalimantan Timur	82,84	16,52	0,64	100,00
Kalimantan Utara	78,15	21,41	0,44	100,00
Sulawesi Utara	66,48	33,17	0,35	100,00
Sulawesi Tengah	53,63	46,26	0,11	100,00
Sulawesi Selatan	71,36	28,31	0,33	100,00
Sulawesi Tenggara	61,81	37,91	0,29	100,00
Gorontalo	75,95	23,87	0,18	100,00
Sulawesi Barat	68,28	31,69	0,03	100,00
Maluku	43,24	56,46	0,30	100,00
Maluku Utara	55,82	43,63	0,55	100,00
Papua Barat	52,40	47,07	0,53	100,00
Papua	29,53	68,88	1,59	100,00
**Indonesia**	**71,92**	**27,81**	**0,27**	**100,00**

Sumber/*Source*: Susenas Maret 2018/*The March 2018 Susenas*

https://www.bps.go.id

Tabel *Table* **2.11.** **Persentase Penduduk Berumur 10 Tahun ke Atas Berstatus Kawin yang Pasangannya Biasa Tinggal di Rumah Tangga menurut Provinsi, Daerah Tempat Tinggal, dan Jenis Kelamin, 2018**
Percentage of Population Aged 10 Years and Over Who Married and Their Spouses Usually Live in The Same Households by Province, Urban Rural Classification, and Sex, 2018

**Provinsi** *Province*	**Perkotaan/***Urban*			**Perdesaan/***Rural*			**Perkotaan+Perdesaan/***Urban+Rural*		
	**Laki-laki** *Male*	**Perempuan** *Female*	**Laki-laki+Perempuan** *Male+Female*	**Laki-laki** *Male*	**Perempuan** *Female*	**Laki-laki+Perempuan** *Male+Female*	**Laki-laki/** *Male*	**Perempuan/** *Female*	**Laki-laki+Perempuan** *Male+Female*
(1)	(2)	(3)	(4)	(5)	(6)	(7)	(8)	(9)	(10)
Aceh	98,31	97,89	98,10	99,06	96,59	97,81	98,83	96,99	97,90
Sumatera Utara	99,18	97,32	98,24	99,42	97,55	98,47	99,29	97,43	98,35
Sumatera Barat	98,93	97,17	98,04	98,90	97,00	97,94	98,91	97,08	97,99
Riau	98,58	97,97	98,27	99,08	98,46	98,77	98,89	98,27	98,58
Jambi	97,09	98,46	97,77	99,25	98,57	98,91	98,59	98,54	98,56
Sumatera Selatan	99,19	97,38	98,27	99,24	98,22	98,73	99,22	97,92	98,57
Bengkulu	98,49	97,28	97,89	99,21	98,19	98,70	98,99	97,91	98,45
Lampung	98,14	97,34	97,74	97,70	97,50	97,60	97,82	97,45	97,64
Kepulauan Bangka Belitung	98,10	98,04	98,07	99,01	99,03	99,02	98,52	98,50	98,51
Kepulauan Riau	96,95	97,76	97,35	99,49	95,10	97,26	97,31	97,36	97,34
DKI Jakarta	96,24	97,19	96,71	-	-	-	96,24	97,19	96,71
Jawa Barat	98,18	96,75	97,47	98,19	94,39	96,28	98,18	96,10	97,14
Jawa Tengah	97,00	93,37	95,16	97,05	91,72	94,33	97,03	92,53	94,74
DI Yogyakarta	98,23	95,23	96,71	97,53	93,84	95,66	98,02	94,81	96,39
Jawa Timur	97,12	95,16	96,13	97,28	94,25	95,74	97,20	94,71	95,94
Banten	97,88	97,91	97,90	98,58	97,27	97,92	98,08	97,73	97,90
Bali	97,16	97,39	97,27	99,14	98,10	98,61	97,86	97,64	97,75
Nusa Tenggara Barat	98,28	92,40	95,22	98,34	90,54	94,20	98,31	91,36	94,65
Nusa Tenggara Timur	95,95	94,33	95,13	98,02	92,88	95,37	97,57	93,19	95,32
Kalimantan Barat	98,26	96,38	97,31	98,93	96,49	97,70	98,71	96,46	97,57
Kalimantan Tengah	97,08	98,01	97,55	96,65	97,63	97,14	96,81	97,77	97,29
Kalimantan Selatan	96,66	97,09	96,88	98,79	97,25	98,02	97,82	97,18	97,50
Kalimantan Timur	96,20	97,20	96,70	97,87	98,21	98,04	96,75	97,54	97,15
Kalimantan Utara	94,69	95,91	95,29	96,12	98,05	97,08	95,30	96,83	96,06
Sulawesi Utara	98,06	95,84	96,94	98,32	96,43	97,37	98,20	96,15	97,16
Sulawesi Tengah	97,48	96,45	96,97	98,68	97,36	98,02	98,37	97,12	97,74
Sulawesi Selatan	97,16	94,98	96,06	98,57	93,96	96,20	98,01	94,36	96,15
Sulawesi Tenggara	97,70	96,06	96,87	98,35	94,44	96,37	98,11	95,02	96,55
Gorontalo	99,05	94,90	96,93	98,81	97,37	98,08	98,90	96,47	97,67
Sulawesi Barat	98,52	96,88	97,70	99,07	96,89	97,97	98,95	96,89	97,91
Maluku	95,16	96,42	95,78	98,53	95,58	97,04	97,16	95,91	96,53
Maluku Utara	94,44	95,74	95,09	98,11	96,62	97,36	97,12	96,38	96,75
Papua Barat	93,17	96,07	94,59	94,33	96,66	95,47	93,87	96,43	95,12
Papua	91,84	96,48	94,08	97,43	98,38	97,91	95,92	97,90	96,90
**Indonesia**	**97,57**	**96,15**	**96,86**	**98,08**	**95,23**	**96,63**	**97,81**	**95,72**	**96,75**

Sumber/*Source*: Susenas Maret 2018/*The March 2018 Susenas*

https://www.bps.go.id

Tabel *Table* **2.12.** **Persentase Penduduk Berumur 5 Tahun ke Atas yang Mempunyai Nomor Induk Kependudukan (NIK) menurut Provinsi, Daerah Tempat Tinggal, dan Jenis Kelamin, 2018**
Percentage of Population Aged 5 Years and Over Who Have National Identity Number by Province, Urban Rural Classification, and Sex, 2018

Provinsi *Province*	Perkotaan/*Urban*			Perdesaan/*Rural*			Perkotaan+Perdesaan/*Urban+Rural*		
	Laki-laki *Male*	Perempuan *Female*	Laki-laki+Perempuan *Male+Female*	Laki-laki *Male*	Perempuan *Female*	Laki-laki+Perempuan *Male+Female*	Laki-laki/ *Male*	Perempuan/ *Female*	Laki-laki+Perempuan *Male+Female*
(1)	(2)	(3)	(4)	(5)	(6)	(7)	(8)	(9)	(10)
Aceh	98,59	98,31	98,45	98,36	98,40	98,38	98,44	98,37	98,40
Sumatera Utara	93,36	93,59	93,47	93,79	92,71	93,25	93,56	93,18	93,37
Sumatera Barat	97,15	96,84	96,99	95,29	95,77	95,53	96,11	96,24	96,18
Riau	95,21	94,85	95,03	94,16	93,52	93,85	94,58	94,06	94,33
Jambi	96,66	96,61	96,64	92,83	92,49	92,66	94,04	93,83	93,94
Sumatera Selatan	96,61	96,20	96,41	94,07	93,64	93,86	95,01	94,61	94,81
Bengkulu	98,17	97,51	97,84	95,23	94,12	94,69	96,17	95,25	95,72
Lampung	96,80	96,29	96,55	97,65	96,83	97,25	97,40	96,67	97,04
Kepulauan Bangka Belitung	98,17	98,21	98,19	96,65	96,62	96,64	97,46	97,48	97,47
Kepulauan Riau	93,15	92,70	92,92	97,86	97,23	97,55	93,84	93,34	93,59
DKI Jakarta	98,15	97,98	98,07	-	-	-	98,15	97,98	98,07
Jawa Barat	95,83	95,99	95,91	96,10	95,58	95,84	95,90	95,89	95,89
Jawa Tengah	98,78	98,52	98,65	98,54	98,15	98,35	98,66	98,34	98,50
DI Yogyakarta	99,47	99,46	99,46	99,82	99,32	99,56	99,56	99,42	99,49
Jawa Timur	98,18	98,04	98,11	96,92	96,22	96,57	97,58	97,17	97,37
Banten	94,19	94,22	94,20	92,93	92,47	92,71	93,83	93,72	93,77
Bali	99,00	98,33	98,67	98,73	98,30	98,51	98,91	98,32	98,62
Nusa Tenggara Barat	99,18	98,61	98,89	97,18	96,75	96,96	98,11	97,62	97,86
Nusa Tenggara Timur	90,18	91,31	90,74	87,24	86,16	86,69	87,93	87,33	87,63
Kalimantan Barat	97,77	97,25	97,51	94,88	94,61	94,75	95,84	95,52	95,68
Kalimantan Tengah	96,72	97,12	96,91	93,33	93,60	93,46	94,62	94,98	94,79
Kalimantan Selatan	99,68	99,43	99,55	99,33	99,28	99,30	99,49	99,35	99,42
Kalimantan Timur	97,11	97,32	97,21	97,67	96,96	97,34	97,30	97,20	97,25
Kalimantan Utara	97,82	98,44	98,11	95,96	95,17	95,59	97,03	97,09	97,05
Sulawesi Utara	92,69	92,55	92,62	92,17	91,73	91,96	92,43	92,14	92,29
Sulawesi Tengah	92,31	92,50	92,41	89,83	90,39	90,10	90,51	91,00	90,75
Sulawesi Selatan	96,85	96,40	96,62	97,22	96,91	97,06	97,07	96,70	96,88
Sulawesi Tenggara	96,58	96,53	96,55	97,53	97,96	97,74	97,16	97,41	97,29
Gorontalo	98,10	97,93	98,01	97,58	97,87	97,72	97,78	97,89	97,83
Sulawesi Barat	95,31	95,86	95,59	96,20	95,87	96,04	96,00	95,87	95,93
Maluku	92,12	92,13	92,13	84,83	84,35	84,59	87,91	87,66	87,78
Maluku Utara	95,63	95,45	95,54	85,17	84,17	84,68	88,15	87,45	87,81
Papua Barat	86,04	89,09	87,49	82,65	82,13	82,41	84,03	84,98	84,48
Papua	84,43	84,63	84,52	43,00	42,29	42,66	54,70	53,75	54,25
**Indonesia**	**96,65**	**96,61**	**96,63**	**94,63**	**94,28**	**94,45**	**95,73**	**95,56**	**95,64**

Sumber/*Source*: Susenas Maret 2018/*The March 2018 Susenas*

https://www.bps.go.id

Tabel *Table* **2.13.** **Persentase Penduduk Berumur 17 Tahun ke Atas yang Mempunyai Nomor Induk Kependudukan (NIK) menurut Provinsi, Daerah Tempat Tinggal, dan Jenis Kelamin, 2018**
Percentage of Population Aged 17 Years and Over Who Have National Identity Number by Province, Urban Rural Classification, and Sex, 2018

Provinsi *Province*	Perkotaan/*Urban*			Perdesaan/*Rural*			Perkotaan+Perdesaan/*Urban+Rural*		
	Laki-laki *Male*	Perempuan *Female*	Laki-laki+Perempuan *Male+Female*	Laki-laki *Male*	Perempuan *Female*	Laki-laki+Perempuan *Male+Female*	Laki-laki/ *Male*	Perempuan/ *Female*	Laki-laki+Perempuan *Male+Female*
(1)	(2)	(3)	(4)	(5)	(6)	(7)	(8)	(9)	(10)
Aceh	98,66	98,40	98,53	98,70	98,48	98,59	98,69	98,45	98,57
Sumatera Utara	93,69	94,09	93,89	94,06	92,92	93,49	93,86	93,56	93,71
Sumatera Barat	97,36	97,09	97,22	95,39	96,03	95,72	96,28	96,51	96,39
Riau	95,65	95,60	95,63	94,72	93,78	94,27	95,10	94,53	94,82
Jambi	97,39	96,98	97,19	92,80	92,76	92,78	94,27	94,15	94,21
Sumatera Selatan	96,88	96,30	96,59	94,29	93,52	93,91	95,25	94,58	94,92
Bengkulu	98,42	97,68	98,05	95,16	93,72	94,46	96,21	95,04	95,64
Lampung	97,03	96,21	96,62	97,94	97,11	97,54	97,67	96,84	97,27
Kepulauan Bangka Belitung	98,13	98,57	98,34	96,71	96,58	96,65	97,47	97,67	97,56
Kepulauan Riau	93,64	93,22	93,43	98,83	97,78	98,32	94,37	93,87	94,12
DKI Jakarta	98,25	97,94	98,09	-	-	-	98,25	97,94	98,09
Jawa Barat	96,30	96,34	96,32	96,74	95,77	96,25	96,41	96,19	96,30
Jawa Tengah	99,03	98,62	98,82	98,79	98,32	98,55	98,91	98,47	98,69
DI Yogyakarta	99,52	99,48	99,50	99,86	99,49	99,67	99,61	99,48	99,54
Jawa Timur	98,47	98,26	98,36	97,40	96,77	97,08	97,96	97,54	97,75
Banten	94,33	94,31	94,32	93,77	93,07	93,42	94,17	93,96	94,07
Bali	99,01	98,57	98,79	99,05	98,49	98,76	99,02	98,54	98,78
Nusa Tenggara Barat	99,28	98,74	98,99	97,70	97,11	97,39	98,44	97,88	98,14
Nusa Tenggara Timur	91,05	92,34	91,70	89,13	88,26	88,68	89,61	89,24	89,42
Kalimantan Barat	97,91	97,58	97,75	95,33	94,84	95,09	96,20	95,80	96,00
Kalimantan Tengah	96,85	97,31	97,07	93,79	94,17	93,97	94,96	95,42	95,18
Kalimantan Selatan	99,71	99,51	99,61	99,27	99,30	99,29	99,48	99,40	99,44
Kalimantan Timur	97,36	97,60	97,47	97,91	97,19	97,58	97,54	97,47	97,51
Kalimantan Utara	97,62	98,44	98,01	96,14	96,23	96,18	97,00	97,55	97,25
Sulawesi Utara	93,47	93,86	93,66	93,19	92,88	93,04	93,33	93,38	93,36
Sulawesi Tengah	92,73	93,20	92,97	90,99	91,21	91,10	91,48	91,79	91,63
Sulawesi Selatan	97,57	97,17	97,36	97,87	97,43	97,64	97,74	97,32	97,52
Sulawesi Tenggara	96,95	97,28	97,12	98,43	99,12	98,78	97,86	98,40	98,13
Gorontalo	98,16	98,38	98,28	98,13	98,17	98,15	98,14	98,25	98,20
Sulawesi Barat	95,84	96,10	95,97	96,12	96,15	96,13	96,05	96,13	96,09
Maluku	93,49	92,94	93,21	85,40	84,18	84,80	88,92	88,02	88,47
Maluku Utara	96,30	96,35	96,33	86,89	84,86	85,89	89,72	88,37	89,06
Papua Barat	86,27	90,08	88,08	84,76	84,92	84,83	85,38	87,09	86,18
Papua	86,04	86,30	86,16	46,11	45,02	45,59	58,07	56,50	57,33
**Indonesia**	**97,00**	**96,92**	**96,96**	**95,29**	**94,85**	**95,07**	**96,24**	**96,00**	**96,12**

Sumber/*Source*: Susenas Maret 2018/*The March 2018 Susenas*

https://www.bps.go.id

Tabel *Table* **2.14.** **Persentase Penduduk Berumur 0-4 Tahun yang Mempunyai Nomor Induk Kependudukan (NIK) menurut Provinsi, Daerah Tempat Tinggal, dan Jenis Kelamin, 2018**
Percentage of Population Aged 0-4 Years Who Have National Identity Number by Province, Urban Rural Classification, and Sex, 2018

**Provinsi** *Province*	**Perkotaan/***Urban*			**Perdesaan/***Rural*			**Perkotaan+Perdesaan/***Urban+Rural*		
	**Laki-laki** *Male*	**Perempuan** *Female*	**Laki-laki+Perempuan** *Male+Female*	**Laki-laki** *Male*	**Perempuan** *Female*	**Laki-laki+Perempuan** *Male+Female*	**Laki-laki/** *Male*	**Perempuan/** *Female*	**Laki-laki+Perempuan** *Male+Female*
**(1)**	**(2)**	**(3)**	**(4)**	**(5)**	**(6)**	**(7)**	**(8)**	**(9)**	**(10)**
Aceh	84,53	85,32	84,91	78,07	79,58	78,81	80,05	81,34	80,68
Sumatera Utara	70,30	67,61	68,97	59,41	59,52	59,46	64,83	63,57	64,21
Sumatera Barat	82,43	80,09	81,33	71,53	71,78	71,65	76,33	75,28	75,83
Riau	67,30	68,36	67,81	65,85	67,02	66,44	66,44	67,54	66,98
Jambi	85,52	81,68	83,69	70,83	73,73	72,27	75,65	76,19	75,91
Sumatera Selatan	82,57	79,96	81,30	70,23	75,06	72,60	74,71	76,81	75,74
Bengkulu	76,92	83,62	80,31	77,84	78,59	78,22	77,54	80,20	78,90
Lampung	77,41	76,58	76,99	78,30	77,51	77,91	78,05	77,23	77,65
Kepulauan Bangka Belitung	89,14	91,93	90,57	82,32	84,14	83,19	85,80	88,38	87,08
Kepulauan Riau	68,18	66,91	67,61	71,11	79,65	75,19	68,53	68,58	68,55
DKI Jakarta	85,14	88,33	86,70	-	-	-	85,14	88,33	86,70
Jawa Barat	76,72	77,82	77,26	72,46	75,38	73,89	75,65	77,21	76,41
Jawa Tengah	88,84	88,74	88,79	87,08	86,32	86,71	87,98	87,57	87,78
DI Yogyakarta	95,43	94,87	95,16	95,45	95,02	95,25	95,44	94,91	95,18
Jawa Timur	82,10	82,00	82,05	78,09	74,81	76,48	80,24	78,61	79,45
Banten	70,68	69,93	70,31	61,83	66,21	63,96	68,03	68,85	68,43
Bali	80,25	77,10	78,70	73,55	74,18	73,87	78,13	76,13	77,14
Nusa Tenggara Barat	77,97	82,30	80,11	65,98	68,65	67,29	71,45	74,93	73,16
Nusa Tenggara Timur	54,43	53,30	53,87	40,57	40,80	40,68	43,52	43,54	43,53
Kalimantan Barat	80,74	82,94	81,83	75,29	71,41	73,39	77,05	75,20	76,14
Kalimantan Tengah	79,54	80,38	79,95	69,96	69,59	69,78	73,53	73,74	73,63
Kalimantan Selatan	92,68	92,48	92,58	81,97	82,43	82,20	86,88	87,12	87,00
Kalimantan Timur	83,05	81,98	82,53	84,64	77,68	81,23	83,58	80,52	82,09
Kalimantan Utara	85,81	82,05	84,01	78,22	75,78	77,03	82,79	79,49	81,20
Sulawesi Utara	66,40	69,82	68,06	58,35	61,46	59,84	62,28	65,60	63,88
Sulawesi Tengah	63,28	64,17	63,69	54,13	58,26	56,19	56,77	59,81	58,26
Sulawesi Selatan	78,03	75,26	76,66	73,94	73,45	73,70	75,61	74,20	74,91
Sulawesi Tenggara	69,41	73,79	71,58	66,57	66,67	66,62	67,61	69,32	68,45
Gorontalo	80,15	86,72	83,35	72,27	73,22	72,73	75,18	78,27	76,67
Sulawesi Barat	80,81	68,92	74,95	72,26	75,87	74,01	74,26	74,21	74,23
Maluku	55,92	57,09	56,50	46,26	44,84	45,58	49,74	49,45	49,60
Maluku Utara	71,43	74,98	73,15	51,92	49,60	50,77	57,29	56,28	56,79
Papua Barat	58,59	57,92	58,27	49,46	51,51	50,45	53,10	54,08	53,57
Papua	52,60	55,59	54,03	24,66	23,36	24,02	32,55	32,06	32,31
**Indonesia**	**78,70**	**78,92**	**78,81**	**70,63**	**70,90**	**70,76**	**74,98**	**75,21**	**75,09**

Sumber/*Source*: Susenas Maret 2018/*The March 2018 Susenas*

https://www.bps.go.id

BAB/CHAPTER 3

PENDIDIKAN

EDUCATION

95,66%

Penduduk 15 tahun ke atas Melek Huruf/
population age 15+ Literate

Sebesar 18,02 % penduduk usia 15 tahun ke atas TIDAK PUNYA IJAZAH

18.02% population age 15+ HAVE NO CERTIFICATE

Angka Partisipasi Murni (APM)
Net Enrollment Ratio (NER)

7-12= 97,58%
13-15= 78,84%
16-18= 60,67%

28,26% penduduk usia 0-6 tahun pernah/sedang mengikuti pendidikan pra sekolah

28,26% population aged 0-6 years still in pre-school

Angka Partisipasi Sekolah (APS)
School Enrollment Ratio (SER)

7-12= 99,22%
13-15= 95,36%
16-18= 71,99%

0,63% penduduk usia 7-24 tahun tidak/belum pernah bersekolah

0,63% population aged 7-24 years never attended school

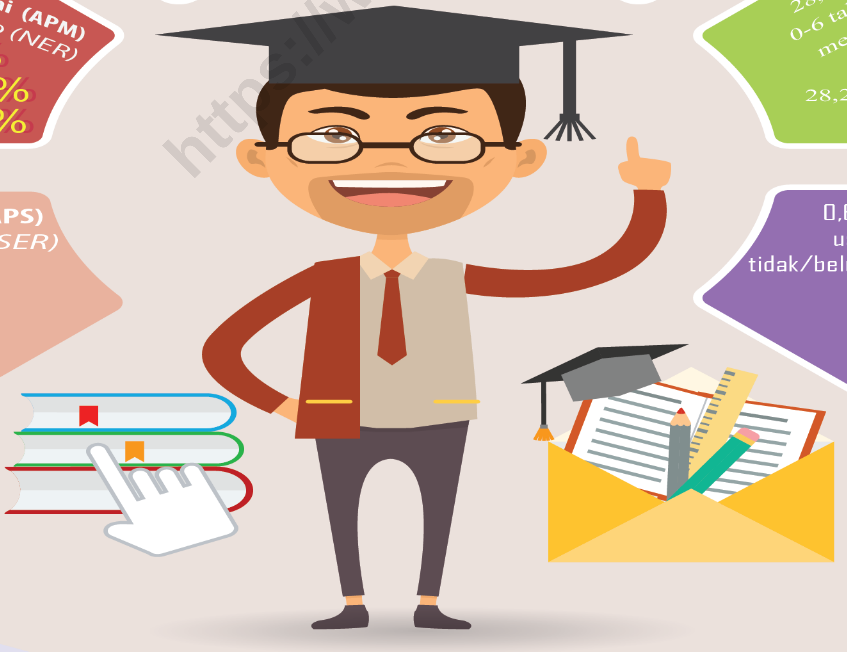

https://www.bps.go.id

https://www.bps.go.id

PENJELASAN TEKNIS

1. **Dapat membaca dan menulis** artinya dapat membaca dan menulis kata-kata/kalimat sederhana dalam huruf latin/alfabet (a-z), huruf arab/hijaiyah, atau huruf lainnya (contoh huruf jawa, kanji, dll).

2. **Angka Buta Huruf** adalah proporsi penduduk kelompok umur tertentu yang tidak dapat membaca dan menulis.

3. **Tidak/belum pernah bersekolah** adalah anggota ruta berumur 5 tahun ke atas yang tidak pernah atau belum pernah terdaftar dan tidak pernah/belum pernah aktif mengikuti pendidikan baik di suatu jenjang pendidikan formal maupun non formal (Paket A/B/C), termasuk juga yang tamat/belum tamat taman kanak-kanak tetapi tidak melanjutkan ke sekolah dasar.

4. **Pendidikan formal** adalah jalur pendidikan yang terstruktur dan berjenjang yang terdiri atas pendidikan dasar, menengah, dan pendidikan tinggi, meliputi SD/MI/sederajat, SMP/MTs/sederajat, SMA/MA/sederajat, dan Perguruan Tinggi.

5. **Pendidikan non formal** adalah jalur pendidikan diluar pendidikan formal yang dapat dilaksanakan secara terstruktur dan berjenjang, meliputi pendidikan kecakapan hidup (kursus), Pendidikan Anak Usia Dini (PAUD), pendidikan kepemudaan, pendidikan pemberdayaan perempuan, pendidikan keaksaraan, pendiidkan keterampilan, dan pelatihan kerja, pendidikan kesetaraan (paket A, B, C), serta pendidikan lainnya untuk mengembangkan kemampuan peserta didik.

TECHNICAL NOTES

1. ***Able to read and write*** *is the ability to read and write at least a simple sentence in any letter of alphabets (a-z), arabic, or other letter (example Java, Japanese, etc).*

2. ***Illiteracy Ratio*** *is the population who are unable to read and write, presented in percentage terms.*

3. ***Not/never attending school*** *is household member aged 5 years and over who has never attended or never been registered in a formal or non formal education (Package A, B, and C). Those who just completed kindergarten are considered as never attended school.*

4. ***Formal education*** *is the hierarchically structured, chronologically graded education system, includes primary school, secondary school, and tertiary school.*

5. ***Non formal education*** *is any organised educational activity outside the established formal system, which can organised structured and graded, includes life skills education, early chilhood education, youth education, empowerment education, literacy education, vocational education, and job training,education equality (Package A, B, C) and other education which objective to develop ability of learners.*

https://www.bps.go.id

6. **Masih bersekolah** adalah anggota ruta berumur 5 tahun ke atas yang terdaftar dan aktif mengikuti pendidikan baik di suatu jenjang pendidikan formal maupun non formal (Paket A/B/C). Termasuk bagi mahasiswa yang sedang cuti dianggap masih bersekolah.

7. **Tidak bersekolah lagi** adalah anggota ruta berumur 5 tahun ke atas yang pernah terdaftar dan aktif mengikuti pendidikan baik di jenjang pendidikan formal maupun non formal (Paket A/B/C), tetapi pada saat pencacahan tidak terdaftar atau tidak aktif mengikuti pendidikan lagi.

8. **Pendidikan tertinggi yang ditamatkan** adalah jenjang pendidikan tertinggi yang ditamatkan oleh seseorang, ditandai dengan sertifikat/ ijazah.

9. **Tamat sekolah** adalah menyelesaikan pelajaran yang ditandai dengan lulus ujian akhir pada kelas atau tingkat terakhir suatu jenjang pendidikan formal dan non formal (Paket A/B/C) di sekolah negeri maupun swasta dengan mendapatkan tanda tamat belajar/ijazah. Seseorang yang belum mengikuti pelajaran pada kelas tertinggi tetapi sudah mengikuti ujian akhir dan lulus, dianggap tamat sekolah.

10. **Angka Partisipasi Sekolah (APS)** adalah proporsi penduduk pada kelompok umur jenjang pendidikan tertentu yang masih bersekolah terhadap penduduk pada kelompok umur tersebut.

11. **Angka Partisipasi Murni (APM)** adalah proporsi penduduk pada kelompok umur jenjang pendidikan tertentu yang masih bersekolah pada jenjang pendidikan yang sesuai dengan kelompok umurnya terhadap penduduk pada kelompok umur tersebut.

*6. **Attending school** is household member aged 5 years and over who is currently attending formal or non formal education (Package A, B, and C). College student who pospones his/her study is considered as attending school.*

*7. **Not attending school anymore** is household member aged 5 years and over who had enrolled and participated in formal or non formal education (Package A, B, and C), but currently does not attend school.*

*8. **Highest Educational Attainment** is the highest education level completed by an individual.*

*9. **Completed particular level of education** is someone who has completed particular level of education in private or public school and accepting graduation certificate. Someone who has never attended the highest grade but passed the final exam is considered as completed particular level of education.*

*10. **School Enrollment Ratio** is the population of a certain age group with the status still school divided by the population age group.*

*11. **Net Enrollment Ratio (NER)** is enrollment of the official age group for a given level of education expressed as a percentage of the corresponding population.*

https://www.bps.go.id

12. **Pendidikan prasekolah** adalah pendidikan yang diselenggarakan sebelum jenjang pendidikan dasar, baik melalui jalur pendidikan formal maupun non formal.

*12. **Preschool education** is education prior primary education, both through formal and non formal education.*

https://www.bps.go.id

https://www.bps.go.id

Tabel *Table* **3.1.1.** **Persentase Penduduk Berumur 15 Tahun ke Atas di Daerah Perkotaan menurut Provinsi, Jenis Kelamin, dan Kemampuan Membaca dan Menulis, 2018**
Percentage of Population Aged 15 Years and Over in Urban Area by Province, Sex, and Literacy, 2018

**Provinsi** *Province*	**Laki-laki/***Male*			**Perempuan/***Female*			**Laki-laki+Perempuan/***Male+Female*		
	**Huruf Latin** *Latin*	**Huruf Arab** *Arabic*	**Huruf Lainnya** *Others*	**Huruf Latin** *Latin*	**Huruf Arab** *Arabic*	**Huruf Lainnya** *Others*	**Huruf Latin** *Latin*	**Huruf Arab** *Arabic*	**Huruf Lainnya** *Others*
**(1)**	**(2)**	**(3)**	**(4)**	**(5)**	**(6)**	**(7)**	**(8)**	**(9)**	**(10)**
Aceh	99,46	36,88	2,27	98,34	36,72	2,09	98,90	36,80	2,18
Sumatera Utara	99,70	41,99	7,60	99,26	44,11	7,43	99,48	43,06	7,52
Sumatera Barat	99,34	30,06	0,77	98,77	31,75	0,91	99,05	30,92	0,84
Riau	99,58	69,98	5,85	98,62	70,52	5,22	99,11	70,25	5,54
Jambi	99,23	44,09	7,44	97,50	42,80	6,94	98,38	43,46	7,19
Sumatera Selatan	99,40	37,03	1,90	98,77	40,14	1,62	99,09	38,59	1,76
Bengkulu	99,22	50,74	4,10	98,53	51,23	4,50	98,87	50,98	4,30
Lampung	98,88	29,15	10,83	96,71	28,50	9,56	97,80	28,83	10,20
Kepulauan Bangka Belitung	98,81	29,40	4,42	96,90	29,71	3,74	97,89	29,55	4,09
Kepulauan Riau	98,91	45,95	9,92	97,90	47,08	7,77	98,42	46,51	8,87
DKI Jakarta	99,64	50,91	9,72	98,74	50,93	9,68	99,18	50,92	9,70
Jawa Barat	98,85	64,52	4,89	96,93	64,30	4,31	97,90	64,41	4,60
Jawa Tengah	97,14	55,16	17,96	92,06	53,94	16,92	94,55	54,53	17,43
DI Yogyakarta	98,07	46,28	23,47	94,71	46,38	19,88	96,37	46,33	21,65
Jawa Timur	96,88	56,12	15,68	92,58	54,09	15,26	94,69	55,09	15,47
Banten	98,56	59,56	7,57	96,44	58,47	7,15	97,51	59,02	7,37
Bali	97,82	14,01	55,13	92,90	11,86	49,86	95,38	12,94	52,51
Nusa Tenggara Barat	93,37	31,39	2,76	85,80	28,98	1,51	89,38	30,12	2,10
Nusa Tenggara Timur	97,50	8,09	1,69	96,38	8,01	2,10	96,93	8,05	1,89
Kalimantan Barat	97,33	25,91	6,53	93,06	25,60	5,17	95,19	25,75	5,85
Kalimantan Tengah	99,55	42,15	3,44	98,44	42,36	2,65	99,01	42,25	3,06
Kalimantan Selatan	99,21	60,78	3,99	97,49	61,52	3,48	98,36	61,14	3,74
Kalimantan Timur	99,17	12,80	2,41	98,10	12,77	1,88	98,66	12,79	2,16
Kalimantan Utara	97,23	19,04	2,46	95,74	20,79	1,43	96,53	19,86	1,98
Sulawesi Utara	99,89	11,21	2,84	99,91	11,93	3,07	99,90	11,57	2,96
Sulawesi Tengah	99,44	44,69	3,32	99,18	49,60	2,66	99,31	47,13	2,99
Sulawesi Selatan	97,09	42,51	22,59	94,59	44,07	23,44	95,80	43,31	23,03
Sulawesi Tenggara	97,49	19,77	1,04	95,23	21,84	1,66	96,35	20,81	1,35
Gorontalo	98,55	33,00	1,43	98,88	42,92	1,54	98,72	38,07	1,49
Sulawesi Barat	95,83	31,18	4,34	93,50	29,87	3,92	94,63	30,50	4,13
Maluku	99,55	21,85	3,93	99,40	22,68	3,83	99,47	22,27	3,88
Maluku Utara	99,89	33,69	1,89	98,90	34,55	1,91	99,40	34,12	1,90
Papua Barat	99,54	19,76	2,57	98,65	21,20	3,40	99,12	20,44	2,96
Papua	98,62	23,10	4,89	97,84	23,97	3,77	98,26	23,49	4,39
**Indonesia**	**98,30**	**50,91**	**10,41**	**95,67**	**50,60**	**9,88**	**96,98**	**50,76**	**10,14**

Sumber/*Source*: Susenas Maret 2018/*The March 2018 Susenas*

https://www.bps.go.id

Tabel 3.1.2. **Persentase Penduduk Berumur 15 Tahun ke Atas di Daerah Perdesaan menurut Provinsi, Jenis Kelamin, dan Kemampuan Membaca dan Menulis, 2018**
Table *Percentage of Population Aged 15 Years and Over in Rural Area by Province, Sex, and Literacy, 2018*

**Provinsi** *Province*	**Laki-laki/***Male*			**Perempuan/***Female*			**Laki-laki+Perempuan/***Male+Female*		
	**Huruf Latin** *Latin*	**Huruf Arab** *Arabic*	**Huruf Lainnya** *Others*	**Huruf Latin** *Latin*	**Huruf Arab** *Arabic*	**Huruf Lainnya** *Others*	**Huruf Latin** *Latin*	**Huruf Arab** *Arabic*	**Huruf Lainnya** *Others*
**(1)**	**(2)**	**(3)**	**(4)**	**(5)**	**(6)**	**(7)**	**(8)**	**(9)**	**(10)**
Aceh	98,19	31,00	1,39	94,91	31,84	1,53	96,53	31,43	1,46
Sumatera Utara	99,02	32,95	2,20	97,08	34,31	1,75	98,04	33,63	1,97
Sumatera Barat	98,71	31,32	0,27	96,78	33,41	0,11	97,73	32,38	0,19
Riau	98,62	58,39	3,95	96,04	60,46	3,25	97,37	59,39	3,61
Jambi	98,07	64,07	4,45	93,90	64,03	3,57	96,04	64,05	4,02
Sumatera Selatan	98,31	34,14	3,27	95,61	35,66	2,67	96,99	34,88	2,97
Bengkulu	97,96	26,96	2,27	94,07	26,06	1,74	96,07	26,52	2,01
Lampung	97,49	34,47	8,17	93,91	33,15	8,00	95,76	33,83	8,09
Kepulauan Bangka Belitung	96,79	29,51	1,91	93,90	30,42	1,70	95,43	29,93	1,81
Kepulauan Riau	94,06	53,38	2,81	90,39	55,75	2,61	92,31	54,51	2,72
DKI Jakarta	-	-	-	-	-	-	-	-	-
Jawa Barat	97,50	63,17	2,50	94,28	62,32	2,16	95,90	62,74	2,33
Jawa Tengah	94,24	52,43	13,27	86,74	49,85	11,32	90,43	51,12	12,28
DI Yogyakarta	93,07	40,56	19,32	84,99	38,76	15,52	88,85	39,62	17,33
Jawa Timur	91,12	52,54	9,81	81,53	48,75	8,44	86,19	50,59	9,11
Banten	97,43	64,65	0,86	91,67	59,09	0,58	94,61	61,93	0,72
Bali	92,93	6,28	59,83	81,21	5,63	47,19	86,99	5,95	53,43
Nusa Tenggara Barat	89,48	27,54	2,31	79,81	20,95	1,60	84,41	24,09	1,94
Nusa Tenggara Timur	91,90	3,28	0,21	88,70	3,23	0,28	90,26	3,26	0,25
Kalimantan Barat	94,19	19,44	1,97	86,57	18,65	1,51	90,48	19,05	1,74
Kalimantan Tengah	98,81	32,20	2,92	98,16	32,32	2,22	98,51	32,26	2,59
Kalimantan Selatan	98,04	42,65	1,44	94,89	42,05	1,16	96,48	42,35	1,30
Kalimantan Timur	97,39	15,73	0,87	94,97	16,19	0,93	96,27	15,94	0,90
Kalimantan Utara	94,32	6,05	1,10	92,00	9,37	1,25	93,26	7,57	1,17
Sulawesi Utara	99,71	9,57	1,12	99,88	10,26	1,16	99,79	9,91	1,14
Sulawesi Tengah	97,34	30,76	2,07	95,25	33,98	1,58	96,33	32,32	1,83
Sulawesi Selatan	89,16	29,97	21,91	84,66	32,55	23,57	86,81	31,32	22,78
Sulawesi Tenggara	95,06	19,37	2,70	89,56	20,58	1,96	92,28	19,98	2,33
Gorontalo	97,55	21,81	0,53	97,57	32,99	0,38	97,56	27,35	0,46
Sulawesi Barat	93,20	18,13	3,03	88,83	20,36	2,70	91,02	19,24	2,86
Maluku	98,78	25,46	1,39	98,07	24,73	0,89	98,42	25,10	1,14
Maluku Utara	98,60	30,84	1,68	96,76	30,54	1,50	97,69	30,69	1,59
Papua Barat	97,27	15,83	3,35	94,59	15,91	3,55	96,02	15,87	3,45
Papua	74,44	5,86	1,11	60,82	4,82	1,02	67,94	5,36	1,07
**Indonesia**	**94,86**	**41,53**	**6,99**	**89,28**	**40,62**	**6,31**	**92,07**	**41,07**	**6,65**

Sumber/*Source*: Susenas Maret 2018/*The March 2018 Susenas*

https://www.bps.go.id

Tabel *Table* **3.1.3.** **Persentase Penduduk Berumur 15 Tahun ke Atas di Daerah Perkotaan dan Perdesaan menurut Provinsi, Jenis Kelamin, dan Kemampuan Membaca dan Menulis, 2018**

Percentage of Population Aged 15 Years and Over in Urban and Rural Area by Province, Sex, and Literacy, 2018

**Provinsi** *Province*	**Laki-laki/***Male*			**Perempuan/***Female*			**Laki-laki+Perempuan/***Male+Female*		
	**Huruf Latin** *Latin*	**Huruf Arab** *Arabic*	**Huruf Lainnya** *Others*	**Huruf Latin** *Latin*	**Huruf Arab** *Arabic*	**Huruf Lainnya** *Others*	**Huruf Latin** *Latin*	**Huruf Arab** *Arabic*	**Huruf Lainnya** *Others*
**(1)**	**(2)**	**(3)**	**(4)**	**(5)**	**(6)**	**(7)**	**(8)**	**(9)**	**(10)**
Aceh	98,60	32,89	1,67	95,99	33,39	1,71	97,28	33,14	1,69
Sumatera Utara	99,39	37,86	5,13	98,27	39,65	4,85	98,82	38,76	4,99
Sumatera Barat	98,99	30,76	0,49	97,67	32,66	0,47	98,32	31,73	0,48
Riau	99,01	63,10	4,72	97,10	64,60	4,06	98,08	63,83	4,40
Jambi	98,44	57,61	5,41	95,08	57,06	4,68	96,80	57,35	5,05
Sumatera Selatan	98,72	35,21	2,76	96,82	37,38	2,26	97,78	36,28	2,51
Bengkulu	98,37	34,69	2,86	95,56	34,49	2,66	97,00	34,59	2,77
Lampung	97,90	32,91	8,95	94,76	31,73	8,48	96,37	32,34	8,72
Kepulauan Bangka Belitung	97,87	29,45	3,25	95,53	30,03	2,82	96,76	29,72	3,04
Kepulauan Riau	98,21	47,03	8,89	96,86	48,29	7,05	97,55	47,64	8,00
DKI Jakarta	99,64	50,91	9,72	98,74	50,93	9,68	99,18	50,92	9,70
Jawa Barat	98,50	64,17	4,27	96,24	63,79	3,75	97,38	63,98	4,01
Jawa Tengah	95,73	53,83	15,67	89,48	51,95	14,20	92,54	52,87	14,92
DI Yogyakarta	96,75	44,77	22,38	92,02	44,28	18,67	94,34	44,52	20,49
Jawa Timur	94,15	54,42	12,90	87,29	51,53	11,99	90,64	52,94	12,44
Banten	98,24	60,99	5,69	95,11	58,64	5,32	96,70	59,84	5,51
Bali	96,19	11,43	56,70	88,90	9,73	48,94	92,55	10,58	52,82
Nusa Tenggara Barat	91,30	29,34	2,52	82,63	24,73	1,56	86,74	26,92	2,02
Nusa Tenggara Timur	93,28	4,47	0,58	90,54	4,38	0,71	91,88	4,42	0,65
Kalimantan Barat	95,25	21,62	3,50	88,84	21,08	2,79	92,09	21,35	3,15
Kalimantan Tengah	99,09	36,00	3,12	98,27	36,28	2,39	98,70	36,13	2,77
Kalimantan Selatan	98,59	51,19	2,64	96,11	51,21	2,25	97,37	51,20	2,45
Kalimantan Timur	98,57	13,77	1,89	97,10	13,87	1,58	97,88	13,82	1,75
Kalimantan Utara	96,01	13,61	1,90	94,21	16,13	1,36	95,18	14,78	1,65
Sulawesi Utara	99,80	10,40	1,99	99,90	11,12	2,14	99,84	10,75	2,06
Sulawesi Tengah	97,94	34,71	2,43	96,40	38,54	1,89	97,19	36,58	2,17
Sulawesi Selatan	92,51	35,27	22,20	88,80	37,35	23,52	90,58	36,36	22,88
Sulawesi Tenggara	96,01	19,53	2,05	91,77	21,07	1,84	93,88	20,31	1,95
Gorontalo	97,93	26,14	0,88	98,10	36,97	0,85	98,02	31,58	0,86
Sulawesi Barat	93,81	21,15	3,33	89,97	22,68	3,00	91,88	21,92	3,16
Maluku	99,11	23,90	2,48	98,65	23,83	2,17	98,88	23,87	2,33
Maluku Utara	98,98	31,69	1,75	97,41	31,76	1,63	98,21	31,73	1,69
Papua Barat	98,21	17,46	3,03	96,29	18,12	3,49	97,31	17,77	3,24
Papua	81,64	11,00	2,24	71,11	10,15	1,78	76,69	10,60	2,03
**Indonesia**	**96,76**	**46,72**	**8,88**	**92,81**	**46,15**	**8,28**	**94,79**	**46,43**	**8,58**

Sumber/*Source*: Susenas Maret 2018/*The March 2018 Susenas*

https://www.bps.go.id

Tabel 3.2.1. **Persentase Penduduk Berumur 15-24 Tahun di Daerah Perkotaan menurut Provinsi, Jenis Kelamin, dan Kemampuan Membaca dan Menulis, 2018**

Table 3.2.1. *Percentage of Population Aged 15-24 Years in Urban Area by Province, Sex, and Literacy, 2018*

**Provinsi** *Province*	**Laki-laki/***Male*			**Perempuan/***Female*			**Laki-laki+Perempuan/***Male+Female*		
	**Huruf Latin** *Latin*	**Huruf Arab** *Arabic*	**Huruf Lainnya** *Others*	**Huruf Latin** *Latin*	**Huruf Arab** *Arabic*	**Huruf Lainnya** *Others*	**Huruf Latin** *Latin*	**Huruf Arab** *Arabic*	**Huruf Lainnya** *Others*
(1)	(2)	(3)	(4)	(5)	(6)	(7)	(8)	(9)	(10)
Aceh	99,81	39,15	1,78	99,94	40,22	1,50	99,87	39,68	1,64
Sumatera Utara	99,98	43,80	5,69	99,77	50,10	6,24	99,87	46,93	5,96
Sumatera Barat	99,86	29,56	0,47	99,81	33,60	1,50	99,83	31,61	0,99
Riau	99,70	72,83	4,08	99,87	77,76	5,09	99,78	75,23	4,57
Jambi	99,77	46,76	5,93	100,00	45,02	5,49	99,89	45,89	5,71
Sumatera Selatan	99,59	40,29	2,36	99,72	47,87	2,15	99,65	44,02	2,26
Bengkulu	100,00	54,35	4,26	100,00	59,47	7,62	100,00	56,84	5,90
Lampung	99,56	29,53	16,42	99,25	34,49	15,17	99,41	32,00	15,80
Kepulauan Bangka Belitung	99,76	31,96	3,73	99,56	34,23	3,62	99,67	33,06	3,68
Kepulauan Riau	99,48	50,75	10,21	99,90	52,21	5,55	99,68	51,45	7,99
DKI Jakarta	99,81	52,91	6,61	99,89	54,25	7,76	99,85	53,59	7,20
Jawa Barat	99,81	68,42	4,31	99,78	71,16	5,22	99,79	69,77	4,76
Jawa Tengah	99,57	66,37	23,27	99,78	71,55	27,75	99,67	68,93	25,48
DI Yogyakarta	100,00	56,67	26,71	99,91	64,38	26,46	99,96	60,49	26,59
Jawa Timur	99,65	66,02	20,17	99,70	67,96	23,84	99,68	66,98	21,99
Banten	99,81	62,79	5,72	99,96	64,94	4,90	99,89	63,85	5,31
Bali	99,84	13,56	62,59	99,83	11,87	64,32	99,83	12,73	63,45
Nusa Tenggara Barat	99,44	32,79	2,07	100,00	44,32	1,70	99,72	38,51	1,89
Nusa Tenggara Timur	99,56	8,41	0,89	99,67	6,76	0,89	99,61	7,58	0,89
Kalimantan Barat	99,84	24,33	4,49	100,00	28,07	3,55	99,92	26,23	4,01
Kalimantan Tengah	100,00	40,69	2,04	99,42	44,46	2,42	99,71	42,56	2,23
Kalimantan Selatan	99,41	67,92	2,59	99,60	67,63	3,39	99,50	67,78	2,98
Kalimantan Timur	99,75	12,42	0,65	100,00	13,75	0,34	99,87	13,07	0,50
Kalimantan Utara	99,47	18,77	0,20	99,47	20,95	0,20	99,47	19,79	0,20
Sulawesi Utara	100,00	13,04	2,47	99,92	14,65	3,38	99,96	13,81	2,91
Sulawesi Tengah	99,57	46,91	1,45	99,76	55,78	0,96	99,66	51,18	1,21
Sulawesi Selatan	99,56	44,94	22,38	99,61	52,25	26,15	99,59	48,56	24,25
Sulawesi Tenggara	99,56	22,15	0,19	99,09	26,58	1,47	99,33	24,32	0,82
Gorontalo	99,32	36,20	1,17	99,92	47,23	2,11	99,62	41,69	1,64
Sulawesi Barat	99,26	32,16	1,75	99,59	31,50	1,30	99,42	31,83	1,53
Maluku	99,82	19,76	2,47	100,00	25,79	2,83	99,91	22,76	2,65
Maluku Utara	100,00	26,02	0,52	99,48	34,08	1,39	99,74	30,00	0,95
Papua Barat	100,00	15,46	1,31	100,00	17,02	2,24	100,00	16,19	1,74
Papua	99,81	18,61	3,49	99,68	22,65	3,46	99,75	20,38	3,48
**Indonesia**	**99,73**	**55,20**	**10,82**	**99,78**	**58,68**	**12,26**	**99,76**	**56,92**	**11,53**

Sumber/*Source*: Susenas Maret 2018/*The March 2018 Susenas*

https://www.bps.go.id

Tabel *Table* **3.2.2.** **Persentase Penduduk Berumur 15-24 Tahun di Daerah Perdesaan menurut Provinsi, Jenis Kelamin, dan Kemampuan Membaca dan Menulis, 2018**
Percentage of Population Aged 15-24 Years in Rural Area by Province, Sex, and Literacy, 2018

**Provinsi** *Province*	**Laki-laki/***Male*			**Perempuan/***Female*			**Laki-laki+Perempuan/***Male+Female*		
	**Huruf Latin** *Latin*	**Huruf Arab** *Arabic*	**Huruf Lainnya** *Others*	**Huruf Latin** *Latin*	**Huruf Arab** *Arabic*	**Huruf Lainnya** *Others*	**Huruf Latin** *Latin*	**Huruf Arab** *Arabic*	**Huruf Lainnya** *Others*
**(1)**	**(2)**	**(3)**	**(4)**	**(5)**	**(6)**	**(7)**	**(8)**	**(9)**	**(10)**
Aceh	99,67	33,64	1,08	99,47	37,65	1,29	99,57	35,60	1,18
Sumatera Utara	99,83	39,43	2,16	99,70	43,36	2,13	99,77	41,32	2,15
Sumatera Barat	99,22	32,62	0,23	99,67	39,01	0,12	99,44	35,73	0,18
Riau	99,60	63,32	2,76	99,60	70,09	3,24	99,60	66,53	2,99
Jambi	99,93	68,20	3,12	99,68	73,53	3,03	99,81	70,78	3,07
Sumatera Selatan	99,70	38,67	1,98	99,39	46,60	2,27	99,55	42,49	2,12
Bengkulu	99,43	29,46	1,27	100,00	33,32	2,01	99,70	31,26	1,62
Lampung	99,48	41,13	15,09	99,88	44,35	18,52	99,67	42,66	16,72
Kepulauan Bangka Belitung	99,56	32,90	0,91	100,00	37,86	1,11	99,77	35,21	1,01
Kepulauan Riau	99,55	57,68	0,62	99,64	77,98	2,58	99,59	66,24	1,45
DKI Jakarta	-	-	-	-	-	-	-	-	-
Jawa Barat	99,48	71,13	2,76	99,87	73,69	3,25	99,67	72,37	3,00
Jawa Tengah	99,27	68,30	20,18	99,51	73,76	23,71	99,39	70,94	21,89
DI Yogyakarta	99,47	66,62	23,30	100,00	72,40	32,71	99,73	69,46	27,92
Jawa Timur	99,44	67,06	14,09	99,55	72,15	15,81	99,49	69,55	14,94
Banten	98,92	66,53	0,61	99,08	67,65	0,46	99,00	67,06	0,54
Bali	99,37	8,11	76,28	97,93	7,87	78,29	98,66	7,99	77,26
Nusa Tenggara Barat	99,79	36,42	2,70	99,36	36,98	1,84	99,58	36,69	2,28
Nusa Tenggara Timur	98,05	3,66	0,21	98,14	4,27	0,18	98,10	3,96	0,20
Kalimantan Barat	99,53	20,14	1,68	99,92	23,58	2,02	99,72	21,80	1,85
Kalimantan Tengah	98,95	33,78	1,10	99,58	38,20	1,09	99,25	35,90	1,09
Kalimantan Selatan	99,48	47,88	0,59	99,82	56,92	1,26	99,64	52,26	0,91
Kalimantan Timur	99,60	16,04	0,19	99,87	16,62	0,01	99,73	16,31	0,11
Kalimantan Utara	98,53	6,08	0,18	99,83	13,20	0,00	99,14	9,39	0,09
Sulawesi Utara	99,71	10,37	0,86	100,00	12,02	1,45	99,84	11,14	1,13
Sulawesi Tengah	98,92	33,81	0,75	98,97	39,51	0,72	98,94	36,58	0,74
Sulawesi Selatan	98,58	39,25	27,31	99,22	46,40	33,45	98,89	42,77	30,33
Sulawesi Tenggara	98,86	21,88	1,61	99,44	24,59	0,76	99,15	23,24	1,18
Gorontalo	99,71	26,07	0,19	99,69	37,57	0,40	99,70	31,65	0,29
Sulawesi Barat	98,88	22,33	1,87	99,53	24,96	2,87	99,19	23,60	2,35
Maluku	99,45	26,59	0,48	99,55	28,70	0,86	99,50	27,60	0,66
Maluku Utara	99,70	30,31	1,59	99,74	34,73	1,75	99,72	32,48	1,67
Papua Barat	99,48	13,23	1,23	98,65	15,74	2,49	99,09	14,40	1,82
Papua	87,69	5,33	0,83	78,41	4,51	1,13	83,32	4,94	0,97
**Indonesia**	**99,12**	**48,38**	**8,70**	**99,10**	**52,82**	**10,15**	**99,11**	**50,53**	**9,40**

Sumber/*Source*: Susenas Maret 2018/*The March 2018 Susenas*

https://www.bps.go.id

Tabel 3.2.3. **Persentase Penduduk Berumur 15-24 Tahun di Daerah Perkotaan dan Perdesaan menurut Provinsi, Jenis Kelamin, dan Kemampuan Membaca dan Menulis, 2018**

Table *Percentage of Population Aged 15-24 Years in Urban and Rural Area by Province, Sex, and Literacy, 2018*

**Provinsi** *Province*	**Laki-laki/***Male*			**Perempuan/***Female*			**Laki-laki+Perempuan/***Male+Female*		
	**Huruf Latin** *Latin*	**Huruf Arab** *Arabic*	**Huruf Lainnya** *Others*	**Huruf Latin** *Latin*	**Huruf Arab** *Arabic*	**Huruf Lainnya** *Others*	**Huruf Latin** *Latin*	**Huruf Arab** *Arabic*	**Huruf Lainnya** *Others*
**(1)**	**(2)**	**(3)**	**(4)**	**(5)**	**(6)**	**(7)**	**(8)**	**(9)**	**(10)**
Aceh	99,72	35,45	1,31	99,62	38,51	1,36	99,67	36,95	1,33
Sumatera Utara	99,91	41,81	4,09	99,74	47,14	4,43	99,82	44,42	4,26
Sumatera Barat	99,51	31,22	0,34	99,74	36,42	0,78	99,62	33,80	0,56
Riau	99,64	67,25	3,31	99,72	73,35	4,03	99,68	70,18	3,65
Jambi	99,88	61,20	4,04	99,79	63,79	3,87	99,84	62,47	3,95
Sumatera Selatan	99,66	39,30	2,13	99,52	47,10	2,22	99,59	43,09	2,17
Bengkulu	99,63	38,00	2,29	100,00	42,74	4,04	99,80	40,25	3,12
Lampung	99,50	37,46	15,51	99,67	41,03	17,39	99,58	39,19	16,42
Kepulauan Bangka Belitung	99,67	32,41	2,36	99,77	35,92	2,45	99,71	34,08	2,40
Kepulauan Riau	99,49	51,74	8,85	99,87	55,22	5,21	99,67	53,37	7,13
DKI Jakarta	99,81	52,91	6,61	99,89	54,25	7,76	99,85	53,59	7,20
Jawa Barat	99,73	69,08	3,93	99,80	71,77	4,75	99,76	70,40	4,33
Jawa Tengah	99,43	67,29	21,79	99,65	72,58	25,86	99,54	69,88	23,78
DI Yogyakarta	99,89	58,69	26,02	99,93	65,97	27,70	99,91	62,29	26,85
Jawa Timur	99,56	66,50	17,35	99,63	69,88	20,16	99,59	68,17	18,73
Banten	99,55	63,88	4,23	99,72	65,69	3,68	99,63	64,76	3,96
Bali	99,69	11,86	66,86	99,24	10,63	68,63	99,47	11,26	67,73
Nusa Tenggara Barat	99,62	34,66	2,39	99,67	40,57	1,77	99,65	37,58	2,09
Nusa Tenggara Timur	98,45	4,93	0,39	98,56	4,95	0,38	98,51	4,94	0,39
Kalimantan Barat	99,64	21,57	2,64	99,95	25,21	2,58	99,79	23,37	2,61
Kalimantan Tengah	99,36	36,48	1,47	99,52	40,75	1,63	99,44	38,55	1,54
Kalimantan Selatan	99,44	57,63	1,56	99,71	62,14	2,30	99,57	59,81	1,92
Kalimantan Timur	99,70	13,64	0,50	99,96	14,68	0,23	99,82	14,14	0,37
Kalimantan Utara	99,09	13,66	0,19	99,62	17,84	0,12	99,34	15,61	0,16
Sulawesi Utara	99,86	11,80	1,72	99,96	13,46	2,51	99,91	12,59	2,09
Sulawesi Tengah	99,14	38,13	0,98	99,22	44,81	0,80	99,18	41,37	0,89
Sulawesi Selatan	99,01	41,77	25,13	99,40	49,01	30,20	99,20	45,34	27,63
Sulawesi Tenggara	99,16	22,00	0,98	99,29	25,43	1,06	99,23	23,71	1,02
Gorontalo	99,55	30,18	0,59	99,78	41,60	1,12	99,67	35,78	0,85
Sulawesi Barat	98,97	24,66	1,84	99,54	26,58	2,48	99,25	25,59	2,15
Maluku	99,61	23,62	1,34	99,75	27,37	1,76	99,68	25,45	1,54
Maluku Utara	99,80	28,90	1,24	99,66	34,51	1,63	99,73	31,67	1,43
Papua Barat	99,70	14,16	1,26	99,22	16,27	2,39	99,47	15,15	1,79
Papua	91,59	9,60	1,69	84,63	9,82	1,82	88,38	9,70	1,75
**Indonesia**	**99,46**	**52,19**	**9,89**	**99,49**	**56,15**	**11,35**	**99,47**	**54,13**	**10,60**

Sumber/*Source*: Susenas Maret 2018/*The March 2018 Susenas*

https://www.bps.go.id

Tabel 3.3.1. **Persentase Penduduk Laki-Laki dan Perempuan Berumur 5 Tahun ke Atas di Daerah Perkotaan menurut Provinsi dan Status Pendidikan, 2018**

Table *Percentage of Male and Female Population Aged 5 Years and Over in Urban Area by Province and Education Status, 2018*

**Provinsi** *Province*	**Tidak/Belum Pernah Sekolah** *No Schooling/Never Attended School*	**Masih Sekolah/***Attending School*					**Tidak Bersekolah Lagi** *Not Attending School Anymore*	**Jumlah** *Total*
		**SD/MI/Paket A** *Primary School*	**SMP/MTs/Paket B** *Junior High School*	**SMA/SMK/MA/Paket C** *Senior High School*	**Diploma I s.d Universitas** *Dipl I to University*	**Jumlah yang Masih Sekolah** *Total Attending School*		
(1)	(2)	(3)	(4)	(5)	(6)	(7)	(8)	(9)
Aceh	3,89	14,74	5,52	5,68	7,56	33,50	62,61	100,00
Sumatera Utara	3,61	14,35	5,78	6,17	3,91	30,21	66,18	100,00
Sumatera Barat	4,69	13,58	5,85	5,36	6,81	31,60	63,71	100,00
Riau	4,48	13,58	5,81	5,63	4,83	29,85	65,67	100,00
Jambi	3,48	12,54	5,20	6,02	5,45	29,21	67,31	100,00
Sumatera Selatan	3,32	13,60	5,54	5,15	4,43	28,72	67,96	100,00
Bengkulu	3,90	13,48	5,67	5,69	5,95	30,79	65,31	100,00
Lampung	4,70	12,90	5,49	5,90	3,98	28,27	67,04	100,00
Kepulauan Bangka Belitung	4,61	12,24	5,13	4,98	1,32	23,67	71,72	100,00
Kepulauan Riau	5,01	14,25	5,54	4,40	2,42	26,61	68,38	100,00
DKI Jakarta	4,39	11,49	4,17	3,38	2,96	22,00	73,60	100,00
Jawa Barat	4,93	12,35	5,16	4,61	2,82	24,94	70,13	100,00
Jawa Tengah	6,02	11,18	4,87	5,05	2,64	23,74	70,24	100,00
DI Yogyakarta	5,71	9,87	3,84	4,35	8,27	26,33	67,97	100,00
Jawa Timur	6,00	10,33	4,74	4,56	3,62	23,25	70,75	100,00
Banten	5,24	12,78	4,87	4,18	4,00	25,83	68,93	100,00
Bali	6,40	10,40	4,82	4,46	3,89	23,57	70,03	100,00
Nusa Tenggara Barat	9,69	13,59	5,78	6,28	3,76	29,41	60,90	100,00
Nusa Tenggara Timur	4,99	14,74	6,79	7,83	6,93	36,29	58,72	100,00
Kalimantan Barat	6,62	13,51	5,27	5,68	4,48	28,94	64,45	100,00
Kalimantan Tengah	4,33	13,28	5,37	4,85	3,44	26,94	68,73	100,00
Kalimantan Selatan	5,38	12,60	4,85	5,04	3,38	25,87	68,75	100,00
Kalimantan Timur	4,65	12,72	4,80	5,54	3,93	26,99	68,36	100,00
Kalimantan Utara	6,01	12,76	5,68	6,30	2,35	27,09	66,90	100,00
Sulawesi Utara	2,69	10,92	5,18	4,83	4,35	25,28	72,03	100,00
Sulawesi Tengah	3,88	11,48	5,58	6,90	7,58	31,54	64,58	100,00
Sulawesi Selatan	5,17	13,46	5,43	5,28	6,31	30,48	64,35	100,00
Sulawesi Tenggara	5,56	14,35	6,35	6,60	8,21	35,51	58,93	100,00
Gorontalo	4,12	12,30	5,03	4,51	5,84	27,68	68,20	100,00
Sulawesi Barat	5,43	15,23	5,15	6,31	4,94	31,63	62,94	100,00
Maluku	3,57	14,35	6,71	6,73	7,28	35,07	61,36	100,00
Maluku Utara	3,56	13,56	6,10	6,41	8,25	34,32	62,12	100,00
Papua Barat	4,31	13,39	5,94	6,69	4,41	30,43	65,26	100,00
Papua	4,80	12,90	6,22	5,94	3,42	28,48	66,72	100,00
**Indonesia**	**5,16**	**12,15**	**5,07**	**4,88**	**3,73**	**25,83**	**69,01**	**100,00**

Sumber/*Source*: Susenas Maret 2018/*The March 2018 Susenas*

https://www.bps.go.id

Tabel 3.3.2. **Persentase Penduduk Laki-Laki dan Perempuan Berumur 5 Tahun ke Atas di Daerah Perdesaan menurut Provinsi dan Status Pendidikan, 2018**

Table 3.3.2. *Percentage of Male and Female Population Aged 5 Years and Over in Rural Area by Province and Education Status, 2018*

**Provinsi** *Province*	**Tidak/Belum Pernah Sekolah** *No Schooling/Never Attended School*	**Masih Sekolah/***Attending School*					**Tidak Bersekolah Lagi** *Not Attending School Anymore*	**Jumlah** *Total*
		**SD/MI/Paket A** *Primary School*	**SMP/MTs/Paket B** *Junior High School*	**SMA/SMK/MA/Paket C** *Senior High School*	**Diploma I s.d Universitas** *Dipl I to University*	**Jumlah yang Masih Sekolah** *Total Attending School*		
(1)	(2)	(3)	(4)	(5)	(6)	(7)	(8)	(9)
Aceh	4,96	15,93	6,10	5,03	2,62	29,68	65,36	100,00
Sumatera Utara	5,69	17,36	6,51	5,76	1,58	31,21	63,10	100,00
Sumatera Barat	5,68	15,56	5,75	4,84	1,33	27,49	66,83	100,00
Riau	5,72	15,28	6,01	4,48	1,91	27,68	66,60	100,00
Jambi	6,17	14,03	5,36	3,91	1,62	24,92	68,91	100,00
Sumatera Selatan	5,07	14,58	5,39	3,97	0,99	24,92	70,01	100,00
Bengkulu	5,83	14,28	5,40	4,25	2,04	25,97	68,20	100,00
Lampung	5,59	14,01	5,32	4,15	0,85	24,33	70,08	100,00
Kepulauan Bangka Belitung	7,15	13,37	4,98	4,15	0,90	23,40	69,45	100,00
Kepulauan Riau	10,81	14,59	6,37	4,90	0,53	26,39	62,80	100,00
DKI Jakarta	-	-	-	-	-	-	-	-
Jawa Barat	6,89	12,46	5,21	3,51	0,75	21,93	71,18	100,00
Jawa Tengah	8,42	11,63	4,73	4,30	0,94	21,60	69,98	100,00
DI Yogyakarta	12,19	10,20	4,31	3,71	1,21	19,43	68,38	100,00
Jawa Timur	11,61	10,61	4,62	3,61	1,17	20,01	68,38	100,00
Banten	6,82	15,35	5,31	3,48	0,77	24,91	68,27	100,00
Bali	12,24	10,75	5,40	4,65	1,17	21,97	65,79	100,00
Nusa Tenggara Barat	13,61	14,46	5,84	5,24	1,69	27,23	59,16	100,00
Nusa Tenggara Timur	9,35	19,57	7,03	4,38	1,45	32,43	58,22	100,00
Kalimantan Barat	10,02	15,33	5,09	4,32	0,80	25,54	64,44	100,00
Kalimantan Tengah	5,31	13,19	6,08	3,90	1,49	24,66	70,03	100,00
Kalimantan Selatan	5,97	13,97	5,19	3,89	1,21	24,26	69,77	100,00
Kalimantan Timur	7,04	13,52	5,68	5,24	1,63	26,07	66,89	100,00
Kalimantan Utara	7,85	13,24	7,21	4,71	1,23	26,39	65,76	100,00
Sulawesi Utara	3,47	12,67	5,24	3,93	1,60	23,44	73,09	100,00
Sulawesi Tengah	5,72	13,66	6,24	3,87	1,80	25,57	68,71	100,00
Sulawesi Selatan	10,17	14,14	5,45	4,76	1,97	26,32	63,50	100,00
Sulawesi Tenggara	7,13	17,11	6,04	4,80	1,85	29,80	63,06	100,00
Gorontalo	4,41	13,64	5,29	4,70	2,17	25,80	69,79	100,00
Sulawesi Barat	9,05	14,78	5,93	5,08	2,01	27,80	63,15	100,00
Maluku	4,36	18,25	7,08	5,27	2,69	33,29	62,35	100,00
Maluku Utara	4,76	17,89	7,07	5,62	2,10	32,68	62,56	100,00
Papua Barat	7,55	16,70	6,16	4,56	2,63	30,05	62,40	100,00
Papua	37,39	14,19	5,40	2,93	1,03	23,55	39,06	100,00
**Indonesia**	**8,57**	**13,60**	**5,38**	**4,21**	**1,28**	**24,47**	**66,96**	**100,00**

Sumber/*Source*: Susenas Maret 2018/*The March 2018 Susenas*

https://www.bps.go.id

Tabel 3.3.3. **Persentase Penduduk Laki-Laki dan Perempuan Berumur 5 Tahun ke Atas di Daerah Perkotaan dan Perdesaan menurut Provinsi dan Status Pendidikan, 2018**
Table *Percentage of Male and Female Population Aged 5 Years and Over in Urban and Rural Area by Province and Education Status, 2018*

**Provinsi** *Province*	**Tidak/Belum Pernah Sekolah** *No Schooling/Never Attended School*	**Masih Sekolah/***Attending School*					**Tidak Bersekolah Lagi** *Not Attending School Anymore*	**Jumlah** *Total*
		**SD/MI/Paket A** *Primary School*	**SMP/MTs/Paket B** *Junior High School*	**SMA/SMK/MA/Paket C** *Senior High School*	**Diploma I s.d Universitas** *Dipl I to University*	**Jumlah yang Masih Sekolah** *Total Attending School*		
(1)	(2)	(3)	(4)	(5)	(6)	(7)	(8)	(9)
Aceh	4,62	15,56	5,92	5,24	4,17	30,89	64,49	100,00
Sumatera Utara	4,58	15,76	6,12	5,98	2,82	30,68	64,74	100,00
Sumatera Barat	5,24	14,69	5,80	5,07	3,75	29,31	65,45	100,00
Riau	5,22	14,59	5,93	4,94	3,09	28,55	66,22	100,00
Jambi	5,31	13,55	5,31	4,59	2,85	26,30	68,39	100,00
Sumatera Selatan	4,42	14,21	5,44	4,42	2,27	26,34	69,24	100,00
Bengkulu	5,20	14,02	5,49	4,72	3,32	27,55	67,25	100,00
Lampung	5,32	13,68	5,37	4,67	1,78	25,50	69,18	100,00
Kepulauan Bangka Belitung	5,78	12,76	5,06	4,59	1,13	23,54	70,67	100,00
Kepulauan Riau	5,85	14,30	5,66	4,47	2,15	26,58	67,57	100,00
DKI Jakarta	4,39	11,49	4,17	3,38	2,96	22,00	73,60	100,00
Jawa Barat	5,44	12,38	5,17	4,32	2,29	24,16	70,40	100,00
Jawa Tengah	7,19	11,40	4,80	4,68	1,81	22,69	70,12	100,00
DI Yogyakarta	7,47	9,96	3,96	4,18	6,35	24,45	68,08	100,00
Jawa Timur	8,67	10,47	4,68	4,11	2,45	21,71	69,62	100,00
Banten	5,69	13,52	5,00	3,98	3,07	25,57	68,74	100,00
Bali	8,38	10,52	5,01	4,52	2,97	23,02	68,60	100,00
Nusa Tenggara Barat	11,78	14,05	5,81	5,73	2,66	28,25	59,97	100,00
Nusa Tenggara Timur	8,35	18,46	6,97	5,18	2,71	33,32	58,33	100,00
Kalimantan Barat	8,87	14,71	5,15	4,78	2,05	26,69	64,44	100,00
Kalimantan Tengah	4,93	13,23	5,80	4,27	2,24	25,54	69,53	100,00
Kalimantan Selatan	5,69	13,33	5,03	4,43	2,22	25,01	69,30	100,00
Kalimantan Timur	5,44	12,99	5,09	5,44	3,16	26,68	67,88	100,00
Kalimantan Utara	6,78	12,96	6,32	5,63	1,88	26,79	66,42	100,00
Sulawesi Utara	3,08	11,79	5,20	4,39	2,98	24,36	72,56	100,00
Sulawesi Tengah	5,20	13,05	6,06	4,72	3,41	27,24	67,55	100,00
Sulawesi Selatan	8,08	13,86	5,44	4,97	3,78	28,05	63,86	100,00
Sulawesi Tenggara	6,53	16,05	6,16	5,50	4,30	32,01	61,47	100,00
Gorontalo	4,30	13,12	5,19	4,62	3,60	26,53	69,17	100,00
Sulawesi Barat	8,20	14,89	5,75	5,37	2,71	28,72	63,10	100,00
Maluku	4,02	16,60	6,92	5,89	4,63	34,04	61,93	100,00
Maluku Utara	4,42	16,64	6,79	5,85	3,87	33,15	62,44	100,00
Papua Barat	6,23	15,35	6,07	5,43	3,35	30,20	63,57	100,00
Papua	28,37	13,84	5,62	3,76	1,69	24,91	46,72	100,00
**Indonesia**	**6,70**	**12,81**	**5,21**	**4,58**	**2,62**	**25,22**	**68,08**	**100,00**

Sumber/*Source*: Susenas Maret 2018/*The March 2018 Susenas*

https://www.bps.go.id

Tabel *Table* **3.3.4.** **Persentase Penduduk Laki-Laki Berumur 5 Tahun ke Atas di Daerah Perkotaan menurut Provinsi dan Status Pendidikan, 2018**
Percentage of Male Population Aged 5 Years and Over in Urban Area by Province and Education Status, 2018

**Provinsi** *Province*	**Tidak/Belum Pernah Sekolah** *No Schooling/Never Attended School*	**Masih Sekolah/***Attending School*					**Tidak Bersekolah Lagi** *Not Attending School Anymore*	**Jumlah** *Total*
		**SD/MI/Paket A** *Primary School*	**SMP/MTs/Paket B** *Junior High School*	**SMA/SMK/MA/Paket C** *Senior High School*	**Diploma I s.d Universitas** *Dipl I to University*	**Jumlah yang Masih Sekolah** *Total Attending School*		
(1)	(2)	(3)	(4)	(5)	(6)	(7)	(8)	(9)
Aceh	3,74	14,87	5,79	5,73	7,10	33,49	62,77	100,00
Sumatera Utara	3,45	14,72	6,04	5,82	3,61	30,19	66,36	100,00
Sumatera Barat	4,48	14,21	5,62	4,99	6,51	31,33	64,19	100,00
Riau	4,35	13,57	5,62	5,18	5,39	29,76	65,89	100,00
Jambi	2,75	12,35	5,50	5,77	4,77	28,39	68,86	100,00
Sumatera Selatan	2,85	14,11	5,76	5,02	3,84	28,73	68,42	100,00
Bengkulu	3,90	13,24	5,99	5,84	5,39	30,46	65,64	100,00
Lampung	3,79	13,40	5,51	5,49	3,69	28,09	68,12	100,00
Kepulauan Bangka Belitung	4,56	12,06	5,05	4,61	1,15	22,87	72,57	100,00
Kepulauan Riau	4,16	13,70	5,57	4,49	2,65	26,41	69,43	100,00
DKI Jakarta	4,18	11,75	4,43	3,29	2,90	22,37	73,45	100,00
Jawa Barat	4,38	12,37	5,27	4,66	2,70	25,00	70,62	100,00
Jawa Tengah	4,73	11,65	4,98	5,33	2,64	24,60	70,67	100,00
DI Yogyakarta	4,05	10,59	4,27	4,38	8,00	27,24	68,71	100,00
Jawa Timur	4,69	10,72	4,90	4,70	3,44	23,76	71,55	100,00
Banten	4,72	12,84	4,76	4,26	3,76	25,62	69,66	100,00
Bali	4,59	10,72	5,12	4,30	3,67	23,81	71,60	100,00
Nusa Tenggara Barat	7,02	14,77	5,98	6,68	3,49	30,92	62,06	100,00
Nusa Tenggara Timur	5,33	15,26	6,72	7,85	6,84	36,67	58,00	100,00
Kalimantan Barat	5,10	14,14	4,82	6,00	4,76	29,72	65,18	100,00
Kalimantan Tengah	3,68	13,05	5,26	4,56	3,49	26,36	69,96	100,00
Kalimantan Selatan	5,16	12,40	5,31	5,27	3,61	26,59	68,25	100,00
Kalimantan Timur	4,15	12,32	4,89	5,66	3,97	26,84	69,01	100,00
Kalimantan Utara	4,69	12,35	5,59	6,37	1,55	25,86	69,45	100,00
Sulawesi Utara	2,94	10,76	4,91	4,79	4,13	24,59	72,47	100,00
Sulawesi Tengah	3,86	11,50	5,02	6,37	6,76	29,65	66,49	100,00
Sulawesi Selatan	4,79	14,16	5,57	5,04	6,21	30,98	64,23	100,00
Sulawesi Tenggara	4,87	15,44	6,53	6,97	7,59	36,53	58,60	100,00
Gorontalo	4,31	12,66	5,16	4,50	5,27	27,59	68,10	100,00
Sulawesi Barat	5,18	15,97	5,07	5,70	4,67	31,41	63,41	100,00
Maluku	3,75	14,48	6,80	6,81	6,46	34,55	61,70	100,00
Maluku Utara	3,73	13,29	6,57	6,34	8,33	34,53	61,74	100,00
Papua Barat	3,48	13,20	6,05	6,75	3,49	29,49	67,03	100,00
Papua	4,13	12,19	6,39	5,87	3,15	27,60	68,27	100,00
**Indonesia**	**4,41**	**12,39**	**5,19**	**4,90**	**3,58**	**26,06**	**69,53**	**100,00**

Sumber/*Source*: Susenas Maret 2018/*The March 2018 Susenas*

https://www.bps.go.id

Tabel *Table* **3.3.5.** **Persentase Penduduk Laki-Laki Berumur 5 Tahun ke Atas di Daerah Perdesaan menurut Provinsi dan Status Pendidikan, 2018**
Percentage of Male Population Aged 5 Years and Over in Rural Area by Province and Education Status, 2018

**Provinsi** *Province*	**Tidak/Belum Pernah Sekolah** *No Schooling/Never Attended School*	**Masih Sekolah/***Attending School*					**Tidak Bersekolah Lagi** *Not Attending School Anymore*	**Jumlah** *Total*
		**SD/MI/Paket A** *Primary School*	**SMP/MTs/Paket B** *Junior High School*	**SMA/SMK/MA/Paket C** *Senior High School*	**Diploma I s.d Universitas** *Dipl I to University*	**Jumlah yang Masih Sekolah** *Total Attending School*		
**(1)**	**(2)**	**(3)**	**(4)**	**(5)**	**(6)**	**(7)**	**(8)**	**(9)**
Aceh	4,24	16,33	6,35	5,03	2,42	30,13	65,63	100,00
Sumatera Utara	5,00	17,60	6,91	5,62	1,57	31,70	63,30	100,00
Sumatera Barat	5,00	16,16	5,81	4,65	1,19	27,81	67,19	100,00
Riau	5,15	15,11	5,54	4,50	1,76	26,91	67,94	100,00
Jambi	4,79	14,27	5,19	3,65	1,58	24,69	70,52	100,00
Sumatera Selatan	4,42	14,69	5,27	3,95	0,92	24,83	70,75	100,00
Bengkulu	4,81	14,60	5,12	4,16	1,64	25,52	69,67	100,00
Lampung	4,78	13,74	5,40	4,07	0,72	23,93	71,29	100,00
Kepulauan Bangka Belitung	5,67	13,17	4,60	4,10	0,70	22,57	71,76	100,00
Kepulauan Riau	9,64	13,55	7,10	5,50	0,45	26,60	63,76	100,00
DKI Jakarta	-	-	-	-	-	-	-	-
Jawa Barat	5,82	12,89	4,95	3,64	0,75	22,23	71,95	100,00
Jawa Tengah	6,09	12,12	4,89	4,55	0,83	22,39	71,52	100,00
DI Yogyakarta	8,06	11,18	4,42	4,42	1,16	21,18	70,76	100,00
Jawa Timur	8,60	10,98	5,13	3,80	1,02	20,93	70,47	100,00
Banten	5,27	15,66	5,06	3,88	0,75	25,35	69,38	100,00
Bali	7,98	11,44	5,64	4,77	1,36	23,21	68,81	100,00
Nusa Tenggara Barat	10,07	15,52	6,03	6,10	1,98	29,63	60,30	100,00
Nusa Tenggara Timur	8,24	20,41	7,33	4,26	1,23	33,23	58,53	100,00
Kalimantan Barat	7,69	15,35	5,17	4,03	0,85	25,40	66,91	100,00
Kalimantan Tengah	4,50	13,32	5,56	3,63	1,23	23,74	71,76	100,00
Kalimantan Selatan	5,08	14,48	5,21	3,88	1,20	24,77	70,15	100,00
Kalimantan Timur	6,03	12,97	6,00	4,81	1,67	25,45	68,52	100,00
Kalimantan Utara	6,57	13,82	7,05	4,34	1,09	26,30	67,13	100,00
Sulawesi Utara	3,46	12,79	5,33	3,73	1,32	23,17	73,37	100,00
Sulawesi Tengah	5,21	13,96	5,98	3,86	1,64	25,44	69,35	100,00
Sulawesi Selatan	9,01	15,01	5,55	4,84	1,50	26,90	64,09	100,00
Sulawesi Tenggara	6,09	17,23	6,40	4,61	2,12	30,36	63,55	100,00
Gorontalo	4,42	13,91	5,15	4,73	1,83	25,62	69,96	100,00
Sulawesi Barat	7,78	14,82	5,91	4,94	1,86	27,53	64,69	100,00
Maluku	4,37	18,06	7,31	5,32	2,55	33,24	62,39	100,00
Maluku Utara	4,38	18,52	6,94	5,43	2,05	32,94	62,68	100,00
Papua Barat	6,26	16,36	5,84	4,97	2,66	29,83	63,91	100,00
Papua	32,97	14,32	5,60	3,27	1,18	24,37	42,66	100,00
**Indonesia**	**6,89**	**13,95**	**5,48**	**4,28**	**1,19**	**24,90**	**68,21**	**100,00**

Sumber/*Source*: Susenas Maret 2018/*The March 2018 Susenas*

https://www.bps.go.id

Tabel *Table* **3.3.6.** **Persentase Penduduk Laki-Laki Berumur 5 Tahun ke Atas di Daerah Perkotaan dan Perdesaan menurut Provinsi dan Status Pendidikan, 2018**
Percentage of Male Population Aged 5 Years and Over in Urban and Rural Area by Province and Education Status, 2018

**Provinsi** *Province*	**Tidak/Belum Pernah Sekolah** *No Schooling/Never Attended School*	**Masih Sekolah**/*Attending School*					**Tidak Bersekolah Lagi** *Not Attending School Anymore*	**Jumlah** *Total*
		**SD/MI/Paket A** *Primary School*	**SMP/MTs/Paket B** *Junior High School*	**SMA/SMK/MA/Paket C** *Senior High School*	**Diploma I s.d Universitas** *Dipl I to University*	**Jumlah yang Masih Sekolah** *Total Attending School*		
**(1)**	**(2)**	**(3)**	**(4)**	**(5)**	**(6)**	**(7)**	**(8)**	**(9)**
Aceh	4,09	15,87	6,17	5,25	3,90	31,19	64,72	100,00
Sumatera Utara	4,18	16,07	6,45	5,72	2,66	30,90	64,92	100,00
Sumatera Barat	4,77	15,30	5,73	4,80	3,53	29,36	65,87	100,00
Riau	4,83	14,49	5,57	4,77	3,22	28,05	67,12	100,00
Jambi	4,15	13,66	5,28	4,32	2,59	25,85	70,00	100,00
Sumatera Selatan	3,84	14,48	5,45	4,34	2,00	26,27	69,89	100,00
Bengkulu	4,52	14,16	5,40	4,70	2,85	27,11	68,37	100,00
Lampung	4,49	13,64	5,43	4,48	1,59	25,14	70,37	100,00
Kepulauan Bangka Belitung	5,08	12,58	4,84	4,37	0,94	22,73	72,19	100,00
Kepulauan Riau	4,96	13,68	5,79	4,64	2,33	26,44	68,60	100,00
DKI Jakarta	4,18	11,75	4,43	3,29	2,90	22,37	73,45	100,00
Jawa Barat	4,75	12,50	5,19	4,40	2,19	24,28	70,97	100,00
Jawa Tengah	5,39	11,88	4,94	4,95	1,76	23,53	71,08	100,00
DI Yogyakarta	5,11	10,75	4,31	4,39	6,19	25,64	69,25	100,00
Jawa Timur	6,55	10,85	5,01	4,27	2,29	22,42	71,03	100,00
Banten	4,88	13,66	4,85	4,15	2,89	25,55	69,58	100,00
Bali	5,73	10,96	5,30	4,46	2,89	23,61	70,66	100,00
Nusa Tenggara Barat	8,66	15,17	6,00	6,37	2,68	30,22	61,12	100,00
Nusa Tenggara Timur	7,56	19,20	7,19	5,10	2,55	34,04	58,40	100,00
Kalimantan Barat	6,82	14,95	5,05	4,69	2,15	26,84	66,34	100,00
Kalimantan Tengah	4,19	13,22	5,45	3,98	2,09	24,74	71,07	100,00
Kalimantan Selatan	5,12	13,51	5,25	4,52	2,33	25,61	69,26	100,00
Kalimantan Timur	4,78	12,54	5,26	5,37	3,20	26,37	68,85	100,00
Kalimantan Utara	5,49	12,97	6,22	5,50	1,36	26,05	68,46	100,00
Sulawesi Utara	3,20	11,78	5,12	4,26	2,71	23,87	72,93	100,00
Sulawesi Tengah	4,84	13,29	5,72	4,54	3,04	26,59	68,57	100,00
Sulawesi Selatan	7,24	14,66	5,56	4,93	3,47	28,62	64,14	100,00
Sulawesi Tenggara	5,62	16,54	6,45	5,52	4,23	32,74	61,64	100,00
Gorontalo	4,38	13,43	5,15	4,65	3,14	26,37	69,25	100,00
Sulawesi Barat	7,18	15,09	5,72	5,11	2,51	28,43	64,39	100,00
Maluku	4,11	16,55	7,09	5,95	4,20	33,79	62,10	100,00
Maluku Utara	4,19	17,03	6,84	5,69	3,84	33,40	62,41	100,00
Papua Barat	5,13	15,08	5,92	5,69	3,00	29,69	65,18	100,00
Papua	24,83	13,72	5,83	4,00	1,73	25,28	49,89	100,00
**Indonesia**	**5,53**	**13,10**	**5,32**	**4,62**	**2,50**	**25,54**	**68,93**	**100,00**

Sumber/*Source*: Susenas Maret 2018/*The March 2018 Susenas*

https://www.bps.go.id

Tabel 3.3.7. **Persentase Penduduk Perempuan Berumur 5 Tahun ke Atas di Daerah Perkotaan menurut Provinsi dan Status Pendidikan, 2018**
Table *Percentage of Female Population Aged 5 Years and Over in Urban Area by Province and Education Status, 2018*

**Provinsi** *Province*	**Tidak/Belum Pernah Sekolah** *No Schooling/Never Attended School*	**Masih Sekolah**/*Attending School*					**Tidak Bersekolah Lagi** *Not Attending School Anymore*	**Jumlah** *Total*
		**SD/MI/Paket A** *Primary School*	**SMP/MTs/Paket B** *Junior High School*	**SMA/SMK/MA/Paket C** *Senior High School*	**Diploma I s.d Universitas** *Dipl I to University*	**Jumlah yang Masih Sekolah** *Total Attending School*		
(1)	(2)	(3)	(4)	(5)	(6)	(7)	(8)	(9)
Aceh	4,05	14,60	5,25	5,64	8,01	33,50	62,45	100,00
Sumatera Utara	3,76	13,98	5,53	6,51	4,21	30,23	66,01	100,00
Sumatera Barat	4,90	12,96	6,07	5,73	7,10	31,86	63,24	100,00
Riau	4,62	13,59	6,02	6,09	4,25	29,95	65,43	100,00
Jambi	4,23	12,73	4,90	6,29	6,13	30,05	65,72	100,00
Sumatera Selatan	3,79	13,09	5,33	5,28	5,01	28,71	67,50	100,00
Bengkulu	3,89	13,73	5,36	5,53	6,51	31,13	64,98	100,00
Lampung	5,62	12,38	5,46	6,32	4,29	28,45	65,93	100,00
Kepulauan Bangka Belitung	4,66	12,44	5,22	5,37	1,51	24,54	70,80	100,00
Kepulauan Riau	5,87	14,81	5,52	4,30	2,19	26,82	67,31	100,00
DKI Jakarta	4,61	11,24	3,91	3,47	3,01	21,63	73,76	100,00
Jawa Barat	5,50	12,33	5,03	4,55	2,96	24,87	69,63	100,00
Jawa Tengah	7,28	10,72	4,77	4,77	2,64	22,90	69,82	100,00
DI Yogyakarta	7,34	9,16	3,40	4,33	8,53	25,42	67,24	100,00
Jawa Timur	7,27	9,95	4,58	4,43	3,80	22,76	69,97	100,00
Banten	5,78	12,71	4,99	4,10	4,25	26,05	68,17	100,00
Bali	8,25	10,08	4,51	4,61	4,11	23,31	68,44	100,00
Nusa Tenggara Barat	12,16	12,48	5,60	5,91	4,03	28,02	59,82	100,00
Nusa Tenggara Timur	4,65	14,23	6,86	7,80	7,01	35,90	59,45	100,00
Kalimantan Barat	8,14	12,87	5,72	5,36	4,20	28,15	63,71	100,00
Kalimantan Tengah	5,03	13,53	5,48	5,15	3,38	27,54	67,43	100,00
Kalimantan Selatan	5,60	12,81	4,38	4,80	3,14	25,13	69,27	100,00
Kalimantan Timur	5,19	13,16	4,71	5,41	3,88	27,16	67,65	100,00
Kalimantan Utara	7,48	13,23	5,78	6,21	3,23	28,45	64,07	100,00
Sulawesi Utara	2,43	11,09	5,45	4,88	4,58	26,00	71,57	100,00
Sulawesi Tengah	3,89	11,45	6,14	7,43	8,39	33,41	62,70	100,00
Sulawesi Selatan	5,53	12,79	5,30	5,50	6,41	30,00	64,47	100,00
Sulawesi Tenggara	6,27	13,24	6,17	6,23	8,83	34,47	59,26	100,00
Gorontalo	3,94	11,96	4,90	4,51	6,39	27,76	68,30	100,00
Sulawesi Barat	5,66	14,53	5,23	6,89	5,21	31,86	62,48	100,00
Maluku	3,39	14,22	6,61	6,65	8,11	35,59	61,02	100,00
Maluku Utara	3,38	13,84	5,61	6,48	8,18	34,11	62,51	100,00
Papua Barat	5,23	13,60	5,81	6,61	5,43	31,45	63,32	100,00
Papua	5,58	13,73	6,01	6,03	3,73	29,50	64,92	100,00
**Indonesia**	**5,92**	**11,90**	**4,96**	**4,85**	**3,89**	**25,60**	**68,48**	**100,00**

Sumber/*Source*: Susenas Maret 2018/*The March 2018 Susenas*

https://www.bps.go.id

Tabel **3.3.8.** **Persentase Penduduk Perempuan Berumur 5 Tahun ke Atas di Daerah Perdesaan menurut Provinsi dan Status Pendidikan, 2018**
Table *Percentage of Female Population Aged 5 Years and Over in Rural Area by Province and Education Status, 2018*

**Provinsi** *Province*	**Tidak/Belum Pernah Sekolah** *No Schooling/Never Attended School*	**Masih Sekolah/***Attending School*					**Tidak Bersekolah Lagi** *Not Attending School Anymore*	**Jumlah** *Total*
		**SD/MI/Paket A** *Primary School*	**SMP/MTs/Paket B** *Junior High School*	**SMA/SMK/MA/Paket C** *Senior High School*	**Diploma I s.d Universitas** *Dipl I to University*	**Jumlah yang Masih Sekolah** *Total Attending School*		
**(1)**	**(2)**	**(3)**	**(4)**	**(5)**	**(6)**	**(7)**	**(8)**	**(9)**
Aceh	5,65	15,55	5,85	5,04	2,82	29,26	65,09	100,00
Sumatera Utara	6,37	17,12	6,11	5,92	1,58	30,73	62,90	100,00
Sumatera Barat	6,35	14,98	5,70	5,03	1,46	27,17	66,48	100,00
Riau	6,32	15,45	6,52	4,46	2,07	28,50	65,18	100,00
Jambi	7,63	13,77	5,55	4,19	1,65	25,16	67,21	100,00
Sumatera Selatan	5,75	14,46	5,51	4,01	1,05	25,03	69,22	100,00
Bengkulu	6,92	13,94	5,70	4,35	2,46	26,45	66,63	100,00
Lampung	6,45	14,30	5,25	4,24	0,98	24,77	68,78	100,00
Kepulauan Bangka Belitung	8,78	13,60	5,39	4,20	1,13	24,32	66,90	100,00
Kepulauan Riau	12,05	15,69	5,59	4,28	0,61	26,17	61,78	100,00
DKI Jakarta	-	-	-	-	-	-	-	-
Jawa Barat	7,99	12,01	5,47	3,39	0,75	21,62	70,39	100,00
Jawa Tengah	10,70	11,16	4,57	4,05	1,04	20,82	68,48	100,00
DI Yogyakarta	16,01	9,29	4,21	3,05	1,26	17,81	66,18	100,00
Jawa Timur	14,50	10,26	4,13	3,43	1,32	19,14	66,36	100,00
Banten	8,45	15,03	5,57	3,04	0,80	24,44	67,11	100,00
Bali	16,48	10,07	5,15	4,53	0,98	20,73	62,79	100,00
Nusa Tenggara Barat	16,93	13,46	5,66	4,43	1,42	24,97	58,10	100,00
Nusa Tenggara Timur	10,43	18,76	6,74	4,50	1,65	31,65	57,92	100,00
Kalimantan Barat	12,48	15,31	5,00	4,61	0,76	25,68	61,84	100,00
Kalimantan Tengah	6,22	13,05	6,66	4,22	1,76	25,69	68,09	100,00
Kalimantan Selatan	6,87	13,44	5,17	3,91	1,23	23,75	69,38	100,00
Kalimantan Timur	8,20	14,15	5,31	5,74	1,57	26,77	65,03	100,00
Kalimantan Utara	9,34	12,57	7,40	5,15	1,40	26,52	64,14	100,00
Sulawesi Utara	3,48	12,55	5,13	4,15	1,90	23,73	72,79	100,00
Sulawesi Tengah	6,26	13,34	6,52	3,89	1,96	25,71	68,03	100,00
Sulawesi Selatan	11,27	13,32	5,36	4,68	2,41	25,77	62,96	100,00
Sulawesi Tenggara	8,19	17,00	5,68	4,99	1,57	29,24	62,57	100,00
Gorontalo	4,39	13,38	5,44	4,65	2,53	26,00	69,61	100,00
Sulawesi Barat	10,34	14,73	5,95	5,22	2,17	28,07	61,59	100,00
Maluku	4,34	18,46	6,85	5,21	2,83	33,35	62,31	100,00
Maluku Utara	5,17	17,23	7,20	5,82	2,14	32,39	62,44	100,00
Papua Barat	9,01	17,08	6,52	4,11	2,59	30,30	60,69	100,00
Papua	42,25	14,05	5,17	2,56	0,87	22,65	35,10	100,00
**Indonesia**	**10,26**	**13,25**	**5,27**	**4,14**	**1,38**	**24,04**	**65,70**	**100,00**

Sumber/*Source*: Susenas Maret 2018/*The March 2018 Susenas*

https://www.bps.go.id

Tabel *Table* **3.3.9.** **Persentase Penduduk Perempuan Berumur 5 Tahun ke Atas di Daerah Perkotaan dan Perdesaan menurut Provinsi dan Status Pendidikan, 2018**
Percentage of Female Population Aged 5 Years and Over in Urban and Rural Area by Province and Education Status, 2018

**Provinsi** *Province*	**Tidak/Belum Pernah Sekolah** *No Schooling/Never Attended School*	**Masih Sekolah/***Attending School*					**Tidak Bersekolah Lagi** *Not Attending School Anymore*	**Jumlah** *Total*
		**SD/MI/Paket A** *Primary School*	**SMP/MTs/Paket B** *Junior High School*	**SMA/SMK/MA/Paket C** *Senior High School*	**Diploma I s.d Universitas** *Dipl I to University*	**Jumlah yang Masih Sekolah** *Total Attending School*		
(1)	(2)	(3)	(4)	(5)	(6)	(7)	(8)	(9)
Aceh	5,15	15,25	5,66	5,23	4,44	30,58	64,27	100,00
Sumatera Utara	4,98	15,45	5,80	6,23	2,98	30,46	64,56	100,00
Sumatera Barat	5,71	14,08	5,87	5,34	3,96	29,25	65,04	100,00
Riau	5,63	14,69	6,32	5,12	2,96	29,09	65,28	100,00
Jambi	6,53	13,43	5,34	4,87	3,11	26,75	66,72	100,00
Sumatera Selatan	5,01	13,94	5,44	4,49	2,55	26,42	68,57	100,00
Bengkulu	5,91	13,86	5,59	4,74	3,82	28,01	66,08	100,00
Lampung	6,20	13,72	5,31	4,86	1,98	25,87	67,93	100,00
Kepulauan Bangka Belitung	6,55	12,97	5,30	4,83	1,34	24,44	69,01	100,00
Kepulauan Riau	6,75	14,94	5,53	4,30	1,96	26,73	66,52	100,00
DKI Jakarta	4,61	11,24	3,91	3,47	3,01	21,63	73,76	100,00
Jawa Barat	6,15	12,25	5,14	4,25	2,38	24,02	69,83	100,00
Jawa Tengah	8,95	10,93	4,67	4,42	1,86	21,88	69,17	100,00
DI Yogyakarta	9,75	9,19	3,63	3,98	6,51	23,31	66,94	100,00
Jawa Timur	10,73	10,10	4,37	3,95	2,61	21,03	68,24	100,00
Banten	6,54	13,38	5,15	3,80	3,26	25,59	67,87	100,00
Bali	11,06	10,08	4,73	4,58	3,04	22,43	66,51	100,00
Nusa Tenggara Barat	14,71	13,00	5,63	5,12	2,64	26,39	58,90	100,00
Nusa Tenggara Timur	9,11	17,73	6,76	5,26	2,87	32,62	58,27	100,00
Kalimantan Barat	10,99	14,47	5,25	4,87	1,94	26,53	62,48	100,00
Kalimantan Tengah	5,75	13,24	6,20	4,58	2,40	26,42	67,83	100,00
Kalimantan Selatan	6,28	13,14	4,80	4,33	2,12	24,39	69,33	100,00
Kalimantan Timur	6,17	13,48	4,90	5,52	3,13	27,03	66,80	100,00
Kalimantan Utara	8,25	12,96	6,45	5,77	2,47	27,65	64,10	100,00
Sulawesi Utara	2,95	11,81	5,29	4,52	3,26	24,88	72,17	100,00
Sulawesi Tengah	5,58	12,80	6,41	4,90	3,81	27,92	66,50	100,00
Sulawesi Selatan	8,89	13,10	5,34	5,02	4,07	27,53	63,58	100,00
Sulawesi Tenggara	7,45	15,55	5,87	5,47	4,36	31,25	61,30	100,00
Gorontalo	4,21	12,82	5,22	4,60	4,06	26,70	69,09	100,00
Sulawesi Barat	9,22	14,68	5,77	5,62	2,90	28,97	61,80	100,00
Maluku	3,94	16,65	6,75	5,83	5,07	34,30	61,76	100,00
Maluku Utara	4,65	16,24	6,74	6,01	3,90	32,89	62,46	100,00
Papua Barat	7,46	15,65	6,23	5,14	3,75	30,77	61,77	100,00
Papua	32,32	13,96	5,40	3,50	1,65	24,51	43,17	100,00
**Indonesia**	**7,88**	**12,51**	**5,10**	**4,53**	**2,75**	**24,89**	**67,23**	**100,00**

Sumber/*Source*: Susenas Maret 2018/*The March 2018 Susenas*

https://www.bps.go.id

Tabel 3.4.1. **Persentase Penduduk Laki-Laki dan Perempuan Berumur 7-24 Tahun di Daerah Perkotaan menurut Provinsi dan Status Pendidikan, 2018**

Table 3.4.1. *Percentage of Male and Female Population Aged 7-24 Years in Urban Area by Province and Education Status, 2018*

**Provinsi** *Province*	**Tidak/Belum Pernah Sekolah** *No Schooling/Never Attended School*	**Masih Sekolah**/*Attending School*					**Tidak Bersekolah Lagi** *Not Attending School Anymore*	**Jumlah** *Total*
		**SD/MI/Paket A** *Primary School*	**SMP/MTs/Paket B** *Junior High School*	**SMA/SMK/MA/Paket C** *Senior High School*	**Diploma I s.d Universitas** *Dipl I to University*	**Jumlah yang Masih Sekolah** *Total Attending School*		
(1)	(2)	(3)	(4)	(5)	(6)	(7)	(8)	(9)
Aceh	0,10	35,31	14,81	15,20	16,11	81,43	18,47	100,00
Sumatera Utara	0,38	34,02	15,27	15,52	8,33	73,14	26,48	100,00
Sumatera Barat	0,30	35,82	16,17	14,84	14,83	81,66	18,04	100,00
Riau	0,29	34,34	15,92	15,41	10,96	76,63	23,08	100,00
Jambi	0,21	33,34	15,25	17,67	10,35	76,61	23,19	100,00
Sumatera Selatan	0,28	34,17	15,68	14,59	8,78	73,22	26,50	100,00
Bengkulu	0,04	35,53	16,39	16,44	12,04	80,40	19,56	100,00
Lampung	0,50	33,68	15,58	16,44	9,01	74,71	24,79	100,00
Kepulauan Bangka Belitung	0,24	34,26	15,79	15,32	3,58	68,95	30,81	100,00
Kepulauan Riau	0,30	39,04	16,21	12,85	5,56	73,66	26,04	100,00
DKI Jakarta	0,24	37,21	14,12	11,46	7,73	70,52	29,24	100,00
Jawa Barat	0,19	33,88	15,00	13,37	7,29	69,54	30,27	100,00
Jawa Tengah	0,28	32,83	15,37	15,89	6,82	70,91	28,80	100,00
DI Yogyakarta	0,04	31,98	12,69	14,42	22,69	81,78	18,18	100,00
Jawa Timur	0,29	32,79	15,74	15,13	8,51	72,17	27,55	100,00
Banten	0,16	34,97	14,39	12,27	8,28	69,91	29,93	100,00
Bali	0,20	31,98	15,61	14,44	11,04	73,07	26,73	100,00
Nusa Tenggara Barat	0,33	34,35	15,69	17,01	8,18	75,23	24,44	100,00
Nusa Tenggara Timur	0,34	32,23	16,34	18,85	12,96	80,38	19,28	100,00
Kalimantan Barat	0,36	34,44	14,81	15,62	10,37	75,24	24,40	100,00
Kalimantan Tengah	0,23	35,45	15,22	13,60	7,71	71,98	27,79	100,00
Kalimantan Selatan	1,05	34,72	14,24	14,71	9,29	72,96	25,98	100,00
Kalimantan Timur	0,24	35,44	14,30	16,36	9,71	75,81	23,95	100,00
Kalimantan Utara	1,04	33,99	15,94	17,67	4,96	72,56	26,40	100,00
Sulawesi Utara	0,34	29,27	15,81	14,76	10,37	70,21	29,45	100,00
Sulawesi Tengah	0,56	29,69	15,69	19,07	14,03	78,48	20,97	100,00
Sulawesi Selatan	0,34	33,74	14,75	14,34	13,98	76,81	22,85	100,00
Sulawesi Tenggara	0,53	31,49	15,86	16,49	15,38	79,22	20,25	100,00
Gorontalo	0,58	31,93	14,05	12,60	14,05	72,63	26,79	100,00
Sulawesi Barat	1,09	36,24	13,30	16,28	8,23	74,05	24,86	100,00
Maluku	0,20	31,81	16,85	16,93	15,28	80,87	18,93	100,00
Maluku Utara	0,47	30,36	15,54	16,35	14,95	77,20	22,33	100,00
Papua Barat	0,91	33,43	15,96	17,98	9,60	76,97	22,12	100,00
Papua	1,86	31,84	15,86	15,70	8,07	71,47	26,67	100,00
**Indonesia**	**0,29**	**33,89**	**15,15**	**14,49**	**8,87**	**72,40**	**27,31**	**100,00**

Sumber/*Source*: Susenas Maret 2018/*The March 2018 Susenas*

https://www.bps.go.id

Tabel **3.4.2.** **Persentase Penduduk Laki-Laki dan Perempuan Berumur 7-24 Tahun di Daerah Perdesaan menurut Provinsi dan Status Pendidikan, 2018**
Table *Percentage of Male and Female Population Aged 7-24 Years in Rural Area by Province and Education Status, 2018*

**Provinsi** *Province*	**Tidak/Belum Pernah Sekolah** *No Schooling/Never Attended School*	**Masih Sekolah/***Attending School*					**Tidak Bersekolah Lagi** *Not Attending School Anymore*	**Jumlah** *Total*
		**SD/MI/Paket A** *Primary School*	**SMP/MTs/Paket B** *Junior High School*	**SMA/SMK/MA/Paket C** *Senior High School*	**Diploma I s.d Universitas** *Dipl I to University*	**Jumlah yang Masih Sekolah** *Total Attending School*		
(1)	(2)	(3)	(4)	(5)	(6)	(7)	(8)	(9)
Aceh	0,32	38,16	16,41	13,53	5,71	73,81	25,87	100,00
Sumatera Utara	0,53	40,23	16,58	14,54	3,74	75,09	24,37	100,00
Sumatera Barat	0,55	41,26	16,15	13,58	3,21	74,20	25,25	100,00
Riau	0,60	38,64	16,32	12,14	4,43	71,53	27,88	100,00
Jambi	0,31	37,36	15,60	11,38	4,09	68,43	31,26	100,00
Sumatera Selatan	0,33	37,40	15,51	11,41	2,24	66,56	33,11	100,00
Bengkulu	0,22	38,79	15,96	12,56	4,71	72,02	27,76	100,00
Lampung	0,29	38,49	15,96	12,42	1,95	68,82	30,89	100,00
Kepulauan Bangka Belitung	0,21	35,84	14,51	12,09	2,45	64,89	34,90	100,00
Kepulauan Riau	0,41	41,44	19,34	14,68	1,45	76,91	22,68	100,00
DKI Jakarta	-	-	-	-	-	-	-	-
Jawa Barat	0,44	35,89	15,83	10,69	2,00	64,41	35,15	100,00
Jawa Tengah	0,42	34,77	15,32	13,93	2,67	66,69	32,88	100,00
DI Yogyakarta	0,23	40,03	17,52	15,00	3,58	76,13	23,64	100,00
Jawa Timur	0,46	34,17	15,83	12,32	3,51	65,83	33,71	100,00
Banten	1,03	38,22	14,66	9,60	1,95	64,43	34,54	100,00
Bali	1,02	35,19	18,45	15,83	3,82	73,29	25,69	100,00
Nusa Tenggara Barat	0,45	36,87	16,25	14,59	3,59	71,30	28,26	100,00
Nusa Tenggara Timur	1,51	43,56	17,11	10,64	2,65	73,96	24,53	100,00
Kalimantan Barat	0,68	38,92	14,22	12,02	1,63	66,79	32,53	100,00
Kalimantan Tengah	0,73	35,36	17,77	11,43	3,68	68,24	31,03	100,00
Kalimantan Selatan	0,40	38,40	15,23	11,33	3,13	68,09	31,51	100,00
Kalimantan Timur	0,44	36,33	16,40	15,15	3,95	71,83	27,73	100,00
Kalimantan Utara	1,60	34,66	20,21	13,23	2,50	70,60	27,80	100,00
Sulawesi Utara	0,59	36,10	16,52	12,40	4,12	69,14	30,27	100,00
Sulawesi Tengah	0,87	36,77	18,22	11,27	4,21	70,47	28,66	100,00
Sulawesi Selatan	0,80	36,67	15,49	13,51	5,06	70,73	28,47	100,00
Sulawesi Tenggara	0,90	40,14	15,65	12,44	3,51	71,74	27,36	100,00
Gorontalo	0,58	35,81	14,85	12,95	4,98	68,59	30,83	100,00
Sulawesi Barat	0,89	35,65	15,59	13,36	4,34	68,94	30,18	100,00
Maluku	0,24	40,00	17,41	12,95	5,75	76,11	23,65	100,00
Maluku Utara	0,61	39,92	17,80	14,16	4,22	76,10	23,29	100,00
Papua Barat	2,13	40,33	15,98	11,87	5,48	73,66	24,21	100,00
Papua	21,84	35,54	13,43	7,64	2,16	58,77	19,39	100,00
**Indonesia**	**1,03**	**37,13**	**15,90**	**12,43**	**3,22**	**68,68**	**30,29**	**100,00**

Sumber/*Source*: Susenas Maret 2018/*The March 2018 Susenas*

https://www.bps.go.id

Tabel *Table* **3.4.3.** **Persentase Penduduk Laki-Laki dan Perempuan Berumur 7-24 Tahun di Daerah Perkotaan dan Perdesaan menurut Provinsi dan Status Pendidikan, 2018**
Percentage of Male and Female Population Aged 7-24 Years in Urban and Rural Area by Province and Education Status, 2018

**Provinsi** *Province*	**Tidak/Belum Pernah Sekolah** *No Schooling/Never Attended School*	**Masih Sekolah/***Attending School*					**Tidak Bersekolah Lagi** *Not Attending School Anymore*	**Jumlah** *Total*
		**SD/MI/Paket A** *Primary School*	**SMP/MTs/Paket B** *Junior High School*	**SMA/SMK/MA/Paket C** *Senior High School*	**Diploma I s.d Universitas** *Dipl I to University*	**Jumlah yang Masih Sekolah** *Total Attending School*		
(1)	(2)	(3)	(4)	(5)	(6)	(7)	(8)	(9)
Aceh	0,25	37,26	15,91	14,06	8,98	76,21	23,54	100,00
Sumatera Utara	0,45	36,99	15,90	15,05	6,14	74,08	25,47	100,00
Sumatera Barat	0,44	38,83	16,16	14,14	8,39	77,52	22,04	100,00
Riau	0,47	36,91	16,16	13,46	7,05	73,58	25,95	100,00
Jambi	0,28	36,08	15,49	13,38	6,09	71,04	28,69	100,00
Sumatera Selatan	0,31	36,18	15,58	12,61	4,70	69,07	30,62	100,00
Bengkulu	0,16	37,71	16,10	13,85	7,15	74,81	25,03	100,00
Lampung	0,36	37,01	15,84	13,66	4,12	70,63	29,01	100,00
Kepulauan Bangka Belitung	0,23	35,01	15,18	13,78	3,05	67,02	32,76	100,00
Kepulauan Riau	0,32	39,37	16,64	13,10	4,99	74,10	25,58	100,00
DKI Jakarta	0,24	37,21	14,12	11,45	7,73	70,51	29,25	100,00
Jawa Barat	0,25	34,39	15,20	12,70	5,96	68,25	31,50	100,00
Jawa Tengah	0,35	33,76	15,35	14,95	4,83	68,89	30,76	100,00
DI Yogyakarta	0,09	33,85	13,82	14,55	18,24	80,46	19,45	100,00
Jawa Timur	0,37	33,43	15,78	13,81	6,17	69,19	30,44	100,00
Banten	0,42	35,95	14,48	11,46	6,37	68,26	31,32	100,00
Bali	0,47	33,03	16,54	14,89	8,68	73,14	26,39	100,00
Nusa Tenggara Barat	0,39	35,68	15,98	15,73	5,76	73,15	26,46	100,00
Nusa Tenggara Timur	1,23	40,92	16,93	12,56	5,05	75,46	23,30	100,00
Kalimantan Barat	0,57	37,41	14,42	13,24	4,58	69,65	29,78	100,00
Kalimantan Tengah	0,53	35,40	16,76	12,28	5,27	69,71	29,76	100,00
Kalimantan Selatan	0,71	36,68	14,77	12,91	6,01	70,37	28,93	100,00
Kalimantan Timur	0,31	35,74	15,01	15,95	7,76	74,46	25,23	100,00
Kalimantan Utara	1,27	34,27	17,74	15,80	3,93	71,74	26,99	100,00
Sulawesi Utara	0,46	32,61	16,16	13,61	7,31	69,69	29,85	100,00
Sulawesi Tengah	0,78	34,73	17,49	13,52	7,03	72,77	26,45	100,00
Sulawesi Selatan	0,60	35,41	15,17	13,87	8,88	73,33	26,07	100,00
Sulawesi Tenggara	0,75	36,73	15,74	14,04	8,18	74,69	24,56	100,00
Gorontalo	0,58	34,30	14,53	12,81	8,52	70,16	29,26	100,00
Sulawesi Barat	0,94	35,79	15,04	14,06	5,27	70,16	28,90	100,00
Maluku	0,22	36,58	17,18	14,61	9,74	78,11	21,67	100,00
Maluku Utara	0,57	37,19	17,15	14,78	7,29	76,41	23,02	100,00
Papua Barat	1,64	37,56	15,98	14,32	7,13	74,99	23,37	100,00
Papua	16,40	34,53	14,09	9,83	3,77	62,22	21,38	100,00
**Indonesia**	**0,63**	**35,36**	**15,49**	**13,55**	**6,30**	**70,70**	**28,67**	**100,00**

Sumber/*Source*: Susenas Maret 2018/*The March 2018 Susenas*

https://www.bps.go.id

Tabel **3.4.4.** **Persentase Penduduk Laki-Laki Berumur 7-24 Tahun di Daerah Perkotaan menurut Provinsi dan Status Pendidikan, 2018**
Table *Percentage of Male Population Aged 7-24 Years in Urban Area by Province and Education Status, 2018*

**Provinsi** *Province*	**Tidak/Belum Pernah Sekolah** *No Schooling/Never Attended School*	**Masih Sekolah/***Attending School*					**Tidak Bersekolah Lagi** *Not Attending School Anymore*	**Jumlah** *Total*
		**SD/MI/Paket A** *Primary School*	**SMP/MTs/Paket B** *Junior High School*	**SMA/SMK/MA/Paket C** *Senior High School*	**Diploma I s.d Universitas** *Dipl I to University*	**Jumlah yang Masih Sekolah** *Total Attending School*		
(1)	(2)	(3)	(4)	(5)	(6)	(7)	(8)	(9)
Aceh	0,12	35,28	15,27	15,09	14,05	79,69	20,19	100,00
Sumatera Utara	0,23	34,30	15,64	14,73	7,06	71,73	28,03	100,00
Sumatera Barat	0,30	37,29	15,42	13,68	13,90	80,29	19,41	100,00
Riau	0,32	34,82	15,38	14,20	12,50	76,90	22,78	100,00
Jambi	0,33	33,14	16,34	17,15	8,12	74,75	24,92	100,00
Sumatera Selatan	0,19	34,52	16,03	14,02	7,17	71,74	28,07	100,00
Bengkulu	0,00	35,40	17,18	16,77	10,73	80,08	19,92	100,00
Lampung	0,33	35,20	15,51	15,21	8,32	74,24	25,44	100,00
Kepulauan Bangka Belitung	0,15	33,91	15,87	14,51	2,99	67,28	32,56	100,00
Kepulauan Riau	0,30	37,75	16,26	13,10	5,80	72,91	26,79	100,00
DKI Jakarta	0,30	38,31	15,00	11,14	7,37	71,82	27,88	100,00
Jawa Barat	0,20	33,83	15,29	13,51	6,88	69,51	30,30	100,00
Jawa Tengah	0,33	33,40	15,27	16,31	6,16	71,14	28,53	100,00
DI Yogyakarta	0,00	33,01	13,65	13,98	20,69	81,33	18,67	100,00
Jawa Timur	0,32	33,16	15,85	15,15	7,97	72,13	27,55	100,00
Banten	0,27	35,54	14,09	12,44	7,24	69,31	30,42	100,00
Bali	0,24	32,46	16,35	13,74	9,63	72,18	27,58	100,00
Nusa Tenggara Barat	0,47	35,59	15,35	17,14	7,05	75,13	24,40	100,00
Nusa Tenggara Timur	0,42	33,30	16,03	18,74	12,36	80,43	19,15	100,00
Kalimantan Barat	0,31	36,24	13,52	16,33	10,23	76,32	23,37	100,00
Kalimantan Tengah	0,12	35,78	15,15	13,16	7,41	71,50	28,38	100,00
Kalimantan Selatan	1,00	34,05	15,47	15,20	9,71	74,43	24,58	100,00
Kalimantan Timur	0,43	34,59	14,74	16,78	9,53	75,64	23,93	100,00
Kalimantan Utara	0,97	32,84	15,83	18,03	3,52	70,22	28,81	100,00
Sulawesi Utara	0,35	29,04	14,80	14,42	9,58	67,84	31,81	100,00
Sulawesi Tengah	0,93	29,78	14,08	17,21	13,35	74,42	24,65	100,00
Sulawesi Selatan	0,44	34,65	14,55	13,17	12,64	75,01	24,55	100,00
Sulawesi Tenggara	0,47	32,77	15,88	16,96	13,05	78,66	20,87	100,00
Gorontalo	1,11	32,38	14,05	12,26	11,51	70,20	28,69	100,00
Sulawesi Barat	1,12	37,58	12,55	14,12	5,77	70,02	28,86	100,00
Maluku	0,38	32,13	16,93	16,97	14,12	80,15	19,47	100,00
Maluku Utara	0,45	29,60	16,66	16,09	12,31	74,66	24,89	100,00
Papua Barat	0,93	33,13	16,29	18,19	6,83	74,44	24,63	100,00
Papua	1,51	29,66	16,14	15,43	7,31	68,54	29,95	100,00
**Indonesia**	**0,30**	**34,24**	**15,30**	**14,39**	**8,17**	**72,10**	**27,60**	**100,00**

Sumber/*Source*: Susenas Maret 2018/*The March 2018 Susenas*

https://www.bps.go.id

Tabel **3.4.5.** **Persentase Penduduk Laki-Laki Berumur 7-24 Tahun di Daerah Perdesaan menurut Provinsi dan Status Pendidikan, 2018**
Table *Percentage of Male Population Aged 7-24 Years in Rural Area by Province and Education Status, 2018*

**Provinsi** *Province*	**Tidak/Belum Pernah Sekolah** *No Schooling/Never Attended School*	**Masih Sekolah**/*Attending School*					**Tidak Bersekolah Lagi** *Not Attending School Anymore*	**Jumlah** *Total*
		**SD/MI/Paket A** *Primary School*	**SMP/MTs/Paket B** *Junior High School*	**SMA/SMK/MA/Paket C** *Senior High School*	**Diploma I s.d Universitas** *Dipl I to University*	**Jumlah yang Masih Sekolah** *Total Attending School*		
(1)	(2)	(3)	(4)	(5)	(6)	(7)	(8)	(9)
Aceh	0,31	37,96	16,65	13,15	5,07	72,83	26,86	100,00
Sumatera Utara	0,48	39,85	17,06	13,67	3,67	74,25	25,27	100,00
Sumatera Barat	0,63	41,69	15,82	12,62	2,84	72,97	26,40	100,00
Riau	0,68	38,68	14,98	12,14	4,07	69,87	29,45	100,00
Jambi	0,33	37,85	15,03	10,58	4,07	67,53	32,13	100,00
Sumatera Selatan	0,24	37,59	15,11	11,27	2,09	66,06	33,70	100,00
Bengkulu	0,31	39,11	14,78	12,01	3,74	69,64	30,05	100,00
Lampung	0,45	37,74	16,09	12,11	1,65	67,59	31,96	100,00
Kepulauan Bangka Belitung	0,38	35,11	13,48	12,00	1,82	62,41	37,21	100,00
Kepulauan Riau	0,38	37,50	20,96	16,09	1,06	75,61	24,01	100,00
DKI Jakarta	-	-	-	-	-	-	-	-
Jawa Barat	0,60	36,82	14,81	10,88	2,06	64,57	34,83	100,00
Jawa Tengah	0,49	34,94	15,26	14,21	2,29	66,70	32,81	100,00
DI Yogyakarta	0,44	40,57	16,73	16,56	3,62	77,48	22,08	100,00
Jawa Timur	0,49	34,12	16,87	12,44	2,91	66,34	33,17	100,00
Banten	1,01	38,76	13,72	10,54	1,90	64,92	34,07	100,00
Bali	0,71	36,47	18,51	15,65	4,31	74,94	24,34	100,00
Nusa Tenggara Barat	0,47	37,59	15,88	16,08	3,87	73,42	26,11	100,00
Nusa Tenggara Timur	1,50	43,82	17,26	9,99	2,10	73,17	25,33	100,00
Kalimantan Barat	0,78	38,87	14,38	11,12	1,77	66,14	33,08	100,00
Kalimantan Tengah	1,02	36,49	16,50	10,77	3,20	66,96	32,02	100,00
Kalimantan Selatan	0,42	39,45	15,06	11,14	3,10	68,75	30,83	100,00
Kalimantan Timur	0,55	34,99	17,72	14,22	4,05	70,98	28,47	100,00
Kalimantan Utara	2,65	36,15	19,70	12,13	1,97	69,95	27,40	100,00
Sulawesi Utara	0,90	35,65	16,51	11,53	3,09	66,78	32,32	100,00
Sulawesi Tengah	0,83	37,96	17,32	11,12	3,98	70,38	28,78	100,00
Sulawesi Selatan	1,00	37,08	15,01	13,09	3,62	68,80	30,20	100,00
Sulawesi Tenggara	1,11	40,36	16,42	11,84	3,96	72,58	26,32	100,00
Gorontalo	0,70	36,46	14,25	12,98	4,31	68,00	31,30	100,00
Sulawesi Barat	1,07	35,47	15,31	12,79	3,95	67,52	31,41	100,00
Maluku	0,19	39,70	17,64	12,83	5,43	75,60	24,22	100,00
Maluku Utara	0,75	41,05	17,42	13,63	3,71	75,81	23,44	100,00
Papua Barat	1,54	40,41	15,34	13,15	5,15	74,05	24,41	100,00
Papua	19,98	35,65	13,59	8,38	2,32	59,94	20,08	100,00
**Indonesia**	**1,06**	**37,33**	**15,80**	**12,34**	**2,90**	**68,37**	**30,57**	**100,00**

Sumber/*Source*: Susenas Maret 2018/*The March 2018 Susenas*

https://www.bps.go.id

Tabel *Table* **3.4.6.** **Persentase Penduduk Laki-Laki Berumur 7-24 Tahun di Daerah Perkotaan dan Perdesaan menurut Provinsi dan Status Pendidikan, 2018**
Percentage of Male Population Aged 7-24 Years in Urban and Rural Area by Province and Education Status, 2018

**Provinsi** *Province*	**Tidak/Belum Pernah Sekolah** *No Schooling/Never Attended School*	**Masih Sekolah/***Attending School*					**Tidak Bersekolah Lagi** *Not Attending School Anymore*	**Jumlah** *Total*
		**SD/MI/Paket A** *Primary School*	**SMP/MTs/Paket B** *Junior High School*	**SMA/SMK/MA/Paket C** *Senior High School*	**Diploma I s.d Universitas** *Dipl I to University*	**Jumlah yang Masih Sekolah** *Total Attending School*		
**(1)**	**(2)**	**(3)**	**(4)**	**(5)**	**(6)**	**(7)**	**(8)**	**(9)**
Aceh	0,24	37,12	16,22	13,76	7,90	75,00	24,76	100,00
Sumatera Utara	0,35	36,97	16,32	14,22	5,43	72,94	26,71	100,00
Sumatera Barat	0,48	39,76	15,65	13,08	7,69	76,18	23,33	100,00
Riau	0,54	37,14	15,14	12,96	7,43	72,67	26,79	100,00
Jambi	0,33	36,38	15,44	12,63	5,33	69,78	29,89	100,00
Sumatera Selatan	0,22	36,44	15,45	12,30	4,00	68,19	31,59	100,00
Bengkulu	0,21	37,92	15,55	13,54	5,99	73,00	26,79	100,00
Lampung	0,42	36,97	15,91	13,05	3,67	69,60	29,98	100,00
Kepulauan Bangka Belitung	0,26	34,49	14,72	13,30	2,42	64,93	34,81	100,00
Kepulauan Riau	0,31	37,72	16,94	13,54	5,11	73,31	26,39	100,00
DKI Jakarta	0,30	38,31	15,00	11,14	7,37	71,82	27,88	100,00
Jawa Barat	0,30	34,59	15,17	12,85	5,65	68,26	31,44	100,00
Jawa Tengah	0,41	34,14	15,27	15,29	4,29	68,99	30,60	100,00
DI Yogyakarta	0,10	34,78	14,37	14,58	16,70	80,43	19,47	100,00
Jawa Timur	0,40	33,61	16,33	13,87	5,59	69,40	30,20	100,00
Banten	0,49	36,53	13,98	11,86	5,60	67,97	31,54	100,00
Bali	0,39	33,78	17,06	14,37	7,88	73,09	26,51	100,00
Nusa Tenggara Barat	0,47	36,65	15,63	16,58	5,37	74,23	25,31	100,00
Nusa Tenggara Timur	1,25	41,38	16,97	12,02	4,48	74,85	23,90	100,00
Kalimantan Barat	0,62	38,00	14,09	12,85	4,58	69,52	29,86	100,00
Kalimantan Tengah	0,67	36,22	15,98	11,69	4,83	68,72	30,61	100,00
Kalimantan Selatan	0,69	36,93	15,25	13,04	6,18	71,40	27,92	100,00
Kalimantan Timur	0,47	34,72	15,76	15,91	7,66	74,05	25,48	100,00
Kalimantan Utara	1,69	34,26	17,50	15,49	2,85	70,10	28,20	100,00
Sulawesi Utara	0,63	32,32	15,64	12,99	6,36	67,31	32,06	100,00
Sulawesi Tengah	0,86	35,67	16,42	12,82	6,60	71,51	27,63	100,00
Sulawesi Selatan	0,76	36,04	14,81	13,12	7,48	71,45	27,78	100,00
Sulawesi Tenggara	0,85	37,34	16,21	13,87	7,58	75,00	24,15	100,00
Gorontalo	0,86	34,89	14,18	12,70	7,08	68,85	30,29	100,00
Sulawesi Barat	1,08	35,98	14,65	13,11	4,38	68,12	30,79	100,00
Maluku	0,27	36,57	17,34	14,54	9,03	77,48	22,25	100,00
Maluku Utara	0,66	37,81	17,21	14,33	6,14	75,49	23,85	100,00
Papua Barat	1,30	37,48	15,72	15,18	5,82	74,20	24,50	100,00
Papua	14,91	34,00	14,29	10,32	3,69	62,30	22,79	100,00
**Indonesia**	**0,65**	**35,65**	**15,53**	**13,45**	**5,76**	**70,39**	**28,96**	**100,00**

Sumber/*Source*: Susenas Maret 2018/*The March 2018 Susenas*

https://www.bps.go.id

Tabel **3.4.7.** **Persentase Penduduk Perempuan Berumur 7-24 Tahun di Daerah Perkotaan menurut Provinsi dan Status Pendidikan, 2018**
Table *Percentage of Female Population Aged 7-24 Years in Urban Area by Province and Education Status, 2018*

**Provinsi** *Province*	**Tidak/Belum Pernah Sekolah** *No Schooling/Never Attended School*	**Masih Sekolah**/*Attending School*					**Tidak Bersekolah Lagi** *Not Attending School Anymore*	**Jumlah** *Total*
		**SD/MI/Paket A** *Primary School*	**SMP/MTs/Paket B** *Junior High School*	**SMA/SMK/MA/Paket C** *Senior High School*	**Diploma I s.d Universitas** *Dipl I to University*	**Jumlah yang Masih Sekolah** *Total Attending School*		
**(1)**	**(2)**	**(3)**	**(4)**	**(5)**	**(6)**	**(7)**	**(8)**	**(9)**
Aceh	0,08	35,34	14,35	15,31	18,24	83,24	16,68	100,00
Sumatera Utara	0,53	33,73	14,89	16,33	9,64	74,59	24,88	100,00
Sumatera Barat	0,30	34,35	16,92	15,98	15,76	83,01	16,69	100,00
Riau	0,26	33,84	16,48	16,68	9,35	76,35	23,39	100,00
Jambi	0,09	33,55	14,16	18,17	12,56	78,44	21,47	100,00
Sumatera Selatan	0,37	33,81	15,32	15,19	10,44	74,76	24,87	100,00
Bengkulu	0,08	35,67	15,59	16,11	13,37	80,74	19,19	100,00
Lampung	0,68	32,10	15,65	17,72	9,72	75,19	24,13	100,00
Kepulauan Bangka Belitung	0,33	34,62	15,70	16,15	4,19	70,66	29,01	100,00
Kepulauan Riau	0,31	40,34	16,15	12,59	5,32	74,40	25,29	100,00
DKI Jakarta	0,17	36,11	13,25	11,76	8,10	69,22	30,61	100,00
Jawa Barat	0,19	33,93	14,69	13,23	7,72	69,57	30,24	100,00
Jawa Tengah	0,24	32,24	15,47	15,47	7,50	70,68	29,08	100,00
DI Yogyakarta	0,08	30,89	11,69	14,88	24,81	82,27	17,65	100,00
Jawa Timur	0,25	32,41	15,62	15,11	9,07	72,21	27,54	100,00
Banten	0,05	34,38	14,71	12,09	9,35	70,53	29,42	100,00
Bali	0,17	31,47	14,83	15,18	12,51	73,99	25,84	100,00
Nusa Tenggara Barat	0,18	33,05	16,04	16,88	9,36	75,33	24,48	100,00
Nusa Tenggara Timur	0,27	31,14	16,66	18,96	13,56	80,32	19,41	100,00
Kalimantan Barat	0,41	32,62	16,13	14,91	10,51	74,17	25,43	100,00
Kalimantan Tengah	0,35	35,11	15,29	14,05	8,02	72,47	27,18	100,00
Kalimantan Selatan	1,10	35,41	12,96	14,20	8,86	71,43	27,47	100,00
Kalimantan Timur	0,04	36,34	13,83	15,90	9,91	75,98	23,97	100,00
Kalimantan Utara	1,11	35,25	16,06	17,28	6,53	75,12	23,77	100,00
Sulawesi Utara	0,32	29,51	16,87	15,12	11,19	72,69	26,98	100,00
Sulawesi Tengah	0,18	29,60	17,30	20,92	14,71	82,53	17,29	100,00
Sulawesi Selatan	0,24	32,80	14,96	15,55	15,36	78,67	21,09	100,00
Sulawesi Tenggara	0,60	30,12	15,84	15,98	17,86	79,80	19,60	100,00
Gorontalo	0,05	31,48	14,04	12,94	16,63	75,09	24,86	100,00
Sulawesi Barat	1,05	34,84	14,07	18,55	10,80	78,26	20,69	100,00
Maluku	0,02	31,48	16,78	16,88	16,46	81,60	18,38	100,00
Maluku Utara	0,49	31,13	14,39	16,62	17,68	79,82	19,69	100,00
Papua Barat	0,89	33,75	15,61	17,75	12,65	79,76	19,35	100,00
Papua	2,26	34,33	15,54	16,01	8,96	74,84	22,90	100,00
**Indonesia**	**0,27**	**33,53**	**15,00**	**14,59**	**9,59**	**72,71**	**27,02**	**100,00**

Sumber/*Source*: Susenas Maret 2018/*The March 2018 Susenas*

https://www.bps.go.id

Tabel *Table* **3.4.8.** **Persentase Penduduk Perempuan Berumur 7-24 Tahun di Daerah Perdesaan menurut Provinsi dan Status Pendidikan, 2018**
Percentage of Female Population Aged 7-24 Years in Rural Area by Province and Education Status, 2018

**Provinsi** *Province*	**Tidak/Belum Pernah Sekolah** *No Schooling/Never Attended School*	**Masih Sekolah/***Attending School*					**Tidak Bersekolah Lagi** *Not Attending School Anymore*	**Jumlah** *Total*
		**SD/MI/Paket A** *Primary School*	**SMP/MTs/Paket B** *Junior High School*	**SMA/SMK/MA/Paket C** *Senior High School*	**Diploma I s.d Universitas** *Dipl I to University*	**Jumlah yang Masih Sekolah** *Total Attending School*		
(1)	(2)	(3)	(4)	(5)	(6)	(7)	(8)	(9)
Aceh	0,33	38,37	16,16	13,93	6,37	74,83	24,83	100,00
Sumatera Utara	0,59	40,64	16,08	15,46	3,82	76,00	23,41	100,00
Sumatera Barat	0,47	40,79	16,50	14,60	3,60	75,49	24,04	100,00
Riau	0,51	38,60	17,77	12,13	4,81	73,31	26,19	100,00
Jambi	0,29	36,84	16,21	12,22	4,11	69,38	30,33	100,00
Sumatera Selatan	0,44	37,20	15,93	11,56	2,39	67,08	32,49	100,00
Bengkulu	0,12	38,43	17,28	13,17	5,81	74,69	25,19	100,00
Lampung	0,12	39,30	15,82	12,75	2,27	70,14	29,74	100,00
Kepulauan Bangka Belitung	0,03	36,62	15,64	12,20	3,14	67,60	32,37	100,00
Kepulauan Riau	0,45	45,87	17,51	13,10	1,90	78,38	21,17	100,00
DKI Jakarta	-	-	-	-	-	-	-	-
Jawa Barat	0,26	34,91	16,92	10,48	1,94	64,25	35,49	100,00
Jawa Tengah	0,35	34,60	15,39	13,63	3,07	66,69	32,96	100,00
DI Yogyakarta	0,00	39,45	18,37	13,33	3,53	74,68	25,32	100,00
Jawa Timur	0,44	34,21	14,74	12,20	4,13	65,29	34,27	100,00
Banten	1,05	37,64	15,68	8,57	2,01	63,90	35,05	100,00
Bali	1,35	33,82	18,39	16,02	3,28	71,51	27,14	100,00
Nusa Tenggara Barat	0,43	36,12	16,64	13,02	3,29	69,07	30,50	100,00
Nusa Tenggara Timur	1,51	43,30	16,96	11,32	3,21	74,79	23,70	100,00
Kalimantan Barat	0,58	38,98	14,05	12,98	1,47	67,48	31,94	100,00
Kalimantan Tengah	0,41	34,14	19,14	12,14	4,20	69,62	29,97	100,00
Kalimantan Selatan	0,39	37,29	15,41	11,53	3,15	67,38	32,24	100,00
Kalimantan Timur	0,32	37,80	14,95	16,17	3,84	72,76	26,91	100,00
Kalimantan Utara	0,36	32,89	20,82	14,53	3,13	71,37	28,27	100,00
Sulawesi Utara	0,25	36,60	16,54	13,36	5,26	71,76	27,99	100,00
Sulawesi Tengah	0,91	35,48	19,19	11,44	4,46	70,57	28,52	100,00
Sulawesi Selatan	0,59	36,25	15,98	13,95	6,55	72,73	26,68	100,00
Sulawesi Tenggara	0,68	39,92	14,87	13,07	3,04	70,90	28,43	100,00
Gorontalo	0,46	35,13	15,46	12,92	5,69	69,20	30,34	100,00
Sulawesi Barat	0,70	35,84	15,88	13,94	4,75	70,41	28,89	100,00
Maluku	0,30	40,32	17,17	13,07	6,09	76,65	23,05	100,00
Maluku Utara	0,47	38,72	18,19	14,71	4,77	76,39	23,14	100,00
Papua Barat	2,77	40,23	16,69	10,48	5,84	73,24	23,99	100,00
Papua	23,91	35,41	13,25	6,81	1,99	57,46	18,63	100,00
**Indonesia**	**1,00**	**36,92**	**16,01**	**12,53**	**3,55**	**69,01**	**29,99**	**100,00**

Sumber/*Source*: Susenas Maret 2018/*The March 2018 Susenas*

https://www.bps.go.id

Tabel *Table* **3.4.9.** **Persentase Penduduk Perempuan Berumur 7-24 Tahun di Daerah Perkotaan dan Perdesaan menurut Provinsi dan Status Pendidikan, 2018**
Percentage of Female Population Aged 7-24 Years in Urban and Rural Area by Province and Education Status, 2018

**Provinsi** *Province*	**Tidak/Belum Pernah Sekolah** *No Schooling/Never Attended School*	**Masih Sekolah/***Attending School*					**Tidak Bersekolah Lagi** *Not Attending School Anymore*	**Jumlah** *Total*
		**SD/MI/Paket A** *Primary School*	**SMP/MTs/Paket B** *Junior High School*	**SMA/SMK/MA/Paket C** *Senior High School*	**Diploma I s.d Universitas** *Dipl I to University*	**Jumlah yang Masih Sekolah** *Total Attending School*		
(1)	(2)	(3)	(4)	(5)	(6)	(7)	(8)	(9)
Aceh	0,25	37,41	15,59	14,37	10,11	77,48	22,26	100,00
Sumatera Utara	0,56	37,00	15,45	15,92	6,89	75,26	24,19	100,00
Sumatera Barat	0,39	37,88	16,69	15,22	9,11	78,90	20,71	100,00
Riau	0,41	36,66	17,24	13,98	6,66	74,54	25,05	100,00
Jambi	0,22	35,77	15,54	14,17	6,87	72,35	27,43	100,00
Sumatera Selatan	0,41	35,91	15,70	12,94	5,45	70,00	29,59	100,00
Bengkulu	0,11	37,48	16,70	14,18	8,40	76,76	23,13	100,00
Lampung	0,30	37,05	15,76	14,31	4,60	71,72	27,98	100,00
Kepulauan Bangka Belitung	0,19	35,56	15,67	14,30	3,70	69,23	30,58	100,00
Kepulauan Riau	0,33	41,07	16,33	12,66	4,87	74,93	24,74	100,00
DKI Jakarta	0,17	36,11	13,25	11,77	8,10	69,23	30,61	100,00
Jawa Barat	0,21	34,18	15,24	12,54	6,28	68,24	31,55	100,00
Jawa Tengah	0,29	33,36	15,43	14,59	5,39	68,77	30,93	100,00
DI Yogyakarta	0,06	32,87	13,24	14,52	19,88	80,51	19,43	100,00
Jawa Timur	0,34	33,25	15,21	13,75	6,77	68,98	30,68	100,00
Banten	0,35	35,34	15,00	11,05	7,18	68,57	31,09	100,00
Bali	0,55	32,23	15,98	15,45	9,53	73,19	26,26	100,00
Nusa Tenggara Barat	0,31	34,67	16,35	14,85	6,17	72,04	27,65	100,00
Nusa Tenggara Timur	1,22	40,45	16,89	13,11	5,63	76,08	22,70	100,00
Kalimantan Barat	0,52	36,79	14,77	13,64	4,58	69,78	29,70	100,00
Kalimantan Tengah	0,39	34,53	17,60	12,90	5,73	70,76	28,85	100,00
Kalimantan Selatan	0,72	36,41	14,26	12,78	5,83	69,28	30,00	100,00
Kalimantan Timur	0,14	36,83	14,20	15,99	7,88	74,90	24,96	100,00
Kalimantan Utara	0,80	34,28	18,01	16,15	5,14	73,58	25,62	100,00
Sulawesi Utara	0,29	32,92	16,71	14,27	8,34	72,24	27,47	100,00
Sulawesi Tengah	0,70	33,74	18,63	14,25	7,49	74,11	25,20	100,00
Sulawesi Selatan	0,44	34,77	15,55	14,63	10,32	75,27	24,29	100,00
Sulawesi Tenggara	0,65	36,10	15,24	14,20	8,82	74,36	24,99	100,00
Gorontalo	0,30	33,69	14,90	12,93	10,00	71,52	28,18	100,00
Sulawesi Barat	0,78	35,60	15,45	15,05	6,20	72,30	26,92	100,00
Maluku	0,18	36,59	17,01	14,68	10,47	78,75	21,07	100,00
Maluku Utara	0,48	36,53	17,09	15,26	8,50	77,38	22,14	100,00
Papua Barat	2,02	37,64	16,26	13,38	8,56	75,84	22,14	100,00
Papua	18,08	35,12	13,87	9,28	3,87	62,14	19,78	100,00
**Indonesia**	**0,60**	**35,06**	**15,45**	**13,66**	**6,87**	**71,04**	**28,36**	**100,00**

Sumber/*Source*: Susenas Maret 2018/*The March 2018 Susenas*

https://www.bps.go.id

Tabel *Table* **3.5.** **Angka Partisipasi Sekolah (APS) Penduduk Berumur 5-18 Tahun menurut Daerah Tempat Tinggal, Jenis Kelamin, dan Kelompok Umur, 2018**
School Enrollment Ratio (SER) of Population Aged 5-18 Years by Urban Rural Classification, Sex, and Age Group, 2018

**Daerah Tempat Tinggal dan Jenis Kelamin** *Urban Rural Classification and Sex*	**APS Formal/***Formal SER*				**APS Formal+Non Formal/***Formal+Non Formal SER*			
	**5-6**	**7-12**	**13-15**	**16-18**	**5-6**	**7-12**	**13-15**	**16-18**
(1)	(2)	(3)	(4)	(5)	(6)	(7)	(8)	(9)
**Perkotaan/***Urban*								
**Laki-laki/***Male*	19,74	99,48	95,57	74,97	19,74	99,61	95,75	75,18
**Perempuan/***Female*	21,86	99,53	97,19	76,84	21,86	99,63	97,22	76,91
**Laki-laki+Perempuan/***Male+Female*	20,77	99,50	96,35	75,90	20,77	99,62	96,46	76,05
**Perkotaan/***Urban*								
**Laki-laki/***Male*	24,05	98,59	92,99	65,88	24,05	98,69	93,14	66,14
**Perempuan/***Female*	26,62	98,78	95,04	68,10	26,62	98,88	95,19	68,26
**Laki-laki+Perempuan/***Male+Female*	25,31	98,68	93,98	66,95	25,31	98,78	94,13	67,16
**Perkotaan dan Perdesaan/***Urban and Rural*								
**Laki-laki/***Male*	21,77	99,05	94,34	70,74	21,77	99,17	94,51	70,98
**Perempuan/***Female*	24,12	99,18	96,17	72,93	24,12	99,27	96,26	73,04
**Laki-laki+Perempuan/***Male+Female*	22,92	99,11	95,23	71,82	22,92	99,22	95,36	71,99

Sumber/*Source*: Susenas Maret 2018/*The March 2018 Susenas*

https://www.bps.go.id

Tabel **3.6.** **Angka Partisipasi Murni (APM) Penduduk menurut Daerah Tempat Tinggal, Jenis Kelamin, dan Jenjang Pendidikan, 2018**

Table *Net Enrollment Ratio (NER) of Population by Urban Rural Classification, Sex, and Educational Level, 2018*

**Daerah Tempat Tinggal dan Jenis Kelamin** *Urban Rural Classification and Sex*	**APM Formal/***Formal NER*			**APM Formal+Non Formal/***Formal+Non Formal NER*		
	**SD** *Primary School*	**SMP** *Junior High School*	**SMA** *Senior High School*	**SD** *Primary School*	**SMP** *Junior High School*	**SMA** *Senior High School*
**(1)**	**(2)**	**(3)**	**(4)**	**(6)**	**(7)**	**(8)**
**Perkotaan/***Urban*						
**Laki-laki/***Male*	97,91	80,34	64,11	98,03	80,45	64,31
**Perempuan/***Female*	97,33	80,76	64,92	97,43	80,78	64,99
**Laki-laki+Perempuan/***Male+Female*	97,63	80,55	64,51	97,74	80,61	64,65
**Perkotaan/***Urban*						
**Laki-laki/***Male*	97,41	75,53	54,29	97,51	75,65	54,47
**Perempuan/***Female*	97,20	78,04	57,39	97,29	78,17	57,50
**Laki-laki+Perempuan/***Male+Female*	97,31	76,75	55,78	97,40	76,86	55,93
**Perkotaan dan Perdesaan/***Urban and Rural*						
**Laki-laki/***Male*	97,67	78,07	59,54	97,79	78,18	59,74
**Perempuan/***Female*	97,27	79,48	61,55	97,37	79,55	61,64
**Laki-laki+Perempuan/***Male+Female*	97,48	78,75	60,53	97,58	78,84	60,67

Sumber/*Source*: Susenas Maret 2018/*The March 2018 Susenas*

https://www.bps.go.id

Tabel 3.7.1. **Persentase Penduduk Laki-Laki dan Perempuan Berumur 15 Tahun ke Atas di Daerah Perkotaan menurut Provinsi dan Ijazah/STTB Tertinggi yang Dimiliki, 2018**
Table *Percentage Percentage of Male and Female Population Aged 15 Years and Over in Urban Area by Province and The Highest School Certificate Owned, 2018*

**Provinsi** *Province*	**Tidak Mempunyai Ijazah** *No Certificate*	**SD/MI** *Primary School*	**SMP/MTs** *Junior High School*	**SMA/MA** *Senior High School*	**SMK/MAK** *Vocational Senior High School*	**Diploma I dan Diploma II** *Diploma I and Diploma II*	**Akademi/Diploma III** *Academy/Diploma III*	**Diploma IV/S1/S2/S3** *Diploma IV/Bachelor/ Master Degree/S3*	**Jumlah** *Total*
(1)	(2)	(3)	(4)	(5)	(6)	(7)	(8)	(9)	(10)
Aceh	9,65	14,65	18,27	35,08	3,28	0,92	4,69	13,46	100,00
Sumatera Utara	6,33	15,63	24,16	34,55	6,65	0,47	2,44	9,77	100,00
Sumatera Barat	12,02	13,83	20,23	29,78	6,94	0,97	3,79	12,44	100,00
Riau	8,18	14,47	20,94	34,38	7,30	0,66	2,99	11,08	100,00
Jambi	10,93	15,28	21,23	34,04	4,96	0,77	2,88	9,91	100,00
Sumatera Selatan	10,72	18,08	20,10	34,31	4,18	0,77	3,13	8,71	100,00
Bengkulu	8,20	16,94	19,53	28,93	5,23	0,74	3,22	17,21	100,00
Lampung	11,59	19,37	23,01	26,62	6,44	0,71	2,34	9,92	100,00
Kepulauan Bangka Belitung	15,09	20,72	21,03	22,86	9,25	0,57	3,11	7,37	100,00
Kepulauan Riau	7,08	14,25	18,42	41,15	8,12	0,53	2,91	7,54	100,00
DKI Jakarta	4,41	13,23	20,73	36,40	8,55	0,55	3,93	12,20	100,00
Jawa Barat	12,95	24,69	21,80	23,13	7,61	0,65	2,35	6,82	100,00
Jawa Tengah	16,71	23,44	22,64	19,03	8,44	0,57	2,36	6,81	100,00
DI Yogyakarta	13,30	12,56	16,49	28,57	12,32	0,48	4,09	12,19	100,00
Jawa Timur	17,60	22,19	20,48	21,48	8,08	0,70	1,57	7,90	100,00
Banten	13,07	19,06	20,53	27,04	8,23	0,58	2,20	9,29	100,00
Bali	13,06	18,00	17,03	29,54	6,69	2,87	1,85	10,96	100,00
Nusa Tenggara Barat	25,64	17,96	18,86	22,39	3,44	0,59	1,68	9,44	100,00
Nusa Tenggara Timur	15,66	16,95	18,86	27,34	4,55	0,90	2,74	13,00	100,00
Kalimantan Barat	17,33	17,73	19,13	27,83	4,03	0,42	3,26	10,27	100,00
Kalimantan Tengah	10,34	19,83	21,33	29,29	3,88	0,93	2,83	11,57	100,00
Kalimantan Selatan	14,16	19,39	19,99	27,58	5,00	0,71	2,71	10,46	100,00
Kalimantan Timur	8,96	16,66	20,59	30,30	8,76	0,88	3,21	10,64	100,00
Kalimantan Utara	11,57	18,27	23,66	29,05	5,25	0,38	2,68	9,14	100,00
Sulawesi Utara	11,69	13,86	19,06	34,26	7,16	0,53	1,90	11,54	100,00
Sulawesi Tengah	11,00	16,94	19,51	30,42	3,87	0,61	2,88	14,78	100,00
Sulawesi Selatan	14,68	17,35	17,80	28,57	3,98	0,66	2,54	14,42	100,00
Sulawesi Tenggara	13,13	12,28	18,11	34,69	2,81	0,57	3,02	15,39	100,00
Gorontalo	23,27	18,82	15,70	22,76	6,21	1,08	2,26	9,90	100,00
Sulawesi Barat	21,21	17,99	18,94	18,76	5,19	0,76	2,89	14,26	100,00
Maluku	8,60	12,97	17,77	40,39	2,96	0,82	2,79	13,70	100,00
Maluku Utara	11,24	11,65	18,68	35,67	4,57	0,54	2,60	15,05	100,00
Papua Barat	8,09	14,44	20,26	33,73	6,21	0,44	3,01	13,82	100,00
Papua	10,46	15,61	19,08	35,74	3,50	0,73	2,55	12,33	100,00
**Indonesia**	**13,09**	**20,10**	**20,94**	**26,31**	**7,36**	**0,69**	**2,50**	**9,01**	**100,00**

Sumber/*Source*: Susenas Maret 2018/*The March 2018 Susenas*

https://www.bps.go.id

Tabel *Table* **3.7.2.** **Persentase Penduduk Laki-Laki dan Perempuan Berumur 15 Tahun ke Atas di Daerah Perdesaan menurut Provinsi dan Ijazah/STTB Tertinggi yang Dimiliki, 2018**
Percentage Percentage of Male and Female Population Aged 15 Years and Over in Rural Area by Province and The Highest School Certificate Owned, 2018

**Provinsi** *Province*	**Tidak Mempunyai Ijazah** *No Certificate*	**SD/MI** *Primary School*	**SMP/MTs** *Junior High School*	**SMA/MA** *Senior High School*	**SMK/MAK** *Vocational Senior High School*	**Diploma I dan Diploma II** *Diploma I and Diploma II*	**Akademi/Diploma III** *Academy/Diploma III*	**Diploma IV/S1/S2/S3** *Diploma IV/Bachelor/ Master Degree/S3*	**Jumlah** *Total*
(1)	(2)	(3)	(4)	(5)	(6)	(7)	(8)	(9)	(10)
Aceh	15,38	26,37	23,96	23,63	1,63	0,78	2,03	6,22	100,00
Sumatera Utara	16,45	25,25	25,66	22,53	4,89	0,29	1,17	3,77	100,00
Sumatera Barat	26,03	23,84	21,54	16,90	4,68	0,50	1,40	5,10	100,00
Riau	18,85	29,29	22,61	19,87	3,39	0,31	1,14	4,54	100,00
Jambi	19,20	30,95	21,93	18,80	3,14	0,54	1,28	4,15	100,00
Sumatera Selatan	21,76	34,21	21,54	16,07	2,43	0,31	0,72	2,96	100,00
Bengkulu	20,62	29,37	22,32	18,97	2,52	0,43	0,99	4,77	100,00
Lampung	21,40	29,72	26,75	14,34	3,93	0,34	0,74	2,78	100,00
Kepulauan Bangka Belitung	29,89	31,38	18,44	12,59	3,77	0,21	1,06	2,67	100,00
Kepulauan Riau	31,83	28,43	17,67	13,49	3,36	0,78	0,35	4,08	100,00
DKI Jakarta	-	-	-	-	-	-	-	-	-
Jawa Barat	19,27	44,96	21,71	8,66	3,12	0,18	0,43	1,66	100,00
Jawa Tengah	24,87	34,38	24,07	8,91	4,79	0,25	0,64	2,08	100,00
DI Yogyakarta	23,17	25,60	23,23	12,17	10,09	0,47	1,40	3,88	100,00
Jawa Timur	27,34	32,91	20,91	11,68	3,96	0,26	0,39	2,55	100,00
Banten	22,46	41,11	20,76	10,66	2,37	0,22	0,12	2,31	100,00
Bali	28,25	26,31	18,87	16,76	4,12	1,39	0,72	3,59	100,00
Nusa Tenggara Barat	35,03	22,55	18,69	14,99	1,87	0,36	1,03	5,49	100,00
Nusa Tenggara Timur	30,48	34,58	15,59	12,11	1,50	0,36	1,01	4,38	100,00
Kalimantan Barat	31,34	31,28	19,13	13,21	1,52	0,27	0,80	2,45	100,00
Kalimantan Tengah	17,77	37,20	21,55	16,03	2,72	0,29	0,96	3,48	100,00
Kalimantan Selatan	24,61	33,57	19,99	14,07	2,76	0,32	0,66	4,02	100,00
Kalimantan Timur	17,83	30,23	22,86	20,26	3,92	0,27	0,80	3,82	100,00
Kalimantan Utara	21,11	27,03	19,33	18,86	3,37	0,34	2,52	7,46	100,00
Sulawesi Utara	20,78	25,31	21,51	20,32	5,27	0,51	1,13	5,17	100,00
Sulawesi Tengah	18,53	35,29	18,90	17,57	2,72	0,86	0,92	5,21	100,00
Sulawesi Selatan	28,81	27,69	18,94	15,07	2,50	0,30	1,14	5,54	100,00
Sulawesi Tenggara	22,77	25,31	21,26	20,32	2,45	0,49	1,46	5,94	100,00
Gorontalo	34,03	30,13	14,75	12,57	3,63	0,29	0,60	4,01	100,00
Sulawesi Barat	27,58	29,45	18,45	13,06	3,91	0,54	1,15	5,87	100,00
Maluku	14,79	29,41	20,37	24,90	2,11	0,71	1,39	6,33	100,00
Maluku Utara	20,15	30,08	19,89	20,05	1,81	0,82	1,38	5,82	100,00
Papua Barat	18,89	22,65	20,03	22,40	2,75	0,50	2,48	10,30	100,00
Papua	49,55	20,78	12,70	10,89	1,37	0,28	0,92	3,52	100,00
**Indonesia**	**24,13**	**32,48**	**21,60**	**13,82**	**3,48**	**0,34**	**0,80**	**3,35**	**100,00**

Sumber/*Source*: Susenas Maret 2018/*The March 2018 Susenas*

https://www.bps.go.id

Tabel 3.7.3. **Persentase Penduduk Laki-Laki dan Perempuan Berumur 15 Tahun ke Atas di Daerah Perkotaan dan Perdesaan menurut Provinsi dan Ijazah/STTB Tertinggi yang Dimiliki, 2018**

Table *Percentage Percentage of Male and Female Population Aged 15 Years and Over in Urban and Rural Area by Province and The Highest School Certificate Owned, 2018*

**Provinsi** *Province*	**Tidak Mempunyai Ijazah** *No Certificate*	**SD/MI** *Primary School*	**SMP/MTs** *Junior High School*	**SMA/MA** *Senior High School*	**SMK/MAK** *Vocational Senior High School*	**Diploma I dan Diploma II** *Diploma I and Diploma II*	**Akademi/Diploma III** *Academy/Diploma III*	**Diploma IV/S1/S2/S3** *Diploma IV/Bachelor/ Master Degree/S3*	**Jumlah** *Total*
(1)	(2)	(3)	(4)	(5)	(6)	(7)	(8)	(9)	(10)
Aceh	13,55	22,63	22,15	27,28	2,16	0,83	2,88	8,53	100,00
Sumatera Utara	10,94	20,02	24,84	29,07	5,85	0,39	1,86	7,03	100,00
Sumatera Barat	19,76	19,37	20,95	22,66	5,69	0,71	2,47	8,38	100,00
Riau	14,49	23,23	21,93	25,80	4,99	0,45	1,89	7,21	100,00
Jambi	16,51	25,85	21,70	23,76	3,73	0,62	1,80	6,02	100,00
Sumatera Selatan	17,59	28,12	20,99	22,96	3,09	0,48	1,63	5,13	100,00
Bengkulu	16,53	25,27	21,40	22,26	3,41	0,53	1,72	8,87	100,00
Lampung	18,47	26,62	25,64	18,01	4,68	0,45	1,22	4,91	100,00
Kepulauan Bangka Belitung	21,91	25,63	19,84	18,13	6,72	0,40	2,16	5,21	100,00
Kepulauan Riau	10,60	16,27	18,31	37,22	7,45	0,57	2,54	7,05	100,00
DKI Jakarta	4,41	13,23	20,73	36,40	8,55	0,55	3,93	12,20	100,00
Jawa Barat	14,59	29,94	21,78	19,38	6,45	0,53	1,85	5,49	100,00
Jawa Tengah	20,68	28,76	23,34	14,10	6,67	0,42	1,52	4,51	100,00
DI Yogyakarta	15,96	16,08	18,31	24,14	11,72	0,48	3,36	9,95	100,00
Jawa Timur	22,24	27,30	20,69	16,81	6,11	0,49	1,01	5,35	100,00
Banten	15,70	25,23	20,59	22,46	6,59	0,48	1,62	7,33	100,00
Bali	18,19	20,80	17,65	25,22	5,82	2,37	1,46	8,47	100,00
Nusa Tenggara Barat	30,62	20,39	18,77	18,46	2,61	0,47	1,33	7,34	100,00
Nusa Tenggara Timur	26,89	30,30	16,38	15,80	2,24	0,49	1,43	6,47	100,00
Kalimantan Barat	26,53	26,64	19,13	18,22	2,39	0,32	1,64	5,13	100,00
Kalimantan Tengah	14,89	30,47	21,47	21,17	3,17	0,54	1,68	6,61	100,00
Kalimantan Selatan	19,69	26,89	19,99	20,43	3,82	0,50	1,63	7,05	100,00
Kalimantan Timur	11,86	21,10	21,33	27,01	7,18	0,68	2,42	8,41	100,00
Kalimantan Utara	15,51	21,89	21,87	24,84	4,47	0,37	2,61	8,44	100,00
Sulawesi Utara	16,16	19,49	20,26	27,41	6,23	0,52	1,52	8,41	100,00
Sulawesi Tengah	16,36	30,01	19,08	21,27	3,05	0,79	1,48	7,96	100,00
Sulawesi Selatan	22,88	23,35	18,46	20,74	3,12	0,45	1,73	9,27	100,00
Sulawesi Tenggara	18,99	20,20	20,02	25,95	2,59	0,52	2,08	9,64	100,00
Gorontalo	29,78	25,67	15,12	16,59	4,65	0,60	1,25	6,33	100,00
Sulawesi Barat	26,07	26,73	18,56	14,42	4,21	0,59	1,56	7,86	100,00
Maluku	12,11	22,29	19,24	31,61	2,48	0,76	2,00	9,52	100,00
Maluku Utara	17,47	24,53	19,53	24,75	2,64	0,74	1,75	8,60	100,00
Papua Barat	14,40	19,23	20,13	27,11	4,19	0,47	2,70	11,77	100,00
Papua	38,27	19,29	14,54	18,06	1,98	0,41	1,39	6,06	100,00
**Indonesia**	**18,02**	**25,63**	**21,24**	**20,73**	**5,63**	**0,53**	**1,74**	**6,48**	**100,00**

Sumber/*Source*: Susenas Maret 2018/*The March 2018 Susenas*

https://www.bps.go.id

Tabel *Table* **3.7.4.** **Persentase Penduduk Laki-Laki Berumur 15 Tahun ke Atas di Daerah Perkotaan menurut Provinsi dan Ijazah/STTB Tertinggi yang Dimiliki, 2018**
Percentage Percentage of Male Population Aged 15 Years and Over in Urban Area by Province and The Highest School Certificate Owned, 2018

**Provinsi** *Province*	**Tidak Mempunyai Ijazah** *No Certificate*	**SD/MI** *Primary School*	**SMP/MTs** *Junior High School*	**SMA/MA** *Senior High School*	**SMK/MAK** *Vocational Senior High School*	**Diploma I dan Diploma II** *Diploma I and Diploma II*	**Akademi/Diploma III** *Academy/Diploma III*	**Diploma IV/S1/S2/S3** *Diploma IV/Bachelor/ Master Degree/S3*	**Jumlah** *Total*
(1)	(2)	(3)	(4)	(5)	(6)	(7)	(8)	(9)	(10)
Aceh	7,98	14,06	17,41	38,43	3,84	0,42	3,27	14,60	100,00
Sumatera Utara	5,03	15,52	24,55	35,42	7,94	0,31	1,81	9,42	100,00
Sumatera Barat	10,71	13,97	21,84	31,42	8,33	0,54	2,65	10,54	100,00
Riau	7,82	13,41	20,50	36,86	8,78	0,31	2,11	10,20	100,00
Jambi	8,85	15,38	20,88	36,83	5,31	0,72	1,94	10,09	100,00
Sumatera Selatan	10,09	17,31	20,02	35,77	5,16	0,41	2,53	8,72	100,00
Bengkulu	7,41	17,10	20,15	28,90	6,16	0,56	2,55	17,18	100,00
Lampung	9,95	20,25	22,29	27,59	7,66	0,45	1,79	10,01	100,00
Kepulauan Bangka Belitung	14,42	21,00	20,62	22,83	10,10	0,63	3,24	7,16	100,00
Kepulauan Riau	5,87	14,29	18,29	41,89	9,08	0,46	2,45	7,67	100,00
DKI Jakarta	3,07	10,96	20,65	39,40	8,99	0,53	3,58	12,81	100,00
Jawa Barat	10,97	23,80	21,52	24,58	9,21	0,54	2,10	7,27	100,00
Jawa Tengah	13,00	23,41	23,79	19,88	10,14	0,61	1,96	7,21	100,00
DI Yogyakarta	10,69	11,62	16,90	29,48	15,13	0,39	3,33	12,46	100,00
Jawa Timur	14,03	21,78	20,79	22,85	10,26	0,61	1,26	8,42	100,00
Banten	11,66	17,22	19,64	28,77	10,00	0,48	1,86	10,37	100,00
Bali	9,16	16,05	17,16	33,22	7,74	3,73	1,70	11,23	100,00
Nusa Tenggara Barat	22,21	17,26	18,63	25,63	4,32	0,44	1,43	10,07	100,00
Nusa Tenggara Timur	14,40	17,21	18,10	28,52	5,15	0,87	2,23	13,51	100,00
Kalimantan Barat	16,16	15,94	19,44	29,96	4,25	0,38	2,84	11,04	100,00
Kalimantan Tengah	7,90	19,10	21,05	31,38	4,59	0,90	2,46	12,63	100,00
Kalimantan Selatan	12,25	18,82	19,33	30,46	5,88	0,52	2,48	10,27	100,00
Kalimantan Timur	7,31	14,46	20,58	32,50	10,69	0,74	2,69	11,02	100,00
Kalimantan Utara	10,44	17,68	23,79	30,37	6,49	0,30	2,33	8,60	100,00
Sulawesi Utara	11,94	13,77	18,46	36,14	6,58	0,41	1,91	10,78	100,00
Sulawesi Tengah	9,60	16,30	18,53	33,82	4,49	0,37	1,35	15,54	100,00
Sulawesi Selatan	12,38	17,52	17,52	30,74	5,05	0,62	1,70	14,47	100,00
Sulawesi Tenggara	11,83	12,07	17,87	35,97	2,96	0,46	2,48	16,36	100,00
Gorontalo	25,69	18,72	15,27	21,37	7,42	0,67	1,95	8,91	100,00
Sulawesi Barat	21,53	19,47	17,26	17,52	5,35	0,94	2,39	15,54	100,00
Maluku	8,71	11,42	17,38	43,64	3,29	0,76	2,40	12,39	100,00
Maluku Utara	10,94	10,12	18,51	37,53	5,30	0,52	1,43	15,66	100,00
Papua Barat	7,50	13,91	19,60	35,92	7,12	0,27	1,82	13,86	100,00
Papua	10,52	14,88	17,94	37,94	4,00	0,56	2,17	11,99	100,00
**Indonesia**	**10,86**	**19,34**	**20,97**	**27,97**	**8,80**	**0,60**	**2,10**	**9,36**	**100,00**

Sumber/*Source*: Susenas Maret 2018/*The March 2018 Susenas*

https://www.bps.go.id

Tabel *Table* **3.7.5.** **Persentase Penduduk Laki-Laki Berumur 15 Tahun ke Atas di Daerah Perdesaan menurut Provinsi dan Ijazah/STTB Tertinggi yang Dimiliki, 2018**
Percentage Percentage of Male Population Aged 15 Years and Over in Rural Area by Province and The Highest School Certificate Owned, 2018

**Provinsi** *Province*	**Tidak Mempunyai Ijazah** *No Certificate*	**SD/MI** *Primary School*	**SMP/MTs** *Junior High School*	**SMA/MA** *Senior High School*	**SMK/MAK** *Vocational Senior High School*	**Diploma I dan Diploma II** *Diploma I and Diploma II*	**Akademi/Diploma III** *Academy/Diploma III*	**Diploma IV/S1/S2/S3** *Diploma IV/Bachelor/ Master Degree/S3*	**Jumlah** *Total*
(1)	(2)	(3)	(4)	(5)	(6)	(7)	(8)	(9)	(10)
Aceh	12,67	26,01	25,01	26,74	2,22	0,44	0,98	5,92	100,00
Sumatera Utara	13,21	25,94	26,84	24,19	5,64	0,21	0,74	3,23	100,00
Sumatera Barat	24,11	25,55	23,19	16,67	5,68	0,29	0,95	3,57	100,00
Riau	16,69	29,40	22,94	21,76	4,23	0,18	0,67	4,13	100,00
Jambi	15,50	32,03	22,23	20,94	3,75	0,51	1,00	4,03	100,00
Sumatera Selatan	19,01	34,60	22,66	17,43	3,01	0,32	0,38	2,59	100,00
Bengkulu	17,93	29,42	23,16	20,94	3,34	0,41	0,70	4,12	100,00
Lampung	18,67	30,90	26,52	15,98	4,59	0,33	0,55	2,47	100,00
Kepulauan Bangka Belitung	28,33	31,73	18,89	13,13	4,08	0,08	1,12	2,64	100,00
Kepulauan Riau	29,48	30,22	19,35	11,76	4,21	1,05	0,24	3,70	100,00
DKI Jakarta	-	-	-	-	-	-	-	-	-
Jawa Barat	16,56	45,12	21,90	9,81	4,34	0,18	0,41	1,67	100,00
Jawa Tengah	20,70	35,59	25,16	9,44	6,09	0,26	0,56	2,20	100,00
DI Yogyakarta	18,30	26,85	24,37	14,29	11,13	0,38	1,43	3,25	100,00
Jawa Timur	22,48	34,27	21,57	13,21	5,20	0,27	0,29	2,69	100,00
Banten	18,74	40,22	22,48	12,99	2,87	0,24	0,15	2,30	100,00
Bali	21,63	26,21	19,47	21,61	5,18	1,16	0,45	4,27	100,00
Nusa Tenggara Barat	31,09	21,74	19,76	16,93	2,41	0,45	1,00	6,63	100,00
Nusa Tenggara Timur	30,17	33,14	15,49	13,49	1,83	0,38	0,78	4,72	100,00
Kalimantan Barat	28,11	32,77	18,73	14,92	1,83	0,30	0,84	2,49	100,00
Kalimantan Tengah	15,43	36,71	21,96	18,17	3,32	0,29	0,84	3,28	100,00
Kalimantan Selatan	20,33	34,27	20,84	16,19	3,62	0,34	0,52	3,87	100,00
Kalimantan Timur	15,99	29,63	22,20	22,83	5,25	0,21	0,68	3,20	100,00
Kalimantan Utara	18,27	25,94	19,73	20,59	4,11	0,47	2,19	8,71	100,00
Sulawesi Utara	20,60	26,29	21,56	21,01	4,83	0,27	0,97	4,47	100,00
Sulawesi Tengah	17,34	34,90	19,20	19,55	3,11	0,50	0,51	4,89	100,00
Sulawesi Selatan	26,83	27,81	19,11	17,55	3,16	0,29	0,52	4,74	100,00
Sulawesi Tenggara	20,10	25,07	21,13	22,95	3,17	0,48	0,79	6,30	100,00
Gorontalo	37,73	29,15	13,52	11,83	3,98	0,13	0,21	3,46	100,00
Sulawesi Barat	26,10	30,47	17,86	14,63	4,54	0,51	0,47	5,41	100,00
Maluku	13,22	28,64	21,32	27,20	2,53	0,41	1,06	5,61	100,00
Maluku Utara	17,66	29,02	20,01	24,20	2,04	0,63	0,74	5,70	100,00
Papua Barat	15,11	22,23	19,93	25,40	3,21	0,38	2,43	11,30	100,00
Papua	42,80	21,44	14,38	13,82	1,78	0,30	0,72	4,77	100,00
**Indonesia**	**20,78**	**33,06**	**22,22**	**15,42**	**4,36**	**0,31**	**0,58**	**3,27**	**100,00**

Sumber/*Source*: Susenas Maret 2018/*The March 2018 Susenas*

https://www.bps.go.id

Tabel *Table* **3.7.6.** **Persentase Penduduk Laki-Laki Berumur 15 Tahun ke Atas di Daerah Perkotaan dan Perdesaan menurut Provinsi dan Ijazah/STTB Tertinggi yang Dimiliki, 2018**
Percentage Percentage of Male Population Aged 15 Years and Over in Urban and Rural Area by Province and The Highest School Certificate Owned, 2018

**Provinsi** *Province*	**Tidak Mempunyai Ijazah** *No Certificate*	**SD/MI** *Primary School*	**SMP/MTs** *Junior High School*	**SMA/MA** *Senior High School*	**SMK/MAK** *Vocational Senior High School*	**Diploma I dan Diploma II** *Diploma I and Diploma II*	**Akademi/Diploma III** *Academy/Diploma III*	**Diploma IV/S1/S2/S3** *Diploma IV/Bachelor/ Master Degree/S3*	**Jumlah** *Total*
(1)	(2)	(3)	(4)	(5)	(6)	(7)	(8)	(9)	(10)
Aceh	11,17	22,17	22,57	30,49	2,74	0,43	1,72	8,71	100,00
Sumatera Utara	8,77	20,28	25,60	30,29	6,89	0,27	1,32	6,59	100,00
Sumatera Barat	18,13	20,39	22,59	23,24	6,86	0,40	1,71	6,68	100,00
Riau	13,09	22,91	21,95	27,90	6,08	0,23	1,25	6,60	100,00
Jambi	13,35	26,65	21,79	26,08	4,26	0,58	1,30	5,99	100,00
Sumatera Selatan	15,69	28,16	21,68	24,26	3,81	0,35	1,18	4,87	100,00
Bengkulu	14,51	25,41	22,18	23,53	4,25	0,46	1,30	8,36	100,00
Lampung	16,12	27,78	25,28	19,38	5,49	0,36	0,92	4,68	100,00
Kepulauan Bangka Belitung	20,91	26,00	19,81	18,30	7,29	0,38	2,25	5,06	100,00
Kepulauan Riau	9,29	16,60	18,44	37,52	8,38	0,54	2,13	7,09	100,00
DKI Jakarta	3,07	10,96	20,65	39,40	8,99	0,53	3,58	12,81	100,00
Jawa Barat	12,42	29,30	21,62	20,76	7,95	0,45	1,66	5,83	100,00
Jawa Tengah	16,76	29,35	24,46	14,79	8,16	0,44	1,28	4,76	100,00
DI Yogyakarta	12,70	15,64	18,87	25,47	14,07	0,39	2,83	10,03	100,00
Jawa Timur	18,04	27,71	21,16	18,28	7,86	0,45	0,80	5,70	100,00
Banten	13,65	23,68	20,44	24,34	8,00	0,41	1,38	8,10	100,00
Bali	13,32	19,44	17,93	29,35	6,89	2,87	1,28	8,91	100,00
Nusa Tenggara Barat	26,93	19,65	19,23	21,00	3,30	0,44	1,20	8,24	100,00
Nusa Tenggara Timur	26,29	29,22	16,13	17,19	2,64	0,50	1,14	6,88	100,00
Kalimantan Barat	24,08	27,10	18,97	19,99	2,64	0,33	1,52	5,37	100,00
Kalimantan Tengah	12,56	30,00	21,61	23,21	3,80	0,52	1,45	6,85	100,00
Kalimantan Selatan	16,53	27,00	20,13	22,91	4,69	0,42	1,44	6,89	100,00
Kalimantan Timur	10,21	19,52	21,12	29,28	8,87	0,57	2,02	8,41	100,00
Kalimantan Utara	13,71	21,13	22,10	26,29	5,50	0,37	2,27	8,65	100,00
Sulawesi Utara	16,24	19,99	20,00	28,63	5,71	0,34	1,44	7,65	100,00
Sulawesi Tengah	15,14	29,62	19,01	23,60	3,50	0,47	0,75	7,91	100,00
Sulawesi Selatan	20,72	23,46	18,44	23,13	3,96	0,43	1,02	8,85	100,00
Sulawesi Tenggara	16,85	19,96	19,85	28,07	3,09	0,47	1,46	10,25	100,00
Gorontalo	33,07	25,11	14,20	15,52	5,31	0,34	0,88	5,57	100,00
Sulawesi Barat	25,04	27,92	17,72	15,30	4,73	0,61	0,92	7,76	100,00
Maluku	11,28	21,24	19,63	34,27	2,86	0,56	1,64	8,53	100,00
Maluku Utara	15,65	23,38	19,56	28,18	3,01	0,60	0,95	8,67	100,00
Papua Barat	11,97	18,79	19,79	29,75	4,83	0,33	2,18	12,36	100,00
Papua	33,18	19,48	15,44	21,00	2,44	0,38	1,16	6,92	100,00
**Indonesia**	**15,29**	**25,47**	**21,53**	**22,36**	**6,82**	**0,47**	**1,42**	**6,63**	**100,00**

Sumber/*Source*: Susenas Maret 2018/*The March 2018 Susenas*

https://www.bps.go.id

Tabel **3.7.7.** **Persentase Penduduk Perempuan Berumur 15 Tahun ke Atas di Daerah Perkotaan menurut Provinsi dan Ijazah/STTB Tertinggi yang Dimiliki, 2018**
Table *Percentage Percentage of Female Population Aged 15 Years and Over in Urban Area by Province and The Highest School Certificate Owned, 2018*

**Provinsi** *Province*	**Tidak Mempunyai Ijazah** *No Certificate*	**SD/MI** *Primary School*	**SMP/MTs** *Junior High School*	**SMA/MA** *Senior High School*	**SMK/MAK** *Vocational Senior High School*	**Diploma I dan Diploma II** *Diploma I and Diploma II*	**Akademi/Diploma III** *Academy/Diploma III*	**Diploma IV/S1/ S2/S3** *Diploma IV/Bachelor/ Master Degree/S3*	**Jumlah** *Total*
(1)	(2)	(3)	(4)	(5)	(6)	(7)	(8)	(9)	(10)
Aceh	11,31	15,23	19,12	31,75	2,74	1,43	6,09	12,32	100,00
Sumatera Utara	7,58	15,75	23,78	33,71	5,41	0,62	3,04	10,11	100,00
Sumatera Barat	13,28	13,70	18,67	28,20	5,61	1,38	4,89	14,28	100,00
Riau	8,56	15,58	21,41	31,77	5,74	1,03	3,91	12,00	100,00
Jambi	13,07	15,17	21,59	31,17	4,61	0,81	3,86	9,72	100,00
Sumatera Selatan	11,36	18,86	20,18	32,85	3,20	1,13	3,73	8,70	100,00
Bengkulu	9,01	16,79	18,90	28,96	4,29	0,93	3,89	17,24	100,00
Lampung	13,24	18,48	23,74	25,65	5,20	0,97	2,89	9,84	100,00
Kepulauan Bangka Belitung	15,81	20,42	21,47	22,90	8,33	0,51	2,98	7,60	100,00
Kepulauan Riau	8,33	14,21	18,55	40,38	7,13	0,60	3,38	7,41	100,00
DKI Jakarta	5,74	15,48	20,81	33,42	8,11	0,58	4,27	11,59	100,00
Jawa Barat	14,97	25,60	22,08	21,65	5,98	0,76	2,60	6,36	100,00
Jawa Tengah	20,26	23,46	21,53	18,21	6,82	0,53	2,75	6,44	100,00
DI Yogyakarta	15,84	13,47	16,10	27,69	9,59	0,56	4,83	11,93	100,00
Jawa Timur	21,04	22,58	20,19	20,16	5,98	0,79	1,86	7,40	100,00
Banten	14,53	20,96	21,44	25,26	6,41	0,68	2,56	8,16	100,00
Bali	17,02	19,97	16,90	25,81	5,63	2,01	1,99	10,68	100,00
Nusa Tenggara Barat	28,71	18,58	19,07	19,48	2,65	0,73	1,91	8,86	100,00
Nusa Tenggara Timur	16,89	16,69	19,60	26,18	3,95	0,94	3,23	12,51	100,00
Kalimantan Barat	18,49	19,52	18,82	25,70	3,83	0,45	3,68	9,50	100,00
Kalimantan Tengah	12,95	20,62	21,63	27,05	3,13	0,97	3,24	10,42	100,00
Kalimantan Selatan	16,13	19,97	20,67	24,62	4,11	0,91	2,94	10,66	100,00
Kalimantan Timur	10,77	19,07	20,59	27,88	6,66	1,03	3,78	10,23	100,00
Kalimantan Utara	12,86	18,93	23,50	27,55	3,84	0,49	3,08	9,75	100,00
Sulawesi Utara	11,45	13,96	19,66	32,35	7,75	0,65	1,89	12,30	100,00
Sulawesi Tengah	12,43	17,60	20,50	26,97	3,23	0,85	4,42	14,00	100,00
Sulawesi Selatan	16,82	17,20	18,06	26,54	2,97	0,70	3,33	14,37	100,00
Sulawesi Tenggara	14,40	12,50	18,34	33,42	2,65	0,69	3,56	14,43	100,00
Gorontalo	20,95	18,92	16,11	24,10	5,05	1,47	2,56	10,84	100,00
Sulawesi Barat	20,91	16,60	20,52	19,94	5,03	0,59	3,36	13,06	100,00
Maluku	8,49	14,51	18,16	37,15	2,62	0,88	3,17	15,01	100,00
Maluku Utara	11,55	13,19	18,85	33,79	3,83	0,58	3,79	14,43	100,00
Papua Barat	8,75	15,04	21,02	31,27	5,18	0,63	4,35	13,78	100,00
Papua	10,38	16,49	20,46	33,09	2,90	0,95	2,99	12,74	100,00
**Indonesia**	**15,31**	**20,85**	**20,92**	**24,65**	**5,92**	**0,77**	**2,91**	**8,67**	**100,00**

Sumber/*Source*: Susenas Maret 2018/*The March 2018 Susenas*

https://www.bps.go.id

Tabel 3.7.8. **Persentase Penduduk Perempuan Berumur 15 Tahun ke Atas di Daerah Perdesaan menurut Provinsi dan Ijazah/STTB Tertinggi yang Dimiliki, 2018**
Table *Percentage Percentage of Female Population Aged 15 Years and Over in Rural Area by Province and The Highest School Certificate Owned, 2018*

**Provinsi** *Province*	**Tidak Mempunyai Ijazah** *No Certificate*	**SD/MI** *Primary School*	**SMP/MTs** *Junior High School*	**SMA/MA** *Senior High School*	**SMK/MAK** *Vocational Senior High School*	**Diploma I dan Diploma II** *Diploma I and Diploma II*	**Akademi/Diploma III** *Academy/Diploma III*	**Diploma IV/S1/S2/S3** *Diploma IV/Bachelor/ Master Degree/S3*	**Jumlah** *Total*
(1)	(2)	(3)	(4)	(5)	(6)	(7)	(8)	(9)	(10)
Aceh	18,01	26,72	22,94	20,61	1,06	1,11	3,05	6,51	100,00
Sumatera Utara	19,63	24,57	24,50	20,91	4,16	0,37	1,58	4,29	100,00
Sumatera Barat	27,90	22,18	19,94	17,14	3,71	0,71	1,84	6,58	100,00
Riau	21,15	29,17	22,27	17,85	2,50	0,45	1,64	4,98	100,00
Jambi	23,09	29,82	21,62	16,54	2,49	0,58	1,58	4,27	100,00
Sumatera Selatan	24,64	33,80	20,36	14,66	1,82	0,30	1,08	3,34	100,00
Bengkulu	23,48	29,32	21,44	16,89	1,66	0,45	1,30	5,46	100,00
Lampung	24,31	28,46	27,01	12,60	3,22	0,36	0,94	3,11	100,00
Kepulauan Bangka Belitung	31,65	30,99	17,93	11,98	3,42	0,35	0,99	2,71	100,00
Kepulauan Riau	34,39	26,48	15,85	15,38	2,43	0,49	0,48	4,49	100,00
DKI Jakarta	-	-	-	-	-	-	-	-	-
Jawa Barat	22,01	44,79	21,52	7,50	1,88	0,19	0,46	1,64	100,00
Jawa Tengah	28,89	33,22	23,02	8,39	3,54	0,24	0,71	1,97	100,00
DI Yogyakarta	27,62	24,45	22,19	10,24	9,12	0,55	1,37	4,46	100,00
Jawa Timur	31,93	31,62	20,28	10,24	2,78	0,25	0,49	2,41	100,00
Banten	26,34	42,03	18,97	8,22	1,84	0,20	0,08	2,31	100,00
Bali	34,70	26,40	18,27	12,03	3,09	1,60	0,97	2,93	100,00
Nusa Tenggara Barat	38,60	23,28	17,72	13,23	1,38	0,27	1,05	4,46	100,00
Nusa Tenggara Timur	30,78	35,94	15,68	10,80	1,19	0,33	1,22	4,06	100,00
Kalimantan Barat	34,73	29,72	19,55	11,42	1,21	0,24	0,75	2,40	100,00
Kalimantan Tengah	20,43	37,75	21,09	13,60	2,04	0,29	1,09	3,70	100,00
Kalimantan Selatan	28,99	32,84	19,12	11,89	1,89	0,29	0,82	4,16	100,00
Kalimantan Timur	19,98	30,92	23,63	17,26	2,38	0,35	0,94	4,54	100,00
Kalimantan Utara	24,46	28,32	18,85	16,81	2,49	0,19	2,91	5,97	100,00
Sulawesi Utara	20,98	24,27	21,45	19,59	5,73	0,76	1,30	5,92	100,00
Sulawesi Tengah	19,78	35,70	18,58	15,48	2,32	1,23	1,36	5,54	100,00
Sulawesi Selatan	30,63	27,58	18,79	12,80	1,90	0,31	1,71	6,27	100,00
Sulawesi Tenggara	25,41	25,55	21,37	17,73	1,74	0,50	2,12	5,58	100,00
Gorontalo	30,26	31,12	16,00	13,31	3,28	0,46	1,00	4,58	100,00
Sulawesi Barat	29,08	28,42	19,04	11,48	3,27	0,56	1,82	6,32	100,00
Maluku	16,38	30,18	19,39	22,56	1,69	1,02	1,72	7,06	100,00
Maluku Utara	22,74	31,18	19,77	15,75	1,57	1,01	2,04	5,95	100,00
Papua Barat	23,24	23,12	20,14	18,94	2,22	0,64	2,54	9,15	100,00
Papua	56,95	20,06	10,85	7,67	0,92	0,25	1,14	2,15	100,00
**Indonesia**	**27,46**	**31,91**	**20,98**	**12,22**	**2,61**	**0,37**	**1,02**	**3,43**	**100,00**

Sumber/*Source*: Susenas Maret 2018/*The March 2018 Susenas*

https://www.bps.go.id

Tabel / *Table* **3.7.9.** **Persentase Penduduk Perempuan Berumur 15 Tahun ke Atas di Daerah Perkotaan dan Perdesaan menurut Provinsi dan Ijazah/STTB Tertinggi yang Dimiliki, 2018**
Percentage Percentage of Female Population Aged 15 Years and Over in Urban and Rural Area by Province and The Highest School Certificate Owned, 2018

**Provinsi** *Province*	**Tidak Mempunyai Ijazah** *No Certificate*	**SD/MI** *Primary School*	**SMP/MTs** *Junior High School*	**SMA/MA** *Senior High School*	**SMK/MAK** *Vocational Senior High School*	**Diploma I dan Diploma II** *Diploma I and Diploma II*	**Akademi/Diploma III** *Academy/Diploma III*	**Diploma IV/S1/S2/S3** *Diploma IV/Bachelor/ Master Degree/S3*	**Jumlah** *Total*
**(1)**	**(2)**	**(3)**	**(4)**	**(5)**	**(6)**	**(7)**	**(8)**	**(9)**	**(10)**
Aceh	15,89	23,08	21,73	24,14	1,59	1,21	4,01	8,35	100,00
Sumatera Utara	13,07	19,76	24,11	27,88	4,84	0,50	2,38	7,47	100,00
Sumatera Barat	21,34	18,38	19,37	22,10	4,56	1,01	3,21	10,04	100,00
Riau	15,97	23,58	21,92	23,58	3,83	0,69	2,57	7,86	100,00
Jambi	19,80	25,01	21,61	21,34	3,19	0,66	2,33	6,06	100,00
Sumatera Selatan	19,55	28,07	20,29	21,63	2,35	0,62	2,10	5,40	100,00
Bengkulu	18,63	25,12	20,59	20,93	2,54	0,61	2,17	9,41	100,00
Lampung	20,93	25,41	26,01	16,58	3,82	0,54	1,54	5,16	100,00
Kepulauan Bangka Belitung	23,01	25,23	19,86	17,93	6,10	0,44	2,07	5,37	100,00
Kepulauan Riau	11,97	15,92	18,18	36,89	6,47	0,59	2,98	7,00	100,00
DKI Jakarta	5,74	15,48	20,81	33,42	8,11	0,58	4,27	11,59	100,00
Jawa Barat	16,80	30,58	21,93	17,98	4,92	0,61	2,04	5,14	100,00
Jawa Tengah	24,46	28,20	22,26	13,44	5,22	0,39	1,76	4,27	100,00
DI Yogyakarta	19,09	16,51	17,78	22,87	9,46	0,56	3,87	9,87	100,00
Jawa Timur	26,25	26,91	20,23	15,41	4,45	0,53	1,21	5,01	100,00
Banten	17,82	26,83	20,75	20,51	5,14	0,55	1,87	6,53	100,00
Bali	23,06	22,17	17,37	21,10	4,76	1,87	1,64	8,03	100,00
Nusa Tenggara Barat	33,95	21,07	18,36	16,17	1,98	0,49	1,45	6,53	100,00
Nusa Tenggara Timur	27,45	31,33	16,62	14,48	1,85	0,48	1,70	6,08	100,00
Kalimantan Barat	29,06	26,16	19,29	16,40	2,12	0,31	1,77	4,88	100,00
Kalimantan Tengah	17,48	30,99	21,30	18,91	2,47	0,56	1,94	6,35	100,00
Kalimantan Selatan	22,94	26,79	19,85	17,88	2,93	0,58	1,81	7,22	100,00
Kalimantan Timur	13,71	22,86	21,56	24,48	5,29	0,81	2,87	8,41	100,00
Kalimantan Utara	17,59	22,76	21,60	23,17	3,29	0,36	3,01	8,20	100,00
Sulawesi Utara	16,08	18,97	20,53	26,15	6,77	0,70	1,60	9,20	100,00
Sulawesi Tengah	17,64	30,41	19,14	18,84	2,58	1,12	2,26	8,01	100,00
Sulawesi Selatan	24,87	23,25	18,48	18,53	2,35	0,47	2,39	9,65	100,00
Sulawesi Tenggara	21,10	20,44	20,19	23,87	2,10	0,58	2,69	9,04	100,00
Gorontalo	26,52	26,22	16,04	17,64	3,99	0,86	1,62	7,09	100,00
Sulawesi Barat	27,09	25,54	19,40	13,54	3,70	0,57	2,20	7,96	100,00
Maluku	12,94	23,34	18,86	28,92	2,10	0,96	2,36	10,53	100,00
Maluku Utara	19,34	25,71	19,49	21,23	2,26	0,88	2,57	8,53	100,00
Papua Barat	17,17	19,74	20,51	24,10	3,46	0,63	3,30	11,09	100,00
Papua	44,00	19,07	13,52	14,74	1,47	0,45	1,66	5,09	100,00
**Indonesia**	**20,74**	**25,79**	**20,94**	**19,10**	**4,44**	**0,59**	**2,07**	**6,33**	**100,00**

Sumber/*Source*: Susenas Maret 2018/*The March 2018 Susenas*

https://www.bps.go.id

Tabel **3.8.1.** **Persentase Penduduk 0-6 Tahun di Daerah Perkotaan menurut Provinsi dan Keikutsertaan Pendidikan Pra Sekolah, 2018**
Table *Percentage Percentage of Population Aged 0-6 Years in Urban Area by Province and The Participation of Pre-School Education, 2018*

**Provinsi** *Province*	**Masih/Pernah Mengikuti Pra Sekolah TA 2017/2018** *Still in Pre-School in This School Year (2017/2018)*	**Pernah Mengikuti Pra Sekolah TA 2017/2018 dan Sebelum TA 2017/2018** *Attended Pre-School During Last School Year (2017/2018) and Before Last School Year*	**Tidak/Belum Pernah Mengikuti Pra Sekolah** *Do Not/Never Attended Pre-School*	**Jumlah** *Total*
**(1)**	**(2)**	**(3)**	**(4)**	**(5)**
Aceh	19,62	10,25	70,13	100,00
Sumatera Utara	15,20	8,41	76,39	100,00
Sumatera Barat	17,02	4,98	78,00	100,00
Riau	15,24	7,47	77,29	100,00
Jambi	15,88	7,99	76,13	100,00
Sumatera Selatan	14,79	8,64	76,57	100,00
Bengkulu	18,24	8,52	73,24	100,00
Lampung	17,96	8,32	73,72	100,00
Kepulauan Bangka Belitung	23,87	8,47	67,66	100,00
Kepulauan Riau	14,25	6,02	79,73	100,00
DKI Jakarta	23,48	5,98	70,54	100,00
Jawa Barat	20,70	6,18	73,12	100,00
Jawa Tengah	30,20	7,57	62,23	100,00
DI Yogyakarta	42,94	3,19	53,87	100,00
Jawa Timur	33,12	6,95	59,93	100,00
Banten	19,44	7,32	73,24	100,00
Bali	21,72	4,93	73,35	100,00
Nusa Tenggara Barat	22,27	4,42	73,31	100,00
Nusa Tenggara Timur	19,39	6,21	74,40	100,00
Kalimantan Barat	13,69	6,52	79,79	100,00
Kalimantan Tengah	21,70	6,44	71,86	100,00
Kalimantan Selatan	26,33	6,77	66,90	100,00
Kalimantan Timur	17,39	6,43	76,18	100,00
Kalimantan Utara	13,39	4,90	81,71	100,00
Sulawesi Utara	15,01	9,58	75,41	100,00
Sulawesi Tengah	19,75	6,22	74,03	100,00
Sulawesi Selatan	17,14	7,03	75,83	100,00
Sulawesi Tenggara	18,76	9,02	72,22	100,00
Gorontalo	25,71	7,30	66,99	100,00
Sulawesi Barat	24,89	4,68	70,43	100,00
Maluku	12,74	7,74	79,52	100,00
Maluku Utara	13,27	7,89	78,84	100,00
Papua Barat	12,82	5,92	81,26	100,00
Papua	10,96	6,74	82,30	100,00
**Indonesia**	**22,53**	**6,81**	**70,66**	**100,00**

Sumber/*Source*: Susenas Maret 2018/*The March 2018 Susenas*

https://www.bps.go.id

Tabel **3.8.2.** **Persentase Penduduk 0-6 Tahun di Daerah Perdesaan menurut Provinsi dan Keikutsertaan Pendidikan Pra Sekolah, 2018**
Table *Percentage Percentage of Population Aged 0-6 Years in Rural Area by Province and The Participation of Pre-School Education, 2018*

**Provinsi** *Province*	**Masih/Pernah Mengikuti Pra Sekolah TA 2017/2018** *Still in Pre-School in This School Year (2017/2018)*	**Pernah Mengikuti Pra Sekolah TA 2017/2018 dan Sebelum TA 2017/2018** *Attended Pre-School During Last School Year (2017/2018) and Before Last School Year*	**Tidak/Belum Pernah Mengikuti Pra Sekolah** *Do Not/Never Attended Pre-School*	**Jumlah** *Total*
(1)	(2)	(3)	(4)	(5)
Aceh	16,05	8,59	75,36	100,00
Sumatera Utara	12,39	6,84	80,77	100,00
Sumatera Barat	15,43	6,16	78,41	100,00
Riau	16,35	6,06	77,59	100,00
Jambi	18,78	8,05	73,17	100,00
Sumatera Selatan	14,44	7,60	77,96	100,00
Bengkulu	15,49	7,43	77,08	100,00
Lampung	18,01	7,49	74,49	100,00
Kepulauan Bangka Belitung	17,85	7,89	74,26	100,00
Kepulauan Riau	21,98	7,76	70,26	100,00
DKI Jakarta	-	-	-	-
Jawa Barat	19,98	4,47	75,55	100,00
Jawa Tengah	26,27	8,44	65,29	100,00
DI Yogyakarta	48,44	6,52	45,04	100,00
Jawa Timur	31,94	6,78	61,28	100,00
Banten	11,55	5,60	82,85	100,00
Bali	17,21	3,62	79,16	100,00
Nusa Tenggara Barat	22,77	5,63	71,60	100,00
Nusa Tenggara Timur	17,90	6,43	75,67	100,00
Kalimantan Barat	11,92	3,68	84,41	100,00
Kalimantan Tengah	21,89	7,42	70,69	100,00
Kalimantan Selatan	26,55	6,96	66,49	100,00
Kalimantan Timur	20,16	7,83	72,01	100,00
Kalimantan Utara	23,91	5,54	70,55	100,00
Sulawesi Utara	19,61	10,08	70,31	100,00
Sulawesi Tengah	24,42	6,99	68,59	100,00
Sulawesi Selatan	18,30	6,50	75,19	100,00
Sulawesi Tenggara	16,68	7,72	75,60	100,00
Gorontalo	30,28	7,57	62,15	100,00
Sulawesi Barat	23,93	6,29	69,79	100,00
Maluku	19,77	6,62	73,62	100,00
Maluku Utara	20,73	8,51	70,76	100,00
Papua Barat	15,41	5,13	79,45	100,00
Papua	6,41	2,02	91,57	100,00
**Indonesia**	**20,38**	**6,64**	**72,98**	**100,00**

Sumber/*Source*: Susenas Maret 2018/*The March 2018 Susenas*

https://www.bps.go.id

Tabel **Persentase Penduduk 0-6 Tahun di Daerah Perkotaan dan Perdesaan menurut Provinsi dan Keikutsertaan Pendidikan Pra Sekolah, 2018**
Table **3.8.3.** *Percentage Percentage of Population Aged 0-6 Years in Urban and Rural Area by Province and The Participation of Pre-School Education, 2018*

**Provinsi** *Province*	**Masih/Pernah Mengikuti Pra Sekolah TA 2017/2018** *Still in Pre-School in This School Year (2017/2018)*	**Pernah Mengikuti Pra Sekolah TA 2017/2018 dan Sebelum TA 2017/2018** *Attended Pre-School During Last School Year (2017/2018) and Before Last School Year*	**Tidak/Belum Pernah Mengikuti Pra Sekolah** *Do Not/Never Attended Pre-School*	**Jumlah** *Total*
(1)	(2)	(3)	(4)	(5)
Aceh	17,14	9,10	73,76	100,00
Sumatera Utara	13,79	7,62	78,59	100,00
Sumatera Barat	16,11	5,65	78,23	100,00
Riau	15,90	6,63	77,47	100,00
Jambi	17,88	8,03	74,09	100,00
Sumatera Selatan	14,57	7,97	77,46	100,00
Bengkulu	16,39	7,78	75,83	100,00
Lampung	18,00	7,73	74,27	100,00
Kepulauan Bangka Belitung	21,08	8,20	70,72	100,00
Kepulauan Riau	15,29	6,25	78,46	100,00
DKI Jakarta	23,48	5,98	70,54	100,00
Jawa Barat	20,52	5,74	73,74	100,00
Jawa Tengah	28,28	8,00	63,73	100,00
DI Yogyakarta	44,39	4,06	51,55	100,00
Jawa Timur	32,57	6,87	60,57	100,00
Banten	17,07	6,80	76,13	100,00
Bali	20,26	4,50	75,24	100,00
Nusa Tenggara Barat	22,55	5,08	72,38	100,00
Nusa Tenggara Timur	18,22	6,38	75,40	100,00
Kalimantan Barat	12,50	4,61	82,89	100,00
Kalimantan Tengah	21,82	7,06	71,12	100,00
Kalimantan Selatan	26,45	6,87	66,68	100,00
Kalimantan Timur	18,33	6,91	74,77	100,00
Kalimantan Utara	17,82	5,17	77,01	100,00
Sulawesi Utara	17,36	9,83	72,81	100,00
Sulawesi Tengah	23,15	6,78	70,08	100,00
Sulawesi Selatan	17,83	6,72	75,45	100,00
Sulawesi Tenggara	17,46	8,21	74,33	100,00
Gorontalo	28,57	7,47	63,96	100,00
Sulawesi Barat	24,15	5,92	69,94	100,00
Maluku	17,10	7,04	75,86	100,00
Maluku Utara	18,73	8,35	72,92	100,00
Papua Barat	14,39	5,44	80,17	100,00
Papua	7,63	3,28	89,09	100,00
**Indonesia**	**21,53**	**6,73**	**71,74**	**100,00**

Sumber/*Source*: Susenas Maret 2018/*The March 2018 Susenas*

https://www.bps.go.id

Tabel *Table* **3.9.1.** **Persentase Penduduk Berumur 0-6 Tahun yang Pernah/Masih Mengikuti Pendidikan Pra Sekolah di Daerah Perkotaan menurut Provinsi dan Jenis Pendidikan Pra Sekolah, 2018**
Percentage of Population Aged 0-6 Years who Has Ever/Still Attended Pre-School Education in Urban Area by Province and Type of Pre-School Education, 2018

**Provinsi** *Province*	**Taman Kanak-Kanak** *Kindergarten*	**Bustanul Athfal/Raudatul Athfal**	**PAUD/PAUD Terintegrasi BKB/Taman Posyandu, dll** *ECD Post, HI ECD, BKB/Posyandu, etc*	**Kelompok Bermain** *Playgroup*	**Taman Penitipan Anak** *Child Day Care*	**Jumlah** *Total*
(1)	(2)	(3)	(4)	(5)	(6)	(7)
Aceh	81,86	2,31	14,21	0,37	1,25	100,00
Sumatera Utara	68,62	12,48	15,87	3,00	0,03	100,00
Sumatera Barat	74,26	2,70	21,64	0,83	0,57	100,00
Riau	79,45	2,38	14,81	2,21	1,15	100,00
Jambi	68,54	1,72	24,15	5,04	0,55	100,00
Sumatera Selatan	74,61	1,95	22,29	0,50	0,65	100,00
Bengkulu	67,79	3,28	23,44	1,81	3,68	100,00
Lampung	75,46	4,33	19,18	1,03	0,00	100,00
Kepulauan Bangka Belitung	74,33	0,98	23,12	1,57	0,00	100,00
Kepulauan Riau	65,14	9,36	24,94	0,30	0,26	100,00
DKI Jakarta	52,60	3,73	39,28	3,33	1,06	100,00
Jawa Barat	49,42	8,29	39,02	2,27	1,00	100,00
Jawa Tengah	66,00	9,42	21,50	2,69	0,39	100,00
DI Yogyakarta	59,47	7,71	24,12	7,09	1,61	100,00
Jawa Timur	65,96	7,49	24,10	2,34	0,11	100,00
Banten	55,18	6,27	35,62	1,03	1,90	100,00
Bali	83,96	1,25	12,98	1,81	0,00	100,00
Nusa Tenggara Barat	63,70	4,31	30,60	1,27	0,12	100,00
Nusa Tenggara Timur	57,11	2,13	34,77	4,30	1,69	100,00
Kalimantan Barat	75,01	2,06	22,93	0,00	0,00	100,00
Kalimantan Tengah	79,13	3,24	12,24	3,73	1,66	100,00
Kalimantan Selatan	82,80	3,61	10,45	1,98	1,16	100,00
Kalimantan Timur	75,20	1,77	19,63	2,75	0,65	100,00
Kalimantan Utara	69,33	3,59	23,61	1,42	2,05	100,00
Sulawesi Utara	83,42	1,97	12,41	1,94	0,26	100,00
Sulawesi Tengah	78,89	1,53	19,05	0,53	0,00	100,00
Sulawesi Selatan	85,32	1,89	10,40	1,92	0,47	100,00
Sulawesi Tenggara	77,22	2,29	17,17	3,32	0,00	100,00
Gorontalo	78,83	0,38	17,70	1,69	1,40	100,00
Sulawesi Barat	66,58	3,09	23,94	6,39	0,00	100,00
Maluku	66,39	0,19	31,35	2,07	0,00	100,00
Maluku Utara	73,60	0,86	23,01	2,53	0,00	100,00
Papua Barat	70,74	4,65	22,21	2,40	0,00	100,00
Papua	71,50	3,75	20,74	3,07	0,94	100,00
**Indonesia**	**63,00**	**6,61**	**27,31**	**2,38**	**0,70**	**100,00**

Sumber/*Source*: Susenas Maret 2018/*The March 2018 Susenas*

https://www.bps.go.id

Tabel **3.9.2.** **Persentase Penduduk Berumur 0-6 Tahun yang Pernah/Masih Mengikuti Pendidikan Pra Sekolah di Daerah Perdesaan menurut Provinsi dan Jenis Pendidikan Pra Sekolah, 2018**
Table *Percentage of Population Aged 0-6 Years who Has Ever/Still Attended Pre-School Education in Rural Area by Province and Type of Pre-School Education, 2018*

**Provinsi** *Province*	**Taman Kanak-Kanak** *Kindergarten*	**Bustanul Athfal/Raudatul Athfal**	**PAUD/PAUD Terintegrasi BKB/ Taman Posyandu, dll** *ECD Post, HI ECD, BKB/Posyandu, etc*	**Kelompok Bermain** *Playgroup*	**Taman Penitipan Anak** *Child Day Care*	**Jumlah** *Total*
(1)	(2)	(3)	(4)	(5)	(6)	(7)
Aceh	78,18	1,93	18,75	1,01	0,12	100,00
Sumatera Utara	52,08	6,16	40,83	0,75	0,18	100,00
Sumatera Barat	67,28	3,31	27,65	0,60	1,15	100,00
Riau	71,18	1,33	25,83	1,32	0,34	100,00
Jambi	56,48	3,11	37,74	1,31	1,36	100,00
Sumatera Selatan	60,54	1,41	37,19	0,37	0,50	100,00
Bengkulu	40,37	2,82	53,96	2,14	0,71	100,00
Lampung	66,78	4,94	27,47	0,68	0,12	100,00
Kepulauan Bangka Belitung	54,59	0,84	43,07	0,70	0,81	100,00
Kepulauan Riau	41,97	2,98	52,14	2,90	0,00	100,00
DKI Jakarta	-	-	-	-	-	-
Jawa Barat	32,40	10,08	55,62	1,08	0,82	100,00
Jawa Tengah	64,64	8,75	24,76	1,69	0,16	100,00
DI Yogyakarta	48,98	8,18	37,40	5,44	0,00	100,00
Jawa Timur	60,89	9,23	26,49	2,92	0,47	100,00
Banten	28,58	1,82	66,29	2,31	0,99	100,00
Bali	83,98	2,92	12,20	0,91	0,00	100,00
Nusa Tenggara Barat	54,26	4,19	39,42	2,13	0,00	100,00
Nusa Tenggara Timur	33,79	1,54	62,64	1,83	0,20	100,00
Kalimantan Barat	31,43	1,89	64,86	1,11	0,70	100,00
Kalimantan Tengah	79,67	0,47	17,30	1,10	1,45	100,00
Kalimantan Selatan	73,53	3,48	21,50	1,31	0,18	100,00
Kalimantan Timur	77,83	1,38	18,52	2,26	0,00	100,00
Kalimantan Utara	46,13	0,59	53,14	0,14	0,00	100,00
Sulawesi Utara	70,21	1,54	26,00	2,04	0,21	100,00
Sulawesi Tengah	64,02	1,11	31,59	2,92	0,36	100,00
Sulawesi Selatan	75,97	4,48	16,90	2,31	0,33	100,00
Sulawesi Tenggara	83,50	1,53	13,80	0,15	1,02	100,00
Gorontalo	53,30	0,69	44,07	1,94	0,00	100,00
Sulawesi Barat	47,81	6,17	42,04	3,82	0,16	100,00
Maluku	44,01	1,64	53,32	0,89	0,14	100,00
Maluku Utara	48,35	1,16	49,36	1,12	0,00	100,00
Papua Barat	48,48	6,23	44,13	1,16	0,01	100,00
Papua	48,95	1,82	47,43	1,11	0,68	100,00
**Indonesia**	**58,20**	**5,64**	**34,02**	**1,73**	**0,41**	**100,00**

Sumber/*Source*: Susenas Maret 2018/*The March 2018 Susenas*

https://www.bps.go.id

Tabel 3.9.3. **Persentase Penduduk Berumur 0-6 Tahun yang Pernah/Masih Mengikuti Pendidikan Pra Sekolah di Daerah Perkotaan dan Perdesaan menurut Provinsi dan Jenis Pendidikan Pra Sekolah, 2018**
Table *Percentage of Population Aged 0-6 Years who Has Ever/Still Attended Pre-School Education in Urban and Rural Area by Province and Type of Pre-School Education, 2018*

**Provinsi** *Province*	**Taman Kanak-Kanak** *Kindergarten*	**Bustanul Athfal/Raudatul Athfal**	**PAUD/PAUD Terintegrasi BKB/ Taman Posyandu, dll** *ECD Post, HI ECD, BKB/Posyandu, etc*	**Kelompok Bermain** *Playgroup*	**Taman Penitipan Anak** *Child Day Care*	**Jumlah** *Total*
(1)	(2)	(3)	(4)	(5)	(6)	(7)
Aceh	79,46	2,07	17,17	0,79	0,52	100,00
Sumatera Utara	61,16	9,63	27,13	1,99	0,09	100,00
Sumatera Barat	70,32	3,04	25,04	0,70	0,90	100,00
Riau	74,51	1,75	21,39	1,68	0,67	100,00
Jambi	59,94	2,71	33,84	2,38	1,13	100,00
Sumatera Selatan	65,75	1,61	31,67	0,41	0,55	100,00
Bengkulu	50,31	2,99	42,90	2,02	1,79	100,00
Lampung	69,33	4,76	25,03	0,79	0,09	100,00
Kepulauan Bangka Belitung	66,28	0,92	31,25	1,21	0,33	100,00
Kepulauan Riau	60,84	8,17	29,99	0,79	0,21	100,00
DKI Jakarta	52,60	3,73	39,28	3,33	1,06	100,00
Jawa Barat	45,38	8,71	42,96	1,99	0,96	100,00
Jawa Tengah	65,36	9,10	23,03	2,22	0,28	100,00
DI Yogyakarta	56,34	7,85	28,08	6,60	1,13	100,00
Jawa Timur	63,61	8,30	25,21	2,61	0,27	100,00
Banten	49,44	5,31	42,24	1,31	1,70	100,00
Bali	83,97	1,70	12,77	1,56	0,00	100,00
Nusa Tenggara Barat	58,40	4,24	35,55	1,75	0,05	100,00
Nusa Tenggara Timur	38,96	1,67	56,45	2,38	0,53	100,00
Kalimantan Barat	48,29	1,96	48,64	0,68	0,43	100,00
Kalimantan Tengah	79,47	1,47	15,47	2,05	1,53	100,00
Kalimantan Selatan	77,77	3,54	16,44	1,62	0,63	100,00
Kalimantan Timur	76,19	1,63	19,22	2,56	0,41	100,00
Kalimantan Utara	56,81	1,97	39,54	0,73	0,94	100,00
Sulawesi Utara	76,05	1,73	19,99	1,99	0,24	100,00
Sulawesi Tengah	67,55	1,21	28,62	2,35	0,28	100,00
Sulawesi Selatan	79,72	3,44	14,30	2,16	0,39	100,00
Sulawesi Tenggara	80,96	1,84	15,16	1,44	0,61	100,00
Gorontalo	62,05	0,58	35,03	1,86	0,48	100,00
Sulawesi Barat	52,06	5,47	37,94	4,40	0,13	100,00
Maluku	51,20	1,17	46,26	1,27	0,10	100,00
Maluku Utara	53,63	1,10	43,85	1,42	0,00	100,00
Papua Barat	56,76	5,64	35,98	1,62	0,01	100,00
Papua	58,73	2,66	35,86	1,96	0,79	100,00
**Indonesia**	**60,86**	**6,18**	**30,29**	**2,09**	**0,57**	**100,00**

Sumber/*Source*: Susenas Maret 2018/*The March 2018 Susenas*

https://www.bps.go.id

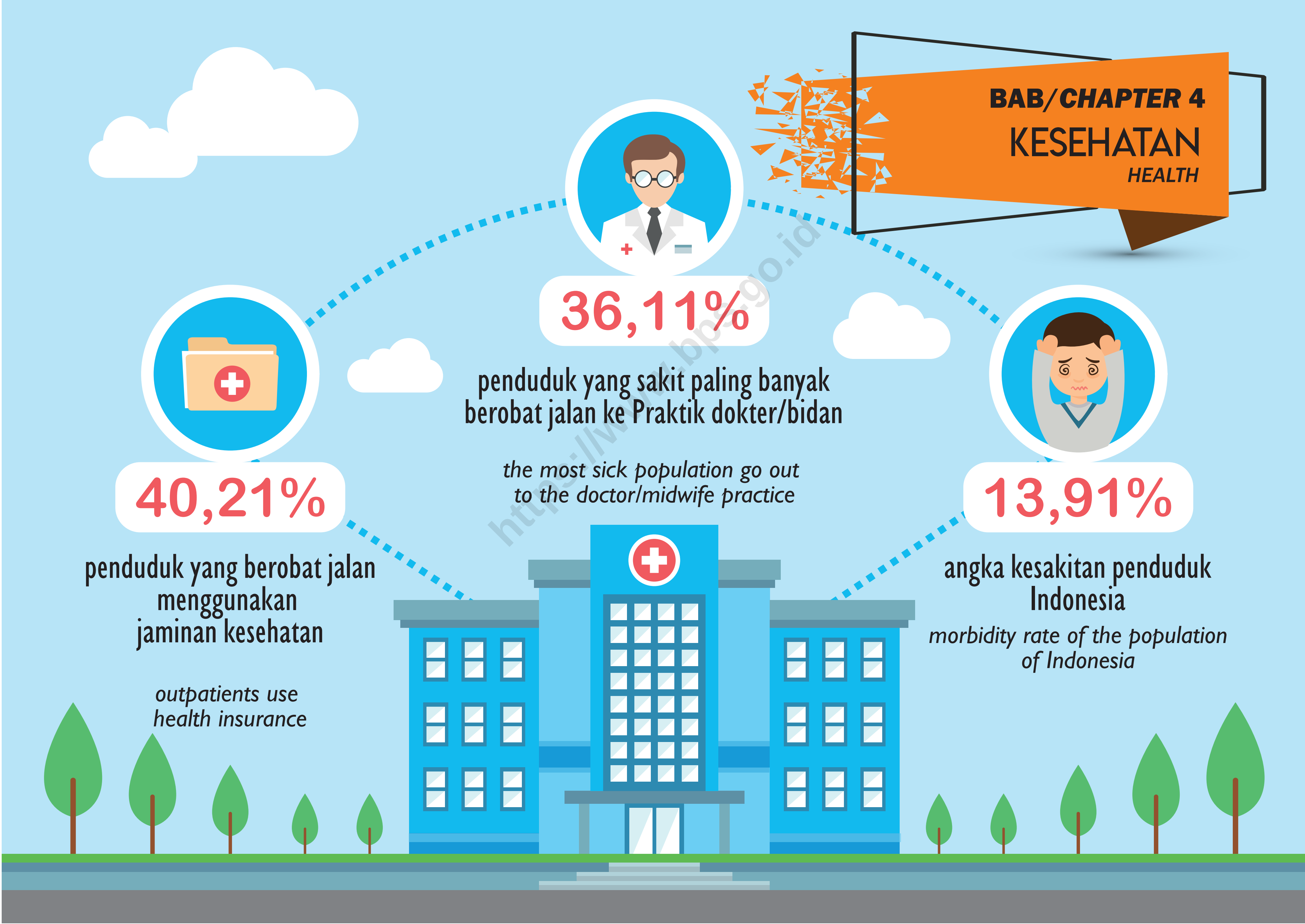

https://www.bps.go.id

IV. KESEHATAN/*HEALTH*

PENJELASAN TEKNIS

1. **Keluhan kesehatan** adalah keadaan seseorang yang mengalami gangguan kesehatan atau kejiwaan, baik karena gangguan/penyakit yang sering dialami penduduk seperti panas, pilek, diare, pusing, sakit kepala, maupun karena penyakit akut, penyakit kronis (meskipun selama sebulan terakhir tidak mempunyai keluhan), kecelakaan, kriminalitas, atau keluhan lainnya.

2. **Menderita sakit** adalah mengalami keluhan kesehatan dan terganggunya pekerjaan, sekolah, atau kegiatan sehari-hari (tidak dapat melakukan kegiatan secara normal seperti bekerja, sekolah, atau kegiatan sehari-hari sebagaimana biasanya).

3. **Berobat jalan** adalah upaya anggota ruta yang mempunyai keluhan kesehatan untuk memeriksakan diri dan mendapatkan pengobatan dengan mendatangi tempat-tempat pelayanan kesehatan modern atau tradisional tanpa menginap, termasuk mendatangkan petugas kesehatan ke rumah anggota ruta.

4. **Jaminan kesehatan** adalah program bantuan sosial untuk pelayanan kesehatan. Menurut UU no. 40 tahun 2004 tentang sistem jaminan sosial nasional, jaminan kesehatan diselenggarakan dengan tujuan menjamin agar peserta memperoleh manfaat pemeliharaan kesehatan dan perlindungan dalam memenuhi kebutuhan dasar kesehatan.

TECHNICAL NOTES

1. ***Health complaint*** *is a state of someone who is experiencing health or psychiatric disorders, either because of disorders/diseases that are often experienced by people such as heat, cold, diarrhea, dizziness, headache, or because of acute illness, chronic disease (although during the past month did not have any complaints), accident, kriminalitas, or other complaints.*

2. ***Fell sick*** *is having health complaints and disruption of work, school or daily activities (can not perform activities such as work, school or daily activities normally as usual).*

3. ***Outpatient*** *are efforts household member who have health complaints for check-ups and treatment by visiting places of modern or traditional health care without a stay, including bringing health workers to the homes of household member.*

4. ***Health insurance*** *is a social assistance program for the health service. According to Law no. 40 of 2004 on the national social security system, health insurance was organized with the aim of ensuring that participants receive the benefits of health care and protection to meet basic health needs.*

https://www.bps.go.id

5. **Rawat inap** adalah upaya penyembuhan keluhan kesehatan dengan menginap 1 malam atau lebih di unit pelayanan kesehatan modern atau tradisional, termasuk dalam kejadian ini adalah rawat inap untuk persalinan.

6. **Merokok** merupakan aktifitas membakar tembakau kemudian menghisap asapnya baik menggunakan rokok maupun pipa pada sebulan terakhir sampai saat pencacahan. Terdapat 2 (dua) cara merokok yang umum dilakukan, yaitu pertama menghisap lalu menelan asap rokok ke dalam paru-paru dan dihembuskan; kedua hanya menghisap sampai mulut lalu dihembuskan melalui mulut atau hidung.

7. **Imunisasi** didefinisikan sebagai suatu upaya untuk menimbulkan/ meningkatkan kekebalan seseorang secara aktif terhadap suatu penyakit, sehingga bila suatu saat terpajan dengan penyakit tersebut tidak akan sakit atau hanya mengalami sakit ringan. Vaksin adalah antigen berupa mikroorganisme yang sudah mati, masih hidup tapi dilemahkan, masih utuh atau bagiannya, yang telah diolah, berupa toksin mikroorganisme yang telah diolah menjadi toksoid, protein rekombinan yang bila diberikan kepada seseorang akan menimbulkan kekebalan spesifik secara aktif terhadap penyakit infeksi tertentu.

*5. **Inpatient** is healing efforts of health complaints by staying one night or more in the modern or traditional health care unit, included in this incident was inpatient for childbirth.*

*6. **Smoking** is an activity to burn tobacco and then inhale the smoke either cigarettes or pipes used in the past month till the time of enumeration. There are two (2) ways of smoking are common, the first suck and swallow the smoke into the lungs and exhaled; second only to suck up to her mouth and exhaled through the mouth or nose.*

*7. **Immunization** is defined as an attempt to induce/enhance a person's active immunity against a disease, so that if one day of exposure to the disease will not get sick or experience only mild illness. The vaccine is an antigen such as microorganisms that are already dead, is still alive but weakened, intact or parts thereof, which have been processed, the form of the toxin microorganisms that have been processed into toxoid, recombinant protein when administered to an individual will generate specific immune active against certain infections.*

https://www.bps.go.id

Tabel *Table* **4.1.** **Persentase Penduduk yang Mempunyai Keluhan Kesehatan selama Sebulan Terakhir menurut Provinsi, Daerah Tempat Tinggal, dan Jenis Kelamin, 2018**
Percentage of Population Who Had Health Complaint during the Last Month by Province, Urban Rural Classification, and Sex, 2018

**Provinsi** *Province*	**Perkotaan/***Urban*			**Perdesaan/***Rural*			**Perkotaan+Perdesaan/***Urban+Rural*		
	**Laki-laki** *Male*	**Perempuan** *Female*	**Laki-laki+Perempuan** *Male+Female*	**Laki-laki** *Male*	**Perempuan** *Female*	**Laki-laki+Perempuan** *Male+Female*	**Laki-laki/** *Male*	**Perempuan/** *Female*	**Laki-laki+Perempuan** *Male+Female*
(1)	(2)	(3)	(4)	(5)	(6)	(7)	(8)	(9)	(10)
Aceh	24,71	29,61	27,15	28,91	34,18	31,56	27,59	32,76	30,18
Sumatera Utara	23,00	26,26	24,64	24,25	25,99	25,12	23,59	26,13	24,86
Sumatera Barat	27,54	32,32	29,94	26,83	31,00	28,92	27,14	31,58	29,37
Riau	27,00	29,77	28,35	29,89	31,07	30,47	28,73	30,54	29,61
Jambi	21,03	21,85	21,43	21,51	24,31	22,88	21,36	23,51	22,41
Sumatera Selatan	28,40	34,17	31,27	25,88	28,09	26,96	26,81	30,38	28,57
Bengkulu	23,86	25,59	24,72	26,73	29,78	28,21	25,81	28,39	27,07
Lampung	29,03	30,61	29,82	29,60	32,51	31,01	29,43	31,94	30,66
Kepulauan Bangka Belitung	26,95	32,34	29,56	26,81	30,73	28,67	26,88	31,60	29,15
Kepulauan Riau	20,92	23,04	21,96	24,49	25,64	25,05	21,43	23,40	22,40
DKI Jakarta	26,92	30,75	28,83	-	-	-	26,92	30,75	28,83
Jawa Barat	28,65	31,87	30,24	31,90	34,75	33,30	29,49	32,62	31,03
Jawa Tengah	32,98	36,88	34,95	31,71	35,72	33,73	32,36	36,32	34,36
DI Yogyakarta	34,19	36,17	35,18	33,88	37,36	35,68	34,11	36,50	35,32
Jawa Timur	31,53	35,12	33,34	33,13	35,45	34,31	32,29	35,28	33,80
Banten	29,58	31,62	30,58	34,02	39,96	36,92	30,87	34,02	32,41
Bali	26,25	28,82	27,52	34,51	37,15	35,83	29,01	31,66	30,33
Nusa Tenggara Barat	38,02	41,13	39,63	35,46	39,93	37,75	36,65	40,49	38,62
Nusa Tenggara Timur	33,60	36,92	35,26	36,79	40,03	38,43	36,05	39,33	37,71
Kalimantan Barat	25,26	28,86	27,06	28,43	30,65	29,51	27,38	30,04	28,68
Kalimantan Tengah	26,71	28,75	27,70	26,64	30,50	28,47	26,67	29,81	28,17
Kalimantan Selatan	30,58	34,66	32,59	31,96	35,76	33,84	31,32	35,25	33,26
Kalimantan Timur	27,00	31,34	29,08	24,40	26,20	25,24	26,13	29,66	27,81
Kalimantan Utara	26,84	31,17	28,90	32,65	35,85	34,14	29,30	33,11	31,09
Sulawesi Utara	25,66	27,69	26,67	30,20	32,62	31,37	27,95	30,12	29,01
Sulawesi Tengah	29,73	34,83	32,27	29,78	32,53	31,12	29,77	33,18	31,44
Sulawesi Selatan	28,79	31,88	30,37	27,32	29,76	28,57	27,94	30,64	29,32
Sulawesi Tenggara	24,55	28,53	26,53	26,57	30,65	28,60	25,80	29,84	27,81
Gorontalo	34,03	36,01	35,04	37,03	39,30	38,15	35,89	38,01	36,95
Sulawesi Barat	31,39	31,38	31,39	27,92	29,85	28,88	28,73	30,22	29,47
Maluku	18,13	20,57	19,34	20,21	20,72	20,46	19,34	20,66	19,99
Maluku Utara	19,17	21,57	20,35	19,04	20,56	19,78	19,07	20,85	19,94
Papua Barat	21,67	27,26	24,33	22,10	24,40	23,18	21,92	25,57	23,65
Papua	19,98	21,04	20,47	16,39	16,62	16,50	17,40	17,82	17,60
**Indonesia**	**28,94**	**32,33**	**30,63**	**29,86**	**32,89**	**31,37**	**29,36**	**32,58**	**30,96**

Sumber/*Source*: Susenas Maret 2018/*The March 2018 Susenas*

https://www.bps.go.id

Tabel **4.2.** **Angka Kesakitan menurut Provinsi, Daerah Tempat Tinggal, dan Jenis Kelamin, 2018**
Table *Morbidity Rate by Province, Urban Rural Classification, and Sex, 2018*

**Provinsi** *Province*	**Perkotaan/***Urban*			**Perdesaan/***Rural*			**Perkotaan+Perdesaan/***Urban+Rural*		
	**Laki-laki** *Male*	**Perempuan** *Female*	**Laki-laki+Perempuan** *Male+Female*	**Laki-laki** *Male*	**Perempuan** *Female*	**Laki-laki+Perempuan** *Male+Female*	**Laki-laki/** *Male*	**Perempuan/** *Female*	**Laki-laki+Perempuan** *Male+Female*
**(1)**	**(2)**	**(3)**	**(4)**	**(5)**	**(6)**	**(7)**	**(8)**	**(9)**	**(10)**
Aceh	11,76	13,58	12,66	15,27	16,99	16,13	14,16	15,92	15,04
Sumatera Utara	9,95	10,68	10,31	12,25	12,91	12,58	11,03	11,73	11,38
Sumatera Barat	11,68	13,07	12,38	14,20	15,90	15,06	13,09	14,65	13,88
Riau	10,50	12,12	11,29	14,13	14,11	14,12	12,67	13,30	12,98
Jambi	8,72	8,46	8,59	10,62	12,42	11,50	10,02	11,14	10,57
Sumatera Selatan	8,17	9,63	8,90	9,98	10,95	10,46	9,32	10,45	9,87
Bengkulu	9,53	10,18	9,85	12,28	14,30	13,26	11,40	12,93	12,15
Lampung	13,30	13,41	13,36	14,10	14,79	14,44	13,87	14,38	14,12
Kepulauan Bangka Belitung	8,84	11,26	10,02	11,97	14,19	13,03	10,31	12,60	11,41
Kepulauan Riau	8,60	9,57	9,07	12,79	13,90	13,32	9,20	10,17	9,68
DKI Jakarta	9,83	10,57	10,20	-	-	-	9,83	10,57	10,20
Jawa Barat	13,43	14,05	13,73	16,57	16,77	16,67	14,24	14,75	14,49
Jawa Tengah	14,13	15,30	14,72	15,17	16,03	15,60	14,63	15,66	15,15
DI Yogyakarta	12,74	13,04	12,89	16,58	19,25	17,96	13,75	14,75	14,26
Jawa Timur	13,25	13,94	13,60	14,89	15,53	15,22	14,03	14,70	14,37
Banten	12,98	13,80	13,38	17,80	20,44	19,09	14,38	15,71	15,03
Bali	11,92	13,29	12,60	18,57	19,06	18,81	14,14	15,26	14,69
Nusa Tenggara Barat	16,99	17,55	17,28	17,23	18,08	17,66	17,12	17,83	17,48
Nusa Tenggara Timur	14,63	14,61	14,62	20,24	22,01	21,14	18,94	20,33	19,64
Kalimantan Barat	12,32	13,58	12,95	13,33	13,63	13,48	13,00	13,61	13,30
Kalimantan Tengah	11,01	12,70	11,83	13,18	14,54	13,82	12,35	13,82	13,05
Kalimantan Selatan	9,34	11,20	10,26	12,83	13,75	13,28	11,20	12,56	11,87
Kalimantan Timur	10,33	11,66	10,96	10,95	11,56	11,23	10,53	11,62	11,05
Kalimantan Utara	10,31	11,13	10,70	14,44	15,27	14,83	12,06	12,84	12,43
Sulawesi Utara	13,65	14,38	14,02	15,63	16,59	16,09	14,65	15,47	15,05
Sulawesi Tengah	15,47	18,76	17,11	17,51	19,12	18,29	16,95	19,02	17,96
Sulawesi Selatan	12,91	13,88	13,40	14,51	14,66	14,59	13,84	14,34	14,09
Sulawesi Tenggara	12,47	13,09	12,78	15,83	17,37	16,60	14,54	15,73	15,13
Gorontalo	17,99	17,11	17,55	19,31	21,06	20,18	18,81	19,51	19,16
Sulawesi Barat	15,72	14,27	14,98	14,19	16,14	15,16	14,54	15,69	15,12
Maluku	8,57	9,42	8,99	12,27	12,62	12,44	10,73	11,28	11,00
Maluku Utara	12,01	11,89	11,95	11,94	12,70	12,31	11,96	12,47	12,21
Papua Barat	11,48	14,15	12,75	11,69	12,34	12,00	11,61	13,08	12,30
Papua	9,15	11,38	10,18	9,03	9,07	9,05	9,07	9,70	9,37
**Indonesia**	**12,48**	**13,36**	**12,92**	**14,65**	**15,56**	**15,10**	**13,46**	**14,36**	**13,91**

Sumber/*Source*: Susenas Maret 2018/*The March 2018 Susenas*

https://www.bps.go.id

Tabel *Table* **4.3.** **Persentase Penduduk yang Mempunyai Keluhan Kesehatan dan Berobat Jalan dalam Sebulan Terakhir menurut Provinsi, Daerah Tempat Tinggal, dan Jenis Kelamin, 2018**
Percentage of Population Who Had Health Complaint Which Treated Outpatient during the Last Month by Province, Urban Rural Classification, and Sex, 2018

**Provinsi** *Province*	**Perkotaan/***Urban*			**Perdesaan/***Rural*			**Perkotaan+Perdesaan/***Urban+Rural*		
	**Laki-laki** *Male*	**Perempuan** *Female*	**Laki-laki+Perempuan** *Male+Female*	**Laki-laki** *Male*	**Perempuan** *Female*	**Laki-laki+Perempuan** *Male+Female*	**Laki-laki/** *Male*	**Perempuan/** *Female*	**Laki-laki+Perempuan** *Male+Female*
(1)	(2)	(3)	(4)	(5)	(6)	(7)	(8)	(9)	(10)
Aceh	51,97	56,51	54,44	64,41	68,42	66,59	60,90	65,06	63,16
Sumatera Utara	47,25	50,01	48,73	46,50	49,20	47,90	46,89	49,63	48,33
Sumatera Barat	55,38	60,44	58,12	61,24	65,17	63,36	58,62	63,03	61,00
Riau	42,96	44,46	43,73	33,66	36,44	35,04	37,17	39,60	38,39
Jambi	42,37	45,45	43,92	38,82	40,21	39,54	39,93	41,78	40,88
Sumatera Selatan	36,75	39,17	38,07	37,73	39,76	38,76	37,34	39,51	38,48
Bengkulu	38,25	44,32	41,39	36,97	42,11	39,61	37,35	42,78	40,14
Lampung	43,96	50,69	47,39	42,46	47,68	45,11	42,89	48,55	45,77
Kepulauan Bangka Belitung	49,22	51,59	50,47	45,60	48,98	47,33	47,53	50,43	49,04
Kepulauan Riau	40,79	47,37	44,18	54,53	58,90	56,70	43,04	49,14	46,17
DKI Jakarta	52,32	55,64	54,08	-	-	-	52,32	55,64	54,08
Jawa Barat	49,42	54,12	51,86	47,98	50,73	49,40	49,02	53,18	51,18
Jawa Tengah	49,05	52,79	51,04	49,31	52,25	50,88	49,17	52,53	50,96
DI Yogyakarta	46,03	49,45	47,80	56,13	57,94	57,11	48,69	51,85	50,34
Jawa Timur	45,71	48,62	47,26	47,93	50,65	49,36	46,79	49,59	48,27
Banten	47,77	51,41	49,62	39,11	40,99	40,11	45,00	47,89	46,49
Bali	59,00	59,85	59,44	63,77	71,47	67,78	60,90	64,50	62,77
Nusa Tenggara Barat	43,07	46,67	45,00	44,97	46,12	45,59	44,06	46,38	45,31
Nusa Tenggara Timur	39,52	39,61	39,57	48,77	53,76	51,40	46,77	50,75	48,86
Kalimantan Barat	47,84	50,36	49,18	38,07	42,00	40,06	41,07	44,75	42,96
Kalimantan Tengah	32,21	33,56	32,89	33,61	34,83	34,23	33,08	34,35	33,72
Kalimantan Selatan	30,29	36,61	33,60	33,66	39,57	36,75	32,13	38,22	35,31
Kalimantan Timur	44,95	48,99	47,04	47,26	55,88	51,45	45,67	50,98	48,36
Kalimantan Utara	49,35	46,61	47,94	40,66	43,49	42,04	45,25	45,21	45,23
Sulawesi Utara	49,60	51,93	50,80	49,56	53,28	51,43	49,58	52,65	51,14
Sulawesi Tengah	34,99	40,40	37,90	40,55	44,59	42,60	39,02	43,34	41,25
Sulawesi Selatan	40,37	45,53	43,12	45,19	49,36	47,42	43,11	47,71	45,57
Sulawesi Tenggara	33,24	34,21	33,76	41,45	45,59	43,66	38,46	41,42	40,04
Gorontalo	52,20	54,64	53,47	39,26	45,86	42,62	43,93	49,13	46,60
Sulawesi Barat	46,38	50,69	48,57	46,82	50,43	48,67	46,71	50,49	48,64
Maluku	37,26	39,25	38,31	41,00	44,21	42,61	39,55	42,14	40,87
Maluku Utara	45,27	51,28	48,41	42,21	45,73	44,00	43,09	47,38	45,29
Papua Barat	43,90	46,28	45,17	54,21	54,88	54,54	50,08	51,13	50,62
Papua	54,44	59,85	57,02	51,56	53,74	52,61	52,49	55,69	54,03
**Indonesia**	**47,23**	**50,94**	**49,18**	**46,25**	**49,70**	**48,05**	**46,78**	**50,37**	**48,66**

Sumber/*Source*: Susenas Maret 2018/*The March 2018 Susenas*

https://www.bps.go.id

Tabel *Table* **4.4.1.** **Persentase Persentase Penduduk yang Sakit tetapi Tidak Berobat Jalan dalam Sebulan Terakhir di Daerah Perkotaan menurut Provinsi dan Alasan Utama Tidak Berobat Jalan, 2018**
Percentage of Population Who Fell Sick but Not Inpatient during the Last Month In Urban Area by Province and Main Reason Not Inpatient, 2018

**Provinsi** *Province*	**Tidak Punya Biaya Berobat** *No Budget for Inpatient*	**Tidak Ada Biaya Transpor** *No Budget for Transportation*	**Tidak Ada Sarana Transportasi** *No Transportation*	**Waktu Tunggu Pelayanan Lama** *Time to Wait is Long*	**Mengobati Sendiri** *Self Treated*	**Tidak Ada yang Mendampingi** *No Company*	**Merasa Tidak Perlu** *No Need*	**Lainnya** *Others*	**Jumlah** *Total*
**(1)**	**(2)**	**(3)**	**(4)**	**(5)**	**(6)**	**(7)**	**(8)**	**(9)**	**(10)**
Aceh	0,05	0,01	0,00	2,00	64,53	0,54	29,03	3,84	100,00
Sumatera Utara	1,98	0,47	0,03	0,53	64,31	0,07	30,11	2,52	100,00
Sumatera Barat	1,02	0,15	0,17	0,56	52,19	0,02	44,22	1,67	100,00
Riau	0,93	0,10	0,01	1,08	65,36	0,13	31,15	1,24	100,00
Jambi	1,15	0,01	0,00	0,14	67,11	0,15	30,74	0,71	100,00
Sumatera Selatan	0,98	0,27	0,00	0,70	69,46	0,00	27,26	1,33	100,00
Bengkulu	2,61	0,40	0,00	0,18	69,45	0,08	26,72	0,57	100,00
Lampung	1,25	0,05	0,05	0,12	67,40	0,16	28,79	2,18	100,00
Kepulauan Bangka Belitung	0,13	0,11	0,22	1,52	72,52	0,28	24,96	0,26	100,00
Kepulauan Riau	2,68	0,00	0,02	1,35	51,06	0,09	42,85	1,96	100,00
DKI Jakarta	0,46	0,16	0,37	1,20	62,51	0,21	32,95	2,14	100,00
Jawa Barat	2,07	0,55	0,05	0,40	69,19	0,29	25,97	1,48	100,00
Jawa Tengah	0,96	0,15	0,11	0,70	67,82	0,23	28,44	1,60	100,00
DI Yogyakarta	0,20	0,00	0,05	1,13	77,13	0,36	19,97	1,17	100,00
Jawa Timur	0,87	0,15	0,13	0,73	70,34	0,13	26,27	1,37	100,00
Banten	1,97	0,39	0,05	0,78	62,26	0,16	33,57	0,82	100,00
Bali	0,81	0,00	0,05	1,14	66,64	0,10	30,29	0,95	100,00
Nusa Tenggara Barat	2,35	0,17	0,00	0,07	67,12	0,03	28,22	2,03	100,00
Nusa Tenggara Timur	0,27	0,13	0,00	0,32	79,57	0,21	18,90	0,61	100,00
Kalimantan Barat	1,68	0,00	0,08	0,00	67,86	0,63	27,57	2,17	100,00
Kalimantan Tengah	1,29	0,00	0,69	0,24	75,49	0,00	21,67	0,62	100,00
Kalimantan Selatan	0,44	0,30	0,04	1,00	80,90	0,18	16,64	0,49	100,00
Kalimantan Timur	0,95	0,14	0,10	1,36	64,44	0,27	31,10	1,64	100,00
Kalimantan Utara	0,97	0,00	0,00	0,69	75,86	0,67	21,09	0,72	100,00
Sulawesi Utara	1,00	0,26	0,00	0,23	58,88	0,40	37,77	1,47	100,00
Sulawesi Tengah	1,23	0,13	0,05	0,42	71,93	0,12	25,39	0,74	100,00
Sulawesi Selatan	0,45	0,35	0,02	0,46	63,86	0,55	32,47	1,84	100,00
Sulawesi Tenggara	3,47	0,00	0,00	0,16	63,56	0,49	31,61	0,70	100,00
Gorontalo	0,34	1,11	0,00	0,40	78,79	0,23	18,05	1,07	100,00
Sulawesi Barat	0,00	0,00	0,00	0,99	70,91	0,00	27,21	0,89	100,00
Maluku	1,49	0,57	0,00	1,16	66,08	0,12	30,28	0,30	100,00
Maluku Utara	1,07	1,79	0,00	0,00	63,51	0,00	31,53	2,11	100,00
Papua Barat	1,82	0,08	0,00	1,22	65,36	0,22	30,52	0,77	100,00
Papua	2,02	0,77	0,00	1,35	66,63	0,54	23,96	4,74	100,00
**Indonesia**	**1,31**	**0,28**	**0,09**	**0,67**	**67,77**	**0,22**	**28,16**	**1,51**	**100,00**

Sumber/*Source*: Susenas Maret 2018/*The March 2018 Susenas*

https://www.bps.go.id

Tabel **4.4.2.** **Persentase Persentase Penduduk yang Sakit tetapi Tidak Berobat Jalan dalam Sebulan Terakhir di Daerah Perdesaan menurut Provinsi dan Alasan Utama Tidak Berobat Jalan, 2018**
Table *Percentage of Population Who Fell Sick but Not Inpatient during the Last Month In Rural Area by Province and Main Reason Not Inpatient, 2018*

**Provinsi** *Province*	**Tidak Punya Biaya Berobat** *No Budget for Inpatient*	**Tidak Ada Biaya Transpor** *No Budget for Transportation*	**Tidak Ada Sarana Transportasi** *No Transportation*	**Waktu Tunggu Pelayanan Lama** *Time to Wait is Long*	**Mengobati Sendiri** *Self Treated*	**Tidak Ada yang Mendampingi** *No Company*	**Merasa Tidak Perlu** *No Need*	**Lainnya** *Others*	**Jumlah** *Total*
**(1)**	**(2)**	**(3)**	**(4)**	**(5)**	**(6)**	**(7)**	**(8)**	**(9)**	**(10)**
Aceh	0,14	0,87	0,46	0,80	66,25	0,12	29,13	2,22	100,00
Sumatera Utara	4,23	0,87	0,37	0,53	62,82	0,18	30,30	0,69	100,00
Sumatera Barat	2,78	0,83	0,64	0,22	43,98	0,65	49,09	1,81	100,00
Riau	1,80	0,09	0,37	0,42	57,13	0,12	39,04	1,03	100,00
Jambi	3,23	0,37	0,35	0,15	60,48	0,07	34,15	1,20	100,00
Sumatera Selatan	0,85	0,63	0,18	0,40	75,71	0,30	21,38	0,55	100,00
Bengkulu	3,17	0,39	0,07	0,02	64,07	0,20	30,55	1,52	100,00
Lampung	2,24	0,48	0,12	0,02	63,94	0,07	31,88	1,24	100,00
Kepulauan Bangka Belitung	1,75	0,38	0,37	0,43	69,13	0,16	27,44	0,33	100,00
Kepulauan Riau	0,41	0,00	0,07	0,31	69,42	0,00	24,91	4,88	100,00
DKI Jakarta	-	-	-	-	-	-	-	-	-
Jawa Barat	3,85	1,13	0,06	0,76	63,78	0,12	28,38	1,93	100,00
Jawa Tengah	0,85	0,44	0,12	0,35	68,35	0,23	28,41	1,25	100,00
DI Yogyakarta	0,01	0,17	0,18	0,00	62,73	0,16	35,55	1,19	100,00
Jawa Timur	1,08	0,15	0,07	0,26	71,45	0,23	25,32	1,42	100,00
Banten	3,81	0,68	0,09	0,37	61,47	0,00	33,15	0,43	100,00
Bali	3,00	0,20	0,00	0,11	66,44	0,24	29,42	0,60	100,00
Nusa Tenggara Barat	2,63	0,67	0,05	0,27	63,61	0,28	30,53	1,95	100,00
Nusa Tenggara Timur	3,66	3,43	0,41	0,78	65,26	0,21	24,19	2,04	100,00
Kalimantan Barat	4,37	0,60	0,47	0,13	58,12	0,27	35,28	0,75	100,00
Kalimantan Tengah	2,69	0,08	0,72	0,14	67,71	0,25	27,60	0,80	100,00
Kalimantan Selatan	0,72	0,36	0,18	0,43	82,20	0,17	15,62	0,32	100,00
Kalimantan Timur	4,51	0,27	0,08	0,46	62,20	0,22	31,34	0,92	100,00
Kalimantan Utara	1,70	0,11	0,45	0,09	58,75	0,00	38,16	0,73	100,00
Sulawesi Utara	2,52	0,91	0,22	0,17	62,84	0,24	32,09	1,01	100,00
Sulawesi Tengah	3,41	0,67	0,16	0,33	69,47	0,24	24,89	0,83	100,00
Sulawesi Selatan	1,17	0,91	0,35	0,44	63,32	0,32	31,36	2,11	100,00
Sulawesi Tenggara	2,25	0,92	0,08	0,07	73,09	0,24	22,36	0,99	100,00
Gorontalo	1,01	0,29	0,04	0,00	89,41	0,15	8,80	0,29	100,00
Sulawesi Barat	2,32	0,62	0,11	0,54	55,76	0,26	38,68	1,71	100,00
Maluku	3,72	0,77	1,07	0,43	69,32	0,30	22,04	2,36	100,00
Maluku Utara	2,64	0,48	0,34	0,06	69,48	0,09	24,45	2,45	100,00
Papua Barat	0,62	0,73	0,43	1,01	61,79	0,46	32,56	2,39	100,00
Papua	0,96	2,27	1,73	0,64	47,57	0,40	38,65	7,77	100,00
**Indonesia**	**2,16**	**0,67**	**0,21**	**0,38**	**66,19**	**0,20**	**28,80**	**1,37**	**100,00**

Sumber/*Source*: Susenas Maret 2018/*The March 2018 Susenas*

https://www.bps.go.id

Tabel *Table* **4.4.3.** **Persentase Persentase Penduduk yang Sakit tetapi Tidak Berobat Jalan dalam Sebulan Terakhir di Daerah Perkotaan dan Perdesaan menurut Provinsi dan Alasan Utama Tidak Berobat Jalan, 2018**

Percentage of Population Who Fell Sick but Not Inpatient during the Last Month In Urban and Rural Area by Province and Main Reason Not Inpatient, 2018

**Provinsi** *Province*	**Tidak Punya Biaya Berobat** *No Budget for Inpatient*	**Tidak Ada Biaya Transpor** *No Budget for Transportation*	**Tidak Ada Sarana Transportasi** *No Transportation*	**Waktu Tunggu Pelayanan Lama** *Time to Wait is Long*	**Mengobati Sendiri** *Self Treated*	**Tidak Ada yang Mendampingi** *No Company*	**Merasa Tidak Perlu** *No Need*	**Lainnya** *Others*	**Jumlah** *Total*
**(1)**	**(2)**	**(3)**	**(4)**	**(5)**	**(6)**	**(7)**	**(8)**	**(9)**	**(10)**
Aceh	0,11	0,57	0,30	1,22	65,65	0,27	29,09	2,78	100,00
Sumatera Utara	3,06	0,66	0,19	0,53	63,60	0,12	30,20	1,64	100,00
Sumatera Barat	1,93	0,50	0,41	0,39	47,94	0,34	46,74	1,75	100,00
Riau	1,49	0,10	0,25	0,65	60,03	0,13	36,25	1,10	100,00
Jambi	2,62	0,26	0,25	0,14	62,41	0,10	33,16	1,06	100,00
Sumatera Selatan	0,90	0,48	0,10	0,53	73,14	0,18	23,79	0,87	100,00
Bengkulu	3,01	0,40	0,05	0,06	65,64	0,17	29,43	1,24	100,00
Lampung	1,97	0,36	0,10	0,05	64,91	0,10	31,02	1,50	100,00
Kepulauan Bangka Belitung	0,89	0,23	0,29	1,01	70,93	0,23	26,13	0,29	100,00
Kepulauan Riau	2,39	0,00	0,03	1,21	53,40	0,08	40,56	2,33	100,00
DKI Jakarta	0,46	0,16	0,37	1,20	62,51	0,21	32,95	2,14	100,00
Jawa Barat	2,58	0,72	0,05	0,50	67,64	0,24	26,67	1,61	100,00
Jawa Tengah	0,90	0,29	0,12	0,53	68,07	0,23	28,42	1,43	100,00
DI Yogyakarta	0,16	0,04	0,08	0,86	73,73	0,31	23,64	1,17	100,00
Jawa Timur	0,97	0,15	0,10	0,51	70,87	0,18	25,82	1,39	100,00
Banten	2,65	0,49	0,07	0,63	61,97	0,10	33,42	0,67	100,00
Bali	1,57	0,07	0,04	0,79	66,57	0,15	29,99	0,83	100,00
Nusa Tenggara Barat	2,50	0,43	0,03	0,18	65,30	0,16	29,42	1,99	100,00
Nusa Tenggara Timur	2,80	2,59	0,31	0,67	68,89	0,21	22,85	1,68	100,00
Kalimantan Barat	3,61	0,43	0,36	0,10	60,88	0,38	33,10	1,15	100,00
Kalimantan Tengah	2,15	0,05	0,71	0,18	70,70	0,16	25,33	0,73	100,00
Kalimantan Selatan	0,59	0,33	0,12	0,70	81,59	0,18	16,10	0,40	100,00
Kalimantan Timur	1,96	0,18	0,09	1,11	63,80	0,26	31,17	1,44	100,00
Kalimantan Utara	1,32	0,06	0,22	0,40	67,54	0,35	29,39	0,73	100,00
Sulawesi Utara	1,81	0,61	0,12	0,20	61,00	0,32	34,73	1,22	100,00
Sulawesi Tengah	2,75	0,51	0,13	0,35	70,21	0,20	25,05	0,80	100,00
Sulawesi Selatan	0,85	0,66	0,20	0,45	63,56	0,43	31,86	1,99	100,00
Sulawesi Tenggara	2,75	0,55	0,05	0,11	69,25	0,34	26,09	0,87	100,00
Gorontalo	0,80	0,56	0,03	0,13	86,02	0,17	11,76	0,54	100,00
Sulawesi Barat	1,73	0,47	0,08	0,65	59,58	0,20	35,78	1,50	100,00
Maluku	2,78	0,69	0,62	0,73	67,95	0,22	25,51	1,49	100,00
Maluku Utara	2,21	0,84	0,24	0,04	67,84	0,07	26,40	2,36	100,00
Papua Barat	1,18	0,43	0,23	1,11	63,45	0,35	31,62	1,63	100,00
Papua	1,28	1,82	1,21	0,86	53,30	0,44	34,23	6,86	100,00
**Indonesia**	**1,71**	**0,46**	**0,15**	**0,53**	**67,04**	**0,21**	**28,46**	**1,45**	**100,00**

Sumber/*Source*: Susenas Maret 2018/*The March 2018 Susenas*

https://www.bps.go.id

Tabel *Table* **4.5.1.** **Persentase Penduduk yang Berobat Jalan dalam Sebulan Terakhir di Daerah Perkotaan menurut Provinsi dan Tempat Berobat Jalan, 2018**
Percentage of Population Who Were Treated as Outpatient during the Last Month in Urban Area by Province and Place of Outpatient, 2018

**Provinsi** *Province*	**Rumah Sakit Pemerintah** *Public Hospital*	**Rumah Sakit Swasta** *Private Hospital*	**Praktek Dokter/Bidan** *Practitioner Doctor/Midwives*	**Klinik/Praktek Dokter Bersama** *Clinics/Practitioner Doctor Centre*	**Puskesmas/ Pustu** *Health Center/ Subsidiary HC*	**UKBM*** *UKBM**	**Praktek Pengobatan Tradisional** *Traditional Healer*	**Lainnya** *Others*
(1)	(2)	(3)	(4)	(5)	(6)	(7)	(8)	(9)
Aceh	18,39	5,37	23,44	18,01	37,98	5,27	1,92	0,79
Sumatera Utara	6,74	11,48	42,42	18,21	21,29	0,86	2,80	0,73
Sumatera Barat	14,37	10,16	36,44	12,68	28,25	1,44	2,88	0,60
Riau	9,56	14,87	23,87	31,08	20,79	0,84	1,45	1,12
Jambi	11,36	10,83	27,86	14,74	38,61	0,68	2,17	0,42
Sumatera Selatan	8,52	12,25	31,98	16,66	32,18	1,03	1,10	0,19
Bengkulu	9,29	6,03	34,15	17,56	33,07	1,20	2,51	1,23
Lampung	5,61	8,93	36,70	13,34	34,17	2,98	2,92	0,98
Kepulauan Bangka Belitung	11,77	15,89	28,86	15,20	29,31	2,28	1,90	0,85
Kepulauan Riau	13,69	10,44	17,75	29,87	28,96	1,48	1,47	1,82
DKI Jakarta	15,34	12,39	7,32	24,61	42,90	1,87	1,05	1,16
Jawa Barat	7,59	12,20	24,61	24,75	32,96	1,77	1,74	0,89
Jawa Tengah	7,42	7,80	39,75	18,02	28,38	2,44	1,17	0,38
DI Yogyakarta	7,31	21,50	26,89	14,56	33,31	2,10	1,19	0,73
Jawa Timur	8,12	10,23	40,71	16,12	25,32	2,75	1,90	0,81
Banten	5,56	11,31	23,03	35,33	27,76	0,75	2,69	0,49
Bali	10,12	6,44	49,70	16,68	20,04	0,14	0,76	0,10
Nusa Tenggara Barat	8,65	1,17	35,47	13,16	44,31	3,00	2,72	1,90
Nusa Tenggara Timur	10,68	4,82	22,20	12,58	49,79	1,26	0,77	0,18
Kalimantan Barat	11,11	5,65	26,49	18,23	37,66	1,89	1,49	1,41
Kalimantan Tengah	20,30	3,85	32,64	11,76	33,14	0,13	0,69	0,93
Kalimantan Selatan	13,45	3,77	30,86	14,94	37,43	1,82	1,53	1,81
Kalimantan Timur	12,40	12,58	16,75	24,11	36,99	0,42	0,90	0,38
Kalimantan Utara	15,48	1,68	26,65	19,83	42,78	0,90	0,64	0,39
Sulawesi Utara	12,45	9,71	29,30	16,31	34,37	1,49	0,47	0,29
Sulawesi Tengah	18,64	2,67	21,42	11,40	48,17	1,63	1,36	0,60
Sulawesi Selatan	14,56	7,45	16,35	16,55	45,77	1,79	0,82	1,03
Sulawesi Tenggara	15,79	3,73	25,58	11,20	44,44	0,47	1,86	0,22
Gorontalo	11,30	1,77	22,26	17,92	52,09	1,23	2,92	0,78
Sulawesi Barat	10,73	1,03	25,80	4,54	56,39	1,63	1,28	5,15
Maluku	8,62	4,56	25,13	10,53	50,54	0,47	1,71	0,32
Maluku Utara	17,82	2,30	28,14	19,28	33,99	1,70	0,18	0,27
Papua Barat	15,36	7,75	12,18	10,88	54,33	1,05	0,69	0,24
Papua	29,07	3,90	12,76	16,22	42,28	0,89	0,41	0,16
**Indonesia**	**9,19**	**10,30**	**29,62**	**20,76**	**31,78**	**1,88**	**1,68**	**0,78**

Catatan/*Note*: *)UKBM terdiri dari Poskesdes, Polindes, Posyandu, Balai Pengobatan/*UKBM consist of Poskesdes, Polindes, Posyandu, Balai Pengobatan or Health Service Centre at Village*
Sumber/*Source*: Susenas Maret 2018/*The March 2018 Susenas*

https://www.bps.go.id

Tabel *Table* **4.5.2.** **Persentase Penduduk yang Berobat Jalan dalam Sebulan Terakhir di Daerah Perdesaan menurut Provinsi dan Tempat Berobat Jalan, 2018**
Percentage of Population Who Were Treated as Outpatient during the Last Month in Rural Area by Province and Place of Outpatient, 2018

**Provinsi** *Province*	**Rumah Sakit Pemerintah** *Public Hospital*	**Rumah Sakit Swasta** *Private Hospital*	**Praktek Dokter/Bidan** *Practitioner Doctor/Midwives*	**Klinik/Praktek Dokter Bersama** *Clinics/Practitioner Doctor Centre*	**Puskesmas/ Pustu** *Health Center/ Subsidiary HC*	**UKBM*** *UKBM**	**Praktek Pengobatan Tradisional** *Traditional Healer*	**Lainnya** *Others*
(1)	(2)	(3)	(4)	(5)	(6)	(7)	(8)	(9)
Aceh	12,72	1,76	31,85	8,92	50,84	4,68	2,87	1,12
Sumatera Utara	6,48	4,85	55,36	6,59	24,50	4,69	3,53	1,48
Sumatera Barat	8,00	2,32	47,33	5,76	33,01	5,10	4,81	0,48
Riau	8,07	6,73	43,52	11,51	30,14	3,75	3,17	1,04
Jambi	9,40	3,82	42,56	7,33	32,48	4,38	3,75	2,84
Sumatera Selatan	6,01	1,54	57,68	6,12	27,06	5,52	4,15	0,45
Bengkulu	7,81	2,48	56,03	10,53	25,82	1,32	2,89	0,16
Lampung	2,87	4,31	58,03	9,51	25,91	1,75	2,24	0,97
Kepulauan Bangka Belitung	5,69	6,16	46,28	7,45	35,32	9,10	2,94	1,35
Kepulauan Riau	7,16	1,41	24,41	5,42	57,82	3,94	2,06	1,07
DKI Jakarta	-	-	-	-	-	-	-	-
Jawa Barat	5,58	2,90	45,35	16,26	31,43	2,86	1,73	1,15
Jawa Tengah	5,62	5,03	51,47	9,98	26,37	3,88	1,76	1,27
DI Yogyakarta	9,06	8,81	34,36	13,37	36,12	1,56	1,50	1,05
Jawa Timur	4,09	3,44	56,74	8,15	24,49	6,27	2,27	1,14
Banten	5,30	2,49	34,68	19,91	39,27	2,16	1,42	1,97
Bali	5,72	2,22	61,46	5,43	28,26	0,52	1,41	0,59
Nusa Tenggara Barat	3,72	0,96	38,07	10,77	45,79	4,25	2,16	1,84
Nusa Tenggara Timur	3,93	2,13	9,34	4,78	72,86	9,97	0,75	0,12
Kalimantan Barat	5,61	2,85	33,74	5,27	44,80	12,60	3,82	0,17
Kalimantan Tengah	11,16	0,57	25,39	7,73	50,51	6,89	2,26	0,48
Kalimantan Selatan	7,54	1,34	41,64	7,55	39,83	6,58	2,47	1,00
Kalimantan Timur	10,49	2,94	21,06	11,36	52,73	3,11	0,77	0,00
Kalimantan Utara	10,40	0,50	13,52	7,60	72,00	0,59	0,39	0,11
Sulawesi Utara	7,21	4,39	40,78	8,80	41,85	1,80	2,14	1,24
Sulawesi Tengah	10,01	0,80	24,30	3,10	54,90	8,63	2,57	0,90
Sulawesi Selatan	8,74	1,04	24,77	7,10	57,74	3,81	1,59	1,66
Sulawesi Tenggara	6,28	0,45	27,74	5,34	55,73	3,99	4,81	1,27
Gorontalo	5,49	0,07	36,06	8,32	53,38	1,26	2,11	1,59
Sulawesi Barat	4,24	0,59	15,53	2,74	71,41	4,27	1,03	3,36
Maluku	7,35	0,94	18,09	3,89	64,58	4,49	1,96	1,44
Maluku Utara	9,67	0,59	12,15	3,67	66,73	7,62	2,29	0,87
Papua Barat	12,90	3,65	10,32	5,18	69,80	1,36	1,33	0,37
Papua	14,22	2,02	3,97	3,70	76,11	1,72	0,99	1,07
**Indonesia**	**6,16**	**3,22**	**43,93**	**9,27**	**36,13**	**4,66**	**2,26**	**1,12**

Catatan/*Note***:** *)UKBM terdiri dari Poskesdes, Polindes, Posyandu, Balai Pengobatan/*UKBM consist of Poskesdes, Polindes, Posyandu, Balai Pengobatan or Health Service Centre at Village*
Sumber/*Source*: Susenas Maret 2018/*The March 2018 Susenas*

https://www.bps.go.id

Tabel *Table* **4.5.3.** **Persentase Penduduk yang Berobat Jalan dalam Sebulan Terakhir di Daerah Perkotaan dan Perdesaan menurut Provinsi dan Tempat Berobat Jalan, 2018**

Percentage of Population Who Were Treated as Outpatient during the Last Month in Urban and Rural Area by Province and Place of Outpatient, 2018

**Provinsi** *Province*	**Rumah Sakit Pemerintah** *Public Hospital*	**Rumah Sakit Swasta** *Private Hospital*	**Praktek Dokter/Bidan** *Practitioner Doctor/Midwives*	**Klinik/Praktek Dokter Bersama** *Clinics/Practitioner Doctor Centre*	**Puskesmas/ Pustu** *Health Center/ Subsidiary HC*	**UKBM*** *UKBM**	**Praktek Pengobatan Tradisional** *Traditional Healer*	**Lainnya** *Others*
(1)	(2)	(3)	(4)	(5)	(6)	(7)	(8)	(9)
Aceh	14,10	2,64	29,81	11,13	47,71	4,82	2,64	1,04
Sumatera Utara	6,62	8,35	48,53	12,73	22,80	2,67	3,14	1,09
Sumatera Barat	10,73	5,67	42,67	8,72	30,97	3,54	3,98	0,53
Riau	8,72	10,31	34,87	20,12	26,03	2,47	2,41	1,08
Jambi	10,04	6,13	37,72	9,77	34,50	3,16	3,23	2,04
Sumatera Selatan	7,03	5,86	47,32	10,37	29,13	3,71	2,92	0,35
Bengkulu	8,27	3,58	49,30	12,70	28,05	1,28	2,77	0,49
Lampung	3,69	5,69	51,68	10,65	28,37	2,12	2,44	0,98
Kepulauan Bangka Belitung	9,09	11,61	36,51	11,80	31,96	5,28	2,36	1,07
Kepulauan Riau	12,42	8,68	19,05	25,10	34,59	1,96	1,58	1,67
DKI Jakarta	15,34	12,39	7,32	24,61	42,90	1,87	1,05	1,16
Jawa Barat	7,05	9,70	30,18	22,48	32,55	2,07	1,74	0,96
Jawa Tengah	6,56	6,48	45,35	14,18	27,42	3,13	1,45	0,81
DI Yogyakarta	7,85	17,57	29,20	14,19	34,18	1,93	1,29	0,83
Jawa Timur	6,12	6,87	48,64	12,18	24,91	4,49	2,08	0,97
Banten	5,48	8,81	26,34	30,95	31,02	1,15	2,33	0,91
Bali	8,23	4,62	54,77	11,83	23,58	0,30	1,04	0,31
Nusa Tenggara Barat	6,05	1,06	36,84	11,90	45,09	3,66	2,42	1,87
Nusa Tenggara Timur	5,10	2,59	11,57	6,14	68,85	8,46	0,75	0,13
Kalimantan Barat	7,62	3,87	31,10	9,99	42,20	8,70	2,97	0,62
Kalimantan Tengah	14,54	1,78	28,07	9,22	44,09	4,39	1,68	0,65
Kalimantan Selatan	10,11	2,40	36,96	10,76	38,79	4,51	2,06	1,35
Kalimantan Timur	11,79	9,49	18,13	20,03	42,03	1,28	0,86	0,26
Kalimantan Utara	13,31	1,17	21,04	14,61	55,26	0,77	0,54	0,27
Sulawesi Utara	9,61	6,83	35,52	12,24	38,42	1,66	1,37	0,80
Sulawesi Tengah	12,28	1,29	23,54	5,28	53,13	6,79	2,25	0,82
Sulawesi Selatan	11,12	3,66	21,33	10,96	52,85	2,99	1,27	1,41
Sulawesi Tenggara	9,21	1,46	27,07	7,14	52,25	2,90	3,90	0,95
Gorontalo	7,93	0,79	30,25	12,37	52,84	1,25	2,45	1,25
Sulawesi Barat	5,87	0,70	18,11	3,20	67,63	3,60	1,09	3,81
Maluku	7,83	2,31	20,75	6,41	59,27	2,97	1,87	1,02
Maluku Utara	12,22	1,13	17,14	8,54	56,51	5,77	1,63	0,68
Papua Barat	13,82	5,18	11,01	7,31	64,02	1,25	1,09	0,32
Papua	19,26	2,66	6,95	7,95	64,62	1,44	0,79	0,76
**Indonesia**	**7,82**	**7,09**	**36,11**	**15,56**	**33,75**	**3,14**	**1,94**	**0,93**

Catatan/*Note***:** *)UKBM terdiri dari Poskesdes, Polindes, Posyandu, Balai Pengobatan/*UKBM consist of Poskesdes, Polindes, Posyandu, Balai Pengobatan or Health Service Centre at Village*

Sumber/*Source*: Susenas Maret 2018/*The March 2018 Susenas*

https://www.bps.go.id

Tabel *Table* **4.6.** **Persentase Penduduk yang Menggunakan Jaminan Kesehatan untuk Berobat Jalan dalam Sebulan Terakhir menurut Provinsi, Daerah Tempat Tinggal, dan Jenis Kelamin, 2018**
Percentage of Population Who Used Health Insurance for Outpatient during the Last Month by Province, Urban Rural Classification, and Sex, 2018

**Provinsi** *Province*	**Perkotaan/***Urban*			**Perdesaan/***Rural*			**Perkotaan+Perdesaan/***Urban+Rural*		
	**Laki-laki** *Male*	**Perempuan** *Female*	**Laki-laki+Perempuan** *Male+Female*	**Laki-laki** *Male*	**Perempuan** *Female*	**Laki-laki+Perempuan** *Male+Female*	**Laki-laki/** *Male*	**Perempuan/** *Female*	**Laki-laki+Perempuan** *Male+Female*
**(1)**	**(2)**	**(3)**	**(4)**	**(5)**	**(6)**	**(7)**	**(8)**	**(9)**	**(10)**
Aceh	65,32	68,39	67,05	64,92	67,89	66,58	65,01	68,01	66,69
Sumatera Utara	38,48	38,23	38,34	25,07	23,84	24,42	32,02	31,56	31,77
Sumatera Barat	42,32	45,09	43,88	31,99	35,54	33,96	36,35	39,67	38,21
Riau	51,31	50,71	51,00	27,67	26,65	27,14	37,99	37,31	37,64
Jambi	56,26	60,13	58,28	24,38	29,32	26,98	34,97	39,38	37,28
Sumatera Selatan	55,45	57,41	56,55	26,02	26,02	26,02	37,32	39,20	38,33
Bengkulu	46,69	51,91	49,58	25,56	27,96	26,87	31,99	35,40	33,86
Lampung	45,87	47,00	46,49	19,88	23,79	21,98	27,52	30,78	29,28
Kepulauan Bangka Belitung	50,38	55,25	53,02	33,33	35,89	34,68	42,76	46,86	44,96
Kepulauan Riau	65,18	60,60	62,65	42,33	50,60	46,59	60,42	58,76	59,51
DKI Jakarta	51,24	59,04	55,51	-	-	-	51,24	59,04	55,51
Jawa Barat	38,36	41,06	39,82	22,76	25,36	24,14	34,09	36,93	35,62
Jawa Tengah	46,83	50,09	48,63	27,29	30,77	29,19	37,45	40,89	39,34
DI Yogyakarta	52,90	59,14	56,23	39,09	48,26	44,11	48,72	55,71	52,48
Jawa Timur	38,16	39,05	38,65	21,82	23,60	22,78	30,01	31,47	30,80
Banten	45,55	47,62	46,64	24,72	26,63	25,75	39,76	41,56	40,71
Bali	35,43	35,72	35,58	22,64	22,68	22,66	30,10	29,95	30,02
Nusa Tenggara Barat	31,60	39,67	36,08	19,76	21,48	20,70	25,33	30,14	27,99
Nusa Tenggara Timur	51,08	59,06	55,25	56,69	58,54	57,71	55,67	58,63	57,29
Kalimantan Barat	42,09	45,95	44,19	27,51	28,49	28,03	32,72	34,95	33,92
Kalimantan Tengah	49,71	45,02	47,30	36,87	36,94	36,90	41,62	39,92	40,75
Kalimantan Selatan	59,15	58,66	58,87	41,25	44,74	43,21	48,93	50,84	50,01
Kalimantan Timur	71,26	72,41	71,88	53,48	54,73	54,14	65,48	66,83	66,20
Kalimantan Utara	64,51	62,57	63,54	64,64	63,79	64,21	64,57	63,09	63,83
Sulawesi Utara	47,93	49,32	48,66	36,71	37,45	37,10	41,83	42,91	42,40
Sulawesi Tengah	46,57	48,53	47,69	45,46	50,34	48,06	45,73	49,84	47,96
Sulawesi Selatan	68,50	70,35	69,54	54,82	60,58	58,02	60,35	64,60	62,73
Sulawesi Tenggara	59,30	54,70	56,81	45,49	46,67	46,15	49,84	49,10	49,43
Gorontalo	58,22	60,94	59,67	39,71	51,15	45,97	47,65	55,21	51,74
Sulawesi Barat	63,87	70,60	67,44	65,79	72,78	69,50	65,31	72,23	68,98
Maluku	31,14	27,83	29,35	41,40	46,97	44,29	37,64	39,52	38,63
Maluku Utara	51,92	58,40	55,51	57,35	57,15	57,24	55,72	57,55	56,70
Papua Barat	66,84	62,05	64,22	68,13	69,01	68,57	67,68	66,26	66,94
Papua	62,46	64,55	63,50	83,14	83,02	83,08	76,19	76,68	76,43
**Indonesia**	**44,59**	**47,24**	**46,04**	**31,79**	**34,38**	**33,19**	**38,75**	**41,44**	**40,21**

Sumber/*Source*: Susenas Maret 2018/*The March 2018 Susenas*

https://www.bps.go.id

Tabel **4.7.1.** **Persentase Penduduk yang Memiliki Jaminan Kesehatan di Daerah Perkotaan menurut Provinsi dan Jenis Jaminan Kesehatan, 2018**
Table *Percentage of Population Who Have Health Insurance in Urban Area by Province and Type of Health Insurance, 2018*

**Provinsi** *Province*	**BPJS Kesehatan** *BPJS Health*	**Jamkesda** *Regional Health Insurance*	**Asuransi Swasta** *Private Health Insurance*	**Perusahaan/Kantor** *Paid by Company*	**Tidak Punya** *Doesn't Have*
(1)	(2)	(3)	(4)	(5)	(6)
Aceh	89,07	5,57	0,45	1,68	5,58
Sumatera Utara	47,56	5,19	1,17	6,09	42,39
Sumatera Barat	61,70	11,10	0,94	2,50	27,28
Riau	46,10	21,42	1,67	5,49	34,35
Jambi	54,33	3,53	1,26	5,69	36,34
Sumatera Selatan	48,40	38,35	1,50	4,42	16,76
Bengkulu	63,44	1,54	0,92	2,43	32,63
Lampung	48,19	25,86	1,09	4,41	30,62
Kepulauan Bangka Belitung	64,40	4,58	0,89	1,86	28,97
Kepulauan Riau	49,28	3,73	1,30	11,78	36,04
DKI Jakarta	76,18	0,99	3,66	6,12	15,95
Jawa Barat	51,56	10,47	2,02	7,66	33,80
Jawa Tengah	56,88	13,60	0,94	3,49	31,11
DI Yogyakarta	70,49	14,20	1,63	3,79	21,64
Jawa Timur	46,53	14,31	1,41	4,77	38,08
Banten	53,98	10,87	3,02	7,28	33,04
Bali	55,23	13,63	3,53	3,53	33,96
Nusa Tenggara Barat	52,51	19,08	0,65	1,74	37,63
Nusa Tenggara Timur	55,46	6,92	0,47	1,51	36,63
Kalimantan Barat	46,67	2,42	2,00	3,86	46,68
Kalimantan Tengah	50,34	12,08	1,16	1,58	36,60
Kalimantan Selatan	46,00	19,18	0,74	5,11	31,50
Kalimantan Timur	65,89	2,88	2,64	9,49	24,28
Kalimantan Utara	71,01	4,48	1,56	4,24	22,28
Sulawesi Utara	66,43	1,93	1,03	3,71	28,21
Sulawesi Tengah	63,27	3,82	0,78	1,44	32,13
Sulawesi Selatan	69,53	2,61	1,43	2,95	25,15
Sulawesi Tenggara	55,27	4,72	0,66	1,89	38,01
Gorontalo	78,48	0,81	0,45	1,02	19,74
Sulawesi Barat	68,94	56,42	0,33	0,88	10,03
Maluku	56,16	2,64	0,39	0,34	40,98
Maluku Utara	53,73	9,90	0,39	1,13	36,90
Papua Barat	63,69	6,32	0,66	3,10	29,90
Papua	56,72	17,24	0,60	2,12	27,09
**Indonesia**	**55,22**	**11,12**	**1,74**	**5,41**	**31,82**

Sumber/*Source*: Susenas Maret 2018/*The March 2018 Susenas*

https://www.bps.go.id

Tabel **4.7.2.** **Persentase Penduduk yang Memiliki Jaminan Kesehatan di Daerah Perdesaan menurut Provinsi dan Jenis Jaminan Kesehatan, 2018**
Table *Percentage of Population Who Have Health Insurance in Rural Area by Province and Type of Health Insurance, 2018*

**Provinsi** *Province*	**BPJS Kesehatan** *BPJS Health*	**Jamkesda** *Regional Health Insurance*	**Asuransi Swasta** *Private Health Insurance*	**Perusahaan/Kantor** *Paid by Company*	**Tidak Punya** *Doesn't Have*
(1)	(2)	(3)	(4)	(5)	(6)
Aceh	85,48	10,15	0,05	0,74	8,05
Sumatera Utara	43,32	4,73	0,30	1,94	51,29
Sumatera Barat	48,21	17,67	0,18	0,83	36,20
Riau	35,93	13,01	0,43	3,36	49,82
Jambi	35,53	6,75	0,24	1,24	58,26
Sumatera Selatan	26,10	69,44	0,42	1,29	14,14
Bengkulu	47,80	1,67	0,03	1,42	49,26
Lampung	40,47	9,02	0,21	1,12	52,50
Kepulauan Bangka Belitung	44,27	18,35	0,38	1,18	37,82
Kepulauan Riau	53,40	17,09	0,26	0,26	30,74
DKI Jakarta	-	-	-	-	-
Jawa Barat	41,89	7,56	0,29	1,68	50,67
Jawa Tengah	50,39	13,22	0,29	1,31	37,99
DI Yogyakarta	72,92	27,89	0,33	1,12	17,21
Jawa Timur	39,47	22,05	0,32	1,07	42,36
Banten	41,77	2,39	0,26	1,79	54,61
Bali	49,87	6,77	1,22	0,99	44,92
Nusa Tenggara Barat	47,50	4,20	0,09	0,31	50,27
Nusa Tenggara Timur	54,00	9,06	0,11	0,12	38,08
Kalimantan Barat	36,65	4,85	0,38	2,64	57,71
Kalimantan Tengah	35,27	15,54	0,40	4,71	46,09
Kalimantan Selatan	33,39	32,26	0,22	2,61	35,99
Kalimantan Timur	52,79	3,22	0,50	5,72	38,98
Kalimantan Utara	67,59	13,30	0,45	1,85	22,45
Sulawesi Utara	59,24	4,06	0,24	1,31	36,84
Sulawesi Tengah	52,13	5,48	0,38	0,67	42,40
Sulawesi Selatan	63,11	5,31	0,29	0,85	31,30
Sulawesi Tenggara	52,57	10,12	0,04	0,39	37,39
Gorontalo	70,26	1,16	0,15	0,19	28,46
Sulawesi Barat	60,42	39,62	0,16	1,01	20,31
Maluku	41,82	6,75	0,02	0,29	51,69
Maluku Utara	35,07	28,82	0,19	0,28	36,77
Papua Barat	62,21	13,91	0,27	0,47	28,03
Papua	23,57	68,18	0,18	0,85	14,21
**Indonesia**	**45,38**	**15,70**	**0,28**	**1,39**	**40,83**

Sumber/*Source*: Susenas Maret 2018/*The March 2018 Susenas*

https://www.bps.go.id

Tabel *Table* **4.7.3.** **Persentase Penduduk yang Memiliki Jaminan Kesehatan di Daerah Perkotaan dan Perdesaan menurut Provinsi dan Jenis Jaminan Kesehatan, 2018**
Percentage of Population Who Have Health Insurance in Urban and Rural Area by Province and Type of Health Insurance, 2018

**Provinsi** *Province*	**BPJS Kesehatan** *BPJS Health*	**Jamkesda** *Regional Health Insurance*	**Asuransi Swasta** *Private Health Insurance*	**Perusahaan/Kantor** *Paid by Company*	**Tidak Punya** *Doesn't Have*
**(1)**	**(2)**	**(3)**	**(4)**	**(5)**	**(6)**
Aceh	86,60	8,71	0,18	1,04	7,28
Sumatera Utara	45,56	4,97	0,76	4,13	46,58
Sumatera Barat	54,15	14,78	0,51	1,57	32,27
Riau	40,03	16,40	0,93	4,22	43,57
Jambi	41,55	5,72	0,57	2,66	51,24
Sumatera Selatan	34,40	57,87	0,82	2,46	15,12
Bengkulu	52,92	1,63	0,32	1,75	43,82
Lampung	42,75	14,00	0,47	2,09	46,03
Kepulauan Bangka Belitung	55,08	10,96	0,65	1,54	33,07
Kepulauan Riau	49,86	5,63	1,15	10,14	35,28
DKI Jakarta	76,18	0,99	3,66	6,12	15,95
Jawa Barat	49,05	9,71	1,57	6,11	38,16
Jawa Tengah	53,72	13,42	0,62	2,42	34,47
DI Yogyakarta	71,15	17,90	1,28	3,07	20,44
Jawa Timur	43,16	18,00	0,89	3,01	40,12
Banten	50,45	8,42	2,22	5,69	39,27
Bali	53,42	11,32	2,75	2,67	37,66
Nusa Tenggara Barat	49,83	11,12	0,35	0,98	44,39
Nusa Tenggara Timur	54,34	8,57	0,19	0,44	37,75
Kalimantan Barat	40,03	4,03	0,93	3,05	53,99
Kalimantan Tengah	41,08	14,21	0,69	3,50	42,43
Kalimantan Selatan	39,26	26,17	0,46	3,78	33,90
Kalimantan Timur	61,55	2,99	1,93	8,24	29,16
Kalimantan Utara	69,58	8,17	1,10	3,24	22,35
Sulawesi Utara	62,85	2,99	0,64	2,52	32,51
Sulawesi Tengah	55,24	5,01	0,49	0,88	39,53
Sulawesi Selatan	65,78	4,19	0,77	1,73	28,74
Sulawesi Tenggara	53,60	8,05	0,28	0,96	37,62
Gorontalo	73,44	1,02	0,27	0,51	25,08
Sulawesi Barat	62,43	43,59	0,20	0,98	17,88
Maluku	47,81	5,03	0,17	0,31	47,22
Maluku Utara	40,41	23,40	0,25	0,52	36,81
Papua Barat	62,82	10,82	0,43	1,54	28,79
Papua	32,74	54,09	0,29	1,20	17,77
**Indonesia**	**50,76**	**13,20**	**1,08**	**3,59**	**35,90**

Sumber/*Source*: Susenas Maret 2018/*The March 2018 Susenas*

https://www.bps.go.id

Tabel 4.8. **Persentase Penduduk yang Pernah Rawat Inap dalam Setahun Terakhir menurut Provinsi, Daerah Tempat Tinggal, dan Jenis Kelamin, 2018**
Table *Percentage of Population Who Had Have Hospitalized during the Last Year by Province, Urban Rural Classification, and Sex, 2018*

**Provinsi** *Province*	**Perkotaan/***Urban*			**Perdesaan/***Rural*			**Perkotaan+Perdesaan/***Urban+Rural*		
	**Laki-laki** *Male*	**Perempuan** *Female*	**Laki-laki+Perempuan** *Male+Female*	**Laki-laki** *Male*	**Perempuan** *Female*	**Laki-laki+Perempuan** *Male+Female*	**Laki-laki/** *Male*	**Perempuan/** *Female*	**Laki-laki+Perempuan** *Male+Female*
**(1)**	**(2)**	**(3)**	**(4)**	**(5)**	**(6)**	**(7)**	**(8)**	**(9)**	**(10)**
Aceh	5,75	9,19	7,46	4,96	7,59	6,28	5,21	8,09	6,65
Sumatera Utara	2,78	5,11	3,95	2,53	3,71	3,12	2,66	4,45	3,56
Sumatera Barat	3,62	7,65	5,65	2,93	5,42	4,18	3,23	6,40	4,83
Riau	3,46	6,32	4,86	2,77	4,00	3,37	3,05	4,94	3,97
Jambi	3,61	6,06	4,82	2,78	4,29	3,52	3,04	4,86	3,93
Sumatera Selatan	3,06	4,88	3,96	2,35	3,68	3,00	2,61	4,13	3,36
Bengkulu	5,39	8,61	7,00	2,93	4,90	3,89	3,72	6,14	4,91
Lampung	4,16	6,11	5,13	2,85	5,28	4,03	3,23	5,53	4,35
Kepulauan Bangka Belitung	3,75	5,56	4,62	2,77	3,88	3,30	3,29	4,79	4,01
Kepulauan Riau	2,41	4,93	3,65	2,19	4,29	3,21	2,38	4,84	3,59
DKI Jakarta	3,33	5,44	4,38	-	-	-	3,33	5,44	4,38
Jawa Barat	3,58	5,78	4,67	3,51	4,56	4,03	3,56	5,47	4,50
Jawa Tengah	5,20	7,23	6,23	4,57	6,86	5,72	4,89	7,05	5,98
DI Yogyakarta	5,20	7,71	6,46	5,71	6,75	6,25	5,34	7,45	6,41
Jawa Timur	4,11	6,02	5,07	3,90	5,20	4,56	4,01	5,63	4,83
Banten	3,05	5,44	4,22	1,50	2,97	2,22	2,60	4,73	3,64
Bali	3,50	5,56	4,52	3,61	4,75	4,18	3,53	5,28	4,40
Nusa Tenggara Barat	4,45	7,11	5,82	5,43	7,23	6,35	4,98	7,17	6,11
Nusa Tenggara Timur	4,12	7,41	5,76	2,53	4,76	3,66	2,90	5,36	4,14
Kalimantan Barat	4,03	6,75	5,38	2,15	3,46	2,78	2,77	4,58	3,66
Kalimantan Tengah	3,38	5,11	4,22	2,39	3,05	2,70	2,77	3,86	3,29
Kalimantan Selatan	2,48	5,16	3,80	2,63	4,05	3,33	2,56	4,57	3,55
Kalimantan Timur	4,04	7,33	5,62	3,48	5,61	4,48	3,85	6,77	5,24
Kalimantan Utara	5,25	9,40	7,22	4,25	6,39	5,24	4,83	8,16	6,39
Sulawesi Utara	5,74	7,15	6,44	5,28	6,61	5,93	5,51	6,88	6,18
Sulawesi Tengah	5,68	9,09	7,38	4,01	5,90	4,93	4,47	6,81	5,61
Sulawesi Selatan	5,10	9,16	7,17	4,08	6,45	5,29	4,50	7,57	6,07
Sulawesi Tenggara	3,53	5,94	4,73	2,96	4,61	3,78	3,18	5,12	4,14
Gorontalo	6,16	9,48	7,84	3,47	6,34	4,89	4,49	7,57	6,03
Sulawesi Barat	5,78	7,45	6,63	2,59	3,55	3,07	3,33	4,49	3,91
Maluku	3,01	4,94	3,98	1,96	1,98	1,97	2,40	3,22	2,81
Maluku Utara	3,67	7,22	5,42	2,26	2,78	2,51	2,66	4,06	3,35
Papua Barat	4,26	7,69	5,89	2,59	5,16	3,80	3,27	6,19	4,65
Papua	4,18	5,89	4,97	1,27	2,13	1,68	2,09	3,14	2,59
**Indonesia**	**3,91**	**6,23**	**5,06**	**3,41**	**5,08**	**4,24**	**3,68**	**5,71**	**4,69**

Sumber/*Source*: Susenas Maret 2018/*The March 2018 Susenas*

https://www.bps.go.id

Tabel *Table* **4.9.1.** **Persentase Penduduk yang Rawat Inap dalam Setahun Terakhir di Daerah Perkotaan menurut Provinsi dan Tempat Rawat Inap, 2018**
Percentage of Population Who Were Inpatient during the Last Year in Urban Area by Province and Place of Inpatient, 2018

**Provinsi** *Province*	**Rumah Sakit Pemerintah** *Public Hospital*	**Rumah Sakit Swasta** *Private Hospital*	**Praktek Dokter/Bidan** *Practitioner Doctor/ Midwives*	**Klinik/Praktek Dokter Bersama** *Clinics/Practitioner Doctor Centre*	**Puskesmas/ Pustu** *Health Center/Subsidiary HC*	**Praktek Pengobatan Tradisional** *Traditional Healer*	**Lainnya** *Others*
(1)	(2)	(3)	(4)	(5)	(6)	(7)	(8)
Aceh	66,12	23,62	2,38	4,27	5,71	0,00	0,00
Sumatera Utara	27,01	58,14	9,89	4,37	1,82	0,58	0,13
Sumatera Barat	45,96	38,75	9,21	3,76	3,45	0,23	0,00
Riau	29,16	55,19	7,53	7,10	2,77	0,00	0,00
Jambi	37,52	52,61	5,37	1,17	4,10	0,42	0,00
Sumatera Selatan	40,65	49,69	5,73	2,67	1,89	0,01	0,00
Bengkulu	54,74	38,41	2,87	1,77	2,36	0,07	0,00
Lampung	27,40	54,45	9,01	4,28	6,28	0,05	0,00
Kepulauan Bangka Belitung	36,03	46,97	9,71	2,13	7,33	0,00	0,00
Kepulauan Riau	35,56	52,57	6,93	3,27	2,97	0,12	0,00
DKI Jakarta	49,75	37,13	5,64	0,29	6,73	0,62	0,71
Jawa Barat	35,40	50,65	5,72	3,88	5,42	0,25	0,94
Jawa Tengah	39,19	47,09	2,82	3,46	9,73	0,39	0,19
DI Yogyakarta	32,64	57,30	3,83	1,75	6,29	0,35	0,00
Jawa Timur	34,62	48,17	3,43	3,24	13,12	0,32	0,40
Banten	25,07	60,00	5,68	3,87	5,76	0,11	0,53
Bali	46,67	47,76	2,70	2,41	1,26	0,00	0,00
Nusa Tenggara Barat	46,24	10,83	2,85	3,91	39,32	0,90	0,49
Nusa Tenggara Timur	57,06	31,78	0,60	0,72	11,79	0,16	0,00
Kalimantan Barat	54,54	32,19	4,65	2,92	6,17	0,00	0,50
Kalimantan Tengah	72,78	13,40	2,57	2,35	8,91	0,01	0,26
Kalimantan Selatan	68,63	21,58	3,81	4,46	4,86	0,00	0,00
Kalimantan Timur	47,22	44,21	4,66	2,37	2,91	0,04	0,00
Kalimantan Utara	77,54	7,77	7,11	0,42	7,89	0,00	0,00
Sulawesi Utara	42,98	51,15	0,23	1,68	5,97	0,00	0,16
Sulawesi Tengah	78,75	15,69	2,15	0,74	5,47	0,41	0,00
Sulawesi Selatan	55,97	30,89	0,88	1,85	11,32	0,00	0,13
Sulawesi Tenggara	62,35	27,95	1,42	1,23	9,67	0,00	0,00
Gorontalo	75,65	18,44	0,00	1,49	5,68	0,00	0,00
Sulawesi Barat	68,92	8,88	0,00	1,17	20,89	0,00	0,15
Maluku	63,87	34,58	0,45	0,87	0,62	0,00	0,00
Maluku Utara	73,59	20,78	2,38	0,00	4,56	0,00	0,00
Papua Barat	71,37	25,92	0,81	0,97	2,27	0,00	0,00
Papua	74,47	17,28	1,66	3,66	2,86	0,00	0,07
**Indonesia**	**40,51**	**45,42**	**4,56**	**3,17**	**7,83**	**0,27**	**0,40**

Sumber/*Source*: Susenas Maret 2018/*The March 2018 Susenas*

https://www.bps.go.id

Tabel **4.9.2.** **Persentase Penduduk yang Rawat Inap dalam Setahun Terakhir di Daerah Perdesaan menurut Provinsi dan Tempat Rawat Inap, 2018**
Table *Percentage of Population Who Were Inpatient during the Last Year in Rural Area by Province and Place of Inpatient, 2018*

**Provinsi** *Province*	**Rumah Sakit Pemerintah** *Public Hospital*	**Rumah Sakit Swasta** *Private Hospital*	**Praktek Dokter/Bidan** *Practitioner Doctor/ Midwives*	**Klinik/Praktek Dokter Bersama** *Clinics/Practitioner Doctor Centre*	**Puskesmas/ Pustu** *Health Center/Subsidiary HC*	**Praktek Pengobatan Tradisional** *Traditional Healer*	**Lainnya** *Others*
(1)	(2)	(3)	(4)	(5)	(6)	(7)	(8)
Aceh	65,71	12,92	3,88	1,72	18,15	0,31	0,28
Sumatera Utara	37,66	40,79	9,52	5,74	8,97	1,35	0,06
Sumatera Barat	51,65	17,28	11,85	3,77	17,52	0,13	0,14
Riau	38,35	40,92	6,73	6,15	11,73	1,29	0,11
Jambi	44,62	27,71	6,95	4,55	18,28	0,00	0,04
Sumatera Selatan	50,66	20,33	14,51	6,36	8,85	0,66	0,17
Bengkulu	60,12	18,73	3,59	6,79	12,79	0,22	0,16
Lampung	20,34	43,07	16,02	11,72	13,14	0,46	0,15
Kepulauan Bangka Belitung	42,80	27,99	3,33	1,08	28,29	0,00	0,55
Kepulauan Riau	63,80	15,90	0,46	1,31	17,92	0,00	3,10
DKI Jakarta	-	-	-	-	-	-	-
Jawa Barat	38,45	26,90	7,46	9,27	19,40	0,79	0,65
Jawa Tengah	35,99	34,39	4,96	5,05	21,42	0,91	0,14
DI Yogyakarta	36,33	45,03	5,47	4,15	10,61	1,15	0,00
Jawa Timur	30,87	31,95	4,31	4,34	31,92	0,40	0,19
Banten	38,73	20,58	8,65	8,51	27,93	0,32	0,32
Bali	55,30	30,47	7,09	3,09	6,70	0,00	0,00
Nusa Tenggara Barat	34,87	6,43	3,56	7,40	48,90	0,41	1,16
Nusa Tenggara Timur	45,17	17,85	0,43	0,78	37,51	0,10	0,00
Kalimantan Barat	58,07	17,73	3,40	2,19	21,91	0,02	0,32
Kalimantan Tengah	74,02	6,05	3,33	3,99	13,71	0,15	0,12
Kalimantan Selatan	70,17	9,45	2,54	3,84	15,35	0,08	0,00
Kalimantan Timur	61,46	11,55	1,93	4,15	21,46	0,44	0,59
Kalimantan Utara	75,22	1,01	0,28	1,37	23,14	0,63	0,00
Sulawesi Utara	46,12	34,65	1,33	1,23	19,73	0,17	0,00
Sulawesi Tengah	65,13	6,59	0,36	0,15	30,60	0,13	0,12
Sulawesi Selatan	54,40	12,31	2,42	1,65	32,12	0,26	0,52
Sulawesi Tenggara	59,24	9,56	1,87	2,34	28,89	0,00	0,00
Gorontalo	67,81	3,50	0,00	0,18	30,19	1,12	0,00
Sulawesi Barat	49,84	9,23	2,08	0,83	40,76	0,00	0,00
Maluku	75,32	12,24	0,00	0,48	12,73	0,00	0,00
Maluku Utara	70,57	2,44	0,94	1,31	24,78	0,14	0,20
Papua Barat	76,52	8,57	1,54	0,53	13,17	0,00	0,07
Papua	58,36	9,08	0,68	3,83	30,81	0,00	0,11
**Indonesia**	**42,53**	**26,14**	**5,60**	**4,94**	**23,16**	**0,54**	**0,26**

Sumber/*Source*: Susenas Maret 2018/*The March 2018 Susenas*

https://www.bps.go.id

Tabel *Table* **4.9.3** **Persentase Penduduk yang Rawat Inap dalam Setahun Terakhir di Daerah Perkotaan dan Perdesaan menurut Provinsi dan Tempat Rawat Inap, 2018**
Percentage of Population Who Were Inpatient during the Last Year in Urban and Rural Area by Province and Place of Inpatient, 2018

**Provinsi** *Province*	**Rumah Sakit Pemerintah** *Public Hospital*	**Rumah Sakit Swasta** *Private Hospital*	**Praktek Dokter/Bidan** *Practitioner Doctor/ Midwives*	**Klinik/Praktek Dokter Bersama** *Clinics/Practitioner Doctor Centre*	**Puskesmas/ Pustu** *Health Center/Subsidiary HC*	**Praktek Pengobatan Tradisional** *Traditional Healer*	**Lainnya** *Others*
(1)	(2)	(3)	(4)	(5)	(6)	(7)	(8)
Aceh	65,86	16,69	3,35	2,61	13,77	0,20	0,18
Sumatera Utara	31,41	50,97	9,73	4,94	4,78	0,90	0,10
Sumatera Barat	48,72	28,34	10,49	3,76	10,26	0,18	0,07
Riau	33,81	47,97	7,13	6,62	7,31	0,65	0,06
Jambi	41,83	37,48	6,33	3,22	12,71	0,16	0,02
Sumatera Selatan	46,26	33,24	10,65	4,74	5,79	0,38	0,09
Bengkulu	57,61	27,90	3,25	4,45	7,93	0,15	0,08
Lampung	22,80	47,03	13,58	9,13	10,75	0,32	0,10
Kepulauan Bangka Belitung	38,61	39,74	7,28	1,74	15,31	0,00	0,21
Kepulauan Riau	39,15	47,91	6,11	3,02	4,87	0,10	0,39
DKI Jakarta	49,75	37,13	5,64	0,29	6,73	0,62	0,71
Jawa Barat	36,10	45,15	6,12	5,13	8,65	0,38	0,87
Jawa Tengah	37,70	41,17	3,82	4,20	15,19	0,63	0,17
DI Yogyakarta	33,61	54,07	4,26	2,38	7,42	0,56	0,00
Jawa Timur	32,93	40,87	3,83	3,74	21,58	0,36	0,30
Banten	27,47	53,07	6,21	4,69	9,66	0,15	0,50
Bali	49,44	42,22	4,11	2,63	3,00	0,00	0,00
Nusa Tenggara Barat	39,91	8,39	3,24	5,85	44,65	0,63	0,86
Nusa Tenggara Timur	48,96	22,29	0,49	0,76	29,30	0,12	0,00
Kalimantan Barat	56,32	24,90	4,02	2,55	14,10	0,01	0,41
Kalimantan Tengah	73,41	9,68	2,96	3,18	11,34	0,08	0,19
Kalimantan Selatan	69,40	15,49	3,17	4,15	10,12	0,04	0,00
Kalimantan Timur	51,26	34,96	3,89	2,87	8,17	0,15	0,17
Kalimantan Utara	76,74	5,45	4,77	0,74	13,12	0,22	0,00
Sulawesi Utara	44,48	43,27	0,76	1,47	12,54	0,08	0,08
Sulawesi Tengah	70,13	9,93	1,02	0,37	21,38	0,24	0,07
Sulawesi Selatan	55,17	21,44	1,66	1,75	21,90	0,13	0,33
Sulawesi Tenggara	60,60	17,60	1,67	1,85	20,49	0,00	0,00
Gorontalo	71,75	11,02	0,00	0,84	17,85	0,56	0,00
Sulawesi Barat	57,49	9,09	1,25	0,96	32,79	0,00	0,06
Maluku	68,55	25,45	0,26	0,71	5,57	0,00	0,00
Maluku Utara	71,97	10,94	1,61	0,70	15,40	0,07	0,11
Papua Barat	73,86	17,51	1,17	0,76	7,55	0,00	0,03
Papua	66,92	13,44	1,20	3,74	15,96	0,00	0,09
**Indonesia**	**41,33**	**37,52**	**4,98**	**3,89**	**14,11**	**0,38**	**0,34**

Sumber/*Source*: Susenas Maret 2018/*The March 2018 Susenas*

https://www.bps.go.id

Tabel 4.10. **Persentase Penduduk yang Menggunakan Jaminan Kesehatan untuk Rawat Inap dalam Setahun Terakhir menurut Provinsi, Daerah Tempat Tinggal, dan Jenis Kelamin, 2018**
Table *Percentage of Population Who Used Health Insurance for Inpatient during The Last Year by Province, Urban Rural Classification, and Sex, 2018*

Provinsi *Province*	Perkotaan/*Urban*			Perdesaan/*Rural*			Perkotaan+Perdesaan/*Urban+Rural*		
	Laki-laki *Male*	Perempuan *Female*	Laki-laki+Perempuan *Male+Female*	Laki-laki *Male*	Perempuan *Female*	Laki-laki+Perempuan *Male+Female*	Laki-laki/ *Male*	Perempuan/ *Female*	Laki-laki+Perempuan *Male+Female*
(1)	(2)	(3)	(4)	(5)	(6)	(7)	(8)	(9)	(10)
Aceh	97,30	92,17	94,15	94,88	97,32	96,36	95,72	95,49	95,58
Sumatera Utara	72,23	64,32	67,08	63,92	57,06	59,85	68,49	61,47	64,09
Sumatera Barat	67,11	69,39	68,67	64,25	64,60	64,48	65,66	67,12	66,64
Riau	62,73	59,70	60,81	46,43	51,21	49,19	53,87	55,61	54,93
Jambi	66,44	75,14	71,84	54,55	53,74	54,07	59,03	62,35	61,04
Sumatera Selatan	78,10	78,92	78,60	51,54	61,47	57,49	63,01	69,23	66,78
Bengkulu	81,77	82,35	82,12	62,60	67,87	65,83	71,51	74,63	73,42
Lampung	80,50	71,80	75,36	52,01	54,78	53,77	62,68	60,43	61,29
Kepulauan Bangka Belitung	69,96	73,13	71,81	63,88	70,60	67,64	67,57	72,19	70,22
Kepulauan Riau	80,75	76,00	77,59	82,95	84,05	83,66	81,04	77,00	78,37
DKI Jakarta	82,75	82,60	82,66	-	-	-	82,75	82,60	82,66
Jawa Barat	63,64	60,62	61,79	49,49	52,24	51,03	60,04	58,80	59,30
Jawa Tengah	59,90	67,70	64,47	51,47	55,89	54,14	56,06	62,11	59,65
DI Yogyakarta	76,69	73,58	74,82	71,72	71,78	71,75	75,28	73,13	74,02
Jawa Timur	56,48	58,57	57,73	45,90	47,55	46,86	51,60	53,70	52,84
Banten	69,89	64,86	66,70	58,39	57,73	57,96	67,96	63,57	65,17
Bali	68,13	63,84	65,52	60,29	53,73	56,55	65,45	60,74	62,65
Nusa Tenggara Barat	60,37	64,32	62,86	49,05	48,98	49,01	53,75	56,06	55,15
Nusa Tenggara Timur	63,96	68,31	66,75	65,92	71,35	69,50	65,28	70,40	68,62
Kalimantan Barat	52,45	58,32	56,12	59,27	63,65	61,92	55,97	60,97	59,04
Kalimantan Tengah	70,04	76,48	73,83	62,73	59,97	61,26	66,12	68,54	67,47
Kalimantan Selatan	73,05	72,00	72,35	65,09	69,52	67,74	68,68	70,82	70,04
Kalimantan Timur	80,73	78,57	79,38	66,11	75,51	71,63	76,29	77,75	77,18
Kalimantan Utara	84,41	86,22	85,52	73,63	88,30	81,92	80,39	86,89	84,29
Sulawesi Utara	73,92	70,51	72,05	68,06	68,66	68,38	71,10	69,63	70,30
Sulawesi Tengah	62,03	69,50	66,62	70,18	76,79	74,02	67,34	74,02	71,30
Sulawesi Selatan	80,49	82,06	81,51	80,56	78,64	79,36	80,52	80,35	80,42
Sulawesi Tenggara	65,96	66,04	66,01	73,46	72,94	73,14	70,27	69,87	70,03
Gorontalo	88,56	89,77	89,30	87,50	86,51	86,87	88,05	88,12	88,09
Sulawesi Barat	65,93	69,57	68,01	77,30	91,42	85,41	72,72	82,69	78,44
Maluku	63,87	54,43	58,01	57,72	61,36	59,53	60,93	56,89	58,63
Maluku Utara	69,74	67,58	68,32	62,28	69,16	65,99	65,20	68,35	67,07
Papua Barat	76,72	76,52	76,60	77,16	72,95	74,47	76,93	74,76	75,57
Papua	75,22	72,28	73,61	82,10	82,94	82,61	78,22	77,54	77,83
**Indonesia**	**66,91**	**67,21**	**67,09**	**57,74**	**60,59**	**59,44**	**63,06**	**64,54**	**63,96**

Sumber/*Source*: Susenas Maret 2018/*The March 2018 Susenas*

https://www.bps.go.id

Tabel *Table* **4.11.1.** **Persentase Penduduk yang Pernah Rawat Inap di Daerah Perkotaan menurut Provinsi, Jumlah Hari Rawat Inap, dan Rata-Rata Lama Rawat Inap (Hari), 2018**
Percentage of Population Who has Hospitalized during the Last Month in Urban Area by Province, Number of Inpatient Days, and Average Length of Inpatient (Days), 2018

Provinsi *Province*	Jumlah Hari Rawat Inap/*Number of Inpatient Days*				Rata-rata Lama Rawat Inap *Average Length of Inpatient*
	≤3	4-6	7-29	≥30	
(1)	(2)	(3)	(4)	(5)	(6)
Aceh	40,58	33,95	23,64	1,83	5,69
Sumatera Utara	40,76	34,75	23,20	1,28	5,48
Sumatera Barat	40,81	31,84	25,35	2,00	5,65
Riau	58,15	21,61	18,53	1,71	5,04
Jambi	50,98	33,39	14,89	0,74	4,56
Sumatera Selatan	49,17	25,66	23,80	1,37	5,24
Bengkulu	61,77	24,90	11,39	1,95	4,60
Lampung	55,70	26,44	17,06	0,80	4,63
Kepulauan Bangka Belitung	60,42	23,76	15,14	0,67	4,67
Kepulauan Riau	63,53	16,69	15,45	4,33	5,91
DKI Jakarta	43,58	29,73	24,65	2,05	6,23
Jawa Barat	49,06	26,70	23,38	0,86	5,00
Jawa Tengah	40,12	33,00	25,49	1,39	5,64
DI Yogyakarta	40,08	38,83	20,57	0,52	5,09
Jawa Timur	42,05	31,65	24,82	1,48	5,59
Banten	50,20	28,39	20,04	1,38	5,00
Bali	44,05	29,32	24,52	2,11	5,36
Nusa Tenggara Barat	53,94	22,97	19,56	3,53	5,88
Nusa Tenggara Timur	59,90	22,41	16,67	1,02	4,39
Kalimantan Barat	46,80	27,42	23,85	1,93	6,24
Kalimantan Tengah	55,75	21,08	21,13	2,04	4,96
Kalimantan Selatan	51,48	26,53	20,99	1,00	4,72
Kalimantan Timur	49,21	26,29	21,37	3,13	6,15
Kalimantan Utara	59,48	24,22	14,63	1,67	4,53
Sulawesi Utara	34,16	28,35	35,48	2,01	6,69
Sulawesi Tengah	40,71	25,70	29,33	4,27	6,82
Sulawesi Selatan	43,62	27,30	27,47	1,61	5,80
Sulawesi Tenggara	51,51	27,01	19,44	2,03	5,34
Gorontalo	33,19	28,71	36,01	2,09	6,43
Sulawesi Barat	54,09	21,24	22,77	1,91	5,24
Maluku	47,84	22,18	29,77	0,21	4,92
Maluku Utara	38,05	37,21	23,37	1,37	5,91
Papua Barat	49,42	28,05	21,43	1,11	4,69
Papua	38,88	29,10	31,04	0,99	5,68
**Indonesia**	**45,76**	**29,24**	**23,55**	**1,46**	**5,43**

Sumber/*Source*: Susenas Maret 2018/*The March 2018 Susenas*

https://www.bps.go.id

Tabel *Table* **4.11.2.** **Persentase Penduduk yang Pernah Rawat Inap di Daerah Perdesaan menurut Provinsi, Jumlah Hari Rawat Inap, dan Rata-Rata Lama Rawat Inap (Hari), 2018**
Percentage of Population Who has Hospitalized during the Last Month in Rural Area by Province, Number of Inpatient Days, and Average Length of Inpatient (Days), 2018

Provinsi *Province*	Jumlah Hari Rawat Inap/*Number of Inpatient Days*				Rata-rata Lama Rawat Inap *Average Length of Inpatient*
	≤3	4-6	7-29	≥30	
(1)	(2)	(3)	(4)	(5)	(6)
Aceh	46,17	28,79	23,78	1,26	5,39
Sumatera Utara	45,64	30,45	21,98	1,92	5,44
Sumatera Barat	50,93	23,60	24,08	1,39	5,46
Riau	57,63	22,60	18,43	1,35	4,93
Jambi	53,76	25,40	20,08	0,76	4,74
Sumatera Selatan	53,25	26,45	19,23	1,07	4,98
Bengkulu	65,25	17,00	16,77	0,98	4,57
Lampung	63,96	22,05	13,14	0,85	4,08
Kepulauan Bangka Belitung	67,74	13,50	17,19	1,57	5,08
Kepulauan Riau	45,30	24,92	26,23	3,55	6,27
DKI Jakarta	-	-	-	-	-
Jawa Barat	56,08	24,44	18,28	1,21	4,56
Jawa Tengah	47,17	30,87	21,16	0,79	5,09
DI Yogyakarta	41,08	36,26	20,29	2,36	5,63
Jawa Timur	48,41	30,31	20,35	0,93	5,00
Banten	54,95	24,27	18,64	2,14	4,79
Bali	41,57	27,58	29,44	1,42	5,72
Nusa Tenggara Barat	65,42	18,97	14,56	1,05	4,36
Nusa Tenggara Timur	59,85	19,31	19,96	0,88	4,52
Kalimantan Barat	53,59	21,87	22,03	2,51	5,66
Kalimantan Tengah	46,96	22,11	28,48	2,44	6,24
Kalimantan Selatan	53,31	23,81	21,80	1,08	5,27
Kalimantan Timur	50,54	20,42	27,84	1,20	5,38
Kalimantan Utara	46,75	30,88	21,64	0,73	4,87
Sulawesi Utara	36,84	31,49	29,60	2,08	6,27
Sulawesi Tengah	48,39	24,12	26,49	1,00	5,08
Sulawesi Selatan	53,31	23,67	22,06	0,96	4,86
Sulawesi Tenggara	51,76	23,02	23,49	1,72	5,32
Gorontalo	44,30	25,78	26,11	3,80	6,28
Sulawesi Barat	52,93	21,48	24,16	1,43	5,30
Maluku	42,51	21,19	32,84	3,46	6,29
Maluku Utara	45,82	22,14	27,13	4,91	6,11
Papua Barat	44,84	22,20	31,54	1,42	5,69
Papua	50,93	23,06	24,57	1,44	5,90
**Indonesia**	**51,36**	**26,60**	**20,84**	**1,19**	**5,02**

Sumber/*Source*: Susenas Maret 2018/*The March 2018 Susenas*

https://www.bps.go.id

Tabel *Table* **4.11.3.** **Persentase Penduduk yang Pernah Rawat Inap di Daerah Perkotaan dan Perdesaan menurut Provinsi, Jumlah Hari Rawat Inap, dan Rata-Rata Lama Rawat Inap (Hari), 2018**
Percentage of Population Who has Hospitalized during the Last Month in Urban and Rural Area by Province, Number of Inpatient Days, and Average Length of Inpatient (Days), 2018

**Provinsi** *Province*	**Jumlah Hari Rawat Inap/***Number of Inpatient Days*				**Rata-rata Lama Rawat Inap** *Average Length of Inpatient*
	**≤3**	**4-6**	**7-29**	**≥30**	
**(1)**	**(2)**	**(3)**	**(4)**	**(5)**	**(6)**
Aceh	44,21	30,60	23,73	1,46	5,49
Sumatera Utara	42,78	32,97	22,70	1,55	5,46
Sumatera Barat	45,72	27,85	24,74	1,70	5,56
Riau	57,88	22,11	18,48	1,53	4,99
Jambi	52,67	28,53	18,04	0,76	4,67
Sumatera Selatan	51,45	26,10	21,24	1,20	5,09
Bengkulu	63,63	20,68	14,26	1,43	4,58
Lampung	61,08	23,58	14,50	0,83	4,27
Kepulauan Bangka Belitung	63,21	19,86	15,92	1,02	4,83
Kepulauan Riau	61,22	17,73	16,82	4,23	5,95
DKI Jakarta	43,58	29,73	24,65	2,05	6,23
Jawa Barat	50,69	26,17	22,20	0,94	4,90
Jawa Tengah	43,41	32,01	23,47	1,11	5,38
DI Yogyakarta	40,34	38,15	20,50	1,00	5,23
Jawa Timur	44,91	31,05	22,81	1,23	5,32
Banten	51,03	27,66	19,79	1,51	4,96
Bali	43,26	28,76	26,10	1,89	5,48
Nusa Tenggara Barat	60,33	20,74	16,77	2,15	5,03
Nusa Tenggara Timur	59,87	20,30	18,91	0,93	4,48
Kalimantan Barat	50,22	24,62	22,93	2,22	5,95
Kalimantan Tengah	51,31	21,60	24,84	2,24	5,60
Kalimantan Selatan	52,40	25,16	21,40	1,04	5,00
Kalimantan Timur	49,59	24,63	23,20	2,58	5,93
Kalimantan Utara	55,11	26,50	17,03	1,35	4,64
Sulawesi Utara	35,44	29,85	32,67	2,04	6,49
Sulawesi Tengah	45,57	24,70	27,53	2,20	5,72
Sulawesi Selatan	48,55	25,46	24,72	1,28	5,32
Sulawesi Tenggara	51,65	24,77	21,72	1,86	5,33
Gorontalo	38,71	27,26	31,09	2,94	6,35
Sulawesi Barat	53,39	21,38	23,60	1,62	5,28
Maluku	45,66	21,77	31,03	1,54	5,48
Maluku Utara	42,21	29,13	25,39	3,27	6,02
Papua Barat	47,20	25,21	26,33	1,26	5,17
Papua	44,52	26,27	28,01	1,20	5,78
**Indonesia**	**48,05**	**28,16**	**22,44**	**1,35**	**5,26**

Sumber/*Source*: Susenas Maret 2018/*The March 2018 Susenas*

https://www.bps.go.id

Tabel *Table* **4.12.** **Persentase Penduduk 5 Tahun ke Atas yang Merokok Tembakau selama Sebulan Terakhir menurut Provinsi, Daerah Tempat Tinggal, dan Kebiasaan Merokok, 2018**
*Percentage of Population 5 Years or over Who Smoked Tobacco during The Last Month by Province, Urban Rural Classification, and Smoking Habit, 2017**

Provinsi *Province*	Perkotaan/*Urban*					Perdesaan/*Rural*				
	Ya, Setiap Hari *Yes, Everyday*	Ya, Tidak Setiap Hari *Yes, Not Everyday*	Tidak *No*	Tidak Tahu *Not Known*	Jumlah *Total*	Ya, Setiap Hari *Yes, Everyday*	Ya, Tidak Setiap Hari *Yes, Not Everyday*	Tidak *No*	Tidak Tahu *Not Known*	Jumlah *Total*
(1)	(2)	(3)	(4)	(5)	(6)	(7)	(8)	(9)	(10)	(11)
Aceh	17,76	1,69	79,68	0,87	100,00	21,52	1,97	75,83	0,67	100,00
Sumatera Utara	19,61	1,58	78,07	0,73	100,00	21,02	1,41	77,27	0,31	100,00
Sumatera Barat	21,35	1,97	75,99	0,70	100,00	24,23	1,48	73,91	0,39	100,00
Riau	19,24	1,54	79,06	0,16	100,00	22,39	1,56	74,76	1,29	100,00
Jambi	19,25	1,49	78,62	0,64	100,00	22,87	1,39	75,11	0,63	100,00
Sumatera Selatan	20,30	1,94	77,60	0,16	100,00	25,71	1,74	72,11	0,44	100,00
Bengkulu	20,85	1,92	77,08	0,14	100,00	26,48	1,80	71,39	0,33	100,00
Lampung	21,58	2,03	76,27	0,12	100,00	26,49	1,55	71,80	0,16	100,00
Kepulauan Bangka Belitung	22,25	1,41	76,18	0,16	100,00	22,69	0,97	76,21	0,13	100,00
Kepulauan Riau	21,70	1,15	76,66	0,50	100,00	23,24	1,06	75,41	0,29	100,00
DKI Jakarta	18,23	2,24	78,88	0,66	100,00	-	-	-	-	100,00
Jawa Barat	23,50	2,00	74,04	0,46	100,00	27,48	2,51	69,59	0,42	100,00
Jawa Tengah	18,30	2,35	79,10	0,26	100,00	22,58	2,35	74,79	0,28	100,00
DI Yogyakarta	16,21	2,35	81,42	0,02	100,00	18,68	2,71	78,50	0,10	100,00
Jawa Timur	18,84	2,20	78,54	0,42	100,00	23,20	2,05	74,27	0,48	100,00
Banten	21,69	2,13	75,68	0,50	100,00	27,42	1,54	68,93	2,11	100,00
Bali	16,11	2,09	81,66	0,14	100,00	16,69	1,80	81,49	0,03	100,00
Nusa Tenggara Barat	20,56	2,60	75,99	0,85	100,00	22,72	1,97	74,93	0,38	100,00
Nusa Tenggara Timur	14,80	3,11	81,93	0,16	100,00	16,58	4,09	79,04	0,29	100,00
Kalimantan Barat	18,78	1,00	79,58	0,64	100,00	22,31	1,62	75,53	0,54	100,00
Kalimantan Tengah	17,96	2,37	79,52	0,15	100,00	22,88	1,69	74,95	0,48	100,00
Kalimantan Selatan	16,67	1,97	81,31	0,05	100,00	19,45	1,32	79,09	0,14	100,00
Kalimantan Timur	16,40	2,13	81,26	0,20	100,00	20,34	1,35	77,62	0,69	100,00
Kalimantan Utara	18,33	2,71	78,40	0,56	100,00	20,33	2,47	77,11	0,09	100,00
Sulawesi Utara	18,59	3,63	73,82	3,96	100,00	21,58	3,57	71,95	2,90	100,00
Sulawesi Tengah	19,62	2,29	77,70	0,38	100,00	24,35	2,07	73,25	0,33	100,00
Sulawesi Selatan	17,30	1,26	80,87	0,58	100,00	19,65	1,55	78,29	0,51	100,00
Sulawesi Tenggara	18,68	1,49	78,99	0,83	100,00	21,17	1,47	77,22	0,14	100,00
Gorontalo	20,71	2,96	76,16	0,17	100,00	25,69	3,68	70,44	0,19	100,00
Sulawesi Barat	17,83	0,95	81,05	0,17	100,00	18,46	1,97	79,06	0,51	100,00
Maluku	14,22	3,74	77,97	4,07	100,00	18,42	4,05	75,35	2,18	100,00
Maluku Utara	18,68	2,40	77,91	1,02	100,00	19,09	4,20	73,78	2,92	100,00
Papua Barat	15,36	3,29	78,27	3,07	100,00	18,03	4,89	75,61	1,46	100,00
Papua	16,75	3,09	75,75	4,40	100,00	15,41	5,33	76,39	2,87	100,00
**Indonesia**	**20,01**	**2,08**	**77,39**	**0,51**	**100,00**	**22,90**	**2,14**	**74,39**	**0,57**	**100,00**

Catatan/*Note***:** *) Data 2018 belum tersedia/*2018 data not available yet*
Sumber/*Source*: Susenas Maret 2017/*The March 2017 Susenas*

https://www.bps.go.id

Lanjutan Tabel/*Table Continued* **4.12.**

Provinsi<br>*Province*	Perkotaan+Perdesaan/*Urban+Rural*				
	Ya, Setiap Hari<br>*Yes, Everyday*	Ya, Tidak Setiap Hari<br>*Yes, Not Everyday*	Tidak<br>*No*	Tidak Tahu<br>*Not Known*	Jumlah<br>*Total*
(1)	(12)	(13)	(14)	(15)	(16)
Aceh	20,39	1,89	76,99	0,73	100,00
Sumatera Utara	20,30	1,50	77,68	0,53	100,00
Sumatera Barat	23,03	1,68	74,77	0,52	100,00
Riau	21,14	1,55	76,47	0,84	100,00
Jambi	21,73	1,43	76,21	0,63	100,00
Sumatera Selatan	23,73	1,81	74,12	0,34	100,00
Bengkulu	24,67	1,84	73,22	0,27	100,00
Lampung	25,14	1,68	73,03	0,15	100,00
Kepulauan Bangka Belitung	22,46	1,20	76,19	0,15	100,00
Kepulauan Riau	21,93	1,13	76,47	0,46	100,00
DKI Jakarta	18,23	2,24	78,88	0,66	100,00
Jawa Barat	24,62	2,15	72,79	0,45	100,00
Jawa Tengah	20,47	2,35	76,91	0,27	100,00
DI Yogyakarta	16,94	2,46	80,55	0,05	100,00
Jawa Timur	20,98	2,13	76,45	0,45	100,00
Banten	23,41	1,96	73,65	0,98	100,00
Bali	16,32	1,98	81,60	0,10	100,00
Nusa Tenggara Barat	21,75	2,26	75,40	0,59	100,00
Nusa Tenggara Timur	16,19	3,87	79,67	0,27	100,00
Kalimantan Barat	21,19	1,43	76,81	0,57	100,00
Kalimantan Tengah	21,11	1,94	76,59	0,36	100,00
Kalimantan Selatan	18,22	1,61	80,07	0,10	100,00
Kalimantan Timur	17,73	1,87	80,03	0,37	100,00
Kalimantan Utara	19,18	2,61	77,85	0,36	100,00
Sulawesi Utara	20,16	3,60	72,84	3,40	100,00
Sulawesi Tengah	23,12	2,13	74,41	0,35	100,00
Sulawesi Selatan	18,72	1,43	79,31	0,54	100,00
Sulawesi Tenggara	20,37	1,47	77,79	0,36	100,00
Gorontalo	23,87	3,41	72,53	0,18	100,00
Sulawesi Barat	18,33	1,76	79,48	0,44	100,00
Maluku	16,69	3,92	76,43	2,96	100,00
Maluku Utara	18,98	3,70	74,92	2,40	100,00
Papua Barat	16,99	4,27	76,66	2,09	100,00
Papua	15,77	4,73	76,22	3,28	100,00
**Indonesia**	**21,37**	**2,11**	**75,98**	**0,54**	**100,00**

Catatan/*Note*: *) Data 2018 belum tersedia/*2018 data not available yet*
Sumber/*Source*: Susenas Maret 2017/*The March 2017 Susenas*

https://www.bps.go.id

Tabel **4.13.1.** **Persentase Penduduk 5 Tahun ke Atas yang Merokok selama Sebulan Terakhir di Daerah Perkotaan menurut Provinsi, dan Jumlah Batang Rokok yang Dihisap per Minggu, 2017***
Table *Percentage of Population 5 Years and Over Smoke during The Last Month in Urban Area by Province and The Number of Cigarettes Smoked per Week, 2017**

**Provinsi** *Province*	**Jumlah Batang Rokok yang Dihisap per Minggu/***Number of Cigarettes Smoked per Week*						**Rata-rata per Minggu (Batang Rokok)** *Average per Week (cigarettes)*
	**1-6 Batang/***1-6 Cigarettes*	**7-14 Batang/***7-14 Cigarettes*	**15-29 Batang/***15-29 Cigarettes*	**30-59 Batang/***30-59 Cigarettes*	**≥ 60 Batang/***≥ 60 Cigarettes*	**Jumlah** *Total*	
(1)	(2)	(3)	(4)	(5)	(6)	(7)	(8)
Aceh	0,96	3,65	8,04	18,64	68,71	100,00	91,35
Sumatera Utara	0,75	3,51	8,54	21,41	65,79	100,00	86,09
Sumatera Barat	0,92	3,92	6,64	18,96	69,56	100,00	93,24
Riau	0,72	2,85	5,48	14,17	76,78	100,00	101,80
Jambi	0,36	1,37	5,53	15,43	77,31	100,00	101,48
Sumatera Selatan	0,98	4,26	9,20	23,93	61,62	100,00	81,06
Bengkulu	0,73	2,87	6,10	19,37	70,93	100,00	90,20
Lampung	0,91	3,41	8,09	22,64	64,94	100,00	85,68
Kepulauan Bangka Belitung	0,44	1,74	3,80	13,66	80,37	100,00	109,38
Kepulauan Riau	0,06	3,49	3,63	15,67	77,14	100,00	103,33
DKI Jakarta	1,01	7,01	8,98	26,26	56,74	100,00	72,83
Jawa Barat	1,04	5,30	9,11	25,67	58,88	100,00	74,06
Jawa Tengah	1,77	6,72	11,30	27,82	52,39	100,00	67,33
DI Yogyakarta	1,54	9,54	12,17	27,96	48,80	100,00	66,01
Jawa Timur	1,47	5,47	10,06	23,90	59,09	100,00	73,95
Banten	0,61	5,23	7,70	17,40	69,05	100,00	83,23
Bali	2,09	6,90	9,55	24,99	56,47	100,00	75,34
Nusa Tenggara Barat	1,38	6,16	9,26	27,39	55,81	100,00	70,53
Nusa Tenggara Timur	4,14	9,40	20,30	23,46	42,70	100,00	60,07
Kalimantan Barat	0,08	3,24	4,02	15,65	77,00	100,00	101,72
Kalimantan Tengah	0,68	2,14	3,85	17,61	75,73	100,00	103,24
Kalimantan Selatan	1,52	2,85	5,83	14,66	75,13	100,00	102,30
Kalimantan Timur	0,72	2,92	6,24	16,84	73,27	100,00	92,57
Kalimantan Utara	1,19	3,58	9,31	22,31	63,61	100,00	91,89
Sulawesi Utara	1,12	4,83	10,16	31,11	52,78	100,00	82,19
Sulawesi Tengah	1,47	3,40	8,56	19,31	67,26	100,00	94,07
Sulawesi Selatan	0,57	4,64	5,92	20,10	68,76	100,00	93,40
Sulawesi Tenggara	0,85	3,48	6,66	21,46	67,55	100,00	93,40
Gorontalo	1,35	4,47	12,49	33,89	47,80	100,00	75,44
Sulawesi Barat	0,00	0,76	4,30	16,53	78,41	100,00	102,87
Maluku	5,00	10,76	18,48	27,04	38,72	100,00	61,54
Maluku Utara	0,81	3,13	8,58	24,09	63,39	100,00	88,81
Papua Barat	1,31	8,94	13,96	21,99	53,80	100,00	75,39
Papua	1,68	3,94	13,20	19,14	62,04	100,00	89,40
**Indonesia**	**1,16**	**5,29**	**9,06**	**23,65**	**60,84**	**100,00**	**78,21**

Catatan/*Note***:** *) Data 2018 belum tersedia/*2018 data not available yet*
Sumber/*Source*: Susenas Maret 2017/*The March 2017 Susenas*

https://www.bps.go.id

Tabel *Table* **4.13.2.** **Persentase Penduduk 5 Tahun ke Atas yang Merokok selama Sebulan Terakhir di Daerah Perdesaan menurut Provinsi, dan Jumlah Batang Rokok yang Dihisap per Minggu, 2017***
*Percentage of Population 5 Years and Over Smoke during The Last Month in Rural Area by Province and The Number of Cigarettes Smoked per Week, 2017**

Provinsi *Province*	Jumlah Batang Rokok yang Dihisap per Minggu/*Number of Cigarettes Smoked per Week*						Rata-rata per Minggu (Batang Rokok) *Average per Week (cigarettes)*
	1-6 Batang/*1-6 Cigarettes*	7-14 Batang/*7-14 Cigarettes*	15-29 Batang/*15-29 Cigarettes*	30-59 Batang/*30-59 Cigarettes*	≥ 60 Batang/*≥ 60 Cigarettes*	Jumlah *Total*	
(1)	(2)	(3)	(4)	(5)	(6)	(7)	(8)
Aceh	0,67	3,02	5,72	17,40	73,19	100,00	92,76
Sumatera Utara	0,27	1,86	3,83	10,50	83,55	100,00	104,01
Sumatera Barat	0,26	2,01	3,66	10,97	83,10	100,00	109,79
Riau	0,18	0,88	2,01	9,51	87,43	100,00	120,44
Jambi	0,20	1,02	2,35	10,25	86,18	100,00	109,43
Sumatera Selatan	0,32	1,73	3,65	13,13	81,16	100,00	90,09
Bengkulu	0,08	1,39	2,36	9,59	86,58	100,00	97,44
Lampung	0,48	2,64	6,00	19,97	70,92	100,00	83,17
Kepulauan Bangka Belitung	0,38	1,02	2,34	8,68	87,57	100,00	114,34
Kepulauan Riau	0,87	1,32	2,21	8,47	87,12	100,00	116,62
DKI Jakarta	-	-	-	-	-	-	-
Jawa Barat	0,92	5,12	8,23	23,25	62,48	100,00	74,24
Jawa Tengah	1,47	5,94	10,55	26,63	55,40	100,00	67,06
DI Yogyakarta	1,65	7,94	12,64	25,56	52,21	100,00	64,53
Jawa Timur	0,80	4,17	8,26	21,69	65,08	100,00	77,86
Banten	0,16	1,11	5,57	13,44	79,72	100,00	86,67
Bali	1,79	5,12	7,99	25,93	59,17	100,00	78,65
Nusa Tenggara Barat	0,86	3,95	9,70	26,08	59,41	100,00	72,55
Nusa Tenggara Timur	3,84	11,61	20,31	30,38	33,86	100,00	51,82
Kalimantan Barat	0,54	0,82	3,31	12,92	82,42	100,00	101,29
Kalimantan Tengah	0,44	1,53	2,44	10,28	85,31	100,00	108,52
Kalimantan Selatan	0,49	1,30	3,35	9,12	85,74	100,00	116,46
Kalimantan Timur	0,11	1,69	4,72	11,70	81,78	100,00	101,41
Kalimantan Utara	1,20	0,71	3,47	16,91	77,71	100,00	100,96
Sulawesi Utara	0,93	4,31	10,19	27,47	57,09	100,00	81,56
Sulawesi Tengah	0,51	2,52	5,01	17,75	74,22	100,00	98,30
Sulawesi Selatan	0,76	1,88	4,20	18,67	74,49	100,00	98,28
Sulawesi Tenggara	0,36	1,59	5,50	14,19	78,36	100,00	101,76
Gorontalo	1,06	5,04	9,41	31,11	53,38	100,00	75,13
Sulawesi Barat	0,29	1,74	3,85	10,66	83,47	100,00	108,93
Maluku	2,39	6,75	17,28	29,52	44,06	100,00	69,55
Maluku Utara	1,20	3,21	12,07	29,04	54,47	100,00	78,02
Papua Barat	1,07	6,31	11,60	28,72	52,31	100,00	71,75
Papua	1,50	7,07	15,20	31,93	44,30	100,00	64,76
**Indonesia**	**0,86**	**3,78**	**7,37**	**19,85**	**68,15**	**100,00**	**84,25**

Catatan/*Note***:** *) Data 2018 belum tersedia/*2018 data not available yet*
Sumber/*Source*: Susenas Maret 2017/*The March 2017 Susenas*

https://www.bps.go.id

Tabel *Table* **4.13.3.** **Persentase Penduduk 5 Tahun ke Atas yang Merokok selama Sebulan Terakhir di Daerah Perkotaan dan Perdesaan menurut Provinsi, dan Jumlah Batang Rokok yang Dihisap per Minggu, 2017***
*Percentage of Population 5 Years and Over Smoke during The Last Month in Urban and Rural Area by Province and The Number of Cigarettes Smoked per Week, 2017**

**Provinsi** *Province*	**Jumlah Batang Rokok yang Dihisap per Minggu/***Number of Cigarettes Smoked per Week*						**Rata-rata per Minggu (Batang Rokok)** *Average per Week (cigarettes)*
	**1-6 Batang/***1-6 Cigarettes*	**7-14 Batang/***7-14 Cigarettes*	**15-29 Batang/***15-29 Cigarettes*	**30-59 Batang/***30-59 Cigarettes*	**≥ 60 Batang/***≥ 60 Cigarettes*	**Jumlah** *Total*	
(1)	(2)	(3)	(4)	(5)	(6)	(7)	(8)
Aceh	0,74	3,18	6,33	17,73	72,02	100,00	92,39
Sumatera Utara	0,51	2,68	6,17	15,94	74,70	100,00	95,08
Sumatera Barat	0,52	2,76	4,83	14,11	77,78	100,00	103,29
Riau	0,38	1,60	3,28	11,22	83,53	100,00	113,62
Jambi	0,24	1,12	3,25	11,71	83,68	100,00	107,19
Sumatera Selatan	0,54	2,54	5,42	16,58	74,93	100,00	87,20
Bengkulu	0,26	1,80	3,39	12,29	82,26	100,00	95,44
Lampung	0,58	2,83	6,51	20,62	69,47	100,00	83,78
Kepulauan Bangka Belitung	0,41	1,40	3,10	11,28	83,81	100,00	111,75
Kepulauan Riau	0,19	3,15	3,41	14,53	78,73	100,00	105,44
DKI Jakarta	1,01	7,01	8,98	26,26	56,74	100,00	72,83
Jawa Barat	1,00	5,24	8,83	24,91	60,02	100,00	74,12
Jawa Tengah	1,61	6,29	10,88	27,16	54,06	100,00	67,18
DI Yogyakarta	1,57	9,01	12,32	27,17	49,92	100,00	65,52
Jawa Timur	1,11	4,77	9,09	22,72	62,30	100,00	76,05
Banten	0,46	3,82	6,97	16,04	72,71	100,00	84,41
Bali	1,98	6,25	8,98	25,33	57,46	100,00	76,55
Nusa Tenggara Barat	1,08	4,90	9,51	26,65	57,86	100,00	71,68
Nusa Tenggara Timur	3,90	11,17	20,31	29,03	35,59	100,00	53,44
Kalimantan Barat	0,41	1,49	3,51	13,68	80,92	100,00	101,41
Kalimantan Tengah	0,52	1,72	2,89	12,60	82,27	100,00	106,84
Kalimantan Selatan	0,92	1,95	4,38	11,43	81,33	100,00	110,58
Kalimantan Timur	0,49	2,46	5,67	14,92	76,45	100,00	95,88
Kalimantan Utara	1,20	2,30	6,70	19,90	69,90	100,00	95,94
Sulawesi Utara	1,02	4,54	10,18	29,09	55,18	100,00	81,84
Sulawesi Tengah	0,72	2,72	5,81	18,10	72,64	100,00	97,34
Sulawesi Selatan	0,69	2,89	4,83	19,19	72,40	100,00	96,50
Sulawesi Tenggara	0,51	2,15	5,85	16,35	75,15	100,00	99,28
Gorontalo	1,15	4,86	10,39	31,99	51,61	100,00	75,23
Sulawesi Barat	0,23	1,55	3,94	11,79	82,49	100,00	107,76
Maluku	3,33	8,19	17,71	28,63	42,15	100,00	66,68
Maluku Utara	1,10	3,19	11,17	27,77	56,77	100,00	80,80
Papua Barat	1,15	7,22	12,41	26,41	52,82	100,00	73,00
Papua	1,55	6,26	14,68	28,62	48,89	100,00	71,13
**Indonesia**	**1,01**	**4,53**	**8,21**	**21,74**	**64,50**	**100,00**	**81,23**

Catatan/*Note***:** *) Data 2018 belum tersedia/*2018 data not available yet*
Sumber/*Source*: Susenas Maret 2017/*The March 2017 Susenas*

https://www.bps.go.id

Tabel **4.14.1.** **Persentase Penduduk Umur 0-59 Bulan (Balita) yang Mempunyai Kartu Imunisasi di Daerah Perkotaan menurut Provinsi dan Jenis Kelamin, 2017***
Table *Percentage of Population Aged 0-59 Months (Under Five Years) Having Immunization Card in Urban Area by Province and Sex, 2017**

**Provinsi** *Province*	**Laki-laki/*Male***			**Perempuan/*Female***			**Laki-laki+Perempuan/*Male+Female***		
	**Ya, Ditunjukkan/** *Yes, Shown*	**Ya, Tidak Dapat Ditunjukkan/***Yes, Can not be Shown*	**Tidak Ada Kartu** *No Card*	**Ya, Ditunjukkan/** *Yes, Shown*	**Ya, Tidak Dapat Ditunjukkan/***Yes, Can not be Shown*	**Tidak Ada Kartu** *No Card*	**Ya, Ditunjukkan/** *Yes, Shown*	**Ya, Tidak Dapat Ditunjukkan/***Yes, Can not be Shown*	**Tidak Ada Kartu** *No Card*
**(1)**	**(2)**	**(3)**	**(4)**	**(5)**	**(6)**	**(7)**	**(8)**	**(9)**	**(10)**
Aceh	12,83	49,58	37,58	16,04	49,13	34,83	14,42	49,36	36,22
Sumatera Utara	19,39	60,50	20,10	17,58	58,66	23,76	18,50	59,60	21,90
Sumatera Barat	24,74	57,62	17,64	25,75	54,99	19,26	25,22	56,36	18,41
Riau	20,63	54,60	24,76	18,50	57,74	23,76	19,60	56,12	24,28
Jambi	21,88	65,97	12,15	19,84	70,22	9,93	20,87	68,09	11,04
Sumatera Selatan	26,11	59,19	14,70	23,52	61,79	14,69	24,83	60,48	14,69
Bengkulu	28,53	58,11	13,36	24,62	60,27	15,12	26,67	59,14	14,20
Lampung	41,93	46,70	11,37	40,69	48,82	10,49	41,35	47,69	10,96
Kepulauan Bangka Belitung	52,80	40,29	6,91	50,14	41,45	8,41	51,48	40,87	7,66
Kepulauan Riau	41,92	36,48	21,60	41,34	42,26	16,39	41,63	39,42	18,95
DKI Jakarta	44,76	47,46	7,78	42,09	50,04	7,87	43,45	48,73	7,82
Jawa Barat	33,80	48,40	17,80	32,38	48,33	19,30	33,11	48,37	18,53
Jawa Tengah	56,28	36,88	6,83	52,07	42,08	5,85	54,24	39,41	6,36
DI Yogyakarta	67,64	28,79	3,57	63,25	34,12	2,64	65,44	31,46	3,10
Jawa Timur	46,02	46,08	7,90	46,14	45,44	8,42	46,08	45,77	8,15
Banten	23,75	56,03	20,22	26,82	51,69	21,49	25,25	53,90	20,84
Bali	44,41	45,78	9,81	45,89	41,96	12,16	45,12	43,94	10,94
Nusa Tenggara Barat	37,38	52,95	9,67	37,83	47,02	15,15	37,60	50,02	12,38
Nusa Tenggara Timur	38,68	49,80	11,52	47,18	43,42	9,40	42,79	46,72	10,50
Kalimantan Barat	30,79	52,55	16,66	36,88	48,82	14,29	33,78	50,72	15,50
Kalimantan Tengah	30,16	57,33	12,50	34,58	52,59	12,83	32,37	54,96	12,67
Kalimantan Selatan	36,43	50,70	12,87	38,95	52,25	8,79	37,58	51,41	11,01
Kalimantan Timur	42,78	48,95	8,27	41,33	47,12	11,56	42,07	48,05	9,88
Kalimantan Utara	33,12	60,35	6,53	41,16	52,00	6,84	37,24	56,07	6,69
Sulawesi Utara	31,21	57,47	11,31	34,28	54,85	10,88	32,68	56,22	11,10
Sulawesi Tengah	29,18	54,97	15,85	41,29	45,81	12,90	35,42	50,25	14,33
Sulawesi Selatan	29,95	53,78	16,27	30,48	52,08	17,44	30,20	52,98	16,82
Sulawesi Tenggara	22,99	51,03	25,99	24,02	59,55	16,43	23,47	55,01	21,53
Gorontalo	45,02	45,90	9,08	46,91	45,86	7,23	45,95	45,88	8,17
Sulawesi Barat	43,33	46,78	9,89	48,07	39,32	12,61	45,76	42,95	11,28
Maluku	22,81	56,79	20,40	22,13	53,79	24,09	22,49	55,39	22,13
Maluku Utara	16,60	71,98	11,43	16,73	71,76	11,51	16,66	71,87	11,47
Papua Barat	27,25	57,31	15,44	27,32	57,44	15,24	27,28	57,37	15,34
Papua	23,53	60,68	15,79	17,64	65,73	16,63	20,71	63,10	16,19
**Indonesia**	**36,89**	**48,99**	**14,12**	**36,15**	**49,14**	**14,71**	**36,53**	**49,06**	**14,41**

Catatan/*Note*: *) Data 2018 belum tersedia/*2018 data not available yet*
Sumber/*Source*: Susenas Maret 2017/*The March 2017 Susenas*

https://www.bps.go.id

Tabel **4.14.2.** **Persentase Penduduk Umur 0-59 Bulan (Balita) yang Mempunyai Kartu Imunisasi di Daerah Perdesaan menurut Provinsi dan Jenis Kelamin, 2017***
Table *Percentage of Population Aged 0-59 Months (Under Five Years) Having Immunization Card in Rural Area by Province and Sex, 2017**

**Provinsi** *Province*	**Laki-laki/***Male*			**Perempuan/***Female*			**Laki-laki+Perempuan/***Male+Female*		
	**Ya, Ditunjukkan/** *Yes, Shown*	**Ya, Tidak Dapat Ditunjukkan/***Yes, Can not be Shown*	**Tidak Ada Kartu** *No Card*	**Ya, Ditunjukkan/** *Yes, Shown*	**Ya, Tidak Dapat Ditunjukkan/***Yes, Can not be Shown*	**Tidak Ada Kartu** *No Card*	**Ya, Ditunjukkan/** *Yes, Shown*	**Ya, Tidak Dapat Ditunjukkan/***Yes, Can not be Shown*	**Tidak Ada Kartu** *No Card*
**(1)**	**(2)**	**(3)**	**(4)**	**(5)**	**(6)**	**(7)**	**(8)**	**(9)**	**(10)**
Aceh	14,60	55,47	29,93	15,52	56,51	27,97	15,05	55,98	28,97
Sumatera Utara	19,56	51,53	28,91	19,34	55,88	24,78	19,45	53,66	26,89
Sumatera Barat	28,80	55,83	15,37	26,53	55,11	18,35	27,69	55,48	16,83
Riau	20,64	52,57	26,79	21,80	57,81	20,39	21,21	55,14	23,66
Jambi	24,33	55,92	19,75	29,10	54,77	16,13	26,63	55,36	18,01
Sumatera Selatan	25,86	59,63	14,51	24,40	60,87	14,73	25,14	60,24	14,62
Bengkulu	30,49	58,36	11,14	27,73	61,55	10,72	29,17	59,89	10,94
Lampung	44,92	44,83	10,24	40,08	49,89	10,03	42,63	47,23	10,14
Kepulauan Bangka Belitung	51,23	42,09	6,68	49,60	40,47	9,93	50,44	41,31	8,25
Kepulauan Riau	40,20	47,66	12,14	34,24	56,35	9,42	37,59	51,47	10,95
DKI Jakarta	-	-	-	-	-	-	-	-	-
Jawa Barat	35,07	46,25	18,68	35,77	46,49	17,74	35,41	46,36	18,22
Jawa Tengah	57,37	35,60	7,03	54,81	37,97	7,22	56,13	36,75	7,12
DI Yogyakarta	69,19	27,32	3,49	75,33	21,56	3,10	72,17	24,53	3,30
Jawa Timur	45,83	44,45	9,72	45,46	43,54	11,01	45,64	44,00	10,35
Banten	25,97	47,35	26,68	22,26	51,73	26,00	24,15	49,50	26,35
Bali	47,00	41,38	11,62	53,29	36,36	10,35	50,19	38,84	10,98
Nusa Tenggara Barat	38,51	46,75	14,75	32,07	56,64	11,29	35,42	51,50	13,09
Nusa Tenggara Timur	31,35	52,12	16,53	30,17	52,99	16,84	30,77	52,55	16,68
Kalimantan Barat	34,24	49,17	16,58	31,79	51,92	16,29	33,06	50,50	16,44
Kalimantan Tengah	29,04	53,11	17,85	31,91	46,98	21,11	30,43	50,12	19,44
Kalimantan Selatan	41,93	47,98	10,09	44,11	45,15	10,74	43,01	46,59	10,41
Kalimantan Timur	34,94	50,73	14,33	37,51	50,70	11,78	36,21	50,72	13,08
Kalimantan Utara	33,69	56,63	9,67	32,13	59,55	8,32	32,91	58,09	9,00
Sulawesi Utara	38,93	51,57	9,50	36,04	54,99	8,97	37,48	53,29	9,23
Sulawesi Tengah	34,59	46,45	18,97	33,90	46,94	19,16	34,24	46,69	19,07
Sulawesi Selatan	34,83	49,94	15,23	36,29	50,58	13,14	35,54	50,25	14,20
Sulawesi Tenggara	23,79	59,86	16,35	25,56	55,48	18,96	24,64	57,76	17,60
Gorontalo	43,24	47,01	9,75	46,45	43,11	10,44	44,82	45,09	10,09
Sulawesi Barat	38,39	51,23	10,38	33,70	54,61	11,70	36,10	52,87	11,02
Maluku	14,17	48,31	37,52	17,08	44,52	38,40	15,57	46,49	37,94
Maluku Utara	18,76	56,95	24,30	18,82	59,62	21,57	18,79	58,29	22,92
Papua Barat	14,80	52,98	32,22	16,63	54,01	29,36	15,69	53,48	30,82
Papua	5,89	54,82	39,30	4,93	57,18	37,89	5,41	55,98	38,60
**Indonesia**	**35,53**	**47,95**	**16,52**	**34,79**	**49,34**	**15,87**	**35,17**	**48,63**	**16,20**

Catatan/*Note*: *) Data 2018 belum tersedia/*2018 data not available yet*
Sumber/*Source*: Susenas Maret 2017/*The March 2017 Susenas*

https://www.bps.go.id

Tabel **4.14.3.** **Persentase Penduduk Umur 0-59 Bulan (Balita) yang Mempunyai Kartu Imunisasi di Daerah Perkotaan dan Perdesaan menurut Provinsi dan Jenis Kelamin, 2017***

Table **4.14.3.** *Percentage of Population Aged 0-59 Months (Under Five Years) Having Immunization Card in Urban and Rural Area by Province and Sex, 2017**

**Provinsi** *Province*	**Laki-laki/***Male*			**Perempuan/***Female*			**Laki-laki+Perempuan/***Male+Female*		
	**Ya, Ditunjukkan/** *Yes, Shown*	**Ya, Tidak Dapat Ditunjukkan/***Yes, Can not be Shown*	**Tidak Ada Kartu** *No Card*	**Ya, Ditunjukkan/** *Yes, Shown*	**Ya, Tidak Dapat Ditunjukkan/***Yes, Can not be Shown*	**Tidak Ada Kartu** *No Card*	**Ya, Ditunjukkan/** *Yes, Shown*	**Ya, Tidak Dapat Ditunjukkan/***Yes, Can not be Shown*	**Tidak Ada Kartu** *No Card*
**(1)**	**(2)**	**(3)**	**(4)**	**(5)**	**(6)**	**(7)**	**(8)**	**(9)**	**(10)**
Aceh	14,08	53,73	32,19	15,67	54,29	30,03	14,86	54,01	31,13
Sumatera Utara	19,48	55,88	24,64	18,48	57,24	24,28	18,99	56,55	24,46
Sumatera Barat	27,15	56,56	16,29	26,23	55,06	18,71	26,70	55,83	17,46
Riau	20,64	53,36	26,00	20,53	57,78	21,69	20,59	55,52	23,90
Jambi	23,56	59,07	17,37	26,06	59,85	14,10	24,78	59,45	15,77
Sumatera Selatan	25,95	59,47	14,58	24,08	61,20	14,72	25,03	60,32	14,65
Bengkulu	29,88	58,28	11,83	26,77	61,15	12,08	28,39	59,66	11,95
Lampung	44,10	45,35	10,55	40,24	49,60	10,16	42,28	47,35	10,37
Kepulauan Bangka Belitung	52,04	41,16	6,80	49,89	40,99	9,12	50,98	41,08	7,94
Kepulauan Riau	41,65	38,25	20,10	40,46	44,01	15,53	41,06	41,12	17,82
DKI Jakarta	44,76	47,46	7,78	42,09	50,04	7,87	43,45	48,73	7,82
Jawa Barat	34,15	47,81	18,04	33,32	47,82	18,86	33,74	47,81	18,44
Jawa Tengah	56,84	36,23	6,93	53,46	39,99	6,55	55,20	38,06	6,75
DI Yogyakarta	68,10	28,35	3,55	66,71	30,52	2,77	67,41	29,43	3,16
Jawa Timur	45,93	45,29	8,78	45,81	44,52	9,67	45,87	44,91	9,22
Banten	24,43	53,36	22,21	25,42	51,70	22,88	24,91	52,55	22,54
Bali	45,28	44,29	10,42	48,56	39,94	11,51	46,89	42,15	10,96
Nusa Tenggara Barat	38,02	49,42	12,56	34,63	52,36	13,01	36,37	50,85	12,78
Nusa Tenggara Timur	32,78	51,66	15,55	33,40	51,18	15,43	33,08	51,42	15,49
Kalimantan Barat	33,17	50,22	16,61	33,40	50,94	15,66	33,28	50,57	16,15
Kalimantan Tengah	29,44	54,62	15,94	32,90	49,05	18,05	31,14	51,88	16,98
Kalimantan Selatan	39,47	49,20	11,33	41,99	48,07	9,94	40,68	48,66	10,67
Kalimantan Timur	40,15	49,55	10,30	40,04	48,33	11,63	40,10	48,95	10,95
Kalimantan Utara	33,36	58,75	7,88	37,41	55,14	7,45	35,41	56,92	7,67
Sulawesi Utara	35,30	54,35	10,35	35,26	54,93	9,82	35,28	54,63	10,09
Sulawesi Tengah	33,28	48,51	18,21	35,78	46,65	17,56	34,54	47,58	17,89
Sulawesi Selatan	32,86	51,49	15,65	34,06	51,15	14,79	33,44	51,33	15,23
Sulawesi Tenggara	23,54	57,06	19,40	25,09	56,73	18,19	24,27	56,90	18,82
Gorontalo	43,85	46,63	9,52	46,61	44,06	9,33	45,21	45,36	9,43
Sulawesi Barat	39,41	50,31	10,28	36,93	51,17	11,90	38,19	50,73	11,08
Maluku	17,41	51,49	31,10	18,91	47,89	33,19	18,12	49,78	32,10
Maluku Utara	18,17	61,01	20,81	18,26	62,84	18,90	18,22	61,93	19,85
Papua Barat	19,48	54,61	25,90	20,68	55,31	24,01	20,07	54,95	24,97
Papua	11,40	56,65	31,95	8,75	59,75	31,50	10,10	58,16	31,73
**Indonesia**	**36,24**	**48,50**	**15,26**	**35,50**	**49,23**	**15,26**	**35,88**	**48,86**	**15,26**

Catatan/*Note*: *) Data 2018 belum tersedia/*2018 data not available yet*
Sumber/*Source*: Susenas Maret 2017/*The March 2017 Susenas*

https://www.bps.go.id

Tabel **4.15.** **Persentase Penduduk Umur 0-59 Bulan (Balita) yang Pernah Mendapat Imunisasi menurut Provinsi, Daerah Tempat Tinggal dan Jenis Imunisasi, 2017***
Table *Percentage of Population Aged 0-59 Months (Under Five Years) Who Ever Been Immunized by Province, Urban Rural Classification, and Type of Immunization, 2017**

**Provinsi** *Province*	**Perkotaan/*Urban***					**Perdesaan/*Rural***				
	**BCG**	**DPT**	**Polio**	**Campak/Morbili**	**Hepatitis B**	**BCG**	**DPT**	**Polio**	**Campak/Morbili**	**Hepatitis B**
**(1)**	**(2)**	**(3)**	**(4)**	**(5)**	**(6)**	**(7)**	**(8)**	**(9)**	**(10)**	**(11)**
Aceh	67,73	60,89	67,22	51,58	58,31	70,85	64,31	74,53	53,92	60,61
Sumatera Utara	84,66	77,90	85,17	67,58	72,04	81,66	73,93	83,85	63,32	68,41
Sumatera Barat	83,12	75,61	82,05	58,19	74,10	84,10	78,07	84,04	63,87	73,47
Riau	84,97	79,71	85,28	66,14	74,35	81,10	74,39	84,08	64,34	67,33
Jambi	89,99	85,00	89,60	69,82	82,91	84,71	78,58	83,76	65,78	74,10
Sumatera Selatan	88,00	84,21	88,57	69,44	80,33	90,96	87,23	89,94	74,21	81,30
Bengkulu	92,49	86,50	92,15	72,22	84,32	90,74	85,55	89,66	73,26	81,06
Lampung	96,08	90,67	90,96	75,31	88,75	92,18	85,54	90,80	74,12	85,16
Kepulauan Bangka Belitung	92,54	87,11	91,13	76,99	88,62	90,33	82,86	89,69	70,05	86,24
Kepulauan Riau	88,82	86,10	89,19	71,53	85,04	89,15	84,04	90,00	69,78	82,30
DKI Jakarta	94,41	89,36	93,41	74,40	88,18	-	-	-	-	-
Jawa Barat	89,82	85,74	90,23	70,72	83,11	87,09	80,34	87,38	66,08	75,50
Jawa Tengah	95,35	91,31	93,80	75,21	91,84	93,64	88,62	92,38	75,75	88,78
DI Yogyakarta	97,12	96,95	95,20	85,42	93,42	97,92	92,72	93,88	85,54	90,75
Jawa Timur	94,56	89,51	92,69	75,48	88,23	89,92	83,44	87,97	70,90	83,30
Banten	87,61	81,45	88,69	68,39	80,39	83,04	74,78	84,78	63,40	67,90
Bali	97,39	93,58	97,02	80,92	92,73	96,40	93,13	95,69	84,09	93,10
Nusa Tenggara Barat	94,66	91,20	95,20	78,36	90,20	92,40	87,93	92,22	75,17	88,67
Nusa Tenggara Timur	95,13	92,10	94,59	81,61	93,06	91,96	88,32	91,55	74,44	85,90
Kalimantan Barat	84,26	74,80	82,16	61,99	74,99	82,09	76,85	83,85	67,00	72,69
Kalimantan Tengah	87,96	82,79	88,76	69,16	83,01	83,10	76,99	83,90	65,85	70,41
Kalimantan Selatan	89,88	83,48	90,13	69,05	85,03	89,97	84,27	90,35	72,72	84,09
Kalimantan Timur	90,85	84,81	91,00	72,12	84,49	90,87	84,98	89,18	74,54	85,55
Kalimantan Utara	92,16	87,21	90,76	73,98	87,17	84,70	80,17	87,39	70,50	74,75
Sulawesi Utara	91,87	86,10	91,03	75,04	85,48	91,93	86,87	90,58	75,02	84,67
Sulawesi Tengah	91,06	83,67	88,14	68,97	82,79	84,65	79,15	85,48	68,45	76,16
Sulawesi Selatan	90,79	85,31	90,36	73,32	85,24	87,21	82,45	86,66	70,13	79,99
Sulawesi Tenggara	87,80	84,75	87,26	69,58	85,82	88,00	84,47	87,17	72,90	83,79
Gorontalo	95,96	90,87	95,62	78,02	93,34	92,11	86,37	92,18	75,96	87,77
Sulawesi Barat	87,95	86,42	84,81	67,00	80,55	86,81	80,70	88,04	69,92	76,90
Maluku	85,49	82,55	87,38	71,43	79,96	81,03	75,61	83,98	65,94	67,88
Maluku Utara	88,38	83,00	84,48	71,37	82,21	82,74	75,31	80,74	67,39	70,51
Papua Barat	90,35	85,97	88,91	69,40	80,76	77,60	68,84	76,02	59,87	63,99
Papua	88,17	84,29	87,92	69,61	83,31	68,17	61,36	67,49	53,97	57,39
**Indonesia**	**90,77**	**85,95**	**90,31**	**71,86**	**84,22**	**87,29**	**81,39**	**87,20**	**69,38**	**78,56**

Catatan/*Note***:** *) Data 2018 belum tersedia/*2018 data not available yet*
Sumber/*Source*: Susenas Maret 2017/*The March 2017 Susenas*

https://www.bps.go.id

Lanjutan Tabel/*Table Continued* **4.15.**

**Provinsi** *Province*	**Perkotaan+Perdesaan/***Urban+Rural*				
	**BCG**	**DPT**	**Polio**	**Campak/Morbili**	**Hepatitis B**
**(1)**	**(12)**	**(13)**	**(14)**	**(15)**	**(16)**
Aceh	69,92	63,29	72,35	53,22	59,92
Sumatera Utara	83,12	75,86	84,49	65,39	70,17
Sumatera Barat	83,71	77,08	83,24	61,59	73,72
Riau	82,60	76,45	84,54	65,04	70,04
Jambi	86,40	80,64	85,64	67,08	76,93
Sumatera Selatan	89,90	86,15	89,45	72,51	80,95
Bengkulu	91,28	85,85	90,43	72,94	82,07
Lampung	93,24	86,93	90,85	74,44	86,13
Kepulauan Bangka Belitung	91,49	85,08	90,44	73,67	87,48
Kepulauan Riau	88,87	85,81	89,30	71,28	84,65
DKI Jakarta	94,41	89,36	93,41	74,40	88,18
Jawa Barat	89,07	84,25	89,44	69,44	81,01
Jawa Tengah	94,48	89,94	93,08	75,49	90,29
DI Yogyakarta	97,35	95,71	94,81	85,45	92,64
Jawa Timur	92,31	86,57	90,41	73,26	85,84
Banten	86,21	79,39	87,49	66,86	76,54
Bali	97,04	93,42	96,56	82,03	92,86
Nusa Tenggara Barat	93,39	89,36	93,52	76,57	89,34
Nusa Tenggara Timur	92,57	89,05	92,14	75,82	87,28
Kalimantan Barat	82,78	76,21	83,32	65,42	73,41
Kalimantan Tengah	84,87	79,10	85,67	67,05	74,99
Kalimantan Selatan	89,94	83,93	90,26	71,14	84,50
Kalimantan Timur	90,85	84,86	90,39	72,94	84,84
Kalimantan Utara	89,01	84,24	89,34	72,51	81,92
Sulawesi Utara	91,90	86,52	90,79	75,03	85,04
Sulawesi Tengah	86,24	80,27	86,14	68,58	77,81
Sulawesi Selatan	88,62	83,58	88,12	71,38	82,06
Sulawesi Tenggara	87,94	84,56	87,20	71,87	84,42
Gorontalo	93,44	87,92	93,37	76,67	89,70
Sulawesi Barat	87,05	81,94	87,34	69,29	77,68
Maluku	82,68	78,18	85,23	67,97	72,35
Maluku Utara	84,25	77,37	81,74	68,46	73,64
Papua Barat	82,42	75,31	80,89	63,47	70,33
Papua	74,30	68,39	73,75	58,77	65,34
**Indonesia**	**89,11**	**83,77**	**88,83**	**70,67**	**81,52**

Catatan/*Note*: *) Data 2018 belum tersedia/*2018 data not available yet*
Sumber/*Source*: Susenas Maret 2017/*The March 2017 Susenas*

https://www.bps.go.id

Tabel *Table* **4.16.** **Persentase Penduduk Umur 0-59 Bulan (Balita) yang Pernah Mendapat Imunisasi Lengkap menurut Provinsi, Daerah Tempat Tinggal dan Jenis Imunisasi, 2017***
*Percentage of Population Aged 0-59 Months (Under Five Years) Who Ever Been Complete Immunized by Province, Urban Rural Classification, and Type of Immunization, 2017**

Provinsi *Province*	**Perkotaan/***Urban*			**Perdesaan/***Rural*			**Perkotaan+Perdesaan/***Urban+Rural*		
	**Laki-laki** *Male*	**Perempuan** *Female*	**Laki-laki+ Perempuan** *Male+Female*	**Laki-laki** *Male*	**Perempuan** *Female*	**Laki-laki+ Perempuan** *Male+Female*	**Laki-laki/** *Male*	**Perempuan/** *Female*	**Laki-laki+ Perempuan** *Male+Female*
(1)	(2)	(3)	(4)	(5)	(6)	(7)	(8)	(9)	(10)
Aceh	31,31	31,95	31,63	23,22	23,72	23,46	25,61	26,19	25,90
Sumatera Utara	37,86	33,07	35,50	26,64	30,75	28,65	32,08	31,88	31,98
Sumatera Barat	37,73	37,10	37,43	36,69	39,15	37,89	37,11	38,34	37,71
Riau	34,23	35,88	35,03	25,71	28,18	26,92	29,02	31,14	30,05
Jambi	43,45	45,38	44,41	34,47	38,33	36,33	37,29	40,64	38,92
Sumatera Selatan	37,50	40,81	39,14	45,54	39,27	42,47	42,68	39,83	41,28
Bengkulu	49,81	45,67	47,84	42,27	42,97	42,61	44,63	43,80	44,23
Lampung	49,11	50,01	49,53	44,74	43,94	44,36	45,94	45,57	45,77
Kepulauan Bangka Belitung	62,06	63,93	62,99	42,26	53,62	47,75	52,45	59,08	55,70
Kepulauan Riau	47,20	47,28	47,24	43,72	44,51	44,06	46,65	46,94	46,80
DKI Jakarta	46,91	47,09	47,00	35,30	37,59	36,43	46,91	47,09	47,00
Jawa Barat	40,94	39,56	40,27	55,81	56,52	56,16	39,40	39,01	39,21
Jawa Tengah	60,30	59,36	59,84	56,32	64,29	60,19	58,02	57,92	57,97
DI Yogyakarta	62,08	67,99	65,04	46,98	49,40	48,17	60,36	66,93	63,62
Jawa Timur	52,08	51,89	51,99	25,36	23,06	24,23	49,62	50,68	50,14
Banten	34,31	33,36	33,84	59,11	68,12	63,68	31,55	30,18	30,88
Bali	66,06	59,04	62,67	49,15	53,00	51,00	63,71	62,32	63,02
Nusa Tenggara Barat	55,64	56,66	56,14	54,02	52,61	53,33	51,95	54,63	53,25
Nusa Tenggara Timur	71,25	65,35	68,41	36,88	39,39	38,09	57,39	55,03	56,23
Kalimantan Barat	39,81	39,78	39,79	36,03	36,98	36,49	37,79	39,51	38,63
Kalimantan Tengah	46,24	43,51	44,87	51,49	52,29	51,89	39,68	39,39	39,54
Kalimantan Selatan	49,22	47,45	48,41	50,88	47,44	49,19	50,47	50,30	50,39
Kalimantan Timur	49,62	49,19	49,41	39,94	44,00	41,96	50,04	48,60	49,33
Kalimantan Utara	64,18	57,60	60,81	49,41	50,40	49,91	53,76	51,95	52,84
Sulawesi Utara	45,19	46,74	45,93	42,56	48,08	45,30	47,43	48,77	48,09
Sulawesi Tengah	49,81	58,41	54,24	46,19	44,32	45,28	44,31	50,72	47,52
Sulawesi Selatan	47,13	43,36	45,36	52,78	48,27	50,62	46,57	43,95	45,31
Sulawesi Tenggara	50,27	58,41	54,07	55,42	52,83	54,14	51,98	51,38	51,69
Gorontalo	72,79	52,82	62,99	46,55	40,06	43,39	61,44	52,83	57,20
Sulawesi Barat	48,85	51,88	50,41	30,48	33,13	31,75	47,03	42,72	44,90
Maluku	47,82	46,74	47,31	34,10	34,23	34,16	36,98	38,08	37,50
Maluku Utara	45,48	49,66	47,55	29,73	30,19	29,95	37,18	38,32	37,75
Papua Barat	38,17	35,89	37,05	22,70	22,56	22,63	32,91	32,35	32,64
Papua	42,27	41,38	41,84	41,74	42,74	42,23	28,81	28,21	28,52
**Indonesia**	**46,35**	**45,48**	**45,93**	**22,08**	**22,02**	**22,05**	**44,16**	**44,17**	**44,16**

Catatan/*Note*: *) Data 2018 belum tersedia/*2018 data not available yet*
Sumber/*Source*: Susenas Maret 2017/*The March 2017 Susenas*

https://www.bps.go.id

Tabel **4.17.** **Persentase Penduduk Umur 0-23 Bulan (Baduta) yang Pernah Diberi ASI menurut Provinsi, Daerah Tempat Tinggal dan Jenis Kelamin, 2017***

Table **4.17.** *Percentage of Population Aged 0-23 Months (Under Two Years) Who Ever Been Breastfeeding by Province, Urban Rural Classification, and Sex, 2017**

**Provinsi** *Province*	**Perkotaan/***Urban*			**Perdesaan/***Rural*			**Perkotaan+Perdesaan/***Urban+Rural*		
	**Laki-laki** *Male*	**Perempuan** *Female*	**Laki-laki+ Perempuan** *Male+Female*	**Laki-laki** *Male*	**Perempuan** *Female*	**Laki-laki+ Perempuan** *Male+Female*	**Laki-laki/** *Male*	**Perempuan/** *Female*	**Laki-laki+ Perempuan** *Male+Female*
**(1)**	**(2)**	**(3)**	**(4)**	**(5)**	**(6)**	**(7)**	**(8)**	**(9)**	**(10)**
Aceh	93,34	97,36	95,17	96,61	96,11	96,37	95,65	96,46	96,03
Sumatera Utara	95,25	89,60	92,22	94,24	97,10	95,59	94,71	93,13	93,91
Sumatera Barat	98,35	94,17	96,22	96,63	96,18	96,41	97,33	95,35	96,33
Riau	96,34	93,90	95,28	96,95	94,43	95,72	96,70	94,24	95,55
Jambi	94,41	91,29	92,88	96,42	95,79	96,11	95,78	94,35	95,07
Sumatera Selatan	93,15	97,78	95,36	95,04	95,53	95,28	94,35	96,31	95,31
Bengkulu	98,79	96,85	97,88	94,24	97,62	95,78	95,65	97,37	96,44
Lampung	94,91	97,81	96,31	94,78	95,69	95,19	94,82	96,31	95,50
Kepulauan Bangka Belitung	93,33	89,53	91,61	93,81	95,14	94,43	93,56	92,19	92,93
Kepulauan Riau	97,17	93,29	95,06	92,36	92,21	92,29	96,29	93,16	94,64
DKI Jakarta	94,03	86,42	90,15	-	-	-	94,03	86,42	90,15
Jawa Barat	91,41	94,70	92,98	93,33	93,96	93,65	91,90	94,49	93,16
Jawa Tengah	95,60	96,60	96,08	97,66	98,38	98,02	96,64	97,54	97,08
DI Yogyakarta	97,87	96,06	96,95	98,13	100,00	99,05	97,95	97,18	97,56
Jawa Timur	92,92	93,44	93,19	94,10	95,03	94,54	93,50	94,16	93,82
Banten	91,64	96,09	93,88	96,40	94,14	95,19	93,01	95,48	94,28
Bali	93,76	96,33	95,05	98,94	96,52	97,64	95,37	96,40	95,90
Nusa Tenggara Barat	96,69	97,69	97,14	95,63	96,28	95,95	96,11	96,87	96,47
Nusa Tenggara Timur	93,04	94,49	93,80	97,78	97,79	97,78	96,91	97,14	97,03
Kalimantan Barat	89,21	91,48	90,30	97,98	97,65	97,83	95,35	95,60	95,46
Kalimantan Tengah	88,51	94,38	91,65	97,53	96,13	96,81	94,09	95,43	94,79
Kalimantan Selatan	94,96	94,38	94,72	94,24	94,34	94,29	94,57	94,36	94,47
Kalimantan Timur	93,35	97,95	95,63	98,15	94,59	96,40	94,82	96,94	95,87
Kalimantan Utara	97,08	94,27	95,61	89,56	96,65	92,92	93,82	95,19	94,51
Sulawesi Utara	88,27	90,52	89,46	91,51	90,25	90,87	90,10	90,37	90,24
Sulawesi Tengah	96,62	91,77	93,90	94,44	95,44	94,93	94,97	94,35	94,65
Sulawesi Selatan	95,32	94,08	94,76	95,24	96,39	95,80	95,27	95,55	95,40
Sulawesi Tenggara	92,02	96,71	94,00	90,09	94,13	92,05	90,76	94,89	92,67
Gorontalo	92,80	84,51	88,65	92,40	92,64	92,52	92,55	89,62	91,08
Sulawesi Barat	99,55	97,06	98,36	97,47	95,42	96,49	97,95	95,80	96,92
Maluku	85,45	90,82	87,85	93,86	96,92	95,33	90,52	94,70	92,47
Maluku Utara	99,50	97,84	98,72	94,37	94,51	94,44	95,94	95,41	95,68
Papua Barat	86,44	85,85	86,17	91,88	91,49	91,69	89,59	89,27	89,44
Papua	88,37	86,79	87,56	96,76	96,51	96,64	93,50	92,62	93,06
**Indonesia**	**93,37**	**93,85**	**93,60**	**95,41**	**95,88**	**95,64**	**94,34**	**94,80**	**94,56**

Catatan/*Note*: *) Data 2018 belum tersedia/*2018 data not available yet*
Sumber/*Source*: Susenas Maret 2017/*The March 2017 Susenas*

https://www.bps.go.id

Tabel **4.18.** **Persentase Penduduk Umur 0-23 Bulan (Baduta) yang Masih Diberi ASI menurut Provinsi, Daerah Tempat Tinggal dan Jenis Kelamin, 2017***
Table *Percentage of Population Aged 0-23 Months (Under Two Years) Who Still Have Breastfeeding by Province, Urban Rural Classification, and Sex, 2017**

Provinsi *Province*	Perkotaan/*Urban*			Perdesaan/*Rural*			Perkotaan+Perdesaan/*Urban+Rural*		
	Laki-laki *Male*	Perempuan *Female*	Laki-laki+ Perempuan *Male+Female*	Laki-laki *Male*	Perempuan *Female*	Laki-laki+ Perempuan *Male+Female*	Laki-laki/ *Male*	Perempuan/ *Female*	Laki-laki+ Perempuan *Male+Female*
(1)	(2)	(3)	(4)	(5)	(6)	(7)	(8)	(9)	(10)
Aceh	82,42	80,79	81,66	87,57	87,86	87,70	86,09	85,84	85,97
Sumatera Utara	66,28	70,18	68,31	79,01	78,66	78,84	73,07	74,34	73,70
Sumatera Barat	79,26	84,08	81,66	87,73	83,70	85,72	84,26	83,86	84,06
Riau	80,66	75,02	78,24	83,68	82,83	83,27	82,44	80,04	81,33
Jambi	80,82	82,03	81,40	87,50	89,63	88,55	85,38	87,27	86,31
Sumatera Selatan	78,17	76,51	77,36	89,97	90,83	90,39	85,75	85,81	85,78
Bengkulu	83,23	80,98	82,19	85,59	89,89	87,59	84,84	87,08	85,88
Lampung	83,83	86,87	85,33	86,14	84,42	85,37	85,54	85,14	85,36
Kepulauan Bangka Belitung	67,42	76,88	71,61	76,06	77,11	76,55	71,44	76,99	73,96
Kepulauan Riau	73,63	78,12	76,03	85,17	65,75	76,85	75,67	76,59	76,15
DKI Jakarta	73,26	70,71	72,01	-	-	-	73,26	70,71	72,01
Jawa Barat	84,21	82,86	83,55	88,99	89,37	89,18	85,44	84,63	85,04
Jawa Tengah	84,96	85,47	85,21	89,37	89,39	89,38	87,22	87,55	87,38
DI Yogyakarta	82,86	80,46	81,65	97,33	97,31	97,32	87,20	85,38	86,29
Jawa Timur	79,19	77,19	78,16	84,19	85,94	85,02	81,66	81,18	81,42
Banten	74,62	77,71	76,21	85,91	84,46	85,14	77,98	79,81	78,93
Bali	74,40	74,35	74,38	89,48	77,54	83,12	79,26	75,46	77,30
Nusa Tenggara Barat	89,21	88,61	88,94	85,10	87,03	86,05	86,99	87,69	87,32
Nusa Tenggara Timur	75,16	76,93	76,09	83,49	80,34	81,92	82,04	79,69	80,86
Kalimantan Barat	71,33	68,51	69,96	87,29	91,65	89,21	82,80	84,30	83,48
Kalimantan Tengah	74,75	77,00	75,99	84,25	91,63	88,00	80,84	85,82	83,45
Kalimantan Selatan	77,02	79,24	77,96	86,21	88,09	87,14	81,98	84,65	83,22
Kalimantan Timur	77,72	82,38	80,09	80,37	82,45	81,37	78,56	82,40	80,48
Kalimantan Utara	80,05	70,30	75,02	81,19	70,83	76,09	80,52	70,51	75,45
Sulawesi Utara	72,76	68,23	70,34	65,85	74,13	70,04	68,80	71,44	70,17
Sulawesi Tengah	63,78	67,53	65,83	82,14	82,46	82,30	77,63	78,14	77,89
Sulawesi Selatan	67,13	75,35	70,81	79,39	83,62	81,48	74,46	80,65	77,41
Sulawesi Tenggara	69,65	78,39	73,44	78,50	81,78	80,12	75,39	80,77	77,95
Gorontalo	63,60	61,83	62,76	74,00	73,19	73,59	70,11	69,22	69,67
Sulawesi Barat	85,46	82,49	84,05	80,94	88,19	84,38	82,00	86,86	84,30
Maluku	64,12	62,57	63,40	77,42	83,48	80,38	72,43	76,17	74,22
Maluku Utara	64,94	71,76	68,12	75,41	79,71	77,61	72,10	77,49	74,78
Papua Barat	66,49	68,06	67,19	79,68	84,09	81,79	74,32	78,04	76,06
Papua	72,07	73,53	72,81	87,85	87,91	87,88	82,06	82,52	82,29
**Indonesia**	**78,87**	**78,86**	**78,86**	**85,13**	**85,77**	**85,44**	**81,86**	**82,14**	**82,00**

Catatan/*Note***:** *) Data 2018 belum tersedia/*2018 data not available yet*
Sumber/*Source*: Susenas Maret 2017/*The March 2017 Susenas*

https://www.bps.go.id

Tabel 4.19. **Persentase Penduduk Umur 0-23 Bulan (Baduta) yang Pernah Diberi ASI menurut Provinsi, Daerah Tempat Tinggal dan Rata-rata Lama Pemberian ASI (Bulan), 2017***

*Table 4.19. Percentage of Population Aged 0-23 Months (Under Two Years) Who Ever Been Breastfeeding by Province, Urban Rural Classification, and Average Durationof Breastfeeding (Months), 2017**

Provinsi *Province*	Perkotaan/*Urban*			Rata-rata Lama Pemberian ASI *Average Duration of Breastfeeding*	Perkotaan/*Urban*			Rata-rata Lama Pemberian ASI *Average Duration of Breastfeeding*	Perkotaan/*Urban*			Rata-rata Lama Pemberian ASI *Average Duration of Breastfeeding*
	<6	6-23	Jumlah/*Total*		<6	6-23	Jumlah/*Total*		<6	6-23	Jumlah/*Total*	
(1)	(2)	(3)	(4)	(6)	(7)	(8)	(9)	(10)	(11)	(12)	(13)	(14)
Aceh	21,17	78,83	100,00	11,04	22,19	77,81	100,00	11,01	21,90	78,10	100,00	11,01
Sumatera Utara	26,23	73,77	100,00	9,65	25,35	74,65	100,00	9,89	25,78	74,22	100,00	9,77
Sumatera Barat	27,46	72,54	100,00	10,88	23,70	76,30	100,00	10,95	25,24	74,76	100,00	10,92
Riau	27,79	72,21	100,00	10,39	24,73	75,27	100,00	10,65	25,91	74,09	100,00	10,55
Jambi	25,93	74,07	100,00	9,98	27,38	72,62	100,00	10,44	26,93	73,07	100,00	10,30
Sumatera Selatan	29,26	70,74	100,00	9,71	22,62	77,38	100,00	10,91	24,97	75,03	100,00	10,49
Bengkulu	28,67	71,33	100,00	9,61	24,11	75,89	100,00	10,77	25,56	74,44	100,00	10,40
Lampung	26,97	73,03	100,00	10,58	27,17	72,83	100,00	10,31	27,11	72,89	100,00	10,38
Kepulauan Bangka Belitung	28,83	71,17	100,00	10,24	29,46	70,54	100,00	9,38	29,13	70,87	100,00	9,83
Kepulauan Riau	31,05	68,95	100,00	9,39	30,54	69,46	100,00	8,74	30,97	69,03	100,00	9,30
DKI Jakarta	29,07	70,93	100,00	9,30	-	-	-	-	29,07	70,93	100,00	9,30
Jawa Barat	26,78	73,22	100,00	10,34	27,22	72,78	100,00	10,30	26,90	73,10	100,00	10,33
Jawa Tengah	23,78	76,22	100,00	10,93	26,09	73,91	100,00	10,78	24,99	75,01	100,00	10,85
DI Yogyakarta	19,37	80,63	100,00	11,64	16,47	83,53	100,00	11,21	18,51	81,49	100,00	11,51
Jawa Timur	24,95	75,05	100,00	10,36	26,62	73,38	100,00	10,48	25,75	74,25	100,00	10,42
Banten	26,87	73,13	100,00	10,10	19,60	80,40	100,00	10,92	24,65	75,35	100,00	10,35
Bali	24,97	75,03	100,00	10,60	23,07	76,93	100,00	10,69	24,34	75,66	100,00	10,63
Nusa Tenggara Barat	26,13	73,87	100,00	10,69	22,81	77,19	100,00	11,36	24,27	75,73	100,00	11,07
Nusa Tenggara Timur	25,54	74,46	100,00	10,74	22,49	77,51	100,00	10,76	23,05	76,95	100,00	10,75
Kalimantan Barat	24,60	75,40	100,00	10,73	23,80	76,20	100,00	10,94	24,04	75,96	100,00	10,88
Kalimantan Tengah	26,56	73,44	100,00	9,99	25,18	74,82	100,00	10,87	25,70	74,30	100,00	10,54
Kalimantan Selatan	26,02	73,98	100,00	10,53	25,55	74,45	100,00	10,87	25,75	74,25	100,00	10,72
Kalimantan Timur	24,52	75,48	100,00	10,41	19,66	80,34	100,00	10,17	23,03	76,97	100,00	10,33
Kalimantan Utara	26,70	73,30	100,00	11,39	29,12	70,88	100,00	10,34	27,67	72,33	100,00	10,97
Sulawesi Utara	34,63	65,37	100,00	9,45	32,91	67,09	100,00	9,36	33,67	66,33	100,00	9,40
Sulawesi Tengah	33,47	66,53	100,00	9,34	25,99	74,01	100,00	10,76	27,99	72,01	100,00	10,38
Sulawesi Selatan	31,86	68,14	100,00	9,49	24,97	75,03	100,00	10,53	27,60	72,40	100,00	10,13
Sulawesi Tenggara	25,26	74,74	100,00	10,58	26,86	73,14	100,00	10,50	26,34	73,66	100,00	10,52
Gorontalo	34,56	65,44	100,00	9,14	32,68	67,32	100,00	9,31	33,36	66,64	100,00	9,25
Sulawesi Barat	16,60	83,40	100,00	10,76	19,66	80,34	100,00	11,03	18,94	81,06	100,00	10,97
Maluku	25,80	74,20	100,00	9,90	26,21	73,79	100,00	9,75	26,06	73,94	100,00	9,80
Maluku Utara	22,72	77,28	100,00	10,00	24,58	75,42	100,00	10,68	24,02	75,98	100,00	10,48
Papua Barat	25,30	74,70	100,00	9,37	19,97	80,03	100,00	10,22	22,06	77,94	100,00	9,89
Papua	23,55	76,45	100,00	9,36	23,64	76,36	100,00	10,05	23,61	76,39	100,00	9,80
**Indonesia**	**26,40**	**73,60**	**100,00**	**10,26**	**25,10**	**74,90**	**100,00**	**10,57**	**25,78**	**74,22**	**100,00**	**10,41**

Catatan/*Note***:** *) Data 2018 belum tersedia/*2018 data not available yet*
Sumber/*Source*: Susenas Maret 2017/*The March 2017 Susenas*

https://www.bps.go.id

Tabel *Table* **4.20.** **Persentase Penduduk Umur 0-23 Bulan (Baduta) menurut Provinsi, Daerah Tempat Tinggal dan Pemberian Makanan/Cairan Tambahan dalam 24 Jam Terakhir, 2017***
*Percentage of Population Aged 0-23 Months (Under Two Years) by Province, Urban Rural Classification, and Weaning Food/Liquid Receiving Status in The Last 24 Hours, 2017**

**Provinsi** *Province*	**Perkotaan/***Urban*			**Perdesaan/***Rural*			**Perkotaan+Perdesaan/***Urban+Rural*		
	**Diberi makanan/ cairan tambahan** *Received Weaning Food/Liquid*	**Tidak Diberi makanan/ cairan tambahan** *Not Received Weaning Food/Liquid*	**Jumlah** *Total*	**Diberi makanan/ cairan tambahan** *Received Weaning Food/Liquid*	**Tidak Diberi makanan/ cairan tambahan** *Not Received Weaning Food/Liquid*	**Jumlah** *Total*	**Diberi makanan/ cairan tambahan** *Received Weaning Food/Liquid*	**Tidak Diberi makanan/ cairan tambahan** *Not Received Weaning Food/Liquid*	**Jumlah** *Total*
**(1)**	**(2)**	**(3)**	**(4)**	**(5)**	**(6)**	**(7)**	**(8)**	**(9)**	**(10)**
Aceh	88,03	11,97	100,00	88,47	11,53	100,00	88,34	11,66	100,00
Sumatera Utara	88,56	11,44	100,00	86,91	13,09	100,00	87,73	12,27	100,00
Sumatera Barat	86,06	13,94	100,00	87,16	12,84	100,00	86,71	13,29	100,00
Riau	87,01	12,99	100,00	89,27	10,73	100,00	88,40	11,60	100,00
Jambi	89,49	10,51	100,00	82,23	17,77	100,00	84,56	15,44	100,00
Sumatera Selatan	86,40	13,60	100,00	85,23	14,77	100,00	85,64	14,36	100,00
Bengkulu	80,73	19,27	100,00	83,88	16,12	100,00	82,90	17,10	100,00
Lampung	84,86	15,14	100,00	85,05	14,95	100,00	85,00	15,00	100,00
Kepulauan Bangka Belitung	89,40	10,60	100,00	91,60	8,40	100,00	90,43	9,57	100,00
Kepulauan Riau	86,29	13,71	100,00	81,35	18,65	100,00	85,53	14,47	100,00
DKI Jakarta	86,58	13,42	100,00	-	-	-	86,58	13,42	100,00
Jawa Barat	85,99	14,01	100,00	84,19	15,81	100,00	85,52	14,48	100,00
Jawa Tengah	84,70	15,30	100,00	82,08	17,92	100,00	83,35	16,65	100,00
DI Yogyakarta	85,58	14,42	100,00	82,72	17,28	100,00	84,74	15,26	100,00
Jawa Timur	87,72	12,28	100,00	86,69	13,31	100,00	87,23	12,77	100,00
Banten	89,51	10,49	100,00	87,38	12,62	100,00	88,87	11,13	100,00
Bali	84,99	15,01	100,00	88,13	11,87	100,00	86,03	13,97	100,00
Nusa Tenggara Barat	79,90	20,10	100,00	82,34	17,66	100,00	81,27	18,73	100,00
Nusa Tenggara Timur	89,42	10,58	100,00	85,39	14,61	100,00	86,16	13,84	100,00
Kalimantan Barat	87,11	12,89	100,00	88,93	11,07	100,00	88,35	11,65	100,00
Kalimantan Tengah	89,71	10,29	100,00	84,48	15,52	100,00	86,53	13,47	100,00
Kalimantan Selatan	87,66	12,34	100,00	85,56	14,44	100,00	86,45	13,55	100,00
Kalimantan Timur	85,72	14,28	100,00	85,65	14,35	100,00	85,70	14,30	100,00
Kalimantan Utara	77,95	22,05	100,00	85,34	14,66	100,00	80,97	19,03	100,00
Sulawesi Utara	87,05	12,95	100,00	87,01	12,99	100,00	87,03	12,97	100,00
Sulawesi Tengah	85,42	14,58	100,00	87,33	12,67	100,00	86,81	13,19	100,00
Sulawesi Selatan	83,48	16,52	100,00	85,30	14,70	100,00	84,60	15,40	100,00
Sulawesi Tenggara	88,20	11,80	100,00	90,23	9,77	100,00	89,58	10,42	100,00
Gorontalo	92,57	7,43	100,00	94,61	5,39	100,00	93,86	6,14	100,00
Sulawesi Barat	92,00	8,00	100,00	82,80	17,20	100,00	84,92	15,08	100,00
Maluku	90,14	9,86	100,00	86,21	13,79	100,00	87,71	12,29	100,00
Maluku Utara	86,95	13,05	100,00	84,93	15,07	100,00	85,51	14,49	100,00
Papua Barat	89,17	10,83	100,00	88,75	11,25	100,00	88,92	11,08	100,00
Papua	90,37	9,63	100,00	84,31	15,69	100,00	86,70	13,30	100,00
**Indonesia**	**86,56**	**13,44**	**100,00**	**85,59**	**14,41**	**100,00**	**86,10**	**13,90**	**100,00**

Catatan/*Note***:** *) Data 2018 belum tersedia/*2018 data not available yet*
Sumber/*Source*: Susenas Maret 2017/*The March 2017 Susenas*

https://www.bps.go.id

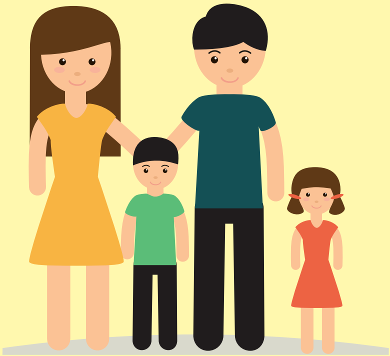

BAB/CHAPTER 5

FERTILITAS & KELUARGA BERENCANA

FERTILITY & FAMILY PLANNING

PEREMPUAN BERUMUR 15-49 TAHUN YANG PERNAH KAWIN:

Ever Married Female Aged 15-49

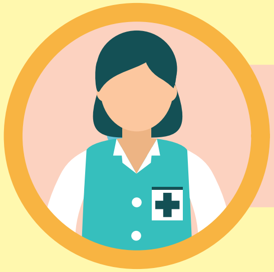

60,00%

yang pernah melahirkan dalam 2 tahun terakhir, penolong proses kelahiran terakhir paling banyak oleh bidan

who ever given birth in the last two years, the most recent birth attendant help by midwives

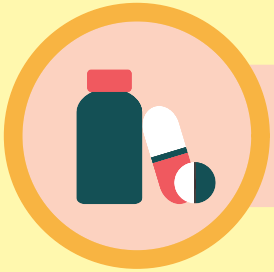

67,80%

pernah/sedang menggunakan alat KB/cara tradisional

ever used/currently use using a contraception/traditional method

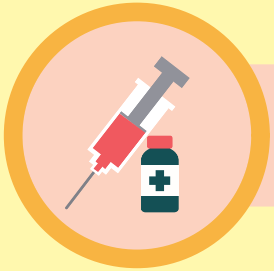

52,03%

alat KB/cara tradisional yang sedang digunakan paling banyak suntikan

contraception/traditional method that most being used is injection

https://www.bps.go.id

https://www.bps.go.id

PENJELASAN TEKNIS

1. **Inisiasi Menyusui Dini (IMD)** adalah meletakkan bayi menempel di dada atau perut ibu segera setelah lahir, membiarkannya merayap mencari puting, kemudian menyusui sampai puas.

2. **Anak lahir hidup** adalah anak yang pada waktu dilahirkan menunjukkan tanda-tanda kehidupan, walaupun mungkin hanya beberapa saat saja, seperti jantung berdenyut, bernafas, dan menangis.

3. **Sterilisasi wanita/tubektomi/MOW** adalah tindakan operasi menyumbat (mengikat dan atau memotong) saluran keluar ovum, yakni tuba, sehingga perjalanan ovum dari ovarium saat ovulasi tidak sampai ke tempat pembuahan di uterus. Dengan demikian, kehadiran sperma tidak mengakibatkan konsepsi, dan tidak terjadi kehamilan.

4. **Sterilisasi pria/vasektomi/MOP** adalah suatu operasi ringan yang dilakukan pada pria dengan maksud untuk mencegah terjadinya kehamilan. Operasi yang dimaksud adalah prosedur klinis untuk menghentikan kapasitas reproduksi pria dengan jalan mengikat/ memotong saluran sperma, sehingga alur transportasi sperma terhambat dan proses pembuahan dengan sel telur tidak terjadi. Vasektomi biasanya bersifat permanen

5. **IUD/AKDR/spiral (alat kontrasepsi dalam rahim)** adalah alat KB dari bahan plastik atau tembaga, dipasang dalam rongga rahim untuk mencegah kehamilan.

TECHNICAL NOTES

1. ***Early Initiation of Breastfeeding (IMD)*** *is putting the baby clinging to mother's chest or abdomen immediately after birth, let it creep latch, then breastfeed until satisfied.*

2. ***Children born alive*** *is a child who at birth showed signs of life, although it may be only a few moments, such as the heart beat, breathing, and cry.*

3. ***Female sterilization/tubektomi/MOW*** *is surgery clog (binding or cutting) the outlet of the ovum, the tube, so traveling ovum from the ovary during ovulation did not get to where fertilization in the uterus. Thus, the presence of sperm does not result in conception, and pregnancy does not occur.*

4. ***Male Sterilisasi/vasectomy/MOP*** *is a minor surgery performed on men with a view to prevent pregnancy. Operation in question is a clinical procedure to stop the male reproductive capacity with the binding/ cutting the vas deferens, so that sperm transport groove inhibited and the process of fertilization with an egg cell does not occur. Vasectomy is usually permanent.*

5. ***IUD/IUD/spiral (intrauterine device)*** *is a contraceptive device of plastic or copper, placed in the cavity of the uterus to prevent pregnancy.*

https://www.bps.go.id

6. **Suntikan** adalah cara KB hormonal dengan cara menyuntikkan hormon progesteron dan atau estrogen. Suntikan diberikan pada masa interval 7 hari setelah haid, segera setelah persalinan atau keguguran atau kapan saja selama yakin tidak sedang hamil.

7. **Susuk KB/implan** adalah alat KB berupa batang susuk, yang tipis dan halus seperti korek api, ditanam di bawah kulit lengan kiri (atau kanan jika kidal) bagian atas perempuan untuk mencegah kehamilan.

8. **Pil** adalah alat KB berupa pil yang mengandung kombinasi progesteron dan estrogen untuk mencegah kehamilan.

9. **Kondom pria/karet KB** adalah alat KB berupa kantong karet tipis dan elastis dipakai oleh pria ketika melakukan hubungan seksual untuk mencegah kehamilan. Kondom berfungsi sebagai penampung sperma agar tidak tumpah ke vagina, sehingga konsepsi tidak terjadi.

10. **Intravag** adalah alat KB berupa tisyu yang dimasukkan pada vagina ketika akan melakukan hubungan seksual.

11. **Diafragma** adalah alat/cara KB yang berbentuk mangkok terbuat dari karet lunak yang dimasukkan ke dalam vagina untuk menutup mulut rahim agar sperma tidak masuk ke dalam rahim dan bertemu dengan sel telur. Diafragma biasanya digunakan bersama *spermisida* (pembunuh sperma) berupa *jelly* atau krim yang berguna untuk menutup mulut rahim (*cervix*) sehingga menghalangi sperma bertemu sel telur.

12. **Kondom wanita** adalah alat/cara KB berupa karet tipis berbentuk tabung yang ujungnya terdapat semacam spong dan dimasukkan ke dalam vagina.

*6. **Injections** is hormonal contraception by injecting progesterone or estrogen. Injections are given at time intervals of 7 days after menstruation, immediately after delivery or miscarriage or anytime for sure not pregnant.*

*7. **Implant** is in the form of rod contraceptive implant, thin and smooth as lighters, implanted under the skin of the left arm (or right if left-handed) the top women to prevent pregnancy.*

*8. **Pill** is a contraceptive form pills containing a combination of progesterone and estrogen to prevent pregnancy.*

*9. **The male condom/rubber KB** is family planning tool in the form of a thin and elastic rubber bag used by men during sexual intercourse to prevent pregnancy. Condoms serve as a reservoir of sperm from spilling into the vagina, so conception does not occur.*

*10. **Intravag** is a contraceptive device in the form of wipes are inserted in the vagina when it would have sexual intercourse.*

*11. **The diaphragm** is a tool/contraception shaped bowl made of soft rubber that is inserted into the vagina to cover the cervix so sperm into the uterus and meet with the egg. The diaphragm is usually used with a spermicide (sperm killer) in the form of jelly or cream that is useful to shut the mouth of the uterus (cervix) thereby blocking the sperm meet the egg.*

*12. **The female condom** is a tool/contraception in the form of a thin rubber tube shaped ends are sort of sponge and inserted into the vagina.*

https://www.bps.go.id

13. **Metode menyusui alami/*Amenorrhea Laktasi* (MAL)** adalah kontrasepsi yang mengandalkan pemberian air susu ibu secara ekskusif (tanpa makanan dan minuman tambahan), belum haid dan bayi berumur kurang dari 6 bulan.

14. **Pantang berkala/kalender** didasarkan pada pemikiran bahwa dengan tidak melakukan senggama pada hari-hari tertentu, yaitu pada masa subur dalam siklus bulanan maka dapat menghindarkan dari kehamilan.

15. **Pelayanan KB di TKBK/TMT/MUYAN** merupakan fasilitas pelayanan KB mobil (bukan statis) yang berfungsi untuk mendekatkan pelayanan KB kepada masyarakat oleh satuan kerja terpadu (KB, Kesehatan, dan pihak lain sesuai keperluan) dan mempunyai kemampuan dan kewenangan memberikan pelayanan alat/cara KB seperti pil KB, kondom, suntik KB, IUD, dan implant.

16. **Penolong persalinan** adalah siapa yang menolong pada saat proses kelahiran anak (balita). Hingga tahun 2014, penolong persalinan ditanyakan untuk anak usia di bawah lima tahun. Namun mulai tahun 2015, penolong persalinan ditanyakan untuk wanita usia 15-49 tahun berstatus pernah kawin yang melahirkan anak dalam 2 tahun terakhir.

*13. **Method of natural breastfeeding/Lactation Amenorrhea (MAL)** is a contraceptive that rely on breastfeeding as ekskusif (without food and drinks extra), yet menstruation and infants aged less than 6 months.*

*14. **Periodic abstinence/calendar** is based on the idea that by not having intercourse on a particular day, which is in the fertile period in a monthly cycle it can prevent pregnancy.*

*15. **Family planning services in TKBK/TMK/Muyan** a facility planning services car (not static) that serves to bring family planning services to the community by working unit integrated (KB, Health, and others as appropriate) and have the ability and authority to provide service tool/method KB such as birth control pills, condoms, injectables, IUDs, and implants.*

*16. **Birth attendant** is who helped during the process of birth of a child (under-five). Until 2014, birth attendant asked for children aged under five years. However, starting in 2015, the birth attendant asked for women aged 15-49 years who have been married that childbearing in the last 2 years.*

https://www.bps.go.id

Tabel *Table* **5.1.** **Persentase Perempuan yang Pernah Kawin Berumur 10 Tahun ke Atas menurut Provinsi, Daerah Tempat Tinggal, dan Umur Perkawinan Pertama, 2018**
Percentage of Ever Married Female Aged 10 Years and Over by Province, Urban Rural Classification, and Age at First Marriage, 2018

Provinsi *Province*	Perkotaan/*Urban*					Perdesaan/*Rural*					Perkotaan+Perdesaan/*Urban+Rural*				
	≤16	17-18	19-20	21+	Jumlah *Total*	≤16	17-18	19-20	21+	Jumlah *Total*	≤16	17-18	19-20	21+	Jumlah *Total*
(1)	(2)	(3)	(4)	(5)	(6)	(7)	(8)	(9)	(10)	(11)	(12)	(13)	(14)	(15)	(16)
Aceh	6,55	13,86	22,76	56,83	100,00	11,50	19,38	23,96	45,16	100,00	10,00	17,70	23,60	48,70	100,00
Sumatera Utara	3,99	12,01	22,09	61,92	100,00	7,26	15,44	26,59	50,71	100,00	5,53	13,63	24,22	56,62	100,00
Sumatera Barat	5,94	11,28	18,50	64,29	100,00	13,13	19,13	25,49	42,25	100,00	10,06	15,78	22,51	51,65	100,00
Riau	5,94	11,99	22,20	59,86	100,00	12,92	22,47	26,86	37,75	100,00	10,16	18,34	25,02	46,48	100,00
Jambi	9,21	15,26	20,70	54,83	100,00	19,11	25,81	25,55	29,54	100,00	16,07	22,57	24,06	37,30	100,00
Sumatera Selatan	8,92	15,19	22,43	53,46	100,00	16,26	25,79	25,14	32,80	100,00	13,66	22,03	24,18	40,13	100,00
Bengkulu	11,23	13,20	21,58	53,99	100,00	19,10	25,39	25,22	30,30	100,00	16,66	21,61	24,09	37,64	100,00
Lampung	10,53	15,59	23,09	50,80	100,00	16,39	23,44	25,58	34,58	100,00	14,74	21,23	24,88	39,14	100,00
Kepulauan Bangka Belitung	7,99	15,99	22,37	53,65	100,00	15,68	25,11	26,04	33,18	100,00	11,55	20,21	24,07	44,17	100,00
Kepulauan Riau	3,99	9,04	18,70	68,26	100,00	11,83	24,37	26,38	37,41	100,00	5,20	11,39	19,88	63,54	100,00
DKI Jakarta	8,12	11,52	21,11	59,25	100,00	-	-	-	-	-	8,12	11,52	21,11	59,25	100,00
Jawa Barat	16,78	20,16	23,04	40,02	100,00	31,74	29,17	21,20	17,89	100,00	20,93	22,66	22,53	33,87	100,00
Jawa Tengah	12,71	18,14	21,92	47,24	100,00	21,03	25,21	22,91	30,85	100,00	16,91	21,71	22,42	38,96	100,00
DI Yogyakarta	4,62	12,19	17,74	65,44	100,00	9,82	20,27	27,98	41,93	100,00	6,21	14,66	20,87	58,26	100,00
Jawa Timur	15,48	17,26	22,21	45,05	100,00	26,04	25,42	22,54	25,99	100,00	20,73	21,32	22,38	35,57	100,00
Banten	11,35	15,86	24,63	48,16	100,00	23,53	25,38	24,61	26,48	100,00	14,97	18,69	24,62	41,71	100,00
Bali	2,79	14,40	21,79	61,01	100,00	4,57	22,64	27,55	45,24	100,00	3,43	17,35	23,85	55,37	100,00
Nusa Tenggara Barat	13,43	21,84	24,89	39,83	100,00	17,65	24,34	23,52	34,49	100,00	15,76	23,22	24,14	36,88	100,00
Nusa Tenggara Timur	3,41	10,65	18,36	67,58	100,00	5,51	14,75	23,49	56,26	100,00	5,06	13,88	22,40	58,66	100,00
Kalimantan Barat	9,60	16,17	23,81	50,41	100,00	15,71	22,63	25,90	35,75	100,00	13,73	20,53	25,23	40,51	100,00
Kalimantan Tengah	11,93	20,56	22,66	44,85	100,00	19,46	26,61	24,26	29,67	100,00	16,63	24,34	23,66	35,37	100,00
Kalimantan Selatan	16,12	19,09	23,47	41,32	100,00	28,16	24,81	22,21	24,82	100,00	22,77	22,25	22,77	32,21	100,00
Kalimantan Timur	9,35	15,98	21,81	52,86	100,00	17,01	24,42	25,76	32,81	100,00	11,91	18,81	23,13	46,14	100,00
Kalimantan Utara	12,95	14,38	24,24	48,42	100,00	16,31	21,22	22,98	39,49	100,00	14,36	17,26	23,71	44,67	100,00
Sulawesi Utara	7,96	13,03	24,62	54,40	100,00	11,03	19,35	26,49	43,14	100,00	9,52	16,25	25,57	48,65	100,00
Sulawesi Tengah	9,63	15,83	21,38	53,15	100,00	14,66	22,91	23,41	39,02	100,00	13,34	21,05	22,88	42,74	100,00
Sulawesi Selatan	12,36	15,28	19,31	53,05	100,00	18,92	21,46	20,41	39,21	100,00	16,37	19,06	19,98	44,59	100,00
Sulawesi Tenggara	11,86	22,25	19,08	46,80	100,00	19,95	24,56	23,44	32,05	100,00	17,07	23,74	21,88	37,31	100,00
Gorontalo	8,52	14,39	26,44	50,65	100,00	15,03	23,62	24,61	36,75	100,00	12,57	20,13	25,30	42,00	100,00
Sulawesi Barat	13,07	18,63	21,44	46,86	100,00	18,49	22,54	23,64	35,32	100,00	17,26	21,65	23,14	37,96	100,00
Maluku	5,05	10,10	21,18	63,67	100,00	7,73	16,12	25,10	51,04	100,00	6,64	13,68	23,51	56,18	100,00
Maluku Utara	6,83	11,98	21,32	59,87	100,00	10,13	22,55	26,20	41,12	100,00	9,21	19,61	24,84	46,34	100,00
Papua Barat	7,11	12,03	23,33	57,53	100,00	11,36	20,68	23,63	44,32	100,00	9,66	17,23	23,51	49,59	100,00
Papua	6,79	14,60	25,08	53,53	100,00	11,25	20,27	28,32	40,16	100,00	10,10	18,80	27,48	43,62	100,00
**Indonesia**	**12,16**	**16,69**	**22,26**	**48,88**	**100,00**	**19,65**	**23,84**	**23,76**	**32,75**	**100,00**	**15,66**	**20,03**	**22,96**	**41,35**	**100,00**

Sumber/*Source*: Susenas Maret 2018/*The March 2018 Susenas*

https://www.bps.go.id

Tabel *Table* **5.2.** **Persentase Perempuan Berumur 15-49 Tahun yang Pernah Hamil menurut Provinsi, Daerah Tempat Tinggal, dan Umur Saat Hamil Pertama Kali, 2017***
*Percentage of Ever Pregnant Female Aged 15-49 Years by Province, Urban Rural Classification, and Age at First Pregnant, 2017**

Provinsi *Province*	Perkotaan/*Urban*					Perdesaan/*Rural*					Perkotaan+Perdesaan/*Urban+Rural*				
	≤16	17-18	19-20	21+	Jumlah *Total*	≤16	17-18	19-20	21+	Jumlah *Total*	≤16	17-18	19-20	21+	Jumlah *Total*
(1)	(2)	(3)	(4)	(5)	(6)	(7)	(8)	(9)	(10)	(11)	(12)	(13)	(14)	(15)	(16)
Aceh	2,99	9,56	17,95	69,51	100,00	5,81	15,59	24,51	54,09	100,00	5,00	13,85	22,61	58,54	100,00
Sumatera Utara	1,76	8,87	17,62	71,75	100,00	3,15	12,88	23,22	60,75	100,00	2,44	10,84	20,37	66,34	100,00
Sumatera Barat	1,94	6,03	14,82	77,21	100,00	4,78	13,31	23,24	58,67	100,00	3,65	10,42	19,89	66,05	100,00
Riau	2,91	9,32	17,95	69,81	100,00	5,50	18,50	25,98	50,01	100,00	4,54	15,09	22,99	57,38	100,00
Jambi	3,73	10,64	20,29	65,35	100,00	11,18	21,89	27,48	39,45	100,00	8,98	18,57	25,35	47,10	100,00
Sumatera Selatan	4,64	11,06	18,24	66,06	100,00	9,43	21,53	26,82	42,22	100,00	7,83	18,03	23,96	50,18	100,00
Bengkulu	4,75	11,30	19,52	64,43	100,00	12,05	23,15	28,15	36,65	100,00	9,89	19,64	25,59	44,87	100,00
Lampung	3,13	9,52	21,84	65,51	100,00	7,02	19,42	27,03	46,52	100,00	6,02	16,86	25,69	51,44	100,00
Kepulauan Bangka Belitung	4,71	13,78	21,60	59,91	100,00	10,40	21,57	30,14	37,89	100,00	7,50	17,60	25,79	49,11	100,00
Kepulauan Riau	1,35	7,56	14,67	76,43	100,00	4,87	22,87	20,68	51,58	100,00	1,88	9,88	15,58	72,66	100,00
DKI Jakarta	2,73	7,95	18,17	71,15	100,00	-	-	-	-	-	2,73	7,95	18,17	71,15	100,00
Jawa Barat	5,30	14,57	24,82	55,31	100,00	10,65	24,55	30,08	34,72	100,00	6,84	17,45	26,34	49,37	100,00
Jawa Tengah	2,76	11,98	20,43	64,83	100,00	6,03	20,33	26,30	47,34	100,00	4,49	16,39	23,53	55,59	100,00
DI Yogyakarta	2,13	5,30	19,94	72,62	100,00	2,44	13,16	22,92	61,48	100,00	2,22	7,72	20,86	69,19	100,00
Jawa Timur	4,61	11,74	21,93	61,72	100,00	8,35	19,70	26,38	45,56	100,00	6,52	15,80	24,20	53,48	100,00
Banten	4,87	14,00	24,28	56,85	100,00	9,75	24,95	27,28	38,02	100,00	6,33	17,29	25,18	51,20	100,00
Bali	2,99	10,36	21,77	64,88	100,00	6,23	20,92	25,19	47,66	100,00	4,19	14,26	23,03	58,52	100,00
Nusa Tenggara Barat	7,01	16,58	27,08	49,33	100,00	7,90	21,37	26,35	44,38	100,00	7,51	19,30	26,67	46,52	100,00
Nusa Tenggara Timur	4,31	10,83	22,03	62,83	100,00	5,31	16,39	25,07	53,22	100,00	5,11	15,28	24,47	55,14	100,00
Kalimantan Barat	4,43	13,40	21,81	60,37	100,00	12,03	22,64	25,24	40,09	100,00	9,77	19,90	24,22	46,11	100,00
Kalimantan Tengah	9,61	16,28	23,09	51,02	100,00	13,60	22,16	25,84	38,40	100,00	12,20	20,10	24,87	42,82	100,00
Kalimantan Selatan	8,32	14,58	21,86	55,24	100,00	13,27	23,89	23,99	38,84	100,00	11,17	19,94	23,09	45,81	100,00
Kalimantan Timur	3,57	12,00	21,31	63,13	100,00	11,49	19,48	25,51	43,51	100,00	6,30	14,58	22,76	56,36	100,00
Kalimantan Utara	6,94	14,33	22,79	55,95	100,00	9,95	21,02	22,12	46,90	100,00	8,21	17,15	22,51	52,13	100,00
Sulawesi Utara	5,30	15,43	21,05	58,23	100,00	8,25	19,10	24,82	47,83	100,00	6,90	17,42	23,10	52,58	100,00
Sulawesi Tengah	6,05	15,06	18,47	60,43	100,00	11,54	20,89	23,68	43,89	100,00	10,20	19,48	22,41	47,91	100,00
Sulawesi Selatan	7,63	13,64	17,55	61,18	100,00	10,58	18,85	22,48	48,10	100,00	9,49	16,92	20,66	52,93	100,00
Sulawesi Tenggara	6,59	16,20	20,46	56,74	100,00	11,81	22,42	24,99	40,78	100,00	10,23	20,53	23,62	45,62	100,00
Gorontalo	6,86	13,88	18,27	60,98	100,00	10,72	20,46	26,69	42,14	100,00	9,41	18,24	23,84	48,51	100,00
Sulawesi Barat	10,54	13,96	19,33	56,17	100,00	12,24	20,77	22,78	44,21	100,00	11,92	19,50	22,13	46,44	100,00
Maluku	4,72	11,53	19,21	64,54	100,00	6,21	16,44	22,95	54,40	100,00	5,61	14,46	21,44	58,49	100,00
Maluku Utara	3,26	16,95	18,33	61,46	100,00	9,31	20,67	23,87	46,14	100,00	7,69	19,68	22,39	50,23	100,00
Papua Barat	4,69	9,13	25,13	61,05	100,00	10,61	17,82	24,31	47,26	100,00	8,34	14,49	24,63	52,55	100,00
Papua	4,86	13,63	21,17	60,33	100,00	7,63	16,99	25,43	49,95	100,00	6,95	16,16	24,38	52,51	100,00
**Indonesia**	**4,29**	**12,16**	**21,64**	**61,90**	**100,00**	**8,27**	**20,10**	**26,10**	**45,53**	**100,00**	**6,21**	**15,99**	**23,79**	**54,01**	**100,00**

Catatan/*Note***:** *) Data 2018 belum tersedia/*2018 data not available yet*
Sumber/*Source*: Susenas Maret 2017/*The March 2017 Susenas*

https://www.bps.go.id

Tabel *Table* **5.3.1.** **Persentase Perempuan Berumur 15-49 Tahun Pernah Kawin yang Pernah Melahirkan dalam 2 Tahun Terakhir di Daerah Perkotaan menurut Provinsi dan Tempat Melahirkan Anak Lahir Hidup yang Terakhir, 2018**
Percentage of Ever Married Female Aged 15-49 Years Who Have Given Birth in The Last Two Years in Urban Area by Province and Place of Last Live Birth, 2018

**Provinsi** *Province*	**Rumah Sakit/RSIA/RS Bersalin** *General Hospital/Mother and Child Hospital/Maternity Hospital*	**Rumah Bersalin/ Klinik** *Maternity Clinic/Clinic*	**Puskesmas** *Community Health Center*	**Puskesmas Pembantu (Pustu)** *Subsidiary Health Center*	**Praktik Tenaga Kesehatan** *Health Workers Clinic*	**Polindes/Poskesdes** *Village Maternity Post/ Village Health Post*	**Rumah** *At Home*	**Lainnya** *Others*	**Jumlah** *Total*
(1)	(2)	(3)	(4)	(5)	(6)	(7)	(8)	(9)	(10)
Aceh	44,94	26,95	3,67	2,12	7,16	7,48	6,90	0,78	100,00
Sumatera Utara	43,51	35,93	2,50	0,04	6,09	0,69	10,82	0,43	100,00
Sumatera Barat	37,38	37,50	3,55	3,01	15,39	1,61	1,07	0,49	100,00
Riau	40,78	40,63	1,87	0,38	7,19	0,40	7,67	1,08	100,00
Jambi	38,34	33,17	1,69	0,00	9,35	2,33	14,60	0,51	100,00
Sumatera Selatan	45,35	27,45	3,09	0,03	15,61	1,67	5,72	1,09	100,00
Bengkulu	47,39	20,78	1,73	0,56	16,69	3,84	7,27	1,73	100,00
Lampung	28,91	39,87	3,46	0,84	18,70	0,00	8,22	0,00	100,00
Kepulauan Bangka Belitung	39,40	30,87	9,55	0,00	9,39	5,19	5,35	0,26	100,00
Kepulauan Riau	43,28	32,30	6,26	0,07	10,40	2,42	3,22	2,07	100,00
DKI Jakarta	45,98	29,90	19,51	0,00	1,54	0,27	0,58	2,23	100,00
Jawa Barat	32,73	27,26	9,58	0,51	16,93	1,50	10,59	0,90	100,00
Jawa Tengah	43,55	20,97	17,83	0,10	14,28	1,56	1,02	0,68	100,00
DI Yogyakarta	52,81	24,31	8,02	0,00	14,45	0,00	0,00	0,40	100,00
Jawa Timur	40,37	24,04	9,26	0,39	17,37	5,06	2,23	1,29	100,00
Banten	34,01	33,02	14,96	0,12	7,79	0,95	7,78	1,37	100,00
Bali	60,30	22,45	5,21	0,29	11,75	0,00	0,00	0,00	100,00
Nusa Tenggara Barat	30,04	6,57	27,83	0,89	4,55	21,73	7,47	0,92	100,00
Nusa Tenggara Timur	52,00	12,18	21,27	4,72	0,13	0,83	8,87	0,00	100,00
Kalimantan Barat	37,15	34,72	7,26	0,00	6,55	2,42	11,90	0,00	100,00
Kalimantan Tengah	26,54	27,11	9,46	0,00	4,88	0,60	31,30	0,12	100,00
Kalimantan Selatan	40,10	29,80	3,52	0,16	12,82	2,14	11,16	0,29	100,00
Kalimantan Timur	49,18	30,16	4,14	0,26	8,57	0,00	6,48	1,21	100,00
Kalimantan Utara	48,98	14,99	21,95	0,00	7,87	0,00	6,21	0,00	100,00
Sulawesi Utara	62,57	9,32	15,78	0,66	0,48	1,29	8,70	1,20	100,00
Sulawesi Tengah	44,93	12,23	16,09	1,89	2,19	3,77	18,90	0,00	100,00
Sulawesi Selatan	52,80	19,79	16,45	2,18	1,26	0,53	5,25	1,74	100,00
Sulawesi Tenggara	28,05	13,90	8,85	3,27	2,18	0,78	41,96	1,01	100,00
Gorontalo	64,06	5,00	14,28	0,96	0,00	10,50	5,19	0,00	100,00
Sulawesi Barat	28,83	9,23	28,21	15,45	2,22	0,00	16,05	0,00	100,00
Maluku	49,82	1,24	2,26	0,24	0,00	0,73	45,41	0,31	100,00
Maluku Utara	64,44	10,29	5,74	0,49	0,61	1,54	16,88	0,00	100,00
Papua Barat	67,78	5,66	4,27	0,40	2,49	0,00	19,41	0,00	100,00
Papua	64,88	9,70	8,49	0,50	3,56	0,17	12,11	0,59	100,00
**Indonesia**	**40,31**	**26,49**	**10,81**	**0,54**	**11,59**	**2,12**	**7,16**	**0,98**	**100,00**

Sumber/*Source*: Susenas Maret 2018/*The March 2018 Susenas*

https://www.bps.go.id

Tabel *Table* **5.3.2.** **Persentase Perempuan Berumur 15-49 Tahun Pernah Kawin yang Pernah Melahirkan dalam 2 Tahun Terakhir di Daerah Perdesaan menurut Provinsi dan Tempat Melahirkan Anak Lahir Hidup yang Terakhir, 2018**
Percentage of Ever Married Female Aged 15-49 Years Who Have Given Birth in The Last Two Years in Rural Area by Province and Place of Last Live Birth, 2018

**Provinsi** *Province*	**Rumah Sakit/RSIA/RS Bersalin** *General Hospital/Mother and Child Hospital/Maternity Hospital*	**Rumah Bersalin/ Klinik** *Maternity Clinic/Clinic*	**Puskesmas** *Community Health Center*	**Puskesmas Pembantu (Pustu)** *Subsidiary Health Center*	**Praktik Tenaga Kesehatan** *Health Workers Clinic*	**Polindes/Poskesdes** *Village Maternity Post/ Village Health Post*	**Rumah** *At Home*	**Lainnya** *Others*	**Jumlah** *Total*
**(1)**	**(2)**	**(3)**	**(4)**	**(5)**	**(6)**	**(7)**	**(8)**	**(9)**	**(10)**
Aceh	30,65	19,54	8,57	3,65	11,34	9,68	15,43	1,14	100,00
Sumatera Utara	21,33	16,55	6,81	2,08	7,29	2,75	42,77	0,43	100,00
Sumatera Barat	25,92	21,78	9,31	7,16	14,89	7,59	12,69	0,66	100,00
Riau	23,89	16,91	5,60	1,44	6,65	2,20	42,15	1,15	100,00
Jambi	18,48	14,36	7,79	0,63	4,09	4,13	50,31	0,21	100,00
Sumatera Selatan	20,16	12,37	6,49	1,70	10,45	12,44	35,47	0,91	100,00
Bengkulu	26,02	9,11	10,05	0,32	2,73	2,98	48,31	0,48	100,00
Lampung	15,90	25,90	6,23	1,07	27,98	5,29	17,56	0,08	100,00
Kepulauan Bangka Belitung	28,50	9,44	10,86	0,96	4,00	26,12	20,14	0,00	100,00
Kepulauan Riau	28,85	10,08	19,08	0,50	13,72	4,05	23,71	0,00	100,00
DKI Jakarta	-	-	-	-	-	-	-	-	-
Jawa Barat	15,47	13,24	14,07	1,10	16,24	3,44	34,80	1,64	100,00
Jawa Tengah	33,37	14,99	24,84	0,88	15,98	5,18	3,82	0,93	100,00
DI Yogyakarta	44,18	28,14	7,12	0,00	17,52	0,16	1,52	1,35	100,00
Jawa Timur	28,57	14,40	11,14	2,29	19,97	14,24	8,12	1,26	100,00
Banten	13,60	12,33	21,76	1,01	3,05	1,09	47,16	0,00	100,00
Bali	48,53	18,93	5,50	0,55	23,85	2,24	0,41	0,00	100,00
Nusa Tenggara Barat	25,21	4,56	23,69	3,85	3,69	27,09	11,52	0,41	100,00
Nusa Tenggara Timur	21,96	0,96	42,68	4,13	0,04	2,75	27,05	0,43	100,00
Kalimantan Barat	15,86	8,37	13,30	1,28	3,04	10,49	47,65	0,00	100,00
Kalimantan Tengah	13,55	7,60	7,57	1,39	1,97	2,68	64,57	0,69	100,00
Kalimantan Selatan	26,94	10,35	9,73	1,12	7,15	11,87	32,25	0,60	100,00
Kalimantan Timur	40,91	7,76	18,39	1,10	6,29	2,46	23,08	0,00	100,00
Kalimantan Utara	38,11	3,84	23,53	1,93	3,26	0,01	29,33	0,00	100,00
Sulawesi Utara	44,62	10,24	15,44	0,93	2,11	4,06	21,83	0,77	100,00
Sulawesi Tengah	22,54	3,86	28,18	5,78	0,08	6,45	32,90	0,21	100,00
Sulawesi Selatan	27,72	6,19	31,01	10,97	0,32	0,39	22,97	0,43	100,00
Sulawesi Tenggara	13,33	3,85	24,58	0,55	0,00	0,00	57,69	0,00	100,00
Gorontalo	23,90	4,99	42,57	4,48	0,00	5,16	18,91	0,00	100,00
Sulawesi Barat	14,30	2,36	18,07	28,04	1,03	2,10	33,14	0,96	100,00
Maluku	12,90	0,70	5,76	0,13	0,19	0,75	78,72	0,85	100,00
Maluku Utara	14,01	1,02	7,00	0,00	0,00	2,78	74,92	0,27	100,00
Papua Barat	34,51	1,61	6,23	1,03	0,01	0,40	54,65	1,56	100,00
Papua	14,84	2,42	17,11	3,40	0,56	1,19	60,03	0,46	100,00
**Indonesia**	**23,93**	**12,56**	**15,85**	**2,63**	**10,66**	**6,52**	**27,09**	**0,76**	**100,00**

Sumber/*Source*: Susenas Maret 2018/*The March 2018 Susenas*

https://www.bps.go.id

Tabel *Table* **5.3.3.** **Persentase Perempuan Berumur 15-49 Tahun Pernah Kawin yang Pernah Melahirkan dalam 2 Tahun Terakhir di Daerah Perkotaan dan Perdesaan menurut Provinsi dan Tempat Melahirkan Anak Lahir Hidup yang Terakhir, 2018**
Percentage of Ever Married Female Aged 15-49 Years Who Have Given Birth in The Last Two Years in Urban and Rural Area by Province and Place of Last Live Birth, 2018

**Provinsi** *Province*	**Rumah Sakit/RSIA/RS Bersalin** *General Hospital/Mother and Child Hospital/Maternity Hospital*	**Rumah Bersalin/ Klinik** *Maternity Clinic/Clinic*	**Puskesmas** *Community Health Center*	**Puskesmas Pembantu (Pustu)** *Subsidiary Health Center*	**Praktik Tenaga Kesehatan** *Health Workers Clinic*	**Polindes/Poskesdes** *Village Maternity Post/ Village Health Post*	**Rumah** *At Home*	**Lainnya** *Others*	**Jumlah** *Total*
(1)	(2)	(3)	(4)	(5)	(6)	(7)	(8)	(9)	(10)
Aceh	34,99	21,79	7,08	3,18	10,07	9,01	12,85	1,03	100,00
Sumatera Utara	32,51	26,31	4,64	1,05	6,69	1,71	26,67	0,43	100,00
Sumatera Barat	30,89	28,59	6,81	5,36	15,11	5,00	7,66	0,59	100,00
Riau	31,00	26,90	4,03	1,00	6,88	1,44	27,63	1,12	100,00
Jambi	24,45	20,01	5,96	0,44	5,67	3,59	39,59	0,30	100,00
Sumatera Selatan	28,52	17,38	5,36	1,15	12,16	8,86	25,59	0,97	100,00
Bengkulu	32,79	12,81	7,41	0,40	7,15	3,25	35,31	0,88	100,00
Lampung	19,54	29,81	5,45	1,00	25,38	3,81	14,94	0,06	100,00
Kepulauan Bangka Belitung	34,63	21,50	10,12	0,42	7,03	14,34	11,81	0,14	100,00
Kepulauan Riau	41,45	29,48	7,89	0,12	10,82	2,62	5,82	1,81	100,00
DKI Jakarta	45,98	29,90	19,51	0,00	1,54	0,27	0,58	2,23	100,00
Jawa Barat	28,37	23,73	10,72	0,66	16,76	1,99	16,70	1,08	100,00
Jawa Tengah	38,44	17,97	21,35	0,50	15,13	3,38	2,42	0,80	100,00
DI Yogyakarta	50,89	25,16	7,82	0,00	15,14	0,03	0,34	0,61	100,00
Jawa Timur	34,74	19,44	10,16	1,30	18,61	9,44	5,04	1,27	100,00
Banten	27,86	26,79	17,01	0,39	6,36	0,99	19,64	0,96	100,00
Bali	56,53	21,32	5,30	0,38	15,62	0,72	0,13	0,00	100,00
Nusa Tenggara Barat	27,33	5,44	25,50	2,55	4,06	24,74	9,74	0,63	100,00
Nusa Tenggara Timur	28,25	3,31	38,19	4,26	0,06	2,35	23,24	0,34	100,00
Kalimantan Barat	23,35	17,64	11,17	0,83	4,28	7,65	35,07	0,00	100,00
Kalimantan Tengah	18,51	15,05	8,29	0,86	3,08	1,88	51,85	0,47	100,00
Kalimantan Selatan	33,16	19,55	6,80	0,66	9,83	7,27	22,28	0,45	100,00
Kalimantan Timur	46,51	22,93	8,74	0,54	7,83	0,79	11,84	0,82	100,00
Kalimantan Utara	44,52	10,41	22,60	0,79	5,97	0,00	15,71	0,00	100,00
Sulawesi Utara	53,81	9,77	15,61	0,79	1,28	2,64	15,11	0,99	100,00
Sulawesi Tengah	28,23	5,99	25,11	4,79	0,62	5,77	29,35	0,15	100,00
Sulawesi Selatan	38,27	11,91	24,89	7,27	0,71	0,45	15,52	0,98	100,00
Sulawesi Tenggara	18,76	7,56	18,78	1,56	0,81	0,29	51,89	0,37	100,00
Gorontalo	40,11	4,99	31,16	3,06	0,00	7,31	13,37	0,00	100,00
Sulawesi Barat	17,49	3,87	20,30	25,28	1,29	1,64	29,38	0,75	100,00
Maluku	27,60	0,92	4,37	0,17	0,12	0,74	65,46	0,63	100,00
Maluku Utara	28,12	3,62	6,65	0,14	0,17	2,43	58,68	0,20	100,00
Papua Barat	48,68	3,34	5,39	0,76	1,06	0,23	39,64	0,90	100,00
Papua	29,07	4,49	14,66	2,57	1,41	0,90	46,40	0,49	100,00
**Indonesia**	**32,73**	**20,04**	**13,14**	**1,51**	**11,16**	**4,16**	**16,39**	**0,87**	**100,00**

Sumber/*Source*: Susenas Maret 2018/*The March 2018 Susenas*

https://www.bps.go.id

Tabel *Table* **5.4.1.** **Persentase Perempuan Berumur 15-49 Tahun Pernah Kawin yang Pernah Melahirkan dalam 2 Tahun Terakhir di Daerah Perkotaan menurut Provinsi dan Penolong Proses Kelahiran Terakhir, 2018**
Percentage of Ever Married Female Aged 15-49 Years Who Have Given Birth in the Last Two Years in Urban Area by Province and Last Birth Attendant, 2018

**Provinsi** *Province*	**Dokter Kandungan** *Obstetrician*	**Dokter Umum** *General Practitioner*	**Bidan** *Midwife*	**Perawat** *Nurse*	**Dukun Beranak/Paraji** *Traditional Birth Attendance*	**Lainnya** *Others*	**Tidak Ada** *None*	**Jumlah** *Total*
(1)	(2)	(3)	(4)	(5)	(6)	(7)	(8)	(9)
Aceh	42,92	1,61	53,87	0,32	0,84	0,44	0,00	100,00
Sumatera Utara	42,85	2,03	53,44	0,55	0,86	0,16	0,11	100,00
Sumatera Barat	39,68	1,14	58,23	0,23	0,47	0,25	0,00	100,00
Riau	40,70	2,63	55,68	0,00	0,99	0,00	0,00	100,00
Jambi	36,32	0,31	59,75	0,36	3,26	0,00	0,00	100,00
Sumatera Selatan	43,23	1,75	52,51	0,21	2,30	0,00	0,00	100,00
Bengkulu	41,45	4,14	52,87	1,48	0,07	0,00	0,00	100,00
Lampung	23,60	0,81	72,93	0,00	2,66	0,00	0,00	100,00
Kepulauan Bangka Belitung	36,71	1,36	59,53	0,47	1,92	0,00	0,00	100,00
Kepulauan Riau	47,43	2,37	48,61	0,37	1,23	0,00	0,00	100,00
DKI Jakarta	49,69	2,39	47,32	0,32	0,28	0,00	0,00	100,00
Jawa Barat	32,40	2,24	60,85	0,18	4,33	0,00	0,00	100,00
Jawa Tengah	41,33	2,18	55,44	0,89	0,15	0,01	0,00	100,00
DI Yogyakarta	54,44	2,95	41,46	1,14	0,00	0,00	0,00	100,00
Jawa Timur	40,71	1,28	56,32	0,36	1,10	0,17	0,06	100,00
Banten	35,90	1,56	58,32	0,64	3,53	0,06	0,00	100,00
Bali	60,08	3,71	36,02	0,18	0,00	0,00	0,00	100,00
Nusa Tenggara Barat	23,42	2,29	68,57	1,56	4,17	0,00	0,00	100,00
Nusa Tenggara Timur	43,78	5,12	45,31	0,33	4,75	0,72	0,00	100,00
Kalimantan Barat	35,55	0,75	57,43	0,62	5,64	0,00	0,00	100,00
Kalimantan Tengah	25,93	1,64	67,53	0,00	4,74	0,00	0,16	100,00
Kalimantan Selatan	32,35	1,16	65,03	0,77	0,70	0,00	0,00	100,00
Kalimantan Timur	42,94	0,04	53,90	0,59	2,17	0,37	0,00	100,00
Kalimantan Utara	28,88	2,00	68,98	0,00	0,00	0,14	0,00	100,00
Sulawesi Utara	54,96	5,65	32,43	0,96	6,00	0,00	0,00	100,00
Sulawesi Tengah	38,83	3,28	49,45	0,55	7,08	0,80	0,00	100,00
Sulawesi Selatan	43,89	1,70	51,12	0,91	1,79	0,28	0,31	100,00
Sulawesi Tenggara	19,88	1,55	65,96	0,30	12,03	0,28	0,00	100,00
Gorontalo	53,59	1,10	43,67	0,30	1,35	0,00	0,00	100,00
Sulawesi Barat	23,66	0,00	70,65	0,00	3,81	1,87	0,00	100,00
Maluku	26,76	2,59	52,32	1,42	16,37	0,36	0,19	100,00
Maluku Utara	44,53	1,84	46,42	0,00	5,35	0,78	1,07	100,00
Papua Barat	33,05	1,56	58,08	1,47	5,17	0,68	0,00	100,00
Papua	38,14	1,63	48,81	4,11	4,19	3,11	0,00	100,00
**Indonesia**	**38,81**	**1,99**	**56,13**	**0,48**	**2,46**	**0,11**	**0,03**	**100,00**

Sumber/*Source*: Susenas Maret 2018/*The March 2018 Susenas*

https://www.bps.go.id

Tabel *Table* **5.4.2.** **Persentase Perempuan Berumur 15-49 Tahun Pernah Kawin yang Pernah Melahirkan dalam 2 Tahun Terakhir di Daerah Perdesaan menurut Provinsi dan Penolong Proses Kelahiran Terakhir, 2018**
Percentage of Ever Married Female Aged 15-49 Years Who Have Given Birth in the Last Two Years in Rural Area by Province and Last Birth Attendant, 2018

**Provinsi** *Province*	**Dokter Kandungan** *Obstetrician*	**Dokter Umum** *General Practitioner*	**Bidan** *Midwife*	**Perawat** *Nurse*	**Dukun Beranak/Paraji** *Traditional Birth Attendance*	**Lainnya** *Others*	**Tidak Ada** *None*	**Jumlah** *Total*
(1)	(2)	(3)	(4)	(5)	(6)	(7)	(8)	(9)
Aceh	26,27	2,01	68,31	0,48	2,87	0,06	0,00	100,00
Sumatera Utara	20,74	2,41	66,19	0,74	8,49	1,23	0,20	100,00
Sumatera Barat	28,56	2,74	63,17	0,49	4,91	0,13	0,00	100,00
Riau	25,01	2,88	60,31	1,14	10,42	0,14	0,10	100,00
Jambi	18,12	2,98	63,67	0,13	14,94	0,05	0,12	100,00
Sumatera Selatan	16,44	2,26	71,08	0,37	9,56	0,09	0,20	100,00
Bengkulu	22,66	1,74	68,32	1,16	5,86	0,14	0,12	100,00
Lampung	16,00	0,89	75,60	0,27	7,24	0,00	0,00	100,00
Kepulauan Bangka Belitung	26,21	1,08	67,56	0,90	3,84	0,41	0,00	100,00
Kepulauan Riau	31,77	1,23	58,09	0,84	8,06	0,00	0,00	100,00
DKI Jakarta	-	-	-	-	-	-	-	-
Jawa Barat	16,46	0,87	67,81	0,33	14,03	0,33	0,17	100,00
Jawa Tengah	32,44	1,61	64,21	0,87	0,76	0,12	0,00	100,00
DI Yogyakarta	31,01	7,99	58,41	2,58	0,00	0,00	0,00	100,00
Jawa Timur	28,13	2,49	62,95	1,24	4,79	0,28	0,13	100,00
Banten	14,94	1,55	58,17	0,99	24,34	0,00	0,00	100,00
Bali	48,82	1,07	49,70	0,00	0,15	0,26	0,00	100,00
Nusa Tenggara Barat	17,84	0,99	73,26	1,86	4,76	0,98	0,31	100,00
Nusa Tenggara Timur	13,67	4,46	59,40	1,25	18,07	2,80	0,35	100,00
Kalimantan Barat	13,50	1,44	63,41	0,93	20,34	0,33	0,05	100,00
Kalimantan Tengah	10,17	1,09	65,84	3,22	19,04	0,47	0,16	100,00
Kalimantan Selatan	21,85	1,47	69,16	0,49	7,03	0,00	0,00	100,00
Kalimantan Timur	34,23	0,16	58,48	1,12	5,86	0,00	0,15	100,00
Kalimantan Utara	19,61	1,74	67,79	1,27	9,59	0,00	0,00	100,00
Sulawesi Utara	38,71	1,98	47,85	0,88	10,43	0,15	0,00	100,00
Sulawesi Tengah	18,20	2,30	62,46	3,15	11,32	1,82	0,75	100,00
Sulawesi Selatan	21,08	2,29	70,36	0,78	4,70	0,56	0,23	100,00
Sulawesi Tenggara	10,13	1,69	73,39	0,42	14,28	0,09	0,00	100,00
Gorontalo	23,95	1,92	64,98	1,65	7,49	0,00	0,00	100,00
Sulawesi Barat	10,89	2,50	74,21	0,13	9,41	2,47	0,39	100,00
Maluku	6,70	1,14	42,48	0,50	48,19	0,76	0,22	100,00
Maluku Utara	8,41	2,41	57,84	0,73	29,39	0,86	0,36	100,00
Papua Barat	19,71	3,93	48,82	1,62	14,20	9,85	1,86	100,00
Papua	8,03	2,53	33,73	4,88	15,47	31,91	3,45	100,00
**Indonesia**	**21,78**	**2,01**	**64,48**	**0,97**	**9,39**	**1,16**	**0,21**	**100,00**

Sumber/*Source*: Susenas Maret 2018/*The March 2018 Susenas*

https://www.bps.go.id

Tabel *Table* **5.4.3.** **Persentase Perempuan Berumur 15-49 Tahun Pernah Kawin yang Pernah Melahirkan dalam 2 Tahun Terakhir di Daerah Perkotaan dan Perdesaan menurut Provinsi dan Penolong Proses Kelahiran Terakhir, 2018**
Percentage of Ever Married Female Aged 15-49 Years Who Have Given Birth in the Last Two Years in Urban and Rural Area by Province and Last Birth Attendant, 2018

**Provinsi** *Province*	**Dokter Kandungan** *Obstetrician*	**Dokter Umum** *General Practitioner*	**Bidan** *Midwife*	**Perawat** *Nurse*	**Dukun Beranak/Paraji** *Traditional Birth Attendance*	**Lainnya** *Others*	**Tidak Ada** *None*	**Jumlah** *Total*
(1)	(2)	(3)	(4)	(5)	(6)	(7)	(8)	(9)
Aceh	31,33	1,89	63,93	0,43	2,25	0,18	0,00	100,00
Sumatera Utara	31,88	2,22	59,77	0,65	4,65	0,69	0,15	100,00
Sumatera Barat	33,37	2,05	61,03	0,37	2,99	0,19	0,00	100,00
Riau	31,62	2,78	58,36	0,66	6,45	0,08	0,06	100,00
Jambi	23,58	2,18	62,49	0,19	11,44	0,04	0,08	100,00
Sumatera Selatan	25,34	2,09	64,91	0,32	7,15	0,06	0,13	100,00
Bengkulu	28,61	2,50	63,43	1,26	4,03	0,09	0,08	100,00
Lampung	18,13	0,87	74,86	0,20	5,96	0,00	0,00	100,00
Kepulauan Bangka Belitung	32,12	1,24	63,04	0,66	2,76	0,18	0,00	100,00
Kepulauan Riau	45,44	2,22	49,81	0,43	2,10	0,00	0,00	100,00
DKI Jakarta	49,69	2,39	47,32	0,32	0,28	0,00	0,00	100,00
Jawa Barat	28,38	1,89	62,60	0,22	6,78	0,08	0,04	100,00
Jawa Tengah	36,87	1,89	59,84	0,88	0,45	0,06	0,00	100,00
DI Yogyakarta	49,24	4,07	45,23	1,46	0,00	0,00	0,00	100,00
Jawa Timur	34,70	1,86	59,48	0,78	2,86	0,22	0,09	100,00
Banten	29,59	1,56	58,27	0,74	9,80	0,04	0,00	100,00
Bali	56,48	2,87	40,40	0,13	0,05	0,08	0,00	100,00
Nusa Tenggara Barat	20,29	1,56	71,20	1,73	4,50	0,55	0,18	100,00
Nusa Tenggara Timur	19,98	4,60	56,45	1,06	15,28	2,36	0,28	100,00
Kalimantan Barat	21,26	1,20	61,31	0,82	15,17	0,21	0,03	100,00
Kalimantan Tengah	16,19	1,30	66,49	1,99	13,57	0,29	0,16	100,00
Kalimantan Selatan	26,81	1,32	67,21	0,62	4,04	0,00	0,00	100,00
Kalimantan Timur	40,13	0,08	55,38	0,76	3,36	0,25	0,05	100,00
Kalimantan Utara	25,07	1,89	68,49	0,52	3,94	0,08	0,00	100,00
Sulawesi Utara	47,03	3,86	39,95	0,92	8,16	0,07	0,00	100,00
Sulawesi Tengah	23,44	2,55	59,15	2,49	10,24	1,56	0,56	100,00
Sulawesi Selatan	30,67	2,04	62,27	0,84	3,48	0,44	0,27	100,00
Sulawesi Tenggara	13,73	1,64	70,65	0,38	13,45	0,16	0,00	100,00
Gorontalo	35,91	1,59	56,38	1,11	5,01	0,00	0,00	100,00
Sulawesi Barat	13,70	1,95	73,43	0,10	8,18	2,34	0,30	100,00
Maluku	14,68	1,72	46,40	0,86	35,52	0,60	0,21	100,00
Maluku Utara	18,51	2,25	54,64	0,53	22,66	0,84	0,56	100,00
Papua Barat	25,39	2,92	52,76	1,55	10,36	5,95	1,07	100,00
Papua	16,60	2,27	38,02	4,66	12,26	23,72	2,47	100,00
**Indonesia**	**30,92**	**2,00**	**60,00**	**0,71**	**5,67**	**0,60**	**0,11**	**100,00**

Sumber/*Source*: Susenas Maret 2018/*The March 2018 Susenas*

https://www.bps.go.id

Tabel 5.5. **Persentase Perempuan Berumur 15-49 Tahun Pernah Kawin yang Pernah Melahirkan dalam 2 Tahun Terakhir menurut Provinsi, Daerah Tempat Tinggal, dan Berat Badan dari Anak Lahir Hidup yang Terakhir Ketika Dilahirkan, 2018**
Table *Percentage of Ever Married Female Aged 15-49 Years Who Have Given Birth in the Last 2 Years by Province, Urban Rural Classification, and Weight of Last Live Birth Child, 2018*

Provinsi *Province*	Perkotaan/*Urban*				Perdesaan/*Rural*				Perkotaan+Perdesaan/*Urban+Rural*			
	< 2,5 kg	≥ 2,5 kg	Tidak Ditimbang *Not Weighted*	Tidak Tahu *Unknown*	< 2,5 kg	≥ 2,5 kg	Tidak Ditimbang *Not Weighted*	Tidak Tahu *Unknown*	< 2,5 kg	≥ 2,5 kg	Tidak Ditimbang *Not Weighted*	Tidak Tahu *Unknown*
(1)	(2)	(3)	(4)	(5)	(6)	(7)	(8)	(9)	(10)	(11)	(12)	(13)
Aceh	16,12	82,87	0,65	0,36	13,10	80,62	3,19	3,10	14,01	81,30	2,42	2,27
Sumatera Utara	10,75	87,82	0,59	0,85	10,88	77,16	8,89	3,07	10,81	82,53	4,71	1,95
Sumatera Barat	9,06	88,89	0,33	1,72	11,01	85,71	1,23	2,04	10,17	87,09	0,84	1,90
Riau	12,99	85,78	0,80	0,43	11,70	83,13	3,08	2,09	12,24	84,24	2,12	1,39
Jambi	10,85	80,82	4,54	3,78	9,78	82,81	4,48	2,94	10,10	82,21	4,50	3,19
Sumatera Selatan	11,01	86,90	0,72	1,37	13,44	81,40	2,91	2,25	12,63	83,23	2,18	1,96
Bengkulu	6,78	91,96	0,15	1,11	9,51	85,59	3,03	1,88	8,64	87,60	2,12	1,63
Lampung	10,08	87,80	0,79	1,34	10,19	86,92	1,86	1,02	10,16	87,17	1,56	1,11
Kepulauan Bangka Belitung	8,47	89,74	1,38	0,41	13,06	86,50	0,45	0,00	10,48	88,32	0,97	0,23
Kepulauan Riau	7,57	91,75	0,49	0,19	14,76	84,07	0,62	0,56	8,48	90,77	0,51	0,24
DKI Jakarta	12,64	85,80	0,56	1,00	-	-	-	-	12,64	85,80	0,56	1,00
Jawa Barat	14,43	83,37	1,07	1,13	14,94	80,97	2,81	1,28	14,56	82,76	1,51	1,17
Jawa Tengah	9,08	90,02	0,39	0,51	11,31	88,13	0,16	0,39	10,20	89,07	0,27	0,45
DI Yogyakarta	12,19	86,58	0,49	0,74	12,81	87,19	0,00	0,00	12,33	86,72	0,38	0,57
Jawa Timur	11,95	85,40	1,22	1,43	13,32	81,62	2,51	2,55	12,61	83,60	1,84	1,96
Banten	15,69	81,52	1,57	1,22	18,37	66,07	8,62	6,94	16,50	76,87	3,69	2,94
Bali	10,29	89,16	0,18	0,38	12,59	87,41	0,00	0,00	11,02	88,60	0,12	0,26
Nusa Tenggara Barat	18,46	79,61	0,79	1,14	14,86	79,70	3,59	1,84	16,44	79,66	2,36	1,54
Nusa Tenggara Timur	12,11	80,11	4,72	3,06	15,96	65,84	14,27	3,93	15,16	68,83	12,27	3,75
Kalimantan Barat	15,31	82,17	1,05	1,47	15,80	73,99	7,23	2,99	15,63	76,87	5,05	2,45
Kalimantan Tengah	10,15	86,17	1,08	2,59	11,99	79,70	5,99	2,33	11,28	82,17	4,12	2,43
Kalimantan Selatan	14,21	85,52	0,27	0,00	9,29	88,96	1,75	0,00	11,61	87,34	1,05	0,00
Kalimantan Timur	13,94	83,94	0,44	1,69	20,18	77,96	0,94	0,92	15,95	82,01	0,60	1,44
Kalimantan Utara	14,00	84,27	0,00	1,73	19,43	75,83	0,01	4,73	16,23	80,80	0,00	2,96
Sulawesi Utara	13,89	81,48	1,74	2,89	13,97	81,19	3,47	1,37	13,93	81,34	2,58	2,15
Sulawesi Tengah	11,66	80,70	2,59	5,05	16,26	73,06	6,54	4,14	15,10	75,00	5,54	4,37
Sulawesi Selatan	14,65	80,26	2,66	2,43	14,32	80,26	4,26	1,16	14,46	80,26	3,58	1,69
Sulawesi Tenggara	15,33	80,74	3,53	0,41	16,21	70,34	10,01	3,45	15,88	74,18	7,62	2,32
Gorontalo	11,14	88,12	0,00	0,74	14,29	79,14	4,90	1,67	13,02	82,77	2,92	1,29
Sulawesi Barat	27,00	73,00	0,00	0,00	15,15	72,49	9,09	3,28	17,75	72,60	7,09	2,56
Maluku	14,94	71,08	12,90	1,09	11,61	48,03	30,38	9,98	12,93	57,20	23,42	6,44
Maluku Utara	15,11	77,43	3,96	3,50	17,72	54,59	20,03	7,66	16,99	60,98	15,53	6,50
Papua Barat	6,11	87,80	1,70	4,39	15,30	62,69	14,63	7,38	11,39	73,38	9,13	6,10
Papua	22,66	66,71	3,22	7,40	8,47	33,80	34,92	22,81	12,51	43,16	25,90	18,42
**Indonesia**	**12,84**	**84,83**	**1,11**	**1,22**	**13,18**	**79,01**	**5,09**	**2,72**	**13,00**	**82,14**	**2,95**	**1,91**

Sumber/*Source*: Susenas Maret 2018/*The March 2018 Susenas*

https://www.bps.go.id

Tabel *Table* **5.6.** **Persentase Perempuan Berumur 15-49 Tahun yang Pernah Melahirkan dalam 2 Tahun Terakhir menurut Provinsi, Daerah Tempat Tinggal, dan Status Inisiasi Menyusui Dini (IMD), 2017***
*Percentage of Ever Birth Women Aged 15-49 Years in the Last 2 Years by Province, Urban Rural Classification, and Initiation of Breast Feeding Status, 2017**

**Provinsi** *Province*	**Perkotaan/***Urban*			**Perdesaan/***Rural*			**Perkotaan+Perdesaan/***Urban+Rural*		
	**Melakukan IMD** *Initiating Breast Feeding*	**Tidak Melakukan IMD** *Not Initiating Breast Feeding*	**Jumlah** *Total*	**Melakukan IMD** *Initiating Breast Feeding*	**Tidak Melakukan IMD** *Not Initiating Breast Feeding*	**Jumlah** *Total*	**Melakukan IMD** *Initiating Breast Feeding*	**Tidak Melakukan IMD** *Not Initiating Breast Feeding*	**Jumlah** *Total*
**(1)**	**(2)**	**(3)**	**(4)**	**(5)**	**(6)**	**(7)**	**(8)**	**(9)**	**(10)**
Aceh	66,56	33,44	100,00	66,10	33,90	100,00	66,23	33,77	100,00
Sumatera Utara	52,10	47,90	100,00	44,07	55,93	100,00	48,02	51,98	100,00
Sumatera Barat	66,86	33,14	100,00	61,96	38,04	100,00	63,87	36,13	100,00
Riau	70,09	29,91	100,00	53,52	46,48	100,00	59,61	40,39	100,00
Jambi	70,50	29,50	100,00	56,14	43,86	100,00	60,81	39,19	100,00
Sumatera Selatan	68,54	31,46	100,00	61,98	38,02	100,00	64,29	35,71	100,00
Bengkulu	66,09	33,91	100,00	65,89	34,11	100,00	65,96	34,04	100,00
Lampung	68,81	31,19	100,00	62,06	37,94	100,00	63,97	36,03	100,00
Kepulauan Bangka Belitung	66,92	33,08	100,00	61,00	39,00	100,00	64,20	35,80	100,00
Kepulauan Riau	70,85	29,15	100,00	75,68	24,32	100,00	71,59	28,41	100,00
DKI Jakarta	78,21	21,79	100,00	-	-	-	78,21	21,79	100,00
Jawa Barat	72,13	27,87	100,00	69,67	30,33	100,00	71,47	28,53	100,00
Jawa Tengah	76,33	23,67	100,00	79,57	20,43	100,00	78,00	22,00	100,00
DI Yogyakarta	78,33	21,67	100,00	79,31	20,69	100,00	78,61	21,39	100,00
Jawa Timur	69,39	30,61	100,00	71,94	28,06	100,00	70,58	29,42	100,00
Banten	70,73	29,27	100,00	58,21	41,79	100,00	66,74	33,26	100,00
Bali	64,10	35,90	100,00	70,26	29,74	100,00	66,13	33,87	100,00
Nusa Tenggara Barat	74,06	25,94	100,00	75,05	24,95	100,00	74,61	25,39	100,00
Nusa Tenggara Timur	68,90	31,10	100,00	67,06	32,94	100,00	67,41	32,59	100,00
Kalimantan Barat	58,95	41,05	100,00	50,86	49,14	100,00	53,38	46,62	100,00
Kalimantan Tengah	54,73	45,27	100,00	51,62	48,38	100,00	52,81	47,19	100,00
Kalimantan Selatan	66,29	33,71	100,00	61,45	38,55	100,00	63,56	36,44	100,00
Kalimantan Timur	73,82	26,18	100,00	61,81	38,19	100,00	70,01	29,99	100,00
Kalimantan Utara	61,18	38,82	100,00	68,35	31,65	100,00	64,13	35,87	100,00
Sulawesi Utara	56,18	43,82	100,00	48,64	51,36	100,00	52,05	47,95	100,00
Sulawesi Tengah	56,31	43,69	100,00	53,45	46,55	100,00	54,22	45,78	100,00
Sulawesi Selatan	70,08	29,92	100,00	63,55	36,45	100,00	66,01	33,99	100,00
Sulawesi Tenggara	64,33	35,67	100,00	48,57	51,43	100,00	53,73	46,27	100,00
Gorontalo	65,08	34,92	100,00	56,28	43,72	100,00	59,54	40,46	100,00
Sulawesi Barat	59,12	40,88	100,00	58,50	41,50	100,00	58,64	41,36	100,00
Maluku	45,14	54,86	100,00	44,26	55,74	100,00	44,58	55,42	100,00
Maluku Utara	80,14	19,86	100,00	45,94	54,06	100,00	55,47	44,53	100,00
Papua Barat	30,55	69,45	100,00	55,99	44,01	100,00	45,71	54,29	100,00
Papua	62,28	37,72	100,00	54,24	45,76	100,00	56,98	43,02	100,00
**Indonesia**	**70,02**	**29,98**	**100,00**	**64,05**	**35,95**	**100,00**	**67,21**	**32,79**	**100,00**

Catatan/*Note***:** *) Data 2018 belum tersedia/*2018 data not available yet*
Sumber/*Source*: Susenas Maret 2017/*The March 2017 Susenas*

https://www.bps.go.id

Tabel *Table* **5.7.** **Persentase Perempuan Berumur 15-49 Tahun yang Pernah Kawin menurut Provinsi, Daerah Tempat Tinggal, dan Status Penggunaan Alat KB atau Cara Tradisional untuk Menunda atau Mencegah Kehamilan, 2018**
Percentage of Ever Married Women Aged 15-49 Years by Province, Urban Rural Classification, and The Used of Contraception or Traditional Method to Prevent or Delay Pregnancy Status, 2018

Provinsi *Province*	Perkotaan/*Urban*			Perdesaan/*Rural*			Perkotaan+Perdesaan/*Urban+Rural*		
	Pernah *Ever Used*	Sedang *Currently Used*	Tidak Menggunakan *Never Used*	Pernah *Ever Used*	Sedang *Currently Used*	Tidak Menggunakan *Never Used*	Pernah *Ever Used*	Sedang *Currently Used*	Tidak Menggunakan *Never Used*
(1)	(2)	(3)	(4)	(5)	(6)	(7)	(8)	(9)	(10)
Aceh	18,23	42,25	39,52	15,93	44,02	40,05	16,63	43,48	39,89
Sumatera Utara	10,85	46,34	42,81	12,31	43,74	43,95	11,54	45,12	43,35
Sumatera Barat	13,95	40,61	45,44	16,82	46,71	36,48	15,59	44,11	40,30
Riau	13,77	42,81	43,42	12,71	56,80	30,49	13,12	51,35	35,53
Jambi	11,51	49,40	39,09	11,60	62,58	25,82	11,57	58,62	29,81
Sumatera Selatan	10,42	52,85	36,73	10,69	67,48	21,83	10,60	62,46	26,94
Bengkulu	16,30	53,92	29,78	12,02	65,80	22,18	13,32	62,20	24,48
Lampung	12,87	56,36	30,76	11,95	66,45	21,60	12,21	63,66	24,13
Kepulauan Bangka Belitung	13,94	57,05	29,01	10,33	67,21	22,45	12,24	61,83	25,93
Kepulauan Riau	10,40	43,29	46,30	17,01	60,00	22,99	11,32	45,62	43,06
DKI Jakarta	8,94	47,51	43,55	-	-	-	8,94	47,51	43,55
Jawa Barat	9,06	58,38	32,56	11,24	64,99	23,76	9,65	60,15	30,21
Jawa Tengah	12,86	53,40	33,74	12,68	62,67	24,64	12,77	58,10	29,13
DI Yogyakarta	10,37	50,02	39,62	12,40	62,78	24,81	10,94	53,59	35,48
Jawa Timur	10,76	56,18	33,06	12,62	62,12	25,26	11,68	59,11	29,22
Banten	10,59	56,51	32,89	14,37	61,79	23,83	11,69	58,04	30,27
Bali	9,38	55,49	35,13	10,11	67,44	22,45	9,62	59,48	30,90
Nusa Tenggara Barat	21,77	51,02	27,22	24,06	50,03	25,91	23,04	50,47	26,49
Nusa Tenggara Timur	12,53	43,54	43,92	18,80	39,76	41,44	17,46	40,57	41,97
Kalimantan Barat	13,53	52,97	33,51	12,77	64,68	22,55	13,01	60,99	26,00
Kalimantan Tengah	15,78	57,48	26,74	12,49	66,52	21,00	13,72	63,14	23,14
Kalimantan Selatan	19,66	59,32	21,02	18,84	65,61	15,55	19,21	62,80	17,99
Kalimantan Timur	10,30	51,16	38,54	10,84	60,68	28,48	10,48	54,36	35,15
Kalimantan Utara	17,51	42,52	39,97	16,51	54,08	29,41	17,09	47,37	35,54
Sulawesi Utara	14,63	53,25	32,12	9,37	66,05	24,58	11,94	59,80	28,26
Sulawesi Tengah	14,65	47,79	37,56	13,02	56,17	30,82	13,43	54,03	32,54
Sulawesi Selatan	18,90	41,54	39,56	17,14	46,81	36,06	17,83	44,72	37,44
Sulawesi Tenggara	17,67	51,74	30,59	22,04	47,81	30,15	20,49	49,21	30,31
Gorontalo	17,91	50,24	31,85	14,18	61,92	23,91	15,53	57,69	26,78
Sulawesi Barat	17,60	44,21	38,19	15,50	48,49	36,01	15,96	47,55	36,49
Maluku	14,61	40,79	44,60	12,91	37,95	49,14	13,59	39,09	47,32
Maluku Utara	16,60	42,57	40,83	13,99	52,13	33,88	14,71	49,47	35,82
Papua Barat	16,66	31,81	51,53	10,70	34,94	54,36	13,05	33,71	53,24
Papua	11,29	35,64	53,06	6,97	18,78	74,25	7,99	22,77	69,24
**Indonesia**	**11,42**	**53,25**	**35,33**	**13,31**	**58,10**	**28,59**	**12,30**	**55,50**	**32,20**

Sumber/*Source*: Susenas Maret 2018/*The March 2018 Susenas*

https://www.bps.go.id

Tabel *Table* **5.8.1.** **Persentase Perempuan Berumur 15-49 Tahun Pernah Kawin yang Tidak Menggunakan Alat KB atau Cara Tradisional untuk Menunda atau Mencegah Kehamilan di Daerah Perkotaan menurut Provinsi dan Alasan Utama Tidak Menggunakan Alat KB atau Cara Tradisional, 2017***
*Percentage of Ever Married Women Aged 15-49 Years Who Currently Not Using Contraception or Traditional Method to Prevent or Delay Pregnancy in Urban Area by Province and The Main Reason of Not Using Contraception or Traditional Method, 2017**

**Provinsi** *Province*	**Alasan Fertilitas** *Fetility Reason*	**Tidak Setuju KB** *Do Not Agree With Family Planning*	**Tidak Tahu Alat atau Cara KB** *Do Not Know About Family Planning Method*	**Takut Efek Samping** *Afraid of Side Effect*	**Lainnya** *Other*	**Tidak Tahu** *Unknown*	**Jumlah** *Total*
**(1)**	**(2)**	**(3)**	**(4)**	**(5)**	**(6)**	**(7)**	**(8)**
Aceh	32,08	7,72	0,00	21,53	36,74	1,93	100,00
Sumatera Utara	23,01	5,37	0,03	25,06	43,12	3,41	100,00
Sumatera Barat	25,51	3,70	0,00	28,92	41,18	0,69	100,00
Riau	22,06	3,64	0,00	30,63	40,96	2,71	100,00
Jambi	18,78	1,52	0,00	18,93	56,72	4,06	100,00
Sumatera Selatan	30,20	3,62	0,33	19,18	44,44	2,22	100,00
Bengkulu	33,14	2,04	0,16	16,90	45,66	2,10	100,00
Lampung	34,57	1,82	0,10	13,70	47,59	2,21	100,00
Kepulauan Bangka Belitung	33,17	1,45	0,00	19,63	42,19	3,56	100,00
Kepulauan Riau	34,13	4,21	0,09	15,57	42,86	3,13	100,00
DKI Jakarta	18,66	2,74	0,09	17,63	54,71	6,17	100,00
Jawa Barat	26,96	1,91	0,12	13,38	53,67	3,97	100,00
Jawa Tengah	33,43	2,24	0,11	18,29	44,22	1,71	100,00
DI Yogyakarta	27,05	0,98	0,00	21,79	49,12	1,06	100,00
Jawa Timur	27,36	2,15	0,00	15,44	50,88	4,17	100,00
Banten	24,67	3,60	0,29	16,08	54,02	1,35	100,00
Bali	29,51	0,65	0,06	26,10	42,92	0,77	100,00
Nusa Tenggara Barat	31,13	0,78	0,17	6,54	61,26	0,11	100,00
Nusa Tenggara Timur	29,48	3,55	0,12	31,19	34,03	1,63	100,00
Kalimantan Barat	25,54	2,47	0,08	19,66	50,35	1,90	100,00
Kalimantan Tengah	39,66	0,65	0,00	12,39	44,00	3,30	100,00
Kalimantan Selatan	34,97	1,84	0,00	10,13	50,36	2,70	100,00
Kalimantan Timur	34,19	2,42	0,00	17,96	41,82	3,61	100,00
Kalimantan Utara	27,85	6,09	0,27	21,46	41,07	3,25	100,00
Sulawesi Utara	31,94	1,26	0,32	19,89	42,90	3,70	100,00
Sulawesi Tengah	36,04	1,50	0,00	26,25	35,14	1,07	100,00
Sulawesi Selatan	33,83	2,78	0,16	21,09	37,64	4,50	100,00
Sulawesi Tenggara	36,22	6,27	0,06	15,86	39,94	1,66	100,00
Gorontalo	33,98	2,47	0,00	16,79	43,03	3,72	100,00
Sulawesi Barat	29,76	2,52	0,00	32,79	33,42	1,51	100,00
Maluku	30,13	7,74	0,19	24,32	34,44	3,17	100,00
Maluku Utara	38,14	4,61	0,00	15,93	38,04	3,28	100,00
Papua Barat	21,45	7,88	0,57	34,91	29,64	5,54	100,00
Papua	28,91	6,67	0,52	17,70	37,67	8,53	100,00
**Indonesia**	**27,78**	**2,69**	**0,10**	**17,60**	**48,55**	**3,27**	**100,00**

Catatan/*Note***:** *) Data 2018 belum tersedia/*2018 data not available yet*
Sumber/*Source*: Susenas Maret 2017/*The March 2017 Susenas*

https://www.bps.go.id

Tabel *Table* **5.8.2.** **Persentase Perempuan Berumur 15-49 Tahun Pernah Kawin yang Tidak Menggunakan Alat KB atau Cara Tradisional untuk Menunda atau Mencegah Kehamilan di Daerah Perdesaan menurut Provinsi dan Alasan Utama Tidak Menggunakan Alat KB atau Cara Tradisional, 2017***

*Percentage of Ever Married Women Aged 15-49 Years Who Currently Not Using Contraception or Traditional Method to Prevent or Delay Pregnancy in Rural Area by Province and The Main Reason of Not Using Contraception or Traditional Method, 2017**

Provinsi *Province*	Alasan Fertilitas *Fetility Reason*	Tidak Setuju KB *Do Not Agree With Family Planning*	Tidak Tahu Alat atau Cara KB *Do Not Know About Family Planning Method*	Takut Efek Samping *Afraid of Side Effect*	Lainnya *Other*	Tidak Tahu *Unknown*	Jumlah *Total*
(1)	(2)	(3)	(4)	(5)	(6)	(7)	(8)
Aceh	25,61	7,05	0,32	20,29	43,40	3,33	100,00
Sumatera Utara	28,03	6,75	0,30	27,77	35,18	1,97	100,00
Sumatera Barat	28,05	5,35	0,18	27,91	37,98	0,53	100,00
Riau	27,00	4,38	0,27	20,17	43,32	4,86	100,00
Jambi	28,44	2,22	0,74	11,99	51,15	5,46	100,00
Sumatera Selatan	42,40	0,88	0,07	13,58	40,41	2,66	100,00
Bengkulu	32,31	1,52	0,11	15,32	48,90	1,84	100,00
Lampung	34,10	1,71	0,00	13,15	47,52	3,51	100,00
Kepulauan Bangka Belitung	35,01	3,30	0,43	10,84	47,78	2,64	100,00
Kepulauan Riau	39,55	4,31	0,35	15,50	35,84	4,45	100,00
DKI Jakarta	-	-	-	-	-	-	-
Jawa Barat	29,05	1,27	0,14	9,56	53,94	6,04	100,00
Jawa Tengah	34,15	1,62	0,06	12,25	49,30	2,62	100,00
DI Yogyakarta	26,66	1,47	0,00	20,93	49,33	1,62	100,00
Jawa Timur	32,39	1,94	0,15	11,68	50,30	3,54	100,00
Banten	32,45	2,79	1,00	12,19	49,86	1,71	100,00
Bali	28,10	0,41	0,00	19,75	49,14	2,59	100,00
Nusa Tenggara Barat	34,34	1,10	0,09	5,52	58,51	0,44	100,00
Nusa Tenggara Timur	33,41	4,22	0,26	26,18	34,41	1,52	100,00
Kalimantan Barat	36,26	1,14	0,24	12,01	46,43	3,92	100,00
Kalimantan Tengah	33,50	2,17	0,41	11,21	47,72	4,99	100,00
Kalimantan Selatan	42,99	1,24	0,08	9,34	45,07	1,28	100,00
Kalimantan Timur	33,45	2,39	0,00	13,44	46,59	4,13	100,00
Kalimantan Utara	36,51	5,85	0,00	17,62	36,68	3,33	100,00
Sulawesi Utara	35,36	2,97	0,00	16,42	40,34	4,91	100,00
Sulawesi Tengah	45,84	2,87	0,11	13,28	34,92	2,97	100,00
Sulawesi Selatan	33,68	3,66	0,37	17,11	41,56	3,61	100,00
Sulawesi Tenggara	40,16	2,75	0,42	18,32	37,20	1,13	100,00
Gorontalo	38,47	1,69	0,00	12,85	44,39	2,59	100,00
Sulawesi Barat	28,04	3,40	0,13	21,36	44,49	2,57	100,00
Maluku	20,32	9,91	0,42	25,26	34,46	9,63	100,00
Maluku Utara	37,17	6,41	0,54	13,23	37,51	5,13	100,00
Papua Barat	33,58	7,32	1,77	16,29	32,75	8,29	100,00
Papua	18,94	18,18	12,02	13,97	22,81	14,07	100,00
**Indonesia**	**31,95**	**3,45**	**0,67**	**15,18**	**45,02**	**3,73**	**100,00**

Catatan/*Note***:** *) Data 2018 belum tersedia/*2018 data not available yet*
Sumber/*Source*: Susenas Maret 2017/*The March 2017 Susenas*

https://www.bps.go.id

Tabel *Table* **5.8.3.** **Persentase Perempuan Berumur 15-49 Tahun Pernah Kawin yang Tidak Menggunakan Alat KB atau Cara Tradisional untuk Menunda atau Mencegah Kehamilan di Daerah Perkotaan dan Perdesaan menurut Provinsi dan Alasan Utama Tidak Menggunakan Alat KB atau Cara Tradisional, 2017***
*Percentage of Ever Married Women Aged 15-49 Years Who Currently Not Using Contraception or Traditional Method to Prevent or Delay Pregnancy in Urban and Rural Area by Province and The Main Reason of Not Using Contraception or Traditional Method, 2017**

**Provinsi** *Province*	**Alasan Fertilitas** *Fetility Reason*	**Tidak Setuju KB** *Do Not Agree With Family Planning*	**Tidak Tahu Alat atau Cara KB** *Do Not Know About Family Planning Method*	**Takut Efek Samping** *Afraid of Side Effect*	**Lainnya** *Other*	**Tidak Tahu** *Unknown*	**Jumlah** *Total*
**(1)**	**(2)**	**(3)**	**(4)**	**(5)**	**(6)**	**(7)**	**(8)**
Aceh	27,56	7,25	0,23	20,66	41,40	2,91	100,00
Sumatera Utara	25,39	6,02	0,16	26,34	39,36	2,73	100,00
Sumatera Barat	26,93	4,62	0,10	28,35	39,39	0,60	100,00
Riau	24,82	4,06	0,15	24,78	42,28	3,91	100,00
Jambi	25,07	1,98	0,48	14,41	53,09	4,97	100,00
Sumatera Selatan	37,15	2,06	0,18	15,99	42,14	2,47	100,00
Bengkulu	32,63	1,72	0,13	15,92	47,65	1,94	100,00
Lampung	34,25	1,75	0,03	13,32	47,55	3,11	100,00
Kepulauan Bangka Belitung	33,94	2,23	0,18	15,94	44,53	3,17	100,00
Kepulauan Riau	34,67	4,22	0,12	15,56	42,17	3,26	100,00
DKI Jakarta	18,66	2,74	0,09	17,63	54,71	6,17	100,00
Jawa Barat	27,51	1,74	0,13	12,36	53,74	4,52	100,00
Jawa Tengah	33,77	1,95	0,09	15,41	46,64	2,14	100,00
DI Yogyakarta	26,95	1,11	0,00	21,56	49,17	1,21	100,00
Jawa Timur	29,70	2,05	0,07	13,69	50,61	3,88	100,00
Banten	26,72	3,38	0,47	15,05	52,92	1,44	100,00
Bali	29,10	0,58	0,04	24,26	44,72	1,30	100,00
Nusa Tenggara Barat	32,95	0,96	0,12	5,96	59,71	0,30	100,00
Nusa Tenggara Timur	32,61	4,09	0,23	27,19	34,34	1,54	100,00
Kalimantan Barat	32,25	1,64	0,18	14,87	47,89	3,17	100,00
Kalimantan Tengah	35,95	1,56	0,25	11,68	46,24	4,32	100,00
Kalimantan Selatan	39,26	1,52	0,04	9,71	47,53	1,94	100,00
Kalimantan Timur	33,98	2,41	0,00	16,67	43,18	3,76	100,00
Kalimantan Utara	31,37	6,00	0,16	19,90	39,29	3,28	100,00
Sulawesi Utara	33,63	2,10	0,16	18,18	41,63	4,30	100,00
Sulawesi Tengah	42,86	2,45	0,08	17,23	34,98	2,39	100,00
Sulawesi Selatan	33,74	3,30	0,28	18,76	39,94	3,98	100,00
Sulawesi Tenggara	38,83	3,94	0,30	17,49	38,13	1,31	100,00
Gorontalo	36,59	2,02	0,00	14,50	43,82	3,06	100,00
Sulawesi Barat	28,42	3,20	0,10	23,88	42,05	2,34	100,00
Maluku	24,15	9,06	0,33	24,90	34,45	7,11	100,00
Maluku Utara	37,47	5,85	0,38	14,06	37,67	4,56	100,00
Papua Barat	28,90	7,53	1,31	23,48	31,55	7,23	100,00
Papua	21,11	15,68	9,52	14,78	26,04	12,87	100,00
**Indonesia**	**29,65**	**3,03**	**0,36**	**16,52**	**46,97**	**3,48**	**100,00**

Catatan/*Note*: *) Data 2018 belum tersedia/*2018 data not available yet*
Sumber/*Source*: Susenas Maret 2017/*The March 2017 Susenas*

https://www.bps.go.id

Tabel 5.9.1. **Persentase Perempuan Berumur 15-49 Tahun yang Pernah Kawin di Daerah Perkotaan menurut Provinsi dan Alat KB atau Cara Tradisional yang Sedang Digunakan, 2018**

Table 5.9.1. *Percentage of Ever Married Female Aged 15-49 Years in Urban Area by Province and The Type of Contraception or Traditional Method Currently Used, 2018*

**Provinsi** *Province*	**Sterilisasi Wanita/ Tubektomi/MOW** *Tubectomy*	**Sterilisasi Pria/ Vasektomi/ MOP** *Vasectomy*	**IUD/ AKDR/ Spiral** *Spiral*	**Suntikan** *Injection*	**Susuk KB/ Implan** *Implants*	**Pil** *Pil*	**Kondom Pria/ Karet KB** *Male Condom*	**Intravag/ Kondom Wanita/ Diafragma** *Female Condom*	**Metode Menyusui Alami** *Lactational Amenorrhoea Method*	**Pantang Berkala/ Kalender** *Period Abstinence/ Rythm*	**Lainnya** *Other*	**Jumlah** *Total*
(1)	(2)	(3)	(4)	(5)	(6)	(7)	(8)	(9)	(10)	(11)	(12)	(13)
Aceh	2,96	0,08	7,41	48,12	2,48	23,33	10,28	0,00	0,26	4,73	0,34	100,00
Sumatera Utara	7,55	0,29	3,99	38,00	8,08	17,14	18,74	0,00	0,64	4,46	1,12	100,00
Sumatera Barat	8,06	0,75	13,37	39,00	8,03	13,76	11,60	0,03	0,58	4,67	0,14	100,00
Riau	5,86	0,74	8,35	41,81	2,78	20,19	12,64	0,11	1,24	4,61	1,68	100,00
Jambi	1,58	0,34	6,43	55,14	5,38	28,34	1,17	0,03	0,20	1,26	0,11	100,00
Sumatera Selatan	2,20	0,48	4,96	53,08	7,84	18,14	8,00	0,16	0,14	4,79	0,21	100,00
Bengkulu	4,29	1,27	10,59	51,96	9,45	12,68	4,56	0,64	0,83	3,37	0,36	100,00
Lampung	1,33	0,32	6,14	53,62	8,18	18,98	7,30	0,03	0,12	3,66	0,32	100,00
Kepulauan Bangka Belitung	4,46	0,48	5,44	50,79	4,10	28,90	2,11	0,40	0,15	2,62	0,53	100,00
Kepulauan Riau	4,86	0,11	9,11	33,40	3,95	24,02	17,47	0,00	1,04	5,96	0,09	100,00
DKI Jakarta	4,70	0,70	16,90	45,03	3,60	15,74	11,73	0,07	0,04	1,33	0,16	100,00
Jawa Barat	3,14	0,54	10,89	50,61	3,44	19,27	10,78	0,09	0,18	0,91	0,15	100,00
Jawa Tengah	6,73	0,50	11,07	48,15	7,59	12,90	5,73	0,06	0,11	6,92	0,25	100,00
DI Yogyakarta	6,19	0,40	28,65	27,18	2,49	9,58	17,07	0,00	0,27	8,16	0,00	100,00
Jawa Timur	5,16	0,45	10,91	47,18	5,68	22,44	5,18	0,07	0,15	2,45	0,32	100,00
Banten	2,23	0,40	8,34	53,91	4,16	16,70	13,44	0,01	0,00	0,69	0,12	100,00
Bali	7,13	0,77	23,55	35,09	2,59	12,85	10,29	0,00	1,42	5,08	1,24	100,00
Nusa Tenggara Barat	3,58	0,19	11,20	53,02	12,49	10,02	8,42	0,00	0,27	0,76	0,06	100,00
Nusa Tenggara Timur	7,03	0,00	10,69	24,91	11,62	10,01	10,62	0,00	0,39	24,61	0,12	100,00
Kalimantan Barat	4,44	1,16	8,83	45,25	1,91	28,18	7,75	0,05	0,19	1,98	0,27	100,00
Kalimantan Tengah	1,19	0,00	4,44	55,79	3,37	28,92	4,56	0,07	0,21	1,24	0,19	100,00
Kalimantan Selatan	2,42	0,33	3,71	39,31	4,41	40,65	2,89	0,23	0,15	5,83	0,06	100,00
Kalimantan Timur	4,09	0,44	9,72	34,24	3,97	29,98	14,77	0,19	0,26	2,17	0,17	100,00
Kalimantan Utara	2,86	0,00	9,69	48,87	4,53	25,79	5,76	0,00	0,88	1,63	0,00	100,00
Sulawesi Utara	3,81	0,80	7,38	46,38	12,10	21,30	5,38	0,07	0,00	2,38	0,40	100,00
Sulawesi Tengah	3,31	0,00	8,54	37,88	9,06	26,70	10,39	0,00	0,37	3,34	0,42	100,00
Sulawesi Selatan	2,20	0,17	7,13	45,08	6,90	20,31	14,70	0,32	0,31	2,63	0,24	100,00
Sulawesi Tenggara	1,32	0,00	5,19	32,63	10,57	28,94	4,75	0,00	0,26	15,82	0,53	100,00
Gorontalo	3,95	0,67	9,22	36,93	20,51	26,35	0,17	0,00	0,00	2,21	0,00	100,00
Sulawesi Barat	0,50	0,00	5,32	40,09	6,04	28,83	15,76	0,00	0,00	1,76	1,71	100,00
Maluku	1,15	0,00	1,75	47,88	9,87	16,04	7,61	0,06	0,91	13,72	1,01	100,00
Maluku Utara	1,46	0,50	5,75	52,67	15,26	11,36	12,06	0,00	0,00	0,21	0,72	100,00
Papua Barat	3,07	0,00	1,42	63,10	5,05	23,13	0,00	0,00	1,09	2,89	0,24	100,00
Papua	3,95	0,28	6,02	47,83	11,60	14,70	9,17	0,00	2,73	2,25	1,47	100,00
**Indonesia**	**4,30**	**0,48**	**10,64**	**47,15**	**5,29**	**18,96**	**9,57**	**0,08**	**0,24**	**3,01**	**0,29**	**100,00**

Sumber/*Source*: Susenas Maret 2018/*The March 2018 Susenas*

https://www.bps.go.id

Tabel 5.9.2. **Persentase Perempuan Berumur 15-49 Tahun yang Pernah Kawin di Daerah Perdesaan menurut Provinsi dan Alat KB atau Cara Tradisional yang Sedang Digunakan, 2018**
Table 5.9.2. *Percentage of Ever Married Female Aged 15-49 Years in Rural Area by Province and The Type of Contraception or Traditional Method Currently Used, 2018*

**Provinsi** *Province*	**Sterilisasi Wanita/ Tubektomi/MOW** *Tubectomy*	**Sterilisasi Pria/ Vasektomi/MOP** *Vasectomy*	**IUD/ AKDR/ Spiral** *Spiral*	**Suntikan** *Injection*	**Susuk KB/ Implan** *Implants*	**Pil** *Pil*	**Kondom Pria/Karet KB** *Male Condom*	**Intravag/ Kondom Wanita/ Diafragma** *Female Condom*	**Metode Menyusui Alami** *Lactational Amenorrhoea Method*	**Pantang Berkala/ Kalender** *Period Abstinence/ Rythm*	**Lainnya** *Other*	**Jumlah** *Total*
(1)	(2)	(3)	(4)	(5)	(6)	(7)	(8)	(9)	(10)	(11)	(12)	(13)
Aceh	1,03	0,41	3,05	66,29	3,61	17,42	5,83	0,09	0,00	2,04	0,22	100,00
Sumatera Utara	6,49	0,24	3,18	44,87	13,19	18,28	9,30	0,13	0,80	1,75	1,79	100,00
Sumatera Barat	4,06	0,70	5,83	57,56	13,95	13,89	2,16	0,17	0,28	1,12	0,28	100,00
Riau	2,00	0,30	2,54	54,08	6,84	25,11	7,29	0,15	0,10	1,24	0,35	100,00
Jambi	0,96	0,47	2,26	62,74	9,47	23,04	0,28	0,00	0,04	0,65	0,09	100,00
Sumatera Selatan	1,01	0,43	1,12	68,88	14,94	11,13	1,25	0,03	0,18	0,96	0,07	100,00
Bengkulu	1,59	0,79	2,49	61,29	16,13	15,76	0,82	0,24	0,20	0,54	0,15	100,00
Lampung	1,24	0,46	2,92	60,29	13,04	16,43	4,10	0,00	0,11	1,12	0,29	100,00
Kepulauan Bangka Belitung	0,79	0,09	2,86	55,63	7,67	31,29	0,60	0,06	0,00	0,80	0,19	100,00
Kepulauan Riau	2,40	0,25	0,68	55,71	9,36	25,79	3,05	0,00	0,00	2,28	0,48	100,00
DKI Jakarta	0,00	0,00	0,00	0,00	0,00	0,00	0,00	0,00	0,00	0,00	0,00	-
Jawa Barat	2,01	0,44	4,46	55,47	5,17	23,30	9,03	0,04	0,00	0,03	0,06	100,00
Jawa Tengah	4,31	0,85	6,69	56,06	11,71	12,42	3,49	0,02	0,01	4,38	0,05	100,00
DI Yogyakarta	4,53	0,53	19,70	36,05	14,34	13,00	7,21	0,00	0,18	3,78	0,69	100,00
Jawa Timur	3,01	0,51	5,49	59,40	8,16	19,20	3,19	0,07	0,07	0,70	0,20	100,00
Banten	0,93	0,00	1,26	70,91	8,40	12,39	6,12	0,00	0,00	0,00	0,00	100,00
Bali	4,42	0,85	25,20	48,71	3,64	11,26	3,56	0,12	0,00	1,70	0,52	100,00
Nusa Tenggara Barat	2,52	0,31	5,35	64,45	14,67	7,43	4,21	0,00	0,13	0,59	0,34	100,00
Nusa Tenggara Timur	4,33	0,01	5,78	51,54	21,37	8,33	1,40	0,04	0,11	6,57	0,53	100,00
Kalimantan Barat	1,86	0,43	2,00	61,13	4,51	28,20	1,15	0,00	0,24	0,34	0,15	100,00
Kalimantan Tengah	0,56	0,15	1,10	63,53	6,02	26,31	1,73	0,04	0,11	0,39	0,05	100,00
Kalimantan Selatan	1,19	0,24	0,86	48,99	6,74	40,61	0,48	0,00	0,00	0,76	0,13	100,00
Kalimantan Timur	1,93	0,05	6,01	44,93	4,99	32,99	7,25	0,35	0,37	0,98	0,16	100,00
Kalimantan Utara	3,68	0,00	3,07	45,72	9,20	30,66	4,46	0,00	0,63	2,57	0,00	100,00
Sulawesi Utara	1,99	0,64	4,27	44,86	22,15	21,31	3,00	0,04	0,10	1,41	0,24	100,00
Sulawesi Tengah	1,69	0,22	3,85	42,59	10,94	34,72	4,50	0,00	0,25	0,77	0,46	100,00
Sulawesi Selatan	0,88	0,14	3,06	57,18	12,56	21,50	2,97	0,00	0,24	1,02	0,45	100,00
Sulawesi Tenggara	1,56	0,55	1,30	50,57	15,59	24,95	1,07	0,00	0,11	3,85	0,46	100,00
Gorontalo	1,18	0,70	5,23	45,40	24,84	22,35	0,03	0,00	0,00	0,16	0,12	100,00
Sulawesi Barat	1,10	0,18	1,85	45,87	15,21	30,65	3,96	0,08	0,10	0,49	0,51	100,00
Maluku	0,46	0,15	0,66	60,88	12,20	11,83	8,16	0,00	0,12	4,62	0,92	100,00
Maluku Utara	0,98	0,18	1,27	63,09	19,12	7,75	7,03	0,13	0,14	0,31	0,00	100,00
Papua Barat	2,07	0,36	4,45	61,83	11,69	18,12	0,65	0,07	0,07	0,15	0,54	100,00
Papua	1,32	0,00	1,40	35,03	5,61	9,93	11,44	0,00	8,53	1,19	25,55	100,00
**Indonesia**	**2,53**	**0,46**	**4,48**	**57,19**	**10,10**	**18,84**	**4,27**	**0,05**	**0,17**	**1,50**	**0,42**	**100,00**

Sumber/*Source*: Susenas Maret 2018/*The March 2018 Susenas*

https://www.bps.go.id

Tabel *Table* **5.9.3.** **Persentase Perempuan Berumur 15-49 Tahun yang Pernah Kawin di Daerah Perkotaan dan Perdesaan menurut Provinsi dan Alat KB atau Cara Tradisional yang Sedang Digunakan, 2018**
Percentage of Married Female Aged 15-49 Years in Urban and Rural Area by Province and The Type of Contraception or Traditional Method Currently Used, 2018

**Provinsi** *Province*	**Sterilisasi Wanita/ Tubektomi/MOW** *Tubectomy*	**Sterilisasi Pria/ Vasektomi/ MOP** *Vasectomy*	**IUD/ AKDR/ Spiral** *Spiral*	**Suntikan** *Injection*	**Susuk KB/ Implan** *Implants*	**Pil** *Pil*	**Kondom Pria/Karet KB** *Male Condom*	**Intravag/ Kondom Wanita/ Diafragma** *Female Condom*	**Metode Menyusui Alami** *Lactational Amenorrhoea Method*	**Pantang Berkala/ Kalender** *Period Abstinence/ Rythm*	**Lainnya** *Other*	**Jumlah** *Total*
(1)	(2)	(3)	(4)	(5)	(6)	(7)	(8)	(9)	(10)	(11)	(12)	(13)
Aceh	1,60	0,31	4,34	60,91	3,27	19,17	7,15	0,06	0,08	2,84	0,26	100,00
Sumatera Utara	7,07	0,27	3,62	41,13	10,41	17,66	14,43	0,06	0,71	3,23	1,42	100,00
Sumatera Barat	5,63	0,72	8,79	50,28	11,63	13,84	5,86	0,12	0,40	2,51	0,23	100,00
Riau	3,25	0,44	4,43	50,09	5,52	23,51	9,03	0,14	0,47	2,33	0,78	100,00
Jambi	1,12	0,44	3,32	60,81	8,43	24,38	0,51	0,01	0,08	0,80	0,10	100,00
Sumatera Selatan	1,36	0,44	2,23	64,29	12,88	13,17	3,21	0,07	0,17	2,07	0,11	100,00
Bengkulu	2,30	0,92	4,62	58,84	14,38	14,95	1,80	0,34	0,36	1,29	0,21	100,00
Lampung	1,26	0,43	3,71	58,65	11,85	17,05	4,88	0,01	0,12	1,75	0,30	100,00
Kepulauan Bangka Belitung	2,59	0,28	4,13	53,26	5,93	30,12	1,34	0,23	0,08	1,69	0,36	100,00
Kepulauan Riau	4,41	0,13	7,57	37,49	4,94	24,34	14,83	0,00	0,85	5,28	0,16	100,00
DKI Jakarta	4,70	0,70	16,90	45,03	3,60	15,74	11,73	0,07	0,04	1,33	0,16	100,00
Jawa Barat	2,81	0,51	9,03	52,02	3,94	20,43	10,27	0,08	0,13	0,65	0,12	100,00
Jawa Tengah	5,41	0,69	8,68	52,47	9,84	12,64	4,51	0,04	0,05	5,53	0,14	100,00
DI Yogyakarta	5,64	0,44	25,72	30,09	6,37	10,70	13,84	0,00	0,24	6,73	0,23	100,00
Jawa Timur	4,05	0,48	8,11	53,51	6,96	20,76	4,15	0,07	0,11	1,54	0,25	100,00
Banten	1,83	0,28	6,16	59,15	5,47	15,37	11,18	0,01	0,00	0,48	0,08	100,00
Bali	6,10	0,80	24,17	40,25	2,99	12,25	7,74	0,04	0,88	3,80	0,97	100,00
Nusa Tenggara Barat	3,00	0,25	7,97	59,32	13,69	8,59	6,10	0,00	0,19	0,66	0,21	100,00
Nusa Tenggara Timur	4,96	0,01	6,91	45,40	19,12	8,71	3,53	0,03	0,17	10,73	0,43	100,00
Kalimantan Barat	2,57	0,63	3,86	56,78	3,80	28,20	2,95	0,01	0,22	0,79	0,18	100,00
Kalimantan Tengah	0,78	0,10	2,24	60,90	5,12	27,19	2,70	0,05	0,14	0,68	0,10	100,00
Kalimantan Selatan	1,71	0,28	2,06	44,91	5,76	40,63	1,50	0,10	0,07	2,90	0,10	100,00
Kalimantan Timur	3,28	0,29	8,33	38,26	4,35	31,11	11,94	0,25	0,30	1,72	0,16	100,00
Kalimantan Utara	3,25	0,00	6,52	47,36	6,76	28,12	5,14	0,00	0,76	2,08	0,00	100,00
Sulawesi Utara	2,78	0,71	5,62	45,52	17,78	21,30	4,03	0,05	0,06	1,83	0,31	100,00
Sulawesi Tengah	2,06	0,17	4,91	41,53	10,51	32,91	5,83	0,00	0,28	1,35	0,45	100,00
Sulawesi Selatan	1,37	0,15	4,56	52,73	10,48	21,06	7,28	0,12	0,27	1,61	0,37	100,00
Sulawesi Tenggara	1,47	0,34	2,75	43,86	13,71	26,44	2,45	0,00	0,16	8,32	0,49	100,00
Gorontalo	2,05	0,69	6,49	42,72	23,47	23,61	0,08	0,00	0,00	0,81	0,08	100,00
Sulawesi Barat	0,97	0,14	2,56	44,69	13,34	30,28	6,37	0,07	0,08	0,75	0,75	100,00
Maluku	0,75	0,09	1,11	55,46	11,23	13,58	7,93	0,03	0,45	8,41	0,96	100,00
Maluku Utara	1,10	0,26	2,34	60,60	18,20	8,61	8,24	0,10	0,10	0,29	0,17	100,00
Papua Barat	2,45	0,23	3,32	62,30	9,21	19,99	0,41	0,04	0,45	1,17	0,43	100,00
Papua	2,29	0,10	3,11	39,77	7,83	11,69	10,60	0,00	6,39	1,58	16,64	100,00
**Indonesia**	**3,44**	**0,47**	**7,64**	**52,03**	**7,63**	**18,90**	**6,99**	**0,06**	**0,21**	**2,27**	**0,36**	**100,00**

Sumber/*Source*: Susenas Maret 2018/*The March 2018 Susenas*

https://www.bps.go.id

Tabel *Table* **5.10.1.** **Persentase Perempuan Berumur 15-49 Tahun Pernah Kawin yang Menggunakan Alat KB Modern di Daerah Perkotaan menurut Provinsi dan Tempat Memperoleh Alat KB Modern yang Terakhir Kali, 2017***

*Percentage of Married Female Aged 15-49 Years Using Moden Contraception in Urban Area by Province and Place Obtains The Last Modern Contraception, 2017**

**Provinsi** *Province*	**Rumah Sakit** *Hospital*	**Puskesmas/ Pustu/Klinik** *PHC/Auxiliary PHC/Clinic*	**TKBK/ TMK/ Muyan**	**Polindes/ Poskesdes** *Village Maternity/ Health Post*	**Posyandu/Pos KB/ PPKBD** *Posyandu/Family Planning Post/PPKBD*	**Rumah Bersalin** *Maternity Hospital*	**Praktik Dokter Umum/Kandungan** *General Practitioner/ Obstetricians*	**Praktik Bidan/Bidan di Desa/Perawat** *Midwife/Nurse Service*	**Apotek/Obat Toko** *Pharmacies/Nurse Service*	**Lainnya** *Other*	**Jumlah** *Total*
**(1)**	**(2)**	**(3)**	**(4)**	**(5)**	**(6)**	**(7)**	**(8)**	**(9)**	**(10)**	**(12)**	**(13)**
Aceh	6,87	24,24	0,41	4,40	0,90	1,23	4,68	43,33	13,43	0,51	100,00
Sumatera Utara	13,48	16,87	0,25	1,44	2,32	1,61	4,56	43,84	15,44	0,20	100,00
Sumatera Barat	12,66	20,89	0,00	3,98	1,57	4,21	6,47	39,24	10,82	0,17	100,00
Riau	11,24	20,37	0,00	0,83	0,47	6,78	4,22	39,74	15,92	0,44	100,00
Jambi	6,96	20,56	0,48	0,52	2,22	3,67	6,33	36,24	22,77	0,25	100,00
Sumatera Selatan	7,10	14,29	0,04	0,88	0,75	1,73	8,24	56,39	10,37	0,21	100,00
Bengkulu	6,94	17,69	0,98	1,05	1,18	0,95	5,91	52,52	12,52	0,26	100,00
Lampung	4,73	13,63	0,09	1,09	1,12	3,53	8,43	56,89	9,76	0,72	100,00
Kepulauan Bangka Belitung	4,75	8,34	0,02	6,28	0,99	1,20	10,55	47,00	20,41	0,46	100,00
Kepulauan Riau	10,77	21,33	0,16	1,90	1,45	2,04	5,70	42,23	14,20	0,21	100,00
DKI Jakarta	16,01	26,02	0,00	0,31	0,65	4,16	6,55	31,92	14,06	0,33	100,00
Jawa Barat	7,29	14,06	0,09	0,83	2,04	1,53	8,32	49,24	15,25	1,34	100,00
Jawa Tengah	12,93	13,25	0,22	1,68	1,16	1,90	6,84	51,32	10,45	0,26	100,00
DI Yogyakarta	20,54	20,19	0,00	0,32	1,74	2,13	6,83	27,71	20,47	0,07	100,00
Jawa Timur	9,80	12,71	0,07	2,44	1,05	1,92	7,28	45,61	18,38	0,74	100,00
Banten	8,05	16,71	0,00	0,21	1,30	2,50	10,03	50,41	10,58	0,20	100,00
Bali	14,93	13,30	0,09	0,06	0,56	1,32	12,79	47,54	9,31	0,12	100,00
Nusa Tenggara Barat	5,58	20,36	0,04	23,45	2,99	0,31	6,15	38,11	1,84	1,17	100,00
Nusa Tenggara Timur	20,73	48,70	0,00	3,18	3,61	0,81	3,28	12,99	6,70	0,00	100,00
Kalimantan Barat	8,30	16,84	0,18	2,14	1,03	1,71	6,55	44,79	18,33	0,14	100,00
Kalimantan Tengah	3,39	19,69	0,00	1,92	0,46	1,72	5,07	42,44	24,53	0,79	100,00
Kalimantan Selatan	4,14	10,87	0,30	1,56	1,64	0,30	5,89	43,08	30,96	1,26	100,00
Kalimantan Timur	9,18	17,63	0,00	0,87	0,59	1,50	6,29	35,07	28,86	0,01	100,00
Kalimantan Utara	6,61	22,29	0,00	0,49	1,19	1,39	12,30	27,83	27,56	0,36	100,00
Sulawesi Utara	9,49	27,93	0,45	1,23	1,39	0,42	13,66	25,21	19,34	0,88	100,00
Sulawesi Tengah	13,73	26,95	0,00	2,88	0,73	1,50	7,68	24,11	22,42	0,01	100,00
Sulawesi Selatan	8,22	30,46	0,06	2,94	2,22	1,64	5,19	30,87	18,31	0,09	100,00
Sulawesi Tenggara	7,00	18,06	0,18	0,33	7,52	0,00	5,65	37,44	23,56	0,25	100,00
Gorontalo	6,28	32,56	0,64	7,05	4,26	0,69	6,44	25,67	14,43	1,97	100,00
Sulawesi Barat	4,55	28,74	0,00	0,00	5,07	0,00	7,70	31,55	18,75	3,65	100,00
Maluku	10,15	28,52	0,00	0,64	2,55	0,05	3,63	41,01	13,30	0,15	100,00
Maluku Utara	6,12	30,65	1,16	5,33	2,17	0,31	6,81	36,95	10,08	0,43	100,00
Papua Barat	13,36	43,11	0,00	0,83	1,36	0,00	3,99	21,96	15,39	0,00	100,00
Papua	17,02	43,32	0,00	0,13	3,34	0,54	6,82	19,70	8,87	0,25	100,00
**Indonesia**	**9,79**	**16,41**	**0,11**	**1,69**	**1,51**	**1,98**	**7,54**	**45,26**	**15,03**	**0,69**	**100,00**

Catatan/*Note***:** *) Data 2018 belum tersedia/*2018 data not available yet*
Sumber/*Source*: Susenas Maret 2017/*The March 2017 Susenas*

https://www.bps.go.id

Tabel *Table* **5.10.2.** **Persentase Perempuan Berumur 15-49 Tahun Pernah Kawin yang Menggunakan Alat KB Modern di Daerah Perdesaan menurut Provinsi dan Tempat Memperoleh Alat KB Modern yang Terakhir Kali, 2017***

*Percentage of Married Female Aged 15-49 Years Using Moden Contraception in Rural Area by Province and Place Obtains The Last Modern Contraception, 2017**

**Provinsi** *Province*	**Rumah Sakit** *Hospital*	**Puskesmas/ Pustu/Klinik** *PHC/Auxiliary PHC/Clinic*	**TKBK/ TMK/ Muyan**	**Polindes/ Poskesdes** *Village Maternity/ Health Post*	**Posyandu/Pos KB/ PPKBD** *Posyandu/Family Planning Post/PPKBD*	**Rumah Bersalin** *Maternity Hospital*	**Praktik Dokter Umum/Kandungan** *General Practitioner/ Obstetricians*	**Praktik Bidan/Bidan di Desa/Perawat** *Midwife/Nurse Service*	**Apotek/Obat Toko** *Pharmacies/Nurse Service*	**Lainnya** *Other*	**Jumlah** *Total*
**(1)**	**(2)**	**(3)**	**(4)**	**(5)**	**(6)**	**(7)**	**(8)**	**(9)**	**(10)**	**(12)**	**(13)**
Aceh	2,36	26,59	0,05	14,10	2,87	0,67	4,41	42,89	5,87	0,20	100,00
Sumatera Utara	9,28	16,60	0,15	3,53	2,62	0,93	6,02	51,43	9,17	0,26	100,00
Sumatera Barat	6,03	27,44	0,06	11,28	1,79	0,89	4,97	43,13	4,18	0,22	100,00
Riau	2,75	20,81	0,00	4,45	1,30	0,89	6,97	52,79	9,40	0,64	100,00
Jambi	1,77	15,94	0,61	5,19	2,58	0,56	7,41	56,60	8,81	0,54	100,00
Sumatera Selatan	0,64	11,12	0,09	13,67	1,43	0,19	4,34	66,36	1,64	0,53	100,00
Bengkulu	1,92	11,84	0,54	4,84	2,04	0,09	5,79	68,69	3,91	0,35	100,00
Lampung	1,55	9,74	0,15	2,15	1,34	0,50	6,70	70,90	5,20	1,77	100,00
Kepulauan Bangka Belitung	1,20	9,90	0,00	30,37	1,00	0,35	4,60	46,45	4,98	1,15	100,00
Kepulauan Riau	4,88	33,07	0,00	15,73	2,56	1,15	4,21	31,68	6,23	0,49	100,00
DKI Jakarta	-	-	-	-	-	-	-	-	-	-	-
Jawa Barat	3,13	9,44	0,14	1,58	5,29	0,61	8,19	58,95	9,77	2,89	100,00
Jawa Tengah	7,55	14,99	0,21	4,71	2,20	0,68	4,64	58,79	5,69	0,54	100,00
DI Yogyakarta	10,83	29,67	0,33	1,51	0,80	0,94	5,51	39,41	11,00	0,00	100,00
Jawa Timur	4,28	11,18	0,09	12,04	1,27	0,34	4,14	54,61	10,89	1,15	100,00
Banten	1,01	16,57	0,00	0,66	6,45	0,69	9,21	60,21	4,69	0,52	100,00
Bali	8,92	18,70	0,11	1,04	1,11	1,23	6,84	59,53	2,14	0,39	100,00
Nusa Tenggara Barat	1,98	20,43	0,05	28,18	3,91	0,19	3,58	39,27	1,20	1,20	100,00
Nusa Tenggara Timur	7,31	63,34	0,00	16,62	5,99	0,00	0,58	5,26	0,83	0,07	100,00
Kalimantan Barat	1,66	16,13	0,16	25,97	2,43	0,38	3,49	45,85	3,01	0,92	100,00
Kalimantan Tengah	1,25	29,16	0,13	13,88	2,38	0,27	1,90	40,65	8,12	2,25	100,00
Kalimantan Selatan	1,67	11,97	0,12	8,19	2,75	0,22	3,35	49,65	18,60	3,49	100,00
Kalimantan Timur	4,76	35,05	0,00	7,63	1,07	0,12	2,37	35,89	11,84	1,26	100,00
Kalimantan Utara	5,98	54,16	0,00	1,06	4,91	0,00	4,70	15,85	12,25	1,08	100,00
Sulawesi Utara	3,87	30,77	0,05	4,18	3,35	0,45	5,68	39,93	7,99	3,72	100,00
Sulawesi Tengah	3,85	25,50	0,10	19,32	4,08	0,03	3,38	31,54	9,78	2,42	100,00
Sulawesi Selatan	2,27	35,23	0,06	9,58	3,12	0,63	3,10	35,10	7,30	3,61	100,00
Sulawesi Tenggara	2,42	20,90	0,60	8,74	4,96	0,14	5,10	45,15	8,97	3,00	100,00
Gorontalo	4,27	31,10	0,13	9,93	7,20	0,00	4,92	31,69	8,23	2,53	100,00
Sulawesi Barat	0,98	60,11	0,00	6,33	3,10	0,12	2,25	18,83	5,41	2,86	100,00
Maluku	0,89	28,69	0,02	4,11	4,35	0,00	5,53	53,34	2,92	0,16	100,00
Maluku Utara	3,61	33,12	0,05	13,76	2,73	0,00	5,10	38,02	2,53	1,07	100,00
Papua Barat	7,99	56,61	0,00	4,62	4,15	0,00	1,39	19,72	5,52	0,00	100,00
Papua	8,86	68,78	0,19	1,68	3,56	0,61	1,36	12,82	2,13	0,00	100,00
**Indonesia**	**4,03**	**17,64**	**0,14**	**8,42**	**2,73**	**0,50**	**5,16**	**52,64**	**7,41**	**1,33**	**100,00**

Catatan/*Note***:** *) Data 2018 belum tersedia/*2018 data not available yet*
Sumber/*Source*: Susenas Maret 2017/*The March 2017 Susenas*

https://www.bps.go.id

Tabel *Table* **5.10.3.** **Persentase Perempuan Berumur 15-49 Tahun Pernah Kawin yang Menggunakan Alat KB Modern di Daerah Perkotaan dan Perdesaan menurut Provinsi dan Tempat Memperoleh Alat KB Modern yang Terakhir Kali, 2017***
*Percentage of Married Female Aged 15-49 Years Using Moden Contraception in Urban and Rural Area by Province and Place Obtains The Last Modern Contraception, 2017**

**Provinsi** *Province*	**Rumah Sakit** *Hospital*	**Puskesmas/ Pustu/Klinik** *PHC/Auxiliary PHC/Clinic*	**TKBK/ TMK/ Muyan**	**Polindes/ Poskesdes** *Village Maternity/ Health Post*	**Posyandu/Pos KB/ PPKBD** *Posyandu/Family Planning Post/PPKBD*	**Rumah Bersalin** *Maternity Hospital*	**Praktik Dokter Umum/Kandungan** *General Practitioner/ Obstetricians*	**Praktik Bidan/Bidan di Desa/Perawat** *Midwife/Nurse Service*	**Apotek/Obat Toko** *Pharmacies/Nurse Service*	**Lainnya** *Other*	**Jumlah** *Total*
**(1)**	**(2)**	**(3)**	**(4)**	**(5)**	**(6)**	**(7)**	**(8)**	**(9)**	**(10)**	**(12)**	**(13)**
Aceh	3,60	25,94	0,15	11,43	2,32	0,82	4,49	43,01	7,95	0,29	100,00
Sumatera Utara	11,32	16,73	0,20	2,52	2,47	1,26	5,31	47,74	12,22	0,23	100,00
Sumatera Barat	8,35	25,15	0,04	8,72	1,71	2,05	5,50	41,77	6,51	0,20	100,00
Riau	5,37	20,67	0,00	3,33	1,04	2,71	6,12	48,77	11,41	0,58	100,00
Jambi	3,11	17,13	0,57	3,99	2,48	1,36	7,13	51,34	12,42	0,47	100,00
Sumatera Selatan	2,41	11,99	0,08	10,16	1,24	0,61	5,41	63,62	4,04	0,44	100,00
Bengkulu	3,12	13,23	0,64	3,94	1,83	0,29	5,82	64,83	5,96	0,33	100,00
Lampung	2,27	10,62	0,14	1,91	1,29	1,18	7,09	67,74	6,23	1,53	100,00
Kepulauan Bangka Belitung	2,83	9,18	0,01	19,30	1,00	0,74	7,33	46,70	12,07	0,83	100,00
Kepulauan Riau	9,52	23,82	0,12	4,84	1,69	1,85	5,39	39,99	12,51	0,27	100,00
DKI Jakarta	16,01	26,02	0,00	0,31	0,65	4,16	6,55	31,92	14,06	0,33	100,00
Jawa Barat	6,01	12,64	0,10	1,06	3,04	1,25	8,28	52,23	13,56	1,82	100,00
Jawa Tengah	9,88	14,24	0,21	3,40	1,75	1,20	5,59	55,56	7,74	0,42	100,00
DI Yogyakarta	17,22	23,43	0,11	0,73	1,42	1,73	6,38	31,71	17,23	0,05	100,00
Jawa Timur	6,80	11,88	0,08	7,65	1,17	1,06	5,57	50,50	14,31	0,96	100,00
Banten	5,73	16,67	0,00	0,36	3,00	1,90	9,76	53,64	8,63	0,31	100,00
Bali	12,46	15,51	0,10	0,46	0,78	1,28	10,35	52,45	6,37	0,23	100,00
Nusa Tenggara Barat	3,53	20,40	0,05	26,15	3,51	0,24	4,69	38,77	1,47	1,19	100,00
Nusa Tenggara Timur	9,61	60,84	0,00	14,32	5,58	0,14	1,04	6,58	1,83	0,06	100,00
Kalimantan Barat	3,32	16,31	0,16	20,02	2,08	0,71	4,25	45,59	6,84	0,72	100,00
Kalimantan Tengah	1,94	26,11	0,09	10,03	1,76	0,73	2,92	41,23	13,40	1,78	100,00
Kalimantan Selatan	2,66	11,53	0,19	5,53	2,31	0,25	4,37	47,02	23,55	2,60	100,00
Kalimantan Timur	7,44	24,48	0,00	3,53	0,78	0,96	4,75	35,39	22,17	0,50	100,00
Kalimantan Utara	6,32	36,72	0,00	0,75	2,88	0,76	8,86	22,40	20,63	0,68	100,00
Sulawesi Utara	6,29	29,55	0,23	2,91	2,51	0,44	9,11	33,60	12,87	2,50	100,00
Sulawesi Tengah	5,73	25,78	0,08	16,19	3,44	0,31	4,20	30,12	12,19	1,96	100,00
Sulawesi Selatan	4,11	33,76	0,06	7,53	2,84	0,94	3,74	33,80	10,70	2,52	100,00
Sulawesi Tenggara	3,64	20,15	0,49	6,50	5,64	0,11	5,25	43,10	12,85	2,27	100,00
Gorontalo	4,86	31,52	0,28	9,09	6,34	0,20	5,37	29,93	10,04	2,37	100,00
Sulawesi Barat	1,53	55,32	0,00	5,36	3,40	0,10	3,08	20,78	7,45	2,98	100,00
Maluku	4,55	28,62	0,01	2,74	3,64	0,02	4,78	48,45	7,03	0,15	100,00
Maluku Utara	4,17	32,57	0,30	11,86	2,61	0,07	5,48	37,78	4,23	0,92	100,00
Papua Barat	9,89	51,84	0,00	3,28	3,17	0,00	2,31	20,51	9,00	0,00	100,00
Papua	12,35	57,88	0,11	1,02	3,47	0,58	3,70	15,77	5,02	0,11	100,00
**Indonesia**	**6,85**	**17,04**	**0,12**	**5,13**	**2,13**	**1,23**	**6,32**	**49,03**	**11,14**	**1,01**	**100,00**

Catatan/*Note***:** *) Data 2018 belum tersedia/*2018 data not available yet*
Sumber/*Source*: Susenas Maret 2017/*The March 2017 Susenas*

https://www.bps.go.id

Tabel *Table* **5.11.** **Persentase Perempuan Berumur 15-49 Tahun Pernah Kawin dan Sedang Menggunakan Alat/Cara KB dan Pernah Berhenti/Berganti Alat/Cara KB menurut Provinsi dan Daerah Tempat Tinggal, 2017***

*Percentage of Ever Married Women Aged 15-49 Years Who Currently Used Contraception and Ever Stopped/Changed Contraception by Province and Urban Rural Classification, 2017**

Provinsi *Province*	Perkotaan/*Urban*			Perdesaan/*Rural*			Perkotaan+Perdesaan/*Urban+Rural*		
	Ya *Yes*	Tidak *No*	Jumlah *Total*	Ya *Yes*	Tidak *No*	Jumlah *Total*	Ya *Yes*	Tidak *No*	Jumlah *Total*
(1)	(2)	(3)	(4)	(5)	(6)	(7)	(8)	(9)	(10)
Aceh	21,17	78,83	100,00	17,82	82,18	100,00	18,75	81,25	100,00
Sumatera Utara	25,33	74,67	100,00	27,91	72,09	100,00	26,64	73,36	100,00
Sumatera Barat	34,06	65,94	100,00	29,98	70,02	100,00	31,42	68,58	100,00
Riau	28,59	71,41	100,00	30,16	69,84	100,00	29,67	70,33	100,00
Jambi	27,33	72,67	100,00	27,83	72,17	100,00	27,70	72,30	100,00
Sumatera Selatan	28,22	71,78	100,00	27,17	72,83	100,00	27,47	72,53	100,00
Bengkulu	35,55	64,45	100,00	30,72	69,28	100,00	31,91	68,09	100,00
Lampung	31,79	68,21	100,00	31,60	68,40	100,00	31,65	68,35	100,00
Kepulauan Bangka Belitung	32,63	67,37	100,00	26,57	73,43	100,00	29,41	70,59	100,00
Kepulauan Riau	30,30	69,70	100,00	31,24	68,76	100,00	30,49	69,51	100,00
DKI Jakarta	23,56	76,44	100,00	-	-	-	23,56	76,44	100,00
Jawa Barat	32,96	67,04	100,00	33,74	66,26	100,00	33,20	66,80	100,00
Jawa Tengah	31,91	68,09	100,00	30,79	69,21	100,00	31,28	68,72	100,00
DI Yogyakarta	34,79	65,21	100,00	36,65	63,35	100,00	35,41	64,59	100,00
Jawa Timur	29,15	70,85	100,00	24,99	75,01	100,00	26,92	73,08	100,00
Banten	24,94	75,06	100,00	29,35	70,65	100,00	26,39	73,61	100,00
Bali	27,17	72,83	100,00	30,92	69,08	100,00	28,70	71,30	100,00
Nusa Tenggara Barat	26,85	73,15	100,00	30,80	69,20	100,00	29,10	70,90	100,00
Nusa Tenggara Timur	31,80	68,20	100,00	28,44	71,56	100,00	29,09	70,91	100,00
Kalimantan Barat	26,75	73,25	100,00	24,83	75,17	100,00	25,32	74,68	100,00
Kalimantan Tengah	23,05	76,95	100,00	29,26	70,74	100,00	27,23	72,77	100,00
Kalimantan Selatan	41,93	58,07	100,00	47,31	52,69	100,00	45,12	54,88	100,00
Kalimantan Timur	27,97	72,03	100,00	28,23	71,77	100,00	28,07	71,93	100,00
Kalimantan Utara	31,99	68,01	100,00	30,13	69,87	100,00	31,15	68,85	100,00
Sulawesi Utara	30,61	69,39	100,00	31,28	68,72	100,00	30,99	69,01	100,00
Sulawesi Tengah	36,01	63,99	100,00	38,03	61,97	100,00	37,64	62,36	100,00
Sulawesi Selatan	29,46	70,54	100,00	27,72	72,28	100,00	28,28	71,72	100,00
Sulawesi Tenggara	31,84	68,16	100,00	37,27	62,73	100,00	35,82	64,18	100,00
Gorontalo	32,62	67,38	100,00	35,61	64,39	100,00	34,74	65,26	100,00
Sulawesi Barat	31,14	68,86	100,00	33,73	66,27	100,00	33,33	66,67	100,00
Maluku	23,65	76,35	100,00	20,77	79,23	100,00	21,96	78,04	100,00
Maluku Utara	20,87	79,13	100,00	22,59	77,41	100,00	22,20	77,80	100,00
Papua Barat	20,68	79,32	100,00	20,63	79,37	100,00	20,65	79,35	100,00
Papua	22,20	77,80	100,00	12,81	87,19	100,00	15,90	84,10	100,00
**Indonesia**	**29,92**	**70,08**	**100,00**	**29,45**	**70,55**	**100,00**	**29,68**	**70,32**	**100,00**

Catatan/*Note***:** *) Data 2018 belum tersedia/*2018 data not available yet*
Sumber/*Source*: Susenas Maret 2017/*The March 2017 Susenas*

https://www.bps.go.id

BAB/CHAPTER 6

PERUMAHAN

HOUSING

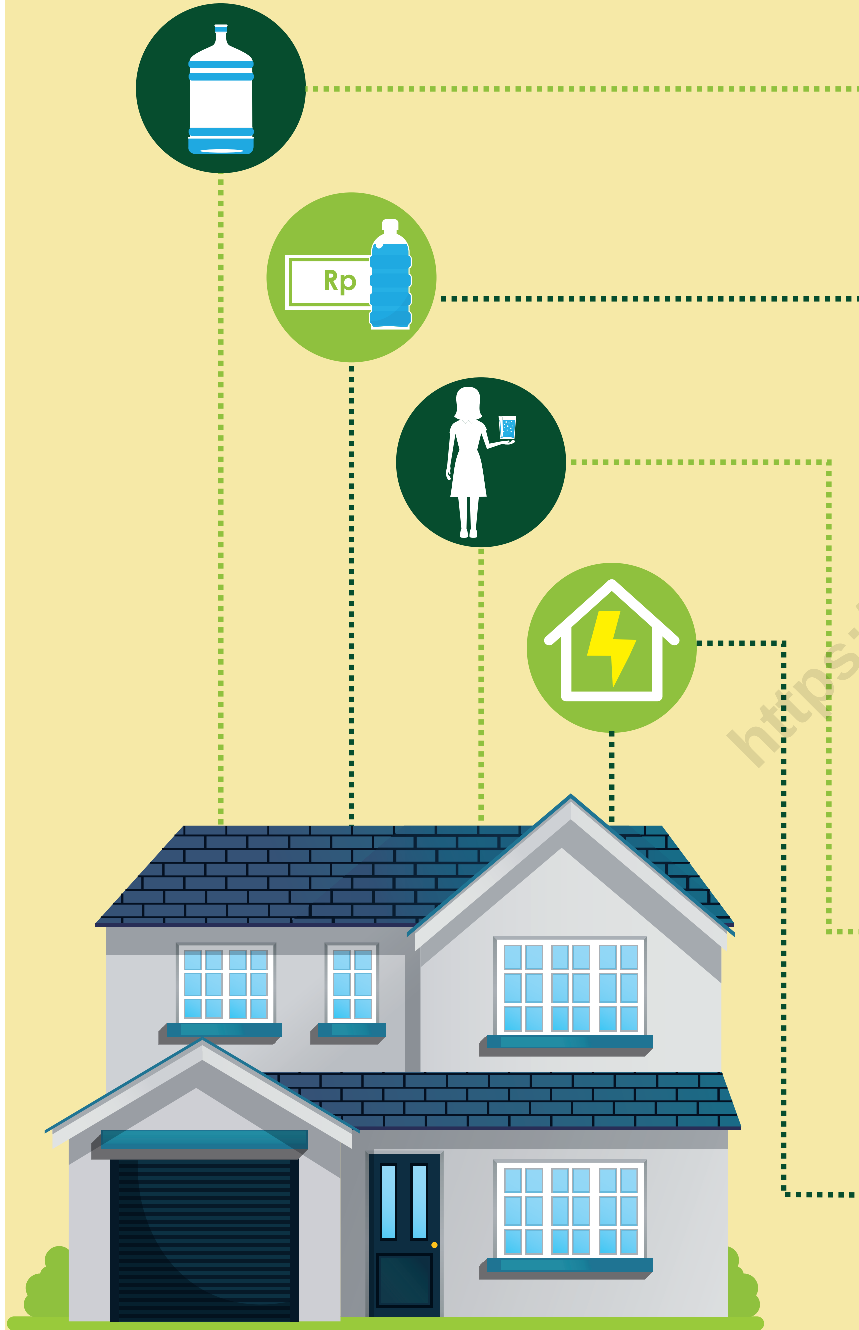

36,28% RUMAH TANGGA MINUM DARI KEMASAN BERMERK/ AIR ISI ULANG

households drink from branded water or refill water

46,72% RUMAH TANGGA MEMPEROLEH AIR MINUM DENGAN CARA MEMBELI

households buy drinking water from retailers

72,99% RUMAH TANGGA MEMILIKI AKSES AIR MINUM BERSIH

households have access to clean drinking water

98,51% RUMAH TANGGA MENGGUNAKAN LISTRIK

households use electricity

https://www.bps.go.id

https://www.bps.go.id

VI. PERUMAHAN/*HOUSING*

PENJELASAN TEKNIS

1. **Keluarga** adalah hubungan yang didasarkan atas ikatan perkawinan, baik yang saat ini statusnya masih kawin atau sudah bercerai.

2. **Kepemilikan bangunan** adalah status penguasaan bangunan tempat tinggal atau rumah yang ditempati dilihat dari sisi anggota ruta yang mendiaminya. Terdiri dari miik sendiri, kontrak sewa, dst.

3. **Luas lantai** adalah luas lantai yang ditempati dan digunakan untuk keperluan sehari-hari (sebatas atap rumah).

4. **Parket (*parquetted*)** adalah menyusun potongan-potongan kayu untuk dijadikan penutup lantai.

5. **MCK Komunal** singkatan dari Mandi, Cuci, Kakus adalah salah satu sarana fasilitas umum yang digunakan bersama oleh beberapa keluargauntuk keperluan mandi, mencuci, dan buang air di lokasi permukiman tertentu yang dinilai berpenduduk cukup padat dan tingkat kemampuan ekonomi rendah.

6. **IPAL** adalah Instalasi Pembuangan Air Limbah (IPAL) terpadu. Dalam sistem pembuangan limbah cair seperti ini, air limbah ruta tidak ditampung dalam tangki atau wadah semacamnya, tetapi langsung dialirkan ke suatu tempat pengolahan limbah cair.

TECHNICAL NOTES

1. ***Family*** *is a relationship based upon marriage, whether the current status is still married or divorced.*

2. ***Ownership of the building*** *was mastering status residential buildings or houses occupied by members of the route in terms of the occupants. It consists of smelling his own, lease, etc.*

3. ***The floor area*** *is the floor area occupied and used for everyday purposes (limited to roofs).*

4. ***Parquet (parquetted)*** *is compile pieces of wood to be used as floor coverings.*

5. ***Communal MCK*** *stands for bathing, washing, toilet is one of the public facilities shared by several families for bathing, washing, and waste water in certain settlements which were quite dense population and low economic level.*

6. ***IPAL*** *is Waste Water Disposal Instalation (IPAL) integrated. In liquid waste disposal systems like this, do not waste water route are accommodated in the tank or container of some kind, but directly discharged into a wastewater treatment plan.*

https://www.bps.go.id

7. **Air minum layak dan bersih** adalah air minum yang terlindung meliputi air ledeng (keran), keran umum, hydrant umum, terminal air, penampungan air hujan (PAH) atau mata air dan sumur terlindung, sumur bor atau sumur pompa, yang jaraknya minimal 10 meter dari pembuangan kotoran, penampungan limbah dan pembuangan sampah. Tidak termasuk air kemasan, air dari penjual keliling, air yang dijual melalui tanki, air sumur dan mata air tidak terlindung.

8. **Perpipaan** adalah bila air yang digunakan disalurkan menggunakan pipa dari sumber air sampai ke rumah.

9. **Hidran umum/terminal air** adalah sarana penyediaan air bersih yang sumbernya berasal dari air permukaan yang dialirkan melalui perpipaan ke tempat atau distribusi yang bersifat komunal.

10. **Sumber air minum bersih** adalah sumber air minum yang terdiri dari air kemasan, air isi ulang, leding, dan [(sumur bor/pompa, sumur terlindung serta mata air terlindung) dengan jarak ke tempat penampungan limbah/kotoran/tinja terdekat ≥ 10 m].

11. **Sumber air minum layak** adalah sumber air minum yang terdiri dari leding, air hujan, dan [(sumur bor/pompa, sumur terlindung serta mata air terlindung) dengan jarak ke tempat penampungan limbah/kotoran/tinja terdekat ≥ 10 m].

12. **Akses air layak** adalah sumber air minumnya terdiri leding, air hujan, dan [(sumur bor/pompa, sumur terlindung serta mata air terlindung) dengan jarak ke tempat penampungan limbah/kotoran/tinja terdekat ≥ 10 ml] dan sumber air minum kemasan/air isi ulang dimana sumber air cuci/masak/mandi/dll menggunakan (sumur bor/pompa, sumur terlindung serta mata air terlindung) dengan jarak ke tempat penampungan limbah/kotoran/tinja terdekat ≥ 10 m.

7. ***Clean drinking water and drinking water** are protected, including tap water (tap), public taps, public hydrant, water terminal, rain water harvesting (PAH) or springs and protected wells, boreholes or well pump, a distance of at least 10 meters of sewage, waste containment and disposal of waste. Excluding bottled water, water from street vendors, who sell water through tanks, water wells and springs are not protected.*

8. ***The piping** is used when the water is channeled through pipes from the water source to the home.*

9. ***Public hydrants/air terminal** is a means of providing clean water source comes from surface water that flows through the piping to the place or distribution is communal.*

10. ***Source of clean water** is drinking water sources consisting of packaged water, refill water, pipe, and [(artesian well/pump, protected well and protected spring) that the distance to the nearest final disposal site of faeces ≥ 10 m].*

11. ***Source of decent water** is drinking water sources consisting of pipe, rain water, and [(artesian well/pump, protected well and protected spring) that the distance to the nearest final disposal site of feces ≥ 10 m].*

12. ***Access of decent water** is the drinking water source consists of pipe, rainwater, and [(artesian well/pump, protected well and protected spring) with the distance to the nearest final disposal site of feces ≥ 10 ml] and bottled water sources/refill water where the water source of washing/cooking/bathing/etc using (artesian well/pump, protected well and protected spring) with the distance to the nearest final disposal site of feces ≥ 10 m.*

https://www.bps.go.id

Tabel 6.1. **Rata-Rata Jumlah Keluarga dalam Bangunan Sensus/Rumah menurut Provinsi dan Daerah Tempat Tinggal, 2018**
Table 6.1. Average of Total Family per Dwelling Unit by Province and Urban Rural Classification, 2018

Provinsi *Province*	Perkotaan *Urban*	Perdesaan *Rural*	Perkotaan+Perdesaan *Urban+Rural*
(1)	(2)	(3)	(4)
Aceh	1,17	1,22	1,20
Sumatera Utara	1,20	1,17	1,19
Sumatera Barat	1,17	1,24	1,21
Riau	1,21	1,15	1,17
Jambi	1,26	1,19	1,21
Sumatera Selatan	1,20	1,20	1,20
Bengkulu	1,12	1,15	1,14
Lampung	1,17	1,14	1,15
Kepulauan Bangka Belitung	1,16	1,13	1,14
Kepulauan Riau	1,08	1,14	1,09
DKI Jakarta	1,23	-	1,23
Jawa Barat	1,26	1,22	1,25
Jawa Tengah	1,35	1,30	1,33
DI Yogyakarta	1,16	1,31	1,20
Jawa Timur	1,33	1,32	1,32
Banten	1,29	1,28	1,29
Bali	1,19	1,30	1,23
Nusa Tenggara Barat	1,16	1,16	1,16
Nusa Tenggara Timur	1,11	1,23	1,20
Kalimantan Barat	1,31	1,27	1,28
Kalimantan Tengah	1,19	1,20	1,20
Kalimantan Selatan	1,24	1,19	1,21
Kalimantan Timur	1,25	1,21	1,23
Kalimantan Utara	1,32	1,30	1,31
Sulawesi Utara	1,48	1,29	1,38
Sulawesi Tengah	1,21	1,24	1,23
Sulawesi Selatan	1,26	1,25	1,26
Sulawesi Tenggara	1,14	1,23	1,19
Gorontalo	1,62	1,30	1,42
Sulawesi Barat	1,20	1,20	1,20
Maluku	1,24	1,27	1,26
Maluku Utara	1,27	1,36	1,34
Papua Barat	1,38	1,31	1,34
Papua	1,37	1,19	1,24
**Indonesia**	**1,26**	**1,25**	**1,26**

Sumber/*Source*: Susenas Maret 2018/*The March 2018 Susenas*

https://www.bps.go.id

Tabel *Table* **6.2.** **Persentase Rumah Tangga menurut Provinsi, Daerah Tempat Tinggal, dan Status Kepemilikan Bangunan Tempat Tinggal yang Ditempati, 2018**
Percentage of Households by Province, Urban Rural Classification, and Ownership Status of Dwelling, 2018

**Provinsi** *Province*	**Perkotaan/***Urban*					**Perdesaan/***Rural*					**Perkotaan+Perdesaan/***Urban+Rural*				
	**Milik Sendiri** *Private Property*	**Kontrak/ Sewa** *Contract/ Rent*	**Bebas Sewa** *Free Use*	**Dinas, Lainnya** *Official, Other*	**Jumlah** *Total*	**Milik Sendiri** *Private Property*	**Kontrak/ Sewa** *Contract/ Rent*	**Bebas Sewa** *Free Use*	**Dinas, Lainnya** *Official, Other*	**Jumlah** *Total*	**Milik Sendiri** *Private Property*	**Kontrak/ Sewa** *Contract/ Rent*	**Bebas Sewa** *Free Use*	**Dinas, Lainnya** *Official, Other*	**Jumlah** *Total*
(1)	(2)	(3)	(4)	(5)	(6)	(7)	(8)	(9)	(10)	(11)	(12)	(13)	(14)	(15)	(16)
Aceh	70,01	16,68	9,69	3,62	100,00	86,30	3,06	9,11	1,54	100,00	81,21	7,31	9,29	2,19	100,00
Sumatera Utara	61,81	19,33	15,82	3,03	100,00	75,51	5,74	14,34	4,41	100,00	68,32	12,87	15,12	3,69	100,00
Sumatera Barat	61,71	22,80	13,82	1,67	100,00	78,72	4,38	15,48	1,43	100,00	71,21	12,51	14,75	1,53	100,00
Riau	55,81	32,15	9,84	2,20	100,00	79,43	5,07	7,57	7,92	100,00	69,90	16,00	8,49	5,61	100,00
Jambi	72,09	17,51	9,07	1,33	100,00	88,89	2,31	7,54	1,27	100,00	83,64	7,06	8,01	1,29	100,00
Sumatera Selatan	68,23	14,38	15,66	1,73	100,00	87,58	1,72	9,40	1,29	100,00	80,65	6,25	11,65	1,45	100,00
Bengkulu	70,89	17,92	9,93	1,26	100,00	89,29	2,71	6,39	1,61	100,00	83,39	7,59	7,52	1,50	100,00
Lampung	77,91	11,65	9,49	0,95	100,00	91,75	0,99	6,40	0,85	100,00	87,89	3,96	7,26	0,88	100,00
Kepulauan Bangka Belitung	80,61	8,30	9,50	1,60	100,00	91,97	1,77	4,80	1,46	100,00	85,91	5,25	7,30	1,53	100,00
Kepulauan Riau	66,43	26,98	5,45	1,14	100,00	88,10	1,80	7,64	2,46	100,00	69,44	23,48	5,75	1,32	100,00
DKI Jakarta	47,85	35,63	15,08	1,44	100,00	-	-	-	-	-	47,85	35,63	15,08	1,44	100,00
Jawa Barat	73,45	13,96	11,64	0,95	100,00	88,97	1,36	9,03	0,64	100,00	77,71	10,50	10,92	0,87	100,00
Jawa Tengah	82,46	5,25	11,61	0,68	100,00	94,05	0,39	5,31	0,26	100,00	88,17	2,85	8,50	0,47	100,00
DI Yogyakarta	70,09	18,61	10,37	0,94	100,00	94,57	0,04	5,14	0,24	100,00	76,54	13,71	8,99	0,75	100,00
Jawa Timur	81,37	10,88	6,97	0,78	100,00	94,08	0,64	4,95	0,32	100,00	87,46	5,97	6,00	0,56	100,00
Banten	75,79	17,17	5,93	1,11	100,00	95,26	0,68	3,58	0,49	100,00	81,33	12,48	5,26	0,93	100,00
Bali	63,02	29,22	7,46	0,30	100,00	89,48	1,23	9,04	0,25	100,00	71,75	19,99	7,98	0,28	100,00
Nusa Tenggara Barat	79,03	6,77	13,32	0,88	100,00	91,01	0,65	7,89	0,45	100,00	85,48	3,47	10,40	0,65	100,00
Nusa Tenggara Timur	65,35	22,74	9,90	2,01	100,00	92,29	1,03	5,65	1,04	100,00	85,91	6,17	6,66	1,27	100,00
Kalimantan Barat	78,96	9,19	9,47	2,38	100,00	93,67	0,56	4,49	1,28	100,00	88,84	3,39	6,13	1,64	100,00
Kalimantan Tengah	69,54	17,47	9,80	3,18	100,00	78,69	2,26	9,15	9,90	100,00	75,24	7,99	9,40	7,37	100,00
Kalimantan Selatan	65,16	19,85	13,82	1,17	100,00	86,83	2,69	8,23	2,25	100,00	76,77	10,66	10,83	1,75	100,00
Kalimantan Timur	63,10	24,18	10,21	2,50	100,00	84,85	5,23	8,58	1,35	100,00	70,31	17,90	9,67	2,12	100,00
Kalimantan Utara	58,73	24,93	12,92	3,42	100,00	79,67	7,09	10,22	3,02	100,00	67,23	17,69	11,82	3,26	100,00
Sulawesi Utara	68,18	11,76	18,49	1,57	100,00	87,20	1,53	9,97	1,30	100,00	77,65	6,66	14,25	1,44	100,00
Sulawesi Tengah	68,55	18,08	11,36	2,01	100,00	90,57	1,21	7,11	1,12	100,00	84,32	5,99	8,32	1,37	100,00
Sulawesi Selatan	70,72	12,22	13,36	3,70	100,00	92,32	0,72	6,41	0,54	100,00	83,61	5,36	9,22	1,81	100,00
Sulawesi Tenggara	71,57	14,05	12,14	2,25	100,00	92,35	0,72	6,14	0,79	100,00	84,43	5,80	8,43	1,35	100,00
Gorontalo	68,89	6,75	23,74	0,62	100,00	86,35	0,44	12,79	0,42	100,00	79,48	2,92	17,10	0,50	100,00
Sulawesi Barat	79,65	7,18	9,72	3,44	100,00	89,04	1,07	6,96	2,93	100,00	86,85	2,49	7,61	3,05	100,00
Maluku	72,21	12,30	9,41	6,08	100,00	86,08	2,22	10,00	1,70	100,00	80,09	6,57	9,74	3,59	100,00
Maluku Utara	66,36	20,94	10,38	2,32	100,00	91,27	1,57	5,61	1,55	100,00	83,87	7,32	7,03	1,78	100,00
Papua Barat	57,15	22,77	14,43	5,66	100,00	77,21	8,86	9,12	4,81	100,00	69,19	14,42	11,24	5,15	100,00
Papua	55,24	29,67	7,94	7,15	100,00	90,39	2,10	4,79	2,73	100,00	81,36	9,18	5,60	3,86	100,00
**Indonesia**	**71,96**	**15,76**	**10,94**	**1,33**	**100,00**	**89,76**	**1,61**	**7,27**	**1,36**	**100,00**	**80,02**	**9,35**	**9,28**	**1,34**	**100,00**

Sumber/*Source*: Susenas Maret 2018/*The March 2018 Susenas*

https://www.bps.go.id

Tabel **6.3.** **Persentase Rumah Tangga menurut Provinsi, Daerah Tempat Tinggal, dan Luas Lantai Rumah (m²), 2018**
Table *Percentage of Households by Province, Urban Rural Classification, and Floor Area (m²), 2018*

**Provinsi** *Province*	**Perkotaan/***Urban*						**Perdesaan/***Rural*						**Perkotaan+Perdesaan/***Urban+Rural*					
	**≤ 19**	**20-49**	**50-99**	**100-149**	**150+**	**Jumlah** *Total*	**≤ 19**	**20-49**	**50-99**	**100-149**	**150+**	**Jumlah** *Total*	**≤ 19**	**20-49**	**50-99**	**100-149**	**150+**	**Jumlah** *Total*
(1)	(2)	(3)	(4)	(5)	(6)	(7)	(8)	(9)	(10)	(11)	(12)	(13)	(14)	(15)	(16)	(17)	(18)	(19)
Aceh	3,36	31,72	33,61	15,46	15,85	100,00	1,79	42,46	41,56	9,92	4,27	100,00	2,28	39,10	39,08	11,65	7,89	100,00
Sumatera Utara	1,87	24,98	47,78	15,44	9,92	100,00	2,58	37,69	48,57	8,36	2,81	100,00	2,21	31,02	48,16	12,07	6,54	100,00
Sumatera Barat	6,63	24,35	37,83	18,91	12,29	100,00	2,41	29,57	48,85	14,24	4,94	100,00	4,27	27,27	43,98	16,30	8,18	100,00
Riau	2,61	32,54	37,06	15,83	11,96	100,00	1,21	32,31	49,06	11,42	6,01	100,00	1,77	32,40	44,22	13,20	8,41	100,00
Jambi	1,55	31,35	40,66	16,67	9,77	100,00	1,39	30,45	51,27	11,90	5,00	100,00	1,44	30,73	47,95	13,39	6,49	100,00
Sumatera Selatan	4,15	36,73	37,78	11,96	9,38	100,00	2,52	38,48	48,35	7,76	2,89	100,00	3,11	37,85	44,56	9,27	5,22	100,00
Bengkulu	3,74	30,51	33,05	14,65	18,04	100,00	2,01	38,67	49,11	6,36	3,84	100,00	2,57	36,05	43,96	9,02	8,40	100,00
Lampung	2,85	18,71	48,56	18,88	11,00	100,00	0,29	16,08	56,65	19,92	7,07	100,00	1,00	16,81	54,39	19,63	8,16	100,00
Kepulauan Bangka Belitung	1,36	29,19	47,20	15,46	6,78	100,00	0,53	27,99	49,92	17,46	4,11	100,00	0,98	28,63	48,47	16,39	5,53	100,00
Kepulauan Riau	6,48	28,56	49,96	8,45	6,54	100,00	0,53	38,72	43,80	12,82	4,14	100,00	5,65	29,97	49,11	9,06	6,21	100,00
DKI Jakarta	19,04	35,47	23,79	11,53	10,18	100,00	-	-	-	-	-	-	19,04	35,47	23,79	11,53	10,18	100,00
Jawa Barat	5,50	30,65	42,33	13,36	8,16	100,00	1,74	41,39	45,65	8,01	3,21	100,00	4,47	33,59	43,24	11,89	6,80	100,00
Jawa Tengah	2,84	14,38	47,95	18,82	16,01	100,00	0,43	11,16	53,83	20,84	13,74	100,00	1,65	12,79	50,84	19,82	14,89	100,00
DI Yogyakarta	12,55	15,94	37,77	18,06	15,69	100,00	0,28	9,13	55,01	23,26	12,32	100,00	9,31	14,14	42,31	19,43	14,80	100,00
Jawa Timur	6,72	18,78	47,00	15,67	11,83	100,00	0,96	18,86	53,32	16,88	9,98	100,00	3,96	18,82	50,03	16,25	10,94	100,00
Banten	4,80	21,29	46,10	16,68	11,13	100,00	0,82	29,01	54,75	9,91	5,50	100,00	3,67	23,49	48,56	14,75	9,53	100,00
Bali	19,10	21,53	31,68	15,18	12,51	100,00	2,33	29,93	48,34	13,84	5,57	100,00	13,57	24,30	37,17	14,74	10,22	100,00
Nusa Tenggara Barat	9,10	42,60	34,14	8,93	5,22	100,00	3,79	55,94	32,85	5,15	2,27	100,00	6,24	49,78	33,45	6,90	3,63	100,00
Nusa Tenggara Timur	13,60	33,06	35,32	11,46	6,56	100,00	1,97	42,86	46,93	6,75	1,49	100,00	4,72	40,54	44,19	7,87	2,69	100,00
Kalimantan Barat	1,35	22,04	40,11	18,45	18,05	100,00	1,31	36,78	47,83	9,50	4,58	100,00	1,32	31,94	45,30	12,44	9,00	100,00
Kalimantan Tengah	4,66	32,59	36,41	13,16	13,19	100,00	2,98	42,40	43,05	8,29	3,28	100,00	3,61	38,70	40,55	10,12	7,01	100,00
Kalimantan Selatan	7,02	33,47	33,97	15,37	10,16	100,00	1,74	31,33	50,47	12,25	4,21	100,00	4,19	32,32	42,81	13,70	6,97	100,00
Kalimantan Timur	4,95	29,38	36,14	14,65	14,88	100,00	2,47	35,21	44,83	11,25	6,24	100,00	4,13	31,31	39,02	13,53	12,02	100,00
Kalimantan Utara	5,05	30,42	35,51	16,53	12,67	100,00	1,69	26,08	45,21	15,16	11,86	100,00	3,69	28,66	39,44	15,87	12,34	100,00
Sulawesi Utara	6,79	32,33	31,53	14,05	15,30	100,00	1,69	43,33	41,84	8,56	4,58	100,00	4,25	37,81	36,67	11,32	9,96	100,00
Sulawesi Tengah	6,28	31,99	35,31	14,52	11,90	100,00	2,48	37,54	45,74	9,50	4,73	100,00	3,56	35,97	42,79	10,92	6,76	100,00
Sulawesi Selatan	5,32	21,92	39,34	18,39	15,03	100,00	1,24	20,85	51,95	18,24	7,71	100,00	2,89	21,28	46,87	18,30	10,66	100,00
Sulawesi Tenggara	9,79	22,11	37,13	16,58	14,39	100,00	1,03	28,61	48,25	15,52	6,59	100,00	4,37	26,14	44,02	15,93	9,56	100,00
Gorontalo	6,72	31,63	35,36	15,72	10,58	100,00	2,60	42,77	40,20	9,89	4,54	100,00	4,22	38,38	38,30	12,19	6,92	100,00
Sulawesi Barat	3,08	25,43	37,88	18,88	14,73	100,00	3,27	31,40	48,20	11,92	5,21	100,00	3,23	30,01	45,80	13,54	7,43	100,00
Maluku	8,84	33,42	37,65	11,80	8,29	100,00	1,51	37,32	46,87	11,04	3,27	100,00	4,68	35,64	42,89	11,36	5,44	100,00
Maluku Utara	12,13	16,74	36,97	21,49	12,66	100,00	0,86	25,72	52,42	14,34	6,66	100,00	4,21	23,05	47,83	16,46	8,45	100,00
Papua Barat	8,26	35,38	31,36	11,40	13,60	100,00	2,80	42,62	43,25	7,91	3,41	100,00	4,99	39,73	38,50	9,30	7,48	100,00
Papua	7,56	43,50	33,77	9,50	5,67	100,00	23,71	61,78	12,35	1,76	0,40	100,00	19,56	57,08	17,85	3,75	1,75	100,00
**Indonesia**	**6,58**	**25,70**	**41,49**	**15,04**	**11,19**	**100,00**	**1,88**	**29,12**	**49,02**	**13,26**	**6,72**	**100,00**	**4,45**	**27,25**	**44,90**	**14,23**	**9,16**	**100,00**

Sumber/*Source*: Susenas Maret 2018/*The March 2018 Susenas*

https://www.bps.go.id

Tabel **6.4.** **Persentase Rumah Tangga menurut Provinsi, Daerah Tempat Tinggal, dan Luas Lantai per Kapita (m^2), 2018**

Table *Percentage of Households by Province, Urban Rural Classification, and Floor Area per Capita (m^2), 2018*

**Provinsi** *Province*	**Perkotaan/***Urban*				**Perdesaan/***Rural*				**Perkotaan+Perdesaan/***Urban+Rural*			
	≤ 7,2	7,3-9,9	≥ 10	**Jumlah** *Total*	≤ 7,2	7,3-9,9	≥ 10	**Jumlah** *Total*	≤ 7,2	7,3-9,9	≥ 10	**Jumlah** *Total*
(1)	(2)	(3)	(4)	(5)	(6)	(7)	(8)	(9)	(10)	(11)	(12)	(13)
Aceh	9,24	12,28	78,49	100,00	12,70	16,24	71,06	100,00	11,62	15,00	73,38	100,00
Sumatera Utara	8,96	11,76	79,28	100,00	14,73	15,59	69,68	100,00	11,70	13,58	74,72	100,00
Sumatera Barat	10,41	11,69	77,90	100,00	10,27	12,45	77,28	100,00	10,33	12,11	77,55	100,00
Riau	7,43	13,40	79,17	100,00	8,15	14,65	77,20	100,00	7,86	14,15	78,00	100,00
Jambi	7,07	11,40	81,53	100,00	6,28	11,86	81,86	100,00	6,52	11,72	81,76	100,00
Sumatera Selatan	15,21	13,24	71,55	100,00	11,66	15,74	72,60	100,00	12,93	14,85	72,23	100,00
Bengkulu	8,69	12,31	79,00	100,00	8,76	14,78	76,46	100,00	8,73	13,99	77,28	100,00
Lampung	5,55	9,39	85,06	100,00	2,03	6,05	91,93	100,00	3,01	6,98	90,01	100,00
Kepulauan Bangka Belitung	5,59	9,97	84,44	100,00	2,88	10,39	86,73	100,00	4,32	10,16	85,51	100,00
Kepulauan Riau	9,11	12,90	77,99	100,00	7,25	12,49	80,26	100,00	8,85	12,84	78,31	100,00
DKI Jakarta	27,02	16,58	56,40	100,00	-	-	-	-	27,02	16,58	56,40	100,00
Jawa Barat	10,71	12,36	76,93	100,00	6,90	12,52	80,58	100,00	9,67	12,40	77,93	100,00
Jawa Tengah	4,01	6,50	89,50	100,00	1,42	4,09	94,50	100,00	2,73	5,31	91,96	100,00
DI Yogyakarta	5,07	8,59	86,35	100,00	0,31	1,68	98,02	100,00	3,81	6,77	89,42	100,00
Jawa Timur	7,22	8,68	84,10	100,00	2,07	6,06	91,87	100,00	4,75	7,42	87,83	100,00
Banten	7,86	13,13	79,00	100,00	4,88	13,94	81,18	100,00	7,01	13,36	79,62	100,00
Bali	14,69	12,47	72,84	100,00	4,86	12,18	82,96	100,00	11,45	12,37	76,18	100,00
Nusa Tenggara Barat	13,42	17,76	68,82	100,00	10,67	18,16	71,17	100,00	11,94	17,97	70,09	100,00
Nusa Tenggara Timur	20,04	18,01	61,95	100,00	14,86	21,16	63,97	100,00	16,09	20,42	63,49	100,00
Kalimantan Barat	5,28	10,30	84,43	100,00	9,75	15,81	74,45	100,00	8,28	14,00	77,73	100,00
Kalimantan Tengah	11,42	12,56	76,02	100,00	10,33	16,09	73,57	100,00	10,74	14,76	74,50	100,00
Kalimantan Selatan	9,89	13,33	76,79	100,00	5,85	12,71	81,44	100,00	7,72	13,00	79,28	100,00
Kalimantan Timur	10,34	11,87	77,79	100,00	8,57	13,84	77,59	100,00	9,76	12,52	77,72	100,00
Kalimantan Utara	13,29	13,10	73,61	100,00	8,14	11,98	79,88	100,00	11,20	12,65	76,15	100,00
Sulawesi Utara	12,64	13,17	74,18	100,00	12,75	15,35	71,90	100,00	12,69	14,26	73,05	100,00
Sulawesi Tengah	11,96	10,30	77,74	100,00	11,47	14,85	73,68	100,00	11,61	13,56	74,83	100,00
Sulawesi Selatan	11,65	11,37	76,98	100,00	6,11	9,60	84,29	100,00	8,34	10,31	81,34	100,00
Sulawesi Tenggara	12,22	11,33	76,44	100,00	9,27	12,20	78,53	100,00	10,40	11,87	77,73	100,00
Gorontalo	11,80	12,96	75,25	100,00	16,84	18,22	64,93	100,00	14,86	16,15	68,99	100,00
Sulawesi Barat	11,38	10,90	77,72	100,00	12,03	13,53	74,45	100,00	11,88	12,92	75,21	100,00
Maluku	19,23	16,56	64,21	100,00	17,88	15,81	66,31	100,00	18,46	16,14	65,40	100,00
Maluku Utara	12,16	10,08	77,76	100,00	9,78	14,77	75,45	100,00	10,49	13,37	76,13	100,00
Papua Barat	18,13	13,53	68,34	100,00	14,59	16,38	69,02	100,00	16,01	15,24	68,75	100,00
Papua	20,54	17,10	62,36	100,00	41,75	21,81	36,44	100,00	36,30	20,60	43,10	100,00
**Indonesia**	**10,34**	**11,41**	**78,25**	**100,00**	**7,25**	**11,02**	**81,73**	**100,00**	**8,94**	**11,24**	**79,83**	**100,00**

Sumber/*Source*: Susenas Maret 2018/*The March 2018 Susenas*

https://www.bps.go.id

Tabel 6.5.1. **Persentase Rumah Tangga di Daerah Perkotaan menurut Provinsi dan Bahan Bangunan Utama Atap Rumah Terluas, 2018**
Table *Percentage of Households in Urban Area by Province and The Main Material of The Widest Part of Roof, 2018*

**Provinsi** *Province*	**Beton** *Concrete*	**Genteng** *Roof*	**Asbes** *Asbestos*	**Seng** *Zinc*	**Bambu/Kayu/Sirap** *Bamboo/Wood*	**Jerami/Ijuk/Daun/ Rumbia** *Hay/Leaves/Rumbia*	**Lainnya** *Others*	**Jumlah** *Total*
(1)	(2)	(3)	(4)	(5)	(6)	(7)	(8)	(9)
Aceh	4,14	5,23	2,90	85,93	0,04	1,75	0,00	100,00
Sumatera Utara	3,15	2,62	5,74	87,42	0,20	0,82	0,05	100,00
Sumatera Barat	1,68	3,19	2,47	92,50	0,01	0,16	0,00	100,00
Riau	3,56	3,72	3,98	88,43	0,09	0,03	0,19	100,00
Jambi	3,45	17,41	2,49	76,09	0,12	0,31	0,13	100,00
Sumatera Selatan	3,04	47,28	8,82	39,93	0,43	0,27	0,23	100,00
Bengkulu	0,85	3,62	1,94	93,02	0,27	0,00	0,30	100,00
Lampung	0,73	82,30	12,52	4,41	0,03	0,00	0,00	100,00
Kepulauan Bangka Belitung	1,61	13,49	50,04	34,49	0,07	0,31	0,00	100,00
Kepulauan Riau	3,73	6,49	49,35	39,15	0,30	0,18	0,79	100,00
DKI Jakarta	3,56	40,37	54,05	1,67	0,25	0,00	0,11	100,00
Jawa Barat	3,58	79,20	16,31	0,60	0,11	0,01	0,20	100,00
Jawa Tengah	2,09	87,35	5,80	4,65	0,03	0,02	0,06	100,00
DI Yogyakarta	0,66	94,78	3,19	1,37	0,00	0,00	0,00	100,00
Jawa Timur	2,73	88,81	7,44	0,76	0,12	0,00	0,14	100,00
Banten	3,02	71,58	24,45	0,40	0,19	0,13	0,23	100,00
Bali	2,43	79,77	9,86	7,87	0,07	0,01	0,00	100,00
Nusa Tenggara Barat	2,98	63,50	10,81	22,44	0,11	0,10	0,07	100,00
Nusa Tenggara Timur	0,44	0,63	0,42	97,42	0,21	0,88	0,00	100,00
Kalimantan Barat	0,85	3,22	1,72	92,35	1,02	0,83	0,00	100,00
Kalimantan Tengah	1,94	16,83	7,97	63,29	8,98	0,51	0,48	100,00
Kalimantan Selatan	1,00	7,38	10,18	71,11	9,36	0,96	0,00	100,00
Kalimantan Timur	1,63	9,97	6,32	79,09	2,90	0,07	0,02	100,00
Kalimantan Utara	1,88	2,07	1,48	94,08	0,48	0,00	0,00	100,00
Sulawesi Utara	3,23	2,17	2,76	91,34	0,19	0,28	0,02	100,00
Sulawesi Tengah	0,82	1,42	1,31	92,79	0,42	3,23	0,00	100,00
Sulawesi Selatan	2,35	4,15	2,16	90,08	0,37	0,77	0,12	100,00
Sulawesi Tenggara	0,99	1,96	4,18	91,13	0,00	1,73	0,00	100,00
Gorontalo	0,32	2,22	0,62	96,50	0,00	0,33	0,00	100,00
Sulawesi Barat	2,61	0,79	2,17	90,17	0,29	3,82	0,15	100,00
Maluku	1,12	1,16	3,11	91,39	0,76	2,43	0,04	100,00
Maluku Utara	3,15	0,00	0,81	95,10	0,43	0,50	0,00	100,00
Papua Barat	1,25	1,48	2,84	94,36	0,06	0,00	0,01	100,00
Papua	2,09	3,57	2,24	90,79	0,43	0,78	0,09	100,00
**Indonesia**	**2,78**	**60,00**	**14,15**	**22,37**	**0,38**	**0,19**	**0,13**	**100,00**

Sumber/*Source*: Susenas Maret 2018/*The March 2018 Susenas*

https://www.bps.go.id

Tabel **6.5.2.** **Persentase Rumah Tangga di Daerah Perdesaan menurut Provinsi dan Bahan Bangunan Utama Atap Rumah Terluas, 2018**
Table *Percentage of Households in Rural Area by Province and The Main Material of The Widest Part of Roof, 2018*

**Provinsi** *Province*	**Beton** *Concrete*	**Genteng** *Roof*	**Asbes** *Asbestos*	**Seng** *Zinc*	**Bambu/Kayu/ Sirap** *Bamboo/Wood*	**Jerami/Ijuk/Daun/ Rumbia** *Hay/Leaves/Rumbia*	**Lainnya** *Others*	**Jumlah** *Total*
(1)	(2)	(3)	(4)	(5)	(6)	(7)	(8)	(9)
Aceh	1,50	1,17	3,22	88,78	0,54	4,77	0,02	100,00
Sumatera Utara	0,97	0,72	2,33	91,90	0,23	3,83	0,02	100,00
Sumatera Barat	0,90	1,78	2,54	93,75	0,33	0,70	0,00	100,00
Riau	0,75	3,29	4,87	89,03	0,09	1,82	0,15	100,00
Jambi	1,84	25,48	5,74	65,93	0,24	0,54	0,24	100,00
Sumatera Selatan	1,34	62,92	5,64	28,06	0,28	1,70	0,08	100,00
Bengkulu	0,81	6,58	4,21	87,89	0,18	0,14	0,18	100,00
Lampung	0,85	86,46	7,93	4,52	0,07	0,14	0,03	100,00
Kepulauan Bangka Belitung	0,63	6,83	57,55	34,56	0,06	0,36	0,00	100,00
Kepulauan Riau	0,93	0,71	54,03	40,60	0,25	3,23	0,24	100,00
DKI Jakarta	-	-	-	-	-	-	-	-
Jawa Barat	1,51	92,60	5,21	0,17	0,19	0,29	0,03	100,00
Jawa Tengah	1,22	87,37	4,58	6,66	0,10	0,03	0,04	100,00
DI Yogyakarta	0,37	97,77	1,45	0,41	0,00	0,00	0,00	100,00
Jawa Timur	1,91	94,97	2,34	0,59	0,16	0,02	0,01	100,00
Banten	1,45	85,56	8,42	0,52	0,26	3,70	0,09	100,00
Bali	2,06	71,70	6,51	19,51	0,07	0,15	0,00	100,00
Nusa Tenggara Barat	1,99	55,26	15,99	26,25	0,00	0,34	0,18	100,00
Nusa Tenggara Timur	0,08	0,37	0,64	88,27	0,50	10,14	0,00	100,00
Kalimantan Barat	0,20	1,94	2,23	87,90	4,46	3,23	0,04	100,00
Kalimantan Tengah	0,39	9,74	12,40	62,07	13,87	1,36	0,16	100,00
Kalimantan Selatan	0,10	5,12	13,43	67,35	8,90	5,09	0,00	100,00
Kalimantan Timur	0,40	4,44	6,16	83,95	4,67	0,34	0,05	100,00
Kalimantan Utara	1,27	0,28	3,53	94,53	0,28	0,11	0,00	100,00
Sulawesi Utara	1,43	1,33	3,00	92,14	0,53	1,40	0,17	100,00
Sulawesi Tengah	0,47	1,75	1,19	85,72	0,50	10,35	0,01	100,00
Sulawesi Selatan	0,78	1,34	2,77	91,74	0,44	2,81	0,11	100,00
Sulawesi Tenggara	0,45	6,13	7,61	79,59	0,17	6,04	0,02	100,00
Gorontalo	0,02	1,28	0,88	92,72	0,00	5,11	0,00	100,00
Sulawesi Barat	0,45	1,28	2,30	87,66	0,92	7,38	0,00	100,00
Maluku	1,09	1,14	8,11	80,23	0,23	9,02	0,18	100,00
Maluku Utara	1,84	0,67	2,86	89,02	0,22	5,29	0,11	100,00
Papua Barat	0,43	0,75	2,36	94,88	0,43	1,06	0,09	100,00
Papua	0,18	0,25	2,01	56,47	1,42	38,78	0,88	100,00
**Indonesia**	**1,19**	**53,55**	**5,05**	**36,91**	**0,75**	**2,48**	**0,07**	**100,00**

Sumber/*Source*: Susenas Maret 2018/*The March 2018 Susenas*

https://www.bps.go.id

Tabel **6.5.3.** **Persentase Rumah Tangga di Daerah Perkotaan dan Perdesaan menurut Provinsi dan Bahan Bangunan Utama Atap Rumah Terluas, 2018**
Table *Percentage of Households in Urban and Rural Area by Province and The Main Material of The Widest Part of Roof, 2018*

**Provinsi** *Province*	**Beton** *Concrete*	**Genteng** *Roof*	**Asbes** *Asbestos*	**Seng** *Zinc*	**Bambu/Kayu/ Sirap** *Bamboo/Wood*	**Jerami/Ijuk/Daun/ Rumbia** *Hay/Leaves/Rumbia*	**Lainnya** *Others*	**Jumlah** *Total*
(1)	(2)	(3)	(4)	(5)	(6)	(7)	(8)	(9)
Aceh	2,33	2,44	3,12	87,89	0,38	3,83	0,01	100,00
Sumatera Utara	2,12	1,72	4,12	89,55	0,21	2,25	0,04	100,00
Sumatera Barat	1,24	2,40	2,51	93,20	0,19	0,46	0,00	100,00
Riau	1,89	3,47	4,51	88,79	0,09	1,10	0,16	100,00
Jambi	2,34	22,96	4,72	69,11	0,20	0,47	0,21	100,00
Sumatera Selatan	1,95	57,31	6,78	32,31	0,33	1,19	0,13	100,00
Bengkulu	0,82	5,63	3,48	89,53	0,21	0,10	0,22	100,00
Lampung	0,82	85,30	9,21	4,49	0,06	0,10	0,02	100,00
Kepulauan Bangka Belitung	1,15	10,38	53,54	34,52	0,07	0,33	0,00	100,00
Kepulauan Riau	3,34	5,69	50,01	39,35	0,30	0,60	0,71	100,00
DKI Jakarta	3,56	40,37	54,05	1,67	0,25	0,00	0,11	100,00
Jawa Barat	3,01	82,87	13,27	0,48	0,13	0,09	0,15	100,00
Jawa Tengah	1,66	87,36	5,20	5,64	0,07	0,02	0,05	100,00
DI Yogyakarta	0,58	95,57	2,73	1,12	0,00	0,00	0,00	100,00
Jawa Timur	2,34	91,76	4,99	0,68	0,14	0,01	0,08	100,00
Banten	2,57	75,56	19,89	0,44	0,21	1,15	0,19	100,00
Bali	2,31	77,10	8,75	11,70	0,07	0,06	0,00	100,00
Nusa Tenggara Barat	2,45	59,06	13,60	24,49	0,05	0,23	0,13	100,00
Nusa Tenggara Timur	0,17	0,43	0,59	90,44	0,43	7,95	0,00	100,00
Kalimantan Barat	0,41	2,36	2,06	89,36	3,33	2,44	0,03	100,00
Kalimantan Tengah	0,98	12,41	10,73	62,53	12,03	1,04	0,28	100,00
Kalimantan Selatan	0,52	6,17	11,92	69,09	9,12	3,17	0,00	100,00
Kalimantan Timur	1,22	8,14	6,27	80,70	3,49	0,16	0,03	100,00
Kalimantan Utara	1,63	1,34	2,31	94,27	0,40	0,04	0,00	100,00
Sulawesi Utara	2,33	1,75	2,88	91,74	0,36	0,84	0,10	100,00
Sulawesi Tengah	0,57	1,66	1,22	87,73	0,48	8,33	0,01	100,00
Sulawesi Selatan	1,42	2,47	2,53	91,07	0,41	1,99	0,12	100,00
Sulawesi Tenggara	0,65	4,54	6,30	83,99	0,11	4,40	0,01	100,00
Gorontalo	0,14	1,65	0,78	94,21	0,00	3,23	0,00	100,00
Sulawesi Barat	0,95	1,17	2,27	88,25	0,77	6,56	0,03	100,00
Maluku	1,10	1,15	5,95	85,05	0,46	6,17	0,12	100,00
Maluku Utara	2,23	0,47	2,25	90,83	0,28	3,86	0,08	100,00
Papua Barat	0,76	1,04	2,55	94,67	0,28	0,64	0,06	100,00
Papua	0,67	1,11	2,07	65,29	1,17	29,02	0,68	100,00
**Indonesia**	**2,06**	**57,08**	**10,03**	**28,96**	**0,55**	**1,23**	**0,10**	**100,00**

Sumber/*Source*: Susenas Maret 2018/*The March 2018 Susenas*

https://www.bps.go.id

Tabel **6.6.1.** **Persentase Rumah Tangga di Daerah Perkotaan menurut Provinsi dan Bahan Bangunan Utama Dinding Rumah Terluas, 2018**
Table *Percentage of Households in Urban Area by Province and The Main Material of The Widest Part of Wall, 2018*

**Provinsi** *Province*	**Tembok** *Concrete*	**Plesteran Anyaman Bambu/Kawat** *Plastering of Woven Bamboo/Wire*	**Kayu, Batang Kayu** *Log, Wood*	**Bambu, Anyaman Bambu** *Bamboo, Woven Bamboo*	**Lainnya** *Others*	**Jumlah** *Total*
(1)	(2)	(3)	(4)	(5)	(6)	(7)
Aceh	67,58	0,09	30,87	1,22	0,23	100,00
Sumatera Utara	78,12	0,26	18,46	2,69	0,47	100,00
Sumatera Barat	82,92	1,41	15,31	0,32	0,04	100,00
Riau	76,98	1,65	21,12	0,11	0,15	100,00
Jambi	70,63	0,79	27,52	0,28	0,78	100,00
Sumatera Selatan	79,44	0,08	19,99	0,24	0,26	100,00
Bengkulu	86,48	2,47	9,85	0,75	0,45	100,00
Lampung	87,51	0,00	7,66	4,77	0,07	100,00
Kepulauan Bangka Belitung	88,78	0,00	11,02	0,00	0,20	100,00
Kepulauan Riau	84,32	0,14	13,89	0,04	1,61	100,00
DKI Jakarta	93,90	0,25	5,65	0,06	0,14	100,00
Jawa Barat	91,81	0,82	1,67	5,00	0,70	100,00
Jawa Tengah	89,18	0,39	8,22	1,63	0,58	100,00
DI Yogyakarta	97,88	0,08	0,99	0,97	0,08	100,00
Jawa Timur	94,79	0,37	2,61	1,81	0,42	100,00
Banten	96,21	0,09	1,27	2,24	0,19	100,00
Bali	97,71	0,02	1,50	0,71	0,06	100,00
Nusa Tenggara Barat	91,66	0,11	3,27	4,95	0,00	100,00
Nusa Tenggara Timur	64,60	1,08	5,99	11,33	17,00	100,00
Kalimantan Barat	71,81	14,69	12,71	0,19	0,60	100,00
Kalimantan Tengah	48,07	0,95	50,06	0,22	0,71	100,00
Kalimantan Selatan	39,67	1,08	58,64	0,29	0,33	100,00
Kalimantan Timur	54,89	0,02	45,04	0,00	0,05	100,00
Kalimantan Utara	54,75	0,00	44,92	0,00	0,33	100,00
Sulawesi Utara	76,67	0,26	20,68	0,89	1,50	100,00
Sulawesi Tengah	72,71	0,03	27,18	0,04	0,04	100,00
Sulawesi Selatan	65,72	0,25	20,90	2,09	11,05	100,00
Sulawesi Tenggara	64,77	0,07	33,76	0,40	1,00	100,00
Gorontalo	87,15	0,00	7,86	4,99	0,00	100,00
Sulawesi Barat	65,24	0,00	25,91	3,24	5,61	100,00
Maluku	83,99	0,73	12,96	0,44	1,88	100,00
Maluku Utara	85,26	0,40	13,42	0,18	0,74	100,00
Papua Barat	82,86	0,04	16,35	0,11	0,63	100,00
Papua	67,06	0,00	31,30	0,23	1,41	100,00
**Indonesia**	**87,30**	**0,62**	**8,83**	**2,41**	**0,84**	**100,00**

Sumber/*Source*: Susenas Maret 2018/*The March 2018 Susenas*

https://www.bps.go.id

Tabel *Table* **6.6.2. Persentase Rumah Tangga di Daerah Perdesaan menurut Provinsi dan Bahan Bangunan Utama Dinding Rumah Terluas, 2018**
Percentage of Households in Rural Area by Province and The Main Material of The Widest Part of Wall, 2018

**Provinsi** *Province*	**Tembok** *Concrete*	**Plesteran Anyaman Bambu/Kawat** *Plastering of Woven Bamboo/Wire*	**Kayu, Batang Kayu** *Log, Wood*	**Bambu, Anyaman Bambu** *Bamboo, Woven Bamboo*	**Lainnya** *Others*	**Jumlah** *Total*
(1)	(2)	(3)	(4)	(5)	(6)	(7)
Aceh	47,44	0,10	49,59	1,87	1,00	100,00
Sumatera Utara	48,90	0,23	45,73	4,64	0,49	100,00
Sumatera Barat	66,84	2,82	28,96	1,17	0,20	100,00
Riau	46,91	1,89	50,42	0,40	0,37	100,00
Jambi	59,81	0,40	39,13	0,20	0,46	100,00
Sumatera Selatan	52,81	0,02	45,55	1,11	0,51	100,00
Bengkulu	56,05	6,36	35,45	1,19	0,95	100,00
Lampung	73,99	0,04	19,36	6,36	0,24	100,00
Kepulauan Bangka Belitung	72,11	0,00	27,56	0,17	0,16	100,00
Kepulauan Riau	50,18	1,68	46,64	0,10	1,40	100,00
DKI Jakarta	-	-	-	-	-	-
Jawa Barat	70,48	0,98	5,27	20,75	2,53	100,00
Jawa Tengah	70,69	0,41	24,17	3,85	0,87	100,00
DI Yogyakarta	85,80	0,00	7,22	6,63	0,35	100,00
Jawa Timur	79,95	0,53	12,46	5,33	1,73	100,00
Banten	70,86	0,04	2,67	25,94	0,50	100,00
Bali	94,74	0,23	2,10	2,85	0,09	100,00
Nusa Tenggara Barat	76,51	0,32	13,16	9,83	0,18	100,00
Nusa Tenggara Timur	34,84	0,44	14,51	28,42	21,78	100,00
Kalimantan Barat	38,21	29,72	31,32	0,36	0,39	100,00
Kalimantan Tengah	23,83	0,45	74,72	0,17	0,83	100,00
Kalimantan Selatan	18,39	0,21	80,53	0,44	0,43	100,00
Kalimantan Timur	22,36	0,09	77,16	0,20	0,19	100,00
Kalimantan Utara	20,72	0,00	78,49	0,00	0,79	100,00
Sulawesi Utara	63,46	0,71	31,78	2,18	1,87	100,00
Sulawesi Tengah	53,05	0,19	44,87	1,54	0,36	100,00
Sulawesi Selatan	34,25	0,14	45,76	6,43	13,41	100,00
Sulawesi Tenggara	37,89	0,05	60,77	0,99	0,30	100,00
Gorontalo	67,61	0,00	24,49	7,90	0,00	100,00
Sulawesi Barat	41,26	0,36	52,13	1,98	4,27	100,00
Maluku	73,54	0,20	23,51	0,68	2,07	100,00
Maluku Utara	69,55	2,18	26,02	1,80	0,46	100,00
Papua Barat	59,79	0,04	37,80	0,22	2,14	100,00
Papua	11,85	0,18	85,79	0,47	1,70	100,00
**Indonesia**	**61,41**	**1,32**	**28,23**	**6,88**	**2,16**	**100,00**

Sumber/*Source*: Susenas Maret 2018/*The March 2018 Susenas*

https://www.bps.go.id

Tabel **6.6.3.** **Persentase Rumah Tangga di Daerah Perkotaan dan Perdesaan menurut Provinsi dan Bahan Bangunan Utama Dinding Rumah Terluas, 2018**
Table *Percentage of Households in Urban and Rural Area by Province and The Main Material of The Widest Part of Wall, 2018*

**Provinsi** *Province*	**Tembok** *Concrete*	**Plesteran Anyaman Bambu/Kawat** *Plastering of Woven Bamboo/Wire*	**Kayu, Batang Kayu** *Log, Wood*	**Bambu, Anyaman Bambu** *Bamboo, Woven Bamboo*	**Lainnya** *Others*	**Jumlah** *Total*
(1)	(2)	(3)	(4)	(5)	(6)	(7)
Aceh	53,73	0,10	43,74	1,67	0,76	100,00
Sumatera Utara	64,24	0,25	31,42	3,62	0,48	100,00
Sumatera Barat	73,94	2,20	22,93	0,80	0,13	100,00
Riau	59,05	1,79	38,59	0,28	0,28	100,00
Jambi	63,19	0,52	35,50	0,23	0,56	100,00
Sumatera Selatan	62,35	0,04	36,40	0,79	0,42	100,00
Bengkulu	65,81	5,11	27,24	1,05	0,79	100,00
Lampung	77,76	0,03	16,10	5,92	0,19	100,00
Kepulauan Bangka Belitung	81,01	0,00	18,73	0,08	0,18	100,00
Kepulauan Riau	79,57	0,35	18,45	0,05	1,58	100,00
DKI Jakarta	93,90	0,25	5,65	0,06	0,14	100,00
Jawa Barat	85,96	0,86	2,66	9,32	1,20	100,00
Jawa Tengah	80,07	0,40	16,08	2,72	0,73	100,00
DI Yogyakarta	94,70	0,06	2,64	2,46	0,15	100,00
Jawa Timur	87,67	0,45	7,33	3,50	1,05	100,00
Banten	89,00	0,07	1,67	8,98	0,28	100,00
Bali	96,73	0,09	1,69	1,41	0,07	100,00
Nusa Tenggara Barat	83,50	0,23	8,60	7,58	0,10	100,00
Nusa Tenggara Timur	41,88	0,59	12,50	24,38	20,65	100,00
Kalimantan Barat	49,24	24,79	25,21	0,31	0,46	100,00
Kalimantan Tengah	32,97	0,64	65,43	0,19	0,78	100,00
Kalimantan Selatan	28,27	0,61	70,37	0,37	0,38	100,00
Kalimantan Timur	44,11	0,05	55,68	0,07	0,10	100,00
Kalimantan Utara	40,95	0,00	58,54	0,00	0,52	100,00
Sulawesi Utara	70,09	0,48	26,21	1,53	1,68	100,00
Sulawesi Tengah	58,63	0,14	39,85	1,11	0,27	100,00
Sulawesi Selatan	46,95	0,18	35,73	4,68	12,46	100,00
Sulawesi Tenggara	48,14	0,05	50,48	0,77	0,56	100,00
Gorontalo	75,30	0,00	17,95	6,76	0,00	100,00
Sulawesi Barat	46,84	0,28	46,02	2,28	4,58	100,00
Maluku	78,05	0,43	18,96	0,58	1,99	100,00
Maluku Utara	74,22	1,65	22,28	1,32	0,54	100,00
Papua Barat	69,01	0,04	29,23	0,18	1,54	100,00
Papua	26,03	0,14	71,79	0,41	1,63	100,00
**Indonesia**	**75,57**	**0,94**	**17,62**	**4,44**	**1,44**	**100,00**

Sumber/*Source*: Susenas Maret 2018/*The March 2018 Susenas*

https://www.bps.go.id

Tabel *Table* **6.7.1.** **Persentase Rumah Tangga di Daerah Perkotaan menurut Provinsi dan Bahan Bangunan Utama Lantai Rumah Terluas, 2018**
Percentage of Households in Urban Area by Province and The Main Material of The Widest Part of Dwelling Floor, 2018

**Provinsi** *Province*	**Marmer/Granit** *Marble/Granite*	**Keramik** *Ceramic*	**Parket/Vinil/Permadani/Ubin/Tegel/Teraso** *Parquet/Vinyle/Rug,Tile/Terrazzo*	**Kayu/Papan** *Plank/Board*	**Semen/Bata Merah** *Cement/Red Brick*	**Bambu** *Bamboo*	**Tanah** *Ground*	**Lainnya** *Others*	**Jumlah** *Total*
(1)	(2)	(3)	(4)	(5)	(5)	(5)	(6)	(7)	(8)
Aceh	2,27	41,67	2,07	4,22	48,65	0,00	1,10	0,02	100,00
Sumatera Utara	1,45	50,68	1,99	4,37	40,64	0,05	0,79	0,02	100,00
Sumatera Barat	1,66	41,02	1,50	6,08	49,16	0,22	0,36	0,00	100,00
Riau	1,67	46,67	2,24	8,54	40,73	0,00	0,14	0,00	100,00
Jambi	1,79	40,30	0,54	16,33	39,77	0,03	1,18	0,07	100,00
Sumatera Selatan	1,63	51,51	2,96	14,32	29,18	0,00	0,40	0,00	100,00
Bengkulu	1,44	56,93	2,37	2,47	36,33	0,02	0,44	0,00	100,00
Lampung	1,69	48,35	2,62	1,07	44,42	0,00	1,85	0,00	100,00
Kepulauan Bangka Belitung	2,33	62,06	3,26	0,97	31,17	0,00	0,19	0,00	100,00
Kepulauan Riau	1,44	64,67	2,38	5,83	25,18	0,00	0,33	0,17	100,00
DKI Jakarta	1,86	84,36	7,13	2,02	4,34	0,03	0,20	0,05	100,00
Jawa Barat	1,41	76,85	7,98	2,45	9,16	0,66	1,41	0,08	100,00
Jawa Tengah	1,16	61,45	11,85	0,32	19,32	0,05	5,78	0,08	100,00
DI Yogyakarta	0,77	69,85	8,50	0,01	19,27	0,00	1,05	0,55	100,00
Jawa Timur	1,21	68,98	10,87	0,15	15,49	0,01	3,21	0,09	100,00
Banten	1,55	86,33	4,91	0,22	5,09	0,10	1,78	0,01	100,00
Bali	1,88	81,78	1,92	0,05	13,56	0,00	0,81	0,00	100,00
Nusa Tenggara Barat	1,05	45,77	2,22	3,42	46,00	0,08	1,46	0,00	100,00
Nusa Tenggara Timur	0,77	41,29	1,26	0,94	50,14	1,35	4,25	0,00	100,00
Kalimantan Barat	0,75	49,96	2,64	32,83	13,82	0,00	0,00	0,00	100,00
Kalimantan Tengah	0,61	39,48	1,19	44,70	13,85	0,00	0,17	0,00	100,00
Kalimantan Selatan	0,35	34,88	1,36	55,79	7,43	0,00	0,19	0,00	100,00
Kalimantan Timur	0,97	47,69	1,41	37,76	11,94	0,00	0,22	0,00	100,00
Kalimantan Utara	0,35	42,87	3,47	33,46	19,61	0,00	0,23	0,00	100,00
Sulawesi Utara	1,29	29,94	20,83	4,37	41,08	0,06	2,36	0,06	100,00
Sulawesi Tengah	1,49	45,61	5,03	7,96	38,75	0,00	1,05	0,11	100,00
Sulawesi Selatan	1,92	43,52	11,58	20,20	21,20	0,20	1,27	0,11	100,00
Sulawesi Tenggara	3,12	41,83	1,54	9,39	42,08	0,38	1,66	0,00	100,00
Gorontalo	0,42	47,88	1,37	1,90	47,90	0,00	0,54	0,00	100,00
Sulawesi Barat	0,55	43,87	6,14	22,65	24,47	0,40	1,76	0,16	100,00
Maluku	1,61	38,61	23,16	4,69	30,31	0,01	1,60	0,00	100,00
Maluku Utara	0,39	57,48	2,66	3,23	34,78	0,00	1,28	0,18	100,00
Papua Barat	1,00	46,40	7,21	10,78	33,84	0,00	0,77	0,00	100,00
Papua	0,67	41,25	9,59	15,34	31,71	0,02	1,42	0,00	100,00
**Indonesia**	**1,38**	**65,92**	**7,34**	**4,83**	**18,25**	**0,21**	1,99	**0,07**	100,00

Sumber/*Source*: Susenas Maret 2018/*The March 2018 Susenas*

https://www.bps.go.id

Tabel *Table* **6.7.2.** **Persentase Rumah Tangga di Daerah Perdesaan menurut Provinsi dan Bahan Bangunan Utama Lantai Rumah Terluas, 2018**
Percentage of Households in Rural Area by Province and The Main Material of The Widest Part of Dwelling Floor, 2018

**Provinsi** *Province*	**Marmer/ Granit** *Marble/Granite*	**Keramik** *Ceramic*	**Parket/Vinil/Permadani/Ubin/Tegel/ Teraso** *Parquet/Vinyle/Rug, Tile/Terrazzo*	**Kayu/Papan** *Plank/Board*	**Semen/Bata Merah** *Cement/Red Brick*	**Bambu** *Bamboo*	**Tanah** *Ground*	**Lainnya** *Others*	**Jumlah** *Total*
(1)	(2)	(3)	(4)	(5)	(5)	(5)	(6)	(7)	(8)
Aceh	1,07	16,48	0,69	9,97	66,12	0,22	5,28	0,17	100,00
Sumatera Utara	0,69	21,99	0,65	10,94	62,26	0,06	3,40	0,01	100,00
Sumatera Barat	0,70	20,13	0,87	11,42	65,55	0,40	0,94	0,00	100,00
Riau	0,99	19,66	2,28	25,57	50,10	0,00	1,39	0,00	100,00
Jambi	0,91	20,17	2,75	21,08	52,78	0,22	2,10	0,00	100,00
Sumatera Selatan	0,54	21,88	2,11	27,05	43,02	0,13	5,28	0,00	100,00
Bengkulu	0,46	29,18	1,92	7,26	57,38	0,22	3,55	0,03	100,00
Lampung	0,58	24,61	2,41	2,47	62,70	0,01	7,21	0,01	100,00
Kepulauan Bangka Belitung	0,85	48,65	0,95	2,34	47,06	0,00	0,15	0,00	100,00
Kepulauan Riau	0,30	21,71	1,72	39,07	36,91	0,00	0,20	0,08	100,00
DKI Jakarta	-	-	-	-	-	-	-	-	-
Jawa Barat	1,03	57,69	8,79	13,78	10,28	4,23	3,91	0,28	100,00
Jawa Tengah	0,90	44,01	10,31	1,55	24,53	0,08	18,41	0,21	100,00
DI Yogyakarta	0,07	36,65	9,26	0,00	43,98	0,00	8,61	1,44	100,00
Jawa Timur	0,81	47,81	8,27	0,22	27,52	0,09	15,12	0,16	100,00
Banten	0,69	63,60	6,90	3,72	9,59	9,44	6,06	0,00	100,00
Bali	0,73	60,21	1,74	0,20	33,63	0,00	3,50	0,00	100,00
Nusa Tenggara Barat	0,22	28,21	0,50	14,05	53,49	0,55	2,84	0,12	100,00
Nusa Tenggara Timur	0,18	8,40	0,54	4,63	52,76	7,23	26,18	0,07	100,00
Kalimantan Barat	0,82	21,64	1,45	53,90	21,70	0,13	0,35	0,02	100,00
Kalimantan Tengah	0,13	13,33	1,21	67,16	17,04	0,01	0,96	0,17	100,00
Kalimantan Selatan	0,35	11,52	1,23	73,90	12,24	0,14	0,62	0,00	100,00
Kalimantan Timur	0,02	17,68	1,07	64,71	15,17	0,01	1,35	0,00	100,00
Kalimantan Utara	0,38	12,42	1,60	72,51	13,10	0,00	0,00	0,00	100,00
Sulawesi Utara	0,60	19,58	8,71	9,50	57,09	0,12	4,37	0,03	100,00
Sulawesi Tengah	0,35	15,17	5,44	14,95	59,43	0,97	3,62	0,08	100,00
Sulawesi Selatan	0,78	16,98	3,08	50,20	27,25	0,52	0,98	0,21	100,00
Sulawesi Tenggara	0,51	16,95	3,31	25,78	49,16	1,47	2,79	0,01	100,00
Gorontalo	0,24	22,40	0,25	3,79	70,66	0,68	1,97	0,00	100,00
Sulawesi Barat	0,84	16,70	2,96	35,67	39,10	1,37	3,16	0,19	100,00
Maluku	0,35	20,70	11,35	5,13	54,60	0,75	7,07	0,05	100,00
Maluku Utara	0,80	19,33	4,81	5,60	60,47	0,66	8,26	0,06	100,00
Papua Barat	0,91	24,00	4,40	20,65	46,50	0,41	1,90	1,22	100,00
Papua	0,19	3,32	1,58	45,86	13,42	2,56	31,77	1,30	100,00
**Indonesia**	**0,72**	**34,09**	**5,32**	**14,81**	**34,87**	**1,17**	**8,87**	**0,16**	**100,00**

Sumber/*Source*: Susenas Maret 2018/*The March 2018 Susenas*

https://www.bps.go.id

Tabel *Table* **6.7.3.** **Persentase Rumah Tangga di Daerah Perkotaan dan Perdesaan menurut Provinsi dan Bahan Bangunan Utama Lantai Rumah Terluas, 2018**
Percentage of Households in Urban and Rural Area by Province and The Main Material of The Widest Part of Dwelling Floor, 2018

**Provinsi** *Province*	**Marmer/Granit** *Marble/Granite*	**Keramik** *Ceramic*	**Parket/Vinil/Permadani/Ubin/ Tegel/Teraso** *Parquet/Vinyle/Rug, Tile/Terrazzo*	**Kayu/Papan** *Plank/Board*	**Semen/Bata Merah** *Cement/Red Brick*	**Bambu** *Bamboo*	**Tanah** *Ground*	**Lainnya** *Others*	**Jumlah** *Total*
(1)	(2)	(3)	(4)	(5)	(5)	(5)	(6)	(7)	(8)
Aceh	1,45	24,35	1,12	8,17	60,66	0,15	3,97	0,12	100,00
Sumatera Utara	1,09	37,04	1,35	7,49	50,92	0,06	2,03	0,02	100,00
Sumatera Barat	1,12	29,35	1,15	9,06	58,32	0,32	0,68	0,00	100,00
Riau	1,27	30,56	2,27	18,70	46,32	0,00	0,89	0,00	100,00
Jambi	1,18	26,46	2,06	19,59	48,71	0,16	1,81	0,02	100,00
Sumatera Selatan	0,93	32,50	2,41	22,49	38,06	0,08	3,53	0,00	100,00
Bengkulu	0,77	38,08	2,07	5,73	50,63	0,16	2,55	0,02	100,00
Lampung	0,89	31,23	2,47	2,08	57,60	0,01	5,71	0,01	100,00
Kepulauan Bangka Belitung	1,64	55,81	2,18	1,61	38,58	0,00	0,17	0,00	100,00
Kepulauan Riau	1,28	58,69	2,29	10,46	26,81	0,00	0,31	0,16	100,00
DKI Jakarta	1,86	84,36	7,13	2,02	4,34	0,03	0,20	0,05	100,00
Jawa Barat	1,31	71,60	8,20	5,55	9,47	1,64	2,09	0,14	100,00
Jawa Tengah	1,03	52,85	11,09	0,93	21,89	0,06	12,01	0,14	100,00
DI Yogyakarta	0,59	61,10	8,70	0,01	25,78	0,00	3,04	0,79	100,00
Jawa Timur	1,02	58,83	9,63	0,18	21,26	0,05	8,92	0,12	100,00
Banten	1,31	79,87	5,48	1,22	6,37	2,76	3,00	0,00	100,00
Bali	1,50	74,66	1,86	0,10	20,18	0,00	1,70	0,00	100,00
Nusa Tenggara Barat	0,60	36,31	1,29	9,15	50,03	0,34	2,21	0,07	100,00
Nusa Tenggara Timur	0,32	16,18	0,71	3,75	52,14	5,84	20,99	0,05	100,00
Kalimantan Barat	0,80	30,94	1,84	46,98	19,11	0,09	0,23	0,01	100,00
Kalimantan Tengah	0,31	23,18	1,20	58,70	15,83	0,00	0,66	0,11	100,00
Kalimantan Selatan	0,35	22,37	1,29	65,50	10,00	0,07	0,42	0,00	100,00
Kalimantan Timur	0,66	37,74	1,30	46,69	13,01	0,00	0,60	0,00	100,00
Kalimantan Utara	0,36	30,52	2,71	49,30	16,97	0,00	0,14	0,00	100,00
Sulawesi Utara	0,95	24,78	14,79	6,92	49,06	0,09	3,36	0,05	100,00
Sulawesi Tengah	0,67	23,80	5,32	12,97	53,56	0,70	2,89	0,09	100,00
Sulawesi Selatan	1,24	27,69	6,51	38,10	24,80	0,39	1,10	0,17	100,00
Sulawesi Tenggara	1,51	26,43	2,63	19,54	46,46	1,06	2,36	0,01	100,00
Gorontalo	0,31	32,43	0,69	3,05	61,70	0,41	1,41	0,00	100,00
Sulawesi Barat	0,78	23,03	3,71	32,64	35,69	1,14	2,84	0,18	100,00
Maluku	0,90	28,43	16,45	4,94	44,11	0,43	4,71	0,03	100,00
Maluku Utara	0,68	30,67	4,17	4,90	52,83	0,47	6,19	0,09	100,00
Papua Barat	0,95	32,96	5,53	16,71	41,44	0,25	1,45	0,73	100,00
Papua	0,31	13,06	3,64	38,02	18,12	1,91	23,97	0,97	100,00
**Indonesia**	**1,08**	**51,51**	**6,43**	**9,35**	**25,77**	**0,65**	**5,10**	**0,11**	**100,00**

Sumber/*Source*: Susenas Maret 2018/*The March 2018 Susenas*

https://www.bps.go.id

Tabel / *Table* **6.8.** **Persentase Rumah Tangga menurut Provinsi, Daerah Tempat Tinggal, dan Kepemilikan serta Penggunaan Fasilitas Buang Air Besar, 2018**
Percentage of Households by Province, Urban Rural Classification, and The Use of Toilet Facilities 2018

Provinsi *Province*	Perkotaan/*Urban*						Perdesaan/*Rural*						Perkotaan+Perdesaan/*Urban+Rural*					
	Ada Fasilitas/*Have Facilities*						Ada Fasilitas/*Have Facilities*						Ada Fasilitas/*Have Facilities*					
	Sendiri *Private*	Bersama *Shared*	Komunal, Umum *Communal, Public Facility*	Tidak Digunakan *Not Used*	Tidak Ada *No Facility*	Jumlah *Total*	Sendiri *Private*	Bersama *Shared*	Komunal, Umum *Communal, Public Facility*	Tidak Digunakan *Not Used*	Tidak Ada *No Facility*	Jumlah *Total*	Sendiri *Private*	Bersama *Shared*	Komunal, Umum *Communal, Public Facility*	Tidak Digunakan *Not Used*	Tidak Ada *No Facility*	Jumlah *Total*
(1)	(2)	(3)	(4)	(5)	(6)	(7)	(8)	(9)	(10)	(11)	(12)	(13)	(14)	(15)	(16)	(17)	(18)	(19)
Aceh	88,55	5,70	1,40	0,01	4,34	100,00	65,52	3,77	4,88	0,40	25,43	100,00	72,72	4,37	3,79	0,28	18,84	100,00
Sumatera Utara	92,95	4,45	0,60	0,03	1,97	100,00	75,51	3,35	3,82	0,12	17,21	100,00	84,66	3,93	2,13	0,07	9,21	100,00
Sumatera Barat	83,03	7,74	2,12	0,29	6,82	100,00	62,90	6,25	4,61	0,31	25,92	100,00	71,79	6,91	3,51	0,30	17,49	100,00
Riau	95,28	3,50	0,48	0,02	0,71	100,00	86,26	3,54	0,97	0,16	9,08	100,00	89,90	3,53	0,77	0,10	5,70	100,00
Jambi	92,96	3,65	0,99	0,00	2,40	100,00	75,68	5,11	1,67	0,03	17,51	100,00	81,09	4,65	1,46	0,02	12,78	100,00
Sumatera Selatan	89,08	6,87	1,87	0,02	2,16	100,00	72,20	8,64	5,01	0,09	14,06	100,00	78,24	8,01	3,88	0,07	9,80	100,00
Bengkulu	92,58	4,71	1,01	0,07	1,63	100,00	74,54	5,39	1,82	0,13	18,13	100,00	80,33	5,17	1,56	0,11	12,83	100,00
Lampung	89,92	6,52	0,81	0,12	2,63	100,00	84,81	7,55	0,66	0,05	6,93	100,00	86,24	7,26	0,70	0,07	5,73	100,00
Kep. Bangka Belitung	92,92	3,28	0,87	0,00	2,94	100,00	82,55	2,25	2,36	0,27	12,58	100,00	88,08	2,80	1,56	0,13	7,43	100,00
Kepulauan Riau	90,22	8,62	0,76	0,00	0,41	100,00	86,83	5,40	1,74	0,04	5,99	100,00	89,75	8,17	0,89	0,01	1,19	100,00
DKI Jakarta	83,13	12,81	3,64	0,02	0,40	100,00	-	-	-	-	-	100,00	83,13	12,81	3,64	0,02	0,40	100,00
Jawa Barat	84,23	9,28	2,85	0,04	3,61	100,00	71,92	8,83	6,45	0,10	12,70	100,00	80,85	9,16	3,83	0,06	6,10	100,00
Jawa Tengah	83,13	9,69	1,82	0,06	5,31	100,00	79,08	8,09	1,56	0,10	11,17	100,00	81,13	8,90	1,69	0,08	8,19	100,00
DI Yogyakarta	78,26	18,80	1,19	0,01	1,74	100,00	87,07	7,99	0,25	0,00	4,69	100,00	80,58	15,95	0,94	0,01	2,52	100,00
Jawa Timur	82,60	9,32	1,57	0,12	6,40	100,00	71,16	10,69	0,84	0,18	17,12	100,00	77,11	9,98	1,22	0,15	11,54	100,00
Banten	89,15	5,47	1,78	0,09	3,51	100,00	62,19	4,22	2,72	0,55	30,33	100,00	81,48	5,11	2,04	0,22	11,14	100,00
Bali	83,28	14,40	0,17	0,04	2,10	100,00	69,24	16,24	0,02	0,20	14,30	100,00	78,65	15,01	0,13	0,09	6,13	100,00
Nusa Tenggara Barat	70,72	15,43	2,39	0,06	11,40	100,00	62,88	12,34	2,42	0,33	22,02	100,00	66,50	13,77	2,41	0,20	17,12	100,00
Nusa Tenggara Timur	76,18	21,09	1,77	0,00	0,96	100,00	69,92	11,39	2,68	0,13	15,89	100,00	71,40	13,68	2,46	0,10	12,35	100,00
Kalimantan Barat	93,73	3,80	0,81	0,07	1,59	100,00	71,11	4,36	2,34	0,15	22,03	100,00	78,54	4,17	1,84	0,13	15,32	100,00
Kalimantan Tengah	88,41	5,24	4,05	0,22	2,08	100,00	66,74	12,79	11,23	0,04	9,19	100,00	74,91	9,95	8,53	0,11	6,51	100,00
Kalimantan Selatan	86,37	9,40	3,78	0,00	0,45	100,00	71,55	15,53	7,81	0,04	5,07	100,00	78,43	12,68	5,94	0,02	2,92	100,00
Kalimantan Timur	94,89	2,45	1,87	0,00	0,78	100,00	84,17	3,23	6,85	0,14	5,61	100,00	91,34	2,71	3,52	0,05	2,38	100,00
Kalimantan Utara	92,63	5,10	1,79	0,00	0,48	100,00	84,28	5,70	3,18	0,00	6,84	100,00	89,24	5,34	2,36	0,00	3,06	100,00
Sulawesi Utara	78,83	15,12	2,62	0,22	3,21	100,00	71,61	8,26	3,10	0,21	16,82	100,00	75,23	11,71	2,86	0,22	9,99	100,00
Sulawesi Tengah	81,21	10,31	1,70	0,06	6,72	100,00	60,51	5,81	4,04	0,05	29,59	100,00	66,38	7,09	3,37	0,05	23,10	100,00
Sulawesi Selatan	85,40	8,22	2,76	0,11	3,51	100,00	78,92	6,61	1,41	0,19	12,88	100,00	81,53	7,26	1,95	0,16	9,10	100,00
Sulawesi Tenggara	82,80	10,66	1,39	0,00	5,16	100,00	70,18	7,96	2,73	0,07	19,07	100,00	74,99	8,99	2,22	0,05	13,77	100,00
Gorontalo	60,72	17,90	9,70	0,00	11,68	100,00	47,04	11,19	16,01	0,04	25,72	100,00	52,42	13,83	13,53	0,03	20,20	100,00
Sulawesi Barat	70,74	11,20	3,95	0,27	13,84	100,00	61,28	8,28	4,36	0,20	25,88	100,00	63,48	8,96	4,27	0,21	23,07	100,00
Maluku	78,08	13,80	2,73	0,09	5,31	100,00	60,37	4,92	8,67	0,13	25,91	100,00	68,02	8,75	6,11	0,11	17,01	100,00
Maluku Utara	76,10	18,74	2,92	0,00	2,24	100,00	56,00	8,81	14,92	0,17	20,10	100,00	61,98	11,76	11,35	0,12	14,79	100,00
Papua Barat	81,34	14,01	3,81	0,00	0,83	100,00	65,83	13,57	12,84	0,14	7,61	100,00	72,03	13,75	9,23	0,08	4,90	100,00
Papua	83,28	10,62	3,17	0,00	2,92	100,00	48,63	7,66	3,47	0,48	39,76	100,00	57,53	8,42	3,40	0,36	30,29	100,00
**Indonesia**	**84,82**	**9,22**	**2,12**	**0,06**	**3,79**	**100,00**	**72,75**	**8,09**	**3,14**	**0,16**	**15,86**	**100,00**	**79,35**	**8,71**	**2,58**	**0,11**	**9,25**	**100,00**

Sumber/*Source*: Susenas Maret 2018/*The March 2018 Susenas*

https://www.bps.go.id

Tabel *Table* **6.9.1.** **Persentase Rumah Tangga* di Daerah Perkotaan menurut Provinsi dan Jenis Kloset yang Digunakan Rumah Tangga, 2018**
Percentage of Households in Urban Area by Province and Type of Toilet Used by Household, 2018

**Provinsi** *Province*	**Leher Angsa** *Swan Trine*	**Plengsengan** *Flushing to Pit Latrine*	**Cemplung/Cubluk** *Plunged Hole*	**Jumlah** *Total*
(1)	(2)	(3)	(4)	(5)
Aceh	98,63	0,38	0,99	100,00
Sumatera Utara	96,80	1,07	2,13	100,00
Sumatera Barat	96,27	1,09	2,64	100,00
Riau	97,20	1,16	1,64	100,00
Jambi	95,86	0,69	3,45	100,00
Sumatera Selatan	97,25	0,60	2,15	100,00
Bengkulu	99,56	0,38	0,06	100,00
Lampung	97,28	0,61	2,11	100,00
Kepulauan Bangka Belitung	99,58	0,24	0,18	100,00
Kepulauan Riau	95,32	1,68	3,00	100,00
DKI Jakarta	98,90	0,79	0,31	100,00
Jawa Barat	97,44	1,75	0,81	100,00
Jawa Tengah	98,13	0,70	1,17	100,00
DI Yogyakarta	99,54	0,03	0,43	100,00
Jawa Timur	96,74	1,41	1,85	100,00
Banten	98,53	0,79	0,68	100,00
Bali	98,85	1,05	0,10	100,00
Nusa Tenggara Barat	98,89	0,43	0,68	100,00
Nusa Tenggara Timur	94,88	3,94	1,19	100,00
Kalimantan Barat	97,01	1,57	1,41	100,00
Kalimantan Tengah	95,83	1,44	2,73	100,00
Kalimantan Selatan	92,54	0,76	6,70	100,00
Kalimantan Timur	97,63	0,68	1,70	100,00
Kalimantan Utara	92,20	2,07	5,74	100,00
Sulawesi Utara	96,70	2,95	0,35	100,00
Sulawesi Tengah	97,18	0,90	1,92	100,00
Sulawesi Selatan	99,27	0,43	0,31	100,00
Sulawesi Tenggara	96,83	1,32	1,86	100,00
Gorontalo	99,26	0,71	0,03	100,00
Sulawesi Barat	98,75	1,09	0,15	100,00
Maluku	96,88	1,18	1,94	100,00
Maluku Utara	99,54	0,46	0,00	100,00
Papua Barat	94,97	1,07	3,96	100,00
Papua	92,17	2,98	4,84	100,00
**Indonesia**	**97,52**	**1,18**	**1,30**	**100,00**

Catatan/*Note*: *)Rumah tangga yang memiliki fasilitas tempat buang air besar dengan penggunaan sendiri atau bersama/*Households that have defecation facilities with their own or shared use*
Sumber/*Source*: Susenas Maret 2018/*The March 2018 Susenas*

https://www.bps.go.id

Tabel **6.9.2.** **Persentase Rumah Tangga* di Daerah Perdesaan menurut Provinsi dan Jenis Kloset yang Digunakan Rumah Tangga, 2018**
Table *Percentage of Households in Rural Area by Province and Type of Toilet Used by Household, 2018*

Provinsi *Province*	Leher Angsa *Swan Trine*	Plengsengan *Flushing to Pit Latrine*	Cemplung/Cubluk *Plunged Hole*	Jumlah *Total*
(1)	(2)	(3)	(4)	(5)
Aceh	91,09	3,24	5,67	100,00
Sumatera Utara	88,16	4,49	7,36	100,00
Sumatera Barat	92,19	2,54	5,28	100,00
Riau	82,61	9,14	8,25	100,00
Jambi	89,55	2,32	8,14	100,00
Sumatera Selatan	80,46	4,08	15,47	100,00
Bengkulu	92,01	2,97	5,02	100,00
Lampung	83,08	2,12	14,81	100,00
Kepulauan Bangka Belitung	97,01	1,00	1,98	100,00
Kepulauan Riau	70,60	6,01	23,39	100,00
DKI Jakarta	-	-	-	-
Jawa Barat	90,60	3,57	5,83	100,00
Jawa Tengah	92,71	2,55	4,73	100,00
DI Yogyakarta	93,17	0,65	6,18	100,00
Jawa Timur	85,14	2,51	12,35	100,00
Banten	96,12	1,42	2,45	100,00
Bali	98,71	0,34	0,95	100,00
Nusa Tenggara Barat	99,58	0,18	0,24	100,00
Nusa Tenggara Timur	68,27	17,40	14,33	100,00
Kalimantan Barat	91,21	3,90	4,88	100,00
Kalimantan Tengah	76,14	2,69	21,17	100,00
Kalimantan Selatan	80,16	1,14	18,69	100,00
Kalimantan Timur	91,54	1,60	6,85	100,00
Kalimantan Utara	89,09	5,62	5,29	100,00
Sulawesi Utara	96,10	3,00	0,90	100,00
Sulawesi Tengah	92,54	1,90	5,56	100,00
Sulawesi Selatan	95,14	1,78	3,08	100,00
Sulawesi Tenggara	88,99	1,02	9,98	100,00
Gorontalo	98,76	0,28	0,96	100,00
Sulawesi Barat	93,88	2,17	3,95	100,00
Maluku	94,71	0,89	4,40	100,00
Maluku Utara	92,46	1,35	6,20	100,00
Papua Barat	88,44	4,15	7,42	100,00
Papua	42,93	10,09	46,98	100,00
**Indonesia**	**87,84**	**3,38**	**8,78**	**100,00**

Catatan/*Note*: *)Rumah tangga yang memiliki fasilitas tempat buang air besar dengan penggunaan sendiri atau bersama/*Households that have defecation facilities with their own or shared use*
Sumber/*Source*: Susenas Maret 2018/*The March 2018 Susenas*

https://www.bps.go.id

Tabel **6.9.3.** **Persentase Rumah Tangga* di Daerah Perkotaan dan Perdesaan menurut Provinsi dan Jenis Kloset yang Digunakan Rumah Tangga, 2018**
Table *Percentage of Households in Urban and Rural Area by Province and Type of Toilet Used by Household, 2018*

**Provinsi** *Province*	**Leher Angsa** *Swan Trine*	**Plengsengan** *Flushing to Pit Latrine*	**Cemplung/Cubluk** *Plunged Hole*	**Jumlah** *Total*
**(1)**	**(2)**	**(3)**	**(4)**	**(5)**
Aceh	93,97	2,15	3,88	100,00
Sumatera Utara	93,15	2,52	4,34	100,00
Sumatera Barat	94,27	1,80	3,94	100,00
Riau	88,83	5,74	5,43	100,00
Jambi	91,77	1,74	6,49	100,00
Sumatera Selatan	87,15	2,69	10,16	100,00
Bengkulu	94,77	2,02	3,21	100,00
Lampung	87,17	1,68	11,15	100,00
Kepulauan Bangka Belitung	98,46	0,57	0,97	100,00
Kepulauan Riau	92,08	2,25	5,67	100,00
DKI Jakarta	98,90	0,79	0,31	100,00
Jawa Barat	95,76	2,20	2,04	100,00
Jawa Tengah	95,55	1,58	2,87	100,00
DI Yogyakarta	97,88	0,19	1,92	100,00
Jawa Timur	91,51	1,91	6,58	100,00
Banten	98,00	0,93	1,07	100,00
Bali	98,81	0,83	0,36	100,00
Nusa Tenggara Barat	99,24	0,30	0,46	100,00
Nusa Tenggara Timur	75,47	13,76	10,78	100,00
Kalimantan Barat	93,46	3,00	3,54	100,00
Kalimantan Tengah	84,33	2,17	13,50	100,00
Kalimantan Selatan	86,20	0,96	12,84	100,00
Kalimantan Timur	95,75	0,96	3,29	100,00
Kalimantan Utara	91,00	3,44	5,56	100,00
Sulawesi Utara	96,42	2,97	0,60	100,00
Sulawesi Tengah	94,18	1,55	4,27	100,00
Sulawesi Selatan	96,90	1,20	1,90	100,00
Sulawesi Tenggara	92,32	1,15	6,54	100,00
Gorontalo	98,99	0,48	0,53	100,00
Sulawesi Barat	95,16	1,88	2,95	100,00
Maluku	95,83	1,04	3,13	100,00
Maluku Utara	95,16	1,01	3,83	100,00
Papua Barat	91,34	2,78	5,88	100,00
Papua	60,94	7,49	31,57	100,00
**Indonesia**	**93,50**	**2,09**	**4,41**	**100,00**

Catatan/*Note*: *)Rumah tangga yang memiliki fasilitas tempat buang air besar dengan penggunaan sendiri atau bersama/*Households that have defecation facilities with their own or shared use*
Sumber/*Source*: Susenas Maret 2018/*The March 2018 Susenas*

https://www.bps.go.id

Tabel 6.10.1. **Persentase Rumah Tangga* di Daerah Perkotaan menurut Provinsi dan Tempat Pembuangan Akhir Tinja, 2018**
Table *Percentage of Households in Urban Area by Province and Final Disposal of Feces, 2018*

**Provinsi** *Province*	**Tangki** *Septic Tank*	**IPAL** *Sewage System*	**Kolam/Sawah/Sungai/Danau/ Laut** *Pond/Rice Field/River/Lake/Sea*	**Lubang Tanah** *Land Hole*	**Pantai/Tanah Lapang/Kebun, Lainnya** *Beach/Open Field/Yard, Others*	**Jumlah** *Total*
(1)	(2)	(3)	(4)	(5)	(6)	(7)
Aceh	94,68	0,26	2,25	2,31	0,50	100,00
Sumatera Utara	89,38	1,23	4,78	2,56	2,05	100,00
Sumatera Barat	80,16	0,28	14,12	4,94	0,50	100,00
Riau	92,45	0,00	2,91	4,01	0,63	100,00
Jambi	85,57	0,00	6,43	6,34	1,66	100,00
Sumatera Selatan	90,89	0,20	4,44	4,20	0,27	100,00
Bengkulu	76,79	0,92	3,31	16,87	2,11	100,00
Lampung	79,56	0,04	5,61	14,17	0,61	100,00
Kepulauan Bangka Belitung	96,59	0,11	1,15	1,99	0,15	100,00
Kepulauan Riau	92,19	0,06	5,37	1,77	0,62	100,00
DKI Jakarta	94,49	0,83	3,28	0,34	1,05	100,00
Jawa Barat	73,61	0,96	19,48	5,35	0,60	100,00
Jawa Tengah	87,04	1,38	7,40	3,85	0,32	100,00
DI Yogyakarta	88,02	8,29	2,30	1,34	0,05	100,00
Jawa Timur	87,76	0,72	4,36	6,75	0,41	100,00
Banten	90,45	0,06	4,58	4,20	0,71	100,00
Bali	97,33	1,58	0,66	0,43	0,00	100,00
Nusa Tenggara Barat	90,32	0,87	6,47	2,24	0,10	100,00
Nusa Tenggara Timur	86,12	0,00	0,00	13,84	0,03	100,00
Kalimantan Barat	86,35	0,63	2,59	10,35	0,08	100,00
Kalimantan Tengah	85,16	0,60	4,85	9,15	0,24	100,00
Kalimantan Selatan	81,59	0,35	8,47	9,36	0,24	100,00
Kalimantan Timur	88,92	1,24	4,59	4,12	1,13	100,00
Kalimantan Utara	87,35	0,00	9,33	1,88	1,44	100,00
Sulawesi Utara	89,77	0,69	2,34	6,62	0,57	100,00
Sulawesi Tengah	93,79	0,32	4,09	1,60	0,20	100,00
Sulawesi Selatan	96,52	0,24	1,03	1,88	0,32	100,00
Sulawesi Tenggara	88,21	0,00	1,70	9,60	0,49	100,00
Gorontalo	97,77	0,20	1,60	0,26	0,17	100,00
Sulawesi Barat	95,81	0,00	1,17	3,02	0,00	100,00
Maluku	89,90	0,49	5,01	4,32	0,29	100,00
Maluku Utara	98,01	0,00	0,54	1,33	0,13	100,00
Papua Barat	91,13	0,47	5,79	1,08	1,54	100,00
Papua	83,09	0,85	5,21	10,18	0,67	100,00
**Indonesia**	**85,29**	**0,96**	**8,46**	**4,69**	**0,61**	**100,00**

Catatan/*Note*: *)Rumah tangga yang memiliki fasilitas tempat buang air besar dengan penggunaan sendiri atau bersama/*Households that have defecation facilities with their own or shared use*
Sumber/*Source*: Susenas Maret 2018/*The March 2018 Susenas*

https://www.bps.go.id

Tabel 6.10.2. **Persentase Rumah Tangga* di Daerah Perdesaan menurut Provinsi dan Tempat Pembuangan Akhir Tinja, 2018**
Table *Percentage of Households in Urban Area by Province and Final Disposal of Feces, 2018*

**Provinsi** *Province*	**Tangki** *Septic Tank*	**IPAL** *Sewage System*	**Kolam/Sawah/Sungai/Danau/Laut** *Pond/Rice Field/River/Lake/Sea*	**Lubang Tanah** *Land Hole*	**Pantai/Tanah Lapang/Kebun, Lainnya** *Beach/Open Field/Yard, Others*	**Jumlah** *Total*
(1)	(2)	(3)	(4)	(5)	(6)	(7)
Aceh	82,66	1,06	3,81	11,61	0,87	100,00
Sumatera Utara	76,91	0,52	4,42	15,89	2,26	100,00
Sumatera Barat	65,31	0,28	17,32	16,86	0,24	100,00
Riau	65,80	0,00	4,19	28,39	1,63	100,00
Jambi	69,32	0,00	6,33	23,84	0,51	100,00
Sumatera Selatan	72,65	0,53	6,31	19,98	0,54	100,00
Bengkulu	37,00	0,25	4,59	57,46	0,69	100,00
Lampung	46,27	0,67	4,00	48,67	0,39	100,00
Kepulauan Bangka Belitung	91,08	0,10	0,43	8,19	0,20	100,00
Kepulauan Riau	58,30	1,11	27,63	8,91	4,05	100,00
DKI Jakarta	-	-	-	-	-	-
Jawa Barat	67,06	0,60	17,35	14,70	0,29	100,00
Jawa Tengah	76,02	0,20	8,88	14,37	0,52	100,00
DI Yogyakarta	80,35	0,08	0,41	18,40	0,75	100,00
Jawa Timur	68,93	0,70	2,57	27,08	0,72	100,00
Banten	50,50	1,64	7,87	39,01	0,98	100,00
Bali	95,79	0,65	1,19	2,10	0,27	100,00
Nusa Tenggara Barat	92,18	0,79	2,00	5,03	0,00	100,00
Nusa Tenggara Timur	57,12	0,00	0,09	42,60	0,19	100,00
Kalimantan Barat	51,87	0,39	5,44	41,81	0,49	100,00
Kalimantan Tengah	46,58	0,51	19,14	33,44	0,34	100,00
Kalimantan Selatan	56,38	0,48	16,19	26,63	0,32	100,00
Kalimantan Timur	72,22	0,00	7,05	20,57	0,16	100,00
Kalimantan Utara	59,94	0,00	4,44	32,21	3,40	100,00
Sulawesi Utara	84,26	1,62	2,07	11,87	0,19	100,00
Sulawesi Tengah	83,70	0,38	3,54	11,56	0,83	100,00
Sulawesi Selatan	85,10	0,02	1,05	13,44	0,39	100,00
Sulawesi Tenggara	80,93	0,02	2,63	15,40	1,02	100,00
Gorontalo	96,63	0,60	1,10	1,63	0,05	100,00
Sulawesi Barat	84,32	0,06	1,58	13,69	0,35	100,00
Maluku	90,41	0,49	1,85	5,76	1,49	100,00
Maluku Utara	88,48	0,00	4,54	5,28	1,70	100,00
Papua Barat	85,12	0,95	5,27	7,74	0,92	100,00
Papua	33,53	1,39	4,41	57,77	2,90	100,00
**Indonesia**	**69,43**	**0,49**	**6,76**	**22,63**	**0,69**	**100,00**

Catatan/*Note*: *)Rumah tangga yang memiliki fasilitas tempat buang air besar dengan penggunaan sendiri atau bersama/*Households that have defecation facilities with their own or shared use*
Sumber/*Source*: Susenas Maret 2018/*The March 2018 Susenas*

https://www.bps.go.id

Tabel **6.10.3.** **Persentase Rumah Tangga* di Daerah Perkotaan dan Perdesaan menurut Provinsi dan Tempat Pembuangan Akhir Tinja, 2018**
Table *Percentage of Households in Urban and Rural Area by Province and Final Disposal of Feces, 2018*

**Provinsi** *Province*	**Tangki** *Septic Tank*	**IPAL** *Sewage System*	**Kolam/Sawah/Sungai/Danau/Laut** *Pond/Rice Field/River/Lake/Sea*	**Lubang Tanah** *Land Hole*	**Pantai/Tanah Lapang/Kebun, Lainnya** *Beach/Open Field/Yard, Others*	**Jumlah** *Total*
(1)	(2)	(3)	(4)	(5)	(6)	(7)
Aceh	87,25	0,75	3,21	8,06	0,73	100,00
Sumatera Utara	84,11	0,93	4,63	8,20	2,14	100,00
Sumatera Barat	72,87	0,28	15,69	10,79	0,37	100,00
Riau	77,17	0,00	3,64	17,98	1,20	100,00
Jambi	75,05	0,00	6,37	17,67	0,91	100,00
Sumatera Selatan	79,92	0,40	5,57	13,69	0,43	100,00
Bengkulu	51,52	0,50	4,12	42,65	1,21	100,00
Lampung	55,85	0,49	4,46	38,75	0,45	100,00
Kepulauan Bangka Belitung	94,20	0,11	0,83	4,69	0,17	100,00
Kepulauan Riau	87,75	0,20	8,28	2,70	1,07	100,00
DKI Jakarta	94,49	0,83	3,28	0,34	1,05	100,00
Jawa Barat	72,00	0,87	18,96	7,65	0,53	100,00
Jawa Tengah	81,78	0,82	8,11	8,87	0,42	100,00
DI Yogyakarta	86,03	6,16	1,81	5,77	0,23	100,00
Jawa Timur	79,28	0,71	3,55	15,91	0,55	100,00
Banten	81,74	0,41	5,30	11,79	0,77	100,00
Bali	96,86	1,30	0,82	0,93	0,08	100,00
Nusa Tenggara Barat	91,26	0,83	4,21	3,65	0,05	100,00
Nusa Tenggara Timur	64,97	0,00	0,07	34,82	0,15	100,00
Kalimantan Barat	65,22	0,48	4,34	29,63	0,33	100,00
Kalimantan Tengah	62,63	0,55	13,19	23,34	0,30	100,00
Kalimantan Selatan	68,68	0,41	12,42	18,20	0,28	100,00
Kalimantan Timur	83,78	0,86	5,35	9,18	0,83	100,00
Kalimantan Utara	76,77	0,00	7,45	13,59	2,19	100,00
Sulawesi Utara	87,24	1,12	2,22	9,02	0,40	100,00
Sulawesi Tengah	87,27	0,36	3,73	8,04	0,60	100,00
Sulawesi Selatan	89,96	0,12	1,04	8,53	0,36	100,00
Sulawesi Tenggara	84,02	0,01	2,23	12,94	0,80	100,00
Gorontalo	97,16	0,41	1,33	0,99	0,10	100,00
Sulawesi Barat	87,35	0,04	1,47	10,88	0,26	100,00
Maluku	90,15	0,49	3,48	5,02	0,87	100,00
Maluku Utara	92,12	0,00	3,01	3,77	1,10	100,00
Papua Barat	87,79	0,74	5,50	4,78	1,20	100,00
Papua	51,66	1,19	4,70	40,36	2,08	100,00
**Indonesia**	**78,69**	**0,76**	**7,75**	**12,15**	**0,64**	**100,00**

Catatan/*Note*: *)Rumah tangga yang memiliki fasilitas tempat buang air besar dengan penggunaan sendiri atau bersama/*Households that have defecation facilities with their own or shared use*
Sumber/*Source*: Susenas Maret 2018/*The March 2018 Susenas*

https://www.bps.go.id

Tabel *Table* **6.11.1.** **Persentase Rumah Tangga* di Daerah Perkotaan menurut Provinsi dan Sumber Air Utama yang Digunakan Rumah Tangga untuk Minum, 2018**
Percentage of Households in Urban Area by Province and Main Source of Drinking Water Consumed by Household, 2018

**Provinsi** *Province*	**Air Kemasan Bermerk, Air Isi Ulang** *Branded Bottled Refill Water*	**Leding** *Top Water*	**Sumur Bor/ Pompa** *Barehole*	**Sumur Terlindung** *Protected Well*	**Sumur Tak Terlindung** *Unprotected Well*	**Mata Air Terlindung, Mata Air Tak Terlindung** *Protected, Unprotected Spring*	**Air Permukaan** *Surface Water*	**Air Hujan** *Rain Water*	**Lainnya** *Others*	**Jumlah** *Total*
(1)	(2)	(3)	(4)	(5)	(6)	(7)	(8)	(9)	(10)	(11)
Aceh	70,54	6,82	4,40	11,86	2,01	3,63	0,15	0,40	0,20	100,00
Sumatera Utara	52,65	19,92	15,27	8,47	1,64	1,67	0,19	0,16	0,03	100,00
Sumatera Barat	56,45	17,00	8,54	10,85	2,48	3,53	0,82	0,28	0,05	100,00
Riau	71,27	0,66	12,26	6,25	0,73	1,49	0,00	7,32	0,00	100,00
Jambi	45,34	17,98	3,86	19,52	3,92	0,24	0,21	8,94	0,00	100,00
Sumatera Selatan	32,98	37,88	4,85	19,42	3,32	0,32	0,76	0,47	0,00	100,00
Bengkulu	33,79	14,61	13,29	20,37	15,06	2,54	0,10	0,11	0,13	100,00
Lampung	43,15	3,17	13,92	25,18	13,82	0,71	0,05	0,00	0,00	100,00
Kepulauan Bangka Belitung	68,01	0,40	8,21	15,61	6,85	0,33	0,08	0,51	0,00	100,00
Kepulauan Riau	78,56	7,88	3,54	6,46	1,60	1,00	0,06	0,50	0,39	100,00
DKI Jakarta	75,52	9,54	14,73	0,20	0,00	0,00	0,00	0,02	0,00	100,00
Jawa Barat	51,56	7,27	18,42	15,56	2,13	4,85	0,06	0,01	0,15	100,00
Jawa Tengah	29,73	22,47	20,76	20,53	1,63	4,64	0,18	0,00	0,05	100,00
DI Yogyakarta	31,71	8,34	17,14	36,86	4,40	1,48	0,00	0,07	0,00	100,00
Jawa Timur	43,35	10,77	22,65	15,67	0,95	6,31	0,05	0,12	0,12	100,00
Banten	63,30	3,82	28,12	3,32	0,69	0,69	0,01	0,00	0,06	100,00
Bali	57,91	19,33	6,81	4,81	0,50	9,94	0,35	0,36	0,00	100,00
Nusa Tenggara Barat	31,44	21,65	10,96	26,68	1,45	7,39	0,23	0,20	0,00	100,00
Nusa Tenggara Timur	30,34	40,78	9,04	15,08	1,38	2,86	0,14	0,38	0,00	100,00
Kalimantan Barat	41,09	5,68	0,63	2,74	1,01	2,64	0,62	45,49	0,11	100,00
Kalimantan Tengah	68,64	9,81	11,61	2,76	0,90	0,22	1,85	4,13	0,07	100,00
Kalimantan Selatan	42,15	41,59	3,84	5,77	4,25	0,03	2,36	0,01	0,00	100,00
Kalimantan Timur	71,18	24,73	1,88	0,85	0,25	0,28	0,40	0,43	0,00	100,00
Kalimantan Utara	83,12	8,08	0,93	0,70	0,00	0,35	0,20	6,63	0,00	100,00
Sulawesi Utara	61,56	14,99	8,79	8,21	1,47	4,05	0,00	0,56	0,36	100,00
Sulawesi Tengah	67,92	9,17	14,90	2,85	0,15	4,25	0,76	0,00	0,00	100,00
Sulawesi Selatan	57,15	24,74	9,06	5,69	0,99	1,07	0,41	0,85	0,04	100,00
Sulawesi Tenggara	53,70	21,88	8,20	7,66	1,17	5,99	0,04	1,36	0,00	100,00
Gorontalo	62,23	16,18	7,70	11,18	1,09	1,60	0,00	0,00	0,04	100,00
Sulawesi Barat	48,24	19,62	15,22	9,07	2,69	3,25	1,91	0,00	0,00	100,00
Maluku	41,97	21,15	9,56	9,94	0,44	15,76	0,28	0,90	0,00	100,00
Maluku Utara	49,93	32,31	4,08	9,15	1,03	0,78	0,02	2,70	0,00	100,00
Papua Barat	74,60	10,98	3,75	4,14	0,20	1,41	0,16	4,78	0,00	100,00
Papua	66,03	16,72	3,42	3,72	1,36	3,58	0,71	4,23	0,23	100,00
**Indonesia**	**50,46**	**13,22**	**16,57**	**13,12**	**1,80**	**3,69**	**0,18**	**0,87**	**0,08**	**100,00**

Catatan/*Note*: *)Rumah tangga yang memiliki fasilitas tempat buang air besar dengan penggunaan sendiri atau bersama/*Households that have defecation facilities with their own or shared use*
Sumber/*Source*: Susenas Maret 2018/*The March 2018 Susenas*

https://www.bps.go.id

Tabel **6.11.2.** **Persentase Rumah Tangga* di Daerah Perdesaan menurut Provinsi dan Sumber Air Utama yang Digunakan Rumah Tangga untuk Minum, 2018**
Table *Percentage of Households in Rural Area by Province and Main Source of Drinking Water Consumed by Household, 2018*

**Provinsi** *Province*	**Air Kemasan Bermerk, Air Isi Ulang** *Branded Bottled Refill Water*	**Leding** *Top Water*	**Sumur Bor/ Pompa** *Barehole*	**Sumur Terlindung** *Protected Well*	**Sumur Tak Terlindung** *Unprotected Well*	**Mata Air Terlindung, Mata Air Tak Terlindung** *Protected, Unprotected Spring*	**Air Permukaan** *Surface Water*	**Air Hujan** *Rain Water*	**Lainnya** *Others*	**Jumlah** *Total*
(1)	(2)	(3)	(4)	(5)	(6)	(7)	(8)	(9)	(10)	(11)
Aceh	25,18	7,07	8,15	31,91	11,20	12,93	2,98	0,45	0,14	100,00
Sumatera Utara	16,70	4,88	27,14	14,90	6,68	20,98	4,74	3,89	0,09	100,00
Sumatera Barat	27,21	9,19	5,30	20,98	8,92	22,95	3,22	2,07	0,15	100,00
Riau	33,81	0,52	15,11	17,14	6,09	2,03	0,61	24,56	0,13	100,00
Jambi	17,61	6,60	4,89	33,05	18,31	3,89	5,31	10,05	0,30	100,00
Sumatera Selatan	11,43	6,45	10,40	39,75	13,95	5,04	4,00	8,91	0,06	100,00
Bengkulu	8,14	9,94	8,43	20,74	36,10	14,38	1,89	0,27	0,12	100,00
Lampung	13,98	1,31	9,60	33,60	31,33	8,82	1,15	0,22	0,00	100,00
Kepulauan Bangka Belitung	45,79	1,41	10,51	24,96	13,51	2,93	0,53	0,36	0,00	100,00
Kepulauan Riau	29,53	7,81	5,25	26,04	16,72	12,49	1,01	1,14	0,00	100,00
DKI Jakarta	-	-	-	-	-	-	-	-	-	-
Jawa Barat	25,09	2,54	15,88	24,40	6,75	24,32	0,77	0,14	0,12	100,00
Jawa Tengah	18,77	9,78	17,39	24,43	3,81	24,26	0,41	1,07	0,07	100,00
DI Yogyakarta	7,03	25,52	8,14	29,08	4,68	11,58	0,19	13,77	0,00	100,00
Jawa Timur	16,67	6,94	27,25	25,11	3,41	19,73	0,58	0,24	0,07	100,00
Banten	26,42	2,58	20,99	20,47	10,92	16,48	1,38	0,30	0,47	100,00
Bali	10,49	27,60	5,15	4,93	1,10	42,73	1,84	6,15	0,00	100,00
Nusa Tenggara Barat	13,99	11,16	19,59	30,17	5,04	19,52	0,53	0,00	0,00	100,00
Nusa Tenggara Timur	1,52	7,16	4,11	18,83	7,30	52,44	3,62	5,02	0,00	100,00
Kalimantan Barat	10,31	2,56	4,76	5,77	6,68	20,43	9,53	39,87	0,08	100,00
Kalimantan Tengah	29,89	4,28	14,00	12,18	6,26	5,17	18,09	10,11	0,03	100,00
Kalimantan Selatan	21,30	16,82	15,72	7,72	17,64	2,37	15,64	2,79	0,00	100,00
Kalimantan Timur	60,34	6,42	4,89	6,47	6,43	5,47	5,79	4,18	0,00	100,00
Kalimantan Utara	47,11	6,26	1,81	3,06	1,05	4,86	9,17	26,68	0,00	100,00
Sulawesi Utara	30,63	7,68	7,99	16,84	5,72	29,02	0,11	1,97	0,04	100,00
Sulawesi Tengah	20,40	7,12	15,46	12,33	3,61	31,02	9,34	0,73	0,00	100,00
Sulawesi Selatan	17,72	8,56	24,26	20,03	7,00	18,46	2,17	1,78	0,02	100,00
Sulawesi Tenggara	18,71	7,82	12,46	26,53	7,53	22,81	0,90	3,25	0,00	100,00
Gorontalo	33,45	8,86	11,86	27,27	4,82	12,83	0,90	0,00	0,00	100,00
Sulawesi Barat	16,88	7,60	9,74	18,96	6,06	29,96	9,58	1,22	0,00	100,00
Maluku	8,88	5,29	5,17	29,53	5,35	38,69	1,91	5,19	0,00	100,00
Maluku Utara	11,45	15,62	5,03	26,94	11,39	20,36	4,43	4,76	0,02	100,00
Papua Barat	26,59	5,20	2,23	13,70	4,86	19,04	9,56	18,82	0,00	100,00
Papua	10,15	0,98	1,71	3,59	4,67	40,74	11,10	27,01	0,06	100,00
**Indonesia**	**19,15**	**6,75**	**16,11**	**22,81**	**8,18**	**19,84**	**2,72**	**4,35**	**0,08**	**100,00**

Catatan/*Note*: *)Rumah tangga yang memiliki fasilitas tempat buang air besar dengan penggunaan sendiri atau bersama/*Households that have defecation facilities with their own or shared use*
Sumber/*Source*: Susenas Maret 2018/*The March 2018 Susenas*

https://www.bps.go.id

Tabel *Table* **6.11.3.** **Persentase Rumah Tangga* di Daerah Perkotaan dan Perdesaan menurut Provinsi dan Sumber Air Utama yang Digunakan Rumah Tangga untuk Minum, 2018**

Percentage of Households in Urban and Rural Area by Province and Main Source of Drinking Water Consumed by Household, 2018

**Provinsi** *Province*	**Air Kemasan Bermerk, Air Isi Ulang** *Branded Bottled Refill Water*	**Leding** *Top Water*	**Sumur Bor/Pompa** *Barehole*	**Sumur Terlindung** *Protected Well*	**Sumur Tak Terlindung** *Unprotected Well*	**Mata Air Terlindung, Mata Air Tak Terlindung** *Protected, Unprotected Spring*	**Air Permukaan** *Surface Water*	**Air Hujan** *Rain Water*	**Lainnya** *Others*	**Jumlah** *Total*
**(1)**	**(2)**	**(3)**	**(4)**	**(5)**	**(6)**	**(7)**	**(8)**	**(9)**	**(10)**	**(11)**
Aceh	39,35	6,99	6,98	25,64	8,33	10,02	2,10	0,43	0,15	100,00
Sumatera Utara	35,57	12,78	20,91	11,53	4,03	10,85	2,35	1,93	0,06	100,00
Sumatera Barat	40,12	12,63	6,73	16,51	6,07	14,38	2,16	1,28	0,11	100,00
Riau	48,93	0,58	13,96	12,74	3,93	1,81	0,36	17,60	0,07	100,00
Jambi	26,28	10,16	4,57	28,82	13,81	2,75	3,71	9,70	0,21	100,00
Sumatera Selatan	19,15	17,71	8,41	32,47	10,14	3,35	2,84	5,89	0,04	100,00
Bengkulu	16,37	11,44	9,99	20,62	29,35	10,58	1,32	0,21	0,12	100,00
Lampung	22,11	1,83	10,81	31,25	26,44	6,56	0,84	0,16	0,00	100,00
Kepulauan Bangka Belitung	57,65	0,87	9,28	19,97	9,96	1,54	0,29	0,44	0,00	100,00
Kepulauan Riau	71,74	7,87	3,78	9,18	3,71	2,60	0,19	0,59	0,34	100,00
DKI Jakarta	75,52	9,54	14,73	0,20	0,00	0,00	0,00	0,02	0,00	100,00
Jawa Barat	44,30	5,97	17,72	17,98	3,39	10,19	0,25	0,05	0,14	100,00
Jawa Tengah	24,33	16,22	19,10	22,45	2,71	14,31	0,29	0,53	0,06	100,00
DI Yogyakarta	25,20	12,87	14,77	34,81	4,48	4,14	0,05	3,68	0,00	100,00
Jawa Timur	30,55	8,94	24,86	20,19	2,13	12,75	0,31	0,18	0,10	100,00
Banten	52,81	3,46	26,09	8,20	3,59	5,18	0,40	0,09	0,18	100,00
Bali	42,27	22,06	6,26	4,85	0,70	20,75	0,84	2,27	0,00	100,00
Nusa Tenggara Barat	22,04	16,00	15,61	28,56	3,38	13,92	0,39	0,09	0,00	100,00
Nusa Tenggara Timur	8,34	15,12	5,27	17,95	5,90	40,71	2,80	3,92	0,00	100,00
Kalimantan Barat	20,42	3,58	3,40	4,78	4,82	14,59	6,61	41,72	0,09	100,00
Kalimantan Tengah	44,49	6,36	13,10	8,63	4,24	3,30	11,97	7,86	0,05	100,00
Kalimantan Selatan	30,98	28,32	10,20	6,81	11,42	1,29	9,47	1,50	0,00	100,00
Kalimantan Timur	67,59	18,66	2,88	2,71	2,30	2,00	2,19	1,68	0,00	100,00
Kalimantan Utara	68,51	7,34	1,29	1,65	0,43	2,18	3,84	14,76	0,00	100,00
Sulawesi Utara	46,15	11,35	8,39	12,51	3,59	16,49	0,06	1,26	0,20	100,00
Sulawesi Tengah	33,88	7,70	15,30	9,64	2,63	23,42	6,91	0,52	0,00	100,00
Sulawesi Selatan	33,63	15,08	18,13	14,24	4,57	11,44	1,46	1,41	0,03	100,00
Sulawesi Tenggara	32,04	13,18	10,83	19,34	5,11	16,40	0,57	2,53	0,00	100,00
Gorontalo	44,78	11,74	10,22	20,94	3,35	8,41	0,55	0,00	0,01	100,00
Sulawesi Barat	24,19	10,40	11,01	16,66	5,28	23,74	7,79	0,94	0,00	100,00
Maluku	23,16	12,14	7,07	21,07	3,23	28,79	1,20	3,34	0,00	100,00
Maluku Utara	22,88	20,58	4,75	21,65	8,31	14,54	3,12	4,15	0,02	100,00
Papua Barat	45,79	7,51	2,83	9,87	3,00	11,99	5,80	13,21	0,00	100,00
Papua	24,50	5,03	2,15	3,62	3,82	31,19	8,43	21,16	0,10	100,00
**Indonesia**	**36,28**	**10,29**	**16,36**	**17,51**	**4,69**	**11,01**	**1,33**	**2,45**	**0,08**	**100,00**

Catatan/*Note*: *)Rumah tangga yang memiliki fasilitas tempat buang air besar dengan penggunaan sendiri atau bersama/*Households that have defecation facilities with their own or shared use*
Sumber/*Source*: Susenas Maret 2018/*The March 2018 Susenas*

https://www.bps.go.id

Tabel **6.12.** **Persentase Rumah Tangga dengan Sumber Air Minum dari Sumur Bor/Pompa, Sumur Terlindung, Sumur Tak Terlindung, Mata Air Terlindung, dan Mata Air Tak Terlindung menurut Provinsi, Daerah Tempat Tinggal, dan Jarak ke Tempat Penampungan Limbah/Kotoran/Tinja Terdekat, 2018**

Table *Percentage of Households Using Artesian Well/Pump, Protected Well, Unprotected Well, Protected Spring, and Unprotected Spring as Source of Drinking Water by Province, Urban Rural Classification, and the Distance to the Nearest Final Disposal Site of Faces, 2018*

**Provinsi** *Province*	**Perkotaan/***Urban*				**Perdesaan/***Rural*				**Perkotaan+Perdesaan/***Urban+Rural*			
	**< 10 m**	**≥ 10 m**	**Tidak Tahu**	**Jumlah** *Total*	**< 10 m**	**≥ 10 m**	**Tidak Tahu**	**Jumlah** *Total*	**< 10 m**	**≥ 10 m**	**Tidak Tahu**	**Jumlah** *Total*
**(1)**	**(2)**	**(3)**	**(4)**	**(5)**	**(6)**	**(7)**	**(8)**	**(9)**	**(10)**	**(11)**	**(12)**	**(13)**
Aceh	38,40	52,86	8,74	100,00	35,97	54,46	9,57	100,00	36,30	54,24	9,46	100,00
Sumatera Utara	43,74	51,25	5,02	100,00	29,61	62,91	7,49	100,00	33,85	59,41	6,75	100,00
Sumatera Barat	39,09	57,52	3,39	100,00	28,02	64,34	7,63	100,00	30,86	62,59	6,55	100,00
Riau	25,90	65,84	8,26	100,00	28,84	67,41	3,75	100,00	28,08	67,00	4,91	100,00
Jambi	24,36	69,00	6,63	100,00	23,42	72,69	3,89	100,00	23,58	72,06	4,36	100,00
Sumatera Selatan	41,41	56,92	1,67	100,00	35,03	61,96	3,01	100,00	36,20	61,04	2,76	100,00
Bengkulu	33,29	62,99	3,72	100,00	33,28	60,91	5,81	100,00	33,28	61,40	5,32	100,00
Lampung	27,86	70,86	1,29	100,00	26,51	73,31	0,18	100,00	26,78	72,82	0,40	100,00
Kepulauan Bangka Belitung	20,24	77,69	2,07	100,00	25,06	67,98	6,96	100,00	23,11	71,92	4,97	100,00
Kepulauan Riau	30,54	65,47	3,99	100,00	10,34	74,84	14,82	100,00	21,71	69,56	8,72	100,00
DKI Jakarta	36,24	55,98	7,78	100,00	-	-	-	100,00	36,24	55,98	7,78	100,00
Jawa Barat	40,99	51,18	7,83	100,00	34,55	57,34	8,11	100,00	38,43	53,63	7,94	100,00
Jawa Tengah	30,51	64,75	4,74	100,00	20,58	73,52	5,90	100,00	24,67	69,91	5,42	100,00
DI Yogyakarta	29,19	67,90	2,91	100,00	11,24	87,13	1,64	100,00	24,84	72,55	2,60	100,00
Jawa Timur	38,18	57,49	4,32	100,00	27,02	67,61	5,37	100,00	31,44	63,60	4,96	100,00
Banten	41,99	53,30	4,71	100,00	41,12	49,59	9,28	100,00	41,60	51,62	6,79	100,00
Bali	19,17	76,28	4,56	100,00	17,66	76,74	5,60	100,00	18,34	76,53	5,12	100,00
Nusa Tenggara Barat	31,23	62,51	6,25	100,00	36,33	58,48	5,19	100,00	34,55	59,88	5,56	100,00
Nusa Tenggara Timur	21,12	73,67	5,21	100,00	10,91	81,63	7,47	100,00	11,89	80,86	7,25	100,00
Kalimantan Barat	19,52	74,22	6,26	100,00	24,63	63,59	11,79	100,00	24,20	64,47	11,32	100,00
Kalimantan Tengah	28,54	65,30	6,16	100,00	25,52	71,34	3,13	100,00	26,12	70,14	3,74	100,00
Kalimantan Selatan	30,68	69,15	0,17	100,00	25,07	73,89	1,04	100,00	26,28	72,87	0,85	100,00
Kalimantan Timur	23,54	67,30	9,15	100,00	17,75	70,95	11,29	100,00	19,03	70,15	10,82	100,00
Kalimantan Utara	40,18	59,82	0,00	100,00	20,50	65,79	13,71	100,00	24,67	64,52	10,81	100,00
Sulawesi Utara	35,92	57,47	6,62	100,00	31,16	60,16	8,68	100,00	32,47	59,42	8,11	100,00
Sulawesi Tengah	28,57	54,49	16,94	100,00	18,61	67,94	13,46	100,00	19,83	66,28	13,88	100,00
Sulawesi Selatan	33,20	63,22	3,57	100,00	19,92	73,26	6,82	100,00	21,78	71,85	6,37	100,00
Sulawesi Tenggara	14,66	76,51	8,83	100,00	12,60	79,53	7,87	100,00	12,95	79,02	8,03	100,00
Gorontalo	49,21	49,69	1,10	100,00	32,96	61,72	5,32	100,00	36,17	59,34	4,49	100,00
Sulawesi Barat	31,21	53,15	15,64	100,00	20,67	68,58	10,76	100,00	21,98	66,66	11,36	100,00
Maluku	17,85	72,64	9,51	100,00	20,19	65,81	14,00	100,00	19,59	67,56	12,85	100,00
Maluku Utara	28,26	50,69	21,05	100,00	33,15	54,69	12,16	100,00	32,71	54,33	12,96	100,00
Papua Barat	25,61	68,19	6,20	100,00	26,74	64,21	9,05	100,00	26,58	64,76	8,66	100,00
Papua	33,03	49,04	17,93	100,00	14,36	68,50	17,13	100,00	15,78	67,02	17,19	100,00
**Indonesia**	**36,25**	**58,08**	**5,67**	**100,00**	**26,67**	**66,94**	**6,39**	**100,00**	**30,39**	**63,50**	**6,11**	**100,00**

Sumber/*Source*: Susenas Maret 2018/*The March 2018 Susenas*

https://www.bps.go.id

Tabel *Table* **6.13.** **Persentase Rumah Tangga Menurut Provinsi, Daerah Tempat Tinggal, dan Cara Memperoleh Air Minum, 2018**
Percentage of Households by Province, Urban Rural Classification, and How to Get Drinking Water, 2018

Provinsi *Province*	Perkotaan/*Urban*			Perdesaan/*Rural*			Perkotaan+Perdesaan/*Urban+Rural*		
	Membeli *Buy*	Tidak Membeli *Do Not Buy*	Jumlah *Total*	Membeli *Buy*	Tidak Membeli *Do Not Buy*	Jumlah *Total*	Membeli *Buy*	Tidak Membeli *Do Not Buy*	Jumlah *Total*
(1)	(2)	(3)	(4)	(5)	(6)	(7)	(8)	(9)	(10)
Aceh	76,00	24,00	100,00	32,67	67,33	100,00	46,21	53,79	100,00
Sumatera Utara	67,91	32,09	100,00	27,07	72,93	100,00	48,51	51,49	100,00
Sumatera Barat	70,95	29,05	100,00	39,41	60,59	100,00	53,33	46,67	100,00
Riau	74,71	25,29	100,00	36,28	63,72	100,00	51,79	48,21	100,00
Jambi	58,98	41,02	100,00	23,99	76,01	100,00	34,93	65,07	100,00
Sumatera Selatan	59,50	40,50	100,00	15,32	84,68	100,00	31,15	68,85	100,00
Bengkulu	46,49	53,51	100,00	17,65	82,35	100,00	26,90	73,10	100,00
Lampung	48,45	51,55	100,00	19,11	80,89	100,00	27,29	72,71	100,00
Kepulauan Bangka Belitung	69,75	30,25	100,00	50,14	49,86	100,00	60,61	39,39	100,00
Kepulauan Riau	85,00	15,00	100,00	41,43	58,57	100,00	78,94	21,06	100,00
DKI Jakarta	77,84	22,16	100,00	-	-	100,00	77,84	22,16	100,00
Jawa Barat	56,41	43,59	100,00	28,30	71,70	100,00	48,71	51,29	100,00
Jawa Tengah	52,48	47,52	100,00	35,01	64,99	100,00	43,87	56,13	100,00
DI Yogyakarta	38,14	61,86	100,00	34,11	65,89	100,00	37,08	62,92	100,00
Jawa Timur	54,13	45,87	100,00	31,13	68,87	100,00	43,10	56,90	100,00
Banten	64,52	35,48	100,00	26,72	73,28	100,00	53,77	46,23	100,00
Bali	78,88	21,12	100,00	53,30	46,70	100,00	70,44	29,56	100,00
Nusa Tenggara Barat	45,52	54,48	100,00	24,24	75,76	100,00	34,06	65,94	100,00
Nusa Tenggara Timur	77,81	22,19	100,00	14,63	85,37	100,00	29,58	70,42	100,00
Kalimantan Barat	42,84	57,16	100,00	12,40	87,60	100,00	22,40	77,60	100,00
Kalimantan Tengah	75,03	24,97	100,00	32,60	67,40	100,00	48,59	51,41	100,00
Kalimantan Selatan	78,11	21,89	100,00	39,49	60,51	100,00	57,42	42,58	100,00
Kalimantan Timur	86,65	13,35	100,00	61,06	38,94	100,00	78,17	21,83	100,00
Kalimantan Utara	87,27	12,73	100,00	49,94	50,06	100,00	72,13	27,87	100,00
Sulawesi Utara	70,95	29,05	100,00	37,81	62,19	100,00	54,43	45,57	100,00
Sulawesi Tengah	75,65	24,35	100,00	36,06	63,94	100,00	47,29	52,71	100,00
Sulawesi Selatan	74,36	25,64	100,00	27,30	72,70	100,00	46,29	53,71	100,00
Sulawesi Tenggara	80,55	19,45	100,00	36,27	63,73	100,00	53,15	46,85	100,00
Gorontalo	71,55	28,45	100,00	39,73	60,27	100,00	52,25	47,75	100,00
Sulawesi Barat	65,76	34,24	100,00	26,38	73,62	100,00	35,55	64,45	100,00
Maluku	63,70	36,30	100,00	14,96	85,04	100,00	36,00	64,00	100,00
Maluku Utara	73,25	26,75	100,00	23,16	76,84	100,00	38,04	61,96	100,00
Papua Barat	80,65	19,35	100,00	28,37	71,63	100,00	49,27	50,73	100,00
Papua	72,49	27,51	100,00	10,09	89,91	100,00	26,12	73,88	100,00
**Indonesia**	**61,24**	**38,76**	**100,00**	**29,18**	**70,82**	**100,00**	**46,72**	**53,28**	**100,00**

Sumber/*Source*: Susenas Maret 2018/*The March 2018 Susenas*

https://www.bps.go.id

Tabel *Table* **6.14.1.** **Persentase Rumah Tangga di Daerah Perkotaan menurut Provinsi dan Sumber Air Utama yang Digunakan Rumah Tangga untuk Memasak/Mandi/ Cuci/dll, 2018**
Percentage of Households in Urban Area by Province and Main Source of Water Used by Household for Cooking/Taking Bath/Washing/etc, 2018

**Provinsi** *Province*	**Air Kemasan Bermerk, Air Isi Ulang** *Branded Bottled Refill Water*	**Leding** *Top Water*	**Sumur Bor/ Pompa** *Barehole*	**Sumur Terlindung, Sumur Tak Terlindung** *Protected Well, Unprotected Well*	**Mata Air Terlindung, Mata Air Tak Terlindung** *Protected, Unprotected Spring*	**Air Permukaan, Air Hujan** *Surface Water, Rain Water*	**Lainnya** *Others*	**Jumlah** *Total*
(1)	(2)	(3)	(4)	(5)	(6)	(7)	(8)	(9)
Aceh	0,51	30,97	17,58	44,12	4,59	1,74	0,50	100,00
Sumatera Utara	1,24	36,13	34,52	24,91	1,74	1,38	0,09	100,00
Sumatera Barat	0,03	38,02	23,86	30,37	4,39	3,31	0,02	100,00
Riau	0,44	8,13	59,52	26,37	0,07	4,91	0,56	100,00
Jambi	0,45	39,48	15,77	38,89	0,53	4,85	0,02	100,00
Sumatera Selatan	0,24	53,14	7,70	33,63	0,38	4,72	0,19	100,00
Bengkulu	0,31	22,25	22,08	52,14	2,51	0,68	0,02	100,00
Lampung	0,00	8,01	33,16	57,12	1,18	0,45	0,08	100,00
Kepulauan Bangka Belitung	0,00	9,22	26,54	61,09	1,16	1,84	0,15	100,00
Kepulauan Riau	3,22	69,48	8,29	15,81	1,51	1,37	0,33	100,00
DKI Jakarta	4,45	35,64	57,81	2,06	0,00	0,04	0,00	100,00
Jawa Barat	2,50	15,42	47,54	27,77	5,66	0,88	0,23	100,00
Jawa Tengah	1,06	25,53	35,79	33,27	3,95	0,32	0,07	100,00
DI Yogyakarta	0,00	12,84	30,79	54,58	1,73	0,06	0,00	100,00
Jawa Timur	1,67	28,11	39,45	25,42	4,52	0,62	0,20	100,00
Banten	1,61	14,25	74,52	8,16	0,88	0,17	0,42	100,00
Bali	0,00	44,01	40,12	10,25	3,69	1,93	0,00	100,00
Nusa Tenggara Barat	0,45	29,49	20,29	40,84	6,56	2,38	0,00	100,00
Nusa Tenggara Timur	0,00	50,82	20,28	25,45	2,77	0,69	0,00	100,00
Kalimantan Barat	0,66	40,11	10,47	21,77	3,18	23,66	0,14	100,00
Kalimantan Tengah	0,26	36,57	41,93	10,54	0,05	10,65	0,00	100,00
Kalimantan Selatan	0,45	63,25	6,93	21,99	0,19	7,19	0,00	100,00
Kalimantan Timur	0,00	78,65	8,69	4,99	0,60	7,07	0,00	100,00
Kalimantan Utara	0,00	63,70	10,36	5,26	0,96	19,71	0,00	100,00
Sulawesi Utara	1,53	27,74	37,59	25,83	5,64	0,87	0,80	100,00
Sulawesi Tengah	0,58	34,89	47,89	8,02	6,50	1,98	0,15	100,00
Sulawesi Selatan	0,14	42,03	36,17	18,46	1,73	1,45	0,02	100,00
Sulawesi Tenggara	0,09	33,88	34,24	20,72	10,01	1,05	0,00	100,00
Gorontalo	0,00	37,70	34,73	25,36	2,13	0,09	0,00	100,00
Sulawesi Barat	0,38	25,37	43,08	26,46	2,68	2,02	0,00	100,00
Maluku	0,31	34,34	28,09	18,75	15,73	1,05	1,73	100,00
Maluku Utara	0,00	68,90	10,42	19,18	0,76	0,74	0,00	100,00
Papua Barat	1,45	23,46	37,42	30,07	4,82	2,65	0,15	100,00
Papua	3,84	26,50	33,81	24,69	4,98	5,60	0,58	100,00
**Indonesia**	**1,64**	**27,43**	**40,51**	**25,17**	**3,53**	**1,54**	**0,17**	**100,00**

Sumber/*Source*: Susenas Maret 2018/*The March 2018 Susenas*

https://www.bps.go.id

Tabel *Table* **6.14.2. Persentase Rumah Tangga di Daerah Perdesaan menurut Provinsi dan Sumber Air Utama yang Digunakan Rumah Tangga untuk Memasak/Mandi/ Cuci/dll, 2018**
Percentage of Households in Rural Area by Province and Main Source of Water Used by Household for Cooking/Taking Bath/Washing/etc, 2018

**Provinsi** *Province*	**Air Kemasan Bermerk, Air Isi Ulang** *Branded Bottled Refill Water*	**Leding** *Top Water*	**Sumur Bor/ Pompa** *Barehole*	**Sumur Terlindung, Sumur Tak Terlindung** *Protected Well, Unprotected Well*	**Mata Air Terlindung, Mata Air Tak Terlindung** *Protected, Unprotected Spring*	**Air Permukaan, Air Hujan** *Surface Water, Rain Water*	**Lainnya** *Others*	**Jumlah** *Total*
(1)	(2)	(3)	(4)	(5)	(6)	(7)	(8)	(9)
Aceh	0,64	9,26	13,03	59,00	12,14	5,71	0,22	100,00
Sumatera Utara	0,52	5,21	35,05	27,92	19,44	11,81	0,05	100,00
Sumatera Barat	0,01	13,84	8,80	42,40	22,32	12,53	0,09	100,00
Riau	0,31	2,16	33,47	45,94	0,83	16,51	0,77	100,00
Jambi	0,21	9,79	9,62	60,67	3,13	16,24	0,35	100,00
Sumatera Selatan	0,05	6,89	13,05	57,16	4,59	18,20	0,07	100,00
Bengkulu	0,18	9,88	9,52	62,72	12,08	5,46	0,15	100,00
Lampung	0,00	1,78	12,67	73,93	8,36	3,22	0,04	100,00
Kepulauan Bangka Belitung	0,00	3,05	16,26	68,31	4,54	7,63	0,21	100,00
Kepulauan Riau	0,45	12,71	9,97	58,81	15,28	2,78	0,00	100,00
DKI Jakarta	-	-	-	-	-	-	-	-
Jawa Barat	1,03	4,17	27,87	39,08	24,27	3,32	0,25	100,00
Jawa Tengah	0,47	10,74	25,31	37,46	23,86	1,99	0,16	100,00
DI Yogyakarta	0,00	26,48	9,41	37,68	12,11	14,32	0,00	100,00
Jawa Timur	0,73	7,97	36,05	33,10	18,92	3,15	0,08	100,00
Banten	0,69	3,84	35,25	38,16	17,49	3,80	0,76	100,00
Bali	0,00	37,87	8,84	9,66	33,20	10,44	0,00	100,00
Nusa Tenggara Barat	0,51	11,29	23,09	43,44	18,23	3,41	0,03	100,00
Nusa Tenggara Timur	0,00	6,75	4,14	28,11	50,99	10,01	0,00	100,00
Kalimantan Barat	0,48	3,53	10,50	23,64	19,80	41,84	0,20	100,00
Kalimantan Tengah	0,23	8,82	21,17	25,98	4,28	39,50	0,02	100,00
Kalimantan Selatan	0,16	15,73	18,21	33,46	2,85	29,60	0,00	100,00
Kalimantan Timur	0,00	21,67	15,86	29,34	6,63	26,49	0,00	100,00
Kalimantan Utara	0,00	20,01	7,65	18,46	7,38	46,50	0,00	100,00
Sulawesi Utara	0,78	10,32	14,04	39,64	31,59	3,41	0,22	100,00
Sulawesi Tengah	0,19	9,57	20,58	23,60	34,53	11,54	0,00	100,00
Sulawesi Selatan	0,14	10,41	32,42	35,69	17,71	3,60	0,03	100,00
Sulawesi Tenggara	0,00	10,80	17,00	42,55	24,15	5,49	0,00	100,00
Gorontalo	0,00	16,31	21,11	45,53	15,08	1,97	0,00	100,00
Sulawesi Barat	0,31	7,27	17,95	37,77	27,36	9,26	0,07	100,00
Maluku	0,17	6,16	8,53	39,91	37,81	7,41	0,00	100,00
Maluku Utara	0,00	17,25	6,13	46,22	20,83	9,57	0,00	100,00
Papua Barat	0,38	8,26	10,66	38,89	20,03	21,65	0,12	100,00
Papua	0,42	1,58	5,05	13,01	35,73	44,09	0,12	100,00
**Indonesia**	**0,46**	**8,27**	**23,51**	**39,32**	**19,23**	**9,06**	**0,15**	**100,00**

Sumber/*Source*: Susenas Maret 2018/*The March 2018 Susenas*

https://www.bps.go.id

Tabel **6.14.3.** **Persentase Rumah Tangga di Daerah Perkotaan dan Perdesaan menurut Provinsi dan Sumber Air Utama yang Digunakan Rumah Tangga untuk Memasak/Mandi/Cuci/dll, 2018**

Table *Percentage of Households in Urban and Rural Area by Province and Main Source of Water Used by Household for Cooking/Taking Bath/Washing/etc, 2018*

**Provinsi** *Province*	**Air Kemasan Bermerk, Air Isi Ulang** *Branded Bottled Refill Water*	**Leding** *Top Water*	**Sumur Bor/ Pompa** *Barehole*	**Sumur Terlindung, Sumur Tak Terlindung** *Protected Well, Unprotected Well*	**Mata Air Terlindung, Mata Air Tak Terlindung** *Protected, Unprotected Spring*	**Air Permukaan, Air Hujan** *Surface Water, Rain Water*	**Lainnya** *Others*	**Jumlah** *Total*
**(1)**	**(2)**	**(3)**	**(4)**	**(5)**	**(6)**	**(7)**	**(8)**	**(9)**
Aceh	0,60	16,05	14,45	54,35	9,78	4,47	0,31	100,00
Sumatera Utara	0,90	21,43	34,77	26,34	10,15	6,33	0,07	100,00
Sumatera Barat	0,02	24,51	15,45	37,09	14,41	8,46	0,06	100,00
Riau	0,37	4,57	43,99	38,04	0,52	11,83	0,68	100,00
Jambi	0,28	19,07	11,54	53,86	2,32	12,68	0,25	100,00
Sumatera Selatan	0,12	23,46	11,13	48,73	3,08	13,37	0,11	100,00
Bengkulu	0,22	13,85	13,55	59,33	9,01	3,93	0,11	100,00
Lampung	0,00	3,51	18,39	69,24	6,36	2,45	0,05	100,00
Kepulauan Bangka Belitung	0,00	6,35	21,74	64,46	2,74	4,54	0,18	100,00
Kepulauan Riau	2,83	61,58	8,52	21,79	3,42	1,56	0,29	100,00
DKI Jakarta	4,45	35,64	57,81	2,06	0,00	0,04	0,00	100,00
Jawa Barat	2,10	12,34	42,15	30,87	10,76	1,55	0,24	100,00
Jawa Tengah	0,77	18,24	30,63	35,34	13,76	1,14	0,12	100,00
DI Yogyakarta	0,00	16,43	25,16	50,13	4,47	3,82	0,00	100,00
Jawa Timur	1,22	18,46	37,82	29,10	11,42	1,84	0,14	100,00
Banten	1,35	11,29	63,35	16,69	5,60	1,21	0,52	100,00
Bali	0,00	41,99	29,80	10,06	13,42	4,74	0,00	100,00
Nusa Tenggara Barat	0,48	19,69	21,80	42,24	12,84	2,93	0,02	100,00
Nusa Tenggara Timur	0,00	17,18	7,96	27,48	39,58	7,80	0,00	100,00
Kalimantan Barat	0,54	15,55	10,49	23,03	14,34	35,87	0,18	100,00
Kalimantan Tengah	0,24	19,28	28,99	20,16	2,68	28,63	0,01	100,00
Kalimantan Selatan	0,29	37,79	12,97	28,14	1,61	19,19	0,00	100,00
Kalimantan Timur	0,00	59,77	11,06	13,06	2,60	13,51	0,00	100,00
Kalimantan Utara	0,00	45,98	9,26	10,62	3,57	30,58	0,00	100,00
Sulawesi Utara	1,15	19,06	25,85	32,71	18,57	2,14	0,51	100,00
Sulawesi Tengah	0,30	16,75	28,32	19,18	26,58	8,83	0,04	100,00
Sulawesi Selatan	0,14	23,17	33,93	28,74	11,26	2,73	0,03	100,00
Sulawesi Tenggara	0,03	19,60	23,57	34,23	18,76	3,80	0,00	100,00
Gorontalo	0,00	24,72	26,47	37,59	9,98	1,23	0,00	100,00
Sulawesi Barat	0,33	11,49	23,80	35,13	21,61	7,57	0,05	100,00
Maluku	0,23	18,32	16,98	30,77	28,28	4,67	0,75	100,00
Maluku Utara	0,00	32,60	7,41	38,19	14,87	6,95	0,00	100,00
Papua Barat	0,81	14,33	21,36	35,36	13,95	14,05	0,13	100,00
Papua	1,30	7,98	12,44	16,01	27,83	34,20	0,24	100,00
**Indonesia**	**1,11**	**18,76**	**32,81**	**31,58**	**10,64**	**4,94**	**0,16**	**100,00**

Sumber/*Source*: Susenas Maret 2018/*The March 2018 Susenas*

https://www.bps.go.id

Tabel *Table* **6.15.** **Persentase Rumah Tangga dengan Sumber Air untuk Memasak/Mandi/Cuci/dll dari Sumur Bor/Pompa, Sumur Terlindung, Sumur Tak Terlindung, Mata Air Terlindung, dan Mata Air Tak Terlindung menurut Provinsi, Daerah Tempat Tinggal, dan Jarak ke Tempat Penampungan Limbah/Kotoran/Tinja Terdekat, 2018**
Percentage of Households Using Artesian Well/Pump, Protected Well, Unprotected Well, Protected Spring, and Unprotected Spring as Source of Water for Cooking/Taking Bath/Washing/ etc by Province, Urban Rural Classification, and the Distance to the Nearest Final Disposal Site of Feces, 2018

**Provinsi** *Province*	**Perkotaan/***Urban*				**Perdesaan/***Rural*				**Perkotaan+Perdesaan/***Urban+Rural*			
	**< 10 m**	**≥ 10 m**	**Tidak Tahu**	**Jumlah** *Total*	**< 10 m**	**≥ 10 m**	**Tidak Tahu**	**Jumlah** *Total*	**< 10 m**	**≥ 10 m**	**Tidak Tahu**	**Jumlah** *Total*
**(1)**	**(2)**	**(3)**	**(6)**	**(7)**	**(8)**	**(9)**	**(10)**	**(11)**	**(12)**	**(13)**	**(14)**	**(15)**
Aceh	36,71	57,15	6,14	100,00	37,04	54,78	8,19	100,00	36,95	55,40	7,65	100,00
Sumatera Utara	43,58	51,20	5,23	100,00	32,12	61,27	6,61	100,00	37,28	56,73	5,99	100,00
Sumatera Barat	38,43	54,38	7,19	100,00	31,73	61,64	6,63	100,00	34,32	58,83	6,85	100,00
Riau	36,87	54,48	8,65	100,00	32,25	62,54	5,21	100,00	34,19	59,15	6,66	100,00
Jambi	22,12	68,02	9,87	100,00	23,81	72,30	3,89	100,00	23,38	71,21	5,41	100,00
Sumatera Selatan	41,28	55,51	3,21	100,00	35,07	61,98	2,95	100,00	36,54	60,45	3,01	100,00
Bengkulu	33,95	60,15	5,89	100,00	32,84	61,77	5,39	100,00	33,17	61,29	5,54	100,00
Lampung	30,53	66,80	2,66	100,00	26,93	72,90	0,17	100,00	27,90	71,25	0,85	100,00
Kepulauan Bangka Belitung	25,60	71,10	3,30	100,00	26,08	67,99	5,93	100,00	25,82	69,64	4,53	100,00
Kepulauan Riau	32,54	60,50	6,95	100,00	19,69	68,44	11,86	100,00	28,09	63,26	8,66	100,00
DKI Jakarta	39,26	44,00	16,74	100,00	-	-	-	100,00	39,26	44,00	16,74	100,00
Jawa Barat	42,30	48,32	9,38	100,00	36,25	55,26	8,49	100,00	40,50	50,39	9,11	100,00
Jawa Tengah	33,21	62,00	4,79	100,00	22,54	72,29	5,17	100,00	27,50	67,51	5,00	100,00
DI Yogyakarta	27,88	65,27	6,85	100,00	11,30	87,22	1,48	100,00	24,63	69,56	5,80	100,00
Jawa Timur	38,60	56,90	4,50	100,00	27,55	67,51	4,94	100,00	32,65	62,62	4,74	100,00
Banten	44,53	48,02	7,45	100,00	41,85	48,74	9,41	100,00	43,72	48,24	8,04	100,00
Bali	28,41	62,10	9,49	100,00	24,97	69,62	5,41	100,00	27,31	64,50	8,19	100,00
Nusa Tenggara Barat	34,16	60,05	5,79	100,00	36,88	58,21	4,91	100,00	35,78	58,95	5,27	100,00
Nusa Tenggara Timur	24,32	65,90	9,78	100,00	11,93	80,29	7,79	100,00	13,82	78,09	8,09	100,00
Kalimantan Barat	29,30	65,86	4,83	100,00	25,51	65,40	9,09	100,00	26,43	65,51	8,05	100,00
Kalimantan Tengah	33,15	60,83	6,02	100,00	26,01	70,54	3,45	100,00	28,74	66,83	4,43	100,00
Kalimantan Selatan	30,17	69,76	0,08	100,00	27,14	72,45	0,41	100,00	28,10	71,60	0,31	100,00
Kalimantan Timur	19,27	68,20	12,53	100,00	20,28	70,23	9,49	100,00	19,92	69,51	10,58	100,00
Kalimantan Utara	40,87	56,96	2,17	100,00	24,40	65,28	10,32	100,00	31,33	61,78	6,89	100,00
Sulawesi Utara	37,11	53,69	9,21	100,00	34,70	57,48	7,82	100,00	35,78	55,78	8,44	100,00
Sulawesi Tengah	36,10	46,83	17,07	100,00	22,38	65,59	12,03	100,00	25,66	61,11	13,24	100,00
Sulawesi Selatan	36,84	53,52	9,64	100,00	21,70	72,02	6,28	100,00	26,35	66,33	7,31	100,00
Sulawesi Tenggara	17,08	72,06	10,86	100,00	14,04	78,27	7,69	100,00	15,02	76,26	8,72	100,00
Gorontalo	44,84	52,22	2,94	100,00	35,69	60,47	3,85	100,00	38,71	57,74	3,54	100,00
Sulawesi Barat	29,23	61,42	9,35	100,00	24,73	66,02	9,25	100,00	25,67	65,06	9,27	100,00
Maluku	19,75	67,96	12,30	100,00	22,33	63,67	14,00	100,00	21,42	65,19	13,39	100,00
Maluku Utara	37,48	42,14	20,39	100,00	34,51	54,02	11,48	100,00	34,95	52,24	12,81	100,00
Papua Barat	31,92	55,66	12,41	100,00	33,07	59,88	7,05	100,00	32,60	58,15	9,24	100,00
Papua	28,33	58,32	13,35	100,00	14,85	70,24	14,90	100,00	18,76	66,79	14,45	100,00
**Indonesia**	**38,10**	**54,19**	**7,70**	**100,00**	**28,31**	**65,65**	**6,04**	**100,00**	**33,25**	**59,87**	**6,88**	**100,00**

Sumber/*Source*: Susenas Maret 2018/*The March 2018 Susenas*

https://www.bps.go.id

Tabel **Persentase Rumah Tangga Menurut Provinsi, Daerah Tempat Tinggal, Sumber Air Minum Bersih, Sumber Air Minum Layak, dan Akses Air Layak, 2018**

Table **6.16.** *Percentage of Households by Province, Urban Rural Classification, Source of Clean Drinking Water, Source of Decent Drinking Water, and Access of Decent Water, 2018*

Provinsi *Province*	Perkotaan/*Urban*			Perdesaan/*Rural*			Perkotaan+Perdesaan/*Urban+Rural*		
	Sumber Air Minum Bersih *Source of Clean Water*	Sumber Air Minum Layak *Source of Decent Water*	Akses Air Layak *Access of Decent Water*	Sumber Air Minum Bersih *Source of Clean Water*	Sumber Air Minum Layak *Source of Decent Water*	Akses Air Layak *Access of Decent Water*	Sumber Air Minum Bersih *Source of Clean Water*	Sumber Air Minum Layak *Source of Decent Water*	Akses Air Layak *Access of Decent Water*
(1)	(2)	(3)	(4)	(5)	(6)	(7)	(8)	(9)	(10)
Aceh	87,36	17,23	85,03	58,51	33,78	58,04	67,53	28,61	66,48
Sumatera Utara	85,17	32,68	82,42	56,65	43,85	60,38	71,62	37,98	71,95
Sumatera Barat	85,99	29,82	82,78	62,02	36,88	59,07	72,60	33,77	69,53
Riau	85,16	21,21	89,96	57,11	47,85	72,73	68,43	37,10	79,68
Jambi	78,81	42,40	84,94	52,24	44,68	58,35	60,55	43,97	66,66
Sumatera Selatan	84,76	52,26	80,11	50,60	48,08	57,06	62,84	49,58	65,31
Bengkulu	71,78	38,10	67,04	42,24	34,37	41,02	51,72	35,57	49,37
Lampung	75,72	32,57	72,08	51,23	37,47	50,85	58,06	36,10	56,78
Kepulauan Bangka Belitung	87,75	20,26	75,15	73,64	28,20	57,31	81,17	23,96	66,83
Kepulauan Riau	93,16	15,11	87,55	68,16	39,77	58,84	89,68	18,54	83,56
DKI Jakarta	93,41	17,91	89,59	-	-	-	93,41	17,91	89,59
Jawa Barat	78,32	26,77	75,86	58,76	33,81	58,35	72,96	28,70	71,06
Jawa Tengah	81,44	51,71	81,40	73,61	55,91	74,83	77,58	53,78	78,16
DI Yogyakarta	77,99	46,36	78,25	72,82	79,56	87,25	76,63	55,11	80,62
Jawa Timur	79,49	36,27	79,74	69,30	52,87	70,27	74,61	44,23	75,20
Banten	84,04	20,74	81,85	51,91	25,78	50,15	74,90	22,17	72,83
Bali	93,46	35,92	94,80	75,80	71,45	82,98	87,64	47,64	90,90
Nusa Tenggara Barat	81,40	50,15	82,20	64,99	51,00	66,25	72,56	50,61	73,61
Nusa Tenggara Timur	90,12	60,17	90,28	60,26	63,76	66,87	67,33	62,91	72,41
Kalimantan Barat	50,70	55,10	90,49	27,23	56,80	64,27	34,94	56,24	72,88
Kalimantan Tengah	87,56	23,05	85,71	54,32	34,54	53,09	66,84	30,21	65,38
Kalimantan Selatan	89,82	47,68	82,81	54,23	35,72	45,23	70,75	41,28	62,67
Kalimantan Timur	97,76	27,01	92,29	77,02	20,86	59,00	90,88	24,98	81,26
Kalimantan Utara	92,31	15,82	96,99	57,76	37,32	75,57	78,30	24,54	88,30
Sulawesi Utara	88,02	27,02	84,61	69,36	40,69	67,74	78,72	33,83	76,20
Sulawesi Tengah	88,34	20,43	85,60	65,79	46,11	65,40	72,19	38,83	71,13
Sulawesi Selatan	91,49	35,19	90,38	67,23	51,29	69,51	77,02	44,79	77,93
Sulawesi Tenggara	92,00	39,67	91,54	71,46	56,00	74,44	79,29	49,78	80,95
Gorontalo	88,69	26,46	88,52	73,03	39,58	72,80	79,20	34,42	78,99
Sulawesi Barat	82,67	34,43	81,65	54,52	38,86	57,31	61,08	37,83	62,98
Maluku	87,88	46,80	88,64	60,26	56,58	67,23	72,19	52,36	76,47
Maluku Utara	89,65	42,42	92,35	54,20	47,51	59,38	64,73	46,00	69,17
Papua Barat	91,82	22,00	92,64	50,99	43,22	66,78	67,31	34,73	77,12
Papua	87,11	25,31	86,11	21,47	38,33	48,75	38,33	34,98	58,35
**Indonesia**	**82,74**	**33,15**	**81,55**	**61,21**	**46,41**	**64,18**	**72,99**	**39,16**	**73,68**

Sumber/*Source*: Susenas Maret 2018/*The March 2018 Susenas*

https://www.bps.go.id

Tabel 6.17. **Persentase Rumah Tangga menurut Provinsi, Daerah Tempat Tinggal, dan Sumber Penerangan Utama, 2018**
Table 6.17. *Percentage of Households by Province, Urban Rural Classification, and The Main Source of Lighting, 2018*

**Provinsi** *Province*	**Perkotaan/***Urban*				**Perdesaan/***Rural*				**Perkotaan+Perdesaan/***Urban+Rural*			
	**Listrik PLN** *Public (PLN) Electricity*	**Listrik Non PLN** *Non Public (PLN) Electricity*	**Bukan Listrik** *No Electricity*	**Jumlah** *Total*	**Listrik PLN** *Public (PLN) Electricity*	**Listrik Non PLN** *Non Public (PLN) Electricity*	**Bukan Listrik** *No Electricity*	**Jumlah** *Total*	**Listrik PLN** *Public (PLN) Electricity*	**Listrik Non PLN** *Non Public (PLN) Electricity*	**Bukan Listrik** *No Electricity*	**Jumlah** *Total*
**(1)**	**(2)**	**(3)**	**(6)**	**(7)**	**(8)**	**(9)**	**(10)**	**(11)**	**(12)**	**(13)**	**(14)**	**(15)**
Aceh	99,47	0,30	0,23	100,00	98,99	0,27	0,74	100,00	99,14	0,28	0,58	100,00
Sumatera Utara	99,57	0,28	0,15	100,00	92,79	2,23	4,98	100,00	96,35	1,20	2,45	100,00
Sumatera Barat	98,95	0,58	0,47	100,00	94,72	2,72	2,56	100,00	96,59	1,77	1,64	100,00
Riau	97,72	1,94	0,34	100,00	84,63	11,39	3,98	100,00	89,91	7,57	2,51	100,00
Jambi	98,31	1,03	0,66	100,00	91,40	5,04	3,55	100,00	93,56	3,79	2,65	100,00
Sumatera Selatan	99,50	0,45	0,05	100,00	92,65	5,41	1,93	100,00	95,10	3,64	1,26	100,00
Bengkulu	99,47	0,12	0,40	100,00	96,09	1,23	2,68	100,00	97,18	0,87	1,95	100,00
Lampung	99,91	0,01	0,08	100,00	93,86	5,54	0,60	100,00	95,55	4,00	0,46	100,00
Kepulauan Bangka Belitung	99,80	0,15	0,05	100,00	98,37	0,69	0,94	100,00	99,13	0,40	0,46	100,00
Kepulauan Riau	99,61	0,15	0,24	100,00	71,28	24,84	3,88	100,00	95,67	3,59	0,74	100,00
DKI Jakarta	100,00	0,00	0,00	100,00	-	-	-	-	100,00	0,00	0,00	100,00
Jawa Barat	99,90	0,09	0,01	100,00	99,63	0,17	0,20	100,00	99,82	0,11	0,06	100,00
Jawa Tengah	99,92	0,02	0,06	100,00	99,89	0,01	0,10	100,00	99,91	0,01	0,08	100,00
DI Yogyakarta	99,94	0,00	0,06	100,00	99,88	0,00	0,12	100,00	99,92	0,00	0,08	100,00
Jawa Timur	99,76	0,11	0,13	100,00	99,41	0,43	0,16	100,00	99,59	0,26	0,15	100,00
Banten	99,84	0,11	0,05	100,00	98,96	0,34	0,70	100,00	99,59	0,18	0,23	100,00
Bali	99,92	0,00	0,08	100,00	99,59	0,05	0,37	100,00	99,81	0,01	0,18	100,00
Nusa Tenggara Barat	99,61	0,13	0,26	100,00	98,68	0,96	0,37	100,00	99,11	0,58	0,32	100,00
Nusa Tenggara Timur	98,89	0,35	0,75	100,00	60,21	16,88	22,91	100,00	69,37	12,97	17,66	100,00
Kalimantan Barat	98,82	0,67	0,50	100,00	76,07	14,04	9,88	100,00	83,54	9,65	6,80	100,00
Kalimantan Tengah	98,52	1,13	0,35	100,00	73,28	22,20	4,53	100,00	82,79	14,26	2,96	100,00
Kalimantan Selatan	99,24	0,48	0,28	100,00	94,82	4,41	0,77	100,00	96,87	2,59	0,54	100,00
Kalimantan Timur	97,91	2,07	0,02	100,00	79,95	18,66	1,39	100,00	91,96	7,57	0,47	100,00
Kalimantan Utara	99,55	0,00	0,45	100,00	78,70	18,77	2,53	100,00	91,09	7,61	1,29	100,00
Sulawesi Utara	99,69	0,20	0,11	100,00	97,64	1,40	0,96	100,00	98,67	0,80	0,53	100,00
Sulawesi Tengah	99,25	0,32	0,43	100,00	85,85	7,92	6,23	100,00	89,65	5,76	4,59	100,00
Sulawesi Selatan	99,56	0,27	0,18	100,00	94,40	3,87	1,73	100,00	96,48	2,42	1,10	100,00
Sulawesi Tenggara	98,98	0,74	0,28	100,00	89,69	7,94	2,37	100,00	93,23	5,20	1,57	100,00
Gorontalo	99,97	0,00	0,03	100,00	93,94	1,26	4,80	100,00	96,31	0,76	2,92	100,00
Sulawesi Barat	98,92	0,47	0,61	100,00	78,40	17,09	4,51	100,00	83,18	13,22	3,60	100,00
Maluku	98,56	0,14	1,31	100,00	81,20	6,83	11,97	100,00	88,69	3,94	7,37	100,00
Maluku Utara	99,05	0,52	0,43	100,00	79,12	14,29	6,59	100,00	85,04	10,20	4,76	100,00
Papua Barat	99,79	0,06	0,15	100,00	70,21	17,07	12,72	100,00	82,04	10,27	7,69	100,00
Papua	95,66	2,94	1,40	100,00	25,49	29,12	45,40	100,00	43,51	22,39	34,10	100,00
**Indonesia**	**99,66**	**0,22**	**0,12**	**100,00**	**92,72**	**4,14**	**3,14**	**100,00**	**96,52**	**1,99**	**1,49**	**100,00**

Sumber/*Source*: Susenas Maret 2018/*The March 2018 Susenas*

https://www.bps.go.id

Tabel **Persentase Rumah Tangga di Daerah Perkotaan menurut Provinsi dan Bahan Bakar Utama yang Digunakan untuk Memasak, 2018**
Table **6.18.1.** *Percentage of Households in Urban Area by Province and Type of Fuel Mainly Used for Cooking, 2018*

**Provinsi** *Province*	**Listrik** *Electricity*	**Gas Elpiji** *LPG*	**Gas Kota/Biogas** *Natural Gas/Biogas*	**Minyak Tanah** *Kerosene*	**Briket, Arang** *Briquettes, Charcoal*	**Kayu Bakar** *Fire Wood*	**Lainnya** *Others*	**Tidak Memasak di Rumah** *Not Cooking in the House*	**Jumlah** *Total*
(1)	(2)	(3)	(4)	(5)	(6)	(7)	(8)	(9)	(10)
Aceh	0,95	89,57	0,42	4,09	0,00	2,33	0,00	2,63	100,00
Sumatera Utara	1,00	88,79	0,84	5,57	0,00	2,32	0,05	1,44	100,00
Sumatera Barat	2,75	74,09	0,00	8,99	0,00	11,08	0,01	3,08	100,00
Riau	0,77	87,22	0,59	7,08	0,24	1,58	0,11	2,39	100,00
Jambi	0,20	84,14	1,50	9,25	0,74	3,15	0,00	1,00	100,00
Sumatera Selatan	0,22	91,51	5,54	1,40	0,00	0,98	0,00	0,36	100,00
Bengkulu	0,36	95,49	0,00	1,39	0,00	2,12	0,00	0,65	100,00
Lampung	0,19	90,24	0,00	0,68	0,01	6,66	0,00	2,23	100,00
Kepulauan Bangka Belitung	0,21	82,22	0,00	15,37	0,00	1,52	0,00	0,68	100,00
Kepulauan Riau	3,56	78,64	1,61	12,71	0,02	0,81	0,00	2,67	100,00
DKI Jakarta	1,07	91,27	0,51	1,12	0,00	0,02	0,00	6,02	100,00
Jawa Barat	0,76	91,51	0,68	0,20	0,00	4,56	0,07	2,22	100,00
Jawa Tengah	0,44	89,13	0,09	0,21	0,09	7,83	0,03	2,19	100,00
DI Yogyakarta	1,59	81,60	0,00	0,20	0,40	8,93	0,11	7,18	100,00
Jawa Timur	0,70	87,60	1,25	0,53	0,01	6,87	0,01	3,03	100,00
Banten	0,93	94,73	0,55	0,26	0,00	2,03	0,05	1,46	100,00
Bali	2,56	83,15	0,05	0,29	0,00	7,59	0,25	6,13	100,00
Nusa Tenggara Barat	1,05	68,60	0,00	13,54	0,00	15,14	0,11	1,56	100,00
Nusa Tenggara Timur	1,54	4,18	0,00	67,92	0,00	25,65	0,00	0,71	100,00
Kalimantan Barat	0,40	97,54	0,00	0,75	0,00	0,85	0,00	0,46	100,00
Kalimantan Tengah	0,08	84,60	0,00	10,63	0,00	4,34	0,00	0,36	100,00
Kalimantan Selatan	0,35	82,05	0,00	10,46	0,00	4,94	0,06	2,14	100,00
Kalimantan Timur	0,42	93,14	2,56	1,46	0,02	0,65	0,00	1,76	100,00
Kalimantan Utara	0,54	52,27	30,94	12,96	0,00	1,72	0,00	1,56	100,00
Sulawesi Utara	1,54	79,49	0,00	12,20	0,00	4,10	0,00	2,67	100,00
Sulawesi Tengah	0,79	73,31	0,00	15,86	0,00	9,18	0,00	0,86	100,00
Sulawesi Selatan	0,57	95,80	0,47	0,49	0,06	1,69	0,00	0,92	100,00
Sulawesi Tenggara	0,08	63,59	0,00	24,97	0,01	10,31	0,00	1,04	100,00
Gorontalo	2,76	87,18	0,00	2,80	0,00	4,76	0,00	2,50	100,00
Sulawesi Barat	0,00	93,18	0,00	0,00	0,00	6,31	0,00	0,51	100,00
Maluku	0,36	1,67	0,00	89,49	0,00	7,19	0,00	1,29	100,00
Maluku Utara	2,41	4,44	0,00	82,88	0,00	7,52	0,13	2,62	100,00
Papua Barat	0,15	7,51	0,00	84,80	0,00	5,24	0,00	2,30	100,00
Papua	0,24	3,74	0,05	86,22	0,00	8,29	0,10	1,36	100,00
**Indonesia**	**0,84**	**86,88**	**0,78**	**3,81**	**0,03**	**5,04**	**0,04**	**2,58**	**100,00**

Sumber/*Source*: Susenas Maret 2018/*The March 2018 Susenas*

https://www.bps.go.id

Tabel 6.18.2. **Persentase Rumah Tangga di Daerah Perdesaan menurut Provinsi dan Bahan Bakar Utama yang Digunakan untuk Memasak, 2018**
Table *Percentage of Households in Rural Area by Province and Type of Fuel Mainly Used for Cooking, 2018*

**Provinsi** *Province*	**Listrik** *Electricity*	**Gas Elpiji** *LPG*	**Gas Kota/Biogas** *Natural Gas/Biogas*	**Minyak Tanah** *Kerosene*	**Briket, Arang** *Briquettes, Charcoal*	**Kayu Bakar** *Fire Wood*	**Lainnya** *Others*	**Tidak Memasak di Rumah** *Not Cooking in the House*	**Jumlah** *Total*
(1)	(2)	(3)	(4)	(5)	(6)	(7)	(8)	(9)	(10)
Aceh	0,39	83,20	0,05	2,03	0,03	13,47	0,03	0,80	100,00
Sumatera Utara	0,38	70,26	0,00	2,06	0,00	26,84	0,02	0,44	100,00
Sumatera Barat	0,42	60,26	0,00	3,91	0,00	35,07	0,00	0,35	100,00
Riau	0,88	85,61	0,00	2,17	1,93	8,99	0,00	0,42	100,00
Jambi	0,34	73,50	0,00	4,09	0,59	20,97	0,00	0,51	100,00
Sumatera Selatan	0,39	84,49	0,43	0,43	0,03	14,04	0,02	0,18	100,00
Bengkulu	0,35	79,76	0,00	0,21	0,00	19,55	0,00	0,13	100,00
Lampung	0,09	73,32	0,00	0,02	0,00	26,49	0,02	0,07	100,00
Kepulauan Bangka Belitung	2,25	80,50	0,00	12,35	0,00	4,71	0,00	0,20	100,00
Kepulauan Riau	0,11	40,11	0,00	44,65	0,24	13,87	0,06	0,98	100,00
DKI Jakarta	-	-	-	-	-	-	-	-	-
Jawa Barat	0,82	70,20	0,03	0,06	0,00	28,01	0,06	0,82	100,00
Jawa Tengah	0,34	70,78	0,01	0,00	0,01	28,12	0,11	0,65	100,00
DI Yogyakarta	0,12	49,56	0,04	0,00	0,00	50,05	0,00	0,23	100,00
Jawa Timur	0,52	66,62	0,03	0,05	0,00	32,06	0,01	0,71	100,00
Banten	0,09	64,65	0,00	0,00	0,00	34,90	0,00	0,36	100,00
Bali	0,47	58,18	0,00	0,03	0,00	41,07	0,00	0,25	100,00
Nusa Tenggara Barat	0,14	50,52	0,00	17,51	0,00	31,50	0,00	0,33	100,00
Nusa Tenggara Timur	0,31	0,07	0,00	9,51	0,00	90,06	0,00	0,05	100,00
Kalimantan Barat	0,43	81,86	0,00	0,15	0,00	17,33	0,12	0,10	100,00
Kalimantan Tengah	0,21	71,93	0,00	5,01	0,00	22,56	0,00	0,29	100,00
Kalimantan Selatan	0,40	69,06	0,00	3,28	0,00	26,95	0,00	0,30	100,00
Kalimantan Timur	0,05	90,87	0,03	0,57	0,21	7,76	0,00	0,52	100,00
Kalimantan Utara	0,18	63,78	0,00	13,80	0,00	21,96	0,00	0,28	100,00
Sulawesi Utara	0,40	65,68	0,00	8,96	0,07	24,63	0,00	0,26	100,00
Sulawesi Tengah	0,20	44,42	0,00	6,35	2,18	46,75	0,00	0,11	100,00
Sulawesi Selatan	0,15	80,51	0,03	0,02	0,68	18,31	0,03	0,27	100,00
Sulawesi Tenggara	0,03	52,81	0,00	11,43	0,43	35,11	0,00	0,18	100,00
Gorontalo	0,22	77,31	0,04	1,17	0,00	21,03	0,00	0,23	100,00
Sulawesi Barat	0,36	68,79	0,00	0,00	0,11	30,47	0,00	0,27	100,00
Maluku	0,07	0,06	0,00	41,83	0,00	57,49	0,03	0,52	100,00
Maluku Utara	0,23	0,54	0,00	30,93	0,00	67,77	0,11	0,43	100,00
Papua Barat	0,03	1,66	0,83	55,04	0,00	41,76	0,00	0,69	100,00
Papua	0,17	0,21	0,00	15,57	0,06	83,75	0,11	0,12	100,00
**Indonesia**	**0,41**	**65,93**	**0,04**	**2,84**	**0,15**	**30,12**	**0,04**	**0,48**	**100,00**

Sumber/*Source*: Susenas Maret 2018/*The March 2018 Susenas*

https://www.bps.go.id

Tabel 6.18.3. **Persentase Rumah Tangga di Daerah Perkotaan dan Perdesaan menurut Provinsi dan Bahan Bakar Utama yang Digunakan untuk Memasak, 2018**

Table 6.18.3. *Percentage of Households in Urban and Rural Area by Province and Type of Fuel Mainly Used for Cooking, 2018*

**Provinsi** *Province*	**Listrik** *Electricity*	**Gas Elpiji** *LPG*	**Gas Kota/Biogas** *Natural Gas/Biogas*	**Minyak Tanah** *Kerosene*	**Briket, Arang** *Briquettes, Charcoal*	**Kayu Bakar** *Fire Wood*	**Lainnya** *Others*	**Tidak Memasak di Rumah** *Not Cooking in the House*	**Jumlah** *Total*
(1)	(2)	(3)	(4)	(5)	(6)	(7)	(8)	(9)	(10)
Aceh	0,57	85,19	0,16	2,67	0,02	9,99	0,02	1,38	100,00
Sumatera Utara	0,70	79,99	0,44	3,90	0,00	13,97	0,04	0,96	100,00
Sumatera Barat	1,45	66,36	0,00	6,15	0,00	24,48	0,00	1,56	100,00
Riau	0,84	86,26	0,24	4,15	1,25	6,00	0,05	1,22	100,00
Jambi	0,30	76,82	0,47	5,71	0,64	15,40	0,00	0,66	100,00
Sumatera Selatan	0,33	87,00	2,26	0,77	0,02	9,36	0,01	0,24	100,00
Bengkulu	0,35	84,80	0,00	0,59	0,00	13,96	0,00	0,30	100,00
Lampung	0,12	78,04	0,00	0,20	0,00	20,95	0,01	0,67	100,00
Kepulauan Bangka Belitung	1,16	81,42	0,00	13,96	0,00	3,01	0,00	0,46	100,00
Kepulauan Riau	3,08	73,28	1,39	17,15	0,05	2,63	0,01	2,43	100,00
DKI Jakarta	1,07	91,27	0,51	1,12	0,00	0,02	0,00	6,02	100,00
Jawa Barat	0,78	85,67	0,50	0,16	0,00	10,98	0,07	1,84	100,00
Jawa Tengah	0,39	80,08	0,05	0,11	0,05	17,83	0,07	1,43	100,00
DI Yogyakarta	1,20	73,15	0,01	0,15	0,29	19,77	0,08	5,35	100,00
Jawa Timur	0,61	77,54	0,67	0,30	0,00	18,95	0,01	1,91	100,00
Banten	0,69	86,18	0,39	0,19	0,00	11,38	0,03	1,14	100,00
Bali	1,87	74,91	0,03	0,20	0,00	18,63	0,16	4,19	100,00
Nusa Tenggara Barat	0,56	58,86	0,00	15,68	0,00	23,95	0,05	0,90	100,00
Nusa Tenggara Timur	0,60	1,04	0,00	23,34	0,00	74,81	0,00	0,21	100,00
Kalimantan Barat	0,42	87,01	0,00	0,35	0,00	11,92	0,08	0,22	100,00
Kalimantan Tengah	0,16	76,71	0,00	7,13	0,00	15,69	0,00	0,31	100,00
Kalimantan Selatan	0,38	75,09	0,00	6,62	0,00	16,73	0,03	1,15	100,00
Kalimantan Timur	0,29	92,39	1,72	1,16	0,08	3,01	0,00	1,35	100,00
Kalimantan Utara	0,39	56,94	18,39	13,30	0,00	9,93	0,00	1,04	100,00
Sulawesi Utara	0,97	72,61	0,00	10,58	0,04	14,33	0,00	1,47	100,00
Sulawesi Tengah	0,36	52,61	0,00	9,05	1,56	36,09	0,00	0,32	100,00
Sulawesi Selatan	0,32	86,68	0,21	0,21	0,43	11,61	0,02	0,53	100,00
Sulawesi Tenggara	0,05	56,92	0,00	16,59	0,27	25,66	0,00	0,51	100,00
Gorontalo	1,22	81,19	0,03	1,81	0,00	14,63	0,00	1,12	100,00
Sulawesi Barat	0,28	74,47	0,00	0,00	0,08	24,84	0,00	0,32	100,00
Maluku	0,19	0,75	0,00	62,41	0,00	35,78	0,02	0,85	100,00
Maluku Utara	0,88	1,70	0,00	46,36	0,00	49,87	0,11	1,08	100,00
Papua Barat	0,08	4,00	0,50	66,94	0,00	27,16	0,00	1,33	100,00
Papua	0,19	1,12	0,01	33,72	0,04	64,37	0,11	0,44	100,00
**Indonesia**	**0,65**	**77,39**	**0,44**	**3,37**	**0,09**	**16,40**	**0,04**	**1,63**	**100,00**

Sumber/*Source*: Susenas Maret 2018/*The March 2018 Susenas*

https://www.bps.go.id

BAB/CHAPTER 7

TEKNOLOGI INFORMASI & KOMUNIKASI

INFORMATION TECHNOLOGY & COMMUNICATION

1

62,41 % penduduk usia 5 tahun ke atas menguasai/memiliki HP

Population aged 5 years and over had/own cellular

2

73,77 % penduduk usia 5 tahun ke atas menggunakan telepon seluler/HP

Population aged 5 years and over using cellular phone

3

19,10 % penduduk usia 5 tahun ke atas menggunakan komputer

Population aged 5 years and over using computer

4

39,90 % penduduk usia 5 tahun ke atas mengakses internet

population aged 5 years and over access internet

5

20,05 % penduduk usia 5 tahun ke atas memiliki komputer

Population aged 5 years and over having/own computer

2,61 % rumah tangga memiliki telepon rumah

household having/own fix line telephone

https://www.bps.go.id

VII. TEKNOLOGI INFORMASI DAN KOMUNIKASI/*INFORMATION TECHNOLOGY AND COMMUNICATION*

PENJELASAN TEKNIS

1. **Telepon seluler (HP)** adalah perangkat telekomunikasi elektronik yang mempunyaikemampuan dasar yang sama dengan telepon tetap kabel, namun dapat dibawa ke mana-mana (portable, mobile) dan tidak perlu disambungkan dengan jaringan telekomunikasi kabel. Selain berfungsi sebagai telepon, telepon selular modern biasanya mendukung layanan tambahan seperti *Short Messages Services* (SMS), *Multimedia Messages Service* (MMS), *e-mail* dan akses Internet, aplikasi bisnis dan permainan, serta fotografi. Saat ini, Indonesia mempunyai dua sistem jaringan telepon bergerak seluler yaitu *Global System for Mobile Telecommunications* (GSM) dan *Code Division Multiple Access* (CDMA).

2. **Komputer** mengacu pada komputer *desktop*, laptop (*portable*) atau tablet (atau komputer genggam yang serupa).
   - ***Personal Computer* (PC)/*Desktop*** adalah komputer yang biasanya tetap di satu tempat,biasanya pengguna ditempatkan di depannya, di belakang keyboard.
   - **Laptop (*Portable*)** adalah komputer yang cukup kecil untuk dibawa dan biasanya memungkinkan tugas yang sama sebagai komputer *desktop*, tetapi juga mencakup *notebook* dan *netbook* tetapi termaksud *tablet* dan sejenisnya komputer genggam.
   - ***Tablet* (atau sejenisnya komputer genggam)** adalah komputer yang terintegrasi ke layar sentuh datar, yang dioperasikan dengan menyentuh layar daripada menggunakan *keyboard* fisik. Dalam hal ini tidak termaksud peralatan dengan beberapa kemampuan komputasi, seperti set TV pintar, dan perangkat dengan telepon

TECHNICAL NOTES

1. ***Cellular phone (HP)*** *is an electronic telecommunication device which has the same basic capabilities with fixed cables, but can be taken anywhere (portable, mobile) and does not need to be connected to the wired telecommunications networks. In addition to functioning as a telephone, modern mobile phone typically supports additional services such as Short Messages Services (SMS), Multimedia Messages Service (MMS), e-mail and access to the Internet, business applications and games, as well as photography. Currently, Indonesia has two cellular mobile telephone network system that are GSM (Global System for Mobile Telecommunications) and Code Division Multiple Access (CDMA).*

2. ***The computer*** *refers to a desktop computer, a laptop (portable) or tablet (or a similar handheld computer).*
   - ***Personal Computer (PC)/Desktop*** *is a computer that typically remain in one place , users are usually placed in front of him, behind the keyboard.*
   - ***Laptop (Portable)*** *is a computer small enough to carry and usually allows the same tasks as a desktop computer, but also includes notebook and netbook but the said tablet and the like handheld computers.*
   - ***Tablet (or similar handheld computer)*** *is a computer integrated into a flat touch screen , which is operated by touching the screen instead of using a physical keyboard it initidak the said equipment with some computing capabilities, such as smart TV sets, and the device with the phone as the primary function they, like smartphones. Tablet*

https://www.bps.go.id

sebagai fungsi utama mereka, seperti *smartphone*. *Tablet* meskipun bisa digunakan untuk menelpon, tidak dimasukan dalam kelompok telepon seluler karena fungsi utama *tablet* adalah sebagai komputer.

3. **Internet** adalah sebuah jaringan komputer publik di seluruh dunia. Internet menyediakan akses ke sejumlah layanan komunikasi termasuk *World Wide Web* dan membuka *email*, berita, hiburan dan *file* data.

4. **Telepon tetap nirkabel atau *Fixed Wireless Acces* (FWA)** merujuk pada jaringan transmisi nirkabel lokal yang menggunakan teknologi selular,gelombang mikro atau radio untuk menghubungkan sinyal kepada pelanggan dilokasi yang tetap ke sentral lokal. Lisensi FWA menggunakan teknologi CDMA (Code Division Multiple Access) yang mengikuti nomor telepon biasa dengan kode area tertentu yang tidak bisa dibawa ke luar area tersebut,kecuali dengan mengganti sementara dengan nomor kode area daerah setempat. Contoh: Smartfren

although it can be used to call, are not included in the group of mobile phone because the main function is as a tablet computer.

3. ***The internet** is a worldwide public computer network. It provides access to a number of communication services including the World Wide Web and carries e-mail, news, entertainment and data files.*

4. ***Fixed wireless or Fixed Wireless Access (FWA)** refers to the local wireless transmission network using cellular technology , micro or radio waves to connect the signal to customers whose fixed location to a local exchange . FWA license using CDMA technology (Code Division Multiple Access), which follow a regular phone number with area code that can not be taken out of the area, but by replacing temporary with local area code numbers setempat. Example: Smartfren*

https://www.bps.go.id

Tabel *Table* **7.1.** **Persentase Penduduk Berumur 5 Tahun ke Atas yang Menggunakan Telepon Seluler (HP)/Nirkabel dalam 3 Bulan Terakhir menurut Provinsi, Daerah Tempat Tinggal, dan Jenis Kelamin, 2018**

Percentage of Population Aged 5 Years and Over who Use Cellular Phone (HP) during The Last 3 Months by Province, Urban Rural Classification, and Sex, 2018

**Provinsi** *Province*	**Perkotaan/***Urban*			**Perdesaan/***Rural*			**Perkotaan+Perdesaan/***Urban+Rural*		
	**Laki-laki** *Male*	**Perempuan** *Female*	**Laki-Laki+Perempuan** *Male+Female*	**Laki-laki** *Male*	**Perempuan/** *Female*	**Laki-Laki+Perempuan** *Male+Female*	**Laki-laki** *Male*	**Perempuan** *Female*	**Laki-Laki+Perempuan** *Male+Female*
(1)	(2)	(3)	(4)	(5)	(6)	(7)	(8)	(9)	(10)
Aceh	81,37	76,94	79,16	71,77	61,72	66,71	74,81	66,48	70,62
Sumatera Utara	83,38	81,45	82,41	75,21	71,11	73,16	79,55	76,62	78,08
Sumatera Barat	83,72	82,03	82,86	76,30	71,64	73,96	79,57	76,25	77,89
Riau	88,17	85,81	87,02	79,78	73,21	76,60	83,15	78,34	80,81
Jambi	86,95	81,73	84,36	77,48	66,99	72,37	80,48	71,77	76,22
Sumatera Selatan	83,16	78,81	80,99	75,66	65,90	70,89	78,42	70,79	74,67
Bengkulu	85,30	81,66	83,49	73,47	64,76	69,26	77,27	70,40	73,92
Lampung	80,83	74,87	77,87	77,83	71,97	75,00	78,70	72,85	75,85
Kepulauan Bangka Belitung	84,91	81,56	83,30	76,84	65,80	71,59	81,16	74,33	77,89
Kepulauan Riau	87,60	82,90	85,26	77,49	71,66	74,66	86,11	81,31	83,73
DKI Jakarta	84,28	79,09	81,69	-	-	-	84,28	79,09	81,69
Jawa Barat	78,86	72,38	75,66	69,86	60,60	65,28	76,52	69,32	72,97
Jawa Tengah	78,85	72,02	75,40	70,49	61,95	66,18	74,77	67,12	70,90
DI Yogyakarta	83,81	79,05	81,42	74,90	66,33	70,45	81,45	75,52	78,44
Jawa Timur	80,24	72,58	76,36	72,93	62,13	67,42	76,76	67,58	72,10
Banten	80,97	73,61	77,36	65,25	53,50	59,51	76,42	67,84	72,22
Bali	85,29	78,15	81,75	75,86	62,64	69,23	82,13	72,85	77,51
Nusa Tenggara Barat	79,05	73,64	76,25	71,52	64,08	67,68	75,02	68,54	71,67
Nusa Tenggara Timur	82,40	80,73	81,57	61,50	57,95	59,69	66,40	63,14	64,75
Kalimantan Barat	84,03	80,12	82,08	66,16	56,45	61,43	72,12	64,59	68,43
Kalimantan Tengah	86,66	82,24	84,52	76,66	67,77	72,46	80,47	73,45	77,12
Kalimantan Selatan	85,14	80,69	82,95	78,24	68,85	73,61	81,46	74,35	77,96
Kalimantan Timur	87,83	85,88	86,90	82,30	75,62	79,19	85,97	82,55	84,35
Kalimantan Utara	85,17	83,95	84,59	79,69	75,28	77,65	82,83	80,37	81,67
Sulawesi Utara	82,24	81,71	81,98	74,11	75,12	74,60	78,16	78,47	78,31
Sulawesi Tengah	83,68	82,53	83,11	65,69	63,85	64,80	70,61	69,21	69,92
Sulawesi Selatan	85,09	83,21	84,13	77,70	75,95	76,80	80,80	78,96	79,86
Sulawesi Tenggara	85,06	84,40	84,73	74,72	73,35	74,04	78,71	77,60	78,16
Gorontalo	81,75	81,09	81,41	74,72	74,59	74,66	77,41	77,16	77,28
Sulawesi Barat	80,73	78,74	79,71	66,79	64,76	65,78	70,02	68,13	69,08
Maluku	78,89	78,16	78,52	60,35	57,04	58,71	68,17	66,02	67,10
Maluku Utara	83,91	81,00	82,47	59,21	56,62	57,95	66,26	63,72	65,02
Papua Barat	83,51	79,88	81,78	67,78	62,25	65,18	74,17	69,48	71,95
Papua	81,36	77,62	79,63	37,79	29,54	33,86	50,09	42,55	46,53
**Indonesia**	**81,39**	**75,81**	**78,61**	**71,77**	**64,03**	**67,91**	**77,04**	**70,49**	**73,77**

Sumber/*Source*: Susenas Maret 2018/*The March 2018 Susenas*

https://www.bps.go.id

Tabel *Table* **7.2.** **Persentase Penduduk Berumur 5 Tahun ke Atas yang Memiliki Telepon Seluler (HP)/Nirkabel dalam 3 Bulan Terakhir menurut Provinsi, Daerah Tempat Tinggal, dan Jenis Kelamin, 2018**

Percentage of Population Aged 5 Years and Over who Had Cellular Phone (HP) during The Last 3 Months by Province, Urban Rural Classification, and Sex, 2018

**Provinsi** *Province*	**Perkotaan/***Urban*			**Perdesaan/***Rural*			**Perkotaan+Perdesaan/***Urban+Rural*		
	**Laki-laki** *Male*	**Perempuan** *Female*	**Laki-Laki+Perempuan** *Male+Female*	**Laki-laki** *Male*	**Perempuan/** *Female*	**Laki-Laki+Perempuan** *Male+Female*	**Laki-laki** *Male*	**Perempuan** *Female*	**Laki-Laki+Perempuan** *Male+Female*
**(1)**	**(2)**	**(3)**	**(4)**	**(5)**	**(6)**	**(7)**	**(8)**	**(9)**	**(10)**
Aceh	73,90	66,89	70,40	62,15	45,64	53,84	65,87	52,29	59,05
Sumatera Utara	70,36	63,74	67,03	57,87	43,84	50,84	64,50	54,44	59,45
Sumatera Barat	74,06	70,74	72,38	60,56	50,06	55,27	66,50	59,22	62,83
Riau	78,65	73,89	76,32	69,03	54,34	61,92	72,90	62,30	67,74
Jambi	78,21	70,07	74,18	68,29	50,49	59,62	71,43	56,84	64,29
Sumatera Selatan	74,98	66,97	70,99	65,20	44,79	55,24	68,81	53,19	61,12
Bengkulu	74,06	70,76	72,42	63,28	44,95	54,42	66,74	53,57	60,31
Lampung	73,78	63,48	68,67	64,33	47,29	56,08	67,08	52,17	59,81
Kepulauan Bangka Belitung	76,62	72,62	74,69	68,43	53,00	61,10	72,81	63,61	68,41
Kepulauan Riau	79,13	73,32	76,25	66,05	56,54	61,44	77,21	70,95	74,11
DKI Jakarta	79,27	73,04	76,16	-	-	-	79,27	73,04	76,16
Jawa Barat	72,85	63,94	68,45	61,49	48,78	55,20	69,90	60,00	65,01
Jawa Tengah	69,85	61,14	65,44	61,45	49,16	55,25	65,75	55,31	60,47
DI Yogyakarta	75,45	69,27	72,34	63,75	51,75	57,53	72,35	64,41	68,32
Jawa Timur	72,59	62,57	67,52	61,57	45,07	53,16	67,35	54,19	60,66
Banten	74,85	65,26	70,14	57,87	41,16	49,71	69,93	58,34	64,25
Bali	78,85	68,90	73,93	65,97	46,87	56,40	74,53	61,38	67,99
Nusa Tenggara Barat	64,70	55,07	59,71	56,40	41,67	48,80	60,26	47,92	53,88
Nusa Tenggara Timur	67,31	64,40	65,86	42,53	32,29	37,32	48,34	39,60	43,91
Kalimantan Barat	76,16	70,58	73,38	57,73	42,89	50,51	63,88	52,42	58,25
Kalimantan Tengah	80,18	73,78	77,07	69,00	55,49	62,61	73,26	62,66	68,20
Kalimantan Selatan	78,00	70,55	74,34	66,69	50,68	58,79	71,97	59,92	66,04
Kalimantan Timur	81,18	77,27	79,31	74,71	65,49	70,42	79,00	73,44	76,36
Kalimantan Utara	80,49	76,21	78,46	70,55	64,66	67,83	76,25	71,43	73,99
Sulawesi Utara	72,12	72,36	72,24	56,52	57,43	56,96	64,28	65,02	64,64
Sulawesi Tengah	74,47	71,65	73,05	53,65	46,74	50,30	59,33	53,88	56,66
Sulawesi Selatan	74,05	70,43	72,20	59,91	55,94	57,86	65,84	61,96	63,85
Sulawesi Tenggara	69,99	69,32	69,65	58,07	50,91	54,50	62,66	57,99	60,34
Gorontalo	67,25	66,97	67,11	52,64	53,44	53,03	58,22	58,79	58,50
Sulawesi Barat	63,52	59,08	61,26	51,51	42,60	47,08	54,29	46,58	50,44
Maluku	72,44	68,72	70,58	48,88	42,06	45,50	58,82	53,40	56,13
Maluku Utara	77,54	72,66	75,12	47,47	42,02	44,81	56,05	50,95	53,55
Papua Barat	77,37	73,28	75,43	62,13	53,25	57,95	68,31	61,46	65,08
Papua	75,71	71,45	73,74	32,89	22,05	27,73	44,98	35,42	40,46
**Indonesia**	**73,60**	**65,70**	**69,66**	**60,33**	**46,85**	**53,61**	**67,59**	**57,19**	**62,41**

Sumber/*Source*: Susenas Maret 2018/*The March 2018 Susenas*

https://www.bps.go.id

Tabel *Table* **7.3.** **Persentase Penduduk Berumur 5 Tahun ke Atas yang Menggunakan Komputer (PC/Desktop, Laptop/Notebook, Tablet) dalam 3 Bulan Terakhir menurut Provinsi, Daerah Tempat Tinggal, dan Jenis Kelamin, 2018**

Percentage of Population Aged 5 Years and Over who Used Computer (PC/Desktop, Laptop/Notebook, Tablet) during The Last 3 Months by Province, Urban Rural Classification, and Sex, 2018

Provinsi *Province*	Perkotaan/*Urban*			Perdesaan/*Rural*			Perkotaan+Perdesaan/*Urban+Rural*		
	Laki-laki *Male*	Perempuan *Female*	Laki-Laki+Perempuan *Male+Female*	Laki-laki *Male*	Perempuan/ *Female*	Laki-Laki+Perempuan *Male+Female*	Laki-laki *Male*	Perempuan *Female*	Laki-Laki+Perempuan *Male+Female*
(1)	(2)	(3)	(4)	(5)	(6)	(7)	(8)	(9)	(10)
Aceh	29,41	26,44	27,92	10,90	9,53	10,21	16,75	14,82	15,78
Sumatera Utara	26,07	24,29	25,17	12,61	11,62	12,12	19,76	18,37	19,06
Sumatera Barat	31,33	32,24	31,79	13,53	15,01	14,28	21,37	22,65	22,01
Riau	33,43	29,82	31,66	12,74	12,65	12,70	21,06	19,64	20,37
Jambi	30,54	27,11	28,84	12,38	12,17	12,28	18,13	17,01	17,59
Sumatera Selatan	26,03	24,13	25,08	9,14	9,03	9,09	15,37	14,75	15,06
Bengkulu	31,08	29,27	30,18	10,88	10,35	10,62	17,36	16,67	17,02
Lampung	25,83	23,97	24,91	9,99	9,67	9,84	14,61	13,98	14,30
Kepulauan Bangka Belitung	25,02	23,04	24,07	9,31	10,40	9,83	17,71	17,24	17,49
Kepulauan Riau	31,51	25,64	28,60	13,37	10,50	11,98	28,84	23,50	26,20
DKI Jakarta	33,56	29,42	31,49	-	-	-	33,56	29,42	31,49
Jawa Barat	25,42	21,41	23,44	10,32	9,03	9,68	21,51	18,19	19,87
Jawa Tengah	23,78	21,33	22,54	12,55	11,47	12,01	18,29	16,53	17,40
DI Yogyakarta	36,61	32,65	34,62	14,70	13,50	14,08	30,80	27,34	29,04
Jawa Timur	24,85	22,53	23,68	12,13	10,51	11,31	18,80	16,78	17,78
Banten	29,93	26,58	28,29	10,37	8,98	9,69	24,27	21,53	22,93
Bali	28,66	25,36	27,02	15,29	11,66	13,47	24,17	20,68	22,44
Nusa Tenggara Barat	23,77	20,95	22,31	12,14	7,83	9,92	17,54	13,95	15,69
Nusa Tenggara Timur	31,18	29,43	30,30	7,92	6,89	7,39	13,38	12,02	12,69
Kalimantan Barat	28,48	23,40	25,95	8,80	6,65	7,75	15,36	12,42	13,92
Kalimantan Tengah	30,45	26,42	28,49	10,77	10,39	10,59	18,26	16,68	17,51
Kalimantan Selatan	34,05	28,56	31,35	12,33	11,54	11,94	22,47	19,46	20,99
Kalimantan Timur	30,87	26,68	28,86	14,37	13,00	13,73	25,31	22,24	23,85
Kalimantan Utara	28,92	26,32	27,69	23,32	20,01	21,79	26,53	23,71	25,21
Sulawesi Utara	24,79	24,29	24,54	11,95	13,28	12,60	18,33	18,88	18,60
Sulawesi Tengah	33,09	32,48	32,78	10,61	10,34	10,48	16,75	16,69	16,72
Sulawesi Selatan	31,65	30,13	30,87	12,68	13,39	13,05	20,64	20,34	20,49
Sulawesi Tenggara	30,78	27,88	29,34	10,58	10,17	10,38	18,37	16,98	17,68
Gorontalo	25,02	26,71	25,88	12,16	13,75	12,95	17,07	18,88	17,98
Sulawesi Barat	27,47	24,57	25,99	11,64	11,12	11,38	15,30	14,37	14,84
Maluku	30,18	29,29	29,74	8,24	8,61	8,42	17,50	17,40	17,45
Maluku Utara	30,42	28,56	29,50	9,18	9,12	9,15	15,24	14,78	15,01
Papua Barat	29,81	24,56	27,31	17,27	15,27	16,33	22,36	19,08	20,81
Papua	27,81	25,61	26,80	5,78	3,91	4,89	12,00	9,78	10,95
**Indonesia**	**27,38**	**24,32**	**25,85**	**11,40**	**10,45**	**10,93**	**20,15**	**18,05**	**19,10**

Sumber/*Source*: Susenas Maret 2018/*The March 2018 Susenas*

https://www.bps.go.id

Tabel 7.4. **Persentase Penduduk Berumur 5 Tahun ke Atas yang Mengakses Internet (Termasuk *Facebook*, *Twitter*, BBM, *Whatsapp*) dalam 3 Bulan Terakhir menurut Provinsi, Daerah Tempat Tinggal, dan Jenis Kelamin, 2018**

Table *Percentage of Population Aged 5 Years and Over who Accessed The Internet (Including Facebook, Twitter, BBM, Whatsapp) during The Last 3 Months by Province, Urban Rural Classification, and Sex, 2018*

Provinsi *Province*	Perkotaan/*Urban*			Perdesaan/*Rural*			Perkotaan+Perdesaan/*Urban+Rural*		
	Laki-laki *Male*	Perempuan *Female*	Laki-Laki+Perempuan *Male+Female*	Laki-laki *Male*	Perempuan/ *Female*	Laki-Laki+Perempuan *Male+Female*	Laki-laki *Male*	Perempuan *Female*	Laki-Laki+Perempuan *Male+Female*
(1)	(2)	(3)	(4)	(5)	(6)	(7)	(8)	(9)	(10)
Aceh	48,51	43,59	46,06	26,45	20,88	23,64	33,43	27,98	30,69
Sumatera Utara	45,07	42,62	43,84	24,97	21,84	23,40	35,64	32,91	34,27
Sumatera Barat	49,13	47,96	48,54	28,20	25,72	26,95	37,42	35,57	36,49
Riau	56,11	51,06	53,64	32,78	28,48	30,70	42,16	37,67	39,98
Jambi	51,89	47,35	49,64	31,96	26,49	29,30	38,28	33,26	35,82
Sumatera Selatan	49,64	46,38	48,02	26,54	22,58	24,60	35,06	31,58	33,35
Bengkulu	51,65	49,45	50,55	25,09	22,74	23,95	33,61	31,66	32,66
Lampung	46,15	43,95	45,06	29,26	24,76	27,08	34,18	30,54	32,41
Kepulauan Bangka Belitung	46,31	44,79	45,58	29,76	25,47	27,72	38,61	35,92	37,33
Kepulauan Riau	59,78	55,58	57,69	33,88	26,40	30,25	55,97	51,45	53,74
DKI Jakarta	68,95	62,83	65,89	-	-	-	68,95	62,83	65,89
Jawa Barat	53,72	48,43	51,11	31,10	26,52	28,84	47,86	42,74	45,33
Jawa Tengah	48,75	43,44	46,06	33,65	27,54	30,57	41,37	35,70	38,51
DI Yogyakarta	65,15	60,44	62,78	38,80	33,00	35,79	58,16	52,83	55,45
Jawa Timur	51,23	45,14	48,15	32,00	25,03	28,45	42,09	35,51	38,75
Banten	59,17	53,38	56,33	30,38	23,64	27,09	50,84	44,85	47,90
Bali	58,45	49,32	53,93	36,47	27,08	31,76	51,07	41,72	46,42
Nusa Tenggara Barat	38,68	33,27	35,87	25,40	18,26	21,71	31,57	25,26	28,31
Nusa Tenggara Timur	49,77	45,61	47,69	14,50	11,75	13,10	22,77	19,46	21,10
Kalimantan Barat	52,79	49,21	51,00	22,76	18,60	20,74	32,78	29,13	30,99
Kalimantan Tengah	51,57	47,66	49,67	29,69	25,43	27,67	38,01	34,15	36,17
Kalimantan Selatan	56,38	50,43	53,46	33,14	26,60	29,92	43,99	37,69	40,89
Kalimantan Timur	59,24	55,37	57,39	37,76	33,60	35,82	52,01	48,30	50,25
Kalimantan Utara	56,02	51,60	53,93	39,19	34,76	37,15	48,84	44,64	46,87
Sulawesi Utara	50,25	51,71	50,98	28,38	31,90	30,09	39,26	41,97	40,59
Sulawesi Tengah	50,05	50,14	50,09	21,07	20,35	20,72	28,98	28,89	28,94
Sulawesi Selatan	52,63	49,60	51,08	27,97	26,24	27,07	38,32	35,93	37,09
Sulawesi Tenggara	47,27	48,08	47,67	25,14	23,35	24,25	33,67	32,86	33,27
Gorontalo	44,25	46,37	45,33	26,44	29,20	27,81	33,24	35,99	34,62
Sulawesi Barat	41,80	41,27	41,53	22,19	20,37	21,29	26,73	25,41	26,07
Maluku	48,97	46,88	47,93	17,75	15,16	16,47	30,92	28,64	29,79
Maluku Utara	51,88	50,10	51,00	16,19	14,98	15,60	26,37	25,21	25,80
Papua Barat	51,80	48,23	50,10	28,93	24,80	26,99	38,22	34,40	36,41
Papua	50,17	44,85	47,72	10,05	7,48	8,83	21,37	17,60	19,59
**Indonesia**	**53,39**	**48,44**	**50,92**	**28,90**	**24,20**	**26,56**	**42,31**	**37,49**	**39,90**

Sumber/*Source*: Susenas Maret 2018/*The March 2018 Susenas*

https://www.bps.go.id

Tabel *Table* **7.5.1.** **Persentase Penduduk Berumur 5 Tahun ke Atas di Daerah Perkotaan yang Mengakses Internet dalam 3 Bulan Terakhir menurut Provinsi, Jenis Kelamin, dan Alat yang Digunakan untuk Mengakses Internet, 2017***
*Percentage of Population Aged 5 Year and Over in Urban Area Who Accessed The Internet during The Last 3 Months by Province, Sex, and Media Used to Access The Internet, 2017**

**Provinsi** *Province*	**Laki-laki/***Male*				**Perempuan/***Female*				**Laki-laki+Perempuan/***Male+Female*			
	**Komputer Desktop** *Computer Desktop*	**Laptop/** *Notebook/* *Tablet*	**HP/Ponsel** *Cellular Phone*	**Lainnya** *Others*	**Komputer Desktop** *Computer Desktop*	**Laptop/** *Notebook/* *Tablet*	**HP/Ponsel** *Cellular Phone*	**Lainnya** *Others*	**Komputer Desktop** *Computer Desktop*	**Laptop/** *Notebook/* *Tablet*	**HP/Ponsel** *Cellular Phone*	**Lainnya** *Others*
**(1)**	**(2)**	**(3)**	**(4)**	**(5)**	**(6)**	**(7)**	**(8)**	**(9)**	**(10)**	**(11)**	**(12)**	**(13)**
Aceh	22,33	40,90	89,69	0,50	15,69	41,19	90,82	0,28	19,22	41,03	90,22	0,40
Sumatera Utara	28,86	28,41	79,21	1,45	23,10	29,03	84,47	0,57	26,05	28,71	81,78	1,02
Sumatera Barat	32,70	40,39	85,40	0,75	29,82	46,88	88,63	0,36	31,28	43,59	86,99	0,55
Riau	21,84	33,87	86,71	0,86	17,78	37,65	88,07	0,63	19,97	35,61	87,34	0,75
Jambi	28,83	43,44	85,00	0,06	23,28	46,36	87,61	0,19	26,14	44,85	86,26	0,12
Sumatera Selatan	19,54	35,39	90,37	0,69	16,79	35,17	90,77	0,00	18,21	35,28	90,57	0,35
Bengkulu	20,39	41,45	89,12	0,33	17,80	45,78	90,17	0,28	19,09	43,62	89,65	0,30
Lampung	20,98	31,12	88,50	0,25	17,74	32,11	89,76	0,19	19,45	31,58	89,09	0,22
Kepulauan Bangka Belitung	15,40	30,40	90,99	0,11	11,93	27,46	92,45	0,32	13,73	28,99	91,69	0,21
Kepulauan Riau	19,30	30,00	89,11	0,37	14,62	30,67	90,42	0,08	17,07	30,31	89,73	0,23
DKI Jakarta	20,26	36,54	92,29	0,94	17,14	34,93	93,52	0,63	18,78	35,78	92,87	0,79
Jawa Barat	21,45	26,60	93,49	0,49	17,08	26,11	94,04	0,57	19,42	26,37	93,74	0,53
Jawa Tengah	19,41	28,55	91,56	0,30	19,42	30,60	91,21	0,26	19,41	29,52	91,40	0,28
DI Yogyakarta	20,24	43,22	94,94	0,69	16,63	42,28	94,39	0,76	18,54	42,78	94,68	0,72
Jawa Timur	18,15	32,36	91,91	0,41	17,89	34,04	91,86	0,60	18,03	33,14	91,89	0,50
Banten	20,06	32,16	94,93	1,17	15,84	30,25	95,18	1,16	18,14	31,29	95,04	1,17
Bali	17,72	32,88	93,85	0,47	15,84	34,19	94,48	0,48	16,88	33,47	94,13	0,47
Nusa Tenggara Barat	19,03	36,37	89,54	0,43	18,24	35,36	89,99	0,63	18,64	35,88	89,76	0,53
Nusa Tenggara Timur	29,98	39,90	85,32	0,64	28,13	34,52	87,24	0,19	29,11	37,38	86,22	0,43
Kalimantan Barat	19,87	33,79	89,85	0,80	13,87	32,01	91,75	0,43	16,99	32,93	90,76	0,62
Kalimantan Tengah	18,54	36,88	92,08	0,47	14,91	33,21	94,80	0,64	16,89	35,21	93,32	0,55
Kalimantan Selatan	15,41	37,30	93,64	0,27	11,33	36,99	93,77	0,33	13,49	37,16	93,70	0,30
Kalimantan Timur	17,66	41,54	92,47	0,53	14,37	38,71	93,10	0,22	16,19	40,27	92,75	0,39
Kalimantan Utara	17,55	34,48	90,22	0,43	14,77	33,29	92,44	0,22	16,30	33,94	91,22	0,34
Sulawesi Utara	17,59	33,18	91,85	0,46	13,81	33,99	94,28	0,32	15,70	33,58	93,06	0,39
Sulawesi Tengah	19,30	39,09	91,24	0,51	16,02	39,48	92,10	0,26	17,68	39,28	91,67	0,38
Sulawesi Selatan	21,14	38,92	88,39	0,45	16,99	40,62	91,70	0,38	19,06	39,77	90,05	0,42
Sulawesi Tenggara	18,20	39,32	91,53	0,00	12,20	36,12	92,99	0,00	15,15	37,69	92,27	0,00
Gorontalo	24,26	35,92	89,74	0,93	25,04	34,37	93,03	0,56	24,66	35,12	91,43	0,74
Sulawesi Barat	17,84	38,85	94,30	0,00	15,68	39,99	92,68	0,00	16,72	39,45	93,46	0,00
Maluku	32,92	32,47	86,14	0,71	24,79	34,81	87,15	0,26	28,91	33,62	86,64	0,49
Maluku Utara	22,12	35,88	90,37	0,33	16,10	33,77	91,74	0,18	19,21	34,86	91,03	0,26
Papua Barat	14,33	25,42	88,58	0,05	9,95	23,94	90,68	0,11	12,39	24,76	89,52	0,08
Papua	12,80	26,35	91,45	0,78	10,66	28,68	92,79	0,34	11,89	27,34	92,02	0,59
**Indonesia**	**20,64**	**32,02**	**91,47**	**0,60**	**17,66**	**32,29**	**92,25**	**0,52**	**19,24**	**32,15**	**91,84**	**0,56**

Catatan/*Note*: *) Data 2018 tidak tersedia/*2018 data not available*
Sumber/*Source*: Susenas Maret 2017/*The March 2017 Susenas*

https://www.bps.go.id

Tabel *Table* **7.5.2.** **Persentase Penduduk Berumur 5 Tahun ke Atas di Daerah Perdesaan yang Mengakes Internet dalam 3 Bulan Terakhir menurut Provinsi, Jenis Kelamin, dan Alat yang Digunakan untuk Mengakses Internet, 2017***
*Percentage of Population Aged 5 Year and Over in Rural Area Who Accessed The Internet during The Last 3 Months by Province, Sex, and Media Used to Access The Internet, 2017**

**Provinsi** *Province*	**Laki-laki/***Male*				**Perempuan/***Female*				**Laki-laki+Perempuan/***Male+Female*			
	**Komputer Desktop** *Computer Desktop*	**Laptop/** *Notebook/* *Tablet*	**HP/Ponsel** *Cellular Phone*	**Lainnya** *Others*	**Komputer Desktop** *Computer Desktop*	**Laptop/** *Notebook/* *Tablet*	**HP/Ponsel** *Cellular Phone*	**Lainnya** *Others*	**Komputer Desktop** *Computer Desktop*	**Laptop/** *Notebook/* *Tablet*	**HP/Ponsel** *Cellular Phone*	**Lainnya** *Others*
(1)	(2)	(3)	(4)	(5)	(6)	(7)	(8)	(9)	(10)	(11)	(12)	(13)
Aceh	11,09	20,63	90,41	0,39	11,53	22,72	91,74	0,16	11,29	21,60	91,03	0,28
Sumatera Utara	27,50	16,62	78,61	0,74	24,50	21,30	79,75	0,49	26,09	18,82	79,15	0,62
Sumatera Barat	23,30	18,03	84,82	0,88	25,80	24,43	84,22	0,71	24,50	21,10	84,53	0,80
Riau	11,08	18,12	91,74	0,42	11,68	20,18	88,08	0,49	11,35	19,03	90,12	0,45
Jambi	10,67	15,93	92,54	0,66	12,19	20,68	91,25	0,36	11,36	18,08	91,96	0,52
Sumatera Selatan	7,88	17,21	92,40	0,24	9,17	20,62	89,61	0,14	8,45	18,71	91,17	0,20
Bengkulu	12,30	19,52	90,59	0,40	15,53	24,27	87,31	0,72	13,75	21,65	89,12	0,54
Lampung	11,98	14,66	89,50	0,14	13,66	18,97	86,44	0,21	12,74	16,61	88,12	0,17
Kepulauan Bangka Belitung	8,12	19,45	92,23	0,00	7,53	23,49	90,28	0,00	7,85	21,31	91,33	0,00
Kepulauan Riau	15,94	18,96	90,13	0,00	10,85	26,13	92,36	0,00	13,56	22,31	91,17	0,00
DKI Jakarta	-	-	-	-	-	-	-	-	-	-	-	-
Jawa Barat	16,51	13,62	92,72	0,43	15,34	13,74	93,29	0,36	15,98	13,67	92,98	0,40
Jawa Tengah	18,55	16,05	90,97	0,41	20,21	20,05	89,46	0,46	19,30	17,86	90,29	0,43
DI Yogyakarta	13,75	18,41	93,95	0,00	14,46	20,23	92,14	0,14	14,09	19,28	93,08	0,07
Jawa Timur	16,22	16,06	91,00	0,52	16,67	21,37	90,51	0,62	16,42	18,36	90,79	0,56
Banten	11,53	12,41	89,48	0,98	9,32	12,92	92,92	0,70	10,64	12,61	90,87	0,87
Bali	12,78	20,78	91,56	0,40	19,47	26,25	91,48	0,40	15,42	22,94	91,53	0,40
Nusa Tenggara Barat	12,28	20,56	91,15	0,30	10,32	16,21	90,57	0,00	11,38	18,57	90,89	0,16
Nusa Tenggara Timur	11,00	22,15	90,51	0,40	14,17	21,73	91,14	0,43	12,45	21,95	90,80	0,41
Kalimantan Barat	9,24	15,78	92,31	0,14	8,35	19,24	93,56	0,18	8,86	17,26	92,84	0,16
Kalimantan Tengah	6,49	19,34	93,99	0,00	5,34	19,30	93,76	0,00	6,03	19,33	93,89	0,00
Kalimantan Selatan	8,26	17,31	94,22	0,17	7,67	22,62	93,11	0,36	8,01	19,59	93,75	0,25
Kalimantan Timur	9,73	17,32	95,88	0,00	6,96	18,99	96,11	0,00	8,51	18,05	95,98	0,00
Kalimantan Utara	9,93	20,01	92,97	0,00	7,91	21,34	91,71	0,00	9,13	20,54	92,47	0,00
Sulawesi Utara	6,90	20,13	93,45	0,63	5,78	19,68	95,15	0,41	6,32	19,90	94,32	0,52
Sulawesi Tengah	12,02	18,12	91,78	0,18	12,56	18,54	90,99	0,10	12,29	18,33	91,39	0,14
Sulawesi Selatan	11,77	20,64	89,70	0,18	13,36	23,20	90,34	0,16	12,57	21,94	90,02	0,17
Sulawesi Tenggara	7,74	17,98	93,93	0,00	5,27	17,02	94,20	0,00	6,54	17,51	94,06	0,00
Gorontalo	9,83	19,72	92,25	0,00	11,08	20,23	93,79	0,10	10,49	19,99	93,06	0,05
Sulawesi Barat	9,65	22,06	92,21	0,40	8,93	21,31	92,46	0,38	9,30	21,70	92,33	0,39
Maluku	7,56	19,29	86,99	0,22	6,86	19,01	88,49	0,18	7,23	19,15	87,69	0,20
Maluku Utara	12,37	20,59	88,72	0,59	8,72	17,15	93,64	0,68	10,71	19,02	90,97	0,63
Papua Barat	12,48	18,93	93,35	0,47	10,78	17,00	93,50	0,00	11,78	18,13	93,41	0,28
Papua	12,83	20,72	92,26	0,11	10,85	24,92	91,34	0,60	12,08	22,31	91,91	0,30
**Indonesia**	**14,81**	**16,78**	**90,72**	**0,41**	**15,16**	**19,78**	**90,18**	**0,39**	**14,97**	**18,13**	**90,48**	**0,40**

Catatan/*Note*: *) Data 2018 tidak tersedia/*2018 data not available*
Sumber/*Source*: Susenas Maret 2017/*The March 2017 Susenas*

https://www.bps.go.id

Tabel *Table* **7.5.3.** **Persentase Penduduk Berumur 5 Tahun ke Atas di Daerah Perkotaan dan Perdesaan yang Mengakes Internet dalam 3 Bulan Terakhir menurut Provinsi, Jenis Kelamin, dan Alat yang Digunakan untuk Mengakses Internet, 2017***
*Percentage of Population Aged 5 Year and Over in Urban and Rural Area Who Accessed The Internet during The Last 3 Months by Province, Sex, and Media Used to Access The Internet, 2017**

Provinsi *Province*	Laki-laki/*Male*				Perempuan/*Female*				Laki-laki+Perempuan/*Male+Female*			
	Komputer Desktop *Computer Desktop*	Laptop/ *Notebook/ Tablet*	HP/Ponsel *Cellular Phone*	Lainnya *Others*	Komputer Desktop *Computer Desktop*	Laptop/ *Notebook/ Tablet*	HP/Ponsel *Cellular Phone*	Lainnya *Others*	Komputer Desktop *Computer Desktop*	Laptop/ *Notebook/ Tablet*	HP/Ponsel *Cellular Phone*	Lainnya *Others*
(1)	(2)	(3)	(4)	(5)	(6)	(7)	(8)	(9)	(10)	(11)	(12)	(13)
Aceh	22,33	40,90	89,69	0,50	15,69	41,19	90,82	0,28	19,22	41,03	90,22	0,40
Sumatera Utara	28,86	28,41	79,21	1,45	23,10	29,03	84,47	0,57	26,05	28,71	81,78	1,02
Sumatera Barat	32,70	40,39	85,40	0,75	29,82	46,88	88,63	0,36	31,28	43,59	86,99	0,55
Riau	21,84	33,87	86,71	0,86	17,78	37,65	88,07	0,63	19,97	35,61	87,34	0,75
Jambi	28,83	43,44	85,00	0,06	23,28	46,36	87,61	0,19	26,14	44,85	86,26	0,12
Sumatera Selatan	19,54	35,39	90,37	0,69	16,79	35,17	90,77	0,00	18,21	35,28	90,57	0,35
Bengkulu	20,39	41,45	89,12	0,33	17,80	45,78	90,17	0,28	19,09	43,62	89,65	0,30
Lampung	20,98	31,12	88,50	0,25	17,74	32,11	89,76	0,19	19,45	31,58	89,09	0,22
Kepulauan Bangka Belitung	15,40	30,40	90,99	0,11	11,93	27,46	92,45	0,32	13,73	28,99	91,69	0,21
Kepulauan Riau	19,30	30,00	89,11	0,37	14,62	30,67	90,42	0,08	17,07	30,31	89,73	0,23
DKI Jakarta	20,26	36,54	92,29	0,94	17,14	34,93	93,52	0,63	18,78	35,78	92,87	0,79
Jawa Barat	21,45	26,60	93,49	0,49	17,08	26,11	94,04	0,57	19,42	26,37	93,74	0,53
Jawa Tengah	19,41	28,55	91,56	0,30	19,42	30,60	91,21	0,26	19,41	29,52	91,40	0,28
DI Yogyakarta	20,24	43,22	94,94	0,69	16,63	42,28	94,39	0,76	18,54	42,78	94,68	0,72
Jawa Timur	18,15	32,36	91,91	0,41	17,89	34,04	91,86	0,60	18,03	33,14	91,89	0,50
Banten	20,06	32,16	94,93	1,17	15,84	30,25	95,18	1,16	18,14	31,29	95,04	1,17
Bali	17,72	32,88	93,85	0,47	15,84	34,19	94,48	0,48	16,88	33,47	94,13	0,47
Nusa Tenggara Barat	19,03	36,37	89,54	0,43	18,24	35,36	89,99	0,63	18,64	35,88	89,76	0,53
Nusa Tenggara Timur	29,98	39,90	85,32	0,64	28,13	34,52	87,24	0,19	29,11	37,38	86,22	0,43
Kalimantan Barat	19,87	33,79	89,85	0,80	13,87	32,01	91,75	0,43	16,99	32,93	90,76	0,62
Kalimantan Tengah	18,54	36,88	92,08	0,47	14,91	33,21	94,80	0,64	16,89	35,21	93,32	0,55
Kalimantan Selatan	15,41	37,30	93,64	0,27	11,33	36,99	93,77	0,33	13,49	37,16	93,70	0,30
Kalimantan Timur	17,66	41,54	92,47	0,53	14,37	38,71	93,10	0,22	16,19	40,27	92,75	0,39
Kalimantan Utara	17,55	34,48	90,22	0,43	14,77	33,29	92,44	0,22	16,30	33,94	91,22	0,34
Sulawesi Utara	17,59	33,18	91,85	0,46	13,81	33,99	94,28	0,32	15,70	33,58	93,06	0,39
Sulawesi Tengah	19,30	39,09	91,24	0,51	16,02	39,48	92,10	0,26	17,68	39,28	91,67	0,38
Sulawesi Selatan	21,14	38,92	88,39	0,45	16,99	40,62	91,70	0,38	19,06	39,77	90,05	0,42
Sulawesi Tenggara	18,20	39,32	91,53	0,00	12,20	36,12	92,99	0,00	15,15	37,69	92,27	0,00
Gorontalo	24,26	35,92	89,74	0,93	25,04	34,37	93,03	0,56	24,66	35,12	91,43	0,74
Sulawesi Barat	17,84	38,85	94,30	0,00	15,68	39,99	92,68	0,00	16,72	39,45	93,46	0,00
Maluku	32,92	32,47	86,14	0,71	24,79	34,81	87,15	0,26	28,91	33,62	86,64	0,49
Maluku Utara	22,12	35,88	90,37	0,33	16,10	33,77	91,74	0,18	19,21	34,86	91,03	0,26
Papua Barat	14,33	25,42	88,58	0,05	9,95	23,94	90,68	0,11	12,39	24,76	89,52	0,08
Papua	12,80	26,35	91,45	0,78	10,66	28,68	92,79	0,34	11,89	27,34	92,02	0,59
**Indonesia**	**20,64**	**32,02**	**91,47**	**0,60**	**17,66**	**32,29**	**92,25**	**0,52**	**19,24**	**32,15**	**91,84**	**0,56**

Catatan/*Note*: *) Data 2018 tidak tersedia/*2018 data not available*
Sumber/*Source*: Susenas Maret 2017/*The March 2017 Susenas*

https://www.bps.go.id

Tabel 7.6.1. **Persentase Persentase Penduduk Berumur 5 Tahun ke Atas di Daerah Perkotaan yang Mengakses Internet dalam 3 Bulan Terakhir menurut Provinsi dan Tempat Mengakses Internet, 2017***

Table *Percentage of Population Aged 5 Years and Over in Urban Area Who Accessed Internet during The Last 3 Months by Province and Places Accessing The Internet, 2017**

**Provinsi** *Province*	**Rumah Sendiri** *Own House*	**Bukan Rumah Sendiri** *Other House*	**Tempat Bekerja/Kantor** *Office/Working Place*	**Gedung Sekolah/ Kampus** *School/Campus*	**Tempat Umum Gratis** *Public Access to Internet (free)*	**Tempat Umum Berbayar** *Public Access to Internet (pay)*	**Kondisi Bergerak** *In Mobile Condition*	**Tempat Lainnya** *Other Places*
(1)	(2)	(3)	(4)	(5)	(6)	(7)	(8)	(9)
Aceh	90,00	30,78	31,79	21,95	27,29	18,09	12,71	0,82
Sumatera Utara	82,98	19,44	24,53	17,48	15,20	20,38	12,39	1,47
Sumatera Barat	88,55	20,41	27,87	22,67	16,79	19,27	13,15	0,08
Riau	91,58	21,41	27,45	18,84	16,59	17,44	10,93	1,48
Jambi	90,46	23,79	31,81	19,74	17,44	12,55	11,71	1,14
Sumatera Selatan	92,34	17,32	28,74	16,51	15,07	12,09	17,71	3,24
Bengkulu	89,06	28,93	28,38	21,64	15,36	15,52	14,80	0,67
Lampung	91,25	21,62	29,19	23,28	18,10	8,91	9,47	0,92
Kepulauan Bangka Belitung	96,19	31,83	33,13	11,71	20,94	8,63	9,18	2,30
Kepulauan Riau	92,54	29,78	33,26	11,41	19,99	14,80	6,83	1,14
DKI Jakarta	92,29	27,73	40,67	13,97	24,91	14,56	22,33	2,09
Jawa Barat	92,45	32,55	33,93	17,09	25,77	13,08	26,65	5,04
Jawa Tengah	92,28	29,18	33,09	20,02	22,43	18,93	17,23	2,03
DI Yogyakarta	96,90	44,10	37,96	26,35	31,93	12,04	15,21	1,93
Jawa Timur	91,39	26,69	31,18	19,67	29,87	12,93	15,22	2,31
Banten	93,38	29,31	36,67	17,50	20,62	13,41	15,80	3,19
Bali	94,88	27,33	41,02	18,45	19,28	12,83	10,03	1,46
Nusa Tenggara Barat	88,47	27,93	27,27	19,92	15,79	14,77	6,72	0,45
Nusa Tenggara Timur	86,80	31,12	32,71	19,92	30,63	26,33	23,29	2,35
Kalimantan Barat	92,98	20,00	28,55	18,04	15,51	18,38	13,98	1,94
Kalimantan Tengah	93,11	24,42	33,85	16,68	22,14	10,50	20,03	1,38
Kalimantan Selatan	95,87	37,31	34,80	16,95	24,94	10,08	19,58	1,42
Kalimantan Timur	94,29	27,89	34,33	16,09	24,33	13,60	17,54	4,74
Kalimantan Utara	92,11	26,03	31,74	17,18	10,33	7,47	12,66	0,77
Sulawesi Utara	90,97	37,11	31,40	19,92	32,25	14,67	25,66	3,53
Sulawesi Tengah	90,94	25,46	30,93	21,39	17,05	14,56	9,89	0,40
Sulawesi Selatan	93,39	28,55	30,48	22,00	18,87	13,75	16,26	0,92
Sulawesi Tenggara	92,29	22,59	27,95	24,20	7,00	12,35	9,38	0,89
Gorontalo	88,23	34,53	31,08	24,47	32,01	17,60	23,99	0,32
Sulawesi Barat	88,73	26,87	33,54	19,27	11,29	11,81	20,81	0,44
Maluku	81,18	20,47	22,82	18,86	24,92	23,43	16,55	0,73
Maluku Utara	90,66	28,57	31,80	18,03	16,51	17,71	11,87	0,54
Papua Barat	83,52	8,62	22,43	16,41	10,23	8,43	7,05	0,38
Papua	90,36	19,07	26,22	14,30	14,33	12,85	13,20	1,52
**Indonesia**	**91,84**	**28,56**	**33,28**	**18,17**	**23,52**	**14,54**	**18,58**	**2,75**

Catatan/*Note*: *) Data 2018 tidak tersedia/*2018 data not available*
Sumber/*Source*: Susenas Maret 2017/*The March 2017 Susenas*

https://www.bps.go.id

Tabel *Table* **7.6.2.** **Persentase Persentase Penduduk Berumur 5 Tahun ke Atas di Daerah Perdesaan yang Mengakses Internet dalam 3 Bulan Terakhir menurut Provinsi dan Tempat Mengakses Internet, 2017***

*Percentage of Population Aged 5 Years and Over in Rural Area Who Accessed Internet during The Last 3 Months by Province and Places Accessing The Internet, 2017**

**Provinsi** *Province*	**Rumah Sendiri** *Own House*	**Bukan Rumah Sendiri** *Other House*	**Tempat Bekerja/Kantor** *Office/Working Place*	**Gedung Sekolah/ Kampus** *School/Campus*	**Tempat Umum Gratis** *Public Access to Internet (free)*	**Tempat Umum Berbayar** *Public Access to Internet (pay)*	**Kondisi Bergerak** *In Mobile Condition*	**Tempat Lainnya** *Other Places*
**(1)**	**(2)**	**(3)**	**(4)**	**(5)**	**(6)**	**(7)**	**(8)**	**(9)**
Aceh	87,52	25,07	21,14	19,42	25,68	13,26	9,91	2,18
Sumatera Utara	80,26	20,39	13,88	16,81	11,14	25,37	7,39	3,26
Sumatera Barat	85,02	20,94	15,38	16,85	14,68	22,34	8,77	0,17
Riau	89,40	31,27	18,07	14,09	13,75	13,06	10,55	2,80
Jambi	90,49	29,58	15,03	17,59	15,73	10,72	8,40	4,27
Sumatera Selatan	92,51	31,48	14,87	15,90	15,86	11,75	11,66	2,52
Bengkulu	89,01	31,20	17,00	14,35	14,26	12,95	6,24	1,24
Lampung	87,01	27,23	13,00	19,51	9,94	8,49	7,10	1,42
Kepulauan Bangka Belitung	96,16	34,96	19,51	17,43	20,95	7,91	8,88	8,10
Kepulauan Riau	85,87	32,80	21,04	17,13	15,28	13,21	11,92	2,16
DKI Jakarta	-	-	-	-	-	-	-	-
Jawa Barat	92,27	39,57	23,74	19,29	17,28	15,92	32,97	5,47
Jawa Tengah	90,32	36,95	23,57	21,94	17,83	17,27	17,64	3,37
DI Yogyakarta	92,54	25,37	28,79	20,91	19,73	8,77	9,24	0,34
Jawa Timur	88,90	29,32	19,36	19,92	26,98	14,50	12,25	2,74
Banten	90,51	36,18	20,36	18,28	15,09	13,59	13,94	2,67
Bali	93,70	29,89	30,00	19,64	15,58	9,50	3,97	1,01
Nusa Tenggara Barat	86,35	26,91	17,77	14,77	10,91	8,98	5,78	1,07
Nusa Tenggara Timur	88,50	51,99	24,92	14,59	15,23	11,24	15,71	4,48
Kalimantan Barat	84,40	25,14	15,95	15,45	20,90	10,81	11,64	3,76
Kalimantan Tengah	92,10	24,54	19,57	12,45	17,91	6,52	12,33	2,32
Kalimantan Selatan	94,23	33,47	22,06	15,23	20,68	11,58	13,19	2,77
Kalimantan Timur	92,60	17,41	19,72	9,33	6,89	12,81	5,43	1,41
Kalimantan Utara	84,77	27,05	22,92	12,81	16,81	13,55	12,49	6,05
Sulawesi Utara	92,94	31,04	18,10	16,55	21,03	5,11	16,02	1,92
Sulawesi Tengah	84,36	29,23	19,78	17,09	13,41	8,72	9,67	1,27
Sulawesi Selatan	87,81	29,59	17,16	21,71	15,30	9,35	11,03	2,24
Sulawesi Tenggara	91,33	27,64	19,88	17,02	11,68	5,94	6,05	1,08
Gorontalo	88,13	34,16	20,26	21,18	23,83	10,94	16,70	0,76
Sulawesi Barat	83,27	25,00	15,33	22,44	18,05	8,23	7,33	1,02
Maluku	81,09	22,28	11,75	14,79	20,43	7,68	11,82	2,05
Maluku Utara	75,74	22,74	24,43	12,79	18,77	13,70	13,67	2,32
Papua Barat	75,29	17,42	19,43	16,04	11,97	8,95	17,63	2,82
Papua	74,75	14,72	18,65	12,04	19,54	10,58	11,35	2,92
**Indonesia**	**89,00**	**31,28**	**19,94**	**18,61**	**18,05**	**14,03**	**14,48**	**2,96**

Catatan/*Note*: *) Data 2018 tidak tersedia/*2018 data not available*
Sumber/*Source*: Susenas Maret 2017/*The March 2017 Susenas*

https://www.bps.go.id

Tabel *Table* **7.6.3.** **Persentase Persentase Penduduk Berumur 5 Tahun ke Atas di Daerah Perkotaan dan Perdesaan yang Mengakses Internet dalam 3 Bulan Terakhir menurut Provinsi dan Tempat Mengakses Internet, 2017***

*Percentage of Population Aged 5 Years and Over in Urban and Rural Area Who Accessed Internet during The Last 3 Months by Province and Places Accessing The Internet, 2017**

**Provinsi** *Province*	**Rumah Sendiri** *Own House*	**Bukan Rumah Sendiri** *Other House*	**Tempat Bekerja/Kantor** *Office/Working Place*	**Gedung Sekolah/ Kampus** *School/Campus*	**Tempat Umum Gratis** *Public Access to Internet (free)*	**Tempat Umum Berbayar** *Public Access to Internet (pay)*	**Kondisi Bergerak** *In Mobile Condition*	**Tempat Lainnya** *Other Places*
(1)	(2)	(3)	(4)	(5)	(6)	(7)	(8)	(9)
Aceh	88,72	27,82	26,27	20,64	26,46	15,59	11,26	1,53
Sumatera Utara	82,11	19,74	21,12	17,26	13,90	21,97	10,79	2,04
Sumatera Barat	87,15	20,62	22,92	20,36	15,95	20,49	11,42	0,12
Riau	90,65	25,63	23,43	16,81	15,37	15,57	10,77	2,04
Jambi	90,48	26,83	23,01	18,61	16,54	11,59	9,98	2,78
Sumatera Selatan	92,41	23,16	23,02	16,26	15,40	11,95	15,21	2,95
Bengkulu	89,04	29,90	23,52	18,53	14,89	14,42	11,15	0,91
Lampung	88,89	24,73	20,19	21,19	13,56	8,68	8,16	1,20
Kepulauan Bangka Belitung	96,18	32,78	29,00	13,44	20,94	8,41	9,09	4,06
Kepulauan Riau	92,07	29,99	32,39	11,82	19,65	14,69	7,19	1,21
DKI Jakarta	92,29	27,73	40,67	13,97	24,91	14,56	22,33	2,09
Jawa Barat	92,42	33,74	32,21	17,46	24,34	13,55	27,71	5,11
Jawa Tengah	91,52	32,21	29,38	20,77	20,64	18,28	17,39	2,56
DI Yogyakarta	96,13	40,81	36,34	25,39	29,78	11,46	14,16	1,65
Jawa Timur	90,54	27,59	27,13	19,75	28,88	13,47	14,20	2,46
Banten	92,98	30,27	34,39	17,61	19,85	13,43	15,54	3,12
Bali	94,61	27,91	38,51	18,72	18,44	12,07	8,65	1,35
Nusa Tenggara Barat	87,59	27,50	23,31	17,78	13,76	12,36	6,33	0,71
Nusa Tenggara Timur	87,61	41,05	29,01	17,38	23,31	19,15	19,68	3,36
Kalimantan Barat	89,50	22,09	23,44	16,99	17,70	15,31	13,03	2,68
Kalimantan Tengah	92,67	24,47	27,64	14,84	20,30	8,77	16,68	1,79
Kalimantan Selatan	95,26	35,87	30,04	16,31	23,35	10,64	17,19	1,93
Kalimantan Timur	93,90	25,48	30,96	14,53	20,31	13,42	14,75	3,97
Kalimantan Utara	89,82	26,35	29,00	15,82	12,35	9,36	12,61	2,42
Sulawesi Utara	91,70	34,85	26,45	18,66	28,07	11,11	22,08	2,93
Sulawesi Tengah	87,70	27,32	25,43	19,27	15,25	11,68	9,78	0,83
Sulawesi Selatan	91,17	28,96	25,19	21,88	17,45	12,00	14,18	1,45
Sulawesi Tenggara	91,81	25,12	23,90	20,60	9,35	9,13	7,71	0,99
Gorontalo	88,19	34,36	26,08	22,95	28,23	14,52	20,62	0,52
Sulawesi Barat	85,17	25,65	21,68	21,33	15,69	9,48	12,04	0,82
Maluku	81,15	21,01	19,47	17,63	23,56	18,66	15,12	1,13
Maluku Utara	84,67	26,23	28,84	15,93	17,42	16,10	12,59	1,25
Papua Barat	79,96	12,43	21,13	16,25	10,98	8,65	11,63	1,44
Papua	85,31	17,66	23,77	13,57	16,01	12,11	12,61	1,97
**Indonesia**	**91,02**	**29,35**	**29,43**	**18,30**	**21,94**	**14,39**	**17,39**	**2,81**

Catatan/*Note*: *) Data 2018 tidak tersedia/*2018 data not available*
Sumber/*Source*: Susenas Maret 2017/*The March 2017 Susenas*

https://www.bps.go.id

Tabel *Table* **7.7.1.** **Persentase Penduduk Berumur 5 Tahun ke Atas di Daerah Perkotaan yang Mengakses Internet dalam 3 Bulan Terakhir menurut Provinsi dan Tujuan Mengakses Internet, 2017***
*Percentage of Population Aged 5 Years and Over in Urban Area Who Accessed Internet during The Last 3 Months by Province and The Purpose for Accessing The Internet, 2017**

**Provinsi** *Province*	**Mendapat Informasi/Berita** *Get Information/ News*	**Mengerjakan Tugas Sekolah** *Do Homework*	**Mengirim/ Menerima Email** *Send/Receive Email*	**Sosial Media/ Jejaring Sosial** *Social Media/Social Network*	**Pembelian Barang/Jasa** *Buy Goods/ Service*	**Penjualan Barang/Jasa** *Sell Goods/Service*	**Hiburan** *Entertainment*	**Fasilitas Finansial** *Financial Facility*	**Mendapat Informasi Barang/Jasa** *Get Information Goods/Service*	**Lainnya** *Others*
(1)	(2)	(3)	(4)	(5)	(6)	(7)	(8)	(9)	(10)	(11)
Aceh	77,83	28,37	33,09	79,25	13,19	3,66	53,63	5,47	12,31	1,05
Sumatera Utara	65,81	30,14	18,98	74,47	5,45	3,33	47,59	3,75	9,01	1,15
Sumatera Barat	68,90	35,40	27,46	75,54	11,32	3,85	54,50	5,29	14,80	2,08
Riau	70,32	28,78	23,34	79,18	8,26	5,44	52,04	6,94	13,93	1,92
Jambi	70,79	30,36	29,72	77,35	12,30	7,77	50,05	8,59	14,49	1,33
Sumatera Selatan	68,84	25,57	25,42	81,47	9,38	4,68	47,07	4,78	10,01	6,17
Bengkulu	66,00	34,21	25,21	77,44	8,87	6,83	43,45	5,64	10,84	0,98
Lampung	65,88	28,25	19,07	75,82	7,97	4,26	38,80	6,57	12,93	1,00
Kepulauan Bangka Belitung	77,44	22,87	20,40	76,75	16,21	5,74	52,76	5,33	17,07	2,73
Kepulauan Riau	69,59	21,91	26,06	73,85	9,90	5,49	45,88	4,27	13,06	1,53
DKI Jakarta	73,49	18,35	31,01	80,92	14,72	6,55	49,58	11,35	16,03	2,29
Jawa Barat	69,06	22,48	22,19	80,28	9,35	5,84	43,55	6,22	12,87	3,22
Jawa Tengah	66,10	27,89	20,30	81,70	7,97	5,84	44,51	4,27	12,47	1,46
DI Yogyakarta	75,11	29,81	28,82	87,22	11,61	7,10	46,89	7,29	16,53	1,41
Jawa Timur	67,29	26,62	21,61	80,03	8,57	5,56	46,67	5,99	13,26	1,34
Banten	70,35	25,81	25,15	80,83	9,20	4,65	42,60	7,80	13,02	2,41
Bali	76,33	26,02	25,16	80,85	9,57	6,21	54,36	5,03	15,42	1,20
Nusa Tenggara Barat	68,64	30,21	21,74	72,79	9,87	4,80	40,90	3,89	12,51	0,72
Nusa Tenggara Timur	66,49	39,22	20,36	75,65	8,01	4,34	48,85	3,60	6,80	1,17
Kalimantan Barat	66,87	27,37	15,46	81,78	10,25	4,50	60,03	4,45	12,94	1,35
Kalimantan Tengah	69,27	25,98	26,44	81,74	11,20	6,99	58,01	8,70	15,73	1,54
Kalimantan Selatan	70,71	25,64	25,52	81,71	11,27	7,13	62,41	6,87	15,40	1,54
Kalimantan Timur	72,23	24,59	29,25	82,88	14,84	7,34	58,48	11,61	19,55	5,35
Kalimantan Utara	66,34	27,41	22,84	77,89	15,38	5,20	48,48	6,10	10,50	0,67
Sulawesi Utara	65,75	23,12	22,44	82,36	10,89	7,00	48,58	5,09	13,20	2,74
Sulawesi Tengah	66,26	30,63	27,14	79,18	14,52	6,04	49,82	7,63	15,01	0,64
Sulawesi Selatan	71,17	30,36	24,68	79,93	11,74	4,28	56,33	7,75	15,64	1,70
Sulawesi Tenggara	63,67	35,61	22,18	73,19	5,56	2,67	38,12	3,01	6,37	0,58
Gorontalo	62,86	33,46	26,89	81,53	11,88	3,03	51,91	4,52	10,66	1,64
Sulawesi Barat	67,78	31,86	29,15	77,11	14,27	7,24	47,95	6,06	14,59	0,94
Maluku	64,94	40,35	18,76	75,21	4,09	3,32	43,70	3,09	6,19	0,96
Maluku Utara	61,94	29,12	22,57	80,27	9,18	1,66	45,86	4,08	5,24	0,52
Papua Barat	55,09	24,86	17,51	68,69	4,51	4,00	31,00	2,30	4,72	0,48
Papua	67,40	22,73	20,32	77,42	7,91	3,02	40,24	5,50	7,36	1,96
**Indonesia**	**69,22**	**25,52**	**23,65**	**80,00**	**9,84**	**5,55**	**46,94**	**6,50**	**13,28**	**2,18**

Catatan/*Note*: *) Data 2018 tidak tersedia/*2018 data not available*
Sumber/*Source*: Susenas Maret 2017/*The March 2017 Susenas*

https://www.bps.go.id

Tabel *Table* **7.7.2.** **Persentase Penduduk Berumur 5 Tahun ke Atas di Daerah Perdesaan yang Mengakses Internet dalam 3 Bulan Terakhir menurut Provinsi dan Tujuan Mengakses Internet, 2017***
*Percentage of Population Aged 5 Years and Over in Rural Area Who Accessed Internet during The Last 3 Months by Province and The Purpose for Accessing The Internet, 2017**

**Provinsi** *Province*	**Mendapat Informasi/Berita** *Get Information/ News*	**Mengerjakan Tugas Sekolah** *Do Homework*	**Mengirim/ Menerima Email** *Send/Receive Email*	**Sosial Media/ Jejaring Sosial** *Social Media/Social Network*	**Pembelian Barang/Jasa** *Buy Goods/ Service*	**Penjualan Barang/Jasa** *Sell Goods/Service*	**Hiburan** *Entertainment*	**Fasilitas Finansial** *Financial Facility*	**Mendapat Informasi Barang/Jasa** *Get Information Goods/Service*	**Lainnya** *Others*
(1)	(2)	(3)	(4)	(5)	(6)	(7)	(8)	(9)	(10)	(11)
Aceh	65,45	23,38	16,99	78,24	3,59	2,83	40,10	1,26	5,17	2,82
Sumatera Utara	59,61	31,63	15,93	68,52	2,88	2,77	43,11	0,86	5,78	1,52
Sumatera Barat	59,23	32,40	15,01	70,25	4,43	2,69	38,12	1,90	6,75	1,20
Riau	61,28	24,65	15,49	75,41	3,18	3,73	44,91	2,59	4,49	1,38
Jambi	63,95	25,50	14,67	81,03	3,75	2,07	37,80	0,96	8,56	0,94
Sumatera Selatan	57,20	22,57	14,55	81,02	3,87	2,30	39,60	1,18	7,33	1,88
Bengkulu	54,54	27,03	12,19	73,09	3,72	2,60	38,84	1,89	9,31	1,32
Lampung	47,73	27,01	11,46	69,64	2,65	2,61	32,52	1,33	4,90	1,14
Kepulauan Bangka Belitung	65,10	25,70	16,75	74,94	6,30	4,21	50,72	1,71	11,98	4,41
Kepulauan Riau	70,95	21,12	16,10	80,90	8,62	4,45	53,23	2,50	6,70	0,72
DKI Jakarta	-	-	-	-	-	-	-	-	-	-
Jawa Barat	57,66	23,39	15,95	80,29	3,64	3,57	40,19	1,46	6,44	2,28
Jawa Tengah	59,13	29,11	14,71	79,47	3,94	3,78	38,85	1,65	7,61	1,75
DI Yogyakarta	61,29	24,51	16,53	83,32	5,78	4,82	37,80	2,02	9,31	1,37
Jawa Timur	56,44	27,63	14,10	77,92	3,51	3,14	42,83	1,54	7,79	1,02
Banten	45,66	28,64	12,50	78,08	2,04	1,48	40,79	0,48	3,62	1,63
Bali	66,61	27,96	10,62	78,26	4,46	2,65	54,34	0,81	8,61	0,57
Nusa Tenggara Barat	57,35	23,23	15,21	72,55	5,39	2,97	37,57	1,33	9,16	1,70
Nusa Tenggara Timur	60,84	26,66	17,63	76,67	3,93	1,87	32,67	1,60	6,70	0,65
Kalimantan Barat	59,24	21,15	15,09	74,88	3,98	2,23	41,85	1,35	7,72	3,20
Kalimantan Tengah	59,88	20,27	15,98	78,31	4,33	1,83	38,34	3,32	5,51	2,04
Kalimantan Selatan	62,16	26,19	15,46	82,50	5,39	2,96	54,48	3,46	9,18	2,31
Kalimantan Timur	67,05	19,20	14,56	77,95	3,13	3,62	38,39	3,04	7,20	3,84
Kalimantan Utara	69,91	20,25	24,34	70,75	5,71	2,40	47,44	2,72	10,99	10,79
Sulawesi Utara	57,07	20,05	11,90	79,38	5,45	3,00	41,45	1,05	5,93	0,47
Sulawesi Tengah	52,81	27,87	13,87	77,01	4,34	2,68	40,43	1,27	7,36	0,37
Sulawesi Selatan	56,39	31,98	14,61	73,64	4,22	2,85	39,25	1,55	6,45	1,00
Sulawesi Tenggara	56,85	27,97	13,74	71,17	3,38	1,87	38,77	1,63	5,67	0,58
Gorontalo	51,81	30,89	15,04	84,86	6,31	3,66	43,00	1,11	9,90	0,40
Sulawesi Barat	55,41	32,05	15,95	71,11	2,49	2,46	33,81	0,76	4,15	1,62
Maluku	42,36	33,88	11,35	70,50	2,96	4,29	23,00	1,05	5,34	1,16
Maluku Utara	54,83	22,58	17,70	72,62	6,01	2,00	33,77	1,96	4,63	1,05
Papua Barat	65,99	23,34	20,76	67,46	2,74	1,41	26,69	0,47	4,26	2,01
Papua	67,14	16,80	19,88	63,72	5,40	3,51	41,87	2,81	8,39	2,83
**Indonesia**	**57,96**	**26,74**	**14,79**	**76,98**	**3,79**	**3,08**	**40,46**	**1,56**	**6,95**	**1,61**

Catatan/*Note*: *) Data 2018 tidak tersedia/*2018 data not available*
Sumber/*Source*: Susenas Maret 2017/*The March 2017 Susenas*

https://www.bps.go.id

Tabel *Table* **7.7.3.** **Persentase Penduduk Berumur 5 Tahun ke Atas di Daerah Perkotaan dan Perdesaan yang Mengakses Internet dalam 3 Bulan Terakhir menurut Provinsi dan Tujuan Mengakses Internet, 2017***
*Percentage of Population Aged 5 Years and Over in Urban and Rural Area Who Accessed Internet during The Last 3 Months by Province and The Purpose for Accessing The Internet, 2017**

**Provinsi** *Province*	**Mendapat Informasi/Berita** *Get Information/ News*	**Mengerjakan Tugas Sekolah** *Do Homework*	**Mengirim/ Menerima Email** *Send/Receive Email*	**Sosial Media/ Jejaring Sosial** *Social Media/Social Network*	**Pembelian Barang/Jasa** *Buy Goods/ Service*	**Penjualan Barang/Jasa** *Sell Goods/Service*	**Hiburan** *Entertainment*	**Fasilitas Finansial** *Financial Facility*	**Mendapat Informasi Barang/Jasa** *Get Information Goods/Service*	**Lainnya** *Others*
(1)	(2)	(3)	(4)	(5)	(6)	(7)	(8)	(9)	(10)	(11)
Aceh	71,42	25,79	24,75	78,73	8,21	3,23	46,62	3,29	8,61	1,97
Sumatera Utara	63,83	30,62	18,00	72,57	4,63	3,15	46,16	2,83	7,97	1,26
Sumatera Barat	65,07	34,21	22,53	73,44	8,59	3,39	48,01	3,95	11,61	1,73
Riau	66,45	27,01	19,98	77,56	6,08	4,71	48,98	5,08	9,89	1,69
Jambi	67,20	27,81	21,83	79,28	7,81	4,78	43,63	4,59	11,38	1,12
Sumatera Selatan	64,04	24,33	20,93	81,28	7,11	3,70	43,99	3,29	8,91	4,40
Bengkulu	61,12	31,15	19,66	75,59	6,67	5,03	41,48	4,04	10,19	1,13
Lampung	55,79	27,56	14,84	72,39	5,01	3,34	35,31	3,66	8,47	1,08
Kepulauan Bangka Belitung	73,69	23,73	19,29	76,20	13,20	5,28	52,14	4,23	15,52	3,24
Kepulauan Riau	69,69	21,86	25,36	74,35	9,81	5,41	46,40	4,14	12,61	1,47
DKI Jakarta	73,49	18,35	31,01	80,92	14,72	6,55	49,58	11,35	16,03	2,29
Jawa Barat	67,14	22,63	21,13	80,28	8,39	5,46	42,98	5,42	11,78	3,06
Jawa Tengah	63,38	28,36	18,12	80,83	6,40	5,04	42,30	3,25	10,58	1,57
DI Yogyakarta	72,68	28,88	26,66	86,53	10,59	6,70	45,29	6,36	15,26	1,40
Jawa Timur	63,57	26,97	19,03	79,30	6,84	4,73	45,35	4,46	11,39	1,23
Banten	66,90	26,21	23,39	80,45	8,20	4,20	42,34	6,78	11,70	2,30
Bali	74,12	26,46	21,84	80,26	8,41	5,40	54,35	4,07	13,87	1,05
Nusa Tenggara Barat	63,94	27,30	19,02	72,69	8,01	4,04	39,51	2,82	11,11	1,13
Nusa Tenggara Timur	63,80	33,24	19,06	76,13	6,07	3,16	41,16	2,65	6,75	0,92
Kalimantan Barat	63,77	24,84	15,31	78,98	7,70	3,58	52,65	3,19	10,82	2,10
Kalimantan Tengah	65,18	23,50	21,89	80,25	8,21	4,74	49,45	6,36	11,28	1,76
Kalimantan Selatan	67,52	25,84	21,76	82,00	9,07	5,57	59,45	5,59	13,07	1,83
Kalimantan Timur	71,04	23,35	25,87	81,74	12,14	6,48	53,85	9,63	16,71	5,00
Kalimantan Utara	67,45	25,18	23,31	75,67	12,37	4,33	48,16	5,05	10,65	3,81
Sulawesi Utara	62,52	21,98	18,52	81,25	8,87	5,51	45,93	3,59	10,50	1,89
Sulawesi Tengah	59,62	29,27	20,59	78,11	9,50	4,38	45,19	4,49	11,23	0,51
Sulawesi Selatan	65,30	31,00	20,68	77,43	8,75	3,71	49,54	5,29	11,99	1,42
Sulawesi Tenggara	60,25	31,77	17,95	72,18	4,46	2,27	38,45	2,32	6,02	0,58
Gorontalo	57,75	32,27	21,41	83,07	9,30	3,32	47,79	2,94	10,31	1,07
Sulawesi Barat	59,72	31,98	20,56	73,20	6,60	4,13	38,75	2,61	7,79	1,38
Maluku	58,10	38,39	16,52	73,78	3,74	3,61	37,43	2,47	5,93	1,02
Maluku Utara	59,08	26,49	20,61	77,20	7,91	1,80	41,01	3,23	5,00	0,73
Papua Barat	59,81	24,21	18,92	68,16	3,75	2,88	29,13	1,51	4,52	1,14
Papua	67,32	20,81	20,17	72,99	7,10	3,18	40,77	4,63	7,69	2,24
**Indonesia**	**65,97**	**25,87**	**21,10**	**79,13**	**8,10**	**4,84**	**45,07**	**5,08**	**11,45**	**2,01**

Catatan/*Note*: *) Data 2018 tidak tersedia/*2018 data not available*
Sumber/*Source*: Susenas Maret 2017/*The March 2017 Susenas*

https://www.bps.go.id

Tabel *Table* **7.8.** **Persentase Rumah Tangga yang Memiliki Telepon Rumah/PSTN dan Komputer/Laptop menurut Provinsi dan Daerah Tempat Tinggal, 2018**
Percentage of Household with Fixed Line Telephone (PSTN) and Computer/Laptop by Province and Urban Rural Classification, 2018

**Provinsi** *Province*	**Telepon Rumah (PSTN)/***Fixed Line Telephone (PSTN)*			**Komputer/Laptop/***Computer/Laptop*		
	**Perkotaan** *Urban*	**Rural** *Rural*	**Perkotaan+Perdesaan** *Urban+Rural*	**Perkotaan** *Urban*	**Perdesaan** *Rural*	**Perkotaan+Perdesaan** *Urban+Rural*
(1)	(2)	(3)	(4)	(5)	(6)	(7)
Aceh	0,96	0,25	0,47	36,68	11,77	19,55
Sumatera Utara	2,65	0,12	1,45	23,80	8,66	16,61
Sumatera Barat	2,48	0,22	1,22	34,48	13,25	22,62
Riau	2,78	0,75	1,57	35,39	14,24	22,78
Jambi	2,90	0,33	1,13	32,36	11,83	18,25
Sumatera Selatan	5,55	0,31	2,19	28,30	8,60	15,66
Bengkulu	4,29	0,12	1,46	39,05	10,97	19,98
Lampung	0,88	0,01	0,25	25,15	7,74	12,60
Kepulauan Bangka Belitung	2,04	0,03	1,10	28,50	14,45	21,95
Kepulauan Riau	4,06	0,28	3,53	34,38	13,24	31,44
DKI Jakarta	11,61	-	11,61	32,24	-	32,24
Jawa Barat	4,39	0,22	3,24	24,96	6,57	19,92
Jawa Tengah	4,80	0,36	2,61	24,73	10,26	17,59
DI Yogyakarta	6,65	0,05	4,91	42,80	13,20	34,99
Jawa Timur	3,48	0,32	1,96	26,59	9,86	18,57
Banten	5,33	0,11	3,84	28,63	5,60	22,08
Bali	7,75	0,95	5,51	33,54	12,51	26,61
Nusa Tenggara Barat	1,32	0,25	0,75	20,21	8,09	13,68
Nusa Tenggara Timur	1,77	0,13	0,52	35,29	6,84	13,57
Kalimantan Barat	4,44	0,09	1,52	33,14	8,70	16,73
Kalimantan Tengah	2,47	0,03	0,95	39,26	14,41	23,78
Kalimantan Selatan	3,45	0,19	1,70	34,85	12,48	22,86
Kalimantan Timur	5,21	0,12	3,52	40,65	18,14	33,19
Kalimantan Utara	2,41	0,17	1,50	35,04	25,30	31,09
Sulawesi Utara	2,59	0,40	1,50	31,61	12,33	22,01
Sulawesi Tengah	1,92	0,05	0,58	35,95	10,63	17,81
Sulawesi Selatan	3,19	0,32	1,48	36,36	14,53	23,34
Sulawesi Tenggara	2,00	0,01	0,77	34,27	11,26	20,03
Gorontalo	0,69	0,06	0,31	28,74	12,09	18,64
Sulawesi Barat	2,22	0,24	0,70	30,15	13,36	17,27
Maluku	2,42	0,13	1,12	37,07	8,75	20,98
Maluku Utara	0,99	0,03	0,32	43,11	11,86	21,15
Papua Barat	1,75	0,61	1,06	34,29	21,02	26,33
Papua	1,76	0,17	0,58	33,99	5,57	12,87
**Indonesia**	**4,57**	**0,26**	**2,61**	**28,43**	**9,93**	**20,05**

Sumber/*Source*: Susenas Maret 2018/*The March 2018 Susenas*

https://www.bps.go.id

LAIN-LAIN

OTHERS

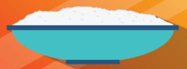

34,74 % RUMAH TANGGA MENERIMA/MEMBELI RASKIN

34,74 % HOUSEHOLDS ACCEPT RICE FOR THE POOR

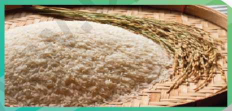

58,37 % MENERIMA/MEMBELI RASKIN DI BAWAH 15 KG

58,37 % ACCEPT/BUY RICE FOR POOR UNDER 15 KG

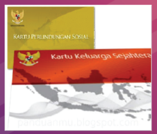

RUMAH TANGGA YANG MENERIMA KPS/KKS SEBESAR 13,87%

HOUSEHOLDS ACCEPTED KPS/KKS WAS 13.87%

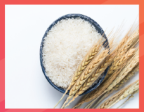

RATA-RATA HARGA RASKIN YANG DIBELI RP. 1 497

PRICE OF AVERAGE PER KG WAS BOUGHT 1 497 RUPIAHS

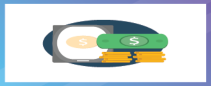

28,73 % RUMAH TANGGA PERNAH MENERIMA KREDIT

28,73 % HOUSEHOLDS ACCEPTED CREDIT

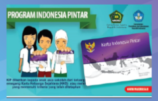

11,11 % RUMAH TANGGA PERNAH MENERIMA PROGRAM INDONESIA PINTAR

11·11 % HOUSEHOLDS ACCEPTED THE SMART INDONESIA PROGRAM

LAIN-LAIN

OTHERS

https://www.bps.go.id

https://www.bps.go.id

PENJELASAN TEKNIS

1. **Korban Kejahatan** adalah seseorang yang diri atau harta bendanya selama setahun terakhir mengalami atau terkena tindak kejahatan atau usaha/percobaan tindak kejahatan.

2. **Beras miskin (raskin)/Beras sejahtera (rastra)** adalah program bantuan dari pemerintah untuk keluarga miskin berupapendistribusian beras khusus kepada keluarga miskin yang harganya telah disubsidi oleh pemerintah.

3. **Program Indonesia Pintar (PIP)** adalah pemberian bantuan tunai pendidikan kepada seluruh anak usia sekolah (6-21 tahun) yang menerima Kartu Indonesia Pintar (KIP), atau yang berasal dari keluarga miskin dan rentan (misalnya dari keluarga/rumah tangga pemegang Kartu Keluarga Sejahtera/KKS) atau anak yang memenuhi kriteria yang telah ditetapkan sebelumnya. Program Indonesia Pintar melalui KIP merupakan bagian penyempurnaan dari Program Bantuan Siswa Miskin (BSM) sejak akhir 2014.

4. **Kartu Perlindungan Sosial (KPS)/Kartu Keluarga Sejahtera (KKS)** adalah kartu yang diterbitkan oleh pemerintah dalam rangka pelaksanaan Program Percepatan dan Perluasan Perlindungan Sosial (P4S) dan BLSM di tahun 2013.

TECHNICAL NOTES

1. ***A Victim of Crime*** *is a person who has possessed or suffered a crime or business/attempted crime.*

2. ***Cheap rice*** *is government assistance program for poor families in the form of a special distribution to poor families whose prices have been subsidized by the government.*

3. ***Smart Indonesia Program (PIP)*** *is the provision of educational cash assistance to all school-age children (6-21 years) who receive the Smart Indonesia Card (KIP), or who come from poor and vulnerable families (for example from family/household Prosperous Family Card/ KKS holders) or children who fulfill predetermined criteria. The Smart Indonesia Program through KIP is an improvement part of the Poor Student Assistance Program (BSM) since the end of 2014.*

4. ***Social Security Card (KPS)/family welfare card (KKS)*** *is a card issued by the government in the implementation of the Program for the Acceleration and Expansion of Social Protection (P4S) and BLSM in 2013.*

https://www.bps.go.id

https://www.bps.go.id

Tabel 8.1. **Persentase Penduduk Menjadi Korban Kejahatan Pencurian, Penganiayaan, Pencurian dengan Kekerasan, Pelecehan Seksual, atau Lainnya sejak 1 Januari-31 Desember 2017 menurut Provinsi, Daerah Tempat Tinggal, dan Jenis Kelamin**
Table 8.1. *Percentage of Population Being Victims of Crime, Theft, Violence, Robbery, Sexual Assault or Others during January 1st - December 31st 2017 by Province, Urban Rural Classification, and Sex*

Provinsi *Province*	Perkotaan/*Urban*			Perdesaan/*Rural*			Perkotaan+Perdesaan/*Urban+Rural*		
	Laki-laki/*Male*	Perempuan/*Female*	Laki-laki+ Perempuan/ *Male+Female*	Laki-laki/*Male*	Perempuan/*Female*	Laki-laki+ Perempuan/ *Male+Female*	Laki-laki/*Male*	Perempuan/*Female*	Laki-laki+ Perempuan/ *Male+Female*
(1)	(2)	(3)	(4)	(5)	(6)	(7)	(8)	(9)	(10)
Aceh	1,45	1,04	1,24	1,22	0,54	0,88	1,29	0,70	0,99
Sumatera Utara	1,66	0,93	1,29	1,06	0,65	0,86	1,37	0,80	1,09
Sumatera Barat	1,07	1,33	1,20	1,08	0,65	0,87	1,08	0,95	1,01
Riau	1,97	1,26	1,63	1,43	0,67	1,06	1,65	0,91	1,29
Jambi	1,44	0,96	1,21	1,53	0,68	1,11	1,50	0,77	1,14
Sumatera Selatan	2,24	1,55	1,90	2,03	0,52	1,29	2,10	0,91	1,52
Bengkulu	2,60	1,24	1,92	1,54	0,39	0,98	1,88	0,68	1,29
Lampung	1,68	1,11	1,40	2,27	0,71	1,51	2,10	0,83	1,48
Kepulauan Bangka Belitung	1,38	0,59	1,00	1,13	0,20	0,69	1,26	0,42	0,86
Kepulauan Riau	1,68	0,88	1,29	0,64	0,71	0,67	1,53	0,86	1,20
DKI Jakarta	1,26	0,95	1,10	-	-	-	1,26	0,95	1,10
Jawa Barat	1,72	0,96	1,34	1,52	0,71	1,12	1,67	0,90	1,29
Jawa Tengah	1,30	1,03	1,16	1,06	0,77	0,92	1,18	0,90	1,04
DI Yogyakarta	2,15	1,53	1,84	0,46	0,70	0,59	1,71	1,30	1,50
Jawa Timur	1,37	0,88	1,12	1,24	0,57	0,90	1,31	0,73	1,02
Banten	1,40	0,86	1,13	1,00	0,22	0,62	1,28	0,68	0,98
Bali	0,99	0,51	0,75	0,47	0,30	0,39	0,82	0,44	0,63
Nusa Tenggara Barat	2,52	2,30	2,41	2,43	1,40	1,90	2,47	1,82	2,14
Nusa Tenggara Timur	1,90	0,89	1,40	1,52	0,61	1,06	1,61	0,68	1,14
Kalimantan Barat	1,79	1,19	1,49	0,62	0,27	0,45	1,01	0,59	0,80
Kalimantan Tengah	1,00	0,77	0,89	0,96	0,38	0,68	0,97	0,53	0,76
Kalimantan Selatan	0,99	0,79	0,89	1,33	0,70	1,02	1,17	0,74	0,96
Kalimantan Timur	2,14	0,80	1,49	1,16	0,57	0,89	1,81	0,72	1,29
Kalimantan Utara	3,38	2,17	2,81	2,04	0,50	1,32	2,81	1,48	2,19
Sulawesi Utara	1,56	0,95	1,25	1,64	0,73	1,20	1,60	0,84	1,23
Sulawesi Tengah	2,73	2,78	2,75	1,83	1,12	1,48	2,07	1,59	1,84
Sulawesi Selatan	2,09	1,90	2,00	1,02	0,61	0,81	1,47	1,15	1,31
Sulawesi Tenggara	2,24	1,70	1,97	1,91	1,03	1,47	2,04	1,29	1,66
Gorontalo	1,64	1,44	1,54	1,06	0,92	0,99	1,28	1,12	1,20
Sulawesi Barat	1,13	0,79	0,96	0,82	0,46	0,64	0,89	0,54	0,72
Maluku	1,74	1,61	1,67	1,28	0,78	1,04	1,48	1,13	1,30
Maluku Utara	3,22	1,89	2,57	1,38	0,66	1,03	1,91	1,01	1,47
Papua Barat	3,17	1,56	2,40	1,87	1,12	1,52	2,40	1,30	1,88
Papua	2,74	1,65	2,24	1,70	0,83	1,29	2,00	1,06	1,55
**Indonesia**	**1,60**	**1,05**	**1,33**	**1,35**	**0,66**	**1,00**	**1,49**	**0,87**	**1,18**

Sumber/*Source*: Susenas Maret 2018/*The March 2018 Susenas*

https://www.bps.go.id

Tabel *Table* **8.2.1.** **Persentase Rumah Tangga yang Membeli/Menerima Beras Miskin (Raskin)/Beras Sejahtera (Rastra) dalam 4 Bulan Terakhir di Daerah Perkotaan menurut Provinsi dan Jumlah Raskin yang Dibeli/Diterima (Kg), 2018**
Percentage of Household That Bought/Received Rice for The Poor (Raskin/Rastra) during the Last 4 Months in Urban Area by Province and Amount of Rice for The Poor Bought/Received (Kg), 2018

**Provinsi** *Province*	**% RT yang Membeli/Menerima Beras Miskin (Raskin)/Beras Sejahtera (Rastra)** *% Household Buying/Receiving Rice for The Poor (Raskin/Rastra)*	**Jumlah Raskin/Rastra yang Dibeli/Diterima (Kg)** *Amount of Rice for The Poor Bought (Kg)*				**Jumlah** *Total*
		**< 15**	**15 - 29**	**30 - 45**	**> 45**	
**(1)**	**(2)**	**(3)**	**(4)**	**(5)**	**(6)**	**(7)**
Aceh	34,75	65,27	25,98	5,70	3,04	100,00
Sumatera Utara	17,32	45,08	34,45	9,76	10,71	100,00
Sumatera Barat	11,36	27,45	25,83	25,69	21,04	100,00
Riau	9,61	46,98	34,25	15,53	3,24	100,00
Jambi	12,08	44,75	28,22	23,91	3,12	100,00
Sumatera Selatan	14,24	41,78	37,49	12,33	8,40	100,00
Bengkulu	21,51	37,02	37,72	14,23	11,02	100,00
Lampung	19,92	53,38	33,33	11,52	1,77	100,00
Kepulauan Bangka Belitung	10,27	15,36	25,27	25,05	34,32	100,00
Kepulauan Riau	4,21	27,08	24,62	31,08	17,21	100,00
DKI Jakarta	0,37	38,84	30,78	23,26	7,13	100,00
Jawa Barat	28,11	87,43	10,15	1,93	0,49	100,00
Jawa Tengah	33,96	65,37	21,16	6,04	7,42	100,00
DI Yogyakarta	19,17	38,01	32,49	24,46	5,05	100,00
Jawa Timur	21,63	65,93	23,12	9,09	1,85	100,00
Banten	14,50	88,57	8,15	2,15	1,13	100,00
Bali	6,88	23,76	26,56	29,36	20,32	100,00
Nusa Tenggara Barat	44,66	79,75	16,18	3,50	0,57	100,00
Nusa Tenggara Timur	17,88	18,45	34,15	27,83	19,57	100,00
Kalimantan Barat	13,24	43,26	26,82	14,72	15,19	100,00
Kalimantan Tengah	6,21	39,85	44,37	9,51	6,27	100,00
Kalimantan Selatan	12,70	29,43	28,38	13,02	29,17	100,00
Kalimantan Timur	9,05	26,65	35,36	17,40	20,59	100,00
Kalimantan Utara	11,82	31,57	29,55	37,39	1,49	100,00
Sulawesi Utara	19,79	25,48	27,76	25,72	21,04	100,00
Sulawesi Tengah	19,11	29,82	36,62	23,60	9,96	100,00
Sulawesi Selatan	10,41	30,35	31,25	18,40	20,00	100,00
Sulawesi Tenggara	25,93	32,54	54,95	11,37	1,14	100,00
Gorontalo	20,83	25,02	19,33	38,09	17,56	100,00
Sulawesi Barat	34,39	68,26	28,33	1,39	2,02	100,00
Maluku	22,28	14,41	46,74	30,52	8,33	100,00
Maluku Utara	10,65	27,02	39,20	19,02	14,75	100,00
Papua Barat	15,34	8,18	58,50	19,23	14,09	100,00
Papua	18,09	8,82	30,25	32,78	28,15	100,00
**Indonesia**	**21,02**	**68,38**	**19,65**	**7,35**	**4,62**	**100,00**

Sumber/*Source*: Susenas Maret 2018/*The March 2018 Susenas*

https://www.bps.go.id

Tabel 8.2.2. **Persentase Rumah Tangga yang Membeli/Menerima Beras Miskin (Raskin)/Beras Sejahtera (Rastra) dalam 4 Bulan Terakhir di Daerah Perdesaan menurut Provinsi dan Jumlah Raskin yang Dibeli/Diterima (Kg), 2018**
Table *Percentage of Household That Bought/Received Rice for The Poor (Raskin/Rastra) during the Last 4 Months in Rural Area by Province and Amount of Rice for The Poor Bought/Received (Kg), 2018*

**Provinsi** *Province*	**% RT yang Membeli/Menerima Beras Miskin (Raskin)/Beras Sejahtera (Rastra)** *% Household Buying/Receiving Rice for The Poor (Raskin/Rastra)*	**Jumlah Raskin/Rastra yang Dibeli/Diterima (Kg)** *Amount of Rice for The Poor Bought (Kg)*				**Jumlah** *Total*
		**< 15**	**15 - 29**	**30 - 45**	**> 45**	
**(1)**	**(2)**	**(3)**	**(4)**	**(5)**	**(6)**	**(7)**
Aceh	70,68	52,95	34,33	9,41	3,32	100,00
Sumatera Utara	44,44	37,81	39,99	15,65	6,55	100,00
Sumatera Barat	30,74	31,37	37,38	22,26	8,99	100,00
Riau	31,78	36,27	44,80	16,79	2,14	100,00
Jambi	34,53	38,49	38,57	14,08	8,86	100,00
Sumatera Selatan	39,67	36,33	44,59	13,94	5,14	100,00
Bengkulu	41,41	18,71	45,40	22,43	13,46	100,00
Lampung	57,36	58,25	32,66	7,54	1,56	100,00
Kepulauan Bangka Belitung	14,94	6,70	21,70	29,78	41,82	100,00
Kepulauan Riau	28,13	23,04	27,13	38,02	11,81	100,00
DKI Jakarta	-	-	-	-	-	-
Jawa Barat	60,21	80,12	15,17	3,66	1,04	100,00
Jawa Tengah	62,35	58,60	27,69	7,28	6,43	100,00
DI Yogyakarta	70,83	21,67	47,84	20,49	10,01	100,00
Jawa Timur	55,89	62,99	24,44	10,06	2,51	100,00
Banten	63,06	73,62	23,10	3,01	0,27	100,00
Bali	22,88	28,22	30,46	31,71	9,61	100,00
Nusa Tenggara Barat	72,25	77,59	19,66	2,51	0,23	100,00
Nusa Tenggara Timur	50,88	6,51	22,58	34,32	36,59	100,00
Kalimantan Barat	37,06	39,29	46,01	11,48	3,22	100,00
Kalimantan Tengah	17,49	46,54	29,92	13,37	10,18	100,00
Kalimantan Selatan	27,78	42,75	22,96	16,92	17,37	100,00
Kalimantan Timur	29,09	20,74	48,64	19,94	10,69	100,00
Kalimantan Utara	23,33	29,07	35,07	28,46	7,41	100,00
Sulawesi Utara	44,52	43,36	39,81	13,26	3,57	100,00
Sulawesi Tengah	46,25	39,79	35,52	17,73	6,96	100,00
Sulawesi Selatan	39,36	27,05	36,08	22,12	14,75	100,00
Sulawesi Tenggara	44,83	38,16	45,95	11,73	4,17	100,00
Gorontalo	46,11	22,37	22,13	44,66	10,84	100,00
Sulawesi Barat	44,21	47,71	47,64	4,17	0,48	100,00
Maluku	70,39	2,67	40,77	39,04	17,52	100,00
Maluku Utara	40,32	16,39	43,37	25,96	14,28	100,00
Papua Barat	46,16	10,69	28,07	30,42	30,82	100,00
Papua	53,95	13,52	40,17	31,29	15,02	100,00
**Indonesia**	**51,31**	**53,41**	**29,16**	**11,58**	**5,85**	**100,00**

Sumber/*Source*: Susenas Maret 2018/*The March 2018 Susenas*

https://www.bps.go.id

Tabel *Table* **8.2.3.** **Persentase Rumah Tangga yang Membeli/Menerima Beras Miskin (Raskin)/Beras Sejahtera (Rastra) dalam 4 Bulan Terakhir di Daerah Perkotaan dan Perdesaan menurut Provinsi dan Jumlah Raskin yang Dibeli/Diterima (Kg), 2018**
Percentage of Household That Bought/Received Rice for The Poor (Raskin/Rastra) during the Last 4 Months in Urban and Rural Area by Province and Amount of Rice for The Poor Bought/Received (Kg), 2018

Provinsi *Province*	% RT yang Membeli/Menerima Beras Miskin (Raskin)/Beras Sejahtera (Rastra) *% Household Buying/Receiving Rice for The Poor (Raskin/Rastra)*	Jumlah Raskin/Rastra yang Dibeli/Diterima (Kg) *Amount of Rice for The Poor Bought (Kg)*				Jumlah *Total*
		< 15	15 - 29	30 - 45	> 45	
(1)	(2)	(3)	(4)	(5)	(6)	(7)
Aceh	59,45	55,20	32,80	8,73	3,27	100,00
Sumatera Utara	30,21	40,00	38,32	13,88	7,80	100,00
Sumatera Barat	22,19	30,48	34,77	23,03	11,72	100,00
Riau	22,83	38,09	43,00	16,58	2,32	100,00
Jambi	27,51	39,35	37,15	15,43	8,07	100,00
Sumatera Selatan	30,56	37,24	43,41	13,67	5,68	100,00
Bengkulu	35,03	22,32	43,89	20,81	12,98	100,00
Lampung	46,92	57,67	32,74	8,01	1,58	100,00
Kepulauan Bangka Belitung	12,45	10,51	23,27	27,70	38,52	100,00
Kepulauan Riau	7,54	24,99	25,93	34,69	14,40	100,00
DKI Jakarta	0,37	38,84	30,78	23,26	7,13	-
Jawa Barat	36,90	84,16	12,40	2,70	0,74	100,00
Jawa Tengah	47,95	61,03	25,34	6,84	6,79	100,00
DI Yogyakarta	32,79	28,70	41,23	22,20	7,87	100,00
Jawa Timur	38,06	63,86	24,05	9,77	2,31	100,00
Banten	28,31	79,10	17,62	2,69	0,58	100,00
Bali	12,16	26,53	28,98	30,82	13,67	100,00
Nusa Tenggara Barat	59,52	78,34	18,46	2,85	0,35	100,00
Nusa Tenggara Timur	43,07	7,68	23,71	33,69	34,92	100,00
Kalimantan Barat	29,24	39,88	43,15	11,97	5,00	100,00
Kalimantan Tengah	13,24	45,36	32,47	12,68	9,49	100,00
Kalimantan Selatan	20,78	38,97	24,50	15,81	20,71	100,00
Kalimantan Timur	15,70	23,02	43,52	18,96	14,51	100,00
Kalimantan Utara	16,49	30,13	32,72	32,26	4,88	100,00
Sulawesi Utara	32,12	37,83	36,08	17,11	8,97	100,00
Sulawesi Tengah	38,55	38,39	35,67	18,55	7,38	100,00
Sulawesi Selatan	27,68	27,55	35,35	21,55	15,55	100,00
Sulawesi Tenggara	37,63	36,68	48,31	11,63	3,37	100,00
Gorontalo	36,16	22,98	21,49	43,17	12,36	100,00
Sulawesi Barat	41,92	51,64	43,95	3,64	0,78	100,00
Maluku	49,62	4,95	41,93	37,39	15,74	100,00
Maluku Utara	31,50	17,46	42,95	25,26	14,33	100,00
Papua Barat	33,84	10,24	33,59	28,39	27,78	100,00
Papua	44,73	13,03	39,14	31,45	16,39	100,00
**Indonesia**	**34,74**	**58,37**	**26,01**	**10,18**	**5,44**	**100,00**

Sumber/*Source*: Susenas Maret 2018/*The March 2018 Susenas*

https://www.bps.go.id

Tabel *Table* **8.3.1.** **Persentase Persentase Rumah Tangga yang Membeli/Menerima Beras Miskin (Raskin)/Beras Sejahtera (Rastra) dalam 4 Bulan Terakhir di Daerah Perkotaan menurut Provinsi, Harga Raskin/Rastra Per Kg, dan Harga Rata-Rata Per Kg (Rp), 2018**
Percentage of Household That Bought/Received Rice for The Poor (Raskin/Rastra) during The Last 4 Months in Urban Area by Province, Price of Rice for The Poor per Kg, and Price of Average per Kg (Rupiahs), 2018

**Provinsi** *Province*	**Harga Raskin/Rastra per Kg (Rupiah)/ Price of Rice for The Poor per Kg (Rupiahs)** *Amount of Rice for The Poor Bought (Kg)*					**Harga Rata-rata Per Kg (Rp)** *Price of Average per Kg (Rupiahs)*
	**< 1 000**	**1 000**	**1 001-2 000**	**> 2 000**	**Jumlah/***Total*	
**(1)**	**(2)**	**(3)**	**(4)**	**(5)**	**(6)**	**(7)**
Aceh	15,07	6,67	53,36	24,90	100,00	851
Sumatera Utara	5,01	11,30	64,58	19,11	100,00	1 702
Sumatera Barat	12,43	3,47	64,21	19,90	100,00	1 641
Riau	3,52	5,10	38,92	52,46	100,00	1 495
Jambi	1,88	1,81	31,11	65,19	100,00	3 372
Sumatera Selatan	9,62	7,65	39,53	43,20	100,00	1 717
Bengkulu	7,41	4,30	39,14	49,15	100,00	1 431
Lampung	13,35	3,26	64,62	18,77	100,00	1 164
Kepulauan Bangka Belitung	79,75	6,37	13,88	0,00	100,00	42
Kepulauan Riau	1,28	0,00	83,43	15,28	100,00	1 549
DKI Jakarta	0,42	0,02	53,12	46,44	100,00	1 820
Jawa Barat	4,55	3,12	37,87	54,46	100,00	1 956
Jawa Tengah	23,56	8,52	56,48	11,44	100,00	1 120
DI Yogyakarta	15,55	2,67	77,83	3,95	100,00	1 347
Jawa Timur	9,88	7,67	70,95	11,50	100,00	1 262
Banten	1,53	0,00	22,99	75,48	100,00	2 310
Bali	22,45	7,68	68,46	1,40	100,00	1 033
Nusa Tenggara Barat	21,74	4,12	53,42	20,72	100,00	847
Nusa Tenggara Timur	1,72	0,24	89,59	8,45	100,00	811
Kalimantan Barat	7,93	0,21	36,47	55,39	100,00	2 169
Kalimantan Tengah	4,55	3,00	61,33	31,12	100,00	1 953
Kalimantan Selatan	24,67	3,03	42,31	29,98	100,00	967
Kalimantan Timur	17,95	10,05	43,58	28,42	100,00	1 455
Kalimantan Utara	3,11	8,11	56,24	32,54	100,00	1 173
Sulawesi Utara	11,58	4,36	66,61	17,45	100,00	1 408
Sulawesi Tengah	6,30	3,13	78,83	11,75	100,00	1 530
Sulawesi Selatan	9,80	3,92	70,08	16,20	100,00	931
Sulawesi Tenggara	2,10	0,00	43,48	54,42	100,00	1 950
Gorontalo	4,94	5,18	77,53	12,35	100,00	958
Sulawesi Barat	4,23	0,28	71,55	23,93	100,00	1 299
Maluku	1,06	0,12	54,28	44,55	100,00	1 959
Maluku Utara	0,21	4,17	59,29	36,33	100,00	2 078
Papua Barat	0,00	0,00	13,63	86,37	100,00	3 374
Papua	0,00	1,68	21,31	77,01	100,00	2 902
**Indonesia**	**10,31**	**5,19**	**50,47**	**34,03**	**100,00**	**1 548**

Sumber/*Source*: Susenas Maret 2018/*The March 2018 Susenas*

https://www.bps.go.id

Tabel **8.3.2.** **Persentase Persentase Rumah Tangga yang Membeli/Menerima Beras Miskin (Raskin)/Beras Sejahtera (Rastra) dalam 4 Bulan Terakhir di Daerah Perdesaan menurut Provinsi, Harga Raskin/Rastra Per Kg, dan Harga Rata-Rata Per Kg (Rp), 2018**

Table *Percentage of Household That Bought/Received Rice for The Poor (Raskin/Rastra) during The Last 4 Months in Rural Area by Province, Price of Rice for The Poor per Kg, and Price of Average per Kg (Rupiahs), 2018*

**Provinsi** *Province*	**Harga Raskin/Rastra per Kg (Rupiah)/ Price of Rice for The Poor per Kg (Rupiahs)** *Amount of Rice for The Poor Bought (Kg)*					**Harga Rata-rata Per Kg (Rp)** *Price of Average per Kg (Rupiahs)*
	**< 1 000**	**1 000**	**1 001-2 000**	**> 2 000**	**Jumlah/***Total*	
**(1)**	**(2)**	**(3)**	**(4)**	**(5)**	**(6)**	**(7)**
Aceh	30,68	6,30	46,50	16,52	100,00	740
Sumatera Utara	6,95	3,07	52,54	37,45	100,00	1 776
Sumatera Barat	8,42	2,35	63,11	26,12	100,00	1 569
Riau	0,76	0,42	46,55	52,27	100,00	2 066
Jambi	1,09	0,44	61,75	36,73	100,00	1 887
Sumatera Selatan	4,18	3,78	43,23	48,81	100,00	1 826
Bengkulu	14,66	5,78	46,38	33,19	100,00	1 304
Lampung	15,76	5,39	62,80	16,05	100,00	1 156
Kepulauan Bangka Belitung	68,85	16,85	5,04	9,27	100,00	76
Kepulauan Riau	0,00	0,00	90,73	9,27	100,00	1 620
DKI Jakarta	-	-	-	-	-	-
Jawa Barat	7,26	4,33	29,25	59,17	100,00	1 909
Jawa Tengah	24,42	8,22	55,57	11,79	100,00	1 136
DI Yogyakarta	20,99	4,30	65,70	9,01	100,00	1 355
Jawa Timur	7,89	5,67	78,34	8,11	100,00	1 351
Banten	0,68	0,14	38,08	61,10	100,00	2 230
Bali	8,30	1,15	82,47	8,08	100,00	1 386
Nusa Tenggara Barat	14,74	6,67	61,43	17,16	100,00	1 000
Nusa Tenggara Timur	0,73	0,47	95,13	3,66	100,00	1 434
Kalimantan Barat	0,08	0,36	14,72	84,84	100,00	3 006
Kalimantan Tengah	2,92	2,99	52,08	42,01	100,00	1 935
Kalimantan Selatan	12,26	7,02	49,66	31,05	100,00	1 039
Kalimantan Timur	2,14	2,72	46,49	48,65	100,00	1 296
Kalimantan Utara	0,00	1,39	66,40	32,20	100,00	1 601
Sulawesi Utara	5,69	1,12	73,37	19,82	100,00	1 695
Sulawesi Tengah	6,09	3,08	74,75	16,08	100,00	1 489
Sulawesi Selatan	10,93	7,05	68,00	14,03	100,00	1 180
Sulawesi Tenggara	5,15	4,66	43,96	46,23	100,00	1 539
Gorontalo	15,86	6,79	66,86	10,50	100,00	1 182
Sulawesi Barat	4,73	1,97	51,00	42,30	100,00	1 655
Maluku	0,78	0,65	47,00	51,57	100,00	2 039
Maluku Utara	0,25	2,95	52,34	44,47	100,00	2 020
Papua Barat	0,16	1,95	53,23	44,66	100,00	2 147
Papua	1,11	5,04	30,15	63,69	100,00	1 036
**Indonesia**	**10,81**	**4,78**	**55,82**	**28,60**	**100,00**	**1 472**

Sumber/*Source*: Susenas Maret 2018/*The March 2018 Susenas*

https://www.bps.go.id

Tabel *Table* **8.3.3.** **Persentase Persentase Rumah Tangga yang Membeli/Menerima Beras Miskin (Raskin)/Beras Sejahtera (Rastra) dalam 4 Bulan Terakhir di Daerah Perkotaan dan Perdesaan menurut Provinsi, Harga Raskin/Rastra Per Kg, dan Harga Rata-Rata Per Kg (Rp), 2018**
Percentage of Household That Bought/Received Rice for The Poor (Raskin/Rastra) during The Last 4 Months in Urban and Rural Area by Province, Price of Rice for The Poor per Kg, and Price of Average per Kg (Rupiahs), 2018

**Provinsi** *Province*	**Harga Raskin/Rastra per Kg (Rupiah)/ Price of Rice for The Poor per Kg (Rupiahs)** *Amount of Rice for The Poor Bought (Kg)*					**Harga Rata-rata Per Kg (Rp)** *Price of Average per Kg (Rupiahs)*
	**< 1 000**	**1 000**	**1 001-2 000**	**> 2 000**	**Jumlah/***Total*	
(1)	(2)	(3)	(4)	(5)	(6)	(7)
Aceh	28,04	6,37	47,66	17,93	100,00	760
Sumatera Utara	6,38	5,49	56,08	32,06	100,00	1 754
Sumatera Barat	9,33	2,60	63,36	24,71	100,00	1 586
Riau	1,13	1,06	45,52	52,29	100,00	1 969
Jambi	1,20	0,64	57,28	40,88	100,00	2 091
Sumatera Selatan	5,08	4,42	42,62	47,89	100,00	1 808
Bengkulu	13,38	5,52	45,11	35,99	100,00	1 329
Lampung	15,49	5,16	63,00	16,35	100,00	1 157
Kepulauan Bangka Belitung	73,55	12,32	8,86	5,26	100,00	61
Kepulauan Riau	0,59	0,00	87,41	12,01	100,00	1 586
DKI Jakarta	0,42	0,02	53,12	46,44	100,00	1 820
Jawa Barat	5,76	3,66	34,02	56,56	100,00	1 935
Jawa Tengah	24,12	8,33	55,89	11,67	100,00	1 130
DI Yogyakarta	18,75	3,63	70,71	6,92	100,00	1 352
Jawa Timur	8,45	6,23	76,25	9,07	100,00	1 325
Banten	1,00	0,09	32,33	66,58	100,00	2 259
Bali	13,36	3,48	77,46	5,70	100,00	1 252
Nusa Tenggara Barat	16,89	5,89	58,96	18,26	100,00	947
Nusa Tenggara Timur	0,78	0,46	94,82	3,94	100,00	1 373
Kalimantan Barat	1,13	0,34	17,63	80,90	100,00	2 881
Kalimantan Tengah	3,21	2,99	53,74	40,05	100,00	1 938
Kalimantan Selatan	15,61	5,95	47,68	30,76	100,00	1 019
Kalimantan Timur	9,28	6,03	45,18	39,52	100,00	1 358
Kalimantan Utara	1,17	3,91	62,60	32,33	100,00	1 419
Sulawesi Utara	7,40	2,07	71,40	19,13	100,00	1 606
Sulawesi Tengah	6,12	3,09	75,36	15,43	100,00	1 495
Sulawesi Selatan	10,79	6,67	68,25	14,29	100,00	1 142
Sulawesi Tenggara	4,30	3,36	43,83	48,51	100,00	1 647
Gorontalo	13,88	6,50	68,79	10,83	100,00	1 132
Sulawesi Barat	4,64	1,68	54,55	39,13	100,00	1 587
Maluku	0,83	0,55	48,27	50,35	100,00	2 024
Maluku Utara	0,24	3,08	53,11	43,57	100,00	2 026
Papua Barat	0,13	1,56	45,29	53,02	100,00	2 370
Papua	0,88	4,34	28,29	66,49	100,00	1 229
**Indonesia**	**10,64**	**4,92**	**54,04**	**30,40**	**100,00**	**1 497**

Sumber/*Source*: Susenas Maret 2018/*The March 2018 Susenas*

https://www.bps.go.id

Tabel **8.4.** **Persentase Rumah Tangga yang Membeli/Menerima Beras Miskin (Raskin)/Beras Sejahtera (Rastra) dalam 4 Bulan Terakhir menurut Provinsi, Daerah Tempat Tinggal, dan Harga Raskin per Kg (Rp) 2018**

Table **8.4.** *Percentage of Household Buying/Receiving Rice for The Poor (Raskin/Rastra) during The Last 4 Months by Province, Urban Rural Classification, and Price of Rice for The Poor per Kg (Rupiahs), 2018*

Provinsi *Province*	Perkotaan/*Urban*				Perdesaan/*Rural*				Perkotaan+Perdesaan/*Urban+Rural*			
	<1 600	1 600	>1 600	Jumlah	<1 600	1 600	>1 600	Jumlah	<1 600	1 600	>1 600	Jumlah
(1)	(2)	(3)	(4)	(5)	(6)	(7)	(8)	(9)	(10)	(11)	(12)	(13)
Aceh	33,12	2,24	64,65	100,00	52,46	4,49	43,04	100,00	49,20	4,11	46,69	100,00
Sumatera Utara	34,04	7,35	58,61	100,00	19,18	6,53	74,29	100,00	23,55	6,77	69,68	100,00
Sumatera Barat	35,14	12,44	52,43	100,00	26,38	14,29	59,33	100,00	28,37	13,87	57,77	100,00
Riau	16,33	4,03	79,64	100,00	7,01	5,29	87,70	100,00	8,27	5,12	86,61	100,00
Jambi	8,78	3,93	87,30	100,00	7,11	6,05	86,84	100,00	7,35	5,74	86,91	100,00
Sumatera Selatan	30,04	7,55	62,41	100,00	18,60	9,64	71,75	100,00	20,49	9,30	70,21	100,00
Bengkulu	35,62	3,13	61,25	100,00	43,07	4,79	52,14	100,00	41,77	4,50	53,74	100,00
Lampung	40,41	1,31	58,28	100,00	51,23	2,07	46,70	100,00	50,04	1,98	47,98	100,00
Kepulauan Bangka Belitung	95,37	0,00	4,63	100,00	88,74	1,99	9,27	100,00	91,60	1,13	7,27	100,00
Kepulauan Riau	1,99	43,60	54,41	100,00	0,25	64,08	35,67	100,00	1,04	54,75	44,21	100,00
DKI Jakarta	10,63	16,93	72,44	100,00	-	-	-	-	10,63	16,93	72,44	100,00
Jawa Barat	18,69	1,02	80,29	100,00	19,26	1,33	79,41	100,00	18,94	1,16	79,90	100,00
Jawa Tengah	60,47	6,25	33,28	100,00	61,75	4,56	33,69	100,00	61,30	5,16	33,54	100,00
DI Yogyakarta	44,46	6,23	49,31	100,00	63,62	2,45	33,93	100,00	55,70	4,01	40,28	100,00
Jawa Timur	30,18	26,79	43,03	100,00	28,97	25,40	45,63	100,00	29,31	25,79	44,90	100,00
Banten	4,69	0,54	94,77	100,00	6,22	0,46	93,33	100,00	5,64	0,49	93,87	100,00
Bali	46,10	28,48	25,42	100,00	20,09	29,45	50,46	100,00	29,39	29,11	41,50	100,00
Nusa Tenggara Barat	45,70	4,11	50,20	100,00	46,64	3,66	49,70	100,00	46,35	3,80	49,85	100,00
Nusa Tenggara Timur	11,05	21,81	67,14	100,00	5,07	38,22	56,71	100,00	5,41	37,28	57,31	100,00
Kalimantan Barat	10,93	16,22	72,85	100,00	0,81	0,92	98,26	100,00	2,17	2,97	94,86	100,00
Kalimantan Tengah	17,57	14,01	68,43	100,00	14,01	7,30	78,69	100,00	14,64	8,50	76,85	100,00
Kalimantan Selatan	45,61	18,49	35,90	100,00	38,54	6,33	55,13	100,00	40,45	9,61	49,94	100,00
Kalimantan Timur	33,33	17,72	48,95	100,00	10,28	25,78	63,93	100,00	20,69	22,14	57,17	100,00
Kalimantan Utara	32,59	19,08	48,33	100,00	15,06	13,86	71,07	100,00	21,63	15,82	62,55	100,00
Sulawesi Utara	33,52	17,44	49,04	100,00	13,39	11,51	75,10	100,00	19,25	13,24	67,52	100,00
Sulawesi Tengah	29,50	17,91	52,59	100,00	20,19	19,22	60,58	100,00	21,59	19,03	59,39	100,00
Sulawesi Selatan	33,83	12,47	53,70	100,00	38,05	12,21	49,74	100,00	37,54	12,24	50,22	100,00
Sulawesi Tenggara	13,82	2,59	83,59	100,00	21,55	5,62	72,82	100,00	19,40	4,78	75,82	100,00
Gorontalo	38,42	33,34	28,24	100,00	48,41	16,69	34,90	100,00	46,60	19,71	33,69	100,00
Sulawesi Barat	41,77	0,00	58,23	100,00	36,30	1,66	62,05	100,00	37,24	1,37	61,39	100,00
Maluku	8,36	5,18	86,46	100,00	4,06	5,17	90,77	100,00	4,81	5,17	90,02	100,00
Maluku Utara	10,66	24,00	65,34	100,00	7,47	5,34	87,19	100,00	7,82	7,41	84,77	100,00
Papua Barat	1,18	1,07	97,75	100,00	5,44	4,18	90,38	100,00	4,59	3,55	91,86	100,00
Papua	1,89	0,00	98,11	100,00	9,55	0,56	89,89	100,00	7,94	0,44	91,62	100,00
**Indonesia**	**31,01**	**7,81**	**61,18**	**100,00**	**31,41**	**9,95**	**58,64**	**100,00**	**31,28**	**9,24**	**59,48**	**100,00**

Sumber/*Source*: Susenas Maret 2018/*The March 2018 Susenas*

https://www.bps.go.id

Tabel 8.5.1. **Persentase Rumah Tangga yang Menerima Kredit dalam Setahun Terakhir di Daerah Perkotaan menurut Provinsi dan Jenis Kredit, 2018**
Table 8.5.1. *Percentage of Household that Received Credit during The Last Year in Urban Area by Province and Type of Credit, 2018*

**Provinsi** *Province*	**% RT yang Menerima Kredit** *% Household Who Received Credit*	**Jenis Kredit/***Type of Credit*									
		**Kredit Usaha Rakyat (KUR)** *People's Business Credit*	**Kredit dari Bank Umum selain KUR** *Other Bank Credit beside KUR*	**Bank Perkreditan Rakyat (BPR)** *Rural bank*	**Kredit dari Koperasi** *Cooperative Credit*	**Perorangan (dengan bunga)** *Individual (with Interest)*	**Pegadaian** *Pownshop*	**Perusahaan Leasing** *Leasing Company*	**KUBE/ KUB** *Business Groups*	**Badan Usaha Milik Desa (Bumdes)** *Village Owned Enterprises*	**Lainnya** *Other*
(1)	(2)	(3)	(4)	(5)	(6)	(7)	(8)	(9)	(10)	(11)	(12)
Aceh	26,37	18,64	32,54	11,41	8,45	3,63	2,30	20,46	0,00	2,88	9,80
Sumatera Utara	19,28	20,40	27,44	6,92	12,78	9,43	3,75	18,46	1,52	0,58	8,58
Sumatera Barat	32,51	26,10	25,31	5,08	17,42	8,23	3,82	23,57	1,61	0,17	8,77
Riau	22,00	15,12	27,75	6,70	8,97	5,60	2,87	33,24	0,72	3,57	9,70
Jambi	25,18	19,08	29,07	4,54	10,39	8,54	6,90	30,93	1,25	0,51	5,87
Sumatera Selatan	22,50	14,27	21,80	5,89	16,91	11,82	3,58	28,04	0,87	0,11	11,35
Bengkulu	40,93	26,66	37,13	2,70	11,36	7,09	1,48	21,09	0,06	0,16	10,05
Lampung	26,03	17,05	25,61	8,42	17,42	8,72	1,47	20,60	0,17	0,82	14,89
Kepulauan Bangka Belitung	21,52	14,79	28,03	5,76	9,82	8,35	2,37	29,85	0,98	0,00	11,48
Kepulauan Riau	22,56	11,29	20,78	11,32	6,74	11,50	16,43	33,38	1,32	0,36	5,40
DKI Jakarta	13,67	11,29	13,07	3,03	9,04	10,83	5,10	46,19	0,30	0,40	13,47
Jawa Barat	30,41	16,74	21,11	6,53	14,36	9,45	3,30	32,05	1,01	0,67	12,17
Jawa Tengah	37,93	24,77	21,90	7,44	20,62	7,62	3,31	16,37	1,20	1,57	15,10
DI Yogyakarta	34,32	25,97	23,07	5,80	18,02	3,78	2,47	11,10	2,74	1,21	26,28
Jawa Timur	33,22	21,10	19,81	7,22	20,94	6,28	4,11	22,85	0,98	1,67	14,10
Banten	23,13	5,99	16,58	4,84	19,33	7,85	2,71	47,51	0,43	0,05	9,88
Bali	42,20	20,96	14,42	6,64	33,26	7,01	2,00	11,00	0,64	5,23	18,90
Nusa Tenggara Barat	31,93	20,59	22,73	5,04	17,97	10,18	13,94	9,52	2,04	1,72	14,15
Nusa Tenggara Timur	31,32	14,64	29,09	3,88	38,58	6,68	15,93	5,99	2,15	0,00	4,07
Kalimantan Barat	22,98	16,06	33,57	7,23	16,36	4,08	7,80	22,69	0,12	0,01	4,12
Kalimantan Tengah	27,46	20,52	33,87	4,83	8,93	5,22	1,27	27,94	0,12	0,44	10,34
Kalimantan Selatan	24,41	22,47	25,41	9,71	6,29	10,27	3,11	28,87	1,34	0,32	8,49
Kalimantan Timur	27,70	18,07	30,40	7,41	5,92	4,27	19,74	21,39	0,23	0,16	6,54
Kalimantan Utara	27,07	15,19	31,45	2,49	10,77	6,91	20,11	23,07	0,26	0,00	5,17
Sulawesi Utara	27,61	19,69	29,07	5,67	15,21	7,59	16,55	23,80	0,24	0,20	3,75
Sulawesi Tengah	32,07	19,06	37,92	11,31	13,46	4,26	12,34	16,08	0,57	0,04	4,50
Sulawesi Selatan	32,43	32,76	24,30	4,11	12,02	7,15	19,98	15,02	0,80	0,16	5,27
Sulawesi Tenggara	36,42	26,80	45,19	2,40	10,71	3,23	5,82	16,04	0,50	0,45	5,32
Gorontalo	35,45	25,05	32,39	3,13	11,33	10,22	12,71	19,60	0,75	2,08	4,32
Sulawesi Barat	37,12	39,45	34,38	5,59	8,51	2,76	16,31	5,36	0,98	0,00	7,49
Maluku	19,62	21,55	40,24	10,95	12,47	2,04	9,56	5,47	0,74	0,15	4,63
Maluku Utara	24,25	15,33	48,96	7,57	12,55	2,36	5,29	10,85	0,42	0,00	3,69
Papua Barat	25,94	27,17	33,07	7,68	13,07	6,98	8,57	5,04	0,00	0,00	7,14
Papua	22,31	35,08	41,33	9,30	9,83	3,76	5,04	3,21	0,14	0,36	3,44
**Indonesia**	**29,14**	**19,58**	**22,31**	**6,57**	**16,89**	**7,87**	**4,85**	**24,95**	**0,99**	**1,10**	**12,44**

Sumber/*Source*: Susenas Maret 2018/*The March 2018 Susenas*

https://www.bps.go.id

Tabel *Table* **8.5.2.** **Persentase Rumah Tangga yang Menerima Kredit dalam Setahun Terakhir di Daerah Perdesaan menurut Provinsi dan Jenis Kredit, 2018**
Percentage of Household that Received Credit during The Last Year in Rural Area by Province and Type of Credit, 2018

**Provinsi** *Province*	**% RT yang Menerima Kredit** *% Household Who Received Credit*	**Jenis Kredit/***Type of Credit*									
		**Kredit Usaha Rakyat (KUR)** *People's Business Credit*	**Kredit dari Bank Umum selain KUR** *Other Bank Credit beside KUR*	**Bank Perkreditan Rakyat (BPR)** *Rural bank*	**Kredit dari Koperasi** *Cooperative Credit*	**Perorangan (dengan bunga)** *Individual (with Interest)*	**Pegadaian** *Pownshop*	**Perusahaan Leasing** *Leasing Company*	**KUBE/ KUB** *Business Groups*	**Badan Usaha Milik Desa (Bumdes)** *Village Owned Enterprises*	**Lainnya** *Other*
(1)	(2)	(3)	(4)	(5)	(6)	(7)	(8)	(9)	(10)	(11)	(12)
Aceh	19,00	20,52	23,41	6,96	11,02	5,64	1,33	15,84	1,04	12,79	10,45
Sumatera Utara	21,81	22,80	22,07	6,17	22,83	11,27	2,05	10,57	1,42	1,50	10,14
Sumatera Barat	30,92	33,16	21,79	5,26	13,50	6,12	0,22	12,57	1,38	1,44	21,68
Riau	28,65	23,22	26,09	4,93	10,88	7,98	2,17	24,67	2,04	13,53	8,44
Jambi	24,52	27,12	25,71	4,54	9,17	3,88	0,47	29,04	0,76	0,91	11,25
Sumatera Selatan	19,41	22,42	22,07	3,95	15,31	15,30	0,79	17,35	1,97	0,76	13,19
Bengkulu	31,31	33,16	22,54	1,45	12,38	11,12	0,88	21,45	1,16	1,41	14,10
Lampung	23,81	23,22	17,89	4,87	16,20	11,85	1,29	15,66	3,37	1,06	20,10
Kepulauan Bangka Belitung	14,34	31,27	19,67	3,78	10,89	10,95	1,78	18,35	2,78	0,65	9,55
Kepulauan Riau	17,19	26,61	21,02	5,10	5,46	8,86	3,21	7,71	8,64	1,31	17,33
DKI Jakarta	0,00	-	-	-	-	-	-	-	-	-	-
Jawa Barat	31,08	23,34	21,63	7,19	11,17	11,55	1,15	17,52	1,38	1,53	19,23
Jawa Tengah	38,31	26,99	18,14	8,14	20,87	7,77	1,70	11,23	2,39	1,94	18,60
DI Yogyakarta	49,92	26,06	22,29	5,13	16,86	3,21	0,54	7,74	4,99	4,57	36,69
Jawa Timur	30,50	24,97	16,67	8,97	24,19	6,45	4,38	9,09	2,18	3,01	18,81
Banten	18,86	8,47	16,02	4,55	17,21	16,87	1,50	18,28	3,07	0,49	25,41
Bali	52,10	24,45	10,53	5,93	20,01	12,98	1,77	1,38	2,46	7,43	36,94
Nusa Tenggara Barat	34,44	20,08	13,95	4,68	14,45	19,80	21,32	6,28	1,25	4,67	17,99
Nusa Tenggara Timur	30,07	12,36	11,44	3,07	44,60	17,85	11,68	1,86	6,65	2,79	7,96
Kalimantan Barat	21,29	18,41	13,89	3,00	42,99	7,53	2,16	12,92	0,35	0,38	10,36
Kalimantan Tengah	18,97	23,63	22,62	5,85	19,56	9,33	1,36	16,28	1,87	2,44	11,04
Kalimantan Selatan	18,05	30,41	20,76	4,57	7,42	13,32	1,11	14,11	3,51	0,96	17,32
Kalimantan Timur	22,34	29,39	22,50	1,29	8,83	5,98	8,31	24,80	2,25	2,35	6,55
Kalimantan Utara	23,30	24,53	36,40	1,65	11,20	2,34	11,29	5,22	0,00	4,68	12,76
Sulawesi Utara	31,81	23,36	23,99	5,24	18,38	19,20	13,01	8,34	1,11	2,45	7,17
Sulawesi Tengah	25,59	30,58	26,21	5,88	11,85	8,57	6,51	11,66	1,91	3,21	6,02
Sulawesi Selatan	26,11	45,61	17,73	4,42	10,71	5,45	19,26	3,96	1,61	1,26	6,94
Sulawesi Tenggara	27,02	33,87	25,61	3,40	15,45	6,42	5,62	9,13	1,22	6,29	8,46
Gorontalo	34,85	37,18	23,26	2,70	12,30	16,42	3,59	10,66	2,37	3,00	7,51
Sulawesi Barat	22,82	38,52	24,62	3,92	15,35	8,37	6,19	6,08	0,67	0,91	7,27
Maluku	11,76	39,50	31,92	7,91	5,65	6,07	1,59	0,99	1,42	6,97	5,48
Maluku Utara	11,95	17,11	36,82	10,09	13,66	8,68	1,73	6,94	1,98	1,72	6,84
Papua Barat	18,02	28,38	42,86	10,21	8,76	4,09	1,63	1,92	0,56	0,22	7,49
Papua	7,05	32,77	37,71	4,78	5,23	8,12	1,47	3,50	2,27	1,02	11,63
**Indonesia**	**28,24**	**25,45**	**19,41**	**6,49**	**18,54**	**9,48**	**3,88**	**12,17**	**2,15**	**2,79**	**16,68**

Sumber/*Source*: Susenas Maret 2018/*The March 2018 Susenas*

https://www.bps.go.id

Tabel **8.5.3.** **Persentase Rumah Tangga yang Menerima Kredit dalam Setahun Terakhir di Daerah Perkotaan dan Perdesaan menurut Provinsi dan Jenis Kredit, 2018**
Table *Percentage of Household that Received Credit during The Last Year in Urban and Rural Area by Province and Type of Credit, 2018*

**Provinsi** *Province*	**% RT yang Menerima Kredit** *% Household Who Received Credit*	**Jenis Kredit/***Type of Credit*									
		**Kredit Usaha Rakyat (KUR)** *People's Business Credit*	**Kredit dari Bank Umum selain KUR** *Other Bank Credit beside KUR*	**Bank Perkreditan Rakyat (BPR)** *Rural bank*	**Kredit dari Koperasi** *Cooperative Credit*	**Perorangan (dengan bunga)** *Individual (with Interest)*	**Pegadaian** *Pownshop*	**Perusahaan Leasing** *Leasing Company*	**KUBE/ KUB** *Business Groups*	**Badan Usaha Milik Desa (Bumdes)** *Village Owned Enterprises*	**Lainnya** *Other*
(1)	(2)	(3)	(4)	(5)	(6)	(7)	(8)	(9)	(10)	(11)	(12)
Aceh	21,30	19,79	26,94	8,68	10,03	4,86	1,70	17,63	0,64	8,95	10,20
Sumatera Utara	20,48	21,61	24,72	6,54	17,87	10,36	2,89	14,47	1,47	1,05	9,37
Sumatera Barat	31,63	29,96	23,39	5,18	15,28	7,08	1,85	17,56	1,48	0,87	15,82
Riau	25,96	20,45	26,66	5,54	10,23	7,17	2,41	27,60	1,59	10,12	8,87
Jambi	24,72	24,56	26,78	4,54	9,56	5,36	2,52	29,64	0,92	0,78	9,54
Sumatera Selatan	20,52	19,22	21,96	4,71	15,94	13,93	1,88	21,55	1,54	0,50	12,47
Bengkulu	34,40	30,68	28,11	1,93	11,99	9,58	1,11	21,31	0,74	0,94	12,55
Lampung	24,43	21,38	20,18	5,92	16,56	10,92	1,34	17,13	2,41	0,99	18,55
Kepulauan Bangka Belitung	18,17	20,86	24,95	5,03	10,21	9,31	2,15	25,62	1,64	0,24	10,77
Kepulauan Riau	21,81	12,97	20,80	10,64	6,60	11,21	14,98	30,56	2,12	0,46	6,70
DKI Jakarta	13,67	11,29	13,07	3,03	9,04	10,83	5,10	46,19	0,30	0,40	13,47
Jawa Barat	30,59	18,58	21,25	6,71	13,47	10,03	2,70	28,00	1,11	0,91	14,13
Jawa Tengah	38,12	25,87	20,04	7,79	20,74	7,70	2,51	13,82	1,79	1,75	16,84
DI Yogyakarta	38,43	26,00	22,81	5,57	17,62	3,59	1,81	9,95	3,51	2,36	29,85
Jawa Timur	31,91	22,87	18,37	8,02	22,43	6,36	4,23	16,55	1,53	2,28	16,26
Banten	21,92	6,60	16,44	4,77	18,81	10,06	2,42	40,35	1,08	0,16	13,68
Bali	45,46	22,28	12,95	6,37	28,25	9,27	1,91	7,36	1,33	6,06	25,72
Nusa Tenggara Barat	33,28	20,31	17,83	4,84	16,01	15,54	18,05	7,71	1,60	3,36	16,29
Nusa Tenggara Timur	30,37	12,92	15,75	3,27	43,13	15,13	12,72	2,87	5,55	2,11	7,01
Kalimantan Barat	21,85	17,60	20,69	4,46	33,79	6,34	4,11	16,29	0,27	0,25	8,20
Kalimantan Tengah	22,17	22,18	27,87	5,38	14,60	7,41	1,32	21,72	1,05	1,51	10,71
Kalimantan Selatan	21,00	26,13	23,27	7,35	6,81	11,67	2,19	22,08	2,34	0,61	12,56
Kalimantan Timur	25,92	21,31	28,14	5,66	6,75	4,76	16,48	22,36	0,81	0,79	6,54
Kalimantan Utara	25,54	18,65	33,28	2,18	10,93	5,22	16,85	16,46	0,16	1,73	7,98
Sulawesi Utara	29,70	21,65	26,36	5,44	16,90	13,79	14,66	15,55	0,70	1,40	5,58
Sulawesi Tengah	27,43	26,76	30,10	7,68	12,39	7,14	8,44	13,12	1,47	2,16	5,52
Sulawesi Selatan	28,66	39,74	20,73	4,28	11,31	6,23	19,59	9,01	1,24	0,76	6,18
Sulawesi Tenggara	30,60	30,66	34,49	2,95	13,30	4,97	5,71	12,26	0,90	3,64	7,03
Gorontalo	35,09	32,35	26,89	2,87	11,91	13,95	7,22	14,22	1,72	2,63	6,24
Sulawesi Barat	26,16	38,83	27,85	4,48	13,09	6,51	9,54	5,84	0,77	0,61	7,34
Maluku	15,15	29,47	36,57	9,61	9,46	3,82	6,04	3,49	1,04	3,16	5,00
Maluku Utara	15,61	16,29	42,43	8,92	13,14	5,76	3,37	8,75	1,26	0,93	5,39
Papua Barat	21,19	27,79	38,07	8,97	10,87	5,51	5,03	3,45	0,29	0,11	7,32
Papua	10,97	33,98	39,60	7,14	7,63	5,85	3,33	3,35	1,16	0,67	7,35
**Indonesia**	**28,73**	**22,20**	**21,02**	**6,54**	**17,62**	**8,59**	**4,42**	**19,26**	**1,51**	**1,85**	**14,33**

Sumber/*Source*: Susenas Maret 2018/*The March 2018 Susenas*

https://www.bps.go.id

Tabel 8.6. **Persentase Rumah Tangga yang Menerima Program Indonesia Pintar (PIP) dalam Bulan Agustus 2017-February 2018 menurut Provinsi dan Daerah Tempat Tinggal**
Table *Percentage of Household who Received Scholarship for The Smart Indonesia Program (PIP) from August 2017 through February 2018 by Province and Urban Rural Classification*

**Provinsi** *Province*	**Perkotaan** *Urban*	**Perdesaan** *Rural*	**Perkotaan+Perdesaan** *Urban+Rural*
**(1)**	**(2)**	**(3)**	**(4)**
Aceh	10,41	21,94	18,34
Sumatera Utara	7,93	14,81	11,20
Sumatera Barat	9,44	17,57	13,98
Riau	5,11	10,30	8,20
Jambi	7,93	9,38	8,93
Sumatera Selatan	7,92	13,08	11,23
Bengkulu	9,25	14,88	13,08
Lampung	12,05	15,32	14,41
Kepulauan Bangka Belitung	4,37	7,41	5,79
Kepulauan Riau	5,58	15,52	6,96
DKI Jakarta	0,94	-	0,94
Jawa Barat	8,71	14,26	10,23
Jawa Tengah	12,46	15,81	14,11
DI Yogyakarta	11,77	20,10	13,97
Jawa Timur	8,16	12,88	10,42
Banten	5,89	12,61	7,80
Bali	5,28	10,74	7,08
Nusa Tenggara Barat	18,59	20,66	19,70
Nusa Tenggara Timur	13,08	22,95	20,61
Kalimantan Barat	7,15	8,98	8,38
Kalimantan Tengah	3,21	7,39	5,82
Kalimantan Selatan	5,45	10,85	8,34
Kalimantan Timur	3,41	8,59	5,13
Kalimantan Utara	6,96	12,57	9,23
Sulawesi Utara	7,01	12,60	9,80
Sulawesi Tengah	9,31	13,98	12,66
Sulawesi Selatan	8,92	19,08	14,98
Sulawesi Tenggara	12,95	26,12	21,10
Gorontalo	12,00	20,24	17,00
Sulawesi Barat	10,83	17,74	16,13
Maluku	6,28	14,35	10,86
Maluku Utara	4,93	9,32	8,02
Papua Barat	12,31	14,43	13,59
Papua	7,30	7,35	7,33
**Indonesia**	**8,27**	**14,54**	**11,11**

Sumber/*Source*: Susenas Maret 2018/*The March 2018 Susenas*

https://www.bps.go.id

Tabel *Table* **8.7.** **Persentase Rumah Tangga yang Menerima Program Indonesia Pintar (PIP) selama Bulan Agustus 2017-February 2018 menurut Provinsi, Daerah Tempat Tinggal, dan Jenis PIP**
Percentage of Household Member who Received Smart Indonesia Program (PIP) from August 2017 through February 2018 by Province, Urban Rural Classification, and Type of PIP

**Provinsi** *Province*	**Perkotaan/***Urban*			**Perdesaan/***Rural*			**Perkotaan+Perdesaan/***Urban+Rural*		
	**SD/***Primary School*	**SMP/***Junior High School*	**SMA/***Senior High School*	**SD/***Primary School*	**SMP/***Junior High School*	**SMA/***Senior High School*	**SD/***Primary School*	**SMP/***Junior High School*	**SMA/***Senior High School*
**(1)**	**(2)**	**(3)**	**(4)**	**(5)**	**(6)**	**(7)**	**(8)**	**(9)**	**(10)**
Aceh	58,74	38,14	29,09	70,01	33,33	22,39	68,01	34,18	23,58
Sumatera Utara	68,30	35,10	26,97	69,77	38,72	23,45	69,22	37,37	24,75
Sumatera Barat	62,26	35,78	30,72	74,08	30,20	20,96	70,56	31,86	23,87
Riau	71,02	32,20	20,64	67,83	33,43	17,94	68,64	33,12	18,62
Jambi	52,24	30,49	33,53	63,57	27,01	17,94	60,43	27,98	22,27
Sumatera Selatan	61,43	45,30	24,98	67,70	31,53	19,19	66,11	35,01	20,66
Bengkulu	62,41	34,75	14,40	72,36	29,35	15,93	70,10	30,58	15,58
Lampung	65,00	36,54	25,91	67,63	27,84	18,05	67,02	29,87	19,88
Kepulauan Bangka Belitung	52,88	39,40	29,56	64,36	30,84	21,27	59,73	34,29	24,61
Kepulauan Riau	74,27	29,45	16,48	53,73	41,54	26,26	67,90	33,20	19,52
DKI Jakarta	55,92	41,23	19,95	-	-	-	55,92	41,23	19,95
Jawa Barat	64,60	32,48	20,04	64,22	33,76	14,61	64,45	32,97	17,97
Jawa Tengah	63,21	32,49	23,22	67,49	29,96	17,02	65,57	31,09	19,80
DI Yogyakarta	70,88	24,04	17,95	67,12	25,77	18,76	69,46	24,70	18,26
Jawa Timur	60,90	29,73	23,11	64,58	28,80	16,64	63,08	29,18	19,28
Banten	73,78	24,87	15,77	75,92	30,19	16,99	74,76	27,31	16,33
Bali	66,10	27,77	24,03	65,74	32,65	22,97	65,92	30,21	23,50
Nusa Tenggara Barat	66,55	32,64	22,89	67,95	29,51	20,46	67,34	30,87	21,52
Nusa Tenggara Timur	57,77	30,79	36,50	69,85	37,03	19,27	68,03	36,09	21,86
Kalimantan Barat	66,17	24,37	26,38	71,28	29,10	17,99	69,85	27,77	20,34
Kalimantan Tengah	54,85	33,39	30,46	59,92	42,92	16,48	58,87	40,94	19,39
Kalimantan Selatan	67,72	28,96	22,16	67,22	28,44	18,88	67,37	28,60	19,88
Kalimantan Timur	65,05	31,05	30,54	70,75	28,97	23,44	68,21	29,90	26,60
Kalimantan Utara	74,30	33,40	29,67	61,57	39,36	30,25	67,27	36,69	29,99
Sulawesi Utara	59,69	35,10	23,94	65,58	37,32	12,21	63,47	36,52	16,42
Sulawesi Tengah	68,73	27,56	26,60	69,44	32,66	15,18	69,30	31,60	17,57
Sulawesi Selatan	73,56	33,35	14,66	69,80	34,20	20,13	70,71	34,00	18,82
Sulawesi Tenggara	68,61	41,04	21,71	72,08	32,42	19,06	71,27	34,43	19,68
Gorontalo	70,63	33,56	16,93	69,62	30,76	18,83	69,90	31,54	18,30
Sulawesi Barat	74,52	25,16	18,56	69,41	37,08	11,58	70,21	35,22	12,67
Maluku	49,95	42,54	31,31	68,95	31,72	24,84	64,21	34,42	26,45
Maluku Utara	64,16	27,14	30,91	57,49	36,97	25,32	58,71	35,17	26,34
Papua Barat	66,31	32,85	22,31	72,95	32,41	20,64	70,54	32,57	21,25
Papua	59,64	36,93	27,93	77,06	36,22	16,11	72,61	36,40	19,13
**Indonesia**	**64,60**	**32,08**	**22,36**	**67,75**	**31,75**	**18,14**	**66,47**	**31,89**	**19,85**

Sumber/*Source*: Susenas Maret 2018/*The March 2018 Susenas*

https://www.bps.go.id

Tabel **8.8.1.** **Persentase Rumah Tangga yang Memiliki atau Menerima Jaminan Sosial dalam Setahun Terakhir di Daerah Perkotaan menurut Provinsi dan Jenis Jaminan Sosial, 2018**
Table *Percentage of Household who Had or Received The Following Social Insurance during The Last One Year in Urban Area by Province and Type of Social Insurance, 2018*

Provinsi<br>*Province*	Jaminan Pensiun/Veteran<br>*Veteran/Pension Insurance*	Jaminan Hari Tua<br>*Pension Saving*	Asuransi Kecelakaan Kerja<br>*Occupational Accident Insurance*	Jaminan/Asuransi Kematian<br>*Life Insurance*	Pesangon Pemutusan Hubungan Kerja (PHK)<br>*Severance of Termination*
(1)	(2)	(3)	(4)	(5)	(6)
Aceh	20,69	17,60	17,71	17,42	5,10
Sumatera Utara	10,96	7,19	8,22	7,30	3,99
Sumatera Barat	15,69	12,76	12,34	11,16	4,52
Riau	7,84	10,00	11,31	8,34	5,63
Jambi	8,49	4,66	6,14	4,60	1,92
Sumatera Selatan	13,66	8,97	8,45	7,73	5,53
Bengkulu	14,43	10,18	8,26	6,73	1,14
Lampung	6,99	3,40	3,94	3,08	1,90
Kepulauan Bangka Belitung	12,75	11,72	12,38	10,52	4,88
Kepulauan Riau	5,72	11,85	12,59	9,04	4,62
DKI Jakarta	5,66	5,05	5,70	3,01	1,15
Jawa Barat	7,19	8,01	9,60	6,72	4,76
Jawa Tengah	8,80	6,75	8,42	5,90	3,91
DI Yogyakarta	14,24	10,40	8,28	7,99	2,18
Jawa Timur	8,23	6,53	6,84	5,44	2,66
Banten	6,29	10,17	11,75	8,28	5,36
Bali	8,49	5,71	7,01	5,61	2,39
Nusa Tenggara Barat	9,45	8,24	8,49	8,30	2,07
Nusa Tenggara Timur	22,47	14,48	14,60	14,91	5,19
Kalimantan Barat	9,09	6,90	6,93	6,26	3,49
Kalimantan Tengah	15,11	10,77	10,93	12,19	3,53
Kalimantan Selatan	16,92	12,93	13,03	12,59	4,81
Kalimantan Timur	9,92	10,87	12,79	9,45	6,05
Kalimantan Utara	11,23	12,36	14,54	9,68	8,89
Sulawesi Utara	10,02	4,13	4,11	4,04	1,22
Sulawesi Tengah	18,81	14,20	13,44	12,84	3,03
Sulawesi Selatan	13,61	8,26	7,00	7,62	2,54
Sulawesi Tenggara	21,27	19,41	18,64	17,36	2,08
Gorontalo	12,61	9,36	9,28	10,69	3,03
Sulawesi Barat	13,67	10,40	9,69	10,69	5,35
Maluku	11,29	4,11	3,89	4,12	0,67
Maluku Utara	21,59	17,97	19,46	18,08	6,11
Papua Barat	12,15	7,69	6,04	5,04	2,94
Papua	17,27	9,64	9,17	9,26	5,07
**Indonesia**	**9,07**	**7,99**	**8,85**	**6,87**	**3,75**

Sumber/*Source*: Susenas Maret 2018/*The March 2018 Susenas*

https://www.bps.go.id

Tabel 8.8.2. **Persentase Rumah Tangga yang Memiliki atau Menerima Jaminan Sosial dalam Setahun Terakhir di Daerah Perdesaan menurut Provinsi dan Jenis Jaminan Sosial, 2018**
Table *Percentage of Household who Had or Received The Following Social Insurance during The Last One Year in Rural Area by Province and Type of Social Insurance, 2018*

**Provinsi** *Province*	**Jaminan Pensiun/Veteran** *Veteran/Pension Insurance*	**Jaminan Hari Tua** *Pension Saving*	**Asuransi Kecelakaan Kerja** *Occupational Accident Insurance*	**Jaminan/Asuransi Kematian** *Life Insurance*	**Pesangon Pemutusan Hubungan Kerja (PHK)** *Severance of Termination*
**(1)**	**(2)**	**(3)**	**(4)**	**(5)**	**(6)**
Aceh	7,59	5,75	6,16	5,74	2,89
Sumatera Utara	6,86	4,87	5,20	4,75	3,26
Sumatera Barat	5,70	4,66	5,45	5,38	2,63
Riau	4,75	4,74	6,38	4,80	4,05
Jambi	4,17	2,83	3,09	2,51	1,23
Sumatera Selatan	2,87	2,41	3,09	2,26	1,47
Bengkulu	4,52	3,56	3,68	3,34	1,93
Lampung	2,63	2,08	2,31	1,70	0,90
Kepulauan Bangka Belitung	2,90	3,64	4,86	3,78	2,58
Kepulauan Riau	3,89	5,29	6,50	4,26	2,24
DKI Jakarta	-	-	-	-	-
Jawa Barat	2,93	2,18	2,77	2,26	1,10
Jawa Tengah	3,02	2,25	3,45	2,14	1,36
DI Yogyakarta	5,32	2,96	4,29	3,50	2,24
Jawa Timur	2,25	1,70	2,23	1,70	0,77
Banten	1,72	1,95	3,59	1,48	2,05
Bali	4,37	3,23	3,77	3,71	1,68
Nusa Tenggara Barat	3,49	2,80	2,97	2,66	0,99
Nusa Tenggara Timur	4,46	2,98	2,78	3,21	1,01
Kalimantan Barat	2,76	2,93	3,29	2,45	2,16
Kalimantan Tengah	7,65	8,89	12,19	9,70	6,81
Kalimantan Selatan	8,12	7,23	8,82	6,60	4,65
Kalimantan Timur	4,16	4,32	6,87	4,92	3,65
Kalimantan Utara	8,44	8,63	11,56	6,90	2,90
Sulawesi Utara	6,27	3,48	2,78	3,32	1,66
Sulawesi Tengah	5,65	4,14	4,70	4,44	1,94
Sulawesi Selatan	4,92	3,04	3,29	3,76	1,39
Sulawesi Tenggara	6,30	4,53	4,52	4,71	0,62
Gorontalo	3,76	2,89	3,66	3,12	0,81
Sulawesi Barat	5,95	5,32	4,57	4,97	2,54
Maluku	5,48	2,15	1,98	1,80	1,09
Maluku Utara	5,29	4,07	3,95	4,27	1,32
Papua Barat	11,37	8,75	9,35	7,49	3,22
Papua	3,77	2,55	1,58	1,27	0,92
**Indonesia**	**3,87**	**3,02**	**3,68**	**2,97**	**1,69**

Sumber/*Source*: Susenas Maret 2018/*The March 2018 Susenas*

https://www.bps.go.id

Tabel *Table* **8.8.3.** **Persentase Rumah Tangga yang Memiliki atau Menerima Jaminan Sosial dalam Setahun Terakhir di Daerah Perkotaan dan Perdesaan menurut Provinsi dan Jenis Jaminan Sosial, 2018**
Percentage of Household who Had or Received The Following Social Insurance during The Last One Year in Urban and Rural Area by Province and Type of Social Insurance, 2018

**Provinsi** *Province*	**Jaminan Pensiun/Veteran** *Veteran/Pension Insurance*	**Jaminan Hari Tua** *Pension Saving*	**Asuransi Kecelakaan Kerja** *Occupational Accident Insurance*	**Jaminan/Asuransi Kematian** *Life Insurance*	**Pesangon Pemutusan Hubungan Kerja (PHK)** *Severance of Termination*
**(1)**	**(2)**	**(3)**	**(4)**	**(5)**	**(6)**
Aceh	11,69	9,45	9,77	9,39	3,58
Sumatera Utara	9,01	6,09	6,79	6,09	3,64
Sumatera Barat	10,11	8,23	8,49	7,93	3,47
Riau	6,00	6,86	8,37	6,22	4,69
Jambi	5,52	3,40	4,04	3,16	1,45
Sumatera Selatan	6,73	4,76	5,01	4,22	2,92
Bengkulu	7,70	5,68	5,15	4,43	1,68
Lampung	3,85	2,44	2,76	2,08	1,18
Kepulauan Bangka Belitung	8,16	7,95	8,87	7,37	3,81
Kepulauan Riau	5,47	10,93	11,74	8,37	4,28
DKI Jakarta	5,66	5,05	5,70	3,01	1,15
Jawa Barat	6,02	6,42	7,72	5,50	3,76
Jawa Tengah	5,95	4,53	5,97	4,04	2,65
DI Yogyakarta	11,89	8,44	7,23	6,81	2,19
Jawa Timur	5,36	4,22	4,63	3,65	1,76
Banten	4,99	7,83	9,43	6,35	4,42
Bali	7,13	4,89	5,94	4,98	2,15
Nusa Tenggara Barat	6,24	5,31	5,52	5,26	1,49
Nusa Tenggara Timur	8,73	5,70	5,58	5,98	2,00
Kalimantan Barat	4,84	4,24	4,49	3,70	2,59
Kalimantan Tengah	10,46	9,60	11,72	10,64	5,57
Kalimantan Selatan	12,20	9,88	10,77	9,38	4,73
Kalimantan Timur	8,01	8,70	10,83	7,95	5,26
Kalimantan Utara	10,09	10,85	13,33	8,56	6,46
Sulawesi Utara	8,15	3,80	3,44	3,68	1,44
Sulawesi Tengah	9,38	6,99	7,18	6,82	2,25
Sulawesi Selatan	8,43	5,15	4,79	5,32	1,85
Sulawesi Tenggara	12,01	10,20	9,90	9,53	1,17
Gorontalo	7,24	5,43	5,87	6,10	1,69
Sulawesi Barat	7,75	6,50	5,76	6,30	3,20
Maluku	7,99	3,00	2,80	2,80	0,91
Maluku Utara	10,13	8,20	8,56	8,37	2,74
Papua Barat	11,68	8,33	8,02	6,51	3,11
Papua	7,24	4,37	3,53	3,32	1,99
**Indonesia**	**6,72**	**5,74**	**6,51**	**5,10**	**2,81**

Sumber/*Source*: Susenas Maret 2018/*The March 2018 Susenas*

https://www.bps.go.id

Tabel *Table* **8.9. Persentase Rumah Tangga yang Menerima Kartu Perlindungan Sosial (KPS)/Kartu Keluarga Sejahtera (KKS) menurut Provinsi dan Daerah Tempat Tinggal, 2018**
Percentage of Household that Received Social Security Card (KPS)/Card for Family Welfare (KKS) by Province and Urban Rural Classification, 2018

Provinsi *Province*	Perkotaan/*Urban*				Perdesaan/*Rural*				Perkotaan+Perdesaan/*Urban+Rural*			
	Dapat Menunjukkan Kartu *Can Show The Cards*	Tidak Dapat Menunjukkan Kartu *Cannot Show The Cards*	Tidak Punya *No Card*	Jumlah *Total*	Dapat Menunjukkan Kartu *Can Show The Cards*	Tidak Dapat Menunjukkan Kartu *Cannot Show The Cards*	Tidak Punya *No Card*	Jumlah *Total*	Dapat Menunjukkan Kartu *Can Show The Cards*	Tidak Dapat Menunjukkan Kartu *Cannot Show The Cards*	Tidak Punya *No Card*	Jumlah *Total*
(1)	(2)	(3)	(4)	(5)	(6)	(7)	(8)	(9)	(10)	(11)	(12)	(13)
Aceh	6,84	2,84	90,32	100,00	14,70	5,32	79,98	100,00	12,24	4,55	83,21	100,00
Sumatera Utara	7,47	2,94	89,59	100,00	14,92	3,82	81,26	100,00	11,01	3,36	85,63	100,00
Sumatera Barat	7,44	2,24	90,32	100,00	13,85	4,12	82,03	100,00	11,02	3,29	85,69	100,00
Riau	4,69	1,54	93,77	100,00	7,50	1,92	90,58	100,00	6,37	1,77	91,87	100,00
Jambi	5,78	2,08	92,14	100,00	7,19	2,23	90,58	100,00	6,75	2,19	91,07	100,00
Sumatera Selatan	6,32	3,41	90,26	100,00	9,95	3,16	86,89	100,00	8,65	3,25	88,10	100,00
Bengkulu	7,09	1,90	91,01	100,00	12,81	4,61	82,59	100,00	10,97	3,74	85,29	100,00
Lampung	10,60	2,61	86,79	100,00	14,71	3,94	81,35	100,00	13,57	3,57	82,86	100,00
Kepulauan Bangka Belitung	2,72	2,67	94,61	100,00	5,25	1,59	93,16	100,00	3,90	2,17	93,93	100,00
Kepulauan Riau	5,35	1,87	92,78	100,00	13,35	4,17	82,48	100,00	6,46	2,19	91,35	100,00
DKI Jakarta	3,41	1,95	94,64	100,00	-	-	-	100,00	3,41	1,95	94,64	100,00
Jawa Barat	7,67	3,27	89,06	100,00	14,39	4,12	81,48	100,00	9,51	3,50	86,99	100,00
Jawa Tengah	10,71	3,04	86,25	100,00	15,34	4,88	79,78	100,00	12,99	3,95	83,06	100,00
DI Yogyakarta	11,37	5,64	82,99	100,00	24,87	8,63	66,50	100,00	14,93	6,43	78,64	100,00
Jawa Timur	7,73	2,54	89,73	100,00	14,50	5,36	80,15	100,00	10,97	3,89	85,14	100,00
Banten	4,17	2,75	93,09	100,00	14,26	5,41	80,33	100,00	7,04	3,50	89,46	100,00
Bali	4,98	1,64	93,38	100,00	15,77	3,34	80,89	100,00	8,54	2,21	89,26	100,00
Nusa Tenggara Barat	14,60	4,84	80,56	100,00	13,92	4,61	81,47	100,00	14,23	4,72	81,05	100,00
Nusa Tenggara Timur	7,59	2,20	90,21	100,00	23,73	6,51	69,76	100,00	19,91	5,49	74,60	100,00
Kalimantan Barat	3,18	2,28	94,54	100,00	6,95	3,26	89,79	100,00	5,71	2,94	91,35	100,00
Kalimantan Tengah	2,04	1,31	96,65	100,00	5,83	2,07	92,10	100,00	4,40	1,78	93,81	100,00
Kalimantan Selatan	4,99	2,07	92,94	100,00	8,74	2,80	88,46	100,00	7,00	2,46	90,54	100,00
Kalimantan Timur	3,03	1,49	95,48	100,00	8,03	3,37	88,60	100,00	4,69	2,11	93,20	100,00
Kalimantan Utara	5,07	3,16	91,78	100,00	8,88	3,47	87,65	100,00	6,61	3,28	90,11	100,00
Sulawesi Utara	8,47	3,62	87,92	100,00	16,47	4,96	78,57	100,00	12,45	4,29	83,26	100,00
Sulawesi Tengah	6,44	2,88	90,68	100,00	15,28	4,37	80,35	100,00	12,77	3,95	83,28	100,00
Sulawesi Selatan	6,67	2,87	90,46	100,00	15,12	5,30	79,58	100,00	11,71	4,32	83,97	100,00
Sulawesi Tenggara	8,32	3,47	88,21	100,00	17,18	5,50	77,32	100,00	13,80	4,72	81,47	100,00
Gorontalo	13,29	3,45	83,26	100,00	20,49	4,17	75,34	100,00	17,66	3,88	78,46	100,00
Sulawesi Barat	9,64	4,70	85,65	100,00	14,10	4,18	81,72	100,00	13,06	4,30	82,63	100,00
Maluku	3,30	1,48	95,22	100,00	15,64	4,96	79,40	100,00	10,32	3,45	86,23	100,00
Maluku Utara	3,01	1,92	95,07	100,00	8,40	2,87	88,73	100,00	6,80	2,59	90,61	100,00
Papua Barat	6,59	5,53	87,88	100,00	9,10	5,90	84,99	100,00	8,10	5,75	86,15	100,00
Papua	2,45	3,01	94,54	100,00	1,79	17,20	81,01	100,00	1,96	13,56	84,48	100,00
**Indonesia**	**7,29**	**2,85**	**89,86**	**100,00**	**13,67**	**4,70**	**81,64**	**100,00**	**10,18**	**3,69**	**86,14**	**100,00**

Sumber/*Source*: Susenas Maret 2018/*The March 2018 Susenas*

https://www.bps.go.id

Tabel 8.10.1. **Persentase Rumah Tangga di Daerah Perkotaan dengan Kepemilikan Aset menurut Provinsi dan Jenis Aset yang Dimiliki, 2018**
Table *Percentage of Households in Urban Area with Assets by Province and Type of Assets Owned, 2018*

**Provinsi** *Province*	**Tabung Gas 5,5 kg atau Lebih** *LPG 5,5 Kgs or More*	**Lemari Es/ Kulkas** *Referigerator*	**AC** *Air Conditioner*	**Pemanas Air** *Water Heater*	**Emas/Perhiasan (Minimal 10 Gram)** *Gold/Jewelery (Min 10 Grams)*	**Sepeda Motor** *Motorbike*	**Perahu** *Boat*	**Perahu Motor** *Motor Boat*	**Mobil** *Car*	**TV Layar Datar** *Flat Monitor TV*	**Tanah/Lahan** *Land*
(1)	(2)	(3)	(4)	(5)	(6)	(7)	(8)	(9)	(10)	(11)	(12)
Aceh	33,72	76,61	22,73	2,51	34,20	85,43	1,36	0,93	19,68	22,13	69,93
Sumatera Utara	12,34	69,94	15,07	3,18	18,91	80,88	0,93	0,52	12,99	22,84	53,99
Sumatera Barat	18,58	68,84	5,88	1,79	27,08	82,60	0,46	0,27	21,51	20,06	44,14
Riau	20,97	77,74	24,02	2,90	23,71	90,37	0,48	0,63	19,65	30,38	55,04
Jambi	25,92	76,90	17,06	3,19	25,17	87,22	2,02	0,55	21,13	28,79	68,65
Sumatera Selatan	12,77	76,24	16,40	2,13	26,33	80,13	0,96	0,33	18,76	26,10	64,29
Bengkulu	30,28	78,47	12,98	2,57	23,98	87,32	0,52	0,63	25,32	23,72	69,08
Lampung	10,87	70,67	11,02	5,24	16,16	82,66	0,59	0,50	16,42	17,43	75,77
Kepulauan Bangka Belitung	46,28	85,78	20,91	3,50	28,85	90,99	1,64	2,70	19,11	27,93	75,22
Kepulauan Riau	12,20	79,78	35,95	1,59	22,98	88,70	1,27	1,74	17,65	40,39	33,86
DKI Jakarta	19,20	78,27	29,36	4,55	26,24	77,81	0,43	0,72	17,13	34,52	38,96
Jawa Barat	13,67	66,79	11,00	3,21	21,37	73,94	0,45	0,28	13,81	20,68	61,67
Jawa Tengah	12,33	60,93	7,05	2,95	21,42	80,48	0,71	0,48	13,69	12,94	76,34
DI Yogyakarta	20,15	59,58	8,46	2,94	27,04	87,05	0,28	0,11	19,13	14,71	65,22
Jawa Timur	8,72	66,10	8,36	3,44	20,17	83,99	0,62	0,44	14,16	13,73	69,36
Banten	16,33	82,10	23,38	4,71	22,72	85,37	0,49	0,61	18,49	29,28	69,49
Bali	23,13	67,91	18,42	4,23	29,18	91,15	0,49	0,25	23,96	21,20	47,82
Nusa Tenggara Barat	6,01	41,64	4,79	1,75	14,95	68,61	0,53	0,80	7,88	8,90	77,47
Nusa Tenggara Timur	5,57	48,74	9,31	2,35	16,34	66,72	0,72	1,00	12,08	17,13	67,80
Kalimantan Barat	21,54	80,79	22,39	2,98	30,33	89,65	3,07	2,16	17,90	31,08	76,64
Kalimantan Tengah	29,93	74,26	18,96	3,43	28,71	89,65	2,07	2,69	21,29	23,09	70,01
Kalimantan Selatan	20,32	74,65	19,23	0,32	29,54	84,85	1,48	0,81	17,13	22,43	59,35
Kalimantan Timur	28,37	86,60	25,88	1,86	33,95	89,75	1,10	1,35	21,93	35,32	64,04
Kalimantan Utara	27,35	78,44	22,66	1,36	29,99	82,92	1,77	0,68	12,88	27,93	61,16
Sulawesi Utara	5,99	67,43	12,53	3,74	17,48	56,75	0,89	1,07	16,30	25,83	57,26
Sulawesi Tengah	14,52	67,21	20,63	0,31	17,02	82,10	2,61	2,56	16,95	19,90	65,72
Sulawesi Selatan	19,99	79,37	20,97	3,88	36,98	79,02	1,65	1,23	19,08	26,69	63,99
Sulawesi Tenggara	12,44	67,10	10,49	0,17	22,16	74,43	2,20	3,26	16,24	15,01	72,28
Gorontalo	4,13	60,67	18,27	0,48	11,84	62,21	3,06	1,66	10,13	17,66	63,14
Sulawesi Barat	13,32	62,25	16,08	1,41	30,03	76,03	4,24	1,46	15,76	13,37	66,25
Maluku	2,44	66,42	14,95	2,04	24,18	53,90	1,98	1,58	7,64	27,58	61,16
Maluku Utara	6,09	64,04	22,87	0,15	22,42	73,20	1,90	2,54	10,10	20,30	75,81
Papua Barat	9,79	67,98	20,85	2,46	21,35	70,44	1,60	3,51	9,58	26,06	56,28
Papua	5,58	63,33	17,65	9,62	25,36	70,03	4,03	3,31	13,17	29,86	47,52
**Indonesia**	**14,56**	**69,03**	**14,17**	**3,27**	**22,83**	**79,88**	**0,75**	**0,62**	**15,53**	**21,17**	**63,22**

Sumber/*Source*: Susenas Maret 2018/*The March 2018 Susenas*

https://www.bps.go.id

Tabel 8.10.2. **Persentase Rumah Tangga di Daerah Perdesaan dengan Kepemilikan Aset menurut Provinsi dan Jenis Aset yang Dimiliki, 2018**
Table **8.10.2.** *Percentage of Households in Rural Area with Assets by Province and Type of Assets Owned, 2018*

**Provinsi** *Province*	**Tabung Gas 5,5 kg atau Lebih** *LPG 5,5 Kgs or More*	**Lemari Es/ Kulkas** *Referigerator*	**AC** *Air Conditioner*	**Pemanas Air** *Water Heater*	**Emas/Perhiasan (Minimal 10 Gram)** *Gold/Jewelery (Min 10 Grams)*	**Sepeda Motor** *Motorbike*	**Perahu** *Boat*	**Perahu Motor** *Motor Boat*	**Mobil** *Car*	**TV Layar Datar** *Flat Monitor TV*	**Tanah/Lahan** *Land*
(1)	(2)	(3)	(4)	(5)	(6)	(7)	(8)	(9)	(10)	(11)	(12)
Aceh	11,02	51,37	3,35	1,11	16,63	80,34	1,50	1,14	6,45	6,24	83,08
Sumatera Utara	3,79	36,85	1,46	0,68	10,53	72,38	0,67	0,85	5,86	7,55	69,95
Sumatera Barat	8,44	48,19	0,87	0,87	15,80	78,64	1,66	0,93	10,31	9,12	57,79
Riau	7,37	57,16	2,75	1,75	15,80	86,88	3,85	3,51	9,10	10,93	74,58
Jambi	12,15	54,40	1,75	1,52	17,19	87,25	3,57	2,40	10,30	8,51	82,89
Sumatera Selatan	3,66	45,19	0,95	0,57	11,66	81,37	4,74	2,88	6,90	5,62	81,22
Bengkulu	6,68	49,79	0,88	1,54	13,30	81,51	0,96	0,62	10,41	5,47	89,27
Lampung	3,42	41,62	1,10	0,71	7,40	85,69	0,68	0,78	7,09	4,73	92,90
Kepulauan Bangka Belitung	29,35	75,30	4,28	1,64	22,15	91,88	3,29	4,10	11,39	11,34	84,53
Kepulauan Riau	9,11	44,66	2,83	0,76	14,93	70,92	14,03	18,70	3,26	14,57	64,38
DKI Jakarta	-	-	-	-	-	-	-	-	-	-	-
Jawa Barat	3,52	41,15	1,40	1,65	11,42	61,38	0,66	0,33	4,15	5,90	71,33
Jawa Tengah	4,13	39,55	1,08	1,14	14,28	77,33	0,62	0,54	7,22	4,91	87,05
DI Yogyakarta	6,12	38,27	1,00	0,47	16,63	78,52	0,15	0,06	8,79	4,05	90,54
Jawa Timur	2,15	39,93	0,63	0,83	13,36	79,74	0,70	0,46	6,83	3,63	81,79
Banten	1,97	46,83	1,25	2,16	11,79	65,26	0,54	1,17	4,86	4,95	81,52
Bali	7,76	39,31	2,20	0,84	17,12	82,94	0,65	1,59	15,19	6,73	64,42
Nusa Tenggara Barat	1,91	28,52	0,47	0,81	7,44	62,09	1,10	1,25	4,00	2,92	88,97
Nusa Tenggara Timur	0,21	9,49	0,37	0,39	2,87	35,47	1,98	1,08	2,12	2,50	85,45
Kalimantan Barat	7,53	38,43	1,40	1,26	11,63	80,89	8,19	4,39	4,04	7,05	84,40
Kalimantan Tengah	20,43	45,23	1,44	1,19	16,92	77,06	6,35	13,51	7,38	6,37	76,94
Kalimantan Selatan	8,82	52,65	1,64	0,02	19,37	80,18	8,01	6,88	7,02	7,00	80,68
Kalimantan Timur	15,46	58,05	2,22	0,59	16,81	85,16	6,81	6,43	11,90	14,29	78,16
Kalimantan Utara	32,63	56,95	5,90	1,01	17,21	78,80	14,54	11,37	10,33	15,80	81,56
Sulawesi Utara	1,76	52,33	1,89	1,72	8,29	53,62	3,39	3,62	8,05	8,47	73,87
Sulawesi Tengah	2,40	38,13	1,97	0,03	6,48	72,25	5,02	4,71	5,41	4,34	85,62
Sulawesi Selatan	9,45	59,53	2,29	1,21	24,34	72,41	1,62	2,37	8,77	7,08	86,26
Sulawesi Tenggara	4,57	44,42	1,52	0,03	11,89	70,52	6,32	5,82	6,63	4,56	88,40
Gorontalo	1,17	42,99	2,70	0,20	3,63	59,66	3,55	1,67	5,35	5,90	79,27
Sulawesi Barat	4,07	31,64	1,12	0,87	7,99	69,77	3,36	2,09	6,06	3,03	84,76
Maluku	0,20	29,14	1,54	0,94	4,91	35,70	12,22	4,68	2,00	4,78	71,90
Maluku Utara	0,95	25,82	1,29	0,01	5,85	45,09	10,20	9,25	3,27	5,63	93,56
Papua Barat	2,99	37,58	5,51	1,41	12,66	56,67	9,53	8,55	7,01	13,44	65,09
Papua	0,44	9,72	0,80	1,81	3,53	18,03	7,61	2,50	1,43	3,68	47,71
**Indonesia**	**4,82**	**41,47**	**1,33**	**1,05**	**12,71**	**72,86**	**2,19**	**1,81**	**6,56**	**5,67**	**80,19**

Sumber/*Source*: Susenas Maret 2018/*The March 2018 Susenas*

https://www.bps.go.id

Tabel 8.10.3. **Persentase Rumah Tangga di Daerah Perkotaan dan Perdesaan dengan Kepemilikan Aset menurut Provinsi dan Jenis Aset yang Dimiliki, 2018**
Table *Percentage of Households in Urban and Rural Area with Assets by Province and Type of Assets Owned, 2018*

**Provinsi** *Province*	**Tabung Gas 5,5 kg atau Lebih** *LPG 5,5 Kgs or More*	**Lemari Es/ Kulkas** *Referigerator*	**AC** *Air Conditioner*	**Pemanas Air** *Water Heater*	**Emas/Perhiasan (Minimal 10 Gram)** *Gold/Jewelery (Min 10 Grams)*	**Sepeda Motor** *Motorbike*	**Perahu** *Boat*	**Perahu Motor** *Motor Boat*	**Mobil** *Car*	**TV Layar Datar** *Flat Monitor TV*	**Tanah/Lahan** *Land*
(1)	(2)	(3)	(4)	(5)	(6)	(7)	(8)	(9)	(10)	(11)	(12)
Aceh	18,11	59,26	9,40	1,55	22,12	81,93	1,46	1,07	10,58	11,21	78,97
Sumatera Utara	8,28	54,21	8,60	1,99	14,93	76,84	0,81	0,68	9,60	15,58	61,57
Sumatera Barat	12,91	57,30	3,08	1,27	20,78	80,39	1,13	0,63	15,26	13,95	51,76
Riau	12,86	65,47	11,34	2,21	18,99	88,29	2,49	2,35	13,36	18,78	66,69
Jambi	16,46	61,43	6,54	2,05	19,68	87,24	3,09	1,82	13,69	14,85	78,44
Sumatera Selatan	6,92	56,31	6,48	1,13	16,92	80,93	3,39	1,97	11,15	12,96	75,16
Bengkulu	14,25	58,99	4,76	1,87	16,73	83,37	0,82	0,62	15,19	11,33	82,79
Lampung	5,50	49,72	3,87	1,98	9,84	84,85	0,66	0,70	9,70	8,27	88,12
Kepulauan Bangka Belitung	38,38	80,89	13,16	2,63	25,73	91,40	2,41	3,35	15,51	20,19	79,56
Kepulauan Riau	11,77	74,90	31,34	1,48	21,86	86,23	3,05	4,10	15,65	36,79	38,11
DKI Jakarta	19,20	78,27	29,36	4,55	26,24	77,81	0,43	0,72	17,13	34,52	38,96
Jawa Barat	10,88	59,76	8,37	2,78	18,64	70,50	0,50	0,29	11,16	16,63	64,32
Jawa Tengah	8,29	50,39	4,11	2,06	17,90	78,93	0,66	0,51	10,50	8,98	81,62
DI Yogyakarta	16,45	53,96	6,50	2,29	24,30	84,80	0,25	0,10	16,40	11,90	71,90
Jawa Timur	5,57	53,56	4,65	2,19	16,90	81,95	0,66	0,45	10,64	8,89	75,32
Banten	12,24	72,07	17,08	3,99	19,61	79,65	0,50	0,77	14,62	22,36	72,91
Bali	18,06	58,48	13,07	3,11	25,21	88,44	0,54	0,69	21,07	16,42	53,29
Nusa Tenggara Barat	3,80	34,58	2,46	1,24	10,91	65,10	0,84	1,04	5,79	5,68	83,66
Nusa Tenggara Timur	1,48	18,78	2,49	0,85	6,06	42,86	1,68	1,06	4,48	5,97	81,28
Kalimantan Barat	12,13	52,34	8,30	1,83	17,77	83,77	6,51	3,66	8,59	14,94	81,85
Kalimantan Tengah	24,01	56,17	8,04	2,03	21,36	81,81	4,74	9,43	12,62	12,67	74,33
Kalimantan Selatan	14,16	62,87	9,80	0,16	24,09	82,35	4,97	4,06	11,71	14,16	70,78
Kalimantan Timur	24,09	77,14	18,04	1,44	28,27	88,23	2,99	3,03	18,61	28,35	68,72
Kalimantan Utara	29,49	69,72	15,86	1,22	24,81	81,25	6,95	5,02	11,85	23,01	69,44
Sulawesi Utara	3,88	59,90	7,23	2,74	12,90	55,19	2,14	2,34	12,19	17,18	65,54
Sulawesi Tengah	5,84	46,38	7,26	0,11	9,47	75,04	4,34	4,10	8,69	8,75	79,98
Sulawesi Selatan	13,70	67,53	9,83	2,29	29,44	75,08	1,63	1,91	12,93	14,99	77,27
Sulawesi Tenggara	7,57	53,06	4,94	0,09	15,80	72,01	4,75	4,84	10,29	8,54	82,26
Gorontalo	2,33	49,95	8,82	0,31	6,86	60,67	3,35	1,66	7,23	10,53	72,93
Sulawesi Barat	6,22	38,77	4,61	0,99	13,13	71,23	3,56	1,94	8,32	5,44	80,44
Maluku	1,17	45,23	7,33	1,41	13,23	43,56	7,80	3,34	4,43	14,62	67,26
Maluku Utara	2,47	37,17	7,70	0,05	10,77	53,44	7,73	7,26	5,30	9,99	88,28
Papua Barat	5,71	49,74	11,64	1,83	16,14	62,18	6,36	6,54	8,04	18,48	61,57
Papua	1,76	23,49	5,13	3,81	9,14	31,39	6,69	2,71	4,45	10,41	47,66
**Indonesia**	**10,15**	**56,55**	**8,36**	**2,26**	**18,25**	**76,70**	**1,40**	**1,16**	**11,47**	**14,15**	**70,90**

Sumber/*Source*: Susenas Maret 2018/*The March 2018 Susenas*

https://www.bps.go.id

ESTIMASI *SAMPLING ERROR*

SAMPLING ERROR ESTIMATE

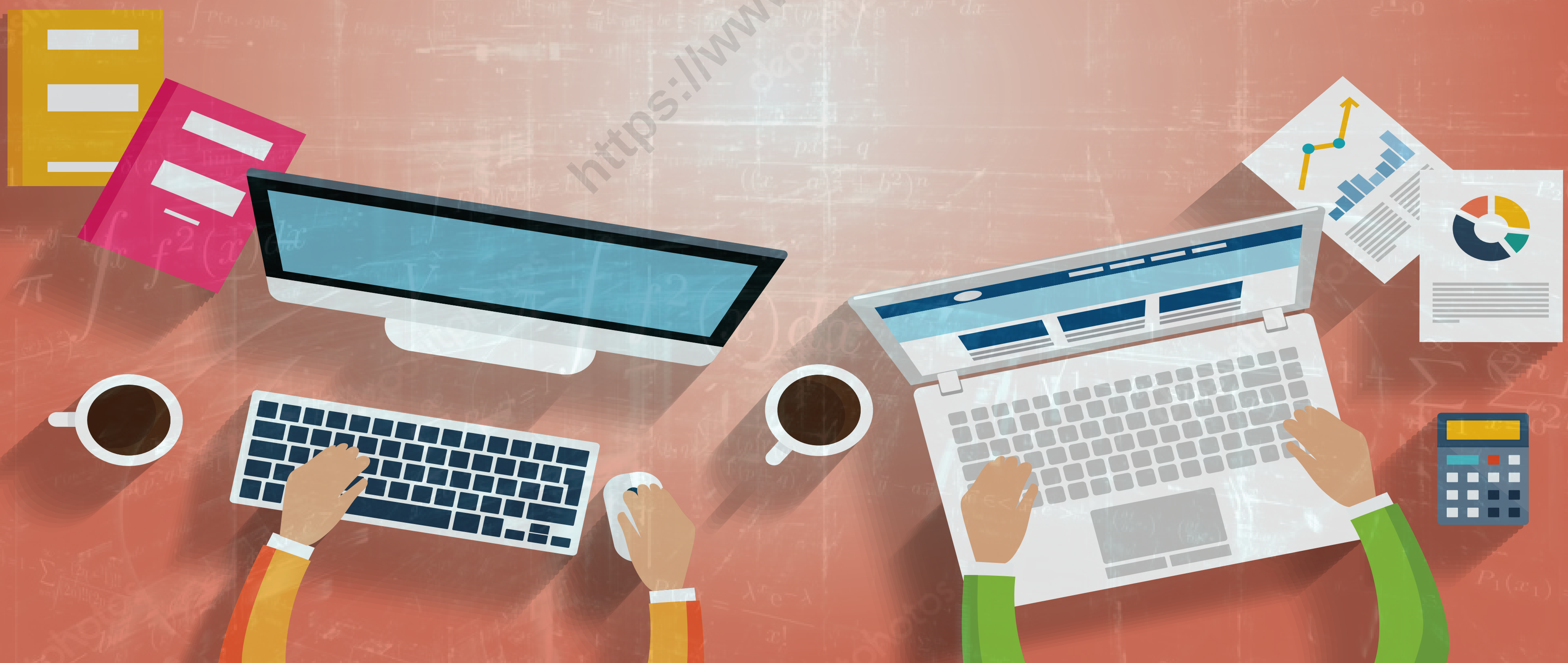

https://www.bps.go.id

https://www.bps.go.id

IX. ESTIMASI *SAMPLING ERROR*
SAMPLING ERROR ESTIMATION

PENJELASAN TEKNIS

Estimasi dari sampel survei dipengaruhi oleh dua jenis *error* (kesalahan) yaitu *sampling error* dan *non-sampling error* (seperti kesalahan dalam wawancara dan kesalahan pengolahan).

Sampling error adalah kesalahan yang ditimbulkan dari penggunaan teknik *sampling* dalam suatu survei. Besarnya *sampling error* secara teori statistik ditunjukan oleh besarnya angka *standard error* dari suatu angka estimasi persentase suatu variabel yang disajikan dari hasil Susenas 2018. Untuk mengukur presisi dari suatu angka estimasi digunakan besarnya *relative standard error*, yaitu rasio dari nilai *standard error* dengan nilai estimasi suatu variabel, yang dinyatakan dalam persentase (%). Dengan menggunakan selang kepercayaan 95 persen, dapat disajikan estimasi interval (*interval estimation*) dengan batas bawah sebesar nilai estimasi dikurangi dua *standard error*.

Penghitungan *sampling error* pada variabel Susenas 2018 menggunakan *software* SPSS versi 20, variabel yang dihitung *sampling error*-nya terbatas dengan rincian seperti terdapat pada tabel berikut:

TECHNICAL NOTES

The estimates from a sample survey are affected by two types of errors, those are: sampling errors and non-sampling errors (such as mistakes made in implementing data collection and data processing).

Sampling errors are mistakes made by sampling technique using in a survey. The value of sampling errors statistically is usually measured in term of the standard error for a particular statistic percentage estimate of variables resulted in the 2018 Susenas. In order to measure the precision of estimate value, the relative standard error, the ratio between standard errors and estimate value of variables, is used and presented in the form of percentage value (%). The standard errors also could be used to calculate interval estimation using 95 percent confidence interval with lower limit equal to value estimation minus two times the standard errors and upper limit equal to value estimation plus two times the standard errors.

Calculation of sampling error in the 2018 Susenas is using statistical software program SPSS version 20, the sampling errors measured only for several variables as listed in the following table:

https://www.bps.go.id

Tabel **9.1.** ***Sampling Error*** **Persentase Penduduk yang Mempunyai Keluhan Kesehatan Selama Sebulan Terakhir menurut Provinsi dan Daerah Tempat Tinggal, 2018**

Table *Sampling Error Percentage of Population Who had Health Complaint during the Last Month by Province and Urban Rural Classification, 2018*

Provinsi / *Province*	Perkotaan/*Urban*					Perdesaan/*Rural*					Perkotaan+Perdesaan/*Urban+Rural*				
	Estimasi / *Estimate*	*Standard Error*	*Relative Standard Error (%)*	Selang Kepercayaan 95% / *Confidence Interval 95%*		Estimasi / *Estimate*	*Standard Error*	*Relative Standard Error (%)*	Selang Kepercayaan 95% / *Confidence Interval 95%*		Estimasi / *Estimate*	*Standard Error*	*Relative Standard Error (%)*	Selang Kepercayaan 95% / *Confidence Interval 95%*	
				Batas Bawah / *Lower Limit*	Batas Atas / *Upper Limit*				Batas Bawah / *Lower Limit*	Batas Atas / *Upper Limit*				Batas Bawah / *Lower Limit*	Batas Atas / *Upper Limit*
(1)	(2)	(3)	(4)	(5)	(6)	(7)	(8)	(9)	(10)	(11)	(12)	(13)	(14)	(15)	(16)
Aceh	27,15	1,02	3,75	25,16	29,15	31,56	0,72	2,27	30,15	32,96	30,18	0,60	1,99	29,00	31,35
Sumatera Utara	24,64	0,75	3,05	23,16	26,11	25,12	0,66	2,61	23,83	26,40	24,86	0,50	2,02	23,88	25,85
Sumatera Barat	29,94	1,08	3,62	27,82	32,07	28,92	0,70	2,43	27,55	30,30	29,37	0,62	2,11	28,16	30,59
Riau	28,35	1,06	3,74	26,27	30,43	30,47	1,00	3,30	28,50	32,44	29,61	0,74	2,50	28,16	31,07
Jambi	21,43	1,52	7,10	18,45	24,42	22,88	0,81	3,54	21,29	24,46	22,41	0,73	3,27	20,98	23,85
Sumatera Selatan	31,27	1,48	4,73	28,37	34,17	26,96	0,78	2,89	25,43	28,49	28,57	0,75	2,63	27,10	30,04
Bengkulu	24,72	1,20	4,84	22,38	27,07	28,21	1,16	4,10	25,94	30,48	27,07	0,87	3,21	25,37	28,78
Lampung	29,82	1,39	4,67	27,09	32,55	31,01	0,75	2,40	29,55	32,47	30,66	0,67	2,17	29,35	31,96
Kep. Bangka Belitung	29,56	1,24	4,20	27,13	31,99	28,67	1,29	4,50	26,15	31,20	29,15	0,89	3,06	27,40	30,90
Kep. Riau	21,96	1,32	6,01	19,37	24,55	25,05	1,45	5,78	22,21	27,88	22,40	1,15	5,14	20,14	24,66
DKI Jakarta	28,83	0,94	3,25	26,99	30,66	-	-	-	-	-	28,83	0,94	3,25	26,99	30,66
Jawa Barat	30,24	0,53	1,76	29,19	31,28	33,30	0,77	2,30	31,80	34,81	31,03	0,44	1,43	30,16	31,90
Jawa Tengah	34,95	0,58	1,66	33,81	36,08	33,73	0,54	1,62	32,66	34,80	34,36	0,40	1,16	33,57	35,14
DI Yogyakarta	35,18	1,21	3,43	32,82	37,55	35,68	2,21	6,21	31,34	40,02	35,32	1,05	2,99	33,25	37,38
Jawa Timur	33,34	0,58	1,73	32,21	34,47	34,31	0,57	1,65	33,20	35,42	33,80	0,41	1,20	33,01	34,60
Banten	30,58	1,13	3,68	28,37	32,79	36,92	1,68	4,54	33,63	40,21	32,41	0,93	2,87	30,59	34,24
Bali	27,52	1,03	3,73	25,51	29,53	35,83	1,96	5,46	32,00	39,66	30,33	0,96	3,16	28,45	32,21
Nusa Tenggara Barat	39,63	1,32	3,32	37,04	42,21	37,75	1,06	2,80	35,68	39,83	38,62	0,84	2,17	36,98	40,27
Nusa Tenggara Timur	35,26	1,69	4,81	31,94	38,58	38,43	0,76	1,96	36,95	39,91	37,71	0,70	1,85	36,34	39,07
Kalimantan Barat	27,06	1,09	4,02	24,93	29,19	29,51	0,93	3,14	27,70	31,33	28,68	0,72	2,52	27,27	30,10
Kalimantan Tengah	27,70	1,29	4,65	25,18	30,22	28,47	1,00	3,50	26,52	30,42	28,17	0,79	2,82	26,62	29,73
Kalimantan Selatan	32,59	1,28	3,94	30,08	35,10	33,84	1,01	2,98	31,86	35,81	33,26	0,81	2,42	31,68	34,84
Kalimantan Timur	29,08	1,25	4,29	26,63	31,53	25,24	1,78	7,04	21,76	28,73	27,81	1,03	3,69	25,80	29,82
Kalimantan Utara	28,90	1,63	5,65	25,70	32,10	34,14	2,96	8,66	28,34	39,93	31,09	1,59	5,12	27,97	34,21
Sulawesi Utara	26,67	1,08	4,04	24,56	28,78	31,37	1,00	3,19	29,41	33,33	29,01	0,74	2,56	27,55	30,47
Sulawesi Tengah	32,27	1,81	5,62	28,72	35,83	31,12	1,01	3,25	29,13	33,10	31,44	0,89	2,82	29,70	33,18
Sulawesi Selatan	30,37	0,93	3,06	28,55	32,19	28,57	0,66	2,30	27,28	29,86	29,32	0,55	1,87	28,24	30,40
Sulawesi Tenggara	26,53	1,59	6,00	23,41	29,65	28,60	0,85	2,96	26,94	30,26	27,81	0,80	2,86	26,25	29,37
Gorontalo	35,04	1,74	4,96	31,64	38,44	38,15	1,77	4,63	34,69	41,61	36,95	1,27	3,44	34,46	39,44
Sulawesi Barat	31,39	3,12	9,94	25,27	37,50	28,88	1,17	4,04	26,59	31,17	29,47	1,19	4,02	27,15	31,80
Maluku	19,34	1,26	6,51	16,88	21,81	20,46	0,90	4,40	18,70	22,22	19,99	0,74	3,70	18,54	21,45
Maluku Utara	20,35	1,39	6,82	17,63	23,07	19,78	0,91	4,59	18,00	21,56	19,94	0,76	3,82	18,45	21,44
Papua Barat	24,33	1,73	7,10	20,94	27,72	23,18	1,15	4,95	20,94	25,43	23,65	0,99	4,17	21,72	25,58
Papua	20,47	1,20	5,87	18,12	22,82	16,50	0,73	4,42	15,07	17,93	17,60	0,63	3,58	16,36	18,83
**Indonesia**	**30,63**	**0,22**	**0,71**	**30,20**	**31,05**	**31,37**	**0,19**	**0,60**	**31,00**	**31,73**	**30,96**	**0,15**	**0,47**	**30,68**	**31,25**

Sumber/*Source*: Susenas Maret 2018/*The March 2018 Susenas*

https://www.bps.go.id

Tabel *Table* **9.2.**

***Sampling Error* Persentase Penduduk Berumur 0-17 Tahun yang tidak Memiliki Akta Kelahiran dari Kantor Catatan Sipil menurut Provinsi dan Daerah Tempat Tinggal, 2018**

Sampling Errors Percentage Population Aged 0-17 Year Old Who does not Have a Birth Certificate From a Civil Registry Office by Province and Urban Rural Classification, 2018

Provinsi *Province*	Perkotaan/*Urban*					Perdesaan/*Rural*					Perkotaan+Perdesaan/*Urban+Rural*				
	Estimasi *Estimate*	*Standard Error*	*Relative Standard Error (%)*	Selang Kepercayaan 95% *Confidence Interval 95%*		Estimasi *Estimate*	*Standard Error*	*Relative Standard Error (%)*	Selang Kepercayaan 95% *Confidence Interval 95%*		Estimasi *Estimate*	*Standard Error*	*Relative Standard Error (%)*	Selang Kepercayaan 95% *Confidence Interval 95%*	
				Batas Bawah *Lower Limit*	Batas Atas *Upper Limit*				Batas Bawah *Lower Limit*	Batas Atas *Upper Limit*				Batas Bawah *Lower Limit*	Batas Atas *Upper Limit*
(1)	(2)	(3)	(4)	(5)	(6)	(7)	(8)	(9)	(10)	(11)	(12)	(13)	(14)	(15)	(16)
Aceh	6,96	0,72	10,37	5,54	8,37	14,21	0,64	4,50	12,95	15,46	12,01	0,50	4,20	11,02	13,00
Sumatera Utara	21,17	0,94	4,43	19,33	23,01	33,15	0,87	2,63	31,45	34,86	27,18	0,65	2,39	25,91	28,45
Sumatera Barat	9,79	0,83	8,42	8,18	11,41	17,52	0,74	4,25	16,06	18,98	14,22	0,57	3,99	13,11	15,34
Riau	17,42	1,39	7,96	14,70	20,14	26,27	1,10	4,19	24,11	28,43	22,78	0,86	3,79	21,09	24,48
Jambi	4,42	0,70	15,79	3,05	5,79	10,88	0,74	6,81	9,43	12,33	8,86	0,57	6,42	7,74	9,97
Sumatera Selatan	7,55	0,73	9,68	6,12	8,98	12,93	0,67	5,18	11,62	14,24	10,98	0,51	4,61	9,99	11,97
Bengkulu	6,32	0,67	10,62	5,01	7,64	10,41	0,68	6,57	9,07	11,75	9,08	0,51	5,67	8,07	10,08
Lampung	10,89	1,17	10,70	8,60	13,17	11,86	0,56	4,70	10,76	12,95	11,58	0,53	4,54	10,54	12,61
Kep. Bangka Belitung	2,61	0,56	21,36	1,52	3,70	7,21	0,88	12,22	5,48	8,94	4,77	0,51	10,80	3,76	5,77
Kep. Riau	6,56	0,80	12,16	4,99	8,12	9,00	1,17	12,96	6,71	11,29	6,91	0,71	10,25	5,52	8,30
DKI Jakarta	3,61	0,37	10,23	2,89	4,34	-	-	-	-	-	3,61	0,37	10,23	2,89	4,34
Jawa Barat	16,58	0,68	4,13	15,24	17,92	23,70	0,87	3,66	21,99	25,40	18,43	0,56	3,02	17,34	19,52
Jawa Tengah	4,64	0,26	5,67	4,13	5,16	7,31	0,43	5,91	6,47	8,16	5,95	0,25	4,29	5,45	6,45
DI Yogyakarta	2,75	0,62	22,57	1,53	3,96	2,00	0,49	24,34	1,04	2,95	2,54	0,47	18,56	1,62	3,47
Jawa Timur	9,10	0,51	5,62	8,10	10,10	18,43	0,63	3,44	17,19	19,68	13,53	0,42	3,10	12,70	14,35
Banten	14,26	1,00	7,02	12,30	16,23	42,77	2,02	4,72	38,81	46,73	23,11	0,98	4,24	21,18	25,03
Bali	6,79	0,59	8,66	5,63	7,94	14,50	1,22	8,40	12,11	16,89	9,44	0,59	6,24	8,29	10,60
Nusa Tenggara Barat	12,99	0,97	7,44	11,10	14,89	25,16	1,19	4,75	22,82	27,50	19,60	0,82	4,18	17,99	21,21
Nusa Tenggara Timur	22,59	1,32	5,85	20,00	25,18	46,60	0,88	1,88	44,89	48,32	41,54	0,80	1,92	39,98	43,11
Kalimantan Barat	6,86	0,85	12,33	5,20	8,51	22,47	1,02	4,53	20,47	24,47	17,36	0,79	4,57	15,81	18,92
Kalimantan Tengah	8,88	0,82	9,23	7,28	10,49	22,28	1,35	6,07	19,63	24,94	17,19	0,96	5,60	15,31	19,08
Kalimantan Selatan	7,83	0,83	10,55	6,21	9,45	13,38	0,89	6,66	11,63	15,12	10,85	0,62	5,73	9,63	12,06
Kalimantan Timur	6,36	0,71	11,18	4,97	7,76	9,48	1,14	12,00	7,25	11,71	7,43	0,61	8,22	6,23	8,63
Kalimantan Utara	6,44	0,96	14,94	4,55	8,33	14,32	1,76	12,28	10,87	17,77	9,83	0,96	9,78	7,95	11,72
Sulawesi Utara	10,85	1,00	9,21	8,89	12,81	17,42	0,93	5,32	15,61	19,24	14,20	0,69	4,85	12,85	15,55
Sulawesi Tengah	19,09	2,17	11,39	14,83	23,35	28,25	1,21	4,29	25,88	30,62	25,77	1,04	4,05	23,73	27,82
Sulawesi Selatan	9,16	0,73	8,00	7,72	10,60	15,09	0,57	3,76	13,98	16,20	12,67	0,46	3,60	11,78	13,56
Sulawesi Tenggara	12,63	1,37	10,87	9,94	15,32	21,30	1,04	4,89	19,26	23,35	18,10	0,87	4,81	16,39	19,81
Gorontalo	5,77	1,11	19,22	3,60	7,94	12,19	1,01	8,32	10,20	14,18	9,80	0,77	7,89	8,28	11,32
Sulawesi Barat	11,53	1,54	13,33	8,51	14,54	15,84	1,05	6,60	13,79	17,89	14,83	0,88	5,95	13,10	16,56
Maluku	17,18	1,73	10,10	13,78	20,58	31,50	1,31	4,16	28,93	34,07	25,96	1,08	4,14	23,85	28,07
Maluku Utara	7,85	0,83	10,63	6,21	9,49	29,02	1,36	4,68	26,36	31,68	23,48	1,12	4,78	21,28	25,68
Papua Barat	16,59	1,47	8,89	13,70	19,48	36,20	2,06	5,69	32,17	40,24	28,49	1,41	4,96	25,72	31,26
Papua	21,05	1,48	7,05	18,14	23,96	76,53	1,16	1,52	74,25	78,81	62,49	1,12	1,79	60,30	64,68
**Indonesia**	**11,42**	**0,22**	**1,95**	**10,99**	**11,86**	**21,48**	**0,20**	**0,92**	**21,09**	**21,87**	**16,13**	**0,15**	**0,94**	**15,84**	**16,43**

Sumber/*Source*: Susenas Maret 2018/*The March 2018 Susenas*

https://www.bps.go.id

Tabel 9.3. ***Sampling Error*** **Persentase Penduduk yang Pernah Rawat Inap dalam Setahun Terakhir menurut Provinsi dan Daerah Tempat Tinggal, 2018**

Table *Sampling Errors Percentage of Population Who Had Have Hospitalized during the Last Year by Province and Urban Rural Classification, 2018*

**Provinsi** *Province*	**Perkotaan/***Urban*					**Perdesaan/***Rural*					**Perkotaan+Perdesaan/***Urban+Rural*				
	**Estimasi** *Estimate*	*Standard Error*	*Relative Standard Error (%)*	**Selang Kepercayaan 95%** *Confidence Interval 95%*		**Estimasi** *Estimate*	*Standard Error*	*Relative Standard Error (%)*	**Selang Kepercayaan 95%** *Confidence Interval 95%*		**Estimasi** *Estimate*	*Standard Error*	*Relative Standard Error (%)*	**Selang Kepercayaan 95%** *Confidence Interval 95%*	
				**Batas Bawah** *Lower Limit*	**Batas Atas** *Upper Limit*				**Batas Bawah** *Lower Limit*	**Batas Atas** *Upper Limit*				**Batas Bawah** *Lower Limit*	**Batas Atas** *Upper Limit*
(1)	(2)	(3)	(4)	(5)	(6)	(7)	(8)	(9)	(10)	(11)	(12)	(13)	(14)	(15)	(16)
Aceh	7,46	0,35	4,70	6,77	8,15	6,28	0,20	3,23	5,88	6,68	6,65	0,18	2,70	6,30	7,00
Sumatera Utara	3,95	0,18	4,54	3,60	4,30	3,12	0,14	4,47	2,85	3,40	3,56	0,12	3,24	3,33	3,79
Sumatera Barat	5,65	0,29	5,05	5,09	6,21	4,18	0,18	4,38	3,82	4,54	4,83	0,16	3,34	4,51	5,14
Riau	4,86	0,27	5,49	4,34	5,38	3,37	0,18	5,31	3,02	3,72	3,97	0,15	3,85	3,67	4,27
Jambi	4,82	0,36	7,48	4,11	5,53	3,52	0,18	5,15	3,16	3,87	3,93	0,17	4,28	3,60	4,26
Sumatera Selatan	3,96	0,23	5,88	3,51	4,42	3,00	0,15	4,98	2,70	3,29	3,36	0,13	3,83	3,10	3,61
Bengkulu	7,00	0,40	5,71	6,21	7,78	3,89	0,26	6,73	3,38	4,40	4,91	0,22	4,51	4,47	5,34
Lampung	5,13	0,34	6,56	4,47	5,79	4,03	0,16	3,98	3,71	4,34	4,35	0,15	3,48	4,06	4,65
Kep. Bangka Belitung	4,62	0,30	6,46	4,04	5,21	3,30	0,29	8,73	2,73	3,86	4,01	0,22	5,44	3,58	4,44
Kep. Riau	3,65	0,36	9,99	2,93	4,36	3,21	0,56	17,52	2,11	4,31	3,59	0,32	9,00	2,95	4,22
DKI Jakarta	4,38	0,22	5,08	3,95	4,82	-	-	-	-	-	4,38	0,22	5,08	3,95	4,82
Jawa Barat	4,67	0,13	2,78	4,41	4,92	4,03	0,16	3,96	3,71	4,34	4,50	0,10	2,33	4,30	4,71
Jawa Tengah	6,23	0,15	2,33	5,94	6,51	5,72	0,14	2,40	5,45	5,99	5,98	0,10	1,68	5,78	6,18
DI Yogyakarta	6,46	0,32	4,92	5,84	7,09	6,25	0,43	6,80	5,41	7,08	6,41	0,26	4,10	5,89	6,92
Jawa Timur	5,07	0,13	2,60	4,82	5,33	4,56	0,12	2,59	4,33	4,79	4,83	0,09	1,85	4,65	5,00
Banten	4,22	0,23	5,37	3,78	4,67	2,22	0,20	8,95	1,83	2,61	3,64	0,17	4,70	3,31	3,98
Bali	4,52	0,22	4,78	4,09	4,94	4,18	0,31	7,30	3,58	4,78	4,40	0,18	4,04	4,05	4,75
Nusa Tenggara Barat	5,82	0,35	5,99	5,14	6,51	6,35	0,34	5,32	5,69	7,01	6,11	0,24	3,96	5,63	6,58
Nusa Tenggara Timur	5,76	0,38	6,59	5,02	6,51	3,66	0,13	3,51	3,41	3,91	4,14	0,13	3,20	3,88	4,40
Kalimantan Barat	5,38	0,31	5,74	4,78	5,99	2,78	0,15	5,24	2,50	3,07	3,66	0,15	4,10	3,37	3,96
Kalimantan Tengah	4,22	0,33	7,80	3,57	4,86	2,70	0,19	6,85	2,34	3,07	3,29	0,17	5,29	2,95	3,63
Kalimantan Selatan	3,80	0,25	6,67	3,30	4,29	3,33	0,18	5,45	2,98	3,69	3,55	0,15	4,29	3,25	3,85
Kalimantan Timur	5,62	0,30	5,36	5,03	6,21	4,48	0,38	8,47	3,73	5,22	5,24	0,24	4,53	4,77	5,71
Kalimantan Utara	7,22	0,62	8,55	6,01	8,43	5,24	0,55	10,52	4,16	6,32	6,39	0,43	6,67	5,56	7,23
Sulawesi Utara	6,44	0,35	5,50	5,74	7,13	5,93	0,26	4,33	5,42	6,43	6,18	0,22	3,55	5,75	6,61
Sulawesi Tengah	7,38	0,57	7,76	6,25	8,50	4,93	0,22	4,43	4,50	5,36	5,61	0,23	4,05	5,17	6,06
Sulawesi Selatan	7,17	0,29	4,10	6,59	7,74	5,29	0,17	3,26	4,96	5,63	6,07	0,16	2,62	5,76	6,39
Sulawesi Tenggara	4,73	0,51	10,69	3,74	5,72	3,78	0,21	5,43	3,38	4,18	4,14	0,23	5,65	3,68	4,60
Gorontalo	7,84	0,53	6,77	6,80	8,88	4,89	0,34	6,87	4,23	5,55	6,03	0,30	4,97	5,44	6,62
Sulawesi Barat	6,63	0,69	10,38	5,28	7,98	3,07	0,24	7,87	2,60	3,54	3,91	0,27	6,87	3,38	4,44
Maluku	3,98	0,33	8,29	3,33	4,62	1,97	0,19	9,56	1,60	2,34	2,81	0,18	6,35	2,46	3,15
Maluku Utara	5,42	0,48	8,79	4,49	6,36	2,51	0,20	7,81	2,13	2,90	3,35	0,20	5,86	2,96	3,73
Papua Barat	5,89	0,56	9,42	4,80	6,98	3,80	0,33	8,68	3,16	4,45	4,65	0,30	6,55	4,06	5,25
Papua	4,97	0,40	8,08	4,19	5,76	1,68	0,14	8,37	1,40	1,95	2,59	0,16	5,99	2,29	2,89
**Indonesia**	**5,06**	**0,05**	**1,02**	**4,96**	**5,16**	**4,24**	**0,04**	**0,94**	**4,16**	**4,32**	**4,69**	**0,03**	**0,72**	**4,62**	**4,75**

Sumber/*Source*: Susenas Maret 2018/*The March 2018 Susenas*

https://www.bps.go.id

Tabel **9.4.** ***Sampling Error*** **Persentase Penduduk yang Memiliki Jaminan Kesehatan Jamkesda menurut Provinsi dan Daerah Tempat Tinggal, 2018**

Table *Sampling Errors Percentage of Population Who Have Health Insurance Jamkesda by Province and Urban Rural Classification, 2018*

**Provinsi** *Province*	**Perkotaan/***Urban*					**Perdesaan/***Rural*					**Perkotaan+Perdesaan/***Urban+Rural*				
	**Estimasi** *Estimate*	*Standard Error*	*Relative Standard Error (%)*	**Selang Kepercayaan 95%** *Confidence Interval 95%*		**Estimasi** *Estimate*	*Standard Error*	*Relative Standard Error (%)*	**Selang Kepercayaan 95%** *Confidence Interval 95%*		**Estimasi** *Estimate*	*Standard Error*	*Relative Standard Error (%)*	**Selang Kepercayaan 95%** *Confidence Interval 95%*	
				**Batas Bawah** *Lower Limit*	**Batas Atas** *Upper Limit*				**Batas Bawah** *Lower Limit*	**Batas Atas** *Upper Limit*				**Batas Bawah** *Lower Limit*	**Batas Atas** *Upper Limit*
(1)	(2)	(3)	(4)	(5)	(6)	(7)	(8)	(9)	(10)	(11)	(12)	(13)	(14)	(15)	(16)
Aceh	2,14	0,91	42,53	0,36	3,92	4,64	0,66	14,19	3,35	5,93	4,03	0,55	13,53	2,96	5,10
Sumatera Utara	1,21	0,30	25,15	0,61	1,81	1,52	0,50	32,51	0,55	2,49	1,36	0,28	20,85	0,80	1,91
Sumatera Barat	3,26	0,57	17,63	2,13	4,38	5,06	0,61	11,96	3,88	6,25	4,29	0,43	9,96	3,45	5,13
Riau	7,14	1,02	14,32	5,14	9,15	5,30	0,85	16,12	3,62	6,97	6,11	0,66	10,73	4,82	7,39
Jambi	1,44	0,71	49,32	0,05	2,84	1,65	0,37	22,08	0,94	2,37	1,58	0,34	21,35	0,92	2,25
Sumatera Selatan	11,59	1,92	16,53	7,84	15,35	12,87	1,40	10,89	10,12	15,62	12,36	1,14	9,22	10,12	14,59
Bengkulu	1,44	1,43	99,22	-1,36	4,24	0,49	0,20	41,71	0,09	0,88	0,78	0,46	59,28	-0,13	1,69
Lampung	9,64	1,54	16,00	6,62	12,66	2,52	0,37	14,71	1,79	3,25	4,64	0,52	11,29	3,61	5,67
Kep. Bangka Belitung	1,46	0,55	37,65	0,38	2,54	4,27	1,12	26,20	2,07	6,46	2,69	0,58	21,34	1,57	3,82
Kep. Riau	3,62	0,70	19,33	2,25	4,99	15,46	2,72	17,59	10,13	20,79	5,93	0,83	13,95	4,31	7,55
DKI Jakarta	0,43	0,13	29,64	0,18	0,67	-	-	-	-	-	0,43	0,13	29,64	0,18	0,67
Jawa Barat	2,83	0,31	10,80	2,23	3,43	2,37	0,32	13,44	1,75	3,00	2,71	0,24	8,85	2,24	3,18
Jawa Tengah	4,65	0,37	7,94	3,92	5,37	2,49	0,28	11,35	1,94	3,05	3,62	0,24	6,54	3,15	4,08
DI Yogyakarta	2,60	0,65	25,04	1,32	3,87	5,72	1,36	23,87	3,04	8,39	3,56	0,62	17,49	2,34	4,78
Jawa Timur	3,78	0,32	8,52	3,15	4,42	4,87	0,48	9,76	3,94	5,80	4,32	0,29	6,65	3,76	4,88
Banten	3,73	0,67	18,00	2,42	5,05	2,29	0,83	36,26	0,66	3,92	3,33	0,53	16,09	2,28	4,37
Bali	3,59	0,60	16,60	2,42	4,76	1,10	0,47	43,12	0,17	2,03	2,52	0,40	15,99	1,73	3,31
Nusa Tenggara Barat	8,00	1,17	14,67	5,70	10,30	1,21	0,34	27,76	0,55	1,88	4,43	0,60	13,54	3,25	5,60
Nusa Tenggara Timur	6,03	1,58	26,13	2,94	9,12	8,97	0,78	8,67	7,45	10,50	8,46	0,71	8,34	7,08	9,85
Kalimantan Barat	4,49	2,20	49,05	0,17	8,81	1,63	0,33	20,23	0,98	2,28	2,67	0,84	31,54	1,02	4,33
Kalimantan Tengah	2,57	0,94	36,64	0,72	4,42	8,52	1,28	15,06	6,01	11,04	6,32	0,90	14,24	4,56	8,09
Kalimantan Selatan	11,62	1,72	14,81	8,25	14,99	15,42	1,51	9,76	12,47	18,37	13,77	1,14	8,25	11,54	16,00
Kalimantan Timur	1,75	0,38	21,73	1,00	2,49	2,35	0,58	24,80	1,21	3,49	1,94	0,32	16,36	1,32	2,56
Kalimantan Utara	1,31	0,68	51,46	-0,01	2,64	3,66	1,66	45,28	0,41	6,91	2,32	0,81	35,06	0,72	3,91
Sulawesi Utara	0,47	0,24	52,40	-0,01	0,94	1,80	0,40	22,50	1,00	2,59	1,19	0,25	21,12	0,70	1,68
Sulawesi Tengah	1,82	0,63	34,81	0,58	3,07	4,38	0,67	15,35	3,06	5,69	3,70	0,53	14,27	2,67	4,74
Sulawesi Selatan	3,25	0,73	22,61	1,81	4,68	2,77	0,38	13,81	2,02	3,52	2,97	0,38	12,72	2,23	3,70
Sulawesi Tenggara	2,22	0,76	34,43	0,72	3,71	5,85	1,32	22,62	3,25	8,44	4,73	0,96	20,27	2,85	6,61
Gorontalo	0,78	0,36	46,73	0,07	1,50	0,29	0,14	47,26	0,02	0,56	0,50	0,18	35,16	0,15	0,84
Sulawesi Barat	21,11	4,85	22,96	11,61	30,61	16,64	2,35	14,14	12,03	21,25	17,76	2,15	12,08	13,56	21,97
Maluku	3,29	0,82	24,82	1,69	4,89	13,09	2,04	15,58	9,09	17,09	9,38	1,36	14,52	6,71	12,05
Maluku Utara	6,41	1,76	27,40	2,97	9,85	23,43	2,33	9,93	18,87	27,99	18,12	1,77	9,75	14,65	21,58
Papua Barat	3,69	1,24	33,65	1,26	6,12	13,02	2,99	23,00	7,15	18,89	9,53	2,00	20,97	5,61	13,45
Papua	14,26	2,08	14,59	10,18	18,34	60,06	2,87	4,79	54,42	65,69	44,50	2,47	5,54	39,67	49,33
**Indonesia**	**3,66**	**0,14**	**3,71**	**3,39**	**3,92**	**4,93**	**0,15**	**3,06**	**4,64**	**5,23**	**4,23**	**0,10**	**2,39**	**4,04**	**4,43**

Sumber/*Source*: Susenas Maret 2018/*The March 2018 Susenas*

https://www.bps.go.id

Tabel 9.5. ***Sampling Error*** **Persentase Penduduk Berumur 5 Tahun ke Atas yang Tidak/Belum Pernah Sekolah menurut Provinsi dan Daerah Tempat Tinggal, 2018**

Table *Sampling Errors Percentage Population Aged 5 Years and Over Who Not Schooling/Never Attended School by Province and Urban Rural Classification, 2018*

**Provinsi** *Province*	**Perkotaan/***Urban*					**Perdesaan/***Rural*					**Perkotaan+Perdesaan/***Urban+Rural*				
	**Estimasi** *Estimate*	*Standard Error*	*Relative Standard Error (%)*	**Selang Kepercayaan 95%** *Confidence Interval 95%*		**Estimasi** *Estimate*	*Standard Error*	*Relative Standard Error (%)*	**Selang Kepercayaan 95%** *Confidence Interval 95%*		**Estimasi** *Estimate*	*Standard Error*	*Relative Standard Error (%)*	**Selang Kepercayaan 95%** *Confidence Interval 95%*	
				**Batas Bawah** *Lower Limit*	**Batas Atas** *Upper Limit*				**Batas Bawah** *Lower Limit*	**Batas Atas** *Upper Limit*				**Batas Bawah** *Lower Limit*	**Batas Atas** *Upper Limit*
(1)	(2)	(3)	(4)	(5)	(6)	(7)	(8)	(9)	(10)	(11)	(12)	(13)	(14)	(15)	(16)
Aceh	3,89	0,22	5,61	3,46	4,32	4,95	0,16	3,24	4,64	5,27	4,62	0,13	2,80	4,37	4,87
Sumatera Utara	3,61	0,15	4,27	3,31	3,91	5,69	0,17	2,91	5,36	6,01	4,58	0,11	2,47	4,36	4,80
Sumatera Barat	4,69	0,25	5,31	4,21	5,18	5,68	0,19	3,40	5,30	6,06	5,24	0,15	2,92	4,94	5,54
Riau	4,48	0,24	5,26	4,02	4,94	5,72	0,23	4,08	5,26	6,17	5,22	0,17	3,25	4,89	5,55
Jambi	3,48	0,26	7,54	2,97	4,00	6,17	0,27	4,43	5,64	6,71	5,31	0,21	3,88	4,91	5,72
Sumatera Selatan	3,32	0,22	6,60	2,89	3,75	5,07	0,24	4,76	4,60	5,55	4,42	0,17	3,94	4,08	4,76
Bengkulu	3,90	0,34	8,78	3,23	4,57	5,83	0,28	4,76	5,29	6,37	5,20	0,22	4,23	4,77	5,63
Lampung	4,70	0,31	6,51	4,10	5,30	5,58	0,19	3,49	5,20	5,97	5,32	0,16	3,08	5,00	5,64
Kep. Bangka Belitung	4,61	0,31	6,72	4,00	5,22	7,15	0,51	7,18	6,15	8,16	5,78	0,30	5,15	5,20	6,37
Kep. Riau	5,01	0,32	6,37	4,38	5,64	10,81	1,05	9,68	8,76	12,86	5,85	0,34	5,74	5,19	6,50
DKI Jakarta	4,39	0,21	4,75	3,98	4,80	-	-	-	-	-	4,39	0,21	4,75	3,98	4,80
Jawa Barat	4,93	0,15	3,12	4,63	5,23	6,90	0,22	3,15	6,47	7,32	5,44	0,13	2,34	5,19	5,69
Jawa Tengah	6,02	0,16	2,67	5,71	6,34	8,42	0,21	2,47	8,01	8,83	7,19	0,13	1,83	6,93	7,45
DI Yogyakarta	5,71	0,35	6,12	5,02	6,39	12,19	0,76	6,26	10,69	13,68	7,47	0,33	4,48	6,81	8,12
Jawa Timur	6,00	0,16	2,61	5,69	6,30	11,61	0,25	2,15	11,12	12,10	8,67	0,15	1,71	8,38	8,96
Banten	5,23	0,28	5,37	4,68	5,79	6,82	0,57	8,38	5,70	7,94	5,69	0,26	4,55	5,18	6,20
Bali	6,40	0,32	4,97	5,78	7,03	12,24	0,64	5,19	11,00	13,49	8,38	0,31	3,69	7,78	8,99
Nusa Tenggara Barat	9,69	0,50	5,18	8,70	10,67	13,61	0,64	4,67	12,36	14,86	11,78	0,42	3,59	10,96	12,61
Nusa Tenggara Timur	4,99	0,36	7,14	4,29	5,69	9,35	0,26	2,80	8,84	9,87	8,35	0,22	2,65	7,91	8,78
Kalimantan Barat	6,62	0,39	5,86	5,86	7,38	10,02	0,32	3,24	9,39	10,66	8,87	0,26	2,92	8,36	9,38
Kalimantan Tengah	4,33	0,29	6,64	3,77	4,90	5,31	0,31	5,80	4,71	5,91	4,93	0,22	4,43	4,50	5,36
Kalimantan Selatan	5,38	0,38	7,15	4,63	6,13	5,97	0,29	4,91	5,39	6,54	5,69	0,24	4,21	5,22	6,16
Kalimantan Timur	4,65	0,30	6,42	4,07	5,24	7,04	0,49	6,93	6,08	8,00	5,44	0,26	4,77	4,93	5,95
Kalimantan Utara	6,01	0,54	8,95	4,96	7,07	7,85	0,65	8,29	6,57	9,12	6,78	0,41	6,10	5,97	7,60
Sulawesi Utara	2,69	0,20	7,54	2,29	3,09	3,47	0,18	5,19	3,12	3,83	3,08	0,14	4,45	2,81	3,35
Sulawesi Tengah	3,88	0,33	8,41	3,24	4,51	5,72	0,28	4,90	5,17	6,27	5,20	0,22	4,27	4,77	5,64
Sulawesi Selatan	5,17	0,23	4,40	4,72	5,61	10,17	0,33	3,24	9,53	10,82	8,08	0,22	2,72	7,65	8,52
Sulawesi Tenggara	5,56	0,56	10,14	4,46	6,67	7,13	0,30	4,27	6,54	7,73	6,53	0,28	4,33	5,98	7,08
Gorontalo	4,12	0,40	9,64	3,34	4,90	4,41	0,35	7,92	3,72	5,09	4,30	0,26	6,13	3,78	4,81
Sulawesi Barat	5,43	0,70	12,94	4,05	6,80	9,05	0,44	4,88	8,19	9,92	8,20	0,40	4,83	7,42	8,97
Maluku	3,57	0,33	9,28	2,92	4,22	4,36	0,30	6,88	3,77	4,94	4,02	0,22	5,57	3,58	4,46
Maluku Utara	3,55	0,39	11,06	2,78	4,33	4,76	0,28	5,93	4,21	5,32	4,42	0,23	5,23	3,96	4,87
Papua Barat	4,31	0,44	10,14	3,45	5,17	7,55	0,51	6,70	6,56	8,54	6,23	0,35	5,60	5,55	6,92
Papua	4,80	0,39	8,16	4,03	5,57	37,39	0,89	2,38	35,64	39,13	28,37	0,73	2,56	26,95	29,79
**Indonesia**	**5,16**	**0,06**	**1,13**	**5,05**	**5,28**	**8,57**	**0,07**	**0,83**	**8,43**	**8,71**	**6,70**	**0,05**	**0,68**	**6,61**	**6,79**

Sumber/*Source*: Susenas Maret 2018/*The March 2018 Susenas*

https://www.bps.go.id

Tabel 9.6. ***Sampling Error* Persentase Penduduk Berumur 5 Tahun ke Atas yang Masih Bersekolah di SD/MI/Paket A menurut Provinsi dan Daerah Tempat Tinggal, 2018**

Table *Sampling Errors Percentage Population Aged 5 Years and Over Who Attending Primary School by Province and Urban-Rural Classification, 2018*

**Provinsi** *Province*	**Perkotaan/***Urban*					**Perdesaan/***Rural*					**Perkotaan+Perdesaan/***Urban+Rural*				
	**Estimasi** *Estimate*	*Standard Error*	*Relative Standard Error (%)*	**Selang Kepercayaan 95%** *Confidence Interval 95%*		**Estimasi** *Estimate*	*Standard Error*	*Relative Standard Error (%)*	**Selang Kepercayaan 95%** *Confidence Interval 95%*		**Estimasi** *Estimate*	*Standard Error*	*Relative Standard Error (%)*	**Selang Kepercayaan 95%** *Confidence Interval 95%*	
				**Batas Bawah** *Lower Limit*	**Batas Atas** *Upper Limit*				**Batas Bawah** *Lower Limit*	**Batas Atas** *Upper Limit*				**Batas Bawah** *Lower Limit*	**Batas Atas** *Upper Limit*
(1)	(2)	(3)	(4)	(5)	(6)	(7)	(8)	(9)	(10)	(11)	(12)	(13)	(14)	(15)	(16)
Aceh	14,74	0,42	2,82	13,92	15,55	15,93	0,24	1,53	15,45	16,41	15,56	0,21	1,35	15,14	15,97
Sumatera Utara	14,35	0,29	2,00	13,79	14,91	17,36	0,23	1,34	16,90	17,82	15,76	0,19	1,19	15,39	16,12
Sumatera Barat	13,58	0,38	2,83	12,83	14,33	15,56	0,27	1,74	15,03	16,09	14,69	0,23	1,54	14,24	15,13
Riau	13,58	0,37	2,76	12,85	14,32	15,28	0,30	1,97	14,69	15,87	14,59	0,23	1,61	14,13	15,05
Jambi	12,54	0,46	3,70	11,63	13,45	14,03	0,28	1,98	13,48	14,57	13,55	0,24	1,78	13,08	14,02
Sumatera Selatan	13,60	0,42	3,12	12,77	14,44	14,58	0,24	1,65	14,10	15,05	14,21	0,22	1,53	13,79	14,64
Bengkulu	13,48	0,56	4,15	12,38	14,58	14,28	0,32	2,24	13,65	14,91	14,02	0,28	2,01	13,46	14,57
Lampung	12,90	0,43	3,37	12,04	13,75	14,01	0,23	1,66	13,56	14,46	13,68	0,21	1,51	13,27	14,09
Kep. Bangka Belitung	12,24	0,45	3,68	11,36	13,12	13,37	0,42	3,14	12,55	14,20	12,76	0,31	2,42	12,16	13,37
Kep. Riau	14,25	0,51	3,58	13,25	15,25	14,59	0,72	4,92	13,18	16,00	14,30	0,45	3,15	13,42	15,18
DKI Jakarta	11,49	0,28	2,47	10,94	12,05	-	-	-	-	-	11,49	0,28	2,47	10,94	12,05
Jawa Barat	12,35	0,16	1,32	12,03	12,67	12,46	0,21	1,68	12,05	12,87	12,38	0,13	1,07	12,12	12,64
Jawa Tengah	11,18	0,16	1,40	10,87	11,48	11,63	0,16	1,37	11,32	11,95	11,40	0,11	0,98	11,18	11,62
DI Yogyakarta	9,87	0,39	3,92	9,11	10,63	10,20	0,49	4,76	9,25	11,15	9,96	0,31	3,12	9,35	10,57
Jawa Timur	10,33	0,16	1,51	10,02	10,63	10,61	0,15	1,45	10,31	10,92	10,46	0,11	1,05	10,25	10,68
Banten	12,78	0,32	2,51	12,15	13,41	15,35	0,37	2,42	14,62	16,08	13,52	0,25	1,87	13,03	14,02
Bali	10,40	0,29	2,81	9,83	10,98	10,75	0,42	3,90	9,93	11,57	10,52	0,24	2,31	10,04	11,00
Nusa Tenggara Barat	13,59	0,39	2,90	12,81	14,36	14,46	0,37	2,56	13,73	15,18	14,05	0,27	1,91	13,52	14,58
Nusa Tenggara Timur	14,74	0,57	3,89	13,62	15,87	19,57	0,24	1,25	19,09	20,05	18,46	0,23	1,26	18,00	18,91
Kalimantan Barat	13,51	0,42	3,08	12,70	14,33	15,33	0,28	1,80	14,79	15,87	14,71	0,23	1,56	14,26	15,16
Kalimantan Tengah	13,29	0,47	3,54	12,36	14,21	13,19	0,27	2,04	12,66	13,72	13,23	0,24	1,85	12,75	13,71
Kalimantan Selatan	12,60	0,38	3,04	11,85	13,35	13,97	0,30	2,15	13,38	14,56	13,33	0,24	1,79	12,86	13,80
Kalimantan Timur	12,72	0,36	2,82	12,02	13,43	13,52	0,52	3,84	12,50	14,53	12,99	0,29	2,27	12,41	13,56
Kalimantan Utara	12,76	0,63	4,95	11,53	14,00	13,24	0,65	4,94	11,96	14,52	12,96	0,45	3,51	12,07	13,86
Sulawesi Utara	10,92	0,35	3,20	10,24	11,61	12,67	0,31	2,44	12,07	13,28	11,79	0,23	1,99	11,33	12,25
Sulawesi Tengah	11,48	0,54	4,66	10,43	12,53	13,66	0,29	2,10	13,10	14,22	13,05	0,25	1,95	12,55	13,55
Sulawesi Selatan	13,47	0,31	2,29	12,86	14,07	14,14	0,23	1,65	13,68	14,60	13,86	0,19	1,34	13,49	14,22
Sulawesi Tenggara	14,35	0,70	4,87	12,98	15,72	17,11	0,37	2,15	16,39	17,83	16,05	0,35	2,16	15,37	16,73
Gorontalo	12,30	0,65	5,26	11,03	13,57	13,64	0,48	3,55	12,70	14,59	13,12	0,39	2,94	12,37	13,88
Sulawesi Barat	15,23	0,98	6,41	13,32	17,15	14,78	0,45	3,08	13,89	15,67	14,89	0,42	2,79	14,07	15,70
Maluku	14,35	0,55	3,81	13,28	15,42	18,25	0,39	2,15	17,48	19,02	16,60	0,32	1,96	15,97	17,24
Maluku Utara	13,56	0,75	5,56	12,09	15,04	17,89	0,43	2,41	17,04	18,74	16,64	0,37	2,22	15,92	17,36
Papua Barat	13,39	0,77	5,74	11,88	14,89	16,70	0,51	3,04	15,71	17,70	15,35	0,44	2,84	14,50	16,21
Papua	12,90	0,58	4,49	11,76	14,04	14,19	0,38	2,66	13,45	14,93	13,84	0,32	2,28	13,22	14,45
**Indonesia**	**12,15**	**0,07**	**0,54**	**12,02**	**12,28**	**13,60**	**0,05**	**0,40**	**13,49**	**13,71**	**12,81**	**0,04**	**0,34**	**12,72**	**12,89**

Sumber/*Source*: Susenas Maret 2018/*The March 2018 Susenas*

https://www.bps.go.id

Tabel *Table* **9.7.** ***Sampling Error*** **Persentase Penduduk Berumur 5 Tahun ke Atas yang Masih Bersekolah di SMP/MTs/Paket B menurut Provinsi dan Daerah Tempat Tinggal, 2018**

Sampling Errors Percentage Population Aged 5 Years and Over Who Attending Junior School by Province and Urban Rural Classification, 2018

Provinsi *Province*	**Perkotaan/***Urban*					**Perdesaan/***Rural*					**Perkotaan+Perdesaan/***Urban+Rural*				
	**Estimasi** *Estimate*	*Standard Error*	*Relative Standard Error (%)*	**Selang Kepercayaan 95%** *Confidence Interval 95%*		**Estimasi** *Estimate*	*Standard Error*	*Relative Standard Error (%)*	**Selang Kepercayaan 95%** *Confidence Interval 95%*		**Estimasi** *Estimate*	*Standard Error*	*Relative Standard Error (%)*	**Selang Kepercayaan 95%** *Confidence Interval 95%*	
				**Batas Bawah** *Lower Limit*	**Batas Atas** *Upper Limit*				**Batas Bawah** *Lower Limit*	**Batas Atas** *Upper Limit*				**Batas Bawah** *Lower Limit*	**Batas Atas** *Upper Limit*
(1)	(2)	(3)	(4)	(5)	(6)	(7)	(8)	(9)	(10)	(11)	(12)	(13)	(14)	(15)	(16)
Aceh	5,52	0,25	4,50	5,04	6,01	6,10	0,16	2,60	5,79	6,41	5,92	0,13	2,25	5,66	6,18
Sumatera Utara	5,78	0,19	3,27	5,41	6,16	6,51	0,15	2,25	6,22	6,80	6,12	0,12	1,98	5,89	6,36
Sumatera Barat	5,85	0,30	5,14	5,26	6,44	5,75	0,18	3,06	5,41	6,10	5,80	0,16	2,80	5,48	6,11
Riau	5,81	0,26	4,55	5,30	6,33	6,01	0,20	3,30	5,62	6,40	5,93	0,16	2,68	5,62	6,24
Jambi	5,20	0,32	6,22	4,57	5,83	5,36	0,20	3,66	4,98	5,75	5,31	0,17	3,18	4,98	5,64
Sumatera Selatan	5,54	0,29	5,19	4,98	6,10	5,39	0,15	2,84	5,09	5,69	5,44	0,14	2,64	5,16	5,73
Bengkulu	5,67	0,39	6,89	4,91	6,44	5,40	0,21	3,94	4,99	5,82	5,49	0,19	3,51	5,11	5,87
Lampung	5,49	0,27	4,98	4,95	6,02	5,32	0,18	3,35	4,97	5,67	5,37	0,15	2,77	5,08	5,66
Kep. Bangka Belitung	5,13	0,31	5,96	4,53	5,73	4,98	0,30	6,05	4,39	5,57	5,06	0,21	4,23	4,64	5,48
Kep. Riau	5,54	0,44	7,93	4,68	6,41	6,37	0,60	9,44	5,19	7,55	5,66	0,38	6,79	4,91	6,42
DKI Jakarta	4,17	0,15	3,69	3,87	4,47	-	-	-	-	-	4,17	0,15	3,69	3,87	4,47
Jawa Barat	5,16	0,11	2,23	4,93	5,38	5,21	0,15	2,86	4,91	5,50	5,17	0,09	1,81	4,99	5,35
Jawa Tengah	4,87	0,11	2,34	4,65	5,10	4,73	0,12	2,57	4,49	4,96	4,80	0,08	1,73	4,64	4,97
DI Yogyakarta	3,84	0,24	6,33	3,36	4,31	4,31	0,42	9,85	3,48	5,14	3,96	0,21	5,27	3,55	4,37
Jawa Timur	4,74	0,11	2,32	4,53	4,96	4,62	0,11	2,47	4,39	4,84	4,68	0,08	1,69	4,53	4,84
Banten	4,87	0,19	3,94	4,50	5,25	5,31	0,28	5,19	4,77	5,85	5,00	0,16	3,16	4,69	5,31
Bali	4,82	0,21	4,26	4,41	5,22	5,40	0,32	5,88	4,77	6,02	5,01	0,17	3,41	4,68	5,35
Nusa Tenggara Barat	5,78	0,28	4,77	5,24	6,32	5,84	0,26	4,41	5,33	6,34	5,81	0,19	3,24	5,44	6,18
Nusa Tenggara Timur	6,79	0,44	6,44	5,93	7,64	7,03	0,17	2,39	6,70	7,36	6,97	0,16	2,35	6,65	7,29
Kalimantan Barat	5,27	0,27	5,18	4,73	5,80	5,09	0,18	3,49	4,74	5,43	5,15	0,15	2,89	4,86	5,44
Kalimantan Tengah	5,37	0,29	5,46	4,79	5,94	6,08	0,26	4,22	5,58	6,58	5,80	0,19	3,34	5,42	6,18
Kalimantan Selatan	4,85	0,25	5,20	4,36	5,34	5,19	0,22	4,20	4,76	5,61	5,03	0,16	3,27	4,71	5,35
Kalimantan Timur	4,80	0,24	5,08	4,32	5,28	5,68	0,35	6,19	4,99	6,37	5,09	0,20	3,94	4,70	5,48
Kalimantan Utara	5,68	0,43	7,54	4,84	6,52	7,21	0,47	6,46	6,30	8,12	6,32	0,32	5,07	5,69	6,95
Sulawesi Utara	5,18	0,30	5,88	4,58	5,77	5,23	0,22	4,18	4,81	5,66	5,20	0,19	3,61	4,84	5,57
Sulawesi Tengah	5,58	0,32	5,73	4,95	6,21	6,24	0,18	2,94	5,88	6,60	6,06	0,16	2,66	5,74	6,37
Sulawesi Selatan	5,43	0,21	3,78	5,03	5,83	5,45	0,15	2,79	5,16	5,75	5,44	0,12	2,27	5,20	5,69
Sulawesi Tenggara	6,35	0,41	6,51	5,54	7,16	6,04	0,24	3,93	5,58	6,51	6,16	0,23	3,68	5,72	6,60
Gorontalo	5,03	0,40	7,87	4,25	5,80	5,29	0,29	5,54	4,72	5,87	5,19	0,24	4,55	4,73	5,65
Sulawesi Barat	5,15	0,56	10,84	4,06	6,25	5,93	0,30	5,03	5,34	6,51	5,75	0,26	4,59	5,23	6,26
Maluku	6,71	0,40	5,99	5,92	7,49	7,08	0,32	4,54	6,45	7,71	6,92	0,25	3,64	6,43	7,42
Maluku Utara	6,09	0,45	7,31	5,22	6,97	7,07	0,28	3,90	6,53	7,61	6,79	0,24	3,46	6,33	7,25
Papua Barat	5,94	0,46	7,79	5,03	6,84	6,16	0,29	4,76	5,58	6,73	6,07	0,26	4,23	5,56	6,57
Papua	6,22	0,33	5,25	5,58	6,86	5,40	0,24	4,46	4,92	5,87	5,62	0,20	3,48	5,24	6,01
**Indonesia**	**5,07**	**0,04**	**0,87**	**4,99**	**5,16**	**5,38**	**0,04**	**0,72**	**5,30**	**5,45**	**5,21**	**0,03**	**0,58**	**5,15**	**5,27**

Sumber/*Source*: Susenas Maret 2018/*The March 2018 Susenas*

https://www.bps.go.id

Tabel 9.8. ***Sampling Error*** **Persentase Penduduk Berumur 5 Tahun ke Atas yang Masih Bersekolah di SMA/SMK/MA/Paket C menurut Provinsi dan Daerah Tempat Tinggal, 2018**

Table *Sampling Errors Percentage Population Aged 5 Years and Over Who Attending Senior High School by Province and Urban Rural Classsification, 2018*

**Provinsi** *Province*	**Perkotaan/***Urban*					**Perdesaan/***Rural*					**Perkotaan+Perdesaan/***Urban+Rural*				
	**Estimasi** *Estimate*	*Standard Error*	*Relative Standard Error (%)*	**Selang Kepercayaan 95%** *Confidence Interval 95%*		**Estimasi** *Estimate*	*Standard Error*	*Relative Standard Error (%)*	**Selang Kepercayaan 95%** *Confidence Interval 95%*		**Estimasi** *Estimate*	*Standard Error*	*Relative Standard Error (%)*	**Selang Kepercayaan 95%** *Confidence Interval 95%*	
				**Batas Bawah** *Lower Limit*	**Batas Atas** *Upper Limit*				**Batas Bawah** *Lower Limit*	**Batas Atas** *Upper Limit*				**Batas Bawah** *Lower Limit*	**Batas Atas** *Upper Limit*
(1)	(2)	(3)	(4)	(5)	(6)	(7)	(8)	(9)	(10)	(11)	(12)	(13)	(14)	(15)	(16)
Aceh	5,68	0,36	6,41	4,97	6,40	5,03	0,15	3,04	4,73	5,33	5,24	0,15	2,95	4,93	5,54
Sumatera Utara	6,17	0,21	3,38	5,76	6,57	5,77	0,15	2,65	5,47	6,07	5,98	0,13	2,20	5,72	6,24
Sumatera Barat	5,36	0,23	4,30	4,91	5,82	4,84	0,15	3,12	4,55	5,14	5,07	0,13	2,61	4,81	5,33
Riau	5,63	0,28	4,98	5,08	6,18	4,48	0,19	4,22	4,11	4,85	4,94	0,16	3,25	4,63	5,26
Jambi	6,03	0,34	5,70	5,35	6,70	3,91	0,17	4,25	3,59	4,24	4,59	0,16	3,48	4,28	4,90
Sumatera Selatan	5,15	0,30	5,91	4,55	5,75	3,98	0,15	3,76	3,68	4,27	4,41	0,15	3,36	4,12	4,70
Bengkulu	5,69	0,40	7,01	4,91	6,47	4,25	0,22	5,09	3,83	4,68	4,72	0,20	4,19	4,33	5,11
Lampung	5,90	0,54	9,17	4,84	6,96	4,15	0,15	3,67	3,85	4,45	4,67	0,19	4,17	4,29	5,05
Kep. Bangka Belitung	4,98	0,33	6,65	4,33	5,63	4,15	0,37	8,83	3,43	4,86	4,59	0,24	5,32	4,11	5,07
Kep. Riau	4,40	0,30	6,84	3,81	4,99	4,91	0,73	14,97	3,47	6,35	4,47	0,27	6,15	3,93	5,01
DKI Jakarta	3,38	0,14	4,22	3,10	3,66	-	-	-	-	-	3,38	0,14	4,22	3,10	3,66
Jawa Barat	4,61	0,11	2,43	4,39	4,83	3,52	0,13	3,73	3,26	3,77	4,32	0,09	2,07	4,15	4,50
Jawa Tengah	5,05	0,12	2,36	4,82	5,28	4,30	0,12	2,76	4,07	4,53	4,68	0,08	1,80	4,52	4,85
DI Yogyakarta	4,35	0,28	6,34	3,81	4,90	3,71	0,39	10,47	2,95	4,47	4,18	0,23	5,59	3,72	4,64
Jawa Timur	4,56	0,12	2,58	4,33	4,80	3,61	0,15	4,24	3,31	3,91	4,11	0,10	2,32	3,92	4,30
Banten	4,18	0,20	4,88	3,78	4,58	3,47	0,24	6,92	3,00	3,95	3,98	0,16	4,04	3,66	4,29
Bali	4,46	0,21	4,82	4,03	4,88	4,65	0,28	5,94	4,11	5,19	4,52	0,17	3,82	4,18	4,86
Nusa Tenggara Barat	6,28	0,33	5,32	5,63	6,94	5,24	0,27	5,10	4,72	5,76	5,72	0,21	3,70	5,31	6,14
Nusa Tenggara Timur	7,83	0,48	6,17	6,88	8,78	4,38	0,17	3,88	4,05	4,72	5,18	0,18	3,45	4,83	5,53
Kalimantan Barat	5,68	0,34	5,94	5,02	6,34	4,32	0,17	4,04	3,98	4,66	4,78	0,17	3,48	4,45	5,11
Kalimantan Tengah	5,00	0,34	6,72	4,34	5,66	3,88	0,20	5,22	3,48	4,28	4,31	0,18	4,18	3,96	4,67
Kalimantan Selatan	5,04	0,26	5,21	4,52	5,55	3,90	0,17	4,32	3,57	4,23	4,43	0,15	3,45	4,13	4,73
Kalimantan Timur	5,54	0,28	5,12	4,98	6,10	5,24	0,37	6,97	4,53	5,96	5,44	0,22	4,12	5,00	5,88
Kalimantan Utara	6,30	0,51	8,02	5,30	7,29	4,71	0,60	12,62	3,55	5,88	5,63	0,39	6,88	4,87	6,39
Sulawesi Utara	4,83	0,27	5,54	4,31	5,36	3,93	0,18	4,54	3,58	4,28	4,39	0,16	3,70	4,07	4,70
Sulawesi Tengah	6,90	0,61	8,87	5,70	8,10	3,87	0,15	3,79	3,58	4,16	4,72	0,21	4,41	4,31	5,13
Sulawesi Selatan	5,28	0,21	4,04	4,86	5,70	4,76	0,15	3,09	4,47	5,05	4,97	0,12	2,48	4,73	5,22
Sulawesi Tenggara	6,60	0,47	7,13	5,68	7,53	4,80	0,20	4,24	4,40	5,20	5,50	0,23	4,12	5,05	5,94
Gorontalo	4,51	0,38	8,54	3,75	5,26	4,70	0,31	6,56	4,09	5,30	4,62	0,24	5,16	4,15	5,09
Sulawesi Barat	6,31	0,77	12,20	4,80	7,81	5,08	0,33	6,43	4,44	5,72	5,37	0,31	5,76	4,76	5,98
Maluku	6,73	0,41	6,07	5,93	7,54	5,27	0,24	4,56	4,80	5,74	5,89	0,23	3,83	5,45	6,33
Maluku Utara	6,41	0,50	7,84	5,43	7,40	5,62	0,29	5,16	5,05	6,19	5,85	0,25	4,31	5,36	6,34
Papua Barat	6,69	0,43	6,42	5,85	7,53	4,56	0,29	6,28	4,00	5,13	5,43	0,25	4,63	4,94	5,92
Papua	5,94	0,35	5,94	5,25	6,63	2,93	0,16	5,44	2,62	3,24	3,76	0,15	4,08	3,46	4,06
**Indonesia**	**4,88**	**0,05**	**0,94**	**4,79**	**4,97**	**4,21**	**0,04**	**0,96**	**4,13**	**4,29**	**4,58**	**0,03**	**0,68**	**4,52**	**4,64**

Sumber/*Source*: Susenas Maret 2018/*The March 2018 Susenas*

https://www.bps.go.id

Tabel 9.9. ***Sampling Error*** **Persentase Penduduk Berumur 5 Tahun ke Atas yang Masih Bersekolah di Diploma I s.d Universitas menurut Provinsi dan Daerah Tempat Tinggal, 2018**

Table *Sampling Errors Percentage Population Aged 5 Years and Over Who Attending Diploma I to University by Province and Urban Rural Classification, 2018*

Provinsi *Province*	**Perkotaan**/*Urban*					**Perdesaan**/*Rural*					**Perkotaan+Perdesaan**/*Urban+Rural*				
	**Estimasi** *Estimate*	*Standard Error*	*Relative Standard Error (%)*	**Selang Kepercayaan 95%** *Confidence Interval 95%*		**Estimasi** *Estimate*	*Standard Error*	*Relative Standard Error (%)*	**Selang Kepercayaan 95%** *Confidence Interval 95%*		**Estimasi** *Estimate*	*Standard Error*	*Relative Standard Error (%)*	**Selang Kepercayaan 95%** *Confidence Interval 95%*	
				**Batas Bawah** *Lower Limit*	**Batas Atas** *Upper Limit*				**Batas Bawah** *Lower Limit*	**Batas Atas** *Upper Limit*				**Batas Bawah** *Lower Limit*	**Batas Atas** *Upper Limit*
(1)	(2)	(3)	(4)	(5)	(6)	(7)	(8)	(9)	(10)	(11)	(12)	(13)	(14)	(15)	(16)
1. Aceh	7,55	0,43	5,70	6,71	8,40	2,62	0,16	6,08	2,31	2,93	4,17	0,19	4,48	3,81	4,54
2. Sumatera Utara	3,91	0,32	8,23	3,28	4,55	1,58	0,12	7,48	1,35	1,81	2,82	0,18	6,42	2,47	3,18
3. Sumatera Barat	6,81	0,52	7,59	5,79	7,82	1,33	0,13	9,59	1,08	1,58	3,75	0,25	6,60	3,26	4,23
4. Riau	4,83	0,38	7,94	4,08	5,58	1,91	0,16	8,31	1,60	2,22	3,09	0,18	5,98	2,73	3,45
5. Jambi	5,45	0,47	8,67	4,52	6,38	1,62	0,15	9,08	1,33	1,90	2,85	0,19	6,70	2,47	3,22
6. Sumatera Selatan	4,42	0,40	9,04	3,64	5,21	0,99	0,10	9,84	0,80	1,18	2,27	0,16	7,12	1,95	2,59
7. Bengkulu	5,95	0,46	7,79	5,04	6,86	2,04	0,20	9,60	1,65	2,42	3,32	0,20	6,12	2,92	3,72
8. Lampung	3,98	0,51	12,82	2,98	4,98	0,85	0,08	9,80	0,69	1,01	1,78	0,17	9,51	1,45	2,11
9. Kep. Bangka Belitung	1,32	0,17	12,64	1,00	1,65	0,90	0,13	14,21	0,65	1,15	1,13	0,11	9,59	0,92	1,34
10. Kep. Riau	2,42	0,31	12,94	1,81	3,04	0,53	0,17	31,37	0,20	0,85	2,15	0,27	12,65	1,62	2,68
11. DKI Jakarta	2,96	0,18	6,05	2,61	3,31	-	-	-	-	-	2,96	0,18	6,05	2,61	3,31
12 Jawa Barat	2,82	0,12	4,14	2,60	3,05	0,75	0,07	9,84	0,60	0,89	2,29	0,09	3,90	2,11	2,46
13. Jawa Tengah	2,64	0,13	4,96	2,38	2,90	0,94	0,07	7,32	0,80	1,07	1,81	0,08	4,19	1,66	1,96
14. DI Yogyakarta	8,27	0,65	7,87	6,99	9,54	1,21	0,27	21,91	0,69	1,73	6,35	0,48	7,58	5,41	7,30
15. Jawa Timur	3,62	0,17	4,73	3,28	3,95	1,17	0,08	6,71	1,02	1,33	2,45	0,10	3,99	2,26	2,64
16. Banten	4,00	0,24	5,95	3,53	4,47	0,77	0,14	18,15	0,50	1,04	3,07	0,18	5,74	2,73	3,42
17. Bali	3,89	0,23	5,88	3,44	4,34	1,17	0,33	28,29	0,52	1,82	2,97	0,18	6,16	2,61	3,33
18. Nusa Tenggara Barat	3,76	0,36	9,46	3,07	4,46	1,69	0,22	13,25	1,25	2,13	2,66	0,20	7,69	2,26	3,06
19. Nusa Tenggara Timur	6,93	0,50	7,17	5,95	7,90	1,45	0,11	7,35	1,24	1,65	2,71	0,15	5,60	2,41	3,01
20. Kalimantan Barat	4,48	0,51	11,36	3,48	5,47	0,80	0,09	11,76	0,62	0,99	2,05	0,19	9,28	1,68	2,42
21. Kalimantan Tengah	3,44	0,32	9,31	2,81	4,07	1,48	0,17	11,62	1,15	1,82	2,24	0,16	7,29	1,92	2,56
22. Kalimantan Selatan	3,38	0,30	8,83	2,80	3,97	1,21	0,15	11,95	0,93	1,50	2,23	0,16	7,18	1,91	2,54
23. Kalimantan Timur	3,93	0,31	7,92	3,32	4,53	1,63	0,25	15,38	1,14	2,12	3,16	0,23	7,14	2,72	3,61
24. Kalimantan Utara	2,35	0,29	12,33	1,78	2,92	1,23	0,29	23,32	0,67	1,80	1,88	0,21	11,01	1,47	2,29
25. Sulawesi Utara	4,35	0,39	8,94	3,59	5,12	1,60	0,18	10,96	1,25	1,94	2,98	0,22	7,26	2,56	3,41
26. Sulawesi Tengah	7,58	0,65	8,61	6,30	8,86	1,80	0,14	8,02	1,51	2,08	3,41	0,22	6,47	2,98	3,85
27. Sulawesi Selatan	6,31	0,43	6,74	5,48	7,14	1,97	0,14	6,91	1,70	2,24	3,78	0,20	5,18	3,40	4,16
28. Sulawesi Tenggara	8,21	0,80	9,79	6,63	9,78	1,85	0,21	11,60	1,43	2,27	4,30	0,35	8,08	3,62	4,98
29. Gorontalo	5,84	0,59	10,10	4,69	7,00	2,17	0,32	14,85	1,54	2,80	3,60	0,30	8,47	3,00	4,20
30. Sulawesi Barat	4,94	0,76	15,29	3,46	6,43	2,01	0,22	11,16	1,57	2,45	2,71	0,25	9,41	2,21	3,20
31. Maluku	7,28	0,63	8,70	6,04	8,52	2,69	0,38	14,25	1,94	3,44	4,63	0,34	7,44	3,96	5,31
32. Maluku Utara	8,25	0,87	10,51	6,55	9,95	2,10	0,25	11,72	1,62	2,58	3,87	0,31	7,94	3,27	4,47
33. Papua Barat	4,41	0,51	11,47	3,42	5,40	2,63	0,35	13,22	1,95	3,31	3,35	0,29	8,73	2,78	3,93
34. Papua	3,42	0,32	9,40	2,79	4,04	1,03	0,10	10,09	0,83	1,24	1,69	0,12	7,09	1,46	1,93
**Indonesia**	**3,73**	**0,06**	**1,53**	**3,62**	**3,84**	**1,28**	**0,03**	**1,99**	**1,23**	**1,33**	**2,62**	**0,03**	**1,28**	**2,56**	**2,69**

Sumber/*Source*: Susenas Maret 2018/*The March 2018 Susenas*

https://www.bps.go.id

Tabel *Table* **9.10.**

Sampling Error **Persentase Perempuan Berumur 15-49 Tahun Pernah Kawin yang Pernah Melahirkan dan Tempat Melahirkan Anak Lahir Hidup yang Terakhir di Rumah Sakit Pemerintah/Rumah Sakit Swasta/RSIA menurut Provinsi dan Daerah Tempat Tinggal, 2018**

Sampling Errors Percentage of Ever Married Female Aged 15-49 Years Who Have Given Birth and Place of Last Live Birth At Government Hospital/Private Hospital/ Mother and Child Hospital by Province and Urban Rural Classification, 2018

**Provinsi** *Province*	**Perkotaan/***Urban*					**Perdesaan/***Rural*					**Perkotaan+Perdesaan/***Urban+Rural*				
	**Estimasi** *Estimate*	*Standard Error*	*Relative Standard Error (%)*	**Selang Kepercayaan 95%** *Confidence Interval 95%*		**Estimasi** *Estimate*	*Standard Error*	*Relative Standard Error (%)*	**Selang Kepercayaan 95%** *Confidence Interval 95%*		**Estimasi** *Estimate*	*Standard Error*	*Relative Standard Error (%)*	**Selang Kepercayaan 95%** *Confidence Interval 95%*	
				**Batas Bawah** *Lower Limit*	**Batas Atas** *Upper Limit*				**Batas Bawah** *Lower Limit*	**Batas Atas** *Upper Limit*				**Batas Bawah** *Lower Limit*	**Batas Atas** *Upper Limit*
(1)	(2)	(3)	(4)	(5)	(6)	(7)	(8)	(9)	(10)	(11)	(12)	(13)	(14)	(15)	(16)
Aceh	44,94	2,75	6,12	39,55	50,33	30,65	1,54	5,03	27,63	33,67	34,99	1,36	3,88	32,33	37,65
Sumatera Utara	43,51	2,31	5,31	38,99	48,04	21,33	1,52	7,13	18,35	24,31	32,51	1,40	4,32	29,75	35,26
Sumatera Barat	37,38	2,98	7,97	31,54	43,22	25,92	1,76	6,78	22,48	29,37	30,89	1,63	5,26	27,70	34,07
Riau	40,78	2,91	7,14	35,07	46,48	23,89	1,83	7,65	20,31	27,48	31,00	1,63	5,27	27,80	34,20
Jambi	38,34	3,88	10,12	30,74	45,95	18,48	1,77	9,55	15,02	21,95	24,45	1,78	7,30	20,95	27,94
Sumatera Selatan	45,35	3,45	7,60	38,59	52,10	20,16	1,57	7,78	17,08	23,23	28,52	1,63	5,70	25,34	31,71
Bengkulu	47,39	4,47	9,43	38,63	56,16	26,02	2,46	9,47	21,19	30,85	32,79	2,18	6,66	28,51	37,07
Lampung	28,91	3,31	11,45	22,42	35,40	15,90	1,47	9,28	13,01	18,79	19,54	1,43	7,31	16,74	22,34
Kep. Bangka Belitung	39,40	3,55	9,01	32,44	46,36	28,50	3,36	11,79	21,91	35,08	34,63	2,47	7,14	29,79	39,48
Kep. Riau	43,28	3,72	8,59	35,99	50,56	28,85	4,85	16,82	19,34	38,37	41,45	3,34	8,05	34,90	47,99
DKI Jakarta	45,98	2,68	5,83	40,73	51,24	-	-	-	-	-	45,98	2,68	5,83	40,73	51,24
Jawa Barat	32,73	1,48	4,51	29,83	35,62	15,47	1,28	8,28	12,96	17,98	28,37	1,16	4,08	26,10	30,64
Jawa Tengah	43,55	1,48	3,40	40,65	46,45	33,37	1,44	4,31	30,56	36,19	38,44	1,03	2,69	36,42	40,47
DI Yogyakarta	52,81	4,03	7,63	44,91	60,71	44,18	5,82	13,18	32,77	55,59	50,89	3,39	6,66	44,25	57,54
Jawa Timur	40,37	1,53	3,79	37,37	43,37	28,57	1,30	4,54	26,03	31,12	34,74	1,02	2,94	32,74	36,74
Banten	34,01	2,44	7,17	29,23	38,79	13,60	2,37	17,46	8,95	18,25	27,86	1,84	6,60	24,26	31,46
Bali	60,30	2,89	4,79	54,63	65,96	48,53	5,27	10,87	38,19	58,87	56,53	2,54	4,50	51,55	61,52
Nusa Tenggara Barat	30,04	2,58	8,59	24,99	35,10	25,21	2,27	8,99	20,76	29,65	27,33	1,71	6,27	23,97	30,68
Nusa Tenggara Timur	52,00	3,79	7,29	44,56	59,43	21,96	1,23	5,62	19,54	24,37	28,25	1,30	4,61	25,70	30,81
Kalimantan Barat	37,15	2,91	7,83	31,45	42,86	15,86	1,47	9,24	12,99	18,74	23,35	1,48	6,33	20,46	26,25
Kalimantan Tengah	26,54	3,08	11,59	20,51	32,56	13,55	1,79	13,21	10,04	17,06	18,51	1,63	8,83	15,31	21,71
Kalimantan Selatan	40,10	2,70	6,73	34,81	45,39	26,94	1,99	7,40	23,03	30,85	33,16	1,67	5,05	29,88	36,44
Kalimantan Timur	49,18	2,90	5,90	43,49	54,87	40,91	4,86	11,87	31,39	50,43	46,51	2,51	5,39	41,59	51,43
Kalimantan Utara	48,98	4,23	8,63	40,69	57,27	38,11	4,54	11,92	29,21	47,01	44,52	3,17	7,12	38,30	50,73
Sulawesi Utara	62,57	3,15	5,04	56,39	68,75	44,62	2,72	6,10	39,28	49,96	53,81	2,15	3,99	49,59	58,02
Sulawesi Tengah	44,93	4,46	9,92	36,19	53,67	22,54	1,89	8,40	18,83	26,25	28,23	1,85	6,54	24,61	31,85
Sulawesi Selatan	52,80	2,73	5,16	47,45	58,14	27,72	1,55	5,58	24,69	30,75	38,27	1,48	3,88	35,36	41,18
Sulawesi Tenggara	28,05	3,82	13,61	20,57	35,54	13,33	1,45	10,85	10,49	16,16	18,76	1,65	8,79	15,53	21,99
Gorontalo	64,06	4,57	7,14	55,10	73,02	23,90	3,25	13,59	17,53	30,27	40,11	2,87	7,15	34,48	45,73
Sulawesi Barat	28,83	6,01	20,83	17,06	40,61	14,30	2,03	14,23	10,31	18,28	17,49	2,07	11,86	13,42	21,56
Maluku	49,82	4,83	9,70	40,34	59,29	12,90	1,80	13,98	9,36	16,43	27,60	2,27	8,24	23,14	32,05
Maluku Utara	64,44	4,14	6,43	56,33	72,56	14,01	1,94	13,83	10,21	17,80	28,12	2,27	8,09	23,66	32,58
Papua Barat	67,78	3,78	5,57	60,38	75,18	34,51	3,67	10,63	27,32	41,69	48,68	2,68	5,50	43,43	53,92
Papua	64,88	2,96	4,56	59,07	70,68	14,84	1,55	10,43	11,81	17,87	29,07	1,57	5,39	26,01	32,14
**Indonesia**	**40,31**	**0,58**	**1,44**	**39,17**	**41,45**	**23,93**	**0,38**	**1,60**	**23,18**	**24,68**	**32,73**	**0,36**	**1,10**	**32,02**	**33,44**

Sumber/*Source*: Susenas Maret 2018/*The March 2018 Susenas*

https://www.bps.go.id

Tabel *Table* **9.11.** ***Sampling Error* Persentase Perempuan Berumur 15-49 Tahun Pernah Kawin yang Pernah Melahirkan dan Tempat Melahirkan Anak Lahir Hidup yang Terakhir di Rumah Bersalin/Klinik menurut Provinsi dan Daerah Tempat Tinggal, 2018**

Sampling Errors Percentage of Ever Married Female Aged 15-49 Years Who Have Given Birth and Place of Last Live Birth At Maternity Clinic/Clinic by Province and Urban Rural Classsification, 2018

Provinsi *Province*	Perkotaan/*Urban*					Perdesaan/*Rural*					Perkotaan+Perdesaan/*Urban+Rural*				
	Estimasi *Estimate*	*Standard Error*	*Relative Standard Error (%)*	Selang Kepercayaan 95% *Confidence Interval 95%*		Estimasi *Estimate*	*Standard Error*	*Relative Standard Error (%)*	Selang Kepercayaan 95% *Confidence Interval 95%*		Estimasi *Estimate*	*Standard Error*	*Relative Standard Error (%)*	Selang Kepercayaan 95% *Confidence Interval 95%*	
				Batas Bawah *Lower Limit*	Batas Atas *Upper Limit*				Batas Bawah *Lower Limit*	Batas Atas *Upper Limit*				Batas Bawah *Lower Limit*	Batas Atas *Upper Limit*
(1)	(2)	(3)	(4)	(5)	(6)	(7)	(8)	(9)	(10)	(11)	(12)	(13)	(14)	(15)	(16)
Aceh	26,95	2,73	10,12	21,61	32,30	19,54	1,44	7,34	16,73	22,36	21,79	1,31	5,99	19,23	24,35
Sumatera Utara	35,93	2,25	6,26	31,52	40,34	16,55	1,51	9,15	13,58	19,52	26,31	1,38	5,26	23,60	29,02
Sumatera Barat	37,50	2,95	7,86	31,72	43,28	21,78	1,74	7,99	18,37	25,20	28,59	1,62	5,67	25,41	31,77
Riau	40,63	2,99	7,35	34,77	46,48	16,91	1,78	10,55	13,41	20,41	26,90	1,69	6,29	23,58	30,21
Jambi	33,17	3,91	11,80	25,50	40,84	14,36	1,51	10,53	11,40	17,33	20,01	1,68	8,40	16,71	23,31
Sumatera Selatan	27,45	2,79	10,16	21,98	32,91	12,37	1,28	10,33	9,87	14,88	17,38	1,29	7,41	14,85	19,90
Bengkulu	20,78	3,55	17,08	13,83	27,74	9,11	1,59	17,49	5,99	12,24	12,81	1,58	12,36	9,71	15,91
Lampung	39,87	3,94	9,89	32,13	47,60	25,90	1,83	7,08	22,30	29,50	29,81	1,72	5,76	26,44	33,17
Kep. Bangka Belitung	30,87	3,55	11,50	23,91	37,83	9,44	2,43	25,72	4,68	14,19	21,50	2,32	10,78	16,96	26,04
Kep. Riau	32,30	3,18	9,84	26,07	38,52	10,08	4,56	45,22	1,15	19,02	29,48	2,87	9,73	23,85	35,10
DKI Jakarta	29,90	2,37	7,92	25,25	34,54	-	-	-	-	-	29,90	2,37	7,92	25,25	34,54
Jawa Barat	27,26	1,36	4,97	24,61	29,92	13,24	1,32	9,94	10,66	15,82	23,73	1,07	4,50	21,63	25,82
Jawa Tengah	20,97	1,28	6,12	18,46	23,49	14,99	1,19	7,94	12,66	17,32	17,97	0,87	4,87	16,26	19,69
DI Yogyakarta	24,31	3,55	14,60	17,35	31,27	28,14	4,79	17,03	18,75	37,54	25,16	2,96	11,76	19,36	30,96
Jawa Timur	24,04	1,40	5,84	21,29	26,79	14,40	1,09	7,59	12,26	16,54	19,44	0,90	4,62	17,68	21,20
Banten	33,02	2,44	7,38	28,24	37,80	12,33	2,14	17,33	8,15	16,52	26,79	1,82	6,79	23,22	30,36
Bali	22,45	2,61	11,63	17,33	27,57	18,93	3,79	20,02	11,50	26,35	21,32	2,17	10,20	17,06	25,59
Nusa Tenggara Barat	6,57	1,47	22,39	3,68	9,45	4,56	1,05	23,03	2,50	6,61	5,44	0,87	16,01	3,73	7,15
Nusa Tenggara Timur	12,18	2,91	23,90	6,48	17,89	0,96	0,27	28,22	0,43	1,49	3,31	0,82	24,75	1,71	4,92
Kalimantan Barat	34,72	3,49	10,04	27,88	41,55	8,37	1,18	14,06	6,06	10,67	17,64	1,53	8,68	14,64	20,64
Kalimantan Tengah	27,11	3,03	11,17	21,17	33,04	7,60	1,51	19,87	4,64	10,55	15,05	1,52	10,12	12,07	18,04
Kalimantan Selatan	29,80	2,88	9,65	24,16	35,44	10,35	1,46	14,09	7,49	13,21	19,55	1,61	8,25	16,39	22,71
Kalimantan Timur	30,16	2,84	9,42	24,59	35,72	7,76	1,98	25,48	3,89	11,64	22,93	2,12	9,24	18,78	27,08
Kalimantan Utara	14,99	3,46	23,06	8,22	21,77	3,84	1,43	37,26	1,04	6,64	10,41	2,18	20,93	6,14	14,68
Sulawesi Utara	9,32	1,73	18,57	5,93	12,71	10,24	1,84	17,91	6,65	13,84	9,77	1,26	12,86	7,31	12,23
Sulawesi Tengah	12,23	2,47	20,20	7,39	17,07	3,86	1,01	26,12	1,89	5,84	5,99	0,98	16,36	4,07	7,91
Sulawesi Selatan	19,79	2,34	11,81	15,21	24,37	6,19	0,89	14,36	4,45	7,93	11,91	1,13	9,52	9,68	14,13
Sulawesi Tenggara	13,90	2,95	21,22	8,12	19,68	3,85	0,85	22,07	2,18	5,51	7,56	1,24	16,43	5,12	9,99
Gorontalo	5,00	1,87	37,46	1,33	8,67	4,99	1,63	32,75	1,79	8,19	4,99	1,25	24,99	2,55	7,44
Sulawesi Barat	9,23	3,88	42,07	1,62	16,85	2,36	1,08	45,77	0,24	4,47	3,87	1,20	31,00	1,52	6,22
Maluku	1,24	0,78	62,78	-0,28	2,76	0,70	0,55	77,97	-0,37	1,78	0,92	0,45	49,25	0,03	1,80
Maluku Utara	10,29	3,27	31,81	3,87	16,71	1,02	0,47	45,90	0,10	1,94	3,62	1,01	27,84	1,64	5,59
Papua Barat	5,66	1,86	32,92	2,01	9,31	1,61	0,83	51,30	-0,01	3,24	3,34	0,93	27,76	1,52	5,15
Papua	9,70	1,94	20,00	5,90	13,51	2,42	0,62	25,73	1,20	3,64	4,49	0,71	15,77	3,10	5,88
**Indonesia**	**26,49**	**0,53**	**2,01**	**25,45**	**27,54**	**12,56**	**0,33**	**2,59**	**11,92**	**13,20**	**20,04**	**0,33**	**1,63**	**19,41**	**20,68**

Sumber/*Source*: Susenas Maret 2018/*The March 2018 Susenas*

https://www.bps.go.id

Tabel *Table* **9.12.** ***Sampling Error*** **Persentase Perempuan Berumur 15-49 Tahun Pernah Kawin yang Pernah Melahirkan dan Penolong Proses Kelahiran Terakhir Dokter Kandungan menurut Provinsi dan Daerah Tempat Tinggal, 2018**

Sampling Errors Percentage of Ever Married Female Aged 15-49 Years Who Have Given Birth and Last Birth Attendant by Obstetrician by Province and Urban Rural Classification, 2018

Provinsi *Province*	Perkotaan/*Urban*					Perdesaan/*Rural*					Perkotaan+Perdesaan/*Urban+Rural*				
	Estimasi *Estimate*	*Standard Error*	*Relative Standard Error (%)*	Selang Kepercayaan 95% *Confidence Interval 95%*		Estimasi *Estimate*	*Standard Error*	*Relative Standard Error (%)*	Selang Kepercayaan 95% *Confidence Interval 95%*		Estimasi *Estimate*	*Standard Error*	*Relative Standard Error (%)*	Selang Kepercayaan 95% *Confidence Interval 95%*	
				Batas Bawah *Lower Limit*	Batas Atas *Upper Limit*				Batas Bawah *Lower Limit*	Batas Atas *Upper Limit*				Batas Bawah *Lower Limit*	Batas Atas *Upper Limit*
(1)	(2)	(3)	(4)	(5)	(6)	(7)	(8)	(9)	(10)	(11)	(12)	(13)	(14)	(15)	(16)
Aceh	42,92	2,76	6,44	37,51	48,34	26,27	1,46	5,57	23,41	29,14	31,33	1,32	4,21	28,74	33,91
Sumatera Utara	42,85	2,30	5,36	38,34	47,36	20,74	1,49	7,19	17,81	23,66	31,88	1,39	4,35	29,16	34,60
Sumatera Barat	39,68	2,99	7,55	33,81	45,55	28,56	1,84	6,45	24,94	32,17	33,37	1,66	4,99	30,11	36,63
Riau	40,70	2,88	7,07	35,06	46,33	25,01	1,81	7,24	21,46	28,56	31,62	1,61	5,10	28,46	34,78
Jambi	36,32	4,06	11,17	28,37	44,27	18,12	1,76	9,71	14,67	21,57	23,58	1,80	7,63	20,06	27,11
Sumatera Selatan	43,23	3,48	8,06	36,40	50,06	16,44	1,44	8,75	13,62	19,26	25,34	1,60	6,32	22,20	28,48
Bengkulu	41,45	4,67	11,27	32,30	50,61	22,66	2,38	10,50	18,00	27,32	28,61	2,17	7,57	24,36	32,86
Lampung	23,60	3,00	12,69	17,73	29,47	16,00	1,49	9,28	13,09	18,91	18,13	1,37	7,54	15,45	20,80
Kep. Bangka Belitung	36,71	4,02	10,94	28,84	44,58	26,21	3,50	13,37	19,34	33,08	32,12	2,69	8,37	26,85	37,39
Kep. Riau	47,43	3,88	8,18	39,82	55,03	31,77	5,38	16,93	21,23	42,31	45,44	3,49	7,67	38,60	52,27
DKI Jakarta	49,69	2,58	5,19	44,63	54,74	-	-	-	-	-	49,69	2,58	5,19	44,63	54,74
Jawa Barat	32,40	1,42	4,39	29,62	35,19	16,46	1,30	7,91	13,91	19,01	28,38	1,12	3,94	26,19	30,58
Jawa Tengah	41,33	1,47	3,55	38,46	44,21	32,44	1,40	4,32	29,69	35,19	36,87	1,02	2,76	34,88	38,86
DI Yogyakarta	54,44	4,13	7,59	46,35	62,54	31,01	5,27	17,00	20,68	41,35	49,24	3,48	7,07	42,42	56,06
Jawa Timur	40,71	1,51	3,71	37,75	43,67	28,13	1,30	4,64	25,57	30,69	34,70	1,01	2,90	32,73	36,68
Banten	35,90	2,53	7,04	30,94	40,85	14,94	2,37	15,88	10,29	19,60	29,59	1,89	6,39	25,88	33,29
Bali	60,08	2,71	4,50	54,78	65,38	48,82	5,31	10,88	38,40	59,23	56,48	2,46	4,36	51,65	61,30
Nusa Tenggara Barat	23,42	2,23	9,52	19,05	27,79	17,84	2,01	11,24	13,91	21,77	20,29	1,50	7,39	17,34	23,23
Nusa Tenggara Timur	43,78	3,90	8,91	36,14	51,43	13,67	1,02	7,43	11,68	15,66	19,98	1,20	5,99	17,64	22,33
Kalimantan Barat	35,55	2,95	8,29	29,78	41,33	13,50	1,36	10,09	10,83	16,17	21,26	1,46	6,85	18,40	24,11
Kalimantan Tengah	25,93	3,22	12,41	19,62	32,23	10,17	1,57	15,42	7,10	13,25	16,19	1,60	9,88	13,06	19,33
Kalimantan Selatan	32,35	2,72	8,41	27,02	37,68	21,85	1,90	8,71	18,12	25,58	26,81	1,64	6,12	23,60	30,03
Kalimantan Timur	42,94	2,86	6,65	37,34	48,54	34,23	4,31	12,60	25,78	42,68	40,13	2,37	5,89	35,49	44,76
Kalimantan Utara	28,88	3,93	13,63	21,16	36,59	19,61	3,83	19,52	12,11	27,11	25,07	2,84	11,31	19,51	30,63
Sulawesi Utara	54,96	3,56	6,47	47,98	61,94	38,71	2,71	7,01	33,39	44,03	47,03	2,28	4,85	42,56	51,50
Sulawesi Tengah	38,83	4,75	12,23	29,53	48,14	18,20	1,85	10,15	14,57	21,82	23,44	1,83	7,81	19,85	27,02
Sulawesi Selatan	43,89	2,63	5,99	38,74	49,04	21,08	1,38	6,53	18,38	23,78	30,67	1,39	4,54	27,94	33,40
Sulawesi Tenggara	19,88	3,25	16,34	13,51	26,25	10,13	1,33	13,12	7,52	12,74	13,73	1,43	10,42	10,92	16,53
Gorontalo	53,59	4,92	9,19	43,94	63,24	23,95	3,09	12,90	17,90	30,01	35,91	2,72	7,58	30,58	41,24
Sulawesi Barat	23,66	5,78	24,42	12,33	34,99	10,89	1,73	15,90	7,50	14,29	13,70	1,84	13,47	10,08	17,31
Maluku	26,76	4,38	16,36	18,18	35,34	6,70	1,38	20,55	4,00	9,40	14,68	1,94	13,22	10,88	18,49
Maluku Utara	44,53	4,68	10,52	35,35	53,72	8,41	1,45	17,21	5,57	11,24	18,51	1,93	10,44	14,73	22,30
Papua Barat	33,05	4,46	13,50	24,30	41,79	19,71	2,83	14,34	14,17	25,25	25,39	2,50	9,85	20,49	30,30
Papua	38,14	3,08	8,08	32,10	44,18	8,03	1,12	13,91	5,84	10,22	16,60	1,22	7,34	14,21	18,98
	**38,81**	**0,57**	**1,47**	**37,68**	**39,93**	**21,78**	**0,37**	**1,72**	**21,04**	**22,51**	**30,92**	**0,35**	**1,15**	**30,23**	**31,62**

Sumber/*Source*: Susenas Maret 2018/*The March 2018 Susenas*

https://www.bps.go.id

Tabel 9.13. ***Sampling Error*** **Persentase Perempuan Berumur 15-49 Tahun Pernah Kawin yang Pernah Melahirkan dan Penolong Proses Kelahiran Terakhir Bidan menurut Provinsi dan Daerah Tempat Tinggal, 2018**

Table 9.13. Sampling Errors Percentage of Ever Married Female Aged 15-49 Years Who Have Given Birth and Last Birth Attendant by Midwife by Province and Urban Rural Classsification, 2018

**Provinsi** *Province*	**Perkotaan/***Urban*					**Perdesaan/***Rural*					**Perkotaan+Perdesaan/***Urban+Rural*				
	**Estimasi** *Estimate*	*Standard Error*	*Relative Standard Error (%)*	**Selang Kepercayaan 95%** *Confidence Interval 95%*		**Estimasi** *Estimate*	*Standard Error*	*Relative Standard Error (%)*	**Selang Kepercayaan 95%** *Confidence Interval 95%*		**Estimasi** *Estimate*	*Standard Error*	*Relative Standard Error (%)*	**Selang Kepercayaan 95%** *Confidence Interval 95%*	
				**Batas Bawah** *Lower Limit*	**Batas Atas** *Upper Limit*				**Batas Bawah** *Lower Limit*	**Batas Atas** *Upper Limit*				**Batas Bawah** *Lower Limit*	**Batas Atas** *Upper Limit*
(1)	(2)	(3)	(4)	(5)	(6)	(7)	(8)	(9)	(10)	(11)	(12)	(13)	(14)	(15)	(16)
Aceh	53,87	2,79	5,18	48,40	59,34	68,31	1,56	2,28	65,26	71,36	63,93	1,38	2,16	61,21	66,64
Sumatera Utara	53,44	2,36	4,41	48,82	58,06	66,19	1,70	2,56	62,87	69,51	59,77	1,46	2,45	56,90	62,63
Sumatera Barat	58,23	3,02	5,18	52,32	64,14	63,17	2,00	3,16	59,25	67,09	61,03	1,72	2,82	57,65	64,41
Riau	55,68	2,99	5,38	49,81	61,55	60,31	2,22	3,69	55,95	64,67	58,36	1,80	3,09	54,83	61,89
Jambi	59,75	4,20	7,03	51,52	67,98	63,67	2,16	3,40	59,42	67,91	62,49	1,98	3,17	58,60	66,38
Sumatera Selatan	52,51	3,54	6,75	45,56	59,45	71,08	1,79	2,52	67,58	74,59	64,91	1,72	2,64	61,55	68,28
Bengkulu	52,87	4,69	8,88	43,67	62,07	68,32	2,61	3,82	63,21	73,43	63,43	2,29	3,62	58,93	67,93
Lampung	72,93	3,16	4,33	66,74	79,13	75,60	1,65	2,19	72,36	78,85	74,86	1,49	1,99	71,93	77,78
Kep. Bangka Belitung	59,53	4,06	6,82	51,58	67,49	67,56	3,68	5,44	60,35	74,77	63,04	2,76	4,37	57,64	68,45
Kep. Riau	48,61	4,01	8,24	40,75	56,46	58,09	8,09	13,93	42,23	73,96	49,81	3,68	7,39	42,60	57,02
DKI Jakarta	47,32	2,58	5,45	42,27	52,37	-	-	-	-	-	47,32	2,58	5,45	42,27	52,37
Jawa Barat	60,85	1,45	2,39	58,00	63,69	67,81	1,78	2,62	64,32	71,30	62,60	1,17	1,88	60,30	64,90
Jawa Tengah	55,44	1,49	2,69	52,52	58,35	64,21	1,44	2,24	61,38	67,03	59,84	1,04	1,74	57,80	61,87
DI Yogyakarta	41,46	4,09	9,87	33,44	49,48	58,41	5,84	10,00	46,96	69,86	45,23	3,46	7,65	38,45	52,01
Jawa Timur	56,32	1,52	2,69	53,35	59,29	62,95	1,39	2,21	60,22	65,67	59,48	1,03	1,74	57,46	61,51
Banten	58,32	2,67	4,57	53,09	63,55	58,17	3,36	5,77	51,59	64,76	58,27	2,12	3,64	54,12	62,43
Bali	36,02	2,66	7,39	30,80	41,24	49,70	5,30	10,66	39,32	60,09	40,40	2,42	6,00	35,65	45,15
Nusa Tenggara Barat	68,57	2,49	3,64	63,68	73,45	73,26	2,26	3,09	68,82	77,69	71,20	1,67	2,35	67,92	74,48
Nusa Tenggara Timur	45,31	4,17	9,21	37,12	53,49	59,40	1,51	2,54	56,45	62,35	56,45	1,48	2,61	53,55	59,34
Kalimantan Barat	57,43	2,94	5,11	51,68	63,19	63,41	2,07	3,27	59,36	67,47	61,31	1,72	2,81	57,94	64,68
Kalimantan Tengah	67,53	3,43	5,09	60,80	74,26	65,84	2,68	4,07	60,58	71,10	66,49	2,12	3,19	62,33	70,64
Kalimantan Selatan	65,03	2,79	4,29	59,56	70,50	69,16	2,07	3,00	65,10	73,22	67,21	1,72	2,56	63,83	70,58
Kalimantan Timur	53,90	3,02	5,60	47,98	59,81	58,48	4,35	7,44	49,96	67,01	55,38	2,46	4,45	50,55	60,21
Kalimantan Utara	68,98	4,12	5,98	60,90	77,07	67,79	5,17	7,63	57,66	77,92	68,49	3,20	4,67	62,23	74,76
Sulawesi Utara	32,43	3,34	10,28	25,89	38,97	47,85	2,81	5,87	42,34	53,35	39,95	2,21	5,53	35,63	44,28
Sulawesi Tengah	49,45	5,03	10,17	39,59	59,31	62,46	2,21	3,55	58,12	66,80	59,15	2,07	3,50	55,09	63,21
Sulawesi Selatan	51,12	2,44	4,78	46,33	55,91	70,36	1,63	2,32	67,15	73,56	62,27	1,42	2,28	59,48	65,05
Sulawesi Tenggara	65,96	3,95	5,99	58,22	73,70	73,39	1,95	2,66	69,57	77,21	70,65	2,01	2,85	66,70	74,59
Gorontalo	43,67	5,16	11,81	33,56	53,78	64,98	3,57	5,50	57,98	71,98	56,38	2,95	5,24	50,59	62,17
Sulawesi Barat	70,65	6,43	9,10	58,05	83,26	74,21	2,47	3,33	69,36	79,05	73,43	2,38	3,24	68,77	78,09
Maluku	52,32	4,44	8,48	43,62	61,01	42,48	2,82	6,63	36,96	48,00	46,40	2,47	5,33	41,55	51,25
Maluku Utara	46,42	4,85	10,45	36,91	55,92	57,84	2,69	4,65	52,57	63,11	54,64	2,44	4,46	49,86	59,43
Papua Barat	58,08	4,50	7,74	49,26	66,89	48,82	2,95	6,05	43,03	54,61	52,76	2,58	4,89	47,71	57,82
Papua	48,81	3,36	6,88	42,23	55,39	33,73	1,94	5,75	29,93	37,53	38,02	1,71	4,50	34,67	41,37
**Indonesia**	**56,13**	**0,58**	**1,03**	**54,99**	**57,27**	**64,48**	**0,43**	**0,67**	**63,63**	**65,33**	**60,00**	**0,37**	**0,62**	**59,27**	**60,72**

Sumber/*Source*: Susenas Maret 2018/*The March 2018 Susenas*

https://www.bps.go.id

Tabel / *Table* **9.14.**

Sampling Error **Persentase Perempuan Berumur 15-49 Tahun yang Pernah Kawin dan Pernah Menggunakan Alat KB atau Cara Tradisional untuk Menunda atau Mencegah Kehamilan menurut Provinsi dan Daerah Tempat Tinggal, 2018**

Sampling Errors Percentage of Ever Married Female Aged 15-49 and The Ever Used of Contraception or a Traditional Method to Prevent or Delay Pregnancy by Province and Urban Rural Classification, 2018

Provinsi *Province*	Perkotaan/*Urban*					Perdesaan/*Rural*					Perkotaan+Perdesaan/*Urban+Rural*				
	Estimasi *Estimate*	*Standard Error*	*Relative Standard Error (%)*	Selang Kepercayaan 95% *Confidence Interval 95%*		Estimasi *Estimate*	*Standard Error*	*Relative Standard Error (%)*	Selang Kepercayaan 95% *Confidence Interval 95%*		Estimasi *Estimate*	*Standard Error*	*Relative Standard Error (%)*	Selang Kepercayaan 95% *Confidence Interval 95%*	
				Batas Bawah *Lower Limit*	Batas Atas *Upper Limit*				Batas Bawah *Lower Limit*	Batas Atas *Upper Limit*				Batas Bawah *Lower Limit*	Batas Atas *Upper Limit*
(1)	(2)	(3)	(4)	(5)	(6)	(7)	(8)	(9)	(10)	(11)	(12)	(13)	(14)	(15)	(16)
Aceh	18,23	1,20	6,59	15,87	20,58	15,93	0,73	4,58	14,50	17,36	16,63	0,62	3,75	15,41	17,85
Sumatera Utara	10,85	0,73	6,71	9,42	12,28	12,31	0,57	4,60	11,20	13,42	11,54	0,47	4,08	10,62	12,46
Sumatera Barat	13,95	1,13	8,09	11,73	16,16	16,82	0,84	4,97	15,18	18,46	15,59	0,69	4,40	14,25	16,94
Riau	13,77	0,96	7,01	11,88	15,66	12,71	0,75	5,93	11,23	14,18	13,12	0,61	4,61	11,93	14,30
Jambi	11,51	1,33	11,58	8,90	14,12	11,60	0,78	6,70	10,07	13,12	11,57	0,68	5,92	10,23	12,91
Sumatera Selatan	10,42	0,95	9,14	8,55	12,29	10,69	0,60	5,65	9,51	11,88	10,60	0,52	4,87	9,59	11,61
Bengkulu	16,30	1,76	10,82	12,84	19,76	12,02	0,87	7,21	10,32	13,72	13,32	0,82	6,13	11,72	14,92
Lampung	12,87	1,10	8,55	10,72	15,03	11,95	0,63	5,31	10,71	13,20	12,21	0,55	4,52	11,13	13,29
Kep. Bangka Belitung	13,94	1,28	9,16	11,43	16,44	10,33	0,96	9,28	8,45	12,21	12,24	0,82	6,73	10,63	13,86
Kep. Riau	10,40	1,05	10,08	8,35	12,46	17,01	3,19	18,78	10,75	23,27	11,32	1,00	8,86	9,36	13,29
DKI Jakarta	8,94	0,60	6,76	7,75	10,12	-	-	-	-	-	8,94	0,60	6,76	7,75	10,12
Jawa Barat	9,06	0,38	4,16	8,32	9,80	11,24	0,54	4,79	10,19	12,30	9,65	0,31	3,24	9,03	10,26
Jawa Tengah	12,86	0,47	3,63	11,95	13,77	12,68	0,47	3,69	11,77	13,60	12,77	0,33	2,59	12,12	13,42
DI Yogyakarta	10,37	0,97	9,35	8,47	12,26	12,40	1,45	11,68	9,56	15,24	10,94	0,83	7,55	9,32	12,55
Jawa Timur	10,76	0,44	4,08	9,90	11,62	12,62	0,45	3,54	11,74	13,49	11,68	0,31	2,69	11,06	12,29
Banten	10,59	0,61	5,77	9,39	11,79	14,37	1,11	7,71	12,20	16,55	11,69	0,54	4,65	10,62	12,75
Bali	9,38	0,74	7,91	7,92	10,83	10,11	0,96	9,46	8,23	11,98	9,62	0,60	6,21	8,45	10,79
Nusa Tenggara Barat	21,77	1,47	6,75	18,89	24,64	24,06	1,43	5,96	21,25	26,87	23,04	1,04	4,50	21,01	25,08
Nusa Tenggara Timur	12,53	1,21	9,63	10,17	14,90	18,80	0,69	3,70	17,44	20,17	17,46	0,62	3,54	16,25	18,67
Kalimantan Barat	13,53	1,29	9,54	11,00	16,06	12,77	0,74	5,82	11,31	14,23	13,01	0,65	5,03	11,73	14,29
Kalimantan Tengah	15,78	1,31	8,32	13,20	18,35	12,49	0,93	7,43	10,67	14,30	13,72	0,77	5,63	12,20	15,23
Kalimantan Selatan	19,66	1,31	6,68	17,08	22,24	18,84	0,93	4,91	17,03	20,66	19,21	0,78	4,08	17,67	20,74
Kalimantan Timur	10,30	0,99	9,63	8,36	12,25	10,84	1,26	11,60	8,37	13,30	10,48	0,79	7,49	8,94	12,02
Kalimantan Utara	17,51	2,27	12,99	13,05	21,97	16,51	1,92	11,62	12,75	20,27	17,09	1,54	9,01	14,07	20,11
Sulawesi Utara	14,63	1,19	8,11	12,31	16,96	9,37	0,77	8,23	7,86	10,88	11,94	0,70	5,90	10,56	13,32
Sulawesi Tengah	14,65	1,61	11,02	11,48	17,82	13,02	0,81	6,23	11,43	14,61	13,43	0,73	5,44	12,00	14,87
Sulawesi Selatan	18,90	1,06	5,59	16,83	20,97	17,14	0,71	4,13	15,75	18,52	17,83	0,60	3,36	16,66	19,01
Sulawesi Tenggara	17,67	1,50	8,48	14,73	20,61	22,04	1,11	5,03	19,86	24,21	20,49	0,94	4,57	18,65	22,32
Gorontalo	17,91	2,17	12,11	13,66	22,16	14,18	1,19	8,38	11,85	16,51	15,53	1,09	7,05	13,38	17,67
Sulawesi Barat	17,60	2,26	12,86	13,16	22,03	15,50	1,20	7,71	13,15	17,84	15,96	1,05	6,59	13,90	18,02
Maluku	14,61	1,55	10,58	11,58	17,64	12,91	1,13	8,74	10,70	15,12	13,59	0,92	6,76	11,79	15,39
Maluku Utara	16,60	1,84	11,08	12,99	20,21	13,99	0,93	6,67	12,16	15,82	14,71	0,84	5,71	13,07	16,36
Papua Barat	16,66	2,09	12,52	12,57	20,74	10,70	1,21	11,34	8,32	13,08	13,05	1,11	8,48	10,89	15,22
Papua	11,29	1,09	9,62	9,16	13,42	6,97	0,52	7,44	5,95	7,99	7,99	0,48	5,95	7,06	8,93
**Indonesia**	**11,42**	**0,16**	**1,43**	**11,10**	**11,74**	**13,31**	**0,15**	**1,16**	**13,01**	**13,61**	**12,30**	**0,11**	**0,92**	**12,08**	**12,52**

Sumber/*Source*: Susenas Maret 2018/*The March 2018 Susenas*

https://www.bps.go.id

Tabel 9.15. ***Sampling Error* Persentase Perempuan Berumur 15-49 Tahun yang Pernah Kawin dan Sedang Menggunakan Alat KB atau Cara Tradisional untuk Menunda atau Mencegah Kehamilan menurut Provinsi dan Daerah Tempat Tinggal, 2018**

Table *Sampling Errors Percentage of Ever Married Female Aged 15-49 and The Currently Use of Contraception or a Traditional Method to Prevent or Delay Pregnancy by Province and Urban Rural Classification, 2018*

**Provinsi** *Province*	**Perkotaan**/*Urban*					**Perdesaan**/*Rural*					**Perkotaan+Perdesaan**/*Urban+Rural*				
	**Estimasi** *Estimate*	*Standard Error*	*Relative Standard Error (%)*	**Selang Kepercayaan 95%** *Confidence Interval 95%*		**Estimasi** *Estimate*	*Standard Error*	*Relative Standard Error (%)*	**Selang Kepercayaan 95%** *Confidence Interval 95%*		**Estimasi** *Estimate*	*Standard Error*	*Relative Standard Error (%)*	**Selang Kepercayaan 95%** *Confidence Interval 95%*	
				**Batas Bawah** *Lower Limit*	**Batas Atas** *Upper Limit*				**Batas Bawah** *Lower Limit*	**Batas Atas** *Upper Limit*				**Batas Bawah** *Lower Limit*	**Batas Atas** *Upper Limit*
(1)	(2)	(3)	(4)	(5)	(6)	(7)	(8)	(9)	(10)	(11)	(12)	(13)	(14)	(15)	(16)
Aceh	42,25	1,41	3,33	39,50	45,00	44,02	0,87	1,97	42,32	45,73	43,48	0,74	1,70	42,03	44,93
Sumatera Utara	46,34	1,08	2,34	44,22	48,46	43,74	0,90	2,06	41,97	45,50	45,12	0,71	1,58	43,72	46,52
Sumatera Barat	40,61	1,58	3,90	37,51	43,71	46,71	1,00	2,14	44,75	48,66	44,11	0,89	2,01	42,37	45,84
Riau	42,81	1,64	3,83	39,60	46,03	56,80	1,09	1,92	54,66	58,94	51,35	0,94	1,84	49,50	53,20
Jambi	49,40	1,97	3,99	45,54	53,27	62,58	1,04	1,66	60,55	64,61	58,62	0,94	1,61	56,77	60,47
Sumatera Selatan	52,85	1,56	2,94	49,80	55,90	67,48	0,85	1,26	65,81	69,15	62,46	0,78	1,25	60,93	63,99
Bengkulu	53,92	2,49	4,63	49,03	58,80	65,80	1,16	1,77	63,52	68,08	62,20	1,12	1,81	60,00	64,41
Lampung	56,36	1,69	3,00	53,05	59,68	66,45	0,82	1,23	64,84	68,06	63,66	0,75	1,18	62,19	65,14
Kep. Bangka Belitung	57,05	1,66	2,91	53,80	60,30	67,21	1,57	2,34	64,13	70,30	61,83	1,17	1,88	59,54	64,11
Kep. Riau	43,29	2,13	4,92	39,12	47,46	60,00	2,95	4,92	54,21	65,78	45,62	1,93	4,23	41,84	49,40
DKI Jakarta	47,51	1,20	2,53	45,16	49,87	-	-	-	-	-	47,51	1,20	2,53	45,16	49,87
Jawa Barat	58,38	0,64	1,09	57,13	59,62	64,99	0,76	1,17	63,51	66,48	60,15	0,51	0,85	59,15	61,15
Jawa Tengah	53,40	0,66	1,24	52,11	54,69	62,67	0,64	1,02	61,42	63,92	58,10	0,46	0,79	57,19	59,00
DI Yogyakarta	50,02	1,66	3,32	46,76	53,28	62,78	2,07	3,30	58,72	66,85	53,59	1,33	2,48	50,99	56,19
Jawa Timur	56,18	0,66	1,17	54,89	57,47	62,12	0,60	0,96	60,95	63,29	59,11	0,45	0,76	58,23	59,98
Banten	56,51	1,12	1,99	54,31	58,72	61,79	1,37	2,22	59,10	64,48	58,04	0,89	1,53	56,30	59,79
Bali	55,49	1,19	2,14	53,16	57,82	67,44	1,82	2,70	63,87	71,00	59,48	1,07	1,80	57,39	61,58
Nusa Tenggara Barat	51,02	1,54	3,03	47,99	54,05	50,03	1,28	2,55	47,53	52,54	50,47	0,99	1,96	48,53	52,41
Nusa Tenggara Timur	43,54	1,97	4,53	39,68	47,41	39,76	0,83	2,08	38,14	41,37	40,57	0,79	1,94	39,02	42,12
Kalimantan Barat	52,97	1,79	3,37	49,46	56,47	64,68	0,91	1,40	62,90	66,46	60,99	0,84	1,38	59,34	62,63
Kalimantan Tengah	57,48	1,58	2,76	54,38	60,59	66,52	1,14	1,71	64,29	68,75	63,14	0,94	1,49	61,31	64,98
Kalimantan Selatan	59,32	1,43	2,41	56,52	62,13	65,61	0,91	1,39	63,82	67,39	62,80	0,82	1,30	61,20	64,41
Kalimantan Timur	51,16	1,36	2,67	48,48	53,83	60,68	1,71	2,81	57,34	64,03	54,36	1,08	1,99	52,24	56,49
Kalimantan Utara	42,52	2,24	5,26	38,14	46,91	54,08	2,29	4,23	49,60	58,55	47,37	1,68	3,54	44,08	50,65
Sulawesi Utara	53,25	1,39	2,62	50,52	55,98	66,05	1,09	1,64	63,92	68,17	59,80	0,90	1,50	58,04	61,56
Sulawesi Tengah	47,79	2,25	4,71	43,38	52,20	56,17	1,00	1,78	54,21	58,13	54,03	0,94	1,74	52,19	55,87
Sulawesi Selatan	41,54	1,29	3,11	39,01	44,07	46,81	0,86	1,83	45,12	48,49	44,72	0,73	1,64	43,29	46,16
Sulawesi Tenggara	51,74	1,97	3,81	47,88	55,61	47,81	1,21	2,53	45,44	50,17	49,21	1,12	2,28	47,00	51,41
Gorontalo	50,24	2,29	4,56	45,75	54,73	61,92	1,70	2,74	58,59	65,24	57,69	1,36	2,36	55,02	60,35
Sulawesi Barat	44,21	2,92	6,61	38,48	49,94	48,49	1,64	3,37	45,29	51,70	47,55	1,44	3,03	44,73	50,37
Maluku	40,79	2,22	5,44	36,44	45,14	37,95	1,78	4,70	34,46	41,45	39,09	1,39	3,55	36,36	41,81
Maluku Utara	42,57	2,58	6,05	37,52	47,61	52,13	1,33	2,55	49,52	54,74	49,47	1,19	2,41	47,14	51,80
Papua Barat	31,81	2,63	8,25	26,67	36,96	34,94	1,86	5,33	31,29	38,59	33,71	1,54	4,56	30,70	36,72
Papua	35,64	1,99	5,58	31,75	39,54	18,78	1,01	5,40	16,79	20,77	22,77	0,91	4,02	20,97	24,56
**Indonesia**	**53,25**	**0,26**	**0,49**	**52,73**	**53,76**	**58,10**	**0,20**	**0,35**	**57,70**	**58,50**	**55,50**	**0,17**	**0,31**	**55,17**	**55,83**

Sumber/*Source*: Susenas Maret 2018/*The March 2018 Susenas*

Tabel 9.16. ***Sampling Error*** **Persentase Perempuan Berumur 15-49 Tahun yang Pernah Kawin dan Tidak Menggunakan Alat KB atau Cara Tradisional untuk Menunda atau Mencegah Kehamilan menurut Provinsi dan Daerah Tempat Tinggal, 2018**

Table *Sampling Errors Percentage of Ever Married Female Aged 15-49 and The Never Used of Contraception or a Traditional Method to Prevent or Delay Pregnancy by Province and Urban Rural Classification, 2018*

Provinsi *Province*	Perkotaan/*Urban*					Perdesaan/*Rural*					Perkotaan+Perdesaan/*Urban+Rural*				
	Estimasi *Estimate*	*Standard Error*	*Relative Standard Error (%)*	Selang Kepercayaan 95% *Confidence Interval 95%*		Estimasi *Estimate*	*Standard Error*	*Relative Standard Error (%)*	Selang Kepercayaan 95% *Confidence Interval 95%*		Estimasi *Estimate*	*Standard Error*	*Relative Standard Error (%)*	Selang Kepercayaan 95% *Confidence Interval 95%*	
				Batas Bawah *Lower Limit*	Batas Atas *Upper Limit*				Batas Bawah *Lower Limit*	Batas Atas *Upper Limit*				Batas Bawah *Lower Limit*	Batas Atas *Upper Limit*
(1)	(2)	(3)	(4)	(5)	(6)	(7)	(8)	(9)	(10)	(11)	(12)	(13)	(14)	(15)	(16)
Aceh	39,52	1,65	4,17	36,29	42,75	40,05	0,99	2,46	38,11	41,98	39,89	0,85	2,13	38,22	41,55
Sumatera Utara	42,81	1,21	2,82	40,44	45,17	43,95	0,89	2,03	42,20	45,70	43,35	0,77	1,77	41,84	44,85
Sumatera Barat	45,44	1,67	3,66	42,18	48,71	36,48	1,05	2,89	34,41	38,54	40,30	0,93	2,31	38,47	42,12
Riau	43,42	1,85	4,26	39,80	47,05	30,49	1,05	3,44	28,44	32,54	35,53	0,98	2,77	33,60	37,46
Jambi	39,09	1,92	4,90	35,33	42,84	25,82	0,97	3,76	23,92	27,73	29,81	0,90	3,03	28,04	31,58
Sumatera Selatan	36,73	1,61	4,39	33,57	39,88	21,83	0,80	3,65	20,27	23,39	26,94	0,77	2,86	25,43	28,45
Bengkulu	29,78	2,19	7,34	25,50	34,07	22,18	1,08	4,86	20,07	24,29	24,48	1,00	4,07	22,53	26,44
Lampung	30,76	1,62	5,27	27,58	33,94	21,60	0,75	3,47	20,13	23,07	24,13	0,71	2,93	22,74	25,52
Kep. Bangka Belitung	29,01	1,76	6,08	25,56	32,47	22,45	1,53	6,79	19,47	25,44	25,93	1,17	4,53	23,63	28,23
Kep. Riau	46,30	2,25	4,86	41,89	50,71	22,99	2,36	10,25	18,37	27,61	43,06	2,04	4,74	39,05	47,06
DKI Jakarta	43,55	1,26	2,89	41,08	46,02	-	-	-	-	-	43,55	1,26	2,89	41,08	46,02
Jawa Barat	32,56	0,70	2,15	31,19	33,93	23,76	0,80	3,37	22,19	25,33	30,21	0,56	1,85	29,11	31,30
Jawa Tengah	33,74	0,67	1,97	32,44	35,05	24,64	0,62	2,50	23,44	25,85	29,13	0,46	1,57	28,24	30,03
DI Yogyakarta	39,62	1,83	4,61	36,04	43,20	24,81	2,35	9,47	20,20	29,42	35,48	1,47	4,14	32,60	38,35
Jawa Timur	33,06	0,66	1,99	31,77	34,35	25,26	0,56	2,22	24,17	26,36	29,22	0,44	1,49	28,36	30,07
Banten	32,89	1,18	3,59	30,58	35,21	23,83	1,33	5,59	21,22	26,45	30,27	0,93	3,06	28,46	32,09
Bali	35,13	1,25	3,55	32,69	37,58	22,45	1,66	7,37	19,21	25,70	30,90	1,05	3,39	28,84	32,95
Nusa Tenggara Barat	27,22	1,53	5,63	24,21	30,22	25,91	1,31	5,04	23,34	28,47	26,49	1,00	3,77	24,53	28,44
Nusa Tenggara Timur	43,92	2,02	4,59	39,97	47,87	41,44	0,88	2,13	39,71	43,17	41,97	0,82	1,94	40,37	43,57
Kalimantan Barat	33,51	1,65	4,91	30,28	36,73	22,55	0,85	3,75	20,89	24,21	26,00	0,78	3,01	24,47	27,54
Kalimantan Tengah	26,74	1,48	5,53	23,84	29,64	21,00	1,01	4,79	19,02	22,97	23,14	0,85	3,68	21,47	24,81
Kalimantan Selatan	21,02	1,22	5,80	18,63	23,40	15,55	0,82	5,27	13,94	17,16	17,99	0,71	3,94	16,60	19,38
Kalimantan Timur	38,54	1,49	3,86	35,63	41,46	28,48	1,82	6,38	24,92	32,04	35,15	1,18	3,35	32,84	37,46
Kalimantan Utara	39,97	2,57	6,43	34,93	45,01	29,41	2,13	7,25	25,24	33,59	35,54	1,78	5,01	32,05	39,03
Sulawesi Utara	32,12	1,35	4,21	29,47	34,77	24,58	1,02	4,14	22,59	26,58	28,26	0,86	3,03	26,58	29,94
Sulawesi Tengah	37,56	2,36	6,28	32,93	42,18	30,82	1,06	3,43	28,75	32,89	32,54	0,99	3,03	30,61	34,47
Sulawesi Selatan	39,56	1,32	3,34	36,97	42,16	36,06	0,87	2,40	34,36	37,75	37,44	0,74	1,98	35,99	38,90
Sulawesi Tenggara	30,59	1,84	6,02	26,98	34,19	30,15	1,16	3,86	27,87	32,44	30,31	1,01	3,34	28,32	32,30
Gorontalo	31,85	2,24	7,04	27,46	36,25	23,91	1,52	6,37	20,92	26,89	26,78	1,26	4,70	24,32	29,25
Sulawesi Barat	38,19	3,35	8,78	31,62	44,76	36,01	1,83	5,08	32,43	39,59	36,49	1,62	4,43	33,32	39,66
Maluku	44,60	2,35	5,27	39,99	49,20	49,14	1,95	3,98	45,31	52,97	47,32	1,50	3,18	44,38	50,27
Maluku Utara	40,83	2,70	6,61	35,54	46,13	33,88	1,31	3,86	31,32	36,45	35,82	1,21	3,38	33,44	38,19
Papua Barat	51,53	2,84	5,52	45,96	57,10	54,36	1,78	3,28	50,86	57,85	53,24	1,58	2,97	50,14	56,34
Papua	53,06	2,04	3,85	49,06	57,07	74,25	1,11	1,50	72,07	76,43	69,24	0,99	1,43	67,30	71,19
**Indonesia**	**35,33**	**0,28**	**0,79**	**34,79**	**35,87**	**28,59**	**0,20**	**0,70**	**28,20**	**28,99**	**32,20**	**0,18**	**0,55**	**31,86**	**32,55**

Sumber/*Source*: Susenas Maret 2018/*The March 2018 Susenas*

https://www.bps.go.id

Tabel *Table* **9.17.** ***Sampling Error* Persentase Penduduk Berumur 5 Tahun ke Atas yang Menggunakan Telepon Seluler (HP)/Nirkabel dalam 3 Bulan Terakhir menurut Provinsi dan Daerah Tempat Tinggal, 2018**

Sampling Errors Percentage of Population Aged 5 Years and Over Who Use Cellular Phone (HP) during The Last 3 Months by Province and Urban Rural Classification, 2018

Provinsi *Province*	**Perkotaan/*Urban***					**Perdesaan/*Rural***					**Perkotaan+Perdesaan/*Urban+Rural***				
	**Estimasi** *Estimate*	*Standard Error*	*Relative Standard Error (%)*	**Selang Kepercayaan 95%** *Confidence Interval 95%*		**Estimasi** *Estimate*	*Standard Error*	*Relative Standard Error (%)*	**Selang Kepercayaan 95%** *Confidence Interval 95%*		**Estimasi** *Estimate*	*Standard Error*	*Relative Standard Error (%)*	**Selang Kepercayaan 95%** *Confidence Interval 95%*	
				**Batas Bawah** *Lower Limit*	**Batas Atas** *Upper Limit*				**Batas Bawah** *Lower Limit*	**Batas Atas** *Upper Limit*				**Batas Bawah** *Lower Limit*	**Batas Atas** *Upper Limit*
(1)	(2)	(3)	(4)	(5)	(6)	(7)	(8)	(9)	(10)	(11)	(12)	(13)	(14)	(15)	(16)
Aceh	79,16	0,80	1,00	77,60	80,72	66,71	0,64	0,97	65,45	67,97	70,62	0,52	0,74	69,60	71,65
Sumatera Utara	82,41	0,67	0,81	81,09	83,72	73,16	0,67	0,92	71,84	74,47	78,08	0,48	0,61	77,14	79,02
Sumatera Barat	82,86	0,83	1,00	81,24	84,49	73,96	0,74	1,00	72,51	75,40	77,89	0,55	0,71	76,81	78,98
Riau	87,02	0,70	0,80	85,65	88,38	76,60	0,74	0,97	75,14	78,06	80,81	0,54	0,67	79,75	81,87
Jambi	84,36	1,05	1,25	82,30	86,42	72,37	0,82	1,13	70,77	73,97	76,22	0,68	0,89	74,88	77,55
Sumatera Selatan	80,99	0,90	1,11	79,22	82,76	70,89	0,69	0,97	69,55	72,24	74,67	0,56	0,75	73,57	75,76
Bengkulu	83,49	0,93	1,11	81,67	85,30	69,26	1,01	1,45	67,29	71,23	73,92	0,78	1,06	72,38	75,45
Lampung	77,87	1,17	1,50	75,58	80,16	75,00	0,66	0,88	73,70	76,29	75,85	0,58	0,76	74,72	76,98
Kep. Bangka Belitung	83,30	0,91	1,09	81,51	85,09	71,59	1,11	1,55	69,41	73,77	77,89	0,77	0,99	76,38	79,40
Kep. Riau	85,26	0,84	0,98	83,62	86,91	74,66	1,64	2,20	71,44	77,88	83,73	0,77	0,91	82,23	85,24
DKI Jakarta	81,69	0,60	0,73	80,51	82,86	-	-	-	-	-	81,69	0,60	0,73	80,51	82,86
Jawa Barat	75,66	0,45	0,59	74,79	76,54	65,28	0,60	0,93	64,09	66,46	72,97	0,37	0,50	72,25	73,68
Jawa Tengah	75,40	0,40	0,53	74,62	76,17	66,18	0,45	0,68	65,30	67,07	70,90	0,31	0,43	70,30	71,51
DI Yogyakarta	81,42	0,73	0,90	79,99	82,85	70,45	1,35	1,91	67,81	73,10	78,44	0,67	0,85	77,14	79,75
Jawa Timur	76,36	0,40	0,53	75,57	77,15	67,42	0,46	0,68	66,53	68,32	72,10	0,31	0,43	71,49	72,70
Banten	77,36	0,81	1,05	75,76	78,95	59,51	1,18	1,98	57,21	61,82	72,22	0,67	0,93	70,90	73,53
Bali	81,75	0,70	0,86	80,38	83,13	69,23	0,96	1,39	67,35	71,12	77,51	0,59	0,76	76,36	78,67
Nusa Tenggara Barat	76,25	1,06	1,40	74,16	78,33	67,68	0,96	1,42	65,80	69,56	71,67	0,74	1,03	70,23	73,11
Nusa Tenggara Timur	81,57	1,06	1,30	79,49	83,64	59,69	0,82	1,38	58,08	61,31	64,75	0,73	1,13	63,31	66,19
Kalimantan Barat	82,08	0,90	1,10	80,31	83,84	61,43	0,89	1,45	59,69	63,18	68,43	0,71	1,04	67,03	69,83
Kalimantan Tengah	84,52	0,91	1,07	82,74	86,29	72,46	0,93	1,28	70,64	74,28	77,12	0,70	0,91	75,74	78,49
Kalimantan Selatan	82,95	0,98	1,18	81,04	84,87	73,61	0,83	1,13	71,98	75,23	77,96	0,65	0,83	76,70	79,23
Kalimantan Timur	86,90	0,59	0,68	85,75	88,05	79,19	1,10	1,39	77,03	81,35	84,35	0,55	0,66	83,26	85,43
Kalimantan Utara	84,59	1,28	1,51	82,09	87,10	77,65	1,67	2,15	74,39	80,92	81,67	1,00	1,23	79,71	83,64
Sulawesi Utara	81,98	0,87	1,07	80,26	83,69	74,60	0,93	1,25	72,77	76,43	78,31	0,65	0,82	77,04	79,58
Sulawesi Tengah	83,11	1,33	1,60	80,50	85,72	64,80	0,99	1,52	62,87	66,73	69,92	0,84	1,20	68,27	71,57
Sulawesi Selatan	84,13	0,68	0,80	82,80	85,45	76,80	0,56	0,73	75,71	77,89	79,86	0,44	0,55	79,00	80,71
Sulawesi Tenggara	84,73	1,30	1,53	82,19	87,27	74,04	0,88	1,19	72,32	75,76	78,16	0,79	1,01	76,60	79,71
Gorontalo	81,41	1,24	1,53	78,98	83,85	74,66	1,32	1,77	72,07	77,25	77,28	0,96	1,24	75,41	79,16
Sulawesi Barat	79,71	1,72	2,16	76,34	83,09	65,78	1,61	2,44	62,64	68,93	69,08	1,34	1,95	66,44	71,71
Maluku	78,52	1,36	1,73	75,87	81,18	58,71	1,31	2,22	56,15	61,27	67,10	0,96	1,44	65,21	68,99
Maluku Utara	82,47	1,23	1,49	80,06	84,89	57,95	1,43	2,47	55,14	60,76	65,02	1,17	1,80	62,73	67,31
Papua Barat	81,78	1,38	1,69	79,08	84,49	65,18	1,63	2,50	61,99	68,36	71,95	1,16	1,61	69,68	74,22
Papua	79,63	1,24	1,56	77,20	82,07	33,86	1,12	3,30	31,67	36,05	46,53	0,97	2,09	44,62	48,44
	**78,61**	**0,17**	**0,21**	**78,29**	**78,93**	**67,91**	**0,16**	**0,23**	**67,60**	**68,22**	**73,77**	**0,12**	**0,16**	**73,54**	**74,00**

Sumber/*Source*: Susenas Maret 2018/*The March 2018 Susenas*

https://www.bps.go.id

Tabel *Table* **9.18.**

Sampling Error **Persentase Penduduk Berumur 5 Tahun ke Atas yang Memiliki/Menguasai Telepon Seluler (HP)/Nirkabel dalam 3 Bulan Terakhir menurut Provinsi dan Daerah Tempat Tinggal, 2018**

Sampling Errors Percentage of Population Aged 5 Years and Over Who Own Cellular Phone (HP) during The Last 3 Months by Province and Urban Rural Classification, 2018

Provinsi *Province*	Perkotaan/*Urban*					Perdesaan/*Rural*					Perkotaan+Perdesaan/*Urban+Rural*				
	Estimasi *Estimate*	*Standard Error*	*Relative Standard Error (%)*	Selang Kepercayaan 95% *Confidence Interval 95%*		Estimasi *Estimate*	*Standard Error*	*Relative Standard Error (%)*	Selang Kepercayaan 95% *Confidence Interval 95%*		Estimasi *Estimate*	*Standard Error*	*Relative Standard Error (%)*	Selang Kepercayaan 95% *Confidence Interval 95%*	
				Batas Bawah *Lower Limit*	Batas Atas *Upper Limit*				Batas Bawah *Lower Limit*	Batas Atas *Upper Limit*				Batas Bawah *Lower Limit*	Batas Atas *Upper Limit*
(1)	(2)	(3)	(4)	(5)	(6)	(7)	(8)	(9)	(10)	(11)	(12)	(13)	(14)	(15)	(16)
Aceh	70,40	0,77	1,09	68,90	71,90	53,84	0,54	1,01	52,77	54,91	59,05	0,47	0,80	58,12	59,97
Sumatera Utara	67,03	0,71	1,06	65,64	68,41	50,84	0,55	1,08	49,76	51,92	59,45	0,46	0,77	58,55	60,35
Sumatera Barat	72,38	0,83	1,14	70,76	74,00	55,27	0,63	1,13	54,04	56,50	62,83	0,53	0,84	61,79	63,87
Riau	76,32	0,84	1,10	74,67	77,97	61,92	0,61	0,99	60,72	63,12	67,74	0,52	0,76	66,73	68,75
Jambi	74,18	1,03	1,39	72,15	76,20	59,62	0,76	1,27	58,13	61,11	64,29	0,65	1,01	63,02	65,55
Sumatera Selatan	70,99	0,90	1,27	69,22	72,75	55,24	0,59	1,07	54,08	56,41	61,12	0,53	0,86	60,09	62,16
Bengkulu	72,42	1,01	1,39	70,44	74,40	54,42	0,76	1,40	52,93	55,92	60,31	0,65	1,09	59,03	61,59
Lampung	68,67	1,03	1,49	66,66	70,69	56,08	0,53	0,95	55,04	57,13	59,81	0,49	0,82	58,85	60,77
Kep. Bangka Belitung	74,69	1,03	1,38	72,67	76,71	61,10	0,97	1,59	59,20	63,00	68,41	0,78	1,15	66,87	69,95
Kep. Riau	76,25	0,96	1,25	74,37	78,12	61,44	1,93	3,14	57,66	65,22	74,11	0,89	1,21	72,36	75,86
DKI Jakarta	76,16	0,60	0,79	74,97	77,34	-	-	-	-	-	76,16	0,60	0,79	74,97	77,34
Jawa Barat	68,45	0,43	0,63	67,61	69,29	55,20	0,53	0,96	54,17	56,24	65,01	0,35	0,54	64,32	65,70
Jawa Tengah	65,44	0,37	0,57	64,71	66,17	55,25	0,37	0,67	54,52	55,98	60,47	0,27	0,45	59,94	61,00
DI Yogyakarta	72,34	0,81	1,12	70,75	73,92	57,53	1,29	2,24	55,00	60,06	68,32	0,70	1,03	66,94	69,69
Jawa Timur	67,52	0,38	0,56	66,77	68,26	53,16	0,38	0,72	52,41	53,90	60,66	0,28	0,47	60,11	61,22
Banten	70,14	0,78	1,11	68,61	71,67	49,71	0,93	1,88	47,88	51,54	64,25	0,61	0,96	63,05	65,46
Bali	73,93	0,66	0,90	72,62	75,23	56,40	0,92	1,64	54,59	58,21	67,99	0,56	0,83	66,88	69,10
Nusa Tenggara Barat	59,71	0,95	1,59	57,85	61,58	48,80	0,74	1,52	47,34	50,26	53,88	0,62	1,15	52,67	55,10
Nusa Tenggara Timur	65,86	1,18	1,80	63,54	68,18	37,32	0,55	1,46	36,25	38,39	43,91	0,58	1,32	42,78	45,05
Kalimantan Barat	73,38	0,86	1,18	71,69	75,07	50,51	0,71	1,42	49,11	51,91	58,25	0,63	1,08	57,02	59,48
Kalimantan Tengah	77,07	1,14	1,47	74,85	79,30	62,61	0,89	1,42	60,88	64,35	68,20	0,75	1,10	66,73	69,66
Kalimantan Selatan	74,34	1,07	1,44	72,24	76,43	58,79	0,74	1,26	57,34	60,25	66,04	0,67	1,02	64,72	67,36
Kalimantan Timur	79,31	0,67	0,85	77,99	80,62	70,42	0,98	1,39	68,50	72,33	76,36	0,58	0,75	75,23	77,49
Kalimantan Utara	78,46	1,18	1,50	76,15	80,77	67,83	1,60	2,35	64,70	70,97	73,99	0,99	1,33	72,06	75,92
Sulawesi Utara	72,24	0,94	1,31	70,39	74,09	56,96	0,77	1,36	55,44	58,48	64,64	0,64	0,98	63,40	65,89
Sulawesi Tengah	73,05	1,46	1,99	70,20	75,91	50,30	0,79	1,58	48,74	51,85	56,66	0,76	1,34	55,17	58,16
Sulawesi Selatan	72,20	0,70	0,97	70,83	73,58	57,86	0,49	0,85	56,90	58,83	63,85	0,43	0,67	63,00	64,69
Sulawesi Tenggara	69,65	1,32	1,90	67,06	72,24	54,50	0,71	1,30	53,11	55,89	60,34	0,68	1,12	59,01	61,66
Gorontalo	67,11	1,32	1,97	64,52	69,69	53,03	0,99	1,86	51,10	54,97	58,50	0,84	1,43	56,87	60,14
Sulawesi Barat	61,26	2,31	3,77	56,73	65,79	47,08	1,23	2,62	44,67	49,50	50,44	1,15	2,29	48,18	52,70
Maluku	70,58	1,42	2,01	67,81	73,36	45,50	1,25	2,74	43,06	47,95	56,13	0,96	1,70	54,25	58,00
Maluku Utara	75,12	1,34	1,78	72,50	77,74	44,81	1,14	2,53	42,59	47,04	53,55	1,04	1,95	51,51	55,59
Papua Barat	75,43	1,54	2,04	72,42	78,44	57,95	1,59	2,75	54,82	61,07	65,08	1,18	1,82	62,76	67,40
Papua	73,74	1,26	1,71	71,27	76,22	27,73	1,00	3,62	25,76	29,70	40,46	0,93	2,29	38,65	42,28
**Indonesia**	**69,66**	**0,16**	**0,23**	**69,35**	**69,98**	**53,61**	**0,13**	**0,25**	**53,35**	**53,87**	**62,41**	**0,11**	**0,17**	**62,19**	**62,62**

Sumber/*Source*: Susenas Maret 2018/*The March 2018 Susenas*

https://www.bps.go.id

Tabel *Table* **9.19.** ***Sampling Error*** **Persentase Penduduk Berumur 5 Tahun ke Atas yang Mengakses Internet (Termasuk Facebook, Twitter, BBM, Whatsapp) dalam 3 Bulan Terakhir menurut Provinsi dan Daerah Tempat Tinggal, 2018**

Sampling Errors Percentage of Population Aged 5 Years and Over Who Accessed The Internet (Including Facebook, Twitter, BBM, Whatsapp) during The Last 3 Months by Province and Urban Rural Classification, 2018

Provinsi *Province*	Perkotaan/*Urban*					Perdesaan/*Rural*					Perkotaan+Perdesaan/*Urban+Rural*				
	Estimasi *Estimate*	*Standard Error*	*Relative Standard Error (%)*	Selang Kepercayaan 95% *Confidence Interval 95%*		Estimasi *Estimate*	*Standard Error*	*Relative Standard Error (%)*	Selang Kepercayaan 95% *Confidence Interval 95%*		Estimasi *Estimate*	*Standard Error*	*Relative Standard Error (%)*	Selang Kepercayaan 95% *Confidence Interval 95%*	
				Batas Bawah *Lower Limit*	Batas Atas *Upper Limit*				Batas Bawah *Lower Limit*	Batas Atas *Upper Limit*				Batas Bawah *Lower Limit*	Batas Atas *Upper Limit*
(1)	(2)	(3)	(4)	(5)	(6)	(7)	(8)	(9)	(10)	(11)	(12)	(13)	(14)	(15)	(16)
Aceh	46,06	0,97	2,10	44,16	47,95	23,64	0,55	2,31	22,58	24,71	30,69	0,53	1,73	29,65	31,73
Sumatera Utara	43,84	0,78	1,78	42,31	45,37	23,40	0,52	2,22	22,38	24,42	34,27	0,50	1,46	33,29	35,25
Sumatera Barat	48,54	1,00	2,07	46,57	50,50	26,95	0,60	2,24	25,77	28,13	36,49	0,59	1,63	35,32	37,65
Riau	53,64	1,02	1,90	51,64	55,64	30,70	0,68	2,23	29,36	32,04	39,98	0,62	1,56	38,75	41,20
Jambi	49,64	1,50	3,03	46,70	52,59	29,30	0,71	2,41	27,91	30,68	35,82	0,74	2,07	34,37	37,27
Sumatera Selatan	48,02	1,16	2,42	45,74	50,29	24,60	0,55	2,22	23,53	25,68	33,35	0,59	1,76	32,20	34,50
Bengkulu	50,55	1,27	2,52	48,05	53,05	23,95	0,78	3,24	22,43	25,47	32,66	0,77	2,37	31,14	34,18
Lampung	45,06	1,21	2,69	42,68	47,43	27,08	0,54	1,99	26,02	28,14	32,41	0,55	1,68	31,34	33,48
Kep. Bangka Belitung	45,58	1,07	2,35	43,48	47,67	27,72	0,96	3,47	25,83	29,61	37,33	0,83	2,21	35,71	38,94
Kep. Riau	57,69	1,62	2,80	54,52	60,86	30,25	2,51	8,28	25,34	35,16	53,74	1,49	2,77	50,81	56,66
DKI Jakarta	65,89	0,76	1,16	64,40	67,39	-	-	-	-	-	65,89	0,76	1,16	64,40	67,39
Jawa Barat	51,11	0,50	0,99	50,12	52,10	28,84	0,51	1,78	27,83	29,84	45,33	0,41	0,90	44,53	46,12
Jawa Tengah	46,06	0,44	0,95	45,21	46,92	30,57	0,37	1,22	29,84	31,30	38,51	0,30	0,79	37,91	39,10
DI Yogyakarta	62,78	0,98	1,57	60,85	64,71	35,79	1,32	3,70	33,19	38,39	55,45	0,84	1,52	53,81	57,10
Jawa Timur	48,15	0,46	0,95	47,25	49,05	28,45	0,38	1,33	27,70	29,19	38,75	0,32	0,82	38,13	39,37
Banten	56,33	1,01	1,79	54,35	58,30	27,09	0,97	3,59	25,18	28,99	47,90	0,76	1,59	46,40	49,40
Bali	53,93	0,86	1,59	52,24	55,61	31,76	1,05	3,30	29,71	33,81	46,42	0,66	1,43	45,12	47,72
Nusa Tenggara Barat	35,87	1,10	3,08	33,71	38,04	21,71	0,70	3,23	20,34	23,09	28,31	0,66	2,34	27,01	29,60
Nusa Tenggara Timur	47,69	1,40	2,93	44,95	50,43	13,10	0,45	3,40	12,23	13,97	21,10	0,63	2,97	19,87	22,32
Kalimantan Barat	51,00	1,17	2,29	48,71	53,30	20,74	0,66	3,19	19,44	22,03	30,99	0,71	2,28	29,60	32,37
Kalimantan Tengah	49,67	1,34	2,71	47,04	52,30	27,67	0,96	3,47	25,79	29,55	36,17	0,86	2,39	34,47	37,86
Kalimantan Selatan	53,46	1,17	2,18	51,17	55,74	29,92	0,80	2,66	28,36	31,48	40,89	0,77	1,88	39,38	42,40
Kalimantan Timur	57,39	0,98	1,71	55,46	59,32	35,82	1,31	3,65	33,26	38,38	50,25	0,86	1,71	48,56	51,93
Kalimantan Utara	53,93	1,34	2,49	51,29	56,56	37,15	2,15	5,78	32,95	41,36	46,87	1,25	2,67	44,42	49,33
Sulawesi Utara	50,98	0,93	1,82	49,16	52,80	30,09	0,78	2,61	28,55	31,62	40,59	0,64	1,59	39,33	41,85
Sulawesi Tengah	50,09	1,72	3,44	46,72	53,47	20,72	0,65	3,15	19,44	22,00	28,94	0,77	2,66	27,43	30,45
Sulawesi Selatan	51,08	0,84	1,65	49,43	52,73	27,07	0,49	1,82	26,11	28,04	37,09	0,50	1,35	36,11	38,07
Sulawesi Tenggara	47,67	1,39	2,92	44,95	50,40	24,25	0,70	2,89	22,87	25,62	33,27	0,75	2,24	31,80	34,73
Gorontalo	45,33	1,67	3,68	42,07	48,60	27,81	1,07	3,84	25,71	29,90	34,62	0,97	2,81	32,71	36,52
Sulawesi Barat	41,53	2,56	6,16	36,51	46,54	21,29	0,97	4,55	19,39	23,18	26,07	1,09	4,19	23,93	28,21
Maluku	47,93	1,53	3,20	44,92	50,93	16,47	0,96	5,82	14,59	18,35	29,79	0,94	3,17	27,94	31,64
Maluku Utara	51,00	1,78	3,48	47,52	54,48	15,60	0,87	5,57	13,89	17,30	25,80	0,97	3,74	23,91	27,69
Papua Barat	50,10	1,91	3,81	46,36	53,84	26,99	1,63	6,06	23,78	30,19	36,41	1,29	3,55	33,88	38,95
Papua	47,72	1,43	3,00	44,91	50,52	8,83	0,70	7,91	7,46	10,19	19,59	0,76	3,90	18,09	21,09
**Indonesia**	**50,92**	**0,19**	**0,38**	**50,54**	**51,30**	**26,56**	**0,13**	**0,49**	**26,30**	**26,81**	**39,90**	**0,13**	**0,32**	**39,66**	**40,15**

Sumber/*Source*: Susenas Maret 2018/*The March 2018 Susenas*

https://www.bps.go.id

Tabel 9.20. ***Sampling Error*** **Persentase Rumah Tangga yang Sumber Air Minumnya Sumur Tak Terlindung menurut Provinsi dan Daerah Tempat Tinggal, 2018**

Table *Sampling Errors Percentage of Households that Source of Drinking Water were Unprotected Well by Province and Urban Rural Classification, 2018*

Provinsi *Province*	Perkotaan/*Urban*					Perdesaan/*Rural*					Perkotaan+Perdesaan/*Urban+Rural*				
	Estimasi *Estimate*	*Standard Error*	*Relative Standard Error (%)*	Selang Kepercayaan 95% *Confidence Interval 95%* Batas Bawah *Lower Limit*	Batas Atas *Upper Limit*	Estimasi *Estimate*	*Standard Error*	*Relative Standard Error (%)*	Selang Kepercayaan 95% *Confidence Interval 95%* Batas Bawah *Lower Limit*	Batas Atas *Upper Limit*	Estimasi *Estimate*	*Standard Error*	*Relative Standard Error (%)*	Selang Kepercayaan 95% *Confidence Interval 95%* Batas Bawah *Lower Limit*	Batas Atas *Upper Limit*
(1)	(2)	(3)	(4)	(5)	(6)	(7)	(8)	(9)	(10)	(11)	(12)	(13)	(14)	(15)	(16)
Aceh	2,01	0,39	19,48	1,25	2,78	11,20	0,76	6,83	9,70	12,70	8,33	0,55	6,61	7,25	9,41
Sumatera Utara	1,64	0,21	12,71	1,23	2,04	6,68	0,44	6,63	5,81	7,55	4,03	0,24	5,93	3,56	4,50
Sumatera Barat	2,48	0,46	18,65	1,57	3,38	8,92	0,64	7,21	7,66	10,18	6,07	0,42	6,85	5,26	6,89
Riau	0,73	0,22	30,29	0,30	1,17	6,09	0,57	9,37	4,97	7,21	3,93	0,36	9,21	3,22	4,64
Jambi	3,92	1,06	27,13	1,83	6,00	18,31	1,12	6,14	16,11	20,51	13,81	0,87	6,27	12,12	15,51
Sumatera Selatan	3,32	0,64	19,34	2,06	4,58	13,95	0,83	5,96	12,32	15,58	10,14	0,59	5,86	8,97	11,30
Bengkulu	15,06	1,80	11,95	11,53	18,59	36,10	1,85	5,12	32,48	39,71	29,35	1,43	4,87	26,54	32,15
Lampung	13,82	1,83	13,23	10,23	17,40	31,33	1,23	3,92	28,92	33,73	26,44	1,02	3,85	24,45	28,44
Kep. Bangka Belitung	6,85	1,05	15,39	4,78	8,91	13,51	1,30	9,62	10,97	16,06	9,96	0,84	8,40	8,32	11,59
Kep. Riau	1,60	0,33	20,47	0,96	2,25	16,72	3,68	22,01	9,51	23,94	3,71	0,63	16,94	2,48	4,94
DKI Jakarta	0,00	0,00	-	0,00	0,00	-	-	-	-	-	0,00	0,00	-	0,00	0,00
Jawa Barat	2,13	0,27	12,77	1,59	2,66	6,75	0,54	7,99	5,70	7,81	3,39	0,25	7,29	2,91	3,88
Jawa Tengah	1,63	0,18	11,12	1,28	1,99	3,81	0,31	8,10	3,21	4,42	2,71	0,18	6,63	2,35	3,06
DI Yogyakarta	4,40	0,77	17,55	2,89	5,92	4,68	1,12	23,91	2,49	6,88	4,48	0,64	14,36	3,22	5,74
Jawa Timur	0,95	0,13	13,50	0,70	1,20	3,41	0,27	8,00	2,88	3,95	2,13	0,15	6,96	1,84	2,42
Banten	0,69	0,27	39,96	0,15	1,22	10,92	1,20	11,01	8,56	13,27	3,59	0,40	11,22	2,80	4,39
Bali	0,50	0,14	27,20	0,23	0,77	1,10	0,39	35,31	0,34	1,87	0,70	0,16	22,51	0,39	1,01
Nusa Tenggara Barat	1,45	0,35	24,45	0,75	2,14	5,04	0,83	16,37	3,42	6,66	3,38	0,48	14,18	2,44	4,32
Nusa Tenggara Timur	1,38	0,39	28,52	0,61	2,15	7,30	0,63	8,58	6,07	8,53	5,90	0,49	8,39	4,93	6,87
Kalimantan Barat	1,01	0,64	63,39	-0,24	2,26	6,68	0,82	12,30	5,07	8,29	4,82	0,60	12,46	3,64	5,99
Kalimantan Tengah	0,90	0,30	33,18	0,31	1,48	6,26	0,76	12,18	4,76	7,75	4,24	0,50	11,73	3,26	5,21
Kalimantan Selatan	4,25	0,63	14,96	3,00	5,49	17,64	1,21	6,85	15,27	20,01	11,42	0,76	6,65	9,93	12,91
Kalimantan Timur	0,25	0,09	35,56	0,08	0,43	6,43	1,07	16,61	4,34	8,53	2,30	0,38	16,48	1,56	3,04
Kalimantan Utara	0,00	0,00	-	0,00	0,00	1,05	0,42	39,60	0,24	1,87	0,43	0,17	40,03	0,09	0,76
Sulawesi Utara	1,47	0,51	34,60	0,47	2,47	5,72	0,67	11,70	4,41	7,04	3,59	0,41	11,53	2,78	4,40
Sulawesi Tengah	0,15	0,09	59,37	-0,03	0,33	3,61	0,50	13,91	2,62	4,59	2,63	0,37	13,94	1,91	3,34
Sulawesi Selatan	0,99	0,19	19,47	0,61	1,37	7,00	0,55	7,81	5,93	8,07	4,57	0,34	7,45	3,91	5,24
Sulawesi Tenggara	1,17	0,61	51,87	-0,02	2,37	7,53	0,76	10,09	6,04	9,02	5,11	0,53	10,44	4,06	6,15
Gorontalo	1,09	0,60	55,34	-0,09	2,27	4,82	0,84	17,40	3,18	6,47	3,35	0,57	16,86	2,24	4,46
Sulawesi Barat	2,69	1,05	38,97	0,64	4,75	6,06	0,80	13,23	4,49	7,63	5,28	0,67	12,70	3,96	6,59
Maluku	0,44	0,16	35,36	0,13	0,74	5,35	0,81	15,08	3,77	6,93	3,23	0,47	14,47	2,31	4,14
Maluku Utara	1,03	0,38	37,29	0,28	1,78	11,39	1,26	11,08	8,92	13,87	8,31	0,91	10,95	6,53	10,10
Papua Barat	0,20	0,12	59,87	-0,03	0,43	4,86	0,90	18,53	3,10	6,63	3,00	0,55	18,35	1,92	4,07
Papua	1,36	0,75	55,44	-0,12	2,84	4,67	0,69	14,75	3,32	6,03	3,82	0,56	14,57	2,73	4,92
**Indonesia**	**1,80**	**0,09**	**4,94**	**1,63**	**1,98**	**8,18**	**0,14**	**1,73**	**7,90**	**8,46**	**4,69**	**0,08**	**1,74**	**4,53**	**4,85**

Sumber/*Source*: Susenas Maret 2018/*The March 2018 Susenas*

https://www.bps.go.id

Tabel 9.21. ***Sampling Error* Persentase Rumah Tangga yang Sumber Air Minumnya Mata Air Tak Terlindung menurut Provinsi dan Daerah Tempat Tinggal, 2018**

Table *Sampling Errors Percentage of Households that Source of Drinking Water were Unprotected Spring by Province and Urban Rural Classification, 2018*

Provinsi/*Province*	Perkotaan/*Urban*: Estimasi/*Estimate*	Perkotaan/*Urban*: *Standard Error*	Perkotaan/*Urban*: *Relative Standard Error (%)*	Perkotaan/*Urban*: Selang Kepercayaan 95%/*Confidence Interval 95%*: Batas Bawah/*Lower Limit*	Perkotaan/*Urban*: Selang Kepercayaan 95%/*Confidence Interval 95%*: Batas Atas/*Upper Limit*	Perdesaan/*Rural*: Estimasi/*Estimate*	Perdesaan/*Rural*: *Standard Error*	Perdesaan/*Rural*: *Relative Standard Error (%)*	Perdesaan/*Rural*: Selang Kepercayaan 95%/*Confidence Interval 95%*: Batas Bawah/*Lower Limit*	Perdesaan/*Rural*: Selang Kepercayaan 95%/*Confidence Interval 95%*: Batas Atas/*Upper Limit*	Perkotaan+Perdesaan/*Urban+Rural*: Estimasi/*Estimate*	Perkotaan+Perdesaan/*Urban+Rural*: *Standard Error*	Perkotaan+Perdesaan/*Urban+Rural*: *Relative Standard Error (%)*	Perkotaan+Perdesaan/*Urban+Rural*: Selang Kepercayaan 95%/*Confidence Interval 95%*: Batas Bawah/*Lower Limit*	Perkotaan+Perdesaan/*Urban+Rural*: Selang Kepercayaan 95%/*Confidence Interval 95%*: Batas Atas/*Upper Limit*
(1)	(2)	(3)	(4)	(5)	(6)	(7)	(8)	(9)	(10)	(11)	(12)	(13)	(14)	(15)	(16)
1. Aceh	0,60	0,27	45,19	0,07	1,13	3,45	0,41	11,82	2,65	4,25	2,56	0,30	11,55	1,98	3,14
2. Sumatera Utara	0,55	0,12	21,78	0,32	0,79	5,97	0,55	9,17	4,90	7,05	3,13	0,27	8,66	2,60	3,66
3. Sumatera Barat	0,66	0,23	34,39	0,22	1,10	8,25	0,76	9,23	6,75	9,74	4,90	0,45	9,15	4,02	5,78
4. Riau	0,17	0,10	60,17	-0,03	0,37	0,22	0,10	46,15	0,02	0,42	0,20	0,07	36,76	0,06	0,35
5. Jambi	0,20	0,20	100,00	-0,19	0,59	2,17	0,53	24,63	1,12	3,22	1,55	0,37	24,12	0,82	2,29
6. Sumatera Selatan	0,06	0,03	56,71	-0,01	0,13	2,28	0,35	15,22	1,60	2,95	1,48	0,23	15,31	1,04	1,93
7. Bengkulu	0,14	0,10	72,39	-0,06	0,35	5,58	0,97	17,41	3,68	7,49	3,84	0,67	17,43	2,53	5,15
8. Lampung	0,19	0,10	50,66	0,00	0,38	4,09	0,52	12,69	3,07	5,11	3,00	0,38	12,64	2,26	3,74
9. Kep. Bangka Belitung	0,04	0,03	69,60	-0,01	0,09	1,20	0,49	40,62	0,24	2,15	0,58	0,23	39,61	0,13	1,03
10. Kep. Riau	0,42	0,11	27,05	0,20	0,65	5,78	1,10	19,07	3,62	7,94	1,17	0,17	14,85	0,83	1,51
11. DKI Jakarta	0,00	0,00	-	0,00	0,00	-	-	-	-	-	0,00	0,00	-	0,00	0,00
12 Jawa Barat	0,83	0,18	22,08	0,47	1,19	8,87	0,76	8,52	7,39	10,35	3,03	0,25	8,34	2,54	3,53
13. Jawa Tengah	0,70	0,19	26,86	0,33	1,06	4,74	0,47	10,01	3,81	5,67	2,69	0,26	9,51	2,19	3,19
14. DI Yogyakarta	0,00	0,00	-	0,00	0,00	2,93	1,07	36,51	0,83	5,03	0,77	0,29	36,93	0,21	1,33
15. Jawa Timur	0,38	0,08	21,14	0,22	0,53	3,75	0,39	10,29	3,00	4,51	2,00	0,19	9,72	1,62	2,38
16. Banten	0,33	0,18	55,40	-0,03	0,68	7,93	1,29	16,26	5,40	10,45	2,49	0,39	15,79	1,72	3,26
17. Bali	0,35	0,13	36,55	0,10	0,60	4,14	0,90	21,76	2,38	5,91	1,60	0,32	19,80	0,98	2,22
18. Nusa Tenggara Barat	0,35	0,15	42,93	0,06	0,65	1,11	0,31	28,18	0,50	1,72	0,76	0,18	24,00	0,40	1,12
19. Nusa Tenggara Timur	0,70	0,35	49,49	0,02	1,39	12,56	0,85	6,75	10,90	14,22	9,75	0,67	6,88	8,44	11,07
20. Kalimantan Barat	0,45	0,26	56,75	-0,05	0,96	8,83	0,94	10,62	6,99	10,67	6,08	0,65	10,66	4,81	7,35
21. Kalimantan Tengah	0,22	0,16	72,45	-0,09	0,53	2,74	0,48	17,56	1,79	3,68	1,79	0,31	17,30	1,18	2,39
22. Kalimantan Selatan	0,03	0,03	100,10	-0,03	0,10	1,68	0,58	34,86	0,53	2,82	0,91	0,32	34,52	0,30	1,53
23. Kalimantan Timur	0,15	0,15	95,79	-0,14	0,45	2,03	0,60	29,82	0,84	3,21	0,78	0,22	28,95	0,34	1,22
24. Kalimantan Utara	0,23	0,18	77,10	-0,12	0,57	2,45	1,41	57,61	-0,32	5,22	1,13	0,59	51,83	-0,02	2,28
25. Sulawesi Utara	0,88	0,35	40,55	0,18	1,57	2,82	0,46	16,45	1,91	3,73	1,84	0,29	15,85	1,27	2,42
26. Sulawesi Tengah	0,91	0,60	65,47	-0,26	2,08	2,45	0,40	16,36	1,66	3,23	2,01	0,34	16,69	1,35	2,67
27. Sulawesi Selatan	0,37	0,13	36,15	0,11	0,64	6,27	0,54	8,67	5,20	7,33	3,89	0,34	8,63	3,23	4,55
28. Sulawesi Tenggara	0,45	0,21	47,30	0,03	0,87	5,08	0,71	13,93	3,69	6,47	3,32	0,46	13,75	2,42	4,21
29. Gorontalo	0,00	0,00	-	0,00	0,00	1,85	0,60	32,49	0,67	3,03	1,12	0,37	33,01	0,40	1,85
30. Sulawesi Barat	0,00	0,00	-	0,00	0,00	14,92	1,46	9,78	12,06	17,78	11,45	1,17	10,25	9,15	13,74
31. Maluku	1,02	0,41	39,81	0,22	1,81	2,83	0,68	24,15	1,49	4,17	2,05	0,43	20,87	1,21	2,89
32. Maluku Utara	0,00	0,00	-	0,00	0,00	3,05	0,77	25,30	1,54	4,56	2,14	0,55	25,45	1,07	3,21
33. Papua Barat	0,11	0,09	76,62	-0,06	0,28	5,30	0,93	17,61	3,47	7,13	3,23	0,56	17,49	2,12	4,33
34. Papua	1,70	0,56	33,02	0,60	2,79	29,50	1,50	5,10	26,56	32,45	22,36	1,15	5,14	20,11	24,61
**Indonesia**	**0,50**	**0,06**	**11,22**	**0,39**	**0,61**	**5,55**	**0,15**	**2,79**	**5,24**	**5,85**	**2,78**	**0,08**	**2,80**	**2,63**	**2,94**

Sumber/*Source*: Susenas Maret 2018/*The March 2018 Susenas*

https://www.bps.go.id

Tabel *Table* **9.22.** ***Sampling Error* Persentase Rumah Tangga yang Tempat Pembuangan Akhir Tinjanya di Tangki Septik menurut Provinsi dan Daerah Tempat Tinggal, 2018**

Sampling Errors Percentage of Households that Final Disposal of Feces were on Septic Tank by Province and Urban Rural Classification, 2018

**Provinsi** *Province*	**Perkotaan/***Urban*					**Perdesaan/***Rural*					**Perkotaan+Perdesaan/***Urban+Rural*				
	**Estimasi** *Estimate*	*Standard Error*	*Relative Standard Error (%)*	**Selang Kepercayaan 95%** *Confidence Interval 95%*		**Estimasi** *Estimate*	*Standard Error*	*Relative Standard Error (%)*	**Selang Kepercayaan 95%** *Confidence Interval 95%*		**Estimasi** *Estimate*	*Standard Error*	*Relative Standard Error (%)*	**Selang Kepercayaan 95%** *Confidence Interval 95%*	
				**Batas Bawah** *Lower Limit*	**Batas Atas** *Upper Limit*				**Batas Bawah** *Lower Limit*	**Batas Atas** *Upper Limit*				**Batas Bawah** *Lower Limit*	**Batas Atas** *Upper Limit*
(1)	(2)	(3)	(4)	(5)	(6)	(7)	(8)	(9)	(10)	(11)	(12)	(13)	(14)	(15)	(16)
Aceh	94,68	0,70	0,74	93,31	96,05	82,66	0,88	1,07	80,93	84,39	87,25	0,63	0,72	86,01	88,49
Sumatera Utara	89,38	0,89	1,00	87,63	91,13	76,91	1,05	1,36	74,86	78,97	84,11	0,69	0,82	82,75	85,46
Sumatera Barat	80,16	1,76	2,20	76,71	83,62	65,31	1,50	2,30	62,36	68,26	72,87	1,17	1,61	70,57	75,17
Riau	92,45	1,07	1,16	90,34	94,56	65,80	1,69	2,57	62,48	69,12	77,17	1,13	1,46	74,97	79,38
Jambi	85,57	1,59	1,86	82,44	88,69	69,32	1,66	2,39	66,08	72,57	75,05	1,25	1,67	72,59	77,50
Sumatera Selatan	90,89	1,34	1,47	88,26	93,51	72,65	1,43	1,97	69,85	75,45	79,92	1,06	1,33	77,83	82,00
Bengkulu	76,79	2,03	2,64	72,82	80,76	37,00	2,15	5,80	32,79	41,21	51,52	1,63	3,17	48,32	54,73
Lampung	79,56	2,08	2,62	75,48	83,64	46,27	1,54	3,34	43,25	49,30	55,85	1,28	2,29	53,34	58,36
Kep. Bangka Belitung	96,59	0,88	0,92	94,86	98,33	91,08	1,77	1,94	87,62	94,54	94,20	0,92	0,98	92,39	96,00
Kep. Riau	92,19	1,77	1,92	88,73	95,66	58,30	6,00	10,29	46,54	70,06	87,75	1,81	2,06	84,20	91,30
DKI Jakarta	94,49	0,85	0,90	92,83	96,15	-	-	-	-	-	94,49	0,85	0,90	92,83	96,15
Jawa Barat	73,61	0,89	1,21	71,86	75,36	67,06	1,28	1,91	64,54	69,57	72,00	0,75	1,04	70,53	73,46
Jawa Tengah	87,04	0,63	0,72	85,81	88,28	76,02	0,75	0,99	74,54	77,50	81,78	0,49	0,60	80,81	82,75
DI Yogyakarta	88,02	1,32	1,50	85,43	90,61	80,35	2,67	3,32	75,11	85,58	86,03	1,21	1,41	83,64	88,41
Jawa Timur	87,76	0,70	0,80	86,38	89,14	68,93	0,88	1,27	67,21	70,65	79,28	0,57	0,72	78,15	80,40
Banten	90,45	1,01	1,12	88,47	92,43	50,50	2,57	5,09	45,47	55,53	81,74	0,98	1,20	79,81	83,67
Bali	97,33	0,68	0,70	95,99	98,66	95,79	0,92	0,96	93,99	97,59	96,86	0,55	0,57	95,78	97,94
Nusa Tenggara Barat	90,32	1,27	1,40	87,83	92,80	92,18	1,33	1,44	89,57	94,78	91,26	0,92	1,01	89,44	93,07
Nusa Tenggara Timur	86,12	2,10	2,43	82,01	90,23	57,12	1,22	2,14	54,73	59,51	64,97	1,13	1,74	62,76	67,18
Kalimantan Barat	86,35	1,74	2,01	82,95	89,76	51,87	1,65	3,19	48,62	55,11	65,22	1,29	1,98	62,69	67,75
Kalimantan Tengah	85,16	1,62	1,90	81,98	88,34	46,58	2,21	4,75	42,24	50,92	62,63	1,67	2,66	59,36	65,89
Kalimantan Selatan	81,59	1,54	1,89	78,57	84,61	56,38	1,86	3,29	52,74	60,02	68,68	1,28	1,87	66,17	71,19
Kalimantan Timur	88,92	1,64	1,84	85,70	92,13	72,22	3,35	4,63	65,67	78,78	83,78	1,55	1,84	80,75	86,80
Kalimantan Utara	87,35	2,59	2,97	82,27	92,43	59,94	4,63	7,72	50,88	69,01	76,77	2,50	3,26	71,87	81,68
Sulawesi Utara	89,77	1,25	1,40	87,31	92,23	84,26	1,66	1,97	81,01	87,51	87,24	1,05	1,20	85,19	89,30
Sulawesi Tengah	93,79	1,26	1,35	91,31	96,27	83,70	1,44	1,72	80,87	86,53	87,27	1,05	1,21	85,20	89,33
Sulawesi Selatan	96,52	0,53	0,55	95,49	97,56	85,10	0,99	1,16	83,16	87,03	89,96	0,62	0,69	88,74	91,18
Sulawesi Tenggara	88,21	1,83	2,07	84,63	91,79	80,93	1,62	2,00	77,76	84,10	84,02	1,27	1,52	81,52	86,52
Gorontalo	97,77	0,75	0,76	96,30	99,23	96,63	0,71	0,74	95,23	98,03	97,16	0,51	0,53	96,15	98,17
Sulawesi Barat	95,81	1,33	1,39	93,21	98,42	84,32	1,93	2,29	80,53	88,11	87,35	1,49	1,70	84,43	90,26
Maluku	89,90	1,83	2,04	86,30	93,49	90,41	2,02	2,23	86,45	94,37	90,15	1,34	1,49	87,52	92,77
Maluku Utara	98,01	0,81	0,83	96,41	99,60	88,48	1,90	2,14	84,77	92,20	92,12	1,23	1,33	89,72	94,53
Papua Barat	91,13	1,93	2,12	87,35	94,90	85,12	1,82	2,14	81,54	88,70	87,79	1,32	1,51	85,20	90,38
Papua	83,09	2,05	2,47	79,07	87,11	33,53	1,70	5,06	30,20	36,86	51,66	1,42	2,75	48,87	54,45
**Indonesia**	**85,29**	**0,29**	**0,34**	**84,71**	**85,86**	**69,43**	**0,31**	**0,45**	**68,82**	**70,04**	**78,69**	**0,22**	**0,28**	**78,27**	**79,12**

Sumber/*Source*: Susenas Maret 2018/*The March 2018 Susenas*

https://www.bps.go.id

Tabel *Table* **9.23.** ***Sampling Error* Persentase Rumah Tangga yang Tempat Pembuangan Akhir Tinjanya di IPAL menurut Provinsi dan Daerah Tempat Tinggal, 2018**

Sampling Errors Percentage of Households that Final Disposal of Feces were on IPAL by Province and Urban-Rural Classification, 2018

**Provinsi** *Province*	**Perkotaan/***Urban*					**Perdesaan/***Rural*					**Perkotaan+Perdesaan/***Urban+Rural*				
	**Estimasi** *Estimate*	*Standard Error*	*Relative Standard Error (%)*	**Selang Kepercayaan 95%** *Confidence Interval 95%*		**Estimasi** *Estimate*	*Standard Error*	*Relative Standard Error (%)*	**Selang Kepercayaan 95%** *Confidence Interval 95%*		**Estimasi** *Estimate*	*Standard Error*	*Relative Standard Error (%)*	**Selang Kepercayaan 95%** *Confidence Interval 95%*	
				**Batas Bawah** *Lower Limit*	**Batas Atas** *Upper Limit*				**Batas Bawah** *Lower Limit*	**Batas Atas** *Upper Limit*				**Batas Bawah** *Lower Limit*	**Batas Atas** *Upper Limit*
(1)	(2)	(3)	(4)	(5)	(6)	(7)	(8)	(9)	(10)	(11)	(12)	(13)	(14)	(15)	(16)
1. Aceh	0,26	0,15	59,21	-0,04	0,56	1,06	0,27	25,62	0,53	1,58	0,75	0,18	23,65	0,40	1,10
2. Sumatera Utara	1,23	0,41	33,73	0,42	2,04	0,52	0,18	34,12	0,17	0,87	0,93	0,25	26,98	0,44	1,42
3. Sumatera Barat	0,28	0,12	42,29	0,05	0,51	0,28	0,16	58,54	-0,04	0,59	0,28	0,10	35,72	0,08	0,47
4. Riau	0,00	0,00	-	0,00	0,00	0,00	0,00	-	0,00	0,00	0,00	0,00	-	0,00	0,00
5. Jambi	0,00	0,00	-	0,00	0,00	0,00	0,00	-	0,00	0,00	0,00	0,00	-	0,00	0,00
6. Sumatera Selatan	0,20	0,15	77,40	-0,10	0,50	0,53	0,27	50,43	0,01	1,05	0,40	0,17	43,07	0,06	0,73
7. Bengkulu	0,92	0,53	57,72	-0,12	1,96	0,25	0,14	55,07	-0,02	0,53	0,50	0,21	43,23	0,08	0,92
8. Lampung	0,04	0,03	69,51	-0,02	0,11	0,67	0,23	34,23	0,22	1,12	0,49	0,16	33,46	0,17	0,81
9. Kep. Bangka Belitung	0,11	0,11	99,87	-0,11	0,34	0,10	0,10	99,96	-0,10	0,31	0,11	0,08	71,70	-0,04	0,26
10. Kep. Riau	0,06	0,04	65,79	-0,02	0,13	1,11	0,69	62,08	-0,24	2,46	0,20	0,10	48,84	0,01	0,38
11. DKI Jakarta	0,83	0,38	46,04	0,08	1,58	-	-	-	-	-	0,83	0,38	46,04	0,08	1,58
12 Jawa Barat	0,96	0,17	17,48	0,63	1,29	0,60	0,21	35,54	0,18	1,02	0,87	0,14	15,74	0,60	1,14
13. Jawa Tengah	1,38	0,22	15,96	0,95	1,81	0,20	0,09	46,02	0,02	0,38	0,82	0,12	15,09	0,58	1,06
14. DI Yogyakarta	8,29	1,15	13,85	6,04	10,54	0,08	0,06	72,56	-0,04	0,20	6,16	0,85	13,85	4,49	7,83
15. Jawa Timur	0,72	0,19	26,40	0,35	1,09	0,70	0,16	23,68	0,37	1,02	0,71	0,13	18,09	0,46	0,96
16. Banten	0,06	0,05	81,58	-0,04	0,16	1,64	0,87	52,98	-0,06	3,34	0,41	0,19	47,96	0,02	0,79
17. Bali	1,58	0,64	40,34	0,33	2,84	0,65	0,34	51,81	-0,01	1,32	1,30	0,46	35,14	0,41	2,20
18. Nusa Tenggara Barat	0,87	0,45	51,51	-0,01	1,75	0,79	0,41	51,68	-0,01	1,59	0,83	0,30	36,39	0,24	1,42
19. Nusa Tenggara Timur	0,00	0,00	-	0,00	0,00	0,00	0,00	-	0,00	0,00	0,00	0,00	-	0,00	0,00
20. Kalimantan Barat	0,63	0,24	38,20	0,16	1,10	0,39	0,15	38,23	0,10	0,69	0,48	0,14	28,49	0,21	0,75
21. Kalimantan Tengah	0,60	0,60	99,33	-0,57	1,78	0,51	0,22	43,34	0,08	0,94	0,55	0,28	51,11	0,00	1,10
22. Kalimantan Selatan	0,35	0,20	59,26	-0,06	0,75	0,48	0,27	55,90	-0,05	1,00	0,41	0,17	41,18	0,08	0,74
23. Kalimantan Timur	1,24	0,60	48,27	0,07	2,42	0,00	0,00	-	0,00	0,00	0,86	0,42	48,48	0,04	1,68
24. Kalimantan Utara	0,00	0,00	-	0,00	0,00	0,00	0,00	-	0,00	0,00	0,00	0,00	-	0,00	0,00
25. Sulawesi Utara	0,69	0,26	38,12	0,18	1,21	1,62	0,39	23,98	0,86	2,38	1,12	0,23	20,51	0,67	1,57
26. Sulawesi Tengah	0,32	0,20	63,75	-0,08	0,72	0,38	0,19	49,30	0,01	0,74	0,36	0,15	40,78	0,07	0,64
27. Sulawesi Selatan	0,24	0,15	63,47	-0,06	0,54	0,02	0,02	99,95	-0,02	0,07	0,12	0,07	57,19	-0,01	0,24
28. Sulawesi Tenggara	0,00	0,00	-	0,00	0,00	0,02	0,02	100,03	-0,02	0,06	0,01	0,01	100,07	-0,01	0,04
29. Gorontalo	0,20	0,15	71,36	-0,08	0,49	0,60	0,28	47,08	0,05	1,15	0,41	0,16	39,77	0,09	0,74
30. Sulawesi Barat	0,00	0,00	-	0,00	0,00	0,06	0,06	99,90	-0,05	0,16	0,04	0,04	99,96	-0,04	0,12
31. Maluku	0,49	0,44	90,23	-0,37	1,34	0,49	0,25	50,62	0,00	0,97	0,49	0,26	52,64	-0,02	0,99
32. Maluku Utara	0,00	0,00	-	0,00	0,00	0,00	0,00	-	0,00	0,00	0,00	0,00	-	0,00	0,00
33. Papua Barat	0,47	0,35	73,11	-0,21	1,15	0,95	0,55	58,13	-0,13	2,03	0,74	0,35	47,09	0,06	1,42
34. Papua	0,85	0,60	70,24	-0,32	2,03	1,39	0,35	25,11	0,71	2,07	1,19	0,31	26,15	0,58	1,80
**Indonesia**	**0,96**	**0,08**	**7,89**	**0,81**	**1,11**	**0,49**	**0,05**	**10,62**	**0,39**	**0,59**	**0,76**	**0,05**	**6,45**	**0,67**	**0,86**

Sumber/*Source*: Susenas Maret 2018/*The March 2018 Susenas*

https://www.bps.go.id

Tabel 9.24. ***Sampling Error*** **Persentase Rumah Tangga yang Tempat Pembuangan Akhir Tinjanya di Pantai/tanah lapang/kebun/lainnya menurut Provinsi dan Daerah Tempat Tinggal, 2018**

Table *Sampling Errors Percentage of Households that Final Disposal of Feces were on Beach/field/garden/other by Province and Urban Rural Classification, 2018*

**Provinsi** *Province*	**Perkotaan/***Urban*					**Perdesaan/***Rural*					**Perkotaan+Perdesaan/***Urban+Rural*				
	**Estimasi** *Estimate*	*Standard Error*	*Relative Standard Error (%)*	**Selang Kepercayaan 95%** *Confidence Interval 95%* **Batas Bawah** *Lower Limit*	**Batas Atas** *Upper Limit*	**Estimasi** *Estimate*	*Standard Error*	*Relative Standard Error (%)*	**Selang Kepercayaan 95%** *Confidence Interval 95%* **Batas Bawah** *Lower Limit*	**Batas Atas** *Upper Limit*	**Estimasi** *Estimate*	*Standard Error*	*Relative Standard Error (%)*	**Selang Kepercayaan 95%** *Confidence Interval 95%* **Batas Bawah** *Lower Limit*	**Batas Atas** *Upper Limit*
(1)	(2)	(3)	(4)	(5)	(6)	(7)	(8)	(9)	(10)	(11)	(12)	(13)	(14)	(15)	(16)
Aceh	0,50	0,16	33,06	0,18	0,82	0,87	0,16	18,66	0,55	1,18	0,73	0,12	16,41	0,49	0,96
Sumatera Utara	2,05	0,43	21,02	1,20	2,89	2,26	0,24	10,45	1,80	2,73	2,14	0,27	12,54	1,61	2,67
Sumatera Barat	0,50	0,16	32,52	0,18	0,82	0,24	0,08	32,17	0,09	0,38	0,37	0,09	24,52	0,19	0,55
Riau	0,63	0,31	49,26	0,02	1,24	1,63	0,50	30,84	0,64	2,61	1,20	0,31	26,20	0,58	1,82
Jambi	1,66	0,49	29,46	0,70	2,62	0,51	0,12	24,24	0,27	0,75	0,91	0,19	20,80	0,54	1,29
Sumatera Selatan	0,27	0,15	54,58	-0,02	0,57	0,54	0,14	25,15	0,27	0,80	0,43	0,10	23,43	0,23	0,63
Bengkulu	2,11	0,59	28,03	0,95	3,27	0,69	0,18	26,24	0,34	1,05	1,21	0,25	20,25	0,73	1,69
Lampung	0,61	0,32	52,75	-0,02	1,24	0,39	0,09	23,48	0,21	0,57	0,45	0,11	24,97	0,23	0,67
Kep. Bangka Belitung	0,15	0,11	73,26	-0,07	0,37	0,20	0,14	71,71	-0,08	0,48	0,17	0,09	51,00	0,00	0,34
Kep. Riau	0,62	0,44	71,39	-0,25	1,49	4,05	1,46	35,97	1,19	6,91	1,07	0,47	43,73	0,15	1,99
DKI Jakarta	1,05	0,41	39,07	0,25	1,86	-	-	-	-	-	1,05	0,41	39,07	0,25	1,86
Jawa Barat	0,60	0,13	21,62	0,35	0,86	0,29	0,10	32,41	0,11	0,48	0,53	0,10	19,23	0,33	0,73
Jawa Tengah	0,32	0,09	28,53	0,14	0,50	0,52	0,13	24,02	0,28	0,77	0,42	0,08	18,29	0,27	0,56
DI Yogyakarta	0,05	0,05	100,02	-0,05	0,15	0,75	0,43	57,18	-0,09	1,59	0,23	0,12	50,70	0,00	0,46
Jawa Timur	0,41	0,09	23,02	0,23	0,60	0,72	0,10	13,21	0,53	0,91	0,55	0,07	12,22	0,42	0,68
Banten	0,71	0,24	34,26	0,23	1,18	0,98	0,34	34,63	0,31	1,64	0,77	0,20	26,52	0,37	1,16
Bali	0,00	0,00	-	0,00	0,00	0,27	0,22	80,94	-0,16	0,70	0,08	0,07	80,92	-0,05	0,21
Nusa Tenggara Barat	0,10	0,08	79,00	-0,06	0,26	0,00	0,00	100,06	0,00	0,01	0,05	0,04	75,54	-0,03	0,13
Nusa Tenggara Timur	0,03	0,03	100,15	-0,03	0,10	0,19	0,05	28,62	0,08	0,30	0,15	0,04	27,59	0,07	0,23
Kalimantan Barat	0,08	0,06	75,99	-0,04	0,20	0,49	0,12	23,71	0,26	0,72	0,33	0,08	22,70	0,18	0,48
Kalimantan Tengah	0,24	0,16	65,06	-0,07	0,54	0,34	0,11	33,02	0,12	0,56	0,30	0,09	30,76	0,12	0,48
Kalimantan Selatan	0,24	0,15	62,15	-0,05	0,52	0,32	0,12	36,40	0,09	0,56	0,28	0,09	33,24	0,10	0,46
Kalimantan Timur	1,13	0,87	77,00	-0,57	2,82	0,16	0,10	62,76	-0,04	0,36	0,83	0,60	72,59	-0,35	2,01
Kalimantan Utara	1,44	0,56	39,14	0,33	2,54	3,40	2,91	85,52	-2,30	9,10	2,19	1,18	53,64	-0,11	4,50
Sulawesi Utara	0,57	0,24	41,21	0,11	1,03	0,19	0,10	52,00	0,00	0,38	0,40	0,14	34,21	0,13	0,66
Sulawesi Tengah	0,20	0,15	73,68	-0,09	0,49	0,83	0,28	34,21	0,27	1,38	0,60	0,19	31,48	0,23	0,98
Sulawesi Selatan	0,32	0,12	35,88	0,10	0,55	0,39	0,11	28,21	0,17	0,60	0,36	0,08	22,12	0,20	0,52
Sulawesi Tenggara	0,49	0,19	39,55	0,11	0,87	1,02	0,30	29,55	0,43	1,61	0,80	0,19	24,22	0,42	1,17
Gorontalo	0,17	0,17	99,82	-0,16	0,49	0,05	0,05	100,02	-0,05	0,14	0,10	0,08	78,93	-0,06	0,26
Sulawesi Barat	0,00	0,00	-	0,00	0,00	0,35	0,26	75,46	-0,17	0,87	0,26	0,20	75,48	-0,12	0,64
Maluku	0,29	0,22	77,47	-0,15	0,72	1,49	0,84	56,67	-0,17	3,14	0,87	0,42	48,55	0,04	1,69
Maluku Utara	0,13	0,13	100,54	-0,13	0,39	1,70	0,62	36,80	0,47	2,92	1,10	0,39	35,57	0,33	1,86
Papua Barat	1,54	0,57	37,36	0,41	2,66	0,92	0,31	33,46	0,32	1,53	1,20	0,31	25,55	0,60	1,80
Papua	0,67	0,26	38,48	0,16	1,17	2,90	0,51	17,63	1,90	3,90	2,08	0,34	16,20	1,42	2,74
**Indonesia**	**0,61**	**0,06**	**9,70**	**0,49**	**0,72**	**0,69**	**0,04**	**5,87**	**0,61**	**0,77**	**0,64**	**0,04**	**5,97**	**0,57**	**0,72**

Sumber/*Source*: Susenas Maret 2018/*The March 2018 Susenas*

https://www.bps.go.id

Tabel *Table* **9.25.** ***Sampling Error* Persentase Rumah Tangga yang Membeli/Menerima Beras Miskin (Raskin) dalam 4 Bulan Terakhir menurut Provinsi dan Daerah Tempat Tinggal, 2018**

Sampling Errors Percentage of Household That Bought/Received Rice for The Poor (Raskin) during the Last 4 Months by Province and Urban Rural Classification, 2018

**Provinsi** *Province*	**Perkotaan/***Urban*					**Perdesaan/***Rural*					**Perkotaan+Perdesaan/***Urban+Rural*				
	**Estimasi** *Estimate*	*Standard Error*	*Relative Standard Error (%)*	**Selang Kepercayaan 95%** *Confidence Interval 95%*		**Estimasi** *Estimate*	*Standard Error*	*Relative Standard Error (%)*	**Selang Kepercayaan 95%** *Confidence Interval 95%*		**Estimasi** *Estimate*	*Standard Error*	*Relative Standard Error (%)*	**Selang Kepercayaan 95%** *Confidence Interval 95%*	
				**Batas Bawah** *Lower Limit*	**Batas Atas** *Upper Limit*				**Batas Bawah** *Lower Limit*	**Batas Atas** *Upper Limit*				**Batas Bawah** *Lower Limit*	**Batas Atas** *Upper Limit*
(1)	(2)	(3)	(4)	(5)	(6)	(7)	(8)	(9)	(10)	(11)	(12)	(13)	(14)	(15)	(16)
Aceh	34,75	1,77	5,09	31,29	38,22	70,68	1,09	1,55	68,53	72,82	59,45	1,02	1,72	57,45	61,45
Sumatera Utara	17,32	0,86	4,94	15,65	19,00	44,44	1,11	2,50	42,26	46,62	30,21	0,73	2,41	28,78	31,64
Sumatera Barat	11,36	0,82	7,21	9,75	12,96	30,74	1,11	3,60	28,57	32,91	22,19	0,74	3,32	20,74	23,63
Riau	9,61	0,96	9,94	7,74	11,49	31,78	1,50	4,72	28,84	34,73	22,83	1,01	4,42	20,85	24,81
Jambi	12,08	1,33	11,00	9,48	14,69	34,53	1,55	4,50	31,48	37,57	27,51	1,18	4,31	25,19	29,83
Sumatera Selatan	14,24	1,14	7,99	12,01	16,47	39,67	1,28	3,23	37,16	42,18	30,56	0,95	3,10	28,70	32,41
Bengkulu	21,51	1,48	6,89	18,60	24,41	41,41	1,55	3,74	38,38	44,45	35,03	1,21	3,45	32,66	37,40
Lampung	19,92	1,73	8,66	16,53	23,30	57,36	1,27	2,21	54,87	59,85	46,92	1,08	2,29	44,81	49,03
Kep. Bangka Belitung	10,27	0,89	8,65	8,53	12,01	14,94	1,10	7,37	12,78	17,10	12,45	0,73	5,86	11,02	13,88
Kep. Riau	4,21	0,51	12,11	3,21	5,21	28,13	3,92	13,92	20,45	35,80	7,54	0,71	9,46	6,14	8,93
DKI Jakarta	0,37	0,09	23,43	0,20	0,54	-	-	-	-	-	0,37	0,09	23,43	0,20	0,54
Jawa Barat	28,11	0,74	2,62	26,66	29,55	60,21	1,06	1,76	58,12	62,29	36,90	0,63	1,70	35,67	38,14
Jawa Tengah	33,96	0,73	2,14	32,54	35,39	62,35	0,84	1,36	60,69	64,00	47,95	0,60	1,24	46,79	49,12
DI Yogyakarta	19,17	1,32	6,90	16,58	21,76	70,83	2,72	3,84	65,50	76,16	32,79	1,39	4,23	30,07	35,51
Jawa Timur	21,63	0,63	2,91	20,40	22,87	55,89	0,89	1,60	54,14	57,64	38,06	0,58	1,53	36,91	39,20
Banten	14,50	1,47	10,15	11,62	17,39	63,06	2,18	3,46	58,79	67,34	28,31	1,30	4,60	25,76	30,86
Bali	6,88	0,65	9,39	5,61	8,15	22,88	1,83	8,00	19,29	26,47	12,16	0,75	6,17	10,69	13,63
Nusa Tenggara Barat	44,66	2,18	4,88	40,39	48,94	72,25	1,81	2,50	68,70	75,79	59,52	1,45	2,44	56,67	62,36
Nusa Tenggara Timur	17,88	1,61	9,00	14,73	21,04	50,88	1,35	2,66	48,23	53,53	43,07	1,20	2,79	40,72	45,43
Kalimantan Barat	13,24	1,43	10,82	10,43	16,05	37,06	1,65	4,44	33,84	40,29	29,24	1,25	4,29	26,78	31,70
Kalimantan Tengah	6,21	0,98	15,86	4,28	8,14	17,49	1,61	9,22	14,33	20,66	13,24	1,11	8,36	11,07	15,41
Kalimantan Selatan	12,70	1,17	9,18	10,41	14,98	27,78	1,25	4,50	25,34	30,23	20,78	0,90	4,32	19,02	22,54
Kalimantan Timur	9,05	0,90	9,89	7,30	10,81	29,09	2,24	7,70	24,70	33,48	15,70	1,02	6,49	13,70	17,69
Kalimantan Utara	11,82	1,54	13,02	8,80	14,84	23,33	2,80	12,01	17,84	28,82	16,49	1,56	9,44	13,44	19,54
Sulawesi Utara	19,79	1,30	6,59	17,24	22,35	44,52	1,57	3,53	41,44	47,60	32,12	1,07	3,33	30,02	34,21
Sulawesi Tengah	19,11	1,99	10,41	15,21	23,00	46,25	1,53	3,31	43,26	49,25	38,55	1,32	3,43	35,97	41,14
Sulawesi Selatan	10,41	0,64	6,16	9,16	11,67	39,36	1,02	2,59	37,36	41,36	27,68	0,72	2,58	26,28	29,08
Sulawesi Tenggara	25,93	2,39	9,22	21,25	30,62	44,83	1,71	3,82	41,48	48,19	37,63	1,52	4,05	34,65	40,62
Gorontalo	20,83	1,94	9,32	17,03	24,64	46,11	2,21	4,79	41,78	50,43	36,16	1,60	4,44	33,02	39,30
Sulawesi Barat	34,39	4,16	12,09	26,24	42,55	44,21	2,07	4,68	40,15	48,26	41,92	1,95	4,66	38,09	45,75
Maluku	22,28	1,92	8,64	18,51	26,05	70,39	2,47	3,51	65,55	75,23	49,62	1,81	3,64	46,08	53,16
Maluku Utara	10,65	1,49	13,95	7,74	13,56	40,32	2,01	4,99	36,37	44,26	31,50	1,60	5,08	28,37	34,64
Papua Barat	15,34	1,70	11,06	12,01	18,66	46,16	2,30	4,98	41,65	50,67	33,84	1,53	4,53	30,83	36,84
Papua	18,09	1,57	8,69	15,01	21,17	53,95	1,51	2,81	50,98	56,91	44,73	1,29	2,88	42,21	47,26
**Indonesia**	**21,02**	**0,26**	**1,25**	**20,50**	**21,54**	**51,31**	**0,29**	**0,57**	**50,74**	**51,89**	**34,74**	**0,21**	**0,60**	**34,33**	**35,15**

Sumber/*Source*: Susenas Maret 2018/*The March 2018 Susenas*

https://www.bps.go.id

Tabel / *Table* **9.26.** ***Sampling Error*** **Persentase Rumah Tangga yang Menerima Program Indonesia Pintar (PIP) menurut Provinsi dan Daerah Tempat Tinggal, 2018**

Sampling Errors Percentage of Household That Received Smart Indonesia Program (PIP) by Province and Urban Rural Classification, 2018

Provinsi *Province*	Perkotaan/*Urban*					Perdesaan/*Rural*					Perkotaan+Perdesaan/*Urban+Rural*				
	Estimasi *Estimate*	*Standard Error*	*Relative Standard Error (%)*	Selang Kepercayaan 95% *Confidence Interval 95%* Batas Bawah *Lower Limit*	Selang Kepercayaan 95% *Confidence Interval 95%* Batas Atas *Upper Limit*	Estimasi *Estimate*	*Standard Error*	*Relative Standard Error (%)*	Selang Kepercayaan 95% *Confidence Interval 95%* Batas Bawah *Lower Limit*	Selang Kepercayaan 95% *Confidence Interval 95%* Batas Atas *Upper Limit*	Estimasi *Estimate*	*Standard Error*	*Relative Standard Error (%)*	Selang Kepercayaan 95% *Confidence Interval 95%* Batas Bawah *Lower Limit*	Selang Kepercayaan 95% *Confidence Interval 95%* Batas Atas *Upper Limit*
(1)	(2)	(3)	(4)	(5)	(6)	(7)	(8)	(9)	(10)	(11)	(12)	(13)	(14)	(15)	(16)
Aceh	10,41	0,82	7,87	8,80	12,02	21,94	0,70	3,20	20,57	23,32	18,34	0,56	3,08	17,23	19,45
Sumatera Utara	7,93	0,55	6,95	6,85	9,01	14,81	0,57	3,88	13,68	15,93	11,20	0,40	3,56	10,42	11,98
Sumatera Barat	9,44	0,83	8,78	7,82	11,07	17,57	0,72	4,11	16,15	18,99	13,98	0,55	3,93	12,91	15,06
Riau	5,11	0,62	12,20	3,89	6,33	10,30	0,63	6,12	9,06	11,53	8,20	0,45	5,54	7,31	9,09
Jambi	7,93	1,01	12,79	5,94	9,92	9,38	0,55	5,81	8,31	10,45	8,93	0,49	5,52	7,96	9,89
Sumatera Selatan	7,92	0,76	9,62	6,43	9,41	13,08	0,63	4,83	11,84	14,31	11,23	0,49	4,38	10,26	12,19
Bengkulu	9,25	0,84	9,06	7,61	10,90	14,88	0,83	5,59	13,25	16,51	13,08	0,63	4,79	11,85	14,31
Lampung	12,05	1,05	8,71	10,00	14,11	15,32	0,61	3,96	14,13	16,51	14,41	0,52	3,64	13,38	15,44
Kep. Bangka Belitung	4,37	0,53	12,23	3,32	5,42	7,41	0,86	11,63	5,72	9,10	5,79	0,50	8,68	4,80	6,77
Kep. Riau	5,58	0,75	13,39	4,12	7,04	15,52	2,24	14,43	11,13	19,91	6,96	0,77	11,04	5,46	8,47
DKI Jakarta	0,94	0,17	17,76	0,61	1,27	-	-	-	-	-	0,94	0,17	17,76	0,61	1,27
Jawa Barat	8,71	0,34	3,92	8,04	9,38	14,26	0,54	3,77	13,20	15,31	10,23	0,29	2,83	9,66	10,80
Jawa Tengah	12,46	0,38	3,05	11,72	13,20	15,81	0,45	2,86	14,93	16,70	14,11	0,30	2,10	13,53	14,69
DI Yogyakarta	11,77	0,89	7,58	10,02	13,52	20,10	1,61	8,01	16,95	23,26	13,97	0,78	5,60	12,43	15,50
Jawa Timur	8,16	0,33	4,01	7,52	8,80	12,88	0,40	3,13	12,09	13,67	10,42	0,26	2,49	9,91	10,93
Banten	5,89	0,64	10,84	4,64	7,15	12,61	1,07	8,48	10,51	14,71	7,80	0,55	7,07	6,72	8,88
Bali	5,28	0,50	9,43	4,30	6,26	10,74	0,91	8,45	8,96	12,52	7,08	0,46	6,48	6,18	7,98
Nusa Tenggara Barat	18,59	1,19	6,42	16,25	20,93	20,66	1,03	4,96	18,65	22,67	19,70	0,78	3,96	18,17	21,23
Nusa Tenggara Timur	13,08	1,27	9,69	10,60	15,57	22,95	0,70	3,03	21,58	24,31	20,61	0,63	3,05	19,38	21,84
Kalimantan Barat	7,15	0,87	12,17	5,44	8,85	8,98	0,59	6,54	7,83	10,13	8,38	0,48	5,79	7,43	9,33
Kalimantan Tengah	3,21	0,48	14,96	2,27	4,16	7,39	0,68	9,26	6,05	8,73	5,82	0,47	8,10	4,89	6,74
Kalimantan Selatan	5,45	0,62	11,34	4,24	6,66	10,85	0,63	5,78	9,62	12,07	8,34	0,45	5,41	7,46	9,23
Kalimantan Timur	3,41	0,53	15,45	2,38	4,45	8,59	1,09	12,69	6,45	10,72	5,13	0,52	10,13	4,11	6,15
Kalimantan Utara	6,96	1,02	14,69	4,96	8,96	12,57	1,69	13,45	9,25	15,88	9,23	0,95	10,27	7,37	11,09
Sulawesi Utara	7,01	0,65	9,20	5,75	8,28	12,60	0,85	6,73	10,93	14,26	9,80	0,54	5,54	8,73	10,86
Sulawesi Tengah	9,31	1,01	10,89	7,32	11,30	13,98	0,65	4,61	12,72	15,25	12,66	0,56	4,46	11,55	13,76
Sulawesi Selatan	8,92	0,70	7,83	7,55	10,29	19,08	0,61	3,18	17,88	20,27	14,98	0,47	3,16	14,05	15,91
Sulawesi Tenggara	12,95	1,23	9,52	10,53	15,36	26,12	0,91	3,50	24,33	27,91	21,10	0,78	3,70	19,57	22,63
Gorontalo	12,00	1,43	11,95	9,19	14,81	20,24	1,47	7,26	17,36	23,12	17,00	1,06	6,24	14,92	19,08
Sulawesi Barat	10,83	1,92	17,70	7,07	14,59	17,74	1,13	6,39	15,52	19,96	16,13	1,01	6,23	14,16	18,10
Maluku	6,28	0,95	15,17	4,41	8,15	14,35	1,11	7,74	12,17	16,52	10,86	0,76	7,03	9,37	12,36
Maluku Utara	4,93	0,88	17,87	3,21	6,66	9,32	0,74	7,89	7,88	10,77	8,02	0,58	7,26	6,88	9,16
Papua Barat	12,31	1,52	12,38	9,32	15,30	14,43	1,02	7,08	12,43	16,44	13,59	0,87	6,38	11,89	15,28
Papua	7,30	0,85	11,65	5,63	8,96	7,35	0,57	7,75	6,23	8,46	7,33	0,48	6,49	6,40	8,26
**Indonesia**	**8,27**	**0,13**	**1,59**	**8,01**	**8,53**	**14,54**	**0,14**	**1,00**	**14,25**	**14,82**	**11,11**	**0,10**	**0,88**	**10,92**	**11,30**

Sumber/*Source*: Susenas Maret 2018/*The March 2018 Susenas*

https://www.bps.go.id

Tabel / *Table* **9.27.** ***Sampling Error* Persentase Rumah Tangga menurut Provinsi, Akses Air Layak, dan Daerah Tempat Tinggal, 2018**
Sampling Errors Percentage of Household by Province, Acces of Decent Water, and Urban Rural Classification, 2018

**Provinsi** *Province*	**Perkotaan/***Urban*					**Perdesaan/***Rural*					**Perkotaan+Perdesaan/***Urban+Rural*				
	**Estimasi** *Estimate*	*Standard Error*	*Relative Standard Error (%)*	**Selang Kepercayaan 95%** *Confidence Interval 95%*		**Estimasi** *Estimate*	*Standard Error*	*Relative Standard Error (%)*	**Selang Kepercayaan 95%** *Confidence Interval 95%*		**Estimasi** *Estimate*	*Standard Error*	*Relative Standard Error (%)*	**Selang Kepercayaan 95%** *Confidence Interval 95%*	
				**Batas Bawah** *Lower Limit*	**Batas Atas** *Upper Limit*				**Batas Bawah** *Lower Limit*	**Batas Atas** *Upper Limit*				**Batas Bawah** *Lower Limit*	**Batas Atas** *Upper Limit*
(1)	(2)	(3)	(4)	(5)	(6)	(7)	(8)	(9)	(10)	(11)	(12)	(13)	(14)	(15)	(16)
Aceh	85,03	1,10	1,29	82,88	87,18	58,04	1,18	2,03	55,73	60,36	66,48	0,93	1,40	64,65	68,30
Sumatera Utara	82,42	0,90	1,09	80,66	84,19	60,38	1,08	1,79	58,26	62,50	71,95	0,71	0,99	70,56	73,34
Sumatera Barat	82,78	1,58	1,91	79,68	85,87	59,07	1,28	2,17	56,55	61,58	69,53	1,02	1,46	67,54	71,53
Riau	89,96	1,02	1,14	87,96	91,96	72,73	1,30	1,79	70,17	75,28	79,68	0,91	1,15	77,89	81,48
Jambi	84,94	2,01	2,37	81,00	88,89	58,35	1,53	2,63	55,35	61,36	66,66	1,26	1,88	64,20	69,13
Sumatera Selatan	80,11	1,68	2,09	76,82	83,39	57,06	1,28	2,24	54,55	59,56	65,31	1,03	1,57	63,30	67,33
Bengkulu	67,04	2,16	3,22	62,81	71,27	41,02	1,83	4,45	37,44	44,60	49,37	1,47	2,98	46,48	52,25
Lampung	72,08	1,98	2,74	68,21	75,96	50,85	1,28	2,51	48,35	53,36	56,78	1,08	1,89	54,67	58,88
Kep. Bangka Belitung	75,15	2,13	2,83	70,98	79,32	57,31	2,10	3,67	53,19	61,43	66,83	1,54	2,30	63,82	69,84
Kep. Riau	87,55	1,44	1,65	84,72	90,39	58,84	4,06	6,90	50,88	66,81	83,56	1,54	1,84	80,55	86,57
DKI Jakarta	89,59	0,74	0,83	88,14	91,04	-	-	-	-	-	89,59	0,74	0,83	88,14	91,04
Jawa Barat	75,86	0,70	0,93	74,48	77,24	58,35	1,07	1,83	56,26	60,44	71,06	0,59	0,83	69,90	72,22
Jawa Tengah	81,40	0,61	0,76	80,19	82,60	74,83	0,80	1,07	73,26	76,39	78,16	0,51	0,65	77,17	79,15
DI Yogyakarta	78,25	1,46	1,87	75,38	81,12	87,25	1,93	2,21	83,46	91,03	80,62	1,21	1,49	78,26	82,98
Jawa Timur	79,74	0,64	0,80	78,49	80,99	70,27	0,83	1,17	68,65	71,88	75,20	0,52	0,69	74,17	76,22
Banten	81,85	1,22	1,49	79,46	84,23	50,15	1,99	3,97	46,25	54,04	72,83	1,05	1,44	70,78	74,89
Bali	94,80	0,56	0,60	93,69	95,91	82,98	1,81	2,18	79,43	86,53	90,90	0,72	0,80	89,48	92,32
Nusa Tenggara Barat	82,20	1,48	1,80	79,30	85,10	66,25	1,92	2,90	62,49	70,02	73,61	1,27	1,73	71,12	76,10
Nusa Tenggara Timur	90,28	1,21	1,34	87,91	92,65	66,87	1,17	1,74	64,58	69,15	72,41	0,98	1,36	70,48	74,33
Kalimantan Barat	90,49	1,11	1,23	88,30	92,67	64,27	1,35	2,11	61,62	66,92	72,88	1,05	1,44	70,82	74,94
Kalimantan Tengah	85,71	1,38	1,61	83,01	88,40	53,09	1,90	3,59	49,36	56,83	65,38	1,42	2,17	62,61	68,16
Kalimantan Selatan	82,81	1,32	1,59	80,23	85,39	45,23	1,70	3,77	41,89	48,57	62,67	1,21	1,93	60,30	65,05
Kalimantan Timur	92,29	1,26	1,37	89,82	94,77	59,00	3,27	5,55	52,58	65,41	81,26	1,47	1,81	78,38	84,14
Kalimantan Utara	96,99	0,94	0,97	95,14	98,83	75,57	3,08	4,08	69,53	81,61	88,30	1,46	1,65	85,44	91,16
Sulawesi Utara	84,61	1,37	1,62	81,93	87,29	67,74	1,51	2,23	64,78	70,70	76,20	1,04	1,36	74,17	78,23
Sulawesi Tengah	85,60	1,81	2,12	82,04	89,15	65,40	1,55	2,37	62,37	68,44	71,13	1,29	1,81	68,61	73,65
Sulawesi Selatan	90,38	0,80	0,88	88,81	91,94	69,51	1,02	1,47	67,51	71,52	77,93	0,72	0,92	76,53	79,33
Sulawesi Tenggara	91,54	1,19	1,30	89,21	93,86	74,44	1,42	1,91	71,65	77,23	80,95	1,09	1,34	78,82	83,09
Gorontalo	88,52	1,55	1,76	85,48	91,57	72,80	1,83	2,52	69,21	76,40	78,99	1,33	1,68	76,39	81,59
Sulawesi Barat	81,65	2,74	3,35	76,29	87,01	57,31	2,06	3,60	53,27	61,35	62,98	1,78	2,83	59,48	66,48
Maluku	88,64	1,67	1,89	85,36	91,93	67,23	2,03	3,02	63,25	71,21	76,47	1,44	1,89	73,65	79,30
Maluku Utara	92,35	1,45	1,57	89,50	95,19	59,38	2,05	3,46	55,35	63,40	69,17	1,59	2,30	66,06	72,28
Papua Barat	92,64	0,98	1,06	90,72	94,57	66,78	2,14	3,21	62,58	70,98	77,12	1,36	1,77	74,45	79,79
Papua	86,11	1,37	1,59	83,42	88,80	48,75	1,53	3,14	45,75	51,75	58,35	1,26	2,16	55,87	60,82
**Indonesia**	**81,55**	**0,26**	**0,31**	**81,05**	**82,05**	**64,18**	**0,28**	**0,44**	**63,63**	**64,74**	**73,68**	**0,19**	**0,26**	**73,31**	**74,06**

https://www.bps.go.id

Tabel *Table* **9.28.**

***Sampling Error* Persentase Rumah Tangga yang Menerima Kartu Perlindungan Sosial (KPS)/Kartu Keluarga Sejahtera (KKS) dan Dapat Menunjukan Kartu menurut Provinsi dan Daerah Tempat Tinggal, 2018**

Sampling Errors Percentage of Household That Received Social Security Card (KPS)/Card for Family Welfare (KKS) and Can Show The Cards by Province and Urban Rural Classification, 2018

Provinsi *Province*	Perkotaan/*Urban*					Perdesaan/*Rural*					Perkotaan+Perdesaan/*Urban+Rural*				
	Estimasi *Estimate*	*Standard Error*	*Relative Standard Error (%)*	Selang Kepercayaan 95% *Confidence Interval 95%*		Estimasi *Estimate*	*Standard Error*	*Relative Standard Error (%)*	Selang Kepercayaan 95% *Confidence Interval 95%*		Estimasi *Estimate*	*Standard Error*	*Relative Standard Error (%)*	Selang Kepercayaan 95% *Confidence Interval 95%*	
				Batas Bawah *Lower Limit*	Batas Atas *Upper Limit*				Batas Bawah *Lower Limit*	Batas Atas *Upper Limit*				Batas Bawah *Lower Limit*	Batas Atas *Upper Limit*
(1)	(2)	(3)	(4)	(5)	(6)	(7)	(8)	(9)	(10)	(11)	(12)	(13)	(14)	(15)	(16)
Aceh	6,84	0,78	11,42	5,31	8,37	14,70	0,69	4,67	13,35	16,04	12,24	0,54	4,43	11,18	13,30
Sumatera Utara	7,47	0,54	7,21	6,41	8,53	14,92	0,58	3,86	13,79	16,04	11,01	0,40	3,62	10,23	11,79
Sumatera Barat	7,44	0,68	9,20	6,10	8,78	13,85	0,65	4,70	12,58	15,13	11,02	0,47	4,28	10,10	11,95
Riau	4,69	0,58	12,28	3,56	5,82	7,50	0,56	7,47	6,40	8,60	6,37	0,41	6,45	5,56	7,18
Jambi	5,78	0,83	14,37	4,15	7,41	7,19	0,50	6,98	6,20	8,17	6,75	0,43	6,41	5,90	7,59
Sumatera Selatan	6,32	0,76	11,97	4,84	7,81	9,95	0,62	6,28	8,73	11,18	8,65	0,49	5,64	7,70	9,61
Bengkulu	7,09	0,91	12,89	5,30	8,88	12,81	0,79	6,14	11,26	14,35	10,97	0,63	5,74	9,74	12,20
Lampung	10,60	1,14	10,79	8,36	12,84	14,71	0,63	4,26	13,48	15,94	13,57	0,55	4,08	12,48	14,65
Kep. Bangka Belitung	2,72	0,46	17,05	1,81	3,63	5,25	0,75	14,27	3,78	6,72	3,90	0,44	11,33	3,03	4,77
Kep. Riau	5,35	0,75	14,03	3,88	6,82	13,35	2,44	18,30	8,56	18,14	6,46	0,78	12,08	4,93	7,99
DKI Jakarta	3,41	0,35	10,28	2,72	4,09	-	-	-	-	-	3,41	0,35	10,28	2,72	4,09
Jawa Barat	7,67	0,30	3,94	7,08	8,26	14,39	0,54	3,78	13,33	15,46	9,51	0,27	2,82	8,99	10,04
Jawa Tengah	10,71	0,38	3,56	9,96	11,45	15,34	0,48	3,11	14,41	16,28	12,99	0,31	2,37	12,39	13,59
DI Yogyakarta	11,37	0,89	7,79	9,63	13,10	24,87	1,93	7,75	21,09	28,65	14,93	0,91	6,11	13,14	16,71
Jawa Timur	7,73	0,32	4,10	7,11	8,35	14,50	0,43	2,98	13,65	15,34	10,97	0,27	2,45	10,44	11,50
Banten	4,17	0,56	13,39	3,07	5,26	14,26	1,09	7,65	12,12	16,40	7,04	0,51	7,22	6,04	8,03
Bali	4,98	0,55	11,14	3,89	6,07	15,77	1,17	7,43	13,47	18,07	8,54	0,56	6,51	7,45	9,63
Nusa Tenggara Barat	14,60	1,12	7,71	12,39	16,80	13,92	0,96	6,93	12,03	15,80	14,23	0,73	5,16	12,79	15,67
Nusa Tenggara Timur	7,59	0,85	11,24	5,92	9,27	23,73	0,79	3,32	22,19	25,28	19,91	0,67	3,39	18,59	21,23
Kalimantan Barat	3,18	0,58	18,40	2,03	4,32	6,95	0,55	7,86	5,88	8,02	5,71	0,41	7,25	4,90	6,52
Kalimantan Tengah	2,04	0,37	18,34	1,31	2,78	5,83	0,63	10,72	4,61	7,06	4,40	0,45	10,20	3,52	5,29
Kalimantan Selatan	4,99	0,61	12,19	3,80	6,18	8,74	0,59	6,78	7,58	9,90	7,00	0,43	6,18	6,15	7,85
Kalimantan Timur	3,03	0,51	16,85	2,03	4,04	8,03	1,09	13,53	5,90	10,16	4,69	0,51	10,82	3,69	5,68
Kalimantan Utara	5,07	0,93	18,33	3,25	6,89	8,88	1,60	18,04	5,74	12,02	6,61	0,85	12,78	4,96	8,27
Sulawesi Utara	8,47	0,67	7,96	7,14	9,79	16,47	0,90	5,48	14,70	18,23	12,45	0,58	4,62	11,32	13,58
Sulawesi Tengah	6,44	1,06	16,52	4,35	8,52	15,28	0,79	5,15	13,74	16,82	12,77	0,65	5,09	11,50	14,05
Sulawesi Selatan	6,67	0,61	9,14	5,47	7,86	15,12	0,63	4,15	13,89	16,35	11,71	0,46	3,95	10,80	12,61
Sulawesi Tenggara	8,32	1,28	15,38	5,81	10,82	17,18	0,82	4,75	15,58	18,78	13,80	0,74	5,40	12,34	15,26
Gorontalo	13,29	1,52	11,43	10,32	16,27	20,49	1,65	8,04	17,26	23,72	17,66	1,18	6,66	15,35	19,96
Sulawesi Barat	9,64	2,03	21,06	5,66	13,63	14,10	1,01	7,14	12,13	16,08	13,06	0,94	7,23	11,21	14,91
Maluku	3,30	0,56	17,09	2,20	4,41	15,64	1,48	9,44	12,75	18,54	10,32	0,91	8,86	8,53	12,11
Maluku Utara	3,01	0,61	20,37	1,81	4,21	8,40	0,79	9,43	6,85	9,96	6,80	0,60	8,89	5,62	7,98
Papua Barat	6,59	1,14	17,22	4,37	8,82	9,10	1,05	11,56	7,04	11,17	8,10	0,78	9,67	6,56	9,63
Papua	2,45	0,59	23,94	1,30	3,61	1,79	0,23	12,96	1,33	2,24	1,96	0,24	11,99	1,50	2,42
**Indonesia**	**7,29**	**0,12**	**1,71**	**7,04**	**7,53**	**13,67**	**0,15**	**1,09**	**13,37**	**13,96**	**10,18**	**0,10**	**0,96**	**9,98**	**10,37**

Sumber/*Source*: Susenas Maret 2018/*The March 2018 Susenas*

https://www.bps.go.id

Tabel *Table* **9.29.** ***Sampling Error*** **Persentase Rumah Tangga yang Menerima Kartu Perlindungan Sosial (KPS)/Kartu Keluarga Sejahtera (KKS) dan Tidak Dapat Menunjukkan Kartu menurut Provinsi dan Daerah Tempat Tinggal, 2018**

Sampling Errors Percentage of Household That Received Social Security Card (KPS)/Card for Family Welfare (KKS) and Can not Show The Cards by Province and Urban Rural Classification, 2018

**Provinsi** *Province*	**Perkotaan/***Urban*					**Perdesaan/***Rural*					**Perkotaan+Perdesaan/***Urban+Rural*				
	**Estimasi** *Estimate*	*Standard Error*	*Relative Standard Error (%)*	**Selang Kepercayaan 95%** *Confidence Interval 95%*		**Estimasi** *Estimate*	*Standard Error*	*Relative Standard Error (%)*	**Selang Kepercayaan 95%** *Confidence Interval 95%*		**Estimasi** *Estimate*	*Standard Error*	*Relative Standard Error (%)*	**Selang Kepercayaan 95%** *Confidence Interval 95%*	
				**Batas Bawah** *Lower Limit*	**Batas Atas** *Upper Limit*				**Batas Bawah** *Lower Limit*	**Batas Atas** *Upper Limit*				**Batas Bawah** *Lower Limit*	**Batas Atas** *Upper Limit*
(1)	(2)	(3)	(4)	(5)	(6)	(7)	(8)	(9)	(10)	(11)	(12)	(13)	(14)	(15)	(16)
Aceh	2,84	0,48	16,82	1,91	3,78	5,32	0,41	7,72	4,52	6,13	4,55	0,32	7,03	3,92	5,17
Sumatera Utara	2,94	0,37	12,62	2,21	3,67	3,82	0,30	7,87	3,23	4,41	3,36	0,24	7,19	2,89	3,83
Sumatera Barat	2,24	0,32	14,37	1,61	2,87	4,12	0,38	9,15	3,38	4,85	3,29	0,26	7,83	2,78	3,79
Riau	1,54	0,33	21,43	0,89	2,18	1,92	0,24	12,74	1,44	2,40	1,77	0,20	11,18	1,38	2,15
Jambi	2,08	0,47	22,60	1,16	3,00	2,23	0,28	12,42	1,69	2,78	2,19	0,24	11,00	1,71	2,66
Sumatera Selatan	3,41	0,57	16,72	2,30	4,53	3,16	0,36	11,44	2,45	3,87	3,25	0,31	9,54	2,64	3,86
Bengkulu	1,90	0,46	23,99	1,01	2,79	4,61	0,48	10,50	3,66	5,56	3,74	0,36	9,63	3,03	4,45
Lampung	2,61	0,51	19,37	1,62	3,60	3,94	0,36	9,01	3,25	4,64	3,57	0,29	8,18	3,00	4,14
Kep. Bangka Belitung	2,67	0,56	20,80	1,58	3,76	1,59	0,33	20,94	0,94	2,25	2,17	0,34	15,45	1,51	2,83
Kep. Riau	1,87	0,43	22,81	1,04	2,71	4,17	1,22	29,22	1,78	6,57	2,19	0,42	18,96	1,38	3,01
DKI Jakarta	1,95	0,28	14,36	1,40	2,50	-	-	-	-	-	1,95	0,28	14,36	1,40	2,50
Jawa Barat	3,27	0,20	6,01	2,88	3,65	4,12	0,32	7,64	3,51	4,74	3,50	0,17	4,76	3,17	3,83
Jawa Tengah	3,04	0,21	6,77	2,64	3,45	4,88	0,28	5,68	4,34	5,42	3,95	0,17	4,38	3,61	4,29
DI Yogyakarta	5,64	0,67	11,84	4,33	6,95	8,63	1,19	13,75	6,30	10,95	6,43	0,59	9,12	5,28	7,58
Jawa Timur	2,54	0,18	7,01	2,19	2,89	5,36	0,29	5,34	4,80	5,92	3,89	0,17	4,29	3,56	4,22
Banten	2,75	0,42	15,44	1,92	3,58	5,41	0,78	14,46	3,87	6,94	3,50	0,38	10,92	2,75	4,25
Bali	1,64	0,24	14,62	1,17	2,12	3,34	0,57	16,98	2,23	4,46	2,21	0,25	11,22	1,72	2,69
Nusa Tenggara Barat	4,84	0,65	13,52	3,56	6,13	4,61	0,55	11,87	3,54	5,69	4,72	0,42	8,94	3,89	5,55
Nusa Tenggara Timur	2,20	0,48	21,72	1,26	3,13	6,51	0,41	6,35	5,70	7,32	5,49	0,34	6,17	4,83	6,16
Kalimantan Barat	2,28	0,58	25,63	1,13	3,42	3,26	0,44	13,66	2,38	4,13	2,94	0,35	12,07	2,24	3,63
Kalimantan Tengah	1,31	0,36	27,68	0,60	2,02	2,07	0,32	15,62	1,44	2,70	1,78	0,24	13,69	1,30	2,26
Kalimantan Selatan	2,07	0,37	17,72	1,35	2,79	2,80	0,31	11,07	2,20	3,41	2,46	0,24	9,74	1,99	2,93
Kalimantan Timur	1,49	0,27	17,83	0,97	2,01	3,37	0,56	16,73	2,26	4,48	2,11	0,26	12,44	1,60	2,63
Kalimantan Utara	3,16	0,83	26,29	1,53	4,78	3,47	0,88	25,49	1,74	5,20	3,28	0,61	18,56	2,09	4,48
Sulawesi Utara	3,62	0,47	13,02	2,69	4,54	4,96	0,46	9,24	4,06	5,86	4,29	0,33	7,61	3,65	4,93
Sulawesi Tengah	2,88	0,59	20,47	1,73	4,04	4,37	0,41	9,46	3,56	5,18	3,95	0,34	8,73	3,27	4,62
Sulawesi Selatan	2,87	0,39	13,41	2,12	3,63	5,30	0,42	7,99	4,47	6,13	4,32	0,30	6,91	3,74	4,91
Sulawesi Tenggara	3,47	0,84	24,20	1,82	5,12	5,50	0,48	8,75	4,55	6,44	4,72	0,42	8,99	3,89	5,56
Gorontalo	3,45	0,65	18,92	2,17	4,73	4,17	0,73	17,44	2,74	5,59	3,88	0,51	13,14	2,88	4,88
Sulawesi Barat	4,70	1,34	28,40	2,08	7,32	4,18	0,71	17,02	2,79	5,58	4,30	0,63	14,61	3,07	5,54
Maluku	1,48	0,38	25,57	0,74	2,21	4,96	0,58	11,61	3,83	6,09	3,45	0,37	10,68	2,73	4,18
Maluku Utara	1,92	0,50	25,97	0,94	2,90	2,87	0,42	14,53	2,05	3,68	2,59	0,33	12,71	1,94	3,23
Papua Barat	5,53	1,07	19,43	3,42	7,63	5,90	0,78	13,14	4,38	7,42	5,75	0,63	11,01	4,51	6,99
Papua	3,01	0,58	19,19	1,88	4,14	17,20	1,20	6,96	14,86	19,55	13,56	0,92	6,76	11,76	15,35
**Indonesia**	**2,85**	**0,08**	**2,77**	**2,69**	**3,00**	**4,70**	**0,09**	**1,99**	**4,52**	**4,88**	**3,69**	**0,06**	**1,65**	**3,57**	**3,81**

Sumber/*Source*: Susenas Maret 2018/*The March 2018 Susenas*

https://www.bps.go.id

KUESIONER VSEN18.K

VSEN18.K QUESIONNAIRE

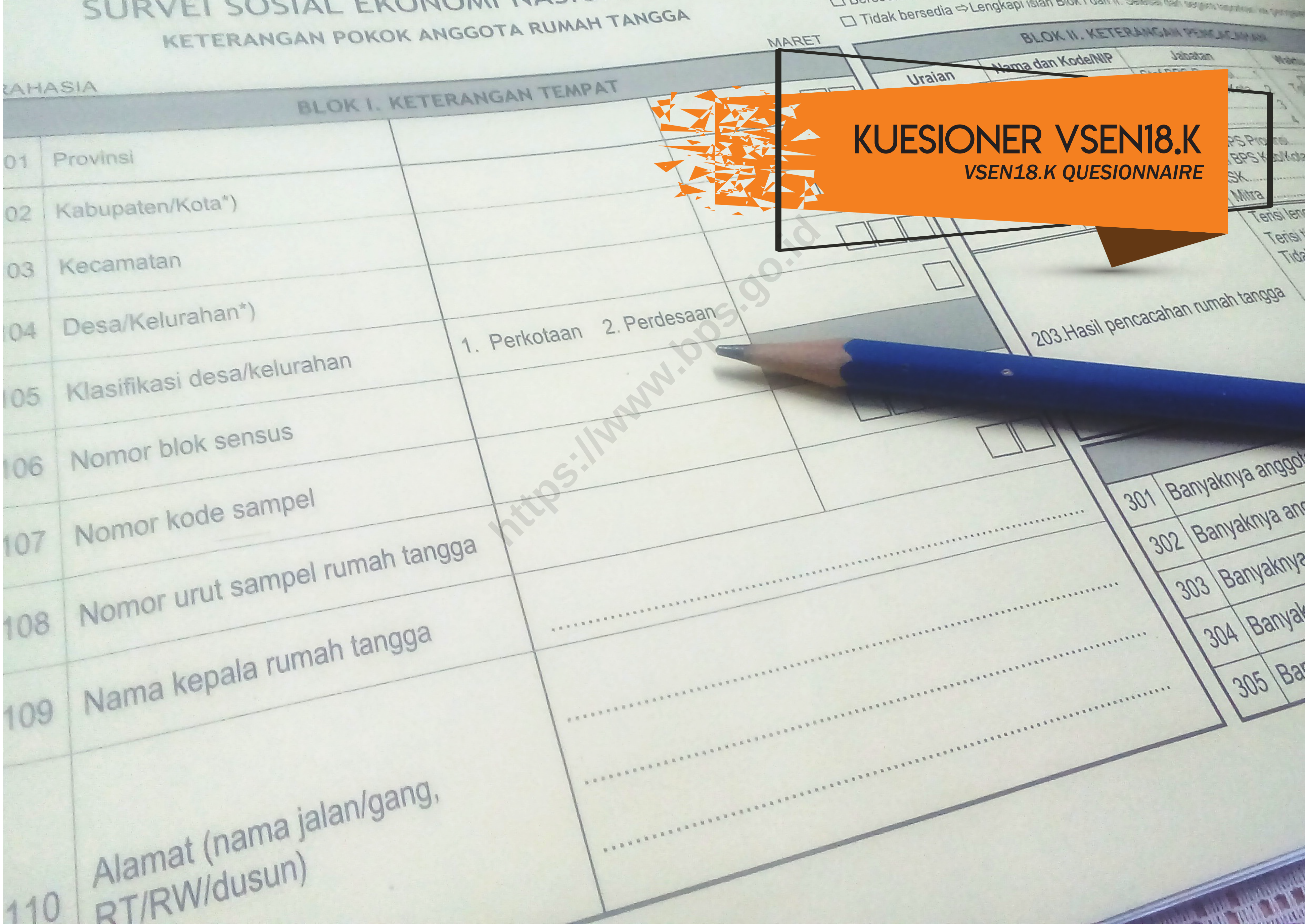

https://www.bps.go.id

https://www.bps.go.id

VSEN18.K
Dibuat 1 set untuk
BPS Kab/Kota

REPUBLIK INDONESIA

SURVEI SOSIAL EKONOMI NASIONAL 2018

KETERANGAN POKOK ANGGOTA RUMAH TANGGA

RAHASIA **MARET**

BLOK I. KETERANGAN TEMPAT			
101	Provinsi		☐☐
102	Kabupaten/Kota*)		☐☐
103	Kecamatan		☐☐☐
104	Desa/Kelurahan*)		☐☐☐
105	Klasifikasi Desa/Kelurahan	1. Perkotaan 2. Perdesaan	☐
106	Nomor Blok Sensus		
107	Nomor Kode Sampel		☐☐☐☐☐
108	Nomor Urut Bangunan Fisik di Sketsa Peta WB		
109	Nomor Urut Sampel Rumah Tangga		☐☐
110	Nama Kepala Rumah Tangga		
111	Alamat (Nama Jalan/Gang, RT/RW/Dusun)	....<br>..	
112	Koordinat Lokasi Rumah Tangga	Latitude (lintang) : .... , ...<br>Longitude (bujur) : .... , ...	

*) *Coret yang tidak perlu*

Selamat pagi/siang/sore/malam. Kami/Saya dari BPS sedang mengumpulkan data/informasi keadaan sosial ekonomi rumah tangga seperti pendidikan, kesehatan, pekerjaan, perumahan dan pengeluaran rumah tangga. Untuk itu Kami/Saya akan mewawancarai Bapak/Ibu beserta anggota rumah tangga (ART) lainnya. Seluruh data yang Bapak/Ibu berikan kepada kami, akan dirahasiakan dan hanya akan digunakan untuk keperluan perencanaan pembangunan. Boleh saya mulai wawancara sekarang?

☐ *Ya bersedia → Mulai wawancara*
☐ *Bersedia dengan perjanjian di lain waktu →* ***Blok XX. Catatan***
☐ *Tidak bersedia → Lengkapi isian Blok I dan II, dan Blok XX Catatan. Lampirkan Berita Acara Nonrespon. Selesai dan segera laporkan ke pengawas*

BLOK II. KETERANGAN PENCACAHAN

Uraian	Nama dan Kode/NIP	Jabatan	Waktu	Tanda Tangan
201. Pencacah	..............................<br>☐☐☐☐☐	Staf BPS Provinsi ....1<br>Staf BPS Kab/Kota ....2<br>KSK ....3<br>Mitra..................................4	Tgl ☐☐<br>Bln ☐☐	
202. Pengawas	..............................<br>☐☐☐☐☐	Staf BPS Provinsi ....1<br>Staf BPS Kab/Kota ....2<br>KSK ....3<br>Mitra..................................4	Tgl ☐☐<br>Bln ☐☐	
203. Hasil pencacahan rumah tangga		Terisi lengkap...1<br>Terisi tidak lengkap ....2<br>Tidak ada ART/responden yang dapat memberi jawaban sampai akhir masa pencacahan .....3<br>Responden menolak ....4<br>Rumah tangga pindah/bangunan sensus sudah tidak ada...5	☐<br>(2–5) ***Blok XX. Catatan***	

BLOK III. RINGKASAN

301	Banyaknya anggota rumah tangga	☐☐
302	Banyaknya anggota rumah tangga berumur 0-4 tahun	☐
303	Banyaknya anggota rumah tangga berumur 5 tahun ke atas	☐☐
304	Banyaknya anggota rumah tangga berumur 10 tahun ke atas	☐☐
305	Banyaknya perempuan berumur 10-54 tahun berstatus pernah kawin	☐

https://www.bps.go.id

1

PETUNJUK PENGISIAN

Dalam pengisian daftar, perlu diperhatikan tata tertib sebagai berikut:

1. Kuasai konsep, definisi, maksud, dan tujuan survei.
2. Tulis isian sejelas-jelasnya dengan pensil hitam pada tempat yang disediakan, agar mudah dibaca.
3. Gunakan blok catatan untuk mencatat hal-hal penting yang perlu diketahui oleh pengawas dan pengolah. Bagian kosong dari kuesioner juga dapat digunakan untuk mencatat hal-hal yang ditemui saat wawancara berlangsung.
4. Pencacah harus meneliti/memeriksa seluruh isian daftar dan memperbaiki setiap kesalahan, sebelum daftar isian diserahkan ke pengawas.
5. Perhatikan dan patuhi tanda-tanda atau alur pertanyaan yang tertera pada daftar isian.
6. Pertanyaan atau pilihan jawaban yang dicetak dengan huruf kapital harus dibacakan, sedangkan pertanyaan atau pilihan jawaban yang dicetak menggunakan huruf kecil tidak perlu dibacakan.
7. Kode pilihan jawaban yang menggunakan huruf kapital seperti A, B, C, dan seterusnya, boleh dilingkari lebih dari satu pilihan jawaban. Kode pilihan jawaban yang menggunakan angka seperti 1, 2, 3, dan seterusnya, hanya boleh dilingkari salah satu.
8. Blok I tentang keterangan tempat diisi sebelum ke lapangan.
9. Isikan Blok IV terlebih dahulu sampai selesai sebagai panduan untuk mengisi pertanyaan dalam format *roster*. Lipat bagian kertas yang ada tanda **garis putus-putus dan tulisan lipat disini** pada Blok IV halaman 2 sebagai panduan mengisi pertanyaan-pertanyaan yang terdapat pada halaman genap. Sementara itu, untuk panduan mengisi pada halaman ganjil, kertas pada halaman 2 tidak perlu dilipat (dilebarkan saja).
10. Pertanyaan dalam format *roster* (nama anggota rumah tangga (ART) per baris) seperti pada Blok V sampai dengan Blok XI diselesaikan dahulu dalam satu *roster* kemudian lanjut ke *roster* berikutnya.
11. Tanda garis tebal pada pertanyaan *roster* menunjukkan batas pertanyaan untuk ART, isikan jawaban pertanyaan di dalam tanda garis tebal untuk seluruh ART, lalu berpindah ke pertanyaan selanjutnya.
12. Tanda garis dua pada pertanyaan *roster* menunjukkan perbedaan tema pertanyaan dari setiap blok.
13. Contoh cara penulisan informasi pembelian/penerimaan Raskin/Rastra adalah menggunakan format rata kanan:

1602. DALAM 4 BULAN TERAKHIR, SEBUTKAN INFORMASI PEMBELIAN/PENERIMAAN RASKIN/RASTRA	*Bulan Februari 2018*
A. BERAPA JUMLAH RASKIN/RASTRA YANG DIBELI (KG)?	A) _1_ _5_,_0_ Kg
B. BERAPA RUPIAH TOTAL YANG DIBAYAR?	B) Rp. __ _2_ _4_._0_ _0_ _0_,-

14. Tabulasi umur responden yang sudah berulang tahun pada bulan Maret 2018:

Tahun lahir	2017	2016	2015	2014	2013	2012	2011	2010	2009	2008	2007	2006	2005	2004	2003
Umur	1	2	3	4	5	6	7	8	9	10	11	12	13	14	15
Tahun lahir	2002	2001	2000	1999	1998	1997	1996	1995	1994	1993	1992	1991	1990	1989	1988
Umur	16	17	18	19	20	21	22	23	24	25	26	27	28	29	30
Tahun lahir	1987	1986	1985	1984	1983	1982	1981	1980	1979	1978	1977	1976	1975	1974	1973
Umur	31	32	33	34	35	36	37	38	39	40	41	42	43	44	45
Tahun lahir	1972	1971	1970	1969	1968	1967	1966	1965	1964	1963	1962	1961	1960	1959	1958
Umur	46	47	48	49	50	51	52	53	54	55	56	57	58	59	60

Lipat di sini

Waktu mulai wawancara: ☐☐ : ☐☐

BLOK IV. KETERANGAN DEMOGRAFI

No. urut ART	NAMA ANGGOTA RUMAH TANGGA (ART), SEBUTKAN SIAPA SAJA YANG BIASA TINGGAL DI RUMAH TANGGA INI DAN KEPENGURUSAN MAKANNYA DIKELOLA DARI SATU DAPUR. MULAI DARI KEPALA RUMAHTANGGA, PASANGANNYA, ANAK YANG BELUM MENIKAH, ANAK YANG SUDAH MENIKAH, MENANTU, CUCU, ORANG TUA/MERTUA, PEMBANTU, FAMILI LAIN, DAN LAINNYA	APAKAH HUBUNGAN (nama) DENGAN KEPALA RUMAH TANGGA? (Kode)	APAKAH STATUS PER-KAWINAN (nama)? 1.Belum kawin 2.Kawin 3.Cerai hidup 4.Cerai mati	APAKAH (nama) LAKI-LAKI ATAU PEREM-PUAN? 1.Laki-laki 2.Perem-puan	KAPAN (nama) DILAHIRKAN? Tgl/Bln/Thn (DD/MM/YYYY)	BERAPA-KAH UMUR (nama)? *Umur harus diisi, Jika≥97 tahun, tulis '97'* (Dalam tahun)	*Jika berstatus kawin (404= 2)* APAKAH SUAMI/ ISTRI (nama) BIASANYA TINGGAL DIRUMAH TANGGA INI? 1.Ya 5.Tidak	*Jika berstatus pernah kawin (404=2,3, atau 4)* PADA UMUR BERAPA (nama) MELANG-SUNGKAN PER-KAWINAN PERTAMA?	No. urut ART pem-beri infor-masi
401	402	403	404	405	406	407	408	409	410
1		1	☐	☐	__/__/____	☐☐	☐	☐☐	___
2		☐	☐	☐	__/__/____	☐☐	☐	☐☐	___
3		☐	☐	☐	__/__/____	☐☐	☐	☐☐	___
4		☐	☐	☐	__/__/____	☐☐	☐	☐☐	___
5		☐	☐	☐	__/__/____	☐☐	☐	☐☐	___
6		☐	☐	☐	__/__/____	☐☐	☐	☐☐	___
7		☐	☐	☐	__/__/____	☐☐	☐	☐☐	___
8		☐	☐	☐	__/__/____	☐☐	☐	☐☐	___
9		☐	☐	☐	__/__/____	☐☐	☐	☐☐	___
10		☐	☐	☐	__/__/____	☐☐	☐	☐☐	___

Pastikan seluruh anggota rumah tangga tercatat dan tidak ada yang terlewat.
Cek sekali lagi, apakah kepengurusan makan seluruh anggota rumah tangga di kolom 402 dikelola dari satu dapur.
Jika terdapat ART yang kepengurusan makannya tidak dari satu dapur, maka keluarkan dari daftar.

Kode 403: Hubungan dengan Kepala Rumah Tangga (KRT)

1. KRT
2. Istri/suami
3. Anak kandung/tiri
4. Anak angkat
5. Menantu
6. Cucu
7. Orang tua/mertua
8. Pembantu/sopir
9. Lainnya (famili lain, orang yang tdk ada hubungan famili dengan KRT)

2

https://www.bps.go.id

KONSEP DAN DEFINISI

- **Pertanyaan 203: Hasil Pencacahan Rumah Tangga**
  - **Terisi lengkap**, apabila petugas berhasil menemui rumah tangga terpilih dan memperoleh informasi secara lengkap.
  - **Terisi tidak lengkap**, apabila petugas berhasil menemui rumah tangga terpilih, tetapi tidak dapat memperoleh informasi secara lengkap. Misalnya sampai batas akhir waktu pencacahan, informasi mengenai rumah tangga tersebut tidak diperoleh secara lengkap karena responden pergi keluar kota.
  - **Tidak ada ART/responden yang dapat memberi jawaban sampai akhir masa pencacahan**, apabila petugas berhasil menemui rumah tangga terpilih, namun tidak ada ART/responden yang dapat memberikan informasi mengenai rumah tangga sampai akhir masa pencacahan.
  - **Responden menolak**, apabila responden menolak untuk diwawancarai.
  - **Rumah tangga pindah/bangunan sensus sudah tidak ada**, apabila petugas tidak berhasil menemukan rumah tangga/bangunan sensus terpilih sampai batas akhir masa pencacahan. Misalnya: rumah tangga pindah keluar blok sensus, bangunan digusur, dan bangunan terbakar/runtuh karena gempa/banjir/bencana lain.
- **Pertanyaan 403: Hubungan dengan Kepala Rumah Tangga**
  Kepala rumah tangga (KRT) adalah salah seorang dari anggota rumah tangga yang bertanggung jawab atas kebutuhan sehari-hari rumah tangga. Pada kasus tertentu, misalnya beberapa anak sekolah mengontrak/menyewa rumah bersama-sama, maka KRT adalah seseorang yang ditunjuk diantara anak sekolah tersebut sebagai KRT.
- **Pertanyaan 408: Apakah Suami/Istri Biasanya Tinggal di Rumah Tangga Ini?**
  Yang dimaksud dengan suami/istri biasanya tinggal di rumah tangga ini adalah jika dalam 6 bulan terakhir, suami/istri tinggal di rumah lebih dari 3 bulan, meskipun tidak berturut-turut.
- **Pertanyaan 607: Apakah Sedang/Pernah Mengikuti Pendidikan Prasekolah?**
  Pendidikan prasekolah adalah pendidikan yang diselenggarakan sebelum jenjang pendidikan dasar, baik melalui jalur pendidikan formal maupun nonformal.
- **Pertanyaan 609, 610, dan 611: Apakah Dapat Membaca dan Menulis Kalimat Sederhana dalam Bahasa Sehari-Hari?**
  Dapat membaca dan menulis yang dimaksud adalah jika seseorang dapat membaca dan menulis kalimat sederhana dalam huruf latin, huruf arab, atau huruf lainnya.
  Kalimat sederhana adalah kalimat yang mengandung kata-kata yang umum dipakai dalam kehidupan sehari-hari dan setidaknya mengandung subjek dan predikat, misalnya "saya membaca".
- **Pertanyaan 612: Apakah Bersekolah (Termasuk Mengikuti Program Paket A/B/C)?**
  Bersekolah: apabila seseorang terdaftar dan aktif mengikuti proses belajar baik di suatu jenjang pendidikan formal maupun nonformal, khususnya program kesetaraan (Paket A/B/C) yang berada di bawah pengawasan Kementerian Pendidikan dan Kebudayaan (Kemdikbud) maupun kementerian lainnya. **Aktif mengikuti paket A, paket B, atau paket C**: apabila dalam **sebulan terakhir** pernah mengikuti proses belajar pada kegiatan paket.
- **Pertanyaan 613: Apa Jenjang Pendidikan Tertinggi yang Sedang/Pernah Diikuti?**
  Jenjang pendidikan tertinggi yang sedang/pernah diduduki: jenjang pendidikan tertinggi yang **sedang** diduduki oleh seseorang yang masih bersekolah atau yang **pernah** diduduki oleh seseorang yang sudah tidak bersekolah lagi, baik jenjang pendidikan formal maupun nonformal kesetaraan (Paket A/B/C).

KONSEP DAN DEFINISI

- **Pertanyaan 614: Apa Tingkat/Kelas Tertinggi yang Sedang/Pernah Diduduki?**
  Tingkat/kelas tertinggi adalah tingkatan/kelas terakhir atau paling tinggi yang dilalui seseorang pada suatu jenjang pendidikan baik formal maupun nonformal (Paket A/B/C) di sekolah negeri maupun swasta.
  Tamat sekolah/satuan pendidikan adalah menyelesaikan pelajaran yang ditandai dengan lulus ujian akhir pada kelas atau tingkat terakhir suatu jenjang baik pendidikan formal maupun nonformal (Paket A/B/C) di sekolah negeri maupun swasta dengan mendapatkan tanda tamat belajar/ijazah. **Seseorang yang belum mengikuti pelajaran pada kelas tertinggi, tetapi sudah mengikuti ujian akhir dan lulus, dianggap tamat sekolah/satuan pendidikan.**
- **Pertanyaan 615: Apa Ijazah/STTB Tertinggi yang Dimiliki?**
  Ijazah/STTB adalah lembaran atau tanda bukti kelulusan yang diberikan kepada seseorang yang sudah menyelesaikan semua persyaratan akademik pada suatu jenjang pendidikan tertentu.
- **Pertanyaan 701: Sejak 1 Januari – 31 Desember 2017, Apakah Pernah Menjadi Korban Kejahatan Pencurian, Penganiayaan, Pencurian dengan Kekerasan, Pelecehan Seksual, atau Lainnya?**
  Korban kejahatan adalah seseorang yang diri atau harta bendanya selama setahun terakhir mengalami atau terkena tindak kejahatan atau usaha/percobaan tindak kejahatan.
- **Pertanyaan 801: Selama Seminggu Terakhir, Apa Saja Kegiatan yang Dilakukan (*nama*)?**
  Bekerja adalah kegiatan melakukan pekerjaan dengan maksud memperoleh atau membantu memperoleh penghasilan atau keuntungan paling sedikit selama satu jam dalam seminggu terakhir. Bekerja selama satu jam tersebut harus dilakukan berturut-turut dan tidak terputus.
  Sekolah adalah kegiatan bersekolah di sekolah formal maupun sekolah non formal (Paket A/B/C), baik pada pendidikan dasar, pendidikan menengah atau pendidikan tinggi. Tidak termasuk yang sedang libur/cuti.
  Mengurus rumah tangga (ruta) adalah kegiatan mengurus ruta/membantu mengurus ruta tanpa mendapat upah/gaji. Anggota rumah tangga yang melakukan kegiatan kerumahtanggaan, seperti memasak, mencuci dsb. digolongkan sebagai mengurus ruta.
  Lainnya selain kegiatan pribadi adalah kegiatan selain bekerja, sekolah, dan mengurus ruta.
- **Pertanyaan 1001: Jaminan Kesehatan Apa Saja yang Dimiliki (*nama*)?**
  Jaminan Kesehatan Nasional (JKN) oleh Badan Penyelenggara Jaminan Sosial (BPJS) Kesehatan:
  Peserta penerima Bantuan Iuran (PBI) Jaminan Kesehatan meliputi orang yang tergolong fakir miskin dan orang tidak mampu yang iurannya dibayar oleh pemerintah.
  Peserta bukan PBI terdiri atas:
  a. **Pekerja Penerima Upah dan anggota keluarganya**, yaitu: a) Pegawai Negeri Sipil; b) Anggota TNI; c) Anggota Polri; d) Pejabat negara; e) Pegawai pemerintah non pegawai negeri; f) Pegawai swasta; dan g) Pekerja yang tidak termasuk huruf a sampai dengan huruf f yang menerima upah.
  b. **Pekerja Bukan Penerima Upah dan anggota keluarganya**, yaitu: a) Pekerja di luar hubungan kerja atau Pekerja mandiri dan b) Pekerja yang tidak termasuk huruf a yang bukan penerima upah. c) Pekerja sebagaimana dimaksud huruf a dan huruf b, termasuk warga negara asing yang bekerja di Indonesia paling singkat 6 (enam) bulan.
  c. **Bukan Pekerja dan anggota keluarganya** terdiri atas: a) Investor; b) Pemberi kerja; c) Penerima pensiun; d) Veteran; e) Perintis kemerdekaan; dan f) Bukan pekerja yang tidak termasuk huruf a sampai dengan huruf e yang mampu membayar Iuran.

BLOK V. KETERANGAN NOMOR INDUK KEPENDUDUKAN			
No. Urut ART	APAKAH (*nama*) MEMPUNYAI NOMOR INDUK KEPENDUDUKAN?<br>1. Ya<br>5. Tidak → ***ART Berikutnya***	Nomor Induk Kependudukan<br>***(Tuliskan Nomor Induk Kependudukan setiap ART)***	Sumber Data Nomor Induk Kependudukan<br>**(Kode)**
401	501	502	503
1	☐	☐☐☐☐ ☐☐☐☐ ☐☐☐☐ ☐☐☐☐	☐
2	☐	☐☐☐☐ ☐☐☐☐ ☐☐☐☐ ☐☐☐☐	☐
3	☐	☐☐☐☐ ☐☐☐☐ ☐☐☐☐ ☐☐☐☐	☐
4	☐	☐☐☐☐ ☐☐☐☐ ☐☐☐☐ ☐☐☐☐	☐
5	☐	☐☐☐☐ ☐☐☐☐ ☐☐☐☐ ☐☐☐☐	☐
6	☐	☐☐☐☐ ☐☐☐☐ ☐☐☐☐ ☐☐☐☐	☐
7	☐	☐☐☐☐ ☐☐☐☐ ☐☐☐☐ ☐☐☐☐	☐
8	☐	☐☐☐☐ ☐☐☐☐ ☐☐☐☐ ☐☐☐☐	☐
9	☐	☐☐☐☐ ☐☐☐☐ ☐☐☐☐ ☐☐☐☐	☐
10	☐	☐☐☐☐ ☐☐☐☐ ☐☐☐☐ ☐☐☐☐	☐

Nomor Induk Kependudukan (NIK) adalah nomor identitas penduduk yang bersifat unik atau khas, tunggal, dan melekat pada seseorang yang terdaftar sebagai penduduk Indonesia.

NIK berlaku seumur hidup dan selamanya, yang diberikan oleh pemerintah dan diterbitkan oleh instansi pelaksana kepada setiap penduduk setelah dilakukan pencatatan biodata.

Kode 503: Sumber Data Nomor Induk Kependudukan

1. KK
2. KTP
3. Lainnya

4

https://www.bps.go.id

BLOK VI. KETERANGAN MIGRASI, AKTA KELAHIRAN, DAN PENDIDIKAN

No. Urut ART	No. Urut Ibu Kandung *Lihat Blok IV* (*Isikan 00 bila ibu kandung tidak tinggal di rumah tangga ini*)	*Untuk ART Semua Umur* — DIMANAKAH TEMPAT LAHIR (*nama*)? *Tuliskan nama tempat (Kode tempat diisi oleh pengawas)* — PROVINSI/NEGARA	KABUPATEN/KOTA *Jika lahir di Kota, tuliskan "Kota" sebelum nama kota*	*Untuk ART berumur 5 tahun ke atas* — DIMANAKAH TEMPAT TINGGAL (*nama*) **5 TAHUN YANG LALU (MARET 2013)**? *Tuliskan nama tempat (Kode tempat diisi oleh pengawas)* — PROVINSI/NEGARA	KABUPATEN/KOTA *Jika di Kota, tuliskan "Kota" sebelum nama kota*	*Untuk ART berumur 0-17 tahun* — APAKAH (*nama*) MEMILIKI **AKTA KELAHIRAN** DARI KANTOR CATATAN SIPIL? BOLEH SAYA MELIHATNYA? **(Kode)**	*Untuk ART berumur 0-10 tahun* — APAKAH (*nama*) SEDANG/ PERNAH MENGIKUTI PENDIDIKAN PRA-SEKOLAH? **(Kode)**	*Jika pernah/ masih (607= 1,2 atau 3)*, APA JENIS PENDIDIKAN PRASEKOLAH NYA? **(Kode)**
401	601	602	603	604	605	606	607	608
1								
2								
3								
4								
5								
6								
7								
8								
9								
10								

Kode 606: Kepemilikan Akta Kelahiran

1. Ya, dapat ditunjukkan
2. Ya, tidak dapat ditunjukkan
5. Tidak memiliki
8. Tidak tahu

Kode 607: Partisipasi Prasekolah

1. Masih mengikuti pendidikan prasekolah tahun ajaran ini (2017/2018)
2. Pernah mengikuti pendidikan prasekolah tahun ajaran ini (2017/2018)
3. Pernah mengikuti pendidikan prasekolah sebelum tahun ajaran 2017/2018
4. Tidak/belum pernah mengikuti pendidikan prasekolah

Kode 608: Jenis Prasekolah

1. Taman Kanak-kanak
2. Bustanul Athfal/Raudatul Athfal
3. PAUD terintegrasi BKB/Taman Posyandu, PAUD-TAAM, PAUD-PAK, PAUD-BIA,TKQ, dll
4. Kelompok Bermain
5. Taman Penitipan Anak

5

https://www.bps.go.id

BLOK VI. KETERANGAN MIGRASI, AKTA KELAHIRAN, DAN PENDIDIKAN

No. urut ART	*Untuk ART berumur 5 tahun ke atas*							*Untuk ART berumur 5-24 tahun*				
	APAKAH (*nama*) DAPAT MEMBACA DAN MENULIS KALIMAT SEDERHANA DALAM BAHASA SEHARI-HARI DENGAN MENGGUNAKAN:			APAKAH (*nama*) BERSEKOLAH? (termasuk mengikuti program paket A/B/C) **(Kode)** ***Jika kode =1 ke 616***	APA JENJANG PENDIDIKAN TERTINGGI YANG SEDANG/ PERNAH DIIKUTI (*nama*)? **(Kode)**	APA TINGKAT/ KELAS TERTINGGI YANG SEDANG/PERNAH DIDUDUKI (*nama*)? **(Kode)**	APA IJAZAH/ STTB TERTINGGI YANG DIMILIKI (*nama*)? **(Kode)**	**DALAM SETAHUN TERAKHIR,** APAKAH (*nama*) MEMILIKI KARTU INDONESIA PINTAR (KIP)? 1. Ya, dapat ditunjukkan 2. Ya, tidak dapat ditunjukkan 5. Tidak	**DALAM SETAHUN TERAKHIR,** APAKAH (*nama*) MEMPEROLEH PROGRAM INDONESIA PINTAR (PIP)? 1. Ya 5. Tidak	***Untuk ART yang masih bersekolah atau tidak bersekolah lagi (612 = 2 atau 3)***		
	HURUF LATIN/ ALFABET? 1.Ya 5.Tidak	HURUF ARAB/ HIJAIYAH? 1.Ya 5.Tidak	HURUFLAINNYA? (*CONTOH: JAWA, KANJI, CINA, DLL*) 1.Ya 5.Tidak							APAKAH (*nama*) BERSEKOLAH PADA TAHUN AJARAN SEBELUMNYA (2016/2017)? **(Kode)** ***Jika kode = 1 atau 3 Ke ART berikutnya***	APA JENJANG PENDIDIKAN YANG DIIKUTI (*nama*) WAKTU ITU? **(Kode)**	APA TINGKAT/ KELAS YANG DIDUDUKI (*nama*) WAKTU ITU? **(Kode)**
401	609	610	611	612	613	614	615	616	617	618	619	620
1												
2												
3												
4												
5												
6												
7												
8												
9												
10												

Kode 612 dan 618: Partisipasi Sekolah
1. Tidak/belum pernah bersekolah
2. Masih bersekolah
3. Tidak bersekolah lagi

Kode 613 dan 619: Jenjang Pendidikan

01.Paket A	06. SMP LB	11.SMA	16.D3
02.SDLB	07. SMP	12.MA	17.D4
03.SD	08. MTs	13.SMK	18.S1
04.MI	09. Paket C	14.MAK	19.S2
05.Paket B	10. SMLB	15.D1/D2	20.S3

Kode 614 dan 620: Tingkat/Kelas

1, 2, 3, 4, 5, 6, 7, **8** (Tamat & Lulus)

Kode 615: Ijazah/STTB

01.Tidak punya ijazah SD	06. Paket B	10. Paket C	14. SMK	18. D4
02.Paket A	07. SMP LB	11. SMLB	15. MAK	19. S1
03.SDLB	08. SMP	12. SMA	16. D1/D2	20. S2
04.SD	09. MTs	13. MA	17. D3	21. S3
05.MI				

6

https://www.bps.go.id

BLOK VII. KETERANGAN KORBAN KEJAHATAN, TEKNOLOGI INFORMASI, KOMUNIKASI, DAN KEPEMILIKAN TABUNGAN

No. urut ART	SEJAK 1 JANUARI - 31 DESEMBER 2017, APAKAH (*nama*) PERNAH MENJADI KORBAN KEJAHATAN PENCURIAN, PENGANIAYAAN, PENCURIAN DENGAN KEKERASAN, PELECEHAN SEKSUAL, ATAU LAINNYA? 1. Ya 5. Tidak → *713*	*Jika 701 = 1*											*Untuk ART berumur 5 tahun ke atas*				
		BERAPA KALI (*nama*) MENJADI KORBAN KEJAHATAN DAN BERAPA KEJADIAN YANG DILAPORKAN KE POLISI?										**(*Jika 703, 705, 707, 709, atau 711>0*)** BERAPA KEJADIAN DALAM PENANGANAN POLISI?	**DALAM 3 BULAN TERAKHIR,** APAKAH (*nama*) **MENGGUNAKAN** TELEPON SELULER (HP)/ NIRKABEL? 1. Ya 5. Tidak	**DALAM 3 BULAN TERAKHIR,** APAKAH (*nama*) **MEMILIKI/ MENGUASAI** TELEPON SELULER (HP)/ NIRKABEL? 1. Ya 5. Tidak	**DALAM 3 BULAN TERAKHIR,** APAKAH (*nama*) MENGGUNAKAN KOMPUTER (*PC/DESKTOP, LAPTOP/ NOTEBOOK, TABLET*)? 1. Ya 5. Tidak	**DALAM 3 BULAN TERAKHIR,** APAKAH (*nama*) PERNAH MENGGUNAKAN INTERNET (TERMASUK *FACEBOOK, TWITTER, BBM, WHATS APP*)? 1. Ya 5. Tidak	APAKAH (*nama*) MEMILIKI REKENING TABUNGAN BAIK ATAS NAMA SENDIRI ATAU BERSAMA-SAMA DI LEMBAGA KEUANGAN (PERBANKAN, KOPERASI)? 1. Ya 5. Tidak
		PENCURIAN		PENGANIAYAAN		PENCURIAN DENGAN KEKERASAN		PELECEHAN SEKSUAL		LAINNYA							
		JUMLAH KEJADIAN	JUMLAH KEJADIAN YANG DILAPOR-KAN KE POLISI	JUMLAH KEJADIAN	JUMLAH KEJADIAN YANG DILAPOR-KAN KE POLISI	JUMLAH KEJADIAN	JUMLAH KEJADIAN YANG DILAPOR-KAN KE POLISI	JUMLAH KEJADIAN	JUMLAH KEJADIAN YANG DILAPOR-KAN KE POLISI	JUMLAH KEJADIAN	JUMLAH KEJADIAN YANG DILAPOR-KAN KE POLISI						
401	701	702	703	704	705	706	707	708	709	710	711	712	713	714	715	716	717
1																	
2																	
3																	
4																	
5																	
6																	
7																	
8																	
9																	
10																	

- **Telepon seluler:** perangkat telekomunikasi elektronik yang mempunyai kemampuan dasar yang sama dengan telepon tetap kabel, namun dapat dibawa kemana-mana (*portable*, *mobile*) dan tidak perlu disambungkan dengan jaringan telekomunikasi kabel. Termasuk telepon seluler adalah *handphone* dan *smartphone*, tetapi tidak termasuk *tablet* meskipun bisa digunakan untuk menelepon.
- **Menggunakan HP** yang dimaksud tidak harus menggunakan HP yang dimiliki sendiri atau dibeli/dibayar sendiri oleh individu yang menggunakan.
- **Memiliki/menguasai HP** apabila anggota rumah tangga tersebut memiliki/menguasai HP dengan minimal 1 kartu yang aktif dalam 3 bulan terakhir.
- **Menggunakan internet:** apabila seseorang meluangkan waktu untuk menggunakan internet, sehingga ia dapat memanfaatkan atau menikmati fasilitas internet. Termasuk menggunakan internet walaupun tidak memiliki kemampuan untuk membuka dan menutup (*log in* dan *log out*) internet. Siapa saja dimasukkan menggunakan meskipun hanya tinggal melanjutkan.

7

https://www.bps.go.id

BLOK VIII. KETERANGAN KETENAGAKERJAAN (UNTUK ANGGOTA RUMAH TANGGA BERUMUR 10 TAHUN KE ATAS)

			Jika tidak bekerja (801 pilihan A tidak dilingkari)	*Jika bekerja atau sementara tidak bekerja (801 = pilihan A terpilih atau 803 =1)*						*Jika 808 = 5 dan 809 = 5*	
No. urut ART	**SELAMA SEMINGGU TERAKHIR,** APA SAJA KEGIATAN YANG DILAKUKAN (*nama*)?<br>***(Pilihan jawaban harus dibacakan)***<br>BEKERJA ....A<br>SEKOLAH..............................B<br>MENGURUS RUMAH TANGGA ... C<br>LAINNYA SELAIN KEGIATAN PRIBADI............. D<br>TIDAK MELAKUKAN KEGIATAN ..X<br>***Jika X terpilih → 803***	**DARI KEGIATAN YANG DILAKUKAN SELAMA SEMINGGU TERAKHIR,** KEGIATAN APAKAH YANG MENGGUNAKAN WAKTU TERBANYAK?<br>BEKERJA.................. 1 → ***804***<br>SEKOLAH .... 2<br>MENGURUS RUMAH TANGGA .... 3<br>LAINNYA SELAIN KEGIATAN PRIBADI.... 4	**SELAMA SEMINGGU TERAKHIR,** APAKAH (*nama*) MEMPUNYAI PEKERJAAN/USAHA, TETAPI SEMENTARA TIDAK BEKERJA?<br>1. Ya<br>5. Tidak → ***808***	**SELAMA SEMINGGU TERAKHIR,** APA LAPANGAN USAHA ATAU BIDANG PEKERJAAN UTAMA DARI TEMPAT PEKERJAAN (*nama*)?<br>***Tuliskan selengkap-lengkapnya***<br>***(Kode lapangan usaha atau bidang pekerjaan utama diisi oleh pengawas)***	**SELAMA SEMINGGU TERAKHIR,** APA STATUS/ KEDUDUKAN (*nama*) DALAM PEKERJAAN UTAMA?<br>***(Kode)***	**SELAMA SEMINGGU TERAKHIR,** BERAPA JUMLAH JAM KERJA DARI **PEKERJAAN UTAMA**?<br>***(Jam)***	**SELAMA SEMINGGU TERAKHIR,** BERAPA JUMLAH JAM KERJA DARI **SELURUH PEKERJAAN**?<br>***(Jam)***	**SELAMA SEMINGGU TERAKHIR,** APAKAH (*nama*) SEDANG MENCARI PEKERJAAN?<br>1. Ya<br>5. Tidak	**SELAMA SEMINGGU TERAKHIR,** APAKAH (*nama*) SEDANG MEMPER-SIAPKAN USAHA BARU?<br>1. Ya<br>5. Tidak	APAKAH ALASAN UTAMA (*nama*) TIDAK MENCARI PEKERJAAN/ MEMPERSIAPKAN USAHA BARU?<br>***(Kode)***<br>***Jika kode 6 atau 7 → ART berikutnya***<br>***Jika kode 8, tuliskan alasan responden***	**JIKA ADA PENAWARAN PEKERJAAN,** APAKAH (*nama*) MASIH MAU MENERIMA?<br>1. Ya<br>5. Tidak
401	801	802	803	804	805	806	807	808	809	810	811
1	A B C D X			....						....	
2	A B C D X			....						....	
3	A B C D X			....						....	
4	A B C D X			....						....	
5	A B C D X			....						....	
6	A B C D X			....						....	
7	A B C D X			....						....	
8	A B C D X			....						....	
9	A B C D X			....						....	
10	A B C D X			....						....	

Kode 805: Kode Status/Kedudukan dalam Pekerjaan Utama
(Jika (803=1), 805 tidak boleh berkode 5 atau 6)

1. Berusaha sendiri
2. Berusaha dibantu buruh tidak tetap/ buruh tidak dibayar
3. Berusaha dibantu buruh tetap/ buruh dibayar
4. Buruh/karyawan/pegawai
5. Pekerja bebas
6. Pekerja keluarga atau tidak dibayar

Kode 810: Alasan Utama Tidak Mencari Pekerjaan/Mempersiapkan Usaha Baru

1. Putus asa: Merasa tidak mungkin mendapatkan pekerjaan (alasan bagi mereka yang berkali-kali mencari pekerjaan, tetapi tidak berhasil mendapatkan pekerjaan sehingga ia merasa tidak mungkin mendapatkan pekerjaan atau mereka yang merasa karena situasi/kondisi/iklim/musim tidak mungkin mendapatkan pekerjaan yang diinginkan)
2. Sudah diterima bekerja, tetapi belum mulai bekerja
3. Sedang bersekolah
4. Mengurus rumah tangga
5. Sudah mempunyai pekerjaan/usaha
6. Merasa sudah cukup
7. Tidak mampu melakukan pekerjaan
8. Lainnya (tuliskan pada tempat yang tersedia)

8

https://www.bps.go.id

BLOK IX. KETERANGAN GANGGUAN FUNGSIONAL

No. urut ART	*Untuk ART berumur 2 tahun ke atas*								*Untuk ART berumur 5 tahun ke atas*
No. urut ART	***Isikan kode 1 jika Umur ≥ 2 atau kode 0 jika Umur < 2*** 1 → **902-909** 0 → ***ART berikutnya***	APAKAH (*nama*) MENGALAMI KESULITAN/GANGGUAN PENGLIHATAN? Ya, sama sekali tidak bisa melihat ...1 Ya, banyak kesulitan..2 Ya, sedikit kesulitan ...3 Tidak mengalami kesulitan 4	APAKAH (*nama*) MENGALAMI KESULITAN/GANGGUAN PENDENGARAN? Ya, sama sekali tidak bisa mendengar...... 1 Ya, banyak kesulitan .. 2 Ya, sedikit kesulitan.... 3 Tidak mengalami kesulitan 4	APAKAH (*nama*) MENGALAMI KESULITAN/GANGGUAN BERJALAN ATAU NAIK TANGGA? Ya, sepenuhnya membutuhkan bantuanoranglain .. 1 Ya, membutuhkan alat bantu............. 2 Ya, sedikit kesulitan.. 3 Tidak mengalami kesulitan 4	APAKAH (*nama*) MENGALAMI KESULITAN MENGGUNAKAN DAN MENGGERAKKAN TANGAN/JARI? Ya, sama sekali tidak bisa menggunakan/ menggerakkan .... 1 Ya, banyak kesulitan.. 2 Ya, sedikit kesulitan ... 3 Tidak mengalami kesulitan 4	APAKAH (*nama*) MENGALAMI KESULITAN/GANGGUAN DALAM HAL MENGINGAT ATAU KONSENTRASI? Ya, selalu mengalami kesulitan..................... 1 Ya, seringkali mengalami kesulitan..................... 2 Ya, sedikit mengalami kesulitan.................... 3 Tidak mengalami kesulitan.................... 4	APAKAH (*nama*) MENGALAMI KESULITAN DALAM MENGENDALIKAN PERILAKU DAN ATAU EMOSIONAL? Ya, selalu mengalami kesulitan ....1 Ya, seringkali mengalami kesulitan ....2 Ya, sedikit mengalami kesulitan ....3 Tidak mengalami kesulitan 4	APAKAH (*nama*) MENGALAMI KESULITAN/ GANGGUAN BERBICARA DAN ATAU MEMAHAMI/ BERKOMUNIKASI DENGAN ORANG LAIN? Ya,sama sekali tidak bisa memahami/dipahami/ berkomunikasi............1 Ya, banyak mengalami kesulitan ....2 Ya sedikit mengalami kesulitan ....3 Tidak mengalami kesulitan 4	APAKAH (*nama*) MENGALAMI KESULITAN/ GANGGUAN UNTUK MENGURUSDIRISENDIRI? (seperti mandi, makan, berpakaian, buang air besar, buang air kecil) Ya, sama sekali tidak bisa mengurus diri sendiri ... 1 Ya, banyak mengalami kesulitan...................... 2 Ya, sedikit mengalami kesulitan...................... 3 Tidak mengalami kesulitan 4
401	901	902	903	904	905	906	907	908	909
1	☐	☐	☐	☐	☐	☐	☐	☐	☐
2	☐	☐	☐	☐	☐	☐	☐	☐	☐
3	☐	☐	☐	☐	☐	☐	☐	☐	☐
4	☐	☐	☐	☐	☐	☐	☐	☐	☐
5	☐	☐	☐	☐	☐	☐	☐	☐	☐
6	☐	☐	☐	☐	☐	☐	☐	☐	☐
7	☐	☐	☐	☐	☐	☐	☐	☐	☐
8	☐	☐	☐	☐	☐	☐	☐	☐	☐
9	☐	☐	☐	☐	☐	☐	☐	☐	☐
10	☐	☐	☐	☐	☐	☐	☐	☐	☐

- **Penyandang disabilitas** adalah setiap orang yang mengalami keterbatasan fisik, intelektual, mental, dan/atau sensorik dalam jangka waktu lama yang dalam berinteraksi dengan lingkungan dapat mengalami hambatan dan kesulitan untuk berpartisipasi secara penuh dan efektif dengan warga negara lainnya berdasarkan kesamaan hak.
- Petugas tidak diperkenankan memutuskan bahwa responden tidak mengalami disabilitas tertentu berdasarkan apa yang dilihat secara kasat mata. Gangguan/keterbatasan fungsi antara lain: kesulitan melihat, kesulitan mendengar, berbicara tidak lancar, kesulitan memahami/hilang ingatan/gangguan jiwa, lambat dalam belajar/memahami pelajaran, keterbatasan berjalan, keterbatasan bergerak, kesulitan mengambil barang kecil menggunakan tangan/jari. **Setiap orang bisa mengalami lebih dari satu jenis gangguan.**

9

https://www.bps.go.id

BLOK X. KETERANGAN KELUHAN KESEHATAN, BEROBAT JALAN, DAN RAWAT INAP

No. urut ART	JAMINAN KESEHATAN APA SAJA YANG DIMILIKI (*nama*)?<br>BPJS Kesehatan<br>Penerima bantuan iuran (PBI).................... A<br>Non PBI.................... B<br>Jamkesda .... C<br>Asuransi swasta.......... D<br>Perusahaan/kantor ..... E<br>Tidak punya X	DALAM SEBULAN TERAKHIR, APAKAH (*nama*) MEMPUNYAI KELUHAN KESEHATAN (PANAS, BATUK, PILEK, DIARE, PUSING, PENYAKIT KRONIS, DSB.)?<br>1.Ya<br>5.Tidak → 1009	APAKAH KELUHAN KESEHATAN TERSEBUT MENGAKIBAT-KAN TERGANGGU-NYA PEKERJAAN, SEKOLAH ATAU KEGIATAN SEHARI-HARI?<br>1.Ya<br>5.Tidak	DALAM SEBULAN TERAKHIR, APAKAH (*nama*) PERNAH MENGOBATI SENDIRI?<br>1.Ya<br>5.Tidak	DALAM SEBULAN TERAKHIR, APAKAH (*nama*) PERNAH BEROBAT JALAN?<br>1.Ya → 1007<br>5.Tidak	APA ALASAN UTAMA (*nama*) TIDAK BEROBAT JALAN?<br>Tidak punya biaya berobat .... 1<br>Tidak ada biaya transpor . 2<br>Tidak ada sarana transportasi .... 3<br>Waktu tunggu pelayanan lama .... 4<br>Mengobati sendiri....... 5<br>Tidak ada yang mendampingi........... 6<br>Merasa tidak perlu...... 7<br>Lainnya....................... 8<br>***(lanjut ke 1009)***	DALAM SEBULAN TERAKHIR, DI MANA SAJA TEMPAT (*nama*) BEROBAT JALAN?<br>RS Pemerintah .... A<br>RS Swasta...................... B<br>Praktik dokter/bidan........ C<br>Klinik/Praktik dokter bersama .... D<br>Puskesmas/Pustu........... E<br>UKBM (Poskesdes, Polindes, Posyandu, Balai Pengobatan) .... F<br>Praktik pengobatan tradisional/alternatif...... G<br>Lainnya.......................... H	JAMINAN KESEHATAN APA SAJA YANG DIGUNAKAN (*nama*) UNTUK BEROBAT JALAN?<br>BPJS Kesehatan<br>Penerima bantuan iuran (PBI)...................... A<br>Non PBI.................... B<br>Jamkesda .... C<br>Asuransi swasta .... D<br>Perusahaan/kantor .... E<br>Tidak menggunakan.. X	DALAM SETAHUN TERAKHIR, APAKAH (*nama*) PERNAH DIRAWAT INAP?<br>1.Ya<br>5.Tidak → ***ART berikutnya***	DALAM SETAHUN TERAKHIR, DI MANA SAJA TEMPAT (*nama*) DIRAWAT INAP?<br>RS Pemerintah .... A<br>RS Swasta .... B<br>Praktik dokter/bidan .... C<br>Klinik/Praktik dokter bersama...................... D<br>Puskesmas/Pustu .... E<br>Praktik pengobatan tradisional/alternatif ...... F<br>Lainnya .... G	DALAM SETAHUN TERAKHIR, BERAPA HARI (*nama*) DIRAWAT INAP?<br>***(Hari)***	JAMINAN KESEHATAN APA SAJA YANG DIGUNAKAN (*nama*) UNTUK RAWAT INAP?<br>BPJS Kesehatan<br>Penerima bantuan iuran (PBI) .... A<br>Non PBI .... B<br>Jamkesda .... C<br>Asuransi swasta.......... D<br>Perusahaan/kantor ..... E<br>Tidak menggunakan ... X
401	1001	1002	1003	1004	1005	1006	1007	1008	1009	1010	1011	1012
1	A B C D E **X**	☐	☐	☐	☐	☐	A B C D E F G H	A B C D E **X**	☐	A B C D E F G	☐☐☐	A B C D E **X**
2	A B C D E **X**	☐	☐	☐	☐	☐	A B C D E F G H	A B C D E **X**	☐	A B C D E F G	☐☐☐	A B C D E **X**
3	A B C D E **X**	☐	☐	☐	☐	☐	A B C D E F G H	A B C D E **X**	☐	A B C D E F G	☐☐☐	A B C D E **X**
4	A B C D E **X**	☐	☐	☐	☐	☐	A B C D E F G H	A B C D E **X**	☐	A B C D E F G	☐☐☐	A B C D E **X**
5	A B C D E **X**	☐	☐	☐	☐	☐	A B C D E F G H	A B C D E **X**	☐	A B C D E F G	☐☐☐	A B C D E **X**
6	A B C D E **X**	☐	☐	☐	☐	☐	A B C D E F G H	A B C D E **X**	☐	A B C D E F G	☐☐☐	A B C D E **X**
7	A B C D E **X**	☐	☐	☐	☐	☐	A B C D E F G H	A B C D E **X**	☐	A B C D E F G	☐☐☐	A B C D E **X**
8	A B C D E **X**	☐	☐	☐	☐	☐	A B C D E F G H	A B C D E **X**	☐	A B C D E F G	☐☐☐	A B C D E **X**
9	A B C D E **X**	☐	☐	☐	☐	☐	A B C D E F G H	A B C D E **X**	☐	A B C D E F G	☐☐☐	A B C D E **X**
10	A B C D E **X**	☐	☐	☐	☐	☐	A B C D E F G H	A B C D E **X**	☐	A B C D E F G	☐☐☐	A B C D E **X**

- **Jaminan kesehatan** yang dimiliki adalah jaminan dalam bentuk kartu atau apapun yang dapat digunakan untuk pembiayaan kesehatan bila nama yang tertera dalam kartu atau lainnya melakukan perawatan kesehatan seperti: ke dokter, puskesmas, rumah sakit, dan sebagainya.
- **Terganggunya pekerjaan, sekolah, atau kegiatan sehari-hari**: tidak dapat melakukan kegiatan secara normal (bekerja, sekolah, atau kegiatan sehari-hari) sebagaimana biasanya.
- **Berobat jalan** adalah upaya anggota rumah tangga yang mempunyai keluhan kesehatan untuk memeriksakan diri dan mendapatkan pengobatan dengan mendatangi tempat-tempat pelayanan kesehatan modern atau tradisional tanpa menginap, termasuk mendatangkan petugas kesehatan ke rumah.
- **Responden yang pernah rawat inap adalah** responden yang telah selesai menjalani rawat inap, tidak termasuk bila pada saat pencacahan sedang menjalani rawat inap.

10

https://www.bps.go.id

BLOK XI. KETERANGAN PEMANFAATAN JAMINAN KESEHATAN

No. urut ART	*Isikan kode 1 jika 1001 berkode A, B, atau C (memiliki JKN/ Jamkesda)*<br>*Lainnya kode 0*<br>**1 → 1102**<br>**0 → ART berikutnya**	DALAM SETAHUN TERAKHIR, APAKAH (*nama*) PERNAH MEMANFAAT-KAN JKN/ JAMKESDA UNTUK PEMERIKSAAN KESEHATAN?<br>1. Ya → **1104**<br>5. Tidak	APAKAH ALASAN (*nama*) TIDAK PERNAH MEMANFAATKAN JKN/JAMKESDA UNTUK PEMERIKSAAN KESEHATAN?<br>Tidak mengalami keluhan kesehatan .... A<br>Mengobati sendiri .... B<br>Tidak tahu cara memanfaatkan jaminan kesehatan...... C<br>Kartu JKN tidak aktif .... D<br>Prosedur/persyaratan sulit dipenuhi .... E<br>Tidak ada faskes yang mudah dijangkau dari rumah responden .... F<br>Tidak ada petugas pemberi pelayanan jaminan kesehatan .... G<br>Tidak ada biaya (transport,dll) .... H<br>Waktu tunggu pelayanan lama/antri panjang .... I<br>Menggunakan asuransi selain JKN/Jamkesda.......... J<br>Lainnya .... K	DALAM SETAHUN TERAKHIR, APAKAH (*nama*) PERNAH DITOLAK PERIKSA KESEHATAN MENGGUNA-KAN JKN/ JAMKESDA?<br>1. Ya<br>5. Tidak → **1106**	APAKAH ALASAN (*nama*) MENGALAMI PENOLAKAN PERIKSA KESEHATAN MENGGUNAKAN JKN/JAMKESDA TERSEBUT?<br>Tidak memenuhi prosedur ...... A<br>Tidak sesuai jadwal pelayanan .. B<br>Jenis pelayanan yang diminta tidak dicakup .... C<br>Tidak ada tenaga medis yang dibutuhkan sebagai pemberi layanan.......... D<br>Tidak ada obat.......... E<br>Tidak memiliki pemeriksaan penunjang...... F<br>Lainnya.......... G	DALAM SETAHUN TERAKHIR, APAKAH (*nama*) PERNAH MEMANFAAT-KAN JKN/ JAMKESDA UNTUK RAWAT INAP?<br>1. Ya → **1108**<br>5. Tidak	APAKAH ALASAN (*nama*) TIDAK PERNAH MEMANFAATKAN JKN/JAMKESDA UNTUK RAWAT INAP?<br>Tidak mengalami gangguan kesehatan yang membutuhkan rawat inap .... A<br>Tidak tahu cara memanfaatkan jaminan kesehatan .... B<br>Prosedur/persyaratan sulit dipenuhi.......... C<br>Kartu JKN tidak aktif.......... D<br>Tidak ada faskes yang mudah dijangkau dari rumah .... E<br>Tidak ada petugas pemberi pelayanan jaminan kesehatan.......... F<br>Tidak ada biaya transportasi & akomodasi.......... G<br>Waktu tunggu pelayanan lama/antri panjang.......... H<br>Menggunakan asuransi selain JKN/Jamkesda .... I<br>Lainnya .... J	DALAM SETAHUN TERAKHIR, APAKAH (*nama*) PERNAH DITOLAK RAWAT INAP MENG-GUNAKAN JKN/ JAMKESDA?<br>1. Ya<br>5. Tidak → ***ART berikutnya***	APAKAH ALASAN (*nama*) MENGALAMI PENOLAKAN RAWAT INAP MENGGUNAKAN JKN/JAMKESDA TERSEBUT?<br>Tidak memenuhi prosedur .... A<br>Tidak sesuai jadwal pelayanan .... B<br>Jenis pelayanan yang diminta tidak dicakup .... C<br>Tidak ada tenaga medis yang dibutuhkan sebagai pemberi layanan.......... D<br>Tidak ada obat.......... E<br>Tidak memiliki pemeriksaan penunjang .... F<br>Tidak ada kamar.......... G<br>Lainnya.......... H
401	1101	1102	1103	1104	1105	1106	1107	1108	1109
1	☐	☐	A B C D E F G H I J K	☐	A B C D E F G	☐	A B C D E F G H I J	☐	A B C D E F G H
2	☐	☐	A B C D E F G H I J K	☐	A B C D E F G	☐	A B C D E F G H I J	☐	A B C D E F G H
3	☐	☐	A B C D E F G H I J K	☐	A B C D E F G	☐	A B C D E F G H I J	☐	A B C D E F G H
4	☐	☐	A B C D E F G H I J K	☐	A B C D E F G	☐	A B C D E F G H I J	☐	A B C D E F G H
5	☐	☐	A B C D E F G H I J K	☐	A B C D E F G	☐	A B C D E F G H I J	☐	A B C D E F G H
6	☐	☐	A B C D E F G H I J K	☐	A B C D E F G	☐	A B C D E F G H I J	☐	A B C D E F G H
7	☐	☐	A B C D E F G H I J K	☐	A B C D E F G	☐	A B C D E F G H I J	☐	A B C D E F G H
8	☐	☐	A B C D E F G H I J K	☐	A B C D E F G	☐	A B C D E F G H I J	☐	A B C D E F G H
9	☐	☐	A B C D E F G H I J K	☐	A B C D E F G	☐	A B C D E F G H I J	☐	A B C D E F G H
10	☐	☐	A B C D E F G H I J K	☐	A B C D E F G	☐	A B C D E F G H I J	☐	A B C D E F G H

- **Pemeriksaan kesehatan** yang dimaksudkan disini adalah pemeriksaan kesehatan baik dalam keadaan memiliki keluhan maupun tidak dalam keadaan memiliki keluhan.
  Contoh pemeriksaan tidak dalam keluhan adalah pemeriksaan kehamilan, imunisasi, keluarga berencana, dan lainnya.
- Responden tergolong **pernah memanfaatkan JKN/Jamkesda untuk pemeriksaan kesehatan** apabila responden pernah memanfaatkan JKN/Jamkesda untuk pemeriksaan kesehatan sampai dengan pemeriksaan kesehatan tersebut selesai.
- Responden tergolong **pernah memanfaatkan JKN/Jamkesda untuk rawat inap** apabila responden pernah memanfaatkan JKN/Jamkesda untuk rawat inap sampai dengan rawat inap tersebut selesai.

11

https://www.bps.go.id

PERTANYAAN	WANITA PERNAH KAWIN (WPK) UMUR 10-54 TAHUN (1)	WANITA PERNAH KAWIN (WPK) UMUR 10-54 TAHUN (2)	WANITA PERNAH KAWIN (WPK) UMUR 10-54 TAHUN (3)
Nama dan no. urut:<br>Umur (*Salin dari Blok IV* 407):<br>Nama & no. urut pemberi informasi:	.... __ __<br>__ __ tahun<br>........................ __ __	.... __ __<br>__ __ tahun<br>........................ __ __	.... __ __<br>__ __ tahun<br>........................ __ __
**BLOK XII. PENOLONG PERSALINAN**			
1201. APAKAH (*nama*) PERNAH MELAHIRKAN ANAK LAHIR HIDUP?	Ya .... 1<br>Tidak .... 5<br>***WPK berikutnya/Blok XIII***	Ya .... 1<br>Tidak .... 5<br>***WPK berikutnya/Blok XIII***	Ya .... 1<br>Tidak .... 5<br>***WPK berikutnya/Blok XIII***
1202. KAPAN MELAHIRKAN ANAK LAHIR HIDUP YANG TERAKHIR?	2 tahun yang lalu atau kurang ....1<br>Lebih dari 2 tahun yang lalu ....2<br>***WPK berikutnya/Blok XIII***	2 tahun yang lalu atau kurang ...1<br>Lebih dari 2 tahun yang lalu..............2<br>***WPK berikutnya/Blok XIII***	2 tahun yang lalu atau kurang ..1<br>Lebih dari 2 tahun yang lalu............2<br>***WPK berikutnya/Blok XIII***
1203.A. DIMANA (*nama*) MELAHIRKAN (*nama anak lahir hidup yang terakhir*)?	RS Pemerintah/RS Swasta/RSIA .... 1<br>Rumah bersalin/Klinik .... 2<br>Puskesmas .... 3<br>Pustu .... 4<br>Praktik nakes .... 5<br>Polindes/Poskesdes .... 6<br>Rumah .... 7<br>Lainnya, ***tuliskan:*** ____________ 8	RS Pemerintah/RS Swasta/RSIA .... 1<br>Rumah bersalin/Klinik .... 2<br>Puskesmas .... 3<br>Pustu .... 4<br>Praktik nakes .... 5<br>Polindes/Poskesdes .... 6<br>Rumah .... 7<br>Lainnya, ***tuliskan:*** ____________ 8	RS Pemerintah/RS Swasta/RSIA .... 1<br>Rumah bersalin/Klinik .... 2<br>Puskesmas .... 3<br>Pustu .... 4<br>Praktik nakes .... 5<br>Polindes/Poskesdes .... 6<br>Rumah .... 7<br>Lainnya, ***tuliskan:*** ____________ 8
B. SIAPA YANG MENOLONG PROSES KELAHIRAN TERAKHIR?<br>(*PROBING*: Jika responden menjawab tidak ada yang menolong, tanyakan APAKAH ADA ORANG DEWASA YANG MENEMANI PADA SAAT MELAHIRKAN?)	Dokter kandungan .... 1<br>Dokter umum .... 2<br>Bidan .... 3<br>Perawat .... 4<br>Dukun beranak/paraji .... 5<br>Lainnya .... 6<br>Tidak ada 7	Dokter kandungan .... 1<br>Dokter umum .... 2<br>Bidan .... 3<br>Perawat .... 4<br>Dukun beranak/paraji .... 5<br>Lainnya .... 6<br>Tidak ada 7	Dokter kandungan .... 1<br>Dokter umum .... 2<br>Bidan .... 3<br>Perawat .... 4<br>Dukun beranak/paraji .... 5<br>Lainnya .... 6<br>Tidak ada 7
C. BERAPA BERAT (*nama anak lahir hidup yang terakhir*) KETIKA DILAHIRKAN?	< 2,5 kg .... 1<br>≥ 2,5 kg .... 2<br>Tidak ditimbang .... 5<br>Tidak tahu 8	< 2,5 kg .... 1<br>≥ 2,5 kg .... 2<br>Tidak ditimbang .... 5<br>Tidak tahu 8	< 2,5 kg .... 1<br>≥ 2,5 kg .... 2<br>Tidak ditimbang .... 5<br>Tidak tahu 8
**BLOK XIII. KELUARGA BERENCANA**			
1301. APAKAH (*nama/pasangan*) **PERNAH/SEDANG** MENGGUNAKAN ALAT KB ATAU CARA TRADISIONAL UNTUK MENUNDA ATAU MENCEGAH KEHAMILAN?	Ya, pernah .... 1→ ***WPK berikutnya/Blok XIV***<br>Ya, sedang .... 2<br>Tidak .... 5→ ***WPK berikutnya/Blok XIV***	Ya, pernah .... 1→ ***WPK berikutnya/Blok XIV***<br>Ya, sedang .... 2<br>Tidak .... 5→ ***WPK berikutnya/Blok XIV***	Ya, pernah ... 1→ ***WPK berikutnya/Blok XIV***<br>Ya, sedang .... 2<br>Tidak .... 5→ ***WPK berikutnya/Blok XIV***
1302. ALAT KB ATAU CARA TRADISIONAL APA YANG SEDANG DIGUNAKAN?<br>***Jika (nama) menyebutkan lebih dari satu, lingkari kode terkecil***	Sterilisasi wanita/ tubektomi/MOW......... 1<br>Sterilisasi pria/vasektomi/MOP .... 2<br>IUD/AKDR/spiral .... 3<br>Suntikan .... 4<br>Susuk KB/implan .... 5<br>Pil .... 6<br>Kondom pria/karet KB .... 7<br>Intravag/kondom wanita/diafragma......... 8<br>Metode menyusui alami .... 9<br>Pantang berkala/kalender .... 10<br>Lainnya, ***tuliskan:*** ____________ 11	Sterilisasi wanita/ tubektomi/MOW .... 1<br>Sterilisasi pria/vasektomi/MOP .... 2<br>IUD/AKDR/spiral .... 3<br>Suntikan .... 4<br>Susuk KB/implan .... 5<br>Pil .... 6<br>Kondom pria/karet KB .... 7<br>Intravag/kondom wanita/diafragma .... 8<br>Metode menyusui alami .... 9<br>Pantang berkala/kalender .... 10<br>Lainnya, ***tuliskan:*** ____________ 11	Sterilisasi wanita/ tubektomi/MOW .... 1<br>Sterilisasi pria/vasektomi/MOP .... 2<br>IUD/AKDR/spiral .... 3<br>Suntikan .... 4<br>Susuk KB/implan .... 5<br>Pil .... 6<br>Kondom pria/karet KB .... 7<br>Intravag/kondom wanita/diafragma .... 8<br>Metode menyusui alami .... 9<br>Pantang berkala/kalender .... 10<br>Lainnya, ***tuliskan:*** ____________ 11

https://www.bps.go.id

BLOK XIV. AKSES TERHADAP MAKANAN (DITANYAKAN PADA KRT/PASANGAN/ART 15 TAHUN KE ATAS)

Nama dan No. urut pemberi informasi: .... ☐☐

SEKARANG SAYA AKAN MENGAJUKAN BEBERAPA PERTANYAAN MENGENAI AKSES TERHADAP MAKANAN. **DALAM SETAHUN TERAKHIR**, APAKAH ADA SAAT DIMANA:

1401. SELAMA SETAHUN TERAKHIR, APAKAH ANDA/ART LAINNYA **KHAWATIR TIDAK AKAN MEMILIKI CUKUP MAKANAN** UNTUK DISANTAP KARENA KURANGNYA UANG ATAU SUMBER DAYA LAINNYA?	Ya .... 1<br>Tidak .... 5<br>Tidak tahu ....8<br>Menolak menjawab 9
1402. SELAMA SETAHUN TERAKHIR, APAKAH ADA SAAT DI MANA ANDA/ART LAINNYA **TIDAK DAPAT MENYANTAP MAKANAN SEHAT DAN BERGIZI** KARENA KURANGNYA UANG ATAU SUMBER DAYA LAINNYA?	Ya .... 1<br>Tidak .... 5<br>Tidak tahu ....8<br>Menolak menjawab 9
1403. SELAMA SETAHUN TERAKHIR, APAKAH ANDA/ART LAINNYA HANYA **MENYANTAP SEDIKIT JENIS MAKANAN** KARENA TIDAK MEMILIKI UANG ATAU SUMBER DAYA LAINNYA?	Ya .... 1<br>Tidak .... 5<br>Tidak tahu ....8<br>Menolak menjawab 9
1404. SELAMA SETAHUN TERAKHIR, APAKAH ANDA/ART LAINNYA PERNAH **MELEWATKAN MAKAN PADA SUATU HARI TERTENTU** KARENA TIDAK MEMILIKI UANG ATAU SUMBER DAYA LAIN YANG CUKUP UNTUK MENDAPATKAN MAKANAN?	Ya .... 1<br>Tidak .... 5<br>Tidak tahu ....8<br>Menolak menjawab 9
1405. SELAMA SETAHUN TERAKHIR, APAKAH ANDA/ART LAINNYA **MAKAN LEBIH SEDIKIT DARIPADA SEHARUSNYA** KARENA KURANGNYA UANG ATAU SUMBER DAYA LAINNYA?	Ya .... 1<br>Tidak .... 5<br>Tidak tahu ....8<br>Menolak menjawab 9
1406. SELAMA SETAHUN TERAKHIR, APAKAH RUMAH TANGGA **KEHABISAN MAKANAN** KARENA KURANGNYA UANG ATAU SUMBER DAYA LAINNYA?	Ya .... 1<br>Tidak .... 5<br>Tidak tahu ....8<br>Menolak menjawab 9
1407. SELAMA SETAHUN TERAKHIR, APAKAH ANDA/ART LAINNYA **MERASA LAPAR TAPI TIDAK MAKAN** KARENA KURANGNYA UANG ATAU SUMBER DAYA LAINNYA UNTUK MENDAPATKAN MAKANAN?	Ya .... 1<br>Tidak .... 5<br>Tidak tahu ....8<br>Menolak menjawab 9
1408. SELAMA SETAHUN TERAKHIR, APAKAH ANDA/ART LAINNYA **TIDAK MAKAN SEHARIAN** KARENA KURANGNYA UANG ATAU SUMBER DAYA LAINNYA?	Ya .... 1<br>Tidak .... 5<br>Tidak tahu ....8<br>Menolak menjawab 9

BLOK XV. KETERANGAN PERUMAHAN

Nama dan No. urut pemberi informasi: .... ☐☐

1501. A. BERAPA JUMLAH RUMAH TANGGA YANG TINGGAL DI DALAM BANGUNAN SENSUS/ RUMAH INI?	☐ *rumah tangga*<br>*(Isikan 7, jika terdapat 7 rumah tangga atau lebih)*
B. BERAPA JUMLAH KELUARGA YANG TINGGAL DI DALAM BANGUNAN SENSUS/ RUMAH INI?	☐ *keluarga*<br>*(Isikan 7, jika terdapat 7 keluarga atau lebih)*
1502. APA STATUS KEPEMILIKAN BANGUNAN TEMPAT TINGGAL YANG DITEMPATI?<br>*(Pilihan jawaban boleh dibacakan)*	Milik sendiri ....1<br>Kontrak/sewa ....2<br>Bebas sewa ....3<br>Dinas..4<br>Lainnya ....5<br>(2–5) → ***1504***
1503. APA JENIS BUKTI KEPEMILIKAN TANAH BANGUNAN TEMPAT TINGGAL INI?<br>*(Pilihan jawaban boleh dibacakan)*	Sertifikat Hak Milik (SHM) atas nama ART ..1<br>SHM bukan atas nama ART dengan perjanjian pemanfaatan tertulis..........................2<br>SHM bukan atas nama ART tanpa perjanjian pemanfaatan tertulis..............3<br>Sertifikat selain SHM (SHGB, SHSRS)...................4<br>Surat bukti lainnya (Girik, *Letter C*, dll) ....5<br>Tidak punya 6
1504. BERAPA LUAS LANTAI RUMAH BANGUNAN TEMPAT TINGGAL?<br>*(Bulatkan dalam meter persegi)*	☐☐☐ m^2
1505. APAKAH KEPALA RUMAH TANGGA/ PASANGANYA/ANAKNYA MEMILIKI RUMAH LAIN, SELAIN RUMAH YANG DITEMPATI SAAT INI?	Ya ....1<br>Tidak.. 5
1506. BERAPA JUMLAH RUANG TIDUR DI RUMAH TANGGA INI?	☐ *ruang*<br>*(Isikan 7, jika terdapat 7 ruangan atau lebih)*

13

https://www.bps.go.id

BLOK XV. KETERANGAN PERUMAHAN

1507. APAKAH BAHAN BANGUNAN UTAMA ATAP RUMAH TERLUAS?<br><br>*(Pilihan jawaban boleh dibacakan)*	Beton ... 1<br>Genteng ... 2<br>Asbes ... 3<br>Seng ... 4<br>Bambu ... 5<br>Kayu/sirap ... 6<br>Jerami/ijuk/daun-daunan/rumbia ... 7<br>Lainnya ... 8
1508. APAKAH BAHAN BANGUNAN UTAMA DINDING RUMAH TERLUAS?	Tembok ... 1<br>Plesteran anyaman bambu/kawat ... 2<br>Kayu/papan ... 3<br>Anyaman bambu ... 4<br>Batang kayu ... 5<br>Bambu ... 6<br>Lainnya ... 7
1509. APAKAH BAHAN BANGUNAN UTAMA LANTAI RUMAH TERLUAS?	Marmer/granit ... 1<br>Keramik ... 2<br>Parket/vinil/karpet ... 3<br>Ubin/tegel/teraso ... 4<br>Kayu/papan ... 5<br>Semen/bata merah ... 6<br>Bambu ... 7<br>Tanah ... 8<br>Lainnya ... 9

BLOK XV. KETERANGAN PERUMAHAN

1510.A. APAKAH MEMILIKI FASILITAS TEMPAT BUANG AIR BESAR DAN SIAPA SAJA YANG MENGGUNAKAN?	Ada, digunakan hanya ART sendiri ... 1<br>Ada, digunakan bersama ART rumah tangga tertentu ... 2<br>Ada di MCK umum/ siapapun menggunakan ... 3<br>Ada, ART tidak menggunakan ... 4 } ***1511.A***<br>Tidak ada fasilitas ... 5
B. (***Jika 1510.A = 1 atau 2***), APAKAH JENIS KLOSET YANG DIGUNAKAN?	Leher angsa ... 1<br>Plengsengan dengan tutup ... 2<br>Plengsengan tanpa tutup ... 3 } ***1510.D***<br>Cemplung/cubluk ... 4
C. (***Jika 1510.B =1***), BERAPA JUMLAH KLOSET LEHER ANGSA YANG DIMILIKI RUMAH TANGGA?	☐ *buah*<br>*(Isikan 7, jika terdapat 7 buah atau lebih)*
D. DIMANAKAH TEMPAT PEMBUANGAN AKHIR TINJA?	Tangki septik ... 1<br>IPAL ... 2<br>Kolam/sawah/sungai/danau/laut ... 3<br>Lubang tanah ... 4 } ***1511.A***<br>Pantai/tanah lapang/kebun ... 5<br>Lainnya ... 6
E. SUDAH BERAPA LAMA TANGKI SEPTIK INI DIBUAT/DIBANGUN?	☐☐ tahun<br>Tidak tahu ... 98
F. **DALAM 5 TAHUN TERAKHIR,** BERAPA KALI TANGKI SEPTIK INI DIKOSONGKAN/DILAKUKAN PENYEDOTAN?	☐ kali (*Isikan 6, jika 6 kali atau lebih)*<br>Tidak pernah ... 7<br>Tidak tahu ... 8

14

https://www.bps.go.id

BLOK XV. KETERANGAN PERUMAHAN

1511.A. APA SUMBER AIR UTAMA YANG DIGUNAKAN RUMAH TANGGA UNTUK MINUM?	Air kemasan bermerk ..... 1<br>Air isi ulang ..... 2<br>Leding ..... 3 } 1512.A<br>Sumur bor/pompa ..... 4<br>Sumur terlindung ..... 5<br>Sumur tak terlindung ..... 6<br>Mata air terlindung ..... 7<br>Mata air tak terlindung ..... 8<br>Air permukaan seperti (sungai/ danau/waduk/kolam/irigasi) ..... 9<br>Air hujan ..... 10<br>Lainnya. ..... 11 } 1512.A
B. [*Jika 1511.A = 4, 5, 6, 7, atau 8 (sumur/pompa/mata air)*], BERAPA JARAK KE TEMPAT PENAMPUNGAN LIMBAH/KOTORAN/TINJA TERDEKAT?	< 10 m ..... 1<br>≥ 10 m ..... 2<br>Tidak tahu 8
1512.A. DI MANAKAH LOKASI SUMBER/FASILITAS AIR MINUM TERSEBUT?	Di rumah/kawasan dalam pagar rumah ..... 1 → ***1513***<br>Di luar kawasan pagar rumah 2
B. BERAPA JARAK DARI RUMAH KE SUMBER/ FASILITAS AIR MINUM?	☐☐☐ meter<br>Tidak tahu 998
C. BERAPA LAMA WAKTU YANG DIBUTUHKAN UNTUK MENGAMBIL AIR KE SUMBER/FASILITAS AIR SAMPAI KEMBALI LAGI KE RUMAH?	☐☐☐ menit<br>Tidak tahu 998

BLOK XV. KETERANGAN PERUMAHAN

1513. **DALAM SETAHUN TERAKHIR**, APAKAH RUMAH TANGGA PERNAH MENGALAMI KEKURANGAN AIR MINUM UNTUK KEBUTUHAN RUMAH TANGGA SELAMA MINIMAL 24 JAM ?	Ya ..... 1<br>Tidak ..... 5<br>Tidak tahu 8
1514. BAGAIMANA KONDISI FISIK AIR BAKU UNTUK MINUM MENURUT ANDA ?	Ya / Tidak<br>A. KERUH ..... 1 / 5<br>B. BERWARNA ..... 1 / 5<br>C. BERASA ..... 1 / 5<br>D. BERBUSA ..... 1 / 5<br>E. BERBAU ..... 1 / 5
1515. BAGAIMANAKAH BIASANYA CARA MEMPEROLEH AIR MINUM?	Membeli ..... 1<br>Tidak membeli 5
1516.A. APA SUMBER AIR UTAMA YANG DIGUNAKAN RUMAH TANGGA UNTUK **MEMASAK/MANDI/CUCI/DLL**?	Air kemasan bermerk ..... 1<br>Air isi ulang ..... 2<br>Leding ..... 3 } 1517.A<br>Sumur bor/pompa ..... 4<br>Sumur terlindung ..... 5<br>Sumur tak terlindung ..... 6<br>Mata air terlindung ..... 7<br>Mata air tak terlindung ..... 8<br>Air permukaan seperti (sungai/ danau/waduk/kolam/irigasi) ..... 9<br>Air hujan ..... 10<br>Lainnya. ..... 11 } 1517.A
B. [*Jika 1516.A = 4, 5, 6, 7, atau 8 (sumur/pompa/mata air)*] BERAPA JAUH JARAK KE TEMPAT PENAMPUNGAN LIMBAH/KOTORAN/TINJA TERDEKAT?	< 10 m ..... 1<br>≥ 10 m ..... 2<br>Tidak tahu 8

https://www.bps.go.id

BLOK XV. KETERANGAN PERUMAHAN

1517.A. BOLEHKAH SAYA MELIHAT TEMPAT DI MANA ANGGOTA RUMAH TANGGA ANDA BIASA MENCUCI TANGAN?	Ya, di dalam rumah ....1<br>Ya, di luar rumah ....2<br>Tidak ada tempat cuci tangan ....3 } 1518.A<br>Tidak diizinkan melihat ....4 } 1518.A
B. *Amati ketersediaan air di tempat mencuci tangan! Verifikasi dengan memeriksa kran/ pompa atau baskom, ember, wadah air atau sejenisnya.*	Tersedia air mengalir........................1<br>Tersedia air tidak mengalir .... 2<br>Tidak tersedia air 3
C. *Cek ketersediaan sabun, deterjen, atau cairan antiseptik di tempat mencuci tangan!*	Tersedia sabun khusus cuci tangan ....1<br>Tersedia cairan antiseptik...................................2<br>Tersedia sabun mandi .... 3<br>Tersedia sabun cuci pakaian/deterjen ....4<br>Tersedia sabun cuci piring......................... 5<br>Tidak ada..6
1518. A. APAKAH SUMBER UTAMA PENERANGAN RUMAH TANGGA INI?	Listrik PLN dengan meteran .... 1<br>Listrik PLN tanpa meteran .... 2 } 1519<br>Listrik non PLN .... 3 } 1519<br>Bukan listrik .... 4 } 1519

BLOK XV. KETERANGAN PERUMAHAN

	Meteran 1	*Meteran 2*	*Meteran 3*
B. BERAPA DAYA TERPASANG DI RUMAH INI?<br>450 *watt* .... 1<br>900 *watt* ....2<br>1.300 *watt* ....3<br>2.200 *watt* ....4<br>> 2.200 *watt* ....5<br>Tidak tahu..................................8	☐	☐	☐
	(Isikan daya untuk setiap meteran yang terpasang)		

1519. APAKAH JENIS BAHAN BAKAR UTAMA YANG DIGUNAKAN UNTUK MEMASAK?	Listrik...1<br>Elpiji 5,5 kg/*blue gaz* .... 2<br>Elpiji 12 kg ....3<br>Elpiji 3 kg .... 4<br>Gas kota ....5<br>Biogas .... 6<br>Minyak tanah ....7<br>Briket .... 8<br>Arang...9<br>Kayu bakar .... 10<br>Lainnya...11<br>Tidak memasak di rumah 0
1520. **DALAM SETAHUN TERAKHIR,** APAKAH RUMAH BAPAK/IBU PERNAH TERGENANG AIR LEBIH TINGGI DARI LUTUT ORANG DEWASA (LEBIH DARI 30 CM) SETELAH 2 (DUA) JAM HUJAN BERHENTI?	Ya ....1<br>Tidak 5

16

https://www.bps.go.id

BLOK XVI. KETERANGAN PERLINDUNGAN SOSIAL				
1601. **DALAM 4 BULAN TERAKHIR**, APAKAH RUMAH TANGGA PERNAH MEMBELI/MENERIMA BERAS MISKIN (RASKIN)/ BERAS SEJAHTERA (RASTRA)?	Ya ....1<br>Tidak .... 5 → ***1604***			
1602. **DALAM 4 BULAN TERAKHIR**, SEBUTKAN INFORMASI PEMBELIAN/PENERIMAAN RASKIN/RASTRA<br>A. BERAPA JUMLAH RASKIN/RASTRA YANG DIBELI (KG)?<br>B. BERAPA RUPIAH TOTAL YANG DIBAYAR?	***Bulan Februari 2018***<br>A) __ __ , __Kg<br>B) Rp. __ __ __ . __ __ __ ,-	***Bulan Januari 2018***<br>A) __ __ , __Kg<br>B) Rp. __ __ __ . __ __ __ ,-	***Bulan Desember 2017***<br>A) __ __ , __Kg<br>B) Rp. __ __ __ . __ __ __ ,-	***Bulan November 2017***<br>A) __ __ , __Kg<br>B) Rp. __ __ __ . __ __ __ ,-
1603. **TERAKHIR KALI** RUMAH TANGGA MENERIMA RASKIN/RASTRA, BAGAIMANA KUALITAS RASKIN/RASTRA YANG DITERIMA/DIBELI?	SANGAT BAIK ....1<br>BAIK ....2<br>CUKUP...3<br>BURUK...4<br>SANGAT BURUK 5			
1604. APAKAH RUMAH TANGGA BAPAK/IBU PERNAH MENJADI PENERIMA BANTUAN PANGAN NON TUNAI (BPNT)?	Ya ....1<br>Tidak .... 5 → ***1606***			
1605. **DALAM 4 BULAN TERAKHIR**, SEBUTKAN INFORMASI PEMBELIAN/PENGGUNAAN BPNT<br>A. APAKAH BAPAK/IBU MENGETAHUI BERAPA NILAI BANTUAN YANG DITERIMA?	***Bulan Februari 2018***<br>A) Ya, (***tuliskan***) .... 1<br>Rp. __ __ __ . __ __ __ ,-<br>Tidak Tahu 8	***Bulan Januari 2018***<br>A) Ya, (***tuliskan***) .... 1<br>Rp. __ __ __ . __ __ __ ,-<br>Tidak Tahu 8	***Bulan Desember 2017***<br>A) Ya, (***tuliskan***) .... 1<br>Rp. __ __ __ . __ __ __ ,-<br>Tidak Tahu 8	***Bulan November 2017***<br>A) Ya, (***tuliskan***) .... 1<br>Rp. __ __ __ . __ __ __ ,-<br>Tidak Tahu 8
B. APAKAH BPNT TERSEBUT DIGUNAKAN UNTUK MEMBELI BAHAN PANGAN?	B) Ya.............. 1<br>Tidak ....5→***Januari'18***	B) Ya.......... 1<br>Tidak ....5→***Desember'17***	B) Ya .... 1<br>Tidak...........5→***November'17***	B) Ya ....1<br>Tidak..........................5→***1606***
C. APA JENIS BAHAN PANGAN YANG DIBELI MENGGUNAKAN BPNT?<br>(i) BERAPA TOTAL RUPIAH YANG DIBELANJAKAN UNTUK MASING-MASING BAHAN PANGAN?<br>(ii) BERAPA KUANTITAS BAHAN PANGAN YANG DIBELI?	C) Beras : (i) Rp. ___ . ___ ,-<br>: (ii) __ __ , __ Kg<br>Telur : (i) Rp. ___ . ___ ,-<br>: (ii) ________ Butir<br>Lainnya : (i) Rp. ___ . ___ ,-<br>: (ii) ______________	C) Beras : (i) Rp. ___ . ___ ,-<br>: (ii) __ __ , __ Kg<br>Telur : (i) Rp. ___ . ___ ,-<br>: (ii) ________ Butir<br>Lainnya : (i) Rp. ___ . ___ ,-<br>: (ii) ______________	C) Beras : (i) Rp. ___ . ___ ,-<br>: (ii) __ __ , __ Kg<br>Telur : (i) Rp. ___ . ___ ,-<br>: (ii) ________ Butir<br>Lainnya : (i) Rp. ___ . ___ ,-<br>: (ii) ______________	C) Beras : (i) Rp. ___ . ___ ,-<br>: (ii) __ __ , __ Kg<br>Telur : (i) Rp. ___ . ___ ,-<br>: (ii) ________ Butir<br>Lainnya : (i) Rp. ___ . ___ ,-<br>: (ii) ______________
D. DI MANA TEMPAT MEMBELI BAHAN PANGAN TERSEBUT?	D) *e-warong* KUBE PKH.....1<br>Kios/warung/toko agen bank...2<br>Rumah Pangan Kita.......3<br>Kantor kelurahan/desa/<br>Kecamatan.............................4<br>Kantor bank ....5<br>Lainnya 6	D) *e-warong* KUBE PKH .... 1<br>Kios/warung/toko agen bank...2<br>Rumah Pangan Kita...... 3<br>Kantor kelurahan/desa/<br>Kecamatan.............................4<br>Kantor bank.................. 5<br>Lainnya 6	D) *e-warong* KUBE PKH ....1<br>Kios/warung/toko agen bank .. 2<br>Rumah Pangan Kita ......3<br>Kantor kelurahan/desa/<br>Kecamatan ....4<br>Kantor bank ....5<br>Lainnya 6	D) *e-warong* KUBE PKH .... 1<br>Kios/warung/toko agen bank .. 2<br>Rumah Pangan Kita ...... 3<br>Kantor kelurahan/desa/<br>Kecamatan .... 4<br>Kantor bank .... 5<br>Lainnya 6
E. BERAPA JAUH JARAK TEMPAT MEMBELI BAHAN PANGAN TERSEBUT DARI RUMAH?	E) ___ , __Km	E) ___ , __Km	E) ___ , __Km	E) ___ , __Km
F. JIKA MEMBELI BERAS [***1605.C nilai (Rp) dan kuantitas (Kg) beras ≠ 0***],<br>BAGAIMANA KUALITAS BERAS YANG DIBELI?	F) SANGAT BAIK ....1<br>BAIK.......................................2<br>CUKUP ....3<br>BURUK.....................................4<br>SANGAT BURUK 5	F) SANGAT BAIK.................... 1<br>BAIK.......................................2<br>CUKUP............................ 3<br>BURUK.....................................4<br>SANGAT BURUK................. 5	F) SANGAT BAIK ....1<br>BAIK.......................................2<br>CUKUP ....3<br>BURUK ....4<br>SANGAT BURUK 5	F) SANGAT BAIK.................... 1<br>BAIK....................................... 2<br>CUKUP .... 3<br>BURUK .... 4<br>SANGAT BURUK................. 5

17

https://www.bps.go.id

BLOK XVI. KETERANGAN PERLINDUNGAN SOSIAL

1606. SELAMA BULAN AGUSTUS 2017 – FEBRUARI 2018, BERAPA JUMLAH PROGRAM INDONESIA PINTAR (PIP) YANG DITERIMA?	JUMLAH ART YANG MENERIMA	JUMLAH UANG YANG DITERIMA (RUPIAH)
i. PIP SD/SEDERAJAT	☐	☐☐.☐☐☐.☐☐☐,-
ii PIP SMP/SEDERAJAT	☐	☐☐.☐☐☐.☐☐☐,-
iii. PIP SM/SEDERAJAT	☐	☐☐.☐☐☐.☐☐☐,-

1607. **DALAM SETAHUN TERAKHIR,** APAKAH ADA ANGGOTA RUMAH TANGGA YANG MEMILIKI ATAU MENERIMA JAMINAN SOSIAL?<br>*(lingkari kode 1 jika "ya", kode 5 bila "tidak")*		Ya	Tidak
	A. JAMINAN PENSIUN/VETERAN	1	5
	B. JAMINAN HARI TUA	1	5
	C. ASURANSI KECELAKAAN KERJA	1	5
	D. JAMINAN/ASURANSI KEMATIAN	1	5
	E. PESANGON PEMUTUSAN HUBUNGAN KERJA (PHK)	1	5

1608. APAKAH RUMAH TANGGA INI MENERIMA KARTU PERLINDUNGAN SOSIAL (KPS)/ KARTU KELUARGA SEJAHTERA (KKS)?	Ya, dapat menunjukkan kartu ....1<br>Ya, tidak dapat menunjukkan kartu ....2<br>Tidak 5
1609. APAKAH RUMAH TANGGA ANDA PERNAH MENJADI PENERIMA PROGRAM KELUARGA HARAPAN (PKH)?	Ya ....1<br>Tidak ....5 → ***1611***
1610. A. APAKAH RUMAH TANGGA ANDA MEMILIKI KARTU PKH?	Ya, dapat menunjukkan kartu ....1<br>Ya, tidak dapat menunjukkan kartu ....2<br>Tidak 5
B. APAKAH SAAT INI RUMAH TANGGA ANDA MASIH TERCATAT/ MENJADI PENERIMA PKH?	Ya ....1<br>Tidak ....5<br>Tidak tahu 8
C. DI MANA RUMAH TANGGA ANDA MENERIMA BANTUAN PKH?	Kantor Pos ....1<br>ATM....2<br>Kantor Bank ....3<br>Agen Bank....4<br>Lainnya 5

18

https://www.bps.go.id

BLOK XVI. KETERANGAN PERLINDUNGAN SOSIAL

1611. **DALAM SETAHUN TERAKHIR,** APAKAH RUMAH TANGGA BAPAK/IBU PERNAH MENERIMA BANTUAN/BANTUAN SOSIAL/SUBSIDI DARI PEMERINTAH DAERAH?	Ya .... 1<br>Tidak .... 5 → ***Blok XVII***	
1612. A. APAKAH RUMAH TANGGA ANDA MENERIMA BANTUAN SOSIAL DARI PEMERINTAH DAERAH BERUPA BANTUAN TUNAI RUTIN?	Ya .... 1 → ***1612.A. i***<br>Tidak .... 5 → ***1612.B***	**Bantuan yang Diterima Setahun Terakhir**<br>(i) BANTUAN TUNAI RUTIN UNTUK ANAK Rp. __ . ___ . ___<br>(ii) BANTUAN TUNAI RUTIN UNTUK LANSIA Rp. __ . ___ . ___<br>(iii) BANTUAN TUNAI RUTIN UNTUK PENYANDANG DISABILITAS Rp. __ . ___ . ___<br>(iv) BANTUAN TUNAI RUTIN LAINNYA Rp. __ . ___ . ___
B. APAKAH RUMAH TANGGA ANDA MENERIMA BANTUAN SOSIAL DARI PEMERINTAH DAERAH BERUPA BANTUAN TUNAI TIDAK RUTIN?	Ya .... 1 → ***1612.B. i***<br>Tidak .... 5 → ***1612.C***	**Bantuan yang Diterima Setahun Terakhir**<br>(i) BANTUAN TUNAI TIDAK RUTIN Rp. __ . ___ . ___
C. APAKAH RUMAH TANGGA ANDA MENERIMA BANTUAN SOSIAL DARI PEMERINTAH DAERAH BERUPA BANTUAN TIDAK TUNAI?	Ya .... 1<br>Tidak 5	

https://www.bps.go.id

BLOK XVII. AKSES TERHADAP LAYANAN KEUANGAN

1701. **DALAM SETAHUN TERAKHIR,** APAKAH ADA ANGGOTA RUMAH TANGGA YANG MENERIMA KREDIT?

(Pilihan jawaban boleh dibacakan, lingkari kode 1 jika menerima, kode 5 bila tidak)

	Ya	Tidak
A. KREDIT USAHA RAKYAT (KUR)	1	5
B. KREDIT DARI BANK UMUM SELAIN KUR	1	5
C. KREDIT DARI BANK PERKREDITAN RAKYAT (BPR)	1	5
D. KREDIT DARI KOPERASI	1	5
E. PERORANGAN (DENGAN BUNGA)	1	5
F. PEGADAIAN	1	5
G. PERUSAHAAN *LEASING*	1	5
H. KELOMPOK USAHA BERSAMA (KUBE/KUB)	1	5
I. BADAN USAHA MILIK DESA (BUMDES)	1	5
J. LAINNYA	1	5

19

BLOK XVIII. KETERANGAN KEPEMILIKAN BARANG

		Ya	Tidak
1801. APAKAH RUMAH TANGGA INI MEMILIKI BARANG-BARANG SEBAGAI BERIKUT? *(lingkari kode 1 jika memiliki, kode 5 bila tidak)*	A. TABUNG GAS 5,5 KG ATAU LEBIH	1	5
	B. LEMARI ES/KULKAS	1	5
	C. AC	1	5
	D. PEMANAS AIR (*WATER HEATER*)	1	5
	E. TELEPON RUMAH (PSTN)	1	5
	F. KOMPUTER/*LAPTOP*	1	5
	G. EMAS/PERHIASAN (MINIMAL 10 GRAM)	1	5
	H. SEPEDA MOTOR	1	5
	I. PERAHU	1	5
	J. PERAHU MOTOR	1	5
	K. MOBIL	1	5
	L. TELEVISI LAYAR DATAR (MINIMAL 30 INCH)	1	5
	M. TANAH/LAHAN	1	5
1802. (***Jika 1801.L = 1***), BERAPA JUMLAH TELEVISI LAYAR DATAR (MINIMAL 30 INCH) YANG DIMILIKI RUMAH TANGGA?	☐ *Buah*		
1803. JIKA MEMILIKI TANAH/LAHAN (***1801.M = 1***), SIAPA PEMILIK TANAH/ LAHAN TERSEBUT?	KRT .... A<br>Pasangan KRT .... B<br>Anak .... C<br>ART lainnya .... D		

BLOK XIX. KETERANGAN SUMBER PENGHASILAN RUMAH TANGGA

1901. A. APAKAH SUMBER TERBESAR PEMBIAYAAN DI RUMAH TANGGA INI?	1. ART YANG BEKERJA → ***1901.B***<br>2. KIRIMAN UANG/BARANG → ***1901.C***<br>3. INVESTASI (DEPOSITO, ROYALTI, SAHAM, BUNGA BANK, DAN SEJENISNYA) → ***BLOK XX***<br>4. PENSIUNAN → ***BLOK XX*** ☐
B. (***Jika 1901.A =1***), SIAPAKAH ART YANG MENANGGUNG PEMBIAYAAN TERBESAR?	Nama: .... /No.urut ART: ☐☐
C. (***Jika 1901.A = 2***), APABILA MENERIMA KIRIMAN UANG/BARANG DARIMANAKAH SUMBER UTAMANYA?	1. ORANG TUA<br>2. ANAK<br>3. FAMILI LAIN<br>4. LAINNYA ☐

20

https://www.bps.go.id

BLOK XX. CATATAN

KUNJUNGAN I	:	TANGGAL:	MULAI: ☐☐ : ☐☐	SELESAI: ☐☐ : ☐☐
KUNJUNGAN II	:	TANGGAL:	MULAI: ☐☐ : ☐☐	SELESAI: ☐☐ : ☐☐
KUNJUNGAN III	:	TANGGAL:	MULAI: ☐☐ : ☐☐	SELESAI: ☐☐ : ☐☐
KUNJUNGAN IV	:	TANGGAL:	MULAI: ☐☐ : ☐☐	SELESAI: ☐☐ : ☐☐

https://www.bps.go.id

Kode 804: Lapangan Usaha

1. Pertanian tanaman padi dan palawija
2. Hortikultura
3. Perkebunan
4. Perikanan
5. Peternakan
6. Kehutanan dan pertanian lainnya
7. Pertambangan dan penggalian
8. Industri pengolahan
9. Pengadaan listrik, gas, uap/air panas, dan udara dingin
10. Pengelolaan air, pengelolaan air limbah, pengelolaan dan daur ulang sampah, dan aktivitas remediasi
11. Konstruksi
12. Perdagangan besar dan eceran, reparasi dan perawatan mobil dan sepeda motor
13. Pengangkutan dan pergudangan
14. Penyediaan akomodasi dan penyediaan makan minum
15. Informasi dan komunikasi
16. Aktivitas keuangan dan asuransi
17. Real estate
18. Aktivitas profesional, ilmiah, dan teknis
19. Aktivitas penyewaan dan sewa guna tanpa hak opsi, ketenagakerjaan, agen perjalanan, dan penunjang usaha lainnya
20. Administrasi pemerintahan, pertahanan, dan jaminan sosial wajib
21. Pendidikan
22. Aktivitas kesehatan manusia dan aktivitas sosial
23. Kesenian, hiburan dan rekreasi
24. Aktivitas jasa lainnya
25. Aktivitas rumah tangga sebagai pemberi kerja
26. Aktivitas badan internasional dan badan ekstra internasional lainnya

Waktu selesai wawancara: ☐☐ : ☐☐

DATA
MENCERDASKAN BANGSA

Enlighten The Nation

https://www.bps.go.id

BADAN PUSAT STATISTIK
BPS-Statistics Indonesia
Jl. dr. Sutomo No. 6-8 Jakarta 10710
Telp. : (021) 3841195, 3842508, 3810291-4, Fax.: (021) 3857046
Homepage : http://www.bps.go.id E-mail : bpshq@bps.go.id

ISSN 2502-7492
9 772502 749008 >
