# Supplementary material for: Urban-rural distinction of potential determinants for prediabetes in Indonesian population aged ≥15 years: a cross-sectional analysis of Indonesian Basic Health Research 2018 among normoglycemic and prediabetic individuals
Source: BMC Public Health. 2020 Oct 6;20:1509. doi: 10.1186/s12889-020-09592-7 (PMC7539503; doi:10.1186/s12889-020-09592-7)
Supplement: Supplementary file 4 — Additional file 4. Supp2 File. LAPORAN NASIONAL RISKESDAS 2018. This is a pdf version for the Official Report of Indonesian Basic Health Research 2018, a nationwide survey conducted by Ministry of Health, Indonesia every 5 years. In addition to showing the main results of the study, this report also provides attachment of survey tools used for data collection, including the questionnaires. The questionnaires used in the survey can be seen in these pages: a. page 595–602 as Attachment 2 (Lampiran 2) for Kuesioner Rumah Tangga (Questionnaire for Household Unit) and. b. page 603–626 as Attachment 3 (Lampiran 3) for Kuesioner Individu (Questionnaire for Individuals/Respondents). The Official Report of RISKESDAS 2018 as well as its attachment including questionnaires used in the National Survey can also be accessed through this link: http://labdata.litbang.kemkes.go.id/images/download/laporan/RKD/2018/Laporan_Nasional_RKD2018_FINAL.pdf. [file 12889_2020_9592_MOESM4_ESM.pdf]

KEMENTERIAN
KESEHATAN
REPUBLIK
INDONESIA

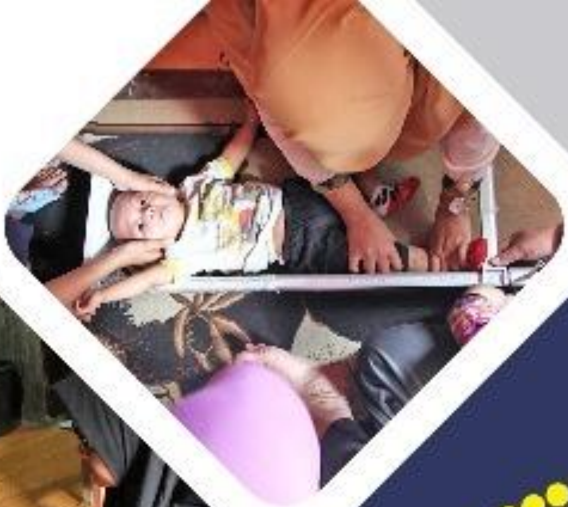

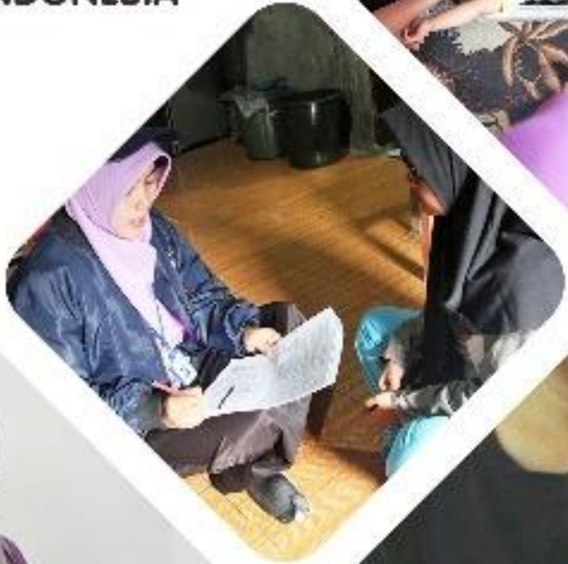

RISKESDAS
2018

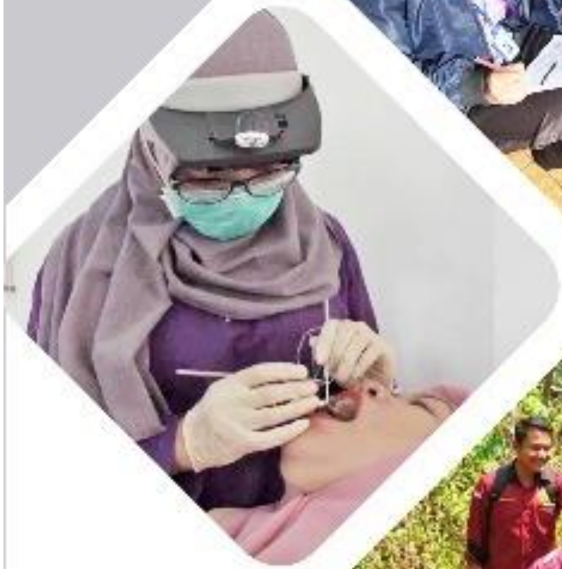

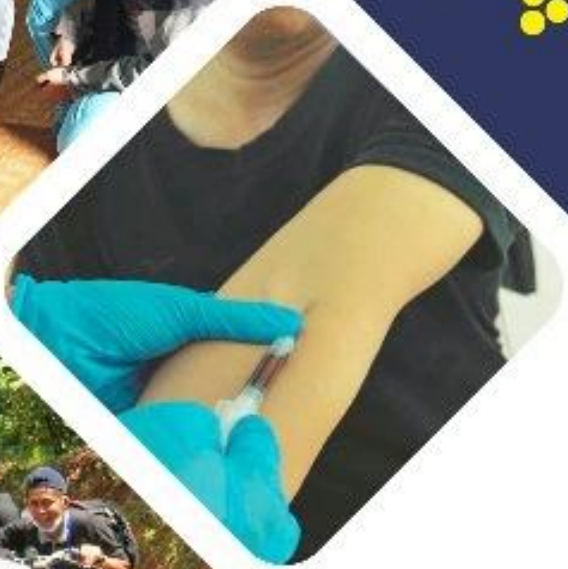

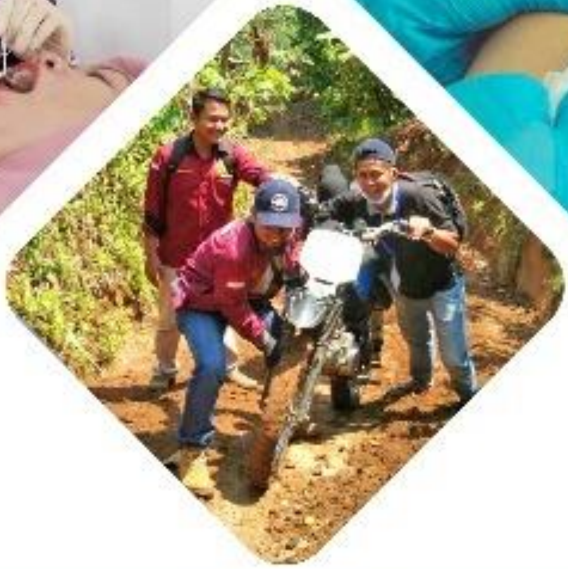

LAPORAN NASIONAL RISKESDAS 2018

Kementerian Kesehatan RI
Badan Penelitian dan Pengembangan Kesehatan

LEMBAGA PENERBIT
BADAN PENELITIAN DAN PENGEMBANGAN KESEHATAN
2019

LAPORAN NASIONAL
RISKESDAS 2018

LAPORAN NASIONAL
RISKESDAS 2018

Penyusun:
Tim Riskesdas 2018

KEMENTERIAN KESEHATAN REPUBLIK INDONESIA
BADAN PENELITIAN DAN PENGEMBANGAN KESEHATAN | 2019

Laporan Nasional Riskesdas 2018

@2019 oleh Lembaga Penerbit Badan Penelitian dan Pengembangan Kesehatan (LPB)

Hal Cipta dan Hak Penerbitan yang dilindungi Undang-undang ada pada Lembaga Penerbit

Badan Penelitian dan Pengembangan Kesehatan (LPB)

Dilarang mengutip dan memperbanyak sebagian atau seluruh isi buku ini tanpa izin

tertulis dari Penerbit

Diterbitkan oleh Lembaga Penerbit Badan Penelitian dan Pengembangan Kesehatan (LPB)

Anggota IKAPI No. 468/DKI/XI/2013

Jalan Percetakan Negara No. 23, Jakarta 10560

Telp. (021) 4261088, ext. 222, 223. Faks. (021) 4243933

Email :lpblitbangkes@gmail.com; website : www.litbang.depkes.go.id

Didistribusikan oleh

Lembaga Penerbit Badan Penelitian dan Pengembangan Kesehatan (LPB)

Katalog Dalam Terbitan

Q 179.9

Bad Badan Penelitian dan Pengembangan Kesehatan

l Laporan Nasional Riskesdas 2018/ Badan Penelitian dan Pengembangan Kesehatan.

Jakarta : Lembaga Penerbit Badan Penelitian dan Pengembangan Kesehatan, 2019.

xliii, 628p. : ilus.; 29 cm.

ISBN 978-602-373-118-3

1. JUDUL I. RESEARCH

DAFTAR ISI

DAFTAR ISI v
DAFTAR TABEL x
DAFTAR GAMBAR xxxv
DAFTAR SINGKATAN xxxvi
KATA PENGANTAR xl
Sambutan Menteri Kesehatan Republik Indonesia xlii
BAB 1 PENDAHULUAN 1
1.1 Latar Belakang 1
1.2 Manfaat dan Luaran Riskesdas 2018 2
A. Manfaat Penelitian 2
B. Luaran Penelitian 3
1.3 Tujuan Riskesdas 4
1.4 Pertanyaan Penelitian 4
1.5 Ruang Lingkup 5
BAB 2 METODE 7
2.1 Desain 7
2.2 Kerangka Konsep 7
2.3 Populasi dan Sampel 8
2.4 Penjamin Mutu Data Riskesdas 2018 10
2.5 Indikator 11
2.6 Pengumpulan Data 12
2.7 Manajemen Data 14
BAB 3 AKSES FASILITAS KESEHATAN 17
3.1 Pengetahuan Akses ke Fasilitas Kesehatan 17

BAB 4 KESEHATAN LINGKUNGAN 25
4.1 Air 25
4.2 Sanitasi 30
4.3 Keadaan Rumah 59
BAB 5 PENYAKIT MENULAR 67
5.1 ISPA 68
5.2 Pneumonia 73
5.3 Tuberculosis Paru 78
5.4 Hepatitis 89
5.5 Diare 92
5.6 Malaria 99
A. Prevalensi Malaria berdasarkan Hasil Wawancara 99
B. Malaria berdasarkan Pemeriksaan RDT dan Pemeriksaan Mikroskopis ... 105
5.7 Filariasis 107
BAB 6 PENYAKIT TIDAK MENULAR 113
6.1 Asma 113
6.2 Kanker 118
6.3 Diabetes 123
6.4 Penyakit Jantung 144
6.5 Hipertensi 152
6.6 Stroke 164
6.7 Penyakit Gagal Ginjal Kronis 169
6.8 Penyakit Sendi 175
BAB 7 KESEHATAN GIGI DAN MULUT 179
7.1 Kesehatan Gigi dan Mulut Berdasarkan Wawancara 179
7.2 Cakupan Layanan Kesehatan Gigi dan Mulut 194
7.3 Kesehatan Gigi dan Mulut berdasarkan Pemeriksaan 197
A. Status gigi geligi 197
B. Status Periodontal 201
C. Status kesehatan gigi dan mulut lainnya 202

7.4 Perilaku Menyikat Gigi 217
BAB 8 KESEHATAN JIWA 221
8.1 Gangguan Jiwa Skizofrenia Dan Psikosis Dalam Keluarga 221
8.2 Depresi 223
8.3 Gangguan Mental Emosional 226
8.4 Cakupan Pengobatan 229
BAB 9 DISABILITAS 235
9.1 Disabilitas Anak (5-17 Tahun) 235
9.2 Disabilitas Dewasa (18-59 Tahun) 238
9.3 Disabilitas Lansia (> 60 Tahun) 244
BAB 10 CEDERA 247
10.1 Gambaran Umum Cedera 247
10.2 Penggunaan Helm 266
BAB 11 PELAYANAN KESEHATAN TRADISIONAL 271
11.1 Pemanfaatan Pelayanan Kesehatan Tradisional 274
11.2 Jenis Pelayanan Kesehatan Tradisional 276
11.3 Pemberi Layanan Kesehatan Tradisional 278
11.4 Pemanfaatan Taman Obat Keluarga (TOGA) 280
BAB 12 PERILAKU KESEHATAN 283
12.1 Pencegahan Penyakit Akibat Gigitan Nyamuk 283
12.2 Konsumsi Makanan Berisiko 288
12.3 Konsumsi Buah dan Sayur 310
12.4 Kebiasaan Buang Air Besar 315
12.5 Perilaku Mencuci Tangan 317
12.6 Konsumsi Rokok dan Tembakau 320
12.7 Aktivitas Fisik 339
12.8 Konsumsi Minuman Beralkohol 342
BAB 13 PENGETAHUAN DAN SIKAP TERHADAP HIV/AIDS 353
13.1 Pengetahuan terhadap HIV/AIDS 353
13.2 Sikap terhadap Penderita HIV/AIDS 358

BAB 14 KESEHATAN IBU 361
14.1 Masa Reproduksi 362
14.2 Masa Kehamilan 365
14.3 Masa Persalinan 385
14.4 Masa Nifas 401
14.5 Penggunaan KB Setelah Melahirkan 410
14.6 Kepemilikan Buku KIA pada Ibu Hamil 416
BAB 15 KESEHATAN BALITA 419
15.1 Riwayat Bayi Baru Lahir 419
A. Prematuritas 419
B. Berat, Panjang dan Lingkar Kepala Bayi Lahir 422
C. Kecacatan Saat Lahir 433
15.2 Perawatan Bayi Baru Lahir 434
A. Perawatan metode kangguru pada bayi BBLR 434
B. Perawatan Tali Pusar 437
C. Pemeriksaan Bayi Baru Lahir 439
D. Pelayanan Kunjungan Neonatal Pertama (KN1) Sesuai Standar 442
E. Pemberian Salep Mata 445
F. Skrining Hipotiroid Kongenital 448
15.3 Kepemilikan dan Pemanfaatan Buku KIA 451
15.4 Imunisasi dan Vitamin A 456
A. Imunisasi 456
B. Imunisasi Dasar Lengkap 459
C. Imunisasi Lanjutan 462
D. Kejadian Ikutan Pasca Imunisasi 465
E. Vitamin A 468
15.5 Pemantauan Pertumbuhan 471
15.6 Perkembangan Anak 478
BAB 16 STATUS GIZI 481
16.1 Tablet Tambah Darah Remaja Putri 481

16.2 Pemberian Makanan Tambahan Ibu Hamil 498
16.3 Tablet Tambah Darah (TTD) Ibu Hamil 509
16.4 Gizi Pada Ibu Hamil (LILA) 517
16.5 Praktek Pemberian Makanan pada Bayi dan Anak 524
A. Inisiasi Menyusu Dini (IMD)..524
B. Waktu Mulai Menyusu 526
C. Perilaku ibu terhadap Kolostrum 528
D. Pemberian Makanan/Minuman Prelakteal 530
E. Riwayat dan Keberlangsungan Pemberian ASI..532
F. Pola Pemberian ASI 537
G. Keragaman Konsumsi Makanan 538
16.6 Pemberian Makanan Tambahan untuk Balita 541
16.7 Status Gizi pada Anak di Bawah Dua Tahun (Baduta) 550
16.8 Status Gizi pada Anak di Bawah Lima Tahun (Balita) 558
16.9 Status Gizi pada Anak dan Remaja 566
16.10 Status Gizi pada Dewasa (IMT dan Obesitas Sentral)...................................580
Lampiran 1 *Respon Rate*..591
Lampiran 2 Kuesioner Rumah Tangga...595
Lampiran 3 Kuesioner Individu 603
Lampiran 4 Tim Penulis 627

DAFTAR TABEL

Tabel 3.1.1 Proporsi Pengetahuan Rumah Tangga terhadap Kemudahan Akses ke Rumah Sakit menurut Provinsi, Riskesdas 2018 19
Tabel 3.1.2 Proporsi Pengetahuan Rumah Tangga terhadap Kemudahan Akses ke Rumah Sakit menurut Karakteristik, Riskesdas 2018 20
Tabel 3.1.3 Proporsi Pengetahuan Rumah Tangga terhadap Kemudahan Akses ke Puskesmas/Pustu/Pusling/Bidan Desa menurut Provinsi, Riskesdas 2018 21
Tabel 3.1.4 Proporsi Pengetahuan Rumah Tangga terhadap Kemudahan Akses ke Puskesmas/Pustu/Pusling/Bidan Desa menurut Karakteristik, Riskesdas 2018 22
Tabel 3.1.5 Proporsi Pengetahuan Rumah Tangga terhadap Kemudahan Akses ke Klinik/Praktek Dokter/Praktek Dokter Gigi/Praktek Bidan Mandiri menurut Provinsi, Riskesdas 2018 23
Tabel 3.1.6 Proporsi Pengetahuan Rumah Tangga terhadap Kemudahan Akses ke Klinik/Praktek Dokter/Praktek Dokter Gigi/Praktek Bidan Mandiri menurut Karakteristik, Riskesdas 2018 24
Tabel 4.1.1 Proporsi Pemakaian Air per Orang per Hari di Rumah Tangga (5 Kategori) menurut Provinsi, Riskesdas 2018 26
Tabel 4.1.2 Proporsi Pemakaian Air per Orang per Hari di Rumah Tangga (5 Kategori) menurut Karakteristik, Riskesdas 2018 27
Tabel 4.1.3 Proporsi Pemakaian Air per Orang per Hari di Rumah Tangga (2 Kategori) menurut Provinsi, Riskesdas 2018 28
Tabel 4.1.4 Proporsi Pemakaian Air per Orang per Hari di Rumah Tangga (2 Kategori) menurut Karakteristik, Riskesdas 2018 29
Tabel 4.2.1 Proporsi Cara Penanganan Tinja Balita oleh Rumah Tangga menurut Provinsi, Riskesdas 2018 39
Tabel 4.2.2 Proporsi Cara Penanganan Tinja Balita oleh Rumah Tangga menurut Karakteristik, Riskesdas 2018 40
Tabel 4.2.3 Proporsi Penanganan Tinja Balita Secara Aman oleh Rumah Tangga menurut Provinsi, Riskesdas 2018 41
Tabel 4.2.4 Proporsi Penanganan Tinja Balita Secara Aman oleh Rumah Tangga menurut Karakteristik, Riskesdas 2018 42
Tabel 4.2.5 Proporsi Tempat Pembuangan Air Limbah Utama dari Kamar Mandi/Tempat Cuci di Rumah Tangga menurut Provinsi, Riskesdas 2018 43
Tabel 4.2.6 Proporsi Tempat Pembuangan Air Limbah Utama dari Kamar Mandi/Tempat Cuci di Rumah Tangga menurut Karakteristik, Riskesdas 2018 44

Tabel 4.2.7 Proporsi Tempat Pembuangan Air Limbah Utama dari Dapur Rumah Tangga menurut Provinsi, Riskesdas 2018 45
Tabel 4.2.8 Proporsi Tempat Pembuangan Air Limbah Utama dari Dapur Rumah Tangga menurut Karakteristik, Riskesdas 2018...46
Tabel 4.2.9 Proporsi Jenis Tempat Pengumpulan/Penampungan Sampah Basah (Organik) di Dalam Rumah menurut Provinsi, Riskesdas 2018 47
Tabel 4.2.10 Proporsi Jenis Tempat Pengumpulan/Penampungan Sampah Basah (Organik) di Dalam Rumah menurut Karakteristik, Riskesdas 2018...............48
Tabel 4.2.11 Proporsi Cara Pengelolaan Sampah Rumah Tangga menurut Provinsi, Riskesdas 2018 49
Tabel 4.2.12 Proporsi Cara Pengelolaan Sampah Rumah Tangga menurut Karakteristik, Riskesdas 2018 50
Tabel 4.2.13 Proporsi Kualitas Pengelolaan Sampah Rumah Tangga menurut Provinsi, Riskesdas 2018 51
Tabel 4.2.14 Proporsi Kualitas Pengelolaan Sampah Rumah Tangga menurut Karakteristik, Riskesdas 2018 52
Tabel 4.2.15 Proporsi Frekuensi Menguras Bak Mandi/Ember Besar/Drum di Rumah Tangga menurut Provinsi, Riskesdas 2018 53
Tabel 4.2.16 Proporsi Frekuensi Menguras Bak Mandi/Ember Besar/Drum di Rumah Tangga menurut Karakteristik, Riskesdas 2018..54
Tabel 4.2.17 Proporsi Upaya Pemberantasan Sarang Nyamuk di Rumah Tangga menurut Provinsi, Riskesdas 2018 55
Tabel 4.2.18 Proporsi Upaya Pemberantasan Sarang Nyamuk di Rumah Tangga menurut Karakteristik, Riskesdas 2018 56
Tabel 4.2.19 Proporsi Upaya Pemberantasan Sarang Nyamuk (3M dan 3M plus) di Rumah Tangga menurut Provinsi, Riskesdas 2018 57
Tabel 4.2.20 Proporsi Upaya Pemberantasan Sarang Nyamuk (3M dan 3M plus) di Rumah Tangga menurut Karakteristik, Riskesdas 2018..................................58
Tabel 4.3.1 Proporsi Keadaan Kamar Tidur Utama di Rumah Tangga menurut Provinsi, Riskesdas 2018 61
Tabel 4.3.2 Proporsi Keadaan Kamar Tidur Utama di Rumah Tangga menurut Karakteristik, Riskesdas 2018 62
Tabel 4.3.3 Proporsi Keadaan Ruang Masak/Dapur di Rumah Tangga menurut Provinsi, Riskesdas 2018 63
Tabel 4.3.4 Proporsi Keadaan Ruang Masak/Dapur di Rumah Tangga menurut Karakteristik, Riskesdas 2018 64
Tabel 4.3.5 Proporsi Keadaan Ruang Keluarga di Rumah Tangga menurut Provinsi, Riskesdas 2018 65
Tabel 4.3.6 Proporsi Keadaan Ruang Keluarga di Rumah Tangga menurut Karakteristik, Riskesdas 2018 66

Tabel 5.1.1 Prevalensi ISPA menurut Provinsi, Riskesdas 2018 69
Tabel 5.1.2 Prevalensi ISPA menurut Karakteristik, Riskesdas 2018 70
Tabel 5.1.3 Prevalensi ISPA pada Balita menurut Provinsi, Riskesdas 2018 71
Tabel 5.1.4 Prevalensi ISPA pada Balita menurut Karakteristik, Riskesdas 2018 72
Tabel 5.2.1 Prevalensi Pneumonia menurut Provinsi, Riskesdas 2018 74
Tabel 5.2.2 Prevalensi Pneumonia menurut Karakteristik, Riskesdas 2018 75
Tabel 5.2.3 Prevalensi Pneumonia pada Balita menurut Provinsi, Riskesdas 2018 76
Tabel 5.2.4 Prevalensi Pneumonia pada Balita menurut Karakteristik, Riskesdas 2018 77
Tabel 5.3.1 Prevalensi TB Paru berdasarkan Riwayat Diagnosis Dokter menurut Provinsi, Riskesdas 2018 79
Tabel 5.3.2 Prevalensi TB Paru berdasarkan Riwayat Diagnosis Dokter menurut Karakteristik, Riskesdas 2018 80
Tabel 5.3.3 Proporsi Pemeriksaan/Diagnosis TB Paru, Jenis Terapi TB Paru yang Diberikan dan Ketersediaan PMO menurut Provinsi, Riskesdas 2018 83
Tabel 5.3.4 Proporsi Pemeriksaan/Diagnosis TB Paru, Jenis Terapi TB Paru yang Diberikan dan Ketersediaan PMO menurut Karakteristik, Riskesdas 2018 84
Tabel 5.3.5 Proporsi Penderita TB (< 6 bulan) yang Minum Obat Secara Rutin menurut Provinsi, Riskesdas 2018 85
Tabel 5.3.6 Proporsi Penderita TB (< 6 bulan) yang Minum Obat Secara Rutin menurut Karakteristik, Riskesdas 2018 86
Tabel 5.3.7 Proporsi Penderita TB (< 6 bulan) menurut Alasan Tidak Rutin Minum Obat menurut Provinsi, Riskesdas 2018 87
Tabel 5.3.8 Proporsi Penderita TB (< 6 bulan) menurut Alasan Tidak Rutin Minum Obat menurut Karakteristik, Riskesdas 2018 88
Tabel 5.4.1 Prevalensi Hepatitis berdasarkan Riwayat Diagnosis Dokter menurut Provinsi, Riskesdas 2018 90
Tabel 5.4.2 Prevalensi Hepatitis berdasarkan Riwayat Diagnosis Dokter menurut Karakteristik, Riskesdas 2018 91
Tabel 5.5.1 Prevalensi Diare menurut Provinsi, Riskesdas 2018 93
Tabel 5.5.2 Prevalensi Diare menurut Karakteristik, Riskesdas 2018 94
Tabel 5.5.3 Prevalensi Diare pada Balita menurut Provinsi, Riskesdas 2018 95
Tabel 5.5.4 Prevalensi Diare pada Balita menurut Karakteristik, Riskesdas 2018 96
Tabel 5.5.5 Proporsi Penggunaan Oralit, Zinc, dan Pengobatan Lainnya pada Penderita Diare Balita menurut Provinsi, Riskesdas 2018 97
Tabel 5.5.6 Proporsi Penggunaan Oralit, Zinc, dan Pengobatan Lainnya pada Penderita Diare Balita menurut Karakteristik, Riskesdas 2018 98
Tabel 5.6.1 Skema Pengobatan Malaria 100

Tabel 5.6.2 Prevalensi Malaria berdasarkan Riwayat Positif Malaria melalui Pemeriksaan Darah oleh Nakes dan Pengobatannya menurut Provinsi, Riskesdas 2018 101
Tabel 5.6.3 Prevalensi Malaria berdasarkan Riwayat Positif Malaria melalui Pemeriksaan Darah oleh Nakes dan Pengobatannya menurut Karakteristik, Riskesdas 2018 102
Tabel 5.6.4 Proporsi Penggunaan ACT untuk Pengobatan Malaria menurut Provinsi, Riskesdas 2018 103
Tabel 5.6.5 Proporsi Penggunaan ACT untuk Pengobatan Malaria menurut Karakteristik, Riskesdas 2018 104
Tabel 5.6.6 Prevalensi Malaria berdasarkan Hasil Pemeriksaan RDT dan Jenis Plasmodium menurut Karakteristik, Riskesdas 2018 106
Tabel 5.6.7 Proporsi Malaria pada Penduduk dengan Riwayat Demam dalam Dua Hari berdasarkan pemeriksaan Mikroskopis, Riskesdas 2018 107
Tabel 5.7.1 Proporsi Pemberian Obat Pencegahan Masal (POPM) Filariasis di Daerah Endemis selama Periode POPM menurut Provinsi, Riskesdas 2018 109
Tabel 5.7.2 Proporsi Pemberian Obat Pencegahan Masal (POPM) Filariasis di Daerah Endemis selama Periode POPM menurut Karakteristik, Riskesdas 2018 110
Tabel 5.7.3 Prevalensi Filariasis berdasarkan Diagnosis Nakes dan Proporsi Minum Obat Sesuai Anjuran Nakes menurut Provinsi, Riskesdas 2018....111
Tabel 5.7.4 Prevalensi Filariasis berdasarkan Diagnosis Nakes dan Proporsi Minum Obat Sesuai Anjuran Nakes menurut Karakteristik, Riskesdas 2018....112
Tabel 6.1.1 Prevalensi Asma berdasarkan Diagnosis Dokter pada Penduduk Semua Umur menurut Provinsi, Riskesdas 2018 114
Tabel 6.1.2 Prevalensi Asma berdasarkan Diagnosis Dokter pada Penduduk Semua Umur menurut Karakteristik, Riskesdas 2018 115
Tabel 6.1.3 Proporsi Kekambuhan Asma dalam 12 Bulan Terakhir pada Penduduk Semua Umur menurut Provinsi, Riskesdas 2018 116
Tabel 6.1.4 Proporsi Kekambuhan Asma dalam 12 Bulan Terakhir pada Penduduk Semua Umur menurut Karakteristik, Riskesdas 2018....117
Tabel 6.2.1 Prevalensi (per mil) Kanker berdasarkan Diagnosis Dokter pada Penduduk Semua Umur menurut Provinsi, Riskesdas 2018 119
Tabel 6.2.2 Prevalensi (per mil) Kanker berdasarkan Diagnosis Dokter pada Penduduk Semua Umur menurut Karakteristik, Riskesdas 2018....120
Tabel 6.2.3 Proporsi Jenis Pengobatan Kanker berdasarkan Diagnosis Dokter pada Penduduk Semua Umur menurut Provinsi, Riskesdas 2018 121
Tabel 6.2.4 Proporsi Jenis Pengobatan Kanker berdasarkan Diagnosis Dokter pada Penduduk Semua Umur menurut Karakteristik, Riskesdas 2018....122

Tabel 6.3.1 Prevalensi Diabetes Melitus berdasarkan Diagnosis Dokter pada Penduduk Semua Umur menurut Provinsi, Riskesdas 2018 127
Tabel 6.3.2 Prevalensi Diabetes Melitus berdasarkan Diagnosis Dokter pada Penduduk Semua Umur menurut Karakteristik, Riskesdas 2018..................128
Tabel 6.3.3 Prevalensi Diabetes Melitus berdasarkan Diagnosis Dokter pada Penduduk Umur ≥15 Tahun menurut Provinsi, Riskesdas 2018...................129
Tabel 6.3.4 Prevalensi Diabetes Melitus berdasarkan Diagnosis Dokter pada Penduduk Umur ≥15 Tahun menurut Karakteristik, Riskesdas 2018 130
Tabel 6.3.5 Prevalensi Diabetes Melitus berdasarkan Pemeriksaan Kadar Gula Darah pada Penduduk Umur ≥15 Tahun menurut Karakteristik, Riskesdas 2018 131
Tabel 6.3.6 Proporsi Gula Darah Puasa Terganggu berdasarkan Pemeriksaan Kadar Gula Darah pada Penduduk Usia ≥15 tahun menurut Karakteristik, Riskesdas 2018 132
Tabel 6.3.7 Proporsi Toleransi Glukosa Terganggu berdasarkan Pemeriksaan Kadar Gula Darah pada Penduduk Umur ≥15 Tahun menurut Karakteristik, Riskesdas 2018 133
Tabel 6.3.8 Proporsi Jenis Pengobatan Diabetes Melitus berdasarkan Diagnosis Dokter pada Penduduk Semua Umur menurut Provinsi, Riskesdas 2018..134
Tabel 6.3.9 Proporsi Jenis Pengobatan Diabetes Melitus berdasarkan Diagnosis Dokter pada Penduduk Semua Umur menurut Karakteristik, Riskesdas 2018 135
Tabel 6.3.10 Proporsi Kepatuhan Minum/Suntik Obat Anti Diabetes pada Penduduk Semua Umur dengan Diabetes Melitus berdasarkan Diagnosis Dokter menurut Provinsi, Riskesdas 2018 136
Tabel 6.3.11 Proporsi Kepatuhan Minum/Suntik Obat Anti Diabetes pada Penduduk Semua Umur dengan Diabetes Melitus berdasarkan Diagnosis Dokter menurut Karakteristik, Riskesdas 2018 137
Tabel 6.3.12 Proporsi Alasan Tidak Minum/Suntik Obat Anti Diabetes Sesuai Petunjuk Dokter pada Penduduk Semua Umur menurut Provinsi, Riskesdas 2018 138
Tabel 6.3.13 Proporsi Alasan Tidak Minum/Suntik Obat Anti Diabetes Sesuai Petunjuk Dokter pada Penduduk Semua Umur menurut Karakteristik, Riskesdas 2018 139
Tabel 6.3.14 Proporsi Jenis Pengendalian Diabetes Melitus berdasarkan Diagnosis Dokter pada Penduduk Semua Umur menurut Provinsi, Riskesdas 2018..140

Tabel 6.3.15 Proporsi Jenis Pengendalian Diabetes Melitus berdasarkan Diagnosis Dokter pada Penduduk Semua Umur menurut Karakteristik, Riskesdas 2018 141
Tabel 6.3.16 Proporsi Kerutinan Memeriksakan Kadar Gula Darah pada Penduduk Semua Umur menurut Provinsi, Riskesdas 2018 142
Tabel 6.3.17 Proporsi Kerutinan Memeriksakan Kadar Gula Darah pada Penduduk Semua Umur menurut Karakteristik, Riskesdas 2018....................143
Tabel 6.4.1 Prevalensi Penyakit Jantung berdasarkan Diagnosis Dokter pada Penduduk Semua Umur menurut Provinsi, Riskesdas 2018 146
Tabel 6.4.2 Prevalensi Penyakit Jantung berdasarkan Diagnosis Dokter pada Penduduk Semua Umur menurut Karakteristik, Riskesdas 2018....................147
Tabel 6.4.3 Proporsi Kadar Kolesterol Total pada Penduduk Umur ≥ 15 Tahun menurut Karakteristik, Riskesdas 2018 148
Tabel 6.4.4 Proporsi Kadar HDL pada Penduduk Umur ≥ 15 Tahun menurut Karakteristik, Riskesdas 2018 149
Tabel 6.4.5 Proporsi Kadar LDL direct pada Penduduk Umur ≥ 15 Tahun menurut Karakteristik, Riskesdas 2018 150
Tabel 6.4.6 Proporsi Kadar Trigliserida pada Penduduk Umur ≥ 15 Tahun Menurut Karakteristik, Riskesdas 2018 151
Tabel 6.5.1 Prevalensi Hipertensi berdasarkan Diagnosis Dokter atau Minum Obat Antihipertensi, pada Penduduk Umur ≥18 Tahun menurut Provinsi, Riskesdas 2018 154
Tabel 6.5.2 Prevalensi Hipertensi berdasarkan Diagnosis Dokter atau Minum Obat Antihipertensi, pada Penduduk Umur ≥18 Tahun Menurut Karakteristik, Riskesdas 2018 155
Tabel 6.5.3 Prevalensi Hipertensi berdasarkan Hasil Pengukuran pada Penduduk Umur ≥18 Tahun menurut Provinsi, Riskesdas 2018 156
Tabel 6.5.4 Prevalensi Hipertensi berdasarkan Hasil Pengukuran pada Penduduk Umur ≥18 Tahun menurut Karakteristik, Riskesdas 2018 157
Tabel 6.5.5 Proporsi Minum Obat Anti Hipertensi secara Rutin pada Penduduk Umur ≥18 Tahun dengan Hipertensi menurut Provinsi, Riskesdas 2018 158
Tabel 6.5.6 Proporsi Minum Obat Anti Hipertensi secara Rutin pada Penduduk Umur ≥18 Tahun dengan Hipertensi menurut Karakteristik, Riskesdas 2018........159
Tabel 6.5.7 Proporsi Alasan Tidak Minum Obat Secara Rutin pada Penduduk Umur ≥18 Tahun dengan Hipertensi menurut Provinsi, Riskesdas 2018 160
Tabel 6.5.8 Proporsi Alasan Tidak Minum Obat Secara Rutin pada Penduduk Umur ≥18 Tahun dengan Hipertensi menurut Karakteristik, Riskesdas 2018........161
Tabel 6.5.9 Proporsi Mengukur Tekanan Darah secara Rutin pada Penduduk Umur ≥18 Tahun menurut Provinsi, Riskesdas 2018 162

Tabel 6.5.10 Proporsi Mengukur Tekanan Darah secara Rutin pada Penduduk Umur ≥18 Tahun menurut Karakteristik, Riskesdas 2018 163
Tabel 6.6.1 Prevalensi (per mil) Stroke berdasarkan Diagnosis Dokter pada Penduduk Umur ≥15 Tahun menurut Provinsi, Riskesdas 2018 165
Tabel 6.6.2 Prevalensi (per mil) Stroke berdasarkan Diagnosis Dokter pada Penduduk Umur ≥15 Tahun menurut Karakteristik, Riskesdas 2018 166
Tabel 6.6.3 Proporsi Kontrol *Stroke* ke Fasilitas Pelayanan Kesehatan pada Penduduk Umur ≥15 Tahun dengan *Stroke* berdasarkan Diagnosis Dokter menurut Provinsi, Riskesdas 2018 167
Tabel 6.6.4 Proporsi Kontrol *Stroke* ke Fasilitas Pelayanan Kesehatan pada Penduduk Umur ≥15 Tahun dengan *Stroke* berdasarkan Diagnosis Dokter menurut Karakteristik, Riskesdas 2018 168
Tabel 6.7.1 Prevalensi Gagal Ginjal Kronis berdasarkan Diagnosis Dokter pada Penduduk Umur ≥15 Tahun menurut Provinsi, Riskesdas 2018 170
Tabel 6.7.2 Prevalensi Gagal Ginjal Kronis berdasarkan Diagnosis Dokter pada Penduduk Umur ≥15 Tahun menurut Karakteristik, Riskesdas 2018 171
Tabel 6.7.3 Proporsi Hemodialisis pada Penduduk Umur ≥15 Tahun dengan Gagal Ginjal Kronis berdasarkan Diagnosis Dokter menurut Provinsi, Riskesdas 2018 172
Tabel 6.7.4 Proporsi Hemodialisis pada Penduduk Umur ≥15 Tahun dengan Gagal Ginjal Kronis berdasarkan Diagnosis Dokter menurut Karakteristik, Riskesdas 2018 173
Tabel 6.7.5 Proporsi Kadar Kreatinin Serum pada Penduduk Umur ≥ 15 Tahun menurut Karakteristik, Riskesdas 2018 174
Tabel 6.8.1 Prevalensi Penyakit Sendi berdasarkan Diagnosis Dokter pada Penduduk Umur ≥15 Tahun menurut Provinsi, Riskesdas 2018 176
Tabel 6.8.2 Prevalensi Penyakit Sendi berdasarkan Diagnosis Dokter pada Penduduk Umur ≥15 Tahun menurut Karakteristik, Riskesdas 2018 177
Tabel 7.1.1 Proporsi Masalah Gigi menurut Provinsi, Riskesdas 2018 182
Tabel 7.1.2 Proporsi Masalah Gigi menurut Karakteristik, Riskesdas 2018........................183
Tabel 7.1.3 Proporsi Masalah Kesehatan Mulut pada Penduduk Umur >3 Tahun menurut Provinsi, Riskesdas 2018 184
Tabel 7.1.4 Proporsi Masalah Kesehatan Mulut pada Penduduk Umur >3 Tahun menurut Karakteristik, Riskesdas 2018 185
Tabel 7.1.5 Proporsi Tindakan untuk Mengatasi Masalah Gigi dan Mulut menurut Provinsi, Riskesdas 2018 186
Tabel 7.1.6 Proporsi Penduduk yang Menerima Tindakan untuk Mengatasi Masalah Gigi dan Mulut menurut Karakteristik, Riskesdas 2018 188
Tabel 7.1.7 Proporsi Pengobat Masalah Kesehatan Gigi dan Mulut menurut Provinsi, Riskesdas 2018 190

Tabel 7.1.8 Proporsi Pengobat Masalah Kesehatan Gigi dan Mulut menurut Karakteristik, Riskesdas 2018 191
Tabel 7.1.9 Proporsi Frekuensi Berobat Ke Tenaga Medis Gigi menurut Provinsi,Riskesdas 2018 192
Tabel 7.1.10 Proporsi Frekuensi Berobat Ke Tenaga Medis Gigi menurut Karakteristik, Riskesdas 2018 193
Tabel 7.2.1 Proporsi Masalah Gigi dan Mulut, Perawatan oleh Tenaga Medis Gigi pada Penduduk Umur >3 Tahun menurut Provinsi, Riskesdas 2018 195
Tabel 7.2.2 Proporsi Masalah Gigi dan Mulut, Perawatan oleh Tenaga Medis Gigi pada Penduduk Umur >3 Tahun menurut Karakteristik, Riskesdas 2018 ...196
Tabel 7.3.1 Prevalensi Karies, Karies Akar dan Periodontitis menurut Karakteristik, Riskesdas 2018 204
Tabel 7.3.2 Rata-rata Indeks *dmf-t* Gigi Sulung pada Penduduk Umur 3-4 Tahun menurut Karakteristik, Riskesdas 2018 205
Tabel 7.3.3 Rata-rata Indeks *dmf-t* Gigi Sulung pada Penduduk Umur 5 Tahun menurut Karakteristik, Riskesdas 2018 205
Tabel 7.3.4 Rata-rata Indeks *DMF-T* Gigi Permanen dan *Root Caries Indeks* menurut Karakteristik, Riskesdas 2018 206
Tabel 7.3.5 Rata-rata Komponen DT/dt, MT/mt, FT/ft, Indeks *DMF-T/dmf-t*, dan Karies Sekunder menurut Karakteristik, Riskesdas 2018......207
Tabel 7.3.6 Proporsi Bebas karies, *Dental Fit* dan *Fissure Sealant* menurut Karakteristik, Riskesdas 2018 208
Tabel 7.3.7 Proporsi *Dentulous, Edentulous,* dan Penggunaan Gigi Tiruan pada Penduduk Umur ≥12 Tahun menurut Karakteristik, Riskesdas 2018 209
Tabel 7.3.8 Rata-rata Komponen *RD, RF, RN*, Karies Sekunder pada Akar Gigi Permanen, menurut Karakteristik, Riskesdas 2018......210
Tabel 7.3.9 Rata-rata Jumlah Gigi dengan Status Penyakit Jaringan Periodontal Umur >15 Tahun menurut Karakteristik, Riskesdas 2018 211
Tabel 7.3.10 Rata-rata Sekstan dengan Kehilangan Perlekatan pada Penduduk Umur >15 Tahun menurut Karakteristik, Riskesdas 2018 212
Tabel 7.3.11 Prevalensi Fluorosis Gigi menurut Karakteristik, Riskesdas 2018 213
Tabel 7.3.12 Proporsi Erosi Gigi Menurut Karakteristik, Riskesdas 2018 214
Tabel 7.3.13 Proporsi Penyakit Lesi Oral Mukosa menurut Karakteristik, Riskesdas 2018......215
Tabel 7.3.14 Proporsi Kebutuhan Perawatan menurut Karakteristik, Riskesdas 2018 216
Tabel 7.4.1 Proporsi Perilaku Menyikat Gigi pada Penduduk Umur >3 Tahun menurut Provinsi, Riskesdas 2018 218
Tabel 7.4.2 Proporsi Perilaku Menyikat Gigi pada Penduduk Umur >3 Tahun menurut menurut Karakteristik, Riskesdas 2018......219

Tabel 8.1.1 Prevalensi (permil) Rumah Tangga dengan ART Gangguan Jiwa Skizofrenia/Psikosis menurut Provinsi, Riskesdas 2018 222
Tabel 8.1.2 Prevalensi (permil) Rumah Tangga dengan ART Gangguan Jiwa Skizofrenia/Psikosis menurut Tempat Tinggal, Riskesdas 2018 223
Tabel 8.1.3 Proporsi Rumah Tangga yang Memiliki ART Gangguan Jiwa Skizofrenia/Psikosis yang Pernah Dipasung menurut Tempat Tinggal, Riskesdas 2018 223
Tabel 8.2.1 Prevalensi Depresi pada Penduduk Umur >15 Tahun menurut Provinsi, Riskesdas 2018 224
Tabel 8.2.2 Prevalensi Depresi pada Penduduk Umur >15 Tahun menurut Karakteristik, Riskesdas 2018 225
Tabel 8.3.1 Prevalensi Gangguan Mental Emosional pada Penduduk Umur >15 Tahun menurut Provinsi, Riskesdas 2018 227
Tabel 8.3.2 Prevalensi Gangguan Mental Emosional Pada Penduduk Umur >15 Tahun menurut Karakteristik, Riskesdas 2018 228
Tabel 8.4.1 Proporsi Pengobatan Rumah Tangga dengan ART Gangguan Jiwa Skizofrenia/Psikosis menurut Provinsi, Riskesdas 2018 230
Tabel 8.4.2 Proporsi Pengobatan Rumah Tangga dengan ART Gangguan Jiwa Skizofrenia/Psikosis menurut Tempat Tinggal, Riskesdas 2018 231
Tabel 8.4.3 Proporsi Alasan Ketidakpatuhan Minum Obat Gangguan Jiwa Skizofrenia/Psikosis pada ART di Rumah Tangga Menurut Tempat Tinggal, Riskesdas 2018 231
Tabel 8.4.4 Proporsi Pengobatan Depresi pada Penduduk Umur ≥15 Tahun menurut Provinsi, Riskesdas 2018...232
Tabel 8.4.5 Proporsi Pengobatan Depresi pada Penduduk Umur ≥15 Tahun menurut Karakteristik, Riskesdas 2018 233
Tabel 9.1.1 Proporsi Disabilitas pada Anak Umur 5-17 Tahun menurut Provinsi, Riskesdas 2018 236
Tabel 9.1.2 Proporsi Disabilitas pada Anak Umur 5-17 Tahun menurut Karakteristik, Riskesdas 2018 237
Tabel 9.2.1 Proporsi Disabilitas pada Penduduk Umur 18-59 Tahun menurut Provinsi, Riskesdas 2018 239
Tabel 9.2.2 Proporsi Disabilitas pada Penduduk Umur 18-59 Tahun menurut Karakteristik, Riskesdas 2018 240
Tabel 9.2.3 Proporsi Tingkat Disabilitas pada Penduduk Umur 18-59 Tahun menurut Provinsi, Riskesdas 2018...242
Tabel 9.2.4 Proporsi Tingkat Disabilitas pada Penduduk Umur 18-59 Tahun menurut Karakteristik, Riskesdas 2018 243
Tabel 9.3.1 Proporsi Tingkat Ketergantungan pada Penduduk Umur >60 Tahun menurut Provinsi, Riskesdas 2018 245

Tabel 9.3.2 Proporsi Tingkat Ketergantungan pada Penduduk Umur >60 Tahun menurut Karakteristik, Riskesdas 2018 246
Tabel 9.3.3 Proporsi Tingkat Ketergantungan pada Penduduk Umur >60 Tahun menurut Penyakit, Riskesdas 2018 246
Tabel 10.1.1 Proporsi Cedera yang Mengakibatkan Kegiatan Sehari-hari Terganggu menurut Provinsi, Riskesdas 2018 250
Tabel 10.1.2 Proporsi Cedera yang Mengakibatkan Kegiatan Sehari-Hari Terganggu menurut Karakteristik, Riskesdas 2018 251
Tabel 10.1.3 Proporsi Bagian Tubuh yang Cedera menurut Provinsi, Riskesdas 2018 252
Tabel 10.1.4 Proporsi Bagian Tubuh yang Cedera menurut Karakteristik, Riskesdas 2018 253
Tabel 10.1.5 Proporsi Jenis Cedera (Jenis Luka, Terkilir, Patah Tulang, Anggota Tubuh Terputus) menurut Provinsi, Riskesdas 2018 254
Tabel 10.1.6 Proporsi Jenis Cedera (Jenis Luka, Terkilir, Patah Tulang, Anggota Tubuh Terputus) menurut Karakteristik, Riskesdas 2018....255
Tabel 10.1.7 Proporsi Jenis Cedera (Cedera Mata, Gegar Otak, Cedera Organ Dalam, Luka Bakar, Lainnya) menurut Provinsi, Riskesdas 2018 256
Tabel 10.1.8 Proporsi Jenis Cedera (Cedera Mata, Gegar Otak, Cedera Organ Dalam, Luka Bakar, Lainnya) menurut Provinsi Karakteristik, Riskesdas 2018 257
Tabel 10.1.9 Proporsi Cedera Mengakibatkan Kecacatan Fisik Permanen menurut Provinsi, Riskesdas 2018 258
Tabel 10.1.10 Proporsi Cedera Mengakibatkan Kecacatan Fisik Permanen menurut Karakteristik, Riskesdas 2018 259
Tabel 10.1.11 Proporsi Tempat Terjadinya Cedera menurut Provinsi, Riskesdas 2018 260
Tabel 10.1.12 Proporsi Tempat Terjadinya Cedera menurut Karakteristik, Riskesdas 2018 261
Tabel 10.1.13 Proporsi Cedera Karena Kecelakaan Lalu Lintas menurut Provinsi, Riskesdas 2018 262
Tabel 10.1.14 Proporsi Cedera Karena Kecelakaan Lalu Lintas menurut Karakteristik, Riskesdas 2018 263
Tabel 10.1.15 Proporsi Kegiatan yang Sedang Dilakukan Saat Kecelakaan Lalu Lintas menurut Provinsi, Riskesdas 2018 264
Tabel 10.1.16 Proporsi Kegiatan yang Sedang Dilakukan Saat Kecelakaan Lalu Lintas menurut Karakteristik, Riskesdas 2018....265
Tabel 10.2.1 Proporsi Kebiasaan Menggunakan Helm Saat Mengendarai atau Membonceng Sepeda Motor pada Penduduk Umur >5 Tahun menurut Provinsi, Riskesdas 2018 267

Tabel 10.2.2 Proporsi Kebiasaan Menggunakan Helm Saat Mengendarai atau Membonceng Sepeda Motor pada Penduduk Umur >5 Tahun menurut Karakteristik, Riskesdas 2018 268
Tabel 10.2.3 Proporsi Cara dan Kondisi Helm yang Digunakan menurut Provinsi, Riskesdas 2018 269
Tabel 10.2.4 Proporsi Cara dan Kondisi Helm yang Digunakan menurut Karakteristik, Riskesdas 2018 270
Tabel 11.1.1 Proporsi Pemanfaatan Pelayanan Kesehatan Tradisional menurut Provinsi, Riskesdas 2018..274
Tabel 11.1.2 Proporsi Pemanfaatan Pelayanan Kesehatan Tradisional menurut Karakteristik, Riskesdas 2018 275
Tabel 11.2.1 Proporsi Jenis Pelayanan Kesehatan Tradisional yang Dimanfaatkan menurut Provinsi, Riskesdas 2018 276
Tabel 11.2.2 Proporsi Jenis Pelayanan Kesehatan Tradisional yang Dimanfaatkan menurut Karakteristik, Riskesdas 2018 277
Tabel 11.3.1 Proporsi Jenis Tenaga yang Dimanfaatkan Menangani Kesehatan Tradisional menurut Provinsi, Riskesdas 2018 278
Tabel 11.3.2 Proporsi Jenis Tenaga yang Dimanfaatkan Menangani Kesehatan Tradisional menurut Karakteristik, Riskesdas 2018..279
Tabel 11.4.1 Proporsi Pemanfaatan TOGA menurut Provinsi, Riskesdas 2018 280
Tabel 11.4.2 Proporsi Pemanfaatan TOGA menurut Karakteristik, Riskesdas 2018 281
Tabel 12.1.1 Proporsi Penggunaan Kelambu *Long Lasting Insecticide Nets* (*LLIN's*) menurut Provinsi, Riskesdas 2018 284
Tabel 12.1.2 Proporsi Penggunaan Kelambu *Long Lasting Insecticide Nets* (*LLIN's*) menurut Karakteristik, Riskesdas 2018 285
Tabel 12.1.3 Proporsi Cara Pencegahan Penyakit Akibat Gigitan Nyamuk menurut Provinsi, Riskesdas 2018..286
Tabel 12.1.4 Proporsi Cara Pencegahan Penyakit Akibat Gigitan Nyamuk menurut Karakteristik, Riskesdas 2018..287
Tabel 12.2.1 Proporsi Kebiasaan Konsumsi Makanan Manis pada Penduduk Umur >3 Tahun menurut Provinsi, Riskesdas 2018..290
Tabel 12.2.2 Proporsi Kebiasaan Konsumsi Makanan Manis pada Penduduk Umur >3 Tahun menurut Karakteristik, Riskesdas 2018 291
Tabel 12.2.3 Proporsi Kebiasaan Konsumsi Minuman Manis pada Penduduk Umur >3 Tahun menurut Provinsi, Riskesdas 2018..292
Tabel 12.2.4 Proporsi Kebiasaan Konsumsi Minuman Manis pada Penduduk Umur >3 Tahun menurut Karakteristik, Riskesdas 2018 293
Tabel 12.2.5 Proporsi Kebiasaan Konsumsi Makanan Asin pada Penduduk Umur >3 Tahun menurut Provinsi, Riskesdas 2018..294

Tabel 12.2.6 Proporsi Kebiasaan Konsumsi Makanan Asin pada Penduduk Umur >3 Tahun menurut Karakteristik, Riskesdas 2018 295
Tabel 12.2.7 Proporsi Kebiasaan Konsumsi Makanan Berlemak/Berkolesterol/Gorengan pada Penduduk Umur >3 Tahun menurut Provinsi, Riskesdas 2018 296
Tabel 12.2.8 Proporsi Kebiasaan Konsumsi Makanan Berlemak/Berkolesterol/Gorengan pada Penduduk Umur >3 Tahun menurut Karakteristik, Riskesdas 2018 297
Tabel 12.2.9 Proporsi Kebiasaan Konsumsi Makanan yang Dibakar pada Penduduk Umur >3 Tahun menurut Provinsi, Riskesdas 2018 298
Tabel 12.2.10 Proporsi Kebiasaan Konsumsi Makanan yang Dibakar pada Penduduk Umur >3 Tahun menurut Karakteristik, Riskesdas 2018 299
Tabel 12.2.11 Proporsi Kebiasaan Konsumsi Makanan Daging/Ayam/Ikan Olahan dengan Pengawet pada Penduduk Umur >3 Tahun menurut Provinsi, Riskesdas 2018 300
Tabel 12.2.12 Proporsi Kebiasaan Konsumsi Makanan Daging/Ayam/Ikan Olahan dengan Pengawet pada Penduduk Umur >3 Tahun menurut Karakteristik, Riskesdas 2018 301
Tabel 12.2.13 Proporsi Kebiasaan Konsumsi Makanan yang Mengandung Bumbu Penyedap pada Penduduk Umur >3 Tahun menurut Provinsi, Riskesdas 2018 302
Tabel 12.2.14 Proporsi Kebiasaan Konsumsi Bumbu Penyedap pada Penduduk Umur >3 Tahun menurut Karakteristik, Riskesdas 2018 303
Tabel 12.2.15 Proporsi Kebiasaan Konsumsi *Soft Drink* atau Minuman Berkarbonasi pada Penduduk Umur >3 Tahun menurut Provinsi, Riskesdas 2018 304
Tabel 12.2.16 Proporsi Kebiasaan Konsumsi *Soft Drink* atau Minuman Berkarbonasi pada Penduduk Umur >3 Tahun menurut Karakteristik, Riskesdas 2018 ...305
Tabel 12.2.17 Proporsi Kebiasaan Konsumsi Minuman Berenergi pada Penduduk Umur >3 Tahun menurut Provinsi, Riskesdas 2018 306
Tabel 12.2.18 Proporsi Kebiasaan Konsumsi Minuman Berenergi pada Penduduk Umur >3 Tahun menurut Karakteristik, Riskesdas 2018 307
Tabel 12.2.19 Proporsi Kebiasaan Konsumsi Mi Instan/Makanan Instan Lainnya pada Penduduk Umur >3 Tahun menurut Provinsi, Riskesdas 2018 308
Tabel 12.2.20 Proporsi Kebiasaan Konsumsi Mi Instan/Makanan Instan Lainnya pada Penduduk Umur >3 Tahun menurut Karakteristik, Riskesdas 2018 ...309
Tabel 12.3.1 Proporsi Konsumsi Buah/Sayur per Hari dalam Seminggu pada Penduduk Umur >5 Tahun menurut Provinsi, Riskesdas 2018 311
Tabel 12.3.2 Proporsi Konsumsi Buah/Sayur per Hari dalam Seminggu pada Penduduk Umur >5 Tahun menurut Karakteristik, Riskesdas 2018 312

Tabel 12.3.3 Proporsi Kurang Makan Buah/Sayur dan Rerata Konsumsi Buah dan Sayur per Hari dalam Seminggu pada Penduduk Umur >5 Tahun menurut Provinsi, Riskesdas 2018 313
Tabel 12.3.4 Proporsi Kurang Makan Buah/Sayur dan Rerata Konsumsi Buah dan Sayur per Hari dalam Seminggu pada Penduduk Umur >5 Tahun menurut karakteristik, Riskesdas 2018 314
Tabel 12.4.1 Proporsi Perilaku Benar Buang Air Besar pada Penduduk Umur >3 Tahun menurut Provinsi, Riskesdas 2018....315
Tabel 12.4.2 Proporsi Perilaku Benar Buang Air Besar pada Penduduk Umur >3 Tahun menurut Karakteristik, Riskesdas 2018....316
Tabel 12.5.1 Proporsi Perilaku Benar dalam Cuci Tangan pada Penduduk Umur >10 Tahun menurut Provinsi, Riskesdas 2018....318
Tabel 12.5.2 Proporsi Perilaku Benar dalam Cuci Tangan pada Penduduk Umur >10 Tahun menurut Karakteristik, Riskesdas 2018....319
Tabel 12.6.1 Proporsi Merokok pada Penduduk Umur ≥10 Tahun menurut Provinsi, Riskesdas 2018 322
Tabel 12.6.2 Proporsi Merokok pada Penduduk Umur ≥10 Tahun menurut Karakteristik, Riskesdas 2018 323
Tabel 12.6.3 Rata-rata Jumlah Batang Rokok (Kretek, Putih, Linting) Perhari dan Perminggu yang Dihisap Penduduk Umur >10 Tahun menurut Provinsi, Riskesdas 2018 324
Tabel 12.6.4 Rata-rata Jumlah Batang Rokok (Kretek, Putih, Linting) Perhari dan Perminggu yang Dihisap Penduduk Umur >10 Tahun menurut karakteristik, Riskesdas 2018....325
Tabel 12.6.5 Proporsi Umur Pertama Kali Merokok Tiap Hari pada Penduduk Umur ≥10 Tahun menurut Provinsi, Riskesdas 2018 326
Tabel 12.6.6 Proporsi Umur Pertama Kali Merokok Tiap Hari pada Penduduk Umur ≥10 Tahun menurut Karakteristik, Riskesdas 2018 327
Tabel 12.6.7 Proporsi Umur Pertama Kali Merokok Pada Penduduk Umur ≥10 Tahun menurut Provinsi, Riskesdas 2018 329
Tabel 12.6.8 Proporsi Umur Pertama Kali Merokok Pada Penduduk Umur ≥10 Tahun menurut Karakteristik, Riskesdas 2018 330
Tabel 12.6.9 Proporsi Jenis Rokok Yang Dihisap Penduduk Umur ≥10 Tahun menurut Provinsi, Riskesdas 2018 331
Tabel 12.6.10 Proporsi Jenis Rokok Yang Dihisap Penduduk Umur ≥10 Tahun menurut Karakteristik, Riskesdas 2018 332
Tabel 12.6.11 Proporsi Merokok Dalam Gedung/Ruangan pada Penduduk Umur ≥10 Tahun menurut Provinsi, Riskesdas 2018....333
Tabel 12.6.12 Proporsi Merokok Dalam Gedung/Ruangan pada Penduduk Umur ≥10 Tahun menurut Karakteristik, Riskesdas 2018....334

Tabel 12.6.13 Proporsi Frekuensi Berada di Dekat Orang yang Merokok di Dalam Ruangan Tertutup pada Penduduk Umur ≥10 Tahun menurut Provinsi, Riskesdas 2018 335
Tabel 12.6.14 Proporsi Frekuensi Berada di Dekat Orang yang Merokok di Dalam Ruangan Tertutup pada Penduduk Umur ≥10 Tahun menurut Karakteristik, Riskesdas 2018 336
Tabel 12.6.15 Proporsi Mengunyah Tembakau pada Penduduk Umur ≥10 Tahun menurut Provinsi, Riskesdas 2018 337
Tabel 12.6.16 Proporsi Mengunyah Tembakau pada Penduduk Umur ≥10 Tahun menurut Karakteristik, Riskesdas 2018 338
Tabel 12.7.1 Proporsi Aktivitas Fisik pada Penduduk Umur >10 Tahun menurut Provinsi, Riskesdas 2018 340
Tabel 12.7.2 ProporsiAktivitas Fisik pada Penduduk Umur >10 Tahun menurut Karakteristik, Riskesdas 2018 341
Tabel 12.8.1 Proporsi Perilaku Konsumsi Minuman Beralkohol dalam 1 Bulan Terakhir pada Penduduk Umur >10 Tahun menurut Provinsi, Riskesdas 2018 344
Tabel 12.8.2 Proporsi Perilaku Konsumsi Minuman Beralkohol dalam 1 Bulan Terakhir pada Penduduk Umur >10 Tahun menurut Karakteristik, Riskesdas 2018 345
Tabel 12.8.3 Proporsi Jenis Minuman Beralkohol yang Biasa Diminum pada Peminum Alkohol Umur >10 Tahun menurut Provinsi, Riskesdas 2018.....346
Tabel 12.8.4 Proporsi Jenis Minuman Beralkohol yang Biasa Diminum pada Peminum Alkohol Umur >10 Tahun menurut Karakteristik, Riskesdas 2018 347
Tabel 12.8.5 Rata-rata Jumlah Satuan Standar Minuman Beralkohol yang Biasa Diminum pada Peminum Alkohol Umur >10 Tahun menurut Provinsi, Riskesdas 2018 348
Tabel 12.8.6 Rata-rata Jumlah Satuan Standar Minuman Beralkohol yang Biasa Diminum pada PeminumAlkohol Umur >10 Tahun menurut Karakteristik, Riskesdas 2018 349
Tabel 12.8.7 Proporsi Konsumsi Minuman Beralkohol yang Berbahaya pada Penduduk Umur>10 Tahun menurut Provinsi, Riskesdas 2018 350
Tabel 12.8.8 Proporsi Konsumsi Minuman Beralkohol yang Berbahaya pada Penduduk Umur>10 Tahun menurut Karakteristik, Riskesdas 2018 351
Tabel 13.1.1 Proporsi Pernah Mendengar *HIV/AIDS* pada Penduduk Umur >15 Tahun menurut Provinsi, Riskesdas 2018 354
Tabel 13.1.2 Proporsi Pernah Mendengar *HIV/AIDS* pada Penduduk Umur >15 Tahun menurut Karakteristik, Riskesdas 2018 355

Tabel 13.1.3 Proporsi Pengetahuan *HIV/AIDS* pada Penduduk >15 Tahun menurut Provinsi, Riskesdas 2018 356
Tabel 13.1.4 Proporsi Pengetahuan *HIV/AIDS* pada Penduduk >15 Tahun menurut Karakteristik, Riskesdas 2018 357
Tabel 13.2.1 Proporsi Sikap terhadap Penderita HIV/AIDS pada Penduduk Umur >15 Tahun menurut Provinsi, Riskesdas 2018 358
Tabel 13.2.2 Proporsi Sikap terhadap Penderita HIV/AIDS pada Penduduk Umur >15 Tahun menurut Karakteristik, Riskesdas 2018 359
Tabel 14.1.1 Proporsi Riwayat Kehamilan pada Perempuan Umur 10-54 tahun yang Pernah Kawin menurut Provinsi, Riskesdas 2018 363
Tabel 14.1.2 Proporsi Riwayat Kehamilan pada Perempuan Umur 10-54 tahun yang Pernah Kawin menurut Karakteristik, Riskesdas 2018 364
Tabel 14.2.1 Proporsi Pemeriksaan Kehamilan (K1, K1 ideal dan K4) pada Perempuan Umur 10-54 Tahun yang Pernah Hamil menurut Provinsi, Riskesdas 2018 367
Tabel 14.2.2 Proporsi Pemeriksaan Kehamilan (K1, K1 ideal dan K4) pada Perempuan Umur 10-54 Tahun yang Pernah Hamil menurut Karakteristik, Riskesdas 2018 368
Tabel 14.2.3 Proporsi Tenaga Pemberi Layanan ANC Selama Kehamilan pada Perempuan Umur 10-54 Tahun yang Pernah Melahirkan menurut Provinsi, Riskesdas 2018 370
Tabel 14.2.4 Proporsi Tenaga Pemberi Layanan ANC Selama Kehamilan pada Perempuan Umur 10-54 Tahun yang Pernah Melahirkan menurut Karakteristik, Riskesdas 2018 371
Tabel 14.2.5 Proporsi Tempat Layanan ANC yang Dimanfaatkan oleh Perempuan Umur 10-54 Tahun Selama Kehamilan menurut Provinsi, Riskesdas 2018 372
Tabel 14.2.6 Proporsi Tempat Layanan ANC yang Dimanfaatkan oleh Perempuan Umur 10-54 Tahun Selama Kehamilan menurut Karakteristik, Riskesdas 2018 373
Tabel 14.2.7 Proporsi Komponen Antenatal Care (ANC) Selama Kehamilan pada Perempuan Umur 10-54 Tahun yang Pernah Melahirkan menurut Provinsi, Riskesdas 2018 374
Tabel 14.2.8 Proporsi Komponen Antenatal Care (ANC) Selama Kehamilan pada Perempuan Umur 10-54 Tahun yang Pernah Melahirkan menurut Karakteristik, Riskesdas 2018 375
Tabel 14.2.9 Proporsi Jenis Pelayanan Pemeriksaan Laboratorium yang Pernah Dimanfaatkan oleh Perempuan Umur 10-54 Tahun Selama Masa Kehamilan menurut Provinsi, Riskesdas 2018 376

Tabel 14.2.10 Proporsi Jenis Pelayanan Pemeriksaan Laboratorium yang Pernah Dimanfaatkan oleh Perempuan Umur 10-54 Tahun Selama Masa Kehamilan menurut Karakteristik, Riskesdas 2018 377
Tabel 14.2.11 Proporsi Gangguan/Komplikasi yang Dialami Selama Kehamilan pada Perempuan Umur 10-54 Tahun menurut Provinsi, Riskesdas 2018.............379
Tabel 14.2.12 Proporsi Gangguan/Komplikasi yang Dialami Selama Kehamilan pada Perempuan Umur 10-54 Tahun menurut Karakteristik, Riskesdas 2018.....380
Tabel 14.2.13 Proporsi Upaya Pertolongan Pertama Komplikasi Kehamilan yang Pernah Dialami pada Perempuan Umur 10-54 Tahun menurut Provinsi, Riskesdas 2018 381
Tabel 14.2.14 Upaya Pertolongan Pertama Komplikasi Kehamilan yang Pernah Dialami pada Perempuan Umur 10-54 Tahun menurut Karakteristik, Riskesdas 2018 382
Tabel 14.2.15 Proporsi Fasilitas Kesehatan Tempat Rujukan Pertama saat Mengalami Komplikasi Kehamilan yang Pernah Dimanfaatkan oleh Perempuan Umur 10-54 Tahun menurut Provinsi, Riskesdas 2018............383
Tabel 14.2.16 Proporsi Fasilitas Kesehatan Tempat Rujukan Pertama saat Mengalami Komplikasi Kehamilan yang Pernah Dimanfaatkan oleh Perempuan Umur 10-54 Tahun menurut Karakteristik, Riskesdas 2018.....384
Tabel 14.3.1 Proporsi Penolong Persalinan dengan Kualifikasi Tertinggi pada Perempuan Umur 10-54 Tahun menurut Provinsi, Riskesdas 2018.............386
Tabel 14.3.2 Proporsi Penolong Persalinan dengan Kualifikasi Tertinggi pada Perempuan Umur 10-54 Tahun menurut Karakteristik, Riskesdas 2018.....387
Tabel 14.3.3 Proporsi Penolong Persalinan dengan Kualifikasi Terendah pada Perempuan Umur 10-54 Tahun menurut Provinsi, Riskesdas 2018.............388
Tabel 14.3.4 Proporsi Penolong Persalinan dengan Kualifikasi Terendah pada Perempuan Umur 10-54 Tahun menurut Karakteristik, Riskesdas 2018.....389
Tabel 14.3.5 Proporsi Tempat Persalinan yang Dimanfaatkan oleh Perempuan Umur 10-54 Tahun menurut Provinsi, Riskesdas 2018 390
Tabel 14.3.6 Proporsi Tempat Persalinan yang Dimanfaatkan oleh Perempuan Umur 10-54 Tahun menurut Karakteristik, Riskesdas 2018 391
Tabel 14.3.7 Proporsi Sumber Pembiayaan Persalinan di Fasilitas Pelayanan Kesehatan pada Perempuan Umur 10-54 Tahun menurut Provinsi, Riskesdas 2018 393
Tabel 14.3.8 Proporsi Sumber Pembiayaan Persalinan di Fasilitas Pelayanan Kesehatan pada Perempuan Umur 10-54 Tahun menurut Karakteristik, Riskesdas 2018 394
Tabel 14.3.9 Proporsi Metode Persalinan pada Perempuan Umur 10-54 Tahun menurut Provinsi, Riskesdas 2018 395

Tabel 14.3.10 Proporsi Metode Persalinan pada Perempuan Umur 10-54 Tahun menurut Karakteristik, Riskesdas 2018 396
Tabel 14.3.11 Proporsi Gangguan/Komplikasi Persalinan pada Perempuan Umur 10-54 Tahun menurut Provinsi, Riskesdas 2018 398
Tabel 14.3.12 Proporsi Gangguan/Komplikasi Persalinan pada Perempuan Umur 10-54 Tahun menurut Karakteristik, Riskesdas 2018 399
Tabel 14.3.13 Proporsi Upaya Pertolongan Pertama untuk Komplikasi Persalinan pada Perempuan Umur 10-54 Tahun menurut Karakteristik, Riskesdas 2018 400
Tabel 14.4.1 Proporsi Pelayanan Masa Nifas pada Perempuan 10-54 Tahun menurut Provinsi, Riskesdas 2018 402
Tabel 14.4.2 Proporsi Pelayanan Masa Nifas pada Perempuan 10-54 Tahun menurut Karakteristik, Riskesdas 2018 403
Tabel 14.4.3 Proporsi Frekuensi Mendapatkan dan Waktu Minum Vitamin A saat Masa Nifas pada Perempuan Umur 10-54 Tahun menurut Provinsi, Riskesdas 2018 405
Tabel 14.4.4 Proporsi Frekuensi Mendapatkan dan Waktu Minum Vitamin A saat Masa Nifas pada Perempuan Umur 10-54 Tahun menurut Karakteristik, Riskesdas 2018 406
Tabel 14.4.5 Proporsi Gangguan atau Komplikasi Masa Nifas pada Perempuan Umur 10 – 54 Tahun menurut Karakteristik, Riskesdas 2018....408
Tabel 14.4.6 Proporsi Upaya Pencarian Pertolongan Pertama Gangguan/Komplikasi Masa Nifas pada Perempuan umur 10-54 Tahun menurut Karakteristik, Riskesdas 2018 409
Tabel 14.5.1 ProporsiJenis Alat/Cara KB Modern yang Digunakan Setelah Melahirkan Anak Terakhir Pada Perempuan Umur 10-54 Tahun menurut Provinsi, Riskesdas 2018 411
Tabel 14.5.2 ProporsiJenis Alat/Cara KB Modern yang Digunakan Setelah Melahirkan Anak Terakhir Pada Perempuan Umur 10-54 Tahun menurut Karakteristik, Riskesdas 2018 412
Tabel 14.5.3 Proporsi Waktu Terima Layanan KB Pertama Kali Setelah Bersalin berdasarkan Tempat Bersalin pada Perempuan Umur 10-54 Tahun menurut Provinsi, Riskesdas 2018 413
Tabel 14.5.4 Proporsi Waktu Terima Layanan KB Pertama Kali Setelah Melahirkan pada Perempuan Umur 10-54 Tahun menurut Karakteristik, Riskesdas 2018 414
Tabel 14.5.5 Proporsi Waktu Terima Layanan KB Pertama Kali Setelah Bersalin pada Perempuan Umur 10-54 Tahun menurut Provinsi, Riskesdas 2018...415
Tabel 14.6.1 Proporsi Kepemilikan Buku KIA pada Ibu Hamil menurut Provinsi, Riskesdas 2018 417

Tabel 14.6.2 Proporsi Kepemilikan Buku KIA pada Ibu Hamil menurut Karakteristik, Riskesdas 2018 418
Tabel 15.1.1 Proporsi Riwayat Kelahiran berdasarkan Umur Kandungan Ibu saat Melahirkan pada Anak Umur 0-59 Bulan menurut Provinsi, Riskesdas 2018 420
Tabel 15.1.2 Proporsi Riwayat Kelahiran berdasarkan Umur Kandungan Ibu saat Melahirkan pada Anak Umur 0-59 Bulan menurut Karakteristik, Riskesdas 2018 421
Tabel 15.1.3 Proporsi Catatan Kondisi Lahir yang Tidak Dimiliki Anak Umur 0-59 Bulan menurut Provinsi, Riskesdas 2018 423
Tabel 15.1.4 Proporsi Catatan Kondisi Lahir yang Tidak Dimiliki Anak Umur 0-59 Bulan menurut Karakteristik, Riskesdas 2018 424
Tabel 15.1.5 Proporsi Berat Badan Lahir pada Anak Umur 0-59 Bulan menurut Provinsi, Riskesdas 2018 425
Tabel 15.1.6 Proporsi Berat Badan Lahir pada Anak Umur 0-59 Bulan menurut karakteristik, Riskesdas 2018 426
Tabel 15.1.7 Proporsi Panjang Badan Lahir pada Anak Umur 0-59 Bulan menurut Provinsi, Riskesdas 2018 427
Tabel 15.1.8 Proporsi Panjang Badan Lahir pada Anak Umur 0-59 Bulan menurut karakteristik, Riskesdas 2018 428
Tabel 15.1.9 Proporsi Berat Badan Lahir <2500 gram dan Panjang Badan Lahir <48 cm pada Anak Umur 0-59 Bulan menurut Provinsi, Riskesdas 2018 429
Tabel 15.1.10 Proporsi Berat Badan Lahir <2500 gram dan Panjang Badan Lahir <48 cm pada Anak Umur 0-59 Bulan menurut Karakteristik, Riskesdas 2018 430
Tabel 15.1.11 Proporsi Lingkar Kepala Lahir pada Anak Umur 0-59 Bulan menurut Provinsi, Riskesdas 2018 431
Tabel 15.1.12 Proporsi Lingkar Kepala Lahir pada Anak Umur 0-59 Bulan menurut karakteristik, Riskesdas 2018 432
Tabel 15.1.13 Proporsi Kelainan/Kecacatan Sejak Lahir pada Anak Umur 24-59 Bulan, Riskesdas 2018 434
Tabel 15.2.1 Proporsi Tindakan yang Dilakukan untuk Bayi Berat Lahir Rendah (<2500 gram) pada Anak Umur 0-59 Bulan menurut Provinsi, Riskesdas 2018 435
Tabel 15.2.2 Proporsi Tindakan yang Dilakukan untuk Bayi Berat Lahir Rendah (<2500 gram) pada Anak Umur 0-59 Bulan menurut Karakteristik, Riskesdas 2018 436
Tabel 15.2.3 Proporsi Cara Perawatan Tali Pusar saat Bayi Baru Lahir pada Anak Umur 0-59 bulan menurut Provinsi, Riskesdas 2018 437

Tabel 15.2.4 Proporsi Cara Perawatan Tali Pusar saat Bayi Baru Lahir pada Anak Umur 0-59 bulan menurut Karakteristik, Riskesdas 2018 438
Tabel 15.2.5 Proporsi Kunjungan Neonatal pada Anak Umur 0-59 Bulan menurut Provinsi, Riskesdas 2018 440
Tabel 15.2.6 Proporsi Kunjungan Neonatal pada Anak Umur 0-59 Bulan menurut karakteristik, Riskesdas 2018 441
Tabel 15.2.7 Proporsi Pelayanan Kunjungan Neonatal Pertama (KN1) Sesuai Standar pada Anak Umur 0-59 Bulan menurut Provinsi, Riskesdas 2018 443
Tabel 15.2.8 Proporsi Pelayanan Kunjungan Neonatal Pertama (KN1) Sesuai Standar pada Anak Umur 0-59 Bulan menurut Karakteristik, Riskesdas 2018 444
Tabel 15.2.9 Proporsi Pemberiaan Salep Mata saat Lahir pada Anak Umur 0-59 Bulan menurut Provinsi, Riskesdas 2018 446
Tabel 15.2.10 Proporsi Pemberiaan Salep Mata saat Lahir pada Anak Umur 0-59 Bulan menurut Karakteristik, Riskesdas 2018 447
Tabel 15.2.11 Proporsi Pemeriksaan Skrining Hipotiroid Kongenital (SHK) saat Lahir pada Anak Umur 0-59 Bulan menurut Provinsi, Riskesdas 2018....449
Tabel 15.2.12 Proporsi Pemeriksaan Skrining Hipotiroid Kongenital (SHK) saat Lahir pada Anak Umur 0-59 Bulan menurut Karakteristik, Indonesia 2018....450
Tabel 15.3.1 Proporsi Kepemilikan Buku KIA pada Anak Umur 0-59 Bulan menurut Provinsi, Riskesdas 2018 452
Tabel 15.3.2 Proporsi Kepemilikan Buku KIA pada Anak Umur 0-59 Bulan menurut Karakteristik, Riskesdas 2018 453
Tabel 15.3.3 Proporsi Pencatatan Buku KIA berdasarkan Isi pada Anak Umur 0-59 Bulan menurut Provinsi, Riskesdas 2018 454
Tabel 15.3.4 Proporsi Pencatatan Buku KIA berdasarkan Isi pada Anak Umur 0-59 Bulan menurut Karakteristik, Riskesdas 2018 455
Tabel 15.4.1 Proporsi Jenis Imunisasi Dasar pada Anak Umur 12-23 Bulan menurut Provinsi, Riskesdas 2018 457
Tabel 15.4.2 Proporsi Jenis Imunisasi Dasar pada Anak Umur 12-23 Bulan menurut Karakteristik, Riskesdas 2018 458
Tabel 15.4.3 Proporsi Imunisasi Dasar Lengkap pada Anak Umur 12-23 Bulan menurut Provinsi, Riskesdas 2018 460
Tabel 15.4.4 Proporsi Imunisasi Dasar Lengkap pada Anak Umur 12-23 Bulan menurut karakteristik, Riskesdas 2018 461
Tabel 15.4.5 Proporsi Imunisasi Lanjutan pada Anak Umur 24-35 Bulan menurut Provinsi, Riskesdas 2018 463
Tabel 15.4.6 Proporsi Imunisasi Lanjutan pada Anak Umur 24-35 Bulan menurut Karakteristik, Riskesdas 2018 464

Tabel 15.4.7 Proporsi Kejadian Ikutan Pasca Imunisasi (KIPI) dan Jenisnya pada Anak Umur 12-23 Bulan menurut Provinsi, Riskesdas 2018 466
Tabel 15.4.8 Proporsi Kejadian Ikutan Pasca Imunisasi (KIPI) dan Jenisnya pada Anak Umur 12-23 Bulan menurut Karakteristik, Riskesdas 2018 467
Tabel 15.4.9 Proporsi Pemberian Kapsul Vitamin A dalam 12 Bulan Terakhir pada Anak Umur 6-59 Bulan menurut Provinsi, Riskesdas 2018 469
Tabel 15.4.10 Proporsi Pemberian Kapsul Vitamin A dalam 12 Bulan Terakhir pada Anak Umur 6-59 Bulan menurut Karakteristik, Riskesdas 2018...................470
Tabel 15.5.1 Proporsi Pemantauan Pertumbuhan dalam 12 Bulan Terakhir pada Anak Umur 0-59 Bulan menurut Provinsi, Riskesdas 2018 472
Tabel 15.5.2 Proporsi Pemantauan Pertumbuhan dalam 12 Bulan Terakhir pada Anak Umur 0-59 Bulan menurut Karakteristik, Riskesdas 2018...................473
Tabel 15.5.3 Proporsi Alasan Utama Tidak Pernah Ditimbang Berat Badan dalam 12 Bulan Terakhir pada Anak Umur 0-59 Bulan Terakhir menurut Provinsi, Riskesdas 2018 474
Tabel 15.5.4 Proporsi Alasan Utama Tidak Pernah Ditimbang Berat Badan dalam 12 Bulan Terakhir pada Anak Umur 0-59 Bulan Terakhir menurut Karakteristik, Riskesdas 2018 475
Tabel 15.5.5 Proporsi Alasan Utama Tidak Pernah Diukur Panjang atau Tinggi Badan dalam 12 Bulan Terakhir pada Anak Umur 0-59 Bulan menurut Provinsi, Riskesdas 2018 476
Tabel 15.5.6 Proporsi Alasan Utama Tidak Pernah Diukur Panjang atau Tinggi Badan dalam 12 Bulan Terakhir pada Anak Umur 0-59 Bulan menurut Karakteristik, Riskesdas 2018 477
Tabel 15.6.1 Proporsi Indeks dan Jenis Perkembangan Anak Umur 36-59 Bulan menurut Provinsi, Riskesdas 2018 479
Tabel 15.6.2 Proporsi Indeks dan Jenis Perkembangan Anak Umur 36-59 Bulan menurut Karakteristik, Riskesdas 2018 480
Tabel 16.1.1 Proporsi Remaja Putri Umur 10-19 Tahun yang Sudah Mendapat Haid/Menstruasi menurut Provinsi, Riskesdas 2018 483
Tabel 16.1.2 Proporsi Remaja Putri Umur 10-19 Tahun yang Memperoleh Tablet Tambah Darah (TTD) menurut Provinsi, Riskesdas 2018............................484
Tabel 16.1.3 Proporsi Remaja Putri Umur 10-19 Tahun yang Memperoleh Tablet Tambah Darah (TTD) menurut Karakteristik, Riskesdas 2018.....................485
Tabel 16.1.4 Proporsi Sumber Perolehan TTD pada Remaja Putri Umur 10-19 Tahun dalam 12 Bulan Terakhir menurut Provinsi, Riskesdas 2018 486
Tabel 16.1.5 Proporsi Sumber Perolehan TTD pada Remaja Putri Umur 10-19 Tahun dalam 12 Bulan Terakhir menurut Karakteristik, Riskesdas 2018................487

Tabel 16.1.6 Proporsi Jumlah Butir TTD yang Diperoleh dan Diminum Remaja Putri umur 10-19 Tahun dalam 12 Bulan Terakhir menurut Provinsi, Riskesdas 2018 488
Tabel 16.1.7 Proporsi Jumlah Butir TTD yang Diperoleh dari Fasilitas Kesehatan dan Diminum Remaja Putri Umur 10-19 Tahun dalam 12 Bulan Terakhir menurut Karakteristik, Riskesdas 2018 489
Tabel 16.1.8 Proporsi Jumlah Butir TTD yang Diperoleh dari Sekolah dan Diminum Remaja Putri Umur 10-19 Tahun dalam 12 Bulan Terakhir menurut Karakteristik, Riskesdas 2018 490
Tabel 16.1.9 Proporsi Jumlah Butir TTD yang Diperoleh dengan Inisiatif Sendiri dan Diminum Remaja Putri Umur 10-19 Tahun dalam 12 Bulan Terakhir menurut Karakteristik, Riskesdas 2018 491
Tabel 16.1.10 Proporsi Alasan Utama Tidak Minum/Menghabiskan TTD yang Diperoleh dari Fasilitas Kesehatan pada Remaja Putri umur 10-19 Tahun menurut Provinsi, Riskesdas 2018 492
Tabel 16.1.11 Proporsi Alasan Utama Tidak Minum/Menghabiskan TTD yang Diperoleh dari Fasilitas Kesehatan pada Remaja Putri umur 10-19 Tahun menurut Karakteristik, Riskesdas 2018 493
Tabel 16.1.12 Proporsi Alasan Utama Tidak Minum/Menghabiskan TTD yang Diperoleh dari Sekolah pada Remaja Putri umur 10 – 19 Tahun menurut Provinsi, Riskesdas 2018 494
Tabel 16.1.13 Proporsi Alasan Utama Tidak Minum/Menghabiskan TTD yang Diperoleh dari Sekolah pada Remaja Putri umur 10 – 19 Tahun menurut Karakteristik, Riskesdas 2018 495
Tabel 16.1.14 Proporsi Alasan Utama Tidak Minum/Menghabiskan TTD yang Diperoleh dengan Inisiatif Sendiri pada Remaja Putri Umur 10-19 Tahun menurut Provinsi, Riskesdas 2018 496
Tabel 16.1.15 Proporsi Alasan Utama Tidak Minum/Menghabiskan TTD yang Diperoleh dengan Inisiatif Sendiri pada Remaja Putri Umur 10-19 Tahun menurut Karakteristik, Riskesdas 2018 497
Tabel 16.2.1 Proporsi Ibu Hamil yang Mendapatkan PMT dan Rata-Rata Umur Kehamilan saat Pertama Mendapatkan PMT menurut Provinsi, Riskesdas 2018 501
Tabel 16.2.2 Proporsi Ibu Hamil yang Mendapatkan PMT dan Rata-Rata Umur Kehamilan saat Pertama Mendapatkan PMT menurut Karakteristik, Riskesdas 2018 502
Tabel 16.2.3 Proporsi Perolehan PMT Program pada Ibu Hamil dan Alasan Utama Tidak Menghabiskan menurut Provinsi, Riskesdas 2018 503
Tabel 16.2.4 Proporsi Perolehan PMT Program pada Ibu Hamil dan Alasan Utama Tidak Menghabiskan menurut Karakteristik, Indonesia 2018 504

Tabel 16.2.5 Proporsi Jenis Makanan Tambahan yang Diperoleh Ibu Hamil menurut Provinsi, Riskesdas 2018 505
Tabel 16.2.6 Proporsi Jenis Makanan Tambahan yang Diperoleh Ibu Hamil menurut Karakteristik, Riskesdas 2018 506
Tabel 16.2.7 Proporsi Alasan Ibu Hamil Memperoleh PMT menurut Provinsi, Riskesdas 2018 507
Tabel 16.2.8 Proporsi Alasan Ibu Hamil Memperoleh PMT menurut Karakteristik, Riskesdas 2018 508
Tabel 16.3.1 Proporsi Riwayat Tablet Tambah Darah (TTD) yang Diterima dan DikonsumsiSelama Kehamilan Anak Terakhir pada Perempuan umur 10-54 tahun menurut Provinsi, Riskesdas 2018 511
Tabel 16.3.2 Proporsi Riwayat Tablet Tambah Darah (TTD) yang Diterima dan DikonsumsiSelama Kehamilan Anak Terakhir pada Perempuan umur 10-54 tahun Menurut Karakteristik, Riskesdas 2018 512
Tabel 16.3.3 Proporsi Alasan Utama Riwayat Tidak Minum/Menghabiskan Tablet Tambah Darah (TTD) Selama Kehamilan Anak Terakhir pada Perempuan umur 10-54 tahun menurut Provinsi, Riskesdas 2018 513
Tabel 16.3.4 Proporsi Alasan Utama Riwayat Tidak Minum/Menghabiskan Tablet Tambah Darah (TTD) Selama Kehamilan Anak Terakhir pada Perempuan umur 10-54 tahun menurut Karakteristik, Riskesdas 2018 514
Tabel 16.3.5 Proporsi Ibu Hamil Memperoleh Tablet Tambah Darah (TTD) dan Jumlah yang Diperoleh menurut Provinsi, Riskesdas 2018 515
Tabel 16.3.6 Proporsi Ibu Hamil Memperoleh Tablet Tambah Darah (TTD) dan Jumlah yang Diperoleh menurut Karakteristik, Riskesdas 2018 516
Tabel 16.4.1 Nilai Rata-rata Lingkar Lengan Atas (LiLA) pada Wanita Usia Subur (WUS) umur 15 - 49 Tahun dan Wanita Hamil menurut Umur, Riskesdas 2018 517
Tabel 16.4.2 Prevalensi Kurang Energi Kronis (KEK) pada Wanita Hamil dan Wanita Tidak Hamil menurut Provinsi, Riskesdas 2018 518
Tabel 16.4.3 Prevalensi Kurang Energi Kronis (KEK) pada Wanita Hamil dan Wanita tidak Hamil menurut Karakteristik, Riskesdas 2018 519
Tabel 16.4.4 Prevalensi Tinggi Badan Berisiko Pada Wanita Hamil menurut Provinsi, Riskesdas 2018 520
Tabel 16.4.5 Prevalensi Tinggi Badan Berisiko Pada Wanita Hamil menurut Karakteristik, Riskesdas 2018 521
Tabel 16.4.6 Prevalensi Anemia pada Ibu Hamil menurut Karakteristik, Riskesdas 2018 522
Tabel 16.4.7 Prevalensi Anemia menurut Karakteristik, Riskesdas 2018 523
Tabel 16.5.1 Proporsi Inisiasi Menyusu Dini (IMD) dan Lama IMD pada Anak Umur 0-23 Bulan menurut Provinsi, Riskesdas 2018 525

Tabel 16.5.2 Proporsi Inisiasi Menyusu Dini (IMD) dan Lama IMD pada Anak Umur 0-23 Bulan menurut Karakteristik, Riskesdas 2018 526
Tabel 16.5.3 Proporsi Waktu Mulai Menyusu pada Anak Umur 0-23 Bulan menurut Provinsi, Riskesdas 2018 527
Tabel 16.5.4 Proporsi Waktu Mulai Menyusu pada Anak Umur 0-23 Bulan menurut Karakteristik, Riskesdas 2018 528
Tabel 16.5.5 Proporsi Perilaku Ibu terhadap Kolostrum pada Anak Umur 0-23 Bulan menurut Provinsi, Riskesdas 2018 529
Tabel 16.5.6 Proporsi Pemberian Makanan Prelakteal pada Bayi Umur 0-11 Bulan menurut Provinsi, Riskesdas 2018 530
Tabel 16.5.7 Proporsi Pemberian Makanan Prelakteal pada Bayi Umur 0-11 Bulan menurut Karakteristik, Riskesdas 2018 531
Tabel 16.5.8 Proporsi Pernah Disusui dan Masih Disusui pada Anak Umur 0-23 Bulan menurut Provinsi, Riskesdas 2018 533
Tabel 16.5.9 Proporsi Pernah Disusui dan Masih Disusui pada Anak Umur 0-23 Bulan menurut Karakteristik, Riskesdas 2018 534
Tabel 16.5.10 Proporsi Alasan Anak Umur 0-23 Bulan Belum/Tidak Pernah Disusui menurut Provinsi, Riskesdas 2018 535
Tabel 16.5.11 Proporsi Alasan Anak Umur 0-23 Bulan Belum/Tidak Pernah Disusui menurut Karakteristik, Riskesdas 2018 536
Tabel 16.5.12 Proporsi Pemberian ASI Saja dalam 24 Jam Terakhir pada Bayi Umur 0-5 Bulan menurut Kelompok Umur, Riskesdas 2018 538
Tabel 16.5.13 Proporsi Pemberian ASI Saja dalam 24 Jam Terakhir pada Bayi Umur 0-5 bulan menurut Karakteristik, Riskesdas 2018 538
Tabel 16.5.14 Proporsi Makanan Beragam yang Dikonsumsi Anak Umur 6-23 Bulan menurut Provinsi, Riskesdas 2018 539
Tabel 16.5.15 Proporsi Makanan Beragam yang Dikonsumsi Anak Umur 6-23 Bulan menurut Karakteristik, Riskesdas 2018 540
Tabel 16.6.1 Proporsi Anak Umur 6-59 Bulan Memperoleh PMT dan PMT Program menurut Provinsi, Riskesdas 2018 543
Tabel 16.6.2 Proporsi Anak Umur 6-59 Bulan Memperoleh PMT dan PMT Program menurut Karakteristik, Riskesdas 2018 544
Tabel 16.6.3 Proporsi Jumlah PMT Program yang Diperoleh dan Alasan Tidak Menghabiskan pada Anak Umur 6-59 Bulan menurut Provinsi, Riskesdas 2018 545
Tabel 16.6.4 Proporsi Jenis Makanan Tambahan yang Dikonsumsi Anak umur 6-59 bulan 546
Tabel 16.6.5 Proporsi Jenis Makanan Tambahan yang Dikonsumsi Anak umur 6-59 bulan 547

Tabel 16.6.6 Proporsi Alasan Anak Umur 6-59 Bulan Memperoleh PMT menurut Provinsi, Riskesdas 2018 548
Tabel 16.6.7 Proporsi Alasan Anak Umur 6-59 Bulan Memperoleh PMT menurut Karakteristik, Riskesdas 2018 549
Tabel 16.7.1 Prevalensi Status Gizi (BB/U) pada Anak Umur 0-23 Bulan (Baduta) menurut Provinsi, Riskesdas 2018 552
Tabel 16.7.2 Prevalensi Status Gizi (BB/U) pada Anak Umur 0-23 Bulan (Baduta) menurut Karakteristik, Riskesdas 2018 553
Tabel 16.7.3 Prevalensi Status Gizi (PB/U) pada Anak Umur 0-23 Bulan (Baduta) menurut Provinsi, Riskesdas 2018 554
Tabel 16.7.4 Prevalensi Status Gizi (PB/U) pada Anak Umur 0-23 Bulan (Baduta) menurut Karakteristik, Riskesdas 2018 555
Tabel 16.7.5 Prevalensi Status Gizi (BB/PB) pada Anak Umur 0-23 Bulan (Baduta) menurut Provinsi, Riskesdas 2018 556
Tabel 16.7.6 Prevalensi Status Gizi (BB/PB) pada Anak Umur 0-23 Bulan (Baduta) menurut Karakteristik, Riskesdas 2018 557
Tabel 16.8.1 Prevalensi Status Gizi (BB/U) Pada Anak Umur 0-59 Bulan (Balita) menurut Provinsi, Riskesdas 2018 560
Tabel 16.8.2 Prevalensi Status Gizi (BB/U) Pada Anak Umur 0-59 Bulan (Balita) menurut Karakteristik, Riskesdas 2018 561
Tabel 16.8.3 Prevalensi Status Gizi (TB/U) pada Anak Umur 0-59 Bulan (Balita) menurut Provinsi, Riskesdas 2018 562
Tabel 16.8.4 Prevalensi Status Gizi (TB/U) pada Anak Umur 0-59 Bulan (Balita) menurut Karakteristik, Riskesdas 2018 563
Tabel 16.8.5 Prevalensi Status Gizi (BB/TB) pada Anak Umur 0-59 Bulan (Balita) menurut Provinsi, Riskesdas 2018 564
Tabel 16.8.6 Prevalensi Status Gizi (BB/TB) pada Anak Umur 0-59 Bulan (Balita) menurut Karakteristik, Riskesdas 2018 565
Tabel 16.9.1 Prevalensi Status Gizi (TB/U) pada Anak Umur 5 - 12 Tahun menurut Provinsi, Riskesdas 2018 569
Tabel 16.9.2 Prevalensi Status Gizi (TB/U) pada Anak Umur 5 - 12 Tahun menurut Karakteristik, ...570
Tabel 16.9.3 Prevalensi Status Gizi (IMT/U) Pada Anak Umur 5- 12 Tahun menurut Provinsi, Riskesdas 2018 571
Tabel 16.9.4 Prevalensi Status Gizi (IMT/U) pada Anak Umur 5- 12 Tahun menurut Karakteristik, ...572
Tabel 16.9.5 Prevalensi Status Gizi (TB/U) Pada Remaja Umur 13 - 15 Tahun menurut Provinsi, ...573
Tabel 16.9.6 Prevalensi Status Gizi (TB/U) pada Remaja Umur 13 - 15 Tahun menurut Karakteristik, Riskesdas 2018 574

Tabel 16.9.7 Prevalensi Status Gizi (IMT/U) pada Remaja Umur 13 - 15 Tahun menurut Karakteristik, Riskesdas 2018 574
Tabel 16.9.8 Prevalensi Status Gizi (IMT/U) pada Remaja Umur 13 - 15 Tahun menurut Provinsi, ...575
Tabel 16.9.9 Prevalensi Status Gizi (TB/U) pada Remaja Umur 16 - 18 Tahun menurut Provinsi, ...576
Tabel 16.9.10 Prevalensi Status Gizi (TB/U) pada Remaja Umur 16 - 18 Tahun menurut Karakteristik, Riskesdas 2018 577
Tabel 16.9.11 Prevalensi Status Gizi (IMT/U) pada Remaja Umur 16 - 18 Tahun menurut Provinsi, ...578
Tabel 16.9.12 Prevalensi Status Gizi (IMT/U) pada Remaja Umur 16 - 18 Tahun menurut Karakteristik, ...579
Tabel 16.10.1 Prevalensi Status Gizi berdasarkan Kategori IMT pada Penduduk Dewasa (umur >18 Tahun) menurut Provinsi, Riskesdas 2018 582
Tabel 16.10.2 Prevalensi Status Gizi berdasarkan Kategori IMT pada Penduduk Dewasa (umur >18 Tahun) menurut Karakteristik, Riskesdas 2018 583
Tabel 16.10.3 Prevalensi Status Gizi berdasarkan Kategori IMT pada Penduduk Laki-Laki Dewasa 584
Tabel 16.10.4 Prevalensi Status Gizi berdasarkan Kategori IMT pada Penduduk Laki-Laki Dewasa 585
Tabel 16.10.5 Prevalensi Status Gizi berdasarkan Kategori IMT pada Penduduk Perempuan Dewasa 586
Tabel 16.10.6 Prevalensi Status Gizi berdasarkan Kategori IMT pada Penduduk Perempuan Dewasa 587
Tabel 16.10.7 Prevalensi Obesitas Sentral pada Penduduk Umur ≥ 15 Tahun menurut Provinsi, ...588
Tabel 16.10.8 Prevalensi Obesitas Sentral pada Penduduk Umur ≥ 15 Tahun menurut karakteristik, Riskesdas 2018 589
Tabel L.1.1 Respon Rate Sampel Wawancara dan Pengukuran, Riskesdas 2018 591
Tabel L.1.2 Jumlah Sampel Hasil Wawancara, Riskesdas 2018 592
Tabel L.1.3 Respon Rate Sampel Pemeriksaan Darah menurut Provinsi, Riskesdas 2018 593
Tabel L.1.4 Respon Rate Sampel Pemeriksaan Gigi dan Mulut menurut Provinsi, Riskesdas 2018 594

DAFTAR GAMBAR

Gambar 2.1.1. Kerangka Konsep Riskesdas 2018 7

DAFTAR SINGKATAN

ACT	:	*Artemisinin Combination Treatment*
ADA	:	American Diabetes Association
ADL	:	*Activities of Daily Living*
AIDS	:	*Acquired Immunodeficiency Syndrome*
ANC	:	*Antenatal Care*
ANC K1 Ideal	:	*Antenatal Care* pada kunjungan kehamilan sejak diketahui atau sejak kurang lebih 6 minggu-12 minggu kehamilan (1-3 bulan)
APN	:	Asuhan Persalinan Normal
ART	:	Anggota Rumah Tangga
ASI	:	Air Susu Ibu
BAB	:	Buang Air Besar
Baduta	:	Bawah Dua Tahun
Balita	:	Bawah Lima Tahun
Battra	:	Pengobat Tradisional
BB	:	Berat Badan
BB/TB	:	Berat Badan menurut Tinggi Badan
BB/U	:	Berat Badan menurut Umur
BBL	:	Berat Badan Lahir
BBLR	:	Berat Badan Lahir Rendah
BCG	:	*Bacillus Calmette-Guerin*
CI	:	*Confidence Interval*
CPI	:	*Community Periodontal Index*
D	:	Diagnosis
D/O	:	Diagnosis /Obat
DAS	:	*Disability Assesment Schedule*
Dentulous	:	kondisi hilangnya gigi asli kurang dari 28 gigi pada individu,(*M-T*<28).
DM	:	*Diabetes melitus*
DMF-T	:	*Decay, missing, filling teeth* untuk gigi tetap
dmf-t	:	*Decay, missing, filling teeth* untuk gigi sulung
DPT-HB	:	Difteri, Pertusis, Tetanus, Hepatitis B
dt	:	*Decay teeth* / gigi sulung yang mengalami karies dan belum diobati atau ditambal (baik dengan tambalan sementara maupun dengan tambalan permanen)
DT	:	*Decay teeth*/ gigi tetap yang mengalami karies dan belum diobati atau ditambal (baik dengan tambalan sementara maupun dengan tambalan permanen)
Edentulous	:	kondisi hilangnya gigi asli lebih dari 28 gigi pada individu,(*M-T*>28)
ESO	:	Efek Samping Obat
Fasyankes	:	Fasilitas Pelayanan Kesehatan
FDC	:	*Fixed Dose Combination*
FT	:	*Filling teeth* gigi tetap yang telah dilakukan penumpatan atau ditambal karena karies

ft	:	*Filling teeth*/ gigi sulung yang telah dilakukan penumpatan atau ditambal karena karies
G	:	Gejala
g/dl	:	*gram /deciliter*
GDP	:	Glukosa Darah Puasa
GDPP	:	Glukosa Darah 2 Jam Pasca Pembebanan
GDPT	:	Glukosa Darah Puasa Terganggu
GDS	:	Glukosa Darah Sewaktu
Germas	:	Gerakan Masyarakat Hidup Sehat
GLP	:	*Good Laboratory Practice*
GPAQ	:	*Global Physical Activity Questionnaire*
Hattra	:	Penyehat Tradisional
Hb	:	*Hemoglobin*
HDL	:	*high-density lipoprotein*
Hib	:	*Hemophilus Influenza* tipe b
HIV	:	*Human Immunodeficiency Virus*
HK	:	Hipotiroid Kongenital
HPHT	:	Hari Pertama Haid Terkahir
ICD	:	*International Classification of Diseases*
ICF	:	*International Classification of Functioning*
IFCC	:	*International Federation of Clinical Chemistry*
IMD	:	Inisiasi Menyusu Dini
IMT	:	Indeks Massa Tubuh
IMT/U	:	Indeks Massa Tubuh menurut Umur
IPKM	:	Indeks Pembangunan Kesehatan Masyarakat
IPV	:	*Inactived Polio Vaccine*
ISPA	:	Infeksi Saluran Pernafasan Akut
ITP	:	*Idiopathic Thrombocytopenic Purpura*
IUD/AKDR	:	*Intra Uterin Device*/Alat Kontrasepsi Dalam Rahim
JNC VII	:	*Joint National Committee VII*
KB	:	Keluarga Berencana
KDT	:	Kombinasi Dosis Tetap
KEK	:	Kurang Energi Kronis
KF	:	Kunjungan Nifas
KIA	:	Kesehatan Ibu dan Anak
KIPI	:	Kejadian Ikutan Pasca Imunisasi
KK	:	Kepala Keluarga
KMS	:	Kartu Menuju Sehat
KN	:	Kunjungan Neonatal
KN PP KIPI	:	Komite Nasional Pengkajian dan Penaggulangan KIPI
LDL	:	*low-density lipoprotein*
LiLA	:	Lingkar Lengan Atas
LP	:	Lingkar Perut
LLINs	:	*Long Lasting Insecticide Nets*
LPCD	:	*Litre per Capita per Day*
MET	:	*Metabolic Equivalent Task*

MICS	:	*Multiple Indicator Cluster Survey*
MINI	:	*Mini International Neuropsychiatric Interview*
MPASI	:	Makanan Pendamping Air Susu Ibu
mt	:	*Missing teeth*/ gigi sulung yang dicabut karena karies atau masih berupa sisa akar
MT	:	*Missing teeth*/ gigi tetap yang dicabut karena karies atau masih berupa sisa akar
NA	:	*Not Available*
Nakes	:	Tenaga Kesehatan
Nakestrad	:	Tenaga Kesehatan Tradisional
NCEP-ATP III	:	*National Cholesterol Education Program—Adult Treatment Panel III*
OAD	:	Obat Anti Diabetes Melitus
OAT	:	Obat Anti Tuberkulosis
PB	:	Panjang Badan
PBL	:	Panjang Badan Lahir
PCA	:	Principal Component Analysis
PD3I	:	Penyakit yang Dapat Dicegah Dengan Imunisasi
PERKENI	:	Perkumpulan Endokrinologi Indonesia
Pf	:	*Plasmodium falciparum*
Pm	:	*Plasmodium malariae*
PMBA	:	Program Pemberian Makan Bayi dan Anak
PMO	:	Pengawas Minum Obat
PMT	:	Pemberian Makanan Tambahan
Po	:	*Plasmpdium ovale*
POCT	:	*point of care test*
Polindes	:	Pondok Bersalin Desa
POPM	:	Pemberian Obat Pencegahan Massal
Poskesdes	:	Pos Kesehatan Desa
Posyandu	:	Pos Pelayanan Terpadu
PPI	:	Program Pengembangan Imunisasi
Puskesmas	:	Pusat Kesehatan Masyarakat
Pusling	:	Puskesmas Keliling
Pustu	:	Puskesmas Pembantu
Pv	:	*Plasmodium vivax*
RCI	:	*Root Caries Index*
RD	:	*Root decay* / gigi dengan karies akar
RDT	:	*Rapid Diagnostic Test*
Renstra	:	Rencana Strategis
RF	:	*Root filling* / gigi dengan karies akar yang sudah ditumpat atau ditambal
RN	:	*Root* Normal, akar yang terbuka namun tidak berkaries
RSV	:	*Respiratory Syncytial Virus*
Ruta	:	Rumah Tangga
SD	:	Standar Deviasi
SDG's	:	*Sustainable Development Goals*
SHK	:	Skrining Hipotiroid Kongenital

SRQ-20	:	*Self Reporting Questionnaire* 20 butir pertanyaan
TB	:	Tuberkulosis
TB	:	Tinggi Badan
TB/U	:	Tinggi Badan menurut Umur
TCM	:	Tes Cepat Molekuler
TGT	:	Toleransi Glukosa Terganggu
TOGA	:	Taman Obat Keluarga
TT	:	*Tetanus Toxoid*
TTD	:	Tablet Tambah Darah
WHO	:	World Health Organization
WUS	:	Wanita Usia Subur
Yankestrad	:	Pelayanan Kesehatan Tradisional

KATA PENGANTAR

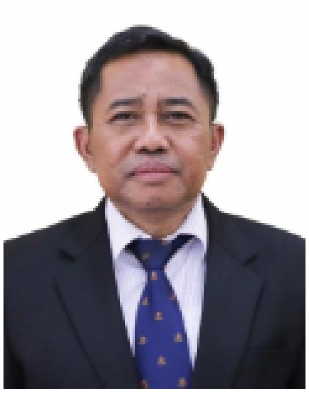

Salam Sehat!

Puji syukur kami panjatkan kepada Allah SWT, berkat rahmat dan karunia-Nya,kegiatan Riset Kesehatan Dasar (Riskesdas) 2018 dapat kami selesaikan. Sebagai riset berkala skala nasional guna masukan kebijakan dan program, Riskesdas menggunakan kerangka konsep HL Blum, dengan melihat determinan status kesehatan, pelayanan kesehatan, perilaku, lingkungan dan biomedis. Dalam buku hasil Riskesdas 2018 ini dapat dilihat capaian indikator *Sustainable Development Goals* (SDGs) dan Rencana Pembangunan Jangka Menengah Nasional (RPJMN) bidang kesehatan, yang merepresentasikan data tingkat nasional dan provinsi.

Proses pengumpulan data sampaikanlah kami menyampaikan penghargaan serta terima kasih untuk seluruh tim yang penuh dedikasi, baik peneliti, litkayasa, dan staf Balitbangkes, mitra kerja dari BPS, para dosen dari Perguruan Tinggi, mitra kerja dari Dinas Kesehatan Provinsi dan Kabupaten/Kota, seluruh enumerator dan masyarakat, yang telah berpartisipasi sebagai responden, serta semua pihak yang telah berpartisipasi menyukseskan Riskesdas tahun 2018. Simpati mendalam diserta idoa, kami haturkan kepada mereka yang mengalami musibah sewaktu melaksanakan Riskesdas tahun 2018.

Secara khusus, ucapan terima kasih kepada Menteri Kesehatan yang telah memberi kepercayaan kepada Badan Litbangkes dalam menunjukkan karya baktinya.Kami menyadari masih banyak kekurangan pada setiap tahapan pelaksanaan Riskesdas, oleh karena itu kami menerima kritik yang bersifat membangun untuk menyempurnakan.

Semoga fakta yang disajikan dalam buku ini, bermanfaat untuk perbaikan perencanaan pembangunan kesehatan.

Jakarta, Desember 2018
Kepala Badan Penelitian dan Pengembangan Kesehatan
Kementerian Kesehatan RI

dr. Siswanto, MHP., DTM

MENTERI KESEHATAN
REPUBLIK INDONESIA

Sambutan

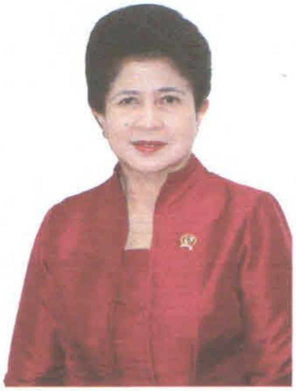

Assalamu'alaikum Wr. Wb

Berkat rahmat dan karunia Allah SWT, Kementerian Kesehatan telah menyelesaikan kegiatan Riset Kesehatan Dasar (Riskesdas) 2018 yang menghasilkan data dasar kesehatan berbasis komunitas. Data tersebut dapat memberikan gambaran capaian indikator kesehatan untuk tingkat nasional, provinsi, dan kabupaten/kota. Riskesdas yang dilaksanakan secara berkala, dapat menjadi data dasar perencanaan, perumusan kebijakan, dan intervensi yang lebih terarah, efektif dan efisien. Hasil Riskesdas juga dapat dimanfaatkan untuk menghitung Indeks Pembangunan Kesehatan Masyarakat, guna mengetahui perubahan pencapaian sasaran pembangunan kesehatan di setiap wilayah, dari tingkat kabupaten/kota, provinsi maupun nasional.

Pelaksanaan Riskesdas Tahun 2018 terintegrasi dengan Survey Sosial Ekonomi Nasional (Susenas) Maret 2018. Terintegrasi dalam kerangka sampel dan kuesioner, sehingga data yang dihasilkan dapat saling melengkapi, dan juga dalam kerangka membangun pendekatan "*one data*". Riskesdas terintegrasi dengan Susenas ini diharapkan mampu menghasilkan informasi yang lengkap, meliputi status kesehatan dengan didukung hasil pengukuran dan pemeriksaan, akses pelayanan kesehatan, perilaku sehat, kesehatan lingkungan serta sanitasi.

Informasi yang bersumber dari Riskesdas ini sangat berharga untuk dimanfaatkan dalam perumusan kebijakan dan program kesehatan yang komprehensif, sebagai upaya mempercepat peningkatan derajat kesehatan masyarakat, guna menciptakan sumber daya manusia yang berkualitas. Perencanaan berbasis bukti untuk tingkat nasional, provinsi, dan kabupaten/kota akan semakin tajam bila tersedia data yang akurat, terkini, dan valid secara ilmiah. Oleh karena itu saya menghimbau agar data hasil Riskesdas ini dapat dimanfaatkan oleh Pemerintah Pusat (penyusunan RPJMN bidang kesehatan dan Renstra Kesehatan) dan Pemerintah Daerah baik provinsi maupun kabupaten/kota (dalam penyusunan RPJMD dan Renstrada bidang kesehatan).

Saya menyampaikan ucapan selamat dan penghargaan yang tinggi kepada Badan Litbangkes dan BPS yang telah bersinergi mulai dari perencanaan, pelaksanaan hingga penyajian hasil Riskesdas ini. Ucapan terimakasih untuk para peneliti, pelatih, para enumerator, para penanggung jawab teknis, para penanggung jawab operasional, para pakar serta semua yang terlibat dalam Riskesdas yang berasal dari Dinas Kesehatan Provinsi dan Kabupaten/Kota, perguruan tinggi, serta tidak lupa jajaran kesehatan di lapangan (Puskesmas).

Jl. H.R. Rasuna Said Blok X5, Kav. 4-9 Jakarta 12950 Telpon/Faxsimile (021) 5201591

Saya juga mengundang para pakar baik dari perguruan tinggi, lintas sektor, pemerhati kesehatan dan semua pihak untuk memanfaatkan dan memberi masukan, serta mengkaji data hasil Riskesdas untuk dianalisis dan dimanfaatkan lebih tajam dan dan analitik, guna perbaikan pembangunan kesehatan di Indonesia.

Khusus untuk para peneliti Balitbangkes, teruslah berkarya dengan tetap menjunjung tinggi etika dan kaidah ilmiah. Tidak bosan mencari terobosan riset dalam lingkup kesehatan masyarakat, kedokteran klinis, maupun biomedis yang bersifat *translating research into policy*.

Semoga Allah SWT selalu memberi petunjuk dan kemudahan buat kita semua.

Wassalamu'alaikum Wr. Wb.

Desember 2018
Menteri Kesehatan,

Prof. Dr. dr. Nila Farid Moeloek, Sp.M (K)

BAB 1
PENDAHULUAN

1.1 Latar Belakang

Badan Penelitian dan Pengembangan Kesehatan (Badan Litbangkes) merupakan salah satu unit eselon 1 di Kementerian Kesehatan yang mempunyai visi sebagai pengawal kebijakan dan legitimator program pembangunan kesehatan berbasis bukti. Visi tersebut memberikan gambaran bahwa melalui kegiatan penelitian, Badan Litbangkes mempunyai peran juga dalam menata arah kebijakan, program, dan kegiatan pembangunan kesehatan. Salah satu misi Badan Litbangkes adalah menghasilkan rekomendasi untuk pembangunan kesehatan dengan menyusun arah pembangunan kesehatan berdasarkan data yang berkualitas.

Badan Litbangkes setiap lima tahun sekali melakukan pengumpulan data berbasis komunitas di seluruh Indonesia, dengan tujuan menilai capaian hasil pembangunan kesehatan dalam kurun waktu lima tahun terakhir. Riset Kesehatan Dasar (Riskesdas) merupakan penelitian bidang kesehatan berbasis komunitas yang indikatornya dapat menggambarkan tingkat nasional sampai dengan tingkat kabupaten/kota. Pelaksanaan lima tahun sekali dianggap interval yang tepat untuk menilai perkembangan status kesehatan masyarakat, faktor risiko, dan perkembangan upaya pembangunan kesehatan.

Hasil Riskesdas 2007 dan 2013 telah dimanfaatkan oleh pelaksana program Kementerian Kesehatan, termasuk pengembangan rencana kebijakan pembangunan kesehatan jangka menengah (RPJMN 2010-2014 dan RPJMN 2015-2019) oleh Bappenas, dan beberapa kabupaten/kota menggunakan data Riskesdas untuk perencanaan, pemantauan, dan mengevaluasi program-program kesehatan dengan berbasis bukti (*evidence-based planning*). Komposit beberapa indikator Riskesdas 2007 dan Riskesdas 2013 juga telah digunakan menyusun Indeks Pembangunan Kesehatan Masyarakat (IPKM) di Indonesia. Nilai IPKM menghasilkan gambaran peringkat Kabupaten/Kota dalam bidang kesehatan, sehingga dapat melihat disparitas pembangunan kesehatan yang terjadi di Indonesia.

Pemilihan indikator dalam Riskesdas 2018, dilakukan dengan mempertimbangkan *Sustainable Development Goals* (*SDGs*), RPJMN, Rencana Strategis (Renstra), Standar Pelayanan Minimal (SPM), Indeks Pembangunan Kesehatan Masyarakat (IPKM), Program Indonesia Sehat – Pendekatan Keluarga (PIS-PK), dan Gerakan Masyarakat Hidup Sehat (Germas), serta masukan berbagai pihak. Pelaksanaan Riskesdas 2018 terintegrasi dengan Susenas Maret 2018 yang dilaksanakan oleh Badan Pusat Statistik (BPS) dalam hal metode dan kerangka sampel.

Proses mengumpulkan data spesifik kesehatan yang dilaksanakan oleh tenaga pengumpul data berlatar belakang pendidikan minimal Diploma3 kesehatan. Metode pengumpulan data dilakukan melalui wawancara, pengukuran, dan pemeriksaan. Indikator kesehatan utama yang diukur dalam Riskesdas 2018 antara lain morbiditas (Penyakit Tidak Menular dan Penyakit Menular), disabilitas, cedera, kesehatan lingkungan (higienis, sanitasi, jamban, air dan perumahan), pengetahuan dan sikap terhadap HIV, perilaku kesehatan (pencarian pengobatan, penggunaan tembakau, minum alkohol, aktivitas fisik, perilaku konsumsi makanan berisiko), berbagai aspek mengenai pelayanan kesehatan (akses dan cakupan kesehatan) dan status gizi, serta status kesehatan gigi dan mulut.

Persiapan Riskesdas 2018 dilakukan mulai tahun 2017, dan pengumpulan data dilaksanakan Tahun 2018 yang diintegrasikan dengan pelaksanaan Susenas Maret 2018 oleh BPS. Integrasi ini untuk mendukung kebijakan *one data* yang akan menghasilkan informasi lengkap terkait bidang kesehatan

Secara umum laporan ini memberikan gambaran kesehatan di Indonesia mengenai indikator yang terkait dengan status kesehatan, pelayanan kesehatan, perilaku kesehatan, dan kesehatan lingkungan.

1.2 Manfaat dan Luaran Riskesdas 2018

A. Manfaat Penelitian

1. Pemegang Program
   a. Pusat

i. Evaluasi capaian program yang telah dijalankan
ii. Informasi dasar dalam menyusun kebijakan strategis
iii. Menyusun perencanaan dan target capaian berbasis data

b. Provinsi
i. Informasi dasar dalam menyusun kebijakan ditingkat provinsi
ii. Dasar evaluasi dan pengembangan program ditingkat provinsi
iii. Menyusun perencanaan dan target capaian berbasis data di tingkat provinsi

c. Kabupaten/Kota
i. Informasi dasar dalam menyusun kebijakan ditingkat kab/kota
ii. Dasar evaluasi dan pengembangan program ditingkat kab/kota
iii. Menyusun perencanaan dan target capaian berbasis data di tingkat kab/kota

2. Badan Penelitian dan Pengembangan Kesehatan
i. Sebagai dasar penyusun Indeks Pembangunan Kesehatan Masyarakat
ii. Sebagai dasar penentuan masalah yang akan diteliti lebih mendalam

3. Akademisi/Institusi Pendidikan

Mendukung menghasilkan sumber daya kesehatan melalui pemanfaatan data oleh institusi pendidikan

B. Luaran Penelitian

Tersedianya data kesehatan berdasarkan karakteristik masyarakat sebagai berikut:

a. Status kesehatan: prevalensi penyakit menular, penyakit tidak menular, penyakit jiwa, cacat bawaan, cedera, status disabilitas, gigi dan mulut, kesehatan reproduksi, kesehatan bayi dan balita, status gizi, hematologi dan kimia darah.
b. Pengetahuan dan perilaku kesehatan: pengetahuan komprehensif dan stigma HIV/AIDS, perilaku higienis, penggunaan tembakau, alkohol, frekuensi makan makanan berisiko, aktivitas fisik, konsumsi buah-sayur,

perilaku penggunaan obat-obat, penggunaan tembakau dan minuman beralkohol.

c. Status sanitasi lingkungan perumahan
d. Upaya pelayanan kesehatan: akses dan pelayanan kesehatan, cakupan pelayanan kesehatan ibu dan anak.

1.3 Tujuan Riskesdas

Tujuan Umum:

Menyediakan informasi derajat kesehatan yang telah dicapai selama kurun waktu 5 tahun terakhir dan informasi besaran masalah faktor risiko terkait derajat kesehatan yang diukur, sebagai bahan pertimbangan dalam merumuskan kebijakan pembangunan kesehatan di Indonesia.

Tujuan Khusus:

1. Menyediakan informasi terkait indikator morbiditas, disabilitas, dan status gizi yang dicapai dari hasil pelaksanaan program selama kurun waktu 5 tahun terakhir pada tingkat nasional, provinsi, dan kabupaten/kota dalam 35 buku laporan yang berbeda.
2. Menyediakan informasi besaran masalah berdasarkan faktor risiko dari indikator morbiditas, disabilitas, dan status gizi pada tingkat nasional, provinsi, kabupaten/kota dalam 35 buku laporan yang berbeda.
3. Memberikan gambaran permasalahan morbiditas dan faktor risiko pada tingkat nasional berdasarkan hasil pengukuran, pemeriksaan darah, serta pemeriksaan gigi dan mulut.

1.4 Pertanyaan Penelitian

1. Bagaimana pencapaian status kesehatan masyarakat pada tingkat nasional, provinsi, dan kabupaten/kota?
2. Bagaimana gambaran karakteristik status kesehatan masyarakat pada tingkat nasional, provinsi dan kabupaten/kota?
3. Bagaimana gambaran faktor risiko yang terkait dengan status kesehatan masyarakat pada tingkat nasional, provinsi dan kabupaten/kota?

1.5 Ruang Lingkup

Riskesdas merupakan penelitian yang dikumpulkan pada tingkat masyarakat. Sebagian besar indikator yang dikumpulkan dapat menggambarkan tingkat kabupaten/kota. Indikator yang dikumpulkan melalui pemeriksaan darah serta pemeriksaan gigi dan mulut, hanya dapat menggambarkan tingkat nasional.

BAB 2
METODE

2.1 Desain

Riskesdas merupakan survei berskala nasional dengan desain potong lintang (*cross-sectional*) dan non-intervensi.

2.2 Kerangka Konsep

Kerangka konsep mengacu pada paradigma kesehatan menurut HL Blum yang menggambarkan keterkaitan antara status kesehatan dan faktor risikonya. Kerangka konsep yang telah menyesuaikan dengan indikator yang dikumpulkan dapat dilihat pada gambar 2.2.1.

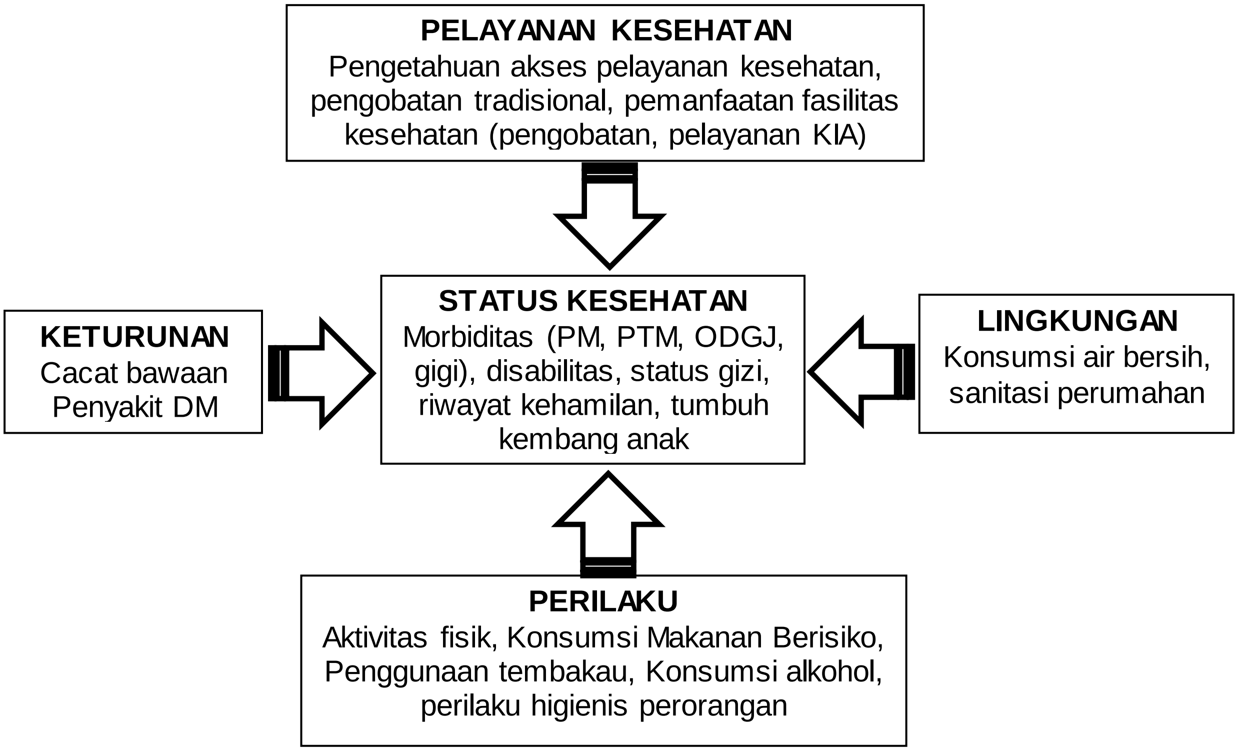

Gambar 2.2.1. Kerangka Konsep Riskesdas 2018 hasil modifikasi dari kerangka teori H.L Blum

2.3 Populasi dan Sampel

Populasi adalah seluruh rumah tangga di Indonesia. Sampel Riskesdas 2018 menggunakan kerangka sampel Susenas 2018 yang dilaksanakan pada bulan Maret 2018.

Target sampel yang dikunjungi 300.000 rumah tangga dari 30.000 Blok Sensus (BS) Susenas yang dilakukan oleh Badan Pusat Statistik (BPS) dengan metode PPS (*probability proportional to size*) menggunakan *linear systematic sampling,* dengan *Two Stage Sampling:*

Tahap 1: Melakukan *implicit stratification* seluruh Blok Sensus (BS) hasil Sensus Penduduk (SP) 2010 berdasarkan strata kesejahteraan. Dari *master frame* 720.000 BS hasil SP 2010 dipilih 180.000 BS (25%) secara PPS untuk menjadi *sampling frame* pemilihan BS. Memilih sejumlah n BS dengan metode PPS di setiap strata urban/rural per Kabupaten/Kota secara ***systematic*** sehingga menghasilkan Daftar Sampel Blok Sensus (DSBS). Jumlah total BS yang dipilih adalah 30.000 BS.

Tahap 2: Memilih 10 rumah tangga di setiap BS hasil pemutakhiran secara *systematic sampling* dengan *implicit stratification* pendidikan tertinggi yang ditamatkan KRT (Kepala Rumah Tangga), untuk menjaga keterwakilan dari nilai keragaman karakteristik rumah tangga.

Individu yang menjadi sampel Riskesdas untuk diwawancarai adalah semua anggota rumah tangga (ART) dalam rumah tangga terpilih. Berbeda dengan individu yang menjadi sampel pemeriksaan adalah sub sampel dengan tingkat keterwakilan nasional. Kriteria sampel masing-masing pemeriksaan:

1. Kadar hemoglobin dilakukan pada responden semua umur.
2. RDT malaria dilakukan pada responden semua umur.
3. Glukosa darah pada responden umur ≥ 15 tahun.
4. Kimia klinis (profil lipid dan kreatinin) pada responden umur ≥ 15 tahun.
5. Mikroskopis malaria dilakukan pada responden semua umur dengan kriteria riwayat demam dalam 2 hari terakhir dan/atau hasil RDT malaria positif.
6. Kesehatan gigi dan mulut pada responden umur ≥ 3 tahun.

Pada Riskesdas 2018, subsampel dengan keterwakilan tingkat nasional (pemeriksaan darah serta pemeriksaan gigi dan mulut) telah ditetapkan sebesar 2.500 BS pada 26 provinsi. Provinsi terpilih dan alokasi jumlah BS serta jumlah rumah tangga disetiap provinsi dapat dilihat pada lampiran 1.

2.4 Penjamin Mutu Data Riskesdas 2018

Kegiatan untuk menjaga kualitas hasil survei yaitu:

1. Penentuan indikator dilaksanakan bersama pemegang program Kemenkes, Bapenas, dan BPS. Indikator tersebut dituangkan menjadi pertanyaan yang disusun dalam instrumen bersama pakar bidang kesehatan (organisasi profesi, perguruan tinggi, dan peneliti senior Badan Litbangkes), serta mendapatkan masukan dari organisasi internasional (WHO, UNICEF, dan World Bank).
2. Melaksanakan uji coba untuk mendapatkan ketepatan 3 hal berikut:
   a. Alur pertanyaan
   b. Materi pertanyaan
   c. Mekanisme pelaksanaan di masyarakat
3. Menyelenggarakan seleksi terbuka untuk pelatih utama dan pelatih nasional melalui sistem online dan wawancara. Materi yang diujikan meliputi pengetahuan umum tentang kesehatan, *psikotest*, dan kemampuan pengoperasian komputer.
4. Menyelenggarakan pelatihan secara berjenjang. Pelatih utama dilatih oleh tim inti Riskedas yang diharapkan dapat membantu melatih pelatih nasional. Pelatih utama dan tim inti Riskesdas melatih Penanggung Jawab Teknis (PJT) provinsi, beberapa Penanggung Jawab Teknis (PJT) Kab/Kota (yang memenuhi syarat sebagai pelatih), serta pelatih nasional yang lulus seleksi untuk dapat menyamakan persepsi dalam melatih enumerator.
5. Melakukan supervisi teknis maupun manajemen pelaksanaan. Supervisi dilakukan oleh PJT Provinsi, penanggung jawab operasional (PJO), maupun tim teknis untuk melihat permasalahan yang ditemukan saat pengumpulan data.
6. Validasi eksternal bersifat independen dilakukan oleh Asosiasi Peneliti Kesehatan Indonesia (APKESI).
7. Kualitas pengumpulan sangat dipengaruhi faktor kemampuan dan integritas enumerator (tenaga pengumpul data), oleh karena itu diberikan syarat latar belakang :

a. Minimal D3 bidang kesehatan bagi tenaga pewawancara
b. Dokter Gigi bagi pemeriksa gigi dan mulut
c. Analis/perawat untuk pengambil sampel darah

8. Melakukan kalibrasi alat yang digunakan untuk melakukan pengukuran dan pemeriksaan.
9. Proses manajemen data dimulai dari pengiriman sampai dengan analisis data meliputi:
   a. *Entry* data dilakukan langsung oleh enumerator saat berada di lokasi penelitian dan dikoreksi oleh PJT Kabupaten/Kota
   b. Pengiriman data oleh PJT Kabupaten/Kota kepada penanggungjawab manajemen data provinsi
   c. Umpan balik terkait kelengkapan dan duplikasi data, langsung diberikan oleh penanggungjawab manajemen data provinsi kepada enumerator yang ada di lokasi penelitian
   d. Pengendalian inkonsistensi data (*cleaning data*) oleh tim pengendali data
   e. Pengendalian kekuatan data untuk dilakukan disagregasi dan inkonsistensi analisis dengan menggunakan beberapa metode analisis
10. Pembahasan *output* analisis dalam penulisan laporan dilakukan bersama antara Tim Teknis, Tim Pakar, dan Penanggung Jawab Laporan Provinsi

2.5 Indikator

Pemilihan indikator berdasarkan: (1) SDGs; (2) RPJMN; (3) Renstra; (4) SPM; (5) IPKM; (6) PIS-PK; (7) Germas. Indikator-indikator utama yang diukur berkaitan dengan:

1. Akses pelayanan kesehatan
2. Pelayanan Kesehatan Tradisional
3. Kesehatan dan Gangguan Jiwa
4. Kesehatan Lingkungan
5. Penyakit Menular
6. Penyakit Tidak Menular
7. Kesehatan Gigi Mulut

8. Disabilitas dan Cedera
9. Perilaku
10. Kesehatan Ibu dan Reproduksi
11. Gizi
12. Kesehatan anak

2.6 Pengumpulan Data

Pengumpulan data dilakukan oleh enumerator setempat dengan pengawasan teknis oleh PJT Kabupaten/kota dan pengawasan administratif oleh PJO Kabupaten/kota. Dalam pengumpulan data 1 tim bertanggungjawab terhadap 11 hingga 12 BS. 1 BS terdiri dari 10 Rumah Tangga (Ruta) sehingga 1 tim bertanggung jawab terhadap 110 hingga 120 Ruta.

Pengumpulan data dimulai dengan PJT Kabupaten/Kota mengambil salinan blok I-IV dari kuesioner Susenas di BPS Kab/Kota. Enumerator, PJT kabupaten, dan PJO kabupaten melakukan identifikasi lokasi sampel. Berdasarkan identifikasi tersebut diharapkan enumerator mendapatkan gambaran lokasi sampel sehingga dapat disusun rencana jadwal pengumpulan data, dan strategi pengumpulan data yang akan dilakukan agar efisien dan efektif.

Pengumpulan data Riskesdas 2018 dilakukan dengan wawancara, pengukuran, dan pemeriksaan. Wawancara menggunakan 2 instrumen yaitu: Instrumen Rumah Tangga dan Instrumen Individu.

Instrumen Rumah Tangga (lampiran 2) terdiri dari 7 blok dengan rincian sebagai berikut:

1. Blok I: Pengenalan tempat
2. Blok II: Keterangan pengumpul data
3. Blok III: Keterangan Rumah Tangga
4. Blok IV: Keterangan Anggota Rumah Tangga
    - Satus pendidikan terakhir hanya ditanyakan kepada ART umur >5 tahun.
    - Status pekerjaan hanya ditanyakan kepada ART umur ≥10 tahun.
5. Blok V: Akses pelayanan kesehatan

6. Blok VI: Gangguang Jiwa Berat
7. Blok VII: Kesehatan lingkungkungan

Instrumen Individu (lampiran 3) terdiri dari 2 blok dengan rincian sebagai berikut:

1. Blok IX Keterangan wawancara individu
2. Blok X Keterangan individu
   a. Blok A Penyakit menular
   b. Blok B Penyakit tidak menular
   c. Blok C Kesehatan Jiwa
   d. Blok D Disabilitas
   e. Blok E Cedera
   f. Blok F Pelayanan kesehatan tradisional
   g. Blok G Perilaku
   h. Blok H Pengetahuan dan sikap terhadap HIV/AIDS
   i. Blok I Pemberian tablet tambah pada remaja putri
   j. Blok J Kesehatan Ibu
   k. Blok K Kesehatan Balita
   l. Blok L Pengukuran dan pemeriksaan

Pengukuran antropometri dilakukan dengan menggunakan timbangan berat badan digital (tingkat ketelitian 0,1 kg), alat ukur tinggi/panjang badan (tingkat ketelitian 1 mm), dan alat ukur LILA (tingkat ketelitian 1 mm). Pengukuran tekanan darah menggunakan alat tensimeter digital.

Pemeriksaan darah dilakukan di lokasi penelitian dan laboratorium. Pemeriksaan yang dilakukan di lokasi penelitian adalah:

1. Pemeriksaan kadar hemoglobin darah berdasarkan panjang gelombang fotometri, dilakukan secara cepat menggunakan alat Hemocue.
2. Pemeriksaan gula darah (puasa dan 2 jam setelah pembebanan, atau sewaktu) berdasarkan reaksi enzimatik perubahan glukosa menjadi

gluconolactone yang dapat dideteksi melalui arus listrik pada alat Accuchek Performa.

3. Pemeriksaan RDT malaria berdasarkan reaksi antigen-antibodi, menggunakan kit komersial.

Pemeriksaan yang dilakukan di Laboratorium Nasional Badan Litbangkes adalah:

1. Pemeriksaan kimia klinis dilakukan secara automatis menggunakan prinsip enzimatik dan berbeda dengan metode *Jaffe-Picrate*. Pemeriksaan kadar kreatinin serum sudah mempertimbangkan metode penghitungan estimasi laju filtrasi glomerulus sehingga hasil yang keluar dapat memberikan gambaran umum terkait fungsi ginjal.
2. Pemeriksaan malaria dengan sediaan apus tebal dilakukan dibawah mikroskop dengan pembesaran 10x100 menggunakan minyak immersi. Pembacaan dilakukan pada seluruh lapangan pandang, sedangkan penentuan spesies dan kepadatan parasit dihitung dalam minimal 200 leukosit.

Pemeriksaan gigi dan mulut dilakukan di lokasi penelitian. Pemeriksaan tersebut dilakukan oleh dokter gigi yang telah dilatih sesuai standar panduan WHO dengan menggunakan formulir dan alat sesuai standar WHO. Pemeriksaan meliputi kelainan pada mahkota gigi, akar gigi, gusi, dan jaringan lunak lainnya pada mulut.

2.7 Manajemen Data

Selain pengumpulan data, tahapan yang cukup penting dalam Riskesdas ini adalah manajemen data. Pemrosesan data dimulai dari edit kuesioner dan pemberian kode dilokasi penelitian yang dilakukan oleh enumerator.Kuesioner yang telah dilakukan edit dan pemberian kode dengan benar,dilanjutkan dengan memasukkan data ke dalam aplikasi yang sudah ditentukan. Setelah data dientri kemudian data dikirim melalui email ditujukan kepada tim manajemen data Badan

Litbangkes untuk dilakukan penggabungan data dan *cleaning* data. C*leaning* data memperhatikan data yang tidak konsisten dan data *outlier*. Data yang tidak konsisten dan *outlier* ditelusuri kembali ke kuesioner untuk melakukan cek kebenaran dari data yang dihasilkan. Dari data yang telah "bersih" (konsisten dan bebas dari *outlier*) diberi nilai penimbang oleh BPS.

Raw data yang sudah bersih dan diberi nilai penimbang merupakan data final yang dapat digunakan analisis. Analisis dapat menggunakan modifikasi data yaitu melakukan komposit beberapa variabel atau mengelompokkan jawaban dari pertanyaan tersebut. Komposit variabel digunakan untuk indikator pengetahuan akses pelayanan kesehatan.Indikator diukur melalui indeks yang dihitung dengan menggunakan *Principal Component Analysis (PCA)* yaitu salah satu teknik statistik yang menyatukan beberapa variable menjadi indicator tunggal. Metode *PCA* digunakan untuk menyederhanakan banyak variable menjadi satu dengan membuat skor variabel-variabel tersebut, skor variabel dibentuk berdasarkan kekuatan korelasi antara variabel. Indeks pengetahuan kemudahan akses pelayanan kesehatan pada Riskesdas 2018 menggunakan tiga jenis akses pelayanan kesehatan yang dihitung yaitu: (1) Akses ke fasilitas Rumah Sakit; (2) Akses ke fasilitas Puskesmas; (3) Akses ke fasilitas Klinik/Praktek Mandiri. Analisis data, sesuai dengan indikator yang direncanakan dan disajikan dalam bentuk tabulasi.

Penyajian hasil dalam laporan Riskesdas menggunakan dua istilah yaitu:

1. prevalensi digunakan untuk indikator yang datanya diperoleh melalui pemeriksaan fisik/laboratorium atau pengukuran atau hasil wawancara tetapi informasi yang diperoleh harus berdasarkan diagnosis dokter atau tenaga kesehatan lainnya
2. proporsi digunakan untuk indikator yang datanya diperoleh melalui hasil wawancara dan informasinya sesuai pengetahuan responden, seperti gejala yang dirasakan responden

BAB 3
AKSES FASILITAS KESEHATAN

3.1 Pengetahuan Akses ke Fasilitas Kesehatan

Indikator pengetahuan akses ke fasilitas kesehatan diukur dengan menggunakan beberapa pertanyaan ditingkat rumah tangga. Indikator dianalisis menggunakan metode *Principal Component Analysis (PCA)* yang dibangun dengan 3 dimensi, yaitu: (1) Jenis alat transportasi yang digunakan ke fasilitas kesehatan; (2) Waktu tempuh pulang pergi dari rumah ke fasilitas kesehatan dan (3) Biaya yang dikeluarkan untuk transportasi pulang pergi ke fasilitas kesehatan. PCA digunakan untuk menyederhanakan beberapa variable menjadi satu variabel yang memiliki makna.

Pada Riskesdas 2018 ini, ada 3 (tiga) jenis akses pelayanan kesehatan yang dihitung yaitu: (1) Akses ke fasilitas Rumah Sakit; (2) Akses ke fasilitas Puskesmas; (3) Akses ke fasilitas Klinik/Praktek Mandiri. Hasil analisis yang diperoleh sebagai berikut:

- Akses ke fasilitas Rumah Sakit, tiga dimensi memberikan penjelasan terhadap skoring indeks sebesar 51,99% dengan korelasi antara 0,18 hingga 0,40
- Akses ke fasilitas Puskesmas/Pustu/Pusling/Bidan Desa, tiga dimensi memberikan penjelasan terhadap skoring indeks sebesar 39,29% dengan korelasi antara 0,02 hingga 0,14
- Akses ke Klinik/Praktek Dokter/Praktek Dokter Gigi/Praktek Bidan Mandiri, tiga dimensi memberikan penjelasan 39,94% dengan korelasi antara 0,03 hingga 0,18.

Tahap selanjutnya, indeks akses ke fasilitas kesehatan yang terbentuk dibagi ke dalam tiga kategori, yaitu: (1) Mudah; (2) Sulit; (3) Sangat Sulit.

FORMULA

$$\text{Pengetahuan Kemudahan Menjangkau Rumah Sakit}$$

$$= \frac{\text{Pengetahuan RuTa terhadap Kemudahan Akses (jenis transportasi, waktu tempuh dan biaya transpor) ke Rumah Sakit}}{\text{Jumlah RuTa yang mengetahui keberadaan Rumah Sakit}}$$

$$\text{Pengetahuan Kemudahan Menjangkau Puskesmas, Pustu, pusling, bidan desa}$$

$$= \frac{\text{Pengetahuan RuTa terhadap Kemudahan Akses (jenis transportasi, waktu tempuh dan biaya transpor) ke Puskesmas/Pustu/Pusling/bidan desa}}{\text{Jumlah RuTa yang mengetahui keberadaan Puskesmas, Pustu, Pusling, Bidan desa}}$$

$$\text{Pengetahuan Kemudahan Menjangkau Klinik/Praktek Dokter/Praktek Dokter Gigi/Praktek Bidan Mandiri}$$

$$= \frac{\text{Pengetahuan RuTa terhadap Kemudahan Akses (jenis transportasi, waktu tempuh dan biaya transpor) ke Klinik/Praktek Dokter/Praktek Dokter Gigi/Praktek Bidan Mandiri}}{\text{Jumlah RuTa yang mengetahui keberadaan Klinik/Praktek Dokter/Praktek Dokter Gigi/Praktek Bidan Mandiri}}$$

Tabel 3.1.1
Proporsi Pengetahuan Rumah Tangga terhadap Kemudahan Akses ke Rumah Sakit
menurut Provinsi, Riskesdas 2018

Provinsi	Akses ke Rumah Sakit									N tertimbang
	Mudah			Sulit			Sangat Sulit			
	%	95% CI		%	95% CI		%	95% CI		
Aceh	29.8	27.9	31.7	41.8	39.6	44.1	28.4	26.3	30.5	4,876
Sumatera Utara	38.8	37.1	40.4	36.7	35.0	38.5	24.5	23.0	26.1	12,528
Sumatera Barat	35.1	32.9	37.4	38.1	35.8	40.4	26.8	24.7	29.0	4,601
Riau	36.6	34.3	38.9	28.0	25.6	30.5	35.4	32.9	38.0	5,901
Jambi	25.3	22.9	27.9	32.7	29.8	35.8	41.9	39.1	44.8	3,377
Sumatera Selatan	24.9	23.0	27.0	33.0	30.4	35.7	42.0	39.6	44.5	7,550
Bengkulu	33.3	30.8	35.9	38.7	35.9	41.7	28.0	25.3	30.8	1,909
Lampung	26.6	24.3	29.1	38.1	35.6	40.7	35.3	32.8	37.9	7,530
Bangka Belitung	46.5	43.3	49.7	30.7	27.4	34.3	22.8	19.8	26.1	1,408
Kepulauan Riau	59.3	54.7	63.7	25.5	21.4	30.1	15.2	12.4	18.6	2,148
DKI Jakarta	52.3	49.9	54.7	31.5	29.4	33.8	16.2	14.3	18.2	11,203
Jawa Barat	32.3	31.0	33.6	38.2	36.9	39.5	29.5	28.3	30.8	49,558
Jawa Tengah	40.9	39.8	42.1	41.3	40.1	42.5	17.8	16.8	18.8	35,293
DI Yogyakarta	70.6	68.0	73.2	21.8	19.7	24.0	7.6	6.2	9.3	4,258
JawaTimur	41.3	40.2	42.5	42.0	40.9	43.2	16.6	15.8	17.5	39,773
Banten	38.4	35.9	40.9	37.9	35.5	40.4	23.7	21.7	25.9	11,021
Bali	60.5	57.8	63.1	29.3	27.0	31.8	10.2	8.7	11.9	4,205
Nusa Tenggara Barat	27.3	24.9	29.7	46.3	43.5	49.2	26.4	24.0	29.0	5,041
Nusa Tenggara Timur	24.5	22.7	26.3	20.9	19.1	22.8	54.6	52.4	56.8	4,149
Kalimantan Barat	28.4	26.1	31.0	25.6	23.0	28.4	45.9	43.4	48.5	3,995
Kalimantan Tengah	28.3	25.6	31.1	24.1	21.1	27.3	47.7	44.6	50.8	2,479
Kalimantan Selatan	39.3	36.9	41.8	39.8	37.2	42.4	20.9	18.9	23.1	4,182
Kalimantan Timur	43.9	40.6	47.2	27.6	24.6	30.8	28.5	25.7	31.5	3,424
Kalimantan Utara	39.1	34.0	44.4	33.6	28.4	39.1	27.4	23.8	31.2	617
Sulawesi Utara	35.8	33.3	38.4	34.9	32.3	37.6	29.3	26.7	32.0	2,362
Sulawesi Tengah	30.3	27.9	32.9	28.0	25.3	30.8	41.7	38.8	44.7	2,550
Sulawesi Selatan	29.8	28.2	31.5	43.2	41.3	45.1	27.0	25.4	28.7	7,919
Sulawesi Tenggara	27.9	25.3	30.6	36.8	33.7	40.0	35.4	32.2	38.7	2,039
Gorontalo	33.1	29.8	36.7	34.1	30.1	38.4	32.8	28.9	36.9	1,065
Sulawesi Barat	21.0	17.3	25.3	28.4	24.1	33.0	50.6	45.7	55.5	1,119
Maluku	26.8	23.2	30.7	28.6	24.9	32.5	44.7	40.7	48.7	1,330
Maluku Utara	31.4	28.3	34.7	26.5	23.1	30.2	42.1	38.0	46.3	899
Papua Barat	34.5	30.3	39.0	25.9	22.2	30.0	39.6	34.8	44.5	845
Papua	19.3	17.4	21.3	21.5	19.2	23.9	59.3	56.6	61.9	2,933
**INDONESIA**	**37.1**	**36.6**	**37.5**	**36.9**	**36.5**	**37.4**	**26.0**	**25.6**	**26.4**	**254,087**

Tabel 3.1.2
Proporsi Pengetahuan Rumah Tangga terhadap Kemudahan Akses ke Rumah Sakit menurut Karakteristik, Riskesdas 2018

Karakteristik	Akses ke Rumah Sakit			N tertimbang
	Mudah	Sulit	Sangat Sulit	
	%	%	%	
**Tempat tinggal**				
Perkotaan	53.9	32.8	13.3	145,312
Perdesaan	14.6	42.4	43.0	108,775
**Pendidikan KRT**				
Tidak/belum pernah sekolah	20.6	42.2	37.1	14,827
Tidak tamat SD/MI	24.3	41.6	34.1	36,032
Tamat SD/MI	26.3	39.8	33.9	68,156
Tamat SLTP/MTS	36.6	37.6	25.7	42,444
Tamat SLTA/MA	51.0	32.6	16.5	69,429
Tamat D1/D2/D3/PT	58.1	29.5	12.4	23,198
**Pekerjaan KRT**				
Tidak Bekerja	38.3	37.9	23.8	27,553
Sekolah	65.8	21.7	12.4	2,141
PNS/TNI/Polri/BUMN/BUMD	55.4	30.0	14.6	12,770
Pegawai swasta	54.7	32.0	13.3	31,381
Wiraswasta	45.9	35.5	18.6	55,325
Petani	15.7	40.1	44.2	66,605
Nelayan	20.7	34.4	44.9	3,709
Buruh/sopir/pembantu ruta	37.7	40.0	22.3	41,172
Lainnya	43.5	36.4	20.1	13,432

Tabel 3.1.3
Proporsi Pengetahuan Rumah Tangga terhadap Kemudahan Akses
ke Puskesmas/Pustu/Pusling/Bidan Desa menurut Provinsi, Riskesdas 2018

Provinsi	Akses ke Puskesmas/Pustu/Pusling/Bidan Desa									N tertimbang
	Mudah			Sulit			Sangat Sulit			
	%	95% CI		%	95% CI		%	95% CI		
Aceh	46.8	44.8	48.7	32.1	30.4	33.8	21.1	19.6	22.8	5,068
Sumatera Utara	36.9	35.3	38.5	33.2	31.8	34.6	30.0	28.5	31.4	13,464
Sumatera Barat	44.5	42.4	46.6	35.2	33.4	37.0	20.3	18.8	21.9	5,072
Riau	48.7	46.4	51.0	26.1	24.3	27.9	25.2	23.4	27.2	6,521
Jambi	45.1	42.4	47.8	27.7	25.4	30.1	27.2	24.8	29.8	3,692
Sumatera Selatan	35.9	33.8	38.2	30.8	28.9	32.6	33.3	31.1	35.6	8,364
Bengkulu	52.0	49.3	54.6	27.9	25.8	30.1	20.1	18.1	22.3	2,057
Lampung	46.4	44.2	48.6	31.1	29.3	32.9	22.5	20.9	24.3	8,654
Bangka Belitung	62.2	58.8	65.5	22.7	20.3	25.3	15.2	12.9	17.8	1,504
Kepulauan Riau	44.1	40.3	48.1	37.9	34.2	41.7	18.0	15.2	21.1	2,169
DKI Jakarta	35.2	32.8	37.7	32.1	29.9	34.3	32.7	30.5	35.0	11,379
Jawa Barat	29.3	28.3	30.4	34.0	32.9	35.1	36.7	35.5	37.9	53,329
Jawa Tengah	44.2	43.2	45.3	33.2	32.3	34.1	22.6	21.7	23.5	37,063
DI Yogyakarta	59.2	56.7	61.7	26.8	24.6	29.1	14.0	12.6	15.5	4,285
JawaTimur	46.4	45.3	47.5	31.9	31.1	32.8	21.7	20.8	22.6	43,066
Banten	29.4	27.5	31.4	34.7	32.8	36.8	35.8	33.6	38.1	12,186
Bali	63.7	61.4	66.0	23.5	21.7	25.4	12.8	11.4	14.2	4,286
Nusa Tenggara Barat	40.8	38.1	43.5	34.0	31.8	36.2	25.2	22.9	27.6	5,511
Nusa Tenggara Timur	18.1	16.7	19.6	25.7	24.2	27.2	56.2	54.4	58.0	4,694
Kalimantan Barat	34.3	32.1	36.6	25.6	23.7	27.6	40.1	37.8	42.3	4,772
Kalimantan Tengah	40.5	37.8	43.3	27.2	25.0	29.5	32.3	29.9	34.8	2,844
Kalimantan Selatan	46.7	44.3	49.1	28.8	27.0	30.7	24.5	22.5	26.5	4,544
Kalimantan Timur	51.3	48.3	54.3	25.6	23.3	28.0	23.1	20.7	25.6	3,715
Kalimantan Utara	45.2	40.8	49.7	31.3	27.8	34.9	23.5	20.5	26.9	688
Sulawesi Utara	34.8	32.5	37.1	40.0	37.8	42.3	25.2	23.2	27.3	2,540
Sulawesi Tengah	37.1	34.7	39.7	33.4	31.4	35.6	29.4	27.2	31.7	3,012
Sulawesi Selatan	34.9	33.3	36.6	33.2	31.8	34.6	31.9	30.2	33.6	8,500
Sulawesi Tenggara	38.5	35.7	41.4	30.7	28.2	33.3	30.8	27.7	34.1	2,509
Gorontalo	38.0	34.7	41.4	40.2	37.2	43.3	21.8	19.1	24.8	1,163
Sulawesi Barat	37.2	33.4	41.1	26.6	23.4	30.1	36.2	32.6	40.0	1,303
Maluku	22.9	19.7	26.4	35.7	32.5	39.0	41.4	38.0	45.0	1,597
Maluku Utara	32.2	28.9	35.6	31.0	28.2	33.9	36.8	33.8	40.0	1,099
Papua Barat	32.5	28.7	36.5	31.5	28.1	35.1	36.0	32.3	39.9	946
Papua	14.4	12.8	16.1	15.4	14.0	17.0	70.2	68.1	72.2	3,822
**INDONESIA**	**39.2**	**38.8**	**39.6**	**31.8**	**31.5**	**32.2**	**29.0**	**28.6**	**29.4**	**275,416**

Tabel 3.1.4
Proporsi Pengetahuan Rumah Tangga terhadap Kemudahan Akses
ke Puskesmas/Pustu/Pusling/Bidan Desa menurut Karakteristik, Riskesdas 2018

Karakteristik	Akses ke Puskesmas/Pustu/Pusling/Bidan Desa			N tertimbang
	Mudah	Sulit	Sangat Sulit	
	%	%	%	
**Tempat tinggal**				
Perkotaan	46.1	31.5	22.5	150,312
Perdesaan	31.0	32.2	36.8	125,104
**Pendidikan KRT**				
Tidak/belumpernahsekolah	26.0	31.4	42.6	18,267
Tidak tamat SD/MI	31.1	32.0	36.9	41,160
Tamat SD/MI	32.9	33.0	34.1	76,422
Tamat SLTP/MTS	41.2	32.2	26.6	45,426
Tamat SLTA/MA	47.8	31.0	21.2	71,022
Tamat D1/D2/D3/PT	54.6	29.3	16.1	23,119
**Pekerjaan KRT**				
Tidak Bekerja	35.5	33.4	31.1	30,289
Sekolah	51.5	30.3	18.2	1,746
PNS/TNI/Polri/BUMN/BUMD	54.4	29.1	16.5	13,072
Pegawaiswasta	47.8	31.4	20.8	31,827
Wiraswasta	47.2	30.9	21.9	58,002
Petani	29.2	31.9	38.9	77,615
Nelayan	30.1	32.9	37.0	4,310
Buruh/sopir/pembantu ruta	37.3	33.0	29.6	44,213
Lainnya	43.0	31.1	26.0	14,343

Tabel 3.1.5
Proporsi Pengetahuan Rumah Tangga terhadap Kemudahan Akses ke Klinik/Praktek Dokter/Praktek Dokter Gigi/Praktek Bidan Mandiri menurut Provinsi, Riskesdas 2018

Provinsi	Akses ke Klinik/Praktek Dokter/Praktek Dokter Gigi/Praktek Bidan Mandiri									N tertimbang
	Mudah			Sulit			Sangat Sulit			
	%	95% CI		%	95% CI		%	95% CI		
Aceh	39.4	37.5	41.3	35.7	33.9	37.6	24.9	23.3	26.5	4,050
Sumatera Utara	37.1	35.5	38.7	26.2	24.8	27.6	36.7	35.1	38.4	11,124
Sumatera Barat	42.6	40.7	44.6	30.8	29.1	32.6	26.5	24.8	28.3	3,992
Riau	49.8	47.6	52.0	29.5	27.6	31.4	20.7	19.0	22.6	5,458
Jambi	39.8	37.0	42.7	34.1	31.5	36.9	26.0	23.6	28.6	2,966
Sumatera Selatan	31.3	29.0	33.7	33.9	31.7	36.1	34.8	32.5	37.3	6,392
Bengkulu	39.7	37.3	42.3	32.8	30.5	35.3	27.4	25.1	29.9	1,663
Lampung	46.8	44.7	49.0	32.4	30.5	34.4	20.8	19.3	22.4	7,508
Bangka Belitung	45.8	42.1	49.6	38.4	34.8	42.1	15.8	13.5	18.3	1,166
Kepulauan Riau	42.2	37.9	46.7	33.8	30.1	37.7	24.0	20.4	28.1	1,835
DKI Jakarta	23.9	21.9	26.1	32.5	30.4	34.7	43.5	41.0	46.1	9,536
Jawa Barat	25.3	24.3	26.4	32.0	30.9	33.1	42.7	41.4	44.0	44,983
Jawa Tengah	48.4	47.3	49.4	26.0	25.2	26.9	25.6	24.7	26.5	31,374
DI Yogyakarta	58.1	55.3	60.8	26.5	24.4	28.8	15.4	13.7	17.3	3,432
JawaTimur	46.6	45.6	47.6	31.0	30.1	31.9	22.4	21.5	23.3	35,985
Banten	27.7	25.7	29.7	34.9	33.0	37.0	37.4	35.2	39.6	10,299
Bali	62.0	59.6	64.3	25.2	23.4	27.1	12.8	11.5	14.3	3,544
Nusa Tenggara Barat	31.9	29.2	34.7	36.3	33.7	38.9	31.9	29.2	34.6	3,627
Nusa Tenggara Timur	20.0	17.8	22.3	27.5	25.1	30.0	52.6	49.7	55.4	1,936
Kalimantan Barat	32.3	29.9	34.7	33.2	31.0	35.5	34.5	32.3	36.7	3,314
Kalimantan Tengah	32.5	29.5	35.7	34.5	31.4	37.7	33.0	30.0	36.1	1,868
Kalimantan Selatan	40.9	38.4	43.4	33.6	31.4	35.8	25.6	23.6	27.7	3,463
Kalimantan Timur	39.3	36.4	42.2	37.4	34.6	40.3	23.3	21.0	25.8	2,852
Kalimantan Utara	34.5	30.6	38.8	39.8	36.0	43.8	25.6	22.1	29.4	516
Sulawesi Utara	24.1	22.1	26.3	32.1	29.7	34.5	43.8	41.1	46.5	1,761
Sulawesi Tengah	33.5	30.5	36.7	30.6	27.9	33.4	35.9	33.0	38.9	1,499
Sulawesi Selatan	26.3	24.5	28.1	35.6	33.7	37.5	38.1	36.1	40.1	5,151
Sulawesi Tenggara	23.1	19.2	27.4	38.8	35.3	42.3	38.1	34.5	41.9	1,313
Gorontalo	29.6	25.9	33.6	40.2	36.3	44.2	30.2	26.6	34.0	710
Sulawesi Barat	29.1	24.7	34.0	33.7	29.1	38.6	37.2	32.2	42.5	659
Maluku	17.2	13.6	21.5	32.2	27.8	36.9	50.6	45.1	56.2	712
Maluku Utara	26.5	22.9	30.5	32.3	28.5	36.3	41.2	36.9	45.6	510
Papua Barat	27.4	22.4	33.1	37.0	32.2	42.1	35.6	30.5	40.9	503
Papua	19.8	17.0	22.9	32.7	29.3	36.4	47.5	43.5	51.6	1,231
**INDONESIA**	**37.3**	**36.9**	**37.8**	**31.1**	**30.7**	**31.5**	**31.5**	**31.1**	**31.9**	**216,934**

Tabel 3.1.6
Proporsi Pengetahuan Rumah Tangga terhadap Kemudahan Akses ke Klinik/Praktek Dokter/Praktek Dokter Gigi/Praktek Bidan Mandiri menurut Karakteristik, Riskesdas 2018

Karakteristik	Akses ke Klinik/Praktek Dokter/Praktek Dokter Gigi/Praktek Bidan Mandiri			N tertimbang
	Mudah	Sulit	Sangat Sulit	
	%	%	%	
**Tempat tinggal**				
Perkotaan	39.1	31.5	29.5	126,522
Perdesaan	34.9	30.7	34.4	90,412
**Pendidikan KRT**				
Tidak/belum pernah sekolah	30.6	30.8	38.6	12,514
Tidak tamat SD/MI	32.4	30.3	37.3	30,323
Tamat SD/MI	34.0	30.9	35.1	58,210
Tamat SLTP/MTS	39.0	30.3	30.7	36,266
Tamat SLTA/MA	41.3	31.7	27.0	59,699
Tamat D1/D2/D3/PT	44.0	33.0	23.0	19,921
**Pekerjaan KRT**				
Tidak Bekerja	32.9	31.4	35.7	23,467
Sekolah	42.2	31.8	26.0	1,577
PNS/TNI/Polri/BUMN/BUMD	44.3	32.7	22.9	10,997
Pegawai swasta	42.0	32.3	25.6	27,650
Wiraswasta	41.3	31.1	27.6	48,567
Petani	33.1	30.7	36.2	54,624
Nelayan	32.2	30.4	37.5	2,842
Buruh/sopir/pembantu ruta	35.4	30.1	34.5	35,935
Lainnya	38.8	31.3	29.9	11,275

BAB 4
KESEHATAN LINGKUNGAN

4.1 Air

Jumlah pemakaian air per orang per hari atau *liters per capita per day* (*LPCD*) adalah jumlah pemakaian air di rumah tangga dalam sehari semalam dibagi dengan jumlah anggota rumah tangga, yang dikelompokkan menjadi 5 kategori, sebagai berikut:

1. Pemakaian air lebih kecil dari 5 liter/orang/hari, menunjukkan akses sangat kurang
2. Pemakaian air antara 5-19,9 liter/orang/hari, menunjukkan akses kurang
3. Pemakaian air antara 20-49,9 liter/orang/hari, menunjukkan akses dasar
4. Pemakaian air antara 50-99,9 liter/orang/hari, menunjukkan akses menengah
5. Pemakaian air lebih besar atau sama dengan 100 liter/orang/hari, menunjukkan akses optimal

Pengelompokan ini mengacu pada kriteria *health concern* yang berhubungan dengan higiene yang digunakan Howard (2003).

Formula:

$$Proporsi\ pemakaian\ air\ per\ orang\ per\ hari\ di\ rumah\ tangga = \frac{\sum Ruta\ dengan\ pemakaian\ air\ per\ orang\ per\ hari\ (5\ kategori)}{\sum Ruta}$$

$$Proporsi\ pemakaian\ air\ per\ orang\ per\ hari\ di\ rumah\ tangga = \frac{\sum Ruta\ dengan\ pemakaian\ air\ per\ orang\ per\ hari\ (2\ kategori)}{\sum Ruta}$$

Tabel 4.1.1
Proporsi Pemakaian Air per Orang per Hari di Rumah Tangga (5 Kategori) menurut Provinsi, Riskesdas 2018

Provinsi	Pemakaian air per orang per hari (dalam liter)					N Tertimbang
	< 5	5-19,9	20-49,9	50-99,9	≥100	
	%	%	%	%	%	
Aceh	0,1	0,8	9,3	43,5	46,3	4.772
Sumatera Utara	0,4	2,2	12,3	40,5	44,5	12.690
Sumatera Barat	1,4	1,7	12,1	43,4	41,4	4.640
Riau	0,2	0,7	7,6	42,6	48,8	6.273
Jambi	0,1	1,2	13,1	47,9	37,8	3.327
Sumatera Selatan	1,0	1,8	15,8	39,0	42,4	7.237
Bengkulu	0,2	1,6	8,8	33,0	56,5	1.951
Lampung	0,5	0,4	3,4	38,5	57,2	8.391
Bangka Belitung	0,1	2,1	10,1	40,7	46,9	1.402
Kepulauan Riau	0,0	1,1	11,6	35,7	51,5	2.264
DKI Jakarta	0,0	0,6	10,6	37,8	51,0	11.557
Jawa Barat	0,8	1,2	10,2	43,7	44,1	52.055
Jawa Tengah	0,3	1,0	9,8	39,4	49,5	35.521
DI Yogyakarta	0,0	0,2	4,8	30,9	64,0	4.392
Jawa Timur	0,4	1,7	11,1	33,8	53,0	41.730
Banten	0,1	0,5	14,0	39,9	45,4	12.167
Bali	0,2	3,6	12,1	35,3	48,8	4.307
Nusa Tenggara Barat	0,3	1,1	6,5	41,0	51,0	5.245
Nusa Tenggara Timur	0,6	13,2	41,4	33,2	11,6	4.413
Kalimantan Barat	1,4	6,1	16,3	37,1	39,2	3.389
Kalimantan Tengah	0,1	2,1	9,5	28,9	59,5	2.053
Kalimantan Selatan	0,3	1,9	12,6	35,3	50,0	3.691
Kalimantan Timur	0,1	2,6	11,8	30,7	54,8	3.320
Kalimantan Utara	0,1	1,5	15,7	37,5	45,2	622
Sulawesi Utara	0,1	1,1	13,5	48,9	36,4	2.418
Sulawesi Tengah	0,4	3,5	20,3	48,2	27,5	2.835
Sulawesi Selatan	0,2	1,7	19,4	40,3	38,4	8.238
Sulawesi Tenggara	0,0	4,2	27,4	39,9	28,5	2.457
Gorontalo	0,2	2,3	14,4	42,1	41,1	1.129
Sulawesi Barat	0,0	1,9	15,5	45,9	36,7	1.208
Maluku	0,1	2,3	23,1	43,3	31,2	1.567
Maluku Utara	0,0	1,8	14,2	61,2	22,8	1.071
Papua Barat	0,4	4,1	28,2	42,2	25,0	869
Papua	3,1	12,2	26,5	29,3	29,0	2.517
**INDONESIA**	**0,5**	**1,8**	**12,0**	**39,3**	**46,5**	**261.718**

Catatan: *Cut off* berdasarkan rekomendasi WHO untuk pemakaian rumah tangga, (Howard G., Bartram J. Domestic Water Quantity, Service Level and Health. WHO; Geneva, Switzerland: 2003)

Tabel 4.1.2
Proporsi Pemakaian Air per Orang per Hari di Rumah Tangga (5 Kategori) menurut Karakteristik, Riskesdas 2018

Karakteristik	Pemakaian air per orang per hari (dalam liter)					N Tertimbang
	< 5	5-19,9	20-49,9	50-99,9	≥100	
	%	%	%	%	%	
**Tempat Tinggal**						
Perkotaan	0,2	1,0	10,0	38,4	50,5	149.263
Perdesaan	0,9	2,8	14,6	40,5	41,2	112.455
**Pendidikan KRT**						
Tidak/belum pernah sekolah	0,6	2,9	14,4	37,9	44,3	16.956
Tidak tamat SD/MI	0,7	2,4	13,6	40,0	43,2	37.970
Tamat SD/MI	0,8	2,1	13,2	40,6	43,4	70.668
Tamat SLTP/MTS	0,4	1,5	11,8	41,2	45,2	42.938
Tamat SLTA/MA	0,2	1,2	10,4	38,5	49,6	69.944
Tamat D1/D2/D3/PT	0,1	1,0	9,1	34,2	55,5	23.241
**Pekerjaan KRT**						
Tidak bekerja	0,4	1,4	10,5	36,7	51,0	29.825
Sekolah	0,1	1,0	10,0	36,1	52,9	2.237
PNS/TNI/Polri/BUMN/BUMD	0,1	1,2	10,1	35,1	53,5	12.739
Pegawai swasta	0,1	0,9	9,8	38,9	50,3	31.768
Wiraswasta	0,3	1,1	10,2	38,5	49,8	56.327
Petani	0,9	3,1	14,9	40,4	40,8	69.010
Nelayan	0,4	2,8	17,4	42,4	37,0	3.967
Buruh/sopir/pembantu ruta	0,6	1,5	12,5	42,3	43,1	41.997
Lainnya	0,3	1,6	12,2	37,8	48,1	13.848
**INDONESIA**	**0,5**	**1,8**	**12,0**	**39,3**	**46,5**	**261.718**

Catatan: *Cut off* berdasarkan rekomendasi WHO untuk pemakaian rumah tangga (Howard G., Bartram J. Domestic Water Quantity, Service Level and Health. WHO; Geneva, Switzerland: 2003)

Tabel 4.1.3
Proporsi Pemakaian Air per Orang per Hari di Rumah Tangga (2 Kategori)
menurut Provinsi, Riskesdas 2018

Provinsi	Pemakaian air per orang per hari				N Tertimbang
	<20 liter		>20 liter		
	%	95%CI	%	95%CI	
Aceh	0,9	0,7 - 1,2	99,1	98,8 - 99,3	4.772
Sumatera Utara	2,7	2,3 - 3,1	97,3	96,9-97,7	12.690
Sumatera Barat	3,1	2,4 - 4,0	96,9	96,0-97,6	4.640
Riau	0,9	0,6 - 1,3	99,1	98,7-99,4	6.273
Jambi	1,2	0,8 - 1,8	98,8	98,2-99,2	3.327
Sumatera Selatan	2,8	2,2 - 3,7	97,2	96,3-97,8	7.237
Bengkulu	1,7	1,2 - 2,6	98,3	97,4-98,8	1.951
Lampung	0,9	0,6 - 1,4	99,1	98,6-99,4	8.391
Bangka Belitung	2,2	1,6 - 3,1	97,8	96,9-98,4	1.402
Kepulauan Riau	1,1	0,7 - 1,8	98,9	98,2-99,3	2.264
DKI Jakarta	0,6	0,4 - 1,0	99,4	99,0-99,6	11.557
Jawa Barat	2,0	1,7 - 2,4	98,0	97,6-98,3	52.055
Jawa Tengah	1,3	1,1 - 1,6	98,7	98,4-98,9	35.521
DI Yogyakarta	0,2	0,1 - 0,7	99,8	99,3-99,9	4.392
Jawa Timur	2,1	1,8 - 2,5	97,9	97,5-98,2	41.730
Banten	0,6	0,4 - 0,9	99,4	99,1-99,6	12.167
Bali	3,8	2,7 - 5,3	96,2	94,7-97,3	4.307
Nusa Tenggara Barat	1,5	1,0 - 2,2	98,5	97,8-99,0	5.245
Nusa Tenggara Timur	13,8	12,5 - 15,2	86,2	84,8-87,5	4.413
Kalimantan Barat	7,4	6,2 - 8,9	92,6	91,1-93,8	3.389
Kalimantan Tengah	2,1	1,6 - 2,9	97,9	97,1-98,4	2.053
Kalimantan Selatan	2,2	1,6 - 3,0	97,8	97,0-98,4	3.691
Kalimantan Timur	2,7	2,0 - 3,8	97,3	96,2-98,0	3.320
Kalimantan Utara	1,6	0,8 - 3,0	98,4	97,0-99,2	622
Sulawesi Utara	1,2	0,8 - 1,7	98,8	98,3-99,2	2.418
Sulawesi Tengah	3,9	3,0 - 5,0	96,1	95,0-97,0	2.835
Sulawesi Selatan	1,9	1,6 - 2,3	98,1	97,7-98,4	8.238
Sulawesi Tenggara	4,2	3,4 - 5,2	95,8	94,8-96,6	2.457
Gorontalo	2,4	1,6 - 3,5	97,6	96,5-98,4	1.129
Sulawesi Barat	1,9	1,3 - 2,7	98,1	97,3-98,7	1.208
Maluku	2,4	1,8 - 3,2	97,6	96,8-98,2	1.567
Maluku Utara	1,8	1,0 - 3,2	98,2	96,8-99,0	1.071
Papua Barat	4,5	3,5 - 5,8	95,5	94,2-96,5	869
Papua	15,2	13,3 - 17,4	84,8	82,6-86,7	2.517
**INDONESIA**	**2,2**	**2,1 - 2,3**	**97,8**	**97,7-97,9**	**261.718**

Catatan: Pemakaian air per orang per hari <20 liter mengindikasikan *health concern* tingkat tinggi (Howard G., Bartram J. Domestic Water Quantity, Service Level and Health. WHO; Geneva, Switzerland: 2003)

Tabel 4.1.4
Proporsi Pemakaian Air per Orang per Hari di Rumah Tangga (2 Kategori) menurut Karakteristik, Riskesdas 2018

Karakteristik	Pemakaian air per orang per hari				N Tertimbang
	<20 liter		>20 liter		
	%	95%CI	%	95%CI	
**Tempat Tinggal**					
Perkotaan	1,1	1,0 - 1,2	98,9	98,8-99,0	149.263
Perdesaan	3,7	3,5 - 3,9	96,3	96,1-96,5	112.455
**Pendidikan KRT**					
Tidak/belum pernah sekolah	3,5	3,2 - 3,9	96,5	96,1-96,8	16.956
Tidak tamat SD/MI	3,1	2,9 - 3,4	96,9	96,6-97,1	37.970
Tamat SD/MI	2,8	2,6 - 3,1	97,2	96,9-97,4	70.668
Tamat SLTP/MTS	1,9	1,7 - 2,1	98,1	97,9-98,3	42.938
Tamat SLTA/MA	1,4	1,3 - 1,5	98,6	98,5-98,7	69.944
Tamat D1/D2/D3/PT	1,1	1,0 - 1,3	98,9	98,7-99,0	23.241
**Pekerjaan KRT**					
Tidak bekerja	1,8	1,6 - 2,0	98,2	98,0-98,4	29.825
Sekolah	1,0	0,6 - 1,8	99,0	98,2-99,4	2.237
PNS/TNI/Polri/BUMN/BUMD	1,3	1,1 - 1,6	98,7	98,4-98,9	12.739
Pegawai swasta	1,0	0,9 - 1,2	99,0	98,8-99,1	31.768
Wiraswasta	1,4	1,3 - 1,6	98,6	98,4-98,7	56.327
Petani	3,9	3,7 - 4,2	96,1	95,8-96,3	69.010
Nelayan	3,1	2,5 - 3,9	96,9	96,1-97,5	3.967
Buruh/sopir/pembantu ruta	2,1	1,8 - 2,3	97,9	97,7-98,2	41.997
Lainnya	1,9	1,6 - 2,3	98,1	97,7-98,4	13.848
**INDONESIA**	**2,2**	**2,1 - 2,3**	**97,8**	**97,7-97,9**	**261.718**

Catatan: Pemakaian air per orang per hari <20 liter mengindikasikan *health concern* tingkat tinggi (Howard G., Bartram J. Domestic Water Quantity, Service Level and Health. WHO; Geneva, Switzerland: 2003)

4.2 Sanitasi

Penanganan tinja balita

Penanganan tinja balita di rumah tangga dapat dilakukan dengan cara berikut:

1. Menggunakan jamban: balita buang air besar (BAB) langsung di jamban.
2. Dibuang di jamban: tinja dari popok/celana dibuang di jamban.
3. Ditanam: tinja dari popok/celana ditanam di tanah atau popoknya ditanam di tanah
4. Dibuang di sembarang tempat (termasuk tempat sampah): tinja dari popok/celana atau popok bersama tinjanya dibuang ke sembarang tempat, termasuk ke tempat sampah.
5. Dibersihkan di sembarang tempat: balita diceboki di tempat tertentu (misalnya di kamar mandi, namun bukan di jamban) dengan sisa tinja yang dialirkan ke sembarang tempat (selokan, kali, atau sungai) atau dialirkan ke penampungan air limbah dari kamar mandi/tempat cuci.

Penanganan tinja balita di rumah tangga dapat dikelompokkan menjadi penanganan tinja balita yang aman dan tidak aman. Disebut aman jika balita selalu menggunakan jamban, atau tinja balita dibuang ke jamban atau ditanam, dan disebut tidak aman jika tinja balita dibuang di sembarang tempat (termasuk ke tempat sampah) atau balita diceboki/dibersihkan di sembarang tempat.

Proporsi rumah tangga dengan penanganan pembuangan tinja balita aman dihitung dengan formula:

$$Proporsi\ ruta\ tinja\ aman = \frac{\sum Ruta\ penanganan\ tinja\ aman}{\sum Ruta\ memiliki\ balita}$$

Pembuangan air limbah dari kamar mandi/tempat cuci dan dari dapur

Sarana pembuangan air limbah dari kamar mandi/tempat cuci adalah tempat pembuangan air limbah yang berasal dari kamar mandi/tempat cuci (tidak berasal dari jamban), sedangkan pembuangan air limbah dari dapur adalah tempat pembuangan air limbah yang berasal dari dapur.

Sarana pembuangan air limbah tersebut dikelompokkan sebagai berikut:

1. Penampungan tertutup, yaitu sarana untuk menampung air limbah yang konstruksinya berupa kolam/sumur dengan atau tanpa dinding beton/plesteran semen dan saringan, serta **tertutup**.
2. Penampungan terbuka: sarana untuk menampung air limbah yang konstruksinya berupa kolam/sumur dengan atau tanpa dinding beton/plesteran semen dan saringan, serta **terbuka**.
3. Tanpa penampungan (di tanah): tidak ada sarana untuk menampung air limbah rumah tangga. Air limbah menggenang di atas tanah.
4. Langsung ke got/kali/sungai: air limbah rumah tangga dibuang langsung ke got/selokan di sekitar rumah menggunakan pipa/paralon atau air limbah dibuang ke kali/sungai menggunakan pipa/paralon.

Formula :

Proporsi rumah tangga dengan penampungan air limbah tertutup dihitung dengan formula:

$$Proporsi\ ruta\ dengan\ penampungan\ tertutup = \frac{\sum Ruta\ dengan\ penampungan\ air\ limbah\ tertutup}{\sum Ruta}$$

Proporsi rumah tangga dengan penampungan air limbah terbuka dihitung dengan formula:

$$Proporsi\ ruta\ dengan\ penampungan\ terbuka = \frac{\sum Ruta\ dengan\ penampungan\ air\ limbah\ terbuka}{\sum Ruta}$$

Proporsi rumah tangga tanpa penampungan air limbah (di tanah) dihitung dengan formula:

$$Proporsi\ ruta\ dengan\ limbah\ ke\ tanah = \frac{\sum Ruta\ dengan\ limbah\ ke\ tanah}{\sum Ruta}$$

Proporsi rumah tangga yang langsung membuang limbah ke got/kali/sungai dihitung dengan formula:

$$Proporsi\ ruta\ dengan\ limbah\ ke\ got\ atau\ kali = \frac{\sum Ruta\ dengan\ limbah\ ke\ got\ atau\ kali}{\sum Ruta}$$

Penanganan Sampah Rumah Tangga

1. **Jenis tempat penampungan sampah organik**

   Tempat penampungan sampah organik adalah kondisi tempat penampungan/pengumpulan sampah rumah tangga yang mudah membusuk/terurai yang disimpan didalam rumah dan berpotensi menarik serangga dan tikus pembawa penyakit serta dapat mencemari udara dalam rumah. Tempat penampungan sampah organik tersebut dikelompokkan menjadi tempat sampah tertutup dan terbuka.

   Formula:

   Proporsi rumah tangga dengan tempat penampungan sampah basah (organik) tertutup dihitung dengan formula:

$$Proporsi\ ruta\ dengan\ TPS\ organik\ tertutup = \frac{\sum Ruta\ dengan\ TPS\ organik\ tertutup}{\sum Ruta}$$

   Proporsi rumah tangga dengan tempat penampungan sampah basah (organik) terbuka dihitung dengan formula:

$$Proporsi\ ruta\ dengan\ TPS\ terbuka = \frac{\sum Ruta\ dengan\ TPS\ terbuka}{\sum Ruta}$$

2. **Pengelolaan sampah rumah tangga**

   Pengelolaan sampah rumah tangga adalah pengumpulan, pengangkutan, pemrosesan, pendaur ulangan, atau pembuangan dari material sampah. Pengelolaan sampah rumah tangga dikelompokkan sebagai berikut :

1. Diangkut: jika sampah dari rumah tangga tersebut secara rutin diangkut oleh petugas kebersihan setempat atau oleh anggota rumah tangga ke tempat penampungan sampah
2. Ditimbun dalam tanah (tertutup): jika sampah dari rumah tangga dibuang ke lubang sampah dan dilakukan penimbunan dengan tanah di sekitar rumah.
3. Dibuat kompos: jika sampah yang dihasilkan tidak dibuang, tetapi langsung ditampung dan diolah untuk dijadikan kompos untuk pupuk atau biogas.
4. Dibakar di sekitar rumah: jika sampah dibakar sendiri atau bersama rumah tangga lainnya.
5. Dibuang ke kali/parit/laut; termasuk dibuang ke selokan
6. Dibuang sembarangan

Upaya pengelolaan sampah rumah tangga dikategorikan baik jika dilakukan dengan cara diangkut (oleh petugas atau art), ditimbun dalam tanah tertutup dan dibuat kompos. Dikategorikan tidak baik jika dibakar dan dibuang kesembarang tempat termasuk ke kali/selokan/laut/sungai.

***Formula**:*

Proporsi rumah tangga yang mengelola sampah rumah tangga dengan cara diangkut, dihitung dengan formula:

$$Proporsi\ ruta\ dengan\ pengelolaan\ sampah\ diangkut = \frac{\sum Ruta\ dengan\ \ pengelolaan\ sampah\ diangkut\ petugas\ \boldsymbol{atau}\ dibuang\ sendiri}{\sum Ruta}$$

Proporsi rumah tangga yang mengelola sampah rumah tangga dengan cara ditimbun dalam tanah, dihitung dengan formula:

$$Proporsi\ ruta\ dengan\ pengelolaan\ sampah\ ditimbun = \frac{\sum Ruta\ dengan\ \ pengelolaan\ sampah\ ditimbun}{\sum Ruta}$$

Proporsi rumah tangga yang mengelola sampah rumah tangga dengan cara dibuat kompos, dihitung dengan formula:

$$Proporsi\ ruta\ dengan\ pengelolaan\ sampah\ dibuat\ kompos = \frac{\sum Ruta\ dengan\ pengelolaan\ sampah\ dibuat\ kompos}{\sum Ruta}$$

Proporsi rumah tangga yang mengelola sampah rumah tangga dengan cara dibakar, dihitung dengan formula:

$$Proporsi\ ruta\ dengan\ pengelolaan\ sampah\ dibakar = \frac{\sum Ruta\ dengan\ pengelolaan\ sampah\ dibakar}{\sum Ruta}$$

Proporsi rumah tangga yang mengelola sampah rumah tangga dengan cara dibuang ke kali/parit/selokan, dihitung dengan formula:

$$Proporsi\ ruta\ dengan\ pengelolaan\ sampah\ dibuang\ ke\ kali\ atau\ selokan = \frac{\sum Ruta\ dengan\ pengelolaan\ sampah\ dibuang\ ke\ kali\ atau\ selokan}{\sum Ruta}$$

Proporsi rumah tangga yang mengelola sampah rumah tangga dengan cara dibuang sembarang, dihitung dengan formula:

$$Proporsi\ ruta\ dengan\ pengelolaan\ sampah\ dibuang\ sembarangan = \frac{\sum Ruta\ dengan\ pengelolaan\ sampah\ dibuang\ sembarangan}{\sum Ruta}$$

Rumah tangga yang mengelola sampah rumah tangga dengan cara baik adalah rumah tangga yang mengelola sampah dengan cara diangkut oleh petugas/sendiri, ditimbun ATAU dibuat kompos. Proporsi rumah tangga yang mengelola sampah rumah tangga dengan cara baik, dihitung dengan formula:

$$Proporsi\ ruta\ dengan\ pengelolaan\ sampah\ yang\ baik = \frac{\sum Ruta\ dengan\ pengelolaan\ sampah\ baik}{\sum Ruta}$$

Proporsi rumah tangga yang mengelola sampah rumah tangga dengan cara tidak baik, dihitung dengan formula:

$$Proporsi\ ruta\ dengan\ pengelolaan\ sampah\ yang\ tidak\ baik = \frac{\sum Ruta\ dengan\ pengelolaan\ sampah\ tidak\ baik}{\sum Ruta}$$

3. **Perilaku menguras bak mandi/ember besar/drum**

Perilaku menguras bak mandi/ember besar/drum adalah perilaku rumah tangga dalam membersihkan bak mandi/ember besar/drum untuk menghindari adanya jentik nyamuk, dikelompokkan sebagai berikut :

a. Lebih dari satu kali dalam seminggu: jika rumah tangga menguras bak mandi/ember/drum lebih dari satu kali dalam seminggu
b. Satu kali dalam seminggu: jika rumah tangga menguras bak mandi/ember/drum satu kali dalam seminggu
c. 1 - 3 kali dalam sebulan: jika rumah tangga menguras bak mandi/emberbesar/drum sebanyak 1-3 kali dalam sebulan. Rumah tangga yang sangat jarang menguras (misalnya dua bulan sekali) atau TIDAK PERNAH SAMA SEKALI masuk dalam kategori ini.

Formula :

Proporsi rumah tangga yang menguras bak mandi/ember besar/drum sebanyak lebih dari satu kali dalam seminggu, dihitung dengan formula:

$$Proporsi\ ruta\ dengan\ perilaku\ menguras > 1 kali\ seminggu = \frac{\sum Ruta\ dengan\ perilaku\ menguras > 1\ kali\ seminggu}{\sum Ruta\ yang\ memiliki\ bak\ atau\ ember\ besar\ atau\ drum}$$

Proporsi rumah tangga yang menguras bak mandi/ember besar/drum sebanyak satu kali dalam seminggu, dihitung dengan formula:

$$Proporsi\ ruta\ dengan\ perilaku\ menguras\ 1 kali\ seminggu = \frac{\sum Ruta\ dengan\ perilaku\ menguras\ 1\ kali\ seminggu}{\sum Ruta\ yang\ memiliki\ bak\ atau\ ember\ besar\ atau\ drum}$$

Proporsi rumah tangga yang menguras bak mandi/ember besar/drum sebanyak 1-3 kali dalam sebulan, dihitung dengan formula:

$$Proporsi\ ruta\ dengan\ perilaku\ menguras\ 1-3 kali\ sebulan = \frac{\sum Ruta\ dengan\ perilaku\ menguras\ 1-3\ kali\ sebulan}{\sum Ruta\ yang\ memiliki\ bak\ atau\ ember\ besar\ atau\ drum}$$

4. Upaya pemberantasan sarang nyamuk

Upaya pemberantasan sarang nyamuk (PSN) melalui kegiatan 3M (menguras, menutup, mengubur dan cara pencegahan plus lainnya), merupakan upaya rumah tangga dalam mencegah berkembangnya vektor nyamuk dalam rumah tangga dan lingkungan. Upaya PSN dikelompokkan sebagai berikut:

a. Memakai obat nyamuk (semprot/bakar/elektrik) yaitu memakai obat nyamuk dengan cara disemprot ke udara dalam ruangan (kamar tidur, ruang keluarga, dll) atau dengan cara dibakar atau disambungkan ke listrik.
b. Menaburkan bubuk larvasida dalam tempat penampungan air : yaitu dengan cara menaburkan bubuk larvasida pada tempat penampungan air yang sulit dibersihkan.
c. Ventilasi rumah dipasang kasa nyamuk : yaitu ventilasi rumah ditambahkan kawat kasa nyamuk, berguna menahan nyamuk agar tidak masuk kedalam rumah.
d. Menguras bak mandi/ember besar/drum : yaitu membersihkan tempat yang sering dijadikan tempat penampungan air seperti bak mandi, ember air, drum, tempat penampungan air minum, penampung air lemari es dan lain-lain
e. Menutup tempat penampungan air di rumah tangga : yaitu menutup rapat-rapat tempat-tempat penampungan air seperti drum, kendi, toren air, dan lain-lain.
f. Memusnahkan barang-barang bekas (kaleng, ban, dll) : memusnahkan barang barang bekas yang memiliki potensi untuk jadi tempat perkembangbiakan nyamuk, seperti kaleng, ban, botol, dan lain-lain.

Formula :

Proporsi rumah tangga yang memakai obat nyamuk, dihitung dengan formula:

$$Proporsi\ ruta\ yang\ memakai\ obat\ nyamuk\ = \frac{\sum Ruta\ yang\ memakai\ obat\ nyamuk}{\sum Ruta}$$

Proporsi rumah tangga yang menaburkan bubuk larvasida, dihitung dengan formula:

$$Proporsi\ ruta\ yang\ menaburkan\ bubuk\ larvasida = \frac{\sum Ruta\ yang\ menaburkan\ bubuk\ larvasida}{\sum Ruta}$$

Proporsi rumah tangga dengan ventilasi rumah dipasang kawat nyamuk, dihitung dengan formula:

$$Proporsi\ ruta\ dengan\ ventilasi\ rumah\ dipasang\ kawat\ nyamuk = \frac{\sum Ruta\ dengan\ ventilasi\ rumah\ dipasang\ kawat\ nyamuk}{\sum Ruta}$$

Proporsi rumah tangga yg menguras bak mandi/ember besar/drum, dihitung dengan formula:

$$Proporsi\ ruta\ yang\ menguras\ bak\ mandi/ember\ besar/drum = \frac{\sum Ruta\ yang\ menguras\ bak\ mandi/ember\ besar/drum}{\sum Ruta}$$

Proporsi rumah tangga yang menutup tempat penampungan, dihitung dengan formula:

$$Proporsi\ ruta\ yang\ menutup\ tempat\ penampungan\ air = \frac{\sum Ruta\ yang\ menutup\ tempat\ penampungan\ air}{\sum Ruta}$$

Proporsi rumah tangga yang memusnahkan barang-barang bekas, dihitung dengan formula:

$$Proporsi\ ruta\ yang\ memusnahkan\ barang-barang\ bekas = \frac{\sum Ruta\ yang\ memusnahkan\ barang-barang\ bekas}{\sum Ruta}$$

Upaya pemberantasan sarang nyamuk melalui kegiatan 3M (menguras, menutup, dan mengubur) atau 3M plus (menguras, menutup, mengubur, dan salah satu cara pencegahan plus lainnya), dihitung dengan formula:

$$Proporsi\ ruta\ yang\ melakukan\ kegiatan\ 3M = \frac{\sum Ruta\ yang\ melakukan\ kegiatan\ menguras, menutup\ dan\ mengubur}{\sum Ruta}$$

$$Proporsi\ ruta\ yang\ melakukan\ kegiatan\ 3M\ Plus = \frac{\sum Ruta\ yang\ melakukan\ kegiatan\ menguras,\ menutup,\ mengubur\ \&\ salah\ satu\ kegiatan\ plus}{\sum Ruta}$$

Tabel 4.2.1
Proporsi Cara Penanganan Tinja Balita oleh Rumah Tangga menurut Provinsi, Riskesdas 2018

Provinsi	Cara Penanganan Tinja Balita						N Tertimbang
	Menggunakan jamban	Dibuang ke jamban	Ditanam ke tanah	Dibuang sembarangan	Dibersihkan di sembarang tempat	Lainnya	
Aceh	29,5	13,3	7,2	44,1	4,2	1,6	1.934
Sumatera Utara	36,3	21,9	2,9	31,3	7,3	0,3	4.736
Sumatera Barat	31,0	22,1	2,4	38,1	5,6	0,7	1.803
Riau	33,9	27,6	4,5	28,9	4,4	0,8	2.430
Jambi	41,1	27,4	4,0	24,0	3,2	0,3	1.162
Sumatera Selatan	38,0	19,1	2,7	29,1	10,7	0,4	2.792
Bengkulu	44,2	28,3	1,9	19,9	5,0	0,7	644
Lampung	39,0	28,5	6,5	22,6	3,1	0,3	2.844
Bangka Belitung	34,5	11,2	5,7	45,0	3,0	0,6	468
Kepulauan Riau	32,1	23,2	2,0	38,5	3,2	0,9	766
DKI Jakarta	35,1	25,0	0,1	37,8	1,2	0,8	3.156
Jawa Barat	46,4	21,0	1,3	28,4	1,5	1,4	16.007
Jawa Tengah	44,3	22,9	4,1	26,2	2,0	0,5	9.682
DI Yogyakarta	50,4	27,7	6,8	13,3	0,8	1,0	990
Jawa Timur	36,8	19,7	6,9	34,0	2,1	0,5	10.375
Banten	33,8	20,0	1,0	41,6	2,6	1,1	4.361
Bali	36,9	15,3	6,2	39,5	1,6	0,5	1.112
Nusa Tenggara Barat	33,0	9,4	6,7	45,4	4,7	0,8	1.817
Nusa Tenggara Timur	25,7	24,6	4,7	28,1	15,9	1,0	1.991
Kalimantan Barat	39,0	16,4	4,4	33,6	5,7	0,9	1.706
Kalimantan Tengah	34,8	17,1	2,7	39,8	4,9	0,7	926
Kalimantan Selatan	31,4	10,9	4,2	49,2	3,3	0,9	1.403
Kalimantan Timur	38,2	12,0	3,8	42,3	1,2	2,5	1.216
Kalimantan Utara	21,5	10,8	0,7	59,0	6,9	1,1	253
Sulawesi Utara	29,1	11,9	4,6	49,8	3,6	0,9	721
Sulawesi Tengah	21,3	16,2	6,3	46,6	9,3	0,2	999
Sulawesi Selatan	33,7	14,9	2,3	43,2	5,7	0,3	2.786
Sulawesi Tenggara	31,2	19,6	5,2	36,6	6,5	0,8	966
Gorontalo	32,6	8,1	3,0	44,3	11,9	0,1	389
Sulawesi Barat	26,5	10,8	5,5	43,7	13,4	0,2	496
Maluku	27,0	11,3	4,6	50,5	4,8	1,8	628
Maluku Utara	31,3	10,9	4,9	47,6	4,7	0,6	463
Papua Barat	25,6	18,5	4,0	44,4	6,6	0,9	365
Papua	17,0	11,8	3,4	48,7	17,2	1,9	1.276
**INDONESIA**	**37,8**	**20,1**	**3,7**	**33,5**	**4,0**	**0,8**	**83.662**

Tabel 4.2.2
Proporsi Cara Penanganan Tinja Balita oleh Rumah Tangga menurut Karakteristik, Riskesdas 2018

Karakteristik	Cara Penanganan Tinja Balita						N Tertimbang
	Menggunakan jamban	Dibuang ke jamban	Ditanam ke tanah	Dibuang sembarangan	Dibersihkan di sembarang tempat	Lainnya	
**Tempat Tinggal**							
Perkotaan	40,6	20,6	1,9	34,0	1,9	1,0	44.787
Perdesaan	34,6	19,7	5,7	33,0	6,4	0,7	38.875
**Pendidikan KRT**							
Tidak/belum pernah sekolah	28,6	16,4	4,8	42,2	7,3	0,7	3.563
Tidak tamat SD/MI	33,8	17,2	4,3	37,3	6,8	0,7	9.984
Tamat SD/MI	36,4	19,6	4,5	33,8	5,1	0,7	21.841
Tamat SLTP/MTS	39,3	20,5	3,8	31,8	3,6	0,9	15.892
Tamat SLTA/MA	40,7	21,3	2,8	31,8	2,5	0,8	24.778
Tamat D1/D2/D3?PT	38,6	23,0	2,5	33,1	1,5	1,4	7.605
**Pekerjaan KRT**							
Tidak bekerja	37,2	19,5	3,5	35,4	3,7	0,7	5.701
Sekolah	35,7	26,4	4,6	27,8	5,0	0,5	160
PNS/TNI/Polri/BUMN/BUMD	39,3	22,5	2,7	32,4	1,9	1,2	3.892
Pegawai swasta	41,8	22,0	2,5	31,2	1,5	1,0	12.052
Wiraswasta	40,6	20,4	3,0	33,0	2,1	0,9	19.067
Petani	32,5	19,2	5,9	33,9	7,9	0,7	21.920
Nelayan	23,9	14,0	3,1	51,8	6,8	0,5	1.704
Buruh/sopir/pembantu ruta	40,4	20,3	2,8	32,4	3,3	0,8	14.996
Lainnya	37,6	19,1	3,4	36,1	2,9	0,9	4.169

Tabel 4.2.3
Proporsi Penanganan Tinja Balita Secara Aman oleh Rumah Tangga
menurut Provinsi, Riskesdas 2018

Provinsi	Penanganan Tinja Balita				N Tertimbang
	Aman		Tidak Aman		
	%	95%CI	%	95%CI	
Aceh	50,0	47,6 - 52,5	50,0	47,5-52,4	1.934
Sumatera Utara	61,1	59,0 - 63,1	38,9	36,9-41,0	4.736
Sumatera Barat	55,5	52,9 - 58,2	44,5	41,8-47,1	1.803
Riau	66,0	63,2 - 68,6	34,0	31,4-36,8	2.430
Jambi	72,5	69,7 - 75,2	27,5	24,8-30,3	1.162
Sumatera Selatan	59,8	56,8 - 62,7	40,2	37,3-43,2	2.792
Bengkulu	74,3	71,1 - 77,3	25,7	22,7-28,9	644
Lampung	74,0	71,6 - 76,2	26,0	23,8-28,4	2.844
Bangka Belitung	51,4	46,9 - 55,8	48,6	44,2-53,1	468
Kepulauan Riau	57,3	50,4 - 63,9	42,7	36,1-49,6	766
DKI Jakarta	60,1	56,2 - 64,0	39,9	36,0-43,8	3.156
Jawa Barat	68,6	66,9 - 70,3	31,4	29,7-33,1	16.007
Jawa Tengah	71,3	69,8 - 72,7	28,7	27,3-30,2	9.682
DI Yogyakarta	84,9	81,3 - 88,0	15,1	12,0-18,7	990
Jawa Timur	63,4	61,9 - 64,9	36,6	35,1-38,1	10.375
Banten	54,8	51,7 - 57,8	45,2	42,2-48,3	4.361
Bali	58,4	54,7 - 62,0	41,6	38,0-45,3	1.112
Nusa Tenggara Barat	49,1	46,1 - 52,1	50,9	47,9-53,9	1.817
Nusa Tenggara Timur	54,9	52,8 - 57,1	45,1	42,9-47,2	1.991
Kalimantan Barat	59,8	57,0 - 62,6	40,2	37,4-43,0	1.706
Kalimantan Tengah	54,6	51,1 - 58,1	45,4	41,9 - 48,9	926
Kalimantan Selatan	46,5	43,7 - 49,4	53,5	50,6 - 56,3	1.403
Kalimantan Timur	54,0	50,0 - 57,9	46,0	42,1 - 50,0	1.216
Kalimantan Utara	33,0	28,5 - 37,8	67,0	62,2 - 71,5	253
Sulawesi Utara	45,7	42,5 - 48,9	54,3	51,1 - 57,5	721
Sulawesi Tengah	43,8	40,7 - 47,0	56,2	53,0 - 59,3	999
Sulawesi Selatan	50,8	48,4 - 53,1	49,2	46,9 - 51,6	2.786
Sulawesi Tenggara	56,0	52,8 - 59,2	44,0	40,8 - 47,2	966
Gorontalo	43,8	39,5 - 48,1	56,2	51,9 - 60,5	389
Sulawesi Barat	42,8	38,4 - 47,2	57,2	52,8 - 61,6	496
Maluku	42,9	39,1 - 46,8	57,1	53,2 - 60,9	628
Maluku Utara	47,1	43,2 - 51,1	52,9	48,9 - 56,8	463
Papua Barat	48,1	43,2 - 53,0	51,9	47,0 - 56,8	365
Papua	32,2	29,4 - 35,2	67,8	64,8 - 70,6	1.276
**INDONESIA**	**61,6**	**61,1 - 62,2**	**38,4**	**37,8 - 38,9**	**83.662**

Catatan: Aman jika menggunakan jamban, dibuang ke jamban atau ditanam di tanah. Tidak aman jika dibuang ke sembarang tempat, dibersihkan di sembarang tempat atau lainnya.

Tabel 4.2.4
Proporsi Penanganan Tinja Balita Secara Aman oleh Rumah Tangga
menurut Karakteristik, Riskesdas 2018

Karakteristik	Kelompok Penanganan Tinja Balita				N Tertimbang
	Aman		Tidak Aman		
	%	95%CI	%	95%CI	
**Tempat Tinggal**					
Perkotaan	63,1	62,3 - 64,0	36,9	36,0 - 37,7	44.787
Perdesaan	59,9	59,2 - 60,6	40,1	39,4 - 40,8	38.875
**Pendidikan KRT**					
Tidak/belum pernah sekolah	49,8	47,4 - 52,3	50,2	47,7 - 52,6	3.563
Tidak tamat SD/MI	55,3	53,9 - 56,7	44,7	43,3 - 46,1	9.984
Tamat SD/MI	60,5	59,4 - 61,4	39,5	38,6 - 40,6	21.841
Tamat SLTP/MTS	63,7	62,6 - 64,9	36,3	35,1 - 37,4	15.892
Tamat SLTA/MA	64,8	63,9 - 65,8	35,2	34,2 - 36,1	24.778
Tamat D1/D2/D3/PT	64,1	62,4 - 65,7	35,9	34,3 - 37,6	7.605
**Pekerjaan KRT**					
Tidak bekerja	60,1	58,1 -62,0	39,9	38,0 - 41,9	5.701
Sekolah	66,7	56,7 - 75,4	33,3	24,6 - 43,3	160
PNS/TNI/Polri/BUMN/BUMD	64,4	62,4 - 66,5	35,6	33,5 - 37,6	3.892
Pegawai swasta	66,3	64,7 -67,9	33,7	32,1 - 35,3	12.052
Wiraswasta	64,0	62,9 - 65,1	36,0	34,9 - 37,1	19.067
Petani	57,5	56,6 - 58,4	42,5	41,6 - 43,4	21.920
Nelayan	40,9	37,8 - 44,1	59,1	55,9 - 62,2	1.704
Buruh/sopir/pembantu ruta	63,5	62,2 - 64,9	36,5	35,1 - 37,8	14.996
Lainnya	60,0	57,7 - 62,3	40,0	37,7 - 42,3	4.169

Catatan: Aman jika menggunakan jamban, dibuang ke jamban atau ditanam di tanah. Tidak aman jika dibuang ke sembarang tempat, dibersihkan di sembarang tempat atau lainnya.

Tabel 4.2.5
Proporsi Tempat Pembuangan Air Limbah Utama dari Kamar Mandi/Tempat Cuci di Rumah Tangga menurut Provinsi, Riskesdas 2018

Provinsi	Tempat pembuangan air limbah dari kamar mandi/tempat cuci				N Tertimbang
	Penampungan tertutup	Penampungan terbuka	Tanpa Penampungan (di tanah)	Langsung ke got/kali/sungai	
Aceh	11,0	18,7	21,6	48,6	5.111
Sumatera Utara	15,8	17,7	15,7	50,8	13.991
Sumatera Barat	20,6	15,6	17,8	46,0	5.184
Riau	12,0	17,1	23,1	47,7	6.792
Jambi	20,4	21,1	19,5	38,9	3.698
Sumatera Selatan	10,3	17,1	22,1	50,5	8.538
Bengkulu	12,0	21,0	27,5	39,5	2.082
Lampung	11,1	27,8	25,4	35,7	8.838
Bangka Belitung	13,9	6,4	39,3	40,3	1.518
Kepulauan Riau	12,3	9,4	9,2	69,1	2.367
DKI Jakarta	18,2	2,6	0,9	78,3	11.849
Jawa Barat	24,0	10,6	4,5	60,9	55.133
Jawa Tengah	20,3	9,9	16,7	53,0	37.516
DI Yogyakarta	53,8	4,8	18,0	23,5	4.514
Jawa Timur	21,9	11,3	21,5	45,2	43.890
Banten	14,7	10,9	9,5	64,9	12.733
Bali	31,6	1,9	16,2	50,3	4.509
Nusa Tenggara Barat	22,5	4,8	25,8	46,9	5.573
Nusa Tenggara Timur	7,9	7,2	75,0	9,9	4.761
Kalimantan Barat	11,9	9,5	32,4	46,2	4.911
Kalimantan Tengah	10,2	9,3	38,4	42,1	2.907
Kalimantan Selatan	12,2	7,9	43,6	36,3	4.648
Kalimantan Timur	17,7	6,4	20,7	55,2	3.794
Kalimantan Utara	18,5	5,0	25,8	50,7	695
Sulawesi Utara	7,9	4,9	23,7	63,5	2.579
Sulawesi Tengah	9,5	9,2	36,5	44,8	3.055
Sulawesi Selatan	10,2	6,3	37,8	45,7	8.677
Sulawesi Tenggara	17,9	16,1	35,4	30,7	2.553
Gorontalo	18,5	9,5	34,8	37,2	1.182
Sulawesi Barat	6,7	12,8	45,4	35,1	1.320
Maluku	13,1	9,1	40,5	37,3	1.633
Maluku Utara	9,6	10,1	41,7	38,6	1.134
Papua Barat	8,7	5,8	25,7	59,9	961
Papua	8,4	8,7	35,8	47,1	4.010
**INDONESIA**	**18,8**	**11,2**	**18,9**	**51,0**	**282.654**

Tabel 4.2.6
Proporsi Tempat Pembuangan Air Limbah Utama dari Kamar Mandi/Tempat Cuci di Rumah Tangga menurut Karakteristik, Riskesdas 2018

Karakteristik	Tempat pembuangan air limbah dari kamar mandi/tempat cuci				N tertimbang
	Penampungan tertutup	Penampungan terbuka	Tanpa Penampungan (di tanah)	Langsung ke got/kali/ sungai	
**Tempat Tinggal**					
Perkotaan	22,2	8,0	8,3	61,4	155.248
Perdesaan	14,7	15,1	31,8	38,4	127.406
**Pendidikan KRT**					
Tidak/belum pernah sekolah	12,6	12,3	31,9	43,2	19.357
Tidak tamat SD/MI	14,6	13,0	26,4	45,9	42.188
Tamat SD/MI	16,8	13,2	22,6	47,4	77.514
Tamat SLTP/MTS	19,1	11,5	17,9	51,4	46.102
Tamat SLTA/MA	21,6	9,2	11,5	57,7	73.398
Tamat D1/D2/D3/PT	28,5	6,6	8,2	56,8	24.095
**Pekerjaan KRT**					
Tidak bekerja	19,1	10,1	16,4	54,4	31.674
Sekolah	27,8	5,8	9,5	56,9	2.351
PNS/TNI/Polri/BUMN/BUMD	27,0	8,1	10,3	54,7	13.302
Pegawai swasta	23,0	7,2	8,1	61,6	33.234
Wiraswasta	22,0	10,6	12,2	55,2	59.238
Petani	13,0	15,2	33,7	38,1	79.010
Nelayan	12,4	10,5	27,7	49,3	4.376
Buruh/sopir/pembantu ruta	18,9	10,3	14,9	55,9	44.842
Lainnya	20,3	10,1	15,9	53,7	14.628

Tabel 4.2.7
Proporsi Tempat Pembuangan Air Limbah Utama dari Dapur Rumah Tangga menurut Provinsi, Riskesdas 2018

Provinsi	Tempat Pembuangan Air Limbah dari Dapur				N tertimbang
	Penampungan tertutup	Penampungan terbuka	Tanpa Penampungan (di tanah)	Langsung ke got/kali/sungai	
Aceh	9,2	19,3	22,8	48,7	5.111
Sumatera Utara	11,5	18,9	17,6	52,0	13.991
Sumatera Barat	16,4	16,3	20,1	47,3	5.184
Riau	8,9	18,2	24,0	48,8	6.792
Jambi	16,2	23,8	20,8	39,3	3.698
Sumatera Selatan	9,1	15,8	23,9	51,2	8.538
Bengkulu	10,9	20,7	28,3	40,2	2.082
Lampung	7,4	29,5	26,3	36,8	8.838
Bangka Belitung	9,8	6,7	44,0	39,5	1.518
Kepulauan Riau	9,6	10,2	10,3	69,8	2.367
DKI Jakarta	12,6	3,0	1,1	83,3	11.849
Jawa Barat	18,4	11,6	4,8	65,2	55.133
Jawa Tengah	16,2	10,2	18,0	55,5	37.516
DI Yogyakarta	46,4	5,1	23,1	25,3	4.514
Jawa Timur	18,7	11,9	23,4	46,0	43.890
Banten	11,1	11,3	9,7	67,9	12.733
Bali	16,9	2,5	24,2	56,4	4.509
Nusa Tenggara Barat	14,4	5,2	32,5	47,9	5.573
Nusa Tenggara Timur	4,6	6,4	80,3	8,7	4.761
Kalimantan Barat	5,9	10,0	36,5	47,6	4.911
Kalimantan Tengah	8,5	9,9	40,6	41,0	2.907
Kalimantan Selatan	10,6	8,7	45,3	35,5	4.648
Kalimantan Timur	8,2	6,9	23,8	61,1	3.794
Kalimantan Utara	8,3	5,0	26,7	60,0	695
Sulawesi Utara	4,2	5,2	24,8	65,8	2.579
Sulawesi Tengah	7,2	9,2	37,9	45,8	3.055
Sulawesi Selatan	5,1	6,6	41,4	46,9	8.677
Sulawesi Tenggara	12,7	17,1	39,0	31,2	2.553
Gorontalo	10,2	10,0	41,4	38,4	1.182
Sulawesi Barat	5,4	12,9	47,4	34,3	1.320
Maluku	7,9	9,6	45,3	37,3	1.633
Maluku Utara	5,5	9,8	46,6	38,2	1.134
Papua Barat	3,5	5,9	29,2	61,5	961
Papua	5,0	9,1	39,8	46,2	4.010
**INDONESIA**	**14,3**	**11,8**	**20,7**	**53,2**	**282.654**

Tabel 4.2.8
Proporsi Tempat Pembuangan Air Limbah Utama dari Dapur Rumah Tangga
menurut Karakteristik, Riskesdas 2018

Karakteristik	Tempat pembuangan air limbah dari dapur				N Tertimbang
	Penampungan tertutup	Penampungan terbuka	Tanpa Penampungan (di tanah)	Langsung ke got/kali/sungai	
**Tempat Tinggal**					
Perkotaan	17,2	8,6	9,3	65,0	155.248
Perdesaan	10,8	15,7	34,7	38,8	127.406
**Pendidikan KRT**					
Tidak/belum pernah sekolah	9,2	12,7	34,8	43,3	19.357
Tidak tamat SD/MI	11,0	13,2	29,0	46,8	42.188
Tamat SD/MI	12,9	13,6	24,6	48,9	77.514
Tamat SLTP/MTS	14,5	12,4	19,5	53,5	46.102
Tamat SLTA/MA	16,3	9,9	12,8	61,0	73.398
Tamat D1/D2/D3/PT	22,4	7,2	9,1	61,3	24.095
**Pekerjaan KRT**					
Tidak bekerja	14,4	10,5	18,2	57,0	31.674
Sekolah	22,1	7,2	10,7	60,0	2.351
PNS/TNI/Polri/BUMN/BUMD	21,0	8,9	11,6	58,6	13.302
Pegawai swasta	17,9	7,8	8,9	65,4	33.234
Wiraswasta	17,2	11,4	13,3	58,1	59.238
Petani	9,5	15,6	36,5	38,3	79.010
Nelayan	8,4	11,1	31,0	49,5	4.376
Buruh/sopir/pembantu ruta	14,0	11,0	16,5	58,5	44.842
Lainnya	15,3	10,7	17,7	56,3	14.628

Tabel 4.2.9
Proporsi Jenis Tempat Pengumpulan/Penampungan Sampah Basah (Organik) di Dalam Rumah menurut Provinsi, Riskesdas 2018

Provinsi	Tempat Penampungan Sampah Organik		N tertimbang
	Tempat sampah tertutup	Tempat sampah terbuka	
Aceh	11,5	80,6	5.111
Sumatera Utara	9,9	81,7	13.991
Sumatera Barat	14,3	77,9	5.184
Riau	11,1	82,7	6.792
Jambi	14,3	84,7	3.698
Sumatera Selatan	15,9	76,7	8.538
Bengkulu	14,9	79,6	2.082
Lampung	9,0	83,2	8.838
Bangka Belitung	18,4	80,9	1.518
Kepulauan Riau	23,9	75,1	2.367
DKI Jakarta	34,2	72,3	11.849
Jawa Barat	21,6	76,7	55.133
Jawa Tengah	14,9	82,8	37.516
DI Yogyakarta	17,1	81,0	4.514
Jawa Timur	20,0	78,8	43.890
Banten	18,3	81,5	12.733
Bali	14,9	77,2	4.509
Nusa Tenggara Barat	9,7	75,0	5.573
Nusa Tenggara Timur	5,0	58,6	4.761
Kalimantan Barat	11,6	73,1	4.911
Kalimantan Tengah	15,6	75,8	2.907
Kalimantan Selatan	22,6	67,2	4.648
Kalimantan Timur	26,5	72,6	3.794
Kalimantan Utara	28,2	72,4	695
Sulawesi Utara	14,2	85,0	2.579
Sulawesi Tengah	9,0	74,3	3.055
Sulawesi Selatan	13,1	78,4	8.677
Sulawesi Tenggara	11,5	75,2	2.553
Gorontalo	7,4	76,3	1.182
Sulawesi Barat	10,1	73,2	1.320
Maluku	10,4	79,6	1.633
Maluku Utara	9,3	74,3	1.134
Papua Barat	13,4	73,9	961
Papua	12,5	57,9	4.010
**INDONESIA**	**17,2**	**77,9**	**282.654**

Tabel 4.2.10
Proporsi Jenis Tempat Pengumpulan/Penampungan Sampah Basah (Organik) di Dalam Rumah menurut Karakteristik, Riskesdas 2018

Karakteristik	Tempat Penampungan Sampah Organik		N Tertimbang
	Tempat sampah tertutup	Tempat sampah terbuka	
**Tempat Tinggal**			
Perkotaan	24,4	76,7	155.248
Perdesaan	8,5	79,4	127.406
**Pendidikan KRT**			
Tidak/belum pernah sekolah	7,6	76,7	19.357
Tidak tamat SD/MI	9,2	78,4	42.188
Tamat SD/MI	11,2	80,2	77.514
Tamat SLTP/MTS	15,7	79,8	46.102
Tamat SLTA/MA	24,4	76,7	73.398
Tamat D1/D2/D3/PT	39,4	71,0	24.095
**Pekerjaan KRT**			
Tidak bekerja	18,5	77,0	31.674
Sekolah	23,1	76,8	2.351
PNS/TNI/Polri/BUMN/BUMD	35,5	72,9	13.302
Pegawai swasta	29,3	74,9	33.234
Wiraswasta	21,1	78,5	59.238
Petani	7,2	78,6	79.010
Nelayan	8,9	75,0	4.376
Buruh/sopir/pembantu ruta	13,9	80,9	44.842
Lainnya	20,2	77,8	14.628

Tabel 4.2.11
Proporsi Cara Pengelolaan Sampah Rumah Tangga menurut Provinsi, Riskesdas 2018

Provinsi	Cara Pengelolaan Sampah Rumah Tangga						N tertimbang
	Diangkut[1]	Ditanam	Dibuat kompos	Dibakar	Dibuang ke kali/selokan	Dibuang sembarang tempat	
Aceh	18,3	1,3	0,2	72,1	5,1	3,0	5.111
Sumatera Utara	29,1	1,0	0,4	58,1	4,6	6,7	13.991
Sumatera Barat	26,4	1,3	0,3	57,4	9,2	5,5	5.184
Riau	27,3	0,9	0,2	64,1	3,9	3,7	6.792
Jambi	25,9	1,8	0,2	59,1	9,7	3,3	3.698
Sumatera Selatan	29,4	2,6	0,1	45,2	13,4	9,2	8.538
Bengkulu	26,9	1,2	0,3	53,0	10,1	8,6	2.082
Lampung	15,0	1,6	0,3	75,6	3,6	3,9	8.838
Bangka Belitung	40,8	1,9	0,4	40,7	2,9	13,2	1.518
Kepulauan Riau	67,9	0,3	0,2	24,2	6,3	1,1	2.367
DKI Jakarta	96,5	0,0	0,0	2,6	0,6	0,2	11.849
Jawa Barat	41,4	0,8	0,2	45,6	8,5	3,5	55.133
Jawa Tengah	25,1	2,4	1,0	58,2	8,1	5,2	37.516
DI Yogyakarta	40,8	2,2	1,9	53,2	0,7	1,3	4.514
Jawa Timur	28,5	2,3	0,5	58,1	6,3	4,2	43.890
Banten	44,2	0,7	0,1	37,6	6,0	11,4	12.733
Bali	57,2	1,6	1,5	33,8	1,9	4,0	4.509
Nusa Tenggara Barat	25,2	1,5	0,1	41,8	22,4	9,0	5.573
Nusa Tenggara Timur	9,4	1,9	1,4	71,6	4,2	11,5	4.761
Kalimantan Barat	20,5	1,2	0,1	58,8	9,6	9,8	4.911
Kalimantan Tengah	33,6	2,2	0,1	40,1	17,8	6,3	2.907
Kalimantan Selatan	45,7	1,5	0,2	35,0	10,6	7,0	4.648
Kalimantan Timur	65,5	0,5	0,1	24,5	7,7	1,7	3.794
Kalimantan Utara	56,4	0,5	0,1	25,1	12,0	5,8	695
Sulawesi Utara	41,7	1,5	0,2	44,9	7,8	3,8	2.579
Sulawesi Tengah	17,6	0,9	0,3	57,3	13,9	10,0	3.055
Sulawesi Selatan	34,0	1,6	0,2	40,5	8,9	14,8	8.677
Sulawesi Tenggara	25,8	2,5	0,3	48,7	9,9	12,8	2.553
Gorontalo	19,9	0,3	0,1	68,5	6,5	4,5	1.182
Sulawesi Barat	15,0	1,5	0,1	48,6	18,1	16,7	1.320
Maluku	31,1	2,0	0,4	29,7	28,9	7,8	1.633
Maluku Utara	27,1	1,7	0,1	24,5	33,6	13,0	1.134
Papua Barat	35,3	2,2	0,3	42,7	14,5	4,9	961
Papua.	21,5	2,1	1,1	33,5	10,8	31,0	4.010
**INDONESIA**	**34,9**	**1,5**	**0,4**	**49,5**	**7,8**	**5,9**	**282.654**

[1] Diangkut artinya diangkut oleh petugas ke TPS atau dibawa sendiri oleh anggota rumah tangga ke TPS

Tabel 4.2.12
Proporsi Cara Pengelolaan Sampah Rumah Tangga menurut Karakteristik, Riskesdas 2018

Karakteristik	Cara Pengelolaan Sampah Rumah Tangga						N Tertimbang
	Diangkut[1]	Ditanam	Dibuat kompos	Dibakar	Dibuang ke kali/selokan/laut	Dibuang sembarang tempat	
**Tempat Tinggal**							
Perkotaan	57,4	0,9	0,2	34,1	4,9	2,5	155.248
Perdesaan	7,4	2,3	0,7	68,2	11,3	10,1	127.406
**Pendidikan KRT**							
Tidak/belum pernah sekolah	13,6	2,1	0,5	58,6	11,7	13,6	19.357
Tidak tamat SD/MI	18,2	2,0	0,6	58,8	11,5	9,0	42.188
Tamat SD/MI	20,9	1,8	0,6	58,9	10,4	7,4	77.514
Tamat SLTP/MTS	33,2	1,5	0,3	53,1	7,2	4,7	46.102
Tamat SLTA/MA	54,7	1,0	0,3	37,2	4,1	2,7	73.398
Tamat D1/D2/D3/PT	68,9	0,9	0,4	25,8	2,2	1,7	24.095
**Pekerjaan KRT**							
Tidak bekerja	40,1	1,3	0,3	45,2	7,2	5,9	31.674
Sekolah	71,3	0,7	0,1	21,8	3,1	3,0	2.351
PNS/TNI/Polri/BUMN/BUMD	59,8	1,2	0,4	33,1	3,3	2,2	13.302
Pegawai swasta	64,6	0,9	0,2	29,5	2,9	1,9	33.234
Wiraswasta	45,1	1,2	0,3	44,3	6,0	3,2	59.238
Petani	7,2	2,4	0,9	67,2	11,3	11,1	79.010
Nelayan	17,5	1,2	0,1	43,2	31,0	7,0	4.376
Buruh/sopir/pembantu ruta	34,2	1,4	0,3	51,5	7,5	5,2	44.842
Lainnya	42,7	1,3	0,4	44,0	7,1	4,5	14.628

[1] Diangkut artinya diangkut oleh petugas ke TPS atau dibawa sendiri oleh anggota rumah tangga ke TPS

Tabel 4.2.13

Proporsi Kualitas Pengelolaan Sampah Rumah Tangga menurut Provinsi, Riskesdas 2018

Provinsi	Pengelolaan Sampah Rumah Tangga				N Tertimbang
	Baik[1]		Tidak Baik[2]		
	%	95% CI	%	95% CI	
Aceh	19,8	18,6-21,1	80,2	78,9-81,4	5.111
Sumatera Utara	30,6	29,1-32,1	69,4	67,9-70,9	13.991
Sumatera Barat	28,0	26,3-29,7	72,0	70,3-73,7	5.184
Riau	28,3	26,3-30,4	71,7	69,6-73,7	6.792
Jambi	27,9	25,9-29,9	72,1	70,1-74,1	3.698
Sumatera Selatan	32,1	30,2-34,1	67,9	65,9-69,8	8.538
Bengkulu	28,4	26,2-30,7	71,6	69,3-73,8	2.082
Lampung	17,0	15,7-18,3	83,0	81,7-84,3	8.838
Bangka Belitung	43,1	40,0-46,3	56,9	53,7-60,0	1.518
Kepulauan Riau	68,4	63,3-73,0	31,6	27,0-36,7	2.367
DKI Jakarta	96,5	95,2-97,5	3,5	2,5-4,8	11.849
Jawa Barat	42,4	40,9-43,9	57,6	56,1-59,1	55.133
Jawa Tengah	28,5	27,5-29,5	71,5	70,5-72,5	37.516
DI Yogyakarta	44,8	41,8-47,9	55,2	52,1-58,2	4.514
Jawa Timur	31,3	30,3-32,4	68,7	67,6-69,7	43.890
Banten	45,0	42,3-47,8	55,0	52,2-57,7	12.733
Bali	60,2	57,9-62,6	39,8	37,4-42,1	4.509
Nusa Tenggara Barat	26,8	24,6-29,0	73,2	71,0-75,4	5.573
Nusa Tenggara Timur	12,7	11,6-13,9	87,3	86,1-88,4	4.761
Kalimantan Barat	21,8	20,3-23,4	78,2	76,6-79,7	4.911
Kalimantan Tengah	35,9	33,4-38,5	64,1	61,5-66,6	2.907
Kalimantan Selatan	47,4	45,2-49,6	52,6	50,4-54,8	4.648
Kalimantan Timur	66,0	63,3-68,5	34,0	31,5-36,7	3.794
Kalimantan Utara	57,0	52,8-61,1	43,0	38,9-47,2	695
Sulawesi Utara	43,5	41,5-45,6	56,5	54,4-58,5	2.579
Sulawesi Tengah	18,8	17,1-20,7	81,2	79,3-82,9	3.055
Sulawesi Selatan	35,8	34,4-37,3	64,2	62,7-65,6	8.677
Sulawesi Tenggara	28,6	26,4-31,0	71,4	69,0 - 73,6	2.553
Gorontalo	20,4	18,5-22,5	79,6	77,5-81,5	1.182
Sulawesi Barat	16,6	13,7-20,1	83,4	79,9-86,3	1.320
Maluku	33,5	30,4-36,9	66,5	63,1-69,6	1.633
Maluku Utara	28,9	26,4-31,6	71,1	68,4-73,6	1.134
Papua Barat	37,9	34,2-41,7	62,1	58,3-65,8	961
Papua.	24,7	22,7-26,7	75,3	73,3-77,3	4.010
**INDONESIA**	**36,8**	**36,4-37,3**	**63,2**	**62,7-63,6**	**282.654**

[1]Jika diangkut oleh petugas atau oleh anggota rumah tangga, ditanam ditanah atau dibuat kompos

[2]Jika dibakar, dibuang ke kali/selokan/laut atau dibuang sembarangan

Tabel 4.2.14

Proporsi Kualitas Pengelolaan Sampah Rumah Tangga menurut Karakteristik, Riskesdas 2018

Karakteristik	Pengelolaan Sampah Rumah Tangga				N Tertimbang
	Baik[1]		Tidak Baik[2]		
	%	95% CI	%	95% CI	
**Tempat Tinggal**					
Perkotaan	58,5	57,7-59,2	41,5	40,8-42,3	155.248
Perdesaan	10,4	10,1-10,8	89,6	89,2-89,9	127.406
**Pendidikan KRT**					
Tidak/belum pernah sekolah	16,2	15,3-17,0	83,8	83,0-84,7	19.357
Tidak tamat SD/MI	20,8	20,1-21,4	79,2	78,6-79,9	42.188
Tamat SD/MI	23,3	22,7-23,8	76,7	76,2-77,3	77.514
Tamat SLTP/MTS	35,0	34,2-35,8	65,0	64,2-65,8	46.102
Tamat SLTA/MA	56,0	55,3-56,7	44,0	43,3-44,7	73.398
Tamat D1/D2/D3/PT	70,2	69,3-71,2	29,8	28,8-30,7	24.095
**Pekerjaan KRT**					
Tidak bekerja	41,7	40,7-42,6	58,3	57,4-59,3	31.674
Sekolah	72,1	68,7-75,3	27,9	24,7-31,3	2.351
PNS/TNI/Polri/BUMN/BUMD	61,4	60,1-62,7	38,6	37,3-39,9	13.302
Pegawai swasta	65,7	64,5-66,8	34,3	33,2-35,5	33.234
Wiraswasta	46,5	45,7-47,3	53,5	52,7-54,3	59.238
Petani	10,4	10,1-10,8	89,6	89,2-89,9	79.010
Nelayan	18,8	16,7-21,2	81,2	78,8-83,3	4.376
Buruh/sopir/pembantu ruta	35,9	34,9-36,8	64,1	63,2-65,1	44.842
Lainnya	44,3	43,0-45,7	55,7	54,3-57,0	14.628

[1]Jika diangkut oleh petugas atau oleh anggota rumah tangga, ditanam ditanah atau dibuat kompos

[2]Jika dibakar, dibuang ke kali/selokan/laut atau dibuang sembarangan

Tabel 4.2.15
Proporsi Frekuensi Menguras Bak Mandi/Ember Besar/Drum di Rumah Tangga menurut Provinsi, Riskesdas 2018

Provinsi	Frekuensi Menguras Bak/Ember besar/Drum			N Tertimbang
	>1 kali dalam seminggu	1x dalam seminggu	1-3 kali dalam sebulan	
Aceh	37,3	40,5	22,2	3.544
Sumatera Utara	48,1	32,4	19,5	10.706
Sumatera Barat	40,7	36,2	23,1	3.839
Riau	39,4	37,0	23,6	4.898
Jambi	23,9	43,5	32,6	2.839
Sumatera Selatan	24,4	37,5	38,0	6.141
Bengkulu	39,6	40,1	20,3	1.638
Lampung	51,8	34,1	14,1	7.149
Bangka Belitung	37,5	36,4	26,1	1.376
Kepulauan Riau	42,0	39,0	19,0	1.846
DKI Jakarta	63,4	28,3	8,3	9.184
Jawa Barat	45,7	34,0	20,3	44.035
Jawa Tengah	46,1	32,3	21,6	30.643
DI Yogyakarta	62,9	26,0	11,1	3.911
Jawa Timur	37,9	34,0	28,2	36.782
Banten	49,7	31,2	19,1	10.202
Bali	46,8	38,8	14,4	3.728
Nusa Tenggara Barat	38,9	30,0	31,1	3.957
Nusa Tenggara Timur	30,3	39,2	30,5	2.962
Kalimantan Barat	19,3	28,3	52,4	3.336
Kalimantan Tengah	32,7	39,7	27,6	2.008
Kalimantan Selatan	34,3	31,5	34,2	3.149
Kalimantan Timur	36,1	38,2	25,7	3.126
Kalimantan Utara	38,1	31,2	30,7	575
Sulawesi Utara	66,6	25,8	7,6	2.172
Sulawesi Tengah	59,1	30,0	10,9	2.395
Sulawesi Selatan	51,9	30,2	17,9	7.470
Sulawesi Tenggara	40,1	33,4	26,4	2.156
Gorontalo	70,5	24,0	5,5	924
Sulawesi Barat	51,1	35,2	13,7	941
Maluku	48,7	35,4	15,8	1.203
Maluku Utara	59,4	32,0	8,6	873
Papua Barat	42,8	32,4	24,8	731
Papua	37,7	33,4	28,9	1.586
**INDONESIA**	**44,1**	**33,5**	**22,4**	**222.030**

Catatan: Hanya ditanyakan pada rumah tangga yang menggunakan bak mandi/ember besar/drum

Tabel 4.2.16
Proporsi Frekuensi Menguras Bak Mandi/Ember Besar/Drum di Rumah Tangga menurut Karakteristik, Riskesdas 2018

Karakteristik	Frekuensi Menguras Bak/Ember besar/Drum			N Tertimbang
	>1 kali dalam seminggu	1x dalam seminggu	1-3 kali dalam sebulan	
**Tempat Tinggal**				
Perkotaan	49,3	33,2	17,5	127.737
Perdesaan	37,0	33,9	29,1	94.293
**Pendidikan KRT**				
Tidak/belum pernah sekolah	37,0	31,3	31,7	13.092
Tidak tamat SD/MI	40,3	32,6	27,1	31.493
Tamat SD/MI	40,8	33,8	25,4	59.840
Tamat SLTP/MTS	45,1	33,2	21,7	36.862
Tamat SLTA/MA	48,8	33,6	17,6	60.784
Tamat D1/D2/D3/PT	48,9	35,6	15,6	19.958
**Pekerjaan KRT**				
Tidak bekerja	44,1	34,0	21,9	25.005
Sekolah	44,0	35,3	20,7	1.782
PNS/TNI/Polri/BUMN/BUMD	47,7	35,4	16,9	11.320
Pegawai swasta	51,3	32,7	15,9	27.466
Wiraswasta	45,0	34,1	20,8	48.761
Petani	36,6	33,7	29,6	56.833
Nelayan	43,7	32,1	24,2	3.279
Buruh/sopir/pembantu ruta	47,5	32,1	20,4	35.769
Lainnya	46,1	32,8	21,1	11.814

Catatan: Hanya ditanyakan pada rumah tangga yang menggunakan bak mandi/ember besar/drum

Tabel 4.2.17
Proporsi Upaya Pemberantasan Sarang Nyamuk di Rumah Tangga
menurut Provinsi, Riskesdas 2018

Provinsi	Upaya Pemberantasan Sarang Nyamuk						N tertimbang
	Memakai obat nyamuk (semprot/bakar/elektrik)	Menaburkan bubuk larvasida pada tempat penampungan air	Ventilasi rumah dipasang kasa nyamuk	Menguras bak mandi/ember besar/drum	Menutup tempat penampungan air di rumah tangga	Memusnahkan barang bekas (kaleng, ban, dll)	
Aceh	59,3	4,9	24,6	73,1	36,2	41,7	5.111
Sumatera Utara	63,4	8,7	20,6	80,3	29,2	38,2	13.991
Sumatera Barat	63,2	5,2	11,4	78,1	38,4	47,6	5.184
Riau	81,0	16,8	23,6	77,3	41,6	54,7	6.792
Jambi	72,9	13,7	13,1	81,5	51,8	52,2	3.698
Sumatera Selatan	68,4	5,8	15,6	76,3	43,5	34,2	8.538
Bengkulu	49,7	7,3	11,8	83,1	43,9	48,6	2.082
Lampung	52,0	7,1	12,6	85,3	41,1	49,4	8.838
Bangka Belitung	77,8	16,6	19,7	94,6	53,7	62,3	1.518
Kepulauan Riau	72,4	9,2	19,8	82,5	30,7	31,2	2.367
DKI Jakarta	57,4	16,8	52,3	83,7	41,1	48,2	11.849
Jawa Barat	62,5	10,0	24,1	85,0	41,4	49,5	55.133
Jawa Tengah	57,5	9,5	9,6	86,7	51,3	61,0	37.516
DI Yogyakarta	50,6	19,5	11,4	91,1	60,2	66,9	4.514
Jawa Timur	60,7	17,3	10,3	89,0	54,1	55,8	43.890
Banten	66,9	11,3	36,2	84,9	41,2	48,7	12.733
Bali	52,1	22,2	15,8	86,2	44,1	56,9	4.509
Nusa Tenggara Barat	49,9	7,8	4,4	74,7	30,1	35,3	5.573
Nusa Tenggara Timur	23,5	14,5	4,8	65,6	55,4	42,5	4.761
Kalimantan Barat	76,6	22,6	12,5	72,3	55,9	51,0	4.911
Kalimantan Tengah	84,7	11,8	14,9	72,7	48,5	47,8	2.907
Kalimantan Selatan	84,7	11,5	20,6	71,8	56,5	52,3	4.648
Kalimantan Timur	80,1	26,2	34,0	86,5	55,1	53,4	3.794
Kalimantan Utara	73,7	31,4	16,6	87,5	54,5	41,3	695
Sulawesi Utara	65,9	3,7	4,3	88,0	54,8	52,2	2.579
Sulawesi Tengah	64,3	7,3	6,9	82,4	51,1	48,6	3.055
Sulawesi Selatan	67,9	14,8	10,5	90,0	52,3	41,0	8.677
Sulawesi Tenggara	72,5	10,9	9,3	88,2	55,9	52,3	2.553
Gorontalo	69,8	3,4	5,8	81,4	49,8	53,1	1.182
Sulawesi Barat	65,5	6,1	5,4	75,5	55,4	40,9	1.320
Maluku	39,6	9,6	6,2	77,5	59,3	42,9	1.633
Maluku Utara	52,9	8,0	6,3	81,7	61,2	44,4	1.134
Papua Barat	44,7	9,0	29,9	79,6	48,9	57,2	961
Papua	31,7	4,4	21,1	42,6	27,6	31,2	4.010
**INDONESIA**	**61,6**	**12,0**	**17,9**	**83,3**	**46,1**	**50,3**	**282.654**

Tabel 4.2.18
Proporsi Upaya Pemberantasan Sarang Nyamuk di Rumah Tangga
menurut Karakteristik, Riskesdas 2018

Karakteristik	Upaya Pemberantasan Sarang Nyamuk						N tertimbang
	Memakai obat nyamuk (semprot/bakar/elektrik)	Menaburkan bubuk larvasida pada tempat penampungan air	Ventilasi rumah dipasang kasa nyamuk	Menguras bak mandi/ember besar/drum	Menutup tempat penampungan air di rumah tangga	Memusnahkan barang bekas (kaleng, ban, dll)	
**Tempat Tinggal**							
Perkotaan	62,1	14,1	25,7	87,2	47,0	53,0	155.248
Perdesaan	60,9	9,5	8,3	78,4	45,0	47,1	127.406
**Pendidikan KRT**							
Tidak/belum pernah sekolah	54,2	7,0	7,5	72.1	40,6	41,9	19.357
Tidak tamat SD/MI	59,4	9,2	9,3	79.2	43,6	47,1	42.188
Tamat SD/MI	61,5	10,4	10,9	81.8	44,8	48,6	77.514
Tamat SLTP/MTS	62,3	12,8	16,8	84.6	46,1	51,0	46.102
Tamat SLTA/MA	63,0	14,7	27,0	87.7	47,7	53,4	73.398
Tamat D1/D2/D3/PT	65,9	16,7	38,0	88.0	53,5	57,8	24.095
**Pekerjaan KRT**							
Tidak bekerja	58,0	12,7	19,6	83,3	45,5	49,1	31.674
Sekolah	51,6	5,5	21,9	81,0	41,1	49,1	2.351
PNS/TNI/Polri/BUMN/BUMD	65,2	18,6	33,3	89,6	54,7	58,7	13.301
Pegawai swasta	65,1	14,7	32,9	88,1	48,4	54,1	33.234
Wiraswasta	64,3	13,3	21,4	87,2	47,7	53,1	59.238
Petani	59,1	9,0	6,8	76,3	43,1	45,2	79.009
Nelayan	67,0	10,4	8,6	79,3	45,4	42,2	4.374
Buruh/sopir/pembantu ruta	60,8	11,3	15,7	84,5	45,1	52,1	44.842
Lainnya	62,8	13,2	20,2	85,4	47,7	50,7	14.628

Tabel 4.2.19
Proporsi Upaya Pemberantasan Sarang Nyamuk (3M dan 3M plus) di Rumah Tangga menurut Provinsi, Riskesdas 2018

Provinsi	Upaya Pemberantasan Sarang Nyamuk				N Tertimbang
	3M[1]		3M plus[2]		
	%	95% CI	%	95% CI	
Aceh	22,5	21,0-24,0	17,6	16,3-18,9	5.111
Sumatera Utara	19,9	18,4-21,5	15,7	14,4-17,1	13.991
Sumatera Barat	25,9	24,3-27,5	18,6	17,3-20,0	5.184
Riau	27,7	25,9-29,7	25,1	23,4-26,9	6.792
Jambi	38,2	35,8-40,6	30,8	28,7-33,0	3.698
Sumatera Selatan	23,3	21,3-25,4	18,6	16,9-20,3	8.538
Bengkulu	30,6	28,1-33,1	19,2	17,5-21,1	2.082
Lampung	30,3	28,2-32,4	19,9	18,4-21,6	8.838
Bangka Belitung	40,5	37,2-43,8	35,8	32,9-38,8	1.518
Kepulauan Riau	16,2	13,9-18,7	13,7	11,6-16,0	2.367
DKI Jakarta	27,3	25,0-29,6	23,6	21,6-25,7	11.849
Jawa Barat	28,7	27,5-29,8	22,3	21,3-23,3	55.133
Jawa Tengah	37,3	36,3-38,3	25,2	24,3-26,0	37.516
DI Yogyakarta	43,6	40,7-46,6	29,3	27,0-31,7	4.514
Jawa Timur	39,9	38,8-41,0	28,9	28,0-29,8	43.890
Banten	26,3	24,3-28,4	22,2	20,4-24,0	12.733
Bali	29,8	27,7-32,1	19,8	18,1-21,6	4.509
Nusa Tenggara Barat	17,7	16,1-19,4	11,0	9,9-12,2	5.573
Nusa Tenggara Timur	28,1	26,5-29,8	14,6	13,3-15,9	4.761
Kalimantan Barat	34,6	32,6-36,6	30,6	28,8-32,5	4.911
Kalimantan Tengah	27,6	25,4-29,9	24,6	22,6-26,8	2.907
Kalimantan Selatan	32,8	30,7-35,0	30,3	28,3-32,4	4.648
Kalimantan Timur	35,4	32,6-38,2	32,8	30,2-35,6	3.794
Kalimantan Utara	27,7	23,7-32,0	24,6	21,1-28,6	695
Sulawesi Utara	39,9	37,4-42,5	27,6	25,7-29,7	2.579
Sulawesi Tengah	32,9	30,8-35,0	24,2	22,4-26,1	3.055
Sulawesi Selatan	27,9	26,4-29,5	21,6	20,2-23,0	8.677
Sulawesi Tenggara	40,1	37,2-43,1	32,7	30,3-35,3	2.553
Gorontalo	37,8	34,0-41,8	28,0	24,7-31,5	1.182
Sulawesi Barat	31,6	27,6-35,8	23,4	20,2-27,0	1.320
Maluku	34,1	30,5-38,0	19,5	17,0-22,3	1.633
Maluku Utara	31,7	29,0-34,6	20,4	18,2-22,9	1.134
Papua Barat	36,5	33,0-40,1	26,3	23,3-29,5	961
Papua	18,8	17,2-20,5	14,1	12,8-15,6	4.010
**INDONESIA**	**31,2**	**30,9-31,6**	**23,4**	**23,1-23,8**	**282.654**

[1] 3M meliputi menguras, menutup dan memusnahkan.
[2] 3M Plus meliputi menguras, menutup, dan memusnahkan, ditambah dengan salah satu upaya plus (menggunakan obat nyamuk atau bubuk larvasida atau kasa pada ventilasi)

Tabel 4.2.20
Proporsi Upaya Pemberantasan Sarang Nyamuk (3M dan 3M plus) di Rumah Tangga menurut Karakteristik, Riskesdas 2018

Karakteristik	Pemberantasan Sarang Nyamuk				N Tertimbang
	3M[1]		3M plus[2]		
	%	95% CI	%	95% CI	
**Tempat Tinggal**					
Perkotaan	32,7	32,2 - 33,3	25,4	24,9 - 25,9	155.248
Perdesaan	29,4	28,9 - 29,9	21,0	20,6 - 21,4	127.406
**Pendidikan KRT**					
Tidak/belum pernah sekolah	25,2	24,2 - 26,2	16,8	16,0 - 17,7	19.357
Tidak tamat SD/MI	28,0	27,3 - 28,7	19,6	19,0 - 20,2	42.188
Tamat SD/MI	30,0	29,4 - 30,5	21,5	21,0 - 22,0	77.514
Tamat SLTP/MTS	31,3	30,6 - 32,0	23,4	22,8 - 24,1	46.102
Tamat SLTA/MA	33,6	32,9 - 34,2	26,6	26,0 - 27,2	73.398
Tamat D1/D2/D3/PT	38,7	37,7 - 39,8	32,3	31,3 - 33,3	24.095
**Pekerjaan KRT**					
Tidak bekerja	31,0	30,1 - 31,8	22,6	21,9 - 23,4	31.674
Sekolah	27,4	24,1 - 31,1	18,7	16,0 - 21,8	2.351
PNS/TNI/Polri/BUMN/BUMD	40,0	38,8 - 41,2	32,9	31,8 - 34,0	13.302
Pegawai swasta	33,8	32,8 - 34,9	27,7	26,7 - 28,7	33.234
Wiraswasta	33,8	33,1 - 34,5	26,1	25,5 - 26,7	59.238
Petani	27,6	27,1 - 28,1	19,3	18,8 - 19,7	79.010
Nelayan	26,5	24,5 - 28,5	19,8	18,1 - 21,6	4.376
Buruh/sopir/pembantu ruta	30,4	29,6 - 31,2	22,3	21,6 - 23,0	44.842
Lainnya	32,1	30,9 - 33,3	24,3	23,2 - 25,4	14.628

[1] 3M meliputi menguras, menutup dan memusnahkan.

[2] 3M Plus meliputi menguras, menutup, dan memusnahkan, ditambah dengan salah satu upaya plus (menggunakan obat nyamuk atau bubuk larvasida atau kasa pada ventilasi)

4.3 Keadaan Rumah

Penilaian keadaan rumah dibedakan pada tiga ruangan yaitu ruang tidur, ruang dapur dan ruang keluarga. Keadaan rumah dinilai berdasarkan kondisi rumah yang dapat mencegah risiko berkembangnya penyakit, yaitu:

a. Jendela dibuka setiap hari : jika pada ruang tidur utama/dapur/ruang keluarga di rumah tangga memiliki jendela yang dibuka setiap hari
b. Ventilasi cukup : jika pada ruang tidur utama/dapur/ruang keluarga di rumah tangga memiliki ventilasi dan luasnya >10% dari luas lantai
c. Pencahayaan cukup : jika pada ruang tidur utama/dapur/ruang keluarga di rumah tangga memiliki pencahayaan yang cukup, ditandai dari kemampuan membaca atau melihat objek kecil di ruangan.

1. Keadaan Ruang Tidur Utama

Proporsi ruang tidur dengan keadaan jendela yang dibuka setiap hari, dihitung dengan formula:

$$Proporsi\ ruta\ dengan\ jendela\ yang\ selalu\ dibuka\ pada\ ruang\ tidur = \frac{\sum Ruta\ dengan\ jendela\ yg\ selalu\ dibuka\ pd\ ruang\ tidur}{\sum Ruta\ yang\ memiliki\ ruang\ tidur}$$

Proporsi ruang tidur dengan ventilasi cukup, dihitung dengan formula:

$$Proporsi\ ruta\ dengan\ luas\ ventilasi > 10\%\ luas\ lantai\ pada\ ruang\ tidur = \frac{\sum Ruta\ dengan\ luas\ ventilasi > 10\%\ luas\ lantai\ pada\ ruang\ tidur}{\sum Ruta\ yang\ memiliki\ ruang\ tidur}$$

Proporsi ruang tidur dengan pencahayaan cukup, dihitung dengan formula:

$$Proporsi\ ruta\ dengan\ pencahayaan\ cukup\ pada\ ruang\ tidur = \frac{\sum Ruta\ dengan\ pencahayaan\ cukup\ pada\ ruang\ tidur}{\sum Ruta\ yang\ memiliki\ ruang\ tidur}$$

2. Keadaan Ruang Dapur

Proporsi ruang dapur dengan keadaan jendela yang dibuka setiap hari, dihitung dengan formula:

$$Proporsi\ ruta\ dengan\ jendela\ yang\ selalu\ dibuka\ pada\ ruang\ dapur = \frac{\sum Ruta\ dengan\ jendela\ yang\ selalu\ dibuka\ pada\ ruang\ dapur}{\sum Ruta\ yang\ memiliki\ ruang\ dapur}$$

Proporsi ruang dapur dengan ventilasi cukup, dihitung dengan formula:

$$Proporsi\ ruta\ dengan\ luas\ ventilasi > 10\%\ luas\ lantai\ pada\ ruang\ dapur = \frac{\sum Ruta\ dengan\ luas\ ventilasi > 10\%\ luas\ lantai\ pada\ ruang\ dapur}{\sum Ruta\ yang\ memiliki\ ruang\ dapur}$$

Proporsi ruang dapur dengan pencahayaan cukup, dihitung dengan formula:

$$Proporsi\ ruta\ dengan\ pencahayaan\ cukup\ pada\ ruang\ dapur = \frac{\sum Ruta\ dengan\ pencahayaan\ cukup\ pd\ ruang\ dapur}{\sum Ruta\ yang\ memiliki\ ruang\ dapur}$$

3. Keadaan Ruang Keluarga

Proporsi ruang keluarga dengan keadaan jendela yang dibuka setiap hari, dihitung dengan formula:

$$Proporsi\ ruta\ dengan\ jendela\ yang\ selalu\ dibuka\ pada\ ruang\ keluarga = \frac{\sum Ruta\ dengan\ jendela\ yang\ selalu\ dibuka\ pd\ ruang\ keluarga}{\sum Ruta\ yang\ memiliki\ ruang\ keluarga}$$

Proporsi ruang keluarga dengan ventilasi cukup, dihitung dengan formula:

$$Proporsi\ ruta\ dengan\ luas\ ventilasi > 10\%\ luas\ lantai\ pada\ ruang\ keluarga = \frac{\sum Ruta\ dengan\ luas\ ventilasi > 10\%\ luas\ lantai\ pada\ ruang\ keluarga}{\sum Ruta\ yang\ memiliki\ ruang\ keluarga}$$

Proporsi ruang keluarga dengan pencahayaan cukup, dihitung dengan formula:

$$Proporsi\ ruta\ dengan\ pencahayaan\ cukup\ pada\ ruang\ keluarga = \frac{\sum Ruta\ dengan\ pencahayaan\ cukup\ pd\ ruang\ keluarga}{\sum Ruta\ yang\ memiliki\ ruang\ keluarga}$$

Tabel 4.3.1
Proporsi Keadaan Kamar Tidur Utama di Rumah Tangga menurut Provinsi, Riskesdas 2018

Provinsi	Keadaan Kamar Tidur Utama (%)			N Tertimbang
	Jendela dibuka tiap hari	Ventilasi cukup	Pencahayaan cukup	
Aceh	54,3	57,4	74,2	5.094
Sumatera Utara	67,8	54,2	73,0	13.874
Sumatera Barat	64,6	53,7	79,2	5.134
Riau	63,8	62,0	78,0	6.764
Jambi	68,5	63,5	77,0	3.686
Sumatera Selatan	62,9	47,6	72,5	8.425
Bengkulu	67,0	55,3	77,2	2.075
Lampung	63,1	57,1	79,8	8.817
Bangka Belitung	65,2	62,1	80,9	1.511
Kepulauan Riau	60,3	56,8	76,3	2.349
DKI Jakarta	35,8	43,4	62,4	11.263
Jawa Barat	40,1	43,2	69,0	54.560
Jawa Tengah	47,4	47,3	74,4	37.383
DI Yogyakarta	52,7	52,7	77,8	4.473
Jawa Timur	54,5	52,4	72,2	43.679
Banten	34,1	42,1	65,8	12.614
Bali	56,5	63,9	80,7	4.498
Nusa Tenggara Barat	44,7	38,8	65,8	5.536
Nusa Tenggara Timur	58,4	47,7	70,5	4.718
Kalimantan Barat	71,4	60,2	83,2	4.894
Kalimantan Tengah	72,8	56,6	81,6	2.868
Kalimantan Selatan	70,1	55,1	79,8	4.503
Kalimantan Timur	70,6	58,9	81,6	3.751
Kalimantan Utara	68,7	54,0	78,0	691
Sulawesi Utara	75,2	60,8	86,1	2.573
Sulawesi Tengah	64,8	47,4	72,5	3.037
Sulawesi Selatan	50,8	40,1	69,1	8.416
Sulawesi Tenggara	61,2	50,6	76,4	2.546
Gorontalo	76,6	66,0	82,0	1.175
Sulawesi Barat	52,2	52,6	74,4	1.306
Maluku	70,2	50,1	76,7	1.628
Maluku Utara	71,9	62,6	83,4	1.127
Papua Barat	72,0	61,2	77,6	952
Papua	43,9	38,1	55,9	3.442
**INDONESIA**	**52,3**	**49,5**	**72,6**	**279.362**

Tabel 4.3.2
Proporsi Keadaan Kamar Tidur Utama di Rumah Tangga menurut Karakteristik, Riskesdas 2018

Karakteristik	Keadaan Kamar Tidur Utama (%)			N Tertimbang
	Jendela dibuka tiap hari	Ventilasi cukup	Pencahayaan cukup	
**Tempat Tinggal**				
Perkotaan	51,6	51,0	73,1	153.570
Perdesaan	53,3	47,8	72,0	125.792
**Pendidikan KRT**				
Tidak/belum pernah sekolah	37,4	36,9	58,5	18.856
Tidak tamat SD/MI	45,6	42,2	65,9	41.571
Tamat SD/MI	49,4	45,5	70,6	76.672
Tamat SLTP/MTS	54,2	49,7	73,5	45.538
Tamat SLTA/MA	57,7	55,2	77,3	72.756
Tamat D1/D2/D3/PT	65,4	67,7	85,6	23.968
**Pekerjaan KRT**				
Tidak bekerja	51,0	49,1	71,4	31.316
Sekolah	57,0	55,5	78,3	2.329
PNS/TNI/Polri/BUMN/BUMD	68,7	68,0	87,5	13.267
Pegawai swasta	55,0	55,7	76,8	32.902
Wiraswasta	55,0	52,9	75,1	58.710
Petani	50,9	45,2	69,5	77.874
Nelayan	55,1	46,1	70,8	4.299
Buruh/sopir/pembantu ruta	44,2	42,4	67,4	44.209
Lainnya	54,5	50,9	74,1	14.453

Tabel 4.3.3
Proporsi Keadaan Ruang Masak/Dapur di Rumah Tangga menurut Provinsi, Riskesdas 2018

Provinsi	Keadaan Ruang Masak/Dapur (%)			N Tertimbang
	Jendela dibuka tiap hari	Ventilasi cukup	Pencahayaan cukup	
Aceh	53,1	54,6	74,2	5.085
Sumatera Utara	62,0	49,9	71,6	13.958
Sumatera Barat	58,1	49,1	77,0	5.045
Riau	62,0	58,2	78,7	6.739
Jambi	68,3	62,1	77,5	3.708
Sumatera Selatan	60,1	44,3	70,8	8.536
Bengkulu	56,1	49,0	73,6	2.072
Lampung	50,4	49,7	74,1	8.816
Bangka Belitung	57,8	57,8	79,9	1.509
Kepulauan Riau	48,5	46,2	69,5	2.297
DKI Jakarta	28,5	37,3	60,3	10.726
Jawa Barat	30,8	36,4	63,7	54.025
Jawa Tengah	35,0	42,9	74,3	37.067
DI Yogyakarta	32,0	47,6	80,4	4.321
Jawa Timur	45,5	49,7	73,7	43.080
Banten	21,5	34,2	59,0	12.571
Bali	46,7	56,8	78,4	4.020
Nusa Tenggara Barat	29,8	31,5	59,1	5.203
Nusa Tenggara Timur	42,1	35,6	57,8	4.573
Kalimantan Barat	67,3	56,4	81,3	4.906
Kalimantan Tengah	67,7	51,9	79,5	2.888
Kalimantan Selatan	63,0	50,8	77,6	4.548
Kalimantan Timur	65,6	56,5	82,7	3.761
Kalimantan Utara	63,6	51,4	77,6	695
Sulawesi Utara	58,9	55,1	81,4	2.579
Sulawesi Tengah	50,4	42,8	69,9	3.025
Sulawesi Selatan	40,0	37,4	67,8	8.611
Sulawesi Tenggara	53,4	47,9	74,3	2.512
Gorontalo	53,7	52,1	75,6	1.162
Sulawesi Barat	45,4	52,8	74,5	1.323
Maluku	50,2	41,1	68,4	1.627
Maluku Utara	54,8	54,2	78,0	1.119
Papua Barat	66,6	56,2	76,3	949
Papua.	40,0	33,8	53,5	3.503
**INDONESIA**	**43,0**	**44,4**	**70,3**	**276.558**

Tabel 4.3.4
Proporsi Keadaan Ruang Masak/Dapur di Rumah Tangga menurut Karakteristik, Riskesdas 2018

Karakteristik	Keadaan Ruang Masak/Dapur (%)			N Tertimbang
	Jendela dibuka tiap hari	Ventilasi cukup	Pencahayaan cukup	
**Tempat Tinggal**				
Perkotaan	40,8	45,3	70,9	150.610
Perdesaan	45,6	43,4	69,6	125.948
**Pendidikan KRT**				
Tidak/belum pernah sekolah	31,7	34,4	58,2	18.719
Tidak tamat SD/MI	37,6	38,0	64,3	41.389
Tamat SD/MI	41,0	41,2	68,4	76.461
Tamat SLTP/MTS	44,3	44,5	71,2	45.053
Tamat SLTA/MA	46,5	48,9	74,5	71.198
Tamat D1/D2/D3/PT	54,9	60,8	82,6	23.736
**Pekerjaan KRT**				
Tidak bekerja	40,7	43,3	69,0	31.072
Sekolah	35,8	43,2	70,7	1.923
PNS/TNI/Polri/BUMN/BUMD	58,8	62,0	84,9	13.268
Pegawai swasta	43,3	48,4	73,2	31.968
Wiraswasta	46,1	48,3	73,4	58.196
Petani	43,7	41,3	67,8	77.989
Nelayan	46,5	41,6	69,9	4.316
Buruh/sopir/pembantu ruta	34,0	37,5	65,0	43.519
Lainnya	44,2	45,5	71,5	14.305

Tabel 4.3.5
Proporsi Keadaan Ruang Keluarga di Rumah Tangga menurut Provinsi, Riskesdas 2018

Provinsi	Keadaan Ruang Keluarga (%)			N Tertimbang
	Jendela dibuka tiap hari	Ventilasi cukup	Pencahayaan cukup	
Aceh	55,7	64,6	80,7	5.037
Sumatera Utara	76,1	64,2	82,3	13.934
Sumatera Barat	66,1	63,9	88,3	4.969
Riau	63,8	68,1	84,0	6.644
Jambi	70,0	69,7	82,5	3.667
Sumatera Selatan	64,8	52,7	77,3	8.263
Bengkulu	61,6	58,9	80,8	2.077
Lampung	55,8	62,0	81,0	8.590
Bangka Belitung	63,3	64,5	85,4	1.503
Kepulauan Riau	61,7	59,7	80,7	2.233
DKI Jakarta	42,9	57,9	77,7	10.425
Jawa Barat	41,3	53,2	79,2	53.382
Jawa Tengah	45,7	55,3	83,4	35.973
DI Yogyakarta	48,1	58,6	85,6	4.215
Jawa Timur	55,4	63,0	83,7	41.941
Banten	35,9	52,7	78,6	12.294
Bali	57,4	71,2	90,2	2.490
Nusa Tenggara Barat	42,6	47,6	76,4	5.196
Nusa Tenggara Timur	61,5	50,9	75,1	4.421
Kalimantan Barat	68,6	62,7	86,2	4.866
Kalimantan Tengah	74,7	61,2	87,2	2.828
Kalimantan Selatan	65,6	60,1	83,6	4.478
Kalimantan Timur	68,1	61,0	85,2	3.691
Kalimantan Utara	74,3	63,1	85,3	681
Sulawesi Utara	74,1	69,4	90,1	2.564
Sulawesi Tengah	61,9	52,9	77,8	2.949
Sulawesi Selatan	52,3	48,0	77,3	8.152
Sulawesi Tenggara	61,2	53,8	78,0	2.398
Gorontalo	74,7	69,6	86,6	1.156
Sulawesi Barat	53,7	54,1	76,3	1.296
Maluku	63,0	50,3	77,4	1.597
Maluku Utara	66,6	65,6	85,4	1.092
Papua Barat	75,2	66,9	82,5	934
Papua	43,8	37,9	55,7	3.645
**INDONESIA**	**53,0**	**57,7**	**81,1**	**269.581**

Tabel 4.3.6

Proporsi Keadaan Ruang Keluarga di Rumah Tangga menurut Karakteristik, Riskesdas 2018

Karakteristik	Keadaan Ruang Keluarga (%)			N Tertimbang
	Jendela dibuka tiap hari	Ventilasi cukup	Pencahayaan cukup	
**Tempat Tinggal**				
Perkotaan	51,7	59,5	82,0	146.154
Perdesaan	54,5	55,6	80,1	123.427
**Pendidikan KRT**				
Tidak/belum pernah sekolah	41,6	47,8	71,1	17.749
Tidak tamat SD/MI	47,9	51,9	77,3	39.797
Tamat SD/MI	50,8	54,9	80,2	73.813
Tamat SLTP/MTS	54,4	57,3	81,5	43.386
Tamat SLTA/MA	57,3	62,5	84,2	68.209
Tamat D1/D2/D3/PT	62,0	71,0	89,1	22.799
**Pekerjaan KRT**				
Tidak bekerja	50,9	57,9	80,3	29.836
Sekolah	48,2	56,8	81,4	1.494
PNS/TNI/Polri/BUMN/BUMD	63,9	70,4	89,5	12.703
Pegawai swasta	56,4	63,5	84,9	30.092
Wiraswasta	54,9	60,6	82,6	56.042
Petani	52,9	53,8	78,6	75.626
Nelayan	56,5	53,6	80,7	3.974
Buruh/sopir/pembantu ruta	45,9	52,9	78,8	41.467
Lainnya	53,1	58,2	81,8	13.5823

BAB 5
PENYAKIT MENULAR

Bab ini berisi beberapa indikator mengenai penyakit menular secara terbatas. yaitu penyakit yang berhubungan dengan indikator SDGs, IPKM, Renstra Kementerian Kesehatan 2014-2019, Program Indonesia Sehat dengan Pendekatan Keluarga (PIS-PK) dan Gerakan Masyarakat Hidup Sehat (Germas). Pengumpulan data ini dilakukan melalui wawancara dan pemeriksaan laboratorium.

Wawancara mengenai penyakit bertujuan untuk mendapatkan informasi prevalensi penyakit menurut riwayat diagnosis oleh tenaga kesehatan dan riwayat pernah mengalami tanda dan gejala penyakit yang didata. Responden ditanya apakah pernah didiagnosis mengalami penyakit tertentu oleh tenaga kesehatan (D: diagnosis). Responden yang menyatakan tidak pernah didiagnosis, ditanyakan lagi apakah pernah/sedang mengalami gejala klinis spesifik penyakit tersebut (G: gejala).

Penyakit yang didata hanya berdasarkan riwayat diagnosis dokter (spesialis dan dokter umum) adalah Tb Paru dan Hepatitis, sedangkan ISPA, Pneumonia, Diare, Malaria dan Filariasis berdasarkan riwayat diagnosis tenaga kesehatan (Dokter spesialis, dokter umum, bidan, dan perawat). Selain melalui riwayat diagnosis nakes, ISPA, Pneumonia, Diare, Malaria dan Filariasis juga diukur melalui gejala-gejala penyakit yang pernah dialami.

Pemeriksaan laboratorium bertujuan untuk mengukur prevalensi penyakit dilakukan dengan 2 tahap, yaitu:

1. Malaria melalui pemeriksaan *rapid diagnostic test* (RDT) dan apusan darah tebal (demam dalam 2 hari sebelum puldata Riskesdas 2018) yang dilakukan pada semua umur responden.
2. Penyakit yang dapat dicegah dengan imunisasi (PD3I) meliputi Difteri, Pertusis, Campak, Rubella, Hepatitis B, serta Tetanus sesuai prioritas kelompok umur.

Hasil analisis disajikan dalam bentuk tabel silang dengan beberapa variabel kunci yaitu Provinsi dan beberapa karakteristik individu (kelompok usia, pendidikan, pekerjaan, dan status perkotaan serta perdesaan). Hal ini bertujuan untuk memberi

gambaran hasil intervensi program dan memberikan opsi intervensi menurut kewilayahan dan karakter penduduk.

5.1 ISPA

Pada Riskesdas 2018, ISPA ditanyakan pada semua responden semua umur dalam kurun waktu 1 bulan sebelum enumerasi. Prevalensi ISPA menurut riwayat diagnosis diukur melalui pertanyaan: “Dalam 1 bulan terakhir, apakah responden pernah didiagnosis ISPA oleh tenaga kesehatan (dokter/perawat/bidan)?” Jika menjawab tidak maka ditanyakan riwayat mengalami gejala ISPA melalui pertanyaan yang menanyakan demam, batuk kurang dari 2 minggu, pilek/hidung tersumbat dan sakit tenggorokan. Jika responden menjawab pernah mengalami gejala demam, batuk kurang dari 2 minggu, pilek/hidung tersumbat dan/atau sakit tenggorokan, maka responden dianggap mengalami ISPA. Prevalensi ISPA dihitung dengan formula sebagai berikut:

$$Prevalensi\ ISPA = \frac{\sum Kasus\ ISPA\ riwayat\ diagnosis\ (D)\ dan/atau\ gejala (DG)}{\sum ART\ Semua\ Umur}$$

$$Prevalensi\ ISPA\ BALITA = \frac{\sum Kasus\ ISPA\ riwayat\ diagnosis\ (D)\ dan/atau\ gejala\ (DG)\ Pada\ Balita}{\sum \text{Balita}}$$

Tabel 5.1.1
Prevalensi ISPA menurut Provinsi, Riskesdas 2018

Provinsi	Infeksi Saluran Pernapasan Akut (ISPA)				
	D[1]		D/G[2]		N tertimbang
	%	95% CI	%	95% CI	
Aceh	4,3	3,8-4,8	9,4	8,7-10,0	20.244
Sumatera Utara	2,8	2,4-3,2	6,8	6,3-7,3	55.351
Sumatera Barat	4,1	3,7-4,6	9,5	8,9-10,1	20.663
Riau	2,2	1,9-2,6	7,1	6,6-7,7	26.085
Jambi	3,2	2,7-3,8	5,5	4,9-6,2	13.692
Sumatera Selatan	3,5	3,1-4,0	6,9	6,4-7,5	32.126
Bengkulu	8,9	8,0-10	11,8	10,8-12,9	7.531
Lampung	4,2	3,8-4,7	7,4	6,8-8,0	32.148
Bangka Belitung	1,5	1,1-1,9	6,9	6,2-7,6	5.592
Kepulauan Riau	3,8	3,1-4,7	6,5	5,6-7,5	8.173
DKI Jakarta	2,7	2,4-3,2	8,5	7,8-9,2	40.210
Jawa Barat	4,7	4,4-5,0	11,2	10,8-11,7	186.809
Jawa Tengah	4,6	4,3-4,9	8,5	8,2-8,8	132.565
DI Yogyakarta	2,8	2,3-3,4	6,9	6,2-7,6	14.602
Jawa Timur	6,0	5,7-6,3	9,5	9,2-9,9	151.878
Banten	5,3	4,6-6,0	11,9	11,1-12,8	48.621
Bali	4,6	4,0-5,3	9,7	9,0-10,4	16.481
Nusa Tenggara Barat	2,7	2,3-3,2	11,7	11-12,5	19.247
Nusa Tenggara Timur	7,3	6,7-7,9	15,4	14,6-16,2	20.599
Kalimantan Barat	3,2	2,8-3,6	8,4	7,8-9,0	19.190
Kalimantan Tengah	6,2	5,5-7,0	8,9	8,1-9,7	10.189
Kalimantan Selatan	2,3	1,9-2,7	7,1	6,6-7,7	16.043
Kalimantan Timur	3,8	3,2-4,6	8,1	7,3-9,1	13.977
Kalimantan Utara	2,1	1,5-2,9	6,8	5,8-7,8	2.733
Sulawesi Utara	2,1	1,8-2,4	6,2	5,7-6,8	9.542
Sulawesi Tengah	2,6	2,2-3,0	9,4	8,8-10,1	11.548
Sulawesi Selatan	1,9	1,6-2,1	8,3	7,8-8,7	33.693
Sulawesi Tenggara	2,8	2,3-3,3	8,1	7,4-8,8	10.167
Gorontalo	2,0	1,7-2,5	9,5	8,4-10,9	4.547
Sulawesi Barat	1,8	1,3-2,3	6,9	6,1-7,9	5.195
Maluku	5,6	4,6-6,7	8,5	7,5-9,7	6.801
Maluku Utara	2,4	2,0-2,9	5,7	5,1-6,3	4.723
Papua Barat	7,5	6,5-8,6	12,3	11,1-13,5	3.588
Papua	10,5	9,5-11,7	13,1	12-14,3	12.736
**INDONESIA**	**4,4**	**4,3-4,5**	**9,3**	**9,2-9,4**	**1.017.290**

1. D: menurut diagnosis oleh tenaga kesehatan (dokter, perawat atau bidan)
2. D/G: menurut diagnosis oleh tenaga kesehatan (dokter, perawat atau bidan) atau gejala yang pernah dialami oleh ART.

Tabel 5.1.2
Prevalensi ISPA menurut Karakteristik, Riskesdas 2018

Karakteristik	ISPA				
	D[1]		D/G[2]		N tertimbang
	%	95% CI	%	95% CI	
**Kelompok umur**					
< 1	7,4	6,8-8,0	9,4	8,8-10,1	18.225
1-4	8,0	7,6-8,3	13,7	13,3-14,1	73.188
5-14	4,9	4,7-5,1	10,6	10,4-10,8	182.338
15-24	3,2	3,0-3,3	7,8	7,6-8,1	165.644
25-34	3,5	3,4-3,7	8,2	7,9-8,4	159.708
35-44	3,7	3,6-3,9	8,6	8,4-8,8	151.539
45-54	4,1	3,9-4,3	9,0	8,8-9,2	124.652
54-64	4,5	4,3-4,8	9,2	8,9-9,5	83.251
65-74	5,0	4,7-5,3	9,6	9,2-10,1	40.180
75+	5,4	4,9-5,9	9,3	8,7-9,9	18.565
**Jenis Kelamin**					
Laki	4,3	4,2-4,5	9,0	8,8 - 9,1	510.714
Perempuan	4,4	4,3-4,5	9,7	9,5 - 9,8	506.576
**Pendidikan**					
Tidak sekolah	5,2	4,9-5,5	11,2	10,8-11,6	70.895
Tidak tamat SD/MI	4,7	4,5-4,9	10,6	10,4-10,9	181.429
Tamat SD/MI	4,0	3,9-4,2	9,2	9,0-9,5	215.967
Tamat SMP/MTS	3,5	3,4-3,7	8,1	7,9-8,3	160.320
Tamat SMA/MA	3,4	3,2-3,5	7,4	7,2-7,6	210.746
Tamat D1-D3/PT	3,4	3,2-3,7	6,9	6,6-7,2	64.093
**Pekerjaan**					
Tidak bekerja	3,9	3,7-4,0	8,9	8,6-9,1	233.629
Sekolah	3,7	3,5-3,9	8,4	8,2-8,7	126.626
Pegawai Negeri/BUMN/TNI	3,5	3,2-3,8	6,1	5,7-6,5	21.931
Pegawai Swasta	3,7	3,4-4,0	7,8	7,4-8,2	75.781
Wiraswasta	3,6	3,4-3,8	7,8	7,6-8,1	105.489
Petani/buruhtani	4,4	4,3-4,6	9,6	9,4-9,8	133.261
Nelayan	4,2	3,5-5,0	10,8	9,7-12,1	5.556
Buruh/sopir/pembantu ruta	3,3	3,1-3,6	8,6	8,2-9,0	75.590
Lainnya	3,9	3,6-4,2	8,6	8,2-9,1	40.644
**Tempat Tinggal**					
Perkotaan	4,1	4,0-4,3	9,0	8,8 - 9,2	556.419
Perdesaan	4,7	4,5-4,8	9,7	9,5 - 9,9	460.871

1. D: menurut diagnosis oleh tenaga kesehatan (dokter, perawat atau bidan)
2. D/G: menurut diagnosis oleh tenaga kesehatan (dokter, perawat atau bidan) atau gejala yang pernah dialami oleh ART.

Tabel 5.1.3
Prevalensi ISPA pada Balita menurut Provinsi, Riskesdas 2018

Provinsi	ISPA Pada Balita				
	D[1]		D/G[2]		N tertimbang
	%	95% CI	%	95% CI	
Aceh	5,9	4,9-7,1	10,4	9,1-11,8	2.250
Sumatera Utara	3,7	2,9-4,7	8,7	7,5-10,0	5.895
Sumatera Barat	8,0	6,7-9,7	12,8	11,2-14,6	2.179
Riau	3,2	2,4-4,3	7,3	6,2-8,7	2.813
Jambi	5,0	3,8-6,4	7,3	6,0-8,9	1.279
Sumatera Selatan	5,5	4,5-6,7	8,6	7,3-10,0	3.079
Bengkulu	14,0	11,7-16,7	16,4	14,1-19,1	716
Lampung	7,7	6,5-9,1	11,3	9,9-12,9	3.094
Bangka Belitung	2,1	1,2-3,6	8,3	6,4-10,8	527
Kepulauan Riau	6,2	4,4-8,8	8,8	6,6-11,7	872
DKI Jakarta	5,4	3,9-7,3	13,2	11,0-15,8	3.582
Jawa Barat	8,2	7,3-9,2	14,7	13,5-16,0	17.228
Jawa Tengah	9,7	8,8-10,7	13,8	12,7-14,8	10.551
DI Yogyakarta	6,0	4,2-8,5	10,6	8,2-13,6	1069
Jawa Timur	12,9	11,8-14,0	17,2	16,0-18,4	11.272
Banten	11,0	9,0-13,3	17,7	15,4-20,2	4.813
Bali	9,9	7,8-12,6	13,7	11,4-16,5	1.275
Nusa Tenggara Barat	5,3	4,1-6,9	11,3	9,7-13,2	1.985
Nusa Tenggara Timur	12,6	11,3-14,1	18,6	17,1-20,3	2.496
Kalimantan Barat	5,5	4,4-6,9	9,7	8,3-11,4	1.928
Kalimantan Tengah	11,8	9,9-13,9	15,1	12,9-17,5	1.000
Kalimantan Selatan	3,3	2,6-4,3	7,2	6,0-8,6	1.563
Kalimantan Timur	6,5	4,9-8,7	10,8	8,8-13,3	1.368
Kalimantan Utara	3,0	1,7-5,5	7,9	5,6-11,0	309
Sulawesi Utara	3,0	2,2-4,0	6,2	5,0-7,8	821
Sulawesi Tengah	3,8	2,9-5,0	11,3	9,7-13,2	1.155
Sulawesi Selatan	2,7	2,1-3,5	8,7	7,7-9,9	3.269
Sulawesi Tenggara	3,6	2,7-4,9	10,1	8,4-12,0	1.169
Gorontalo	2,6	1,7-3,9	6,6	4,8-9,0	445
Sulawesi Barat	2,3	1,4-3,7	7,3	5,6-9,6	584
Maluku	6,9	5,3-9,0	9,0	7,2-11,3	779
Maluku Utara	3,6	2,5-5,1	6,0	4,6-7,8	536
Papua Barat	9,2	7,4-11,4	13,2	10,8-16,1	415
Papua	11,9	10,1-14,1	14,0	12,1-16,3	1.302
**INDONESIA**	**7,8**	**7,5-8,2**	**12,8**	**12,5-13,2**	**93.620**

1. D: menurut diagnosis oleh tenaga kesehatan (dokter, perawat atau bidan)
2. D/G: menurut diagnosis oleh tenaga kesehatan (dokter, perawat atau bidan) atau gejala yang pernah dialami oleh ART.

Tabel 5.1.4
Prevalensi ISPA pada Balita menurut Karakteristik, Riskesdas 2018

Karakteristik	ISPA pada Balita				N tertimbang
	D[1]		D/G[2]		
	%	95% CI	%	95% CI	
**Kelompok Usia Balita (Bulan)**					
0-11	7,4	6,8-8,0	9,4	8,8-10,1	18.665
12-23	9,4	8,7-10,1	14,4	13,6-15,3	18.333
24-35	8,5	7,9-9,1	13,8	13,0-14,6	19.112
36-47	7,3	6,7-7,9	13,1	12,4-13,9	18.821
48-59	6,7	6,2-7,3	13,5	12,7-14,3	18.688
**Jenis Kelamin**					
Laki – laki	8,1	7,8-8,5	13,2	12,7-13,7	47.764
Perempuan	7,5	7,1-8,0	12,4	11,9-12,9	45.856
**Tempat Tinggal**					
Perkotaan	7,6	7,1-8,1	12,8	12,2-13,4	50.361
Perdesaan	8,1	7,8-8,5	12,9	12,5-13,4	43.259

1. D: menurut diagnosis oleh tenaga kesehatan (dokter, perawat atau bidan)
2. D/G: menurut diagnosis oleh tenaga kesehatan (dokter, perawat atau bidan) atau gejala yang pernah dialami oleh ART.

5.2 Pneumonia

Prevalensi diagnosis dan gejala pneumoni diperoleh melalui wawancara dengan pertanyaan: 1) “Dalam 1 tahun terakhir, apakah responden pernah didiagnosis menderita radang paru (Pneumonia) dengan atau tanpa dilakukan foto dada (foto rontgen) oleh tenaga kesehatan (dokter/perawat/bidan)?”; 2) “Dalam 1 tahun terakhir, apakah [NAMA] mengalami gejala penyakit sebagai berikut” dengan opsi jawaban: “demam tinggi”, “batuk”, “kesulitan bernapas”. Jika menjawab ketiganya maka dikatakan pneumonia. Prevalens pneumonia dihitung dengan formula sebagai berikut:

$$Prevalensi\ Pneumoni\ = \frac{\sum\ Kasus\ pneumoni\ (riwayat\ diagnosis\ dan/atau\ gejala)}{\sum ART\ Semua\ Umur}$$

$$Prevalensi\ Pneumoni\ Balita\ = \frac{\begin{array}{c}\sum Kasus\ pneumoni\ (riwayat\ \ diagnosis\ dan/atau\ gejala)\\ Pada\ Balita\ (0-59\ Bulan)\end{array}}{\sum Balita\ (0-59\ Bulan)}$$

Tabel 5.2.1
Prevalensi Pneumonia menurut Provinsi, Riskesdas 2018

Provinsi	Pneumonia				
	D[1]		D/G[2]		N tertimbang
	%	95% CI	%	95% CI	
Aceh	2,5	2,3-2,7	3,8	3,5-4,1	20.244
Sumatera Utara	2,1	1,9-2,3	4,0	3,6-4,3	55.351
Sumatera Barat	1,7	1,4-2,1	3,2	2,9-3,6	20.663
Riau	1,5	1,3-1,7	3,0	2,7-3,3	26.085
Jambi	1,9	1,6-2,2	2,6	2,3-3,0	13.692
Sumatera Selatan	2,3	2,0-2,6	3,7	3,3-4,1	32.126
Bengkulu	3,4	2,7-4,1	5,3	4,5-6,1	7.531
Lampung	2,0	1,7-2,3	3,1	2,8-3,4	32.148
Kep. Bangka Belitung	1,4	1,1-1,9	3,3	2,8-3,9	5.592
Kepulauan Riau	1,7	1,3-2,3	2,6	2,1-3,4	8.173
DKI Jakarta	2,2	1,9-2,5	3,8	3,4-4,3	40.210
Jawa Barat	2,6	2,4-2,8	4,7	4,5-5,0	186.809
Jawa Tengah	1,8	1,7-1,9	3,4	3,2-3,5	132.565
DI Yogyakarta	1,3	1,1-1,6	3,7	3,3-4,2	14.602
Jawa Timur	1,8	1,7-2,0	3,4	3,2-3,5	151.878
Banten	2,0	1,7-2,3	4,9	4,5-5,3	48.621
Bali	1,0	0,8-1,2	3,3	3,0-3,7	16.481
Nusa Tenggara Barat	1,4	1,2-1,6	4,6	4,2-5,0	19.247
Nusa Tenggara Timur	1,1	1,0-1,3	6,9	6,5-7,4	20.599
Kalimantan Barat	2,1	1,9-2,4	4,4	4,0-4,8	19.190
Kalimantan Tengah	1,9	1,6-2,2	3,7	3,3-4,2	10.189
Kalimantan Selatan	1,7	1,5-2,0	3,5	3,1-3,8	16.043
Kalimantan Timur	1,8	1,5-2,2	3,4	3,0-3,9	13.977
Kalimantan Utara	2,5	2,0-3,1	4,0	3,3-4,8	2.733
Sulawesi Utara	1,8	1,5-2,1	4,0	3,7-4,4	9.542
Sulawesi Tengah	1,7	1,5-1,9	5,9	5,4-6,4	11.548
Sulawesi Selatan	1,6	1,5-1,8	5,1	4,7-5,4	33.693
Sulawesi Tenggara	1,6	1,4-1,9	4,3	3,9-4,8	10.167
Gorontalo	1,8	1,5-2,2	6,1	5,4-6,9	4.547
Sulawesi Barat	2,2	1,7-2,7	4,9	4,2-5,6	5.195
Maluku	1,9	1,6-2,3	4,2	3,7-4,8	6.801
Maluku Utara	2,1	1,7-2,5	4,3	3,9-4,9	4.723
Papua Barat	2,9	2,4-3,5	6,1	5,4-7,0	3.588
Papua	3,6	3,1-4,0	7,0	6,4-7,6	12.736
**INDONESIA**	**2,0**	**2,0-2,1**	**4,0**	**4,0-4,1**	**1.017.290**

1. D: menurut diagnosis oleh tenaga kesehatan (dokter, perawat atau bidan)
2. D/G: menurut diagnosis oleh tenaga kesehatan (dokter, perawat atau bidan) atau gejala yang pernah dialami oleh ART.

Tabel 5.2.2
Prevalensi Pneumonia menurut Karakteristik, Riskesdas 2018

Karakteristik	Prevalens Pneumonia				N tertimbang
	D[1]		D/G[2]		
	%	95% CI	%	95% CI	
**Kelompok umur**					
< 1	2,1	1,9-2,5	3,8	3,4-4,3	18.225
1-4	2,1	1,9-2,3	5,0	4,7-5,3	73.188
5-14	1,7	1,6-1,8	3,5	3,4-3,7	182.338
15-24	1,8	1,7-1,9	3,7	3,5-3,9	165.644
25-34	1,9	1,8-2,0	3,6	3,4-3,8	159.708
35-44	1,9	1,8-2,0	3,7	3,6-3,9	151.539
45-54	2,2	2,1-2,3	4,3	4,2-4,5	124.652
54-64	2,5	2,3-2,6	4,8	4,6-5,1	83.251
65-74	3,0	2,7-3,2	5,8	5,5-6,1	40.180
75+	2,9	2,6-3,3	5,7	5,3-6,2	18.565
**Jenis Kelamin**					
Laki	2,1	2,1-2,2	4,2	4,1-4,3	510.714
Perempuan	1,9	1,8-1,9	3,9	3,8-4,0	506.576
**Pendidikan**					
Tidak sekolah	2,1	2,0-2,3	5,1	4,8-5,3	70.895
Tidak tamat SD/MI	1,9	1,8-2,1	4,5	4,3-4,6	181.429
Tamat SD/MI	2,1	2,0-2,2	4,2	4,1-4,4	215.967
Tamat SMP/MTS	1,9	1,8-2,0	3,6	3,4-3,7	160.320
Tamat SMA/MA	2,0	1,9-2,1	3,5	3,3-3,6	210.746
Tamat D1-D3/PT	2,0	1,9-2,2	3,1	2,9-3,3	64.093
**Pekerjaan**					
Tidak bekerja	2,2	2,1-2,3	4,3	4,1-4,4	233.629
Sekolah	1,6	1,5-1,8	3,3	3,1-3,4	126.626
PNS/TNI/Polri/BUMN/BUMD	2,2	2,0-2,5	3,2	2,9-3,5	21.931
Pegawai Swasta	2,0	1,8-2,2	3,4	3,1-3,6	75.781
Wiraswasta	2,1	2,0-2,3	3,7	3,5-3,9	105.489
Nelayan	2,1	1,6-2,6	5,6	4,9-6,4	5.556
Petani/Buruh tani	2,1	2,0-2,2	4,8	4,7-5,0	133.261
Lainnya	2,0	1,8-2,1	4,0	3,8-4,2	116.233
**Tempat Tinggal**					
Perkotaan	2,0	1,9-2,1	3,8	3,7-3,9	556.419
Perdesaan	2,0	1,9-2,1	4,3	4,2-4,4	460.871

1. D: menurut diagnosis oleh tenaga kesehatan (dokter, perawat atau bidan)
2. D/G: menurut diagnosis oleh tenaga kesehatan (dokter, perawat atau bidan) atau gejala yang pernah dialami oleh ART.

Tabel 5.2.3
Prevalensi Pneumonia pada Balita menurut Provinsi, Riskesdas 2018

Provinsi	Prevalensi Pneumonia Balita				N tertimbang
	D[1]		D/G[2]		
	%	95% CI	%	95% CI	
Aceh	1,9	1,4 - 2,5	3,2	2,6 - 3,9	2.250
Sumatera Utara	2,0	1,6 - 2,6	4,2	3,5 - 5,1	5.895
Sumatera Barat	1,3	0,9 - 1,9	3,8	3,1 - 4,7	2.179
Riau	1,2	0,8 - 1,7	2,8	2,2 - 3,6	2.813
Jambi	1,4	0,9 - 2,4	2,9	2,0 - 4,2	1.279
Sumatera Selatan	2,6	1,9 - 3,6	4,6	3,7 - 5,8	3.079
Bengkulu	3,5	2,4 - 5,1	5,4	4,0 - 7,2	716
Lampung	2,1	1,5 - 2,8	3,6	2,8 - 4,6	3.094
Bangka Belitung	1,4	0,7 - 2,7	4,7	3,4 - 6,5	527
Kepulauan Riau	1,6	0,8 - 3,2	2,7	1,5 - 4,6	872
DKI Jakarta	2,0	1,3 - 3,1	4,2	3,1 - 5,6	3.582
Jawa Barat	2,8	2,3 - 3,4	5,5	4,8 - 6,3	17.228
Jawa Tengah	2,1	1,7 - 2,7	3,9	3,3 - 4,5	10.551
DI Yogyakarta	3,7	2,5 - 5,5	8,3	6,3 -10,8	1.069
Jawa Timur	2,0	1,6 - 2,5	4,4	3,8 – 5,0	11.272
Banten	1,5	1,0 - 2,2	5,5	4,4 - 6,8	4.813
Bali	1,6	1,0 - 2,5	5,1	3,9 - 6,7	1.275
Nusa Tenggara Barat	1,5	1,0 - 2,2	5,3	4,3 - 6,6	1.985
Nusa Tenggara Timur	1,6	1,2 - 2,1	8,8	7,7-10,0	2.496
Kalimantan Barat	1,7	1,2 - 2,5	4,8	3,8 – 6,0	1.928
Kalimantan Tengah	2,1	1,4 - 3,2	4,3	3,2 - 5,8	1.000
Kalimantan Selatan	1,2	0,8 - 1,8	2,8	2,1 - 3,6	1.563
Kalimantan Timur	2,0	1,2 - 3,3	3,7	2,6 - 5,2	1.368
Kalimantan Utara	3,1	1,8 - 5,3	5,0	3,4 - 7,4	309
Sulawesi Utara	1,6	1,0 - 2,6	4,1	3,1 - 5,5	821
Sulawesi Tengah	2,1	1,4 - 3,1	7,3	6,0 - 8,7	1.155
Sulawesi Selatan	1,2	0,8 - 1,7	5,3	4,5 - 6,3	3.269
Sulawesi Tenggara	1,4	0,9 – 2,0	5,5	4,5 - 6,8	1.169
Gorontalo	2,2	1,3 - 3,6	6,6	4,9 - 8,7	445
Sulawesi Barat	2,1	1,3 - 3,4	6,0	4,5 - 7,9	584
Maluku	2,1	1,2 - 3,6	5,0	3,8 - 6,7	779
Maluku Utara	2,5	1,6 - 3,7	5,0	3,7 - 6,8	536
Papua Barat	2,2	1,3 - 3,7	5,9	4,3 - 8,1	415
Papua	3,9	3,0 - 5,2	7,7	6,4 - 9,4	1.302
**INDONESIA**	**2,1**	**1,9 - 2,2**	**4,8**	**4,5 – 5,0**	**93.619**

1. D: menurut diagnosis oleh tenaga kesehatan (dokter, perawat atau bidan)
2. D/G: menurut diagnosis oleh tenaga kesehatan (dokter, perawat atau bidan) atau gejala yang pernah dialami oleh ART.

Tabel 5.2.4
Prevalensi Pneumonia pada Balita menurut Karakteristik, Riskesdas 2018

Karakteristik	Pneumonia				N tertimbang
	D[1]		D/G[2]		
	%	95% CI	%	95% CI	
**Kelompok Usia (Bulan)**					
0-11	2,1	1,9-2,5	3,8	3,4-4,3	18.665
12-23	2,5	2,2-3,0	6,0	5,4-6,6	18.333
24-35	2,2	1,9-2,6	5,3	4,8-5,9	19.112
36-47	1,8	1,5-2,1	4,5	4,1-5,0	18.821
48-59	1,8	1,5-2,1	4,1	3,7-4,6	18.688
**Jenis Kelamin**					
Laki	2,1	1,9-2,4	5,0	4,7-5,4	47.764
Perempuan	2,0	1,8-2,3	4,5	4,2-4,8	45.855
**Tempat Tinggal**					
Perkotaan	2,2	1,9-2,4	4,7	4,3-5,0	50.360
Perdesaan	2,0	1,8-2,2	4,9	4,6-5,2	43.259

1. D: menurut diagnosis oleh tenaga kesehatan (dokter, perawat atau bidan)
2. D/G: menurut diagnosis oleh tenaga kesehatan (dokter, perawat atau bidan) atau gejala yang pernah dialami oleh ART.

5.3 Tuberculosis Paru

Penyakit TB paru ditanyakan pada responden untuk kurun waktu ≤1 tahun berdasarkan riwayat diagnosis tenaga kesehatan melalui pemeriksaan dahak, foto toraks atau keduanya, berbeda dibandingkan dengan Riskesdas sebelumnya, penyakit TB paru ditanyakan pada responden untuk kurun waktu ≤1 tahun berdasarkan diagnosis yang ditegakkan oleh dokter melalui pemeriksaan dahak, foto toraks atau keduanya (Riskesdas sebelumnya melalui riwayat diagnosis tenaga kesehatan).

Prevalensi TB Paru adalah persentase responden yang pernah didiagnosis menderita TB Paru oleh dokter terhadap jumlah total responden dengan formula sebagai berikut:

$$\boldsymbol{Prevalensi\ Tb\ Paru} = \frac{\sum \boldsymbol{kasus\ Tb\ Paru\ (riwayat\ diagnosis\ dokter)}}{\sum \boldsymbol{ART\ Semua\ Umur}}$$

Tabel 5.3.1
Prevalensi TB Paru berdasarkan Riwayat Diagnosis Dokter menurut Provinsi, Riskesdas 2018

Provinsi	Prevalensi TB Paru		
	%	95% CI	N tertimbang
Aceh	0,49	0,4 - 0,6	20.244
Sumatera Utara	0,30	0,2 - 0,4	55.351
Sumatera Barat	0,31	0,2 - 0,4	20.663
Riau	0,22	0,2 - 0,3	26.085
Jambi	0,27	0,2 - 0,4	13.692
Sumatera Selatan	0,53	0,4 - 0,8	32.126
Bengkulu	0,41	0,3 - 0,5	7.531
Lampung	0,33	0,3 - 0,4	32.148
Bangka Belitung	0,09	0,1 - 0,2	5.592
Kepulauan Riau	0,29	0,2 - 0,5	8.173
DKI Jakarta	0,51	0,4 - 0,7	40.210
Jawa Barat	0,63	0,6 - 0,7	186.809
Jawa Tengah	0,36	0,3 - 0,4	132.565
DI Yogyakarta	0,16	0,1 - 0,2	14.602
Jawa Timur	0,29	0,3 - 0,3	151.878
Banten	0,76	0,6 - 0,9	48.621
Bali	0,13	0,1 - 0,2	16.481
Nusa Tenggara Barat	0,32	0,2 - 0,4	19.247
Nusa Tenggara Timur	0,27	0,2 - 0,3	20.599
Kalimantan Barat	0,36	0,3 - 0,5	19.190
Kalimantan Tengah	0,39	0,3 - 0,5	10.189
Kalimantan Selatan	0,41	0,3 - 0,5	16.043
Kalimantan Timur	0,33	0,2 - 0,5	13.977
Kalimantan Utara	0,52	0,3 - 0,8	2.733
Sulawesi Utara	0,39	0,3 - 0,5	9.542
Sulawesi Tengah	0,39	0,3 - 0,5	11.548
Sulawesi Selatan	0,36	0,3 - 0,4	33.693
Sulawesi Tenggara	0,41	0,3 - 0,5	10.167
Gorontalo	0,42	0,3 - 0,6	4.547
Sulawesi Barat	0,31	0,2 - 0,5	5.195
Maluku	0,39	0,2 - 0,7	6.801
Maluku Utara	0,30	0,2 - 0,5	4.723
Papua Barat	0,53	0,4 - 0,8	3.588
Papua	0,77	0,6 - 0,9	12.736
**INDONESIA**	**0,42**	**0,4 - 0,4**	**1.017.290**

Tabel 5.3.2
Prevalensi TB Paru berdasarkan Riwayat Diagnosis Dokter menurut Karakteristik, Riskesdas 2018

Karakteristik	Prevalensi TB Paru		
	%	95% CI	N tertimbang
**Kelompok umur**			
< 1	0,1	0,0 - 0,2	18.225
1-4	0,3	0,3 - 0,4	73.188
5-14	0,2	0,2 - 0,2	182.338
15-24	0,2	0,2 - 0,3	165.644
25-34	0,4	0,3 - 0,4	159.708
35-44	0,4	0,4 - 0,5	151.539
45-54	0,6	0,5 - 0,6	124.652
55-64	0,8	0,7 - 0,9	83.251
65-74	1,0	0,9 - 1,2	40.180
75+	0,8	0,7 - 1,0	18.565
**Jenis kelamin**			
Laki-laki	0,5	0,4 - 0,5	510.714
Perempuan	0,4	0,3 - 0,4	506.576
**Pendidikan**			
Tidak/belum pernah sekolah	0,6	0,5 - 0,7	70.895
Tidak tamat SD/MI	0,5	0,4 - 0,5	181.429
Tamat SD/MI	0,5	0,5 - 0,6	215.967
Tamat SLTP/MTS	0,4	0,3 - 0,4	160.320
Tamat SLTA/MA	0,4	0,3 - 0,4	210.746
Tamat D1/D2/D3/PT	0,2	0,2 - 0,3	64.093
**Pekerjaan**			
Tidak bekerja	0,6	0,5 - 0,6	233.629
Sekolah	0,2	0,1 - 0,2	126.626
PNS/TNI/Polri/BUMN/BUMD	0,2	0,2 - 0,3	21.931
Pegawai swasta	0,3	0,2 - 0,4	75.781
Wiraswasta	0,9	0,4 - 0,6	105.489
Petani/buruh tani	0,5	0,5 - 0,6	133.261
Nelayan	0,6	0,7 - 1,2	5.556
Lainnya	0,5	0,4 - 0,6	116.233
**Tempat tinggal**			
Perkotaan	0,4	0,4 - 0,5	556.419
Perdesaan	0,4	0,4 - 0,4	460.871

Pemeriksaan TB Paru yang ditanyakan pada penelitian ini meliputi pemeriksan: 1) Laboratoris Dahak seperti *smear microscopis* (sputum BTA), biakan/kultur bakteriologis, pemeriksaan Xpert MTB/RIF/Tes Cepat Molekuler (TCM), pemeriksaan molekuler lainnya (LPA); 2) Pemeriksaan radiologis, rontgen dada/foto thoraks; dan 3) Tuberkulin test, biasanya dilakukan pada kasus TB paru anak. Formula yang digunakan adalah sebagai berikut:

$$\text{Cara pemeriksaan TB paru} = \frac{Jenis\ Pemeriksaan}{\text{Total yang didiagnosis TB Paru}}$$

Dalam pengendalian TB Nasional, diagnosis TB paru pada orang dewasa harus ditegakan terlebih dahulu dengan pemeriksaan bakteriologis (pemeriksaan *smear microscopis*, biakan maupun tes cepat). Jika hasil pemeriksaan bakteriologis negatif, maka diagnosis TB Paru dapat dilakukan dengan secara klinis baik pemeriksaan klinis maupun penunjang (foto thoraks) dan ditetapkan oleh dokter terlatih TB. Diagnosis TB tidak dibenarkan hanya menggunakan pemeriksaan serologis saja, foto thoraks saja, atau tuberkulin saja.

Selain cara melakukan diagnosis pada Riskesdas 2018 juga dapat menggambarkan proporsi pengobatan yang pernah atau sedang diterima oleh responden. Saat ini, paduan Obat Anti Tb (OAT) yang digunakan di Indonesia mengikuti rekomendasi World Health Organization (WHO) dan *International Standard for TB Care* (ISTC). Paduan obat Program Nasional Pengendalian TB di Indonesia meliputi: 1) *Fixed Dose Combination* (FDC) atau kombinasi dosis tetap (KDT) yaitu paket obat untuk satu periode pengobatan; 2) Kombipak yaitu paket obat lepas yang terdiri dari Isoniasid (H), Rifampisin (R), Pirazinamid (Z) dan Etambutol (E) yang dikemas dalam bentuk blister dan dikatagorikan sebagai obat lepasan; dan 3) Obat Lepasan yaitu Sediaan obat tunggal/bukan paket, diberikan oleh tenaga kesehatan dalam bentuk terpisah dengan dosis berdasarkan keputusan klinis.

OAT disediakan dalam bentuk paket (KDT), bertujuan untuk memudahkan pemberian obat dan menjamin kontinuitas pengobatan sampai selesai dengan prinsip satu paket untuk satu pasien dalam satu periode pengobatan. Terapi yang

diberikan adalah persentase responden yang mendapatkan obat baik Kombinasi Dosis Tetap (KDT) maupun lepasan terhadap jumlah responden pernah didiagnosis menderita TB Paru.

$$\text{Jenis obat yang diberikan} = \frac{\text{Jenis Obat yang diberikan (KDT atau Lepasan)}}{\sum \text{Kasus Tb Paru (ART yang didiagnosis menderita TB Paru)}}$$

Pengawas Minum Obat (PMO) adalah seseorang yang ditunjuk dan dipercaya untuk mengawasi dan memantau penderita tuberkulosis dalam meminum obatnya secara teratur dan tuntas. PMO bisa berasal dari keluarga, tetangga, kader, tokoh masyarakat atau petugas kesehatan. PMO merupakan kegiatan yang dilakukan untuk menjamin kepatuhan penderita untuk minum obat sesuai dengan dosis dan jadwal mencegah terjadinya resistensi obat. Pada Riskesdas 2018, keberadaan PMO diukur menurut pengakuan responden. Proporsi keberadaan PMO diukur dengan formula:

$$\text{Proporsi keberadaan PMO} = \frac{\sum \text{kasus TB Paru dengan PMO}}{\text{jumlah kasus TB Paru}}$$

Proporsi penderita TB Paru yang berobat rutin adalah persentase responden penderita TB Paru yang berobat rutin terhadap yang pernah didiagnosis menderita TB Paru oleh tenaga kesehatan

$$\text{Rutinitas minum obat} = \frac{\sum \text{ART yang rutin minum obat secara rutin}}{\sum \text{Kasus Tb Paru (ART yang didiagnosis menderita TB Paru)}}$$

Tabel 5.3.3
Proporsi Pemeriksaan/Diagnosis TB Paru, Jenis Terapi TB Paru yang Diberikan dan Ketersediaan PMO menurut Provinsi, Riskesdas 2018

Provinsi	Pemeriksaan/diagnosis (%)			Terapi yang diberikan (%)		Ketersediaan PMO (%)	N Tertimbang[3]
	Dahak	Rontgen dada	*Mantoux Test*[1]	KDT[2]	Lepasan		
Aceh	73,5	82,0	36,5	78,7	51,9	53,8	93
Sumatera Utara	69,4	78,9	15,6	77,9	54,1	68,5	154
Sumatera Barat	76,5	70,6	49,6	87,8	54,7	70,3	61
Riau	64,9	78,2	0,0	74,3	44,0	57,1	55
Jambi	77,7	65,7	6,6	87,0	49,7	54,8	34*
Sumatera Selatan	50,6	49,8	15,4	52,0	29,3	77,7	162
Bengkulu	56,5	59,4	37,6	68,8	51,8	74,3	29*
Lampung	74,1	69,8	40,2	75,4	52,6	78,6	100
Bangka Belitung	78,5	61,5	0,0	59,1	46,0	46,0	5*
Kepulauan Riau	69,5	76,2	9,0	80,5	40,7	60,6	23*
DKI Jakarta	79,4	86,7	69,0	82,9	45,9	70,0	194
Jawa Barat	62,2	88,2	55,5	79,7	52,4	69,9	1.105
Jawa Tengah	63,5	83,5	26,2	81,0	53,4	68,9	452
DI Yogyakarta	60,7	80,0	19,9	94,1	39,5	64,1	22*
Jawa Timur	73,7	82,4	49,0	80,4	48,3	66,9	414
Banten	69,0	83,3	33,7	84,5	51,8	54,1	351
Bali	87,9	74,5	94,1	82,1	40,3	41,6	20*
Nusa Tenggara Barat	86,1	78,6	36,8	88,6	35,0	78,1	58
Nusa Tenggara Timur	75,0	76,2	38,6	82,1	51,3	63,3	52
Kalimantan Barat	64,6	76,0	39,0	71,8	49,4	57,3	65
Kalimantan Tengah	70,9	81,2	48,3	81,6	47,6	64,8	37*
Kalimantan Selatan	80,4	68,7	55,7	89,4	53,9	62,7	62
Kalimantan Timur	77,3	69,9	34,5	71,9	49,9	69,1	43*
Kalimantan Utara	72,2	87,6	20,0	55,5	55,4	52,7	14*
Sulawesi Utara	81,8	66,0	22,4	75,3	45,0	58,3	35*
Sulawesi Tengah	83,3	69,1	32,1	85,4	56,3	51,5	43*
Sulawesi Selatan	88,0	80,5	43,8	81,1	38,7	58,3	114
Sulawesi Tenggara	73,3	65,7	0,0	74,5	48,7	57,6	40*
Gorontalo	81,1	80,1	46,5	86,6	50,1	60,8	18*
Sulawesi Barat	82,1	74,9	40,8	79,6	53,1	54,7	15*
Maluku	77,7	53,7	12,2	62,2	46,1	68,3	25*
Maluku Utara	73,4	65,1	0,7	83,8	62,5	73,3	13*
Papua Barat	79,8	68,7	12,5	66,2	49,4	64,4	18*
Papua	75,0	68,6	46,0	71,9	41,2	61,5	93
**INDONESIA**	**68,9**	**80,2**	**43,8**	**79,0**	**49,4**	**66,2**	**4.021**

1. Hanya untuk umur 15 tahun ke bawah
2. Kombinasi dosis tetap (KDT) yaitupaket obat untuk satu periode pengobatan
3. N tertimbang tidak untuk *Mantoux test*

* N tertimbang <50

Tabel 5.3.4

Proporsi Pemeriksaan/Diagnosis TB Paru, Jenis Terapi TB Paru yang Diberikan dan Ketersediaan PMO menurut Karakteristik, Riskesdas 2018

**Karakteristik**	**Pemeriksaan/diagnosis (%)**			**Terapi yang diberikan (%)**		**Ketersediaan PMO (%)**	**N Tertimbang[3]**
	Dahak	Rontgen dada	*Mantoux Test*[1]	KDT[2]	Lepasan		
**Kelompok umur**							
< 1	30,2	50,8	17,3	55,7	33,9	76,0	13*
1-4	27,7	83,5	39,6	72,2	43,5	87,6	229
5-14	43,9	79,3	46,4	75,2	41,4	83,3	361
15-24	69,4	80,2	56,2	78,8	54,4	70,2	382
25-34	74,3	75,5	N/A	83,7	48,2	62,9	558
35-44	75,6	77,5	N/A	78,6	48,5	63,2	610
45-54	73,2	83,3	N/A	80,9	52,8	59,9	680
55-64	72,6	82,7	N/A	80,9	51,2	61,8	651
65-74	78,8	83,6	N/A	77,4	51,4	61,4	393
75+	84,9	75,5	N/A	73,0	47,1	66,8	144
**Jenis kelamin**							
Laki-laki	68,6	80,8	37,9	77,9	51,3	65,2	2.261
Perempuan	69,2	79,6	49,6	80,5	47,1	67,6	1.760
**Tempat tinggal**							
Perkotaan	68,1	83,1	51,6	79,3	50,7	69,0	2.214
Perdesaan	69,9	76,8	31,6	78,7	47,9	62,8	1.807
**Pendidikan**							
Tidak sekolah	66,9	76,5	59,2	77,3	43,3	69,6	419
Tidak tamat SD	66,5	75,8	41,1	74,9	46,8	65,0	819
Tamat SD	75,2	81,8	49,7	82,1	52,1	64,5	1.091
Tamat SLTP	70,9	81,4	76,3	81,0	48,4	54,7	568
Tamat SLTA	74,2	82,4	N/A	80,5	53,8	68,3	734
Tamat D1/D2/D3/PT	81,8	87,0	N/A	81,1	56,2	71,3	139
**Pekerjaan**							
Tidak bekerja	75,3	81,3	48,0	80,1	52,4	67,3	1.257
Sekolah	56,3	75,7	52,4	73,8	37,7	83,3	227
PNS/TNI/Polri/BUMN/BUMD	82,4	89,4	N/A	75,3	46,6	72,3	50
Pegawai Swasta	80,9	83,9	N/A	87,0	48,4	63,2	212
Wiraswasta	74,6	82,3	N/A	77,4	56,8	62,5	515
Nelayan	74,8	82,6	N/A	84,6	61,8	51,7	49*
Petani/Buruh tani	72,2	73,3	N/A	78,5	46,1	57,5	755
Lainnya	70,8	83,9	N/A	81,7	51,1	59,0	165

1. Hanya untuk umur 15 tahun ke bawah
2. Kombinasi dosis tetap (KDT) yaitupaket obat untuk satu periode pengobatan
3. N tertimbang tidak untuk *Mantoux test*

   * N tertimbang <50

Tabel 5.3.5
Proporsi Penderita TB (< 6 bulan) yang Minum Obat Secara Rutin menurut Provinsi, Riskesdas 2018

Provinsi	Minum obat Rutin[1]		
	%	95% CI	N tertimbang
Aceh	55,5	44,8 - 65,7	45*
Sumatera Utara	72,6	60,6 – 82,0	78
Sumatera Barat	54,8	35,1 - 73,1	22*
Riau	54,9	39,1 - 69,8	29*
Jambi	68,0	45,9 - 84,2	13*
Sumatera Selatan	58,7	40,5 - 74,7	46*
Bengkulu	79,3	65,7 - 88,5	10*
Lampung	68,7	50,0 - 82,9	40*
Bangka Belitung	51,6	51,6 - 51,6	2*
Kepulauan Riau	60,3	30,3 - 84,2	4*
DKI Jakarta	71,5	48,0 - 87,2	80
Jawa Barat	71,1	62,3 - 78,5	447
Jawa Tengah	77,7	68,2 - 85,0	174
DI Yogyakarta	70,0	19,1 - 95,8	7*
Jawa Timur	72,9	64,2 - 80,1	163
Banten	58,3	41,8 - 73,1	151
Bali	75,4	67,8 - 81,6	11*
Nusa Tenggara Barat	54,9	31,9 - 75,9	14*
Nusa Tenggara Timur	57,7	45,0 - 69,6	14*
Kalimantan Barat	69,0	56,6 - 79,2	22*
Kalimantan Tengah	57,3	39,1 - 73,7	16*
Kalimantan Selatan	67,8	52,0 - 80,3	27*
Kalimantan Timur	78,8	53,4 - 92,4	17*
Kalimantan Utara	76,6	43,2 - 93,4	4*
Sulawesi Utara	60,2	40,7 - 77,0	19*
Sulawesi Tengah	71,4	55,4 - 83,4	19*
Sulawesi Selatan	69,9	56,1 - 80,8	45*
Sulawesi Tenggara	80,0	66,9 - 88,7	19*
Gorontalo	84,0	56,3 - 95,5	10*
Sulawesi Barat	59,3	31,2 - 82,4	7*
Maluku	71,6	49,1 - 86,9	12*
Maluku Utara	58,1	39,9 - 74,3	7*
Papua Barat	65,3	43,8 - 82,0	9*
Papua	78,3	63,9 - 88,1	32*
**INDONESIA**	**69,2**	**65,7 - 72,5**	**1.616**

[1]selalu minum obat dalam 1 periode pengobatan tanpa terlewat
* N tertimbang <50

Tabel 5.3.6
Proporsi Penderita TB (< 6 bulan) yang Minum Obat Secara Rutin menurut Karakteristik, Riskesdas 2018

Karakteristik	Minum obat Rutin[1]		
	%	95% CI	N tertimbang
**Kelompok umur**			
< 1	94,0	66,8-99,2	7*
1-4	78,6	62,9-88,8	120
5-9	80,0	67,3-88,7	93
10-14	81,5	71,4-88,6	39*
15-24	70,7	58,8-80,3	157
25-34	63,4	54,6-71,4	251
35-44	66,3	58,7-73,1	214
45-54	59,9	51,5-67,8	261
55-64	65,3	55,0-74,3	263
65-74	69,0	53,6-81,1	159
75+	69,2	65,7-72,5	51
**Jenis kelamin**			
Laki-laki	68,5	63,8 - 72,7	930
Perempuan	70,1	64,9 - 74,9	686
**Tempat tinggal**			
Perkotaan	72,0	66,5 - 76,9	887
Perdesaan	65,7	61,5 - 69,7	729
**Pendidikan**			
Tidak sekolah	66,3	55,1-76,0	155
Tidak tamat SD	60,9	52,1-69,0	331
Tamat SD	65,5	58,6-71,7	415
Tamat SLTP	76,2	67,9-82,9	224
Tamat SLTA	74,4	67,1-80,6	312
Tamat D1/D2/D3/PT	68,2	49,3-82,5	69
**Pekerjaan**			
Tidak bekerja	68,3	62,2-73,9	526
Sekolah	86,0	76,7-92,0	74
PNS/TNI/Polri/BUMN/BUMD	68,1	43,3-85,6	19*
Pegawai Swasta	71,8	56,3-83,4	89
Wiraswasta	66,3	56,6-74,9	204
Nelayan	61,0	40,6-78,2	24*
Petani/Buruh tani	59,6	52,8-66,1	298
Lainnya	70,2	57,7-80,3	209

[1]selalu minum obat dalam 1 periode pengobatan tanpa terlewat
* N tertimbang <50

Tabel 5.3.7
Proporsi Penderita TB (< 6 bulan) menurut Alasan Tidak Rutin Minum Obat
menurut Provinsi, Riskesdas 2018

Provinsi	Alasan tidak rutin minum obat (%)								N ter-timbang
	Sering lupa	Tidak tersedia di fasyankes	Tidak tahan efek samping	Masa pengobatan terasa lama	Tidak mampu membeli obat TB secara rutin	Tidak rutin berobat	Merasa sudah sehat	Lainnya	
Aceh	11,96	4,02	21,04	26,33	13,8	38,54	50,33	23,15	22*
Sumatera Utara	6,54	5,00	33,92	8,38	12,4	27,28	33,73	18,42	23*
Sumatera Barat	27,63	21,75	24,54	6,43	0,00	18,80	67,63	0,00	11*
Riau	11,29	0,00	11,00	8,34	16,7	16,97	44,73	27,42	14*
Jambi	0,00	0,00	9,06	24,07	19,7	48,46	33,88	9,06	4*
Sumatera Selatan	15,54	0,00	12,57	19,65	21,7	16,31	31,44	25,81	21*
Bengkulu	18,41	23,17	12,94	23,17	20,1	30,29	17,35	21,68	2*
Lampung	11,34	27,91	10,83	37,83	1,5	19,94	55,16	15,41	14*
Bangka Belitung	0,00	0,00	0,00	0,00	0,00	49,68	50,32	0,00	1*
Kepulauan Riau	0,00	16,70	38,55	26,41	0,00	77,62	82,78	0,53	2*
DKI Jakarta	0,00	0,00	7,46	0,00	10,7	19,29	23,81	50,18	25*
Jawa Barat	2,39	0,00	7,04	11,69	23,9	22,11	33,43	26,70	141
Jawa Tengah	8,67	0,00	19,46	15,67	10,6	24,52	27,26	24,86	42*
DI Yogyakarta	30,96	30,96	0,00	30,96	0,00	30,96	100,00	0,00	2*
Jawa Timur	3,70	8,71	18,97	16,27	32,6	26,40	47,59	30,20	48*
Banten	9,41	6,33	36,75	35,16	10,9	57,98	39,98	15,44	68
Bali	0,00	23,86	0,00	0,00	45,3	20,55	20,55	30,88	3*
Nusa Tenggara Barat	0,00	0,00	3,91	13,31	20,5	5,99	19,04	56,55	7*
Nusa Tenggara Timur	17,67	1,62	2,72	8,06	23,1	46,55	26,84	23,47	6*
Kalimantan Barat	20,32	0,00	10,10	27,75	29,8	55,83	27,42	1,54	7*
Kalimantan Tengah	9,11	9,20	5,66	9,20	19,6	10,59	22,88	43,07	7*
Kalimantan Selatan	9,26	5,01	20,05	5,01	16,9	31,54	15,16	55,71	10*
Kalimantan Timur	0,00	0,00	0,00	0,00	0,00	0,00	42,37	57,63	4*
Kalimantan Utara	0,00	0,00	0,00	0,00	0,00	0,00	0,00	35,62	1*
Sulawesi Utara	7,80	0,00	0,65	0,65	16,8	4,01	42,35	29,79	8*
Sulawesi Tengah	17,62	0,00	5,94	36,62	0,00	55,16	43,27	22,40	6*
Sulawesi Selatan	20,77	12,13	13,17	12,03	7,6	15,72	59,26	24,15	15*
Sulawesi Tenggara	18,63	0,00	0,00	0,00	8,5	34,37	32,47	25,01	4*
Gorontalo	0,00	0,00	0,00	0,00	0,00	0,00	17,55	6,94	2*
Sulawesi Barat	0,00	0,00	0,00	0,00	0,00	40,79	18,58	8,25	3*
Maluku	15,22	12,04	34,91	7,94	7,9	26,81	37,94	0,00	4*
Maluku Utara	19,87	6,74	6,74	0,00	0,00	0,00	65,90	7,48	3*
Papua Barat	13,21	14,45	0,17	46,45	7,9	23,73	25,82	14,99	3*
Papua	35,56	30,77	14,13	33,95	31,6	29,76	51,43	12,70	8*
**INDONESIA**	**8,12**	**4,72**	**15,66**	**16,54**	**17,3**	**28,42**	**37,51**	**24,95**	**541**

* N tertimbang <50

Tabel 5.3.8
Proporsi Penderita TB (< 6 bulan) menurut Alasan Tidak Rutin Minum Obat
menurut Karakteristik, Riskesdas 2018

Karakteristik	Alasan tidak rutin minum obat (%)								N ter-timbang
	Sering lupa	Tidak tersedia di fasyankes	Tidak tahan efek samping	Masa pengobatan terasa lama	Tidak mampu membeli obat TB secara rutin	Tidak rutin berobat	Merasa sudah sehat	Lainnya	
**Kelompok umur**									
< 1									
1-4	5,3	2,0	1,1	5,3	3,6	40,6	21,6	38,9	28*
5-14	9,5	0,9	4,6	21,6	9,1	17,6	54,6	28,7	29*
15-24	11,2	0,7	15,1	30,3	8,8	24,7	53,8	17,3	32*
25-34	8,7	2,8	22,1	16,0	26,2	24,9	27,2	21,6	80
35-44	11,2	3,0	15,3	9,2	19,8	22,5	47,2	23,8	85
45-54	4,1	9,7	17,2	17,7	26,0	33,0	35,3	27,6	96
55-64	4,2	7,0	20,3	19,5	8,9	23,3	29,3	30,3	115
65-74	14,8	4,1	7,4	17,0	23,2	40,0	46,7	9,9	60
75+	11,7	0,0	20,0	12,6	1,2	48,3	38,4	34,0	17*
**Jenis kelamin**									
Laki-laki	7,9	5,4	18,1	22,1	18,2	31,6	39,7	25,0	319
Perempuan	8,4	3,7	12,2	8,6	15,9	23,9	34,4	24,9	222
**Tempat tinggal**									
Perkotaan	4,3	2,4	15,3	15,0	14,4	22,0	36,2	31,7	270
Perdesaan	11,9	7,0	16,0	18,1	20,1	34,8	38,8	18,3	271
Pendidikan									
Tidak sekolah	14,5	6,7	9,7	9,2	19,0	26,1	34,8	22,0	56
Tidak tamat SD	6,1	3,8	21,6	20,0	22,5	35,3	33,4	21,6	139
Tamat SD	8,0	4,8	14,2	18,3	24,4	29,0	36,3	22,5	154
Tamat SLTP	0,9	4,9	9,2	10,0	9,7	24,0	33,5	30,7	57
Tamat SLTA	14,2	6,9	25,3	23,9	9,2	22,3	55,4	25,1	86
Tamat D1/D2/D3/PT	3,3	0,0	3,7	5,6	1,1	8,4	38,7	36,4	24*
**Pekerjaan**									
Tidak bekerja	4,8	2,3	14,5	13,0	17,3	28,0	37,4	26,5	177
Sekolah	12,3	2,0	12,0	18,3	5,5	22,2	27,5	33,9	11*
PNS/TNI/Polri/BUMN/BUMD	5,6	17,1	0,0	20,5	18,5	18,5	23,5	47,2	6*
Pegawai Swasta	26,0	12,5	17,4	20,3	14,4	30,9	35,3	29,1	27*
Wiraswasta	10,0	0,3	8,8	14,0	2,5	12,6	40,6	26,3	73
Nelayan	2,0	23,8	0,0	19,9	24,0	13,2	45,1	24,6	10*
Petani/Buruh tani	12,2	5,5	20,4	21,5	24,5	30,9	36,3	15,8	128
Lainnya	1,9	10,0	32,1	21,4	31,2	46,4	34,9	25,2	66
**INDONESIA**	**8,12**	**4,72**	**15,66**	**16,54**	**17,3**	**28,42**	**37,51**	**24,95**	**541**

* N tertimbang <50

5.4 Hepatitis

Pada Riskesdas 2018, kejadian hepatitis diukur dengan wawancara dengan pertanyaan “**Dalam 1 tahun terakhir**, apakah [NAMA] pernah didiagnosis menderita Hepatitis melalui pemeriksaan darah oleh dokter?” jika menjawab “Ya“ maka dianggap Hepatitis.

Prevalensi hepatitis adalah persentase ART yang mengaku menderita hepatitis yang didiagnosis oleh dokter terhadap total responden, dihitung dengan formula sebagai berikut:

$$\text{Prevalensi Hepatitis} = \frac{\sum \text{Kasus Hepatitis menurut riwayat diagnosis dokter}}{\sum \text{ART } \textit{semua umur}}$$

Tabel 5.4.1
Prevalensi Hepatitis berdasarkan Riwayat Diagnosis Dokter menurut Provinsi, Riskesdas 2018

Provinsi	Diagnosis Hepatitis		
	%	95% CI	N tertimbang
Aceh	0,41	0,3 - 0,5	20.244
Sumatera Utara	0,37	0,3 - 0,5	55.351
Sumatera Barat	0,36	0,3 - 0,4	20.663
Riau	0,39	0,3 - 0,5	26.085
Jambi	0,39	0,3 - 0,5	13.692
Sumatera Selatan	0,33	0,3 - 0,4	32.126
Bengkulu	0,43	0,3 - 0,6	7.531
Lampung	0,30	0,2 - 0,4	32.148
Bangka Belitung	0,18	0,1 - 0,3	5.592
Kepulauan Riau	0,25	0,1 - 0,5	8.173
DKI Jakarta	0,48	0,4 - 0,6	40.210
Jawa Barat	0,45	0,4 - 0,5	186.809
Jawa Tengah	0,28	0,2 - 0,3	132.565
DI Yogyakarta	0,35	0,2 - 0,5	14.602
Jawa Timur	0,40	0,3 - 0,5	151.878
Banten	0,42	0,3 - 0,5	48.621
Bali	0,36	0,3 - 0,5	16.481
Nusa Tenggara Barat	0,56	0,5 - 0,7	19.247
Nusa Tenggara Timur	0,35	0,3 - 0,4	20.599
Kalimantan Barat	0,29	0,2 - 0,4	19.190
Kalimantan Tengah	0,40	0,3 - 0,5	10.189
Kalimantan Selatan	0,33	0,3 - 0,4	16.043
Kalimantan Timur	0,39	0,3 - 0,5	13.977
Kalimantan Utara	0,33	0,2 - 0,5	2.733
Sulawesi Utara	0,47	0,4 - 0,6	9.542
Sulawesi Tengah	0,62	0,5 - 0,8	11.548
Sulawesi Selatan	0,43	0,4 - 0,5	33.693
Sulawesi Tenggara	0,39	0,3 - 0,5	10.167
Gorontalo	0,55	0,4 - 0,8	4.547
Sulawesi Barat	0,57	0,4 - 0,8	5.195
Maluku	0,34	0,2 - 0,5	6.801
Maluku Utara	0,33	0,2 - 0,5	4.723
Papua Barat	0,42	0,3 - 0,6	3.588
Papua	0,66	0,5 - 0,9	12.736
**INDONESIA**	**0,39**	**0,37 - 0,41**	**1.017.290**

Tabel 5.4.2
Prevalensi Hepatitis berdasarkan Riwayat Diagnosis Dokter menurut Karakteristik, Riskesdas 2018

Karakteristik	Diagnosis Hepatitis		
	%	95% CI	N tertimbang
**Kelompok umur**			
< 1	0,45	0,3-0,6	18.225
1-4	0,36	0,3-0,4	73.188
5-14	0,30	0,3-0,3	182.338
15-24	0,38	0,3-0,4	165.644
25-34	0,42	0,4-0,5	159.708
35-44	0,44	0,4-0,5	151.539
45-54	0,46	0,4-0,5	124.652
55-64	0,38	0,3-0,4	83.251
65-74	0,44	0,4-0,5	40.180
75+	0,41	0,3-0,6	18.565
**Jenis kelamin**			
Laki-laki	0,40	0,4-0,4	510.714
Perempuan	0,39	0,4-0,4	506.576
**Pendidikan**			
Tidak sekolah	0,36	0,3-0,4	70.895
Tidak tamat SD	0,39	0,4-0,4	181.429
Tamat SD	0,40	0,4-0,5	215.967
Tamat SLTP	0,39	0,4-0,5	160.320
Tamat SLTA	0,41	0,4-0,5	210.746
Tamat D1/D2/D3/PT	0,36	0,3-0,4	64.093
**Pekerjaan**			
Tidak bekerja	0,43	0,4-0,5	233.629
Sekolah	0,35	0,3-0,4	126.626
PNS/TNI/Polri/BUMN/BUMD	0,40	0,3-0,5	21.931
Pegawai Swasta	0,35	0,3-0,4	75.781
Wiraswasta	0,42	0,4-0,5	105.489
Nelayan	0,44	0,2-0,7	5.556
Petani/Buruh tani	0,39	0,4-0,5	133.261
Lainnya	0,39	0,3-0,5	116.233
**Tempat tinggal**			
Perkotaan	0,40	0,4-0,4	556.419
Perdesaan	0,38	0,4-0,4	460.871

5.5 Diare

Diare adalah buang air besar (BAB) dengan konsistensi feces lebih cair dengan frekuensi >3 kali sehari, kecuali pada neonatus (bayi < 1 bulan) yang mendapatkan ASI biasanya buang air besar dengan frekuensi lebih sering (5-6 kali sehari) dengan konsistensi baik dianggap normal. Pada Riskesdas 2018, kasus diare diukur dengan wawancara kepada responden dengan pertanyaan sebagai berikut: **"Dalam 1 bulan terakhir,** apakah [NAMA] pernah didiagnosis menderita diare oleh tenaga kesehatan (dokter/perawat/bidan)?". Pada responden yang menjawab tidak, ditanyakan gejala diare yang pernah dialami dengan pertanyaan "**Dalam 1 bulan terakhir**, apakah [NAMA] pernah mengalami: Buang Air Besar (BAB) 3 – 6 kali sehari BAB > 6 kali sehari, Kotoran/tinja lembek atau cair".

Prevalensi diare menurut gejala dihitung dengan menggabungkan kasus diare baik diagnosis maupun hanya memiliki gejala. Pada bayi usia 0-28 hari (neonatus), dikatakan kasus diare jika responden mengaku didiagnosis diare oleh tenaga kesehatan atau jika pernah mengalami gejala diare meliputi diare meliputi BAB > 6 kali perhari dan dengan konsistensi lembek atau cair. Selain neonatus jika responden menjawab lebih dari 3 kali dengan konsistensi lembek/cair, maka dianggap diare.

$$\text{Prevalensi Diare} = \frac{\sum \text{Kasus diare} \begin{pmatrix}\text{diagnosis dan atau gejala} \\ \text{dalam 1 bln terakhir}\end{pmatrix}}{\sum \text{ART Semua Umur} \begin{pmatrix}\text{diagnosis dan atau} \\ \text{gejala dalam 1 bln terakhir}\end{pmatrix}}$$

$$\text{Prevalensi Diare (Balita)} = \frac{\begin{matrix}\sum \text{Kasus daire (diagnosis dan atau gejala)} \\ \text{pada Balita dalam 1 bulan terakhir } (0-59 \text{ bulan})\end{matrix}}{\sum \text{Balita}}$$

$$\text{Proporsi penggunaan oralit} = \frac{\begin{matrix}\sum \text{Kasus daire (diagnosis dan atau gejala)} \\ \text{yang menggunakan oralit}\end{matrix}}{\sum \text{Kasus daire (diagnosis dan atau gejala)}}$$

Tabel 5.5.1
Prevalensi Diare menurut Provinsi, Riskesdas 2018

Provinsi	Diare				N tertimbang
	D[1]		D/G[2]		
	%	95% CI	%	95% CI	
Aceh	8,5	8,0 - 9,1	9,1	8,6-9,6	20.244
Sumatera Utara	8,1	7,7 - 8,6	9,1	8,6-9,6	55.351
Sumatera Barat	8,3	7,8 - 8,8	9,3	8,7-9,8	20.663
Riau	6,4	6,0 - 6,9	7,5	7,0-8,0	26.085
Jambi	4,1	3,7 - 4,5	4,5	4,1-5,0	13.692
Sumatera Selatan	5,0	4,6 - 5,5	5,5	5,1-6,0	32.126
Bengkulu	8,9	8,1 - 9,8	9,4	8,5-10,2	7.531
Lampung	4,5	4,2 - 4,9	4,9	4,6-5,3	32.148
Bangka Belitung	3,2	2,8 - 3,6	4,4	3,9-5,0	5.592
Kepulauan Riau	3,9	3,3 - 4,7	4,3	3,6-5,1	8.173
DKI Jakarta	5,7	5,2 - 6,3	7,2	6,6-7,8	40.210
Jawa Barat	7,4	7,1 - 7,8	8,6	8,2-9,0	186.809
Jawa Tengah	7,2	6,9 - 7,4	8,4	8,1-8,6	132.565
DI Yogyakarta	6,1	5,5 - 6,8	8,5	7,7-9,3	14.602
Jawa Timur	6,5	6,2 - 6,8	7,6	7,3-7,9	151.878
Banten	7,6	7,0 - 8,2	9,2	8,6-9,8	48.621
Bali	6,6	6,1 - 7,1	8,3	7,8-8,9	16.481
Nusa Tenggara Barat	8,4	7,8 – 9,0	10,2	9,5-10,9	19.247
Nusa Tenggara Timur	5,1	4,7 - 5,5	6,6	6,1-7,0	20.599
Kalimantan Barat	7,4	6,9 - 7,9	8,7	8,1-9,2	19.190
Kalimantan Tengah	4,6	4,2 - 5,2	5,5	5-6,1,0	10.189
Kalimantan Selatan	5,6	5,2 – 6,0	6,5	6-6,9,0	16.043
Kalimantan Timur	5,1	4,6 - 5,7	6,1	5,5-6,8	13.977
Kalimantan Utara	6,6	5,5 - 7,9	7,7	6,5-9,1	2.733
Sulawesi Utara	5,4	5,0 - 5,9	6,7	6,2-7,3	9.542
Sulawesi Tengah	7,3	6,7 – 8,0	10,3	9,6-11,1	11.548
Sulawesi Selatan	7,0	6,6 - 7,5	9,2	8,7-9,7	33.693
Sulawesi Tenggara	5,6	5,1 - 6,3	6,9	6,3-7,5	10.167
Gorontalo	6,4	5,6 - 7,2	8,8	7,9-9,8	4.547
Sulawesi Barat	6,9	6,1 - 7,8	8,4	7,5-9,3	5.195
Maluku	5,4	4,8 - 6,1	6,6	5,9-7,5	6.801
Maluku Utara	4,4	3,8 – 5,0	5,4	4,8-6,1	4.723
Papua Barat	6,7	5,8 - 7,7	7,8	6,9-8,8	3.588
Papua	8,3	7,5 - 9,1	9,4	8,6-10,3	12.736
**INDONESIA**	**6,8**	**6,7 - 6,9**	**8,0**	**7,8-8,1**	**1.017.290**

1. D: menurut diagnosis oleh tenaga kesehatan (dokter, perawat atau bidan)
2. D/G: menurut diagnosis oleh tenaga kesehatan (dokter, perawat atau bidan) atau gejala yang pernah dialami oleh ART.

Tabel 5.5.2
Prevalensi Diare menurut Karakteristik, Riskesdas 2018

Karakteristik	Diare				N tertimbang
	D[1]		D/G[2]		
	%	95% CI	%	95% CI	
**Kelompok Umur**					
< 1	9,0	8,4-9,7	10,6	9,9-11,3	18.225
1-4	11,5	11,1-11,9	12,8	12,4-13,2	73.188
5-14	6,2	6,0-6,4	7,0	6,8-7,2	182.338
15-24	6,7	6,5-6,9	8,1	7,9-8,4	165.644
25-34	6,0	5,8-6,2	7,2	7,0-7,4	159.708
35-44	6,0	5,8-6,1	7,1	6,9-7,3	151.539
45-54	6,5	6,3-6,8	7,8	7,6-8,1	124.652
55-64	6,6	6,4-6,9	7,9	7,6-8,2	83.251
65-74	6,9	6,5-7,3	8,0	7,6-8,4	40.180
75+	7,2	6,7-7,8	8,1	7,5-8,6	18.565
**Jenis kelamin**					
Laki-laki	6,5	6,3 - 6,6	7,6	7,5-7,7	510.714
Perempuan	7,1	6,9 - 7,2	8,3	8,2-8,5	506.576
**Tempat tinggal**					
Perkotaan	6,6	6,4 - 6,7	7,9	7,7-8,0	556.419
Perdesaan	7,0	6,8 - 7,1	8,1	7,9-8,2	460.871
**Pendidikan Tertinggi**					
Tidak sekolah	6,7	6,4-7,0	7,8	7,5-8,1	70.895
Tidak tamat SD	6,8	6,6-7,0	7,9	7,7-8,1	181.429
Tamat SD	6,7	6,5-6,9	7,9	7,7-8,1	215.967
Tamat SLTP	6,5	6,3-6,7	7,7	7,5-8,0	160.320
Tamat SLTA	5,7	5,5-5,9	6,9	6,8-7,1	210.746
Tamat D1/D2/D3/PT	5,0	4,7-5,3	6,0	5,7-6,3	64.093
**Pekerjaan**					
Tidak bekerja	6,7	6,6-6,9	8,0	7,8-8,2	233.629
Sekolah	6,3	6,1-6,5	7,5	7,3-7,8	126.626
PNS/TNI/Polri/BUMN/BUMD	4,7	4,4-5,1	5,4	5,0-5,9	21.931
Pegawai Swasta	5,5	5,2-5,8	6,7	6,4-7,1	75.781
Wiraswasta	6,1	5,9-6,4	7,3	7,1-7,6	105.489
Nelayan	8,9	7,8-10,1	10,5	9,4-11,8	5.556
Petani/Buruh tani	6,4	6,2-6,6	7,7	7,5-7,9	133.261
Lainnya	6,5	6,2-6,7	7,7	7,5-8,0	116.233

1. D: menurut diagnosis oleh tenaga kesehatan (dokter, perawat atau bidan)
2. D/G: menurut diagnosis oleh tenaga kesehatan (dokter, perawat atau bidan) atau gejala yang pernah dialami oleh ART.

Tabel 5.5.3
Prevalensi Diare pada Balita menurut Provinsi, Riskesdas 2018

Provinsi	Diare pada Balita				
	D[1]		D/G[2]		N
	%	95% CI	%	95% CI	tertimbang
Aceh	13,8	12,5 - 15,3	14,5	13,2-16,0	2.250
Sumatera Utara	14,2	13,0 - 15,5	15,4	14,2-16,8	5.895
Sumatera Barat	12,9	11,5 - 14,6	13,8	12,3-15,4	2.179
Riau	9,5	8,3 – 11,0	10,6	9,3-12,0	2.813
Jambi	7,7	6,4 - 9,3	8,6	7,2-10,3	1.279
Sumatera Selatan	10,1	8,8 - 11,6	10,7	9,4-12,3	3.079
Bengkulu	13,6	11,6 - 15,9	14,3	12,3-16,6	716
Lampung	8,8	7,5 - 10,2	9,1	7,9-10,6	3.094
Bangka Belitung	6,0	4,5 – 8,0	7,5	5,8-9,6	527
Kepulauan Riau	5,1	3,5 - 7,3	6,0	4,3-8,2	872
DKI Jakarta	9,1	7,3 - 11,3	11,0	9,1-13,4	3.582
Jawa Barat	12,8	11,7 - 14,1	14,4	13,2-15,8	17.228
Jawa Tengah	11,1	10,3 – 12,0	12,4	11,5-13,3	10.551
DI Yogyakarta	7,4	5,4 - 10,1	8,7	6,5-11,6	1.069
Jawa Timur	9,9	9,1 - 10,8	10,7	9,9-11,6	11.272
Banten	12,3	10,6 - 14,2	14,2	12,4-16,2	4.813
Bali	8,0	6,4 – 10,0	9,6	7,9-11,7	1.275
Nusa Tenggara Barat	13,4	11,7 - 15,4	15,1	13,3-17,1	1.985
Nusa Tenggara Timur	9,4	8,3 - 10,6	11,0	9,8-12,3	2.496
Kalimantan Barat	13,1	11,5 - 14,9	14,5	12,8-16,3	1.928
Kalimantan Tengah	8,0	6,6 - 9,7	9,0	7,4-10,8	1.000
Kalimantan Selatan	8,6	7,3 - 10,1	10,0	8,6-11,7	1.563
Kalimantan Timur	8,9	7,0 - 11,2	9,8	7,8-12,1	1.368
Kalimantan Utara	10,2	7,9 - 13,2	11,8	9,2-15,1	309
Sulawesi Utara	8,0	6,5 - 9,7	8,9	7,3-10,7	821
Sulawesi Tengah	11,5	10,0 - 13,3	14,4	12,8-16,3	1.155
Sulawesi Selatan	9,4	8,2 - 10,7	11,8	10,5-13,2	3.269
Sulawesi Tenggara	7,5	6,1 - 9,2	9,7	8,1-11,5	1.169
Gorontalo	10,7	8,5 - 13,3	12,6	10,3-15,4	445
Sulawesi Barat	10,3	8,2 - 12,7	12,6	10,4-15,3	584
Maluku	7,6	6,0 - 9,5	10,1	8,3-12,4	779
Maluku Utara	7,3	5,9 – 9,0	8,1	6,6-9,8	536
Papua Barat	8,8	6,8 - 11,2	9,7	7,7-12,1	415
Papua	13,9	12,0 – 16,0	15,8	13,7-18,0	1.302
**INDONESIA**	**11,0**	**10,7 - 11,3**	**12,3**	**12,0-12,7**	**93.619**

1. D: menurut diagnosis oleh tenaga kesehatan (dokter, perawat atau bidan)
2. D/G: menurut diagnosis oleh tenaga kesehatan (dokter, perawat atau bidan) atau gejala yang pernah dialami oleh ART.

Tabel 5.5.4
Prevalensi Diare pada Balita menurut Karakteristik, Riskesdas 2018

Karakteristik	Diare pada Balita				
	D[1]		D/G[2]		N tertimbang
	%	95% CI	%	95% CI	
**Jenis kelamin**					
Laki-laki	11,4	11-11,9	12,8	12,3-13,3	47.764
Perempuan	10,5	10,1-11,0	11,9	11,4-12,4	45.855
**Tempat tinggal**					
Perkotaan	10,5	10,0- 11,0	11,9	11,3-12,5	50.360
Perdesaan	11,6	11,2 – 12,0	12,9	12,5-13,3	43.259
**Kelompok Umur (bulan)**					
0-11	9,0	8,4-9,7	10,6	9,9-11,3	18.665
12-23	15,0	14,2-15,8	16,6	15,7-17,5	18.333
24-35	12,8	12,0-13,6	14,3	13,5-15,1	19.112
36-47	10,2	9,5-10,9	11,2	10,5-11,9	18.821
48-59	8,0	7,4-8,6	9,1	8,4-9,7	18.688

1. D: menurut diagnosis oleh tenaga kesehatan (dokter, perawat atau bidan)
2. D/G: menurut diagnosis oleh tenaga kesehatan (dokter, perawat atau bidan) atau gejala yang pernah dialami oleh ART.

Tabel 5.5.5
Proporsi Penggunaan Oralit, Zinc, dan Pengobatan Lainnya pada Penderita Diare Balita menurut Provinsi, Riskesdas 2018

Provinsi	Pengobatan diare pada Balita						N tertimbang
	Oralit/LGG		Zinc	Obat anti diare	Antibiotik	Herbal/ tradisional	
	%	95% CI					
Aceh	29,9	24,8-35,4	27,6	56,1	43,1	17,0	297
Sumatera Utara	26,7	23,0-30,8	18,3	65,0	42,6	12,8	798
Sumatera Barat	42,5	37,0-48,3	22,5	56,0	40,7	17,3	269
Riau	36,5	30,0-43,5	25,0	48,8	31,2	13,3	257
Jambi	42,0	32,8-51,7	27,9	41,7	32,3	15,3	94
Sumatera Selatan	40,5	33,9-47,4	33,4	40,4	35,2	14,6	298
Bengkulu	36,7	29,2-45,0	30,9	47,0	33,0	17,0	93
Lampung	41,6	35,2-48,2	28,0	51,4	38,3	16,1	259
Bangka Belitung	35,7	22,0-52,2	31,1	52,6	53,7	10,6	30*
Kepulauan Riau	34,2	18,0-55,2	9,9	48,3	23,2	2,3	42*
DKI Jakarta	39,8	29,8-50,7	30,9	47,8	21,9	6,4	311
Jawa Barat	33,8	29,6-38,3	25,6	61,3	38,5	12,4	2.115
Jawa Tengah	26,6	23,3-30,3	22,1	62,9	38,2	9,2	1.120
DI Yogyakarta	39,1	25,0-55,3	26,3	51,5	30,6	11,7	75
Jawa Timur	37,1	32,9-41,5	22,7	60,6	34,3	7,5	1.071
Banten	37,9	30,9-45,5	31,2	60,3	40,2	12,2	566
Bali	43,7	33,1-54,9	27,3	41,8	43,3	6,4	98
Nusa Tenggara Barat	43,7	36,8-50,8	29,3	34,4	25,5	15,7	255
Nusa Tenggara Timur	45,6	40,0-51,4	32,0	28,1	22,6	14,7	223
Kalimantan Barat	40,1	33,4-47,1	28,7	54,8	43,0	12,3	242
Kalimantan Tengah	33,6	25,2-43,2	33,1	62,3	34,5	16,2	77
Kalimantan Selatan	23,1	16,8-30,9	34,0	46,6	25,1	10,6	128
Kalimantan Timur	39,6	27,3-53,3	28,1	38,3	29,3	11,8	116
Kalimantan Utara	37,9	25,8-51,6	27,5	34,4	25,9	18,0	30*
Sulawesi Utara	32,0	23,6-41,9	19,9	59,7	35,4	16,1	63
Sulawesi Tengah	32,5	26,2-39,4	29,4	52,0	38,3	17,2	127
Sulawesi Selatan	25,2	19,6-31,8	29,3	38,7	25,7	8,8	293
Sulawesi Tenggara	37,7	30,5-45,4	31,4	28,4	20,2	17,3	84
Gorontalo	35,1	26,1-45,2	40,2	51,1	53,5	11,3	45*
Sulawesi Barat	28,1	19,7-38,3	18,0	29,1	23,3	31,8	57
Maluku	31,7	23,2-41,6	13,9	32,1	21,1	13,8	56
Maluku Utara	39,1	29,4-49,6	26,3	33,6	15,1	15,5	37*
Papua Barat	25,9	17,3-36,8	23,0	38,4	16,9	15,3	35*
Papua	58,9	52,6-64,9	45,4	39,2	36,7	13,8	173
**INDONESIA**	**34,8**	**33,4-36,2**	**26,1**	**54,8**	**35,8**	**12,1**	**9.838**

* N Tertimbang <50

Tabel 5.5.6
Proporsi Penggunaan Oralit, Zinc, dan Pengobatan Lainnya pada Penderita Diare Balita menurut Karakteristik, Riskesdas 2018

Karakteristik	Pengobatan diare pada Balita						N tertimbang
	Oralit/LGG		Zinc	Obat anti diare	Anti biotik	Herbal/ tradisional	
	%	95% CI					
**Jenis kelamin**							
Laki-laki	34,7	32,8-36,7	26,2	56,2	36,8	12,1	5.221
Perempuan	34,9	32,8-37,1	25,9	53,2	34,7	12,0	4.617
**Tempat tinggal**							
Perkotaan	34,6	32,3-36,9	26,5	56,5	35,2	10,8	5.054
Perdesaan	35,0	33,5-36,7	25,6	53,1	36,5	13,5	4.784
**Kelompok Umur (bulan)**							
0-11	28,0	25,0-31,3	30,2	42,3	33,2	8,6	1.612
12-23	37,8	34,9-40,7	28,1	51,8	38,2	11,8	2.626
24-35	37,4	34,4-40,6	27,4	59,4	39,9	13,6	2.338
36-47	33,0	29,9-36,3	21,7	60,3	31,7	13,1	1.829
48-59	35,0	31,5-38,6	21,1	60,1	33,2	12,8	1.433

* N Tertimbang <50

5.6 Malaria

A. Prevalensi Malaria berdasarkan Hasil Wawancara

Malaria adalah penyakit yang umumnya ditandai dengan panas tinggi yang dapat naik turun secara berkala disertai dengan salah satu atau lebih gejala lain seperti menggigil, muka pucat, kepala sakit, pusing, tidak nafsu makan, mual, muntah, nyeri otot atau pegal-pegal. Penyakit ini disebabkan oleh parasite malaria dan ditularkan melalui gigitan nyamuk *Anopheles sp*. Diagnostik pasti malaria harus ditegakkan dengan pemeriksaan sediaan darah secara mikroskopis maupun dengan tes diagnostik cepat/RDT. Pada daerah endemik, biasanya responden mengetahui penyakit ini disertai dengan pembesaran limpa (Splenomegali).

Artemisinin Combination Treatment (ACT) adalah obat malaria yang merupakan kombinasi dari obat malaria golongan Artemisinin.Jenis artemisinin: *Dihydroartemisin*, *Artesunate*, *Artemether*, kombinasi obat anti malaria golongan lainnya seperti *Piperaquin*, *Amodiaquine*, *Meflokuin*. Saat ini ACT yang beredar di Indonesia mempunyai sediaan yaitu:

1. Dihidroartemisinin+Piperaquin
2. Artesunate+Amodiaquine
3. Artemether+Lumefantrine

Artemisinin (ACT) 3 hari + primaquin 1 hari diberikan untuk penderita malaria akibat *Plasmodium falcifarum* dengan lama pemberian 3 hari, sedangkan Artemisinin (ACT) 3 hari + primaquin 14 hari diberikan untuk penderita malaria akibat *Plasmodium vivax*. Dengan demikian lama pemeberian ACT dan Primaquin bisa menjadi proksi untuk mengetahui penyebab Malaria yang pernah diderita oleh responden.

Tabel 5.6.1
Skema Pengobatan Malaria

Nama Obat	Hari ke-1	Hari ke-2	Hari ke-3	Hari ke-n	Hari ke-14
**Plasmodium palcifarum**					
- ACT	✓	✓	✓		
- Primaquin	✓	-	-		
**Plasmodium Vivax**					
- ACT	✓	✓	✓		
- Primaquin	✓	✓	✓	✓	✓

Pada Riskesdas 2018 kasus malaria diukur melalui 2 pertanyaan. Pertanyaan pertama “**Dalam 1 tahun terakhir**, apakah [NAMA] pernah diambil darah untuk pemeriksaan malaria oleh tenaga kesehatan (dokter/perawat/bidan)?” jika menjawab “Ya” maka dilanjutkan dengan pertanyaan kedua “Apakah [NAMA] dinyatakan positif menderita malaria setelah pemeriksaan tersebut oleh tenaga kesehatan (dokter/perawat/bidan)?”. Dikatakan Malaria jika pertanyaan pertama menjawab Ya dan hasilnya positif (pertanyaan ke-2 dijawab “Ya”), dihitung dengan formula:

$$Prevalensi\ malaria\ = \frac{\sum Kasus\ positif\ malaria\ melalui\ Riwayat\ pemeriksaan}{Total\ \ ART}$$

$$Proporsi\ Jenis\ Pengobatan\ = \frac{Jenis\ obat\ yang\ diberikan\ dokter}{\sum\ kasus\ Malaria\ yang\ diobati}$$

Tabel 5.6.2
Prevalensi Malaria berdasarkan Riwayat Positif Malaria melalui Pemeriksaan Darah oleh Nakes dan Pengobatannya menurut Provinsi, Riskesdas 2018

Provinsi	Malaria			Pengobatan			
				ACT3 hari +Primaquin		Lainnya	N tertimbang
	%	95% CI	N ter-timbang	1 hari	14 hari		
Aceh	0,20	0,15-0,27	20.244	61,46	22,86	20,95	88
Sumatera Utara	0,20	0,16-0,26	55.351	45,38	37,02	13,75	242
Sumatera Barat	0,14	0,09-0,22	20.663	32,16	44,18	7,43	62
Riau	0,12	0,09-0,17	26.085	67,10	10,59	18,52	69
Jambi	0,26	0,20-0,35	13.692	61,40	40,02	15,07	78
Sumatera Selatan	0,24	0,18-0,32	32.126	54,58	26,45	11,49	164
Bengkulu	1,54	1,28-1,86	7.531	51,73	16,60	24,69	252
Lampung	0,25	0,20-0,32	32.148	49,80	28,23	18,23	175
Bangka Belitung	1,07	0,78-1,47	5.592	47,16	28,14	24,72	130
Kepulauan Riau	0,32	0,17-0,61	8.173	51,21	37,08	19,06	56
DKI Jakarta	0,04	0,01-0,13	40.210	90,52	0,50	0,00	37*
Jawa Barat	0,04	0,03-0,08	186.809	19,76	19,34	10,19	180
Jawa Tengah	0,03	0,02-0,04	132.565	38,58	25,01	9,08	75
DI Yogyakarta	0,08	0,03-0,18	14.602	63,44	61,92	0,00	25*
Jawa Timur	0,02	0,02-0,04	151.878	23,41	16,50	12,90	81
Banten	0,09	0,05-0,16	48.621	17,02	32,49	7,65	95
Bali	0,04	0,01-0,16	16.481	25,35	65,90	8,75	14*
Nusa Tenggara Barat	0,65	0,51-0,81	19.247	54,31	29,36	13,53	270
Nusa Tenggara Timur	1,99	1,80-2,20	20.599	54,59	31,94	18,51	888
Kalimantan Barat	0,38	0,29-0,50	19.190	57,82	17,18	24,06	159
Kalimantan Tengah	0,18	0,12-0,26	10.189	43,25	45,54	19,03	39
Kalimantan Selatan	0,12	0,08-0,18	16.043	24,63	22,36	28,23	41
Kalimantan Timur	0,20	0,13-0,30	13.977	37,31	54,23	8,88	59
Kalimantan Utara	0,16	0,08-0,33	2.733	56,20	6,48	11,30	10*
Sulawesi Utara	0,47	0,34-0,65	9.542	43,12	32,58	29,17	97
Sulawesi Tengah	0,74	0,60-0,92	11.548	45,84	42,82	16,11	186
Sulawesi Selatan	0,13	0,10-0,18	33.693	35,96	32,00	30,29	97
Sulawesi Tenggara	0,22	0,15-0,32	10.167	48,47	38,29	26,58	48
Gorontalo	0,12	0,06-0,25	4.547	63,62	68,34	20,50	12*
Sulawesi Barat	0,21	0,13-0,32	5.195	49,69	36,63	21,43	23*
Maluku	1,21	0,94-1,56	6.801	34,17	33,64	28,71	178
Maluku Utara	1,36	1,13-1,64	4.723	52,43	29,28	20,45	139
Papua Barat	8,64	7,60-9,81	3.588	38,99	53,00	19,15	672
Papua	12,07	11,14-13,08	12.736	54,05	35,13	27,68	3.334
**INDONESIA**	**0,37**	**0,35-0,38**	**1.017.290**	**49,63**	**34,00**	**22,09**	**8.076**

*N Tertimbang <50

Tabel 5.6.3
Prevalensi Malaria berdasarkan Riwayat Positif Malaria melalui Pemeriksaan Darah oleh Nakes dan Pengobatannya menurut Karakteristik, Riskesdas 2018

Karakteristik	Malaria			Pengobatan			
				ACT3 hari + Primaquin		Lainnya	N tertimbang
	%	95% CI	N ter-timbang	1 hari	14 hari		
**Kelompok umur**							
< 1	0,1	0,1-0,1	18.225	40,5	14,7	48,5	30
1-4	0,3	0,3-0,3	73.188	47,9	31,9	29,1	474
5-14	0,4	0,3-0,4	182.338	49,7	35,5	23,2	1.402
15-24	0,4	0,3-0,4	165.644	52,8	32,3	20,6	1.347
25-34	0,5	0,4-0,5	159.708	46,3	35,2	21,6	1.616
35-44	0,4	0,4-0,5	151.539	54,7	35,1	18,8	1.421
45-54	0,4	0,3-0,4	124.652	45,6	35,2	24,2	1.006
55-64	0,3	0,3-0,4	83.251	47,5	29,2	23,4	572
65-74	0,2	0,2-0,2	40.180	50,8	34,2	16,5	164
75+	0,1	0,1-0,2	18.565	51,2	31,2	19,0	42*
**Jenis kelamin**							
Laki-laki	0,42	0,40-0,45	510.714	49,24	34,37	21,61	4679
Perempuan	0,31	0,29-0,33	506.576	50,17	33,49	22,75	3397
**Pendidikan**							
Tidak sekolah	0,50	0,43-0,58	70.895	59,88	30,52	18,50	749
Tidak tamat SD	0,41	0,38-0,44	181.429	49,12	33,11	24,02	1.567
Tamat SD	0,31	0,28-0,34	215.967	50,15	34,84	17,93	1.424
Tamat SLTP	0,35	0,32-0,38	160.320	48,51	36,29	21,28	1.186
Tamat SLTA	0,41	0,38-0,44	210.746	47,29	34,13	24,17	1.833
Tamat D1/D2/D3/PT	0,39	0,33-0,47	64.093	49,51	34,37	19,63	534
**Pekerjaan**							
Tidak bekerja	0,63	0,57-0,7	234.405	49,94	34,10	20,04	1.484
Sekolah	0,80	0,73-0,88	127.157	47,96	35,49	21,88	1.014
PNS/TNI/Polri/ BUMN/BUMD	1,48	1,27-1,74	22.103	48,25	37,53	22,42	328
Pegawai Swasta	0,52	0,42-0,64	75.987	45,12	39,78	15,49	395
Wiraswasta	0,63	0,55-0,73	105.839	45,63	32,65	23,82	670
Nelayan	2,27	1,73-2,97	5.623	41,19	38,55	31,05	128
Petani/Buruh tani	1,34	1,24-1,46	134.205	54,46	33,77	18,80	1.805
Lainnya	0,58	0,5-0,66	116.584	46,82	31,17	26,93	671
**Tempat tinggal**							
Perkotaan	0,24	0,22-0,26	556.419	44,19	31,93	26,04	2871
Perdesaan	0,52	0,49-0,55	460.871	52,63	35,14	19,92	5205

*N Tertimbang <50

Tabel 5.6.4
Proporsi Penggunaan ACT untuk Pengobatan Malaria menurut Provinsi, Riskesdas 2018

Provinsi	Penggunaan ACT		N tertimbang
	%	95% CI	
Aceh	73,3	61,3-82,7	88
Sumatera Utara	74,4	60,7-84,6	242
Sumatera Barat	72,6	50,7-87,2	62
Riau	75,3	58,5-86,8	69
Jambi	88,2	79,4-93,5	78
Sumatera Selatan	72,0	58,5-82,5	164
Bengkulu	66,5	54,9-76,4	252
Lampung	74,3	66,4-80,9	175
Bangka Belitung	74,2	62,1-83,5	130
Kepulauan Riau	83,6	63,9-93,6	56
DKI Jakarta	91,0	91,0-91,0	37*
Jawa Barat	39,1	16,2-68,0	180
Jawa Tengah	63,4	45,7-78,1	75
DI Yogyakarta	89,9	49,4-98,8	25*
Jawa Timur	39,9	23,4-59,1	81
Banten	48,1	26,6-70,3	95
Bali	91,3	79,8-96,5	14*
Nusa Tenggara Barat	81,9	72,7-88,5	270
Nusa Tenggara Timur	83,1	77,6-87,5	888
Kalimantan Barat	72,3	61,5-81,0	159
Kalimantan Tengah	83,7	68,5-92,4	39*
Kalimantan Selatan	43,8	27,5-61,6	41*
Kalimantan Timur	90,4	71,8-97,2	59
Kalimantan Utara	62,2	25,2-88,9	10*
Sulawesi Utara	69,1	54,4-80,8	97
Sulawesi Tengah	82,5	74,6-88,4	186
Sulawesi Selatan	60,2	44,9-73,7	97
Sulawesi Tenggara	79,4	64,3-89,2	48*
Gorontalo	79,6	45,9-94,7	12*
Sulawesi Barat	64,5	48,7-77,7	23*
Maluku	63,6	48,3-76,6	178
Maluku Utara	76,9	67,0-84,5	139
Papua Barat	88,4	83,2-92,2	672
Papua	82,5	79,2-85,4	3.334
**INDONESIA**	**78,3**	**76,4-80,2**	**8.076**

*N Tertimbang <50

Tabel 5.6.5
Proporsi Penggunaan ACT untuk Pengobatan Malaria menurut Karakteristik, Riskesdas 2018

Karakteristik	Penggunaan ACT		N tertimbang
	%	95% CI	
**Kelompok Umur**			
< 1	50,6	31,2-69,9	30*
1-4	74,6	69,3-79,2	474
5-14	79,0	75,4-82,1	1.402
15-24	80,6	76,9-83,8	1.347
25-34	77,2	72,0-81,6	1.616
35-44	82,4	79,5-85,0	1.421
45-54	76,2	72,1-79,9	1.006
55-64	72,1	66,5-77,2	572
65-74	81,1	72,9-87,2	164
75+	79,4	60,9-90,5	42*
**Jenis kelamin**			
Laki-laki	78,7	76,4-80,8	4.679
Perempuan	77,9	75,4-80,2	3.397
**Pendidikan**			
Tidak sekolah	80,9	75,0-85,6	749
Tidak tamat SD	76,6	73,3-79,5	1.567
Tamat SD	80,4	77,3-83,1	1.424
Tamat SLTP	79,2	75,1-82,7	1.186
Tamat SLTA	77,5	73,6-81,0	1.833
Tamat D1/D2/D3/PT	80,2	71,1-86,9	534
**Pekerjaan**			
Tidak bekerja	78,1	74,2-81,5	1.484
Sekolah	78,5	74,8-81,8	1.014
PNS/TNI/Polri/BUMN/BUMD	80,5	73,8-85,8	328
Pegawai Swasta	82,7	70,9-90,4	395
Wiraswasta	74,9	67,9-80,9	670
Nelayan	82,0	79,4-84,4	1.805
Petani/Buruh tani	78,2	66,6-86,5	128
Lainnya	70,5	60,5-78,8	359
**Tempat tinggal**			
Perkotaan	72,7	68,6-76,4	2.871
Perdesaan	81,5	79,4-83,4	5.205

*N Tertimbang <50

B. Malaria berdasarkan Pemeriksaan RDT dan Pemeriksaan Mikroskopis

Prevalensi angka positif malaria berdasarkan hasil pemeriksaan *Rapid Diagnostic Test* (RDT) Malaria yang dapat memberikan salah satu dari 5 hasil: Negatif (RDT=1), positif untuk spesies *Plasmodium falciparum*/Pf (RDT=2), positif untuk spesies Plasmodium lainnya di luar spesies *Plasmodium falciparum* (Pan, RDT=3), positif untuk campuran spesies *Plasmodium falciparum* dan spesies lain (Pan+Pf, RDT=4) dan hasil tidak sah /invalid (RDT=5).

$$RDT\ positif = \frac{\begin{array}{c} ART\ semua\ umur\ dengan\ hasil\ pemeriksaan\ positif\ P.falciparum\ (RDT = 2) \\ atau\ positif\ untuk\ spesies\ selain\ P.falciparum\ (RDT = 3) \\ atau\ positif\ untuk\ campuran\ P.falciparum\ dan\ spesies\ lain\ (RDT = 4) \end{array}}{ART\ semua\ umur\ yang\ menjalani\ pemeriksaan\ RDT}$$

Proporsi angka positif malaria berdasarkan pemeriksaan mikroskopis hanya dilakukan pada penduduk yang mempunyai riwayat demam dalam dua hari terakhir. Sampel dinyatakan negatif apabila tidak ditemukan parasit pada seluruh lapangan pandang Sampel dinyatakan positif apabila didapatkan minimal satu jenis parasit malaria yang dapat menginfeksi manusia yaitu *Plasmodium falciparum, P.vivax, P. Malariae, P.ovale* atau *P.knowlesi*. Kepadatan parasit dihitung per 200 leukosit, jika jumlah parasit yang ditemukan kurang dari 10 maka perhitungan dilanjutkan sampai 500 leukosit.

$$Malaria\ positif = \frac{\begin{array}{c} ART\ semua\ umur\ yang\ mempunyai\ riwayat\ demam\ dalam\ dua\ hari\ terakhir\ dengan\ hasil \\ pemeriksaan\ positif\ P.falciparum\ atau\ P.vivax\ atau\ P.malariae\ atau\ P.ovale\ atau\ P.knowlesi \\ atau\ positif\ infeksi\ campuran\ antar\ spesies\ tersebut \end{array}}{\begin{array}{c} ART\ semua\ umur\ yang\ mempunyai\ riwayat\ demam\ dalam\ dua\ hari\ terakhir\ dan \\ menjalani\ pemeriksaan\ darah\ secara\ mikroskopis \end{array}}$$

Tabel 5.6.6
Prevalensi Malaria berdasarkan Hasil Pemeriksaan RDT dan Jenis Plasmodium menurut Karakteristik, Riskesdas 2018

Karakteristik	*Positif malaria*		*Jenis Plasmodium*						Ntertimbang
			P. falciparum (Pf)		*Non Pf*(PAN)		Mix (Pf+Non Pf)		
	%	95%CI	%	95%CI	%	95%CI	%	95%CI	
**Kelompok Umur**									
0-11 bulan	1,0	0,1-6,6	0,98	0,14-6,56	0,00	-	0,00	-	337
12-59 bulan	0,6	0,2-1,9	0,60	0,18-1,92	0,00	-	0,03	0,03-0,03	2.114
5-9 tahun	1,0	0,7-1,4	0,92	0,65-1,32	0,02	0,00-0,16	0,02	0,02-0,02	3.670
10-14 tahun	0,5	0,3-0,7	0,45	0,28-0,71	0,02	0,02-0,02	0	0-0,01	3.819
≥ 15 tahun	0,6	0,5-0,8	0,58	0,45-0,75	0,04	0,02-0,06	0,01	0,01-0,04	37.047
**Wanita hamil**	0,5	0,5-0,5	0,5	0,5-0,5	-	-	-	-	525
**Jenis kelamin**									
Laki-laki	0,7	0,5-0,9	0,63	0,48-0,83	0,04	0,02-0,08	0,01	0-0,02	23.609
Perempuan	0,6	0,5-0,8	0,57	0,41-0,79	0,03	0,01-0,04	0,02	0,01-0,05	23.378
**Pendidikan**									
Tidak Sekolah	0,7	0,5-0,9	0,62	0,45-0,86	0,02	0,00-0,17	0,01	0,01-0,01	3.800
Tidak Tamat SD	0,8	0,6-1,0	0,71	0,55-0,93	0,04	0,02-0,08	0,01	0,01-0,01	9.273
Tamat SD	0,7	0,5-0,9	0,60	0,45-0,81	0,03	0,01-0,08	0,02	0,01-0,06	12.978
Tamat SMP	0,7	0,5-1,0	0,60	0,41-0,89	0,05	0,02-0,17	0,02	0-0,1	7.498
Tamat SMA	0,4	0,2-0,8	0,42	0,21-0,81	0,02	0,00-0,05	0,00	-	8.321
Tamat D1-D3/PT	0,8	0,5-1,3	0,68	0,39-1,16	0,07	0,01-0,46	0,04	0,01-0,17	2.099
**Pekerjaan**									
Tidak Bekerja	0,6	0,4-0,8	0,51	0,35-0,75	0,05	0,03-0,09	0,01	0-0,03	13.168
Sekolah	0,5	0,3-0,7	0,47	0,31-0,7	0,02	0,02-0,02	0,01	0,01-0,01	4.963
PNS/TNI/Polri/BUMN/BUMD	0,9	0,4-2,1	0,92	0,4-2,11	0,00	0,00	0,00	0,00	545
Pegawai Swasta	0,6	0,3-1,1	0,58	0,32-1,06	0,00	0,00	0,00	0,00	2.986
Wiraswasta	0,6	0,4-0,9	0,53	0,35-0,82	0,03	0,01-0,11	0,01	0,01-0,01	5.506
Petani/Buruh Tani	0,8	0,7-1,0	0,72	0,58-0,9	0,06	0,02-0,19	0,03	0,01-0,21	7.442
Nelayan	-	-	0,00	0,00	0,00	0,00	0,00	0,00	148
Buruh/Supir/Pembantu Ruta	0,5	0,2-1,1	0,42	0,17-1,08	0,02	0,00-0,17	0,02	0-0,16	4.240
Lainnya	1,0	0,6-1,5	0,98	0,63-1,53	0,00	0,00	0,00	0,00	1.963
**Tempat Tinggal**									
Perkotaan	0,6	0,4-1,0	0,60	0,37-0,98	0,03	0,02-0,06	0,01	0-0,05	25.722
Perdesaan	0,6	0,5-0,8	0,60	0,5-0,72	0,03	0,02-0,07	0,01	0,01-0,01	21.265
**INDONESIA**	**0,6**	**0,5-0,8**	**0,57**	**0,45-0,72**	**0,04**	**0,02-0,06**	**0,01**	**0,01-0,03**	**46.987**

Catatan: 0,0 adalah proporsi yang sangat kecil mendekati nol. tanda “–“ menyatakan tidak ada kasus pada kelompok tersebut

Tabel 5.6.7
Proporsi Malaria pada Penduduk dengan Riwayat Demam dalam Dua Hari
berdasarkan pemeriksaan Mikroskopis, Riskesdas 2018

Jenis Plasmodium	Positif
Pf	0,5
Pv	0,3
Pm	0,1
Pf + Pv	0,3
**Malaria**	**1,2**
N Sampel	730

5.7 Filariasis

Penyakit kaki gajah (Filariasis) adalah penyakit zoonosis. Di Indonesia, filariasis merupakan salah satu penyakit endemis. Gejala yang timbul biasanya berupa pembengkakan (edema) di daerah tertentu (pada aliran pembuluh limfa di dalam tubuh manusia). Gejala ini dapat berupa pembesaran tungkai/kaki (kaki gajah) atau lengan dan pembesaran skrotum/vagina yang pembengkakan (edema)nya bersifat permanen. Filariasis bersifat menahun (kronis) dan jarang menimbulkan kematian pada penderitanya. Pada fase awal bisa juga menunjukan tanpa gejala (asimtomatis).

Program Pemberian Obat Pencegahan Massal (POPM) Filariasis **sekali setahun** selama 5 tahun berturut-turut bertujuan untuk memutuskan mata rantai penularan filariasis di daerah endemis. Pada tahun 2002, pencanangan eliminasi filariasis di Sumsel. Pada tahun 2014, 46 kabupaten/kota sudah melaksanakan POPM dari 241 kabupaten/kota yang merupakan daerah endemis. Periode tahun 2015 hingga tahun 2019, 195 kab/kota lainnya direncanakan melakukan POPM.

Pada Riskesdas 2018, untuk menilai keberhasilan POPM ditanyakan kepada responden “Apakah [NAMA] pernah diberikan obat pencegahan filariasis (*diethylcarbamazine citrate* dan *albendasol*) oleh petugas kesehatan?”

$$\text{Cakupan obat POPM Filariasis di daerah endemis} = \frac{\sum \text{ART yang mendapatkan POPM Filariasis } \textit{di daerah endemis dan berusia } 2 \textit{ tahun ke atas}}{\sum \text{ART di daerah endemis yang berusia 2 tahun ke atas}}$$

Prevalensi Filariasis diukur berdasarkan riwayat diagnosis oleh tenaga kesehatan (dokter/perawat/bidan) dengan pertanyaan "Apakah [NAMA] pernah didiagnosis menderita kaki gajah (filariasis) oleh tenaga kesehatan (dokter/perawat/bidan)?" Jika menjawab "Ya pada 2017" maka dihitung sebagai kasus Filariasis yang diukur. Formula yang dipakai adalah:

$$\textbf{Prevalensi Filariasis} = \frac{\textbf{Jumlah kasus Filariasis (riwayat diagnosis pada 2017)}}{\sum \textbf{ART Semua Umur}}$$

Tabel 5.7.1
Proporsi Pemberian Obat Pencegahan Masal (POPM) Filariasis di Daerah Endemis selama Periode POPM menurut Provinsi, Riskesdas 2018

Provinsi	Pernah menerima POPM Filariasis		
	%	95% CI	N Tertimbang
Aceh	60,3	58,2-62,3	14.471
Sumatera Utara	28,1	26,4-29,8	20.184
Sumatera Barat	59,1	57,2-60,9	14.929
Riau	59,7	57,8-61,5	20.598
Jambi	47,6	44,0-51,3	6.713
Sumatera Selatan	71,9	69,7-73,9	20.388
Bengkulu	68,9	66,1-71,6	3.964
Lampung	68,9	64,8-72,8	4.273
Kep. Bangka Belitung	57,8	55,7-60,0	6.020
Kepulauan Riau	59,6	55,6-63,4	6.427
Jawa Barat	53,8	52,3-55,3	112.129
Jawa Tengah	59,3	57,3-61,4	39.247
Banten	16,3	14,5-18,2	39.239
Nusa Tenggara Timur	50,6	49,2-52,1	17.784
Kalimantan Barat	72,6	70,6-74,6	13.958
Kalimantan Tengah	69,2	67,2-71,2	8.733
Kalimantan Selatan	78,9	77,2-80,6	8.474
Kalimantan Timur	71,3	69,0-73,5	4.937
Kalimantan Utara	66,4	62,8-69,9	1.851
Sulawesi Tengah	46,8	44,8-48,8	8.975
Sulawesi Selatan	40,3	37,2-43,5	4.646
Sulawesi Tenggara	50,9	48,3-53,5	8.133
Gorontalo	20,0	17,7-22,5	4.872
Sulawesi Barat	39,6	36,8-42,5	4.290
Maluku	65,3	62,3-68,2	6.131
Maluku Utara	51,6	48,3-54,8	2.594
Papua Barat	60,2	57,9-62,5	3.695
Papua	31,0	28,8-33,4	10.684
**INDONESIA**	**51,8**	**51,2-52,4**	**418.340**

Tabel 5.7.2
Proporsi Pemberian Obat Pencegahan Masal (POPM) Filariasis di Daerah Endemis selama Periode POPM menurut Karakteristik, Riskesdas 2018

Karakteristik	Pernah menerima POPM Filariasis		
	%	95% CI	N tertimbang
**Kelompok umur**			
2-4	31,8	30,7-32,8	25.660
5-14	54,3	53,6-55,1	82.599
15-24	50,5	49,7-51,4	72.598
25-34	51,4	50,5-52,3	71.638
35-44	56,7	55,9-57,5	65.489
45-54	57,6	56,7-58,5	49.502
55-64	52,6	51,5-53,7	30.851
65-74	44,9	43,4-46,4	13.970
75+	34,4	32,4-36,4	6.032
**Jenis kelamin**			
Laki-laki	49,8	49,2-50,4	211.837
Perempuan	53,9	53,3-54,5	206.503
**Pendidikan**			
Tidak/belum pernah sekolah	46,9	45,7-48,1	28.504
Tidak tamat SD/MI	54,5	53,7-55,2	80.511
Tamat SD/MI	57,8	57,0-58,6	93.437
Tamat SLTP/MTS	55,3	54,4-56,2	67.884
Tamat SLTA/MA	49,7	48,8-50,7	87.431
Tamat D1/D2/D3/PT	46,7	45,2-48,1	25.112
**Pekerjaan**			
Tidak bekerja	53,6	52,8-54,4	102.192
Sekolah	55,8	54,9-56,7	54.278
PNS/TNI/Polri/BUMN/BUMD	53,7	51,9-55,4	9.274
Pegawai Swasta	44,3	42,7-45,8	29.588
Wiraswasta	49,9	48,8-51,1	39.262
Nelayan	60,1	59,3-60,9	57.715
Petani/Buruh tani	50,9	47,5-54,2	3.007
Lainnya	48,6	47,1-50,1	30.564
**Tempat tinggal**			
Perkotaan	44,8	43,9-45,8	212.666
Perdesaan	59,0	58,4-59,7	205.674

Tabel 5.7.3

Prevalensi Filariasis berdasarkan Diagnosis Nakes dan Proporsi Minum Obat Sesuai Anjuran Nakes menurut Provinsi, Riskesdas 2018

Provinsi	Diagnosis			Minum obat sesuai anjuran tenaga kesehatan	
	%	95% CI	N tertimbang	%	N tertimbang
Aceh	1,3	1,1-1,6	20.244	53,1	261
Sumatera Utara	0,9	0,8-1,1	55.351	16,9	520
Sumatera Barat	0,7	0,5-0,8	20.663	10,6	140
Riau	0,6	0,5-0,8	26.085	20,1	158
Jambi	0,7	0,6-0,9	13.692	13,0	97
Sumatera Selatan	1,0	0,7-1,3	32.126	25,6	308
Bengkulu	0,9	0,7-1,2	7.531	31,6	70
Lampung	0,7	0,6-0,9	32.148	11,3	230
Bangka Belitung	0,6	0,5-0,9	5.592	34,9	36
Kepulauan Riau	1,4	0,8-2,3	8.173	36,3	112
DKI Jakarta	0,6	0,5-0,8	40.210	3,0	253
Jawa Barat	1,0	0,8-1,2	186.809	22,9	1.792
Jawa Tengah	0,8	0,7-0,9	132.565	25,5	1.038
DI Yogyakarta	0,5	0,3-0,7	14.602	6,9	72
Jawa Timur	0,6	0,5-0,7	151.878	5,5	893
Banten	0,7	0,6-0,9	48.621	20,0	356
Bali	0,4	0,3-0,5	16.481	0,9	61
Nusa Tenggara Barat	0,6	0,5-0,8	19.247	0,0	123
Nusa Tenggara Timur	0,7	0,5-0,8	20.599	49,2	135
Kalimantan Barat	0,8	0,7-1,0	19.190	32,9	161
Kalimantan Tengah	0,9	0,7-1,3	10.189	40,7	95
Kalimantan Selatan	0,8	0,6-1,0	16.043	37,8	130
Kalimantan Timur	0,7	0,5-0,9	13.977	29,2	91
Kalimantan Utara	0,8	0,5-1,2	2.733	50,2	22
Sulawesi Utara	0,5	0,3-0,6	9.542	4,4	43
Sulawesi Tengah	0,7	0,6-0,9	11.548	34,6	82
Sulawesi Selatan	0,5	0,4-0,6	33.693	9,2	169
Sulawesi Tenggara	1,3	0,9-1,9	10.167	55,8	134
Gorontalo	0,5	0,4-0,8	4.547	10,7	24
Sulawesi Barat	0,9	0,6-1,3	5.195	29,2	48
Maluku	1,8	1,4-2,4	6.801	51,8	124
Maluku Utara	0,8	0,6-1,0	4.723	22,5	38
Papua Barat	1,4	1,1-1,9	3.588	51,0	51
Papua	1,4	1,2-1,8	12.736	55,1	184
**INDONESIA**	**0,8**	**0,7-0,8**	**1.017.290**	**23,1**	**8.051**

Tabel 5.7.4
Prevalensi Filariasis berdasarkan Diagnosis Nakes dan Proporsi Minum Obat Sesuai Anjuran Nakes menurut Karakteristik, Riskesdas 2018

Karakteristik	Diagnosis			Minum obat sesuai dengan anjuran tenaga kesehatan	
	%	95% CI	N tertimbang	%	N tertimbang
**Kelompok umur**					
< 1	1,1	0,8-1,4	18.225	2,2	199
1-4	0,9	0,8-1,0	73.188	17,1	637
5-14	0,9	0,8-0,9	182.338	27,7	1.552
15-24	0,7	0,7-0,8	165.644	22,4	1.212
25-34	0,8	0,7-0,9	159.708	24,6	1.266
35-44	0,8	0,8-0,9	151.539	25,1	1.265
45-54	0,7	0,7-0,8	124.652	25,1	904
55-64	0,8	0,7-0,9	83.251	20,7	643
65-74	0,6	0,5-0,7	40.180	16,2	245
75+	0,7	0,5-0,9	18.565	11,2	128
**Jenis kelamin**					
Laki-laki	0,8	0,8-0,9	510.714	21,2	4.154
Perempuan	0,8	0,7-0,8	506.576	25,1	3.897
**Pendidikan**					
Tidak/belum pernah sekolah	0,8	0,7-0,9	70.895	21,5	602
Tidak tamat SD/MI	0,8	0,7-0,9	181.429	27,1	1.542
Tamat SD/MI	0,7	0,7-0,8	215.967	24,1	1.584
Tamat SLTP/MTS	0,7	0,7-0,8	160.320	25,7	1.172
Tamat SLTA/MA	0,8	0,7-0,9	210.746	22,8	1.716
Tamat D1/D2/D3/PT	0,8	0,7-0,9	64.093	18,1	523
**Pekerjaan**					
Tidak bekerja	0,8	0,7-0,9	233.629	24,1	1.879
Sekolah	0,8	0,7-0,9	126.626	27,3	1.022
PNS/TNI/Polri/BUMN/BUMD	1,0	0,8-1,2	21.931	29,3	219
Pegawai Swasta	0,7	0,6-0,8	75.781	18,1	503
Wiraswasta	0,7	0,6-0,8	105.489	18,5	766
Nelayan	1,0	0,8-1,4	5.556	32,2	59
Petani/Buruh tani	0,8	0,7-0,9	133.261	26,6	1.077
Lainnya	0,8	0,7-0,9	116.233	21,2	917
**Tempat tinggal**					
Perkotaan	0,8	0,7-0,9	556.419	19,8	4.473
Perdesaan	0,8	0,7-0,8	460.871	27,2	3.578

BAB 6
PENYAKIT TIDAK MENULAR

6.1 Asma

Prevalensi asma dihitung menggunakan formula:

$$Prevalensi\ Asma = \frac{ART\ yang\ pernah\ didiagnosis\ asma\ oleh\ dokter}{ART\ semua\ umur}$$

Proporsi kekambuhan asma dalam 12 bulan terakhir dihitung menggunakan formula:

$$Proporsi\ kekambuhan\ asma\ dalam\ 12\ bulan\ terakhir = \frac{ART\ yang\ pernah\ kambuh\ asmanya\ dalam\ 12\ bulan\ terakhir}{ART\ yang\ pernah\ didiagnosis\ asma\ oleh\ dokter}$$

Tabel 6.1.1
Prevalensi Asma berdasarkan Diagnosis Dokter pada Penduduk Semua Umur menurut Provinsi, Riskesdas 2018

Provinsi	Asma		N tertimbang
	%	95% CI	
Aceh	2,3	2,1 - 2,5	20.244
Sumatera Utara	1,0	0,9 - 1,2	55.351
Sumatera Barat	2,0	1,8 - 2,2	20.663
Riau	2,2	2,0 - 2,5	26.085
Jambi	1,7	1,5 - 1,9	13.692
Sumatera Selatan	1,9	1,7 - 2,2	32.126
Bengkulu	2,4	2,1 - 2,8	7.531
Lampung	1,6	1,4 - 1,8	32.148
Bangka Belitung	2,8	2,5 - 3,2	5.592
Kepulauan Riau	2,4	2,0 - 2,9	8.173
DKI Jakarta	2,6	2,3 - 2,9	40.210
Jawa Barat	2,8	2,6 –3,0	186.809
Jawa Tengah	1,8	1,7 - 1,9	132.565
DI Yogyakarta	4,5	4,0 - 5,1	14.602
Jawa Timur	2,6	2,4 - 2,7	151.878
Banten	2,5	2,2 - 2,8	48.621
Bali	3,9	3,5 - 4,3	16.481
Nusa Tenggara Barat	3,1	2,8 - 3,5	19.247
Nusa Tenggara Timur	1,5	1,4 - 1,7	20.599
Kalimantan Barat	3,2	2,9 - 3,5	19.190
Kalimantan Tengah	3,4	3,0 - 3,8	10.189
Kalimantan Selatan	2,8	2,5 - 3,0	16.043
Kalimantan Timur	4,0	3,6 - 4,4	13.977
Kalimantan Utara	3,3	2,8 –4,0	2.733
Sulawesi Utara	2,1	1,8 - 2,3	9.542
Sulawesi Tengah	3,0	2,7 - 3,3	11.548
Sulawesi Selatan	2,5	2,3 - 2,8	33.693
Sulawesi Tenggara	2,4	2,1 - 2,8	10.167
Gorontalo	2,8	2,4 - 3,2	4.547
Sulawesi Barat	1,5	1,2 - 1,8	5.195
Maluku	1,9	1,6 - 2,3	6.801
Maluku Utara	1,8	1,5 - 2,1	4.723
Papua Barat	2,3	1,8 - 3,1	3.588
Papua	1,9	1,7 - 2,3	12.736
**INDONESIA**	**2,4**	**2,3 - 2,4**	**1.017.290**

Tabel 6.1.2
Prevalensi Asma berdasarkan Diagnosis Dokter pada Penduduk Semua Umur menurut Karakteristik, Riskesdas 2018

Karakteristik	Asma		N tertimbang
	%	95% CI	
**Kelompok umur**			
< 1	0,4	0,3 - 0,5	18.225
1-4	1,6	1,4 - 1,7	73.188
5-14	1,9	1,8 - 2,1	182.338
15-24	2,2	2,1 - 2,3	165.644
25-34	2,2	2,1 - 2,3	159.708
35-44	2,3	2,2 - 2,4	151.539
45-54	2,6	2,5 - 2,7	124.652
55-64	3,4	3,3 - 3,6	83.251
65-74	4,5	4,2 - 4,8	40.180
75+	5,1	4,6 - 5,6	18.565
**Jenis kelamin**			
Laki-laki	2,3	2,2- 2,4	510.714
Perempuan	2,5	2,4 - 2,6	506.576
**Pendidikan**			
Tidak/belum pernah sekolah	3,0	2,8 - 3,1	70.895
Tidak tamat SD/MI	2,5	2,4 - 2,6	181.429
Tamat SD/MI	2,5	2,4 - 2,6	215.967
Tamat SLTP/MTS	2,2	2,1 - 2,4	160.320
Tamat SLTA/MA	2,4	2,3 - 2,5	210.746
Tamat D1/D2/D3/PT	3,0	2,8 - 3,2	64.093
**Pekerjaan**			
Tidak bekerja	3,1	2,9 - 3,2	233.629
Sekolah	2,2	2,0 - 2,3	126.626
PNS/TNI/Polri/BUMN/BUMD	2,7	2,4–3,0	21.931
Pegawai swasta	2,4	2,2 - 2,6	75.781
Wiraswasta	2,5	2,4 - 2,6	105.489
Petani/buruh tani	2,4	2,3 - 2,5	133.261
Nelayan	2,5	2,0–3,0	5.556
Buruh/sopir/pembantu ruta	2,3	2,1 - 2,5	75.590
Lainnya	2,5	2,3 - 2,7	40.644
**Tempat tinggal**			
Perkotaan	2,6	2,5 - 2,7	556.419
Perdesaan	2,2	2,1 - 2,2	460.871

Tabel 6.1.3
Proporsi Kekambuhan Asma dalam 12 Bulan Terakhir pada Penduduk Semua Umur menurut Provinsi, Riskesdas 2018

Provinsi	Kekambuhan asma dalam 12 bulan terakhir		N tertimbang
	%	95% CI	
Aceh	68,9	64,7 - 72,9	449
Sumatera Utara	55,2	49,7 - 60,6	560
Sumatera Barat	66,2	60,9 - 71,2	404
Riau	62,6	57,7 - 67,3	558
Jambi	62,0	56,4 - 67,3	228
Sumatera Selatan	63,6	57,6 - 69,2	613
Bengkulu	65,2	58,6 - 71,2	176
Lampung	64,7	59,1 - 69,9	503
Bangka Belitung	55,0	48,4 - 61,6	154
Kepulauan Riau	59,6	50,2 - 68,4	194
DKI Jakarta	52,7	46,6 - 58,7	1.001
Jawa Barat	57,4	54,6 - 60,1	5.101
Jawa Tengah	55,1	52,3 - 57,9	2.289
DI Yogyakarta	46,1	39,8 - 52,6	640
Jawa Timur	58,7	56,3 - 61,0	3.811
Banten	57,2	52,2 - 62,1	1.179
Bali	53,5	48,7 - 58,2	628
Nusa Tenggara Barat	59,2	54,5 - 63,8	590
Nusa Tenggara Timur	62,5	57,6 - 67,2	304
Kalimantan Barat	59,6	55,6 - 63,5	597
Kalimantan Tengah	51,4	45,8 - 56,9	337
Kalimantan Selatan	55,0	50,5 - 59,5	432
Kalimantan Timur	51,8	47,6 - 56,0	543
Kalimantan Utara	53,8	46,1 - 61,4	89
Sulawesi Utara	48,6	42,7 - 54,6	191
Sulawesi Tengah	58,1	52,9 - 63,2	338
Sulawesi Selatan	60,6	57,0 - 64,1	835
Sulawesi Tenggara	55,9	50,7 - 60,9	241
Gorontalo	61,8	54,8 - 68,4	123
Sulawesi Barat	56,8	44,4 - 68,4	74
Maluku	61,7	53,1 - 69,6	125
Maluku Utara	56,3	48,3 - 64,0	81
Papua Barat	56,9	47,3 - 66,0	82
Papua	48,2	42,3 - 54,0	241
**INDONESIA**	**57,5**	**56,5 - 58,46**	**23.713**

Tabel 6.1.4
Proporsi Kekambuhan Asma dalam 12 Bulan Terakhir pada Penduduk Semua Umur menurut Karakteristik, Riskesdas 2018

Karakteristik	Kekambuhan asma dalam 12 bulan terakhir		N tertimbang
	%	95% CI	
**Kelompok umur**			
< 1	66,8	48,4 - 81,1	66
1-4	68,2	64,1 - 72,1	1.118
5-14	53,9	51,4 - 56,4	3.464
15-24	50,1	47,5 - 52,7	3.544
25-34	50,5	47,8 - 53,2	3.451
35-44	56,2	53,7 - 58,6	3.439
45-54	58,7	56,3 - 61,2	3.169
55-64	61,7	59,2 - 64,2	2.779
65-74	72,3	69,2 - 75,2	1.763
75+	71,6	67,5 - 75,4	920
**Jenis kelamin**			
Laki-laki	56,1	54,7 - 57,5	11.446
Perempuan	58,8	57,5 - 60,1	12.267
**Pendidikan**			
Tidak/belum pernah sekolah	64,4	61,4 - 67,3	2.054
Tidak tamat SD/MI	60,6	58,6 - 62,7	4.481
Tamat SD/MI	61,0	59,0 - 62,9	5.235
Tamat SLTP/MTS	55,3	52,8 - 57,8	3.527
Tamat SLTA/MA	52,3	50,1 - 54,5	4.976
Tamat D1/D2/D3/PT	43,7	40,3 - 47,1	1.906
**Pekerjaan**			
Tidak bekerja	61,5	59,7 - 63,2	7.041
Sekolah	48,1	45,4 - 50,9	2.691
PNS/TNI/Polri/BUMN/BUMD	45,6	40,3 - 51,0	575
Pegawai swasta	46,1	42,3 - 49,9	1.772
Wiraswasta	56,7	53,8 - 59,5	2.596
Petani/buruh tani	63,6	61,4 - 65,7	3.162
Nelayan	59,9	50,3 - 68,8	135
Buruh/sopir/pembantu ruta	55,5	51,5 - 59,5	1.685
Lainnya	58,1	53,7 - 62,3	990
**Tempat tinggal**			
Perkotaan	54,5	53,1 - 55,9	14.049
Perdesaan	61,9	60,6 - 63,1	9.664

6.2 Kanker

Kanker yang dimaksud adalah semua jenis kanker yang didiagnosis oleh dokter. Prevalensi kanker (dalam permil) dihitung menggunakan formula:

$$Prevalensi\ Kanker = \frac{ART\ yang\ pernah\ didiagnosis\ kanker\ oleh\ dokter}{ART\ semua\ umur}$$

Jenis pengobatan kanker (pembedahan/operasi, radiasi/penyinaran, kemoterapi, lainnya) dihitung menggunakan formula:

$$Jenis\ pengobatan\ kanker = \frac{\begin{matrix} Masing - masing\ jenis\ pengobatan\ kanker\ yang\ dijalani \\ (bedah, radiasi, kemoterapi, lainnya) \end{matrix}}{ART\ yang\ pernah\ didiagnosis\ kanker\ oleh\ dokter}$$

Tabel 6.2.1
Prevalensi (per mil) Kanker berdasarkan Diagnosis Dokter pada Penduduk Semua Umur menurut Provinsi, Riskesdas 2018

Provinsi	Kanker		N tertimbang
	‰	95% CI (‰)	
Aceh	2,00	1,56 - 2,58	20.244
Sumatera Utara	1,55	1,10 - 2,19	55.351
Sumatera Barat	2,47	1,93 - 3,17	20.663
Riau	1,67	1,21 - 2,30	26.085
Jambi	1,32	0,91 - 1,93	13.692
Sumatera Selatan	1,54	1,10 - 2,17	32.126
Bengkulu	1,37	0,86 - 2,16	7.531
Lampung	1,40	0,97 - 2,03	32.148
Bangka Belitung	1,49	0,89 - 2,50	5.592
Kepulauan Riau	1,87	1,05 - 3,32	8.173
DKI Jakarta	2,33	1,65 - 3,29	40.210
Jawa Barat	1,41	1,12 - 1,78	186.809
Jawa Tengah	2,11	1,79 - 2,48	132.565
DI Yogyakarta	4,86	3,72 - 6,34	14.602
Jawa Timur	2,17	1,85 - 2,55	151.878
Banten	1,39	0,90 - 2,15	48.621
Bali	2,27	1,63 - 3,15	16.481
Nusa Tenggara Barat	0,85	0,50 - 1,44	19.247
Nusa Tenggara Timur	1,49	1,04 - 2,14	20.599
Kalimantan Barat	1,55	1,04 - 2,31	19.190
Kalimantan Tengah	1,36	0,86 - 2,14	10.189
Kalimantan Selatan	2,13	1,49 - 3,05	16.043
Kalimantan Timur	1,45	0,94 - 2,22	13.977
Kalimantan Utara	2,16	1,13 - 4,16	2.733
Sulawesi Utara	1,71	1,17 - 2,50	9.542
Sulawesi Tengah	2,23	1,61 - 3,10	11.548
Sulawesi Selatan	1,59	1,23 - 2,05	33.693
Sulawesi Tenggara	1,31	0,76 - 2,25	10.167
Gorontalo	2,44	1,45 - 4,11	4.547
Sulawesi Barat	1,58	0,90 - 2,78	5.195
Maluku	0,90	0,42 - 1,93	6.801
Maluku Utara	0,94	0,47 – 1,90	4.723
Papua Barat	1,32	0,58 - 2,99	3.588
Papua	1,58	0,80 - 3,11	12.736
**INDONESIA**	**1,79**	**1,68 - 1,92**	**1.017.290**

Tabel 6.2.2
Prevalensi (per mil) Kanker berdasarkan Diagnosis Dokter pada Penduduk Semua Umur menurut Karakteristik, Riskesdas 2018

Karakteristik	Kanker		N tertimbang
	‰	95%CI (‰)	
**Kelompok umur**			
< 1	0,03	0,00 - 0,22	18.225
1-4	0,08	0,04 - 0,17	73.188
5-14	0,31	0,22 - 0,44	182.338
15-24	0,47	0,35 - 0,64	165.644
25-34	1,21	0,99 – 1,48	159.708
35-44	2,58	2,22 – 3,00	151.539
45-54	4,03	3,56 – 4,56	124.652
55-64	4,62	4,00 – 5,33	83.251
65-74	3,52	2,87 – 4,32	40.180
75+	3,84	2,76 – 5,33	18.565
**Jenis kelamin**			
Laki-laki	0,74	0,64 – 0,86	510.714
Perempuan	2,85	2,65 – 3,07	506.576
**Pendidikan**			
Tidak/belum pernah sekolah	1,93	1,56 – 2,40	70.895
Tidak tamat SD/MI	1,44	1,22 – 1,69	181.429
Tamat SD/MI	2,25	1,98 – 2,56	215.967
Tamat SLTP/MTS	1,68	1,43 – 1,97	160.320
Tamat SLTA/MA	2,03	1,76 – 2,34	210.746
Tamat D1/D2/D3/PT	3,57	2,89 – 4,40	64.093
**Pekerjaan**			
Tidak bekerja	3,48	3,15 – 3,86	233.629
Sekolah	0,41	0,30 – 0,57	126.626
PNS/TNI/Polri/BUMN/BUMD	4,10	3,22 – 5,22	21.931
Pegawai swasta	1,55	1,09 – 2,19	75.781
Wiraswasta	2,15	1,81 – 2,55	105.489
Petani/buruh tani	1,93	1,66 – 2,26	133.261
Nelayan	1,65	0,77 – 3,53	5.556
Buruh/sopir/pembantu ruta	1,28	0,99 – 1,67	75.590
Lainnya	2,39	1,81 – 3,16	40.644
**Tempat tinggal**			
Perkotaan	2,06	1,89 – 2,26	556.419
Perdesaan	1,47	1,34 – 1,61	460.871

Tabel 6.2.3
Proporsi Jenis Pengobatan Kanker berdasarkan Diagnosis Dokter
pada Penduduk Semua Umur menurut Provinsi, Riskesdas 2018

Provinsi	Jenis pengobatan kanker (%)				N Tertimbang
	Pembedahan/operasi	Radiasi/penyinaran	Kemoterapi	Lainnya	
Aceh	66,6	24,3	19,1	26,8	42*
Sumatera Utara	48,5	14,9	25,3	30,0	88
Sumatera Barat	58,0	17,4	20,4	29,2	52
Riau	70,1	17,5	22,7	21,4	45*
Jambi	92,1	19,8	31,3	21,9	19*
Sumatera Selatan	60,4	14,0	19,1	30,2	51
Bengkulu	75,6	24,1	21,0	20,0	11*
Lampung	68,0	10,5	24,9	23,4	46*
Bangka Belitung	21,0	12,4	9,5	63,1	9*
Kepulauan Riau	77,2	13,3	25,9	11,1	16*
DKI Jakarta	66,4	25,2	25,8	31,3	96
Jawa Barat	62,8	14,7	35,8	20,6	270
Jawa Tengah	68,0	22,1	26,6	21,2	287
DI Yogyakarta	71,8	19,5	23,4	20,9	73
Jawa Timur	65,1	14,1	24,9	20,7	339
Banten	44,3	32,1	21,1	21,0	69
Bali	63,4	20,2	41,8	15,4	38*
Nusa Tenggara Barat	71,7	19,6	7,5	25,6	17*
Nusa Tenggara Timur	29,8	10,2	11,9	59,4	32*
Kalimantan Barat	49,2	11,2	12,8	32,9	31*
Kalimantan Tengah	52,9	5,7	12,7	25,7	14*
Kalimantan Selatan	41,9	21,3	21,8	9,8	35*
Kalimantan Timur	41,4	16,6	34,0	31,6	21*
Kalimantan Utara	60,2	0,1	4,6	31,6	6*
Sulawesi Utara	30,6	24,8	19,4	27,2	17*
Sulawesi Tengah	50,9	3,6	18,7	26,3	26*
Sulawesi Selatan	62,1	6,4	10,3	34,1	55
Sulawesi Tenggara	58,0	4,9	18,3	46,0	14*
Gorontalo	70,2	1,8	9,0	17,4	11*
Sulawesi Barat	65,7	36,1	13,1	8,0	8*
Maluku	55,6	11,6	11,6	35,1	6*
Maluku Utara	59,9	16,9	13,8	0,0	5*
Papua Barat	60,3	0,9	0,6	45,6	5*
Papua	61,9	39,2	28,8	21,7	21*
**INDONESIA**	**61,8**	**17,3**	**24,9**	**24,1**	**1.872**

*N Tertimbang <50

Tabel 6.2.4
Proporsi Jenis Pengobatan Kanker berdasarkan Diagnosis Dokter
pada Penduduk Semua Umur menurut Karakteristik, Riskesdas 2018

Karakteristik	Jenis pengobatan kanker (%)				N tertimbang
	Pembedahan/operasi	Radiasi/penyinaran	Kemoterapi	Lainnya	
**Kelompok umur**					
< 1	100,0	0,0	0,0	0,0	1*
1-4	48,4	11,3	6,4	15,6	6*
5-14	47,8	13,1	18,6	21,2	58
15-24	52,8	5,1	11,3	15,9	80
25-34	55,1	12,4	9,2	22,8	198
35-44	57,4	14,1	26,2	30,6	401
45-54	69,2	20,0	29,3	26,7	515
55-64	63,9	22,4	34,7	19,4	394
65-74	65,1	18,7	17,7	22,1	145
75+	55,7	17,3	12,8	15,3	73
**Jenis kelamin**					
Laki-laki	48,5	20,7	18,8	20,0	389
Perempuan	65,3	16,5	26,5	25,2	1.483
**Pendidikan**					
Tidak/belum pernah sekolah	62,6	23,8	19,5	21,2	141
Tidak tamat SD/MI	60,4	19,3	20,3	24,3	268
Tamat SD/MI	55,4	14,4	22,6	26,3	500
Tamat SLTP/MTS	62,6	18,3	17,8	26,3	277
Tamat SLTA/MA	65,0	15,6	29,5	26,0	439
Tamat D1/D2/D3/PT	69,6	19,8	38,6	15,1	235
**Pekerjaan**					
Tidak bekerja	63,9	17,2	28,3	23,1	848
Sekolah	61,0	10,3	15,8	11,4	55
PNS/TNI/Polri/BUMN/BUMD	70,7	23,0	36,2	21,1	94
Pegawai swasta	69,4	13,3	26,2	18,6	122
Wiraswasta	63,3	21,4	27,6	27,0	236
Petani/buruh tani	55,2	13,9	16,1	26,1	268
Nelayan	35,0	19,2	0,7	53,4	10*
Buruh/sopir/pembantu ruta	53,1	19,7	12,4	29,6	101
Lainnya	58,7	22,0	25,4	26,9	101
**Tempat tinggal**					
Perkotaan	62,1	19,0	29,0	23,4	1.178
Perdesaan	61,2	14,6	18,0	25,3	694

*N Tertimbang <50

6.3 Diabetes

Prevalensi diabetes melitus semua umur menurut diagnosis dokter dihitung dengan formula:

$$\mathit{Prevalensi\ diabetes\ melitus\ berdasarkan\ diagnosis\ dokter}\ (\mathit{semua\ umur}) = \frac{\mathit{ART\ yang\ pernah\ didiagnosis\ diabetes\ melitus}}{\mathit{ART\ semua\ umur}}$$

Prevalensi diabetes melitus umur ≥ 15 tahun menurut diagnosis dokter dihitung dengan formula:

$$\mathit{Prevalensi\ diabetes\ melitus\ berdasarkan\ diagnosis\ dokter}\ (\mathit{ART} \geq 15\ \mathit{tahun}) = \frac{\mathit{ART\ yang\ pernah\ didiagnosis\ diabetes\ melitus}}{\mathit{ART}\ \text{umur}\ \geq\ 15\ \text{tahun}}$$

Prevalensi diabetes melitus berdasarkan pemeriksaan darah mengikuti kriteria diabetes melitus dari konsensus PERKENI yang mengadopsi kriteria American Diabetes Association (ADA). Pada laporan ini, terdapat 2 versi, yaitu versi terbaru (Konsensus PERKENI dan ADA 2015) dan versi 2011.

Menurut kriteria 2015, diabetes melitus ditegakkan bila kadar glukosa darah puasa (GDP) ≥ 126 mg/dL; atau glukosa darah 2 jam pasca pembebanan (GDPP) ≥ 200 mg/dL; atau glukosa darah sewaktu (GDS) ≥ 200 mg/dL dengan gejala sering lapar, sering haus, sering buang air kecil & jumlah banyak, dan berat badan turun. Pada Riskesdas 2018, pemeriksaan kadar gula darah dilakukan pada ART berumur ≥ 15 tahun.

$$\mathit{Prevalensi\ DM}\ (\mathit{PERKENI}\ 2015) = \frac{\begin{array}{c}\mathit{ART}\ \geq 15\ \mathit{tahun\ dengan}\ \text{"}\mathit{kadar\ GDP}\ \geq 126\ \mathit{mg\ per\ dl}\text{"}\\ \mathit{atau}\ \text{"}\mathit{GDPP}\ \geq 200\ \mathit{mg\ per\ dl}\text{"}\\ \mathit{atau}\ \text{"}\mathit{GDS}\ \geq 200\ \mathit{mg\ per\ dl\ disertai}\ 4\ \mathit{gejala\ khas\ DM}\text{"}\end{array}}{\begin{array}{c}\mathit{ART\ umur}\ \geq 15\ \mathit{tahun\ yang\ menjalani\ pemeriksaan\ kadar\ gula\ darah}\\ (\mathit{GDP\ atau\ GDPP\ atau\ GDS})\end{array}}$$

Sedangkan menurut Konsensus PERKENI 2011, diabetes melitus ditegakkan sebagai berikut:

$$Prevalensi\ DM\ (PERKENI\ 2011) = \frac{\begin{gathered}ART\ \geq 15\ tahun\ dengan\ "kadar\ GDP\ \geq 126\ mg\ per\ dl\ disertai\ 4\ gejala\ khas\ DM" \\ atau\ "GDPP\ \geq 200\ mg\ per\ dl" \\ atau\ "GDS\ \geq 200\ mg\ per\ dl\ disertai\ 4\ gejala\ khas\ DM"\end{gathered}}{\begin{gathered}ART\ umur\ \geq 15\ tahun\ yang\ menjalani\ pemeriksaan\ kadar\ gula\ darah \\ (GDP\ atau\ GDPP\ atau\ GDS)\end{gathered}}$$

Proporsi glukosa darah puasa terganggu (GDPT) berdasarkan pemeriksaan darah mengikuti kriteria ADA 2011, yaitu kadar GDP antara 100-125 mg/dl.

$$Proporsi\ GDP\ terganggu = \frac{\begin{gathered}ART\ \geq 15\ tahun\ dengan\ kadar\ glukosa\ darah\ puasa\ (GDP) \\ 100 - 125\ mg\ per\ dl\end{gathered}}{ART\ \geq 15\ tahun\ yang\ menjalani\ pemeriksaan\ kadar\ GDP}$$

$$Proporsi\ GDP\ terganggu = \frac{\begin{gathered}ART\ \geq 15\ tahun\ dengan\ kadar\ GDP\ 100 - 125\ mg\ per\ dl\ dan \\ kadar\ GDPP < 140\ mg\ per\ dl\end{gathered}}{ART\ \geq 15\ tahun\ yang\ menjalani\ pemeriksaan\ kadar\ GDP\ dan\ GDPP}$$

Proporsi toleransi glukosa terganggu (TGT) berdasarkan pemeriksaan darah mengikuti kriteria ADA 2011, yaitu kadar glukosa darah 2 jam PP antara 140-199 mg/dl.

$$Proporsi\ TGT = \frac{\begin{gathered}ART\ \geq 15\ tahun\ dengan\ kadar\ glukosa\ darah\ 2\ jam\ pasca\ pembebanan\ (GDPP) \\ 140 - 199\ mg\ per\ dl\end{gathered}}{ART\ \geq 15\ tahun\ yang\ menjalani\ pemeriksaan\ kadar\ GDPP}$$

Proporsi toleransi glukosa terganggu (TGT) berdasarkan pemeriksaan biomedis mengikuti kriteria **ADA dan PERKENI 2015**, yaitu kadar GDPP antara 140-199 mg/dl dan GDP <100 mg/dl.

$$Proporsi\ TGT = \frac{\begin{gathered}ART\ \geq 15\ tahun\ dengan\ kadar\ GDPP\ 140 - 199\ mg\ per\ dl\ dan \\ kadar\ GDP < 100\ mg\ per\ dl\end{gathered}}{ART\ \geq 15\ tahun\ yang\ menjalani\ pemeriksaan\ kadar\ GDP\ dan\ GDPP}$$

Jenis pengobatan diabetes melitus= masing-masing jenis pengobatan yang dijalani (obat anti diabetes melitus/OAD dari tenaga medis, injeksi insulin, OAD dari tenaga medis dan injeksi insulin, tidak diobati)/ART semua umur yang pernah didiagnosis DM oleh dokter

$$Jenis\ pengobatan\ diabetes\ melitus = \frac{Masing - masing\ jenis\ pengobatan\ yang\ dijalani\ (obat\ dari\ tenaga\ medis,\ injeksi,\ obat\ dari\ tenaga\ medis\ dan\ injeksi, tidak\ diobati)\ (B08)}{ART\ semua\ umur\ yang\ pernah\ didiagnosis\ diabetes\ melitus\ oleh\ dokter\ (B06 = 1)}$$

Proporsi kerutinanminum/suntik obat antidiabetes sesuai petunjuk dokter : ART semua umur yang minum obat atau suntik obat antidiabetes secara rutin /ART yang pernah didiagnosis diabetes melitus oleh dokter dan mendapat obat atau injeksi antidiabetes

$$Proporsi\ minum\ obat\ dan\ atau\ suntik\ obat\ antidiabetes\ secara\ rutin\ sesuai\ petunjuk\ dokter = \frac{Minum\ obat\ dan\ atau\ suntik\ obat\ antidiabetes\ secara\ rutin\ sesuai\ petunjuk\ dokter}{ART\ semua\ umur\ yang\ mendapat\ obat\ dan\ atau\ injeksi\ insulin}$$

Proporsi alasan tidak minum atau suntik obat antidiabetes secara rutin sesuai petunjuk dokter= masing-masing alasan (sering lupa, obat tidak tersedia di fasyankes, minum obat tradisional, tidak tahan efek samping, tidak mampu beli obat, tidak rutin ke fasyankes, merasa sudah sehat, lainnya) /ART semua umur yang pernah didiagnosis diabetes melitus oleh dokter, mendapat obat/suntik antidiabetes tapi tidak minum atau suntik antidiabetes secara rutin.

$$Proporsi\ alasan\ tidak\ minum\ atau\ suntik\ obat\ antidiabetes\ secara\ rutin = \frac{Masing - masing\ alasan\ tidak\ minum\ atau\ suntik\ obat\ antidiabetes\ secara\ rutin}{ART\ semua\ umur\ yang\ tidak\ minum\ atau\ suntik\ obat\ antidiabetes\ secara\ rutin}$$

Proporsi jenis pengendalian diabetes mellitus adalah masing-masing jenis pengendalian diabetes melitus (pengaturan makan, olahraga, alternatif herbal) /ART semua umur yang pernah didiagnosis diabetes melitus oleh dokter.

$$Proporsi\ jenis\ pengendalian\ diabetes\ melitus\ (pengaturan\ makan, olahraga, alternatif\ herbal)$$

$$= \frac{Masing - masing\ jenis\ pengendalian\ diabetes\ melitus\ (pengaturan\ makan, olahraga, alternatif\ herbal)}{ART\ semua\ umur\ yang\ pernah\ didiagnosis\ diabetes\ melitus\ oleh\ dokter}$$

Proporsi kerutinan memeriksakan kadar gula darah. Disebut “Rutin” jika:

- ART memeriksakan kadar gula darah sesuai petunjuk dokter (bagi ART yang pernah didiagnosis diabetes mellitus oleh dokter) atau
- ART memeriksakan kadar gula darah minimal 1 kali per tahun (bagi ART yang belum pernah didiagnosisdiabetes mellitus oleh dokter)

$$Proporsi\ kerutinan\ memeriksakan\ kadar\ gula\ darah$$

$$= \frac{ART\ yang\ periksa\ kadar\ gula\ darah\ secara\ rutin}{ART\ semua\ umur}$$

Tabel 6.3.1
Prevalensi Diabetes Melitus berdasarkan Diagnosis Dokter pada Penduduk Semua Umur menurut Provinsi, Riskesdas 2018

Provinsi	Diagnosis diabetes melitus		N Tertimbang
	%	95% CI	
Aceh	1,7	1,5 - 1,9	20.244
Sumatera Utara	1,4	1,3 - 1,5	55.351
Sumatera Barat	1,2	1,0 - 1,3	20.663
Riau	1,3	1,1 - 1,5	26.085
Jambi	1,0	0,9 - 1,2	13.692
Sumatera Selatan	0,9	0,8 - 1,1	32.126
Bengkulu	0,9	0,8 - 1,1	7.531
Lampung	1,0	0,9 - 1,1	32.148
Bangka Belitung	1,8	1,6 - 2,1	5.592
Kepulauan Riau	1,2	0,9 - 1,6	8.173
DKI Jakarta	2,6	2,3 - 2,9	40.210
Jawa Barat	1,3	1,2 - 1,4	186.809
Jawa Tengah	1,6	1,5 - 1,7	132.565
DI Yogyakarta	2,4	2,1 - 2,8	14.602
Jawa Timur	2,0	1,9 - 2,1	151.878
Banten	1,6	1,4 - 1,8	48.621
Bali	1,3	1,1 - 1,5	16.481
Nusa Tenggara Barat	1,2	1,0 - 1,3	19.247
Nusa Tenggara Timur	0,6	0,5 - 0,7	20.599
Kalimantan Barat	1,1	1,0 - 1,3	19.190
Kalimantan Tengah	1,1	1,0 - 1,3	10.189
Kalimantan Selatan	1,3	1,1 - 1,5	16.043
Kalimantan Timur	2,3	2,0 - 2,6	13.977
Kalimantan Utara	1,6	1,3 - 2,0	2.733
SulawesiUtara	2,3	2,0 - 2,5	9.542
SulawesiTengah	1,5	1,3 - 1,8	11.548
SulawesiSelatan	1,3	1,2 - 1,5	33.693
SulawesiTenggara	0,9	0,7 - 1,0	10.167
Gorontalo	1,7	1,5 - 2,1	4.547
SulawesiBarat	0,9	0,6 - 1,1	5.195
Maluku	0,7	0,6 - 1,0	6.801
Maluku Utara	1,0	0,8 - 1,2	4.723
Papua Barat	1,3	1,0 - 1,7	3.588
Papua	0,8	0,6 - 0,9	12.736
**INDONESIA**	**1,5**	**1,5 - 1,5**	**1.017.290**

Tabel 6.3.2
Prevalensi Diabetes Melitus berdasarkan Diagnosis Dokter pada Penduduk Semua Umur menurut Karakteristik, Riskesdas 2018

Karakteristik	Diagnosis diabetes melitus		N tertimbang
	%	95% CI	
**Kelompok Umur**			
< 1	0,01	0,00 - 0,03	18.225
1-4	0,00	0,00 - 0,01	73.188
5-14	0,00	0,00 - 0,01	182.338
15-24	0,05	0,04 - 0,07	165.644
25-34	0,22	0,18 - 0,26	159.708
35-44	1,08	1,01 - 1,16	151.539
45-54	3,88	3,73 - 4,03	124.652
55-64	6,29	6,05 - 6,54	83.251
65-74	6,03	5,69 - 6,38	40.180
75+	3,32	2,97 - 3,71	18.565
**Jenis kelamin**			
Laki-laki	1,21	1,16 - 1,25	510.714
Perempuan	1,78	1,73 - 1,84	506.576
**Pendidikan**			
Tidak/belum pernah sekolah	1,64	1,50 - 1,78	70.895
Tidak tamat SD/MI	1,36	1,29 - 1,44	181.429
Tamat SD/MI	1,83	1,75 - 1,92	215.967
Tamat SLTP/MTS	1,41	1,33 – 151	160.320
Tamat SLTA/MA	1,64	1,56 - 1,72	210.746
Tamat D1/D2/D3/PT	2,84	2,67 - 3,04	64.093
**Pekerjaan**			
Tidak bekerja	2,90	2,80 - 3,00	233.629
Sekolah	0,05	0,04 - 0,08	126.626
PNS/TNI/Polri/BUMN/BUMD	4,17	3,81 - 4,56	21.931
Pegawai swasta	1,09	0,98 - 1,21	75.781
Wiraswasta	2,59	2,45 - 2,74	105.489
Petani/buruh tani	1,21	1,14 - 1,29	133.261
Nelayan	1,26	0,97 - 1,65	5.556
Buruh/sopir/pembantu ruta	1,12	1,01 - 1,24	75.590
Lainnya	2,64	2,43 - 2,87	40.644
**Tempat tinggal**			
Perkotaan	1.89	1.83 - 1,95	556.419
Perdesaan	1.01	0.98 - 1,05	460.871

Tabel 6.3.3
Prevalensi Diabetes Melitus berdasarkan Diagnosis Dokter pada Penduduk Umur ≥15 Tahun menurut Provinsi, Riskesdas 2018

**Provinsi**	**Diabetes Melitus Diagnosis dokter**		**N Tertimbang**
	**%**	**95% CI**	
Aceh	2,4	2,2 – 2,7	13.389
Sumatera Utara	2,0	1,9 – 2,2	36.410
Sumatera Barat	1,6	1,4 – 1,9	13.834
Riau	1,9	1,7 – 2,1	17.258
Jambi	1,4	1,2 – 1,7	9.511
Sumatera Selatan	1,3	1,1 – 1,5	22.013
Bengkulu	1,3	1,1 – 1,5	5.175
Lampung	1,4	1,2 – 1,6	22.171
Bangka Belitung	2,5	2,2 – 2,9	3.915
Kepulauan Riau	1,7	1,2 – 2,3	5.463
DKI Jakarta	3,4	3,1 – 3,8	28.985
Jawa Barat	1,7	1,6 – 1,9	131.846
Jawa Tengah	2,1	2,0 – 2,2	96.794
DI Yogyakarta	3,1	2,7 – 3,6	10.975
Jawa Timur	2,6	2,5 – 2,7	113.045
Banten	2,2	2,0 – 2,6	33.587
Bali	1,7	1,5 – 2,0	12.092
Nusa Tenggara Barat	1,6	1,4 – 1,9	13.036
Nusa Tenggara Timur	0,9	0,7 – 1,0	12.777
Kalimantan Barat	1,6	1,4 - 1,9	13.035
Kalimantan Tengah	1,6	1,3 – 1,9	7.031
Kalimantan Selatan	1,8	1,5 - 2,1	11.068
Kalimantan Timur	3,1	2,7 – 3,6	9.696
Kalimantan Utara	2,3	1,8 – 2,9	1.838
SulawesiUtara	3,0	2,7 – 3,4	6.827
SulawesiTengah	2,2	1,9 – 2,5	7.847
SulawesiSelatan	1,8	1,6 – 2,0	23.069
SulawesiTenggara	1,3	1,1 – 1,5	6.510
Gorontalo	2,4	2,0 – 2,8	3.144
SulawesiBarat	1,3	0,9 - 1,7	3.408
Maluku	1,1	0,9 - 1,4	4.351
Maluku Utara	1,5	1,2 - 1,8	3.005
Papua Barat	1,9	1,5 - 2,5	2.363
Papua	1,1	0,9 - 1,4	8.317
**INDONESIA**	**2,0**	**2,0 - 2,1**	**713.783**

Tabel 6.3.4
Prevalensi Diabetes Melitus berdasarkan Diagnosis Dokter pada Penduduk Umur ≥15 Tahun menurut Karakteristik, Riskesdas 2018

Karakteristik	Diagnosis Dokter			N Tertimbang
	%	95% CI		
**Kelompok umur**				
15-24	0,1	0,0	0,1	159.014
25-34	0,2	0,2	0,3	153.316
35-44	1,1	1,0	1,2	145.474
45-54	3,9	3,7	4,0	119.663
55-64	6,3	6,0	6,5	79.919
65-74	6,0	5,7	6,4	38.572
75+	3,3	3,0	3,7	17.821
**Jenis kelamin**				
Laki-laki	1,7	1,6	1,7	355.726
Perempuan	2,4	2,4	2,5	358.056
**Pendidikan**				
Tidak/belum pernah sekolah	2,8	2,5	3,0	40.429
Tidak tamatSD/MI	2,9	2,7	3,0	83.023
Tamat SD/MI	2,2	2,1	2,3	174.011
Tamat SLTP/MTS	1,4	1,4	1,5	151.413
Tamat SLTA/MA	1,6	1,6	1,7	203.127
Tamat D1/D2/D3/PT	2,8	2,7	3,0	61.776
**Pekerjaan**				
Tidak Bekerja	3,2	3,1	3,3	209.273
Sekolah	0,1	0,1	0,2	57.439
PNS/TNI/Polri/BUMN/BUMD	4,2	3,8	4,6	21.428
Pegawaiswasta	1,1	1,0	1,2	73.962
Wiraswasta	2,6	2,5	2,7	102.967
Petani/buruh tani	1,2	1,1	1,3	130.042
Nelayan	1,3	1,0	1,7	5.402
Buruh/sopir/pembantu ruta	1,1	1,0	1,2	73.720
Lainnya	2,6	2,4	2,9	39.545
**Tempat tinggal**				
Perkotaan	2,6	2,5	2,6	394.850
Perdesaan	1,4	1,4	1,5	318.932

Tabel 6.3.5
Prevalensi Diabetes Melitus berdasarkan Pemeriksaan Kadar Gula Darah pada Penduduk Umur ≥15 Tahun menurut Karakteristik, Riskesdas 2018

Karakteristik	DM menurut ADA & konsensus Perkeni 2011		DM menurut ADA & konsensus Perkeni 2015		N tertimbang
	%	95%CI	%	95%CI	
**Kelompok Umur**					
15-24 tahun	1,6	1,4 – 2,0	2,0	1,7 – 2,4	5.591
25-34 tahun	2,8	2,4 – 3,3	4,1	3,6 – 4,6	6.102
35-44 tahun	6,6	6,1 – 7,2	8,6	8,0 – 9,3	8.152
45-54 tahun	11,5	10,8 – 12,3	14,4	13,6 – 15,3	8.112
55-64 tahun	15,6	14,6 – 16,6	19,6	18,6 – 20,7	5.804
65-74 tahun	15,1	13,7 – 16,5	19,6	18,1 – 21,3	2.674
≥ 75 tahun	13,6	11,9 – 15,6	17,0	15,0 – 19,2	1.025
**Jenis Kelamin**					
Laki-laki	6,7	6,3 – 7,1	9,0	8,5 – 9,5	18.699
Perempuan	10,3	9,9 – 10,7	12,7	12,3 – 13,2	18.761
**Pendidikan**					
Tidak Sekolah	13,6	12,2 – 15,1	17,2	15,7 – 18,7	2.471
Tidak Tamat SD	11,0	10,2 – 11,8	14,4	13,6 – 15,4	5.343
Tamat SD	9,5	8,9 – 10,1	11,9	11,2 – 12,6	11.454
Tamat SMP	6,2	5,7 – 6,9	7,8	7,1 – 8,5	7.451
Tamat SMA	6,3	5,7 – 6,9	8,3	7,6 – 9,0	8.511
Tamat D1-D3/PT	8,0	6,9 – 9,2	10,3	9,1 – 11,7	2.229
**Pekerjaan**					
Tidak Bekerja	10,6	10,0 – 11,1	12,8	12,2 – 13,4	12.045
Sekolah	1,3	0,9 – 1,8	1,7	1,2 – 2,4	2.115
PNS/TNI/Polri/BUMN/BUMD	10,1	7,7 – 13,1	13,5	10,8 – 16,9	579
P.Swasta	5,7	4,7 – 6,7	7,6	6,6 – 8,8	3.171
Wiraswasta	8,2	7,5 – 9,0	11,0	10,2 – 11,9	5.555
Petani/Buruh tani	9,8	9,1 – 10,5	12,6	11,8 – 13,5	7.628
Nelayan	4,7	2,6 – 8,3	8,5	5,1 – 13,8	152
Buruh/sopir/pembantu Ruta	6,1	5,3 – 6,9	8,5	7,6 – 9,4	4.251
Lainnya	9,3	8,0 – 10,7	11,6	10,1 – 13,2	1.965
**Tempat Tinggal**					
Perkotaan	8,1	7,7 – 8,5	10,6	10,1 – 11,1	20.701
Perdesaan	9,0	8,5 - 9,4	11,2	10,7 – 11,8	16.759
**INDONESIA**	**8,5**	**8,2 – 8,8**	**10,9**	**10,5 – 11,2**	**37.460**

Tabel 6.3.6
Proporsi Gula Darah Puasa Terganggu berdasarkan Pemeriksaan Kadar Gula Darah pada Penduduk Usia ≥15 tahun menurut Karakteristik, Riskesdas 2018

Karakteristik	GDP terganggu menurut ADA & konsensus Perkeni 2011		N tertimbang	GDP terganggu menurut ADA & konsensus Perkeni 2015		N tertimbang
	%	95%CI		%	95%CI	
**Umur**						
15-24 tahun	14,9	13,8 – 16,1	3.644	10,7	9,6 - 11,9	3.336
25-34 tahun	18,8	17,5 – 20,1	4.115	12,1	11,0 - 13,2	3.851
35-44 tahun	24,7	23,6 – 25,9	5.826	12,6	11,7 - 13,5	5.548
45-54 tahun	29,8	28,6 – 31,0	6.031	14,1	13,1 - 15,1	5.777
55-64 tahun	34,5	33,0 – 35,9	4.377	14,6	13,6 - 15,7	4.238
65-74 tahun	35,3	33,3 – 37,4	1.959	13,2	11,8 - 14,8	1.885
≥ 75 tahun	36,5	33,1 – 40,0	683	15,4	12,9 - 18,3	639
**Jenis Kelamin**						
Laki-laki	27,3	26,3 – 28,3	13.293	14,9	14,2 - 15,7	12.601
Perempuan	25,3	24,6 – 26,0	13.343	11,2	10,6 - 11,7	12.673
**Pendidikan**						
Tidak Sekolah	36,5	34,3 – 38,8	1.763	16,6	15,0 - 18,3	1.692
Tidak Tamat SD	30,7	29,2 – 32,2	3.935	14,0	12,9 - 15,2	3.796
Tamat SD	27,9	26,8 – 28,9	8.241	14,0	13,2 - 14,8	7.929
Tamat SMP	21,2	20,0 – 22,5	5.112	11,0	10,0 - 12,0	4.809
Tamat SMA	22,7	21,5 – 24,0	5.998	12,2	11,2 - 13,2	5.608
Tamat D1-D3/PT	25,7	23,4 – 28,1	1.586	11,5	10,0 - 13,3	1.439
**Pekerjaan**						
Tidak Bekerja	25,8	24,9 – 26,7	8.533	11,1	10,4 - 11,8	8.164
Sekolah	14,9	13,1 – 16,9	1.428	11,0	9,3 - 13,1	1.282
PNS/TNI/Polri/BUMN/BUMD	28,7	24,4 – 33,4	445	12,8	10,1 - 16,2	404
P.Swasta	21,1	19,0 – 23,4	2.216	11,6	9,9 - 13,5	2.024
Wiraswasta	25,6	24,2 – 27,1	3.945	12,9	11,7 - 14,1	3.732
Petani/Buruh tani	31,6	30,3 – 32,9	5.583	15,8	14,9 - 16,8	5.379
Nelayan	28,8	19,5 – 40,3	109	19,5	11,4 - 31,3	101
Buruh/sopir/pembantu Ruta	26,9	25,1 – 28,8	2.998	15,0	13,5 - 16,7	2.872
Lainnya	27,8	25,4 – 30,3	1.379	13,9	12,2 - 15,8	1.316
**Tempat Tinggal**						
Perkotaan	25,1	24,2 – 26,1	14.735	12,3	11,6 - 13,1	13.992
Perdesaan	27,7	26,9 – 28,6	11.901	14,0	13,3 - 14,7	11.282
**INDONESIA**	**26,3**	**25,6 – 27,0**	**26.636**	**13,1**	**12,5 - 13,6**	**25.274**

Tabel 6.3.7
Proporsi Toleransi Glukosa Terganggu berdasarkan Pemeriksaan Kadar Gula Darah pada Penduduk Umur ≥15 Tahun menurut Karakteristik, Riskesdas 2018

Karakteristik	TGT menurut ADA & konsensus Perkeni 2011		N tertimbang	TGT menurut ADA & konsensus Perkeni 2015		N tertimbang
	%	95%CI		%	95%CI	
**Umur**						
15-24 tahun	21,2	19,9 – 22,6	3.339	16,8	15,6 - 18,1	3.336
25-34 tahun	27,2	25,8 – 28,7	3.852	20,8	19,5 - 22,1	3.851
35-44 tahun	31,9	30,7 – 33,1	5.553	21,6	20,5 - 22,7	5.548
45-54 tahun	32,4	31,2 – 33,7	5.781	19,7	18,7 - 20,8	5.777
55-64 tahun	34,2	32,8 – 35,6	4.244	18,4	17,2 - 19,5	4.238
65-74 tahun	37,5	35,2 – 39,8	1.884	20,0	18,3 - 21,9	1.885
≥ 75 tahun	34,5	30,9 – 38,3	639	21,0	18,0 - 24,4	639
**Jenis Kelamin**						
Laki-laki	26,8	26,0 – 27,7	12.613	16,5	15,8 – 17,2	12.601
Perempuan	34,7	33,9 – 35,4	12.679	23,0	22,3 – 23,6	12.673
**Pendidikan**						
Tidak Sekolah	33,0	31,2 – 34,9	1.691	17,3	15,8 - 18,9	1.692
Tidak Tamat SD	34,4	32,9 – 35,9	3.799	19,9	18,7 - 21,2	3.796
Tamat SD	32,3	31,3 – 33,3	7.934	21,1	20,2 - 22,0	7.929
Tamat SMP	28,5	27,3 – 29,8	4.814	19,7	18,6 - 20,8	4.809
Tamat SMA	28,3	27,0 – 29,6	5.615	19,3	18,2 - 20,5	5.608
Tamat D1-D3/PT	27,4	24,9 – 30,0	1.439	16,2	14,3 - 18,3	1.439
**Pekerjaan**						
Tidak Bekerja	33,9	32,9 – 34,9	8.170	22,2	21,3 - 23,1	8.164
Sekolah	20,5	18,5 – 22,7	1.283	16,1	14,3 - 18,1	1.282
PNS/TNI/Polri/BUMN/BUMD	27,8	23,4 – 32,8	404	13,1	10,1 - 16,8	404
P.Swasta	26,9	24,8 – 29,1	2.024	19,2	17,4 - 21,3	2.024
Wiraswasta	29,7	28,1 – 31.3	3.737	18,3	17,1 - 19,6	3.732
Petani/Buruh tani	32,4	31,2 – 33,5	5.383	19,4	18,4 - 20,3	5.379
Nelayan	32,3	22,7 – 43,7	101	24,6	16,6 - 34,7	101
Buruh/sopir/pembantu Ruta	27,1	25,4 – 28,9	2.872	16,9	15,5 - 18,4	2.872
Lainnya	32,6	30,2 – 35,1	1.318	22,1	20,0 - 24,4	1.316
**Tempat Tinggal**						
Perkotaan	28,8	28,0 – 29,7	14.001	17,9	17,2-18,7	13.992
Perdesaan	33,1	32,3 – 34,0	11.291	21,9	21,2-22,7	11.282
**INDONESIA**	**30,8**	30,2 – 31,4	**25.292**	**19,7**	19,2 – 20,2	**25.274**

Tabel 6.3.8
Proporsi Jenis Pengobatan Diabetes Melitus berdasarkan Diagnosis Dokter pada Penduduk Semua Umur menurut Provinsi, Riskesdas 2018

Provinsi	Jenis Pengobatan Diabetes Melitus (%)				N tertimbang
	Obat anti DM/OAD dari tenaga medis	Injeksi insulin	Obat anti DM/OAD dari tenaga medis dan injeksi insulin	Tidak diobati	
Aceh	75,9	8,6	8,7	6,9	335
Sumatera Utara	69,0	7,6	12,6	10,7	759
Sumatera Barat	73,9	5,9	8,7	11,6	234
Riau	77,0	5,4	9,7	7,9	332
Jambi	76,3	7,3	9,8	6,6	137
Sumatera Selatan	69,5	4,7	11,1	14,6	286
Bengkulu	69,0	10,5	10,6	9,9	67
Lampung	82,6	2,2	8,6	6,5	312
Kep.Bangka Belitung	81,4	2,8	10,8	5,0	100
Kepulauan Riau	63,9	10,7	12,2	13,2	94
DKI Jakarta	74,3	5,2	11,2	9,3	1.018
Jawa Barat	79,6	3,6	7,6	9,2	2.354
Jawa Tengah	75,9	4,4	11,0	8,8	2.074
DI Yogyakarta	66,3	8,4	13,0	12,2	351
Jawa Timur	76,3	4,6	11,3	7,7	3.015
Banten	74,8	5,4	11,5	8,4	774
Bali	64,6	15,7	15,3	4,4	215
Nusa Tenggara Barat	74,1	4,7	14,0	7,2	219
Nusa Tenggara Timur	67,2	4,0	11,5	17,3	115
Kalimantan Barat	74,8	4,9	10,5	9,8	216
Kalimantan Tengah	72,1	4,2	11,5	12,2	114
Kalimantan Selatan	68,8	4,7	15,4	11,1	204
Kalimantan Timur	71,5	4,2	10,0	14,3	311
Kalimantan Utara	70,6	1,9	12,0	15,5	43*
Sulawesi Utara	70,2	6,7	6,6	16,4	213
Sulawesi Tengah	69,0	6,3	12,9	11,8	175
Sulawesi Selatan	69,8	6,2	13,0	11,0	432
Sulawesi Tenggara	74,4	4,9	15,9	4,9	87
Gorontalo	79,8	2,0	13,8	4,4	78
Sulawesi Barat	74,3	4,1	14,5	7,0	44*
Maluku	72,7	6,5	10,2	10,5	50
Maluku Utara	70,6	7,9	9,5	12,0	46*
Papua Barat	76,4	2,5	8,7	12,5	47*
Papua	62,8	3,2	17,0	16,9	96
**INDONESIA**	**74,8**	**5,1**	**10,8**	**9,3**	**14.951**

*N Tertimbang <50

Tabel 6.3.9
Proporsi Jenis Pengobatan Diabetes Melitus berdasarkan Diagnosis Dokter pada Penduduk Semua Umur menurut Karakteristik, Riskesdas 2018

Karakteristik	Jenis Pengobatan Diabetes Melitus (%)				N Tertimbang
	Obat anti DM/OADdari tenaga medis	Injeksi insulin	Obat anti DM/OADdari tenaga medis dan injeksi insulin	Tidak diobati	
**Kelompok Umur**					
< 1	0,0	0,0	0,0	100,0	1*
1-4	100,0	0,0	0,0	0,0	2*
5-14	33,0	21,4	16,9	28,7	8*
15-24	48,7	18,7	7,1	25,5	87
25-34	64,2	6,4	14,6	14,7	346
35-44	70,0	4,8	9,9	15,3	1.613
45-54	75,7	4,8	10,5	9,0	4.754
55-64	75,3	5,0	11,5	8,1	5.151
65-74	77,6	5,6	10,1	6,7	2.383
75+	75,8	4,0	9,7	10,5	606
**Jenis kelamin**					
Laki-laki	71,8	5,8	10,6	11,7	6.063
Perempuan	76,9	4,6	10,9	7,6	8.888
**Pendidikan**					
Tidak/belum pernah	75,5	3,3	11,6	9,6	1.145
Tidak tamat SD/MI	77,0	3,5	10,9	8,6	2.440
Tamat SD/MI	77,7	4,1	10,2	8,0	3.908
Tamat SLTP/MTS	73,6	5,0	11,9	9,5	2.239
Tamat SLTA/MA	72,4	6,5	10,5	10,5	3.412
Tamat D1/D2/D3/PT	71,2	8,3	10,2	10,3	1.801
**Pekerjaan**					
Tidak Bekerja	74,3	5,9	11,9	7,9	6.782
Sekolah	53,7	16,5	9,0	20,9	69
PNS/TNI/Polri/BUMN/BUM	72,3	9,0	11,0	7,7	915
Pegawai swasta	71,9	4,7	9,1	14,3	828
Wiraswasta	75,3	3,7	10,3	10,7	2.738
Petani/buruh tani	76,5	3,1	10,7	9,7	1.618
Nelayan	82,6	3,0	2,4	12,1	70
Buruh/sopir/pembantu ruta	81,7	1,6	6,8	9,8	849
Lainnya	74,3	6,2	10,1	9,5	1.074
**Tempat tinggal**					
Perkotaan	74,5	5,2	10,9	9,3	10.351
Perdesaan	75,5	4,9	10,4	9,2	4.600

*N Tertimbang <50

Tabel 6.3.10
Proporsi Kepatuhan Minum/Suntik Obat Anti Diabetes pada Penduduk Semua Umur dengan Diabetes Melitus berdasarkan Diagnosis Dokter menurut Provinsi, Riskesdas 2018

Provinsi	Kepatuhan Minum/Suntik Obat Anti Diabetes Sesuai Petunjuk Dokter		N tertimbang
	%	95 % CI	
Aceh	91,62	88,04-94,19	311
Sumatera Utara	88,24	84,72-91,03	675
Sumatera Barat	88,84	83,75-92,48	206
Riau	91,91	87,68-94,78	305
Jambi	91,81	86,13-95,29	128
Sumatera Selatan	91,03	83,51-95,32	244
Bengkulu	92,72	86,41-96,22	60
Lampung	90,78	86,56-93,78	291
Bangka Belitung	89,11	82,96-93,23	95
Kepulauan Riau	93,24	84,96-97,12	81
DKI Jakarta	89,77	85,67-92,79	920
Jawa Barat	89,94	87,08-92,23	2.129
Jawa Tengah	92,17	90,40-93,64	1.886
DI Yogyakarta	89,87	84,83-93,37	307
Jawa Timur	88,81	86,89-90,47	2.772
Banten	92,94	88,23-95,86	707
Bali	86,98	80,67-91,45	205
Nusa Tenggara Barat	95,38	91,60-97,50	203
Nusa Tenggara Timur	95,90	92,19-97,89	95
Kalimantan Barat	88,12	81,63-92,53	194
Kalimantan Tengah	90,84	85,90-94,16	100
Kalimantan Selatan	93,03	88,35-95,91	181
Kalimantan Timur	89,65	83,23-93,80	266
Kalimantan Utara	89,79	72,56-96,69	36*
Sulawesi Utara	92,79	89,98-94,86	177
Sulawesi Tengah	89,64	84,83-93,05	154
Sulawesi Selatan	92,88	89,85-95,06	383
Sulawesi Tenggara	96,76	93,70-98,36	82
Gorontalo	91,82	85,30-95,59	74
Sulawesi Barat	97,00	87,89-99,31	41*
Maluku	90,50	80,24-95,72	45*
Maluku Utara	98,66	96,45-99,50	41*
Papua Barat	91,58	81,11-96,50	41*
Papua	92,15	86,36-95,60	79
**INDONESIA**	**90,56**	**89,79-91,28**	**13.515**

*N Tertimbang <50

Tabel 6.3.11
Proporsi Kepatuhan Minum/Suntik Obat Anti Diabetes pada Penduduk Semua Umur dengan Diabetes Melitus berdasarkan Diagnosis Dokter menurut Karakteristik, Riskesdas 2018

Karakteristik	Kepatuhan Minum/Suntik Obat Anti Diabetes Sesuai Petunjuk Dokter		N Tertimbang
	%	95 % CI	
**Kelompok Umur**			
< 1 th	NA	NA	NA
1-4	100,00	100,00-100,00	2*
5-14	100,00	100,00-100,00	5*
15-24	87,59	72,04-95,08	64
25-34	89,21	81,49-93,95	294
35-44	87,39	84,43-89,86	1.362
45-54	89,61	88,18-90,88	4.313
55-64	91,27	89,95-92,42	4.719
65-74	92,69	91,00-94,08	2.215
75+	92,13	88,32-94,78	541
**Jenis Kelamin**			
Laki-Laki	88,93	87,54-90,18	5.333
Perempuan	91,62	90,70-92,45	8.182
**Pendidikan**			
Tidak/Belum Pernah Sekolah	91,80	89,12-93,87	1.032
Tidak Tamat SD/MI	90,60	88,64-92,26	2.224
Tamat SD/MI	91,18	89,73-92,45	3.584
Tamat SLTP/MTS	90,34	88,35-92,02	2.020
Tamat SLTA/MA	89,02	87,11-90,68	3.042
Tamat D1/D2/D3/PT	91,47	89,25-93,26	1.610
**Pekerjaan**			
Tidak Bekerja	91,52	90,42-92,51	6.222
Sekolah	87,38	75,24-94,04	54
PNS/TNI/Polri/BUMN/BUMD	91,73	88,98-93,84	842
Pegawai Swasta	87,04	81,85-90,90	707
Wiraswasta	89,92	88,07-91,51	2.436
Petani/Buruh Tani	90,64	88,43-92,47	1.457
Nelayan	87,49	76,17-93,87	62
Buruh/Sopir/Pembantu Ruta	88,69	84,75-91,71	763
Lainnya	89,20	85,46-92,07	968
**Tempat Tinggal**			
Perkotaan	90,31	89,30-91,23	9.354
Perdesaan	91,12	89,98-92,14	4.161

*N Tertimbang <50

Tabel 6.3.12
Proporsi Alasan Tidak Minum/Suntik Obat Anti Diabetes Sesuai Petunjuk Dokter pada Penduduk Semua Umur menurut Provinsi, Riskesdas 2018

Provinsi	Alasan tidak minum/suntik obat anti diabetes sesuai petunjuk dokter (%)								N ter-timbang
	Sering Lupa	Obat tidak tersedia di fasyankes	Minum obat tradisional	Tidak tahan efek samping obat	Tidak mampu membeli obat	Tidak rutin berobat ke fasilitas kesehatan	Merasa sudah sehat	Lainnya	
Aceh	24,04	7,38	34,69	10,32	9,34	24,16	47,32	13,30	25*
Sumatera Utara	22,10	2,82	31,93	4,62	10,32	23,22	41,08	15,10	76
Sumatera Barat	29,52	0,56	39,65	14,07	3,96	39,25	43,36	12,08	22*
Riau	14,48	0,00	26,87	24,54	13,60	32,02	17,75	33,03	23*
Jambi	9,46	0,00	60,49	43,03	0,00	43,10	36,36	27,61	10*
Sumatera Selatan	40,09	0,00	10,11	20,82	0,00	38,32	49,91	4,26	21*
Bengkulu	12,25	17,94	12,25	0,00	4,13	4,13	43,46	22,23	4*
Lampung	17,78	0,00	45,04	13,16	11,11	57,02	40,63	7,73	25*
Bangka Belitung	15,25	0,00	31,67	32,23	8,53	23,14	52,38	17,76	10*
Kepulauan Riau	33,80	0,00	36,62	25,04	0,00	13,30	21,03	12,31	5*
DKI Jakarta	19,26	0,34	23,53	5,79	2,97	17,06	43,61	27,32	90
Jawa Barat	20,00	0,81	25,27	13,97	9,98	45,18	51,28	19,26	204
Jawa Tengah	14,22	4,74	20,25	15,51	6,30	29,64	61,51	18,37	140
DI Yogyakarta	28,17	0,00	3,42	6,20	16,65	9,90	49,44	11,98	30*
Jawa Timur	12,09	1,58	17,54	9,52	7,91	29,06	55,00	20,20	295
Banten	12,61	1,56	27,51	20,96	20,26	17,12	55,37	18,50	47*
Bali	30,53	4,58	28,69	13,69	8,15	28,03	45,10	8,32	25*
Nusa Tenggara Barat	0,00	0,00	44,45	5,36	25,95	26,77	24,16	20,96	9*
Nusa Tenggara Timur	17,85	0,00	38,06	0,00	0,00	16,21	60,55	12,59	4*
Kalimantan Barat	34,44	6,43	39,88	20,37	15,16	46,40	61,83	23,74	22*
Kalimantan Tengah	9,06	3,48	19,37	18,24	3,00	48,71	54,19	13,25	9*
Kalimantan Selatan	6,47	0,00	30,11	20,24	6,47	17,54	62,76	5,44	12*
Kalimantan Timur	38,35	1,39	43,25	12,33	1,39	24,93	44,63	4,85	26*
Kalimantan Utara	52,47	0,00	13,54	0,00	0,00	25,40	100,00	13,54	4*
Sulawesi Utara	35,21	0,00	34,70	5,69	7,57	13,80	57,15	13,41	12*
Sulawesi Tengah	20,37	0,00	30,88	20,11	14,55	22,72	44,17	5,35	15*
Sulawesi Selatan	21,85	7,94	34,11	13,73	3,95	19,69	45,21	17,72	26*
Sulawesi Tenggara	13,68	13,68	69,34	13,68	21,82	56,10	29,77	4,52	3*
Gorontalo	20,38	15,94	12,88	8,92	6,09	12,83	28,97	19,02	6*
Sulawesi Barat	0,00	0,00	100,00	10,44	0,00	89,56	18,68	0,00	1*
Maluku	27,36	0,00	35,41	32,48	10,98	27,14	7,74	30,37	4*
Maluku Utara	25,52	24,12	24,12	0,00	49,63	24,12	74,48	0,00	1*
Papua Barat	56,98	0,00	56,15	4,40	0,00	31,23	54,51	43,51	3*
Papua	45,79	1,17	67,55	11,89	0,00	33,30	41,24	21,98	6*
**INDONESIA**	**18,77**	**2,11**	**25,29**	**12,58**	**8,45**	**30,24**	**50,40**	**18,20**	**1.213**

*N Tertimbang <50

Tabel 6.3.13
Proporsi Alasan Tidak Minum/Suntik Obat Anti Diabetes Sesuai Petunjuk Dokter pada Penduduk Semua Umur menurut Karakteristik, Riskesdas 2018

Karakteristik	Alasan tidak minum/suntik obat anti diabetes sesuai petunjuk dokter (%)								N ter-timbang
	Sering Lupa	Obat tidak tersedia di fasyankes	Minum obat tradisional	Tidak tahan efek samping obat	Tidak mampu membeli obat	Tidak rutin berobat ke fasilitas kesehatan	Merasa Sudahsehat	Lainnya	
**Kelompok Umur**									
1	NA	NA	NA	NA	NA	NA	NA	NA	NA
1-4	NA	NA	NA	NA	NA	NA	NA	NA	NA
5-14	NA	NA	NA	NA	NA	NA	NA	NA	NA
15-24	34,09	34,09	16,62	0,00	0,00	0,00	23,91	76,09	8*
25-34	4,05	0,23	15,54	14,16	0,00	30,13	70,03	39,93	30*
35-44	26,18	3,38	20,20	8,66	6,25	30,99	72,24	11,37	163
45-54	16,85	1,14	28,89	12,13	6,44	32,95	45,57	21,14	426
55-64	15,45	1,12	27,20	12,81	12,07	27,09	50,22	16,46	392
65-74	26,42	3,09	19,52	17,14	9,78	29,68	35,59	14,68	154
75+	20,30	8,46	20,29	14,82	6,42	37,03	61,49	17,81	40*
**Jenis kelamin**									
Laki-laki	19,24	2,44	24,16	10,76	8,35	30,70	57,57	20,08	561
Perempuan	18,36	1,83	26,26	14,15	8,54	29,83	44,22	16,58	652
**Pendidikan**									
Tidak/belum pernah sekolah	12,38	4,67	29,68	6,50	11,73	41,35	57,79	10,52	80
Tidak tamat SD/MI	13,46	2,01	25,74	13,55	12,96	37,16	44,50	13,47	199
Tamat SD/MI	24,46	2,96	28,76	17,30	14,77	32,90	49,77	14,21	300
Tamat SLTP/MTS	18,35	3,32	26,13	8,23	8,09	24,04	50,36	25,08	185
Tamat SLTA/MA	18,57	0,58	21,18	11,98	1,24	27,31	52,92	20,95	318
Tamat D1/D2/D3/PT	18,79	0,76	22,73	11,65	3,10	22,64	50,21	22,81	131
**Pekerjaan**									
Tidak bekerja	17,88	1,71	25,82	15,04	9,49	28,31	42,19	20,52	501
Sekolah	0,00	9,26	19,52	10,13	15,69	21,28	26,00	52,73	7*
PNS/TNI/Polri/BUMN/BUMD	15,99	0,58	23,78	9,38	0,00	16,99	50,00	29,07	66
Pegawai swasta	31,05	6,95	20,05	2,80	1,80	25,11	71,34	9,42	87
Wiraswasta	11,77	0,84	22,15	12,66	2,75	25,30	53,54	20,01	233
Petani/buruh tani	22,93	1,41	31,08	12,63	23,48	47,85	55,58	9,90	130
Nelayan	18,83	21,99	9,59	17,78	3,46	0,00	80,42	4,38	7*
Buruh/sopir/pembantu ruta	27,69	1,42	19,11	6,27	2,95	40,57	76,62	14,84	82
Lainnya	19,26	3,44	34,69	15,61	12,92	36,19	37,33	14,96	99
**Tempat tinggal**									
Perkotaan	19,33	1,11	24,31	12,93	6,07	27,26	49,70	18,90	862
Perdesaan	17,39	4,56	27,69	11,73	14,30	37,54	52,10	16,47	351

*N Tertimbang <50

Tabel 6.3.14
Proporsi Jenis Pengendalian Diabetes Melitus berdasarkan Diagnosis Dokter pada Penduduk Semua Umur menurut Provinsi, Riskesdas 2018

Provinsi	Jenis Pengendalian Diabetes Melitus			N Tertimbang
	Pengaturan makan	Olahraga	Alternatif herbal	
	%	%	%	
Aceh	82,3	40,4	39,3	335
Sumatera Utara	78,3	46,1	37,1	759
Sumatera Barat	75,0	51,0	45,8	234
Riau	77,0	46,3	41,1	332
Jambi	81,4	37,9	44,6	137
Sumatera Selatan	79,1	42,3	44,3	286
Bengkulu	83,9	43,6	49,6	67
Lampung	85,5	50,2	42,6	312
Bangka Belitung	83,6	32,5	38,3	100
Kepulauan Riau	82,4	45,9	50,3	94
DKI Jakarta	81,0	53,3	28,5	1.018
Jawa Barat	75,3	47,1	35,0	2.354
Jawa Tengah	82,1	49,7	28,4	2.074
DI Yogyakarta	87,0	57,4	27,4	351
Jawa Timur	81,3	49,7	28,4	3.015
Banten	77,4	53,0	38,4	774
Bali	79,4	54,0	25,7	215
Nusa Tenggara Barat	78,6	53,3	48,7	219
Nusa Tenggara Timur	78,5	41,3	47,2	115
Kalimantan Barat	84,0	43,4	39,7	216
Kalimantan Tengah	84,4	40,3	46,0	114
Kalimantan Selatan	80,4	36,8	46,3	204
Kalimantan Timur	76,7	42,8	47,5	311
Kalimantan Utara	74,2	41,3	36,4	43*
SulawesiUtara	84,4	46,2	40,5	213
SulawesiTengah	81,7	39,9	56,4	175
SulawesiSelatan	84,1	40,4	61,5	432
SulawesiTenggara	89,0	52,2	56,5	87
Gorontalo	79,9	42,6	47,9	78
SulawesiBarat	91,0	44,5	55,7	44*
Maluku	90,2	43,8	56,6	50
Maluku Utara	88,4	54,1	55,9	46*
Papua Barat	75,3	42,5	58,1	47*
Papua	81,6	52,7	28,9	96
**INDONESIA**	**80,2**	**48,1**	**35,7**	**14.951**

*N Tertimbang <50

Tabel 6.3.15
Proporsi Jenis Pengendalian Diabetes Melitus berdasarkan Diagnosis Dokter pada Penduduk Semua Umur menurut Karakteristik, Riskesdas 2018

Karakteristik	Jenis Pengendalian Diabetes Melitus			N Tertimbang
	Pengaturan Makan	Olahraga	Alternatif Herbal	
	%	%	%	
**Kelompok Umur**				
< 1	0	0	0	1*
1-4	34,9	0	12,0	2*
5-14	91,1	22,1	36,1	8*
15-24	59,2	28,6	21,4	87
25-34	80,8	40,0	32,6	346
35-44	82,3	47,7	39,0	1.613
45-54	81,7	52,3	39,6	4.754
55-64	78,7	48,4	34,7	5.151
65-74	81,1	45,7	31,0	2.383
75+	75,4	30,8	27,1	606
**Jenis kelamin**				
Laki-laki	78,3	54,6	37,8	6.063
Perempuan	81,5	43,6	34,2	8.888
**Pendidikan**				
Tidak/belum pernah sekolah	74,2	36,4	30,6	1.145
Tidak tamat SD/MI	78,0	38,2	32,7	2.440
Tamat SD/MI	77,6	43,0	33,8	3.908
Tamat SLTP/MTS	80,9	48,2	36,2	2.239
Tamat SLTA/MA	82,2	56,1	39,0	3.412
Tamat D1/D2/D3/PT	88,1	64,7	40,0	1.801
**Pekerjaan**				
Tidak Bekerja	80,6	43,5	34,4	6.782
Sekolah	72,9	45,2	29,2	69
PNS/TNI/Polri/BUMN/BUMD	87,6	66,9	42,5	915
Pegawai swasta	81,9	61,1	34,5	828
Wiraswasta	77,9	50,8	37,9	2.738
Petani/buruh tani	79,4	42,3	36,9	1.618
Nelayan	74,6	38,3	31,9	70
Buruh/sopir/pembantu ruta	75,7	47,4	31,3	849
Lainnya	82,0	54,6	35,7	1.074
**Tempat tinggal**				
Perkotaan	80,6	50,7	34,3	10.351
Perdesaan	79,4	42,2	38,9	4.600

*N Tertimbang <50

Tabel 6.3.16
Proporsi Kerutinan Memeriksakan Kadar Gula Darah pada Penduduk Semua Umur menurut Provinsi, Riskesdas 2018

Provinsi	Kerutinan Memeriksakan Kadar Gula Darah						N tertimbang
	Rutin		Tidak rutin		Tidak pernah		
	%	95 % CI	%	95 % CI	%	95 % CI	
Aceh	1,3	1,2 - 1,5	12,0	11,5-12,6	86,7	86,0-87,2	20.244
Sumatera Utara	1,2	1,1 - 1,4	11,9	11,3-12,5	86,9	86,3-87,5	55.351
Sumatera Barat	1,1	0,9 - 1,2	11,3	10,7-12,0	87,6	86,9-88,2	20.663
Riau	1,4	1,2 - 1,6	13,6	12,8-14,5	85,0	84,1-85,9	26.085
Jambi	0,9	0,7 - 1,1	9,9	9,0-11,0	89,2	88,1-90,2	13.692
Sumatera Selatan	0,8	0,6 - 0,9	8,9	8,2-9,8	90,3	89,5-91,1	32.126
Bengkulu	0,8	0,7 - 1,0	8,3	7,5-9,1	90,9	90,1-91,7	7.531
Lampung	0,9	0,7 - 1,0	10,1	9,4-10,9	89,0	88,3-89,7	32.148
Bangka Belitung	2,1	1,8 - 2,4	12,5	11,6-13,4	85,4	84,4-86,4	5.592
Kepulauan Riau	2,2	1,8 - 2,8	14,8	13,2-16,5	83,0	81,1-84,7	8.173
DKI Jakarta	4,1	3,7 - 4,6	23,4	22,2-24,6	72,5	71,1-73,8	40.210
Jawa Barat	1,8	1,6 - 1,9	12,0	11,6-12,5	86,2	85,7-86,7	186.809
Jawa Tengah	1,8	1,7 - 1,9	13,1	12,7-13,5	85,1	84,7-85,5	132.565
DI Yogyakarta	3,7	3,2 - 4,2	20,3	19,2-21,4	76,0	74,8-77,2	14.602
Jawa Timur	2,4	2,3 - 2,5	13,7	13,3-14,1	83,9	83,5-84,3	151.878
Banten	1,9	1,7 - 2,2	11,7	11,0-12,5	86,4	85,5-87,2	48.621
Bali	2,0	1,8 - 2,3	12,3	11,6-13,1	85,6	84,8-96/4	16.481
Nusa Tenggara Barat	0,8	0,7 - 1,0	8,2	7,5-8,9	91,0	90,2-91,7	19.247
Nusa Tenggara Timur	0,5	0,4 - 0,6	5,2	4,8-5,6	94,3	93,9-94,7	20.599
Kalimantan Barat	1,3	1,1 - 1,5	11,1	10,4-11,8	87,6	86,8-88,3	19.190
Kalimantan Tengah	1,3	1,1 - 1,5	12,7	11,8-13,6	86,0	85,1-86,9	10.189
Kalimantan Selatan	2,0	1,7 - 2,3	15,7	15,0-16,5	82,3	81,4-83,1	16.043
Kalimantan Timur	2,9	2,5 - 3,3	19,9	18,8-21,1	77,2	76,0-78,4	13.977
Kalimantan Utara	5,2	4,2 - 6,5	12,2	10,5-14,1	82,5	80,4-84,5	2.733
Sulawesi Utara	1,8	1,6 - 2,1	14,0	13,3-14,8	84,1	83,3-84,9	9.542
Sulawesi Tengah	1,3	1,1 - 1,5	12,1	11,3-12,8	86,6	85,8-87,4	11.548
Sulawesi Selatan	1,5	1,3 - 1,6	13,6	13,0-14,1	85,0	84,4-85,5	33.693
Sulawesi Tenggara	1,4	1,1 - 1,7	11,2	10,4-12,1	87,4	86,5-88,3	10.167
Gorontalo	2,1	1,7 - 2,5	10,7	9,6-11,9	87,2	86,0-88,4	4.547
Sulawesi Barat	1,0	0,8 - 1,3	12,2	10,9-13,6	86,8	85,3-88,1	5.195
Maluku	0,9	0,7 - 1,1	10,3	9,3-11,5	88,8	87,6-89,9	6.801
Maluku Utara	1,0	0,8- 1,3	10,7	9,9-11,6	88,2	87,3-89,1	4.723
Papua Barat	1,3	1,0 - 1,8	10,3	9,3-11,3	88,4	87,2-89,4	3.588
Papua	0,8	0,6 - 1,0	9,3	8,5-10,1	90,0	89,1-90,8	12.736
**INDONESIA**	**1,8**	**1,7 - 1,8**	**12,8**	**12,6-12,9**	**85,5**	**85,3-85,6**	**1.017.290**

Tabel 6.3.17
Proporsi Kerutinan Memeriksakan Kadar Gula Darah pada Penduduk Semua Umur menurut Karakteristik, Riskesdas 2018

Karakteristik	Kerutinan Memeriksakan Kadar Gula Darah						N tertimbang
	Rutin		Tidak rutin		Tidak pernah		
	%	95 % CI	%	95 % CI	%	95 % CI	
**Kelompok umur**							
< 1	0,0	0,0-0,1	4,2	3,7-4,6	95,8	95,3-96,2	18.225
1-4	0,1	0,0-0,1	4,0	3,7-4,2	96,0	95,8-96,2	73.188
5-14	0,1	0,1-0,1	4,0	3,9-4,2	95,9	95,7-96,0	182.338
15-24	0,3	0,3-0,4	7,2	6,9-7,4	92,5	92,3-92,8	165.644
25-34	1,0	0,9-1,1	12,8	12,5-13,1	86,2	85,9-86,5	159.708
35-44	1,8	1,7-2,0	17,0	16,7-17,3	81,1	80,8-81,5	151.539
45-54	4,0	3,9-4,2	22,2	21,8-22,6	73,8	73,4-74,2	124.652
55-64	5,9	5,6-6,1	24,1	23,7-24,6	70,0	69,5-70,5	83.251
65-74	5,9	5,5-6,2	23,1	22,5-23,7	71,0	70,3-71,7	40.180
75+	3,5	3,2-4,0	19,7	18,9-20,6	76,8	75,8-77,7	18.565
**Jenis kelamin**							
Laki-laki	1,5	1,4-1,5	11,2	11,1-11,4	87,3	87,1-87,5	510.714
Perempuan	2,1	2,0-2,2	14,3	14,1-14,4	83,6	83,4-83,8	506.576
**Pendidikan**							
Tidak/belum pernah sekolah	1,1	1,0-1,3	10,4	10,0-10,7	88,5	88,1-88,9	70.895
Tidak tamat SD/MI	1,1	1,0-1,1	9,1	8,9-9,3	89,8	89,6-90,0	181.429
Tamat SD/MI	1,6	1,5-1,7	11,9	11,7-12,2	86,5	86,2-86,7	215.967
Tamat SLTP/MTS	1,5	1,4-1,6	12,1	11,9-12,4	86,4	86,1-86,7	160.320
Tamat SLTA/MA	2,5	2,4-2,6	17,2	16,9-17,5	80,3	80,0-80,6	210.746
Tamat D1/D2/D3/PT	6,3	6,0-6,6	30,5	29,9-31,2	63,2	62,5-63,9	64.093
**Pekerjaan**							
Tidak bekerja	2,9	2,8-3,0	16,9	16,6-17,1	80,2	79,9-80,5	233.629
Sekolah	0,2	0,2-0,2	5,4	5,2-5,6	94,4	94,2-94,6	126.626
PNS/TNI/Polri/BUMN/BUMD	9,0	8,4-9,6	34,8	33,9-35,7	56,3	55,2-57,3	21.931
Pegawai swasta	3,0	2,8-3,2	19,8	19,2-20,4	77,2	76,6-77,9	75.781
Wiraswasta	2,9	2,7-3,0	19,2	18,8-19,6	77,9	77,5-78,3	105.489
Petani/buruh tani	0,9	0,9-1,0	10,9	10,6-11,1	88,2	87,9-88,5	133.261
Nelayan	1,1	0,8-1,5	11,6	10,4-12,9	87,3	86,0-88,5	5.556
Buruh/sopir/pembantu ruta	1,0	0,9-1,2	11,6	11,2-12,0	87,4	87,0-87,8	75.590
Lainnya	3,2	2,9-3,5	18,1	17,5-18,7	78,7	78,0-79,3	40.644
**Tempat tinggal**							
Perkotaan	2,5	2,4-2,6	15,3	15,1-15,6	82,1	81,9-82,4	556.419
Perdesaan	0,9	0,9-0,9	9,6	9,5-9,8	89,5	89,3-89,7	460.871

6.4 Penyakit Jantung

Penyakit jantung adalah semua jenis penyakit jantung termasuk kelainan jantung bawaan yang didiagnosis oleh dokter.

Prevalensi penyakit jantung yang didiagnosis dokter

$$Prevalensi\ penyakit\ jantung\ berdasarkan\ diagnosis\ dokter = \frac{ART\ yang\ pernah\ didiagnosis\ penyakit\ jantung\ oleh\ dokter}{ART\ semua\ umur}$$

Parameter kimia klinis yang diperiksa pada Riskesdas 2018 meliputi pemeriksaan kadar kolesterol total, *high-density lipoprotein* (*HDL*), *low-density lipoprotein* (*LDL*) *direct*, dan trigliserida. Penentuan *cut off point* abnormalitas keempat parameter tersebut merujuk pada pedoman *National Cholesterol Education Program - Adult Treatment Panel* III (*NCEP-ATP* III) 2001.

$$Proporsi\ Kolesterol\ Total\ Borderline = \frac{ART\ \geq 15\ tahun\ dengan\ kadar\ Kolesterol\ Total\ 200 - 239\ mg\ per\ dl}{ART\ \geq 15\ tahun\ yang\ diperiksa\ kadar\ Kolesterol\ Totalnya\ dalam\ serum}$$

$$Proporsi\ Kolesterol\ Total\ Tinggi = \frac{ART\ \geq 15\ tahun\ dengan\ kadar\ Kolesterol\ Total\ \geq 240\ mg\ per\ dl}{ART\ \geq 15\ tahun\ yang\ diperiksa\ kadar\ Kolesterol\ Totalnya\ dalam\ serum}$$

$$Proporsi\ HDL\ Rendah = \frac{ART\ \geq 15\ tahun\ dengan\ kadar\ HDL < 40\ mg\ per\ dl}{ART\ \geq 15\ tahun\ yang\ diperiksa\ kadar\ HDLnya\ dalam\ serum}$$

$$Proporsi\ HDL\ Tinggi = \frac{ART\ \geq 15\ tahun\ dengan\ kadar\ HDL\ \geq 60\ mg\ per\ dl}{ART\ \geq 15\ tahun\ yang\ diperiksa\ kadar\ HDLnya\ dalam\ serum}$$

$$Proporsi\ LDL\ Near\ Optimal = \frac{ART\ \geq 15\ tahun\ dengan\ kadar\ LDL\ 100 - 129\ mg\ per\ dl}{ART\ \geq 15\ tahun\ yang\ diperiksa\ kadar\ LDLnya\ dalam\ serum}$$

$$Proporsi\ LDL\ Borderline = \frac{ART\ \geq 15\ tahun\ dengan\ kadar\ LDL\ 130 - 159\ mg\ per\ dl}{ART\ \geq 15\ tahun\ yang\ diperiksa\ kadar\ LDLnya\ dalam\ serum}$$

$$Proporsi\ LDL\ Tinggi = \frac{ART\ \geq 15\ tahun\ dengan\ kadar\ LDL\ 160 - 189\ mg\ per\ dl}{ART\ \geq 15\ tahun\ yang\ diperiksa\ kadar\ LDLnya\ serum}$$

$$Proporsi\ LDL\ Sangat\ Tinggi = \frac{ART\ \geq 15\ tahun\ dengan\ kadar\ LDL\ \geq 190\ mg\ per\ dl}{ART\ \geq 15\ tahun\ yang\ diperiksa\ kadar\ LDLnya\ dalam\ serum}$$

$$Proporsi\ Trigliserida\ Borderline\ Tinggi = \frac{ART\ \geq 15\ tahun\ dengan\ kadar\ Trigliserida\ 150 - 199\ mg\ per\ dl}{ART\ \geq 15\ tahun\ yang\ diperiksa\ kadar\ Trigliseridanya\ dalam\ serum}$$

$$Proporsi\ Trigliserida\ Tinggi = \frac{ART\ \geq 15\ tahun\ dengan\ kadar\ Trigliserida\ 200 - 499\ mg\ per\ dl}{ART\ \geq 15\ tahun\ yang\ diperiksa\ kadar\ Trigliseridanya\ dalam\ serum}$$

$$Proporsi\ Trigliserida\ Sangat\ Tinggi = \frac{ART\ \geq 15\ tahun\ dengan\ kadar\ Trigliserida\ Total\ \geq 500\ mg\ per\ dl}{ART\ \geq 15\ tahun\ yang\ diperiksa\ kadar\ Trigliseridanya\ dalam\ serum}$$

Tabel 6.4.1
Prevalensi Penyakit Jantung berdasarkan Diagnosis Dokter pada Penduduk Semua Umur menurut Provinsi, Riskesdas 2018

Provinsi	Penyakit jantung		N tertimbang
	%	95% CI	
Aceh	1,6	1,5 - 1,8	20.244
Sumatera Utara	1,3	1,2 - 1,5	55.351
Sumatera Barat	1,6	1,4 - 1,8	20.663
Riau	1,1	0,9 - 1,2	26.085
Jambi	0,9	0,7 - 1,1	13.692
Sumatera Selatan	1,2	1,0 - 1,4	32.126
Bengkulu	1,3	1,1 - 1,5	7.531
Lampung	1,2	1,1 - 1,4	32.148
Bangka Belitung	1,5	1,2 - 1,8	5.592
Kepulauan Riau	1,5	1,2 - 1,9	8.173
DKI Jakarta	1,9	1,7 - 2,2	40.210
Jawa Barat	1,6	1,5 - 1,7	186.809
Jawa Tengah	1,6	1,5 - 1,7	132.565
DI Yogyakarta	2,0	1,7 - 2,3	14.602
Jawa Timur	1,5	1,4 - 1,6	151.878
Banten	1,4	1,2 - 1,5	48.621
Bali	1,3	1,2 - 1,5	16.481
Nusa Tenggara Barat	0,9	0,7 - 1,1	19.247
Nusa Tenggara Timur	0,7	0,6 - 0,8	20.599
Kalimantan Barat	1,3	1,2 - 1,5	19.190
Kalimantan Tengah	1,3	1,1 - 1,5	10.189
Kalimantan Selatan	1,3	1,1 - 1,5	16.043
Kalimantan Timur	1,9	1,6 - 2,2	13.977
Kalimantan Utara	2,2	1,8 - 2,7	2.733
Sulawesi Utara	1,8	1,5 - 2,0	9.542
Sulawesi Tengah	1,9	1,7 - 2,2	11.548
Sulawesi Selatan	1,5	1,3 - 1,6	33.693
Sulawesi Tenggara	1,4	1,2 - 1,6	10.167
Gorontalo	2,0	1,6 - 2,4	4.547
Sulawesi Barat	1,5	1,2 - 2,0	5.195
Maluku	1,5	1,2 - 1,7	6.801
Maluku Utara	1,1	0,9 - 1,4	4.723
Papua Barat	1,2	0,9 - 1,5	3.588
Papua	0,9	0,7 - 1,0	12.736
**INDONESIA**	**1,5**	**1,4 - 1,5**	**1.017.290**

Tabel 6.4.2
Prevalensi Penyakit Jantung berdasarkan Diagnosis Dokter pada Penduduk Semua Umur menurut Karakteristik, Riskesdas 2018

Karakteristik	Penyakit jantung		N tertimbang
	%	95% CI	
**Kelompok umur**			
< 1	0,1	0,1 - 0,2	18.225
1-4	0,4	0,3 - 0,4	73.188
5-14	0,7	0,6 - 0,7	182.338
15-24	0,7	0,6 - 0,8	165.644
25-34	0,8	0,8 - 0,9	159.708
35-44	1,3	1,2 - 1,4	151.539
45-54	2,4	2,3 - 2,6	124.652
55-64	3,9	3,7 - 4,1	83.251
65-74	4,6	4,3 - 4,9	40.180
75+	4,7	4,3 - 5,2	18.565
**Jenis kelamin**			
Laki-laki	1,3	1,3 - 1,4	510.714
Perempuan	1,6	1,6 - 1,7	506.576
**Pendidikan**			
Tidak/belum pernah sekolah	1,8	1,6 - 1,9	70.895
Tidak tamat SD/MI	1,5	1,4 - 1,6	181.429
Tamat SD/MI	1,8	1,7 - 1,8	215.967
Tamat SLTP/MTS	1,4	1,3 - 1,5	160.320
Tamat SLTA/MA	1,5	1,4 - 1,6	210.746
Tamat D1/D2/D3/PT	2,1	2,0 - 2,3	64.093
**Pekerjaan**			
Tidak bekerja	2,3	2,2 - 2,4	233.629
Sekolah	0,6	0,6 - 0,7	126.626
PNS/TNI/Polri/BUMN/BUMD	2,7	2,5 - 3,0	21.931
Pegawai swasta	1,2	1,1 - 1,3	75.781
Wiraswasta	1,9	1,8 - 2,0	105.489
Petani/buruh tani	1,5	1,5 - 1,6	133.261
Nelayan	1,3	1,0 - 1,7	5.556
Buruh/sopir/pembantu ruta	1,2	1,1 - 1,3	75.590
Lainnya	2,3	2,1 - 2,5	40.644
**Tempat tinggal**			
Perkotaan	1,6	1,6 - 1,7	556.419
Perdesaan	1,3	1,2 - 1,3	460.871

Tabel 6.4.3
Proporsi Kadar Kolesterol Total pada Penduduk Umur ≥ 15 Tahun menurut Karakteristik, Riskesdas 2018

Karakteristik	*Borderline*		Tinggi		N tertimbang
	%	95% CI	%	95% CI	
**Kelompok umur**					
15-24	7,2	6,5 - 8,0	1,9	1,5 - 2,3	4.968
25-34	14,7	13,7 - 15,8	4,4	3,8 - 5,0	5.689
35-44	20,6	19,6 - 21,7	6,5	5,8 - 7,1	7.812
45-54	27,4	26,3 - 28,6	10,5	9,8 - 11,3	7.535
55-64	29,2	27,9 - 30,5	12,6	11,7 - 13,6	5.425
65-74	27,6	25,7 - 29,5	10,6	9,4 - 11,9	2.443
75+	25,1	22,2 - 28,2	7,8	6,2 - 9,8	948
**Jenis kelamin**					
Laki-laki	18,3	17,5 – 19,0	5,4	5,0 - 5,8	17.382
Perempuan	24,0	23,4 – 24,7	9,9	9,4 - 10,8	17.438
**Pendidikan**					
Tidak/belum pernah sekolah	27,4	25,7 - 29,3	10,0	8,9 - 11,2	2.347
Tidak tamat SD/MI	23,6	22,3 - 24,9	9,0	8,2 - 9,9	5.085
Tamat SD/MI	21,6	20,7 - 22,5	7,6	7,0 - 8,1	10.550
Tamat SLTP/MTS	17,4	16,4 - 18,4	6,0	5,4 - 6,6	6.872
Tamat SLTA/MA	19,4	18,4 - 20,4	7,0	6,4 - 7,7	7.973
Tamat D1/D2/D3/PT	25,1	23,0 - 27,4	9,8	8,5 - 11,4	1.993
**Pekerjaan**					
Tidak bekerja	22,7	21,8 - 23,6	9,4	8,8 - 10,0	11183
Sekolah	6,8	5,6 - 8,2	1,4	0,9 - 2,2	1.738
PNS/TNI/Polri/BUMN/BUMD	27,6	23,5 - 32,2	13,4	10,6 - 16,8	515
Pegawai swasta	19,8	18,1 - 21,6	7,0	5,9 - 8,2	2.913
Wiraswasta	22,7	21,5 - 24,1	7,8	7,0 - 8,7	5.285
Petani/buruh tani	21,0	19,9 - 22,0	6,6	6,0 - 7,3	7.064
Nelayan	15,7	9,9 - 24,1	9,7	5,7 - 16,1	144
Buruh/sopir/pembantu ruta	19,7	18,2 - 21,2	6,4	5,6 - 7,4	4.133
Lainnya	25,5	23,3 - 27,9	7,8	6,6 - 9,3	1.845
**Tempat tinggal**					
Perkotaan	22,1	21,4 – 22,8	8,3	7,8 – 8,7	19.267
Perdesaan	20,0	19,3 – 20,7	6,8	6,4 – 7,3	15.553
**Indonesia**	**21,2**	**20,6 – 21,7**	**7,6**	**7,3 – 8,0**	**34.820**

Catatan: berdasarkan pemeriksaan darah

Tabel 6.4.4

Proporsi Kadar HDL pada Penduduk Umur ≥ 15 Tahun menurut Karakteristik, Riskesdas 2018

Karakteristik	Rendah		Tinggi		N tertimbang
	%	95% CI	%	95% CI	
**Kelompok umur**					
15-24	24,8	23,3 - 26,2	10,8	9,9 - 11,7	4.968
25-34	24,5	23,3 - 25,8	12,7	11,8 - 13,6	5.689
35-44	26,7	25,5 - 27,9	11,7	11,0 - 12,5	7.812
45-54	24,6	23,4 - 25,8	14,1	13,3 - 15,0	7.535
55-64	22,5	21,2 - 23,8	16,6	15,5 - 17,7	5.425
65-74	19,3	17,5 - 21,2	18,8	17,2 - 20,6	2.443
75+	22,4	19,5 - 25,6	21,4	18,8 - 24,3	948
**Jenis kelamin**					
Laki-laki	33,7	32,7 – 34,6	6,8	6,4 – 7,3	17.382
Perempuan	15,0	14,4 -15,6	20,7	20,0 -21,4	17.438
**Pendidikan**					
Tidak/belum pernah sekolah	20,8	19,0 - 22,8	17,6	16,0 - 19,2	2.347
Tidak tamat SD/MI	23,5	22,2 - 24,9	16,0	14,9 - 17,2	5.085
Tamat SD/MI	23,7	22,7 - 24,7	13,6	12,9 - 14,4	10.550
Tamat SLTP/MTS	25,2	24,0 - 26,5	11,7	10,9 - 12,6	6.872
Tamat SLTA/MA	25,4	24,2 - 26,6	13,0	12,3 - 13,8	7.973
Tamat D1/D2/D3/PT	26,2	24,1 - 28,3	14,6	13,2 - 16,2	1.993
**Pekerjaan**					
Tidak bekerja	19,2	18,4 - 20,0	17,5	16,8 - 18,3	11.183
Sekolah	23,0	20,8 - 25,4	11,5	10,1 - 13,1	1.738
PNS/TNI/Polri/BUMN/BUMD	33,5	29,0 - 38,4	12,1	9,5 - 15,4	515
Pegawai swasta	28,5	26,6 - 30,4	10,3	9,2 - 11,6	2.913
Wiraswasta	30,1	28,6 - 31,6	10,5	9,6 - 11,4	5.285
Petani/buruh tani	23,6	22,5 - 24,8	13,7	12,8 - 14,7	7.064
Nelayan	40,9	31,5 - 51,1	7,3	3,8 - 13,8	144
Buruh/sopir/pembantu ruta	28,6	26,8 - 30,5	11,0	10,0 - 12,2	4.133
Lainnya	22,5	20,4 - 24,7	15,4	13,8 - 17,1	1.845
**Tempat tinggal**					
Perkotaan	24,5	23,7 – 25,4	14,0	13,4 – 14,6	19.267
Perdesaan	24,1	23,3 – 24,9	13,5	12,9 – 14,2	15.553
**Indonesia**	**24,3**	**23,7 – 24,9**	**13,8**	**13,3 -14,2**	**34.820**

Catatan: berdasarkan pemeriksaan darah

Tabel 6.4.5
Proporsi Kadar LDL direct pada Penduduk Umur ≥ 15 Tahun menurut Karakteristik, Riskesdas 2018

Karakteristik	*Near Optimal*		*Borderline*		Tinggi		Sangat Tinggi		N tertimbang
	%	95% CI	%	95% CI	%	95% CI	%	95% CI	
**Kelompok umur**									
15-24	34,6	33,1 - 36,1	11,5	10,5 - 12,5	2,6	2,2 - 3,2	0,9	0,6 - 1,2	4.968
25-34	39,9	38,5 - 41,3	21,4	20,2 - 22,6	6,4	5,7 - 7,1	1,6	1,3 - 2,0	5.689
35-44	38,6	37,4 - 39,8	26,2	25,1 - 27,4	8,3	7,7 - 9,1	3,1	2,7 - 3,5	7.812
45-54	35,5	34,3 - 36,7	29,8	28,6 - 31,0	11,9	11,1 - 12,7	4,8	4,3 - 5,4	7.535
55-64	33,6	32,2 - 35,0	30,0	28,6 - 31,5	13,0	12,0 - 14,0	5,8	5,2 - 6,5	5.425
65-74	35,2	33,2 - 37,3	28,8	26,8 - 30,9	11,1	9,9 - 12,4	4,7	3,9 - 5,7	2.443
75+	35,0	31,7 - 38,4	25,4	22,5 - 28,4	11,2	9,2 - 13,6	3,0	0,6 - 1,2	948
**Jenis kelamin**									
Laki-laki	37,5	36,7 – 38,4	23,3	22,5 -24,1	7,6	7,1 – 8,1	2,4	2,2 – 2,7	17382
Perempuan	35,4	34,7 – 36,1	26,4	25,8 – 27,1	10,3	9,9 – 10,8	4,4	4,1 – 4,8	17438
**Pendidikan**									
Tidak/belum pernah sekolah	34,3	32,3 - 36,5	28,2	26,3 - 30,2	11,5	10,3 - 12,9	4,5	3,8 - 5,5	2.347
Tidak tamat SD/MI	36,0	34,5 - 37,5	26,1	24,7 - 27,4	9,6	8,8 - 10,5	4,0	3,4 - 4,7	5.085
Tamat SD/MI	36,8	35,8 - 37,9	25,6	24,6 - 26,6	8,7	8,1 - 9,3	3,4	3,0 - 3,8	10.550
Tamat SLTP/MTS	36,4	35,2 - 37,7	21,5	20,4 - 22,6	7,9	7,2 - 8,7	2,6	2,2 - 3,1	6.872
Tamat SLTA/MA	36,9	35,7 - 38,1	24,2	23,2 - 25,2	8,7	8,0 - 9,5	3,1	2,7 - 3,6	7.973
Tamat D1/D2/D3/PT	36,2	33,9 - 38,6	28,1	26,0 - 30,4	10,3	9,0 - 11,9	4,8	3,9 - 6,0	1.993
**Pekerjaan**									
Tidak bekerja	34,8	33,9 - 35,7	25,4	24,6 - 26,3	10,1	9,5 - 10,7	4,2	3,8 - 4,6	11.183
Sekolah	32,8	30,4 - 35,2	11,0	9,5 - 12,8	2,6	1,9 - 3,5	0,5	0,2 - 0,9	1.738
PNS/TNI/Polri/BUMN/BUMD	34,0	29,6 - 38,7	30,5	26,6 - 34,8	11,7	9,0 - 15,0	7,1	5,1 - 9,7	515
Pegawai swasta	37,5	35,4 - 39,6	25,4	23,6 - 27,2	9,3	8,0 - 10,7	3,3	2,6 - 4,1	2.913
Wiraswasta	36,7	35,2 - 38,2	27,1	25,8 - 28,6	9,8	8,9 - 10,8	3,6	3,1 - 4,2	5.285
Petani/buruh tani	38,6	37,4 - 39,8	24,6	23,5 - 25,8	8,2	7,5 - 8,9	2,8	2,4 - 3,2	7.064
Nelayan	38,5	30,7 - 47,0	24,5	18,8 - 31,2	7,3	4,0 - 12,8	6,5	3,2 - 12,6	144
Buruh/sopir/pembantu ruta	37,8	36,0 - 39,6	24,5	22,9 - 26,1	7,9	7,0 - 8,9	2,9	2,4 - 3,6	4.133
Lainnya	36,9	34,5 - 39,3	27,1	24,9 - 29,4	10,0	8,6 - 11,6	3,8	3,0 - 4,8	1.845
**Tempat tinggal**									
Perkotaan	35,6	34,8 – 36,4	25,5	24,8 – 26,3	9,6	9,1 – 10,2	3,8	3,4 – 4,1	19.267
Perdesaan	37,5	36,8 – 38,3	24,0	23,2 – 24,8	8,1	7,7 - 8,6	3,0	2,7 – 3,3	15.553
**Indonesia**	**36,5**	**35,9 – 37,0**	**24,9**	**24,3 – 25,4**	**9,0**	**8,6 – 9,3**	**3,4**	**3,2 – 3,7**	**34.820**

Catatan: berdasarkan pemeriksaan darah

Tabel 6.4.6
Proporsi Kadar Trigliserida pada Penduduk Umur ≥ 15 Tahun Menurut Karakteristik, Riskesdas 2018

Karakteristik	*Borderline* tinggi		Tinggi		Sangat Tinggi		N tertimbang
	%	95% CI	%	95% CI	%	95% CI	
**Kelompok umur**							
15-24	8,7	7,9 - 9,6	7,1	6,3 - 7,9	0,2	0,1 - 0,5	4.968
25-34	10,4	9,6 - 11,3	12,5	11,6 - 13,5	0,5	0,3 - 0,7	5.689
35-44	13,7	12,9 - 14,6	14,8	13,9 - 15,8	1,1	0,9 - 1,5	7.812
45-54	15,3	14,4 - 16,2	16,3	15,3 - 17,2	1,2	0,9 - 1,5	7.535
55-64	16,4	15,3 - 17,5	16,9	15,8 - 18,1	0,7	0,5 - 1,0	5.425
65-74	14,3	12,8 - 15,8	14,3	12,8 - 15,9	0,7	0,4 - 1,2	2.443
75+	14,6	12,3 - 17,2	11,3	9,2 - 13,7	0,4	0,1 - 1,4	948
**Jenis kelamin**							
Laki-laki	14,6	13,9 – 15,2	16,3	15,6 – 17,0	1,1	1,0 -1,3	17.382
Perempuan	12,0	11,5 – 12,5	11,4	10,9 – 11,9	0,4	0,3 – 0,6	17.438
**Pendidikan**							
Tidak/belum pernah sekolah	16,0	14,5 - 17,6	13,8	12,4 - 15,4	0,4	0,2 - 0,7	2.347
Tidak tamat SD/MI	14,1	13,1 - 15,2	14,0	12,9 - 15,1	0,6	0,4 - 0,9	5.085
Tamat SD/MI	13,8	13,1 - 14,5	13,8	13,0 - 14,6	0,8	0,6 - 1,0	10.550
Tamat SLTP/MTS	11,4	10,6 - 12,3	12,4	11,6 - 13,3	1,0	0,7 - 1,3	6.872
Tamat SLTA/MA	12,3	11,5 - 13,2	14,3	13,4 - 15,2	0,8	0,6 - 1,0	7.973
Tamat D1/D2/D3/PT	15,4	13,8 - 17,3	17,3	15,5 - 19,2	1,3	0,8 - 2,1	1.993
**Pekerjaan**							
Tidak bekerja	12,2	11,6 - 12,9	12,7	12,1 - 13,4	0,5	0,4 - 0,7	11.183
Sekolah	8,2	6,9 - 9,8	5,4	4,3 - 6,7	0,2	0,1 - 0,6	1.738
PNS/TNI/Polri/BUMN/BUMD	18,2	14,8 - 22,2	21,8	17,9 - 26,2	0,9	0,4 - 2,3	515
Pegawai swasta	14,2	12,7 - 15,8	15,7	14,1 - 17,3	0,9	0,6 - 1,5	2.913
Wiraswasta	14,3	13,2 - 15,4	17,2	16,1 - 18,5	1,5	1,2 - 2,0	5.285
Petani/buruh tani	13,7	12,8 - 14,5	12,5	11,6 - 13,4	0,5	0,3 - 0,7	7.064
Nelayan	13,9	8,6 - 21,6	19,8	13,7 - 27,8	0,7	0,1 - 4,8	144
Buruh/sopir/pembantu ruta	14,6	13,4 - 16,0	14,9	13,6 - 16,3	1,2	0,9 - 1,8	4.133
Lainnya	14,2	12,5 - 16,0	16,1	14,3 - 18,2	0,6	0,3 - 1,3	1.845
**Tempat tinggal**							
Perkotaan	13,6	13,1 - 14,2	14,5	13,9 – 15,1	0,9	0,8 – 1,1	19.267
Perdesaan	12,8	12,3 – 13,4	13,0	12,4 – 13,7	0,6	0,5 – 0,7	15.553
**Indonesia**	**13,3**	**12,9 – 13,7**	**13,8**	**13,4 – 14,3**	**0,8**	**0,7 – 0,9**	**34.820**

Catatan: berdasarkan pemeriksaan darah

6.5 Hipertensi

Hipertensi hasil pengukuran mengikuti kriteria JNC VII yaitu bila tekanan darah sistolik ≥140 mmHg dan atau tekanan darah diastolik ≥90 mmHg. Prevalensi hipertensi berdasarkan diagnosis dokter dihitung dengan formula:

$$Prevalensi\ hipertensi\ berdasarkan\ diagnosis\ dokter = \frac{ART\ yang\ pernah\ \ didiagnosis\ hipertensi\ oleh\ dokter}{ART\ umur\ \geq 18\ th}$$

Prevalensi hipertensi menurut diagnosis dokter atau sedang minum obat antihipertensi dihitung dengan formula:

$$Prevalensi\ \ hipertensi\ menurut\ diagnosis\ dokter\ atau\ sedang\ minum\ obat\ antihipertensi = \frac{\begin{array}{c}ART\ yang\ pernah\ didiagnosis\ hipertensi\ oleh\ dokter\ \ atau\ sedang\ minum\ obat\ antihipertensi\\ rutin\ setiap\ hari\end{array}}{ART\ umur\ \geq 18\ th}$$

Prevalensi hipertensi berdasarkan hasil pengukuran tekanan darah dihitung dengan formula:

$$Prevalensi\ hipertensi\ berdasarkan\ pengukuran = \frac{\begin{array}{c}ART\ dengan\ rata-rata\ hasil\ pengukuran\ tekanan\ darah\\ sistolik\ \geq 140\ mmHg\ dan\ atau\ diastolik\ \geq 90\ mmHg\end{array}}{ART\ umur\ \geq\ 18\ th\ \ yang\ diukur\ tekanan\ darah}$$

Proporsi kepatuhan minum obat antihipertensi secara rutin dihitung dengan formula:

$$Proporsi\ minum\ obat\ antihipertensi\ secara\ rutin = \frac{\begin{array}{c}Minum\ obat\ \ antihipertensi\ secara\ rutin\ sesuai\ petunjuk\ dokter\ atau\ minum\\ obat\ antihipertensi\ setiap\ hari\\ (inisiatif\ sendiri)\end{array}}{ART\ umur\ \geq 18\ th\ \ yang\ pernah\ didiagnosis\ hipertensi\ oleh\ dokter}$$

Proporsi alasan tidak minum obat antihipertensi setiap hari dihitung dengan formula:

$$Proporsi\ alasan\ tidak\ minum\ obat\ antihipertensi\ setiap\ hari = \frac{Jenis\ alasan\ tidak\ minum\ obat\ antihipertensi\ setiap\ hari}{ART\ umur\ \geq 18\ th\ yang\ tidak\ minum\ obat\ antihipertensi\ secara\ rutin}$$

Proporsi kerutinan mengukur tekanan darah

Disebut “Rutin” jika:

- ART menjalani pengukuran tekanan darah sesuai petunjuk dokter (bagi ART yang pernah didiagnosis hipertensi oleh dokter) atau minimal 1 kali per bulan (bagi ART dengan hipertensi bukan didiagnosis oleh dokter)
- ART menjalani pengukuran tekanan darah, minimal 1 kali per tahun (bagi ART yang tidak pernah didiagnosis atau tidak tahu apakah menderita hipertensi/tidak)

$$Proporsi\ kerutinan\ mengukur\ tekanan\ darah = \frac{ART\ yang\ mengukur\ tekanan\ darah\ secara\ rutin}{ART\ umur\ \geq 18\ th}$$

Tabel 6.5.1
Prevalensi Hipertensi berdasarkan Diagnosis Dokter atau Minum Obat Antihipertensi, pada Penduduk Umur ≥18 Tahun menurut Provinsi, Riskesdas 2018

Provinsi	Hipertensi				N tertimbang
	Diagnosis dokter (D)		Diagnosis/Obat[1] (DO)		
	%	95% CI	%	95% CI	
Aceh	9,32	8,89 - 9,77	9,52	9,09 - 9,98	12.259
Sumatera Utara	5,52	5,20 - 5,86	6,07	5,73 - 6,42	32.944
Sumatera Barat	7,27	6,83 - 7,73	7,69	7,24 - 8,16	12.650
Riau	8,44	7,94 - 8,96	8,83	8,32 - 9,38	15.807
Jambi	7,43	6,92 - 7,98	7,59	7,07 - 8,15	8.760
Sumatera Selatan	7,34	6,88 - 7,82	7,76	7,29 - 8,25	20.231
Bengkulu	8,36	7,74 - 9,02	8,74	8,12 - 9,41	4.777
Lampung	7,95	7,48 - 8,45	8,49	8,00 - 9,00	20.484
Bangka Belitung	8,32	7,63 - 9,05	9,06	8,36 - 9,81	3.605
Kepulauan Riau	8,62	7,69 - 9,66	8,93	7,95 - 10,03	5.052
DKI Jakarta	10,17	9,48 - 10,90	10,47	9,76 - 11,22	27.195
Jawa Barat	9,67	9,32 - 10,03	9,97	9,62 - 10,34	121.153
Jawa Tengah	8,17	7,90 - 8,44	8,61	8,34 - 8,88	89.648
DI Yogyakarta	10,68	9,93 - 11,49	11,01	10,25 - 11,82	10.318
Jawa Timur	8,01	7,76 - 8,27	8,59	8,33 - 8,86	105.380
Banten	8,61	8,07 - 9,17	8,94	8,40 - 9,51	31.052
Bali	9,57	8,96 - 10,22	9,91	9,29 - 10,57	11.242
Nusa Tenggara Barat	7,19	6,64 - 7,78	7,80	7,23 - 8,41	11.881
Nusa Tenggara Timur	5,36	5,01 - 5,74	5,99	5,62 - 6,38	11.505
Kalimantan Barat	8,16	7,62 - 8,72	8,80	8,24 - 9,38	11.924
Kalimantan Tengah	8,38	7,77 - 9,03	9,23	8,58 - 9,92	6.470
Kalimantan Selatan	9,98	9,40 - 10,59	10,81	10,21 - 11,43	10.162
Kalimantan Timur	10,57	9,84 - 11,34	11,07	10,33 - 11,85	8.957
Kalimantan Utara	10,46	9,25 - 11,80	10,59	9,40 - 11,92	1.675
Sulawesi Utara	13,21	12,48 - 13,97	13,53	12,79 - 14,31	6.307
Sulawesi Tengah	8,69	8,09 - 9,33	10,55	9,92 - 11,21	7.221
Sulawesi Selatan	7,22	6,81 - 7,65	7,77	7,36 - 8,21	21.142
Sulawesi Tenggara	6,25	5,71 - 6,84	7,23	6,65 - 7,86	5.902
Gorontalo	10,11	9,22 - 11,07	11,10	10,18 - 12,10	2.898
Sulawesi Barat	6,58	5,84 - 7,40	7,78	6,97 - 8,67	3.068
Maluku	5,01	4,41 - 5,68	6,30	5,62 - 7,05	3.914
Maluku Utara	5,71	4,99 - 6,53	6,63	5,87 - 7,48	2.725
Papua Barat	7,45	6,54 - 8,48	7,83	6,83 - 8,96	2.163
Papua	4,39	3,83 - 5,02	4,75	4,17 - 5,40	7.730
**INDONESIA**	**8,36**	**8,26 - 8,47**	**8,84**	**8,73 - 8,94**	**658.201**

[1]minum obat bagi penduduk yang diwawancara

Tabel 6.5.2
Prevalensi Hipertensi berdasarkan Diagnosis Dokter atau Minum Obat Antihipertensi, pada Penduduk Umur ≥18 Tahun Menurut Karakteristik, Riskesdas 2018

Karakteristik	Hipertensi				N tertimbang
	Diagnosis dokter (D)		Diagnosis /Obat[1] (DO)		
	%	95% CI	%	95% CI	
**Kelompok Umur**					
18-24	0,79	0,71 - 0,89	0,85	0,76 - 0,95	106.849
25-34	2,07	1,95 - 2,20	2,24	2,11 - 2,36	152.373
35-44	5,73	5,55 - 5,91	6,10	5,92 - 6,29	144.578
45-54	12,62	12,35 - 12,90	13,30	13,03 - 13,59	118.927
55-64	18,31	17,92 - 18,71	19,30	18,90 - 19,70	79.427
65-74	23,31	22,70 - 23,94	24,53	23,91 - 25,16	38.335
75+	24,04	23,14 - 24,95	25,26	24,35 - 26,19	17.712
**Jenis kelamin**					
Laki-laki	5,74	5,62 - 5,86	6,07	5,94 - 6,19	327.150
Perempuan	10,95	10,80 - 11,11	11,57	11,41 - 11,74	331.051
**Pendidikan**					
Tidak/belum pernah sekolah	14,88	14,37 - 15,41	15,80	15,28 - 16,34	39.556
Tidak tamat SD/MI	12,98	12,64 - 13,34	13,77	13,42 - 14,13	80.199
Tamat SD/MI	10,29	10,06 - 10,52	10,94	10,71 - 11,18	159.357
Tamat SLTP/MTS	6,39	6,18 - 6,61	6,71	6,50 - 6,94	119.569
Tamat SLTA/MA	5,27	5,12 - 5,42	5,53	5,37 - 5,68	198.150
Tamat D1/D2/D3/PT	6,94	6,65 - 7,24	7,25	6,96 - 7,55	61.371
**Pekerjaan**					
Tidak bekerja	12,70	12,47 - 12,93	13,30	13,06 - 13,53	196.220
Sekolah	1,31	1,10 - 1,58	1,40	1,17 - 1,66	21.093
PNS/TNI/Polri/BUMN/BUMD	10,22	9,67 - 10,79	10,71	10,15 - 11,29	21.228
Pegawai swasta	4,03	3,79 - 4,27	4,20	3,97 - 4,45	72.970
Wiraswasta	7,62	7,37 - 7,87	8,03	7,77 - 8,29	101.834
Petani/buruh tani	7,46	7,28 - 7,65	8,12	7,93 - 8,32	128.377
Nelayan	4,66	4,03 - 5,38	5,07	4,43 - 5,81	5.259
Buruh/sopir/pembantu ruta	5,14	4,89 - 5,40	5,39	5,13 - 5,66	72.315
Lainnya	8,84	8,44 - 9,25	9,45	9,04 - 9,87	38.905
**Tempat tinggal**					
Perkotaan	9,10	8,94 - 9,26	9,46	9,30 - 9,63	364.630
Perdesaan	7,45	7,32 - 7,57	8,06	7,93 - 8,19	293.571

[1]minum obat bagi penduduk yang diwawancara

Tabel 6.5.3
Prevalensi Hipertensi berdasarkan Hasil Pengukuran pada Penduduk Umur ≥18 Tahun menurut Provinsi, Riskesdas 2018

Provinsi	Hipertensi (Pengukuran)		N tertimbang
	%	95% CI	
Aceh	26,45	25,67 - 27,24	12.259
Sumatera Utara	29,19	28,46 - 29,94	32.944
Sumatera Barat	25,16	24,29 - 26,04	12.650
Riau	29,14	28,19 - 30,12	15.807
Jambi	28,99	27,91 - 30,09	8.760
Sumatera Selatan	30,44	29,50 - 31,40	20.231
Bengkulu	28,14	27,08 - 29,22	4.777
Lampung	29,94	29,10 - 30,80	20.484
Bangka Belitung	29,90	28,55 - 31,30	3.605
Kepulauan Riau	25,84	24,17 - 27,59	5.052
DKI Jakarta	33,43	32,13 - 34,75	27.195
Jawa Barat	39,60	38,93 - 40,27	121.153
Jawa Tengah	37,57	37,02 - 38,12	89.648
DI Yogyakarta	32,86	31,59 - 34,15	10.318
Jawa Timur	36,32	35,81 - 36,84	105.380
Banten	29,47	28,34 - 30,61	31.052
Bali	29,97	28,97 - 30,99	11.242
Nusa Tenggara Barat	27,80	26,69 - 28,92	11.881
Nusa Tenggara Timur	27,72	26,92 - 28,54	11.505
Kalimantan Barat	36,99	35,91 - 38,09	11.924
Kalimantan Tengah	34,47	33,26 - 35,70	6.470
Kalimantan Selatan	44,13	42,91 - 45,35	10.162
Kalimantan Timur	39,30	37,81 - 40,81	8.957
Kalimantan Utara	33,02	30,97 - 35,13	1.675
Sulawesi Utara	33,12	32,09 - 34,16	6.307
Sulawesi Tengah	29,75	28,76 - 30,76	7.221
Sulawesi Selatan	31,68	30,84 - 32,53	21.142
Sulawesi Tenggara	29,75	28,68 - 30,85	5.902
Gorontalo	29,64	28,14 - 31,20	2.898
Sulawesi Barat	34,77	33,03 - 36,55	3.068
Maluku	28,96	27,52 - 30,45	3.914
Maluku Utara	24,65	23,30 - 26,04	2.725
Papua Barat	25,90	23,90 - 28,00	2.163
Papua	22,22	20,96 - 23,53	7.730
**INDONESIA**	**34,11**	**33,91 - 34,32**	**658.201**

Tabel 6.5.4

Prevalensi Hipertensi berdasarkan Hasil Pengukuran pada Penduduk Umur ≥18 Tahun menurut Karakteristik, Riskesdas 2018

Karakteristik	Hipertensi (Pengukuran)		N tertimbang
	%	95% CI	
**Kelompok Umur**			
18-24	13,22	12,87 - 13,57	106.849
25-34	20,13	19,79 - 20,48	152.373
35-44	31,61	31,24 - 31,98	144.578
45-54	45,32	44,91 - 45,74	118.927
55-64	55,23	54,72 - 55,73	79.427
65-74	63,22	62,53 - 63,90	38.335
75+	69,53	68,58 - 70,47	17.712
**Jenis kelamin**			
Laki-laki	31,34	31,06 - 31,61	327.150
Perempuan	36,85	36,60 - 37,11	331.051
**Pendidikan**			
Tidak/belum pernah sekolah	51,55	50,80 - 52,31	39.556
Tidak tamat SD/MI	46,25	45,73 - 46,78	80.199
Tamat SD/MI	39,99	39,60 - 40,38	159.357
Tamat SLTP/MTS	29,07	28,65 - 29,49	119.569
Tamat SLTA/MA	25,92	25,60 - 26,25	198.150
Tamat D1/D2/D3/PT	28,30	27,74 - 28,87	61.371
**Pekerjaan**			
Tidak bekerja	39,73	39,39 - 40,08	196.220
Sekolah	14,84	14,10 - 15,61	21.093
PNS/TNI/Polri/BUMN/BUMD	36,91	36,04 - 37,79	21.228
Pegawai swasta	24,37	23,81 - 24,95	72.970
Wiraswasta	34,03	33,56 - 34,50	101.834
Petani/buruh tani	36,14	35,78 - 36,51	128.377
Nelayan	27,85	26,36 - 29,38	5.259
Buruh/sopir/pembantu ruta	30,22	29,63 - 30,82	72.315
Lainnya	34,79	34,08 - 35,52	38.905
**Tempat tinggal**			
Perkotaan	34,43	34,13 - 34,74	364.630
Perdesaan	33,72	33,46 - 33,98	293.571

Tabel 6.5.5

Proporsi Minum Obat Anti Hipertensi secara Rutin pada Penduduk Umur ≥18 Tahun dengan Hipertensi menurut Provinsi, Riskesdas 2018

Provinsi	Kerutinan minum obat anti hipertensi						N tertimbang
	Rutin		Tidak rutin		Tidak minum obat		
	%	95% CI	%	95% CI	%	95% CI	
Aceh	47,10	44,28 – 49,94	41,84	39,21 - 44,52	11,06	9,49 - 12,86	1.217
Sumatera Utara	51,98	48,67 – 55,26	34,58	31,64 - 37,64	13,44	11,48 - 15,69	1.938
Sumatera Barat	43,35	40,42 – 46,33	43,65	40,75 - 46,59	13,00	10,99 - 15,31	979
Riau	44,03	40,63 – 47,49	41,05	37,98 - 44,19	14,92	12,86 - 17,24	1.421
Jambi	44,35	40,47 – 48,31	44,66	40,91 - 48,48	10,98	8,97 - 13,39	694
Sumatera Selatan	52,40	48,66 – 56,10	33,29	30,17 - 36,57	14,31	11,92 - 17,09	1.582
Bengkulu	46,44	42,52 – 50,41	36,39	32,79 - 40,16	17,16	14,17 - 20,64	425
Lampung	49,52	46,48 – 52,56	38,90	36,10 - 41,77	11,58	9,80 - 13,64	1.735
Bangka Belitung	60,71	56,03 – 65,19	27,08	23,21 - 31,34	12,21	9,40 - 15,71	319
Kepulauan Riau	56,71	51,02 – 62,23	31,20	26,56 - 36,26	12,09	8,64 - 16,65	464
DKI Jakarta	59,91	56,47 – 63,25	26,57	23,77 - 29,58	13,52	11,35 - 16,03	2.946
Jawa Barat	55,12	53,21 – 57,01	32,54	30,78 - 34,36	12,34	11,12 - 13,67	12.480
Jawa Tengah	55,77	54,10 – 57,44	29,97	28,50 - 31,48	14,26	13,14 - 15,45	7.798
DI Yogyakarta	50,29	46,31 – 54,26	33,87	30,67 - 37,23	15,84	13,12 - 19,01	1.174
Jawa Timur	56,07	54,45 – 57,68	29,23	27,75 - 30,76	14,70	13,57 - 15,90	8.991
Banten	54,62	51,10 – 58,10	31,73	28,62 - 35,02	13,64	11,44 - 16,20	2.847
Bali	46,05	42,67 – 49,46	42,70	39,31 - 46,16	11,25	9,24 - 13,63	1.146
Nusa Tenggara Barat	53,99	49,93 – 58,00	32,06	28,35 - 36,01	13,95	11,51 - 16,80	910
Nusa Tenggara Timur	56,75	53,34 – 60,10	28,58	25,62 - 31,73	14,68	12,50 - 17,16	657
Kalimantan Barat	55,37	52,17 - 58,53	31,29	28,39 - 34,34	13,34	11,38 - 15,57	1.036
Kalimantan Tengah	46,70	42,79 - 50,65	41,37	37,68 - 45,16	11,93	9,60 - 14,73	577
Kalimantan Selatan	60,86	57,56 - 64,07	27,56	24,87 - 30,43	11,58	9,84 - 13,57	1.080
Kalimantan Timur	52,46	48,42 - 56,46	32,01	28,37 - 35,90	15,53	12,76 - 18,77	1.008
Kalimantan Utara	52,87	47,05 - 58,60	29,79	24,33 - 35,89	17,34	12,97 - 22,79	187
Sulawesi Utara	54,80	52,02 - 57,55	32,07	29,46 - 34,79	13,14	11,29 - 15,23	887
Sulawesi Tengah	61,93	58,56 - 65,19	30,72	27,72 - 33,89	7,35	5,94 - 9,06	668
Sulawesi Selatan	57,90	55,11 - 60,64	29,73	27,09 - 32,51	12,37	10,72 - 14,23	1.626
Sulawesi Tenggara	58,06	53,67 - 62,32	30,85	26,97 - 35,02	11,10	8,84 - 13,85	393
Gorontalo	62,75	57,88 - 67,38	30,91	26,41 - 35,80	6,34	4,63 - 8,62	312
Sulawesi Barat	60,79	54,99 - 66,31	31,19	25,79 - 37,15	8,02	5,18 - 12,21	215
Maluku	60,30	54,50 - 65,81	24,80	19,91 - 30,42	14,91	11,24 - 19,51	209
Maluku Utara	64,51	58,30 - 70,27	25,11	20,55 - 30,29	10,38	7,14 - 14,85	166
Papua Barat	54,96	48,63 - 61,14	28,01	22,13 - 34,76	17,03	13,11 - 21,82	172
Papua	51,08	45,37 - 56,76	31,51	26,79 - 36,65	17,41	13,65 - 21,95	361
**INDONESIA**	**54,40**	**53,74 - 55,05**	**32,27**	**31,68 - 32,88**	**13,33**	**12,89 - 13,78**	**58.621**

Tabel 6.5.6

Proporsi Minum Obat Anti Hipertensi secara Rutin pada Penduduk Umur ≥18 Tahun dengan Hipertensi menurut Karakteristik, Riskesdas 2018

Karakteristik	Kerutinan minum obat anti hipertensi						N tertimbang
	Rutin		Tidak rutin		Tidak minum obat		
	%	95% CI	%	95% CI	%	95% CI	
**Kelompok Umur**							
18-24	38,85	33,05 - 44,99	30,02	25,10 - 35,46	31,12	25,91 - 36,86	903
25-34	41,16	38,35 - 44,03	34,55	31,91 - 37,30	24,29	21,92 - 26,82	3.361
35-44	45,92	44,28 - 47,56	36,06	34,55 - 37,60	18,03	16,79 - 19,33	8.820
45-54	55,34	54,16 - 56,51	32,66	31,57 - 33,76	12,01	11,23 - 12,83	15.989
55-64	57,66	56,51 - 58,81	31,33	30,26 - 32,42	11,01	10,30 - 11,76	15.491
65-74	60,11	58,62 - 61,58	29,80	28,44 - 31,20	10,09	9,22 - 11,03	9.521
75+	57,35	55,19 - 59,48	30,75	28,83 - 32,73	11,90	10,50 - 13,47	4.535
**Jenis kelamin**							
Laki-laki	52,86	51,80 - 53,93	32,09	31,11 - 33,09	15,04	14,26 - 15,86	19.994
Perempuan	55,19	54,40 - 55,98	32,37	31,65 - 33,09	12,44	11,93 - 12,97	38.627
**Pendidikan**							
Tidak/belum pernah sekolah	54,65	52,80 - 56,50	32,61	30,92 - 34,34	12,74	11,51 - 14,08	6.272
Tidak tamat SD/MI	55,04	53,65 - 56,43	33,36	32,05 - 34,70	11,59	10,75 - 12,49	11.090
Tamat SD/MI	54,10	52,92 - 55,27	33,21	32,13 - 34,31	12,70	11,94 - 13,49	17.460
Tamat SLTP/MTS	54,03	52,30 - 55,74	32,16	30,57 - 33,79	13,82	12,66 - 15,06	8.141
Tamat SLTA/MA	53,34	51,90 - 54,76	31,30	30,00 - 32,64	15,36	14,34 - 16,44	11.122
Tamat D1/D2/D3/PT	56,90	54,66 - 59,12	28,14	26,26 - 30,10	14,96	13,41 - 16,65	4.537
**Pekerjaan**							
Tidak bekerja	57,04	56,06 - 58,02	30,50	29,62 - 31,39	12,46	11,82 - 13,12	26.540
Sekolah	51,83	42,74 - 60,81	31,69	23,52 - 41,16	16,48	11,05 - 23,85	295
PNS/TNI/Polri/BUMN/BUMD	57,63	54,80 - 60,42	30,40	27,96 - 32,96	11,96	10,02 - 14,22	2.310
Pegawai swasta	49,32	46,36 - 52,30	32,21	29,55 - 34,99	18,46	16,26 - 20,90	3.130
Wiraswasta	53,01	51,40 - 54,62	32,77	31,24 - 34,33	14,22	13,08 - 15,44	8.262
Petani/buruh tani	50,20	48,89 - 51,50	36,61	35,36 - 37,87	13,20	12,39 - 14,05	10.203
Nelayan	53,69	46,67 - 60,56	31,76	25,93 - 38,22	14,55	9,89 - 20,91	261
Buruh/sopir/pembantu ruta	51,81	49,16 - 54,44	32,92	30,60 - 35,33	15,27	13,44 - 17,31	3.958
Lainnya	55,41	53,04 - 57,75	32,56	30,41 - 34,78	12,03	10,56 - 13,69	3.662
**Tempat tinggal**							
Perkotaan	56,46	55,54 - 57,38	29,95	29,11 - 30,79	13,59	12,97 - 14,24	35.329
Perdesaan	51,27	50,41 - 52,12	35,80	35,00 - 36,61	12,93	12,38 - 13,50	23.292

Tabel 6.5.7

Proporsi Alasan Tidak Minum Obat Secara Rutin pada Penduduk Umur ≥18 Tahun dengan Hipertensi menurut Provinsi, Riskesdas 2018

Provinsi	Alasan tidak minum obat sesuai petunjuk (%)								N tertimbang
	sering lupa	obat tidak tersedia	Minum obat tradisional	tidak tahan ESO	tidak mampu beli obat rutin	tidak rutin berobat	merasa sudah sehat	Lain-nya	
Aceh	11,8	2,9	25,3	5,4	6,0	26,9	66,8	7,2	647
Sumatera Utara	15,7	1,7	16,7	7,7	10,2	24,0	64,4	12,1	935
Sumatera Barat	10,4	2,7	22,1	9,3	5,4	32,2	62,5	8,1	557
Riau	10,9	1,8	17,0	4,8	8,2	24,6	55,5	14,3	799
Jambi	19,2	4,0	36,9	6,0	7,3	39,3	65,6	8,1	388
Sumatera Selatan	19,3	1,2	21,4	7,9	13,7	43,8	54,3	7,1	756
Bengkulu	10,4	1,9	27,9	5,4	8,8	29,0	54,7	7,6	229
Lampung	12,4	1,7	18,2	4,6	9,8	38,1	62,2	8,4	880
Kep.Bangka Belitung	9,8	2,6	9,9	6,2	5,7	29,1	57,0	16,0	126
Kepulauan Riau	11,8	1,7	14,5	3,9	2,8	20,3	53,7	20,1	202
DKI Jakarta	17,3	2,2	6,4	4,4	3,0	22,6	58,4	23,7	1.186
Jawa Barat	10,2	2,1	13,2	2,9	11,2	37,7	55,2	13,8	5.626
Jawa Tengah	8,0	1,6	9,0	3,3	6,9	34,4	61,1	11,9	3.464
DI Yogyakarta	6,5	0,3	7,8	4,1	3,1	30,1	63,4	19,8	586
Jawa Timur	10,3	1,4	11,7	4,5	7,4	28,5	64,1	12,4	3.967
Banten	9,5	2,7	12,0	4,4	8,8	26,9	61,2	13,3	1.298
Bali	5,3	1,2	7,6	4,2	5,8	34,7	66,3	7,5	621
Nusa Tenggara Barat	9,1	2,7	14,9	2,4	11,1	33,1	58,7	8,9	420
Nusa Tenggara Timur	16,7	5,2	20,1	6,1	12,9	38,8	41,4	9,3	286
Kalimantan Barat	12,0	4,4	16,2	5,7	9,0	33,3	57,4	10,9	464
Kalimantan Tengah	13,3	2,6	18,6	5,1	7,6	27,6	65,5	8,2	309
Kalimantan Selatan	7,7	0,9	12,8	5,1	3,7	21,9	62,0	16,1	424
Kalimantan Timur	13,1	1,8	16,5	4,7	3,3	25,2	57,8	16,0	481
Kalimantan Utara	12,8	0,6	23,6	4,7	2,5	16,8	67,6	10,2	88
Sulawesi Utara	17,1	1,8	17,6	4,7	5,9	18,1	68,6	6,7	403
Sulawesi Tengah	13,5	1,4	20,2	7,7	8,3	24,4	64,3	8,0	255
Sulawesi Selatan	15,3	2,8	28,5	7,3	6,9	22,0	55,7	9,3	687
Sulawesi Tenggara	16,1	2,6	29,0	3,8	8,9	23,4	59,5	6,9	166
Gorontalo	13,2	2,8	15,7	4,2	2,6	40,0	67,2	8,6	117
Sulawesi Barat	21,9	3,5	32,3	4,5	1,8	18,5	48,0	12,3	85
Maluku	29,5	3,6	24,8	7,7	5,4	21,0	43,2	11,8	83
Maluku Utara	22,7	5,2	23,0	2,7	4,4	25,0	55,2	11,9	59
Papua Barat	18,2	3,0	23,1	5,8	6,6	25,1	50,1	12,2	78
Papua	37,7	6,0	16,1	7,1	9,1	35,5	57,5	18,4	177
**INDONESIA**	**11,5**	**2,0**	**14,5**	**4,5**	**8,1**	**31,3**	**59,8**	**12,5**	**26.848**

Tabel 6.5.8

Proporsi Alasan Tidak Minum Obat Secara Rutin pada Penduduk Umur ≥18 Tahun dengan Hipertensi menurut Karakteristik, Riskesdas 2018

Karakteristik	Alasan tidak minum obat sesuai petunjuk (%)								N tertimbang
	sering lupa	obat tidak tersedia	Minum obat tradisional	tidak tahan ESO	tidak mampu beli obat rutin	tidak rutin berobat	merasa sudah sehat	Lainnya	
**Kelompok Umur**									
18-24	9,3	2,0	6,3	4,5	7,3	24,8	62,7	27,0	555
25-34	10,0	1,9	9,6	3,8	2,9	20,3	60,6	21,0	1.987
35-44	11,0	1,9	15,1	4,4	5,6	27,2	60,9	13,9	4.791
45-54	12,3	2,0	15,6	4,8	7,9	28,8	61,0	11,2	7.172
55-64	10,5	2,0	15,5	4,4	8,5	33,4	60,7	10,7	6.587
65-74	13,0	2,4	14,2	4,3	11,6	38,2	57,6	10,0	3.814
75+	12,8	1,6	13,4	4,7	12,0	43,1	52,5	11,4	555
**Jenis kelamin**									
Laki-laki	13,0	2,0	13,9	4,1	7,7	31,1	61,9	12,3	9.465
Perempuan	10,7	2,0	14,9	4,7	8,3	31,4	58,7	12,5	17.383
**Pendidikan**									
Tidak/belum pernah sekolah	11,3	2,5	14,3	4,7	16,4	40,9	54,8	9,4	2.856
Tidak tamat SD/MI	11,8	1,9	16,2	4,0	11,9	38,8	56,6	9,2	5.007
Tamat SD/MI	11,2	2,3	14,6	4,2	9,4	34,8	60,6	11,6	8.049
Tamat SLTP/MTS	11,4	1,9	14,4	4,6	4,7	27,2	60,7	13,7	3.759
Tamat SLTA/MA	11,6	1,6	13,3	4,9	2,4	21,5	62,2	16,6	5.212
Tamat D1/D2/D3/PT	12,4	1,4	13,5	5,5	2,3	18,3	64,3	15,6	1.964
**Pekerjaan**									
Tidak bekerja	11,2	2,0	14,7	4,8	9,6	32,9	57,0	13,6	11.450
Sekolah	2,5	1,4	5,5	0,7	8,7	29,4	68,6	13,8	143
PNS/TNI/Polri/BUMN/BUMD	12,2	1,4	16,5	4,8	2,5	17,7	64,2	16,4	983
Pegawai swasta	14,9	1,7	10,3	4,2	1,7	20,3	63,9	15,4	1.593
Wiraswasta	10,6	1,6	14,5	4,9	4,1	27,1	63,1	13,4	3.899
Petani/buruh tani	12,2	2,5	17,0	3,8	11,4	39,8	59,4	7,4	5.103
Nelayan	13,8	6,0	13,8	5,4	12,0	24,5	60,7	15,3	121
Buruh/sopir/pembantu ruta	9,5	2,0	10,1	3,9	8,2	29,8	63,9	12,2	1.916
Lainnya	12,8	2,3	14,7	4,4	5,8	25,2	61,2	12,7	1.640
**Tempat tinggal**									
Perkotaan	11,4	1,8	12,8	4,5	6,1	26,6	60,6	15,1	15.448
Perdesaan	11,7	2,2	16,8	4,5	10,8	37,7	58,7	8,9	11.400

Tabel 6.5.9
Proporsi Mengukur Tekanan Darah secara Rutin pada Penduduk Umur ≥18 Tahun menurut Provinsi, Riskesdas 2018

Provinsi	Mengukur Tekanan Darah						N tertimbang
	Rutin		Kadang-kadang		Tidak		
	%	95% CI	%	95% CI	%	95% CI	
Aceh	8,1	7,6 - 8,6	49,1	47,9 - 50,3	42,8	41,6 - 44,0	12.259
Sumatera Utara	7,4	7,0 - 7,9	44,0	42,8 - 45,1	48,7	47,5- 49,8	32.944
Sumatera Barat	9,5	8,9 - 10,2	60,4	58,9 - 61,9	30,2	28,8 - 31,6	12.650
Riau	10,6	9,9 - 11,4	52,9	51,3 - 54,4	36,5	35,0 - 38,0	15.807
Jambi	7,2	6,5 - 7,9	37,8	36,0 - 39,6	55,0	53,1 - 56,9	8.760
Sumatera Selatan	8,4	7,8 - 9,1	38,6	37,1 - 40,2	53,0	51,4 - 54,5	20.231
Bengkulu	10,3	9,5 - 11,1	41,5	39,7 - 43,4	48,2	46,2 - 50,3	4.777
Lampung	9,4	8,8 - 10,0	43,3	42,0 - 44,5	47,3	46,0 - 48,7	20.484
Kep.Bangka Belitung	15,8	14,7- 17,0	45,1	43,4 - 46,9	39,0	37,1 - 41,0	3.605
Kepulauan Riau	14,7	13,4 - 16,1	49,1	46,5 - 51,8	36,2	33,4 - 39,0	5.052
DKI Jakarta	15,9	14,8 - 17,0	51,4	49,5 - 53,3	32,7	30,8 - 34,7	27.195
Jawa Barat	14,0	13,6 - 14,5	44,9	44,0 - 45,8	41,0	40,1 - 41,9	121.153
Jawa Tengah	13,1	12,7 - 13,5	50,2	49,4 - 50,9	36,7	36,0 - 37,5	89.648
DI Yogyakarta	18,4	17,2 - 19,6	60,1	58,4 - 61,9	21,5	19,9 - 23,2	10.318
Jawa Timur	12,3	12,0 - 12,7	43,5	42,8 - 44,2	44,2	43,5 - 44,9	105.380
Banten	12,8	12,0 - 13,7	46,8	45,3 - 48,3	40,4	38,8 - 41,9	31.052
Bali	13,5	12,6 - 14,3	59,4	57,7 - 61,1	27,1	25,5 - 28,9	11.242
Nusa Tenggara Barat	8,9	8,2 - 9,7	54,6	53,1 - 56,2	36,4	34,8 - 38,1	11.881
Nusa Tenggara Timur	7,1	6,6 - 7,6	44,8	43,6 - 46,0	48,1	46,9 - 49,3	11.505
Kalimantan Barat	13,1	12,3 - 13,9	41,5	40,1 - 42,8	45,5	44,0 - 46,9	11.924
Kalimantan Tengah	10,2	9,4 - 11,0	43,9	42,1 - 45,8	45,9	43,9 - 48,0	6.470
Kalimantan Selatan	14,5	13,7 - 15,3	50,8	49,4 - 52,1	34,8	33,3 - 36,2	10.162
Kalimantan Timur	13,6	12,6 - 14,7	48,8	47,0 - 50,7	37,6	35,7 - 39,4	8.957
Kalimantan Utara	26,3	22,8 - 30,1	30,1	26,8 - 33,7	43,6	40,1 - 47,2	1.675
Sulawesi Utara	10,7	9,9 - 11,4	50,3	48,7 - 51,8	39,1	37,6 - 40,6	6.307
Sulawesi Tengah	10,5	9,7 - 11,3	51,7	50,2 - 53,1	37,9	36,4 - 39,3	7.221
Sulawesi Selatan	10,0	9,4 - 10,6	52,9	51,8 - 53,9	37,1	36,1- 38,2	21.142
Sulawesi Tenggara	10,6	9,5 - 11,7	49,0	47,3 - 50,7	40,4	38,7- 42,3	5.902
Gorontalo	14,2	12,6 - 16,0	45,2	43,0 - 47,5	40,6	38,4 - 42,7	2.898
Sulawesi Barat	9,0	8,0 - 10,0	50,8	48,6 - 53,1	40,2	37,9 - 42,5	3.068
Maluku	5,5	4,9 - 6,1	51,0	48,7 - 53,3	43,5	41,2 - 45,8	3.914
Maluku Utara	8,3	7,4 - 9,2	48,7	46,8 - 50,7	43,0	41,0 - 45,0	2.725
Papua Barat	9,2	8,1 - 10,4	45,1	42,8 - 47,4	45,7	43,2 - 48,2	2.163
Papua	3,6	3,2 - 4,2	34,2	32,3- 36,2	62,1	60,1 - 64,2	7.730
**INDONESIA**	**12,0**	**11,8 - 12,1**	**47,0**	**46,7- 47,3**	**41,0**	**40,7 - 41,3**	**658.201**

Tabel 6.5.10

Proporsi Mengukur Tekanan Darah secara Rutin pada Penduduk Umur ≥18 Tahun menurut Karakteristik, Riskesdas 2018

Karakteristik	Mengukur Tekanan Darah						N tertimbang
	Rutin		Kadang-kadang		Tidak		
	%	95% CI	%	95% CI	%	95% CI	
**Kelompok Umur**							
18-24	6,3	6,1 - 6,6	38,3	37,8 - 38,9	55,3	54,8 - 55,9	106.849
25-34	10,4	10,1 - 10,7	45,4	44,9 - 45,8	44,2	43,8 - 44,7	152.373
35-44	11,1	10,8 - 11,3	49,0	48,5 - 49,4	40,0	39,5 - 40,4	144.578
45-54	13,7	13,4 - 14,0	50,8	50,3 - 51,2	35,5	35,1 - 36,0	118.927
55-64	17,0	16,6 - 17,4	50,6	50,1 - 51,1	32,4	31,9 - 32,9	79.427
65-74	19,3	18,7 - 19,9	50,0	49,2 - 50,7	30,7	30,0 - 31,4	38.335
75+	16,9	16,1 - 17,7	49,7	48,6 - 50,7	33,4	32,5 - 34,4	17.712
**Jenis kelamin**							
Laki-laki	6,7	6,6 - 6,9	42,4	42,1 - 42,7	50,9	50,5 - 51,2	327.150
Perempuan	17,2	16,9 - 17,4	51,6	51,2 - 51,9	31,3	30,9 - 31,6	331.051
**Pendidikan**							
Tidak/belum pernah sekolah	9,9	9,5 - 10,3	45,5	44,7 - 46,3	44,6	43,8 - 45,4	39.556
Tidak tamat SD/MI	11,6	11,3 - 11,9	46,8	46,2 - 47,3	41,6	41,1 - 42,2	80.199
Tamat SD/MI	11,9	11,6 - 12,1	46,3	45,9 - 46,8	41,8	41,3 - 42,2	159.357
Tamat SLTP/MTS	11,5	11,2 - 11,8	44,8	44,3 - 45,3	43,7	43,2 - 44,2	119.569
Tamat SLTA/MA	11,2	11,0 - 11,4	47,3	46,8- 47,7	41,6	41,1 - 42,0	198.150
Tamat D1/D2/D3/PT	17,6	17,1 - 18,1	53,5	52,9 - 54,2	28,8	28,2 - 29,5	61.371
**Pekerjaan**							
Tidak bekerja	17,5	17,2-17,8	49,1	48,7- 49,5	33,4	33,0 - 33,8	196.220
Sekolah	4,2	3,7 - 4,6	39,5	38,4 - 40,6	56,3	55,2 - 57,5	21.093
PNS/TNI/Polri/BUMN/BUMD	21,9	21,1 - 22,8	53,3	52,4 - 54,3	24,7	23,9 - 25,6	21.228
Pegawai swasta	10,6	10,2 - 11,0	49,1	48,3 - 49,8	40,3	39,6 - 41,1	72.970
Wiraswasta	11,3	11,0 - 11,6	47,5	46,9 - 48,0	41,3	40,7 - 41,8	101.834
Petani/buruh tani	7,2	7,0 - 7,4	44,5	44,0 - 45,0	48,3	47,8 - 48,8	128.377
Nelayan	5,6	4,8 - 6,5	39,9	38,0 - 41,9	54,5	52,5 - 56,5	5.259
Buruh/sopir/pembantu ruta	6,5	6,2 - 6,8	42,7	41,9 - 43,4	50,8	50,1 - 51,6	72.315
Lainnya	14,3	13,8 - 14,9	49,4	48,6 - 50,2	36,3	35,5 - 37,1	38.905
**Tempat tinggal**							
Perkotaan	13,7	13,5 - 14,0	47,7	47,3 - 48,1	38,6	38,2 - 39,0	364.630
Perdesaan	9,8	9,6 - 10,0	46,2	45,8 - 46,5	44,0	43,6 - 44,4	293.571

6.6 Stroke

Stroke adalah kerusakan pada otak yang muncul mendadak, progresif, dan cepat akibat gangguan peredaran darah otak non traumatik. Gangguan tersebut secara mendadak menimbulkan gejala antara lain kelumpuhan sesisi wajah atau anggota badan, bicara tidak lancar, bicara tidak jelas (pelo), perubahan kesadaran, gangguan penglihatan, dan lain-lain.

Prevalensi stroke menurut diagnosis dokter dihitung dengan formula:

$$Prevalensi\ stroke\ menurut\ diagnosis\ dokter = \frac{ART\ yang\ pernah\ didiagnosis\ stroke\ oleh\ dokter}{ART\ umur\ \geq 15\ th}$$

Proporsi kepatuhan kontrol stroke ke fasilitas pelayanan kesehatan dihitung dengan formula:

$$Proporsi\ kepatuhan\ kontrol\ stroke\ ke\ fasyankes = \frac{ART\ yang\ pernah\ didiagnosis\ stroke\ oleh\ dokter\ yang\ rutin\ memeriksakan\ ulang\ (kontrol)\ ke\ fasyankes}{ART\ umur\ \geq 15\ th\ yang\ pernah\ didiagnosis\ stroke\ oleh\ dokter}$$

Tabel 6.6.1
Prevalensi (per mil) Stroke berdasarkan Diagnosis Dokter pada Penduduk Umur ≥15 Tahun menurut Provinsi, Riskesdas 2018

Provinsi	Stroke		N tertimbang
	‰	95% CI(‰)	
Aceh	7,8	6,7-9,1	13.389
Sumatera Utara	9,3	8,0-10,7	36.410
Sumatera Barat	10,8	9,3-12,7	13.834
Riau	8,3	6,9-10,1	17.258
Jambi	6,8	5,5-8,5	9.511
Sumatera Selatan	10,0	8,5-11,8	22.013
Bengkulu	9,5	7,6-12,0	5.175
Lampung	8,3	7,0-9,8	22.171
Bangka Belitung	12,6	10,2-15,7	3.915
Kepulauan Riau	12,9	10,1-16,5	5.463
DKI Jakarta	12,2	10,2-14,7	28.985
Jawa Barat	11,4	10,4-12,6	131.846
Jawa Tengah	11,8	10,9-12,7	96.794
DI Yogyakarta	14,6	12,1-17,6	10.975
Jawa Timur	12,4	11,5-13,4	113.045
Banten	11,0	9,1-13,3	33.587
Bali	10,7	9,0-12,7	12.092
Nusa Tenggara Barat	8,8	7,0-11,0	13.036
Nusa Tenggara Timur	6,1	5,1-7,2	12.777
Kalimantan Barat	9,8	8,3-11,6	13.035
Kalimantan Tengah	12,1	10,1-14,4	7.031
Kalimantan Selatan	12,7	10,9-14,7	11.068
Kalimantan Timur	14,7	11,9-18,0	9.696
Kalimantan Utara	12,7	8,9-18,3	1.838
Sulawesi Utara	14,2	12,1-16,7	6.827
Sulawesi Tengah	10,4	8,7-12,5	7.847
Sulawesi Selatan	10,6	9,2-12,2	23.069
Sulawesi Tenggara	8,3	6,5-10,5	6.510
Gorontalo	10,9	8,6-13,8	3.144
Sulawesi Barat	7,0	4,9-9,9	3.408
Maluku	9,0	6,8-11,8	4.351
Maluku Utara	4,6	3,4-6,2	3.005
Papua Barat	6,4	4,3-9,6	2.363
Papua	4,1	2,9-5,8	8.317
**INDONESIA**	**10,9**	**10,6-11,3**	**713.783**

Tabel 6.6.2
Prevalensi (per mil) Stroke berdasarkan Diagnosis Dokter pada Penduduk Umur ≥15 Tahun menurut Karakteristik, Riskesdas 2018

Karakteristik	Stroke		N Tertimbang
	‰	95% CI(‰)	
**Kelompok Umur**			
15-24	0,6	0,5 - 0,9	159.014
25-34	1,4	1,1 – 1,8	153.316
35-44	3,7	3,3 - 4,2	145.474
45-54	14,2	13,2 – 15,2	119.663
55-64	32,4	30,7 – 34,2	79.919
65-74	45,3	42,4 – 48,3	38.572
75+	50,2	45,8 - 55,0	17.821
**Jenis kelamin**			
Laki-laki	11,0	10,5 – 11,5	355.726
Perempuan	10,9	10,4 -11,4	358.056
**Pendidikan**			
Tidak/belum pernah sekolah	21,2	19,3 - 23,3	40.430
Tidak tamat SD/MI	18,6	17,3 - 20,1	83.024
Tamat SD/MI	13,2	12,5 – 14,0	174.012
Tamat SLTP/MTS	6,8	6,2 - 7,5	151.414
Tamat SLTA/MA	7,4	6,8 - 7,9	203.128
Tamat D1/D2/D3/PT	9,1	8,0 - 10,2	61.776
**Pekerjaan**			
Tidak bekerja	21,8	20,9 – 22,7	209.274
Sekolah	1,1	0,7 – 1,5	57.440
PNS/TNI/Polri/BUMN/BUMD	12,2	10,4 – 14,4	21.429
Pegawai swasta	3,4	2,8 - 4,2	73.962
Wiraswasta	8,5	7,7 – 9,4	102.968
Petani/buruh tani	7,3	6,8 – 7,9	130.042
Nelayan	6,3	4,2 – 9,5	5.402
Buruh/sopir/pembantu ruta	4,8	4,1 - 5,6	73.720
Lainnya	11,1	9,8 -12,6	39.546
**Tempat tinggal**			
Perkotaan	12,6	12,1 – 13,2	394.850
Perdesaan	8,8	8,4 -9,2	318.933

Tabel 6.6.3

Proporsi Kontrol *Stroke* ke Fasilitas Pelayanan Kesehatan pada Penduduk Umur ≥15 Tahun dengan *Stroke* berdasarkan Diagnosis Dokter menurut Provinsi, Riskesdas 2018

Provinsi	Pemeriksaan ulang (kontrol) *stroke* ke fasilitas pelayanan kesehatan						N tertimbang
	Rutin		kadang-kadang/tidak rutin		Tidak memeriksakan ulang		
	%	95 % CI	%	95 % CI	%	95 % CI	
Aceh	41,0	33,3 - 49,1	41,8	34,0 - 50,0	17,2	11,5 - 25,0	108
Sumatera Utara	36,4	29,8 - 43,5	45,6	38,9 - 52,5	18,0	13,3 - 23,8	350
Sumatera Barat	44,4	36,6 - 52,6	35,3	28,2 - 43,0	20,3	15,5 - 26,1	155
Riau	33,0	24,9 - 42,3	42,5	33,9 - 51,5	24,5	17,7 - 32,8	148
Jambi	41,3	31,8 - 51,5	43,9	34,0 - 54,4	14,8	9,1 - 23,0	67
Sumatera Selatan	38,5	30,1 - 47,7	35,1	27,4 - 43,6	26,4	19,3 - 35,1	227
Bengkulu	33,8	24,7 - 44,2	44,4	34,1 - 55,2	21,9	14,7 - 31,3	51
Lampung	37,0	29,2 - 45,6	40,8	32,1 - 50,1	22,1	16,0 - 29,8	189
Bangka Belitung	33,5	24,2 - 44,2	49,7	39,4 - 60,1	16,8	10,9 - 24,9	51
Kepulauan Riau	40,3	28,9 - 52,8	40,3	30,3 - 51,3	19,4	10,8 - 32,4	73
DKI Jakarta	51,0	41,5 - 60,4	33,8	25,0 - 43,9	15,2	9,5 - 23,3	367
Jawa Barat	36,4	31,7 - 41,5	38,9	34,2 - 43,9	24,6	20,4 - 29,4	1.557
Jawa Tengah	40,9	37,0 - 44,9	38,5	34,6 - 42,5	20,6	17,7 - 24,0	1.176
DI Yogyakarta	47,1	37,6 - 56,9	29,5	21,0 - 39,8	23,3	15,3 - 33,8	166
Jawa Timur	40,0	36,5 - 43,6	39,5	35,9 - 43,2	20,5	17,7 - 23,7	1.452
Banten	43,9	36,4 - 51,8	27,2	20,1 - 35,6	28,9	21,9 - 37,1	381
Bali	44,0	36,0 - 52,3	40,3	32,6 - 48,5	15,8	10,8 - 22,4	133
Nusa Tenggara Barat	35,0	25,6 - 45,9	35,6	26,8 - 45,6	29,3	20,9 - 39,5	118
Nusa Tenggara Timur	33,2	25,8 - 41,5	40,2	32,4 - 48,6	26,6	19,8 - 34,8	80
Kalimantan Barat	38,6	30,7 - 47,1	39,6	32,2 - 47,4	21,9	15,6 - 29,7	132
Kalimantan Tengah	40,3	31,4 - 49,8	42,7	33,7 - 52,2	17,0	11,9 - 23,7	88
Kalimantan Selatan	37,5	30,5 - 44,9	41,2	33,8 - 48,9	21,4	16,0 - 28,0	145
Kalimantan Timur	33,1	25,4 - 41,9	36,0	27,5 - 45,3	30,9	22,5 - 40,9	147
Kalimantan Utara	55,8	39,2 - 71,2	33,5	19,0 - 52,0	10,7	4,2 - 24,8	24*
Sulawesi Utara	39,8	32,8 - 47,2	39,8	33,0 - 47,1	20,4	15,2 - 26,9	100
Sulawesi Tengah	27,3	19,6 - 36,7	45,0	36,0 - 54,4	27,7	19,9 - 37,0	84
Sulawesi Selatan	42,7	36,9 - 48,8	38,3	32,7 - 44,3	18,9	14,6 - 24,2	253
Sulawesi Tenggara	26,1	18,9 - 34,9	50,9	39,7 - 61,9	23,0	15,0 - 33,7	56
Gorontalo	28,9	18,9 - 41,5	53,6	41,5 - 65,2	17,5	10,8 - 27,1	35*
Sulawesi Barat	49,0	33,6 - 64,5	42,0	27,5 - 58,0	9,1	3,5 - 21,8	24*
Maluku	23,2	15,4 - 33,5	50,7	37,4 - 64,0	26,0	16,5 - 38,5	40*
Maluku Utara	37,2	22,9 - 54,2	45,9	30,1 - 62,6	16,9	9,1 - 29,0	14*
Papua Barat	30,4	14,6 - 52,7	36,2	19,0 - 57,7	33,5	16,9 - 55,3	16*
Papua	36,1	23,5 - 51,0	47,0	32,5 - 62,1	16,8	10,5 - 25,8	35*
**INDONESIA**	**39,4**	**37,8 - 41,0**	**38,7**	**37,1 - 40,3**	**21,9**	**20,6 - 23,3**	**8.042**

*N Tertimbang <50

Tabel 6.6.4

Proporsi Kontrol *Stroke* ke Fasilitas Pelayanan Kesehatan pada Penduduk Umur ≥15 Tahun dengan *Stroke* berdasarkan Diagnosis Dokter menurut Karakteristik, Riskesdas 2018

Karakteristik	Pemeriksaan ulang (kontrol) *stroke* ke fasilitas pelayanan kesehatan						N tertimbang
	Rutin		kadang-kadang/ tidak rutin		Tidak memeriksakan ulang		
	%	95 % CI	%	95 % CI	%	95 % CI	
**Kelompok Umur**							
15-24	22,0	12,7 - 35,3	67,6	54,0 - 78,7	10,4	5,5 - 18,9	106
25-34	18,4	12,2 - 26,8	63,5	52,8 - 73,0	18,1	11,3 - 27,8	225
35-44	33,6	28,2 - 39,4	41,4	35,8 - 47,2	25,0	20,3 - 30,5	557
45-54	45,5	42,0 - 49,1	34,3	31,1 - 37,6	20,2	17,6 - 23,1	1.755
55-64	42,3	39,6 - 45,1	38,0	35,3 - 40,7	19,7	17,5 - 22,1	2.673
65-74	39,7	36,5 - 43,0	37,3	34,2 - 40,5	23,0	20,0 - 26,2	1.803
75+	29,4	25,4 - 33,7	41,0	36,5 - 45,7	29,6	25,4 - 34,3	924
**Jenis kelamin**							
Laki-laki	41,0	38,7 - 43,3	37,9	35,7 - 40,1	21,1	19,4 - 23,0	4.023
Perempuan	37,8	35,6 - 40,0	39,6	37,3 - 41,8	22,6	20,7 - 24,7	4.019
**Pendidikan**							
Tidak/belum pernah sekolah	29,3	25,0 - 34,1	41,6	37,1 - 46,2	29,1	25,0 - 33,5	885
Tidak tamat SD/MI	35,2	31,9 - 38,7	37,5	34,1 - 41,1	27,3	24,1 - 30,7	1.597
Tamat SD/MI	37,2	34,5 - 40,1	41,0	38,2 - 43,9	21,7	19,4 - 24,2	2.374
Tamat SLTP/MTS	43,6	39,1 - 48,1	36,1	31,9 - 40,6	20,3	16,8 - 24,3	1.063
Tamat SLTA/MA	44,9	41,3 - 48,6	38,1	34,5 - 41,8	17,0	14,3 - 20,0	1.545
Tamat D1/D2/D3/PT	52,8	47,0 - 58,5	34,5	29,0 - 40,4	12,7	9,4 - 17,0	578
**Pekerjaan**							
Tidak bekerja	39,4	37,3 - 41,5	37,7	35,6 - 39,8	22,9	21,0 - 24,8	4.708
Sekolah	36,9	22,0 - 54,7	58,2	40,6 - 73,9	5,0	1,4 - 15,9	63
PNS/TNI/Polri/BUMN/BUMD	53,3	45,2 - 61,3	33,7	26,3 - 42,0	13,0	8,4 - 19,6	270
Pegawai swasta	36,5	27,6 - 46,6	48,1	38,7 - 57,7	15,3	9,6 - 23.5	261
Wiraswasta	42,7	38,2 - 47,3	37,9	33,4 - 42,7	19,4	15,9 - 23,3	905
Petani/buruh tani	34,0	30,3 - 37,9	41,1	37,4 - 44,9	24,9	21,8 - 28,4	982
Nelayan	41,2	23,1 - 62,0	39,8	22,2 - 60,4	19,1	8,0 - 38,8	35*
Buruh/sopir/pembantu ruta	31,7	25,1 - 39,2	44,3	36,3 - 52,5	24,0	17,7 - 31,6	365
Lainnya	44,1	37,9 - 50,5	35,9	30,1 - 42,0	20,0	15,6 - 25,3	452
**Tempat tinggal**							
Perkotaan	42,4	40,2 - 44,6	36,5	34,4 - 38,7	21,1	19,3 - 23,0	5.152
Perdesaan	34,1	32,0 - 36,2	42,6	40,4 - 44,8	23,3	21,5 - 25,2	2.890

*N Tertimbang <50

6.7 Penyakit Gagal Ginjal Kronis

Penyakit ginjal adalah gangguan organ ginjal yang timbul akibat berbagai faktor, misalnya infeksi, tumor, kelainan bawaan, penyakit metabolik atau degeneratif dan lain-lain.

Prevalensi gagal ginjal kronis yang didiagnosis dokter dihitung dengan formula:

$$Prevalensi\ gagal\ ginjal\ kronis\ berdasarkan\ diagnosis\ dokter = \frac{ART\ yang\ pernah\ didiagnosis\ gagal\ ginjal\ kronis\ oleh\ dokter}{ART\ umur\ \geq 15\ th}$$

Proporsi hemodialisis pada ART yang pernah didiagnosis gagal ginjal kronis oleh dokter dihitung dengan formula:

$$Proporsi\ hemodialisis\ pada\ ART\ dengan\ gagal\ ginjal\ kronis\ berdasarkan\ diagnosis\ dokter = \frac{ART\ yang\ pernah\ didiagnosis\ gagal\ ginjal\ kronis\ oleh\ dokter\ dan\ menjalani\ cuci\ darah}{ART\ umur\ \geq 15\ th\ yang\ pernah\ didiagnosis\ gagal\ ginjal\ kronis\ oleh\ dokter}$$

Parameter kimia klinis yang diperiksa pada Riskesdas 2018 meliputi pemeriksaan kadar kreatinin serum. Penentuan *cut off point* kreatinin serum abnormal merujuk pada nilai common reference interval di atas persentil 97,5 menurut International Federation of Clinical Chemistry (IFCC), Mauro Panteghini 2008.

$$Proporsi\ Kreatinin\ Serum\ Abnormal = \frac{ART\ Pria \geq 15\ tahun\ dengan\ kadar\ Kreatinin\ Serum > 1{,}18\ mg\ per\ dl\ dan\ ART\ Wanita \geq 15\ tahun\ dengan\ kadar\ Kreatinin\ Serum > 1{,}02\ mg\ per\ dl}{ART\ \geq 15\ tahun\ yang\ diperiksa\ kadar\ Kreatininnya\ dalam\ serum}$$

Tabel 6.7.1
Prevalensi Gagal Ginjal Kronis berdasarkan Diagnosis Dokter pada Penduduk Umur ≥15 Tahun menurut Provinsi, Riskesdas 2018

Provinsi	Gagal ginjal kronis		N tertimbang
	%	95% CI	
Aceh	0,49	0,39 -0,61	13.389
Sumatera Utara	0,33	0,26 - 0,42	36.410
Sumatera Barat	0,40	0,31-0,50	13.834
Riau	0,26	0,20 -0,35	17.258
Jambi	0,32	0,21 -0,48	9.511
Sumatera Selatan	0,27	0,20 -0,37	22.013
Bengkulu	0,43	0,32 -0,59	5.175
Lampung	0,39	0,29 -0,51	22.171
Bangka Belitung	0,29	0,19- 0,43	3.915
Kepulauan Riau	0,32	0,13- 0,83	5.463
DKI Jakarta	0,45	0,32- 0,63	28.985
Jawa Barat	0,48	0,40- 0,56	131.846
Jawa Tengah	0,42	0,36- 0,49	96.794
DI Yogyakarta	0,43	0,27- 0,70	10.975
Jawa Timur	0,29	0,25- 0,34	113.045
Banten	0,25	0,18- 0,35	33.587
Bali	0,44	0,32- 0,60	12.092
Nusa Tenggara Barat	0,52	0,39- 0,68	13.036
Nusa Tenggara Timur	0,33	0,25 -0,44	12.777
Kalimantan Barat	0,43	0,34 -0,55	13.035
Kalimantan Tengah	0,31	0,22 -0,42	7.031
Kalimantan Selatan	0,32	0,23 -0,43	11.068
Kalimantan Timur	0,42	0,27 -0,65	9.696
Kalimantan Utara	0,64	0,43 -0,96	1.838
Sulawesi Utara	0,53	0,41 -0,68	6.827
Sulawesi Tengah	0,52	0,40 -0,66	7.847
Sulawesi Selatan	0,37	0,29 -0,49	23.069
Sulawesi Tenggara	0,35	0,25 -0,48	6.510
Gorontalo	0,52	0,36 -0,76	3.144
Sulawesi Barat	0,18	0,10 -0,35	3.408
Maluku	0,47	0,31 -0,71	4.351
Maluku Utara	0,56	0,38 -0,84	3.005
Papua Barat	0,33	0,19 -0,56	2.363
Papua	0,36	0,25 -0,52	8.317
**INDONESIA**	**0,38**	**0,36 -0,41**	**713.783**

Tabel 6.7.2
Prevalensi Gagal Ginjal Kronis berdasarkan Diagnosis Dokter pada Penduduk Umur ≥15 Tahun menurut Karakteristik, Riskesdas 2018

Karakteristik	Gagal ginjal kronis		N tertimbang
	%	95% CI	
**Kelompok Umur**			
15-24	0,13	0,11 -0,17	159.015
25-34	0,23	0,18 -0,28	153.317
35-44	0,33	0,29 -0,37	145.474
45-54	0,56	0,51 -0,63	119.664
55-64	0,72	0,64 -0,81	79.919
65-74	0,82	0,70 -0,97	38.572
75+	0,75	0,58 -0,96	17.822
**Jenis kelamin**			
Laki-laki	0,42	0,39 - 0,45	355.726
Perempuan	0,35	0,32 – 0,38	358.057
**Pendidikan**			
Tidak/belum pernah sekolah	0,57	0,48 -0,68	40.430
Tidak tamat SD/MI	0,53	0,46 - 0,60	83.024
Tamat SD/MI	0,44	0,40 - 0,49	174.012
Tamat SLTP/MTS	0,28	0,25 - 0,32	151.414
Tamat SLTA/MA	0,31	0,27 - 0,35	203.128
Tamat D1/D2/D3/PT	0,41	0,33 - 0,50	61.776
**Pekerjaan**			
Tidak bekerja	0,48	0,43-0,53	209.274
Sekolah	0,15	0,11-0,21	57.440
PNS/TNI/Polri/BUMN/BUMD	0,46	0,34-0,62	21.429
Pegawai swasta	0,23	0,17-0,30	73.962
Wiraswasta	0,35	0,30-0,40	102.968
Petani/buruh tani	0,46	0,42-0,51	130.042
Nelayan	0,41	0,22-0,76	5.402
Buruh/sopir/pembantu ruta	0,37	0,30-0,45	73.720
Lainnya	0,35	0,28-0,44	39.546
**Tempat tinggal**			
Perkotaan	0,38	0,35 – 0,42	394.850
Perdesaan	0,38	0,36 - 0,41	318.933

Tabel 6.7.3
Proporsi Hemodialisis pada Penduduk Umur ≥15 Tahun dengan Gagal Ginjal Kronis berdasarkan Diagnosis Dokter menurut Provinsi, Riskesdas 2018

Provinsi	Hemodialisis			N tertimbang
	%	95% CI		
Aceh	14,09	8,53	22,38	67
Sumatera Utara	11,57	6,56	19,62	125
Sumatera Barat	15,00	9,88	22,12	57
Riau	25,57	13,16	43,79	47*
Jambi	18,79	6,30	44,35	31*
Sumatera Selatan	17,79	9,91	29,86	62
Bengkulu	20,26	10,54	35,42	23*
Lampung	16,64	10,39	25,59	89
Bangka Belitung	25,98	11,68	48,22	12*
Kepulauan Riau	5,24	1,62	15,64	18*
DKI Jakarta	38,71	23,21	56,90	135
Jawa Barat	19,34	13,99	26,11	651
Jawa Tengah	16,15	11,97	21,44	422
DI Yogyakarta	35,51	18,44	57,29	49*
Jawa Timur	23,14	17,04	30,62	340
Banten	28,47	15,02	47,27	86
Bali	37,04	26,16	49,42	55
Nusa Tenggara Barat	25,69	14,21	41,91	70
Nusa Tenggara Timur	9,94	4,68	19,89	44*
Kalimantan Barat	17,86	11,78	26,16	58
Kalimantan Tengah	7,72	4,25	13,65	22*
Kalimantan Selatan	24,34	13,42	40,04	36*
Kalimantan Timur	15,20	7,88	27,31	42*
Kalimantan Utara	8,89	1,83	33,84	12*
Sulawesi Utara	13,68	7,04	24,90	37*
Sulawesi Tengah	7,34	2,17	22,06	42*
Sulawesi Selatan	8,17	3,10	19,83	90
Sulawesi Tenggara	1,99	0,43	8,75	24*
Gorontalo	21,11	8,32	44,10	17*
Sulawesi Barat	18,74	8,38	36,77	7*
Maluku	6,21	1,67	20,53	21*
Maluku Utara	4,88	1,87	12,12	18*
Papua Barat	7,73	5,27	11,20	8*
Papua	12,73	5,69	26,07	31*
**INDONESIA**	**19,33**	**17,18**	**21,67**	**2.850**

*N Tertimbang <50

Tabel 6.7.4
Proporsi Hemodialisis pada Penduduk Umur ≥15 Tahun dengan Gagal Ginjal Kronis berdasarkan Diagnosis Dokter menurut Karakteristik, Riskesdas 2018

Karakteristik	Hemodialisis		N tertimbang
	%	95% CI	
**Kelompok Umur**			
15-24	24,06	16,45 - 33,76	219
25-34	19,29	12,43 - 28,69	363
35-44	14,99	10,99 - 20,11	500
45-54	18,85	15,03 - 23,39	701
55-64	22,91	18,21 - 28,40	599
65-74	20,08	14,57 - 27,00	330
75+	12,68	6,92 - 22,10	138
**Jenis kelamin**			
Laki-laki	17,08	14,13 - 20,48	1.542
Perempuan	21,98	18,94 - 25,35	1.308
**Pendidikan**			
Tidak/belum pernah sekolah	16,87	11,67 - 23,76	241
Tidak tamat SD/MI	21,21	16,28 - 27,14	453
Tamat SD/MI	14,93	11,61 - 19,01	798
Tamat SLTP/MTS	13,28	9,77 - 17,80	445
Tamat SLTA/MA	22,27	17,67 - 27,67	653
Tamat D1/D2/D3/PT	34,69	25,08 - 45,73	261
**Pekerjaan**			
Tidak bekerja	22,19	18,68 - 26,14	1.034
Sekolah	37,64	22,64 - 55,46	89
PNS/TNI/Polri/BUMN/BUMD	30,85	17,04 - 49,23	102
Pegawai swasta	22,94	13,02 - 37,20	175
Wiraswasta	18,49	13,27 - 25,16	373
Petani/buruh tani	11,32	8,79 - 14,45	627
Nelayan	3,74	1,35 - 9,93	23*
Buruh/sopir/pembantu ruta	15,65	10,20 - 23,25	281
Lainnya	21,55	13,54 - 32,53	144
**Tempat tinggal**			
Perkotaan	22,36	19,01 - 26,11	1.576
Perdesaan	15,57	13,31 - 18,13	1.273

*N Tertimbang <50

Tabel 6.7.5
Proporsi Kadar Kreatinin Serum pada Penduduk Umur ≥ 15 Tahun menurut Karakteristik, Riskesdas 2018

Karakteristik	Tinggi		N tertimbang
	%	95% CI	
**Kelompok umur**			
15-24	1,9	1,5 - 2,4	4.968
25-34	3,0	2,5 - 3,6	5.689
35-44	4,2	3,7 - 4,8	7.812
45-54	8,1	7,4 - 8,9	7.535
55-64	11,0	10,0 - 12,0	5.425
65-74	15,0	13,5 - 16,7	2.443
75+	18,9	16,3 - 21,9	948
**Jenis kelamin**			
Laki-laki	9,7	9,1 - 10,3	17.382
Perempuan	3,8	3,5 - 4,1	17.438
**Pendidikan**			
Tidak/belum pernah sekolah	7,7	6,6 - 9,0	2.347
Tidak tamat SD/MI	7,8	7,0 - 8,7	5.085
Tamat SD/MI	6,2	5,7 - 6,8	10.550
Tamat SLTP/MTS	5,0	4,4 - 5,7	6.872
Tamat SLTA/MA	6,9	6,3 - 7,6	7.973
Tamat D1/D2/D3/PT	10,5	9,1 - 12,3	1.993
**Pekerjaan**			
Tidak bekerja	6,3	5,8 - 6,8	11.183
Sekolah	2,0	1,4 - 2,9	1.738
PNS/TNI/Polri/BUMN/BUMD	12,3	9,5 - 15,8	515
Pegawai swasta	7,8	6,7 - 9,1	2.913
Wiraswasta	7,8	6,9 - 8,7	5.285
Petani/buruh tani	6,2	5,6 - 6,9	7.064
Nelayan	9,5	5,7 - 15,5	144
Buruh/sopir/pembantu ruta	7,3	6,3 - 8,3	4.133
Lainnya	7,9	6,6 - 9,6	1.845
**Tempat tinggal**			
Perkotaan	7,7	7,3 - 8,3	19.267
Perdesaan	5,5	5,1 - 5,9	15.553
**Indonesia**	**6,7**	**6,4 – 7,1**	**34.820**

Catatan: berdasarkan pemeriksaan darah

6.8 Penyakit Sendi

Penyakit sendi adalah gangguan nyeri pada persendian yang disertai kekakuan, merah, dan pembengkakan yang bukan disebabkan karena benturan/kecelakaan. Penyakit sendi yang dimaksud termasuk *osteoarthritis*, nyeri akibat asam urat yang tinggi/hiperurisemia akut maupun kronis, dan rematoid artritis. Prevalensi penyakit sendi berdasarkan diagnosis dokter dihitung dengan formula:

$$Prevalensi\ penyakit\ sendi\ berdasarkan\ diagnosis\ dokter = \frac{ART\ yang\ pernah\ didiagnosis\ penyakit\ sendi\ oleh\ dokter}{ART\ umur\ \geq 15\ th}$$

Tabel 6.8.1
Prevalensi Penyakit Sendi berdasarkan Diagnosis Dokter pada Penduduk Umur ≥15 Tahun menurut Provinsi, Riskesdas 2018

Provinsi	Penyakit sendi		N tertimbang
	%	95% CI	
Aceh	13,26	12,55 -14,00	13.389
Sumatera Utara	5,35	4,99 -5,74	36.410
Sumatera Barat	7,21	6,69 -7,77	13.834
Riau	7,10	6,56 -7,69	17.258
Jambi	8,67	7,94 -9,47	9.511
Sumatera Selatan	6,48	5,93 -7,07	22.013
Bengkulu	12,11	11,18 -13,10	5.175
Lampung	7,61	7,01 -8,26	22.171
Bangka Belitung	4,51	3,92 -5,18	3.915
Kepulauan Riau	5,45	4,62 -6,41	5.463
DKI Jakarta	6,76	6,15 -7,44	28.985
Jawa Barat	8,86	8,47 -9,26	131.846
Jawa Tengah	6,78	6,47 -7,10	96.794
DI Yogyakarta	5,93	5,37 -6,55	10.975
Jawa Timur	6,72	6,41 -7,04	113.045
Banten	6,15	5,66 -6,68	33.587
Bali	10,46	9,75 -11,21	12.092
Nusa Tenggara Barat	5,30	4,79 -5,87	13.036
Nusa Tenggara Timur	5,13	4,72 -5,58	12.777
Kalimantan Barat	9,57	8,85 -10,33	13.035
Kalimantan Tengah	7,61	6,89 -8,40	7.031
Kalimantan Selatan	4,79	4,32 -5,31	11.068
Kalimantan Timur	8,12	7,24 -9,10	9.696
Kalimantan Utara	5,63	4,79 -6,61	1.838
Sulawesi Utara	8,35	7,68 -9,08	6.827
Sulawesi Tengah	7,72	7,01 -8,51	7.847
Sulawesi Selatan	6,39	5,95 -6,85	23.069
Sulawesi Tenggara	5,63	5,03 -6,31	6.510
Gorontalo	6,85	6,08 -7,71	3.144
Sulawesi Barat	3,16	2,62 - 3,81	3.408
Maluku	5,08	4,43 -5,82	4.351
Maluku Utara	4,73	4,16 -5,38	3.005
Papua Barat	8,15	7,29 -9,11	2.363
Papua	10,43	9,33 -11,65	8.317
**INDONESIA**	**7,30**	**7,18 -7,42**	**713.783**

Tabel 6.8.2
Prevalensi Penyakit Sendi berdasarkan Diagnosis Dokter pada Penduduk Umur ≥15 Tahun menurut Karakteristik, Riskesdas 2018

Karakteristik	Penyakit sendi		N
	%	95% CI	tertimbang
**Kelompok Umur**			
15-24	1,23	1,15-1,32	159.015
25-34	3,10	2,96-3,26	153.317
35-44	6,27	6,07-6,48	145.474
45-54	11,08	10,80-11,37	119.664
55-64	15,55	15,17-15,94	79.919
65-74	18,63	18,04-19,22	38.572
75+	18,95	18,15-19,77	17.822
**Jenis kelamin**			
Laki-laki	6,13	5,99 -6,27	355.726
Perempuan	8,46	8,30 - 8,61	358.057
**Pendidikan**			
Tidak/belum pernah sekolah	13,66	13,13 -14,21	40.430
Tidak tamat SD/MI	12,00	11,65 -12,36	83.024
Tamat SD/MI	9,56	9,32 -9,81	174.012
Tamat SLTP/MTS	5,06	4,89 -5,24	151.414
Tamat SLTA/MA	4,49	4,35 -4,64	203.128
Tamat D1/D2/D3/PT	5,13	4,87 -5,39	61.776
**Pekerjaan**			
Tidak bekerja	9,12	8,92 -9,34	209.274
Sekolah	1,09	0,97 -1,24	57.440
PNS/TNI/Polri/BUMN/BUMD	7,46	7,00 -7,95	21.429
Pegawai swasta	3,53	3,30 -3,77	73.962
Wiraswasta	7,31	7,05 -7,58	102.968
Petani/buruh tani	9,86	9,60 -10,13	130.042
Nelayan	7,36	6,42 -8,43	5.402
Buruh/sopir/pembantu ruta	6,12	5,82 -6,45	73.720
Lainnya	7,31	6,93 -7,70	39.546
**Tempat tinggal**			
Perkotaan	6,87	6,71 -7,04	394.850
Perdesaan	7,83	7,66 -8,00	318.933

BAB 7
KESEHATAN GIGI DAN MULUT

7.1 Kesehatan Gigi dan Mulut Berdasarkan Wawancara

Mengidentifikasi responden yang mempunyai masalah dengan gigi/mulut dalam 12 bulan terakhir, dan menentukan apakah mereka menerima perawatan yang tepat sesuai dengan masalah yang dihadapi.

Gigi yang rusak, berlubang atau cenderung menyebabkan sakit (menurut pendapat masyarakat awam) adalah rongga pada gigi yang rusak secara permanen di wilayah permukaan keras gigi yang berkembang mulai dari lubang kecil sampai menjadi lubang yang merusak gigi. **Pengertian gigi berlubang**, biasa juga disebut **masalah gigi berlubang** karena kerusakan gigi atau karies gigi, dimana hal tersebut disebabkan oleh kombinasi dari berbagai faktor.

Gigi yang hilang karena dicabut atau tanggal sendiri adalah hilangnya gigi dari rongga mulut secara sengaja ataupun tidak sengaja.

Gigi yang ditambal atau ditumpat karena berlubang adalah bentuk perawatan terhadap gigi berlubang berupa penutupan lubang gigi dengan bahan tambal setelah jaringan gigi yang rusak dibersihkan. Pertanyaan ini bertujuan untuk mengidentifikasi responden yang mempunyai masalah dengan gigi/mulut dalam 12 bulan terakhir, dan apakah mereka menerima perawatan yang tepat sesuai dengan masalah yang dihadapi.

Gigi goyah adalah kondisi gigi yang dapat goyah baik digerakkan oleh tangan ataupun ketika mengunyah.

Gusi bengkak dan/atau keluar bisul (abses) adalah benjolan atau bisul pada gusi, disertai dengan ada atau tidaknya jalan keluar nanah, terjadi karena peradangan menahun (kronis) dan pernanahan pada daerah akar gigi (*periapical abcess*). Asal mula penyakit ini adalah terjadinya karies (lubang) pada gigi, biasanya pada gigi geraham, namun bisa saja terjadi pada semua gigi.

Gusi mudah berdarah adalah keadaan gusi yang mudah berdarah, seperti pada saat menyikat gigi atau saat menggigit.

Sariawan berulang minimal 4 kali adalah Sariawan atau *Stomatitis Apthousa Recurrent* (SAR) merupakan istilah untuk menerangkan berbagai macam lesi/luka dalam rongga mulut. Luka tersebut dapat berbentuk oval atau bulat berwarna putih

atau kuning dengan tepian merah. Lokasi sariawan dapat terjadi di bagian dalam pipi atau bibir, permukaan gusi dan lidah. Sariawan yang tumbuh dapat berjumlah satu atau lebih. Gejalanya berupa rasa sakit atau rasa terbakar satu sampai dua hari yang kemudian dapat timbul luka (ulser) di rongga mulut. Rasa sakit dan rasa panas pada sariawan ini sering mengganggu dan tidak nyaman, terutama ketika makan. Sariawan ini dapat timbul berulang kali, biasanya dalam 3-4 hari akan sembuh.

Sariawan menetap dan tidak pernah sembuh minimal 1 bulan adalah sariawan di dalam rongga mulut yang tidak pernah kunjung sembuh, sakit selama lebih dari 2 minggu, bentuknya kadang semakin membesar serta muncul rasa terbakar di rongga mulut, sering disertai rasa lemah pada penderitanya.

Riwayat responden yang melakukan pemeriksaan dan perawatan pada tenaga medis gigi, dengan alasan apapun di semua jenis pelayanan kesehatan (RS, Puskesmas, klinik swasta, praktek mandiri, kerja sosial kesehatan atau bakti sosial).

Masalah kesehatan gigi/mulut dan telah mendapat perawatan profesional dari perawat gigi, dokter gigi atau dokter gigi spesialis dalam 12 bulan terakhir. Maksudnya adalah untuk menentukan jenis perawatan atau pengobatan yang diterima. Hal ini menggambarkan proporsi penduduk dengan masalah gigi dan mulut yang menerima perawatan dari tenaga medis gigi dalam 12 bulan terakhir. Meliputi beberapa hal sebagai berikut:

- **Pengobatan/minum obat** adalah pengobatan yang berupa pil, sirup atau bentuk obat lainnya yang diresepkan/diberikan langsung oleh tenaga profesional kedokteran gigi untuk mengobati masalah gigi, mulut, lidah ataupun gusi, **tidak termasuk pengobatan tradisional**.
- **Konseling perawatan, kebersihan, dan kesehatan gigi dan mulut** adalah kegiatan penyuluhan tentang perawatan kebersihan gigi dan mulut, tanpa dilakukan tindakan klinis terhadap gigi dan mulut.
- **Pencabutan gigi** adalah tindakan menghilangkan gigi dari rongga mulut
- **Bedah gigi/mulut** adalah tindakan bedah dalam area rongga mulut dan sekitarnya, seperti pada kasus gigi geraham bungsu yang terbenam (*impacted*), gigi Molar tiga (3) malposisi, tumor lidah, patah rahang, dan lain-lain yang memerlukan tindakan bedah.
- **Pemasangan gigi palsu** adalah gigi tiruan, dapat sebagian (beberapa gigi) ataupun seluruh gigi, yang dapat dilepas dan dipasang sendiri oleh responden, atau gigi palsu yang tidak bisa dilepas sendiri oleh responden dan hanya bisa dilepas oleh tenaga profesional kedokteran gigi.

- **Pemasangan gigi tanam (*Implant denture*)** adalah gigi tiruan yang ditanam pada tulang rahang responden dan tidak bisa dilepas sendiri oleh responden, dilakukan melalui prosedur bedah, dan hanya dapat dipasang atau dilepas oleh dokter gigi.
- **Perawatan orthodonti (behel/kawat gigi)** adalah perawatan untuk merapikan gigi berjejal dengan menggunakan alat orthodonti.
- **Pembersihan karang gigi (*scalling*)** adalah tindakan untuk membersihkan karang gigi dengan menggunakan alat manual atau alat elektrik.
- **Perawatan gusi (*Periodontal treatment*)** adalah perawatan jaringan penyangga gigi (yang mengalami gingivitis, periodontitis dll).

Pengobat masalah kesehatan gigi dan mulut adalah orang yang dicari dan dianggap mampu untuk mengatasi masalah kesehatan gigi dan mulut oleh responden, meliputi:

- **Dokter gigi spesialis** adalah tenaga profesional kedokteran gigi yang sudah mendapatkan sertifikat spesialisasi tertentu dalam bidang kedokteran gigi.
- **Dokter gigi** adalah adalah tenaga profesional kedokteran gigi yang sudah mendapatkan sertifikat sebagai dokter gigi.
- **Perawat gigi** adalah tenaga profesional kedokteran gigi yang sudah mendapatkan sertifikat sebagai perawat gigi.
- **Dokter umum/paramedik lain** adalah tenaga profesional kedokteran yang sudah mendapatkan sertifikat sebagai dokter umum atau perawat umum.
- **Tukang gigi** adalah tenaga non-profesional atau non-formal dalam memberikan pelayanan pembuatan gigi tiruan lepasan, tetapi sebagian masyarakat mempercayainya dapat memberikan pelayanan untuk mengatasi masalah gigi dan mulut.
- **Pengobatan sendiri** adalah tindakan untuk menyembuhkan penyakit yang dilakukan oleh responden sendiri tanpa adanya pengawasan tenaga profesional.

Tabel 7.1.1
Proporsi Masalah Gigi menurut Provinsi, Riskesdas 2018

Provinsi	Gigi rusak/berlubang/sakit		Gigi hilang karena dicabut/tanggal sendiri		Gigi telah ditambal atau ditumpat karena berlubang		Gigi goyah		N tertimbang
	%	95%CI	%	95%CI	%	95%CI	%	95%CI	
Aceh	47,0	46,0-48,0	19,8	19,1-20,6	4,6	4,2-5,1	9,3	8,9-9,8	18.855
Sumatera Utara	43,1	42,1-44,1	17,7	17,0-18,5	2,7	2,4-3,0	10,1	9,6-10,6	51.977
Sumatera Barat	43,9	42,8-44,9	19,6	18,9-20,4	3,5	3,2-3,9	10,4	9,9-11,0	19.399
Riau	45,6	44,4-46,8	19,6	18,8-20,5	3,3	3,0-3,6	11,9	11,2-12,7	24.405
Jambi	37,7	36,1-39,4	15,6	14,5-16,7	2,7	2,3-3,1	6,0	5,5-6,6	12.936
Sumatera Selatan	45,1	43,5-46,7	17,9	17,0-18,9	3,0	2,7-3,4	7,5	6,9-8,1	30.341
Bengkulu	44,0	42,5-45,5	18,0	17,0-19,1	2,7	2,3-3,0	7,9	7,2-8,6	7.123
Lampung	47,2	45,9-48,5	17,3	16,4-18,2	2,6	2,4-2,9	7,8	7,3-8,3	30.300
Bangka Belitung	45,9	44,1-47,6	19,2	18,1-20,4	2,5	2,1-2,9	11,3	10,4-12,2	5.301
Kepulauan Riau	36,5	34,3-38,8	13,0	11,7-14,3	3,2	2,4-4,1	8,0	6,8-9,3	7.679
DKI Jakarta	41,3	39,8-42,7	18,4	17,4-19,4	7,0	6,4-7,6	11,9	11,1-12,9	38.124
Jawa Barat	45,7	44,9-46,4	19,9	19,3-20,4	4,8	4,6-5,1	10,7	10,3-11,2	176.728
Jawa Tengah	43,4	42,8-44,1	17,3	16,9-17,8	3,5	3,3-3,7	11,1	10,8-11,5	126.225
DI Yogyakarta	47,7	46,2-49,1	20,0	19,1-21,0	5,5	4,9-6,1	15,8	14,8-16,8	13.989
Jawa Timur	42,4	41,8-43,1	18,0	17,5-18,5	3,8	3,6-4,0	8,5	8,2-8,8	145.173
Banten	48,5	47,1-49,8	19,7	18,8-20,8	5,4	5,0-5,9	11,5	10,7-12,3	45.831
Bali	41,1	39,9-42,3	18,0	17,1-18,9	6,4	5,9-7,0	10,2	9,6-10,9	15.737
Nusa Tenggara Barat	41,4	40,1-42,7	19,8	18,7-20,8	4,2	3,8-4,6	11,9	11,1-12,6	18.058
Nusa Tenggara Timur	43,9	42,9-44,8	18,2	17,5-18,9	1,8	1,6-2,0	12,2	11,6-12,8	19.115
Kalimantan Barat	49,6	48,3-50,8	22,3	21,3-23,3	3,8	3,5-4,2	9,6	9,0-10,2	18.073
Kalimantan Tengah	42,6	40,9-44,2	15,7	14,8-16,6	3,9	3,6-4,4	7,9	7,2-8,7	9.615
Kalimantan Selatan	46,9	45,6-48,2	17,8	16,9-18,8	7,0	6,4-7,6	8,7	8,1-9,3	15.077
Kalimantan Timur	48,0	46,5-49,6	19,1	17,9-20,3	6,0	5,5-6,6	9,8	9,1-10,6	13.195
Kalimantan Utara	54,4	52,1-56,7	21,5	19,7-23,5	6,6	5,9-7,4	11,0	9,8-12,4	2.547
Sulawesi Utara	55,5	54,1-56,9	19,9	19,0-21,0	2,6	2,3-2,9	13,3	12,5-14,1	9.055
Sulawesi Tengah	60,4	59,2-61,5	24,2	23,2-25,3	3,4	3,0-3,8	15,4	14,5-16,3	10.837
Sulawesi Selatan	55,5	54,6-56,4	24,5	23,8-25,2	4,0	3,7-4,3	12,0	11,4-12,6	31.703
Sulawesi Tenggara	51,9	50,6-53,3	23,3	22,2-24,4	4,5	4,0-5,1	11,1	10,3-11,9	9.464
Gorontalo	51,1	49,2-53,0	22,9	21,5-24,3	2,7	2,3-3,2	12,1	11,0-13,2	4.286
Sulawesi Barat	56,2	54,3-58,2	20,3	19,0-21,7	2,8	2,4-3,4	8,0	7,0-9,0	4.831
Maluku	56,3	54,7-57,8	24,6	22,9-26,4	3,3	2,9-3,9	12,6	11,5-13,7	6.334
Maluku Utara	47,9	46,1-49,7	19,5	18,3-20,8	2,9	2,5-3,4	15,0	13,8-16,4	4.404
Papua Barat	53,7	51,7-55,7	21,9	20,3-23,6	3,9	3,3-4,6	12,7	11,5-14,0	3.341
Papua	40,9	39,2-42,6	21,5	20,3-20,3	3,3	2,9-3,7	11,2	10,3-12,1	11.987
**Indonesia**	**45,3**	**45,0-45,5**	**19,0**	**18,8-19,2**	**4,1**	**4,0-4,2**	**10,4**	**10,2-10,5**	**956.045**

Tabel 7.1.2
Proporsi Masalah Gigi menurut Karakteristik, Riskesdas 2018

Karakteristik	Gigi rusak, berlubang ataupun sakit		Gigi hilang karena dicabut atau tanggal sendiri		Gigi telah ditambal atau ditumpat karena berlubang		Gigi goyah		N ter- timbang
	%	95%CI	%	95%CI	%	95%CI	%	95%CI	
**Kelompok Umur**									
3 - 4	36,4	35,6-37,1	8,0	7.6-8,5	1,1	0,9-1,3	3,6	3,3-3,8	36.608
5 - 9	54,0	53,5-54,5	33,2	32,7-33,7	3,0	2,8-3,1	21,7	21,3-22,2	92.746
10 -14	41,4	40,9- 41,9	20,0	19,6-20,5	3,0	2,8-3,1	13,0	12,6-13,3	89.506
15 - 24	38,1	37,7- 38,6	8,4	8,1-8,6	4,1	3,9-4,2	4,7	4,5-4,9	165.565
25 - 34	44,9	44,4-45,3	12,1	11,9-12,4	5,0	4,8-5,2	5,4	5,2-5,6	159.633
35 - 44	48,8	48,3-49,2	17,5	17,2-17,8	5,0	4,8-5,2	8,2	8,0-8,4	151.467
45 - 54	50,8	50,3-51,2	23,6	23,2-24,0	4,9	4,7-5,1	12,5	12,2-12,7	124.593
55 - 64	48,5	47,9-49,0	29,0	28,5-29,5	4,2	4,0-4,4	15,9	15,5-16,3	83.211
65 +	38,6	38,0-39,2	30,6	30,0-31,2	3,1	2,9-3,3	15,5	15,1-16,0	58.717
**Kelompok umur (WHO)**									
5	51,2	50,1-52,2	16,9	16,1-17,	2,4	2,1-2,8	9,9	9,3-10,6	19.529
12	39,9	38,8-40,9	18,4	17,6-19,3	3,0	2,7-3,5	11,7	11,0-12,4	17980
15	36,2	35,2-37,2	8,6	8,0-9,2	3,4	3,0-3,9	5,7	5,2-6,2	19.390
35-44	48,8	48,3-49,2	17,5	17,2-17,8	5,0	4,8-5,2	8,2	8,0-8,4	157.550
≥65	38,6	38,0-39,2	30,6	30,0-31,2	3,1	2,9-3,3	15,5	15,1-16,0	61.076
**Jenis kelamin**									
Laki – laki	44,8	44,5-45,1	19,0	18,8-19,2	3,6	3,5-3,7	10,8	10,6-10,9	482.729
Perempuan	45,7	45,4-46,0	19,0	18,8-19,2	4,6	4,5-4,7	10,0	9,8-10,1	479.316
**Pendidikan**									
Tidak sekolah	48,0	47,4-48,6	29,5	28,9-30,0	3,0	2,8-3,2	17,0	16,6-17,4	70.895
Tidak tamat SD/MI	49,2	48,8-49,6	28,6	28,2-28,9	3,1	2,9-3,2	17,5	17,2-17,8	181.429
Tamat SD/MI	46,6	46,2-47,0	19,5	19,2-19,7	3,1	3,0-3,3	10,3	10,1-10,5	215.967
Tamat SMP/MTS	44,5	44,0-44,9	14,3	14,0-14,6	3,8	3,6-3,9	7,4	7,2-7,7	160.320
Tamat SMA/MA	42,9	42,5-43,3	13,9	13,7-14,2	5,5	5,3-5,7	6,9	6,7-7,0	210.746
Tamat D1-D3/PT	39,6	39,0-40,2	14,1	13,6-14,5	9,9	9,5-10,3	5,9	5,7-6,2	64.093
**Pekerjaan**									
Tidak bekerja	44,1	43,7-44,5	18,6	18,3-18,9	4,4	4,2-4,5	9,3	9,1-9,5	233.629
Sekolah	39,4	38,9-39,8	14,4	14,1-14,7	3,6	3,5-3,8	9,3	9,0-9,5	126.626
PNS/TNI/Polri/BUMN/BUMD	41,8	40,9-42,7	17,7	17,0-18,4	9,1	8,5-9,6	7,7	7,3-8,2	21.931
Pegawai swasta	41,1	40,4-41,8	12,4	12,0-12,9	6,8	6,5-7,1	6,2	5,8-6,5	75.781
Wiraswasta	46,0	45,5-46,5	17,8	17,5-18,2	5,0	4,8-5,3	9,1	8,8-9,4	105.489
Petani/buruh tani	50,0	49,5-50,4	23,1	22,8-23,5	2,7	2,6-2,9	11,8	11,6-12,1	133.261
Nelayan	53,9	52,1-55,7	22,3	20,9-23,7	3,1	2,6-3,8	13,4	12,3-14,7	5.556
Buruh/sopir/pembantu ruta	47,3	46,6-48,0	17,5	17,0-17,9	3,4	3,1-3,6	9,6	9,3-10,0	75.590
Lainnya	45,8	45,1-46,6	18,4	17,8-19,0	5,0	4,6-5,3	8,8	8,4-9,3	40.644
**Tempat tinggal**									
Perkotaan	43,5	43,2-43,9	18,5	18,2-18,8	5,2	5,1-5,3	10,4	10,2-10,6	526.861
Perdesaan	47,3	47,0-47,0	19,6	19,4-19,8	2,8	2,7-2,9	10,4	10,2-10,5	435.184

Tabel 7.1.3

Proporsi Masalah Kesehatan Mulut pada Penduduk Umur >3 Tahun menurut Provinsi, Riskesdas 2018

Provinsi	Gusi bengkak dan/atau keluar bisul (abses)		Gusi mudah berdarah (seperti saat menyikat gigi)		Sariawan berulang minimal 4x		Sariawan menetap dan tidak pernah sembuh, minimal 1 bulan		N ter-timbang
	%	95%CI	%	95%CI	%	95%CI	%	95%CI	
Aceh	11,1	10,5-11,7	13,8	13,1-14,5	7,9	7,3-8,4	1,0	0,9-1,2	18.855
Sumatera Utara	11,8	11,1-12,5	15,6	14,8-16,4	9,1	8,4-9,7	1,0	0,9-1,2	51.977
Sumatera Barat	15,2	14,5-16,0	17,1	16,3-17,9	11,6	10,9-12,4	1,0	0,8-1,1	19.399
Riau	15,1	14,3-16,0	16,4	15,5-17,4	9,8	9,1-10,5	0,9	0,8-1,1	24.405
Jambi	7,8	7,1-8,7	6,2	5,5-6,9	6,7	5,9-7,6	0,9	0,6-1,2	12.936
Sumatera Selatan	9,3	8,7-10,0	7,7	7,1-8,2	5,2	4,7-5,8	0,9	0,7-1,3	30.341
Bengkulu	12,4	11,6-13,3	10,5	9,7-11,4	11,0	9,9-12,1	1,2	1,0-1,5	7.123
Lampung	11,2	10,6-12,0	8,6	8,0-9,3	6,0	5,5-6,6	0,7	0,6-0,8	30.300
Bangka Belitung	14,3	13,2-15,5	14,8	13,8-15,9	6,9	6,1-7,8	1,0	0,9-1,3	5.301
Kepulauan Riau	9,8	8,6-11,2	11,2	9,8-12,8	6,4	5,4-7,5	0,7	0,5-1,0	7.679
DKI Jakarta	15,4	14,4-16,5	16,0	14,9-17,2	7,6	6,8-8,4	1,0	0,8-1,3	38.124
Jawa Barat	15,4	14,8-16,0	13,8	13,3-14,4	7,6	7,2-8,0	0,9	0,8-1,1	176.728
Jawa Tengah	13,9	13,6-14,3	11,6	11,2-12,0	7,3	6,9-7,6	0,8	0,7-0,9	126.225
DI Yogyakarta	15,4	14,5-16,4	17,1	16,0-18,3	8,7	8,0-9,5	0,8	0,7-1,0	13.989
Jawa Timur	11,5	11,2-11,9	10,5	10,1-10,9	7,1	6,7-7,4	0,7	0,6-0,7	145.173
Banten	17,5	16,5-18,5	17,0	16,1-18,0	10,8	9,9-11,7	1,0	0,9-1,3	45.831
Bali	14,0	13,2-14,9	14,6	13,6-15,6	10,0	9,3-10,9	0,7	0,6-0,9	15.737
Nusa Tenggara Barat	11,4	10,6-12,2	15,5	14,6-16,6	8,3	7,5-9,1	0,8	0,6-1,0	18.058
Nusa Tenggara Timur	15,7	15,0-16,5	17,4	16,6-18,3	6,4	6,0-6,9	0,8	0,6-0,9	19.115
Kalimantan Barat	15,6	14,7-16,5	16,2	15,3-17,2	9,9	9,2-10,7	0,8	0,7-1,0	18.073
Kalimantan Tengah	12,9	11,9-13,9	11,2	10,2-12,2	9,0	8,1-9,9	0,8	0,6-1,0	9.615
Kalimantan Selatan	15,3	14,5-16,1	14,2	13,3-15,1	6,6	6,0-7,2	0,8	0,7-1,0	15.077
Kalimantan Timur	15,3	14,2-16,5	14,6	13,4-15,8	7,3	6,6-8,1	0,8	0,6-1,0	13.195
Kalimantan Utara	12,9	11,5-14,6	14,2	12,5-16,2	5,6	4,6-6,7	0,9	0,6-1,3	2.547
Sulawesi Utara	18,2	17,2-19,3	20,4	19,3-21,5	10,4	9,6-11,2	1,3	1,1-1,6	9.055
Sulawesi Tengah	23,4	22,4-24,5	28,1	26,9-29,3	14,2	13,3-15,0	1,5	1,3-1,7	10.837
Sulawesi Selatan	17,5	16,9-18,2	22,7	21,9-23,5	9,9	9,4-10,4	1,0	0,9-1,2	31.703
Sulawesi Tenggara	14,8	13,7-16,0	19,4	18,1-20,7	9,6	8,9-10,5	1,1	0,9-1,3	9.464
Gorontalo	19,6	18,0-21,2	20,1	18,3-22,1	10,4	9,3-11,6	1,4	1,1-1,8	4.286
Sulawesi Barat	15,5	14,0-17,2	17,3	15,6-19,1	10,2	9,1-11,4	1,1	0,8-1,4	4.831
Maluku	15,2	13,9-16,7	21,4	19,9-23,0	7,9	6,9-9,0	0,9	0,7-1,1	6.334
Maluku Utara	16,7	15,5-17,9	21,3	19,6-23,1	6,9	6,2-7,7	1,2	0,9-1,4	4.404
Papua Barat	15,8	14,7-17,0	23,3	21,8-24,9	7,4	6,3-8,7	0,7	0,5-1,0	3.341
Papua	11,2	10,3-12,25	10,8	9,9-11,8	4,3	3,8-5,0	0,7	0,5-0,8	11.987
**INDONESIA**	**14,0**	**13,8-14,1**	**13,9**	**13,7-14,1**	**8,0**	**7,8-8,1**	**0,9**	**0,8-0,9**	**956.993**

Tabel 7.1.4
Proporsi Masalah Kesehatan Mulut pada Penduduk Umur >3 Tahun menurut Karakteristik, Riskesdas 2018

Karakteristik	Gusi bengkak dan/atau keluar bisul (abses)		Gusi mudah berdarah (seperti saat menyikat gigi)		Sariawan berulang minimal 4x		Sariawan menetap dan tidak pernah sembuh, minimal 1 bulan		N Tertimbang
	%	95%CI	%	95%CI	%	95%CI	%	95%CI	
**Kelompok Umur**									
3 - 4	5,5	5,1-5,8	2,8	2,6-3,1	3,7	3,4-4,0	0,3	0,2-0,4	36.608
5 - 9	11,0	10,7-11,3	9,6	9,3-9,9	6,3	6,1-6,6	0,7	0,6-0,8	92.746
10 -14	11,3	11,0-11,6	14,3	14,0-14,7	8,3	8,0-8,6	0,8	0,7-0,9	89.506
15 - 24	14,6	14,3-14,9	19,0	18,6-19,4	9,6	9,3-9,9	0,9	0,9-1,0	165.565
25 - 34	15,8	15,4-16,1	17,8	17,4-18,1	9,0	8,7-9,3	0,9	0,8-1,0	159.633
35 - 44	16,6	16,2-16,9	15,4	15,1-15,7	8,6	8,4-8,9	1,0	0,9-1,1	151.467
45 - 54	16,6	16,2-16,9	13,0	12,7-13,3	8,0	7,8-8,3	1,0	0,9-1,1	124.593
55 - 64	14,1	13,7-14,5	9,9	9,6-10,3	6,9	6,7--7,2	1,1	1,0-1,2	83.211
65 +	8,9	8,5-9,2	5,5	5,2-5,8	4,9	4,7-5,2	0,7	0,6-0,8	58.717
**Kelompok umur (WHO)**									
5	9,5	8,9-10,2	5,8	5,3-6,3	5,0	4,5-5,5	0,6	0,4-0,8	19.529
12	11,0	10,4-11,7	14,3	13,6-15,1	8,4	7,8-9,1	1,0	0,8-1,3	17.980
15	12,3	11,6-13,15	17,2	16,4-18,1	9,1	8,5-9,8	0,7	0,6-0,9	19.390
35-44	16,6	16,2-16,9	15,4	15,1-15,7	8,6	8,4-8,9	1,0	0,9-1,1	157.550
≥65	8,9	8,5-9,2	5,5	5,2-5,8	4,9	4,7-5,2	0,7	0,6-0,8	61.076
**Jenis kelamin**									
Laki – laki	13,7	13,5-13,9	13,5	13,3-13,7	7,4	7,3-7,6	0,8	0,8-0,9	482.729
Perempuan	14,3	14,1-14,4	14,2	14,0-14,4	8,5	8,4-8,7	0,9	0,9-1,0	479.316
**Pendidikan**									
Tidak sekolah	11,6	11,3-12,0	9,9	9,6-10,3	6,6	6,3-6,9	1,0	0,9-1,1	70.895
Tidak tamat SD/MI	13,6	13,3-13,8	13,0	12,8-13,3	7,8	7,6-8,0	0,9	0,9-1,0	181.429
Tamat SD/MI	15,4	15,1-15,7	14,6	14,3-14,9	8,6	8,4-8,9	1,0	0,9-1,0	215.967
Tamat SMP/MTS	15,6	15,3-15,9	16,4	16,1-16,7	9,0	8,7-9,2	0,9	0,9-1,0	160.320
Tamat SMA/MA	14,9	14,6-15,2	15,9	15,6-16,2	8,4	8,1-8,6	0,9	0,8-0,9	210.746
Tamat D1-D3/PT	12,0	11,6-12,4	14,0	13,5-14,5	7,1	6,8-7,5	0,6	0,6-0,7	64.093
**Pekerjaan**									
Tidak bekerja	14,4	14,1-14,7	14,5	14,3-14,8	8,5	8,3-8,8	1,0	0,9-1,1	233.629
Sekolah	12,6	12,3-12,9	16,5	16,1-16,8	8,8	8,5-9,1	0,8	0,7-0,9	126.626
PNS/TNI/Polri/BUMN/BUMD	11,6	11,1-12,2	11,8	11,2-12,4	6,2	5,8-6,6	0,6	0,5-0,7	21.931
Pegawai swasta	14,3	13,8-14,8	15,4	14,9-15,9	8,0	7,6-8,4	0,7	0,6-0,9	75.781
Wiraswasta	15,6	15,2-16,0	13,9	13,5-14,3	8,6	8,3-8,9	1,0	0,9-1,1	105.489
Petani/buruh tani	15,5	15,3-15,8	14,2	13,9-14,5	7,9	7,7-8,1	1,0	1,0-1,1	133.261
nelayan	19,4	17,9-21,0	19,2	17,7-20,8	9,1	8,1-10,3	0,9	0,6-1,2	5.556
Buruh/sopir/pembantu ruta	16,8	16,3-17,4	15,3	14,8-15,8	8,0	7,6-8,4	0,9	0,8-1,0	75.590
Lainnya	15,1	14,6-15,7	15,5	14,9-16,0	9,0	8,5-9,4	1,0	0,9-1,2	40.644
**Tempat tinggal**									
Perkotaan	13,7	13,5-14,0	13,6	13,3-13,8	7,8	7,6-8,1	0,9	0,8-0,9	526.861
Perdesaan	14,3	14,1-14,5	14,3	14,1-14,5	8,1	8,0-8,3	0,9	0,9-1,0	435.183

Tabel 7.1.5

Proporsi Tindakan untuk Mengatasi Masalah Gigi dan Mulut menurut Provinsi, Riskesdas 2018

Provinsi	Jenis tindakan yang diterima untuk mengatasi masalah gigi & mulut										N Tertimbang
	Pengobatan/ minum obat		Konseling perawatan kebersihan dan kesehatan gigi & mulut		Penumpatan/p enambalan		Pencabutan gigi		Bedah mulut		
	%	95%CI	%	95%CI	%	95%CI	%	95%CI	%	95%CI	
Aceh	64,7	63,3-66,0	6,4	5,8-7,1	4,0	3,5-4,5	9,7	9,0-10,4	0,3	0,3-0,5	10.480
Sumatera Utara	55,1	53,6-56,6	3,1	2,8-3,5	2,2	1,9-2,5	6,4	6,0-6,9	0,1	0,1-0,25	28.482
Sumatera Barat	47,3	45,7-48,9	5,2	4,7-5,7	3,3	2,9-3,8	7,4	6,9-8,1	0,3	0,2-0,5	11.401
Riau	52,9	51,3-54,5	4,9	4,3-5,5	3,1	2,8-3,5	8,2	7,6-8,9	0,2	0,2-0,3	14.403
Jambi	70,3	68,1-72,5	4,4	3,8-5,1	3,1	2,6-3,8	12,4	11,3-13,7	0,3	0,1-0,4	5.848
Sumatera Selatan	48,0	46,0-50,0	3,5	3,0-4,1	2,2	1,8-2,6	6,1	5,5-6,8	0,3	0,2-0,4	15.972
Bengkulu	56,7	54,4-59,0	2,3	1,9-2,8	2,2	1,8-2,7	6,4	5,7-7,3	0,2	0,1-0,3	3.951
Lampung	51,0	49,1-52,8	2,7	2,4-3,1	1,8	1,4-2,2	5,6	5,1-6,2	0,2	0,1-0,3	17.114
Bangka Belitung	52,5	49,9-55,1	3,7	3,1-4,4	2,6	2,2-3,2	16,6	15,2-18,1	0,2	0,1-0,3	3.129
Kepulauan Riau	46,4	43,2-49,6	6,5	5,2-8,1	4,2	3,0-5,7	11,9	10,0-14,1	0,3	0,1-0,7	3.734
DKI Jakarta	46,0	44,2-47,9	12,3	11,3-13,4	9,6	8,7-10,6	9,6	8,8-10,5	0,6	0,4-0,9	22.642
Jawa Barat	58,4	57,3-59,5	8,7	8,3-9,2	5,2	4,8-5,5	7,6	7,2-8,0	0,3	0,2-0,4	102.936
Jawa Tengah	47,3	46,4-48,1	6,6	6,3-6,9	3,7	3,5-3,9	5,2	4,9-5,4	0,2	0,2-0,3	71.83
DI Yogyakarta	35,4	33,9-37,0	14,0	12,8-15,2	6,4	5,6-7,3	8,6	7,8-9,5	0,6	0,4-0,9	9.216
Jawa Timur	54,3	53,4-55,3	7,1	6,8-7,5	4,6	4,3-4,8	7,8	7,4-8,1	0,2	0,2-0,3	79.064
Banten	53,8	51,9-55,8	8,1	7,3-8,8	4,9	4,4-5,5	6,6	6,0-7,2	0,3	0,2-0,4	28.895
Bali	39,5	37,9-41,2	10,9	10,0-12,0	8,9	8,2-9,7	11,1	10,2-12,0	0,3	0,2-0,5	9.238
Nusa Tenggara Barat	50,3	48,5-52,1	7,7	6,9-8,5	4,1	3,6-4,7	6,7	6,0-7,4	0,2	0,1-0,3	10.085
Nusa Tenggara Timur	46,2	44,7-47,6	4,1	3,7-4,6	1,6	1,3-1,9	3,8	3,5-4,3	0,1	0,1-0,2	10.547
Kalimantan Barat	54,6	52,9-56,2	4,7	4,1-5,2	3,3	2,9-3,8	8,9	8,2-9,8	0,4	0,2-0,5	11.040
Kalimantan Tengah	65,0	63,0-66,9	4,8	4,2-5,4	4,4	3,8-5,1	6,7	6,0-7,6	0,1	0,1-0,3	5.184
Kalimantan Selatan	61,6	59,8-63,3	6,6	5,9-7,4	7,3	6,5-8,1	8,5	7,9-9,1	0,1	0,1-0,2	9.025
Kalimantan Timur	48,7	46,8-50,7	8,1	7,2-9,0	6,7	5,9-7,4	10,3	9,4-11,3	0,3	0,2-0,5	8.153
Kalimantan Utara	46,8	44,1-49,5	8,6	7,2-10,2	7,5	6,6-8,6	12,6	11,3-14,1	0,4	0,2-0,7	1.620
Sulawesi Utara	59,0	57,2-60,8	3,9	3,4-4,4	1,9	1,6-2,2	7,5	6,9-8,2	0,3	0,2-0,4	6.045
Sulawesi Tengah	60,5	59,0-62,0	2,7	2,3-3,1	2,1	1,8-2,5	10,5	9,8-11,3	0,1	0,1-0,2	8.005
Sulawesi Selatan	47,0	45,6-48,4	4,9	4,5-5,3	3,6	3,3-3,9	14,7	14,0-15,4	0,3	0,2-0,4	21.925
Sulawesi Tenggara	52,8	50,8-54,8	4,1	3,5-4,7	3,6	3,1-4,3	16,8	15,6-18,2	0,2	0,1-0,3	6.023
Gorontalo	50,8	47,9-53,7	4,1	3,3-5,0	1,6	1,3-2,1	5,8	4,9-7,0	0,2	0,1-0,5	2.743
Sulawesi Barat	53,7	51,1-56,2	2,4	1,9-3,1	2,1	1,7-2,7	11,0	9,8-12,4	0,2	0,1-0,4	3.140
Maluku	61,4	58,8-63,9	2,2	1,8-2,7	2,0	1,5-2,6	8,4	7,2-9,8	0,2	0,1-0,3	4.244
Maluku Utara	60,8	58,2-63,3	3,2	2,7-3,9	2,3	1,8-2,8	6,1	5,3-7,1	0,3	0,2-0,5	2.602
Papua Barat	50,7	48,1-53,3	4,0	3,2-5,1	2,3	1,8-3,0	6,8	5,7-8,1	0,2	0,1-0,5	2.199
Papua	50,3	47,7-52,9	6,7	5,7-7,9	3,5	2,9-4,1	13,5	11,8-15,3	0,3	0,2-0,5	6.007
**INDONESIA**	**52,9**	**52,5-53,2**	**6,7**	**6,6-6,8**	**4,3**	**4,2-4,4**	**7,9**	**7,8-8,0**	**0,3**	**0,2-0,3**	**556.921**

Tabel 7.1.5 (Lanjutan)
Proporsi Tindakan untuk Mengatasi Masalah Gigi dan Mulut menurut Provinsi, Riskesdas 2018

Provinsi	Jenis tindakan yang diterima untuk mengatasi masalah gigi dan mulut										N Tertimbang
	Pemasangan gigi palsu		Pemasangan gigi tanam (implant denture)		Perawatan orthodonti (behel/kawat gigi)		Pembersihan karang gigi (scaling)		Perawatan gusi/periodontal treatment		
	%	95%CI	%	95%CI	%	95%CI	%	95%CI	%	95%CI	
Aceh	1,7	1,5-2,0	0,2	0,1-0,3	0,5	0,4-0,7	1,2	1,0-1,5	0,4	0,3-0,5	10.480
Sumatera Utara	1,4	1,2-1,6	0,1	0,1-0,1	0,4	0,3-0,6	1,0	0,8-1,3	0,3	0,2-0,4	28.482
Sumatera Barat	1,5	1,3-1,8	0,1	0,0-0,2	0,5	0,4-0,6	1,3	1,0-1,5	0,2	0,2-0,3	11.401
Riau	1,4	1,2-1,7	0,1	0,1-0,2	0,3	0,2-0,4	1,2	0,9-1,4	0,2	0,1-0,3	14.403
Jambi	2,1	1,8-2,6	0,2	0,1-0,3	0,2	0,1-0,3	0,7	0,5-1,0	0,3	0,1-0,4	5.848
Sumatera Selatan	1,5	1,3-1,8	0,1	0,1-0,3	0,2	0,1-0,3	0,4	0,3-0,6	0,3	0,2-0,4	15.972
Bengkulu	1,1	0,9-1,5	0,0	0,0-0,1	0,1	0,1-0,3	0,5	0,4-0,8	0,3	0,2-0,4	3.951
Lampung	1,0	0,8-1,2	0,1	0,1-0,2	0,1	0,1-0,1	0,4	0,3-0,5	0,1	0,1-0,2	17.114
Bangka Belitung	2,2	1,6-3,0	0,1	0,0-0,2	0,3	0,2-0,5	0,8	0,6-1,1	0,1	0,1-0,3	3.129
Kepulauan Riau	1,5	1,1-2,0	0,1	0,0-0,4	0,3	0,1-0,7	1,3	0,9-1,9	0,4	0,2-0,8	3.734
DKI Jakarta	1,4	1,1-1,7	0,2	0,1-0,3	0,7	0,5-1,0	4,0	3,5-4,6	0,5	0,4-0,7	22.642
Jawa Barat	1,1	1,0-1,2	0,2	0,1-0,2	0,4	0,3-0,5	1,7	1,5-1,9	0,5	0,4-0,6	102.936
Jawa Tengah	1,0	0,9-1,1	0,2	0,1-0,2	0,2	0,2-0,3	1,1	0,9-1,2	0,3	0,2-0,3	71.830
DI Yogyakarta	1,5	1,2-1,8	0,1	0,0-0,2	0,7	0,5-1,0	4,2	3,6-4,9	0,7	0,5-1,0	9.216
Jawa Timur	1,8	1,6-1,95	0,2	0,2-0,3	0,3	0,2-0,4	1,3	1,1-1,4	0,3	0,3-0,4	79.064
Banten	1,0	0,9-1,3	0,1	0,1-0,3	0,5	0,4-0,7	2,1	1,7-2,4	0,4	0,3-0,6	28.895
Bali	1,4	1,1-1,6	0,4	0,2-0,5	0,6	0,4-0,8	3,6	3,1-4,2	0,5	0,3-0,8	9.238
Nusa Tenggara Barat	0,8	0,6-1,1	0,2	0,1-0,3	0,3	0,2-0,4	0,8	0,6-1,0	0,3	0,2-0,5	10.085
Nusa Tenggara Timur	0,4	0,3-0,5	0,1	0,0-0,2	0,1	0,0-0,1	0,5	0,4-0,7	0,2	0,1-0,3	10.547
Kalimantan Barat	2,6	2,3-3,1	0,2	0,1-0,3	0,1	0,0-0,2	1,2	0,9-1,4	0,2	0,2-0,4	11.040
Kalimantan Tengah	1,8	1,5-2,2	0,2	0,1-0,4	0,2	0,1-0,3	0,7	0,5-0,9	0,3	0,2-0,5	5.184
Kalimantan Selatan	3,3	2,9-3,7	0,1	0,1-0,2	0,3	0,2-0,5	1,0	0,7-1,3	0,3	0,2-0,4	9.025
Kalimantan Timur	1,6	1,3-2,0	0,2	0,1-0,4	0,2	0,1-0,4	2,2	1,8-2,7	0,5	0,4-0,7	8.153
Kalimantan Utara	2,2	1,5-3,1	0,2	0,1-0,5	0,4	0,3-0,8	2,3	1,8-3,0	0,4	0,2-0,7	1.620
Sulawesi Utara	2,0	1,7-2,3	0,2	0,1-0,3	0,2	0,1-0,3	0,9	0,7-1,1	0,3	0,2-0,4	6.045
Sulawesi Tengah	1,7	1,5-2,0	0,1	0,1-0,2	0,1	0,1-0,2	0,8	0,7-1,0	0,3	0,2-0,4	8.005
Sulawesi Selatan	2,1	1,9-2,3	0,2	0,2-0,3	0,5	0,4-0,6	1,9	1,7-2,2	0,4	0,3-0,5	21.925
Sulawesi Tenggara	1,7	1,4-2,0	0,3	0,2-0,4	0,3	0,2-0,5	1,2	0,9-1,5	0,3	0,2-0,5	6.023
Gorontalo	1,3	0,9-1,8	0,2	0,1-0,5	0,1	0,0-0,3	0,5	0,3-0,8	0,3	0,1-0,5	2.743
Sulawesi Barat	1,8	1,4-2,3	0,2	0,1-0,45	0,2	0,1-0,4	0,5	0,3-0,8	0,2	0,1-0,4	3.140
Maluku	1,1	0,8-1,5	0,1	0,0-0,2	0,1	0,0-0,3	0,5	0,4-0,8	0,3	0,2-0,5	4.244
Maluku Utara	1,2	0,9-1,6	0,3	0,1-0,5	0,2	0,1-0,4	0,9	0,7-1,3	0,3	0,2-0,4	2.602
Papua Barat	1,1	0,7-1,6	0,1	0,1-0,3	0,1	0,0-0,3	0,8	0,5-1,25	0,5	0,3-0,8	2.199
Papua	0,6	0,4-0,8	0,2	0,1-0,3	0,2	0,1-0,3	0,7	0,5-1,0	0,4	0,3-0,6	6.007
**INDONESIA**	**1,4**	**1,4-1,4**	**0,2**	**0,2-0,2**	**0,3**	**0,3-0,4**	**1,4**	**1,4-1,5**	**0,4**	**0,3-0,4**	**556.921**

Tabel 7.1.6

Proporsi Penduduk yang Menerima Tindakan untuk Mengatasi Masalah Gigi dan Mulut menurut Karakteristik, Riskesdas 2018

Karakteristik	Jenis tindakan yang diterima untuk mengatasi masalah gigi dan mulut										N ter-timbang
	Pengobatan/minum obat		Konseling perawatan kebersihan dan kesehatan gigi & mulut		Penumpatan/ penambalan		Pencabutan gigi		Bedah mulut		
	%	95%CI	%	95%CI	%	95%CI	%	95%CI	%	95%CI	
**Kelompok Umur**											
3 – 4	39,8	38,6-41,0	5,4	4,9-6,0	0,8	0,8-1,4	1,9	1,6-2,2	0,1	0,0-0,2	15.107
5 – 9	47,9	47,248,5	8,3	7,9-8,6	2,0	2,0-2,4	13,6	13,2-14,1	0,2	0,1-0,3	62.710
10 -14	49,1	48,4-49,8	5,9	5,6-6,2	3,1	3,1-3,6	9,3	8,9-9,7	0,2	0,1-0,3	49.957
15 – 24	51,5	50,9-52,1	6,1	5,8-6,4	5,1	5,1-5,6	4,7	4,5-4,9	0,3	0,2-0,3	86.383
25 – 34	56,0	55,4-56,6	7,3	7,0-7,7	5,4	5,4-6,0	6,7	6,5-7,0	0,4	0,3-0,4	90.794
35 – 44	58,6	58,1-59,2	7,4	7,1-7,7	5,1	5,1-5,6	7,8	7,6-8,1	0,4	0,3-0,4	90.646
45 – 54	57,5	57,0-58,1	7,1	6,8-7,4	4,3	4,3-4,8	8,8	8,5-9,1	0,3	0,2-0,3	77.655
55 – 64	52,4	51,7-53,0	5,7	5,4-6,0	3,2	3,2-3,7	8,3	8,0-8,7	0,2	0,2-0,3	51.711
65 +	42,8	41,9-43,6	4,3	4,0-4,7	2,0	2,0-2,5	6,8	6,4-7,3	0,2	0,2-0,3	31.958
**Kelompok umur (WHO)**											
5	48,9	47,5-50,3	7,2	6,4-8,0	2,0	1,5-2,5	5,1	4,5-5,8	0,1	0,0-0,2	11.352
12	50,9	49,4-52,4	4,8	4,2-5,4	3,7	3,1-4,4	9,1	8,2-10,1	0,1	0,0-0,2	9.648
15	49,6	48,1-51,2	5,2	4,6-6,0	5,2	4,5-6,0	5,1	4,4-5,,9	0,1	0,1-0,35	9.618
35-44	58,6	58,1-59,2	7,4	7,1-7,7	5,4	5,1-5,6	7,8	7,6-8,1	0,4	0,3-0,4	94.294
>65	42,8	41,9-43,6	4,3	4,0-4,7	2,2	2,0-2,5	6,8	6,4-7,3	0,2	0,2-0,3	33.244
**Jenis kelamin**											
Laki – laki	52,6	52,2-53,0	5,8	5,6-5,9	3,6	3,5-3,8	7,6	8,8-9,2	0,2	0,2-0,3	275.496
Perempuan	53,1	52,7-53,5	7,6	7,4-7,8	4,9	4,8-5,1	8,2	6,5-6,8	0,3	0,3-0,3	281.425
**Pendidikan**											
Tidak sekolah	49,1	48,3-49,9	5,4	5,0-5,7	1,9	1,7-2,1	9,1	8,7-9,5	0,2	0,1-0,2	43.380
Tidak tamat SD/MI	51,0	50,5-51,5	5,9	5,6-6,1	2,3	2,1-2,4	10,4	10,1-10,6	0,2	0,1-0,25	115.135
Tamat SD/MI	56,0	55,4-56,5	4,6	4,4-4,8	2,7	2,5-2,8	6,4	6,2-6,6	0,2	0,2-0,2	126.89
Tamat SMP/MTS	55,5	54,9-54,0	5,7	5,4-5,9	3,9	3,7-4,1	6,3	6,1-6,6	0,2	0,2-0,3	91.543
Tamat SMA/MA	53,4	52,9-51,6	8,7	8,4-9,1	6,8	6,5-7,1	7,9	7,7-8,2	0,4	0,3-0,5	118.673
Tamat D1-D3/PT	50,7	49,8-51,6	15,2	14,5-15,8	14,6	14,0-15,2	11,5	11,0-12,0	0,8	0,7-1,0	34.500
**Pekerjaan**											
Tidak bekerja	53,7	53,2-54,2	6,8	6,6-7,1	4,6	4,4-4,8	7,6	7,3-7,8	0,3	0,2-0,3	134.937
Sekolah	49,0	48,4-49,6	6,4	6,1-6,75	4,7	4,5-5,0	7,3	7,0-7,6	0,2	0,2-0,3	68.432
PNS/TNI/Polri/BUMN/ BUMD	53,3	52,0-54,5	14,7	13,8-15,7	12,6	11,7-13,4	13,6	12,8-14,4	0,8	0,6-1,2	11.999
Pegawai swasta	52,1	51,2-53,0	11,1	10,5-11,6	9,3	8,7-9,8	7,8	7,3-8,2	0,6	0,5-0,7	41.398
Wiraswasta	56,3	55,6-57,0	7,3	7,0-7,7	5,4	5,1-5,7	8,5	8,2-8,9	0,3	0,2-0,4	61.772
Petani/buruh tani	55,8	55,2-56,4	3,1	2,9-3,3	1,7	1,6-1,8	6,0	5,7-6,2	0,1	0,1-0,1	81.311
nelayan	61,0	58,7-63,3	2,9	2,3-3,6	2,4	1,7-3,3	9,2	8,1-10,4	0,2	0,1-0,4	3.545
Buruh/sopir/pembantu ruta	56,3	55,4-57,1	4,8	4,5-5,2	2,8	2,5-3,1	5,2	4,9-5,5	0,2	0,2-0,3	44.924
Lainnya	55,4	54,4-56,4	6,6	6,2-7,1	5,1	4,7-5,5	8,2	7,7-8,7	0,4	0,3-0,5	24.222
**Tempat tinggal**											
Perkotaan	51,4	50,9-51,9	8,9	8,7-9,1	6,1	5,9-6,3	9,0	8,8-9,2	0,4	0,3-0,4	302.662
Perdesaan	54,6	54,2-55,1	4,1	4,0-4,2	2,1	2,0-2,2	6,6	6,5-6,8	0,2	0,1-0,2	254.259

Tabel 7.1.6 (Lanjutan)
Proporsi Penduduk yang Menerima Tindakan untuk Mengatasi Masalah Gigi dan Mulut menurut Karakteristik, Riskesdas 2018

Karakteristik	Jenis tindakan yang diterima untuk mengatasi masalah gigi & mulut										N ter-timbang
	Pemasangan gigi palsu		Pemasangan gigi tanam (implant denture)		Perawatan orthodonti (behel/kawat gigi)		Pembersihan karang gigi (scaling)		Perawatan gusi/periodontal teratment		
	%	95%CI	%	95%CI	%	95%CI	%	95%CI	%	95%CI	
**Kelompok Umur**											
3 – 4	0,1	0,0-0,2	0,0	0,0-0,0	0,0	0,0-0,0	0,2	0,1-0,4	0,1	0,1-0,2	15.107
5 – 9	0,1	0,1-0,2	0,1	0,0-0,1	0,1	0,0-0,1	0,5	0,4-0,5	0,3	0,2-0,3	62.71
10 -14	0,1	0,1-0,2	0,0	0,0-0,1	0,3	0,2-0,3	0,7	0,6-0,8	0,2	0,2-0,3	49.957
15 – 24	0,4	0,4-0,5	0,1	0,1-0,2	1,1	1,0-1,2	2,4	2,3-2,6	0,4	0,3-0,5	86.383
25 – 34	0,8	0,7-0,9	0,1	0,1-0,1	0,4	0,3-0,5	2,2	2,0-2,4	0,4	0,3-0,5	90.794
35 – 44	1,4	1,3-1,5	0,2	0,1-0,2	0,2	0,2-0,2	1,7	1,6-1,9	0,4	0,4-0,5	90.646
45 – 54	2,5	2,4-2,7	0,3	0,3-0,4	0,2	0,1-0,2	1,4	1,3-1,5	0,4	0,3-0,4	77.655
55 – 64	3,8	3,6-4,1	0,4	0,3-0,5	0,1	0,1-0,1	0,9	0,8-1,1	0,3	0,3-0,4	51.711
65 +	4,1	3,8-4,4	0,3	0,2-0,3	0,1	0,0-0,1	0,6	0,4-0,7	0,3	0,2-0,4	31.958
**Kelompok umur (WHO)**											
5	0,1	0,0-0,5	0,0	0,0-0,2	0,0	0,0-0,2	0,3	0,2-0,5	0,3	0,2-0,6	11.352
12	0,1	0,1-0,3	0,1	0,0-0,2	0,2	0,1-0,3	1,0	0,7-1,5	0,3	0,2-0,5	9.648
15	0,3	0,2-0,5	0,1	0,1-0,3	1,0	0,7-1,4	1,7	1,3-2,2	0,4	0,2-0,7	9.618
35-44	1,4	1,3-1,5	0,2	0,1-0,2	0,2	0,2-0,2	1,7	1,6-1,9	0,4	0,4-0,5	94.294
65 +	4,1	3,8-4,4	0,3	0,2-0,3	0,1	0,0-0,1	0,6	0,4-0,7	0,3	0,2-0,4	33.244
**Jenis kelamin**											
Laki – laki	1,2	1,1-1,2	0,2	0,1-0,2	0,2	0,1-0,2	1,2	1,1-1,3	0,3	0,3-0,4	275.496
Perempuan	1,6	1,6-1,7	0,2	0,2-0,2	0,5	0,5-0,5	1,7	1,6-1,8	0,4	0,3-0,4	281.425
**Pendidikan**											
Tidak sekolah	1,4	1,3-1,6	0,2	0,1-0,2	0,1	0,1-0,2	0,4	0,3-0,5	0,3	0,2-0,3	43.38
Tidak tamat SD/MI	1,1	1,0-1,2	0,1	0,1-0,1	0,1	0,1-0,1	0,5	0,4-0,5	0,2	0,2-0,3	115.135
Tamat SD/MI	1,6	1,6-1,8	0,2	0,1-0,2	0,2	0,1-0,2	0,6	0,5-0,7	0,2	0,2-0,3	126.89
Tamat SMP/MTS	1,4	1,3-1,5	0,2	0,1-0,2	0,4	0,3-0,4	1,3	1,1-1,4	0,3	0,2-0,4	91.543
Tamat SMA/MA	1,5	1,4-1,6	0,2	0,2-0,3	0,6	0,6-0,7	2,6	2,4-2,7	0,5	0,5-0,6	118.673
Tamat D1-D3/PT	2,2	2,0-2,4	0,3	0,3-0,4	1,1	1,0-1,3	6,8	6,3-7,2	1,1	0,9-1,2	34.5
**Pekerjaan**											
Tidak bekerja	2,0	1,9-2,1	0,2	0,2-0,2	0,4	0,3-0,4	1,5	1,3-1,65	0,3	0,3-0,4	134.937
Sekolah	0,3	0,2-0,3	0,1	0,1-0,1	0,7	0,6-0,8	1,7	1,5-1,8	0,3	0,3-0,4	68.432
PNS/TNI/Polri/BUMN/ BUMD	3,1	2,7-3,5	0,5	0,4-0,8	0,7	0,5-0,9	5,8	5,2-6,5	1,1	0,9-1,5	11.999
Pegawai swasta	1,0	0,9-1,2	0,3	0,2-0,3	0,7	0,6-0,8	4,0	3,7-4,4	0,8	0,6-0,9	41.398
Wiraswasta	2,1	1,9-2,2	0,2	0,2-0,3	0,3	0,2-0,3	1,9	1,8-2,1	0,4	0,3-0,5	61.772
Petani/buruh tani	1,9	1,8-2,1	0,2	0,2-0,2	0,1	0,0-0,1	0,3	0,2-0,3	0,2	0,1-0,2	81.311
nelayan	1,6	1,2-2,4	0,0	0,0-0,1	0,1	0,0-0,3	0,3	0,1-0,65	0,2	0,1-0,4	3.545
Buruh/sopir/ pembantu ruta	1,0	0,9-1,2	0,1	0,1-0,2	0,2	0,1-0,3	0,7	0,6-0,9	0,2	0,2-0,3	44.924
Lainnya	2,4	2,1-2,6	0,3	0,2-0,4	0,4	0,3-0,5	1,6	1,4-1,9	0,4	0,3-0,6	24.222
**Tempat tinggal**											
Perkotaan	1,4	1,3-1,5	0,2	0,2-0,2	0,5	0,4-0,5	2,2	2,1-2,3	0,5	0,4-0,5	302.662
Perdesaan	1,4	1,3-1,4	0,2	0,1-0,2	0,2	0,1-0,2	0,6	0,6-0,6	0,2	0,2-0,2	254.259

Tabel 7.1.7
Proporsi Pengobat Masalah Kesehatan Gigi dan Mulut menurut Provinsi, Riskesdas 2018

Provinsi	Dokter gigi spesialis		Dokter gigi		Perawat gigi		Dokter umum/paramedik lain		Tukang gigi		Pengobatan sendiri		N Ter-timbang
	%	95%CI	%	95%CI	%	95%CI	%	95%CI	%	95%CI	%	95%CI	
Aceh	2,5	2,1-2,8	13,9	13,1-14,9	13,1	12,1-14,2	13,9	12,7-15,1	2,2	1,9-2,5	41,6	40,2-43,0	10,480
Sumatera Utara	2,0	1,8-2,4	9,6	8,9-10,3	1,3	1,1-1,5	5,0	4,5-5,5	1,0	0,8-1,2	50,9	49,4-52,5	28,482
Sumatera Barat	2,2	1,7-2,8	12,5	11,8-13,4	2,3	2,0-2,6	7,2	6,5-7,8	1,2	1,0-1,4	37,0	35,5-38,5	11,401
Riau	1,8	1,6-2,1	12,5	11,7-13,4	1,0	0,8-1,3	5,2	4,7-5,8	1,6	1,4-2,0	41,9	40,3-43,6	14,403
Jambi	1,9	1,3-2,6	14,3	13,2-15,6	8,1	6,9-9,5	7,6	6,5-8,9	2,4	2,0-2,9	58,8	56,7-60,8	5,848
Sumatera Selatan	1,3	1,0-1,6	7,5	6,7-8,4	3,4	3,0-4,0	6,4	5,8-7,1	1,1	0,9-1,4	47,0	45,0-49,1	15,972
Bengkulu	1,2	0,9-1,5	9,0	8,1-10,0	1,3	1,0-1,7	7,4	6,5-8,3	0,8	0,6-1,1	49,8	47,7-52,0	3,951
Lampung	1,1	0,9-1,3	7,2	6,4-8,0	3,6	3,1-4,1	8,7	7,9-9,5	1,0	0,8-1,2	44,6	42,746,4-	17,114
Bangka Belitung	2,7	2,2-3,4	16,0	14,5-17,5	3,1	2,5-3,8	3,1	2,7-3,7	2,6	1,9-3,6	46,4	43,7-49,1	3,129
Kepulauan Riau	3,3	2,4-4,5	17,1	15,1-19,4	1,0	0,7-1,5	4,1	3,4-5,1	1,6	1,2-2,1	41,1	37,6-44,7	3,734
DKI Jakarta	5,4	4,8-6,2	23,1	21,6-24,5	1,2	1,0-1,6	2,6	2,2-3,1	0,8	0,6-1,1	32,6	30,6-34,5	22,642
Jawa Barat	2,7	2,4-3,0	16,7	16,0-17,3	2,8	2,5-3,1	5,1	4,7-5,5	0,9	0,7-1,0	43,1	42,0-44,3	102,936
Jawa Tengah	2,4	2,2-2,6	12,9	12,4-13,3	1,8	1,7-2,0	5,4	5,1-5,7	0,8	0,7-0,9	36,2	35,3-37,1	71,830
DI Yogyakarta	5,4	4,7-6,2	20,3	19,1-21,6	2,7	2,1-3,4	3,5	2,9-4,1	0,7	0,5-1,0	22,7	21,4-24,0	9,216
Jawa Timur	2,3	2,1-2,5	15,2	14,7-15,7	1,7	1,6-1,9	4,4	4,1-4,7	1,5	1,3-1,6	43,1	42,1-44,0	79,064
Banten	2,4	2,0-2,8	15,4	14,4-16,5	1,3	1,0-1,6	6,4	5,6-7,2	0,8	0,6-1,0	42,4	40,5-44,2	28,895
Bali	2,2	1,8-2,6	24,2	23,1-25,4	2,0	1,7-2,5	3,7	3,2-4,3	0,7	0,5-0,9	29,4	27,7-31,1	9,238
Nusa Tenggara Barat	1,7	1,4-2,1	10,6	9,8-11,5	6,0	5,3-6,8	4,3	3,7-5,0	0,8	0,6-1,1	40,8	38,9-42,8	10,085
Nusa Tenggara Timur	1,0	0,8-1,2	5,1	4,6-5,6	4,2	3,8-4,8	5,6	5,0-6,1	0,4	0,3-0,5	42,9	41,4-44,4	10,547
Kalimantan Barat	1,3	1,0-1,7	8,8	8,0-9,7	7,6	6,9-8,4	4,4	3,8-5,0	2,1	1,7-2,4	43,7	42,0-45,5	11,040
Kalimantan Tengah	1,2	0,9-1,6	9,6	8,5-10,8	4,7	3,9-5,6	6,3	5,5-7,1	1,9	1,6-2,3	58,6	56,8-60,5	5,184
Kalimantan Selatan	2,7	2,3-3,2	13,0	12,0-14,1	9,1	8,3-10,0	2,8	2,4-3,3	3,3	2,9-3,7	51,3	49,5-53,0	9,025
Kalimantan Timur	3,3	2,7-4,0	19,2	17,9-20,5	1,1	0,8-1,5	3,7	3,2-4,4	1,6	1,2-2,1	39,1	37,2-41,2	8,153
Kalimantan Utara	3,0	2,3-4,0	21,6	19,8-23,5	2,0	1,5-2,8	2,9	2,3-3,8	1,6	1,0-2,3	35,7	32,7-38,9	1,620
Sulawesi Utara	2,2	1,8-2,6	9,5	8,6-10,5	3,9	3,4-4,5	5,9	5,2-6,6	0,8	0,7-1,1	49,0	47,1-50,9	6,045
Sulawesi Tengah	0,9	0,7-1,1	8,0	7,3-8,7	3,1	2,6-3,7	4,5	4,0-5,1	3,3	2,9-3,7	52,6	50,8-54,5	8,005
Sulawesi Selatan	1,9	1,6-2,2	15,2	14,6-15,9	3,7	3,3-4,1	2,8	2,4-3,2	2,9	2,7-3,3	38,8	37,3-40,4	21,925
Sulawesi Tenggara	1,6	1,3-2,1	11,5	10,5-12,7	4,6	4,0-5,2	2,2	1,8-2,7	4,8	4,3-5,5	46,8	44,7-48,9	6,023
Gorontalo	2,1	1,6-2,7	6,8	5,8-7,8	2,5	1,8-3,4	4,7	4,0-5,5	1,5	1,2-2,0	54,3	51,5-57,1	2,743
Sulawesi Barat	1,2	0,8-1,8	9,7	8,5-11,1	2,2	1,6-2,9	6,0	5,1-7,0	2,8	2,2-3,5	42,4	39,8-45,1	3,140
Maluku	1,6	1,1-2,2	6,2	5,4-7,2	3,8	2,9-4,8	3,2	2,6-3,9	2,4	1,8-3,2	55,5	52,8-58,2	4,244
Maluku Utara	2,7	2,0-3,5	6,6	5,9-7,5	1,4	1,0-1,9	2,4	2,0-3,0	2,6	2,1-3,3	50,5	48,0-53,0	2,602
Papua Barat	2,3	1,6-3,4	9,5	8,4-10,8	2,4	1,8-3,2	7,6	6,5-8,9	0,9	0,6-1,4	43,7	41,0-46,5	2,199
Papua	3,4	2,6-4,5	11,8	10,4-13,5	4,1	3,3-4,9	14,2	12,5-16,1	1,4	1,0-2,2	39,6	37,1-42,1	6,007
**INDONESIA**	**2,4**	**2,3-2,5**	**13,9**	**13,8-14,1**	**2,9**	**2,8-2,9**	**5,2**	**5,1-5,3**	**1,3**	**1,3-1,3**	**42,2**	**41,8-42,5**	**556,921**

Tabel 7.1.8

Proporsi Pengobat Masalah Kesehatan Gigi dan Mulut menurut Karakteristik, Riskesdas 2018

Karakteristik	Dokter gigi spesialis		Dokter Gigi		Perawat gigi		Dokter umum/paramedik lain		Tukang gigi		Pengobatan sendiri		N Ter-timbang
	%	95%CI	%	95%CI	%	95%CI	%	95%CI	%	95%CI	%	95%CI	
**Kelompok Umur**													
3 – 4	1,0	0,8-1,3	8,3	7,6-9,0	2,1	1,8-2,4	7,0	6,4-7,6	0,2	0,1-0,4	31,8	30,7-33,0	15.107
5 – 9	2,0	1,9-2,2	17,8	17,3-18,3	3,7	3,5-3,9	6,0	5,7-6,2	0,4	0,3-0,5	36,6	36,0-37,3	62.710
10 -14	1,6	1,51,8-	13,5	13,1-14,0	3,0	2,8-3,2	4,8	4,5-5,0	0,5	0,4-0,6	40,2	39,5-40,9	49.957
15 – 24	2,6	2,4-2,8	13,1	12,7-13,5	2,5	2,3-2,6	4,4	4,2-4,7	0,9	0,8-0,9	42,3	41,7-42,9	86.383
25 – 34	3,0	2,8-3,2	14,5	14,1-14,9	2,8	2,7-3,0	5,1	4,9-5,4	1,1	1,0-1,3	43,8	43,2-44,4	90.794
35 – 44	2,6	2,5-2,8	15,1	14,7-15,5	3,1	2,9-3,2	5,6	5,4-5,9	1,4	1,3-1,5	45,4	44,8-45,9	90.646
45 – 54	2,6	2,4-2,8	14,4	14,0-14,8	2,9	2,8-3,1	5,4	5,2-5,7	2,0	1,9-2,1	45,3	44,8-45,9	77.655
55 – 64	2,2	2,0-2,4	12,3	11,9-12,8	2,6	2,4-2,8	5,3	5,0-5,6	2,5	2,3-2,7	42,9	42,2-43,6	51.711
65+	1,9	1,6-2,1	8,8	8,4-9,3	2,1	1,9-2,4	4,3	3,9-4,6	2,5	2,3-2,7	37,8	36,9-38,6	31.958
**Kelompok umur (WHO)**													
5	1,3	1,0-1,7	13,5	12,4-14,6	2,6	2,2-3,0	7,1	6,4-7,8	0,3	0,2-0,5	36,7	35,4-38,1	11.352
12	1,4	1,1-1,9	13,3	12,3-14,5	3,0	2,6-3,5	5,0	4,4-5,6	0,4	0,3-0,6	42,5	41,1-44,0	9.648
15	2,1	1,6-2,6	12,4	11,4-13,5	2,6	2,2-3,0	4,3	3,8-4,9	0,8	0,6-1,2	39,9	38,5-41,4	9.618
35-44	2,6	2,5-2,8	15,1	14,7-15,5	3,1	2,9-3,2	5,6	5,4-5,9	1,4	1,3-1,5	45,4	44,8-45,9	94.294
65 +	1,9	1,6-2,1	8,8	8,4-9,3	2,1	1,9-2,4	4,3	3,9-4,6	2,5	2,3-2,7	37,8	36,9-38,6	33.244
**Jenis kelamin**													
Laki – laki	2,1	2,0-2,3	12,4	12,2-12,6	2,6	2,5-2,7	5,0	4,8-5,1	1,2	1,1-1,3	43,4	43,0-43,8	275.496
Perempuan	2,6	2,5-2,7	15,4	15,2-15,7	3,1	3,0-3,2	5,5	5,3-5,6	1,4	1,3-1,5	40,9	40,5-41,3	281.425
**Pendidikan**													
Tidak sekolah	1,2	1,1-1,4	11,4	10,9-11,9	2,8	2,6-3,0	5,6	5,2-5,9	1,5	1,4-1,7	41,6	40,8-42,4	43.380
Tidak tamat SD/MI	1,5	1,4-1,6	13,0	12,7-13,3	3,1	3,0-3,3	5,4	5,2-5,6	1,2	1,1-1,2	42,2	41,7-42,7	115.135
Tamat SD/MI	1,2	1,1-1,3	10,0	9,7-10,3	2,7	2,6-2,9	5,5	5,2-5,7	1,7	1,6-1,8	46,9	46,4-47,5	126.890
Tamat SMP/MTS	1,9	1,7-2,0	12,5	12,2-12,9	2,7	2,6-2,9	5,1	4,9-5,4	1,3	1,2-1,5	45,2	44,6-45,8	91.543
Tamat SMA/MA	3,7	3,5-3,9	17,7	17,3-18,1	2,8	2,6-2,9	4,7	4,5-4,9	1,2	1,1-1,3	39,8	39,3-40,4	118.673
Tamat D1/D2/D3/PT	9,2	8,6-9,7	28,0	27,3-28,8	3,7	3,4-4,0	4,1	3,8-4,4	1,1	1,0-1,2	31,3	30,4-32,1	34.500
**Pekerjaan**													
Tidak bekerja	2,4	2,2-2,5	14,2	13,8-14,5	2,8	2,7-2,9	5,2	5,0-5,4	1,6	1,5-1,7	41,7	41,1-42,2	134.937
Sekolah	2,3	2,2-2,5	14,0	13,5-14,4	2,7	2,6-2,9	4,3	4,1-4,5	0,6	0,5-0,6	39,9	39,2-40,5	68.432
PNS/TNI/Polri/BUMN/BUMD	8,6	7,8-9,4	28,4	27,3-29,5	4,5	4,0-4,9	4,7	4,2-5,2	1,4	1,2-1,7	32,3	31,1-33,4	11.999
Pegawai swasta	5,1	4,7-5,5	20,9	20,1-21,6	2,3	2,1-2,5	4,6	4,2-4,9	0,8	0,7-0,9	37,1	36,2-38,0	41.398
Wiraswasta	3,1	2,9-3,4	15,9	15,4-16,4	2,8	2,7-3,0	4,7	4,4-5,0	1,6	1,5-1,8	43,5	42,8-44,2	61.772
Petani/buruh tani	0,7	0,6-0,8	6,5	6,3-6,8	2,8	2,6-2,9	6,5	6,3-6,8	2,1	2,0-2,3	50,0	49,5-50,6	81.311
nelayan	0,9	0,5-1,3	10,2	8,7-11,9	2,9	2,4-3,6	5,4	4,4-6,6	2,6	2,1-3,3	52,6	50,4-54,8	3.545
Buruh/sopir/pembantu ruta	1,2	1,0-1,4	10,3	9,8-10,8	2,3	2,1-2,5	4,3	4,0-4,7	1,2	1,0-1,3	47,8	46,9-48,7	44.924
Lainnya	2,9	2,5-3,2	14,6	13,9-15,3	3,6	3,3-3,9	5,2	4,8-5,6	1,8	1,6-2,1	43,3	42,3-44,4	24.222
**Tempat tinggal**													
Perkotaan	3,5	3,4-3,7	18,3	18,0-18,6	2,5	2,4-2,6	4,0	3,8-4,2	1,0	1,0-1,1	38,4	37,9-39,0	302.662
Perdesaan	1,0	1,0-1,1	8,8	8,6-9,0	3,3	3,1-3,4	6,7	6,5-6,9	1,6	1,6-1,7	46,6	46,1-47,0	254.259

Tabel 7.1.9

Proporsi Frekuensi Berobat Ke Tenaga Medis Gigi menurut Provinsi,Riskesdas 2018

Provinsi	Frekuensi berobat ke tenaga medis gigi								N ter-timbang
	1 – 3x		4 – 6 x		≥ 7x		Tidak pernah berobat		
	%	95%CI	%	95%CI	%	95%CI	%	95%CI	
Aceh	0,9	0,7-1,1	2,8	2,5 - 3,1	1,5	1,2-1,8	94,9	94,4-95,3	8.370
Sumatera Utara	0,7	0,6-0,9	2,3	2,0-2,6	1,1	0,9-1,3	95,9	95,5-96,3	23.477
Sumatera Barat	1,0	0,8-1,2	2,1	1,7-2,5	0,9	0,7-1,2	96,0	95,5-96,5	8.000
Riau	1,0	0,7-1,5	2,2	1,8-2,7	1,0	0,8-1,2	95,8	95,1-96,4	10.005
Jambi	0,8	0,6-1,1	2,2	1,8-2,7	2,0	1,5-2,6	95,1	94,2-95,9	7.071
Sumatera Selatan	0,9	0,6-1,2	3,5	2,9-4,1	1,5	1,1-1,9	94,2	93,4-95,0	14.353
Bengkulu	0,9	0,6-1,2	2,9	2,4-3,5	1,4	1,1-1,8	94,8	94,0-95,4	3.170
Lampung	0,5	0,4-0,7	2,0	1,5-2,8	1,1	0,8-1,4	96,4	95,6-97,0	13.182
Bangka Belitung	1,3	1,0-1,8	1,3	0,9-1,8	0,6	0,4-0,9	96,9	96,1-97,5	2.172
Kepulauan Riau	1,5	1,0-2,2	3,1	2,2-4,4	0,9	0,6-1,5	94,5	92,9-95,7	3.937
DKI Jakarta	3,1	2,5-3,9	2,0	1,5-2,5	0,9	0,6-1,2	94,1	93,2-94,9	15.488
Jawa Barat	1,7	1,4-1,9	2,3	2,0-2,6	1,2	1,1-1,4	94,8	94,4-95,2	73.799
Jawa Tengah	1,2	1,0-1,3	1,9	1,7-2,1	0,9	0,8-1,1	96,1	95,7-96,4	54.383
DI Yogyakarta	3,9	3,2-4,8	1,9	1,4-2,5	0,7	0,5-1,1	93,5	92,4-94,4	4.784
Jawa Timur	1,5	1,3-1,8	1,7	1,5-1,9	1,0	0,9-1,1	95,7	95,4-96,1	66.058
Banten	1,8	1,4-2,2	1,8	1,5-2,2	0,9	0,7-1,1	95,6	94,9-96,1	16.960
Bali	2,7	2,2-3,2	0,9	0,7-1,2	0,7	0,5-1,0	95,7	95,0-96,3	6.500
Nusa Tenggara Barat	0,7	0,5-1,0	1,8	1,4-2,2	1,0	0,7-1,4	96,5	95,8-97,1	7.970
Nusa Tenggara Timur	0,5	0,4-0,7	1,5	1,3-1,8	0,8	0,7-1,0	97,2	96,8-97,5	8.563
Kalimantan Barat	1,2	0,9-1,5	2,0	1,7-2,4	1,5	1,2-1,8	95,4	94,8-95,9	7.039
Kalimantan Tengah	1,2	0,9-1,7	1,7	1,4-2,2	1,0	0,7-1,3	96,1	95,4-96,7	4.427
Kalimantan Selatan	1,7	1,3-2,2	2,0	1,6-2,3	0,9	0,7-1,2	95,4	94,8-96,0	6.055
Kalimantan Timur	2,3	1,8-3,0	1,6	1,2-2,2	0,8	0,5-1,1	95,3	94,2-96,2	5.047
Kalimantan Utara	2,5	1,8-3,6	3,0	2,2-4,1	1,5	1,0-2,2	92,9	91,2-94,3	929
Sulawesi Utara	1,0	0,7-1,5	2,0	1,6-2,6	0,8	0,6-1,1	96,2	95,4-96,8	3.018
Sulawesi Tengah	1,3	1,0-1,8	1,7	1,3-2,3	0,9	0,7-1,3	96,1	95,2-96,8	2.850
Sulawesi Selatan	1,6	1,3-1,9	2,0	1,7-2,4	0,9	0,7-1,1	95,5	95,0-96,0	9.815
Sulawesi Tenggara	1,4	1,1-1,9	1,6	1,2-2,0	0,9	0,7-1,3	96,1	95,4-96,7	3.446
Gorontalo	0,8	0,5-1,2	1,6	1,1-2,2	0,7	0,5-1,1	96,9	96,2-97,6	1.545
Sulawesi Barat	1,4	1,0-2,0	2,0	1,4-2,8	1,1	0,7-1,6	95,6	94,4-96,5	1.695
Maluku	0,8	0,5-1,2	2,5	1,9-3,3	1,1	0,8-1,7	95,6	94,5-96,5	2.096
Maluku Utara	0,9	0,6-1,4	2,2	1,7-2,8	1,1	0,7-1,5	95,8	95,0-96,5	1.803
Papua Barat	1,3	0,8-2,0	2,9	2,2-3,8	0,7	0,5-1,1	95,1	94,0-96,0	1.145
Papua	0,8	0,5-1,3	2,5	2,1-2,9	1,3	1,0-1,7	95,4	94,7-96,0	5.971
**INDONESIA**	**1,4**	**1,3-1,5**	**2,1**	**2,0-2,1**	**1,1**	**1,0-1,1**	**95,5**	**95,4-95,6**	**405.124**

Tabel 7.1.10
Proporsi Frekuensi Berobat Ke Tenaga Medis Gigi menurut Karakteristik, Riskesdas 2018

Karakteristik	Frekuensi berobat ke tenaga medis gigi								N Tertimbang
	1 – 3x		4 – 6 x		≥ 7x		Tidak pernah berobat ke tenaga medis gigi		
	%	95%CI	%	95%CI	%	95%CI	%	95%CI	
**Kelompok Umur**									
3 – 4	0,7	0,5-0,9	2,4	2,1-2,8	0,6	0,4-0,8	96,3	95,9-96,7	21.438
5 – 9	1,3	1,1-1,5	1,7	1,5-2,0	0,5	0,4-0,6	96,5	96,1-96,7	30.129
10 -14	1,4	1,2-1,6	1,7	1,5-1,9	0,9	0,7-1,0	96,1	95,8-96,3	39.530
15 - 24	1,5	1,4-1,7	2,0	1,8-2,1	1,5	1,4-1,7	95,0	94,7-95,2	79.085
25 - 34	1,6	1,5-1,8	2,2	2,0-2,4	1,3	1,1-1,4	94,9	94,6-95,2	68.822
35 - 44	1,4	1,2-1,5	2,1	2,0-2,3	1,1	1,0-1,3	95,3	95,1-95,6	60.851
45 - 54	1,5	1,3-1,6	2,1	1,9-2,3	0,8	0,7-1,0	95,6	95,3-95,9	46.994
55 - 64	1,4	1,2-1,6	2,2	2,0-2,5	0,9	0,8-1,1	95,4	95,1-95,8	31.536
65+	1,0	0,9-1,2	2,2	2,0-2,5	0,8	0,7-1,0	96,0	95,6-96,3	26.739
**Kelompok umur (WHO)**									
5	1,2	0,9-1,7	1,5	1,2-1,9	0,3	0,2-0,5	97,0	96,4-97,5	8.179
12	1,5	1,1-2,1	1,7	1,4-2,1	0,7	0,6-1,0	96,0	95,4-96,6	8.325
15	1,0	0,8-1,4	1,5	1,2-2,0	1,7	1,4-2,1	95,7	95,1-96,3	9.757
35-44	1,4	1,2-1,5	2,1	2,0-2,3	1,1	1,0-1,3	95,3	95,1-95,6	63.296
65 +	1,0	0,9-1,2	2,2	2,0-2,5	0,8	0,7-1,0	96,0	95,6-96,3	27.813
**Jenis kelamin**									
Laki – laki	1,1	1,0-1,2	1,9	1,8-2,1	1,1	1,0-1,2	95,8	95,7-96,0	207.192
Perempuan	1,7	1,6-1,8	2,2	2,1-2,3	1,0	1,0-1,1	95,1	94,9-95,3	197.932
**Pendidikan**									
Tidak sekolah	0,9	0,7-1,0	2,0	1,8-2,3	1,0	0,8-1,1	96,2	95,8-96,5	27.542
Tidak tamat SD/MI	1,0	0,9-1,1	1,8	1,6-1,9	0,9	0,8-1,0	96,4	96,2-96,6	66.428
Tamat SD/MI	0,9	0,8-1,0	2,1	1,9-2,2	1,1	1,0-1,2	96,0	95,7-96,2	89.080
Tamat SMP/MTS	1,0	0,9-1,1	2,0	1,8-2,2	1,3	1,2-1,4	95,8	95,5-96,0	68.738
Tamat SMA/MA	1,7	1,6-1,9	2,1	1,9-2,2	1,1	1,0-1,3	95,1	94,8-95,3	91.996
Tamat D1/D2/D3/PT	5,0	4,6-5,6	2,8	2,5-3,2	1,2	1,0-1,4	91,0	90,4-91,6	29.546
**Pekerjaan**									
Tidak bekerja	1,4	1,3-1,6	2,2	2,1-2,4	1,1	1,0-1,2	95,3	95,1-95,5	98.693
Sekolah	1,5	1,4-1,7	1,8	1,6-2,0	1,2	1,1-1,4	95,5	95,2-95,8	58.110
PNS/TNI/Polri/BUMN/BUMD	5,0	4,4-5,7	2,7	2,3-3,2	1,3	1,0-1,6	91,0	90,2-91,8	9.920
Pegawai swasta	2,7	2,4-3,0	2,1	1,9-2,4	1,2	1,0-1,4	94,0	93,5-94,4	34.341
Wiraswasta	1,5	1,3-1,7	2,0	1,8-2,2	1,0	0,9-1,2	95,4	95,1-95,7	43.733
Petani/buruh tani	0,4	0,3-0,5	2,1	1,9-2,2	1,2	1,1-1,4	96,3	96,1-96,5	52.029
Nelayan	0,2	0,1-0,6	2,1	1,4-3,1	0,9	0,6-1,4	96,8	95,7-97,6	2.017
Buruh/sopir/pembantu ruta	0,6	0,5-0,8	1,8	1,6-2,0	1,1	1,0-1,4	96,4	96,1-96,8	30.689
Lainnya	1,6	1,4-1,9	2,3	1,9-2,7	1,1	0,9-1,3	95,1	94,5-95,5	16.436
**Tempat tinggal**									
Perkotaan	2,0	1,8-2,1	2,1	1,9-2,2	1,0	1,0-1,1	94,9	94,7-95,1	224.175
Perdesaan	0,7	0,7-0,8	2,0	1,9-2,2	1,1	1,0-1,1	96,2	96,0-96,3	180.949

7.2 Cakupan Layanan Kesehatan Gigi dan Mulut

Proporsi penduduk yang bermasalah gigi dan mulutnya adalah orang yang mengeluh atau merasa bermasalah dengan kesehatan gigi dan mulutnya seperti gigi berlubang, gigi telah dicabut, mengalami kegoyahan gigi, pernah mengalami pembengkakan dan atau pernah ada bisul pada gusi, sariawan (*perceived need*), dalam 12 bulan terakhir

Proporsi penduduk telah menerima perawatan (utilisasi) atau pengobatan gigi dari tenaga medis gigi adalah penduduk yang telah menerima perawatan dari tenaga medis gigi (dokter gigi spesialis, dokter gigi, perawat gigi) dalam 12 bulan terakhir.

Tabel 7.2.1

Proporsi Masalah Gigi dan Mulut, Perawatan oleh Tenaga Medis Gigi pada Penduduk Umur ≥3 Tahun menurut Provinsi, Riskesdas 2018

Provinsi	Bermasalah gigi & Mulut		Menerima perawatan oleh tenaga medis gigi		N Tertimbang
	%	95%CI	%	95%CI	
Aceh	55,3	54,3-56,4	13,9	13,2-14,6	18.855
Sumatera Utara	54,6	53,5-55,7	6,7	6,3-7,1	51.977
Sumatera Barat	58,5	57,3-59,7	9,3	8,8-9,9	19.399
Riau	58,8	57,3-60,2	8,6	8,1-9,2	24.405
Jambi	45,0	43,2-46,8	9,5	8,8-10,4	12.936
Sumatera Selatan	52,4	50,8-54,1	5,8	5,3-6,3	30.341
Bengkulu	55,2	53,7-56,8	6,0	5,4-6,7	7.123
Lampung	56,2	54,8-57,6	6,2	5,7-6,8	30.300
Bangka Belitung	58,8	56,9-60,6	11,8	10,9-12,9	5.301
Kepulauan Riau	48,4	45,6-51,3	10,0	8,6-11,5	7.679
DKI Jakarta	59,1	57,4-60,8	16,4	15,5-17,3	38.124
Jawa Barat	58,0	57,1-58,9	11,9	11,5-12,3	176.728
Jawa Tengah	56,7	56,0-57,3	9,0	8,7-9,3	126.225
DI Yogyakarta	65,6	64,2-67,0	16,4	15,5-17,4	13.989
Jawa Timur	54,2	53,5-54,9	9,8	9,4-10,1	145.173
Banten	62,8	61,3-64,2	11,3	10,6-12,1	45.831
Bali	58,4	57,2-59,7	16,2	15,4-17,0	15.737
Nusa Tenggara Barat	55,6	54,2-57,0	9,0	8,4-9,6	18.058
Nusa Tenggara Timur	54,9	53,9-56,0	5,1	4,7-5,5	19.115
Kalimantan Barat	60,8	59,4-62,2	10,0	9,3-10,8	18.073
Kalimantan Tengah	53,7	51,9-55,5	7,6	7,0-8,4	9.615
Kalimantan Selatan	59,6	58,1-61,1	12,3	11,6-13,1	15.077
Kalimantan Timur	61,5	59,9-63,1	13,8	12,9-14,7	13.195
Kalimantan Utara	63,3	60,6-65,9	16,0	14,7-17,3	2.547
Sulawesi Utara	66,5	65,1-67,8	9,4	8,7-10,2	9.055
Sulawesi Tengah	73,5	72,5-74,6	8,2	7,5-8,9	10.837
Sulawesi Selatan	68,9	68,0-69,7	13,0	12,5-13,6	31.703
Sulawesi Tenggara	63,4	61,8-64,9	10,4	9,5-11,4	9.464
Gorontalo	63,7	61,6-65,8	6,6	5,8-7,5	4.286
Sulawesi Barat	64,7	62,6-66,7	8,1	7,2-9,1	4.831
Maluku	66,7	65,0-68,3	7,2	6,3-8,3	6.334
Maluku Utara	58,8	56,8-60,8	5,8	5,2-6,5	4.404
Papua Barat	65,5	63,6-67,4	8,5	7,5-8,9	3.341
Papua	49,9	48,1-51,7	7,9	7,0-8,9	11.987
**INDONESIA**	**57,6**	**57,4-57,9**	**10,2**	**10,1-10,3**	**962.045**

Tabel 7.2.2
Proporsi Masalah Gigi dan Mulut, Perawatan oleh Tenaga Medis Gigi pada Penduduk Umur >3 Tahun menurut Karakteristik, Riskesdas 2018

Karakteristik	Bermasalah gigi & Mulut		Menerima perawatan dari tenaga medis gigi		N tertimbang
	%	95%CI	%	95%CI	
**Kelompok Umur**					
3 – 4	41,1	40,3-41,9	4,3	4,0-4,6	36.608
5 – 9	67,3	66,8-67,8	14,6	14,3-15,0	92.746
10 -14	55,6	55,1-56,1	9,4	9,1-9,7	89.506
15 - 24	51,9	51,5-52,4	8,7	8,5-9,0	165.565
25 - 34	56,6	56,2-57,1	10,6	10,3-10,9	159.633
35 - 44	59,6	59,1-60,0	11,4	11,2-11,7	151.467
45 - 54	62,1	61,6-62,5	11,3	11,1-11,6	124.593
55 - 64	61,9	61,3-62,4	9,8	9,4-10,1	83.211
65+	54,2	53,5-54,8	6,4	6,1-6,7	58.717
**Kelompok umur (WHO)**					
5	57,9	56,8-58,9	9,5	8,8-10,2	19.529
12	53,4	52,3-54,5	9,0	8,3-9,6	17.980
15	49,4	48,3-50,5	7,8	7,2-8,4	19.390
35-44	59,6	59,1-60,0	11,4	11,2-11,7	157.550
65 +	54,2	53,5-54,8	6,4	6,1-6,7	61.076
**Jenis kelamin**					
Laki – laki	56,8	56,5-57,1	9,1	8,9-13,1	482.729
Perempuan	58,5	58,2-58,2	11,4	11,2-7,1	479.316
**Pendidikan**					
Tidak sekolah	60,8	60,2-61,4	8,7	8,3-9,0	70.895
Tidak tamat SD/MI	63,1	62,7-63,4	10,2	10,0-10,5	181.429
Tamat SD/MI	58,4	58,0-58,8	7,5	7,3-7,7	215.967
Tamat SMP/MTS	56,7	56,3-57,2	9,0	8,7-9,2	160.320
Tamat SMA/MA	55,9	55,5-56,4	12,5	12,3-12,8	210.746
Tamat D1/D2/D3/PT	53,5	52,8-54,2	20,0	19,5-20,5	64.093
**Pekerjaan**					
Tidak bekerja	57,3	56,9-57,7	10,2	10,0-10,4	233.629
Sekolah	53,6	53,1-54,1	9,5	9,3-9,8	126.626
PNS/TNI/Polri/BUMN/BUMD	54,3	53,4-55,2	20,4	19,7-21,2	21.931
Pegawai swasta	54,2	53,5-55,2	14,2	13,8-14,7	75.781
Wiraswasta	58,1	57,6-58,6	11,7	11,4-12,1	105.489
Petani/buruh tani	60,5	60,1-61,0	5,5	5,4-5,7	133.261
nelayan	63,3	61,5-65,1	8,2	7,1-9,4	5.556
Buruh/sopir/pembantu ruta	59,0	58,3-59,7	7,6	7,2-7,9	75.590
Lainnya	59,1	58,3-59,9	11,2	10,8-11,7	40.644
**Tempat tinggal**					
Perkotaan	57,2	56,8-57,6	12,9	12,7-13,1	526.861
Perdesaan	58,2	57,8-58,5	6,9	6,8-7,1	435.184

7.3 Kesehatan Gigi dan Mulut berdasarkan Pemeriksaan

A. Status gigi geligi

Pemeriksaan status gigi geligi untuk mengetahui terjadinya penyakit gigi dan mulut termasuk prevalensi karies gigi. Pemeriksaan status gigi geligi dilakukan pada semua gigi termasuk permukaan akar pada gigi tetap atau dewasa. Pada gigi sulung tidak dilakukan pencatatan untuk status akar.

Decay/D/d

Jumlah gigi permanen/gigi sulung yang mengalami karies dan belum diobati atau ditambal (baik dengan tambalan sementara maupun dengan tambalan permanen) dibagi jumlah orang yang diperiksa;

DT/dt rata-rata adalah rata-rata jumlah gigi dengan karies dan sekunder karies dari populasi yang dihitung dengan membagi jumlah skor D/d per orang dengan jumlah orang yang diperiksa.

$$DT/dt\ rata-rata = \frac{Jumlah\ skor\ DT/dt}{Jumlah\ ART\ yang\ diperiksa}$$

Missing/M/m

Jumlah gigi permanen/gigi sulung yang dicabut karena karies atau masih berupa sisa akar dibagi jumlah orang yang diperiksa;

MT/mt rata-rata adalah rata-rata jumlah gigi yang hilang karena karies dari populasi yang dihitung dengan membagi jumlah skor M/m per orang dengan jumlah orang yang diperiksa.

$$MT/mt\ rata-rata = \frac{Jumlah\ skor\ MT/mt}{Jumlah\ ART\ yang\ diperiksa}$$

Filling/F/f

Jumlah gigi permanen/gigi sulung yang telah dilakukan penumpatan atau ditambal karena karies dibagi jumlah orang yang diperiksa

$$FT/ft\ rata-rata = \frac{Jumlah\ skor\ FT/ft}{Jumlah\ ART\ yang\ diperiksa}$$

Indeks *DMF-T* Gigi Permanen

Menggambarkan tingkat keparahan kerusakan gigi permanen. Jumlah rata-rata D + M + F dibagi jumlah orang yang diperiksa.

Indeks *dmf-t* Gigi Sulung

Menggambarkan tingkat keparahan kerusakan gigi sulung. Jumlah rata-rata d + m + f dibagi jumlah orang yang diperiksa.

$$Indeks\ DMFT/dmft = \frac{Jumlah\ skor\ DT/dt\ +\ Mt/mt + \ Ft/ft}{Jumlah\ ART\ yang\ diperiksa}$$

Prevalensi Karies

Prevalensi Karies = Jumlah orang yang mempunyai (D/M/F)T dibagi jumlah orang yang diperiksa

$$Prevalensi\ karies = \frac{Jumlah\ orang\ yang\ mempunyai\ (D/M/F)T}{Jumlah\ ART\ yang\ diperiksa}$$

Karies sekunder adalah adanya kerusakan atau lubang baru pada gigi yang telah ditambal dibagi jumlah orang yang diperiksa.

Prevalensi karies sekunder adalah pembagian jumlah gigi yang memiliki karies sekunder (kode gigi 2 untuk gigi tetap) dengan jumlah orang yang diperiksa.

$$Prevalensi\ karies\ sekunder = \frac{Jumlah\ gigi\ dengan\ karies\ sekunder\ \ perorang}{Jumlah\ ART\ yang\ diperiksa}$$

Karies akar adalah kerusakan gigi atau lubang gigi yang terjadi di daerah sekitar akar gigi (gigi permanen).

Prevalensi Karies akar adalah pembagian jumlah gigi yang memiliki karies akar (kode gigi 1,2 dan 3 untuk gigi tetap) dengan jumlah orang yang diperiksa.

Pengalaman karies atau riwayat karies adalah orang dengan riwayat/pengalaman karies dimana (*DMF-T/dmf-t*> 0).

Prevalensi pengalaman karies atau riwayat karies adalah pembagian jumlah orang yang memiliki riwayat/pengalaman karies dengan jumlah orang yang diperiksa.

Bebas karies *(caries free)* adalah keadaan dimana *DMF-T/dmf-t* = 0, tidak ada karies, tidak ada pencabutan, tidak ada penambalan atau penumpatan.
Proporsi bebas karies *(caries free)* adalah pembagian jumlah orang bebas karies dengan dengan jumlah orang yang diperiksa.

Dental fit (*cavity free*) adalah keadaan dimana tidak ada lubang gigi (D=0), termasuk jika ada penggunaan protesa cekat.
Proporsi Dental fit (*cavity free*) adalah pembagian jumlah orang yang memiliki dental fit dengan jumlah orang yang diperiksa.

Fissure Sealant: Tindakan pencegahan karies gigi berupa penutupan pit & fissure mahkota gigi dengan menggunakan bahan GIC atau resin, baik pada gigi sulung maupun pada gigi tetap.
Proporsi Fissure Sealantadalah pembagian jumlah orang yang memiliki fissure sealant dengan jumlah orang yang diperiksa.

Dentulous adalah kondisi hilangnya gigi asli kurang dari 28 gigi pada individu, (*M-T*<28). Kehilangan gigi dapat disebabkan oleh kerusakan gigi, periodontitis, atau kecelakaan.
Prevalensi dentulous adalah pembagian jumlah orang dentulous dengan jumlah orang yang diperiksa.

$$Prevalensi\ Dentulous = \frac{Jumlah\ orang\ yang\ kehilangan\ gigi\ asli\ kurang\ dari\ 28}{Jumlah\ ART\ yang\ diperiksa}$$

Edentulous adalah kondisi hilangnya gigi asli lebih dari 28 gigi pada individu, (*M-T*>28). Kehilangan gigi dapat disebabkan oleh kerusakan gigi, periodontitis, atau kecelakaan.
Prevalensi edentulous adalah pembagian jumlah orang edentulous dengan jumlah orang yang diperiksa.

$$Prevalensi\ Edentulous = \frac{Jumlah\ orang\ yang\ kehilangan\ gigi\ asli\ 28\ atau\ lebih}{Jumlah\ ART\ yang\ diperiksa}$$

Proporsi gigi tiruan sebagian adalah jumlah pengguna gigi tiruan sebagian (kode gigi tiruan 1).

$$Proporsi\ Pengguna\ Gigi\ Tiruan\ sebagian = \frac{Jumlah\ pengguna\ gigi\ tiruan\ sebagian}{Jumlah\ ART\ yang\ diperiksa}$$

Proporsi gigi tiruan penuh adalah jumlah pengguna gigi tiruan penuh (kode gigi tiruan 2).

$$Proporsi\ Pengguna\ Gigi\ Tiruan\ Penuh = \frac{Jumlah\ pengguna\ gigi\ tiruan\ penuh}{Jumlah\ orang\ yang\ diperiksa}$$

Catatan: Gigi tiruan Penuh ATAU Gigi Tiruan Sebagian bisa pada rahang atas atau rahang bawah saja atau rahang bawah dan rahang atas atau rahang atas dan rahang bawah.

Proporsi gigi tiruan cekat adalah jumlah pengguna gigi tiruan cekat (kode gigi 7).

$$Proporsi\ Pengguna\ Gigi\ Tiruan\ Cekat = \frac{Jumlah\ \ pengguna\ gigi\ tiruan\ cekat}{Jumlah\ ART\ yang\ diperiksa}$$

Root Decay (**RD*****)*** adalah rata-rata jumlah gigi dengan karies akar dari populasi yang dihitung dengan membagi jumlah gigi dengan kode akar 1 dengan jumlah orang yang diperiksa.[1]

$$RD = \frac{Jumlah\ gigi\ dengan\ karies\ akar\ dan\ sekunder\ karies\ akar\ per - orang}{Jumlah\ ART\ yang\ diperiksa}$$

Root Filling (**RF*****)*** adalah rata-rata jumlah gigi dengan karies akar yang sudah ditumpat dari populasi yang dihitung dengan membagi jumlah gigi dengan kode akar 3 dengan jumlah orang yang diperiksa.

$$RF = \frac{Jumlah\ gigi\ dengan\ karies\ akar\ yang\ sudah\ ditumpat\ \ per - orang}{Jumlah\ orang\ yang\ diperiksa/ART}$$

[1] *Estimating Rates of New Roots Caries in Older Adults.* Griffin SO. J Dent Res. 2004 Aug; 83(8): 634-8

Root Normal **(RN)** adalah rata-rata jumlah gigi dengan permukaan akar yang terbuka namun tidak berkaries dari populasi yang dihitung dengan membagi jumlah gigi dengan kode akar 0 dengan jumlah orang yang diperiksa.

$$RN = \frac{Jumlah\ gigi\ dengan\ akar\ terbuka\ dan\ tidak\ karies\ \ per - orang}{Jumlah\ orang\ yang\ diperiksa/ART}$$

Karies sekunder pada akar adalah akar gigi dengan karies setelah ditumpat, dihitung dengan cara membagi jumlah akar gigi dengan karies sekunder (kode gigi 2 untuk gigi tetap, gigi sulung tidak diperiksa) dibagi dengan jumlah orang yang diperiksa.

Root Caries Indeks**(RCI)** adalah persentase gigi dengan karies akar, dihitung dengan cara membagi jumlah gigi dengan karies akar (RD) dan karies akar yang sudah ditambal (RF) dibagi dengan semua akar gigi yang terekspos.

$$RCI = \frac{(RD) + (RF)}{(RD) + (RF) + (RN)}$$

B. Status Periodontal

Status periodontal adalah gambaran yang menunjukkan kondisi jaringan pendukung gigi, yang diukur dengan Indeks *Community Periodontal Indeks* (indeks CPI). Pemeriksaan status periodontal menggunakan probe WHO. Pemeriksaan dilakukan pada semua gigi dengan menginput ukuran poket terdalam setelah dilakukan *walking probing*.

Pendarahan gingiva (*bleeding on probing*) adalah perdarahan yang terjadi pada gingiva ketika dilakukan *walking probing* dengan menggunakan *Probe* CPI WHO, diamati satu menit setelah dilakukan probing. Perdarahan gingiva dicatat dengan skor 0 (Tidak ada perdarahan) dan 1 (Ada perdarahan).

Sekstan adalah kelompok gigi untuk pemeriksaan, meliputi gigi 18-14, 13-23, 24-28, 38-34, 33-43, dan 44-48.

Gigi indeks adalah gigi yang dipergunakan untuk pemeriksaan kehilangan perlekatan yang mewakili tiap sekstan. Gigi yang diperiksa adalah gigi 17/16, 11, 26/27, 47/46, 31, 36/37.Jika tidak terdapat gigi indeks pada sekstan tersebut, maka yang dicatat adalah gigi dengan skor tertinggi pada sekstan.

Kedalaman Poket diukur dengan melakukan *walking probing* menggunakan *ProbeCPI WHO,* dicatat dengan skor 0 (Tidak ada poket), 1 (Poket 4-5 mm), 2 (Poket 6 mm atau lebih).

Kehilangan perlekatan *(loss of attachment)* dilakukan pemeriksaan dengan menggunakan gigi indeks. Pengukuran kehilangan perlekatan pada gigi indeks dinilai berdasarkan nilai tertinggi saat dilakukan probing, dengan menggunakan kriteria 0 (0-3 mm), 1 (4-5 mm), 2 (6-8 mm), 3 (9-11 mm), 4 (12 mm atau lebih).

Prevalensi gingivitis adalah pembagian jumlah orang yang menderita perdarahan pada gusi dibagi jumlah orang yang diperiksa.

$$Prevalensi\ gingivitis = \frac{Jumlah\ penderita\ yang\ mengalami\ perdarahan\ gingiva}{Jumlah\ orang\ yang\ diperiksa}$$

Prevalensi periodontitis adalah pembagian jumlah orang yang menderita sedikitnya satu gejala dengan (bleeding (+) atau pocket (+) atau attachment loss (+)) dibagi jumlah orang yang diperiksa.

$$Prevalensi\ Periodontitis = \frac{Jumlah\ penderita\ yang\ mengalami\ (\text{bleeding }(+), \text{pocket }(+)\text{ dan attachment loss }(+))}{Jumlah\ orang\ yang\ diperiksa}$$

C. Status kesehatan gigi dan mulut lainnya

Fluorosis adalah perubahan warna permukaan gigi karena terlalu banyak fluor, biasanya bilateral simetris dan tampak sebagai garis halus horizontal pada gigi, diukur dengan Indeks Dean.

Indeks Dean untuk mengukur keparahan fluorosis.

0 : permukaan enamel halus, mengkilat, warna keputih-putihan

1 : enamel menunjukkan sedikit kelainan dari enamel yang normal/translucent, yang berkisar antara bintik-bintik halus berwarna keputihan sampai ada kalanya berbentuk noda yang lebih besar/spots

2 : bintik-bintik putih kecil yang lebih jelas terpencar secara tidak teratur meliputi permukaan gigi kurang dari 25% permukaan gigi sebelah labial

3 : bintik-bintik putih lebih meluas akan tetapi meliputi permukaan gigi kurang dari 50%

4 : permukaan enamel gigi tampak tidak rata, seringkali berwarna coklat,

5 : permukaan enamel yang terkena berakibat jelek sekali dan hipoplasia tampak jelas sekali, sehingga bentuk gigi tampak berubah, bercak-bercak coklat tersebar luas

Erosi gigi adalah kerusakan jaringan keras gigi yang terlihat licin dan mengkilat, terjadi pada enamel dengan atau tanpa melibatkan dentin yang bukan disebabkan infeksi bakteri. Pemeriksaan erosi gigi berdasarkan tingkat keparahan daerah yang terlibat (mulai dari email sampai pulpa), dicatat dengan kriteria 0 (Tidak ada erosi), 1 (Erosi pada bagian email), 2 (Erosi pada bagian dentin), 3 (Erosi sudah mencapai pulpa)

Lesi mukosa oral dinilai berdasarkan bentuk lesi pada jaringan lunak yang terdapat dalam rongga mulut. Pemeriksaan berdasarkan kondisi lesi yang terdapat dalam rongga mulut. Lesi pada rongga mulut berupa lesi datar, lesi putih, tonjolan padat, kantung berisi cairan bening, ulser berupa lesi cekung pada mukosa mulut, lesi Ptekie berupa bercak merah dibawah epitel mukosa mulut. Dicatat dengan menggunakan kriteria 0 (TIdak ada lesi), 1 (Terdapat Stomatitis Aphtosa Rekuren), 2 (Suspek Kanker Mulut), 3 (Lesi Lain).

Status gigi tiruan digunakan untuk mengetahui individu dalam populasi yang menggunakan gigi tiruan *removable* sebagai salah satu indikator dalam penggunaan akses pelayanan kesehatan.

Kebutuhan perawatan segera dapat dilihat berdasarkan tingkat urgensi jenis perawatan gigi. Salah satu contoh dari perlunya dilakukan perawatan segera

adalah abses periapikal, *Acute Necrotizing Ulceration Gingivitis* (ANUG), *Acute Necrotizing Ulceration Periodontitis* (ANUP), dll.

Tabel 7.3.1
Prevalensi Karies, Karies Akar dan Periodontitis menurut Karakteristik, Riskesdas 2018

Karakteristik	Karies			Karies akar			Periodontitis		
	%	95% CI	N Ter-timbang	%	95% CI	N Ter-timbang	%	95% CI	N Ter-Timbang
**Kelompok umur**									
3 – 4*	81,5	78,8-84,0	1.141	13,3	11,8-15,0	2.236	[redacted]		
5 – 9	92,6	91,4-93,6	4.149	28,5	27,0-30,0	5.629			
10 -14	73,4	71,7-75,0	3.903	48,1	46,5-49,7	6.211			
15 – 24	75,3	73,8-76,6	5.640	61,1	59,7-62,5	8.277	67,8	66,0-69,5	5.627
25 – 34	87,0	85,9-88,0	6.223	70,0	68,6-71,4	7.883	74,1	72,5-75,6	6.209
35 – 44	92,2	91,4-92,9	8.293	75,6	74,1-77,1	5.551	77,0	75,6-78,3	8.274
45 – 54	94,5	93,8-95,1	7.898	73,5	71,7-75,2	3.518	77,8	76,5-79,1	7.880
55 – 64	96,8	96,2-97,3	5.562	13,3	11,8-15,0	2.236	75,9	74,4-77,3	5.550
65 +	95,0	94,1-95,8	3.524	28,5	27,0-30,0	5.629	66,0	64,1-67,9	3.517
**Kelompok umur (WHO)**									
5*	90,2	87,1-92,7	711	[redacted]					
12	72,0	68,4-75,4	740	11,6	9,3-14,4	738			
15	68,5	64,4-72,3	697	20,8	17,6-24,3	696	60,2	55,9-64,2	695
35-44	92,2	91,4-92,9	8.339	61,1	59,7-62,5	8.327	77,0	75,6-78,3	8.312
65 +	95,0	94,1-95,8	3.544	73,5	71,7-75,2	3.539	66,0	64,1-67,9	3.532
**Jenis kelamin**									
Laki – laki	87,2	86,5-87,9	19.815	56,7	55,6-57,7	16.182	73,2	72,0-74,4	15.034
Perempuan	89,9	89,4-90,4	26.518	56,6	55,6-57,6	23.123	74,7	73,6-75,7	22.023
**Pendidikan**									
Tidak sekolah	92,1	91,1-93,1	4.000	68,0	65,5-70,4	2.636	72,8	70,5-74,9	2.566
Tidak tamat SD/MI	90,5	89,8-91,2	9.618	63,7	62,0-65,4	5.994	74,6	72,9-76,3	5.397
Tamat SD/MI	89,0	88,4-89,7	12.961	59,2	57,8-60,5	12.818	77,7	76,4-78,9	11.357
Tamat SMP/MTS	86,6	85,7-87,5	7.484	50,4	48,9-51,9	7.474	73,7	72,3-75,1	7.356
Tamat SMA/MA	87,5	86,5-88,5	8.301	51,5	50,1-52,9	8.289	71,5	70,0-72,9	8.287
Tamat D1-D3/PT	88,7	86,7-90,4	2.096	48,8	46,4-51,3	2.094	66,1	63,4-68,6	2.093
**Pekerjaan**									
Tidak bekerja	89,7	89,1-90,3	14.284	56,6	55,3-57,8	13.816	74,0	72,8-75,2	13.314
Sekolah	73,9	72,4-75,5	4.978	20,8	19,3-22,3	3.781	63,2	60,6-65,8	2.076
PNS/TNI/Polri/BUMN/BUMD	89,8	86,2-92,6	515	55,5	50,9-60,1	514	68,1	63,6-72,4	514
Pegawai swasta	86,4	84,8-88,0	2.832	50,6	48,3-52,9	2.828	67,9	65,6-70,2	2.825
Wiraswasta	91,5	90,5-92,3	5.268	61,2	59,5-62,8	5.264	74,1	72,3-75,7	5.256
Petani/buruh tani	93,3	92,5-93,9	7.098	70,6	68,9-72,2	7.092	78,3	76,7-79,8	7.087
Nelayan	91,1	82,2-95,7	141	70,4	59,8-79,2	141	79,6	69,8-86,8	140
Buruh/sopir/pembantu ruta	90,4	89,2-91,5	3.885	62,1	60,0-64,1	3.883	76,6	74,7-78,4	3.868
Lainnya	91,7	90,3-93,0	1.991	60,4	57,8-62,9	1.986	75,9	73,6-78,1	1.977
**Tempat tinggal**									
Perkotaan	87,9	87,1-88,7	25.430	53,7	52,5-54,9	21.505	71,5	70,2-72,9	20.229
Perdesaan	89,8	89,3-90,3	20.903	60,1	58,7-61,5	17.800	77,1	75,7-78,5	16.828
**INDONESIA**	**88,8**	**88,3-89,3**	**46.333**	**56,6**	**55,7-57,5**	**39.305**	**74,1**	**73,1-75,0**	**37.057**

*Pada usia tersebut yang diperiksa adalah gigi sulung

Tabel 7.3.2
Rata-rata Indeks *dmf-t* Gigi Sulung pada Penduduk Umur 3-4 Tahun menurut Karakteristik, Riskesdas 2018

Karakteristik	Indeks dmf-t		
	$\bar{x}$	95% CI	N Tertimbang
**Jenis kelamin**			
Laki – laki	6,7	6,2-7,2	609
Perempuan	5,6	5,1-6,1	532
**Tempat tinggal**			
Perkotaan	5,9	5,4-6,3	680
Perdesaan	6,6	6,1-7,1	461
**INDONESIA**	**6,2**	**5,8-6,5**	**1.141**

Tabel 7.3.3
Rata-rata Indeks *dmf-t* Gigi Sulung pada Penduduk Umur 5 Tahun menurut Karakteristik, Riskesdas 2018

Karakteristik	Indeks dmf-t (WHO)		
	$\bar{x}$	95% CI	N Tertimbang
**Jenis kelamin**			
Laki – laki	8,3	7,6-8,9	374
Perempuan	8,0	7,4-8,6	357
**Tempat tinggal**			
Perkotaan	8,2	7,5-8,8	400
Perdesaan	8,0	7,5-8,6	331
**INDONESIA**	**8,1**	**7,7-8,6**	**731**

Tabel 7.3.4
Rata-rata Indeks *DMF-T* Gigi Permanen dan *Root Caries Indeks* menurut Karakteristik, Riskesdas 2018

Karakteristik	Indeks *DMF-T*			*Root Caries Indeks*		
	$\bar{x}$	95% CI	N Tertimbang	$\bar{x}$	95% CI	N Tertimbang
**Kelompok umur**						
5 – 9	0,7	0,7-0,8	3.440	[redacted]		
10 -14	1,8	1,7-1,8	3.901	0,2	0,2-0,2	2.236
15 – 24	3,1	3,0-3,2	5.637	0,6	0,5-0,6	5.629
25 – 34	5,1	4,9-5,2	6.219	1,0	1,4-1,6	6.211
35 – 44	6,9	6,8-7,1	8.288	2,4	2,3-2,5	8.277
45 – 54	9,2	9,0-9,4	7.894	3,5	3,4-3,6	7.883
55 – 64	12,6	12,3-12,8	5.559	4,4	4,3-4,6	5.551
65 +	16,8	16,3-17,2	3.522	4,6	4,4-4,8	3.518
**Kelompok umur (WHO)**						
12	1,9	1,7-2,1	738	0,2	0,1-0,2	738
15	2,4	2,1-2,6	696	0,3	0,3-0,4	696
35-44	6,9	6,8-7,1	8.327	2,4	2,3-2,5	8.327
65 +	16,8	16,3-17,2	3.539	4,6	4,4-4,8	3.539
**Jenis kelamin**						
Laki – laki	7,0	6,9-7,2	18.835	2,8	2,7-2,9	16.182
Perempuan	7,2	7,1-7,3	25.626	2,4	2,4-2,5	23.123
**Pendidikan***						
Tidak sekolah	8,5	8,1-8,9	2.838	4,1	3,8-4,3	2.636
Tidak tamat SD/MI	6,5	6,3-6,7	7.340	3,4	3,2-3,5	5.994
Tamat SD/MI	8,2	8,0-8,3	12.961	2,9	2,7-3,0	12.818
Tamat SMP/MTS	6,2	6,0-6,3	7.484	1,9	1,8-2,0	7.474
Tamat SMA/MA	6,5	6,3-6,7	8.301	1,9	1,8-2,0	8.289
Tamat D1-D3/PT	7,1	6,8-7,4	2.096	1,7	1,5-1,8	2.094
**Pekerjaan****						
Tidak bekerja	8,1	7,9-8,2	14.284	2,5	2,4-2,5	13.816
Sekolah	2,4	2,3-2,5	4.978	0,5	0,4-0,5	3.781
PNS/TNI/Polri/BUMN/BUMD	7,7	7,1-8,3	515	2,2	1,9-2,5	514
Pegawai swasta	6,0	5,7-6,2	2.832	1,7	1,6-1,9	2.828
Wiraswasta	8,2	8,0-8,5	5.268	2,8	2,7-2,9	5.264
Petani/buruh tani	10,5	10,2-10,8	7.098	4,0	3,8-4,1	7.092
Nelayan	8,3	6,9-9,6	141	3,2	2,4-3,9	141
Buruh/sopir/ pembantu ruta	7,9	7,6-8,2	3.885	2,8	2,7-3,0	3.883
Lainnya	8,4	8,0-8,8	1.991	2,7	2,5-2,9	1.986
**Tempat tinggal**						
Perkotaan	6,8	6,7-6,9	24.349	2,3	2,2-2,4	21.505
Perdesaan	7,5	7,4-7,7	20.112	2,9	2,8-3,0	17.800
**INDONESIA**	**7,1**	**7,0-7,2**	**44.461**	**2,6**	**2,5-2,6**	**39.305**

*Untuk Nilai *DMF-T* dihitung mulai usia 5 tahun ke atas (N=41.021)
** Untuk Nilai *DMF-T* dihitung mulai usia 10 tahun ke atas (N=40.991)

Tabel 7.3.5
Rata-rata Komponen DT/dt, MT/mt, FT/ft, Indeks *DMF-T/dmf-t*, dan Karies Sekunder menurut Karakteristik, Riskesdas 2018

Karakteristik	DT/dt		MT/mt		FT/ft		Karies Sekunder		N tertimbang
	$\bar{x}$	95% CI	$\bar{x}$	95% CI	$\bar{x}$	95% CI	$\bar{x}$	95% CI	
**Kelompok Umur**									
10 -14	1,7	1,6-1,8	0,0	0,0-0,1	0,0	0,0-0,0	0,0	-0,3-0,3	3.056
15 – 24	2,9	2,8-3,0	0,1	0,1-0,2	0,0	0,0-0,0	0,0	-0,3-0,3	5.640
25 – 34	4,4	4,2-4,5	0,7	0,6-0,7	0,0	0,0-0,1	0,0	-0,5-0,6	6.223
35 – 44	5,2	5,0-5,3	1,7	1,6-1,7	0,1	0,0-0,1	0,0	-0,6-0,7	8.293
45 – 54	5,9	5,8-6,0	3,2	3,1-3,3	0,1	0,1-0,1	0,0	-0,5-0,6	7.898
55 – 64	6,6	6,4-6,7	5,9	5,7-6,2	0,1	0,0-0,1	0,0	-0,5-0,5	5.562
65 +	6,6	6,4-6,8	10,1	9,7-10,5	0,0	0,0-0,1	0,0	-0,6-0,6	3.524
**Kelompok umur (WHO)**									
12	1,8	1,6-2,0	0,1	0,0-0,1	0,0	0,0-0,0	0,0	-0,5-0,6	740
15	2,3	2,1-2,5	0,0	0,0-0,1	0,0	0,0-0,0	0,0	-0,2-0,2	697
35-44	5,2	5,0-5,3	1,7	1,6-1,7	0,1	0,1-0,1	0,0	-0,6-0,7	8.339
65 +	6,6	6,4-6,8	10,1	9,7-10,5	0,0	0,0-0,1	0,0	-0,6-0,6	3.544
**Jenis kelamin**									
Laki – laki	4,4	4,4-4,5	2,5	2,4-2,6	0,0	0,0-0,0	0,0	-0,4-0,5	16.622
Perempuan	4,6	4,5-4,7	2,5	2,5-2,6	0,1	0,0-0,1	0,0	-0,5-0,6	23.575
**Pendidikan**									
Tidak sekolah	4,4	4,2-4,6	4,1	3,8-4,4	0,0	0,0-0,0	0,0	-0,3-0,3	2.725
Tidak tamat SD/MI	3,9	3,8-4,1	2,5	2,4-2,7	0,0	0,0-0,0	0,0	-0,3-0,3	6.649
Tamat SD/MI	5,2	5,1-5,3	3,0	2,8-3,1	0,0	0,0-0,0	0,0	-0,3-0,3	12.919
Tamat SMP/MTS	4,5	4,3-4,6	1,7	1,6-1,8	0,0	0,0-0,0	0,0	-0,5-0,5	7.484
Tamat SMA/MA	4,4	4,3-4,6	2,0	1,9-2,1	0,1	0,1-0,1	0,1	-0,7-0,8	8.301
Tamat D1-D3/PT	4,2	4,0-4,4	2,5	2,3-2,7	0,4	0,4-0,5	0,2	-1,0-1,4	2.096
**Pekerjaan**									
Tidak bekerja	4,9	4,8-5,0	3,1	3,0-3,2	0,1	0,1-0,1	0,0	-0,6-0,7	14.031
Sekolah	2,2	2,1-2,3	0,2	0,1-0,2	0,0	0,0-0,0	0,0	-0,3-0,4	4.374
PNS/TNI/Polri/BUMN/BUMD	4,4	4,0-4,7	3,0	2,6-3,5	0,3	0,2-0,4	0,1	-0,9-1,2	514
Pegawai swasta	4,3	4,1-4,5	1,5	1,4-1,7	0,2	0,1-0,2	0,1	-0,7-0,8	2.828
Wiraswasta	5,4	5,2-5,5	2,8	2,7-3,0	0,1	0,1-0,1	0,0	-0,5-0,6	5.264
Petani/buruh tani	6,2	6,1-6,4	4,3	4,0-4,5	0,0	0,0-0,0	0,0	-0,3-0,3	7.093
Nelayan	5,3	4,5-6,1	3,0	2,0-3,9	0,0	0,0-0,0	0,0	-0,1-0,2	141
Buruh/sopir/ pembantu ruta	5,3	5,1-5,5	2,6	2,4-2,8	0,0	0,0-0,0	0,0	-0,3-0,3	3.882
Lainnya	5,1	4,9-5,3	3,2	2,9-3,5	0,1	0,0-0,1	0,0	-0,5-0,5	1.990
**Tempat tinggal**									
Perkotaan	4,4	4,3-4,4	2,4	2,3-2,5	0,1	0,1-0,1	0,1	-0,6-0,7	22.002
Perdesaan	4,8	4,7-4,9	2,8	2,6-2,9	0,0	0,0-0,0	0,0	-0,3-0,3	18.195
**INDONESIA**	**4,5**	**4,5-4,6**	**2,5**	**2,5-2,6**	**0,1**	**0,0-0,1**	**0,0**	**-0,5-0,6**	**40.196**

Tabel 7.3.6
Proporsi Bebas karies, *Dental Fit* dan *Fissure Sealant* menurut Karakteristik, Riskesdas 2018

Karakteristik	Bebas Karies			*Dental fit (cavity free)*			*Fissure Sealant*		
	%	95% CI	N tertimbang	%	95% CI	N tertimbang	%	95% CI	N tertimbang
**Kelompok Umur**									
3 – 4*	19,0	16,5-21,7	1.141	19,1	16,6-21,8	1.141	0,0	0,0-0,0	1.141
5 – 9*	9,9	8,7-11,2	4.149	10,7	9,5-12,0	4.149	0,0	0,0-0,1	4.149
10 -14	37,3	35,5-39,2	3.903	37,8	35,9-39,6	3.903	0,1	0,0-0,3	3.903
15 - 24	25,2	23,8-26,6	5.640	26,5	25,1-28,0	5.640	0,1	0,0-0,2	5.640
25 - 34	13,1	12,1-14,2	6.223	15,5	14,4-16,6	6.223	0,0	0,0-0,1	6.223
35 - 44	7,9	7,2-8,6	8.293	10,5	9,8-11,4	8.293	0,0	0,0-0,1	8.293
45 - 54	5,5	5,0-6,2	7.898	8,2	7,5-8,9	7.898	0,1	0,0-0,2	7.898
55 - 64	3,2	2,7-3,8	5.562	5,7	5,1-6,5	5.562	0,1	0,0-0,3	5.562
65 +	5,0	4,2-5,9	3.524	6,5	5,6-7,4	3.524	0,0	0,0-0,2	3.524
**Kelompok umur (WHO)**									
5*	9,9	7,4-13,0	711	10,2	7,7-13,3	711	0,0	0,0-0,1	711
12	34,5	30,9-38,2	740	34,7	31,1-38,4	740	0,0	0,0-0,0	740
15	32,9	29,0-36,9	697	33,7	29,9-37,8	697	0,1	0,0-0,9	697
35-44	7,9	7,2-8,6	8.339	10,5	9,8-11,4	8.339	0,0	0,0-0,1	8.339
65 +	5,0	4,2-5,9	3.544	6,5	5,6-7,4	3.544	0,0	0,0-0,2	3.544
**Jenis kelamin**									
Laki – laki	19,0	18,3-19,8	19.815	25,8	25,0-26,6	19.815	0,0	0,0-0,1	19.815
Perempuan	13,8	13,3-14,3	26.518	19,0	18,4-19,6	26.518	0,1	0,1-0,1	26.518
**Pendidikan**									
Tidak sekolah	27,9	26,2-29,7	4.000	29,3	27,5-31,0	4.000	0,1	0,0-0,3	4.000
Tidak tamat SD/MI	26,2	25,1-27,3	9.618	27,4	26,3-28,5	9.618	0,1	0,0-0,2	9.618
Tamat SD/MI	11,5	10,8-12,2	12.961	13,0	12,3-13,8	12.961	0,1	0,0-0,1	12.961
Tamat SMP/MTS	13,6	12,7-14,5	7.484	15,6	14,7-16,6	7.484	0,1	0,0-0,2	7.484
Tamat SMA/MA	12,6	11,6-13,6	8.301	15,4	14,4-16,4	8.301	0,1	0,0-0,1	8.301
Tamat D1-D3/PT	11,3	9,6-13,3	2.096	17,2	15,2-19,3	2.096	0,2	0,1-0,5	2.096
**Pekerjaan**									
Tidak bekerja	11,1	10,5-11,8	14.284	13,0	12,3-13,7	14.284	0,1	0,1-0,2	14.284
Sekolah	32,4	30,8-34,1	4.978	33,4	31,8-35,1	4.978	0,1	0,0-0,3	4.978
PNS/TNI/Polri/ BUMN/BUMD	10,3	7,5-14,0	515	15,9	12,5-19,9	515	0,0	0,0-0,0	515
Pegawai swasta	13,6	12,1-15,3	2.832	17,3	15,6-19,1	2.832	0,0	0,0-0,0	2.832
Wiraswasta	8,6	7,7-9,5	5.268	11,2	10,2-12,2	5.268	0,0	0,0-0,1	5.268
Petani/buruh tani	6,8	6,2-7,6	7.098	8,6	7,9-9,5	7.098	0,1	0,0-0,2	7.098
Nelayan	8,9	4,3-17,8	141	9,8	4,9-18,7	141	0,0	0,0-0,0	141
Buruh/sopir/pembantu ruta	9,6	8,5-10,8	3.885	11,9	10,7-13,3	3.885	0,0	0,0-0,0	3.885
Lainnya	8,4	7,2-9,8	1.991	11,2	9,8-12,8	1.991	0,0	0,0-0,2	1.991
**Tempat tinggal**									
Perkotaan	16,5	15,8-17,3	25.430	22,8	22,0-23,6	25.430	0,1	0,1-0,1	25.430
Perdesaan	15,4	14,8-16,0	20.903	20,8	20,1-21,5	20.903	0,1	0,0-0,1	20.903
**INDONESIA**	**16,0**	**15,5-16,5**	**46.333**	**21,9**	**21,3-22,5**	**46.333**	**0,1**	**0,0-0,1**	**46.333**

*berdasarkan penilaian terhadap gigi sulung

Tabel 7.3.7

Proporsi *Dentulous, Edentulous,* dan Penggunaan Gigi Tiruan pada Penduduk Umur ≥12 Tahun menurut Karakteristik, Riskesdas 2018

Karakteristik	Dentulous		Edentulous		Penggunaan Gigi Tiruan						N Tertimbang
					Gigi Tiruan Sebagian		Gigi Tiruan Penuh		Gigi Tiruan Cekat		
	%	95% CI	%	95% CI	%	95% CI	%	95% CI	%	95% CI	
**Kelompok umur**											
15 - 24	2,8	2,1-3,8	0,0	0,0-0,0	0,6	0,4-0,9	0,0	0,0-0,0	0,3	0,2-0,5	5.629
25 - 34	11,1	10,2-12,1	0,0	0,0-0,0	1,9	1,5-2,3	0,1	0,0-0,2	0,5	0,4-0,8	6.211
35 - 44	35,2	33,9-36,7	0,1	0,0-0,1	3,0	2,6-3,5	0,2	0,1-0,4	0,8	0,6-1,0	8.277
45 - 54	55,6	54,3-56,9	0,5	0,4-0,7	4,9	4,4-5,5	1,0	0,8-1,2	1,1	0,9-1,4	7.883
55 - 64	70,2	68,9-71,4	2,6	2,2-3,1	6,5	5,8-7,3	2,9	2,4-3,4	1,1	0,8-1,4	5.551
65 +	79,8	78,5-81,0	9,0	8,0-10,0	6,4	5,5-7,3	5,8	5,0-6,7	1,1	0,8-1,6	3.518
**Kelompok umur (WHO)**											
15	4,1	2,7-6,0	0,0	0,0-0,0	0,1	0,0-0,7	0,0	0,0-0,0	0,1	0,0-0,4	696
35-44	55,6	54,3-56,9	0,1	0,0-0,1	3,0	2,6-3,5	0,2	0,1-0,4	0,8	0,6-1,0	8.327
65 +	78,2	76,5-79,9	9,0	8,0-10,0	6,4	5,5-7,3	5,8	5,0-6,7	1,1	0,8-1,6	3.539
**Jenis kelamin**											
Laki – laki	49,6	48,7-50,6	1,6	1,4-1,8	2,7	2,4-3,0	1,2	1,0-1,3	0,6	0,5-0,8	16.182
Perempuan	52,6	51,8-53,5	1,1	1,0-1,3	4,1	3,8-4,4	1,2	1,1-1,4	0,9	0,7-1,0	23.123
**Pendidikan**											
Tidak sekolah	68,8	66,6-70,9	4,9	4,1-5,8	3,6	2,8-4,5	3,1	2,4-3,9	0,7	0,4-1,2	2.636
Tidak tamat SD/MI	58,1	56,6-59,7	2,3	1,9-2,7	3,0	2,6-3,6	1,6	1,3-2,0	0,5	0,4-0,8	5.994
Tamat SD/MI	51,6	50,5-52,7	1,3	1,1-1,5	2,9	2,6-3,3	1,1	0,9-1,3	0,5	0,3-0,6	12.818
Tamat SMP/MTS	40,5	39,2-41,8	0,4	0,3-0,6	3,0	2,6-3,5	0,7	0,5-0,9	0,5	0,3-0,7	7.474
Tamat SMA/MA	48,7	47,4-50,1	0,5	0,4-0,7	4,3	3,9-4,9	0,9	0,7-1,1	1,1	0,9-1,4	8.289
Tamat D1-D3/PT	58,3	55,8-60,8	0,5	0,3-0,9	6,7	5,6-8,0	1,2	0,8-1,9	2,8	2,2-3,6	2.094
**Pekerjaan**											
Tidak bekerja	52,6	51,5-53,7	1,8	1,6-2,0	4,0	3,7-4,4	1,6	1,4-1,8	0,8	0,6-1,0	13.816
Sekolah	8,3	7,3-9,4	0,0	0,0-0,1	0,4	0,2-0,7	0,1	0,0-0,2	0,2	0,1-0,4	3.781
PNS/TNI/Polri/ BUMN/BUMD	66,5	61,9-70,8	0,7	0,3-1,6	8,2	6,1-10,9	1,5	0,7-2,8	3,2	1,9-5,2	514
Pegawai swasta	47,8	45,6-50,0	0,2	0,1-0,5	3,4	2,7-4,2	0,2	0,1-0,5	1,3	0,9-1,8	2.828
Wiraswasta	59,6	58,061,2	0,8	0,6-1,1	4,8	4,2-5,5	1,1	0,9-1,5	1,0	0,8-1,4	5.264
Petani/buruh tani	63,7	62,2-65,1	2,1	1,8-2,5	3,0	2,5-3,5	1,6	1,3-2,0	0,5	0,3-0,7	7.092
Nelayan	55,7	45,6-65,3	0,3	0,0-2,0	2,3	0,7-7,8	1,0	0,3-3,1	1,3	0,3-5,3	141
Buruh/sopir/ pembantu ruta	53,2	51,3-55,2	0,8	0,6-1,2	2,3	1,8-2,9	0,4	0,2-0,7	0,6	0,4-0,9	3.883
Lainnya	56,6	54,1-59,1	1,4	0,9-2,0	5,3	4,3-6,4	1,5	1,0-2,2	0,9	0,5-1,4	1.986
**Tempat tinggal**											
Perkotaan	51,2	50,2-52,3	0,9	0,-8-1,1	4,0	3,7-4,4	1,1	1,0-1,3	0,9	0,8-1,1	21.505
Perdesaan	51,6	50,6-52,5	1,7	1,5-1,9	2,8	2,5-3,2	1,3	1,1-1,5	0,5	0,4-0,7	17.800
**INDONESIA**	**51,4**	**50,7-52,1**	**1,3**	**1,2-1,4**	**3,5**	**3,3-3,7**	**1,2**	**1,1-1,3**	**0,8**	**0,7-0,9**	**39.305**

Tabel 7.3.8
Rata-rata Komponen *RD, RF, RN*, Karies Sekunder pada Akar Gigi Permanen, menurut Karakteristik, Riskesdas 2018

Karakteristik	*RD*		*RF*		*RN*		Karies Sekunder pada Akar Gigi		N Tertimbang
	$\bar{x}$	SD	$\bar{x}$	SD	$\bar{x}$	SD	$\bar{x}$	SD	
**Kelompok umur**									
10 -14	0,2	0,6	0,0000	0,0000	1,5	5,3	0.0	5,3	2.236
15 - 24	0,6	1,3	0,0007	0,0426	2,5	6,2	0.0	6,2	5.629
25 - 34	1,5	2,5	0,0016	0,0657	4,4	7,3	0.0	7,3	6.211
35 - 44	2,4	3,4	0,0048	0,0946	6,2	7,6	0.0	7,6	8.277
45 - 54	3,5	4,3	0,0075	0,1433	7,6	7,6	0.0	7,6	7.883
55 - 64	4,4	5,1	0,0063	0,1181	7,3	7,2	0.0	7,2	5.551
65 +	4,6	5,0	0,0121	0,1921	5,7	6,6	0.0	6,6	3.518
**Kelompok umur (WHO)**									
12	0,2	0,5	0,0000	0,0000	1,3	5,1	1,3	5,1	738
15	0,3	0,8	0,0000	0,0000	2,1	6,2	2,1	6,2	696
35-44	2,4	3,4	0,0048	0,0946	6,2	7,6	6,2	7,6	8.327
65 +	4,6	5,0	0,0121	0,1921	5,7	6,6	5,7	6,6	3.539
**Jenis kelamin**									
Laki – laki	2,8	4,3	0,0051	0,1242	6,0	7,7	0.0	7,7	16.182
Perempuan	2,4	3,6	0,0047	0,1000	5,1	7,1	0.0	7,1	23.123
**Pendidikan**									
Tidak sekolah	4,1	5,0	0,0002	0,0151	5,9	7,2	0.0	7,2	2.636
Tidak tamat SD/MI	3,4	4,6	0,0017	0,0460	6,1	7,6	0.0	7,6	5.994
Tamat SD/MI	2,9	4,1	0,0021	0,0602	5,7	7,4	0.0	7,4	12.818
Tamat SMP/MTS	1,9	3,3	0,0012	0,0434	4,8	7,2	0.0	7,2	7.474
Tamat SMA/MA	1,9	3,1	0,0095	0,1506	5,2	7,4	0.0	7,4	8.289
Tamat D1-D3/PT	1,7	2,9	0,0311	0,3219	5,5	7,3	0.0	7,3	2.094
**Pekerjaan**									
Tidak bekerja	2,4	3.7	0,0056	0,1143	5,1	7,1	0.0	7,1	13.816
Sekolah	0,5	1,5	0,0011	0,0397	2,0	5,9	0.0	5,9	3.781
PNS/TNI/Polri/BUMN/ BUMD	2,2	3,3	0,0071	0,1345	6,4	7,7	0.0	7,7	514
Pegawai swasta	1,7	2,9	0,0126	0,2087	5,3	7,3	0.0	7,3	2.828
Wiraswasta	2,8	4,1	0,0065	0,1359	6,5	7,7	0.0	7,7	5.264
Petani/buruh tani	3,9	4,8	0,0006	0,0242	6,7	7,7	0.0	7,7	7.092
Nelayan	3,2	3,9	0,0000	0,0000	7,4	8,1	0.0	8,1	141
Buruh/sopir/pembantu ruta	2,8	4,0	0,0031	0,0593	6,4	7,6	0.0	7,6	3.883
Lainnya	2,7	3,8	0,0095	0,1529	6,2	7,4	0.0	7,4	1.986
**Tempat tinggal**									
Perkotaan	2,3	3,6	0,0083	0,1463	5,2	7,0	0.0	7,0	21.505
Perdesaan	2,9	4,2	0,0006	0,0335	5,9	7,8	0.0	7,8	17.800
**INDONESIA**	**2,6**	**3,9**	**0,0062**	**0,1242**	**5,5**	**7,4**	**0.0**	**7,4**	**39.305**

Tabel 7.3.9

Rata-rata Jumlah Gigi dengan Status Penyakit Jaringan Periodontal Umur ≥15 Tahun menurut Karakteristik, Riskesdas 2018

Karakteristik	Kedalaman Poket						Perdarahan gusi		N Tertimbang
	Sehat /tidak ada poket		Poket Dangkal		Poket Dalam				
	$\bar{x}$	95% CI	$\bar{x}$	95% CI	$\bar{x}$	95% CI	$\bar{x}$	95% CI	
**Kelompok Umur**									
15 - 24	2,5	2,5-2,6	0,2	0,2-0,3	0,01	0,00-0,01	0,69	0,67-0,71	5.627
25 - 34	3,1	3,1-3,2	0,4	0,4-0,5	0,02	0,01-0,02	0,74	0,73-0,76	6.209
35 - 44	2,7	2,6-2,7	0,5	0,4-0,5	0,03	0,03-0,04	0,76	0,74-0,77	8.274
45 - 54	2,1	2,0-2,2	0,4	0,4-0,5	0,04	0,03-0,05	0,76	0,74-0,77	7.880
55 - 64	1,4	1,4-1,5	0,4	0,3-0,4	0,04	0,03-0,05	0,75	0,74-0,77	5.550
65 +	2,5	2,5-2,6	0,2	0,2-0,2	0,02	0,02-0,03	0,73	0,71-0,75	3.517
**Kelompok umur (WHO)**									
15	1,8	1,7-1,9	0,12	0,08-0,16	0,00	0,00-0,01	0,62	0,58-0,67	695
35-44	2,7	2,6-2,7	0,48	0,45-0,51	0,03	0,03-0,04	0,76	0,74-0,77	8.312
65 +	0,9	0,8-0,9	0,22	0,19-0,24	0,02	0,02-0,03	0,73	0,71-0,75	3.532
**Jenis kelamin**									
Laki – laki	2,2	2,2-2,3	0,4	0,4-0,4	0,04	0,03-0,04	0,73	0,72-0,75	15.034
Perempuan	2,3	2,2-2,3	0,4	0,3-0,4	0,02	0,02-0,03	0,74	0,73-0,75	22.023
**Pendidikan**									
Tidak sekolah	1,5	1,4-1,5	0,3	0,3-0,4	0,04	0,03-0,05	0,76	0,74-0,78	2.566
Tidak tamat SD/MI	1,9	1,8-1,9	0,4	0,4-0,4	0,03	0,03-0,04	0,75	0,73-0,76	5.397
Tamat SD/MI	2,2	2,1-2,2	0,4	0,4-0,5	0,03	0,02-0,03	0,77	0,76-0,78	11.357
Tamat SMP/MTS	2,5	2,4-2,5	0,4	0,3-0,4	0,02	0,02-0,03	0,73	0,71-0,74	7.356
Tamat SMA/MA	2,6	2,5-2,6	0,4	0,3-0,4	0,03	0,02-0,04	0,72	0,71-0,74	8.287
Tamat D1-D3/PT	2,5	2,4-2,6	0,4	0,3-0,4	0,03	0,02-0,03	0,67	0,65-0,70	2.093
**Pekerjaan**									
Tidak bekerja	2,2	2,2-2,3	0,4	0,3-0,4	0,02	0,02-0,03	0,74	0,73-0,76	13.314
Sekolah	2,1	2,1-2,2	0,2	0,1-0,2	0,00	0,00-0,01	0,65	0,63-0,68	2.076
PNS/TNI/Polri/BUMN/ BUMD	2,3	2,2-2,5	0,4	0,3-0,4	0,05	0,03-0,08	0,68	0,64-0,73	514
Pegawai swasta	2,7	2,6-2,8	0,3	0,3-0,4	0,02	0,01-0,03	0,69	0,67-0,72	2.825
Wiraswasta	2,4	2,3-2,4	0,4	0,4-0,4	0,03	0,03-0,04	0,73	0,71-0,75	5.256
Petani/buruh tani	2,0	1,9-2,0	0,5	0,4-0,5	0,04	0,03-0,05	0,78	0,76-0,80	7.087
nelayan	2,3	1,9-2,6	0,4	0,2-0,7	0,02	0,00-0,05	0,82	0,74-0,90	140
Buruh/sopir/pembantu ruta	2,4	2,3-2,5	0,5	0,4-0,5	0,04	0,03-0,05	0,75	0,73-0,77	3.868
Lainnya	2,3	2,2-2,4	0,4	0,3-0,4	0,03	0,02-0,03	0,75	0,73-0,78	1.977
**Tempat tinggal**									
Perkotaan	2,2	2,2-2,3	0,4	0,3-0,4	0,03	0,02-0,03	0,72	0,71-0,74	20.229
Perdesaan	2,3	2,2-2,3	0,4	0,4-0,4	0,03	0,03-0,04	0,76	0,75-0,78	16.828
**INDONESIA**	**2,2**	**2,2**	**0,4**	**0,4-0,4**	**0,03**	**0,03-0,03**	**0,74**	**0,73-0,75**	**37.057**

Tabel 7.3.10

Rata-rata Sekstan dengan Kehilangan Perlekatan pada Penduduk Umur ≥15 Tahun menurut Karakteristik, Riskesdas 2018

Karakteristik	Rata-rata Sekstan dengan KehilanganPerlekatan										N Tertimbang
	0-3mm		4-5mm		6-8mm		9-11mm		≥12mm		
	$\bar{x}$	SD	$\bar{x}$	SD	$\bar{x}$	SD	$\bar{x}$	SD	$\bar{x}$	SD	
**Kelompokumur**											
15 – 24	5,6	1,0	0,3	0,8	0,0	0,1	0,0	0,0	0,0	0,0	5.454
25 – 34	5,2	1,3	0,4	1,0	0,0	0,2	0,0	0,1	0,0	0,0	6.036
35 – 44	4,7	1,6	0,6	1,2	0,1	0,4	0,0	0,1	0,0	0,1	8.065
45 – 54	4,0	1,9	0,8	1,3	0,1	0,5	0,0	0,2	0,0	0,1	7.523
55 – 64	3,3	2,0	0,8	1,3	0,2	0,6	0,0	0,2	0,0	0,1	5.120
65 +	2,5	2,0	0,8	1,2	0,3	0,7	0,1	0,4	0,0	0,2	2.813
**Kelompok umur (WHO)**											
15	5,8	0,8	0,1	0,6	0,0	0,1	0,0	0,0	0,0	0,0	663
35-44	4,7	1,6	0,6	1,2	0,1	0,4	0,0	0,1	0,0	0,1	8.065
65 +	2,5	2,0	0,8	1,2	0,3	0,7	0,1	0,4	0,0	0,2	2.813
**Jeniskelamin**											
Laki – laki	4,3	2,0	0,7	1,2	0,1	0,5	0,0	0,2	0,0	0,1	14.158
Perempuan	4,5	1,8	0,6	1,1	0,1	0,4	0,0	0,1	0,0	0,1	20.853
**Pendidikan**											
Tidak sekolah	3,4	2,1	0,7	1,2	0,2	0,6	0,0	0,3	0,0	0,1	2.307
Tidak tamat SD/MI	3,7	2,1	0,8	1,3	0,2	0,5	0,0	0,2	0,0	0,1	4.992
Tamat SD/MI	4,2	1,9	0,7	1,2	0,1	0,5	0,0	0,2	0,0	0,1	10.372
Tamat SMP/MTS	4,8	1,6	0,5	1,1	0,1	0,3	0,0	0,1	0,0	0,1	7.069
Tamat SMA/MA	4,8	1,6	0,5	1,0	0,1	0,4	0,0	0,1	0,0	0,1	8.110
Tamat D1-D3/PT	4,8	1,7	0,5	1,1	0,1	0,3	0,0	0,1	0,0	0,1	2.161
**Pekerjaan**											
Tidak bekerja	4,4	1,9	0,6	1,1	0,1	0,4	0,0	0,2	0,0	0,1	12.050
Sekolah	5,5	1,1	0,3	0,8	0,0	0,1	0,0	0,0	0,0	0,0	2.022
PNS/TNI/Polri/BUMN/BUMD	4,5	1,8	0,6	1,1	0,1	0,4	0,0	0,2	0,0	0,1	576
Pegawai swasta	4,9	1,6	0,5	1,0	0,1	0,4	0,0	0,1	0,0	0,1	2.618
Wiraswasta	4,4	1,8	0,6	1,1	0,1	0,4	0,0	0,2	0,0	0,1	5.042
Petani/buruh tani	3,8	2,1	0,8	1,3	0,2	0,5	0,0	0,2	0,0	0,1	7.211
nelayan	3,9	2,0	0,9	1,4	0,2	0,5	0,1	0,2	0,1	0,2	153
Buruh/sopir/pembantu ruta	4,4	1,9	0,7	1,2	0,1	0,5	0,0	0,2	0,0	0,1	3.420
Lainnya	4,3	1,9	0,6	1,1	0,1	0,4	0,0	0,2	0,0	0,1	1.919
**Tempat tinggal**											
Perkotaan	4,5	1,8	0,5	1,1	0,1	0,4	0,0	0,2	0,0	0,1	17.787
Perdesaan	4,3	2,0	0,7	1,2	0,1	0,4	0,0	0,2	0,0	0,1	17.224
**INDONESIA**	**4,4**	**1,9**	**0,6**	**1,1**	**0,1**	**0,4**	**0,0**	**0,2**	**0,0**	**0,1**	**35.011**

Tabel 7.3.11
Prevalensi Fluorosis Gigi menurut Karakteristik, Riskesdas 2018

Karakteristik	Flourosis Gigi												N Tertimbang
	Normal		Meragukan		Sangat Ringan		Ringan		Sedang		Berat		
	%	95% CI	%	95% CI	%	95% CI	%	95% CI	%	95% CI	%	95% CI	
**Kelompok umur**													
3 – 4*	95,8	94,4-96,9	1,6	1,0-2,6	2,0	1,3-3,1	0,6	0,3-1,3	0,0	0,0-0,0	0,0	0,0-0,0	1113
5 - 9	94,0	93,1-94,8	2,6	2,1-3,2	2,5	2,0-3,2	0,6	0,4-1,0	0,2	0,1-0,5	0,0	0,0-0,0	4.057
10 -14	91,5	90,4-92,6	3,6	3,0-4,4	3,4	2,8-4,1	1,1	0,8-1,6	0,3	0,1-0,6	0,0	0,0-0,1	3.822
15 - 24	94,8	94,0-95,4	2,4	1,9-2,9	2,1	1,7-2,6	0,6	0,4-1,0	0,1	0,1-0,3	0,0	0,0-0,0	5.530
25 - 34	96,2	95,5-96,8	1,8	1,4-2,2	1,5	1,2-2,0	0,3	0,2-0,5	0,2	0,1-0,3	0,0	0,0-0,1	6.131
35 – 44	96,1	95,4-96,6	1,8	1,4-2,3	1,5	1,2-1,8	0,5	0,3-0,7	0,2	0,1-0,3	0,0	0,0-0,1	8.213
45 – 54	96,1	95,5-96,7	1,7	1,4-2,1	1,4	1,1-1,7	0,6	0,4-0,8	0,2	0,1-0,3	0,0	0,0-0,1	7.781
55 – 64	97,2	96,5-97,7	1,4	1,1-1,9	0,8	0,6-1,1	0,5	0,3-0,7	0,1	0,0-0,2	0,0	0,0-0,1	5.406
65+	97,8	97,1-98,4	1,2	0,8-1,7	0,6	0,4-0,9	0,4	0,2-0,8	0,0	0,0-0,0	0,0	0,0-0,0	3.269
**Kelompok umur (WHO)**													
5	95,2	93,2-96,7	1,9	1,1-3,4	2,0	1,1-3,5	0,6	0,2-1,7	0,3	0,1-0,9	0,0	0,0-0,0	691
12	92,2	89,8-94,0	2,8	1,8-4,3	3,8	2,6-5,7	0,9	0,4-2,0	0,3	0,1-1,6	0,0	0,0-0,0	725
15	94,2	91,9-95,8	1,8	1,0-3,2	3,3	2,1-5,1	0,6	0,2-1,7	0,2	0,0-1,5	0,0	0,0-0,0	680
35-44	96,1	95,4-96,6	1,8	1,4-2,3	1,5	1,2-1,8	0,5	0,3-0,7	0,2	0,1-0,3	0,0	0,0-0,1	8.245
65 +	97,8	97,1-98,4	1,2	0,8-1,7	0,6	0,4-0,9	0,4	0,2-0,8	0,0	0,0-0,0	0,0	0,0-0,0	3.282
**Jenis kelamin**													
Laki – laki	95,6	95,1-96,1	1,9	1,7-2,2	1,7	1,4-1,9	0,6	0,5-0,8	0,2	0,1-0,2	0,0	0,0-0,1	19.293
Perempuan	95,6	95,2-96,0	2,1	1,8-2,4	1,7	1,5-1,9	0,5	0,4-0,6	0,2	0,1-0,2	0,0	0,0-0,0	26.028
**Pendidikan**													
Tidak sekolah	96,0	95,1-96,7	1,7	1,3-2,3	1,6	1,2-2,1	0,6	0,4-0,9	0,1	0,0-0,4	0,0	0,0-0,0	3.827
Tidak tamat SD/MI	94,4	93,8-95,1	2,6	2,2-3,1	2,0	1,7-2,3	0,7	0,6-1,0	0,3	0,2-0,4	0,0	0,0-0,1	9.395
Tamat SD/MI	95,4	94,7-95,9	2,0	1,6-2,3	1,8	1,5-2,2	0,7	0,5-0,9	0,2	0,1-0,3	0,0	0,0-0,1	12.776
Tamat SMP/MTS	95,8	95,1-96,4	1,9	1,5-2,4	1,7	1,4-2,0	0,5	0,3-0,8	0,2	0,1-0,3	0,0	0,0-0,0	7.378
Tamat SMA/MA	96,7	96,2-97,2	1,6	1,3-2,0	1,2	1,0-1,5	0,3	0,2-0,5	0,1	0,0-0,2	0,0	0,0-0,1	8.093
Tamat D1-D3/PT	97,0	95,8-97,8	2,0	1,3-3,1	0,9	0,6-1,5	0,1	0,0-0,5	0,0	0,0-0,0	0,0	0,0-0,0	2.017
**Pekerjaan**													
Tidak bekerja	96,0	95,5-96,4	1,8	1,-5-2,2	1,6	1,4-1,9	0,5	0,4-0,6	0,1	0,1-0,2	0,0	0,0-0,0	13.937
Sekolah	92,8	91,7-93,7	3,2	2,7-3,9	2,9	2,4-3,5	0,9	0,6-1,3	0,2	0,1-0,4	0,0	0,0-0,1	4.862
PNS/TNI/Polri/BUMN/BUMD	96,1	93,9-97,6	1,8	1,0-3,5	1,4	0,6-3,2	0,4	0,1-1,4	0,2	0,0-1,2	0,0	0,0-0,0	493
Pegawai swasta	97,5	96,8-98,1	1,3	0,9-1,9	0,9	0,5-1,4	0,2	0,1-0,4	0,1	0,0-0,2	0,0	0,0-0,0	2.738
Wiraswasta	96,4	95,6-97,0	1,8	1,4-2,3	1,3	1,0-1,8	0,4	0,2-0,6	0,1	0,1-0,3	0,0	0,0-0,1	5.166
Petani/buruh tani	96,3	95,4-97,0	1,7	1,3-2,2	1,2	0,9-1,6	0,7	0,4-1,0	0,2	0,1-0,4	0,0	0,0-0,1	6.962
nelayan	93,4	85,2-97,2	4,3	1,3-13,3	1,2	0,4-4,0	1,0	0,1-6,9	0,0	0,0-0,0	0,0	0,0-0,0	137
Buruh/sopir/pembantu ruta	95,6	94,7-96,4	1,9	1,4-2,5	1,6	1,1-2,2	0,8	0,5-1,2	0,2	0,1-0,5	0,0	0,0-0,0	3.819
Lainnya	96,7	95,5-97,5	1,8	1,2-2,8	1,0	0,6-1,6	0,3	0,1-0,7	0,2	0,1-0,6	0,0	0,0-0,0	1.962
**Tempat tinggal**													
Perkotaan	95,7	95,1-96,1	2,2	1,9-2,6	1,6	1,4-1,8	0,5	0,4-0,6	0,1	0,1-0,2	0,0	0,0-0,0	24.641
Perdesaan	95,6	94,9-96,2	1,8	1,5-2,1	1,8	1,5-2,1	0,7	0,5-0,8	0,2	0,1-0,3	0,0	0,0-0,1	20.680
**Indonesia**	**95,6**	**95,2-96,0**	**2,0**	**1,8-2,2**	**1,7**	**1,5-1,9**	**0,6**	**0,5-0,7**	**0,2**	**0,1-0,2**	**0,0**	**0,0-0,0**	**45.321**

Tabel 7.3.12
Proporsi Erosi Gigi Menurut Karakteristik, Riskesdas 2018

Karakteristik	Erosi Gigi								N Tertimbang
	Tanpa Erosi		Pada Email		Pada Dentin		Keterlibatan Pulpa		
	%	95% CI	%	95% CI	%	95% CI	%	95% CI	
**Kelompok umur**									
3 – 4	98,9	98,0-99,4	0,8	0,4-1,6	0,3	0,1-1,0	0,0	0,0-0,0	1.141
5 - 9	98,7	98,2-99,0	0,9	0,6-1,4	0,4	0,2-0,6	0,0	0,0-0,1	4.149
10 -14	98,8	98,3-99,1	1,1	0,8-1,5	0,1	0,0-0,3	0,0	0,0-0,1	3.903
15 - 24	95,6	94,8-96,3	4,0	3,3-4,8	0,3	0,2-0,6			5.640
25 - 34	92,6	91,7-93,4	6,5	5,8-7,4	0,8	0,6-1,2	0,0	0,0-0,2	6.223
35 - 44	89,2	88,2-90,1	9,2	8,3-10,1	1,6	1,3-2,0	0,1	0,0-0,2	8.293
45 - 54	85,0	83,7-86,2	11,8	10,8-13,0	3,0	2,6-3,6	0,1	0,1-0,2	7.898
55 - 64	82,8	81,3-84,1	12,6	11,5-13,9	4,4	3,7-5,1	0,2	0,1-0,4	5.562
65 +	84,7	83,2-86,1	10,0	8,9-11,3	4,7	4,0-5,6	0,6	0,3-0,9	3.524
**Kelompok umur (WHO)**									
5	97,9	96,4-98,8	1,2	0,6-2,5	0,9	0,4-2,1	0,0	0,0-0,0	711
12	98,9	97,8-99,5	1,0	0,5-2,1	0,1	0,0-0,5	0,0	0,0-0,0	740
15	97,3	95,7-98,4	2,6	1,6-4,3	0,1	0,0-0,4	0,0	0,0-0,0	697
35-44	89,2	88,2-90,1	9,2	8,3-10,1	1,6	1,3-2,0	0,1	0,0-0,2	8.339
65 +	84,7	83,2-86,1	10,0	8,9-11,3	4,7	4,0-5,6	0,6	0,3-0,9	3.544
**Jenis kelamin**									
Laki – laki	89,7	89,0-90,4	7,9	7,3-8,5	2,3	2,0-2,6	0,1	0,1-0,2	19.815
Perempuan	91,1	90,5-91,7	7,2	6,7-7,8	1,6	1,4-1,8	0,1	0,1-0,2	26.518
**Pendidikan**									
Tidak sekolah	87,9	86,4-89,3	8,9	7,7-10,2	2,9	2,3-3,7	0,3	0,1-0,4	4.000
Tidak tamat SD/MI	90,4	89,5-91,3	7,2	6,5-8,0	2,2	1,8-2,6	0,2	0,1-0,3	9.618
Tamat SD/MI	89,1	88,1-90,0	8,7	7,9-9,5	2,1	1,8-2,5	0,1	0,1-0,2	12.961
Tamat SMP/MTS	91,1	90,2-92,0	7,5	6,7-8,4	1,4	1,1-1,7	0,0	0,0-0,1	7.484
Tamat SMA/MA	91,7	90,8-92,5	6,8	6,1-7,6	1,5	1,2-1,8	0,1	0,0-0,1	8.301
Tamat D1-D3/PT	90,7	88,9-92,2	7,6	6,3-9,1	1,7	1,1-2,4	0,1	0,0-0,2	2.096
**Pekerjaan**									
Tidak bekerja	90,4	89,6-91,2	7,7	7,0-8,4	1,8	1,6-2,1	0,1	0,1-0,2	14.284
Sekolah	97,8	97,2-98,2	2,0	1,6-2,5	0,3	0,2-0,5	0,0	0,0-0,1	4.978
PNS/TNI/Polri/BUMN/ BUMD	87,5	84,0-90,3	10,4	7,7-13,8	1,9	1,0-3,6	0,2	0,1-0,9	515
Pegawai swasta	91,0	89,5-92,3	7,2	6,1-8,6	1,6	1,2-2,3	0,1	0,0-0,4	2.832
Wiraswasta	88,1	86,8-89,2	9,2	8,1-10,3	2,7	2,2-3,3	0,1	0,0-0,3	5.268
Petani/buruh tani	84,1	82,5-85,6	12,1	10,9-13,5	3,5	3,0-4,2	0,2	0,2-0,4	7.098
nelayan	79,5	66,8-88,2	19,7	11,1-32,6	0,5	0,1-2,2	0,3	0,0-2,1	141
Buruh/sopir/pembant u ruta	86,5	84,7-88,1	11,2	9,8-12,8	2,2	1,7-2,9	0,1	0,0-0,3	3.885
Lainnya	89,4	87,4-91,1	8,4	6,9-10,2	2,1	1,5-3,0	0,1	0,0-0,5	1.991
**Tempat tinggal**									
Perkotaan	91,0	90,1-91,7	7,2	6,5-7,9	1,8	1,5-2,1	0,1	0,0-0,1	25.430
Perdesaan	89,9	89,0-90,8	7,9	7,2-8,7	2,0	1,7-2,3	0,2	0,1-0,2	20.903
**Indonesia**	**90,5**	**89,9-91,1**	**7,5**	**7,0-8,0**	**1,9**	**1,7-2,1**	**0,1**	**0,1-0,1**	**46.333**

Tabel 7.3.13
Proporsi Penyakit Lesi Oral Mukosa menurut Karakteristik, Riskesdas 2018

Karakteristik	Lesi oral mukosa						N Tertimbang
	SAR		Suspek Kanker Mulut		Lesi lain		
	%	95% CI	%	95% CI	%	95% CI	
**Kelompok umur**							
3 – 4	1,0	0,5-1,8	0,0	0,0-0,0	1,3	0,7-2,2	1.141
5 - 9	1,6	1,2-2,1	0,0	0,0-0,0	3,1	2,5-3,9	4.149
10 -14	2,2	1,7-2,7	0,0	0,0-0,2	3,7	3,0-4,5	3.903
15 - 24	3,7	3,2-4,4	0,0	0,0-0,1	5,2	4,5-6,0	5.640
25 - 34	3,2	2,8-3,8	0,1	0,0-0,2	5,7	5,0-6,5	6.223
35 - 44	3,2	2,8-3,6	0,1	0,0-0,2	5,7	5,1-6,5	8.293
45 - 54	2,9	2,6-3,4	0,1	0,0-0,2	6,0	5,3-6,8	7.898
55 - 64	3,1	2,6-3,6	0,3	0,1-0,5	5,9	5,1-6,7	5.562
65 +	1,9	1,5-2,4	0,1	0,0-0,4	6,4	5,5-7,4	3.524
**Kelompok umur (WHO)**							
5	0,7	0,3-1,7	0,0	0,0-0,0	3,0	1,8-4,7	711
12	2,7	1,7-4,4	0,0	0,0-0,0	3,0	1,9-4,7	740
15	2,3	1,4-3,8	0,0	0,0-0,0	4,8	3,4-6,9	697
35-44	3,2	2,8-3,6	0,1	0,0-0,2	5,7	5,1-6,5	8.339
65 +	1,9	1,5-2,4	0,1	0,0-0,4	6,4	5,5-7,4	3.544
**Jenis kelamin**							
Laki – laki	2,6	2,4-2,9	0,1	0,1-0,2	5,4	5,0-6,0	19.815
Perempuan	3,0	2,7-3,3	0,1	0,0-0,1	5,1	4,7-5,6	26.518
**Pendidikan**							
Tidak sekolah	2,3	1,9-2,9	0,1	0,0-0,3	4,2	3,6-5,0	4.000
Tidak tamat SD/MI	2,3	2,0-2,7	0,2	0,1-0,3	4,7	4,2-5,4	9.618
Tamat SD/MI	2,9	2,5-3,2	0,1	0,0-0,2	5,7	5,1-6,4	12.961
Tamat SMP/MTS	3,5	3,0-4,0	0,1	0,0-0,2	5,9	5,2-6,6	7.484
Tamat SMA/MA	3,3	2,9-3,8	0,0	0,0-0,1	5,8	5,1-6,6	8.301
Tamat D1-D3/PT	3,6	2,8-4,5	0,1	0,0-0,3	5,5	4,4-6,9	2.096
**Pekerjaan**							
Tidak bekerja	3,0	2,7-3,3	0,1	0,1-0,2	5,9	5,4-6,6	14.284
Sekolah	3,1	2,6-3,7	0,0	0,0-0,1	3,9	3,2-4,7	4.978
PNS/TNI/Polri/BUMN/BUMD	4,4	2,8-6,7	0,0	0,0-0,2	4,6	2,9-7,3	515
Pegawai swasta	3,3	2,6-4,1	0,1	0,1-0,4	5,8	4,8-7,0	2.832
Wiraswasta	3,6	3,0-4,2	0,1	0,1-0,3	6,3	5,4-7,3	5.268
Petani/buruh tani	2,3	1,9-2,7	0,0	0,0-0,1	5,0	4,3-5,8	7.098
nelayan	3,9	1,4-10,4	0,0	0,0-0,0	7,3	2,9-17,5	141
Buruh/sopir/pembantu ruta	3,5	2,9-4,2	0,2	0,1-0,5	6,0	5,1-7,1	3.885
Lainnya	2,6	1,9-3,5	0,2	0,1-0,6	6,5	5,1-8,1	1.991
**Tempat tinggal**							
Perkotaan	3,0	2,7-3,3	0,1	0,1-0,2	5,6	5,0-6,1	25.430
Perdesaan	2,6	2,3-2,9	0,1	0,0-0,1	4,9	4,3-5,6	20.903
**INDONESIA**	**2,8**	**2,6-3,0**	**0,1**	**0,1-0,1**	**5,3**	**4,8-5,7**	**46.333**

Tabel 7.3.14
Proporsi Kebutuhan Perawatan menurut Karakteristik, Riskesdas 2018

Karakteristik	Kebutuhan Perawatan						N Tertimbang
	Tidak Perlu Perawatan		Perlu, tidak segera		Perlu, segera		
	%	95% CI	%	95% CI	%	95% CI	
**Kelompok Umur**							
3 - 4	46,0	42,6-49,5	51,9	48,4-55,3	2,1	1,3-3,3	1.141
5 - 9	33,8	31,7-36,0	61,6	59,4-63,8	4,6	3,9-5,4	4.149
10 -14	40,3	38,1-42,4	55,5	53,4-57,6	4,2	3,5-5,0	3.903
15 - 24	29,7	27,9-31,5	66,9	65,1-68,7	3,4	2,8-4,1	5.640
25 - 34	23,2	21,7-24,9	73,1	71,4-74,8	3,6	3,0-4,3	6.223
35 - 44	20,0	18,6-21,5	75,9	74,4-77,4	4,1	3,6-4,7	8.293
45 - 54	19,0	17,5-20,5	76,4	74,8-78,0	4,6	4,0-5,2	7.898
55 - 64	17,1	15,7-18,7	77,5	75,9-79,0	5,4	4,7-6,2	5.562
65 +	20,0	18,3-21,9	74,5	72,5-76,3	5,5	4,7-6,5	3.524
**Kelompok umur (WHO)**							
5	46,0	42,6-49,5	51,9	48,4-55,3	2,1	1,3-3,3	1.141
12	33,8	31,7-36,0	61,6	59,4-63,8	4,6	3,9-5,4	4.149
15	40,3	38,1-42,4	55,5	53,4-57,6	4,2	3,5-5,0	3.903
35-44	29,7	27,9-31,5	66,9	65,1-68,7	3,4	2,8-4,1	5.640
65 +	23,2	21,7-24,9	73,1	71,4-74,8	3,6	3,0-4,3	6.223
**Jenis kelamin**							
Laki – laki	25,3	24,1-26,6	70,5	69,2-71,8	4,1	3,7-4,5	19.815
Perempuan	24,2	23,0-25,4	71,4	70,1-72,6	4,4	4,0-4,9	26.518
**Pendidikan**							
Tidak sekolah	25,3	23,2-27,5	69,8	67,6-71,9	4,9	4,1-5,8	4.000
Tidak tamat SD/MI	26,2	24,7-27,9	69,1	67,4-70,7	4,7	4,1-5,3	9.618
Tamat SD/MI	22,0	20,6-23,5	73,6	72,0-75,1	4,4	3,9-4,9	12.961
Tamat SMP/MTS	22,6	21,1-24,1	73,4	71,8-75,0	4,0	3,5-4,7	7.484
Tamat SMA/MA	24,1	22,6-25,8	71,6	70,0-73,2	4,2	3,7-4,9	8.301
Tamat D1-D3/PT	26,8	24,2-29,5	69,8	67,1-72,4	3,4	2,6-4,5	2.096
**Pekerjaan**							
Tidak bekerja	22,8	21,5-24,2	72,4	71,0-73,8	4,8	4,3-5,3	14.284
Sekolah	36,4	34,5-38,3	59,6	57,6-61,5	4,0	3,4-4,7	4.978
PNS/TNI/Polri/BUMN/BUMD	26,0	21,8-30,8	70,4	65,7-74,8	3,6	2,3-5,5	515
Pegawai swasta	25,5	23,3-27,8	70,7	68,3-73,0	3,8	3,1-4,7	2.832
Wiraswasta	20,1	18,4-21,9	75,5	73,7-77,3	4,4	3,7-5,2	5.268
Petani/buruh tani	17,6	15,8-19,5	78,7	76,7-80,5	3,7	3,2-4,4	7.098
nelayan	15,7	9,3-25,2	80,0	70,0-87,3	4,3	2,0-9,0	141
Buruh/sopir/pembantu ruta	20,5	18,4-22,7	74,9	72,6-77,0	4,7	3,9-5,6	3.885
Lainnya	22,2	19,4-25,2	73,6	70,5-76,4	4,3	3,3-5,5	1.991
**Tempat tinggal**							
Perkotaan	26,9	25,3-28,5	68,3	66,6-69,9	68,3	66,6-69,9	25.430
Perdesaan	22,0	20,4-23,7	74,3	72,6-76,0	74,3	72,6-76,0	20.903
**INDONESIA**	**24,7**	**23,5-25,9**	**71,0**	**69,8-72,2**	**4,3**	**3,9-4,7**	**46.333**

7.4 Perilaku Menyikat Gigi

Untuk mencegah terjadinya karies gigi dan agar higiene mulut terjaga baik, seseorang perlu menjaga kebersihan gigi dan mulutnya dengan cara menyikat gigi dengan baik dan teratur.

Menyikat gigi adalah kegiatan membersihkan gigi menggunakan sikat gigi atau alat lain (misalnya serabut kelapa) dengan atau tanpa pasta gigi.

Menyikat gigi setiap hari adalah kegiatan membersihkan gigi yang dilakukan secara rutin tiap hari di luar keadaan darurat (sakit, kecelakaan, retak/patah rahang, trismus (tidak dapat membuka mulut), dan keadaan lain yg tidak memungkinkan seseorang menyikat gigi).

Perilaku benar dalam menyikat gigi mengacu pada FDI (Fédération Dentaire Internationale), adalah kebiasaan menyikat gigi setiap hari, minimal dua kali sehari, sesudah makan pagi dan sebelum tidur malam.

Tabel 7.4.1
Proporsi Perilaku Menyikat Gigi pada Penduduk Umur ≥3 Tahun menurut Provinsi, Riskesdas 2018

Provinsi	Menyikat gigi setiap hari			Waktu menyikat gigi yang benar[1]		
	%	95%CI	N tertimbang	%	95%CI	N tertimbang
Aceh	93,6	93,2-94,0	18.855	2,8	2,4-3,1	17.323
Sumatera Utara	92,9	92,5-93,3	51.977	1,6	1,4-1,8	47.386
Sumatera Barat	95,3	94,9-95,6	19.399	1,2	1,0-1,4	18.144
Riau	95,8	95,4-96,2	24.405	1,6	1,4-1,9	22.958
Jambi	96,4	95,9-96,9	12.936	1,0	0,7-1,4	12.246
Sumatera Selatan	96,0	95,7-96,4	30.341	1,4	1,1-1,7	28.603
Bengkulu	97,0	96,7-97,4	7.123	1,7	1,3-2,2	6.786
Lampung	96,5	96,3-96,8	30.3	1,1	0,9-1,3	28.717
Bangka Belitung	96,0	95,5-96,4	5.301	4,5	4,0-5,2	4.994
Kepulauan Riau	97,6	97,1-98,0	7.679	2,4	1,9-3,0	7.357
DKI Jakarta	97,5	97,0-97,9	38.124	2,7	2,3-3,2	36.481
Jawa Barat	96,8	96,6-97,0	176.728	2,8	2,6-3,1	167.911
Jawa Tengah	95,4	95,2-95,4	126.225	2,0	1,8-2,1	118.164
DI Yogyakarta	94,9	94,4-95,4	13.989	6,0	5,2-6,8	13.033
Jawa Timur	94,5	94,3-94,7	145.173	1,8	1,7-2,0	134.727
Banten	95,3	94,9-95,8	45.831	2,3	2,0-2,7	42.895
Bali	92,9	92,3-93,4	15.737	5,3	4,8-6,0	14.349
Nusa Tenggara Barat	94,5	93,9-95,0	18.058	4,5	4,0-5,1	16.744
Nusa Tenggara Timur	84,4	83,6-85,2	19.115	3,7	3,4-4,1	15.845
Kalimantan Barat	95,3	94,9-95,7	18.073	3,9	3,4-4,5	16.913
Kalimantan Tengah	96,9	96,6-97,3	9.615	3,3	2,8-3,8	9.149
Kalimantan Selatan	96,0	95,7-96,4	15.077	5,0	4,4-5,6	14.213
Kalimantan Timur	97,5	97,1-97,7	13.195	3,6	3,1-4,2	12.628
Kalimantan Utara	95,6	94,5-96,6	2.547	3,0	2,5-3,7	2.391
Sulawesi Utara	96,3	95,9-96,6	9.055	3,5	3,1-4,2	8.556
Sulawesi Tengah	93,1	92,5-93,6	10.837	5,5	4,8-6,3	9.903
Sulawesi Selatan	93,5	93,1-93,8	31.703	8,8	8,3-9,4	29.085
Sulawesi Tenggara	93,6	92,9-94,3	9.464	8,2	7,3-9,1	8.698
Gorontalo	95,9	95,3-96,4	4.286	4,6	4,0-5,4	4.034
Sulawesi Barat	93,0	92,3-93,7	4.831	7,1	6,1-8,3	4.412
Maluku	96,1	95,7-96,5	6.334	4,0	3,4-4,7	5.977
Maluku Utara	91,5	90,6-92,3	4.404	5,7	4,8-6,8	3.955
Papua Barat	90,3	88,9-9,5	3.341	4,9	4,1-5,8	2.962
Papua	54,4	52,2-56,7	11.987	5,6	4,8-6,6	6.407
**INDONESIA**	**94,7**	**94,6-94,7**	**962.045**	**2,8**	**2,8-2,9**	**893.948**

[1]menyikat gigi setelah sarapan dan sebelum tidur

Tabel 7.4.2

Proporsi Perilaku Menyikat Gigi pada Penduduk Umur >3 Tahun menurut menurut Karakteristik, Riskesdas 2018

Karakteristik	Menyikat gigi setiap hari			Waktu menyikat gigi yang benar[1]		
	%	95%CI	N tertimbang	%	95%CI	N tertimbang
**Kelompok Umur**						
3 - 4	86,7	86,2-87,2	36.608	1,1	1,0-1,3	31.167
5 - 9	93,2	92,9-93,4	92.746	1,4	1,3-1,5	84.810
10 -14	96,5	96,3-96,7	89.506	2,1	1,9-2,2	84.762
15 - 24	98,5	98,4-98,6	165.565	3,3	3,2-3,5	160.102
25 - 34	98,3	98,2-98,4	159.633	3,2	3,1-3,4	153.997
35 - 44	97,8	97,7-97,9	151.467	3,2	3,1-3,4	145.435
45 - 54	96,7	96,5-96,8	124.593	3,1	3,0-3,3	118.232
55 - 64	91,2	91,0-91,5	83.211	2,9	2,7-3,1	74.513
65+	71,0	70,5-71,6	58.717	2,9	2,7-3,2	40.931
**Jenis kelamin**						
Laki – laki	94,0	93,8-94,1	482.729	2,5	2,4-2,6	445.213
Perempuan	95,4	95,3-95,5	479.316	3,1	3,1-3,2	448.735
**Pendidikan**						
Tidak sekolah	82,1	81,6-82,5	57.171	1,7	1,6-1,9	57.171
Tidak tamat SD/MI	91,8	91,6-92,0	163.61	2,0	1,9-2,1	163.61
Tamat SD/MI	95,1	95,0-95,2	201.794	2,5	2,4-2,6	201.794
Tamat SMP/MTS	98,0	97,8-98,1	154.304	2,7	2,6-2,8	154.304
Tamat SMA/MA	98,7	98,6-98,8	204.35	3,6	3,5-3,7	204.35
Tamat D1/D2/D3/PT	99,2	99,1-99,3	62.496	6,2	5,9-6,5	62.496
**Pekerjaan**						
Tidak bekerja	93,1	93,0-93,3	213.855	3,3	3,1-3,4	213.855
Sekolah	97,6	97,5-97,8	121.505	2,7	2,6-2,9	121.505
PNS/TNI/Polri/BUMN/BUMD	99,0	98,8-99,1	21.331	7,1	6,6-7,6	21.331
Pegawai swasta	99,2	99,1-99,3	73.883	3,7	3,5-3,9	73.883
Wiraswasta	98,0	97,9-98,1	101.596	3,2	3,0-3,4	101.596
Petani/buruh tani	89,8	89,5-90,0	117.533	2,1	2,0-2,2	117.533
nelayan	93,0	92,1-93,8	5.077	3,1	2,6-3,8	5.077
Buruh/sopir/pembantu ruta	97,2	97,0-97,4	72.203	2,2	2,0-2,3	72.203
Lainnya	96,6	96,3-96,8	38.58	3,7	3,5-4,0	38.580
**Tempat tinggal**						
Perkotaan	96,4	96,3-96,5	498.771	3,1	3,0-3,2	498.771
Perdesaan	92,5	92,4-92,6	395.177	2,5	2,4-2,6	395.177

[1]menyikat gigi setelah sarapan dan sebelum tidur

BAB 8
KESEHATAN JIWA

8.1 Gangguan Jiwa Skizofrenia Dan Psikosis Dalam Keluarga

Prevalensi rumah tangga dengan ART gangguan jiwa skizofrenia/psikosis dihitung dengan formula:

$$Prevalensi\ rumah\ tangga\ dengan\ ART\ gangguan\ jiwa\ skizofrenia/psikosis = \frac{Jumlah\ rumah\ tangga\ yang\ memiliki\ ART\ gangguan\ jiwa\ skizofrenia/psikosis}{Jumlah\ seluruh\ Rumah\ Tangga\ yang\ diwawancara}$$

Catatan: Riskesdas 2018 melaporkan prevalensi rumah tangga yang mempunyai ART gangguan jiwa skizofrenia atau psikosis. Berbeda dengan Riskesdas 2013 yang melaporkan prevalensi gangguan jiwa skizofrenia atau psikosis.

Proporsi rumah tangga yang memiliki ART gangguan jiwa skizofrenia/psikosis yang pernah dipasung dihitung dengan formula:

$$Proporsi\ rumah\ tangga\ yang\ memiliki\ ART\ gangguan\ jiwa\ skizofrenia/psikosis\ yang\ pernah\ dipasung = \frac{Jumlah\ \ rumah\ tangga\ yang\ memiliki\ ART\ gangguan\ jiwa\ skizofrenia/psikosis\ yang\ pernah\ dipasung}{Jumlah\ \ rumah\ tangga\ yang\ memiliki\ ART\ gangguan\ jiwa\ skizofrenia/psikosis}$$

Proporsi rumah tangga yang memiliki ART gangguan jiwa skizofrenia/psikosis yang pernah dipasung 3 bulan dihitung dengan formula:

$$Proporsi\ rumah\ tangga\ yang\ memiliki\ ART gangguan\ jiwa\ skizofrenia/psikosis\ yang\ pernah\ dipasung\ 3\ bulan = \frac{Jumlah\ Rumah\ tangga\ yang\ memiliki\ ART gangguan\ jiwa\ skizofrenia/psikosis\ yang\ pernah\ dipasung\ 3\ bulan\ terakhir}{jumlah\ rumah\ tangga\ yang\ memiliki\ ART gangguan\ jiwa\ skizofrenia/psikosis\ pernah\ dipasung}$$

Tabel 8.1.1
Prevalensi (permil) Rumah Tangga dengan ART Gangguan Jiwa Skizofrenia/Psikosis menurut Provinsi, Riskesdas 2018

Provinsi	Gangguan jiwa skizofrenia/psikosis		N Tertimbang
	(‰)	95%CI(‰)	
Aceh	8,7	6,7 - 11,2	5.111
Sumatera Utara	6,3	4,7 - 8,4	13.991
Sumatera Barat	9,1	7,0 - 11,8	5.184
Riau	6,1	4,3 - 8,6	6.792
Jambi	6,6	4,6 - 9,3	3.698
Sumatera Selatan	8,0	5,9 - 11,0	8.538
Bengkulu	5,3	3,6 - 7,9	2.082
Lampung	6,0	4,2 - 8,7	8.838
Bangka Belitung	6,7	4,2 - 10,5	1.518
Kepulauan Riau	2,8	1,6 - 5,1	2.367
DKI Jakarta	6,6	4,2 - 10,2	11.849
Jawa Barat	5,0	3,9 - 6,3	55.133
Jawa Tengah	8,7	7,5 - 10,2	37.516
DI Yogyakarta	10,4	7,0 - 15,3	4.514
Jawa Timur	6,4	5,4 - 7,6	43.890
Banten	5,7	3,7 - 8,8	12.733
Bali	11,1	7,8 - 15,8	4.509
Nusa Tenggara Barat	9,6	6,9 - 13,4	5.573
Nusa Tenggara Timur	3,6	2,6 - 5,0	4.761
Kalimantan Barat	7,9	5,9 - 10,6	4.911
Kalimantan Tengah	4,4	2,6 - 7,5	2.907
Kalimantan Selatan	5,1	3,6 - 7,2	4.648
Kalimantan Timur	5,1	3,1 - 8,2	3.794
Kalimantan Utara	6,8	3,0 - 15,1	695
Sulawesi Utara	7,4	5,1 - 10,9	2.579
Sulawesi Tengah	8,2	5,6 - 11,9	3.055
Sulawesi Selatan	8,8	7,2 - 10,8	8.677
Sulawesi Tenggara	5,6	3,6 - 8,7	2.553
Gorontalo	6,6	3,8 - 11,4	1.182
Sulawesi Barat	7,7	4,8 - 12,2	1.320
Maluku	3,9	2,3 - 6,6	1.633
Maluku Utara	4,8	2,8 - 8,1	1.134
Papua Barat	6,8	3,0 - 15,0	961
Papua	4,5	2,2 - 9,3	4.010
**INDONESIA**	**6,7**	**6,2 - 7,1**	**282.654**

Tabel 8.1.2
Prevalensi (permil) Rumah Tangga dengan ART Gangguan Jiwa Skizofrenia/Psikosis menurut Tempat Tinggal, Riskesdas 2018

Tempat Tinggal	Gangguan jiwa skizofrenia/psikosis		N tertimbang
	(‰)	95%CI (‰)	
Perkotaan	6,4	5,8 - 7,1	155.248
Perdesaan	7,0	6,4 - 7,5	127.406
**INDONESIA**	6,7	6,2 - 7,1	282.654

Tabel 8.1.3
Proporsi Rumah Tangga yang Memiliki ART Gangguan Jiwa Skizofrenia/Psikosis yang Pernah Dipasung menurut Tempat Tinggal, Riskesdas 2018

Tempat Tinggal	Pernah pasung (seumur hidup)		N tertimbang	Pasung 3 bulan terakhir		N tertimbang
	(%)	95% CI		(%)	95% CI	
Perkotaan	10,7	8,3 - 13,6	1.021	31,1	26,3 - 36,2	125
Perdesaan	17,7	14,9 - 21,0	907	31,8	26,9 - 37,1	183
**INDONESIA**	**14,0**	**12,1 - 16,2**	**1.929**	**31,5**	**28,0 - 35,2**	**309**

8.2 Depresi

Prevalensi depresi dihitung dengan formula:

$$Prevalensi\ depresi = \frac{Jumlah\ ART\ umur\ 15\ tahun\ ke\ atas\ yang\ saat\ ini\ mengalami\ gangguan\ depresi\ (menurut\ MINI)}{Jumlah\ seluruh\ ART\ berumur\ \geq 15\ tahun}$$

Tabel 8.2.1
Prevalensi Depresi pada Penduduk Umur ≥15 Tahun menurut Provinsi, Riskesdas 2018

Provinsi	Depresi[1]				N tertimbang
	(%)	95% CI			
Aceh	4,4	4,0	-	4,8	13.285
Sumatera Utara	7,9	7,3	-	8,5	36.147
Sumatera Barat	8,2	7,6	-	8,8	13.683
Riau	6,6	6,1	-	7,2	17.165
Jambi	1,8	1,5	-	2,1	9.439
Sumatera Selatan	3,4	3,0	-	3,8	21.889
Bengkulu	4,8	4,2	-	5,5	5.144
Lampung	3,2	3,0	-	3,6	21.994
Bangka Belitung	6,5	5,7	-	7,3	3.884
Kepulauan Riau	3,7	3,0	-	4,5	5.431
DKI Jakarta	5,9	5,2	-	6,7	28.747
Jawa Barat	7,8	7,3	-	8,2	130.528
Jawa Tengah	4,4	4,2	-	4,6	95.461
DI Yogyakarta	5,5	4,92	-	6,1	10.811
Jawa Timur	4,5	4,3	-	4,8	111.879
Banten	8,7	8,0	-	9,5	33.269
Bali	5,1	4,6	-	5,7	11.885
Nusa Tenggara Barat	8,8	8,0	-	9,7	12.945
Nusa Tenggara Timur	9,7	9,0	-	10,4	12.666
Kalimantan Barat	6,2	5,6	-	6,8	12.876
Kalimantan Tengah	3,9	3,3	-	4,5	6.981
Kalimantan Selatan	4,8	4,4	-	5,3	10.982
Kalimantan Timur	6,2	5,4	-	7,1	9.602
Kalimantan Utara	5,7	4,7	-	7,0	1.816
Sulawesi Utara	6,6	6,0	-	7,4	6.754
Sulawesi Tengah	12,3	11,4	-	13,2	7.763
Sulawesi Selatan	7,8	7,3	-	8,4	22.798
Sulawesi Tenggara	6,3	5,6	-	7,1	6.440
Gorontalo	10,3	9,1	-	11,6	3.117
Sulawesi Barat	4,3	3,3	-	5,5	3.380
Maluku	5,3	4,6	-	6,2	4.329
Maluku Utara	9,3	8,3	-	10,5	2.976
Papua Barat	7,4	6,3	-	8,7	2.343
Papua	4,0	3,5	-	4,5	8.279
**INDONESIA**	**6,1**	**6,0**	**-**	**6,2**	**706.689**

[1]berdasarkan *Mini International Neuropsychiatric Interview*

Tabel 8.2.2
Prevalensi Depresi pada Penduduk Umur >15 Tahun menurut Karakteristik, Riskesdas 2018

Karakteristik	Depresi[1]		N tertimbang
	%	95% CI	
**Kelompok Umur**			
15 – 24	6,2	5,9 - 6,4	157.695
25 – 34	5,4	5,2 - 5,6	152.522
35 – 44	5,6	5,4 - 5,8	144.800
45 – 54	6,1	5,9 - 6,3	119.070
55 – 64	6,5	6,2 - 6,8	79.170
65 – 74	8,0	7,6 - 8,4	37.491
75+	8,9	8,3 - 9,5	15.941
**Jenis kelamin**			
Laki-laki	4,7	4,6 - 4,9	352.269
Perempuan	7,4	7,2 - 7,6	354.420
**Pendidikan**			
Tidak/belum pernah sekolah	8,2	7,8 - 8,7	38.204
Tidak tamat SD/MI	8,1	7,8 - 8,4	81.510
Tamat SD/MI	7,0	6,7 - 7,2	172.323
Tamat SLTP/MTS	6,0	5,8 - 6,2	150.634
Tamat SLTA/MA	5,0	4,9 - 5,2	202.438
Tamat D1/D2/D3/PT	3,1	2,9 - 3,4	61.579
**Pekerjaan**			
Tidak bekerja	8,1	7,9 - 8,3	204.063
Sekolah	6,0	5,7 - 6,3	56.924
PNS/TNI/POLRI/BUMN/BUMD	2,4	2,1 - 2,7	21.374
Pegawai swasta	4,3	4,0 - 4,6	73.840
Wiraswasta	5,1	4,8 - 5,3	102.763
Petani/buruh tani	5,5	5,3 - 5,6	129.477
Nelayan	6,9	6,0 - 7,9	5.386
Buruh/sopir/pembantu ruta	5,8	5,5 - 6,2	73.472
Lainnya	5,9	5,5 - 6,3	39.389
**Tempat tinggal**			
Perkotaan	6,3	6,1 - 6,5	391.028
Perdesaan	5,8	5,7 - 5,9	315.661
**INDONESIA**	**6,1**	**6,0 - 6,2**	**706.689**

[1]berdasarkan *Mini International Neuropsychiatric Interview*

8.3 Gangguan Mental Emosional

Prevalensi gangguan mental emosional dihitung dengan formula:

$$Prevalensi\ gangguan\ mental\ emosional = \frac{\begin{array}{c}Jumlah\ ART\ umur\ \geq\ 15\ tahun\ yang\ saat\ ini\ mengalami\\ gangguan\ mental\ emosional\ (menurut\ SRQ - 20)\end{array}}{Jumlah\ seluruh\ ART\ umur\ \geq 15\ tahun}$$

Tabel 8.3.1
Prevalensi Gangguan Mental Emosional pada Penduduk Umur ≥15 Tahun menurut Provinsi, Riskesdas 2018

Provinsi	Gangguan Mental Emosional[1]		N tertimbang
	%	95% CI	
Aceh	9,0	8,3 - 9,7	13.285
Sumatera Utara	11,6	10,9 - 12,3	36.146
Sumatera Barat	13,0	12,2 - 13,9	13.682
Riau	10,4	9,7 - 11,3	17.165
Jambi	3,6	3,2 - 4,2	9.438
Sumatera Selatan	6,3	5,8 - 6,9	21.888
Bengkulu	7,4	6,6 - 8,3	5.144
Lampung	5,6	5,1 - 6,0	21.993
Bangka Belitung	11,0	9,9 - 12,1	3.884
Kepulauan Riau	5,5	4,6 - 6,7	5.431
DKI Jakarta	10,1	9,1 - 11,1	28.746
Jawa Barat	12,1	11,6 - 12,7	130.528
Jawa Tengah	7,7	7,4 - 8,1	95.460
DI Yogyakarta	10,1	9,3 - 10,9	10.810
Jawa Timur	6,8	6,5 - 7,2	111.878
Banten	14,0	12,9 - 15,0	33.269
Bali	8,4	7,7 - 9,2	11.885
Nusa Tenggara Barat	12,8	11,8 - 14,0	12.945
Nusa Tenggara Timur	15,7	14,8 - 16,7	12.666
Kalimantan Barat	10,9	10,0 - 11,8	12.876
Kalimantan Tengah	7,4	6,4 - 8,5	6.980
Kalimantan Selatan	7,8	7,1 - 8,5	10.981
Kalimantan Timur	9,6	8,6 - 10,8	9.602
Kalimantan Utara	10,2	8,7 - 12,1	1.816
Sulawesi Utara	10,9	10,0 - 11,8	6.753
Sulawesi Tengah	19,8	18,6 - 21,1	7.762
Sulawesi Selatan	12,8	12,1 - 13,6	22.798
Sulawesi Tenggara	11,0	10,0 - 12,1	6.440
Gorontalo	17,7	15,8 - 19,7	3.116
Sulawesi Barat	8,5	7,1 - 10,2	3.379
Maluku	11,6	10,2 - 13,1	4.328
Maluku Utara	13,2	11,8 - 14,7	2.976
Papua Barat	11,3	10,0 - 12,8	2.342
Papua	8,5	7,6 - 9,4	8.279
**INDONESIA**	**9,8**	**9,7 - 10,0**	**706.688**

[1]berdasarkan *Self Reporting Questionnaire-20;* Nilai Batas Pisah *(Cut off Point)* ≥ 6

Tabel 8.3.2
Prevalensi Gangguan Mental Emosional Pada Penduduk Umur ≥15 Tahun menurut Karakteristik, Riskesdas 2018

Karakteristik	Gangguan Mental Emosional[1]		N tertimbang
	(%)	95% CI	
**Kelompok Umur**			
15 – 24	10,0	9,7 - 10,3	157.695
25 – 34	8,5	8,2 - 8,8	152.522
35 – 44	9,0	8,8 - 9,3	144.800
45 – 54	10,0	9,7 - 10,2	119.070
55 – 64	11,0	10,7 - 11,3	79.170
65 – 74	12,8	12,3 - 13,3	37.491
75+	15,8	14,9 - 16,6	15.941
**Jenis kelamin**			
Laki-laki	7,6	7,5 - 7,8	352.269
Perempuan	12,1	11,8 - 12,3	354.420
**Pendidikan**			
Tidak/belum pernah sekolah	13,9	13,3 - 14,4	38,204
Tidak tamat SD/MI	13,5	13,1 - 13,9	81,510
Tamat SD/MI	11,4	11,1 - 11,7	172,323
Tamat SLTP/MTS	9,6	9,3 - 9,8	150,634
Tamat SLTA/MA	8,0	7,8 - 8,2	202,438
Tamat D1/D2/D3/PT	5.1	4,8 - 5,3	61,579
**Pekerjaan**			
Tidak bekerja	13,0	12,7 - 13,3	204.063
Sekolah	9,8	9,4 - 10,1	56.924
PNS/TNI/POLRI/BUMN/BUMD	3,9	3,6 - 4,3	21.374
Pegawai swasta	6,3	6,0 - 6,7	73.840
Wiraswasta	7,9	7,6 - 8,2	102.763
Petani/buruh tani	9,7	9,4 - 9,9	129.477
Nelayan	10,8	9,7 - 12,0	5.386
Buruh/sopir/pembantu ruta	9,7	9,3 - 10,1	73.472
Lainnya	9,4	8,9 - 9,8	39.389
**Tempat tinggal**			
Perkotaan	9,8	9,5 - 10,0	391.028
Perdesaan	10,0	9,8 - 10,2	315.661
**INDONESIA**	**9,9**	**9,7 - 10,0**	**706.689**

[1]berdasarkan *Self Reporting Questionnaire-20;* Nilai Batas Pisah *(Cut off Point)* ≥6

8.4 Cakupan Pengobatan

Cakupan pengobatan skizofrenia/psikosis (pernah/seumur hidup) dihitung dengan formula:

$$Proporsi\ pengobatan\ skizofrenia/psikosis\ (pernah/seumur\ hidup) = \frac{\begin{array}{c} jumlah\ Rumah\ tangga\ yang\ memiliki\ ART\ gangguan\ jiwa\ skizofrenia/psikosis\ semua\ umur \\ pernah\ berobat\ medis\ dalam\ seumur\ hidupnya\ (dulu\ dan\ atau\ sekarang) \end{array}}{Jumlah\ rumah\ tangga\ yang\ memiliki\ ART\ gangguan\ jiwa\ skizofrenia/psikosis}$$

Cakupan pengobatan rumah tangga dengan ART skizofrenia/psikosis (minum obat rutin 1 bulan terakhir) dihitung dengan formula:

$$Proporsi\ pengobatan\ rumah\ tangga\ dengan\ ART\ skizofrenia/psikosis\ (minum\ obat\ rutin\ 1\ bulan\ terakhir) = \frac{\begin{array}{c} Jumlah\ Rumah\ tangga\ yang\ memiliki\ ART\ gangguan\ jiwa\ skizofrenia/psikosis \\ minum\ obat\ rutin\ 1\ bulan\ terakhir \end{array}}{\begin{array}{c} Jumlah\ rumah\ tangga\ yang\ memiliki\ ART\ gangguan\ jiwa\ skizofrenia/psikosis \\ dan\ pernah/seumur\ hidup\ berobat\ medis \end{array}}$$

Cakupan pengobatan depresi dihitung dengan formula:

$$Proporsi\ pengobatan\ depresi = \frac{\begin{array}{c} Jumlah\ ART\ umur\ \geq 15\ tahun\ yang\ saat\ ini\ mengalami \\ gejala\ depresi\ (menurut\ MINI)\ dan\ berobat\ medis \end{array}}{Jumlah\ ART\ umur\ \geq 15\ tahun\ yang\ saat\ ini\ mengalami\ gejala\ depresi\ (menurut\ MINI)}$$

Proporsi alasan ketidakpatuhan minum obat dihitung dengan formula:

$$Proporsi\ alasan\ ketidakpatuhan\ minum\ obat = \frac{Jumlah\ masing-masing\ opsi\ alasan\ tidak\ rutin\ minum\ obat}{Jumlah\ total\ ART\ yang\ tidak\ rutin\ minum\ obat}$$

Tabel 8.4.1
Proporsi Pengobatan Rumah Tangga dengan ART Gangguan Jiwa Skizofrenia/Psikosis menurut Provinsi, Riskesdas 2018

Provinsi	Berobat ke RS Jiwa/Fasyankes/Nakes									
	Pernah (seumur hidup)					Minum obat rutin1 bulan terakhir				
	(%)	95%CI			N tertimbang	(%)	95%CI			N tertimbang
Aceh	83,5	72,9	-	90,4	45*	57,0	44,4	-	68,8	37*
Sumatera Utara	88,1	78,4	-	93,8	90	38,2	26,9	-	50,9	77
Sumatera Barat	87,5	77,9	-	93,3	48*	57,7	45,8	-	68,7	41*
Riau	90,4	74,6	-	96,8	42*	45,0	28,9	-	62,3	37*
Jambi	88,8	76,4	-	95,1	25*	52,0	36,3	-	67,4	21*
Sumatera Selatan	73,9	63,5	-	82,2	70	35,4	19,8	-	54,8	50
Bengkulu	85,2	64,7	-	94,8	11*	45,3	26,8	-	65,2	9*
Lampung	75,9	57,8	-	87,9	54	42,8	27,6	-	59,5	40*
Bangka Belitung	92,2	72,0	-	98,2	10*	29,9	12,7	-	55,7	9*
Kepulauan Riau	83,3	60,0	-	94,3	7*	40,6	16,4	-	70,5	6*
DKI Jakarta	92,9	75,3	-	98,2	80	84,1	68,2	-	92,9	72
Jawa Barat	83,0	73,6	-	89,6	281	55,8	43,3	-	67,7	226
Jawa Tengah	88,9	84,0	-	92,5	335	45,7	37,4	-	54,2	288
DI Yogyakarta	100	100,0	-	100,0	48*	51,1	30,6	-	71,2	46*
Jawa Timur	81,4	73,6	-	87,3	288	47,9	39,1	-	56,9	227
Banten	92,4	78,9	-	97,5	75	22,4	10,3	-	42,0	67
Bali	97,9	91,4	-	99,5	51	53,8	42,1	-	65,0	49*
Nusa Tenggara Barat	77,6	61,5	-	88,3	55	59,4	40,8	-	75,6	41*
Nusa Tenggara Timur	55,5	40,7	-	69,4	18*	11,5	3,9	-	29,4	9*
Kalimantan Barat	89,5	80,1	-	94,8	40*	40,6	27,1	-	55,7	34*
Kalimantan Tengah	75,5	59,8	-	86,4	13*	74,3	58,1	-	85,8	10*
Kalimantan Selatan	83,9	64,1	-	93,8	24*	53,2	37,8	-	68,1	20*
Kalimantan Timur	79,3	67,5	-	87,7	20*	58,1	30,2	-	81,6	15*
Kalimantan Utara	94,4	67,0	-	99,3	5*	38,4	14,3	-	69,9	4*
Sulawesi Utara	92,9	87,3	-	96,2	20*	65,6	42,5	-	83,2	18*
Sulawesi Tengah	86,4	67,3	-	95,1	26*	42,2	27,8	-	58,0	21*
Sulawesi Selatan	79,2	69,2	-	86,6	79	44,8	34,5	-	55,5	60
Sulawesi Tenggara	83,2	64,0	-	93,3	15*	64,0	44,7	-	79,7	12*
Gorontalo	85,6	71,2	-	93,5	8*	43,1	13,8	-	78,2	7*
Sulawesi Barat	84,5	58,8	-	95,4	10*	47,3	21,0	-	75,2	8*
Maluku	61,9	41,1	-	79,0	7*	50,2	19,7	-	80,6	4*
Maluku Utara	64,6	42,9	-	81,6	6*	45,5	29,7	-	62,3	3*
Papua Barat	97,8	85,0	-	99,7	7*	39,1	8,2	-	82,2	6*
Papua	65,6	37,9	-	85,7	18*	20,7	6,5	-	49,7	12*
**INDONESIA**	**85,0**	**82,8**	**-**	**86,9**	**1.929**	**48,9**	**45,5**	**-**	**52,2**	**1588**

*N Tertimbang <50

Tabel 8.4.2
Proporsi Pengobatan Rumah Tangga dengan ART Gangguan Jiwa Skizofrenia/Psikosis menurut Tempat Tinggal, Riskesdas 2018

Tempat tinggal	Berobat ke RS Jiwa/Fasyankes/Nakes								N Tertimbang
	Pernah (Seumur hidup)			NTertimbang	Minum obat rutin 1 bulan terakhir				
	(%)	95%CI			(%)	96% CI			
Perkotaan	80,1	76,1 -	83,6	1.021	54,9	49,9 -	59,7		880
Perdesaan	67,8	64,2 -	71,3	907	41,4	37,2 -	45,7		707
**INDONESIA**	**74,3**	**71,7 -**	**76,8**	**1.929**	**48,9**	**45,5 -**	**52,2**		**1.588**

Tabel 8.4.3
Proporsi Alasan Ketidakpatuhan Minum Obat Gangguan Jiwa Skizofrenia/Psikosis pada ART di Rumah Tangga Menurut Tempat Tinggal, Riskesdas 2018

Tempat Tinggal	Alasan tidak rutin minum obat (%)								N Tertimbang
	Sering lupa	Tidak mampu beli obat rutin	Obat tidak tersedia	Tidak rutin berobat	Tidak tahan ESO	Merasa dosis tidak sesuai	Merasa sudah sehat	Lainnya	
Perkotaan	3,5	22,3	1,1	32,5	5,4	4,4	37,5	36,2	407
Perdesaan	8,6	24,9	3,7	34,9	8,5	7,8	34,7	28,0	425
**INDONESIA**	**6,1**	**23,6**	**2,4**	**33,7**	**7,0**	**6,1**	**36,1**	**32,0**	**832**

Tabel 8.4.4
Proporsi Pengobatan Depresi pada Penduduk Umur ≥15 Tahun menurut Provinsi, Riskesdas 2018

Provinsi	Minum Obat/ Menjalani Pengobatan Medis		N Tertimbang
	(%)	95% CI	
Aceh	11,7	9,5 - 14,3	575
Sumatera Utara	8,5	7,2 - 10,1	2.831
Sumatera Barat	8,6	7,0 - 10,5	1.109
Riau	7,3	5,8 - 9,2	1.130
Jambi	12,2	8,5 - 17,2	164
Sumatera Selatan	13,0	10,0 - 16,6	737
Bengkulu	11,2	8,6 - 14,5	246
Lampung	11,3	8,6 - 14,8	703
Bangka Belitung	10,6	8,0 - 14,0	249
Kepulauan Riau	9,9	5,7 - 16,5	198
DKI Jakarta	10,7	8,0 - 14,1	1.689
Jawa Barat	9,0	7,8 - 10,4	10.054
Jawa Tengah	8,8	7,7 - 10,1	4.170
DI Yogyakarta	8,9	6,4 - 12,4	590
Jawa Timur	10,1	9,0 - 11,5	5.038
Banten	8,3	6,6 - 10,3	2.866
Bali	6,0	4,4 - 8,1	600
Nusa Tenggara Barat	8,0	6,4 - 10,0	1.130
Nusa Tenggara Timur	5,9	4,8 - 7,3	1.215
Kalimantan Barat	9,0	7,0 - 11,4	792
Kalimantan Tengah	11,1	7,9 - 15,3	268
Kalimantan Selatan	11,2	9,2 - 13,6	526
Kalimantan Timur	8,1	5,8 - 11,1	594
Kalimantan Utara	7,9	5,1 - 12,1	104
Sulawesi Utara	7,7	6,1 - 9,8	445
Sulawesi Tengah	7,3	6,1 - 8,7	946
Sulawesi Selatan	6,7	5,6 - 7,9	1.766
Sulawesi Tenggara	7,3	5,2 - 10,0	405
Gorontalo	18,2	14,8 - 22,3	318
Sulawesi Barat	13,2	8,5 - 20,0	143
Maluku	9,2	6,2 - 13,7	230
Maluku Utara	7,4	5,6 - 9,7	276
Papua Barat	9,8	5,9 - 15,7	173
Papua	11,1	8,4 - 14,6	327
**INDONESIA**	**9,0**	**8,6 - 9,5**	**42.606**

Tabel 8.4.5
Proporsi Pengobatan Depresi pada Penduduk Umur ≥15 Tahun menurut Karakteristik, Riskesdas 2018

Karakteristik	Minum Obat/ Menjalani Pengobatan Medis		N Tertimbang
	%	(95% CI)	
**Kelompok Umur**			
15 – 24	5,25	4,51-6,09	9.316
25 – 34	8,25	7,27-9,34	7.873
35 – 44	9,62	8,73-10,59	7.736
45 – 54	10,64	9,65-11,72	7.004
55 – 64	12,26	10,89-13,78	4.894
65 – 74	11,89	10,34-13,63	2.816
75+	10,05	8,12-12,38	1.303
**Jenis kelamin**			
Laki-laki	8,27	7,63-8,96	16.532
Perempuan	9,51	8,95-10,09	26.074
**Pendidikan**			
Tidak/belum pernah sekolah	9,09	7,6-10,84	2.947
Tidak tamat SD/MI	10,98	9,87-12,19	6.269
Tamat SD/MI	10,07	9,20-11,00	11.519
Tamat SLTP/MTS	7,55	6,73-8,46	8.705
Tamat SLTA/MA	8,04	7,25-8,90	9.778
Tamat D1/D2/D3/PT	8,04	6,5-9,90	1.786
**Pekerjaan**			
Tidak bekerja	10,53	9,77-11,35	16.013
Sekolah	3,29	2,58-4,20	3.216
PNS/TNI/POLRI/BUMN/BUMD	10,38	7,92-13,48	449
Pegawai swasta	6,62	5,20-8,40	2.905
Wiraswasta	9,19	8-10,53	4.926
Petani/buruh tani	9,59	8,74-10,5	6.774
Nelayan	5,29	3,27-8,43	312
Buruh/sopir/pembantu ruta	7,69	6,49-9,09	4.020
Lainnya	10,7	8,93-12,77	2.154
**Tempat tinggal**			
Perkotaan	8,57	7,94-9,25	23.777
Perdesaan	9,64	9,10-10,22	17.787
**INDONESIA**	**9,03**	**8,59-9,48**	**41.844**

BAB 9
DISABILITAS

Disabilitas/ketidakmampuan dalam Riskesdas 2018 diukur pada 3 kelompok usia

1. Disabilitas pada anak (5-17 tahun)
2. Disabilitas pada penduduk dewasa (18-59 tahun)
3. Disabilitias pada penduduk lanjut usia (≥60 tahun)

Pengukuran disabilitas bertujuan untuk mendapatkan informasi hambatan yang dialami penduduk Indonesia usia 5 tahun keatas.

9.1 Disabilitas Anak (5-17 Tahun)

Disabilitas pada anak ditujukan untuk mengukur pencapaian SDGs pada butir 1.3.5 tentang jumlah anak penyandang disabilitas dalam keluarga. Pertanyaan disabilitas pada anak mengadopsi pertanyaan *Module UN Washington Group*, yang tercantum dalam *Multiple Indicator Cluster Surveys (MICS)* yang dikembangkan oleh UNICEF. Untuk mengukur disabilitas pada anak digunakan 10 pertanyaan dengan 5 opsi jawaban: 1) Tidak ada; 2) Ringan; 3) Sedang; 4) Berat; 5) Sangat Berat. Anak dikatakan disabilitas bila bila menjawab berat atau sangat berat dari 10 pertanyaan yang diajukan.

Pertanyan disabilitas pada anak ditujukan untuk mengukur fungsi:

1. Penglihatan
2. Pendengaran
3. Mobilitas
4. Komunikasi
5. Mempelajari suatu hal
6. Daya ingat
7. Konsentrasi
8. Menerima perubahan
9. Menjalin pertemanan
10. Mengontrol tingkah laku

Proposi disabilitas pada anak mengacu pada:

$$\frac{\textit{Jumlah anak usia 5-17 tahun yang mengalami disability}}{\textit{Jumlah penduduk 5-17 tahun}}$$

Tabel 9.1.1
Proporsi Disabilitas pada Anak Umur 5-17 Tahun menurut Provinsi, Riskesdas 2018

Provinsi	Disabilitas		
	%	95%CI	N Tertimbang
Aceh	1,8	1,5 - 2,2	5.848
Sumatera Utara	3,3	2,8 - 3,8	16.947
Sumatera Barat	5,0	4,3 - 5,9	5.935
Riau	2,9	2,5 - 3,4	7.617
Jambi	1,4	1,0 - 1,9	3.669
Sumatera Selatan	1,6	1,3 - 2,0	8.903
Bengkulu	2,9	2,2 - 3,7	2.052
Lampung	1,4	1,1 - 1,8	8.613
Bangka Belitung	3,8	3,0 - 4,8	1.461
Kepulauan Riau	2,6	1,7 - 4,0	2.280
DKI Jakarta	4,8	3,9 - 5,8	9.234
Jawa Barat	2,8	2,5 - 3,2	48.372
Jawa Tengah	2,9	2,6 - 3,2	31.834
DI Yogyakarta	4,8	3,9 - 6,1	3.086
Jawa Timur	3,2	2,9 - 3,6	34.219
Banten	5,0	4,2 - 5,9	12.797
Bali	3,4	2,8 - 4,2	3.884
Nusa Tenggara Barat	2,7	2,2 - 3,3	5.462
Nusa Tenggara Timur	3,4	2,9 - 3,9	6.865
Kalimantan Barat	2,7	2,2 - 3,3	5.410
Kalimantan Tengah	2,5	1,9 - 3,3	2.734
Kalimantan Selatan	3,6	3,0 - 4,3	4.347
Kalimantan Timur	4,1	3,4 - 4,9	3.659
Kalimantan Utara	5,4	4,2 - 6,8	760
Sulawesi Utara	3,3	2,7 - 4,0	2.396
Sulawesi Tengah	7,0	6,1 - 8,1	3.208
Sulawesi Selatan	5,3	4,7 - 6,0	9.384
Sulawesi Tenggara	3,4	2,9 - 4,1	3.194
Gorontalo	5,4	4,3 - 6,8	1.210
Sulawesi Barat	1,4	1,0 - 2,0	1.585
Maluku	4,6	3,6 - 5,8	2.179
Maluku Utara	3,4	2,7 - 4,1	1.512
Papua Barat	2,7	1,9 - 3,7	1.030
Papua	3,1	2,4 - 4,0	3.784
**INDONESIA**	**3,3**	**3,1 - 3,4**	**265.469**

Tabel 9.1.2
Proporsi Disabilitas pada Anak Umur 5-17 Tahun menurut Karakteristik, Riskesdas 2018

Karakteristik	Disabilitas		
	%	95% CI	N Tertimbang
**Kelompok Umur**			
5- 9	2,5	2,3 - 2,6	104.381
10-14	3,5	3,4 - 3,7	100.735
15-17	4,2	3,9 - 4,4	60.353
**Jenis Kelamin**			
Laki-Laki	3,4	3,3 - 3,6	130.396
Perempuan	3,1	2,9 - 3,2	123.980
**Tempat tinggal**			
Perkotaan	3,6	3,4 - 3,8	134.047
Perdesaan	2,9	2,8 - 3,0	120.125

9.2 Disabilitas Dewasa (18-59 Tahun)

Kuesioner disabilitas dikembangkan oleh WHO untuk mendapatkan informasi sejauh mana seseorang dapat memenuhi perannya di rumah, tempat kerja, sekolah atau area sosiallain. Pertanyaan disabilitas pada penduduk dewasa ini ditujukan untuk mengukur fungsi dan kemampuan penduduk dalam 1 bulan terakhir, yang meliputi:

1. Mobilitas/berpindah tempat
2. Melakukan aktivitas sehari-hari
3. Mengurus diri sendiri
4. Daya ingat
5. Bersosialisasi
6. Pengendalian emosi
7. Konsentrasi
8. Adaptasi lingkungan dan sosial.

Kuesioner disabilitas pada Riskesdas 2018 diadaptasi dari WHODAS 2 terkait ***Disabilily Assesment Schedule (DAS)*** sebagai operasionalisasi dari konsep *International classification of functioning* (ICF), yang terdiri dari 12 pernyataan/komponen untuk mendapatkan informasi tentang status disabilitas seseorang. Disabilitas pada Riskesdas 2018 ini mengacu pada ketidakmampuan fisik dan mental yang diukur dalam kurun waktu satu bulan terakhir sebelum survei. Terdapat lima opsi jawaban untuk responden untuk mengukur disabilitas, yaitu 1) tidak ada kesulitan, 2) sedikit kesulitan/ringan, 3) cukup mengalamikesulitan/sedang, 4) kesulitan berat, dan 5) sangat berat/tidak mampu melakukan kegiatan.

Seseorang dikatakan mengalami disabilitas bila salah satu jawaban dari 11 pertanyaan yang diajukan untuk mengukur ketidak mampuan fisik menjawab 3, 4 atau 5.

Proporsi orang yang mengalami disabilitas berumur 18-59 tahun =

$$\frac{\sum \text{ART umur 18-59 tahun yang mengalami disabilitas}}{\sum \text{ARTumur 18-59 tahun}}$$

Tabel 9.2.1
Proporsi Disabilitas pada Penduduk Umur 18-59 Tahun menurut Provinsi, Riskesdas 2018

Provinsi	Disabilitas				N Tertimbang
	%	95% CI			
Aceh	18,1	16,8	-	19,4	10.765
Sumatera Utara	20,1	18,9	-	21,4	28.452
Sumatera Barat	32,4	30,4	-	34,5	10.584
Riau	22,7	21,2	-	24,2	14.213
Jambi	14,2	12,7	-	15,9	7.669
Sumatera Selatan	15,4	14,1	-	16,7	17.578
Bengkulu	15,9	14,3	-	17,5	4.200
Lampung	13,8	12,7	-	15,0	17.546
Bangka Belitung	26,6	24,4	-	29,0	3.148
Kepulauan Riau	14,0	11,7	-	16,6	4.619
DKI Jakarta	22,1	20,2	-	24,2	23.897
Jawa Barat	23,8	22,8	-	24,8	103.328
Jawa Tengah	20,2	19,4	-	20,9	71.956
DI Yogyakarta	33,2	31,0	-	35,5	8.216
JawaTimur	17,7	17,0	-	18,5	85.652
Banten	22,2	20,6	-	23,8	27.740
Bali	19,2	17,7	-	20,8	9.336
Nusa Tenggara Barat	27,6	25,7	-	29,6	10.203
Nusa Tenggara Timur	27,3	25,8	-	28,8	9.790
Kalimantan Barat	23,2	21,5	-	24,9	10.337
Kalimantan Tengah	21,2	19,4	-	23,2	5.811
Kalimantan Selatan	24,3	22,5	-	26,1	8.909
Kalimantan Timur	25,4	23,2	-	27,8	8.001
Kalimantan Utara	26,5	23,4	-	29,8	1.482
Sulawesi Utara	21,6	20,1	-	23,1	5.230
Sulawesi Tengah	40,6	38,5	-	42,6	6.248
Sulawesi Selatan	33,6	32,1	-	35,2	17.801
Sulawesi Tenggara	28,9	26,6	-	31,2	5.149
Gorontalo	27,9	25,1	-	30,9	2.520
Sulawesi Barat	22,7	19,8	-	25,9	2.694
Maluku	24,9	22,1	-	28,1	3.402
Maluku Utara	26,0	23,8	-	28,5	2.404
Papua Barat	22,3	20,0	-	24,9	1.965
Papua	24,1	22,2	-	26,2	7.201
**INDONESIA**	**22,0**	**21,7**	**-**	**22,3**	**558.048**

Tabel 9.2.2

Proporsi Disabilitas pada Penduduk Umur 18-59 Tahun menurut Karakteristik, Riskesdas 2018

Karakteristik	Disabilitas				N Tertimbang
	%	95 CI			
**Kelompok Umur**					
18-24	21.1	20,6	-	21,7	104.915
25-34	20.6	20,1	-	21,0	149.683
35-44	20.3	19,9	-	20,7	142.104
45-54	23,8	23,4	-	24,2	116.853
55-59	29,6	29,0	-	30,2	44.493
**Jenis Kelamin**					
Laki-laki	18,8	18,5	-	19,1	278.933
Perempuan	25,2	24,8	-	25,6	279.115
**Pendidikan**					
Tidak sekolah	30,7	29,7	-	31,8	19.697
Tidak tamat SD/MI	28,2	27,6	-	28,9	54.339
Tamat SD/MI	24,0	23,5	-	24,5	129.271
Tamat SLTP/MTS	21,2	20,7	-	21,7	110.888
Tamat SLTA/MA	19,7	19,3	-	20,1	187.159
Tamat Diploma/PT	17,6	17,0	-	18,1	56.693
**Pekerjaan**					
PNS/TNI/Polri/BUMD	26,7	26,3	-	27,2	151.403
Pegawai Swasta	21,0	20,1	-	21,9	20.459
Wiraswasta	16,5	15,8	-	17,3	19.806
Petani	17,6	17,0	-	18,2	70.766
Nelayan	19,8	19,3	-	20,3	91.667
Petani/Buruhtani	21,6	21,2	-	22,1	100.077
Nelayan	23,9	21,8	-	26,1	4.697
Buruh/sopir/pembantu ruta	21,0	20,4	-	21,7	66.210
Lainnya	22,5	21,7	-	23,3	32.961
**Tempat tinggal**					
Perkotaan	21,9	21,5	-	22,4	312.569
Perdesaan	22,1	21,7	-	22,5	245.479

Riskesdas 2018 ini tidak hanya mendapatkan proprosi ART yang mengalami disabilitas tapi juga dapat diperoleh informasi tingkat disabilitas yang dialaminya dengan melihat tingkat ketidakmampuan fisik dan mental. Untuk mengukur tingkat disabilitas (ketidakmampuan fisik dan mental) digunakan skoring berdasarkan 11 pertanyaan yang diajukan.

Penilaian hasil jawaban berupa skor dengan kategori :

- Untuk skor 0-<5 adalah tidak ada kesulitan
- Untuk skor 5-<25 adalah ada kesulitan ringan
- Untuk skor 25-<50 adalah ada kesulitan sedang
- Untuk skor 50-100 adalah ada kesulitan berat/tidak mampu.

Tabel 9.2.3
Proporsi Tingkat Disabilitas pada Penduduk Umur 18-59 Tahun menurut Provinsi, Riskesdas 2018

Provinsi	Tingkat Disabilitas												N tertimbang
	Tidak ada kesulitan			Kesulitan ringan			Kesulitan sedang			Kesulitan berat/tidak mampu			
	%	95% CI		%	95% CI		%	95% CI		%	95% CI		
Aceh	69,9	68,2	71,5	18,4	17,3	19,7	10,5	9,6	11,4	1,2	1,0	1,5	10.765
Sumatera Utara	69,4	67,9	70,9	18,2	17,1	19,3	11,0	10,3	11,8	1,4	1,2	1,6	28.452
Sumatera Barat	58,4	56,0	60,7	23,9	22,7	25,3	16,1	14,3	18,1	1,6	1,3	1,8	10.584
Riau	66,7	64,8	68,6	22,4	21,1	23,8	9,8	8,7	10,9	1,1	0,9	1,3	14.213
Jambi	74,4	71,9	76,8	15,4	13,8	17,1	9,3	8,1	10,7	0,8	0,7	1,1	7.669
Sumatera Selatan	74,2	72,6	75,8	15,7	14,5	16,9	9,3	8,4	10,3	0,8	0,6	1,0	17.578
Bengkulu	76,3	74,2	78,3	13,5	12,3	14,7	9,0	7,8	10,5	1,2	0,9	1,6	4.200
Lampung	77,7	76,1	79,3	14,0	12,9	15,1	7,2	6,5	8,1	1,1	0,9	1,3	17.546
Bangka Belitung	67,0	64,5	69,5	23,4	21,5	25,4	8,9	7,8	10,1	0,7	0,5	0,9	3.148
Kepulauan Riau	79,7	76,6	82,4	13,4	11,6	15,5	6,3	5,0	7,9	0,6	0,3	0,9	4.619
DKI Jakarta	69,1	66,7	71,3	24,3	22,4	26,2	6,1	5,2	7,1	0,6	0,4	0,8	23.897
Jawa Barat	67,5	66,3	68,6	23,3	22,4	24,2	8,5	7,9	9,1	0,8	0,7	0,9	103.328
Jawa Tengah	71,7	70,7	72,6	19,7	19,0	20,3	7,8	7,3	8,3	0,9	0,8	1,0	71.956
DI Yogyakarta	61,0	58,6	63,3	32,4	30,5	34,4	6,2	5,3	7,2	0,4	0,2	0,6	8.216
JawaTimur	73,7	72,8	74,6	17,3	16,7	18,0	8,2	7,6	8,8	0,8	0,7	0,9	85.652
Banten	70,3	68,4	72,1	23,4	22,0	25,0	5,7	5,0	6,5	0,5	0,4	0,7	27.740
Bali	75,3	73,5	77,1	19,6	18,2	21,1	4,4	3,9	5,1	0,6	0,5	0,8	9.336
Nusa Tenggara Barat	62,7	60,3	65,0	22,3	20,9	23,9	13,1	11,8	14,5	1,9	1,6	2,2	10.203
Nusa Tenggara Timur	61,8	60,1	63,5	24,4	23,2	25,6	12,3	11,3	13,3	1,6	1,3	1,8	9.790
Kalimantan Barat	65,7	63,7	67,7	23,7	22,2	25,3	9,5	8,6	10,5	1,1	0,9	1,4	10.337
Kalimantan Tengah	64,6	62,0	67,1	21,4	19,6	23,4	12,9	11,3	14,7	1,1	0,8	1,4	5.811
Kalimantan Selatan	67,5	65,3	69,6	20,0	18,5	21,5	10,9	9,8	12,1	1,7	1,4	2,0	8.909
Kalimantan Timur	65,6	63,2	68,0	24,3	22,4	26,2	9,4	8,2	10,7	0,7	0,5	1,0	8.001
Kalimantan Utara	62,8	58,9	66,6	25,1	22,1	28,2	11,4	9,1	14,3	0,7	0,4	1,0	1.482
Sulawesi Utara	65,9	64,0	67,7	19,6	18,3	21,0	13,2	12,0	14,5	1,3	1,1	1,6	5.230
Sulawesi Tengah	48,0	45,8	50,2	30,5	29,0	32,0	19,1	17,8	20,5	2,4	2,1	2,8	6.248
Sulawesi Selatan	51,6	49,9	53,3	27,1	25,9	28,3	18,9	17,6	20,3	2,4	2,1	2,8	17.801
Sulawesi Tenggara	61,5	58,9	64,0	22,4	20,9	24,0	14,4	12,9	15,9	1,8	1,4	2,2	5.149
Gorontalo	62,2	59,0	65,4	23,9	21,5	26,5	12,1	10,5	13,9	1,7	1,2	2,3	2.520
Sulawesi Barat	64,9	60,7	68,8	14,1	12,4	16,0	18,8	15,7	22,5	2,1	1,6	2,9	2.694
Maluku	60,7	57,1	64,2	20,8	19,1	22,6	16,0	13,7	18,5	2,5	1,6	3,8	3.402
Maluku Utara	60,6	57,7	63,3	20,0	18,4	21,7	18,1	16,3	20,0	1,4	1,0	1,9	2.404
Papua Barat	66,0	62,5	69,3	21,3	19,0	23,8	11,4	9,8	13,3	1,3	0,9	1,8	1.965
Papua	59,3	56,9	61,7	22,4	20,7	24,2	16,4	14,9	18,0	1,9	1,4	2,6	7.201
**INDONESIA**	68,7	68,3	69,0	20,9	20,6	21,2	9,4	9,2	9,6	1,0	1,0	1,1	558.048

Tabel 9.2.4
Proporsi Tingkat Disabilitas pada Penduduk Umur 18-59 Tahun menurut Karakteristik, Riskesdas 2018

Karakteristik	Tingkat Disabilitas								N Tertimbang
	Tidak ada kesulitan		Kesulitan ringan		Kesulitan sedang		Kesulitan berat/tidak mampu		
	%	95 CI	%	95 CI	%	95 CI	%	95 CI	
**Kelompok Umur**									
18-24	70,5	69,9 - 71,1	20,2	19,8 - 20,7	8,6	8,3 - 9,0	0,7	0,6 - 0,7	104.915
25-34	70,8	70,3 - 71,3	20,1	19,7 - 20,5	8,5	8,2 - 8,8	0,6	0,5 - 0,7	149.683
35-44	70,2	69,7 - 70,6	20,3	20,0 - 20,7	8,7	8,4 - 9,0	0,8	0,7 - 0,8	142.104
45-54	65,9	65,5 - 66,4	21,9	21,5 - 22,3	10,7	10,4 - 11,0	1,4	1,3 - 1,5	116.853
55-59	59,5	58,8 - 60,1	24,2	23,6 - 24,8	13,4	12,9 - 13,9	3,0	2,8 - 3,2	44.493
**Jenis Kelamin**									
Laki-laki	72,1	71,7 - 72,5	18,4	18,1 - 18,7	8,6	8,4 - 8,9	0,8	0,8 - 0,9	278.933
Perempuan	65,2	64,8 - 65,6	23,4	23,1 - 23,7	10,2	10,0 - 10,5	1,2	1,1 - 1,2	279.115
**Pendidikan**									
Tidak sekolah	57,6	56,4 - 58,8	23,6	22,7 - 24,6	15,0	14,2 - 15,8	3,7	3,4 - 4,1	19.697
Tidak tamat SD/MI	60,7	59,9 - 61,4	23,7	23,1 - 24,2	13,6	13,1 - 14,1	2,0	1,9 - 2,2	54.339
Tamat SD/MI	65,7	65,1 - 66,2	22,4	21,9 - 22,8	10,7	10,4 - 11,1	1,3	1,2 - 1,3	129.271
Tamat SLTP/MTS	69,7	69,2 - 70,3	20,7	20,2 - 21,1	8,8	8,5 - 9,1	0,8	0,7 - 0,9	110.888
Tamat SLTA/MA	71,9	71,4 - 72,3	19,7	19,4 - 20,1	7,8	7,6 - 8,1	0,6	0,5 - 0,6	187.159
Tamat Diploma/PT	74,4	73,7 - 75,0	18,2	17,6 - 18,8	7,0	6,7 - 7,4	0,4	0,4 - 0,5	56.693
**Pekerjaan**									
PNS/TNI/Polri/ BUMD	64,0	63,4 - 64,5	23,4	23,0 - 23,8	10,8	10,5 - 11,1	1,8	1,7 - 1,9	151.403
Pegawai Swasta	70,9	69,8 - 72,0	20,4	19,5 - 21,4	8,1	7,6 - 8,7	0,5	0,4 - ,7	20.459
Wiraswasta	74,7	73,8 - 75,6	17,2	16,5 - 18,0	7,5	7,1 - 8,0	0,5	0,4 - 0,7	19.806
Petani	75,1	74,3 - 75,8	18,4	17,8 - 19,0	6,2	5,9 - 6,6	0,3	0,3 - 0,4	70.766
Nelayan	71,3	70,7 - 71,9	19,8	19,3 - 20,3	8,3	7,9 - 8,6	0,7	0,6 - 0,7	91.667
Petani/Buruh tani	66,6	66,1 - 67,2	20,3	19,9 - 20,7	11,9	11,5 - 12,3	1,1	1,1 - 1,2	100.077
Nelayan	66,6	64,3 - 68,9	20,0	18,3 - 21,8	12,2	10,9 - 13,6	1,2	0,9 - 1,7	4.697
Buruh/sopir/pe mbantu ruta	70,3	69,5 - 71,1	21,2	20,6 - 21,9	8,0	7,6 - 8,4	0,5	0,4 - 0,6	66.210
Lainnya	67,4	66,5 - 68,3	21,4	20,7 - 22,2	10,3	9,8 - 10,9	0,8	0,7 - 0,9	32.961
**Tempat Tinggal**									
Perkotaan	69,7	69,2 - 70,3	21,3	20,9 - 21,7	8,1	7,8 - 8,4	0,8	0,8 - 0,9	312.569
Perdesaan	67,3	66,8 - 67,8	20,4	20,0 - 20,7	11,1	10,8 - 11,4	1,3	1,2 - 1,3	245.479

9.3 Disabilitas Lansia (≥ 60 Tahun)

Pertanyaan disabilitas ART umur ≥ 60 tahun ini mengacu pada ***Barthel Index of Activities of Daily Living*** (ADL).Tujuan dari pertanyaan ini yaitu: (1) Menilai tingkat kemandirian responden umur ≥ 60 tahun dalam melakukan aktivitas sehari-hari; (2) Menilai kemajuan responden dengan penyakit kronis sebelum dan sesudah terapi; (3) Menentukan seberapa besar bantuan perawatan yang dibutuhkan responden umur ≥ 60 tahun.

Penilaian dalam disabilitas pada lansia dihitung menggunakan skoring dari jawaban dengan memodifikasi *Barthel Index* mengikuti kriteria sebagai berikut :

1. ≥ 20 : Mandiri
2. 12-19 : Ketergantungan ringan
3. 9-12 : Ketergantungan sedang
4. 5-8 : Ketergantungan berat
5. 0-4 : Ketergantungan total

Tabel 9.3.1
Proporsi Tingkat Ketergantungan pada Penduduk Umur ≥60 Tahun menurut Provinsi, Riskesdas 2018

Provinsi	Tingkat ketergantungan										N Ter-timbang
	Mandiri		Tergantung Ringan		Tergantung Sedang		Tergantung Berat		Tergantung Total		
	%	95% CI	%	95% CI	%	95% CI	%	95% CI	%	95% CI	
Aceh	70,33	67,90 -72,60	25,25	23,10 – 27,52	1,68	1,17 -2,42	1,25	0,84 -1.85	1,50	1,03 -2.17	1.414
Sumatera Utara	69,19	67,26 -71,06	26,66	24,89 – 28,51	1,34	0,94 -1,90	1,14	0,79 - 1.66	1,67	1,18 - 2.34	4.331
Sumatera Barat	76,53	74,33 -78,59	20,22	18,25 – 22,34	1,18	0,70 -1,98	1,01	0,69 - 1.47	1,06	0,74 -1.53	2.026
Riau	71,59	68,70 – 74,30	25,11	22,54 – 27,88	1,16	0,71 -1,89	0,98	0,58 - 1.66	1,16	0,72 -1.86	1.476
Jambi	72,16	69,24 -74,90	24,13	21,53 – 26,93	1,58	1,02 – 2,46	1,07	0,59 –1.91	1,07	0,63 -1.79	1.031
Sumatera Selatan	72,15	69,63 – 74,52	24,02	21,79 – 26,40	1,48	1,03 – 2,13	0,62	0,31 –1.23	1,74	1,19 - 2.53	2.555
Bengkulu	73,30	70,22 – 76,17	21,96	19,27 – 24,92	1,00	0,58 – 1,73	0,99	0,57 –1.72	2,75	1,84 -4.07	556
Lampung	77,79	75,80 – 79,67	18,41	16,72 – 20,23	1,24	0,84 -1,84	1,05	0,66 –1.66	1,51	1,05 -2.18	2.837
Bangka Belitung	75,37	71,75 -78,67	19,56	16,57 – 22,94	1,52	0,85 – 2,70	1,22	0,67 –2.24	2,33	1,42 -3.78	435
Kepulauan Riau	73,03	67,11 – 78,24	23,27	18,06 – 29,43	1,16	0,41 – 3,29	1,53	0,68 - 3.39	1,01	0,54 -1.87	386
DKI Jakarta	71,69	68,52 – 74,65	24,66	21,83 – 27,72	1,14	0,64- 2,02	1,26	0,73 –2.18	1,25	0,68 -2.32	3.114
Jawa Barat	72,42	70,94 – 73,85	24,12	22,74 – 25,57	0,87	0,67 – 1,13	0,81	0,59 –1.12	1,78	1,42 -2.23	17.203
Jawa Tengah	76,11	75,11 -77,08	19,90	18,98 – 20,85	1,17	0,96 – 1,43	1,14	0,95 –1.36	1,68	1,42 - 2.00	17.675
DI Yogyakarta	75,73	73,31 – 77,99	20,88	18,76 – 23,16	0,75	0,41 – 1,34	1,18	0,72 - 1.93	1,47	0,92 -2.34	2.108
Jawa Timur	76,10	75,12 – 77,05	20,18	19,29 – 21,11	1,11	0,92 -1,34	0,99	0,81 - 1.22	1,61	1,37 -1.90	19.663
Banten	69,07	66,07 – 71,91	27,08	24,45 – 29,89	0,75	0,39 – 1,46	1,53	0,82 –2.82	1,57	0,97 -2.54	3.039
Bali	78,90	76,85 – 80,81	16,85	15,12 – 18,73	1,45	0,98 -2,15	1,28	0,82 - 1.99	1,52	1,03 -2.24	1.861
Nusa Tenggara Barat	76,16	73,38 – 78,74	20,73	18,32 – 23,37	1,47	0,94 – 2,29	0,39	0,16 - 0.93	1,25	0,75 -2.07	1.626
Nusa Tenggara Timur	78,34	76,45 – 80,11	18,78	17,11 – 20,57	0,98	0,71 – 1,36	0,73	0,48 - 1.12	1,18	0,82 -1.68	1.667
Kalimantan Barat	75,50	73,12 – 77,72	20,61	18,55 – 22,83	1,26	0,85 -1,87	1,30	0,80 - 2.10	1,34	0,89 -2.00	1.491
Kalimantan Tengah	70,38	67,00 – 73,56	24,78	21,78 – 28,03	1,73	0,97 -3,05	1,29	0,77 - 2.17	1,82	1,09 -3.03	609
Kalimantan Selatan	72,16	69,44 -74,72	23,95	21,51 – 26,56	1,35	0,81 -2,22	0,88	0,47 - 1.63	1,67	1,06 -2.62	1.189
Kalimantan Timur	70,37	66,41 – 74,04	26,45	22,87 – 30,37	1,75	0,92 – 3,30	0,53	0,20 - 1.38	0,90	0,39 -2.05	872
Kalimantan Utara	69,45	63,04 -75,19	27,65	22,19 - 33,88	0,98	0,39 -2,46	1,01	0,38 -2.67	0,91	0,30 -2.67	175
Sulawesi Utara	76,42	74,26 – 78,44	19,74	17,94 – 21,68	1,61	1,06 -2,44	0,93	0,60 -1.45	1,30	0,86 -1.95	1.058
Sulawesi Tengah	70,98	68,27 – 73,54	24,79	22,28 – 27,47	1,05	0,64 -1,72	2,00	1,19 -3.34	1,19	0,73 -1.91	928
Sulawesi Selatan	74,75	73,11 – 76,33	21,73	20,25 – 23,29	1,20	0,87 -1,64	0,84	0,57 -1.23	1,48	1,10 -1.98	3.239
Sulawesi Tenggara	76,90	73,41 -80,05	20,22	17,36 – 23,42	0,82	0,45 -1,50	0,58	0,28 -1.22	1,48	0,85 -2.56	710
Gorontalo	71,77	67,08 -76,04	24,89	20,90 – 29,37	1,42	0,61 -3,27	0,84	0,30 -2.30	1,08	0,57 -2.04	363
Sulawesi Barat	74,82	70,12 – 79,00	19,82	16,14 – 24,09	2,19	1,13 -4,18	1,96	0,99 -3.85	1,21	0,62 -2.37	351
Maluku	70,19	66,17 – 73,92	25,74	22,11 – 29,73	1,44	0,71 – 2,91	1,13	0,58- 2.22	1,50	0,84 -2.66	495
Maluku Utara	70,05	66,32 -73,54	25,12	21,98 – 28,53	2,05	1,17 -3,60	1,29	0,63- 2.62	1,49	0,80 -2.76	304
Papua Barat	72,60	67,20 -77,41	24,86	20,13 – 30,27	0,85	0,34 -2,10	0,31	0,10 -0.98	1,38	0,65 -2.90	171
Papua	69,70	64,64 – 74,32	26,01	21,67 – 30,87	2,18	1,13 -4,18	0,52	0,22 -1.20	1,60	0,75 -3.36	449
**INDONESIA**	**74,25**	**73,81 -74,69**	**22,01**	**21,59 – 22,43**	**1,15**	**1,06 -1,24**	**1,02**	**0,93 -1.11**	**1,58**	**1,46 -1.71**	**97.407**

Tabel 9.3.2

Proporsi Tingkat Ketergantungan pada Penduduk Umur >60 Tahun menurut Karakteristik, Riskesdas 2018

Karakteristik	Tingkat Ketergantungan										N Tertimbang
	Mandiri		Tergantung ringan		Tergantung sedang		Tergantung berat		Tergantung total		
	%	95% CI	%	95% CI	%	95% CI	%	95% CI	%	95% CI	
**Kelompok Umur**											
60-69	80,30	79,81-80,78	17,51	17,05 -17,98	0,60	0,52 -0,69	0,53	0,45 -0,63	1,07	0,95 – 1,20	61.767
70-79	68,09	67,25 -68,93	27,22	26,41 -28,04	1,50	1,32 -1,72	1,33	1,16 -1,52	1,86	1,62 -2,13	27.123
80+	50,04	48,49 – 51,58	38,03	36,54 -39,56	3,97	3,47 -4,56	3,56	3,01 -4,20	4,39	3,81 -5,07	8.517
**Jenis kelamin**											
Laki-laki	77,78	77,20 -78,35	19,07	18,53 -19,62	0,94	0,83 -1,07	0,78	0,67 -0,90	1,43	1,28 -1,61	46.332
Perempuan	71,05	70,45 -71,64	24,67	24,10 -25,24	1,33	1,21 -1,47	1,23	1,10 -1,39	1,71	1,55 -1,90	51.075
**Pendidikan**											
Tidak/belum pernah sekolah	68,11	67,11 -69,09	26,67	25,74 -27,63	1,71	1,49 -1,97	1,45	1,21 -1,73	2,06	1,79 -2,37	20.315
Tidak tamat SD/MI	73,92	73,12 -74,71	22,43	21,69 -23,20	1,08	0,93 -1,26	0,95	0,81 -1,12	1,61	1,38 – 1,88	26.637
Tamat SD/MI	75,96	75,20 – 76,69	20,54	19,84 -21,25	0,99	0,84 -1,15	0,99	0,83 -1,17	1,53	1,34 – 1,76	30.098
Tamat SLTP/MTS	77,63	76,19 – 79,01	19,70	18,38 -21,10	0,89	0,66 -1,21	0,83	0,58 -1,20	0,94	0,70 -1,26	7.443
Tamat SLTA/MA	77,75	76,40 – 79,04	19,37	1814 -20,66	0,91	0,67 -1,22	0,74	0,54 -0,01	1,23	0,92 -1,65	8.762
Tamat D1/D2/D3/PT	80,66	78,72 -82,47	16,76	15,06 -18,61	0,90	0,55 – 1,47	0,45	0,26 -0,78	1,23	0,84 -1,79	4.152
**Tempat tinggal**											
Perkotaan	73,27	72,57 -73,95	22,82	22,17 -23,49	1,11	0,98 -1,26	1,06	0,92 -1,21	1,75	1,56 -1,95	50.275
Perdesaan	75,30	74,77 -75,83	21,13	20,63 -21,65	1,19	1,08 -1,31	0,97	0,87 -1,09	1,40	1,28 -1,55	47.132

Tabel 9.3.3

Proporsi Tingkat Ketergantungan pada Penduduk Umur >60 Tahun menurut Penyakit, Riskesdas 2018

Penyakit[1]	Tingkat Ketergantungan										N Tertimbang
	Mandiri		Tergantung ringan		Tergantung sedang		Tergantung berat		Tergantung total		
	%	95% CI	%	95% CI	%	95% CI	%	95% CI	%	95% CI	
Jantung	63,98	61,89 -66,03	30,22	28,28 -32,24	1,80	1,29 – 2,50	2,09	1,51 -2,89	1,90	1,41 – 2,56	4,365
DM	63,59	61,66 -65,48	30,56	28,78 -32,41	1,63	1,23 – 2,16	2,08	1,57 – 2,74	2,14	1,60 – 2,86	5,579
Stroke	36,33	34,19 -38,53	33,25	31,20 -35,37	7,10	6,08 -8,28	9,43	8,16 -0,88	13,88	12,25-15,69	4,270
Sendi	67,45	66,38 -68,49	28,44	27,43 -29,47	1,52	1,29 -1,79	1,13	0,92 -1,38	1,46	1,22 -1,76	17,547
Cedera	63,23	61,60-64,84	29,67	28,14 -31,24	2,24	1,83 -2,74	2,10	1,70 -2,60	2,75	2,30 -3,30	7,976

[1]Penyakit berdasarkan diagnosis dokter (kecuali untuk cedera berdasarkan pengakuan)

BAB 10
CEDERA

10.1 Gambaran Umum Cedera

Cedera

Proporsi cedera dalam 12 bulan terakhir yang mengakibatkan kegiatan sehari-hari terganggu (pada semua umur) dihitung dengan formula:

$$Proporsi\ cedera\ dalam\ 12\ bulan\ terakhir\ yang\ mengakibatkan\ kegiatan\ sehari-hari\ terganggu$$

$$= \frac{\sum ART semua\ umur\ yang\ pernah\ cedera\ \ dalam\ 12\ bulan\ terakhir\ yang\ mengakibatkan\ kegiatan\ sehari-hari\ terganggu}{\sum ART semua\ umur}$$

Bagian tubuh yang terkena cedera

Bagian tubuh yang terkena cedera dapat lebih dari satu bagian (*multiple injury*). Klasifikasi bagian tubuh yang cedera menurut ICD-10, dikelompokkan menjadi:

1. Kepala meliputi indera (mata, hidung, telinga, mulut), bagian muka, dan leher.
2. Dada meliputi tubuh bagian depan dari atas pinggang sampai bawah leher termasuk tulang dada.
3. Punggung meliputi tubuh bagian belakang dari atas pinggang sampai bawah leher termasuk tulang belakang.
4. Perut meliputi tubuh dari bawah pinggang, bagian depan dan belakang, termasuk alat kelamin dan organ dalam.
5. Anggota gerak atas **(**meliputi lengan atas, lengan bawah, punggung tangan, telapak dan jari tangan.
6. Anggota gerak bawah meliputi paha, betis, telapak dan jari kaki.

Proporsi bagian tubuh yang terkena cedera dihitung dengan formula:

$$Proporsi\ bagian\ tubuh\ yang\ terkena\ cedera$$

$$= \frac{\sum ART\ semua\ umur\ dengan\ bagian\ tubuh\ yang\ terkena\ cedera\ sehingga\ mengakibatkan\ kegiatan\ sehari-hari\ terganggu}{\sum ART\ semua\ umur\ yang\ pernah\ cedera\ yang\ mengakibatkan\ kegiatan\ sehari-hari\ terganggu}$$

Jenis Cedera

Jenis cedera yang dialami dapat berupa luka (lecet, robek), terkilir, patah tulang, anggota tubuh terputus, mata, gegar otak, cedera organ dalam, luka bakar, lainnya. Proporsi jenis cedera dihitung dengan formula:

$$Proporsi\ jenis\ cedera = \frac{\sum ART\ semua\ umur\ yang\ mengalami\ jenis\ cedera}{\begin{array}{c}\sum ART\ semua\ umur\ yang\ pernah\ cedera\ dalam\ 12\ bulan\ terakhir\\ yang\ mengakibatkan\ kegiatan\ sehari-hari\ terganggu\end{array}}$$

Cedera mengakibatkan kecacatan fisik yang permanen

Cacat fisik akibat cedera adalah kondisi seseorang yang mempunyai gangguan fisik seperti hilangnya sebagian atau kurang berfungsinya anggota badan sebagai akibat dari cedera yang pernah dialami. Proporsi cedera mengakibatkan kecacatan fisik yang permanen pada bagian tubuh dihitung dengan formula:

$$Proporsi\ cedera\ mengakibatkan\ kecacatan\ fisik\ yang\ permanen\ pada\ bagian\ tubuh$$
$$= \frac{\begin{array}{c}\sum ART\ dengan\ cedera\ mengakibatkan\ kecacatan\ fisik\ yang\ permanen\\ pada\ bagian\ tubuh\ yang\ dialami\ ART\end{array}}{\begin{array}{c}\sum ART\ yang\ pernah\ cedera\ dalam\ 12\ bulan\ terakhir\\ yang\ mengakibatkan\ kegiatan\ sehari-hari\ terganggu\end{array}}$$

Tempat terjadinya cedera

Tempat terjadinya cedera adalah lokasi/area dimana peristiwa/kejadian yang mengakibatkan cedera. Tempat terjadinya cedera dikelompokkan menjadi:

1. Jalan raya (jalan yang dilalui kendaraan).
2. Rumah dan lingkungannya (*indoor* maupun *outdoor*).
3. Sekolah dan lingkungannya (dalam kelas maupun halaman sekolah).
4. Tempat bekerja (tempat kerja responden yang berupa ruangan/bangunan tertutup/terbuka termasuk halamannya: contoh pabrik, pertokoan, perkantoran, pasar, pelabuhan, dll).
5. Lainnya seperti perairan/sungai/laut, sawah, ladang, hutan, tambang, dll.

Proporsi tempat terjadinya cedera dihitung dengan formula:

$$Proporsi\ tempat\ terjadinya\ cedera = \frac{\sum ART\ yang\ cedera\ di\ tempat\ terjadinya\ cedera}{\sum ART\ yang\ pernah\ cedera\ yang\ mengakibatkan\ kegiatan\ sehari-hari\ terganggu\ dalam\ 12\ bulan\ terakhir}$$

Cedera karena kecelakaan lalu lintas

Kecelakaan lalu lintas adalah suatu peristiwa di jalan raya yang tidak diduga dan tidak disengaja, melibatkan kendaraan dengan atau tanpa pengguna jalan lain yang mengakibatkan korban manusia dan/atau kerugian harta benda (UU RI No. 22 Tahun 2009).

Proporsi cedera karena kecelakaan lalu lintas dihitung dengan formula:

$$Proporsi\ cedera\ karena\ kecelakaan\ lalu\ lintas = \frac{\sum ART\ yang\ mengalami\ cedera\ disebabkan\ karena\ kecelakaan\ lalu\ lintas}{\sum ART\ semua\ umur}$$

Kecelakaan lalu lintas yang terjadi melibatkan kendaraan bermotor atau tidak bermotor. Kendaraan bermotor dapat berupa kendaraan roda dua atau sepeda motor, roda tiga seperti bemo, roda empat atau lebih dari 4 seperti mobil, truk, tronton, dll. Kendaraan tidak bermotor dapat berupa sepeda, dokar, dll. Responden yang mengalami cedera berkaitan dengan kecelakaan lalu lintas dapat sebagai pengemudi atau penumpang kendaraan yang terlibat kecelakaan, atau sebagai pejalan kaki.

Proporsi kegiatan yang sedang dilakukan ART saat terjadi kecelakaan lalu lintas dihitung dengan formula:

$$Proporsi\ kegiatan\ yang\ sedang\ dilakukan\ ART\ saat\ terjadi\ kecelakaan\ lalu\ lintas = \frac{\sum ART\ yang\ sedang\ melakukan\ kegiatan\ pada\ saat\ terjadi\ cedera\ karena\ kecelakaan\ lalu\ lintas}{\sum ART\ yang\ mengalami\ cedera\ karena\ kecelakaan\ lalu\ lintas}$$

Tabel 10.1.1
Proporsi Cedera yang Mengakibatkan Kegiatan Sehari-hari Terganggu menurut Provinsi, Riskesdas 2018

Provinsi	Cedera		N Tertimbang
	%	(95%CI)	
Aceh	8,1	7,7-8,5	20.244
Sumatera Utara	9,9	9,3-10,4	55.351
Sumatera Barat	8,7	8,2-9,3	20.663
Riau	8,2	7,7-8,9	26.085
Jambi	5,6	5,0-6,2	13.692
Sumatera Selatan	7,1	6,5-7,7	32.126
Bengkulu	9,0	8,3-9,8	7.531
Lampung	8,1	7,5-8,7	32.148
Bangka Belitung	7,3	6,5-8,2	5.592
Kepulauan Riau	8,1	7,0-9,3	8.173
DKI Jakarta	10,1	9,3-10,9	40.210
Jawa Barat	8,7	8,4-9,1	186.809
Jawa Tengah	9,3	9,0-9,6	132.565
DI Yogyakarta	10,6	9,8-11,5	14.602
Jawa Timur	9,1	8,8-9,5	151.878
Banten	11,1	10,3-11,8	48.621
Bali	8,8	8,2-9,5	16.481
Nusa Tenggara Barat	8,6	8,0-9,2	19.247
Nusa Tenggara Timur	10,3	9,7-11,0	20.599
Kalimantan Barat	9,0	8,4-9,7	19.190
Kalimantan Tengah	7,2	6,6-7,9	10.189
Kalimantan Selatan	8,8	8,2-9,5	16.043
Kalimantan Timur	10,5	9,6-11,4	13.977
Kalimantan Utara	8,1	7,2-9,2	2.733
Sulawesi Utara	11,6	10,8-12,4	9.542
Sulawesi Tengah	13,8	12,9-14,9	11.548
Sulawesi Selatan	11,0	10,4-11,5	33.693
Sulawesi Tenggara	11,3	10,3-12,4	10.167
Gorontalo	6,9	6,2-7,6	4.547
Sulawesi Barat	9,6	8,4-11,0	5.195
Maluku	9,2	8,3-10,2	6.801
Maluku Utara	9,0	8,2-9,9	4.723
Papua Barat	12,6	11,3-14,1	3.588
Papua	10,1	9,2-11,1	12.736
**INDONESIA**	**9,2**	**9,1-9,4**	**1.017.290**

Tabel 10.1.2
Proporsi Cedera yang Mengakibatkan Kegiatan Sehari-Hari Terganggu menurut Karakteristik, Riskesdas 2018

Karakteristik	Cedera		N Tertimbang
	%	95% CI	
**Kelompok umur**			
< 1	0,0	0,0-0,0	1.255
1 – 4	8,2	7,8-8,4	91.413
5 – 14	12,1	11,9-12,4	182.338
15 – 24	12,2	12-12,5	165.644
25 – 34	7,9	7,7-8,1	159.708
35 – 44	7,4	7,2-7,6	151.539
45 – 54	7,1	6,9-7,3	124.652
55 – 64	7,7	7,5-8,0	83.251
65 – 74	8,1	7,7-8,5	40.180
75+	9,2	8,6-9,8	18.565
**Jenis Kelamin**			
Laki-laki	11,0	10,9-11,2	510.714
Perempuan	7,4	7,3-7,6	506.576
**Pendidikan**			
Tidak sekolah	9,8	9,5-10,2	70.895
Tidak tamat SD/MI	10,4	10,1-10,6	181.429
Tamat SD/MI	9,1	8,9-9,3	215.967
Tamat SMP/MTS	9,6	9,4-9,9	160.320
Tamat SMA/MA	8,8	8,6-9,0	210.746
Tamat Diploma/PT	7,3	7,0-7,7	64.093
**Status pekerjaan**			
Tidak bekerja	7,9	7,7-8,0	233.629
Sekolah	13,0	12,7-13,3	126.626
PNS/TNI/Polri/BUMN/BUMD	6,4	5,9-6,9	21.931
Pegawai swasta	9,4	9,0-9,8	75.781
Wiraswasta	8,2	7,9-8,4	105.489
Petani/buruh tani	8,2	7,9-8,4	133.261
Nelayan	9,5	8,5-10,5	5.556
Buruh/sopir/pembantu ruta	10,1	9,7-10,5	75.590
Lainnya	7,9	7,5-8,3	40.644
**Tempat tinggal**			
Perkotaan	9,4	9,2-9,6	556.419
Perdesaan	9,0	8,8-9,1	460.871

Tabel 10.1.3

Proporsi Bagian Tubuh yang Cedera menurut Provinsi, Riskesdas 2018

Provinsi	Bagian tubuh yang cedera (%)						N Tertimbang
	Kepala	Dada	Punggung	Perut	Anggota gerak atas	Anggota gerak bawah	
Aceh	13,3	4,1	7,4	2,1	38,2	62,6	1.619
Sumatera Utara	10,3	2,3	5,4	3,0	32,9	70,2	5.401
Sumatera Barat	14,3	3,0	6,7	2,3	33,8	65,7	1.785
Riau	11,4	2,8	5,6	2,4	36,1	64,0	2.129
Jambi	10,7	2,1	5,5	2,8	36,0	64,4	756
Sumatera Selatan	13,9	3,0	6,5	3,9	37,2	67,5	2.256
Bengkulu	10,7	2,1	6,1	3,7	35,8	69,4	673
Lampung	12,1	2,8	6,2	1,8	32,9	68,8	2.575
Bangka Belitung	14,2	4,5	4,2	3,2	38,7	62,7	405
Kepulauan Riau	12,5	1,8	3,5	1,3	34,3	67,9	655
DKI Jakarta	11,8	1,7	5,0	1,7	29,1	71,9	4.017
Jawa Barat	12,3	2,6	6,0	2,1	33,1	68,8	16.150
Jawa Tengah	10,6	1,9	6,4	2,0	30,7	68,3	12.213
DI Yogyakarta	11,0	2,3	9,1	2,4	33,6	64,5	1.537
Jawa Timur	11,1	2,1	6,6	1,7	32,3	68,1	13.726
Banten	10,5	2,5	6,2	1,5	30,6	70,5	5.333
Bali	10,7	2,7	5,3	1,2	32,1	67,4	1.436
Nusa Tenggara Barat	15,2	2,6	7,7	2,9	33,7	66,8	1.640
Nusa Tenggara Timur	15,1	4,4	8,9	1,8	32,4	66,3	2.109
Kalimantan Barat	11,3	3,1	6,6	2,3	32,8	69,5	1.718
Kalimantan Tengah	11,3	3,4	6,6	2,7	33,1	66,7	730
Kalimantan Selatan	8,6	2,1	9,9	2,1	32,6	68,6	1.404
Kalimantan Timur	10,2	2,1	5,8	1,3	31,7	64,6	1.447
Kalimantan Utara	12,8	3,5	5,6	1,6	33,7	62,9	220
Sulawesi Utara	15,5	6,6	8,8	2,4	32,3	65,6	1.093
Sulawesi Tengah	14,5	4,5	8,3	3,9	35,2	66,5	1.583
Sulawesi Selatan	15,0	2,8	6,6	2,3	32,4	65,6	3.659
Sulawesi Tenggara	11,4	2,9	7,0	3,0	32,4	65,8	1.139
Gorontalo	17,9	5,3	6,3	3,3	31,8	66,3	309
Sulawesi Barat	12,7	1,7	5,0	2,7	38,8	62,4	495
Maluku	12,8	4,6	6,9	3,7	34,0	64,1	620
Maluku Utara	14,3	4,4	9,8	3,5	27,7	65,3	421
Papua Barat	12,6	3,5	8,1	2,8	33,0	64,8	449
Papua	16,5	3,8	10,6	3,8	37,8	64,7	1.276
**INDONESIA**	**11,9**	**2,6**	**6,5**	**2,2**	**32,7**	**67,9**	**92.976**

Tabel 10.1.4
Proporsi Bagian Tubuh yang Cedera menurut Karakteristik, Riskesdas 2018

Karakteristik	Bagian tubuh yang cedera (%)						N Tertimbang
	Kepala	Dada	Punggung	Perut	Anggota gerak atas	Anggota gerak bawah	
**Kelompok umur**							
< 1	-	-	-	-	-	-	-
1– 4	29,5	1,8	5,3	1,7	24,1	61,9	7.287
5 – 14	11,4	1,6	2,5	1,7	27,4	75,5	21.883
15 – 24	9,0	2,4	5,1	2,4	37,6	72,5	20.063
25 – 34	8,7	2,6	7,2	1,9	37,7	66,9	12.517
35 – 44	10,1	3,4	8,2	2,3	35,2	62,8	11.125
45 – 54	10,6	3,7	9,8	2,4	34,3	62,6	8.809
55 – 64	11,2	4,0	11,8	2,9	30,7	60,7	6.376
65 – 74	16,0	4,2	12,9	2,7	29,4	58,1	3.225
75+	17,1	2,4	13,8	2,9	31,9	55,9	1.691
**Jenis Kelamin**							
Laki-laki	12,2	2,9	6,3	2,1	34,6	69,1	51.909
Perempuan	11,5	2,2	6,7	2,3	29,8	66,5	41.067
**Pendidikan**							
Tidak sekolah	13,8	2,2	7,7	2,3	28,5	68,0	6.930
Tidak tamat SD/MI	11,4	2,3	5,9	2,3	29,3	70,6	18.682
Tamat SD/MI	10,2	3,1	6,9	2,3	33,8	67,1	19.436
Tamat SMP/MTS	9,3	2,7	6,7	2,3	37,6	68,1	15.345
Tamat SMA/MA	9,2	2,8	6,9	2,1	36,6	67,8	18.487
Tamat Diploma/PT	7,5	2,3	6,6	1,9	34,1	68,1	4.662
**Status pekerjaan**							
Tidak bekerja	11,4	2,6	7,8	2,7	32,6	67,0	18.243
Sekolah	8,2	1,8	3,6	2,0	32,9	75,3	16.379
PNS/TNI/Polri/ BUMN/BUMD	9,6	2,8	8,3	2,6	36,0	63,3	1.391
Pegawai swasta	7,5	2,9	6,2	1,5	36,7	68,0	7.078
Wiraswasta	10,4	3,0	7,5	2,3	36,9	64,4	8.559
Petani/buruh tani	10,4	3,8	11,1	3,0	33,8	62,7	10.788
Nelayan	12,2	6,2	9,1	3,4	38,8	56,0	522
Buruh/sopir/ pembantu ruta	9,9	3,3	7,9	1,9	37,7	63,8	7.554
Lainnya	11,0	3,2	8,8	1,5	36,3	66,2	3.201
**Tempat tinggal**							
Perkotaan	11,8	2,3	6,0	1,9	32,3	69,1	51.909
Perdesaan	12,1	3,0	7,2	2,5	33,2	66,5	41.067

Tabel 10.1.5
Proporsi Jenis Cedera (Jenis Luka, Terkilir, Patah Tulang, Anggota Tubuh Terputus) menurut Provinsi, Riskesdas 2018

Provinsi	Jenis cedera yang dialami (%)					N Tertimbang
	Lecet/lebam / memar	Luka iris/robek/ tusuk	Terkilir	Patah tulang	Anggota tubuh terputus	
Aceh	60,9	19,2	43,7	7,9	0,6	1,619
Sumatera Utara	63,1	23,9	33,5	3,7	0,6	5,401
Sumatera Barat	54,5	22,0	43,2	5,6	0,5	1,785
Riau	55,3	24,7	36,2	6,0	0,5	2,129
Jambi	64,8	23,7	31,6	5,1	0,5	756
Sumatera Selatan	69,2	17,6	34,6	4,2	0,4	2,256
Bengkulu	62,3	21,5	33,5	4,9	0,4	673
Lampung	65,1	14,8	36,2	4,5	0,5	2,575
Bangka Belitung	57,7	22,7	35,2	9,1	0,3	405
Kepulauan Riau	66,1	18,0	23,1	5,0	1,0	655
DKI Jakarta	67,7	17,0	38,7	4,3	0,2	4,017
Jawa Barat	63,1	21,4	36,2	6,4	0,6	16,150
Jawa Tengah	64,7	15,4	32,8	5,8	0,4	12,213
DI Yogyakarta	56,1	19,7	36,1	7,2	0,5	1,537
Jawa Timur	67,4	18,2	30,5	5,8	0,6	13,726
Banten	61,3	14,3	36,9	6,0	0,4	5,333
Bali	56,4	20,6	29,8	7,5	0,5	1,436
Nusa Tenggara Barat	68,2	23,8	29,8	7,2	0,5	1,640
Nusa Tenggara Timur	64,9	29,3	20,2	5,6	0,7	2,109
Kalimantan Barat	65,1	21,3	28,8	4,0	0,8	1,718
Kalimantan Tengah	63,3	16,4	33,6	4,3	0,4	730
Kalimantan Selatan	57,2	18,0	40,9	4,2	0,4	1,404
Kalimantan Timur	62,0	19,0	33,1	3,5	0,4	1,447
Kalimantan Utara	61,5	28,4	25,7	8,1	0,4	220
Sulawesi Utara	75,6	16,5	24,2	4,5	0,6	1,093
Sulawesi Tengah	65,4	25,8	30,6	4,2	0,3	1,583
Sulawesi Selatan	67,0	27,8	20,9	4,0	0,6	3,659
Sulawesi Tenggara	66,1	23,1	27,7	4,4	0,2	1,139
Gorontalo	61,8	22,3	31,9	6,0	0,7	309
Sulawesi Barat	65,4	29,3	15,0	3,9	0,7	495
Maluku	67,6	21,8	23,3	6,6	0,2	620
Maluku Utara	64,3	20,9	20,2	6,5	0,3	421
Papua Barat	61,5	20,6	28,0	4,9	0,5	449
Papua	62,1	38,5	23,0	6,3	0,4	1,276
**INDONESIA**	**64,1**	**20,1**	**32,8**	**5,5**	**0,5**	**92,976**

Tabel 10.1.6
Proporsi Jenis Cedera (Jenis Luka, Terkilir, Patah Tulang, Anggota Tubuh Terputus) menurut Karakteristik, Riskesdas 2018

Karakteristik	Jenis cedera yang dialami (%)					N Tertimbang
	Lecet/lebam/memar	Luka iris/robek/tusuk	Terkilir	Patah tulang	Anggota tubuh terputus	
**Kelompok Umur**						
< 1	0,0	0,0	0,0	0,0	0,0	0
1-4	78,9	12,1	17,2	1,4	0,3	7,011
5 – 14	73,7	18,4	23,1	3,2	0,2	2,401
15 – 24	65,9	21,1	37,5	5,3	0,5	19,585
25 – 34	59,0	22,3	38,3	6,2	0,8	12,130
35 – 44	56,2	24,0	37,4	6,5	0,7	10,800
45 – 54	54,9	22,7	38,7	7,9	0,8	8,548
55 – 64	53,3	20,8	38,5	8,4	0,5	6,149
65 – 74	53,3	16,9	37,2	9,3	0,8	3,063
75+	53,9	12,0	38,3	14,5	0,5	1,571
**Jenis Kelamin**						
Laki-laki	62,9	23,1	32,6	6,2	0,6	55,749
Perempuan	65,9	15,5	33,1	4,5	0,4	37,227
**Pendidikan**						
Tidak sekolah	64,4	19,0	27,9	6,5	0,5	6,930
Tidak tamat SD/MI	67,8	20,3	28,7	4,7	0,4	18,682
Tamat SD/MI	60,5	22,3	35,7	6,1	0,6	19,436
Tamat SMP/MTS	61,5	21,8	36,5	6,6	0,5	15,345
Tamat SMA/MA	60,4	21,0	38,7	6,4	0,8	18,487
Tamat Diploma/PT	57,8	17,1	40,9	6,5	0,3	4,662
**Status pekerjaan**						
Tidak bekerja	61,3	16,7	37,8	7,1	0,6	18,243
Sekolah	68,4	19,0	33,2	4,2	0,3	16,379
PNS/TNI/Polri/BUMN/BUMD	56,7	19,2	39,1	7,5	0,9	1,391
Pegawai swasta	60,7	19,5	38,0	5,9	0,7	7,078
Wiraswasta	58,2	22,4	37,9	7,3	0,7	8,559
Petani/buruh tani	53,9	28,1	36,7	6,1	0,7	10,788
Nelayan	52,3	30,7	29,0	7,4	0,9	522
Buruh/sopir/pembantu ruta	57,4	26,7	36,0	8,3	0,9	7,554
Lainnya	59,7	20,6	37,9	7,0	0,3	3,201
**Tempat tinggal**						
Perkotaan	64,4	18,8	33,5	5,7	0,5	51,909
Perdesaan	63,7	21,8	31,9	5,3	0,6	41,067

Tabel 10.1.7

Proporsi Jenis Cedera (Cedera Mata, Gegar Otak, Cedera Organ Dalam, Luka Bakar, Lainnya) menurut Provinsi, Riskesdas 2018

Provinsi	Jenis cedera yang dialami (%)					N Tertimbang
	Cedera mata	Gegar otak	Cedera organ dalam	Luka bakar	Lainnya	
Aceh	0,6	0,2	1,2	0,9	1,6	1.619
Sumatera Utara	0,3	0,1	0,7	1,0	1,9	5.401
Sumatera Barat	0,9	0,1	0,9	1,8	1,8	1.785
Riau	0,5	0,0	0,9	1,7	2,4	2.129
Jambi	0,1	0,1	1,1	1,0	2,8	756
Sumatera Selatan	0,6	0,1	1,7	1,4	1,4	2.256
Bengkulu	0,5	0,2	1,6	1,3	2,0	673
Lampung	0,7	0,0	0,9	1,4	2,2	2.575
Bangka Belitung	1,6	0,1	1,1	0,7	2,2	405
Kepulauan Riau	0,3	1,1	0,9	1,5	1,3	655
DKI Jakarta	0,5	0,2	0,8	1,7	3,3	4.017
Jawa Barat	0,5	0,2	1,6	1,6	2,9	16.150
Jawa Tengah	0,6	0,3	1,1	1,0	2,8	12.213
DI Yogyakarta	0,5	0,9	2,0	1,3	5,6	1.537
Jawa Timur	0,6	0,4	0,9	1,1	2,8	13.726
Banten	0,2	0,1	1,0	1,2	3,0	5.333
Bali	0,4	0,2	0,7	1,5	2,9	1.436
Nusa Tenggara Barat	0,4	0,4	0,9	1,0	2,9	1.640
Nusa Tenggara Timur	0,8	0,4	1,2	1,1	1,5	2.109
Kalimantan Barat	0,8	0,1	1,0	1,2	2,1	1.718
Kalimantan Tengah	0,8	0,1	2,4	1,0	1,5	730
Kalimantan Selatan	0,7	0,0	1,9	1,9	2,4	1.404
Kalimantan Timur	0,2	0,2	1,0	1,2	4,5	1.447
Kalimantan Utara	0,9	0,1	2,0	1,8	2,0	220
Sulawesi Utara	0,7	0,1	1,4	0,5	1,6	1.093
Sulawesi Tengah	0,4	0,1	1,8	1,5	1,4	1.583
Sulawesi Selatan	0,7	0,2	1,4	1,3	1,7	3.659
Sulawesi Tenggara	0,2	0,2	0,7	1,0	1,5	1.139
Gorontalo	0,8	0,1	2,4	1,5	2,5	309
Sulawesi Barat	0,6	0,1	1,0	1,2	2,5	495
Maluku	0,8	0,0	1,3	0,8	2,2	620
Maluku Utara	0,8	0,1	1,8	1,0	2,7	421
Papua Barat	0,5	0,5	2,1	1,4	2,0	449
Papua	0,9	0,4	3,0	2,1	2,2	1.276
**INDONESIA**	**0,5**	**0,2**	**1,2**	**1,3**	**2,6**	**92.976**

Tabel 10.1.8
Proporsi Jenis Cedera (Cedera Mata, Gegar Otak, Cedera Organ Dalam, Luka Bakar, Lainnya) menurut Provinsi Karakteristik, Riskesdas 2018

Karakteristik	Jenis cedera yang dialami (%)					N Tertimbang
	Cedera mata	Gegar otak	Cedera organ dalam	Luka bakar	Lainnya	
**Kelompok Umur**						
< 1	-	-	-	-	-	-
1-4	0,3	0,1	0,3	1,4	1,3	7.287
5 – 14	0,3	0,1	0,4	0,9	1,3	21.883
15 – 24	0,5	0,3	1,0	1,3	2,0	20.063
25 – 34	0,5	0,2	1,1	1,8	2,8	12.517
35 – 44	0,8	0,3	1,8	1,4	3,4	11.125
45 – 54	0,8	0,4	2,1	1,3	4,2	8.809
55 – 64	1,0	0,3	2,6	1,3	4,3	6.376
65 – 74	0,7	0,4	2,1	1,3	5,2	3.225
75+	0,4	0,3	2,8	0,6	4,4	1.691
**Jenis Kelamin**						
Laki-laki	0,7	0,2	1,2	1,2	2,4	55,749
Perempuan	0,4	0,2	1,1	1,4	2,8	37,227
**Pendidikan**						
Tidak sekolah	0,5	0,2	1,4	1,1	2,7	6,930
Tidak tamat SD/MI	0,5	0,2	0,8	1,1	2,0	18,682
Tamat SD/MI	0,7	0,2	1,7	1,2	2,6	19,436
Tamat SMP/MTS	0,6	0,4	1,2	1,5	2,4	15,345
Tamat SMA/MA	0,6	0,3	1,4	1,4	3,2	18,487
Tamat Diploma/PT	0,4	0,3	1,8	1,4	5,1	4,662
**Status pekerjaan**						
Tidak bekerja	0,5	0,3	1,7	1,4	3,4	18,243
Sekolah	0,4	0,2	0,8	1,1	1,7	16,379
PNS/TNI/Polri/BUMN/BUMD	0,6	0,2	2,2	1,0	5,2	1,391
Pegawai swasta	0,5	0,2	1,0	1,5	3,6	7,078
Wiraswasta	0,9	0,6	1,4	1,8	3,3	8,559
Petani/buruh tani	0,8	0,2	1,7	1,3	2,6	10,788
Nelayan	0,5	0,2	2,2	0,9	2,7	522
Buruh/sopir/pembantu ruta	0,8	0,2	1,9	1,1	3,4	7,554
Lainnya	0,6	0,2	1,6	1,6	2,8	3,201
**Tempat tinggal**						
Perkotaan	0,5	0,2	1,2	1,3	3,0	51,909
Perdesaan	0,6	0,2	1,2	1,2	2,1	41,067

Tabel 10.1.9

Proporsi Cedera Mengakibatkan Kecacatan Fisik Permanen menurut Provinsi, Riskesdas 2018

Provinsi	Cedera mengakibatkan kecacatan fisik permanen (%)			N Tertimbang
	Panca indera tidak berfungsi (buta/tuli/bisu dll)	Kehilangan sebagian anggota badan (jari/tangan/kaki putus dll)	Bekas luka permanen yang mengganggu kenyamanan	
Aceh	0,7	0,9	6,6	1,619
Sumatera Utara	0,4	0,5	7,6	5,401
Sumatera Barat	1,1	0,6	8,6	1,785
Riau	0,4	0,7	12,5	2,129
Jambi	0,6	0,4	8,2	756
Sumatera Selatan	0,7	0,2	8,8	2,256
Bengkulu	0,9	0,8	8,8	673
Lampung	0,6	0,7	6,8	2,575
Bangka Belitung	0,2	0,2	13,2	405
Kepulauan Riau	0,4	0,5	9,5	655
DKI Jakarta	0,5	0,3	9,3	4,017
Jawa Barat	0,5	0,6	9,8	16,150
Jawa Tengah	0,5	0,5	6,8	12,213
DI Yogyakarta	0,7	0,7	9,0	1,537
Jawa Timur	0,5	0,5	9,0	13,726
Banten	0,5	0,7	6,8	5,333
Bali	0,4	0,4	9,6	1,436
Nusa Tenggara Barat	0,7	0,5	12,9	1,640
Nusa Tenggara Timur	0,6	0,8	13,6	2,109
Kalimantan Barat	0,5	0,8	11,0	1,718
Kalimantan Tengah	0,6	0,3	8,6	730
Kalimantan Selatan	0,5	0,3	7,2	1,404
Kalimantan Timur	0,6	0,7	10,1	1,447
Kalimantan Utara	1,0	0,8	9,6	220
Sulawesi Utara	1,0	0,8	10,7	1,093
Sulawesi Tengah	0,7	0,8	11,9	1,583
Sulawesi Selatan	0,6	0,6	12,4	3,659
Sulawesi Tenggara	0,4	0,2	8,9	1,139
Gorontalo	0,6	0,5	12,5	309
Sulawesi Barat	0,9	0,6	8,9	495
Maluku	0,6	0,3	10,5	620
Maluku Utara	0,7	0,5	15,0	421
Papua Barat	0,6	1,2	12,3	449
Papua	1,0	1,1	14,9	1,276
**INDONESIA**	**0,5**	**0,6**	**9,2**	**92,976**

Tabel 10.1.10
Proporsi Cedera Mengakibatkan Kecacatan Fisik Permanen menurut Karakteristik, Riskesdas 2018

Karakteristik	Cedera mengakibatkan kecacatan fisik permanen (%)			N Tertimbang
	Panca indera tidak berfungsi (buta/tuli/bisu dll)	Kehilangan sebagian anggota badan (jari/tangan/kaki putus dll)	Bekas luka permanen yang mengganggu kenyamanan	
**Kelompok Umur**				
< 1	-	-	-	-
1-4	0,1	0,2	3,3	7,287
5 – 14	0,3	0,3	6,5	21,883
15 – 24	0,4	0,4	10,5	20,063
25 – 34	0,6	0,7	10,4	12,517
35 – 44	0,7	0,8	10,9	11,125
45 – 54	0,9	1,0	11,3	8,809
55 – 64	1,0	0,7	11,0	6,376
65 – 74	0,9	0,6	10,9	3,225
75+	2,2	0,7	11,2	1,691
**Jenis Kelamin**				
Laki-laki	0,6	0,7	9,7	55,749
Perempuan	0,4	0,4	8,4	37,227
**Pendidikan**				
Tidak sekolah	0,7	0,6	8,1	6,930
Tidak tamat SD/MI	0,6	0,5	8,3	18,682
Tamat SD/MI	0,7	0,6	10,5	19,436
Tamat SMP/MTS	0,5	0,6	10,4	15,345
Tamat SMA/MA	0,5	0,6	10,8	18,487
Tamat Diploma/PT	0,6	0,3	9,6	4,662
**Status pekerjaan**				
Tidak bekerja	0,8	0,6	10,8	18,243
Sekolah	0,3	0,3	8,8	16,379
PNS/TNI/Polri/BUMN/ BUMD	0,2	0,7	9,5	1,391
Pegawai swasta	0,5	0,4	9,9	7,078
Wiraswasta	0,6	0,8	10,8	8,559
Petani/buruh tani	0,9	0,8	11,0	10,788
Nelayan	0,7	1,1	10,8	522
Buruh/sopir/ pembantu ruta	0,6	0,9	10,1	7,554
Lainnya	0,7	0,7	12,4	3,201
**Tempat tinggal**				
Perkotaan	0,5	0,5	8,8	51,909
Perdesaan	0,6	0,7	9,7	41,067

Tabel 10.1.11
Proporsi Tempat Terjadinya Cedera menurut Provinsi, Riskesdas 2018

Provinsi	Tempat terjadinya cedera (%)					N Tertimbang
	Jalan Raya	Rumah dan lingkungannya	Sekolah dan lingkungannya	Tempat bekerja	Lainnya	
Aceh	31,1	50,9	4,7	7,7	5,6	1.619
Sumatera Utara	27,3	51,7	6,5	9,8	4,7	5.401
Sumatera Barat	34,8	41,3	5,3	8,7	10	1.785
Riau	32,2	39,4	6,4	13,0	8,9	2.129
Jambi	25,6	47,3	8,6	11,3	7,2	756
Sumatera Selatan	26,9	48,9	6,4	8,8	9	2.256
Bengkulu	36,1	37,7	6,7	11,4	8,1	673
Lampung	28,0	47,6	5,7	10,9	7,8	2.575
Bangka Belitung	37,7	33,9	6,3	12,7	9,5	405
Kepulauan Riau	31,2	48,1	6,9	6,9	7	655
DKI Jakarta	29,8	47,7	7,8	6,4	8,3	4.017
Jawa Barat	32,1	43,2	6,8	9,0	9	16.150
Jawa Tengah	31,7	44,7	6,4	9,2	8	12.213
DI Yogyakarta	31,0	40,2	6,4	10,7	11,6	1.537
Jawa Timur	31,8	44,8	7,4	9,0	7	13.726
Banten	28,7	47,5	6,1	6,7	11,1	5.333
Bali	34,8	39,0	5,8	9,4	11	1.436
Nusa Tenggara Barat	39,8	39,3	4,1	7,2	9,6	1.640
Nusa Tenggara Timur	35,9	44,1	5,8	5,9	8,3	2.109
Kalimantan Barat	30,7	42,5	6,8	12,7	7,3	1.718
Kalimantan Tengah	31,6	40,6	8,0	13,4	6,5	730
Kalimantan Selatan	28,2	44,7	7,5	10,3	9,4	1.404
Kalimantan Timur	29,2	39,9	7,4	11,1	12,5	1.447
Kalimantan Utara	38,3	38,2	7,0	9,8	6,7	220
Sulawesi Utara	39,2	43,0	4,8	8,6	4,5	1.093
Sulawesi Tengah	31,2	41,5	5,2	11,3	10,9	1.583
Sulawesi Selatan	36,1	43,1	6,1	7,5	7,2	3.659
Sulawesi Tenggara	32,4	43,8	5,3	9,6	8,9	1.139
Gorontalo	45,2	34,0	4,2	8,9	7,8	309
Sulawesi Barat	28,2	48,3	4,7	12,9	5,9	495
Maluku	30,8	48,9	5,5	6,2	8,7	620
Maluku Utara	32,7	43,3	2,6	6,4	15	421
Papua Barat	29,7	46,2	7,1	6,4	10,6	449
Papua	20,0	49,8	7,7	15,2	7,3	1.276
**INDONESIA**	**31,4**	**44,7**	**6,5**	**9,1**	**8,3**	**92.976**

Tabel 10.1.12
Proporsi Tempat Terjadinya Cedera menurut Karakteristik, Riskesdas 2018

Karakteristik	Tempat terjadinya cedera (%)					N Tertimbang
	Jalan Raya	Rumah dan lingkungannya	Sekolah dan lingkungannya	Tempat bekerja	Lainnya	
**Kelompok Umur**						
< 1	-	-	-	-	-	-
1-4	5,6	91,2	1,6	0,0	1,6	7.287
5 – 14	17,4	58,9	18,5	0,0	5,3	21.883
15 – 24	49,5	24,6	8,4	5,4	12,1	20.063
25 – 34	43,4	28,1	0,6	17,0	10,8	12.517
35 – 44	37,9	32,3	0,5	21,1	8,2	11.125
45 – 54	33,4	39,1	0,5	18,4	8,7	8.809
55 – 64	26,6	48,5	0,4	15,7	8,8	6.376
65 – 74	18,7	63,9	0,8	7,8	8,8	3.225
75+	9,4	81,9	0,8	2,3	5,7	1.691
**Jenis Kelamin**						
Laki-laki	33,3	37,2	6,5	12,8	10,3	55.749
Perempuan	28,6	56,0	6,6	3,6	5,2	37.227
**Pendidikan**						
Tidak sekolah	15,5	63,8	8,4	6,7	5,7	6.930
Tidak tamat SD/MI	20,0	54,0	12,7	6,3	7,0	18.682
Tamat SD/MI	33,0	38,0	7,0	13,2	8,9	19.436
Tamat SMP/MTS	43,9	29,1	6,6	11,1	9,3	15.345
Tamat SMA/MA	46,4	28,4	2,4	11,7	11,0	18.487
Tamat Diploma/PT	44,1	32,7	1,4	8,4	13,4	4.662
**Status pekerjaan**						
Tidak bekerja	32,6	54,0	4,5	-	8,9	18.243
Sekolah	35,6	34,7	19,5	0,0	10,1	16.379
PNS/TNI/Polri/BUMN/BUMD	48,9	27,5	1,9	10,7	11,1	1.391
Pegawai swasta	48,1	21,9	1,0	17,7	11,3	7.078
Wiraswasta	45,1	33,2	0,8	13,8	7,2	8.559
Petani/buruh tani	27,3	32,0	0,4	27,9	12,4	10.788
Nelayan	32,0	17,1	1,1	33,4	16,4	522
Buruh/sopir/pembantu ruta	39,7	23,5	0,5	29,1	7,2	7.554
Lainnya	42,6	35,1	1,1	11,8	9,4	3.201
**Tempat tinggal**						
Perkotaan	32,4	44,5	7,1	7,8	8,2	51.909
Perdesaan	30,2	45,0	5,8	10,7	8,3	41.067

Tabel 10.1.13

Proporsi Cedera Karena Kecelakaan Lalu Lintas menurut Provinsi, Riskesdas 2018

Provinsi	Cedera karena kecelakaan lalu lintas						N Tertimbang
	Cedera karena kecelakaan lalulintas		Cedera tidak karena kecelakaan lalulintas		Tidak pernah cedera dalam 1 tahun terakhir		
	%	95% CI	%	95% CI	%	95% CI	
Aceh	1,9	1,7-2,1	0,6	0,5-0,7	97,5	97,3-97,7	20.244
Sumatera Utara	1,9	1,7-2,1	0,8	0,7-1,0	97,3	97,1-97,6	55.351
Sumatera Barat	2,4	2,1-2,6	0,7	0,6-0,8	97,0	96,7-97,2	20.663
Riau	1,8	1,6-2,0	0,9	0,7-1,0	97,4	97,1-97,6	26.085
Jambi	1,1	0,9-1,2	0,4	0,3-0,5	98,6	98,4-98,7	13.692
Sumatera Selatan	1,5	1,3-1,7	0,4	0,4-0,6	98,1	97,8-98,3	32.126
Bengkulu	2,7	2,4-3,1	0,5	0,4-0,7	96,7	96,3-97,1	7.531
Lampung	1,7	1,5-1,9	0,6	0,5-0,7	97,7	97,5-98,0	32.148
Bangka Belitung	2,4	2,0-2,7	0,4	0,3-0,6	97,2	96,8-97,6	5.592
Kepulauan Riau	2,0	1,5-2,5	0,6	0,4-0,9	97,5	96,8-98,0	8.173
DKI Jakarta	2,2	2,0-2,6	0,8	0,6-1,0	97,0	96,6-97,3	40.210
Jawa Barat	2,2	2,0-2,4	0,6	0,5-0,7	97,2	97,0-97,4	186.809
Jawa Tengah	2,3	2,1-2,4	0,7	0,6-0,8	97,1	96,9-97,2	132.565
DI Yogyakarta	2,5	2,2-2,9	0,8	0,6-1,0	96,7	96,3-97,1	14.602
Jawa Timur	2,2	2,1-2,3	0,7	0,6-0,8	97,1	97,0-97,2	151.878
Banten	2,4	2,1-2,7	0,8	0,6-0,9	96,8	96,5-97,1	48.621
Bali	2,5	2,2-2,8	0,6	0,4-0,7	96,9	96,6-97,2	16.481
Nusa Tenggara Barat	2,8	2,5-3,1	0,7	0,5-0,8	96,6	96,2-96,9	19.247
Nusa Tenggara Timur	2,7	2,5-3,0	1,0	0,9-1,2	96,3	96,0-96,6	20.599
Kalimantan Barat	2,1	1,8-2,3	0,7	0,6-0,8	97,2	96,9-97,5	19.190
Kalimantan Tengah	1,5	1,3-1,7	0,8	0,6-1,0	97,7	97,4-98,0	10.189
Kalimantan Selatan	1,8	1,6-2,1	0,7	0,5-0,8	97,5	97,2-97,8	16.043
Kalimantan Timur	2,3	1,9-2,7	0,8	0,6-1,0	97,0	96,5-97,3	13.977
Kalimantan Utara	2,6	2,1-3,1	0,6	0,4-0,8	96,9	96,3-97,4	2.733
Sulawesi Utara	3,6	3,2-4,0	1,0	0,8-1,2	95,5	95,0-95,9	9.542
Sulawesi Tengah	3,2	2,9-3,5	1,1	1,0-1,3	95,7	95,3-96,0	11.548
Sulawesi Selatan	3,3	3,0-3,5	0,7	0,6-0,8	96,0	95,8-96,3	33.693
Sulawesi Tenggara	2,9	2,5-3,3	0,8	0,6-1,0	96,3	95,9-96,7	10.167
Gorontalo	2,5	2,2-2,9	0,6	0,4-0,8	96,9	96,5-97,3	4.547
Sulawesi Barat	2,1	1,7-2,7	0,6	0,4-0,8	97,3	96,7-97,7	5.195
Maluku	1,8	1,5-2,2	1,0	0,8-1,3	97,2	96,7-97,6	6.801
Maluku Utara	2,1	1,8-2,5	0,9	0,6-1,2	97,1	96,5-97,5	4.723
Papua Barat	2,3	1,9-2,7	1,5	1,1-2,0	96,3	95,5-96,9	3.588
Papua	1,5	1,3-1,8	0,5	0,4-0,6	98,0	97,7-98,2	12.736
**INDONESIA**	**2.2**	**2,2-2,3**	**0,7**	**0,7-0,7**	**97,1**	**97,0-97,2**	**1.017.290**

Tabel 10.1.14
Proporsi Cedera Karena Kecelakaan Lalu Lintas menurut Karakteristik, Riskesdas 2018

Karakteristik	Cedera karena kecelakaan lalu lintas						N Tertimbang
	Cedera karena kecelakaan lalulintas		Cedera tidak karena kecelakaan lalulintas		Tidak pernah cedera dalam 1 tahun terakhir		
	%	95% CI	%	95% CI	%	95% CI	
**Kelompok Umur**							
< 1	0,0	0,0-0,0	0,0	0,0-0,0	100.0	100.0- 100.0	1.368
1-4	0,2	0,2-0,3	0,2	0,2-0,3	99,6	99,5-99,6	90.045
5 – 14	1,2	1,2-1,3	0,9	0,8-0,9	97,9	97,8-98,0	182.338
15 – 24	4,9	4,7-5,1	1,2	1,1-1,2	93,9	93,7-94,1	165.644
25 – 34	2,8	2,6-2,9	0,7	0,6-0,7	96,6	96,4-96,7	159.708
35 – 44	2,2	2,1-2,3	0,6	0,5-0,6	97,2	97,1-97,3	151.539
45 – 54	1,8	1,7-1,9	0,6	0,5-0,6	97,6	97,5-97,7	124.652
55 – 64	1,5	1,4-1,6	0,6	0,5-0,6	97,9	97,8-98,1	83.251
65 – 74	1,2	1,0-1,3	0,4	0,3-0,4	98,5	98,3-98,6	40.180
75+	0,5	0,4-0,7	0,4	0,3-0,5	99,1	98,9-99,3	18.565
**Jenis Kelamin**							
Laki-laki	2,9	2,8-2,9	0,8	0,8-0,9	96,3	96,2-96,4	510.714
Perempuan	1,6	1,5-1,6	0,6	0,5-0,6	97,9	97,8-97,9	506.576
**Pendidikan**							
Tidak sekolah	1,0	0,9-1,1	0,6	0,5-0,6	98,5	98,3-98,6	70.895
Tidak tamat SD/MI	1,3	1,2-1,4	0,8	0,7-0,8	97,9	97,8-98,0	181.429
Tamat SD/MI	2,2	2,1-2,3	0,8	0,7-0,8	97,0	96,9-97,1	215.967
Tamat SMP/MTS	3,4	3,3-3,6	0,8	0,7-0,9	95,8	95,6-95,9	160.320
Tamat SMA/MA	3,3	3,2-3,4	0,8	0,7-0,9	95,9	95,8-96,0	210.746
Tamat Diploma/PT	2,7	2,5-2,9	0,5	0,5-0,6	96,8	96,6-97,0	64.093
**Status pekerjaan**							
Tidak bekerja	1,9	1,8-2,0	0,6	0,6-0,7	97,4	97,3-97,5	233.629
Sekolah	3,5	3,3-3,6	1,2	1,1-1,3	95,4	95,2-95,5	126.626
PNS/TNI/Polri/BUMN/ BUMD	2,6	2,3-2,9	0,5	0,4-0,7	96,9	96,6-97,2	21.931
Pegawai swasta	3,7	3,4-3,9	0,8	0,7-1,0	95,5	95,2-95,7	75.781
Wiraswasta	3,0	2,8-3,1	0,7	0,6-0,8	96,3	96,1-96,5	105.489
Petani/buruh tani	1,7	1,6-1,7	0,6	0,5-0,6	97,8	97,7-97,9	133.261
Nelayan	2,4	2,0-2,9	0,6	0,4-0,9	97,0	96,4-97,5	5.556
Buruh/sopir/pembantu ruta	3,3	3,1-3,6	0,7	0,6-0,8	96,0	95,7-96,2	75.590
Lainnya	2,6	2,3-2,8	0,8	0,7-1,0	96,6	96,4-96,9	40.644
**Tempat tinggal**							
Perkotaan	2,4	2,3-2,4	0,7	0,6-0,7	97,0	96,9-97,0	556.419
Perdesaan	2,0	2,0-2,1	0,7	0,7-0,7	97,3	97,2-97,4	460.871

Tabel 10.1.15
Proporsi Kegiatan yang Sedang Dilakukan Saat Kecelakaan Lalu Lintas menurut Provinsi, Riskesdas 2018

Provinsi	Kegiatan yang sedang dilakukan saat kecelakaan lalu lintas (%)						N Tertimbang
	Mengendarai sepeda motor	Membonceng/penumpang sepeda motor	Mengendarai mobil (sopir)	Menumpang/penumpang mobil	Naik kendaraan tidak bermesin	Jalan kaki	
Aceh	76,0	19,8	0,8	0,8	0,8	2,6	373
Sumatera Utara	69,1	22,5	1,5	1,6	2,8	3,7	985
Sumatera Barat	69,2	21,5	1,7	2,0	2,1	5,1	463
Riau	76,8	17,2	0,9	1,1	2,6	4,1	449
Jambi	71,9	24,3	1,5	1,7	0,0	3,4	139
Sumatera Selatan	71,2	17,3	2,5	2,6	1,3	5,0	450
Bengkulu	75,0	17,3	2,7	1,6	2,8	2,9	196
Lampung	79,5	14,9	1,1	1,2	1,5	3,1	522
Bangka Belitung	75,3	16,5	2,4	0,8	3,3	2,0	126
Kepulauan Riau	80,1	17,5	0,5	3,4	0,1	1,3	153
DKI Jakarta	74,4	15,8	1,0	0,4	2,8	6,9	862
Jawa Barat	70,5	20,8	1,6	1,3	2,0	4,9	3.912
Jawa Tengah	73,8	17,0	0,7	1,1	3,9	4,3	2.865
DI Yogyakarta	78,5	11,0	1,1	2,0	3,6	3,8	352
Jawa Timur	74,1	18,3	1,1	1,1	4,8	4,1	3.204
Banten	70,2	20,7	1,2	1,1	1,1	3,8	1.124
Bali	77,4	16,5	0,4	1,0	1,2	3,4	394
Nusa Tenggara Barat	71,4	22,8	1,1	0,6	1,1	3,9	507
Nusa Tenggara Timur	68,4	22,5	0,8	2,8	0,8	5,1	533
Kalimantan Barat	74,2	18,9	0,5	2,1	4,0	3,1	381
Kalimantan Tengah	72,5	19,5	2,8	0,3	2,1	4,6	144
Kalimantan Selatan	76,3	19,7	0,1	1,5	2,4	2,7	281
Kalimantan Timur	81,6	15,1	0,2	0,1	1,4	2,6	306
Kalimantan Utara	77,6	15,0	1,3	1,5	2,8	3,3	67
Sulawesi Utara	72,2	22,1	1,8	1,5	1,1	2,7	324
Sulawesi Tengah	69,2	24,0	1,4	0,8	3,5	4,8	351
Sulawesi Selatan	71,6	19,5	1,5	1,8	2,9	4,8	1.047
Sulawesi Tenggara	71,4	21,9	1,6	0,6	2,0	3,8	279
Gorontalo	65,3	26,5	1,6	0,8	4,2	3,9	110
Sulawesi Barat	72,7	23,9	0,4	1,0	0,9	2,0	106
Maluku	71,3	23,6	0,6	0,7	2,3	2,8	118
Maluku Utara	66,5	24,1	0,7	2,7	2,6	3,4	94
Papua Barat	76,3	19,4	0,1	3,1	1,2	1,7	78
Papua	64,2	22,4	1,1	2,2	0,6	7,4	187
**INDONESIA**	**72,7**	**19,2**	**1,2**	**1,3**	**2,7**	**4,3**	**21.483**

Tabel 10.1.16

Proporsi Kegiatan yang Sedang Dilakukan Saat Kecelakaan Lalu Lintas menurut Karakteristik, Riskesdas 2018

Karakteristik	Kegiatan yang sedang dilakukan saat kecelakaan lalu lintas (%)						N Tertimbang
	Mengendarai sepeda motor	Membonceng/ penumpang sepeda motor	Mengendarai mobil (sopir)	Menumpang/ penumpang mobil	Naik kendaraan tidak bermesin	Jalan kaki	
**Kelompok Umur**							
< 1	0,0	0,0	0,0	0,0	0,0	0,0	0
1-4	0,8	75,6	0,0	3,0	2,3	21,2	191
5 – 14	38,2	36,3	0,9	2,5	13,1	12,4	2.134
15 – 24	79,4	18,0	0,9	0,7	0,7	1,9	7.771
25 – 34	82,5	13,0	1,7	1,2	0,6	2,5	4.252
35 – 44	78,5	15,7	1,2	1,2	1,1	2,5	3.219
45 – 54	72,5	18,8	1,8	2,2	1,9	3,5	2.187
55 – 64	64,7	19,0	1,1	2,2	4,7	8,7	1.195
65 – 74	47,1	21,4	0,2	1,1	13,4	15,4	448
75+	31,3	23,9	1,4	3,9	19,0	31,6	86
**Jenis Kelamin**							
Laki-laki	80,9	11,8	1,3	1,2	2,5	3,2	13.919
Perempuan	57,6	33,0	0,9	1,5	3,0	6,2	7.564
**Pendidikan**							
Tidak sekolah	42,7	32,9	0,8	2,7	10,3	13,4	660
Tidak tamat SD/MI	50,0	29,2	0,9	2,2	9,5	9,8	2.288
Tamat SD/MI	69,6	21,8	1,1	1,6	3,6	4,4	4.599
Tamat SMP/MTS	77,7	18,6	1,2	0,8	0,9	2,4	5.260
Tamat SMA/MA	82,4	12,9	1,4	1,0	0,6	2,3	6.660
Tamat Diploma/PT	82,8	11,2	1,3	1,2	0,9	2,7	1.657
**Status pekerjaan**							
Tidak bekerja	65,0	27,1	0,9	1,5	2,4	5,0	4.334
Sekolah	69,7	22,8	0,9	1,3	3,8	4,2	4.226
PNS/TNI/Polri/BUMN/BUMD	82,9	10,4	0,9	1,7	1,0	1,4	549
Pegawai swasta	86,4	8,6	1,0	0,6	0,2	2,3	2.694
Wiraswasta	82,3	12,0	1,7	1,2	1,8	2,9	3.025
Petani/buruh tani	74,4	18,0	1,1	1,9	3,0	4,3	2.122
Nelayan	82,4	11,7	1,1	1,0	0,6	3,7	129
Buruh/sopir/pembantu ruta	81,7	11,1	2,7	1,2	1,6	2,4	2.434
Lainnya	73,3	18,5	0,2	1,5	2,5	3,3	1.004
**Tempat tinggal**							
Perkotaan	73,4	17,6	1,3	1,3	2,8	4,4	12.576
Perdesaan	71,7	21,5	1,0	1,4	2,5	4,0	8.907

10.2 Penggunaan Helm

Proporsi kebiasaan menggunakan helm saat mengendarai atau membonceng sepeda motor pada penduduk umur ≥5 tahun dihitung dengan formula:

$$Proporsi\ kebiasaan\ menggunakan\ helm\ saat\ mengendarai\ atau\ membonceng\ sepeda\ motor$$
$$= \frac{\sum ART\ umur\ \geq 5\ tahun\ dengan\ kebiasaan\ menggunakan\ helm\ saat\ mengendarai\ atau\ membonceng\ sepeda\ motor}{\sum ART\ umur\ \geq\ 5\ tahun\ yang\ selalu\ dan\ kadang-kadang\ menggunakan\ helm\ saat\ mengendarai\ atau\ membonceng\ sepeda\ motor}$$

Cara dan kondisi helm yang digunakan pada saat mengendarai atau membonceng sepeda motor dikelompokkan menjadi:

1. Memakai helm standar terkancing.
2. Memakai helm standar tidak terkancing.
3. Memakai helm tidak standar (helm: sepeda, proyek, tentara).

Proporsi cara dan kondisi helm yang digunakan pada saat mengendarai atau membonceng sepeda motor penduduk yang berumur ≥5 tahun dihitung dengan formula:

$$Proporsi\ cara\ dan\ kondisi\ helm\ yang\ digunakan\ pada\ saat\ mengendarai\ atau\ membonceng\ sepeda\ motor$$
$$= \frac{\sum ART\ umur\ \geq 5\ tahun\ dengan\ cara\ dan\ kondisi\ helm\ yang\ digunakan\ saat\ mengendarai\ atau\ membonceng\ sepeda\ motor}{\sum ART\ umur\ \geq 5\ tahun\ yang\ selalu\ atau\ kadang-kadang\ menggunakan\ helm\ saat\ mengendarai\ atau\ membonceng\ sepeda\ motor}$$

Tabel 10.2.1
Proporsi Kebiasaan Menggunakan Helm Saat Mengendarai atau Membonceng Sepeda Motor pada Penduduk Umur >5 Tahun menurut Provinsi, Riskesdas 2018

Provinsi	Kebiasaan menggunakan helm (%)			N Tertimbang
	Selalu	Kadang-kadang	Tidak pernah	
Aceh	30,0	39,1	30,9	14.887
Sumatera Utara	22,4	36,9	40,7	41.984
Sumatera Barat	25,1	46,6	28,4	17.376
Riau	29,3	37,4	33,3	21.136
Jambi	35,0	38,9	26,1	11.207
Sumatera Selatan	26,0	37,0	37,0	26.569
Bengkulu	36,4	46,1	17,5	6.353
Lampung	24,8	44,9	30,3	26.797
Bangka Belitung	41,9	42,4	15,6	4.598
Kepulauan Riau	50,2	26,9	22,9	6.324
DKI Jakarta	40,5	46,3	13,2	33.546
Jawa Barat	37,0	39,7	23,3	148.908
Jawa Tengah	34,2	49,3	16,5	112.887
DI Yogyakarta	41,8	49,5	8,7	12.736
Jawa Timur	38,5	45,5	16,1	127.041
Banten	30,3	42,3	27,3	40.079
Bali	32,8	54,1	13,1	14.206
Nusa Tenggara Barat	18,2	35,1	46,7	15.812
Nusa Tenggara Timur	21,8	32,9	45,2	15.937
Kalimantan Barat	37,8	38,2	24,1	15.547
Kalimantan Tengah	41,0	37,3	21,7	8.342
Kalimantan Selatan	43,6	41,8	14,6	12.983
Kalimantan Timur	40,5	45,5	14,0	11.674
Kalimantan Utara	55,4	20,5	24,1	2.162
Sulawesi Utara	27,9	49,0	23,2	7.891
Sulawesi Tengah	17,2	51,2	31,6	9.707
Sulawesi Selatan	37,3	42,0	20,7	27.909
Sulawesi Tenggara	32,8	34,0	33,3	8.357
Gorontalo	26,6	47,8	25,7	3.743
Sulawesi Barat	35,1	39,1	25,8	4.122
Maluku	28,3	31,1	40,7	4.972
Maluku Utara	24,1	25,5	50,4	3.546
Papua Barat	37,4	29,3	33,3	2.667
Papua	27,0	21,8	51,2	7.547
**INDONESIA**	**33,7**	**42,4**	**23,9**	**829.552**

Tabel 10.2.2

Proporsi Kebiasaan Menggunakan Helm Saat Mengendarai atau Membonceng Sepeda Motor pada Penduduk Umur ≥5 Tahun menurut Karakteristik, Riskesdas 2018

Karakteristik	Kebiasaan menggunakan helm (%)			N Tertimbang
	Selalu	Kadang-kadang	Tidak pernah	
**Kelompok Umur**				
5 – 14	12,4	29,3	58,2	156.648
15 – 24	37,9	46,0	16,1	156.566
25 – 34	42,2	45,5	12,3	150.821
35 – 44	41,4	45,2	13,4	141.006
45 – 54	39,7	45,8	14,5	113.333
55 – 64	33,7	46,0	20,3	70.876
65 – 74	26,5	43,7	29,8	29.765
75+	19,0	37,8	43,1	10.538
**Jenis Kelamin**				
Laki-laki	38,3	41,4	20,3	424.193
Perempuan	28,9	43,5	27,7	405.359
**Pendidikan**				
Tidak sekolah	15,6	36,5	48,5	55.424
Tidak tamat SD/MI	17,3	37,7	45,3	154.443
Tamat SD/MI	26,2	48,4	25,8	192.349
Tamat SMP/MTS	36,1	48,8	15,6	150.334
Tamat SMA/MA	51,2	41,8	7,5	200.173
Tamat Diploma/PT	64,1	32,5	4,2	60.088
**Status pekerjaan**				
Tidak bekerja	29,7	46,7	23,7	201.412
Sekolah	25,4	39,4	35,2	115.638
PNS/TNI/Polri/BUMN/BUMD	68,1	27,3	4,6	20.732
Pegawai swasta	62,4	34,1	3,5	72.955
Wiraswasta	48,2	43,3	8,5	99.610
Petani/buruh tani	20,2	53,5	26,4	118.275
Nelayan	25,4	49,6	25,0	4.972
Buruh/sopir/pembantu ruta	42,2	47,1	10,7	70.862
Lainnya	41,9	44,0	14,1	37.459
**Tempat tinggal**				
Perkotaan	44,2	38,9	17,0	459.078
Perdesaan	20,7	46,8	32,4	370.474

Tabel 10.2.3
Proporsi Cara dan Kondisi Helm yang Digunakan menurut Provinsi, Riskesdas 2018

Provinsi	Cara dan kondisi helm yang digunakan (%)			N Tertimbang
	Helm standar terkancing	Helm standar tidak terkancing	Helm tidak standar (helm sepeda, proyek, tentara)	
Aceh	90,6	8,9	0,5	9.540
Sumatera Utara	89,5	9,4	1,1	23.115
Sumatera Barat	90,4	9,1	0,5	11.550
Riau	96,2	3,2	0,6	13.082
Jambi	90,8	8,1	1,1	7.685
Sumatera Selatan	86,6	12,6	0,8	15.532
Bengkulu	93,1	6,2	0,7	4.862
Lampung	88,9	10,7	0,5	17.320
Bangka Belitung	96,8	3,1	0,1	3.599
Kepulauan Riau	95,9	3,5	0,6	4.523
DKI Jakarta	93,4	6,3	0,4	27.030
Jawa Barat	93,1	6,4	0,6	105.931
Jawa Tengah	90,8	8,8	0,4	87.470
DI Yogyakarta	94,1	5,4	0,6	10.792
Jawa Timur	92,6	7,0	0,4	98.941
Banten	91,9	7,6	0,5	27.019
Bali	97,8	2,0	0,3	11.456
Nusa Tenggara Barat	96,5	2,7	0,8	7.818
Nusa Tenggara Timur	92,6	6,5	0,9	8.098
Kalimantan Barat	94,9	4,4	0,7	10.949
Kalimantan Tengah	91,4	8,0	0,6	6.060
Kalimantan Selatan	77,3	22,3	0,4	10.287
Kalimantan Timur	94,8	4,8	0,4	9.319
Kalimantan Utara	87,7	11,5	0,8	1.522
Sulawesi Utara	81,9	17,4	0,7	5.626
Sulawesi Tengah	92,5	6,6	1,0	6.164
Sulawesi Selatan	83,7	15,8	0,6	20.538
Sulawesi Tenggara	89,6	9,5	1,0	5.176
Gorontalo	86,4	12,3	1,3	2.581
Sulawesi Barat	91,6	7,5	0,9	2.837
Maluku	83,1	16,1	0,9	2.737
Maluku Utara	90,8	8,4	0,8	1.632
Papua Barat	88,3	11,0	0,8	1.650
Papua	85,2	13,9	0,9	3.416
**INDONESIA**	**91,4**	**8,0**	**0,6**	**585.858**

Tabel 10.2.4

Proporsi Cara dan Kondisi Helm yang Digunakan menurut Karakteristik, Riskesdas 2018

Karakteristik	Cara dan kondisi helm yang digunakan (%)			N Tertimbang
	Helm standar terkancing	Helm standar tidak terkancing	Helm tidak standar (helm sepeda, proyek, tentara)	
**Kelompok Umur**				
5 – 14	88,9	9,9	1,2	60.706
15 – 24	92,8	6,8	0,4	121.943
25 – 34	92,4	7,2	0,4	122.688
35 – 44	91,4	8,2	0,4	113.290
45 – 54	91,3	8,3	0,5	89.899
55 – 64	90,6	8,8	0,6	52.395
65 – 74	89,5	9,8	0,7	19.377
75+	86,8	12,0	1,2	5.559
**Jenis Kelamin**				
Laki-laki	92,0	7,4	0,6	313.752
Perempuan	90,8	8,7	0,6	272.106
**Pendidikan**				
Tidak sekolah	88,2	10,7	1,1	26.531
Tidak tamat SD/MI	88,9	10,1	1,0	78.548
Tamat SD/MI	90,2	9,2	0,7	132.696
Tamat SMP/MTS	91,9	7,7	0,4	117.976
Tamat SMA/MA	93,1	6,6	0,3	172.013
Tamat Diploma/PT	93,8	5,9	0,2	53.500
**Status pekerjaan**				
Tidak bekerja	90,6	8,9	0,5	144.394
Sekolah	91,4	8,0	0,7	70.342
PNS/TNI/Polri/BUMN/BUMD	93,6	6,1	0,3	18.578
Pegawai swasta	94,7	5,0	0,4	66.143
Wiraswasta	92,1	7,6	0,4	85.637
Petani/buruh tani	89,3	10,0	0,7	81.761
Nelayan	89,3	10,2	0,5	3.504
Buruh/sopir/pembantu ruta	92,3	7,3	0,5	59.442
Lainnya	92,1	7,5	0,4	30.216
**Tempat tinggal**				
Perkotaan	92,3	7,2	0,5	353.571
Perdesaan	90,2	9,2	0,7	232.287

BAB 11
PELAYANAN KESEHATAN TRADISIONAL

Pelayanan Kesehatan Tradisional (yankestrad) adalah pengobatan dan/atau perawatan dengan cara dan obat berdasarkan pengalaman dan keterampilan turun-temurun secara empirik, yang dapat dipertanggungjawabkan**,** dan diterapkan sesuai dengan norma yang berlaku di masyarakat (UU No.36 Tahun 2009 tentang kesehatan). Termasuk pelayanan kesehatan tradisional (yankestrad) adalah:

1. Yankestrad ramuan, baik ramuan kemasan maupun ramuan buatan sendiri dengan menggunakan bahan yang berasal dari: tanaman; hewan; mineral; dan/atau sediaan sarian (galenik) atau campuran dari bahan-bahan.
2. Yankestrad keterampilan manual adalah teknik pengobatan yang berdasarkan manipulasi dan gerakan dari satu atau beberapa bagian tubuh misalnya pijat urut, refleksi, akupresur.
3. Yanskestrad keterampilan olah pikir adalah adalah teknik pengobatan yang bertujuan untuk memanfaatkan kemampuan pikiran untuk memperbaiki fungsi tubuh misalnya hipnoterapi.
4. Yankestrad keterampilan energi adalah teknik pengobatan dengan menggunakan lapangan energi baik dari luar maupun dari dalam tubuh itu sendiri misalnya tenaga dalam dan prana.

Definisi Operasional dan Formula :

1. Pemanfaatan yankestrad yaitu Anggota Rumah Tangga (ART) yang pernah memanfaatkan pelayanan kesehatan tradisional dalam satu tahun terakhir.
   ART pernah memanfaatkan yankestrad dengan mendatangi panti sehat/fasilitas yankestrad/fasilitas yankes atau mendatangkan penyehat tradisional/nakestrad/ terapi. ART melakukan upaya sendiri meliputi: melakukan pijat atau akupresur tanpa bantuan penyehat tradisional/nakestrad/terapis, membuat ramuan tradisional, membeli jamu gendong, jamu godok, jamu dan obat tradisional lainnya atas inisiatif sendiri, serta memanfaatkan Taman Obat Keluarga (TOGA).

$$Proporsi\ Pemanfaatan\ Yankestrad = \frac{ART\ Pernah\ memanfaatkan\ yankestrad\ dalam\ satu\ tahun\ terakhir}{ART\ semua\ umur}$$

2. Jenis yankestrad yang dimanfaatkan dalam satu tahun terakhir meliputi:
   - **Ramuan jadi**, merupakan ramuan yang diperoleh dalam bentuk sediaan jadi, yang beredar di pasar dan terdaftar di Badan POM atau diberikan langsung oleh praktisi, digunakan sesuai aturan yang berlaku.

- **Ramuan buatan sendiri,** merupakan ramuan yang dibuat secara mandiri berdasarkan pengalaman sendiri atau mengacu pada buku resmi atau informasi yang dapat dipercaya dengan bahan yang diperoleh dari taman obat keluarga atau membeli di pasar, baik dalam bentuk segar, kering atau bentuk simplisia (serbuk). Ramuan dapat digunakan untuk diminum atau pemakaian luar (misalnya balur atau oles)
- **Keterampilan manual** (pijat, tusuk jarum), merupakan bagian dari pelayanan kesehatan tradisional yang dalam pelaksanaannya menggunakan keterampilan dengan ataupun tanpa alat bantu. (PP No. 103 Tahun 2014 tentang Pelayanan Kesehatan Tradisional) dan dapat dilakukan oleh Hattra ataupun nakestrad. Contoh: pijat urut dewasa/bayi, patah tulang, refleksi, akupuntur, chiropractic, kop/bekam, apiterapi, ceragem, akupresur dll.
- **Keterampilan olah pikir/hipnoterapi** merupakan bagian dari pelayanan kesehatan tradisional yang dalam pelaksanaannya menggunakan teknik keterampilan olah pikir. (PP No. 103 Tahun 2014 tentang Pelayanan Kesehatan Tradisional) dan dapat dilakukan oleh Hattra ataupun nakestrad. Contoh: hipnoterapi, meditasi.

$$Proporsi\ jenis\ yankestrad\ yang\ dimanfaatkan = \frac{Jenis\ yankestrad\ yang\ dimanfaatkan}{ART\ Pernah\ memanfaatkan\ yankestrad\ dalam\ satu\ tahun\ terakhir}$$

3. Jenis tenaga kesehatan tradisional (nakestrad) meliputi:
   - **Dokter atau tenaga kesehatan**: Jika ART pernah memanfaatkan yankestrad dengan mendatangi fasilitas yankestrad atau mendatangkan terapis dengan latar belakang pendidikan dokter atau tenaga kesehatan lainnya. Tenaga pendidikan dokter termasuk dokter umum, dokter gigi, dan dokter spesialis. Tenaga kesehatan adalah tenaga yang ilmu dan keterampilannya diperoleh melalui pendidikan tinggi di bidang kesehatan, dan memiliki kewenangan dalam melakukan upaya kesehatan (UU No.36 Tahun 2014 tentang Tenaga Kesehatan).
   - **Penyehat tradisional (Hattra/Battra):** Jika ART pernah memanfaatkan yankestrad dengan mendatangi panti sehat atau mendatangkan terapis yang bukan seorang dokter atau tenaga kesehatan. Penyehat tradisional adalah seseorang yang ilmu dan keterampilannya diperoleh melalui turun-temurun atau pendidikan nonformal.

$$proporsi\ \ jenis\ nakestrad = \frac{Jenis\ tenaga\ kesehatan\ tradisional\ yang\ memberikan\ yankestrad}{ART\ Pernah\ memanfaatkan\ yankestrad\ dalam\ satu\ tahun\ terakhir}$$

4. Pemanfaatan TOGA yaitu ART pernah memanfaatkan TOGA milik keluarga atau lingkungan sekitar dalam 1 tahun terakhir pada ART yang pernah memanfaatkan pelayanan kesehatan tradisional dalam satu tahun terakhir.
   TOGA atau taman obat keluarga adalah sekumpulan tanaman berkhasiat obat untuk kesehatan keluarga. TOGA pada hakekatnya adalah sebidang tanah, baik di halaman rumah, kebun ataupun ladang yang digunakan untuk membudidayakan tanaman yang berkhasiat sebagai obat, termasuk TOGA milik RT/RW atau tetangga. Tidak termasuk pemanfaatan TOGA jika mengambil tumbuhan liar di hutan atau di sembarang tempat.

$$proporsi\ \ pemanfaatan\ TOGA$$
$$= \frac{ART\ pernah\ memanfaatkan\ TOGA\ milik\ keluarga\ atau\ lingkungan\ sekitar\ dalam\ 1\ tahun\ terakhir}{ART\ Pernah\ memanfaatkan\ yankestrad\ dalam\ satu\ tahun\ terakhir}$$

11.1 Pemanfaatan Pelayanan Kesehatan Tradisional

Tabel 11.1.1
Proporsi Pemanfaatan Pelayanan Kesehatan Tradisional menurut Provinsi, Riskesdas 2018

Provinsi	Memanfaatkan Yankestrad (%)	95%CI	Melakukan upaya sendiri (%)	95%CI	N Tertimbang
Aceh	15,9	15,1 - 16,7	8,6	8,0 - 9,2	20.244
Sumatera Utara	35,2	34,0 - 36,4	9,6	8,9 - 10,3	55.351
Sumatera Barat	31,3	29,9 - 32,8	12,3	11,4 - 13,3	20.663
Riau	31,5	30,2 - 32,9	8,7	8,0 - 9,5	26.085
Jambi	24,2	22,4 - 26,1	10,5	9,3 - 11,8	13.692
Sumatera Selatan	29,5	27,8 - 31,2	9,9	8,9 - 10,9	32.126
Bengkulu	31,2	29,3 - 33,2	8,1	7,3 - 8,9	7.531
Lampung	42,7	41,3 - 44,1	9,6	8,8 - 10,5	32.148
Kep.Bangka Belitung	28,4	26,6 - 30,3	11,1	9,9 - 12,4	5.592
Kepulauan Riau	20,6	18,2 - 23,3	13,6	10,8 - 16,9	8.173
DKI Jakarta	39,2	37,3 - 41,2	11,0	9,7 - 12,4	40.210
Jawa Barat	23,3	22,5 - 24,2	9,2	8,8 - 9,7	186.809
Jawa Tengah	33,0	32,2 - 33,7	13,9	13,4 - 14,5	132.565
DI Yogyakarta	48,1	46,3 – 50,0	13,4	12,2 - 14,6	14.602
Jawa Timur	48,3	47,4 - 49,2	15,5	14,9 - 16,2	151.878
Banten	35,5	33,8 - 37,3	14,1	12,7 - 15,6	48.621
Bali	19,2	18,1 - 20,5	23,6	22,2 - 25,1	16.481
Nusa Tenggara Barat	31,5	29,9 - 33,2	13,1	12,0 - 14,3	19.247
Nusa Tenggara Timur	17,1	16,0 - 18,3	23,0	21,8 - 24,2	20.599
Kalimantan Barat	19,7	18,3 - 21,1	8,9	8,1 - 9,7	19.190
Kalimantan Tengah	28,1	26,1 - 30,1	12,3	11,1 - 13,6	10.189
Kalimantan Selatan	54,1	52,1 - 56,2	9,6	8,5 - 10,7	16.043
Kalimantan Timur	29,7	28,0 - 31,5	16,6	15,3 - 18,1	13.977
Kalimantan Utara	17,4	15,2 - 19,8	11,9	10,1 – 14,0	2.733
Sulawesi Utara	24,7	23,3 - 26,1	19,1	17,7 - 20,7	9.542
Sulawesi Tengah	22,0	20,4 - 23,7	23,9	22,7 - 25,3	11.548
Sulawesi Selatan	9,3	8,7 – 10,0	18,8	18,0 - 19,7	33.693
Sulawesi Tenggara	19,5	17,7 - 21,3	12,4	11,2 - 13,7	10.167
Gorontalo	37,8	34,7 - 40,9	11,4	9,8 - 13,3	4.547
Sulawesi Barat	8,5	7,2 - 9,9	20,5	18,4 - 22,7	5.195
Maluku	14,5	13,1 – 16,0	19,5	17,9 - 21,2	6.801
Maluku Utara	24,1	22,5 - 25,8	20,2	18,5 – 22,0	4.723
Papua Barat	15,1	13,3 – 17,0	18,1	16,3 - 20,1	3.588
Papua	9,5	8,4 - 10,6	18,3	16,4 - 20,4	12.736
**INDONESIA**	**31,4**	**31,1 - 31,6**	**12,9**	**12,7 - 13,2**	**1.017.290**

Tabel 11.1.2
Proporsi Pemanfaatan Pelayanan Kesehatan Tradisional menurut Karakteristik, Riskesdas 2018

Karakteristik	Memanfaatkan Yankestrad (%)	95%CI	Melakukan upaya sendiri (%)	95%CI	N Tertimbang
**Kelompok Umur**					
< 1	8,0	5,8 - 10,7	2,8	1,8 - 4,6	1.368
1-4	27,8	27,3 - 28,3	9,0	8,7 - 9,3	90.045
5-14	22,4	22,1 - 22,8	9,8	9,6 - 10,1	182.338
15-24	25,9	25,5 - 26,3	10,4	10,1 - 10,7	165.644
25-34	33,7	33,2 - 34,2	12,8	12,5 - 13,2	159.708
35-44	36,5	36,0 - 36,9	14,8	14,5 - 15,2	151.539
45-54	38,4	37,9 - 38,9	16,6	16,2 - 16,9	124.652
55-64	38,5	37,9 – 39,0	17,5	17,0 - 17,9	83.251
65-74	36,7	36,0 - 37,5	17,7	17,1 - 18,2	40.180
>=75	34,0	33,0 – 35,0	17,2	16,4 - 17,9	18.565
**Jenis Kelamin**					
Laki-laki	30,9	30,6 - 31,2	12,1	11,8 - 12,3	510.714
Perempuan	31,8	31,5 - 32,1	13,9	13,6 - 14,1	506.576
**Pendidikan**					
Tidak/belum pernah sekolah	30,4	29,8 - 31,1	14,4	14,0 - 14,9	70.895
Tidak tamat SD/MI	29,0	28,6 - 29,4	13,3	13,0 - 13,6	181.429
Tamat SD/MI	31,7	31,2 - 32,1	14,5	14,2 - 14,8	215.967
Tamat SLTP/MTS	31,9	31,5 - 32,4	12,7	12,4 – 13,0	160.320
Tamat SLTA/MA	33,7	33,2 - 34,1	12,7	12,4 - 13,1	210.746
Tamat D1/D2/D3/PT	36,2	35,5 - 36,9	13,0	12,5 - 13,4	64.093
**Pekerjaan**					
Tidak bekerja	31,9	31,5 - 32,3	13,9	13,6 - 14,2	233.629
Sekolah	22,4	22 ,0- 22,8	10,1	9,8 - 10,4	126.626
PNS/TNI/Polri/BUMN/BUMD	37,1	36,1 - 38,1	14,2	13,6 - 14,9	21.931
Pegawai swasta	35,3	34,6 - 36,1	11,8	11,3 - 12,3	75.781
Wiraswasta	38,4	37,8 – 39,0	13,3	12,9 - 13,7	105.489
Petani/buruh tani	35,4	34,9 – 36,0	17,9	17,5 - 18,3	133.261
Nelayan	33,8	31,4 - 36,4	14,6	13,3 – 16,0	5.556
Buruh/sopir/pembantu ruta	34,8	34,1 - 35,5	13,7	13,2 - 14,3	75.590
Lainnya	33,2	32,4 – 34,0	14,9	14,4 - 15,5	40.644
**Tempat Tinggal**					
Perkotaan	32,1	31,7 - 32,6	11,6	11,4 - 11,9	556.419
Perdesaan	30,4	30,1 - 30,8	14,5	14,2 - 14,8	460.871

11.2 Jenis Pelayanan Kesehatan Tradisional

Tabel 11.2.1
Proporsi Jenis Pelayanan Kesehatan Tradisional yang Dimanfaatkan menurut Provinsi, Riskesdas 2018

Provinsi	Ramuan Jadi	Ramuan buatan sendiri	keterampilan manual	Keterampilan olah pikir	Keterampilan energi	N Tertimbang
	%	%	%	%	%	
Aceh	37,0	60,3	45,2	2,9	3,6	4.812
Sumatera Utara	48,9	28,4	72,8	2,0	2,0	24.033
Sumatera Barat	22,8	55,7	66,0	1,8	2,4	8.748
Riau	43,7	27,5	69,2	2,0	1,8	10.193
Jambi	50,5	45,2	59,2	1,4	2,8	4.613
Sumatera Selatan	34,6	24,4	79,9	1,7	1,8	12.257
Bengkulu	29,5	41,8	67,0	2,1	2,4	2.871
Lampung	48,2	37,0	72,7	1,6	1,4	16.320
Kep.Bangka Belitung	43,5	21,6	73,1	3,3	3,5	2.141
Kepulauan Riau	51,7	28,6	55,5	1,5	2,8	2.712
DKI Jakarta	59,6	18,7	68,6	2,0	2,3	19.587
Jawa Barat	51,9	28,1	59,3	2,0	2,4	58.997
Jawa Tengah	55,5	18,5	64,0	1,9	1,7	60.323
DI Yogyakarta	56,1	26,0	62,7	1,7	1,6	8.713
Jawa Timur	51,1	28,7	78,4	1,9	2,0	94.054
Banten	55,6	20,8	70,1	1,6	1,8	23.406
Bali	37,5	49,4	46,6	2,2	2,3	6.848
Nusa Tenggara Barat	33,9	36,5	57,5	1,5	3,3	8.328
Nusa Tenggara Timur	28,9	69,3	40,6	1,2	1,9	8.013
Kalimantan Barat	47,5	40,4	56,6	2,3	2,4	5.312
Kalimantan Tengah	54,7	30,4	69,4	2,0	1,9	3.988
Kalimantan Selatan	58,4	17,9	83,3	1,6	1,8	9.914
Kalimantan Timur	53,8	27,9	62,3	2,2	1,9	6.288
Kalimantan Utara	49,8	29,5	50,9	2,6	1,6	777
Sulawesi Utara	22,4	70,5	49,4	1,6	1,5	4.056
Sulawesi Tengah	25,5	60,8	50,5	1,9	1,7	5.149
Sulawesi Selatan	31,2	70,5	15,6	1,8	2,7	9.207
Sulawesi Tenggara	25,5	55,8	52,2	2,4	2,8	3.145
Gorontalo	25,8	42,6	76,5	2,3	2,4	2.172
Sulawesi Barat	21,9	85,5	9,3	1,6	1,5	1.458
Maluku	21,0	78,2	31,0	1,6	2,1	2.241
Maluku Utara	25,2	75,6	28,0	1,5	2,1	2.032
Papua Barat	24,9	70,8	25,3	1,1	0,8	1.154
Papua	18,9	81,7	17,2	1,7	3,0	3.432
**INDONESIA**	**48,0**	**31,8**	**65,3**	**1,9**	**2,1**	**437.291**

Tabel 11.2.2
Proporsi Jenis Pelayanan Kesehatan Tradisional yang Dimanfaatkan menurut Karakteristik, Riskesdas 2018

Karakteristik	Ramuan Jadi	Ramuan buatan sendiri	keterampilan manual	Keterampilan olah pikir	Keterampilan energi	N Tertimbang
	%	%	%	%	%	
**Umur**						
< 1	0,5	7,4	93,9	0,0	0,0	143
1-4	22,6	19,3	79,7	1,3	1,5	32.126
5-14	42,4	25,8	64,3	1,5	1,8	57.026
15-24	47,4	27,5	65,7	2,1	2,1	58.355
25-34	50,4	29,0	67,4	1,9	2,2	72.085
35-44	52,0	33,1	64,7	2,0	2,0	75.454
45-54	52,7	37,8	64,0	2,0	2,3	66.489
55-64	53,0	40,1	61,1	1,9	2,1	45.186
65-74	53,6	42,9	57,6	2,0	2,2	21.206
>=75	53,7	41,4	55,9	1,8	2,1	9.221
**Jenis Kelamin**						
Laki-laki	46,4	29,5	68,4	1,9	2,2	212.964
Perempuan	49,6	33,9	62,4	1,8	1,9	224.327
**Pendidikan**						
Tidak/belum pernah sekolah	51,3	35,9	60,4	1,8	2,1	31.033
Tidak tamat SD/MI	49,7	34,3	62,1	1,8	1,9	74.960
Tamat SD/MI	52,0	35,7	62,4	1,9	2,1	97.277
Tamat SLTP/MTS	51,0	31,2	65,0	1,9	2,1	69.829
Tamat SLTA/MA	49,9	30,0	66,3	2,1	2,2	95.456
Tamat D1/D2/D3/PT	44,4	30,9	69,2	2,1	2,4	30.738
**Pekerjaan**						
Tidak bekerja	35,6	35,6	60,3	2,0	2,1	105.004
Sekolah	27,6	27,6	62,9	1,7	1,8	40.339
PNS/TNI/Polri/BUMN/BUMD	35,3	35,3	67,6	2,6	2,6	11.048
Pegawai swasta	23,0	23,0	70,9	2,2	2,2	35.035
Wiraswasta	29,4	29,4	68,7	2,0	2,3	53.513
Petani/buruh tani	43,5	43,5	62,1	1,9	2,1	69.744
Nelayan	39,7	39,7	62,4	1,9	2,2	2.641
Buruh/sopir/pembantu ruta	27,2	27,2	66,0	1,9	1,9	36.035
Lainnya	36,9	36,9	62,9	1,9	2,1	19.195
**Tempat Tinggal**						
Perkotaan	51,7	25,9	65,7	1,9	2,0	236.263
Perdesaan	43,7	38,7	65,0	1,8	2,1	201.028

11.3 Pemberi Layanan Kesehatan Tradisional

Tabel 11.3.1
Proporsi Jenis Tenaga yang Dimanfaatkan Menangani Kesehatan Tradisional
menurut Provinsi, Riskesdas 2018

Provinsi	Dokter/nakes (%)	Penyehat Tradisional (%)	N Tertimbang
Aceh	7,0	97,5	2.925
Sumatera Utara	2,6	98,7	17.718
Sumatera Barat	2,3	99,0	5.891
Riau	2,8	98,7	7.485
Jambi	6,9	97,9	3.018
Sumatera Selatan	3,2	98,4	8.614
Bengkulu	6,6	97,4	2.141
Lampung	1,4	99,1	12.492
Kep.Bangka Belitung	5,3	97,6	1.446
Kepulauan Riau	3,4	98,4	1.533
DKI Jakarta	3,0	98,4	14.356
Jawa Barat	3,4	97,8	39.642
Jawa Tengah	2,2	98,5	39.790
DI Yogyakarta	2,4	98,5	6.398
Jawa Timur	1,9	99,1	66.752
Banten	2,3	98,4	15.710
Bali	3,9	97,2	2.886
Nusa Tenggara Barat	1,4	99,1	5.522
Nusa Tenggara Timur	3,8	97,1	3.210
Kalimantan Barat	5,0	97,3	3.435
Kalimantan Tengah	1,9	98,6	2.602
Kalimantan Selatan	1,9	98,9	7.904
Kalimantan Timur	2,7	98,2	3.782
Kalimantan Utara	4,4	96,0	432
Sulawesi Utara	4,4	96,7	2.142
Sulawesi Tengah	2,6	98,5	2.313
Sulawesi Selatan	9,3	94,5	2.862
Sulawesi Tenggara	3,5	97,7	1.800
Gorontalo	1,8	99,4	1.564
Sulawesi Barat	3,8	96,7	400
Maluku	4,3	96,8	895
Maluku Utara	3,0	98,3	1.037
Papua Barat	5,9	96,8	492
Papua	5,6	96,9	1.096
**INDONESIA**	**2,7**	**98,5**	**290.285**

Tabel 11.3.2
Proporsi Jenis Tenaga yang Dimanfaatkan Menangani Kesehatan Tradisional menurut Karakteristik, Riskesdas 2018

Karakteristik	Dokter /nakes (%)	Penyehat Tradisional (%)	N Tertimbang
**Umur**			
< 1	3,9	96,1	67
1-4	2,6	98,3	22.313
5-14	2,2	98,7	36.558
15-24	2,4	98,6	38.338
25-34	2,5	98,6	48.102
35-44	2,6	98,5	49.540
45-54	3,1	98,3	42.883
55-64	3,1	98,4	28.638
65-74	3,8	98,0	13.086
>=75	3,9	97,8	5.532
**Jenis Kelamin**			
Laki-laki	2,8	98,5	143.726
Perempuan	2,7	98,5	146.559
**Pendidikan**			
Tidak/belum pernah sekolah	2,5	98,5	19.759
Tidak tamat SD/MI	2,5	98,6	48.235
Tamat SD/MI	2,7	98,5	62.627
Tamat SLTP/MTS	2,6	98,6	46.896
Tamat SLTA/MA	2,7	98,4	65.020
Tamat D1/D2/D3/PT	4,0	97,6	21.244
**Pekerjaan**			
Tidak bekerja	3,0	98,3	67.668
Sekolah	2,1	98,7	25.563
PNS/TNI/Polri/BUMN/BUMD	4,5	97,3	7.227
Pegawai swasta	2,9	98,3	24.000
Wiraswasta	2,8	98,5	36.583
Petani/buruh tani	2,6	98,7	42.717
Nelayan	2,5	98,8	1.543
Buruh/sopir/pembantu ruta	2,2	98,7	23.629
Lainnya	2,9	98,4	12.023
**Tempat Tinggal**			
Perkotaan	2,7	98,4	162.644
Perdesaan	2,7	98,6	127.641

11.4 Pemanfaatan Taman Obat Keluarga (TOGA)

Tabel 11.4.1
Proporsi Pemanfaatan TOGA menurut Provinsi, Riskesdas 2018

Provinsi	Pernah memanfaatkan TOGA		N Tertimbang
	%	95% CI	
Aceh	36,3	34,3 - 38,5	4.812
Sumatera Utara	22,2	20,9 - 23,5	24.033
Sumatera Barat	50,4	48,5 - 52,3	8.748
Riau	26,7	25,1 - 28,5	10.193
Jambi	39,4	36,3 - 42,5	4.613
Sumatera Selatan	24,1	22,1 - 26,3	12.257
Bengkulu	41,9	38,8 - 45,1	2.871
Lampung	31,6	29,7 - 33,6	16.320
Kep.Bangka Belitung	18,1	16,2 - 20,1	2.141
Kepulauan Riau	22,0	18,7 - 25,6	2.712
DKI Jakarta	9,1	8,1 - 10,3	19.587
Jawa Barat	20,3	19,3 - 21,3	58.997
Jawa Tengah	16,9	16,2 - 17,6	60.323
DI Yogyakarta	25,1	23,3 - 27,0	8.713
Jawa Timur	21,8	21,0 - 22,5	94.054
Banten	17,4	16,0 - 18,9	23.406
Bali	40,3	38,2 - 42,5	6.848
Nusa Tenggara Barat	19,6	17,8 - 21,6	8.328
Nusa Tenggara Timur	55,1	52,9 - 57,3	8.013
Kalimantan Barat	34,7	32,6 - 36,9	5.312
Kalimantan Tengah	26,7	24,7 - 28,9	3.988
Kalimantan Selatan	17,7	16,5 - 19,0	9.914
Kalimantan Timur	24,2	22,3 - 26,3	6.288
Kalimantan Utara	23,5	20,7 - 26,6	777
Sulawesi Utara	55,6	53,5 - 57,7	4.056
Sulawesi Tengah	44,7	42,4 - 47,1	5.149
Sulawesi Selatan	49,4	47,6 - 51,2	9.207
Sulawesi Tenggara	31,2	28,5 - 34,0	3.145
Gorontalo	38,1	35,5 - 40,7	2.172
Sulawesi Barat	54,4	50,2 - 58,5	1.458
Maluku	39,0	35,6 - 42,5	2.241
Maluku Utara	37,8	34,9 - 40,7	2.032
Papua Barat	28,7	25,3 - 32,4	1.154
Papua	44,1	40,1 - 48,3	3.432
**INDONESIA**	**24,6**	**24,2 - 24,9**	**437.291**

Tabel 11.4.2
Proporsi Pemanfaatan TOGA menurut Karakteristik, Riskesdas 2018

Karakteristik	Pernah memanfaatkan TOGA		N Tertimbang
	%	95% CI	
**Umur**			
< 1	12,4	6,2 – 23,4	143
1-4	17,5	16,9 – 18,1	32.126
5-14	19,7	19,2 – 20,2	57.026
15-24	20,2	19,6 – 20,7	58.355
25-34	22,2	21,6 – 22,7	72.085
35-44	25,9	25,4 – 26,4	75.454
45-54	29,2	28,7 – 29,7	66.489
55-64	31,0	30,4 – 31,7	45.186
65-74	33,0	32,0 – 33,9	21.206
>=75	30,8	29,5 – 32,1	9.221
**Jenis Kelamin**			
Laki-laki	22,7	22,4 – 23,1	212.964
Perempuan	26,3	25,9 – 26,7	224.327
**Pendidikan**			
Tidak/belum pernah sekolah	25,5	24,7 – 26,4	31.033
Tidak tamat SD/MI	25,3	24,8 – 25,8	74.960
Tamat SD/MI	26,9	26,4 – 27,4	97.277
Tamat SLTP/MTS	24,3	23,7 – 24,8	69.829
Tamat SLTA/MA	23,6	23,1 – 24,1	95.456
Tamat D1/D2/D3/PT	26,8	26,0 – 27,6	30.738
**Pekerjaan**			
Tidak bekerja	27,4	26,9 – 27,9	105.004
Sekolah	20,2	19,6 – 20,8	40.339
PNS/TNI/Polri/BUMN/BUMD	31,3	30,1 – 32,4	11.048
Pegawai swasta	18,6	17,9 – 19,3	35.035
Wiraswasta	22,1	21,5 – 22,7	53.513
Petani/buruh tani	33,6	33,0 – 34,2	69.744
Nelayan	21,3	19,2 – 23,5	2.641
Buruh/sopir/pembantu ruta	19,8	19,1 – 20,6	36.035
Lainnya	28,9	28,0 – 29,9	19.195
**Tempat Tinggal**			
Perkotaan	19,4	19,0 – 19,8	236.263
Perdesaan	30,6	30,1 – 31,1	201.028

BAB 12
PERILAKU KESEHATAN

Indikator perilaku berisiko kesehatan yang disajikan dalam bab ini adalah beberapa perilaku yang berkaitan dengan penyakit tidak menular dan penyakit menular. Indikator yang termasuk dalam faktor risiko perilaku terkait penyakit tidak menular mencakup perilaku konsumsi makanan berisiko kesehatan, kurang konsumsi sayur dan buah, kebiasaan merokok dan konsumsi tembakau, kurang aktivitas fisik, dan konsumsi minuman beralkohol. Sedangkan untuk faktor risiko perilaku terkait penyakit menular mencakup pencegahan penyakit akibat gigitan nyamuk, kebiasaan mencuci tangan dengan benar, dan buang air besar di jamban.

Khusus untuk individu dengan umur kurang dari 15 tahun wawancara dapat dilakukan dengan pendampingan orang tua atau wali, dan untuk individu balita (3-5 tahun) wawancara dilakukan dengan diwakili oleh orang tua atau wali yang mengetahui perilaku terkait.

12.1 Pencegahan Penyakit Akibat Gigitan Nyamuk

Program pengendalian vektor malaria yang telah dilakukan dengan cara mengendalikan populasi nyamuk dewasa melalui penyemprotan dalam rumah (*Indoor Residual Spray*) dan kelambu berinsektisida (*Long Lasting Insecticide Nets*), larvasidasi serta modifikasi/manipulasi habitat perkembangbiakan nyamuk. Penyemprotan dalam rumah dan pemakaian kelambu berinsektisida bertujuan untuk memperpendek umur nyamuk sehingga penyebaran dan penularan malaria dapat terputus. Pada Riskesdas 2018, juga dikumpulkan data cara mengendalikan populasi nyamuk dewasa untuk menjawab salah satu indikator program. Pengendalian Penyakit Menular yaitu proporsi responden yang menggunakan kelambu LLINs, Indikator ini dihitung dengan formula sebagai berikut:

$$\text{Proporsi ART yang menggunakan kelambu LLIN's} = \frac{\sum \text{ART yang memakai kelambu LLIN's}}{\sum \text{ART Semua Umur}}$$

Untuk menggambarkan cara masyarakat melakukan pencegahan gigitan nyamuk dihitung dengan formula sebagai berikut:

$$\text{Proporsi pencegahan penyakit akibat gigitan nyamuk} = \frac{\sum \text{ART menurut cara pencegahan gigtan nyamuk}}{\sum \text{ART Semua Umur}}$$

Tabel 12.1.1
Proporsi Penggunaan Kelambu *Long Lasting Insecticide Nets* (*LLIN's*) menurut Provinsi, Riskesdas 2018

Provinsi	Penggunaan kelambu LLIN			Penggunaan kelambu LLIN pada balita			Penggunaan kelambu LLIN pada balita didaerah endemis		
	%	95% CI	N ter-timbang	%	95% CI	N ter-tim bang	%	95% CI	N ter-timbang
Aceh	4,2	3,8-4,7	20.244	4,9	4,1-5,8	2.250	7,8	5,8-10,3	549
Sumatera Utara	2,1	1,8-2,4	55.351	2,9	2,4-3,6	5.895	6,1	4,8-7,6	2.992
Sumatera Barat	1,0	0,8-1,3	20.663	2,1	1,5-2,8	2.179	8,4	5,9-12,0	557
Riau	1,0	0,8-1,3	26.085	1,4	1,0-2,0	2.813	1,6	1,0-2,6	1.981
Jambi	5,4	4,6-6,3	13.692	12,6	10,6-14,8	1.279	15,3	12,8-18,1	1.468
Sumatera Selatan	2,1	1,8-2,5	32.126	3,8	3,0-4,9	3.079	6,9	5,3-8,9	2.168
Bengkulu	8,7	7,6-10	7.531	15,3	12,7-18,3	716	19,5	16,2-23,3	805
Lampung	1,5	1,3-1,8	32.148	2,8	2,2-3,5	3.094	4,6	3,5-5,9	2.454
Bangka Belitung	6,7	5,7-7,8	5.592	15,5	12,7-18,7	527	20,9	16,0-26,8	235
Kepulauan Riau	2,5	1,9-3,2	8.173	2,6	1,9-3,5	872	14,0	10,4-18,6	190
DKI Jakarta	0,2	0,1-0,3	40.210	0,4	0,2-1,0	3.582	N/A	N/A	N/A
Jawa Barat	0,2	0,2-0,3	186.809	0,5	0,3-0,9	17.228	0,1	0-0,6	2.716
Jawa Tengah	0,4	0,4-0,5	132.565	0,6	0,4-0,8	10.551	0,7	0,4-1,4	3.361
DI Yogyakarta	0,2	0,1-0,4	14.602	1,0	0,4-2,5	1.069	1,8	0,4-7,2	184
Jawa Timur	0,3	0,2-0,4	151.878	0,5	0,3-0,7	11.272	N/A	N/A	N/A
Banten	0,5	0,4-0,7	48.621	0,7	0,4-1,2	4.813	1,2	0,5-2,9	1.474
Bali	0,2	0,1-0,3	16.481	0,1	0,0-0,3	1.275	N/A	N/A	N/A
Nusa Tenggara Barat	3,1	2,5-3,8	19.247	5,6	4,5-7,1	1.985	5,7	4,3-7,4	1.875
Nusa Tenggara Timur	56,3	54,6-58	20.599	65,7	63,2-68	2.496	65,7	63,2-68,0	3.733
Kalimantan Barat	6,4	5,8-7,1	19.190	10,7	9,1-12,6	1.928	11,8	9,9-13,9	2.549
Kalimantan Tengah	4,3	3,6-5,2	10.189	5,5	4,3-7,1	1.000	7,1	5,3-9,5	981
Kalimantan Selatan	2,4	1,9-3,0	16.043	4,0	3,0-5,4	1.563	5,8	4,2-8,0	1.429
Kalimantan Timur	2,7	2,2-3,3	13.977	4,7	3,4-6,4	1.368	7,4	5,3-10,3	1.105
Kalimantan Utara	2,1	1,5-2,8	2.733	2,7	1,7-4,2	309	4,3	2,7-6,8	284
Sulawesi Utara	5,8	4,9-6,7	9.542	8,3	6,7-10,2	821	8,7	6,7-11,2	895
Sulawesi Tengah	9,8	8,7-10,9	11.548	12,2	10,3-14,3	1.155	15,3	12,8-18,1	1.271
Sulawesi Selatan	1,1	0,9-1,2	33.693	2,2	1,8-2,8	3.269	3,4	1,6-7,3	381
Sulawesi Tenggara	4,1	3,3-5,1	10.167	5,9	4,0-8,6	1.169	10,8	6,6-17,2	721
Gorontalo	6,1	4,8-7,6	4.547	8,9	6,3-12,6	445	10,4	6,9-15,4	483
Sulawesi Barat	4,5	3,6-5,6	5.195	7,5	5,7-9,8	584	11,7	8,6-15,8	411
Maluku	31,2	29,1-33,4	6.801	40,5	36,5-44,6	779	40,5	36,5-44,6	1.166
Maluku Utara	33,4	31,2-35,7	4.723	41,2	37,6-44,9	536	41,2	37,6-44,9	802
Papua Barat	44,0	40,8-47,3	3.588	48,4	43,6-53,2	415	48,4	43,6-53,2	621
Papua	36,1	33,8-38,4	12.736	43,8	40,2-47,4	1.302	43,8	40,2-47,4	1.947
**INDONESIA**	**3,4**	**3,4-3,5**	**1.017.290**	**5,4**	**5,3-5,6**	**93.620**	**15,8**	**15,3-16,3**	**41.789**

N/A : Provinsi Bali, Jatim dan DKI, seluruh (100%) Kabupaten/Kota sudah mendapat sertifikat eliminasi malaria

Tabel 12.1.2
Proporsi Penggunaan Kelambu *Long Lasting Insecticide Nets* (*LLIN's*) menurut Karakteristik, Riskesdas 2018

Karakteristik	Penggunaan kelambu			Penggunaan kelambu (balita)		
	%	95% CI	N tertimbang	%	95% CI	N tertimbang
**Kelompok Umur**						
<1	6,3	5,9-6,6	18.225	[redacted]		
1-4	5,2	5,1-5,4	73.188			
5-14	4,1	4-4,2,0	182.338			
15-24	2,9	2,8-3,1	165.644			
25-34	3,6	3,5-3,7	159.708			
35-44	3,3	3,2-3,4	151.539			
45-54	2,8	2,7-2,9	124.652			
55-64	2,6	2,5-2,7	83.251			
65-74	2,6	2,4-2,7	40.180			
75+	2,2	2-2,4,0	18.565			
**Kelompok Umur Balita (bulan)**						
0-11	[redacted]			6,3	5,9-6,6	18.665
12-23				6,0	5,7-6,4	18.333
24-35				5,5	5,2-5,8	19.112
36-47				4,8	4,5-5,1	18.821
48-59				4,7	4,4-5,0	18.688
**Pendidikan**						
Tidak/belum pernah sekolah	4,1	3,9-4,4	70.895	[redacted]		
Tidak tamat SD/MI	4,1	4,4-3,0	181.429			
Tamat SD/MI	3,5	3,4-3,6	215.967			
Tamat SLTP/MTS	2,9	2,8-3,0	160.320			
Tamat SLTA/MA	2,4	2,3-2,4	210.746			
Tamat D1/D2/D3/PT	2,3	2,2-2,5	64.093			
**Pekerjaan**						
Tidak bekerja	2,8	2,7-2,9	233.629			
Sekolah	3,4	3,3-3,5	126.626			
PNS/TNI/Polri/BUMN/BUMD	3,4	3,2-3,6	21.931			
Pegawai Swasta	1,2	1,1-1,3	75.781			
Wiraswasta	1,6	1,5-1,7	105.489			
Nelayan	6,3	6,1-6,5	133.261			
Petani/Buruh tani	8,7	7,7-9,8	5.556			
Lainnya	1,3	1,2-1,4	75.590			
**Tempat Tinggal**						
Perkotaan	1,2	1,1-1,3	556.419	2,1	1,9-2,3	50.361
Perdesaan	6,1	6-6,3,0	460.871	9,4	9-9,7	43.259

Tabel 12.1.3

Proporsi Cara Pencegahan Penyakit Akibat Gigitan Nyamuk menurut Provinsi, Riskesdas 2018

Provinsi	Cara pencegahan penyakit akibat gigitan nyamuk (%)					N tertimbang
	Cara[1]	Cara[2]	Cara[3]	Cara[4]	Cara[5]	
Aceh	50,4	4,2	5,6	38,6	9,7	20.244
Sumatera Utara	36,6	2,1	1,7	51,7	8,8	55.351
Sumatera Barat	16,2	1,0	1,7	46,1	12,1	20.663
Riau	24,3	1,0	1,2	62,0	9,4	26.085
Jambi	33,9	5,4	3,7	52,6	11,6	13.692
Sumatera Selatan	44,2	2,1	3,1	49,9	5,6	32.126
Bengkulu	38,4	8,7	10,1	30,0	12,8	7.531
Lampung	58,3	1,5	2,5	32,0	5,6	32.148
Bangka Belitung	10,2	6,7	8,4	42,2	10,1	5.592
Kepulauan Riau	8,2	2,5	2,1	62,0	13,0	8.173
DKI Jakarta	2,1	0,2	0,4	52,6	10,1	40.210
Jawa Barat	4,1	0,2	0,4	53,2	12,6	186.809
Jawa Tengah	14,8	0,4	0,6	49,0	9,5	132.565
DI Yogyakarta	4,2	0,2	0,4	31,8	11,8	14.602
Jawa Timur	9,8	0,3	0,5	52,0	11,9	151.878
Banten	9,4	0,5	0,5	64,5	8,6	48.621
Bali	0,8	0,2	0,1	38,1	10,4	16.481
Nusa Tenggara Barat	19,2	3,1	2,2	35,9	7,7	19.247
Nusa Tenggara Timur	19,1	56,3	16,3	13,2	4,6	20.599
Kalimantan Barat	39,0	6,4	15,6	48,6	10,6	19.190
Kalimantan Tengah	61,6	4,3	4,1	43,5	8,3	10.189
Kalimantan Selatan	53,5	2,4	2,4	51,4	11,9	16.043
Kalimantan Timur	20,4	2,7	2,2	62,8	14,6	13.977
Kalimantan Utara	16,6	2,1	3,6	48,6	16,9	2.733
Sulawesi Utara	5,7	5,8	5,8	45,7	10,0	9.542
Sulawesi Tengah	29,6	9,8	12,8	43,2	6,6	11.548
Sulawesi Selatan	52,1	1,1	1,3	50,1	6,0	33.693
Sulawesi Tenggara	51,8	4,1	4,8	49,2	8,7	10.167
Gorontalo	7,2	6,1	7,9	46,6	18,1	4.547
Sulawesi Barat	66,1	4,5	4,5	46,7	5,4	5.195
Maluku	10,6	31,2	13,0	22,5	5,5	6.801
Maluku Utara	13,5	33,4	11,6	36,3	6,6	4.723
Papua Barat	15,1	44,0	12,2	33,5	8,6	3.588
Papua	19,1	36,1	13,1	22,7	5,0	12.736
**INDONESIA**	**19,5**	**3,4**	**2,4**	**48,9**	**10,1**	**1.017.290**

1. Tidur menggunakan kelambu tanpa insektisida
2. Tidur menggunakan kelambu dengan berinsektisida ≤3 tahun
3. Tidur menggunakan kelambu dengan berinsektisida >3 tahun
4. Menggunakan repelen/bahan-bahan pencegah gigitan nyamuk
5. Menggunakan alat pembasmi nyamuk elektrik

Tabel 12.1.4
Proporsi Cara Pencegahan Penyakit Akibat Gigitan Nyamuk menurut Karakteristik, Riskesdas 2018

Karakteristik	Cara pencegahan penyakit akibat gigitan nyamuk (%)					N tertimbang
	Cara[1]	Cara[2]	Cara[3]	Cara[4]	Cara[5]	
**Kelompok Umur**						
<1	35,4	6,3	2,4	31,4	11,0	18.225
1-4	23,8	5,2	2,9	45,7	11,0	73.188
5-14	20,4	4,1	2,8	50,9	9,4	182.338
15-24	15,8	2,9	1,8	49,3	8,9	165.644
25-34	19,2	3,6	2,4	48,7	11,6	159.708
35-44	19,3	3,3	2,5	50,8	11,1	151.539
45-54	18,3	2,8	2,3	51,2	10,6	124.652
55-64	19,1	2,6	2,2	48,2	9,6	83.251
65-74	21,0	2,6	2,2	43,8	7,7	40.180
75+	21,6	2,2	1,8	39,0	6,1	18.565
**Pendidikan**						
Tidak/belum pernah sekolah	23,1	4,1	2,7	47,4	7,0	70.895
Tidak tamat SD/MI	22,2	4,1	2,9	49,8	7,9	181.429
Tamat SD/MI	21,5	3,5	2,6	49,9	7,8	215.967
Tamat SLTP/MTS	19,0	2,9	2,2	49,8	9,7	160.320
Tamat SLTA/MA	13,8	2,4	1,7	50,0	12,5	210.746
Tamat D1/D2/D3/PT	10,4	2,3	1,5	46,4	18,9	64.093
**Pekerjaan**						
Tidak kerja	18,8	2,8	2,1	49,9	10,0	233.629
Sekolah	16,4	3,4	2,3	50,2	9,0	126.626
PNS/TNI/Polri/BUMN/BUMD	12,0	3,4	2,0	45,5	19,7	21.931
P,SWASTA	8,5	1,2	1,1	51,1	14,2	75.781
Wiraswasta	14,1	1,6	1,4	51,2	12,7	105.489
Petani/buruh tani	33,3	6,3	4,3	43,9	5,5	133.261
Nelayan	25,7	8,7	4,6	52,1	6,4	5.556
Buruh/sopir/pembantu ruta	12,1	1,3	1,3	52,3	7,9	75.590
Lainnya	19,5	3,6	2,9	49,3	7,8	40.644
**Tempat Tinggal**						
Perkotaan	9,5	1,2	1,0	51,6	12,7	556.419
Perdesaan	31,5	6,1	4,0	45,6	6,9	460.871

1. Tidur menggunakan kelambu tanpa insektisida
2. Tidur menggunakan kelambu dengan berinsektisida ≤3 tahun
3. Tidur menggunakan kelambu dengan berinsektisida >3 tahun
4. Menggunakan repelen/bahan-bahan pencegah gigitan nyamuk
5. Menggunakan alat pembasmi nyamuk elektrik

12.2 Konsumsi Makanan Berisiko

Indikator yang dikumpulkan untuk mendapatkan gambaran konsumsi makanan berisiko pada penduduk umur 3 tahun ke atas meliputi konsumsi makanan/minuman manis, makanan asin, makanan berlemak/kolesterol/gorengan, makanan yang dibakar, makanan daging/ayam/ikan olahan dengan pengawet, bumbu penyedap, soft drink atau minuman berkarbonasi, minuman berenergi, mie instant/makanan instant lainnya. kebiasaan konsumsi dikelompokkan menjadi $\geq$1 kali per hari, 1-6 kali per minggu dan $\leq$3 kali per bulan.

Konsumsi Makanan/Minuman Manis

$$Proporsi\ kebiasaan\ konsumsi\ makanan\ manis = \frac{ART\ umur\ \geq 3\ tahun\ dengan\ kebiasaan\ konsumsi\ makanan\ manis}{Semua\ ART\ umur\ \geq 3\ tahun}$$

Konsumsi Makanan Asin

$$Proporsi\ kebiasaan\ konsumsi\ makanan\ asin = \frac{ART\ umur\ \geq 3\ tahun\ dengan\ kebiasaan\ konsumsi\ makanan\ asin}{Semua\ ART\ umur\ \geq 3\ tahun}$$

Konsumsi Makanan Berlemak/Berkolestrol/Gorengan

$$Proporsi\ kebiasaan\ konsumsi\ makanan\ berlemak\ atau\ berkolesterol\ atau\ gorengan = \frac{ART\ umur\ \geq 3\ tahun\ dengan\ kebiasaan\ konsumsi\ makanan\ berlemak\ atau\ berkolesterol\ atau\ gorengan}{Semua\ ART\ umur\ \geq 3\ tahun}$$

Konsumsi Makanan Yang Dibakar

$$Proporsi\ kebiasaan\ konsumsi\ makanan\ yang\ dibakar = \frac{ART\ umur\ \geq 3\ tahun\ dengan\ kebiasaan\ konsumsi\ makanan\ yang\ dibakar}{Semua\ ART\ umur\ \geq 3\ tahun}$$

Konsumsi Makanan Daging/Ayam/Ikan Olahan dengan Pengawet

$$Proporsi\ kebiasaan\ konsumsi\ makanan\ daging\ atau\ ayam\ atau\ ikan\ olahan\ dengan\ pengawet = \frac{ART\ umur\ \geq 3\ tahun\ dengan\ kebiasaan\ konsumsi\ makanan\ daging\ atau\ ayam\ atau\ ikan\ olahan\ dengan\ pengawet}{Semua\ ART\ umur\ \geq 3\ tahun}$$

Konsumsi Bumbu Penyedap

$$Proporsi\ kebiasaan\ konsumsi\ bumbu\ penyedap = \frac{ART\ umur\ \geq\ 3\ tahun\ dengan\ kebiasaan\ konsumsi\ bumbu\ penyedap}{Semua\ ART\ umur\ \geq\ 3\ tahun}$$

Konsumsi *Soft Drink* atau Minuman Berkarbonasi

$$Proporsi\ kebiasaan\ konsumsi\ minuman\ ringan\ berkarbonasi = \frac{ART\ umur\ \geq\ 3\ tahun\ dengan\ kebiasaan\ konsumsi\ minuman\ berkarbonasi}{Semua\ ART\ umur\ \geq\ 3\ tahun}$$

Kebiasaan Konsumsi Minuman Berenergi

$$Proporsi\ kebiasaan\ konsumsi\ minuman\ berenergi = \frac{ART\ umur\ \geq\ 3\ tahun\ dengan\ kebiasaan\ konsumsi\ minuman\ berenergi}{Semua\ ART\ umur\ \geq\ 3\ tahun}$$

Konsumsi Mi Instan/Makanan Instan Lainnya

$$Proporsi\ kebiasaan\ konsumsi\ mi\ instan\ atau\ makanan\ instan\ lain = \frac{ART\ umur\ \geq\ 3\ tahun\ dengan\ kebiasaan\ konsumsi\ mi\ instan\ atau\ makanan\ instan\ lain}{Semua\ ART\ umur\ \geq\ 3\ tahun}$$

Tabel 12.2.1
Proporsi Kebiasaan Konsumsi Makanan Manis pada Penduduk Umur ≥3 Tahun menurut Provinsi, Riskesdas 2018

Provinsi	Kebiasaan Konsumsi Makanan Manis[1]			N tertimbang
	≥1 kali per hari (%)	1 - 6 kali per minggu (%)	≤3 kali perbulan (%)	
Aceh	44,1	47,6	8,3	18.855
Sumatera Utara	34,3	51,8	13,9	51.977
Sumatera Barat	43,4	44,5	12,1	19.399
Riau	37,0	52,7	10,2	24.405
Jambi	36,3	55,6	8,1	12.936
Sumatera Selatan	40,3	51,5	8,2	30.341
Bengkulu	33,2	55,2	11,6	7.123
Lampung	40,2	48,1	11,7	30.300
Bangka Belitung	38,2	50,8	11,0	5.301
Kepulauan Riau	41,9	49,5	8,6	7.679
DKI Jakarta	36,4	49,0	14,6	38.124
Jawa Barat	51,1	41,2	7,7	176.728
Jawa Tengah	40,5	47,9	11,7	126.225
DI Yogyakarta	41,1	46,6	12,3	13.989
Jawa Timur	31,3	51,6	17,1	145.173
Banten	40,2	47,3	12,6	45.831
Bali	30,3	45,8	23,8	15.737
Nusa Tenggara Barat	33,3	50,9	15,8	18.058
Nusa Tenggara Timur	26,5	49,2	24,3	19.115
Kalimantan Barat	37,6	52,1	10,2	18.073
Kalimantan Tengah	47,1	46,4	6,5	9.615
Kalimantan Selatan	54,8	38,9	6,2	15.077
Kalimantan Timur	40,6	48,4	11,0	13.195
Kalimantan Utara	48,1	42,3	9,6	2.547
Sulawesi Utara	40,5	51,0	8,5	9.055
Sulawesi Tengah	38,9	52,3	8,8	10.837
Sulawesi Selatan	40,1	52,1	7,8	31.703
Sulawesi Tenggara	42,0	47,9	10,0	9.464
Gorontalo	40,9	50,9	8,1	4.286
Sulawesi Barat	46,9	46,9	6,3	4.831
Maluku	49,6	39,9	10,5	6.334
Maluku Utara	43,1	45,4	11,6	4.404
Papua Barat	38,7	46,2	15,1	3.341
Papua	33,9	40,2	25,9	11.987
**INDONESIA**	**40,1**	**47,8**	**12,0**	**962.045**

[1]Makanan manis yaitu makanan mengandung gula yang tinggi termasuk yang lengket

Tabel 12.2.2
Proporsi Kebiasaan Konsumsi Makanan Manis pada Penduduk Umur >3 Tahun
menurut Karakteristik, Riskesdas 2018

Karakteristik	kebiasaan konsumsi makanan manis[1]			N tertimbang
	≥1 kali per hari (%)	1 - 6 kali per minggu (%)	≤3 kali perbulan (%)	
**Kelompok Umur**				
3 – 4	59,6	35,7	4,6	36.608
5 – 9	59,0	36,7	4,3	92.746
10-14	50,4	44,0	5,6	89.506
15-19	41,0	50,6	8,5	83.422
20-24	37,8	51,7	10,6	82.143
25-29	37,4	51,1	11,5	81.351
30-34	35,9	51,4	12,7	78.281
35-39	35,4	50,9	13,6	79.035
40-44	34,7	50,3	15,0	72.432
45-49	34,4	49,8	15,8	67.116
50-54	33,7	49,0	17,2	57.477
55-59	32,9	48,7	18,4	47.552
60-64	31,7	48,8	19,5	35.659
65 +	30,9	47,9	21,2	58.717
**Jenis Kelamin**				
Laki-laki	40,5	47,4	12,1	482.729
Perempuan	39,8	48,2	12,0	479.316
**Pendidikan**				
Tidak sekolah	40,3	43,6	16,1	70.895
Tidak tamat SD	44,3	44,7	10,9	181.429
Tamat SD	37,8	49,1	13,2	215.967
Tamat SLTP	37,8	50,5	11,7	160.320
Tamat SLTA	36,5	51,0	12,5	210.746
Tamat D1/D2/D3 PT	36,8	50,5	12,7	64.093
**Pekerjaan**				
Tidak Bekerja	36,5	49,7	13,8	233.629
Sekolah	46,0	47,3	6,8	126.626
PNS/TNI/Polri/BUMN/BUMD	35,4	50,4	14,2	21.931
Pegawai swasta	36,8	50,4	12,8	75.781
Wiraswasta	37,9	48,3	13,8	105.489
Petani/Buruh tani	30,4	52,0	17,6	133.261
Nelayan	39,8	47,7	12,5	5.556
Buruh/sopir/pembantu ruta	36,8	49,3	14,0	75.590
Lainnya	36,1	50,5	13,4	40.644
**Tempat tinggal**				
Perkotaan	41,8	46,5	11,6	526.861
Perdesaan	38,1	49,3	12,6	435.184

[1]Makanan manis yaitu makanan mengandung gula yang tinggi termasuk yang lengket

Tabel 12.2.3
Proporsi Kebiasaan Konsumsi Minuman Manis pada Penduduk Umur ≥3 Tahun menurut Provinsi, Riskesdas 2018

Provinsi	Kebiasaan Konsumsi Minuman Manis[1]			N tertimbang
	≥1 kali per hari (%)	1 - 6 kali per minggu (%)	≤3 kali perbulan (%)	
Aceh	59,81	34,01	6,18	18.855
Sumatera Utara	58,51	33,02	8,46	51.977
Sumatera Barat	60,63	29,52	9,85	19.399
Riau	62,39	30,25	7,35	24.405
Jambi	54,46	38,54	7,00	12.936
Sumatera Selatan	63,82	30,46	5,72	30.341
Bengkulu	55,81	34,48	9,71	7.123
Lampung	65,95	26,72	7,33	30.300
Bangka Belitung	57,26	32,15	10,59	5.301
Kepulauan Riau	59,58	33,56	6,86	7.679
DKI Jakarta	61,72	28,58	9,69	38.124
Jawa Barat	63,91	29,03	7,06	176.728
Jawa Tengah	68,82	24,47	6,71	126.225
DI Yogyakarta	71,01	21,38	7,61	13.989
Jawa Timur	57,00	32,52	10,48	145.173
Banten	61,46	29,79	8,76	45.831
Bali	42,90	35,69	21,42	15.737
Nusa Tenggara Barat	51,73	36,76	11,51	18.058
Nusa Tenggara Timur	53,60	31,58	14,82	19.115
Kalimantan Barat	59,05	34,54	6,41	18.073
Kalimantan Tengah	64,57	30,98	4,45	9.615
Kalimantan Selatan	70,82	23,95	5,23	15.077
Kalimantan Timur	61,57	30,38	8,05	13.195
Kalimantan Utara	67,53	26,19	6,28	2.547
Sulawesi Utara	60,94	30,83	8,22	9.055
Sulawesi Tengah	58,67	31,43	9,90	10.837
Sulawesi Selatan	56,29	34,88	8,83	31.703
Sulawesi Tenggara	52,66	36,36	10,98	9.464
Gorontalo	56,16	35,72	8,11	4.286
Sulawesi Barat	58,25	33,66	8,10	4.831
Maluku	62,06	29,76	8,19	6.334
Maluku Utara	59,40	30,92	9,68	4.404
Papua Barat	59,18	31,17	9,66	3.341
Papua	53,45	34,10	12,44	11.987
**INDONESIA**	**61,27**	**30,22**	**8,51**	**962.045**

[1]Minuman manis yaitu minuman mengandung gula yang tinggi

Tabel 12.2.4
Proporsi Kebiasaan Konsumsi Minuman Manis pada Penduduk Umur ≥3 Tahun menurut Karakteristik, Riskesdas 2018

Karakteristik	Kebiasaan Konsumsi Minuman Manis[1] ≥1 kali per hari (%)	1 - 6 kali per minggu (%)	≤3 kali perbulan (%)	N tertimbang
**Kelompok Umur**				
3 – 4	68,57	26 ,9	4,55	36.608
5 – 9	66,50	29 ,3	4,21	92.746
10-14	61,86	33 ,5	4,67	89.506
15-19	56,43	37 ,0	6,57	83.422
20-24	56,43	35 ,7	7,90	82.143
25-29	57,87	33 ,2	8,89	81.351
30-34	60,24	30 ,9	8,90	78.281
35-39	61,52	29 ,2	9,32	79.035
40-44	62,58	27 ,9	9,52	72.432
45-49	62,71	27 ,1	10,16	67.116
50-54	62,24	26 ,5	11,26	57.477
55-59	63,04	25 ,3	11,70	47.552
60-64	62,23	25 ,5	12,25	35.659
65 +	60,73	24 ,9	14,40	58.717
**Jenis Kelamin**				
Laki-laki	67,32	26,48	6,20	482.729
Perempuan	55,18	33,98	10,84	479.316
**Pendidikan**				
Tidak sekolah	60,31	28,97	10,72	70.895
Tidak tamat SD	63,15	29,54	7,31	181.429
Tamat SD	61,98	29,20	8,81	215.967
Tamat SLTP	61,05	30,89	8,05	160.320
Tamat SLTA	59,58	31,49	8,92	210.746
Tamat D1/D2/D3 PT	54,56	33,68	11,76	64.093
**Pekerjaan**				
Tidak Bekerja	54,11	33,71	12,17	233.629
Sekolah	59,09	35,43	5,48	126.626
PNS/TNI/Polri/BUMN/BUMD	57,54	30,59	11,88	21.931
Pegawai swasta	61,35	30,67	7,98	75.781
Wiraswasta	64,48	26,77	8,74	105.489
Petani/Buruh tani	64,41	26,47	9,12	133.261
Nelayan	67,94	25,00	7,06	5.556
Buruh/sopir/pembantu ruta	69,45	24,05	6,49	75.590
Lainnya	58,12	31,27	10,61	40.644
**Tempat tinggal**				
Perkotaan	62,02	29,56	8,42	526.861
Perdesaan	60,37	31,01	8,63	435.184

[1]Minuman manis yaitu minuman mengandung gula yang tinggi

Tabel 12.2.5
Proporsi Kebiasaan Konsumsi Makanan Asin pada Penduduk Umur ≥3 Tahun menurut Provinsi, Riskesdas 2018

Provinsi	Kebiasaan Konsumsi Makanan Asin[1]			N tertimbang
	≥1 kali per hari (%)	1 - 6 kali per minggu (%)	≤3 kali perbulan (%)	
Aceh	13,2	53,8	33,0	18.855
Sumatera Utara	15,8	50,2	34,0	51.977
Sumatera Barat	11,2	47,1	41,7	19.399
Riau	16,3	55,2	28,5	24.405
Jambi	27,7	54,6	17,8	12.936
Sumatera Selatan	39,8	48,8	11,4	30.341
Bengkulu	27,3	44,8	27,9	7.123
Lampung	29,4	47,2	23,3	30.300
Bangka Belitung	13,4	48,9	37,7	5.301
Kepulauan Riau	13,6	49,8	36,6	7.679
DKI Jakarta	25,5	43,6	30,9	38.124
Jawa Barat	54,1	34,4	11,4	176.728
Jawa Tengah	32,0	41,7	26,3	126.225
DI Yogyakarta	30,3	40,3	29,3	13.989
Jawa Timur	28,7	43,3	28,0	145.173
Banten	33,1	45,0	21,9	45.831
Bali	12,3	37,8	49,9	15.737
Nusa Tenggara Barat	10,0	42,6	47,4	18.058
Nusa Tenggara Timur	7,0	38,5	54,5	19.115
Kalimantan Barat	22,2	53,6	24,2	18.073
Kalimantan Tengah	20,7	54,1	25,2	9.615
Kalimantan Selatan	22,4	52,4	25,2	15.077
Kalimantan Timur	23,9	46,9	29,2	13.195
Kalimantan Utara	22,6	42,6	34,9	2.547
Sulawesi Utara	10,1	30,8	59,1	9.055
Sulawesi Tengah	11,5	40,7	47,8	10.837
Sulawesi Selatan	20,2	49,8	30,0	31.703
Sulawesi Tenggara	13,2	41,2	45,6	9.464
Gorontalo	10,6	30,9	58,5	4.286
Sulawesi Barat	28,0	54,8	17,2	4.831
Maluku	15,3	36,6	48,1	6.334
Maluku Utara	21,1	36,2	42,7	4.404
Papua Barat	14,6	31,1	54,3	3.341
Papua	13,2	25,4	61,4	11.987
**INDONESIA**	**29,7**	**43,0**	**27,3**	**962.045**

[1]Makanan asin adalah makanan yang lebih dominan rasa asin atau mengandung garam yang tinggi

Tabel 12.2.6

Proporsi Kebiasaan Konsumsi Makanan Asin pada Penduduk Umur >3 Tahun menurut Karakteristik, Riskesdas 2018

Karakteristik	Kebiasaan Konsumsi Makanan Asin[1] ≥1 kali per hari (%)	1 - 6 kali per minggu (%)	≤3 kali perbulan (%)	N tertimbang
**Kelompok Umur**				
3 – 4	31,1	39,4	29,5	36.608
5 – 9	32,9	41,0	26,1	92.746
10-14	31,4	43,1	25,5	89.506
15-19	30,5	43,8	25,7	83.422
20-24	30,2	44,1	25,6	82.143
25-29	29,9	45,0	25,1	81.351
30-34	29,8	44,3	25,9	78.281
35-39	29,0	44,7	26,3	79.035
40-44	29,6	44,0	26,5	72.432
45-49	29,3	43,2	27,5	67.116
50-54	28,1	43,3	28,6	57.477
55-59	28,0	41,9	30,1	47.552
60-64	26,9	41,1	32,0	35.659
65 +	25,1	39,2	35,6	58.717
**Jenis Kelamin**				
Laki-laki	28,9	43,4	27,7	482.729
Perempuan	30,5	42,6	26,9	479.316
**Pendidikan**				
Tidak sekolah	29,4	41,1	29,5	70.895
Tidak tamat SD	30,8	42,4	26,8	181.429
Tamat SD	32,4	42,7	24,9	215.967
Tamat SLTP	30,3	44,1	25,6	160.320
Tamat SLTA	26,6	44,3	29,0	210.746
Tamat D1/D2/D3 PT	25,0	43,5	31,5	64.093
**Pekerjaan**				
Tidak Bekerja	30,3	42,2	27,5	233.629
Sekolah	30,2	43,7	26,1	126.626
PNS/TNI/Polri/BUMN/BUMD	22,3	42,9	34,8	21.931
Pegawai swasta	27,8	44,5	27,7	75.781
Wiraswasta	28,7	43,6	27,7	105.489
Petani/Buruh tani	28,2	44,6	27,2	133.261
Nelayan	22,1	45,1	32,8	5.556
Buruh/sopir/pembantu ruta	32,5	43,0	24,5	75.590
Lainnya	27,4	43,6	29,0	40.644
**Tempat tinggal**				
Perkotaan	30,5	42,0	27,5	526.861
Perdesaan	28,7	44,2	27,1	435.184

[1]Makanan asin adalah makanan yang lebih dominan rasa asin atau mengandung garam yang tinggi

Tabel 12.2.7

Proporsi Kebiasaan Konsumsi Makanan Berlemak/Berkolesterol/Gorengan pada Penduduk Umur ≥3 Tahun menurut Provinsi, Riskesdas 2018

Provinsi	Kebiasaan Konsumsi Makanan Berlemak/Berkolesterol/Gorengan[1]			N tertimbang
	≥1 kali per hari (%)	1 - 6 kali per minggu (%)	≤3 kali perbulan (%)	
Aceh	20,7	59,3	19,9	18.855
Sumatera Utara	21,4	55,3	23,3	51.977
Sumatera Barat	38,1	50,1	11,8	19.399
Riau	36,0	51,9	12,1	24.405
Jambi	19,0	60,6	20,3	12.936
Sumatera Selatan	32,0	54,5	13,5	30.341
Bengkulu	24,7	61,3	14,1	7.123
Lampung	36,6	50,7	12,7	30.300
Bangka Belitung	19,1	59,3	21,6	5.301
Kepulauan Riau	23,3	57,6	19,1	7.679
DKI Jakarta	39,4	47,9	12,7	38.124
Jawa Barat	52,5	39,8	7,8	176.728
Jawa Tengah	58,4	36,0	5,6	126.225
DI Yogyakarta	50,7	42,5	6,8	13.989
Jawa Timur	48,5	38,5	12,9	145.173
Banten	49,5	40,6	9,9	45.831
Bali	31,8	44,4	23,8	15.737
Nusa Tenggara Barat	32,4	46,1	21,5	18.058
Nusa Tenggara Timur	10,3	43,4	46,3	19.115
Kalimantan Barat	26,2	54,5	19,3	18.073
Kalimantan Tengah	36,5	49,4	14,1	9.615
Kalimantan Selatan	35,3	50,1	14,6	15.077
Kalimantan Timur	35,8	49,0	15,2	13.195
Kalimantan Utara	34,7	51,1	14,2	2.547
Sulawesi Utara	39,3	51,8	8,8	9.055
Sulawesi Tengah	34,0	55,0	11,0	10.837
Sulawesi Selatan	28,4	56,1	15,5	31.703
Sulawesi Tenggara	24,5	58,8	16,7	9.464
Gorontalo	49,7	44,3	6,1	4.286
Sulawesi Barat	26,9	58,1	15,0	4.831
Maluku	32,3	52,8	14,8	6.334
Maluku Utara	39,7	51,2	9,1	4.404
Papua Barat	26,7	51,9	21,5	3.341
Papua	18,7	43,3	38,0	11.987
**INDONESIA**	**41,7**	**45,0**	**13,2**	**962.045**

[1]Makanan berlemak adalah makanan mengandung lemak yang tinggi, termasuk lemak jenuh, dan makanan yang mengandung kolesterol

Tabel 12.2.8
Proporsi Kebiasaan Konsumsi Makanan Berlemak/Berkolesterol/Gorengan pada Penduduk Umur ≥3 Tahun menurut Karakteristik, Riskesdas 2018

Karakteristik	Kebiasaan Konsumsi Makanan Berlemak/Berkolesterol/Gorengan[1] ≥1 kali per hari (%)	1 - 6 kali per minggu (%)	≤3 kali perbulan (%)	N tertimbang
**Kelompok Umur**				
3 – 4	35,0	46,5	18,5	36.608
5 – 9	42,3	44,7	13,0	92.746
10-14	44,2	45,1	10,6	89.506
15-19	43,8	45,7	10,5	83.422
20-24	41,8	47,0	11,2	82.143
25-29	41,1	47,0	11,9	81.351
30-34	41,8	46,4	11,9	78.281
35-39	42,0	45,8	12,2	79.035
40-44	43,1	44,4	12,5	72.432
45-49	42,9	44,1	13,0	67.116
50-54	42,2	43,5	14,3	57.477
55-59	41,5	42,7	15,8	47.552
60-64	39,6	42,7	17,7	35.659
65 +	36,9	42,0	21,1	58.717
**Jenis Kelamin**				
Laki-laki	40,7	45,8	13,5	482.729
Perempuan	42,8	44,3	12,9	479.316
**Pendidikan**				
Tidak sekolah	40,3	42,0	17,6	70.895
Tidak tamat SD	42,1	44,2	13,6	181.429
Tamat SD	44,0	43,4	12,6	215.967
Tamat SLTP	43,9	44,8	11,3	160.320
Tamat SLTA	40,6	47,0	12,3	210.746
Tamat D1/D2/D3 PT	37,4	49,2	13,4	64.093
**Pekerjaan**				
Tidak Bekerja	41,9	44,6	13,5	233.629
Sekolah	44,0	45,7	10,3	126.626
PNS/TNI/Polri/BUMN/BUMD	36,0	49,3	14,8	21.931
Pegawai swasta	42,9	46,1	11,0	75.781
Wiraswasta	43,9	43,8	12,3	105.489
Petani/Buruh tani	36,7	45,9	17,4	133.261
Nelayan	36,0	48,4	15,6	5.556
Buruh/sopir/pembantu ruta	47,8	42,0	10,2	75.590
Lainnya	40,2	46,8	13,0	40.644
**Tempat tinggal**				
Perkotaan	43,6	44,6	11,8	526.861
Perdesaan	39,5	45,5	14,9	435.184

[1]Makanan berlemak adalah makanan mengandung lemak yang tinggi, termasuk lemak jenuh, dan makanan yang mengandung kolesterol

Tabel 12.2.9

Proporsi Kebiasaan Konsumsi Makanan yang Dibakar pada Penduduk Umur $\geq$3 Tahun menurut Provinsi, Riskesdas 2018

Provinsi	Kebiasaan Konsumsi Makanan Yang Dibakar[1]			N tertimbang
	≥1 kali per hari (%)	1 - 6 kali per minggu (%)	≤3 kali perbulan (%)	
Aceh	4,6	42,7	52,8	18.855
Sumatera Utara	5,3	30,3	64,4	51.977
Sumatera Barat	5,6	40,8	53,6	19.399
Riau	4,9	40,7	54,3	24.405
Jambi	3,0	39,6	57,3	12.936
Sumatera Selatan	4,9	34,5	60,7	30.341
Bengkulu	3,4	35,0	61,7	7.123
Lampung	3,6	24,0	72,4	30.300
Bangka Belitung	3,9	48,8	47,3	5.301
Kepulauan Riau	3,7	42,6	53,7	7.679
DKI Jakarta	3,6	33,8	62,5	38.124
Jawa Barat	4,7	32,8	62,5	176.728
Jawa Tengah	3,3	26,2	70,5	126.225
DI Yogyakarta	2,7	30,6	66,7	13.989
Jawa Timur	2,8	24,6	72,6	145.173
Banten	6,6	36,5	56,9	45.831
Bali	3,0	33,4	63,6	15.737
Nusa Tenggara Barat	5,0	34,2	60,8	18.058
Nusa Tenggara Timur	4,8	32,2	63,0	19.115
Kalimantan Barat	2,5	23,9	73,6	18.073
Kalimantan Tengah	5,0	45,0	50,0	9.615
Kalimantan Selatan	4,9	48,4	46,7	15.077
Kalimantan Timur	4,5	39,9	55,6	13.195
Kalimantan Utara	4,6	35,8	59,6	2.547
Sulawesi Utara	9,2	60,4	30,4	9.055
Sulawesi Tengah	13,8	59,5	26,7	10.837
Sulawesi Selatan	8,6	57,0	34,3	31.703
Sulawesi Tenggara	9,8	57,6	32,6	9.464
Gorontalo	9,2	70,3	20,5	4.286
Sulawesi Barat	10,9	60,3	28,8	4.831
Maluku	15,0	55,9	29,1	6.334
Maluku Utara	17,2	56,1	26,7	4.404
Papua Barat	12,4	45,5	42,1	3.341
Papua	37,4	33,6	29,1	11.987
**INDONESIA**	**5.1**	**33.9**	**61,0**	**956.993**

[1] Makanan yang diproses dengan cara dibakar di atas api secara langsung

Tabel 12.2.10

Proporsi Kebiasaan Konsumsi Makanan yang Dibakar pada Penduduk Umur ≥3 Tahun menurut Karakteristik, Riskesdas 2018

Karakteristik	Kebiasaan Konsumsi Makanan Yang Dibakar[1]			N tertimbang
	≥1 kali per hari (%)	1 - 6 kali per minggu (%)	≤3 kali perbulan (%)	
**Kelompok Umur**				
3 – 4	5,7	33,5	60,8	36.608
5 – 9	7,4	37,8	54,8	92.746
10-14	7,1	38,4	54,4	89.506
15-19	5,5	38,3	56,2	83.422
20-24	5,0	38,0	56,9	82.143
25-29	5,0	36,9	58,1	81.351
30-34	4,6	35,3	60,0	78.281
35-39	4,6	34,0	61,3	79.035
40-44	4,6	32,2	63,2	72.432
45-49	4,3	31,2	64,5	67.116
50-54	4,3	30,0	65,7	57.477
55-59	4,0	27,7	68,3	47.552
60-64	3,5	25,5	71,0	35.659
65 +	3,1	22,2	74,8	58.717
**Jenis Kelamin**				
Laki-laki	5,3	34,7	60,0	482.729
Perempuan	4,9	33,1	62,0	479.316
**Pendidikan**				
Tidak sekolah	6,5	28,4	65,1	70.895
Tidak tamat SD	6,1	32,7	61,2	181.429
Tamat SD	4,7	30,5	64,8	215.967
Tamat SLTP	4,7	33,6	61,7	160.320
Tamat SLTA	4,4	37,4	58,2	210.746
Tamat D1/D2/D3 PT	4,6	43,5	51,9	64.093
**Pekerjaan**				
Tidak Bekerja	4,6	32,0	63,4	233.629
Sekolah	6,2	39,2	54,6	126.626
PNS/TNI/Polri/BUMN/BUMD	5,0	44,2	50,8	21.931
Pegawai swasta	3,9	38,0	58,1	75.781
Wiraswasta	4,4	34,6	61,0	105.489
Petani/Buruh tani	5,2	26,9	68,0	133.261
Nelayan	13,0	45,9	41,1	5.556
Buruh/sopir/pembantu ruta	3,4	28,5	68,1	75.590
Lainnya	4,8	36,5	58,7	40.644
**Tempat tinggal**				
Perkotaan	4,6	35,4	60,1	526.861
Perdesaan	5,7	32,2	62,1	435.184

[1]Makanan yang diproses dengan cara dibakar di atas api secara langsung

Tabel 12.2.11

Proporsi Kebiasaan Konsumsi Makanan Daging/Ayam/Ikan Olahan dengan Pengawet pada Penduduk Umur $\geq$3 Tahun menurut Provinsi, Riskesdas 2018

Provinsi	Kebiasaan Konsumsi Makanan Daging/Ayam/Ikan Olahan dengan Pengawet[1]			N tertimbang
	≥1 kali per hari (%)	1 - 6 kali per minggu (%)	≤3 kali perbulan (%)	
Aceh	3,7	19,4	76,9	18.855
Sumatera Utara	3,2	17,3	79,5	51.977
Sumatera Barat	3,8	15,8	80,4	19.399
Riau	3,9	19,0	77,1	24.405
Jambi	3,1	24,6	72,3	12.936
Sumatera Selatan	4,1	20,8	75,1	30.341
Bengkulu	2,9	18,9	78,1	7.123
Lampung	3,5	18,0	78,5	30.300
Bangka Belitung	3,0	14,9	82,1	5.301
Kepulauan Riau	4,2	27,2	68,6	7.679
DKI Jakarta	6,1	27,5	66,4	38.124
Jawa Barat	6,3	31,1	62,6	176.728
Jawa Tengah	4,7	23,2	72,1	126.225
DI Yogyakarta	3,4	25,2	71,4	13.989
Jawa Timur	4,8	21,7	73,5	145.173
Banten	6,5	28,3	65,2	45.831
Bali	5,1	19,7	75,2	15.737
Nusa Tenggara Barat	4,7	18,9	76,5	18.058
Nusa Tenggara Timur	1,2	6,7	92,0	19.115
Kalimantan Barat	3,5	23,3	73,2	18.073
Kalimantan Tengah	6,6	25,4	68,0	9.615
Kalimantan Selatan	4,1	20,1	75,8	15.077
Kalimantan Timur	4,8	19,5	75,7	13.195
Kalimantan Utara	7,6	23,4	69,1	2.547
Sulawesi Utara	3,2	27,1	69,8	9.055
Sulawesi Tengah	3,5	13,4	83,1	10.837
Sulawesi Selatan	5,5	21,6	72,9	31.703
Sulawesi Tenggara	4,3	17,0	78,6	9.464
Gorontalo	2,4	18,2	79,4	4.286
Sulawesi Barat	4,8	21,3	73,9	4.831
Maluku	12,8	14,2	73,1	6.334
Maluku Utara	5,7	14,0	80,3	4.404
Papua Barat	4,0	19,1	76,9	3.341
Papua	6,3	21,7	72,0	11.987
**INDONESIA**	**4,9**	**23,0**	**72,1**	**962.045**

[1] makanan yang berasal dari hewan, melalui proses pengolahan dan ditambahkan bahan pengawet.

Tabel 12.2.12
Proporsi Kebiasaan Konsumsi Makanan Daging/Ayam/Ikan Olahan dengan Pengawet pada Penduduk Umur >3 Tahun menurut Karakteristik, Riskesdas 2018

Karakteristik	Kebiasaan Konsumsi Makanan Daging/Ayam/Ikan Olahan dengan Pengawet[1]			N tertimbang
	≥1 kali per hari (%)	1 - 6 kali per minggu (%)	≤3 kali perbulan (%)	
**Kelompok Umur**				
3 – 4	8,4	32,5	59,1	36.608
5 – 9	10,3	33,6	56,2	92.746
10-14	8,8	32,6	58,6	89.506
15-19	6,1	29,4	64,5	83.422
20-24	4,4	26,1	69,5	82.143
25-29	4,0	23,0	73,0	81.351
30-34	3,6	21,2	75,2	78.281
35-39	3,4	20,1	76,6	79.035
40-44	3,4	19,0	77,6	72.432
45-49	3,0	17,3	79,7	67.116
50-54	2,7	15,9	81,3	57.477
55-59	2,4	14,1	83,5	47.552
60-64	1,9	12,8	85,3	35.659
65 +	2,0	11,4	86,7	58.717
**Jenis Kelamin**				
Laki-laki	4,8	22,8	72,3	482.729
Perempuan	4,9	23,3	71,8	479.316
**Pendidikan**				
Tidak sekolah	5,8	21,1	73,2	70.895
Tidak tamat SD	6,4	24,6	69,0	181.429
Tamat SD	4,0	20,4	75,7	215.967
Tamat SLTP	4,3	22,6	73,2	160.320
Tamat SLTA	3,9	22,9	73,2	210.746
Tamat D1/D2/D3 PT	4,0	23,8	72,2	64.093
**Pekerjaan**				
Tidak Bekerja	4,0	20,8	75,2	233.629
Sekolah	7,4	31,8	60,7	126.626
PNS/TNI/Polri/BUMN/BUMD	3,9	20,4	75,8	21.931
Pegawai swasta	4,2	24,9	70,9	75.781
Wiraswasta	3,7	20,2	76,0	105.489
Petani/Buruh tani	2,2	14,2	83,5	133.261
Nelayan	4,5	14,2	81,4	5.556
Buruh/sopir/pembantu ruta	3,0	19,3	77,7	75.590
Lainnya	3,8	19,3	77,0	40.644
**Tempat tinggal**				
Perkotaan	5,5	25,9	68,6	526.861
Perdesaan	4,1	19,7	76,2	435.184

[1]makanan yang berasal dari hewan, melalui proses pengolahan dan ditambahkan bahan pengawet.

Tabel 12.2.13
Proporsi Kebiasaan Konsumsi Makanan yang Mengandung Bumbu Penyedap pada Penduduk Umur ≥3 Tahun menurut Provinsi, Riskesdas 2018

Provinsi	Kebiasaan Konsumsi Makanan yang Mengandung Bumbu Penyedap[1]			N tertimbang
	≥1 kali per hari (%)	1 - 6 kali per minggu (%)	≤3 kali perbulan (%)	
Aceh	30,0	22,0	48,0	18.855
Sumatera Utara	45,6	15,5	38,9	51.977
Sumatera Barat	57,9	16,8	25,3	19.399
Riau	81,7	8,0	10,3	24.405
Jambi	71,1	14,3	14,6	12.936
Sumatera Selatan	82,5	9,4	8,1	30.341
Bengkulu	86,1	5,0	9,0	7.123
Lampung	84,5	8,9	6,6	30.300
Bangka Belitung	81,5	10,3	8,2	5.301
Kepulauan Riau	75,5	12,2	12,2	7.679
DKI Jakarta	79,2	9,4	11,4	38.124
Jawa Barat	87,2	7,9	4,9	176.728
Jawa Tengah	83,3	8,4	8,3	126.225
DI Yogyakarta	77,4	10,8	11,8	13.989
Jawa Timur	79,0	10,3	10,7	145.173
Banten	86,0	8,9	5,1	45.831
Bali	77,6	9,9	12,5	15.737
Nusa Tenggara Barat	87,0	6,2	6,8	18.058
Nusa Tenggara Timur	76,2	11,3	12,5	19.115
Kalimantan Barat	68,3	14,5	17,2	18.073
Kalimantan Tengah	72,3	18,7	9,0	9.615
Kalimantan Selatan	73,2	16,2	10,6	15.077
Kalimantan Timur	78,7	11,3	10,0	13.195
Kalimantan Utara	70,0	17,6	12,4	2.547
Sulawesi Utara	70,0	19,6	10,4	9.055
Sulawesi Tengah	81,5	10,3	8,2	10.837
Sulawesi Selatan	77,7	14,9	7,4	31.703
Sulawesi Tenggara	77,5	13,2	9,3	9.464
Gorontalo	77,2	15,2	7,6	4.286
Sulawesi Barat	68,2	20,0	11,7	4.831
Maluku	71,6	15,0	13,5	6.334
Maluku Utara	69,3	18,0	12,8	4.404
Papua Barat	66,5	15,8	17,7	3.341
Papua	59,5	16,2	24,4	11.987
**INDONESIA**	**77,6**	**10,8**	**11,6**	**962.045**

[1]Bumbu penyedap seperti vetsin, kaldu instan dan bumbu masak lainnya

Tabel 12.2.14
Proporsi Kebiasaan Konsumsi Bumbu Penyedap pada Penduduk Umur >3 Tahun menurut Karakteristik, Riskesdas 2018

Karakteristik	Kebiasaan Konsumsi Makanan yang Mengandung Bumbu Penyedap[1]			N tertimbang
	≥1 kali per hari (%)	1 - 6 kali per minggu (%)	≤3 kali perbulan (%)	
**Kelompok Umur**				
3 – 4	76,0	10,4	13,6	36.608
5 – 9	77,4	11,0	11,5	92.746
10-14	78,5	11,0	10,6	89.506
15-19	78,3	11,1	10,5	83.422
20-24	79,0	10,8	10,2	82.143
25-29	78,5	10,6	10,8	81.351
30-34	79,0	10,8	10,2	78.281
35-39	78,8	10,5	10,7	79.035
40-44	78,9	10,5	10,6	72.432
45-49	78,0	10,7	11,3	67.116
50-54	77,0	10,7	12,3	57.477
55-59	76,0	10,4	13,6	47.552
60-64	74,5	10,8	14,6	35.659
65 +	71,6	11,1	17,2	58.717
**Jenis Kelamin**				
Laki-laki	77,3	11,0	11,7	482.729
Perempuan	77,9	10,5	11,5	479.316
**Pendidikan**				
Tidak sekolah	77,1	10,8	12,1	70.895
Tidak tamat SD	78,4	10,7	10,9	181.429
Tamat SD	80,6	10,0	9,4	215.967
Tamat SLTP	80,0	10,2	9,8	160.320
Tamat SLTA	76,1	11,4	12,5	210.746
Tamat D1/D2/D3 PT	65,8	13,5	20,7	64.093
**Pekerjaan**				
Tidak Bekerja	77,9	10,7	11,5	233.629
Sekolah	78,1	11,1	10,8	126.626
PNS/TNI/Polri/BUMN/BUMD	64,5	13,9	21,6	21.931
Pegawai swasta	78,0	10,6	11,4	75.781
Wiraswasta	77,0	10,8	12,2	105.489
Petani/Buruh tani	77,4	10,6	11,9	133.261
Nelayan	74,6	12,8	12,6	5.556
Buruh/sopir/pembantu ruta	82,8	9,2	8,0	75.590
Lainnya	76,0	12,1	11,9	40.644
**Tempat tinggal**				
Perkotaan	77,3	10,8	11,9	526.861
Perdesaan	78,0	10,8	11,2	435.184

[1]Bumbu penyedap seperti vetsin, kaldu instan dan bumbu masak lainnya

Tabel 12.2.15

Proporsi Kebiasaan Konsumsi *Soft Drink* atau Minuman Berkarbonasi pada Penduduk Umur >3 Tahun menurut Provinsi, Riskesdas 2018

Provinsi	Kebiasaan Konsumsi *Soft Drink* atau Minuman Berkarbonasi[1]			N tertimbang
	≥1 kali per hari (%)	1 - 6 kali per minggu (%)	≤3 kali perbulan (%)	
Aceh	2,1	13,1	84,8	18.855
Sumatera Utara	2,2	10,9	87,0	51.977
Sumatera Barat	1,6	10,5	87,9	19.399
Riau	2,2	11,4	86,4	24.405
Jambi	2,2	13,4	84,4	12.936
Sumatera Selatan	2,6	8,9	88,5	30.341
Bengkulu	2,2	9,4	88,4	7.123
Lampung	2,2	6,8	91,0	30.300
Bangka Belitung	1,8	11,8	86,4	5.301
Kepulauan Riau	3,3	13,2	83,5	7.679
DKI Jakarta	1,9	9,8	88,3	38.124
Jawa Barat	2,9	12,9	84,2	176.728
Jawa Tengah	1,4	7,8	90,8	126.225
DI Yogyakarta	1,4	9,3	89,3	13.989
Jawa Timur	1,4	7,9	90,6	145.173
Banten	2,4	13,6	83,9	45.831
Bali	2,1	17,8	80,1	15.737
Nusa Tenggara Barat	2,9	13,8	83,4	18.058
Nusa Tenggara Timur	1,3	5,6	93,1	19.115
Kalimantan Barat	3,0	12,6	84,4	18.073
Kalimantan Tengah	3,6	16,0	80,3	9.615
Kalimantan Selatan	1,9	12,1	86,0	15.077
Kalimantan Timur	1,7	10,4	87,9	13.195
Kalimantan Utara	3,3	13,9	82,9	2.547
Sulawesi Utara	2,7	17,9	79,3	9.055
Sulawesi Tengah	3,0	12,1	84,8	10.837
Sulawesi Selatan	2,1	15,9	81,9	31.703
Sulawesi Tenggara	2,7	11,6	85,7	9.464
Gorontalo	3,2	13,4	83,4	4.286
Sulawesi Barat	2,6	14,4	83,0	4.831
Maluku	6,3	15,1	78,6	6.334
Maluku Utara	4,1	14,2	81,7	4.404
Papua Barat	5,3	20,3	74,4	3.341
Papua	5,0	16,7	78,3	11.987
**INDONESIA**	**2,2**	**11,0**	**86,8**	**962.045**

[1]minuman bersoda atau berkarbonasi.

Tabel 12.2.16
Proporsi Kebiasaan Konsumsi *Soft Drink* atau Minuman Berkarbonasi
pada Penduduk Umur ≥3 Tahun menurut Karakteristik, Riskesdas 2018

Karakteristik	Kebiasaan Konsumsi *Soft Drink* atau Minuman Berkarbonasi[1]			N tertimbang
	≥1 kali per hari (%)	1 - 6 kali per minggu (%)	≤3 kali perbulan (%)	
**Kelompok Umur**				
3 – 4	1,8	5,5	92,7	36.608
5 – 9	2,9	9,5	87,7	92.746
10-14	3,2	14,7	82,1	89.506
15-19	3,4	20,4	76,2	83.422
20-24	3,0	18,3	78,7	82.143
25-29	2,4	14,1	83,5	81.351
30-34	2,2	11,7	86,1	78.281
35-39	1,8	10,2	88,0	79.035
40-44	1,7	9,2	89,1	72.432
45-49	1,6	7,8	90,6	67.116
50-54	1,4	6,6	92,0	57.477
55-59	1,4	4,9	93,7	47.552
60-64	1,2	3,9	94,9	35.659
65 +	1,1	2,6	96,3	58.717
**Jenis Kelamin**				
Laki-laki	2,7	14,7	82,7	482.729
Perempuan	1,8	7,2	91,0	479.316
**Pendidikan**				
Tidak sekolah	2,2	6,8	91,0	70.895
Tidak tamat SD	2,4	9,8	87,9	181.429
Tamat SD	2,4	10,6	87,1	215.967
Tamat SLTP	2,3	13,5	84,1	160.320
Tamat SLTA	2,2	13,4	84,5	210.746
Tamat D1/D2/D3 PT	1,6	10,0	88,3	64.093
**Pekerjaan**				
Tidak Bekerja	1,9	8,5	89,6	233.629
Sekolah	3,1	17,0	79,9	126.626
PNS/TNI/Polri/BUMN/BUMD	1,5	9,0	89,5	21.931
Pegawai swasta	2,2	14,4	83,4	75.781
Wiraswasta	2,1	11,7	86,2	105.489
Petani/Buruh tani	1,8	8,1	90,1	133.261
Nelayan	3,5	17,8	78,7	5.556
Buruh/sopir/pembantu ruta	2,4	14,3	83,3	75.590
Lainnya	2,1	9,6	88,3	40.644
**Tempat tinggal**				
Perkotaan	2,2	11,4	86,4	526.861
Perdesaan	2,3	10,4	87,2	435.184

[1]minuman bersoda atau berkarbonasi

Tabel 12.2.17

Proporsi Kebiasaan Konsumsi Minuman Berenergi pada Penduduk Umur ≥3 Tahun menurut Provinsi, Riskesdas 2018

Provinsi	Kebiasaan Konsumsi Minuman Berenergi[1]			N tertimbang
	≥1 kali per hari (%)	1 - 6 kali per minggu (%)	≤3 kali perbulan (%)	
Aceh	1,7	9,5	88,7	18.855
Sumatera Utara	1,4	5,4	93,3	51.977
Sumatera Barat	1,0	5,3	93,7	19.399
Riau	1,5	6,7	91,8	24.405
Jambi	1,3	9,7	89,0	12.936
Sumatera Selatan	1,8	5,9	92,3	30.341
Bengkulu	1,2	5,3	93,5	7.123
Lampung	1,3	4,1	94,6	30.300
Bangka Belitung	2,3	6,8	90,9	5.301
Kepulauan Riau	2,6	7,4	90,1	7.679
DKI Jakarta	1,4	4,1	94,5	38.124
Jawa Barat	1,7	6,8	91,6	176.728
Jawa Tengah	1,2	3,9	94,9	126.225
DI Yogyakarta	2,1	3,3	94,6	13.989
Jawa Timur	1,3	4,3	94,4	145.173
Banten	2,3	7,2	90,5	45.831
Bali	1,2	5,4	93,5	15.737
Nusa Tenggara Barat	2,4	8,9	88,7	18.058
Nusa Tenggara Timur	1,1	4,7	94,2	19.115
Kalimantan Barat	2,5	10,0	87,5	18.073
Kalimantan Tengah	3,0	10,3	86,7	9.615
Kalimantan Selatan	2,0	7,8	90,3	15.077
Kalimantan Timur	1,4	4,7	93,9	13.195
Kalimantan Utara	1,9	6,2	91,9	2.547
Sulawesi Utara	1,8	7,6	90,6	9.055
Sulawesi Tengah	2,7	8,2	89,1	10.837
Sulawesi Selatan	1,8	8,7	89,4	31.703
Sulawesi Tenggara	2,0	7,4	90,6	9.464
Gorontalo	3,5	14,2	82,3	4.286
Sulawesi Barat	2,4	9,3	88,3	4.831
Maluku	6,5	14,4	79,1	6.334
Maluku Utara	5,2	15,2	79,6	4.404
Papua Barat	5,3	14,0	80,7	3.341
Papua	5,9	13,3	80,9	11.987
**INDONESIA**	**1,7**	**6,1**	**92,1**	**962.045**

[1]Minuman yang mengandung kafein sebagai sumber energi.

Tabel 12.2.18
Proporsi Kebiasaan Konsumsi Minuman Berenergi pada Penduduk Umur ≥3 Tahun menurut Karakteristik, Riskesdas 2018

Karakteristik	kebiasaan konsumsi minuman berenergi[1] ≥1 kali per hari (%)	1 - 6 kali per minggu (%)	≤3 kali perbulan (%)	N tertimbang
**Kelompok Umur**				
3 – 4	1,0	1,8	97,3	36.608
5 – 9	1,2	2,8	95,9	92.746
10-14	1,5	4,9	93,6	89.506
15-19	2,4	9,8	87,8	83.422
20-24	2,5	10,8	86,6	82.143
25-29	2,3	9,3	88,4	81.351
30-34	2,0	8,2	89,8	78.281
35-39	1,8	7,1	91,1	79.035
40-44	1,8	6,4	91,8	72.432
45-49	1,6	5,6	92,8	67.116
50-54	1,3	4,6	94,0	57.477
55-59	1,2	3,7	95,1	47.552
60-64	1,0	2,9	96,1	35.659
65 +	0,9	1,9	97,2	58.717
**Jenis Kelamin**				
Laki-laki	2,4	9,7	87,9	482.729
Perempuan	1,1	2,5	96,4	479.316
**Pendidikan**				
Tidak sekolah	1,5	3,7	94,8	70.895
Tidak tamat SD	1,5	4,7	93,8	181.429
Tamat SD	1,9	6,7	91,4	215.967
Tamat SLTP	2,0	8,2	89,7	160.320
Tamat SLTA	1,8	7,7	90,5	210.746
Tamat D1/D2/D3 PT	1,3	4,6	94,1	64.093
**Pekerjaan**				
Tidak Bekerja	1,3	4,2	94,5	233.629
Sekolah	1,7	6,5	91,8	126.626
PNS/TNI/Polri/BUMN/BUMD	1,5	4,7	93,8	21.931
Pegawai swasta	1,6	7,6	90,8	75.781
Wiraswasta	2,0	7,6	90,4	105.489
Petani/Buruh tani	2,0	7,6	90,4	133.261
Nelayan	4,4	16,7	78,9	5.556
Buruh/sopir/pembantu ruta	2,9	11,2	85,9	75.590
Lainnya	1,8	6,1	92,1	40.644
**Tempat tinggal**				
Perkotaan	1,6	5,5	92,9	526.861
Perdesaan	1,8	6,9	91,3	435.184

[1]Minuman yang mengandung kafein sebagai sumber energi.

Tabel 12.2.19
Proporsi Kebiasaan Konsumsi Mi Instan/Makanan Instan Lainnya
pada Penduduk Umur >3 Tahun menurut Provinsi, Riskesdas 2018

Provinsi	Kebiasaan Konsumsi Mi Instan/Makanan Instan Lainnya[1]			N tertimbang
	≥1 kali per hari (%)	1 - 6 kali per minggu (%)	≤3 kali perbulan (%)	
Aceh	7,5	55,3	37,2	18.855
Sumatera Utara	4,6	48,7	46,7	51.977
Sumatera Barat	3,0	46,5	50,6	19.399
Riau	5,6	61,0	33,4	24.405
Jambi	5,6	61,0	33,4	12.936
Sumatera Selatan	14,7	63,8	21,5	30.341
Bengkulu	5,6	60,5	33,9	7.123
Lampung	5,4	60,4	34,2	30.300
Bangka Belitung	9,1	63,2	27,7	5.301
Kepulauan Riau	6,0	62,2	31,8	7.679
DKI Jakarta	6,3	60,3	33,4	38.124
Jawa Barat	10,8	65,1	24,0	176.728
Jawa Tengah	3,8	58,9	37,3	126.225
DI Yogyakarta	3,9	56,8	39,3	13.989
Jawa Timur	4,6	52,7	42,7	145.173
Banten	10,6	64,1	25,3	45.831
Bali	4,4	47,2	48,4	15.737
Nusa Tenggara Barat	8,5	61,3	30,3	18.058
Nusa Tenggara Timur	6,0	52,8	41,2	19.115
Kalimantan Barat	9,8	61,5	28,7	18.073
Kalimantan Tengah	13,1	61,1	25,8	9.615
Kalimantan Selatan	8,9	60,1	31,1	15.077
Kalimantan Timur	10,7	61,4	28,0	13.195
Kalimantan Utara	10,4	56,3	33,3	2.547
Sulawesi Utara	5,2	49,4	45,4	9.055
Sulawesi Tengah	10,4	59,3	30,2	10.837
Sulawesi Selatan	15,3	59,4	25,3	31.703
Sulawesi Tenggara	20,7	58,3	21,0	9.464
Gorontalo	4,7	55,2	40,1	4.286
Sulawesi Barat	17,3	56,1	26,7	4.831
Maluku	12,9	59,1	28,0	6.334
Maluku Utara	17,8	54,5	27,7	4.404
Papua Barat	15,0	52,6	32,4	3.341
Papua	13,1	51,8	35,1	11.987
**INDONESIA**	**7,8**	**58,5**	**33,8**	**962.045**

[1]Termasuk makanan instan adalah mi instan, bubur instan, dan makanan instan lainnya.

Tabel 12.2.20
Proporsi Kebiasaan Konsumsi Mi Instan/Makanan Instan Lainnya
pada Penduduk Umur ≥3 Tahun menurut Karakteristik, Riskesdas 2018

Karakteristik	Kebiasaan Konsumsi Mi Instan/Makanan Instan Lainnya[1]			N tertimbang
	≥1 kali per hari (%)	1 - 6 kali per minggu (%)	≤3 kali perbulan (%)	
**Kelompok Umur**				
3 – 4	7,6	58,7	33,7	36.608
5 – 9	10,4	65,4	24,2	92.746
10-14	11,6	68,3	20,2	89.506
15-19	11,2	67,6	21,2	83.422
20-24	9,5	64,8	25,7	82.143
25-29	8,2	63,4	28,3	81.351
30-34	7,6	62,2	30,2	78.281
35-39	7,3	59,9	32,8	79.035
40-44	6,7	57,7	35,6	72.432
45-49	5,8	53,9	40,4	67.116
50-54	4,7	48,5	46,8	57.477
55-59	4,2	44,0	51,8	47.552
60-64	3,6	39,9	56,5	35.659
65 +	3,1	35,7	61,1	58.717
**Jenis Kelamin**				
Laki-laki	8,4	59,8	31,8	482.729
Perempuan	7,2	57,1	35,7	479.316
**Pendidikan**				
Tidak sekolah	7,8	53,0	39,2	70.895
Tidak tamat SD	8,9	59,2	31,9	181.429
Tamat SD	8,4	58,2	33,4	215.967
Tamat SLTP	8,4	62,0	29,6	160.320
Tamat SLTA	6,8	59,1	34,2	210.746
Tamat D1/D2/D3 PT	4,1	50,9	45,0	64.093
**Pekerjaan**				
Tidak Bekerja	7,4	55,5	37,0	233.629
Sekolah	10,7	68,3	21,0	126.626
PNS/TNI/Polri/BUMN/BUMD	4,3	45,2	50,6	21.931
Pegawai swasta	6,1	59,9	34,0	75.781
Wiraswasta	6,4	55,0	38,6	105.489
Petani/Buruh tani	6,3	53,2	40,5	133.261
Nelayan	11,4	57,6	31,0	5.556
Buruh/sopir/pembantu ruta	8,3	60,6	31,2	75.590
Lainnya	7,0	56,0	37,0	40.644
**Tempat tinggal**				
Perkotaan	7,4	58,4	34,1	526.861
Perdesaan	8,2	58,5	33,3	435.184

[1]Termasuk makanan instan adalah mi instan, bubur instan, dan makanan instan lainnya

12.3 Konsumsi Buah dan Sayur

Perilaku penduduk dalam mengonsumsi buah dan sayur diukur berdasarkan frekuensi dan porsi konsumsi buah dan sayur pada ART umur 5 tahun ke atas, dengan menghitung jumlahhari konsumsi dalam seminggu dan jumlah porsi rata-rata dalam sehari. Instrumen yang digunakan untuk mengumpulkan data konsumsi sayur dan buah adalah instrumen *STEP wise* dari World Health Organization (WHO). Penduduk dikategorikan ‘cukup’ konsumsi sayur dan buah apabila mengonsumsi sayur dan/atau buah (kombinasi sayur dan buah) minimal 5 porsi per hari selama 7 hari dalam seminggu. Dikategorikan ’kurang’ apabila konsumsi sayur dan buah kurang dari ketentuan di atas.

$$Proporsi\ penduduk\ dengan\ tingkat\ konsumsi\ buah\ dan\ sayur\ kurang = \frac{ART\ umur\ \geq\ 5\ tahun\ dengan\ konsumsi\ buah\ dan/atau\ sayur\ kurang\ dari\ 5\ porsi\ sehari}{Semua\ ART\ umur\ \geq\ 5\ tahun}$$

Tabel 12.3.1

Proporsi Konsumsi Buah/Sayur per Hari dalam Seminggu pada Penduduk Umur ≥5 Tahun menurut Provinsi, Riskesdas 2018

Provinsi	Porsi Makan Buah/Sayur Per Hari Dalam Seminggu[1]								N Tertimbang
	Tidak konsumsi		1 - 2 Porsi		3 - 4 Porsi		≥5 Porsi		
	%	95% CI	%	95% CI	%	95% CI	%	95% CI	
Aceh	13,4	12,6-14,3	70,4	69,2-71,5	13,1	12,3-14,0	3,1	2,7-3,6	18.004
Sumatera Utara	10,4	9,7-11,1	68,0	66,7-69,3	17,5	16,4-18,7	4,1	3,6-4,6	49.477
Sumatera Barat	19,4	18,5-20,4	66,6	65,5-67,8	10,7	9,9-11,5	3,2	2,8-3,7	18.490
Riau	12,1	11,2-13	68,5	67,1-69,9	15,1	14,0-16,2	4,3	3,8-4,9	23.283
Jambi	10,8	9,7-12	75,9	74,1-77,5	10,8	9,7-12,1	2,5	2,0-3,1	12.413
Sumatera Selatan	10,3	9,3-11,4	76,8	75,4-78,1	9,9	9,1-10,7	3,0	2,6-3,5	29.050
Bengkulu	6,8	6,2-7,6	67,9	65,9-69,7	19,2	17,6-20,8	6,1	5,4-7,0	6.815
Lampung	6,5	5,9-7,1	64,1	62,4-65,8	23,2	21,7-24,7	6,2	5,5-7,0	29.057
Bangka Belitung	16,6	15,1-18,2	70,6	68,7-72,4	9,9	8,6-11,5	2,9	2,4-3,5	5.066
Kepulauan Riau	11,5	10,1-13	61,8	59,0-64,5	17,8	15,5-20,3	9,0	7,1-11,2	7.304
DKI Jakarta	13,2	12,2-14,4	64,3	62,9-65,7	17,6	16,4-18,8	4,9	4,4-5,5	36.625
Jawa Barat	14,2	13,7-14,8	73,2	72,5-73,9	10,6	10,1-11,2	1,9	1,7-2,2	169.581
Jawa Tengah	5,2	5,0-5,5	63,6	62,8-64,5	25,8	25,1-26,6	5,3	5,0-5,6	121.971
DI Yogyakarta	6,2	5,6-6,9	57,5	55,7-59,3	27,0	25,6-28,5	9,2	8,3-10,2	13.526
Jawa Timur	8,6	8,2-9,0	60,8	60,0-61,5	24,6	23,9-25,2	6,1	5,7-6,4	140.536
Banten	17,6	16,5-18,8	66,1	64,8-67,3	13,3	12,3-14,3	3,1	2,6-3,6	43.817
Bali	8,1	7,4-8,8	63,8	62,2-65,3	21,9	20,6-23,2	6,3	5,6-7,0	15.200
Nusa Tenggara Barat	7,1	6,4-7,7	66,6	64,8-68,4	22,0	20,5-23,5	4,3	3,7-5,0	17.267
Nusa Tenggara Timur	4,1	3,7-4,6	56,1	54,5-57,6	32,2	30,8-33,6	7,6	6,9-8,4	18.119
Kalimantan Barat	9,0	8,3-9,8	63,2	61,6-64,7	20,0	18,8-21,4	7,8	6,9-8,7	17.266
Kalimantan Tengah	11,6	10,6-12,7	71,3	69,6-72,9	13,2	12,0-14,5	3,9	3,3-4,6	9.190
Kalimantan Selatan	19,8	18,7-20,8	69,5	68,2-70,7	8,9	8,0-9,8	1,9	1,6-2,3	14.481
Kalimantan Timur	11,5	10,4-12,7	65,6	63,8-67,4	18,5	16,9-20,1	4,4	3,8-5,2	12.611
Kalimantan Utara	9,5	8,3-10,9	58,1	55,2-61,0	24,9	22,9-27,0	7,5	6,1-9,1	2.426
Sulawesi Utara	10,0	9,3-10,9	70,8	69,6-72,1	14,7	13,8-15,7	4,4	3,9-4,9	8.720
Sulawesi Tengah	9,8	9,0-10,6	62,8	61,3-64,3	21,2	20,0-22,4	6,3	5,7-6,9	10.395
Sulawesi Selatan	11,8	11,1-12,5	68,8	67,9-69,8	14,8	14,1-15,5	4,6	4,2-5,1	30.429
Sulawesi Tenggara	11,1	10,0-12,3	65,6	64,0-67,1	16,9	15,8-18,2	6,3	5,3-7,6	9.004
Gorontalo	17,1	15,6-18,8	62,5	60,5-64,3	14,2	12,8-15,7	6,2	5,3-7,3	4.103
Sulawesi Barat	10,1	9,0-11,3	72,0	70,2-73,8	14,4	13,0-16,0	3,5	2,8-4,2	4.614
Maluku	8,8	7,6-10,2	62,2	59,3-64,9	21,1	19,0-23,3	8,0	6,7-9,6	6.025
Maluku Utara	15,7	14,1-17,5	63,1	61,1-65,1	14,2	13,0-15,4	7,0	5,8-8,3	4.190
Papua Barat	4,8	3,9-5,8	56,2	53,6-58,8	28,7	26,7-30,8	10,4	8,9-12,0	3.175
Papua	9,7	8,1-11,4	58,6	56,1-61,0	25,6	23,6-27,6	6,2	5,5-7,0	11.438
**INDONESIA**	10,7	10,5-10,9	66,5	66,2-66,7	18,3	18,1-18,5	4,6	4,5-4,7	923.670

[1] Rata-rata porsi buah/sayur yang dikonsumsi per hari

Tabel 12.3.2
Proporsi Konsumsi Buah/Sayur per Hari dalam Seminggu pada Penduduk Umur >5 Tahun menurut Karakteristik, Riskesdas 2018

Karakteristik	Porsi Makan Buah/Sayur Per Hari Dalam Seminggu[1]								N Tertimbang
	Tidak konsumsi		1 - 2 Porsi		3 - 4 Porsi		≥5 Porsi		
	%	95% CI	%	95% CI	%	95% CI	%	95% CI	
**Kelompok Umur**									
5-9	17,0	16,6-17,4	67,1	66,6-67,5	12,8	12,5-13,2	3,1	3,0-3,3	92.569
10-14	15,3	15,0-15,7	67,4	66,9-67,9	14,1	13,7-14,4	3,2	3,0-3,4	89.335
15-19	13,3	12,9-13,7	67,9	67,4-68,5	15,2	14,8-15,6	3,6	3,4-3,7	83.263
20-24	10,4	10,0-10,8	67,7	67,2-68,3	17,6	17,1-18,0	4,3	4,1-4,5	81.986
25-29	8,8	8,5-9,2	66,9	66,3-67,5	19,6	19,2-20,1	4,6	4,4-4,8	81.196
30-34	8,5	8,2-8,9	67,1	66,5-67,6	19,4	18,9-19,8	5,0	4,8-5,3	78.132
35-39	7,9	7,6-8,3	66,5	65,9-67,0	20,4	19,9-20,8	5,2	5,0-5,5	78.884
40-44	7,9	7,6-8,2	66,3	65,7-66,9	20,5	20,0-20,9	5,3	5,1-5,6	72.293
45-49	7,7	7,3-8,0	65,3	64,7-65,8	21,5	21,1-22,0	5,5	5,3-5,8	66.988
50-54	7,8	7,4-8,1	64,5	63,9-65,1	21,9	21,4-22,4	5,9	5,6-6,2	57.367
55-59	8,5	8,2-8,9	64,1	63,4-64,7	21,6	21,0-22,2	5,8	5,5-6,1	47.461
60-64	9,6	9,1-10,1	65,2	64,4-65,9	20,2	19,6-20,8	5,1	4,8-5,4	35.591
65 +	11,7	11,3-12,1	65,1	64,5-65,7	18,7	18,2-19,2	4,5	4,3-4,8	58.605
**Jenis Kelamin**									
Laki-laki	11,8	11,6-12,0	66,5	66,2-66,8	17,4	17,1-17,6	4,3	4,2-4,4	462.970
Perempuan	9,6	9,4-9,7	66,4	66,1-66,7	19,2	18,9-19,5	4,8	4,7-5,0	460.700
**Pendidikan**									
Tidak sekolah	15,0	14,5-15,5	66,2	65,6-66,8	15,4	15,0-15,9	3,4	3,2-3,6	70.895
Tidak tamat SD	14,3	14,0-14,6	67,0	66,6-67,4	15,2	14,9-15,5	3,5	3,4-3,6	181.429
Tamat SD	11,1	10,9-11,4	67,5	67,1-67,9	17,5	17,1-17,8	3,9	3,7-4,0	215.967
Tamat SLTP	9,6	9,4-9,9	67,2	66,7-67,6	18,8	18,4-19,1	4,4	4,2-4,6	160.320
Tamat SLTA	7,8	7,5-8,0	66,3	65,9-66,7	20,4	20,1-20,8	5,5	5,4-5,7	210.746
Tamat D1/D2/D3 PT	4,4	4,1-4,7	60,5	59,8-61,2	26,3	25,7-26,9	8,8	8,4-9,2	64.093
**Pekerjaan**									
Tidak Bekerja	10,5	10,3-10,8	66,7	66,3-67,1	18,3	18,0-18,6	4,5	4,3-4,6	233.629
Sekolah	13,7	13,4-14,1	67,7	67,2-68,1	15,0	14,7-15,4	3,5	3,4-3,7	126.626
PNS/TNI/Polri/BUMN/BUMD	3,6	3,2-4,0	59,3	58,3-60,3	27,3	26,5-28,2	9,8	9,2-10,4	21.931
Pegawai swasta	7,6	7,2-8,0	65,6	64,9-66,3	21,2	20,6-21,9	5,6	5,3-5,9	75.781
Wiraswasta	7,7	7,4-8,0	65,3	64,8-65,9	21,2	20,7-21,6	5,8	5,5-6,0	105.489
Petani/Buruh tani	8,9	8,7-9,2	66,2	65,8-66,7	20,2	19,8-20,6	4,6	4,5-4,8	133.261
Nelayan	18,8	17,4-20,4	63,1	61,2-65,0	14,6	13,1-16,2	3,5	2,9-4,1	5.556
Buruh/sopir/pembantu ruta	11,5	11,0-11,9	68,2	67,6-68,9	16,8	16,3-17,4	3,5	3,3-3,7	75.590
Lainnya	8,5	8,1-9,0	66,5	65,7-67,2	19,7	19,0-20,4	5,3	5,0-5,7	40.644
**Tempat tinggal**									
Perkotaan	10,7	10,5-11,0	66,5	66,1-66,9	18,1	17,8-18,5	4,7	4,5-4,8	506.036
Perdesaan	10,6	10,4-10,9	66,5	66,1-66,8	18,5	18,2-18,8	4,4	4,3-4,6	417.634

[1] Rata-rata porsi buah/sayur yang dikonsumsi per hari

Tabel 12.3.3
Proporsi Kurang Makan Buah/Sayur dan Rerata Konsumsi Buah dan Sayur per Hari dalam Seminggu pada Penduduk Umur ≥5 Tahun menurut Provinsi, Riskesdas 2018

Provinsi	Kurang konsumsi sayur buah [1]		Rata-rata konsumsi sayur dan buah			Rata-rata konsumsi sayur			Rata-rata konsumsi buah			N Tertimbang
	%	95%CI	$\bar{x}$	min-max	sd	$\bar{x}$	min-max	sd	$\bar{x}$	min-max	sd	
Aceh	96,9	96,4-97,3	1,5	0-14	1,2	1,0	0-9	0,8	0,6	0-8	0,7	18.004
Sumatera Utara	95,9	95,4-96,4	1,8	0-16	1,3	1,2	0-9	1,0	0,5	0-9	0,7	49.477
Sumatera Barat	96,8	96,3-97,2	1,4	0-14	1,3	0,8	0-9	0,9	0,6	0-9	0,7	18.490
Riau	95,7	95,1-96,2	1,7	0-14	1,4	1,1	0-9	1,1	0,6	0-9	0,7	23.283
Jambi	97,5	96,9-98,0	1,5	0-14	1,2	1,0	0-8	0,9	0,5	0-9	0,6	12.413
Sumatera Selatan	97,0	96,5-97,4	1,5	0-15,5	1,3	1,1	0-9	1,0	0,5	0-9	0,6	29.050
Bengkulu	93,9	93,0-94,6	2,0	0-15,9	1,6	1,3	0-9	1,2	0,7	0-9	0,8	6.815
Lampung	93,8	93,0-94,5	2,0	0-15	1,5	1,5	0-9	1,1	0,6	0-9	0,7	29.057
Bangka Belitung	97,1	96,5-97,6	1,4	0-15	1,2	0,8	0-9	0,8	0,6	0-9	0,7	5.066
Kepulauan Riau	91,0	88,8-92,9	2,1	0-14	2,0	1,3	0-9	1,4	0,8	0-8	1,1	7.304
DKI Jakarta	95,1	94,5-95,6	1,8	0-13	1,4	1,1	0-9	1,0	0,7	0-9	0,8	36.625
Jawa Barat	98,1	97,8-98,3	1,4	0-15,4	1,1	0,9	0-9	0,8	0,6	0-9	0,6	169.581
Jawa Tengah	94,7	94,4-95,0	2,1	0-17	1,4	1,4	0-9	1,0	0,7	0-9	0,8	121.971
DI Yogyakarta	90,8	89,8-91,7	2,3	0-12	1,6	1,5	0-9	1,2	0,8	0-9	0,9	13.526
Jawa Timur	93,9	93,6-94,3	2,1	0-17	1,5	1,4	0-9	1,1	0,6	0-9	0,8	140.536
Banten	96,9	96,4-97,4	1,5	0-14	1,3	1,0	0-9	0,9	0,6	0-8	0,7	43.817
Bali	93,7	93,0-94,4	2,0	0-14	1,5	1,4	0-9	1,1	0,7	0-9	0,9	15.200
Nusa Tenggara Barat	95,7	95,0-96,3	1,9	0-14	1,3	1,4	0-8	1,0	0,6	0-9	0,7	17.267
Nusa Tenggara Timur	92,4	91,6-93,1	2,4	0-17	1,5	1,9	0-9	1,2	0,5	0-9	0,7	18.119
Kalimantan Barat	92,2	91,3-93,1	2,0	0-15	1,7	1,5	0-9	1,3	0,6	0-9	0,8	17.266
Kalimantan Tengah	96,1	95,4-96,7	1,7	0-14,4	1,3	1,2	0-9	1,0	0,5	0-9	0,7	9.190
Kalimantan Selatan	98,1	97,7-98,4	1,3	0-14	1,1	0,9	0-8	0,8	0,5	0-9	0,6	14.481
Kalimantan Timur	95,6	94,8-96,2	1,8	0-14	1,4	1,2	0-9	1,0	0,6	0-9	0,7	12.611
Kalimantan Utara	92,5	90,9-93,9	2,2	0-14	1,6	1,6	0-9	1,2	0,6	0-9	0,8	2.426
Sulawesi Utara	95,6	95,1-96,1	1,8	0-15	1,4	1,1	0-9	1,0	0,6	0-8	0,8	8.720
Sulawesi Tengah	93,7	93,1-94,3	2,0	0-14	1,5	1,3	0-9	1,1	0,7	0-9	0,9	10.395
Sulawesi Selatan	95,4	94,9-95,8	1,7	0-15	1,4	1,1	0-9	1,1	0,6	0-9	0,8	30.429
Sulawesi Tenggara	93,7	92,4-94,7	1,9	0-16	1,7	1,3	0-9	1,2	0,6	0-9	0,8	9.004
Gorontalo	93,8	92,7-94,7	1,7	0-14	1,6	1,2	0-9	1,4	0,5	0-9	0,7	4.103
Sulawesi Barat	96,5	95,8-97,2	1,7	0-14	1,3	1,2	0-9	1,0	0,5	0-8	0,7	4.614
Maluku	92,0	90,4-93,3	2,1	0-14	1,6	1,4	0-9	1,1	0,7	0-9	0,9	6.025
Maluku Utara	93,0	91,7-94,2	1,8	0-14	1,8	1,1	0-9	1,3	0,7	0-9	1,0	4.190
Papua Barat	89,6	88,0-91,1	2,5	0-15	1,8	1,8	0-9	1,3	0,7	0-9	1,0	3.175
Papua	93,8	93,0-94,5	2,1	0-15	1,5	1,6	0-9	1,1	0,5	0-9	0,7	11.438
**INDONESIA**	95,4	95,3-95,5	1,8	0-17	1,4	1,2	0-9	1,0	0,6	0-9	0,7	923.670

[1] porsi makan buah/sayur < 5 porsi per hari dalam seminggu (WHO)

Tabel 12.3.4
Proporsi Kurang Makan Buah/Sayur dan Rerata Konsumsi Buah dan Sayur per Hari dalam Seminggu pada Penduduk Umur ≥5 Tahun menurut karakteristik, Riskesdas 2018

Karakteristik	Kurang konsumsi sayur buah [1]		Rata-rata konsumsi sayur dan buah			Rata-rata konsumsi sayur			Rata-rata konsumsi buah			N Tertimbang
	%	95% CI	$\bar{x}$	min-max	sd	$\bar{x}$	min-max	sd	$\bar{x}$	min-max	sd	
**Kelompok Umur**												
5 – 9	96,9	96,7-97,0	1,5	0-15,5	1,3	0,9	0-9	1,0	0,6	0-9	0,7	92.569
10-14	96,8	96,6-97,0	1,6	0-16	1,3	1,0	0-9	1,0	0,5	0-9	0,7	89.335
15-19	96,4	96,3-96,6	1,6	0-15,4	1,3	1,1	0-9	1,0	0,6	0-9	0,7	83.263
20-24	95,7	95,5-95,9	1,8	0-15	1,4	1,2	0-9	1,0	0,6	0-9	0,7	81.986
25-29	95,4	95,2-95,6	1,9	0-15,9	1,4	1,3	0-9	1,1	0,6	0-9	0,7	81.196
30-34	95,0	94,7-95,2	1,9	0-17	1,4	1,3	0-9	1,1	0,6	0-9	0,8	78.132
35-39	94,8	94,5-95,0	1,9	0-16	1,4	1,3	0-9	1,1	0,6	0-9	0,8	78.884
40-44	94,7	94,4-94,9	2,0	0-17	1,5	1,3	0-9	1,1	0,6	0-9	0,8	72.293
45-49	94,5	94,2-94,7	2,0	0-14,2	1,5	1,3	0-9	1,1	0,7	0-9	0,8	66.988
50-54	94,1	93,8-94,4	2,0	0-16	1,5	1,3	0-9	1,1	0,7	0-9	0,8	57.367
55-59	94,2	93,9-94,5	2,0	0-15,5	1,5	1,3	0-9	1,1	0,7	0-9	0,8	47.461
60-64	94,9	94,6-95,2	1,9	0-15	1,5	1,3	0-9	1,1	0,6	0-9	0,8	35.591
65 +	95,5	95,2-95,7	1,8	0-17	1,4	1,2	0-9	1,0	0,6	0-9	0,7	58.605
**Jenis Kelamin**												
Laki-laki	95,7	95,6-95,8	1,8	0-17	1,4	1,2	0-9	1,0	0,6	0-9	0,7	462.970
Perempuan	95,2	95,0-95,3	1,9	0-17	1,4	1,2	0-9	1,1	0,6	0-9	0,7	460.700
**Pendidikan**												
Tidak sekolah	96,6	96,4-96,8	1,6	0-15,9	1,3	1,6	0-15,9	1,3	0,5	0-9	0,6	70.895
Tidak tamat SD	96,5	96,4-96,6	1,6	0-17	1,3	1,6	0-17	1,3	0,5	0-9	0,7	181.429
Tamat SD	96,1	96,0-96,3	1,7	0-16	1,4	1,7	0-16	1,4	0,5	0-9	0,7	215.967
Tamat SLTP	95,6	95,4-95,8	1,8	0-17	1,4	1,8	0-17	1,4	0,6	0-9	0,7	160.320
Tamat SLTA	94,5	94,3-94,6	2,0	0-17	1,4	2,0	0-17	1,4	0,7	0-9	0,8	210.746
Tamat D1/D2/D3 PT	91,2	90,8-91,6	2,3	0-17	1,6	2,3	0-17	1,6	1,0	0-9	0,9	64.093
**Pekerjaan**												
Tidak Bekerja	95,5	95,4-95,7	1,8	0-16	1,4	1,8	0-16	1,4	0,6	0-9	0,7	233.629
Sekolah	96,5	96,3-96,6	1,6	0-16	1,3	1,6	0-16	1,3	0,6	0-9	0,7	126.626
PNS/TNI/Polri/BUMN/BUMD	90,2	89,6-90,8	2,4	0-17	1,7	2,4	0-17	1,7	1,0	0-9	1,0	21.931
Pegawai swasta	94,4	94,1-94,7	2,0	0-15	1,4	2,0	0-15	1,4	0,7	0-9	0,8	75.781
Wiraswasta	94,2	94,0-94,5	2,0	0-17	1,5	2,0	0-17	1,5	0,7	0-9	0,8	105.489
Petani/Buruh tani	95,4	95,2-95,5	1,9	0-17	1,4	1,9	0-17	1,4	0,5	0-9	0,7	133.261
Nelayan	96,5	95,9-97,1	1,6	0-14	1,4	1,6	0-14	1,4	0,5	0-9	0,7	5.556
Buruh/sopir/pembantu ruta	96,5	96,3-96,7	1,7	0-15,4	1,3	1,7	0-15,4	1,3	0,5	0-9	0,6	75.590
Lainnya	94,7	94,3-95,0	1,9	0-16	1,5	1,9	0-16	1,5	0,7	0-9	0,8	40.644
**Tempat tinggal**												
Perkotaan	95,3	95,2-95,5	1,8	0-17	1,4	1,2	0-9	1,0	0,7	0-9	0,8	506.036
Perdesaan	95,6	95,4-95,7	1,8	0-17	1,4	1,3	0-9	1,1	0,5	0-9	0,7	417.634

[1]porsi makan buah/sayur < 5 porsi per hari dalam seminggu (WHO)

12.4 Kebiasaan Buang Air Besar

Informasi perilaku BAB dikumpulkan pada penduduk usia >3 tahun. Perilaku BAB yang dianggap benar adalah bila ART buang air besar di jamban.

$$Proporsi\ kebiasaan\ BAB\ dengan\ benar = \frac{ART\ umur\ \geq\ 3\ tahun\ dengan\ kebiasaan\ BAB\ di\ jamban}{Semua\ ART\ umur\ \geq\ 3\ tahun}$$

Tabel 12.4.1
Proporsi Perilaku Benar Buang Air Besar pada Penduduk Umur >3 Tahun menurut Provinsi, Riskesdas 2018

Provinsi	Perilaku Benar Buang Air Besar[1]		N Tertimbang
	%	95% CI	
Aceh	82,5	81,1-83,8	18.855
Sumatera Utara	87,3	86,3-88,2	51.977
Sumatera Barat	76,5	74,8-78,1	19.399
Riau	91,1	89,7-92,3	24.405
Jambi	88,4	86,7-89,8	12.936
Sumatera Selatan	82,6	80,9-84,1	30.341
Bengkulu	88,8	87,1-90,4	7.123
Lampung	88,3	87,0-89,4	30.300
Bangka Belitung	93,4	92,1-94,5	5.301
Kepulauan Riau	91,1	88,3-93,3	7.679
DKI Jakarta	97,6	96,4-98,4	38.124
Jawa Barat	90,9	90,1-91,6	176.728
Jawa Tengah	89,3	88,6-90,0	126.225
DI Yogyakarta	96,7	95,9-97,4	13.989
Jawa Timur	86,9	86,2-87,6	145.173
Banten	89,3	87,9-90,6	45.831
Bali	94,1	93,1-94,9	15.737
Nusa Tenggara Barat	87,0	85,3-88,5	18.058
Nusa Tenggara Timur	85,8	84,5-87,0	19.115
Kalimantan Barat	85,0	83,4-86,4	18.073
Kalimantan Tengah	75,8	73,1-78,2	9.615
Kalimantan Selatan	82,1	80,1-83,9	15.077
Kalimantan Timur	90,2	87,5-92,3	13.195
Kalimantan Utara	88,5	84,8-91,4	2.547
Sulawesi Utara	95,1	94,4-95,8	9.055
Sulawesi Tengah	80,3	78,3-82,2	10.837
Sulawesi Selatan	92,5	91,8-93,2	31.703
Sulawesi Tenggara	87,3	85,6-88,9	9.464
Gorontalo	86,2	83,7-88,4	4.286
Sulawesi Barat	82,7	79,8-85,3	4.831
Maluku	86,1	83,7-88,1	6.334
Maluku Utara	89,1	87,0-91,0	4.404
Papua Barat	91,0	88,9-92,8	3.341
Papua	55,8	53,3-58,3	11.987
**INDONESIA**	**88,2**	**87,9-88,5**	**962.045**

[1]Perilaku Benar Buang Air Besar adalah kebiasaan buang air besar di jamban.

Tabel 12.4.2

Proporsi Perilaku Benar Buang Air Besar pada Penduduk Umur $\geq$3 Tahun menurut Karakteristik, Riskesdas 2018

Karakteristik	Perilaku Benar Buang Air Besar[1]		N tertimbang
	%	(95%CI)	
**Kelompok Umur**			
3 – 4	87.5	87.0-87,9	36.608
5 – 9	87.0	86,7-87,4	92.746
10 – 14	87.3	86,9-87,6	89.506
15-19	88.6	88,2-88,9	83.422
20-24	89.6	89,2-90,0	82.143
25-29	89.4	89,0-89,8	81.351
30-34	88.7	88,3-89,0	78.281
35-39	88.5	88,1-88,9	79.035
40-44	88.6	88,2-89,0	72.432
45-49	88.6	88,2-89,0	67.116
50-54	88.7	88,3-89,1	57.477
55-59	87.7	87,3-88,2	47.552
60-64	87.3	86,8-87,8	35.659
65 +	86.3	85,8-86,8	58.717
**Jenis Kelamin**			
Laki-laki	88,0	87,8-88,3	482.729
Perempuan	88,4	88,1-88,6	479.316
**Pendidikan**			
Tidak sekolah	79,6	78,9-80,2	69.843
Tidak tamat SD	83,7	83,2-84,1	179.797
Tamat SD	84,7	84,3-85,1	213.903
Tamat SLTP	89,8	89,5-90,1	158.737
Tamat SLTA	94,8	94,6-95,0	208.642
Tamat D1/D2/D3 PT	97,7	97,5-97,9	62.864
**Pekerjaan**			
Tidak Bekerja	88,6	88,3-89,0	233.629
Sekolah	89,6	89,2-89,9	126.626
PNS/TNI/Polri/BUMN/BUMD	97,8	97,5-98,0	21.931
Pegawai swasta	96,3	96,0-96,6	75.781
Wiraswasta	93,5	93,2-93,8	105.489
Petani/Buruh tani	76,5	76,0-77,1	133.261
Nelayan	70,2	67,8-72,5	5.556
Buruh/sopir/pembantu ruta	88,7	88,2-89,3	75.590
Lainnya	90,4	89,9-90,9	40.644
**Tempat tinggal**			
Perkotaan	94,2	93,9-94,5	526.861
Perdesaan	80,9	80,5-81,3	435.184

[1]Perilaku Benar Buang Air Besar adalah kebiasaan buang air besar di jamban.

12.5 Perilaku Mencuci Tangan

Perilaku cuci tangan ditanyakan pada penduduk usia 10 tahun ke atas. Perilaku cuci tangan yang dianggap benar, jika penduduk melakukannya sebelum menyiapkan makanan, setiap kali tangan kotor (memegang uang, binatang dan berkebun), setelah buang air besar, setelah menceboki bayi/anak, setelah menggunakan pestisida/insektisida, sebelum menyusui bayi, dan sebelum makan dengan menggunakan sabun dan air mengalir.

$$Proporsi\ penduduk\ dengan\ perilaku\ cuci\ tangan\ yang\ benar$$
$$= \frac{ART\ umur\ \geq 10\ tahun\ dengan\ kebiasaan\ mencuci\ tangan\ memakai\ air\ bersih\ mengalir,\ dan\ memakai\ sabun}{Semua\ ART\ umur\ \geq 10\ tahun}$$

Tabel 12.5.1

Proporsi Perilaku Benar dalam Cuci Tangan pada Penduduk Umur >10 Tahun menurut Provinsi, Riskesdas 2018

Provinsi	Perilaku Benar dalam Cuci Tangan[1]		N tertimbang
	%	95% CI	
Aceh	31,6	30,1-33,1	15.622
Sumatera Utara	37,8	36,5-39,1	42.787
Sumatera Barat	37,9	36,4-39,5	16.176
Riau	39,9	38,1-41,7	20.198
Jambi	43,7	41,5-45,9	10.987
Sumatera Selatan	42,1	40,3-44,0	25.509
Bengkulu	40,2	37,8-42,6	5.978
Lampung	48,4	46,5-50,3	25.593
Bangka Belitung	56,2	53,9-58,4	4.489
Kepulauan Riau	58,3	55,2-61,3	6.374
DKI Jakarta	54,8	52,5-57,0	32.563
Jawa Barat	56,8	55,6-57,9	150.646
Jawa Tengah	53,6	52,8-54,4	109.680
DI Yogyakarta	52,3	50,4-54,2	12.242
Jawa Timur	54,3	53,455,2	127.176
Banten	46,9	45,0-48,8	38.535
Bali	67,4	65,8-68,9	13.705
Nusa Tenggara Barat	46,5	44,4-48,6	15.129
Nusa Tenggara Timur	20,4	19,2-21,7	15.531
Kalimantan Barat	46,5	44,4-48,6	15.135
Kalimantan Tengah	49,5	47,0-51,9	8.121
Kalimantan Selatan	51,7	49,8-53,6	12.710
Kalimantan Timur	58,3	56,0-60,5	11.131
Kalimantan Utara	64,1	61,3-66,8	2.133
Sulawesi Utara	48,5	46,4-50,5	7.795
Sulawesi Tengah	43,0	41,2-44,8	9.134
Sulawesi Selatan	50,1	48,8-51,5	26.719
Sulawesi Tenggara	42,3	39,9-44,7	7.750
Gorontalo	51,6	48,9-54,3	3.627
Sulawesi Barat	46,7	43,6-49,9	4.017
Maluku	35,5	32,9-38,1	5.182
Maluku Utara	31,8	29,5-34,2	3.604
Papua Barat	45,2	42,5-48,0	2.766
Papua	26,7	24,8-28,7	9.764
**INDONESIA**	**49,8**	**49,4-50,1**	**818.507**

[1]Cuci tangan dengan benar adalah cuci tangan pakai sabun dengan air mengalir sebelum menyiapkan makanan, setiapkali tangan kotor (memegang uang, binatang dan berkebun), setelah buang air besar, setelah menceboki bayi/anak, setelah menggunakan pestisida/insektisida, sebelum menyusui bayi, dan sebelum makan.

Tabel 12.5.2

Proporsi Perilaku Benar dalam Cuci Tangan pada Penduduk Umur ≥10 Tahun menurut Karakteristik, Riskesdas 2018

Karakteristik	Perilaku Benar dalam Cuci Tangan[1]		N tertimbang
	%	95% CI	
**Kelompok Umur**			
10 – 14	43,0	42,4-43,5	87.981
15-19	47,2	46,6-47,8	82.001
20-24	50,4	49,7-51,1	80.744
25-29	48,9	48,3-49,6	79.965
30-34	50,1	49,4-50,7	76.948
35-39	51,2	50,6-51,8	77.689
40-44	53,2	52,6-53,8	71.198
45-49	54,0	53,3-54,6	65.973
50-54	53,7	53,0-54,3	56.498
55-59	53,7	53,0-54,4	46.742
60-64	50,9	50,151,7	35.052
65 +	45,2	44,6-45,9	87.981
**Jenis Kelamin**			
Laki-laki	48,0	47,6-48,4	409.223
Perempuan	51,6	51,2-51,9	409.284
**Pendidikan**			
Tidak sekolah	39,4	38,6-40,2	46.843
Tidak tamat SD	43,8	43,3-44,3	129.354
Tamat SD	46,9	46,4-47,3	213.043
Tamat SLTP	49,2	48,7-49,7	158.149
Tamat SLTA	54,9	54,4-55,4	207.893
Tamat D1/D2/D3 PT	64,1	63,4-64,9	63.225
**Pekerjaan**			
Tidak Bekerja	49,1	48,7-49,6	233.629
Sekolah	46,0	45,4-46,5	126.626
PNS/TNI/Polri/BUMN/BUMD	65,4	64,5-66,4	21.931
Pegawai swasta	59,8	59,0-60,6	75.781
Wiraswasta	55,7	55,2-56,3	105.489
Petani/Buruh tani	41,1	40,6-41,7	133.261
Nelayan	37,7	35,7-39,8	5.556
Buruh/sopir/pembantu ruta	49,5	48,7-50,3	75.590
Lainnya	53,4	52,5-54,3	40.644
**Tempat tinggal**			
Perkotaan	55,5	55,0-56,1	450.011
Perdesaan	42,7	42,3-43,2	368.496

[1]Cuci tangan dengan benar adalah cuci tangan pakai sabun dengan air mengalir sebelum menyiapkan makanan, setiapkali tangan kotor (memegang uang, binatang dan berkebun), setelah buang air besar, setelah menceboki bayi/anak, setelah menggunakan pestisida/insektisida, sebelum menyusui bayi, dan sebelum makan.

12.6 Konsumsi Rokok dan Tembakau

Perilaku merokok dan konsumsi tembakau ditanyakan pada ART umur >10 tahun. Pada bagian ini akan menyajikan indikator perilaku merokok dan perokok sekunder/pasif. Indikator terkait rokok dan tembakau termasuk sebagai berikut: perilaku merokok, umur pertama merokok, umur mulai berhenti merokok (bagi mantan perokok), jenis rokok, rata-rata batang rokok yang dikonsumsi, dan perilaku mengunyah tembakau. Perilaku konsumsi tembakau termasuk kebiasaan konsumsi rokok hisap, rokok elektronik, shisha dan tembakau kunyah. Sedangkan perokok pasif mencakup perilaku merokok di dalam rumah atau dalam gedung bagi ART yang masih merokok dan berada di dekat orang yang merokok bagi ART yang tidak merokok.

Perilaku merokok saat ini mencakup kebiasaan merokok setiap hari atau kadang-kadang dalam sebulan terakhir. Perilaku merokok di masa lalu mencakup merokok setiap hari atau kadang-kadang di masa lalu. Tidak pernah merokok yaitu termasuk tidak pernah mencoba merokok sampai dengan saat pengumpulan data**.**

Indikator terkait rokok dan tembakau dihitung dengan formula sebagai berikut:

$$Proporsi\ merokok\ pada\ umur\ 10\ s.d\ 18\ tahun = \frac{ART\ \text{umur}10\ -\ 18\ tahun\ yang\ merokok\ dalam\ 1\ bulan\ terakhir}{Semua\ ART\ \text{umur}\ 10\ -\ 18\ tahun}$$

$$Proporsi\ merokok\ pada\ umur \geq\ 15\ tahun = \frac{ART\ \text{umur} \geq\ 15\ tahun\ yang\ merokok\ dalam\ 1\ bulan\ terakhir)}{Semua\ ART\ \text{umur} \geq\ 15\ tahun}$$

$$Proporsi\ merokok\ pada\ umur \geq\ 10\ tahun = \frac{ART\ \text{umur} \geq\ 10\ tahun\ yang\ merokok\ dalam\ 1\ bulan\ terakhir}{Semua\ ART\ \text{umur} \geq\ 10\ tahun}$$

$$Proporsi\ konsumsi\ tembakau\ umur \geq 15\ tahun = \frac{ART\ \text{umur} \geq 15\ tahun\ yang\ menghisap\ rokok\ dan\ atau\ mengunyah\ tembakau\ dalam\ satu\ bulan\ terakhir\ (tiap\ hari\ atau\ kadang - kadang)}{Semua\ ART\ \text{umur} \geq\ 15\ tahun}$$

$$Proporsi\ mantan\ perokok = \frac{ART\ umur \geq 10\ yang\ pernah\ merokok\ setiap\ hari\ atau\ kadang-kadang}{ART\ umur \geq 10\ tahun}$$

$$Proporsi\ kebiasaan\ menghisap\ rokok\ kretek = \frac{ART\ \text{umur} \geq 10\ tahun\ yang\ mempunyai\ kebiasaan\ merokok\ kretek\ (tiap\ hari\ dan\ kadang - kadang)}{Semua\ ART\ \text{umur} \geq\ 10\ tahun}$$

$$Proporsi\ kebiasaan\ menghisap\ rokok\ putih = \frac{ART\ umur \geq 10\ tahun\ yang\ mempunyai\ kebiasaan\ merokok\ putih\ (tiap\ hari\ dan\ kadang - kadang)}{Semua\ ART\ umur \geq\ 10\ tahun}$$

$$Proporsi\ kebiasaan\ menghisap\ rokok\ elektronik = \frac{ART\ \text{umur} \geq 10\ tahun\ yang\ mempunyai\ kebiasaan\ merokok\ elektronik}{Semua\ ART\ \text{umur} \geq\ 10\ tahun}$$

$$Proporsi\ konsumsi\ tembakau\ kunyah = \frac{ART\ umur \geq 10\ tahun\ yang\ mempunyai\ kebiasaan\ konsumsi\ tembakau\ kunyah\ (tiap\ hari\ dan\ kadang - kadang)}{Semua\ ART\ umur \geq\ 10\ tahun}$$

$$Proporsi\ kebiasaan\ merokok\ di\ dalam\ gedung = \frac{ART\ umur \geq\ 10\ tahun\ yang\ mempunyai\ kebiasaan\ merokok\ (tiap\ hari\ dan\ kadang - kadang)\ di\ dalam\ gedung}{Semua\ ART\ umur \geq\ 10\ tahun}$$

Tabel 12.6.1
Proporsi Merokok pada Penduduk Umur ≥10 Tahun menurut Provinsi, Riskesdas 2018

Provinsi	Perokok saat ini				Tidak merokok				N Tertimbang
	Perokok setiap hari		Perokok kadang-kadang		Mantan perokok		Bukan perokok		
	%	95%CI	%	95%CI	%	95%CI	%	95%CI	
Aceh	24,0	23,4-24,6	4,1	3,7-4,4	2,3	2,0-2,5	69,7	69,1-70,3	15.622
Sumatera Utara	22,4	21,8-23,0	4,8	4,5-5,1	4,9	4,6-5,3	67,9	67,3-68,5	42.787
Sumatera Barat	26,9	26,3-27,6	3,9	3,6-4,3	6,0	5,6-6,5	63,1	62,4-63,8	16.176
Riau	25,3	24,5-26,0	3,4	3,2-3,8	6,0	5,5-6,5	65,3	64,5-66,0	20.198
Jambi	21,5	20,7-22,3	3,8	3,4-4,2	2,5	2,2-2,8	72,3	71,4-73,1	10.987
Sumatera Selatan	25,3	24,6-26,0	4,1	3,7-4,4	3,0	2,7-3,3	67,7	66,9-68,4	25.509
Bengkulu	27,8	26,9-28,7	3,6	3,2-4,0	2,8	2,4-3,2	65,8	64,9-66,7	5.978
Lampung	28,1	27,5-28,8	3,6	3,3-3,9	4,5	4,2-4,9	63,8	63,1-64,4	25.593
Bangka Belitung	24,8	23,7-25,9	3,8	3,4-4,4	4,5	4,0-5,1	66,9	65,6-68,0	4.489
Kepulauan Riau	22,3	20,7-23,9	4,2	3,5-5,1	5,3	4,5-6,3	68,2	66,4-69,4	6.374
DKI Jakarta	22,9	22,1-23,7	5,4	4,9-5,9	10,7	9,9-11,4	61,1	60,0-62,1	32.563
Jawa Barat	27,1	26,6-27,6	4,9	4,7-5,2	5,1	4,8-5,4	62,8	62,3-63,4	150.646
Jawa Tengah	23,2	22,8-23,6	4,8	4,6-5,0	6,5	6,2-6,7	65,6	65,2-66,0	109.680
DI Yogyakarta	19,5	18,5-20,5	4,4	3,9-5,0	14,4	13,5-15,5	61,6	60,6-62,7	12.242
Jawa Timur	23,9	23,5-24,3	4,2	4,0-4,4	4,2	4,0-4,4	67,7	67,3-68,1	127.176
Banten	26,8	25,9-27,6	4,7	4,3-5,1	5,6	5,0-6,1	63,0	62,1-63,9	38.535
Bali	18,9	18,1-19,7	4,6	4,2-5,0	7,3	6,7-7,9	69,2	68,3-70,2	13.705
Nusa Tenggara Barat	26,3	25,5-27,1	4,0	3,6-4,3	3,2	2,9-3,6	66,6	65,8-67,3	15.129
Nusa Tenggara Timur	19,0	18,5-19,6	7,3	6,9-7,7	2,8	2,5-3,1	70,9	70,4-71,5	15.531
Kalimantan Barat	23,5	22,7-24,2	4,1	3,8-4,5	3,2	2,9-3,6	69,2	68,4-70,0	15.135
Kalimantan Tengah	24,3	23,4-25,3	4,7	4,2-5,2	3,0	2,7-3,4	67,9	66,9-69,0	8.121
Kalimantan Selatan	20,6	19,9-21,3	3,9	3,5-4,2	4,6	4,25-5,0	71,0	70,2-71,7	12.710
Kalimantan Timur	21,9	20,7-23,1	4,0	3,6-4,5	7,8	7,1-8,7	66,2	64,8-67,6	11.131
Kalimantan Utara	22,5	21,0-24,1	3,8	3,2-4,5	7,1	6,1-8,3	66,6	64,9-68,2	2.133
Sulawesi Utara	23,5	22,7-24,3	6,2	5,7-6,6	5,7	5,2-6,2	64,7	63,7-65,7	7.795
Sulawesi Tengah	26,1	25,3-26,8	5,2	4,7-5,6	6,0	5,5-6,6	62,7	61,8-63,7	9.134
Sulawesi Selatan	22,0	21,5-22,6	3,9	3,6-4,2	5,8	5,5-6,2	68,3	67,7-68,9	26.719
Sulawesi Tenggara	22,3	21,4-23,1	3,7	3,3-4,1	4,0	3,6-4,5	70,0	69,1-70,9	7.750
Gorontalo	27,4	26,3-28,5	4,4	3,9-5,0	4,4	3,9-5,1	63,8	62,6-64,9	3.627
Sulawesi Barat	21,8	20,8-22,8	3,5	3,0-4,0	2,9	2,3-3,5	71,9	70,7-73,0	4.017
Maluku	22,1	20,9-23,2	6,2	5,5-6,9	2,5	2,1-3,0	69,3	68,1-70,4	5.182
Maluku Utara	23,9	22,9-24,9	6,0	5,3-6,9	3,5	3,1-4,0	66,5	65,4-67,6	3.604
Papua Barat	22,1	20,8-23,5	5,8	5,1-6,6	3,9	3,3-4,5	68,3	66,7-69,8	2.766
Papua	18,8	17,8-19,9	6,7	6,1-7,3	2,2	1,9-2,6	72,2	71,1-73,4	9.764
**INDONESIA**	**24,3**	**24,1-24,4**	**4,6**	**4,5-4,6**	**5,3**	**5,2-5,3**	**65,9**	**65,8-66,1**	**818. 507**

Tabel 12.6.2
Proporsi Merokok pada Penduduk Umur ≥10 Tahun menurut Karakteristik, Riskesdas 2018

Karakteristik	Perokok saat ini (%,95%CI)		Tidak merokok (%,95%CI)		N Tertimbang
	Perokok setiap hari	Perokok kadang-kadang	Mantan perokok	Bukan perokok	
**Kelompok Umur**					
10-14	0,7 (0,6-0,8)	1,4 (1,3-1,5)	2,0 (1,9-2,1)	95,9 (95,7-96,1)	87.981
15-19	12,7 (12,3-13,1)	6,9 (6,6-7,2)	4,0 (3,8-4,2)	76,4 (75,9-76,8)	82.001
20-24	27,3 (26,8-27,8)	5,9 (5,6-6,2)	3,5 (3,3-3,8)	63,3 (62,7-63,8)	80.744
25-29	30,4 (29,9-30,9)	4,8 (4,5-5,0)	3,8 (3,6-4,1)	61,0 (60,5-61,5)	79.965
30-34	32,2 (31,6-32,7)	4,5 (4,2-4,7)	4,1 (3,9-4,4)	59,3 (58,7-59,8)	76.948
35-39	32,0 (31,5-32,5)	4,5 (4,3-4,7)	4,5 (4,3-4,8)	59,0 (58,5-59,5)	77.689
40-44	31,2 (30,8-31,7)	4,6 (4,3-4,8)	5,0 (4,8-5,3)	59,2 (58,7-59,7)	71.198
45-49	29,6 (29,1-30,1)	4,9 (4,6-5,1)	6,0 (5,7-6,2)	59,6 (59,1-60,1)	65.973
50-54	28,7 (28,2-29,3)	4,5 (4,3-4,8)	6,8 (6,5-7,1)	60,0 (59,4-60,5)	56.498
55-59	27,8 (27,3-28,4)	4,5 (4,2-4,7)	8,1 (7,8-8,5)	59,6 (59,0-60,2)	46.742
60-64	25,7 (25,0-26,3)	4,4 (4,1-4,7)	10,0 (9,5-10,5)	59,9 (59,2-60,6)	35.052
65 +	20,1 (19,6-20,6)	4,2 (4,0-4,4)	11,6 (11,3-12,0)	64,1 (63,6-64,6)	57.717
**Kelompok Umur (Khusus)**					
10-18	5,3 (5,1-5,5)	3,8 (3,6-3,9)	2,9 (2,8-3,0)	88,0 (87,7-88,2)	176.556
15+	24,3 (24,1-24,4)	4,6 (4,5-4,6)	5,3 (5,2-5,3)	65,9 (65,8-66,1)	818.507
10+	24,3 (24,1-24,4)	4,6 (4,5-4,6)	5,3 (5,2-5,3)	65,9 (65,8-66,1)	799.747
**Jenis Kelamin**					
Laki-laki	47,3 (47,1-47,6)	8,5 (8,3-8,6)	9,2 (9,0-9,3)	35,0 (34,7-35,3)	409.223
Perempuan	1,2 (1,1-1,2)	0,7 (0,6-0,7)	1,3 (1,3-1,4)	96,8 (96,7-96,9)	409.284
**Pendidikan**					
Tidak sekolah	19,1 (18,6-19,6)	3,4 (3,2-3,7)	4,5 (4,2-4,7)	73,0 (72,5-73,6)	46.843
Tidak tamat SD	19,3 (19,0-19,6)	3,2 (3,0-3,3)	4,1 (3,9-4,3)	73,4 (73,1-73,8)	129.354
Tamat SD	24,8 (24,5-25,0)	4,2 (4,1-4,3)	4,6 (4,5-4,8)	66,4 (66,1-66,7)	213.043
Tamat SLTP	26,2 (25,9-26,6)	5,2 (5,0-5,4)	4,7 (4,5-4,8)	63,9 (63,5-64,3)	158.149
Tamat SLTA	28,7 (28,4-29,0)	5,8 (5,6-5,9)	6,3 (6,1-6,5)	59,2 (58,9-59,6)	207.893
Tamat D1/D2/D3 PT	17,0 (16,5-17,5)	4,1 (3,9-4,4)	8,3 (8,0-8,7)	70,5 (70,0-71,1)	63.225
**Pekerjaan**					
Tidak Bekerja	8,2 (8,0-8,4)	2,6 (2,5-2,7)	3,9 (3,8-4,0)	85,3 (85,0-85,5)	233.629
Sekolah	4,7 (4,5-4,9)	3,9 (3,8-4,1)	3,3 (3,2-3,5)	88,0 (87,7-88,3)	126.626
PNS/TNI/Polri/BUMN/BUMD	23,3 (22,5-24,0)	4,4 (4,1-4,8)	9,2 (8,7-9,8)	63,1 (62,2-63,9)	21.931
Pegawai swasta	32,4 (31,7-33,0)	6,3 (6,0-6,7)	7,5 (7,1-7,8)	53,8 (53,2-54,5)	75.781
Wiraswasta	36,0 (35,6-36,5)	5,5 (5,3-5,7)	7,0 (6,8-7,3)	51,5 (51,0-51,9)	105.489
Nelayan	63,7 (61,9-65,4)	6,7 (5,8-7,6)	5,6 (4,9-6,3)	24,1 (22,6-25,7)	5.556
Petani/Buruh tani	40,4 (40,1-40,8)	5,7 (5,5-5,8)	5,5 (5,3-5,6)	48,4 (48,1-48,8)	133.261
Lainnya	41,6 (41,1-42,1)	5,9 (5,7-6,1)	6,1 (5,8-6,3)	46,4 (46,0-46,9)	116.233
**Tempat tinggal**					
Perkotaan	23,0 (22,8-23,2)	4,6 (4,5-4,8)	6,2 (6,1-6,4)	66,2 (65,9-66,4)	450.011
Perdesaan	25,8 (25,6-26,0)	4,5 (4,4-4,6)	4,1 (4,0-4,2)	65,6 (65,4-65,8)	368.496

Tabel 12.6.3
Rata-rata Jumlah Batang Rokok (Kretek, Putih, Linting) Perhari dan Perminggu yang Dihisap Penduduk Umur >10 Tahun menurut Provinsi, Riskesdas 2018

Provinsi	Jumlah Batang perhari		Jumlah Batang perminggu	
	Rata-rata (+ SD)	N Tertimbang	Rata-rata (+ SD)	N Tertimbang
Aceh	16,35 (+ 9,44)	3.643	10.60 (+ 9.35)	704
Sumatera Utara	14,73 (+ 8,59)	9.571	9.59 (+ 9.42)	3.020
Sumatera Barat	16,67(+ 9,08)	4.365	9.55 (+ 10.14)	1.109
Riau	16,80 (+ 9,23)	5.102	9.47 (+ 8,26)	1.332
Jambi	16,52 (+ 7,82)	2.346	13.59 (+ 10.75)	449
Sumatera Selatan	13,53 (+ 7,02)	6.300	9.39 (+ 7.13)	1.266
Bengkulu	15,22 (+ 8,22)	1.604	9.92 (+ 9.25)	270
Lampung	12,47 (+ 6,85)	6.958	9.18 (+ 10,26)	1.555
Bangka Belitung	18,32 (+ 9,83)	1.095	11.18 (+ 11.29)	269
Kepulauan Riau	16,97 (+ 9,50)	1.411	9.77 (+11.31)	446
DKI Jakarta	12,66 (+ 9,23)	7.828	6.94 (+8.46)	3.752
Jawa Barat	11,25 (+ 7,27)	40.392	7.53 (+9.31)	10.869
Jawa Tengah	10,94 (+ 7,08)	25.283	8.28 (+9.75)	9.252
DI Yogyakarta	10,88 (+ 7,53)	2.751	7.65 (+11.10)	1.539
Jawa Timur	11,90 (+ 7,63)	29.862	9.04 (+9.89)	7.750
Banten	13,19 (+ 7,86)	10.238	8.86 (+9.51)	2.820
Bali	12,32 (+ 8,00)	2.895	8.85 (+10.12)	944
Nusa Tenggara Barat	11,71 (+ 7,82)	3.822	6.98 (+7.82)	813
Nusa Tenggara Timur	10,91 (+ 7,35)	2.962	7.00 (+6.37)	1.173
Kalimantan Barat	15,29 (+ 8,49)	3.493	10.19 (+11.24)	770
Kalimantan Tengah	16,11 (+ 8,29)	1.907	9.96 (+10.18)	479
Kalimantan Selatan	16,72 (+ 9,03)	2.580	10.30 (+9.94)	779
Kalimantan Timur	15,91(+ 9,48)	2.499	11.41 (+13.34)	932
Kalimantan Utara	15,64 (+ 9,12)	488	9.78 (+10.73)	163
Sulawesi Utara	13,41 (+ 8,09)	1.870	10.05 (+9.70)	650
Sulawesi Tengah	14,72 (+ 9,12)	2.373	7.77 (+8.42)	746
Sulawesi Selatan	14,61 (+ 8,82)	5.984	9.01 (+11.11)	1.772
Sulawesi Tenggara	14,86 (+ 8,50)	1.728	9.33 (+11.93)	407
Gorontalo	13,01 (+ 8,28)	965	7.88 (+10.16)	240
Sulawesi Barat	15,57 (+ 8,30)	852	11.59 (+10.10)	183
Maluku	12,14 (+ 9,43)	1.110	7.69 (+7.19)	352
Maluku Utara	13,32 (+ 9,33)	863	6.74 (+7.31)	243
Papua Barat	14,66 (+ 9,57)	610	10.35 (+8.66)	195
Papua	13,63 (+ 8,12)	1.782	10.72 (+9.41)	696
**INDONESIA**	**12,8 (+ 8,14)**	**197,532**	**8.6 (+9.67)**	**57.940**

Tabel 12.6.4
Rata-rata Jumlah Batang Rokok (Kretek, Putih, Linting) Perhari dan Perminggu yang Dihisap Penduduk Umur >10 Tahun menurut karakteristik, Riskesdas 2018

Karakteristik	Jumlah Batang perhari		Jumlah Batang perminggu	
	Rata-rata (+ SD)	N Tertimbang	Rata-rata (+ SD)	N Tertimbang
**Kelompok Umur**				
10-14	6,75 (+ 6,27)	549	3,54 (+ 5,00)	2.637
15-19	9,26 (+ 6,52)	9.721	6,26 (+ 7,19)	7.885
20-24	11,93 (+ 7,34)	20.730	8,45 (+ 9,82)	6.385
25-29	12,90 (+ 7,58)	23.262	8,60 (+ 9,02)	5.276
30-34	13,27 (+ 7,77)	23.856	9,84 (+ 10,83)	4.893
35-39	13,71 (+ 8,16)	24.169	9,66 (+ 10,32)	5.051
40-44	13,75 (+ 8,43)	21,784	9,45 (+ 10,02)	4.880
45-49	13,78 (+ 8,68)	19.491	9,18 (+ 9,46)	4.960
50-54	13,43 (+ 8,63)	16.508	9,07 (+ 9,76)	4.236
55-59	13,04 (+ 8,59)	13.607	9,60 (+ 10,51)	3.711
60-64	12,43 (+ 8,32)	9.931	9,93 (+ 10,95)	2.968
65 +	11,02 (+ 8,21)	13.924	9,52 (+ 10,36)	5.057
**Kelompok umur khusus**				
10-18	8,65 (+ 6,38)	7.916	5,29 (+6,44)	10.267
15 +	12,84 (+ 8,14)	196.837	9,32 (+ 9,86)	54.859
10+	12,82 (+ 8,14)	197.532	8,60 (+ 9,67)	57.940
**Jenis Kelamin**				
Laki-laki	12,94 (+ 8,13)	192.173	9,04 (+ 9,83)	51.493
Perempuan	8,50 (+ 7,42)	5.359	5,08 (+ 7,34)	6.447
**Pendidikan**				
Tidak sekolah	12,08 (+ 8,08)	9.115	9,04 (+ 9,2)	2.459
Tidak tamat SD	12,70 (+ 8,21)	25.127	8,75 (+ 9,85)	6.358
Tamat SD	12,72 (+ 7,82)	52.063	8,32 (+ 9,06)	13.501
Tamat SLTP	12,83 (+ 8,14)	40.442	8,52 (+ 9,92)	11.858
Tamat SLTA	12,95 (+ 8,25)	59.282	8,84 (+ 9,97)	18.287
Tamat D1/D2/D3 PT	13,49 (+ 8,76)	11.503	8,31 (+ 9,54)	5.477
**Pekerjaan**				
Tidak Bekerja	10,92 (+ 7,88)	20.715	7,60 (+ 9,38)	10.661
Sekolah	8,74 (+ 6,38)	5.692	5,26 (+ 6,75)	8.004
PNS/TNI/Polri/BUMN/BUMD	14,91 (+ 9,37)	5.422	9,17 (+ 9,82)	1.988
Pegawai swasta	12,51 (+ 7,96)	24.255	9,06 (+ 10,39)	7.708
Wiraswasta	13,90 (+ 8,71)	37.971	9,66 ((+ 10,39)	8.995
Nelayan	15,41 (+ 8,72)	3.397	10,88 (+ 10,30)	473
Petani/Buruh tani	12,97 (+ 7,74)	52.660	9,92 (+ 9,75)	10.412
Lainnya	12,86 (+ 7,96)	47.419	9,46 (+ 9,86)	9.699
**Tempat tinggal**				
Perkotaan	12,55 (+ 8,26)	104.750	8,26 (+ 9.69)	34.535
Perdesaan	13,13 (+ 7,99)	92.782	9,10 (+ 9.61)	23.405

Tabel 12.6.5

Proporsi Umur Pertama Kali Merokok Tiap Hari pada Penduduk Umur ≥10 Tahun menurut Provinsi, Riskesdas 2018

Provinsi	Umur pertama kali merokok tiap hari (tahun) (%,95%CI)						N Tertimbang
	5-9 tahun	10-14 tahun	15-19 tahun	20-24 tahun	25-29 tahun	>=30 tahun	
Aceh	0,5 (0,3-0,9)	7,9 (7,0-8,8)	53,3 (51,4-55,1)	28,3 (26,7-30,1)	7,1 (6,3-7,9)	3,0 (2,5-3,6)	2.761
Sumatera Utara	0,5 (0,3-0,7)	8,0 (7,1-9,0)	48,8 (47,3-50,3)	29,6 (28,2-31,0)	8,2 (7,4-9,0)	5,0 (4,5-5,6)	8.028
Sumatera Barat	1,3 (1,0-1,6)	14,3 (13,1-15,5)	47,9 (46,3-49,4)	24,3 (23,0-25,7)	7,2 (6,5-8,0)	5,1 (4,5-5,8)	3.844
Riau	0,8 (0,5-1,2)	9,5 (8,5-10,6)	47,1 (45,3-49,0)	29,5 (27,9-31,2)	8,2 (7,4-9,2)	4,8 (4,1-5,5)	4.249
Jambi	0,4 (0,2-0,7)	9,1 (7,8-10,6)	53,2 (50,7-55,7)	27,3 (25,2-29,6)	6,2 (5,2-7,3)	3,9 (3,1-4,8)	1.687
Sumatera Selatan	0,9 (0,6-1,4)	13,4 (12,2-14,6)	53,3 (51,3-55,2)	21,5 (19,9-23,1)	7,7 (6,7-9,0)	3,2 (2,7-3,8)	5.052
Bengkulu	0,9 (0,6-1,3)	11,7 (10,3-13,2)	51,6 (49,4-53,7)	24,4 (22,7-26,2)	7,6 (6,5-8,7)	3,9 (3,2-4,9)	1.360
Lampung	0,8 (0,6-1,2)	10,3 (9,3-11,4)	52,0 (50,4-53,5)	24,4 (23,1-25,8)	7,8 (7,0-8,8)	4,6 (4,0-5,3)	5.830
Bangka Belitung	1,1 (0,7-1,7)	10,7 (9,2-12,4)	47,3 (44,6-50,1)	28,9 (26,5-31,4)	7,0 (5,9-8,3)	4,9 (4,1-5,9)	980
Kepulauan Riau	0,5 (0,3-1,2)	9,2 (7,3-11,5)	47,1 (42,8-51,5)	30,1 (26,2-34,3)	7,5 (5,9-9,6)	5,5 (4,0-7,5)	1.183
DKI Jakarta	0,6 (0,3-0,9)	9,9 (8,6-11,4)	44,3 (42,1-46,5)	31,0 (28,9-33,2)	8,5 (7,4-9,9)	5,7 (4,8-6,7)	7.036
Jawa Barat	0,6 (0,5-0,8)	9,4 (8,7-10,1)	49,2 (48,0-50,3)	26,8 (25,8-27,8)	8,2 (7,6-8,8)	5,8 (5,3-6,3)	33.783
Jawa Tengah	1,2 (1,0-1,4)	11,8 (11,1-12,4)	45,8 (44,9-46,8)	25,9 (25,0-26,7)	9,0 (8,4-9,5)	6,4 (5,9-6,9)	21.198
DI Yogyakarta	1,1 (0,7-1,8)	11,3 (9,8-13,6)	47,1 (44,3-49,9)	23,6 (21,4-25,9)	8,5 (7,2-9,9)	8,4 (7,1-9,9)	2.471
Jawa Timur	1,2 (1,0-1,4)	11,0 (10,5-11,6)	47,3 (46,4-48,3)	25,7 (24,9-26,6)	8,5 (8,1-9,0)	6,2 (5,8-6,6)	26.325
Banten	0,7 (0,4-1,1)	11,0 (9,8-12,4)	50,1 (48,1-52,1)	25,9 (24,1-27,7)	7,5 (6,5-8,5)	4,9 (4,2-5,8)	8.520
Bali	0,5 (0,3-0,8)	7,9 (6,9-9,0)	45,9 (43,7-48,1)	27,9 (26,0-30,0)	10,0 (8,8-11,4)	7,8 (6,8-8,9)	2.676
Nusa Tenggara Barat	1,0 (0,7-1,5)	14,7 (13,2-16,3)	52,2 (50,1-54,3)	21,8 (20,3-23,5)	5,8 (4,9-6,8)	4,5 (3,8-5,3)	3.183
Nusa Tenggara Timur	0,5 (0,3-0,9)	8,7 (7,7-9,8)	42,9 (41,2-44,7)	31,2 (29,6-32,8)	10,5 (9,6-11,5)	6,1 (5,4-6,9)	2.572
Kalimantan Barat	0,9 (0,7-1,3)	10,9 (9,8-12,2)	49,4 (47,5-51,4)	27,0 (25,3-28,8)	7,1 (6,2-8,1)	4,6 (3,9-5,5)	2.688
Kalimantan Tengah	0,6 (0,4-1,0)	10,5 (9,1-12,1)	46,9 (44,5-49,4)	29,2 (27,0-31,6)	7,7 (6,6-9,1)	5,0 (4,1-6,0)	1.428
Kalimantan Selatan	0,7 (0,4-1,1)	10,6 (9,3-12,0)	45,0 (42,9-47,2)	28,6 (26,7-30,5)	8,5 (7,4-9,6)	6,6 (5,7-7,7)	2.174
Kalimantan Timur	1,2 (0,8-1,9)	9,9 (8,5-11,6)	45,5 (42,8-48,2)	29,1 (26,8-31,6)	8,4 (7,0-10,0)	5,8 (4,7-7,1)	2.229
Kalimantan Utara	1,7 (0,9-3,1)	10,0 (8,0-12,5)	48,7 (45,2-52,2)	28,6 (25,4-32,0)	7,1 (5,5-9,3)	3,8 (2,7-5,5)	403
Sulawesi Utara	0,3 (0,2-0,5)	8,8 (7,6-10,0)	50,8 (48,8-52,8)	25,9 (24,3-27,7)	7,5 (6,7-8,5)	6,7 (5,8-7,7)	1.551
Sulawesi Tengah	0,9 (0,6-1,4)	11,4 (10,2-12,8)	44,7 (42,7-46,7)	27,1 (25,4-28,9)	9,6 (8,6-10,8)	6,2 (5,4-7,1)	2.092
Sulawesi Selatan	1,2 (0,9-1,5)	13,8 (12,8-14,9)	48,9 (47,4-50,5)	23,4 (22,1-24,7)	7,1 (6,3-8,0)	5,6 (5,0-6,4)	5.073
Sulawesi Tenggara	0,8 (0,5-1,4)	11,2 (9,8-12,8)	50,9 (48,1-53,7)	24,3 (22,4-26,2)	7,8 (6,4-9,5)	5,0 (3,9-6,3)	1.404
Gorontalo	0,3 (0,-0,6)	9,8 (8,4-11,4)	51,5 (48,9-54,1)	25,6 (23,2-28,1)	7,3 (6,1-8,8)	5,5 (4,5-6,7)	784
Sulawesi Barat	0,8 (0,3-2,0)	17,9 (14,7-21,5)	45,2 (41,6-48,8)	24,4 (21,6-27,4)	7,4 (5,7-9,5)	4,5 (3,3-6,0)	603
Maluku	0,5 (0,2-1,0)	7,2 (5,8-9,0)	49,3 (46,3-52,3)	29,2 (26,6-31,8)	7,8 (6,2-9,6)	6,1 (4,7-7,8)	935
Maluku Utara	0,3 (0,1-1,1)	7,2 (6,0-8,7)	46,9 (44,5-49,4)	29,9 (27,7-32,3)	9,3 (8,0-10,7)	6,3 (5,2-7,5)	735
Papua Barat	0,7 (0,3-1,3)	9,8 (8,0-12,0)	45,0 (41,2-48,8)	30,1 (26,6-33,9)	9,0 (7,2-11,1)	5,4 (4,0-7,3)	522
Papua	1,1 (0,7-1,8)	13,8 (11,6-16,3)	44,5 (41,3-47,7)	25,9 (23,2-28,9)	8,7 (7,2-10,4)	6,0 (4,8-7,6)	1.204
**INDONESIA**	**0,9 (0,8-0,9)**	**10,6 (10,4-10,9)**	**48,2 (47,8-48,6)**	**26,5 (26,2-26,8)**	**8,2 (8,0-8,4)**	**5,6 (5,5-5,8)**	**166.563**

Tabel 12.6.6

Proporsi Umur Pertama Kali Merokok Tiap Hari pada Penduduk Umur ≥10 Tahun menurut Karakteristik, Riskesdas 2018

Karakteristik	Umur pertama kali merokok tiap hari (%,95%CI)						N Tertimbang
	5-9 tahun	10-14 tahun	15-19 tahun	20-24 tahun	25-29 tahun	>=30 tahun	
**Kelompok Umur**							
10-14	3,8 (2,3-6,3)	96.2 (93.7-97.7)	[redacted]	[redacted]	[redacted]	[redacted]	502
15-19	0,7 (0,5-0,9)	23.2 (22.0-24.5)	76,1 (74,8-77,4)	[redacted]	[redacted]	[redacted]	9.114
20-24	0,5 (0,4-0,7)	11.3 (10.7-12.1)	68,7 (67,7-69,8)	19,4 (18,5-20,3)	[redacted]	[redacted]	19.041
25-29	0,4 (0,3-0,5)	9.6 (9.0-10.3)	56,0 (54,9-57,1)	29,7 (28,7-30,8)	4,2 (3,8-4,7)	[redacted]	20.849
30-34	0,7 (0,5-0,9)	9.0 (8.4-9.6)	50,3 (49,2-51,4)	30,0 (29,1-31,0)	8,7 (8,1-9,3)	1,3 (1,1-1,5)	20.772
35-39	0,6 (0,4-0,7)	8.8 (8.3-9.4)	45,0 (44,0-46,0)	31,0 (30,1-32,0)	10,8 (10,2-11,4)	3,8 (3,5-4,2)	20.680
40-44	0,7 (0,6-0,9)	8.3 (7.7-8.9)	41,7 (40,7-42,8)	30,3 (29,3-31,2)	11,5 (10,9-12,2)	7,5 (6,9-8,0)	18.320
45-49	0,8 (0,6-1,1)	8.6 (8.1-9.2)	39,9 (38,8-40,9)	29,7 (28,7-30,6)	12,1 (11,5-12,9)	8,8 (8,2-9,5)	16.188
50-54	1,2 (0,9-1,4)	9.4 (8.7-10.1)	38,2 (37,0-39,3)	28,0 (27,0-29,1)	11,8 (11,1-12,6)	11,5 (10,7-12,2)	13.446
55-59	1,5 (1,2-1,9)	10.0 (9.3-10.8)	37,2 (36,0-38,5)	27,7 (26,6-28,8)	10,8 (10,0-11,6)	12,7 (11,9-13,6)	10.650
60-64	1,7 (1,4-2,2)	11.4 (10.5-12.4)	34,8 (33,3-36,3)	26,8 (25,4-28,2)	11,6 (10,7-12,5)	13,7 (12,7-14,8)	7.522
65 +	2,3 (1,8-2,7)	12.0 (11.2-13.0)	31,5 (30,2-32,8)	26,5 (25,2-27,7)	11,3 (10,4-12,2)	16,5 (15,4-17,5)	9.480
**Kelompok Umur Khusus**							
10-18	1,0 (0,7-1,3)	31.2 (29.6-32.8)	67,9 (66,3-69,4)	[redacted]	[redacted]	[redacted]	7.686
15 +	0,9 (0,8-0,9)	10.4 (10.1-10.6)	48,3 (48,0-48,7)	26,6 (26,2-26,9)	8,2 (8,0-8,4)	5,6 (5,5-5,8)	167.390
10+	0,9 (0,8-0,9)	10.6 (10.4-10.9)	48,2 (47,8-48,6)	26,5 (26,2-26,8)	8,2 (8,0-8,4)	5,6 (5,5-5,8)	168.041
**Jenis Kelamin**							
Laki-laki	0,9 (0,8-0,9)	10.7 (10.5-10.9)	48,7 (48,3-49,1)	26,6 (26,3-26,9)	8,2 (8,0-8,4)	5,0 (4,9-5,2)	162.546
Perempuan	1,3 (0,8-2,1)	8.0 (6.9-9.3)	28,5 (26,3-30,7)	22,9 (21,0-24,8)	10,4 (9,2-11,9)	28,9 (26,9-31,1)	4.017
**Tempat tinggal**							
Perkotaan	0,7 (0,6-0,8)	9.8 (9.5-10.2)	48,2 (47,7-48,8)	27,0 (26,5-27,6)	8,4 (8,1-8,7)	5,7 (5,5-6,0)	90.768
Perdesaan	1,0 (0,9-1,1)	11.6 (11.3-11.9)	48,1 (47,7-48,6)	25,9 (25,5-26,3)	8,0 (7,7-8,2)	5,5 (5,3-5,7)	75.795

Tabel 12.6.6 (lanjutan)
Proporsi Umur Pertama Kali Merokok Tiap Hari pada Penduduk Umur ≥10 Tahun menurut Karakteristik, Riskesdas 2018

Karakteristik	Umur pertama kali merokok tiap hari (%,95%CI)						N Tertimbang
	5-9 tahun	10-14 tahun	15-19 tahun	20-24 tahun	25-29 tahun	>=30 tahun	
**Pendidikan**							
Tidak sekolah	2,5 (2,0-3,2)	15.6 (14.4-17.0)	41,0 (39,3-42,8)	23,2 (21,8-24,6)	7,9 (7,1-8,7)	9,8 (8,9-10,8)	6.338
Tidak tamat SD	1,9 (1,7-2,2)	13.8 (13.1-14.4)	42,9 (41,9-43,9)	24,5 (23,7-25,4)	8,6 (8,1-9,2)	8,3 (7,8-8,8)	19.615
Tamat SD	1,1 (1,0-1,3)	13.4 (12.9-13.9)	44,4 (43,7-45,1)	25,4 (24,8-26,0)	8,9 (8,6-9,4)	6,8 (6,4-7,1)	42.189
Tamat SLTP	0,5 (0,5-0,7)	11.3 (10.7-11.8)	52,1 (51,3-52,9)	24,9 (24,3-25,6)	7,1 (6,7-7,5)	4,0 (3,7-4,3)	35.208
Tamat SLTA	0,4 (0,3-0,5)	7.2 (6.8-7.5)	52,6 (51,9-53,2)	28,2 (27,6-28,8)	7,6 (7,3-7,9)	4,1 (3,9-4,3)	52.884
Tamat D1/D2/D3 PT	0,4 (0,3-0,6)	5.8 (5.2-6.5)	42,3 (41,0-43,7	33,7 (32,4-35,1)	11,4 (10,5-12,2)	6,4 (5,8-7,1)	10.330
**Pekerjaan**							
Tidak Bekerja	1,1 (0,9-1,4)	13.1 (12.3-13.9)	51,6 (50,3-52,8)	21,5 (20,5-22,5)	5,1 (4,6-5,6)	7,7 (7,1-8,3)	16.790
Sekolah	0,7 (0,5-1,0)	23.6 (21.9-25.3)	67,5 (65,5 – 69,4)	6,8 (5,8-8,1)	0,8 (0,5-1,2)	0,6 (0,3-1,0)	5.331
PNS/TNI/Polri/BUMN/ BUMD	0,4 (0,3-0,7)	5.2 (4.4-6.1)	36,4 (34,6-38,1)	36,0 (34,3-37,8)	13,0 (11,8-14,3)	9,0 (7,9-10,2)	4.771
Pegawai swasta	0,4 (0,3-0,5)	7.0 (6.4-7.6)	50,7 (49,6-51,9)	30,5 (29,4-31,6)	7,7 (7,1-8,3)	3,7 (3,4-4,1)	21.875
Wiraswasta	0,6 (0,5-0,8)	9.3 (8.8-9.8)	46,8 (45,9-47,6)	28,3 (27,6-29,0)	9,5 (9,1-9,8)	5,5 (5,1-5,8)	32.746
Petani/Buruh tani	1,2 (1,1-1,4)	11.4 (11.0-11.8)	44,7 (44,0-45,3)	26,5 (25,9-27,0)	9,4 (9,1-9,8)	6,8 (6,5-7,1)	41.710
Nelayan	0,7 (0,4-1,1)	12.2 (10.8-13.8)	50,7 (48,3-53,1)	24,2 (22,3-26,1)	7,4 (6,2-8,9)	4,8 (4,0-5,7)	2.851
Buruh/sopir/pembantu ruta	0,9 (0,7-1,1)	10.8 (10.2-11.4)	49,1 (48,2-50,0)	26,8 (26,0-27,6)	7,7 (7,3-8,2)	4,7 (4,3-5,0)	32.234
Lainnya	1,0 (0,7-1,3)	10.1 (9.2-11.2)	47,8 (46,2-49,3)	26,2 (24,9-27,5)	8,7 (7,8-9,6)	6,3 (5,6-7,1)	8.255

Tabel 12.6.7
Proporsi Umur Pertama Kali Merokok Pada Penduduk Umur ≥10 Tahun menurut Provinsi, Riskesdas 2018

Provinsi	Umur pertama kali merokok												N Ter-timbang
	5-9 tahun		10-14 tahun		15-19 tahun		20-24 tahun		25-29 tahun		>=30 tahun		
	%	95% CI	%	95% CI	%	95% CI	%	95% CI	%	95% CI	%	95% CI	
Aceh	1,6	1,3-2,1	19,4	18,2-20,7	61,7	60,0-63,4	13,0	11,8-14,2	2,8	2,4-3,3	1,5	1,1-1,9	3.297
Sumatera Utara	2,1	1,8-2,5	21,6	20,4-22,8	55,2	53,8-56,6	14,6	13,7-15,6	3,3	2,8-3,7	3,2	2,8-3,6	10.721
Sumatera Barat	5,0	4,4-5,6	32,4	30,9-33,8	46,5	45,0-48,0	10,4	9,6-11,3	3,0	2,6-3,5	2,7	2,4-3,1	4.822
Riau	3,4	2,9-4,0	25,1	23,7-26,5	51,7	50,1-53,3	14,0	12,9-15,2	3,1	2,7-3,6	2,7	2,3-3,2	5.437
Jambi	1,7	1,3-2,2	23,0	21,2-24,9	58,6	56,4-60,7	11,7	10,5-13,0	2,9	2,3-3,6	2,1	1,6-2,7	2.011
Sumatera Selatan	2,3	1,8-2,8	24,5	22,9-26,1	55,4	53,7-57,2	12,5	11,4-13,8	3,2	2,7-3,8	2,1	1,7-2,5	6.130
Bengkulu	3,0	2,4-3,7	24,9	23,2-26,7	55,3	53,4-57,1	11,7	10,6-12,8	2,9	2,3-3,7	2,2	1,7-2,8	1.594
Lampung	2,5	2,1-2,9	23,5	22,2-25,0	54,1	52,6-55,6	13,2	12,3-14,2	3,4	3,0-4,0	3,3	2,8-3,8	7.156
Bangka Belitung	4,0	3,1-5,2	25,5	23,3-27,8	53,7	51,2-56,2	11,2	10,0-12,5	3,6	3,0-4,5	1,9	1,5-2,6	1.216
Kepulauan Riau	2,9	2,0-4,3	24,2	21,2-27,4	51,2	47,6-54,9	15,1	13,1-17,5	3,5	2,5-4,9	3,0	2,2-4,2	1.587
DKI Jakarta	2,3	1,8-2,9	25,6	24,0-27,2	51,5	49,6-53,3	14,5	13,2-15,8	3,7	3,1-4,4	2,5	2,1-3,0	10.521
Jawa Barat	1,7	1,5-2,0	20,8	19,9-21,6	53,9	52,8-54,9	15,6	14,9-16,3	4,3	3,9-4,7	3,8	3,5-4,1	43.552
Jawa Tengah	3,1	2,8-3,4	24,6	23,9-25,4	48,5	47,6-49,3	15,3	14,8-15,9	4,7	4,4-5,1	3,7	3,5-4,1	29.353
DI Yogyakarta	5,4	4,5-6,4	30,6	28,7-32,5	44,1	42,1-46,2	12,2	10,9-13,7	4,0	3,3-4,8	3,7	3,1-4,4	3.922
Jawa Timur	2,7	2,4-3,0	20,9	20,2-21,6	51,1	50,3-52,0	16,5	15,9-17,1	5,2	4,8-5,5	3,7	3,4-4,0	33.368
Banten	2,3	1,8-2,8	25,9	24,3-27,5	54,4	52,7-56,1	11,7	10,7-12,8	3,4	2,9-4,0	2,3	1,9-2,8	11.030
Bali	1,5	1,2-2,1	17,0	15,6-18,4	52,5	50,7-54,4	18,4	17,0-19,8	5,7	4,9-6,6	4,9	4,2-5,7	3.553
Nusa Tenggara Barat	2,8	2,2-3,6	26,9	25,0-28,9	52,6	50,5-54,6	11,8	10,7-13,0	3,0	2,5-3,7	2,8	2,3-3,5	3.867
Nusa Tenggara Timur	1,6	1,2-2,0	18,6	17,4-19,9	53,0	51,6-54,5	19,3	18,2-20,5	4,4	3,9-5,0	3,1	2,7-3,6	3.569
Kalimantan Barat	2,2	1,8-2,7	20,1	18,6-21,7	52,1	50,5-53,7	17,0	15,7-18,4	4,8	4,1-5,6	3,9	3,3-4,6	3.340
Kalimantan Tengah	2,2	1,7-2,8	20,9	19,2-22,7	54,1	51,8-56,4	15,1	13,7-16,6	4,7	3,9-5,6	3,0	2,4-3,7	1.813
Kalimantan Selatan	2,8	2,3-3,4	24,3	22,8-25,9	49,3	47,5-51,1	16,2	14,9-16,6	4,1	3,4-4,8	3,4	2,8-4,1	2.858
Kalimantan Timur	3,0	2,4-3,8	23,8	21,8-26,0	50,2	47,9-52,5	15,5	13,9-17,3	4,0	3,2-5,0	3,4	2,6-4,4	3.100
Kalimantan Utara	4,1	3,1-5,5	24,2	21,4-27,1	52,3	48,8-55,8	14,2	12,0-16,8	2,8	2,0-3,8	2,5	1,7-3,6	542
Sulawesi Utara	1,3	1,0-1,7	20,7	19,2-22,2	56,0	54,3-57,6	14,0	12,9-15,1	4,2	3,6-4,8	3,9	3,3-4,7	2.109
Sulawesi Tengah	3,1	2,6-3,6	25,4	23,9-26,8	49,4	47,8-51,1	14,1	13,0-15,4	4,2	3,6-5,0	3,8	3,2-4,4	2.788
Sulawesi Selatan	3,7	3,2-4,2	27,1	25,8-28,4	48,1	46,7-49,5	13,3	12,4-14,3	4,2	3,7-4,7	3,7	3,2-4,1	6.588
Sulawesi Tenggara	2,4	1,8-3,2	21,5	19,8-23,4	53,2	51,1-55,3	15,7	14,2-17,3	4,2	3,2-5,4	3,0	2,3-3,9	1.759
Gorontalo	1,7	1,2-2,4	21,6	19,8-23,6	57,6	55,2-59,9	12,8	11,2-14,5	3,4	2,7-4,3	2,9	2,3-3,7	989
Sulawesi Barat	1,5	0,9-2,6	28,2	25,5-31,2	47,4	44,2-50,6	15,7	13,5-18,1	4,6	3,4-6,1	2,5	1,7-3,7	766
Maluku	0,9	0,6-1,4	19,5	17,2-21,9	58,1	55,5-60,7	15,3	13,5-17,3	4,1	3,4-5,1	2,1	1,6-2,7	1.263
Maluku Utara	1,1	0,7-1,7	16,3	14,6-18,1	58,7	56,3-60,9	16,5	14,9-18,1	3,9	3,1-4,9	3,5	2,9-4,4	944
Papua Barat	2,9	1,9-4,2	21,8	19,2-24,6	50,8	47,5-54,1	18,2	15,7-20,9	3,7	2,8-5,0	2,7	2,0-3,5	698
Papua	2,8	2,1-3,7	26,5	24,0-29,2	50,1	47,5-52,7	13,8	12,2-15,6	4,1	3,3-5,0	2,7	2,1-3,6	1.657
**INDONESIA**	**2,5**	**2,4-2,6**	**23,1**	**22,8-23,3**	**52,1**	**51,8-52,4**	**14,8**	**14,6-15,0**	**4,2**	**4,1-4,3**	**3,3**	**3,2-3,4**	**217.919**

Tabel 12.6.8
Proporsi Umur Pertama Kali Merokok Pada Penduduk Umur ≥10 Tahun menurut Karakteristik, Riskesdas 2018

Karakteristik	Umur pertama kali merokok (%,95%CI)						N Ter-timbang
	5-9 tahun	10-14 tahun	15-19 tahun	20-24 tahun	25-29 tahun	>=30 tahun	
**Kelompok Umur**							
10-14	12,5 (11,1-14,1)	87,5 (85,9-88,9)					3.064
15-19	3,0 (2,7-3,4)	42,8 (41,7-43,9)	54,2 (53,1-55,3)				16.714
20-24	2,2 (1,9-2,4)	26,1 (25,2-27,0)	64,1 (63,1-65,0)	7,7 (7,2-8,3)			25.159
25-29	2,0 (1,8-2,3)	22,9 (22,0-23,7)	60,7 (59,7-61,7)	12,3 (11,7-13,0)	2,1 (1,8-2,4)		25.676
30-34	2,2 (1,9-2,5)	22,0 (21,2-22,8)	57,3 (56,4-58,3)	14,3 (13,7-15,0)	3,4 (3,1-3,7)	0,7 (0,6-0,9)	25.285
35-39	1,9 (1,7-2,1)	19,5 (18,8-20,3)	55,9 (55,1-56,8)	16,4 (15,8-17,1)	4,3 (4,0-4,7)	1,9 (1,7-2,1)	25.264
40-44	2,1 (1,9-2,3)	18,2 (17,5-18,9)	51,9 (51,0-52,9)	18,7 (18,0-19,4)	5,5 (5,1-5,9)	3,6 (3,3-4,0)	22.587
45-49	2,2 (1,9-2,5)	18,1 (17,3-18,8)	48,2 (47,3-49,2)	20,0 (19,2-20,8)	6,5 (6,0-7,0)	5,0 (4,6-5,4)	20.591
50-54	2,4 (2,1-2,7)	17,8 (17,0-18,5)	46,5 (45,4-47,5)	19,5 (18,7-20,3)	7,0 (6,5-7,5)	6,9 (6,4-7,5)	17.033
55-59	2,9 (2,5-3,3)	18,5 (17,6-19,4)	42,8 (41,6-43,9)	21,0 (20,1-22,0)	6,9 (6,4-7,5)	8,0 (7,4-8,6)	13.746
60-64	3,5 (3,0-4,1)	18,6 (17,619,7)	40,0 (38,7-41,3)	20,5 (19,4-21,6)	8,0 (7,3-8,7)	9,5 (8,7-10,3)	9.867
65 +	3,5 (3,0-4,0)	17,5 (16,6-18,4)	36,6 (35,5-37,8)	22,0 (21,0-23,0)	8,6 (7,9-9,3)	11,9 (11,1-12,7)	12.932
**Kelompok umur khusus**							
10-18	5,0 (4,6-5,5)	54,5 (53,4-55,6)	40,5 (39,4-41,5)				17.894
15 +	2,4 (2,3-2,5)	22,1 (21,8-22,4)	52,8 (52,5-53,2)	15,0 (14,8-15,3)	4,2 (4,1-4,4)	3,4 (3,3-3,5)	215.993
10+	2,5 (2,4-2,6)	23,1 (22,8-23,3)	52,1 (51,8-52,4)	14,8 (14,6-15,0)	4,2 (4,1-4,3)	3,3 (3,2-3,4)	219.702
**Jenis Kelamin**							
Laki-laki	2,5 (2,4-2,6)	23,6 (23,3-23,9)	52,9 (52,5-53,2)	14,6 (14,4-14,9)	4,0 (3,9-4,1)	2,4 (2,3-2,5)	208.525
Perempuan	2,3 (1,9-2,9)	11,6 (10,6-12,6)	34,9 (33,4-36,4)	19,2 (18,0-20,5)	8,3 (7,5-9,1)	23,7 (22,5-25,0)	9.394
**Pendidikan**							
Tidak sekolah	4,0 (3,4-4,7)	21,4 (20,2-22,7)	41,9 (40,3-43,5)	18,3 (17,2-19,5)	5,6 (5,0-6,3)	8,8% (8,0-9,6)	7.977
Tidak tamat SD	4,5 (4,2-4,9)	23,1 (22,4-23,8)	43,9 (43,0-44,8)	16,7 (16,1-17,4)	5,6 (5,2-6,0)	6,1 (5,7-6,6)	24.699
Tamat SD	3,0 (2,8-3,2)	25,9 (25,4-26,5)	45,8 (45,2-46,5)	16,0 (15,5-16,4)	4,9 (4,6-5,1)	4,4 (4,2-4,7)	53.822
Tamat SLTP	2,0 (1,8-2,1)	26,4 (25,8-27,0)	54,0 (53,3-54,6)	12,3 (11,9-12,7)	3,4 (3,2-3,7)	2,0 (1,8-2,1)	46.136
Tamat SLTA	1,8 (1,6-1,9)	20,0 (19,5-20,5)	59,4 (58,8-60,0)	13,6 (13,3-14,0)	3,3 (3,1-3,5)	1,9 (1,7-2,0)	69.884
Tamat D1/D2/D3/PT	2,2 (1,9-2,5)	17,5 (16,6-18,4)	53,8 (52,6-55,0)	18,9 (18,0-19,8)	4,7 (4,3-5,2)	2,9 (2,6-3,3)	15.400
**Pekerjaan**							
Tidak Bekerja	3,0 (2,7-3,35)	24,8 (23,9-25,6)	49,0 (48,0-49,9)	13,2 (12,6-13,9)	3,8 (3,4-4,1)	6,3 (5,9-6,8)	25.727
Sekolah	4,7 (4,3-5,2)	49,8 (48,6-51,1)	42,9 (41,6-44,1)	2,1 (1,8-2,5)	0,3 (0,2-0,5)	0,2 (0,1-0,3)	13.090
PNS/TNI/Polri/BUMN/BUMD	1,8 (1,5-2,2)	15,4 (14,3-16,6)	49,9 (48,3-51,4)	22,3 (21,0-23,6)	6,3 (5,6-7,1)	4,4 (3,7-5,2)	6.569
Pegawai swasta	1,9 (1,7-2,2)	20,4 (19,6-21,3)	57,9 (56,9-58,9)	14,8 (14,1-15,5)	3,4 (3,1-3,7)	1,5 (1,4-1,8)	29.099
Wiraswasta	2,1 (1,9-2,3)	20,2 (19,6-20,8)	54,3 (53,6-55,0)	16,1 (15,6-16,6)	4,5 (4,2-4,8)	2,8 (2,6-3,1)	40.932
Petani/Buruh tani	2,7 (2,5-2,9)	20,4 (20,0-20,9)	49,0 (48,5-49,6)	17,5 (17,0-17,9)	5,6 (5,4-5,9)	4,8 (4,5-5,0)	49.994
Nelayan	2,5 (1,9-3,3)	23,6 (22,0-25,4)	53,9 (51,8-56,0)	14,3 (13,0-15,8)	3,9 (3,2-4,6)	1,8 (1,4-2,3)	3.269
Buruh/sopir/pembantu ruta	2,3 (2,1-2,6)	23,2 (22,4-23,9)	54,4 (53,6-55,3)	14,2 (13,6-14,8)	3,7 (3,5-4,0)	2,2 (2,0-2,4)	38.480
Lainnya	2,4 (2,0-2,8)	20,8 (19,7-22,0)	53,3 (52,0-54,7)	15,0 (14,1-16,0)	4,3 (3,8-4,9)	4,1 (3,6-4,7)	10.759
**Tempat tinggal**							
Perkotaan	2,4 (2,3-2,6)	23,4 (23,0-23,9)	52,6 (52,1-53,1)	14,4 (14,1-14,8)	4,0 (3,8-4,2)	3,1 (2,9-3,2)	122.279
Perdesaan	2,6 (2,5-2,8)	22,6 (22,2-23,0)	51,4 (51,0-51,9)	15,3 (15,0-15,6)	4,4 (4,2-4,5)	3,7 (3,5-3,8)	95.640

Tabel 12.6.9
Proporsi Jenis Rokok Yang Dihisap Penduduk Umur ≥10 Tahun menurut Provinsi, Riskesdas 2018

Provinsi	jenis rokok yang dihisap (%,95%CI)					N Ter-timbang
	Kretek	Rokok putih	Rokok linting	Elektrik	Shisha	
Aceh	69,2 (67,3-71,0)	43,4 (41,5-45,4)	6,8 (6,1-7,6)	0,7 (0,6-1,0)	0,5 (0,4-0,7)	4.351
Sumatera Utara	77,4 (76,0-78,8)	30,9 (29,3-32,5)	3,5 (3,0-4,0)	0,7 (0,5-1,0)	0,6 (0,4-0,8)	12.611
Sumatera Barat	75,1 (73,4-76,7)	37,3 (35,3-39,4)	7,1 (6,4-7,9)	1,9 (1,5-2,3)	0,8 (0,6-1,1)	5.487
Riau	75,4 (73,6-77,2)	33,1 (31,1-35,2)	6,2 (5,4-7,1)	1,6 (1,2-2,0)	0,5 (0,4-0,8)	6.442
Jambi	64,4 (62,0-66,7)	40,8 (38,2-43,4)	3,1 (2,5-3,9)	0,8 (0,6-1,2)	0,5 (0,3-0,8)	2.799
Sumatera Selatan	74,6 (72,5-76,5)	36,3 (33,9-33,8)	10,3 (9,3-11,4)	1,5 (1,0-2,2)	1,2 (0,9-1,7)	7.577
Bengkulu	67,5 (65,3-69,7)	46,5 (44,0-49,1)	9,0 (7,7-10,4)	1,0 (0,7-1,4)	0,3 (0,2-0,5)	1.876
Lampung	71,0 (69,4-72,6)	41,9 (40,1-43,8)	21,1 (19,8-22,5)	1,0 (0,8-1,3)	0,6 (0,5-0,9)	8.517
Bangka Belitung	56,4 (53,0-59,8)	57,8 (54,1-61,5)	5,5 (4,6-6,6)	3,0 (2,3-4,2)	3,0 (2,3-3,9)	1.367
Kepulauan Riau	56,9 (52,9-60,7)	55,4 (51,2-59,4)	4,9 (3,4-6,9)	3,2 (2,2-4,7)	1,5 (1,0-2,5)	1.864
DKI Jakarta	61,2 (58,6-63,7)	50,4 (47,9-52,9)	4,7 (3,9-5,6)	5,9 (5,1-6,8)	4,3 (3,6-5,0)	11.645
Jawa Barat	68,1 (66,9-69,3)	45,8 (44,4-47,1)	10,3 (9,7-10,9)	2,7 (2,4-3,1)	1,9 (1,7-2,2)	51.428
Jawa Tengah	61,0 (60,0-62,1)	49,3 (48,1-50,4)	22,8 (22,0-23,6)	2,9 (2,7-3,2)	1,1 (1,0-1,3)	34.651
DI Yogyakarta	55,7 (53,1-58,3)	60,4 (58,0-62,7)	26,4 (24,4-28,5)	7,4 (6,1-8,8)	4,0 (3,1-5,2)	4.314
Jawa Timur	71,6 (70,6-72,5)	36,6 (35,5-37,7)	23,1 (22,3-23,9)	3,0 (2,7-3,3)	1,6 (1,4-1,9)	37.701
Banten	76,1 (74,0-78,0)	33,2 (31,2-35,4)	4,3 (3,7-5,0)	3,3 (2,6-4,1)	2,4 (1,9-3,0)	13.092
Bali	44,1 (41,7-46,5)	60,6 (58,2-62,9)	5,8 (5,0-6,7)	4,2 (3,,5-5,0)	0,8 (0,5-1,2)	3.872
Nusa Tenggara Barat	73,8 (72,0-75,6)	33,4 (30,9-35,9)	38,1 (35,9-40,3)	3,0 (2,3-4,0)	1,3 (0,9-1,9)	4.647
Nusa Tenggara Timur	78,5 (77,2-79,8)	33,1 (31,2-35,0)	24,2 (22,8-25,8)	0,8 (0,5-1,1)	0,4 (0,2-0,5)	4.144
Kalimantan Barat	52,1 (49,8-54,4)	54,0 (51,6-56,4)	14,3 (13,0-15,7)	1,9 (1,5-2,5)	0,8 (0,6-1,2)	4.278
Kalimantan Tengah	53,6 (51,1-56,2)	58,1 (55,3-60,9)	8,0 (6,6-9,7)	2,4 (1,9-3,2)	1,3 (0,9-1,8)	2.391
Kalimantan Selatan	67,7 (65,0-70,2)	42,7 (39,7-45,7)	4,9 (4,2-5,8)	4,9 (4,1-5,8)	3,4 (2,7-4,2)	3.390
Kalimantan Timur	58,2 (54,9-61,5)	56,3 (53,0-59,6)	8,5 (7,1-10,1)	6,0 (4,8-7,5)	5,2 (4,0-6,8)	3.453
Kalimantan Utara	60,9 (56,8-64,8)	46,3 (42,3-50,4)	6,4 (4,9-8,2)	2,7 (1,9-3,9)	1,7 (1,0-3,0)	655
Sulawesi Utara	67,4 (65,3-69,5)	54,5 (52,2-56,8)	12,0 (10,8-13,4)	2,1 (1,7-2,6)	0,6 (0,4-0,9)	2.528
Sulawesi Tengah	62,3 (59,7-64,8)	54,8 (51,9-57,7)	16,8 (15,2-18,5)	2,7 (2,3-3,3)	1,4 (1,0-1,9)	3.125
Sulawesi Selatan	71,0 (69,2-727)	45,9 (44,0-47,9)	16,4 (15,3-17,6)	4,0 (3,5-4,6)	1,2 (0,9-1,5)	7.788
Sulawesi Tenggara	62,2 (59,4-65,0)	50,7 (47,8-53,6)	11,3 (10,0-12,8)	2,1 (1,6-2,8)	0,7 (0,4-1,1)	2.136
Gorontalo	58,8 (56,0-61,5)	57,5 (54,3-60,5)	26,7 (24,1-29,4)	2,9 (2,3-3,8)	0,6 (0,4-1,1)	1.207
Sulawesi Barat	67,1 (62,8-71,2)	46,3 (41,6-51,1)	6,6 (5,2-8,3)	2,4 (1,5-3,6)	0,7 (0,3-1,3)	1.038
Maluku	70,6 (68,0-73,1)	35,6 (33,0-38,4)	19,2 (16,4-22,3)	1,8 (1,2-2,7)	0,9 (0,5-1,7)	1.463
Maluku Utara	71,9 (68,6-75,1)	40,7 (37,5-43,9)	22,0 (19,9-14,3)	1,1 (0,7-1,6)	0,4 (0,2-0,7)	1.108
Papua Barat	75,1 (72,0-78,0)	41,2 (37,4-45,1)	12,5 (10,8-14,5)	2,3 (1,4-3,7)	0,8 (0,4-1,5)	806
Papua	71,7 (69,1-74,1)	40,9 (38,3-43,6)	26,6 (24,4-29,0)	1,2 (0,7-1,9)	0,8 (0,5-1,3)	2.489
**INDONESIA**	**67,8 (67,4-68,2)**	**43,4 (43,0-43,9)**	**14,4 (14,2-14,7)**	**2,8 (2,7-2,9)**	**1,6 (1,5-1,7)**	**256.242**

Tabel 12.6.10
Proporsi Jenis Rokok Yang Dihisap Penduduk Umur ≥10 Tahun menurut Karakteristik, Riskesdas 2018

Karakteristik	jenis rokok yang dihisap (%,95%CI)					N Ter-timbang
	Kretek	Rokok putih	Rokok linting	Elektrik	Shisha	
**Kelompok Umur**						
10-14	53,6 (51,3-55,9)	51,2 (48,9-53,5)	4,3 (3,6-5,3)	10,6 (9,1-12,2)	2,8 (2,0-3,8)	3,295
15-19	57,2 (56,0-58,4)	55,6 (54,5-56,8)	6,2 (5,7-6,7)	10,5 (9,8-11,3)	5,1 (4,6-5,7)	17,795
20-24	58,6 (57,6-59,7)	56,9 (55,9-58,0)	7,1 (6,6-7,6)	7,0 (6,5-7,5)	3,9 (3,5-4,3)	27,242
25-29	61,2 (60,2-62,2)	54,4 (53,4-55,4)	8,8 (8,3-9,4)	4,3 (3,9-4,8)	2,6 (2,3-2,9)	28,641
30-34	65,3 (64,3-66,2)	49,5 (48,5-50,5)	9,6 (9,1-10,1)	2,2 (1,9-2,5)	1,6 (1,4-1,9)	28,793
35-39	67,5 (66,6-68,4)	46,4 (45,5-47,4)	10,6 (10,1-11,1)	1,5 (1,3-1,7)	0,7 (0,6-0,9)	29,246
40-44	71,2 (70,4-72,0)	42,1 (41,2-43,0)	11,9 (11,4-12,4)	0,8 (0,7-1,0)	0,7 (0,5-0,8)	26,700
45-49	74,2 (73,4-74,9)	38,2 (37,3-39,1)	14,1 (13,5-14,7)	0,8 (0,7-1,0)	0,5 (0,4-0,7)	24,479
50-54	75,1 (74,3-76,0)	35,0 (34,0-35,9)	17,1 (16,4-17,8)	0,7 (0,5-0,9)	0,6 (0,4-0,8)	20,764
55-59	76,2 (75,3-77,0)	30,6 (29,6-31,5)	22,2 (21,4-23,0)	0,5 (0,4-0,7)	0,4 (0,3-0,6)	17,338
60-64	77,4 (76,4-78,3)	26,6 (25,6-27,7)	29,0 (28,0-30,1)	0,2 (0,2-0,4)	0,2 (0,1-0,3)	12,917
65 +	72,3 (71,5-73,2)	22,0 (21,1-22,8)	40,1 (39,1-41,1)	0,3 (0,2-0,4)	0,3 (0,2-0,4)	19,032
**Kelompok Umur khusus**						
10-18	56,9 (55,8-58,1)	54,1 (53,0-55,3)	5,7 (5,3-6,2)	10,9 (10,2-11,7)	4,8 (4,3-5,4)	18,706
15 +	68,0 (67,6-68,4)	43,3 (42,9-43,8)	14,6 (14,3-14,8)	2,7 (2,6-2,8)	1,6 (1,5-1,7)	254,260
10+	67,8 (67,4-68,2)	43,4 (43,0-43,9)	14,4 (14,2-14,7)	2,8 (2,7-2,9)	1,6 (1,5-1,7)	258,149
**Jenis Kelamin**						
Laki-laki	68,2 (67,8-68,6)	43,7 (43,3-44,2)	14,4 (14,2-14,7)	2,8 (2,7-2,9)	1,6 (1,5-1,6)	244,303
Perempuan	58,8 (57,3-60,2)	37,2 (35,8-38,6)	14,9 (14,0-15,9)	2,7 (2,3-3,2)	2,4 (1,9-2,9)	11,939
**Pendidikan**						
Tidak sekolah	68,0 (66,8-69,2)	25,1 (24,0-26,3)	38,1 (36,8-39,4)	0,8 (0,6-1,1)	0,5 (0,4-0,7)	11,595
Tidak tamat SD	73,2 (72,5-74,0)	32,0 (31,1-32,8)	27,6 (26,9-28,4)	1,1 (1,0-1,3)	0,7 (0,6-0,9)	31,567
Tamat SD	72,6 (72,1-73,2)	37,7 (37,0-38,4)	19,2 (18,8-19,7)	1,6 (1,5-1,8)	0,8 (0,7-0,9)	65,702
Tamat SLTP	67,5 (66,9-68,2)	47,0 (46,3-47,8)	10,2 (9,8-10,6)	3,3 (3,0-3,5)	1,8 (1,6-2,0)	52,443
Tamat SLTA	63,3 (62,6-64,0)	51,0 (50,3-51,7)	6,1 (5,9-6,4)	4,0 (3,7-4,2)	2,3 (2,2-2,6)	77,830
Tamat D1/D2/D3 PT	60,2 (59,0-61,4)	53,5 (52,3-54,6)	6,4 (5,9-7,0)	4,9 (4,4-5,5)	3,0 (2,6-3,5)	17,105
**Pekerjaan**						
Tidak Bekerja	66,0 (65,1-66,9)	40,9 (40,0-41,9)	15,4 (14,8-16,0)	3,9 (3,5-4,2)	2,3 (2,0-2,6)	31,559
Sekolah	55,6 (54,3-57,0)	54,5 (53,1-55,8)	5,4 (4,9-6,0)	12,1 (11,3-13,1)	5,4 (4,8-6,1)	13,961
PNS/TNI/Polri/BUMN/BUMD	64,8 (63,3-66,3)	49,0 (47,4-50,5)	5,9 (5,2-6,6)	2,3 (1,9-2,8)	1,2 (0,8-1,6)	7,434
Pegawai swasta	58,4 (57,3-59,5)	55,3 (54,2-56,4)	5,0 (4,6-5,4)	4,6 (4,3-5,1)	2,7 (2,4-3,1)	32,122
Wiraswasta	67,8 (67,0-68,5)	46,9 (46,1-47,4)	8,1 (7,7-8,5)	2,0 (1,8-2,2)	1,3 (1,1-1,5)	47,023
Petani/buruh tani	73,8 (73,3-74,3)	32,5 (31,9-33,1)	29,3 (28,8-29,9)	0,7 (0,6-0,8)	0,5 (0,4-0,6)	63,106
Nelayan	73,5 (71,2-75,7)	40,8 (38,3-43,3)	10,6 (9,5-11,9)	1,0 (0,7-1,5)	0,5 (0,3-1,0)	3,872
Buruh/sopir/pembantu ruta	70,6 (69,7-71,4)	44,0 (43,1-45,0)	11,7 (11,2 – 12,2)	1,9 (1,7-2,1)	1,1 (1,0-1,3)	44,637
Lainnya	70,0 (68,7-71,2)	44,3 (42,9-45,7)	11,0 (10,2-11,8)	2,5 (2,1-3,0)	1,7 (1,3-2,1)	12,529
**Tempat tinggal**						
Perkotaan	65,8 (65,2-66,5)	46,9 (46,2-47,5)	8,6 (8,3-8,9)	3,8 (3,6-4,0)	2,3 (2,1-2,4)	139,845
Perdesaan	70,1 (69,7-70,6)	39,3 (38,7-39,8)	21,5 (21,1-21,9)	1,6 (1,5-1,7)	0,8 (0,7-0,9)	116,397

Tabel 12.6.11
Proporsi Merokok Dalam Gedung/Ruangan pada Penduduk Umur ≥10 Tahun
menurut Provinsi, Riskesdas 2018

Provinsi	merokok dalam gedung/ruangan		N Tertimbang
	%	95% CI	
Aceh	87,0	85,8-88,2	4.045
Sumatera Utara	86,6	85,5-87,7	10.722
Sumatera Barat	89,7	88,5-90,8	4.610
Riau	88,7	87,4-89,8	5.353
Jambi	87,1	85,4-88,7	2.560
Sumatera Selatan	87,4	85,8-88,8	6.904
Bengkulu	89,6	87,9-91,1	1.730
Lampung	88,7	87,4-89,8	7.487
Bangka Belitung	86,4	84,1-88,4	1.186
Kepulauan Riau	77,5	73,5-81,1	1.560
DKI Jakarta	59,0	56,3-61,6	8.497
Jawa Barat	75,1	74,0-76,1	44.544
Jawa Tengah	83,4	82,7-84,2	28.280
DI Yogyakarta	59,9	57,0-62,7	2.702
Jawa Timur	81,8	81,0-82,7	32.986
Banten	74,0	72,1-75,8	11.176
Bali	51,6	49,1-54,2	2.968
Nusa Tenggara Barat	78,7	76,7-80,6	4.218
Nusa Tenggara Timur	88,0	86,8-89,0	3.767
Kalimantan Barat	89,7	88,2-90,9	3.851
Kalimantan Tengah	86,3	84,6-87,9	2.175
Kalimantan Selatan	85,0	83,2-86,5	2.864
Kalimantan Timur	80,5	78,2-82,7	2.662
Kalimantan Utara	75,6	71,4-79,3	517
Sulawesi Utara	83,5	81,7-85,1	2.131
Sulawesi Tengah	88,3	87,0-89,5	2.632
Sulawesi Selatan	88,3	87,2-89,3	6.388
Sulawesi Tenggara	92,1	90,5-93,4	1.857
Gorontalo	88,7	86,5-90,5	1.064
Sulawesi Barat	93,6	91,6-95,2	936
Maluku	88,5	86,3-90,4	1.350
Maluku Utara	87,9	86,0-89,5	995
Papua Barat	85,1	81,9-87,8	712
Papua	80,5	78,2-82,6	2.298
**INDONESIA**	**80,6**	**80,3-81,0**	**217.728**

Tabel 12.6.12
Proporsi Merokok Dalam Gedung/Ruangan pada Penduduk Umur ≥10 Tahun menurut Karakteristik, Riskesdas 2018

Karakteristik	merokok dalam gedung/ruangan		N Tertimbang
	%	95% CI	
**Kelompok Umur**			
10 – 14	63,3	60,1-66,3	1.683
15-19	75,3	74,2-76,4	14.833
20-24	81,2	80,3-82,1	24.742
25-29	80,7	79,8-81,5	25.944
30-34	80,0	79,1-80,5	26.002
35-39	80,3	79,5-81,1	26.129
40-44	79,9	79,1-80,7	23.520
45-49	80,9	80,1-81,7	20.965
50-54	82,3	81,4-83,1	17.329
55-59	82,6	81,6-83,5	13.916
60-64	83,7	82,6-84,7	9.741
65 +	84,2	83,3-85,1	12.925
**Kelompok Umur Khusus**			
10-18	72,8	71,6-73,9	14.138
15 +	80,8	80,4-81,1	215.702
10+	80,6	80,3-81,0	219.440
**Jenis Kelamin**			
Laki-laki	80,8	80,5-81,2	210.762
Perempuan	74,4	72,8-76,0	6.966
**Pendidikan**			
Tidak sekolah	86,4	85,4-87,3	9.721
Tidak tamat SD	85,6	84,9-86,3	26.813
Tamat SD	84,5	84,0-85,0	56.935
Tamat SLTP	81,2	80,6-81,8	45.871
Tamat SLTA	76,1	75,4-76,7	66.068
Tamat D1/D2/D3 PT	69,9	68,5-71,2	12.320
**Pekerjaan**			
Tidak Bekerja	78,3	77,4-79,2	23.305
Sekolah	69,6	68,3-71,0	10.130
PNS/TNI/Polri/BUMN/BUMD	74,7	73,0-76,3	5.599
Pegawai swasta	69,4	68,3-70,4	27.056
Wiraswasta	80,3	79,6-81,0	40.416
Petani/buruh tani	88,6	88,1-88,9	55.925
Nelayan	89,3	87,8-90,7	3.361
Buruh/sopir/pembantu ruta	81,9	81,2-82,6	39.733
Lainnya	79,5	78,1-80,8	9.993
**Tempat tinggal**			
Perkotaan	74,7	74,2-75,3	114.686
Perdesaan	87,2	86,9-87,6	103.042

Tabel 12.6.13
Proporsi Frekuensi Berada di Dekat Orang yang Merokok di Dalam Ruangan Tertutup pada Penduduk Umur ≥10 Tahun menurut Provinsi, Riskesdas 2018

Provinsi	Frekuensi berada di dekat orang yang merokok di dalam ruangan tertutup[1]						N Tertimbang
	Setiap hari		Kadang-kadang		Tidak pernah		
	%	95% CI	%	95% CI	%	95% CI	
Aceh	25,8	24,6-27,1	51,6	50,2-53,0	22,6	21,5-31,8	11.591
Sumatera Utara	31,3	30,1-32,5	47,1	45,7-48,5	21,6	20,5-22,8	32.143
Sumatera Barat	45,8	44,4-47,2	39,1	37,7-40,4	15,1	14,1-16,3	11.531
Riau	38,8	37,2-40,5	44,2	42,5-45,9	17,0	15,6-18,4	14.847
Jambi	24,5	22,9-26,2	55,1	53,2-57,1	20,3	18,6-22,2	8.470
Sumatera Selatan	31,8	30,2-33,5	45,9	44,1-47,7	22,3	20,8-23,9	18.591
Bengkulu	37,5	35,5-39,5	43,6	41,7-45,6	18,9	17,4-20,5	4.232
Lampung	39,8	38,2-41,4	42,8	41,2-44,4	17,4	16,2-18,7	18.026
Bangka Belitung	36,5	34,4-38,7	43,0	40,9-45,1	20,5	18,6-22,6	3.304
Kepulauan Riau	28,0	25,5-30,6	45,0	42,1-47,9	27,0	24,4-29,9	4.830
DKI Jakarta	27,7	26,0-29,4	43,9	42,0-45,9	28,4	26,4-30,4	24.086
Jawa Barat	33,7	32,7-34,8	42,1	41,1-43,1	24,2	23,3-25,1	105.571
Jawa Tengah	32,9	32,1-33,6	42,4	41,5-43,2	24,8	24,1-25,5	81.506
DI Yogyakarta	26,2	24,6-28,0	42,4	40,6-44,3	31,3	29,4-33,4	9.605
Jawa Timur	30,8	30,0-31,5	39,4	38,6-40,2	29,9	29,0-30,7	94.289
Banten	38,2	36,4-40,1	36,5	35,0-38,0	25,3	23,7-26,9	27.250
Bali	20,3	18,9-21,8	35,0	33,5-36,6	44,7	42,7-46,7	10.817
Nusa Tenggara Barat	32,8	31,0-34,6	44,2	42,5-45,9	23,0	21,5-24,6	10.888
Nusa Tenggara Timur	28,3	27,2-29,5	55,5	54,2-56,9	16,2	15,1-17,3	11.807
Kalimantan Barat	33,0	31,4-34,7	46,8	45,1-48,6	20,1	18,8-21,5	11.305
Kalimantan Tengah	32,1	30,1-34,2	46,0	43,8-48,1	22,0	20,2-23,8	5.944
Kalimantan Selatan	27,6	26,3-28,9	44,4	42,9-45,8	28,0	26,6-29,5	9.907
Kalimantan Timur	29,6	27,6-31,7	45,9	44,0-47,9	24,5	22,6-26,5	8.504
Kalimantan Utara	30,7	27,7-33,9	42,6	39,6-45,6	26,7	24,2-29,4	1.621
Sulawesi Utara	30,4	28,8-32,1	52,9	50,9-54,8	16,7	15,2-18,4	5.656
Sulawesi Tengah	43,5	41,7-45,2	40,0	38,5-41,5	16,6	15,3-17,9	6.478
Sulawesi Selatan	33,3	32,1-34,5	45,9	44,6-47,2	20,9	19,8-22,0	20.416
Sulawesi Tenggara	32,7	30,8-34,7	43,5	41,3-45,6	23,8	22,0-25,7	5.917
Gorontalo	48,7	45,9-51,5	32,5	30,0-35,0	18,9	17,1-20,8	2.552
Sulawesi Barat	33,5	30,9-36,2	47,5	44,8-50,3	19,0	17,2-21,1	3.097
Maluku	29,3	26,9-31,8	48,0	45,4-50,5	22,8	20,6-25,1	3.836
Maluku Utara	32,5	30,4-34,8	45,2	42,9-47,4	22,3	20,3-24,4	2.604
Papua Barat	28,5	26,4-30,7	46,0	43,4-48,5	25,5	23,3-27,9	2.057
Papua	20,6	18,8-22,5	48,3	45,9-50,6	31,2	28,6-33,8	7.501
**INDONESIA**	**32,4**	**32,1-32,7**	**43,1**	**42,8-43,4**	**24,5**	**24,3-24,8**	**600.799**

[1]Ruangan tertutup termasuk rumah, tempat kerja, dan sarana transportasi

Tabel 12.6.14

Proporsi Frekuensi Berada di Dekat Orang yang Merokok di Dalam Ruangan Tertutup pada Penduduk Umur ≥10 Tahun menurut Karakteristik, Riskesdas 2018

Karakteristik	Frekuensi berada di dekat orang yang merokok di dalam ruangan tertutup[1]						N Tertimbang
	Setiap hari		Kadang-kadang		Tidak pernah		
	%	95% CI	%	95% CI	%	95% CI	
**Kelompok Umur**							
10-14	32,9	32,4-33,4	43,6	43,1-44,1	23,5	23,0-24,0	88.856
15-19	34,1	33,5-34,7	45,5	44,9-46,1	20,4	19,9-20,9	67.991
20-24	35,9	35,2-36,6	44,2	43,4-44,9	19,9	19,3-20,6	55.618
25-29	35,2	34,5-35,9	42,8	42,0-43,5	22,0	21,4-22,7	53.472
30-34	35,5	34,8-36,2	41,6	40,9-42,4	22,9	22,2-23,6	50.295
35-39	34,3	33,7-35,0	41,8	41,1-42,5	23,9	23,2-24,5	50.917
40-44	33,6	32,9-34,2	42,5	41,8-43,2	23,9	23,4-24,6	47.134
45-49	33,0	32,4-33,7	42,3	41,6-43,0	24,7	24,1-25,3	44.606
50-54	31,5	30,8-32,3	43,1	42,3-43,8	25,4	24,7-26,1	38.899
55-59	28,8	28,1-29,6	43,9	43,1-44,7	27,2	26,5-28,0	32.652
60-64	25,3	24,5-26,2	43,2	42,2-44,1	31,5	30,6-32,4	25.262
65 +	20,8	20,2-21,4	41,1	40,4-41,8	38,1	37,4-38,8	45.078
**Kelompok Umur Khusus**							
10-18	33,3	32,8-33,7	44,4	44,0-44,9	22,3	21,9-22,7	162.656
15 +	32,3	32,0-32,6	43,0	42,7-43,3	24,7	24,4-25,0	498.081
10+	32,4	32,1-32,7	43,1	42,8-43,4	24,5	24,3-24,8	600.799
**Jenis Kelamin**							
Laki-laki	27,1	26,7-27,5	48,8	48,4-49,2	24,1	23,7-24,6	45.892
Perempuan	34,8	34,4-35,1	40,5	40,2-40,8	24,7	24,4-25,0	103.831
**Pendidikan**							
Tidak sekolah	29,6	28,9-30,4	40,0	39,2-40,8	30,4	29,6-31,4	36.763
Tidak tamat SD	33,7	33,2-34,2	41,9	41,4-42,4	24,4	23,9-24,8	102.347
Tamat SD	35,8	35,3-36,3	41,8	41,3-42,2	22,4	22,1-22,8	154.576
Tamat SLTP	35,0	34,5-35,5	43,5	43,0-44,0	21,5	21,1-22,0	110.644
Tamat SLTA	30,4	30,0-30,9	44,4	43,9-45,0	25,1	24,6-25,6	139.013
Tamat D1/D2/D3 PT	21,1	20,5-21,7	46,9	46,2-47,7	32,0	31,2-32,8	50.380
**Pekerjaan**							
Tidak Bekerja	34,0	33,5-34,4	40,2	39,8-40,6	25,8	25,4-26,2	214.898
Sekolah	32,1	31,7-32,6	45,3	44,7-45,8	22,6	22,2-23,1	119.270
PNS/TNI/Polri/BUMN/BUMD	22,2	21,4-23,1	47,3	46,2-48,4	30,5	29,4-31,6	16.360
Pegawai swasta	28,5	27,7-29,3	45,0	44,1-45,9	26,6	25,7-27,4	47.913
Wiraswasta	29,7	29,1-30,3	44,3	43,7-45,0	26,0	25,3-26,6	63.619
Petani/buruh tani	33,8	33,3-34,4	44,4	43,8-44,9	21,8	21,3-22,3	74.080
Nelayan	30,5	27,6-33,6	47,8	44,5-51,0	21,7	18,6-25,3	1.701
Buruh/sopir/pembantu ruta	34,3	33,4-35,3	44,0	43,0-45,0	21,7	20,9-22,5	32.583
Lainnya	33,8	32,9-34,7	42,4	41,5-43,3	23,8	23,0-24,6	30.355
**Tempat tinggal**							
Perkotaan	29,4	28,9-29,8	43,1	42,6-43,5	27,6	27,1-28,0	335.913
Perdesaan	36,2	35,8-36,6	43,1	42,7-43,5	20,7	20,4-21,1	264.866

[1]Ruangan tertutup termasuk rumah, tempat kerja, dan sarana transportasi

Tabel 12.6.15
Proporsi Mengunyah Tembakau pada Penduduk Umur ≥10 Tahun menurut Provinsi, Riskesdas 2018

Provinsi	Mengunyah tembakau saat ini				Tidak mengunyah tembakau				N tertimbang
	Setiap hari		Kadang-kadang		Mantan		Tidak pernah		
	%	95% CI	%	95% CI	%	95% CI	%	95% CI	
Aceh	1,5	1,3-1,6	6,5	6,0-7,0	0,9	0,8-1,2	91,1	90,5-91,7	15.622
Sumatera Utara	2,1	1,9-2,4	3,0	2,7-3,2	1,1	1,0-1,3	93,7	93,3-94,1	42.787
Sumatera Barat	0,8	0,6-1,0	3,0	2,7-3,4	2,0	1,7-2,4	94,2	93,5-94,7	16.176
Riau	0,5	0,4-0,7	1,7	1,5-1,9	1,0	0,8-1,2	96,8	96,4-97,1	20.198
Jambi	0,4	0,3-0,5	0,9	0,8-1,2	0,5	0,4-0,6	98,2	97,9-98,5	10.987
Sumatera Selatan	0,6	0,4-0,7	0,8	0,6-0,9	0,6	0,4-0,8	98,1	97,8-98,4	25.509
Bengkulu	0,8	0,6-1,0	1,2	0,9-1,4	0,5	0,4-0,7	97,5	97,1-97,8	5.978
Lampung	0,6	0,5-0,7	0,8	0,7-1,0	0,5	0,4-0,6	98,1	97,9-98,4	25.593
Bangka Belitung	0,6	0,4-0,9	1,3	1,0-1,8	0,6	0,4-0,8	97,5	96,9-98,0	4.489
Kepulauan Riau	0,4	0,3-0,7	1,1	0,8-1,5	0,9	0,6-1,2	97,6	97,0-98,0	6.374
DKI Jakarta	0,2	0,1-0,3	0,6	0,5-0,8	1,0	0,8-1,2	98,2	97,9-98,5	32.563
Jawa Barat	0,4	0,3-0,5	0,8	0,7-0,9	0,7	0,6-0,8	98,1	97,9-98,2	150.646
Jawa Tengah	0,6	0,6-0,7	0,6	0,5-0,7	0,8	0,7-0,8	98,0	97,8-98,1	109.680
DI Yogyakarta	0,7	0,5-0,9	0,7	0,6-0,9	2,2	1,8-2,5	96,5	96,0-96,9	12.242
Jawa Timur	0,5	0,4-0,6	0,4	0,4-0,5	0,5	0,4-0,5	98,6	98,5-98,7	127.176
Banten	0,5	0,4-0,7	1,0	0,8-1,3	0,9	0,8-1,2	97,5	97,2-97,8	38.535
Bali	2,5	2,2-2,9	1,2	1,0-1,4	1,1	0,9-1,3	95,2	94,7-95,7	13.705
Nusa Tenggara Barat	1,6	1,3-1,9	2,3	1,9-2,7	1,1	0,9-1,4	95,0	94,4-95,5	15.129
Nusa Tenggara Timur	11,7	11,0-12,3	11,0	10,3-11,7	1,0	0,8-1,3	76,3	75,2-77,4	15.531
Kalimantan Barat	1,8	1,5-2,1	2,8	2,5-3,2	0,9	0,7-1,1	94,5	93,8-95,0	15.135
Kalimantan Tengah	1,8	1,4-2,2	3,4	2,7-4,2	0,8	0,6-1,0	94,1	92,9-95,0	8.121
Kalimantan Selatan	0,4	0,3-0,5	0,6	0,5-0,7	0,9	0,7-1,1	98,1	97,9-98,4	12.710
Kalimantan Timur	0,5	0,3-0,7	0,9	0,8-1,2	0,8	0,6-1,0	97,8	97,4-98,1	11.131
Kalimantan Utara	0,6	0,4-1,0	1,2	0,9-1,6	0,6	0,4-0,9	97,6	96,9-98,1	2.133
Sulawesi Utara	0,4	0,3-0,5	1,0	0,9-1,2	0,9	0,7-1,1	97,7	97,4-98,0	7.795
Sulawesi Tengah	0,7	0,5-0,9	1,3	1,1-1,6	1,0	0,8-1,2	97,1	96,6-97,5	9.134
Sulawesi Selatan	0,5	0,4-0,5	0,9	0,8-1,0	0,7	0,6-0,8	98,0	97,8-98,2	26.719
Sulawesi Tenggara	0,7	0,5-0,9	1,0	0,8-1,3	0,4	0,3-0,6	97,9	97,5-98,2	7.750
Gorontalo	0,3	0,2-0,5	1,0	0,8-1,3	0,8	0,6-1,1	97,9	97,4-98,2	3.627
Sulawesi Barat	0,5	0,3-0,7	0,8	0,5-1,1	0,6	0,4-0,8	98,2	97,7-98,6	4.017
Maluku	2,5	2,1-3,0	4,2	3,4-5,0	1,4	1,0-2,0	91,9	90,6-93,1	5.182
Maluku Utara	4,3	3,7-5,1	9,7	8,8-10,7	1,6	1,2-2,0	84,4	82,9-85,7	3.604
Papua Barat	3,2	2,5-4,2	4,3	3,5-5,4	1,0	0,7-1,4	91,5	89,7-93,0	2.766
Papua	5,6	4,8-6,6	9,2	8,2-10,4	2,5	1,7-3,6	82,7	80,7-84,5	9.764
**INDONESIA**	**1,0**	**1,0-1,0**	**1,5**	**1,5-1,5**	**0,8**	**0,8-0,9**	**96,7**	**96,6-96,7**	**818.507**

Tabel 12.6.16

Proporsi Mengunyah Tembakau pada Penduduk Umur ≥10 Tahun menurut Karakteristik, Riskesdas 2018

Karakteristik	Mengunyah tembakau saat ini				Tidak mengunyah tembakau				N Tertimbang
	Setiap hari		Kadang-kadang		Mantan		Tidak pernah		
	%	95% CI	%	95% CI	%	95% CI	%	95% CI	
**Kelompok Umur**									
10-14	0,15	0,12-0,19	0,85	0,78-0,92	0,24	0,20-0,28	98,76	98,67-98,85	87.981
15-19	0,28	0,23-0,34	1,05	0,96-1,14	0,36	0,30-0,43	98,32	98,19-98,44	82.001
20-24	0,40	0,34-0,47	1,27	1,17-1,39	0,46	0,40-0,54	97,87	97,71-98,02	80.744
25-29	0,62	0,54-0,70	1,35	1,24-1,46	0,58	0,50-0,67	97,46	97,29-97,61	79.965
30-34	0,66	0,60-0,74	1,47	1,36-1,58	0,60	0,52-0,69	97,27	97,11-97,42	76.948
35-39	0,87	0,79-0,95	1,57	1,47-1,69	0,78	0,69-0,89	96,78	96,60-96,94	77.689
40-44	0,94	0,86-1,02	1,64	1,53-1,76	0,76	0,68-0,86	96,66	96,48-96,83	71.198
45-49	1,10	1,01-1,20	1,72	1,60-1,85	0,91	0,81-1,03	96,26	96,07-96,45	65.973
50-54	1,22	1,12-1,32	1,78	1,65-1,92	1,06	0,94-1,20	95,94	95,72-96,15	56.498
55-59	1,61	1,49-1,75	1,90	1,74-2,07	1,17	1,05-1,31	95,31	95,06-95,55	46.742
60-64	2,06	1,90-2,24	1,95	1,78-2,14	1,60	1,43-1,79	94,38	94,07-94,68	35.052
65 +	4,06	3,85-4,28	2,25	2,10-2,41	2,58	2,40-2,78	91,11	90,77-91,43	57.717
**Kelompok Umur Khusus**									
10-18	0,20	0,18-0,24	0,93	0,87-1,00	0,29	0,25-0,34	98,57	98,48-98,65	176.556
10 +	1,11	1,08-1,15	1,57	1,53-1,62	0,90	0,86-0,93	96,42	96,34-96,49	818.507
15 +	1,11	1,08-1,15	1,57	1,53-1,62	0,90	0,86-0,93	96,42	96,34-96,49	713.783
**Jenis Kelamin**									
Laki-laki	0,79	0,75-0,83	1,43	1,37-1,48	0,79	0,75-0,84	96,99	96,90-97,08	409.223
Perempuan	1,23	1,18-1,28	1,57	1,51-1,62	0,86	0,81-0,90	96,35	96,26-96,43	409.284
**Pendidikan**									
Tidak sekolah	4,14	3,91-4,39	2,51	2,33-2,70	1,94	1,76-2,13	91,41	91,04-91,77	46.843
Tidak tamat SD	1,50	1,42-1,59	1,69	1,60-1,79	0,97	0,89-1,05	95,84	95,68-95,99	129.354
Tamat SD	1,05	0,99-1,11	1,49	1,42-1,56	0,77	0,72-0,83	96,69	96,57-96,80	213.043
Tamat SLTP	0,58	0,53-0,63	1,33	1,26-1,41	0,61	0,55-0,68	97,48	97,36-97,59	158.149
Tamat SLTA	0,49	0,45-0,54	1,30	1,23-1,37	0,73	0,68-0,80	97,47	97,37-97,58	207.893
Tamat D1/D2/D3 PT	0,33	0,28-0,38	1,41	1,30-1,54	0,72	0,64-0,82	97,54	97,37-97,70	63.225
**Pekerjaan**									
Tidak bekerja	1,09	1,03-1,15	1,46	1,39-1,53	0,97	0,91-1,04	96,48	96,37-96,59	233.629
Sekolah	0,22	0,19-0,26	0,95	0,89-1,03	0,33	0,29-0,39	98,49	98,40-98,59	126.626
PNS/TNI/Polri/BUMN/BUMD	0,62	0,52-0,75	1,82	1,63-2,03	0,75	0,63-0,90	96,80	96,51-97,07	21.931
Pegawai swasta	0,28	0,23-0,34	0,93	0,82-1,04	0,63	0,55-0,74	98,16	97,99-98,31	75.781
Wiraswasta	0,54	0,48-0,60	1,26	1,17-1,36	0,93	0,84-1,02	97,28	97,13-97,42	105.489
Petani/buruh tani	2,72	2,61-2,84	2,73	2,62-2,86	1,05	0,97-1,13	93,50	93,29-93,70	133.261
Nelayan	1,51	1,21-1,89	1,93	1,59-2,33	0,92	0,65-1,28	95,65	94,99-96,22	5.556
Buruh/sopir/pembantu ruta	0,56	0,49-0,65	0,97	0,87-1,08	0,80	0,70-0,92	97,66	97,48-97,83	75.590
Lainnya	0,96	0,85-1,08	1,77	1,62-1,93	0,93	0,81-1,08	96,34	96,09-96,57	40.644
**Tempat tinggal**									
Perkotaan	0,48	0,45-0,51	1,04	0,99-1,09	0,80	0,75-0,85	97,68	97,6-97,77	450.011
Perdesaan	1,66	1,60-1,72	2,06	1,99-2,13	0,85	0,81-0,90	95,43	95,32-95,54	368.496

12.7 Aktivitas Fisik

Pengukuran aktivitas fisik dilakukan menggunakan pertanyaan yang merupakan modifikasi dari Global Physical Activity Questionnaire (GPAC) dari WHO yang menjadi bagian dari instrument STEPS WHO untuk mengukur dan monitoring faktor risiko penyakit tidak menular. Gambaran perilaku aktivitas fisik yang dikumpulkan mencakup kegiatan aktivitas fisik berat dan sedang pada kegiatan sehari-hari (gabungan saat bekerja atau di rumah, waktu senggang dan transportasi) dalam jumlah hari per minggu dan jumlah menit per hari, yang ditanyakan pada ART umur 10 tahun ke atas.

Aktivitas fisik berat adalah aktivitas fisik yang dilakukan selama ≥3 hari per minggu dan MET minute per minggu ≥1500 (nilai MET minute aktivitas fisik berat= 8). MET merupakan satuan pengeluaran energi dan digunakan untuk mengukur aktivitas fisik dalam menit. MET minute merupakan satuan yang digunakan dalam mengukur *volume* aktivitas fisik individu.

Aktivitas fisik sedang adalah aktivitas fisik sedang dilakukan selama ≥5 hari dalam seminggu dengan rata-rata lama aktivitas tersebut ≥150 menit dalam seminggu (atau ≥30 menit per hari).

$$Proporsi\ penduduk\ dengan\ aktivitas\ fisik\ kurang = \frac{ART\ umur\ \geq\ 10\ tahun\ dengan\ aktivitas\ fisik\ kurang\ (selain\ sedang\ atau\ berat)}{Semua\ ART\ umur\ \geq\ 10\ tahun}$$

$$Proporsi\ penduduk\ dengan\ aktivitas\ fisik\ cukup = \frac{ART\ umur\ \geq\ 10\ tahun\ dengan\ aktivitas\ fisik\ sedang\ atau\ berat}{Semua\ ART\ umur\ \geq\ 10\ tahun}$$

Tabel 12.7.1
Proporsi Aktivitas Fisik pada Penduduk Umur >10 Tahun menurut Provinsi, Riskesdas 2018

Provinsi	Aktivitas fisik				N Tertimbang
	Cukup		Kurang		
	%	95% CI	%	95% CI	
Aceh	64,2	62,9-65,4	35,8	34,6-37,1	15.622
Sumatera Utara	66,3	65,1-67,4	33,7	32,6-34,9	42.787
Sumatera Barat	60,6	59,4-61,8	39,4	38,2-40,6	16.176
Riau	67,4	65,8-68,9	32,6	31,1-34,2	20.198
Jambi	57,6	56,0-59,2	42,4	40,8-44,0	10.987
Sumatera Selatan	64,0	62,2-65,7	36,0	34,3-37,8	25.509
Bengkulu	72,8	71,5-74,1	27,2	25,9-28,5	5.978
Lampung	71,4	69,9-72,8	28,6	27,2-30,1	25.593
Bangka Belitung	65,7	63,7-67,6	34,3	32,4-36,3	4.489
Kepulauan Riau	58,2	55,8-60,5	41,8	39,3-44,2	6.374
DKI Jakarta	52,2	50,2-54,2	47,8	45,8-49,8	32.563
Jawa Barat	62,5	61,6-63,5	37,5	36,5-38,4	150.646
Jawa Tengah	70,5	69,8-71,2	29,5	28,8-30,2	109.680
DI Yogyakarta	71,9	69,9-73,9	28,1	26,1-30,1	12.242
Jawa Timur	73,5	72,8-74,2	26,5	25,8-27,2	127.176
Banten	58,8	57,2-60,4	41,2	39,6-42,8	38.535
Bali	74,0	72,7-75,3	26,0	24,7-27,3	13.705
Nusa Tenggara Barat	68,8	67,2-70,4	31,2	29,6-32,8	15.129
Nusa Tenggara Timur	74,8	73,8-75,8	25,2	24,2-26,2	15.531
Kalimantan Barat	69,2	67,8-70,6	30,8	29,4-32,2	15.135
Kalimantan Tengah	65,7	64,2-67,2	34,3	32,8-35,8	8.121
Kalimantan Selatan	66,3	64,9-67,7	33,7	32,3-35,1	12.710
Kalimantan Timur	58,9	56,9-60,9	41,1	39,1-43,1	11.131
Kalimantan Utara	53,9	51,0-56,7	46,1	43,3-49,0	2.133
Sulawesi Utara	66,3	64,6-68,0	33,7	32,0-35,4	7.795
Sulawesi Tengah	67,0	65,6-68,4	33,0	31,6-34,4	9.134
Sulawesi Selatan	66,6	65,5-67,6	33,4	32,4-34,5	26.719
Sulawesi Tenggara	64,5	62,6-66,4	35,5	33,6-37,4	7.750
Gorontalo	66,0	64,0-68,0	34,0	32,0-36,0	3.627
Sulawesi Barat	70,4	68,2-72,6	29,6	27,4-31,8	4.017
Maluku	57,5	55,3-59,6	42,5	40,4-44,7	5.182
Maluku Utara	61,7	59,5-63,9	38,3	36,1-40,5	3.604
Papua Barat	58,8	56,3-61,2	41,2	38,8-43,7	2.766
Papua	66,1	64,3-67,8	33,9	32,2-35,7	9.764
**INDONESIA**	**66,5**	**66,2-66,7**	**33,5**	**33,3-33,8**	**818.507**

Tabel 12.7.2

ProporsiAktivitas Fisik pada Penduduk Umur ≥10 Tahun menurut Karakteristik, Riskesdas 2018

Karakteristik	Aktivitas fisik (%,95%CI)				N Tertimbang
	Cukup		Kurang		
	%	95% CI	%	95% CI	
**Kelompok Umur**					
10-14	35,6	35,1-36,2	64,4	63,8-64,9	87.981
15-19	50,4	49,8-51,0	49,6	49,0-50,2	82.001
20-24	66,8	66,2-67,4	33,2	32,6-33,8	80.744
25-29	73,0	72,4-73,6	27,0	26,4-27,6	79.965
30-34	76,2	75,6-76,7	23,8	23,3-24,4	76.948
35-39	77,9	77,3-78,4	22,1	21,6-22,7	77.689
40-44	78,9	78,4-79,4	21,1	20,6-21,6	71.198
45-49	78,1	77,6-78,7	21,9	21,3-22,4	65.973
50-54	76,8	76,2-77,3	23,2	22,7-23,8	56.498
55-59	74,2	73,6-74,8	25,8	25,2-26,4	46.742
60-64	68,6	67,8-69,3	31,4	30,7-32,2	35.052
65 +	52,1	51,4-52,7	47,9	47,3-48,6	57.717
**Jenis Kelamin**					
Laki-laki	63,6	63,2-63,9	36,4	36,1-36,8	409.223
Perempuan	69,3	69,0-69,6	30,7	30,4-31,0	409.284
**Pendidikan**					
Tidak sekolah	61,5	60,8-62,3	38,5	37,7-39,2	46.843
Tidak tamat SD	60,1	59,6-60,6	39,9	39,4-40,4	129.354
Tamat SD	69,4	69,0-69,8	30,6	30,2-31,0	213.043
Tamat SLTP	68,9	68,4-69,3	31,1	30,7-31,6	158.149
Tamat SLTA	68,0	67,5-68,5	32,0	31,5-32,5	207.893
Tamat D1/D2/D3 PT	62,2	61,4-62,9	37,8	37,1-38,6	63.225
**Pekerjaan**					
Tidak Bekerja	62,9	62,5-63,3	37,1	36,7-37,5	233.629
Sekolah	40,9	40,3-41,4	59,1	58,6-59,7	126.626
PNS/TNI/Polri/BUMN/BUMD	63,5	62,5-64,5	36,5	35,5-37,5	21.931
Pegawai swasta	65,7	64,9-66,5	34,3	33,5-35,1	75.781
Wiraswasta	72,8	72,3-73,3	27,2	26,7-27,7	105.489
Petani/Buruh tani	84,6	84,2-84,9	15,4	15,1-15,8	133.261
Nelayan	77,1	75,2-79,0	22,9	21,0-24,8	5.556
Buruh/sopir/pembantu ruta	78,0	77,4-78,7	22,0	21,3-22,6	75.590
Lainnya	70,6	69,8-71,4	29,4	28,6-30,2	40.644
**Tempat tinggal**					
Perkotaan	62,2	61,8-62,7	37,8	37,3-38,2	450.011
Perdesaan	71,6	71,3-71,9	28,4	28,1-28,7	368.496

12.8 Konsumsi Minuman Beralkohol

Data terkait perilaku konsumsi minuman beralkohol berdasarkan konsumsi ART dalam sebulan terakhir, yang mencakup gambaran konsumsi minuman beralkohol saat ini dan konsumsi minuman beralkohol yang berlebihan, serta rata-rata satuan standar minuman beralkohol dan jenis minuman beralkohol yang biasa diminum.

Konsumsi minuman beralkohol yang berlebihan dihitung berdasarkan jumlah satuan standar minuman, yaitu sebanyak $\geq$5 satuan standar pada laki-laki dan $\geq$4 satuan standar pada perempuan (STEPS analysis guide WHO).

Rata-rata satuan standar minuman beralkohol dihitung berdasarkan jenis minuman dan kemasan yang digunakan (botol/kaleng/gelas/sloki/lainnya) yang biasa digunakan pada mereka yang mengonsumsi minuman beralkohol.

Istilah ”minuman standar” menggambarkan intensitas konsumsi alkohol, yang dapat dihitung dari jenis dan volume minuman beralkohol yang dikonsumsi.

Satu minuman standar rata-rata mengandung 10 g (antara 8 – 13 g) etanol murni, yang terdapat dalam:

- Minuman dengan kadar alkohol rendah seperti bir:1 gelas bir/botol kecil/kaleng (285 – 330 ml)
- Minuman dengan kadar alkohol sedang, seperti *white wine*, *champagne*, *sparkling wine*: 1 gelas *wine* (biasanya diisi 120 ml)
- Minuman dengan kadar alkohol tinggi, seperti *whiskey*, *vodka*, *tequilla*: 1 sloki (30 ml)
- Minuman tradisional beralkohol bening: $^1/_2$ gelas minum (100 ml)
- Minuman tradisional beralkohol keruh: 1 gelas minum (200 ml)
- Minuman oplosan mengandung kadar alkohol sekitar 20% atau lebih

Wawancara dilakukan dengan menggunakan gambar peraga kemasan minuman beralkohol untuk menyamakan persepsi ukuran yang digunakan responden. Ukuran satuan standar minuman beralkohol ditetapkan berdasarkan jenis minuman beralkohol dan volume kemasannya.

$$Proporsi\ perilaku\ konsumsi\ alkohol = \frac{ART\ umur\ \geq\ 10\ tahun\ yang\ mengonsumsi\ minimal\ 1\ standar\ alkohol\ dalam\ 1\ bulan\ terakhir}{ART\ umur\ \geq\ 10\ tahun}$$

$$Proporsi\ jenis\ minuman\ beralkohol\ yang\ biasa\ dikonsumsi = \frac{\begin{array}{c}ART\ umur\ \geq 10\ tahun\ dengan\ kebiasaan\ konsumsi\ minuman\ beralkohol\ minimal\ 1\ satuan\ standar \\ berdasarkan\ jenis\ minuman\ beralkohol\end{array}}{ART\ umur\ \geq 10\ tahun\ yang\ pernah\ konsumsi\ minuman\ beralkohol\ minimal\ satu\ satuan\ standar}$$

Tabel 12.8.1

Proporsi Perilaku Konsumsi Minuman Beralkohol dalam 1 Bulan Terakhir pada Penduduk Umur >10 Tahun menurut Provinsi, Riskesdas 2018

Provinsi	Konsumsi minuman beralkohol				N Tertimbang
	Ya		Tidak		
	%	95% CI	%	95% CI	
Aceh	0,4	0,3-0,5	99,6	99,5-99,7	15.622
Sumatera Utara	5,5	5,1-5,9	94,5	94,1-94,9	42.787
Sumatera Barat	1,5	1,3-1,8	98,5	98,2-98,7	16.176
Riau	2,2	1,9-2,5	97,8	97,5-98,1	20.198
Jambi	0,7	0,6-0,9	99,3	99,1-99,4	10.987
Sumatera Selatan	1,2	1,0-1,4	98,8	98,6-99,0	25.509
Bengkulu	1,7	1,4-2,0	98,3	98,0-98,6	5.978
Lampung	1,8	1,6-2,1	98,2	97,9-98,4	25.593
Bangka Belitung	3,0	2,6-3,5	97,0	96,5-97,4	4.489
Kepulauan Riau	3,3	2,7-4,1	96,7	95,9-97,3	6.374
DKI Jakarta	3,0	2,6-3,4	97,0	96,6-97,4	32.563
Jawa Barat	1,7	1,5-1,8	98,3	98,2-98,5	150.646
Jawa Tengah	1,9	1,7-2,0	98,1	98,0-98,3	109.680
DI Yogyakarta	3,2	2,7-3,8	96,8	96,2-97,3	12.242
Jawa Timur	1,8	1,7-2,0	98,2	98,0-98,3	127.176
Banten	2,1	1,8-2,4	97,9	97,6-98,2	38.535
Bali	14,0	13,2-14,8	86,0	85,2-86,8	13.705
Nusa Tenggara Barat	3,4	3,0-3,9	96,6	96,1-97,0	15.129
Nusa Tenggara Timur	15,6	15,0-16,3	84,4	83,7-85,0	15.531
Kalimantan Barat	7,5	6,7-8,3	92,5	91,7-93,3	15.135
Kalimantan Tengah	4,2	3,5-5,0	95,8	95,0-96,5	8.121
Kalimantan Selatan	1,3	1,1-1,6	98,7	98,4-98,9	12.710
Kalimantan Timur	2,9	2,5-3,4	97,1	96,6-97,5	11.131
Kalimantan Utara	4,5	3,7-5,4	95,5	94,6-96,3	2.133
Sulawesi Utara	16,0	15,2-16,9	84,0	83,1-84,8	7.795
Sulawesi Tengah	8,8	8,1-9,6	91,2	90,4-91,9	9.134
Sulawesi Selatan	6,3	5,9-6,7	93,7	93,3-94,1	26.719
Sulawesi Tenggara	6,2	5,6-6,9	93,8	93,1-94,4	7.750
Gorontalo	11,3	10,3-12,4	88,7	87,6-89,7	3.627
Sulawesi Barat	4,1	3,4-4,9	95,9	95,1-96,6	4.017
Maluku	11,1	10,0-12,2	88,9	87,8-90,0	5.182
Maluku Utara	7,2	6,4-8,1	92,8	91,9-93,6	3.604
Papua Barat	7,5	6,5-8,6	92,5	91,4-93,5	2.766
Papua	4,9	4,3-5,6	95,1	94,4-95,7	9.764
**INDONESIA**	**3,3**	**3,2-3,3**	**96,7**	**96,7-96,8**	**818.507**

Tabel 12.8.2
Proporsi Perilaku Konsumsi Minuman Beralkohol dalam 1 Bulan Terakhir pada Penduduk Umur ≥10 Tahun menurut Karakteristik, Riskesdas 2018

Karakteristik	Konsumsi minuman beralkohol				N Tertimbang
	Ya		Tidak		
	%	95% CI	%	95% CI	
**Kelompok Umur**					
10-14	0,3	0,3-0,4	99,7	99,6-99,7	87.981
15-19	3,7	3,5-3,9	96,3	96,1-96,5	82.001
20-24	6,4	6,1-6,6	93,6	93,4-93,9	80.744
25-29	5,6	5,3-5,8	94,4	94,2-94,7	79.965
30-34	4,3	4,1-4,5	95,7	95,5-95,9	76.948
35-39	4,0	3,8-4,1	96,0	95,9-96,2	77.689
40-44	3,2	3,1-3,4	96,8	96,6-96,9	71.198
45-49	2,8	2,7-3,0	97,2	97,0-97,3	65.973
50-54	2,3	2,2-2,5	97,7	97,5-97,8	56.498
55-59	1,9	1,7-2,0	98,1	98,0-98,3	46.742
60-64	1,6	1,4-1,7	98,4	98,3-98,6	35.052
65 +	1,0	0,9-1,0	99,0	99,0-99,1	57.717
**Jenis Kelamin**					
Laki-laki	6,1	6,0-6,2	93,9	93,8-94,0	409.223
Perempuan	0,4	0,4-0,5	99,6	99,5-99,6	409.284
**Pendidikan**					
Tidak sekolah	1,8	1,7-2,0	98,2	98,0-98,3	46.843
Tidak tamat SD	2,0	1,9-2,1	98,0	97,9-98,1	129.354
Tamat SD	2,7	2,6-2,8	97,3	97,2-97,4	213.043
Tamat SLTP	3,9	3,8-4,1	96,1	95,9-96,2	158.149
Tamat SLTA	4,5	4,3-4,6	95,5	95,4-95,7	207.893
Tamat D1/D2/D3 PT	3,3	3,1-3,6	96,7	96,4-96,9	63.225
**Pekerjaan**					
Tidak Bekerja	1,4	1,3-1,5	98,6	98,5-98,7	233.629
Sekolah	1,5	1,4-1,6	98,5	98,4-98,6	126.626
PNS/TNI/Polri/BUMN/BUMD	3,5	3,2-3,8	96,5	96,2-96,8	21.931
Pegawai swasta	5,1	4,8-5,4	94,9	94,6-95,2	75.781
Wiraswasta	3,8	3,6-4,0	96,2	96,0-96,4	105.489
Petani/Buruh tani	5,1	5,0-5,3	94,9	94,7-95,1	133.261
Nelayan	10,5	9,4-11,7	89,5	88,3-90,6	5.556
Buruh/sopir/pembantu ruta	5,6	5,3-5,8	94,4	94,2-94,7	75.590
Lainnya	3,1	2,9-3,4	96,9	96,6-97,1	40.644
**Tempat tinggal**					
Perkotaan	3,0	2,9-3,1	97,0	96,9-97,1	450.011
Perdesaan	3,6	3,5-3,7	96,4	96,3-96,5	368.496

Tabel 12.8.3
Proporsi Jenis Minuman Beralkohol yang Biasa Diminum pada Peminum Alkohol Umur >10 Tahun menurut Provinsi, Riskesdas 2018

Provinsi	Jenis minuman berlakohol (%,95%CI)							N tertimbang
	Bir	Anggur/arak	Whisky	Minuman tradisional keruh	Minuman tradisional bening	Minuman oplosan	Lainnya	
Aceh	11,7 (7,0-18,8)	20,1 (14,3-27,5)	3,4 (1,4-8,4)	45,2 (34,8-55,9)	7,2 (3,2-15,6)	7,7 (2,4-21,8)	4,7 (2,4-9,0)	80
Sumatera Utara	10,6 (8,8-12,7)	5,9 (4,3-7,9)	0,9 (0,5-1,7)	71,1 (67,9-74,1)	7,5 (6,3-09,0)	1,2 (0,7-2,2)	2,8 (1,9-4,1)	2.975
Sumatera Barat	63,2 (55,8-70,0)	7,8 (4,8-12,7)	5,4 (3,2-9,0)	15,7 (11,0-21,9)	2,4 (0,8-6,5)	1,8 (0,8-4,2)	3,6 (2,1-6,3)	306
Riau	39,0 (33,6-44,7)	5,2 (3,3-8,1)	1,3 (0,6-2,8)	39,2 (33,0-45,7)	3,8 (2,1-6,7)	2,5 (1,1-5,6)	9,0 (5,9-13,6)	564
Jambi	50,0 (39,9-60,1)	9,9 (5,6-17,0)	1,5 (0,4-5,4)	28,5 (19,4-39,8)	2,7 (0,7-10,0)	1,1 (0,2-7,7)	6,2 (2,6-14,1)	103
Sumatera Selatan	58,1 (50,9-65,0)	21,6 (16,6-27,6)	6,7 (3,8-11,6)	5,3 (2,9-9,4)	0,9 (0,2-3,7)	1,7 (0,7-4,3)	5,7 (2,9-10,9)	393
Bengkulu	36,6 (28,2-45,8)	18,4 (12,1-27,1)	4,5 (2,5-7,9)	22,0 (14,6-31,7)	2,7 (0,9-7,5)	6,6 (3,2-12,9)	9,3 (4,8-17,4)	127
Lampung	50,5 (44,7-56,2)	27,0 (22,3-32,4)	1,5 (0,5-4,2)	11,4 (8,3-15,4)	0,8 (0,3-2,1)	2,9 (1,3-06,3)	5,8 (3,6-9,3)	592
Bangka Belitung	80,3 (73,8-85,6)	17,2 (12,2-23,7)	0,5 (0,1-2,3)	0,1 (0,0-0,8)	1,1 (0,2-4,4)	0,4 (0,1-2,6)	0,3 (0,0-2,3)	170
Kepulauan Riau	67,9 (58,5-76,1)	10,3 (5,9-17,4)	4,2 (1,8-9,6)	7,7 (3,6-15,8)	0,1 (0,0-0,8)	5,3 (1,5-17,3)	4,4 (1,5-12,3)	268
DKI Jakarta	49,8 (43,2-56,4)	33,8 (27,9-40,4)	8,0 (5,4-11,8)	1,3 (0,5-3,3)	1,8 (0,3-9,5)	1,4 (0,5-3,7)	3,9 (2,2-6,8)	1.223
Jawa Barat	33,6 (29,4-38,0)	46,1 (41,7-50,6)	6,0 (4,3-8,4)	3,5 (2,2-5,5)	3,3 (2,1-5,1)	3,6 (2,2-5,9)	3,9 (2,5-6,0)	3.187
Jawa Tengah	38,0 (34,9-41,3)	38,8 (35,6-42,0)	4,5 (3,3-6,1)	4,1 (2,9-5,7)	4,3 (3,2-5,7)	6,6 (5,1-8,6)	3,7 (2,7-5,2)	2.606
DI Yogyakarta	26,9 (20,7-34,1)	57,0 (50,3-63,4)	7,1 (4,2-11,7)	0,9 (0,2-4,1)	0,5 (0,1-2,2)	1,9 (0,5-6,3)	5,7 (2,8-11,3)	494
Jawa Timur	44,7 (41,2-48,3)	29,6 (26,4-32,9)	7,3 (5,8-9,3)	7,3 (5,8-9,1)	3,4 (2,4-4,8)	5,7 (4,3-7,5)	2,0 (1,2-3,4)	2.914
Banten	45,4 (37,8-53,3)	40,6 (33,6-48,0)	2,8 (1,3-5,7)	3,7 (1,9-6,9)	1,3 (0,4-4,1)	2,0 (0,9-4,4)	4,2 (2,4-7,4)	1.012
Bali	39,6 (36,4-42,9)	17,9 (15,7-20,4)	4,2 (3,2-5,5)	31,3 (28,3-34,6)	5,4 (94,1-7,2)	0,1 (0,0-0,7)	1,4 (0,8-2,4)	2.420
Nusa Tenggara Barat	30,4 (26,1-35,1)	8,0 (5,7-11,2)	1,9 (0,8-4,2)	41,4 (35,6-47,4)	14,1 (10,5-18,8)	1,7 (0,9-3,5)	2,5 (1,1-5,4)	649
Nusa Tenggara Timur	8,7 (7,6-10,1)	10,8 (9,4-12,4)	1,0 (0,7-1,4)	24,8 (22,5-27,3)	49,0 (46,3-51,8)	3,4 (2,6-2,4)	2,3 (1,7-3,1)	3.067
Kalimantan Barat	11,6 (9,4-14,2)	50,4 (44,9-55,8)	0,6 (03-1,1)	27,1 (21,9-33,2)	8,7 (6,7-11,4)	0,7 (0,4-1,4)	0,8 (0,4-1,6)	1.430
Kalimantan Tengah	40,9 (34,0-48,2)	24,9 (19,9-30,5)	0,9 (0,4-2,1)	23,6 (19,2-28,7)	7,9 (3,2-18,2)	1,4 (0,4-4,4)	0,4 (0,1-1,4)	429
Kalimantan Selatan	32,6 (25,3-40,7)	19,9 (14,4-26,8)	19,6 (14,3-26,3)	5,7 (3,0-10,5)	0,8 (0,1-5,4)	16,4 (11,1-23,5)	5,1 (2,5-10,1)	216
Kalimantan Timur	39,2 (32,3-46,5)	30,2 (23,5-37,8)	8,2 (5,1-12,7)	15,1 (9,6-22,8)	2,7 (1,1-6,1)	1,3 (0,5-3,1)	3,4 (1,8-6,3)	407
Kalimantan Utara	46,1 (38,5-53,8)	3,6 (1,8-7,1)	11,3 (6,4-19,2)	18,5 (13,2-25,3)	16,7 (12,7-21,6)	1,6 (0,6-4,1)	2,3 (0,9-5,5)	122
Sulawesi Utara	14,1 (12,2-16,3)	6,5 (4,5-9,5)	0,4 (0,2-0,7)	5,2 (4,0-6,7)	65,1 (61,6-68,5)	7,4 (5,9-9,2)	1,3 (0,8-2,0)	1.576
Sulawesi Tengah	18,6 (15,8-21,8)	2,9 (2,0-4,2)	1,0 (0,5-1,9)	27,5 (23,9-31,4)	41,1 (37,1-45,2)	6,9 (5,0-9,3)	2,0 (1,3-3,1)	1.019
Sulawesi Selatan	21,5 (19,2-24,0)	3,6 (2,7-4,8)	0,8 (0,4-1,6)	59,3 (55,9-62,7)	12,6 (10,1-15,6)	0,5 (0,2-0,9)	1,7 (0,9-3,1)	2.119
Sulawesi Tenggara	25,8 (21,9-30,1)	16,2 (13,5-19,4)	3,8 (2,4-6,1)	41,6 (36,6-46,7)	10,8 (8,4-13,8)	0,2 (0,1-0,5)	1,6 (0,9-2,8)	609
Gorontalo	33,6 (29,7-37,7)	2,0 (1,1-3,7)	1,2 (0,4-3,8)	12,9 (10,4-15,9)	34,0 (30,3-37,9)	9,4 (7,2-12,2)	7,0 (5,2-9,3)	518
Sulawesi Barat	17,7 (12,6-24,3)	4,4 (2,6-7,4)	1,7 (0,5-5,2)	62,7 (54,7-70,0)	5,9 (3,4-10,0)	0,8 (0,2-03,1)	6,8 (3,8-12,1)	206
Maluku	19,6 (16,5-23,1)	4,4 (2,1-9,0)	0,7 (0,3-1,7)	13,8 (10,5-18,1)	56,3 (50,8-61,6)	2,1 (1,1-3,8)	3,2 (1,8-5,5)	723
Maluku Utara	12,3 (8,3-17,9)	2,6 (1,3-5,4)	0,5 (0,2-1,6)	23,5 (18,6-29,3)	44,4 (38,6-50,2)	6,4 (4,3-9,5)	10,3 (6,1-16,8)	327
Papua Barat	28,7 (23,7-34,3)	4,4 (2,4-7,1)	11,8 (8,9-15,5)	15,4 (11,4-20,4)	24,8 (18,8-32,0)	3,6 (1,8-7,0)	11,4 (7,5-16,5)	261
Papua	28,9 (24,2-34,1)	9,2 (7,0-12,0)	24,2 (20,0-29,1)	23,6 (18,8-29,3)	4,4 (2,5-7,8)	3,5 (2,1-5,7)	6,1 (4,1-9,1)	607
**INDONESIA**	**29,5 (28,7-30,4)**	**21,6 (20,8-22,4)**	**3,8 (3,5-4,2)**	**23,4 (22,6-24,2)**	**15,3 (14,7-15,9)**	**3,3 (2,9-3,6)**	**3,1 (2,8-3,5)**	**33.721**

Tabel 12.8.4

Proporsi Jenis Minuman Beralkohol yang Biasa Diminum pada Peminum Alkohol Umur >10 Tahun menurut Karakteristik, Riskesdas 2018

Karakteristik	Jenis minuman beralkohol														N Tertimbang
	Bir		Anggur/arak		Whisky		Minuman tradisional keruh		Minuman tradisional bening		Minuman oplosan		Lainnya		
	%	95% CI	%	95% CI	%	95% CI	%	95% CI	%	95% CI	%	95% CI	%	95% CI	
**Kelompok Umur**															
10 – 14	20,9	15,7-27,4	15,8	10,8-22,5	1,2	0,3-4,1	35,3	29,0-42,0	13,9	9,5-20,0	8,4	4,5-15,1	4,5	1,9-10,3	342
15-19	29,4	27,1-31,8	28,4	25,9-31,0	3,9	3,0-5,2	16,2	14,6-18,0	11,9	10,6-13,4	5,6	4,4-7,2	4,5	3,6-5,6	3.787
20-24	34,5	32,5-36,6	24,6	22,7-26,6	5,1	4,3-6,1	15,5	14,0-17,1	13,1	11,9-14,3	4,3	3,5-5,2	2,9	2,3-3,7	6.482
25-29	33,4	31,2-35,7	23,0	21,0-25,2	5,1	4,2-6,1	17,3	15,9-18,8	14,4	13,2-15,7	3,1	2,4-4,0	3,7	2,9-4,7	5.618
30-34	31,5	29,2-33,8	19,1	17,1-21,3	4,3	3,3-5,4	24,9	23,1-26,8	14,7	13,3-16,2	2,5	2,0-3,1	3,1	2,3-4,1	4.176
35-39	31,4	29,2-33,7	19,8	17,8-21,9	3,6	2,7-4,6	25,3	23,6-27,1	15,0	13,8-16,3	2,5	1,9-3,2	2,5	1,9-3,4	3.880
40-44	26,2	23,9-28,7	18,7	16,6-21,0	2,9	2,0-4,1	29,0	27,0-31,1	17,8	16,3-19,4	2,6	1,9-3,7	2,7	2,0-3,8	2.918
45-49	26,3	23,8-28,9	18,5	16,3-20,9	2,0	1,3-3,0	30,0	27,8-32,4	18,1	16,4-19,9	2,3	1,7-3,1	2,8	1,8-4,3	2.373
50-54	22,0	19,3-25,0	17,3	15,0-19,9	2,9	1,6-5,0	33,9	31,2-36,6	19,5	17,5-21,7	2,0	1,4-2,7	2,4	1,7-3,4	1.661
55-59	16,5	13,9-19,5	17,6	14,8-20,6	1,2	0,6-2,1	39,4	36,1-42,8	20,6	18,2-23,1	1,6	1,1-2,3	3,2	2,0-5,1	1.095
60-64	15,4	12,0-19,6	16,8	13,1-21,3	1,7	0,7-4,1	41,2	37,0-45,6	21,4	18,3-24,8	1,7	0,8-3,7	1,8	0,9-3,7	696
65 +	12,6	9,4-16,6	16,2	12,6-20,5	0,3	0,1-0,8	44,1	39,7-48,6	22,4	19,4-25,7	2,3	1,3-4,2	2,2	0,9-5,5	693
**Jenis Kelamin**															
Laki-laki	29,9	29,0-30,8	20,5	19,7-21,4	3,9	3,6-4,3	23,2	22,5-24,0	15,8	15,2-16,4	3,4	3,1-3,8	3,2	2,9-3,6	31.469
Perempuan	24,9	22,1-28,0	36,0	32,5-39,7	2,5	1,6-3,9	25,1	22,5-27,9	8,6	7,3-10,1	1,0	0,5-1,9	1,8	1,1-2,9	2.252
**Pendidikan**															
Tidak sekolah	13,9	11,1-17,1	17,4	14,2-21,1	1,6	0,8-3,0	42,4	38,4-46,4	20,0	17,3-23,0	2,2	1,4-3,4	2,6	1,6-4,3	1.078
Tidak tamat SD	18,3	16,4-20,4	19,4	17,3-21,7	1,4	0,9-2,0	33,5	31,3-35,7	21,1	19,5-22,9	3,5	2,8-4,4	2,8	2,0-3,8	3.231
Tamat SD	23,4	21,8-25,0	22,0	20,4-23,7	2,0	1,6-2,6	27,1	25,7-28,6	18,0	16,9-19,2	4,0	3,4-4,8	3,4	2,8-4,2	7.172
Tamat SLTP	29,0	27,3-30,8	24,8	23,1-26,5	3,2	2,6-4,0	22,4	21,1-23,7	13,7	12,7-14,8	3,8	3,1-4,6	3,1	2,5-3,7	7.884
Tamat SLTA	36,0	34,5-37,5	20,3	19,0-21,7	5,2	4,5-5,9	19,1	17,9-20,3	13,7	12,9-14,6	2,8	2,3-3,4	2,9	2,5-3,5	11.700
Tamat D1/D2/D3 PT	39,4	36,0-42,8	20,8	18,0-23,8	8,4	6,7-10,5	15,0	13,1-17,1	10,6	9,2-12,2	1,7	1,1-2,9	4,0	2,8-5,8	2.657
**Pekerjaan**															
Tidak Bekerja	28,7	26,4-31,1	26,7	24,2-29,4	4,1	3,2-5,2	17,8	16,2-19,5	14,9	13,4-16,7	5,0	4,1-6,2	2,7	2,1-3,5	4.170
Sekolah	31,1	28,1-34,2	24,8	22,1-27,8	4,7	3,4-6,6	16,3	14,4-18,4	13,9	11,9-16,0	5,2	3,8-7,0	4,1	3,0-5,5	2.353
PNS/TNI/Polri/BUMN/BUMD	33,8	29,9-37,8	17,6	14,1-21,8	5,4	3,6-7,8	19,7	16,6-23,2	18,1	15,3-21,2	1,9	1,1-3,4	3,6	2,3-5,7	958
Pegawai swasta	45,4	42,8-48,1	24,1	21,9-26,5	6,1	5,0-7,4	10,4	8,9-12,1	8,6	7,5-9,8	1,9	1,4-2,6	3,4	2,5-4,7	4.880
Wiraswasta	37,0	34,7-39,4	21,4	19,4-23,5	5,4	4,4-6,6	20,0	18,3-21,9	9,9	8,9-11,0	2,3	1,7-3,0	4,0	3,2-5,1	5.088
Petani/Buruh tani	13,7	12,7-14,7	14,9	13,8-16,1	1,2	0,9-1,5	41,1	39,5-42,7	23,8	22,6-25,0	2,9	2,5-3,4	2,4	2,0-2,9	8.609
Nelayan	28,7	23,4-34,8	13,7	9,3-19,7	3,9	1,9-7,6	25,8	21,3-30,9	22,2	18,4-26,4	3,3	2,1-5,1	2,4	1,4-4,1	735
Buruh/sopir/pembantu ruta	32,2	29,8-34,6	26,3	24,0-28,8	3,6	2,8-4,7	19,0	17,2-21,0	12,1	10,9-13,4	3,9	3,0-5,2	2,9	2,1-3,9	5.313
Lainnya	31,9	28,7-35,3	22,2	18,9-26,0	4,1	2,8-5,9	18,5	16,1-21,2	15,9	13,8-18,4	3,7	2,6-5,3	3,7	2,6-5,2	1.615
**Tempat tinggal**															
Perkotaan	38,1	36,7-39,5	25,4	24,0-26,7	5,4	4,8-6,0	13,6	12,6-14,7	10,4	9,7-11,2	3,4	2,9-4,0	3,7	3,2-4,3	16.955
Perdesaan	20,9	20,0-21,8	17,7	16,8-18,7	2,2	2,0-2,6	33,2	32,1-34,4	20,2	19,3-21,1	3,1	2,8-3,5	2,6	2,2-2,9	16.766

Tabel 12.8.5
Rata-rata Jumlah Satuan Standar Minuman Beralkohol yang Biasa Diminum pada Peminum Alkohol Umur ≥10 Tahun menurut Provinsi, Riskesdas 2018

Provinsi	jumlah minuman beralkohol[1]		SD	N tertimbang
	Rata-rata	min – maks		
Aceh	7,8	0,2-71,4	11,3	80
Sumatera Utara	4,0	0,1-62,5	5,5	2.975
Sumatera Barat	4,4	0,1-60	7,0	306
Riau	5,8	0,1-108,9	8,6	564
Jambi	5,6	0,1-33,6	7,6	103
Sumatera Selatan	4,6	0,1-91,6	7,2	393
Bengkulu	6,5	0,1-106	10,9	127
Lampung	5,8	0,1-53,4	7,9	592
Bangka Belitung	3,0	0,1-27,2	3,5	170
Kepulauan Riau	5,8	0,1-53,4	7,0	268
DKI Jakarta	7,3	0,1-140	13,7	1.223
Jawa Barat	7,5	0,1-116,2	11,0	3.187
Jawa Tengah	6,4	0,1-150	11,0	2.606
DI Yogyakarta	5,0	0,1-60	8,1	494
Jawa Timur	7,3	0,1-210	15,9	2.914
Banten	5,5	0,1-83,1	9,7	1.012
Bali	4,0	0,1-140	7,3	2.420
Nusa Tenggara Barat	3,7	0,1-60	6,0	649
Nusa Tenggara Timur	4,2	0,1-124,8	7,0	3.067
Kalimantan Barat	4,0	0,1-66,7	6,7	1.430
Kalimantan Tengah	6,8	0,1-200	11,7	429
Kalimantan Selatan	8,7	0,1-90,8	15,2	216
Kalimantan Timur	6,6	0,1-103,8	11,3	407
Kalimantan Utara	6,6	0,1-112,5	13,2	122
Sulawesi Utara	3,4	0,1-140	7,3	1.576
Sulawesi Tengah	5,5	0,1-89,9	9,9	1.019
Sulawesi Selatan	4,2	0,1-64	5,1	2.119
Sulawesi Tenggara	5,0	0,1-66,7	7,1	609
Gorontalo	3,8	0,1-80	6,5	518
Sulawesi Barat	4,0	0,1-38,8	5,4	206
Maluku	4,5	0,1-90,8	8,4	723
Maluku Utara	5,6	0,1-66,4	8,4	327
Papua Barat	8,1	0,1-160	15,0	261
Papua	9,9	0,1-204	19,6	607
**INDONESIA**	**5,4**	**0,1-210**	**9,9**	**33.721**

[1] Rata-rata satuan standard minuman beralkohol yang biasa dikonsumsi dalam satu bulan terakhir. Satuan standar mengacu pada referensi WHO

Tabel 12.8.6
Rata-rata Jumlah Satuan Standar Minuman Beralkohol yang Biasa Diminum pada PeminumAlkohol Umur >10 Tahun menurut Karakteristik, Riskesdas 2018

Karakteristik	jumlah minuman beralkohol[1]		SD	N tertimbang
	Rata-rata	min – maks		
**Kelompok Umur**				
10 – 14	4,0	0.1-80.0	9.1	342
15-19	5,9	0.1-200.0	11.7	3.787
20-24	5,9	0.1-160.0	10.9	6.482
25-29	5,6	0.1-120.0	8.8	5.618
30-34	5,8	0.1-210.0	11.5	4.176
35-39	5,4	0.1-200.0	9.9	3.880
40-44	5,2	0.1-90.8	8.7	2.918
45-49	4,4	0.1-140.0	8.1	2.373
50-54	4,3	0.1-86.6	7.2	1.661
55-59	4,3	0.1-80.0	7.5	1.095
60-64	5,3	0.1-140.0	12.0	696
65 +	3,5	0.1-64.0	5.4	693
**Jenis Kelamin**				
Laki-laki	5,3	0.1-210.0	9.8	31.469
Perempuan	7,6	0.1-140.0	11.8	2.252
**Pendidikan**				
Tidak sekolah	4,7	0.1-106.0	7.3	1.078
Tidak tamat SD	4,9	0.1-160.0	9.4	3.231
Tamat SD	5,2	0.1-160.0	8.3	7.172
Tamat SLTP	5,6	0.1-210.0	10.6	7.884
Tamat SLTA	5,3	0.1-204.0	10.2	11.700
Tamat D1/D2/D3 PT	6,8	0.1-140.0	12.3	2.657
**Pekerjaan**				
Tidak Bekerja	6,6	0.1-140.0	11.0	4.170
Sekolah	5,2	0.1-150.0	9.6	2.353
PNS/TNI/Polri/BUMN/BUMD	5,2	0.1-200.0	10.1	958
Pegawai swasta	5,6	0.1-200.0	11.3	4.880
Wiraswasta	5,2	0.1-210.0	9.1	5.088
Petani/Buruh tani	4,5	0.1-140.0	7.4	8.609
Nelayan	5,9	0.1-150.0	13.6	735
Buruh/sopir/pembantu ruta	5,9	0.1-150.0	10.7	5.313
Lainnya	6,3	0.1-150.0	12.3	1.615
**Tempat tinggal**				
Perkotaan	5,9	0.1-204.0	11.0	16.955
Perdesaan	5,0	0.1-210.0	8.7	16.766

[1] Rata-rata satuan standard minuman beralkohol yang biasa dikonsumsi dalam satu bulan terakhir. Satuan standarmengacu pada referensi WHO

Tabel 12.8.7
Proporsi Konsumsi Minuman Beralkohol yang Berbahaya pada Penduduk Umur≥10 Tahun menurut Provinsi, Riskesdas 2018

Provinsi	konsumsi minuman beralkohol yang berbahaya[1]		N tertimbang
	%	95% CI	
Aceh	0,1	0,1-0,2	15.622
Sumatera Utara	1,1	1,0-1,3	42.787
Sumatera Barat	0,3	0,2-0,4	16.176
Riau	0,7	0,5-0,9	20.198
Jambi	0,2	0,2-0,3	10.987
Sumatera Selatan	0,3	0,2-0,4	25.509
Bengkulu	0,6	0,4-0,4	5.978
Lampung	0,6	0,5-0,7	25.593
Bangka Belitung	0,5	0,3-0,6	4.489
Kepulauan Riau	1,1	0,8-1,6	6.374
DKI Jakarta	0,7	0,5-0,9	32.563
Jawa Barat	0,6	0,5-0,7	150.646
Jawa Tengah	0,6	0,5-0,7	109.680
DI Yogyakarta	0,8	0,6-1,1	12.242
Jawa Timur	0,6	0,5-0,6	127.176
Banten	0,5	0,4-0,6	38.535
Bali	2,9	2,5-3,3	13.705
Nusa Tenggara Barat	0,6	0,5-0,9	15.129
Nusa Tenggara Timur	3,2	2,9-3,5	15.531
Kalimantan Barat	1,4	1,2-1,7	15.135
Kalimantan Tengah	1,5	1,2-1,9	8.121
Kalimantan Selatan	0,5	0,4-0,7	12.710
Kalimantan Timur	0,9	0,7-1,1	11.131
Kalimantan Utara	1,5	1,1-1,9	2.133
Sulawesi Utara	2,4	2,1-2,8	7.795
Sulawesi Tengah	2,3	2,0-2,7	9.134
Sulawesi Selatan	1,7	1,5-1,9	26.719
Sulawesi Tenggara	1,8	1,5-2,1	7.750
Gorontalo	2,1	1,8-2,6	3.627
Sulawesi Barat	0,9	0,7-1,3	4.017
Maluku	2,4	2,0-3,0	5.182
Maluku Utara	2,2	1,7-2,7	3.604
Papua Barat	2,3	1,9-2,8	2.766
Papua	2,0	1,7-2,3	9.764
**INDONESIA**	**0,8**	**0,8-0,9**	**818.507**

[1]Konsumsi minuman beralkohol yang berbahaya jika minum sebanyak ≥5 satuan standar untuk laki-laki dan ≥4 satuan standar untuk perempuan

Tabel 12.8.8
Proporsi Konsumsi Minuman Beralkohol yang Berbahaya pada Penduduk Umur≥10 Tahun menurut Karakteristik, Riskesdas 2018

Karakteristik	konsumsi minuman beralkohol yang berbahaya[1]		N tertimbang
	%	95% CI	
**Kelompok Umur**			
10-14	0,1	0,0-0,1	87.981
15-19	1,0	0,9-1,1	82.001
20-24	1,8	1,7-2,0	80.744
25-29	1,6	1,4-1,7	79.965
30-34	1,2	1,0-1,3	76.948
35-39	1,0	0,9-1,1	77.689
40-44	0,8	0,7-0,9	71.198
45-49	0,6	0,5-0,7	65.973
50-54	0,5	0,4-0,5	56.498
55-59	0,4	0,3-0,4	46.742
60-64	0,3	0,3-0,4	35.052
65 +	0,2	0,1-0,2	87.981
**Jenis Kelamin**			
Laki-laki	1,7	1,6-1,8	409.223
Perempuan	0,0	0,0-0,0	409.284
**Pendidikan**			
Tidak sekolah	0,5	0,4-0,6	46.843
Tidak tamat SD	0,5	0,4-0,5	129.354
Tamat SD	0,7	0,7-0,8	213.043
Tamat SLTP	1,1	1,0-1,1	158.149
Tamat SLTA	1,1	1,1-1,2	207.893
Tamat D1/D2/D3 PT	0,8	0,7-0,9	63.225
**Pekerjaan**			
Tidak Bekerja	0,4	0,3-0,4	233.629
Sekolah	0,3	0,3-0,4	126.626
PNS/TNI/Polri/BUMN/BUMD	0,8	0,6-0,9	21.931
Pegawai swasta	1,3	1,2-1,5	75.781
Wiraswasta	1,0	0,9-1,1	105.489
Petani/Buruh tani	1,2	1,1-1,3	133.261
Nelayan	3,1	2,5-3,9	5.556
Buruh/sopir/pembantu ruta	1,6	1,5-1,8	75.590
Lainnya	0,8	0,7-1,0	40.644
**Tempat tinggal**			
Perkotaan	0,8	0,7-0,8	450.011
Perdesaan	0,9	0,9-1,0	368.496

[1]Konsumsi minuman beralkohol yang berbahaya jika minum sebanyak ≥5 satuan standar untuk laki-laki dan ≥4 satuan standar untuk perempuan

BAB 13
PENGETAHUAN DAN SIKAP TERHADAP HIV/AIDS

13.1 Pengetahuan terhadap HIV/AIDS

Human immunodeficiency virus (HIV) adalah Virus yang memperlemah sistem kekebalan tubuh dan pada akhirnya menyebabkan *Acquired immunodeficiency syndrome* (*AIDS*). *AIDS* merupakan sekelompok kondisi medis yang menunjukkan lemahnya kekebalan tubuh, sering berwujud infeksi ikutan (infeksi oportunistik) dan kanker. Hingga saat ini, *AIDS* belum bisa disembuhkan.

Data pengetahuan tentang *HIV/AIDS* diperoleh dari wawancara langsung kepada ART umur ≥15 tahun dan tidak boleh diwakilkan. Pengetahuan komprehensif dibangun dari 24 pertanyaan mengenai cara penularan, cara pencegahan, dan cara mengetahui seseorang mendarita *HIV/AIDS*.

Proporsi pernah mendengar *HIV/AIDS* dihitung dengan formula:

$$Proporsi\ pernah\ mendengar\ HIV/AIDS = \frac{ART\ Umur\ \geq 15\ tahun\ yang\ pernah\ mendengar\ tentang\ HIV/AIDS}{ART\ Umur\ \geq 15}$$

Proporsi pengetahuan tentang *HIV AIDS* dihitung dengan formula:

$$Proporsi\ pengetahuan\ tentang\ HIV/AIDS = \frac{ART\ Umur\ \geq 15\ tahun\ yang\ memiliki\ pengetahuan\ tentang\ HIV/AIDS}{ART\ Umur\ \geq 15\ tahun\ yang\ pernah\ mendengar\ HIV/AIDS}$$

Tabel 13.1.1
Proporsi Pernah Mendengar *HIV/AIDS* pada Penduduk Umur >15 Tahun menurut Provinsi, Riskesdas 2018

Provinsi	pernah mendengar tentang HIV/AIDS		
	%	95% CI	N tertimbang
Aceh	50,1	48,9 - 51,4	13.285
Sumatera Utara	61,0	60,0 - 62,0	36.147
Sumatera Barat	60,2	58,9 - 61,5	13.683
Riau	62,6	61,2 - 64,0	17.165
Jambi	55,1	53,4 - 56,8	9.439
Sumatera Selatan	41,2	39,6 - 42,8	21.889
Bengkulu	53,6	52,0 - 55,2	5.144
Lampung	51,0	49,6 - 52,3	21.994
Bangka Belitung	68,7	66,8 - 70,6	3.884
Kepulauan Riau	74,7	71,6 - 77,7	5.431
DKI Jakarta	79,3	77,8 - 80,7	28.747
Jawa Barat	57,9	56,9 - 58,9	130.528
Jawa Tengah	56,7	56,0 - 57,4	95.461
DI Yogyakarta	74,4	73,0 - 75,8	10.811
Jawa Timur	54,2	53,5 - 54,9	111.879
Banten	58,1	56,3 - 59,9	33.269
Bali	73,4	72,0 - 74,7	11.885
Nusa Tenggara Barat	47,0	45,3 - 48,6	12.945
Nusa Tenggara Timur	53,2	51,9 - 54,5	12.666
Kalimantan Barat	54,4	52,7 - 56,1	12.876
Kalimantan Tengah	47,7	45,7 - 49,6	6.981
Kalimantan Selatan	57,9	56,2 - 59,5	10.982
Kalimantan Timur	68,3	66,5 - 70,1	9.602
Kalimantan Utara	65,5	62,7 - 68,2	1.816
Sulawesi Utara	62,7	61,2 - 64,2	6.754
Sulawesi Tengah	51,7	50,0 - 53,4	7.763
Sulawesi Selatan	48,7	47,5 - 49,9	22.798
Sulawesi Tenggara	53,4	51,6 - 55,3	6.440
Gorontalo	43,2	40,7 - 45,7	3.117
Sulawesi Barat	39,2	36,7 - 41,8	3.380
Maluku	61,5	59,1 - 63,8	4.329
Maluku Utara	48,7	46,6 - 50,8	2.976
Papua Barat	75,2	73,1 - 77,1	2.343
Papua	61,4	59,1 - 63,6	8.279
**INDONESIA**	**57,4**	**57,1 - 57,7**	**706.689**

Tabel 13.1.2
Proporsi Pernah Mendengar *HIV/AIDS* pada Penduduk Umur >15 Tahun menurut Karakteristik, Riskesdas 2018

Karakteristik	pernah mendengar tentang HIV/AIDS		
	%	95% CI	N tertimbang
**Kelompok Umur**			
15-24	72,0	71,6 - 72,4	157.695
25-34	70,9	70,5 - 71,4	152.523
35-44	62,5	62,0 - 62,9	144.800
45-54	49,8	49,3 - 50,3	119.070
54-64	32,1	31,5 - 32,6	79.170
65-74	19,8	19,2 - 20,5	37.491
75+	8,6	8,0 - 9,3	15.941
**Pendidikan**			
Tidak/belum pernah sekolah	12,4	11,8 - 12,9	38.204
Tidak tamat SD/MI	23,1	22,6 - 23,6	81.510
Tamat SD/MI	36,3	35,9 - 36,8	172.323
Tamat SLTP/MTS	64,6	64,1 - 65,0	150.634
Tamat SLTA/MA	81,2	80,8 - 81,5	202.439
Tamat D1/D2/D3/PT	93,8	93,5 - 94,1	61.579
**Pekerjaan**			
Tidak bekerja	53,1	52,6 - 53,5	204.063
Sekolah	74,9	74,4 - 75,5	56.924
PNS/TNI/Polri/BUMN/BUMD	92,0	91,5 - 92,5	21.374
Pegawai swasta	85,0	84,4 - 85,5	73.840
Wiraswasta	65,4	64,8 - 65,9	102.763
Petani	29,2	28,7 - 29,6	129.477
Nelayan	39,2	37,2 - 41,2	5.386
Buruh/sopir/pembantu ruta	55,2	54,5 - 56,0	73.472
Lainnya	62,4	61,6 - 63,2	39.389
**Jenis Kelamin**			
Laki-laki	59,2	58,9 - 59,6	352.269
Perempuan	55,6	55,2 - 55,9	354.420
**Tempat Tinggal**			
Perkotaan	68,0	67,6 - 68,5	391.028
Perdesaan	44,2	43,9 - 44,6	315.661
**Status Perkawinan**			
Belum Kawin	72,9	72,5 - 73,3	163.766
Kawin	55,6	55,3 - 55,9	484.012
Cerai hidup	52,5	51,3 - 53,8	13.773
Cerai mati	22,1	21,5 - 22,6	45.138

Tabel 13.1.3
Proporsi Pengetahuan *HIV/AIDS* pada Penduduk ≥15 Tahun menurut Provinsi, Riskesdas 2018

Provinsi	Pengetahuan HIV/AIDS (jumlah jawaban benar)				N tertimbang
	Tidak tahu	0-7	8-15	16-24	
	%	%	%	%	
Aceh	2,9	66,6	28,7	1,9	6.197
Sumatera Utara	2,2	66,4	30,3	1,0	20.515
Sumatera Barat	2,7	67,3	28,5	1,6	7.666
Riau	2,7	64,4	31,8	1,1	10.004
Jambi	1,8	54,2	42,8	1,3	4.839
Sumatera Selatan	1,9	63,9	32,5	1,7	8.386
Bengkulu	3,2	64,6	31,3	1,0	2.567
Lampung	3,2	65,7	30,0	1,2	10.429
Bangka Belitung	1,5	66,3	31,6	0,6	2.484
Kepulauan Riau	1,5	64,3	33,1	1,2	3.778
DKI Jakarta	0,5	68,3	30,7	0,5	21.211
Jawa Barat	1,6	68,3	29,5	0,7	70.360
Jawa Tengah	1,6	65,3	32,3	0,8	50.396
DI Yogyakarta	1,5	60,7	37,2	0,6	7.487
Jawa Timur	1,6	64,6	32,9	0,9	56.444
Banten	1,7	68,9	28,8	0,6	18.001
Bali	1,4	58,3	39,8	0,6	8.118
Nusa Tenggara Barat	2,3	66,0	30,5	1,2	5.660
Nusa Tenggara Timur	4,8	60,5	32,7	2,0	6.276
Kalimantan Barat	2,9	62,2	34,0	0,9	6.519
Kalimantan Tengah	1,5	67,0	30,4	1,1	3.096
Kalimantan Selatan	2,1	65,8	31,1	1,0	5.913
Kalimantan Timur	1,0	66,9	31,6	0,5	6.105
Kalimantan Utara	3,6	61,3	33,7	1,5	1.107
Sulawesi Utara	3,4	67,9	27,1	1,6	3.941
Sulawesi Tengah	4,5	64,8	28,8	2,0	3.738
Sulawesi Selatan	3,7	62,4	32,4	1,5	10.331
Sulawesi Tenggara	3,9	65,0	29,4	1,7	3.203
Gorontalo	4,8	61,8	31,0	2,4	1.252
Sulawesi Barat	6,6	61,2	29,7	2,5	1.234
Maluku	3,6	52,9	40,9	2,6	2.476
Maluku Utara	6,2	61,0	29,1	3,8	1.349
Papua Barat	4,0	50,2	44,1	1,7	1.639
Papua	4,2	48,2	45,6	2,1	4.728
**INDONESIA**	**2,0**	**65,2**	**31,8**	**1,0**	**377.449**

Tabel 13.1.4
Proporsi Pengetahuan *HIV/AIDS* pada Penduduk >15 Tahun menurut Karakteristik, Riskesdas 2018

Karakteristik	Pengetahuan HIV/AIDS (jumlah jawaban benar)				N tertimbang
	Tidak tahu	0-7	8-15	16-24	
	%	%	%	%	
**Kelompok Umur**					
15-24	1,0	64,8	33,2	1,0	105.659
25-34	1,5	64,5	33,1	1,0	100.678
35-44	2,2	64,4	32,5	0,9	84.153
45-54	2,9	67,1	29,1	1,1	55.135
54-64	4,6	68,0	26,4	1,0	23.638
65-74	6,1	67,8	24,9	1,2	6.911
75+	8,5	64,9	25,7	1,0	1.276
**Pendidikan**					
Tidak/belum pernah sekolah	8,2	64,4	24,8	2,7	4.392
Tidak tamat SD/MI	7,0	68,0	23,1	1,9	17.536
Tamat SD/MI	4,7	69,4	24,6	1,4	58.284
Tamat SLTP/MTS	2,0	67,8	29,2	1,1	90.532
Tamat SLTA/MA	0,9	65,4	33,0	0,8	152.941
Tamat D1/D2/D3/PT	0,3	55,0	44,2	0,5	53.765
**Pekerjaan**					
Tidak bekerja	2,5	68,1	28,5	0,9	100.777
Sekolah	0,9	62,6	35,6	0,9	39.701
PNS/TNI/Polri/BUMN/BUMD	0,5	55,2	43,8	0,6	18.307
Pegawai swasta	0,5	62,1	36,9	0,6	58.401
Wiraswasta	1,8	65,4	31,8	1,0	62.529
Petani	5,5	65,6	27,0	2,0	35.128
Nelayan	4,2	66,7	26,4	2,7	1.966
Buruh/sopir/pembantu ruta	2,1	68,9	27,9	1,1	37.775
Lainnya	2,1	65,4	31,6	0,9	22.866
**Tempat Tinggal**					
Perkotaan	1,4	65,2	32,7	0,7	247.513
Perdesaan	3,2	65,2	30,1	1,5	129.936
**Status Perkawinan**					
Belum Kawin	0,9	63,4	34,7	1,0	111.102
Kawin	2,4	65,8	30,8	1,0	250.349
Cerai	4,3	68,3	26,5	1,0	15.998

13.2 Sikap terhadap Penderita HIV/AIDS

Tabel 13.2.1
Proporsi Sikap terhadap Penderita HIV/AIDS pada Penduduk Umur ≥15 Tahun menurut Provinsi, Riskesdas 2018

Provinsi	Sikap terhadap penderita HIV/AIDS (%)					N tertimbang
	Sikap[1]	Sikap[2]	Sikap[3]	Sikap[4]	Sikap[5]	
Aceh	51,4	85,9	17,9	28,9	36,6	6.197
Sumatera Utara	43,6	79,5	16,5	33,5	39,6	20.515
Sumatera Barat	42,3	92,2	17,4	38,0	44,0	7.666
Riau	43,1	90,3	14,3	40,1	41,3	10.004
Jambi	51,7	89,4	15,5	34,0	33,0	4.839
Sumatera Selatan	56,6	83,2	18,3	37,8	41,6	8.386
Bengkulu	41,3	90,2	17,4	38,7	38,1	2.567
Lampung	48,0	89,6	15,1	46,4	42,1	10.429
Bangka Belitung	39,5	87,0	15,5	36,6	41,8	2.484
Kepulauan Riau	45,9	91,3	12,0	40,6	37,5	3.778
DKI Jakarta	50,6	89,5	11,7	42,3	39,5	21.211
Jawa Barat	50,3	88,1	14,2	42,5	38,7	70.360
Jawa Tengah	48,2	92,7	12,5	46,3	37,7	50.396
DI Yogyakarta	41,6	93,7	8,4	48,5	32,0	7.487
Jawa Timur	46,9	91,2	15,0	39,5	39,6	56.444
Banten	50,3	90,0	11,6	45,8	41,4	18.001
Bali	40,0	91,4	13,8	39,4	36,8	8.118
Nusa Tenggara Barat	54,4	91,1	16,1	40,0	42,1	5.660
Nusa Tenggara Timur	24,6	80,9	20,5	29,5	40,0	6.276
Kalimantan Barat	41,2	82,2	19,4	36,0	40,6	6.519
Kalimantan Tengah	49,3	88,0	14,4	37,5	33,4	3.096
Kalimantan Selatan	50,4	83,8	16,2	34,0	38,9	5.913
Kalimantan Timur	38,4	89,2	13,6	41,8	43,6	6.105
Kalimantan Utara	38,2	86,0	13,1	33,9	41,6	1.107
Sulawesi Utara	30,8	88,8	19,2	30,4	41,3	3.941
Sulawesi Tengah	36,7	86,9	17,4	31,9	42,8	3.738
Sulawesi Selatan	36,4	84,2	17,2	33,6	41,4	10.331
Sulawesi Tenggara	31,3	82,2	16,4	29,7	37,8	3.203
Gorontalo	37,9	82,9	19,8	26,0	39,6	1.252
Sulawesi Barat	33,0	81,0	15,8	28,7	36,9	1.234
Maluku	40,0	78,7	22,0	29,0	39,9	2.476
Maluku Utara	38,5	81,4	20,8	31,3	40,6	1.349
Papua Barat	55,5	82,5	23,3	45,9	44,1	1.639
Papua	52,0	82,4	16,6	48,0	36,3	4.728
**INDONESIA**	**46,5**	**88,5**	**14,7**	**40,4**	**39,3**	**377.449**

1. Merahasiakan apabila ada ART yang tertular *HIV/AIDS*
2. Bersedia merawat ART yang menderita *HIV/AIDS*
3. Mengucilkan Tetangga yang menderita *HIV/AIDS*
4. Membeli sayuran segar dari petani atau penjual yang diketahui terinfeksi HIV/AIDS
5. Menyetujui guru yang menderita *HIV/AIDS* tidak diperkenankan mengajar

Tabel 13.2.2
Proporsi Sikap terhadap Penderita HIV/AIDS pada Penduduk Umur ≥15 Tahun menurut Karakteristik, Riskesdas 2018

Karakteristik	Sikap terhadap penderita HIV/AIDS (%)					N tertimbang
	Sikap[1]	Sikap[2]	Sikap[3]	Sikap[4]	Sikap[5]	
**Kelompok Umur**						
15-24	53,5	87,6	14,8	35,7	39,7	105.659
25-34	48,7	89,3	14,0	41,5	38,3	100.678
35-44	43,5	89,6	14,0	44,9	38,8	84.153
45-54	39,2	89,0	15,6	42,5	39,7	55.135
54-64	37,6	86,6	16,5	37,7	41,4	23.638
65-74	37,1	83,5	17,0	35,1	41,2	6.911
75+	34,3	77,8	19,3	31,7	43,3	1.276
**Pendidikan**						
Tidak/belum pernah sekolah	45,7	82,9	19,6	35,6	38,3	4.392
Tidak tamat SD/MI	42,9	85,7	20,0	37,1	41,8	17.536
Tamat SD/MI	43,8	87,3	17,3	38,8	40,4	58.284
Tamat SLTP/MTS	47,3	88,1	15,1	39,4	40,4	90.532
Tamat SLTA/MA	47,4	89,1	13,7	40,6	38,9	152.941
Tamat D1/D2/D3/PT	47,0	90,5	11,9	44,7	36,7	53.765
**Pekerjaan**						
Tidak bekerja	46,8	86,9	15,5	38,7	39,9	100.777
Sekolah	54,3	87,4	14,2	35,2	39,6	39.701
PNS/TNI/Polri/BUMN/BUMD	42,8	90,4	12,8	45,1	37,7	18.307
Pegawai swasta	49,0	91,3	12,0	43,1	37,3	58.401
Wiraswasta	44,6	88,7	14,6	41,5	39,1	62.529
Petani	42,4	87,5	18,4	38,8	40,7	35.128
Nelayan	43,1	86,7	20,5	35,5	40,8	1.966
Buruh/sopir/pembantu ruta	44,9	90,1	14,6	43,4	39,6	37.775
Lainnya	43,5	88,2	14,6	41,2	40,0	22.866
**Tempat Tinggal**						
Perkotaan	47,1	88,9	13,4	41,5	38,7	247.513
Perdesaan	45,5	87,9	17,1	38,4	40,5	129.936
**Status Kawin**						
Belum Kawin	53,2	88,3	14,4	37,4	39,1	111.102
Kawin	43,8	88,8	14,7	41,8	39,3	250.349
Cerai	42,2	85,7	16,2	39,6	39,9	15.998

1. Merahasiakan apabila ada ART yang tertular *HIV/AIDS*
2. Bersedia merawat ART yang menderita *HIV/AIDS*
3. Mengucilkan Tetangga yang menderita *HIV/AIDS*
4. Membeli sayuran segar dari petani atau penjual yang diketahui terinfeksi HIV/AIDS
5. Menyetujui guru yang menderita *HIV/AIDS* tidak diperkenankan mengajar

BAB 14
KESEHATAN IBU

Data kesehatan ibu dalam Riskesdas 2018 bertujuan untuk menyediakan informasi tentang pelayanan kesehatan ibu dan morbiditas maternal (gangguan/komplikasi) sejak masa kehamilan hingga masa nifas. Informasi yang dikumpulkan dari responden perempuan umur 10-54 tahun yang pernah kawin adalah pengalaman reproduksi dalam kurun waktu lima tahun terakhir. Sedangkan bagi perempuan umur 10-54 tahun yang sudah pernah hamil, informasi yang dikumpulkan mengenai riwayat kehamilan untuk anak terakhir (lahir hidup/lahir mati/keguguran), mencakup:

1. Masa kehamilan terdiri dari; pelayanan kesehatan ibu hamil (ANC K1, K1 ideal dan K4), tenaga dan tempat pemberi layanan ANC, komponen ANC, tes laboratorium untuk ibu hamil, serta gangguan/komplikasi selama masa kehamilan.
2. Saat persalinan terdiri dari; penolong persalinan, tempat persalinan, metode persalinan, sumber biaya persalinan dan gangguan/komplikasi pada saat bersalin.
3. Masa nifas terdiri dari; layanan ibu nifas (KF1, KF2, KF3 dan KF lengkap), pemberian vitamin A, dan gangguan/komplikasi pada masa nifas.
4. Layanan KB setelah bersalin

Bagi perempuan umur 10-54 tahun yang sedang hamil ditanyakan informasi terkait kepemilikan buku KIA.

14.1 Masa Reproduksi

Definisi ‘pernah hamil’

Setiap kejadian kehamilan yang dialami seumur hidup perempuan umur 10-54 tahun yang pernah kawin, tanpa memperhitungkan hasil kehamilannya (lahir hidup, lahir mati atau keguguran, termasuk yang sedang hamil saat wawancara).

$$Proporsi\ riwayat\ kehamilan\ "pernah\ hamil" = \frac{\Sigma\ perempuan\ pernah\ hamil}{\Sigma\ perempuan\ umur\ 10-54\ tahun\ yang\ pernah\ kawin}$$

Definisi ‘sedang hamil’

Responden perempuan umur 10-54 tahun yang pernah kawin dan menyatakan pernah hamil, sedang dalam kondisi hamil pada saat wawancara.

$$Proporsi\ kehamilan\ 'sedang\ hamil' = \frac{\Sigma\ perempuan\ sedang\ hamil}{\Sigma\ perempuan\ umur\ 10-54\ tahun\ yang\ pernah\ hamil}$$

Tabel 14.1.1
Proporsi Riwayat Kehamilan pada Perempuan Umur 10-54 tahun yang Pernah Kawin menurut Provinsi, Riskesdas 2018

Provinsi	Riwayat Kehamilan[1]					
	Pernah hamil[2]			Sedang hamil[3]		
	%	95% CI	N tertimbang	%	95% CI	N tertimbang
Aceh	92,6	91,8-93,3	4.386	6,0	5,3-6,7	4.073
Sumatra Utara	93,4	92,8-94,0	11.285	5,0	4,4-5,7	10.570
Sumatra Barat	94,5	93,7-95,2	4.228	5,7	5,1-6,4	4.006
Riau	94,2	93,4-94,9	5.935	5,8	5,0-6,6	5.607
Jambi	93,7	92,7-94,5	3.245	3,7	3,1-4,4	3.048
Sumatera Selatan	94,2	93,4-94,9	7.341	4,9	4,2-5,6	6.935
Bengkulu	94,9	94,1-95,6	1.780	4,7	3,9-5,6	1.694
Lampung	94,9	94,1-95,5	7.494	4,8	4,2-5,5	7.130
Bangka Belitung	95,3	94,2-96,2	1.273	5,3	4,4-6,4	1.217
Kepulauan Riau	90,9	88,5-92,8	1.908	6,6	4,9-9,0	1.739
DKI Jakarta	91,8	90,6-92,9	9.429	5,5	4,7-6,6	8.683
Jawa Barat	93,8	93,3-94,2	43.970	5,3	4,8-5,8	41.345
Jawa Tengah	93,8	93,4-94,2	30.456	4,2	3,9-4,6	28.660
DI Yogyakarta	93,0	91,6-94,2	3.195	4,5	3,5-5,8	2.979
Jawa Timur	92,5	92,0-92,9	36.820	4,1	3,7-4,4	34.147
Banten	94,2	93,4-94,9	11.508	4,7	4,0-5,5	10.870
Bali	93,7	92,4-94,8	3.751	5,0	4,3-5,9	3.526
Nusa Tenggara Barat	94,1	93,1-94,9	4.653	5,6	4,8-6,5	4.389
Nusa Tenggara Timur	94,2	93,4-94,8	3.998	5,4	4,8-6,1	3.775
Kalimantan Barat	93,1	92,3-93,9	4.422	4,8	4,2-5,5	4.130
Kalimantan Tengah	93,3	92,3-94,2	2.499	4,5	3,8-5,5	2.338
Kalimantan Selatan	93,9	93,1-94,6	3.778	4,6	3,9-5,4	3.558
Kalimantan Timur	92,2	90,8-93,3	3.226	4,9	4,0-6,0	2.982
Kalimantan Utara	92,4	90,5-93,9	601	5,0	3,8-6,5	557
Sulawesi Utara	94,5	93,6-95,2	2.138	3,8	3,1-4,6	2.025
Sulawesi Tengah	93,5	92,7-94,3	2.637	5,8	5,0-6,8	2.474
Sulawesi Selatan	91,9	91,2-92,6	7.340	5,3	4,7-6,0	6.768
Sulawesi Tenggara	94,2	93,2-95,2	2.212	6,2	5,3-7,2	2.091
Gorontalo	93,4	92,2-94,5	1.046	4,0	3,2-5,1	980
Sulawesi Barat	91,9	90,3-93,2	1.146	5,5	4,5-6,7	1.056
Maluku	92,5	90,7-94,0	1.354	5,2	4,2-6,5	1.256
Maluku Utara	93,9	92,8-94,8	1.023	5,7	4,7-6,9	963
Papua Barat	90,2	88,2-91,9	790	5,3	4,1-6,9	715
Papua	84,2	82,6-85,7	3.135	3,0	2,3-3,8	2.648
**Indonesia**	**93,3**	**93,1-93,5**	**234.004**	**4,8**	**4,7-5,0**	**218.938**

[1] Riwayat kehamilan di antara perempuan pernah kawin 10-54 tahun, pengalaman seumur hidup responden

[2] Di antara perempuan pernah kawin 10-54 tahun yang melaporkan pernah hamil (termasuk yang sedang hamil)

[3] Di antara perempuan pernah kawin 10-54 tahun yang pernah hamil.

Tabel 14.1.2

Proporsi Riwayat Kehamilan pada Perempuan Umur 10-54 tahun yang Pernah Kawin menurut Karakteristik, Riskesdas 2018

Karakteristik	Riwayat Kehamilan[1]					
	Pernah hamil[2]			Sedang hamil[3]		
	%	95% CI	N Tertimbang	%	95% CI	N Tertimbang
**Umur saat wawancara**						
10-14	4,1	2,3-7,2	359	38,6	16,7-66,3	15*
15 – 19	63,2	60,8-65,4	4.503	25,1	22,7-27,8	2.852
20 – 24	85,6	84,8-86,3	21.950	14,3	13,4-15,3	18.839
25 – 29	91,8	91,3-92,3	35.251	9,6	9,1-10,2	32.462
30 – 34	95,0	94,7-95,4	37.244	7,0	6,5-7,4	35.495
35 – 39	96,1	95,8-96,3	38.584	3,5	3,2-3,7	37.174
40 – 44	96,0	95,7-96,3	35.266	0,8	0,6-0,9	33.953
45 – 49	96,0	95,7-96,2	32.589	0,1	0,1-0,1	31.364
50 – 54	94,5	94,1-94,9	28.259	0,0	0,0-0,0	26.785
**Kelompok Umur (Khusus)**						
10 - 19 (Remaja)	58,8	56,5-61,0	4.861	25,2	22,7-27,8	2.867
15 - 49 (WUS)	93,3	93,1-93,5	205.386	5,5	5,3-5,7	192.139
**Pendidikan**						
Tidak pernah sekolah	91,4	90,7-92,2	8.463	1,7	1,3-2,1	7.761
Tidak tamat SD/MI	94,0	93,5-94,4	23.671	2,3	2,0-2,7	22.307
Tamat SD/MI	95,0	94,7-95,2	62.602	3,1	2,8-3,3	59.628
Tamat SLTP/MTS	93,6	93,3-94,0	51.257	5,3	4,9-5,6	48.132
Tamat SLTA/MA	92,4	92,0-92,7	65.203	6,5	6,2-6,9	60.404
Tamat D1/D2/D3/PT	90,5	89,9-91,1	22.809	7,9	7,3-8,5	20.706
**Pekerjaan**						
Tidak bekerja	94,4	94,2-94,6	115	5,9	5,7-6,2	109
Sekolah	55,9	52,6-59,1	2.054	8,6	6,4-11,6	1.151
PNS/TNI/Polri/BUMN/BUMD	94,2	93,5-94,8	6.866	3,8	3,2-4,3	6.484
Pegawai swasta	87,2	86,3-88,1	16.951	7,5	6,8-8,3	14.831
Wiraswasta	94,1	93,6-94,5	29.59	2,9	2,6-3,2	27.915
Petani/buruh tani	94,1	93,7-94,4	32.268	1,9	1,7-2,1	30.444
Nelayan	90,7	84,2-94,6	282	5,0	2,7-9,2	257
Buruh/supir/pembantu ruta	92,7	91,9-93,4	13.161	2,7	2,2-3,3	12.235
Lainnya	93,8	93,3-94,3	17.152	5,7	5,2-6,3	16.131
**Tempat tinggal**						
Perkotaan	93,1	92,9-93,4	126.001	5,0	4,8-5,3	117.700
Perdesaan	93,5	93,3-93,7	108.003	4,6	4,5-4,8	101.238

[1] Riwayat kehamilan dari pengalaman seumur hidup responden perempuan pernah kawin 10-54 tahun,

[2] Di antara perempuan pernah kawin 10-54 tahun yang melaporkan pernah hamil (termasuk yang sedang hamil)

[3] Di antara perempuan pernah kawin 10-54 tahun yang pernah hamil.

*N Tertimbang <50

14.2 Masa Kehamilan

Pemeriksaan Kehamilan (Antenatal Care)

Definisi: ANC adalah pelayanan kesehatan kehamilan yang diterima ibu pada masa kehamilan anak terakhir dan diberikan oleh tenaga kesehatan, meliputi dokter (dokter umum dan/atau dokter kandungan), bidan dan perawat.

Cakupan ANC K1

Definisi: Pelayanan kesehatan yang diterima pada masa kehamilan anak terakhir oleh tenaga kesehatan, minimal 1 kali tanpa memperhitungkan periode waktu pemeriksaan.

$$Proporsi\ ANC\ K1 = \frac{\begin{gathered}\Sigma\,Perempuan\ pernah\ kawin\ 10-54\ tahun\ yang\ mendapat\\ layanan\ pemeriksaan\ kehamilan\ oleh\ tenaga\ kesehatan\end{gathered}}{\begin{gathered}\Sigma\ perempuan\ pernah\ kawin\ 10-54\ tahun\ yang\\ bersalin\ anak\ terakhir\ pada\ periode\\ 1\ Januari\ 2013\ sd\ wawancara\end{gathered}}$$

Cakupan ANC K1 ideal

Definisi: Pelayanan kesehatan yang diterima pada masa kehamilan anak terakhir oleh tenaga kesehatan, dan pemeriksaan kehamilan tersebut pertama kali dilakukan pada masa kehamilan trimester 1.

$$Proporsi\ ANC\ K1\ ideal$$
$$= \frac{\begin{gathered}\Sigma\,Perempuan\ umur\ 10-54\ tahun\ yang\ mendapat\ layanan\\ pemeriksaan\ kehamilan\ anak\ terakhir\ oleh\ tenaga\ kesehatan\\ dan\ pertama\ kali\ diperiksa\ pada\ masa\ kehamilan\ trimester\ 1\end{gathered}}{\begin{gathered}\Sigma\ perempuan\ umur\ 10-54\ tahun\ yang\ bersalin\\ anak\ terakhir\ pada\ periode\ \ 1\ Januari\ 2013\ sd\ wawancara\\ dan\ mendapat\ layanan\ ANC\ (K1)\ selama\ hamil\end{gathered}}$$

<u>Cakupan ANC K4</u>

Definisi: Pelayanan pemeriksaan kesehatan kehamilan oleh tenaga kesehatan dengan frekuensi ANC selama masa kehamilan anak terakhir minimal 4 kali sesuai kriteria yaitu minimal 1 kali pada masa kehamilan trimester 1, 1 kali pada trimester 2 dan 2 kali pada trimester 3.

$$Proporsi\ ANC\ K4 := \frac{\begin{array}{c}\Sigma\ Perempuan\ umur\ 10-54\ tahun\ yang\ mendapat\\ layanan\ pemeriksaan\ kehamilan\ oleh\ tenaga\ kesehatan\\ dengan\ frekuensi\ ANC\ selama\ masa\ kehamilan\ anak\ terakhir\\ minimal\ 4\ kali\ sesuai\ kriteria\ minimal\ 1-1-2\end{array}}{\begin{array}{c}\Sigma\ perempuan\ pernah\ kawin\ umur\ 10-54\ tahun\ yang\ bersalin\\ anak\ terakhir\ pada\ periode\ 1\ Januari\ 2013\ sd\ wawancara\\ dan\ mendapat\ layanan\ ANC\ (K1)\ selama\ hamil\end{array}}$$

Tabel 14.2.1
Proporsi Pemeriksaan Kehamilan (K1, K1 ideal dan K4)
pada Perempuan Umur 10-54 Tahun yang Pernah Hamil menurut Provinsi,
Riskesdas 2018

Provinsi	ANC (K1)[1]		N ter-timbang	K1 ideal[2]		ANC K4[3]		N ter-timbang
	%	95% CI		%	95% CI	%	95% CI	
Aceh	96,7	95,8-97,4	1.791	87,3	85,6-88,8	66,2	63,8-68,5	1.700
Sumatera Utara	91,8	90,8-92,7	4.466	84,0	82,4-85,5	61,4	59,2-63,5	4.025
Sumatera Barat	98,7	98,1-99,1	1.797	86,1	84,3-87,8	72,8	70,6-74,9	1.741
Riau	94,7	93,5-95,7	2.402	84,1	82,2-85,9	69,9	67,5-72,2	2.232
Jambi	93,4	91,8-94,8	1.104	85,7	83,6-87,6	67,1	63,9-70,1	1.013
Sumatera Selatan	94,1	92,8-95,1	2.582	86,3	84,4-88,0	65,1	62,3-67,9	2.383
Bengkulu	96,1	94,1-97,4	604	83,7	81,2-85,9	71,2	68,1-74,1	569
Lampung	97,5	96,8-98,1	2.702	89,5	88,0-90,8	74,1	71,7-76,4	2.586
Bangka Belitung	97,1	95,4-98,1	461	84,8	81,8-87,3	71,7	67,9-75,1	439
Kepulauan Riau	96,0	92,7-97,9	718	91,8	89,5-93,7	77,7	73,2-81,7	677
DKI Jakarta	98,3	97,2-98,9	3.184	90,6	88,1-92,7	84,2	81,3-86,7	3.070
Jawa Barat	97,4	96,8-98,0	15.325	88,7	87,6-89,7	79,7	78,3-81,1	14.655
Jawa Tengah	98,8	98,4-99,0	9.537	89,5	88,5-90,3	82,0	80,8-83,1	9.244
DI Yogyakarta	98,7	97,4-99,4	1.007	94,4	92,2-96,0	90,2	87,6-92,3	976
Jawa Timur	98,1	97,6-98,4	10.089	88,3	87,3-89,2	80,2	79,0-81,4	9.711
Banten	94,7	93,4-95,7	4.398	88,3	86,4-90,0	74,8	72,3-77,2	4.087
Bali	98,6	97,5-99,3	1.163	90,3	88,2-92,0	82,0	79,4-84,4	1.126
Nusa Tenggara Barat	98,1	97,2-98,7	1.801	87,2	85,3-88,9	79,1	76,6-81,3	1.733
Nusa Tenggara Timur	94,0	93,0-94,9	1.865	73,9	71,9-75,8	64,3	62,2-66,3	1.721
Kalimantan Barat	93,2	91,8-94,3	1.604	79,3	77,0-81,5	65,1	62,4-67,7	1.467
Kalimantan Tengah	91,6	89,6-93,2	871	78,0	75,2-80,5	60,7	57,5-63,8	783
Kalimantan Selatan	97,2	96,0-98,0	1.384	86,7	84,9-88,4	74,9	72,5-77,1	1.320
Kalimantan Timur	97,7	96,2-98,6	1.176	85,4	82,9-87,5	72,4	69,1-75,4	1.128
Kalimantan Utara	97,4	95,7-98,4	250	79,1	75,3-82,5	67,4	63,0-71,4	239
Sulawesi Utara	96,0	94,7-97,0	696	72,1	68,9-75,0	61,0	57,8-64,1	656
Sulawesi Tengah	93,1	91,5-94,5	986	71,3	68,5-73,9	58,7	55,8-61,6	901
Sulawesi Selatan	96,8	95,9-97,5	2.637	79,5	77,6-81,3	61,3	59,0-63,6	2.505
Sulawesi Tenggara	92,9	91,3-94,3	931	63,5	60,6-66,3	48,9	45,9-52,0	849
Gorontalo	96,9	95,6-97,8	373	65,6	61,6-69,4	47,4	43,4-51,5	355
Sulawesi Barat	94,7	92,6-96,2	442	78,1	74,4-81,4	60,5	56,2-64,6	411
Maluku	85,9	82,6-88,6	594	70,8	67,0-74,3	47,9	43,8-51,9	500
Maluku Utara	91,0	88,7-92,9	452	74,0	70,8-77,0	55,6	51,9-59,2	404
Papua Barat	86,7	83,3-89,5	329	69,8	65,2-74,1	48,1	42,8-53,4	281
Papua	66,8	63,5-70,0	927	68,6	64,6-72,4	43,8	39,9-47,7	608
**INDONESIA**	**96,1**	**95,9-96,3**	**80.648**	**86,0**	**85,6-86,4**	**74,1**	**73,6-74,5**	**76.093**

[1] ANC K1 adalah pemeriksaan kehamilan yang diberikan oleh tenaga kesehatan . minimal 1 kali tanpa memperhitungkan periode waktu pemeriksaan pada perempuan umur 10-54 tahun yang pernah hamil.

[2] ANC K1 ideal adalah pemeriksaan kehamilan oleh tenaga kesehatan dan pemeriksaan kehamilan tersebut pertama kali dilakukan pada trimester 1 pada perempuan umur 10-54 tahun yang pernah melakukan ANC.

[3] ANC K4 adalah pemeriksaan kehamilan yang diberikan oleh tenaga kesehatan minimal empat kali sesuai kriteria minimal 1-1-2, yaitu minimal satu kali pada trimester 1, 1 kali pada trimester 2 dan 2 kali pada trimester 3 pada perempuan umur 10-54 tahun yang pernah melakukan ANC.

Tabel 14.2.2
Proporsi Pemeriksaan Kehamilan (K1, K1 ideal dan K4)
pada Perempuan Umur 10-54 Tahun yang Pernah Hamil menurut Karakteristik,
Riskesdas 2018

Karakteristik	ANC (K1)[1]		N ter-timbang	K1 ideal[2]		ANC K4[3]		N ter-timbang
	%	95% CI		%	95% CI	%	95% CI	
**Umur saat bersalin**								
<15	85,3	70,2-93,4	63	72,9	34,4-72,9	44,7	27,0-63,9	53
15-19	95,5	94,7-96,1	6.807	81,2	78,3-81,2	67,3	65,5-69,1	6.378
20-24	96,6	96,2-96,9	19.548	87,3	85,8-87,3	74,8	73,8-75,7	18.527
25-29	97,0	96,7-97,3	23.118	89,5	88,4-89,5	77,8	77,0-78,6	22.004
30-34	96,6	96,3-96,9	18.080	88,0	86,7-88,0	75,5	74,6-76,4	17.142
35-39	95,0	94,4-95,6	9.738	84,0	81,9-84,0	70,1	68,9-71,3	9.084
40-44	91,1	89,6-92,4	2.907	78,2	73,9-78,2	60,2	57,7-62,6	2.600
45-49	82,1	76,0-86,9	345	77,0	63,4-77,0	55,4	47,7-62,9	278
50-54	67,1	46,6-82,6	41*	90,1	54,2-90,1	62,5	37,5-82,2	27*
**Kelompok Umur (Khusus)**								
10 - 19 (Remaja)	95,4	94,6-96,0	6.870	81,0	78,1-81,0	67,1	65,3-68,9	6.431
15 - 49 (WUS)	96,2	96,0-96,3	80.544	86,4	85,7-86,4	74,1	73,6-74,5	76.013
**Pendidikan**								
Tidak pernah sekolah	78,3	75,5-81,0	1.308	77,7	74,1-80,9	58,3	54,3-62,2	1.006
Tidak tamat SD/MI	90,2	89,1-91,2	4.806	78,9	77,3-80,5	65,4	63,4-67,3	4.254
Tamat SD/MI	94,0	93,5-94,6	16.654	80,1	79,1-81,0	67,2	66,1-68,3	15.371
Tamat SLTP/MTS	96,9	96,6-97,2	20.384	85,6	84,8-86,3	74,3	73,3-75,2	19.387
Tamat SLTA/MA	97,8	97,5-98,0	27.523	88,5	87,9-89,1	76,8	76,1-77,6	26.414
Tamat D1/D2/D3/PT	98,7	98,4-99,0	9.973	93,4	92,8-94,0	82,5	81,4-83,5	9.661
**Pekerjaan**								
Tidak kerja	96,7	96,5-96,9	47.363	85,2	84,7-85,7	73,7	73,1-74,3	44.951
Sekolah	93,1	88,0-96,2	566	81,3	75,6-85,9	68,9	62,7-74,5	518
PNS/TNI/Polri/BUMN/BUMD	98,6	97,9-99,0	2.027	93,4	92,0-94,6	81,2	79,1-83,2	1.961
Pegawai swasta	98,4	97,8-98,8	6.705	92,8	91,8-93,7	82,3	80,8-83,7	6.472
Wiraswasta	97,5	97,0-97,9	7.667	90,1	89,2-91,0	78,6	77,3-79,9	7.336
Petani/buruh tani	87,1	86,2-88,0	6.641	77,4	76,2-78,6	61,2	59,8-62,7	5.677
Nelayan	87,6	79,1-93,0	85	82,6	72,9-89,4	70,0	59,0-79,1	73
Buruh/sopir/pembantu ruta	96,0	94,7-97,1	3.071	86,4	84,3-88,3	74,8	72,2-77,3	2.895
Lainnya	97,0	96,4-97,5	6.524	85,2	84,1-86,3	72,3	70,9-73,8	6.211
**Tempat Tinggal**								
Perkotaan	97,8	97,6-98,1	43.650	88,6	88,1-89,1	77,9	77,2-78,6	41.917
Perdesaan	94,1	93,8-94,4	36.998	82,8	88,1-89,1	69,3	68,7-69,9	34.176

1 ANC K1 adalah pemeriksaan kehamilan yang diberikan oleh tenaga kesehatan . minimal 1 kali tanpa memperhitungkan periode waktu pemeriksaan pada perempuan umur 10-54 tahun yang pernah hamil.

2 ANC K1 ideal adalah pemeriksaan kehamilan oleh tenaga kesehatan dan pemeriksaan kehamilan tersebut pertama kali dilakukan pada trimester 1 pada perempuan umur 10-54 tahun yang pernah melakukan ANC.

3 ANC K4 adalah pemeriksaan kehamilan yang diberikan oleh tenaga kesehatan minimal empat kali sesuai kriteria minimal 1-1-2, yaitu minimal satu kali pada trimester 1, 1 kali pada trimester 2 dan 2 kali pada trimester 3 pada perempuan umur 10-54 tahun yang pernah melakukan ANC.

*N Tertimbang < 50

Tenaga Pemberi Layanan Antenatal (ANC)

Definisi: Tenaga pemberi layanan ANC adalah tenaga kesehatan melakukan pemeriksaan kehamilan, meliputi dokter ahli kebidanan dan kandungan, dokter umum, bidan dan perawat.

$$Proporsi\ Tenaga\ Pemberi\ Layanan\ Antenatal\ (ANC):$$

$$= \frac{\begin{array}{c}\Sigma\ Perempuan\ umur\ 10-54\ tahun\ yang\ mendapat\ layanan \\ pemeriksaan\ kehamilan\ anak\ terakhir\ oleh\ tenaga\ kesehatan \\ menurut\ jenis\ tenaga\ kesehatan\ yang\ memberi\ layanan\end{array}}{\begin{array}{c}\Sigma\ Perempuan\ umur\ 10-54\ tahun\ yang\ bersalin \\ anak\ terakhir\ pada\ periode\ 1\ Januari\ 2013\ sd\ wawancara \\ yang\ mendapat\ layanan\ ANC\ (K1)\ selama\ hamil\end{array}}$$

Tempat Pemberi Layanan Antenatal (ANC)

Definisi: Tempat ibu hamil mendapat layanan ANC, mencakup fasilitas pelayanan kesehatan (Rumah Sakit, klinik, Puskesmas/pustu/pusling dan praktik tenaga kesehatan), atau tempat pelayanan lain (poskesdes, polindes dan posyandu) yang menyediakan layanan pemeriksaan kehamilan.

$$Proporsi\ Tempat\ Pemberi\ Layanan\ Antenatal\ (ANC)$$

$$= \frac{\begin{array}{c}\Sigma\ Perempuan\ umur\ 10-54\ tahun\ yang\ mendapat\ layanan \\ pemeriksaan\ kehamilan\ anak\ terakhir\ oleh\ tenaga\ kesehatan \\ menurut\ tempat\ layanan\ ANC\end{array}}{\begin{array}{c}\Sigma\ Perempuan\ umur\ 10-54\ tahun\ yang\ pernah\ kawin\ yang\ bersalin \\ anak\ terakhir\ pada\ periode\ 1\ Januari\ 2013\ sd\ wawancara \\ yang\ mendapat\ layanan\ ANC\ (K1)\ selama\ hamil\end{array}}$$

Komponen pelayanan antenatal (ANC)

Definisi: Jenis pemeriksaan kesehatan yang diterima ibu pada pelayanan antenatal (ANC), yang meliputi pengukuran tinggi badan, penimbangan berat badan, pengukuran tekanan darah, pengukuran denyut jantung janin, pengukuran tinggi fundus/rahim, penentuan letak janin, pengukuran lingkar lengan atas (LILA), pemberian imunisasi tetanus toksoid (TT), pemberian tablet tambah darah (TTD), pemberian konseling dan tindakan.

$$Proporsi\ Komponen\ Pelayanan\ Antenatal\ (ANC)$$

$$= \frac{\begin{array}{c}\Sigma\ Perempuan\ umur\ 10-54\ tahun\ yang\ mendapat\ layanan \\ pemeriksaan\ kehamilan\ anak\ terakhir\ oleh\ tenaga\ kesehatan \\ menurut\ jenis\ komponen\ ANC\ yang\ diterima\end{array}}{\begin{array}{c}\Sigma\ Perempuan\ umur\ 10-54\ tahun\ yang\ melahirkan \\ anak\ terakhir\ pada\ periode\ 1\ Januari\ 2013\ sd\ wawancara \\ yang\ mendapat\ layanan\ ANC\ selama\ hamil\end{array}}$$

Tabel 14.2.3

Proporsi Tenaga Pemberi Layanan ANC Selama Kehamilan pada Perempuan Umur 10-54 Tahun yang Pernah Melahirkan menurut Provinsi, Riskesdas 2018

Provinsi	Jenis tenaga yang memberi pelayanan ANC (%)				N Tertimbang
	Dokter spesialis kebidanan & kandungan	Dokter umum	Bidan	Perawat	
Aceh	19,6	0,7	79,6	0,1	1.700
Sumatera Utara	13,5	0,8	85,6	0,1	4.025
Sumatera Barat	13,0	1,1	85,8	0,2	1.741
Riau	18,0	1,5	80,3	0,2	2.232
Jambi	10,0	0,6	88,9	0,5	1.013
Sumatera Selatan	13,1	1,3	84,8	0,8	2.383
Bengkulu	12,3	0,9	86,5	0,4	569
Lampung	7,0	0,6	92,3	0,1	2.586
Bangka Belitung	16,6	0,4	82,9	0,0	439
Kepulauan Riau	30,5	3,7	65,6	0,3	677
DKI Jakarta	20,6	1,5	77,6	0,3	3.070
Jawa Barat	12,9	0,9	86,1	0,1	14.655
Jawa Tengah	13,2	0,8	86,0	0,1	9.244
DI Yogyakarta	27,3	0,8	71,9	0,0	976
Jawa Timur	13,3	0,6	86,0	0,2	9.711
Banten	13,4	0,5	85,9	0,2	4.087
Bali	39,4	0,8	59,8	0	1.126
Nusa Tenggara Barat	6,2	0,3	93,4	0,1	1.733
Nusa Tenggara Timur	5,3	0,7	93,5	0,5	1.721
Kalimantan Barat	9,7	0,6	88,7	1,0	1.467
Kalimantan Tengah	9,8	0,7	88,8	0,7	783
Kalimantan Selatan	9,5	0,6	89,6	0,3	1.320
Kalimantan Timur	25,9	0,8	73,3	0,0	1.128
Kalimantan Utara	25,3	1,8	72,9	0,1	239
Sulawesi Utara	30,6	4,5	64,2	0,7	656
Sulawesi Tengah	9,3	0,8	89,7	0,2	901
Sulawesi Selatan	13,5	2,0	84,3	0,2	2.505
Sulawesi Tenggara	11,5	0,6	87,6	0,2	849
Gorontalo	16,9	1,5	80,4	1,2	355
Sulawesi Barat	3,9	1,2	94,6	0,2	411
Maluku	14,5	2,1	82,8	0,6	500
Maluku Utara	15,8	1,2	82,9	0,0	404
Papua Barat	26,3	0,9	71,7	1,1	281
Papua	13,8	2,4	81,8	2,0	608
**INDONESIA**	**14,1**	**0,9**	**84,8**	**0,2**	**76.093**

Tabel 14.2.4
Proporsi Tenaga Pemberi Layanan ANC Selama Kehamilan pada Perempuan Umur 10-54 Tahun yang Pernah Melahirkan menurut Karakteristik, Riskesdas 2018

Karakteristik	Jenis tenaga pemberi layanan ANC (%)				N tertimbang
	Dokter spesialis kebidanan & kandungan	Dokter umum	Bidan	Perawat	
**Umur saat Bersalin**					
10 – 14	2,7	0	97,3	0	53
15 - 19	3,7	0,8	95,3	0,2	6.378
20 - 24	10,0	1,0	88,8	0,2	18.527
25 - 29	17,7	0,8	81,3	0,2	22.004
30 - 34	16,6	1,1	82,1	0,2	17.142
35 - 39	16,2	0,8	82,7	0,2	9.084
40 - 44	15,0	1,3	83,5	0,2	2.600
45 - 49	11,9	2,0	85,7	0,4	278
50 – 54	3,5	16,8	79,7	0	27*
**Kelompok Umur (Khusus)**					
10 - 19 (Remaja)	3,7	0,8	95,3	0,2	6.431
15 - 49 (WUS)	14,1	0,9	84,7	0,2	76.013
**Pendidikan**					
Tidak pernah sekolah	4,8	1,3	92,9	1,0	1.006
Tidak tamat SD/MI	3,4	0,6	95,6	0,3	4.254
Tamat SD/MI	3,8	0,9	94,9	0,3	15.371
Tamat SLTP/MTS	5,2	0,7	93,9	0,2	19.387
Tamat SLTA/MA	16,4	1,1	82,3	0,2	26.414
Tamat D1/D2/D3/PT	47,6	1,1	51,2	0,2	9.661
**Pekerjaan**					
Tidak bekerja	11,1	0,9	87,8	0,2	44.951
Sekolah	18,5	0,8	80,0	0,7	518
PNS/TNI/Polri/BUMN/BUMD	50,2	1,4	48,1	0,3	1.961
Pegawai swasta	33,3	1,2	65,3	0,2	6.472
Wiraswasta	16,8	0,7	82,4	0,1	7.336
Petani/buruh tani	2,7	0,8	96,0	0,5	5.677
Nelayan	17,7	0	82,3	0,1	73
Buruh/supir/pembantu ruta	8,8	1,3	89,6	0,3	2.895
Lainnya	13,5	0,9	85,4	0,1	6.211
**Tempat Tinggal**					
Perkotaan	20,7	1,1	78,0	0,2	41.917
Perdesaan	5,9	0,8	93,0	0,3	34.176

*N Tertimbang <50

Tabel 14.2.5
Proporsi Tempat Layanan ANC yang Dimanfaatkan oleh Perempuan Umur 10-54 Tahun Selama Kehamilan menurut Provinsi, Riskesdas 2018

Provinsi	Tempat mendapatkan pelayanan ANC (%)								N tertimbang
	Rumah sakit	Puskes-mas/ Pustu	Praktik dokter/Klinik	Praktik bidan	Praktik perawat	Poskesdes/ Polindes	Posyandu	Lainnya	
Aceh	8,0	14,1	19,3	36,2	0,0	14,0	7,1	1,3	1.700
Sumatera Utara	8,3	10,3	25,8	44,6	0,1	4,1	4,6	2,2	4.025
Sumatera Barat	8,0	20,1	9,9	40,0	0,1	10,3	11,2	0,5	1.741
Riau	11,6	14,3	15,9	40,5	0,1	3,6	12,5	1,5	2.232
Jambi	5,8	24,9	11,5	39,1	0	11,8	4,6	2,3	1.013
Sumatera Selatan	9,0	10,4	10,9	40,9	0,2	17,8	9,3	1,5	2.383
Bengkulu	6,9	10,6	11,4	44,0	0,2	5,5	20,0	1,5	569
Lampung	4,7	6,2	5,9	69,6	0,1	2,3	10,7	0,4	2.586
Bangka Belitung	7,8	12,2	16,8	30,3	0,2	28,6	3,3	0,9	439
Kepulauan Riau	18,4	13,2	24,3	39,3	0	2,5	2,2	0,2	677
DKI Jakarta	18,3	31,8	9,1	39,7	0,3	0,1	0,4	0,3	3.070
Jawa Barat	9,8	11,6	9,6	53,5	0,0	2,1	12,9	0,6	14.655
Jawa Tengah	9,1	13,5	9,7	58,3	0,1	5,3	3,8	0,3	9.244
DI Yogyakarta	19,8	22,1	15,3	42,3	0	0,4	0,1	0	976
Jawa Timur	9,5	10,1	7,6	56,2	0,0	10,5	5,9	0,2	9.711
Banten	12,3	11,4	10,7	45,2	0	1,7	18,2	0,5	4.087
Bali	16,1	13,1	27,1	42,8	0	0,5	0,3	0,1	1.126
Nusa Tenggara Barat	3,1	17,0	4,1	4,2	0	22,8	48,6	0,1	1.733
Nusa Tenggara Timur	3,8	60,8	5,1	0,6	0	14,6	14,7	0,6	1.721
Kalimantan Barat	7,3	24,8	8,6	22,7	0,3	19,2	14,8	2,2	1.467
Kalimantan Tengah	3,5	34,2	14,7	26,3	0,4	9,0	9,5	2,3	783
Kalimantan Selatan	5,1	22,9	8,0	32,1	0,2	14,6	16,0	1,1	1.320
Kalimantan Timur	14,7	29,6	21,4	25,0	0,0	2,0	6,4	1,0	1.128
Kalimantan Utara	7,9	49,0	22,4	13,8	0	0,2	6,4	0,3	239
Sulawesi Utara	13,4	36,0	23,3	7,5	0,1	3,6	15,0	1,1	656
Sulawesi Tengah	3,6	27,0	7,8	4,5	0,0	16,6	38,9	1,5	901
Sulawesi Selatan	8,5	55,7	9,7	10,4	0,0	7,7	6,9	1,1	2.505
Sulawesi Tenggara	4,4	22,0	9,3	5,9	0,2	3,0	53,1	2,2	849
Gorontalo	3,8	42,7	14,9	0,9	0	5,7	31,4	0,5	355
Sulawesi Barat	3,1	70,2	5,5	5,2	0,2	7,3	5,5	3,0	411
Maluku	5,3	44,9	12,2	4,3	0,6	3,8	23,0	5,9	500
Maluku Utara	6,0	29,1	13,4	1,6	0,1	5,4	42,1	2,4	404
Papua Barat	8,7	50,7	21,1	4,9	0,0	3,3	10,1	1,2	281
Papua	10,5	67,2	9,7	4,3	0,4	1,7	4,9	1,4	608
**INDONESIA**	**9,4**	**18,4**	**11,3**	**42,5**	**0,1**	**6,6**	**10,9**	**0,8**	**76.093**

Tabel 14.2.6

Proporsi Tempat Layanan ANC yang Dimanfaatkan oleh Perempuan Umur 10-54 Tahun Selama Kehamilan menurut Karakteristik, Riskesdas 2018

Karakteristik	Tempat mendapatkan pelayanan ANC (%)								N ter-timbang
	Rumah sakit	Puskesmas/ Pustu	Praktik dokter/Klinik	Praktik bidan	Praktik perawat	Poskesdes/Polindes	Posyandu	Lainnya	
**Umur saat Bersalin**									
10 – 14	0,7	24,6	6,2	35,1	0,0	7,5	21,5	4,4	53
15 - 19	3,2	19,4	6,6	44,2	0,1	9,6	16,1	0,9	6.378
20 - 24	6,2	17,9	10,6	45,8	0,1	6,7	12,0	0,7	18.527
25 - 29	11,8	17,0	12,6	41,9	0,1	6,1	9,8	0,7	22.004
30 - 34	11,1	18,8	11,9	41,8	0,1	6,2	9,4	0,8	17.142
35 - 39	10,9	20,9	11,7	39,1	0,0	6,4	10,1	0,9	9.084
40 - 44	10,1	22,3	11,0	37,1	0,2	7,0	11,1	1,2	2.600
45 - 49	12,7	17,9	9,2	39,8	0,2	8,1	11,6	0,5	278
50 – 54	5,9	21,1	26,2	17,1	0,0	21,6	8,0	0,0	27*
**Kelompok Umur (Khusus)**									
10 - 19 (Remaja)	3,2	19,4	6,6	44,1	0,1	9,5	16,1	1,0	6.431
15 - 49 (WUS)	9,4	18,4	11,3	42,5	0,1	6,6	10,9	0,8	76.013
**Pendidikan**									
Tidak pernah sekolah	3,1	27,9	6,2	32,5	0,0	12,0	16,6	1,7	1.006
Tidak tamat SD/MI	2,8	25,3	5,6	34,9	0,1	10,6	19,3	1,4	4.254
Tamat SD/MI	2,7	22,1	5,3	40,9	0,1	9,5	18,1	1,3	15.371
Tamat SLTP/MTS	3,8	18,5	8,3	49,7	0,1	7,2	11,7	0,8	19.387
Tamat SLTA/MA	11,2	17,3	13,4	45,4	0,1	5,0	7,0	0,5	26.414
Tamat D1/D2/D3/PT	29,7	11,8	24,1	27,0	0,0	3,0	3,9	0,4	9.661
**Pekerjaan**									
Tidak bekerja	7,8	19,1	10,0	44,2	0,1	6,2	11,8	0,8	44.951
Sekolah	10,3	21,5	12,4	42,7	0,0	5,6	6,3	1,1	518
PNS/TNI/Polri/BUMN/BUMD	27,5	14,2	29,8	21,3	0,0	3,3	3,2	0,7	1.961
Pegawai swasta	22,5	10,7	18,3	41,8	0,0	3,1	3,1	0,3	6.472
Wiraswasta	10,1	14,2	14,3	48,8	0,1	5,3	6,7	0,5	7.336
Petani/buruh tani	2,4	25,7	4,0	30,0	0,1	16,6	19,3	1,9	5.677
Nelayan	12,4	44,9	14,2	13,2	0,0	4,1	9,6	1,7	73
Buruh/supir/pembantu ruta	7,5	17,2	10,0	51,1	0,0	5,6	8,4	0,2	2.895
Lainnya	8,0	21,4	11,0	37,5	0,1	7,3	13,3	1,3	6.211
**Tempat Tinggal**									
Perkotaan	14,0	16,4	14,8	46,2	0,1	2,5	5,7	0 ,4	41.917
Perdesaan	3,8	20,9	6,9	37,9	0,1	11,8	17,3	1 ,3	34.176

*N Tertimbang < 50

Tabel 14.2.7
Proporsi Komponen Antenatal Care (ANC) Selama Kehamilan pada Perempuan Umur 10-54 Tahun yang Pernah Melahirkan menurut Provinsi, Riskesdas 2018

Provinsi	Jenis komponen ANC yang diterima (%)											N ter-timbang
	Ukur BB	Ukur TB	Ukur Tekanan Darah	Ukur LILA	Ukur Tinggi Rahim	Penentuan Letak Janin	Hitung DJJ	Konsumsi TTD	Imunisasi TT	Konseling	Tata Laksana Kasus	
Aceh	94,2	64,6	95,4	76,5	83,2	88,0	88,3	83,7	79,1	90,6	94,6	1.700
Sumatera Utara	89,8	41,5	96,4	56,8	69,0	87,1	88,7	76,4	58,5	87,9	93,4	4.025
Sumatera Barat	97,1	68,7	98,2	88,4	91,4	96,9	97,4	92,5	74,6	93,6	95,9	1.741
Riau	94,0	50,9	97,1	66,8	72,0	89,8	90,5	82,8	66,4	91,0	94,7	2.232
Jambi	98,0	76,8	98,1	91,7	93,1	95,2	95,9	90,4	89,2	92,7	95,1	1.013
Sumatera Selatan	97,3	65,9	97,9	80,4	83,6	89,5	90,4	78,3	80,0	91,9	96,3	2.383
Bengkulu	97,4	64,0	97,5	80,5	86,2	93,6	94,6	87,2	86,0	87,6	94,3	569
Lampung	98,6	65,8	98,9	84,0	90,4	94,3	95,0	91,5	83,0	92,4	97,5	2.586
Bangka Belitung	98,2	66,8	98,9	81,1	92,7	96,2	97,5	89,8	74,6	95,3	97,6	439
Kepulauan Riau	99,0	73,3	99,5	75,8	87,6	94,2	96,0	87,6	71,7	89,0	98,3	677
DKI Jakarta	98,8	72,7	99,7	76,8	90,9	96,3	96,8	92,1	81,6	94,3	96,9	3.070
Jawa Barat	97,9	63,2	98,8	72,5	89,3	95,6	96,8	91,9	86,2	92,4	96,0	14.655
Jawa Tengah	99,0	74,9	99,4	87,4	94,9	96,9	97,3	95,1	82,4	94,5	97,5	9.244
DI Yogyakarta	99,3	81,2	99,5	90,7	94,1	96,8	98,2	96,2	79,3	94,1	98,0	976
Jawa Timur	98,8	82,6	99,2	87,6	94,3	96,8	96,9	90,8	66,8	93,9	97,1	9.711
Banten	97,6	55,6	98,5	69,6	86,1	92,9	95,7	92,4	76,9	89,8	95,1	4.087
Bali	98,9	68,0	98,7	74,2	83,6	93,2	96,2	92,4	80,2	91,6	95,8	1.126
Nusa Tenggara Barat	98,1	78,6	99,1	92,5	97,5	97,7	96,9	96,9	87,1	95,5	97,4	1.733
Nusa Tenggara Timur	97,1	85,7	98,0	94,2	95,5	96,3	96,9	96,1	85,6	94,4	96,7	1.721
Kalimantan Barat	97,4	69,1	98,0	83,8	87,4	93,6	94,8	87,2	79,4	91,7	95,2	1.467
Kalimantan Tengah	96,5	65,9	98,0	82,7	90,7	94,0	92,9	84,4	84,1	91,0	94,9	783
Kalimantan Selatan	98,5	79,5	98,7	88,8	94,4	96,9	96,5	91,3	82,8	93,3	97,7	1.320
Kalimantan Timur	98,6	74,6	98,7	84,4	87,6	94,5	95,9	88,4	75,0	92,6	97,6	1.128
Kalimantan Utara	96,9	72,9	98,5	84,5	89,6	94,4	95,6	86,8	84,0	92,0	94,0	239
Sulawesi Utara	97,6	69,2	98,3	81,1	85,2	91,8	92,7	90,4	95,2	91,9	95,0	656
Sulawesi Tengah	96,9	74,6	98,0	88,7	89,6	94,2	95,1	93,2	90,3	91,0	95,5	901
Sulawesi Selatan	97,7	80,7	98,6	88,9	91,9	95,8	95,5	94,0	90,0	91,2	95,5	2.505
Sulawesi Tenggara	94,0	56,1	98,2	89,0	89,7	94,9	95,2	91,2	88,7	92,4	95,5	849
Gorontalo	98,4	87,2	98,9	93,9	96,6	97,6	97,9	95,3	93,9	91,3	96,5	355
Sulawesi Barat	98,0	90,2	98,9	95,1	93,5	93,9	93,6	95,1	89,4	92,5	95,3	411
Maluku	89,8	62,4	90,7	76,9	78,8	86,7	87,0	92,0	86,5	83,9	92,5	500
Maluku Utara	95,7	75,9	96,9	90,2	90,8	93,1	93,2	94,6	91,2	92,5	95,2	404
Papua Barat	94,2	67,5	94,6	82,3	86,9	93,9	93,5	93,7	85,9	85,8	90,4	281
Papua	96,6	79,5	95,1	89,6	91,3	93,5	93,8	89,9	89,6	90,5	95,7	608
**INDONESIA**	**97,4**	**69,1**	**98,5**	**80,3**	**89,1**	**94,6**	**95,4**	**90,5**	**79,5**	**92,4**	**96,2**	**76.093**

Tabel 14.2.8

Proporsi Komponen Antenatal Care (ANC) Selama Kehamilan pada Perempuan Umur 10-54 Tahun yang Pernah Melahirkan menurut Karakteristik, Riskesdas 2018

Karakteristik	Jenis Komponen ANC yang diterima (%)											N tertimbang
	Ukur BB	Ukur TB	Ukur Tekanan Darah	Ukur LILA	Ukur Tinggi Rahim	Penentuan Letak Janin	Hitung DJJ	Konsumsi TTD	Imunisasi TT	Konseling	Tata Laksana Kasus	
**Umur saat Bersalin**												
10 – 14	98,7	78,1	98,7	77,0	82,1	82,4	95,2	90,4	61,7	82,0	82,0	53
15 - 19	97,7	71,1	98,6	84,2	91,4	95,0	95,7	91,8	81,4	92,0	95,3	6378
20 - 24	97,4	70,1	98,6	81,8	89,8	95,2	95,9	91,3	80,9	92,6	96,4	18527
25 - 29	97,6	68,7	98,5	79,5	89,1	94,9	95,7	90,8	79,2	92,5	96,3	22004
30 - 34	97,7	68,5	98,5	79,4	88,7	94,5	95,4	90,0	79,2	92,9	96,5	17142
35 - 39	96,7	68,3	98,2	79,3	88,6	94,1	94,6	89,4	77,9	91,9	95,9	9084
40 - 44	95,4	66,7	97,9	75,7	84,5	91,0	91,8	86,4	75,5	90,1	95,1	2600
45 - 49	95,9	68,6	96,3	78,3	86,4	89,4	91,4	82,7	73,6	85,0	92,2	278
50 – 54	100,0	68,4	100,0	71,0	74,1	95,3	95,8	89,2	95,0	95,5	95,8	27*
**Kelompok Umur (Khusus)**												
10 - 19 (Remaja)	97,7	71,2	98,6	84,1	91,3	94,9	95,7	91,8	81,3	92,0	95,2	6431
15 - 49 (WUS)	97,4	69,1	98,5	80,3	89,1	94,6	95,4	90,5	79,5	92,4	96,2	76013
**Pendidikan**												
Tidak pernah sekolah	95,3	69,4	97,1	81,7	88,5	91,7	92,2	88,9	77,2	86,5	92,1	1.006
Tidak tamat SD/MI	95,2	65,8	97,5	80,0	87,4	93,6	93,7	89,0	78,4	90,5	94,6	4.254
Tamat SD/MI	96,5	68,0	97,9	80,5	89,1	93,8	94,0	90,1	81,0	90,9	95,5	15.371
Tamat SLTP/MTS	97,5	70,1	98,6	82,2	90,6	95,1	95,8	92,4	81,4	92,7	96,2	19.387
Tamat SLTA/MA	98,0	69,7	98,7	80,4	89,1	95,0	95,9	90,8	78,9	93,1	96,6	26.414
Tamat D1/D2/D3/PT	98,3	68,9	99,0	75,8	87,0	94,7	96,4	87,3	75,3	93,8	97,1	9.661
**Pekerjaan**												
Tidak bekerja	97,5	68,2	98,5	79,7	89,2	94,8	95,7	91,1	79,5	92,4	96,1	44.951
Sekolah	98,2	72,5	97,7	83,8	87,2	94,6	95,1	89,4	81,7	92,9	97,1	518
PNS/TNI/Polri/BUMN/BUMD	98,3	73,5	99,1	79,5	89,3	95,3	96,5	88,0	78,6	94,6	97,5	1.961
Pegawai swasta	98,7	69,0	99,2	77,5	88,0	94,3	95,9	89,5	75,9	93,1	96,7	6.472
Wiraswasta	97,4	70,8	98,5	80,7	89,7	95,1	95,8	89,4	78,3	92,9	96,4	7.336
Petani/buruh/tani	95,1	68,8	97,1	83,3	87,7	91,9	91,8	88,5	80,2	90,9	95,1	5.677
Nelayan	98,1	90,7	98,5	87,9	96,1	97,0	91,4	96,7	89,4	90,1	96,8	73
Buruh/supir/pembantu ruta	97,2	70,3	98,7	80,7	89,7	95,5	95,1	91,7	81,5	91,0	96,1	2.895
Lainnya	97,5	71,5	98,8	83,8	90,3	94,9	95,5	90,5	82,8	92,7	96,4	6.211
**Tempat tinggal**												
Perkotaan	98,0	67,5	98,9	77,2	88,4	94,9	96,2	90,0	77,2	92,6	96,4	41.917
Perdesaan	96,7	71,1	98,0	84,1	90,0	94,3	94,4	91,1	82,2	92,2	95,9	34.176

*N Tertimbang < 50

Tabel 14.2.9

Proporsi Jenis Pelayanan Pemeriksaan Laboratorium yang Pernah Dimanfaatkan oleh Perempuan Umur 10-54 Tahun Selama Masa Kehamilan menurut Provinsi, Riskesdas 2018

Provinsi	Jenis pelayanan pemeriksaan laboratorium (%)					N Tertimbang
	Golongan darah	Gluko-protein urin	Hemoglobin (Hb)	HIV	Lainnya	
Aceh	30,6	24,9	42,3	6,5	0,7	1.791
Sumatera Utara	5,9	7,1	7,5	2,5	1,6	4.466
Sumatera Barat	35,3	28,5	49,0	9,3	1,8	1.797
Riau	17,8	11,7	21,1	5,0	1,5	2.402
Jambi	37,9	29,8	49,3	10,2	2,9	1.104
Sumatera Selatan	17,9	9,6	16,8	3,4	0,4	2.582
Bengkulu	19,9	9,9	22,4	7,8	1,1	604
Lampung	33,5	19,1	36,9	9,1	2,7	2.702
Kep. Bangka Belitung	38,9	27,8	52,2	15,8	7,7	461
Kepulauan Riau	36,9	26,8	34,6	25,8	2,2	718
DKI Jakarta	61,2	60,1	74,3	41,9	8,8	3.184
Jawa Barat	34,0	31,0	45,4	12,2	2,0	15.325
Jawa Tengah	68,2	55,1	79,1	30,9	13,1	9.537
DI Yogyakarta	75,7	62,4	89,9	32,8	24,1	1.007
Jawa Timur	51,7	43,8	56,1	30,7	6,2	10.089
Banten	30,7	28,9	39,6	12,1	2,9	4.398
Bali	53,0	40,8	52,8	45,2	5,4	1.163
Nusa Tenggara Barat	74,8	55,0	80,5	13,5	11,9	1.801
Nusa Tenggara Timur	36,7	37,1	59,0	12,4	11,8	1.865
Kalimantan Barat	29,7	23,7	31,2	14,1	3,8	1.604
Kalimantan Tengah	27,4	14,5	29,3	8,2	4,0	871
Kalimantan Selatan	55,8	44,8	63,4	20,2	4,4	1.384
Kalimantan Timur	44,3	26,0	56,1	23,9	9,7	1.176
Kalimantan Utara	41,1	40,5	50,8	30,7	3,1	250
Sulawesi Utara	25,5	19,4	22,3	15,9	2,0	696
Sulawesi Tengah	32,7	19,5	33,4	6,5	2,2	986
Sulawesi Selatan	51,3	50,7	59,2	23,7	6,1	2.637
Sulawesi Tenggara	20,4	13,8	22,1	7,5	2,0	931
Gorontalo	44,4	27,9	42,9	16,2	6,4	373
Sulawesi Barat	30,9	35,3	49,5	7,0	4,1	442
Maluku	16,2	14,4	22,8	11,4	3,2	594
Maluku Utara	23,1	20,4	32,0	8,8	4,1	452
Papua Barat	41,1	29,0	52,8	35,1	12,4	329
Papua	25,3	18,9	34,8	29,9	5,2	927
**INDONESIA**	**40,8**	**34,0**	**48,7**	**18,3**	**5,4**	**80.648**

Tabel 14.2.10
Proporsi Jenis Pelayanan Pemeriksaan Laboratorium yang Pernah Dimanfaatkan oleh Perempuan Umur 10-54 Tahun Selama Masa Kehamilan menurut Karakteristik, Riskesdas 2018

Karakteristik	Jenis pelayanan pemeriksaan laboratorium (%)					N Tertimbang
	Golongan darah	Gluko-protein urin	Hemoglobin (Hb)	HIV	Lainnya	
**Umur saat bersalin**						
10 - 14	34,4	23,0	34,0	11,4	5,3	63
15 - 19	38,1	30,0	44,9	14,8	3,9	6.807
20 - 24	40,8	32,9	49,1	18,5	5,6	19.548
25 - 29	42,5	35,7	50,6	19,9	5,6	23.118
30 - 34	40,5	34,9	49,1	18,4	5,6	18.080
35 - 39	41,2	34,7	47,6	18,0	5,4	9.738
40 - 44	34,8	31,6	41,9	15,1	3,9	2.907
45 - 49	31,4	25,3	34,9	9,6	3,3	345
50 - 54	19,8	27,9	34,6	14,7	1,2	41*
**Kelompok Umur (Khusus)**						
10 - 19 (Remaja)	38,0	29,9	44,8	14,8	3,9	6.870
15 - 49 (WUS)	40,8	34,1	48,7	18,3	5,4	80.544
**Pendidikan**						
Tidak pernah sekolah	26,1	20,5	30,9	10,3	2,4	1.308
Tidak tamat SD/MI	29,7	25,0	34,8	10,6	3,2	4.806
Tamat SD/MI	34,3	29,7	42,1	13,3	4,0	16.654
Tamat SLTP/MTS	39,9	32,6	47,8	16,9	4,7	20.384
Tamat SLTA/MA	44,2	36,6	52,6	21,5	6,0	27.523
Tamat D1/D2/D3/PT	51,1	43,4	59,7	25,7	8,5	9.973
**Pekerjaan**						
Tidak bekerja	40,2	33,6	48,4	17,8	5,0	47.363
Sekolah	42,7	39,5	53,7	21,5	6,0	566
PNS/TNI/Polri/BUMN/BUMD	48,6	41,7	58,7	27,0	7,5	2.027
Pegawai swasta	51,9	43,4	60,0	26,7	8,3	6.705
Wiraswasta	44,3	36,2	51,2	20,6	5,6	7.667
Petani/buruh tani	25,3	21,2	31,0	8,7	3,6	6.641
Nelayan	51,1	33,4	51,1	10,6	3,5	85
Buruh/supir/pembantu ruta	42,6	35,5	50,6	18,7	7,2	3.071
Lainnya	41,3	34,6	49,3	18,0	5,2	6.524
**Tempat tinggal**						
Perkotaan	45,3	38,7	53,7	22,3	5,9	43.650
Perdesaan	35,4	28,5	42,8	13,6	4,7	36.998

*N tertimbang <50

<u>Riwayat gangguan/komplikasi kehamilan</u>

Definisi: Gangguan atau masalah kesehatan yang pernah dialami oleh ibu selama kehamilan anak terakhir. Jenis komplikasi kehamilan dapat berupa muntah atau diare terus menerus, demam tinggi, bengkak kaki disertai kejang perdarahan pada jalan lahir, ketuban keluar sebelum waktunya dan janin kurang bergerak.

$$Proporsi\ Jenis\ Gangguan\ atau\ Komplikasi\ Kehamilan = \frac{\begin{gathered}\Sigma\, Perempuan\ umur\ 10-54\ tahun\ yang\ pernah \\ mengalami\ gangguan\ atau\ komplikasi\ kehamilan\ menurut\ jenis \\ gangggguan\ selama\ kehamilan\ \ anak\ terakhir\end{gathered}}{\begin{gathered}\Sigma\, Perempuan\ umur\ 10-54\ tahun\ yang\ bersalin \\ anak\ terakhir\ pada\ periode\ 1\ Januari\ 2013\ sampai\ dengan\ wawancara\end{gathered}}$$

Tabel 14.2.11
Proporsi Gangguan/Komplikasi yang Dialami Selama Kehamilan pada Perempuan Umur 10-54 Tahun menurut Provinsi, Riskesdas 2018

Provinsi	Jenis gangguan/komplikasi selama kehamilan (%)										Mengalami salah satu gangguan/komplikasi Kehamilan		N ter-timbang
	Muntah/diare terus menerus	Demam tinggi	Hipertensi	Janin kurang bergerak	Pendarahan pada jalan lahir	Keluar air ketuban	Bengkak kaki disertai kejang	Batuk lama	Nyeri dada/jantung berdebar	Lainnya	%	95% CI	
Aceh	21,5	4,1	2,7	1,0	2,0	1,6	2,4	2,0	2,0	5,7	27,6	25,5-29,9	1.791
Sumatera Utara	16,7	2,5	2,7	1,2	2,5	1,8	3,2	2,3	2,0	3,7	23,9	21,9-25,9	4.466
Sumatera Barat	23,9	3,6	4,0	2,2	2,6	1,9	2,5	3,1	2,8	6,4	33,0	30,6-35,4	1.797
Riau	22,5	2,8	2,9	0,7	2,4	1,6	2,8	2,5	2,5	7,4	29,2	26,8-31,8	2.402
Jambi	14,4	2,8	2,2	0,5	1,9	1,5	1,2	1,6	0,9	3,1	19,2	16,9-21,8	1.104
Sumatera Selatan	17,0	3,1	3,0	0,6	1,8	1,4	2,4	1,6	1,2	4,6	22,1	19,9-24,6	2.582
Bengkulu	19,6	3,4	1,8	0,8	1,5	1,2	2,8	1,9	1,6	5,9	24,8	21,8-28,1	604
Lampung	17,2	1,8	2,6	0,6	2,2	1,6	1,5	0,9	0,8	5,8	23,0	20,8-25,3	2.702
Kep. Bangka Belitung	20,7	3,3	4,2	1,3	1,5	2,6	1,5	1,9	2,2	8,9	29,2	25,7-32,9	461
Kepulauan Riau	22,1	2,6	4,3	0,9	3,8	2,5	6,4	2,8	1,3	7,3	30,7	25,7-36,1	718
DKI Jakarta	20,7	1,8	3,7	0,1	3,1	3,6	4,0	2,3	2,0	7,8	29,8	26,6-33,2	3.184
Jawa Barat	18,0	2,5	3,6	1,0	2,5	3,0	2,4	2,3	1,5	7,3	26,6	24,9-28,4	15.325
Jawa Tengah	19,6	1,5	3,5	0,7	3,0	2,8	1,6	2,4	0,9	9,0	28,2	26,8-29,6	9.537
DI Yogyakarta	24,5	0,5	4,5	0,9	4,4	4,9	3,3	1,5	0,7	11,4	36,5	32,4-41,0	1.007
Jawa Timur	22,5	1,9	3,9	0,4	3,0	4,1	2,5	2,3	1,2	8,2	31,9	30,4-33,4	10.089
Banten	20,3	1,8	3,3	0,9	2,9	2,6	2,0	2,0	0,9	7,7	29,8	27,1-32,6	4.398
Bali	16,1	1,1	2,4	1,2	3,4	3,4	1,5	1,8	0,9	8,1	24,6	21,8-27,7	1.163
Nusa Tenggara Barat	25,2	3,0	2,9	1,3	2,4	3,6	2,8	2,9	1,4	9,7	31,3	28,4-34,3	1.801
Nusa Tenggara Timur	20,0	3,5	3,1	1,3	1,4	2,4	4,7	3,5	2,2	9,5	27,2	25,2-29,2	1.865
Kalimantan Barat	16,1	2,7	2,9	1,2	2,0	1,5	2,6	2,0	1,7	5,1	22,7	20,3-25,2	1.604
Kalimantan Tengah	14,6	2,5	2,9	0,7	2,1	0,9	2,0	1,7	0,8	6,5	20,7	18,0-23,7	871
Kalimantan Selatan	17,0	1,1	3,2	0,8	2,5	0,9	1,6	1,8	0,8	6,4	23,1	20,7-25,6	1.384
Kalimantan Timur	21,9	1,7	4,7	1,1	2,8	2,8	2,8	1,9	1,1	5,8	30,4	27,0-34,0	1.176
Kalimantan Utara	19,5	1,7	4,0	0,8	2,0	2,4	3,3	1,4	1,0	6,3	28,2	24,0-32,8	250
Sulawesi Utara	30,9	3,5	4,2	0,9	2,5	3,9	6,4	3,3	3,6	3,5	38,8	35,6-42,1	696
Sulawesi Tengah	24,9	4,4	3,8	2,0	2,9	2,7	3,5	4,4	4,7	9,3	33,0	30,0-36,2	986
Sulawesi Selatan	24,3	2,5	3,6	1,4	2,1	2,7	5,0	2,5	3,5	8,0	32,3	30,0-34,6	2.637
Sulawesi Tenggara	18,3	2,0	2,1	1,3	2,1	1,0	1,7	1,5	2,5	5,0	25,3	22,6-28,2	931
Gorontalo	22,6	4,3	5,2	2,4	2,2	3,1	5,9	3,0	3,9	5,1	32,1	27,9-36,7	373
Sulawesi Barat	24,2	2,1	2,0	0,7	1,0	0,4	3,0	1,0	0,9	7,6	28,2	24,3-32,5	442
Maluku	30,4	4,5	2,3	0,7	2,1	5,3	6,3	3,2	2,4	5,1	37,2	33,4-41,3	594
Maluku Utara	23,3	3,7	1,3	1,0	1,2	2,3	4,5	3,2	2,7	2,7	28,1	24,8-31,5	452
Papua Barat	22,8	6,6	2,3	0,7	2,2	1,7	5,9	2,1	1,2	7,1	29,6	25,0-34,7	329
Papua	17,5	3,1	0,7	0,9	1,0	1,6	4,0	2,5	2,0	3,4	22,4	19,2-26,0	927
**Indonesia**	**20,0**	**2,4**	**3,3**	**0,9**	**2,6**	**2,7**	**2,7**	**2,3**	**1,6**	**7,2**	**28,0**	**27,5-28,6**	**80.648**

*N Tertimbang <50

Tabel 14.2.12
Proporsi Gangguan/Komplikasi yang Dialami Selama Kehamilan pada Perempuan Umur 10-54 Tahun menurut Karakteristik, Riskesdas 2018

Karakteristik	Jenis gangguan/komplikasi selama kehamilan (%)										Mengalami salah satu gangguan/komplikasi Kehamilan		N ter-timbang
	Muntah/diare terus menerus	Demam tinggi	Hipertensi	Janin kurang bergerak	Pendarahan pada jalan lahir	Keluar air ketuban	Bengkak kaki disertai kejang	Batuk lama	Nyeri dada/jantung berdebar	Lainnya	%	95% CI	
**Umur saat bersalin**													
10 – 14	15,0	5,7	0,5	0,6	4,7	12,1	7,6	2,4	12,4	2,7	38,5	21,9-58,2	63
15 – 19	21,1	3,3	1,9	0,5	1,4	2,6	2,5	1,9	1,7	6,5	27,7	26,0-29,6	6.807
20 – 24	21,5	2,8	2,0	0,9	2,0	3,1	2,6	2,4	1,6	6,9	28,2	27,1-29,3	19.548
25 – 29	20,8	2,3	2,7	0,8	2,4	2,6	2,6	2,2	1,5	7,4	27,8	26,9-28,8	23.118
30 – 34	19,7	2,1	3,7	0,9	3,0	2,7	2,8	2,2	1,6	7,0	28,2	27,2-29,2	18.080
35 – 39	17,4	1,9	6,1	1,1	3,4	2,4	3,0	2,4	1,4	8,0	28,1	26,9-29,4	9.738
40 – 44	14,3	1,5	9,0	1,2	4,4	1,9	2,6	2,6	1,7	7,5	27,8	25,8-30,0	2.907
45 – 49	11,5	2,4	7,1	2,1	7,0	4,0	3,2	3,5	2,0	6,1	26,4	20,7-33,0	345
50 – 54	7,8	0	0	1,1	3,5	6,4	1,1	0	0,5	0	17,7	7,3-37,1	41*
**Kelompok Umur (Khusus)**													
10 -19 (Remaja)	21,1	3,4	1,9	0,5	1,4	2,6	2,5	1,9	1,8	6,5	27,8	26,1-29,7	6.870
15 - 49 (WUS)	20,1	2,4	3,3	0,9	2,6	2,7	2,7	2,3	1,6	7,2	28,0	27,5-28,6	80.544
**Pendidikan**													
Tidak pernah sekolah	17,5	3,0	2,7	0,9	1,9	1,1	2,7	2,4	1,6	3,8	23,9	21,1-27,0	1.308
Tidak tamat SD/MI	19,8	3,5	4,0	1,2	2,5	3,4	2,9	2,9	2,4	6,2	28,9	27,2-30,8	4.806
Tamat SD/MI	19,8	2,7	3,7	0,9	2,2	2,5	2,6	2,4	1,6	6,8	27,5	26,5-28,6	16.654
Tamat SLTP/MTS	19,4	2,3	3,1	0,9	2,1	2,6	2,5	2,1	1,5	6,6	26,8	25,8-27,8	20.384
Tamat SLTA/MA	21,0	2,2	3,3	0,8	3,0	2,9	2,9	2,2	1,6	7,7	29,2	28,3-30,1	27.523
Tamat D1/D2/D3/PT	19,6	1,7	3,1	0,9	3,1	2,7	2,6	2,1	1,2	8,3	28,2	26,9-29,6	9.973
**Pekerjaan**													
Tidak bekerja	20,7	2,4	3,6	0,9	2,4	2,8	2,8	2,4	1,7	7,1	28,7	28,0-29,4	47.363
Sekolah	17,9	3,1	1,7	0,5	1,5	2,6	2,4	1,5	1,8	9,1	25,3	20,0-31,5	566
PNS/TNI/Polri/BUMN/BUMD	16,3	1,4	3,3	0,5	2,6	2,2	2,1	1,0	1,0	7,9	24,5	22,2-26,9	2.027
Pegawai swasta	20,1	1,8	3,5	0,7	3,3	3,4	2,9	2,2	1,2	7,4	29,2	27,4-31,0	6.705
Wiraswasta	19,9	2,1	2,9	1,0	3,0	2,3	2,7	1,7	1,0	8,3	27,5	26,0-29,0	7.667
Petani/buruh tani	16,9	2,8	2,3	0,9	2,1	1,7	2,4	2,3	1,7	5,4	23,3	22,1-24,5	6.641
Nelayan	21,6	1,7	0,8	0	0	0,5	2,1	1,9	1,4	2,9	23,4	13,7-36,9	85
Buruh/sopir/pembantu ruta	17,4	2,3	3,4	1,3	3,5	3,6	2,1	2,3	1,4	6,5	27,4	24,8-30,2	3.071
Lainnya	21,3	3,0	3,3	1,3	2,6	2,6	2,5	2,3	1,8	7,6	28,9	27,3-30,6	6.524
**Tempat tinggal**													
Perkotaan	20,4	2,2	3,8	0,9	2,9	3,1	2,8	2,3	1,5	7,6	29,5	28,6-30,3	43.650
Perdesaan	19,6	2,5	2,8	0,9	2,2	2,2	2,5	2,2	1,6	6,7	26,3	25,7-26,9	36.998
**Indonesia**	**20,0**	**2,4**	**3,3**	**0,9**	**2,6**	**2,7**	**2,7**	**2,3**	**1,6**	**7,2**	**28,0**	**27,5-28,6**	**80.648**

*N Tertimbang <50

Tabel 14.2.13
Proporsi Upaya Pertolongan Pertama Komplikasi Kehamilan yang Pernah Dialami pada Perempuan Umur 10-54 Tahun menurut Provinsi, Riskesdas 2018

Provinsi	Upaya pertolongan pertama		Tidak dirujuk	N Tertimbang
	Segera dirujuk	Ada jeda waktu		
	%	%	%	
Aceh	59,7	17,5	22,8	543
Sumatera Utara	44,9	20,8	34,3	1.140
Sumatera Barat	53,7	19,7	26,6	643
Riau	46,5	17,6	36,0	787
Jambi	59,5	12,9	27,6	230
Sumatera Selatan	51,8	19,7	28,5	625
Bengkulu	50,3	16,7	33,0	167
Lampung	58,7	18,3	23,0	724
Kep. Bangka Belitung	57,8	21,1	21,0	154
Kepulauan Riau	59,5	13,3	27,2	244
DKI Jakarta	55,7	18,2	26,1	1.071
Jawa Barat	57,5	18,7	23,8	4.677
Jawa Tengah	66,5	16,9	16,6	3.156
DI Yogyakarta	61,9	18,7	19,4	422
Jawa Timur	64,6	16,8	18,5	3.633
Banten	56,1	19,2	24,7	1.486
Bali	63,4	17,2	19,4	333
Nusa Tenggara Barat	42,8	20,3	36,9	655
Nusa Tenggara Timur	30,2	27,4	42,4	597
Kalimantan Barat	48,5	21,0	30,5	401
Kalimantan Tengah	50,0	21,0	29,0	209
Kalimantan Selatan	63,8	18,9	17,3	365
Kalimantan Timur	55,4	17,4	27,2	387
Kalimantan Utara	42,0	14,6	43,4	78
Sulawesi Utara	42,4	17,4	40,1	270
Sulawesi Tengah	41,4	22,1	36,5	367
Sulawesi Selatan	40,6	20,0	39,4	948
Sulawesi Tenggara	42,1	19,0	38,9	259
Gorontalo	38,8	25,4	35,8	127
Sulawesi Barat	37,4	33,5	29,1	149
Maluku	26,4	18,9	54,7	232
Maluku Utara	35,8	17,7	46,5	130
Papua Barat	45,6	9,7	44,7	111
Papua	26,5	20,9	52,6	218
**Indonesia**	**55,3**	**18,6**	**26,1**	**25.538**

Tabel 14.2.14
Upaya Pertolongan Pertama Komplikasi Kehamilan yang Pernah Dialami pada Perempuan Umur 10-54 Tahun menurut Karakteristik, Riskesdas 2018

Karakteristik	Upaya pertolongan pertama		Tidak dirujuk	N Tertimbang
	Segera dirujuk	Ada jeda waktu		
	%	%	%	
**Umur saat bersalin**				
10 - 14	34,8	33,2	32,0	25*
15 - 19	47,4	19,5	33,0	2.109
20 - 24	53,8	18,8	27,4	6.156
25 - 29	55,0	19,4	25,7	7.299
30 - 34	57,4	18,2	24,3	5.720
35 - 39	58,9	17,3	23,8	3.181
40 - 44	58,6	18,1	23,3	941
45 - 49	62,2	7,7	30,1	100
50 - 54	61,3	0	38,7	7*
**Kelompok Umur (Khusus)**				
10 - 19 (Remaja)	47,3	19,7	33,0	1.989
15 - 49 (WUS)	55,3	18,6	26,1	25.506
**Pendidikan**				
Tidak pernah sekolah	43,7	17,3	39,1	337
Tidak tamat SD/MI	51,2	18,1	30,8	1.528
Tamat SD/MI	52,0	18,8	29,2	5.198
Tamat SLTP/MTS	56,0	18,4	25,6	6.152
Tamat SLTA/MA	56,3	19,1	24,5	9.099
Tamat D1/D2/D3/PT	59,2	17,7	23,1	3.225
Tamat PT	43,7	17,3	39,1	337
**Pekerjaan**				
Tidak bekerja	54,6	19,5	25,9	15.325
Sekolah	62,0	12,6	25,4	172
PNS/TNI/Polri/BUMN/BUMD	61,0	16,7	22,3	586
Pegawai swasta	62,7	14,7	22,5	2.194
Wiraswasta	58,9	16,8	24,4	2.470
Petani/buruh tani	44,4	19,9	35,7	1.732
Buruh/supir/pembantu ruta	43,9	27,2	28,9	21*
Lainnya	55,7	19,6	24,7	939
**Tempat tinggal**				
Perkotaan	57,6	17,8	24,6	14.529
Perdesaan	52,2	19,8	28,1	11.009

*N Tertimbang <50

Tabel 14.2.15
Proporsi Fasilitas Kesehatan Tempat Rujukan Pertama saat Mengalami Komplikasi Kehamilan yang Pernah Dimanfaatkan oleh Perempuan Umur 10-54 Tahun menurut Provinsi, Riskesdas 2018

Provinsi	Fasilitas kesehatan rujukan pertama			Polindes/bidan desa	N Tertimbang
	Rumah sakit	Puskesmas/Pustu	Praktik nakes		
	%	%	%	%	
Aceh	12,6	21,8	50,8	13,7	400
Sumatera Utara	14,5	10,8	69,7	3,4	716
Sumatera Barat	15,9	21,5	50,0	12,1	451
Riau	21,4	20,4	53,2	3,9	481
Jambi	14,9	27,3	47,9	9,3	159
Sumatera Selatan	15,9	16,2	48,4	18,4	427
Bengkulu	16,2	18,5	57,1	6,7	107
Lampung	11,9	7,7	77,0	2,3	533
Kep. Bangka Belitung	13,9	16,6	38,2	30,0	116
Kepulauan Riau	29,2	11,5	56,7	2,3	170
DKI Jakarta	28,6	26,2	44,3	0,0	756
Jawa Barat	16,1	16,0	64,3	2,3	3.407
Jawa Tengah	17,8	15,8	59,4	5,8	2.513
DI Yogyakarta	26,9	16,2	56,4	0,0	325
Jawa Timur	17,2	11,9	60,1	9,9	2.828
Banten	19,9	16,2	60,0	1,5	1.069
Bali	24,8	10,4	62,8	1,4	256
Nusa Tenggara Barat	9,1	32,8	15,8	38,6	394
Nusa Tenggara Timur	11,8	66,3	5,6	15,8	329
Kalimantan Barat	12,4	32,1	35,9	18,4	266
Kalimantan Tengah	15,6	36,9	37,1	9,4	142
Kalimantan Selatan	15,2	25,7	44,7	12,1	288
Kalimantan Timur	32,3	26,2	39,1	1,5	269
Kalimantan Utara	20,1	46,3	33,6	0,0	42*
Sulawesi Utara	24,8	36,0	34,2	4,1	155
Sulawesi Tengah	10,0	44,9	16,8	25,2	223
Sulawesi Selatan	18,0	55,4	21,5	3,6	548
Sulawesi Tenggara	16,1	49,6	23,4	7,0	151
Gorontalo	8,5	63,8	20,9	6,0	78
Sulawesi Barat	5,2	79,5	8,7	5,5	101
Maluku	11,0	62,1	16,6	6,8	101
Maluku Utara	19,3	47,1	19,2	12,2	66
Papua Barat	26,8	40,7	29,8	2,7	58
Papua	21,5	61,8	15,2	1,4	99
**Indonesia**	**17,5**	**21,0**	**53,2**	**7,0**	**18.026**

*N Tertimbang <50

Tabel 14.2.16

Proporsi Fasilitas Kesehatan Tempat Rujukan Pertama saat Mengalami Komplikasi Kehamilan yang Pernah Dimanfaatkan oleh Perempuan Umur 10-54 Tahun menurut Karakteristik, Riskesdas 2018

Karakteristik	Fasilitas kesehatan rujukan pertama			Polindes/bidan desa	N Tertimbang
	Rumah sakit	Puskesmas/Pustu	Praktik nakes		
	%	%	%	%	
**Umur saat bersalin**					
10 – 14	5,4	66,2	24,4	4,0	16*
15 - 19	8,5	25,1	53,8	10,7	1.349
20 - 24	12,7	21,0	57,3	7,4	4.269
25 - 29	18,7	18,8	55,4	6,2	5.183
30 - 34	20,7	20,5	51,1	6,4	4.135
35 - 39	21,8	23,1	47,0	6,9	2.315
40 - 44	21,4	25,4	44,9	6,7	690
45 - 49	30,3	18,9	45,5	5,3	66
50 – 54			100,0		4*
**Kelompok Umur (Khusus)**					
10 - 19 (Remaja)	8,5	25,6	53,4	10,6	1.365
15- 49 (WUS)	17,5	21,0	53,2	7,0	18.006
**Pendidikan**					
Tidak pernah sekolah	9,2	37,8	40,8	11,6	196
Tidak tamat SD/MI	8,3	31,7	46,8	10,8	1.010
Tamat SD/MI	9,6	26,7	52,0	10,3	3.516
Tamat SLTP/MTS	11,1	22,6	57,8	7,3	4.373
Tamat SLTA/MA	20,1	18,2	55,1	5,2	6.561
Tamat D1/D2/D3/PT	38,6	11,7	45,0	4,2	2.369
**Pekerjaan**					
Tidak bekerja	15,2	22,2	54,7	6,5	10.851
Sekolah	28,0	21,3	38,6	10,7	123
PNS/TNI/Polri/BUMN/BUM	41,6	15,2	39,3	3,8	436
Pegawai swasta	33,1	11,1	51,9	3,3	1.623
Wiraswasta	19,2	16,4	57,6	5,9	1.785
Petani/buruh tani	9,2	30,9	40,4	18,3	1.065
Buruh/supir/pembantu ruta	1,6	35,7	62,7		14*
Lainnya	17,0	16,9	58,7	6,6	675
**Tempat tinggal**					
Perkotaan	22,9	17,0	56,3	2,8	10.460
Perdesaan	10,0	26,6	48,9	12,8	7.566

*N Tertimbang <50

14.3 Masa Persalinan

Penolong persalinan

Definisi: Tenaga yang membantu proses persalinan, meliputi tenaga kesehatan (dokter umum, dokter kandungan bidan dan perawat terlatih), tenaga non kesehatan (dukun, keluarga/lainnya).

$$Proporsi\ Penolong\ Persalinan = \frac{\Sigma Perempuan\ Umur\ 10-54\ tahun\ yang\ pernah\ bersalin\ menurut\ jenis\ tenaga\ penolong\ persalinan}{\Sigma Perempuan\ Umur\ 10-54\ tahun\ yang\ bersalin\ anak\ terakhir\ pada\ periode\ 1\ Januari\ 2013\ sd\ wawancara}$$

Penolong persalinan oleh tenaga kesehatan

Definisi: Tenaga kesehatan yang membantu proses persalinan, meliputi dokter umum, dokter kandungan (dokter spesialis kandungan dan kebidanan), bidan dan perawat.

Jika penolong lebih dari satu, untuk kualifikasi tertinggi dipilih jenis tenaga penolong persalinan dengan strata yang paling tinggi. Dan sebaliknya untuk kualifikasi terendah.

$$Proporsi\ Penolong\ Persalinan\ oleh\ Tenaga\ Kesehatan = \frac{\Sigma Perempuan\ Umur\ 10-54\ tahun\ yang\ pernah\ bersalin\ dibantu\ oleh\ tenaga\ kesehatan}{\Sigma Perempuan\ Umur\ 10-54\ tahun\ yang\ bersalin\ anak\ terakhir\ pada\ periode\ 1\ Januari\ 2013\ sd\ wawancara}$$

Tempat persalinan

Definisi: Tempat yang dijadikan lokasi persalinan anak terakhir.

Tempat persalinan dapat terjadi di fasilitas pelayanan kesehatan (Rumah Sakit, klinik, puskesmas/pustu/pusling dan praktik tenaga kesehatan), tempat pelayanan lain (poskesdes, polindes), rumah, atau lainnya (contoh: alat transportasi).

Cakupan persalinan di Fasilitas Pelayanan Kesehatan (Fasyankes)

Fasyankes menurut Peraturan Pemerintah No 47 Tahun 2016 yang dapat dikaitkan dengan pelayanan persalinan yaitu Rumah Sakit, Klinik, Puskesmas/Pustu/Pusling dan Praktek Nakes.

$$Cakupan\ persalinan\ di\ Fasyankes = \frac{\Sigma Perempuan\ Umur\ 10-54\ yang\ saat\ bersalin\ anak\ terakhir\ di\ Fasyankes}{\Sigma Perempuan\ Umur\ 10-54\ tahun\ yang\ bersalin\ anak\ terakhir\ pada\ periode\ 1\ Januari\ 2013\ sd\ wawancara}$$

Tabel 14.3.1
Proporsi Penolong Persalinan dengan Kualifikasi Tertinggi
pada Perempuan Umur 10-54 Tahun menurut Provinsi, Riskesdas 2018

Provinsi	Penolong persalinan kualifikasi tertinggi[1] (%)							Pertolongan persalinan oleh Nakes[2]		N ter-timbang
	dr kandungan	dr umum	bidan	perawat	dukun	keluarga/ lainnya	Tidak ada penolong	%	95% CI	
Aceh	31,7	0,5	65,7	0,2	1,7	0,1	0,0	98,2	97,5 - 98,6	1.758
Sumatera Utara	28,9	0,8	64,6	0,4	4,7	0,4	0,1	94,8	94,0 - 95,5	4.379
Sumatera Barat	35,7	1,4	58,9	0,4	3,4	0,1	0,0	96,4	95,4 - 97,2	1.770
Riau	29,2	1,4	61,1	0,4	7,4	0,2	0,3	92,2	90,7 - 93,5	2.348
Jambi	20,5	1,0	70,2	0,1	8,0	0,1	0,1	91,8	89,6 - 93,5	1.087
Sumatera Selatan	20,7	1,6	71,4	0,2	5,9	0,2	0,0	93,9	92,5 – 95,0	2.529
Bengkulu	22,4	1,5	71,1	0,2	4,4	0,3	0,1	95,3	93,2 - 96,7	599
Lampung	19,7	1,3	72,9	0,2	5,7	0,1	0,1	94,1	92,9 - 95,1	2.644
Kep. Bangka Belitung	30,6	1,1	65,3	0,7	2,3	0,0	0,0	97,7	96,4 - 98,5	452
Kepulauan Riau	38,9	1,6	58,1	0,3	1,0	0,0	0,0	99,0	98,0 - 99,5	693
DKI Jakarta	46,8	1,5	51,4	0,0	0,3	0,0	0,0	99,7	99,0 - 99,9	3.058
Jawa Barat	26,0	0,9	63,8	0,2	8,9	0,1	0,1	90,9	89,4 - 92,2	15.043
Jawa Tengah	33,9	0,8	64,5	0,2	0,4	0,1	0,0	99,4	99,1 - 99,6	9.291
DI Yogyakarta	49,8	1,2	48,4	0,1	0,3	0,2	0,0	99,5	98,1 - 99,9	971
Jawa Timur	32,7	1,1	63,5	0,1	2,3	0,1	0,1	97,4	96,8 - 97,8	9.832
Banten	29,9	0,8	55,3	0,1	13,5	0,2	0,1	86,2	84,2 – 88,0	4.265
Bali	54,4	0,7	43,9	0,0	0,6	0,2	0,1	99,1	98,2 - 99,5	1.115
Nusa Tenggara Barat	19,2	1,5	74,2	0,3	4,5	0,2	0,1	95,2	93,6 - 96,3	1.752
Nusa Tenggara Timur	16,0	2,8	61,4	0,6	16,1	2,7	0,4	80,8	78,9 - 82,5	1.835
Kalimantan Barat	16,4	1,5	67,2	1,3	13,5	0,1	0,1	86,3	84,2 - 88,1	1.584
Kalimantan Tengah	14,0	1,2	69,3	1,6	13,5	0,3	0,1	86,1	83,5 - 88,4	848
Kalimantan Selatan	26,3	0,5	67,0	0,3	5,5	0,2	0,2	94,1	92,5 - 95,4	1.353
Kalimantan Timur	34,6	0,9	60,5	0,2	3,1	0,4	0,2	96,2	94,8 - 97,2	1.144
Kalimantan Utara	28,0	1,0	64,4	0,4	4,9	1,2	0,1	93,8	91,3 - 95,6	242
Sulawesi Utara	45,0	3,6	44,4	0,6	6,1	0,4	0,0	93,5	91,9 - 94,9	676
Sulawesi Tengah	22,2	2,2	62,7	0,5	11,2	1,0	0,2	87,6	85,4 - 89,5	949
Sulawesi Selatan	29,8	1,6	63,4	0,1	3,4	1,5	0,3	94,8	93,7 - 95,7	2.576
Sulawesi Tenggara	12,6	1,4	71,5	0,5	12,9	0,8	0,3	85,9	83,1 - 88,4	890
Gorontalo	32,6	1,6	60,0	0,5	4,9	0,3	0,1	94,8	92,7 - 96,3	364
Sulawesi Barat	13,3	1,1	73,4	0,8	10,2	1,1	0,1	88,6	85,6 – 91,0	427
Maluku	12,1	1,4	51,8	0,4	33,4	0,6	0,3	65,6	61,2 - 69,8	581
Maluku Utara	16,6	1,9	53,7	0,4	26,1	1,0	0,2	72,7	69,2 - 75,9	443
Papua Barat	25,0	1,2	54,1	0,8	11,5	6,7	0,6	81,1	77,3 - 84,5	321
Papua	15,5	2,3	39,0	1,1	17,7	21,3	3,1	57,9	54,1 - 61,6	916
**INDONESIA**	**28,9**	**1,2**	**62,7**	**0,3**	**6,2**	**0,6**	**0,1**	**93,1**	**92,8-93,5**	**78.736**

[1] Kualifikasi tertinggi bila terdapat lebih dari satu penolong persalinan, maka yang dipilih adalah tenaga penolong yang paling tinggi kualifikasinya
[2] Penolong persalinan oleh tenaga kesehatan (Nakes) adalah bila persalinan ditolong oleh dokter spesialis kandungan dan kebidanan, dokter umum, bidan/perawat

Tabel 14.3.2
Proporsi Penolong Persalinan dengan Kualifikasi Tertinggi
pada Perempuan Umur 10-54 Tahun menurut Karakteristik, Riskesdas 2018

Karakteristik	Penolong persalinan kualifikasi tertinggi[1] (%)							Pertolongan persalinan oleh Nakes[2]		N ter-timbang
	dr kandungan	dr umum	bidan	perawat	dukun	keluarga/ lainnya	Tidak ada penolong	%	95% CI	
**Umur saat Bersalin**										
10-14	21,3	0,7	66,1	0,0	6,7	5,2	0,0	88,1	76,2-94,5	59
15-19	18,2	1,3	70,4	0,3	8,9	0,7	0,2	90,2	88,8-91,5	6.725
20-24	24,6	1,2	67,5	0,3	5,9	0,4	0,1	93,5	92,9-94,1	19.211
25-29	30,5	1,0	62,1	0,3	5,5	0,5	0,1	93,8	93,3-94,3	22.708
30-34	31,5	1,2	60,6	0,3	5,7	0,6	0,2	93,6	93,0-94,1	17.657
35-39	34,9	1,4	56,3	0,2	6,4	0,6	0,2	92,9	92,1-93,6	9.366
40-44	36,3	0,8	52,9	0,2	8,2	1,3	0,3	90,2	88,6-91,6	2.670
45-49	35,8	0,9	51,8	0,2	9,1	1,5	0,8	88,6	84,7-91,7	301
50-54	20,0	0,0	54,8	0,0	22,8	2,3	0,0	74,8	51,3-89,3	38*
**Kelompok Umur (Khusus)**										
10 - 19 (Remaja)	18,3	1,3	70,4	0,3	8,9	0,7	0,2	90,2	88,7-91,5	6.519
15 - 49 (WUS)	29,0	1,2	62,7	0,3	6,2	0,6	0,1	93,4	92,8-93,5	78.639
**Pendidikan**										
Tidak pernah sekolah	15,5	1,2	53,4	0,6	20,3	8,2	0,9	70,6	67,3-73,8	1.285
Tidak tamat SD/MI	15,6	1,1	64,3	0,4	16,7	1,5	0,3	81,4	79,8-83,0	4.700
Tamat SD/MI	17,8	1,0	67,0	0,4	12,8	0,7	0,3	86,2	85,2-87,2	16.286
Tamat SLTP/MTS	22,2	1,0	70,7	0,3	5,3	0,4	0,1	94,3	93,7-94,7	19.989
Tamat SLTA/MA	34,3	1,3	61,7	0,2	2,3	0,3	0,1	97,4	97,1-97,7	26.787
Tamat D1/D2/D3/PT	55,0	1,4	42,6	0,2	0,7	0,1	0,0	99,2	99,0-99,3	9.688
**Pekerjaan**										
Tidak bekerja	27,3	1,1	64,5	0,3	6,4	0,4	0,1	93,1	92,6-93,6	46.404
Sekolah	29,4	0,6	62,7	0,0	7,0	0,0	0,3	92,7	88,5-95,4	550
PNS/TNI/Polri/BUMN/BUMD	54,5	1,2	42,9	0,1	1,1	0,2	0,0	98,7	97,9-99,2	1.964
Pegawai swasta	47,4	1,0	50,0	0,2	1,1	0,2	0,1	98,7	98,1-99,1	6.460
Wiraswasta	33,7	1,2	62,1	0,2	2,6	0,1	0,1	97,2	96,7-97,7	7.441
Petani/buruh tani	12,1	1,4	66,2	0,5	16,1	3,1	0,7	80,1	79,0-81,3	6.503
Nelayan	16,8	2,2	64,3	0,0	12,2	4,5	0,0	83,3	73,7-89,9	85
Buruh/sopir/pembantu ruta	25,3	0,8	67,2	0,5	5,7	0,4	0,1	93,8	91,9-95,3	2.968
Lainnya	28,0	1,4	64,5	0,3	5,2	0,5	0,1	94,1	93,3-94,9	6.360
**Tempat tinggal**										
Perkotaan	36,5	1,0	59,0	0,1	3,0	0,2	0,1	96,7	96,2-97,2	42.461
Perdesaan	20,1	1,3	67,1	0,5	9,9	1,0	0,2	88,9	88,4-89,3	36.275

[1] Kualifikasi tertinggi bila terdapat lebih dari satu penolong persalinan, maka yang dipilih adalah tenaga penolong yang paling tinggi kualifikasinya
[2] Penolong persalinan oleh tenaga kesehatan (Nakes) adalah bila persalinan ditolong oleh dokter spesialis kandungan dan kebidanan, dokter umum, bidan/perawat
*N Tertimbang <50

Tabel 14.3.3
Proporsi Penolong Persalinan dengan Kualifikasi Terendah
pada Perempuan Umur 10-54 Tahun menurut Provinsi, Riskesdas 2018

Provinsi	Penolong persalinan kualifikasi terendah[1] (%)							Pertolongan persalinan oleh Nakes[2]		N Ter-timbang
	dr kandungan	dr umum	bidan	perawat	dukun	keluarga/ lainnya	Tidak ada penolong	%	95% CI	
Aceh	19,6	0,4	73,5	2,8	3,5	0,1	0,0	96,3	95,4-97,1	1.758
Sumatera Utara	15,7	0,8	71,8	4,7	6,2	0,7	0,1	93,0	92,0-93,9	4.379
Sumatera Barat	19,5	0,7	66,8	8,2	4,5	0,2	0,0	95,2	94,0-96,2	1.770
Riau	17,8	0,8	60,3	6,8	13,3	0,7	0,3	85,8	83,9-87,5	2.348
Jambi	12,1	0,2	63,9	2,8	20,7	0,2	0,1	79,0	76,2-81,7	1.087
Sumatera Selatan	14,3	0,9	70,7	2,9	10,8	0,3	0,0	88,8	86,9-90,5	2.529
Bengkulu	14,6	0,7	71,2	2,9	10,1	0,3	0,1	89,4	87,0-91,5	599
Lampung	11,6	0,6	75,0	2,0	10,5	0,2	0,1	89,2	87,6-90,5	2.644
Kep. Bangka Belitung	9,7	0,5	71,3	9,5	9,0	0,0	0,0	91,0	88,7-93,0	452
Kepulauan Riau	25,0	0,5	60,6	10,9	2,9	0,0	0,0	97,0	95,7-98,0	693
DKI Jakarta	20,8	0,5	63,2	14,0	1,0	0,6	0,0	98,4	97,4-99,1	3.058
Jawa Barat	12,6	0,7	65,5	4,1	16,1	0,9	0,1	82,9	81,2-84,5	15.043
Jawa Tengah	14,8	0,4	74,5	7,2	2,4	0,6	0,0	96,9	96,2-97,5	9.291
DI Yogyakarta	22,6	0,4	61,3	14,5	0,9	0,2	0,0	98,9	97,5-99,5	971
Jawa Timur	16,2	0,6	75,2	4,3	3,2	0,4	0,1	96,3	95,6-96,8	9.832
Banten	12,1	0,4	59,6	5,3	21,8	0,7	0,1	77,4	74,9-79,7	4.265
Bali	33,3	0,3	60,1	4,9	0,8	0,5	0,1	98,7	97,7-99,2	1.115
Nusa Tenggara Barat	4,9	0,7	77,7	4,0	12,2	0,4	0,1	87,3	85,2-89,1	1.752
Nusa Tenggara Timur	7,3	0,5	64,7	3,4	19,3	4,4	0,4	75,9	73,9-77,7	1.835
Kalimantan Barat	8,6	0,4	61,5	3,6	25,4	0,4	0,1	74,1	71,4-76,7	1.584
Kalimantan Tengah	6,8	0,3	63,9	4,7	23,7	0,5	0,1	75,7	72,8-78,4	848
Kalimantan Selatan	9,7	0,3	73,4	6,6	9,4	0,5	0,2	90,0	88,1-91,6	1.353
Kalimantan Timur	21,8	0,7	65,9	5,6	5,0	0,7	0,2	94,1	92,4-95,5	1.144
Kalimantan Utara	18,3	0,4	67,6	5,2	5,9	2,4	0,1	91,6	88,8-93,8	242
Sulawesi Utara	16,7	0,8	52,0	19,8	9,5	1,2	0,0	89,3	87,3-90,9	676
Sulawesi Tengah	6,8	0,6	63,7	6,6	20,5	1,6	0,2	77,8	75,2-80,2	949
Sulawesi Selatan	7,9	1,1	67,7	12,9	7,9	2,2	0,3	89,6	88,3-90,9	2.576
Sulawesi Tenggara	6,1	0,7	55,3	2,0	34,4	1,2	0,3	64,1	60,7-67,3	890
Gorontalo	10,5	0,3	58,2	6,2	24,2	0,6	0,1	75,1	71,1-78,8	364
Sulawesi Barat	6,8	0,1	59,1	2,1	30,5	1,4	0,1	68,0	63,6-72,2	427
Maluku	7,6	0,5	44,0	1,6	42,9	3,0	0,3	53,7	49,0-58,3	581
Maluku Utara	7,4	0,7	49,1	4,0	37,0	1,7	0,2	61,1	57,6-64,6	443
Papua Barat	10,5	0,5	60,4	4,9	15,7	7,4	0,6	76,3	71,9-80,1	321
Papua	5,1	1,0	43,6	5,3	18,0	24,0	3,1	54,9	51,2-58,5	916
**INDONESIA**	**14,0**	**0,6**	**67,7**	**5,7**	**10,9**	**1,1**	**0,1**	**87,9**	**87,5-88,3**	**78.736**

[1] Kualifikasi terendah bila terdapat lebih dari satu penolong persalinan, maka yang dipilih adalah tenaga penolong yang paling rendah kualifikasinya

[2] Penolong persalinan oleh tenaga kesehatan (Nakes) adalah bila persalinan ditolong oleh dokter spesialis kandungan dan kebidanan, dokter umum, bidan/perawat.

Tabel 14.3.4
Proporsi Penolong Persalinan dengan Kualifikasi Terendah
pada Perempuan Umur 10-54 Tahun menurut Karakteristik, Riskesdas 2018

Karakteristik	Penolong Persalinan Kualifikasi Terendah[1] (%)							Persalinan oleh Tenaga Kesehatan[2]		N ter-timbang
	dr kandungan	dr umum	bidan	perawat	dukun	keluarga/ lainnya	Tidak ada penolong	%	95% CI	
**Umur saat bersalin**										
10-14	8,9	na	50,7	3,8	31,4	5,2	0,0	63,4	42,7-80,1	59
15-19	6,9	0,6	69,5	4,1	17,4	1,2	0,2	81,2	79,5-82,8	6.725
20-24	10,6	0,5	71,6	5,1	11,2	1,0	0,1	87,8	87,0-88,5	19.211
25-29	15,5	0,6	67,1	5,7	10,0	1,0	0,1	88,9	88,2-89,5	22.708
30-34	15,9	0,6	66,6	6,5	9,3	1,1	0,2	89,5	88,8-90,1	17.657
35-39	17,4	0,8	64,6	6,2	9,8	1,0	0,2	89,0	88,1-89,8	9.366
40-44	18,9	0,5	59,1	6,8	12,7	1,6	0,3	85,4	83,6-87,0	2.670
45-49	15,4	0,4	62,4	6,6	12,5	2,0	0,8	84,8	80,3-88,4	301
50-54	4,2	0,0	60,1	na	33,3	2,4	0,0	64,3	41,3-82,2	38*
**Kelompok Umur (Khusus)**										
10 - 19 (Remaja)	7,0	0,6	69,4	4,1	17,5	1,2	0,2	81,1	79,4-82,7	6.784
15 - 49 (WUS)	14,0	0,6	67,7	5,7	10,8	1,1	0,1	88,0	87,5-88,4	78.639
**Pendidikan**										
Tidak pernah sekolah	7,3	0,7	53,6	2,9	25,7	9,0	0,9	64,4	60,8-67,9	1.285
Tidak tamat SD/MI	5,7	0,5	64,2	3,2	24,0	2,0	0,3	73,7	71,8-75,4	4.700
Tamat SD/MI	7,5	0,4	66,3	3,5	20,8	1,2	0,3	77,7	76,5-78,8	16.286
Tamat SLTP/MTS	9,5	0,4	73,8	4,7	10,5	0,9	0,1	88,5	87,8-89,2	19.989
Tamat SLTA/MA	16,1	0,8	70,1	7,0	5,2	0,8	0,1	93,9	93,5-94,4	26.787
Tamat D1/D2/D3/PT	32,9	0,9	54,1	9,5	2,2	0,4	0,0	97,5	97,1-97,8	9.688
**Pekerjaan**										
Tidak bekerja	12.5	0,6	68,9	5,7	11,4	0,9	0,1	87,7	87,1-88,2	46.404
Sekolah	15.9	0,5	65,5	5,6	10,9	1,4	0,3	87,4	82,7-91,1	550
PNS/TNI/Polri/BUMN/BUMD	34.2	0,6	52,8	9,0	3,0	0,4	0,0	96,6	95,6-97,4	1.964
Pegawai swasta	25.8	0,8	62,3	7,5	2,9	0,6	0,1	96,4	95,5-97,1	6.460
Wiraswasta	16.3	0,7	70,1	6,6	5,6	0,5	0,1	93,8	93,0-94,6	7.441
Petani/buruh tani	5.6	0,4	63,5	2,3	23,6	3,8	0,7	71,9	70,6-73,2	6.503
Nelayan	9.0	0,0	61,8	6,4	18,1	4,8	0,0	77,1	66,6-85,1	85
Buruh/sopir/pembantu ruta	12.0	0,3	71,1	4,3	11,2	1,0	0,1	87,8	85,3-89,9	2.968
Lainnya	13.4	0,6	68,8	5,5	10,6	1,0	0,1	88,3	87,2-89,3	6.360
**Tempat tinggal**										
Perkotaan	18.2	0,7	67,4	7,5	5,4	0,7	0,1	93,8	93,2-94,4	42.461
Perdesaan	9.0	0,5	68,0	3,6	17,3	1,4	0,2	81,0	80,5-81,6	36.275

[1] Kualifikasi terendah bila terdapat lebih dari satu penolong persalinan, maka yang dipilih adalah tenaga penolong yang paling rendah kualifikasinya

[2] Penolong persalinan oleh tenaga kesehatan (Nakes) adalah bila persalinan ditolong oleh dokter spesialis kandungan dan kebidanan, dokter umum, bidan/perawat.

*N Tertimbang <50

Tabel 14.3.5
Proporsi Tempat Persalinan yang Dimanfaatkan oleh Perempuan Umur 10-54 Tahun menurut Provinsi, Riskesdas 2018

Provinsi	Tempat persalinan (%)								Fasilitas Pelayanan Kesehatan[1]		N Tertimbang
	RS Pemerintah	RS Swasta	Klinik	Puskesmas/ Pustu/ Pusling	Praktek nakes	Poskesdes Polindes	Rumah	Lainnya	%	95% CI	
Aceh	20,8	13,3	5,7	7,5	29,8	9,8	12,8	0,2	77,1	75,3-78,8	1.758
Sumatera Utara	7,4	22,1	12,5	6,4	24,3	1,9	25,3	0,1	72,7	71,0-74,4	4.379
Sumatera Barat	15,3	18,5	7,6	10,6	32,4	5,7	9,7	0,2	84,3	82,5-86,1	1.770
Riau	11,0	19,4	7,4	4,6	23,7	1,1	32,4	0,3	66,1	63,6-68,6	2.348
Jambi	9,9	12,7	6,4	8,1	19,7	5,4	37,6	0,2	56,8	53,6-60,0	1.087
Sumatera Selatan	13,2	10,7	5,0	5,0	31,6	9,5	24,7	0,3	65,5	62,8-68,1	2.529
Bengkulu	17,1	10,4	3,1	9,6	28,3	2,4	29,1	0,1	68,4	65,1-71,5	599
Lampung	4,7	16,7	2,0	4,3	54,6	1,3	16,0	0,2	82,5	80,5-84,3	2.644
Bangka Belitung	20,9	13,7	7,6	8,4	23,2	12,5	13,2	0,4	73,8	70,5-77,0	452
Kepulauan Riau	16,2	25,2	12,7	6,9	30,7	0,9	7,2	0,3	91,6	88,8-93,8	693
DKI Jakarta	18,5	29,0	5,2	18,5	26,8	0,1	1,9	0,0	98,0	96,7-98,7	3.058
Jawa Barat	11,6	16,3	4,6	8,2	40,8	1,2	17,3	0,1	81,4	79,8-83,0	15.043
Jawa Tengah	16,4	20,9	4,9	15,5	37,1	2,4	2,6	0,0	95,0	94,3-95,6	9.291
DI Yogyakarta	23,2	32,7	6,3	6,7	29,0	0,7	1,4	0,0	97,9	95,9-98,9	971
Jawa Timur	13,6	24,0	2,4	8,4	40,1	7,1	4,3	0,2	88,5	87,4-89,4	9.832
Banten	9,4	22,7	6,5	10,9	26,6	0,7	23,0	0,2	76,1	73,6-78,5	4.265
Bali	23,1	29,1	7,8	6,0	32,5	0,1	1,4	0,0	98,5	97,6-99,1	1.115
Nusa Tenggara Barat	20,8	5,5	1,5	23,2	8,1	30,7	10,0	0,3	59,0	55,9-62,0	1.752
Nusa Tenggara Timur	20,3	7,3	2,1	42,0	1,0	2,7	24,3	0,3	72,7	70,7-74,7	1.835
Kalimantan Barat	11,5	10,6	6,2	12,3	15,0	7,6	36,7	0,1	55,5	52,6-58,4	1.584
Kalimantan Tengah	14,7	2,2	7,5	9,0	11,6	1,7	52,9	0,4	45,0	41,7-48,3	848
Kalimantan Selatan	23,6	7,5	3,7	7,2	24,9	7,5	25,5	0,2	66,8	64,1-69,5	1.353
Kalimantan Timur	28,3	20,7	9,0	7,8	21,1	0,8	12,2	0,0	86,9	84,3-89,1	1.144
Kalimantan Utara	41,7	2,7	1,6	19,2	15,3	0,0	19,5	0,0	80,5	77,0-83,6	242
Sulawesi Utara	23,2	25,9	8,4	18,7	5,4	1,7	16,5	0,1	81,7	79,1-84,0	676
Sulawesi Tengah	21,8	7,1	1,5	25,9	3,0	7,8	32,5	0,3	59,4	56,5-62,2	949
Sulawesi Selatan	24,6	14,4	2,1	31,5	8,4	3,3	15,3	0,5	80,9	79,1-82,6	2.576
Sulawesi Tenggara	11,6	6,5	2,4	26,1	4,9	1,5	46,8	0,2	51,5	48,2-54,8	890
Gorontalo	31,9	8,4	1,4	32,3	3,0	5,8	16,8	0,4	77,0	73,3-80,4	364
Sulawesi Barat	14,5	4,6	1,8	43,7	3,9	3,7	27,2	0,5	68,5	64,1-72,6	427
Maluku	16,0	7,3	0,3	5,5	1,0	0,9	68,6	0,4	30,1	26,5-34,0	581
Maluku Utara	22,9	4,2	2,6	11,3	2,1	2,2	54,3	0,4	43,1	39,8-46,5	443
Papua Barat	34,1	8,4	0,9	14,1	5,1	1,3	35,3	0,9	62,6	58,2-66,8	321
Papua	19,9	4,6	2,1	15,5	3,1	0,3	54,0	0,5	45,2	41,8-48,6	916
**INDONESIA**	**14,9**	**17,8**	**4,9**	**12,1**	**29,6**	**3,8**	**16,7**	**0,2**	**79,3**	**78,8-79,7**	**78.736**

[1] Fasilitas Pelayanan Kesehatan terdiri dari RS pemerintah, RS swasta, klinik, Puskesmas/Pustu/Pusling, dan praktik nakes

Tabel 14.3.6
Proporsi Tempat Persalinan yang Dimanfaatkan oleh Perempuan Umur 10-54 Tahun menurut Karakteristik, Riskesdas 2018

Karakteristik	Tempat bersalin (%)								Fasilitas Pelayanan Kesehatan[1]		N ter-timbang
	RS Peme-rintah	RS Swas ta	Klinik	Puskes-mas/ Pustu/ Pusling	Prak-tek nakes	Poskes-des/Poli ndes	Rumah	Lain-nya	%	95% CI	
**Umur saat bersalin**											
10-14	20,2	6.7	5,2	14,0	9,1	4,8	38,7	1,4	55,2	36,3-72,7	59
15-19	13,4	9.1	3,7	14,6	30,4	5,7	23,0	0,2	71,0	69,2-72,8	6.725
20-24	13,8	14.8	5,0	13,4	31,9	4,1	16,9	0,1	78,9	78,0-79,7	22.708
25-29	13,4	20.0	5,8	11,6	30,1	3,7	15,2	0,2	80,9	80,2-81,6	17.657
30-34	15,0	20.1	4,8	11,3	29,7	3,4	15,7	0,1	80,8	80,0-81,6	9.366
35-39	19,2	20.4	4,4	10,9	25,3	3,2	16,5	0,2	80,2	79,1-81,1	2.670
40-44	22,0	19.0	3,4	9,5	23,2	3,2	19,4	0,3	77,1	75,1-79,0	301
45-49	21,1	15.2	6,9	13,4	19,7	3,1	20,6	0,0	76,3	70,8-81,0	38*
50-54	8,6	0.8	7,1	0,8	26,4	2,8	53,4	0,0	43,8	23,7-66,2	59
**Kelompok Umur (Khusus)**											
10 - 19 (Remaja)	13,4	9,1	3,7	14,6	30,2	5,7	23,2	0,2	79,3	78,8-79,7	6.784
15 - 49 (WUS)	14,9	17,8	4,9	12,1	29,6	3,8	16,7	0,2	70,9	69,1-72,6	78.638
**Pendidikan**											
Tidak pernah sekolah	10,2	6,8	2,0	13,8	18,0	6,3	42,3	0,5	50,9	47,2-54,6	1.285
Tidak tamat SD/MI	12,2	7,3	3,1	15,0	23,2	5,9	32,8	0,5	60,8	58,8-62,7	4.700
Tamat SD/MI	13,2	7,8	2,9	14,9	27,8	5,2	28,1	0,2	66,6	65,4-67,7	16.286
Tamat SLTP/MTS	13,6	12,2	4,8	13,4	35,5	4,1	16,3	0,2	79,5	78,6-80,3	19.989
Tamat SLTA/MA	15,9	22,5	6,2	10,7	31,3	3,1	10,2	0,1	86,6	86,1-87,1	26.787
Tamat D1/D2/D3/PT	19.2	39.6	6.7	7.0	20.3	1.7	5.5	0.1	86,6	92,1-93,2	9.688
**Pekerjaan**											
Tidak bekerja	14,6	16.5	4,9	12,5	30,9	3,7	16,7	0,2	79,4	78,8-80,0	46.404
Sekolah	15,3	18.1	6,1	9,9	31,4	3,6	15,7	0,0	80,7	75,7-84,9	550
PNS/TNI/Polri/BUMN/BUMD	27,3	32.9	6,4	6,8	15,7	1,7	8,9	0,2	89,2	87,7-90,6	1.964
Pegawai swasta	16,1	34.7	6,7	7,3	27,7	1,8	5,5	0,1	92,6	91,6-93,5	6.460
Wiraswasta	15,4	21.3	6,1	9,1	35,1	3,4	9,7	0.0	86,9	85,8-87,9	7.441
Petani/buruh tani	10,1	4.9	2,2	16,3	19,1	8,1	39,0	0,3	52,6	51,2-54,0	6.503
Nelayan	14,8	6.9	2,8	21,6	14,9	6,0	33,0	0,0	61	50,1-70,8	85
Buruh/supir/pembantu ruta	14,3	15.6	4,9	12,0	36,6	2,8	13,3	0,4	83,5	81,0-85,6	2.968
Lainnya	16,4	15.5	4,3	14,5	27,7	3,9	17,6	0,2	78,4	77,0-79,7	6.360
**Tempat tinggal**											
Perkotaan	16,4	24.4	6,2	9,4	33,8	1,7	8,0	0,1	90,2	89,5-90,7	42.461
Perdesaan	13,0	10.1	3,5	15,2	24,8	6,3	27,0	0,2	66,5	65,9-67,2	36.275

[1] Fasilitas Pelayanan Kesehatan terdiri dari RS pemerintah, RS swasta, klinik, Puskesmas/Pustu/Pusling, dan praktik nakes
*N Tertimbang <50

Sumber pembiayaan persalinan

Definisi: Sumber biaya digunakan ibu saat bersalin di fasilitas pelayanan kesehatan, yang terdiri dari BPJS/KIS, asuransi swasta, biaya kantor, biaya orang lain, biaya sendiri, jampersal, atau jamperda.

$$Proporsi\ Sumber\ Pembiayaan\ Persalinan = \frac{\Sigma\ Perempuan\ Umur\ 10-54\ tahun\ menurut\ jenis\ sumber\ dana\ untuk\ biaya\ persalinan\ anak\ terakhir}{\Sigma\ Perempuan\ Umur\ 10-54\ tahun\ yang\ bersalin\ anak\ terakhir\ di\ Fasyankes\ pada\ periode\ 1\ Januari\ 2013\ sampai\ saat\ wawancara}$$

Metode persalinan

Definisi: Metode atau cara ibu melahirkan bayi, yaitu normal, operasi dan lainnya (vakum, forsep, atau lainnya).

$$Persentase\ Metode\ Bersalin = \frac{\Sigma\ Perempuan\ Umur\ 10-54\ tahun\ menurut\ metode\ persalinan\ saat\ melahirkan\ anak\ terakhir}{\Sigma\ Perempuan\ Umur\ 10-54\ tahun\ yang\ bersalin\ anak\ terakhir\ pada\ periode\ 1\ Januari\ 2013\ sampai\ saat\ wawancara}$$

Tabel 14.3.7
Proporsi Sumber Pembiayaan Persalinan di Fasilitas Pelayanan Kesehatan pada Perempuan Umur 10-54 Tahun menurut Provinsi, Riskesdas 2018

Provinsi	Sumber pembiayaan persalinan (%)								N ter-timbang
	BPJS/ KIS	Asuransi Swasta	Biaya kantor	Biaya orang lain	Biaya sendiri	Jam-persal	Jam-perda	Tidak menge-luarkan biaya	
Aceh	80,5	0,5	1,4	0,0	18,0	2,1	0,1	0,9	1.230
Sumatera Utara	39,6	1,4	3,1	0,5	52,8	3,8	0,2	0,5	2.889
Sumatera Barat	48,2	0,9	0,4	0,4	51,2	2,7	0,5	0,4	1.355
Riau	31,7	2,4	4,0	0,9	62,7	1,8	2,7	0,9	1.409
Jambi	48,1	1,1	1,0	0,3	50,7	4,6	0,7	0,4	561
Sumatera Selatan	28,7	1,9	1,5	0,5	62,8	4,8	1,7	1,1	1.503
Bengkulu	56,6	0,4	1,1	0,0	41,5	4,0	0,5	0,1	372
Lampung	38,6	0,8	0,7	0,1	60,8	3,6	1,3	0,1	1.978
Bangka Belitung	41,5	1,2	1,9	0,6	58,6	3,6	3,7	0,5	303
Kepulauan Riau	45,2	2,5	6,1	0,3	45,2	2,2	1,4	1,1	576
DKI Jakarta	44,5	1,1	7,0	0,6	49,9	2,0	0,3	0,1	2.718
Jawa Barat	34,8	1,7	3,8	0,2	59,9	4,1	0,9	0,8	11.114
Jawa Tengah	40,5	1,1	1,1	0,4	53,6	6,0	1,4	0,6	8.005
DI Yogyakarta	50,8	1,6	2,1	0,2	43,6	7,1	5,5	0,1	863
Jawa Timur	31,1	0,9	1,8	0,2	63,8	5,3	1,1	0,4	7.892
Banten	32,6	2,7	4,3	0,4	59,8	3,6	1,6	0,2	2.947
Bali	36,3	1,4	3,4	0,2	58,5	5,4	3,8	0,7	997
Nusa Tenggara Barat	58,3	0,5	1,3	0,5	36,7	6,0	0,2	3,0	937
Nusa Tenggara Timur	61,2	0,3	0,2	0,4	26,3	3,7	7,6	4,3	1.211
Kalimantan Barat	41,2	1,4	2,0	0,6	56,7	3,6	0,9	0,4	798
Kalimantan Tengah	34,2	1,1	3,4	0,7	57,0	6,2	1,4	0,7	346
Kalimantan Selatan	32,5	2,2	2,9	0,2	54,7	7,3	4,2	1,4	821
Kalimantan Timur	43,0	2,7	4,8	0,2	49,5	2,1	3,5	1,2	902
Kalimantan Utara	67,0	0,1	0,3	0,0	35,3	0,2	0,7	2,5	177
Sulawesi Utara	59,4	1,4	0,6	0,5	40,7	1,6	0,9	1,2	501
Sulawesi Tengah	60,1	0,8	0,8	0,3	33,4	6,0	5,3	2,1	511
Sulawesi Selatan	66,1	0,8	1,1	0,4	28,0	3,8	3,3	2,2	1.891
Sulawesi Tenggara	58,3	0,3	0,6	0,1	36,7	7,2	1,2	1,7	416
Gorontalo	81,5	0,7	0,0	0,4	21,5	2,6	0,1	2,4	255
Sulawesi Barat	74,6	1,5	0,3	0,0	19,5	2,2	0,0	5,8	265
Maluku	61,5	0,5	1,5	0,2	37,2	4,7	1,4	2,4	159
Maluku Utara	49,1	0,2	0,6	0,9	44,3	5,8	2,0	3,7	173
Papua Barat	63,2	0,1	0,9	0,6	41,3	0,1	0,4	4,3	182
Papua	54,4	1,2	1,5	0,4	33,0	2,2	4,9	10,8	376
**INDONESIA**	**41,2**	**1,3**	**2,5**	**0,3**	**53,3**	**4,3**	**1,5**	**0,9**	**56.632**

Tabel 14.3.8
Proporsi Sumber Pembiayaan Persalinan di Fasilitas Pelayanan Kesehatan pada Perempuan Umur 10-54 Tahun menurut Karakteristik, Riskesdas 2018

Karakteristik	Sumber pembiayaan persalinan (%)								N ter-timbang
	BPJS/ KIS	Asuransi Swasta	Biaya kantor	Biaya orang lain	Biaya sendiri	Jamper-sal	Jam-perda	Tidak keluar biaya	
**Umur saat Bersalin**									
10-14	28,7	0,0	3,7	4,7	56,3	5,8	1,3	9,4	29*
15-19	34,8	0,4	0,9	0,1	59,2	5,2	2,2	1,3	4.334
20-24	39,4	0,4	2,0	0,5	56,2	4,5	1,5	0,8	13.749
25-29	40,5	1,9	3,5	0,2	53,2	3,8	1,4	0,9	16.668
30-34	42,2	1,7	2,8	0,3	52,4	4,2	1,5	0,8	12.947
35-39	46,2	1,5	2,2	0,3	48,1	4,6	1,6	0,9	6.812
40-44	50,4	0,7	1,8	0,3	45,2	4,5	1,8	1,3	1.868
45-49	49,6	0,5	0,7	1,7	47,1	3,5	0,7	0,4	208
50-54	50,6	0,0	0,0	0,0	49,4	0,0	0,0	0,0	15*
**Kelompok Umur (Khusus)**									
10 - 19 (Remaja)	34,7	0,7	0,9	0,2	59,2	5,2	2,2	1,3	4.364
15 - 49 (WUS)	41,2	1,3	2,5	0,3	53,3	4,3	1,5	0,9	56.587
**Pendidikan**									
Tidak pernah sekolah	41,5	0,4	0,2	0,2	51,9	3,7	1,7	3,7	593
Tidak tamat SD/MI	39,8	0,2	0,6	0,4	53,4	5,7	2,1	2,2	2.593
Tamat SD/MI	39,9	0,3	0,8	0,4	53,2	6,0	2,1	1,3	9.837
Tamat SLTP/MTS	38,0	0,5	1,3	0,3	57,3	4,9	1,7	1,0	14.411
Tamat SLTA/MA	42,3	1,4	2,8	0,4	53,1	4,0	1,4	0,6	21.050
Tamat D1/D2/D3/PT	46,3	4,3	6,9	0,2	46,9	1,7	0,6	0,5	8.147
**Pekerjaan**									
Tidak bekerja	40,8	1,1	2,4	0,3	53,9	4,3	1,6	0,8	33.426
Sekolah	36,8	0,9	0,5	0,0	59,8	3,0	0,9	2,0	402
PNS/TNI/Polri/BUMN/BUMD	58,9	3,6	4,9	0,1	38,2	1,8	0,5	0,3	1.590
Pegawai swasta	43,9	3,5	6,1	0,3	50,4	1,9	1,2	0,4	5.426
Wiraswasta	35,4	1,1	1,3	0,4	60,2	4,3	1,3	0,6	5.867
Petani/buruh tani	40,3	0,2	0,4	0,2	50,1	7,1	2,8	2,9	3.103
Nelayan	50,2	0,0	0,7	1,8	45,9	5,2	1,7	2,0	47*
Buruh/supir/pembantu ruta	39,6	1,4	2,5	0,3	51,8	7,2	1,9	0,8	2.248
Lainnya	44,0	1,1	1,9	0,5	50,5	4,5	1,3	1,2	4.523
**Tempat tinggal**									
Perkotaan	41,3	1,8	3,6	0,4	53,5	3,7	1,3	0,6	34.732
Perdesaan	41,2	0,6	0,9	0,3	53,0	5,3	1,9	1,4	21.900

*N Tertimbang <50

Tabel 14.3.9
Proporsi Metode Persalinan pada Perempuan Umur 10-54 Tahun menurut Provinsi, Riskesdas 2018

Provinsi	Metode persalinan (%)			N Tertimbang
	Normal	Operasi	Lainnya[1]	
Aceh	77,4	22,2	0,4	1.758
Sumatera Utara	75,9	23,9	0,2	4.379
Sumatera Barat	74,6	23,6	1,7	1.770
Riau	78,8	20,2	0,9	2.348
Jambi	85,3	14,3	0,3	1.087
Sumatera Selatan	90,2	9,4	0,4	2.529
Bengkulu	84,5	14,9	0,5	599
Lampung	86,0	13,2	0,9	2.644
Bangka Belitung	82,4	16,8	0,8	452
Kepulauan Riau	75,8	23,7	0,5	693
DKI Jakarta	67,8	31,1	1,1	3.058
Jawa Barat	83,4	15,5	1,1	15.043
Jawa Tengah	81,5	17,1	1,4	9.291
DI Yogyakarta	74,2	23,1	2,7	971
Jawa Timur	76,9	22,4	0,7	9.832
Banten	79,6	19,3	1,2	4.265
Bali	67,9	30,2	1,9	1.115
Nusa Tenggara Barat	87,4	11,6	1,0	1.752
Nusa Tenggara Timur	89,4	10,0	0,6	1.835
Kalimantan Barat	89,7	9,9	0,4	1.584
Kalimantan Tengah	91,8	7,6	0,6	848
Kalimantan Selatan	86,3	13,5	0,1	1.353
Kalimantan Timur	80,2	19,5	0,3	1.144
Kalimantan Utara	82,6	17,4	0,0	242
Sulawesi Utara	81,3	18,1	0,6	676
Sulawesi Tengah	84,2	15,3	0,5	949
Sulawesi Selatan	86,1	13,6	0,3	2.576
Sulawesi Tenggara	91,6	7,7	0,7	890
Gorontalo	80,1	18,9	0,9	364
Sulawesi Barat	90,4	9,6	0,1	427
Maluku	91,6	8,2	0,2	581
Maluku Utara	90,4	9,4	0,2	443
Papua Barat	88,1	11,7	0,3	321
Papua	91,2	6,7	2,0	916
**INDONESIA**	**81,5**	**17,6**	**0,9**	**78.736**

[1] Lainya misal: vakum, forsep atau lainnya

Tabel 14.3.10

Proporsi Metode Persalinan pada Perempuan Umur 10-54 Tahun menurut Karakteristik, Riskesdas 2018

Karakteristik	Metode Persalinan (%)			N tertimbang
	Normal	Operasi	Lainnya[1]	
**Umur saat Bersalin**				
10-14	88,6	11,4	na	59
15-19	89,6	9,4	1,0	6.725
20-24	84,7	14,1	1,2	22.708
25-29	81,0	18,2	0,7	17.657
30-34	79,4	19,8	0,8	9.366
35-39	75,8	23,3	1,0	2.670
40-44	74,7	24,7	0,5	301
45-49	78,2	20,8	1,0	38*
50-54	95,6	4,4	0,0	59
**Kelompok Umur (Khusus)**				
10 - 19 (Remaja)	89,6	9,4	1,0	19.211
15 - 49 (WUS)	81,4	17,7	0,9	78.638
**Pendidikan**				
Tidak pernah sekolah	89,0	10,0	1,1	1.285
Tidak tamat SD/MI	90,2	8,9	0,8	4.700
Tamat SD/MI	88,7	10,6	0,7	16.286
Tamat SLTP/MTS	85,8	13,5	0,6	19.989
Tamat SLTA/MA	77,6	21,3	1,1	26.787
Tamat D1/D2/D3/PT	65,7	33,2	1,2	9.688
**Pekerjaan**				
Tidak bekerja	82,3	16,8	0,9	46.404
Sekolah	82,9	16,4	0,7	550
PNS/TNI/Polri/BUMN/BUMD	65,6	33,6	0,7	1.964
Pegawai swasta	71,3	27,7	1,0	6.460
Wiraswasta	78,2	20,7	1,1	7.441
Petani/buruh tani	92,2	7,2	0,6	6.503
Nelayan	86,0	14,0		85
Buruh/supir/pembantu ruta	84,5	14,3	1,2	2.968
Lainnya	81,6	17,4	1,1	6.360
**Tempat tinggal**				
Perkotaan	76,9	22,1	1,0	42.461
Perdesaan	86,8	12,4	0,8	36.275
**INDONESIA**	**81,5**	**17,6**	**0,9**	**78.736**

[1] Lainya misal: vakum, forsep atau lainnya

*N Tertimbang <50

Riwayat gangguan/komplikasi persalinan

Definisi: Gangguan atau masalah kesehatan yang pernah dialami oleh ibu saat persalinan. Jenis komplikasi persalinan dapat berupa kejang, perdarahan pada jalan lahir, ketuban keluar sebelum waktunya, posisi janin sungsang, partus lama, plasenta letak rendah (*plasenta previa*), dan hipertensi.

$$Proporsi\ Jenis\ Gangguan\ atau\ Komplikasi\ Kehamilan = \frac{\begin{array}{c}\Sigma\ Perempuan\ Umur\ 10-54\ tahun\\ menurut\ jenis\ ganggguan\ atau\ komplikasi\ persalinan\end{array}}{\begin{array}{c}\Sigma\ Perempuan\ Umur\ 10-54\ tahun\ yang\ bersalin\\ anak\ terakhir\ pada\ periode\ 1\ Januari\ 2013\ sd\ wawancara\end{array}}$$

Upaya pertolongan pertama saat mengalami gangguan persalinan

Definisi: Upaya diberikan pertama kali kepada ibu saat mengalami gangguan atau masalah kesehatan dalam kehamilan. Upaya pertolongan pertama yang segera diberikan dalam jangka waktu < 30 menit setelah mengalami komplikasi persalinan.

$$Proporsi\ Upaya\ Pertolongan\ Pertama\ saat\ Mengalami\ Gangguan\ atau\ Komplikasi\ persalinan = \frac{\begin{array}{c}\Sigma\ Perempuan\ Umur\ 10-54\ tahun\\ menurut\ upaya\ pertolongan\ pertama\ saat\\ mengalami\ ganggguan\ atau\ komplikasi\ persalinan\ \ anak\ terakhir\end{array}}{\begin{array}{c}\Sigma\ Perempuan\ Umur\ 10-54\ tahun\ yang\ bersalin\\ anak\ terakhir\ ada\ gangguan\ persalinan\end{array}}$$

Tabel 14.3.11
Proporsi Gangguan/Komplikasi Persalinan pada Perempuan Umur 10-54 Tahun menurut Provinsi, Riskesdas 2018

Provinsi	Mengalami Gangguan/Komplikasi Persalinan (%)	Jenis gangguan/komplikasi Persalinan (%)										N Ter-timbang
		Posisi janin melintang/ sunsang	Perdarahan	Kejang	Ketuban pecah dini	Partus lama	Lilitan tali pusat	Placenta previa	Placenta tertinggal	Hipertensi	Lainnya	
Aceh	18,1	2,2	2,0	0,2	3,2	3,9	2,2	0,6	0,3	1,5	4,7	1.758
Sumatera Utara	18,2	2,7	1,6	0,2	4,3	3,7	3,4	0,9	0,7	1,6	2,9	4.379
Sumatera Barat	27,4	4,4	2,6	0,1	4,4	4,8	3,5	0,6	1,4	3,5	6,2	1.770
Riau	20,2	3,2	2,0	0,1	3,7	3,4	3,7	0,6	0,9	2,6	3,4	2.348
Jambi	15,1	2,4	1,9	0,4	2,8	2,3	1,6	0,8	1,3	1,3	2,9	1.087
Sumatera Selatan	13,1	1,1	2,0	0,2	2,6	2,9	2	0,5	0,5	2,1	1,9	2.529
Bengkulu	18,4	3,5	1,9	0,2	4,5	3,5	2,2	0,7	1,4	0,8	2,6	599
Lampung	19,2	2,3	2,6	0,2	4,2	3,7	2	0,6	0,9	1,7	3,8	2.644
Kep.Bangka Belitung	25,2	2,7	2,5	0,2	6,2	4,5	3,3	0,1	1,6	3,9	5,9	452
Kepulauan Riau	22,5	3,0	1,7	0,2	5,3	2,9	3,8	0,9	0,1	2,6	4,5	693
DKI Jakarta	28,7	3,8	3,0	0	7,0	4,9	4,7	1,9	0,6	3,7	6,1	3.058
Jawa Barat	24,6	3,6	2,8	0,2	6,3	4,1	3,3	0,8	1,0	3,1	4,6	15.043
Jawa Tengah	26,3	3,3	2,3	0,1	6,4	5,2	2,9	0,6	0,6	3,1	5,9	9.291
DI Yogyakarta	37,8	4,7	3,3	0,2	10,1	7,9	5,1	0,9	0,9	3,9	8,2	971
Jawa Timur	29,5	3,5	2,4	0,1	8,3	5	2,8	0,9	0,5	3,6	7,0	9.832
Banten	28,1	4,1	2,8	0,4	7	4,4	3,6	0,7	0,8	3,9	4,7	4.265
Bali	24,3	3,2	2,6	0,1	5,6	3,5	2,1	0,9	0,2	2,3	5,9	1.115
Nusa Tenggara Barat	20,5	2,4	2,4	0,4	5,4	4,5	0,9	0,5	0,3	2,2	4,3	1.752
Nusa Tenggara Timur	15,7	2,4	2,5	0,5	3,2	4,4	2,4	0,3	0,5	1,4	2,2	1.835
Kalimantan Barat	16,6	1,8	2,0	0,3	3,1	3	2,7	0,4	0,9	1,9	3,1	1.584
Kalimantan Tengah	16,4	2,0	2,1	0,1	2,7	3,3	2,1	0,8	1	2,5	2,1	848
Kalimantan Selatan	20,9	3,0	3,1	0,2	3,6	2,2	2,4	0,6	1,1	2,7	4,4	1.353
Kalimantan Timur	24,6	3,5	1,9	0,1	4,7	3,3	3,2	0,4	1,5	2,7	5,6	1.144
Kalimantan Utara	22,5	3,3	1,9	0,3	4,8	3,9	4,5	0,3	0,7	2,1	4,9	242
Sulawesi Utara	22,3	3,5	3,4	0,4	5,5	5,8	2,9	1	0,4	2,7	2,2	676
Sulawesi Tengah	21,3	2,3	4,3	0,5	4,3	4,7	3,5	0,7	1,3	2,1	3,9	949
Sulawesi Selatan	20,1	2,5	2,6	0,3	4,9	5,2	2,2	0,8	1,3	1,8	3,6	2.576
Sulawesi Tenggara	14,4	2,2	2,1	0,8	4,1	3,3	2	0,2	0,8	0,8	2,3	890
Gorontalo	18,7	3,8	2,8		3,8	3,7	2,1	0,6	0,7	2,3	3,2	364
Sulawesi Barat	15,1	1,0	1,8	0,2	2,9	4,1	0,9	0,1	0,5	1,8	3,6	427
Maluku	14,3	1,9	1,4	0,3	3,6	4,7	3,6	0	1,1	0,7	1,4	581
Maluku Utara	13,0	1,4	2,1	0,2	3,6	4,2	1,1	0,2	1,5	0,3	2,2	443
Papua Barat	14,2	2,4	2,3	0,2	3,3	3,5	3,4	0,2	0,4	1,1	1,3	321
Papua	9,6	0,8	1,4	0,1	2,7	2,7	1,7	0,2	0,6	0,8	1,0	916
**INDONESIA**	**23,2**	**3,1**	**2,4**	**0,2**	**5,6**	**4,3**	**2,9**	**0,7**	**0,8**	**2,7**	**4,6**	**78.736**

Tabel 14.3.12
Proporsi Gangguan/Komplikasi Persalinan pada Perempuan Umur 10-54 Tahun
menurut Karakteristik, Riskesdas 2018

Karakteristik	Mengalami Gangguan/Komplikasi Persalinan (%)	Jenis gangguan/komplikasi Persalinan (%)										N Tertimbang
		Sungsang	Perdarahan	Kejang-kejang	KPD	Partus lama	Lilitan tali pusat	Plasenta Previa	Plasenta tertinggal	Hipertensi	Lainnya	
**Umur saat Bersalin**												
<15	35,8	0,7	2,2	1,2	22,9	10,3	0,7	0,7	0,7	0,0	7,3	59
15-19	19,6	2,2	2,2	0,4	5,1	4,7	2,5	0,3	0,3	1,0	4,0	6.725
20-24	22,2	2,4	2,2	0,2	6,3	5,1	3,0	0,4	0,6	1,5	4,3	19.211
25-29	23,2	2,9	2,4	0,2	5,8	4,6	3,3	0,6	0,6	1,9	5,0	22.708
30-34	23,4	3,7	2,4	0,2	5,2	3,5	3,2	1,2	1,0	3,0	4,4	17.657
35-39	27,0	4,3	3,1	0,2	5,1	3,6	2,1	1,1	1,2	6,1	5,3	9.366
40-44	26,5	3,8	3,0	0,3	4,7	2,5	1,8	0,9	1,3	7,7	5,2	2.670
45-49	18,6	1,5	2,5	0,0	3,4	2,3	0,4	0,8	1,1	7,7	2,1	301
50-54	16,5	0,2	0,0	0,0	4,2	6,8	0,0	0,0	1,7	0,0	3,7	38*
**Kelompok Umur (Khusus)**												
10 - 19 (Remaja)	19,8	2,1	2,2	0,4	5,3	4,7	2,5	0,3	0,3	1,0	4,0	6.784
15 - 49 (WUS)	23,2	3,1	2,4	0,2	5,6	4,3	2,9	0,7	0,8	2,7	4,6	78.639
**Pendidikan**												
Tidak pernah sekolah	15,0	2,8	2,2	0,3	3,8	2,2	1,4	0,1	0,9	1,9	1,9	1.285
Tidak tamat SD/MI	18,7	2,3	2,8	0,2	4,6	3,6	1,9	0,3	0,9	2,6	3,0	4.700
Tamat SD/MI	20,2	2,9	2,9	0,3	4,6	3,2	2,4	0,6	1,0	2,9	3,5	16.286
Tamat SLTP/MTS	22,1	2,9	2,5	0,2	5,4	4,3	2,6	0,5	0,7	2,5	4,2	19.989
Tamat SLTA/MA	25,2	3,3	2,2	0,2	6,3	4,9	3,4	0,9	0,8	2,8	5,1	26.787
Tamat D1/D2/D3/PT	28,3	3,6	2,2	0,1	6,5	4,9	4,0	1,4	0,5	2,8	7,4	9.688
**Pekerjaan**												
Tidak bekerja	23,4	3,1	2,6	0,2	5,9	4,3	3,0	0,7	0,8	2,8	4,5	46.404
Sekolah	24,6	1,5	1,6	0,2	5,3	5,0	4,3	0,3	0,6	3,3	6,8	550
PNS/TNI/Polri/BUMN/BUMD	24,5	2,7	2,3	0,1	4,3	4,2	3,4	1,1	0,4	3,1	6,8	1.964
Pegawai swasta	29,0	3,2	2,3	0,1	7,5	5,4	3,9	0,9	0,7	2,9	6,6	6.460
Wiraswasta	24,5	4,0	2,2	0,3	5,5	4,3	2,8	0,8	0,7	2,5	5,4	7.441
Petani/buruh tani	13,9	1,5	2,1	0,2	3,0	2,8	1,8	0,3	0,7	1,5	2,5	6.503
Nelayan	17,4	1,6	0,0	0,0	5,9	5,6	2,7	0,1	0,7	0,8	0,9	85
Buruh/supir/pembantu ruta	24,4	3,9	2,0	0,1	5,9	4,1	3,0	0,6	0,8	3,0	5,1	2.968
Lainnya	23,2	3,4	2,5	0,3	4,9	4,8	3,0	1,0	0,9	2,7	4,1	6.360
**Tempat tinggal**												
Perkotaan	25,8	3,6	2,6	0,2	6,4	4,5	3,4	0,9	0,7	3,2	5,2	42.461
Perdesaan	20,2	2,5	2,3	0,2	4,7	4,0	2,4	0,5	0,9	2,1	4,0	36.275

*N Tertimbang <50

Tabel 14.3.13
Proporsi Upaya Pertolongan Pertama untuk Komplikasi Persalinan
pada Perempuan Umur 10-54 Tahun menurut Karakteristik, Riskesdas 2018

Karakteristik	Mengalami gangguan/komplikasi pada saat bersalin			Upaya mencari faskes untuk pertolongan pertama gangguan/komplikasi persalinan			
	%	95% CI	N ter-timbang	Segera dirujuk	Ada jeda waktu	Tidak dirujuk	N ter-timbang
**Umur saat Bersalin**							
<15	35,8	18,3-58,0	59	55,5	36,8	7,7	19*
15-19	19,6	18,1-21,2	6.725	52,3	8,0	39,7	1.172
20-24	22,2	21,2-23,1	19.211	48,1	9,7	42,1	3.782
25-29	23,2	22,3-24,1	22.708	45,2	7,9	46,8	4.672
30-34	23,4	22,4-24,3	17.657	47,2	6,9	45,8	3.661
35-39	27,0	25,8-28,3	9.366	54,6	7,1	38,3	2.247
40-44	26,5	24,4-28,7	2.670	54,7	6,8	38,5	628
45-49	18,6	13,1-25,8	301	64,4	13,8	21,7	50
50-54	16,5	5,8-39,0	38*	47,8	0,0	52,2	6*
**Kelompok Umur (Khusus)**							
10 - 19 (Remaja)	19,8	18,3-21,4	6.784	52,4	8,4	39,2	1.191
15 - 49 (WUS)	23,2	22,7-23,7	78.639	48,6	8,0	43,4	16.211
**Pendidikan**							
Tidak pernah sekolah	15,0	12,6-17,8	1.285	57,8	4,9	37,3	171
Tidak tamat SD/MI	18,7	17,2-20,4	4.700	48,3	10,5	41,2	782
Tamat SD/MI	20,2	19,2-21,2	16.286	51,5	7,9	40,6	2.919
Tamat SLTP/MTS	22,1	21,3-23,1	19.989	52,1	8,1	39,8	3.930
Tamat SLTA/MA	25,2	24,4-26,1	26.787	48,4	8,6	43,0	5.999
Tamat D1/D2/D3/PT	28,3	26,9-29,7	9.688	39,4	6,0	54,5	2.434
**Pekerjaan**							
Tidak bekerja	23,4	22,7-24,0	46.404	48,6	8,2	43,2	9.634
Sekolah	24,6	19,2-30,9	550	42,8	16,9	40,3	120
PNS/TNI/Polri/BUMN/BUMD	24,5	22,3-26,9	1.964	44,6	4,5	51,0	428
Pegawai swasta	29,0	27,2-30,9	6.460	46,9	6,6	46,5	1.663
Wiraswasta	24,5	23,1-26,0	7.441	50,8	7,3	41,9	1.620
Petani/buruh tani	13,9	13,0-14,9	6.503	48,0	10,9	41,2	805
Nelayan	17,4	8,7-31,6	85	47,3	0,0	52,7	13*
Buruh/supir/pembantu ruta	24,4	21,9-27,1	2.968	49,0	7,2	43,7	643
Lainnya	23,2	21,7-24,7	6.360	49,8	8,6	41,6	1.308
**Tempat tinggal**							
Perkotaan	25,8	25,0-26,6	42.461	46,5	7,1	46,4	9.732
Perdesaan	20,2	19,7-20,7	36.275	51,7	9,5	38,8	6.503
**INDONESIA**	**23,2**	**22,7-23,7**	**78.736**	**48,6**	**8,0**	**43,4**	**16.235**

*N Tertimbang <50

14.4 Masa Nifas

Pelayanan Masa Nifas (KF)

Definisi: Pelayanan kesehatan ibu yang diperoleh selama 42 hari setelah proses persalinan, minimal 3 kali meliputi:KF 1 (6 jam sampai 3 hari setelah melahirkan), KF 2 (4 sampai 28 hari setelah melahirkan), dan KF 3 (29 sampai 42 hari setelah melahirkan).

$$Proporsi\ Pelayanan\ KF1 = \frac{\Sigma\ Perempuan\ Umur\ 10-54\ tahun\ yang\ mendapat\ Pelayanan\ Masa\ Nifas\ pada\ periode\ 6\ jam\ sampai\ 3\ hari\ setelah\ melahirkan\ anak\ terakhir}{\Sigma\ Perempuan\ Umur\ 10-54\ tahun\ yang\ bersalin\ pada\ periode\ 1\ Januari\ 2013\ sd\ wawancara\ yang\ mendapatkan\ pelayanan\ masa\ nifas}$$

$$Proporsi\ Pelayanan\ KF2 = \frac{\Sigma\ Perempuan\ Umur\ 10-54\ tahun\ yang\ mendapat\ Pelayanan\ Masa\ Nifas\ pada\ periode\ 4\ sampai\ 28\ hari\ setelah\ melahirkan\ anak\ terakhir}{\Sigma\ Perempuan\ Umur\ 10-54\ tahun\ yang\ bersalin\ pada\ periode\ 1\ Januari\ 2013\ sd\ wawancara\ yang\ mendapatkan\ pelayanan\ masa\ nifas}$$

$$Proporsi\ Pelayanan\ KF3 = \frac{\Sigma\ Perempuan\ Umur\ 10-54\ tahun\ yang\ mendapat\ Pelayanan\ Masa\ Nifas\ pada\ periode\ 29\ sampai\ 42\ hari\ setelah\ melahirkan\ anak\ terakhir}{\Sigma\ Perempuan\ Umur\ 10-54\ tahun\ yang\ bersalin\ pada\ periode\ 1\ Januari\ 2013\ sd\ wawancara\ yang\ mendapatkan\ pelayanan\ masa\ nifas}$$

$$Proporsi\ Pelayanan\ KF\ Lengkap = \frac{\Sigma\ Perempuan\ pernah\ kawin\ 10-54\ tahun\ yang\ mendapat\ Pelayanan\ Masa\ Nifas\ pada\ periode\ 6\ jam\ sampai\ 42\ hari\ setelah\ melahirkan\ anak\ terakhir\ selama\ 3\ kali\ sesuai\ \ KF1-KF2\ dan\ KF3}{\Sigma\ Perempuan\ pernah\ kawin\ 10-54\ tahun\ yang\ bersalin\ pada\ periode\ 1\ Januari\ 2013\ sd\ wawancara\ yang\ mendapatkan\ pelayanan\ masa\ nifas}$$

Tabel 14.4.1
Proporsi Pelayanan Masa Nifas pada Perempuan 10-54 Tahun menurut Provinsi, Riskesdas 2018

Provinsi	Periode mendapatkan pelayanan masa nifas (KF) 6 jam-3 hr (KF1)[1]	4-28 hr (KF2)	29-42 hr (KF3)	KF lengkap[2] %	KF lengkap[2] 95% CI	tidak pernah diperiksa, setelah bersalin	N Tertimbang
Aceh	96,7	65,8	30,1	29,2	27,2 - 31,3	2,5	1,758
Sumatera Utara	93,1	58,7	18,6	17,5	15,9 - 19,2	6,1	4,379
Sumatera Barat	97,2	52,5	34,4	28,6	26,4 - 30,9	1,8	1,770
Riau	92,7	53,9	23,2	20,5	18,5 - 22,8	6,7	2,348
Jambi	91,6	56,4	36,1	33,4	30,3 - 36,7	7,5	1,087
Sumatera Selatan	93,0	51,9	40,4	33,9	31,1 - 36,8	5,2	2,529
Bengkulu	95,1	56,5	29,9	28,3	25,0 - 31,9	4,3	599
Lampung	95,5	64,2	42,7	37,2	34,7 - 39,7	3,0	2,644
Kep. Bangka Belitung	97,4	56,3	34,1	30,8	27,1 - 34,8	1,6	452
Kepulauan Riau	97,1	64,9	40,9	37,3	31,8 - 43,2	1,9	693
DKI Jakarta	98,9	85,2	65,8	62,4	58,9 - 65,7	0,4	3,058
Jawa Barat	92,0	74,6	56,9	50,5	48,7 - 52,2	4,8	15,043
Jawa Tengah	98,4	76,4	47,6	43,6	42,0 - 45,1	0,7	9,291
DI Yogyakarta	99,6	88,6	55,2	53,5	48,9 - 57,9	0,0	971
Jawa Timur	97,2	80,4	54,4	51,3	49,6 - 53,0	1,5	9,832
Banten	89,4	69,1	53,3	46,0	42,9 - 49,2	6,5	4,265
Bali	98,6	78,7	57,8	51,5	48,0 - 55,1	0,5	1,115
Nusa Tenggara Barat	96,1	64,1	50,7	43,2	40,1 - 46,3	2,5	1,752
Nusa Tenggara Timur	84,5	60,8	50,7	41,3	39,0 - 43,6	10,4	1,835
Kalimantan Barat	86,5	43,2	30,0	25,0	22,8 - 27,4	10,6	1,584
Kalimantan Tengah	84,8	49,3	28,1	22,1	19,4 - 25,1	11,7	848
Kalimantan Selatan	95,0	60,3	32,1	30,4	27,7 - 33,3	4,0	1,353
Kalimantan Timur	95,5	67,5	47,3	42,2	38,5 - 45,9	2,7	1,144
Kalimantan Utara	94,3	63,9	43,6	37,2	32,1 - 42,6	3,6	242
Sulawesi Utara	91,5	62,5	51,3	44,3	41,0 - 47,6	5,1	676
Sulawesi Tengah	88,4	53,7	35,1	29,1	26,3 - 32,0	9,2	949
Sulawesi Selatan	94,6	36,3	26,1	19,0	17,3 - 20,7	4,1	2,576
Sulawesi Tenggara	86,2	42,5	24,3	18,8	16,4 - 21,4	12,0	890
Gorontalo	90,9	49,2	36,4	30,2	26,0 - 34,8	6,7	364
Sulawesi Barat	90,4	53,6	37,2	32,9	28,9 - 37,0	7,7	427
Maluku	67,7	39,3	27,6	22,7	19,3 - 26,5	27,1	581
Maluku Utara	77,3	45,3	35,6	27,9	24,4 - 31,7	16,3	443
Papua Barat	80,3	30,7	23,2	16,8	13,6 - 20,5	15,6	321
Papua	56,3	36,7	33,4	22,3	19,5 - 25,3	33,2	916
**INDONESIA**	**93,3**	**66,9**	**45,2**	**40,3**	**39,8 - 40,9**	**4,6**	**78,736**

[1] KF1 termasuk ibu yang melahirkan di fasyankes
[2] KF lengkap = menerima KF 1 (6 jam - 3 hari), KF 2 (4 - 28 hari) dan KF 3 (29 - 42 hari)

Tabel 14.4.2
Proporsi Pelayanan Masa Nifas pada Perempuan 10-54 Tahun menurut Karakteristik, Riskesdas 2018

Karakteristik	Periode mendapatkan pelayanan masa nifas (KF) 6 jam-3 hr (KF1)[1]	4-28 hr (KF2)	29-42 hr (KF3)	KF lengkap[2] %	KF lengkap[2] 95% CI	tidak pernah diperiksa, setelah bersalin	N Tertimbang
**Umur saat Bersalin**							
10 – 14	82,6	50,6	29,9	26,3	13,7 - 44,5	15,3	59
15 – 19	90,4	62,3	43,0	36,7	34,8 - 38,6	6,4	6.725
20 – 24	93,8	66,5	44,7	39,9	38,7 - 41,0	4,3	22.708
25 – 29	94,2	68,2	45,8	41,2	40,2 - 42,2	4,0	17.657
30 – 34	93,8	67,7	46,1	41,3	40,2 - 42,4	4,3	9.366
35 – 39	92,7	68,1	46,2	41,6	40,2 - 42,9	5,0	2.670
40 – 44	89,7	63,2	41,5	35,8	33,5 - 38,2	7,5	301
45 – 49	89,2	60,4	42,8	36,5	29,3 - 44,3	8,1	38*
50 – 54	74,5	63,4	65,4	50,6	29,4 - 71,5	14,7	59
**Kelompok Umur (Khusus)**							
10 - 19 (Remaja)	90,3	62,2	42,9	36,6	34,8 - 38,5	6,5	19.211
15 - 49 (WUS)	93,4	66,9	45,2	40,3	39,8 - 40,9	4,6	78.638
**Pendidikan**							
Tidak pernah sekolah	72,6	45,7	33,5	28,2	24,9 - 31,7	23,7	1.285
Tidak tamat SD/MI	83,8	52,8	36,7	30,0	28,1 - 31,9	12,8	4.700
Tamat SD/MI	87,5	60,7	41,1	35,5	34,3 - 36,7	8,9	16.286
Tamat SLTP/MTS	94,6	67,9	45,4	40,3	39,2 - 41,4	3,5	19.989
Tamat SLTA/MA	96,8	71,2	47,9	43,4	42,5 - 44,4	1,9	26,787
Tamat D1/D2/D3/PT	98,0	73,5	50,2	46,6	45,1 - 48,1	0,9	9.688
**Pekerjaan**							
Tidak bekerja	93,5	66,9	44,6	39,6	38,8 - 40,3	4,3	46.404
Sekolah	91,6	65,1	41,5	38,9	32,6 - 45,6	6,4	550
PNS/TNI/Polri/BUMN/BUMD	98,1	71,3	51,4	48,5	45,8 - 51,3	1,2	1.964
Pegawai swasta	97,7	76,5	54,4	50,4	48,4 - 52,5	1,2	6.460
Wiraswasta	96,7	71,2	48,8	44,3	42,6 - 46,0	2,1	7.441
Petani/buruh tani	81,7	53,2	35,8	29,9	28,6 - 31,3	14,6	6.503
Nelayan	78,9	51,8	33,8	30,3	19,6 - 43,7	19,3	85
Buruh/supir/pembantu ruta	93,7	71,2	50,6	45,3	42,4 - 48,4	3,9	2.968
Lainnya	94,0	63,8	42,3	37,0	35,3 - 38,7	4,1	6.360
**Tempat Tinggal**							
Perkotaan	72,1	72,1	50,5	45,7	44,8 - 46,6	2,2	42.461
Perdesaan	60,9	60,9	39,1	34,0	33,4 - 34,7	7,5	36.275
**INDONESIA**	**93,3**	**66,9**	**45,2**	**40,3**	**39,8 - 40,9**	**4,6**	**78.736**

[1] KF1 termasuk ibu yang melahirkan di fasyankes
[2] KF lengkap = menerima KF 1 (6 jam - 3 hari), KF 2 (4 - 28 hari) dan KF 3 (29 - 42 hari)
*N Tertimbang <50

Pemberian Vitamin A pada Ibu Nifas

Definisi: Pemberian kapsul merah vitamin A dosis tinggi (200.000 IU) kepada ibu nifas sebanyak dua buah, yaitu 1 (satu) kapsul diminum segera setelah persalinan dan 1 (satu) kapsul diminum 24 jam sesudah pemberian kapsul pertama.

$$Proporsi\ Pemberian\ Vitamin\ A: = \frac{\begin{array}{c}\Sigma\ Perempuan\ Umur\ 10-54\ tahun\ yang\ mendapat\\ Vitamin\ A\ setelah\ bersalin\ menurut\ frekuensi\ pemberian\end{array}}{\begin{array}{c}\Sigma\ Perempuan\ Umur\ 10-54\ tahun\ yang\ melahirkan\\ pada\ Periode\ 1\ Januari\ 2013\ sampai\ saat\ wawancara\end{array}}$$

$$Proporsi\ Waktu\ Pemberian\ Vitamin\ A: = \frac{\begin{array}{c}\Sigma\ Perempuan\ Umur\ 10-54\ tahun\ yang\ mendapat\\ Vitamin\ A\ setelah\ bersalin\ menurut\ waktu\ pemberian\end{array}}{\begin{array}{c}\Sigma\ Perempuan\ Umur\ 10-54\ tahun\ yang\ melahirkan\\ pada\ Periode\ 1\ Januari\ 2013\ sampai\ saat\ wawancara\end{array}}$$

Tabel 14.4.3
Proporsi Frekuensi Mendapatkan dan Waktu Minum Vitamin A saat Masa Nifas
pada Perempuan Umur 10-54 Tahun menurut Provinsi, Riskesdas 2018

Provinsi	Mendapat vitamin A (%)				N Ter-timbang	Waktu mendapatkan vitamin A (%)			N Ter-timbang
	1 kali	2 kali	> 2 kali	Tidak mendapat vitamin A		Sesaat setelah melahiran	24jam setelah melahirkan	Lebih 48 jam setelah melahirkan	
Aceh	32,8	18,2	4,6	44,3	1.758	43,1	35,6	21,3	980
Sumatera Utara	24,7	12,1	4,0	59,2	4.379	35,5	45,2	19,3	1.789
Sumatera Barat	32,5	13,4	3,7	50,5	1.770	43,5	38,1	18,4	878
Riau	32,4	12,9	3,3	51,4	2.348	48,6	37,8	13,6	1.143
Jambi	38,3	23,4	2,7	35,5	1.087	50,8	36,4	12,9	702
Sumatera Selatan	41,3	12,9	2,6	43,1	2.529	48,2	38,9	12,9	1.442
Bengkulu	33,0	14,6	4,2	48,2	599	41,0	39,3	19,8	311
Lampung	40,8	20,4	2,4	36,3	2.644	50,8	39,9	9,3	1.687
Kep. Bangka Belitung	40,2	17,3	2,3	40,2	452	57,1	32,7	10,2	271
Kepulauan Riau	41,9	17,3	4,5	36,3	693	68,0	22,1	9,9	442
DKI Jakarta	32,7	22,9	3,7	40,6	3.058	55,8	31,2	13,0	1.820
Jawa Barat	32,6	21,1	3,3	43,0	15.043	51,1	34,9	13,9	8.595
Jawa Tengah	37,5	27,5	3,8	31,1	9.291	52,9	34,8	12,2	6.410
DI Yogyakarta	40,1	22,2	3,6	34,1	971	55,2	26,6	18,3	641
Jawa Timur	32,5	20,5	4,0	43,0	9.832	50,0	35,0	14,9	5.614
Banten	32,3	15,8	2,9	48,9	4.265	54,5	30,6	15,0	2.183
Bali	30,8	11,6	2,5	55,2	1.115	52,0	32,1	16,0	501
Nusa Tenggara Barat	38,4	21,2	2,5	37,9	1.752	56,5	35,5	8,0	1.089
Nusa Tenggara Timur	39,0	23,2	2,4	35,4	1.835	55,0	33,5	11,6	1.188
Kalimantan Barat	35,9	21,8	3,0	39,3	1.584	53,1	31,1	15,8	962
Kalimantan Tengah	29,4	19,4	5,6	45,6	848	48,5	38,8	12,7	462
Kalimantan Selatan	33,8	17,8	2,6	45,8	1.353	50,7	36,4	12,9	735
Kalimantan Timur	35,2	20,3	2,4	42,1	1.144	57,4	33,0	9,6	664
Kalimantan Utara	47,1	18,6	2,7	31,6	242	55,0	39,3	5,7	166
Sulawesi Utara	41,5	15,5	7,5	35,5	676	43,3	41,7	15,0	437
Sulawesi Tengah	33,2	14,3	4,2	48,3	949	53,7	32,9	13,4	491
Sulawesi Selatan	38,0	12,6	3,7	45,7	2.576	55,6	32,4	12,1	1.400
Sulawesi Tenggara	34,1	12,4	2,8	50,7	890	52,6	35,7	11,7	439
Gorontalo	45,2	16,8	2,4	35,6	364	59,1	29,6	11,3	235
Sulawesi Barat	37,4	19,1	3,8	39,7	427	62,6	27,9	9,6	258
Maluku	31,3	15,1	4,6	49,1	581	43,8	36,7	19,6	296
Maluku Utara	28,4	21,8	5,5	44,2	443	49,0	34,1	16,9	247
Papua Barat	34,4	21,5	3,6	40,6	321	50,0	30,2	19,8	191
Papua	28,2	14,6	2,9	54,2	916	40,9	34,9	24,2	420
**INDONESIA**	**34,2**	**19,4**	**3,5**	**42,8**	**78.736**	**51,1**	**35,0**	**13,9**	**45.090**

Tabel 14.4.4
Proporsi Frekuensi Mendapatkan dan Waktu Minum Vitamin A saat Masa Nifas pada Perempuan Umur 10-54 Tahun menurut Karakteristik, Riskesdas 2018

Karakteristik	Mendapat vitamin A (%)				N ter-timbang	Waktu mendapat vitamin A (%)			N ter-timbang
	1 kali	2 kali	> 2 kali	Tidak mendapat vitamin A		Sesaat setelah melahirkan	24 jam setelah melahirkan	Lebih 48 jam setelah melahirkan	
**Umur saat Bersalin**									
10 – 14	17,4	40,7	2,9	39,0	59	32,2	31,7	36,1	36*
15 – 19	34,1	20,3	3,4	42,2	6.725	49,8	35,1	15,1	3.892
20 – 24	35,1	20,0	3,4	41,6	22.708	52,0	34,3	13,8	11.247
25 – 29	34,3	19,3	3,6	42,8	17.657	51,9	34,4	13,7	13.014
30 – 34	34,2	18,6	3,6	43,5	9.366	50,5	36,0	13,6	9.987
35 – 39	33,9	19,5	3,2	43,5	2.670	49,9	35,6	14,4	5.302
40 – 44	30,7	19,2	3,9	46,2	301	49,4	37,9	12,7	1.439
45 – 49	26,0	21,6	2,9	49,5	38*	55,4	35,0	9,7	152
50 – 54	32,2	22,5	0,0	45,3	59	16,4	70,9	12,8	21*
**Kelompok Umur (Khusus)**									
10 - 19 (Remaja)	33,9	20,4	3,4	42,2	19.211	49,6	35,1	15,3	3.927
15 - 49 (WUS)	34,3	19,4	3,5	42,8	78.638	51,1	35,0	13,9	45.033
**Pendidikan**									
Tidak pernah sekolah	31,2	13,8	2,7	52,3	1.285	49,1	33,9	17,0	615
Tidak tamat SD/MI	32,8	16,4	3,6	47,2	4.700	54,2	31,4	14,4	2.486
Tamat SD/MI	33,5	18,8	3,6	44,1	16.286	52,9	33,0	14,2	9.123
Tamat SLTP/MTS	35,1	21,1	3,3	40,5	19.989	51,5	35,3	13,3	11.913
Tamat SLTA/MA	34,7	19,2	3,6	42,4	26.787	50,4	35,6	14,0	15.446
Tamat D1/D2/D3/PT	33,5	19,8	3,5	43,2	9.688	48,0	38,1	13,9	5.507
**Pekerjaan**									
Tidak bekerja	33,8	19,3	3,4	43,5	46.404	51,4	34,6	14,0	26.260
Sekolah	32,6	20,0	3,7	43,8	550	54,7	26,9	18,4	309
PNS/TNI/Polri/BUMN/BUMD	35,4	22,0	4,0	38,6	1.964	47,2	36,7	16,1	1.209
Pegawai swasta	34,0	20,4	3,4	42,1	6.460	49,0	37,8	13,2	3.744
Wiraswasta	35,6	20,7	4,3	39,4	7.441	50,4	36,5	13,1	4.514
Petani	33,0	17,4	3,3	46,3	6.503	48,7	36,2	15,2	3.495
Nelayan	39,8	16,4	5,9	37,9	85	53,7	25,4	20,9	53
Buruh	36,2	20,8	2,7	40,4	2.968	52,9	35,0	12,1	1.773
Lainnya	36,0	18,4	4,2	41,4	6.360	53,7	33,0	13,3	3.733
**Tempat Tinggal**									
Perkotaan	33,6	19,3	3,6	43,5	42.461	51,8	34,5	13,6	24.035
Perdesaan	35,0	19,6	3,4	42,1	36.275	50,2	35,6	14,2	21.055

*N Tertimbang <50

Riwayat Komplikasi Masa Nifas

Definisi: Gangguan atau masalah kesehatan yang pernah dialami oleh ibu saat masa nifas atau setelah bersalin. Jenis komplikasi masa nifas dapat berupa perdarahan pada jalan lahir, bengkak (di wajah, tangan dan kaki), kejang, demam lebih dari 2 hari, atau payudara bengkak dan merah disertai rasa sakit.

$$Proporsi\ komplikasi\ masa\ nifas: = \frac{\begin{array}{c}\Sigma\, Perempuan\ Umur\ 10-54\ tahun\ yang\ pernah\\ mengalami\ gangguan\ atau\ komplikasi\ pada\ masa\ nifas\end{array}}{\begin{array}{c}\Sigma\, Perempuan\ Umur\ 10-54\ tahun\ yang\ melahirkan\\ pada\ Periode\ 1\ Januari\ 2013\ sampai\ saat\ wawancara\end{array}}$$

Upaya Pertolongan Pertama Saat Mengalami Komplikasi Masa Nifas

Definisi: Upaya pertolongan yang diberikan pertama kali kepada ibu dalam jangka waktu <30 menit setelah mengalami komplikasi masa nifas.

$$Proporsi\ Upaya\ Pertolongan\ Pertama\ saat\ mengalami\ komplikasi\ masa\ nifas = \frac{\begin{array}{c}\Sigma\, Perempuan\ Umur\ 10-54\ tahun\ yang\ pernah\\ mengalami\ gangguan\ atau\ komplikasi\ pada\ masa\ nifas\\ setelah\ melahirkan\ anak\ terakhir\ dan\ menurut\ jenis\\ upaya\ pertolongan\end{array}}{\begin{array}{c}\Sigma\, Perempuan\ Umur\ 10-54\ tahun\ yang\ melahirkan\\ pada\ Periode\ 1\ Januari\ 2013\ sampai\ saat\ wawancara\end{array}}$$

Tabel 14.4.5
Proporsi Gangguan atau Komplikasi Masa Nifas pada Perempuan Umur 10 – 54 Tahun menurut Karakteristik, Riskesdas 2018

Karakteristik	Mengalami Gangguan/Komplikasi Masa Nifas (%)	Jenis gangguan/komplikasi pada masa nifas (%)										N Ter-timbang
		perdarahan byk pd jalan lahir	Keluar cairan berbau dr jalan lahir	bengkak kaki, tangan dan wajah	Sakit kepala	kejang kejang	Demam >2 hari	Payudara bengkak	*Baby blues*	Hipertensi	Lainnya	
**Umur saat Bersalin**												
10-14	6,9	3,4	3,4	1,9	3,3	0	2,2	1,0	2,2	1,9	0,1	59
15-19	13,1	1,8	1,0	1,5	3,5	0,3	1,6	7,2	0,8	0,5	1,0	6.725
20-24	12,1	1,5	0,7	1,4	3,4	0,2	1,6	6,3	1,0	0,4	1,3	19.211
25-29	10,8	1,5	0,6	1,2	3,3	0,2	1,5	4,9	0,9	0,8	1	22.708
30-34	10,6	1,4	0,6	1,0	3,0	0,2	1,2	4,2	0,7	1,3	1,2	17.657
35-39	11,3	1,7	0,6	1,0	3,4	0,2	1,2	3,6	0,9	2,2	1,4	9.366
40-44	12,0	1,5	0,4	1,1	3,7	0,2	1,7	2,4	1,2	3,3	1,7	2.670
45-49	7,6	1,2	0,9	0,4	2,7	0	0,4	1,1	0,7	1,9	0,5	301
50-54	9,1	0	0	0	2,3	0	6,7	9	0	0	0	38*
**Kelompok Umur (Khusus)**												
**10-19 (Remaja)**	13,1	1,8	1,1	1,5	3,5	0,3	1,6	7,1	0,9	0,5	1,0	6.784
**15-49 (WUS)**	11,4	1,5	0,6	1,2	3,3	0,2	1,4	5	0,9	1,0	1,2	78.639
**Pendidikan**												
Tidak pernah sekolah	8,8	1,6	1,0	0,9	3,8	0,3	1,1	3,2	0,7	0,7	1,2	1.285
Tidak tamat SD/MI	11,7	1,8	1,0	1,3	4,4	0,2	1,7	4,3	0,9	1,2	1,0	4.700
Tamat SD/MI	11,3	1,4	0,9	1,3	3,7	0,3	1,5	4,6	0,8	1,2	1,1	16.286
Tamat SLTP/MTS	11,3	1,7	0,6	1,2	3,4	0,1	1,4	4,8	0,9	1,1	1,0	19.989
Tamat SLTA/MA	11,7	1,5	0,6	1,2	3,1	0,2	1,5	5,6	0,9	0,9	1,4	26.787
Tamat D1/D2/D3/PT	10,9	1,2	0,3	1,2	2,5	0,1	1,3	5,1	0,9	0,9	1,5	9.688
**Pekerjaan**												
Tidak bekerja	11,8	1,6	0,7	1,3	3,4	0,2	1,5	5,3	1,0	1,2	1,1	46.404
Sekolah	9,5	2,2	0,1	0,5	1,4	0,1	0,9	5,9	0,2	0,8	0,3	550
PNS/TNI/Polri/BUMN/BUMD	8,5	1,1	0,1	0,6	1,5	0	0,8	3	0,5	1,0	1,8	1.964
Pegawai swasta	11,7	1,2	0,4	1,3	2,4	0,1	1,4	5,6	1,3	0,8	1,6	6.460
Wiraswasta	11,3	1,3	0,5	1,4	3,2	0,2	1,1	5,5	0,7	0,9	1,5	7.441
Petani/buruh tani	8,8	1,3	0,9	0,8	3,5	0,3	1,2	3,1	0,5	0,7	0,8	6.503
Nelayan	8,2	0,5	1,6	1,9	0,9	0,7	0,4	3,9	0	0,6	0	85
Buruh/supir/pembantu ruta	12,4	2,2	0,8	1,2	4,1	0,2	2,3	4,6	0,6	1,1	1,9	2.968
Lainnya	10,8	1,4	0,7	1,0	3,2	0,1	1,5	4,9	0,9	0,8	1,2	6.360
**Tempat Tinggal**												
Perkotaan	12,3	1,6	0,5	1,2	3,3	0,1	1,4	5,6	1,1	1,2	1,3	42.461
Perdesaan	10,3	1,4	0,8	1,2	3,3	0,2	1,5	4,3	0,7	0,8	1,0	36.275
**INDONESIA**	**11,4**	**1,5**	**0,6**	**1,2**	**3,3**	**0,2**	**1,5**	**5,0**	**0,9**	**1,0**	**1,2**	**78.736**

*N Tertimbang <50

Tabel 14.4.6
Proporsi Upaya Pencarian Pertolongan Pertama Gangguan/Komplikasi Masa Nifas pada Perempuan umur 10-54 Tahun menurut Karakteristik, Riskesdas 2018

Karakteristik	Mengalami gangguan/komplikasi pada masa nifas			Upaya mencari faskessaat mengalami gangguan/komplikasi masa nifas			
	%	95% CI	N ter-timbang	Segera (%)	Ada jeda waktu (%)	Tidak Mencari (%)	N ter-timbang
**Umur saat Bersalin**							
10-14	6,9	3,0-15,3	59	9,6	0,0	90,4	4*
15-19	13,1	11,8-14,6	6.725	36,6	7,7	55,7	813
20-24	12,1	11,3-12,9	19.211	37,6	10,4	52,0	2.134
25-29	10,8	10,2-11,5	22.708	38,5	10,7	50,8	2.262
30-34	10,6	9,9-11,3	17.657	41,9	10,8	47,3	1.715
35-39	11,3	10,5-12,3	9.366	43,0	12,0	45,0	977
40-44	12,0	10,5-13,8	2.670	38,2	14,3	47,5	296
45-49	7,6	4,8-11,7	301	33,7	10,5	55,8	21*
50-54	9,1	2,0-33,3	38*	0,0	74,3	25,7	3*
**Kelompok Umur (Khusus)**							
10 - 19 (Remaja)	13,1	11,7-14,6	6.784	36,5	7,7	55,8	817
15 - 49 (WUS)	11,4	11,0-11,7	78.639	39,3	10,6	50,1	8.218
**Pendidikan**							
Tidak pernah sekolah	8,8	7,0-11,1	1.285	33,0	8,4	58,6	104
Tidak tamat SD/MI	11,7	10,4-13,1	4.700	34,0	14,9	51,1	506
Tamat SD/MI	11,3	10,5-12,1	16.286	38,9	12,6	48,6	1.691
Tamat SLTP/MTS	11,3	10,6-12,0	19.989	38,7	9,8	51,5	2.078
Tamat SLTA/MA	11,7	11,0-12,3	26.787	40,7	10,1	49,2	2.876
Tamat D1/D2/D3/PT	10,9	10,0-11,9	9.688	40,4	8,8	50,8	971
**Pekerjaan**							
Tidak bekerja	11,8	11,3-12,4	46.404	39,1	10,9	50,0	5.057
Sekolah	9,5	6,3-14,0	550	30,9	2,3	66,8	48*
PNS/TNI/Polri/BUMN/BUMD	8,5	7,2-10,1	1.964	47,2	13,5	39,3	154
Pegawai swasta	11,7	10,4-13,1	6.460	41,8	6,8	51,4	695
Wiraswasta	11,3	10,2-12,4	7.441	42,5	9,7	47,8	771
Petani/buruh tani	8,8	8,0-9,6	6.503	30,9	12,3	56,8	524
Nelayan	8,2	3,9-16,6	85	15,6	20,7	63,6	6*
Buruh/supir/pembantu ruta	12,4	10,5-14,6	2.968	35,7	14,3	50,0	338
Lainnya	10,8	9,8-11,9	6.360	42,2	10,0	47,8	632
**Tempat Tinggal**							
Perkotaan	12,3	11,7-12,9	42.461	39,2	9,7	51,1	4.791
Perdesaan	10,3	9,9-10,7	36.275	39,4	11,9	48,7	3.434
**INDONESIA**	**11,4**	**11,0-11,7**	**78.736**	**39,3**	**10,6**	**50,1**	**8.225**

* N tertimbang< 50

14.5 Penggunaan KB Setelah Melahirkan

Definisi: Penggunaan alat Keluarga Berencana (KB) modern yang pertama diterima oleh ibu setelah melahirkan anak terakhir.

$$Proporsi\ Jenis\ alat\ atau\ cara\ KB\ yang\ digunakan\ Setelah\ Melahirkan = \frac{\begin{array}{c}\Sigma\ Perempuan\ umur\ 10-54\ tahun\\ menurut\ jenis\ KB\ modern\ yang\ digunakan\\ pertama\ kali\ setelah\ melahirkan\ anak\ yang\ terakhir\end{array}}{\begin{array}{c}\Sigma\ Perempuan\ umur\ 10-54\ tahun\ yang\ melahirkan\\ pada\ Periode\ 1\ Januari\ 2013\ sampai\ saat\ wawancara\end{array}}$$

$$Proporsi\ Waktu\ Terima\ Layanan\ KB\ Pertama\ Kali\ Setelah\ Melahirkan = \frac{\begin{array}{c}\Sigma\ Perempuan\ umur\ 10-54\ tahun\\ menurut\ periode\ waktu\ saat\ mendapat\ pelayanan\ KB\\ modern\ yang\ pertama\ kali\ setelah\ melahirkan\ anak\ yang\ terakhir\end{array}}{\begin{array}{c}\Sigma\ Perempuan\ umur\ 10-54\ tahun\ yang\ melahirkan\\ pada\ Periode\ 1\ Januari\ 2013\ sampai\ saat\ wawancara\end{array}}$$

Tabel 14.5.1

ProporsiJenis Alat/Cara KB Modern yang Digunakan Setelah Melahirkan Anak Terakhir Pada Perempuan Umur 10-54 Tahun menurut Provinsi, Riskesdas 2018

Provinsi	Sterilisasi wanita	Sterilisasi pria	IUD/ AKDS/spiral	Suntikan 3 bln	Suntikan 1 bln	Implant /Susuk KB	Pil	Kondom pria	N ter-timbang
	%	%	%	%	%	%	%	%	
Aceh	3,1	0,3	4,4	32,0	14,0	2,1	11,2	1,8	1.755
Sumatera Utara	4,9	0,2	2,1	17,3	10,1	6,5	9,4	1,2	4.369
Sumatera Barat	4,5	0,1	5,6	30,9	8,3	7,1	6,5	1,6	1.768
Riau	3,2	0,3	3,9	34,7	10,7	3,2	10,8	2,2	2.345
Jambi	2,4	0,2	3,9	47,1	10,6	4,5	12,0	0,5	1.084
Sumatera Selatan	1,8	0,5	2,9	51,0	5,1	6,2	8,1	1,0	2.525
Bengkulu	2,2	0,6	4,6	47,6	7,2	7,5	7,6	1,7	598
Lampung	2,6	0,2	3,6	54,4	3,1	7,1	7,1	0,6	2.636
Bangka Belitung	3,3	0,2	3,2	38,2	13,7	3,9	14,8	0,8	452
Kepulauan Riau	4,9	0,6	4,6	21,3	8,9	1,5	11,0	2,2	693
DKI Jakarta	4,6	0,1	13,9	34,8	5,3	2,4	5,9	1,1	3.055
Jawa Barat	2,5	0,1	8,4	52,9	6,4	1,8	8,5	1,0	15.024
Jawa Tengah	3,9	0,2	8,5	45,0	3,8	7,3	4,3	1,4	9.263
DI Yogyakarta	3,3	0,1	17,6	24,9	2,4	4,7	3,8	6,0	969
Jawa Timur	3,4	0,2	8,6	43,8	6,2	3,7	8,4	1,3	9.820
Banten	2,5	0,2	4,9	53,8	4,9	3,5	8,3	1,2	4.254
Bali	5,5	0,1	17,1	31,4	6,6	2,1	6,8	1,0	1.112
Nusa Tenggara Barat	1,3	0,1	7,3	53,0	2,1	8,6	3,7	0,3	1.747
Nusa Tenggara Timur	4,2	0,1	3,2	29,4	0,9	12,3	3,6	0,1	1.831
Kalimantan Barat	2,6	0,2	4,6	44,2	6,4	2,2	17,0	0,8	1.582
Kalimantan Tengah	2,1	0,4	2,3	49,1	9,1	3,4	15,7	0,8	846
Kalimantan Selatan	2,1	0,1	2,1	44,7	9,6	2,5	21,7	0,7	1.352
Kalimantan Timur	3,0	0,0	7,3	35,2	7,3	2,1	16,3	2,2	1.138
Kalimantan Utara	2,2	0,0	6,8	36,4	4,2	3,5	11,5	1,4	241
Sulawesi Utara	5,1	0,1	5,7	36,8	8,4	9,9	10,3	0,7	675
Sulawesi Tengah	2,8	0,1	5,3	36,6	5,3	6,7	18,7	0,2	947
Sulawesi Selatan	1,9	0,2	4,5	38,3	4,8	6,7	10,2	0,6	2.572
Sulawesi Tenggara	1,2	0,4	2,3	28,7	4,8	6,0	14,1	0,2	889
Gorontalo	2,2	0,3	5,2	37,6	4,6	18,8	9,5	0,0	363
Sulawesi Barat	2,4	0,0	2,6	32,3	2,8	6,0	14,3	0,8	425
Maluku	0,9	0,1	2,0	37,3	3,1	7,3	5,2	0,1	573
Maluku Utara	1,2	0,4	1,8	46,0	2,7	13,2	4,4	0,0	442
Papua Barat	2,0	0,1	2,0	32,1	5,7	5,9	6,6	0,2	319
Papua	1,5	0,1	1,0	18,5	4,3	3,5	2,5	0,0	904
**INDONESIA**	**3,1**	**0,2**	**6,6**	**42,4**	**6,1**	**4,7**	**8,5**	**1,1**	**78.568**

Tabel 14.5.2
ProporsiJenis Alat/Cara KB Modern yang Digunakan Setelah Melahirkan Anak Terakhir Pada Perempuan Umur 10-54 Tahun menurut Karakteristik, Riskesdas 2018

Karakteristik	Sterilisasi wanita	Sterili-sasi pria	IUD/AKDS/ spiral	Suntikan 3 bln	Suntikan 1 bln	Implant / Susuk KB	Pil	Kondom pria	N ter-timbang
	%	%	%	%	%	%	%	%	
**Umur saat Bersalin**									
10 – 14	0,0	0,0	3,1	40,3	6,0	9,0	6,4	0,0	59
15 – 19	1,0	0,2	4,3	56,4	6,1	5,2	6,8	0,2	6.725
20 – 24	0,6	0,1	5,8	48,8	7,7	4,5	7,5	0,7	22.708
25 – 29	1,2	0,2	7,0	41,3	6,5	4,7	8,6	1,5	17.657
30 – 34	3,7	0,2	7,9	38,7	5,4	4,7	9,9	1,5	9.366
35 – 39	9,6	0,3	7,0	33,8	4,4	4,6	8,7	1,3	2.670
40 – 44	14,0	0,1	6,8	28,3	3,0	5,0	9,7	0,9	301
45 – 49	18,3	0,6	5,4	26,8	2,1	5,0	5,2	1,5	38*
50 – 54	0,0	0,0	1,1	41,0	2,5	0,0	12,4	0,0	59
**Kelompok Umur (Khusus)**									
10 - 19 (Remaja)	1,0	0,2	4,3	56,3	6,1	5,2	6,8	0,2	19.211
15 - 49 (WUS)	3,1	0,2	6,6	42,4	6,1	4,7	8,5	1,1	78.638
**Pendidikan**									
Tidak pernah sekolah	2,3	0,0	3,6	38,9	4,1	0,0	7,5	0,2	1.285
Tidak tamat SD/MI	2,6	0,2	4,1	48,5	4,0	0,1	9,4	0,2	4.700
Tamat SD/MI	3,1	0,2	4,4	51,6	4,3	0,1	10,4	0,4	16.286
Tamat SLTP/MTS	2,7	0,1	5,6	50,0	6,1	0,1	8,6	0,6	19.989
Tamat SLTA/MA	3,3	0,2	7,0	38,1	7,5	0,0	8,2	1,5	26.787
Tamat D1/D2/D3/PT	3,5	0,2	13,1	21,0	6,7	0,0	6,0	3,0	4.185
**Pekerjaan**									
Tidak bekerja	3,0	0,2	5,9	45,1	6,0	0,0	9,0	1,0	46.404
Sekolah	1,9	0,0	4,5	38,6	10,0	0,0	6,4	1,0	550
PNS/TNI/Polri/BUMN/BUMD	7,4	0,1	12,9	19,8	7,3	0,0	5,8	2,9	1.964
Pegawai swasta	3,0	0,1	11,7	30,9	7,1	0,0	6,2	1,9	6.460
Wiraswasta	4,0	0,2	8,1	39,4	7,0	0,0	8,2	1,4	7.441
Petani/buruh tani	2,7	0,2	3,3	44,6	3,4	0,1	7,7	0,4	6.503
Nelayan	2,0	0,0	1,9	31,3	4,1	0,0	8,7	1,7	85
Buruh/supir/pembantu ruta	2,3	0,2	7,2	47,8	6,3	0,0	8,6	0,7	2.968
Lainnya	2,4	0,2	6,5	40,8	6,8	0,1	9,2	1,4	6.360
**Tempat Tinggal**									
Perkotaan	3,6	0,2	8,4	38,5	6,6	0,0	8,1	1,6	42.461
Perdesaan	2,4	0,2	4,6	47,1	5,5	0,0	9,0	0,6	36.275

*N tertimbang <50

Tabel 14.5.3
Proporsi Waktu Terima Layanan KB Pertama Kali Setelah Bersalin berdasarkan Tempat Bersalin pada Perempuan Umur 10-54 Tahun menurut Provinsi, Riskesdas 2018

Provinsi	Bersalin di Fasyankes (%)				Bersalin di non-fasyankes (%)	
	Bersamaan dengan proses persalinan	Setelah persalinan selesai di fasyankes	≤ 42 hari/periode masa nifas sepulang dari fasyankes	> 42 hari setelah bersalin	≤ 42 hari setelah persalinan	>42 hari setelah persalinan
Aceh	6,2	4,8	9,1	79,9	10,7	89,3
Sumatera Utara	10,0	7,2	13,9	68,9	18,5	81,5
Sumatera Barat	9,5	5,8	12,0	72,7	14,0	86,0
Riau	7,0	4,5	10,6	78,0	11,3	88,7
Jambi	5,6	2,9	17,4	74,0	16,6	83,4
Sumatera Selatan	1,7	5,6	19,2	73,5	16,1	83,9
Bengkulu	3,3	3,5	16,8	76,4	20,9	79,1
Lampung	4,5	5,0	20,2	70,3	24,2	75,8
Bangka Belitung	4,2	3,9	12,7	79,2	16,6	83,4
Kepulauan Riau	8,2	6,2	18,4	67,2	28,3	71,7
DKI Jakarta	15,2	6,2	26,6	52,0	27,9	72,1
Jawa Barat	5,7	4,7	25,5	64,1	21,9	78,1
Jawa Tengah	7,9	6,1	18,2	67,8	23,3	76,7
DI Yogyakarta	19,0	4,2	14,5	62,3	43,4	56,6
Jawa Timur	8,5	4,0	15,8	71,7	19,1	80,9
Banten	5,7	3,8	19,4	71,1	19,8	80,2
Bali	13,8	5,2	26,2	54,9	30,2	69,8
Nusa Tenggara Barat	4,5	6,4	12,3	76,8	14,8	85,2
Nusa Tenggara Timur	6,9	6,2	19,3	67,6	16,2	83,8
Kalimantan Barat	7,6	6,5	19,8	66,1	20,4	79,6
Kalimantan Tengah	4,3	7,2	21,3	67,3	21,9	78,1
Kalimantan Selatan	3,3	4,3	23,8	68,6	27,3	72,7
Kalimantan Timur	7,4	3,4	22,6	66,6	16,3	83,7
Kalimantan Utara	6,4	11,4	23,6	58,5	18,0	82,0
Sulawesi Utara	11,7	9,3	22,3	56,7	26,5	73,5
Sulawesi Tengah	5,9	4,3	28,0	61,8	17,7	82,3
Sulawesi Selatan	3,8	8,9	27,3	59,9	20,1	79,9
Sulawesi Tenggara	3,0	2,6	11,0	83,5	9,1	90,9
Gorontalo	9,7	4,5	17,9	67,9	19,7	80,3
Sulawesi Barat	6,1	7,3	11,2	75,4	15,9	84,1
Maluku	4,1	6,3	17,0	72,6	20,7	79,3
Maluku Utara	5,5	5,3	18,0	71,2	12,8	87,2
Papua Barat	6,0	6,4	30,7	56,9	17,5	82,5
Papua	6,0	8,4	31,4	54,2	22,3	77,7
**INDONESIA**	**7,3**	**5,2**	**20,0**	**67,5**	**19,0**	**81,0**

Tabel 14.5.4
Proporsi Waktu Terima Layanan KB Pertama Kali Setelah Melahirkan
pada Perempuan Umur 10-54 Tahun menurut Karakteristik, Riskesdas 2018

Karakteristik	Bersalin di Fasyankes (%)				Bersalin di non-fasyankes (%)	
	Bersama-an dengan proses persalinan	Setelah persalinan selesai di fasyankes	≤ 42 hari/periode masa nifas sepulang dari fasyankes	> 42 hari setelah bersalin	≤ 42 hari setelah persalinan	>42 hari setelah persalinan
**Umur saat Bersalin**						
10 – 14	na	1,0	23,0	76,0	6,6	93,4
15 - 19	2,8	4,7	23,8	68,7	20,8	79,2
20 - 24	3,5	4,4	21,0	71,1	20,0	80,0
25 - 29	5,1	4,6	20,4	69,9	18,7	81,3
30 - 34	8,6	5,5	19,1	66,8	19,1	80,9
35 - 39	16,5	7,1	17,2	59,2	17,4	82,6
40-44	24,1	9,4	15,5	51,1	13,6	86,4
45 – 49	24,9	9,0	9,5	56,5	12,9	87,1
50 – 54	0,0	9,8	13,9	76,3	2,9	97,1
**Kelompok Umur (Khusus)**						
10 - 19 (Remaja)	2,8	4,7	23,8	68,8	20,7	79,3
15 - 49 (WUS)	7,3	5,2	20,0	67,5	19,1	80,9
**Pendidikan**						
Tidak pernah sekolah	8,4	6,2	20,4	65,0	21,2	78,8
Tidak tamat SD/MI	6,3	5,0	23,1	65,6	17,4	82,6
Tamat SD/MI	6,5	4,7	20,7	68,1	19,2	80,8
Tamat SLTP/MTS	5,5	5,1	20,8	68,6	20,5	79,5
Tamat SLTA/MA	7,9	5,4	19,3	67,4	18,0	82,0
Tamat D1/D2/D3/PT	11,4	6,1	17,3	65,1	16,8	83,2
**Pekerjaan**						
Tidak bekerja	6,8	5,1	20,4	67,7	19,0	81,0
Sekolah	4,1	5,3	28,1	62,5	25,5	74,5
PNS/TNI/Polri/BUMN/BUMD	15,6	7,2	17,1	60,2	11,0	89,0
Pegawai swasta	9,8	5,7	18,1	66,4	19,5	80,5
Wiraswasta	8,8	5,2	20,5	65,5	19,8	80,2
Petani/buruh tani	5,6	5,1	19,3	70,0	18,8	81,2
Nelayan	6,6	6,4	27,4	59,6	24,3	75,7
Buruh/supir/pembantu ruta	5,9	5,3	20,4	68,4	20,3	79,7
Lainnya	6,1	5,2	18,3	70,3	19,2	80,8
**Tempat Tinggal**						
Perkotaan	8,4	5,4	21,1	65,0	21,7	78,3
Perdesaan	5,7	5,0	18,3	71,1	18,1	81,9

Tabel 14.5.5
Proporsi Waktu Terima Layanan KB Pertama Kali Setelah Bersalin
pada Perempuan Umur 10-54 Tahun menurut Provinsi, Riskesdas 2018

Provinsi	≤ 42 hari setelah persalinan	>42 hari setelah persalinan	N Tertimbang
	%	%	
Aceh	17,7	82,3	1,158
Sumatera Utara	27,5	72,5	2,165
Sumatera Barat	25,1	74,9	1,092
Riau	18,2	81,8	1,548
Jambi	21,8	78,2	844
Sumatera Selatan	22,6	77,4	1,855
Bengkulu	22,7	453	453
Lampung	28,7	71,3	1,987
Bangka Belitung	19,6	80,4	338
Kepulauan Riau	32,2	67,8	364
DKI Jakarta	47,6	52,4	1,993
Jawa Barat	33,3	66,7	11,749
Jawa Tengah	31,7	68,3	6,609
DI Yogyakarta	38,0	62,0	583
Jawa Timur	27,2	72,8	7,110
Banten	26,5	73,5	3,237
Bali	44,8	55,2	755
Nusa Tenggara Barat	19,6	80,4	1,277
Nusa Tenggara Timur	28,2	71,8	943
Kalimantan Barat	27,5	72,5	1,181
Kalimantan Tengah	26,5	73,5	672
Kalimantan Selatan	30,0	70,0	1,079
Kalimantan Timur	30,8	69,2	805
Kalimantan Utara	36,4	63,6	152
Sulawesi Utara	39,9	60,1	497
Sulawesi Tengah	29,5	70,5	689
Sulawesi Selatan	36,0	64,0	1,653
Sulawesi Tenggara	12,9	87,1	491
Gorontalo	29,0	71,0	272
Sulawesi Barat	21,9	78,1	250
Maluku	22,5	77,5	309
Maluku Utara	19,6	80,4	295
Papua Barat	34,3	65,7	167
Papua	40,3	59,7	273
**INDONESIA**	**29,6**	**70,4**	**54,846**

14.6 Kepemilikan Buku KIA pada Ibu Hamil

Kepemilikan Buku KIA

Definisi: Status kepemilikan buku Kesehatan Ibu dan Anak (KIA) pada ibu yang sedang hamil. Status kepemilikan berdasarkan pengakuan dan observasi fisik buku KIA.

$$Proporsi\ Kepemilikan\ Buku\ KIA = \frac{\Sigma\ Perempuan\ Umur\ 10-54\ tahun\ yang\ sedang\ hamil\ saat\ wawancara\ menurut\ kepemilikan\ buku\ KIA}{\Sigma\ Perempuan\ pernah\ kawin\ 10-54\ tahun\ yang\ melahirkan\ pada\ Periode\ 1\ Januari\ 2013\ sampai\ saat\ wawancara}$$

$$Proporsi\ Pernah\ Memiliki\ Buku\ KIA = \frac{\Sigma\ Perempuan\ Umur\ 10-54\ tahun\ yang\ sedang\ hamil\ saat\ wawancara\ menurut\ pengakuan\ memiliki\ buku\ KIA\ tetapi\ tidak\ dapat\ menunjukkan}{\Sigma\ Perempuan\ pernah\ kawin\ 10-54\ tahun\ yang\ melahirkan\ pada\ Periode\ 1\ Januari\ 2013\ sampai\ saat\ wawancara}$$

Tabel 14.6.1

Proporsi Kepemilikan Buku KIA pada Ibu Hamil menurut Provinsi, Riskesdas 2018

Provinsi	Kepemilikan Buku KIA (%)			N Tertimbang
	Bisa menunjukkan	Tidak bisa menunjukkan	Tidak memiliki	
Aceh	49,5	15,5	35,0	204
Sumatera Utara	37,7	14,2	48,1	446
Sumatera Barat	73,8	9,5	16,6	193
Riau	54,4	10,0	35,6	271
Jambi	70,9	8,9	20,2	96
Sumatera Selatan	46,5	10,8	42,7	283
Bengkulu	54,2	17,5	28,3	66
Lampung	77,6	6,7	15,7	290
Bangka Belitung	73,1	6,1	20,9	55
Kepulauan Riau	48,1	7,8	44,1	97
DKI Jakarta	71,9	6,6	21,5	405
Jawa Barat	60,7	9,7	29,6	1,826
Jawa Tengah	81,3	7,9	10,8	1,016
DI Yogyakarta	70,4	10,4	19,2	113
Jawa Timur	75,7	6,5	17,8	1,167
Banten	74,2	6,3	19,6	431
Bali	67,6	2,2	30,2	149
Nusa Tenggara Barat	74,5	5,5	20,0	205
Nusa Tenggara Timu	67,5	12,9	19,6	172
Kalimantan Barat	63,5	9,0	27,6	167
Kalimantan Tengah	67,6	7,3	25,1	89
Kalimantan Selatan	69,4	9,2	21,4	137
Kalimantan Timur	75,6	4,8	19,6	124
Kalimantan Utara	68,3	8,0	23,7	23*
Sulawesi Utara	56,2	16,4	27,5	64
Sulawesi Tengah	61,7	7,0	31,3	121
Sulawesi Selatan	69,5	10,8	19,7	301
Sulawesi Tenggara	56,3	11,6	32,1	109
Gorontalo	76,4	4,6	18,9	33*
Sulawesi Barat	71,2	7,8	20,9	49*
Maluku	46,7	13,8	39,5	55
Maluku Utara	59,2	12,0	28,8	46*
Papua Barat	52,7	14,9	32,4	32*
Papua	36,7	18,9	44,3	66
**INDONESIA**	66,2	9,0	24,9	8.902

*N Tertimbang <50

Tabel 14.6.2
Proporsi Kepemilikan Buku KIA pada Ibu Hamil menurut Karakteristik, Riskesdas 2018

Karakteristik	Kepemilikan Buku KIA (%)			N Tertimbang
	Bisa menunjukkan	Tidak bisa menunjukkan	Tidak memiliki	
**Kelompok Umur**				
10 –14	77,1	5,7	17,2	5*
15 - 19	66,4	8,7	24,9	602
20 - 24	67,0	9,0	24,0	2.269
25 - 29	66,6	9,3	24,1	2.626
30 - 34	66,4	9,2	24,4	2,075
35 - 39	65,0	7,4	27,7	1.083
40-44	54,6	12,1	33,3	217
45 – 49	56,6		37,5	23*
50 – 54	93,4	5,9	6,6	2*
**Kelompok Umur (Khusus)**				
10 – 19 (Remaja)	66,5	8,7	24,8	607
15 – 49 (WUS)	64,6	9,0	24,9	8.896
**Pendidikan**				
Tidak pernah sekolah	52,8	11,1	36,1	109
Tidak tamat SD/MI	60,3	9,2	30,5	437
Tamat SD/MI	68,1	7,9	24,0	1.543
Tamat SLTP/MTS	69,9	8,7	21,4	2.127
Tamat SLTA/MA	66,5	9,3	24,2	3.313
Tamat D1/D2/D3/PT	60,1	9,6	30,2	1.373
**Pekerjaan**				
Tidak bekerja	68,0	8,4	23,7	5.459
Sekolah	61,8	1,5	36,7	84
PNS/TNI/Polri/BUMN/BUMD	62,9	12,0	25,2	204
Pegawai swasta	67,1	6,6	26,2	935
Wiraswasta	61,7	10,9	27,4	675
Petani/buruh tani	59,4	11,4	29,2	485
Nelayan	68,6	14,6	16,9	11*
Buruh/supir/pembantu ruta	54,7	15,7	29,5	274
Lainnya	65,5	10,6	24,0	776
**Tempat tinggal**				
Perkotaan	63,2	8,9	27,8	4.964
Perdesaan	69,8	9,0	21,1	3.938

*N Tertimbang <50

BAB 15
KESEHATAN BALITA

Responden untuk menjawab pertanyaan terkait kesehatan balita adalah ibu dari balita yang bersangkutan atau anggota rumah tangga lain yang paling mengetahui tentang kesehatan dan tumbuh kembang balita tersebut. Pertanyaan dikategorikan sebagai berikut ini:

Kategori umur	Pertanyaan
**0-59 bulan**	Kondisi saat lahir
	Perawatan bayi baru lahir
	Kepemilikan dan pemanfaatan buku KIA
	Imunisasi
	Pemantauan pertumbuhan
	Kecacatan
**6-59 bulan**	Pemberian makanan tambahan
**36-59 bulan**	Perkembangan anak

15.1 Riwayat Bayi Baru Lahir

Informasi usia kehamilan saat melahirkan dikumpulkan berdasarkan catatan ataupun pengakuan. Catatan dapat berupa catatan usia kehamilan pada saat anak dilahirkan, catatan selisih Hari Pertama Haid Terakhir (HPHT) dan tanggal lahir anak.

A. Prematuritas

Masa kehamilan normal yaitu jika bayi dilahirkan saat usia kehamilan ibu mencapai 37-42 minggu.

Definsi prematuritas: anak dilahirkan kurang dari 37 minggu

$$Proporsi\ kelahiran\ prematur = \frac{Anak\ umur\ \ 0-59\ bulan\ \ yang\ memiliki\ riwayat\ kelahiran\ pada\ umur\ kehamilan < 37\ minggu}{Jumlah\ anak\ umur\ 0-59\ bulan}$$

Tabel 15.1.1
Proporsi Riwayat Kelahiran berdasarkan Umur Kandungan Ibu saat Melahirkan pada Anak Umur 0-59 Bulan menurut Provinsi, Riskesdas 2018

Provinsi	Umur kandungan ibu saat melahirkan (%)			N Tertimbang
	Premature/kurang bulan (<37 minggu)	Cukup bulan (37-42 minggu)	Lebih bulan (> 42 minggu)	
Aceh	51,4	48,6	0,0	2.250
Sumatera Utara	56,6	43,2	0,1	5.895
Sumatera Barat	36,7	62,9	0,4	2.179
Riau	51,3	48,4	0,1	2.813
Jambi	24,0	75,6	0,3	1.279
Sumatera Selatan	43,0	57,0	0,0	3.079
Bengkulu	35,9	64,1	0,0	716
Lampung	24,8	74,9	0,3	3.094
Bangka Belitung	31,4	68,6	0,0	527
Kepulauan Riau	27,9	71,7	0,4	872
DKI Jakarta	19,5	80,5	0,0	3.582
Jawa Barat	23,5	76,2	0,4	17.228
Jawa Tengah	19,0	80,5	0,4	10.551
DI Yogyakarta	15,2	84,5	0,3	1.069
Jawa Timur	23,3	76,5	0,2	11.272
Banten	23,9	75,9	0,2	4.813
Bali	16,2	83,4	0,4	1.275
Nusa Tenggara Barat	20,6	79,0	0,4	1.985
Nusa Tenggara Timur	30,8	68,9	0,2	2.496
Kalimantan Barat	25,0	74,8	0,2	1.928
Kalimantan Tengah	29,6	70,2	0,2	1.000
Kalimantan Selatan	17,7	82,2	0,1	1.563
Kalimantan Timur	16,3	83,4	0,3	1.368
Kalimantan Utara	15,0	85,0	0,0	309
Sulawesi Utara	50,5	49,2	0,2	821
Sulawesi Tengah	38,0	61,5	0,5	1.155
Sulawesi Selatan	45,3	54,4	0,2	3.269
Sulawesi Tenggara	32,4	66,8	0,7	1.169
Gorontalo	33,3	66,4	0,0	445
Sulawesi Barat	25,9	73,2	0,9	584
Maluku	51,3	48,5	0,1	779
Maluku Utara	56,2	43,6	0,1	536
Papua Barat	40,4	59,1	0,3	415
Papua	44,6	55,0	0,4	1.302
**INDONESIA**	**29,5**	**70,2**	**0,3**	**93.620**

Tabel 15.1.2
Proporsi Riwayat Kelahiran berdasarkan Umur Kandungan Ibu saat Melahirkan pada Anak Umur 0-59 Bulan menurut Karakteristik, Riskesdas 2018

Karakteristik	Umur kandungan ibu saat anak dilahirkan (%)			N Tertimbang
	Premature/kurang bulan (<37 minggu)	Cukup bulan (37-42 minggu)	Premature/kurang bulan (<42 minggu)	
**Kelompok umur (bulan)**				
0-5	27,0	72,7	0,2	8.908
6-11	28,5	71,2	0,2	9.757
12-23	28,9	70,7	0,4	18.333
24-35	30,0	69,7	0,2	19.112
36-47	30,4	69,3	0,3	18.821
48-59	30,6	69,1	0,2	18.688
**Jenis kelamin**				
Laki-laki	29,5	70,2	0,3	47.764
Perempuan	29,6	70,1	0,3	45.856
**Pendidikan KRT**				
Tidak/belum pernah sekolah	30,1	69,6	0,3	3.855
Tidak tamat SD/MI	30,2	69,6	0,1	11.148
Tamat SD/MI	29,4	70,2	0,3	24.131
Tamat SLTP/MTS	29,5	70,1	0,4	17.658
Tamat SLTA/MA	30,2	69,6	0,2	27.939
Tamat D1/D2/D3/PT	27,0	72,7	0,3	8.888
**Pekerjaan KRT**				
Tidak bekerja	29,4	70,3	0,2	6.378
Sekolah	38,4	61,6	0,0	184
PNS/TNI/Polri/BUMN/BUMD	30,8	69,0	0,2	4.542
Pegawai swasta	25,4	74,3	0,3	13.498
Wiraswasta	29,1	70,7	0,2	21.185
Petani/buruh tani	33,1	66,5	0,3	24.382
Nelayan	37,5	62,2	0,2	1.966
Buruh/sopir/pembantu ruta	26,8	72,9	0,3	16.707
Lainnya	30,2	69,3	0,5	4.778
**Tempat tinggal**				
Perkotaan	26,8	72,9	0,3	50.361
Perdesaan	32,7	67,0	0,2	43.259

B. Berat, Panjang dan Lingkar Kepala Bayi Lahir

Informasi berat badan, panjang badan, dan lingkar kepala saat lahir diperoleh dari catatan yang dimiliki oleh responden. Sumber catatan atau dokumen berat badan lahir dapat berupa buku KIA, KMS, buku catatan kelahiran, atau buku catatan kesehatan anak.

Berat Badan Lahir Rendah (BBLR)

Definisi berat badan lahir rendah jika lahir dengan berat badan di bawah 2500 gram, Berat lahir normal berkisar 2500-4000 gram (Kementerian Kesehatan, 2010).

$$Proporsi\ Berat\ Badan\ Lahir\ Rendah = \frac{Jumlah\ anak\ umur\ 0-59\ bulan\ yang\ memiliki\ catatan\ berat\ badan\ lahir\ < 2500\ gram}{Jumlah\ anak\ umur\ 0-59\ bulan\ yang\ memiliki\ catatan\ berat\ badan\ lahir}$$

Panjang Badan Lahir Pendek

Panjang badan lahir yang normal berada pada rentang 48-52 cm (Kementerian Kesehatan, 2010).

Definisi panjang badan lahir pendek: saat lahir mempunyai panjang badan di bawah 48 cm

$$Proporsi\ Panjang\ Badan\ Lahir\ Pendek = \frac{Jumlah\ anak\ umur\ 0-59\ bulan\ yang\ memiliki\ catatan\ panjang\ lahir\ < 48\ cm}{jumlah\ anak\ umur\ 0-59\ bulan\ yang\ memiliki\ catatan\ panjang\ badan\ lahir}$$

Lingkar Kepala Lahir Kecil (<33 cm)

lingkar kepala lahir yang normal berada pada rentang 33-37 cm (Kementerian Kesehatan, 2010).

Definisi lingkar kepala saat lahir dianggap kecil: jika lingkar kepala saat lahir di bawah 33 cm.

$$Proporsi\ Lingkar\ Kepala\ Lahir\ Kecil = \frac{Jumlah\ anak\ umur\ 0-59\ bulan\ yang\ memiliki\ catatan\ lingkar\ kepala < 33cm}{Jumlah\ anak\ umur\ 0-59\ bulan\ yang\ memiliki\ catatan\ lingkar\ kepala\ lahir}$$

Tabel 15.1.3
Proporsi Catatan Kondisi Lahir yang Tidak Dimiliki Anak Umur 0-59 Bulan menurut Provinsi, Riskesdas 2018

Provinsi	Tidak ada catatan (%)			N Tertimbang
	Berat badan lahir	Panjang badan lahir	Lingkar kepala lahir	
Aceh	59,3	81,0	94,9	2.250
Sumatera Utara	57,7	67,1	96,3	5.895
Sumatera Barat	51,6	58,1	90,7	2.179
Riau	52,4	65,4	95,4	2.813
Jambi	45,0	55,2	78,4	1.279
Sumatera Selatan	48,1	64,7	94,2	3.079
Bengkulu	42,4	61,3	82,6	716
Lampung	42,4	50,4	84,2	3.094
Bangka Belitung	46,8	52,9	83,6	527
Kepulauan Riau	40,1	45,0	87,6	872
DKI Jakarta	27,9	32,0	71,4	3.582
Jawa Barat	42,5	50,7	83,7	17.228
Jawa Tengah	33,0	39,2	66,4	10.551
DI Yogyakarta	22,6	25,9	46,5	1.069
Jawa Timur	29,2	36,0	69,4	11.272
Banten	51,6	58,2	87,9	4.813
Bali	30,5	36,7	62,7	1.275
Nusa Tenggara Barat	38,1	46,8	72,9	1.985
Nusa Tenggara Timur	56,0	67,3	84,0	2.496
Kalimantan Barat	39,6	50,1	77,0	1.928
Kalimantan Tengah	49,2	62,9	84,8	1.000
Kalimantan Selatan	39,1	46,8	75,2	1.563
Kalimantan Timur	35,8	42,3	74,5	1.368
Kalimantan Utara	33,2	40,4	76,8	309
Sulawesi Utara	47,4	57,4	91,6	821
Sulawesi Tengah	60,8	70,5	86,0	1.155
Sulawesi Selatan	51,6	62,3	86,6	3.269
Sulawesi Tenggara	64,9	80,2	96,2	1.169
Gorontalo	46,3	53,1	86,1	445
Sulawesi Barat	51,9	59,9	78,9	584
Maluku	69,7	83,0	96,4	779
Maluku Utara	71,8	83,2	95,0	536
Papua Barat	65,3	77,0	94,5	415
Papua	73,6	78,9	96,4	1.302
**INDONESIA**	**43,4**	**52,1**	**81,0**	**93.620**

Tabel 15.1.4
Proporsi Catatan Kondisi Lahir yang Tidak Dimiliki Anak Umur 0-59 Bulan menurut Karakteristik, Riskesdas 2018

Karakteristik	Tidak ada catatan (%)			N Tertimbang
	Berat badan lahir	Panjang badan lahir	Lingkar kepala lahir	
**Kelompok umur (bulan)**				
0-5	27,8	35,6	70,6	8.908
6-11	32,4	39,7	73,0	9.757
12-23	37,2	45,8	76,7	18.333
24-35	44,5	53,7	82,2	19.112
36-47	50,7	59,8	86,1	18.821
48-59	54,3	63,4	88,1	18.688
**Jenis kelamin**				
Laki-laki	43,4	51,9	81,0	47.764
Perempuan	43,4	52,3	81,0	45.856
**Pendidikan KRT**				
Tidak/belum pernah sekolah	50,4	59,4	80,7	3.855
Tidak tamat SD/MI	46,4	55,7	81,1	11.148
Tamat SD/MI	46,4	56,0	81,0	24.131
Tamat SLTP/MTS	43,7	52,8	80,7	17.658
Tamat SLTA/MA	40,4	48,5	81,9	27.939
Tamat D1/D2/D3/PT	37,6	43,9	79,1	8.888
**Pekerjaan KRT**				
Tidak bekerja	42,4	49,3	80,3	6.378
Sekolah	50,7	60,0	82,8	184
PNS/TNI/Polri/BUMN/BUMD	41,8	49,5	82,8	4.542
Pegawai swasta	35,0	41,7	78,2	13.498
Wiraswasta	41,5	50,2	81,0	21.185
Petani/buruh tani	50,3	61,1	82,6	24.382
Nelayan	53,4	65,0	86,4	1.966
Buruh/sopir/pembantu ruta	41,9	50,2	79,6	16.707
Lainnya	44,0	51,9	83,2	4.778
**Tempat tinggal**				
Perkotaan	38,2	45,4	79,9	50.361
Perdesaan	49,5	60,0	82,3	43.259

Tabel 15.1.5

Proporsi Berat Badan Lahir pada Anak Umur 0-59 Bulan menurut Provinsi, Riskesdas 2018

Provinsi	Berat Badan Lahir[1] (%)					N Tertimbang
	< 2500 gram		2500-2999 gram	3000-3999 gram	≥4000 gram	
	%	95% CI				
Aceh	6,5	5,1 - 8,2	25,8	60,1	7,7	812
Sumatera Utara	4,2	3,1 - 5,7	22,6	64,5	8,7	2.211
Sumatera Barat	4,6	3,5 - 6,1	25,1	65,9	4,4	935
Riau	6,1	4,7 - 7,9	28,4	58,0	7,4	1.188
Jambi	2,6	1,6 – 4,0	34,3	60,2	3,0	625
Sumatera Selatan	6,8	5,0 - 9,2	30,6	59,3	3,4	1.417
Bengkulu	4,9	3,5 - 6,8	29,5	61,2	4,3	366
Lampung	4,9	3,9 - 6,2	29,4	61,4	4,2	1.582
Bangka Belitung	5,9	4,0 - 8,5	26,1	64,5	3,6	249
Kepulauan Riau	7,2	4,4 - 11,7	26,7	61,8	4,2	464
DKI Jakarta	6,1	4,5 - 8,2	29,0	61,6	3,3	2.290
Jawa Barat	6,3	5,3 - 7,5	29,4	60,8	3,4	8.784
Jawa Tengah	6,1	5,3 – 7,0	28,0	62,8	3,1	6.272
DI Yogyakarta	8,3	6,1 - 11,2	32,4	57,7	1,6	734
Jawa Timur	6,6	5,8 - 7,5	30,1	60,9	2,3	7.083
Banten	6,9	5,1 - 9,2	30,8	59,2	3,0	2.066
Bali	5,6	4,1 - 7,8	24,9	64,4	5,1	786
Nusa Tenggara Barat	5,3	3,9 - 7,3	30,8	61,1	2,7	1.091
Nusa Tenggara Timur	8,4	7,1 – 10,0	37,3	51,4	2,9	974
Kalimantan Barat	6,0	4,8 - 7,6	36,8	54,7	2,5	1.032
Kalimantan Tengah	5,8	4,3 - 7,9	32,7	57,7	3,8	451
Kalimantan Selatan	5,9	4,4 - 7,7	31,6	59,3	3,2	845
Kalimantan Timur	7,1	5,0– 10,0	29,9	59,0	4,0	779
Kalimantan Utara	6,2	4,2 - 9,1	32,2	57,0	4,6	183
Sulawesi Utara	5,9	4,4 - 7,8	30,0	59,7	4,4	383
Sulawesi Tengah	8,9	6,9 - 11,4	32,8	53,3	5,0	402
Sulawesi Selatan	7,1	5,7 - 8,7	31,0	58,4	3,5	1.404
Sulawesi Tenggara	5,8	4,2 – 8,0	24,6	62,5	7,2	365
Gorontalo	8,6	6,2 - 11,8	30,8	56,4	4,2	212
Sulawesi Barat	6,2	4,1 - 9,1	31,8	59,5	2,6	249
Maluku	5,8	3,5 - 9,4	24,7	61,7	7,7	209
Maluku Utara	8,7	6,2 - 12,1	23,0	60,2	8,1	134
Papua Barat	5,4	3,1 - 9,3	25,7	65,0	3,8	128
Papua	5,6	3,6 - 8,5	32,9	57,3	4,2	305
**INDONESIA**	**6,2**	**5,9 - 6,5**	**29,4**	**60,7**	**3,7**	**47.011**

[1] Berdasarkan 56,6% balita yang memiliki catatan berat lahir

Tabel 15.1.6
Proporsi Berat Badan Lahir pada Anak Umur 0-59 Bulan menurut karakteristik, Riskesdas 2018

Karakteristik	Berat Badan Lahir[1] (%)					N Tertimbang
	< 2500 gram		2500-2999 gram	3000-3999 gram	≥4000 gram	
	%	95% CI				
**Kelompok umur (bulan)**						
0-5	5,8	5,0 - 6,7	29,4	61,1	3,7	5.706
6-11	6,4	5,5 - 7,4	29,8	60,8	3,0	5.853
12-23	6,2	5,5 - 6,9	30,8	59,9	3,1	10.211
24-35	6,3	5,6 - 7,2	28,0	61,6	4,0	9.417
36-47	5,6	4,9 - 6,4	29,4	60,6	4,3	8.239
48-59	6,8	6,0 - 7,7	28,9	60,2	4,1	7.585
**Jenis kelamin**						
Laki-laki	5,7	5,3 - 6,1	26,7	63,4	4,2	23.996
Perempuan	6,7	6,3 - 7,2	32,2	57,9	3,2	23.015
**Pendidikan KRT**						
Tidak/belum pernah sekolah	7,2	5,7 - 9,1	33,1	56,9	2,8	1.696
Tidak tamat SD/MI	7,2	6,3 - 8,2	31,5	57,9	3,4	5.298
Tamat SD/MI	7,0	6,3 - 7,7	29,8	59,6	3,6	11.481
Tamat SLTP/MTS	5,9	5,2 - 6,7	29,5	60,5	4,2	8.826
Tamat SLTA/MA	5,7	5,1 - 6,4	28,4	62,1	3,8	14.786
Tamat D1/D2/D3/PT	4,9	4,1 - 5,9	27,8	63,7	3,7	4.924
**Pekerjaan KRT**						
Tidak bekerja	7,8	6,5 - 9,4	30,8	57,8	3,5	3.259
Sekolah	6,2	2,5 - 14,7	35,8	56,9	1,1	80
PNS/TNI/Polri/BUMN/BUMD	3,7	2,9 - 4,8	27,1	64,9	4,3	2.346
Pegawai swasta	6,4	5,4 - 7,6	28,7	61,8	3,2	7.778
Wiraswasta	5,5	4,9 - 6,2	28,8	61,8	3,9	10.995
Petani/buruh tani	6,5	5,9 - 7,1	30,3	59,4	3,8	10.753
Nelayan	6,0	4,5 - 8,0	29,3	59,5	5,2	812
Buruh/sopir/pembantu ruta	6,4	5,7 - 7,3	29,7	60,2	3,7	8.614
Lainnya	6,9	5,5 - 8,6	29,8	59,8	3,5	2.373
**Tempat tinggal**						
Perkotaan	6,1	5,6 - 6,6	29,1	61,2	3,5	27.631
Perdesaan	6,3	5,9 - 6,8	29,8	59,9	4,0	19.380

[1] Berdasarkan 56,6% balita yang memiliki catatan berat lahir

Tabel 15.1.7
Proporsi Panjang Badan Lahir pada Anak Umur 0-59 Bulan menurut Provinsi, Riskesdas 2018

Provinsi	Panjang Badan Lahir[1] (%)				N Tertimbang
	< 48 cm		48-52 cm	>52 cm	
	%	95% CI			
Aceh	17,5	14,0 - 21,7	77,1	5,4	359
Sumatera Utara	18,6	15,8 - 21,7	78,0	3,4	1.635
Sumatera Barat	19,8	17,1 - 22,8	79,1	1,1	770
Riau	21,4	18,0 - 25,2	75,0	3,6	819
Jambi	21,2	17,8 - 25,1	75,7	3,0	482
Sumatera Selatan	29,2	25,2 - 33,6	67,9	2,9	915
Bengkulu	15,5	12,4 - 19,2	81,7	2,8	234
Lampung	24,3	21,6 - 27,2	74,7	1,0	1.292
Bangka Belitung	30,7	26,3 - 35,5	67,0	2,3	209
Kepulauan Riau	21,1	16,2 - 27,1	75,5	3,4	404
DKI Jakarta	26,2	22,8 - 30,0	71,8	2,0	2.052
Jawa Barat	22,4	20,4 - 24,5	74,1	3,5	7.151
Jawa Tengah	28,5	27,0 - 30,2	69,7	1,7	5.407
DI Yogyakarta	33,0	28,6 - 37,7	65,8	1,1	667
Jawa Timur	17,4	16,0 - 18,8	80,2	2,4	6.074
Banten	24,4	21,1 – 28,0	73,9	1,7	1.695
Bali	8,4	6,4 - 10,9	86,2	5,4	679
Nusa Tenggara Barat	14,0	11,5 – 17,0	83,7	2,3	890
Nusa Tenggara Timur	25,9	22,9 - 29,2	71,8	2,3	688
Kalimantan Barat	26,1	23,0 - 29,5	70,5	3,4	809
Kalimantan Tengah	24,2	20,0 - 28,9	71,8	4,1	313
Kalimantan Selatan	12,0	9,9 - 14,5	81,4	6,6	701
Kalimantan Timur	19,4	16,0 - 23,3	77,3	3,3	665
Kalimantan Utara	22,9	17,9 - 28,7	73,3	3,9	155
Sulawesi Utara	29,5	25,6 - 33,7	67,8	2,7	294
Sulawesi Tengah	30,8	26,6 - 35,5	67,2	2,0	286
Sulawesi Selatan	27,1	24,2 - 30,2	71,5	1,4	1.037
Sulawesi Tenggara	17,7	13,8 - 22,5	78,4	3,9	195
Gorontalo	36,1	30,5 - 42,0	62,6	1,3	176
Sulawesi Barat	25,2	20,5 - 30,4	72,8	2,0	197
Maluku	11,7	8,0 - 17,0	83,7	4,6	112
Maluku Utara	30,0	23,0 - 38,0	65,2	4,8	76
Papua Barat	22,5	16,5 - 30,0	74,3	3,1	81
Papua	25,8	21,0 - 31,3	68,8	5,5	232
**INDONESIA**	**22,6**	**22,0 - 23,3**	**74,7**	**2,7**	**37.754**

[1] Berdasarkan 47,9% balita yang memiliki catatan panjang lahir

Tabel 15.1.8
Proporsi Panjang Badan Lahir pada Anak Umur 0-59 Bulan menurut karakteristik, Riskesdas 2018

Karakteristik	Panjang Badan Lahir[1] (%)				N Tertimbang
	< 48 cm		48-52 cm	>52 cm	
	%	95% CI			
**Kelompok umur (bulan)**					
0-5	22,7	21,0 - 24,5	74,7	2,5	4.831
6-11	23,2	21,5 - 25,0	74,4	2,4	4.959
12-23	23,5	22,1 - 24,9	73,7	2,9	8.370
24-35	23,0	21,6 - 24,6	74,4	2,5	7.457
36-47	21,3	19,9 - 22,8	75,3	3,4	6.374
48-59	21,8	20,3 - 23,5	75,9	2,3	5.763
**Jenis kelamin**					
Laki-laki	20,5	19,7 - 21,4	76,5	3,0	19.336
Perempuan	24,9	23,9 - 25,8	72,8	2,3	18.418
**Pendidikan Kepala Keluarga**					
Tidak/belum pernah sekolah	25,0	22,0 - 28,2	73,2	1,9	1.320
Tidak tamat SD/MI	23,2	21,4 - 25,1	74,2	2,6	4.161
Tamat SD/MI	23,4	22,2 - 24,7	74,3	2,2	8.935
Tamat SLTP/MTS	24,3	22,8 - 25,8	73,2	2,5	7.018
Tamat SLTA/MA	21,8	20,7 - 23,1	75,3	2,8	12.121
Tamat D1/D2/D3/PT	19,3	17,5 - 21,3	76,9	3,8	4.199
**Pekerjaan KRT**					
Tidak bekerja	24,3	21,9 - 26,9	72,7	3,0	2.721
Sekolah	35,7	22,2 - 51,9	64,3	0,0	62
PNS/TNI/Polri/BUMN/BUMD	16,8	14,6 - 19,3	80,5	2,7	1.932
Pegawai swasta	21,9	20,1 - 23,7	75,1	3,0	6.630
Wiraswasta	21,9	20,6 - 23,3	75,5	2,6	8.891
Petani/buruh tani	23,1	22,0 - 24,3	74,8	2,1	7.992
Nelayan	22,0	18,2 - 26,4	75,4	2,6	580
Buruh/sopir/pembantu ruta	24,8	23,3 - 26,5	72,3	2,8	7.011
Lainnya	21,9	19,4 - 24,7	74,5	3,6	1.935
**Tempat tinggal**					
Perkotaan	22,3	21,5 - 23,3	74,6	3,0	23.175
Perdesaan	23,1	22,3 - 24,0	74,7	2,2	14.579

[1]Berdasarkan 47,9% balita yang memiliki catatan panjang lahir

Tabel 15.1.9
Proporsi Berat Badan Lahir <2500 gram dan Panjang Badan Lahir <48 cm pada Anak Umur 0-59 Bulan menurut Provinsi, Riskesdas 2018

Provinsi	BBL <2500 gram dan PBL<48 cm (%)		N tertimbang
	%	95% CI	
Aceh	2,4	1,4 - 4,2	355
Sumatera Utara	2,0	1,2 - 3,3	1.620
Sumatera Barat	3,7	2,6 - 5,2	760
Riau	2,3	1,5 - 3,6	815
Jambi	1,3	0,7 - 2,6	481
Sumatera Selatan	5,1	3,3 - 7,8	903
Bengkulu	2,1	1,1 - 3,7	232
Lampung	3,7	2,8 - 5,0	1.286
Bangka Belitung	5,0	3,2 - 7,6	209
Kepulauan Riau	4,6	2,2 - 9,4	404
DKI Jakarta	4,6	3,2 - 6,6	2.038
Jawa Barat	4,2	3,3 - 5,4	7.096
Jawa Tengah	4,3	3,6 - 5,1	5.378
DI Yogyakarta	7,6	5,4 - 10,6	665
Jawa Timur	3,9	3,2 - 4,7	6.054
Banten	4,4	2,9 - 6,7	1.686
Bali	3,2	2,1 - 4,9	678
Nusa Tenggara Barat	2,7	1,7 - 4,3	888
Nusa Tenggara Timur	4,9	3,6 - 6,5	686
Kalimantan Barat	4,2	3,2 - 5,5	806
Kalimantan Tengah	2,7	1,5 - 5,0	310
Kalimantan Selatan	2,5	1,7 - 3,8	697
Kalimantan Timur	4,1	2,6 - 6,4	659
Kalimantan Utara	4,8	2,9 - 7,9	155
Sulawesi Utara	3,3	2,0 - 5,3	291
Sulawesi Tengah	6,9	4,8 - 9,9	285
Sulawesi Selatan	4,3	3,2 - 5,9	1.030
Sulawesi Tenggara	3,6	2,1 - 6,2	191
Gorontalo	5,9	4,0 - 8,7	175
Sulawesi Barat	5,1	3,2 - 8,2	196
Maluku	1,3	0,5 - 3,6	112
Maluku Utara	4,8	2,5 - 9,0	75
Papua Barat	3,2	1,3 - 7,6	80
Papua	4,7	2,6 - 8,1	228
**INDONESIA**	**4,0**	**3,7 - 4,3**	**37.524**

Tabel 15.1.10

Proporsi Berat Badan Lahir <2500 gram dan Panjang Badan Lahir <48 cm pada Anak Umur 0-59 Bulan menurut Karakteristik, Riskesdas 2018

Karakteristik	BBL<2500 gram dan PBL<48 cm (%)		N
	%	95% CI	
**Kelompok umur (bulan)**			
0-5	4,1	3,4 - 4,9	4.818
6-11	4,4	3,6 - 5,4	4.925
12-23	3,9	3,3 - 4,6	8.328
24-35	4,1	3,4 - 4,9	7.411
36-47	3,5	2,9 - 4,3	6.329
48-59	4,2	3,5 - 5,1	5.712
**Jenis kelamin**			
Laki-laki	3,6	3,2 – 4,0	19.209
Perempuan	4,4	4,0 - 4,9	18.315
**Pendidikan KRT**			
Tidak/belum pernah sekolah	5,2	3,8 - 7,2	1.311
Tidak tamat SD/MI	4,4	3,7 - 5,4	4.135
Tamat SD/MI	4,3	3,7 - 4,9	8.868
Tamat SLTP/MTS	4,3	3,6 - 5,1	6.993
Tamat SLTA/MA	3,7	3,2 - 4,4	12.040
Tamat D1/D2/D3/PT	2,9	2,2 - 3,8	4.177
**Pekerjaan KRT**			
Tidak bekerja	4,9	3,8 - 6,3	2.702
Sekolah	6,8	2,5 - 17,4	62
PNS/TNI/Polri/BUMN/BUMD	1,8	1,3 - 2,7	1.924
Pegawai swasta	4,6	3,6 - 5,7	6.590
Wiraswasta	3,5	3,0 - 4,2	8.825
Petani/buruh tani	4,1	3,6 - 4,7	7.948
Nelayan	4,1	2,7 - 6,4	577
Buruh/sopir/pembantu ruta	4,2	3,5 - 5,0	6.972
Lainnya	3,7	2,6 - 5,3	1.924
**Tempat tinggal**			
Perkotaan	3,9	3,5 - 4,4	23.040
Perdesaan	4,1	3,7 - 4,5	14.484

Tabel 15.1.11
Proporsi Lingkar Kepala Lahir pada Anak Umur 0-59 Bulan menurut Provinsi, Riskesdas 2018

Provinsi	Lingkar Kepala Lahir[1] (%)			N Tertimbang
	< 33 cm (microcephalus)	33-37 cm (normal)	>37 cm (macrocephalus)	
Aceh	42,5	55,8	1,6	96
Sumatera Utara	38,0	60,4	1,6	182
Sumatera Barat	32,0	67,6	0,5	171
Riau	37,8	61,2	1,0	109
Jambi	32,1	64,5	3,4	232
Sumatera Selatan	37,9	58,6	3,5	150
Bengkulu	54,5	45,5	0,0	105
Lampung	41,1	57,5	1,4	411
Bangka Belitung	39,6	58,8	1,6	73
Kepulauan Riau	30,4	67,7	1,9	90
DKI Jakarta	37,2	62,0	0,8	861
Jawa Barat	40,4	58,4	1,3	2.361
Jawa Tengah	40,7	58,4	0,9	2.977
DI Yogyakarta	35,8	64,2	0,0	480
Jawa Timur	40,8	58,5	0,7	2.899
Banten	37,8	62,2	0,0	490
Bali	37,4	62,0	0,6	399
Nusa Tenggara Barat	48,1	51,8	0,0	452
Nusa Tenggara Timur	45,8	51,3	2,9	335
Kalimantan Barat	48,3	50,1	1,6	371
Kalimantan Tengah	60,4	39,6	0,0	128
Kalimantan Selatan	48,7	50,4	0,9	325
Kalimantan Timur	36,5	62,8	0,8	293
Kalimantan Utara	39,5	60,1	0,5	60
Sulawesi Utara	27,1	71,0	1,9	58
Sulawesi Tengah	47,9	50,0	2,0	136
Sulawesi Selatan	40,0	58,4	1,6	367
Sulawesi Tenggara	53,1	46,9	0,0	37*
Gorontalo	34,5	65,5	0,0	52
Sulawesi Barat	33,8	65,0	1,2	104
Maluku	51,3	39,0	9,8	23*
Maluku Utara	41,6	56,9	1,4	23*
Papua Barat	33,1	66,9	0,0	19*
Papua	32,7	62,0	5,4	40*
**INDONESIA**	**40,6**	**58,4**	**1,0**	**14.908**

[1] Berdasarkan 19% balita yang memiliki catatan lingkar kepala lahir
* N tertimbang <50

Tabel 15.1.12
Proporsi Lingkar Kepala Lahir pada Anak Umur 0-59 Bulan menurut karakteristik, Riskesdas 2018

Karakteristik	Lingkar Kepala Lahir[1] (%)			N Tertimbang
	< 33 cm	33-37 cm	>37 cm	
**Kelompok umur (bulan)**				
0-5	39,6	59,5	0,9	2.195
6-11	41,4	57,3	1,3	2.211
12-23	39,8	59,2	1,0	3.592
24-35	40,7	58,6	0,7	2.858
36-47	40,8	58,0	1,3	2.192
48-59	41,8	57,2	1,0	1.860
**Jenis kelamin**				
Laki-laki	38,0	60,9	1,1	7.599
Perempuan	43,3	55,8	1,0	7.309
**Pendidikan KRT**				
Tidak/belum pernah sekolah	43,4	55,9	0,7	624
Tidak tamat SD/MI	42,9	55,8	1,3	1.767
Tamat SD/MI	43,2	55,9	0,9	3.849
Tamat SLTP/MTS	43,3	55,6	1,0	2.862
Tamat SLTA/MA	38,1	61,1	0,9	4.249
Tamat D1/D2/D3/PT	32,0	66,4	1,6	1.557
**Pekerjaan KRT**				
Tidak bekerja	40,3	58,8	0,9	1.053
Sekolah	55,2	44,8	0,0	27*
PNS/TNI/Polri/BUMN/BUMD	33,8	65,3	0,9	654
Pegawai swasta	37,0	61,9	1,1	2.467
Wiraswasta	38,7	60,4	0,9	3.379
Petani/buruh tani	44,7	54,1	1,1	3.569
Nelayan	45,7	53,4	1,0	225
Buruh/sopir/pembantu ruta	42,2	56,8	1,0	2.864
Lainnya	38,6	60,0	1,4	672
**Tempat tinggal**				
Perkotaan	38,4	60,6	1,0	8.486
Perdesaan	43,4	55,5	1,1	6.422

[1] Berdasarkan 19% balita yang memiliki catatan lingkar kepala lahir
* N tertimbang <50

C. Kecacatan Saat Lahir

Kecacatan yang dimaksud adalah semua kecacatan sejak lahir yang dapat diobservasi yaitu:

1. Tuna netra (buta) adalah sejak lahir tidak memiliki pengalaman penglihatan atau cacat penglihatan.
2. Tuna rungu (tuli) adalah sejak lahir tidak mampu mendengar atau memiliki hambatan permanen maupun tidak permanen dalam pendengaran.
3. Tuna wicara (berbicara/bisu) adalah sejak lahir memiliki hambatan dalam berbicara, biasanya juga memiliki hambatan permanen maupun tidak permanen dalam pendengaran.
4. Tuna daksa (tubuh/cacat anggota badan) adalah tidak memiliki salah satu atau keseluruhan alat gerak, atau memiliki gangguan gerak yang disebabkan oleh kelainan neuromuskular (syaraf otot) dan struktur tulang yang bersifat bawaan.
5. Bibir sumbing adalah suatu ketidaksempurnaan pada penyambungan bibir bagian atas yang biasanya berlokasi tepat dibawah hidung hingga celah langit-langit mulut menuju ke saluran udara di hidung sehingga membentuk celah pada bibir atas diantara mulut dan hidung.
6. Down syndrome adalah kelainan genetik yang terjadi pada masa pertumbuhan janin (pada kromosom 21/trisomi 21) dengan gejala yang sangat bervariasi dari gejala minimal sampai muncul tanda khas berupa keterbelakangan mental dengan tingkat IQ kurang dari 70 serta bentuk muka (Mongoloid). Ciri-ciri down syndrome antara lain bentuk kepala anak relative lebih kecil dari ukuran kepala anak normal dan bagian kepala belakang yang tampak datar, ukuran hidung kecil dan datar (pesek), ukuran mulut kecil, menguncup, dengan lidah yang tebal dan pangkal mulut yang cenderung dangkal, yang dapat mengakibatkan lidah sering menjulur keluar, bentuk mata yang miring dan tidak punya lipatan di kelopak matanya, letak telinga lebih rendah dari posisi normal dan ukuran telinga lebih kecil, rambut lurus dan halus atau tipis dan jarang, kulit yang kering, tangan dan jari kaki yang pendek dan ruas kedua jari kelingking miring atau bahkan tidak ada sama sekali, pada telapak tangan terdapat garis melintang yang disebut Simian Crease yang juga terdapat di kaki mereka, diantara telunjuk dan ibu jari yang jaraknya cenderung lebih jauh dari pada kaki orang normal.

Tabel 15.1.13
Proporsi Kelainan/Kecacatan Sejak Lahir pada Anak Umur 24-59 Bulan, Riskesdas 2018

Jenis Kelainan/Kecacatan	kelainan/kecacatan sejak lahir		
	%	95%CI	N tertimbang
Tuna netra	0,10	0,05 - 0,19	57.361
Tuna wicara	0,15	0,09 - 0,26	57.361
Tuna rungu	0,11	0,05 - 0,26	57.361
Tuna daksa	0,16	0,09 - 0,28	57.361
Bibir sumbing	0,12	0,06 - 0,23	57.361
Down syndrome	0,21	0,13 - 0,34	57.361
**Minimal satu jenis cacat**	**0,41**	**0,34 - 0,50**	**57.361**

15.2 Perawatan Bayi Baru Lahir

A. Perawatan metode kangguru pada bayi BBLR

Perawatan metode kanguru merupakan bagian dari perawatan atau tindakan yang dilakukan kepada bayi yang lahir dengan berat badan kurang dari 2500 gram. Data perawatan metode kanguru diperoleh dari pengakuan responden.

$$Proporsi\ tindakan\ metode\ kanguru = \frac{Jumlah\ anak\ umur\ 0-59\ bulan\ dengan\ riwayat\ BBLR\ yang\ melakukan\ perawatan\ metode\ kanguru}{Jumlah\ anak\ umur\ 0-59\ bulan\ dengan\ riwayat\ BBLR}$$

Tabel 15.2.1
Proporsi Tindakan yang Dilakukan untuk Bayi Berat Lahir Rendah (<2500 gram) pada Anak Umur 0-59 Bulan menurut Provinsi, Riskesdas 2018

Provinsi	Tidak dilakukan apa-apa	Di inkubator	Perawatan metode kangguru	Lainnya	N Tertimbang
	%	%	%	%	
Aceh	24,4	42,1	31,5	2,0	54
Sumatera Utara	34,8	44,6	15,7	4,9	95
Sumatera Barat	27,4	47,0	18,5	7,1	44*
Riau	27,9	55,9	12,7	3,5	74
Jambi	16,5	73,6	10,0	0,0	16*
Sumatera Selatan	28,7	46,5	22,6	2,2	99
Bengkulu	36,9	45,6	12,7	4,8	18*
Lampung	28,3	43,6	25,7	2,5	80
Bangka Belitung	26,9	38,1	32,8	2,3	15*
Kepulauan Riau	36,8	24,3	38,9	0,0	34
DKI Jakarta	22,2	60,4	14,8	2,5	142
Jawa Barat	31,0	44,3	17,3	7,5	567
Jawa Tengah	34,1	39,3	21,7	5,0	391
DI Yogyakarta	25,9	47,5	17,7	8,9	62
Jawa Timur	25,9	46,7	21,7	5,6	479
Banten	25,1	40,9	19,8	14,2	145
Bali	27,0	51,2	13,4	8,4	45*
Nusa Tenggara Barat	24,2	37,5	36,7	1,7	60
Nusa Tenggara Timur	28,1	31,9	37,6	2,4	84
Kalimantan Barat	44,0	30,6	25,4	0,0	64
Kalimantan Tengah	24,2	36,1	35,3	4,4	27*
Kalimantan Selatan	35,1	31,4	29,4	4,1	51
Kalimantan Timur	42,2	40,2	17,5	0,0	57
Kalimantan Utara	24,1	34,3	34,3	7,3	12*
Sulawesi Utara	46,0	45,4	7,6	1,1	23*
Sulawesi Tengah	38,3	28,0	28,4	5,4	37*
Sulawesi Selatan	32,4	40,9	25,9	0,9	102
Sulawesi Tenggara	27,4	39,6	27,8	5,2	22*
Gorontalo	53,0	15,9	31,1	0,0	19*
Sulawesi Barat	28,6	21,6	45,7	4,1	16*
Maluku	20,6	37,7	41,7	0,0	12*
Maluku Utara	41,2	27,9	28,2	2,8	12*
Papua Barat	41,3	36,6	22,1	0,0	7*
Papua	38,2	28,7	26,4	6,7	17*
**INDONESIA**	**30,1**	**43,0**	**21,7**	**5,1**	**2.980**

*N Tertimbang <50

Tabel 15.2.2
Proporsi Tindakan yang Dilakukan untuk Bayi Berat Lahir Rendah (<2500 gram) pada Anak Umur 0-59 Bulan menurut Karakteristik, Riskesdas 2018

Karakteristik	Tidak dilakukan apa-apa	Di inkubator	Perawatan metode kangguru	Lainnya	N Tertimbang
	%	%	%	%	
**Kelompok umur (bulan)**					
0-5	32,5	37,3	28,2	2,0	339
6-11	31,9	42,6	20,8	4,7	382
12-23	30,6	39,2	23,5	6,7	647
24-35	35,1	40,1	20,2	4,7	610
36-47	27,2	51,1	17,5	4,2	475
48-59	23,6	47,9	21,8	6,7	528
**Jenis Kelamin**					
Laki-laki	30,6	44,1	20,5	4,8	1.396
Perempuan	29,7	42,1	22,8	5,4	1.584
**Pendidikan KRT**					
Tidak/belum pernah sekolah	32,6	34,1	24,8	8,5	125
Tidak tamat SD/MI	35,1	32,0	26,3	6,7	390
Tamat SD/MI	29,0	42,5	23,8	4,7	819
Tamat SLTP/MTS	32,6	45,0	18,6	3,8	532
Tamat SLTA/MA	28,8	47,6	20,3	3,3	869
Tamat D1/D2/D3/PT	24,1	46,5	17,8	11,6	246
**Pekerjaan KRT**					
Tidak bekerja	33,2	44,1	18,4	4,3	260
Sekolah	55,0	13,7	31,3	N/A	5*
PNS/TNI/Polri/BUMN/BUMD	28,6	41,3	19,9	10,2	89
Pegawai swasta	29,2	48,3	19,9	2,6	508
Wiraswasta	28,3	46,1	20,9	4,8	621
Petani/buruh tani	29,5	38,2	27,5	4,7	712
Nelayan	31,1	27,3	29,6	12,1	50
Buruh/sopir/pembantu ruta	30,7	41,5	19,5	8,3	568
Lainnya	35,3	46,3	16,9	1,6	167
**Tempat tinggal**					
Perkotaan	27,4	46,0	20,8	5,8	1.723
Perdesaan	33,9	39,0	23,0	4,2	1.257

*N Tertimbang <50

B. Perawatan Tali Pusar

Menurut Asuhan Persalinan Normal (APN), cara perawatan tali pusar bayi baru lahir yang benar yaitu tali pusar yang telah dipotong dan diikat, tidak diberi apa-apa. Dalam Riskesdas 2018, data perawatan tali pusar didapatkan dari pengakuan ibu.

$$Proporsi\ cara\ perawatan\ tali\ pusar = \frac{Anak\ umur\ \ 0-59\ bulan\ berdasarkan\ perawatan\ tali\ pusar}{Jumlah\ anak\ umur\ 0-59\ bulan}$$

Tabel 15.2.3
Proporsi Cara Perawatan Tali Pusar saat Bayi Baru Lahir pada Anak Umur 0-59 bulan menurut Provinsi, Riskesdas 2018

Provinsi	Tidak diberi apa-apa dan kering	Diberi Betadin/ Alkohol	Diberi Obat Tabur	Diberi Ramuan/ Obat tradisional	Tidak tahu	N Ter-timbang
	%	%	%	%	%	
Aceh	53,7	35,4	2,2	1,8	6,9	2.250
Sumatera Utara	17,3	68,8	2,2	2,2	9,5	5.895
Sumatera Barat	29,9	62,3	0,4	1,0	6,4	2.179
Riau	19,6	67,6	1,1	5,9	5,8	2.813
Jambi	43,3	43,9	0,4	4,8	7,7	1.279
Sumatera Selatan	21,7	67,2	1,6	4,4	5,1	3.079
Bengkulu	39,0	51,1	0,4	3,3	6,2	716
Lampung	41,0	50,3	1,1	2,3	5,2	3.094
Kep.Bangka Belitung	39,0	55,8	0,5	1,7	3,1	527
Kepulauan Riau	46,7	44,4	0,9	2,6	5,4	872
DKI Jakarta	53,1	41,3	0,3	0,7	4,6	3.582
Jawa Barat	45,5	45,6	0,6	1,8	6,5	17.228
Jawa Tengah	39,3	55,2	0,5	0,1	4,9	10.551
DI Yogyakarta	66,4	28,6	0,1	0,2	4,6	1.069
Jawa Timur	57,9	35,7	1,0	0,7	4,7	11.272
Banten	27,2	61,8	1,3	5,0	4,7	4.813
Bali	63,3	28,3	0,4	2,5	5,4	1.275
Nusa Tenggara Barat	62,9	22,0	1,7	5,5	7,8	1.985
Nusa Tenggara Timur	56,9	25,5	3,2	7,3	7,1	2.496
Kalimantan Barat	32,3	46,7	2,3	13,2	5,5	1.928
Kalimantan Tengah	42,8	32,1	3,9	15,4	5,9	1.000
Kalimantan Selatan	54,1	38,1	1,1	2,8	3,8	1.563
Kalimantan Timur	44,8	46,5	1,2	1,3	6,1	1.368
Kalimantan Utara	69,7	22,2	0,5	0,6	7,0	309
Sulawesi Utara	5,1	89,6	0,5	0,9	4,0	821
Sulawesi Tengah	31,0	53,0	3,9	6,5	5,6	1.155
Sulawesi Selatan	35,3	51,8	1,5	1,2	10,2	3.269
Sulawesi Tenggara	36,6	38,3	8,6	6,9	9,6	1.169
Gorontalo	28,2	50,0	11,0	3,5	7,3	445
Sulawesi Barat	48,2	29,6	2,0	5,4	14,8	584
Maluku	15,7	65,0	1,7	14,6	3,0	779
Maluku Utara	24,3	42,1	4,3	21,4	7,9	536
Papua Barat	32,0	48,4	1,2	10,7	7,7	415
Papua	47,8	29,2	0,8	11,5	10,7	1.302
**INDONESIA**	**41,5**	**48,0**	**1,3**	**3,0**	**6,2**	**93.620**

Tabel 15.2.4
Proporsi Cara Perawatan Tali Pusar saat Bayi Baru Lahir pada Anak Umur 0-59 bulan menurut Karakteristik, Riskesdas 2018

Karakteristik	Tidak diberi apa-apa dan kering	Betadin/ Alkohol	Obat Tabur	Ramuan Obat tradisional	Tidak tahu	N Tertimbang
	%	%	%	%	%	
**Kelompok umur (bulan)**						
0-5	55,0	37,8	1,3	2,1	3,8	8.908
6-11	49,4	41,7	1,4	2,7	4,8	9.757
12-23	44,4	46,1	1,3	3,0	5,3	18.333
24-35	39,3	49,7	1,3	3,2	6,5	19.112
36-47	37,0	51,2	1,4	3,2	7,1	18.821
48-59	34,8	53,1	1,2	3,2	7,6	18.688
**Jenis kelamin**						
Laki-laki	41,4	48,0	1,3	2,9	6,3	47.764
Perempuan	41,5	48,1	1,3	3,1	6,1	45.856
**Pendidikan KRT**						
Tidak/belum pernah sekolah	40,1	42,6	2,2	7,0	8,0	3.855
Tidak tamat SD/MI	39,9	46,5	1,7	5,0	6,9	11.148
Tamat SD/MI	39,4	48,6	1,4	3,9	6,7	24.131
Tamat SLTP/MTS	40,2	49,8	1,4	2,8	5,8	17.658
Tamat SLTA/MA	42,4	49,3	1,0	1,6	5,6	27.939
Tamat D1/D2/D3/PT	46,6	46,3	1,0	0,9	5,2	2.805
**Pekerjaan KRT**						
Tidak bekerja	41,3	48,8	1,2	2,0	6,7	6.378
Sekolah	43,3	44,3	1,1	3,8	7,6	184
PNS/TNI/Polri/BUMN/BUMD	47,2	45,4	0,6	1,1	5,7	4.542
Pegawai swasta	46,3	46,6	0,8	1,2	5,2	13.498
Wiraswasta	42,5	48,5	1,2	1,6	6,2	21.185
Petani/buruh tani	39,0	45,7	1,8	6,2	7,3	24.382
Nelayan	31,6	51,5	3,2	6,6	7,2	1.966
Buruh/sopir/pembantu ruta	39,3	52,2	1,1	2,1	5,4	16.707
Lainnya	42,8	47,5	1,3	2,8	5,6	4.778
**Tempat tinggal**						
Perkotaan	43,1	49,1	0,9	1,3	5,7	50.361
Perdesaan	39,6	46,8	1,7	5,0	6,8	43.259

C. Pemeriksaan Bayi Baru Lahir

Pemeriksaan bayi baru lahir umur 0-28 hari (neonatus) oleh tenaga kesehatan merupakan salah satu upaya mengurangi kematian pada usia neonatal. Pada Riskesdas 2018 dilakukan pengumpulan data kunjungan neonatus yang meliputi kunjungan pertama saat berumur 6-48 jam (KN1), kunjungan kedua saat berumur 3-7 hari (KN2), dan kunjungan ketiga saat berumur 8-28 hari (KN3). Cakupan Kunjungan Neonatal Pertama atau KN1 merupakan indikator yang menggambarkan upaya kesehatan yang dilakukan untuk mengurangi risiko kematian pada periode neonatal yaitu 6-48 jam.

Definisi kunjungan neonatal lengkap: pada saat umur 0-28 hari telah mendapatkan minimal 1 kali pelayanan neonatal saat umur 6-48 jam (KN1), minimal 1 kali pelayanan neonatal saat umur 3-7 hari (KN2), dan minimal 1 kali pelayanan neonatal saat umur 8-28 hari (KN3)

$$Proporsi\ kunjungan\ neonatal\ lengkap = \frac{Jumlah\ anak\ umur\ 0-59\ bulan\ yang\ pada\ saat\ umur\ \ 0-28\ hari\ mendapatkan\ kunjungan\ neonatal\ ke\ 1,2\ dan\ 3}{Jumlah\ anak\ umur\ 0-59\ bulan}$$

Tabel 15.2.5
Proporsi Kunjungan Neonatal pada Anak Umur 0-59 Bulan menurut Provinsi, Riskesdas 2018

Provinsi	Kunjungan Neonatal							
	6-48 jam (KN1)		3-7 hari (KN2)		8-28 hari (KN3)		KN Lengkap	
	%	N	%	N	%	N	%	N
Aceh	88,3	2.250	71,4	2.232	35,4	2.199	33,5	2.250
Sumatera Utara	83,2	5.895	67,6	5.757	23,7	5.736	21,6	5.895
Sumatera Barat	89,6	2.179	56,3	2.166	36,1	2.158	29,8	2.179
Riau	86,3	2.813	63,8	2.774	27,1	2.768	23,3	2.813
Jambi	87,6	1.279	66,7	1.264	41,6	1.256	37,3	1.279
Sumatera Selatan	84,1	3.079	58,4	3.039	41,0	2.961	34,8	3.079
Bengkulu	90,0	716	73,2	708	36,6	705	34,5	716
Lampung	85,0	3.094	66,5	3.071	45,8	3.051	38,4	3.094
Bangka Belitung	87,6	527	61,6	524	37,5	522	32,5	527
Kepulauan Riau	84,6	872	67,5	864	44,5	861	38,2	872
DKI Jakarta	93,5	3.582	86,6	3.564	77,5	3.559	71,3	3.582
Jawa Barat	81,9	17.228	77,9	16.964	62,8	16.883	53,2	17.228
Jawa Tengah	90,0	10.551	81,4	10.485	59,6	10.446	53,5	10.551
DI Yogyakarta	95,9	1.069	91,1	1.066	74,2	1.062	68,2	1.069
Jawa Timur	87,3	11.272	82,8	11.208	63,8	11.177	56,5	11.272
Banten	77,0	4.813	70,6	4.728	53,5	4.702	44,1	4.813
Bali	95,3	1.275	81,5	1.271	70,2	1.269	61,3	1.275
Nusa Tenggara Barat	83,8	1.985	64,8	1.969	53,9	1.965	42,8	1.985
Nusa Tenggara Timur	74,1	2.496	58,3	2.409	49,3	2.402	38,7	2.496
Kalimantan Barat	76,2	1.928	52,4	1.863	30,9	1.841	23,8	1.928
Kalimantan Tengah	77,9	1.000	59,8	958	30,2	947	26,0	1.000
Kalimantan Selatan	89,4	1.563	75,1	1.549	40,3	1.546	37,0	1.563
Kalimantan Timur	86,1	1.368	72,4	1.353	54,8	1.344	47,7	1.368
Kalimantan Utara	86,2	309	70,1	307	51,3	306	45,1	309
Sulawesi Utara	85,9	821	70,5	813	57,5	807	49,3	821
Sulawesi Tengah	81,7	1.155	59,9	1.143	39,0	1.138	33,1	1.155
Sulawesi Selatan	86,9	3.269	46,5	3.238	26,8	3.158	20,0	3.269
Sulawesi Tenggara	76,3	1.169	49,7	1.136	25,2	1.121	20,0	1.169
Gorontalo	81,9	445	49,2	442	31,6	441	27,2	445
Sulawesi Barat	79,1	584	61,1	579	38,9	579	35,0	584
Maluku	60,6	779	45,1	738	27,7	727	22,5	779
Maluku Utara	65,4	536	45,0	528	33,5	514	24,8	536
Papua Barat	65,8	415	35,6	397	19,9	391	14,7	415
Papua	46,2	1.302	36,5	1.147	31,8	1.134	19,2	1.302
**INDONESIA**	**84,1**	**93.620**	**71,1**	**92.254**	**50,6**	**91.673**	**43,5**	**93.620**

Tabel 15.2.6
Proporsi Kunjungan Neonatal pada Anak Umur 0-59 Bulan menurut karakteristik, Riskesdas 2018

Karakteristik	Kunjungan Neonatal							
	6-48 jam (KN1)		3-7 hari (KN2)		8-28 hari (KN3)		KN Lengkap	
	%	N	%	N	%	N	%	N
**Kelompok umur (bulan)**								
0-5	86,1	8.908	69,8	8.799	47,4	8.660	39,6	8.908
6-11	86,4	9.757	72,9	9.657	52,2	9.601	44,7	9.757
12-23	85,5	18.333	72,2	18.085	51,7	17.984	44,9	18.333
24-35	82,9	19.112	71,1	18.826	50,0	18.749	43,0	19.112
36-47	83,3	18.821	70,5	18.519	50,1	18.414	43,0	18.821
48-59	82,5	18.688	70,4	18.368	51,1	18.265	44,4	18.688
**Jenis kelamin**								
Laki-laki	84,1	47.764	71,5	47.050	51,0	46.776	43,8	47.764
Perempuan	84,0	45.856	70,8	45.204	50,1	44.897	43,1	45.856
**Pendidikan KRT**								
Tidak/belum pernah sekolah	73,4	3.855	60,4	3.702	43,9	3.680	36,1	3855
Tidak tamat SD/MI	78,1	11.148	65,1	10.932	44,9	10.878	37,8	11.148
Tamat SD/MI	80,1	24.131	67,7	23.706	47,3	23.557	39,7	24.131
Tamat SLTP/MTS	85,3	17.658	72,5	17.437	50,3	17.351	43,3	17.658
Tamat SLTA/MA	88,5	27.939	74,8	27.658	54,1	27.452	47,1	27.939
Tamat D1/D2/D3/PT	90,8	8.888	78,1	8.818	58,8	8.755	53,0	8.888
**Pekerjaan KRT**								
Tidak bekerja	84,5	6.378	70,6	6.280	51,1	6.232	44,4	6.378
Sekolah	72,4	184	66,0	175	37,8	173	31,2	184
PNS/TNI/Polri/BUMN/BUMD	90,5	4.542	75,4	4.497	55,7	4.451	50,0	4.542
Pegawai swasta	90,1	13.498	79,1	13.386	60,7	13.300	53,3	13.498
Wiraswasta	86,8	21.185	74,5	20.973	52,2	20.851	45,9	21.185
Petani/buruh tani	77,8	24.382	63,2	23.780	42,1	23.627	34,7	24.382
Nelayan	76,6	1.966	60,9	1.930	39,0	1.918	33,8	1.966
Buruh/sopir/pembantu ruta	83,5	16.707	71,8	16.513	52,7	16.425	44,6	16.707
Lainnya	85,9	4.778	72,2	4.720	49,7	4.697	43,1	4.778
**Tempat tinggal**								
Perkotaan	87,6	50.361	75,6	49.885	56,5	49.540	49,2	50.361
Perdesaan	80,0	43.259	65,9	42.369	43,7	42.133	36,8	43.259

D. Pelayanan Kunjungan Neonatal Pertama (KN1) Sesuai Standar

Definisi pelayanan kunjungan neonatal pertama (6-48jam) sesuai standar, jika mendapatkan pelayanan pengukuran berat badan, pengukuran panjang badan, pengukuran suhu tubuh, tenaga kesehatan menanyakan riwayat sakit, riwayat diare, perawatan tali pusat, menanyakan status kesehatan, dan masalah dalam pemberian ASI, tenaga kesehatan memberitahu cara pemberian ASI yang baik, dan tanda bahaya pada bayi baru lahir, selain itu jika bayi belum menerima maka diberikan imunisasi HB-0 dan memberikan vitamin K.

Pertanyaan tentang jenis pemeriksaan saat kunjungan neonatal pertama hanya diajukan jika Ibu melakukan pemeriksaan neonatus (bayi baru lahir) ke tenaga kesehatan pada 6-48 jam setelah lahir. Informasi ini akan menjawab kualitas pelayanan kesehatan neonatus pada saat pemeriksaan bayi usia 6-48 jam. Kualitas pemeriksaan mengacu pada prosedur Asuhan Persalinan Normal (APN). Data dikumpulkan berdasarkan observasi pada dokumen buku KIA/buku catatan kesehatan lainnya atau pengakuan responden.

$$\textit{Proporsi Pelayanan Kunjungan Neonatal pertama} = \frac{\begin{array}{c}\textit{Anak umur } 0-59 \textit{ bulan yang pada saat umur } 6-48 \textit{ jam yang mendapatkan} \\ \textit{minimal } 1 \textit{ kali pelayanan KN dengan jenis layanan sesuai standar}\end{array}}{\begin{array}{c}\textit{Jumlah anak } 0-59 \textit{ bulan yang melakukan pemeriksaan bayi baru lahir} \\ \textit{pada saat umur } 6-48 \textit{ jam}\end{array}}$$

Tabel 15.2.7
Proporsi Pelayanan Kunjungan Neonatal Pertama (KN1) Sesuai Standar pada Anak Umur 0-59 Bulan menurut Provinsi, Riskesdas 2018

Provinsi	Kunjungan Neonatal pertama (KN1) sesuai standar		N Tertimbang
	%	95% CI	
Aceh	25,1	22,9 - 27,4	1.928
Sumatera Utara	13,6	12,1 - 15,3	4.758
Sumatera Barat	31,7	28,7 - 34,8	1.896
Riau	21,3	18,9 - 23,9	2.355
Jambi	48,5	45,0 - 51,9	1.088
Sumatera Selatan	32,4	29,7 - 35,4	2.512
Bengkulu	30,3	26,7 - 34,1	626
Lampung	35,5	32,6 - 38,5	2.553
Bangka Belitung	49,3	44,3 - 54,3	448
Kepulauan Riau	36,4	29,9 - 43,3	716
DKI Jakarta	47,0	43,1 - 50,9	3.252
Jawa Barat	38,3	36,4 - 40,3	13.696
Jawa Tengah	42,2	40,6 - 43,9	9.218
DI Yogyakarta	51,4	46,5 - 56,2	995
Jawa Timur	45,0	43,2 - 46,8	9.551
Banten	34,1	30,5 - 37,8	3.597
Bali	50,1	45,9 - 54,2	1.179
Nusa Tenggara Barat	45,3	41,8 - 48,7	1.615
Nusa Tenggara Timur	56,3	53,6 - 58,9	1.795
Kalimantan Barat	34,7	31,3 - 38,3	1.426
Kalimantan Tengah	31,6	28,0 - 35,5	756
Kalimantan Selatan	50,7	47,2 - 54,2	1.356
Kalimantan Timur	46,3	41,7 – 51,0	1.143
Kalimantan Utara	36,4	31,4 - 41,8	259
Sulawesi Utara	38,8	35,0 - 42,6	684
Sulawesi Tengah	38,2	34,7 - 41,8	916
Sulawesi Selatan	37,6	35,1 - 40,2	2.756
Sulawesi Tenggara	24,4	21,1 – 28,0	866
Gorontalo	43,5	38,6 - 48,5	354
Sulawesi Barat	49,2	44,3 - 54,1	448
Maluku	25,1	20,7 – 30,0	458
Maluku Utara	28,9	25,2 – 33,0	340
Papua Barat	19,2	15,6 - 23,4	265
Papua	32,0	28,0 - 36,2	583
**INDONESIA**	**37,9**	**37,2 - 38,5**	**76.392**

Tabel 15.2.8
Proporsi Pelayanan Kunjungan Neonatal Pertama (KN1) Sesuai Standar pada Anak Umur 0-59 Bulan menurut Karakteristik, Riskesdas 2018

Karakteristik	Kunjungan Neonatal pertama (KN1) sesuai standar		N Tertimbang
	%	95% CI	
**Kelompok umur (bulan)**			
0-5	39,0	37,3 - 40,7	7.441
6-11	40,9	39,2 - 42,6	8.183
12-23	38,3	37,1 - 39,5	15.211
24-35	37,4	36,2 - 38,6	15.382
36-47	36,3	35,2 - 37,5	15.209
48-59	37,3	36,1 - 38,5	14.965
**Jenis kelamin**			
Laki-laki	38,1	37,3 - 38,9	38.986
Perempuan	37,6	36,8 - 38,4	37.406
**Pendidikan KRT**			
Tidak/belum pernah sekolah	37,6	35,0 - 40,2	2.746
Tidak tamat SD/MI	36,0	34,5 - 37,6	8.453
Tamat SD/MI	37,3	36,3 - 38,4	18.752
Tamat SLTP/MTS	36,7	35,5 – 38,0	14.615
Tamat SLTA/MA	37,9	36,8 – 39,0	23.995
Tamat D1/D2/D3/PT	43,2	41,4 - 45,1	7.831
**Pekerjaan KRT**			
Tidak bekerja	38,3	36,2 - 40,4	5.231
Sekolah	36,1	25,1 - 48,8	129
PNS/TNI/Polri/BUMN/BUMD	44,0	41,7 - 46,3	3.989
Pegawai swasta	39,3	37,7 – 41,0	11.805
Wiraswasta	38,9	37,7 - 40,1	17.846
Petani/buruh tani	35,4	34,5 - 36,4	18.411
Nelayan	30,0	27,2 - 32,9	1.461
Buruh/sopir/pembantu ruta	37,6	36,2 - 39,1	13.537
Lainnya	37,2	34,8 - 39,7	3.982
**Tempat tinggal**			
Perkotaan	39,2	38,3 - 40,1	42.812
Perdesaan	36,2	35,4 - 36,9	33.580

E. Pemberian Salep Mata

Pemberian salep mata antibiotik dilakukan pada saat bayi baru lahir yang bertujuan untuk mencegah terjadinya infeksi pada mata bayi akibat proses kelahirannya.

$$Proporsi\ pemberian\ salep\ mata\ saat\ lahir\ = \frac{Anak\ umur\ 0-59\ bulan\ yang\ mendapatkan\ salep\ mata}{Jumlah\ anak\ 0-59\ bulan}$$

Tabel 15.2.9
Proporsi Pemberiaan Salep Mata saat Lahir pada Anak Umur 0-59 Bulan menurut Provinsi, Riskesdas 2018

Provinsi	Diberikan salep mata (%)		Tidak diberikan salep mata (%)	Tidak Tahu (%)	N Tertimbang
	berdasarkan dokumen	berdasarkan ingatan ibu			
Aceh	5,4	25,4	37,0	32,1	2.250
Sumatera Utara	2,8	15,7	47,9	33,5	5.895
Sumatera Barat	8,8	23,6	39,1	28,4	2.179
Riau	4,7	22,5	47,0	25,8	2.813
Jambi	15,1	33,6	23,2	28,2	1.279
Sumatera Selatan	5,4	33,8	34,8	26,1	3.079
Bengkulu	9,8	32,4	32,3	25,6	716
Lampung	16,7	36,6	22,8	23,9	3.094
Bangka Belitung	16,6	36,7	23,1	23,6	527
Kepulauan Riau	9,3	41,6	20,8	28,3	872
DKI Jakarta	18,3	33,1	23,3	25,3	3.582
Jawa Barat	13,7	42,1	23,8	20,4	17.228
Jawa Tengah	27,0	36,0	17,0	19,9	10.551
DI Yogyakarta	42,0	30,4	15,7	11,9	1.069
Jawa Timur	28,1	32,9	17,1	21,9	11.272
Banten	9,1	32,6	35,8	22,5	4.813
Bali	24,1	29,0	21,4	25,5	1.275
Nusa Tenggara Barat	24,0	33,0	26,3	16,7	1.985
Nusa Tenggara Timur	15,0	39,1	28,5	17,4	2.496
Kalimantan Barat	19,7	26,1	32,6	21,6	1.928
Kalimantan Tengah	9,8	40,3	27,8	22,2	1.000
Kalimantan Selatan	19,6	39,7	20,3	20,4	1.563
Kalimantan Timur	27,9	27,2	19,7	25,1	1.368
Kalimantan Utara	20,2	36,6	21,4	21,8	309
Sulawesi Utara	7,7	32,2	31,7	28,5	821
Sulawesi Tengah	11,6	30,5	34,3	23,7	1.155
Sulawesi Selatan	11,6	28,2	29,8	30,4	3.269
Sulawesi Tenggara	3,8	22,0	46,9	27,2	1.169
Gorontalo	11,1	24,3	35,4	29,2	445
Sulawesi Barat	19,0	30,5	23,6	26,8	584
Maluku	4,8	23,9	60,2	11,2	779
Maluku Utara	6,8	30,5	40,5	22,2	536
Papua Barat	6,7	36,9	35,5	20,9	415
Papua	4,2	21,3	49,9	24,6	1.302
**INDONESIA**	**16,0**	**32,8**	**27,8**	**23,4**	**93.620**

Tabel 15.2.10
Proporsi Pemberiaan Salep Mata saat Lahir pada Anak Umur 0-59 Bulan menurut Karakteristik, Riskesdas 2018

Karakteristik	Diberikan Salep Mata (%)		Tidak diberikan salep mata (%)	Tidak tahu (%)	N tertimbang
	Berdasarkan dokumen	Berdasarkan ingatan ibu			
**Kelompok umur (bulan)**					
0-5	24,7	31,2	25,2	18,9	8.908
6-11	21,4	32,0	26,2	20,4	9.757
12-23	19,4	33,1	26,5	21,0	18.333
24-35	14,8	32,6	28,7	23,9	19.112
36-47	12,2	33,1	29,0	25,7	18.821
48-59	10,7	33,8	29,0	26,5	18.688
**Jenis kelamin**					
Laki-laki	16,0	32,6	27,9	23,5	47.764
Perempuan	16,0	33,2	27,6	23,2	45.856
**Pendidikan KRT**					
Tidak/belum pernah sekolah	16,4	26,4	32,8	24,5	3.855
Tidak tamat SD/MI	16,5	29,7	31,3	22,6	11.148
Tamat SD/MI	17,0	31,2	28,4	23,4	24.131
Tamat SLTP/MTS	16,9	33,3	26,9	23,0	17.658
Tamat SLTA/MA	14,7	34,8	26,8	23,7	27.939
Tamat D1/D2/D3/PT	14,7	37,1	24,7	23,5	8.888
**Pekerjaan KRT**					
Tidak bekerja	16,4	30,6	27,7	25,3	6.378
Sekolah	13,1	34,7	19,6	32,7	184
PNS/TNI/Polri/BUMN/BUMD	12,9	38,1	24,6	24,3	4.542
Pegawai swasta	16,7	37,2	24,4	21,7	13.498
Wiraswasta	16,2	33,9	26,0	23,9	21.185
Petani/buruh tani	15,8	28,7	32,0	23,5	24.382
Nelayan	11,2	26,1	36,8	25,9	1.966
Buruh/sopir/pembantu ruta	17,5	34,1	26,5	21,9	16.707
Lainnya	13,7	33,1	27,7	25,6	4.778
**Tempat tinggal**					
Perkotaan	16,0	34,9	25,6	23,5	50.361
Perdesaan	16,0	30,5	30,4	23,2	43.259

F. Skrining Hipotiroid Kongenital

Skrining Hipotiroid Kongenital (SHK) adalah uji saring untuk memilah bayi yang menderita HK dengan bayi yang bukan menderita HK. Hipotiroid Kongenital (HK) adalah kelainan akibat kekurangan hormon tiroid yang dialami sejak lahir berupa gangguan dan perkembangan baik fisik maupun mental, Program ini sudah dimulai sejak tahun 2000, tapi sampai dengan tahun 2014 baru kurang dari 1 % bayi diskrining. Skrining hipotyroid paling baik dilakukan pada bayi baru lahir usia 48-72 jam atau sebelum bayi pulang dari rumah sakit atau fasilitas kesehatan. Data dikumpulkan berdasarkan observasi pada dokumen buku KIA/buku catatan kesehatan lainnya atau pengakuan responden.

$$Proporsi\ skrining\ hipotiroid\ kongenital$$

$$= \frac{Anak\ umur\ 0-59\ bulan\ yang\ mendapatkan\ skrining\ hipotiroid\ kongenital\ pada\ saat\ umur\ 48-72\ jam}{Jumlah\ anak\ umur\ 0-59\ bulan}$$

Tabel 15.2.11
Proporsi Pemeriksaan Skrining Hipotiroid Kongenital (SHK) saat Lahir pada Anak Umur 0-59 Bulan menurut Provinsi, Riskesdas 2018

Provinsi	Pemeriksaan Skrining Hipotiroid Kongenital					N Tertimbang
	Periksa		Tidak Periksa (%)	Tidak Berlaku (%)	Tidak tahu (%)	
	%	95% CI				
Aceh	3,2	2,4 - 4,2	61,2	6,8	28,8	2.199
Sumatera Utara	1,7	1,2 - 2,5	65,5	2,7	30,1	5.736
Sumatera Barat	5,5	4,7 - 6,5	71,3	0,8	22,4	2.158
Riau	1,9	1,3 - 2,6	77,7	0,9	19,5	2.768
Jambi	4,4	3,3 - 5,9	63,2	2,3	30,1	1.256
Sumatera Selatan	4,0	3,1 - 5,2	65,5	3,1	27,5	2.961
Bengkulu	3,3	2,3 - 4,5	61,0	6,5	29,3	705
Lampung	2,5	1,9 - 3,3	68,7	3,3	25,5	3.051
Bangka Belitung	2,7	1,7 - 4,1	69,3	5,4	22,7	522
Kepulauan Riau	5,1	3,4 - 7,4	59,5	1,7	33,7	861
DKI Jakarta	12,3	10,0 - 14,9	54,4	0,8	32,5	3.559
Jawa Barat	5,4	4,7 - 6,3	61,5	2,9	30,2	16.883
Jawa Tengah	5,1	4,5 - 5,8	59,7	2,4	32,9	10.446
DI Yogyakarta	14,6	11,4 - 18,6	60,4	0,4	24,6	1.062
Jawa Timur	4,4	3,7 - 5,1	61,3	2,7	31,6	11.177
Banten	5,8	4,6 - 7,4	67,4	2,0	24,8	4.702
Bali	5,2	4,0 - 6,7	67,3	0,5	27,1	1.269
Nusa Tenggara Barat	3,4	2,4 - 4,7	70,1	3,2	23,3	1.965
Nusa Tenggara Timur	4,3	3,5 - 5,3	65,7	2,9	27,1	2.402
Kalimantan Barat	3,4	2,5 - 4,6	64,7	8,0	23,9	1.841
Kalimantan Tengah	2,6	1,9 - 3,6	62,8	6,8	27,8	947
Kalimantan Selatan	3,1	2,1 - 4,7	69,5	2,7	24,7	1.546
Kalimantan Timur	2,3	1,5 - 3,4	66,4	4,2	27,1	1.344
Kalimantan Utara	3,7	2,3 - 5,8	69,1	1,2	26,0	306
Sulawesi Utara	7,5	6,0 - 9,4	56,8	2,7	33,0	807
Sulawesi Tengah	3,3	2,5 - 4,4	71,8	1,7	23,2	1.138
Sulawesi Selatan	3,4	2,6 - 4,3	56,9	2,2	37,6	3.158
Sulawesi Tenggara	2,1	1,4 - 3,1	75,6	2,5	19,8	1.121
Gorontalo	5,1	3,7 – 7,0	56,3	5,3	33,3	441
Sulawesi Barat	2,4	1,6 - 3,5	52,6	4,6	40,3	579
Maluku	3,8	2,5 - 5,7	73,9	5,5	16,7	727
Maluku Utara	5,0	3,6 - 6,9	66,0	3,0	26,1	514
Papua Barat	2,4	1,4 - 4,2	64,1	7,7	25,7	391
Papua	2,3	1,6 - 3,2	55,1	9,2	33,5	1.134
**INDONESIA**	**4,6**	**4,4 - 4,9**	**63,4**	**2,9**	**29,1**	**91.673**

Tabel 15.2.12
Proporsi Pemeriksaan Skrining Hipotiroid Kongenital (SHK) saat Lahir
pada Anak Umur 0-59 Bulan menurut Karakteristik, Indonesia 2018

Karakteristik	Pemeriksaan Skrining Hipotiroid Kongenital					N Tertimbang
	Periksa		Tidak Periksa (%)	Tidak Berlaku (%)	Tidak tahu (%)	
	%	95% CI				
**Kelompok umur (bulan)**						
0-5	5,1	4,4 - 5,9	64,2	3,2	27,5	8.660
6-11	6,3	5,5 - 7,2	63,7	3,1	26,9	9.601
12-23	5,0	4,5 - 5,5	64,5	2,8	27,7	17.984
24-35	4,4	3,9 - 4,9	63,4	2,8	29,4	18.749
36-47	4,2	3,7 - 4,7	62,9	2,8	30,2	18.414
48-59	3,8	3,4 - 4,3	62,4	2,9	30,9	18.265
**Jenis kelamin**						
Laki-laki	4,6	4,3 - 4,9	63,0	3,1	29,4	46.776
Perempuan	4,7	4,3 – 5,0	63,9	2,7	28,8	44.897
**Pendidikan KRT**						
Tidak/belum pernah sekolah	2,8	2,2 - 3,7	62,5	3,5	31,2	3.680
Tidak tamat SD/MI	3,7	3,2 - 4,4	64,5	3,5	28,2	10.878
Tamat SD/MI	3,9	3,5 - 4,4	64,3	3,1	28,7	23.557
Tamat SLTP/MTS	4,4	3,9 - 4,9	63,5	2,7	29,4	17.351
Tamat SLTA/MA	5,1	4,6 - 5,6	62,8	2,6	29,6	27.452
Tamat D1/D2/D3/PT	7,4	6,5 - 8,4	62,0	2,6	28,0	8.755
**Pekerjaan KRT**						
Tidak bekerja	6,0	5,0- 7,1	61,4	2,8	29,8	6.232
Sekolah	6,6	2,8 - 15,1	64,0	1,2	28,2	173
PNS/TNI/Polri/BUMN/BUMD	6,2	5,2 - 7,3	62,0	3,1	28,7	4.451
Pegawai swasta	6,0	5,2 - 6,8	62,0	2,0	30,0	13.300
Wiraswasta	5,0	4,5 - 5,6	62,7	2,6	29,7	20.851
Petani/buruh tani	3,4	3,2 - 3,8	64,7	3,7	28,1	23.627
Nelayan	2,8	2,1 - 3,8	64,9	3,5	28,7	1.918
Buruh/sopir/pembantu ruta	4,2	3,7 - 4,8	64,1	2,8	28,9	16.425
Lainnya	3,9	3,2 - 4,8	65,2	2,5	28,4	4.697
**Tempat tinggal**						
Perkotaan	5,7	5,3 - 6,1	62,0	2,4	29,8	49.540
Perdesaan	3,3	3,1 - 3,6	65,0	3,4	28,2	42.133

15.3 Kepemilikan dan Pemanfaatan Buku KIA

Kepemilikan buku KIA

Kepemilikan buku KIA pada anak umur 0-59 bulan dihitung menggunakan formula:

$$Proporsi\ Kepemilikan\ Buku\ KIA = \frac{Anak\ umur\ 0-59\ bulan\ yang\ memiliki\ buku\ KIA}{Jumlah\ anak\ umur\ 0-59\ bulan}$$

Pemanfaatan Buku KIA

Pemanfaatan buku KIA dinilai berdasarkan observasi isi pencatatan pada buku KIA. Kelengkapan pencatatan buku KIA terdiri dari lima komponen yaitu:

1. Catatan riwayat bayi baru lahir. Catatan riwayat bayi baru lahir terisi lengkap jika setiap isian dalam halaman catatan kesehatan ‘Bayi Saat Lahir’ terisi lengkap, kecuali bagian isian ‘Keterangan tambahan’ (Hal 24 pada buku KIA baru/model 2015 atau Hal 18 pada buku KIA lama).
2. Pemantauan pertumbuhan.Catatan pemantauan pertumbuhan terisi lengkap jika berat badan tertulis dan titik di plot pada KMS sesuai waktu [NAMA] melakukan pengukuran/penimbangan. (Antara hal 64-65 pada buku KIA baru/model 2015 atau Hal 49-56 pada buku KIA lama).
3. Pemantauan perkembangan. Catatan pemantauan perkembangan terisi lengkap jika pada kolom KPSP terdapat catatan hasil pengukuran berupa hasil penilaian ‘S=Sesuai, M=Meragukan, P=Penyimpangan’ (Hal 66 Buku KIA baru/model 2015) atau pada kolom ‘anjuran pemberian rangsangan oleh petugas’ terisi catatan(Hal 50 Buku KIA lama/model <2015).
4. Riwayat imunisasi. Catatan riwayat imunisasi Terisi lengkap jika semua catatan imunisasi terisi sesuai dengan jenis imunisasi yang telah diterima sesuai umur. (Hal 38 pada buku KIA baru/model 2015 atau Hal 53-56 pada buku KIA lama)
5. Pemeriksaan pada saat sakit. Catatan pemeriksaan pada saat sakit terisi lengkap jika Terisi lengkap jika terisi pada setiap kolom isian (bukan baris). (Hal 80-83 pada buku KIA baru/model 2015).

Formula:

$$Proporsi\ pemanfaatan\ buku\ KIA = \frac{Anak\ umur\ 0-59\ bulan\ yang\ mempunyai\ catatan\ riwayat\ dalam\ buku\ KIA}{Jumlah\ anak\ umur\ 0-59\ bulan\ yang\ dapat\ menunjukkan\ buku\ KIA}$$

Tabel 15.3.1
Proporsi Kepemilikan Buku KIA pada Anak Umur 0-59 Bulan menurut Provinsi, Riskesdas 2018

Provinsi	Kepemilikan Buku KIA (%)				N Tertimbang
	Memiliki		Tidak Memiliki		
	dapat menunjukkan	tidak dapat menunjukkan	Pernah memiliki	Tidak pernah memiliki	
Aceh	30,3	20,6	22,9	26,3	2.250
Sumatera Utara	24,1	13,3	34,8	27,7	5.895
Sumatera Barat	47,1	15,7	30,2	6,9	2.179
Riau	27,4	20,6	26,8	25,2	2.813
Jambi	47,2	18,8	26,4	7,5	1.279
Sumatera Selatan	29,4	17,0	35,6	17,9	3.079
Bengkulu	48,4	16,4	25,7	9,5	716
Lampung	56,6	15,9	22,0	5,6	3.094
Bangka Belitung	62,0	10,0	22,3	5,7	527
Kepulauan Riau	39,1	14,6	16,0	30,3	872
DKI Jakarta	58,5	13,3	17,9	10,3	3.582
Jawa Barat	47,5	16,0	23,7	12,9	17.228
Jawa Tengah	68,4	13,0	16,6	2,0	10.551
DI Yogyakarta	76,4	14,3	6,9	2,4	1.069
Jawa Timur	64,3	20,9	11,4	3,4	11.272
Banten	39,6	13,8	29,6	16,9	4.813
Bali	59,8	8,2	18,5	13,5	1.275
Nusa Tenggara Barat	59,0	13,8	22,5	4,7	1.985
Nusa Tenggara Timur	42,7	29,2	16,8	11,3	2.496
Kalimantan Barat	55,3	12,7	21,6	10,4	1.928
Kalimantan Tengah	42,9	13,3	30,8	13,0	1.000
Kalimantan Selatan	59,3	14,4	20,1	6,2	1.563
Kalimantan Timur	63,8	13,8	16,7	5,7	1.368
Kalimantan Utara	55,8	11,9	22,8	9,4	309
Sulawesi Utara	45,6	16,9	30,8	6,8	821
Sulawesi Tengah	48,6	15,7	23,9	11,9	1.155
Sulawesi Selatan	50,4	16,5	24,4	8,7	3.269
Sulawesi Tenggara	34,8	15,8	31,2	18,2	1.169
Gorontalo	61,0	14,5	21,1	3,5	445
Sulawesi Barat	50,6	20,9	22,8	5,7	584
Maluku	32,1	13,5	25,1	29,3	779
Maluku Utara	35,6	15,7	32,3	16,4	536
Papua Barat	32,4	15,3	28,0	24,3	415
Papua	22,3	18,8	24,4	34,5	1.302
**INDONESIA**	**49,7**	**16,2**	**22,4**	**11,7**	**93.620**

Tabel 15.3.2
Proporsi Kepemilikan Buku KIA pada Anak Umur 0-59 Bulan menurut Karakteristik, Riskesdas 2018

Karakteristik	Kepemilikan Buku KIA (%)				N Tertimbang
	Memiliki		Tidak Memiliki		
	dapat menunjukkan	tidak dapat menunjukkan	Pernah memiliki	Tidak pernah memiliki	
**Kelompok umur (bulan)**					
0-5	73,8	11,8	3,2	11,1	8.908
6-11	70,9	12,6	6,8	9,8	9.757
12-23	61,5	15,3	12,8	10,4	18.333
24-35	46,7	17,0	23,2	13,0	19.112
36-47	38,4	17,4	31,9	12,3	18.821
48-59	30,1	18,9	38,6	12,4	18.688
**Jenis kelamin**					
Laki-laki	49,9	16,0	22,4	11,7	47.764
Perempuan	49,5	16,4	22,4	11,7	45.856
**Pendidikan KRT**					
Tidak/belum pernah sekolah	48,5	15,9	20,2	15,5	3.855
Tidak tamat SD/MI	51,9	15,4	20,8	11,9	11.148
Tamat SD/MI	52,6	16,0	20,7	10,7	24.131
Tamat SLTP/MTS	52,1	16,1	21,8	10,1	17.658
Tamat SLTA/MA	47,9	16,3	24,3	11,4	27.939
Tamat D1/D2/D3/PT	40,2	17,7	25,2	16,9	8.888
**Pekerjaan KRT**					
Tidak bekerja	51,1	15,5	22,0	11,4	6.378
Sekolah	42,8	12,8	30,1	14,3	184
PNS/TNI/Polri/BUMN/BUMD	41,1	18,1	27,1	13,7	4.542
Pegawai swasta	49,5	15,6	22,3	12,6	13.498
Wiraswasta	48,8	15,8	24,3	11,1	21.185
Petani/buruh tani	49,8	18,1	19,5	12,6	24.382
Nelayan	44,4	15,7	23,7	16,2	1.966
Buruh/sopir/pembantu ruta	53,9	14,1	22,5	9,4	16.707
Lainnya	48,0	16,1	23,6	12,3	4.778
**Tempat tinggal**					
Perkotaan	49,4	15,0	23,7	11,9	50.361
Perdesaan	50,0	17,6	20,8	11,5	43.259

Tabel 15.3.3
Proporsi Pencatatan Buku KIA berdasarkan Isi pada Anak Umur 0-59 Bulan menurut Provinsi, Riskesdas 2018

Provinsi	Pencatatan Buku KIA (%)				N Tertimbang
	Pemantauan pertumbuhan	Pemantauan perkembangan	Riwayat imunisasi	Pemeriksaan kesehatan pada saat sakit	
Aceh	37,0	34,9	36,6	14,9	625
Sumatera Utara	44,5	40,5	54,3	14,6	1.305
Sumatera Barat	48,0	41,2	56,1	19,4	942
Riau	48,4	41,4	57,9	12,3	706
Jambi	58,8	53,6	76,2	33,3	554
Sumatera Selatan	47,7	44,6	61,7	19,4	830
Bengkulu	56,1	49,7	72,0	25,3	318
Lampung	59,2	49,0	71,6	19,1	1.606
Bangka Belitung	63,7	53,6	74,6	17,5	299
Kepulauan Riau	58,4	53,4	75,9	22,2	313
DKI Jakarta	59,8	48,1	81,2	27,5	1.921
Jawa Barat	55,6	46,3	63,7	20,4	7.505
Jawa Tengah	63,4	46,8	77,4	24,3	6.620
DI Yogyakarta	60,3	44,3	89,7	23,6	748
Jawa Timur	65,0	49,1	80,5	26,3	6.644
Banten	37,1	29,9	55,0	14,6	1.749
Bali	58,9	32,5	81,0	18,4	700
Nusa Tenggara Barat	68,1	50,4	69,0	29,5	1.074
Nusa Tenggara Timur	56,6	47,9	60,9	25,0	977
Kalimantan Barat	61,1	48,8	68,8	24,4	977
Kalimantan Tengah	51,1	39,2	64,4	16,1	394
Kalimantan Selatan	63,8	51,3	74,8	18,2	851
Kalimantan Timur	50,8	41,0	72,8	18,6	800
Kalimantan Utara	57,1	53,6	73,8	17,6	158
Sulawesi Utara	54,2	50,2	68,3	17,0	343
Sulawesi Tengah	57,4	39,3	56,6	14,3	514
Sulawesi Selatan	50,5	41,7	64,9	16,1	1.511
Sulawesi Tenggara	55,1	48,8	55,6	16,4	373
Gorontalo	46,1	41,3	56,4	18,2	249
Sulawesi Barat	58,0	49,3	63,1	26,7	271
Maluku	44,7	45,5	51,2	16,7	230
Maluku Utara	44,3	37,4	50,7	11,5	175
Papua Barat	50,0	46,2	64,3	18,6	123
Papua	52,8	44,3	60,5	20,7	266
**INDONESIA**	**57,2**	**45,6**	**69,7**	**21,7**	**42.671**

Tabel 15.3.4
Proporsi Pencatatan Buku KIA berdasarkan Isi pada Anak Umur 0-59 Bulan
menurut Karakteristik, Riskesdas 2018

Karakteristik	Pencatatan Buku KIA (%)				N Tertimbang
	Pemantauan pertumbuhan	Pemantauan perkembangan	Riwayat imunisasi	Pemeriksaan kesehatan pada saat sakit	
**Kelompok umur (bulan)**					
0-5	51,0	40,6	65,6	21,3	6.033
6-11	58,3	46,4	73,6	21,4	6.342
12-23	59,2	46,9	72,1	23,1	10.335
24-35	57,0	46,1	69,6	20,6	8.182
36-47	57,9	46,7	68,5	22,3	6.624
48-59	58,4	45,9	66,4	20,9	5.156
**Jenis kelamin**					
Laki-laki	57,1	45,2	69,7	21,8	21.845
Perempuan	57,4	46,0	69,7	21,6	20.826
**Pendidikan KRT**					
Tidak/belum pernah sekolah	55,2	43,4	66,5	20,5	1.713
Tidak tamat SD/MI	55,4	44,3	66,3	21,8	5.309
Tamat SD/MI	57,5	45,7	67,3	21,0	11.645
Tamat SLTP/MTS	56,6	45,4	69,3	21,8	8.440
Tamat SLTA/MA	58,6	46,3	73,3	21,7	12.285
Tamat D1/D2/D3/PT	56,5	46,5	72,3	24,8	3.279
**Pekerjaan KRT**					
Tidak bekerja	57,4	46,2	70,3	22,3	2.987
Sekolah	54,9	46,1	64,4	20,3	72
PNS/TNI/Polri/BUMN/BUMD	56,3	47,2	73,7	23,3	1.712
Pegawai swasta	58,3	46,7	75,5	22,7	6.133
Wiraswasta	56,8	45,1	70,7	22,3	9.475
Petani/buruh tani	56,8	45,2	67,0	21,3	11.127
Nelayan	51,7	42,1	56,0	19,2	800
Buruh/sopir/pembantu ruta	57,3	44,8	68,4	20,8	8.261
Lainnya	60,0	49,8	68,1	21,2	2.104
**Tempat tinggal**					
Perkotaan	57,1	45,6	72,2	22,5	22.822
Perdesaan	57,3	45,6	66,8	20,9	19.849

15.4 Imunisasi dan Vitamin A

A. Imunisasi

Program imunisasi dilaksanakan di Indonesia sejak tahun 1956. Kementerian Kesehatan melaksanakan Program Pengembangan Imunisasi (PPI) pada anak dalam upaya menurunkan kejadian penyakit yang dapat dicegah dengan imunisasi (PD3I), yaitu tuberkulosis, difteri, pertusis, campak, polio, tetanus serta hepatitis B.

Informasi cakupan imunisasi pada Riskesdas 2018 ditanyakan kepada ibu yang mempunyai balita umur 0-59 bulan. Informasi imunisasi dikumpulkan berdasarkan dua sumber informasi, yaitu wawancara kepada ibu balita atau anggota rumah tangga yang mengetahui, serta catatan dalam KMS atau catatan dalam buku kesehatan anak lainnya. Apabila salah satu dari kedua sumber tersebut menyatakan bahwa anak sudah diimunisasi, disimpulkan bahwa anak tersebut sudah diimunisasi untuk jenis yang ditanyakan.

Tabel 15.4.1
Proporsi Jenis Imunisasi Dasar pada Anak Umur 12-23 Bulan menurut Provinsi, Riskesdas 2018

Provinsi	Jenis imunisasi dasar (%)							N Ter-timbang
	HB-0	BCG	DPT-HB/DPT-HB-Hib 1	DPT-HB/DPT-HB-Hib 2	DPT-HB/DPT-HB-Hib 3	Polio 1-4 atau IPV 1-3	Campak	
Aceh	53,9	50,7	26,9	24,9	22,0	25,9	37,9	464
Sumatera Utara	62,7	75,8	44,3	43,1	39,2	47,1	65,4	1.068
Sumatera Barat	76,2	81,2	53,7	51,4	45,4	50,5	63,9	419
Riau	74,8	79,6	40,7	39,3	36,7	45,8	68,6	528
Jambi	84,7	86,3	63,7	62,9	60,5	69,0	80,3	268
Sumatera Selatan	79,7	85,8	54,1	53,3	51,1	59,7	78,4	607
Bengkulu	81,1	88,1	68,2	68,4	67,6	72,6	85,1	137
Lampung	84,9	90,7	72,0	71,2	70,1	79,4	83,0	602
Bangka Belitung	88,9	87,7	73,5	72,4	72,0	80,4	81,9	103
Kepulauan Riau	92,7	89,6	79,7	76,0	74,9	77,9	80,0	185
DKI Jakarta	97,2	97,4	77,9	76,9	72,9	78,4	82,1	688
Jawa Barat	86,7	89,7	66,7	65,3	61,9	67,8	78,1	3.184
Jawa Tengah	94,3	94,8	80,3	79,2	77,1	82,6	86,6	2.003
DI Yogyakarta	97,8	99,2	91,0	91,0	91,0	86,9	96,9	228
Jawa Timur	92,1	92,4	75,2	73,7	72,0	76,8	82,8	2.293
Banten	76,6	79,7	58,4	54,8	52,6	57,0	63,8	875
Bali	99,5	98,0	90,8	90,8	90,6	96,0	94,4	237
Nusa Tenggara Barat	90,7	94,0	71,0	71,1	69,2	78,2	87,9	393
Nusa Tenggara Timur	78,6	86,8	49,7	48,5	46,8	65,5	82,5	493
Kalimantan Barat	71,2	77,6	68,2	66,5	61,9	60,3	68,1	388
Kalimantan Tengah	68,4	76,0	60,1	57,9	54,2	56,4	67,0	198
Kalimantan Selatan	86,8	88,7	73,2	70,9	68,6	76,1	81,0	338
Kalimantan Timur	87,1	92,2	75,0	72,8	71,6	80,9	84,1	302
Kalimantan Utara	90,3	93,9	78,2	77,2	75,2	84,8	85,4	63
Sulawesi Utara	81,2	91,9	64,0	62,7	61,8	71,3	84,1	154
Sulawesi Tengah	74,3	80,7	60,0	56,2	53,3	61,2	75,7	250
Sulawesi Selatan	86,5	86,9	67,2	65,8	63,7	69,8	79,8	660
Sulawesi Tenggara	68,1	84,5	54,6	53,6	49,8	59,8	80,2	223
Gorontalo	87,1	91,4	69,2	66,9	66,3	72,8	81,3	93
Sulawesi Barat	78,7	82,6	65,6	63,9	56,9	64,2	75,3	124
Maluku	52,1	75,8	46,3	45,4	42,8	51,9	68,9	165
Maluku Utara	68,9	75,8	54,6	52,0	48,8	50,2	66,0	106
Papua Barat	68,7	75,6	55,0	52,4	50,7	55,3	73,0	83
Papua	42,0	62,5	37,0	36,0	34,0	39,7	60,7	245
**INDONESIA**	**83,1**	**86,9**	**65,4**	**63,9**	**61,3**	**67,6**	**77,3**	**18.165**

Tabel 15.4.2
Proporsi Jenis Imunisasi Dasar pada Anak Umur 12-23 Bulan menurut Karakteristik, Riskesdas 2018

Karakteristik	Jenis imunisasi dasar (%)							N Ter-timbang
	HB-0	BCG	DPT-HB/DPT-HB-Hib 1	DPT-HB/DPT-HB-Hib 2	DPT-HB/DPT-HB-Hib 3	Polio 1-4 atau IPV 1-3	Campak	
**Jenis kelamin**								
Laki-laki	83,4	87,0	65,2	63,9	60,8	66,9	77,0	9.188
Perempuan	82,7	86,7	65,5	63,9	61,9	68,3	77,6	8.977
**Pendidikan KRT**								
Tidak/belum pernah sekolah	74,3	75,9	59,5	57,9	53,7	57,6	67,5	760
Tidak tamat SD/MI	76,5	81,0	60,8	59,1	57,4	62,1	71,9	2.112
Tamat SD/MI	80,2	84,7	65,5	64,0	61,2	66,6	74,9	4.635
Tamat SLTP/MTS	84,5	89,1	66,3	64,9	62,0	69,0	79,2	3.412
Tamat SLTA/MA	86,5	89,9	67,4	66,0	63,6	71,1	79,8	5.497
Tamat D1/D2/D3/PT	88,5	90,3	64,8	63,4	61,3	67,4	82,7	1.750
**Pekerjaan KRT**								
Tidak bekerja	81,8	83,4	65,8	63,7	60,7	67,4	72,6	1.309
Sekolah	67,4	64,9	47,5	44,8	44,8	52,5	57,9	30*
PNS/TNI/Polri/BUMN/BUMD	89,5	90,1	65,5	65,3	63,0	73,4	86,8	918
Pegawai swasta	91,7	93,5	70,7	69,1	67,0	74,0	83,4	2.695
Wiraswasta	84,2	87,7	65,7	64,2	61,5	67,9	76,2	4.042
Petani/buruh tani	76,6	82,8	61,3	59,8	57,5	63,6	75,1	4.787
Nelayan	70,2	74,2	51,2	47,8	45,5	52,9	62,3	367
Buruh/sopir/pembantu ruta	84,4	88,2	68,7	67,6	64,5	68,5	77,3	3.160
Lainnya	82,6	87,9	63,3	61,9	59,1	66,2	80,3	858
**Tempat tinggal**								
Perkotaan	88,4	89,9	68,7	67,1	64,5	70,6	78,9	9.723
Perdesaan	76,9	83,4	61,5	60,2	57,7	64,1	75,5	8.442

*N Tertimbang <50

B. Imunisasi Dasar Lengkap

Menurut Peraturan Menteri Kesehatan Nomor 12 Tahun 2017 tentang penyelenggaraan imunisasi, seorang anak dinyatakan telah memperoleh imunisasi dasar lengkap apabila telah mendapatkan satu kali imunisasi HB-0, satu kali imunisasi BCG, tiga kali imunisasi DPT-HB/DPT-HB-HiB, empat kali imunisasi polio atau tiga kali imunisasi IPV, dan satu kali imunisasi campak (Kementerian Kesehatan, 2017).

Informasi imunisasi dasar lengkap diperoleh dari catatan imunisasi maupun pengakuan ibu atau orang yang paling mengetahui riwayat imunisasi baduta.

$$Proporsi\ imunisasi\ dasar\ lengkap = \frac{Anak\ umur\ 12-23\ bulan\ yang\ menerima\ imunisasi\ dasar\ lengkap}{Jumlah\ anak\ umur\ 12-23\ bulan}$$

Tabel 15.4.3
Proporsi Imunisasi Dasar Lengkap pada Anak Umur 12-23 Bulan menurut Provinsi, Riskesdas 2018

Provinsi	Kelengkapan Imunisasi Dasar				N Tertimbang
	Lengkap		Tidak lengkap (%)	Tidak imunisasi (%)	
	%	95% CI			
Aceh	19,5	16,8 - 22,6	39,6	40,9	464
Sumatera Utara	32,7	29,0 - 36,5	48,5	18,8	1.068
Sumatera Barat	38,7	34,4 - 43,2	48,1	13,2	419
Riau	34,4	29,7 - 39,4	51,3	14,3	528
Jambi	62,6	56,9 - 67,9	26,8	10,6	268
Sumatera Selatan	48,3	43,1 - 53,5	42,4	9,3	607
Bengkulu	62,6	56,0 - 68,7	28,1	9,3	137
Lampung	67,3	62,2 - 72,0	25,8	6,9	602
Bangka Belitung	75,2	66,9 - 81,9	16,7	8,1	103
Kepulauan Riau	71,2	59,8 - 80,5	23,3	5,4	185
DKI Jakarta	68,0	60,9 - 74,4	30,4	1,5	688
Jawa Barat	58,3	54,3 - 62,1	35,3	6,5	3.184
Jawa Tengah	75,0	72,1 - 77,8	22,3	2,7	2.003
DI Yogyakarta	83,7	76,0 - 89,3	16,3	0,0	228
Jawa Timur	69,2	66,2 - 72,0	26,3	4,6	2.293
Banten	47,0	40,8 - 53,3	37,4	15,6	875
Bali	92,1	86,8 - 95,3	7,4	0,5	237
Nusa Tenggara Barat	70,8	65,4 - 75,6	24,8	4,5	393
Nusa Tenggara Timur	51,6	47,3 - 55,8	40,5	7,9	493
Kalimantan Barat	48,0	42,8 - 53,3	35,4	16,6	388
Kalimantan Tengah	47,5	41,2 - 53,8	34,2	18,3	198
Kalimantan Selatan	68,7	63,4 - 73,6	24,3	6,9	338
Kalimantan Timur	73,6	66,8 - 79,5	19,9	6,4	302
Kalimantan Utara	73,3	64,8 - 80,4	22,2	4,5	63
Sulawesi Utara	56,9	50,2 - 63,4	37,1	6,0	154
Sulawesi Tengah	47,9	42,3 - 53,5	39,9	12,3	250
Sulawesi Selatan	60,8	56,4 - 65,1	31,7	7,4	660
Sulawesi Tenggara	45,6	39,9 - 51,5	43,0	11,4	223
Gorontalo	61,6	53,3 - 69,4	33,7	4,7	93
Sulawesi Barat	50,2	41,8 - 58,5	39,6	10,2	124
Maluku	33,1	27,4 - 39,4	49,5	17,4	165
Maluku Utara	38,1	31,8 - 44,8	43,8	18,1	106
Papua Barat	47,6	39,2 - 56,1	34,1	18,3	83
Papua	29,2	24,2 - 34,7	37,6	33,2	245
**INDONESIA**	**57,9**	**56,9 - 59,0**	**32,9**	**9,2**	**18.165**

Tabel 15.4.4
Proporsi Imunisasi Dasar Lengkap pada Anak Umur 12-23 Bulan menurut karakteristik, Riskesdas 2018

Karakteristik	Kelengkapan imunisasi dasar				N Tertimbang
	Lengkap		Tidak lengkap (%)	Tidak imunisasi (%)	
	%	95% CI			
**Jenis kelamin**					
Laki-laki	57,2	55,7 - 58,8	33,8	9,0	9.188
Perempuan	58,7	57,1 - 60,2	31,9	9,4	8.977
**Pendidikan KRT**					
Tidak/belum pernah sekolah	50,3	45,5 - 55,2	31,5	18,2	760
Tidak tamat SD/MI	52,5	49,4 - 55,6	33,7	13,8	2.112
Tamat SD/MI	55,5	53,5 - 57,5	34,1	10,4	4.635
Tamat SLTP/MTS	58,5	56,0 - 61,0	33,9	7,6	3.412
Tamat SLTA/MA	61,9	59,8 - 64,1	31,2	6,9	5.497
Tamat D1/D2/D3/PT	60,6	57,1 - 64,0	32,5	6,9	1.750
**Pekerjaan KRT**					
Tidak bekerja	56,5	52,4 - 60,6	32,8	10,7	1.309
Sekolah	45,0	24,7 - 67,0	33,2	21,8	30*
PNS/TNI/Polri/BUMN/BUMD	68,5	64,4 - 72,2	25,4	6,1	918
Pegawai swasta	66,4	63,0 - 69,6	29,2	4,4	2.695
Wiraswasta	57,0	54,7 - 59,3	34,3	8,6	4.042
Petani/buruh tani	53,4	51,7 - 55,2	33,7	12,8	4.787
Nelayan	42,0	36,3 - 47,9	39,8	18,3	367
Buruh/sopir/pembantu ruta	58,9	55,9 - 61,9	32,9	8,2	3.160
Lainnya	55,4	50,5 - 60,3	37,5	7,1	858
**Tempat tinggal**					
Perkotaan	61,5	59,8 - 63,2	31,9	6,6	9.723
Perdesaan	53,8	52,5 - 55,1	34,0	12,2	8.442

*N Tertimbang <50

C. Imunisasi Lanjutan

Imunisasi lanjutan merupakan kegiatan yang bertujuan untuk menjamin terjaganya tingkat imunitas terhadap penyakit tertentu pada anak umur di bawah dua tahun (Baduta). Seorang anak dinyatakan telah mendapatkan imunisasi lanjutan apabila telah lengkap imunisasi dasar dan mendapatkan imunisasi DPT-HB-HiB dan campak lanjutan dalam rentang usia 18-24 bulan. Interval pemberian imunisasi DPT-HB-HiB minimal 12 bulan dari imunisasi DPT-HB-HiB 3 dan pemberian imuniasi campak minimal 6 bulan dari imunisasi campak dosis pertama.

Informasi imunisasi lanjutan diperoleh dari anak umur 24-35 bulan yang menerima imunisasi satu dosis vaksin DPT-HB-HiB lanjutan dan satu dosis vaksin campak lanjutan, baik menurut catatan imunisasi maupun pengakuan.

$$Proporsi\ imunisasi\ lanjutan = \frac{Jumlah\ anak\ umur\ 24-35\ bulan\ yang\ menerima\ imunisasi\ lanjutan}{Jumlah\ anak\ umur\ 24-35\ bulan}$$

Tabel 15.4.5
Proporsi Imunisasi Lanjutan pada Anak Umur 24-35 Bulan menurut Provinsi, Riskesdas 2018

Provinsi	Jenis imunisasi lanjutan (%)		N Tertimbang
	DPT/HB/Hib lanjutan	Campak lanjutan	
Aceh	16,7	19,8	467
Sumatera Utara	20,4	24,0	1.227
Sumatera Barat	22,1	23,5	429
Riau	23,3	25,8	607
Jambi	40,6	40,0	256
Sumatera Selatan	26,5	27,9	622
Bengkulu	49,5	50,3	134
Lampung	39,1	39,0	651
Bangka Belitung	43,4	38,5	99
Kepulauan Riau	34,8	35,9	170
DKI Jakarta	51,8	45,5	705
Jawa Barat	40,6	39,7	3.639
Jawa Tengah	48,1	44,8	2.144
DI Yogyakarta	74,6	73,0	206
Jawa Timur	47,3	43,9	2.170
Banten	29,2	29,0	994
Bali	76,0	59,3	256
Nusa Tenggara Barat	46,7	44,7	402
Nusa Tenggara Timur	36,6	35,7	506
Kalimantan Barat	39,3	36,4	355
Kalimantan Tengah	28,2	32,7	186
Kalimantan Selatan	46,6	44,3	316
Kalimantan Timur	52,8	45,9	242
Kalimantan Utara	44,6	45,5	60
Sulawesi Utara	51,5	56,0	172
Sulawesi Tengah	38,1	38,0	233
Sulawesi Selatan	47,6	44,8	658
Sulawesi Tenggara	29,4	34,0	229
Gorontalo	47,9	48,1	85
Sulawesi Barat	33,5	39,5	120
Maluku	23,3	26,1	151
Maluku Utara	37,3	37,4	124
Papua Barat	28,7	33,6	83
Papua	25,5	25,4	289
**INDONESIA**	**39,4**	**38,3**	**18.986**

Tabel 15.4.6
Proporsi Imunisasi Lanjutan pada Anak Umur 24-35 Bulan menurut Karakteristik, Riskesdas 2018

Karakteristik	Jenis imunisasi lanjutan (%)		N Tertimbang
	DPT/HB/Hib lanjutan	Campak lanjutan	
**Jenis kelamin**			
Laki-laki	38,4	37,4	9.655
Perempuan	40,5	39,2	9.331
**Pendidikan KRT**			
Tidak/belum pernah sekolah	34,5	31,0	789
Tidak tamat SD/MI	35,2	35,2	2.349
Tamat SD/MI	39,0	37,4	4.929
Tamat SLTP/MTS	40,3	40,0	3.510
Tamat SLTA/MA	39,9	39,7	5.649
Tamat D1/D2/D3/PT	44,9	40,2	1.761
**Pekerjaan KRT**			
Tidak bekerja	42,0	38,8	1.322
Sekolah	40,9	37,4	32*
PNS/TNI/Polri/BUMN/BUMD	45,4	41,4	972
Pegawai swasta	42,5	39,3	2.685
Wiraswasta	38,7	38,5	4.274
Petani/buruh tani	36,5	37,0	4.949
Nelayan	26,5	29,4	410
Buruh/sopir/pembantu ruta	40,3	38,5	3.425
Lainnya	41,5	40,6	918
**Tempat tinggal**			
Perkotaan	41,4	39,4	10.288
Perdesaan	37,0	37,0	8.698

*N Tertimbang <50

D. Kejadian Ikutan Pasca Imunisasi

Menurut Komite Nasional Pengkajian dan Penanggulangan KIPI (KN PP KIPI), yang dimaksud dengan Kejadian Ikutan Pasca Imunisasi (KIPI) adalah semua kejadian sakit dan/atau kematian yang terjadi dalam masa 1 bulan setelah imunisasi yang diduga berhubungan dengan imunisasi. Dalam Riskesdas 2018, seorang anak umur 12-23 bulan dinyatakan pernah mengalami KIPI apabila dalam periode 1 bulan setelah imunisasi pernah mengalami demam tinggi, bernanah/abses dan/atau kejang. Kondisi bernanah/abses setelah imunisasi BCG tidak termasuk dalam KIPI kecuali bernanah/abses yang berkepanjangan (misal >3 minggu).

Formula:

$$Proporsi\ Kejadian\ Ikutan\ Pasca\ Imunisasi = \frac{Jumlah\ anak\ umur\ 12-23\ bulan\ yang\ mengalami\ KIPI}{Jumlah\ anak\ umur\ 12-23\ bulan\ yang\ pernah\ diimunisasi}$$

$$Proporsi\ Jenis\ Keluhan\ Kejadian\ Ikutan\ Pasca\ Imunisasi = \frac{Jumlah\ anak\ umur\ 12-23\ bulan\ berdasarkan\ jenis\ keluhan\ KIPI}{Jumlah\ anak\ umur\ 12-23\ bulan\ yang\ pernah\ diimunisasi}$$

Tabel 15.4.7
Proporsi Kejadian Ikutan Pasca Imunisasi (KIPI) dan Jenisnya pada Anak Umur 12-23 Bulan menurut Provinsi, Riskesdas 2018

Provinsi	Pernah mengalami KIPI	Keluhan kejadian ikutan pasca imunisasi (KIPI)				N Ter-timbang
		Demam tinggi	Bernanah/ abses	Kejang	Lainnya	
	%	%	%	%	%	
Aceh	45,1	40,4	19,6	0,5	0,0	194
Sumatera Utara	42,7	37,4	14,7	0,3	0,5	599
Sumatera Barat	37,8	27,9	19,8	0,2	0,6	141
Riau	40,3	31,0	13,6	2,2	0,6	337
Jambi	44,8	39,6	8,6	0,0	0,0	107
Sumatera Selatan	46,7	42,0	14,4	0,9	1,0	363
Bengkulu	47,0	44,3	8,8	0,8	0,0	48
Lampung	32,6	29,0	8,4	1,0	0,6	190
Bangka Belitung	39,8	22,2	19,3	1,0	0,0	25
Kepulauan Riau	38,2	31,2	15,3	2,9	0,0	112
DKI Jakarta	33,4	29,8	7,7	2,2	0,0	231
Jawa Barat	46,4	42,0	11,4	0,3	0,7	1.417
Jawa Tengah	30,5	27,2	6,8	0,8	1,7	475
DI Yogyakarta	40,8	28,5	4,3	0,0	8,0	42
Jawa Timur	36,6	34,1	4,5	1,3	0,8	638
Banten	44,5	42,5	9,9	0,8	1,4	388
Bali	32,7	23,6	16,0	0,0	0,0	66
Nusa Tenggara Barat	50,3	43,5	21,5	3,0	2,4	116
Nusa Tenggara Timur	49,5	43,7	15,9	1,3	1,0	253
Kalimantan Barat	40,5	37,1	10,5	0,8	0,0	119
Kalimantan Tengah	46,2	39,0	24,4	7,8	2,3	76
Kalimantan Selatan	39,8	34,8	16,3	0,3	0,2	106
Kalimantan Timur	36,3	30,3	2,8	0,6	4,9	82
Kalimantan Utara	38,4	27,5	19,7	0,6	0,0	22*
Sulawesi Utara	36,7	32,2	10,5	3,2	0,1	67
Sulawesi Tengah	44,9	34,5	21,2	2,4	0,5	95
Sulawesi Selatan	55,2	51,0	16,4	1,1	0,4	245
Sulawesi Tenggara	38,8	32,1	12,8	1,5	0,9	110
Gorontalo	58,4	55,7	12,4	0,0	0,0	32
Sulawesi Barat	38,4	34,2	12,4	0,7	2,1	46
Maluku	32,6	28,9	7,3	1,4	0,3	89
Maluku Utara	43,6	41,1	11,2	5,6	1,5	42
Papua Barat	43,3	40,8	17,1	1,5	1,6	42
Papua	56,3	54,5	17,3	1,3	0,2	117
**INDONESIA**	**42,3**	**37,5**	**12,0**	**1,0**	**0,8**	**7.033**

*N Tertimbang <50

Tabel 15.4.8
Proporsi Kejadian Ikutan Pasca Imunisasi (KIPI) dan Jenisnya pada Anak Umur 12-23 Bulan menurut Karakteristik, Riskesdas 2018

Karakteristik	Pernah mengalami KIPI	Keluhan kejadian ikutan pasca imunisasi (KIPI)				N Ter-timbang
		Demam tinggi	Bernanah/abses	Kejang	Lainnya	
	%	%	%	%	%	
**Jenis kelamin**						
Laki-laki	41,8	37,3	12,3	0,9	0,6	3.596
Perempuan	42,8	37,6	11,7	1,2	1,1	3.437
**Pendidikan KRT**						
Tidak/belum pernah sekolah	44,1	40,1	9,6	0,3	0,8	239
Tidak tamat SD/MI	41,5	37,8	12,8	0,8	0,8	749
Tamat SD/MI	43,3	38,8	12,6	0,9	0,9	1.618
Tamat SLTP/MTS	44,5	38,2	13,8	1,2	1,0	1.317
Tamat SLTA/MA	42,9	38,1	12,2	1,5	0,8	2.223
Tamat D1/D2/D3/PT	35,6	31,7	7,7	0,5	0,6	887
**Pekerjaan KRT**						
Tidak bekerja	45,0	41,5	13,1	0,9	0,0	494
Pegawai	38,6	34,3	10,3	1,7	1,0	1.567
Wiraswasta	40,9	37,7	9,3	0,5	0,8	1.654
Petani/nelayan/buruh	45,0	39,0	14,0	1,1	0,9	2.973
Lainnya	37,7	31,7	13,8	0,4	0,6	345
**Tempat tinggal**						
Perkotaan	40,9	36,6	11,3	1,1	1,1	3.879
Perdesaan	43,9	38,6	12,8	1,0	0,5	3.154

E. Vitamin A

Dalam PMK Nomor 43 Tahun 2016 tentang Standar Pelayanan Minimal Bidang Kesehatan tercantum bahwa setiap balita mendapatkan pelayanan standar diantaranya pemberian kapsul vitamin A sebanyak 2 kali setahun.

Pemberian kapsul vitamin A sebagaimana dimaksud dalam Peraturan Menteri Kesehatan Nomor 25 Tahun 2014 Tentang Upaya Kesehatan Anak, dilakukan satu kali untuk anak usia 6 (enam) bulan sampai 11 (sebelas) bulan dan 2 (dua) kali dalam setahun untuk anak usia 12 (dua belas) bulan.

Definisi anak usia 6-59 bulan yang menerima kapsul vitamin A sesuai standar yaitu satu kali untuk anak usia 6 (enam) bulan sampai 11 (sebelas) bulan dan 2 (dua) kali dalam setahun untuk anak usia 12 (dua belas) bulan.

$$Proporsi\ Pemberian\ Kapsul\ Vitamin\ A = \frac{Anak\ umur\ 6-59\ bulan\ yang\ menerima\ kapsul\ vitamin\ A\ sesuai\ standar\ dalam\ 12\ bulan\ terakhir}{Jumlah\ anak\ umur\ 6-59\ bulan}$$

Tabel 15.4.9
Proporsi Pemberian Kapsul Vitamin A dalam 12 Bulan Terakhir pada Anak Umur 6-59 Bulan menurut Provinsi, Riskesdas 2018

Provinsi	Pemberian kapsul Vitamin A (%)			N Tertimbang
	Sesuai standar	Tidak sesuai standar	Tidak pernah mendapat	
Aceh	43,2	29,9	26,9	2.030
Sumatera Utara	32,9	30,9	36,2	5.407
Sumatera Barat	52,4	29,4	18,2	1.994
Riau	50,0	26,3	23,7	2.573
Jambi	51,2	33,2	15,6	1.183
Sumatera Selatan	47,4	31,8	20,8	2.819
Bengkulu	54,1	29,0	16,8	658
Lampung	54,5	28,7	16,8	2.798
Bangka Belitung	53,5	29,8	16,7	482
Kepulauan Riau	49,1	34,9	16,0	815
DKI Jakarta	57,4	29,5	13,1	3.258
Jawa Barat	57,8	29,6	12,6	15.632
Jawa Tengah	65,1	24,7	10,3	9.532
DI Yogyakarta	62,5	29,9	7,7	985
Jawa Timur	61,6	25,8	12,6	10.251
Banten	45,4	30,3	24,3	4.374
Bali	59,8	26,5	13,7	1.169
Nusa Tenggara Barat	57,3	28,6	14,2	1.794
Nusa Tenggara Timur	52,1	27,1	20,8	2.256
Kalimantan Barat	44,7	33,0	22,3	1.754
Kalimantan Tengah	44,5	32,3	23,2	918
Kalimantan Selatan	56,5	29,2	14,2	1.407
Kalimantan Timur	55,7	30,3	14,1	1.249
Kalimantan Utara	53,2	31,6	15,2	279
Sulawesi Utara	46,4	34,8	18,8	745
Sulawesi Tengah	52,2	27,1	20,7	1.048
Sulawesi Selatan	48,3	31,0	20,6	2.942
Sulawesi Tenggara	47,8	30,7	21,6	1.032
Gorontalo	48,4	33,8	17,8	413
Sulawesi Barat	53,1	28,8	18,0	523
Maluku	39,5	29,7	30,8	707
Maluku Utara	43,5	33,2	23,3	493
Papua Barat	43,3	30,6	26,1	375
Papua	32,4	30,0	37,7	1.201
**INDONESIA**	**53,5**	**28,8**	**17,6**	**85.095**

Tabel 15.4.10
Proporsi Pemberian Kapsul Vitamin A dalam 12 Bulan Terakhir pada Anak Umur 6-59 Bulan menurut Karakteristik, Riskesdas 2018

Karakteristik	Pemberian kapsul Vitamin A (%)			N Tertimbang
	Sesuai standar	Tidak sesuai standar	Tidak pernah mendapat	
**Kelompok umur (bulan)**				
6-11	71,5	42,4	28,5	9.801
12-23	43,3	31,1	14,3	18.416
24-35	54,2	29,1	14,7	19.199
36-47	54,0	28,1	16,9	18.906
48-59	53,0	28,8	18,9	18.773
**Jenis kelamin**				
Laki-laki	53,5	28,9	17,7	43.414
Perempuan	53,6	28,8	17,6	41.681
**Pendidikan KRT**				
Tidak/belum pernah sekolah	49,5	25,8	24,7	3.473
Tidak tamat SD/MI	51,2	29,0	19,7	10.097
Tamat SD/MI	53,7	28,7	17,6	21.895
Tamat SLTP/MTS	54,3	29,4	16,4	16.036
Tamat SLTA/MA	54,2	29,6	16,2	25.486
Tamat D1/D2/D3/PT	54,1	27,0	18,9	8.107
**Pekerjaan KRT**				
Tidak bekerja	54,6	27,2	18,2	5.733
Sekolah	53,4	26,9	19,7	164
PNS/TNI/Polri/BUMN/BUMD	55,6	28,2	16,1	4.190
Pegawai swasta	56,0	29,6	14,4	12.356
Wiraswasta	52,7	29,2	18,1	19.310
Petani	51,0	28,8	20,2	22.079
Nelayan	42,8	31,6	25,6	1.766
Buruh/sopir/pembantu ruta	56,3	28,6	15,1	15.207
Lainnya	54,4	28,0	17,6	4.289
**Tempat tinggal**				
Perkotaan	54,1	29,3	16,6	45.823
Perdesaan	52,9	28,3	18,8	39.272

15.5 Pemantauan Pertumbuhan

Berdasarkan PMK Nomor 43 Tahun 2016 tentang Standar Pelayanan Minimal Bidang Kesehatan bahwa selain pemberian kapsul vitamin A sebanyak 2 kali setahun dan pemberian imunisasi dasar lengkap, setiap balita juga mendapatkan penimbangan minimal 8 kali setahun dan pengukuran panjang/tinggi badan minimal 2 kali setahun.

Definisi penimbangan pada Riskesdas 2018 yaitu dihitung telah melakukan 1 (satu) kali, jika minimal 1 kali atau lebih pada bulan yang sama melakukan penimbangan berat badan dan pengukuran panjang/tinggi badan. Penimbangan tidak termasuk penimbangan yang dilakukan pada saat pengumpulan data Riskesdas dilakukan.

Definisi penimbangan BB sesuai standar yaitu anak umur 0-59 bulan ditimbang minimal 8 kali setahun.

$$\mathit{Proporsi\ penimbangan\ berat\ badan\ sesuai\ standar} = \frac{\mathit{Jumlah\ anak\ umur}\ 0-59\ \mathit{bulan\ melakukan\ penimbangan\ berat\ badan\ minimal}\ 8\ \mathit{kali\ setahun\ dalam}\ 12\ \mathit{bulan\ terakhir}}{\mathit{Jumlah\ anak\ umur}\ 0-59\ \mathit{bulan}}$$

Definisi pengukuran TB sesuai standar yaitu anak umur 0-59 bulan diukur panjang/tinggi badan minimal 2 kali setahun.

$$\mathit{Proporsi\ pemantauan\ pertumbuhan\ tinggi\ badan} = \frac{\mathit{Jumlah\ anak\ umur}\ 0-59\ \mathit{bulan\ melakukan\ pengukuran\ tinggi\ atau\ panjang\ badan\ minimal}\ 2\ \mathit{kali\ setahun\ dalam}\ 12\ \mathit{bulan\ terakhir}}{\mathit{Jumlah\ anak\ umur}\ 0-59\ \mathit{bulan}}$$

Tabel 15.5.1

Proporsi Pemantauan Pertumbuhan dalam 12 Bulan Terakhir pada Anak Umur 0-59 Bulan menurut Provinsi, Riskesdas 2018

Provinsi	Ditim-bang BB	N Tertimbang	Frekuensi penimbangan		N Ter-timbang	diukur PB atau TB	N Ter-timbang	Frekuensi pengukuran		N Tertim bang
			< 8kali	≥ 8 kali				1 kali	≥ 2 kali	
	%		%	%		%		%	%	
Aceh	71,8	2.250	49,9	40,3	1.530	48,3	2.250	15,2	72,1	1.014
Sumatera Utara	58,0	5.895	66,5	22,6	3.235	27,5	5.895	26,2	61,0	1.511
Sumatera Barat	82,0	2.179	45,4	49,8	1.692	64,1	2.179	19,0	75,6	1.303
Riau	71,6	2.813	57,8	37,3	1.907	42,2	2.813	20,2	74,4	1.107
Jambi	70,3	1.279	66,2	24,6	851	37,8	1.279	19,8	67,6	451
Sumatera Selatan	61,3	3.079	56,5	35,9	1.785	30,4	3.079	22,2	68,2	873
Bengkulu	72,3	716	52,7	39,9	491	33,1	716	19,4	72,7	222
Lampung	78,5	3.094	45,5	50,3	2.300	34,0	3.094	22,7	73,9	983
Bangka Belitung	78,4	527	42,9	51,0	391	60,6	527	13,1	80,8	298
Kepulauan Riau	80,2	872	49,6	47,2	662	66,3	872	13,2	83,2	540
DKI Jakarta	89,8	3.582	40,2	56,9	3.044	69,8	3.582	10,1	83,9	2.334
Jawa Barat	88,6	17.228	32,9	62,1	14.440	62,7	17.228	14,0	80,9	10.079
Jawa Tengah	90,0	10.551	28,9	67,2	8.989	63,3	10.551	12,1	83,2	6.232
DI Yogyakarta	95,6	1.069	21,9	77,6	968	85,2	1.069	6,2	91,6	850
Jawa Timur	89,3	11.272	28,2	69,0	9.532	70,2	11.272	13,1	83,4	7.379
Banten	78,3	4.813	50,7	39,5	3.566	31,5	4.813	19,2	65,2	1.415
Bali	90,6	1.275	46,8	50,4	1.093	33,2	1.275	33,7	62,6	395
Nusa Tenggara Barat	88,6	1.985	30,9	65,5	1.664	58,5	1.985	34,7	57,9	1.083
Nusa Tenggara Timu	81,4	2.496	29,1	66,8	1.923	53,1	2.496	25,3	69,5	1.236
Kalimantan Barat	70,1	1.928	47,3	45,3	1.279	48,0	1.928	12,9	78,4	310
Kalimantan Tengah	63,0	1.000	50,7	36,2	596	33,2	1.000	19,1	61,0	887
Kalimantan Selatan	79,9	1.563	49,1	45,8	1.183	60,8	1.563	13,0	80,6	687
Kalimantan Timur	77,5	1.368	44,3	51,5	1.003	53,8	1.368	13,7	79,3	188
Kalimantan Utara	76,6	309	55,1	40,1	224	65,1	309	12,2	82,8	863
Sulawesi Utara	75,6	821	50,9	41,7	588	35,3	821	25,8	61,9	271
Sulawesi Tengah	77,4	1.155	47,8	47,4	846	56,0	1.155	16,6	78,2	604
Sulawesi Selatan	77,3	3.269	51,5	43,5	2.391	50,5	3.269	19,8	73,8	1.541
Sulawesi Tenggara	67,3	1.169	50,4	42,2	745	24,3	1.169	27,2	62,0	265
Gorontalo	77,2	445	48,1	42,7	326	67,0	445	10,9	78,2	278
Sulawesi Barat	78,9	584	41,4	47,8	436	62,4	584	15,8	73,2	340
Maluku	67,5	779	48,3	46,1	498	39,4	779	19,0	72,6	287
Maluku Utara	71,8	536	41,4	48,9	364	39,0	536	15,1	72,1	195
Papua Barat	59,9	415	48,9	42,4	235	29,8	415	18,3	72,5	115
Papua	52,4	1.302	53,2	29,3	646	27,1	1.302	17,9	61,4	329
**INDONESIA**	**80,6**	**93.620**	**40,0**	**54,6**	**71.422**	**53,2**	**93.620**	**15,9**	**77,8**	**46.461**

Tabel 15.5.2

Proporsi Pemantauan Pertumbuhan dalam 12 Bulan Terakhir pada Anak Umur 0-59 Bulan menurut Karakteristik, Riskesdas 2018

Karakteristik	ditimbang BB	N Ter-timbang	Frekuensi penimbangan		N Ter-timbang	diukur PB atau TB	N Ter-timbang	Frekuensi pengukuran		N Ter-timbang
			< 8kali	≥ 8 kali				1 kali	≥ 2 kali	
	%		%	%		%		%	%	
**Kelompok umur (bulan)**										
0-5	88,9	7.922	96,0	2,2	7.498	61,9	5.514	47,3	50,4	5.144
6-11	91,3	8.910	50,6	45,9	8.434	59,0	5.757	17,2	77,7	5.371
12-23	86,8	15.913	27,5	67,3	15.062	56,2	10.299	12,0	82,0	9.609
24-35	81,1	15.508	30,0	64,3	14.679	53,1	10.151	10,8	82,7	9.470
36-47	75,0	14.111	32,8	60,8	13.356	49,0	9.225	11,4	81,2	8.607
48-59	70,1	13.093	33,8	58,7	12.393	47,4	8.853	10,6	80,9	8.260
**Jenis kelamin**										
Laki-laki	80,2	38.301	40,5	54,2	36.253	53,2	25.398	15,7	78,1	23.696
Perempuan	81,0	37.156	39,5	55,0	35.169	53,2	24.400	16,1	77,5	22.765
**Pendidikan KRT**										
Tidak/belum pernah sekolah	73,5	2.833	38,1	56,1	2.682	46,1	1.777	16,9	76,0	1.658
Tidak tamat SD/MI	78,5	8.746	38,4	56,2	8.279	51,3	5.720	17,9	75,3	5.337
Tamat SD/MI	80,2	19.364	37,1	57,8	18.328	52,7	12.713	15,8	78,2	11.861
Tamat SLTP/MTS	81,3	14.361	40,3	54,1	13.593	53,3	9.404	15,3	77,7	8.774
Tamat SLTA/MA	81,7	22.833	41,8	53,0	21.612	54,7	15.277	15,5	78,4	14.253
Tamat D1/D2/D3/PT	82,4	7.321	44,2	50,0	6.929	55,2	4.908	15,7	78,6	4.579
**Pekerjaan KRT**										
Tidak bekerja	82,0	5.231	39,8	54,6	4.951	55,3	3.527	15,8	77,2	3.290
Sekolah	67,4	124	42,3	49,7	117	43,3	80	21,8	74,9	74
PNS/TNI/Polri/BUMN/ BUMD	81,1	3.684	44,3	50,1	3.487	54,3	2.466	16,0	78,2	2.300
Pegawai swasta	85,7	11.569	40,4	55,3	10.950	58,6	7.913	14,4	79,9	7.383
Wiraswasta	80,0	16.950	41,5	52,9	16.043	52,4	11.108	15,5	78,2	10.363
Petani/buruh tani	76,6	18.684	38,7	55,0	17.685	49,3	12.028	17,5	75,4	11.222
Nelayan	72,4	1.422	45,7	47,1	1.346	44,8	881	16,4	75,3	822
Buruh/sopir/pembantu ruta	83,6	13.961	37,5	58,0	13.214	55,5	9.280	15,3	79,3	8.658
Lainnya	80,2	3.833	41,9	53,0	3.628	52,7	2.517	16,8	77,0	2.349
**Tempat tinggal**										
Perkotaan	83,0	41.799	41,1	54,1	39.563	55,9	28.143	14,8	79,4	26.257
Perdesaan	77,8	33.658	38,7	55,3	31.859	50,1	21.655	17,3	75,7	20.204

Tabel 15.5.3

Proporsi Alasan Utama Tidak Pernah Ditimbang Berat Badan dalam 12 Bulan Terakhir pada Anak Umur 0-59 Bulan Terakhir menurut Provinsi, Riskesdas 2018

Provinsi	Alasan utama tidak ditimbang (%)									N Ter-timbang
	anak sudah besar (≥1 tahun)	anak sudah imunisasi	anak tidak mau ditimbang	malas	lupa/tidak tahu jadwalnya	tidak ada tempat penim bangan	Tempat -nya jauh	Sibuk/r epot	alat timbang badan tidak tersedia	
Aceh	7,2	6,8	7,9	17,3	18,8	3,2	6,4	31,1	1,4	775
Sumatera Utara	13,3	13,1	4,9	14,7	12,2	2,8	6,8	30,1	2,2	3.027
Sumatera Barat	11,5	9,7	8,3	21,0	12,0	1,4	3,8	30,5	1,7	479
Riau	15,4	13,4	6,6	21,1	6,8	2,5	10,0	22,9	1,3	976
Jambi	11,7	20,0	7,8	19,0	9,5	1,0	1,8	28,8	0,5	464
Sumatera Selatan	16,7	20,5	4,7	14,1	10,4	1,5	5,3	26,6	0,1	1.458
Bengkulu	13,1	16,1	8,5	13,1	9,5	3,3	5,8	27,4	3,3	242
Lampung	17,9	15,8	8,9	16,7	6,3	1,2	6,5	26,1	0,6	812
Bangka Belitung	14,0	18,5	8,5	21,1	8,6	0,8	6,1	21,1	1,3	139
Kepulauan Riau	22,7	8,7	4,7	12,0	11,4	0,9	2,8	29,4	7,5	211
DKI Jakarta	11,2	8,3	6,5	11,1	20,2	3,3	1,8	35,5	2,2	447
Jawa Barat	12,5	7,2	13,5	12,3	18,3	2,5	6,6	26,2	0,9	2.411
Jawa Tengah	15,5	8,6	16,5	9,8	13,2	3,6	3,2	29,3	0,3	1.288
DI Yogyakarta	7,7	12,0	13,2	5,2	31,5	1,4	7,6	21,3	0,0	57
Jawa Timur	10,2	8,1	9,1	14,5	14,5	1,0	8,6	33,2	0,8	1.469
Banten	8,4	4,9	10,4	19,4	13,6	5,3	6,0	30,3	1,8	1.277
Bali	14,7	10,3	11,0	9,5	12,7	6,1	3,8	29,4	2,4	147
Nusa Tenggara Barat	17,2	13,9	12,4	10,4	10,6	1,3	5,1	29,0	0,0	277
Nusa Tenggara Timur	11,8	8,2	4,3	16,7	11,1	2,5	9,8	35,1	0,5	568
Kalimantan Barat	17,3	5,7	5,7	19,3	10,9	1,0	9,5	29,6	1,0	453
Kalimantan Tengah	20,9	11,8	6,9	10,3	8,5	2,6	8,0	28,7	2,4	383
Kalimantan Selatan	16,5	13,6	10,2	14,7	10,1	1,4	5,8	27,1	0,6	377
Kalimantan Timur	18,4	7,9	6,2	9,7	12,8	4,5	4,3	35,7	0,5	89
Kalimantan Utara	18,3	9,0	6,8	12,9	19,7	1,3	2,9	28,7	0,3	704
Sulawesi Utara	13,2	14,0	7,1	13,2	7,9	7,8	1,2	34,1	1,5	245
Sulawesi Tengah	17,4	13,1	6,6	16,0	12,8	2,5	6,6	24,2	0,8	319
Sulawesi Selatan	21,0	10,8	6,6	16,4	9,0	2,6	8,6	23,8	1,3	908
Sulawesi Tenggara	19,1	20,7	4,7	15,9	12,0	2,0	4,4	19,7	1,5	467
Gorontalo	9,0	11,5	3,5	18,1	23,4	0,0	4,4	27,3	2,8	124
Sulawesi Barat	22,2	10,2	14,9	8,4	11,6	3,6	5,6	21,5	2,0	150
Maluku	12,6	5,9	9,2	15,7	15,1	7,5	6,0	26,5	1,5	310
Maluku Utara	19,2	4,0	3,7	17,3	14,9	1,5	4,8	33,4	1,2	185
Papua Barat	17,0	10,6	4,0	15,3	10,9	6,4	6,3	29,0	0,4	204
Papua	12,3	4,0	1,7	17,3	15,8	7,0	20,8	19,9	1,3	757
**INDONESIA**	**14,1**	**10,8**	**8,1**	**15,1**	**12,8**	**2,8**	**6,8**	**28,3**	**1,3**	**22.198**

Tabel 15.5.4
Proporsi Alasan Utama Tidak Pernah Ditimbang Berat Badan dalam 12 Bulan Terakhir pada Anak Umur 0-59 Bulan Terakhir menurut Karakteristik, Riskesdas 2018

Karakteristik	Alasan utama tidak ditimbang (%)									N Ter-timbang
	anak sudah besar (≥1 tahun)	anak sudah imunisasi	anak tidak mau ditimbang	malas	Lupa/tidak tahu jadwalnya	tidak ada tempat penimbangan	tempatnya jauh	sibuk/ repot	alat timbang badan tidak tersedia	
**Kelompok umur (bulan)**	1,4	6,3	3,2	15,0	22,4	7,4	12,7	26,7	4,8	1.205
0-5	1,7	5,6	3,7	21,8	19,4	3,0	10,9	32,4	1,5	1.035
6-11	7,5	6,8	4,5	18,4	16,3	3,3	8,7	33,2	1,4	2.958
12-23	12,5	11,0	8,4	17,2	12,3	2,4	5,8	29,5	0,9	4.405
24-35	16,0	11,2	10,7	13,6	11,4	2,1	6,4	27,5	1,1	5.756
36-47	20,6	13,7	8,8	12,5	10,1	2,5	5,3	25,7	0,9	6.838
48-59	1,4	6,3	3,2	15,0	22,4	7,4	12,7	26,7	4,8	1.205
**Jenis kelamin**										
Laki-laki	13,8	11,2	8,7	14,4	13,0	2,8	6,6	28,1	1,3	11.566
Perempuan	14,5	10,3	7,5	15,8	12,6	2,7	7,0	28,5	1,2	10.632
**Pendidikan KRT**										
Tidak/belum pernah sekolah	9,4	7,2	6,4	17,2	14,9	4,5	14,6	24,5	1,1	1.249
Tidak tamat SD/MI	12,2	9,0	8,5	16,5	13,9	3,1	10,2	25,3	1,3	2.936
Tamat SD/MI	13,8	10,8	8,9	16,9	13,2	2,9	7,6	24,7	1,3	5.828
Tamat SLTP/MTS	15,3	10,0	9,6	16,5	12,8	2,4	6,0	26,6	0,9	4.030
Tamat SLTA/MA	15,6	12,7	7,7	12,8	11,7	2,9	4,5	30,5	1,5	6.240
Tamat D1/D2/D3/PT	14,0	11,7	4,2	10,2	11,8	1,2	3,2	42,7	1,1	1.916
**Pekerjaan KRT**										
Tidak bekerja	13,6	10,6	10,6	14,0	12,3	1,4	5,9	30,6	1,1	1.401
Sekolah	13,5	13,3	1,6	11,3	7,9	2,8	13,6	36,1	0,0	73
PNS/TNI/Polri/BUMN/BUMD	16,5	13,1	7,3	9,4	11,1	2,2	3,6	36,1	0,8	1.049
Pegawai swasta	13,6	11,7	7,8	13,4	11,4	3,3	3,4	34,2	1,3	2.358
Wiraswasta	15,2	11,4	8,2	13,7	12,9	1,8	4,3	31,0	1,5	5.177
Petani/buruh tani	13,4	10,9	6,8	15,8	13,1	3,2	10,8	24,8	1,3	6.964
Nelayan	11,6	7,2	7,1	22,4	13,6	2,7	10,1	24,2	1,3	664
Buruh/sopir/pembantu ruta	14,1	9,5	10,3	17,9	13,2	3,9	5,3	24,7	1,1	3.357
Lainnya	14,6	10,0	8,0	14,4	14,2	2,9	6,9	28,0	1,1	1.155
**Tempat tinggal**										
Perkotaan	14,7	10,9	8,4	13,7	12,7	2,5	3,7	32,3	1,2	10.464
Perdesaan	13,6	10,7	7,8	16,3	12,9	3,1	9,5	24,8	1,3	11.734

Tabel 15.5.5
Proporsi Alasan Utama Tidak Pernah Diukur Panjang atau Tinggi Badan
dalam 12 Bulan Terakhir pada Anak Umur 0-59 Bulan menurut Provinsi, Riskesdas 2018

Provinsi	Alasan utama tidak pernah diukur panjang/tinggi badannya (%)									N Ter-timbang
	anak sudah besar (≥1 tahun)	anak sudah imuni-sasi	anak tidak mau diukur	lupa/tidak tahu jadwal-nya	tidak ada tempat penguku ran	Temp at-nya jauh	sibuk/ repot	malas	Tidak ada alat ukur PB/TB	
Aceh	4,8	3,6	10,1	14,1	22,4	4,3	21,3	12,2	7,1	1.252
Sumatera Utara	10,1	7,4	6,2	9,3	19,4	4,4	20,9	10,4	12,0	4.601
Sumatera Barat	8,6	5,9	7,6	9,8	21,8	2,7	19,5	14,2	9,8	843
Riau	10,7	7,1	7,7	5,8	19,6	6,2	16,2	13,3	13,4	1.751
Jambi	7,1	11,0	7,9	7,3	26,0	1,8	19,1	10,8	9,1	856
Sumatera Selatan	11,0	12,2	6,4	8,3	23,6	3,5	19,5	8,7	6,8	2.306
Bengkulu	8,7	6,8	9,7	6,2	29,9	2,8	15,3	7,1	13,4	515
Lampung	7,1	6,5	5,5	3,9	39,6	2,6	11,9	5,7	17,3	2.196
Bangka Belitung	9,9	11,2	8,8	6,8	23,3	4,0	19,9	13,5	2,7	223
Kepulauan Riau	16,2	5,8	11,6	9,3	10,8	2,0	26,1	7,8	10,6	316
DKI Jakarta	6,7	3,3	12,0	9,2	34,5	1,0	16,7	4,9	11,7	1.162
Jawa Barat	5,9	2,9	12,2	9,1	35,1	3,1	12,6	5,3	13,8	6.915
Jawa Tengah	5,7	3,2	11,1	6,4	40,2	1,3	11,1	3,6	17,4	4.166
DI Yogyakarta	6,3	1,9	7,7	11,2	50,8	2,5	10,5	2,5	6,5	170
Jawa Timur	5,3	3,8	10,6	7,7	38,4	3,4	15,3	6,0	9,4	3.619
Banten	3,6	1,7	6,9	5,8	39,2	2,7	12,9	7,1	20,0	3.547
Bali	6,7	2,6	4,6	4,7	56,5	0,6	7,6	2,1	14,5	916
Nusa Tenggara Barat	7,3	3,2	6,6	8,3	45,9	1,8	13,1	3,5	10,3	887
Nusa Tenggara Timur	7,1	3,3	3,5	6,0	36,6	4,4	16,3	7,4	15,4	1.260
Kalimantan Barat	13,1	3,3	5,7	9,8	23,0	5,7	19,5	12,6	7,4	1.080
Kalimantan Tengah	14,3	6,3	7,4	6,6	28,1	4,7	20,0	5,3	7,2	719
Kalimantan Selatan	10,6	7,1	12,9	7,9	23,4	3,6	19,0	9,6	5,9	660
Kalimantan Timur	10,6	4,2	5,1	10,6	27,1	2,9	20,4	5,4	13,6	679
Kalimantan Utara	11,2	6,7	8,0	16,3	14,4	2,0	26,8	9,8	4,9	116
Sulawesi Utara	7,1	5,7	4,7	6,8	33,8	1,1	16,9	6,1	17,7	571
Sulawesi Tengah	10,4	6,5	6,4	8,9	25,2	3,7	18,1	11,4	9,5	546
Sulawesi Selatan	11,5	5,8	7,2	7,1	24,5	4,2	16,4	9,6	13,7	1.741
Sulawesi Tenggara	8,9	9,6	3,9	5,5	32,5	2,2	10,4	7,0	20,0	953
Gorontalo	6,7	6,3	4,2	18,1	16,0	4,1	23,0	12,4	9,2	158
Sulawesi Barat	15,9	5,1	16,6	9,8	12,4	4,6	19,2	6,5	9,8	236
Maluku	7,9	3,0	7,4	9,0	26,5	3,6	19,5	9,7	13,3	508
Maluku Utara	10,1	2,7	4,9	10,5	35,1	2,4	19,3	7,9	7,1	352
Papua Barat	10,7	5,6	2,8	7,4	29,5	4,0	19,8	9,2	10,9	314
Papua	8,3	3,1	3,4	12,8	22,2	14,5	15,1	12,6	8,1	1.022
**INDONESIA**	**7,8**	**5,0**	**8,3**	**8,0**	**31,5**	**3,4**	**15,7**	**7,6**	**12,9**	**47.159**

Tabel 15.5.6
Proporsi Alasan Utama Tidak Pernah Diukur Panjang atau Tinggi Badan
dalam 12 Bulan Terakhir pada Anak Umur 0-59 Bulan menurut Karakteristik, Riskesdas 2018

Karakteristik	Alasan utama tidak diukur panjang/tinggi badannya (%)									N Ter-timbang
	anak sudah besar (≥1 tahun)	anak sudah imuni-sasi	anak tidak mau diukur	lupa/tidak tahu jadwal-nya	tidak ada tempat pengukuran	Tempat-nya jauh	sibuk/repot	malas	Tidak ada alat ukur PB/TB	
**Kelompok umur (bulan)**										
0-5	0,7	1,6	3,2	9,9	46,1	4,0	12,6	5,5	16,4	3.653
6-11	1,0	1,2	3,6	8,8	46,8	3,3	11,6	6,2	17,4	4.305
12-23	3,8	2,6	8,2	7,8	37,4	3,4	14,1	7,3	15,4	8.646
24-35	6,7	5,8	9,7	7,6	30,5	3,0	15,5	8,1	12,9	9.644
36-47	10,3	6,2	10,2	8,1	25,5	3,7	17,7	7,7	10,7	10.327
48-59	14,7	7,7	8,7	7,3	21,9	3,4	17,9	8,5	9,9	10.584
**Jenis kelamin**										
Laki-laki	8,1	5,2	8,8	8,3	30,7	3,4	16,1	7,1	12,4	24.069
Perempuan	7,5	4,7	7,7	7,6	32,3	3,5	15,3	8,1	13,4	23.090
**Pendidikan KRT**										
Tidak/belum pernah sekolah	6,2	3,2	7,2	8,6	30,8	7,8	14,6	9,5	12,0	2.237
Tidak tamat SD/MI	6,5	4,4	8,4	9,0	30,5	5,4	13,6	8,9	13,3	5.842
Tamat SD/MI	7,4	4,7	8,3	8,4	32,7	4,0	13,8	8,3	12,3	12.288
Tamat SLTP/MTS	8,3	4,7	8,6	8,2	31,6	2,9	14,0	8,3	13,5	8.883
Tamat SLTA/MA	8,2	5,9	8,4	7,1	31,9	2,4	17,1	6,2	12,8	13.626
Tamat D1/D2/D3/PT	8,9	5,1	7,3	7,2	27,8	1,5	23,3	5,4	13,3	4.283
**Pekerjaan KRT**										
Tidak bekerja	7,1	4,6	8,6	8,2	32,5	2,5	15,6	7,6	13,3	3.068
Sekolah	12,7	9,4	3,0	6,0	21,8	8,8	24,7	8,2	5,5	112
PNS/TNI/Polri/BUMN/BUMD	9,9	6,1	7,8	6,9	28,4	1,8	20,9	4,6	13,5	2.234
Pegawai swasta	7,1	4,8	9,0	6,5	34,2	1,6	16,9	5,8	14,0	6.010
Wiraswasta	8,2	5,2	8,9	8,1	30,2	2,3	17,3	7,5	12,5	10.845
Petani/buruh tani	7,9	5,4	7,3	8,5	30,5	5,9	14,4	8,4	11,7	13.295
Nelayan	7,2	3,8	7,0	9,0	26,5	5,4	16,0	11,5	13,6	1.167
Buruh/sopir/pembantu ruta	6,8	4,1	8,9	7,7	34,2	2,8	12,8	8,0	14,8	7.993
Lainnya	9,1	4,5	7,5	9,0	31,1	3,1	16,9	7,4	11,4	2.433
**Tempat tinggal**										
Perkotaan	7,8	4,6	8,9	7,8	31,6	1,9	17,2	6,6	13,6	23.910
Perdesaan	7,7	5,4	7,6	8,2	31,3	5,0	14,1	8,6	12,1	23.249

15.6 Perkembangan Anak

Kuesioner perkembangan yang digunakan pada Riskesdas 2018 ini diadaptasi dan dimodifikasi dari study *Multiple Indicator Cluster Survey* (*MICS*) 2016 untuk menghitung Indeks Perkembangan Anak Usia Dini (*Early child Development Index*/*ECDI*). Berbagai pertanyaan yang ada digunakan untuk menentukan perkembangan anak sesuai dengan 4 (empat) domain yaitu:

- Literasi dan numerasi: anak dikategorikan perkembangannya sesuai jika dua diantara 3 (tiga) pernyataan berikut adalah benar (dijawab "Ya"): (1) dapat mengenali/atau menyebut paling tidaky10 huruf abjad/alphabet, (2) dapat membaca setidaknya 4 kata sederhana/populer,(3) mengetahui dan mengenali simbol angka 1-10. Pertanyaan K51, K52, dan K55 yang termasuk dalam domain ini.
- Physical – fisik: jika anak (1) dapat mengambil benda kecil seperti batu atau pensil dari lantai dengan menggunakan 2 ujung jari (telunjuk dan ibu jari) dan/atau (2) tidak diindikasi kadang-kadang sakit untuk bermain (pertanyaan apakah anak kadang-kadang terlalu sakit untuk bermain dijawab "Tidak"), maka perkembangan anak dikategorikan sesuai. Pertanyaan K53 dan K54 yang termasuk dalam domain in.
- Sosial emosional: anak dikategorikan perkembangannya sesuai jika dua diantara 3 (tiga) pernyataan berikut adalah benar (dijawab "Ya") yaitu (1) anak dapat bersosialisasi dengan baik bersama anak-anak lainnya, (2) anak tidak menendang, menggigit, atau memukul orang lain, dan (3) anak tidak mudah terganggu konsentrasinya dalam melakukan sesuatu. Pertanyaan K58, K59 dan K60 yang termasuk dalam domain ini.
- Learning – belajar: jika anak (1) dapat mengikuti perintah-perintah sederhana untuk melakukan sesuatu dengan benar dan atau (2) pada saat anak disuruh, anak dapat melakukannya sendiri tanpa dibantu, maka perkembangan anak dikategorikan sesuai (*on track*). Pertanyaan K56 dan K57 yang termasuk dalam domain ini.

Indeks Perkembangan Anak Usia Dini (umur 36-59 bulan) dianggap perkembangannya sesuai minimal 3 dari 4 domain diatas.

$$Proporsi\ indeks\ perkembangan\ anak\ usia\ dini = \frac{Anak\ umur\ 36-59\ bulan\ yang\ perkembangannya\ sesuai\ umur}{Jumlah\ anak\ umur\ 36-59\ bulan}$$

Tabel 15.6.1
Proporsi Indeks dan Jenis Perkembangan Anak Umur 36-59 Bulan menurut Provinsi, Riskesdas 2018

Provinsi	Indeks Perkembangan Anak Usia Dini (%)	Perkembangan anak (%)				N Tertimbang
		Literasi numerasi	Kemampuan fisik	Kemampuan sosial emosional	Kemampuan belajar	
Aceh	88,6	66,3	97,8	74,7	91,6	856
Sumatera Utara	86,2	54,3	96,8	76,0	92,2	2.521
Sumatera Barat	85,2	58,1	97,4	66,0	96,1	915
Riau	91,3	58,6	98,6	78,7	95,9	1.130
Jambi	90,2	72,1	96,9	63,3	94,7	523
Sumatera Selatan	88,5	66,6	98,0	68,0	92,0	1.295
Bengkulu	91,7	65,8	98,8	72,5	94,0	310
Lampung	87,4	59,5	97,2	77,9	93,3	1.244
Bangka Belitung	91,1	65,4	98,3	71,6	96,1	236
Kepulauan Riau	87,9	60,7	96,5	82,5	94,5	378
DKI Jakarta	89,4	74,3	97,9	69,7	97,5	1.498
Jawa Barat	90,0	71,6	98,3	65,3	96,1	7.152
Jawa Tengah	90,6	67,2	98,4	72,2	96,9	4.215
DI Yogyakarta	86,6	63,1	99,6	72,1	98,4	459
Jawa Timur	91,5	70,4	98,5	71,9	95,7	4.578
Banten	81,9	59,2	95,0	66,8	95,0	2.023
Bali	87,1	48,6	99,7	74,0	97,5	533
Nusa Tenggara Barat	90,4	62,2	98,6	68,4	97,1	795
Nusa Tenggara Timur	80,7	53,2	98,6	61,7	94,4	1.007
Kalimantan Barat	87,4	62,3	98,0	66,5	95,7	812
Kalimantan Tengah	88,4	67,9	96,4	73,7	92,6	428
Kalimantan Selatan	91,6	75,6	99,5	67,9	95,9	595
Kalimantan Timur	92,0	70,0	98,1	72,0	98,2	586
Kalimantan Utara	89,1	60,6	97,7	71,2	97,5	122
Sulawesi Utara	86,8	59,2	97,4	68,3	93,3	334
Sulawesi Tengah	74,8	54,8	94,2	58,3	91,8	442
Sulawesi Selatan	84,6	51,4	95,9	74,5	95,3	1.274
Sulawesi Tenggara	89,8	62,0	97,7	72,8	95,5	464
Gorontalo	84,4	64,7	97,8	56,2	96,0	184
Sulawesi Barat	87,4	59,0	96,0	66,6	92,8	218
Maluku	82,4	62,6	94,7	57,9	90,1	311
Maluku Utara	82,9	66,1	94,8	59,7	93,2	217
Papua Barat	83,5	54,3	98,1	64,7	93,4	168
Papua	79,2	43,8	97,0	67,4	89,9	553
**INDONESIA**	**88,3**	**64,6**	**97,8**	**69,9**	**95,2**	**38.375**

Tabel 15.6.2
Proporsi Indeks dan Jenis Perkembangan Anak Umur 36-59 Bulan menurut Karakteristik, Riskesdas 2018

Karakteristik	Indeks Perkembangan Anak Usia Dini (%)	Perkembangan anak (%)				N Tertimbang
		Literasi numerasi	Kemampuan Fisik	Kemampuan Sosial emosional	Kemampuan belajar	
**Kelompok umur (bulan)**						
36-47	85,9	57,3	97,3	71,1	94,3	19.255
48-59	90,6	71,9	98,2	68,6	96,2	19.120
**Jenis kelamin**						
Laki-laki	87,3	63,1	97,8	68,8	95,2	19.784
Perempuan	89,3	66,2	97,7	70,9	95,2	18.591
**Pendidikan KRT**						
Tidak/belum pernah sekolah	83,5	58,5	96,6	67,0	93,5	1.503
Tidak tamat SD/MI	85,9	58,6	97,3	69,5	94,9	4.444
Tamat SD/MI	87,4	62,1	97,9	68,9	94,7	9.828
Tamat SLTP/MTS	88,5	64,4	97,5	69,4	94,9	7.328
Tamat SLTA/MA	89,2	67,3	98,0	71,2	95,8	11.615
Tamat D1/D2/D3/PT	91,7	73,1	98,5	70,7	96,5	3.657
**Pekerjaan KRT**						
Tidak bekerja	87,5	63,7	97,4	70,2	95,6	2.391
Sekolah	86,6	77,2	95,2	68,2	99,4	90
PNS/TNI/Polri/BUMN/BUMD	91,0	73,3	98,8	69,6	96,5	1.829
Pegawai swasta	90,7	70,0	97,8	71,5	95,7	5.683
Wiraswasta	89,9	67,9	98,0	70,4	96,1	8.886
Petani/buruh tani	85,4	57,9	97,6	69,0	93,5	9.806
Nelayan	87,1	61,7	97,7	71,9	92,5	761
Buruh/sopir/pembantu ruta	87,4	63,4	97,5	68,9	95,6	6.980
Lainnya	89,8	65,6	98,0	70,0	96,1	1.949
**Tempat tinggal**						
Perkotaan	89,6	68,1	97,9	70,5	96,2	20.815
Perdesaan	86,7	60,5	97,7	69,2	94,1	17.560

BAB 16
STATUS GIZI

16.1 Tablet Tambah Darah Remaja Putri

Tablet Tambah Darah (TTD) adalah suplemen gizi penambah darah berbentuk **tablet/kaplet/kapsul** yang dapat diperoleh dari Program atau mandiri. TTD Program disediakan oleh Pemerintah dan didistribusikan ke kelompok sasaran melalui fasilitas pelayanan kesehatan pemerintah. TTD mandiri diperoleh berdasarkan resep/instruksi tenaga kesehatan, inisiatif membeli sendiri di fasilitas kesehatan swasta/apotik/toko obat, atau diperoleh dari pemberian keluarga/orang lain.

JUMLAH TTD YANG DIPEROLEH

Proporsi remaja putri umur 10-19 tahun yang pernah mendapatkan TTD dan mendapatkan TTD dalam 12 bulan terakhir, dihitung menggunakan formula:

$$Proporsi\ remaja\ putri\ yang\ memperoleh\ TTD = \frac{Jumlah\ remaja\ putri\ umur\ 10-19\ tahun\ yang\ memperoleh\ TTD}{Jumlah\ remaja\ putri\ umur\ 10-19\ tahun}$$

SUMBER PEROLEHAN TTD

Sumber TTD:

1. Fasilitas Kesehatan (Puskesmas, Rumah Sakit) dan Tenaga Kesehatan (Dokter praktek, Bidan praktek, perawat praktek)
2. Sekolah,
3. Inisiatif sendiri, yaitu jika responden membeli suplemen/TTD atas kemauan sendiri, tanpa resep dari dokter/tenaga kesehatan lainnya, termasuk TTD yang diperoleh dari keluarga/orang lain

$$Proporsi\ sumber\ perolehan\ TTD\ pada\ remaja\ putri = \frac{Jumlah\ remaja\ putri\ umur\ 10-19\ tahun\ yang\ memperoleh\ TTD\ menurut\ sumber\ perolehan}{Jumlah\ remaja\ putri\ umur\ 10-19\ tahun\ yang\ memperoleh\ TTD}$$

JUMLAH TTD YANG DIPEROLEH DAN DIMINUM

Pemerintah mempunyai program untuk remaja putri di sekolah yaitu memberikan satu butir TTD setiap minggu sepanjang tahun (total 52 butir)

$$Proporsi\ jumlah\ butir\ TTD\ yang\ diperoleh\ remaja\ putri = \frac{Jumlah\ remaja\ putri\ umur\ 10-19\ tahun\ menurut\ jumlah\ perolehan\ TTD}{Jumlah\ remaja\ putri\ umur\ 10-19\ tahun\ yang\ memperoleh\ TTD}$$

$$Proporsi\ jumlah\ butir\ TTD\ yang\ diminum\ remaja\ putri = \frac{\begin{matrix}Jumlah\ remaja\ putri\ umur\ 10-19\ tahun\\ menurut\ jumlah\ TTD\ yang\ diminum\end{matrix}}{Jumlah\ remaja\ putri\ umur\ 10-19\ tahun\ yang\ memperoleh\ TTD}$$

$$Proporsi\ alasan\ utama\ remaja\ putri\ tidak\ minum/menghabiskan\ TTD = \frac{\begin{matrix}Jumlah\ remaja\ putri\ umur\ 10-19\ tahun\ tidak\ menghabiskan\ TTD\\ menurut\ alasan\end{matrix}}{Jumlah\ remaja\ putri\ umur\ 10-19\ tahun\ yang\ memperoleh\ TTD}$$

Tabel 16.1.1

Proporsi Remaja Putri Umur 10-19 Tahun yang Sudah Mendapat Haid/Menstruasi menurut Provinsi, Riskesdas 2018

Provinsi	Sudah mendapat haid/menstruasi				N Ter-timbang
	Ya		Tidak		
	%	95% CI	%	95% CI	
Aceh	63,9	62,0 - 65,9	36,1	34,1 – 38,0	1.953
Sumatera Utara	68,8	67,2 - 70,3	31,2	29,7 - 32,8	5.642
Sumatera Barat	68,1	66,2 – 70,0	31,9	30,0 - 33,8	2.038
Riau	67,1	65,0 - 69,2	32,9	30,8 – 35,0	2.481
Jambi	66,7	64,3 - 69,1	33,3	30,9 - 35,7	1.281
Sumatera Selatan	66,5	64,2 - 68,8	33,5	31,2 - 35,8	2.969
Bengkulu	69,5	66,6 - 72,3	30,5	27,7 - 33,4	675
Lampung	68,6	66,4 - 70,7	31,4	29,3 - 33,6	2.884
Bangka Belitung	69,4	66,1 - 72,6	30,6	27,4 - 33,9	497
Kepulauan Riau	66,0	60,8 - 70,7	34,0	29,3 - 39,2	736
DKI Jakarta	72,8	69,6 - 75,7	27,2	24,3 - 30,4	3.008
Jawa Barat	72,1	70,7 - 73,6	27,9	26,4 - 29,3	16.725
Jawa Tengah	72,2	71,0 - 73,3	27,8	26,7 – 29,0	11.273
DI Yogyakarta	72,8	68,9 - 76,4	27,2	23,6 - 31,1	1.084
Jawa Timur	73,6	72,4 - 74,8	26,4	25,2 - 27,6	12.368
Banten	71,0	68,5 - 73,4	29,0	26,6 - 31,5	4.325
Bali	72,4	69,6 - 75,1	27,6	24,9 - 30,4	1.385
Nusa Tenggara Barat	67,3	64,6 - 69,8	32,7	30,2 - 35,4	1.866
Nusa Tenggara Timur	58,7	56,9 - 60,4	41,3	39,6 - 43,1	2.308
Kalimantan Barat	70,1	67,9 - 72,1	29,9	27,9 - 32,1	1.818
Kalimantan Tengah	70,5	67,7 - 73,1	29,5	26,9 - 32,3	929
Kalimantan Selatan	72,7	70,5 - 74,8	27,3	25,2 - 29,5	1.441
Kalimantan Timur	71,1	67,7 - 74,3	28,9	25,7 - 32,3	1.236
Kalimantan Utara	69,9	65,0 - 74,3	30,1	25,7 – 35,0	260
Sulawesi Utara	70,7	68,1 - 73,1	29,3	26,9 - 31,9	822
Sulawesi Tengah	70,9	68,5 - 73,2	29,1	26,8 - 31,5	1.081
Sulawesi Selatan	66,9	65,1 - 68,7	33,1	31,3 - 34,9	3.191
Sulawesi Tenggara	68,6	65,8 - 71,2	31,4	28,8 - 34,2	1.067
Gorontalo	74,2	70,9 - 77,2	25,8	22,8 - 29,1	426
Sulawesi Barat	65,7	62,2 - 69,1	34,3	30,9 - 37,8	539
Maluku	61,0	57,9 - 64,1	39,0	35,9 - 42,1	718
Maluku Utara	63,9	61,3 - 66,4	36,1	33,6 - 38,7	501
Papua Barat	62,7	58,5 - 66,7	37,3	33,3 - 41,5	348
Papua	57,5	54,0 – 61,0	42,5	39,0 – 46,0	1.134
**INDONESIA**	**70,1**	**69,7 - 70,6**	**29,9**	**29,4 - 30,3**	**91.009**

Tabel 16.1.2
Proporsi Remaja Putri Umur 10-19 Tahun yang Memperoleh Tablet Tambah Darah (TTD) menurut Provinsi, Riskesdas 2018

Provinsi	Pernah memperoleh TTD			Memperoleh TTD dalam 12 bulan terakhir		
	%	95% CI	N Tertimbang	%	95% CI	N Tertimbang
Aceh	12,8	11,4-14,3	1.941	68,1	63,0-72,9	262
Sumatera Utara	9,7	8,7-10,8	5.595	72,8	68,0-77,2	572
Sumatera Barat	30,4	28,4-32,6	2.021	77,3	73,8-80,4	649
Riau	18,1	16,2-20,1	2.459	78,5	73,5-82,8	469
Jambi	22,9	20,5-25,5	1.244	71,6	65,7-76,8	301
Sumatera Selatan	14,4	12,7-16,3	2.872	78,8	73,5-83,2	437
Bengkulu	27,5	24,3-30,8	655	79,8	74,6-84,1	190
Lampung	20,9	18,8-23,0	2.781	69,8	65,0-74,2	612
Bangka Belitung	47,7	43,9-51,5	478	87,9	84,0-90,9	240
Kepulauan Riau	28,1	24,1-32,5	735	81,3	74,7-86,5	218
DKI Jakarta	17,9	15,5-20,6	2.994	82,6	75,8-87,8	566
Jawa Barat	16,4	15,2-17,7	16.263	71,7	68,0-75,1	2.819
Jawa Tengah	30,9	29,5-32,3	10.966	78,1	75,9-80,1	3.579
DI Yogyakarta	35,9	31,6-40,3	1.079	71,8	65,3-77,5	408
Jawa Timur	29,7	28,3-31,2	11.953	75,7	73,4-77,9	3.748
Banten	17,7	15,7-19,8	4.233	69,6	63,4-75,2	790
Bali	45,9	42,7-49,0	1.357	84,1	80,8-87,0	657
Nusa Tenggara Barat	27,8	25,2-30,6	1.762	77,2	71,9-81,7	517
Nusa Tenggara Timur	16,3	14,7-18,0	2.268	77,7	73,4-81,5	390
Kalimantan Barat	17,2	15,2-19,3	1.742	69,4	62,9-75,3	316
Kalimantan Tengah	26,5	23,3-29,9	875	83,6	78,3-87,8	244
Kalimantan Selatan	31,9	29,2-34,6	1.405	71,2	66,8-75,2	473
Kalimantan Timur	24,8	21,6-28,4	1.216	75,4	69,2-80,7	319
Kalimantan Utara	19,0	15,1-23,6	253	77,2	67,5-84,7	51
Sulawesi Utara	17,7	15,7-19,9	791	70,5	63,8-76,4	148
Sulawesi Tengah	37,1	34,2-40,1	1.021	79,5	75,8-82,8	400
Sulawesi Selatan	30,8	29,0-32,6	3.102	85,9	83,2-88,3	1.007
Sulawesi Tenggara	34,4	31,6-37,4	1.026	79,4	74,9-83,2	373
Gorontalo	27,6	24,3-31,1	407	78,4	72,1-83,5	118
Sulawesi Barat	25,5	22,6-28,6	520	84,2	78,1-88,8	140
Maluku	18,2	15,5-21,3	705	67,2	58,1-75,2	135
Maluku Utara	22,9	20,2-25,9	480	77,6	72,5-82,0	116
Papua Barat	13,2	10,3-16,8	333	51,6	39,3-63,8	46*
Papua	10,6	8,9-12,5	1.087	68,0	59,3-75,6	121
**INDONESIA**	**22,9**	**22,5-23,4**	**88.616**	**76,2**	**75,3-77,1**	**21.434**

*N Tertimbang <50

Tabel 16.1.3
Proporsi Remaja Putri Umur 10-19 Tahun yang Memperoleh Tablet Tambah Darah (TTD) menurut Karakteristik, Riskesdas 2018

Karakteristik	Pernah memperoleh TTD			Memperoleh TTD dalam 12 bulan terakhir		
	%	95% CI	N Ter-timbang	%	95 %CI	N Ter-timbang
**Kelompok Umur**						
10-11	2,7	2,4- 3,0	19.886	77,9	73,0-82,2	564
12-15	25,1	24,5-25,8	36.798	84,4	83,3-85,4	9.765
16-18	34,6	33,7-35,5	26.054	72,4	71,0-73,8	9.522
19	25,5	23,8-27,3	5.878	47,7	43,9-51,5	1.582
**Pendidikan**						
Tidak/belum tamat sekolah	4,9	4,0-5,9	3.163	77,4	68,5-84,4	162
Tidak tamat SD/MI	6,1	5,7-6,5	24.498	79,9	77,0-82,6	1.574
Tamat SD/MI	26,0	25,1-26,8	26.790	84,0	82,7-85,3	7.342
Tamat SLTP/MTs	36,0	35,0-37,0	23.485	75,7	74,4-77,0	8.925
Tamat SLTA/MA	30,5	29,1-31,8	10.602	58,8	56,2-61,3	3.408
Tamat D1-D3/PT	27,1	15,7 - 42,6	77	61,3	35,7 - 81,9	22*
**Tempat tinggal**						
Perkotaan	22,9	22,3-23,6	48.309	76,2	74,9-77,5	11.686
Perdesaan	22,9	22,3-23,5	40.307	76,1	75,0-77,3	9.748

*N Tertimbang <50

Tabel 16.1.4
Proporsi Sumber Perolehan TTD pada Remaja Putri Umur 10-19 Tahun
dalam 12 Bulan Terakhir menurut Provinsi, Riskesdas 2018

Provinsi	Fasilitas kesehatan		Sekolah		Inisiatif sendiri		N Tertimbang
	%	95% CI	%	95% CI	%	95 %CI	
Aceh	26,6	20,8-33,4	69,7	63,4-75,2	9,5	6,2-14,3	183
Sumatera Utara	19,6	15,1-25,1	72,3	67,1-77,0	12,3	9,1-16,5	427
Sumatera Barat	15,0	12,1-18,4	84,1	80,3-87,4	6,8	4,5-9,9	514
Riau	20,6	16,2-25,8	77,7	72,2-82,4	9,4	6,2-13,9	377
Jambi	13,9	10,0-18,9	84,0	78,6-88,2	6,1	3,7-9,8	221
Sumatera Selatan	14,2	9,5-20,7	84,7	77,7-89,8	4,7	2,2-9,9	353
Bengkulu	26,7	21,0-33,4	78,1	71,6-83,5	2,4	1,1-5,1	155
Lampung	26,9	20,9-33,8	70,6	63,6-76,8	10,4	7,1-15,1	438
Bangka Belitung	10,8	7,6-15,1	88,2	83,8-91,6	6,3	4,1-9,5	216
Kepulauan Riau	9,5	6,0-14,5	89,9	82,6-94,4	4,8	1,6-13,4	182
DKI Jakarta	15,5	10,3-22,5	66,3	57,6-73,9	27,2	20,1-35,6	479
Jawa Barat	17,9	14,7-21,6	76,1	72,2-79,7	10,4	8,1-13,2	2.068
Jawa Tengah	11,2	9,4-13,1	84,3	82,1-86,2	11,7	9,9-13,8	2.862
DI Yogyakarta	12,7	9,0-17,7	81,7	75,5-86,7	12,7	8,4-18,9	300
Jawa Timur	9,7	8,1-11,6	83,7	81,2-85,8	11,3	9,5-13,3	2.906
Banten	14,8	10,1-21,1	78,1	70,7-84,1	13,8	9,2-20,4	563
Bali	6,1	4,3-8,6	91,6	88,5-93,8	5,4	3,4-8,4	566
Nusa Tenggara Barat	10,0	7,2-13,7	89,9	86,4-92,6	6,4	3,9-10,2	409
Nusa Tenggara Timur	36,9	31,5-42,6	62,9	57,1-68,3	6,5	4,5-9,4	311
Kalimantan Barat	15,9	11,6-21,5	77,2	71,9-81,7	12,8	9,2-17,4	224
Kalimantan Tengah	15,4	11,7-20,1	82,8	78,1-86,6	8,8	5,9-13,0	209
Kalimantan Selatan	21,4	15,9-28,1	76,8	70,6-82,1	6,6	4,3-9,9	345
Kalimantan Timur	14,8	9,1-23,1	79,1	70,3-85,8	11,5	6,9-18,4	246
Kalimantan Utara	9,1	4,6-17,3	89,3	79,8-94,6	2,7	0,6-10,4	40*
Sulawesi Utara	23,5	17,8-30,3	75,7	69,4-81,1	5,8	3,1-10,4	107
Sulawesi Tengah	24,5	19,7-30,1	76,2	70,7-81,0	3,2	1,9-5,4	326
Sulawesi Selatan	13,0	10,8-15,5	87,6	85,1-89,8	3,5	2,3-5,3	886
Sulawesi Tenggara	12,0	9,2-15,4	89,1	85,4-91,9	2,1	1,0-4,4	303
Gorontalo	29,7	23,4-36,9	74,9	68,2-80,6	3,4	1,3-8,5	95
Sulawesi Barat	21,6	15,5-29,1	80,1	73,0-85,8	1,7	0,5-5,2	120
Maluku	19,8	12,4-30,1	72,8	61,8-81,6	9,4	5,2-16,4	93
Maluku Utara	32,4	26,0-39,6	70,3	63,2-76,5	3,0	1,2-7,4	92
Papua Barat	43,8	28,1-61,0	52,6	38,2-66,6	12,9	4,9-29,7	25*
Papua	23,7	16,6-32,6	77,6	69,1-84,3	8,4	4,7-14,7	85
**INDONESIA**	**14,9**	**14,1-15,7**	**80,9**	**80,0-81,8**	**9,7**	**9,0-10,4**	**16.726**

*N Tertimbang <50

Tabel 16.1.5
Proporsi Sumber Perolehan TTD pada Remaja Putri Umur 10-19 Tahun
dalam 12 Bulan Terakhir menurut Karakteristik, Riskesdas 2018

Karakteristik	Fasilitas kesehatan		Sekolah		Inisiatif sendiri		N Tertimbang
	%	95% CI	%	95% CI	%	95% CI	
**Kelompok Umur**							
10-11	15,0	10,9-20,2	81,1	75,5-85,6	5,8	3,6-9,1	450
12-15	12,0	11,1-13,0	87,6	86,5-88,5	4,8	4,1-5,7	8.439
16-18	16,9	15,6-18,3	77,6	76,1-79,1	12,3	11,2-13,6	7.064
19	28,8	24,0-34,1	37,6	32,6-42,9	41,2	35,9-46,7	773
**Pendidikan**							
Tidak/belum tamat sekolah	18,9	11,5-29,5	80,1	69,0-87,9	5,2	2,1-12,1	129
Tidak tamat SD/MI	14,4	11,9-17,3	83,8	80,6-86,5	6,2	4,4-8,5	1.289
Tamat SD/MI	11,5	10,5-12,7	86,9	85,6-88,1	5,3	4,5-6,3	6.319
Tamat SLTP/MTs	15,9	14,7-17,2	81,5	80,1-82,8	9,2	8,2-10,3	6.924
Tamat SLTA/MA	22,1	19,4-25,1	58,5	55,3-61,8	27,2	24,3-30,2	2.052
Tamat D1/D2/D3/PT	0,0	0,0 – 0,0	80,9	21,7 – 84,0	45,3	16,0 – 78,3	14*
**Tempat tinggal**							
Perkotaan	13,5	12,4-14,7	79,9	78,5-81,2	12,6	11,5-13,8	9.125
Perdesaan	16,7	15,6-17,8	82,1	80,9-83,2	6,2	5,5-7,1	7.601

*N Tertimbang <50

Tabel 16.1.6

Proporsi Jumlah Butir TTD yang Diperoleh dan Diminum Remaja Putri umur 10-19 Tahun dalam 12 Bulan Terakhir menurut Provinsi, Riskesdas 2018

Provinsi	Jumlah yang didapat (butir)						Jumlah yang diminum (butir)					
	Fasilitas Kesehatan		Sekolah		Inisiatif Sendiri		Fasilitas Kesehatan		Sekolah		Inisiatif Sendiri	
	< 52	≥ 52	< 52	≥ 52	< 52	≥ 52	< 52	≥ 52	< 52	≥ 52	< 52	≥ 52
	%	%	%	%	%	%	%	%	%	%	%	%
Aceh	96,2	3,8	99,4	0,6	93,2	6,8	98,3	1,7	99,8	0,2	93,2	6,8
Sumatera Utara	98,0	2,0	99,0	1,0	99,4	0,6	98,0	2,0	99,6	0,4	99,4	0,6
Sumatera Barat	98,6	1,4	96,1	3,9	97,1	2,9	99,2	0,8	99,0	1,0	97,1	2,9
Riau	97,1	2,9	99,4	0,6	100,0	0	100,0	0	99,8	0,2	100,0	0
Jambi	99,7	0,3	97,5	2,5	100,0	0	99,7	0,3	99,0	1,0	100,0	0
Sumatera Selatan	99,0	1,0	97,9	2,1	100,0	0	100,0	0	99,6	0,4	100,0	0
Bengkulu	100,0	0	96,5	3,5	90,8	9,2	100,0	0	97,8	2,2	90,8	9,2
Lampung	95,8	4,2	96,9	3,1	98,1	1,9	97,6	2,4	99,8	0,2	98,1	1,9
Bangka Belitung	100,0	0	96,4	3,6	100,0	0	100,0	0	98,4	1,6	100,0	0
Kepulauan Riau	96,9	3,1	97,8	2,2	98,9	1,1	96,9	3,1	99,4	0,6	98,9	1,1
DKI Jakarta	97,4	2,6	95,3	4,7	100,0	0,0	100,0	0	98,2	1,8	100,0	0
Jawa Barat	97,5	2,5	95,3	4,7	95,8	4,2	97,5	2,5	98,8	1,2	96,2	3,8
Jawa Tengah	97,4	2,6	93,9	6,1	90,7	9,3	97,9	2,1	97,5	2,5	91,7	8,3
DI Yogyakarta	93,7	6,3	97,7	2,3	87,2	12,8	93,7	6,3	98,9	1,1	100,0	0
Jawa Timur	93,5	6,5	95,6	4,4	98,3	1,7	96,4	3,6	98,0	2,0	98,6	1,4
Banten	100,0	0	94,5	5,5	100,0	0	100,0	0	98,5	1,5	100,0	0
Bali	91,6	8,4	96,4	3,6	96,8	3,2	97,4	2,6	98,4	1,6	99,1	0,9
Nusa Tenggara Barat	98,7	1,3	98,0	2,0	94,0	6,0	98,7	1,3	98,8	1,2	94,0	6,0
Nusa Tenggara Timur	99,5	0,5	99,3	0,7	98,2	1,8	99,5	0,5	99,9	0,1	100,0	0
Kalimantan Barat	100,0	0	98,6	1,4	87,8	12,2	100,0	0	99,5	0,5	95,9	4,1
Kalimantan Tengah	99,6	0,4	98,4	1,6	98,2	1,8	99,7	0,3	98,9	1,1	100,0	0
Kalimantan Selatan	93,4	6,6	92,8	7,2	83,2	16,8	96,3	3,7	97,7	2,3	83,2	16,8
Kalimantan Timur	100,0	0	99,3	0,7	100,0	0	100,0	0	99,7	0,3	100,0	0
Kalimantan Utara	100,0	0	99,2	0,8	100,0	0	100,0	0	100,0	0	100,0	0
Sulawesi Utara	97,9	2,1	98,8	1,2	100,0	0	97,9	2,1	99,8	0,2	100,0	0
Sulawesi Tengah	93,0	7,0	99,3	0,7	100,0	0	95,9	4,1	99,8	0,2	100,0	0
Sulawesi Selatan	98,6	1,4	98,7	1,3	100,0	0	99,4	0,6	99,4	0,6	100,0	0
Sulawesi Tenggara	96,1	3,9	99,4	0,6	100,0	0	100,0	0	99,8	0,2	100,0	0
Gorontalo	100,0	0	100,0	0	100,0	0	100,0	0	100,0	0	100,0	0
Sulawesi Barat	100,0	0	99,5	0,5	100,0	0	100,0	0	99,7	0,3	100,0	0
Maluku	100,0	0	98,4	1,6	100,0	0	100,0	0	99,8	0,2	100,0	0
Maluku Utara	97,5	2,5	98,6	1,4	100,0	0	98,6	1,4	99,2	0,8	100,0	0
Papua Barat	100,0	0	98,7	1,3	100,0	0	100,0	0	98,7	1,3	100,0	0
Papua	100,0	0,0	96,4	3,6	100,0	0	100,0	0,0	98,6	1,4	100,0	0
**INDONESIA**	**97,1**	**2,9**	**96,3**	**3,7**	**96,0**	**4,0**	**98,2**	**1,8**	**98,6**	**1,4**	**96,8**	**3,2**

Tabel 16.1.7

Proporsi Jumlah Butir TTD yang Diperoleh dari Fasilitas Kesehatan dan Diminum Remaja Putri Umur 10-19 Tahun dalam 12 Bulan Terakhir menurut Karakteristik, Riskesdas 2018

Karakteristik	Jumlah butir TTD yang diperoleh dari Fasilitas Kesehatan			Jumlah butir TTD yang diminum		
	%		N	%		N
	< 52	≥ 52	Tertimbang	< 52	≥ 52	Tertimbang
**Kelompok Umur**						
10-11	100,0	0,0	73	100,0	0,0	73
12-15	97,0	3,0	1.099	97,9	2,1	1.099
16-18	97,3	2,7	1.298	98,8	1,2	1.298
19	96,2	3,8	241	96,2	3,8	241
**Pendidikan**						
Tidak/belum tamat sekolah	97,9	2,1	26	97,9	2,1	26*
Tidak tamat SD/MI	96,1	3,9	201	97,5	2,5	201
Tamat SD/MI	97,3	2,7	791	97,8	2,2	791
Tamat SLTP/MTs	97,1	2,9	1.199	98,6	1,4	1.199
Tamat SLTA/MA	97,2	2,8	493	98,2	1,8	493
Tamat D1/D2/D3/PT	N/A	N/A	0*	N/A	N/A	0*
**Tempat tinggal**						
Perkotaan	98,2	1,8	1.336	99,0	1,0	1.336
Perdesaan	96,0	4,0	1.375	97,5	2,5	1.375

*N Tertimbang <50

Tabel 16.1.8
Proporsi Jumlah Butir TTD yang Diperoleh dari Sekolah dan Diminum Remaja Putri Umur 10-19 Tahun dalam 12 Bulan Terakhir menurut Karakteristik, Riskesdas 2018

Karakteristik	Jumlah butir TTD yang diperoleh dari Sekolah			Jumlah butir TTD yang diminum		
	%		N tertimbang	%		N tertimbang
	< 52	≥ 52		< 52	≥ 52	
**Kelompok Umur**						
10-11	97,4	2,6	370	98,8	1,2	370
12-15	96,6	3,4	7.477	98,6	1,4	7.477
16-18	95,7	4,3	5.549	98,6	1,4	5.549
19	96,5	3,5	294	98,3	1,7	294
**Pendidikan**						
Tidak/belum tamat sekolah	97,7	2,3	104	98,4	1,6	104
Tidak tamat SD/MI	97,0	3,0	1.093	98,4	1,6	1.093
Tamat SD/MI	96,1	3,9	5.559	98,5	1,5	5.559
Tamat SLTP/MTs	96,2	3,8	5.711	98,6	1,4	5.711
Tamat SLTA/MA	97,0	3,0	1.215	98,9	1,1	1.215
Tamat D1/D2/D3/PT	100,0	0,0	8*	100,0	0,0	8*
**Tempat tinggal**						
Perkotaan	95,9	4,0	7.375	98,6	1,4	7.375
Perdesaan	96,7	3,3	6.315	98,6	1,4	6.315

*N Tertimbang <50

Tabel 16.1.9

Proporsi Jumlah Butir TTD yang Diperoleh dengan Inisiatif Sendiri dan Diminum Remaja Putri Umur 10-19 Tahun dalam 12 Bulan Terakhir menurut Karakteristik, Riskesdas 2018

Karakteristik	Jumlah butir TTD yang diperolehdenganinisiatifsendiri			Jumlah butir TTD yang diminum		
	%		N tertimbang	%		N tertimbang
	< 52	≥ 52		< 52	≥ 52	
**Kelompok Umur**						
10-11	87,0	13,0	20*	87,0	13,0	20*
12-15	94,4	5,6	306	94,6	5,4	306
16-18	96,7	3,3	651	97,5	2,5	651
19	96,8	3,2	238	98,7	1,3	238
**Pendidikan**						
Tidak/belum tamat sekolah	100,0	0,0	5*	100,0	0,0	5*
Tidak tamat SD/MI	85,7	14,3	60	87,0	7,748	60
Tamat SD/MI	96,4	3,6	250	96,4	9,065	250
Tamat SLTP/MTs	96,7	3,3	478	97,4	12,452	478
Tamat SLTA/MA	96,3	3,7	418	97,8	9,190	418
Tamat D1/D2/D3/PT	100,0	0,0	5*	100,0	0,0	5*
**Tempat tinggal**						
Perkotaan	95,2	4,8	861	96,0	4,0	861
Perdesaan	97,8	2,2	354	98,8	1,2	354

*N Tertimbang <50

Tabel 16.1.10
Proporsi Alasan Utama Tidak Minum/Menghabiskan TTD yang Diperoleh dari Fasilitas Kesehatan pada Remaja Putri umur 10-19 Tahun menurut Provinsi, Riskesdas 2018

Provinsi	Alasan utama tidak minum/menghabiskan TTD (%)							N Ter-timbang
	Hanya diminum ketika haid	Lupa	Rasa dan bau tidak enak	Ada efek samping	Belum waktunya habis	Merasa tidak perlu	Lain-nya	
Aceh	4,7	29,2	10,5	12,2	3,6	13,5	26,2	27*
Sumatera Utara	11,3	15,5	21,8	6,3	13,4	22,2	9,5	39*
Sumatera Barat	0,3	21,6	22,6	13,2	0,0	40,7	1,5	44*
Riau	6,7	25,4	6,8	11,1	4,1	21,4	24,5	30*
Jambi	4,5	18,8	25,0	0,0	0,0	16,8	35,0	7*
Sumatera Selatan	6,7	18,4	7,6	5,3	4,0	50,4	7,6	25*
Bengkulu	6,2	44,6	18,0	13,1	0,0	7,5	10,6	20*
Lampung	6,5	27,7	26,2	10,0	8,7	16,2	4,7	69
Bangka Belitung	17,2	11,6	36,8	4,5	2,8	12,6	14,6	13*
Kepulauan Riau	0,0	10,6	34,1	16,6	3,6	31,7	3,4	6*
DKI Jakarta	5,5	9,0	19,7	0,0	0,0	53,7	12,0	57
Jawa Barat	4,6	22,8	20,0	10,9	3,5	28,4	9,8	197
Jawa Tengah	15,5	11,5	27,9	10,7	2,3	20,2	12,0	196
DI Yogyakarta	7,9	16,6	44,0	0,0	0,0	20,8	10,7	29*
Jawa Timur	3,7	18,3	20,3	5,2	3,9	37,0	11,6	137
Banten	0,0	9,3	5,9	0,0	2,9	60,1	21,7	31*
Bali	0,0	63,5	2,6	12,1	8,4	13,4	0,0	21*
Nusa Tenggara Barat	10,4	4,1	35,3	3,1	0,0	28,5	18,5	22*
Nusa Tenggara Timur	6,8	16,5	37,4	28,3	1,1	9,4	0,5	22*
Kalimantan Barat	0,0	16,8	32,6	9,7	5,9	18,5	16,5	22*
Kalimantan Tengah	8,9	36,6	17,9	6,7	5,2	14,9	9,9	16*
Kalimantan Selatan	7,2	18,1	17,4	11,6	12,9	23,5	9,2	45*
Kalimantan Timur	0,0	0,0	8,9	13,0	0,0	78,1	0,0	13*
Kalimantan Utara	0,0	50,7	24,6	0,0	0,0	24,7	0,0	1*
Sulawesi Utara	12,8	23,0	23,6	4,3	0,0	28,5	7,9	18*
Sulawesi Tengah	6,9	17,2	28,6	13,4	2,9	6,5	24,5	52
Sulawesi Selatan	0,2	26,3	26,2	11,3	5,6	17,2	13,3	68
Sulawesi Tenggara	0,8	16,9	27,6	9,4	3,9	16,6	24,8	25*
Gorontalo	8,3	13,3	40,7	12,4	2,1	15,6	7,6	20*
Sulawesi Barat	11,0	25,4	22,7	5,1	6,7	21,6	7,5	16*
Maluku	3,9	14,7	47,2	1,8	0,0	19,3	13,0	10*
Maluku Utara	0,0	49,0	10,5	1,1	1,8	30,1	1,3	13*
Papua Barat	0,0	12,6	48,0	0,0	0,0	35,9	3,6	6*
Papua	3,0	52,5	6,2	0,0	5,5	16,0	16,7	12*
**INDONESIA**	**6,6**	**20**	**22,9**	**8,9**	**3,8**	**26,1**	**11,7**	**1.332**

*N Tertimbang <50

Tabel 16.1.11
Proporsi Alasan Utama Tidak Minum/Menghabiskan TTD yang Diperoleh dari Fasilitas Kesehatan pada Remaja Putri umur 10-19 Tahun menurut Karakteristik, Riskesdas 2018

Karakteristik	Alasan utama tidak minum/menghabiskan TTD (%)							N tertimbang
	Hanya diminum ketika haid	Lupa	Rasa dan bau tidak enak	Ada efek samping	Belum waktunya habis	Merasa tidak perlu	Lainnya	
**Kelompok Umur**								
10-11	12,4	12,2	17,6	4,7	0,0	53,2	0,0	17*
12-15	4,4	19,8	27,1	5,8	6,1	21,1	15,6	499
16-18	8,2	20,5	20,4	11,3	1,7	27,8	10,0	710
19	5,0	18,1	20,9	7,5	8,0	34,1	6,4	106
**Pendidikan**								
Tidak/belum tamat sekolah	3,3	7,5	43,8	0,0	7,8	20,2	17,4	16*
Tidak tamat SD/MI	4,4	26,9	17,3	7,1	1,0	38,6	4,9	72
Tamat SD/MI	5,1	20,3	27,1	7,9	7,3	18,9	13,4	353
Tamat SLTP/MTs	6,6	19,1	23,6	10,0	2,6	26,2	12,0	629
Tamat SLTA/MA	9,3	20,5	16,0	8,3	2,8	32,8	10,3	262
Tamat D1/D2/D3/PT	0,0	0,0	0,0	0,0	0,0	0,0	0,0	0*
**Tempat tinggal**								
Perkotaan	7,8	18,6	19,3	7,4	3,3	31,9	11,7	674
Perdesaan	5,3	21,3	26,7	10,4	4,4	20,3	11,6	658

*N Tertimbang <50

Tabel 16.1.12
Proporsi Alasan Utama Tidak Minum/Menghabiskan TTD yang Diperoleh dari Sekolah pada Remaja Putri umur 10 – 19 Tahun menurut Provinsi, Riskesdas 2018

Provinsi	Alasan utama tidak minum/menghabiskan TTD (%)							N Tertimbang
	Hanya diminum ketika haid	Lupa	Rasa dan bau tidak enak	Ada efek samping	Belum waktunya habis	Merasa tidak perlu	Lainnya	
Aceh	6,3	24,0	22,9	5,9	3,0	28,6	9,3	70
Sumatera Utara	6,7	23,3	20,6	11,0	4,1	16,3	18,0	148
Sumatera Barat	1,4	19,4	30,7	11,8	0,7	26,1	10,0	277
Riau	0,6	19,1	28,4	9,1	3,2	23,8	15,8	102
Jambi	2,9	20,3	48,1	7,1	0,6	18,5	2,6	77
Sumatera Selatan	1,4	22,6	20,0	14,6	5,4	29,0	6,9	182
Bengkulu	3,5	17,9	31,4	11,9	0,0	26,5	8,6	50
Lampung	6,6	12,6	38,4	5,2	3,9	22,7	10,7	191
Bangka Belitung	0,7	21,7	27,1	12,8	1,1	22,2	14,4	78
Kepulauan Riau	8,0	16,4	40,9	8,8	6,1	13,6	6,1	90
DKI Jakarta	3,8	27,9	22,7	16,5	2,0	23,4	3,8	144
Jawa Barat	1,6	12,5	41,0	16,2	1,6	8,1	19,1	886
Jawa Tengah	3,9	17,1	34,9	9,1	1,7	18,5	14,9	1.310
DI Yogyakarta	2,9	20,6	37,5	15,4	0,3	14,2	9,0	133
Jawa Timur	3,7	19,7	29,1	7,7	1,9	26,8	11,1	1.288
Banten	3,5	20,3	33,0	8,3	1,9	17,5	15,4	251
Bali	4,7	26,7	27,9	9,2	2,1	20,1	9,4	256
Nusa Tenggara Barat	4,6	12,4	45,6	7,3	0,9	17,5	11,7	163
Nusa Tenggara Timur	2,0	34,0	16,1	14,9	3,5	22,1	7,4	33*
Kalimantan Barat	3,3	17,0	30,4	9,4	7,1	22,9	9,9	114
Kalimantan Tengah	7,6	15,9	24,8	10,5	3,6	25,7	11,8	80
Kalimantan Selatan	8,9	13,7	24,1	13,5	5,7	22,6	11,4	166
Kalimantan Timur	1,6	18,4	40,3	12,8	5,6	9,0	12,3	88
Kalimantan Utara	0,2	30,9	14,6	10,1	4,2	29,8	10,1	25*
Sulawesi Utara	6,2	37,3	23,8	5,9	0,5	19,4	7,0	57
Sulawesi Tengah	3,9	23,0	26,9	16,0	3,6	17,3	9,2	109
Sulawesi Selatan	2,0	20,9	22,2	6,7	2,7	29,6	16,0	466
Sulawesi Tenggara	2,3	28,9	21,4	6,6	1,5	26,4	12,9	161
Gorontalo	3,0	19,5	37,7	10,2	2,4	14,8	12,3	35*
Sulawesi Barat	0,4	28,8	40,3	2,4	5,9	11,2	11,0	42*
Maluku	2,9	30,4	21,4	3,5	5,2	20,5	16,2	48*
Maluku Utara	2,8	30,4	33,7	7,7	3,6	7,6	14,3	28*
Papua Barat	0,0	30,3	27,2	0,0	0,0	29,1	13,4	4*
Papua	4,0	45,5	16,0	1,2	4,9	21,5	7,0	34*
**INDONESIA**	**3,5**	**19,2**	**31,5**	**10,0**	**2,3**	**20,5**	**12,9**	**7.188**

*N Tertimbang <50

Tabel 16.1.13

Proporsi Alasan Utama Tidak Minum/Menghabiskan TTD yang Diperoleh dari Sekolah pada Remaja Putri umur 10 – 19 Tahun menurut Karakteristik, Riskesdas 2018

Karakteristik	Alasan utama tidak minum/menghabiskan TTD (%)							N Tertimbang
	Hanya diminum ketika haid	Lupa	Rasa dan bau tidak enak	Ada efek samping	Belum waktunya habis	Merasa tidak perlu	Lainnya	
**Kelompok Umur**								
10-11	1,5	17,1	41,0	7,2	3,2	18,7	11,2	114
12-15	3,2	20,2	32,1	9,3	2,7	20,1	12,3	3.679
16-18	3,9	18,4	30,1	11,2	1,8	20,9	13,7	3.242
19	0,2	14,4	38,6	6,5	4,7	24,6	11,0	153
**Pendidikan**								
Tidak/belum tamat sekolah	0,7	29,6	23,2	7,9	0	14,4	6,5	38*
Tidak tamat SD/MI	2,5	18,8	30,3	9,0	2,7	23,9	6,3	415
Tamat SD/MI	3,3	20,1	29,7	9,5	2,8	19,3	12,7	2.732
Tamat SLTP/MTs	3,5	18,4	28,6	10,7	2,0	20,6	13,8	3.292
Tamat SLTA/MA	4,5	18,8	23,6	9,4	2,1	23,4	13,9	706
Tamat D1/D2/D3/PT	0,0	38,3	20,0	23,2	0,0	0,0	18,5	4*
**Tempat tinggal**								
Perkotaan	2,8	18,8	29,4	11,0	2,2	21,7	14,0	4.086
Perdesaan	4,3	19,8	34,2	8,7	2,5	19,0	11,5	3.102

*N Tertimbang <50

Tabel 16.1.14
Proporsi Alasan Utama Tidak Minum/Menghabiskan TTD yang Diperoleh dengan Inisiatif Sendiri pada Remaja Putri Umur 10-19 Tahun menurut Provinsi, Riskesdas 2018

Provinsi	Alasan utama tidak minum/menghabiskan TTD (%)							N Ter-timbang
	Hanya diminum ketika haid	Lupa	Rasa dan bau tidak enak	Ada efek samping	Belum waktunya habis	Merasa tidak perlu	Lainnya	
Aceh	19,0	19,7	44,6	6,7	0,0	10,0	0,0	4*
Sumatera Utara	26,4	16,5	0,7	15,6	13,4	27,0	0,5	17*
Sumatera Barat	7,4	19,5	11,6	25,0	0,0	23,3	13,2	11*
Riau	32,6	7,9	14,7	0,0	9,4	30,5	4,9	15*
Jambi	19,8	0,0	0,0	9,9	0,0	70,3	0,0	2*
Sumatera Selatan	0,0	34,7	0,0	27,1	0,0	38,2	0,0	3*
Bengkulu	0,0	0,0	89,7	0,0	0,0	10,3	0,0	1*
Lampung	13,5	7,5	9,1	9,9	10,9	41,2	7,9	18*
Bangka Belitung	17,7	41,5	9,3	0,0	0,0	31,5	0,0	5*
Kepulauan Riau	39,4	56,6	0,0	0,0	0,0	4,0	0,0	5*
DKI Jakarta	14,1	14,6	11,8	0,0	0,7	35,7	23,1	53
Jawa Barat	6,1	14,2	6,7	7,4	20,3	38,3	7,1	72
Jawa Tengah	27,2	3,7	10,7	0,9	1,8	42,0	13,6	107
DI Yogyakarta	7,9	24,8	14,8	0,0	0,0	45,2	7,4	16*
Jawa Timur	19,0	15,4	4,1	2,0	4,0	43,0	12,6	110
Banten	5,5	22,4	0,0	0,0	0,0	56,2	15,9	42*
Bali	37,7	17,0	8,3	0,0	0,0	29,9	7,1	14*
Nusa Tenggara Barat	22,2	0,0	19,3	0,0	7,6	29,3	21,5	7*
Nusa Tenggara Timur	34,4	25,6	0,0	8,8	0,0	31,2	0,0	5*
Kalimantan Barat	34,4	14,9	9,9	0,0	13,6	16,2	11,1	8*
Kalimantan Tengah	15,7	22,3	18,9	0,0	0,0	38,9	4,3	6*
Kalimantan Selatan	15,4	18,1	11,5	0,0	2,4	28,4	24,2	9*
Kalimantan Timur	6,8	11,8	13,0	0,8	0,0	61,9	5,7	13*
Kalimantan Utara	0,0	21,1	0,0	0,0	0,0	78,9	0,0	1*
Sulawesi Utara	7,1	11,1	0,0	0,0	0,0	54,8	27,0	2*
Sulawesi Tengah	34,6	19,0	10,4	0,0	0,0	36,0	0,0	3*
Sulawesi Selatan	37,5	14,7	0,7	8,8	0,0	38,3	0,0	14*
Sulawesi Tenggara	4,4	32,8	0,0	0,0	0,0	62,8	0,0	3*
Gorontalo	0,0	66,4	0,0	33,6	0,0	0,0	0,0	1*
Sulawesi Barat	50,5	38,4	0,0	0,0	11,1	0,0	0,0	1*
Maluku	0,0	0,0	13,0	0,0	0,0	53,8	33,2	3*
Maluku Utara	0,0	10,6	0,0	0,0	0,0	46,3	43,1	1*
Papua Barat	0,0	27,1	26,2	0,0	21,2	9,9	15,7	1*
Papua	27,5	23,9	0,0	0,0	0,0	37,1	11,5	2*
**INDONESIA**	**18,2**	**14,3**	**7,9**	**3,3**	**5,1**	**39,7**	**11,5**	**575**

*N Tertimbang <50

Tabel 16.1.15
Proporsi Alasan Utama Tidak Minum/Menghabiskan TTD yang Diperoleh dengan Inisiatif Sendiri pada Remaja Putri Umur 10-19 Tahun menurut Karakteristik, Riskesdas 2018

Karakteristik	Alasan utama tidak minum/menghabiskan TTD (%)							NTer-timbang
	Hanya diminum ketika haid	Lupa	Rasa dan bau tidak enak	Ada efek samping	Belum waktunya habis	Merasa tidak perlu	Lainnya	
**Kelompok Umur**								
10-11	35,6	16,6	11,3	0,0	0,0	34,6	1,8	9*
12-15	27,2	15,0	10,4	2,1	9,6	26,8	8,9	143
16-18	15,8	14,0	7,2	3,8	3,9	45,8	9,4	302
19	12,3	13,8	6,8	3,7	3,0	40,2	20,2	122
**Pendidikan**								
Tidak/belum tamat sekolah	0,0	0,0	100,0	0,0	0,0	0,0	0,0	2*
Tidak tamat SD/MI	18,9	18,6	18,5	0	1,6	39,7	2,7	30
Tamat SD/MI	22,2	16,6	8,3	3,0	10,4	29,9	9,6	115
Tamat SLTP/MTs	21,4	15,5	6,5	3,2	3,2	44,1	6,2	215
Tamat SLTA/MA	11,9	11,4	7,1	4,2	4,7	41,2	19,4	210
Tamat D1/D2/D3/PT	72,1	0,0	0,0	0,0	0,0	27,9	0,0	4*
**Tempat tinggal**								
Perkotaan	18,4	15,1	7,3	3,8	3,6	39,8	12,0	403
Perdesaan	17,8	12,3	9,5	2,2	8,4	39,6	10,2	172

*N Tertimbang <50

16.2 Pemberian Makanan Tambahan Ibu Hamil

Ibu hamil (bumil) merupakan kelompok rawan gizi yang menjadi salah satu sasaran program Pemberian Makanan Tambahan (PMT). Program ini bertujuan untuk mengatasi gizi kurang pada bumil dengan fokus zat gizi makro maupun mikro yang diperlukan untuk mencegah Bayi Berat Lahir Rendah (BBLR). Makanan tambahan yang diberikan dapat berbentuk makanan keluarga berbasis pangan lokal dengan resep–resep yang dianjurkan maupun makanan tambahan pabrikan yang lebih praktis dengan komposisi zat gizi yang sudah baku sesuai Permenkes nomor 51 Tahun 2016.

Makanan tambahan yang termasuk di dalam pertanyaan ini yaitu:

1. Makanan tambahan yang hanya diberikan setiap kali posyandu (PMT penyuluhan).
2. Makanan tambahan yang khusus diberikan untuk ibu hamil KEK, biasanya diberikan selama 90 hari makan (PMT pemulihan). Biasa diberikan di Posyandu atau melalui kader/bidan/petugas puskesmas.
3. Makanan tambahan yang diperoleh dari bantuan pihak lain, contoh: sumbangan dari LSM/perusahaan atau pihak tertentu yang sedang melakukan kampanye atau promosi produk tertentu.

Makanan tambahan yang diperoleh dari **membeli sendiri tidak termasuk** dalamkategori PMT dalam pertanyaan subbab ini.

IBU HAMIL MENDAPATKAN PMT

Definisi: Ibu hamil saat ini yang mendapatkan makanan tambahan (PMT) dengan jenis makanan tambahan yaitu biskuit program; biskuit selain program, susu bubuk, susu cair, bahan makanan mentah, dan bahan makanan matang.

$$Proporsi\ ibu\ hamil\ mendapatkan\ PMT = \frac{Jumlah\ perempuan\ umur\ 10-54\ tahun\ sedang\ hamil\ dan\ mendapat\ PMT}{Jumlah\ perempuan\ 10-54\ tahun\ sedang\ hamil}$$

RATA-RATA UMUR KEHAMILAN PERTAMA KALI MENDAPATKA PMT

Definisi: Jumlah bulan kehamilan saat pertama kali Ibu hamil mendapatkan makanan tambahan (PMT) dengan jenis makanan tambahan yaitu biskuit program; biskuit selain program, susu bubuk, susu cair, bahan makanan mentah, dan bahan makanan matang.

$$Rata-rata\ umur\ kehamilan\ ibu\ saat\ pertama\ mendapatkan\ PMT = \frac{Jumlah\ bulan\ kehamilan\ seluruh\ ibu\ hamil\ saat\ pertama\ kali\ mendapat\ PMT}{Jumlah\ ibu\ hamil}$$

IBU HAMIL MENDAPATKAN PMT PROGRAM

Definisi: Ibu hamil yang mendapatkan PMT Biskuit Program Kementerian Kesehatan pada kehamilaan saat wawancara.

$$Proporsi\ ibu\ hamil\ mendapatkan\ PMT\ Program = \frac{Ibu\ hamil\ yang\ mendapat\ PMT\ Program}{Total\ ibu\ hamil\ yang\ mendapat\ PMT}$$

JUMLAH PMT PROGRAM YANG DIDAPAT IBU HAMIL

Definisi: Jumlah PMT Biskuit Program Kementerian Kesehatan yang diperoleh ibu hamil dalam satuan bungkus, dengan kategori (1) 0-30 bungkus; (2) 31-89 bungkus; dan (3) ≥ 90 bungkus.

$$Proporsi\ ibu\ hamil\ mendapatkan\ PMT\ Program = \frac{Jumlah\ PMT\ Program\ (bungkus)\ yang\ diperoleh\ Ibu\ hamil}{Total\ ibu\ hamil\ yang\ mendapat\ PMT}$$

IBU HAMIL YANG TIDAK MENGHABISKAN PMT PROGRAM

Definisi: Ibu hamil tidak menghabiskan PMT Biskuit Program Kementerian Kesehatan dihitung dengan formula.

$$Proporsi\ ibu\ hamil\ yang\ tidak\ menghabiskan\ PMT\ Program = \frac{Ibu\ hamil\ yang\ tidak\ menghabiskan\ PMT\ Program}{Total\ ibu\ hamil\ yang\ mendapat\ PMT\ Program}$$

ALASAN TIDAK MENGHABISKAN PMT PROGRAM

Definisi: Ibu hamil dengan alasan tidak menghabiskan PMT Biskuit Program Kementerian Kesehatan, yaitu (1) rasa tidak enak; (2) rasa kurang bervariasi; (3) terlalu manis; (4) tidak suka aroma; (5) ada fek samping; (6) lupa; (7) dimakan ART lain; dan (8) lainnya.

$$Proporsi\ alasan\ ibu\ hamil\ tidak\ menghabiskan\ PMT\ Program = \frac{Jumlah\ ibu\ hamil\ menurut\ alasan\ tidak\ menghabiskan\ PMT\ Program}{Total\ ibu\ hamil\ yang\ tidak\ menghabiskan\ PMT\ Program}$$

JENIS PMT YANG DIDAPAT IBU HAMIL

Definisi: Jenis PMT yang didapat oleh ibu hamil, yaitu (1) biskuit program; (2) biskuit lainnya; (3) susu bubuk; (4) susu cair; (5) bahan makanan mentah; dan (6) makanan matang.

$$Proporsi\ jenis\ PMT\ yang\ didapat\ ibu\ hamil = \frac{Jumlah\ ibu\ hamil\ menurut\ jenis\ PMT\ yang\ diperoleh}{Total\ ibu\ hamil\ yang\ mendapat\ PMT}$$

ALASAN MENDAPATKAN MAKANAN TAMBAHAN

Definisi: Ibu hamil dengan alasan mendapatkan makanan tambahan, yaitu (1) ibu kurang gizi (KEK); (2) keluarga miskin; (3) periksa hamil ke posyandu; (4) berat badan hamil tidak pernah naik; (5) anemia; dan (8) lainnya.

$$Proporsi\ alasan\ ibu\ hamil\ mendapatkan\ makanan\ tambahan = \frac{Jumlah\ ibu\ hamil\ menurut\ alasan\ mendapatkan\ makanan\ tambahan}{Total\ ibu\ hamil\ yang\ mendapat\ PMT}$$

Tabel 16.2.1
Proporsi Ibu Hamil yang Mendapatkan PMT dan Rata-Rata Umur Kehamilan saat Pertama Mendapatkan PMT menurut Provinsi, Riskesdas 2018

Provinsi	Ibu hamil mendapatkan PMT		N ter-timbang	Umur kehamilan saat pertama mendapatkan PMT		N ter-timbang
	%	95% CI		Rerata	SD	
Aceh	37,7	32,3 – 43,5	204	3,0	1,72	77
Sumatera Utara	19,1	14,9 – 24,0	446	3,9	1,68	85
Sumatera Barat	31,0	25,4 – 37,2	193	3,9	2,00	60
Riau	30,4	24,5 – 36,9	271	3,1	1,71	82
Jambi	29,9	23,2 – 37,6	96	3,6	1,80	29*
Sumatera Selatan	19,9	14,8 – 26,3	283	4,4	1,85	56
Bengkulu	23,5	16,7 – 32,0	66	3,5	1,28	16*
Lampung	30,2	24,6 – 36,4	290	2,9	1,66	87
Bangka Belitung	18,0	12,1 – 26,0	55	3,3	1,48	10*
Kepulauan Riau	17,5	9,1 – 31,1	97	3,4	1,20	17*
DKI Jakarta	14,7	9,2 – 22,9	405	3,6	2,05	60
Jawa Barat	29,6	25,3 – 34,3	1,826	3,5	1,95	541
Jawa Tengah	34,3	30,3 – 38,7	1,016	3,8	1,90	349
DI Yogyakarta	36,8	24,2 – 51,5	113	3,0	1,98	41*
Jawa Timur	17,3	14,3 – 20,7	1,167	3,5	1,98	201
Banten	24,0	18,4 – 30,7	431	3,6	2,20	104
Bali	18,5	12,9 – 25,7	149	3,2	1,67	28*
Nusa Tenggara Barat	19,9	13,9 – 27,5	205	3,3	1,78	41*
Nusa Tenggara Timur	22,0	17,5 – 27,3	172	4,4	1,83	38*
Kalimantan Barat	23,8	18,1 – 30,7	167	3,2	1,93	40*
Kalimantan Tengah	24,4	17,4 – 33,1	89	3,5	1,80	22*
Kalimantan Selatan	16,3	11,3 – 23,0	137	3,6	1,73	22*
Kalimantan Timur	19,2	11,9 – 29,5	124	3,7	2,56	24*
Kalimantan Utara	15,5	8,5 – 26,6	23*	3,1	2,37	4*
Sulawesi Utara	27,5	19,3 – 37,5	64	3,4	1,65	18*
Sulawesi Tengah	26,9	20,3 – 34,6	121	3,5	1,86	32*
Sulawesi Selatan	24,8	19,5 – 31,0	301	3,3	1,83	75
Sulawesi Tenggara	18,3	13,3 – 26,0	109	3,8	1,57	20*
Gorontalo	27,4	17,7 – 39,8	33*	4,3	1,68	9*
Sulawesi Barat	25,5	17,4 – 37,5	49*	3,8	1,67	12*
Maluku	28,9	20,8 – 38,5	55	4,2	1,76	16*
Maluku Utara	22,6	14,9 – 32,8	46*	4,5	1,81	10*
Papua Barat	30,9	19,1 – 45,8	32*	4,0	1,47	10*
Papua	11,8	7,6 – 17,9	66	3,3	2,02	8*
**INDONESIA**	**25,2**	**23,8 – 26,6**	**8,902**	**3.6**	**1.91**	**2.243**

*N Tertimbang <50

Tabel 16.2.2
Proporsi Ibu Hamil yang Mendapatkan PMT dan Rata-Rata Umur Kehamilan saat Pertama Mendapatkan PMT menurut Karakteristik, Riskesdas 2018

Karakteristik	Ibu hamil mendapatkan PMT		Umur kehamilan saat pertama mendapatkan PMT		N ter-timbang
	%	95 % CI	Rerata	SD	
**Kelompok Umur (tahun)**					
10–14	59,1	22,7 – 87,7	4,0	2,12	3*
15–19	31,8	26,4 – 37,8	3,7	1,90	192
20-24	26,6	23,8 – 29,6	3,6	2,01	604
25-29	252	22,7 – 27,8	3,3	1,68	661
30-34	23,2	20,6 – 25,9	3,8	2,01	480
35-39	24,8	21,5 – 28,4	3,5	1,96	268
40-44	13,0	8,9 – 18,5	3,7	1,92	28*
45-49	27,7	13,8 – 47,8	3,0	1,69	6*
50-54	6,6	0,6 – 45,6	2,0	0,00	0*
**Kelompok Umur (Khusus)**					
10 - 19 (Remaja)	32,1	26,7 – 38,0	3,7	1,90	195
15 - 49 (WUS)	25,2	23,8 – 26,6	3,6	1,91	2.240
**Pendidikan**					
Tidak/belum pernah sekolah	27,3	18,8 – 37,9	4,2	2,00	30*
Tidak tamat SD/MI	27,5	21,7 – 34,2	3,8	2,00	120
Tamat SD/MI	29,6	26,2 – 33,3	3,3	1,80	457
Tamat SLTP/MTS	31,5	28,6 – 34,6	3,6	1,94	671
Tamat SLTA/MA	22,4	20,3 – 24,6	3,6	1,92	742
Tamat D1/D2/D3/PT	16,2	13,8 – 18,9	3,4	1,89	223
**Pekerjaan**					
Tidak bekerja	27,2	25,4 – 29,1	3,6	1,90	1.485
Sekolah	24,1	13,7 – 39,0	4,6	2,17	20*
PNS/TNI/Polri/BUMN/BUMD	16,2	11,6 – 22,1	3,2	1,86	33*
Pegawai swasta	15,2	12,1 – 18,9	3,7	1,89	142
Wiraswasta	24,5	19,9 – 29,7	3,7	2,13	165
Petani	27,5	23,5 – 31,9	3,7	1,92	133
Nelayan	27,4	9,7 – 57,2	3,0	1,53	3*
Buruh/sopir/pembantu ruta	26,1	18,3 – 35,8	2,6	1,88	72
Lainnya	24,4	20,5 – 28,7	3,6	2,00	189
**Tempat Tinggal**					
Perkotaan	20,8	18,9 – 22,8	3,6	2,01	1.030
Perdesaan	30,8	29,0 – 32,7	3,6	1,81	1.213

*N tertimbang < 50

Tabel 16.2.3
Proporsi Perolehan PMT Program pada Ibu Hamil dan Alasan Utama Tidak Menghabiskan menurut Provinsi, Riskesdas 2018

Provinsi	Di antara bumil		Di antara bumil yang mendapat PMT					Di antara bumil yang tidak menghabiskan PMT: Alasan utama tidak menghabiskan								
	Bumil mendapat PMT Program	N tertimbang	Jumlah yang didapatkan (bungkus			Bumil tidak menghabiskan	N tertimbang	Rasa tidak enak	Rasa kurang bervariasi	Terlalu manis	Tidak suka aroma	Ada efek samping	Lupa	Dimakan ART lain	Lainnya	N tertimbang
			0–30	31–89	≥90											
	%		%	%	%	%		%	%	%	%	%	%	%	%	
Aceh	79,2	78	94,9	3,8	1,3	35,7	60	17,1	15,2	2,8	16,8	6,7	3,2	28,4	9,7	21 *
Sumatera Utara	87,6	86	96,5	3,5	0,0	37,7	73	12,4	10,4	12,4	16,8	1,6	0,0	44,2	2,3	27*
Sumatera Barat	91,8	60	88,6	9,4	2,0	36,7	54	38,2	10,8	9,6	9,0	11,3	2,8	10,0	8,2	19 *
Riau	84,9	83	90,4	5,6	3,9	40,2	69	31,5	4,2	1,7	30,2	7,0	3,9	15,4	6,0	27*
Jambi	98,6	29	97,4	2,6	0,0	26,9	28	30,6	0,0	10,5	17,3	0,0	3,9	16,8	20,9	7 *
Sumatera Selatan	93,9	57	97,6	2,4	0,0	28,9	52	23,8	11,4	16,7	4,9	0,0	5,8	33,0	4,3	15 *
Bengkulu	86,7	16 *	95,1	4,9	0,0	25,8	13	28,9	0,0	10,1	23,9	0,0	0,0	31,3	5,7	3 *
Lampung	96,6	88	95,1	4,9	0,0	33,9	83	16,2	6,6	10,4	17,1	8,8	0,0	33,1	7,9	27*
Bangka Belitung	93,0	10 *	83,7	3,0	13,2	28,9	9 *	43,4	0,0	0,0	12,3	0,0	0,0	11,7	32,5	3 *
Kepulauan Riau	100,0	17 *	99,2	0,0	0,8	53,0	17 *	74,3	0,0	3,5	0,0	0,0	8,0	11,9	2,5	9 *
DKI Jakarta	74,9	60	72,8	27,2	0,0	49,5	44	55,0	0,0	0,3	27,9	0,0	0,2	0,6	16,1	21 *
Jawa Barat	96,9	544	93,7	4,7	1,6	27,0	516	15,5	7,4	16,1	4,1	12,1	0,1	15,4	29,2	135
Jawa Tengah	90,7	351	91,0	6,5	2,5	34,9	312	23,7	6,7	17,3	6,2	5,2	3,4	20,9	16,5	105
DI Yogyakarta	100,0	42	89,3	2,5	8,2	72,3	41	28,6	11,3	13,4	7,7	9,8	1,7	13,3	14,2	28*
Jawa Timur	77,5	203	93,8	4,6	1,6	42,2	154	32,7	6,2	12,6	5,2	3,9	4,2	16,5	18,8	63
Banten	95,1	104	95,6	4,4	0,0	29,1	97	14,6	19,5	6,6	25,0	4,0	3,1	0,0	27,2	27*
Bali	83,6	28 *	85,0	12,7	2,4	47,2	23 *	8,3	21,0	24,4	0,1	12,5	0,0	0,0	33,6	10 *
Nusa Tenggara Barat	70,7	41	83,8	5,9	10,3	47,6	28	16,2	0,0	25,5	5,7	4,7	0,0	45,3	2,4	13 *
Nusa Tenggara Timur	88,9	38	90,8	7,7	1,5	25,0	33	0,0	5,5	6,0	17,8	11,6	0,0	59,1	0,0	8 *
Kalimantan Barat	83,2	40	87,5	12,5	0,0	25,8	33	51,2	0,0	1,4	3,2	14,0	23,9	6,4	0,0	8 *
Kalimantan Tengah	83,5	22 *	95,5	1,2	3,3	31,9	18 *	19,1	0,0	43,9	13,5	0,0	0,0	11,8	11,7	6 *
Kalimantan Selatan	94,5	22 *	77,0	12,9	10,2	33,3	21 *	46,6	0,0	18,1	0,0	17,5	0,0	0,0	17,7	7 *
Kalimantan Timur	87,9	24 *	98,3	0,5	1,2	45,1	21 *	27,6	1,4	23,2	2,3	27,0	0,0	18,5	0,0	9 *
Kalimantan Utara	68,5	4 *	91,4	0,0	8,6	22,2	2 *	76,6	23,4	0,0	0,0	0,0	0,0	0,0	0,0	1 *
Sulawesi Utara	95,6	18 *	96,1	3,9	0,0	45,8	17 *	43,6	8,8	3,2	0,0	6,5	6,7	1,7	29,4	7 *
Sulawesi Tengah	66,6	33 *	90,1	7,4	2,4	25,9	21 *	5,6	10,4	10,0	12,6	15,4	0,0	23,3	22,7	5 *
Sulawesi Selatan	88,5	75	86,7	6,5	6,8	50,9	65	19,1	9,4	26,5	13,8	0,0	5,5	15,1	10,7	32*
Sulawesi Tenggara	99,1	20 *	86,9	13,1	0,0	40,9	19 *	14,6	0,0	27,1	12,3	0,0	5,3	29,8	10,9	8 *
Gorontalo	91,1	9 *	96,9	3,1	0,0	36,8	8 *	15,4	0,0	3,9	10,7	0,0	0,0	22,7	47,3	3 *
Sulawesi Barat	91,1	12 *	94,8	5,2	0,0	23,4	11 *	18,4	0,0	10,3	0,0	0,0	57,2	14,2	0,0	3 *
Maluku	96,4	16 *	98,4	1,6	0,0	33,5	15 *	5,9	7,1	31,5	42,1	0,0	0,0	10,3	3,2	5 *
Maluku Utara	78,7	10 *	94,3	5,7	0,0	12,8	8 *	0,0	40,8	14,7	0,0	0,0	0,0	0,0	44,5	1 *
Papua Barat	79,3	10 *	80,8	19,2	0,0	47,6	8 *	6,5	0,0	50,9	11,7	0,0	0,0	12,8	18,1	4 *
Papua	92,3	8 *	100,0	0,0	0,0	7,7	7 *	37,9	0,0	0,0	0,0	0,0	0,0	62,1	0,0	1 *
**INDONESIA**	**89,7**	**2.256**	**92,1**	**5,9**	**2,0**	**34,8**	**1,981**	**23,7**	**7,5**	**14,1**	**10,2**	**6,8**	**2,7**	**18,4**	**16,6**	**664**

*N Tertimbang <50

Tabel 16.2.4
Proporsi Perolehan PMT Program pada Ibu Hamil dan Alasan Utama Tidak Menghabiskan menurut Karakteristik, Indonesia 2018

Karakteristik	DI antara bumil:		Di antara bumil yang mendapat PMT:					Di antara bumil yang tidak menghabiskan PMT: alasan utama tidak menghabiskan								
	Bumil mendapat PMT	N ter-timbang	Jumlah yang didapatkan (bungkus)			Bumil tidak habis-kan	N ter-timbang	Rasa tidak enak	Rasa kurang bervariasi	Terlalu manis	Tidak suka aroma	Ada efek samping	Lupa	Dimakan ART lain	Lain-nya	N ter-timbang
			0-30	31-89	≥90											
	%		%	%	%	%		%	%	%	%	%	%	%	%	
**Kelompok Umur**																
10–14	100,0	3*	100,0	0,0	0,0	52.4	3*	100,0	0,0	0,0	0,0	0,0	0,0	0,0	0,0	1*
15–19	96,0	193	87,1	8,6	4,3	28.9	181	20,4	0,7	10,8	16,9	4,7	5,2	20,3	21,0	51*
20-24	90,9	607	91,9	5,7	2,4	35.2	540	31,7	7,8	11,9	7,6	8,5	3,4	14,4	14,6	183
25-29	88,6	665	91,5	6,6	1,9	42.5	577	22,2	10,2	17,1	11,1	5,0	1,2	19,2	14,1	236
30-34	88,1	483	95,3	4,3	0,4	28.4	417	12,9	5,4	15,6	7,6	8,7	3,3	19,6	26,9	114
35-39	89,3	270	91,7	5,7	2,6	31.9	236	26,3	6,8	10,0	14,2	7,6	3,2	21,8	10,1	73
40-44	81,7	28	97,2	2,8	0,0	23.3	23*	24,4	0,0	16,8	1,7	1,4	0,0	24,1	31,6	5*
45-49	61,3	6*	100,0	0,0	0,0	23.7	4*	7,3	0,0	0,0	0,0	0,0	0,0	92,7	0,0	1*
50-54	100,0	0*	100,0	0,0	0,0	0,0	0*	0,0	0,0	0,0	0,0	0,0	0,0	0,0	0,0	0*
**Kelompok Umur (Khusus)**																
10 - 19 (Remaja)	96,1	196	87,3	8,5	4,2	29.2	184	22,5	0,7	10,5	16,5	4,6	5,0	19,7	20,5	52
15 - 49 (WUS)	89,7	2.253	92,1	5,9	2,0	34,7	1.978	23,5	7,5	14,1	10,2	6,8	2,7	18,4	16,6	663
**Pendidikan**																
Tidakpernah sekolah	87,8	30	96,1	2,0	1,9	23.	26	20,0	0,0	0,0	9,3	0,0	8,6	35,4	26,6	6*
Tidak tamat SD/MI	94,4	121	90,8	8,6	0,6	27.9	112	18,3	0,0	21,9	8,7	9,7	0,0	22,8	18,6	30*
Tamat SD/MI	93,1	460	93,5	5,7	0,7	24.5	419	20,3	5,2	21,1	15,3	4,0	3,3	18,5	12,4	99
Tamat SLTP/MTS	91,1	675	92,0	6,6	1,4	30.5	602	24,0	8,4	12,4	7,3	8,5	1,4	21,5	16,5	177
Tamat SLTA/MA	87,3	746	91,5	4,9	3,6	44.1	638	24,2	9,0	10,5	10,9	6,4	3,2	16,2	19,4	271
Tamat D1/D2/D3/PT	83,7	224	91,8	6,0	2,2	45.7	184	27,7	6,4	19,4	8,7	7,2	3,5	16,0	11,0	81
**Pekerjaan**																
Tidak bekerja	90,1	1,494	93,2	5,1	1,7	32.4	1,318	25,2	9,0	12,7	8,3	5,5	2,8	17,7	18,8	412
Sekolah	83,6	20*	100,0	0,0	0,0	53.8	17*	9,8	30,7	0,0	0,0	0,0	0,0	35,3	24,2	9*
PNS/TNI/Polri/BUMN	87,0	33	95,9	1,6	2,5	54.7	28	28,1	3,7	28,0	22,0	4,7	4,5	7,4	1,4	15*
Pegawai swasta	88,2	143	89,9	5,2	4,9	43.4	123	28,7	7,5	22,3	7,3	5,7	3,4	16,4	8,7	52
Wiraswasta	90,2	166	93,7	5,1	1,3	31.7	147	15,2	0,7	20,1	18,8	7,1	0,4	27,3	10,4	45*
Petani	85,7	134*	91,9	6,9	1,2	23.5	113	20,6	10,0	12,9	6,4	6,4	3,6	33,5	6,5	25*
Nelayan	93,2	3*	100,0	0,0	0,0	39.7	3*	0,0	0,0	68,5	0,0	0,0	0,0	31,5	0,0	1*
Buruh/sopir/pembantu ruta	94,9	72	77,2	22,8	0,00	58.4	67	24,6	0,0	5,7	31,0	18,7	4,7	15,3	0,0	38*
Lainnya	88,7	190	88,6	6,9	4,5	42.8	165	18,3	4,0	15,4	6,9	10,3	1,4	14,4	29,3	68
**Tempat Tinggal**																
Perkotaan	88,6	1,036	92,6	5,9	1,5	38.5	899	26,8	8,5	11,8	9,4	5,9	1,2	17,1	19,3	333
Perdesaan	90,6	1,220	91,7	5,8	2,4	31.7	1,082	20,6	6,5	16,4	11,0	7,7	4,2	19,7	13,9	331

*N Tertimbang <50

Tabel 16.2.5

Proporsi Jenis Makanan Tambahan yang Diperoleh Ibu Hamil menurut Provinsi, Riskesdas 2018

Provinsi	Jenis makanan tambahan (%)						N ter-timbang
	Biskuit program	Biskuit lainnya	Susu bubuk	Susu cair	Bahan makanan mentah	Makanan matang	
Aceh	79,2	20,3	35,9	8,6	7,4	33,0	78
Sumatera Utara	87,6	8,0	21,4	1,9	1,4	10,8	86
Sumatera Barat	91,8	5,0	10,9	0,8	5,0	16,7	60
Riau	84,9	4,9	15,5	4,3	2,2	13,9	83
Jambi	98,6	1,4	8,6	2,0	6,8	13,9	29*
Sumatera Selatan	93,9	5,6	8,3	1,5	2,7	9,1	57
Bengkulu	86,7	10,9	13,1	3,5	3,7	21,1	16 *
Lampung	96,6	2,5	7,5	1,7	2,7	3,1	88
Bangka Belitung	93,0	0,0	14,3	1,4	0,0	11,0	10 *
Kepulauan Riau	100,0	1,8	3,4	0,0	1,4	2,8	17 *
DKI Jakarta	74,9	14,8	42,2	7,3	6,0	23,4	60
Jawa Barat	96,9	3,1	8,1	1,7	0,4	7,8	544
Jawa Tengah	90,7	9,4	19,9	3,1	0,0	12,7	351
DI Yogyakarta	100,0	9,1	4,8	0,0	0,0	10,3	42
Jawa Timur	77,5	11,1	28,5	4,9	1,2	20,3	203
Banten	95,1	9,7	10,5	3,5	0,0	10,6	104
Bali	83,6	0,0	38,8	1,2	1,2	1,2	28*
Nusa Tenggara Barat	70,7	0,9	2,0	0,8	3,3	42,1	41*
Nusa Tenggara Timur	88,9	3,2	14,1	0,4	2,6	9,0	38*
Kalimantan Barat	83,2	12,2	20,0	10,4	10,2	20,0	40*
Kalimantan Tengah	83,5	1,1	20,2	0,0	0,0	14,6	22 *
Kalimantan Selatan	94,5	4,5	18,2	2,1	0,0	7,1	22 *
Kalimantan Timur	87,9	2,7	9,2	0,0	0,0	15,9	24 *
Kalimantan Utara	68,5	5,7	28,4	28,3	13,7	45,0	4 *
Sulawesi Utara	95,6	16,1	27,5	3,6	12,2	21,4	18 *
Sulawesi Tengah	66,6	4,1	17,5	2,1	11,1	44,2	33
Sulawesi Selatan	88,5	2,3	17,4	0,0	1,1	10,4	75
Sulawesi Tenggara	99,1	0,0	6,4	0,0	1,2	6,6	20 *
Gorontalo	91,1	0,0	21,7	3,8	0,0	4,6	9 *
Sulawesi Barat	91,1	4,0	10,1	2,9	0,0	2,8	12 *
Maluku	96,4	4,7	13,6	6,9	6,9	16,0	16 *
Maluku Utara	78,7	11,0	14,2	0,7	0,0	18,9	10 *
Papua Barat	79,3	4,1	35,5	3,1	7,8	6,8	10 *
Papua	92,3	0,0	43,6	26,8	0,0	12,2	8 *
**INDONESIA**	**89,7**	**6,6**	**16,3**	**2,9**	**1,9**	**13,5**	**2.256**

*N Tertimbang < 50

Tabel 16.2.6
Proporsi Jenis Makanan Tambahan yang Diperoleh Ibu Hamil menurut Karakteristik, Riskesdas 2018

Karakteristik	Jenis makanan tambahan (%)						N tertimbang
	Biskuit program	Biskuit lainnya	Susu bubuk	Susu cair	Bahan makanan mentah	Makanan matang	
**Kelompok Umur**							
10–14	100,0	28,1	0,0	0,0	0,0	0,0	3 *
15–19	96,0	7,3	17,2	5,1	0,0	0,0	193
20-24	90,9	5,2	15,7	3,1	2,1	13,0	607
25-29	88,6	6,8	16,6	1,6	1,7	15,1	665
30-34	88,1	7,4	17,7	4,4	3,1	14,3	483
35-39	89,3	6,3	13,1	1,8	1,3	11,4	270
40-44	81,7	18,1	29,3	4,4	0,8	12,5	28*
45-49	61,3	0,0	12,4	0,0	0,0	26,2	6 *
50-54	100,0	0,0	0,0	0,0	0,0	0,0	0 *
**Kelompok Umur (Khusus)**							
10 - 19 (Remaja)	96,1	7,6	16,9	5,1	0,0	10,1	196
15 - 49 (WUS)	89,7	6,6	16,4	2,9	1,9	13,5	2,253
**Pendidikan**							
Tidakpernah sekolah	87,8	14,4	13,7	0,0	4,2	8,3	30*
Tidak tamat SD/MI	94,4	3,7	13,8	5,9	3,6	11,0	121
Tamat SD/MI	93,1	8,4	11,9	2,5	1,6	11,7	460
Tamat SLTP/MTS	91,1	4,2	16,0	2,4	1,4	12,2	675
Tamat SLTA/MA	87,3	6,9	18,5	2,8	2,1	16,5	746
Tamat D1/D2/D3/PT	83,7	10,1	20,7	4,7	2,0	13,3	224
**Pekerjaan**							
Tidak bekerja	90,1	5,7	16,3	3,1	1,8	13,5	1,494
Sekolah	83,6	5,4	6,5	5,4	1,2	27,5	20*
PNS/TNI/Polri/BUMN/BUMD	87,0	12,3	23,7	1,0	1,4	16,6	33*
Pegawai swasta	88,2	6,9	22,4	2,1	1,1	9,4	143
Wiraswasta	90,2	6,1	17,9	1,1	2,0	8,9	166
Petani	85,7	17,0	16,2	7,3	3,4	19,0	134
Nelayan	93,2	6,8	11,7	0,0	0,0	0,0	3 *
Buruh/sopir/pembantu ruta	94,9	7,9	19,2	1,4	2,2	2,9	72
Lainnya	88,7	5,6	9,7	1,5	2,3	18,8	190
**Tempat Tinggal**							
Perkotaan	88,6	6,3	17,4	3,1	1,4	11,9	1.036
Perdesaan	90,6	6,9	15,4	2,8	2,3	14,9	1.220

*N Tertimbang <50

Tabel 16.2.7
Proporsi Alasan Ibu Hamil Memperoleh PMT menurut Provinsi, Riskesdas 2018

Provinsi	Alasan memperoleh PMT (%)						N ter-timbang
	Ibu kurang gizi/KEK	Keluarga miskin	Periksa hamil di Posyandu	Berat badan hamil tidak pernah naik	Anemia	Lain–nya	
Aceh	8,2	4,0	74,6	6,2	12,8	8,0	78
Sumatera Utara	1,7	0,0	67,3	2,0	1,0	20,5	86
Sumatera Barat	10,4	7,3	75,0	5,8	7,6	6,5	60
Riau	11,6	7,1	60,3	3,9	6,8	17,4	83
Jambi	9,1	7,9	69,1	7,8	2,6	9,1	29*
Sumatera Selatan	10,1	5,4	79,3	7,5	12,3	8,3	57
Bengkulu	8,3	2,9	64,2	5,7	8,3	5,6	16*
Lampung	7,0	4,3	68,3	11,6	1,1	18,0	88
Bangka Belitung	19,8	6,1	65,2	0,0	7,7	14,9	10*
Kepulauan Riau	25,1	12,3	65,3	1,5	1,7	9,8	17*
DKI Jakarta	17,2	8,6	38,5	12,1	5,1	20,9	60
Jawa Barat	12,2	2,4	72,7	5,5	6,4	16,5	544
Jawa Tengah	19,0	1,6	58,1	5,3	5,0	22,4	351
DI Yogyakarta	10,7	0,0	24,7	0,0	12,0	58,8	42*
Jawa Timur	15,4	7,4	65,3	7,6	9,6	16,0	203
Banten	7,8	0,0	78,3	5,3	6,7	15,8	104
Bali	27,4	3,4	35,1	0,0	4,2	29,9	28*
Nusa Tenggara Barat	29,7	11,3	59,7	0,0	16,9	1,3	41*
Nusa Tenggara Timur	40,4	6,4	55,9	13,5	6,6	0,6	38*
Kalimantan Barat	20,5	1,9	52,9	8,3	3,4	7,9	40*
Kalimantan Tengah	6,6	0,8	72,1	4,6	4,2	19,1	22*
Kalimantan Selatan	27,6	4,9	49,5	4,3	12,6	27,8	22*
Kalimantan Timur	30,0	0,0	57,3	14,3	14,2	20,3	24*
Kalimantan Utara	10,7	0,0	73,6	16,8	10,7	5,9	4*
Sulawesi Utara	3,9	3,2	75,7	2,6	0,0	19,6	18*
Sulawesi Tengah	13,4	6,5	70,0	7,7	13,6	8,9	33*
Sulawesi Selatan	32,0	0,8	46,1	13,1	6,4	10,6	75
Sulawesi Tenggara	49,7	0,0	61,6	3,1	2,2	5,3	20*
Gorontalo	8,3	0,0	77,9	9,4	0,0	11,2	9*
Sulawesi Barat	23,6	13,1	49,1	10,2	13,7	11,4	12*
Maluku	8,3	0,0	60,5	10,8	15,9	4,9	16*
Maluku Utara	31,1	13,5	54,6	22,1	4,6	0,0	10*
Papua Barat	11,4	3,0	88,9	1,8	20,1	6,4	10*
Papua	19,6	5,5	68,0	12,4	15,4	8,0	8*
**INDONESIA**	**15,1**	**3,6**	**64,5**	**6,3**	**6,9**	**16,4**	**2.256**

*N Tertimbang <50

Tabel 16.2.8
Proporsi Alasan Ibu Hamil Memperoleh PMT menurut Karakteristik, Riskesdas 2018

Karakteristik	Alasan memperoleh PMT (%)						N ter-timbang
	Ibu kurang gizi/KEK	Keluarga miskin	Periksa hamil di Posyandu	Berat badan hamil tidak pernah naik	Anemia	Lainnya	
**Kelompok Umur**							
10–14	28,1	0,0	71,9	0,0	0,0	0,0	3 *
15–19	29,5	8,6	63,0	5,7	8,2	11,1	193
20-24	17,1	3,4	62,2	5,7	6,4	15,5	607
25-29	13,9	3,4	64,1	7,1	9,4	16,6	665
30-34	13,3	3,4	67,3	5,9	5,5	18,6	483
35-39	7,2	1,2	68,2	6,1	4,4	17,3	270
40-44	7,9	5,7	55,8	17,7	0,0	24,2	28*
45-49	20,7	6,7	39,3	0,0	0,0	30,8	6 *
50-54	0,0	100,0	100,0	0,0	0,0	0,0	0 *
**Kelompok Umur (Khusus)**							
10 - 19 (Remaja)	29,5	8,4	63,2	5,6	8,1	10,9	196
15 - 49 (WUS)	15,1	3,6	64,5	6,3	6,9	16,5	2,253
**Pendidikan**							
Tidak/belum pernah sekolah	16,6	9,9	71,7	4,6	6,4	7,6	30*
Tidak tamat SD/MI	9,5	7,5	77,5	7,7	7,6	15,8	121
Tamat SD/MI	18,5	6,0	70,0	6,0	6,4	11,0	460
Tamat SLTP/MTS	14,8	3,0	66,0	6,2	6,3	16,1	675
Tamat SLTA/MA	15,6	2,7	60,7	7,3	8,2	16,9	746
Tamat D1/D2/D3/PT	10,1	1,0	53,7	3,3	5,0	28,5	224
**Pekerjaan**							
Tidak bekerja	16,1	4,1	67,6	6,2	7,1	14,2	1,494
Sekolah	0,6	0,0	40,3	0,0	1,5	41,7	20*
PNS/TNI/Polri/BUMN/BUMD	3,3	0,2	51,9	4,5	9,2	33,5	33*
Pegawai swasta	14,4	3,2	53,6	2,7	1,6	23,9	143
Wiraswasta	8,2	2,2	63,6	7,0	3,7	31,1	166
Petani	16,7	4,6	67,7	8,3	9,7	10,5	134
Nelayan	41,6	14,6	33,5	14,6	6,8	25,4	3 *
Buruh/sopir/pembantu ruta	9,1	2,8	55,6	4,2	12,5	24,8	72
Lainnya	18,0	2,2	55,8	9,4	8,3	11,0	190
**Tempat Tinggal**							
Perkotaan	14,2	2,8	61,0	7,0	6,5	20,0	1.036
Perdesaan	15,9	4,4	67,5	5,7	7,2	13,4	1.220

*N Tertimbang <50

16.3 Tablet Tambah Darah (TTD) Ibu Hamil

Pemberian tablet tambah darah sebagai salah satu upaya penting dan merupakan cara yang efektif karena dapat mencegah dan menanggulangi anemia akibat kekurangan zat besi dan atau asam folat. Tablet tambah darah diberikan kepada wanita usia subur dan ibu hamil. Ibu hamil diberikan tablet tambah darah setiap hari selama masa kehamilannya atau minimal 90 (sembilan puluh) tablet (Kemenkes, 2014).

Data pemberian tablet tambah darah (TTD) pada ibu hamil bertujuan untuk mengetahui gambaran konsumsi TTD ibu hamil sebagai faktor risiko anemia pada ibu hamil serta mengetahui capaian indikator cakupan ibu hamil yang mendapat Tablet Tambah Darah yang telah ditetapkan dalam Renstra Kementerian Kesehatan Tahun 2015 – 2019. Pada Riskesdas 2018, ada dua kelompok ibu hamil yang mendapatkan tablet tambah darah yaitu:

1. Riwayat ibu hamil yang mendapatkan TTD yaitu ibu hamil yang mendapatkan TTD dalam kurun waktu 5 tahun terakhir (1 Januari 2013 sampai saat wawancara) untuk kehamilan terakhir
2. Saat pengumpulan data, ibu sedang dalam kondisi hamil.

RIWAYAT IBU HAMIL YANG PERNAH MENERIMA TABLET TAMBAH DARAH

$$\textit{Proporsi Riwayat Ibu Hamil Menerima TTD}:$$

$$= \frac{\Sigma\, \textit{Perempuan umur } 10-54 \textit{ tahun pada kehamilan anak terakhir menerima tablet tambah darah}}{\Sigma\, \textit{Perempuan umur } 10-54 \textit{ tahun yang bersalin anak terakhir pada periode } 1 \textit{ Januari } 2013 \textit{ sd wawancara}}$$

JUMLAH TTD YANG DIPEROLEH SELAMA KEHAMILAN

Jumlah TTD dikelompokkan dalam 2 kategori Jumlah TTD yang diperoleh yaitu < 90 tablet dan ≥90 tablet.

$$\textit{Proporsi Jumlah TTD yang diterima Ibu Hamil}:$$

$$= \frac{\Sigma\, \textit{Perempuan umur } 10-54 \textit{ tahun menurut kategori jumlah TTD yang diterima selama kehamilan terakhir}}{\Sigma\, \textit{Perempuan umur } 10-54 \textit{ tahun yang bersalin anak terakhir pada periode } 1 \textit{ Januari } 2013 \textit{ sd wawancara dan pernah mendapat TTD selama hamil}}$$

JUMLAH TTD YANG DIMINUM SELAMA KEHAMILAN

Jumlah TTD yang diminum oleh ibu selama kehamilan anak terakhir dikelompokkan dalam 2 kategori yaitu <90 tablet dan ≥90 tablet.

$$Proporsi\ Jumlah\ TTD\ yang\ diminum\ Ibu\ Hamil = \frac{\Sigma\ Perempuan\ umur\ 10-54\ tahun\ yang\ mendapat\ TTD\ selama\ kehamilan\ anak\ terakhir\ menurut\ kategori\ jumlah\ TTD\ yang\ diminum}{\Sigma\ Perempuan\ umur\ 10-54\ tahun\ yang\ selama\ kehamilan\ anak\ terakhir\ mendapat\ TTD}$$

ALASAN UTAMA TIDAK MINUM/MENGHABISKAN TTD

Definisi: Alasan utama ibu hamil yang tidak meminum/menghabiskan TTD selama kehamilan anak terakhir. Ragam alasan utama tidak mengonsumsi TTD adalah tidak suka, mual/muntah karena proses kehamilan, bosan, lupa, efek samping, belum waktunya habis.

$$Proporsi\ alasan\ utama\ ibu\ hamil\ tidak\ minum\ TTD = \frac{\Sigma\ Perempuan\ umur\ 10-54\ tahun\ yang\ tidak\ minum/menghabiskan\ TTD\ selama\ kehamilan\ anak\ terakhir\ menurut\ alasan\ utama}{\Sigma\ Perempuan\ umur\ 10-54\ tahun\ yang\ selama\ kehamilan\ anak\ terakhir\ tidak\ mengonsumsi\ TTD}$$

IBU MENDAPATKAN TTD UNTUK KEHAMILAN SAAT INI

Definisi: Pada saat wawancara, ibu sedang dalam kondisi hamil yang mendapatkan Tablet Tambah Darah pada kehamilan saat ini.

$$Proporsi\ ibu\ hamil\ yang\ mendapat\ TTD = \frac{Jumlah\ perempuan\ umur\ 10-54\ tahun\ sedang\ hamil\ saat\ wawancara\ dan\ selama\ hamil\ mendapatkan\ TTD}{Jumlah\ perempuan\ umur\ 10-54\ tahun\ sedang\ hamil\ saat\ wawancara}$$

JUMLAH TTD YANG DIDAPAT DAN DIMINUM

Definisi: Menurut program, jumlah minimum Tablet Tambah Darah yang diminum sebanyak 90 tablet

$$Proporsi\ \ ibu\ hamil\ yang\ mendapat\ TTD\ (butir) = \frac{Jumlah\ perempuan\ sedang\ hamil\ yang\ mendapatkan\ TTD\ (butir)}{Jumlah\ perempuan\ 10-54\ tahun\ yang\ sedang\ hamil\ saat\ wawancara}$$

Tabel 16.3.1
Proporsi Riwayat Tablet Tambah Darah (TTD) yang Diterima dan DikonsumsiSelama Kehamilan Anak Terakhir pada Perempuan umur 10-54 tahun menurut Provinsi, Riskesdas 2018

Provinsi	Pernah mendapat TTD			Jumlah TTD yang didapat				Jumlah TTD yang diminum			
				< 90 tablet		≥ 90 tablet		< 90 tablet		≥ 90 tablet	
	%	95% CI	N Ter-timbang	%	N Ter-timbang	%	N Ter-timbang	%	N Ter-timbang	%	N Ter-timbang
Aceh	81,6	79,7-83,4	1.462	63,8	915	36,2	520	79,5	1.140	20,5	294
Sumatera Utara	70,9	68,9-72,9	3.167	68,5	2.092	31,5	963	78,3	2.391	21,7	663
Sumatera Barat	91,5	90,1-92,8	1.646	50,3	811	49,7	802	65,9	1.063	34,1	550
Riau	79,2	77,2-81,0	1.902	60,2	1.124	39,8	743	71,5	1.336	28,5	531
Jambi	85,6	83,4-87,6	945	51,6	480	48,4	450	66,4	618	33,6	312
Sumatera Selatan	74,2	71,7-76,5	1.915	65,7	1.239	34,3	647	77,2	1.455	22,8	430
Bengkulu	84,7	81,9-87,1	511	57,9	292	42,1	212	72,6	366	27,4	138
Lampung	89,8	88,1-91,3	2.426	55,8	1.332	44,2	1.056	67,5	1.611	32,5	777
Bangka Belitung	87,6	85,0-89,8	404	61,8	244	38,2	151	72,9	289	27,1	107
Kepulauan Riau	85,6	81,3-89,1	615	41,1	248	58,9	355	51,5	311	48,5	293
DKI Jakarta	90,5	88,3-92,4	2.883	34,0	948	66,0	1.837	44,1	1.228	55,9	1.557
Jawa Barat	90,2	89,1-91,1	13.817	41,5	5.588	58,5	7.888	56,0	7.551	44,0	5.923
Jawa Tengah	94,2	93,5-94,9	8.987	40,3	3.542	59,7	5.239	53,4	4.693	46,6	4.087
DI Yogyakarta	95,0	93,0-96,5	957	24,4	228	75,6	708	35,8	335	64,2	602
Jawa Timur	89,4	88,4-90,3	9.019	50,2	4.402	49,8	4.375	64,4	5.654	35,6	3.122
Banten	88,4	86,5-90,0	3.887	42,5	1.618	57,5	2.190	56,4	2.151	43,6	1.665
Bali	91,5	89,3-93,3	1.064	36,9	386	63,1	660	44,4	465	55,6	583
Nusa Tenggara Barat	95,4	94,2-96,4	1.718	41,7	705	58,3	987	63,1	1.067	36,9	625
Nusa Tenggara Timur	91,4	90,2-92,5	1.705	37,4	628	62,6	1.051	49,1	824	50,9	855
Kalimantan Barat	82,5	80,4-84,4	1.323	59,5	775	40,5	527	70,9	923	29,1	379
Kalimantan Tengah	77,8	74,5-80,7	677	59,5	396	40,5	270	70,0	466	30,0	200
Kalimantan Selatan	89,5	87,6-91,2	1.239	40,3	491	59,7	728	56,1	684	43,9	535
Kalimantan Timur	87,0	84,3-89,3	1.024	40,1	403	59,9	602	55,5	558	44,5	448
Kalimantan Utara	85,2	81,6-88,1	213	75,5	158	24,5	51	85,8	180	14,2	30*
Sulawesi Utara	87,8	85,7-89,6	611	74,0	441	26,0	155	83,7	499	16,3	97
Sulawesi Tengah	87,7	85,7-89,6	865	71,5	609	28,5	243	84,2	717	15,8	134
Sulawesi Selatan	91,6	90,3-92,8	2.416	67,7	1.610	32,3	768	80,8	1.921	19,2	456
Sulawesi Tenggara	85,8	83,7-87,7	799	69,4	546	30,6	241	84,5	665	15,5	122
Gorontalo	93,1	90,8-94,8	347	75,8	257	24,2	82	86,1	292	13,9	47*
Sulawesi Barat	91,4	88,9-93,3	404	52,0	207	48,0	191	70,3	279	29,7	118
Maluku	81,7	78,7-84,4	485	71,8	343	28,2	135	81,3	389	18,7	89
Maluku Utara	87,9	85,2-90,2	398	62,8	246	37,2	146	77,3	302	22,7	89
Papua Barat	83,1	79,4-86,2	274	78,7	212	21,3	57	86,0	232	14,0	38*
Papua	63,0	59,6-66,3	584	56,1	321	43,9	251	70,7	404	29,3	168
**INDONESIA**	**87,6**	**87,3-88,0**	**70.687**	**49,0**	**33.837**	**51,0**	**35.284**	**62,3**	**43.060**	**37,7**	**26.065**

*N Tertimbang <50

Tabel 16.3.2
Proporsi Riwayat Tablet Tambah Darah (TTD) yang Diterima dan DikonsumsiSelama Kehamilan Anak Terakhir pada Perempuan umur 10-54 tahun Menurut Karakteristik, Riskesdas 2018

Karakteristik	Pernah mendapat TTD			Jumlah yang didapat				Jumlah yang diminum			
				< 90 tablet		≥ 90 tablet		< 90 tablet		≥ 90 tablet	
	%	95% CI	N Ter-timbang	%	N Ter-timbang	%	N Ter-timbang	%	N Ter-timbang	%	N Ter-timbang
**Pendidikan**											
Belum pernah sekolah	71,2	68,1-74,2	932	57,5	523	42,5	387	71,0	645	29,0	264
Tidak tamat SD/MI	81,7	80,2-83,0	3.924	54,2	2.088	45,8	1.762	68,9	2.654	31,1	1.196
Tamat SD/MI	85,6	84,8-86,4	14.259	52,4	7.318	47,6	6.643	65,1	9.095	34,9	4.867
Tamat SLTP/MTS	90,1	89,5-90,7	18.373	50,1	8.996	49,9	8.964	64,1	11.519	35,9	6.445
Tamat SLTA/MA	89,3	88,7-89,9	24.581	46,9	11.257	53,1	12.767	60,2	14.466	39,8	9.558
Tamat D1/D2/D3/PT	86,4	85,4-87,4	8.618	43,4	3.655	56,6	4.761	55,6	4.681	44,4	3.734
**Pekerjaan**											
Tidak bekerja	88,7	88,3-89,2	42.020	49,5	20.346	50,5	20.736	62,7	25.770	37,3	15.313
Sekolah	87,1	81,9-91,0	494	50,7	246	49,3	239	67,4	327	32,6	158
PNS/TNI/Polri/BUMN/BUMD	87,1	85,2-88,7	1.765	43,3	749	56,7	981	54,7	946	45,3	784
Pegawai swasta	88,3	87,0-89,5	5.923	42,1	2.430	57,9	3.337	55,5	3.204	44,5	2.567
Wiraswasta	87,6	86,5-88,7	6.717	45,6	2.994	54,4	3.574	59,5	3.907	40,5	2.660
Petani	78,5	77,3-79,6	5.210	55,0	2.813	45,0	2.306	67,8	3.473	32,2	1.646
Nelayan	87,1	78,8-92,5	74	52,5	38	47,5	35	70,0	51	30,0	22*
Buruh/sopir/pembantu ruta	88,6	86,5-90,3	2.720	48,7	1.296	51,3	1.364	62,7	1.669	37,3	991
Lainnya	88,4	87,2-89,4	5.765	51,9	2.925	48,1	2.711	65,9	3.712	34,1	1.923
**Tempat tinggal**											
Perkotaan	88,5	88,0-89,0	38.645	45,6	17.177	54,4	20.533	58,6	22.095	41,4	15.623
Perdesaan	86,6	86,2-87,0	32.042	53,0	16.660	47,0	14.751	66,7	20.964	33,3	10.443

*N Tertimbang <50

Tabel 16.3.3

Proporsi Alasan Utama Riwayat Tidak Minum/Menghabiskan Tablet Tambah Darah (TTD) Selama Kehamilan Anak Terakhir pada Perempuan umur 10-54 tahun menurut Provinsi, Riskesdas 2018

Provinsi	Alasan utama tidak minum/menghabiskan TTD (%)						N Ter-timbang
	Tidak suka	Mual/muntah karena proses kehamilan	Bosan	Lupa	Efek samping (mual,sem-belit)	Belum waktunya habis	
Aceh	22,6	12,5	21,8	27,4	12,6	3,3	712
Sumatera Utara	20,6	9,2	31,0	30,3	7,2	1,8	1.160
Sumatera Barat	22,8	16,5	19,7	23,2	15,6	2,3	798
Riau	20,4	18,4	27,8	19,5	11,5	2,4	804
Jambi	22,8	23,2	22,0	18,0	12,4	1,6	346
Sumatera Selatan	23,8	16,0	17,5	21,8	17,3	3,5	645
Bengkulu	26,1	24,2	12,9	16,1	18,9	1,7	234
Lampung	22,0	23,8	22,5	13,4	15,1	3,2	915
Bangka Belitung	32,1	15,1	24,3	11,9	14,7	2,0	150
Kepulauan Riau	16,5	19,8	18,8	27,2	15,1	2,6	207
DKI Jakarta	13,9	17,3	22,0	26,1	14,6	6,1	1.049
Jawa Barat	16,5	23,7	17,2	20,3	19,1	3,2	5.877
Jawa Tengah	18,5	18,7	24,7	19,2	13,2	5,6	3.270
DI Yogyakarta	13,9	19,0	17,3	24,9	14,9	10,0	350
Jawa Timur	23,0	16,4	23,5	16,6	16,9	3,7	3.664
Banten	15,1	22,8	18,5	17,3	20,5	5,8	1.898
Bali	11,6	13,9	17,6	32,9	17,4	6,6	286
Nusa Tenggara Barat	34,2	16,6	17,7	12,2	14,6	4,7	914
Nusa Tenggara Timur	19,8	20,8	10,4	25,1	17,5	6,4	479
Kalimantan Barat	24,0	19,6	15,0	15,6	23,7	2,0	474
Kalimantan Tengah	21,4	16,9	17,4	21,1	19,4	3,8	233
Kalimantan Selatan	20,8	15,6	14,8	20,6	23,4	4,7	486
Kalimantan Timur	19,2	20,0	14,7	24,2	11,6	10,4	427
Kalimantan Utara	31,2	18,4	10,1	18,9	15,5	6,0	107
Sulawesi Utara	28,8	15,0	14,8	24,0	12,8	4,6	274
Sulawesi Tengah	29,9	20,4	16,9	12,7	17,1	2,9	419
Sulawesi Selatan	35,0	9,7	19,1	19,8	14,2	2,1	1.281
Sulawesi Tenggara	33,7	16,5	16,3	10,6	19,5	3,5	462
Gorontalo	39,6	12,2	11,3	12,7	23,0	1,2	171
Sulawesi Barat	33,4	14,8	13,7	19,8	16,3	2,0	184
Maluku	22,8	20,4	15,2	25,3	14,4	1,9	202
Maluku Utara	26,6	15,6	20,4	21,6	14,2	1,6	204
Papua Barat	25,4	15,7	20,0	22,4	14,5	2,0	107
Papua	21,7	16,1	13,6	33,8	11,2	3,5	266
**INDONESIA**	**21,2**	**18,6**	**20,1**	**20,0**	**16,2**	**3,9**	**29.056**

Tabel 16.3.4
Proporsi Alasan Utama Riwayat Tidak Minum/Menghabiskan Tablet Tambah Darah (TTD) Selama Kehamilan Anak Terakhir pada Perempuan umur 10-54 tahun menurut Karakteristik, Riskesdas 2018

Karakteristik	Alasan utama tidak minum/menghabiskan TTD (%)						N ter-timbang
	Tidak suka	Mual/muntah karena proses kehamilan	Bosan	Lupa	Efek samping (mual,sem-belit)	Belum waktunya habis	
**Pendidikan**							
Tidak pernah sekolah	24,1	21,5	14,9	19,2	17,6	2,9	353
Tidak tamat SD/MI	23,9	21,0	19,2	15,8	17,4	2,8	1.656
Tamat SD/MI	22,1	19,6	20,0	15,9	19,0	3,6	5.864
Tamat SLTP/MTS	20,9	18,3	20,6	19,2	17,2	3,8	7.934
Tamat SLTA/MA	20,6	17,9	20,2	21,8	15,0	4,4	10.014
Tamat D1/D2/D3/PT	20,7	17,8	19,7	26,0	11,4	4,4	3.235
**Pekerjaan**							
Tidak bekerja	20,6	18,6	20,6	19,2	17,0	4,0	17.568
Sekolah	23,8	21,8	20,6	21,6	8,4	3,7	207
PNS/TNI/Polri/BUMN/BUMD	20,3	16,4	20,4	29,0	10,6	3,3	643
Pegawai swasta	20,7	20,2	18,8	23,3	12,6	4,5	2.382
Wiraswasta	21,9	17,9	20,7	20,4	15,0	4,1	2.731
Petani	24,9	20,1	16,8	18,7	16,0	3,4	1.912
Nelayan	21,7	12,7	28,1	27,2	4,7	5,7	32*
*Buruh/sopir/pembantu ruta	22,3	16,8	20,5	17,6	19,0	3,9	1.176
Lainnya	22,0	17,6	19,3	21,8	15,9	3,4	2.406
**Tempat tinggal**							
Perkotaan	20,0	18,2	20,8	21,6	15,2	4,2	15.845
Perdesaan	22,7	19,0	19,2	18,0	17,4	3,7	13.211

*N Tertimbang <50

Tabel 16.3.5

Proporsi Ibu Hamil Memperoleh Tablet Tambah Darah (TTD) dan Jumlah yang Diperoleh menurut Provinsi, Riskesdas 2018

Provinsi	Memperoleh TTD pada kehamilan saat wawancara			Jumlah TTD yang diperoleh			
				< 90 tablet		≥ 90 tablet	
	%	95% CI	N Ter-timbang	%	N Ter-timbang	%	N Ter-timbang
Aceh	65,9	60,4-71,0	135	83,6	110	16,4	22*
Sumatera Utara	56,4	50,4-62,3	252	92,8	228	7,0	17*
Sumatera Barat	81,2	75,8-85,6	156	71,5	109	28,2	43*
Riau	68,8	62,4-74,6	187	82,8	151	17,2	31*
Jambi	73,1	64,7-80,1	70	79,9	55	20,1	14*
Sumatera Selatan	56,7	48,7-64,3	161	87,9	138	12,1	19*
Bengkulu	70,1	60,5-78,1	46*	79,1	36*	20,9	9*
Lampung	72,6	66,2-78,2	211	85,1	175	14,9	31*
Bangka Belitung	72,2	62,3-80,4	40*	75,6	29*	24,4	9*
Kepulauan Riau	67,3	52,5-79,3	65	59,0	38*	41,0	26*
DKI Jakarta	73,6	64,7-80,8	298	66,7	194	33,3	97
Jawa Barat	74,1	69,4-78,3	1.352	73,7	973	26,2	347
Jawa Tengah	80,0	76,6-83,0	813	75,7	601	24,3	193
DI Yogyakarta	63,0	46,9-76,6	71	66,3	46*	33,7	23*
Jawa Timur	77,3	73,6-80,6	902	79,0	696	20,4	180
Banten	81,5	75,0-86,6	351	58,6	201	41,4	142
Bali	79,2	70,8-85,7	118	56,5	65	43,5	50
Nusa Tenggara Barat	82,2	75,4-87,5	169	71,3	118	28,7	47*
Nusa Tenggara Timur	75,3	69,6-80,2	130	68,1	86	31,9	40*
Kalimantan Barat	65,6	57,5-72,9	110	87,9	94	12,1	13*
Kalimantan Tengah	69,6	59,5-78,0	62	71,4	43*	28,6	17*
Kalimantan Selatan	70,8	62,9-77,6	97	72,2	68	27,8	26*
Kalimantan Timur	68,6	59,0-76,9	85	65,3	54	34,7	29*
Kalimantan Utara	78,1	64,8-87,4	18*	75,9	13*	24,1	4*
Sulawesi Utara	71,1	62,8-78,2	46*	88,0	39*	12,0	5*
Sulawesi Tengah	66,7	59,1-73,5	80	86,7	68	13,3	10*
Sulawesi Selatan	78,9	73,9-83,2	238	86,7	201	13,3	31*
Sulawesi Tenggara	59,8	51,5-67,6	65	88,3	56	11,7	7*
Gorontalo	72,2	59,2-82,3	24*	87,3	20*	12,7	3*
Sulawesi Barat	81,9	72,1-88,8	40*	85,0	33*	15,0	6*
Maluku	62,3	51,2-72,3	34*	89,5	30*	10,5	4*
Maluku Utara	74,1	64,6-81,8	34*	79,7	27*	20,3	7*
Papua Barat	66,5	54,0-77,1	21*	92,3	19*	7,7	2*
Papua	57,6	48,5-66,2	38*	67,1	25*	32,9	12*
**INDONESIA**	**73,2**	**71,8-74,6**	**6.518**	**76,0**	**4.841**	**23,8**	**1.518**

*N Tertimbang <50

Tabel 16.3.6

Proporsi Ibu Hamil Memperoleh Tablet Tambah Darah (TTD) dan Jumlah yang Diperoleh menurut Karakteristik, Riskesdas 2018

Karakteristik	Mendapat TTD pada kehamilan saat wawancara			Jumlah yang didapat			
				< 90 tablet		≥ 90 tablet	
	%	95% CI	N Ter-timbang	%	N Ter-timbang	%	N Ter-timbang
**Kelompok Umur**							
10-14	61,0	24,1-88,5	3*	100,0	3*	0,0*	0
15-19	77,4	72,1-81,9	466	76,5	348	23,5	107
20-24	74,0	70,8-77,0	1.680	77,7	1.274	22,3	366
25-29	72,6	69,9-75,2	1.908	76,5	1.425	23,3	435
30-34	74,1	71,4-76,6	1.537	73,3	1.101	26,6	399
35-39	70,3	66,6-73,7	761	76,6	570	23,3	173
40-44	68,0	60,4-74,7	147	75,0	108	24,9	36*
45-49	68,4	49,3-82,9	15*	78,1	12*	21,9	3*
50-54	33,8	3,9-86,7	1*	100,0	1*	0,0	0*
**10 - 19 (Remaja)**	77,2	72,0-81,7	469	76,6	351	23,4	107
**15 - 49 (WUS)**	73,2	71,8-74,5	6.515	76,0	4.838	23,9	1.518
**Pendidikan**							
Belum pernah sekolah	58,1	47.8-67.7	63	83,3	51	16,7*	10*
Tidak tamat SD/MI	67,4	61.0-73.3	295	77,6	223	22,4	65
Tamat SD/MI	73,4	69.8-76.8	1.133	81,1	897	18,5	205
Tamat SLTP/MTS	76,2	73.5-78.8	1.622	75,5	1.196	24,4	386
Tamat SLTA/MA	74,3	71.9-76.5	2.461	74,8	1.798	25,2	606
Tamat D1/D2/D3/PT	68,8	65.3-72.2	945	73,2	676	26,7	247
**Pekerjaan**							
Tidak bekerja	74,9	73,0-76,6	4.086	75,8	3.024	24,2	967
Sekolah	64,9	49,6-77,7	54	88,3	47*	11,7*	6*
PNS/TNI/Polri/BUMN/BUMD	69,5	61,9-76,1	142	73,3	102	26,6	37*
Pegawai swasta	71,3	66,6-75,6	666	72,1	469	27,9	182
Wiraswasta	71,1	66,4-75,3	480	77,6	363	21,9	103
Petani	67,5	63,1-71,6	327	80,8	258	17,8	57
Nelayan	57,3	25,3-84,2	6*	74,3	5*	25,7	2*
Buruh/sopir/pembantu ruta	64,3	53,6-73,7	176	83,6	144	16,4	28*
Lainnya	74,8	70,2-78,9	580	75,9	430	24,1	137
**Tempat tinggal**							
Perkotaan	72,0	69.8-74.1	3.575	73,4	2.562	26,5	925
Perdesaan	74,7	73.1-76.3	2.943	79,3	2.279	20,6	593

*N Tertimbang <50

16.4 Gizi Pada Ibu Hamil (LILA)

Tabel 16.4.1
Nilai Rata-rata Lingkar Lengan Atas (LiLA) pada Wanita Usia Subur (WUS) umur 15 - 49 Tahun dan Wanita Hamil menurut Umur, Riskesdas 2018

Umur (tahun)	Nilai LiLA (cm)			
	Hamil		Tidak Hamil	
	Rata-rata	SD	Rata-rata	SD
15	23,8	2,7	24,3	3,2
16	24,5	2,6	24,6	3,2
17	24,9	3,0	24,8	3,3
18	25,1	2,9	25,0	3,3
19	24,8	3,4	25,3	3,5
20	25,5	3,3	25,6	3,6
21	25,5	3,5	25,7	3,6
22	25,6	3,5	26,1	3,7
23	26,1	3,2	26,3	3,7
24	26,4	3,7	26,6	3,7
25	26,9	3,7	26,9	3,8
26	26,5	3,5	27,1	3,8
27	26,7	3,5	27,5	3,9
28	26,9	3,6	27,6	3,9
29	27,9	3,9	27,7	3,7
30	27,1	3,4	28,0	3,9
31	27,2	3,8	28,0	3,9
32	27,5	3,7	28,2	3,7
33	27,4	4,0	28,4	3,8
34	27,5	3,4	28,5	3,8
35	28,1	3,9	28,5	3,8
36	27,9	3,6	28,8	3,8
37	28,3	3,6	28,8	3,8
38	28,5	4,3	29,0	3,8
39	28,6	4,2	29,0	3,7
40	28,6	3,9	29,1	3,8
41	28,3	3,5	29,2	3,8
42	26,9	3,3	29,1	3,8
43	28,3	2,6	29,3	3,8
44	27,8	4,0	29,2	3,8
45	26,7	3,8	29,0	3,8
46	28,7	2,7	29,1	3,9
47	30,3	3,7	29,0	3,9
48	28,3	4,9	28,9	3,9
49	36,0	0,0	28,9	4,0
**Total**	**26,8**	**3,7**	**27,6**	**4,0**

Tabel 16.4.2
Prevalensi Kurang Energi Kronis (KEK) pada Wanita Hamil dan Wanita Tidak Hamil menurut Provinsi, Riskesdas 2018

Provinsi	KEK (LiLA < 23,5 cm)			
	Wanita Hamil		Wanita Tidak Hamil	
	%	95% CI	%	95% CI
Aceh	11,3	8,1-15,5	11,8	10,9-12,8
Sumatera Utara	14,8	10,8-19,8	10,8	10.0-11,7
Sumatera Barat	16,7	12,2-22,4	14,3	13,3-15,4
Riau	12,9	9,0-18,2	11,4	10,5-12,4
Jambi	15,9	10,4-23,7	10,8	9,7-12,0
Sumatera Selatan	17,2	12,0-24,1	12,8	11,8-13,9
Bengkulu	12,1	7,4-19,3	11,1	10,0-12,3
Lampung	13,6	9,8-18,6	12,8	11,9-13,7
Bangka Belitung	13,7	7,8-23,1	10,8	9,5-12,2
Kepulauan Riau	18,3	9,2-33,1	13,1	11,2-15,4
DKI Jakarta	13,2	7,7-21,7	11,2	10,0-12,5
Jawa Barat	14,1	11,0-17,9	12,5	11,8-13,2
Jawa Tengah	20,0	16,7-23,8	18,2	17,6-18,9
DI Yogyakarta	24,1	14,4-37,5	19,1	17,2-21,2
Jawa Timur	19,6	16,2-23,4	13,9	13,3-14,5
Banten	18,6	12,9-26,0	12,2	11,2-13,3
Bali	13,8	8,7-21,2	11,6	10,6-12,8
Nusa Tenggara Barat	21,5	15,5-29,0	21,0	19,4-22,7
Nusa Tenggara Timur	36,8	31,0-43,0	32,5	31,3-33,8
Kalimantan Barat	13,9	9,3-20,2	14,1	13,0-15,2
Kalimantan Tengah	18,2	11,8-27,0	16,1	14,2-18,2
Kalimantan Selatan	19,5	13,9-26,5	14,4	13,4-15,5
Kalimantan Timur	11,5	5,5-22,4	12,4	11,2-13,7
Kalimantan Utara	1,7	0,5-6,2	14,4	12,5-16,6
Sulawesi Utara	14,8	9,5-22,5	11,0	9,8-12,2
Sulawesi Tengah	22,7	16,9-29,9	16,5	15,0-18,1
Sulawesi Selatan	16,9	12,9-21,8	17,7	16,8-18,7
Sulawesi Tenggara	28,0	22,2-34,7	20,5	18,8-22,4
Gorontalo	8,8	4,1-18,0	16,0	14,3-17,9
Sulawesi Barat	17,5	10,3-28,2	15,4	13,7-17,4
Maluku	30,7	22,3-40,6	21,4	19,6-23,4
Maluku Utara	29,9	21,4-39,9	19,4	17,6-21,3
Papua Barat	23,2	14,4-35,3	20,8	18,2-23,7
Papua	25,8	17,2-36,8	18,1	16,2-20,1
**INDONESIA**	**17,3**	**16,1-18,5**	**14,5**	**14,3-14,7**

Tabel 16.4.3

Prevalensi Kurang Energi Kronis (KEK) pada Wanita Hamil dan Wanita tidak Hamil menurut Karakteristik, Riskesdas 2018

Karakteristik	KEK (LiLA < 23,5 cm)			
	Wanita Hamil		Wanita Tidak Hamil	
	%	95% CI	%	95% CI
**Kelompok Umur**				
15 - 19	33,5	28,0-39,5	36,3	35,6-37,1
20 - 24	23,3	20,7-26,2	23,3	22,6-24,0
25 - 29	16,7	14,5-19,1	13,5	12,9-14,0
30 - 34	12,3	10,4-14,5	8,4	8-8,9,0
35 -39	8,5	6,8-10,5	6,0	5,7-6,3
40 - 44	6,5	4,0-10,5	5,2	4,9-5,5
45 - 49	11,1	4,4-25,5	6,0	5,6-6,3
**Pendidikan**				
Tidak sekolah	15,7	9,7-24,2	15,6	14,3-16,9
Tidak Tamat SD	13,2	9,5-18,0	10,9	10,3-11,5
Tamat SD	17,2	14,2-20,6	11,5	11,1-11,9
Tamat SLTP	17,6	15,3-20,2	18,3	17,8-18,7
Tamat SLTA	19,4	17,4-21,6	15,1	14,7-15,5
Tamat D1-D3/PT	13,0	10,8-15,6	11,9	11,3-12,5
**Pekerjaan**				
Tidak bekerja	18,3	16,7-20,0	12,4	12,1-12,7
Sekolah	25,9	15,0-40,9	36,0	35,2-36,9
PNS/TNI/Polri/BUMN/BUMD	12,1	8,1-17,8	6,6	5,8-7,4
Pegawai swasta	14,4	11,4-18,0	15,2	14,4-16,0
Wiraswasta	12,6	9,4-16,8	8,0	7,5-8,5
Petani/buruh tani	18,5	15,2-22,3	10,4	9,9-10,8
Nelayan	9,2	2,2-31,0	19,9	10,1-35,3
Buruh/Supir/pembantu ruta	20,9	13,4-31,2	13,3	12,4-14,3
Lainnya	15,7	12,8-19,1	11,5	10,9-12,3
**Tempat Tinggal**				
Perkotaan	15,7	14,0-17,5	13,8	13,5-14,2
Perdesaan	19,3	17,7-20,9	15,3	15,0-15,6

Tabel 16.4.4
Prevalensi Tinggi Badan Berisiko Pada Wanita Hamil menurut Provinsi, Riskesdas 2018

Provinsi	Tinggi Badan Berisiko (< 150 cm)	
	%	95% CI
Aceh	30,8	25,8 – 36,2
Sumatera Utara	28,3	23,4 – 33,7
Sumatera Barat	37,2	31,0 – 44,0
Riau	30,9	24,7 – 37,9
Jambi	33,6	26,7 – 41,3
Sumatera Selatan	28,3	22,9 – 34,5
Bengkulu	32,1	24,3 – 41,0
Lampung	27,3	21,9 – 33,4
Bangka Belitung	33,8	25,6 – 43,0
Kepulauan Riau	15,5	6,8 – 31,4
DKI Jakarta	21,6	14,8 – 30,2
Jawa Barat	29,9	25,6 – 34,6
Jawa Tengah	31,4	27,5 – 35,5
DI Yogyakarta	21,9	12,7 – 35,0
Jawa Timur	34,7	30,8 – 38,9
Banten	27,9	20,5 – 36,7
Bali	17,0	11,2 – 24,9
Nusa Tenggara Barat	32,5	25,3 – 40,7
Nusa Tenggara Timur	34,3	28,8 – 40,2
Kalimantan Barat	33,3	26,4 – 41,1
Kalimantan Tengah	40,4	30,9 – 50,6
Kalimantan Selatan	36,7	29,3 – 44,8
Kalimantan Timur	38,2	28,5 – 48,9
Kalimantan Utara	30,3	19,9 – 43,3
Sulawesi Utara	21,2	15,1 – 29,0
Sulawesi Tengah	36,7	30,1 – 43,8
Sulawesi Selatan	28,9	23,7 – 34,7
Sulawesi Tenggara	30,9	23,7 – 39,2
Gorontalo	47,0	34,8 – 59,5
Sulawesi Barat	55,3	44,9 – 65,4
Maluku	29,1	20,2 – 40,0
Maluku Utara	22,5	15,6 – 31,5
Papua Barat	22,2	14,3 – 32,9
Papua	30,4	21,3 – 41,4
**INDONESIA**	**30,5**	**29,1 – 32,0**

Tabel 16.4.5

Prevalensi Tinggi Badan Berisiko Pada Wanita Hamil menurut Karakteristik, Riskesdas 2018

Karakteristik	Tinggi Badan Berisiko (< 150 cm)	
	%	95% CI
**Pendidikan**		
Tidak sekolah	43,2	32,7-54,4
Tidak Tamat SD	38,4	32,4-44,8
Tamat SD	40,7	36,8-44,7
Tamat SLTP	33,1	30,3-36,2
Tamat SLTA	26,8	24,6-29,1
Tamat D1-D3/PT	20,3	17,7-23,2
**Pekerjaan**		
Tidak bekerja	32,1	30,2-34,1
Sekolah	23,0	13,7-35,9
PNS/TNI/Polri/BUMN/BUMD	21,6	16,2-28,3
Pegawai swasta	23,4	19,1-28,4
Petani/buruh tani	24,0	20,2-28,1
Wiraswasta	38,5	34,0-43,2
Nelayan	28,6	10,4-57,9
Buruh/Supir/pembantu ruta	33,1	24,6-42,9
Lainnya	30,5	26,4-34,8
**Tempat Tinggal**		
Perkotaan	27,6	25,5-29,8
Perdesaan	34,2	32,4-36,0

Tabel 16.4.6
Prevalensi Anemia pada Ibu Hamil menurut Karakteristik, Riskesdas 2018

Karateristik	Anemia		Tidak Anemia		N Tertimbang
	%	95% CI	%	95% CI	
**Umur (tahun)**					
15-24	84,6	82,8 – 86,3	15,4	13,7 – 17,2	161
25-34	33,7	30,9 – 36,6	66,3	63,4 – 69,1	280
35-44	33,6	28,0 – 39,7	66,4	60,3 – 72,0	95
45-54	24,0	24,0 – 24,0	76,0	76,0 – 76,0	2*
**Pendidikan**					
Tidak/ belum pernah sekolah	41.5	41.5 – 41,5	58.5	58.5 – 58,5	7*
Tidak tamat SD/MI	42.9	33.1 – 53,2	57.1	46.8 – 66,9	27*
Tamat SD/MI	50.8	46.2 – 55,5	49.2	44.5 – 53,8	126
Tamat SLTP/MTS	51.5	47.8 – 55,2	48.5	44.8 – 52,2	156
Tamat SLTA/MA	50.6	45.5 – 55,6	49.4	44.4 – 54,4	181
Tamat D1/D2/D3/PT	31.0	24.5 – 38,5	69.0	61.5 – 75,5	41*
**Pekerjaan**					
Tidak Bekerja	53.0	50.4 – 55,6	47.0	44.4 – 49,6	372
Sekolah	54.4	13.9 – 89,8	45.6	10.2 – 86,1	4*
PNS/TNI/Polri/BUMN/BUMD	27.0	27.0 – 27,0	73.0	73.0 – 73,0	3*
Pegawai swasta	38.5	31.9 – 45,6	61.5	54.4 – 68,1	35*
Wiraswasta	29.8	20.6 – 40,8	70.2	59.2 – 79,4	35*
Petani/buruh tani	43.0	35.3 – 50,9	57.0	49.1 – 64,7	28*
Buruh/sopir/pembantu ruta	55.5	45.2 – 65,3	44.5	34.7 – 54,8	19*
Lainnya	39.0	29.7 – 49,2	61.0	50.8 – 70,3	41*
**Tempat Tinggal**					
Perkotaan	48,3	45,0 – 51,7	51.7	48.3 – 55,0	291
Perdesaan	49,5	47,4 – 51,7	50.5	48.3 – 52,6	247
**INDONESIA**	**48,9**	**46,9 – 50,9**	**51,1**	**49,1 – 53,1**	**538**

*N Tertimbang <50

Tabel 16.4.7
Prevalensi Anemia menurut Karakteristik, Riskesdas 2018

Karateristik	Anemia		Tidak Anemia		N Tertimbang
	%	95% CI	%	95% CI	
**Umur (tahun)**					
0-59 bulan	38.5	36.6 – 40,4	61.5	59.6 – 63,4	2.513
5-14 tahun	26.8	25.8 – 27,9	73.2	72.1 – 74,2	7.587
15-24 tahun	32.0	30.7 – 33,3	68.0	66.7 – 69,3	5.671
25-34 tahun	15.1	14.2 – 16,0	84.9	84.0 – 85,8	6.122
35-44 tahun	16.7	15.9 – 17,6	83.3	82.4 – 84,1	8.304
45-54 tahun	18.8	17.9 – 19,7	81.2	80.3 – 82,1	7.977
55-64 tahun	24.5	23.4 – 25,6	75.5	74.4 – 76,6	5.689
65-74 tahun	31.7	30.0 – 33,4	68.3	66.6 – 70,0	2.589
>75 tahun	42.3	39.5 – 45,1	57.7	54.9 – 60,5	1.014
**Jenis Kelamin**					
Laki-laki	20.3	19.7 – 21,0	79.7	79.0 – 80,3	23.849
Perempuan	27.2	26.6 – 27,8	72.8	72.2 – 73,4	23.616
**Pendidikan**					
Tidak/ belum pernah sekolah	30.6	29.1 – 32,1	69.4	67.9 – 70,9	3.821
Tidak tamat SD/MI	24.9	24.0 – 25,8	75.1	74.2 – 76,0	9.400
Tamat SD/MI	21.4	20.6 – 22,2	78.6	77.8 – 79,4	13.092
Tamat SLTP/MTS	21.4	20.5 – 22,3	78.6	77.7 – 79,5	7.563
Tamat SLTA/MA	20.3	19.4 – 21,3	79.7	78.7 – 80,6	8.397
Tamat D1/D2/D3/PT	17.7	16.1 – 19,5	82.3	80.5 – 83,9	2.117
**Tempat Tinggal**					
Perkotaan	22.7	22.0 – 23,5	77.3	76.5 – 78,0	25.984
Perdesaan	25.0	24.3 – 25,7	75.0	74.3 – 75,7	21.481
**INDONESIA**	**23,7**	**23,2 – 24,3**	**76,3**	**75,7 – 76,8**	**47.465**

16.5 Praktek Pemberian Makanan pada Bayi dan Anak

Pada Riskesdas 2018 dikumpulkan data mengenai Praktek Pemberian Makan pada Bayi dan Anak (PMBA) yang meliputi praktek inisiasi menyusu dini (IMD), proses mulai menyusu, perilaku terhadap kolostrum, pemberian makanan/minuman prelakteal, riwayat dan keberlangsungan pemberian ASI, pola pemberian ASI, pemberian makanan pendamping ASI (MP ASI) serta keragaman konsumsi makan.

A. Inisiasi Menyusu Dini (IMD)

IMD adalah kontak antara kulit ibu dengan kulit bayi sesegera mungkin dalam jangka waktu 1 (satu) jam setelah bayi dilahirkan. Bayi yang baru lahir diletakkan di dada/perut ibu dengan kulit ibu melekat pada kulit bayi (tanpa penghalang apapun). Indikator ini didasarkan pada riwayat *(historic recall)*.

Praktek IMD anak usia 0-23 bulan dihitung menggunakan formula:

$$Proporsi\ IMD = \frac{\begin{matrix}Anak\ umur\ 0-23\ bulan\ yang\ mempunyai\ riwayat\ diletakkan\ kedada/perut\ ibu\ segera\ setelah\ lahir\\ (dalam\ jangka\ waktu\ 1\ jam\ setelah\ dilahirkan)\end{matrix}}{Jumlah\ anak\ usia\ 0-23\ bulan}$$

Tabel 16.5.1
Proporsi Inisiasi Menyusu Dini (IMD) dan Lama IMD pada Anak Umur 0-23 Bulan menurut Provinsi, Riskesdas 2018

Provinsi	IMD		N Tertimbang	Lama IMD		N Tertimbang
	%	95%CI		< 1 jam	≥ 1 jam	
Aceh	59,3	56,2-62,3	925	89,8	10,2	513
Sumatera Utara	39,9	36,9-43,0	2.152	90,7	9,3	804
Sumatera Barat	62,7	59,2-66,0	836	86,8	13,2	490
Riau	54,8	51,0-58,6	1.075	88,9	11,1	551
Jambi	62,0	57,9-65,9	500	90,4	9,6	290
Sumatera Selatan	55,5	51,6-59,4	1.164	86,3	13,7	605
Bengkulu	56,0	51,2-60,7	273	92,2	7,8	143
Lampung	54,3	50,8-57,8	1.198	90,1	9,9	609
Bangka Belitung	57,3	51,7-62,7	193	91,0	9,0	103
Kepulauan Riau	66,6	58,8-73,6	324	90,2	9,8	202
DKI Jakarta	74,1	69,4-78,4	1.38	79,2	20,8	957
Jawa Barat	61,2	58,6-63,8	6.443	82,9	17,1	3.690
Jawa Tengah	64,9	62,7-67,0	4.188	82,3	17,7	2.541
DI Yogyakarta	68,5	61,9-74,7	405	62,6	37,4	260
Jawa Timur	63,7	61,4-65,9	452	81,9	18,1	2.692
Banten	54,6	50,0-59,2	1.798	88,9	11,1	918
Bali	41,9	36,6-47,4	487	81,9	18,1	191
Nusa Tenggara Barat	57,9	53,8-61,8	788	75,6	24,4	427
Nusa Tenggara Timur	57,6	54,4-60,7	982	77,1	22,9	529
Kalimantan Utara	63,3	56,2-69,9	127	88,1	11,9	75
Kalimantan Barat	51,1	47,1-55,1	761	83,9	16,1	364
Kalimantan Tengah	43,0	37,9-48,2	387	82,1	17,9	156
Kalimantan Selatan	60,1	56,2-63,8	651	87,0	13,0	366
Kalimantan Timur	66,1	61,1-70,8	540	82,2	17,8	334
Sulawesi Utara	47,1	42,2-52,1	315	93,4	6,6	139
Sulawesi Tengah	50,9	46,6-55,1	478	82,8	17,2	227
Sulawesi Selatan	59,7	56,3-63,0	1.333	88,8	11,2	744
Sulawesi Tenggara	44,3	39,6-49,0	475	92,2	7,8	197
Gorontalo	57,9	51,4-64,2	177	88,2	11,8	96
Sulawesi Barat	52,8	47,0-58,4	245	81,6	18,4	121
Maluku	38,0	33,5-42,7	317	88,3	11,7	113
Maluku Utara	40,4	35,5-45,5	196	86,4	13,6	74
Papua Barat	34,3	28,6-40,4	164	87,6	12,4	53
Papua	40,5	36,2-45,0	461	84,8	15,2	175
**INDONESIA**	**58,2**	**57,4-59,0**	**36.259**	**84,1**	**15,9**	**19.748**

Tabel 16.5.2
Proporsi Inisiasi Menyusu Dini (IMD) dan Lama IMD pada Anak Umur 0-23 Bulan menurut Karakteristik, Riskesdas 2018

Karakteristik	IMD		N Tertimbang	Lama IMD		N Tertimbang
	%	95%CI		< 1jam	≥ 1 jam	
**Kelompok Umur**						
0 – 5 bulan	56,7	55,1-58,3	8,730	83,8	16,2	4.630
6 – 11 bulan	58,3	56,8-59,8	9,562	84,1	15,9	5.218
12 – 23 bulan	58,9	57,8-60,0	17.967	84,3	15,7	9.900
**Jenis kelamin**						
Laki-laki	58,0	56,9-59,1	18.333	84,4	15,6	9.943
Perempuan	58,5	57,4-59,6	17.926	83,9	16,1	9.805
**Pendidikan KRT**						
Tidak/belum pernah sekolah	54,2	50,6-57,7	1.560	82,9	17,1	790
Tidak tamat SD/MI	53,5	51,3-55,6	4.351	85,1	14,9	2.175
Tamat SD/MI	56,3	54,9-57,8	9.372	84,1	15,9	4.937
Tamat SLTP/MTS	58,7	56,9-60,5	6.823	85,0	15,0	3.748
Tamat SLTA/MA	60,5	59,1-62,0	10.682	84,0	16,0	6.049
Tamat D1/D2/D3/PT	63,1	60,6-65,5	3.471	82,6	17,4	2.049
**Pekerjaan KRT**						
Tidak bekerja	58,1	55,2-61,0	2.656	83,9	16,1	1.443
Sekolah	52,6	36,3-68,3	63	83,3	16,7	31
PNS/TNI/Polri/BUMN/BUMD	60,4	57,3-63,5	1.740	83,5	16,5	983
Pegawai swasta	63,5	61,1-65,8	5.136	83,1	16,9	3.050
Wiraswasta	60,3	58,7-61,9	8.034	84,7	15,3	4.531
Petani/buruh tani	52,6	51,3-53,9	9.620	84,6	15,4	4.734
Nelayan	50,6	46,2-55,0	792	84,7	15,3	375
Buruh/sopir/pembantu ruta	60,4	58,4-62,5	6.308	84,4	15,6	3.566
Lainnya	57,9	54,3-61,3	1.910	82,6	17,4	1.034
**Tempat tinggal**						
Perkotaan	61,9	60,7-63,1	19.266	83,4	16,6	11.155
Perdesaan	54,1	53,1-55,0	16.993	85,2	14,8	8.593

B. Waktu Mulai Menyusu

Proporsi waktu mulai menyusu dihitung dengan formula:

$$Proporsi\ waktu\ mulai\ menyusu\ pada\ anak\ 0-23\ bulan = \frac{Anak\ usia\ 0-23\ bulan\ berdasarkan\ waktu\ mulai\ menyusu}{Jumlah\ seluruh\ anak\ usia\ 0-23\ bulan}$$

Tabel 16.5.3
Proporsi Waktu Mulai Menyusu pada Anak Umur 0-23 Bulan menurut Provinsi, Riskesdas 2018

Provinsi	Waktu Mulai Menyusu (%)					N Tertimbang
	< 1 Jam	1-6 Jam	7-23 Jam	24-47 jam	≥ 48 jam	
Aceh	33,5	41,6	5,0	5,3	14,6	825
Sumatera Utara	17,4	31,9	6,6	10,8	33,3	1.874
Sumatera Barat	30,2	49,6	6,5	6,2	7,4	794
Riau	26,7	40,2	4,6	9,5	19,1	981
Jambi	27,8	45,8	7,8	7,2	11,5	475
Sumatera Selatan	24,9	49,8	4,4	6,2	14,7	1.053
Bengkulu	27,0	41,2	7,4	7,1	17,4	252
Lampung	24,9	46,0	4,2	6,3	18,7	1.106
Bangka Belitung	36,5	36,7	2,2	7,0	17,5	169
Kepulauan Riau	25,3	45,3	5,9	2,7	20,8	298
DKI Jakarta	26,5	49,0	7,6	6,7	10,3	1.290
Jawa Barat	27,5	45,3	4,4	7,6	15,2	5.973
Jawa Tengah	33,0	42,5	5,2	6,4	13,0	3.958
DI Yogyakarta	25,0	46,2	13,3	5,4	10,1	395
Jawa Timur	28,3	42,5	4,9	8,1	16,4	4.191
Banten	29,1	38,0	6,8	6,1	20,0	1.642
Bali	37,8	40,3	6,4	7,7	7,8	459
Nusa Tenggara Barat	38,5	46,5	2,4	4,2	8,3	741
Nusa Tenggara Timur	34,3	50,7	3,8	5,0	6,1	920
Kalimanatan Utara	32,6	49,6	5,5	4,8	7,6	118
Kalimantan Barat	25,6	50,9	6,8	5,4	11,3	700
Kalimantan Tengah	27,4	43,6	7,3	7,3	14,5	348
Kalimantan Selatan	28,7	43,5	6,0	6,5	15,3	591
Kalimantan Timur	32,0	48,2	4,8	5,1	9,9	503
Sulawesi Utara	24,5	41,3	3,9	10,5	19,8	270
Sulawesi Tengah	24,5	40,9	3,9	8,2	22,5	430
Sulawesi Selatan	29,3	39,1	4,1	9,2	18,2	1.206
Sulawesi Tenggara	31,3	37,5	3,1	6,6	21,4	428
Gorontalo	36,7	43,8	1,3	5,9	12,3	160
Sulawesi Barat	35,9	37,8	3,8	6,4	16,1	221
Maluku	25,4	46,0	4,5	6,2	17,9	270
Maluku Utara	25,0	43,0	3,8	7,6	20,7	170
Papua Barat	18,8	55,8	7,0	4,4	14,1	142
Papua	23,2	54,3	6,2	8,9	7,4	423
**INDONESIA**	**28,4**	**43,5**	**5,2**	**7,2**	**15,7**	**33.376**

Tabel 16.5.4
Proporsi Waktu Mulai Menyusu pada Anak Umur 0-23 Bulan menurut Karakteristik, Riskesdas 2018

Karakteristik	Kategori Proses Mulai Menyusu (%)					N Tertimbang
	< 1 Jam	1-6 Jam	7-23 Jam	24-47 jam	≥ 48 jam	
**Kelompok Umur**						
0 – 5 bulan	27,6	42,2	4,9	7,6	17,8	8.132
6 – 11 bulan	28,6	43,0	5,2	6,8	16,4	8.817
12 – 23 bulan	28,7	44,5	5,4	7,2	14,2	16.428
**Jenis kelamin**						
Laki-laki	28,3	42,6	5,2	7,5	16,4	16.842
Perempuan	28,5	44,5	5,2	6,8	14,9	16.534
**Pendidikan KRT**						
Tidak/belum pernah sekolah	28,6	44,9	4,3	5,4	16,8	1.423
Tidak tamat SD/MI	30,3	42,9	4,0	7,7	15,2	3.975
Tamat SD/MI	29,4	43,1	5,3	6,5	15,7	8.566
Tamat SLTP/MTS	27,8	44,0	5,4	6,6	16,3	6.285
Tamat SLTA/MA	27,8	43,1	5,5	7,7	15,9	9.895
Tamat D1/D2/D3/PT	26,5	45,5	5,9	8,6	13,6	3.233
**Pekerjaan KRT**						
Tidak bekerja	26,9	43,2	5,0	7,8	17,1	2.437
Sekolah	33,0	39,6	4,7	9,7	12,9	54
PNS/TNI/Polri/BUMN/BUMD	26,2	44,3	6,0	8,8	14,7	1.61
Pegawai swasta	27,6	44,0	5,4	7,7	15,3	4.758
Wiraswasta	28,3	42,9	6,4	7,0	15,4	7.394
Petani/buruh tani	28,7	44,3	4,9	6,6	15,5	8.815
Nelayan	25,7	40,6	4,6	7,7	21,5	720
Buruh/sopir/pembantu ruta	29,7	42,9	4,3	7,1	16,0	5.82
Lainnya	30,6	44,4	4,0	6,6	14,3	1.768
**Tempat tinggal**						
Perkotaan	28,1	43,3	5,6	7,5	15,6	17.759
Perdesaan	28,8	43,8	4,8	6,8	15,8	15.617

C. Perilaku ibu terhadap Kolostrum

Proporsi perilaku ibu terhadap kolostrum pada anak umur 0-23 bulan dihitung dengan formula:

$$Proporsi\ perilaku\ ibu\ terhadap\ kolostrum\ pada\ anak\ umur\ 0-23\ bulan$$
$$= \frac{Jumlah\ anak\ umur\ 0-23\ bulan\ berdasarkan\ perilaku\ ibu\ terhadap\ kolostrum}{Jumlah\ anak\ umur\ 0-23\ bulan}$$

Tabel 16.5.5
Proporsi Perilaku Ibu terhadap Kolostrum pada Anak Umur 0-23 Bulan menurut Provinsi, Riskesdas 2018

Provinsi	Perilaku ibu terhadap kolostrum (%)			N Tertimbang
	Diberikan semua	Dibuang sebagian	Dibuang semua	
Aceh	83,2	9,9	2,4	825
Sumatera Utara	72,8	11,7	8,5	1.874
Sumatera Barat	88,6	5,7	3,8	794
Riau	81,4	7,8	5,7	981
Jambi	84,9	9,3	4,0	475
Sumatera Selatan	78,9	9,0	4,6	1.053
Bengkulu	88,3	8,2	1,8	252
Lampung	89,0	5,3	2,2	1.106
Bangka Belitung	80,8	10,7	3,9	169
Kepulauan Riau	90,6	3,9	2,1	298
DKI Jakarta	89,8	4,6	1,9	1.29
Jawa Barat	86,9	6,8	2,9	5.973
Jawa Tengah	92,5	3,9	1,4	3.958
DI Yogyakarta	97,7	0,7	0,2	395
Jawa Timur	89,3	5,3	2,3	4.191
Banten	75,4	10,2	8,8	1.642
Bali	92,8	3,4	2,0	459
Nusa Tenggara Barat	88,1	3,6	2,1	741
Nusa Tenggara Timur	87,4	5,2	5,4	920
Kalimantan Utara	86,9	5,2	2,1	118
Kalimantan Barat	81,8	8,9	4,6	700
Kalimantan Tengah	71,2	15,2	7,4	348
Kalimantan Selatan	87,0	6,2	3,0	591
Kalimantan Timur	91,5	4,5	1,1	503
Sulawesi Utara	77,9	12,0	4,3	270
Sulawesi Tengah	82,0	6,6	7,8	430
Sulawesi Selatan	82,1	7,4	4,8	1.206
Sulawesi Tenggara	77,5	9,1	4,6	428
Gorontalo	77,5	10,8	8,1	160
Sulawesi Barat	84,4	6,7	2,4	221
Maluku	80,0	14,1	3,2	270
Maluku Utara	73,1	13,7	10,9	170
Papua Barat	79,4	7,3	7,9	142
Papua	68,8	13,7	5,2	423
**INDONESIA**	**85,4**	**6,9**	**3,7**	**33.376**

D. Pemberian Makanan/Minuman Prelakteal

$$Proporsi\ pemberian\ makanan\ prelakteal\ pada\ bayi\ 0-11\ bulan$$
$$=\frac{Jumlah\ Anak\ usia\ 0-11\ bulan\ berdasarkan\ jenis\ makanan\ prelakteal}{Jumlah\ anak\ usia\ 0-23\ bulan}$$

Tabel 16.5.6
Proporsi Pemberian Makanan Prelakteal pada Bayi Umur 0-11 Bulan menurut Provinsi, Riskesdas 2018

Provinsi	Diberikan makanan prelakteal (%)	N Ter-timbang	Jenis Makanan Prelakteal (%)											N Ter-timbang
			Susu formula	Susu non-formula	Madu/Madu+ air	Air gula	Air tajin	Air kelapa	Teh manis	Air putih	Bubur tepung/bubur saring	Pisang dihalus-kan	Nasi dihalus-kan	
Aceh	35,4	825	85,6	1,6	9,0	4,5	1,1	0,5	2,9	13,9	3,0	5,5	4,4	291
Sumatera Utara	59,3	1.874	91,8	1,4	6,2	2,2	0,7	0,7	1,4	12,1	2,0	1,4	2,0	1.105
Sumatera Barat	27,1	794	85,1	0,8	10,2	3,1	1,6	0,0	3,2	17,2	3,3	2,5	2,3	214
Riau	42,1	981	88,2	1,7	19,4	3,3	2,9	3,0	3,0	15,8	3,6	4,2	2,4	411
Jambi	30,4	475	73,1	2,4	21,4	4,3	2,4	1,3	1,0	22,5	2,4	2,7	3,4	144
Sumatera Selatan	31,0	1.053	77,7	2,4	18,5	4,0	4,5	2,8	3,7	16,4	4,3	2,1	5,3	324
Bengkulu	38,7	252	80,8	0,8	24,6	1,6	0,0	0,8	2,1	11,5	1,9	1,6	3,5	97
Lampung	40,1	1.106	85,9	2,5	19,2	1,6	1,9	0,9	0,6	8,9	2,9	3,0	1,5	441
Kep, Bangka Belitung	28,0	169	88,6	0,0	19,9	1,7	0,0	0,0	0,9	19,2	1,9	0,3	1,9	47*
Kepulauan Riau	34,1	298	91,5	0,2	16,9	2,0	1,6	0,0	2,0	9,8	0,2	0,0	2,8	101
DKI Jakarta	24,3	1.290	75,9	0,0	10,5	2,4	0,0	1,7	3,9	25,3	4,1	14,7	9,3	312
Jawa Barat	30,5	5.973	77,9	3,0	11,7	1,5	1,5	1,1	2,8	17,6	2,8	3,1	2,8	1.815
Jawa Tengah	26,6	3.958	81,2	1,7	13,4	3,0	1,5	0,8	3,1	13,0	4,2	5,9	5,0	1.049
DI Yogyakarta	16,8	395	66,9	0,0	15,7	3,4	1,8	0,0	1,8	18,3	0,0	0,0	5,2	66
Jawa Timur	38,5	4.191	90,3	1,7	9,7	3,7	1,2	2,4	1,1	6,4	2,6	2,9	2,2	1.605
Banten	31,5	1.642	72,1	1,2	21,9	0,9	1,0	0,0	1,1	17,5	3,2	8,4	2,4	515
Bali	38,1	459	97,6	0,0	1,6	0,0	0,0	0,4	0,4	0,4	0,8	1,6	0,3	174
Nusa Tenggara Barat	23,2	741	60,4	3,7	26,2	5,8	3,8	5,6	2,1	19,4	3,7	4,5	6,1	171
Nusa Tenggara Timur	13,2	920	63,1	1,1	8,2	0,0	2,9	0,0	4,2	26,9	4,0	1,5	3,7	121
Kalimantan Utara	19,4	118	76,8	0,2	28,7	5,3	4,0	0,0	0,0	11,9	2,8	6,5	3,7	23*
Kalimantan Barat	28,9	700	71,9	1,5	16,5	3,6	3,0	1,8	3,1	20,3	7,2	2,6	3,2	201
Kalimantan Tengah	34,3	348	87,6	0,9	17,2	4,4	1,1	3,7	2,8	12,2	2,0	2,5	1,3	119
Kalimantan Selatan	37,5	591	63,2	4,5	26,2	5,3	0,3	0,8	1,4	23,8	4,1	4,2	2,5	221
Kalimantan Timur	23,9	503	79,8	5,5	24,8	3,5	3,4	3,0	3,3	11,5	3,0	4,5	2,1	119
Sulawesi Utara	43,0	270	90,5	1,0	5,0	1,1	1,1	0,0	2,4	7,5	4,2	2,0	1,4	115
Sulawesi Tengah	41,8	430	76,8	2,6	17,5	7,7	0,4	0,2	1,2	7,3	2,3	2,9	1,2	179
Sulawesi Selatan	34,0	1.206	77,0	0,3	11,6	1,1	2,5	0,0	0,9	19,5	1,4	0,6	0,9	408
Sulawesi Tenggara	40,7	428	78,5	0,1	18,3	4,6	1,0	0,0	0,0	8,0	1,0	2,9	0,8	173
Gorontalo	45,9	160	55,6	1,6	42,7	16,4	0,0	0,6	2,9	7,5	4,4	2,4	1,7	73
Sulawesi Barat	21,4	221	66,9	1,4	18,8	3,7	4,0	0,0	2,2	12,2	2,9	2,5	4,9	47*
Maluku	42,7	270	73,6	1,5	9,1	6,0	0,8	0,0	2,7	7,3	2,8	0,9	0,2	115
Maluku Utara	35,0	170	40,2	2,9	18,7	4,9	0,7	2,4	2,9	13,5	6,9	3,6	3,6	59
Papua Barat	34,5	142	86,4	6,9	10,1	2,8	2,4	2,4	2,9	9,8	4,3	3,4	3,7	49*
Papua	21,5	423	74,4	6,7	7,8	3,8	1,2	1,1	7,7	24,9	5,2	7,4	5,8	91
**INDONESIA**	**33,1**	**33.376**	**81,4**	**1,9**	**13,5**	**3,0**	**1,5**	**1,2**	**2,1**	**13,9**	**3,0**	**3,6**	**2,9**	**10.997**

*N Tertimbang <50

Tabel 16.5.7
Proporsi Pemberian Makanan Prelakteal pada Bayi Umur 0-11 Bulan menurut Karakteristik, Riskesdas 2018

Karakteristik	Diberikan makanan prelakteal (%)	N Ter-timbang	Jenis Makanan Prelakteal (%)											N Ter-timbang
			Susu formula	Susu non-formula	Madu/Madu+air	Air gula	Air tajin	Air kelapa	Teh manis	Air putih	Bubur tepung /bubur saring	Pisang dihaluskan	Nasi dihaluskan	
**Kelompok Umur**														
0 – 5 bulan	33,3	8.132	84,5	1,7	11,3	1,8	0,4	0,6	1,0	9,3	1,0	1,7	0,3	2.698
6 – 11 bulan	35,2	8.817	81,4	1,7	12,3	3,3	1,9	1,5	1,9	15,0	3,7	4,8	4,2	3.093
12 – 23 bulan	31,8	16.428	79,9	2,1	15,3	3,5	1,9	1,4	2,9	15,7	3,6	3,9	3,5	5.205
**Jenis kelamin**														
Laki-laki	33,9	16.842	81,6	1,7	13,3	3,0	1,6	1,4	2,1	14,6	3,2	4,1	3,1	5.679
Perempuan	32,3	16.534	81,3	2,1	13,7	3,0	1,4	1,1	2,2	13,2	2,8	3,1	2,8	5.318
**Pendidikan KRT**														
Tidak/belum pernah sekolah	32,2	1.423	75,0	0,8	15,7	3,1	2,8	1,7	2,9	19,2	5,7	4,8	4,6	456
Tidak tamat SD/MI	32,2	3.975	77,7	1,8	18,0	4,5	2,8	1,6	1,9	16,2	3,7	4,7	3,5	1.275
Tamat SD/MI	33,2	8.566	78,3	2,0	15,3	3,4	1,4	1,4	2,5	15,7	2,9	4,2	3,2	2.831
Tamat SLTP/MTS	34,4	6.285	81,7	1,9	14,6	3,4	1,5	1,3	2,6	13,6	2,2	2,9	2,1	2.151
Tamat SLTA/MA	33,4	9.895	85,0	1,8	10,7	2,3	1,3	1,1	1,8	12,4	2,9	3,4	2,8	3.289
Tamat D1/D2/D3/PT	30,9	3.233	85,7	2,6	8,4	1,2	0,4	0,5	1,2	9,2	2,9	2,3	3,1	995
**Pekerjaan KRT**														
Tidak bekerja	34,7	2.437	83,9	1,8	15,4	4,1	1,9	2,5	1,8	16,8	4,5	7,6	5,2	841
Sekolah	33,1	54	81,4	0,0	2,9	2,7	0,0	0,0	0,0	19,3	3,6	3,6	0,0	18*
PNS/TNI/Polri/BUMN /BUMD	34,0	1.61	88,4	1,5	9,0	1,4	0,4	0,3	1,1	6,4	1,3	1,2	1,9	545
Pegawai swasta	31,6	4.758	85,0	2,0	10,1	2,1	0,7	1,4	1,7	13,8	3,1	2,9	3,1	1.496
Wiraswasta	33,5	7.394	84,9	1,5	13,3	1,7	1,3	0,9	1,6	12,3	2,4	2,7	2,1	2.463
Petani/buruh tani	33,3	8.815	76,1	2,0	16,9	4,5	2,0	1,6	2,8	15,9	3,7	3,9	3,2	2.923
Nelayan	36,7	720	78,5	2,7	14,8	3,9	2,0	0,5	0,5	15,0	3,3	0,4	0,6	263
Buruh/sopir/pembantu ruta	32,5	5.82	80,5	2,7	12,3	3,2	2,0	1,1	2,3	14,1	2,1	4,0	3,0	1.881
Lainnya	32,2	1.768	78,6	0,6	10,4	2,2	0,2	0,4	3,7	12,6	3,4	5,0	3,2	567
**Tempat tinggal**														
Perkotaan	32,3	17.759	85,1	1,7	10,7	2,3	1,4	1,1	2,2	13,5	2,9	3,6	2,7	5.714
Perdesaan	34,0	15.617	77,5	2,1	16,5	3,8	1,6	1,4	2,1	14,4	3,1	3,6	3,2	5.283

*N Tertimbang <50

E. Riwayat dan Keberlangsungan Pemberian ASI

Riwayat pemberian ASI

Proporsi anak umur 0-23 bulan yang pernah diberikan Air Susu Ibu (ASI) dihitung dengan formula:

$$Proporsi\ \ anak\ umur\ 0-23\ bulan\ yang\ pernah\ diberikan\ Air\ Susu\ Ibu = \frac{\text{Jumlah anak umur}\ \ 0-23\ \text{bulan yang pernah diberi ASI}}{Jumlah\ anak\ umur\ 0-23\ bulan}$$

Keberlangsungan menyusui

Proporsi anak umur 0-23 bulan yang masih diberi Air Susu Ibu dihitung dengan formula:

$$Proporsi\ anak\ umur\ 0-23\ bulan\ yang\ masih\ diberi\ Air\ Susu\ Ibu = \frac{Jumlah\ anak\ umur\ 0-23\ bulan\ yang\ masih\ diberi\ ASI}{Jumlah\ anak\ umur\ 0-23\ bulan\ yang\ pernah\ diberi\ ASI}$$

Alasan Utama Tidak Diberi ASI

Proporsi alasan utama anak usia 0-23 bulan tidak diberi ASI bayi dihitung dengan formula:

$$Proporsi\ anak\ umur\ 0-23\ bulan\ berdasarkan\ alasan\ utama\ tidak\ diberi\ ASI = \frac{Jumlah\ anak\ umur\ 0-23\ bulan\ berdasarkan\ alasan\ utama\ tidak\ diberi\ ASI}{Jumlah\ anak\ umur\ 0-23\ bulan}$$

Tabel 16.5.8

Proporsi Pernah Disusui dan Masih Disusui pada Anak Umur 0-23 Bulan menurut Provinsi, Riskesdas 2018

Provinsi	Pernah Disusui		Masih Disusui	
	%	N Tertimbang	%	N Tertimbang
Aceh	90,1	925	80,6	825
Sumatera Utara	88,0	2.152	75,0	1.874
Sumatera Barat	96,0	836	83,2	794
Riau	92,2	1.075	78,9	981
Jambi	96,1	500	79,2	475
Sumatera Selatan	91,4	1.164	78,3	1.053
Bengkulu	93,2	273	81,8	252
Lampung	93,3	1.198	80,4	1.106
Bangka Belitung	88,5	193	71,8	169
Kepulauan Riau	92,9	324	63,2	298
DKI Jakarta	94,4	1.380	73,7	1.290
Jawa Barat	93,7	6.443	80,5	5.973
Jawa Tengah	95,5	4.188	86,7	3.958
DI Yogyakarta	98,6	405	83,8	395
Jawa Timur	93,7	4.520	76,9	4.191
Banten	92,3	1.798	76,3	1.642
Bali	95,3	487	74,6	459
Nusa Tenggara Barat	95,0	788	85,9	741
Nusa Tenggara Timur	94,6	982	77,7	920
Kalimantan Utara	93,7	761	69,9	118
Kalimantan Barat	92,9	387	80,9	700
Kalimantan Tengah	90,8	651	81,4	348
Kalimantan Selatan	91,8	540	76,6	591
Kalimantan Timur	94,0	127	70,5	503
Sulawesi Utara	86,5	315	68,6	270
Sulawesi Tengah	90,9	478	75,8	430
Sulawesi Selatan	91,4	1.333	75,3	1.206
Sulawesi Tenggara	91,1	475	73,5	428
Gorontalo	91,5	177	60,2	160
Sulawesi Barat	91,2	245	84,3	221
Maluku	86,1	317	70,8	270
Maluku Utara	87,9	196	73,1	170
Papua Barat	87,3	164	74,1	142
Papua	92,7	461	77,7	423
**INDONESIA**	**93,0**	**36.259**	**78,8**	**33.376**

Tabel 16.5.9
Proporsi Pernah Disusui dan Masih Disusui pada Anak Umur 0-23 Bulan
menurut Karakteristik, Riskesdas 2018

Karakteristik	Pernah Disusui		Masih Disusui	
	%	N Tertimbang	%	N Tertimbang
**Kelompok Umur (bulan)**				
0 – 5	94,1	8.730	93,0	8.132
6 – 11	93,2	9.562	84,9	8.817
12 – 15	92,3	5.897	79,4[a]	5.388
16 – 19	91,9	5.762	70,5	5.243
20 – 23	92,9	6.308	56,8[b]	5.797
**Jenis kelamin**				
Laki-laki	92,8	18.333	77,9	16.842
Perempuan	93,2	17.926	79,7	16.534
**Pendidikan KRT**				
Tidak/belum pernah sekolah	92,2	1.560	80,8	1.423
Tidak tamat SD/MI	92,3	4.351	81,1	3.975
Tamat SD/MI	92,3	9.372	82,0	8.566
Tamat SLTP/MTS	93,1	6.823	79,3	6.285
Tamat SLTA/MA	93,6	10.682	75,6	9.895
Tamat D1/D2/D3/PT	94,1	3.471	75,8	3.233
**Pekerjaan KRT**				
Tidak bekerja	92,7	2.656	76,5	2.437
Sekolah	86,4	63	75,6	54
PNS/TNI/Polri/BUMN/BUMD	93,5	1.740	71,5	1.610
Pegawai swasta	93,6	5.136	75,0	4.758
Wiraswasta	93,0	8.034	78,1	7.394
Petani/buruh tani	92,6	9.620	82,8	8.815
Nelayan	91,8	792	80,5	720
Buruh/sopir/pembantu ruta	93,2	6.308	79,9	5.820
Lainnya	93,5	1.910	77,9	1.768
**Tempat tinggal**				
Perkotaan	93,1	19.266	76,0	17.759
Perdesaan	92,9	16.993	82,0	15.617

Tabel 16.5.10
Proporsi Alasan Anak Umur 0-23 Bulan Belum/Tidak Pernah Disusui menurut Provinsi, Riskesdas 2018

Provinsi	Alasan belum/tidak pernah disusui (%)								N Tertimbang
	ASI tdk keluar	Anak tdk bisa menyusu	Repot	Rawat pisah	Alasan medis	Anak terpisah dari ibu nya	Ibu meninggal	Lainnya	
Aceh	86,3	4,6	0,0	2,9	3,7	0,9	0,0	1,7	92
Sumatera Utara	70,1	4,5	1,2	3,6	12,7	1,9	0,3	5,6	232
Sumatera Barat	42,2	20,7	0,0	21,7	0,0	4,7	6,0	4,7	34*
Riau	69,1	6,3	0,6	3,8	0,0	15,7	0,5	4,0	75
Jambi	0,0	0,0	0,0	0,0	0,0	0,0	0,0	0,0	19*
Sumatera Selatan	76,2	15,9	0,4	0,5	2,9	2,7	0,0	1,5	76
Bengkulu	0,0	0,0	0,0	0,0	0,0	0,0	0,0	0,0	17*
Lampung	55,4	11,3	1,2	11,1	5,0	2,8	4,2	9,2	68
Bangka Belitung	0,0	0,0	0,0	0,0	0,0	0,0	0,0	0,0	23*
Kepulauan Riau	0,0	0,0	0,0	0,0	0,0	0,0	0,0	0,0	23*
DKI Jakarta	75,2	0,0	0,0	15,3	0,0	6,8	0,0	2,7	70
Jawa Barat	57,0	6,6	1,7	17,5	5,4	4,8	4,0	3,0	408
Jawa Tengah	60,4	4,4	8,7	8,3	2,2	7,9	4,4	3,7	172
DI Yogyakarta	0,0	0,0	0,0	0,0	0,0	0,0	0,0	0,0	4*
Jawa Timur	70,2	3,9	2,7	8,8	4,4	4,6	0,0	5,4	277
Banten	76,9	0,0	2,5	6,2	8,4	1,1	1,6	3,3	117
Bali	0,0	0,0	0,0	0,0	0,0	0,0	0,0	0,0	24*
Nusa Tenggara Barat	78,5	3,1	0,0	4,4	4,2	3,6	1,4	4,7	38*
Nusa Tenggara Timur	59,1	6,1	0,0	3,3	8,4	9,7	0,0	13,4	50
Kalimantan Utara	0,0	0,0	0,0	0,0	0,0	0,0	0,0	0,0	19*
Kalimantan Barat	38,2	15,4	3,7	10,3	12,2	8,1	0,0	12,1	45*
Kalimantan Tengah	69,9	7,5	0,0	11,1	0,5	2,6	0,0	8,4	29*
Kalimantan Selatan	65,3	7,3	4,8	3,7	8,8	4,4	1,4	4,4	54
Kalimantan Timur	77,9	3,3	8,7	4,8	4,1	0,0	0,0	1,2	31*
Sulawesi Utara	0,0	0,0	0,0	0,0	0,0	0,0	0,0	0,0	8*
Sulawesi Tengah	74,8	3,7	1,4	7,0	5,3	1,2	1,6	4,9	41*
Sulawesi Selatan	67,8	8,0	0,2	5,9	6,8	8,0	0,2	3,1	42*
Sulawesi Tenggara	76,6	5,1	0,0	8,9	3,0	0,8	1,3	4,2	116
Gorontalo	59,5	17,4	0,0	3,9	14,2	3,2	0,0	1,8	41*
Sulawesi Barat	0,0	0,0	0,0	0,0	0,0	0,0	0,0	0,0	13*
Maluku	0,0	0,0	0,0	0,0	0,0	0,0	0,0	0,0	19*
Maluku Utara	58,8	16,0	2,3	2,9	7,5	8,5	1,2	2,8	40*
Papua Barat	0,0	0,0	0,0	0,0	0,0	0,0	0,0	0,0	21*
Papua	0,0	0,0	0,0	0,0	0,0	0,0	0,0	0,0	19*
**INDONESIA**	**65,7**	**6,6**	**2,2**	**8,4**	**5,7**	**5,4**	**1,5**	**4,5**	**2.357**

*N Tertimbang <50

Tabel 16.5.11
Proporsi Alasan Anak Umur 0-23 Bulan Belum/Tidak Pernah Disusui menurut Karakteristik, Riskesdas 2018

Karakteristik	Alasan belum/tidak pernah disusui (%)								N Tertimbang
	ASI tdk keluar	Anak tdk bisa menyusu	Repot	Rawat pisah	Alasan medis	Anak terpisah dari ibunya	Ibu meninggal	Lainnya	
**Kelompok Umur**									
0 – 5 bulan	68,3	6,5	3,4	8,1	3,5	4,5	2,0	3,7	523
6 – 11 bulan	63,9	6,4	2,5	9,3	5,7	4,8	2,0	5,5	623
12 – 23 bulan	65,4	6,8	1,5	8,0	6,7	6,2	1,1	4,4	1.211
**Jenis kelamin**									
Laki-laki	65,4	7,4	2,1	8,4	4,9	6,6	1,1	4,1	1.243
Perempuan	66,0	5,6	2,2	8,4	6,7	4,1	2,0	5,0	1.114
**Pendidikan KRT**									
Tidak/belum pernah sekolah	65,6	6,9	2,5	10,1	5,7	1,8	0,3	7,0	113
Tidak tamat SD/MI	63,4	8,2	3,4	5,8	6,5	8,3	1,5	2,9	314
Tamat SD/MI	67,4	8,3	0,9	4,7	4,9	6,5	2,1	5,3	668
Tamat SLTP/MTS	64,9	4,4	2,7	9,9	6,6	4,2	2,1	5,2	436
Tamat SLTA/MA	70,0	5,7	1,1	7,6	5,9	4,8	1,0	3,9	635
Tamat D1/D2/D3/PT	50,9	5,7	6,8	23,4	4,9	4,1	0,6	3,5	192
**Pekerjaan KRT**									
Tidak bekerja	72,5	6,8	1,4	4,3	3,4	5,2	2,8	3,7	177
Sekolah	0,0	0,0	0,0	0,0	0,0	0,0	0,0	0,0	6*
PNS/TNI/Polri/BUMN/BUMD	61,4	7,5	0,2	11,0	10,4	5,2	0,0	4,3	99
Pegawai swasta	65,5	4,5	2,0	14,8	6,2	3,1	0,1	3,9	315
Wiraswasta	66,3	7,1	3,4	8,2	4,3	6,4	0,6	3,7	531
Petani/buruh tani	65,3	6,1	1,2	5,9	6,1	6,4	2,5	6,6	619
Nelayan	65,8	8,4	0,0	10,1	1,4	4,7	6,4	3,2	60
Buruh/sopir/pembantu ruta	65,6	6,7	3,0	6,6	7,2	5,2	1,6	4,2	425
Lainnya	60,3	8,1	3,3	14,8	5,6	5,0	0,7	2,3	124
**Tempat tinggal**									
Perkotaan	65,7	6,4	2,1	9,3	6,9	4,6	0,9	4,1	1.258
Perdesaan	65,6	6,8	2,3	7,3	4,4	6,5	2,3	4,9	1.099

*N Tertimbang <50

F. Pola Pemberian ASI

Pola pemberian ASI pada Riskesdas 2018 merujuk pada praktek pemberian ASI pada 6 bulan pertama, Pola pemberian ASI dibedakan menjadi ASI eksklusif, ASI predominan dan ASI parsial (WHO, 2008).

ASI eksklusif 0-5 bulan

Proporsi bayi yang mendapatkan ASI Eksklusif dihitung dengan formula:

$$Proporsi\ bayi\ yang\ mendapatkan\ ASI\ Eksklusif = \frac{\begin{matrix} jumlah\ anak\ usia\ 0-5\ bulan\ yang\ hanya\ menerima\ Air\ Susu\ Ibu\ saja, \\ tidak\ diberi\ makanan\ atau\ minuman\ lain, termasuk\ air\ putih \\ (kecuali\ obat-obatan\ dan\ vitamin\ atau\ mineral\ tetes;\ ASI\ perah)\ dalam\ 24\ jam\ terakhir \end{matrix}}{jumlah\ seluruh\ bayi\ usia\ 0-5\ bulan}$$

ASI eksklusif 0-5 bulan pada Riskesdas 2018 merupakan komposit dari pertanyaan apakah bayi/anak masih disusui, selama 24 jam terakhir hanya diberi ASI saja, serta tidak diberi makanan/minuman lain.

Tabel 16.5.12
Proporsi Pemberian ASI Saja dalam 24 Jam Terakhir pada Bayi Umur 0-5 Bulan menurut Kelompok Umur, Riskesdas 2018

Kelompok Umur	Dalam 24 Jam Terakhir Hanya Diberi ASI			N tertimbang
	%	CI 95%		
0 bulan	81.0	77.4	84.1	1,242
1 bulan	78.4	74.7	81.8	1,198
2 bulan	79.7	76.0	83.0	1,125
3 bulan	74.4	70.4	78.0	1,313
4 bulan	72.4	68.8	75.8	1,244
5 bulan	62.2	58.2	65.9	1,293
**0-5 bulan**	**74.5**	**73.0**	**76.0**	**7,415**

Tabel 16.5.13
Proporsi Pemberian ASI Saja dalam 24 Jam Terakhir pada Bayi Umur 0-5 bulan menurut Karakteristik, Riskesdas 2018

Karakteristik	Dalam 24 Jam Terakhir Hanya Diberi ASI			N tertimbang
	%	CI 95%		
**Jenis Kelamin**				
Laki-laki	74.1	71.9	76.2	3,772
Perempuan	74.9	72.7	76.9	3,643
**Tempat Tinggal**				
Perkotaan	72.7	70.2	75.0	3,914
Perdesaan	76.6	74.9	78.2	3,501
**Indonesia**	**74.5**	**73.0**	**76.0**	**7,415**

G. Keragaman Konsumsi Makanan

Proporsi konsumsi makanan yang beragam pada anak umur 6-23 bulan dihitung dengan formula:

$$\text{Proporsi konsumsi makanan yang beragam pada anak umur } 6-23 \text{ bulan} = \frac{\text{jumlah anak umur } 6-23 \text{ bulan yang mengonsumsi } 4 \text{ atau lebih kelompok makanan dari } 7 \text{ kelompok makanan pada waktu } 24 \text{ jam sebelumnya}}{\text{jumlah anak umur } 6-23 \text{ bulan}}$$

Tujuh kelompok makanan tersebut yaitu : 1) serealia dan umbi-umbian, 2) kacang-kacangan, 3) susu dan olahannya (yogurt, susu, keju dll), 4) makanan daging (termasuk ikan, ayam, daging, hati dll), 5) telur, 6) sayur dan buah sumber vitamin A dan 7) sayur dan buah lainnya.

Tabel 16.5.14
Proporsi Makanan Beragam yang Dikonsumsi Anak Umur 6-23 Bulan menurut Provinsi, Riskesdas 2018

Provinsi	Makanan Beragam		N Tertimbang
	%	95%CI	
Aceh	41,0	35,93-46,17	331
Sumatera Utara	35,6	30,97-40,55	750
Sumatera Barat	53,0	48,11-57,82	369
Riau	38,7	33,06-44,72	454
Jambi	39,2	32,84-45,97	212
Sumatera Selatan	44,1	38,01-50,35	415
Bengkulu	45,9	38,36-53,72	106
Lampung	47,3	41,69-52,98	458
Bangka Belitung	45,6	36,01-55,48	62
Kepulauan Riau	29,4	17,86-44,33	96
DKI Jakarta	54,3	45,04-63,23	394
Jawa Barat	55,4	50,66-59,98	2.199
Jawa Tengah	55,0	51,53-58,46	1.697
DI Yogyakarta	69,2	58,51-78,1	164
Jawa Timur	48,1	44,37-51,79	1.573
Banten	58,5	50,48-66,1	570
Bali	49,3	41,48-57,2	172
Nusa Tenggara Barat	47,3	39,98-54,81	297
Nusa Tenggara Timur	26,3	22,16-30,85	315
Kalimantan Barat	41,7	35,27-48,42	261
Kalimantan Tengah	37,9	30,82-45,49	133
Kalimantan Selatan	38,2	31,79-45,04	228
Kalimantan Timur	47,4	37,26-57,82	144
Kalimantan Utara	31,2	20,1-44,92	39*
Sulawesi Utara	30,4	23,86-37,87	102
Sulawesi Tengah	34,6	29,15-40,47	184
Sulawesi Selatan	32,7	28,05-37,69	442
Sulawesi Tenggara	24,7	18,8-31,83	148
Gorontalo	28,3	19,47-39,11	50
Sulawesi Barat	30,1	22,69-38,66	85
Maluku	18,2	11,98-26,63	85
Maluku Utara	16,7	11,03-24,47	51
Papua Barat	34,1	22,83-47,44	48*
Papua	27,3	20,10-35,85	145
**INDONESIA**	**46,6**	**45,34-47,96**	**12.776**

*N Tertimbang <50

Tabel 16.5.15
Proporsi Makanan Beragam yang Dikonsumsi Anak Umur 6-23 Bulan menurut Karakteristik, Riskesdas 2018

Karakteristik	Makanan Beragam		N Tertimbang
	%	95% CI	
**Kelompok umur (bulan)**			
6-11	29,8	27,99-31,76	5.048
12-15	54,9	52,32-57,53	2.886
16-19	58,1	55,21-60,89	2.577
20-23	60,5	57,39-63,58	2.265
**Jenis kelamin**			
Laki-laki	47,0	45,15-48,80	6.404
Perempuan	46,3	44,49-48,17	6.372
**Pendidikan KRT**			
Tidak/belum pernah sekolah	41,9	36,11-47,96	561
Tidak tamat SD/MI	44,8	41,31-48,28	1.523
Tamat SD/MI	42,4	40,06-44,76	3.441
Tamat SLTP/MTS	43,9	41,00-46,79	2.399
Tamat SLTA/MA	50,4	47,74-52,99	3.687
Tamat D1/D2/D3/PT	57,9	53,50-62,15	1.165
**Pekerjaan KRT**			
Tidak bekerja	49,8	44,88-54,82	855
Sekolah	40,3	18,09-67,41	26*
PNS/TNI/Polri/BUMN/BUMD	53,6	48,00-59,15	548
Pegawai swasta	52,9	48,68-57,13	1.779
Wiraswasta	48,3	45,52-51,01	2.746
Petani/buruh tani	39,0	37,03-40,98	3.614
Nelayan	37,1	30,43-44,31	295
Buruh/sopir/pembantu ruta	49,5	46,08-52,94	2.240
Lainnya	49,8	44,03-55,48	673
**Tempat tinggal**			
Perkotaan	52,2	50,26-54,56	6.453
Perdesaan	40,8	39,28-42,25	6.323

*N Tertimbang <50

16.6 Pemberian Makanan Tambahan untuk Balita

Pemberian makanan tambahan (PMT) untuk balita merupakan suplementasi gizi dalam bentuk makanan tambahan dengan formulasi khusus dan difortifikasi dengan vitamin dan mineral dengan sasaran kelompok balita untuk pemulihan atau pemenuhan status gizi (Kementerian Kesehatan RI, 2017). Dalam Riskesdas 2018, makanan tambahan untuk balita merujuk pada seluruh makanan tambahan yang diberikan ketika posyandu (PMT penyuluhan); makanan tambahan khusus diberikan untuk balita kurus yang diberikan selama 90 hari makan (PMT pemulihan); makanan tambahan yang diperoleh dari bantuan pihak lain, seperti sumbangan dari LSM/perusahaan atau pihak tertentu yang sedang melakukan kegiatan tertentu atau promosi produk tertentu.

1. ANAK UMUR 6-59 BULAN MEMPEROLEH PMT

Dalam 12 bulan terakhir, anak umur 6-59 bulan yang memperoleh makanan tambahan (PMT) berupa biskuit program; biskuit selain program, susu bubuk, susu cair, bahan makanan mentah, dan bahan makanan matang, dihitung menggunakan f*ormula:*

$$Proporsi\ anak\ umur\ 6-59\ bulan\ mendapat\ PMT = \frac{\begin{array}{c} jumlah\ anak\ umur\ 6-59\ bulan\ \ yang\ memperoleh\ PMT \\ dalam\ 12\ bulan\ terakhir \end{array}}{jumlah\ anak\ umur\ 6-59\ bulan}$$

2. ANAK UMUR 6-59 BULAN MEMPEROLEH PMT PROGRAM

Anak 6-59 bulan yang memperoleh PMT Biskuit Program Kementerian Kesehatan dalam periode 12 bulan terakhir, dihitung dengan formula:

$$Proporsi\ anak\ umur\ 6-59\ bulan\ mendapat\ PMT\ program = \frac{\begin{array}{c} jumlah\ anak\ umur\ 6-59\ bulan\ \ yang\ memperoleht\ PMT\ program \\ dalam\ 12\ bulan\ terakhir \end{array}}{jumlah\ anak\ umur\ 6-59\ bulan\ yang\ memperoleh\ PMT}$$

3. JUMLAH PMT PROGRAM YANG DIPEROLEH ANAK UMUR 6-59 BULAN

Jumlah PMT Biskuit Program Kementerian Kesehatan yang diperoleh anak 6-59 bulan dengan kategori (1) 0-30 bungkus; (2) 31-89 bungkus; dan (3) ≥ 90 bungkus, dihitung dengan formula:

$$Proporsi\ jumlah\ PMT\ program\ yang\ diperoleh\ anak\ umur\ 6-59\ bulan$$

$$= \frac{\begin{matrix} jumlah\ anak\ umur\ 6-59\ bulan\ \ yang\ mendapat\ PMT \\ dalam\ 12\ bulan\ terakhir\ menurut\ kategori\ jumlah\ PMT \end{matrix}}{jumlah\ anak\ umur\ 6-59\ bulan\ yang\ memperoleh\ PMT\ program}$$

4. PMT PROGRAM TIDAK DIHABISKAN

Jumlah anak umur 6-59 bulan yang tidak menghabiskan PMT Biskuit Program Kementerian Kesehatan, dihitung menggunakan formula:

$$Proporsi\ anak\ umur\ 6-59\ bulan\ yang\ tidak\ menghabiskan\ PMT\ program$$

$$= \frac{Jumlah\ anak\ umur\ 6-59\ bulan\ \ yang\ tidak\ menghabiskan\ PMT\ program}{jumlah\ anak\ umur\ 6-59\ bulan\ yang\ memperoleh\ PMT\ program}$$

5. ALASAN TIDAK MENGHABISKAN PMT PROGRAM

Alasan anak umur 6-59 bulan tidak menghabiskan PMT Biskuit Program Kementerian Kesehatan, yaitu (1) anak tidak mau; (2) ibu lupa memberikan; (3) ada efek samping; (4) dimakan anggota rumah tangga lainnya; dan (5) lainnya.

$$Proporsi\ alasan\ anak\ umur\ 6-59\ bulan\ tidak\ menghabiskan\ PMT\ program$$

$$= \frac{Jumlah\ anak\ umur\ 6-59\ bulan\ menurut\ alasan\ tidak\ menghabiskan\ PMT\ program}{jumlah\ anak\ umur\ 6-59\ bulan\ yang\ tidak\ menghabiskan\ PMT\ program}$$

6. ALASAN MEMPEROLEH MAKANAN TAMBAHAN

Alasan anak 6-59 bulan mendapatkan makanan tambahan, yaitu (1) gizi buruk; (2) gizi kurang/BGM; (3) kurus; (4) berat badan tidak pernah naik; (5) sakit-sakitan; (6) ikut penimbangan di posyandu; (7) keluarga miskin; dan (8) lainnya.

$$Proporsi\ alasan\ anak\ umur\ 6-59\ bulan\ mendapatkan\ PMT$$

$$= \frac{Jumlah\ anak\ umur\ 6-59\ bulan\ \ menurut\ alasan\ memperoleh\ PMT}{jumlah\ anak\ umur\ 6-59\ bulan\ yang\ memperoleh\ PMT}$$

Tabel 16.6.1
Proporsi Anak Umur 6-59 Bulan Memperoleh PMT dan PMT Program menurut Provinsi, Riskesdas 2018

Provinsi	Memperoleh PMT			Memperoleh PMT Program		
	%	95% CI	N tertimbang	%	95% CI	N tertimbang
Aceh	43,1	40,6 - 45,6	2.030	73,2	70,1 - 76,1	789
Sumatera Utara	22,7	20,9 - 24,6	5.407	60,7	56,8 - 64,6	1.105
Sumatera Barat	37,2	34,4 - 40,1	1.994	51,5	47,1 - 55,9	668
Riau	26,2	23,8 - 28,7	2.573	68,0	63,1 - 72,6	607
Jambi	29,2	26,0 - 32,5	1.183	69,7	63,6 - 75,1	311
Sumatera Selatan	23,1	20,8 - 25,7	2.819	74,4	68,7 - 79,3	588
Bengkulu	27,4	24,1 - 31,0	658	80,9	74,9 - 85,6	163
Lampung	38,4	35,7 - 41,2	2.798	91,7	88,9 - 93,8	968
Bangka Belitung	28,7	24,8 - 33,0	482	51,6	44,3 - 58,9	125
Kepulauan Riau	18,9	14,3 - 24,7	815	67,1	53,2 - 78,5	139
DKI Jakarta	46,6	42,4 - 51,0	3.258	40,8	34,7 - 47,2	1.369
Jawa Barat	49,0	46,8 - 51,1	15.632	66,7	63,8 - 69,4	6.901
Jawa Tengah	56,2	54,4 - 58,0	9.532	58,6	56,4 - 60,9	4.829
DI Yogyakarta	72,4	67,7 - 76,7	985	41,0	35,4 - 46,9	643
Jawa Timur	54,4	52,6 - 56,2	10.250	33,7	31,5 - 36,0	5.026
Banten	43,5	40,2 - 46,9	4.374	74,9	70,6 - 78,7	1.715
Bali	51,3	47,4 - 55,2	1.169	46,4	41,5 - 51,4	541
Nusa Tenggara Barat	33,0	29,8 - 36,3	1.794	37,0	32,0 - 42,3	533
Nusa Tenggara Timur	28,7	26,6 - 31,0	2.256	60,7	56,3 - 64,9	584
Kalimantan Barat	24,0	21,5 - 26,7	1.754	66,8	61,4 - 71,7	379
Kalimantan Tengah	26,2	23,1 - 29,4	918	57,7	50,6 - 64,5	216
Kalimantan Selatan	19,7	17,3 - 22,2	1.407	41,7	35,9 - 47,7	249
Kalimantan Timur	27,0	23,0 - 31,6	1.249	55,5	47,6 - 63,0	304
Kalimantan Utara	14,9	11,3 - 19,3	279	58,3	47,2 - 68,6	37*
Sulawesi Utara	31,8	28,6 - 35,1	745	68,8	63,5 - 73,6	213
Sulawesi Tengah	35,2	32,1 - 38,5	1.048	50,7	45,3 - 56,1	333
Sulawesi Selatan	37,0	34,8 - 39,3	2.942	70,1	66,5 - 73,4	981
Sulawesi Tenggara	22,5	19,6 - 25,7	1.032	70,1	63,5 - 75,9	209
Gorontalo	37,5	33,1 - 42,0	413	82,2	76,7 - 86,7	139
Sulawesi Barat	31,5	27,5 - 35,9	523	65,8	58,0 - 72,8	149
Maluku	37,3	33,3 - 41,4	707	62,1	55,6 - 68,2	237
Maluku Utara	21,6	18,5 - 25,1	493	66,7	58,6 - 73,9	96
Papua Barat	28,5	24,9 - 32,3	375	76,5	69,8 - 82,1	96
Papua	20,3	17,9 - 22,9	1.201	69,9	64,0 - 75,2	220
**INDONESIA**	**41,0**	**40,4 - 41,7**	**85.094**	**58,3**	**57,3 - 59,3**	**31.464**

*Ntertimbang< 50

Tabel 16.6.2
Proporsi Anak Umur 6-59 Bulan Memperoleh PMT dan PMT Program menurut Karakteristik, Riskesdas 2018

Karakteristik	Memperoleh PMT			Memperoleh PMT Program		
	%	95% CI	N tertimbang	%	95% CI	N tertimbang
**Umur (bulan)**						
6-11	37,0	35,5 - 38,5	9.801	55,5	52,9 - 57,9	3.266
12-23	44,6	43,4 - 45,7	18.416	59,2	57,4 - 60,9	7.397
24-35	45,3	44,2 - 46,5	19.199	60,6	59,0 - 62,3	7.844
36-47	40,2	39,1 - 41,3	18.906	58,6	56,8 - 60,4	6.848
48-59	36,1	35,0 - 37,3	18.773	55,5	53,5 - 57,4	6.109
**Jenis kelamin**						
Laki-laki	41,1	40,4 - 41,9	43.414	58,2	56,9 - 59,4	16.101
Perempuan	40,9	40,1 - 41,7	41.680	58,4	57,2 - 59,7	15.363
**Pendidikan KRT**						
Tidak/belum pernah sekolah	38,4	36,0 - 41,0	3.473	60,3	56,1 - 64,4	1.203
Tidak tamat SD/MI	41,2	39,7 - 42,7	10.097	61,9	59,6 - 64,3	3.751
Tamat SD/MI	43,2	42,1 - 44,3	21.895	62,5	60,9 - 64,2	8.519
Tamat SLTP/MTS	43,3	42,0 - 44,5	16.036	60,8	58,8 - 62,7	6.251
Tamat SLTA/MA	40,1	39,0 - 41,2	25.486	53,4	51,6 - 55,1	9.210
Tamat D1/D2/D3/PT	34,6	32,8 - 36,5	8.107	49,6	46,2 - 53,1	2.530
**Pekerjaan KRT**						
Tidak bekerja	41,2	39,1 - 43,3	5.733	57,2	53,9 - 60,5	2.128
Sekolah	24,4	16,4 - 34,6	164	70,0	45,2 - 86,9	36*
PNS/TNI/Polri/BUMN/BUMD	34,0	31,8 - 36,3	4.190	49,5	45,3 - 53,6	1.285
Pegawai swasta	42,8	41,1 - 44,6	12.356	51,2	48,4 - 53,9	4.768
Wiraswasta	39,1	37,9 - 40,3	19.310	57,4	55,4 - 59,3	6.810
Petani	40,3	39,4 - 41,2	22.079	63,8	62,3 - 65,2	8.018
Nelayan	35,8	32,8 - 38,9	1.766	66,9	62,2 - 71,3	569
Buruh/sopir/pembantu ruta	46,2	44,8 - 47,7	15.206	59,9	57,7 - 62,0	6.339
Lainnya	39,1	36,7 - 41,4	4.289	55,0	51,1 - 58,7	1.510
**Tempat tinggal**						
Perkotaan	41,8	40,8 - 42,8	45.823	54,5	53,0 - 56,0	17.260
Perdesaan	40,1	39,4 - 40,9	39.271	63,0	61,9 - 64,1	14.204

*NTertimbang< 50.

Tabel 16.6.3

Proporsi Jumlah PMT Program yang Diperoleh dan Alasan Tidak Menghabiskan pada Anak Umur 6-59 Bulan menurut Provinsi, Riskesdas 2018

Provinsi	Jumlah PMT program yang diperoleh (bungkus)			tidak meng-habis-kan	N Ter-timbang	Alasan tidak menghabiskan PMT Program					N Ter-timbang
	0-30	31-89	≥90			Anak tidak mau	Ibu lupa mem-berikan	Ada efek sam-ping	Dima-kan ART lain	Lain-nya	
	%	%	%	%		%	%	%	%	%	
Aceh	98,2	1,2	0,6	32,5	604	54,3	2,8	1,0	40,9	1,1	184
Sumatera Utara	98,9	1,0	0,1	34,4	702	54,2	3,6	0,2	41,1	0,9	226
Sumatera Barat	96,5	2,2	1,3	39,7	360	63,6	3,1	0,7	30,6	2,0	134
Riau	94,2	4,6	1,2	42,5	432	55,0	3,1	0	35,5	6,4	172
Jambi	96,6	2,6	0,8	22,4	227	54,2	5,5	0	35,1	5,2	48*
Sumatera Selatan	98,8	1,2	0	26,8	457	62,1	0	0	37,3	0,6	115
Bengkulu	97,8	2,2	0	38,2	137	59,4	0,4	0,4	39,7	0	49*
Lampung	97,4	1,8	0,8	43,0	928	43,1	2,7	0,1	52,2	1,8	375
Bangka Belitung	95,9	2,0	2,1	43,3	67	69,8	2,0	0	23,4	4,8	27*
Kepulauan Riau	98,4	0,8	0,8	48,8	98	61,2	0,6	0	37,5	0,7	45*
DKI Jakarta	97,8	1,6	0,6	36,1	584	71,9	0	0	21,2	6,9	198
Jawa Barat	97,7	1,0	1,3	30,9	4.813	64,6	2,5	1,2	27,7	4,0	1.393
Jawa Tengah	97,2	2,1	0,7	41,6	2.961	64,3	3,3	0,2	28,4	3,8	1.156
DI Yogyakarta	93,5	5,0	1,4	63,0	276	59,5	1,4	0	25,9	13,2	163
Jawa Timur	97,1	2,4	0,6	36,0	1.771	66,6	3,9	0,5	23,4	5,6	597
Banten	97,9	1,1	1,0	45,5	1.343	60,1	4,1	0,4	32,6	2,8	573
Bali	96,9	2,2	0,9	30,4	262	56,8	3,8	0,4	36,7	2,2	75
Nusa Tenggara Barat	91,4	7,4	1,2	36,5	206	46,5	9,0	0	39,8	4,8	71
Nusa Tenggara Timur	94,1	4,3	1,7	24,9	371	43,1	0,2	1,5	53,1	2,1	86
Kalimantan Barat	98,1	1,9	0	31,3	265	62,6	5,3	0	32,1	0	78
Kalimantan Tengah	96,7	2,9	0,4	33,5	131	69,9	4,2	1,1	23,2	1,6	41*
Kalimantan Selatan	92,8	5,8	1,4	45,2	109	63,6	0	2,4	27,8	6,2	46*
Kalimantan Timur	98,0	1,4	0,6	26,6	177	40,0	8,9	0	47,6	3,5	44*
Kalimantan Utara	98,5	0	1,5	45,4	23*	34,5	5,9	0	57,8	1,8	10*
Sulawesi Utara	97,3	1,1	1,6	35,0	154	71,9	7,2	1,7	18,9	0,3	50
Sulawesi Tengah	94,8	4,9	0,3	34,1	176	42,3	4,2	0	51,0	2,6	56
Sulawesi Selatan	95,0	3,9	1,1	39,5	719	39,4	3,4	0,5	52,3	4,4	266
Sulawesi Tenggara	97,2	2,7	0,1	34,7	153	50,7	4,9	0,8	39,0	4,6	50
Gorontalo	98,3	1,7	0	29,7	120	52,7	7,0	0	38,0	2,4	33*
Sulawesi Barat	98,0	1,8	0,2	24,3	102	54,5	2,9	1,5	41,1	0	23*
Maluku	98,7	1,1	0,3	17,7	154	31,4	7,1	1,3	58,4	1,8	26*
Maluku Utara	94,6	4,6	0,8	25,1	67	45,4	1,2	5,4	39,6	8,4	16*
Papua Barat	98,4	1,1	0,5	31,3	77	44,8	2,3	0,7	52,2	0,1	23*
Papua	89,4	5,6	5,0	25,0	160	24,4	4,5	0	71,1	0	38*
**INDONESIA**	**97,1**	**2,0**	**0,9**	**36,0**	**19.186**	**59,5**	**3,1**	**0,5**	**33,1**	**3,8**	**6.488**

*Ntertimbang< 50.

Tabel 16.6.4
Proporsi Jenis Makanan Tambahan yang Dikonsumsi Anak umur 6-59 bulan
menurut Provinsi, Riskesdas 2018

Provinsi	Jenis makanan tambahan (%)						N Ter-timbang
	Biskuit program	Biskuit lainnya	Susu bubuk	Susu cair	Bahan makanan mentah	Makanan matang	
Aceh	73,2	29,0	21,7	10,1	14,1	61,9	789
Sumatera Utara	60,7	21,3	20,5	5,2	6,5	55,2	1.105
Sumatera Barat	51,5	18,8	7,0	4,0	7,3	72,8	668
Riau	68,0	16,9	9,8	7,2	6,7	57,0	607
Jambi	69,7	37,7	32,6	14,2	11,6	51,0	311
Sumatera Selatan	74,4	24,4	14,5	8,3	6,7	31,9	588
Bengkulu	80,9	18,9	14,9	8,8	7,9	37,6	163
Lampung	91,7	11,3	5,2	9,1	5,4	28,1	968
Bangka Belitung	51,6	31,8	26,4	8,7	6,0	55,5	125
Kepulauan Riau	67,1	25,8	18,4	14,8	12,2	44,0	139
DKI Jakarta	40,8	47,2	16,7	48,5	7,5	79,6	1.369
Jawa Barat	66,7	31,2	7,3	20,8	5,7	52,0	6.901
Jawa Tengah	58,6	26,3	5,0	11,2	4,0	72,2	4.829
DI Yogyakarta	41,0	10,9	4,0	11,2	5,0	90,4	643
Jawa Timur	33,7	33,1	6,2	16,0	3,8	80,1	5.026
Banten	74,9	29,2	11,6	16,8	4,0	43,8	1.715
Bali	46,4	23,8	12,6	16,5	3,2	83,5	541
Nusa Tenggara Barat	37,0	16,2	3,2	5,5	6,1	79,2	533
Nusa Tenggara Timur	60,7	8,0	5,2	7,3	5,1	51,7	584
Kalimantan Barat	66,8	25,8	20,4	18,8	6,7	52,4	379
Kalimantan Tengah	57,7	16,8	16,4	12,6	5,3	56,7	216
Kalimantan Selatan	41,7	18,2	14,2	6,9	3,4	68,0	249
Kalimantan Timur	55,5	26,8	18,1	17,9	6,9	55,7	304
Kalimantan Utara	58,3	13,6	13,8	6,9	12,1	50,0	37*
Sulawesi Utara	68,8	25,4	31,9	11,7	11,7	50,7	213
Sulawesi Tengah	50,7	17,2	13,6	7,6	7,2	69,7	333
Sulawesi Selatan	70,1	19,1	9,8	7,4	5,6	47,5	981
Sulawesi Tenggara	70,1	3,8	4,9	3,6	5,4	37,7	209
Gorontalo	82,2	23,8	21,3	6,6	2,3	36,9	139
Sulawesi Barat	65,8	22,3	10,1	10,3	5,8	43,8	149
Maluku	62,1	9,8	5,4	5,2	3,0	61,1	237
Maluku Utara	66,7	7,6	5,7	4,4	2,3	48,1	96
Papua Barat	76,5	9,5	18,4	4,3	5,3	36,8	96
Papua	69,9	16,3	23,4	9,3	7,6	48,1	220
**INDONESIA**	**58,3**	**26,9**	**9,6**	**15,1**	**5,5**	**61,8**	**31.464**

*N tertimbang< 50

Tabel 16.6.5
Proporsi Jenis Makanan Tambahan yang Dikonsumsi Anak umur 6-59 bulan
menurut Karakteristik, Riskesdas 2018

Karakteristik	Jenis makanan tambahan (%)						N Tertimbang
	Biskuit program	Biskuit lainnya	Susu bubuk	Susu cair	Bahan makanan mentah	Makanan matang	
**Kelompok Umur (bulan)**							
6-11	55,5	26,8	7,9	7,8	3,9	54,6	3.104
12-23	59,2	28,1	9,3	11,9	4,7	60,4	7.157
24-35	60,6	26,3	10,6	17,1	6,3	62,3	7.585
36-47	58,6	26,1	9,7	17,1	6,0	62,8	6.612
48-59	55,5	27,0	9,3	18,4	5,7	65,8	5.878
**Jenis kelamin**							
Laki-laki	58,2	26,3	9,7	15,4	5,8	62,1	15.759
Perempuan	58,4	27,5	9,4	14,8	5,1	61,5	15.019
**Pendidikan KRT**							
Tidak/belum pernah sekolah	60,3	20,1	7,0	11,4	3,6	60,7	1.099
Tidak tamat SD/MI	61,9	24,3	9,1	13,2	4,6	56,8	3.576
Tamat SD/MI	62,5	24,6	7,6	12,7	5,1	58,5	8.245
Tamat SLTP/MTS	60,8	27,0	8,4	14,8	6,0	60,6	6.014
Tamat SLTA/MA	53,4	29,5	11,5	17,4	5,6	66,6	8.917
Tamat D1/D2/D3/PT	49,6	31,7	13,9	20,7	7,4	66,7	2.361
**Pekerjaan KRT**							
Tidak bekerja	57,2	25,7	10,2	14,9	4,2	63,5	1.985
Sekolah	70,0	42,9	19,9	6,6	16,3	67,6	20*
PNS/TNI/Polri/BUMN/BUMD	49,5	27,4	15,8	16,1	7,2	68,2	1.178
Pegawai swasta	51,2	31,8	10,9	21,7	5,6	68,7	4.528
Wiraswasta	57,4	27,5	9,6	15,7	5,8	61,8	6.551
Petani	63,8	22,8	8,3	10,7	5,3	56,9	7.797
Nelayan	66,9	23,2	10,3	14,5	4,7	53,9	507
Buruh/sopir/pembantu ruta	59,9	28,1	8,3	14,8	5,6	61,6	6.078
Lainnya	55,0	26,8	10,9	16,6	4,5	62,5	1.394
**Tempat tinggal**							
Perkotaan	54,5	29,5	10,3	19,2	5,6	66,1	16.866
Perdesaan	63,0	23,7	8,6	10,2	5,3	56,6	13.975

*Ntertimbang< 50

Tabel 16.6.6
Proporsi Alasan Anak Umur 6-59 Bulan Memperoleh PMT menurut Provinsi, Riskesdas 2018

Provinsi	Alasan memperoleh PMT (%)								N Tertimbang
	Gizi buruk	Gizi kurang/BGM	Kurus	Berat badan tidak pernah naik (2T)	Sakit-sakitan	Ikut penim-bangan di posyandu	Keluarga miskin	Lainnya	
Aceh	0,7	2,3	4,0	3,7	1,4	82,0	3,8	6,5	789
Sumatera Utara	0,7	1,8	6,3	3,4	2,4	72,2	2,8	10,2	1.105
Sumatera Barat	0,3	2,5	1,7	3,7	1,5	85,3	2,4	5,6	668
Riau	0,4	3,1	4,8	3,1	0,5	80,2	1,2	8,8	607
Jambi	0,3	2,0	4,2	1,7	1,3	64,9	3,4	7,8	311
Sumatera Selatan	1,4	3,6	3,9	1,4	1,3	71,0	6,7	5,7	588
Bengkulu	0,8	3,7	1,1	2,4	0,2	67,5	1,4	7,5	163
Lampung	0,5	3,8	4,4	3,7	1,4	85,5	1,4	3,1	968
Bangka Belitung	0	4,0	6,1	5,3	1,3	56,4	2,8	21,0	125
Kepulauan Riau	0,7	4,0	2,8	4,6	0	67,8	4,5	5,2	139
DKI Jakarta	0,8	3,9	5,4	5,2	1,6	84,9	1,8	4,1	1.369
Jawa Barat	0,5	1,7	2,8	2,4	1,4	87,2	1,9	4,7	6.901
Jawa Tengah	0,6	1,9	2,9	2,3	0,7	88,3	1,4	3,5	4.829
DI Yogyakarta	0,3	1,8	2,9	1,9	1,2	85,0	0,7	6,2	643
Jawa Timur	0,5	2,1	2,1	1,6	0,8	88,6	1,3	2,5	5.026
Banten	0,4	1,3	2,9	3,1	1,1	86,7	1,3	5,3	1.715
Bali	0,4	1,4	1,7	2,5	0,7	88,5	1,6	3,4	541
Nusa Tenggara Barat	1,1	8,8	4,9	4,7	1,2	88,4	2,9	3,7	533
Nusa Tenggara Timur	4,6	9,6	4,7	7,1	2,5	79,8	2,0	4,6	584
Kalimantan Barat	0,7	3,9	4,7	6,4	0,7	64,6	2,8	6,4	379
Kalimantan Tengah	0,3	5,0	3,7	3,8	1,8	74,7	1,9	7,5	216
Kalimantan Selatan	2,5	7,4	11,0	9,1	3,5	65,7	3,2	12,3	249
Kalimantan Timur	0	3,5	2,6	1,4	0,7	71,5	0,3	12,6	304
Kalimantan Utara	1,1	7,3	0,4	5,7	0,6	57,3	1,6	11,4	37*
Sulawesi Utara	1,1	2,3	7,1	4,1	2,0	69,7	2,0	11,4	213
Sulawesi Tengah	1,7	4,0	4,2	4,6	1,3	77,6	5,4	7,0	333
Sulawesi Selatan	1,3	6,3	10,1	9,4	1,7	66,5	3,6	8,6	981
Sulawesi Tenggara	1,0	4,9	10,3	4,4	1,7	79,6	3,1	4,8	209
Gorontalo	2,5	5,5	7,4	7,9	6,2	68,0	4,0	5,0	139
Sulawesi Barat	1,4	10,3	11,9	8,8	5,5	68,8	8,1	12,4	149
Maluku	1,4	5,0	5,2	4,1	1,8	78,3	1,7	4,9	237
Maluku Utara	2,7	8,2	8,8	11,3	4,6	77,4	4,3	4,0	96
Papua Barat	0,4	2,3	2,1	8,8	3,7	75,0	3,0	5,1	96
Papua	1,5	2,5	6,3	5,4	2,1	69,6	10,2	9,2	220
**INDONESIA**	**0,7**	**2,8**	**3,7**	**3,2**	**1,3**	**83,5**	**2,1**	**5,0**	**31.464**

*Ntertimbang< 50

Tabel 16.6.7
Proporsi Alasan Anak Umur 6-59 Bulan Memperoleh PMT menurut Karakteristik, Riskesdas 2018

Karakteristik	Alasan memperoleh PMT (%)								N Ter-timbang
	Gizi buruk	Gizi kurang/ BGM	Kurus	Berat badan tidak pernah naik (2T)	Sakit-sakitan	Ikut penimba-ngan di posyandu	Kelu-arga miskin	Lain-nya	
**Kelompok Umur (bulan)**									
6-11	0,4	1,6	1,7	1,9	1,1	82,6	1,6	5,1	3.266
12-23	0,7	3,0	4,5	3,5	1,4	83,2	2,3	4,7	7.397
24-35	0,9	3,6	4,1	3,5	1,3	84,1	2,0	4,8	7.844
36-47	0,9	2,8	3,9	3,5	1,4	83,3	2,4	5,6	6.848
48-59	0,6	2,1	2,8	2,8	1,0	84,0	1,9	5,0	6.109
**Jenis kelamin**									
Laki-laki	0,7	2,5	3,5	3,1	1,4	83,7	2,1	5,3	16.101
Perempuan	0,7	3,1	3,8	3,2	1,2	83,3	2,0	4,8	15.363
**Pendidikan KRT**									
Tidak/belum pernah sekolah	1,1	3,0	3,3	2,7	1,2	84,9	2,8	3,2	1.203
Tidak tamat SD/MI	1,0	3,4	4,1	4,2	1,9	83,5	3,5	4,1	3.751
Tamat SD/MI	0,8	3,4	4,5	3,7	1,5	83,8	3,0	4,1	8.519
Tamat SLTP/MTS	0,7	2,3	3,5	2,9	1,1	84,4	1,8	4,6	6.251
Tamat SLTA/MA	0,6	2,5	3,4	2,9	1,1	82,6	1,2	6,0	9.210
Tamat D1/D2/D3/PT	0,5	1,4	1,9	2,0	0,7	83,1	0,4	7,7	2.530
**Pekerjaan KRT**									
Tidak bekerja	0,9	4,2	4,5	3,0	1,6	83,8	2,4	4,8	2.128
Sekolah	0,7	2,9	18,5	2,1	0,7	89,2	4,7	6,2	36*
PNS/TNI/Polri/BUMN/BUMD	0,4	1,6	1,6	2,0	0,8	80,9	0,5	8,8	1.285
Pegawai swasta	0,5	1,9	2,2	2,0	0,7	84,9	0,5	5,0	4.768
Wiraswasta	0,6	2,3	3,3	3,0	1,1	82,8	1,5	5,4	6.810
Petani	0,9	3,4	4,1	3,5	1,4	83,2	3,1	4,1	8.018
Nelayan	1,6	5,3	5,6	6,7	2,3	78,2	4,1	6,1	569
Buruh/sopir/pembantu ruta	0,8	2,5	4,4	3,8	1,6	84,9	2,5	4,6	6.339
Lainnya	0,4	3,2	3,8	3,0	1,8	82,1	2,1	6,6	1.510
**Tempat tinggal**									
Perkotaan	0,6	2,5	3,7	3,1	1,3	83,3	1,6	5,6	17.260
Perdesaan	0,8	3,1	3,7	3,3	1,3	83,8	2,6	4,4	14.204

*NTertimbang< 50.

16.7 Status Gizi pada Anak di Bawah Dua Tahun (Baduta)

Status gizi anak baduta (bawah dua tahun) diukur berdasarkan umur, berat badan (BB) dan panjang badan (PB). Berat badan anak baduta ditimbang menggunakan timbangan digital yang memiliki presisi 0,1 kg, sedangkan panjang badan diukur menggunakan alat ukur panjang badan dengan presisi 0,1 cm.

Variabel BB dan PB anak baduta disajikan dalam bentuk tiga indeks antropometri, yaitu BB/U, PB/U, dan BB/PB. Untuk menilai status gizi anak baduta, maka angka berat badan dan panjang badan setiap anak baduta dikonversikan dalam nilai terstandar (*Z-score*) menggunakan baku antropometri anak balita WHO 2005. Selanjutnya berdasarkan nilai *Z-score* dari masing-masing indikator tersebut ditentukan status gizi anak baduta dengan batasan sebagai berikut :

a. Klasifikasi status gizi berdasarkan indeks BB/U :

Gizi Buruk	:	*Z-score* < -3,0
Gizi Kurang	:	*Z-score* ≥ -3,0 s/d Z-*score* < -2,0
Gzi Baik	:	*Z-score* ≥ -2,0 s/d *Z-score* ≤ 2,0
Gizi Lebih	:	*Z-score* > 2,0

b. Klasifikasi status gizi berdasarkan indikator PB/U:

Sangat pendek	:	*Z-score* < -3,0
Pendek	:	*Z-score* ≥ -3,0 s/d *Z-score* < -2,0
Normal	:	*Z-score* ≥ -2,0

c. Klasifikasi status gizi berdasarkan indikator BB/PB:

Sangat kurus	:	*Z-score* < -3,0
Kurus	:	*Z-score* ≥ -3,0 s/d *Z-score* < -2,0
Normal	:	*Z-score* ≥ -2,0 s/d *Z-score* ≤ 2,0
Gemuk	:	*Z-score* > 2,0

Berdasarkan indikator BB/U:

Prevalensi Baduta Gizi Buruk	:	(∑Baduta gizi buruk/∑Baduta ditimbang BB) x 100%
Prevalensi Baduta Gizi Kurang	:	(∑Baduta gizi kurang/∑Baduta ditimbang BB) x 100%
Prevalensi Baduta Gizi Baik	:	(∑Baduta gizi baik/∑Baduta ditimbang BB) x 100%

Berdasarkan indikator PB/U

Prevalensi Baduta Sangat Pendek	:	(∑Baduta sangat pendek/∑Baduta diukur PB) x 100%
Prevalensi Baduta Pendek	:	(∑Baduta pendek/∑Baduta diukur PB) x 100%
Prevalensi Baduta Normal	:	(∑Baduta normal/∑Baduta diukur PB) x 100%

Berdasarkan indikator BB/PB:

Prevalensi Baduta Sangat Kurus	:	(∑Baduta sangat kurus/∑Baduta diukur BB dan PB) x 100%
Prevalensi Baduta Kurus	:	(∑Baduta kurus/∑Baduta diukur BB dan PB) x 100%
Prevalensi Baduta Normal	:	(∑Baduta normal/∑Baduta diukur BB dan PB) x 100%
Prevalensi Baduta Gemuk	:	(∑Baduta gemuk/∑Baduta diukur BB dan PB) x 100%

Tabel 16.7.1
Prevalensi Status Gizi (BB/U) pada Anak Umur 0-23 Bulan (Baduta) menurut Provinsi, Riskesdas 2018

Provinsi	Status Gizi Menurut BB/U								N Ter-timbang
	Gizi Buruk		Gizi Kurang		Gizi Baik		Gizi Lebih		
	%	95% CI	%	95% CI	%	95% CI	%	95% CI	
Aceh	6,3	5,1-7,9	14,4	12,4-16,6	76,3	73,7-78,7	3,0	2,1-4,2	880
Sumatera Utara	5,8	4,7-7,2	12,9	11,0-15,0	76,5	74,0-78,8	4,9	3,7-6,3	2.099
Sumatera Barat	3,9	2,8-5,4	13,0	10,7-15,7	82,1	79,2-84,7	1,0	0,6-1,7	819
Riau	4,4	3,1-6,2	11,2	9,2-13,6	80,7	77,7-83,4	3,6	2,5-5,2	1.053
Jambi	4,9	3,3-7,1	8,2	6,2-10,6	81,9	78,5-84,8	5,1	3,5-7,3	480
Sumatera Selatan	3,9	2,7-5,7	8,4	6,6-10,6	83,6	80,7-86,2	4,0	2,9-5,6	1.136
Bengkulu	2,4	1,4-4,2	10,4	7,9-13,6	81,7	77,8-85.0	5,5	3,6-8,3	266
Lampung	2,7	1,8-4,0	10,2	8,2-12,5	84,7	82,1-87,1	2,4	1,6-3,6	1.181
Bangka Belitung	4,6	2,6-8,1	12,5	9,4-16,5	80,3	75,5-84,4	2,5	1,3-5,0	190
Kepulauan Riau	2,7	1,2-5,9	8,8	5,6-13,5	84,4	78,9-88,7	4,1	1,9-8,8	315
DKI Jakarta	1,5	0,7-3,4	11,7	8,6-15,6	84,1	79,5-87,8	2,7	1,2-6,3	1.358
Jawa Barat	2,5	1,8-3,4	8,1	6,8-9,6	87,2	85,4-88,8	2,2	1,5-3,2	6.259
Jawa Tengah	3,5	2,7-4,5	11,5	10,1-13,0	83,0	81,2-84,5	2,1	1,5-2,7	4.043
DI Yogyakarta	1,4	0,6-3,6	9,9	6,7-14,4	87,5	82,7-91,1	1,2	0,3-4,3	400
Jawa Timur	3,6	2,8-4,5	11,6	10,2-13,1	82,2	80,4-84,0	2,6	2-3,5,0	4.437
Banten	3,0	1,9-4,6	10,6	8,2-13,7	83,3	79,7-86,3	3,1	1,9-5,2	1.770
Bali	1,6	0,6-4,1	11,2	8,0-15,4	85,0	80,5-88,6	2,1	1,2-3,9	476
Nusa Tenggara Barat	4,4	3-6,5,0	14,4	11,7-17,6	78,8	74,9-82,2	2,3	1,4-3,8	776
Nusa Tenggara Timur	6,9	5,7-8,4	17,6	15,4-20,0	74,0	71,2-76,5	1,5	1,0-2,2	977
Kalimantan Barat	5,2	3,8-7,2	14,3	12,0-17,1	77,5	74,1-80,5	3,0	1,9-4,5	738
Kalimantan Tengah	4,7	3,2-7,0	13,8	10,6-17,7	77,3	72,6-81,4	4,2	2,6-6,8	380
Kalimantan Selatan	3,9	2,6-5,8	14,5	11,9-17,6	80,3	76,8-83,4	1,3	0,6-2,7	632
Kalimantan Timur	3,4	1,9-6,2	7,4	5,3-10,3	85,9	81,7-89,2	3,3	1,5-7,1	511
Kalimantan Utara	1,7	0,7-4,2	15,8	11,4-21,5	80,6	74,5-85,5	2,0	0,8-4,6	124
Sulawesi Utara	6,3	3,6-10,8	10,9	8,4-14,2	80,0	75,4-83,9	2,8	1,6-4,8	303
Sulawesi Tengah	3,8	2,6-5,7	15,5	12,9-18,4	79,1	75,6-82,2	1,6	0,9-2,7	468
Sulawesi Selatan	4,8	3,6-6,2	14,8	12,8-17,1	77,5	74,8-80,0	2,9	2,0-4,2	1.300
Sulawesi Tenggara	6,3	4,1-9,7	13,2	10,9-16	78,8	74,4-82,6	1,7	0,9-3,0	465
Gorontalo	8,1	5,4-12,1	16,0	11,7-21,3	71,3	64,9-76,9	4,7	2,7-7,8	172
Sulawesi Barat	5,7	3,7-8,7	14,2	10,6-18,7	79,5	74,4-83,8	0,6	0,2-1,7	234
Maluku	10,3	7,2-14,4	12,5	9,8-15,8	74,4	69,8-78,5	2,8	1,6-4,9	309
Maluku Utara	6,5	4,6-9,2	14,4	11,2-18,3	74,8	70,4-78,8	4,3	2,5-7,2	187
Papua Barat	4,1	2,6-6,2	12,1	8,5-17	80,9	75,5-85,3	2,9	1,3-6,3	159
Papua	4,5	3,2-6,2	11,7	9,4-14,5	77,7	74,1-80,9	6,1	4,6-8,1	425
**INDONESIA**	**3,8**	**3,6-4,1**	**11,4**	**10,9-11,9**	**82,0**	**81,5-82,6**	**2,7**	**2,5-3,0**	**35.321**

Catatan:

Gizi Buruk : *Zscore* < -3,0

Gizi Kurang : *Zscore* ≥ -3,0 s/d Zscore < -2,0

Gizi Baik : *Zscore* ≥ -2,0 s/d Zscore ≤ 2,0

Gizi Lebih : Zscore > 2,0

Tabel 16.7.2

Prevalensi Status Gizi (BB/U) pada Anak Umur 0-23 Bulan (Baduta) menurut Karakteristik, Riskesdas 2018

Karakteristik	Status Gizi Menurut BB/U								
	Gizi Buruk		Gizi Kurang		Gizi Baik		Gizi Lebih		N Ter-timbang
	%	95% CI	%	95% CI	%	95% CI	%	95% CI	
**Kelompok umur (bln)**									
0 - 5	4,6	4-5,2	8,1	7,3-9,0	85,1	84,0-86,2	2,2	1,8-2,8	8.388
6 - 11	2,9	2,5-3,5	9,2	8,4-10,1	85,2	84,2-86,2	2,6	2,2-3,1	9.381
12 - 23	3,9	3,6-4,3	14,1	13,4-14,9	78,9	78,0-79,7	3,0	2,7-3,5	17.552
**Jenis Kelamin**									
Laki-laki	5,0	4,6-5,5	12,7	12,0-13,4	79,8	78,9-80,6	2,5	2,2-2,9	17.836
Perempuan	2,6	2,3-3,0	10,1	9,5-10,8	84,3	83,5-85,1	3,0	2,6-3,4	17.485
**Pendidikan KRT**									
Tidak sekolah	5,2	3,9-6,9	12,4	10,4-14,8	79,2	76,4-81,7	3,2	2,3-4,4	1.524
Tidak Tamat SD/MI	4,4	3,7-5,3	12,8	11,4-14,4	80,1	78,3-81,7	2,7	2,1-3,4	4.233
Tamat SD/MI	4,6	4,0-5,2	12,1	11,3-13,0	80,8	79,7-81,8	2,5	2,1-3,0	9.127
Tamat SLTP/MTS	3,9	3,3-4,7	11,7	10,6-12,9	81,3	79,8-82,7	3,0	2,4-3,8	6.645
Tamat SLTA/MA	3,0	2,6-3,5	10,6	9,7-11,6	83,6	82,5-84,7	2,7	2,3-3,3	10.422
Tamat D1-D3/PT	2,7	2,1-3,5	8,8	7,5-10,4	85,8	83,9-87,5	2,7	1,9-3,7	3.370
**Pekerjaan KRT**									
Tidak bekerja	3,8	2,9-5,0	11,3	9,5-13,4	82,4	80,0-84,5	2,5	1,8-3,4	2.605
Sekolah	9,7	3,1-16,3	15,3	7,0-30,2	74,9	58,2-86,4	0,2	0,0-1,5	62
PNS/TNI/Polri/BUMN/BUMD	2,1	1,4-3,0	9,6	7,7-12,0	84,8	82,2-87,1	3,5	2,5-4,8	1.692
Pegawai swasta	2,6	2,0-3,3	10,4	9,0-11,9	84,3	82,5-86,0	2,7	2,0-3,7	4.993
Wiraswasta	3,8	3,2-4,5	10,7	9,7-11,7	82,9	81,7-84,1	2,6	2,1-3,2	7.807
Petani/buruh tani	5,0	4,5-5,5	12,5	11,7-13,3	79,6	78,6-80,5	3,0	2,6-3,4	9.358
Nelayan	5,7	3,6-8,8	13,8	11,5-16,6	77,6	73,8-80,9	2,9	1,7-5,0	777
Buruh/Supir/pembantu ruta	3,4	2,8-4,1	11,4	10,2-12,7	83,0	81,4-84,4	2,3	1,7-3,0	6.155
Lainnya	3,6	2,7-4,9	12,3	10,3-14,6	80,8	77,7-83,4	3,3	1,9-5,7	1.872
**Tempat Tinggal**									
Perkotaan	3,2	2,8-3,6	10,6	9,9-11,3	83,7	82,8-84,6	2,5	2,1-2,9	18.760
Perdesaan	4,6	4,2-5,0	12,3	11,7-12,9	80,1	79,4-80,8	3,0	2,7-3,3	16.561

Catatan:

Gizi Buruk : *Zscore* < -3,0

Gizi Kurang : *Zscore* ≥ -3,0 s/d Zscore < -2,0

Gizi Baik : *Zscore* ≥ -2,0 s/d Zscore ≤ 2,0

Gizi Lebih : Zscore > 2,0

Tabel 16.7.3
Prevalensi Status Gizi (PB/U) pada Anak Umur 0-23 Bulan (Baduta) menurut Provinsi, Riskesdas 2018

Provinsi	Status Gizi Menurut PB/U						
	Sangat Pendek		Pendek		Normal		N Ter-timbang
	%	95% CI	%	95% CI	%	95% CI	
Aceh	18,9	16,6-21,3	19,0	16,6-21,6	62,1	59,0-65,2	815
Sumatera Utara	13,6	11,7-15,7	18,6	16,3-21,2	67,9	64,8-70,8	2.004
Sumatera Barat	10,6	8,7-13,0	18,2	15,7-21,1	71,1	67,7-74,4	808
Riau	9,8	7,8-12,2	15,0	12,7-17,6	75,2	72,0-78,2	1.036
Jambi	15,7	12,8-19,2	14,6	12,0-17,7	69,7	65,6-73,4	451
Sumatera Selatan	13,7	11,3-16,5	16,1	13,5-19,1	70,2	66,6-73,5	1.062
Bengkulu	10,4	8,0-13,5	18,6	15,1-22,6	71,0	66,8-74,9	249
Lampung	12,2	9,8-15,1	15,2	12,9-17,8	72,6	69,3-75,7	1.144
Bangka Belitung	9,5	6,8-13,1	13,8	10,3-18,3	76,7	71,6-81,2	188
Kepulauan Riau	8,6	5,0-14,5	21,5	15,4-29,1	69,9	61,6-77,1	307
DKI Jakarta	7,0	4,6-10,4	9,2	6,6-12,6	83,8	79,6-87,4	1.313
Jawa Barat	13,2	11,5-15,1	15,9	14,1-17,8	70,9	68,5-73,3	5.974
Jawa Tengah	13,9	12,4-15,5	19,4	17,6-21,3	66,8	64,5-68,9	3.876
DI Yogyakarta	8,3	5,3-12,7	12,2	8,6-16,9	79,5	73,9-84,2	392
Jawa Timur	15,2	13,6-16,9	18,4	16,7-20,2	66,4	64,2-68,5	4.251
Banten	8,7	6,7-11,2	14,6	11,6-18,2	76,7	72,7-80,3	1.674
Bali	9,2	6,2-13,5	15,4	12,0-19,6	75,3	70,4-79,6	478
Nusa Tenggara Barat	8,1	6,2-10,5	17,0	14,2-20,3	74,9	71,2-78,2	759
Nusa Tenggara Timur	17,4	15,2-19,8	18,5	16,3-21,0	64,1	61,2-66,9	954
Kalimantan Barat	13,1	10,7-16,0	18,3	15,5-21,5	68,5	64,9-72,0	716
Kalimantan Tengah	15,9	12,5-20,0	18,3	15,0-22,1	65,9	61,4-70,1	357
Kalimantan Selatan	13,3	10,8-16,2	18,2	15,5-21,3	68,5	64,8-72,1	618
Kalimantan Timur	11,8	8,8-15,8	18,2	14,3-22,8	70,0	65,1-74,5	482
Kalimantan Utara	8,7	6,0-12,4	22,1	16,8-28,7	69,2	62,0-75,5	123
Sulawesi Utara	11,9	9,1-15,5	14,7	11,6-18,5	73,4	68,9-77,4	293
Sulawesi Tengah	11,7	9,4-14,5	16,1	13,5-19,1	72,2	68,5-75,6	461
Sulawesi Selatan	13,3	11,4-15,4	20,6	18,2-23,1	66,2	63,2-69,0	1.242
Sulawesi Tenggara	10,3	8,3-12,8	16,0	13,4-19,1	73,6	69,9-77,0	455
Gorontalo	13,1	9,4-18,0	15,4	11,6-20,1	71,6	65,7-76,7	169
Sulawesi Barat	12,6	9,1-17,2	24,5	19,9-29,9	62,9	57,1-68,3	229
Maluku	14,1	10,4-18,9	17,8	13,9-22,5	68,1	63,0-72,8	283
Maluku Utara	12,3	9,0-16,6	19,6	15,8-24,0	68,1	63,2-72,6	182
Papua Barat	12,5	9,0-17,1	17,7	13,0-23,7	69,7	63,3-75,5	151
Papua	15,1	12,4-18,4	18,9	15,5-22,9	66,0	61,5-70,2	388
**INDONESIA**	**12,8**	**12,3-13,4**	**17,1**	**16,5-17,7**	**70,1**	**69,4-70,8**	**33.882**

Catatan:

Sangat pendek : *Zscore* < -3,0
Pendek : *Zscore* ≥ -3,0 s/d *Zscore* < -2,0
Normal : *Zscore* ≥ -2,0

Tabel 16.7.4
Prevalensi Status Gizi (PB/U) pada Anak Umur 0-23 Bulan (Baduta) menurut Karakteristik, Riskesdas 2018

Karakteristik	Status Gizi Menurut PB/U						N Ter-timbang
	Sangat Pendek		Pendek		Normal		
	%	95% CI	%	95% CI	%	95% CI	
**Kelompok umur (bln)**							
0 - 5	11,7	10,7-12,8	11,4	10,4-12,5	76,8	75,4-78,2	8.190
6 - 11	9,2	8,4-10,1	12,3	11,4-13,2	78,6	77,3-79,7	8.948
12 - 23	15,3	14,5-16,1	22,4	21,5-23,4	62,3	61,2-63,4	16.743
**Jenis Kelamin**							
Laki-laki	14,3	13,6-15,1	17,8	17,0-18,7	67,9	66,8-68,9	17.084
Perempuan	11,3	10,6-12,1	16,3	15,5-17,2	72,3	71,3-73,3	16.798
**Pendidikan KRT**							
Tidak sekolah	14,4	12,1-17,1	18,7	16,2-21,5	66,9	63,5-70,1	1.455
Tidak Tamat SD	14,2	12,8-15,7	16,1	14,7-17,7	69,7	67,7-71,6	4.062
Tamat SD	14,9	13,9-16'0	18,5	17,4-19,7	66,5	65,1-67,9	8.767
Tamat SLTP	13,5	12,3-14,8	18,5	17,1-20,0	68,0	66,2-69,7	6.414
Tamat SLTA	10,6	9,7-11,5	15,5	14,4-16,6	73,9	72,6-75,3	9.951
Tamat D1-D3/PT	10,3	8,9-11,9	15,8	13,9-17,8	74,0	71,6-76,2	3.233
**Pekerjaan KRT**							
Tidak bekerja	12,4	10,7-14,5	16,0	14,1-18,1	71,6	68,9-74,1	2.504
Sekolah	18,4	8,6-35,0	7,8	3,6-15,8	73,8	57,9-85,2	62
PNS/TNI/Polri/BUMN/BUMD	10,6	8,6-12,9	17,0	14,6-19,7	72,5	69,3-75,4	1.635
Pegawai swasta	9,9	8,6-11,4	15,8	14,2-17,7	74,2	72,1-76,2	4.762
Wiraswasta	12,4	11,4-13,6	16,8	15,5-18,1	70,8	69,2-72,3	7.492
Petani/buruh tani	15,0	14,1-16,0	18,1	17,2-19,1	66,8	65,6-68,0	8.963
Nelayan	13,9	10,8-17,7	18,3	15,2-21,8	67,8	63,3-72,0	750
Buruh/Supir/pembantu ruta	13,2	11,9-14,7	17,6	16,1-19,2	69,2	67,2-71,1	5.917
Lainnya	11,7	9,8-14,0	16,3	13,9-19,0	72,0	68,8-75,0	1.797
**Tempat Tinggal**							
Perkotaan	11,2	10,4-12,0	16,2	15,3-17,1	72,7	71,5-73,7	18.022
Perdesaan	14,7	14,0-15,4	18,1	17,4-18,9	67,2	66,3-68,1	15.860

Catatan:

Sangat pendek	:	*Zscore* < -3,0
Pendek	:	*Zscore* ≥ -3,0 s/d *Zscore* < -2,0
Normal	:	*Zscore* ≥ -2,0

Tabel 16.7.5

Prevalensi Status Gizi (BB/PB) pada Anak Umur 0-23 Bulan (Baduta) menurut Provinsi, Riskesdas 2018

Provinsi	Status Gizi menurut BB/PB								N Ter-timbang
	SangatKurus		Kurus		Normal		Gemuk		
	%	95% CI	%	95% CI	%	95% CI	%	95% CI	
Aceh	6,7	5,2-8,6	7,8	6,2-9,7	74,2	71,3-76,9	11,3	9,5-13,5	588
Sumatera Utara	6,3	5,0-7,9	8,3	6,8-10,0	74,7	72,0-77,2	10,7	8,9-12,8	1.457
Sumatera Barat	4,9	3,6-6,6	9,7	7,7-12,1	79,9	76,9-82,7	5,5	4,2-7,2	639
Riau	5,4	4,0-7,2	10,4	8,1-13,2	76,7	73,2-79,8	7,6	5,8-9,8	775
Jambi	7,2	4,9-10,3	7,0	5,2-9,5	73,6	69,3-77,5	12,2	9,5-15,3	329
Sumatera Selatan	5,9	4,4-7,9	5,4	4,0-7,2	77,5	74,4-80,4	11,2	9,1-13,7	809
Bengkulu	3,4	2,0-5,6	6,3	4,2-9,2	78,4	73,8-82,3	12,0	9,0-15,9	189
Lampung	4,9	3,5-6,8	6,9	5,2-9,2	79,3	76,0-82,2	8,9	7,1-11,1	879
Bangka Belitung	3,4	1,9-6,0	8,4	5,6-12,3	82,3	77,8-86,1	6,0	3,8-9,2	152
Kepulauan Riau	3,4	1,5-7,6	5,7	3,4-9,4	82,0	75,8-87,0	8,8	5,2-14,6	251
DKI Jakarta	4,5	2,6-7,8	8,1	5,3-12,1	81,2	75,4-85,9	6,2	3,5-10,8	1.053
Jawa Barat	4,0	3,1-5,2	5,4	4,3-6,7	80,8	78,6-82,8	9,8	8,3-11,5	4.743
Jawa Tengah	3,5	2,7-4,5	6,1	5,1-7,3	80,2	78,3-82,0	10,2	8,8-11,7	3.050
DI Yogyakarta	0,7	0,2-2,2	7,4	4,5-12,0	88,3	83,1-92,0	3,6	1,7-7,5	346
JawaTimur	3,6	2,9-4,6	7,1	6,0-8,5	78,6	76,7-80,5	10,6	9,3-12,1	3.297
Banten	7,2	5,1-10,1	6,3	4,6-8,7	79,1	75,2-82,5	7,4	5,5-9,8	1.311
Bali	2,4	1,1-4,9	4,6	3,0-7,1	84,7	80,9-87,8	8,3	6,0-11,4	396
Nusa Tenggara Barat	4,6	3,1-6,7	10,3	7,9-13,3	80,6	76,9-83,9	4,5	3,0-6,8	602
Nusa Tenggara Timur	6,6	5,0-8,8	8,2	6,8-10,0	79,9	77,3-82,3	5,2	4,1-6,7	747
Kalimantan Barat	4,9	3,4-7,0	11,9	9,5-14,7	75,1	71,4-78,5	8,1	6,1-10,7	524
Kalimantan Tengah	4,2	2,6-6,7	8,6	6,4-11,5	76,5	72,4-80,1	10,7	8,1-14	269
Kalimantan Selatan	3,4	2,3-5,1	8,7	6,6-11,4	80,6	77,3-83,5	7,3	5,4-9,7	493
Kalimantan Timur	3,7	1,9-6,9	5,1	3,3-7,7	82,3	77,7-86,1	9,0	6,2-12,8	388
Kalimantan Utara	1,3	0,6-3,1	3,5	1,9-6,4	89,5	85,4-92,5	5,7	3,5-9,1	109
Sulawesi Utara	4,1	2,6-6,3	8,0	5,9-10,7	78,9	74,8-82,6	9,0	6,5-12,2	229
Sulawesi Tengah	4,0	2,7-5,9	10,5	8,2-13,3	80,8	77,4-83,8	4,7	3,3-6,6	364
Sulawesi Selatan	4,0	2,8-5,5	8,3	6,6-10,4	79,2	76,5-81,7	8,5	7-10,3	960
Sulawesi Tenggara	5,4	3,2-8,8	8,5	6,4-11,1	79,0	74,3-83,0	7,2	5,5-9,3	348
Gorontalo	5,3	3,3-8,4	10,3	7,0-14,8	78,7	73,0-83,5	5,7	3,3-9,7	129
Sulawesi Barat	3,7	2,2-6,4	8,6	5,6-12,9	82,5	77,0-86,9	5,2	3,3-8	184
Maluku	6,3	4,4-8,9	11,1	8,1-15,1	77,1	72,4-81,2	5,5	3,6-8,3	215
Maluku Utara	3,9	2,4-6,2	10,2	7,7-13,5	78,1	74,0-81,8	7,7	5,3-11	139
Papua Barat	5,9	4,0-8,7	6,0	3,9-9,2	79,7	74,3-84,2	8,4	5,3-13,1	118
Papua	5,0	3,2-7,8	8,2	6,2-10,9	71,8	67,4-75,8	15,0	11,9-18,6	275
**INDONESIA**	**4,5**	**4,2-4,9**	**7,2**	**6,8-7,7**	**79,2**	**78,6-79,9**	**9,0**	**8,5-9,5**	**26.357**

Catatan:

Sangat Kurus : *Zscore* < -3,0

Kurus : *Zscore* ≥ -3,0 s/d Zscore < -2,0

Normal : *Zscore* ≥ -2,0 s/d Zscore ≤ 2,0

Gemuk : Zscore > 2,0

Tabel 16.7.6

Prevalensi Status Gizi (BB/PB) pada Anak Umur 0-23 Bulan (Baduta) menurut Karakteristik, Riskesdas 2018

Karakteristik	Status Gizi menurut BB/PB								
	Sangat Kurus		Kurus		Normal		Gemuk		N Ter-timbang
	%	95% CI	%	95% CI	%	95% CI	%	95% CI	
**Kelompok umur (bln)**									
0 - 5	5,1	4,4-5,9	7,7	6,9-8,6	73,6	72,1-75,1	13,6	12,5-14,8	7.765
6 - 11	4,8	4,2-5,5	8,1	7,4-9,0	78,9	77,6-80,1	8,2	7,3-9,1	8.924
12 - 23	4,1	3,7-4,6	6,6	6,0-7,1	82,0	81,2-82,9	7,3	6,7-7,9	16.578
**Jenis Kelamin**									
Laki-laki	5,3	4,8-5,8	7,8	7,2-8,4	77,8	76,8-78,7	9,2	8,5-9,9	16.815
Perempuan	3,8	3,4-4,3	6,7	6,2-7,3	80,7	79,8-81,6	8,8	8,2-9,5	16.452
**Pendidikan KRT**									
Tidak sekolah	3,7	2,7-5,1	7,9	6,2-9,9	80,1	77,2-82,8	8,3	6,5-10,5	1.425
Tidak tamat SD	5,3	4,3-6,5	7,5	6,5-8,7	79,1	77,3-80,8	8,0	7,0-9,3	3.956
Tamat SD	4,3	3,7-4,9	7,4	6,7-8,2	78,6	77,3-79,8	9,7	8,8-10,7	8.619
Tamat SLTP	4,8	4,1-5,6	6,8	6,0-7,7	79,5	78,0-80,9	8,9	7,9-10,0	6.281
Tamat SLTA	4,4	3,8-5,1	7,3	6,5-8,1	79,7	78,4-81,0	8,6	7,7-9,5	9.806
Tamat D1-D3/PT	4,6	3,4-6,0	7,0	5,7-8,6	78,5	76,2-80,7	9,9	8,5-11,6	3.181
**Pekerjaan KRT**									
Tidak Bekerja	4,4	3,3-5,5	7,0	5,7-8,6	80,3	77,9-82,5	8,2	6,8-10,0	2.470
Sekolah	8,8	3,4-13,2	10,3	4,8-20,6	73,2	57,8-84,6	7,7	2,6-12,8	59
PNS/TNI/Polri/BUMN/BUMD	3,4	2,4-4,9	6,1	4,6-8,0	81,3	78,5-83,7	9,3	7,5-11,3	1.604
Pegawai swasta	4,5	3,5-5,7	5,9	4,9-7,2	81,2	79,2-83,1	8,4	7,1-9,9	4.703
Wiraswasta	4,7	4,0-5,5	7,0	6,2-8,0	78,6	77,1-80,1	9,6	8,6-10,8	7.350
Petani/buruh tani	4,9	4,3-5,5	7,7	7,0-8,4	78,1	77,0-79,2	9,3	8,6-10,1	8.779
Nelayan	5,1	3,5-7,2	9,5	7,4-12,2	77,0	73,4-80,3	8,4	6,4-11,0	744
Buruh/Supir/pembantu ruta	4,0	3,2-4,8	7,4	6,4-8,5	80,0	78,3-81,6	8,6	7,5-9,9	5.805
Lainnya	5,0	3,7-6,9	9,1	7,2-11,4	77,2	73,9-80,1	8,7	6,7-11,1	1.755
**Tempat Tinggal**									
Perkotaan	4,5	4,0-5,0	7,1	6,4-7,7	79,8	78,8-80,8	8,6	7,9-9,4	17.701
Perdesaan	4,6	4,2-5,0	7,5	7,0-8,0	78,5	77,8-79,3	9,4	8,8-10	15.566

Catatan:

Sangat Kurus	:	*Zscore* < -3,0
Kurus	:	*Zscore* ≥ -3,0 s/d Zscore < -2,0
Normal	:	*Zscore* ≥ -2,0 s/d Zscore ≤ 2,0
Gemuk	:	Zscore > 2,0

16.8 Status Gizi pada Anak di Bawah Lima Tahun (Balita)

Status gizi anak balita (bawah lima tahun) diukur berdasarkan umur, berat badan (BB) dan tinggi badan (TB). Berat badan anak balita ditimbang menggunakan timbangan digital yang memiliki presisi 0,1 kg, sedangkan tinggi badan diukur menggunakan alat ukur tinggi badan dengan presisi 0,1 cm. Variabel BB dan TB anak balita disajikan dalam bentuk tiga indeks antropometri, yaitu BB/U, TB/U, dan BB/TB. Untuk menilai status gizi anak balita, maka angka berat badan dan tinggi badan setiap anak balita dikonversikan dalam nilai terstandar (*Z-score*) menggunakan baku antropometri anak balita WHO 2005. Selanjutnya berdasarkan nilai *Z-score* dari masing-masing indikator tersebut ditentukan status gizi anak balita dengan batasan sebagai berikut :

a. Klasifikasi status gizi berdasarkan indeks BB/U :

Gizi Buruk	:	*Z-score* < -3,0
Gizi Kurang	:	*Z-score* ≥ -3,0 s/d Z-*score* < -2,0
Gzi Baik	:	*Z-score* ≥ -2,0 s/d *Z-score* ≤ 2,0
Gizi Lebih	:	*Z-score* > 2,0

b. Klasifikasi status gizi berdasarkan indikator PB/U:

Sangat pendek	:	*Z-score* < -3,0
Pendek	:	*Z-score* ≥ -3,0 s/d *Z-score* < -2,0
Normal	:	*Z-score* ≥ -2,0

c. Klasifikasi status gizi berdasarkan indikator BB/PB:

Sangat kurus	:	*Z-score* < -3,0
Kurus	:	*Z-score* ≥ -3,0 s/d *Z-score* < -2,0
Normal	:	*Z-score* ≥ -2,0 s/d *Z-score* ≤ 2,0
Gemuk	:	*Z-score* > 2,0

Berdasarkan indikator BB/U:

Prevalensi Balita Gizi Buruk	:	(∑Balita gizi buruk/∑Balita ditimbang BB) x 100%
Prevalensi Balita Gizi Kurang	:	(∑Balita gizi kurang/∑Balita ditimbang BB) x 100%
Prevalensi Balita Gizi Baik	:	(∑Balita gizi baik/∑Balita ditimbang BB) x 100%

Berdasarkan indikator TB/U

Prevalensi Balita Sangat Pendek	:	(∑ Balita sangat pendek/∑Balita diukur TB) x 100%
Prevalensi Balita Pendek	:	(∑ Balita pendek/∑ Balita diukur TB) x 100%
Prevalensi Balita Normal	:	(∑ Balita normal/∑Balita diukur TB) x 100%

Berdasarkan indikator BB/TB:

Prevalensi Balita Sangat Kurus	:	(∑ Balita sangat kurus/∑ Balita diukur BB dan TB) x 100%
Prevalensi Balita Kurus	:	(∑ Balita kurus/∑ Balita diukur BB dan TB) x 100%
Prevalensi Balita Normal	:	(∑ Balita normal/∑ Balita diukur BB dan TB) x 100%
Prevalensi Balita Gemuk	:	(∑ Balita gemuk/∑ Balita diukur BB dan TB) x 100%

Tabel 16.8.1
Prevalensi Status Gizi (BB/U) Pada Anak Umur 0-59 Bulan (Balita) menurut Provinsi, Riskesdas 2018

Provinsi	Status Gizi BB/U								
	Gizi Buruk		Gizi Kurang		Gizi Baik		Gizi Lebih		N Ter-timbang
	%	95% CI	%	95% CI	%	95% CI	%	95% CI	
Aceh	6,7	5,8-7,7	16,8	15,5-18,3	73,6	71,9-75,2	2,9	2,3-3,6	2.148
Sumatera Utara	5,4	4,7-6,2	14,3	13,0-15,7	76,3	74,8-77,8	4,0	3,4-4,8	5.774
Sumatera Barat	3,5	2,8-4,4	15,4	13,9-17,1	79,5	77,6-81,2	1,6	1,1-2,3	2.144
Riau	4,3	3,5-5,2	14,0	12,6-15,6	78,1	76,2-79,9	3,5	2,8-4,5	2.756
Jambi	3,8	2,9-5,0	11,9	10,4-13,6	80,0	77,8-82,1	4,2	3,2-5,6	1.238
Sumatera Selatan	4,9	3,9-6,0	12,3	10,9-13,9	78,6	76,6-80,5	4,2	3,4-5,1	3.002
Bengkulu	2,8	2,0-3,9	10,4	8,7-12,4	82,3	79,9-84,6	4,5	3,4-5,8	696
Lampung	3,1	2,5-3,9	12,8	11,4-14,3	81,2	79,4-82,9	2,8	2,1-3,7	3.052
Bangka Belitung	3,4	2,2-5,3	13,6	11,4-16,1	78,7	75,5-81,6	4,3	3,1-5,9	520
Kepulauan Riau	3,2	1,8-5,6	9,8	7,8-12,3	82,6	79,1-85,7	4,4	2,8-6,8	846
DKI Jakarta	2,3	1,5-3,5	12,0	10,0-14,3	80,9	78,1-83,5	4,8	3,5-6,7	3.498
Jawa Barat	2,6	2,1-3,2	10,6	9,6-11,6	84,3	83,1-85,5	2,5	2,0-3,0	16.715
Jawa Tengah	3,1	2,6-3,6	13,7	12,8-14,7	80,6	79,4-81,7	2,7	2,3-3,1	10.260
DI Yogyakarta	2,5	1,5-4,3	13,0	10,4-16,2	82,3	78,8-85,3	2,2	1,3-3,6	1.045
Jawa Timur	3,3	2,9-3,9	13,4	12,5-14,4	79,8	78,6-80,9	3,4	2,9-4,0	11.075
Banten	3,6	2,8-4,7	12,6	10,9-14,5	79,9	77,6-82,0	3,9	2,9-5,1	4.699
Bali	2,0	1,3-3,3	11,1	9,1-13,4	83,8	81,0-86,2	3,1	2,2-4,3	1.258
Nusa Tenggara Barat	5,9	4,9-7,1	20,5	18,4-22,7	71,5	69,0-73,8	2,1	1,4-3,1	1.965
Nusa Tenggara Timur	7,3	6,5-8,3	22,2	20,8-23,7	69,3	67,6-71,0	1,1	0,8-1,6	2.486
Kalimantan Barat	5,2	4,3-6,4	18,6	16,8-20,5	73,1	71,0-75,2	3,0	2,3-3,9	1.884
Kalimantan Tengah	5,5	4,3-6,9	16,3	14,3-18,6	74,1	71,5-76,5	4,1	3,1-5,5	981
Kalimantan Selatan	5,5	4,3-6,8	19,0	16,9-21,3	73,0	70,4-75,5	2,5	1,7-3,5	1.516
Kalimantan Timur	3,2	2,2-4,6	11,5	9,6-13,7	80,8	77,9-83,4	4,5	3,2-6,3	1.319
Kalimantan Utara	2,4	1,5-3,7	14,4	11,6-17,7	80,3	76,7-83,4	2,9	1,8-4,9	305
Sulawesi Utara	4,2	3,0-5,9	11,2	9,6-13,1	81,6	79,2-83,8	2,9	2,1-4,1	801
Sulawesi Tengah	4,8	3,9-6,0	18,6	16,7-20,8	74,7	72,3-77,0	1,8	1,2-2,7	1.136
Sulawesi Selatan	4,6	3,9-5,4	18,4	16,9-20,0	74,2	72,4-75,9	2,9	2,3-3,7	3.190
Sulawesi Tenggara	5,6	4,4-7,2	16,4	14,3-18,7	76,2	73,1-79,1	1,8	1,3-2,5	1.153
Gorontalo	6,8	5,2-9,0	19,3	16,4-22,7	69,1	65,3-72,6	4,7	3,4-6,7	435
Sulawesi Barat	6,3	4,8-8,2	18,4	15,5-21,7	74,1	70,3-77,6	1,2	0,7-2,0	566
Maluku	7,4	5,6-9,6	17,5	15,4-19,9	72,4	69,2-75,3	2,7	1,8-4,1	764
Maluku Utara	5,6	4,4-7,1	16,6	14,3-19,2	74,8	71,9-77,4	3,0	2,2-4,2	520
Papua Barat	5,1	3,9-6,8	14,1	11,7-16,9	77,5	74,2-80,6	3,2	2,1-4,9	401
Papua	5,1	4,2-6,3	11,4	9,8-13,3	76,0	73,5-78,4	7,4	6,0-9,1	1.214
**INDONESIA**	**3,9**	**3,7-4,1**	**13,8**	**13,5-14,1**	**79,2**	**78,8-79,6**	**3,1**	**3,0-3,3**	**91.365**

Catatan:

Gizi Buruk	:	*Zscore* < -3,0
Gizi Kurang	:	*Zscore* ≥ -3,0 s/d Zscore < -2,0
Gizi Baik	:	*Zscore* ≥ -2,0 s/d Zscore ≤ 2,0
Gizi Lebih	:	Zscore > 2,0

Tabel 16.8.2
Prevalensi Status Gizi (BB/U) Pada Anak Umur 0-59 Bulan (Balita) menurut Karakteristik, Riskesdas 2018

Karakteristik	Status Gizi Menurut BB/U								
	Gizi Buruk		Gizi Kurang		Gizi Baik		Gizi Lebih		N Ter-timbang
	%	95% CI	%	95% CI	%	95% CI	%	95% CI	
**Kelompok umur (bln)**									
0 - 5	4,6	4,0-5,2	8,1	7,3-9,0	85,1	84,0-86,2	2,2	1,8-2,8	8.572
6 - 11	2,9	2,5-3,5	9,2	8,4-10,1	85,2	84,2-86,2	2,6	2,2-3,1	9.587
12 - 23	3,9	3,6-4,3	14,1	13,4-14,9	78,9	78,0-79,7	3,0	2,7-3,5	17.937
24 - 35	4,5	4,1-4,9	16,2	15,4-17,0	76,4	75,5-77,2	3,0	2,6-3,4	18.639
36 - 47	3,7	3,3-4,1	15,5	14,7-16,3	77,6	76,7-78,5	3,2	2,8-3,6	18.326
48 - 59	3,6	3,2-4,0	14,5	13,7-15,2	78,0	77,1-78,8	4,0	3,6-4,4	18.304
**Jenis Kelamin**									
Laki-laki	4,5	4,2-4,7	14,3	13,8-14,8	78,1	77,5-78,6	3,2	2,9-3,4	46.535
Perempuan	3,3	3,0-3,5	13,3	12,9-13,8	80,3	79,8-80,9	3,1	2,8-3,3	44.830
**Pendidikan KRT**									
Tidak sekolah	3,6	4,7-6,8	16,5	15,0-18,2	74,3	72,3-76,1	3,6	2,9-4,5	3.741
Tidak Tamat SD/MI	0,3	4,3-5,3	16,6	15,6-17,7	75,9	74,8-77,1	2,6	2,3-3,1	10.880
Tamat SD/MI	5,1	4,3-5,0	15,2	14,6-15,8	77,6	76,8-78,4	2,6	2,3-2,9	23.561
Tamat SLTP/MTS	3,7	3,7-4,7	14,3	13,6-15,1	78,7	77,8-79,6	2,8	2,4-3,2	17.242
Tamat SLTA/MA	3,1	2,6-3,2	12,0	11,5-12,7	81,5	80,8-82,2	3,6	3,2-3,9	27.303
Tamat D1-D3/PT	2,8	2,1-3,0	9,8	8,9-10,7	83,3	82,1-84,4	4,4	3,8-5,1	8.638
**Pekerjaan KRT**									
Tidak bekerja	4,0	3,4-4,8	12,0	10,8-13,2	80,4	78,9-81,8	3,6	3,0-4,3	6.244
Sekolah	5,0	2,1-11,4	16,3	10,4-24,7	78,3	69,3-85,2	0,3	0,1-1,1	180
PNS/TNI/Polri/BUMN/BUMD	2,0	1,6-2,5	9,3	8,2-10,6	83,6	82,0-85,0	5,1	4,3-6,1	4.406
Pegawai swasta	2,6	2,1-3,0	10,8	10,0-11,8	83,0	81,8-84,0	3,7	3,1-4,3	13.140
Wiraswasta	3,8	3,4-4,2	13,0	12,3-13,7	80,2	79,3-81,0	3,1	2,7-3,4	20.616
Petani/buruh tani	4,8	4,5-5,2	15,8	15,3-16,4	76,5	75,8-77,1	2,8	2,6-3,1	23.788
Nelayan	6,6	5,3-8,3	19,2	17,3-21,3	71,8	69,3-74,2	2,4	1,7-3,4	1.934
Buruh/Supir/pembantu ruta	3,8	3,4-4,2	15,3	14,4-16,2	78,4	77,4-79,4	2,5	2,1-3,0	16.349
Lainnya	3,8	3,1-4,6	14,7	13,3-16,2	78,1	76,3-79,8	3,5	2,6-4,5	4.707
**Tempat Tinggal**									
Perkotaan	3,3	3,0-3,5	12,4	11,9-12,9	80,9	80,3-81,5	3,4	3,2-3,7	49.091
Perdesaan	4,6	4,4-4,8	15,4	15,0-15,8	77,2	76,7-77,7	2,8	2,6-3,0	42.274

Catatan:

Gizi Buruk : *Zscore* < -3,0
Gizi Kurang : *Zscore* ≥ -3,0 s/d Zscore < -2,0
Gizi Baik : *Zscore* ≥ -2,0 s/d Zscore ≤ 2,0
Gizi Lebih : Zscore > 2,0

Tabel 16.8.3

Prevalensi Status Gizi (TB/U) pada Anak Umur 0-59 Bulan (Balita) menurut Provinsi, Riskesdas 2018

Provinsi	Status Gizi Menurut TB/U						
	Sangat Pendek		Pendek		Normal		N Ter-timbang
	%	95% CI	%	95% CI	%	95% CI	
Aceh	16,0	14,7-17,6	21,1	19,6-22,9	62,9	60,7-64,7	2.006
Sumatera Utara	13,2	12,1-14,5	19,2	17,7-20,5	67,6	65,9-69,4	5.470
Sumatera Barat	9,6	8,3-11,0	20,3	18,7-22,3	70,1	67,8-72,2	2.093
Riau	10,3	8,9-11,8	17,1	15,6-18,9	72,6	70,5-74,6	2.695
Jambi	13,4	11,4-15,4	16,8	14,8-18,9	69,9	67,2-72,6	1.165
Sumatera Selatan	14,4	12,9-16,3	17,2	15,8-19,2	68,3	65,8-70,3	2.839
Bengkulu	9,8	8,2-11,8	18,2	16,1-20,8	72,0	69,0-74,5	660
Lampung	9,6	8,2-11,0	17,7	16,2-19,5	72,7	70,7-74,6	2.962
Bangka Belitung	7,3	5,8-9,4	16,1	13,8-19,0	76,6	73,1-79,4	511
Kepulauan Riau	8,5	5,9-12,3	15,1	11,9-18,5	76,4	71,6-80,8	812
DKI Jakarta	6,1	4,8-7,9	11,5	9,6-13,8	82,4	79,7-84,7	3.315
Jawa Barat	11,7	10,5-12,8	19,4	18,1-20,8	68,9	67,3-70,5	15.965
Jawa Tengah	11,2	10,2-12,0	20,1	19,0-21,3	68,8	67,5-70,2	9.839
DI Yogyakarta	6,3	4,4-9,0	15,1	12,5-18,1	78,6	75,0-81,7	1.029
Jawa Timur	12,9	12,0-14,0	19,9	18,7-21,0	67,2	65,8-68,6	10.704
Banten	9,6	8,1-11,1	17,0	15,4-19,1	73,4	71,1-75,6	4.536
Bali	5,6	4,1-7,5	16,3	13,9-18,8	78,2	75,4-80,9	1.246
Nusa Tenggara Barat	9,2	7,9-10,8	24,3	22,3-26,7	66,5	63,7-68,9	1.914
Nusa Tenggara Timur	16,0	14,6-17,5	26,7	25,0-28,3	57,4	55,5-59,3	2.437
Kalimantan Barat	11,4	10,0-13,0	21,9	20,0-24,3	66,7	64,0-68,8	1.816
Kalimantan Tengah	12,7	10,8-15,1	21,3	19,0-24,1	66,0	62,8-68,6	936
Kalimantan Selatan	12,0	10,5-13,8	21,1	19,2-23,3	66,9	64,2-69,3	1.474
Kalimantan Timur	10,2	8,2-12,6	19,0	16,6-22,0	70,8	67,6-73,5	1.251
Kalimantan Utara	6,8	5,2-9,1	20,1	16,8-23,8	73,1	68,8-77,0	299
Sulawesi Utara	9,8	8,1-11,9	15,7	13,7-18,0	74,5	71,6-77,1	776
Sulawesi Tengah	11,9	10,2-13,6	20,4	18,6-22,6	67,7	65,2-70,0	1.110
Sulawesi Selatan	12,5	11,2-13,8	23,2	21,6-24,9	64,3	62,4-66,2	3.064
Sulawesi Tenggara	10,1	8,5-11,9	18,6	16,7-20,8	71,3	68,6-73,8	1.129
Gorontalo	12,7	10,1-15,6	19,8	16,9-23,1	67,5	63,7-71,2	426
Sulawesi Barat	16,2	13,2-19,3	25,4	22,7-29,2	58,4	53,9-62,3	544
Maluku	12,5	10,2-15,1	21,5	18,9-24,6	66,0	62,5-69,2	722
Maluku Utara	11,0	9,1-13,1	20,4	18,1-23,1	68,6	65,6-71,4	504
Papua Barat	11,7	9,5-14,4	16,1	13,6-19,1	72,3	68,6-75,5	379
Papua	15,3	13,4-17,5	17,8	15,6-19,9	66,9	64,1-69,8	1.110
**INDONESIA**	**11,5**	**11,1-11,8**	**19,3**	**19,0-19,7**	**69,2**	**68,7-69,7**	**87.737**

Catatan:

Sangat pendek : *Zscore* < -3,0

Pendek : *Zscore* ≥ -3,0 s/d *Zscore* < -2,0

Normal : *Zscore* ≥ -2,0

Tabel 16.8.4
Prevalensi Status Gizi (TB/U) pada Anak Umur 0-59 Bulan (Balita) menurut Karakteristik, Riskesdas 2018

Karakteristik	Status Gizi Menurut TB/U						N Ter-timbang
	Sangat Pendek		Pendek		Normal		
	%	95% CI	%	95% CI	%	95% CI	
**Kelompok umur (bln)**							
0 - 5	11,7	10,7-12,8	11,4	10,4-12,5	76,8	75,4-78,2	8.380
6 - 11	9,2	8,4-10,1	12,3	11,4-13,2	78,6	77,3-79,7	9.155
12 - 23	15,3	14,5-16,1	22,4	21,5-23,4	62,3	61,2-63,4	17.130
24 - 35	13,5	12,7-14,3	22,1	21,2-23,0	64,5	63,4-65,5	17.946
36 - 47	10,7	10,0-11,4	20,9	20,0-21,8	68,4	67,3-69,4	17.955
48 - 59	7,7	7,2-8,3	19,2	18,3-20,0	73,1	72,2-74,0	18.110
**Jenis Kelamin**							
Laki-laki	12,1	11,7-12,6	19,6	19,1-20,2	68,2	67,6-68,9	45.138
Perempuan	10,8	10,4-11,3	18,9	18,4-19,5	70,0	69,6-70,9	43.538
**Pendidikan KRT**							
Tidak sekolah	14,2	12,7-15,8	21,5	19,7-23,4	64,3	62,2-66,5	3.619
Tidak Tamat SD	13,5	12,6-14,4	21,6	20,6-22,7	64,9	63,6-66,1	10.557
Tamat SD	13,3	12,7-14,0	22,4	21,6-23,2	64,3	63,4-65,2	22.841
Tamat SLTP	11,9	11,2-12,7	20,4	19,5-21,3	67,7	66,6-68,8	16.791
Tamat SLTA	9,5	9,0-10,1	16,4	15,7-17,1	74,1	73,3-75,0	26.470
Tamat D1-D3/PT	8,2	7,4-9,1	14,2	13,1-15,3	77,6	76,3-78,9	8.399
**Pekerjaan KRT**							
Tidak bekerja	10,9	9,8-12,1	16,7	15,4-18,1	72,3	70,6-74	5.798
Sekolah	11,9	6,9-19,6	18,2	11,7-27,1	70,0	60,2-78,2	137
PNS/TNI/Polri/BUMN/BUMD	8,0	7,0-9,2	15,4	14,0-17,0	76,6	74,8-78,3	4.087
Pegawai swasta	8,7	7,9-9,6	15,8	14,7-16,9	75,5	74,2-76,8	12.301
Wiraswasta	11,0	10,3-11,7	18,2	17,4-19,0	70,8	69,8-71,8	19.614
Petani/buruh tani	14,1	13,5-14,7	22,1	21,5-22,8	63,8	63,0-64,6	22.639
Nelayan	14,8	12,9-16,9	22,7	20,5-25,0	62,5	59,8-65,2	1.737
Buruh/Supir/pembantu ruta	11,7	10,9-12,6	21,3	20,3-22,4	67,0	65,7-68,2	15.379
Lainnya	10,3	9,0-11,7	18,4	16,9-20,1	71,3	69,3-73,1	4.331
**Tempat Tinggal**							
Perkotaan	9,9	9,4-10,3	17,4	16,8-18,0	72,8	72,0-73,4	47.711
Perdesaan	13,4	13,0-13,8	21,5	21,0-22,0	65,0	64,5-65,7	40.965

Catatan:

Sangat pendek	:	*Zscore* < -3,0
Pendek	:	*Zscore* ≥ -3,0 s/d *Zscore* < -2,0
Normal	:	*Zscore* ≥ -2,0

Tabel 16.8.5

Prevalensi Status Gizi (BB/TB) pada Anak Umur 0-59 Bulan (Balita) menurut Provinsi, Riskesdas 2018

Provinsi	Status GiziMenurut BB/TB								
	Sangat Kurus		Kurus		Normal		Gemuk		N Ter-timbang
	%	95% CI	%	95% CI	%	95% CI	%	95% CI	
Aceh	5,0	4,2-5,9	6,9	6,0-8,0	79,3	77,6-80,9	8,8	7,7-10,0	1.966
Sumatera Utara	4,6	3,9-5,4	7,5	6,6-8,5	79,1	77,6-80,5	8,9	7,9-9,9	5.399
Sumatera Barat	2,9	2,2-3,7	8,4	7,2-9,8	83,3	81,6-84,9	5,4	4,5-6,5	2.084
Riau	4,2	3,4-5,2	8,0	6,8-9,5	80,0	78,1-81,8	7,8	6,6-9,0	2.666
Jambi	5,7	4,4-7,4	6,3	5,1-7,8	77,1	74,6-79,5	10,8	9,3-12,6	1.155
Sumatera Selatan	4,7	3,8-5,8	6,7	5,7-8,0	77,8	75,7-79,7	10,8	9,3-12,5	2.802
Bengkulu	3,5	2,5-4,8	4,8	3,6-6,3	81,4	78,8-83,7	10,4	8,6-12,4	646
Lampung	3,9	3,1-4,9	6,8	5,7-8,0	82,2	80,3-83,9	7,2	6,1-8,4	2.915
Bangka Belitung	2,8	1,9-4,1	7,1	5,3-9,4	82,2	79,4-84,6	8,0	6,4-9,9	502
Kepulauan Riau	4,5	3,0-6,9	6,7	4,8-9,2	79,5	75,7-82,9	9,2	6,8-12,4	805
DKI Jakarta	3,9	2,6-5,6	6,2	4,7-8,2	82,0	78,9-84,8	7,9	6,1-10,1	3.304
Jawa Barat	3,2	2,7-3,8	5,2	4,5-6,0	82,9	81,6-84,1	8,7	7,8-9,7	15.848
Jawa Tengah	2,7	2,3-3,2	5,8	5,1-6,5	83,9	82,8-85,0	7,6	6,8-8,4	9.745
DI Yogyakarta	1,2	0,6-2,4	7,2	5,3-9,7	86,9	83,9-89,3	4,7	3,3-6,8	1.028
JawaTimur	2,9	2,4-3,4	6,3	5,6-7,0	81,6	80,4-82,7	9,3	8,5-10,2	10.538
Banten	4,6	3,6-5,9	5,9	4,8-7,1	81,0	78,6-83,1	8,6	7,1-10,3	4.477
Bali	1,9	1,2-3,0	4,4	3,2-5,9	85,9	83,6-87,8	7,9	6,4-9,6	1.231
Nusa Tenggara Barat	4,4	3,5-5,5	10,0	8,5-11,8	82,3	80,2-84,2	3,3	2,5-4,4	1.899
Nusa Tenggara Timur	4,6	3,7-5,6	8,2	7,4-9,2	83,4	82,0-84,7	3,8	3,1-4,5	2.411
Kalimantan Barat	4,0	3,1-5,2	10,3	8,6-12,1	77,7	75,3-79,9	8,0	6,8-9,5	1.795
Kalimantan Tengah	4,0	3,0-5,5	9,9	8,3-11,7	76,2	73,7-78,6	9,8	8,2-11,8	919
Kalimantan Selatan	3,9	2,9-5,3	9,2	7,7-11,0	79,7	77,4-81,8	7,1	5,8-8,7	1.457
Kalimantan Timur	2,0	1,2-3,4	5,5	4,1-7,4	83,0	80,2-85,5	9,4	7,6-11,6	1.234
Kalimantan Utara	1,1	0,6-2,0	3,5	2,3-5,4	87,8	84,8-90,2	7,6	5,6-10,2	297
Sulawesi Utara	2,9	2,1-3,9	6,7	5,4-8,2	82,8	80,5-84,9	7,7	6,3-9,3	765
Sulawesi Tengah	3,7	2,7-4,9	9,2	7,7-10,8	83,7	81,7-85,5	3,5	2,7-4,6	1.094
Sulawesi Selatan	2,5	2,0-3,2	7,5	6,5-8,7	82,9	81,3-84,3	7,1	6,1-8,2	3.024
Sulawesi Tenggara	3,4	2,4-4,9	8,5	7,2-10,1	81,9	79,5-84,1	6,1	5,1-7,3	1.116
Gorontalo	3,8	2,7-5,4	10,6	8,5-13	80,2	77,0-83,0	5,4	3,9-7,5	415
Sulawesi Barat	3,2	2,2-4,7	7,3	5,6-9,5	84,0	81,0-86,6	5,5	4,1-7,4	547
Maluku	4,0	3,0-5,4	9,1	7,5-11,0	81,8	79,2-84,2	5,0	3,8-6,7	714
Maluku Utara	4,0	2,9-5,4	7,9	6,4-9,7	82,5	79,9-84,7	5,7	4,3-7,3	498
Papua Barat	3,9	2,7-5,5	8,3	6,6-10,5	80,0	76,9-82,8	7,8	5,9-10,2	374
Papua	4,8	3,6-6,4	5,5	4,4-6,9	76,5	73,6-79,1	13,2	11,1-15,6	1.081
**INDONESIA**	**3,5**	**3,3-3,7**	**6,7**	**6,4-6,9**	**81,8**	**81,4-82,2**	**8,0**	**7,8-8,3**	**86.753**

Catatan:

Sangat Kurus : *Zscore* < -3,0

Kurus : *Zscore* ≥ -3,0 s/d Zscore < -2,0

Normal : *Zscore* ≥ -2,0 s/d Zscore ≤ 2,0

Gemuk : Zscore > 2,0

Tabel 16.8.6

Prevalensi Status Gizi (BB/TB) pada Anak Umur 0-59 Bulan (Balita) menurut Karakteristik, Riskesdas 2018

Karakteristik	Status Gizi Menurut BB/TB								
	Sangat Kurus		Kurus		Normal		Gemuk		N Ter-timbang
	%	95% CI	%	95% CI	%	95% CI	%	95% CI	
**Kelompokumur (bln)**									
0 - 5	5,1	4,4-5,9	7,7	6,9-8,6	73,6	72,1-75,1	13,6	12,5-14,8	7.927
6 - 11	4,8	4,2-5,5	8,1	7,4-9	78,9	77,6-80,1	8,2	7,3-9,1	9.110
12 - 23	4,1	3,7-4,6	6,6	6-7,1	82,0	81,2-82,9	7,3	6,7-7,9	16.924
24 - 35	3,7	3,3-4,1	6,8	6,3-7,4	82,5	81,6-83,3	7,1	6,5-7,7	17.706
36 - 47	2,6	2,3-2,9	5,9	5,4-6,4	83,9	83-84,6	7,7	7,1-8,3	17.525
48 - 59	2,4	2,1-2,7	6,2	5,7-6,7	83,9	83,1-84,7	7,6	7-8,2	17.561
**JenisKelamin**									
Laki-laki	3,9	3,7-4,2	7,2	6,9-7,6	80,5	79,9-81,1	8,4	8-8,8	44.238
Perempuan	3,1	2,9-3,4	6,1	5,8-6,4	83,1	82,5-83,6	7,7	7,3-8,1	42.515
**Pendidikan KRT**									
Tidak sekolah	4,6	3,7-5,6	7,6	6,5-8,9	80,2	78,3-81,9	7,7	6,5-9	3.519
Tidak tamat SD	4,0	3,5-4,7	7,2	6,5-7,9	81,8	80,7-82,9	6,9	6,3-7,7	10.264
Tamat SD	3,6	3,2-3,9	6,9	6,4-7,3	81,6	80,9-82,3	7,9	7,4-8,5	22.419
Tamat SLTP	3,4	3-3,8	6,5	6-7,1	82,8	82-83,7	7,2	6,6-7,8	16.418
Tamat SLTA	3,3	2,9-3,6	6,5	6-6,9	81,8	81,1-82,6	8,5	7,9-9	25.941
Tamat D1-D3/PT	3,4	2,8-4,1	6,0	5,2-6,8	80,4	79-81,8	10,2	9,2-11,3	8.192
**Pekerjaan KRT**									
Tidak Bekerja	3,7	3,1-4,5	7,2	6,2-8,2	80,4	78,8-81,9	8,7	7,7-9,9	5.954
Sekolah	5,7	2-15,3	6,0	3,3-10,5	78,5	68,6-85,9	9,8	5-18,3	175
PNS/TNI/Polri/BUMN/BUMD	2,8	2,2-3,6	5,0	4,2-6	82,3	80,6-83,9	9,9	8,7-11,2	4.186
Pegawaiswasta	3,0	2,6-3,6	5,8	5,2-6,6	82,4	81,2-83,6	8,7	7,8-9,6	12.465
Wiraswasta	3,4	3,1-3,8	6,3	5,8-6,9	81,7	80,9-82,6	8,5	7,9-9,2	19.619
Petani/buruhtani	3,9	3,6-4,3	7,1	6,7-7,5	81,2	80,6-81,9	7,7	7,3-8,2	22.493
Nelayan	3,7	2,9-4,8	9,3	7,9-11	80,0	77,6-82,1	7,0	5,6-8,6	1.859
Buruh/Supir/pembantu ruta	3,2	2,8-3,8	6,9	6,3-7,5	83,2	82,2-84,1	6,7	6,1-7,4	15.542
Lainnya	4,5	3,6-5,6	7,7	6,6-8,9	80,0	78,1-81,8	7,9	6,7-9,2	4.460
**TempatTinggal**									
Perkotaan	3,4	3,1-3,7	6,4	6-6,8	82,0	81,4-82,6	8,2	7,8-8,6	46.727
Perdesaan	3,7	3,5-3,9	7,0	6,7-7,3	81,5	81-81,9	7,9	7,5-8,2	40.026

Catatan:

Sangat Kurus	:	*Zscore* < -3,0
Kurus	:	*Zscore* ≥ -3,0 s/d Zscore < -2,0
Normal	:	*Zscore* ≥ -2,0 s/d Zscore ≤ 2,0
Gemuk	:	Zscore > 2,0

16.9 Status Gizi pada Anak dan Remaja

Status gizi anak umur 5-18 tahun dikelompokan menjadi tiga kelompok umur yaitu 5-12 tahun, 13-15 tahun dan 16-18 tahun. Indikator status gizi yang digunakan untuk kelompok umur ini didasarkan pada pengukuran antropometri berat badan (BB) dan tinggi badan (TB) yang disajikan dalam bentuk indeks tinggi badan menurut umur (TB/U) dan Indeks Massa Tubuh menurut umur (IMT/U). Indeks massa tubuh dihitung berdasarkan rumus berikut:

$$IMT = \frac{BB\ (kg)}{TB^2\ (meter)}$$

Dengan menggunakan baku antropometri anak 5-19 tahun WHO 2007 dihitung nilai *Z-score* TB/U dan IMT/U masing-masing anak. Selanjutnya berdasarkan nilai *Z-score* ini status gizi anak dikategorikan sebagai berikut:

Berdasarkan indikator TB/U:

Sangat pendek	: Z-*score* < -3,0
Pendek	: Z-*score* $\geq$ -3,0 s/d < -2,0
Normal	: *Z-score* $\geq$ -2,0

Berdasarkan indikator IMT/U:

Sangat kurus	: *Z-score* < -3,0
Kurus	: *Z-score* $\geq$ -3,0 s/d < -2,0
Normal	: *Z-score* $\geq$ -2,0 s/d $\leq$ 1,0
Gemuk	: *Z-score* > 1,0 s/d $\leq$ 2,0
Obesitas	: *Z-score* > 2,0

<u>Status gizi anak 5-12 tahun</u>

Berdasarkan indikator TB/U:

Prevalensi anak 5-12 Tahun Sangat Pendek	:	(∑Anak 5-12 tahun sangat pendek/∑anak 5-12 tahun diukur TB) x 100%
Prevalensi anak 5-12 Tahun Pendek	:	(∑Anak 5-12 tahun pendek/∑anak 5-12 tahun diukur TB) x 100%
Prevalensi anak 5-12 Tahun Normal	:	(∑Anak 5-12 tahun normal/∑anak 5-12 tahun diukur TB) x 100%

Berdasarkan indikator IMT/U:

Prevalensi anak 5-12 Tahun Sangat Kurus	:	(∑Anak 5-12 tahun sangat kurus/∑anak 5-12 tahun diukur BB dan TB) x 100%
Prevalensi anak 5-12 Tahun Kurus	:	(∑Anak 5-12 tahun kurus/∑anak 5-12 tahun diukur BB dan TB) x 100%
Prevalensi anak 5-12 Tahun Normal	:	(∑Anak 5-12 tahun normal/∑anak 5-12 tahun diukur BB dan TB) x 100%
Prevalensi anak 5-12 Tahun Gemuk		(∑Anak 5-12 tahun gemuk/∑anak 5-12 tahun diukur BB dan TB) x 100%

Status gizi anak 13-15 tahun

Berdasarkan indikator TB/U:

Prevalensi anak 13-15 Tahun Sangat Pendek	:	(∑Anak 13-15 tahun sangat pendek/∑anak 13-15 tahun diukur TB) x 100%
Prevalensi anak 13-15 Tahun Pendek	:	(∑Anak 13-15 tahun pendek/∑anak 13-15 tahun diukur TB) x 100%
Prevalensi anak 13-15 Tahun Normal	:	(∑Anak 13-15 tahun normal/∑anak 13-15 tahun diukur TB) x 100%

Berdasarkan indikator IMT/U:

Prevalensi anak 13-15 Tahun Sangat Kurus	:	(∑Anak 13-15 tahun sangat kurus/∑anak 13-15 tahun diukur BB dan TB) x 100%
Prevalensi anak 13-15 Tahun Kurus	:	(∑Anak 13-15 tahun kurus/∑anak 13-15 tahun diukur BB dan TB) x 100%
Prevalensi anak 13-15 Tahun Normal	:	(∑Anak 13-15 tahun normal/∑anak 13-15 tahun diukur BB dan TB) x 100%
Prevalensi anak 13-15 Tahun Gemuk		(∑Anak 13-15 tahun gemuk/∑anak 13-15 tahun diukur BB dan TB) x 100%

Status gizi anak 16-18 tahun

Berdasarkan indikator TB/U:

Prevalensi anak 16-18 Tahun Sangat Pendek	:	(∑Anak 16-18 tahun sangat pendek/∑anak 16-18 tahun diukur TB) x 100%
Prevalensi anak 16-18 Tahun Pendek	:	(∑Anak 16-18 tahun pendek/∑anak 16-18 tahun diukur TB) x 100%
Prevalensi anak 16-18 Tahun Normal	:	(∑Anak 16-18 tahun normal/∑anak 16-18 tahun diukur TB) x 100%

Berdasarkan indikator IMT/U:

Prevalensi anak 16-18 Tahun Sangat Kurus	:	(∑Anak 16-18 tahun sangat kurus/∑anak 16-18 tahun diukur BB dan TB) x 100%
Prevalensi anak 16-18 Tahun Kurus	:	(∑Anak 16-18 tahun kurus/∑anak 16-18 tahun diukur BB dan TB) x 100%
Prevalensi anak 16-18 Tahun Normal	:	(∑Anak 16-18 tahun normal/∑anak 16-18 tahun diukur BB dan TB) x 100%
Prevalensi anak 16-18 Tahun Gemuk		(∑Anak 16-18 tahun gemuk/∑anak 16-18 tahun diukur BB dan TB) x 100%

Tabel 16.9.1

Prevalensi Status Gizi (TB/U) pada Anak Umur 5 - 12 Tahun menurut Provinsi, Riskesdas 2018

Provinsi	Status Gizi Menurut TB/U						N Ter-timbang
	Sangat Pendek		Pendek		Normal		
	%	95% CI	%	95% CI	%	95% CI	
Aceh	11,5	10,4-12,7	20,8	19,6-22,1	67,7	66,1-69,3	3.777
Sumatera Utara	9,2	8,5-10,0	19,2	18,2-20,1	71,6	70,5-72,8	10.580
Sumatera Barat	6,0	5,2-6,8	18,5	17,2-19,9	75,5	73,8-77,1	3.744
Riau	6,5	5,6-7,6	16,6	15,4-17,9	76,9	75,3-78,4	4.961
Jambi	8,9	7,8-10,2	17,5	16,0-19,1	73,6	71,6-75,5	2.286
Sumatera Selatan	9,7	8,6-10,9	16,7	15,5-18,0	73,6	71,9-75,2	5.615
Bengkulu	6,7	5,6-8,1	15,6	14,1-17,3	77,7	75,5-79,6	1.311
Lampung	5,2	4,5-6,0	16,1	14,8-17,4	78,8	77,2-80,2	5.535
Bangka Belitung	3,7	2,7-5,0	15,0	13,1-17,1	81,3	78,9-83,6	929
Kepulauan Riau	5,0	3,6-7,0	13,5	11,0-16,6	81,5	77,6-84,8	1.497
DKI Jakarta	2,7	1,9-3,6	8,1	6,9-9,5	89,2	87,5-90,7	6.178
Jawa Barat	6,4	5,8-7,1	16,4	15,5-17,4	77,1	75,9-78,3	29.855
Jawa Tengah	5,6	5,1-6,2	15,2	14,5-16	79,2	78,3-80,1	19.549
DI Yogyakarta	2,4	1,7-3,5	11,7	9,9-13,9	85,8	83,6-87,8	1.989
Jawa Timur	6,9	6,4-7,5	16,7	15,9-17,5	76,4	75,4-77,4	21.051
Banten	6,1	5,2-7,2	16,4	14,9-17,9	77,5	75,7-79,2	8.310
Bali	2,0	1,5-2,8	9,5	8,2-11,0	88,4	86,7-89,9	2.388
Nusa Tenggara Barat	6,3	5,3-7,3	21,1	19,4-22,9	72,6	70,7-74,5	3.435
Nusa Tenggara Timur	11,3	10,4-12,2	29,8	28,6-31,1	58,8	57,4-60,3	4.371
Kalimantan Barat	8,9	7,9-10,1	17,4	16,1-18,8	73,7	71,9-75,4	3.388
Kalimantan Tengah	7,6	6,4-8,9	16,9	15,3-18,6	75,6	73,6-77,4	1.723
Kalimantan Selatan	6,5	5,6-7,6	19,2	17,7-20,8	74,3	72,4-76,1	2.730
Kalimantan Timur	4,4	3,4-5,6	16,0	14,0-18,2	79,6	77,1-81,9	2.389
Kalimantan Utara	3,9	2,6-5,8	16,2	13,9-18,8	79,9	77,0-82,4	476
Sulawesi Utara	4,4	3,5-5,4	12,3	11,0-13,8	83,3	81,6-84,9	1.455
Sulawesi Tengah	6,6	5,6-7,7	19,7	18,0-21,5	73,7	71,6-75,7	2.010
Sulawesi Selatan	5,8	5,2-6,5	20,2	19,0-21,4	73,9	72,6-75,3	5.863
Sulawesi Tenggara	5,9	5,0-6,8	19,8	18,1-21,5	74,4	72,3-76,3	2.039
Gorontalo	5,7	4,6-7,2	19,9	17,7-22,4	74,3	71,6-76,9	759
Sulawesi Barat	11,6	9,9-13,4	26,3	24,0-28,7	62,2	59,3-65,0	982
Maluku	8,6	7,3-10,0	20,4	18,4-22,5	71,1	68,4-73,6	1.383
Maluku Utara	6,0	5,0-7,3	17,1	15,5-18,8	76,9	74,8-78,8	955
Papua Barat	6,5	5,0-8,3	16,8	14,7-19,1	76,7	74,1-79,2	643
Papua	13,3	11,5-15,2	16,8	15,1-18,5	70,0	67,5-72,3	2.405
**INDONESIA**	**6,7**	**10,4-12,7**	**16,9**	**16,7-17,2**	**76,3**	**76,0-76,6**	**166.561**

Catatan:

Sangat pendek : *Zscore* < -3,0

Pendek : *Zscore* ≥ -3,0 s/d *Zscore* < -2,0

Normal : *Zscore* ≥ -2,0

Tabel 16.9.2
Prevalensi Status Gizi (TB/U) pada Anak Umur 5 - 12 Tahun menurut Karakteristik, Riskesdas 2018

Karakteristik	Status Gizi Menurut TB/U						N Ter-timbang
	Sangat Pendek		Pendek		Normal		
	%	95% CI	%	95% CI	%	95 CI	
**Jenis Kelamin**							
Laki-laki	7,0	6,8-7,3	17,5	17,1-17,8	75,5	75,0-76,0	85.336
Perempuan	6,4	6,2-6,7	16,4	16,0-16,8	77,2	76,7-77,6	81.225
**Tempat Tinggal**							
Perkotaan	5,0	4,8-5,3	14,0	13,6-14,5	80,9	80,4-81,4	87.805
Perdesaan	8,6	8,4-8,9	20,2	19,8-20,6	71,2	70,7-71,6	78.756

Catatan:

Sangat pendek : *Zscore* < -3,0
Pendek : *Zscore* ≥ -3,0 s/d *Zscore* < -2,0
Normal : *Zscore* ≥ -2,0

Tabel 16.9.3
Prevalensi Status Gizi (IMT/U) Pada Anak Umur 5- 12 Tahun menurut Provinsi, Riskesdas 2018

Provinsi	Status Gizi Menurut IMT/U										
	Sangat Kurus		Kurus		Normal		Gemuk		Obesitas		N Ter- timbang
	%	95% CI	%	95% CI	%	95% CI	%	95% CI	%	95% CI	
Aceh	3,1	2,7-3,7	7,4	6,6-8,3	68,6	67-70,2	10,7	9,8-11,8	10,1	9,1-11,3	3.782
Sumatera Utara	2,1	1,7-2,4	5,6	5,0-6,3	72,7	71,4-74	10,6	9,8-11,4	9,1	8,3-9,9	10.530
Sumatera Barat	2,1	1,7-2,7	7,5	6,7-8,4	76,6	75,1-78,1	7,8	7,0-8,8	5,9	5,2-6,7	3.732
Riau	2,7	2,2-3,2	7,2	6,3-8,1	72,7	71,2-74,3	9,1	8,2-10,1	8,4	7,4-9,5	4.938
Jambi	3,0	2,4-3,8	6,0	5,1-7,1	67,3	65,2-69,4	12,2	11,0-13,6	11,4	10,1-12,8	2.284
Sumatera Selatan	3,7	3,1-4,4	6,3	5,4-7,2	70,7	68,9-72,4	10,2	9,3-11,3	9,1	8,0-10,2	5.550
Bengkulu	2,2	1,6-2,9	5,2	4,3-6,3	71,3	69,1-73,4	10,9	9,5-12,5	10,4	9,0-12,1	1.302
Lampung	2,4	1,9-3,0	7,0	6,1-8,0	71,7	70,1-73,4	10,5	9,5-11,6	8,4	7,4-9,4	5.512
Bangka Belitung	1,7	1,2-2,5	7,6	6,2-9,2	69,9	67,4-72,3	10,8	9,1-12,8	10,0	8,6-11,5	927
Kepulauan Riau	3,3	2,2-4,9	7,1	5,5-9,0	66,8	63,4-70	10,5	8,4-13,1	12,3	10,2-14,7	1.495
DKI Jakarta	1,9	1,3-2,7	6,0	4,9-7,3	62,9	60,3-65,4	15,2	13,4-17,2	14,0	12,4-15,8	6.144
Jawa Barat	1,9	1,6-2,3	5,2	4,6-5,8	71,6	70,4-72,7	11,7	10,9-12,5	9,6	8,9-10,4	29.658
Jawa Tengah	2,1	1,8-2,4	6,5	6,0-7,0	71,3	70,3-72,3	11,1	10,4-11,7	9,1	8,5-9,7	19.458
DI Yogyakarta	1,8	1,1-3,0	6,5	5,1-8,2	70,5	67,8-73,1	10,9	9,2-13,0	10,2	8,5-12,2	1.979
Jawa Timur	2,2	1,9-2,6	5,8	5,3-6,3	67,7	66,6-68,7	13,2	12,5-13,9	11,1	10,4-11,8	20.935
Banten	2,7	2,1-3,4	7,4	6,5-8,5	69,9	67,9-71,7	10,1	9,0-11,3	10,0	8,8-11,5	8.263
Bali	1,2	0,8-1,9	5,0	4,1-6,1	70,2	67,9-72,5	13,0	11,3-14,9	10,6	9,2-12,1	2.383
Nusa Tenggara Barat	3,0	2,5-3,8	13,2	11,9-14,7	74,5	72,6-76,2	5,4	4,5-6,4	3,9	3,1-4,8	3.424
Nusa Tenggara Timur	4,6	4-5,3,0	13,9	12,9-14,9	75,4	74,1-76,7	3,7	3,2-4,3	2,4	1,9-2,9	4.346
Kalimantan Barat	3,2	2,6-4,0	8,4	7,5-9,5	69,2	67,4-70,9	10,3	9,2-11,4	8,9	7,9-10,1	3.357
Kalimantan Tengah	3,0	2,4-3,9	8,2	7,0-9,6	66,5	64,2-68,6	13,0	11,7-14,6	9,3	8,1-10,6	1.705
Kalimantan Selatan	2,7	2,2-3,5	10,0	8,8-11,3	68,5	66,6-70,4	9,8	8,7-11,0	9,0	7,8-10,3	2.700
Kalimantan Timur	1,7	1,2-2,4	6,7	5,6-8,1	67,5	65,1-69,8	12,6	11,1-14,3	11,4	9,9-13,1	2.374
Kalimantan Utara	1,9	1,2-2,8	6,8	5,3-8,6	72,1	69,1-74,9	9,6	7,6-12,0	9,7	7,9-11,8	475
Sulawesi Utara	1,9	1,4-2,6	4,5	3,7-5,5	72,1	70,2-73,9	12,1	10,8-13,4	9,4	8,2-10,8	1.454
Sulawesi Tengah	2,6	2,0-3,3	9,5	8,4-10,8	77,4	75,6-79	6,6	5,7-7,7	3,9	3,2-4,8	2.006
Sulawesi Selatan	3,0	2,4-3,6	8,6	7,8-9,6	74,0	72,6-75,4	7,8	7,0-8,7	6,5	5,7-7,5	5.851
Sulawesi Tenggara	2,8	2,3-3,5	8,4	7,2-9,9	76,2	74,1-78,2	7,2	6,1-8,4	5,4	4,2-6,8	2.024
Gorontalo	2,7	1,8-3,9	8,4	7,1-10,1	73,7	71-76,1	8,9	7,5-10,6	6,3	5,0-7,8	756
Sulawesi Barat	2,6	1,8-3,7	5,4	4,3-6,8	78,9	76,5-81,1	7,6	6,4-9,1	5,5	4,4-6,8	980
Maluku	3,7	2,7-4,9	11,5	9,7-13,6	74,6	71,9-77,1	5,8	4,8-7,1	4,4	3,3-5,8	1.379
Maluku Utara	2,7	2,1-3,5	8,2	7,1-9,6	75,8	73,9-77,7	7,0	5,9-8,3	6,2	5,1-7,4	955
Papua Barat	2,6	1,9-3,7	8,3	6,7-10,2	73,2	69,6-76,6	9,3	7,3-11,7	6,5	5,1-8,3	639
Papua	2,5	1,9-3,3	4,7	3,8-5,8	62,2	59,2-65,1	15,3	13,6-17,3	15,3	13,0-17,9	2.388
**INDONESIA**	**2,4**	**2,3-2,5**	**6,8**	**6,0,6-7**	**70,8**	**70,4-71,1**	**10,8**	**10,6-11,1**	**9,2**	**9,0-9,5**	**165.682**

Catatan:

Sangat kurus	:	*Zscore* < -3,0
Kurus	:	*Zscore* ≥ -3,0 s/d *Zscore* < -2,0
Normal	:	*Zscore* ≥ -2,0 s/d *Zscore* ≤ 1,0
Gemuk	:	*Zscore* > 1,0 s/d *Zscore* ≤ 2,0
Obesitas	:	*Zscore* > 2,0

Tabel 16.9.4
Prevalensi Status Gizi (IMT/U) pada Anak Umur 5- 12 Tahun menurut Karakteristik, Riskesdas 2018

Karakteristik	Status Gizi Menurut IMT/U										N Ter-timbang
	Sangat Kurus		Kurus		Normal		Gemuk		Obesitas		
	%	95% CI	%	95% CI	%	95% CI	%	95% CI	%	95% CI	
**Jenis Kelamin**											
Laki-laki	2,8	2,6-2,9	7,3	7,1-7,6	68,8	68,3-69,3	10,4	10,1-10,8	10,7	10,3-11	84.599
Perempuan	2,0	1,9-2,2	6,2	6,0-6,5	72,8	72,3-73,3	11,2	10,9-11,6	7,7	7,4-8,0	81.083
**Tempat Tinggal**											
Perkotaan	2,3	2,1-2,5	6,5	6,3-6,8	68,8	68,3-69,4	11,9	11,5-12,3	10,5	10,1-10,9	87.338
Perdesaan	2,5	2,4-2,7	7,1	6,8-7,3	73,0	72,5-73,4	9,6	9,4-9,9	7,8	7,6-8,1	78.344

Catatan:

Sangat kurus : *Zscore* < -3,0
Kurus : *Zscore* ≥ -3,0 s/d *Zscore* < -2,0
Normal : *Zscore* ≥ -2,0 s/d *Zscore* ≤ 1,0
Gemuk : *Zscore* > 1,0 s/d *Zscore* ≤ 2,0
Obesitas : *Zscore* > 2,0

Tabel 16.9.5

Prevalensi Status Gizi (TB/U) Pada Remaja Umur 13 - 15 Tahun menurut Provinsi, Riskesdas 2018

Provinsi	Status Gizi menurut TB/U						N Ter-timbang
	Sangat Pendek		Pendek		Normal		
	%	95% CI	%	95% CI	%	95% CI	
Aceh	12,4	10,7-14,5	23,9	21,7-26,2	63,6	60,9-66,3	1.184
Sumatera Utara	9,2	8,2-10,4	20,3	18,7-21,9	70,5	68,6-72,3	3.835
Sumatera Barat	7,2	6,0-8,7	18,4	16,5-20,5	74,4	71,9-76,7	1.367
Riau	6,1	4,8-7,8	17,9	15,8-20,3	75,9	73,3-78,4	1.666
Jambi	12,9	10,6-15,5	21,7	19,2-24,4	65,5	62,1-68,6	862
Sumatera Selatan	9,5	7,9-11,3	19,0	16,9-21,3	71,5	68,8-74,0	2.049
Bengkulu	4,3	3,1-5,8	17,4	14,8-20,4	78,3	75,0-81,3	463
Lampung	6,4	5,1-8,1	20,3	18,1-22,7	73,3	70,6-75,9	1.908
Bangka Belitung	4,3	2,8-6,4	15,9	13,0-19,2	79,9	76,2-83,1	327
Kepulauan Riau	4,1	2,3-7,1	10,3	7,6-13,8	85,7	81,4-89,1	510
DKI Jakarta	2,9	1,8-4,6	7,1	5,3-9,6	90,0	87,0-92,3	1.884
Jawa Barat	6,7	5,7-7,8	17,4	16,0-19,0	75,9	74,0-77,6	11.036
Jawa Tengah	5,1	4,4-5,9	16,5	15,2-17,9	78,4	76,9-79,9	7.193
DI Yogyakarta	1,1	0,4-2,6	8,2	5,8-11,7	90,7	87,2-93,3	623
Jawa Timur	6,4	5,5-7,3	18,8	17,5-20,3	74,8	73,1-76,4	7.792
Banten	7,7	6,1-9,6	16,7	14,3-19,4	75,6	72,5-78,5	2.759
Bali	1,0	0,5-1,7	8,2	6,4-10,3	90,9	88,7-92,7	934
Nusa Tenggara Barat	6,3	4,8-8,1	23,4	20,3-26,7	70,4	66,8-73,7	1.211
Nusa Tenggara Timur	17,0	15,4-18,7	29,7	27,7-31,7	53,3	51,0-55,7	1.666
Kalimantan Barat	7,7	6,4-9,2	20,7	18,6-23,0	71,6	68,9-74,1	1.244
Kalimantan Tengah	8,6	6,8-10,8	21,0	18,2-24,1	70,4	66,9-73,7	617
Kalimantan Selatan	7,3	5,8-9,1	24,0	21,4-26,7	68,8	65,8-71,6	973
Kalimantan Timur	4,8	3,2-7,3	12,7	10,3-15,7	82,4	79,1-85,3	771
Kalimantan Utara	4,7	2,8-7,7	20,8	16,3-26,2	74,5	69,0-79,3	166
Sulawesi Utara	5,1	3,8-6,6	18,2	15,8-20,9	76,7	73,9-79,4	586
Sulawesi Tengah	6,6	5,3-8,3	22,0	19,6-24,6	71,4	68,5-74,1	760
Sulawesi Selatan	6,2	5,2-7,4	22,1	20,2-24,0	71,8	69,7-73,8	2.195
Sulawesi Tenggara	8,8	7,1-10,8	20,8	18,4-23,5	70,4	67,2-73,4	731
Gorontalo	9,3	7,0-12,2	24,3	20,8-28,2	66,4	62,1-70,4	289
Sulawesi Barat	10,0	7,7-12,9	31,8	28,0-36,0	58,2	53,9-62,3	369
Maluku	11,4	9,4-13,8	22,6	19,3-26,3	66,0	61,7-70,0	507
Maluku Utara	8,0	6,2-10,4	20,1	17,2-23,4	71,9	68,5-75,0	361
Papua Barat	8,0	5,6-11,1	21,1	17,6-25,1	70,9	66,0-75,5	235
Papua	26,0	21,4-31,1	20,0	16,6-23,8	54,1	49,6-58,5	697
**INDONESIA**	**7,2**	**6,9-7,5**	**18,5**	**18,1-19,0**	**74,3**	**73,7-74,8**	**59.771**

Tabel 16.9.6
Prevalensi Status Gizi (TB/U) pada Remaja Umur 13 - 15 Tahun menurut Karakteristik, Riskesdas 2018

Karakteristik	Status Gizi menurut TB/U						N Ter-timbang
	Sangat Pendek		Pendek		Normal		
	%	95% CI	%	95% CI	%	95% CI	
**Jenis Kelamin**							
Laki-laki	9,0	8,5-9,5	17,5	16,9-18,1	73,5	72,8-74,3	30.925
Perempuan	5,3	5-5,7	19,6	19-20,3	75,4	74,3-75,8	28.846
**Pendidikan**							
Tidak sekolah	19,9	16,1-24,5	21,6	18,3-25,3	58,5	53,8-63	1.040
Tidak Tamat SD	13,2	12,1-14,4	23,8	22,3-25,4	63,0	61,2-64,7	7.009
Tamat SD	6,7	6,4-7,1	18,2	17,6-18,8	75,1	74,4-75,7	39.505
Tamat SLTP	4,4	3,9-5	16,2	15,2-17,2	79,5	78,4-80,5	11.838
Tamat SLTA	3,3	1,6-6,6	17,8	12,4-24,9	78,9	71,6-84,7	380
**Tempat Tinggal**							
Perkotaan	4,9	4,5-5,4	15,2	14,6%	15,9	79,1-80,7	31.696
Perdesaan	9,9	9,4-10,3	22,2	21,6%	22,9	67,2-68,6	28.075

Tabel 16.9.7
Prevalensi Status Gizi (IMT/U) pada Remaja Umur 13 - 15 Tahun menurut Karakteristik, Riskesdas 2018

Karakteristik	Status Gizi Menurut IMT/U										N Ter-timbang
	Sangat Kurus		Kurus		Normal		Gemuk		Obesitas		
	%	95% CI	%	95% CI	%	95% CI	%	95% CI	%	95% CI	
**Jenis Kelamin**											
Laki-laki	2,8	2,5-3,1	8,9	8,4-9,4	72,3	71,5-73,1	10,7	10,2-11,3	5,3	4,9-5,7	31.039
Perempuan	0,9	0,8-1,0	4,5	4,1-4,9	78,6	77,8-79,3	11,7	11,2-12,3	4,3	4,0-4,7	28.981
**Pendidikan**											
Tidak sekolah	3,2	2,0-5,0	6,9	5,2-9,1	70,8	66,6-74,8	13,8	10,9-17,2	5,3	3,4-8,2	1.056
Tidak Tamat SD	2,8	2,3-3,3	8,6	7,6-9,7	73,9	72,4-75,4	11,0	10,0-12,1	3,7	3,2-4,4	7.049
Tamat SD	1,8	1,6-2,1	6,8	6,5-7,2	75,4	74,7-76	11,0	10,5-11,5	5,0	4,7-5,3	39.666
Tamat SLTP	1,3	1,0-1,6	5,6	5,0-6,3	76,3	75,1-77,5	12,1	11,2-13,1	4,7	4,1-5,3	11.867
Tamat SLTA	2,0	0,7-5,3	3,4	1,6-7,1	75,4	67,1-82,2	9,0	5,6-14,0	10,3	5,4-18,5	382
**Tempat Tinggal**											
Perkotaan	1,9	1,6-2,2	6,7	6,3-7,3	73,2	72,4-74,1	12,3	11,7-12,9	5,9	5,4-6,3	31.780
Perdesaan	1,8	1,7-2,0	6,8	6,5-7,2	77,7	77,1-78,3	10,0	9,6-10,5	3,6	3,4-3,9	28.240

Tabel 16.9.8
Prevalensi Status Gizi (IMT/U) pada Remaja Umur 13 - 15 Tahun menurut Provinsi, Riskesdas 2018

Provinsi	Status Gizi Menurut IMT/U										N Ter-timbang
	Sangat Kurus		Kurus		Normal		Gemuk		Obesitas		
	%	95% CI	%	95% CI	%	95% CI	%	95% CI	%	95% CI	
Aceh	1,9	1,3-2,7	6,5	5,3-7,9	73,1	70,7-75,4	13,3	11,7-15,2	5,2	4,2-6,5	1.197
Sumatera Utara	1,6	1,2-2,2	5,7	4,7-6,9	75,0	73,2-76,7	12,9	11,6-14,3	4,8	3,9-5,9	3.856
Sumatera Barat	2,1	1,5-3,1	6,0	4,8-7,4	77,3	75,0-79,5	10,3	8,8-12,1	4,3	3,4-5,3	1.368
Riau	1,2	0,8-2,0	6,2	5,0-7,6	76,4	74,0-78,6	12,0	10,2-14,1	4,2	3,2-5,4	1.677
Jambi	2,1	1,4-3,2	5,5	4,3-7,0	78,1	75,5-80,6	10,5	8,7-12,6	3,8	2,7-5,4	868
Sumatera Selatan	1,6	1,0-2,3	7,2	5,9-8,8	76,9	74,6-79,2	10,9	9,2-12,8	3,4	2,6-4,5	2.063
Bengkulu	1,0	0,5-1,9	6,5	4,7-8,9	75,9	72,7-78,9	12,6	10,4-15,4	3,9	2,8-5,6	467
Lampung	2,3	1,5-3,5	6,5	5,3-8,0	79,3	76,8-81,5	8,9	7,4-10,7	3,0	2,1-4,2	1.918
Bangka Belitung	1,0	0,4-2,1	7,8	5,9-10,3	72,5	68,3-76,3	13,6	10,8-16,8	5,2	3,5-7,7	329
Kepulauan Riau	1,9	0,9-3,7	6,3	3,7-10,4	72,1	67,0-76,6	15,1	11,0-20,3	4,8	2,9-7,7	510
DKI Jakarta	1,9	1,0-3,6	6,2	4,2-8,8	66,8	62,6-70,8	15,1	12,4-18,3	10,0	7,9-12,6	1.890
Jawa Barat	1,7	1,3-2,4	6,1	5,1-7,3	75,3	73,5-77,0	12,0	10,7-13,4	4,9	4,1-5,8	11.081
Jawa Tengah	1,8	1,4-2,3	6,6	5,8-7,6	77,0	75,5-78,4	10,1	9,1-11,2	4,5	3,9-5,3	7.207
DI Yogyakarta	0,8	0,3-1,8	8,0	5,6-11,4	70,6	65,5-75,3	12,6	9,5-16,4	8,0	5,7-11,2	625
Jawa Timur	1,5	1,1-2,0	5,7	4,9-6,5	73,5	71,9-75,0	13,3	12,1-14,6	6,0	5,3-6,9	7.843
Banten	1,6	1,0-2,5	7,5	6,0-9,4	77,0	74,0-79,7	9,7	7,9-12,0	4,2	3,0-5,9	2.760
Bali	1,1	0,5-2,1	5,2	3,4-8,0	71,9	68,5-75,0	12,5	10,4-14,9	9,3	7,2-12,1	934
Nusa Tenggara Barat	2,5	1,6-3,9	12,0	9,8-14,6	76,1	73,0-78,9	7,2	5,5-9,3	2,3	1,5-3,5	1.211
Nusa Tenggara Timur	4,6	3,8-5,6	14,1	12,7-15,5	76,8	75,0-78,5	3,6	2,9-4,5	0,9	0,6-1,4	1.671
Kalimantan Barat	1,9	1,3-2,9	7,4	5,9-9,2	77,8	75,2-80,1	8,7	7,3-10,4	4,1	3,2-5,4	1.252
Kalimantan Tengah	1,4	0,8-2,6	6,2	4,3-8,7	78,8	75,5-81,7	9,7	7,9-11,8	4,0	2,8-5,6	618
Kalimantan Selatan	2,4	1,6-3,5	8,4	6,8-10,3	75,1	72,3-77,7	10,2	8,5-12,2	3,9	2,8-5,4	973
Kalimantan Timur	2,2	1,3-3,8	5,2	3,6-7,4	73,4	69,1-77,3	12,0	9,6-15,0	7,1	5,1-9,9	772
Kalimantan Utara	1,2	0,5-2,7	8,8	5,8-13,1	70,2	64,4-75,4	12,7	9,3-17,2	7,1	4,5-11,1	167
Sulawesi Utara	1,5	0,8-2,8	4,0	3,0-5,4	74,9	72,0-77,5	13,0	10,9-15,4	6,6	5,1-8,5	586
Sulawesi Tengah	2,4	1,6-3,8	7,5	6,1-9,2	78,9	76,1-81,4	8,1	6,6-10,0	3,1	2,2-4,3	766
Sulawesi Selatan	2,5	1,8-3,3	8,3	7,1-9,6	74,7	72,6-76,6	10,5	9,1-12,0	4,1	3,3-5,3	2.194
Sulawesi Tenggara	1,8	1,1-2,8	8,1	6,3-10,2	80,0	77,2-82,5	7,4	5,8-9,3	2,8	1,9-4,2	734
Gorontalo	1,5	0,7-3,1	10,8	7,8-14,8	73,1	68,4-77,3	10,4	8,1-13,2	4,2	2,9-6,0	289
Sulawesi Barat	1,0	0,5-2,1	6,9	5,0-9,4	79,9	76,3-83,1	8,5	6,4-11,4	3,7	2,4-5,5	371
Maluku	4,5	3,2-6,2	10,6	8,5-13,1	75,5	71,7-78,8	7,1	5,1-9,8	2,4	1,3-4,5	510
Maluku Utara	2,0	1,0-3,9	7,3	5,5-9,7	77,4	73,7-80,8	9,6	7,6-12,2	3,6	2,3-5,6	361
Papua Barat	2,3	1,2-4,5	7,0	4,4-11,0	75,6	70,2-80,3	10,0	7,2-13,7	5,1	3,2-7,9	237
Papua	2,1	1,2-3,8	4,4	3,2-6,2	71,1	67,3-74,6	15,6	13,0-18,7	6,7	5,0-8,9	715
**INDONESIA**	**1,9**	**1,7-2,0**	**6,8**	**6,5-7,1**	**75,3**	**74,8-75,9**	**11,2**	**10,8-11,6**	**4,8**	**4,6-5,1**	**60.020**

Tabel 16.9.9
Prevalensi Status Gizi (TB/U) pada Remaja Umur 16 - 18 Tahun menurut Provinsi, Riskesdas 2018

Provinsi	Status Gizi Menurut TB/U						
	Sangat Pendek		Pendek		Normal		N Ter-timbang
	%	95% CI	%	95% CI	%	95% CI	
Aceh	7,8	6,5-9,4	28,6	26,0-31,4	63,6	60,5-66,5	1.091
Sumatera Utara	5,7	4,8-6,7	23,5	21,7-25,4	70,8	68,9-72,7	3.218
Sumatera Barat	3,7	2,8-5,0	23,1	20,7-25,6	73,2	70,6-75,7	1.079
Riau	4,5	3,4-6,0	23,3	20,8-26,0	72,2	69,2-74,9	1.318
Jambi	5,7	4,0-8,1	21,5	18,8-24,6	72,8	69,3-76,0	672
Sumatera Selatan	4,7	3,7-5,9	19,0	16,6-21,6	76,3	73,5-79,0	1.667
Bengkulu	3,6	2,4-5,3	19,9	16,8-23,4	76,5	72,9-79,8	368
Lampung	3,5	2,5-4,8	22,4	20,0-25,0	74,2	71,4-76,7	1.603
Bangka Belitung	2,3	1,3-4,0	23,7	19,9-27,9	74,0	69,7-77,9	283
Kepulauan Riau	2,6	1,4-4,7	14,1	10,4-19,0	83,3	78,4-87,2	361
DKI Jakarta	1,2	0,6-2,5	16,2	13,3-19,7	82,5	79,1-85,5	1.596
Jawa Barat	4,5	3,7-5,4	21,4	19,7-23,1	74,2	72,3-76,0	9.819
Jawa Tengah	3,0	2,5-3,7	19,7	18,2-21,2	77,3	75,7-78,9	6.782
DI Yogyakarta	2,3	0,9-5,6	14,9	11,5-19,1	82,8	77,7-87,0	650
Jawa Timur	4,6	3,9-5,4	24,5	23-26,1	70,8	69,2-72,5	7.352
Banten	3,6	2,5-5,1	19,8	17,0-22,9	76,6	73,4-79,5	2.401
Bali	0,4	0,1-1,0	10,0	7,9-12,6	89,6	87,0-91,7	765
Nusa Tenggara Barat	3,9	2,7-5,4	23,1	20,2-26,3	73,0	69,7-76,1	1.098
Nusa Tenggara Timur	8,9	7,5-10,6	31,4	29,3-33,7	59,7	57,2-62,1	1.142
Kalimantan Barat	6,7	5,2-8,7	24,8	22,3-27,4	68,5	65,5-71,4	997
Kalimantan Tengah	4,5	3,2-6,3	27,1	23,6-31,0	68,4	64,4-72,1	518
Kalimantan Selatan	6,1	4,8-7,8	27,3	24,4-30,5	66,6	63,3-69,7	830
Kalimantan Timur	3,3	2,1-5,2	20,9	17,2-25,2	75,8	71,3-79,8	701
Kalimantan Utara	3,4	1,7-6,5	22,0	17,1-27,9	74,6	68,5-79,8	152
Sulawesi Utara	3,3	2,2-4,9	21,7	19,1-24,6	75,0	71,9-77,8	466
Sulawesi Tengah	6,7	5,1-8,7	29,3	26,2-32,7	64,0	60,2-67,6	583
Sulawesi Selatan	5,0	4,0-6,2	25,4	23,3-27,7	69,6	67,2-71,9	1.781
Sulawesi Tenggara	4,7	3,4-6,4	26,1	21,8-30,8	69,3	64,8-73,4	573
Gorontalo	5,9	4,0-8,5	33,7	29,4-38,2	60,5	55,4-65,3	225
Sulawesi Barat	9,2	6,8-12,3	35,3	31,1-39,8	55,5	50,6-60,2	290
Maluku	7,0	4,2-11,3	26,2	22,7-30,1	66,8	61,6-71,6	377
Maluku Utara	4,1	2,6-6,3	25,3	21,7-29,3	70,6	66,4-74,5	248
Papua Barat	5,9	3,7-9,3	29,3	24,2-35,1	64,8	59,0-70,1	190
Papua	15,1	11,6-19,6	25,2	22,0-28,6	59,7	54,7-64,4	497
**INDONESIA**	**4,5**	**4,2-4,8**	**22,4**	**21,9-22,9**	**73,1**	**72,5-73,7**	**51.694**

Tabel 16.9.10
Prevalensi Status Gizi (TB/U) pada Remaja Umur 16 - 18 Tahun menurut Karakteristik, Riskesdas 2018

Karakteristik	Status Gizi Menurut TB/U						
	Sangat Pendek		Pendek		Normal		N Ter-timbang
	%	95% CI	%	95% CI	%	95% CI	
**Jenis Kelamin**							
Laki-laki	5,5	5,1-5,9	23,3	22,5-24,1	71,2	70,4-72,0	26.295
Perempuan	3,5	3,2-3,8	21,5	20,7-22,2	75,0	74,2-75,8	25.399
**Pendidikan**							
Tidak sekolah	17,7	13,6-22,7	26,8	21,9-32,5	55,5	49,3-61,5	519
Tidak Tamat SD	10,2	8,5-12,2	29,6	26,6-32,7	60,2	56,9-63,4	1.753
Tamat SD	8,2	7,2-9,3	29,3	27,6-31,0	62,5	60,6-64,4	6.398
Tamat SLTP	3,8	3,5-4,1	21,4	20,8-22,1	74,8	74,1-75,5	32.242
Tamat SLTA	2,9	2,5-3,4	19,9	18,8-21,1	77,1	75,9-78,3	10.702
Tamat D1-D3/PT	0,0		13,7	5,4-30,8	86,3	69,2-94,6	80
**Pekerjaan**							
Tidak bekerja	5,3	4,8-5,9	23,7	22,6-24,7	71,0	69,8-72,1	14.562
Sekolah	3,9	3,6-4,2	20,9	20,2-21,5	75,3	74,5-76,0	30.380
PNS/TNI/Polri/BUMN/BUMD	9,4	1,5-40,7	10,8	5,4-20,4	79,9	59,5-91,5	58
Pegawai swasta	2,6	1,5-4,5	22,0	18,6-25,7	75,4	71,6-78,8	1.480
Wiraswasta	4,7	3,1-7,0	26,7	22,6-31,2	68,6	64,0-72,9	1.031
Petani/buruh tani	7,5	6,1-9,3	30,9	28,1-33,9	61,5	58,3-64,6	1.374
Nelayan	9,0	4,2-18,4	29,2	20,9-39,1	61,8	51,3-71,3	155
Buruh/Supir/pembantu ruta	5,6	4,1-7,6	26,7	23,2-30,4	67,7	63,8-71,4	1.894
Lainnya	5,8	3,8-8,8	28,2	23,7-33,2	65,9	60,7-70,8	761
**Tempat Tinggal**							
Perkotaan	3,2	2,9-3,6	18,9	18,1-19,7	77,9	77,1-78,7	28.267
Perdesaan	6,0	5,7-6,4	26,7	25,9-27,4	67,3	66,5-68,1	23.427

Tabel 16.9.11
Prevalensi Status Gizi (IMT/U) pada Remaja Umur 16 - 18 Tahun menurut Provinsi, Riskesdas 2018

Provinsi	Status Gizi Menurut IMT/U										N Ter-timbang
	Sangat Kurus		Kurus		Normal		Gemuk		Obesitas		
	%	95% CI	%	95% CI	%	95% CI	%	95% CI	%	95% CI	
Aceh	1,3	0,8-2,1	4,7	3,7-6,1	79,0	76,7-81,1	11,4	9,7-13,4	3,5	2,7-4,6	1.094
Sumatera Utara	0,7	0,4-1,1	4,4	3,5-5,5	80,0	78,1-81,8	10,9	9,6-12,4	4,0	3,2-5,1	3.233
Sumatera Barat	1,8	1,2-2,8	7,6	6,1-9,5	79,1	76,4-81,5	7,8	6,4-9,4	3,7	2,8-5,0	1.082
Riau	1,5	1,0-2,5	6,5	5,0-8,2	80,4	77,8-82,9	7,1	5,6-8,9	4,5	3,4-6,0	1.328
Jambi	1,5	0,9-2,5	6,5	5,0-8,3	81,6	78,7-84,2	7,8	6,2-9,8	2,7	1,8-4,0	673
Sumatera Selatan	1,9	1,2-3,1	6,3	5,0-8,0	82,5	80,2-84,6	7,2	5,7-9,0	2,0	1,4-3,0	1.668
Bengkulu	0,6	0,2-1,5	4,2	3,0-6,0	84,8	81,8-87,4	7,5	5,6-9,8	2,9	1,8-4,5	369
Lampung	0,7	0,3-1,4	6,8	5,5-8,4	80,9	78,4-83,3	9,4	7,7-11,5	2,2	1,4-3,3	1.610
Bangka Belitung	0,8	0,3-2,0	7,7	5,6-10,5	76,1	71,9-79,8	9,1	6,6-12,5	6,3	4,4-8,8	283
Kepulauan Riau	0,7	0,3-1,8	10,2	6,3-16,0	76,6	70,6-81,6	8,5	5,7-12,4	4,0	2,2-7,3	361
DKI Jakarta	1,9	1,1-3,3	7,0	5,1-9,7	70,0	65,8-74,0	12,8	10,0-16,1	8,3	6,1-11,2	1.610
Jawa Barat	1,4	1,0-2,0	5,6	4,8-6,6	77,6	75,8-79,2	10,9	9,6-12,3	4,5	3,7-5,5	9.823
Jawa Tengah	1,6	1,1-2,2	8,1	7,2-9,2	78,7	77,2-80,2	7,9	7,0-8,9	3,7	3,1-4,4	6.790
DI Yogyakarta	1,2	0,5-2,8	6,4	4,4-9,2	78,0	73,4-82,0	8,2	6,1-11,1	6,2	3,8-9,9	651
Jawa Timur	1,1	0,8-1,5	6,8	6,0-7,8	75,7	74,2-77,2	11,3	10,3-12,4	5,1	4,4-5,9	7.373
Banten	1,2	0,6-2,3	7,9	6,3-9,9	77,6	74,4-80,4	9,7	7,6-12,3	3,6	2,4-5,3	2.397
Bali	0,9	0,4-1,9	5,1	3,7-6,9	76,5	73,1-79,7	11,9	9,6-14,5	5,6	4,1-7,5	768
Nusa Tenggara Barat	2,2	1,3-3,8	8,2	6,6-10,2	81,7	78,7-84,3	6,4	4,7-8,5	1,5	0,8-2,6	1.103
Nusa Tenggara Timur	2,7	2,0-3,8	9,5	8,1-11,2	83,3	81,2-85,2	3,4	2,6-4,5	1,0	0,6-1,6	1.145
Kalimantan Barat	1,5	0,8-2,7	5,7	4,3-7,5	81,8	79,2-84,1	7,6	6,1-9,5	3,4	2,4-4,9	999
Kalimantan Tengah	1,9	1,0-3,4	5,5	3,9-7,7	81,2	78,0-84,1	7,1	5,5-9,3	4,2	2,9-6,2	520
Kalimantan Selatan	2,6	1,8-3,9	9,7	7,9-11,8	75,6	72,5-78,5	8,9	7,3-10,9	3,2	2,1-4,7	830
Kalimantan Timur	2,6	1,5-4,2	7,2	5,1-10	71,5	67,5-75,2	12,9	10,0-16,5	5,8	4,0-8,4	703
Kalimantan Utara	1,8	0,8-3,9	6,1	3,9-9,3	78,8	73,6-83,3	9,3	6,3-13,6	4,0	2,2-6,9	153
Sulawesi Utara	0,7	0,3-1,5	4,9	3,7-6,4	78,0	74,9-80,8	11,4	9,3-13,9	5,0	3,5-7	467
Sulawesi Tengah	2,2	1,3-3,8	7,0	5,5-8,9	79,7	76,8-82,4	8,1	6,5-10,0	3,0	2-4,4	582
Sulawesi Selatan	2,4	1,6-3,4	8,0	6,8-9,5	79,1	77,0-81,0	7,8	6,5-9,3	2,7	2-3,7	1.786
Sulawesi Tenggara	1,0	0,5-2,3	5,9	4,2-8,1	85,6	82,9-87,9	5,7	4,4-7,3	1,8	1,2-2,9	575
Gorontalo	0,8	0,3-2,5	7,7	5,4-10,8	79,1	74,9-82,8	8,6	6,1-11,9	3,8	2,4-6,1	225
Sulawesi Barat	0,0	0,0-0,1	6,3	4,2-9,3	80,5	76,4-84,1	10,3	7,6-13,6	2,9	1,7-4,9	292
Maluku	1,3	0,5-3,1	8,8	6,4-12,0	83,0	79,6-85,9	4,4	3,0-6,3	2,6	1,6-4,3	378
Maluku Utara	1,2	0,5-2,6	3,9	2,6-5,8	86,2	83,0-88,9	6,0	4,3-8,4	2,7	1,4-5,1	250
Papua Barat	2,4	1,0-5,7	6,3	3,2-11,9	76,6	70,7-81,6	11,6	8,1-16,5	3,1	1,7-5,5	190
Papua	0,6	0,2-1,8	2,9	1,8-4,9	78,4	73,9-82,3	13,5	10,3-17,6	4,5	2,9-7,1	513
**INDONESIA**	**1,4**	**1,3-1,6**	**6,7**	**6,4-7,0**	**78,3**	**77,8-78,9**	**9,5**	**9,1-9,9**	**4,0**	**3,8-4,3**	**51.826**

Tabel 16.9.12

Prevalensi Status Gizi (IMT/U) pada Remaja Umur 16 - 18 Tahun menurut Karakteristik, Riskesdas 2018

Karakteristik	Status Gizi Menurut IMT/U										N Ter-timbang
	Sangat Kurus		Kurus		Normal		Gemuk		Obesitas		
	%	95% CI	%	95% CI	%	95% CI	%	95% CI	%	95% CI	
**Jenis Kelamin**											
Laki-laki	2,3	2,0-2,6	9,5	9,0-10,0	77,0	76,2-77,7	7,7	7,2-8,2	3,6	3,3-3,9	26.363
Perempuan	0,5	0,4-0,7	3,8	3,4-4,1	79,8	79,0-80,5	11,4	10,8-12,1	4,5	4,1-4,9	25.463
**Pendidikan**											
Tidak sekolah	2,4	1,4-4,1	5,9	4,1-8,4	78,1	73,3-82,2	10,0	7,1-13,8	3,6	2,1-6,1	521
Tidak Tamat SD	2,9	1,9-4,3	7,0	5,5-8,8	76,8	73,7-79,5	8,8	7-11	4,6	3,3-6,3	1.777
Tamat SD	1,7	1,2-2,3	6,8	6,0-7,8	79,2	77,6-80,7	8,6	7,6-9,8	3,7	3-4,5	6.412
Tamat SLTP	1,4	1,2-1,6	6,7	6,3-7,1	78,4	77,8-79,1	9,5	9-10	4,0	3,6-4,3	32.311
Tamat SLTA	1,3	1,0-1,7	6,4	5,7-7,1	77,8	76,6-79	10,1	9,3-11,1	4,4	3,8-5	10.726
Tamat D1-D3/PT	0,0	0,0-0,0	5,4	1,0-23,5	78,0	58,9-89,8	14,0	5,3-32,1	2,6	0,6-11,3	80
**Pekerjaan**											
Tidak bekerja	1,6	1,3-2,0	6,4	5,8-7	77,6	76,5-78,6	10,1	9,4-10,9	4,3	3,8-4,9	14.598
Sekolah	1,5	1,3-1,7	7,0	6,6-7,4	78,0	77,3-78,7	9,4	8,9-9,9	4,1	3,8-4,5	30.472
PNS/TNI/Polri/BUMN/BUMD	1,5	0,2-10,3	5,1	0,9-24,6	78,8	62,5-89,2	6,2	2,3-16,1	8,3	2,5-24	58
Pegawai swasta	0,9	0,5-1,7	5,6	4-7,6	77,4	73,3-81	12,6	9,7-16,2	3,5	2,3-5,4	1.483
Wiraswasta	0,8	0,4-1,7	7,1	4,5-10,9	80,4	76,2-84,1	7,9	5,9-10,6	3,7	2,4-5,8	1.033
Petani/buruh tani	0,7	0,3-1,4	5,6	4,4-7,3	83,8	81,2-86,1	7,5	5,8-9,6	2,4	1,6-3,6	1.375
Nelayan	2,5	0,9-6,9	3,9	1,9-7,7	91,3	86,1-94,6	1,7	0,7-3,8	0,6	0,2-1,8	155
Buruh/Supir/pembantu ruta	1,0	0,4-2,4	5,8	4,2-7,9	84,4	81,5-86,9	6,7	5,1-8,8	2,1	1,3-3,4	1.891
Lainnya	0,7	0,3-1,9	5,8	3,8-8,8	76,5	71,5-80,9	12,0	8,6-16,5	5,0	3,1-7,8	761
**Tempat Tinggal**											
Perkotaan	1,6	1,4-1,9	7,1	6,6-7,6	75,9	75-76,7	10,7	10,1-11,3	4,8	4,4-5,2	28.327
Perdesaan	1,2	1,1-1,4	6,2	5,8-6,6	81,3	80,7-82	8,1	7,7-8,6	3,1	2,8-3,4	23.499

16.10 Status Gizi pada Dewasa (IMT dan Obesitas Sentral)

Indeks Massa Tubuh (IMT):

Status gizi dewasa adalah penilaian status gizi penduduk di atas 18 tahun yang dinilai dengan Indeks Massa Tubuh (IMT). Indikator status gizi yang digunakan untuk kelompok umur ini didasarkan pada pengukuran antropometri berat badan (BB) dan tinggi badan (TB) yang disajikan dalam bentuk Indeks Massa Tubuh (IMT).

Indeks massa tubuh dihitung berdasarkan formula berikut:

$$IMT = \frac{BB\ (kg)}{TB^2\ (meter)}$$

Berikut adalah batasan IMT yang digunakan untuk menilai status gizi penduduk dewasa (> 18 tahun) sebagai berikut :

- Kategori kurus :IMT < 18,5
- Kategori normal :IMT ≥ 18,5 - < 25,0
- Kategori BB lebih :IMT ≥ 25,0 - < 27,0
- Kategori obesitas :IMT ≥ 27,0

Berdasarkan indikator IMT:

Prevalensi penduduk > 18 Tahun Kurus	:	(∑Penduduk > 18 tahun kurus/∑penduduk > 18 tahun diukur BB dan TB) x 100%
Prevalensi penduduk > 18 Tahun Normal	:	(∑Penduduk > 18 tahun normal/∑penduduk > 18 tahun diukur BB dan TB) x 100%
Prevalensi penduduk > 18 Tahun BB Lebih	:	(∑Penduduk > 18 tahun BB lebih/∑penduduk > 18 tahun diukur BB dan TB) x 100%
Prevalensi penduduk > 18 Tahun obese	:	(∑Penduduk > 18 tahun obese/∑penduduk > 18 tahun diukur BB dan TB) x 100%

Obesitas Sentral:

Obesitas sentral atau obesitas abdominal adalah kumpulan lemak abdominal berlebih yang terdapat di daerah abdomen atau perut. Pengukuran lingkar perut dilakukan pada penduduk dewasa usia ≥ 15 tahun.

Batasan obesitas sentral adalah jika nilai lingkar perut pada:

- Laki-laki > 90 cm
- Perempuan > 80 cm

Formula Perhitungan:

$$\text{Prevalensi obesitas sentral pada penduduk} \geq 15 \text{ tahun} = \frac{\sum \text{Penduduk} \geq 15 \text{ tahun dengan nilai lingkar perut laki-laki} > 90 \text{ cm atau perempuan} > 80 \text{ cm)}}{\sum \text{Penduduk} \geq 15 \text{ tahun diukur lingkar perut}} \text{X100\%}$$

Tabel 16.10.1
Prevalensi Status Gizi berdasarkan Kategori IMT pada Penduduk Dewasa (umur >18 Tahun) menurut Provinsi, Riskesdas 2018

Provinsi	Status Gizi Menurut IMT								
	Kurus		Normal		BB Lebih		Obesitas		N Ter-timbang
	%	95% CI	%	95% CI	%	95% CI	%	95% CI	
Aceh	7,2	6,7-7,8	54,5	53,6-55,3	13,9	13,4-14,5	24,4	23,6-25,2	11.530
Sumatera Utara	6,0	5,6-6,4	53,4	52,6-54,3	14,8	14,3-15,4	25,8	25,0-26,5	31.201
Sumatera Barat	10,8	10,2-11,5	55,7	54,8-56,6	13,1	12,5-13,8	20,4	19,6-21,2	11.977
Riau	8,1	7,5-8,7	53,8	52,8-54,9	14,0	13,3-14,7	24,1	23,1-25,0	15.018
Jambi	8,3	7,7-8,9	60,8	59,7-62,0	13,3	12,6-14,0	17,6	16,7-18,5	8.387
Sumatera Selatan	9,3	8,7-9,8	60,4	59,4-61,3	12,9	12,3-13,5	17,4	16,7-18,2	19.259
Bengkulu	8,3	7,7-9,0	58,7	57,5-59,9	13,2	12,4-14,0	19,9	19,0-20,7	4.564
Lampung	9,0	8,4-9,5	61,4	60,5-62,3	12,3	11,8-13,0	17,3	16,6-18,0	19.519
Bangka Belitung	7,4	6,6-8,2	55,2	53,8-56,6	13,7	12,8-14,7	23,7	22,6-24,9	3.428
Kepulauan Riau	7,9	6,8-9,3	52,2	50,3-54,1	13,6	12,2-15,2	26,2	24,4-28,1	4.784
DKI Jakarta	7,9	7,2-8,7	46,6	45,5-47,8	15,6	14,8-16,6	29,8	28,7-30,9	25.926
Jawa Barat	9,2	8,9-9,7	54,1	53,4-54,7	13,7	13,2-14,1	23,0	22,5-23,5	114.678
Jawa Tengah	10,4	10,1-10,8	56,3	55,7-56,8	13,0	12,6-13,3	20,4	19,9-20,8	84.712
DI Yogyakarta	11,4	10,5-12,3	54,0	52,5-55,5	13,3	12,4-14,2	21,4	20,1-22,6	9.812
Jawa Timur	9,3	9,0-9,6	54,6	54,1-55,1	13,7	13,4-14,1	22,4	21,9-22,8	100.419
Banten	9,9	9,3-10,6	54,8	53,7-56,0	13,2	12,5-14,0	22,1	21,2-23,0	29.550
Bali	8,3	7,7-9,0	52,9	51,7-54,2	15,5	14,7-16,3	23,3	22,3-24,3	10.701
Nusa Tenggara Barat	14,0	13,2-14,9	59,2	58,1-60,3	11,8	11,1-12,5	14,9	14,2-15,7	11.248
Nusa Tenggara Timur	18,1	17,3-18,9	62,9	62,0-63,7	8,8	8,3-9,3	10,3	9,7-10,9	10.842
Kalimantan Barat	9,7	9,0-10,4	60,1	59,0-61,1	13,2	12,5-13,9	17,0	16,2-17,8	11.302
Kalimantan Tengah	9,1	8,4-9,8	59,1	58,0-60,1	13,2	12,5-14,0	18,7	17,8-19,6	6.174
Kalimantan Selatan	10,7	10,1-11,4	56,3	55,3-57,4	13,4	12,8-14,1	19,5	18,6-20,4	9.638
Kalimantan Timur	7,1	6,3-8,0	48,7	47,4-50,1	15,5	14,6-16,4	28,7	27,4-30,1	8.498
Kalimantan Utara	6,2	5,3-7,2	53,5	51,5-55,5	14,2	12,8-15,6	26,1	24,4-28,0	1.590
Sulawesi Utara	5,0	4,6-5,5	48,5	47,4-49,6	16,3	15,6-17,0	30,2	29,2-31,3	6.025
Sulawesi Tengah	9,7	9,0-10,5	55,8	54,7-56,9	13,8	13,1-14,5	20,7	19,8-21,7	6.842
Sulawesi Selatan	10,8	10,2-11,3	57,2	56,4-57,9	13,0	12,5-13,5	19,1	18,5-19,8	19.983
Sulawesi Tenggara	7,8	7,1-8,6	59,7	58,5-60,9	13,1	12,2-14,1	19,3	18,4-20,3	5.551
Gorontalo	8,3	7,4-9,2	52,5	50,9-54,1	14,9	13,9-15,9	24,4	23,0-25,8	2.769
Sulawesi Barat	9,3	8,4-10,2	59,4	57,8-60,9	12,7	11,7-13,8	18,7	17,3-20,1	2.921
Maluku	10,0	9,0-11,1	57,2	55,6-58,7	12,9	11,9-14,0	19,9	18,7-21,3	3.715
Maluku Utara	6,6	5,8-7,4	55,4	53,9-56,9	13,4	12,3-14,6	24,6	23,4-25,9	2.574
Papua Barat	6,6	5,6-7,8	53,7	51,7-55,6	13,3	12,0-14,6	26,4	24,8-28,2	2.044
Papua	5,1	4,4-5,8	59,9	58,2-61,5	14,9	14,0-15,8	20,2	18,9-21,5	7.382
**INDONESIA**	**9,3**	**9,2-9,5**	**55,3**	**55,1-55,5**	**13,6**	**13,4-13,7**	**21,8**	**21,7-22,0**	**624.563**

Catatan:

Kurus	:	IMT <18,5
Normal	:	IMT ≥18,5 s/d IMT <25,0
BB Lebih	:	IMT ≥ 25,0 s/d IMT <27,0
Obesitas	:	IMT ≥ 27,0

Tabel 16.10.2

Prevalensi Status Gizi berdasarkan Kategori IMT pada Penduduk Dewasa (umur >18 Tahun) menurut Karakteristik, Riskesdas 2018

Karakteristik	Status Gizi Menurut IMT								N Ter-timbang
	Kurus		Normal		BB Lebih		Obesitas		
	%	95% CI	%	95% CI	%	95% CI	%	95% CI	
**Kelompok Umur**									
19	20,7	19,7-21,8	63,8	62,5-65,1	6,6	5,9-7,2	8,9	8,1-9,7	12.123
20 - 24	15,8	15,4-16,3	63,6	63,0-64,2	8,4	8,1-8,7	12,1	11,7-12,5	75.441
25 - 29	9,8	9,4-10,1	59,5	58,9-60,0	12,0	11,6-12,4	18,8	18,3-19,2	74.393
30 - 34	7,2	6,9-7,5	55,5	54,9-56,0	14,4	14,0-14,8	23,0	22,5-23,5	72.074
35 -39	5,0	4,8-5,3	52,4	51,9-53,0	15,5	15,2-15,9	27,0	26,5-27,5	73.916
40 - 44	4,4	4,1-4,6	49,8	49,2-50,3	16,3	15,9-16,7	29,6	29,1-30,1	68.584
45 - 49	4,6	4,4-4,8	49,9	49,4-50,5	16,8	16,4-17,2	28,6	28,1-29,1	63.689
50 - 54	6,3	6,0-6,5	51,5	50,9-52,1	16,2	15,8-16,6	26,0	25,5-26,6	54.467
55 - 59	8,3	8,0-8,7	53,6	52,9-54,2	15,0	14,5-15,5	23,1	22,6-23,7	44.817
60 - 64	11,7	11,2-12,2	56,0	55,3-56,8	12,9	12,5-13,5	19,3	18,7-19,9	33.322
65 +	20,7	20,2-21,2	57,8	57,2-58,4	9,7	9,3-10,0	11,9	11,5-12,3	51.737
**Jenis Kelamin**									
Laki-laki	10,8	10,7-11	62,6	62,3-62,9	12,1	11,9-12,3	14,5	14,3-14,7	315.623
Perempuan	7,8	7,6-7,9	47,8	47,6-48,1	15,1	14,9-15,2	29,3	29,1-29,6	308.940
**Pendidikan**									
Tidak sekolah	17,6	17-18,1	57,9	57,2-58,7	10,2	9,8-10,7	14,2	13,7-14,8	37.149
Tidak Tamat SD	12,1	11,8-12,5	57,4	56,9-57,9	11,9	11,6-12,3	18,5	18,1-18,9	77.335
Tamat SD	8,4	8,2-8,6	56,9	56,6-57,3	13,1	12,9-13,4	21,5	21,2-21,8	154.261
Tamat SLTP	8,1	7,8-8,3	55,4	54,9-55,8	13,8	13,5-14,1	22,8	22,4-23,2	109.725
Tamat SLTA	9,2	8,9-9,4	54,4	54,0-54,7	14,0	13,7-14,2	22,5	22,2-22,8	186.701
Tamat D1-D3/PT	5,6	5,4-5,9	49,3	48,6-49,9	17,0	16,6-17,5	28,1	27,5-28,6	59.393
**Pekerjaan**									
Tidak bekerja	9,9	9,7-10,1	48,6	48,2-48,9	14,3	14,1-14,6	27,3	26,9-27,6	178.590
Sekolah	17,3	16,3-18,3	60,0	58,7-61,3	9,0	8,2-9,7	13,8	12,8-14,7	14.380
PNS/TNI/Polri/BUMN/BUMD	2,8	2,5-3,1	43,6	42,7-44,5	20,0	19,2-20,7	33,7	32,8-34,6	20.844
Pegawai swasta	8,3	7,9-8,7	55,6	55,0-56,3	14,3	13,8-14,7	21,8	21,3-22,3	70.756
Wiraswasta	6,7	6,5-7,0	51,6	51,1-52,1	15,5	15,2-15,9	26,2	25,8-26,6	100.056
Petani/buruh tani	11,3	11,1-11,5	65,4	65,0-65,7	10,7	10,5-10,9	12,7	12,4-12,9	126.577
Nelayan	8,8	7,9-9,9	67,3	65,8-68,8	11,2	10,3-12,2	12,7	11,5-13,9	5.164
Buruh/Supir/pembantu ruta	10,2	9,8-10,6	62,1	61,5-62,7	12,0	11,7-12,4	15,7	15,2-16,1	70.793
Lainnya	8,0	7,6-8,4	52,8	52,1-53,5	14,4	13,9-14,9	24,8	24,2-25,5	37.404
**Tempat Tinggal**									
Perkotaan	8,7	8,5-8,9	51,6	51,3-51,9	14,6	14,4-14,8	25,1	24,8-25,3	345.890
Perdesaan	10,1	10,0-10,3	59,8	59,8-59,6	12,2	12,2-12,1	17,8	17,6-18,0	278.673

Catatan:

Kurus	:	IMT <18,5
Normal	:	IMT ≥18,5 s/d IMT <25,0
BB Lebih	:	IMT ≥ 25,0 s/d IMT <27,0
Obesitas	:	IMT ≥ 27,0

Tabel 16.10.3
Prevalensi Status Gizi berdasarkan Kategori IMT pada Penduduk Laki-Laki Dewasa (umur >18 Tahun) menurut Provinsi, Riskesdas 2018

Provinsi	Status Gizi Berdasarkan IMT								
	Kurus		Normal		BB Lebih		Obesitas		N Ter-timbang
	%	95% CI	%	95% CI	%	95% CI	%	95% CI	
Aceh	7,9	7,2-8,7	63,9	62,7-65,0	13,1	12,4-13,9	15,1	14,2-16,0	374
Sumatera Utara	6,7	6,1-7,3	60,0	58,8-61,2	14,6	13,8-15,4	18,7	17,8-19,7	819
Sumatera Barat	13,4	12,4-14,5	63,4	62,0-64,7	10,6	9,8-11,5	12,6	11,7-13,6	494
Riau	9,8	8,9-10,7	61,2	59,8-62,5	12,9	12,0-13,8	16,2	15,1-17,3	450
Jambi	9,1	8,3-10,0	67,4	65,9-68,9	11,9	11,0-13,0	11,5	10,5-12,7	301
Sumatera Selatan	11,0	10,2-11,9	67,3	66,0-68,6	10,9	10,0-11,8	10,8	9,9-11,7	694
Bengkulu	9,9	9,0-10,9	67,6	66,1-69,2	11,4	10,3-12,6	11,1	10,1-12,1	144
Lampung	10,8	10,0-11,6	70,2	69,1-71,4	9,6	8,8-10,3	9,4	8,8-10,2	656
Bangka Belitung	8,9	7,8-10,1	64,1	62,3-65,9	12,2	10,9-13,5	14,8	13,6-16,2	89
Kepulauan Riau	9,7	8,0-11,7	56,6	54,0-59,3	13,1	11,1-15,3	20,6	18,5-22,9	138
DKI Jakarta	9,5	8,4-10,6	52,1	50,4-53,7	15,3	14,1-16,5	23,2	21,8-24,7	808
Jawa Barat	11,8	11,2-12,4	62,5	61,6-63,3	11,5	11,0-12,1	14,2	13,6-14,9	3.687
Jawa Tengah	11,9	11,4-12,4	63,5	62,7-64,2	11,6	11,1-12,1	13,1	12,6-13,6	3.845
DI Yogyakarta	13,4	12,2-14,7	57,0	54,7-59,2	12,6	11,2-14,1	17,1	15,5-18,8	465
Jawa Timur	10,6	10,2-11,1	62,1	61,4-62,8	12,6	12,1-13,0	14,7	14,2-15,2	4.090
Banten	12,4	11,4-13,4	62,0	60,5-63,5	11,0	10,1-12,0	14,5	13,6-15,6	1.039
Bali	8,5	7,6-9,4	55,9	54,3-57,6	15,3	14,2-16,5	20,3	19,1-21,6	431
Nusa Tenggara Barat	16,3	15,0-17,6	67,0	65,5-68,5	9,0	8,1-10,0	7,7	6,9-8,5	703
Nusa Tenggara Timur	16,9	15,9-18,0	68,8	67,6-70,0	7,1	6,4-7,8	7,2	6,5-7,9	1.056
Kalimantan Barat	10,9	9,9-12,0	66,5	65,1-67,9	11,5	10,6-12,4	11,1	10,2-12,0	460
Kalimantan Tengah	10,6	9,6-11,6	65,9	64,4-67,3	11,5	10,5-12,6	12,0	11,0-13,1	211
Kalimantan Selatan	12,2	11,3-13,2	62,8	61,3-64,2	12,3	11,4-13,2	12,7	11,7-13,8	431
Kalimantan Timur	7,9	6,7-9,3	56,4	54,3-58,4	14,2	13,0-15,4	21,6	19,9-23,4	243
Kalimantan Utara	6,7	5,4-8,2	60,6	57,9-63,3	13,0	11,5-14,7	19,7	17,7-21,8	41
Sulawesi Utara	4,8	4,2-5,5	55,3	53,8-56,8	16,8	15,9-17,9	23,0	21,8-24,3	152
Sulawesi Tengah	10,8	9,8-11,8	64,4	62,9-65,8	11,2	10,4-12,2	13,6	12,6-14,8	281
Sulawesi Selatan	12,4	11,5-13,3	64,6	63,5-65,7	10,9	10,2-11,5	12,2	11,4-13,0	956
Sulawesi Tenggara	8,2	7,2-9,4	66,8	65,0-68,5	11,9	10,8-13,0	13,1	12,1-14,2	203
Gorontalo	10,0	8,8-11,4	61,5	59,3-63,6	13,2	11,9-14,7	15,3	13,8-17,0	90
Sulawesi Barat	9,5	8,3-10,8	68,4	66,3-70,4	10,4	9,2-11,8	11,7	10,3-13,3	132
Maluku	9,3	8,0-10,7	65,9	63,8-68,0	11,1	9,8-12,5	13,7	12,3-15,3	198
Maluku Utara	5,9	5,0-7,0	64,7	62,6-66,7	13,6	12,1-15,3	15,8	14,2-17,4	91
Papua Barat	6,3	5,0-8,0	61,0	58,4-63,5	13,6	11,9-15,5	19,1	17,3-21,0	66
Papua	4,2	3,5-5,1	64,6	62,7-66,6	15,1	13,9-16,5	16,0	14,6-17,5	207
**INDONESIA**	**10,8**	**10,7-11,0**	**62,6**	**62,3-62,9**	**12,1**	**11,9-12,3**	**14,5**	**14,3-14,7**	**24.045**

Tabel 16.10.4
Prevalensi Status Gizi berdasarkan Kategori IMT pada Penduduk Laki-Laki Dewasa (umur >18 Tahun) menurut Karakteristik, Riskesdas 2018

Karakteristik	Status Gizi Berdasarkan IMT								N Ter-timbang
	Kurus		Normal		BB Lebih		Obesitas		
	%	95% CI	%	95% CI	%	95% CI	%	95% CI	
**Kelompok Umur**									
19	22,5	21,0-24,1	64,5	62,8-66,3	5,8	5,1-6,7	7,2	6,2-8,2	6.108
20 - 24	17,3	16,6-17,9	65,9	65,1-66,7	7,4	6,9-7,8	9,4	9,0-10,0	39.829
25 - 29	11,9	11,3-12,5	64,4	63,6-65,2	10,4	9,9-10,9	13,3	12,8-13,9	38.718
30 - 34	9,5	9,0-10,0	63,2	62,3-64,0	12,3	11,7-12,8	15,1	14,5-15,7	37.101
35 -39	6,9	6,5-7,3	62,6	61,8-63,3	13,8	13,3-14,4	16,8	16,2-17,3	37.125
40 - 44	5,8	5,5-6,2	61,0	60,3-61,8	14,6	14,0-15,1	18,6	18,0-19,2	34.308
45 - 49	5,8	5,5-6,2	59,8	59,0-60,5	15,6	15,1-16,2	18,8	18,2-19,4	31.957
50 - 54	7,4	6,9-7,8	59,9	59,1-60,8	15,3	14,6-15,9	17,4	16,8-18,1	27.019
55 - 59	9,4	8,9-10,0	61,5	60,6-62,4	13,7	13,0-14,3	15,4	14,7-16,1	22.325
60 - 64	12,4	11,7-13,1	63,5	62,5-64,6	11,5	10,8-12,2	12,6	11,8-13,3	16.727
65 +	21,6	20,8-22,3	62,2	61,3-63,0	8,3	7,8-8,8	8,0	7,5-8,5	24.405
**Pendidikan**									
Tidak sekolah	18,7	17,8-19,6	67,1	65,9-68,2	7,3	6,7-7,9	7,0	6,4-7,6	13.667
Tidak Tamat SD	14,7	14,2-15,3	67,5	66,8-68,2	9,2	8,7-9,6	8,7	8,2-9,1	36.032
Tamat SD	10,8	10,5-11,2	68,6	68,1-69,1	10,1	9,8-10,5	10,4	10,1-10,8	74.647
Tamat SLTP	10,5	10,1-10,9	65,0	64,4-65,6	11,5	11,1-12,0	13,0	12,5-13,4	56.052
Tamat SLTA	10,3	9,9-10,6	58,5	58,0-59,0	13,6	13,3-13,9	17,6	17,3-18,0	105.678
Tamat D1-D3/PT	5,3	4,9-5,7	49,3	48,5-50,2	18,3	17,6-19,0	27,1	26,3-27,9	29.548
**Pekerjaan**									
Tidak bekerja	19,1	18,3-19,8	60,4	59,5-61,3	9,1	8,6-9,7	11,4	10,9-12,0	30.810
Sekolah	17,0	15,7-18,4	61,3	59,4-63,0	9,2	8,1-10,3	12,5	11,3-13,9	7.346
PNS/TNI/Polri/BUMN/BUMD	2,9	2,5-3,4	45,9	44,7-47,1	20,7	19,7-21,7	30,5	29,4-31,6	12.522
Pegawai swasta	8,3	7,9-8,8	56,7	55,9-57,5	14,6	14,0-15,1	20,4	19,8-21,1	46.702
Wiraswasta	8,0	7,6-8,3	57,6	57,0-58,2	15,2	14,7-15,6	19,2	18,7-19,7	63.377
Petani/buruh tani	12,3	12,0-12,6	71,5	71,1-71,9	8,7	8,5-8,9	7,5	7,3-7,7	80.178
Nelayan	8,8	7,9-9,8	68,6	67,0-70,2	11,2	10,2-12,2	11,4	10,3-12,7	4.824
Buruh/Supir/pembantu ruta	11,0	10,6-11,5	65,8	65,1-66,5	11,2	10,7-11,6	12,0	11,6-12,5	53.882
Lainnya	9,7	9,0-10,4	60,1	58,9-61,2	13,0	12,3-13,8	17,1	16,3-18,1	15.981
**Tempat Tinggal**									
Perkotaan	10,5	10,2-11,0	57,9	57,5-58,3	13,7	13,4-14,0	17,9	17,6-18,2	175.081
Perdesaan	11,3	11,0-11,5	68,4	68,1-68,8	10,1	9,9-10,3	10,2	10,0-10,5	140.542

Tabel 16.10.5
Prevalensi Status Gizi berdasarkan Kategori IMT pada Penduduk Perempuan Dewasa (umur >18 Tahun) menurut Provinsi, Riskesdas 2018

Provinsi	Status Gizi Berdasarkan IMT								N Ter-timbang
	Kurus		Normal		BB Lebih		Obesitas		
	%	95% CI	%	95% CI	%	95% CI	%	95% CI	
Aceh	6,5	5,9-7,1	45,0	43,8-46,2	14,7	13,9-15,6	33,7	32,6-34,9	5.748
Sumatera Utara	5,2	4,7-5,8	46,9	45,8-47,9	15,1	14,4-15,8	32,8	31,7-33,9	15.616
Sumatera Barat	8,2	7,5-9,0	48,1	46,8-49,3	15,6	14,6-16,6	28,1	27,0-29,3	5.999
Riau	6,3	5,6-7,0	45,8	44,4-47,2	15,2	14,3-16,2	32,7	31,4-34,1	7.147
Jambi	7,4	6,7-8,2	53,8	52,3-55,2	14,8	13,8-15,7	24,0	22,8-25,3	4.055
Sumatera Selatan	7,4	6,8-8,1	53,1	51,8-54,4	15,0	14,1-15,9	24,5	23,3-25,7	9.350
Bengkulu	6,5	5,8-7,4	49,1	47,5-50,7	15,0	14,0-16,2	29,3	27,9-30,8	2.201
Lampung	7,0	6,4-7,6	51,8	50,7-53,0	15,4	14,5-16,3	25,8	24,7-27,0	9.380
Bangka Belitung	5,6	4,7-6,6	45,0	43,0-47,0	15,5	14,2-17,0	33,9	32,0-35,7	1.599
Kepulauan Riau	6,0	4,9-7,5	47,3	44,1-50,5	14,3	12,2-16,6	32,4	29,7-35,2	2.286
DKI Jakarta	6,3	5,6-7,2	41,0	39,3-42,7	16,0	14,9-17,2	36,6	35,1-38,2	12.758
Jawa Barat	6,6	6,2-7,0	45,3	44,4-46,2	15,9	15,3-16,5	32,2	31,4-33,0	56.000
Jawa Tengah	9,0	8,6-9,4	49,2	48,5-49,8	14,3	13,8-14,8	27,5	26,9-28,2	42.733
DI Yogyakarta	9,4	8,4-10,5	51,1	49,2-53,0	13,9	12,8-15,2	25,5	24,0-27,2	4.953
Jawa Timur	8,0	7,7-8,4	47,2	46,5-47,9	14,9	14,5-15,4	29,8	29,2-30,5	50.868
Banten	7,3	6,5-8,0	47,2	45,6-48,7	15,5	14,6-16,5	30,0	28,6-31,5	14.318
Bali	8,2	7,3-9,1	49,8	48,2-51,5	15,6	14,6-16,7	26,4	24,9-27,8	5.287
Nusa Tenggara Barat	12,0	11,0-13,0	52,1	50,6-53,6	14,4	13,4-15,4	21,6	20,3-22,9	5.862
Nusa Tenggara Timur	19,2	18,3-20,2	57,1	56,0-58,2	10,4	9,7-11,1	13,3	12,5-14,2	5.496
Kalimantan Barat	8,4	7,7-9,2	53,3	51,9-54,7	15,0	14,1-16,0	23,3	22,1-24,5	5.478
Kalimantan Tengah	7,4	6,6-8,2	51,1	49,6-52,7	15,1	14,1-16,3	26,3	25,0-27,7	2.866
Kalimantan Selatan	9,2	8,4-10,0	49,6	48,2-51,0	14,6	13,7-15,5	26,6	25,4-28,0	4.705
Kalimantan Timur	6,2	5,4-7,2	39,8	38,1-41,5	17,0	15,7-18,4	37,0	35,2-38,9	3.914
Kalimantan Utara	5,7	4,6-7,0	45,0	42,4-47,7	15,5	13,6-17,6	33,8	31,2-36,6	725
Sulawesi Utara	5,2	4,6-5,9	41,3	39,8-42,8	15,7	14,8-16,8	37,8	36,2-39,4	2.929
Sulawesi Tengah	8,5	7,7-9,5	46,6	45,0-48,1	16,5	15,4-17,6	28,4	27,0-29,8	3.289
Sulawesi Selatan	9,3	8,7-9,9	50,2	49,2-51,2	14,9	14,2-15,7	25,6	24,7-26,5	10.309
Sulawesi Tenggara	7,4	6,5-8,4	52,5	50,9-54,1	14,4	13,2-15,7	25,7	24,2-27,3	2.746
Gorontalo	6,5	5,6-7,6	43,4	41,3-45,6	16,5	15,2-17,9	33,5	31,6-35,5	1.376
Sulawesi Barat	9,1	7,9-10,4	50,2	47,9-52,5	15,0	13,5-16,6	25,7	23,7-27,8	1.450
Maluku	10,7	9,5-12,1	48,3	46,1-50,5	14,7	13,1-16,5	26,2	24,2-28,3	1.847
Maluku Utara	7,3	6,3-8,4	45,6	43,9-47,3	13,2	11,9-14,7	33,9	32,2-35,7	1.251
Papua Barat	6,9	5,6-8,6	45,2	42,6-47,9	12,9	11,3-14,6	35,0	32,4-37,7	946
Papua	6,0	5,1-7,0	54,5	52,3-56,7	14,6	13,4-15,9	24,9	23,2-26,7	3.456
**INDONESIA**	**7,8**	**7,6-7,9**	**47,8**	**47,6-48,1**	**15,1**	**14,9-15,2**	**29,3**	**29,1-29,6**	**308.940**

Tabel 16.10.6
Prevalensi Status Gizi berdasarkan Kategori IMT pada Penduduk Perempuan Dewasa (umur >18 Tahun) menurut Karakteristik, Riskesdas 2018

Karakteristik	Status Gizi Berdasarkan IMT								
	Kurus		Normal		BB Lebih		Obesitas		N Ter-timbang
	%	95% CI	%	95% CI	%	95% CI	%	95% CI	
**Kelompok Umur**									
19	19,0	17,6-20,4	63,1	61,2-64,9	7,3	6,4-8,4	10,6	9,4-12,0	640
20 - 24	14,2	13,7-14,8	61,1	60,3-61,9	9,6	9,1-10,1	15,1	14,5-15,7	5.375
25 - 29	7,5	7,1-7,9	54,1	53,3-54,9	13,7	13,1-14,3	24,7	24,--25,4	8.807
30 - 34	4,7	4,4-5,0	47,3	46,5-48,1	16,6	16,1-17,2	31,4	30,6-32,1	10.971
35 -39	3,2	2,9-3,4	42,2	41,5-42,9	17,3	16,7-17,8	37,3	36,6-38,0	13.735
40 - 44	2,9	2,6-3,1	38,5	37,8-39,2	18,0	17,4-18,6	40,6	39,9-41,4	13.923
45 - 49	3,4	3,2-3,7	40,1	39,3-40,8	18,1	17,5-18,7	38,5	37,7-39,2	12.210
50 - 54	5,2	4,8-5,5	43,2	42,4-44,0	17,1	16,5-17,8	34,5	33,7-35,3	9.476
55 - 59	7,2	6,8-7,7	45,7	44,8-46,6	16,3	15,6-17,0	30,8	30,0-31,7	6.931
60 - 64	11,0	10,4-11,6	48,5	47,4-49,5	14,4	13,7-15,2	26,1	25,2-27,1	4.339
65 +	19,9	19,2-20,5	53,9	53,0-54,7	10,9	10,4-11,4	15,4	14,8-16,0	4.203
**Pendidikan**									
Tidak sekolah	16,9	16,3-17,6	52,6	51,8-53,5	12,0	11,4-12,5	18,5	17,8-19,2	23.482
Tidak Tamat SD	9,9	9,5-10,3	48,6	48,0-49,3	14,4	13,9-14,8	27,1	26,5-27,7	41.303
Tamat SD	6,2	6,0-6,4	46,0	45,5-46,5	15,9	15,5-16,3	31,9	31,4-32,4	79.614
Tamat SLTP	5,5	5,3-5,8	45,3	44,7-46,0	16,1	15,7-16,6	33,0	32,4-33,6	53.674
Tamat SLTA	7,8	7,5-8,1	48,9	48,4-49,5	14,5	14,2-14,9	28,8	28,3-29,3	81.023
Tamat D1-D3/PT	6,0	5,6-6,4	49,2	48,4-50,0	15,8	15,2-16,4	29,0	28,3-29,8	29.844
**Pekerjaan**									
Tidak bekerja	8,0	7,8-8,2	46,1	45,7-46,5	15,4	15,1-15,7	30,6	30,2-31,0	147.780
Sekolah	17,5	16,2-19	58,7	56,9-60,5	8,7	7,8-9,8	15,0	13,7-16,4	7.034
PNS/TNI/Polri/BUMN/BUMD	2,5	2,1-3,0	40,1	38,8-41,5	18,9	17,8-20,0	38,5	37,2-39,8	8.322
Pegawai swasta	8,3	7,7-8,9	53,6	52,5-54,6	13,7	13,0-14,4	24,4	23,5-25,4	24.054
Wiraswasta	4,5	4,2-4,9	41,1	40,4-41,9	16,1	15,6-16,7	38,2	37,5-39,0	36.678
Petani/buruh tani	9,6	9,3-9,9	54,7	54,1-55,2	14,1	13,8-14,5	21,6	21,1-22,0	46.399
Nelayan	9,5	5,3-16,2	49,0	43,3-54,7	11,6	8,4-15,8	30,0	23,9-36,7	340
Buruh/Supir/pembantu ruta	7,4	6,8-8,0	50,4	49,3-51,6	14,9	14,1-15,7	27,3	26,2-28,4	16.911
Lainnya	6,7	6,3-7,2	47,3	46,4-48,2	15,4	14,8-16,1	50,3	29,7-31,4	21.423
**Tempat Tinggal**									
Perkotaan	6,8	6,6-7,0	45,2	44,8-45,6	15,6	15,3-15,9	32,4	32,0-32,8	170.809
Perdesaan	9,0	8,8-7,9	51,1	50,7-51,4	14,4	14,2-14,6	25,5	25,2-25,8	138.131

Tabel 16.10.7
Prevalensi Obesitas Sentral pada Penduduk Umur ≥ 15 Tahun menurut Provinsi, Riskesdas 2018

Provinsi	Obesitas Sentral[1]		
	%	95% CI	N Tertimbang
Aceh	30,2	29,4-31,1	12.841
Sumatera Utara	34,9	34,1-35,8	35.399
Sumatera Barat	32,8	31,9-33,7	13.447
Riau	32,5	31,6-33,5	16.811
Jambi	24,6	23,6-25,7	9.278
Sumatera Selatan	27,1	26,2-28,0	21.467
Bengkulu	29,3	28,2-30,4	5.047
Lampung	26,0	25,2-26,8	21.646
Bangka Belitung	33,8	32,6-35,1	3.823
Kepulauan Riau	33,7	31,9-35,5	5.221
DKI Jakarta	41,9	40,6-43,1	27.645
Jawa Barat	32,0	31,4-32,6	127.351
Jawa Tengah	28,8	28,3-29,3	93.894
DI Yogyakarta	32,0	30,5-33,4	10.678
Jawa Timur	30,4	29,9-30,9	110.288
Banten	30,6	29,6-31,6	32.588
Bali	36,9	35,7-38,1	11.795
Nusa Tenggara Barat	25,3	24,3-26,3	12.645
Nusa Tenggara Timur	19,3	18,6-20,1	12.323
Kalimantan Barat	25,6	24,7-26,6	12.574
Kalimantan Tengah	24,7	23,7-25,7	6.833
Kalimantan Selatan	29,2	28,3-30,2	10.714
Kalimantan Timur	37,3	36,0-38,7	9.426
Kalimantan Utara	32,5	30,7-34,3	1.787
Sulawesi Utara	42,5	41,4-43,5	6.640
Sulawesi Tengah	32,9	31,9-34,0	7.615
Sulawesi Selatan	31,6	30,8-32,4	22.383
Sulawesi Tenggara	30,4	29,0-31,8	6.308
Gorontalo	36,6	35,1-38,2	3.076
Sulawesi Barat	27,1	25,7-28,5	3.324
Maluku	31,8	30,2-33,3	4.185
Maluku Utara	32,1	30,7-33,6	2.917
Papua Barat	34,6	32,7-36,5	2.287
Papua	31,7	30,2-33,3	7.749
**Indonesia**	**31,0**	**30,8-31,2**	**692.007**

[1]Lingkar Perut Laki-laki (> 90cm), Lingkar perut Perempuan (> 80 cm)

Tabel 16.10.8

Prevalensi Obesitas Sentral pada Penduduk Umur ≥ 15 Tahun menurut karakteristik, Riskesdas 2018

Karakteristik	Obesitas Sentral[1]		
	%	95 % CI	N Tertimbang
**Kelompok Umur**			
15 - 24	12,6	12,3-12,9	153.711
25 - 34	29,6	29,2-30,0	146.439
35 - 44	39,2	38,8-39,6	142.829
45 - 54	42,3	41,9-42,7	118.436
55 - 64	39,1	38,6-39,6	78.203
65 - 74	31,8	31,1-32,5	36.761
75 +	22,3	21,4-23,2	15.627
**Jenis Kelamin**			
Laki-laki	15,7	15,5 - 15,9	350.013
Perempuan	46,7	46,4 - 46,9	341.994
**Pendidikan**			
Tidak sekolah	28,5	27,8-29,2	37.936
Tidak Tamat SD	31,2	30,7-31,6	80.460
Tamat SD	31,1	30,8-31,5	169.428
Tamat SLTP	26,8	26,5-27,2	147.378
Tamat SLTA	31,2	30,8-31,6	197.330
Tamat D1-D3/PT	41,7	41,1-42,4	59.475
**Pekerjaan**			
Tidak bekerja	42,9	42,5-43,2	196.373
Sekolah	10,4	10,0-10,9	56.608
PNS/TNI/Polri/BUMN/BUMD	48,5	47,6-49,4	20.968
Pegawai swasta	28,8	28,2-29,4	72.275
Wiraswasta	36,0	35,5-36,5	101.188
Petani/buruh tani	20,6	20,3-20,9	128.203
Nelayan	14,3	13,2-15,4	5.329
Buruh/Supir/pembantu ruta	20,9	20,4-21,4	72.898
Lainnya	38,4	37,7-39,1	38.165
**Tempat Tinggal**			
Perkotaan	35,1	34,8 - 35,4	382.533
Perdesaan	25,9	25,7 - 26,1	309.474

[1]Lingkar Perut Laki-laki (> 90cm), Lingkar perut Perempuan (> 80 cm)

Lampiran 1
Respon Rate

Tabel L.1. 1

Respon Rate Sampel Wawancara dan Pengukuran, Riskesdas 2018

Kode	Provinsi	Jumlah Kabupaten/Kota	Blok Sensus					Rumah Tangga					Individu		
			Target	Di-kunjungi	*Respon Rate*	Wa-wancara	*Respon Rate*	Target	Di-kunjungi	*Respon Rate*	Wa-wancara	*Respon Rate*	Di-kunjungi	Wa-wancara	*Respon Rate*
(1)	(2)	(3)	(4)	(5)	(6) = (5)/(4)	(7)	(8)=(7)/(5)	(9)	(10)	(11)=(10)/(9)	(12)	(13)=(12)/(10)	(14)	(15)	(16)=(15)/(14)
			n	n	%	n	%	n	n	%	n	%	n	n	%
11	Aceh	23	1168	1168	100,00	1168	100,00	11680	11518	98,61	11061	96,03	44458	41596	93,56
12	Sumatera Utara	33	1896	1895	99,95	1895	100,00	18960	18799	99,15	18014	95,82	73525	69517	94,55
13	Sumatera Barat	19	1020	1020	100,00	1020	100,00	10200	10136	99,37	9764	96,33	39093	37063	94,81
14	Riau	12	764	764	100,00	764	100,00	7.640	7.563	98,99	7.134	94,33	28.532	26.778	93,85
15	Jambi	11	628	628	100,00	628	100,00	6.280	6.262	99,71	6.011	95,99	22.639	21.602	95,42
16	Sumatera Selatan	17	980	980	100,00	980	100,00	9.800	9.770	99,69	9.291	95,10	35.746	33.566	93,90
17	Bengkulu	10	512	512	100,00	512	100,00	5.120	5.105	99,71	4.909	96,16	18.275	17.419	95,32
18	Lampung	15	932	932	100,00	932	100,00	9.320	9.261	99,37	8.969	96,85	33.235	31.462	94,67
19	Bangka Belitung	7	360	360	100,00	360	100,00	3.600	3.534	98,17	3.483	98,56	12.914	12.567	97,31
21	Kepulauan Riau	7	360	360	100,00	360	100,00	3.600	3.520	97,78	3.302	93,81	12.388	11.698	94,43
31	DKI Jakarta	6	520	520	100,00	520	100,00	5.200	5.004	96,23	4.536	90,65	16.690	15.170	90,89
32	Jawa Barat	27	2.396	2.396	100,00	2.395	99,96	23.960	23.654	98,72	22.489	95,07	79.970	73.285	91,64
33	Jawa Tengah	35	2.752	2.752	100,00	2.752	100,00	27.520	27.374	99,47	26.842	98,06	96.450	91.161	94,52
34	DI Yogyakarta	5	376	376	100,00	376	100,00	3.760	3.747	99,65	3.564	95,12	11.916	11.319	94,99
35	Jawa Timur	38	2.996	2.996	100,00	2.996	100,00	29.960	29.823	99,54	29.228	98,00	102.159	98.566	96,48
36	Banten	8	676	676	100,00	676	100,00	6.760	6.680	98,82	6.327	94,72	25.380	23.262	91,65
51	Bali	9	576	576	100,00	576	100,00	5.760	5.758	99,97	5.559	96,54	21.090	20.560	97,49
52	Nusa Tenggara Barat	10	632	632	100,00	632	100,00	6.320	6.288	99,49	6.153	97,85	22.126	21.308	96,30
53	Nusa Tenggara Timur	22	1.088	1.088	100,00	1.088	100,00	10.880	10.788	99,15	10.620	98,44	47.762	44.782	93,76
61	Kalimantan Barat	14	804	804	100,00	804	100,00	8.040	7.990	99,38	7.582	94,89	30.379	28.343	93,30
62	Kalimantan Tengah	14	692	692	100,00	692	100,00	6.920	6.836	98,79	6.407	93,72	24.130	22.092	91,55
63	Kalimantan Selatan	13	752	752	100,00	752	100,00	7.520	7.484	99,52	7.156	95,62	25.145	23.915	95,11
64	Kalimantan Timur	10	524	524	100,00	524	100,00	5.240	5.206	99,35	4.801	92,22	19.205	17.490	91,07
65	Kalimantan Utara	5	208	208	100,00	208	100,00	2.080	2.032	97,69	1.917	94,34	8.248	7.574	91,83
71	Sulawesi Utara	15	740	740	100,00	740	100,00	7.400	7.395	99,93	7.089	95,86	27.000	25.661	95,04
72	Sulawesi Tengah	13	620	620	100,00	620	100,00	6.200	6.146	99,13	5.828	94,83	23.585	21.904	92,87
73	Sulawesi Selatan	24	1.384	1.384	100,00	1.384	100,00	13.840	13.811	99,79	13.356	96,71	54.316	50.127	92,29
74	Sulawesi Tenggara	17	616	616	100,00	616	100,00	6.160	6.152	99,87	5.931	96,41	24.670	22.982	93,16
75	Gorontalo	6	304	304	100,00	304	100,00	3.040	3.026	99,54	2.910	96,17	11.832	10.997	92,94
76	Sulawesi Barat	6	296	296	100,00	296	100,00	2.960	2.910	98,31	2.784	95,67	11.765	10.843	92,16
81	Maluku	11	496	496	100,00	496	100,00	4.960	4.910	98,99	4.598	93,65	21.944	19.019	98,99
82	Maluku Utara	10	416	416	100,00	416	100,00	4.160	4.124	99,13	3.834	92,97	17.496	15.381	87,91
91	Papua Barat	13	412	411	99,76	411	100,00	4.120	4.064	98,64	3.505	86,25	16.158	13.656	84,52
94	Papua	29	1.104	930	84,24	928	99,78	11.040	9.050	81,97	7.700	85,08	31.307	24.625	78,66
	**INDONESIA**	**514**	**30.000**	**29.824**	**99,41**	**29.821**	**99,99**	**300.000**	**295.720**	**98,57**	**282.654**	**95,58**	**1.091.528**	**1.017.290**	**93,20**

Tabel L.1. 2
Jumlah Sampel Hasil Wawancara, Riskesdas 2018

Provinsi	Jumlah Sampel					
	0-59 BLN	0-23 BLN	12-23 BLN	>10 TAHUN	>15 TAHUN	HAMIL
Aceh	4.393	1.780	886	32.496	28.316	440
Sumatera Utara	6.668	2.401	1.222	54.512	45.972	555
Sumatera Barat	3.493	1.340	678	29.584	25.532	347
Riau	2.696	1.022	502	20.972	17.965	264
Jambi	1.936	774	404	17.540	15.469	166
Sumatera Selatan	3.000	1.127	590	27.130	23.688	299
Bengkulu	1.572	607	293	14.049	12.322	149
Lampung	2.963	1.158	572	25.286	22.345	301
Kep. Bangka Belitung	1.095	408	212	10.208	8.971	124
Kep. Riau	1.130	430	223	9.298	8.060	112
Dki Jakarta	1.240	470	240	12.600	11.226	143
Jawa Barat	6.611	2.507	1.248	59.571	52.511	651
Jawa Tengah	7.364	2.856	1.356	75.804	67.977	733
Di Yogyakarta	731	270	151	9.712	8.848	73
Jawa Timur	7.275	2.880	1.437	83.171	75.490	761
Banten	2.185	812	382	18.749	16.452	208
BALI	1.470	561	276	17.337	15.591	170
NTB	2.149	849	418	16.936	14.825	224
NTT	4.824	1.845	921	34.521	28.430	395
Kalimantan Barat	2.616	1.029	525	22.703	19.738	228
Kalimantan Tengah	2.086	813	410	17.621	15.281	189
Kalimantan Selatan	2.087	871	443	19.441	17.160	222
Kalimantan Timur	1.737	693	363	13.747	11.919	182
Kalimantan Utara	862	344	170	5.859	5.005	75
Sulawesi Utara	1.989	755	372	21.408	18.890	165
Sulawesi Tengah	2.121	893	466	17.495	14.918	219
Sulawesi Selatan	4.543	1.807	900	40.466	34.958	434
Sulawesi Tenggara	2.485	1.035	499	17.815	15.006	255
Gorontalo	974	372	197	8.953	7.792	84
Sulawesi Barat	1.080	443	217	8.544	7.109	112
Maluku	2.112	819	402	14.488	11.991	165
Maluku Utara	1.614	597	317	11.918	9.907	156
Papua Barat	1.774	683	336	10.080	8.374	125
Papua	2.745	1.008	537	18.493	15.745	176
**INDONESIA**	**93.620**	**36.259**	**18.165**	**818.507**	**713.783**	**8.902**

Tabel L.1. 3

Respon Rate Sampel Pemeriksaan Darah menurut Provinsi, Riskesdas 2018

No	Provinsi	Blok Sensus			Rumah Tangga		
		Target	Kunjungan	*Respon Rate*	Target	Kunjungan	*Respon Rate*
(1)	(2)	(3)	(4)	(5) = (4)/(3)	(6)	(7)	(8)=(7)/(6)
		n	n	%	n	n	%
1	ACEH	21	21	100,00	210	150	71,4
2	SUMUT	21	21	100,00	210	168	80,0
3	SUMBAR	23	23	100,00	230	204	88,7
4	RIAU	36	36	100,00	360	273	75,8
5	SUMSEL	65	65	100,00	650	473	72,8
6	BENGKULU	3	3	100,00	30	23	76,7
7	LAMPUNG	38	38	100,00	380	328	86,3
8	DKI	205	205	100,00	2.050	1.263	61,6
9	JABAR	761	760	99,87	7.600	5.831	76,7
10	JATENG	369	369	100,00	3.690	3.172	86,0
11	DIY	23	23	100,00	230	178	77,4
12	JATIM	559	558	99,82	5.580	4.480	80,3
13	BANTEN	125	125	100,00	1.250	956	76,5
14	BALI	25	25	100,00	250	139	55,6
15	NTB	50	50	100,00	500	433	86,6
16	NTT	5	5	100,00	50	47	94,0
17	KALBAR	10	10	100,00	100	88	88,0
18	KALSEL	25	25	100,00	250	177	70,8
19	KALTIM	30	30	100,00	300	222	74,0
20	SULUT	11	11	100,00	110	84	76,4
21	SULTENG	9	9	100,00	90	80	88,9
22	SULSEL	73	73	100,00	730	535	73,3
23	SULTRA	1	1	100,00	10	9	90,0
24	MALUKU	7	7	100,00	70	60	85,7
25	MALUT	1	1	100,00	10	10	100,0
26	PAPUA	4	4	100,00	40	35	87,5
	**Total**	**2.500**	**2.498**	**99,92**	**24.980**	**19.418**	**77,7**

Tabel L.1. 4

Respon Rate Sampel Pemeriksaan Gigi dan Mulut menurut Provinsi, Riskesdas 2018

No	Provinsi	Blok Sensus			Rumah Tangga		
		Target	Kunjungan	*Respon Rate*	Target	Kunjungan	*Respon Rate*
(1)	(2)	(3)	(4)	(5) = (4)/(3)	(6)	(7)	(8)=(7)/(6)
		n	n	%	n	n	%
1	ACEH	21	21	100,00	210	155	73,8
2	SUMUT	21	21	100,00	210	174	82,9
3	SUMBAR	23	23	100,00	230	205	89,1
4	RIAU	36	36	100,00	360	281	78,1
5	SUMSEL	65	65	100,00	650	475	73,1
6	BENGKULU	3	3	100,00	30	24	80,0
7	LAMPUNG	38	38	100,00	380	332	87,4
8	DKI	205	205	100,00	2.050	1273	62,1
9	JABAR	761	760	99,87	7.600	5830	76,7
10	JATENG	369	369	100,00	3.690	3198	86,7
11	DIY	23	23	100,00	230	179	77,8
12	JATIM	559	559	100,00	5.590	4521	80,9
13	BANTEN	125	125	100,00	1.250	972	77,8
14	BALI	25	25	100,00	250	142	56,8
15	NTB	50	50	100,00	500	436	87,2
16	NTT	5	5	100,00	50	50	100,0
17	KALBAR	10	10	100,00	100	89	89,0
18	KALSEL	25	25	100,00	250	178	71,2
19	KALTIM	30	30	100,00	300	223	74,3
20	SULUT	11	11	100,00	110	84	76,4
21	SULTENG	9	9	100,00	90	82	91,1
22	SULSEL	73	73	100,00	730	536	73,4
23	SULTRA	1	1	100,00	10	9	90,0
24	MALUKU	7	7	100,00	70	60	85,7
25	MALUT	1	1	100,00	10	10	100,0
26	PAPUA	4	4	100,00	40	35	87,5
	**Total**	**2.500**	**2.499**	**99,96**	**24.990**	**19.553**	**78,2**

Lampiran 2
Kuesioner Rumah Tangga

KEMENTERIAN KESEHATAN REPUBLIK INDONESIA

KEMENTERIAN KESEHATAN REPUBLIK INDONESIA
BADAN PENELITIAN DAN PENGEMBANGAN KESEHATAN

RISKESDAS 2018

RISET KESEHATAN DASAR 2018

RAHASIA	PERTANYAAN RUMAH TANGGA	RKD18. RT

I. PENGENALAN TEMPAT

NO 1-9 SALIN DARI BLOK I VSEN18.K

1	Provinsi		☐☐
2	Kabupaten/Kota*)		☐☐
3	Kecamatan		☐☐☐
4	Desa/Kelurahan*)		☐☐☐
5	Klasifikasi Desa/Kelurahan	1. Perkotaan 2. Perdesaan	☐
6	Nomor Blok Sensus		
7	Nomor Kode Sampel		☐☐☐☐☐
8	No. Urut Sampel Rumah Tangga		☐☐
9	Nama Kepala Rumah Tangga		
10	Alamat rumah		
11	Terpilih sampel biomedis	1. Ya 2. Tidak	☐

*) coret yang tidak perlu

II. KETERANGAN PENGUMPUL DATA

1	Nama Pengumpul Data:		5.	Nama Ketua Tim:
2	Tanggal Pengumpulan data: (tgl-bln)	☐☐-☐☐-2018		
3	Tanda tangan Pengumpul Data		6.	Tanggal. Pengecekan: (tgl-bln) : ☐☐-☐☐-2018
4	Hasil pengumpulan data<br>1. Semua ART dapat diwawancarai<br>2. Tidak semua/ sebagian ART yang dapat diwawancarai<br>3. Semua ART tidak dapat diwawancarai sampai akhir pencacahan<br>4. Rumah Tangga sampel Susenas menolak<br>5. Rumah Tangga sampel Susenas pindah<br>6. Bangunan Sensus atau Blok Sensus sampel Susenas sudah tidak ada	☐	7.	Tanda tangan Ketua Tim

III. KETERANGAN RUMAH TANGGA

ISIKAN SESUAI KONDISI SAAT WAWANCARA RISKESDAS

1	Banyaknya Anggota Rumah Tangga:		☐☐
2	Banyaknya balita (0-59 bulan)		☐☐
3	Banyaknya Anggota Rumah Tangga yang diwawancarai:		☐☐
	**KETERANGAN KONDISI BANGUNAN SENSUS**		
4	Banyaknya **Rumah Tangga** dalam **Bangunan Sensus**		☐
5	Banyaknya **orang** dalam **Bangunan Sensus**		☐☐

HAL 1 dari 8 KUESIONER RUTA RISKESDAS 2018(2)

IV. KETERANGAN ANGGOTA RUMAH TANGGA

No. urut ART	Nama Anggota Rumah Tangga (ART)	Hubungan dengan kepala rumah tangga [KODE]	Jenis Kelamin 1. Laki 2. Perem-puan	Verifikasi: Status 1. Perubahan 2. Tidak 3. Meninggal 4. ART pindah 5. Lahir 6. ART baru	Verifikasi: Hubungan dengan kepala rumah tangga	Verifikasi: Jenis Kelamin 1. Laki 2. Perem-puan	Status Kawin [KODE]	Tanggal Lahir	Umur Jika umur < 1 bln isikan dalam kotak"Hari" Jika umur < 5 thn isikan dlm kotak"Bulan" Jika umur ≥ 5 thn isikan dlm kotak "Tahun"	Khusus ART >5 tahun Status Pendidikan tertinggi yang ditamatkan [KODE]	Khusus ART ≥ 10 tahun Status Pekerjaan [KODE]	ART diwawancarai? 1.Ya 2.Ya, didampingi 3.Ya, diwakili 4.Tidak
(1)	(2)	(3)	(4)	(5)	(6)	(7)	(8)	(9)	(10)	(11)	(12)	(13)
1		☐☐	☐	☐	☐☐	☐	☐	Tgl: ☐☐<br>Bln: ☐☐<br>Thn: ☐☐☐☐	a. ☐☐ Hr<br>b. ☐☐ Bln<br>c. ☐☐☐ Thn	☐	☐	☐
2		☐☐	☐	☐	☐☐	☐	☐	Tgl: ☐☐<br>Bln: ☐☐<br>Thn: ☐☐☐☐	a. ☐☐ Hr<br>b. ☐☐ Bln<br>c. ☐☐☐ Thn	☐	☐	☐
3		☐☐	☐	☐	☐☐	☐	☐	Tgl: ☐☐<br>Bln: ☐☐<br>Thn: ☐☐☐☐	a. ☐☐ Hr<br>b. ☐☐ Bln<br>c. ☐☐☐ Thn	☐	☐	☐
4		☐☐	☐	☐	☐☐	☐	☐	Tgl: ☐☐<br>Bln: ☐☐<br>Thn: ☐☐☐☐	a. ☐☐ Hr<br>b. ☐☐ Bln<br>c. ☐☐☐ Thn	☐	☐	☐

GUNAKAN HALAMAN 3 APABILA JUMLAH ART > 4 ORANG

Kode kolom (3) dan (6): Hubungan dgn kepala rumah tangga	Kode kolom (8):Status Kawin	Kode kolom (11): Pendidikan Tertinggi	Kode Kolom (12): Status Pekerjaan
01= Kepala RT<br>02= Istri/suami<br>03= Anak kandung<br>04= Anak angkat/tiri<br>05= Menantu<br>06= Cucu<br>07= Orang tua/ mertua<br>08= Pembantu rumah tangga/ sopir<br>09= Famili lain<br>10= Lainnya	1= Belum Kawin<br>2= Kawin<br>3= Cerai hidup<br>4= Cerai mati	1= Tidak/ belum pernah sekolah<br>2= Tidak tamat SD/MI<br>3= Tamat SD/MI<br>4= Tamat SLTP/MTS<br>5= Tamat SLTA/MA<br>6= Tamat D1/D2/D3<br>7= Tamat PT	1= Tidak bekerja<br>2= Sekolah<br>3= PNS/ TNI/ Polri/ BUMN/ BUMD<br>4= Pegawai swasta<br>5= Wiraswasta<br>6= Petani/buruh tani<br>7= Nelayan<br>8= Buruh/ sopir/ pembantu ruta<br>9= Lainnya

HAL 2 dari 8 KUESIONER RUTA RISKESDAS 2018(2)

IV. KETERANGAN ANGGOTA RUMAH TANGGA

No. urut ART	Nama Anggota Rumah Tangga (ART)	Hubungan dengan kepala rumah tangga [KODE]	Jenis Kelamin 1. Laki 2. Perem-puan	Verifikasi: Status 1. Perubahan 2. Tidak 3. Meninggal 4. ART pindah 5. Lahir 6. ART baru	Verifikasi: Hubungan dengan kepala rumah tangga	Verifikasi: Jenis Kelamin 1. Laki 2. Perem-puan	Status Kawin [KODE]	Tanggal Lahir	Umur Jika umur < 1 bln isikan dalam kotak "Hari" Jika umur < 5 thn isikan dlm kotak "Bulan" Jika umur ≥ 5 thn isikan dlm kotak "Tahun"	Khusus ART >5 tahun Status Pendidikan tertinggi yang ditamatkan [KODE]	Khusus ART ≥ 10 tahun Status Pekerjaan [KODE]	ART diwawancarai? 1.Ya 2.Ya, didampingi 3.Ya, diwakili 4.Tidak
(1)	(2)	(3)	(4)	(5)	(6)	(7)	(8)	(9)	(10)	(11)	(12)	(13)
5		□□	□	□	□□	□	□	Tgl: □□ Bln: □□ Thn: □□□□	a. □□ Hr b. □□ Bln c. □□□ Thn	□	□	□
6		□□	□	□	□□	□	□	Tgl: □□ Bln: □□ Thn: □□□□	a. □□ Hr b. □□ Bln c. □□□ Thn	□	□	□
7		□□	□	□	□□	□	□	Tgl: □□ Bln: □□ Thn: □□□□	a. □□ Hr b. □□ Bln c. □□□ Thn	□	□	□
8		□□	□	□	□□	□	□	Tgl: □□ Bln: □□ Thn: □□□□	a. □□ Hr b. □□ Bln c. □□□ Thn	□	□	□

GUNAKAN HALAMAN 4 APABILA JUMLAH ART > 8 ORANG

Kode kolom (3) dan (6): Hubungan dgn kepala rumah tangga
01= Kepala RT
02= Istri/suami
03= Anak kandung
04= Anak angkat/tiri
05= Menantu
06= Cucu
07= Orang tua/ mertua
08= Pembantu rumah tangga/ sopir
09= Famili lain
10= Lainnya

Kode kolom (8): Status Kawin
1= Belum Kawin
2= Kawin
3= Cerai hidup
4= Cerai mati

Kode kolom (11): Pendidikan Tertinggi
1= Tidak/ belum pernah sekolah
2= Tidak tamat SD/MI
3= Tamat SD/MI
4= Tamat SLTP/MTS
5= Tamat SLTA/MA
6= Tamat D1/D2/D3
7= Tamat PT

Kode Kolom (12): Status Pekerjaan
1= Tidak bekerja
2= Sekolah
3= PNS/ TNI/ Polri/ BUMN/ BUMD
4= Pegawai swasta
5= Wiraswasta
6= Petani/buruh tani
7= Nelayan
8= Buruh/ sopir/ pembantu ruta
9= Lainnya

HAL 3 dari 8 KUESIONER RUTA RISKESDAS 2018(2)

IV. KETERANGAN ANGGOTA RUMAH TANGGA

No. urut ART	Nama Anggota Rumah Tangga (ART)	Hubungan dengan kepala rumah tangga [KODE]	Jenis Kelamin 1. Laki 2. Perem-puan	Verifikasi			Status Kawin [KODE]	Tanggal Lahir	Umur Jika umur < 1 bln isikan dalam kotak"Hari" Jika umur < 5 thn isikan dlm kotak"Bulan" Jika umur ≥ 5 thn isikan dlm kotak "Tahun"	Khusus ART >5 tahun Status Pendidikan tertinggi yang ditamatkan [KODE]	Khusus ART ≥ 10 tahun Status Pekerjaan [KODE]	ART diwawancarai? 1.Ya 2.Ya, didampingi 3.Ya, diwakili 4.Tidak
				Status 1. Perubahan 2. Tidak 3. Meninggal 4. ART pindah 5. Lahir 6. ART baru	Hubungan dengan kepala rumah tangga	Jenis Kelamin 1. Laki 2. Perem-puan						
(1)	(2)	(3)	(4)	(5)	(6)	(7)	(8)	(9)	(10)	(11)	(12)	(13)
9		☐☐	☐	☐	☐☐	☐	☐	Tgl: ☐☐ Bln: ☐☐ Thn: ☐☐☐☐	a. ☐☐ Hr b. ☐☐ Bln c. ☐☐☐ Thn	☐	☐	☐
10		☐☐	☐	☐	☐☐	☐	☐	Tgl: ☐☐ Bln: ☐☐ Thn: ☐☐☐☐	a. ☐☐ Hr b. ☐☐ Bln c. ☐☐☐ Thn	☐	☐	☐
11		☐☐	☐	☐	☐☐	☐	☐	Tgl: ☐☐ Bln: ☐☐ Thn: ☐☐☐☐	a. ☐☐ Hr b. ☐☐ Bln c. ☐☐☐ Thn	☐	☐	☐
12		☐☐	☐	☐	☐☐	☐	☐	Tgl: ☐☐ Bln: ☐☐ Thn: ☐☐☐☐	a. ☐☐ Hr b. ☐☐ Bln c. ☐☐☐ Thn	☐	☐	☐

GUNAKAN HALAMAN 5 APABILA JUMLAH ART > 12 ORANG

Kode kolom (3) dan (6): Hubungan dgn kepala rumah tangga	Kode kolom (8):Status Kawin	Kode kolom (11): Pendidikan Tertinggi	Kode Kolom (12): Status Pekerjaan
01= Kepala RT<br>02= Istri/suami<br>03= Anak kandung<br>04= Anak angkat/tiri<br>05= Menantu<br>06= Cucu<br>07= Orang tua/ mertua<br>08= Pembantu rumah tangga/ sopir<br>09= Famili lain<br>10= Lainnya	1= Belum Kawin<br>2= Kawin<br>3= Cerai hidup<br>4= Cerai mati	1= Tidak/ belum pernah sekolah<br>2= Tidak tamat SD/MI<br>3= Tamat SD/MI<br>4= Tamat SLTP/MTS<br>5= Tamat SLTA/MA<br>6= Tamat D1/D2/D3<br>7= Tamat PT	1= Tidak bekerja<br>2= Sekolah<br>3= PNS/ TNI/ Polri/ BUMN/ BUMD<br>4= Pegawai swasta<br>5= Wiraswasta<br>6= Petani/buruh tani<br>7= Nelayan<br>8= Buruh/ sopir/ pembantu ruta<br>9= Lainnya

HAL 4 dari 8 KUESIONER RUTA RISKESDAS 2018(2)

IV. KETERANGAN ANGGOTA RUMAH TANGGA

No. urut ART	Nama Anggota Rumah Tangga (ART)	Hubungan dengan kepala rumah tangga [KODE]	Jenis Kelamin 1. Laki 2. Perem-puan	Verifikasi: Status 1. Perubahan 2. Tidak 3. Meninggal 4. ART pindah 5. Lahir 6. ART baru	Verifikasi: Hubungan dengan kepala rumah tangga	Verifikasi: Jenis Kelamin 1. Laki 2. Perem-puan	Status Kawin [KODE]	Tanggal Lahir	Umur Jika umur < 1 bln isikan dalam kotak "Hari" Jika umur < 5 thn isikan dlm kotak "Bulan" Jika umur ≥ 5 thn isikan dlm kotak "Tahun"	Khusus ART >5 tahun Status Pendidikan tertinggi yang ditamatkan [KODE]	Khusus ART ≥ 10 tahun Status Pekerjaan [KODE]	ART diwawancarai? 1.Ya 2.Ya, didampingi 3.Ya, diwakili 4.Tidak
(1)	(2)	(3)	(4)	(5)	(6)	(7)	(8)	(9)	(10)	(11)	(12)	(13)
13		☐☐	☐	☐	☐☐	☐	☐	Tgl: ☐☐ Bln: ☐☐ Thn: ☐☐☐☐	a. ☐☐ Hr b. ☐☐ Bln c. ☐☐☐ Thn	☐	☐	☐
14		☐☐	☐	☐	☐☐	☐	☐	Tgl: ☐☐ Bln: ☐☐ Thn: ☐☐☐☐	a. ☐☐ Hr b. ☐☐ Bln c. ☐☐☐ Thn	☐	☐	☐
15		☐☐	☐	☐	☐☐	☐	☐	Tgl: ☐☐ Bln: ☐☐ Thn: ☐☐☐☐	a. ☐☐ Hr b. ☐☐ Bln c. ☐☐☐ Thn	☐	☐	☐
16		☐☐	☐	☐	☐☐	☐	☐	Tgl: ☐☐ Bln: ☐☐ Thn: ☐☐☐☐	a. ☐☐ Hr b. ☐☐ Bln c. ☐☐☐ Thn	☐	☐	☐

Kode kolom (3) dan (6): Hubungan dgn kepala rumah tangga
01= Kepala RT 02= Istri/suami 03= Anak kandung 04= Anak angkat/tiri 05= Menantu 06= Cucu 07= Orang tua/ mertua 08= Pembantu rumah tangga/ sopir 09= Famili lain 10= Lainnya

Kode kolom (8):Status Kawin
1= Belum Kawin 2= Kawin 3= Cerai hidup 4= Cerai mati

Kode kolom (11): Pendidikan Tertinggi
1= Tidak/ belum pernah sekolah 2= Tidak tamat SD/MI 3= Tamat SD/MI 4= Tamat SLTP/MTS 5= Tamat SLTA/MA 6= Tamat D1/D2/D3 7= Tamat PT

Kode Kolom (12): Status Pekerjaan
1= Tidak bekerja 2= Sekolah 3= PNS/ TNI/ Polri/ BUMN/ BUMD 4= Pegawai swasta 5= Wiraswasta 6= Petani/buruh tani 7= Nelayan 8= Buruh/ sopir/ pembantu ruta 9= Lainnya

HAL 5 dari 8 KUESIONER RUTA RISKESDAS 2018(2)

V. AKSES FASILITAS KESEHATAN			
Sekarang kami akan menanyakan jenis fasilitas kesehatan terdekat dan kemudahan akses untuk setiap pelayanan kesehatan tersebut: **(Pengertian dekat: bisa dalam satu atau beda kabupaten/kota, kecamatan, kelurahan, desa dimana rumah tangga berada)**			
1	Apakah **[RUMAH TANGGA]** mengetahui keberadaan rumah sakit yang terdekat?	1. Ada dalam kab/kota 2. Ada di kab/kota terdekat 3. Tidak ada → **V.6** 8. Tidak tahu → **V.6**	☐
2	Alat transportasi apa yang digunakan sekali jalan dari rumah ke rumah sakit? **Bila jawaban lebih dari 1 jumlahkan kode jawaban alat transportasi yang digunakan** 1.Kendaraan pribadi bermotor 2.Kendaraan umum bermotor 4. Kendaraan pribadi tidak bermotor 8. Kendaraan umum tidak bermotor 16. Jalan kaki 32.Transportasi air 64.Transportasi udara 128. Lainnya		☐☐☐
3	Berapa waktu tempuh dari rumah ke rumah sakit (sekali Jalan)?	Jam : Menit	☐☐:☐☐
4	Berapa jumlah uang (Rp) yang dikeluarkan untuk transportasi pulang-pergi?		☐.☐☐☐.☐☐☐
5	Apakah biaya transportasi tersebut terjangkau?	1. Terjangkau 2. Tidak terjangkau	☐
6	Apakah **[RUMAH TANGGA]** mengetahui keberadaan puskesmas/ pustu/ pusling/ bidan desa yang terdekat?	1. Ada dalam kab/kota 2. Ada di kab/kota terdekat 3. Tidak ada → **V.11** 8. Tidak tahu → **V.11**	☐
7	Alat transportasi apa yang digunakan sekali jalan dari rumah ke puskesmas/ pustu/ pusling/ bidan desa? **Bila jawaban lebih dari 1 jumlahkan kode jawaban alat transportasi yang digunakan** 1.Kendaraan pribadi bermotor 2.Kendaraan umum bermotor 4. Kendaraan pribadi tidak bermotor 8. Kendaraan umum tidak bermotor 16. Jalan kaki 32.Transportasi air 64.Transportasi udara 128. Lainnya		☐☐☐
8	Berapa waktu tempuh dari rumah ke puskesmas/ pustu/ pusling/ bidan desa (sekali Jalan)?	Jam : Menit	☐☐:☐☐
9	Berapa jumlah uang (Rp) yang dikeluarkan untuk transportasi pulang-pergi?		☐.☐☐☐.☐☐☐
10	Apakah biaya transportasi tersebut terjangkau?	1.Terjangkau 2. Tidak terjangkau	☐
11	Apakah **[RUMAH TANGGA]** mengetahui keberadaan klinik/ praktek dokter/ praktek dokter gigi/ praktek bidan mandiri yang terdekat?	1. Ada dalam kab/kota 2. Ada di kab/kota terdekat 3. Tidak ada → **VI** 8. Tidak tahu → **VI**	☐
12	Alat transportasi apa yang digunakan sekali jalan dari rumah ke klinik/ praktek dokter/ praktek dokter gigi/ praktek bidan mandiri? **Bila jawaban lebih dari 1 jumlahkan kode jawaban alat transportasi yang digunakan** 1.Kendaraan pribadi bermotor 2.Kendaraan umum bermotor 4. Kendaraan pribadi tidak bermotor 8. Kendaraan umum tidak bermotor 16. Jalan kaki 32.Transportasi air 64.Transportasi udara 128. Lainnya		☐☐☐
13	Berapa waktu tempuh dari rumah ke klinik/praktek dokter/ praktek dokter gigi/ praktek bidan mandiri (sekali Jalan)?	Jam : Menit	☐☐:☐☐
14	Berapa jumlah uang (Rp) yang dikeluarkan untuk transportasi pulang-pergi?		☐.☐☐☐.☐☐☐
15	Apakah biaya transportasi tersebut terjangkau?	1.Terjangkau 2. Tidak terjangkau	☐

VI. GANGGUAN JIWA DALAM RUMAH TANGGA			
1	Apakah ada Anggota Rumah Tangga yang pernah menderita gangguan jiwa?	1. Ya 2. Tidak → **BLOK VII**	☐
2	Apakah Anggota Rumah Tangga tersebut ada yang didiagnosa gangguan jiwa **Skizofrenia/ Psikosis oleh tenaga kesehatan**?	1. Ya 2. Tidak → **VI.7**	☐
3	Berapa Anggota Rumah Tangga yang mengalami hal tersebut?	------------- orang	☐☐
4	Apakah Anggota Rumah Tangga tersebut pernah berobat ke Rumah Sakit Jiwa/ fasilitas kesehatan/ tenaga kesehatan? 1. Ya, semua pernah berobat 2.Ya, tidak semua pernah berobat 3. Tidak ada yang berobat → **VI.7**		☐
5	Apakah 1 bulan terakhir ini Anggota Rumah Tangga tersebut minum obat rutin?	1. Ya → **VI.7** 2. Tidak	☐
6	Bila tidak, apa alasannya? **(POINT a-g DIBACAKAN) ISIKAN KODE JAWABAN 1=YA ATAU 2=TIDAK**		
	a. Sering lupa ☐	e. Tidak tahan efek samping obat	☐
	b. Tidak mampu membeli obat secara rutin ☐	f. Merasa dosis tidak sesuai	☐
	c. Obat tidak tersedia di fasilitas pelayanan kesehatan ☐	g. Merasa sudah sehat/ Tidak merasa sakit	☐
	d. Tidak rutin berobat ke fasilitas pelayanan kesehatan ☐	h. Lainnya, sebutkan__________________	☐
7	Apakah ada yang pernah dipasung/diasingkan/dikekang atau tindakan mirip dipasung?	1. Ya 2. Tidak → **BLOK VII**	☐
8	Apakah **dalam 3 bulan** ini ada yang dipasung?	1. Ya 2. Tidak	☐

HAL 6 dari 8 KUESIONER RUTA RISKESDAS 2018(2)

VII. KESEHATAN LINGKUNGAN

1	Dimana tempat pembuangan air limbah utama dari kamar mandi / tempat cuci dan dapur?		
	a. Kamar Mandi/ Tempat Cuci	1.Penampungan tertutup<br>2. Penampungan terbuka<br>3.Tanpa Penampungan (di tanah)<br>4.Langsung ke got/ kali/ sungai	☐
	b. Dapur	1.Penampungan tertutup<br>2. Penampungan terbuka<br>3.Tanpa Penampungan (di tanah)<br>4.Langsung ke got/ kali/ sungai	☐
2	Untuk rumah tangga yang memiliki balita, bagaimana cara pembuangan tinja balita?<br>1. Menggunakan jamban<br>2. Dibuang di jamban<br>3. Ditanam<br>4. Dibuang di sembarang tempat/ tempat sampah<br>5. Dibersihkan di sembarang tempat<br>6. Lainnya, sebutkan ....<br>7. Tidak **ada balita**		☐
3	Apa jenis tempat pengumpulan/ penampungan sampah basah (organik) di dalam rumah?<br>**(BACAKAN POINT a DAN b)**	a. Tempat sampah tertutup — 1.Ya 2. Tidak	☐
		b. tempat sampah terbuka — 1.Ya 2. Tidak	☐
4	Bagaimana cara utama dalam menangani sampah rumah tangga :	1. Diangkut petugas<br>2. Dibuang sendiri ke TPS<br>3. Ditimbun dalam tanah<br>4. Dibuat kompos<br>5. Dibakar<br>6. Dibuang ke kali/ parit/ laut<br>7. Dibuang sembarangan	☐
5	Apa yang biasa [RUMAH TANGGA] lakukan selama ini untuk mencegah penularan penyakit akibat gigitan nyamuk?<br>**(ISIKAN KODE JAWABAN: 1.YA ATAU 2.TIDAK) POIN a S/D f DIBACAKAN**		
	a. Memakai obat nyamuk (semprot/bakar/elektrik) ☐	d. Menguras bak mandi/ ember besar/ drum	☐
	b. Menaburkan bubuk larvasida pada tempat penampungan air ☐	e. Menutup tempat penampungan air di Rumah Tangga	☐
	c. Ventilasi rumah dipasang kasa nyamuk ☐	f. Memusnahkan barang-barang bekas (kaleng, ban, dll)	☐
6	Berapa kali [RUMAH TANGGA] menguras bak mandi/ember besar/ drum?<br>1. > 1 kali dalam seminggu<br>2. Satu kali dalam seminggu<br>3. 1-3 kali dalam sebulan<br>7. Tidak berlaku		☐
7	Lakukan observasi terhadap keadaan ruangan dalam rumah.		

Jenis Ruangan	Jendela<br>1. Ada, dibuka tiap hari;<br>2. Ada, jarang dibuka;<br>3.Tidak ada<br>7. Tidak berlaku/ tidak ada ruangan	Ventilasi<br>1=Ada, luasnya>=10% luas lantai;<br>2=Ada, luasnya <10% luas lantai;<br>3=Tidak ada	Pencahayaan<br>1=Cukup<br>2=Tidak cukup
	(a)	(b)	(c)
a.Kamar Tidur Utama	☐	☐	☐
b.Masak/dapur	☐	☐	☐
c.Ruang keluarga	☐	☐	☐

8	Apakah jenis sarana air utama yang digunakan rumah tangga untuk keperluan minum?<br>1. Air kemasan bermerk<br>2. Air isi ulang<br>3. Air ledeng/PDAM<br>4. Air ledeng eceran/membeli<br>5. Sumur bor/pompa<br>6.Sumur gali terlindung<br>7. Sumur gali tak terlindung<br>8. Mata air terlindung<br>9. Mata air tidak terlindung<br>10. Penampungan air hujan<br>11. Air permukaan (sungai/ danau/ irigasi<br>12. Lainnya, sebutkan	☐☐
9	Apakah jenis sarana air utama yang digunakan rumah tangga untuk keperluan masak, kebersihan pribadi dan mencuci (pakaian dan peralatan masak/makan)?<br>1. Air kemasan bermerk<br>2. Air isi ulang<br>3. Air ledeng/PDAM<br>4. Air ledeng eceran/membeli<br>5. Sumur bor/pompa<br>6. Sumur gali terlindung<br>7. Sumur gali tidak terlindung<br>8. Mata air terlindung<br>9. Mata air tidak terlindung<br>10. Penampungan air hujan<br>11. Air permukaan (sungai/ danau/ irigasi)<br>12. Lainnya, sebutkan	☐☐
10	Berapa jumlah pemakaian air (dalam liter) untuk keperluan minum, masak, mandi dan mencuci (pakaian dan peralatan masak/makan) seluruh anggota rumah tangga dalam sehari semalam? ....liter	☐☐☐☐

HAL 7 dari 8 KUESIONER RUTA RISKESDAS 2018(2)

CATATAN

LEMBAR BANTU UNTUK MENGHITUNG PEMAKAIAN AIR (BLOK VII-P.10)

No	Aktivitas	Ukuran	Jumlah Konsumsi								Total
			ART-1	ART-2	ART-3	ART-4	ART-5	ART-6	ART-7	ART-8	Σ (ART)
1	Minum										
	- Gelas	200 ml; 250 ml									
	- Teko	1 L									
2	Mandi										
	- Shower	20 ltr (5 menit) *									
	- Ember										
3	Masak										
	- Panci	D 20 cm, 1,5L									
4	Mencuci pakaian										
	- Mesin cuci	50 L (1x putar) *									
	- Ember										
	- Baskom										
5	Mencuci alat masak/makan										
	- Keran	10 L (5 menit)									
	- Ember										
	TOTAL										

Catatan :

1. Tanyakan berapa gelas setiap ART minum dalam 24 jam (gunakan gelas ukur standar)
2. Tanyakan berapa kali setiap ART mandi dalam 24 jam (menggunakan shower atau ember, tanyakan volume ember yg digunakan)
3. Tanyakan berapa kali RT masak dalam 24 jam
4. Tanyakan berapa kali RT mencuci pakaian dalam 24 jam (jika menggunakan mesin cuci tanyakan berapa kali mengisi air dalam mesin cuci, jika menggunakan ember/baskom tanyakan berapa ember/baskom yang digunakan)
5. Tanyakan berapa kali RT mencuci piring (yang utama). Jika menggunakan keran, tanyakan berapa menit lama mencuci, jika menggunakan ember tanyakan berapa ember yg digunakan.
6. Jika aktivitas mencuci atau masak dilakukan tidak setiap hari (misalnya 2 kali dalam seminggu), konversikan ke dalam hari.
   Konversi minggu menjadi hari : 2 kali per minggu = 2/7 atau 0,286 (per hari)
   Misalnya: RT A mencuci pakaian 3 kali dalam seminggu menggunakan mesin cuci, setiap kali mencuci perlu 4kali load/putar, sehingga pemakaian air untuk mencuci pakaian di RT A menjadi :
   = 4 load * 50 L/load * (3/7 per hari)
   = 85,7 L/hari

Lampiran 3
Kuesioner Individu

KEMENTERIAN KESEHATAN REPUBLIK INDONESIA
BADAN PENELITIAN DAN PENGEMBANGAN KESEHATAN

RISET KESEHATAN DASAR 2018

RAHASIA | **RKD18. IND**

PERTANYAAN INDIVIDU

VIII.PENGENALAN TEMPAT						
Prov	Kab/Kota	Kec	Desa/Kel	D/K	Nomor Kode Sampel	No.Urut RT

Kutip dari Blok I PENGENALAN TEMPAT RKD18.RT

IX.KETERANGAN WAWANCARA INDIVIDU					
1	Tanggal kunjungan pertama:Tgl -Bln	☐☐-☐☐	3	Nama Pengumpul data	
2	Tanggal kunjungan akhir: Tgl -Bln	☐☐-☐☐	4	Tanda tangan Pengumpul data	

X. KETERANGAN INDIVIDU			
IDENTIFIKASI RESPONDEN			
1	Tuliskan nama dan nomor urut Anggota Rumah Tangga (ART)	Nama ART	Nomor urut ART: ☐☐
NO URUT ART UNTUK PERTANYAAN P.2, P,3, P.4 JIKA BUKAN ART DALAM RUTA INI ISIKAN KODE '00'			
2	Tuliskan nama dan nomor urut Ayah kandung	Nama ART	Nomor urut ART: ☐☐
3	Tuliskan nama dan nomor urut Ibu kandung	Nama ART	Nomor urut ART: ☐☐
4	Untuk ART < 15 tahun/ kondisi sakit/ orang tua yang perlu didampingi, tuliskan nama dan nomor urut ART yang mendampingi	Nama ART	Nomor urut ART: ☐☐

A. PENYAKIT MENULAR			
[NAMA] pada pertanyaan di bawah ini merujuk pada NAMA yang tercatat pada pertanyaan Blok X P.1			
**INFEKSI SALURAN PERNAPASAN AKUT (ISPA) [ART SEMUA UMUR]**			
A01	**Dalam 1 bulan terakhir**, apakah [NAMA] pernah didiagnosis ISPA oleh tenaga kesehatan (dokter/ perawat/ bidan)?	1. Ya → **A03**<br>2. Tidak	☐
A02	Dalam 1 bulan terakhir [NAMA] mengalami gejala sebagai berikut:		
	a. Demam — 1. Ya 2. Tidak ☐	c. Pilek/ hidung tersumbat — 1. Ya 2. Tidak	☐
	b. Batuk kurang dari 2 minggu — 1. Ya 2. Tidak ☐	d. Sakit tenggorokan — 1. Ya 2. Tidak	☐
**PNEUMONIA/RADANG PARU [ART SEMUA UMUR]**			
A03	**Dalam 1 tahun terakhir**, apakah [NAMA] pernah didiagnosis menderita radang paru (Pneumonia) dengan atau tanpa **dilakukan foto dada (foto rontgen)** oleh tenaga kesehatan (dokter/ perawat/ bidan)?	1. Ya, kurang dari 1 bulan terakhir → **A05**<br>2. Ya,1 – 12 bulan yang lalu →**A05**<br>3. Tidak<br>4. Tidak tahu	☐
A04	**Dalam 1 tahun terakhir**, apakah [NAMA] mengalami gejala penyakit sebagai berikut:		
	a. Demam tinggi	1.Ya 2.Tidak	☐
	b. Batuk	1.Ya 2.Tidak	☐
	c. Kesulitan bernafas dengan atau tanpa nyeri dada	1.Ya 2.Tidak	☐
**ART UMUR 5 TAHUN KE ATAS → KE A05**			
	**POIN d,e,f UNTUK ART UMUR 0 – 59 BLN**		
	d. Nafas cepat	1.Ya 2.Tidak	☐
	e. Nafas cuping hidung	1.Ya 2.Tidak	☐
	f. Tarikan dinding dada bagian bawah ke dalam **(PERLIHATKAN GAMBAR PERAGA)**	1.Ya 2.Tidak	☐

HAL 1 dari 24 - KUESIONER INDIVIDU RISKESDAS 2018 (2)

TUBERKULOSIS PARU (TB PARU) [ART SEMUA UMUR]			
A05	**Dalam 1 tahun terakhir**, apakah [NAMA] pernah didiagnosis TB Paru oleh dokter/ perawat/ bidan?	1. Ya, dalam 6 bulan terakhir<br>2. Ya, lebih dari 6 bulan<br>3. Tidak → **A12**	☐
A06	Pemeriksaan apa yang digunakan untuk menegakkan diagnosis tersebut?		
	a. Pemeriksaan tuberkulin/ mantoux **[KHUSUS ART UMUR ≤ 15 TAHUN]**	1. Ya 2. Tidak	☐
	b. Pemeriksaan dahak **[ART SEMUA UMUR]**	1. Ya 2. Tidak	☐
	c. Pemeriksaan foto dada/ rontgen **[ART SEMUA UMUR]**	1. Ya 2. Tidak	☐
A07	Apakah mendapat obat TB Kombinasi Dosis Tetap (KDT)? **(PERLIHATKAN GAMBAR PERAGA)**	1. Ya 2. Tidak	☐
A08	Apakah mendapat obat TB lepasan? **(PERLIHATKAN GAMBAR PERAGA)**	1. Ya 2. Tidak	☐
A09	Apakah ada anggota keluarga atau lainnya yang bertugas sebagai Pengawas Minum Obat (PMO)?	1. Ya 2. Tidak	☐

JIKA ART DIDIAGNOSIS TB PARU > 6 BULAN (A05 = 2) → A12
JIKA ART DIDIAGNOSIS TB PARU DALAM 6 BULAN TERAKHIR (A05 = 1) → A10

A10	Apakah saat ini masih menggunakan obat TB secara rutin (diagnosis dalam 6 bulan terakhir)	1. Ya→**A12** 2. Tidak	☐
A11	Mengapa tidak minum obat secara rutin **(DIBACAKAN) ISIKAN KODE JAWABAN: 1.YA ATAU 2.TIDAK**		

a. Sering lupa	☐	e. Tidak mampu membeli obat TB secara rutin	☐
b. Obat tidak tersedia di fasilitas pelayanan kesehatan	☐	f. Tidak rutin berobat ke fasilitas pelayanan kesehatan	☐
c. Tidak tahan efek samping	☐	g. Merasa sudah sehat	☐
d. Masa pengobatan terasa lama	☐	h. Lainnya, sebutkan	☐

HEPATITIS/ SAKIT LIVER/ SAKIT KUNING [ART SEMUA UMUR]			
A12	**Dalam 1 tahun terakhir**, apakah [NAMA] pernah didiagnosis menderita Hepatitis melalui pemeriksaan darah oleh dokter?	1. Ya 2. Tidak	☐

DIARE/ MENCRET [ART SEMUA UMUR]			
A13	**Dalam 1 bulan terakhir**, apakah [NAMA] pernah didiagnosis menderita diare oleh tenaga kesehatan (dokter/perawat/ bidan)?<br>1. Ya, dalam ≤ 2 minggu terakhir→**A15** 2. Ya, > 2 minggu – 1 bulan→**A15** 3. Tidak 8.Tidak tahu		☐
A14	**Dalam 1 bulan terakhir**, apakah [NAMA] pernah mengalami:		
	a. Buang Air Besar (BAB) 3 – 6 kali sehari	1. Ya, dalam ≤ 2 minggu terakhir<br>2. Ya, > 2 minggu – 1 bulan<br>3. Tidak<br>8.Tidak tahu	☐
	b. BAB > 6 kali sehari	1. Ya, dalam ≤ 2 minggu terakhir<br>2. Ya, > 2 minggu – 1 bulan<br>3. Tidak<br>8.Tidak tahu	☐
	c.Kotoran/ tinja lembek atau cair	1. Ya, dalam ≤ 2 minggu terakhir<br>2. Ya, > 2 minggu – 1 bulan<br>3. Tidak<br>8.Tidak tahu	☐

JIKA JAWABAN A13 DAN A14 SEMUA BERKODE "3" ATAU "8", LANJUT KE A16

A15	Apakah [NAMA] minum obat untuk penyakit/ keluhan diare tersebut?		
	a. Oralit/ Larutan Gula Garam (LGG)	1. Ya 2. Tidak 8. Tidak Tahu	☐
	b. Obat anti diare	1. Ya 2. Tidak 8. Tidak Tahu	☐
	c. Antibiotik	1. Ya 2. Tidak 8. Tidak Tahu	☐
	d. Obat herbal/ tradisional	1. Ya 2. Tidak 8. Tidak Tahu	☐
	e. Obat Zinc **(Khusus untuk balita) PERLIHATKAN GAMBAR PERAGA**	1. Ya 2. Tidak 8. Tidak Tahu	☐

MALARIA [ART SEMUA UMUR]			
A16	**Dalam 1 tahun terakhir**, apakah [NAMA] pernah diambil darah untuk pemeriksaan malaria oleh tenaga kesehatan (dokter/ perawat/ bidan)?	1. Ya, < 1 bulan terakhir<br>2. Ya, 1 – 12 bulan<br>3. Tidak → **A19**	☐
A17	Apakah [NAMA] dinyatakan positif menderita malaria setelah pemeriksaan tersebut oleh tenaga kesehatan (dokter/ perawat/ bidan)?	1. Ya<br>2. Tidak→**A19**	☐

HAL 2 dari 24 – KUESIONER INDIVIDU RISKESDAS 2018 (2)

A18	Apakah [NAMA] diberi obat malaria sebagai berikut? **(PERLIHATKAN GAMBAR PERAGA)**		
	a. Artemisinin(ACT) 3 hari + Primaquin 1 hari	1.Ya 2.Tidak	☐
	b. Artemisinin(ACT) 3 hari + Primaquin 14 hari	1.Ya 2.Tidak	☐
	c. Obat lain, sebutkan............	1. Ya 2.Tidak	☐
**FILARIASIS/ KAKI GAJAH [ART SEMUA UMUR]**			
A19	Apakah [NAMA] pernah diberikan obat pencegahan filariasis (diethylcarbamazine citrate dan albendasol) oleh petugas kesehatan? **(PERLIHATKAN GAMBAR PERAGA)**	1. Ya 2.Tidak	☐
A20	Apakah [NAMA] pernah didiagnosis menderita kaki gajah (filariasis) oleh tenaga kesehatan (dokter/perawat/bidan)?	1. Ya, sebelum tahun 2017 2. Ya, pada tahun 2017 3. Ya, pada tahun 2018 4. Tidak → **BLOK B**	☐
A21	Apakah [NAMA] minum obat sesuai dengan anjuran tenaga kesehatan (dokter/perawat/bidan)?	1. Ya 2.Tidak	☐
**B. PENYAKIT TIDAK MENULAR**			
**ASMA/ MENGI/ BENGEK [ART SEMUA UMUR]**			
B01	Apakah [NAMA] pernah didiagnosis asma oleh dokter?	1. Ya 2. Tidak→ **B04**	☐
B02	Umur berapa pertama kali didiagnosis asma? **(ISIKAN "98" JIKA TIDAK INGAT)**	..............tahun	☐☐
B03	Apakah asma [NAMA] pernah kambuh dalam 12 bulan terakhir?	1. Ya 2. Tidak	☐
**KANKER [ART SEMUA UMUR]**			
B04	Apakah [NAMA] pernah didiagnosis menderita penyakit kanker oleh dokter?	1.Ya 2.Tidak → **B06**	☐
B05	Apakah [NAMA] telah menjalani pengobatan kanker seperti di bawah ini :		
	a.pembedahan/operasi	1. Ya 2. Tidak	☐
	b.Radiasi/penyinaran	1. Ya 2. Tidak	☐
	c.Kemoterapi	1. Ya 2. Tidak	☐
	d. Lainnya, Sebutkan	1. Ya 2. Tidak	☐
**DIABETES MELLITUS/ KENCING MANIS [ART SEMUA UMUR]**			
B06	Apakah [NAMA] pernah didiagnosis diabetes mellitus/ kencing manis oleh dokter?	1.Ya 2.Tidak →**B12**	☐
B07	Umur berapa pertama kali didiagnosis diabetes mellitus/ kencing manis? **(ISIKAN "98" JIKA TIDAK INGAT)**	.........tahun	☐☐
B08	Jenis pengobatan apa yang diperoleh [NAMA]? 1. Obat Anti DM (OAD) dari tenaga medis 2. Injeksi insulin 3.Obat Anti DM (OAD) dari tenaga medis dan Injeksi insulin 4.Tidak diobati → **B11**		☐
B09	Apakah [NAMA] minum/suntik obat anti diabetes sesuai petunjuk dokter?	1.Ya, sesuai petunjuk dokter → **B11** 2. Tidak sesuai petunjuk dokter	☐
B10	Mengapa [NAMA] tidak minum obat sesuai petunjuk dokter? **(ISIKAN KODE JAWABAN: 1.YA ATAU 2.TIDAK) POIN a s/d g DIBACAKAN**		
	a. Sering lupa		☐
	b. Obat tidak tersedia di fasyankes (RS/Puskesmas/Apotek)		☐
	c. Minum obat tradisional		☐
	d. Tidak tahan efek samping obat		☐
	e.Tidak mampu membeli obat		☐
	f. Tidak rutin berobat ke fasilitas pelayanan kesehatan		☐
	g.Merasa sudah sehat		☐
	h.Lainnya, sebutkan		☐
B11	Apa yang [NAMA] lakukan untuk mengendalikan diabetes mellitus?		
	a.Pengaturan makan	1. Ya 2. Tidak	☐
	b.Olah raga	1. Ya 2. Tidak	☐
	c.Alternatif herbal	1. Ya 2. Tidak	☐
B12	Apakah [NAMA] dalam 1 bulan terakhir mengalami gejala: **(BACAKAN POINT a - d)**		
	a. Sering lapar	1. Ya 2. Tidak	☐
	b. sering haus	1. Ya 2. Tidak	☐
	c. sering buang air kecil & jumlah banyak	1. Ya 2. Tidak	☐
	d. Berat badan turun	1. Ya 2. Tidak	☐
B13	Apakah [NAMA] memeriksakan kadar gula darah?	1. Ya, rutin 2. Ya, kadang-kadang 3.Tidak pernah	☐
**PENYAKIT JANTUNG [ART SEMUA UMUR]**			
B14	Apakah [NAMA] pernah didiagnosis menderita sakit jantung oleh dokter?	1.Ya 2.Tidak	☐

HAL 3 dari 24 – KUESIONER INDIVIDU RISKESDAS 2018 (2)

JIKA ART UMUR ≤ 2 TAHUN→BLOK E<br>JIKA ART UMUR ≥ 3 TAHUN → B15				
**KESEHATAN GIGI DAN MULUT [ART UMUR ≥ 3 TAHUN]**				
B15	Dalam **1 tahun terakhir**, apakah [NAMA] mempunyai masalah:			
	a. Gigi rusak, berlubang ataupun sakit?	1.Ya	2. Tidak	☐
	b. Gigi hilang karena dicabut atau tanggal sendiri?	1.Ya	2. Tidak	☐
	c. Gigi telah ditambal atau ditumpat karena berlubang?	1.Ya	2. Tidak	☐
	d. Gigi goyah?	1.Ya	2. Tidak	☐

B16	Dalam **1 tahun terakhir**, apakah [NAMA] mempunyai masalah mulut: **ISIKAN KODE JAWABAN: 1.YA ATAU 2.TIDAK**			
	a. Gusi bengkak dan/atau keluar bisul (abses)	☐	c. Sariawan berulang minimal 4 kali	☐
	b. Gusi mudah berdarah (seperti saat menyikat gigi)	☐	d. Sariawan menetap dan tidak pernah sembuh minimal 1 bulan	☐

JIKA B15 DAN B16 SELURUHNYA BERKODE "2" (TIDAK), LANJUT KE B19

B17	Dalam **1 tahun terakhir**, jenis tindakan apa saja yang diterima [NAMA] untuk mengatasi masalah gigi dan mulut?<br>**ISIKAN KODE JAWABAN: 1.YA ATAU 2. TIDAK**			
	a. Pengobatan/ minum obat	☐	f. Pemasangan gigi palsu	☐
	b. Konseling perawatan kebersihan dan kesehatan gigi dan mulut	☐	g. Pemasangan gigi tanam (*implant denture*)	☐
	c. Penumpatan / penambalan	☐	h. Perawatan orthodonsi (behel/ kawat gigi)	☐
	d. Pencabutan gigi	☐	i. Pembersihan karang gigi (*scaling*)	☐
	e. Bedah mulut	☐	j. Perawatan gusi/ periodontal treatment	☐

B18	Dalam **1 tahun terakhir**, kemana biasanya [NAMA] mencari pengobatan?							
	a. Dokter gigi spesialis	1. Ya	2. Tidak	☐	d. Dokter umum/ Paramedik lain	1. Ya	2. Tidak	☐
	b. Dokter gigi	1. Ya	2. Tidak	☐	e. Tukang gigi	1. Ya	2. Tidak	☐
	c. Perawat gigi	1. Ya	2. Tidak	☐	f. Pengobatan sendiri	1. Ya	2. Tidak	☐

B19	Dalam **1 tahun terakhir**, seberapa sering [NAMA] berobat ke tenaga medis gigi?<br>1. 1 – 3 kali 2. 4-6 kali 3. ≥ 7 kali 4. Tidak pernah berobat ke tenaga medis gigi	☐

	JIKA ART UMUR 3 - 4 TAHUN → BLOK E<br>JIKA ART UMUR 5 - 14 TAHUN → D01	JIKA ART UMUR ≥ 15 TAHUN → B20

HIPERTENSI [ART UMUR ≥ 15 TAHUN]

B20	Apakah [NAMA] pernah memeriksakan tekanan darah?	1. Ya, rutin 2. Ya, kadang-kadang 3. Tidak→**B25**	☐
B21	Apakah hasil pemeriksaan menunjukkan [NAMA] mengalami tekanan darah tinggi?	1. Ya 2. Tidak	☐
B22	Apakah [NAMA] pernah didiagnosis menderita hipertensi/ penyakit tekanan darah tinggi oleh dokter?	1. Ya 2. Tidak	☐

JIKA B21 BERKODE "1" ATAU B22 BERKODE "1", LANJUT KE B23
JIKA B21 BERKODE "2" DAN B22 BERKODE "2", LANJUT KE B25

B23	Apakah [NAMA] minum obat anti hipertensi?	1.Ya, rutin → **B25** 3. Tidak minum obat<br>2. Tidak rutin	☐

B24	Mengapa [NAMA] tidak minum obat secara rutin setiap hari? **(ISIKAN KODE JAWABAN: 1.YA ATAU 2.TIDAK)**<br>**POIN a S/D g DIBACAKAN**			
	a. Sering lupa	☐	e. Tidak mampu membeli obat secara rutin	☐
	b. Obat tidak tersedia di fasyankes (RS/Puskesmas/Apotek)	☐	f. Tidak rutin berobat ke fasilitas pelayanan kesehatan	☐
	c. Minum obat tradisional	☐	g. Merasa sudah sehat	☐
	d. Tidak tahan efek samping obat	☐	h. Lainnya, sebutkan _____________	☐

HAL 4 dari 24 – KUESIONER INDIVIDU RISKESDAS 2018 (2)

STROKE [ART UMUR ≥ 15 TAHUN]			
B25	Apakah [NAMA] pernah didiagnosis menderita penyakit *stroke* oleh dokter?	1. Ya 2. Tidak →**B27**	☐
B26	Apakah [NAMA] memeriksakan ulang (kontrol) penyakit *stroke* yang dialami ke fasilitas pelayanan kesehatan?	1.Ya, rutin 2. Ya, kadang-kadang 3. Tidak	☐
B27	Apakah [NAMA] pernah mengalami **keluhan secara mendadak** seperti di bawah ini? **(ISIKAN KODE JAWABAN: 1.YA 2.TIDAK)**		
	a. Kelumpuhan pada satu sisi tubuh ☐	d.Bicara pelo	☐
	b. Kesemutan/ baal satu sisi tubuh ☐	e.Sulit bicara/ komunikasi dan/ atau tidak mengerti pembicaraan	☐
	c. Mulut menjadi mencong tanpa kelumpuhan otot mata ☐		

PENYAKIT GAGAL GINJAL KRONIS [ART UMUR ≥ 15 TAHUN]			
B28	Apakah [NAMA] pernah didiagnosis oleh dokter, menderita penyakit gagal ginjal kronis (minimal ginjal sakit selama 3 bulan berturut-turut)?	1. Ya 2. Tidak→**B30**	☐
B29	Apakah [NAMA] pernah/ sedang menjalani cuci darah (haemodialisa)?	1. Ya 2. Tidak	☐

PENYAKIT SENDI [ART UMUR ≥ 15 TAHUN]			
B30	Apakah [NAMA] pernah didiagnosis menderita penyakit sendi oleh dokter?	1. Ya 2. Tidak	☐

C. KESEHATAN JIWA

DEPRESI [KHUSUS UNTUK ART UMUR ≥ 15 TAHUN DAN "TIDAK DIWAKILI"]			
C01	**Selama 2 minggu terakhir,** Apakah [NAMA] secara terus menerus merasa sedih, depresif atau murung, hampir sepanjang hari, hampir setiap hari?	1. Ya 2. Tidak	☐
C02	**Selama 2 minggu terakhir,** Apakah [NAMA] hampir sepanjang waktu kurang berminat terhadap banyak hal atau kurang bisa menikmati hal-hal yang biasanya [NAMA] nikmati?	1. Ya 2. Tidak	☐
C03	**Selama 2 minggu terakhir,** Apakah [NAMA] merasa lelah atau tidak bertenaga, hampir sepanjang waktu?	1. Ya 2. Tidak	☐
C04	**Selama 2 minggu terakhir,** Apakah nafsu makan [NAMA] berubah secara mencolok atau apakah berat badan [NAMA] meningkat atau menurun tanpa upaya yang disengaja?	1. Ya 2. Tidak	☐
C05	**Selama 2 minggu terakhir,** Apakah [NAMA] mengalami gangguan tidur hampir setiap malam (kesulitan untuk mulai tidur, terbangun tengah malam, terbangun lebih dini, tidur berlebihan)?	1. Ya 2. Tidak	☐
C06	**Selama 2 minggu terakhir,** Apakah [NAMA] berbicara atau bergerak lebih lambat daripada biasanya, gelisah, tidak tenang atau mengalami kesulitan untuk tetap diam?	1. Ya 2. Tidak	☐
C07	**Selama 2 minggu terakhir,** Apakah [NAMA] kehilangan kepercayaan diri, atau apakah [Nama] merasa tidak berharga atau bahkan lebih rendah daripada orang lain?	1. Ya 2. Tidak	☐
C08	**Selama 2 minggu terakhir,** Apakah [NAMA] merasa bersalah atau mempersalahkan diri sendiri?	1. Ya 2. Tidak	☐
C09	**Selama 2 minggu terakhir,** Apakah [NAMA] mengalami kesulitan berpikir atau berkonsentrasi, atau apakah mempunyai kesulitan untuk mengambil keputusan?	1. Ya 2. Tidak	☐
C10	**Selama 2 minggu terakhir,** Apakah [NAMA] berniat untuk menyakiti diri sendiri, ingin bunuh diri atau berharap bahwa [NAMA] mati?	1. Ya 2. Tidak	☐
**JIKA SALAH SATU JAWABAN C01 S/D C10 BERKODE "1", LANJUT KE C11**<br>**JIKA JAWABAN C01 S/D C10 SEMUA BERKODE "2" TIDAK, LANJUT KE C12**			
C11	Untuk semua keluhan yang disebutkan di atas (C01 s/d C10), apakah [NAMA] minum obat atau menjalani pengobatan medis?	1. Ya 2. Tidak	☐

KESEHATAN MENTAL EMOSIONAL [KHUSUS UNTUK ART UMUR ≥ 15 TAHUN DAN "TIDAK DIWAKILI"]			
Kami akan mengajukan 20 pertanyaan. Kalau kurang mengerti kami akan membacakan sekali lagi, namun kami tidak akan menjelaskan/ mendiskusikan. Jika ada pertanyaan akan kita bicarakan setelah selesai menjawab ke 20 pertanyaan.			
C12	**Dalam 1 bulan terakhir**, apakah [NAMA] sering menderita sakit kepala?	1. Ya 2. Tidak	☐
C13	**Dalam 1 bulan terakhir**, apakah [NAMA] tidak nafsu makan?	1. Ya 2. Tidak	☐
C14	**Dalam 1 bulan terakhir**, apakah [NAMA] sulit tidur?	1. Ya 2. Tidak	☐
C15	**Dalam 1 bulan terakhir**, apakah [NAMA] mudah takut?	1. Ya 2. Tidak	☐
C16	**Dalam 1 bulan terakhir**, apakah [NAMA] merasa tegang, cemas atau kuatir?	1. Ya 2. Tidak	☐

HAL 5 dari 24 – KUESIONER INDIVIDU RISKESDAS 2018 (2)

C17	**Dalam 1 bulan terakhir,** apakah tangan [NAMA] gemetar?	1. Ya 2. Tidak	☐
C18	**Dalam 1 bulan terakhir**, apakah pencernaan [NAMA] terganggu/ buruk?	1. Ya 2. Tidak	☐
C19	**Dalam 1 bulan terakhir**, apakah [NAMA] sulit untuk berpikir jernih?	1. Ya 2. Tidak	☐
C20	**Dalam 1 bulan terakhir**, apakah [NAMA] merasa tidak bahagia?	1. Ya 2. Tidak	☐
C21	**Dalam 1 bulan terakhir**, apakah [NAMA] menangis lebih sering?	1. Ya 2. Tidak	☐
C22	**Dalam 1 bulan terakhir**, apakah [NAMA] merasa sulit untuk menikmati kegiatan sehari-hari?	1. Ya 2. Tidak	☐
C23	**Dalam 1 bulan terakhir**, apakah [NAMA] sulit untuk mengambil keputusan?	1. Ya 2. Tidak	☐
C24	**Dalam 1 bulan terakhir**, apakah pekerjaan [NAMA] sehari-hari terganggu?	1. Ya 2. Tidak	☐
C25	**Dalam 1 bulan terakhir**, apakah [NAMA] tidak mampu melakukan hal-hal yang bermanfaat dalam hidup?	1. Ya 2. Tidak	☐
C26	**Dalam 1 bulan terakhir**, apakah [NAMA] kehilangan minat pada berbagai hal?	1. Ya 2. Tidak	☐
C27	**Dalam 1 bulan terakhir**, apakah [NAMA] merasa tidak berharga?	1. Ya 2. Tidak	☐
C28	**Dalam 1 bulan terakhir**, apakah [NAMA] mempunyai pikiran untuk mengakhiri hidup?	1. Ya 2. Tidak	☐
C29	**Dalam 1 bulan terakhir**, apakah [NAMA] merasa lelah sepanjang waktu?	1. Ya 2. Tidak	☐
C30	**Dalam 1 bulan terakhir**, apakah [NAMA] mengalami rasa tidak enak di perut?	1. Ya 2. Tidak	☐
C31	**Dalam 1 bulan terakhir**, apakah [NAMA] mudah lelah?	1. Ya 2. Tidak	☐

JIKA ART UMUR 15-17 TAHUN → BLOK D01
JIKA ART UMUR 18-59 TAHUN → BLOK D11
JIKA ART UMUR ≥ 60 TAHUN →D24

D. DISABILITAS/ KETIDAKMAMPUAN

KESULITAN/ HAMBATAN FUNGSI PADA ART UMUR 5 – 17 TAHUN

UNTUK PERTANYAAN D01 – D10, BACAKAN PERTANYAAN & ALTERNATIF JAWABAN. ISIKAN KODE PILIHAN JAWABAN:
1. TIDAK ADA 2. RINGAN 3. SEDANG 4. BERAT 5. SANGAT BERAT

D01	Apakah [NAMA] mempunyai hambatan penglihatan?	☐
D02	Apakah [NAMA] mempunyai hambatan pendengaran?	☐
D03	Apakah [NAMA] mempunyai hambatan berjalan?	☐
D04	Dibandingkan teman seusianya, apakah [NAMA] saat berbicara sulit dimengerti dan [NAMA] mempunyai kesulitan untuk mengerti pembicaraan orang dalam keluarganya atau diluar keluarganya?	☐
D05	Dibandingkan dengan teman seusianya, apakah [NAMA] mempunyai kesulitan untuk belajar sesuatu?	☐
D06	Dibandingkan teman seusianya apakah [NAMA] memiliki kesulitan untuk mengingat sesuatu?	☐
D07	Dibandingkan teman seusianya apakah [NAMA] memiliki kesulitan untuk konsentrasi dalam melakukan aktifitas?	☐
D08	Dibandingkan teman seusianya apakah [NAMA] memiliki kesulitan bermain/bergaul dengan teman seusianya?	☐
D09	Apakah [NAMA] memiliki kesulitan menerima perubahan rutinitas?	☐
D10	Apakah [NAMA] memiliki kesulitan dalam mengendalikan perilakunya?	☐

LANJUT KE BLOK E

KETIDAKMAMPUAN FISIK DAN MENTAL PADA ART UMUR 18-59 TAHUN [KHUSUS UNTUK ART YANG "TIDAK DIWAKILI"]

Sekarang saya akan menanyakan keadaan kesehatan menurut penilaian [NAMA] sendiri. Keadaan kesehatan yang dimaksud disini adalah keadaan fisik dan mental [NAMA]

D11	**Selama 1 bulan terakhir**, secara umum, bagaimana kondisi kesehatan [NAMA]?	1. Baik 2. Cukup 3. Buruk	☐

HAL 6 dari 24 – KUESIONER INDIVIDU RISKESDAS 2018 (2)

UNTUK PERTANYAAN D12 – D23, BACAKAN PERTANYAAN & ALTERNATIF JAWABAN. ISIKAN KODE PILIHAN JAWABAN: 1. TIDAK ADA 2. RINGAN 3. SEDANG 4. BERAT 5. SANGAT BERAT		
D12	**Dalam 1 bulan terakhir,** seberapa sulit [NAMA] untuk berdiri dalam waktu lama misalnya 30 menit?	☐
D13	**Dalam 1 bulan terakhir,** seberapa sulit [NAMA] untuk melaksanakan atau mengerjakan kegiatan rumah tangga yang menjadi tanggung jawabnya?	☐
D14	**Dalam 1 bulan terakhir**, seberapa sulit [NAMA] mempelajari/ mengerjakan hal-hal baru, seperti untuk menemukan tempat/alamat baru?	☐
D15	**Dalam 1 bulan terakhir**, seberapa sulit [NAMA] dapat berperan serta dalam kegiatan kemasyarakatan (misalnya dalam kegiatan arisan, pengajian, keagamaan, atau kegiatan lain) seperti orang lain dapat melakukan?	☐
D16	**Dalam 1 bulan terakhir**, seberapa besar masalah kesehatan yang dialami mempengaruhi keadaan emosi [NAMA]?	☐
D17	**Dalam 1 bulan terakhir**, seberapa sulit [NAMA] memusatkan pikiran dalam melakukan sesuatu selama 10 menit?	☐
D18	**Dalam 1 bulan terakhir**, seberapa sulit [NAMA] dapat berjalan jarak jauh misalnya 1 kilometer?	☐
D19	**Dalam 1 bulan terakhir**, seberapa sulit [NAMA] membersihkan seluruh tubuhnya/ mandi?	☐
D20	**Dalam 1 bulan terakhir**, seberapa sulit [NAMA] mengenakan pakaian?	☐
D21	**Dalam 1 bulan terakhir**, seberapa sulit [NAMA] berinteraksi/ bergaul dengan orang yang belum dikenal sebelumnya?	☐
D22	**Dalam 1 bulan terakhir**, seberapa sulit [NAMA] memelihara persahabatan?	☐
D23	**Dalam 1 bulan terakhir**, seberapa sulit [NAMA] mengerjakan pekerjaan sehari-hari?	☐
**LANJUT KE BLOK E**		
**KETIDAKMAMPUAN FISIK PADA ART UMUR > 60 TAHUN**		
D24	**Dalam 1 bulan terakhir**, apakah [NAMA] dapat mengendalikan keinginan buang air besar?<br>1. Tidak terkendali/ tak teratur atau perlu pencahar<br>2. Kadang-kadang tak terkendali (1x/ minggu)<br>3. Terkendali teratur	☐
D25	**Dalam 1 bulan terakhir**, apakah [NAMA] dapat mengendalikan keinginan kencing/ buang air kecil?<br>1. Tak terkendali atau pakai kateter<br>2. Kadang-kadang tak terkendali (hanya 1x/24 jam)<br>3. Terkendali teratur	☐
D26	**Dalam 1 bulan terakhir**, apakah [NAMA] dapat membersihkan diri sendiri (seperti: mencuci wajah, menyisir rambut, mencukur kumis, sikat gigi)?<br>1. Butuh pertolongan orang lain<br>2. Mandiri	☐
D27	**Dalam 1 bulan terakhir**, apakah [NAMA] dapat menggunakan WC sendiri (seperti: keluar masuk WC, melepas/ memakai celana, cebok, menyiram)?<br>1. Tergantung pertolongan orang lain<br>2. Perlu pertolongan pada beberapa kegiatan tetapi dapat mengerjakan sendiri beberapa kegiatan yang lain<br>3. Mandiri	☐
D28	**Dalam 1 bulan terakhir**, apakah [NAMA] dapat makan minum sendiri? (jika makan harus berupa potongan, dianggap dibantu)<br>1. Tidak mampu<br>2. Perlu ditolong memotong makanan<br>3. Mandiri	☐
D29	**Dalam 1 bulan terakhir**, apakah [NAMA] dapat berpindah dari kursi ke tempat tidur dan dari tempat tidur ke kursi (termasuk duduk di tempat tidur)?<br>1. Tidak mampu/ tidak dapat duduk dengan seimbang (diangkat oleh dua orang)<br>2. Perlu dibantu oleh minimal dua orang untuk bisa duduk<br>3. Memerlukan bantuan ringan, minimal oleh satu orang<br>4. Mandiri	☐
D30	**Dalam 1 bulan terakhir**, apakah [NAMA] dapat berjalan di tempat rata (bagi pengguna kursi roda, mampu menjalankan kursi roda tanpa bantuan)?<br>1. Tidak mampu<br>2. Bisa (pindah) menggunakan kursi roda<br>3. Berjalan dengan dibantu oleh 1 orang (bantuan fisik atau lisan)<br>4. Mandiri (walaupun menggunakan tongkat)	☐
D31	**Dalam 1 bulan terakhir**, apakah [NAMA] dapat berpakaian sendiri (termasuk memasang tali sepatu, mengencangkan sabuk)?<br>1. Tergantung orang lain<br>2. Sebagian dibantu (mis: mengancingkan baju)<br>3. Mandiri	☐
D32	**Dalam 1 bulan terakhir**, apakah [NAMA] dapat naik turun tangga sendiri?<br>1. Tidak mampu<br>2. Butuh pertolongan<br>3. Mandiri	☐
D33	**Dalam 1 bulan terakhir**, apakah [NAMA] dapat mandi sendiri?<br>1. Tergantung orang lain<br>2. Mandiri	☐

HAL 7 dari 24 – KUESIONER INDIVIDU RISKESDAS 2018 (2)

E. CEDERA [ART SEMUA UMUR]							
E01	**Dalam 1 tahun terakhir,** apakah [NAMA] pernah mengalami cedera, yang mengakibatkan kegiatan sehari-hari terganggu?				1. Ya 2. Tidak→ F		☐
E02	Bagian tubuh yang terkena:						
	a. Kepala	1. Ya 2. Tidak	☐	d. Perut	1. Ya 2. Tidak		☐
	b. Dada	1. Ya 2. Tidak	☐	e. Anggota gerak atas	1. Ya 2. Tidak		☐
	c. Punggung	1. Ya 2. Tidak	☐	f. Anggota gerak bawah	1. Ya 2. Tidak		☐
E03	Jenis cedera yang dialami:						
	a. Lecet/lebam/memar	1. Ya 2. Tidak	☐	f. Cedera mata	1. Ya 2. Tidak		☐
	b. Luka iris/robek/tusuk	1. Ya 2. Tidak	☐	g. Gegar otak	1. Ya 2. Tidak		☐
	c. Terkilir	1. Ya 2. Tidak	☐	h. Cedera organ dalam	1. Ya 2. Tidak		☐
	d. Patah tulang	1. Ya 2. Tidak	☐	i. Luka Bakar	1. Ya 2. Tidak		☐
	e. Anggota tubuh terputus	1. Ya 2. Tidak	☐	j. Lainnya, sebutkan	1. Ya 2. Tidak		☐
E04	Apakah cedera mengakibatkan kecacatan fisik yang permanen pada bagian tubuh dibawah ini: **(ISIKAN KODE JAWABAN: 1.YA ATAU 2.TIDAK)**						
	a. Panca indera tidak berfungsi (buta/tuli/bisu dll)		☐	c. Bekas luka permanen yang mengganggu kenyamanan			☐
	b. Kehilangan sebagian anggota badan (jari/tangan/kaki putus dll)		☐				
E05	Tempat terjadinya cedera	1. Jalan Raya →**E06**<br>2. Rumah dan lingkungannya →**F**<br>3. Sekolah dan lingkungannya →**F**		4. Tempat bekerja →**F**<br>5. Lainnya, sebutkan........................ →**F**			☐
E06	Apakah cedera disebabkan karena kecelakaan lalu lintas				1. Ya 2. Tidak→**F**		☐
E07	Bila ya apakah cedera terjadi saat: **(ISIKAN KODE JAWABAN: 1.YA ATAU 2.TIDAK)**						
	a. Mengendarai sepeda motor (pengendara)		☐	d. Menumpang mobil (penumpang mobil)			☐
	b. Membonceng sepeda motor (penumpang sepeda motor)		☐	e. Naik kendaraan tidak bermesin			☐
	c. Mengendarai mobil (sopir)		☐	f. Jalan kaki			☐

F. PELAYANAN KESEHATAN TRADISIONAL [ART SEMUA UMUR]				
F01	Apakah pernah memanfaatkan pelayanan kesehatan tradisional dalam satu tahun terakhir?		1.Ya<br>2. Tidak, tetapi melakukan upaya sendiri → **F03**<br>3.Tidak sama sekali →**G**	☐
F02	Siapa saja yang memberikan pelayanan kesehatan tradisional tersebut? **ISIKAN KODE JAWABAN: 1.YA ATAU 2.TIDAK**			
	a. Dokter atau tenaga kesehatan	☐	b. Penyehat tradisional	☐
F03	Apa saja jenis pelayanan kesehatan tradisional yang dimanfaatkan? **ISIKAN KODE JAWABAN: 1.YA ATAU 2.TIDAK**			
	a. Ramuan jadi	☐	d. Keterampilan olah pikir/ hipnoterapi	☐
	b. Ramuan buatan sendiri	☐	e. Keterampilan energi/ tenaga dalam	☐
	c. Keterampilan Manual (pijat, tusuk jarum)	☐		
F04	**Dalam satu tahun terakhir**, apakah [NAMA] pernah memanfaatkan Taman Obat Keluarga (TOGA)		1.Ya 2.Tidak	☐

G. PERILAKU				
PENCEGAHAN PENYAKIT AKIBAT GIGITAN NYAMUK [ART SEMUA UMUR]				
G01	Apa yang [NAMA] lakukan untuk menghindari gigitan nyamuk? **(ISIKAN KODE JAWABAN: 1.YA ATAU 2.TIDAK)**			
	a. Tidur menggunakan kelambu tanpa insektisida	☐	d. Menggunakan repelen/ bahan-bahan pencegah gigitan nyamuk	☐
	b. Tidur menggunakan kelambu dengan berinsektisida ≤ 3 tahun	☐	e. Menggunakan alat pembasmi nyamuk elektrik (contoh: raket nyamuk elektrik)	☐
	c. Tidur menggunakan kelambu dengan berinsektisida > 3 tahun	☐		

HAL 8 dari 24 – KUESIONER INDIVIDU RISKESDAS 2018 (2)

JIKA ART UMUR ≥ 3 TAHUN → G02<br>JIKA ART UMUR ≤ 2 TAHUN → BLOK K			
**KONSUMSI MAKANAN BERISIKO [ART UMUR ≥ 3 TAHUN]**			
Tanyakan frekuensi dalam satu hari. Jika tidak dikonsumsi setiap hari, tanyakan per minggu atau per bulan			
G02	**Dalam satu bulan terakhir,** berapa kali [NAMA] **biasanya** mengonsumsi makanan berikut:<br>**ISIKAN KODE: 1. > 1 kali per hari 2. 1 kali per hari 3. 3 – 6 kali per minggu 4. 1 – 2 kali per minggu 5. < 3 kali per bulan 6. Tidak pernah**		
	a. Makanan manis	☐	f. Makanan daging/ ayam/ ikan olahan dengan pengawet ☐
	b. Minuman manis	☐	g. Bumbu penyedap ☐
	c. Makanan asin	☐	h. Soft drink atau minuman berkarbonasi ☐
	d. Makanan berlemak/ berkolesterol/ gorengan	☐	i. Minuman berenergi ☐
	e. Makanan yang dibakar	☐	j. Mie instant/ makanan instant lainnya ☐

**PERILAKU HIGIENIS [ART UMUR ≥ 3 TAHUN]**						
G03	Apakah [NAMA] biasa menyikat gigi setiap hari?	1. Ya	2. Tidak → **G05**	7. Tidak Berlaku → **G05**		☐
G04	Kapan saja [NAMA] menyikat gigi?					
	a. Sebelum makan pagi	1. Ya	2. Tidak	3. Kadang-Kadang	7. Tidak berlaku/ tidak pernah makan pagi	☐
	b. Setelah makan pagi	1. Ya	2. Tidak	3. Kadang-Kadang	7. Tidak berlaku/ tidak pernah makan pagi	☐
	c. Sesudah makan siang	1. Ya	2. Tidak	3. Kadang-Kadang	7. Tidak berlaku/ tidak pernah makan siang	☐
	d. Saat mandi pagi	1. Ya	2. Tidak	3. Kadang-Kadang	7. Tidak berlaku/ tidak pernah mandi pagi	☐
	e. Saat mandi sore	1. Ya	2. Tidak	3. Kadang-Kadang	7. Tidak berlaku/ tidak pernah mandi sore	☐
	f. Sebelum tidur malam	1. Ya	2. Tidak	3. Kadang-Kadang	7. Tidak berlaku/ tidak pernah tidur malam	☐
G05	Dimana [NAMA] biasa buang air besar?<br>**(JAWABAN TIDAK DIBACAKAN)**	1. Jamban<br>2. Sungai/danau/laut<br>3. Pantai/ tanah lapang/ kebun/ halaman		4. Kolam/sawah/selokan<br>5. Lubang tanah		☐
JIKA ART UMUR ≥ 5 TAHUN → G06<br>JIKA ART UMUR 3-4 TAHUN → BLOK K						

**PERILAKU PENGGUNAAN HELM [ART UMUR ≥5 TAHUN]**			
G06	Apakah [NAMA] biasa menggunakan helm saat mengendarai atau membonceng sepeda motor?<br>1. Ya, selalu<br>2. Ya, kadang-kadang<br>3. Tidak pernah menggunakan helm → **G08**<br>7. Tidak pernah mengendarai/ membonceng sepeda motor → **G08**		☐
G07	Bila menggunakan helm, bagaimana cara dan kondisi helm yang digunakan?<br>**(PERLIHATKAN GAMBAR PERAGA JENIS HELM)**	1. Memakai helm standar terkancing<br>2. Memakai helm standar tidak terkancing<br>3. Memakai helm tidak standar (helm: sepeda, proyek, tentara)	☐
**KONSUMSI BUAH DAN SAYUR (GUNAKAN GAMBAR PERAGA) [ART UMUR ≥5 TAHUN]**			
G08	**Biasanya dalam 1 minggu**, berapa hari [NAMA] makan buah-buahan segar?<br>**JIKA TIDAK PERNAH ISIKAN 0 → LANJUT KE G10**	...........hari	☐
G09	**Berapa porsi rata-rata** [NAMA] mengkonsumsi buah-buahan segar dalam **satu hari** dari hari-hari tersebut?	.... porsi	☐.☐
G10	**Biasanya dalam 1 minggu,** berapa hari [NAMA] mengkonsumsi sayur-sayuran?<br>**JIKA TIDAK PERNAH ISIKAN 0 → LANJUT KE G12**	...........hari	☐
G11	**Berapa porsi rata-rata** [NAMA] mengkonsumsi sayur-sayuran dalam **satu hari** dari hari-hari tersebut?	........porsi	☐.☐
G12	**JIKA ART UMUR ≥ 10 TAHUN → G13**<br>**JIKA ART UMUR 5-9 TAHUN → L01**		
**PERILAKU CUCI TANGAN [ART UMUR ≥ 10 TAHUN]**			
G13	Apakah [NAMA] biasa mencuci tangan?	1. Ya 2. Tidak → **G17**	☐
G14	Apakah [NAMA] **selalu** mencuci tangan pakai sabun?	1. Ya 2. Tidak	☐

HAL 9 dari 24 – KUESIONER INDIVIDU RISKESDAS 2018 (2)

G15	Apakah [NAMA] **selalu** mencuci tangan menggunakan air bersih mengalir?	1.Ya 2. Tidak	☐
G16	Kapan [NAMA] biasa mencuci tangan? **(POIN a S/D c: ISIKAN KODE JAWABAN: 1.YA ATAU 2.TIDAK)**		
	a. Sebelum menyiapkan makanan/ sebelum makan ☐	c.Setelah buang air besar	☐
	b. Setiap kali tangan kotor (memegang uang, binatang, berkebun) ☐		
	**POIN d S/D f: ISIKAN KODE JAWABAN 1=YA ATAU 2=TIDAK ATAU 7=TIDAK BERLAKU)**		
	d.Setelah menggunakan pestisida/ insektisida ☐	f.Sebelum menyusui bayi	☐
	e.Setelah menceboki balita ☐		
**MEROKOK DAN PENGGUNAAN TEMBAKAU [ART UMUR ≥10 TAHUN]**			
G17	Apakah [NAMA] **pernah** merokok?	1. Ya, setiap hari 2. Ya,tidak setiap hari→**G19** 3. Tidak pernah merokok→**G26**	☐
G18	Berapa umur [NAMA] mulai merokok setiap hari? **ISIKAN DENGAN "98" JIKA TIDAK INGAT**		☐☐ tahun
G19	Berapa umur [NAMA] ketika pertama kali merokok? **ISIKAN DENGAN "98" JIKA TIDAK INGAT**		☐☐ tahun
G20	Sebutkan jenis rokok yang biasa [NAMA] hisap: **(BACAKAN POINT a SAMPAI DENGAN e)**		
	a. Rokok kretek 1. Ya 2. Tidak ☐	d. Elektrik 1. Ya 2. Tidak	☐
	b. Rokok putih 1. Ya 2. Tidak ☐	e.Shisha 1. Ya 2. Tidak	☐
	c. Rokok linting 1. Ya 2. Tidak ☐		
**JIKA G20a=1 ATAU G20b=1 ATAU G20c=1, LANJUT KE G21<br>JIKA G20a=2 DAN G20b=2 DAN G20c = 2, LANJUT KE G22**			
G21	**JIKA G17= 1, G21a DIISI KODE 1 DAN G21b ISI JUMLAH RATA-RATA PER HARI<br>JIKA G17=2, G21a DIISI KODE 2 DAN G21b ISI JUMLAH RATA-RATA PER MINGGU**		
	Rata-rata berapa batang rokok kretek/putih/linting yang [NAMA] hisap perhari atau per minggu?	a. Satuan: 1. Batang/hari 2.Batang/minggu	☐
		b. Jumlah	☐☐
G22	Apakah [NAMA] merokok **selama 1 bulan terakhir? (BACAKAN JAWABAN)**	1. Ya, setiap hari<br>2. Ya, tidak setiap hari<br>3. Sudah berhenti merokok→ **G25**	☐
G23	Apakah biasanya [NAMA] merokok di dalam gedung/ruangan (tempat umum, sekolah, tempat kerja, gedung/ ruang lainnya)	1. Ya 2. Tidak	☐
G24	Apakah [NAMA] biasa merokok di dalam rumah?	1. Ya → **G27** 2. Tidak → **G27**	☐
G25	Berapa umur [NAMA] ketika berhenti/ tidak merokok sama sekali?<br>**ISIKAN DENGAN "98" JIKA [NAMA] MENJAWAB TIDAK INGAT**	.... tahun	☐☐
G26	Seberapa sering orang lain merokok di dekat **[NAMA]** dalam ruangan tertutup (termasuk di rumah, tempat kerja, dan sarana transportasi)	1. Ya, setiap hari<br>2. Ya, tidak setiap hari<br>3. Tidak pernah sama sekali	☐
G27	Apakah [NAMA] **MENGUNYAH TEMBAKAU** (nginang, nyirih, susur) selama 1 bulan terakhir? **(BACAKAN JAWABAN)**<br>1. Ya, setiap hari 3. Tidak, tapi sebelumnya pernah mengunyah tembakau<br>2. Ya, kadang-kadang 4. Tidak pernah sama sekali → **G29**		☐
G28	Apakah [NAMA] sebelumnya pernah mengunyah tembakau tiap hari?	1. Ya 2. Tidak	☐
**AKTIFITAS FISIK [ART UMUR ≥10 TAHUN]<br>(PERLIHATKAN GAMBAR PERAGA SESUAI DENGAN JENIS AKTIFITAS FISIK YANG DITANYAKAN)**			
G29	Apakah [NAMA] biasa melakukan **aktivitas fisik berat**, yang dilakukan terus-menerus paling sedikit selama 10 menit setiap kali melakukannya?	1.Ya 2.Tidak →**G32**	☐
G30	Biasanya berapa hari dalam seminggu, **[NAMA]** melakukan **aktivitas fisik berat** tersebut?	.........hari	☐
G31	Biasanya dalam sehari, berapa lama **[NAMA]** melakukan **aktivitas fisik berat** tersebut?	........Jam	☐☐
		.........menit	☐☐

HAL 10 dari 24 – KUESIONER INDIVIDU RISKESDAS 2018 (2)

G32	Apakah [NAMA] biasa melakukan **aktivitas fisik sedang**, yang dilakukan terus-menerus paling sedikit selama 10 menit setiap kali melakukannya?	1. Ya 2.Tidak → **G35**	☐
G33	Biasanya berapa hari dalam seminggu, [**NAMA**] melakukan **aktivitas fisik sedang** tersebut?	.........hari	☐
G34	Biasanya dalam sehari, berapa lama [**NAMA**] melakukan **aktivitas fisik sedang** tersebut?	........Jam	☐☐
		.........menit	☐☐

MINUMAN BERALKOHOL [ART UMUR ≥10 TAHUN]

G35	Apakah [NAMA] mengonsumsi minuman beralkohol dalam 1 bulan terakhir?	1. Ya 2. Tidak→**G39**	☐
G36	Dalam 1 bulan terakhir, jenis minuman beralkohol yang **biasa** [NAMA] minum dalam satu waktu?<br>1.Bir 3. Whisky 5. Minuman tradisional bening 7.Lainnya, sebutkan............<br>2.Anggur/ arak 4. Minuman tradisional keruh 6. Minuman oplosan		☐
G37	Selama 1 bulan terakhir, berapa hari [NAMA] mengonsumsi minuman beralkohol?		☐☐
G38	Berapa jumlah rata-rata minuman beralkohol biasanya dikonsumsi per hari?<br>a.Satuan: 1. gelas bir (330ml) 2. gelas belimbing (200-250 ml) 3.gelas anggur (200-250 ml) 4.botol kecil (250-300 ml) 5.botol besar (750-800 ml) 6.kaleng (330 ml) 7.sloki/gelas kecil (30-40 ml)		☐
	b.Jumlah rata-rata per hari:		☐☐,☐
G39	**JIKA ART UMUR ≥ 15 TAHUN → BLOK H**	**JIKA ART LAKI-LAKI UMUR 10-14 TAHUN → BLOK L**<br>**JIKA ART PEREMPUAN UMUR 10-14 TAHUN → BLOK I**	

H. PENGETAHUAN DAN SIKAP TERHADAP HIV/ AIDS [KHUSUS ART UMUR ≥ 15 TAHUN DAN "TIDAK DIWAKILI"]

H01	Apakah [NAMA] pernah mendengar tentang HIV/AIDS?	1.Ya 2. Tidak→**H07**	☐
H02	Menurut [NAMA], HIV/ AIDS termasuk penyakit apa? **(POIN a SAMPAI DENGAN e DIBACAKAN)**		
	a. Penyakit keturunan	1.Ya 2. Tidak 8. Tidak Tahu	☐
	b. Penyakit kutukan	1.Ya 2. Tidak 8. Tidak Tahu	☐
	c. Penyakit menular	1. Ya 2. Tidak 8. Tidak Tahu	☐
	d. Penyakit kelamin	1. Ya 2. Tidak 8. Tidak Tahu	☐
	e. Penyakit menurunnya kekebalan tubuh	1. Ya 2. Tidak 8. Tidak Tahu	☐

H03	Menurut [NAMA], bagaimana cara penularan HIV/AIDS? **(POIN a SAMPAI DENGAN j DIBACAKAN)**<br>**(ISIKAN KODE JAWABAN: 1.YA ATAU 2.TIDAK ATAU 8.TIDAK TAHU)**			
	a. Melalui batuk atau bersin	☐	f. Menggunakan toilet umum	☐
	b. Berenang di dalam kolam yang sama dengan penderita HIV/AIDS	☐	g. Melakukan hubungan seksual dengan penderita HIV/ AIDS	☐
	c. Menggunakan jarum suntik bekas penderita HIV/AIDS	☐	h. Dari ibu hamil terhadap janinnya	☐
	d. Makan/minum sepiring dengan penderita HIV/AIDS	☐	i. Menerima donor darah dari penderita HIV/AIDS	☐
	e. Hubungan seksual sesama pria	☐	j. Bersentuhan kulit dengan penderita HIV/AIDS	☐
H04	Menurut [NAMA], bagaimana cara pencegahan HIV/AIDS? **(POIN a SAMPAI DENGAN f DIBACAKAN)**<br>**(ISIKAN KODE JAWABAN: 1.YA ATAU 2.TIDAK ATAU 8.TIDAK TAHU)**			
	a. Memiliki pasangan seksual hanya satu orang	☐	d. Tidak tinggal di lingkungan penderita HIV/AIDS	☐
	b. Menggunakan kondom saat berhubungan	☐	e. Tidak bertukar pakaian dengan penderita HIV/AIDS	☐
	c. Menggunakan jarum suntik sekali pakai	☐	f. Tidak menjalani "hubungan" sesama jenis	☐
H05	Menurut [NAMA], bagaimana cara mengetahui seseorang menderita HIV/AIDS? **(POIN a SAMPAI DENGAN c DIBACAKAN)**<br>**(ISIKAN KODE JAWABAN: 1.YA ATAU 2.TIDAK ATAU 8.TIDAK TAHU)**			
	a. Dengan mengenali fisik orang tersebut	☐	c. Dengan tes darah sukarela (VCT)	☐
	b. Dengan mengenali perilaku orang tersebut	☐	d. Lainnya, Sebutkan.........................	☐

HAL 11 dari 24 – KUESIONER INDIVIDU RISKESDAS 2018 (2)

H06	Bagaimana sikap [NAMA] terhadap penderita HIV/AIDS? **(ISIKAN KODE JAWABAN: 1.YA ATAU 2.TIDAK)**	
	a. Jika salah satu anggota keluarga [NAMA] tertular HIV/AIDS, apakah [NAMA] akan merahasiakannya?	☐
	b. Jika salah satu anggota keluarga [NAMA] menderita HIV/AIDS, apakah [NAMA] bersedia untuk merawatnya?	☐
	c. Jika salah satu tetangga [NAMA] diketahui menderita HIV/AIDS, apakah [NAMA] akan mengucilkan orang tersebut?	☐
	d. Apakah [NAMA] akan membeli sayuran segar dari petani atau penjual yang diketahui terinfeksi HIV/AIDS?	☐
	e. Apakah [NAMA] setuju jika seorang guru menderita HIV/AIDS, tidak diperkenankan untuk mengajar?	☐
H07	• **JIKA ART PEREMPUAN UMUR 15-19 TAHUN → BLOK I**<br>• **JIKA ART PEREMPUAN UMUR 20-54 TAHUN STATUS KAWIN/CERAI HIDUP/CERAI MATI→ BLOK J**<br>• **JIKA ART PEREMPUAN UMUR ≥ 20 TAHUN BELUM KAWIN/ ART PEREMPUAN UMUR > 54 TAHUN/ ART LAKI-LAKI → BLOK L**	

I. PEMBERIAN TABLET TAMBAH DARAH (TTD) REMAJA PUTRI [ART PEREMPUAN UMUR 10-19 TAHUN]

I01	Apakah [NAMA] sudah mendapat haid/ menstruasi?	1. Ya 2. Tidak →**I04**	☐
I02	Sejak usia berapa [NAMA] mendapatkan haid/menstruasi?	.... tahun	☐☐
I03	Apakah [NAMA] sudah pernah hamil?	1. Ya → **J01b** 2. Tidak	☐
I04	Apakah [NAMA] pernah mendapat/membeli TTD? **(LIHAT GAMBAR PERAGA)**	1. Ya 2. Tidak → **I07**	☐
I05	Apakah dalam 12 bulan terakhir [NAMA] pernah mendapat/membeli TTD? **(LIHAT GAMBAR PERAGA)**	1. Ya 2. Tidak → **I07**	☐

I06	Sumber TTD dalam 12 bulan terakhir	Isikan kode:<br>1. Ya<br>2. Tidak→ **ke baris berikutnya**	Total butir TTD yang didapat/ dibeli	Jumlah yang diminum	Alasan utama tidak minum/ tidak menghabiskan TTD? **[LIHAT KODE]**
	(1)	(2)	(3)	(4)	(5)
	a.Fasilitas kesehatan	☐	☐☐☐	☐☐☐	☐
	b.Sekolah	☐	☐☐☐	☐☐☐	☐
	c. Inisiatif sendiri	☐	☐☐☐	☐☐☐	☐
	**KODE KOL. (5)**	1.Hanya diminum ketika haid<br>2.Lupa<br>3. Rasa dan bau tidak enak	4. Ada efek samping (mual, sembelit)<br>5. Belum waktunya habis<br>6. Merasa tidak perlu	7. Lainnya	
I07	**LANJUT KE BLOK L**				

J. KESEHATAN IBU

ART PEREMPUAN UMUR 10-54 TAHUN STATUS KAWIN/ CERAI HIDUP/ CERAI MATI

MASA REPRODUKSI

J01	a. Apakah [NAMA] pernah hamil?	1. Ya 2. Tidak →**BLOK L**	☐
	b. Berapa umur [NAMA] ketika pertama kali hamil?	.... tahun	☐☐
J02	Selama hidup, berapa kali [NAMA] **hamil**, **keguguran** dan **bersalin**?		
	a. Gravida (kehamilan) ☐☐	b. Partus (melahirkan) ☐☐	c.Aborsi (keguguran) ☐☐
J03	a. Apakah pada periode **1 Januari 2013** sampai saat wawancara pernah melahirkan yang berakhir dengan lahir hidup atau lahir mati atau keguguran?	1. Ya 2. Tidak →**J48**	☐
	b. Kapan persalinan/keguguran dari kehamilan terakhir? (Tuliskan tgl,bulan dan tahun)<br>**BILA LEBIH DARI 1 KEHAMILAN, PILIH KEHAMILAN PALING TERAKHIR YANG SUDAH BERAKHIR DENGAN PERSALINAN/ KEGUGURAN**	☐☐/☐☐/☐☐☐☐	

Selanjutnya saya akan menanyakan riwayat masa kehamilan, saat persalinan, dan masa nifas dari kehamilan terakhir tersebut. Mohon [NAMA] mengingat-ingat kembali pengalaman selama kehamilan terakhir tersebut

RIWAYAT KEHAMILAN, PERSALINAN, DAN MASA NIFAS

"KEHAMILAN TERAKHIR YANG SUDAH BERAKHIR" PADA PERIODE 1 JANUARI 2013- SAAT WAWANCARA

J04	NAMA ANAK (JIKA BELUM DIBERI NAMA TULISKAN NN)		
J05	Nomor urut [NAMA ANAK] dalam rumah tangga	**JIKA [NAMA ANAK] TIDAK ADA PADA BLOK IV TULISKAN 00**	☐☐
J06	Dari seluruh kehamilan, urutan kehamilan keberapa saat mengandung [NAMA ANAK]?		☐☐

HAL 12 dari 24 – KUESIONER INDIVIDU RISKESDAS 2018 (2)

J07	Apakah kehamilan tunggal atau kembar?	1. Tunggal 2. Kembar 3. Belum diketahui	☐
J08	Bagaimana hasil kehamilan?	1. Lahir hidup 2. Lahir mati 3. Lahir hidup & lahir mati 4. Keguguran	☐
J09	Jenis kelamin [NAMA ANAK]:	1. Laki-laki 2. Perempuan 3. Belum diketahui	☐
J10	Umur kandungan saat kehamilan berakhir?	...............bulan	☐☐
J11	Pada saat hamil [NAMA ANAK], apakah kehamilan tersebut diinginkan saat itu/ diinginkan kemudian/ tidak diinginkan?<br>1. Diinginkan saat itu 2. Diinginkan kemudian 3. Tidak diinginkan		☐
	**JIKA J08 BERKODE 1 ATAU 3, LANJUT KE J12**<br>**JIKA J08 BERKODE 2 ATAU 4, LANJUT KE J14**		
J12	Apakah [NAMA ANAK] masih hidup?	1. Masih Hidup → **J14** 2. Sudah meninggal	☐
J13	Umur waktu meninggal<br>**(JIKA UMUR < 30 HARI ISIKAN DALAM HARI, JIKA UMUR ≥ 30 HARI ISIKAN DALAM BULAN)**	a. Satuan: 1. Bulan 2.Hari	☐
		b.Umur	☐☐
**MASA KEHAMILAN**			
J14	Apakah melakukan pemeriksaan kehamilan ke tenaga kesehatan (dokter spesialis, dokter umum, bidan atau perawat)?	1. Ya 2. Tidak → **J21**	☐
J15	Berapa bulan umur kandungan [NAMA] saat memeriksakan kehamilan pertama kali?	________ bulan	☐☐

J16 Berapa kali [NAMA] melakukan **pemeriksaan kehamilan oleh tenaga kesehatan?**

Umur kehamilan (1)	Tenaga yang melakukan ANC (2)		Frekuensi ANC (3)
a. 0-3 bulan	1.Tenaga Kesehatan 2.Tenaga Non Kesehatan→**J16b** 3.Tidak ANC→**J16b**	☐	☐☐ kali
b. 4-6 bulan	1. Tenaga Kesehatan 2. Tenaga Non Kesehatan→**J16c** 3. Tidak ANC→**J16c** 7. Tidak Berlaku→**J17a**	☐	☐☐ kali
c. 7-melahirkan	1. Tenaga Kesehatan 2. Tenaga Non Kesehatan→**J17a** 3. Tidak ANC→**J17a** 7. Tidak Berlaku→**J17a**	☐	☐☐ kali

J17a	**JIKA J16a=1 ATAU J16b=1 ATAU J16c=1, LANJUT KE J17**<br>**JIKA J16a≠1 DAN J16b≠1 DAN J16c≠1, LANJUT KE J21**		
J17	Siapa yang paling sering memeriksa kehamilan [NAMA]?	1. dokter spesialis 2. dokter umum 3. bidan 4. perawat	☐
J18	Dimana [NAMA] paling sering melakukan pemeriksaan kehamilan (ANC)?<br>1. RS Pemerintah 2. RS Swasta 3. Klinik 4. Puskesmas/Pustu/Pusling 5. Praktek dokter mandiri 6. Praktek bidan mandiri 7. Poskesdes/Polindes 8. Posyandu 9. Praktek Perawat 10. **Rumah**		☐☐
J19	Selama kehamilan [NAMA ANAK], apakah [NAMA] mendapat pemeriksaan:		
	a. Pengukuran tinggi badan	1. Ya 2. Tidak	☐
	b. Penimbangan berat badan	1. Ya 2. Tidak	☐
	c. Pengukuran tekanan darah	1. Ya 2. Tidak	☐
	d. Pengukuran lingkar lengan atas (LILA) → **GUNAKAN GAMBAR PERAGA**	1. Ya 2. Tidak	☐
	e. Pengukuran tinggi rahim → **GUNAKAN GAMBAR PERAGA**	1. Ya 2. Tidak	☐
	f. Penentuan letak janin → **GUNAKAN GAMBAR PERAGA**	1. Ya 2. Tidak	☐
	g. Perhitungan denyut jantung janin (DJJ) → **GUNAKAN GAMBAR PERAGA**	1. Ya 2. Tidak	☐
	h. Konseling dan tata laksana kasus (tindakan) **JIKA JAWABAN > 1, JUMLAHKAN KODE JAWABAN**<br>1.Konseling/ penjelasan/ nasehat 2. Tindakan/ tata laksana kasus 4. Tidak memperoleh keduanya		☐
J20	Selama kehamilan [NAMA ANAK], apakah [NAMA] mendapat imunisasi TT (biasanya suntikan pada lengan atas ibu untuk mencegah bayi dari penyakit tetanus, atau kejang-kejang setelah lahir)?<br>1. Ya 2. Tidak diimunisasi 3. Dinyatakan oleh nakes tidak perlu imunisasi TT		☐

HAL 13 dari 24 – KUESIONER INDIVIDU RISKESDAS 2018 (2)

J21	a. Apakah selama kehamilan [NAMA ANAK], [NAMA] mendapat tablet tambah darah (TTD)?	1.Ya 2.Tidak→ J23	☐
	b. Berapa total butir TTD yang diperoleh/ dibeli?		☐☐☐ butir
	c. Berapa jumlah tablet tambah darah (tablet Fe) yang [NAMA] minum selama kehamilan?		☐☐☐ butir
J22	Jika yang diminum lebih sedikit dari yang diperoleh, apa alasan utama TTD tidak minum/ tidak menghabiskan?<br>1. Tidak suka 3. Bosan 5. Efek samping (mual, sembelit)<br>2. Mual/ muntah karena proses kehamilan 4. Lupa 6. Belum waktunya habis		☐
J23	Apakah saat hamil [NAMA ANAK], [NAMA] melakukan pemeriksaan laboratorium?		
	a. Tes gluko-protein urin	1. Ya 2. Tidak 3. Tidak Tahu	☐
	b. Tes darah hemoglobin (Hb)	1. Ya 2. Tidak 3. Tidak tahu	☐
	c. Tes HIV	1. Ya 2. Tidak 3. Tidak tahu	☐
	d. Tes golongan darah	1. Ya 2. Tidak 3. Tidak tahu 4. Sudah pernah tes	☐
	e. Tes lainnya, sebutkan________	1. Ya 2. Tidak 3. Tidak tahu	☐
J24	Apakah selama hamil, [NAMA] mengalami masalah/gangguan kehamilan seperti:<br>**(JAWABAN BISA > 1, TULISKAN JAWABAN BERDASARKAN URUTAN ABJAD)**<br>A. Muntah atau diare terus menerus G. Terasa sakit saat kencing<br>B. Demam tinggi (3 hari sebelum melahirkan) H. Batuk lama (> 2 minggu)<br>C. Hipertensi I. Jantung berdebar-debar dan nyeri dada<br>D. Janin kurang bergerak J. Bengkak kaki disertai kejang<br>E. Perdarahan banyak pada jalan lahir K. Lainnya________________(SEBUTKAN)<br>F. Keluar air ketuban sebelum waktunya **Z. Tidak ada masalah/ gangguan →J27**	----------------------	
J25	Apakah [NAMA] segera mencari pertolongan ke tenaga kesehatan ketika mengalami keluhan?	1. Ya, segera 2. Ya, ada jeda waktu 3. Tidak→J27	☐
J26	Fasilitas kesehatan apakah yang pertama kali [NAMA] manfaatkan?	1. RS 4. Praktek tenaga kesehatan<br>2. Puskesmas/Pustu 5. Lainnya , sebutkan<br>3. Polindes ____________________	☐
J27	**JIKA J08 BERKODE "1" ATAU "2" ATAU "3", LANJUT KE J28**<br>**JIKA J08 BERKODE "4", LANJUT KE J48**		
**MASA PERSALINAN**			
J28	Siapa saja yang membantu [NAMA] saat persalinan?<br>**(JAWABAN BISA > 1, TULISKAN JAWABAN BERDASARKAN KRONOLOGIS PENOLONG)**<br>A. Dokter Kandungan D. Perawat **Z. Tidak ada yang menolong**<br>B. Dokter Umum E. Dukun<br>C. Bidan F. Lainnya................................	____________________	
J29	Dimana tempat persalinan [NAMA ANAK]?<br>1. RS Pemerintah 4. Puskesmas/ Pustu/ Pusling 7. Poskesdes/ Polindes<br>2. RS Swasta 5. Praktek dokter mandiri 8. Rumah →J31<br>3. Klinik 6. Praktek bidan mandiri 9. Lainnya, sebutkan____________ →J31		☐
J30	Berapa jam [NAMA IBU] dirawat di fasilitas pelayanan kesehatan (fasyankes) **setelah bersalin sampai pulang?**	.... jam	☐☐☐
J31	Darimana sumber pembiayaan persalinan?<br>**(JAWABAN BISA > 1, TULISKAN JAWABAN BERDASARKAN URUTAN ABJAD)**<br>A. BPJS/KIS C. Biaya kantor E. Biaya sendiri G. Jamperda<br>B. Asuransi swasta D. Biaya orang lain F. Jampersal H.Tidak mengeluarkan biaya	____________________	
J32	Bagaimana metode persalinan [NAMA ANAK]?	1. Normal 4. Forceps (memakai alat)<br>2. Operasi sesar 5. Lainnya,sebutkan ______________<br>3. Vacuum	☐
J33	Apakah pada saat melahirkan [NAMA] mengalami masalah/gangguan sebagai berikut:<br>**(JAWABAN BISA > 1, TULISKAN JAWABAN BERDASARKAN URUTAN ABJAD)**<br>A. Posisi janin melintang/sungsang F. Lilitan tali pusar<br>B. Perdarahan G. Plasenta letak rendah/*Placenta Previa*<br>C. Kejang H. Plasenta tertinggal<br>D. Ketuban pecah dini I. Hipertensi<br>E. Partus Lama X. Lainnya __________(SEBUTKAN)<br>**Z. Tidak ada masalah/ gangguan →J40**	____________________	
J34	Apakah [NAMA] dirujuk ke fasilitas kesehatan terkait masalah yang dialami pada saat bersalin tersebut?	1.Ya, segera 3.Tidak →J39<br>2.Ya, ada jeda waktu	☐

J35	a. Berapa lama waktu yang diperlukan dari tempat persalinan pertama untuk mencapai fasilitas pelayanan rujukan pertama?	jam : menit	☐☐:☐☐
	b. Berapa lama waktu untuk memperoleh tindakan/penanganan saat di fasilitas kesehatan?	jam : menit	☐☐:☐☐
J36	Ke fasilitas pelayanan kesehatan mana saja [NAMA] dirujuk?<br>**JAWABAN DAPAT > 1 TULISKAN KODE JAWABAN BERDASARKAN URUTAN RUJUKAN**<br>A. Praktek Nakes C. Klinik E. Rumah Sakit Swasta<br>B. Puskesmas D. Rumah Sakit Bersalin F. Rumah Sakit Pemerintah	____________	
J37	Berapa lama waktu yang diperlukan dari fasilitas kesehatan pertama sampai rujukan terakhir?	Jam:menit	☐☐:☐☐
J38	Darimana saja sumber pembiayaan rujukan?<br>**(JAWABAN BISA > 1, TULISKAN JAWABAN BERDASARKAN URUTAN ABJAD)**<br>A. BPJS/KIS C. Biaya kantor E. Biaya sendiri G. Lainnya<br>B. Asuransi swasta D. Biaya orang lain F. Jampersal	________	

LANJUT KE J40

J39	Alasan [NAMA] tidak dirujuk ke fasilitas kesehatan terkait masalah yang dialami pada saat bersalin tersebut?<br>**JIKA JAWABAN > 1, JUMLAHKAN KODE JAWABAN**<br>1.Merasa tidak perlu 4.Masalah biaya 16.Jarak Faskes jauh<br>2.Tidak diijinkan keluarga 8.Tidak ada transportasi 32.Masalah keluarga yang ditinggal<br>64. Lainnya, sebutkan	☐☐☐

MASA NIFAS

J40	Apakah setelah melahirkan, [NAMA] diperiksa kesehatannya oleh tenaga kesehatan (mengunjungi/dikunjungi nakes)?		
	a. Periode **setelah melahirkan sampai 3 hari setelah melahirkan** [NAMA ANAK]	1. Ya 2. Tidak	☐
	b. Periode **4 hari sampai 28 hari setelah melahirkan** [NAMA ANAK]	1. Ya 2. Tidak	☐
	c. Periode **29 hari sampai 42 hari setelah melahirkan** [NAMA ANAK]	1. Ya 2. Tidak	☐
J41	Pada waktu sesaat setelah bersalin sampai 42 hari setelah melahirkan, berapa kali [NAMA IBU] mendapat kapsul vitamin A? **(TUNJUKKAN GAMBAR PERAGA)**	1.satu kali 3.lebih dari 2 kali<br>2.dua kali 4.Tidak pernah → **J43**	☐
J42	Kapan saja waktu minum vitamin A setelah bersalin? **JIKA JAWABAN > 1, JUMLAH KODE JAWABAN**<br>1. Sesaat setelah melahirkan 2. 24 jam setelah melahirkan 4. Lebih dari 48 jam setelah melahirkan		☐
J43	Apakah pada periode nifas (0-42 hari setelah melahirkan) mengalami hal sebagai berikut:<br>**(JAWABAN BISA > 1, TULISKAN JAWABAN BERDASARKAN URUTAN ABJAD)**<br>A. Perdarahan banyak pada jalan lahir G.Payudara bengkak, merah disertai rasa sakit<br>B. Keluar cairan berbau dari jalan lahir H.Ibu terlihat sedih, murung/ menangis tanpa sebab (depresi)<br>C. Bengkak diwajah, tangan dan kaki<br>D. Sakit kepala I. Hipertensi<br>E. Kejang-kejang X. Lainnya<br>F. Demam lebih dari 2 hari **Z. Tidak ada masalah/ gangguan →J46**	________	
J44	Apakah [NAMA] mencari pertolongan ke tenaga kesehatan ketika mengalami keluhan?	1. Ya, segera 2. Ya, ada jeda waktu 3. Tidak→**J46**	☐
J45	Fasilitas kesehatan apakah yang pertama kali [NAMA] manfaatkan?	1. RS 4. Praktek tenaga kesehatan<br>2. Puskesmas/Pustu 5. Lainnya , sebutkan ____________<br>3. Polindes/ Poskesdes	☐

PELAYANAN KB PASCA SALIN

J46	Apakah setelah melahirkan, [NAMA]/ pasangan menggunakan alat/ cara kontrasepsi modern?<br>1. Sterilisasi wanita 4.Suntikan 3 bln 7. Pil<br>2. Sterilisasi pria 5.Suntikan 1 bln 8. Kondom pria<br>3. IUD/AKDR/Spiral 6.*Implant*/ Susuk KB 9. **Tidak menggunakan → J48**	☐
J47	Kapan [NAMA]/ pasangan menggunakan alat/ cara kontrasepsi tersebut?<br>1. Bersamaan dengan proses persalinan<br>2. Setelah persalinan selesai, tetapi sebelum pulang dari Fasilitas Kesehatan<br>3. Setelah pulang dari Fasilitas Kesehatan sampai dengan 42 hari setelah persalinan<br>4. Di atas 42 hari setelah persalinan	☐

PEMBERIAN MAKANAN TAMBAHAN (PMT) PADA IBU HAMIL

J48	Apakah **saat ini** [NAMA] sedang hamil? **[TANYAKAN KONDISI SAAT WAWANCARA]**	1. Ya 2. Tidak → **BLOK L**	☐
J49	Berapa usia kehamilan [NAMA] **saat ini**?	.... bulan	☐
J50	Apakah selama **kehamilan saat ini**, ibu mendapatkan PMT?	1. Ya 2. Tidak → **J54**	☐

HAL 15 dari 24 – KUESIONER INDIVIDU RISKESDAS 2018 (2)

J51	Isikan bentuk dan jumlah PMT yang diperoleh selama **kehamilan saat ini**, yang dihabiskan, dan alasan utama tidak dihabiskan				
	Bentuk PMT	Bentuk PMT yang diperoleh Isikan kode<br>1. Ya<br>2. Tidak→**ke baris berikutnya**	Jumlah total PMT yang diperoleh	Apakah PMT dihabiskan oleh [NAMA]?<br>1. Ya→**ke baris berikutnya**<br>2. Tidak	**Alasan utama** PMT tidak dihabiskan?<br>1.Rasanya tidak enak<br>2.Rasa kurang bervariasi<br>3.Terlalu manis<br>4.Tidak suka aromanya/ baunya<br>5.Ada efek samping (mual, alergi, dll)<br>6. Lupa<br>7. Dimakan ART lain<br>8. Lainnya, sebutkan....
		(1)	(2)	(3)	(4)
	a. Biskuit Program **(LIHAT GAMBAR PERAGA)**	☐	☐☐ bungkus	☐	☐ ....
	b. Biskuit lainnya	☐		☐	☐ ....
	c. Susu Bubuk	☐		☐	☐ ....
	d. Susu Cair	☐		☐	☐ ....
	e. Bahan Makanan Mentah	☐		☐	☐ ....
	f. Makanan Matang	☐		☐	☐ ....

J52	Alasan ibu mendapatkan PMT untuk **kehamilan saat ini**? **POIN a S/D e TIDAK DIBACAKAN (ISIKAN KODE JAWABAN: 1.YA ATAU 2.TIDAK)**			
	a. Ibu kurang gizi/ KEK	☐	d. Berat Badan selama hamil tidak pernah naik	☐
	b. Keluarga miskin	☐	e. Anemia	☐
	c. Periksa hamil di posyandu	☐	f. Lainnya, sebutkan ________________	☐

J53	Pada **kehamilan saat ini**, sejak umur kehamilan berapa bulan ibu mulai mendapatkan PMT?	............bulan	☐

PEMBERIAN TABLET TAMBAH DARAH PADA IBU HAMIL

J54	Apakah selama **kehamilan saat ini**, [NAMA] pernah mendapatkan TTD?	1. Ya 2. Tidak →**J58**	☐
J55	Berapa total butir TTD yang didapat/ dibeli selama **kehamilan saat ini**?		☐☐☐ Butir
J56	Berapa jumlah tablet tambah darah (TTD) yang [NAMA] minum selama **kehamilan saat ini**?		☐☐☐ Butir
J57	Alasan utama tidak minum/ tidak menghabiskan TTD selama **kehamilan saat ini**?<br>1. Tidak suka 3. Bosan 5. Efek samping (mual, sembelit)<br>2. Mual/ muntah karena proses kehamilan 4. Lupa 6. Belum waktunya habis		☐

KEPEMILIKAN BUKU KIA-IBU

J58	Apakah ibu memiliki buku KIA untuk **kehamilan saat ini**?<br>1. Ya, dapat menunjukkan 2. Ya, tidak dapat menunjukkan 3. Tidak memiliki	☐

LANJUT KE BLOK L

K. KESEHATAN BALITA

BUKU KIA-IBU [ART UMUR 0 – 59 BULAN]

K00	Apakah ibu memiliki buku KIA pada saat hamil [NAMA]?<br>1. Ya, dapat menunjukkan 2. Ya, tidak dapat menunjukkan 3. Tidak memiliki	☐

PENOLONG DAN TEMPAT PERSALINAN [ART UMUR 0 – 59 BULAN]

K01	Siapa saja yang membantu persalinan? **(JAWABAN BISA > 1. TULISKAN JAWABAN BERDASARKAN URUTAN YANG MENOLONG)**<br>A. Dokter Kandungan D. Perawat Z. Tidak ada yang menolong<br>B. Dokter Umum E. Dukun<br>C. Bidan F. Lainnya..................................	__________________
K02	Dimana tempat persalinan [NAMA ANAK] (saat bayi lahir sampai 6 jam setelah persalinan)<br>1. RS Pemerintah 4. Puskesmas/ Pustu/ Pusling 7. Poskesdes/ Polindes<br>2. RS Swasta 5. Praktek dokter mandiri 8. Rumah<br>3. Klinik 6. Praktek bidan mandiri 9. Lainnya, sebutkan_____________	☐

KONDISI SAAT LAHIR [ART UMUR 0 – 59 BULAN]

K03	Berapa usia kehamilan ibu pada saat [NAMA] dilahirkan?	.... minggu	☐☐
K04	Apakah [NAMA] mempunyai catatan/dokumen berat badan lahir? (Berat badan lahir adalah berat badan yang ditimbang dalam kurun waktu 24 jam setelah dilahirkan)	1. Ya 2. Tidak → **K07**	☐
K05	Salin dari catatan/dokumen berat badan lahir [NAMA] **(JIKA ≥ 2500 GRAM → K07)**	.... gram	☐☐☐☐
K06	Jika berat lahir [NAMA] kurang dari 2500 gram, tindakan apa yang dilakukan pada saat itu?<br>1. Tidak dilakukan apa-apa 3. Didekap didada dengan kulit ***bayi*** menyentuh kulit ***ibu*** dan terbungkus (metode kanguru)<br>2. Di inkubator 4. Lainnya, sebutkan ________________		☐
K07	Apakah [NAMA] mempunyai catatan/dokumen panjang badan lahir? (Panjang badan lahir adalah panjang badan yang diukur dalam kurun waktu 24 jam setelah dilahirkan)	1.Ya 2. Tidak → **K09**	☐
K08	Salin dari catatan/dokumen panjang badan lahir [NAMA]	...... cm	☐☐,☐
K09	Apakah [NAMA] mempunyai catatan/ dokumen lingkar kepala?	1.Ya 2. Tidak → **K11**	☐
K10	Salin dari catatan/dokumen lingkar kepala [NAMA]	......cm	☐☐,☐

K11 Apakah [NAMA] mempunyai kelainan/cacat sejak lahir? **PERLIHATKAN GAMBAR PERAGA**

a. Tuna netra (penglihatan)	1. Ya 2. Tidak	☐	d. Tuna daksa (bagian tubuh)	1. Ya 2. Tidak	☐
b. Tuna rungu (pendengaran)	1. Ya 2. Tidak	☐	e. Bibir Sumbing	1. Ya 2. Tidak	☐
c. Tuna wicara (berbicara)	1. Ya 2. Tidak	☐	*f. Down Syndrome*	1. Ya 2. Tidak	☐

PERAWATAN BAYI BARU LAHIR/ NEONATUS [ART UMUR 0 – 59 BULAN]

K12	Apa yang dilakukan dalam perawatan tali pusar [NAMA] saat baru lahir?<br>1. Tidak diberi apa-apa dan kering 3. Obat tabur (berbentuk bubuk) 8. Tidak tahu<br>2. Betadin/alkohol 4. Ramuan/ obat tradisional		☐
K13	Apakah pada saat (NAMA) baru lahir diberikan salep mata antibiotika?	1. Ya, catatan dokumen 3. Tidak<br>2. Ya, ingatan ibu 8. Tidak tahu	☐

K14 Apakah [NAMA] dilakukan pemeriksaan bayi baru lahir oleh tenaga kesehatan?

	(1)		Diperiksa di mana? (KODE JAWABAN) (2)	Siapa yang memeriksa? (KODE JAWABAN) (3)
a. 6–48 jam setelah lahir	1. Ya 7. Tidak berlaku→**K17**<br>2. Tidak→**K14b** 8. Tidak Tahu→**K14b**	☐	☐	☐
b. 3–7 hari setelah lahir	1. Ya 7. Tidak berlaku→**K17**<br>2. Tidak→**K14c** 8. Tidak Tahu→**K14c**	☐	☐	☐
c. 8–28 hari setelah lahir	1. Ya 7. Tidak berlaku<br>2. Tidak 8. Tidak Tahu	☐	☐	☐

KODE KOLOM (2) TEMPAT PEMERIKSAAN	KODE KOLOM (3) TENAGA PEMERIKSA
1. Rumah Sakit Pemerintah 4. Puskesmas/Pustu/Pusling 7. Tempat Praktik Mandiri Tenaga Kesehatan<br>2. Rumah Sakit Swasta 5. Posyandu/Poskesdes/Polindes<br>3. Rumah Sakit Bersalin 6. Klinik 8. Rumah	1. Dokter spesialis 4. Perawat/ nakes lainnya<br>2. Dokter umum<br>3. Bidan 5. Lainnya

JIKA K14a kolom (1) BERKODE "1", LANJUT KE K15
JIKA K14a kolom (1) BERKODE "2" ATAU "8" DAN K14b kolom (1) BERKODE "1", LANJUT KE K16
JIKA K14a kolom (1) ≠ "1" DAN K14b kolom (1) ≠ "1", LANJUT KE K17

K15 Apakah petugas kesehatan melakukan hal-hal berikut kepada [NAMA] pada saat pemeriksaan **6-48 jam setelah lahir**?
KODE ISIAN a-i: 1. YA 2. TIDAK 8. TIDAK TAHU

a. Pengukuran berat badan	☐	f. Nakes menanyakan [NAMA] mengalami diare atau tidak	☐
b. Pengukuran panjang badan	☐	g. Menanyakan masalah dalam pemberian ASI	☐
c. Pengukuran suhu tubuh	☐	h. Memberitahu cara pemberian ASI yang baik	☐
d. Perawatan tali pusar	☐	i. Memberitahukan mengenai tanda bahaya pada bayi	☐
e. Nakes menanyakan status kesehatan [NAMA]	☐		

KODE ISIAN j-k: 1. YA 2. TIDAK 7. SUDAH PERNAH 8. TIDAK TAHU

j. Memberikan imunisasi Hb-0	☐	k. Vitamin K	☐

HAL 17 dari 24 – KUESIONER INDIVIDU RISKESDAS 2018 (2)

K16	Apakah **[NAMA]** pada **usia 48-72 jam** dilakukan pemeriksaaan skrining hipotiroid kongenital (darah diambil dari tumit)?	1. Ya<br>2. Tidak<br>7. Tidak berlaku<br>8. Tidak tahu	☐

BUKU KIA-ANAK [ART UMUR 0 – 59 BULAN]

K17	Apakah [NAMA] memiliki Buku KIA (Buku Kesehatan Ibu dan Anak)?<br>1. Ya, dapat menunjukkan buku model tahun 2015<br>2. Ya, dapat menunjukkan buku model sebelum tahun 2015<br>3. Ya, tidak dapat menunjukkan (disimpan kader/ bidan/ di posyandu)→**K19**<br>4. Pernah memiliki, tetapi hilang → **K19**<br>5. Tidak pernah memiliki →**K19**	☐

K18	Cek isi buku KIA:					
	a. Riwayat kehamilan	1. Terisi lengkap	2. Terisi tidak lengkap	3. Tidak terisi	7. Tidak berlaku	☐
	b. Riwayat persalinan	1. Terisi lengkap	2. Terisi tidak lengkap	3. Tidak terisi	7. Tidak berlaku	☐
	c. Riwayat bayi baru lahir	1. Terisi lengkap	2. Terisi tidak lengkap	3. Tidak terisi	7. Tidak berlaku	☐
	d. Pemeriksaan kesehatan pada saat sakit	1. Terisi lengkap	2. Terisi tidak lengkap	3. Tidak terisi	7. Tidak berlaku	☐
	e. Pemantauan pertumbuhan	1. Terisi lengkap	2. Terisi tidak lengkap	3. Tidak terisi		☐
	f. Pemantauan perkembangan	1. Terisi lengkap	2. Terisi tidak lengkap	3. Tidak terisi		☐
	g. Riwayat pemberian imunisasi	1. Terisi lengkap	2. Terisi tidak lengkap	3. Tidak terisi		☐

JIKA K18g BERKODE "1" ATAU BERKODE "2"→K21
JIKA K18g BERKODE "3" →K19

K19	Apakah [NAMA] mempunyai KMS/ Buku Catatan Kesehatan Anak yang berisi catatan imunisasi?	1.Ya→**K21** 2.Tidak	☐
K20	Apakah [NAMA] pernah diimunisasi?	1. Ya 2. Tidak→**K24**	☐

K21	Apakah setelah mendapat imunisasi [NAMA] pernah mengalami keluhan *Kejadian Ikutan Pasca Imunisasi (KIPI)* seperti berikut:								
	a. Demam tinggi	1. Ya	2. Tidak	☐	c. Kejang	1.Ya	2.Tidak	☐	
	b. Bernanah/abses	1. Ya	2. Tidak	☐	d. Lainnya	1. Ya	2. Tidak	☐	

JIKA K18g BERKODE "1" ATAU "2" ATAU K19 BERKODE "1"→K22
JIKA K18g BERKODE "3" DAN K19 BERKODE "2" DAN K20 BERKODE "1"→K23
JIKA K17 BERKODE "3" ATAU "4" ATAU "5" DAN K19 BERKODE "2" DAN K20 BERKODE "1"→K23

IMUNISASI [ART UMUR 0 – 59 BULAN]

K22 **Salin dari KMS/Buku KIA/Buku Catatan Kesehatan Anak**, tanggal/ bulan/ tahun, untuk setiap jenis imunisasi

KODE KOLOM (2):
1. Diberikan imunisasi
2. Tidak diberikan imunisasi → KE JENIS IMUNISASI BERIKUTNYA
7. Belum waktunya diberikan karena umur anak → KE JENIS IMUNISASI BERIKUTNYA
8. Ditulis diberi imunisasi tetapi tgl/ bln/ thn tidak ada → KE JENIS IMUNISASI BERIKUTNYA

Jenis Imunisasi	Ket	Tgl/Bln/Thn Imunisasi	Jenis Imunisasi	Ket	Tgl/Bln/Thn Imunisasi
(1)	(2)	(3)	(4)	(5)	(6)
a. Hepatitis B 0	☐	☐☐/☐☐/☐☐	i. Polio 1	☐	☐☐/☐☐/☐☐
b. BCG	☐	☐☐/☐☐/☐☐	j. Polio 2	☐	☐☐/☐☐/☐☐
c. DPT-HB Combo 1/ DPT-HB-HiB 1	☐	☐☐/☐☐/☐☐	k. Polio 3	☐	☐☐/☐☐/☐☐
d. DPT-HB Combo 2/ DPT-HB-HiB 2	☐	☐☐/☐☐/☐☐	l. Polio 4	☐	☐☐/☐☐/☐☐
e. DPT-HB Combo 3/ DPT-HB-HiB 3	☐	☐☐/☐☐/☐☐	m.Campak	☐	☐☐/☐☐/☐☐
f. IPV 1	☐	☐☐/☐☐/☐☐	n. DPT-HB Combo Lanjutan/ DPT-HB-HiB Lanjutan	☐	☐☐/☐☐/☐☐
g. IPV 2	☐	☐☐/☐☐/☐☐			
h. IPV 3	☐	☐☐/☐☐/☐☐	o. Campak Lanjutan	☐	☐☐/☐☐/☐☐

JIKA CATATAN TANGGAL IMUNISASI ART TIDAK LENGKAP (KODE KOLOM 2 = 2,7,8), LANJUT KE K23
JIKA CATATAN IMUNISASI ART LENGKAP (SEMUA BERKODE 1), LANJUT KE K24

K23	Apakah [NAMA] pernah mendapat imunisasi berikut: **(INFORMASI DAPAT DIPEROLEH DARI BERBAGAI SUMBER)**		
	a. **Imunisasi Hepatitis B-0**, diberikan sesaat setelah bayi lahir sampai bayi berumur 7 hari yang disuntikkan di paha bayi?	1. Ya 2. Tidak→ **K23c** 8. Tidak tahu→**K23c**	☐
	b. Pada umur berapa hari [NAMA] diimunisasi Hepatitis B0?	1. 0 - 24 jam 2. >24 jam - 7 hari 8. Tidak tahu	☐
	c. **Imunisasi BCG**, mulai diberikan umur 1 bulan dan disuntikkan di lengan (kanan) atas (biasanya meninggalkan bekas (**scar**) di bawah kulit)?	1. Ya 2. Tidak→**K23e** 8. Tidak tahu→**K23e**	☐
	d. Pada umur berapa [NAMA] diimunisasi BCG?	1. 0 – 29 hari 2. ≥ 1 bulan 8. Tidak tahu	☐
	e. **Imunisasi polio**, cairan merah muda atau putih yang biasanya mulai diberikan pada umur 1 bulan dan diteteskan ke mulut?	1. Ya 2. Tidak→**K23h** 7. Belum waktunya (umur ≤ 1 bulan) →**K23h** 8. Tidak Tahu →**K23h**	☐
	f. Pada umur berapa [NAMA] pertama kali diimunisasi polio? **JIKA TIDAK TAHU ISIKAN KODE "88" UNTUK BULAN**	.... bulan	☐☐
	g. Berapa kali **[NAMA]** diimunisasi polio? **JIKA TIDAK TAHU ISIKAN KODE "8"**	.... Kali	☐
	h. Imunisasi IPV/ polio suntik	1. Ya 2. Tidak→**K23k** 7. Belum waktunya (umur ≤ 1 bulan) →**K23k** 8. Tidak Tahu →**K23k**	☐
	i. Pada umur berapa [NAMA] pertama kali diimunisasi IPV/ polio suntik? **JIKA TIDAK TAHU ISIKAN KODE "88"**	.... bulan	☐☐
	j. Berapa kali **[NAMA]** diimunisasi IPV/ polio suntik? **JIKA TIDAK TAHU ISIKAN KODE "8"**	.... Kali	☐
	k. **Imunisasi DPT-HB** combo (Diphteri Pertusis Tetanus-Hepatitis B combo) / DPT-HB-HiB yang biasanya disuntikkan di paha dan biasanya mulai diberikan pada saat anak berusia 2 bulan bersama dengan Polio 2?	1. Ya 2. Tidak →**K23n** 7. Belum waktunya (umur ≤ 2 bulan)→ **K23n** 8. Tidak Tahu →**K23n**	☐
	l. Pada umur berapa (NAMA) pertama kali diimunisasi DPT-HB Combo/ DPT-HB-HiB **JIKA TIDAK TAHU ISIKAN KODE "88"**	.... bulan	☐☐
	m. Berapa kali [NAMA] diimunisasi DPT-HB Combo / DPT-HB-HiB? **JIKA TIDAK TAHU ISIKAN KODE "8"**	.... kali	☐
	n. **Imunisasi campak** yang biasanya diberikan umur 9 bulan dan disuntikkan di paha atau lengan kiri atas serta diberikan satu kali?	1. Ya 2. Tidak 7. Belum waktunya (umur <9 bulan) 8. Tidak Tahu	☐
	o. **Imunisasi campak lanjutan** yang biasanya mulai diberikan umur 18-24 bulan	1. Ya 2. Tidak 7. Belum waktunya (umur <18 bln) 8. Tidak Tahu	☐
	p. Imunisasi DPT-HB Combo lanjutan / DPT-HB-HiB lanjutan yang biasanya mulai diberikan umur 18 bulan	1. Ya 2. Tidak 7. Belum waktunya (umur <18 bln) 8. Tidak Tahu	☐
**PEMANTAUAN PERTUMBUHAN [ART UMUR 0 – 59 BULAN]**			
K24	Apakah dalam 12 bulan terakhir [NAMA] ditimbang berat badannya?	1. Ya 2. Tidak → **K26** 8. Tidak tahu→**K26**	☐
K25	Dalam 12 bulan terakhir, berapa kali [NAMA] ditimbang berat badannya? **JIKA "TIDAK TAHU", ISI KODE "88"**	.... Kali	☐☐
**LANJUT KE K27**			
K26	**Alasan utama** dalam 12 bulan terakhir [NAMA] tidak pernah ditimbang:<br>1. Anak sudah besar (≥1 tahun) 4. Lupa/tidak tahu jadwalnya 7. Sibuk/repot<br>2. Anak sudah selesai imunisasi 5. Tidak ada tempat penimbangan 8. Malas<br>3. Anak tidak mau ditimbang 6. Tempatnya jauh 9. Alat timbang badan tidak tersedia		☐
K27	Apakah dalam 12 bulan terakhir [NAMA] diukur panjang/tinggi badannya?	1. Ya 2. Tidak →**K29** 8. Tidak tahu→**K29**	☐
K28	Dalam 12 bulan terakhir, berapa kali [NAMA] diukur panjang/ tinggi badannya? **JIKA "TIDAK TAHU", ISI KODE "88"**	.... Kali	☐☐
**LANJUT KE K30**			
K29	Alasan utama dalam 12 bulan terakhir [NAMA] tidak pernah diukur panjang/ tinggi badannya?<br>1. Anak sudah besar (≥1 tahun) 4. Lupa/tidak tahu jadwalnya 7. Sibuk/repot<br>2. Anak sudah selesai imunisasi 5. Tidak ada kegiatan pengukuran 8. Malas<br>3. Anak tidak mau diukur 6. Tempatnya jauh 9. Alat ukur panjang badan tidak tersedia		☐
K30	Apakah dalam 12 bulan terakhir [NAMA] pernah mendapatkan kapsul vitamin A? **(PERLIHATKAN GAMBAR PERAGA)**	1. Ya, 1 kali 2. Ya, 2 kali 3. Tidak pernah 7. Belum waktunya (umur < 6 bulan)	☐
**JIKA ART UMUR 0-23 BULAN →K31**<br>**JIKA ART UMUR 24-59 BULAN →K46**			

HAL 19 dari 24 – KUESIONER INDIVIDU RISKESDAS 2018 (2)

ASI DAN MP-ASI [ART UMUR 0 – 23 BULAN]			
K31	a. Apakah sesaat setelah [NAMA] lahir, diletakkan di dada/perut ibu dengan kulit ibu melekat pada kulit bayi?	1. Ya 2. Tidak→ **K32**	☐
	b. Kapan [NAMA] mulai diletakkan di dada/perut ibu setelah dilahirkan?	..................menit	☐☐
	c. Berapa lama proses pelekatan bayi pada dada/ perut ibu setelah dilahirkan?	1. < 1 jam 2. ≥ 1 jam	☐
K32	Apakah [NAMA] pernah disusui atau diberi ASI (Air Susu Ibu)?	1. Ya→**K34** 2. Tidak 8. Tidak tahu →**K44**	☐
K33	Jika belum/ tidak pernah diberi ASI, apa alasan utamanya?<br>1. ASI tidak keluar 3. Repot 5. Alasan medis 7.Ibu meninggal<br>2. Anak tidak bisa menyusu 4. Rawat pisah 6. Anak terpisah dari ibunya 8. Lainnya		☐

LANJUT KE K43

K34	Kapan ibu mulai melakukan proses menyusui untuk yang pertama kali, setelah **[NAMA]** dilahirkan? **JIKA KURANG DARI 1 JAM, TULIS 00; JIKA KURANG DARI 24 JAM, TULIS DALAM JAM; JIKA 24 JAM ATAU LEBIH TULIS DALAM HARI**	a.Satuan: 1. Jam 2. Hari	☐
		b.Jumlah jam atau hari	☐☐
K35	Apa yang dilakukan IBU terhadap kolostrum (ASI yang pertama keluar,biasanya encer, bening dan atau berwarna kekuningan)?	1. Diberikan semua kepada bayi 3. Dibuang semua<br>2. Dibuang sebagian 8. Tidak tahu	☐
K36	Apakah sebelum disusui yang pertama kali atau sebelum ASI keluar/ lancar, [NAMA] pernah diberi minuman (cairan) atau makanan selain ASI?	1.Ya 8. Tidak tahu→**K38**<br>2. Tidak→ **K38**	☐

K37 Apa jenis minuman/makanan yang pernah diberikan kepada [NAMA] sebelum mulai disusui atau sebelum ASI keluar/lancar?

a.Susu formula	1. Ya	2. Tidak	☐	g. Teh Manis	1. Ya	2. Tidak	☐
b.Susu non formula	1. Ya	2. Tidak	☐	h. Air putih	1. Ya	2. Tidak	☐
c. Madu/ Madu + air	1. Ya	2. Tidak	☐	i. Bubur tepung/bubur saring	1. Ya	2. Tidak	☐
d. Air gula	1. Ya	2. Tidak	☐	j. Pisang dihaluskan	1. Ya	2. Tidak	☐
e.Air Tajin	1. Ya	2. Tidak	☐	k. Nasi dihaluskan	1. Ya	2. Tidak	☐
f. Air kelapa	1. Ya	2. Tidak	☐	l. Lainnya, sebutkan________	1. Ya	2. Tidak	☐

K38	Apakah saat ini [NAMA] masih disusui/ diberi ASI?	1. Ya →**K40** 2. Tidak	☐
K39	Pada umur berapa bulan [NAMA] disapih/ mulai tidak disusui lagi?	.... bulan **(BILA TIDAK TAHU TULIS 88)**	☐☐

LANJUT KE K42

K40	Apakah dalam 24 jam terakhir [NAMA] **hanya mendapatkan air susu ibu** (ASI) saja dan tidak diberi minuman (cairan) dan atau makanan selain ASI?	1.Ya 2. Tidak	☐
K41	Apakah **sejak lahir sampai dengan sebelum 24 jam terakhir** [NAMA] **pernah diberi minuman (cairan) dan/ atau makanan**?	1.Ya 2. Tidak	☐
K42	Pada saat [NAMA] umur berapa, IBU pertama kali mulai memberikan minuman (cairan) atau makanan selain ASI?<br>1. 0 – 7 hari 4. 2 – < 3 bulan 7. ≥ 6 bulan<br>2. 8 – 28 hari 5. 3 – < 4 bulan 8. Tidak tahu<br>3. 29 hari – < 2 bulan 6. 4 – < 6 bulan 9. Tidak berlaku **(JIKA K40=1 DAN K41=2)→K45**		☐

K43 Apa jenis minuman (cairan) atau makanan selain ASI, **yang pertama kali mulai** diberikan kepada [NAMA] pada umur tersebut?

a. Susu formula	1. Ya	2. Tidak	☐	f. Air tajin	1. Ya	2. Tidak	☐
b. Susu non-formula	1. Ya	2. Tidak	☐	g.Buah dihaluskan (Pisang, dll)	1. Ya	2. Tidak	☐
c. Bubur formula	1. Ya	2. Tidak	☐	h.Bubur nasi/ nasi tim/nasi dihaluskan	1. Ya	2. Tidak	☐
d. Biskuit	1. Ya	2. Tidak	☐	i. Sari buah	1. Ya	2. Tidak	☐
e. Bubur tepung/bubur saring	1. Ya	2. Tidak	☐	j. Lainnya, sebutkan____________	1. Ya	2. Tidak	☐

HAL 20 dari 24 – KUESIONER INDIVIDU RISKESDAS 2018 (2)

K44	**Dalam 24 jam terakhir** (mulai dari kemarin pagi hingga pagi tadi), makanan apa sajakah yang dimakan [NAMA]? **(ISIKAN KODE JAWABAN 1 = YA ATAU 2 = TIDAK)**			
	a. Air putih	☐	h. Telur	☐
	b. Air tajin, madu, teh, kopi, air gula, jus buah	☐	i. Sayuran sumber vitamin A (daun, hijau, wortel, bayam, tomat, dll)	☐
	c. Bubur nasi/ nasi/ roti/ mie/ ketela/ ubi/ kentang/ biskuit	☐	j. Buah sumber vitamin A (tomat, pepaya, buah naga, apel dll)	☐
	d. Kacang-kacangan/ tempe/ tahu	☐	k. Sayuran lainnya (tauge, kubis putih, mentimun, dll)	☐
	e. Susu selain ASI	☐	l. Buah lainnya (nanas, melon, dll)	☐
	f. Keju/ yoghurt	☐	m. Lainnya..	☐
	g. Daging sapi/ daging ayam/ daging unggas lain/ ikan/ jerohan	☐		
K45	**JIKA ART UMUR 0-5 BULAN → LANJUT KE BLOK L**<br>**JIKA ART UMUR 6-23 BULAN → LANJUT KE K46**			

PEMBERIAN MAKANAN TAMBAHAN (PMT) PERIODE 12 BULAN TERAKHIR [ART UMUR 6-59 BULAN]

K46	Apakah selama 12 bulan terakhir [NAMA] pernah diberi PMT (makanan tambahan)?	1. Ya 2. Tidak → **K49**	☐

K47 Pada kolom berikut,isikan: bentuk dan jumlah PMT yang diperoleh, PMT yang dihabiskan, dan alasan utama tidak dihabiskan **(selama 12 bulan terakhir)**

Bentuk PMT	Bentuk PMT yang diperoleh<br>Isikan kode<br>1. Ya<br>2. Tidak → **ke baris berikutnya**	Jumlah total PMT yang diperoleh<br><br>(bungkus)	Apakah PMT dihabiskan oleh [NAMA]<br>1. Ya → **ke baris berikutnya**<br>2. Tidak	Alasan utama PMT tidak dihabiskan?<br>1. Anak tidak mau<br>2. Ibu Lupa memberikan<br>3. Ada efek samping (diare, muntah, alergi dll)<br>4. Dimakan ART lain<br>5. Lainnya.......
	(1)	(2)	(3)	(4)
a. Biskuit Program **(LIHAT BUKU PERAGA)**	☐	☐☐	☐	☐...........
b. Biskuit lainnya	☐		☐	☐...........
c. Susu Bubuk	☐		☐	☐...........
d. Susu Cair	☐		☐	☐...........
e. Bahan Makanan Mentah	☐		☐	☐...........
f. Makanan Matang	☐		☐	☐...........

K48	Alasan [NAMA] mendapatkan PMT? **POIN a S/D g TIDAK DIBACAKAN (ISIKAN KODE JAWABAN: 1. YA ATAU 2. TIDAK)**			
	a. Gizi buruk	☐	e. Sakit – sakitan	☐
	b. Gizi Kurang/BGM	☐	f. Karena ikut penimbangan di Posyandu	☐
	c. Kurus	☐	g. Keluarga Miskin (gakin)	☐
	d. Berat badan tidak pernah naik (2T)	☐	h. Lainnya	☐
K49	**JIKA ART UMUR 6-35 BULAN →BLOK L**<br>**JIKA ART UMUR 36-59 BULAN →K50**			

PERKEMBANGAN ANAK [ART UMUR 36-59 BULAN]

K50	Apakah [NAMA] dapat menyebutkan namanya sendiri tanpa dibantu?	1. Ya	2. Tidak	☐
K51	Apakah [NAMA] dapat membaca setidaknya 4 kata sederhana/ populer?	1. Ya	2. Tidak	☐
K52	Apakah [NAMA] mengetahui dan mengenali simbol angka 1-10	1. Ya	2. Tidak	☐
K53	Apakah [NAMA] dapat mengambil benda kecil (seperti kerikil atau tongkat kayu kecil) dengan menggunakan 2 jari (ibu jari dan telunjuk)	1. Ya	2. Tidak	☐
K54	Apakah [NAMA] kadang-kadang sakit sampai tidak bisa bermain?	1. Ya	2. Tidak	☐

HAL 21 dari 24 – KUESIONER INDIVIDU RISKESDAS 2018 (2)

K55	Apakah [NAMA] mengenali atau dapat menyebutkan setidaknya 10 huruf alphabet/abjad?	1.Ya	2. Tidak	☐
K56	Apakah [NAMA] dapat mengikuti perintah sederhana untuk melakukan sesuatu dengan benar?	1.Ya	2. Tidak	☐
K57	Saat diperintah/diberi tahu sesuatu, apakah [NAMA] mampu melakukannya sendiri tanpa dibantu?	1.Ya	2. Tidak	☐
K58	Apakah [NAMA] mampu bermain/bersosialisasi dengan baik bersama anak-anak lainnya?	1.Ya	2. Tidak	☐
K59	Apakah [NAMA] suka menendang, menggigit, atau memukul anak lain atau orang dewasa?	1.Ya	2. Tidak	☐
K60	Apakah [NAMA] mudah terganggu konsentrasinya dalam melakukan suatu hal?	1.Ya	2. Tidak	☐

L. PENGUKURAN DAN PEMERIKSAAN				
**TINGGI BADAN/ PANJANG BADAN [ART SEMUA UMUR]**				
L01	a. Apakah [NAMA] diukur Tinggi/ Panjang Badan?	1. Ya	2. Tidak→**L02**	☐
	b. Tinggi/Panjang Badan (Cm)	.... cm		☐☐☐,☐
	c. **KHUSUS UNTUK BALITA**, (Posisi pengukuran TB/PB)	1. Berdiri	2. Telentang	☐
	d. Kondisi [NAMA] saat diukur	1. Dapat berdiri tegak/ telentang lurus	2. Tidak dapat berdiri tegak/ telentang lurus	☐
**BERAT BADAN [ART SEMUA UMUR]**				
L02	a. Apakah [NAMA] ditimbang?	1. Ya	2. Tidak→ **L03**	☐
	b. Berat Badan (kg)	.... kg		☐☐☐,☐
	c. Kondisi [NAMA] saat ditimbang	1. Sehat	2. Sakit	☐
**LINGKAR PERUT [ART UMUR ≥ 15 TAHUN, KECUALI PEREMPUAN HAMIL (J48="1")]**				
L03	a. Apakah [NAMA] diukur Lingkar Perut	1. Ya	2. Tidak → **L04**	☐
	b. Lingkar Perut (Cm)	.... cm		☐☐☐,☐
**TEKANAN DARAH DIUKUR DI LENGAN KIRI [ART UMUR ≥ 15 TAHUN]**				
L04	**a. Apakah dilakukan pengukuran tekanan darah yang pertama:**	1. Ya	2. Tidak→**L07**	☐
	b. Tekanan darah sistolik (mmHg) ☐☐☐	c. Tekanan darah diastolik (mmHg)		☐☐☐
L05	**a. Apakah dilakukan pengukuran tekanan darah yang kedua :**	1. Ya	2. Tidak→**L07**	☐
	b. Tekanan darah sistolik (mmHg) ☐☐☐	c. Tekanan darah diastolik (mmHg)		☐☐☐
L06	**a. Apakah dilakukan pengukuran tekanan darah yang ketiga :**	1. Ya	2. Tidak→**L07**	☐
	b. Tekanan darah sistolik (mmHg) ☐☐☐	c. Tekanan darah diastolik (mmHg)		☐☐☐
**LINGKAR LENGAN ATAS (LILA) [ART PEREMPUAN USIA SUBUR (15-49 TAHUN) ATAU PEREMPUAN HAMIL (J48="1")]**				
L07	a. Apakah [NAMA] diukur Lingkar Lengan Atas (LILA)	1. Ya	2. Tidak→**L08**	☐
	b. Lingkar Lengan Atas (cm)	.... cm		☐☐,☐

CATATAN

HAL 22 dari 24 – KUESIONER INDIVIDU RISKESDAS 2018 (2)

PENGAMBILAN SPESIMEN DARAH			
L08	a. Apakah [NAMA] mempunyai riwayat hemofili/ ITP/ minum obat antikoagulan/ sakit berat?	1. Ya, jelaskan__________________ 2. Tidak	☐
	b. Apakah [NAMA] dilakukan pengambilan darah? JUMLAHKAN KODE JAWABAN, JIKA JAWABAN > 1	1. Ya, darah vena 2. Ya, darah kapiler 4. Tidak →SELESAI	☐
	c. Waktu pengambilan darah (jam:menit)	☐☐:☐☐	
L09	STIKER NOMOR DARAH	TEMPEL STIKER DI SINI (6 digit)	

PEMERIKSAAN Hb [ART SEMUA UMUR]			
L10	a. Apakah [NAMA] dilakukan pemeriksaan Hb	1. Ya 2. Tidak →L11	☐
	b. Nilai Hb (g/dl)	.... g/dl	☐☐,☐

PEMERIKSAAN MALARIA [ART SEMUA UMUR]			
L11	a. Apakah [NAMA] dilakukan pemeriksaan (RDT) Malaria?	1. Ya 2. Tidak →L12	☐
	b. Apakah [NAMA] mengalami riwayat demam/ panas dalam 2 hari terakhir?	1. Ya 2. Tidak	☐
	c. Hasil RDT malaria	1. Negatif 2. *P.falciparum (Pf)* 3. *PAN* (non *P.falciparum*) 4. *Pf* dan *PAN* (mix) 5. Hasil tidak sahih	☐

PEMERIKSAAN KADAR GULA DARAH [ART UMUR ≥ 15 TAHUN]			
L12	a. Apakah [NAMA] mempunyai riwayat menderita diabetes (berdasarkan hasil dokter pemeriksa)?	1. Ya 2. Tidak	☐
	b. Apakah [NAMA] minum obat oral anti diabetes/ injeksi insulin (berdasarkan hasil dokter pemeriksa)?	1. Ya 2. Tidak	☐
	c. Apakah [NAMA] berpuasa?	1. Ya 2. Tidak	☐
	d. Kapan [NAMA] terakhir makan?	1. malam 2. pagi	☐
	e. Pukul berapa terakhir makan/ minum (kecuali air tawar)? (jam:menit)	☐☐:☐☐	
	f. Apakah [NAMA] dilakukan pemeriksaan kadar gula darah sewaktu?	1. Ya 2. Tidak →L12h	☐
	g. Kadar glukosa darah sewaktu	☐☐☐ mg/dl	
	h. Apakah [NAMA] dilakukan pemeriksaan kadar gula darah puasa?	1. Ya 2. Tidak →L12j	☐
	i. Kadar glukosa darah puasa	☐☐☐ mg/dl	
	j. Apakah [NAMA] dilakukan pembebanan glukosa?	1. Ya 2. Tidak →SELESAI	☐
	k. Pukul berapa mulai dilakukan pembebanan glukosa? (jam:menit)	☐☐:☐☐	
	l. Apakah dilakukan pemeriksaan kadar gula darah pada 2 jam setelah pembebanan?	1. Ya 2. Tidak →SELESAI	☐
	m. Pukul berapa dilakukan pengambilan darah setelah pembebanan? (jam:menit)	☐☐:☐☐	
	n. Kadar glukosa darah 2 jam setelah pembebanan	☐☐☐ mg/dl	

CATATAN

HAL 23 dari 24 – KUESIONER INDIVIDU RISKESDAS 2018 (2)

LEMBAR BANTU UNTUK MENGHITUNG AKTIVITAS FISIK (G29-G34)

TULISKAN SECARA RINCI SEMUA AKTIFITAS FISIK YANG BIASA DILAKUKAN SEHARI-HARI BERDASARKAN JENIS DAN LAMANYA KEGIATAN:

1. Pagi: (duduk/berdiri/jalan) [*KRITERIA BERAT/ SEDANG]

JAM (_:_-_:_)	JML (MENIT)		KRITERIA*

2. Siang (duduk, berdiri/jalan) [*KRITERIA BERAT/ SEDANG]

JAM (_:_-_:_)	JML (MENIT)		KRITERIA *

3. Malam (duduk/berdiri/jalan) [*KRITERIA BERAT/ SEDANG]

JAM (_:_-_:_)	JML (MENIT)		KRITERIA *

4. Waktu lain (duduk/berdiri/jalan) [*KRITERIA BERAT/ SEDANG]

JAM (_:_-_:_)	JML (MENIT)		KRITERIA *

Lampiran 4
Tim Penulis

Penyakit Menular dan Pengetahuan HIV
- Miko Hananto
- Asep Hermawan
- Rita Marleta
- Khariri
- Puti Sari Hidayangsih
- Made Agus Nurjana
- Heny Lestary
- Yusniar Ariati
- Anton Suryatma

Kesehatan Jiwa
- Sri Idaiani
- Indri Yunita Suryaputri
- Lely Indrawati

Disabilitas
- Siti Isfandari
- Sri Poedji Hastoeti Djaiman
- Milana Salim

Status Gizi
- Yunita Diana Sari
- Rika Rachmalina
- Yurista Permanasari
- Dwi Sisca Kumalasari
- Agus Triwinarto
- Dyah Santi Puspitasari
- Prisca Petty Arfines
- Ina Kusrini

Kesehatan Anak
- Kencana Sari
- Suparmi
- Bunga Christita Rosa
- Nurilah Amaliah
- Nur Handayani
- Khadijah Azhar

Penyakit Tidak Menular
- Julianty Pradono
- Delima
- Frans Dany
- Sarwo Handayani
- Yuana Wiryawan
- Antonius Yudi Kristanto

Cedera
- Lusianawaty Tana
- Tri Wurisastuti

Farmasi dan Pelayanan Kesehatan Tradisional
- Andi Leny Susyanti
- Yuyun Yuniar
- Raharni
- Lusi Kristiana
- Lucie Widowati

Perilaku Kesehatan
- Nunik Kusumawardani
- Rofingatul Mubasyiroh
- Olwin Nainggolan
- Astridya Paramitha

Pengetahuan Akses Pelayanan Kesehatan
- Ingan Ukur Tarigan
- Tati Suryati
- Zainul Khaqiqi Nantabah

Kesehatan Gigi dan Mulut
- Lely Andayasari
- Masagus Zainuri
- Melissa Adiatman
- Tince A. Jovina
- Grace Monica Santoso
- Nurhayati

Kesehatan Ibu
- Tin Afifah
- Annisa Riskianti
- Aprildah Nur Sapardin
- Novianti
- Djunaedi

Kesehatan Lingkungan
- Zahra
- Athena Anwar
- Tities Puspita
- Cahyorini
- Ika Dharmayanti

TIM ANALISIS

- Diana Andriyani Pratamawati
- Revi Rosavika Kinansi
- Merry Budiarti
- Yuniarti Suryatinah
- Marya Ulfa
- Mutiara Widawati
- Zumrotus Sholichah
- Totih Ratna Sondari Setiadi
- Nur Rahmawati Wijaya
- Abidah Nur
- Windarti Fauziah
- Irawati Wike
- Ritawati
- Meiske Elisabeth Koraag
- Dewi Rahayu
- Monika Noshirma
- Sri Muljati
- Mara Ipa
- Sri Prihatini
- Arif Musoddaq
- Narendro Arifia
- Amir Suudi
- Hadi Ashar
- Dwi Priyanto
- Eddy Purwanto
- Bhakti Samsu Adi
- Rianto Purnama
- Yusup
- Akhmad Wahyudin
- Rika Mayasari

TIM PENGARAH
- Siswanto
- Nana Mulyana
- Agus Suprapto
- Irmansyah
- Vivi Setiawaty
- Sugianto
- Trihono
- Joko Waluyo
- Akhmad Saikhu
- Sri Irianti
- Ekowati Rahajeng
- Dede Anwar Musadad
- Abbas Basuni Jahari
- Iwan Ariawan
- Purnawan Junadi
- Suzanna Immanuel
- Anton Rahardjo
- Emiliana Tjitra
- Wasis Budiarto
- Lestari Handayani
- Niniek L. Pratiwi

TIM PENYUNTING/EDITOR

- Agus Suprapto
- Joko Irianto
- Dwi Hapsari Tjandrarini
- Bayu Ari Bowo

TIM DESIGN
- Achmad Firmansyah
- Eka Sri Setyaningsih

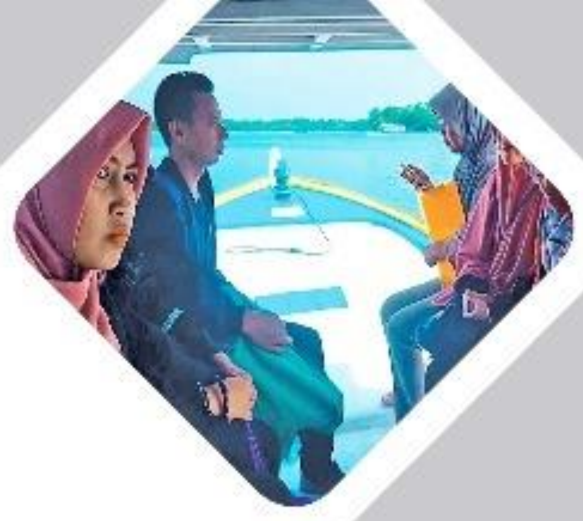

Diterbitkan oleh :

LEMBAGA PENERBIT
BADAN PENELITIAN DAN PENGEMBANGAN KESEHATAN
Jalan Percetakan Negara No. 23, Jakarta 10560
Telp. (021) 4261088, ext. 222, 223 . Fax. (021) 4243933

ISBN 978-602-373-118-3
